# Effects of microgravity on human iPSC-derived neural organoids on the International Space Station

**DOI:** 10.1093/stcltm/szae070

**Published:** 2024-10-23

**Authors:** Davide Marotta, Laraib Ijaz, Lilianne Barbar, Madhura Nijsure, Jason Stein, Nicolette Pirjanian, Ilya Kruglikov, Twyman Clements, Jana Stoudemire, Paula Grisanti, Scott A Noggle, Jeanne F Loring, Valentina Fossati

**Affiliations:** The New York Stem Cell Foundation Research Institute, New York, NY 10019, United States; The New York Stem Cell Foundation Research Institute, New York, NY 10019, United States; The New York Stem Cell Foundation Research Institute, New York, NY 10019, United States; The New York Stem Cell Foundation Research Institute, New York, NY 10019, United States; Department of Molecular Medicine, Scripps Research, La Jolla, CA 92037, United States; The New York Stem Cell Foundation Research Institute, New York, NY 10019, United States; The New York Stem Cell Foundation Research Institute, New York, NY 10019, United States; Space Tango, Lexington, KY 40505, United States; Space Tango, Lexington, KY 40505, United States; National Stem Cell Foundation, Louisville, KY 40202, United States; The New York Stem Cell Foundation Research Institute, New York, NY 10019, United States; Department of Molecular Medicine, Scripps Research, La Jolla, CA 92037, United States; National Stem Cell Foundation, Louisville, KY 40202, United States; The New York Stem Cell Foundation Research Institute, New York, NY 10019, United States

**Keywords:** microgravity, organoids, induced pluripotent stem cells, neurons, microglia, Parkinson’s disease, multiple sclerosis

## Abstract

Research conducted on the International Space Station (ISS) in low-Earth orbit (LEO) has shown the effects of microgravity on multiple organs. To investigate the effects of microgravity on the central nervous system, we developed a unique organoid strategy for modeling specific regions of the brain that are affected by neurodegenerative diseases. We generated 3-dimensional human neural organoids from induced pluripotent stem cells (iPSCs) derived from individuals affected by primary progressive multiple sclerosis (PPMS) or Parkinson’s disease (PD) and non-symptomatic controls, by differentiating them toward cortical and dopaminergic fates, respectively, and combined them with isogenic microglia. The organoids were cultured for a month using a novel sealed cryovial culture method on the International Space Station (ISS) and a parallel set that remained on Earth. Live samples were returned to Earth for analysis by RNA expression and histology and were attached to culture dishes to enable neurite outgrowth. Our results show that both cortical and dopaminergic organoids cultured in LEO had lower levels of genes associated with cell proliferation and higher levels of maturation-associated genes, suggesting that the cells matured more quickly in LEO. This study is continuing with several more missions in order to understand the mechanisms underlying accelerated maturation and to investigate other neurological diseases. Our goal is to make use of the opportunity to study neural cells in LEO to better understand and treat neurodegenerative disease on Earth and to help ameliorate potentially adverse neurological effects of space travel.

## Graphical Abstract

**Graphical Abstract:**
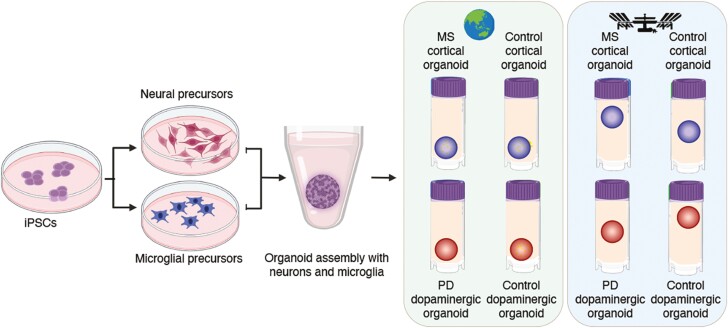

**Significance statement**

To investigate the effects of microgravity on the brain, we developed simplified models of parts of the human brain affected by multiple sclerosis and Parkinson’s disease. Neuronal organoids, tiny aggregates of cells, were made from induced pluripotent stem cells and maintained in a novel static culture system for 30 days on the International Space Station (ISS). We observed that the neurons on the ISS developed faster than those on Earth, and future missions will investigate the underlying causes. This is the first report of using disease-associated neural organoids on the ISS to better understand microgravity’s effects on the brain and to investigate treatments for neurodegenerative diseases.

## Introduction

Experimental biological research on the International Space Station (ISS) in low-Earth orbit (LEO) is intended to both understand the effects of microgravity on human health and to investigate the use of the ISS as a platform for improvements in disease modeling and drug development. The studies performed so far have indicated that microgravity causes changes in musculoskeletal systems and the peripheral immune system, and can affect vestibular function and cognition.^1^ Cell types derived from human induced pluripotent stem cells (iPSCs) can be used to build relevant in vitro models of human organs to study functional and structural changes that may be important to human health in space and on Earth. Because of our interest in degenerative diseases of the central nervous system (CNS), we included iPSC-derived neural cells from individuals with primary progressive multiple sclerosis (PPMS) and Parkinson’s disease (PD). We created 3-dimensional aggregates of cells, known as organoids, that contained iPSC-derived cortical or dopaminergic neurons to model the most vulnerable neuronal populations in MS and PD, respectively. To improve the utility of these simple neuronal models, we added microglia, which are part of the CNS resident immune system, to a portion of the organoids.

**Figure 1.**
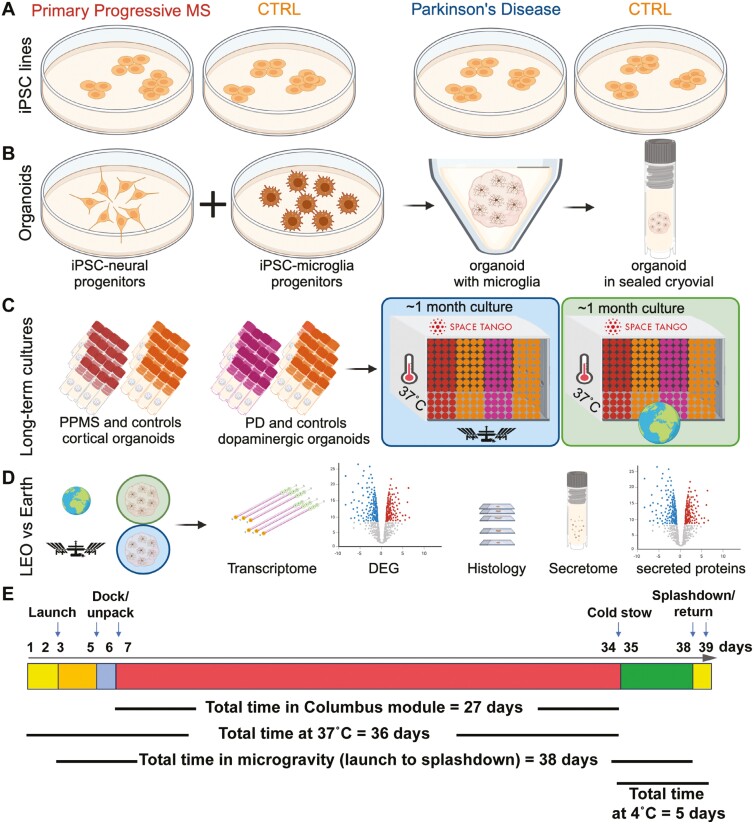
Experimental design. A. iPSCs from 4 individuals were selected for experiments: 2 controls and 2 with the neurodegenerative diseases Parkinson’s disease or primary progressive multiple sclerosis. B. iPSCs were differentiated into cortical or dopaminergic neuron precursors, aggregated to form organoids, and matched microglia derived from each of the cell lines were added to half of the organoids. Each organoid was placed in a separate cryovial with 1 mL of culture medium and sealed for the duration of the experiment. One set of cryovials was transported to the ISS on the SpX CRS-19 mission while the other replicate set remained on ground. C. Matching sets of cryovial cultures were incubated for a month at 37 °C on ground and onboard the ISS. D. Upon return to Earth, both sets of organoid cultures were analyzed by multiple methods. E. Timeline of the mission.

We developed new protocols specifically for the experiments on the ISS, including a novel cell culture system, transport methods, and post-flight recovery and analysis. We generated the organoids in our lab, transported them to the Space Station Processing Facility at Kennedy Space Center (KSC), and placed each organoid in a cryovial containing 1 mL of culture medium; the vials were loaded into flight hardware that maintained temperature control during transport to and return from the ISS. Station radiation monitoring logs were used to report radiation levels during the mission. The cultures were launched to the ISS on the SpaceX 19th Commercial Resupply Services mission for NASA (SpX CRS-19) and maintained in static culture without medium exchange onboard the ISS for 30 days, followed by a live return for post-flight analysis (Figure 1). We maintained a parallel ground control experiment at the KSC laboratory.

The ground and LEO samples were analyzed by multiple methods: post-flight viability, immunocytochemistry, whole genome RNA sequencing, and analysis of proteins secreted into the culture medium. The returning organoids were alive and extended neurites when plated onto culture dishes. We observed significant differences in gene expression associated with LEO samples in both cortical and dopaminergic organoids, suggesting changes in cell-cell signaling. The radiation monitoring and gene expression analysis indicate that microgravity, not radiation, was the primary cause of these differences. This pioneering experiment demonstrates that complex cultures of iPSC-derived CNS cells can be successfully maintained in LEO for long periods of time, establishing the foundation for future studies. These findings from transcriptomic and secretome analyses support the hypothesis that microgravity affects CNS cells and warrants additional investigations.

## Materials and methods

### Cell lines

This study was performed using 4 iPSC lines (Table 1). iPSC line 051121-01-MR-017 and AK003-01-MR-008 were previously reprogrammed from dermal fibroblasts with an mRNA/miRNA method^2,3^ iPSC line HDF410iPS504^4^ and iPSC line UEC741iPS517 were previously reprogrammed from dermal fibroblasts and urine epithelial cells, respectively using non-integrating Sendai virus.^5^ iPSC lines were expanded and maintained in mTeSR1 medium, following published methods,^6^ assessed for pluripotency using RNAseq and PluriTest^®7^ and for genomic integrity with single nucleotide polymorphism microarrays.^8^

**Table 1.** Cell lines and characteristics.

Subject	Cell Line ID	Characteristic	Sex	Ethnicity	Somatic cell type	Reprogramming method
Subject 1	051121-01-MR-017	No known diseases	Female	Caucasian	Biopsy-derived fibroblast	mRNA
Subject 2	AK003-01-MR-008	Primary progressive multiple sclerosis	Female	Caucasian	Biopsy-derived fibroblast	mRNA
Subject 3	UEC741iPS517	No known diseases	Male	Vietnamese	Epithelial cells from urine	Sendai
Subject 4	HDF410iPS504	Sporadic Parkinson’s disease	Male	Caucasian	Biopsy-derived fibroblast	Sendai

### Comparison of organoid culture methods

To validate our novel culture method, we used lines 051121-01-MR-017 and HDF410iPS504, differentiated into cortical and dopaminergic progenitors respectively (Supplementary Figure S1). We compared organoids cultured for a month either conventionally with medium changes or in static culture in cryovials. Cells were assembled into spheres using ultra-low attachment (ULA) 96-well V-bottom plates (PrimeSurface 3D culture spheroid plates) and then half of them were transferred into cryovials with 1 mL of medium for static culture and the other half maintained in wells of a 96-well plate with 100 µL culture medium per well for 1 month. All organoids were kept in the same incubator at 37 ^o^C in 5% CO_2_, but organoids in 96-well plates were fed 3 times a week with either cortical or dopaminergic medium. After 30 days, organoids were retrieved, fixed with 4% paraformaldehyde (PFA) in PBS, embedded in OCT (optimal cutting temperature) compound, and flash frozen as described.^9^ Organoids were cryosectioned at 20 µm. Sections were stained with Hoechst for visualizing nuclei. Images were acquired using a Nikon Ti2-E High Content Analysis System (Supplementary Figure S2).

### Differentiation and assembly of organoids and microglia

Figure 1 and Supplementary Figure S1 illustrate the steps involved in the experiment from culture of the organoids to post-flight recovery and analysis. iPSC lines 051121-01-MR-017 and AK003-01-MR-008 were differentiated into cortical neural precursors using established protocols^10^ and iPSC lines HDF410iPS504 and UEC741iPS517 were differentiated into dopaminergic neural precursors.^4^ Twenty-five days after differentiation was initiated, both cortical and dopaminergic precursors were dissociated and cryopreserved. Microglia were separately derived from all 4 cell lines.^11^ A week before the estimated launch date, cryopreserved neural precursors were thawed, washed in DMEM/F12, and spun at 300*g* for 5 minutes. Cell pellets were resuspended in cortical neuron medium^10^ or dopaminergic neuron medium^4^ and plated at a density of 1 × 10^5^ cells/well onto ULA V-bottom plates (Sbio MS-9096VZ) and incubated overnight at 37 °C, 5% CO_2,_ to allow cell aggregation into spherical organoids. For half of the samples, 10^4^ iPSC-derived microglia were added to the matched organoids after 24 hours, and IL-34 (100 ng/mL) and GM-CSF (10 ng/mL) were included in the medium to promote microglial maturation. Cultures were incubated for an additional 24 hours before being shipped at 4 °C to the Kennedy Space Center (KSC) for payload preparation and launch.

### Payload preparation, launch, and splashdown

Each organoid was transferred into a cryovial (NUNC Coded Cryobank Vial, Thermo Fisher Scientific #374088), containing 1 mL of cortical or dopaminergic neuron medium with the addition of IL-34 (100 ng/mL) and GM-CSF (10 ng/mL) for those containing microglia. The basal medium was DMEM/F-12 buffered with 15 mM HEPES (Thermo Fisher Scientific #11330). The cryovials were closed and loaded into a CubeLab (Space Tango), a miniaturized incubator that maintains a stable temperature of 37 ^o^C. The CubeLab was then transferred to the rocket and after 2 days, launched into low-Earth orbit onboard the SpaceX 19th Commercial Resupply Services mission (SpX CRS-19) to the ISS for a 30-day stay in microgravity. Under similar conditions, ground control samples were kept at the KSC laboratory. After 30 days, the Dragon capsule carrying the organoids back to Earth splashed down in the Pacific Ocean, and the CubeLab was opened six days post-undock from the ISS (see Figure 1).

### Post-flight culture of organoids

Immediately after the CubeLab was opened, the viability of the organoids was assessed by plating 4 organoids separately on culture dishes coated with polyornithine/laminin/fibronectin and cultured for 2 weeks at 37 ^o^C in differentiation medium conventionally buffered for CO_2_.

### RNA extraction and sequencing

Twenty organoids cultured in LEO and 22 from ground control, both with and without added microglia, were snap-frozen and stored at −80 °C. RNA was extracted using the Qiagen RNeasy Micro kit (QIAGEN #74004). To maximize the yield, the RNA was eluted into 12 μL of ultrapure DI water. The RNA concentrations were assessed with the RNA 6000 Pico assay (Bioanalyzer; Agilent) and RNA Integrity Numbers were reported (Supplementary Table S1). The RNA was sequenced by an outside provider (Novogene; https://www.novogene.com/us-en/) that used an ultra-low input preparation of samples with a minimum of 10 ng of RNA and the Illumina NovaSeq Platform for high-throughput sequencing. A pilot subset of samples was cDNA-amplified before sequencing with the Clontech SMART kit employing Clontech SMART CDS Primer II A. Raw FASTQ data were filtered for adapters with fastp v0.23.0 software and aligned to the human genome using STAR v 2.7.10a with indexes generated from the Homo sapiens GRCh38 primary assembly and the GRCh38.107.gtf build ensemble annotations. Initial quality control suggested mouse sequence contamination in some raw FASTQ sample files. Sequence reads were separately aligned to the human genome using STAR v 2.7.10a with indexes generated from the Homo sapiens GRCh38 primary assembly with the GRCh38.107.gtf build ensemble annotations and to the Mus musculus GRCm39 primary assembly with the Mus musculus GRCm39.107.gtf build ensemble annotations. The XenofilteR R-package software^12^ was then used to generate human.bam files filtered for mouse sequence contamination. The featureCounts v2.0.3 software was used to generate a counts matrix for differential expression analysis. The organoids were analyzed individually, and after datasets were filtered, data from organoids composed of the same cell types were combined to increase the power of the analysis.

### Differential gene expression, gene set enrichment analysis (GSEA), gene ontology (GO) terms, transcription factor enrichment analysis

The RNA sequencing data were analyzed using the R packages DESeq2,^13^ gene set enrichment analysis (GSEA), and gProfiler^14^ and ggplot2^15^ for GO terms. TFEA^16^ was performed on gene sets that were differentially expressed in LEO compared to the parallel samples on the ground.

### Analysis of secreted proteins using proximity extension assay

Culture medium from vials cultured in LEO or on ground (Supplementary Figure S5) was removed and frozen at −20 °C. Proteomic analysis was performed by Olink (Olink Proteomics AB, Uppsala, Sweden) using the Olink Explore 1536 Olink protocol technology based on the proximity extension assay (PEA).^17^ Olink Explore 1536 included a combination of 4 separate Olink Explore 384 panels: Olink Explore 384 Inflammation (Product Code 91101), Olink Explore 384 Oncology (Product Code 91102), Olink Explore 384 Cardiometabolic (Product Code 91103), and Olink Explore 384 Neurology (Product Code 91104). Each panel used 384 pairs of antibodies conjugated to unique DNA sequences that hybridize and amplify upon binding to their target proteins. The resulting DNA amplicons were then quantified by sequencing using the Illumina NovaSeq system. The output data were reported as Normalized Protein eXpression (NPX) values, which are log2-transformed relative quantification values derived from optimized standard curves for each assay that are normalized for inter-plate variation and internal controls. Each sample was assessed for its overall quality based on the number of detected proteins and the percentage of missing values. Samples with fewer than 46% of the possible detected proteins or more than 25% missing values were excluded from further analysis. The secretome data were analyzed using the OlinkAnalyze R-package v3.4.1. As with the RNAseq data, we analyzed cortical organoids and dopaminergic organoids separately. We used the olink_lmer function to obtain estimated means for each assay, fitting a linear mixed model with NPX as the dependent variable, microglia presence and ground vs LEO treatment condition as the fixed effect, and cell line as random effect. Post-hoc analysis with olink_lmer_posthoc was used to obtain relative NPX expression estimates for the ground vs LEO treatment conditions for the cortical and dopaminergic organoids.

### Multi-electrode array recordings from organoids after cryovial culture

Cortical organoids from a healthy control line were cultured in cryovials for 30 days, then collected with a wide-orifice 1 mL pipette and transferred onto polyethylenimine (PEI)/laminin pre-coated multi-electrode array (MEA) 48-well plates (Axion Biosystems #M768-tMEA-48B). PEI was diluted to 0.1% in 0.1M borate buffer (pH 8.4) and then filtered. MEA plates were coated by incubation in PEI for 4 hours, washed 5 times with distilled H_2_O, then incubated in 10 μg/mL laminin for 4 hours. To allow organoids to attach to the MEA wells, single organoids were plated in each well in a small volume of culture medium to cover the organoid and the electrodes, and left undisturbed for up to 1 hour at 37 °C. After ensuring organoid attachment, medium was added to fill the well. The cultures were fed twice a week and measurements were collected 4 hours after the medium change at 2 timepoints after plating (3 and 4 weeks). Recordings were performed using a Maestro MEA system (Axion Biosystems) and AxIS software in “spontaneous neural activity” configuration. Spikes were detected with AxIS software using an adaptive threshold crossing set to 5.5 times the standard deviation of the estimated noise for each electrode (channel). For the MEA analysis, the electrodes that detected at least 5 spikes/minute were classified as active electrodes. Weighted mean firing rates were calculated as averages for the active electrodes. Microscopic images were captured to assess cell density and electrode coverage. A custom MATLAB routine was used for generating a continuous raster plot of activity in each well (Supplementary Figure S2).

### Immunocytochemistry

Low-Earth orbit and ground controls from the 30-day mission were fixed with 4% PFA in PBS for 30 minutes and embedded for cryosectioning. Sections were stained with markers of neural rosettes using antibodies to NCAD (Cadherin2, N-cadherin; 1:100, BioLegend 350802) and PAX6 (Paired box protein 6; 1:200, BioLegend 901301).

### Radiation monitoring

Radiation environmental monitoring (REM) and hybrid electronic radiation assessor devices are currently operational on the ISS.^17^ Station monitoring logs adjacent to the payload storage location on the ISS were provided after the flight to estimate radiation exposure during the mission. The payload was stowed in the European Space Agency (ESA) Columbus Laboratory during the mission. Timestamp-associated REM measurements from radiation logs provided by NASA for this location were reported as daily Galactic Cosmic Radiation (GCR) and Southern Atlantic Anomaly (SAA) doses; GCR and SAA doses were combined to report averaged daily exposures (mGy/d). Cumulative exposures were calculated for the period defined as the time from docking of the Dragon vehicle to the ISS through the day of vehicle undocking (Table 2).

## Results

### Neural organoid cultures successfully maintained for 30 days onboard the ISS

**Figure 2.**
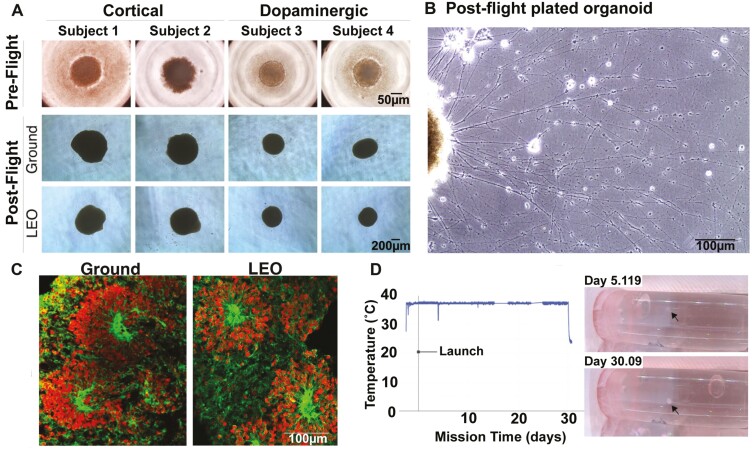
Neural organoids cultured in the static system without medium exchange were viable after 30 days. A. Phase-contrast images of cortical and dopaminergic neural organoids, from preflight stage to the post-flight stage in low-Earth orbit (LEO) and on ground. B. Organoid cultured after 30 days in LEO shows outgrowth of neurites with growth cones. C. Immunocytochemistry of cryosectioned cortical organoid shows the neural rosettes typical of cortical organoids (red: PAX6; green: CDH2 [NCAD]) for ground and LEO samples. D. Telemetry shows the temperature maintained inside the CubeLab onboard the ISS and the images show the organoid (arrows) cultured in the static system at mission days 5.119 and 30.09 (images taken onboard the ISS).

As a model to investigate the effects of microgravity on the brain, we developed methods to maintain human iPSC-derived neural organoids in LEO onboard the ISS. We differentiated cortical or dopaminergic neurons from 4 different donors (Table 1), choosing cell lines that are in use in our laboratories for projects studying PPMS and PD. Since the affected individuals had sporadic neurogenerative disease without a known genetic basis, our studies were not powered to detect disease-associated differences among the iPSCs from PPMS and PD and the controls. Because neurons originate from ectoderm, and microglia from mesodermal extraembryonic yolk sac during the early stages of embryogenesis, we derived microglia from each of the iPSC lines separately and added the matching cells to half of the organoids (Figure 1). The spherical organoids, about 200-500 µm in diameter, were formed in microwells on the ground, then transferred to individual cryovials containing 1 mL of buffered culture medium. Duplicate sets of organoids were cultured in parallel in LEO or on Earth for approximately 1 month without medium changes. The culture medium in the vials maintained constant pH, there was no evaporation, and the organoids maintained their morphology both in LEO and on ground (Figure 2A). Upon their return to Earth, several of the organoids from the ISS were transferred to culture dishes, where they attached to the surface and rapidly elaborated networks of neurites (Figure 2B). The cortical organoids showed the typical neural rosettes of this neuronal subtype (Figure 2C). There was a brief drop in temperature when the Dragon capsule reached the ISS and incubators were transferred to ISS power. Monitors in the incubator showed that the temperature then remained constant at 37 ^o^C before lowering to 4 ^o^C for the return to Earth (Figure 2D). The organoids could be visualized in the cryotubes during their time on the ISS (Figure 2D). These data show that CNS organoids cultured in a static system remain viable without medium exchange during long-term cultures in LEO and on Earth. The viability of the organoids in static culture was separately confirmed by directly comparing organoids cultured in conventional conditions in 96-well dishes with regular medium exchange in parallel with static cryovial cultures (Supplementary Figure S2). In addition, we confirmed neuronal activity by collecting multielectrode array (MEA) recordings from organoids after a month of cryovial culture (Supplementary Figure S2).

### Enhanced expression of maturation-associated genes in LEO

**Figure 3.**
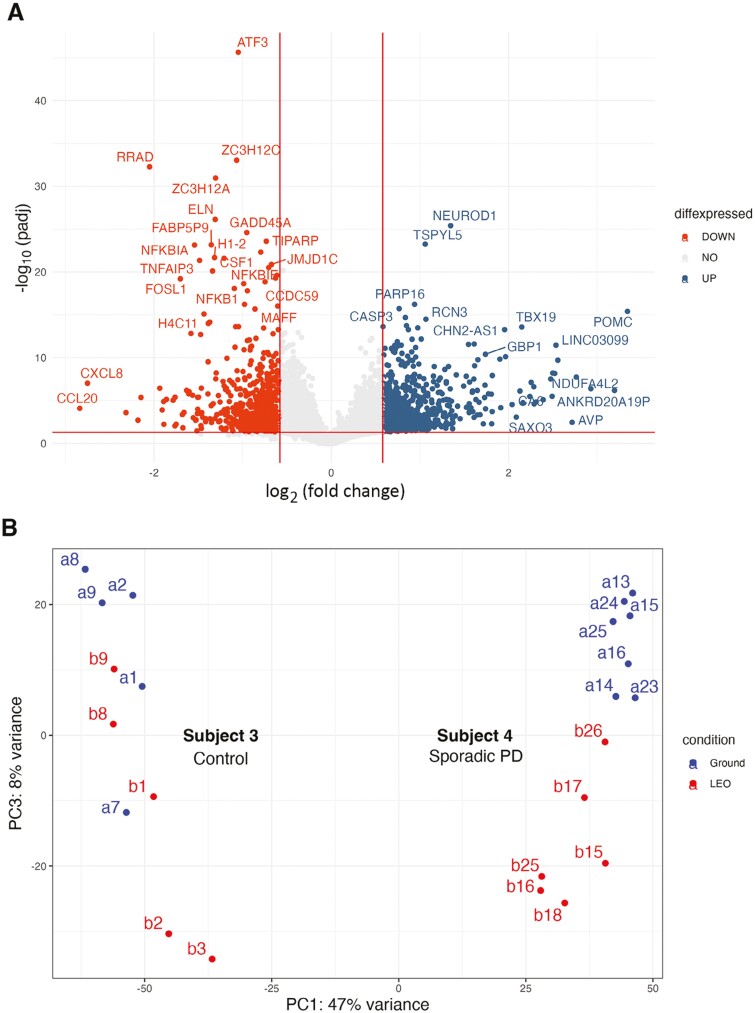

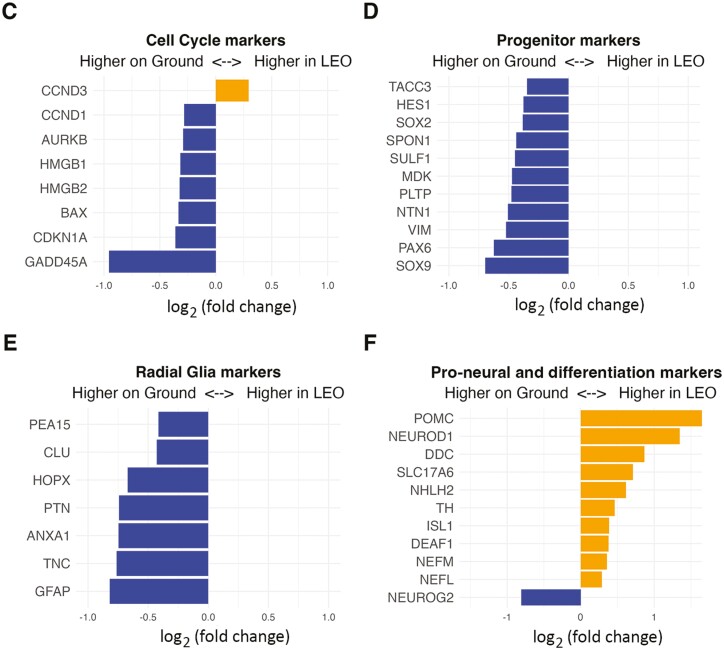
Microgravity alters gene expression in neural organoids: Dopaminergic organoids. A. Volcano plot showing the differentially expressed genes (DEGs) between iPSC-derived cortical organoids in low-Earth orbit (LEO) and ground. Expression levels of genes with significant enrichment (padj < .05) are plotted as red (lower in LEO, “down”) or blue (higher in LEO, “up”), NO = no difference. B. PCA plot showing the differences between LEO (red) and ground (blue) of the dopaminergic organoids derived from the 2 iPSC lines. Labels refer to the samples from the 2 subjects listed in Supplementary Table S1. C-F. Upregulated and downregulated gene sets. C. Cell cycle markers; D. Progenitors; E. Radial glia markers; F. Pro-neural and post-mitotic differentiation markers.

**Figure 4.**
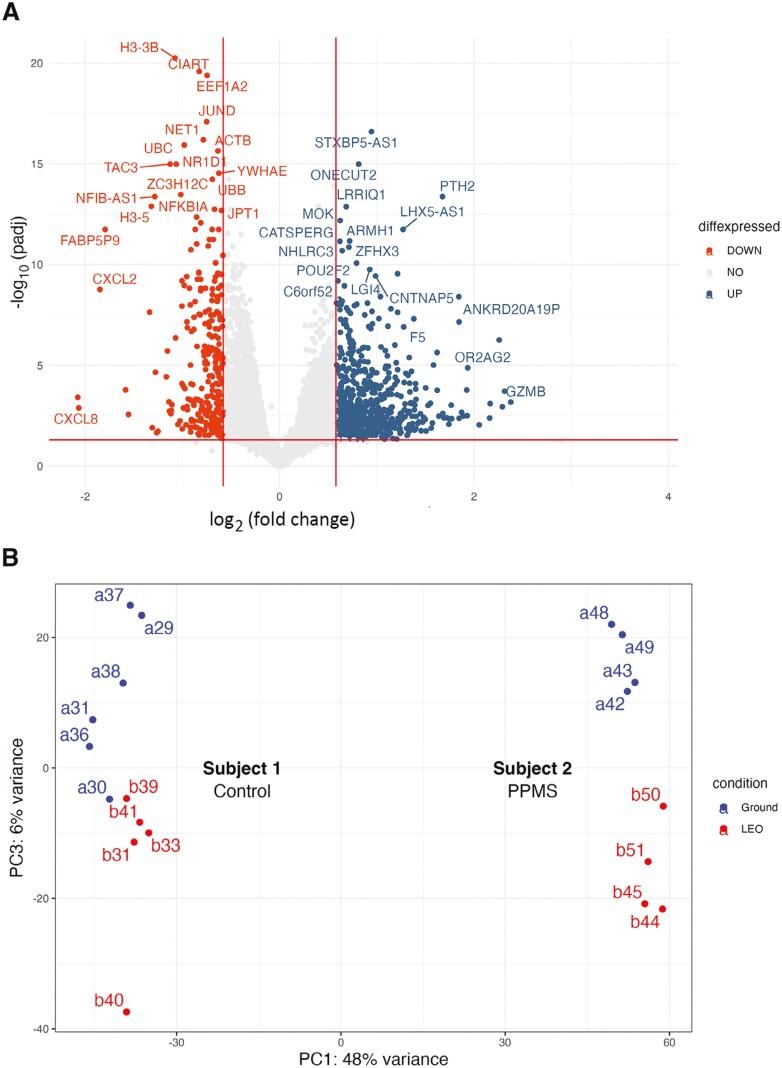

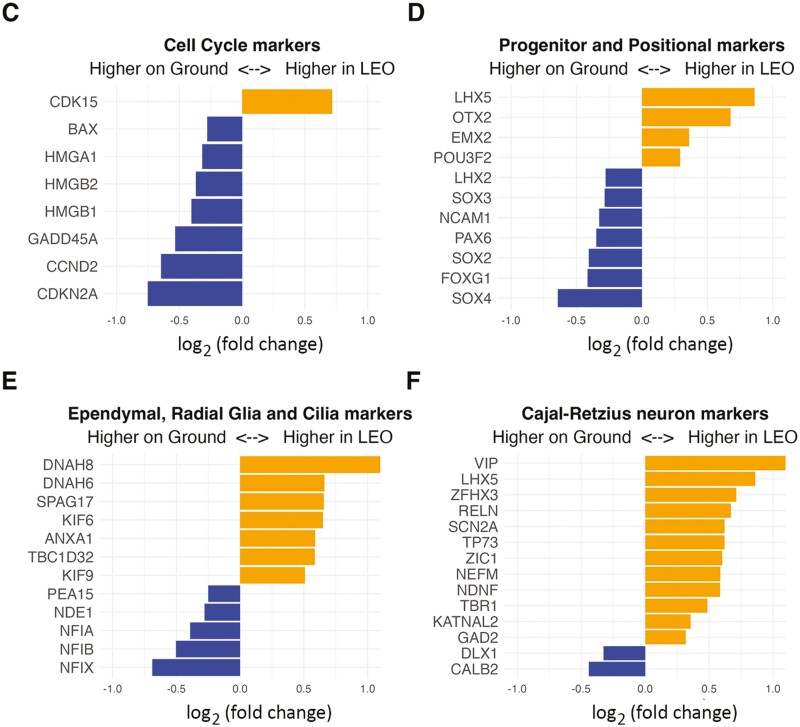
Microgravity alters gene expression in neural organoids: Cortical organoids. A. Volcano plot showing the differentially expressed genes (DEGs) between iPSC-derived cortical organoids in low-Earth orbit (LEO) and ground. NO = no difference. B. PCA plot showing the differences between LEO (red) and ground (blue) of the cortical organoids derived from the 2 iPSC lines. Labels refer to the samples from the 2 subjects, listed in Supplementary Table S1. C-F. Upregulated and downregulated gene sets. C. Cell Cycle markers; D. Progenitor and positional markers; E. Ependymal, Radial glia, and Cilia markers; F. Cajal-Retzius neuron markers.

Supplementary Table S1 lists the individual organoids that were analyzed by RNAseq. Figures 3 and 4 illustrate the results of global gene expression analysis of organoids from dopaminergic neurons (Figure 3) and cortical neurons (Figure 4). After quality control and filtering, principal component analysis (PCA) of all organoids demonstrated a clear distinction based on organoid type (dopaminergic vs cortical organoids) and the cell line used to generate the organoids. These variables accounted for the majority of the data’s variance. However, distinctions based on ground vs LEO conditions were also identified by PCA and clustering analysis (Figs. 3B, 4B). To investigate the effects of LEO on each organoid type, we performed separate analyses for each organoid type (cortical and dopaminergic). After controlling for the sequencing library preparation method, organoid type, and cell line, we conducted a differential gene expression analysis between organoids cultured on Earth (ground condition) and those cultured in LEO on the ISS. For the dopaminergic organoids, we found 1183 DEGs (627 higher in LEO vs 556 lower in LEO, at adjusted *P*-value < .05). For the cortical organoids, we found 926 differentially expressed genes (DEGs) between LEO and ground conditions (671 higher in LEO vs 255 lower in LEO, at adjusted *P*-value < .05) (Figure 3A), (Supplementary Tables S2a, 2b). These results suggest a significant impact of microgravity on both organoid types during the month in culture.

To understand the biological processes impacted by microgravity, we performed gene ontology (GO) analysis and gene set enrichment analysis (GSEA) on the ground vs LEO DEGs (Supplementary Tables S3a and 3b, Supplementary Figs. S3 and 4). To assess how cell types in dopaminergic organoids changed during the month in culture in microgravity, we applied GSEA using gene sets representing expected cell types found in the midbrain during fetal development.^18^ This analysis (Supplementary Figure S3A) suggested that medial and medial-lateral neuroblasts, red nucleus neurons, differentiated dopaminergic neurons, and oculomotor and trochlear nucleus neuronal cell types were enriched in LEO while progenitors and radial glia cell types were depleted. Further analysis of panels of genes marking stages of differentiation from progenitors to maturing cell types indicated that organoids in LEO had lower expression of early progenitor markers (eg, *VIM*, *SOX2*, *PAX6*; Figure 3D) as well as a panel of cell cycle genes (eg, *CCND1*, *CDKN1A*, *GADD45A*; Figure 3C). Additionally, radial glia markers (*HOPX*, *ANXA1*; Figure 3E) were decreased in the dopaminergic organoids in LEO. Enriched in LEO were genes associated with neuronal maturity, including pro-neural and post-mitotic markers (eg, *NEUROD1*, *SLC17A6*, and the neurofilament genes; Figure 3F). Notably, later markers of neuronal maturity, including tyrosine hydroxylase (*TH*) and dopa decarboxylase (*DDC*), were significantly increased in LEO and consistent between the dopaminergic organoids made from 2 cell lines (Figure 3F and Supplementary Figure S3B). We also observed some cell line differences, including in expression of the hypothalamic differentiation marker *POMC,* which was more highly enriched in the subject 3 cell line (Supplementary Figure S3B).

Like the dopaminergic organoids, in cortical organoids cell cycle markers (eg, *GADD45A*, *CND2*, *CDKN2A*; Figure 4C), early neural plate markers and general neural precursor markers (eg, *SOX2*, *PAX6*, *NCAM1*; Figure 4D) were lower in LEO and behaved consistently between the 2 cell lines. In contrast to dopaminergic organoids, we observed higher levels of radial glial and ependymal cell markers in LEO. In the cortical differentiation protocol used here,^10^ radial glial markers are expected to differentiate from neural progenitor cells later in the protocol (equivalent to our ending timepoint, after ~1 month in LEO). We observed an enrichment of the ventricular radial glia marker *ANXA1* (Figure 4E) in LEO, suggesting accelerated differentiation of the radial glia that remain attached to the ventricular wall. Consistent with this, in LEO we observed an enrichment for markers of motile cilia (eg, *SPAG17*, *DNAH6,* and *DNAH9*, *KIF6*; Figure 4E), which are also markers of ependymal cells that differentiate from the radial glia on the ventricular wall. While other radial glial markers (eg, *HOPX*, *FAM107A*, *TNC*) were expressed but not enriched in LEO, upregulation of these ciliary genes is consistent with early differentiation of the radial glia into ependymal cells or their precursors. Additionally, we consistently observed markers of early-born Cajal-Retzius neurons enriched in both lines in LEO (Figure 4F). For example, *RELN*, *LHX5,* and *TP73* were all significantly enriched in LEO. *TBR1* was also expressed in the post-mitotic neurons, including Cajal-Retzius neurons enriched in LEO. Together these results suggest an accelerated differentiation of the cortical organoids in microgravity.

Transcription factor enrichment analysis of the DEGs highlighted the common transcription factor binding sites of sets of differentially expressed genes (Supplementary Table S4). This analysis revealed co-regulated sets of genes associated with neural differentiation, and confirmed the dominant involvement of Wnt signaling pathways associated with neural development that are underlying the differences between organoids in LEO and on the ground. These results, increases in differentiation-associated markers and decreases in proliferation-associated genes, are consistent with the idea that neuronal maturation is enhanced and modified in LEO compared to ground controls.

### Protein secretion in organoid cultures

To assess the effects of microgravity on secretion of proteins, culture medium was collected from vials containing single organoids and analyzed using Olink Explore 1536. We used Olink Explore 1536, a high-throughput proteomics platform based on the Proximity Extension Assay (PEA) technology, to measure the expression levels of 1536 human proteins in 30-day conditioned supernatants from ground and LEO organoid samples. Overall, we found that NPX levels were lower in LEO compared to ground. However, both of the organoid types showed LEO vs ground differentially secreted proteins that were either upregulated or downregulated. For example, in cortical organoids, SFRP1, a modulator of the Wnt signaling pathway, was lower in LEO, whereas NTF3, a neurotrophic factor, was upregulated in LEO. Multiple microglia markers, including CXCL8 and CD14, were found to be consistently upregulated in the samples that had been loaded with microglia (Supplementary Figure S3). Additionally, we found that several proteins were upregulated in LEO but only in the presence of microglia, including the inflammatory mediator IL1R1 (Supplementary Figure S3). The data lacked the power to conclude that there were consistent differences, but suggest that there were complex alterations in differentiation and inflammatory pathways in response to the microgravity environment in LEO.

### Radiation exposure of organoids onboard the ISS

The radiation field inside the ISS fluctuates due to different local shielding thicknesses and the orbital changes of the ISS. The cultures were located in the European Space Agency (ESA) Columbus Laboratory. Timestamp-associated Radiation Environmental Monitoring (REM) measurements from radiation logs provided by NASA for this location were reported as the amount of radiation in mGy (milligrays) per day from Galactic Cosmic Radiation (GCR) and Southern Atlantic Anomaly (SAA) doses combined. The station radiation logs provided by NASA for this location reported a GCR daily average dose of 0.153 mGy per day and an SAA daily average dose of 0.231 mGy/d, for a combined daily radiation exposure dose of 0.384 mGy/d. Cumulative radiation exposure using this combined daily average dose would be 11.5 mGy (approximately 12 mGy) over the 30 days the payload was stowed in the Columbus module. The calculation of effective radiation exposure, measured in millisieverts (mSV), is dependent on many variables, including the amount of shielding of the object and tissue-specific radiation sensitivity. Table 2 provides a list of approximate radiation exposures on Earth and on the ISS. Organoid-specific exposure factors relative to whole body exposure have not been determined.

**Table 2.** Comparison of radiation dosage levels.

Location	Estimated Radiation	Source
Earth (annual total)	Total 3–4 mSv/year	Natural sources (rocks, cosmic rays penetrating atmosphere)
Pilot (long-haul polar routes; annual total)	Total 6 mSv/year	Cosmic radiation
Astronaut on ISS (daily; 6-month mission)	0.5 mSv/day Total 90 mSv/6 months	GCR/SAA (Galactic Cosmic Radiation (GCR)/Southern Atlantic Anomaly (SAA))
Payload SpX—19 (30-day mission)	0.4 mSv/day Total 12 mSv/30 days	GCR/SAA
NASA threshold for astronauts (lifetime)	Total 600 mSv/lifetime	GCR/SAA
Russian and Canadian threshold for astronauts (lifetime)	Total 1000 mSv/lifetime	GCR/SAA

On Earth, humans are exposed to 3-4 mSV of radiation per year, mostly from natural sources. Crew and pilots serving on long-haul polar routes, however, may receive an annual effective dose of up to 6 mSv.^19^ We consider the estimated 12 mGy of cumulative dose over the 30-day mission to be indicative of relatively low exposure levels during the time of the mission. While it is unlikely that there could have been meaningful acute exposure during the 5 days between launch and payload transfer to the Columbus module, the remaining 30 days would likely be representative of the consistent effects. While cumulative radiation exposure on long-duration space missions is an area of intensive interest for NASA, the limited exposure of payloads during short-duration missions is more similar to risks posed by common radiological procedures (Supplementary Table S5). This suggests that the differences observed between flight and ground control samples were unlikely to be meaningfully caused by exposure to radiation.

## Discussion

To determine the effects of culture in microgravity on the ISS, we compared human neural organoids cultured on the ISS with organoids cultured on Earth. We developed a novel static approach for organoid culture, placing single organoids into cryovials containing 1 mL culture medium. This strategy was undertaken to avoid potential problems with leakage of other culture vessels under the changes in gravity. We found that after 30 days of culture in sealed cryovials, the organoids not only remained healthy but also rapidly extended dense webs of neurites when plated onto adherent surfaces.

These organoids were designed to be simplified representations of different parts of the brain, containing either iPSC-derived cortical or dopaminergic neurons with or without incorporation of small populations of matched microglia derived separately from the same cell lines. We chose these 2 neuronal cell types because they are affected by different neurodegenerative diseases; cortical neurons are damaged in multiple sclerosis and dopamine neurons are lost in Parkinson’s disease. We incorporated microglia, which are a major immune cell type in the CNS, in order to monitor possible immunological effects of long-term culture in microgravity. The microglia were derived from the same cell lines separately because they arise from an independent mesodermal lineage and migrate to the brain during embryogenesis.^20^

Individual organoids were analyzed separately for gene expression, protein secretion, and immunocytochemistry. Remarkably, all of the organoid types from the 4 individuals showed significant differences depending on whether they were cultured in LEO or on Earth. We found minimal evidence of stress, inflammation, or apoptosis, and no signs of hypoxia in either group. Interestingly, the organoids cultured on Earth, not the LEO samples, had slightly elevated levels of stress-associated genes. Our most surprising result was that the organoids cultured in microgravity had higher levels of genes associated with neural maturity and lower levels of proliferation-associated genes compared to the ground controls. This accelerated maturation is consistent with the observed differences in elements of Wnt signaling pathways. Wnt signaling is highly complex and controls cell proliferation, migration, and differentiation, especially in the brain.^21^ The results are likely due to microgravity rather than other possible factors such as radiation exposure; the level of radiation exposure is relatively low and comparable to the exposure levels of astronauts over the same time period.

During the month on the ISS, the neural progenitor cells in the organoids differentiated more quickly than those on Earth. However, it is important to note that this is evidence of accelerated maturation, not of accelerated aging. Agingmanifests differently in different organs and cell types, and there is no uniform method for determining their biological age. It will be of interest to assess the effects of microgravity on cellular aging using a relative cell-type-agnostic method such as DNA methylation profiling.^22^

Research studying human iPSC-derived organoids in microgravity is in its early stages and there are not yet established methods that allow comparisons among studies. At this time, published reports are inconsistent. For example, it has been reported that cardiac organoids had higher rates of proliferation, or enhanced maturity, or both on the ISS or in simulated microgravity.^23-27^ As more research studies are reported, there should be a convergence of methods that will aid in the interpretation of the effects of microgravity on multiple human cell types.

Our additional missions are designed to further investigate the mechanisms underlying microgravity’s effect on neural organoids with increasingly complex designs. With each flight, we are expanding the experiments, by including additional cell lines, some with genetic forms of neurodegenerative disease, additional neuronal cell types, and more sophisticated methods of analysis. We hope to use the perturbation of microgravity to better understand neurological disorders and the data reported here form the basis for further explorations. Our goal is to make use of the opportunity to study neural cells in LEO to better understand and treat neurodegenerative disease on Earth, and to help ameliorate potentially adverse neurological effects of space travel.

## Supplementary material

Supplementary material is available at *Stem Cells Translational Medicine* online.

szae070_suppl_Supplementary_Materials

## Data Availability

The data underlying this article are available in GEO (Gene Expression Omnibus) at https://www.ncbi.nlm.nih.gov/geo/, and can be accessed with GEO accession GSE259421.
