# Supplementary material for: Effects of microgravity on human iPSC-derived neural organoids on the International Space Station
Source: Stem Cells Transl Med. 2024 Oct 23;13(12):1186–97. doi: 10.1093/stcltm/szae070 (PMC11631337; doi:10.1093/stcltm/szae070)
Supplement: szae070_suppl_Supplementary_Materials [file szae070_suppl_supplementary_materials.zip › R1Table S1 List of RNAseq samples.pdf]

Supplementary Table 1. Samples analyzed by RNAseq						
Sample reference ID	Ground/LEO	Subject ID	Cell Line ID	Microglia	Library prep	RNA RIN
**Cortical organoids**						
b31	LEO	Subject1	051121-01-MR-017	-	Nextera	8.3
b33	LEO	Subject1	051121-01-MR-017	-	Nextera	7.6
b39	LEO	Subject1	051121-01-MR-017	+	Nextera	7.4
b40	LEO	Subject1	051121-01-MR-017	+	Pilot-TruSeq	7.8
b41	LEO	Subject1	051121-01-MR-017	+	Nextera	8.9
b44	LEO	Subject2	AK003-01-MR-008	-	Nextera	7.6
b45	LEO	Subject2	AK003-01-MR-008	-	Nextera	7.7
b50	LEO	Subject2	AK003-01-MR-008	+	Nextera	7.5
b51	LEO	Subject2	AK003-01-MR-008	+	Nextera	7.9
a29	Ground	Subject1	051121-01-MR-017	-	Nextera	6.6
a30	Ground	Subject1	051121-01-MR-017	-	Pilot-TruSeq	6.4
a31	Ground	Subject1	051121-01-MR-017	-	Nextera	7.1
a36	Ground	Subject1	051121-01-MR-017	+	Pilot-TruSeq	7.0
a37	Ground	Subject1	051121-01-MR-017	+	Nextera	6.9
a38	Ground	Subject1	051121-01-MR-017	+	Nextera	7.1
a42	Ground	Subject2	AK003-01-MR-008	-	Nextera	7.2
a43	Ground	Subject2	AK003-01-MR-008	-	Nextera	7.7
a48	Ground	Subject2	AK003-01-MR-008	+	Nextera	7.4
a49	Ground	Subject2	AK003-01-MR-008	+	Nextera	7.3
**Dopaminergic organoids**						
b1	LEO	Subject3	UEC741iPS517	-	Nextera	7.8
b2	LEO	Subject3	UEC741iPS517	-	Pilot-TruSeq	7.2
b3	LEO	Subject3	UEC741iPS517	-	Nextera	7.8
b8	LEO	Subject3	UEC741iPS517	+	Nextera	7.1
b9	LEO	Subject3	UEC741iPS517	+	Nextera	7.4
b15	LEO	Subject4	HDF410iPS504	-	Pilot-TruSeq	7.0
b16	LEO	Subject4	HDF410iPS504	-	Nextera	7.4
b17	LEO	Subject4	HDF410iPS504	-	Nextera	7.4
b18	LEO	Subject4	HDF410iPS504	-	Nextera	7.7
b25	LEO	Subject4	HDF410iPS504	+	Nextera	7.6
b26	LEO	Subject4	HDF410iPS504	+	Nextera	7.7
a1	Ground	Subject3	UEC741iPS517	-	Nextera	7.1
a2	Ground	Subject3	UEC741iPS517	-	Nextera	7.6
a7	Ground	Subject3	UEC741iPS517	+	Pilot-TruSeq	7.4
a8	Ground	Subject3	UEC741iPS517	+	Nextera	7.5
a9	Ground	Subject3	UEC741iPS517	+	Nextera	7.6
a13	Ground	Subject4	HDF410iPS504	-	Nextera	7.4
a14	Ground	Subject4	HDF410iPS504	-	Pilot-TruSeq	7.2
a15	Ground	Subject4	HDF410iPS504	-	Nextera	7.4
a16	Ground	Subject4	HDF410iPS504	-	Nextera	7.9
a23	Ground	Subject4	HDF410iPS504	+	Pilot-TruSeq	7.3
a24	Ground	Subject4	HDF410iPS504	+	Nextera	7.4
a25	Ground	Subject4	HDF410iPS504	+	Nextera	7.1
