# Supplementary material for: Effects of microgravity on human iPSC-derived neural organoids on the International Space Station
Source: Stem Cells Transl Med. 2024 Oct 23;13(12):1186–97. doi: 10.1093/stcltm/szae070 (PMC11631337; doi:10.1093/stcltm/szae070)
Supplement: szae070_suppl_Supplementary_Materials [file szae070_suppl_supplementary_materials.zip › R1Table S2a Cortical-List of differentially expressed transcripts.pdf]

CORTICAL NEURON ORGANOIDS				
SYMBOL (NA: uncharacterized lncRNA or pseudogene)	log2FoldChange	pvalue	padj	ENSEMBL
HIGHER IN LEO THAN GROUND: LARGEST TO SMALLEST DIFFERENCE (BLUE)				
GZMB	2.3792	3.5010E-05	6.7350E-04	ENSG00000100453
AC126175.1	2.3155	7.4812E-06	1.9384E-04	ENSG00000277738
ADAM7	2.2926	6.9359E-05	1.1562E-03	ENSG00000069206
BX005019.1	2.2596	6.2761E-09	5.5548E-07	ENSG00000259946
AL592463.1	2.1664	4.3637E-05	7.9947E-04	ENSG00000232896
AC108474.1	2.1613	3.6660E-04	4.1832E-03	ENSG00000250971
AIF1	2.0537	1.0021E-03	9.0713E-03	ENSG00000204472
OR2AG2	1.9359	2.7654E-07	1.3342E-05	ENSG00000188124
AC107973.1	1.9305	2.5731E-04	3.1962E-03	ENSG00000254734
AC025278.2	1.9256	6.2632E-06	1.6758E-04	ENSG00000269761
CSF3R	1.8912	2.6891E-04	3.3047E-03	ENSG00000119535
DNAH8	1.8732	1.5816E-04	2.1625E-03	ENSG00000124721
NA	1.8476	3.7878E-04	4.2830E-03	ENSG00000288923
AC134043.2	1.8471	5.9177E-10	7.1027E-08	ENSG00000279204
ANKRD20A19P	1.8441	1.6893E-11	3.9586E-09	ENSG00000196593
ADAM28	1.7707	4.0580E-04	4.5220E-03	ENSG00000042980
TINAG	1.7660	5.4489E-05	9.5153E-04	ENSG00000137251
CFAP47	1.7486	4.2233E-04	4.6661E-03	ENSG00000165164
AQP7B	1.7421	9.1646E-04	8.4870E-03	ENSG00000259916
AL133387.1	1.7204	3.4558E-04	3.9990E-03	ENSG00000225208
LINC02154	1.6919	3.5721E-04	4.1032E-03	ENSG00000235385
AC006058.1	1.6800	1.8697E-04	2.4649E-03	ENSG00000261786
PTH2	1.6764	3.0860E-17	4.2183E-14	ENSG00000142538
AC091979.1	1.6393	2.7388E-04	3.3493E-03	ENSG00000254350
LOC124902532	1.6219	6.5471E-06	1.7359E-04	ENSG00000287560
TNFRSF9	1.6212	3.4138E-08	2.3140E-06	ENSG00000049249
NCKAP1L	1.5840	1.9151E-07	9.7357E-06	ENSG00000123338
LINC02751	1.5727	7.6350E-04	7.4048E-03	ENSG00000254946
LINC02409	1.5599	1.6761E-03	1.3235E-02	ENSG00000257920
LINC02473	1.5519	4.2197E-05	7.7831E-04	ENSG00000261121
AC090409.1	1.5274	5.6901E-05	9.8484E-04	ENSG00000267279
EXOC1L	1.5077	2.5690E-03	1.8140E-02	ENSG00000250821
LOC124904294	1.5032	4.6303E-06	1.3278E-04	ENSG00000289168
VTCN1	1.4981	4.4420E-04	4.8446E-03	ENSG00000134258
ADAMTS7P1	1.4827	3.2714E-04	3.8385E-03	ENSG00000274376
AF230666.1	1.4729	6.5752E-04	6.6270E-03	ENSG00000223697
LOC124900869	1.4567	2.4956E-03	1.7759E-02	ENSG00000286580
AL137127.1	1.4497	1.9555E-04	2.5471E-03	ENSG00000272084
TRAV18	1.4378	6.2899E-04	6.3979E-03	ENSG00000211798
C1QTNF9B	1.4232	7.7128E-04	7.4597E-03	ENSG00000205863

AL590399.5	1.4055	2.8801E-04	3.4772E-03	ENSG00000233651
AC126755.3	1.4040	2.3941E-05	4.9502E-04	ENSG00000257527
NA	1.4013	1.7331E-03	1.3545E-02	ENSG00000288321
AC011840.1	1.4005	5.5175E-06	1.5168E-04	ENSG00000231421
LINC02199	1.3990	2.5574E-06	8.1508E-05	ENSG00000250198
NA	1.3987	1.8954E-03	1.4470E-02	ENSG00000287968
AC007406.3	1.3914	5.2240E-05	9.2273E-04	ENSG00000256540
AP004607.4	1.3898	2.2566E-03	1.6495E-02	ENSG00000254916
MSLN	1.3869	1.0941E-04	1.6516E-03	ENSG00000102854
LRP2	1.3816	3.8503E-10	4.9600E-08	ENSG00000081479
LINC01589	1.3765	4.6164E-05	8.3214E-04	ENSG00000238120
OTOP2	1.3682	8.2727E-04	7.8530E-03	ENSG00000183034
ACTN4P2	1.3680	1.5782E-03	1.2701E-02	ENSG00000214141
AL157373.2	1.3640	2.7845E-04	3.3900E-03	ENSG00000229896
LINC02522	1.3574	4.6305E-07	2.0130E-05	ENSG00000231056
LINC02293	1.3531	3.7627E-05	7.1658E-04	ENSG00000257185
NA	1.3459	2.3098E-03	1.6783E-02	ENSG00000285534
TMEM273	1.3451	4.1884E-05	7.7427E-04	ENSG00000204161
FAM9A	1.3412	8.3339E-06	2.1183E-04	ENSG00000183304
FAM81B	1.3383	2.4427E-06	7.8566E-05	ENSG00000153347
LOC105378644	1.3353	9.6949E-04	8.8382E-03	ENSG00000284640
AC010894.3	1.3345	1.3865E-04	1.9572E-03	ENSG00000236449
SLC12A1	1.3338	6.7187E-08	4.1329E-06	ENSG00000074803
RFPL4B	1.3303	2.6344E-03	1.8478E-02	ENSG00000251258
LINC01111	1.3244	1.4962E-03	1.2233E-02	ENSG00000254300
LINC00207	1.3244	1.8116E-04	2.4029E-03	ENSG00000187012
CD163L1	1.3224	7.9206E-07	3.0934E-05	ENSG00000177675
AL365434.2	1.3184	4.4342E-03	2.7468E-02	ENSG00000273124
AC006460.1	1.3128	5.0555E-03	3.0347E-02	ENSG00000228509
KRT75	1.3087	1.7428E-03	1.3596E-02	ENSG00000170454
LINC02202	1.2995	6.1840E-07	2.5275E-05	ENSG00000245812
ZNF648	1.2905	4.6457E-05	8.3497E-04	ENSG00000179930
AC114316.2	1.2903	3.0488E-04	3.6414E-03	ENSG00000250049
AC093772.1	1.2876	1.0329E-04	1.5841E-03	ENSG00000248491
BMP3	1.2845	1.1458E-04	1.7055E-03	ENSG00000152785
IL15	1.2820	2.1523E-06	7.0800E-05	ENSG00000164136
F5	1.2773	1.1433E-09	1.2392E-07	ENSG00000198734
LHX5-AS1	1.2706	2.2192E-15	1.8135E-12	ENSG00000257935
ISCA2P1	1.2697	3.7163E-04	4.2216E-03	ENSG00000286769
NA	1.2506	4.9683E-04	5.2806E-03	ENSG00000226912
CCDC13-AS2	1.2506	1.7852E-04	2.3730E-03	ENSG00000225611
TWIST2	1.2494	3.2485E-03	2.1744E-02	ENSG00000233608
GPC5-AS2	1.2486	1.5640E-05	3.4825E-04	ENSG00000232885
AL591848.2	1.2433	1.9283E-04	2.5251E-03	ENSG00000235021
AL008638.2	1.2403	3.6788E-03	2.3997E-02	ENSG00000236858
AF131215.7	1.2389	1.7214E-03	1.3484E-02	ENSG00000270076
GSTA1	1.2373	2.5197E-03	1.7856E-02	ENSG00000243955
NA	1.2317	8.0489E-04	7.7000E-03	ENSG00000287097
FGFBP2	1.2200	4.7422E-04	5.0997E-03	ENSG00000137441

AC010247.2	1.2177	3.7725E-04	4.2749E-03	ENSG00000259436
HTR3B	1.2147	3.7179E-07	1.6693E-05	ENSG00000149305
SAMD3	1.2143	1.5561E-10	2.3490E-08	ENSG00000164483
LOC100507336	1.2141	7.4593E-13	2.8678E-10	ENSG00000228793
LOC101928335	1.2105	1.7737E-06	6.0446E-05	ENSG00000236064
IDO1	1.2092	1.2458E-05	2.9027E-04	ENSG00000131203
GUCA1C	1.2088	6.5709E-04	6.6262E-03	ENSG00000138472
GNGT1	1.2051	8.7175E-03	4.5862E-02	ENSG00000127928
AC010300.1	1.2005	1.3955E-07	7.5136E-06	ENSG00000267934
GJD2	1.1903	1.0891E-06	3.9936E-05	ENSG00000159248
ESPNL	1.1896	7.6229E-04	7.3989E-03	ENSG00000144488
NPS	1.1885	1.7020E-03	1.3367E-02	ENSG00000214285
AC127024.2	1.1867	2.9471E-06	9.1557E-05	ENSG00000263603
LOC124904138	1.1865	1.2275E-08	9.9415E-07	ENSG00000278048
ENOX1-AS1	1.1817	1.3443E-03	1.1329E-02	ENSG00000233821
LINC00504	1.1782	1.0480E-03	9.4075E-03	ENSG00000248360
OR10A2	1.1772	1.3564E-03	1.1402E-02	ENSG00000170790
SLC49A3	1.1760	9.1919E-04	8.5025E-03	ENSG00000169026
AL033504.1	1.1754	7.9350E-04	7.6178E-03	ENSG00000227681
ZNF750	1.1660	8.0994E-03	4.3267E-02	ENSG00000141579
AL354733.2	1.1584	7.1243E-05	1.1796E-03	ENSG00000231616
NA	1.1583	4.0773E-04	4.5415E-03	ENSG00000226521
LOC101928906	1.1554	5.0671E-06	1.4249E-04	ENSG00000205611
AL672277.1	1.1516	1.4872E-03	1.2194E-02	ENSG00000237531
PRDM16-DT	1.1512	1.1663E-05	2.7674E-04	ENSG00000177133
NA	1.1498	4.7076E-04	5.0736E-03	ENSG00000224769
LOC124903183	1.1486	7.5291E-05	1.2326E-03	ENSG00000285572
C6	1.1471	1.5203E-04	2.1038E-03	ENSG00000039537
NA	1.1456	9.1420E-11	1.5621E-08	ENSG00000237637
NA	1.1442	6.1929E-04	6.3122E-03	ENSG00000235526
LOC105374042	1.1411	9.3572E-04	8.6133E-03	ENSG00000239482
NANOGP8	1.1399	3.5509E-03	2.3311E-02	ENSG00000255192
ACSL6-AS1	1.1318	3.2966E-03	2.1988E-02	ENSG00000223548
PTPRH	1.1249	5.6887E-07	2.3684E-05	ENSG00000080031
ADAMTS16-DT	1.1230	1.1160E-04	1.6733E-03	ENSG00000250579
NA	1.1215	1.1372E-06	4.1454E-05	ENSG00000288758
ABCA13	1.1202	1.9915E-04	2.5858E-03	ENSG00000179869
LOC101927245	1.1193	2.2895E-07	1.1358E-05	ENSG00000283959
VWA3B	1.1190	6.5169E-06	1.7316E-04	ENSG00000168658
PHEX-AS1	1.1188	1.2111E-05	2.8461E-04	ENSG00000224204
NA	1.1159	5.0811E-03	3.0465E-02	ENSG00000223511
LOC124902439	1.1155	2.0330E-04	2.6217E-03	ENSG00000228566
NA	1.1148	1.0406E-09	1.1481E-07	ENSG00000286542
AMY2A	1.1139	1.5403E-03	1.2462E-02	ENSG00000243480
SAMMSON	1.1091	1.1208E-03	9.8706E-03	ENSG00000240405
SLITRK6	1.1090	5.3226E-08	3.3928E-06	ENSG00000184564
HK2	1.1082	1.3092E-06	4.6753E-05	ENSG00000159399
VIP	1.0987	3.2850E-04	3.8476E-03	ENSG00000146469
AL359762.1	1.0968	7.5281E-04	7.3387E-03	ENSG00000284882

LINC00992	1.0964	1.8630E-03	1.4275E-02	ENSG00000248663
STC2	1.0938	2.2852E-07	1.1358E-05	ENSG00000113739
AL354751.1	1.0897	6.6252E-03	3.7251E-02	ENSG00000234537
AL359232.1	1.0891	3.1581E-07	1.4773E-05	ENSG00000258561
AL645728.2	1.0881	1.9600E-06	6.5347E-05	ENSG00000284740
PEX5L	1.0876	2.6227E-08	1.8543E-06	ENSG00000114757
KRT18P61	1.0857	4.0909E-03	2.5991E-02	ENSG00000267083
FCGBP	1.0857	1.1247E-05	2.6945E-04	ENSG00000275395
NA	1.0807	8.8978E-03	4.6630E-02	ENSG00000286474
LINC01579	1.0769	7.8333E-04	7.5390E-03	ENSG00000258754
LINC01978	1.0769	6.9566E-04	6.9131E-03	ENSG00000262188
POSTN	1.0753	2.0077E-05	4.2956E-04	ENSG00000133110
AC079610.3	1.0748	1.0055E-04	1.5512E-03	ENSG00000272519
LINC01471	1.0704	6.2326E-03	3.5515E-02	ENSG00000239921
AC099521.1	1.0682	1.3118E-04	1.8903E-03	ENSG00000274698
MIR3179-4	1.0670	1.3159E-03	1.1159E-02	ENSG00000277014
CFAP95	1.0641	2.9836E-04	3.5794E-03	ENSG00000204711
NXF2	1.0632	7.5806E-04	7.3636E-03	ENSG00000269405
LOC105375233	1.0609	1.6631E-03	1.3153E-02	ENSG00000232930
ARHGEF33	1.0576	7.4053E-04	7.2477E-03	ENSG00000214694
LINC02334	1.0537	3.1292E-04	3.7178E-03	ENSG00000274317
ID3	1.0506	3.7808E-04	4.2771E-03	ENSG00000117318
LINC01982	1.0505	6.2030E-03	3.5379E-02	ENSG00000263317
AMPD3	1.0493	2.4240E-05	4.9910E-04	ENSG00000133805
LINC00867	1.0489	1.5962E-04	2.1746E-03	ENSG00000232139
GHRH	1.0443	3.5225E-06	1.0544E-04	ENSG00000118702
ARHGDIB	1.0436	1.5783E-08	1.2289E-06	ENSG00000111348
AL354977.2	1.0436	1.6203E-03	1.2915E-02	ENSG00000237359
AC008035.1	1.0425	2.3908E-04	3.0075E-03	ENSG00000272369
AC116348.2	1.0411	3.0092E-03	2.0550E-02	ENSG00000261332
CFAP144	1.0382	1.2456E-05	2.9027E-04	ENSG00000186973
AC006058.3	1.0379	1.6448E-11	3.9291E-09	ENSG00000272121
AC107886.1	1.0342	8.9647E-04	8.3552E-03	ENSG00000255372
PDC	1.0327	1.6472E-03	1.3062E-02	ENSG00000116703
LHX1-DT	1.0260	5.1610E-06	1.4365E-04	ENSG00000277268
RASGRP3	1.0217	3.6956E-10	4.8112E-08	ENSG00000152689
AANAT	1.0210	5.5343E-04	5.7570E-03	ENSG00000129673
AP006547.1	1.0202	3.7899E-04	4.2835E-03	ENSG00000261044
LINC01886	1.0190	7.9384E-05	1.2876E-03	ENSG00000224568
NA	1.0161	1.3725E-04	1.9442E-03	ENSG00000288571
ETV7	1.0158	1.9555E-04	2.5471E-03	ENSG00000010030
RPL23AP32	1.0133	1.0330E-03	9.2934E-03	ENSG00000237887
LGR6	1.0116	1.5159E-03	1.2337E-02	ENSG00000133067
NA	1.0100	1.8160E-03	1.4007E-02	ENSG00000287677
AL132857.2	1.0095	6.8026E-04	6.8067E-03	ENSG00000283098
NA	1.0057	1.1054E-03	9.7629E-03	ENSG00000287402
LINC01511	1.0052	3.5430E-05	6.7999E-04	ENSG00000250584
VWA3A	1.0047	2.5306E-05	5.1758E-04	ENSG00000175267
FAM138B	1.0031	3.3209E-03	2.2090E-02	ENSG00000226516

CYP21A1P	1.0019	2.4437E-04	3.0599E-03	ENSG00000204338
ANKRD20A9P	0.9989	3.1998E-03	2.1510E-02	ENSG00000206192
LINC01091	0.9987	5.6556E-07	2.3626E-05	ENSG00000249464
KCNH5	0.9980	3.2743E-06	9.9955E-05	ENSG00000140015
AC068790.7	0.9966	1.5820E-04	2.1625E-03	ENSG00000270130
AC127070.3	0.9943	2.8105E-05	5.6589E-04	ENSG00000255703
LINC02754	0.9932	3.8701E-03	2.4934E-02	ENSG00000287312
RPS8P7	0.9932	2.9061E-05	5.7993E-04	ENSG00000229721
LINC02388	0.9927	3.9992E-03	2.5562E-02	ENSG00000257259
ZBTB20-AS5	0.9884	4.8497E-03	2.9375E-02	ENSG00000242290
CNTNAP5	0.9874	1.0384E-12	3.7027E-10	ENSG00000155052
LOC102724482	0.9872	4.5759E-05	8.2685E-04	ENSG00000287687
ALDH1A2	0.9868	4.4173E-06	1.2712E-04	ENSG00000128918
LGALS7	0.9820	1.2043E-03	1.0430E-02	ENSG00000178934
PHEX	0.9804	9.1911E-06	2.2825E-04	ENSG00000102174
VIM-AS1	0.9800	4.0522E-04	4.5218E-03	ENSG00000229124
LCP1	0.9792	5.6192E-04	5.8166E-03	ENSG00000136167
ZNF547	0.9781	5.5696E-07	2.3386E-05	ENSG00000152433
NA	0.9779	1.8512E-03	1.4222E-02	ENSG00000285863
AL136964.1	0.9759	4.5198E-03	2.7893E-02	ENSG00000274718
LINC00951	0.9732	8.3512E-06	2.1205E-04	ENSG00000226070
MEI1	0.9724	1.5204E-03	1.2358E-02	ENSG00000167077
CFAP69	0.9718	5.6948E-06	1.5586E-04	ENSG00000105792
ROPN1L	0.9697	2.9461E-06	9.1557E-05	ENSG00000145491
UPK3B	0.9696	1.9607E-03	1.4855E-02	ENSG00000243566
LINC02669	0.9674	8.7721E-04	8.2107E-03	ENSG00000233321
PPFIA2-AS1	0.9673	1.3257E-04	1.8997E-03	ENSG00000257467
AC068418.2	0.9641	2.4832E-03	1.7715E-02	ENSG00000265043
LINC00698	0.9632	9.8733E-05	1.5308E-03	ENSG00000244342
GRM1	0.9601	4.9145E-07	2.1214E-05	ENSG00000152822
AL359922.3	0.9598	1.4095E-03	1.1709E-02	ENSG00000279670
TMEM261P1	0.9587	6.2577E-05	1.0700E-03	ENSG00000270814
AL354809.1	0.9570	8.0251E-04	7.6832E-03	ENSG00000273507
NA	0.9565	3.7265E-06	1.1034E-04	ENSG00000286176
FGF7P3	0.9565	2.0190E-03	1.5178E-02	ENSG00000204837
RNU4ATAC	0.9558	7.6280E-10	8.7296E-08	ENSG00000264229
IHO1	0.9557	1.3883E-04	1.9587E-03	ENSG00000173421
LINC01630	0.9544	9.1932E-06	2.2825E-04	ENSG00000227115
ONECUT1	0.9516	3.6996E-07	1.6641E-05	ENSG00000169856
NA	0.9512	1.3186E-10	2.1067E-08	ENSG00000289009
CHODL	0.9505	1.0399E-04	1.5892E-03	ENSG00000154645
AC012254.3	0.9472	1.2110E-04	1.7805E-03	ENSG00000267724
C11orf97	0.9469	2.4462E-03	1.7517E-02	ENSG00000257057
LINC02250	0.9469	2.5841E-03	1.8197E-02	ENSG00000235731
Z83843.1	0.9467	3.3099E-04	3.8707E-03	ENSG00000271533
STXBP5-AS1	0.9457	5.0828E-21	2.5013E-17	ENSG00000233452
RNU4-2	0.9456	3.8585E-05	7.3142E-04	ENSG00000202538
LOC122455341	0.9453	5.0417E-03	3.0301E-02	ENSG00000284294
AL137796.1	0.9444	2.8015E-05	5.6455E-04	ENSG00000273384

MIR210	0.9426	3.1440E-03	2.1244E-02	ENSG00000199038
SUSD2	0.9410	2.6499E-05	5.3797E-04	ENSG00000099994
NA	0.9401	2.6121E-03	1.8352E-02	ENSG00000287633
NA	0.9397	2.2200E-04	2.8217E-03	ENSG00000286863
AP1S3	0.9389	1.4455E-09	1.5200E-07	ENSG00000152056
CFAP119	0.9373	8.0529E-07	3.1401E-05	ENSG00000196118
DHRS2	0.9361	4.3207E-06	1.2448E-04	ENSG00000100867
AC104009.1	0.9332	4.1583E-05	7.7061E-04	ENSG00000254768
DYNLRB2	0.9311	2.0539E-06	6.8106E-05	ENSG00000168589
OVOL1-AS1	0.9299	1.8148E-03	1.4007E-02	ENSG00000255120
LGI4	0.9280	3.9427E-13	1.7638E-10	ENSG00000153902
FOXI3	0.9252	1.8546E-03	1.4224E-02	ENSG00000214336
FCER1G	0.9247	1.1041E-03	9.7546E-03	ENSG00000158869
TLK2P2	0.9240	1.6030E-04	2.1816E-03	ENSG00000228828
NA	0.9210	5.2137E-04	5.4799E-03	ENSG00000286861
SCML4	0.9190	9.6491E-07	3.6081E-05	ENSG00000146285
ADAMTS19-AS1	0.9182	3.7131E-03	2.4157E-02	ENSG00000249421
OR2L2	0.9180	1.2308E-03	1.0588E-02	ENSG00000203663
CFAP210	0.9167	1.1036E-09	1.2069E-07	ENSG00000154479
CYP4A11	0.9131	4.5504E-03	2.8019E-02	ENSG00000187048
LOC124903324	0.9130	4.4871E-03	2.7719E-02	ENSG00000286257
FABP4	0.9108	2.1474E-03	1.5852E-02	ENSG00000170323
MUC1	0.9094	1.8544E-08	1.3911E-06	ENSG00000185499
AP005229.2	0.9087	1.2375E-07	6.8576E-06	ENSG00000273119
LOC100506271	0.9074	2.0812E-03	1.5499E-02	ENSG00000237862
LINC02458	0.9071	2.2223E-03	1.6303E-02	ENSG00000246363
SNORD3A	0.9046	4.2703E-11	8.2255E-09	ENSG00000263934
XACT	0.9044	5.0911E-05	9.0380E-04	ENSG00000241743
ALB	0.9037	3.1835E-03	2.1460E-02	ENSG00000163631
NA	0.9034	4.0220E-05	7.5467E-04	ENSG00000287360
RF00019	0.9001	4.7958E-03	2.9136E-02	ENSG00000207525
RSPO2	0.8996	5.3900E-04	5.6315E-03	ENSG00000147655
P3H2-AS1	0.8992	5.5567E-06	1.5242E-04	ENSG00000225764
PEBP4	0.8989	4.0820E-04	4.5447E-03	ENSG00000134020
AL035446.1	0.8949	6.1065E-05	1.0500E-03	ENSG00000234147
AC091982.1	0.8947	5.6338E-03	3.2918E-02	ENSG00000253921
LINC01201	0.8934	3.6960E-06	1.0970E-04	ENSG00000228659
FAM27E3	0.8890	5.9319E-05	1.0214E-03	ENSG00000274026
LINC00487	0.8868	2.3240E-06	7.5240E-05	ENSG00000205837
NA	0.8868	3.2012E-05	6.2512E-04	ENSG00000285922
AC103591.3	0.8853	4.8910E-07	2.1155E-05	ENSG00000273338
BARHL2	0.8850	1.0301E-03	9.2742E-03	ENSG00000143032
NA	0.8813	1.2329E-04	1.8067E-03	ENSG00000273373
LOC124906789	0.8812	3.1477E-03	2.1260E-02	ENSG00000248690
NEK10	0.8791	1.6874E-06	5.8311E-05	ENSG00000163491
CALHM1	0.8778	4.7560E-03	2.8956E-02	ENSG00000185933
EFHB	0.8766	2.3034E-10	3.1840E-08	ENSG00000163576
NOVA1-DT	0.8764	3.4939E-07	1.5950E-05	ENSG00000257842
PDE7B-AS1	0.8753	7.0695E-03	3.9095E-02	ENSG00000237596

ASTILCS	0.8748	5.4919E-03	3.2304E-02	ENSG00000244998
LOHAN2	0.8728	9.7107E-03	4.9674E-02	ENSG00000258779
EEF1AKMT4	0.8716	2.3892E-06	7.7046E-05	ENSG00000284753
NA	0.8710	1.1511E-07	6.4810E-06	ENSG00000287720
AC233266.2	0.8704	1.3439E-04	1.9127E-03	ENSG00000261600
MACROD2-AS1	0.8702	4.7747E-04	5.1280E-03	ENSG00000235914
UTS2	0.8701	5.7822E-03	3.3594E-02	ENSG00000049247
NA	0.8700	2.4909E-04	3.1112E-03	ENSG00000289192
LINC02985	0.8697	1.0198E-04	1.5713E-03	ENSG00000256546
IFITM2	0.8683	3.4570E-04	3.9990E-03	ENSG00000185201
FRZB	0.8680	1.6712E-08	1.2931E-06	ENSG00000162998
AC040168.1	0.8660	1.1992E-05	2.8309E-04	ENSG00000279523
CFC1B	0.8654	1.6441E-04	2.2300E-03	ENSG00000152093
AL390957.1	0.8654	7.0582E-05	1.1711E-03	ENSG00000285280
CAVIN3	0.8630	2.5952E-03	1.8260E-02	ENSG00000170955
PCSK1	0.8619	6.9362E-06	1.8273E-04	ENSG00000175426
AC093010.2	0.8615	1.1089E-04	1.6668E-03	ENSG00000241490
LHX5	0.8605	9.8467E-05	1.5276E-03	ENSG00000089116
NPY1R	0.8539	7.9933E-05	1.2922E-03	ENSG00000164128
UGT3A2	0.8527	2.3255E-03	1.6876E-02	ENSG00000168671
ARHGAP29	0.8523	1.9081E-08	1.4227E-06	ENSG00000137962
MIR6719	0.8505	2.3523E-03	1.7004E-02	ENSG00000277759
IGFBP7	0.8501	9.9672E-04	9.0329E-03	ENSG00000163453
AP002336.1	0.8482	8.3791E-04	7.9143E-03	ENSG00000254484
AL354732.1	0.8467	1.1654E-05	2.7674E-04	ENSG00000231407
SPNS3	0.8457	5.6574E-04	5.8416E-03	ENSG00000182557
NA	0.8441	5.3755E-06	1.4845E-04	ENSG00000285896
AC092114.1	0.8434	3.9158E-03	2.5143E-02	ENSG00000260848
AL356804.1	0.8433	5.4947E-08	3.4755E-06	ENSG00000259033
COL28A1	0.8433	9.1108E-04	8.4530E-03	ENSG00000215018
AC139792.1	0.8433	5.8266E-03	3.3788E-02	ENSG00000271771
FER1L6-AS2	0.8417	2.2235E-04	2.8245E-03	ENSG00000253868
IFITM1	0.8416	3.2324E-04	3.7976E-03	ENSG00000185885
SHROOM4	0.8406	9.0508E-05	1.4275E-03	ENSG00000158352
NA	0.8400	1.5423E-03	1.2475E-02	ENSG00000289148
ADD3-AS1	0.8393	1.6679E-04	2.2548E-03	ENSG00000203876
NA	0.8392	8.6443E-04	8.1274E-03	ENSG00000280241
AVP	0.8389	4.7678E-03	2.9009E-02	ENSG00000101200
LINC02562	0.8383	5.2866E-04	5.5422E-03	ENSG00000260265
TNNI1	0.8373	3.3248E-04	3.8826E-03	ENSG00000159173
CDH18	0.8371	4.3538E-07	1.9130E-05	ENSG00000145526
LINC01943	0.8368	6.2051E-05	1.0639E-03	ENSG00000280721
PRTG	0.8342	3.9213E-05	7.3990E-04	ENSG00000166450
UGT2B7	0.8337	7.0773E-04	7.0103E-03	ENSG00000171234
APRG1	0.8326	4.2055E-05	7.7626E-04	ENSG00000198590
RTP1	0.8281	1.4027E-03	1.1671E-02	ENSG00000175077
NA	0.8275	1.1493E-07	6.4810E-06	ENSG00000186301
RSPO1	0.8269	7.6919E-04	7.4423E-03	ENSG00000169218
AC023090.2	0.8266	6.1602E-03	3.5176E-02	ENSG00000279077

AL161804.1	0.8264	5.1414E-04	5.4154E-03	ENSG00000259039
RAD9B	0.8250	7.5767E-04	7.3636E-03	ENSG00000151164
CDH9	0.8237	2.4972E-04	3.1174E-03	ENSG00000113100
VWC2	0.8219	1.0943E-05	2.6378E-04	ENSG00000188730
DNAAF1	0.8216	2.6960E-05	5.4553E-04	ENSG00000154099
RNU1-16P	0.8213	5.6489E-04	5.8375E-03	ENSG00000202347
FAM95B1	0.8213	5.3866E-03	3.1895E-02	ENSG00000223839
LINC01783	0.8207	9.0376E-04	8.4009E-03	ENSG00000233421
MMP1	0.8205	1.9113E-03	1.4555E-02	ENSG00000196611
LOC100506551	0.8204	1.8219E-03	1.4044E-02	ENSG00000257279
AC004156.1	0.8196	1.0035E-03	9.0775E-03	ENSG00000267666
ADAMTS16	0.8189	1.7179E-04	2.3059E-03	ENSG00000145536
AL031708.1	0.8186	9.3724E-05	1.4660E-03	ENSG00000261732
LOC100419928	0.8161	2.9139E-08	2.0083E-06	ENSG00000255836
NAT1	0.8152	5.0111E-06	1.4189E-04	ENSG00000171428
ONECUT2	0.8142	4.5623E-19	1.0305E-15	ENSG00000119547
DNAJC15	0.8118	2.5052E-05	5.1409E-04	ENSG00000120675
NA	0.8115	4.1735E-04	4.6173E-03	ENSG00000286379
CABCOCO1	0.8106	2.0811E-07	1.0472E-05	ENSG00000183346
AC131212.2	0.8071	2.8252E-05	5.6839E-04	ENSG00000279700
C22orf42	0.8059	4.5438E-07	1.9788E-05	ENSG00000205856
LOC124909362	0.8059	1.0002E-03	9.0614E-03	ENSG00000289460
S100A4	0.8057	4.0754E-05	7.6023E-04	ENSG00000196154
NA	0.8054	1.1292E-04	1.6883E-03	ENSG00000289319
AC023905.1	0.8054	1.6455E-03	1.3053E-02	ENSG00000259221
NA	0.8041	7.5768E-03	4.1145E-02	ENSG00000290065
LDB3	0.8024	4.5915E-04	4.9768E-03	ENSG00000122367
NA	0.8022	6.4707E-03	3.6575E-02	ENSG00000259001
CFC1	0.8012	1.1958E-04	1.7672E-03	ENSG00000136698
COL19A1	0.7997	4.5008E-04	4.8950E-03	ENSG00000082293
NA	0.7994	4.0000E-03	2.5562E-02	ENSG00000288074
VSTM2A-OT1	0.7984	1.6287E-03	1.2969E-02	ENSG00000224223
AL162726.3	0.7964	4.2150E-03	2.6504E-02	ENSG00000228430
LOC105375798	0.7949	1.5639E-04	2.1462E-03	ENSG00000254812
AC090774.3	0.7945	5.3213E-05	9.3405E-04	ENSG00000266369
SH3D21	0.7944	5.0256E-11	9.2973E-09	ENSG00000214193
GJD2-DT	0.7943	3.5687E-03	2.3409E-02	ENSG00000250007
NA	0.7934	4.5492E-04	4.9397E-03	ENSG00000249150
NA	0.7925	2.6465E-06	8.3590E-05	ENSG00000287530
POU2F2	0.7920	1.6863E-13	8.4679E-11	ENSG00000028277
STK31	0.7918	3.5173E-03	2.3140E-02	ENSG00000196335
KLF3P1	0.7915	9.0427E-03	4.7113E-02	ENSG00000253218
LOC102723670	0.7912	2.2746E-03	1.6587E-02	ENSG00000259868
SEMA3E	0.7905	5.6141E-07	2.3492E-05	ENSG00000170381
PTPRD-DT	0.7896	1.8534E-04	2.4478E-03	ENSG00000226717
LOC100506606	0.7895	2.0456E-04	2.6365E-03	ENSG00000257176
SLC11A1	0.7881	2.3892E-04	3.0069E-03	ENSG00000018280
NA	0.7879	5.3391E-03	3.1672E-02	ENSG00000272788
FAM138F	0.7863	5.4169E-03	3.2024E-02	ENSG00000282591

MYH7	0.7860	2.3236E-04	2.9305E-03	ENSG00000092054
CNTN5	0.7856	1.7834E-04	2.3719E-03	ENSG00000149972
DMRTA1	0.7851	1.6408E-07	8.5352E-06	ENSG00000176399
LRGUK	0.7849	7.3927E-05	1.2167E-03	ENSG00000155530
NA	0.7834	2.1527E-03	1.5882E-02	ENSG00000286739
FRMPD2	0.7816	1.0639E-03	9.5151E-03	ENSG00000170324
TGFBR3	0.7814	1.3799E-04	1.9512E-03	ENSG00000069702
CYP2A6	0.7805	5.6710E-03	3.3097E-02	ENSG00000255974
FN1	0.7786	2.5159E-06	8.0498E-05	ENSG00000115414
AL033530.1	0.7778	1.3136E-04	1.8912E-03	ENSG00000285407
AC020917.4	0.7775	8.6814E-04	8.1498E-03	ENSG00000280332
AL110292.1	0.7775	7.0151E-03	3.8849E-02	ENSG00000258081
LINC00626	0.7743	4.2522E-03	2.6690E-02	ENSG00000225826
CCDC13	0.7730	9.4687E-04	8.6770E-03	ENSG00000244607
PNOC	0.7725	9.6407E-05	1.5023E-03	ENSG00000168081
CXCR4	0.7708	6.1211E-11	1.0873E-08	ENSG00000121966
KLKP1	0.7706	6.4375E-05	1.0916E-03	ENSG00000197588
NPIPB10P	0.7680	8.5553E-05	1.3641E-03	ENSG00000196796
AC092384.1	0.7679	8.7573E-04	8.2054E-03	ENSG00000205018
DCDC2	0.7672	1.4565E-03	1.2026E-02	ENSG00000146038
SND1-DT	0.7669	2.3949E-04	3.0111E-03	ENSG00000240790
NA	0.7660	2.2503E-03	1.6464E-02	ENSG00000288076
LAMA2	0.7654	6.9345E-05	1.1562E-03	ENSG00000196569
RNFT1P3	0.7645	2.0539E-03	1.5368E-02	ENSG00000187870
LEKR1	0.7628	4.4685E-07	1.9495E-05	ENSG00000197980
LOC124904138	0.7625	4.0553E-03	2.5830E-02	ENSG00000276596
LRRC46	0.7624	6.3876E-04	6.4677E-03	ENSG00000141294
FAM47E	0.7621	1.1237E-07	6.3709E-06	ENSG00000189157
AC066612.1	0.7612	8.1593E-03	4.3521E-02	ENSG00000259235
PPP1R42	0.7603	1.6574E-07	8.5853E-06	ENSG00000178125
AC063926.3	0.7600	7.7686E-04	7.5004E-03	ENSG00000279905
NPTN-IT1	0.7597	1.0169E-08	8.4814E-07	ENSG00000281183
CFAP251	0.7580	1.3392E-05	3.0747E-04	ENSG00000158023
AMY1C	0.7576	3.7254E-03	2.4224E-02	ENSG00000187733
RCN3	0.7570	1.7566E-08	1.3368E-06	ENSG00000142552
ALK	0.7562	1.8300E-06	6.1850E-05	ENSG00000171094
AC100774.1	0.7557	1.1111E-04	1.6681E-03	ENSG00000261529
NA	0.7554	1.6392E-03	1.3032E-02	ENSG00000286529
OTOF	0.7551	5.4410E-04	5.6728E-03	ENSG00000115155
EBF1	0.7550	2.7374E-08	1.9135E-06	ENSG00000164330
LINC01902	0.7548	8.4437E-07	3.2666E-05	ENSG00000283503
KRTCAP3	0.7534	1.4354E-04	2.0113E-03	ENSG00000157992
CRYGS	0.7527	3.7736E-03	2.4434E-02	ENSG00000213139
IRS4	0.7509	6.3062E-06	1.6847E-04	ENSG00000133124
FYB2	0.7503	1.0530E-03	9.4423E-03	ENSG00000187889
ATP8B4	0.7501	9.0645E-03	4.7183E-02	ENSG00000104043
OTX2-AS1	0.7493	1.6997E-03	1.3357E-02	ENSG00000248550
TBC1D22A-AS1	0.7468	7.0471E-03	3.9000E-02	ENSG00000280080
TCHH	0.7453	7.1316E-09	6.2224E-07	ENSG00000159450

IRGM	0.7448	1.6972E-03	1.3346E-02	ENSG00000237693
AP000347.1	0.7443	4.5033E-04	4.8950E-03	ENSG00000272578
NA	0.7433	1.2568E-04	1.8309E-03	ENSG00000288966
AL445309.1	0.7432	1.4787E-03	1.2148E-02	ENSG00000272320
KCNH8	0.7413	3.6644E-08	2.4702E-06	ENSG00000183960
MUC20	0.7394	8.7724E-08	5.1886E-06	ENSG00000176945
COL13A1	0.7382	6.0185E-04	6.1522E-03	ENSG00000197467
LOC101927827	0.7378	5.5752E-03	3.2661E-02	ENSG00000229311
KHDC3L	0.7368	2.0609E-05	4.3865E-04	ENSG00000203908
LINC02941	0.7354	1.9185E-03	1.4605E-02	ENSG00000236013
LOC124901321	0.7333	4.2862E-05	7.8761E-04	ENSG00000289609
HS3ST5	0.7326	1.7083E-04	2.2944E-03	ENSG00000249853
AC140725.1	0.7325	4.7077E-08	3.0482E-06	ENSG00000259553
AC010148.1	0.7312	2.1966E-05	4.6241E-04	ENSG00000235726
AL354733.1	0.7303	8.3995E-03	4.4531E-02	ENSG00000226877
PLCXD3	0.7302	1.2605E-04	1.8351E-03	ENSG00000182836
LOC105372436	0.7294	1.8050E-10	2.6356E-08	ENSG00000287001
LOC101929473	0.7292	4.8530E-03	2.9375E-02	ENSG00000255087
SIAH3	0.7269	1.8869E-08	1.4112E-06	ENSG00000215475
AC069224.1	0.7268	3.4295E-05	6.6278E-04	ENSG00000260572
SEMA6A-AS1	0.7262	4.7195E-03	2.8836E-02	ENSG00000248445
HSD52	0.7261	7.0372E-04	6.9818E-03	ENSG00000224609
CCT6B	0.7258	9.0264E-09	7.6321E-07	ENSG00000132141
IGIP	0.7256	2.3034E-04	2.9109E-03	ENSG00000182700
IL1RAP	0.7248	1.8603E-09	1.9072E-07	ENSG00000196083
LINC02930	0.7239	7.7378E-04	7.4779E-03	ENSG00000274461
SEMA3B	0.7232	5.9898E-09	5.3206E-07	ENSG00000012171
FTCD	0.7226	1.5864E-04	2.1662E-03	ENSG00000160282
MIR137HG	0.7219	1.5839E-04	2.1639E-03	ENSG00000225206
TCP11X1	0.7213	5.5099E-03	3.2379E-02	ENSG00000268235
ARMH1	0.7210	1.0198E-14	6.7816E-12	ENSG00000198520
BPIFC	0.7210	1.5062E-03	1.2292E-02	ENSG00000184459
RPS27AP12	0.7206	2.6731E-04	3.2869E-03	ENSG00000225224
LINC01109	0.7205	1.3665E-03	1.1452E-02	ENSG00000270866
TDO2	0.7198	1.2192E-05	2.8544E-04	ENSG00000151790
SP6	0.7188	1.3803E-03	1.1525E-02	ENSG00000189120
CDK15	0.7162	3.7540E-03	2.4345E-02	ENSG00000138395
LIMS2	0.7162	3.0584E-03	2.0776E-02	ENSG00000072163
C4A	0.7160	3.0131E-07	1.4230E-05	ENSG00000244731
FAM138A	0.7145	6.9793E-03	3.8735E-02	ENSG00000237613
AC015849.3	0.7144	7.9484E-05	1.2883E-03	ENSG00000270871
AC068790.5	0.7144	3.8690E-05	7.3228E-04	ENSG00000270061
ZFHX4	0.7141	4.7733E-09	4.3499E-07	ENSG00000091656
ZFHX3	0.7133	2.3488E-14	1.3760E-11	ENSG00000140836
BCO1	0.7124	7.0651E-03	3.9082E-02	ENSG00000135697
CALCRL	0.7122	1.1769E-03	1.0254E-02	ENSG00000064989
AL158063.1	0.7121	6.8807E-03	3.8320E-02	ENSG00000276957
BX322562.1	0.7121	6.3244E-04	6.4223E-03	ENSG00000273796
OPRM1	0.7118	1.6547E-03	1.3114E-02	ENSG00000112038

CLEC18B	0.7110	1.2427E-03	1.0678E-02	ENSG00000140839
RPL11P3	0.7109	1.0254E-03	9.2487E-03	ENSG00000213613
NOX4	0.7094	1.8983E-04	2.4978E-03	ENSG00000086991
NA	0.7090	4.8204E-03	2.9242E-02	ENSG00000287580
NA	0.7089	3.3795E-09	3.2230E-07	ENSG00000289612
LOC105369147	0.7079	3.9460E-08	2.6312E-06	ENSG00000247081
CLRN1-AS1	0.7076	3.6977E-03	2.4088E-02	ENSG00000239265
RMST	0.7066	1.2019E-10	1.9585E-08	ENSG00000255794
C21orf62	0.7051	4.7154E-04	5.0776E-03	ENSG00000205929
AK8	0.7049	5.0563E-06	1.4235E-04	ENSG00000165695
FER1L5	0.7047	4.0558E-05	7.5830E-04	ENSG00000249715
NA	0.7047	1.5249E-03	1.2379E-02	ENSG00000289968
TEKT4P2	0.7042	1.3304E-03	1.1253E-02	ENSG00000188681
ERICH2	0.7040	9.6790E-06	2.3863E-04	ENSG00000204334
CDH12P3	0.7036	4.3296E-05	7.9402E-04	ENSG00000253492
MMRN1	0.7024	4.3806E-05	8.0137E-04	ENSG00000138722
AHSG	0.7020	1.3435E-03	1.1328E-02	ENSG00000145192
DAB2	0.7004	1.5321E-04	2.1154E-03	ENSG00000153071
PDC-AS1	0.6995	4.0406E-05	7.5604E-04	ENSG00000229739
LOC105375423	0.6994	1.5898E-06	5.5484E-05	ENSG00000286923
AMY1A	0.6992	5.1788E-03	3.0936E-02	ENSG00000237763
NA	0.6973	4.9881E-04	5.2940E-03	ENSG00000247372
NA	0.6968	4.6664E-04	5.0359E-03	ENSG00000283415
NA	0.6952	4.8525E-03	2.9375E-02	ENSG00000279428
PIERCE1	0.6907	1.1300E-09	1.2303E-07	ENSG00000160345
CLEC18A	0.6902	9.0891E-04	8.4417E-03	ENSG00000157322
MAP3K20-AS1	0.6894	5.9424E-04	6.0852E-03	ENSG00000238133
RAPGEF6	0.6894	8.3240E-11	1.4322E-08	ENSG00000158987
TTC39C-AS1	0.6883	8.0854E-04	7.7263E-03	ENSG00000264745
NA	0.6874	2.4308E-04	3.0468E-03	ENSG00000290011
CDH6	0.6870	4.4643E-07	1.9495E-05	ENSG00000113361
TLL1	0.6869	4.1125E-03	2.6080E-02	ENSG00000038295
CCL25	0.6869	1.4398E-04	2.0152E-03	ENSG00000131142
FUT3	0.6868	1.4126E-04	1.9832E-03	ENSG00000171124
LZTS1-AS1	0.6867	1.8389E-03	1.4148E-02	ENSG00000253733
RNU5B-1	0.6866	1.0463E-04	1.5940E-03	ENSG00000200156
HTR1E	0.6859	3.6639E-03	2.3909E-02	ENSG00000168830
LRRIQ1	0.6856	1.1434E-16	1.3397E-13	ENSG00000133640
RPE65	0.6849	2.0724E-03	1.5457E-02	ENSG00000116745
GTF2IP14	0.6846	6.2581E-03	3.5611E-02	ENSG00000226002
LOC105376654	0.6845	4.5915E-03	2.8229E-02	ENSG00000254746
NA	0.6844	8.4253E-06	2.1350E-04	ENSG00000287562
DMRT1	0.6827	1.3600E-03	1.1419E-02	ENSG00000137090
GREB1L	0.6822	8.1954E-10	9.3355E-08	ENSG00000141449
AC005041.1	0.6822	5.3327E-03	3.1663E-02	ENSG00000159239
AL137003.2	0.6813	1.5058E-04	2.0888E-03	ENSG00000272341
TMEM45A	0.6808	3.7915E-05	7.2094E-04	ENSG00000181458
AL513314.2	0.6804	4.3472E-03	2.7107E-02	ENSG00000276997
CSRP1-AS1	0.6788	6.9486E-03	3.8585E-02	ENSG00000224536

AL627309.2	0.6787	5.9033E-03	3.4132E-02	ENSG00000239906
OTX2	0.6784	1.0155E-06	3.7699E-05	ENSG00000165588
SPEF2	0.6782	4.3781E-10	5.5527E-08	ENSG00000152582
CFAP57	0.6778	5.4539E-03	3.2144E-02	ENSG00000243710
GALP	0.6770	1.2955E-05	2.9958E-04	ENSG00000197487
AC114980.1	0.6769	6.0697E-03	3.4818E-02	ENSG00000251023
EMB	0.6749	2.7379E-04	3.3493E-03	ENSG00000170571
BST1	0.6746	1.8358E-06	6.1961E-05	ENSG00000109743
CELA2B	0.6740	3.5730E-03	2.3425E-02	ENSG00000215704
LOC107986742	0.6730	5.7381E-03	3.3401E-02	ENSG00000287815
GALR1	0.6730	5.2784E-03	3.1410E-02	ENSG00000166573
NA	0.6729	9.0715E-08	5.3144E-06	ENSG00000260563
CDH12P1	0.6697	4.5770E-05	8.2685E-04	ENSG00000254335
RELN	0.6695	1.8082E-06	6.1281E-05	ENSG00000189056
NA	0.6694	2.0952E-04	2.6920E-03	ENSG00000264940
NA	0.6684	2.3072E-03	1.6780E-02	ENSG00000267429
NA	0.6680	1.7345E-05	3.8276E-04	ENSG00000260285
NA	0.6672	3.8857E-12	1.1418E-09	ENSG00000131797
GPR18	0.6656	1.2670E-04	1.8425E-03	ENSG00000125245
TH	0.6656	3.0771E-07	1.4449E-05	ENSG00000180176
NA	0.6655	2.2838E-05	4.7743E-04	ENSG00000289866
CDH12P4	0.6653	2.1219E-03	1.5738E-02	ENSG00000253900
AL358473.1	0.6643	1.7808E-06	6.0603E-05	ENSG00000229191
NA	0.6642	1.4544E-05	3.2981E-04	ENSG00000289244
AC015922.4	0.6633	1.5537E-04	2.1357E-03	ENSG00000276855
DNAH6	0.6627	1.5752E-06	5.5054E-05	ENSG00000115423
SLC2A1-DT	0.6625	1.2364E-04	1.8097E-03	ENSG00000227533
SLC13A4	0.6618	2.1149E-03	1.5707E-02	ENSG00000164707
SLC26A2	0.6611	7.3672E-10	8.6319E-08	ENSG00000155850
KCNK2	0.6606	1.4915E-04	2.0734E-03	ENSG00000082482
NA	0.6601	6.6785E-05	1.1247E-03	ENSG00000287710
MOV10L1	0.6585	1.4171E-03	1.1760E-02	ENSG00000073146
IGFBP7-AS1	0.6571	4.7567E-03	2.8956E-02	ENSG00000245067
SPAG17	0.6568	1.0124E-03	9.1512E-03	ENSG00000155761
C1orf94	0.6560	9.7580E-04	8.8793E-03	ENSG00000142698
CTSW	0.6558	4.6517E-03	2.8514E-02	ENSG00000172543
ARID3A	0.6557	2.2573E-07	1.1243E-05	ENSG00000116017
NA	0.6552	4.4413E-04	4.8446E-03	ENSG00000273188
MSANTD1	0.6551	3.3640E-04	3.9210E-03	ENSG00000188981
NA	0.6545	5.0132E-04	5.3076E-03	ENSG00000288952
TRPV1	0.6540	1.9599E-05	4.2117E-04	ENSG00000196689
NA	0.6536	6.8768E-03	3.8307E-02	ENSG00000226644
CEMIP	0.6532	1.2015E-03	1.0410E-02	ENSG00000103888
NA	0.6524	5.3925E-03	3.1908E-02	ENSG00000224944
KIF6	0.6505	1.3337E-04	1.9086E-03	ENSG00000164627
LINC01829	0.6491	5.5417E-03	3.2527E-02	ENSG00000236780
CRYGEP	0.6488	4.1073E-04	4.5654E-03	ENSG00000229150
RAB11FIP1P1	0.6486	5.0276E-03	3.0231E-02	ENSG00000228492
RBKS	0.6475	2.0165E-06	6.6957E-05	ENSG00000171174

DGKK	0.6462	2.6886E-03	1.8788E-02	ENSG00000274588
AC022126.1	0.6459	6.8130E-03	3.8038E-02	ENSG00000248898
DIRC3	0.6447	5.0574E-05	8.9870E-04	ENSG00000231672
NHLRC3	0.6445	3.6491E-14	2.0406E-11	ENSG00000188811
PRSS23	0.6442	3.2360E-11	6.5802E-09	ENSG00000150687
LINC02363	0.6435	2.8910E-05	5.7769E-04	ENSG00000180712
SEPSECS	0.6431	4.1531E-08	2.7610E-06	ENSG00000109618
LOC102546299	0.6429	1.0898E-03	9.6737E-03	ENSG00000241956
MIR1302-2HG	0.6420	1.5116E-05	3.3934E-04	ENSG00000243485
LOC124906016	0.6415	1.4833E-05	3.3408E-04	ENSG00000204929
AC007952.4	0.6413	2.8880E-05	5.7769E-04	ENSG00000262202
TTN	0.6401	2.2285E-06	7.2722E-05	ENSG00000155657
NABP1	0.6395	5.9251E-05	1.0209E-03	ENSG00000173559
NA	0.6394	8.9859E-07	3.3807E-05	ENSG00000261889
CD109	0.6384	3.5370E-04	4.0667E-03	ENSG00000156535
IFITM3	0.6375	7.0168E-06	1.8426E-04	ENSG00000142089
LOC100505978	0.6357	8.9903E-03	4.6955E-02	ENSG00000257711
NA	0.6347	4.0570E-04	4.5220E-03	ENSG00000286329
AGBL4	0.6347	3.4443E-06	1.0386E-04	ENSG00000186094
PCDH10-DT	0.6344	2.9994E-08	2.0500E-06	ENSG00000250241
ZIC4	0.6343	3.5070E-05	6.7415E-04	ENSG00000174963
AC009407.1	0.6327	8.5340E-04	8.0348E-03	ENSG00000261829
NA	0.6321	4.0931E-06	1.1933E-04	ENSG00000286964
AL807742.1	0.6320	3.6350E-04	4.1561E-03	ENSG00000283380
DOP1A	0.6318	5.2698E-11	9.6763E-09	ENSG00000083097
PCAT1	0.6316	6.3271E-03	3.5911E-02	ENSG00000253438
NA	0.6311	2.8100E-06	8.8301E-05	ENSG00000285646
CNTNAP5-DT	0.6307	5.0990E-05	9.0455E-04	ENSG00000228400
ELMO3	0.6288	8.5851E-03	4.5359E-02	ENSG00000102890
WASH5P	0.6286	4.0554E-10	5.1952E-08	ENSG00000282458
HERC2P11	0.6285	5.3393E-03	3.1672E-02	ENSG00000261581
CHRNA9	0.6275	1.7287E-03	1.3525E-02	ENSG00000174343
LOC101928626	0.6275	3.1478E-05	6.1617E-04	ENSG00000230021
KCNMB2-AS1	0.6272	1.1734E-03	1.0227E-02	ENSG00000237978
HDAC1P2	0.6265	1.0047E-04	1.5512E-03	ENSG00000233012
LURAP1L-AS1	0.6264	8.2662E-04	7.8499E-03	ENSG00000235448
CACNB2	0.6258	2.3004E-09	2.3293E-07	ENSG00000165995
NA	0.6250	2.5130E-03	1.7835E-02	ENSG00000274363
NA	0.6237	1.1364E-04	1.6957E-03	ENSG00000289881
SNORD3B-1	0.6236	7.0026E-06	1.8408E-04	ENSG00000265185
CDH12P2	0.6236	1.0345E-04	1.5841E-03	ENSG00000249230
MOK	0.6233	6.9296E-16	6.5578E-13	ENSG00000080823
LINC01971	0.6230	1.7670E-08	1.3377E-06	ENSG00000278200
PDGFRA	0.6227	3.1745E-03	2.1411E-02	ENSG00000134853
CLDN4	0.6221	1.0147E-03	9.1681E-03	ENSG00000189143
FBXO15	0.6217	2.9334E-07	1.3998E-05	ENSG00000141665
BBS12	0.6210	6.7919E-05	1.1399E-03	ENSG00000181004
NA	0.6209	6.2240E-04	6.3360E-03	ENSG00000288778
SCN2A	0.6206	1.0359E-10	1.7458E-08	ENSG00000136531

CATSPERG	0.6205	1.0806E-14	6.9966E-12	ENSG00000099338
B4GALT6	0.6203	9.2818E-07	3.4867E-05	ENSG00000118276
TP73	0.6200	1.5242E-03	1.2379E-02	ENSG00000078900
CFAP96	0.6186	8.8098E-07	3.3400E-05	ENSG00000205129
INHA	0.6186	6.8511E-04	6.8365E-03	ENSG00000123999
NA	0.6179	4.4171E-03	2.7404E-02	ENSG00000288916
NA	0.6171	3.0856E-05	6.0617E-04	ENSG00000287733
TRPV3	0.6161	3.8387E-03	2.4758E-02	ENSG00000167723
NA	0.6155	2.3465E-04	2.9578E-03	ENSG00000288012
LOC124906232	0.6154	2.7306E-03	1.8993E-02	ENSG00000287629
DNAI7	0.6154	1.9452E-04	2.5391E-03	ENSG00000118307
ASIC3	0.6151	1.1556E-04	1.7180E-03	ENSG00000213199
TENM3	0.6139	6.7160E-08	4.1329E-06	ENSG00000218336
CLEC18C	0.6134	3.4453E-03	2.2745E-02	ENSG00000157335
RFX3-DT	0.6131	2.3505E-11	5.2188E-09	ENSG00000232104
MARCHF10	0.6129	6.2378E-03	3.5536E-02	ENSG00000173838
CCN5	0.6123	1.6117E-03	1.2859E-02	ENSG00000064205
TTLL6	0.6122	9.3462E-03	4.8261E-02	ENSG00000170703
FAM178B	0.6109	1.8519E-03	1.4222E-02	ENSG00000168754
NA	0.6104	7.6585E-03	4.1451E-02	ENSG00000287590
ZCCHC14-DT	0.6098	2.7490E-03	1.9093E-02	ENSG00000288568
ANKRD31	0.6086	5.6269E-05	9.7707E-04	ENSG00000145700
NA	0.6081	2.5031E-03	1.7787E-02	ENSG00000280441
ENPEP	0.6072	5.3709E-03	3.1820E-02	ENSG00000138792
AK3P3	0.6070	4.9735E-04	5.2815E-03	ENSG00000230042
KCTD8	0.6070	7.0804E-04	7.0106E-03	ENSG00000183783
AC144831.1	0.6069	3.6849E-03	2.4018E-02	ENSG00000261888
AC026356.1	0.6059	1.2140E-03	1.0492E-02	ENSG00000274964
LINC00642	0.6059	6.8144E-03	3.8038E-02	ENSG00000233208
CFAP70	0.6047	2.2764E-05	4.7629E-04	ENSG00000156042
AC093525.7	0.6041	4.9940E-04	5.2941E-03	ENSG00000269937
NA	0.6031	3.5882E-03	2.3506E-02	ENSG00000287207
NA	0.6031	5.0071E-11	9.2973E-09	ENSG00000232931
LINC00539	0.6029	4.9335E-04	5.2595E-03	ENSG00000224429
ZIC1	0.6025	1.4590E-05	3.3055E-04	ENSG00000152977
HES1	0.6023	3.4735E-11	6.8924E-09	ENSG00000114315
NA	0.6021	3.2315E-03	2.1689E-02	ENSG00000289578
AL158206.1	0.6017	1.3580E-04	1.9270E-03	ENSG00000260912
MISFA	0.6013	2.5885E-04	3.2102E-03	ENSG00000247595
C4B	0.6003	1.1476E-05	2.7308E-04	ENSG00000224389
SNORD3B-2	0.6003	9.7451E-06	2.4002E-04	ENSG00000262074
C6orf52	0.6001	2.0242E-12	6.4683E-10	ENSG00000137434
LINC01001	0.6001	4.2730E-07	1.8876E-05	ENSG00000230724
**LOWER IN LEO THAN GROUND: LARGEST TO SMALLEST DIFFERENCE (GREEN)**				
NA	-2.0762	1.7620E-05	3.8778E-04	ENSG00000289224
CXCL8	-2.0668	8.1598E-05	1.3131E-03	ENSG00000169429
CXCL2	-1.8487	6.6392E-12	1.7195E-09	ENSG00000081041
AC104046.1	-1.7947	2.3024E-15	1.8135E-12	ENSG00000259630

SCGN	-1.5842	6.1889E-06	1.6606E-04	ENSG00000079689
MIR6087	-1.5543	2.1755E-04	2.7793E-03	ENSG00000275110
CXCL3	-1.3377	1.5138E-10	2.2992E-08	ENSG00000163734
H3F3C	-1.3194	1.0429E-16	1.2831E-13	ENSG00000188375
NA	-1.3102	1.5522E-03	1.2538E-02	ENSG00000287974
AL136366.1	-1.2839	3.0244E-17	4.2183E-14	ENSG00000225472
AC026470.2	-1.2775	5.2173E-07	2.2287E-05	ENSG00000261267
AC007731.4	-1.2648	3.3765E-03	2.2369E-02	ENSG00000236003
AC147067.1	-1.2507	2.7137E-03	1.8905E-02	ENSG00000244459
LINC03040	-1.1648	1.0176E-06	3.7709E-05	ENSG00000181577
GADD45G	-1.1538	2.8247E-08	1.9633E-06	ENSG00000130222
ATP5PBP1	-1.1379	3.3726E-05	6.5340E-04	ENSG00000224451
PPIAP2	-1.1334	1.3452E-04	1.9132E-03	ENSG00000227379
TAC3	-1.1256	4.3862E-19	1.0305E-15	ENSG00000166863
AC007191.1	-1.1178	1.9702E-04	2.5622E-03	ENSG00000279407
AC008764.10	-1.1138	1.2066E-04	1.7785E-03	ENSG00000279977
AC091488.1	-1.1078	5.0587E-05	8.9870E-04	ENSG00000271320
H3F3B	-1.0769	2.2734E-25	5.5937E-21	ENSG00000132475
ACTBP11	-1.0701	4.8007E-09	4.3587E-07	ENSG00000188460
NR1D1	-1.0629	4.6069E-19	1.0305E-15	ENSG00000126368
AL391839.2	-1.0456	9.4634E-04	8.6754E-03	ENSG00000233825
AL731563.2	-1.0243	5.5838E-04	5.7921E-03	ENSG00000237768
ZC3H12C	-1.0170	2.1760E-17	3.3463E-14	ENSG00000149289
MDFI	-1.0051	6.5899E-11	1.1582E-08	ENSG00000112559
RN7SL5P	-1.0042	2.5235E-06	8.0638E-05	ENSG00000265735
H1-3	-1.0040	1.9988E-12	6.4683E-10	ENSG00000124575
NA	-1.0006	1.8893E-03	1.4432E-02	ENSG00000285857
BCAP29	-0.9924	3.5219E-06	1.0544E-04	ENSG00000075790
HSPE1P27	-0.9900	8.2327E-05	1.3205E-03	ENSG00000235112
KLF9	-0.9815	3.6165E-12	1.0937E-09	ENSG00000119138
UBC	-0.9804	3.2933E-20	1.1576E-16	ENSG00000150991
AC139143.1	-0.9746	5.1508E-06	1.4365E-04	ENSG00000230080
CALM1P1	-0.9621	9.9384E-04	9.0201E-03	ENSG00000223467
INSM1	-0.9557	1.1269E-12	3.9561E-10	ENSG00000173404
AC013394.1	-0.9536	5.8641E-10	7.0729E-08	ENSG00000279765
RNU6-415P	-0.9528	6.5456E-05	1.1062E-03	ENSG00000252061
AL162430.1	-0.9484	1.2753E-09	1.3673E-07	ENSG00000233406
HES2	-0.9481	1.0811E-04	1.6359E-03	ENSG00000069812
LINC01235	-0.9401	2.2347E-05	4.6875E-04	ENSG00000270547
DUSP10	-0.9278	5.4867E-12	1.5000E-09	ENSG00000143507
HIST1H4C	-0.9183	5.4703E-11	9.9701E-09	ENSG00000197061
PMF1-BGLAP	-0.9169	1.4577E-03	1.2032E-02	ENSG00000260238
AC002543.1	-0.9164	1.9406E-07	9.8451E-06	ENSG00000235945
FABP5P1	-0.9158	2.6328E-05	5.3582E-04	ENSG00000236972
ZEB2-AS1	-0.9146	3.0447E-03	2.0708E-02	ENSG00000238057
RGS10	-0.9125	3.2168E-14	1.8407E-11	ENSG00000148908
ACTA1	-0.9108	2.6360E-06	8.3365E-05	ENSG00000143632
AC025442.1	-0.9082	4.5025E-04	4.8950E-03	ENSG00000253744
SLC18A2	-0.9073	5.8816E-04	6.0324E-03	ENSG00000165646

CRLF1	-0.9060	2.0543E-09	2.0887E-07	ENSG00000006016
AP000529.1	-0.8925	2.9634E-06	9.1716E-05	ENSG00000241838
PROKR2	-0.8828	3.9252E-05	7.4008E-04	ENSG00000101292
MSRA	-0.8822	1.6145E-09	1.6691E-07	ENSG00000175806
UBBP2	-0.8812	5.6018E-05	9.7408E-04	ENSG00000228247
HIST1H2BC	-0.8802	3.1260E-10	4.1353E-08	ENSG00000180596
AC016027.2	-0.8786	5.7030E-04	5.8811E-03	ENSG00000234913
AL358075.3	-0.8771	1.3716E-03	1.1475E-02	ENSG00000233114
OSTN	-0.8735	1.3496E-03	1.1361E-02	ENSG00000188729
N4BP2L1	-0.8699	3.5247E-11	6.9379E-09	ENSG00000139597
TMEM54	-0.8677	2.1815E-15	1.8135E-12	ENSG00000121900
MEF2C-AS1	-0.8609	2.3117E-05	4.8142E-04	ENSG00000248309
TIPARP	-0.8536	4.4690E-16	4.3983E-13	ENSG00000163659
CRABP1	-0.8527	1.5003E-14	9.4652E-12	ENSG00000166426
RF00019	-0.8495	1.5021E-03	1.2268E-02	ENSG00000252367
ATF3	-0.8494	1.4670E-08	1.1569E-06	ENSG00000162772
PPP1R14A	-0.8455	1.6045E-12	5.3729E-10	ENSG00000167641
AL049844.2	-0.8381	3.8947E-03	2.5047E-02	ENSG00000270890
SH3RF3-AS1	-0.8378	2.0140E-04	2.6053E-03	ENSG00000259863
RN7SL1	-0.8360	2.2939E-04	2.9004E-03	ENSG00000276168
TNFAIP8L3	-0.8354	2.5525E-05	5.2077E-04	ENSG00000183578
TLE2	-0.8312	5.7456E-13	2.3961E-10	ENSG00000065717
HMX1	-0.8304	6.5922E-13	2.6591E-10	ENSG00000215612
MGARP	-0.8280	2.0667E-10	2.9565E-08	ENSG00000137463
OR2AK2	-0.8276	4.7843E-03	2.9087E-02	ENSG00000187080
CIART	-0.8270	2.0873E-24	2.5679E-20	ENSG00000159208
AC005014.2	-0.8231	5.3894E-04	5.6315E-03	ENSG00000272361
SPR	-0.8157	2.2278E-10	3.1091E-08	ENSG00000116096
BHMT	-0.8148	2.0541E-03	1.5368E-02	ENSG00000145692
HIST1H1C	-0.8131	1.6159E-12	5.3729E-10	ENSG00000187837
HNRNPCP2	-0.8111	7.9966E-12	2.0077E-09	ENSG00000204253
ARRDC3	-0.8110	9.2437E-16	8.4238E-13	ENSG00000113369
LMNB1	-0.8102	5.4260E-12	1.5000E-09	ENSG00000113368
NA	-0.8074	3.9853E-04	4.4653E-03	ENSG00000288754
NA	-0.8073	3.1623E-05	6.1851E-04	ENSG00000288876
AC087521.1	-0.8050	1.0258E-04	1.5775E-03	ENSG00000244953
AC105137.1	-0.8047	2.2814E-03	1.6618E-02	ENSG00000261714
POTEKP	-0.8016	5.0462E-06	1.4235E-04	ENSG00000204434
AL031428.1	-0.8010	5.8023E-04	5.9635E-03	ENSG00000240553
GALNT14	-0.7988	1.2099E-04	1.7805E-03	ENSG00000158089
AC010834.3	-0.7972	6.4701E-05	1.0956E-03	ENSG00000253854
NET1	-0.7842	1.5645E-20	6.4157E-17	ENSG00000173848
ACTBP2	-0.7828	3.0864E-08	2.1036E-06	ENSG00000213763
CERS3-AS1	-0.7766	2.6215E-03	1.8403E-02	ENSG00000259430
NME1-NME2	-0.7759	5.2338E-08	3.3536E-06	ENSG00000011052
SYNPR	-0.7702	1.4247E-08	1.1308E-06	ENSG00000163630
AC097717.1	-0.7702	2.2010E-03	1.6185E-02	ENSG00000232732
HIST1H2AI	-0.7700	1.1396E-04	1.6984E-03	ENSG00000196747
AC032044.2	-0.7677	4.7488E-03	2.8943E-02	ENSG00000280408

LEPR	-0.7670	1.5657E-07	8.2142E-06	ENSG00000116678
CHRM1	-0.7662	1.1831E-07	6.6159E-06	ENSG00000168539
TCF15	-0.7661	7.4496E-10	8.6558E-08	ENSG00000125878
SPRN	-0.7639	1.9969E-04	2.5883E-03	ENSG00000203772
TUBB2BP1	-0.7634	1.4548E-10	2.2372E-08	ENSG00000216819
HSPB1	-0.7627	5.5655E-12	1.5048E-09	ENSG00000106211
EPHA6	-0.7615	3.5838E-07	1.6239E-05	ENSG00000080224
AC008060.1	-0.7613	1.0904E-07	6.2247E-06	ENSG00000218672
ADRB1	-0.7596	1.3357E-04	1.9086E-03	ENSG00000043591
ACTG1P9	-0.7581	7.2370E-04	7.1227E-03	ENSG00000229349
SLC7A10	-0.7566	1.4416E-04	2.0165E-03	ENSG00000130876
FBXO32	-0.7526	1.1239E-03	9.8908E-03	ENSG00000156804
CDKN2A	-0.7526	3.6775E-05	7.0144E-04	ENSG00000147889
JUND	-0.7502	1.3167E-21	8.0994E-18	ENSG00000130522
NA	-0.7481	2.1999E-04	2.8046E-03	ENSG00000286689
MYC	-0.7464	1.3496E-04	1.9184E-03	ENSG00000136997
POTEM	-0.7462	1.6463E-05	3.6493E-04	ENSG00000222036
EEF1A2	-0.7456	4.9271E-24	4.0410E-20	ENSG00000101210
MICB	-0.7435	1.6107E-07	8.4145E-06	ENSG00000204516
SETP14	-0.7434	2.5767E-04	3.1988E-03	ENSG00000240489
AC023886.2	-0.7404	2.2665E-08	1.6450E-06	ENSG00000250966
AC020663.3	-0.7387	3.9856E-05	7.5088E-04	ENSG00000275056
KCNK12	-0.7370	1.7603E-08	1.3368E-06	ENSG00000184261
TUBA1B	-0.7348	1.9468E-14	1.1975E-11	ENSG00000123416
HSPA5	-0.7346	4.3023E-08	2.8079E-06	ENSG00000044574
SRGAP2D	-0.7344	1.9287E-06	6.4478E-05	ENSG00000270872
CHRDL1	-0.7340	1.6172E-08	1.2552E-06	ENSG00000101938
WNT16	-0.7294	4.8513E-03	2.9375E-02	ENSG00000002745
NTS	-0.7272	1.2553E-04	1.8297E-03	ENSG00000133636
FTH1P10	-0.7271	6.2325E-10	7.3727E-08	ENSG00000223361
AC055811.3	-0.7255	6.8726E-05	1.1507E-03	ENSG00000266498
KCNIP3	-0.7253	1.0295E-10	1.7458E-08	ENSG00000115041
GPD1	-0.7247	1.0396E-03	9.3461E-03	ENSG00000167588
FTH1P16	-0.7224	3.1261E-09	3.0046E-07	ENSG00000227376
FKBP4	-0.7219	8.2998E-15	5.7350E-12	ENSG00000004478
TUBAP2	-0.7217	1.1558E-09	1.2473E-07	ENSG00000214391
SLC30A3	-0.7217	8.9071E-07	3.3665E-05	ENSG00000115194
ENTPD3	-0.7190	6.6061E-05	1.1141E-03	ENSG00000168032
H4C11	-0.7077	2.3245E-10	3.1952E-08	ENSG00000197238
ACVR1C	-0.7052	1.1125E-03	9.8078E-03	ENSG00000123612
ECHDC2	-0.7041	2.6454E-08	1.8651E-06	ENSG00000121310
SLC9A3	-0.7032	1.9374E-03	1.4718E-02	ENSG00000066230
AL020997.2	-0.7030	3.4027E-04	3.9548E-03	ENSG00000269971
CRYM	-0.7023	1.4220E-05	3.2457E-04	ENSG00000103316
IKZF1	-0.7018	8.3525E-04	7.9005E-03	ENSG00000185811
ARHGAP42	-0.6992	4.0315E-04	4.5048E-03	ENSG00000165895
HMGN2P3	-0.6975	4.8531E-10	6.0308E-08	ENSG00000230330
PDLIM1	-0.6972	8.6491E-07	3.3025E-05	ENSG00000107438
FTH1P7	-0.6960	1.8389E-08	1.3837E-06	ENSG00000232187

CFLAR	-0.6952	2.9141E-11	6.0350E-09	ENSG00000003402
CSRNP1	-0.6945	4.1626E-09	3.9092E-07	ENSG00000144655
NA	-0.6944	2.9223E-03	2.0085E-02	ENSG00000285658
HNRNPC	-0.6939	2.3585E-15	1.8135E-12	ENSG00000092199
AC011939.1	-0.6929	1.5474E-03	1.2508E-02	ENSG00000259924
POTEG	-0.6929	5.8689E-04	6.0219E-03	ENSG00000187537
UBB	-0.6919	3.1828E-18	5.7829E-15	ENSG00000170315
SFRP1	-0.6919	3.3794E-03	2.2372E-02	ENSG00000104332
CCSAP	-0.6914	1.2324E-11	3.0630E-09	ENSG00000154429
HIST1H2AD	-0.6913	6.5053E-06	1.7304E-04	ENSG00000196866
HIST1H1E	-0.6906	6.1475E-12	1.6264E-09	ENSG00000168298
SEZ6	-0.6902	1.0023E-06	3.7310E-05	ENSG00000063015
NFIX	-0.6893	3.2898E-06	1.0030E-04	ENSG00000008441
AL078596.1	-0.6848	1.4206E-03	1.1781E-02	ENSG00000279398
NA	-0.6835	1.9271E-04	2.5248E-03	ENSG00000288994
STMN1	-0.6824	8.3910E-15	5.7350E-12	ENSG00000117632
AC079174.1	-0.6821	1.6037E-03	1.2820E-02	ENSG00000258355
AC017007.2	-0.6792	1.6456E-03	1.3053E-02	ENSG00000234841
HIST1H4K	-0.6770	2.2365E-10	3.1091E-08	ENSG00000273542
HRH1	-0.6765	1.7805E-05	3.9082E-04	ENSG00000196639
NFKBIA	-0.6700	1.6057E-16	1.7959E-13	ENSG00000100906
FTH1P20	-0.6691	2.5514E-08	1.8196E-06	ENSG00000226564
RPL36AP26	-0.6689	9.3370E-04	8.6043E-03	ENSG00000235828
TUBB2B	-0.6684	2.0811E-12	6.5650E-10	ENSG00000137285
TICAM1	-0.6684	1.8222E-04	2.4118E-03	ENSG00000127666
TBXA2R	-0.6666	1.1263E-03	9.9041E-03	ENSG00000006638
FOXN2	-0.6640	5.7140E-08	3.5965E-06	ENSG00000170802
RN7SL4P	-0.6633	3.8441E-04	4.3367E-03	ENSG00000263740
TCERG1L	-0.6612	6.3585E-04	6.4465E-03	ENSG00000176769
AL354793.1	-0.6609	3.6624E-09	3.4793E-07	ENSG00000230105
PDE2A	-0.6597	1.3539E-07	7.3377E-06	ENSG00000186642
AC009060.1	-0.6596	1.1899E-03	1.0335E-02	ENSG00000247228
FTH1	-0.6593	1.4539E-09	1.5222E-07	ENSG00000167996
CERS6	-0.6587	1.1777E-12	4.0246E-10	ENSG00000172292
NUDT3	-0.6586	1.6151E-13	8.2791E-11	ENSG00000272325
CFD	-0.6584	3.1735E-07	1.4789E-05	ENSG00000197766
NA	-0.6575	4.1887E-04	4.6299E-03	ENSG00000287673
TUBA1C	-0.6563	1.5628E-13	8.1814E-11	ENSG00000167553
ARMCX5-GPRASP2	-0.6554	2.4971E-07	1.2215E-05	ENSG00000271147
AC106772.2	-0.6534	2.9309E-03	2.0115E-02	ENSG00000250385
NR4A2	-0.6521	2.4796E-06	7.9439E-05	ENSG00000153234
ITPRIPL1	-0.6520	8.8593E-08	5.2274E-06	ENSG00000198885
H1F0	-0.6518	2.2252E-08	1.6246E-06	ENSG00000189060
AC008060.4	-0.6499	1.6132E-03	1.2867E-02	ENSG00000260426
NDP	-0.6483	4.1276E-05	7.6765E-04	ENSG00000124479
GRASP	-0.6473	3.8811E-07	1.7331E-05	ENSG00000161835
CCND2	-0.6457	1.4012E-10	2.1960E-08	ENSG00000118971
HIST1H2AJ	-0.6454	1.0497E-04	1.5972E-03	ENSG00000276368
SOWAHA	-0.6447	1.8834E-05	4.0794E-04	ENSG00000198944

SOX4	-0.6424	1.6865E-11	3.9586E-09	ENSG00000124766
LINC01578	-0.6414	3.3067E-11	6.6689E-09	ENSG00000272888
FTH1P11	-0.6409	7.7330E-06	1.9882E-04	ENSG00000237264
HMGB1P10	-0.6378	4.7449E-08	3.0642E-06	ENSG00000213707
RN7SL751P	-0.6365	4.4467E-03	2.7518E-02	ENSG00000240964
MXD1	-0.6361	6.7352E-13	2.6729E-10	ENSG00000059728
NBL1	-0.6352	2.3835E-11	5.2363E-09	ENSG00000158747
AC009779.2	-0.6349	5.5291E-07	2.3337E-05	ENSG00000258056
ACTB	-0.6343	7.4026E-20	2.2768E-16	ENSG00000075624
EFS	-0.6338	2.6645E-05	5.3958E-04	ENSG00000100842
H1FX	-0.6335	5.5432E-07	2.3355E-05	ENSG00000184897
RND1	-0.6326	2.9828E-08	2.0443E-06	ENSG00000172602
AL035458.2	-0.6321	1.2447E-03	1.0690E-02	ENSG00000250917
GLCE	-0.6316	4.9134E-09	4.4446E-07	ENSG00000138604
AL080243.1	-0.6310	8.0559E-05	1.3006E-03	ENSG00000213857
RAC3	-0.6299	3.2874E-05	6.3992E-04	ENSG00000169750
AC026124.2	-0.6293	2.8537E-06	8.9219E-05	ENSG00000276853
HMGN2	-0.6293	1.5380E-09	1.5967E-07	ENSG00000198830
CDC42EP1	-0.6291	6.2167E-07	2.5311E-05	ENSG00000128283
SPOCK1	-0.6291	5.4179E-12	1.5000E-09	ENSG00000152377
PRR19	-0.6275	1.3041E-05	3.0101E-04	ENSG00000188368
RPS4XP16	-0.6269	1.2429E-03	1.0678E-02	ENSG00000224892
ZC3HAV1	-0.6265	3.5358E-07	1.6081E-05	ENSG00000105939
STMN4	-0.6263	2.2769E-15	1.8135E-12	ENSG00000015592
PLPP2	-0.6261	5.6621E-04	5.8418E-03	ENSG00000141934
YWHAE	-0.6254	1.4313E-18	2.9347E-15	ENSG00000108953
CAPG	-0.6241	7.8036E-13	2.9540E-10	ENSG00000042493
ACSS1	-0.6241	7.4227E-08	4.4984E-06	ENSG00000154930
PCDHAC2	-0.6237	1.0019E-06	3.7310E-05	ENSG00000243232
AC093525.8	-0.6234	9.3839E-05	1.4669E-03	ENSG00000279520
PIM1	-0.6224	1.8102E-10	2.6356E-08	ENSG00000137193
FOXD1	-0.6220	1.4461E-05	3.2825E-04	ENSG00000251493
SLC25A25	-0.6219	1.0652E-08	8.8417E-07	ENSG00000148339
TBX1	-0.6217	5.4243E-03	3.2044E-02	ENSG00000184058
FTH1P2	-0.6215	9.3435E-06	2.3175E-04	ENSG00000234975
FTH1P8	-0.6211	8.1084E-06	2.0739E-04	ENSG00000219507
LINC02321	-0.6165	4.4046E-03	2.7361E-02	ENSG00000258884
NA	-0.6156	1.6701E-03	1.3196E-02	ENSG00000285587
AES	-0.6146	2.6686E-10	3.6077E-08	ENSG00000104964
LAMP5	-0.6142	5.5494E-04	5.7682E-03	ENSG00000125869
ANO4	-0.6141	5.1683E-05	9.1486E-04	ENSG00000151572
ALOX5	-0.6121	1.8613E-04	2.4569E-03	ENSG00000012779
C11orf96	-0.6118	2.5164E-03	1.7844E-02	ENSG00000187479
TUBB2A	-0.6111	4.4046E-10	5.5577E-08	ENSG00000137267
FTH1P23	-0.6108	1.2271E-06	4.4398E-05	ENSG00000242960
KCNK6	-0.6100	5.2700E-04	5.5296E-03	ENSG00000099337
AL590139.1	-0.6088	2.2757E-06	7.4067E-05	ENSG00000179101
OPN3	-0.6069	4.4344E-04	4.8446E-03	ENSG00000054277
GPR21	-0.6069	1.3772E-04	1.9497E-03	ENSG00000188394

GSEC	-0.6058	2.2190E-06	7.2606E-05	ENSG00000280832
PIGHP1	-0.6055	1.2149E-05	2.8496E-04	ENSG00000259657
FO681548.1	-0.6047	3.9800E-03	2.5489E-02	ENSG00000229492
DUSP16	-0.6046	2.6254E-06	8.3332E-05	ENSG00000111266
NA	-0.6025	9.1780E-04	8.4929E-03	ENSG00000289721
AL589740.1	-0.6012	3.3269E-05	6.4664E-04	ENSG00000271860
**NO SIGNIFICANT DIFFERENCE BETWEEN LEO AND GROUND (Alphabetical)**				
A1BG	-0.1331	1.2442E-01	2.9424E-01	ENSG00000121410
A1BG-AS1	-0.0563	5.2437E-01	7.0992E-01	ENSG00000268895
A1CF	-0.0069	8.6072E-01	9.3013E-01	ENSG00000148584
A2M	0.3570	1.2084E-02	5.8271E-02	ENSG00000175899
A2M-AS1	0.0766	3.7852E-01	5.8703E-01	ENSG00000245105
A2ML1	0.0462	4.3820E-01	6.3860E-01	ENSG00000166535
A3GALT2	-0.0097	8.9254E-01		ENSG00000184389
A4GALT	-0.3604	1.6024E-02	7.1155E-02	ENSG00000128274
AAAS	0.0082	8.9828E-01	9.4974E-01	ENSG00000094914
AACS	-0.2500	3.0678E-04	3.6589E-03	ENSG00000081760
AADAT	0.0853	2.2695E-01	4.3117E-01	ENSG00000109576
AAGAB	0.1804	2.0243E-02	8.3626E-02	ENSG00000103591
AAK1	0.2930	1.2429E-06	4.4709E-05	ENSG00000115977
AAMDC	-0.0474	5.3589E-01	7.1900E-01	ENSG00000087884
AAMP	-0.0193	7.2191E-01	8.4757E-01	ENSG00000127837
AAR2	-0.1267	5.1607E-02	1.6290E-01	ENSG00000131043
AARD	-0.0052	9.4888E-01	9.7468E-01	ENSG00000205002
AARS1	-0.2002	1.1863E-02	5.7539E-02	ENSG00000090861
AARS2	-0.0149	8.4348E-01	9.1973E-01	ENSG00000124608
AARSD1	0.1468	1.0972E-01	2.7073E-01	ENSG00000266967
AARSD1P1	0.0169	8.4423E-01	9.2003E-01	ENSG00000234969
AASDH	0.2362	1.0470E-03	9.4024E-03	ENSG00000157426
AASDHPPT	-0.0358	6.1241E-01	7.7442E-01	ENSG00000149313
AASS	0.0397	6.0401E-01	7.6833E-01	ENSG00000008311
AATBC	0.0431	6.1272E-01	7.7472E-01	ENSG00000215458
AATF	-0.1583	3.3093E-02	1.1922E-01	ENSG00000275700
AATK	0.1209	1.2986E-01	3.0243E-01	ENSG00000181409
ABALON	-0.0403	4.5956E-02		ENSG00000281376
ABAT	0.2537	4.3444E-04	4.7650E-03	ENSG00000183044
ABCA1	-0.1042	1.4137E-01	3.1824E-01	ENSG00000165029
ABCA10	0.2555	3.3260E-02	1.1964E-01	ENSG00000154263
ABCA11P	-0.0303	6.0271E-01	7.6738E-01	ENSG00000251595
ABCA12	0.1204	9.7147E-02	2.5043E-01	ENSG00000144452
ABCA17P	0.3536	1.3953E-03	1.1634E-02	ENSG00000238098
ABCA2	0.1223	1.1068E-01	2.7237E-01	ENSG00000107331
ABCA3	0.0718	2.5222E-01	4.6034E-01	ENSG00000167972
ABCA4	0.0587	4.0521E-01	6.0966E-01	ENSG00000198691
ABCA5	0.0196	7.8102E-01	8.8386E-01	ENSG00000154265
ABCA6	0.0518	1.7134E-01	3.6050E-01	ENSG00000154262
ABCA7	0.1215	1.5733E-01	3.4109E-01	ENSG00000064687
ABCA8	-0.0663	3.7089E-01	5.7984E-01	ENSG00000141338

ABCA9	-0.0208	4.4359E-01		ENSG00000154258
ABCB1	-0.0286	7.3153E-01	8.5317E-01	ENSG00000085563
ABCB10	0.2633	3.8232E-04	4.3153E-03	ENSG00000135776
ABCB10P3	0.0133	8.9787E-01		ENSG00000261524
ABCB10P4	0.0313	6.0415E-01		ENSG00000260053
ABCB11	0.0418	5.2184E-01	7.0850E-01	ENSG00000073734
ABCB4	-0.2427	2.5460E-02	9.8776E-02	ENSG00000005471
ABCB5	-0.0387	5.3870E-01	7.2098E-01	ENSG00000004846
ABCB6	0.2606	3.6253E-02	1.2712E-01	ENSG00000115657
ABCB7	-0.1457	9.0603E-02	2.3914E-01	ENSG00000131269
ABCB8	0.0240	7.0446E-01	8.3699E-01	ENSG00000197150
ABCB9	-0.1193	1.2076E-01	2.8864E-01	ENSG00000150967
ABCC1	0.0449	5.5495E-01	7.3317E-01	ENSG00000103222
ABCC10	0.3035	2.0562E-04	2.6488E-03	ENSG00000124574
ABCC11	0.1146	8.8590E-02	2.3506E-01	ENSG00000121270
ABCC12	0.0093	7.8041E-01		ENSG00000140798
ABCC2	0.0762	3.6808E-01	5.7725E-01	ENSG00000023839
ABCC3	0.0697	5.2291E-01	7.0893E-01	ENSG00000108846
ABCC4	0.0765	3.5110E-01	5.6122E-01	ENSG00000125257
ABCC5	-0.0920	1.1978E-01	2.8698E-01	ENSG00000114770
ABCC5-AS1	-0.0162	7.0379E-01		ENSG00000223882
ABCC6	0.1426	1.3898E-01	3.1497E-01	ENSG00000091262
ABCC8	-0.0837	3.1067E-01	5.2094E-01	ENSG00000006071
ABCC9	0.0424	2.8492E-01	4.9552E-01	ENSG00000069431
ABCD1	-0.0056	9.4271E-01	9.7154E-01	ENSG00000101986
ABCD2	-0.3653	5.2526E-03	3.1293E-02	ENSG00000173208
ABCD3	-0.0742	2.6526E-01	4.7436E-01	ENSG00000117528
ABCD4	0.1154	4.9978E-02	1.5968E-01	ENSG00000119688
ABCE1	0.0350	5.7710E-01	7.4907E-01	ENSG00000164163
ABCF1	0.0336	5.2641E-01	7.1158E-01	ENSG00000204574
ABCF2	-0.0280	7.0333E-01	8.3621E-01	ENSG00000033050
ABCF3	0.0377	6.1829E-01	7.7892E-01	ENSG00000161204
ABCG1	-0.0247	6.9168E-01	8.2897E-01	ENSG00000160179
ABCG2	0.0281	4.1783E-01		ENSG00000118777
ABCG4	0.0040	9.5996E-01	9.8006E-01	ENSG00000172350
ABCG5	0.0295	7.1911E-01	8.4556E-01	ENSG00000138075
ABCG8	0.0180	7.5876E-01		ENSG00000143921
ABHD1	0.1446	1.2947E-01	3.0194E-01	ENSG00000143994
ABHD10	-0.1605	1.6045E-02	7.1211E-02	ENSG00000144827
ABHD11	-0.1417	1.3560E-02	6.3264E-02	ENSG00000106077
ABHD12	0.0359	5.5692E-01	7.3477E-01	ENSG00000100997
ABHD12B	0.1534	6.8976E-02	1.9859E-01	ENSG00000131969
ABHD13	-0.1094	9.5962E-02	2.4857E-01	ENSG00000139826
ABHD14A	0.0093	9.0678E-01	9.5437E-01	ENSG00000248487
ABHD14A-ACY1	0.0238	6.8332E-01	8.2333E-01	ENSG00000114786
ABHD14B	-0.0055	9.4423E-01	9.7218E-01	ENSG00000114779
ABHD15	-0.1932	4.1975E-02	1.4138E-01	ENSG00000168792
ABHD16A	-0.0935	3.0013E-01	5.0986E-01	ENSG00000204427
ABHD17A	-0.1036	9.4728E-02	2.4629E-01	ENSG00000129968

ABHD17B	-0.1705	5.9038E-03	3.4132E-02	ENSG00000107362
ABHD17C	-0.0248	7.4810E-01	8.6312E-01	ENSG00000136379
ABHD18	0.2483	2.4772E-03	1.7688E-02	ENSG00000164074
ABHD2	-0.1843	3.1910E-03	2.1486E-02	ENSG00000140526
ABHD3	-0.5537	1.9517E-05	4.1978E-04	ENSG00000158201
ABHD4	-0.1563	7.8072E-02	2.1615E-01	ENSG00000100439
ABHD5	-0.0016	9.6999E-01	9.8504E-01	ENSG00000011198
ABHD6	-0.0041	9.5852E-01	9.7936E-01	ENSG00000163686
ABHD8	-0.0664	3.0730E-01	5.1753E-01	ENSG00000127220
ABI1	-0.2469	5.3526E-04	5.6043E-03	ENSG00000136754
ABI2	-0.0931	8.4041E-02	2.2649E-01	ENSG00000138443
ABI3	0.0911	2.4909E-02		ENSG00000108798
ABI3BP	0.0825	2.5794E-01	4.6663E-01	ENSG00000154175
ABITRAM	0.4244	4.0751E-10	5.1952E-08	ENSG00000119328
ABL1	-0.1645	3.3220E-03	2.2092E-02	ENSG00000097007
ABL2	0.2063	8.0299E-03	4.3017E-02	ENSG00000143322
ABLIM1	0.0758	3.7980E-01	5.8830E-01	ENSG00000099204
ABLIM2	-0.0880	2.8832E-01	4.9874E-01	ENSG00000163995
ABLIM3	-0.0746	3.7109E-01	5.7998E-01	ENSG00000173210
ABO	0.1884	7.7319E-02	2.1482E-01	ENSG00000175164
ABR	-0.0210	7.2467E-01	8.4905E-01	ENSG00000159842
ABRACL	0.0122	9.0553E-01	9.5378E-01	ENSG00000146386
ABRAXAS1	0.1137	2.1036E-01	4.1079E-01	ENSG00000163322
ABRAXAS2	-0.0214	7.2127E-01	8.4722E-01	ENSG00000165660
ABT1	0.1308	3.4160E-02	1.2165E-01	ENSG00000146109
ABTB1	-0.5720	2.0800E-07	1.0472E-05	ENSG00000114626
ABTB2	-0.2579	1.2228E-02	5.8717E-02	ENSG00000166016
ABTB3	-0.0759	3.7948E-01	5.8795E-01	ENSG00000151136
ACAA1	0.1283	3.5484E-02	1.2525E-01	ENSG00000060971
ACAA2	-0.1213	7.5852E-02	2.1218E-01	ENSG00000167315
ACAA2P1	0.0595	3.9581E-01	6.0197E-01	ENSG00000233585
ACACA	-0.0166	7.9505E-01	8.9166E-01	ENSG00000278540
ACACB	-0.0841	3.0811E-01	5.1839E-01	ENSG00000076555
ACAD10	0.2273	3.1628E-03	2.1344E-02	ENSG00000111271
ACAD11	-0.0042	9.5093E-01	9.7575E-01	ENSG00000240303
ACAD8	0.0818	2.1199E-01	4.1293E-01	ENSG00000151498
ACAD9	-0.0727	2.9383E-01	5.0390E-01	ENSG00000177646
ACADL	-0.0385	5.4564E-01	7.2606E-01	ENSG00000115361
ACADM	-0.0714	2.5451E-01	4.6257E-01	ENSG00000117054
ACADS	-0.0517	4.9029E-01	6.8299E-01	ENSG00000122971
ACADSB	0.2372	1.5831E-03	1.2728E-02	ENSG00000196177
ACADVL	-0.0209	7.6350E-01	8.7275E-01	ENSG00000072778
ACAN	0.0035	9.6026E-01	9.8016E-01	ENSG00000157766
ACAP1	0.4716	3.1070E-07	1.4561E-05	ENSG00000072818
ACAP2	0.3317	4.0403E-06	1.1821E-04	ENSG00000114331
ACAP3	-0.0824	2.7084E-01	4.8008E-01	ENSG00000131584
ACAT1	-0.4201	3.1849E-11	6.5305E-09	ENSG00000075239
ACAT2	-0.1388	6.8329E-02	1.9733E-01	ENSG00000120437
ACBD3	-0.0942	1.3696E-01	3.1237E-01	ENSG00000182827

ACBD3-AS1	0.0464	3.7702E-01	5.8556E-01	ENSG00000234478
ACBD4	-0.0370	6.3888E-01	7.9356E-01	ENSG00000181513
ACBD5	-0.0522	4.1146E-01	6.1553E-01	ENSG00000107897
ACBD6	-0.1018	2.3768E-02	9.4219E-02	ENSG00000230124
ACBD7	-0.5395	9.6899E-08	5.6099E-06	ENSG00000176244
ACCS	-0.0335	6.9949E-01	8.3382E-01	ENSG00000110455
ACD	-0.0275	6.2070E-01	7.8065E-01	ENSG00000102977
ACE	0.0646	4.5039E-01	6.4965E-01	ENSG00000159640
ACE2	-0.0014	9.5353E-01		ENSG00000130234
ACER1	0.0104	5.7284E-01		ENSG00000167769
ACER2	0.1456	1.3824E-01	3.1416E-01	ENSG00000177076
ACER3	0.0998	1.4100E-01	3.1769E-01	ENSG00000078124
ACHE	0.0407	6.3225E-01	7.8880E-01	ENSG00000087085
ACIN1	-0.1141	1.1377E-01	2.7744E-01	ENSG00000100813
ACKR1	0.5232	3.8508E-06	1.1339E-04	ENSG00000213088
ACKR2	0.0798	2.0055E-01	3.9896E-01	ENSG00000144648
ACKR3	0.0468	5.7312E-01	7.4620E-01	ENSG00000144476
ACKR4	-0.0288	5.2336E-01	7.0918E-01	ENSG00000129048
ACLY	-0.0643	2.2995E-01	4.3461E-01	ENSG00000131473
ACMSD	0.1715	1.5739E-02	7.0320E-02	ENSG00000153086
ACO1	-0.0729	2.9901E-01	5.0876E-01	ENSG00000122729
ACO2	-0.2825	4.1989E-03	2.6464E-02	ENSG00000100412
ACOT1	0.1416	1.3808E-01	3.1404E-01	ENSG00000184227
ACOT11	0.0615	4.6183E-01	6.5900E-01	ENSG00000162390
ACOT12	0.0330	5.2923E-01	7.1386E-01	ENSG00000172497
ACOT13	-0.0941	2.1945E-01	4.2177E-01	ENSG00000112304
ACOT2	0.1809	6.7698E-02	1.9622E-01	ENSG00000119673
ACOT4	0.0139	7.9426E-01	8.9126E-01	ENSG00000177465
ACOT6	0.0250	6.9018E-01	8.2782E-01	ENSG00000205669
ACOT7	-0.1583	5.3751E-02	1.6775E-01	ENSG00000097021
ACOT8	-0.3310	3.3907E-04	3.9464E-03	ENSG00000101473
ACOT9	-0.1239	5.6217E-02	1.7323E-01	ENSG00000123130
ACOX1	0.0076	9.0745E-01	9.5461E-01	ENSG00000161533
ACOX2	0.0635	4.0255E-01	6.0701E-01	ENSG00000168306
ACOX3	0.2864	8.7335E-05	1.3873E-03	ENSG00000087008
ACOXL	0.0732	3.9697E-01	6.0282E-01	ENSG00000153093
ACOXL-AS1	0.1009	9.5906E-02	2.4848E-01	ENSG00000204581
ACP1	-0.0605	2.5318E-01	4.6111E-01	ENSG00000143727
ACP2	-0.0839	2.1404E-01	4.1517E-01	ENSG00000134575
ACP3	0.0451	4.1205E-01	6.1601E-01	ENSG00000014257
ACP4	0.0460	5.5277E-01	7.3128E-01	ENSG00000142513
ACP5	0.0520	3.3416E-01	5.4428E-01	ENSG00000102575
ACP6	0.3042	4.5840E-03	2.8190E-02	ENSG00000162836
ACP7	0.0076	7.9718E-01		ENSG00000183760
ACR	0.0120	6.8245E-01		ENSG00000100312
ACRBP	0.0074	9.2142E-01	9.6140E-01	ENSG00000111644
ACRV1	0.0834	2.7135E-01	4.8053E-01	ENSG00000134940
ACSBG1	0.5669	5.0215E-03	3.0201E-02	ENSG00000103740
ACSBG2	0.0520	4.2318E-01	6.2585E-01	ENSG00000130377

ACSF2	-0.0064	9.2767E-01	9.6456E-01	ENSG00000167107
ACSF3	0.1370	8.6844E-02	2.3171E-01	ENSG00000176715
ACSL1	-0.2094	3.3040E-03	2.2024E-02	ENSG00000151726
ACSL3	-0.1155	4.6126E-02	1.5117E-01	ENSG00000123983
ACSL4	0.0340	6.4495E-01	7.9731E-01	ENSG00000068366
ACSL5	0.0402	4.9269E-01	6.8480E-01	ENSG00000197142
ACSL6	0.2583	5.8229E-03	3.3787E-02	ENSG00000164398
ACSM1	0.0707	1.5391E-01		ENSG00000166743
ACSM3	0.0166	8.4549E-01	9.2087E-01	ENSG00000005187
ACSM4	-0.0046	8.4590E-01		ENSG00000215009
ACSM5	0.0276	6.1607E-01		ENSG00000183549
ACSM6	0.0115	8.1229E-01	9.0204E-01	ENSG00000173124
ACSS2	-0.0603	3.0725E-01	5.1752E-01	ENSG00000131069
ACSS3	-0.0819	3.5056E-01	5.6067E-01	ENSG00000111058
ACTA2	0.0832	2.7077E-01	4.8008E-01	ENSG00000107796
ACTA2-AS1	-0.0040	9.5284E-01		ENSG00000180139
ACTBP1	-0.0786	1.7703E-01	3.6824E-01	ENSG00000229145
ACTBP14	-0.0419	1.9812E-01		ENSG00000229001
ACTC1	0.0270	6.9480E-01	8.3093E-01	ENSG00000159251
ACTE1P	0.0366	2.1169E-01		ENSG00000172900
ACTG1	-0.4453	2.2765E-11	5.1387E-09	ENSG00000184009
ACTG1P12	-0.0060	8.6357E-01		ENSG00000226642
ACTG1P14	-0.3535	1.2275E-02	5.8863E-02	ENSG00000230581
ACTG1P15	-0.0349	2.4718E-01		ENSG00000259904
ACTG1P16	-0.0005	9.6192E-01		ENSG00000260298
ACTG1P17	0.0590	3.5345E-01	5.6354E-01	ENSG00000259315
ACTG1P19	-0.3592	1.1480E-02	5.6134E-02	ENSG00000237999
ACTG1P23	-0.0304	6.5088E-01	8.0209E-01	ENSG00000230362
ACTG1P24	-0.0477	3.9210E-01	5.9911E-01	ENSG00000226359
ACTG1P25	0.0212	7.9032E-01	8.8919E-01	ENSG00000234996
ACTG2	-0.3901	3.6300E-03	2.3710E-02	ENSG00000163017
ACTL10	0.0644	3.4221E-01	5.5185E-01	ENSG00000288649
ACTL11P	0.0137	5.3748E-01		ENSG00000234667
ACTL6A	0.0957	1.4371E-01	3.2175E-01	ENSG00000136518
ACTL6B	-0.1962	3.1296E-02	1.1469E-01	ENSG00000077080
ACTL7A	-0.0040	8.2652E-01		ENSG00000187003
ACTL7B	-0.0038	5.5453E-01		ENSG00000148156
ACTL8	0.0206	7.4997E-01		ENSG00000117148
ACTMAP	0.0076	9.1363E-01	9.5757E-01	ENSG00000188493
ACTN1	0.0844	1.8486E-01	3.7842E-01	ENSG00000072110
ACTN1-DT	-0.0006	8.5983E-01		ENSG00000259062
ACTN2	0.0819	2.6667E-01	4.7595E-01	ENSG00000077522
ACTN3	-0.0044	4.1146E-01	6.1553E-01	ENSG00000248746
ACTN4	-0.0532	4.8906E-01	6.8220E-01	ENSG00000130402
ACTR10	-0.3048	1.5963E-06	5.5632E-05	ENSG00000131966
ACTR1A	-0.2159	3.0151E-05	5.9616E-04	ENSG00000138107
ACTR1B	0.0212	7.8585E-01	8.8674E-01	ENSG00000115073
ACTR2	-0.1951	7.0503E-04	6.9892E-03	ENSG00000138071
ACTR3	-0.4578	1.6744E-12	5.4932E-10	ENSG00000115091

ACTR3B	-0.0192	6.8973E-01	8.2764E-01	ENSG00000133627
ACTR3C	-0.0259	7.5290E-01	8.6591E-01	ENSG00000106526
ACTR5	0.0842	2.5908E-01	4.6768E-01	ENSG00000101442
ACTR6	-0.1323	3.0408E-02	1.1229E-01	ENSG00000075089
ACTR8	-0.0101	8.6023E-01	9.2963E-01	ENSG00000113812
ACTRT3	0.1541	8.4270E-02	2.2688E-01	ENSG00000184378
ACVR1	-0.2755	1.1892E-03	1.0332E-02	ENSG00000115170
ACVR1B	0.0459	4.9118E-01	6.8348E-01	ENSG00000135503
ACVR2A	0.0800	2.5567E-01	4.6388E-01	ENSG00000121989
ACVR2B	0.5027	2.5777E-07	1.2510E-05	ENSG00000114739
ACVR2B-AS1	0.0466	5.8305E-01	7.5366E-01	ENSG00000229589
ACVRL1	0.0016	9.2813E-01		ENSG00000139567
ACY1	-0.0490	3.8366E-01	5.9181E-01	ENSG00000243989
ACYP1	-0.2898	1.7065E-06	5.8724E-05	ENSG00000119640
ACYP2	0.1229	4.7268E-02	1.5364E-01	ENSG00000170634
ADA	-0.2307	2.1601E-02	8.7691E-02	ENSG00000196839
ADA2	-0.0195	7.9566E-01	8.9205E-01	ENSG00000093072
ADAD2	0.0295	3.3848E-01		ENSG00000140955
ADAL	0.1614	2.3222E-02	9.2592E-02	ENSG00000168803
ADAM10	-0.0752	2.7417E-01	4.8389E-01	ENSG00000137845
ADAM11	-0.0010	9.8837E-01	9.9358E-01	ENSG00000073670
ADAM12	0.0531	5.3277E-01	7.1644E-01	ENSG00000148848
ADAM15	0.2410	2.7129E-04	3.3275E-03	ENSG00000143537
ADAM17	-0.0125	8.1968E-01	9.0638E-01	ENSG00000151694
ADAM19	0.0003	9.9758E-01	9.9860E-01	ENSG00000135074
ADAM1B	-0.0612	2.5344E-01		ENSG00000226469
ADAM20	0.0421	1.6126E-01		ENSG00000134007
ADAM21	0.1180	1.1632E-01	2.8127E-01	ENSG00000139985
ADAM21P1	-0.0013	7.4425E-01		ENSG00000235812
ADAM22	-0.0764	3.1803E-01	5.2826E-01	ENSG00000008277
ADAM23	0.1964	4.5469E-03	2.8004E-02	ENSG00000114948
ADAM29	0.0234	6.6870E-01	8.1432E-01	ENSG00000168594
ADAM32	-0.0324	5.9460E-01	7.6167E-01	ENSG00000197140
ADAM33	-0.0105	4.2273E-01	6.2545E-01	ENSG00000149451
ADAM7-AS2	0.0083	8.2004E-01		ENSG00000253643
ADAM8	0.0311	7.1272E-01	8.4237E-01	ENSG00000151651
ADAM9	-0.0731	3.3466E-01	5.4471E-01	ENSG00000168615
ADAMDEC1	0.0145	3.4526E-01		ENSG00000134028
ADAMTS1	0.0613	4.5559E-01	6.5386E-01	ENSG00000154734
ADAMTS10	-0.0669	3.6384E-01	5.7295E-01	ENSG00000142303
ADAMTS12	0.0296	7.2264E-01	8.4795E-01	ENSG00000151388
ADAMTS13	0.1168	2.0145E-01	4.0006E-01	ENSG00000160323
ADAMTS14	-0.0908	2.4360E-01	4.5142E-01	ENSG00000138316
ADAMTS15	0.0807	3.0960E-01	5.1984E-01	ENSG00000166106
ADAMTS17	-0.0541	4.3193E-01	6.3331E-01	ENSG00000140470
ADAMTS18	0.2830	2.6650E-02	1.0208E-01	ENSG00000140873
ADAMTS19	0.4733	1.0044E-02	5.0942E-02	ENSG00000145808
ADAMTS2	0.3248	1.0246E-03	9.2450E-03	ENSG00000087116
ADAMTS20	0.2167	5.2892E-02	1.6566E-01	ENSG00000173157

ADAMTS3	0.4797	1.0324E-04	1.5841E-03	ENSG00000156140
ADAMTS4	-0.3678	1.3010E-02	6.1336E-02	ENSG00000158859
ADAMTS5	0.3283	1.3973E-03	1.1639E-02	ENSG00000154736
ADAMTS6	0.0404	6.1608E-01	7.7733E-01	ENSG00000049192
ADAMTS7	0.0153	8.2373E-01	9.0843E-01	ENSG00000136378
ADAMTS7P3	0.0102	9.0786E-01	9.5461E-01	ENSG00000261143
ADAMTS7P4	-0.0093	8.5628E-01		ENSG00000218052
ADAMTS8	-0.0469	5.7011E-01	7.4394E-01	ENSG00000134917
ADAMTS9	0.0189	8.1652E-01	9.0449E-01	ENSG00000163638
ADAMTS9-AS1	0.0551	1.0577E-01		ENSG00000241158
ADAMTS9-AS2	0.0224	7.9696E-01	8.9286E-01	ENSG00000241684
ADAMTSL1	-0.0715	4.1340E-01	6.1699E-01	ENSG00000178031
ADAMTSL2	0.0002	9.9567E-01	9.9755E-01	ENSG00000197859
ADAMTSL3	-0.0027	9.6540E-01	9.8290E-01	ENSG00000156218
ADAMTSL4	0.0061	9.2611E-01	9.6390E-01	ENSG00000143382
ADAMTSL5	-0.0080	9.0144E-01	9.5173E-01	ENSG00000185761
ADAP1	0.0317	6.9557E-01	8.3149E-01	ENSG00000105963
ADAP2	0.0286	7.2874E-01	8.5120E-01	ENSG00000184060
ADAR	0.0118	8.2731E-01	9.1051E-01	ENSG00000160710
ADARB1	0.0330	6.5882E-01	8.0745E-01	ENSG00000197381
ADARB2	0.1834	7.9883E-02	2.1932E-01	ENSG00000185736
ADARB2-AS1	0.0590	4.1733E-01	6.2078E-01	ENSG00000205696
ADAT1	0.1699	5.7950E-03	3.3654E-02	ENSG00000065457
ADAT2	0.2250	9.0305E-03	4.7095E-02	ENSG00000189007
ADAT3	0.5285	2.7709E-04	3.3752E-03	ENSG00000213638
ADCK1	-0.1360	6.1024E-02	1.8244E-01	ENSG00000063761
ADCK2	-0.1369	9.4605E-02	2.4612E-01	ENSG00000133597
ADCK5	-0.0846	2.9723E-01	5.0720E-01	ENSG00000173137
ADCY1	-0.0259	7.5243E-01	8.6565E-01	ENSG00000164742
ADCY10	0.0190	7.3348E-01	8.5431E-01	ENSG00000143199
ADCY2	0.1605	1.0928E-01	2.6987E-01	ENSG00000078295
ADCY3	-0.0713	3.7084E-01	5.7981E-01	ENSG00000138031
ADCY4	0.0460	4.3118E-01	6.3289E-01	ENSG00000129467
ADCY5	0.0758	2.9611E-01	5.0607E-01	ENSG00000173175
ADCY6	0.2282	9.0674E-03	4.7188E-02	ENSG00000174233
ADCY6-DT	0.0111	8.5306E-01	9.2528E-01	ENSG00000257660
ADCY7	-0.5019	6.9495E-04	6.9088E-03	ENSG00000121281
ADCY8	0.0683	4.0960E-01	6.1388E-01	ENSG00000155897
ADCY9	0.1867	3.3263E-02	1.1964E-01	ENSG00000162104
ADCYAP1	0.3340	1.3330E-02	6.2535E-02	ENSG00000141433
ADCYAP1R1	-0.4081	2.8849E-04	3.4795E-03	ENSG00000078549
ADD1	-0.1274	1.8989E-02	7.9992E-02	ENSG00000087274
ADD2	-0.1340	6.5800E-02	1.9251E-01	ENSG00000075340
ADD3	-0.0276	7.2101E-01	8.4711E-01	ENSG00000148700
ADGB	0.0062	8.8835E-01		ENSG00000118492
ADGRA1	0.1122	1.8453E-01	3.7800E-01	ENSG00000197177
ADGRA2	-0.3428	9.4399E-03	4.8618E-02	ENSG00000020181
ADGRA3	-0.0332	6.3741E-01	7.9226E-01	ENSG00000152990
ADGRB1	-0.0826	3.6174E-01	5.7107E-01	ENSG00000181790

ADGRB2	-0.1311	9.7795E-02	2.5120E-01	ENSG00000121753
ADGRB3	-0.0665	2.6631E-01	4.7564E-01	ENSG00000135298
ADGRD1	0.1755	6.2360E-02	1.8545E-01	ENSG00000111452
ADGRD2	-0.1145	1.7714E-01	3.6832E-01	ENSG00000180264
ADGRE1	0.0141	3.7936E-01		ENSG00000174837
ADGRE2	0.0528	2.4049E-01	4.4729E-01	ENSG00000127507
ADGRE5	0.4671	2.2378E-04	2.8396E-03	ENSG00000123146
ADGRF1	0.0318	3.5319E-01		ENSG00000153292
ADGRF2	0.0132	5.9391E-01		ENSG00000164393
ADGRF3	-0.1030	2.1229E-01	4.1327E-01	ENSG00000173567
ADGRF4	0.0242	3.2365E-01		ENSG00000153294
ADGRF5P1	-0.0787	2.2980E-01	4.3443E-01	ENSG00000227582
ADGRG1	-0.0335	6.0639E-01	7.7039E-01	ENSG00000205336
ADGRG2	-0.0153	7.4994E-01		ENSG00000173698
ADGRG3	0.0183	8.2511E-01	9.0922E-01	ENSG00000182885
ADGRG4	0.0520	1.3599E-01		ENSG00000156920
ADGRG5	0.0068	9.1688E-01	9.5877E-01	ENSG00000159618
ADGRG6	0.0257	6.2077E-01	7.8065E-01	ENSG00000112414
ADGRG7	-0.0135	6.5279E-01		ENSG00000144820
ADGRL1	0.1335	1.0254E-01	2.5907E-01	ENSG00000072071
ADGRL1-AS1	-0.0155	8.5456E-01	9.2596E-01	ENSG00000267169
ADGRL2	0.1361	9.8234E-02	2.5191E-01	ENSG00000117114
ADGRL3	0.5164	3.1998E-07	1.4883E-05	ENSG00000150471
ADGRL4	0.0594	2.2433E-01	4.2815E-01	ENSG00000162618
ADGRV1	0.3469	1.2503E-04	1.8236E-03	ENSG00000164199
ADH1B	0.0146	5.2242E-01		ENSG00000196616
ADH4	0.0085	8.8006E-01		ENSG00000198099
ADH5	0.0130	8.6586E-01	9.3228E-01	ENSG00000197894
ADH5P3	0.0159	6.4143E-01		ENSG00000223694
ADH6	0.0139	8.3096E-01	9.1267E-01	ENSG00000172955
ADHFE1	0.0241	7.6655E-01	8.7479E-01	ENSG00000147576
ADI1	-0.1633	3.4627E-02	1.2298E-01	ENSG00000182551
ADIPOR1	-0.0146	8.0713E-01	8.9868E-01	ENSG00000159346
ADIPOR2	0.0178	8.1884E-01	9.0583E-01	ENSG00000006831
ADISSP	-0.0839	1.9464E-01	3.9135E-01	ENSG00000101220
ADK	0.0403	4.9357E-01	6.8562E-01	ENSG00000156110
ADM	0.0771	3.4175E-01	5.5139E-01	ENSG00000148926
ADM2	-0.5604	6.4118E-04	6.4869E-03	ENSG00000128165
ADM5	0.1424	1.4734E-01	3.2731E-01	ENSG00000224420
ADNP	0.0122	8.1350E-01	9.0281E-01	ENSG00000101126
ADNP-AS1	-0.2259	4.5977E-02	1.5082E-01	ENSG00000259456
ADNP2	-0.1360	4.1256E-02	1.3982E-01	ENSG00000101544
ADO	0.0676	2.8597E-01	4.9669E-01	ENSG00000181915
ADORA1	-0.0795	2.7651E-01	4.8646E-01	ENSG00000163485
ADORA2A	0.0157	7.0685E-01	8.3870E-01	ENSG00000128271
ADORA2A-AS1	-0.0587	9.6499E-02		ENSG00000178803
ADORA2B	-0.1027	2.5953E-01	4.6792E-01	ENSG00000170425
ADPGK	0.1196	5.5339E-02	1.7136E-01	ENSG00000159322
ADPGK-AS1	-0.1469	1.0658E-01	2.6552E-01	ENSG00000260898

ADPRH	0.0313	5.6417E-01	7.4015E-01	ENSG00000144843
ADPRHL1	-0.0301	7.0932E-01	8.4029E-01	ENSG00000153531
ADPRM	-0.0835	1.8380E-01	3.7715E-01	ENSG00000170222
ADPRS	0.0901	1.7418E-01	3.6446E-01	ENSG00000116863
ADRA1A	-0.3887	4.7399E-04	5.0995E-03	ENSG00000120907
ADRA1B	0.0272	7.2359E-01	8.4829E-01	ENSG00000170214
ADRA1D	0.0196	6.9532E-01	8.3135E-01	ENSG00000171873
ADRA2A	-0.2621	1.1785E-02	5.7285E-02	ENSG00000150594
ADRA2B	0.0592	1.6176E-01		ENSG00000274286
ADRA2C	-0.0371	6.4014E-01	7.9412E-01	ENSG00000184160
ADRB2	-0.0687	4.2306E-01	6.2576E-01	ENSG00000169252
ADRB3	-0.0370	3.0576E-01		ENSG00000188778
ADRM1	-0.0286	6.0150E-01	7.6659E-01	ENSG00000130706
ADSL	-0.0427	5.9597E-01	7.6275E-01	ENSG00000239900
ADSS1	0.0859	2.6858E-01	4.7794E-01	ENSG00000185100
ADSS2	-0.1731	1.0632E-03	9.5128E-03	ENSG00000035687
ADTRP	0.0248	2.6184E-01		ENSG00000111863
AEBP1	-0.3552	2.1753E-04	2.7793E-03	ENSG00000106624
AEBP2	-0.1266	8.4187E-02	2.2670E-01	ENSG00000139154
AEN	0.0488	4.7810E-01	6.7341E-01	ENSG00000181026
AFAP1	0.0598	3.8811E-01	5.9566E-01	ENSG00000196526
AFAP1-AS1	-0.0545	3.8355E-01	5.9176E-01	ENSG00000272620
AFAP1L1	-0.0137	8.3997E-01	9.1765E-01	ENSG00000157510
AFAP1L2	0.0493	5.6282E-01	7.3940E-01	ENSG00000169129
AFDN	-0.0342	6.3927E-01	7.9381E-01	ENSG00000130396
AFF1	-0.0842	2.4556E-01	4.5333E-01	ENSG00000172493
AFF2	-0.1498	5.0391E-02	1.6042E-01	ENSG00000155966
AFF3	-0.0922	1.3371E-01	3.0760E-01	ENSG00000144218
AFF4	-0.2408	1.1892E-05	2.8135E-04	ENSG00000072364
AFG1L	-0.0189	8.0928E-01	8.9970E-01	ENSG00000135537
AFG2A	0.1107	1.6770E-01	3.5599E-01	ENSG00000145375
AFG2B	-0.1853	9.8021E-03	4.9986E-02	ENSG00000171763
AFG3L1P	0.2792	9.0605E-05	1.4275E-03	ENSG00000223959
AFG3L2	0.0032	9.5813E-01	9.7936E-01	ENSG00000141385
AFMID	-0.1203	7.5146E-02	2.1073E-01	ENSG00000183077
AFP	0.0100	8.4557E-01		ENSG00000081051
AFTPH	0.1572	5.4937E-02	1.7048E-01	ENSG00000119844
AFTPH-DT	-0.0130	8.6967E-01	9.3437E-01	ENSG00000260101
AGA	0.3891	3.0992E-06	9.5200E-05	ENSG00000038002
AGA-DT	0.0052	9.4429E-01	9.7218E-01	ENSG00000250131
AGAP1	0.1002	2.1372E-01	4.1477E-01	ENSG00000157985
AGAP1-IT1	-0.0234	5.4198E-01		ENSG00000235529
AGAP2	-0.0273	7.1981E-01	8.4612E-01	ENSG00000135439
AGAP3	-0.1505	4.4700E-02	1.4779E-01	ENSG00000133612
AGAP4	0.3621	4.3694E-05	7.9992E-04	ENSG00000188234
AGAP5	0.0367	3.2836E-01		ENSG00000172650
AGAP6	0.0327	2.1416E-01	4.1527E-01	ENSG00000204149
AGAP9	0.1803	5.8343E-02	1.7725E-01	ENSG00000204172
AGBL1	0.2011	7.2307E-02	2.0539E-01	ENSG00000273540

AGBL1-AS1	0.0544	3.1062E-02		ENSG00000260125
AGBL2	-0.0083	9.0964E-01	9.5525E-01	ENSG00000165923
AGBL3	0.3329	1.6853E-03	1.3286E-02	ENSG00000146856
AGBL4-AS1	0.0004	9.8387E-01		ENSG00000230114
AGBL5	0.0118	8.5308E-01	9.2528E-01	ENSG00000084693
AGBL5-AS1	0.0425	5.6967E-01	7.4363E-01	ENSG00000231636
AGER	-0.0380	6.5497E-01	8.0515E-01	ENSG00000204305
AGFG1	0.1048	5.8139E-02	1.7694E-01	ENSG00000173744
AGFG2	0.2739	2.5200E-02	9.8077E-02	ENSG00000106351
AGGF1	0.2356	1.8703E-04	2.4649E-03	ENSG00000164252
AGK	-0.0031	9.5944E-01	9.7976E-01	ENSG00000006530
AGL	0.3103	4.5544E-05	8.2459E-04	ENSG00000162688
AGMAT	0.0632	2.8140E-01	4.9174E-01	ENSG00000116771
AGMO	0.0335	6.2716E-01	7.8515E-01	ENSG00000187546
AGO1	0.0704	3.1433E-01	5.2456E-01	ENSG00000092847
AGO2	0.0552	4.0670E-01	6.1130E-01	ENSG00000123908
AGO3	0.1000	1.8463E-01	3.7815E-01	ENSG00000126070
AGO4	-0.0266	6.8072E-01	8.2147E-01	ENSG00000134698
AGPAT1	-0.2244	6.5428E-04	6.6005E-03	ENSG00000204310
AGPAT2	-0.1683	7.9371E-03	4.2621E-02	ENSG00000169692
AGPAT3	-0.0227	7.0703E-01	8.3887E-01	ENSG00000160216
AGPAT4	0.0713	2.9857E-01	5.0836E-01	ENSG00000026652
AGPAT5	-0.0333	5.4449E-01	7.2527E-01	ENSG00000155189
AGPS	-0.1471	5.2826E-02	1.6554E-01	ENSG00000018510
AGR2	-0.0038	6.5998E-01		ENSG00000106541
AGR3	0.0052	8.0040E-01		ENSG00000173467
AGRN	0.0618	4.3721E-01	6.3776E-01	ENSG00000188157
AGRP	0.0335	3.8848E-01		ENSG00000159723
AGT	-0.1123	2.0022E-01	3.9864E-01	ENSG00000135744
AGTPBP1	0.1210	4.5903E-02	1.5067E-01	ENSG00000135049
AGTR1	0.0192	6.7946E-01		ENSG00000144891
AGTRAP	0.0520	4.8293E-01	6.7718E-01	ENSG00000177674
AGXT	-0.0074	5.6148E-01		ENSG00000172482
AGXT2	0.0042	9.7618E-01		ENSG00000113492
AHCTF1	0.0189	7.8460E-01	8.8624E-01	ENSG00000153207
AHCY	0.0487	4.2360E-01	6.2602E-01	ENSG00000101444
AHCYL1	-0.0815	1.7742E-01	3.6864E-01	ENSG00000168710
AHCYL2	-0.0157	8.0697E-01	8.9856E-01	ENSG00000158467
AHCYP3	0.0258	4.8185E-01		ENSG00000233955
AHCYP7	0.0084	4.8114E-01		ENSG00000259465
AHDC1	-0.1055	1.6507E-01	3.5225E-01	ENSG00000126705
AHI1	0.5588	7.6765E-18	1.2592E-14	ENSG00000135541
AHI1-DT	0.4749	1.9640E-04	2.5554E-03	ENSG00000231028
AHNAK	0.2368	3.0649E-02	1.1296E-01	ENSG00000124942
AHNAK2	0.2026	2.6036E-02	1.0044E-01	ENSG00000185567
AHR	-0.0380	6.4437E-01	7.9692E-01	ENSG00000106546
AHRR	-0.0517	4.0890E-01	6.1323E-01	ENSG00000063438
AHSA1	-0.2116	1.1730E-03	1.0227E-02	ENSG00000100591
AHSA2P	0.0422	5.9110E-01	7.5904E-01	ENSG00000173209

AHSP	0.0279	4.2391E-01		ENSG00000169877
AICDA	0.0001	9.6180E-01		ENSG00000111732
AIDA	-0.1292	6.5194E-02	1.9121E-01	ENSG00000186063
AIDAP2	-0.0119	7.1822E-01		ENSG00000251429
AIF1L	-0.0389	6.1254E-01	7.7453E-01	ENSG00000126878
AIFM1	-0.1394	2.0491E-02	8.4366E-02	ENSG00000156709
AIFM2	-0.0606	4.8813E-01	6.8129E-01	ENSG00000042286
AIFM3	-0.1939	7.1213E-02	2.0325E-01	ENSG00000183773
AIG1	0.0007	9.9011E-01	9.9452E-01	ENSG00000146416
AIM2	0.2048	3.1734E-02	1.1574E-01	ENSG00000163568
AIMP1	-0.0114	7.9811E-01	8.9364E-01	ENSG00000164022
AIMP1P1	0.0111	8.3967E-01		ENSG00000234187
AIMP2	-0.0462	5.0602E-01	6.9575E-01	ENSG00000106305
AIP	-0.0705	2.6756E-01	4.7692E-01	ENSG00000110711
AIPL1	0.0362	2.1130E-01		ENSG00000129221
AIRE	0.0071	7.2068E-01		ENSG00000160224
AIRIM	0.0850	2.3296E-01	4.3810E-01	ENSG00000116922
AIRN	-0.0468	3.6269E-01	5.7192E-01	ENSG00000268257
AJAP1	0.0823	2.9759E-01	5.0754E-01	ENSG00000196581
AJM1	0.0413	6.2621E-01	7.8444E-01	ENSG00000232434
AJUBA	0.3508	1.2752E-02	6.0489E-02	ENSG00000129474
AK1	-0.2731	4.2072E-03	2.6489E-02	ENSG00000106992
AK2	-0.0052	9.1710E-01	9.5883E-01	ENSG00000004455
AK2P1	0.0497	3.2735E-01	5.3779E-01	ENSG00000185839
AK3	-0.0570	3.3087E-01	5.4112E-01	ENSG00000147853
AK3P7	0.0193	2.8363E-01		ENSG00000270941
AK4	-0.1808	3.7680E-02	1.3108E-01	ENSG00000162433
AK5	-0.1060	2.2380E-01	4.2766E-01	ENSG00000154027
AK6	-0.0823	1.8271E-01	3.7583E-01	ENSG00000085231
AK7	0.0946	2.7036E-01	4.7989E-01	ENSG00000140057
AK9	0.0823	2.3179E-01	4.3673E-01	ENSG00000155085
AKAIN1	0.1783	7.9786E-02	2.1918E-01	ENSG00000231824
AKAP1	0.0068	8.9018E-01	9.4564E-01	ENSG00000121057
AKAP10	0.0789	2.4427E-01	4.5185E-01	ENSG00000108599
AKAP11	0.2260	1.2996E-03	1.1061E-02	ENSG00000023516
AKAP12	0.1710	1.6613E-02	7.2901E-02	ENSG00000131016
AKAP13	0.1142	7.9840E-02	2.1925E-01	ENSG00000170776
AKAP14	-0.0800	3.5426E-01	5.6418E-01	ENSG00000186471
AKAP17A	0.0144	8.2663E-01	9.1015E-01	ENSG00000197976
AKAP3	0.1745	8.5914E-02	2.2980E-01	ENSG00000111254
AKAP5	-0.1228	1.8421E-01	3.7770E-01	ENSG00000179841
AKAP6	0.2162	8.7730E-04	8.2107E-03	ENSG00000151320
AKAP7	0.0019	9.8570E-01	9.9239E-01	ENSG00000118507
AKAP8	-0.0642	3.0860E-01	5.1884E-01	ENSG00000105127
AKAP8L	-0.0713	3.0701E-01	5.1732E-01	ENSG00000011243
AKAP9	0.3622	1.3568E-10	2.1539E-08	ENSG00000127914
AKIP1	-0.0592	2.8270E-01	4.9317E-01	ENSG00000166452
AKIRIN1	-0.1996	1.4071E-03	1.1693E-02	ENSG00000174574
AKIRIN2	-0.3529	7.6136E-10	8.7296E-08	ENSG00000135334

AKNA	-0.0689	4.2954E-01	6.3112E-01	ENSG00000106948
AKNAD1	-0.0361	6.5198E-01	8.0290E-01	ENSG00000162641
AKR1A1	-0.1010	7.4803E-02	2.1006E-01	ENSG00000117448
AKR1B1	-0.1040	8.6172E-02	2.3031E-01	ENSG00000085662
AKR1B10	0.0654	1.7669E-01	3.6798E-01	ENSG00000198074
AKR1B15	0.0354	1.2023E-01		ENSG00000227471
AKR1B1P1	-0.0013	9.4305E-01		ENSG00000229991
AKR1C1	-0.1999	4.7591E-02	1.5442E-01	ENSG00000187134
AKR1C2	-0.2370	1.6560E-02	7.2759E-02	ENSG00000151632
AKR1C3	-0.0289	5.8376E-01	7.5412E-01	ENSG00000196139
AKR1C4	0.0381	7.3424E-01	8.5491E-01	ENSG00000198610
AKR1C7P	0.0091	5.8419E-01		ENSG00000215267
AKR1C8	0.0097	4.7211E-01		ENSG00000264006
AKR1D1	0.0034	8.4842E-01		ENSG00000122787
AKR1E2	0.0250	7.5910E-01	8.6999E-01	ENSG00000165568
AKR7A2	-0.3006	4.0615E-03	2.5856E-02	ENSG00000053371
AKR7A3	0.0989	2.5537E-01	4.6351E-01	ENSG00000162482
AKR7L	0.2721	2.7636E-02	1.0478E-01	ENSG00000211454
AKT1	-0.1785	2.9240E-02	1.0914E-01	ENSG00000142208
AKT1S1	0.0137	8.3867E-01	9.1701E-01	ENSG00000204673
AKT2	0.0249	7.3343E-01	8.5431E-01	ENSG00000105221
AKT3	-0.1387	5.5858E-02	1.7253E-01	ENSG00000117020
AKT3-IT1	0.0053	9.6733E-01	9.8377E-01	ENSG00000228939
AKTIP	0.1035	1.0354E-01	2.6056E-01	ENSG00000166971
ALAD	-0.1111	1.3669E-01	3.1207E-01	ENSG00000148218
ALAS1	-0.0997	1.8307E-01	3.7628E-01	ENSG00000023330
ALAS2	-0.0101	4.4173E-01		ENSG00000158578
ALCAM	0.0896	3.0783E-01	5.1814E-01	ENSG00000170017
ALDH16A1	0.0531	5.0677E-01	6.9620E-01	ENSG00000161618
ALDH18A1	-0.2144	3.0037E-03	2.0546E-02	ENSG00000059573
ALDH1A1	-0.0125	8.4196E-01	9.1870E-01	ENSG00000165092
ALDH1A3	0.0003	9.3011E-01	9.6551E-01	ENSG00000184254
ALDH1A3-AS1	-0.0592	2.3138E-01	4.3622E-01	ENSG00000259583
ALDH1B1	-0.2963	5.3803E-04	5.6261E-03	ENSG00000137124
ALDH1L1	-0.0778	3.7901E-01	5.8752E-01	ENSG00000144908
ALDH1L1-AS1	0.0116	3.3258E-01		ENSG00000250218
ALDH1L1-AS2	-0.0083	7.4061E-01		ENSG00000246022
ALDH1L2	0.0669	3.8213E-01	5.9030E-01	ENSG00000136010
ALDH2	-0.1600	6.8607E-03	3.8226E-02	ENSG00000111275
ALDH3A1	0.1031	1.8369E-01	3.7705E-01	ENSG00000108602
ALDH3A2	-0.1303	7.0905E-02	2.0251E-01	ENSG00000072210
ALDH3B1	-0.3797	2.7989E-03	1.9372E-02	ENSG00000006534
ALDH4A1	-0.5402	1.7977E-06	6.1093E-05	ENSG00000159423
ALDH5A1	-0.0811	2.9956E-01	5.0913E-01	ENSG00000112294
ALDH6A1	-0.2684	6.1391E-03	3.5088E-02	ENSG00000119711
ALDH7A1	-0.0642	2.5505E-01	4.6314E-01	ENSG00000164904
ALDH7A1P1	0.0252	6.1355E-01	7.7529E-01	ENSG00000251400
ALDH8A1	0.0853	2.8432E-01	4.9496E-01	ENSG00000118514
ALDH9A1	-0.1192	7.6635E-02	2.1349E-01	ENSG00000143149

ALDOA	0.0523	4.6444E-01	6.6139E-01	ENSG00000149925
ALDOB	0.0244	4.9012E-01		ENSG00000136872
ALDOC	0.2892	1.1279E-03	9.9150E-03	ENSG00000109107
ALG1	0.0876	1.9140E-01	3.8676E-01	ENSG00000033011
ALG10	0.0886	3.1784E-01	5.2805E-01	ENSG00000139133
ALG10B	-0.0219	7.8862E-01	8.8805E-01	ENSG00000175548
ALG11	-0.0226	7.4493E-01	8.6115E-01	ENSG00000253710
ALG12	-0.2689	2.2732E-03	1.6582E-02	ENSG00000182858
ALG13	0.1926	1.8564E-02	7.8730E-02	ENSG00000101901
ALG14	0.3483	7.8426E-06	2.0122E-04	ENSG00000172339
ALG14-AS1	0.0027	5.2520E-01		ENSG00000230427
ALG1L10P	-0.0589	2.2830E-01		ENSG00000254016
ALG1L11P	-0.0443	2.5017E-01		ENSG00000249889
ALG1L2	0.0044	4.2506E-01		ENSG00000251287
ALG1L8P	0.0006	8.2124E-01		ENSG00000227620
ALG1L9P	0.5962	8.1213E-04	7.7481E-03	ENSG00000248671
ALG2	0.1141	1.0091E-01	2.5632E-01	ENSG00000119523
ALG3	0.0165	8.1491E-01	9.0369E-01	ENSG00000214160
ALG5	0.0895	1.5487E-01	3.3794E-01	ENSG00000120697
ALG6	-0.0836	2.6548E-01	4.7473E-01	ENSG00000088035
ALG8	0.0564	4.2505E-01	6.2738E-01	ENSG00000159063
ALG9	0.2317	5.9307E-03	3.4216E-02	ENSG00000086848
ALKAL1	0.1923	4.6536E-02	1.5206E-01	ENSG00000196711
ALKAL2	-0.2661	2.9737E-02	1.1046E-01	ENSG00000189292
ALKBH1	0.1530	4.1406E-02	1.4015E-01	ENSG00000100601
ALKBH2	0.3120	4.7606E-06	1.3602E-04	ENSG00000189046
ALKBH3	0.0817	2.1331E-01	4.1434E-01	ENSG00000166199
ALKBH3-AS1	0.0038	9.7351E-01	9.8713E-01	ENSG00000244926
ALKBH4	-0.2325	1.2077E-03	1.0452E-02	ENSG00000160993
ALKBH5	-0.0802	3.2644E-01	5.3655E-01	ENSG00000091542
ALKBH6	0.0401	6.3437E-01	7.9040E-01	ENSG00000239382
ALKBH7	-0.0520	3.9200E-01	5.9901E-01	ENSG00000125652
ALKBH8	0.1000	2.4224E-01	4.4970E-01	ENSG00000137760
ALLC	0.0257	3.1936E-01		ENSG00000151360
ALMS1	0.1388	5.7213E-02	1.7522E-01	ENSG00000116127
ALMS1-IT1	0.0197	8.0143E-01	8.9522E-01	ENSG00000230002
ALOX12	0.5438	2.4833E-03	1.7715E-02	ENSG00000108839
ALOX12-AS1	0.0366	6.2241E-01	7.8178E-01	ENSG00000215067
ALOX12B	0.3377	1.2250E-02	5.8776E-02	ENSG00000179477
ALOX15	0.0056	9.1519E-01	9.5818E-01	ENSG00000161905
ALOX15B	0.0540	2.0023E-01		ENSG00000179593
ALOX5AP	0.0585	1.4351E-01	3.2147E-01	ENSG00000132965
ALOXE3	0.1177	2.0636E-01	4.0606E-01	ENSG00000179148
ALPI	-0.0057	8.2061E-01		ENSG00000163295
ALPK1	-0.0238	7.7590E-01	8.8072E-01	ENSG00000073331
ALPK2	0.0951	1.2349E-01	2.9306E-01	ENSG00000198796
ALPK3	0.2111	6.2308E-02	1.8544E-01	ENSG00000136383
ALPL	-0.2373	3.9040E-02	1.3451E-01	ENSG00000162551
ALS2	0.0236	6.8333E-01	8.2333E-01	ENSG00000003393

ALS2CL	-0.0676	4.3770E-01	6.3813E-01	ENSG00000178038
ALX1	0.0338	6.0728E-01	7.7108E-01	ENSG00000180318
ALX3	-0.1294	1.0647E-01	2.6528E-01	ENSG00000156150
ALX4	-0.0400	8.8779E-02		ENSG00000052850
ALYREF	-0.4156	2.7554E-08	1.9206E-06	ENSG00000183684
AMACR	0.0233	5.3592E-01		ENSG00000242110
AMBN	-0.3826	5.2435E-04	5.5088E-03	ENSG00000178522
AMBP	0.0605	3.7348E-01	5.8205E-01	ENSG00000106927
AMBRA1	0.0688	3.5255E-01	5.6288E-01	ENSG00000110497
AMD1	-0.1516	5.1724E-02	1.6309E-01	ENSG00000123505
AMD1P1	-0.0071	9.9378E-01		ENSG00000228339
AMDHD1	0.1767	7.8030E-02	2.1606E-01	ENSG00000139344
AMDHD2	0.1061	1.9999E-01	3.9838E-01	ENSG00000162066
AMELX	0.0190	4.1195E-01		ENSG00000125363
AMER1	0.0104	8.8227E-01	9.4106E-01	ENSG00000184675
AMER2	-0.0539	4.3533E-01	6.3641E-01	ENSG00000165566
AMER3	0.0346	6.7629E-01	8.1858E-01	ENSG00000178171
AMFR	-0.0965	7.8428E-02	2.1675E-01	ENSG00000159461
AMH	0.0895	2.8290E-01	4.9333E-01	ENSG00000104899
AMHR2	0.0949	8.5858E-02		ENSG00000135409
AMIGO1	-0.1478	7.9160E-02	2.1811E-01	ENSG00000181754
AMIGO2	0.0931	3.0129E-01	5.1129E-01	ENSG00000139211
AMMECR1	-0.0035	9.6816E-01	9.8424E-01	ENSG00000101935
AMMECR1L	-0.0047	9.7678E-01	9.8837E-01	ENSG00000144233
AMN	0.0115	8.4049E-01	9.1794E-01	ENSG00000166126
AMN1	-0.1715	3.6814E-03	2.4007E-02	ENSG00000151743
AMOT	-0.1041	1.5310E-01	3.3528E-01	ENSG00000126016
AMOTL1	-0.0369	5.5996E-01	7.3725E-01	ENSG00000166025
AMOTL2	-0.0982	2.1899E-01	4.2144E-01	ENSG00000114019
AMPD2	0.1995	1.7368E-02	7.5216E-02	ENSG00000116337
AMPH	-0.1649	5.5538E-02	1.7180E-01	ENSG00000078053
AMT	-0.0205	7.4264E-01	8.5987E-01	ENSG00000145020
AMY1B	0.5994	6.9834E-03	3.8735E-02	ENSG00000174876
AMY2B	0.5967	3.7564E-07	1.6835E-05	ENSG00000240038
AMYP1	0.0203	7.1996E-01		ENSG00000227408
AMZ1	-0.0398	6.4830E-01	8.0013E-01	ENSG00000174945
AMZ2	-0.1288	1.6579E-02	7.2777E-02	ENSG00000196704
ANAPC1	-0.0513	4.0183E-01	6.0655E-01	ENSG00000153107
ANAPC10	0.3601	3.3484E-05	6.4974E-04	ENSG00000164162
ANAPC11	-0.1574	5.1371E-02	1.6240E-01	ENSG00000141552
ANAPC13	-0.0853	9.0770E-02	2.3940E-01	ENSG00000129055
ANAPC15	0.0303	7.1210E-01	8.4207E-01	ENSG00000110200
ANAPC16	-0.1024	5.5529E-02	1.7179E-01	ENSG00000166295
ANAPC1P2	-0.0264	7.5079E-01	8.6466E-01	ENSG00000285793
ANAPC1P3	-0.0006	5.9104E-01		ENSG00000230395
ANAPC2	0.2253	2.4870E-02	9.7288E-02	ENSG00000176248
ANAPC4	0.0270	6.9389E-01	8.3040E-01	ENSG00000053900
ANAPC5	-0.0074	8.8769E-01	9.4426E-01	ENSG00000089053
ANAPC7	0.0410	5.8566E-01	7.5545E-01	ENSG00000196510

ANCV1RP	0.0049	5.2394E-01		ENSG00000287794
ANG	-0.0275	7.3858E-01	8.5765E-01	ENSG00000214274
ANGEL1	-0.0217	7.9102E-01	8.8958E-01	ENSG00000013523
ANGEL2	0.0105	8.8588E-01	9.4315E-01	ENSG00000174606
ANGPT1	-0.0156	8.2883E-01	9.1135E-01	ENSG00000154188
ANGPT2	0.0426	6.0950E-01	7.7223E-01	ENSG00000091879
ANGPT4	0.0160	7.4057E-01	8.5871E-01	ENSG00000101280
ANGPTL1	-0.1320	1.6393E-01	3.5067E-01	ENSG00000116194
ANGPTL2	-0.5753	7.4629E-05	1.2247E-03	ENSG00000136859
ANGPTL3	-0.0013	9.7715E-01	9.8848E-01	ENSG00000132855
ANGPTL4	-0.1045	2.3388E-01	4.3905E-01	ENSG00000167772
ANGPTL5	-0.0122	8.6416E-01	9.3150E-01	ENSG00000187151
ANGPTL6	0.4344	7.1525E-03	3.9468E-02	ENSG00000130812
ANGPTL7	0.0822	1.1860E-01	2.8509E-01	ENSG00000171819
ANGPTL8	0.1969	5.7439E-02	1.7572E-01	ENSG00000130173
ANK1	0.0779	3.6812E-01	5.7728E-01	ENSG00000029534
ANK2	0.1634	3.9840E-02	1.3639E-01	ENSG00000145362
ANK2-AS1	0.2996	9.6424E-03	4.9403E-02	ENSG00000248152
ANK3	0.2505	2.7692E-03	1.9214E-02	ENSG00000151150
ANKAR	0.2244	2.9758E-02	1.1050E-01	ENSG00000151687
ANKDD1A	-0.1642	2.2578E-02	9.0624E-02	ENSG00000166839
ANKDD1B	0.0392	5.4776E-01	7.2734E-01	ENSG00000189045
ANKEF1	-0.0148	8.4352E-01	9.1973E-01	ENSG00000132623
ANKFN1	0.5742	4.2755E-04	4.7090E-03	ENSG00000153930
ANKFY1	0.0859	1.8088E-01	3.7314E-01	ENSG00000185722
ANKH	0.0678	3.9040E-01	5.9767E-01	ENSG00000154122
ANKHD1	-0.1047	2.4273E-01	4.5047E-01	ENSG00000131503
ANKHD1-EIF4EBP3	0.0319	6.9939E-01	8.3379E-01	ENSG00000254996
ANKIB1	0.1014	1.5791E-01	3.4192E-01	ENSG00000001629
ANKK1	0.0358	4.7110E-01		ENSG00000170209
ANKLE1	-0.2008	5.1950E-02	1.6352E-01	ENSG00000160117
ANKLE2	-0.1300	2.7170E-02	1.0352E-01	ENSG00000176915
ANKMY1	0.5191	1.7047E-04	2.2908E-03	ENSG00000144504
ANKMY2	-0.0347	6.2161E-01	7.8115E-01	ENSG00000106524
ANKRA2	-0.1001	1.0402E-01	2.6141E-01	ENSG00000164331
ANKRD1	1.3658	4.6074E-03		ENSG00000148677
ANKRD10	0.0651	2.7090E-01	4.8008E-01	ENSG00000088448
ANKRD11	0.2475	1.7463E-06	5.9730E-05	ENSG00000167522
ANKRD12	0.2625	9.4367E-10	1.0554E-07	ENSG00000101745
ANKRD13A	0.2208	1.7391E-03	1.3576E-02	ENSG00000076513
ANKRD13B	-0.1401	7.7543E-02	2.1521E-01	ENSG00000198720
ANKRD13C	-0.0530	4.0789E-01	6.1222E-01	ENSG00000118454
ANKRD13C-DT	-0.1792	9.6881E-03	4.9579E-02	ENSG00000197568
ANKRD13D	-0.0743	2.5138E-01	4.5965E-01	ENSG00000172932
ANKRD16	-0.0564	4.3652E-01	6.3728E-01	ENSG00000134461
ANKRD17	0.2869	1.9236E-05	4.1482E-04	ENSG00000132466
ANKRD18A	-0.2709	1.8658E-02	7.9003E-02	ENSG00000180071
ANKRD18B	-0.0072	9.1420E-01	9.5780E-01	ENSG00000230453
ANKRD18DP	0.0866	1.9786E-01	3.9565E-01	ENSG00000226435

ANKRD18EP	-0.1165	2.0563E-01	4.0506E-01	ENSG00000217165
ANKRD18FP	0.0061	8.2231E-01		ENSG00000218577
ANKRD2	-0.0488	4.5401E-02		ENSG00000165887
ANKRD20A1	0.4121	3.1458E-04	3.7302E-03	ENSG00000260691
ANKRD20A18P	0.0017	9.6789E-01		ENSG00000249493
ANKRD20A8P	0.0583	2.8940E-01	4.9983E-01	ENSG00000229089
ANKRD22	0.0554	2.2214E-01	4.2551E-01	ENSG00000152766
ANKRD23	0.0837	2.8304E-01	4.9343E-01	ENSG00000163126
ANKRD24	-0.0524	5.3882E-01	7.2101E-01	ENSG00000089847
ANKRD26	0.3202	2.0821E-05	4.4051E-04	ENSG00000107890
ANKRD26P4	-0.0035	7.7113E-01		ENSG00000229427
ANKRD27	0.3471	6.3478E-05	1.0806E-03	ENSG00000105186
ANKRD28	-0.1032	1.2392E-01	2.9362E-01	ENSG00000206560
ANKRD29	-0.1125	2.0544E-01	4.0480E-01	ENSG00000154065
ANKRD30A	0.0092	1.1776E-01	2.8353E-01	ENSG00000148513
ANKRD30B	0.0637	1.0998E-01		ENSG00000180777
ANKRD30BL	0.0424	3.9680E-01		ENSG00000163046
ANKRD33	-0.0130	4.4652E-01		ENSG00000167612
ANKRD33B	-0.3262	1.0406E-03	9.3513E-03	ENSG00000164236
ANKRD33BP1	-0.0044	7.8592E-01		ENSG00000229689
ANKRD34A	-0.1792	8.8633E-02	2.3510E-01	ENSG00000272031
ANKRD34B	-0.1085	2.2752E-01	4.3192E-01	ENSG00000189127
ANKRD34C	-0.0059	8.6840E-01		ENSG00000235711
ANKRD34C-AS1	-0.0630	1.8809E-01	3.8293E-01	ENSG00000259234
ANKRD35	0.0585	4.8456E-01	6.7829E-01	ENSG00000198483
ANKRD36	0.3281	4.1526E-06	1.2067E-04	ENSG00000135976
ANKRD36B	0.2853	4.5079E-05	8.1857E-04	ENSG00000196912
ANKRD36C	0.2844	8.9760E-05	1.4176E-03	ENSG00000174501
ANKRD37	0.1834	4.4503E-02	1.4729E-01	ENSG00000186352
ANKRD39	0.0834	1.9905E-01	3.9731E-01	ENSG00000213337
ANKRD40	-0.1578	8.1887E-02	2.2263E-01	ENSG00000154945
ANKRD40CL	-0.0176	6.8094E-01	8.2170E-01	ENSG00000167117
ANKRD42	0.4127	1.9890E-05	4.2631E-04	ENSG00000137494
ANKRD42-DT	0.3721	6.3773E-04	6.4600E-03	ENSG00000247137
ANKRD44	0.0432	5.3220E-01	7.1607E-01	ENSG00000065413
ANKRD44-IT1	-0.1820	8.5039E-02	2.2818E-01	ENSG00000236977
ANKRD45	0.0711	3.9624E-01	6.0223E-01	ENSG00000183831
ANKRD46	-0.0571	3.4788E-01	5.5811E-01	ENSG00000186106
ANKRD49	-0.1258	3.6194E-02	1.2696E-01	ENSG00000168876
ANKRD49P2	0.0121	6.4029E-01		ENSG00000225349
ANKRD50	0.1573	1.6449E-02	7.2414E-02	ENSG00000151458
ANKRD52	0.1332	6.4611E-02	1.9016E-01	ENSG00000139645
ANKRD53	-0.1171	1.7124E-01	3.6042E-01	ENSG00000144031
ANKRD54	0.0963	1.5781E-01	3.4181E-01	ENSG00000100124
ANKRD55	0.3855	5.4369E-03	3.2088E-02	ENSG00000164512
ANKRD6	-0.0879	2.7069E-01	4.8008E-01	ENSG00000135299
ANKRD61	-0.0184	8.2289E-01	9.0841E-01	ENSG00000157999
ANKRD62	0.0018	9.0402E-01		ENSG00000181626
ANKRD63	-0.1728	1.5676E-02	7.0182E-02	ENSG00000230778

ANKRD65	-0.1225	1.7376E-01	3.6398E-01	ENSG00000235098
ANKRD66	0.0126	3.9596E-01		ENSG00000230062
ANKRD7	0.0244	6.9929E-01	8.3370E-01	ENSG00000106013
ANKRD9	-0.0831	2.4799E-01	4.5577E-01	ENSG00000156381
ANKS1A	0.4727	1.4459E-07	7.7339E-06	ENSG00000064999
ANKS1B	0.0369	6.1806E-01	7.7882E-01	ENSG00000185046
ANKS3	0.2110	4.0943E-03	2.5998E-02	ENSG00000168096
ANKS4B	0.0004	9.6648E-01		ENSG00000175311
ANKS6	0.0303	6.8560E-01	8.2489E-01	ENSG00000165138
ANKUB1	0.0544	1.3015E-01		ENSG00000206199
ANKZF1	0.1904	1.7108E-02	7.4383E-02	ENSG00000163516
ANLN	-0.1349	1.1437E-01	2.7840E-01	ENSG00000011426
ANO1	0.0047	8.5328E-01		ENSG00000131620
ANO10	0.0375	5.8085E-01	7.5220E-01	ENSG00000160746
ANO2	0.0316	6.2530E-01	7.8361E-01	ENSG00000047617
ANO3	0.1358	5.6155E-02	1.7315E-01	ENSG00000134343
ANO5	-0.0661	4.1207E-01	6.1601E-01	ENSG00000171714
ANO6	0.0140	8.6090E-01	9.3024E-01	ENSG00000177119
ANO7	0.0899	2.3194E-01	4.3688E-01	ENSG00000146205
ANO8	-0.0778	3.6705E-01	5.7634E-01	ENSG00000074855
ANO9	0.0352	2.0543E-01		ENSG00000185101
ANOS1	-0.0365	6.6085E-01	8.0888E-01	ENSG00000011201
ANP32A	-0.4824	1.2619E-07	6.9459E-06	ENSG00000140350
ANP32AP1	0.0043	9.6293E-01		ENSG00000259516
ANP32B	-0.2194	4.3317E-03	2.7044E-02	ENSG00000136938
ANP32BP1	-0.0061	5.7846E-01		ENSG00000259790
ANP32E	-0.1761	2.8134E-02	1.0617E-01	ENSG00000143401
ANPEP	-0.0224	2.9841E-01		ENSG00000166825
ANTKMT	0.0769	1.8898E-01	3.8390E-01	ENSG00000103254
ANTXR1	0.1435	4.5105E-02	1.4863E-01	ENSG00000169604
ANTXR2	0.0995	2.7852E-01	4.8835E-01	ENSG00000163297
ANXA1	0.5893	3.4616E-03	2.2835E-02	ENSG00000135046
ANXA11	-0.0659	3.2284E-01	5.3288E-01	ENSG00000122359
ANXA13	-0.0963	5.1830E-02	1.6331E-01	ENSG00000104537
ANXA2	-0.0531	5.2796E-01	7.1270E-01	ENSG00000182718
ANXA2R	-0.3822	1.0491E-02	5.2507E-02	ENSG00000177721
ANXA2R-AS1	-0.0646	3.8121E-01	5.8940E-01	ENSG00000215068
ANXA2R-OT1	-0.0799	2.6954E-01	4.7902E-01	ENSG00000177738
ANXA3	0.1533	9.3546E-02	2.4419E-01	ENSG00000138772
ANXA4	-0.1272	1.6913E-01	3.5762E-01	ENSG00000196975
ANXA5	-0.1450	4.1971E-02	1.4138E-01	ENSG00000164111
ANXA6	-0.5055	3.8912E-05	7.3534E-04	ENSG00000197043
ANXA7	-0.0189	7.5291E-01	8.6591E-01	ENSG00000138279
ANXA8	0.0190	6.3975E-01		ENSG00000265190
ANXA8	0.0195	9.2457E-01		ENSG00000276850
ANXA9	0.1415	1.2718E-01	2.9863E-01	ENSG00000143412
AOAH	-0.0099	8.9074E-01	9.4581E-01	ENSG00000136250
AOC1	-0.0019	8.1487E-01		ENSG00000002726
AOC2	-0.0850	3.3283E-01	5.4313E-01	ENSG00000131480

AOC3	0.0569	4.8651E-01	6.8018E-01	ENSG00000131471
AOPEP	-0.0800	2.7887E-01	4.8883E-01	ENSG00000148120
AOX1	0.2061	3.5906E-02	1.2636E-01	ENSG00000138356
AP1AR	0.1088	9.4756E-02	2.4633E-01	ENSG00000138660
AP1B1	0.0019	9.8652E-01	9.9247E-01	ENSG00000100280
AP1G1	0.0313	5.8282E-01	7.5356E-01	ENSG00000166747
AP1G2	0.1821	3.3516E-02	1.2025E-01	ENSG00000213983
AP1M1	-0.0600	3.4851E-01	5.5845E-01	ENSG00000072958
AP1M2	0.0798	3.0179E-01	5.1180E-01	ENSG00000129354
AP1S1	-0.1467	1.0936E-01	2.7004E-01	ENSG00000106367
AP1S2	-0.0452	5.6901E-01	7.4329E-01	ENSG00000182287
AP2A1	-0.0118	8.5747E-01	9.2791E-01	ENSG00000196961
AP2A2	0.0309	6.2906E-01	7.8649E-01	ENSG00000183020
AP2B1	-0.2953	5.1580E-05	9.1379E-04	ENSG00000006125
AP2B1P1	-0.0151	4.2227E-01		ENSG00000234130
AP2M1	-0.1614	2.1362E-02	8.7021E-02	ENSG00000161203
AP2S1	-0.1446	4.2314E-02	1.4219E-01	ENSG00000042753
AP3B1	0.0594	3.8618E-01	5.9390E-01	ENSG00000132842
AP3B2	-0.0462	4.9593E-01	6.8762E-01	ENSG00000103723
AP3D1	0.0144	7.8156E-01	8.8427E-01	ENSG00000065000
AP3M1	0.0387	5.9039E-01	7.5864E-01	ENSG00000185009
AP3M2	-0.0003	9.9928E-01	9.9948E-01	ENSG00000070718
AP3S1	-0.2513	7.7645E-04	7.5004E-03	ENSG00000177879
AP3S2	0.0369	5.7498E-01	7.4775E-01	ENSG00000157823
AP4B1	0.0467	5.3886E-01	7.2101E-01	ENSG00000134262
AP4B1-AS1	-0.0042	7.5617E-01		ENSG00000226167
AP4E1	0.0153	7.9967E-01	8.9430E-01	ENSG00000081014
AP4M1	-0.0187	7.8179E-01	8.8438E-01	ENSG00000221838
AP4S1	-0.0236	7.6244E-01	8.7215E-01	ENSG00000100478
AP5B1	0.0592	4.5549E-01	6.5383E-01	ENSG00000254470
AP5M1	-0.0661	2.9955E-01	5.0913E-01	ENSG00000053770
AP5S1	-0.0133	8.5685E-01	9.2762E-01	ENSG00000125843
AP5Z1	-0.0092	8.6472E-01	9.3172E-01	ENSG00000242802
APAF1	-0.0642	3.4049E-01	5.5018E-01	ENSG00000120868
APBA1	0.0461	5.5655E-01	7.3441E-01	ENSG00000107282
APBA2	-0.1594	2.2505E-02	9.0478E-02	ENSG00000034053
APBA3	-0.0688	2.9481E-01	5.0482E-01	ENSG00000011132
APBB1	-0.0580	3.9958E-01	6.0503E-01	ENSG00000166313
APBB1IP	0.0022	9.9557E-01		ENSG00000077420
APBB2	-0.0940	1.6851E-01	3.5691E-01	ENSG00000163697
APBB3	-0.0642	4.3072E-01	6.3244E-01	ENSG00000113108
APC	-0.0851	2.1719E-01	4.1904E-01	ENSG00000134982
APC2	0.1112	1.1082E-01	2.7258E-01	ENSG00000115266
APCDD1	0.1399	3.6090E-02	1.2675E-01	ENSG00000154856
APCDD1L	0.0504	2.3665E-01	4.4242E-01	ENSG00000198768
APCDD1L-DT	0.1608	4.7993E-02	1.5505E-01	ENSG00000231290
APEH	0.0344	6.2273E-01	7.8201E-01	ENSG00000164062
APELA	0.0047	6.4081E-01		ENSG00000248329
APEX1	-0.1842	1.2397E-04	1.8134E-03	ENSG00000100823

APEX2	0.3957	1.9066E-03	1.4528E-02	ENSG00000169188
APH1A	-0.1263	1.8863E-02	7.9624E-02	ENSG00000117362
APH1B	0.1165	1.0757E-01	2.6698E-01	ENSG00000138613
API5	-0.1547	3.4287E-02	1.2195E-01	ENSG00000166181
APIP	0.3010	1.1462E-03	1.0048E-02	ENSG00000149089
APLF	-0.1424	1.4500E-01	3.2376E-01	ENSG00000169621
APLN	0.0860	2.8865E-01	4.9908E-01	ENSG00000171388
APLNR	0.0126	5.3702E-01		ENSG00000134817
APLP1	-0.1364	5.9630E-02	1.7952E-01	ENSG00000105290
APLP2	0.1239	3.4687E-02	1.2316E-01	ENSG00000084234
APMAP	0.0378	5.6130E-01	7.3831E-01	ENSG00000101474
APOA1	0.1413	1.0214E-01	2.5831E-01	ENSG00000118137
APOA1-AS	0.0380	5.3719E-01	7.2010E-01	ENSG00000235910
APOB	0.0139	4.3542E-01		ENSG00000084674
APOBEC2	0.0619	4.1798E-01	6.2126E-01	ENSG00000124701
APOBEC3A	0.0013	7.8087E-01		ENSG00000128383
APOBEC3AP1	0.0032	8.8411E-01		ENSG00000248822
APOBEC3B	0.3706	6.1140E-03	3.4985E-02	ENSG00000179750
APOBEC3C	-0.0099	9.0063E-01	9.5128E-01	ENSG00000244509
APOBEC3D	0.0260	8.3225E-01	9.1348E-01	ENSG00000243811
APOBEC3F	-0.0585	4.9101E-01	6.8345E-01	ENSG00000128394
APOBEC3G	0.0008	8.5552E-01	9.2662E-01	ENSG00000239713
APOBEC3H	-0.0024	9.2428E-01		ENSG00000100298
APOBEC4	-0.0071	7.8905E-01		ENSG00000173627
APOC1	0.1813	7.8859E-02	2.1753E-01	ENSG00000130208
APOD	0.5576	8.0479E-03	4.3085E-02	ENSG00000189058
APOE	-0.0245	7.6461E-01	8.7349E-01	ENSG00000130203
APOF	0.0265	5.4207E-01		ENSG00000175336
APOH	0.0117	7.0851E-01		ENSG00000091583
APOL1	0.0070	8.9194E-01		ENSG00000100342
APOL2	0.0830	3.3651E-01	5.4652E-01	ENSG00000128335
APOL3	0.0477	1.1846E-01		ENSG00000128284
APOL4	0.1886	7.0439E-02	2.0141E-01	ENSG00000100336
APOL5	0.0040	9.3136E-01		ENSG00000128313
APOL6	0.0160	7.3852E-01	8.5765E-01	ENSG00000221963
APOLD1	-0.0083	9.1024E-01	9.5568E-01	ENSG00000178878
APOM	0.3947	9.1243E-05	1.4354E-03	ENSG00000204444
APOO	-0.1561	1.5245E-03	1.2379E-02	ENSG00000184831
APOOL	-0.0291	6.5200E-01	8.0290E-01	ENSG00000155008
APP	-0.1697	2.6343E-02	1.0124E-01	ENSG00000142192
APPBP2	0.0105	8.8272E-01	9.4124E-01	ENSG00000062725
APPL1	0.0690	2.2538E-01	4.2915E-01	ENSG00000157500
APPL2	-0.0272	6.5819E-01	8.0707E-01	ENSG00000136044
APRT	-0.0488	4.5098E-01	6.4998E-01	ENSG00000198931
APTR	0.2229	1.1005E-02	5.4362E-02	ENSG00000214293
APTX	-0.0558	3.3502E-01	5.4508E-01	ENSG00000137074
AQP1	-0.1370	1.0726E-01	2.6649E-01	ENSG00000240583
AQP10	0.0081	7.1777E-01		ENSG00000143595
AQP11	-0.0406	5.9214E-01	7.5986E-01	ENSG00000178301

AQP12A	0.0461	1.0913E-01		ENSG00000184945
AQP12B	0.0759	4.0184E-02		ENSG00000185176
AQP2	0.0062	7.5317E-01		ENSG00000167580
AQP3	-0.0820	3.4833E-01	5.5839E-01	ENSG00000165272
AQP4	-0.2070	6.0750E-02	1.8187E-01	ENSG00000171885
AQP4-AS1	0.3354	2.1661E-02	8.7875E-02	ENSG00000260372
AQP5	0.0146	8.5207E-01	9.2462E-01	ENSG00000161798
AQP5-AS1	-0.0990	2.7805E-01	4.8808E-01	ENSG00000257588
AQP6	-0.5188	7.1357E-03	3.9419E-02	ENSG00000086159
AQP7	0.0064	6.7397E-01		ENSG00000165269
AQP7P2	0.0250	5.1110E-01		ENSG00000181997
AQP8	0.0492	5.5018E-01	7.2920E-01	ENSG00000103375
AQR	-0.0037	9.3885E-01	9.6983E-01	ENSG00000021776
AR	-0.0039	9.5853E-01	9.7936E-01	ENSG00000169083
ARAF	0.0038	9.2957E-01	9.6530E-01	ENSG00000078061
ARAP1	0.0023	9.7655E-01	9.8837E-01	ENSG00000186635
ARAP1-AS2	0.0000	9.7709E-01		ENSG00000245148
ARAP2	0.0593	4.8074E-01	6.7548E-01	ENSG00000047365
ARAP3	0.0286	7.1991E-01	8.4619E-01	ENSG00000120318
ARB2A	-0.2022	4.8967E-04	5.2293E-03	ENSG00000113391
ARC	-0.1222	1.5655E-01	3.4030E-01	ENSG00000198576
ARCN1	-0.0411	5.5032E-01	7.2925E-01	ENSG00000095139
AREG	0.1321	2.1857E-02	8.8524E-02	ENSG00000109321
AREL1	0.0876	1.3482E-01	3.0926E-01	ENSG00000119682
ARF1	-0.2254	8.4105E-04	7.9349E-03	ENSG00000143761
ARF3	-0.1689	3.7116E-02	1.2956E-01	ENSG00000134287
ARF4	0.1714	1.0523E-01	2.6353E-01	ENSG00000168374
ARF4-AS1	-0.0616	2.4579E-01	4.5353E-01	ENSG00000272146
ARF5	-0.1690	5.3173E-03	3.1602E-02	ENSG00000004059
ARF6	-0.3087	9.1587E-05	1.4390E-03	ENSG00000165527
ARFGAP1	0.1865	2.0569E-02	8.4575E-02	ENSG00000101199
ARFGAP2	-0.1290	3.8202E-02	1.3239E-01	ENSG00000149182
ARFGAP3	-0.1500	2.8940E-03	1.9940E-02	ENSG00000242247
ARFGEF1	0.1787	1.2046E-02	5.8186E-02	ENSG00000066777
ARFGEF2	0.1019	1.7233E-01	3.6190E-01	ENSG00000124198
ARFGEF3	0.3628	2.3440E-06	7.5788E-05	ENSG00000112379
ARFIP1	0.2312	1.1030E-02	5.4437E-02	ENSG00000164144
ARFIP2	0.1148	5.1669E-02	1.6301E-01	ENSG00000132254
ARFRP1	-0.0387	5.9053E-01	7.5874E-01	ENSG00000101246
ARG1	0.0033	9.6704E-01	9.8355E-01	ENSG00000118520
ARG2	0.1042	1.3701E-01	3.1238E-01	ENSG00000081181
ARGFX	-0.0043	7.5165E-01		ENSG00000186103
ARGLU1	-0.1783	4.1460E-03	2.6204E-02	ENSG00000134884
ARGLU1-DT	-0.0427	3.9723E-01	6.0306E-01	ENSG00000272274
ARHGAP1	-0.0208	7.3475E-01	8.5528E-01	ENSG00000175220
ARHGAP10	0.0504	5.5624E-01	7.3416E-01	ENSG00000071205
ARHGAP11A	-0.0551	5.2038E-01	7.0740E-01	ENSG00000198826
ARHGAP11A-SCG5	0.0177	8.1024E-01		ENSG00000288864
ARHGAP11B	0.0989	5.7221E-02	1.7522E-01	ENSG00000285077

ARHGAP12	-0.1314	5.4456E-02	1.6926E-01	ENSG00000165322
ARHGAP15	0.1716	6.1634E-02	1.8384E-01	ENSG00000075884
ARHGAP17	-0.0599	3.1631E-01	5.2637E-01	ENSG00000140750
ARHGAP18	0.0297	7.2580E-01	8.4951E-01	ENSG00000146376
ARHGAP19	-0.0205	7.9582E-01	8.9207E-01	ENSG00000213390
ARHGAP19-SLIT1	0.0453	4.7560E-01	6.7115E-01	ENSG00000269891
ARHGAP20	0.3618	5.2459E-03	3.1268E-02	ENSG00000137727
ARHGAP21	0.0492	4.5641E-01	6.5469E-01	ENSG00000107863
ARHGAP22	0.1689	9.2394E-02	2.4212E-01	ENSG00000128805
ARHGAP23	0.0021	9.9780E-01	9.9870E-01	ENSG00000275832
ARHGAP23P1	-0.0007	7.7811E-01		ENSG00000260781
ARHGAP24	0.0596	4.7161E-01	6.6777E-01	ENSG00000138639
ARHGAP25	0.1218	1.7331E-01	3.6326E-01	ENSG00000163219
ARHGAP26	-0.1551	5.1620E-02	1.6292E-01	ENSG00000145819
ARHGAP26-IT1	0.0012	9.5229E-01		ENSG00000230789
ARHGAP27	0.2957	2.5686E-02	9.9467E-02	ENSG00000159314
ARHGAP27P1-BPTFP1-KI	0.0002	9.9622E-01	9.9789E-01	ENSG00000215769
ARHGAP28	0.4612	1.0202E-05	2.4953E-04	ENSG00000088756
ARHGAP30	0.0443	5.9806E-01	7.6415E-01	ENSG00000186517
ARHGAP31	0.0323	6.8443E-01	8.2406E-01	ENSG00000031081
ARHGAP31-AS1	-0.0247	6.0428E-01	7.6833E-01	ENSG00000241155
ARHGAP32	0.0655	3.1929E-01	5.2957E-01	ENSG00000134909
ARHGAP33	-0.0935	2.4947E-01	4.5729E-01	ENSG00000004777
ARHGAP35	0.1090	1.0387E-01	2.6120E-01	ENSG00000160007
ARHGAP36	0.1428	1.2610E-01	2.9692E-01	ENSG00000147256
ARHGAP39	0.2614	3.0282E-03	2.0628E-02	ENSG00000147799
ARHGAP4	0.4301	1.7924E-05	3.9271E-04	ENSG00000089820
ARHGAP40	-0.0226	6.8854E-01	8.2690E-01	ENSG00000124143
ARHGAP42-AS1	0.0235	7.3102E-01	8.5293E-01	ENSG00000248027
ARHGAP44	0.2007	1.4896E-02	6.7600E-02	ENSG00000006740
ARHGAP44-AS1	0.0046	8.7540E-01		ENSG00000265489
ARHGAP45	-0.1490	9.1915E-02	2.4136E-01	ENSG00000180448
ARHGAP5	-0.0261	6.0209E-01	7.6698E-01	ENSG00000100852
ARHGAP6	0.3285	1.8000E-02	7.6998E-02	ENSG00000047648
ARHGAP8	0.0417	6.2196E-01	7.8146E-01	ENSG00000241484
ARHGAP9	-0.5135	3.6643E-03	2.3909E-02	ENSG00000123329
ARHGDIA	-0.2530	7.9124E-04	7.6019E-03	ENSG00000141522
ARHGDIG	-0.4887	4.5441E-05	8.2333E-04	ENSG00000242173
ARHGEF1	0.0126	8.6271E-01	9.3079E-01	ENSG00000076928
ARHGEF10	0.3042	2.0183E-02	8.3570E-02	ENSG00000104728
ARHGEF10L	0.0959	2.3183E-01	4.3677E-01	ENSG00000074964
ARHGEF11	0.0760	2.9339E-01	5.0348E-01	ENSG00000132694
ARHGEF12	0.1595	2.6214E-02	1.0092E-01	ENSG00000196914
ARHGEF15	0.0793	1.3562E-01	3.1034E-01	ENSG00000198844
ARHGEF16	0.2236	4.6559E-02	1.5208E-01	ENSG00000130762
ARHGEF17	0.0986	1.9280E-01	3.8877E-01	ENSG00000110237
ARHGEF17-AS1	0.0135	4.3502E-01		ENSG00000257038
ARHGEF18	0.0556	4.0172E-01	6.0655E-01	ENSG00000104880
ARHGEF19	0.5762	9.5024E-09	7.9526E-07	ENSG00000142632

ARHGEF2	-0.1255	8.9302E-02	2.3639E-01	ENSG00000116584
ARHGEF2-AS1	-0.0336	2.8610E-01		ENSG00000224276
ARHGEF2-AS2	0.0560	3.9958E-01	6.0503E-01	ENSG00000273002
ARHGEF25	0.0210	7.1849E-01	8.4517E-01	ENSG00000240771
ARHGEF26	-0.0712	3.8013E-01	5.8854E-01	ENSG00000114790
ARHGEF26-AS1	-0.4918	1.3797E-04	1.9512E-03	ENSG00000243069
ARHGEF28	-0.0147	8.6378E-01	9.3139E-01	ENSG00000214944
ARHGEF3	-0.1529	8.0802E-02	2.2098E-01	ENSG00000163947
ARHGEF35	0.0027	9.3757E-01	9.6914E-01	ENSG00000213214
ARHGEF37	0.1544	9.8076E-02	2.5166E-01	ENSG00000183111
ARHGEF38	-0.1156	2.0884E-01	4.0887E-01	ENSG00000236699
ARHGEF39	0.0147	8.4846E-01	9.2267E-01	ENSG00000137135
ARHGEF4	-0.1630	5.2519E-02	1.6489E-01	ENSG00000136002
ARHGEF40	0.2459	1.2964E-03	1.1041E-02	ENSG00000165801
ARHGEF5	-0.0209	6.5482E-01	8.0512E-01	ENSG00000050327
ARHGEF6	0.0311	6.6946E-01	8.1482E-01	ENSG00000129675
ARHGEF7	0.0075	9.2632E-01	9.6393E-01	ENSG00000102606
ARHGEF7-AS2	0.0127	8.4625E-01	9.2116E-01	ENSG00000235875
ARHGEF9	-0.1205	1.2780E-02	6.0567E-02	ENSG00000131089
ARHGEF9-IT1	-0.0131	6.0555E-01		ENSG00000231729
ARID1A	-0.0278	6.7917E-01	8.2049E-01	ENSG00000117713
ARID1B	-0.0192	6.9777E-01	8.3294E-01	ENSG00000049618
ARID2	0.1748	1.2085E-02	5.8271E-02	ENSG00000189079
ARID3B	0.0828	2.1501E-01	4.1628E-01	ENSG00000179361
ARID3C	-0.0042	9.5268E-01	9.7678E-01	ENSG00000205143
ARID4A	0.2050	2.0538E-03	1.5368E-02	ENSG00000032219
ARID4B	-0.0603	2.2370E-01	4.2754E-01	ENSG00000054267
ARID5A	-0.3590	1.1638E-03	1.0158E-02	ENSG00000196843
ARID5B	-0.3777	1.4561E-11	3.5125E-09	ENSG00000150347
ARIH1	0.1758	1.6117E-03	1.2859E-02	ENSG00000166233
ARIH2	-0.0747	1.7886E-01	3.7029E-01	ENSG00000177479
ARK2C	0.0649	4.1698E-01	6.2042E-01	ENSG00000141622
ARK2N	0.0615	2.9729E-01	5.0724E-01	ENSG00000152242
ARL1	0.1001	1.8215E-01	3.7504E-01	ENSG00000120805
ARL10	-0.0257	6.9528E-01	8.3135E-01	ENSG00000175414
ARL13A	0.0228	4.5342E-01		ENSG00000174225
ARL13B	0.3013	5.2729E-05	9.2871E-04	ENSG00000169379
ARL14EP	0.0405	5.1209E-01	7.0032E-01	ENSG00000152219
ARL14EPL	-0.0027	7.6734E-01		ENSG00000268223
ARL14EPP1	-0.0472	1.0581E-01		ENSG00000224891
ARL15	-0.1569	5.4630E-02	1.6974E-01	ENSG00000185305
ARL16	-0.2025	1.7669E-04	2.3559E-03	ENSG00000214087
ARL17A	-0.0734	2.5180E-01	4.6011E-01	ENSG00000185829
ARL17A	-0.0747	2.2844E-01	4.3305E-01	ENSG00000228696
ARL2	-0.1603	1.0940E-02	5.4119E-02	ENSG00000213465
ARL2-SNX15	-0.0201	2.5448E-01		ENSG00000273003
ARL2BP	-0.1974	1.9606E-02	8.1805E-02	ENSG00000102931
ARL3	-0.1977	9.8139E-04	8.9269E-03	ENSG00000138175
ARL4A	-0.0800	2.8629E-01	4.9708E-01	ENSG00000122644

ARL4C	-0.4442	5.1754E-08	3.3248E-06	ENSG00000188042
ARL4D	-0.1667	7.9236E-02	2.1825E-01	ENSG00000175906
ARL5A	-0.0214	7.1290E-01	8.4243E-01	ENSG00000162980
ARL5AP3	0.0218	6.2065E-01		ENSG00000228431
ARL5B	0.1859	3.5658E-03	2.3397E-02	ENSG00000165997
ARL5C	0.0037	8.4895E-01		ENSG00000141748
ARL6	-0.0441	5.3598E-01	7.1907E-01	ENSG00000113966
ARL6IP1	-0.2576	1.8206E-04	2.4110E-03	ENSG00000170540
ARL6IP4	0.5255	3.2385E-03	2.1712E-02	ENSG00000182196
ARL6IP5	-0.3042	7.8147E-08	4.6784E-06	ENSG00000144746
ARL6IP6	-0.0175	7.7828E-01	8.8198E-01	ENSG00000177917
ARL8A	-0.4446	5.5277E-05	9.6188E-04	ENSG00000143862
ARL8B	-0.1020	8.7997E-02	2.3401E-01	ENSG00000134108
ARL8BP1	0.0104	5.6269E-01		ENSG00000228413
ARL9	0.1191	1.8689E-01	3.8119E-01	ENSG00000196503
ARMC1	-0.0404	4.1003E-01	6.1426E-01	ENSG00000104442
ARMC10	-0.2095	2.3339E-03	1.6915E-02	ENSG00000170632
ARMC12	0.1897	6.8890E-02	1.9846E-01	ENSG00000157343
ARMC2	-0.1149	1.5184E-01	3.3355E-01	ENSG00000118690
ARMC3	0.1698	3.3224E-02	1.1955E-01	ENSG00000165309
ARMC5	-0.0514	4.8386E-01	6.7787E-01	ENSG00000140691
ARMC6	-0.1187	1.2791E-01	2.9962E-01	ENSG00000105676
ARMC7	0.2927	1.5592E-02	6.9929E-02	ENSG00000125449
ARMC8	-0.1733	3.3140E-03	2.2068E-02	ENSG00000114098
ARMC9	0.2439	3.5012E-03	2.3046E-02	ENSG00000135931
ARMCX1	-0.0456	4.9629E-01	6.8781E-01	ENSG00000126947
ARMCX2	0.3796	1.7313E-04	2.3201E-03	ENSG00000184867
ARMCX3	0.0299	6.1067E-01	7.7309E-01	ENSG00000102401
ARMCX4	-0.0758	2.9410E-01	5.0396E-01	ENSG00000196440
ARMCX5	0.1564	1.4700E-02	6.7067E-02	ENSG00000125962
ARMCX5-GPRASP2	0.0038	8.5551E-01		ENSG00000286237
ARMCX6	-0.1020	1.0818E-01	2.6815E-01	ENSG00000198960
ARMH3	-0.2404	8.8412E-04	8.2620E-03	ENSG00000120029
ARMH4	-0.0535	4.0195E-01	6.0655E-01	ENSG00000139971
ARMS2	-0.0231	2.8336E-01		ENSG00000254636
ARMT1	-0.1894	2.3343E-02	9.2997E-02	ENSG00000146476
ARNT	0.0278	7.1291E-01	8.4243E-01	ENSG00000143437
ARNT2	0.0482	5.2943E-01	7.1398E-01	ENSG00000172379
ARNT2-DT	0.1048	1.9886E-01	3.9705E-01	ENSG00000259495
ARPC1A	-0.2152	1.2859E-03	1.0965E-02	ENSG00000241685
ARPC1B	0.1501	1.2393E-01	2.9362E-01	ENSG00000130429
ARPC2	-0.2431	1.2987E-06	4.6446E-05	ENSG00000163466
ARPC3	-0.2315	1.7277E-03	1.3521E-02	ENSG00000111229
ARPC4	-0.2418	6.6021E-07	2.6414E-05	ENSG00000241553
ARPC4-TTLL3	-0.0154	7.2472E-01	8.4905E-01	ENSG00000250151
ARPC5	-0.1484	1.5003E-02	6.7966E-02	ENSG00000162704
ARPC5L	-0.1440	1.5123E-02	6.8388E-02	ENSG00000136950
ARPIN	-0.3164	8.4677E-05	1.3529E-03	ENSG00000242498
ARPIN-AP3S2	-0.0437	2.3156E-01		ENSG00000250021

ARPP19	-0.2116	3.5652E-05	6.8372E-04	ENSG00000128989
ARPP21	-0.0452	5.8034E-01	7.5181E-01	ENSG00000172995
ARR3	0.0621	3.8035E-01	5.8873E-01	ENSG00000120500
ARRB1	-0.1285	1.3666E-01	3.1206E-01	ENSG00000137486
ARRB2	-0.3729	7.1079E-06	1.8605E-04	ENSG00000141480
ARRDC1	-0.0035	9.5338E-01	9.7697E-01	ENSG00000197070
ARRDC1-AS1	0.0046	9.6252E-01	9.8133E-01	ENSG00000203993
ARRDC2	-0.3382	8.9757E-03	4.6899E-02	ENSG00000105643
ARRDC3-AS1	0.4780	2.0096E-03	1.5140E-02	ENSG00000281357
ARRDC4	0.0993	2.6851E-01	4.7792E-01	ENSG00000140450
ARSA	0.1279	5.1540E-02	1.6274E-01	ENSG00000100299
ARSB	0.0427	5.2283E-01	7.0893E-01	ENSG00000113273
ARSD	0.0958	1.9145E-01	3.8676E-01	ENSG00000006756
ARSF	-0.1167	1.8603E-01	3.8015E-01	ENSG00000062096
ARSG	-0.0134	8.5952E-01	9.2921E-01	ENSG00000141337
ARSH	0.1219	2.4862E-02	9.7285E-02	ENSG00000205667
ARSI	0.0103	8.4836E-01	9.2265E-01	ENSG00000183876
ARSJ	-0.0272	6.9433E-01	8.3061E-01	ENSG00000180801
ARSK	-0.1159	9.8246E-02	2.5191E-01	ENSG00000164291
ARSL	0.0358	6.7180E-01	8.1641E-01	ENSG00000157399
ART1	0.0179	2.0755E-01		ENSG00000129744
ART3	-0.0375	3.9059E-01	5.9778E-01	ENSG00000156219
ART4	0.0443	6.0487E-02		ENSG00000111339
ART5	-0.0841	3.4351E-01	5.5311E-01	ENSG00000167311
ARTN	0.0287	5.9986E-01	7.6571E-01	ENSG00000117407
ARV1	0.1995	9.6472E-03	4.9403E-02	ENSG00000173409
ARVCF	-0.2039	3.0629E-03	2.0801E-02	ENSG00000099889
ARX	-0.3326	1.4032E-03	1.1671E-02	ENSG00000004848
AS3MT	-0.0028	7.5723E-01	8.6856E-01	ENSG00000214435
ASAH1	0.0519	4.1679E-01	6.2025E-01	ENSG00000104763
ASAH1-AS1	-0.0374	6.5668E-01	8.0619E-01	ENSG00000245281
ASAH2	0.0001	9.9844E-01	9.9913E-01	ENSG00000188611
ASAH2B	0.1074	1.8995E-01	3.8502E-01	ENSG00000204147
ASAP1	0.0257	7.5230E-01	8.6560E-01	ENSG00000153317
ASAP1-IT2	0.3937	1.2068E-02	5.8233E-02	ENSG00000280543
ASAP2	-0.3617	1.1201E-06	4.0891E-05	ENSG00000151693
ASAP3	-0.0754	2.4877E-01	4.5649E-01	ENSG00000088280
ASB1	0.2109	1.1160E-02	5.4906E-02	ENSG00000065802
ASB12	-0.1695	8.4336E-02	2.2697E-01	ENSG00000198881
ASB13	0.1506	8.0520E-02	2.2058E-01	ENSG00000196372
ASB14	0.1492	1.2882E-01	3.0087E-01	ENSG00000239388
ASB15	0.0138	6.3598E-01		ENSG00000146809
ASB16	-0.0255	6.0254E-01		ENSG00000161664
ASB16-AS1	-0.3141	1.3302E-06	4.7435E-05	ENSG00000267080
ASB18	0.0269	5.9949E-01		ENSG00000182177
ASB2	0.0161	8.3976E-01	9.1755E-01	ENSG00000100628
ASB3	0.0345	5.4584E-01	7.2621E-01	ENSG00000115239
ASB4	0.0926	1.6297E-01	3.4926E-01	ENSG00000005981
ASB5	0.1152	1.2380E-01	2.9362E-01	ENSG00000164122

ASB6	-0.2034	7.5722E-03	4.1138E-02	ENSG00000148331
ASB7	-0.2299	4.8676E-03	2.9449E-02	ENSG00000183475
ASB8	-0.0264	5.9458E-01	7.6167E-01	ENSG00000177981
ASB9	0.0777	5.6984E-03		ENSG00000102048
ASCC1	0.0561	3.4532E-01	5.5508E-01	ENSG00000138303
ASCC2	0.1041	1.0621E-01	2.6497E-01	ENSG00000100325
ASCC3	0.0998	1.9465E-01	3.9135E-01	ENSG00000112249
ASCL1	-0.4139	9.9629E-03	5.0575E-02	ENSG00000139352
ASCL2	0.0345	6.6841E-01	8.1417E-01	ENSG00000183734
ASCL4	-0.0073	8.2283E-01		ENSG00000187855
ASCL5	0.0202	6.4687E-01		ENSG00000232237
ASF1A	0.0404	6.1964E-01	7.7991E-01	ENSG00000111875
ASF1B	-0.0575	4.9224E-01	6.8446E-01	ENSG00000105011
ASGR1	0.1510	2.5393E-02	9.8579E-02	ENSG00000141505
ASGR2	0.0230	2.0185E-01		ENSG00000161944
ASH1L	0.0722	2.7021E-01	4.7977E-01	ENSG00000116539
ASH1L-AS1	0.0161	8.3470E-01	9.1500E-01	ENSG00000235919
ASH2L	-0.0413	4.9662E-01	6.8809E-01	ENSG00000129691
ASIC1	-0.2107	5.6836E-02	1.7448E-01	ENSG00000110881
ASIC2	0.0541	5.2084E-01	7.0760E-01	ENSG00000108684
ASIC4	0.2593	3.3305E-02	1.1975E-01	ENSG00000072182
ASIC5	0.0186	3.3031E-01		ENSG00000256394
ASIP	0.0314	5.5797E-01		ENSG00000101440
ASL	0.4179	9.0684E-06	2.2653E-04	ENSG00000126522
ASMT	0.0227	5.7790E-01		ENSG00000196433
ASMTL	-0.2877	6.7367E-04	6.7566E-03	ENSG00000169093
ASNS	0.0008	9.9231E-01	9.9567E-01	ENSG00000070669
ASNSD1	-0.1127	4.6877E-02	1.5289E-01	ENSG00000138381
ASNSP3	-0.0004	8.6774E-01		ENSG00000236554
ASPA	0.0247	7.3591E-01		ENSG00000108381
ASPDH	0.2008	6.5081E-02	1.9091E-01	ENSG00000204653
ASPG	0.0211	9.1570E-01	9.5844E-01	ENSG00000166183
ASPH	0.2715	8.1183E-06	2.0743E-04	ENSG00000198363
ASPHD1	-0.1601	3.0754E-02	1.1325E-01	ENSG00000174939
ASPHD2	-0.0144	8.3313E-01	9.1405E-01	ENSG00000128203
ASPM	0.0289	7.2426E-01	8.4867E-01	ENSG00000066279
ASPN	0.0257	6.9837E-01	8.3330E-01	ENSG00000106819
ASPSCR1	0.0517	3.9418E-01	6.0050E-01	ENSG00000169696
ASRGL1	-0.0360	4.6197E-01	6.5902E-01	ENSG00000162174
ASS1	-0.1320	1.4873E-01	3.2923E-01	ENSG00000130707
ASS1P1	-0.0059	9.8736E-01		ENSG00000220517
ASS1P10	0.0181	4.5816E-01		ENSG00000215325
ASS1P2	0.1846	7.8014E-02	2.1606E-01	ENSG00000223922
ASTE1	0.1742	5.9732E-02	1.7972E-01	ENSG00000034533
ASTL	-0.0198	5.6329E-01		ENSG00000188886
ASTN1	-0.2457	2.4734E-04	3.0940E-03	ENSG00000152092
ASTN2	-0.0194	8.0471E-01	8.9755E-01	ENSG00000148219
ASTN2-AS1	-0.0085	8.6492E-01		ENSG00000229105
ASXL1	0.1638	5.5986E-03	3.2767E-02	ENSG00000171456

ASXL2	0.3061	7.9759E-03	4.2802E-02	ENSG00000143970
ASXL3	0.2163	9.7657E-03	4.9852E-02	ENSG00000141431
ATAD1	0.0022	9.7470E-01	9.8758E-01	ENSG00000138138
ATAD2	-0.2638	6.5275E-03	3.6829E-02	ENSG00000156802
ATAD2B	0.0903	2.1705E-01	4.1880E-01	ENSG00000119778
ATAD3A	0.0327	6.5868E-01	8.0739E-01	ENSG00000197785
ATAD3B	0.4596	1.0350E-07	5.9499E-06	ENSG00000160072
ATAD3C	0.0362	6.4681E-01	7.9886E-01	ENSG00000215915
ATAD5	0.1913	1.6576E-02	7.2777E-02	ENSG00000176208
ATAT1	-0.0630	2.8416E-01	4.9483E-01	ENSG00000137343
ATCAY	-0.1176	5.0147E-02	1.5999E-01	ENSG00000167654
ATE1	0.1071	4.3924E-02	1.4581E-01	ENSG00000107669
ATE1-AS1	-0.0369	6.4620E-01	7.9834E-01	ENSG00000226864
ATF1	0.2400	3.0091E-03	2.0550E-02	ENSG00000123268
ATF2	-0.1921	6.8146E-03	3.8038E-02	ENSG00000115966
ATF4	-0.2197	3.0053E-03	2.0546E-02	ENSG00000128272
ATF5	-0.0347	6.7606E-01	8.1840E-01	ENSG00000169136
ATF6	-0.1148	4.7006E-02	1.5315E-01	ENSG00000118217
ATF6B	0.1844	1.5665E-02	7.0156E-02	ENSG00000213676
ATF7	0.0321	6.8805E-01	8.2675E-01	ENSG00000170653
ATF7IP	-0.0441	4.8230E-01	6.7666E-01	ENSG00000171681
ATF7IP2	-0.2222	1.4737E-04	2.0556E-03	ENSG00000166669
ATG10	0.2105	1.7130E-03	1.3436E-02	ENSG00000152348
ATG101	0.0343	6.6866E-01	8.1432E-01	ENSG00000123395
ATG12	-0.0296	6.0615E-01	7.7013E-01	ENSG00000145782
ATG13	-0.0537	3.6404E-01	5.7312E-01	ENSG00000175224
ATG14	-0.5121	9.0188E-10	1.0179E-07	ENSG00000126775
ATG16L1	0.0974	1.7275E-01	3.6252E-01	ENSG00000085978
ATG16L2	0.0134	8.6960E-01	9.3437E-01	ENSG00000168010
ATG2A	-0.0486	5.5029E-01	7.2925E-01	ENSG00000110046
ATG2B	0.4198	7.5784E-06	1.9525E-04	ENSG00000066739
ATG3	-0.1456	5.3904E-03	3.1905E-02	ENSG00000144848
ATG4A	-0.0902	2.2593E-01	4.2979E-01	ENSG00000101844
ATG4B	0.0276	5.8724E-01	7.5641E-01	ENSG00000168397
ATG4C	-0.0385	5.8231E-01	7.5322E-01	ENSG00000125703
ATG4D	-0.0189	7.6786E-01	8.7557E-01	ENSG00000130734
ATG5	0.0791	2.4807E-01	4.5579E-01	ENSG00000057663
ATG7	0.1274	1.1927E-01	2.8632E-01	ENSG00000197548
ATG9A	-0.0539	4.8054E-01	6.7541E-01	ENSG00000198925
ATG9B	0.4925	1.2730E-05	2.9576E-04	ENSG00000181652
ATIC	-0.1889	1.8013E-02	7.7014E-02	ENSG00000138363
ATL1	-0.1326	8.0521E-02	2.2058E-01	ENSG00000198513
ATL2	-0.1114	1.0484E-01	2.6276E-01	ENSG00000119787
ATL3	0.1222	1.2648E-01	2.9765E-01	ENSG00000184743
ATM	0.0932	1.7053E-01	3.5938E-01	ENSG00000149311
ATMIN	-0.1316	3.4754E-02	1.2327E-01	ENSG00000166454
ATN1	-0.0863	2.4956E-01	4.5739E-01	ENSG00000111676
ATOH1	0.0115	7.6533E-01		ENSG00000172238
ATOH7	-0.0595	4.1224E-01	6.1603E-01	ENSG00000179774

ATOH8	-0.0918	2.8302E-01	4.9342E-01	ENSG00000168874
ATOSA	0.1891	3.2287E-02	1.1702E-01	ENSG00000047346
ATOSB	-0.0316	6.6284E-01	8.1036E-01	ENSG00000005238
ATOX1	-0.0750	2.1926E-01	4.2175E-01	ENSG00000177556
ATP10A	-0.0678	4.3223E-01	6.3365E-01	ENSG00000206190
ATP10B	-0.0319	6.9184E-01	8.2904E-01	ENSG00000118322
ATP10D	-0.2180	1.8367E-02	7.8085E-02	ENSG00000145246
ATP11A	-0.0303	6.5863E-01	8.0739E-01	ENSG00000068650
ATP11A-AS1	-0.0492	4.9939E-01	6.9047E-01	ENSG00000232684
ATP11B	-0.0215	7.5639E-01	8.6801E-01	ENSG00000058063
ATP11C	0.2540	3.6645E-04	4.1832E-03	ENSG00000101974
ATP12A	0.0124	7.9469E-01	8.9153E-01	ENSG00000075673
ATP13A1	0.0882	2.5881E-01	4.6754E-01	ENSG00000105726
ATP13A2	0.0062	9.1972E-01	9.6048E-01	ENSG00000159363
ATP13A3	0.2363	7.6773E-04	7.4311E-03	ENSG00000133657
ATP13A4	0.0174	7.8060E-01	8.8359E-01	ENSG00000127249
ATP13A5	0.4801	2.0679E-02	8.4929E-02	ENSG00000187527
ATP1A1	-0.3566	4.5051E-06	1.2934E-04	ENSG00000163399
ATP1A1-AS1	0.0986	2.6777E-01	4.7718E-01	ENSG00000203865
ATP1A2	0.3794	1.6305E-02	7.1936E-02	ENSG00000018625
ATP1A3	0.0024	9.6466E-01	9.8256E-01	ENSG00000105409
ATP1A4	0.0705	2.5841E-01	4.6721E-01	ENSG00000132681
ATP1B1	-0.5524	2.6959E-11	5.7183E-09	ENSG00000143153
ATP1B2	0.0496	5.4064E-01	7.2261E-01	ENSG00000129244
ATP1B3	-0.1882	1.4902E-03	1.2205E-02	ENSG00000069849
ATP1B4	0.0013	9.1764E-01		ENSG00000101892
ATP23	-0.0616	3.1399E-01	5.2428E-01	ENSG00000166896
ATP2A1	0.1696	9.6216E-02	2.4902E-01	ENSG00000196296
ATP2A1-AS1	-0.1275	1.4392E-01	3.2193E-01	ENSG00000260442
ATP2A2	-0.1998	3.3788E-03	2.2372E-02	ENSG00000174437
ATP2A3	-0.1499	9.6919E-02	2.5005E-01	ENSG00000074370
ATP2B1	0.1180	1.6032E-01	3.4556E-01	ENSG00000070961
ATP2B1-AS1	0.4692	1.2670E-06	4.5444E-05	ENSG00000271614
ATP2B2	0.0222	7.4071E-01	8.5875E-01	ENSG00000157087
ATP2B3	-0.0814	3.0154E-01	5.1157E-01	ENSG00000067842
ATP2B4	-0.0740	3.1072E-01	5.2095E-01	ENSG00000058668
ATP2C1	-0.0823	1.4745E-01	3.2746E-01	ENSG00000017260
ATP2C2	0.1410	1.3125E-01	3.0423E-01	ENSG00000064270
ATP2C2-AS1	-0.0541	3.0668E-01		ENSG00000261286
ATP4A	0.1205	8.4563E-02	2.2730E-01	ENSG00000105675
ATP4B	0.0094	7.7056E-01		ENSG00000186009
ATP5F1A	-0.2764	1.7415E-08	1.3368E-06	ENSG00000152234
ATP5F1AP2	-0.0124	5.2486E-01		ENSG00000227682
ATP5F1AP3	0.0159	7.4962E-01	8.6410E-01	ENSG00000263232
ATP5F1B	-0.3937	1.9859E-08	1.4718E-06	ENSG00000110955
ATP5F1BP1	-0.0151	3.3289E-01		ENSG00000231635
ATP5F1C	-0.3263	1.1568E-08	9.4564E-07	ENSG00000165629
ATP5F1CP1	-0.0543	1.3734E-01	3.1278E-01	ENSG00000224004
ATP5F1D	-0.1258	4.5574E-02	1.4991E-01	ENSG00000099624

ATP5F1E	-0.1247	7.2658E-02	2.0610E-01	ENSG00000124172
ATP5IF1	-0.1136	9.7770E-02	2.5117E-01	ENSG00000130770
ATP5MC1	-0.0231	7.2806E-01	8.5083E-01	ENSG00000159199
ATP5MC2	-0.2188	2.0756E-03	1.5471E-02	ENSG00000135390
ATP5MC3	-0.2774	8.1889E-05	1.3163E-03	ENSG00000154518
ATP5ME	-0.2425	1.8286E-03	1.4082E-02	ENSG00000169020
ATP5MF	-0.3585	1.2849E-05	2.9769E-04	ENSG00000241468
ATP5MF-PTCD1	-0.0305	3.6720E-01		ENSG00000248919
ATP5MFP1	-0.0266	3.5381E-01		ENSG00000224421
ATP5MG	-0.1409	2.3558E-02	9.3629E-02	ENSG00000167283
ATP5MGL	-0.0786	2.5212E-01	4.6034E-01	ENSG00000249222
ATP5MGP3	0.0096	8.4488E-01		ENSG00000249256
ATP5MJ	-0.2230	2.6199E-03	1.8397E-02	ENSG00000156411
ATP5MK	-0.0151	7.4826E-01	8.6323E-01	ENSG00000173915
ATP5PB	-0.1708	2.7969E-03	1.9363E-02	ENSG00000116459
ATP5PD	-0.1107	4.7678E-02	1.5458E-01	ENSG00000167863
ATP5PDP2	-0.0020	6.4867E-01		ENSG00000229184
ATP5PF	-0.1953	2.4744E-03	1.7673E-02	ENSG00000154723
ATP5PO	-0.3075	1.4718E-04	2.0541E-03	ENSG00000241837
ATP6	0.0054	9.4645E-01	9.7347E-01	ENSG00000198899
ATP6AP1	-0.0187	7.6074E-01	8.7130E-01	ENSG00000071553
ATP6AP1-DT	0.1618	4.3204E-02	1.4426E-01	ENSG00000197180
ATP6AP2	-0.0606	3.8967E-01	5.9714E-01	ENSG00000182220
ATP6V0A1	-0.0580	3.7853E-01	5.8703E-01	ENSG00000033627
ATP6V0A2	0.2786	2.0833E-04	2.6809E-03	ENSG00000185344
ATP6V0A4	0.1032	3.9232E-02		ENSG00000105929
ATP6V0B	-0.2227	1.2573E-03	1.0764E-02	ENSG00000117410
ATP6V0C	-0.2695	3.2093E-02	1.1650E-01	ENSG00000185883
ATP6V0CP4	-0.0124	4.1290E-01		ENSG00000284138
ATP6V0D1	-0.0641	2.5969E-01	4.6810E-01	ENSG00000159720
ATP6V0D1-DT	0.0975	2.8173E-01	4.9216E-01	ENSG00000270049
ATP6V0D2	0.0335	4.3884E-01		ENSG00000147614
ATP6V0E1	-0.1067	2.0668E-01	4.0621E-01	ENSG00000113732
ATP6V0E1P1	-0.0453	4.4840E-01	6.4759E-01	ENSG00000225364
ATP6V0E2	-0.1406	2.0520E-02	8.4430E-02	ENSG00000171130
ATP6V0E2-AS1	-0.0001	9.9821E-01	9.9899E-01	ENSG00000204934
ATP6V1A	-0.0541	4.3375E-01	6.3492E-01	ENSG00000114573
ATP6V1B1	-0.0163	4.2498E-01		ENSG00000116039
ATP6V1B2	-0.0447	5.0573E-01	6.9547E-01	ENSG00000147416
ATP6V1C1	0.1252	1.2399E-01	2.9372E-01	ENSG00000155097
ATP6V1C2	0.0552	4.7911E-01	6.7432E-01	ENSG00000143882
ATP6V1D	-0.2890	4.0241E-05	7.5467E-04	ENSG00000100554
ATP6V1E1	-0.1253	7.5880E-02	2.1219E-01	ENSG00000131100
ATP6V1E2	-0.1150	1.2444E-01	2.9424E-01	ENSG00000250565
ATP6V1F	-0.1865	9.4200E-03	4.8560E-02	ENSG00000128524
ATP6V1G1	-0.2672	2.8817E-04	3.4774E-03	ENSG00000136888
ATP6V1G1P2	-0.0148	4.3165E-01		ENSG00000253502
ATP6V1G1P4	0.0216	4.6947E-01		ENSG00000233343
ATP6V1G2	-0.2727	1.5684E-03	1.2636E-02	ENSG00000213760

ATP6V1G3	0.0301	1.2062E-01		ENSG00000151418
ATP6V1H	-0.1774	6.6137E-03	3.7203E-02	ENSG00000047249
ATP7A	0.2283	9.0824E-03	4.7256E-02	ENSG00000165240
ATP7B	0.0867	2.8127E-01	4.9160E-01	ENSG00000123191
ATP8	-0.0171	8.3537E-01	9.1524E-01	ENSG00000228253
ATP8A1	-0.3500	1.0733E-04	1.6262E-03	ENSG00000124406
ATP8A2	-0.0462	5.4352E-01	7.2458E-01	ENSG00000132932
ATP8B1	0.0338	5.5563E-01	7.3361E-01	ENSG00000081923
ATP8B1-AS1	-0.0678	3.3740E-01	5.4743E-01	ENSG00000267040
ATP8B2	0.1699	5.7866E-02	1.7631E-01	ENSG00000143515
ATP8B3	-0.0938	2.6693E-01	4.7620E-01	ENSG00000130270
ATP9A	0.0419	5.5919E-01	7.3659E-01	ENSG00000054793
ATP9B	0.0055	9.5921E-01	9.7963E-01	ENSG00000166377
ATPAF1	-0.2011	8.2233E-04	7.8181E-03	ENSG00000123472
ATPAF2	-0.1335	4.7427E-02	1.5401E-01	ENSG00000171953
ATPSCKMT	-0.0016	9.6900E-01	9.8478E-01	ENSG00000150756
ATR	0.2703	1.1000E-03	9.7355E-03	ENSG00000175054
ATRAID	-0.2097	4.3359E-03	2.7063E-02	ENSG00000138085
ATRIP	-0.2032	1.1503E-02	5.6214E-02	ENSG00000164053
ATRN	0.3096	2.5029E-04	3.1229E-03	ENSG00000088812
ATRNL1	-0.0875	1.6790E-01	3.5617E-01	ENSG00000107518
ATRX	0.1217	6.2560E-02	1.8579E-01	ENSG00000085224
ATXN1	-0.1090	1.2769E-01	2.9943E-01	ENSG00000124788
ATXN10	-0.2091	5.3981E-04	5.6376E-03	ENSG00000130638
ATXN1L	-0.1046	1.6199E-01	3.4802E-01	ENSG00000224470
ATXN2	0.0261	6.9864E-01	8.3342E-01	ENSG00000204842
ATXN2-AS	0.5806	4.8739E-04	5.2094E-03	ENSG00000258099
ATXN2L	-0.3205	1.8686E-06	6.2811E-05	ENSG00000168488
ATXN3	0.1374	5.3279E-02	1.6657E-01	ENSG00000066427
ATXN7	0.1713	2.2417E-02	9.0209E-02	ENSG00000163635
ATXN7L1	-0.0044	9.4985E-01	9.7513E-01	ENSG00000146776
ATXN7L2	-0.3460	1.8884E-05	4.0867E-04	ENSG00000162650
ATXN7L3	-0.0824	2.7289E-01	4.8241E-01	ENSG00000087152
ATXN7L3-AS1	-0.1109	1.9634E-01	3.9369E-01	ENSG00000260793
ATXN7L3B	-0.1698	4.4694E-03	2.7631E-02	ENSG00000253719
AUH	-0.1088	1.5659E-01	3.4030E-01	ENSG00000148090
AUNIP	-0.1278	1.2598E-01	2.9667E-01	ENSG00000127423
AUP1	-0.0900	7.6528E-02	2.1337E-01	ENSG00000115307
AURKA	0.0799	2.7809E-01	4.8808E-01	ENSG00000087586
AURKAIP1	0.0178	8.0717E-01	8.9868E-01	ENSG00000175756
AURKB	-0.0728	3.9227E-01	5.9920E-01	ENSG00000178999
AURKC	0.0606	4.5201E-01	6.5100E-01	ENSG00000105146
AUTS2	0.1497	5.0434E-02	1.6049E-01	ENSG00000158321
AVEN	0.0711	3.8931E-01	5.9670E-01	ENSG00000169857
AVIL	-0.2697	1.8237E-02	7.7705E-02	ENSG00000135407
AVL9	0.1884	6.8522E-03	3.8191E-02	ENSG00000105778
AVPI1	0.1665	7.6985E-02	2.1406E-01	ENSG00000119986
AVPR1A	-0.1042	7.9091E-02	2.1795E-01	ENSG00000166148
AVPR1B	-0.0430	4.4139E-01	6.4096E-01	ENSG00000198049

AWAT1	0.0060	3.2914E-01		ENSG00000204195
AWAT2	0.0006	9.8149E-01		ENSG00000147160
AXDND1	0.5486	5.2923E-03	3.1476E-02	ENSG00000162779
AXIN1	-0.0749	3.1779E-01	5.2800E-01	ENSG00000103126
AXIN2	0.0055	9.3521E-01	9.6814E-01	ENSG00000168646
AXL	-0.0306	7.0642E-01	8.3829E-01	ENSG00000167601
AZGP1	0.0444	3.6870E-01	5.7783E-01	ENSG00000160862
AZGP1P2	0.0180	3.6481E-01		ENSG00000214252
AZI2	-0.0892	8.9750E-02	2.3735E-01	ENSG00000163512
AZIN1	-0.4402	3.7293E-10	4.8295E-08	ENSG00000155096
AZIN2	0.1846	2.5029E-02	9.7719E-02	ENSG00000142920
AZU1	0.0438	6.0055E-01	7.6606E-01	ENSG00000172232
B2M	-0.0839	3.4267E-01	5.5221E-01	ENSG00000166710
B3GALNT1	-0.3558	1.4001E-03	1.1656E-02	ENSG00000169255
B3GALNT2	-0.1006	1.2684E-01	2.9818E-01	ENSG00000162885
B3GALT1	-0.1661	5.1100E-02	1.6175E-01	ENSG00000172318
B3GALT1-AS1	0.0477	4.1892E-01		ENSG00000235335
B3GALT2	0.0894	3.1328E-01	5.2355E-01	ENSG00000162630
B3GALT4	-0.0946	2.5323E-01	4.6115E-01	ENSG00000235863
B3GALT5	0.2221	4.1752E-02	1.4086E-01	ENSG00000183778
B3GALT6	0.2196	1.3677E-03	1.1458E-02	ENSG00000176022
B3GALT9	0.3566	7.8112E-04	7.5370E-03	ENSG00000214654
B3GAT1	0.0815	3.4111E-01	5.5097E-01	ENSG00000109956
B3GAT1-DT	0.4304	2.8469E-04	3.4455E-03	ENSG00000255545
B3GAT2	-0.0356	6.5086E-01	8.0209E-01	ENSG00000112309
B3GAT3	0.1704	2.7721E-02	1.0502E-01	ENSG00000149541
B3GLCT	-0.1598	4.8651E-02	1.5658E-01	ENSG00000187676
B3GNT2	-0.3265	1.1108E-04	1.6681E-03	ENSG00000170340
B3GNT3	-0.0274	3.2553E-01		ENSG00000179913
B3GNT4	0.0207	8.0180E-01	8.9537E-01	ENSG00000176383
B3GNT5	-0.1148	1.8907E-01	3.8400E-01	ENSG00000176597
B3GNT6	-0.0012	7.4204E-01		ENSG00000198488
B3GNT7	-0.0793	1.3993E-01	3.1639E-01	ENSG00000156966
B3GNT8	0.0642	4.5510E-01	6.5346E-01	ENSG00000177191
B3GNT9	-0.2508	3.4735E-02	1.2326E-01	ENSG00000237172
B3GNTL1	-0.0286	7.2729E-01	8.5032E-01	ENSG00000175711
B4GALNT1	-0.3303	3.2139E-04	3.7855E-03	ENSG00000135454
B4GALNT3	0.0869	3.2846E-01	5.3892E-01	ENSG00000139044
B4GALNT4	0.0372	6.4295E-01	7.9601E-01	ENSG00000182272
B4GALT1	0.2060	5.4799E-02	1.7018E-01	ENSG00000086062
B4GALT2	-0.0854	2.9182E-01	5.0208E-01	ENSG00000117411
B4GALT3	-0.0474	4.2718E-01	6.2915E-01	ENSG00000158850
B4GALT4	0.2432	5.6760E-03	3.3118E-02	ENSG00000121578
B4GALT4-AS1	0.0466	3.4550E-01		ENSG00000240254
B4GALT5	-0.1093	1.2983E-01	3.0243E-01	ENSG00000158470
B4GALT7	-0.0789	1.4969E-01	3.3054E-01	ENSG00000027847
B4GAT1	-0.0275	6.7523E-01	8.1782E-01	ENSG00000174684
B4GAT1-DT	0.0441	6.0132E-01	7.6645E-01	ENSG00000255468
B9D1	0.1791	1.4234E-02	6.5498E-02	ENSG00000108641

B9D2	0.0683	3.4882E-01	5.5864E-01	ENSG00000123810
BAALC	0.1967	3.4223E-02	1.2177E-01	ENSG00000164929
BAAT	0.0692	4.1355E-01	6.1714E-01	ENSG00000136881
BABAM1	-0.1689	3.6327E-03	2.3722E-02	ENSG00000105393
BABAM2	0.0928	1.1543E-01	2.7998E-01	ENSG00000158019
BACE1	0.0661	3.6129E-01	5.7083E-01	ENSG00000186318
BACE2	-0.4447	2.0509E-03	1.5361E-02	ENSG00000182240
BACH1	-0.1873	4.9896E-04	5.2940E-03	ENSG00000156273
BACH2	0.0258	7.4099E-01	8.5887E-01	ENSG00000112182
BAD	-0.0927	9.4347E-02	2.4565E-01	ENSG00000002330
BAG1	-0.0455	4.0179E-01	6.0655E-01	ENSG00000107262
BAG2	0.3664	4.9251E-03	2.9745E-02	ENSG00000112208
BAG3	-0.4102	1.3348E-06	4.7530E-05	ENSG00000151929
BAG4	-0.2154	1.1230E-03	9.8859E-03	ENSG00000156735
BAG5	-0.1961	4.1212E-03	2.6114E-02	ENSG00000166170
BAG6	-0.0343	6.4406E-01	7.9685E-01	ENSG00000204463
BAHCC1	0.1481	1.1611E-01	2.8090E-01	ENSG00000266074
BAHD1	-0.0714	3.6392E-01	5.7304E-01	ENSG00000140320
BAIAP2	-0.1410	3.7999E-02	1.3195E-01	ENSG00000175866
BAIAP2-DT	-0.0985	1.9794E-01	3.9576E-01	ENSG00000226137
BAIAP2L1	-0.1164	2.1127E-01	4.1207E-01	ENSG00000006453
BAIAP2L2	0.2596	1.5221E-02	6.8669E-02	ENSG00000128298
BAIAP3	0.2033	2.8752E-02	1.0781E-01	ENSG00000007516
BAK1	-0.0009	9.8595E-01	9.9239E-01	ENSG00000030110
BAMBI	-0.0784	2.6801E-01	4.7744E-01	ENSG00000095739
BANCR	0.0326	1.2454E-02		ENSG00000278910
BANF1	-0.2512	5.5885E-04	5.7946E-03	ENSG00000175334
BANK1	0.0171	7.0007E-01	8.3420E-01	ENSG00000153064
BANP	-0.1533	3.6189E-02	1.2696E-01	ENSG00000172530
BAP1	-0.0497	3.9719E-01	6.0304E-01	ENSG00000163930
BARD1	-0.2827	7.5217E-03	4.0954E-02	ENSG00000138376
BARX1	-0.1100	2.3083E-01	4.3566E-01	ENSG00000131668
BARX1-DT	0.0314	2.9690E-01		ENSG00000235601
BASP1	-0.4787	4.6155E-13	2.0270E-10	ENSG00000176788
BASP1-AS1	0.1780	2.2096E-02	8.9257E-02	ENSG00000215196
BATF2	0.0527	2.0544E-01		ENSG00000168062
BATF3	-0.2173	4.3913E-02	1.4580E-01	ENSG00000123685
BAX	-0.2769	2.5540E-04	3.1786E-03	ENSG00000087088
BAZ1A	-0.0079	9.0857E-01	9.5481E-01	ENSG00000198604
BAZ1A-AS1	-0.1357	1.5125E-01	3.3263E-01	ENSG00000258738
BAZ1B	-0.1946	7.3429E-04	7.2067E-03	ENSG00000009954
BAZ2A	-0.0024	9.6354E-01	9.8180E-01	ENSG00000076108
BAZ2B	0.1735	4.3031E-03	2.6931E-02	ENSG00000123636
BAZ2B	0.1431	1.0395E-01	2.6131E-01	ENSG00000226266
BAZ2B-AS1	-0.2062	5.8191E-02	1.7701E-01	ENSG00000224152
BBC3	-0.5619	8.1812E-09	7.0631E-07	ENSG00000105327
BBIP1	0.0708	2.4642E-01	4.5414E-01	ENSG00000214413
BBLN	0.0945	1.7379E-01	3.6398E-01	ENSG00000171159
BBOF1	0.0657	3.9603E-01	6.0206E-01	ENSG00000119636

BBOX1	0.0376	6.3063E-01	7.8728E-01	ENSG00000129151
BBOX1-AS1	-0.0319	6.9837E-01	8.3330E-01	ENSG00000254560
BBS1	0.1335	1.6774E-01	3.5602E-01	ENSG00000174483
BBS10	0.1308	1.6413E-01	3.5099E-01	ENSG00000179941
BBS2	0.0416	6.0102E-01	7.6634E-01	ENSG00000125124
BBS4	0.1569	2.2941E-02	9.1755E-02	ENSG00000140463
BBS5	0.1098	1.6580E-01	3.5336E-01	ENSG00000163093
BBS7	0.0839	1.7726E-01	3.6843E-01	ENSG00000138686
BBS9	0.0412	5.3024E-01	7.1477E-01	ENSG00000122507
BBX	0.0953	5.3903E-02	1.6814E-01	ENSG00000114439
BCAM	0.0501	5.6307E-01	7.3964E-01	ENSG00000187244
BCAN	-0.0995	2.4530E-01	4.5305E-01	ENSG00000132692
BCAP31	0.0043	9.4616E-01	9.7334E-01	ENSG00000185825
BCAR1	-0.2546	6.2526E-03	3.5588E-02	ENSG00000050820
BCAR3	0.0250	7.4634E-01	8.6199E-01	ENSG00000137936
BCAR4	-0.0101	4.9887E-01		ENSG00000262117
BCAS1	0.1326	9.1305E-02	2.4032E-01	ENSG00000064787
BCAS2	-0.2787	3.5909E-05	6.8758E-04	ENSG00000116752
BCAS3	-0.0735	3.0564E-01	5.1579E-01	ENSG00000141376
BCAS4	-0.2634	9.1820E-05	1.4408E-03	ENSG00000124243
BCAT1	0.3302	4.0263E-04	4.5010E-03	ENSG00000060982
BCAT2	-0.0374	6.0847E-01	7.7161E-01	ENSG00000105552
BCCIP	-0.0810	8.1903E-02	2.2263E-01	ENSG00000107949
BCDIN3D	0.1116	1.2280E-01	2.9181E-01	ENSG00000186666
BCDIN3D-AS1	-0.0793	3.8100E-01	5.8923E-01	ENSG00000258057
BCHE	-0.1412	5.8180E-02	1.7701E-01	ENSG00000114200
BCKDHA	-0.0035	9.3031E-01	9.6563E-01	ENSG00000248098
BCKDHB	0.0568	4.8965E-01	6.8252E-01	ENSG00000083123
BCKDK	-0.0398	5.2237E-01	7.0877E-01	ENSG00000103507
BCL10	-0.1863	3.0354E-03	2.0671E-02	ENSG00000142867
BCL11A	-0.3449	2.2500E-04	2.8478E-03	ENSG00000119866
BCL11B	-0.1252	1.6824E-01	3.5654E-01	ENSG00000127152
BCL2	-0.2293	1.8603E-02	7.8822E-02	ENSG00000171791
BCL2A1	-0.0839	6.8793E-05	1.1507E-03	ENSG00000140379
BCL2L1	-0.0984	1.5654E-01	3.4030E-01	ENSG00000171552
BCL2L10	-0.0884	1.0476E-01	2.6264E-01	ENSG00000137875
BCL2L11	0.1211	1.7452E-01	3.6487E-01	ENSG00000153094
BCL2L12	0.1019	2.5953E-01	4.6792E-01	ENSG00000126453
BCL2L13	-0.0036	9.7031E-01	9.8509E-01	ENSG00000099968
BCL2L14	0.0125	9.6999E-01	9.8504E-01	ENSG00000121380
BCL2L15	0.1911	7.2632E-02	2.0608E-01	ENSG00000188761
BCL2L2	0.0541	5.1261E-01	7.0055E-01	ENSG00000129473
BCL2L2-PABPN1	-0.0309	6.0043E-01	7.6598E-01	ENSG00000258643
BCL3	-0.2602	6.5108E-03	3.6760E-02	ENSG00000069399
BCL6	-0.2442	3.1063E-02	1.1415E-01	ENSG00000113916
BCL6B	0.0522	4.3954E-01	6.3954E-01	ENSG00000161940
BCL7A	-0.2121	2.0190E-03	1.5178E-02	ENSG00000110987
BCL7B	-0.0398	5.0350E-01	6.9392E-01	ENSG00000106635
BCL7C	-0.1592	7.8223E-03	4.2180E-02	ENSG00000099385

BCL9	0.0169	8.0266E-01	8.9599E-01	ENSG00000116128
BCL9L	-0.3328	3.3894E-03	2.2418E-02	ENSG00000186174
BCLAF1	-0.1440	2.6727E-02	1.0230E-01	ENSG00000029363
BCLAF1P2	-0.1967	1.3690E-02	6.3772E-02	ENSG00000279800
BCLAF3	0.3950	2.0525E-07	1.0370E-05	ENSG00000173681
BCO2	0.1209	2.0085E-01	3.9939E-01	ENSG00000197580
BCOR	-0.3147	1.1989E-04	1.7696E-03	ENSG00000183337
BCORL1	0.3559	7.0787E-05	1.1734E-03	ENSG00000085185
BCR	0.0000	9.9553E-01	9.9752E-01	ENSG00000186716
BCRP7	-0.0357	1.5583E-01		ENSG00000215544
BCRP8	0.0682	3.4055E-01	5.5026E-01	ENSG00000236794
BCS1L	-0.0911	1.3726E-01	3.1266E-01	ENSG00000074582
BCYRN1	-0.0323	6.8827E-01	8.2684E-01	ENSG00000236824
BDH1	-0.3442	1.8506E-04	2.4455E-03	ENSG00000161267
BDH2	0.1462	6.6042E-02	1.9308E-01	ENSG00000164039
BDKRB1	0.0091	9.3469E-01		ENSG00000100739
BDKRB2	-0.1682	9.9619E-02	2.5400E-01	ENSG00000168398
BDNF	0.3028	1.7497E-02	7.5580E-02	ENSG00000176697
BDNF-AS	0.0389	6.2500E-01	7.8348E-01	ENSG00000245573
BDP1	0.2817	4.6326E-05	8.3331E-04	ENSG00000145734
BEAN1	-0.0818	3.4609E-01	5.5612E-01	ENSG00000166546
BECN1	-0.0984	1.6761E-01	3.5589E-01	ENSG00000126581
BEGAIN	-0.0818	3.2468E-01	5.3476E-01	ENSG00000183092
BEND2	0.0012	9.8279E-01		ENSG00000177324
BEND3	0.0082	9.2027E-01	9.6091E-01	ENSG00000178409
BEND4	-0.0727	3.8194E-01	5.9016E-01	ENSG00000188848
BEND5	-0.0337	6.1040E-01	7.7294E-01	ENSG00000162373
BEND6	-0.1905	3.1447E-02	1.1506E-01	ENSG00000151917
BEND7	-0.1692	2.3042E-02	9.2051E-02	ENSG00000165626
BEST1	-0.0951	2.7732E-01	4.8738E-01	ENSG00000167995
BEST2	0.0747	2.9243E-01	5.0249E-01	ENSG00000039987
BEST3	0.0882	2.7925E-01	4.8928E-01	ENSG00000127325
BEST4	0.1492	5.7639E-02	1.7606E-01	ENSG00000142959
BET1	0.4087	4.4863E-05	8.1585E-04	ENSG00000105829
BET1L	0.1772	1.7541E-02	7.5731E-02	ENSG00000177951
BEX1	-0.1386	9.4708E-02	2.4628E-01	ENSG00000133169
BEX2	-0.3824	1.2455E-07	6.8868E-06	ENSG00000133134
BEX3	-0.4500	6.7823E-07	2.7003E-05	ENSG00000166681
BEX4	-0.4257	7.3200E-11	1.2684E-08	ENSG00000102409
BEX5	0.0731	3.4419E-01	5.5391E-01	ENSG00000184515
BFAR	0.0610	3.5145E-01	5.6166E-01	ENSG00000103429
BFSP1	0.0326	7.0082E-01	8.3468E-01	ENSG00000125864
BFSP2	-0.0361	5.5105E-01	7.2986E-01	ENSG00000170819
BFSP2-AS1	0.0384	2.2340E-01		ENSG00000249993
BGLAP	0.0129	5.2759E-01		ENSG00000242252
BGLT3	0.0096	3.5983E-01		ENSG00000260629
BGN	0.1592	1.0370E-01	2.6089E-01	ENSG00000182492
BHLHA15	-0.1179	2.7969E-02		ENSG00000180535
BHLHE22	-0.0765	2.9182E-01	5.0208E-01	ENSG00000180828

BHLHE22-AS1	0.0392	6.5438E-01	8.0476E-01	ENSG00000254102
BHLHE23	-0.0826	3.4797E-01	5.5811E-01	ENSG00000125533
BHLHE40	-0.1086	2.3783E-01	4.4393E-01	ENSG00000134107
BHLHE40-AS1	0.0717	3.6969E-01	5.7894E-01	ENSG00000235831
BHLHE41	-0.0736	3.9408E-01	6.0049E-01	ENSG00000123095
BHMT2	0.0624	1.0764E-01		ENSG00000132840
BICC1	-0.0233	7.7548E-01	8.8055E-01	ENSG00000122870
BICD1	-0.2439	1.5840E-03	1.2728E-02	ENSG00000151746
BICD1P1	0.0098	3.6885E-01		ENSG00000224346
BICD2	-0.1135	1.0633E-01	2.6512E-01	ENSG00000185963
BICDL1	0.3269	2.5112E-04	3.1301E-03	ENSG00000135127
BICDL2	0.0826	3.3644E-01	5.4649E-01	ENSG00000162069
BICRA	-0.0129	8.7839E-01	9.3937E-01	ENSG00000063169
BICRAL	0.0569	4.4509E-01	6.4409E-01	ENSG00000112624
BID	-0.3451	3.0049E-05	5.9481E-04	ENSG00000015475
BIK	-0.4312	6.8278E-04	6.8208E-03	ENSG00000100290
BIN1	-0.0830	2.5280E-01	4.6074E-01	ENSG00000136717
BIN2	0.0088	2.8642E-01		ENSG00000110934
BIN3	-0.0034	9.5173E-01	9.7623E-01	ENSG00000147439
BIRC2	-0.0006	9.6966E-01	9.8504E-01	ENSG00000110330
BIRC3	-0.0570	4.5019E-01	6.4945E-01	ENSG00000023445
BIRC5	-0.0659	4.2474E-01	6.2715E-01	ENSG00000089685
BIRC6	0.2223	1.0154E-03	9.1685E-03	ENSG00000115760
BIRC7	0.0463	7.3555E-02		ENSG00000101197
BISPR	-0.0301	6.4516E-01	7.9748E-01	ENSG00000282851
BIVM	0.1489	4.7084E-02	1.5328E-01	ENSG00000134897
BLACAT1	0.1121	2.2175E-01	4.2500E-01	ENSG00000281406
BLCAP	0.0815	2.3392E-01	4.3905E-01	ENSG00000166619
BLK	0.0944	1.1543E-01	2.7998E-01	ENSG00000136573
BLM	-0.1675	7.3519E-02	2.0773E-01	ENSG00000197299
BLMH	-0.2221	5.8168E-04	5.9734E-03	ENSG00000108578
BLNK	0.0211	6.0553E-01		ENSG00000095585
BLOC1S1	-0.4037	7.7638E-05	1.2626E-03	ENSG00000135441
BLOC1S2	-0.1116	3.6248E-02	1.2712E-01	ENSG00000196072
BLOC1S3	0.0074	9.2853E-01	9.6486E-01	ENSG00000189114
BLOC1S4	0.0022	9.7181E-01	9.8589E-01	ENSG00000186222
BLOC1S5	0.2401	1.8515E-03	1.4222E-02	ENSG00000188428
BLOC1S6	-0.1815	9.6621E-05	1.5038E-03	ENSG00000104164
BLTP1	0.2008	5.1293E-03	3.0685E-02	ENSG00000138688
BLTP2	0.0855	2.0214E-01	4.0092E-01	ENSG00000007202
BLTP3A	-0.1339	4.7209E-02	1.5355E-01	ENSG00000065060
BLTP3B	-0.0845	2.0409E-01	4.0315E-01	ENSG00000111647
BLVRA	-0.2350	1.6217E-03	1.2922E-02	ENSG00000106605
BLVRB	-0.0810	3.2468E-01	5.3476E-01	ENSG00000090013
BLVRBP1	0.0560	2.5214E-01		ENSG00000273682
BLZF1	0.3664	4.4349E-04	4.8446E-03	ENSG00000117475
BMAL1	-0.1206	1.2197E-01	2.9071E-01	ENSG00000133794
BMAL2	-0.0938	2.7846E-01	4.8835E-01	ENSG00000029153
BMERB1	-0.2217	1.1104E-03	9.8031E-03	ENSG00000166780

BMF	-0.0054	9.4993E-01	9.7516E-01	ENSG00000104081
BMI1	-0.1899	1.9917E-02	8.2754E-02	ENSG00000168283
BMP1	0.2213	3.9068E-02	1.3457E-01	ENSG00000168487
BMP2	0.0395	6.4096E-01	7.9458E-01	ENSG00000125845
BMP2K	0.3353	4.6711E-03	2.8598E-02	ENSG00000138756
BMP2KL	0.0244	6.6695E-01		ENSG00000204113
BMP4	0.0411	5.9330E-01	7.6055E-01	ENSG00000125378
BMP5	0.0762	1.7422E-01	3.6452E-01	ENSG00000112175
BMP6	0.1113	1.9705E-01	3.9468E-01	ENSG00000153162
BMP7	-0.3265	2.8790E-04	3.4772E-03	ENSG00000101144
BMP8A	-0.0001	9.9603E-01	9.9774E-01	ENSG00000183682
BMP8B	-0.0982	2.7851E-01	4.8835E-01	ENSG00000116985
BMPER	0.4741	4.9071E-03	2.9644E-02	ENSG00000164619
BMPR1A	0.0213	7.7871E-01	8.8226E-01	ENSG00000107779
BMPR1AP1	0.0285	2.9049E-01		ENSG00000219642
BMPR1B	0.2046	5.2789E-02	1.6550E-01	ENSG00000138696
BMPR1B-DT	-0.0367	3.7406E-01		ENSG00000249599
BMPR2	-0.1098	1.0642E-01	2.6523E-01	ENSG00000204217
BMS1	0.1645	7.5828E-03	4.1169E-02	ENSG00000165733
BMS1P10	-0.2612	1.8402E-03	1.4154E-02	ENSG00000237238
BMS1P11	-0.2944	5.6938E-03	3.3208E-02	ENSG00000225883
BMS1P13	-0.1580	9.5528E-02	2.4778E-01	ENSG00000231701
BMS1P15	0.0063	9.3579E-01	9.6833E-01	ENSG00000258780
BMS1P16	0.0375	6.0547E-01	7.6942E-01	ENSG00000258684
BMS1P4	-0.0068	8.1836E-01		ENSG00000271816
BMS1P4-AGAP5	-0.0112	7.2338E-01		ENSG00000242288
BMS1P9	-0.2851	1.1506E-02	5.6217E-02	ENSG00000198312
BMT2	-0.1638	2.3479E-02	9.3372E-02	ENSG00000164603
BMX	0.0049	9.5994E-01		ENSG00000102010
BNC1	0.0416	1.6994E-01		ENSG00000169594
BNC2	0.1081	2.2833E-01	4.3305E-01	ENSG00000173068
BNC2-AS1	0.0009	9.4762E-01		ENSG00000234779
BNIP1	-0.0827	1.4073E-01	3.1746E-01	ENSG00000113734
BNIP2	-0.0764	1.7474E-01	3.6515E-01	ENSG00000140299
BNIP3	0.0503	5.3836E-01	7.2078E-01	ENSG00000176171
BNIP3L	0.0089	8.6600E-01	9.3231E-01	ENSG00000104765
BNIP3P1	0.0520	5.0303E-01	6.9351E-01	ENSG00000197358
BNIP3P10	-0.0016	6.6924E-01		ENSG00000270270
BNIP3P11	0.0120	6.3884E-01		ENSG00000271550
BNIP3P16	-0.0065	6.2546E-01		ENSG00000267641
BNIP3P17	-0.0267	2.6329E-01		ENSG00000271524
BNIP3P22	0.0425	2.2612E-01		ENSG00000268174
BNIP3P24	0.0135	4.1667E-01		ENSG00000269040
BNIP3P25	-0.0985	6.3717E-02	1.8823E-01	ENSG00000268322
BNIP3P28	-0.1684	1.4561E-02	6.6579E-02	ENSG00000271095
BNIP3P30	-0.0048	9.2250E-01	9.6166E-01	ENSG00000268079
BNIP3P36	-0.0082	5.9724E-01		ENSG00000271661
BNIP3P37	-0.0169	7.8091E-01		ENSG00000270479
BNIP3P38	0.0070	6.5708E-01		ENSG00000268469

BNIP3P40	-0.0005	6.6535E-01		ENSG00000268058
BNIP3P42	0.0370	4.0243E-01		ENSG00000223974
BNIP3P47	-0.0245	5.1757E-01		ENSG00000273791
BNIP5	0.0194	5.0175E-01		ENSG00000189325
BNIPL	-0.1939	4.9807E-02	1.5922E-01	ENSG00000163141
BOC	-0.0722	3.8525E-01	5.9312E-01	ENSG00000144857
BOD1	-0.0989	1.7541E-01	3.6620E-01	ENSG00000145919
BOD1L1	0.3209	9.9210E-10	1.1046E-07	ENSG00000038219
BOD1L2	0.0029	7.2119E-01		ENSG00000228075
BOK	-0.4351	1.9627E-04	2.5551E-03	ENSG00000176720
BOLA1	-0.1589	2.0401E-02	8.4083E-02	ENSG00000178096
BOLA2	0.0862	1.8264E-01	3.7574E-01	ENSG00000169627
BOLA2-SMG1P6	-0.0618	4.6098E-01	6.5837E-01	ENSG00000261740
BOLA2P2	-0.0020	8.3166E-01		ENSG00000271161
BOLA2P3	-0.0599	4.4004E-01	6.4006E-01	ENSG00000220771
BOLA3	0.2515	7.8342E-04	7.5390E-03	ENSG00000163170
BOLA3-DT	-0.0261	6.6074E-01	8.0883E-01	ENSG00000225439
BOLL	-0.0584	7.5480E-02		ENSG00000152430
BOP1	-0.1528	2.0177E-02	8.3563E-02	ENSG00000261236
BORA	0.0533	5.3083E-01	7.1508E-01	ENSG00000136122
BORCS5	-0.0693	2.8854E-01	4.9906E-01	ENSG00000165714
BORCS6	-0.0881	3.1138E-01	5.2141E-01	ENSG00000196544
BORCS7	-0.3249	4.4667E-10	5.5788E-08	ENSG00000166275
BORCS8	0.1011	1.5659E-01	3.4030E-01	ENSG00000254901
BORCS8-MEF2B	-0.0143	5.6040E-01		ENSG00000213999
BPGM	-0.0475	4.3111E-01	6.3288E-01	ENSG00000172331
BPHL	-0.2024	3.3862E-04	3.9431E-03	ENSG00000137274
BPIFB1	-0.0002	7.1795E-01		ENSG00000125999
BPIFB9P	-0.0251	6.3361E-01	7.8999E-01	ENSG00000125997
BPNT1	-0.0244	7.3118E-01	8.5293E-01	ENSG00000162813
BPNT2	-0.0157	7.6690E-01	8.7499E-01	ENSG00000104331
BPTF	0.2546	9.3933E-04	8.6246E-03	ENSG00000171634
BRAF	0.0443	5.1650E-01	7.0415E-01	ENSG00000157764
BRAFP1	-0.0528	1.9877E-01	3.9701E-01	ENSG00000224775
BRAP	-0.1521	1.7138E-02	7.4458E-02	ENSG00000089234
BRAT1	0.0223	7.3914E-01	8.5776E-01	ENSG00000106009
BRCA1	-0.0429	6.0070E-01	7.6611E-01	ENSG00000012048
BRCA2	-0.0637	4.4751E-01	6.4695E-01	ENSG00000139618
BRCC3	-0.0713	2.2892E-01	4.3343E-01	ENSG00000185515
BRCC3P1	0.0256	5.1456E-01		ENSG00000251667
BRD1	0.2856	5.4834E-05	9.5552E-04	ENSG00000100425
BRD2	-0.2731	2.4671E-06	7.9246E-05	ENSG00000204256
BRD3	-0.1466	2.7731E-03	1.9224E-02	ENSG00000169925
BRD3OS	0.0101	8.9389E-01	9.4737E-01	ENSG00000235106
BRD4	0.0293	6.7305E-01	8.1708E-01	ENSG00000141867
BRD7	-0.1412	1.7959E-02	7.6955E-02	ENSG00000166164
BRD7P2	-0.0061	9.2859E-01	9.6486E-01	ENSG00000184100
BRD7P5	0.0007	9.9961E-01		ENSG00000235768
BRD8	0.0145	8.3745E-01	9.1605E-01	ENSG00000112983

BRD9	0.1623	2.9703E-03	2.0352E-02	ENSG00000028310
BRDT	-0.0046	9.4673E-01		ENSG00000137948
BRF1	0.0228	7.4128E-01	8.5899E-01	ENSG00000185024
BRF2	-0.0157	8.4420E-01	9.2003E-01	ENSG00000104221
BRI3	-0.0466	5.4341E-01	7.2458E-01	ENSG00000164713
BRI3BP	0.1257	1.0596E-01	2.6468E-01	ENSG00000184992
BRICD5	0.2507	3.9131E-02	1.3470E-01	ENSG00000182685
BRINP1	0.0052	9.5840E-01	9.7936E-01	ENSG00000078725
BRINP2	-0.0258	7.2909E-01	8.5153E-01	ENSG00000198797
BRINP3	-0.2012	2.5972E-02	1.0026E-01	ENSG00000162670
BRIP1	-0.0662	4.3627E-01	6.3710E-01	ENSG00000136492
BRIX1	-0.2127	1.3668E-04	1.9372E-03	ENSG00000113460
BRK1	-0.0888	1.3289E-01	3.0662E-01	ENSG00000254999
BRME1	0.2257	1.5506E-02	6.9661E-02	ENSG00000132016
BRMS1	-0.1559	2.1298E-02	8.6848E-02	ENSG00000174744
BRMS1L	-0.0858	2.5406E-01	4.6204E-01	ENSG00000100916
BROX	-0.0937	1.7612E-01	3.6720E-01	ENSG00000162819
BRPF1	-0.2871	3.6925E-04	4.2023E-03	ENSG00000156983
BRPF3	-0.0407	5.7390E-01	7.4682E-01	ENSG00000096070
BRS3	-0.0682	4.1418E-01	6.1785E-01	ENSG00000102239
BRSK1	-0.2441	3.3326E-02	1.1981E-01	ENSG00000160469
BRSK2	-0.1336	8.3870E-02	2.2635E-01	ENSG00000174672
BRWD1	-0.0032	9.5776E-01	9.7921E-01	ENSG00000185658
BRWD1-AS1	0.0242	5.2001E-01		ENSG00000238141
BRWD1-AS2	0.5881	2.3263E-05	4.8344E-04	ENSG00000255568
BRWD3	0.1529	7.1163E-02	2.0318E-01	ENSG00000165288
BSCL2	-0.1168	1.3470E-01	3.0916E-01	ENSG00000168000
BSDC1	0.0112	7.9309E-01	8.9076E-01	ENSG00000160058
BSG	-0.1537	2.0561E-03	1.5368E-02	ENSG00000172270
BSG-AS1	0.2393	4.0559E-02	1.3812E-01	ENSG00000267751
BSN	0.2666	1.4358E-03	1.1883E-02	ENSG00000164061
BSN-DT	-0.0973	2.7318E-01	4.8280E-01	ENSG00000226913
BSND	0.0151	3.1860E-01		ENSG00000162399
BSPRY	-0.0113	8.7448E-01	9.3710E-01	ENSG00000119411
BST2	-0.1746	8.6941E-02	2.3184E-01	ENSG00000130303
BSX	-0.0056	8.6998E-01		ENSG00000188909
BTAF1	0.3259	1.1122E-05	2.6699E-04	ENSG00000095564
BTBD1	-0.1387	7.9762E-02	2.1918E-01	ENSG00000064726
BTBD10	0.0338	5.5797E-01	7.3555E-01	ENSG00000148925
BTBD10P1	0.0009	8.6155E-01		ENSG00000257675
BTBD16	0.0403	3.5023E-01		ENSG00000138152
BTBD17	-0.0373	6.4352E-01	7.9647E-01	ENSG00000204347
BTBD18	-0.2747	3.3650E-02	1.2050E-01	ENSG00000233436
BTBD19	-0.0599	4.6436E-01	6.6132E-01	ENSG00000222009
BTBD2	-0.2784	2.8926E-05	5.7769E-04	ENSG00000133243
BTBD3	0.1402	9.2316E-02	2.4203E-01	ENSG00000132640
BTBD6	-0.2032	1.6596E-03	1.3137E-02	ENSG00000184887
BTBD7	0.0811	2.2000E-01	4.2266E-01	ENSG00000011114
BTBD8	-0.0185	7.5097E-01	8.6466E-01	ENSG00000189195

BTBD9	-0.0442	5.7127E-01	7.4485E-01	ENSG00000183826
BTBD9-AS1	0.0349	5.7518E-01	7.4788E-01	ENSG00000226533
BTC	0.0338	5.9853E-01	7.6455E-01	ENSG00000174808
BTD	0.0583	3.3816E-01	5.4806E-01	ENSG00000169814
BTF3	-0.3647	8.0725E-07	3.1428E-05	ENSG00000145741
BTF3L4	-0.1686	4.9547E-04	5.2798E-03	ENSG00000134717
BTF3P6	-0.0093	4.9758E-01		ENSG00000233956
BTG1	-0.1882	3.1591E-02	1.1536E-01	ENSG00000133639
BTG2	-0.0070	9.2848E-01	9.6486E-01	ENSG00000159388
BTG2-DT	0.1377	1.5699E-01	3.4064E-01	ENSG00000233791
BTG3	-0.2904	5.1356E-06	1.4359E-04	ENSG00000154640
BTG3-AS1	0.0540	5.2864E-01	7.1322E-01	ENSG00000280594
BTG4	0.0355	3.7436E-01		ENSG00000137707
BTK	0.4415	1.2547E-02	5.9782E-02	ENSG00000010671
BTLA	0.0255	2.4557E-01		ENSG00000186265
BTN1A1	0.0100	4.8524E-01		ENSG00000124557
BTN1A1P1	-0.0031	9.3537E-01		ENSG00000284688
BTN2A1	0.0915	1.7689E-01	3.6813E-01	ENSG00000112763
BTN2A2	-0.1140	1.6316E-01	3.4957E-01	ENSG00000124508
BTN3A1	-0.0664	3.8748E-01	5.9508E-01	ENSG00000026950
BTN3A2	-0.2106	5.0499E-02	1.6063E-01	ENSG00000186470
BTN3A3	0.1699	9.8709E-02	2.5262E-01	ENSG00000111801
BTNL2	-0.0183	3.9088E-01	5.9796E-01	ENSG00000204290
BTNL9	-0.0512	5.0876E-01	6.9782E-01	ENSG00000165810
BTRC	0.2678	2.4076E-04	3.0224E-03	ENSG00000166167
BUB1	-0.1030	2.3435E-01	4.3946E-01	ENSG00000169679
BUB1B	0.0213	7.8851E-01	8.8805E-01	ENSG00000156970
BUB3	-0.1917	1.1790E-03	1.0269E-02	ENSG00000154473
BUD13	0.0200	7.6636E-01	8.7469E-01	ENSG00000137656
BUD23	-0.0209	6.7190E-01	8.1641E-01	ENSG00000071462
BUD31	-0.2226	2.6131E-04	3.2293E-03	ENSG00000106245
BVES	0.1027	2.6047E-01	4.6896E-01	ENSG00000112276
BVES-AS1	0.0090	9.0721E-01	9.5454E-01	ENSG00000203808
BYSL	0.1951	1.7226E-02	7.4704E-02	ENSG00000112578
BZW1	-0.0966	4.6560E-02	1.5208E-01	ENSG00000082153
BZW2	-0.2658	5.9109E-05	1.0196E-03	ENSG00000136261
C10orf105	0.0229	2.7584E-01		ENSG00000214688
C10orf126	0.0139	7.1346E-01	8.4284E-01	ENSG00000232624
C10orf143	0.0896	3.0295E-01	5.1304E-01	ENSG00000237489
C10orf53	0.0297	7.3341E-01	8.5431E-01	ENSG00000178645
C10orf55	0.0671	1.8702E-01		ENSG00000222047
C10orf67	0.1093	1.3523E-01	3.0990E-01	ENSG00000179133
C10orf67-AS1	0.0083	9.3089E-01		ENSG00000224215
C10orf88	0.4679	4.4953E-06	1.2921E-04	ENSG00000119965
C10orf88B	0.0709	2.9576E-01	5.0578E-01	ENSG00000255624
C10orf90	0.0054	8.4946E-01	9.2331E-01	ENSG00000154493
C10orf95	0.0142	6.0299E-01		ENSG00000120055
C10orf95-AS1	0.1906	1.1221E-02	5.5175E-02	ENSG00000269609
C11orf16	0.0066	8.5177E-01		ENSG00000176029

C11orf21	-0.0029	7.4860E-01		ENSG00000110665
C11orf24	0.2855	6.2660E-06	1.6758E-04	ENSG00000171067
C11orf40	0.0039	6.1980E-01		ENSG00000171987
C11orf42	0.0016	8.8728E-01		ENSG00000180878
C11orf54	0.0376	5.9985E-01	7.6571E-01	ENSG00000182919
C11orf58	0.0195	7.1937E-01	8.4572E-01	ENSG00000110696
C11orf65	0.2152	3.4121E-02	1.2153E-01	ENSG00000166323
C11orf68	0.2310	2.1429E-03	1.5829E-02	ENSG00000175573
C11orf71	0.1327	4.3659E-02	1.4526E-01	ENSG00000180425
C11orf86	0.0298	2.6091E-01		ENSG00000173237
C11orf87	0.1152	1.6845E-01	3.5683E-01	ENSG00000185742
C11orf91	0.0240	6.3933E-01		ENSG00000205177
C11orf98	-0.0667	1.4279E-01	3.2041E-01	ENSG00000278615
C11orf98P1	0.0185	3.5749E-01		ENSG00000232158
C11orf98P3	-0.0010	9.7833E-01		ENSG00000228171
C12orf4	-0.0244	7.0817E-01	8.3965E-01	ENSG00000047621
C12orf40	0.0082	9.2985E-01		ENSG00000180116
C12orf42	-0.0427	3.1447E-01	5.2456E-01	ENSG00000179088
C12orf43	-0.2142	1.0997E-03	9.7355E-03	ENSG00000157895
C12orf50	0.0014	9.4426E-01		ENSG00000165805
C12orf54	1.9195	1.3312E-03		ENSG00000177627
C12orf56	0.0440	2.4309E-01		ENSG00000185306
C12orf57	-0.0370	5.9329E-01	7.6055E-01	ENSG00000111678
C12orf60	0.3680	5.3612E-04	5.6110E-03	ENSG00000182993
C12orf71	0.0069	4.5367E-01		ENSG00000214700
C12orf75	-0.2674	1.3969E-03	1.1639E-02	ENSG00000235162
C12orf76	0.2638	1.5687E-04	2.1479E-03	ENSG00000174456
C13orf42	0.0131	6.4669E-01		ENSG00000226792
C13orf46	-0.1211	1.5820E-01	3.4236E-01	ENSG00000283199
C14orf119	0.1183	4.8337E-02	1.5589E-01	ENSG00000179933
C14orf132	-0.1350	8.0905E-02	2.2113E-01	ENSG00000227051
C14orf178	0.0338	2.2856E-01		ENSG00000197734
C14orf180	-0.0080	5.2886E-01		ENSG00000184601
C14orf28	0.2239	2.9363E-02	1.0945E-01	ENSG00000179476
C14orf39	0.1427	1.3969E-01	3.1606E-01	ENSG00000179008
C14orf93	-0.2126	1.4304E-02	6.5713E-02	ENSG00000100802
C15orf32	-0.0102	6.0338E-01		ENSG00000183643
C15orf39	-0.0418	6.1965E-01	7.7991E-01	ENSG00000167173
C15orf40	0.1352	3.7217E-02	1.2977E-01	ENSG00000169609
C15orf48	-0.0661	2.7694E-01	4.8690E-01	ENSG00000166920
C15orf61	-0.1043	1.6884E-01	3.5726E-01	ENSG00000189227
C15orf62	0.4112	1.5157E-02	6.8455E-02	ENSG00000188277
C16orf46	0.0349	5.8971E-01	7.5820E-01	ENSG00000166455
C16orf74	-0.4498	1.5413E-07	8.1379E-06	ENSG00000154102
C16orf82	0.0560	1.4335E-01	3.2131E-01	ENSG00000234186
C16orf86	0.2403	3.9765E-02	1.3631E-01	ENSG00000159761
C16orf87	-0.3817	1.5028E-08	1.1776E-06	ENSG00000155330
C16orf89	-0.0270	5.7952E-01	7.5104E-01	ENSG00000153446
C16orf90	0.0190	7.1853E-01		ENSG00000215131

C16orf92	0.0191	7.7730E-01	8.8144E-01	ENSG00000167194
C16orf95	-0.0218	8.0718E-01	8.9868E-01	ENSG00000260456
C16orf95-DT	-0.0037	9.6212E-01	9.8110E-01	ENSG00000270006
C16orf96	-0.0300	6.0911E-01	7.7208E-01	ENSG00000205832
C17orf100	-0.0445	5.7198E-01	7.4542E-01	ENSG00000256806
C17orf107	0.0531	5.2331E-02		ENSG00000205710
C17orf113	0.0851	3.3641E-01	5.4649E-01	ENSG00000267221
C17orf114	0.1078	2.2007E-01	4.2270E-01	ENSG00000262165
C17orf49	-0.0041	9.5699E-01	9.7875E-01	ENSG00000258315
C17orf50	0.0515	5.4023E-01	7.2221E-01	ENSG00000270806
C17orf58	-0.2198	2.1286E-03	1.5761E-02	ENSG00000186665
C17orf67	0.0178	8.2922E-01	9.1166E-01	ENSG00000214226
C17orf75	0.0569	3.7459E-01	5.8323E-01	ENSG00000108666
C17orf78	0.0353	1.1740E-01		ENSG00000278505
C17orf80	0.4354	2.8729E-04	3.4736E-03	ENSG00000141219
C17orf99	0.0109	9.6144E-01		ENSG00000187997
C18orf15	0.0807	1.3848E-01		ENSG00000279020
C18orf21	0.2168	3.3471E-03	2.2216E-02	ENSG00000141428
C18orf32	-0.1063	1.7162E-01	3.6079E-01	ENSG00000177576
C18orf54	0.0541	5.2370E-01	7.0933E-01	ENSG00000166845
C19orf12	-0.1097	7.1276E-02	2.0333E-01	ENSG00000131943
C19orf18	0.0947	2.7610E-01	4.8589E-01	ENSG00000177025
C19orf25	0.1199	7.8228E-02	2.1637E-01	ENSG00000119559
C19orf38	0.3329	1.7760E-02	7.6318E-02	ENSG00000214212
C19orf44	0.0233	7.7868E-01	8.8226E-01	ENSG00000105072
C19orf47	0.1074	1.5924E-01	3.4381E-01	ENSG00000160392
C19orf48P	0.1975	3.3815E-03	2.2378E-02	ENSG00000167747
C19orf53	-0.1849	3.3300E-03	2.2126E-02	ENSG00000104979
C19orf67	0.0484	2.1953E-01		ENSG00000188032
C19orf73	-0.0751	3.5502E-01	5.6473E-01	ENSG00000221916
C19orf81	0.2363	5.8547E-03	3.3919E-02	ENSG00000235034
C19orf84	-0.0126	6.6065E-01	8.0883E-01	ENSG00000262874
C19orf85	-0.0173	7.7062E-01	8.7734E-01	ENSG00000283567
C1D	-0.1442	1.3817E-02	6.4157E-02	ENSG00000197223
C1GALT1	-0.1281	4.9346E-02	1.5807E-01	ENSG00000106392
C1GALT1C1	-0.0917	1.3271E-01	3.0630E-01	ENSG00000171155
C1GALT1C1L	0.0052	9.4857E-01	9.7460E-01	ENSG00000223658
C1orf105	0.0208	6.5662E-01		ENSG00000180999
C1orf115	-0.3937	4.4680E-05	8.1313E-04	ENSG00000162817
C1orf116	-0.0516	2.8296E-01	4.9336E-01	ENSG00000182795
C1orf122	-0.1091	1.3406E-01	3.0813E-01	ENSG00000197982
C1orf127	0.0517	1.5268E-01		ENSG00000175262
C1orf131	0.1133	1.2785E-01	2.9962E-01	ENSG00000143633
C1orf141	0.0086	5.0765E-01		ENSG00000203963
C1orf146	0.0546	1.6184E-01		ENSG00000203910
C1orf159	0.1617	1.6917E-02	7.3789E-02	ENSG00000131591
C1orf162	-0.0391	6.2262E-01	7.8193E-01	ENSG00000143110
C1orf167	-0.0511	1.8824E-01		ENSG00000215910
C1orf174	-0.2720	6.3587E-04	6.4465E-03	ENSG00000198912

C1orf185	0.0026	7.3402E-01		ENSG00000204006
C1orf198	-0.0236	7.5224E-01	8.6560E-01	ENSG00000119280
C1orf202	0.0072	7.3424E-01		ENSG00000284188
C1orf21	-0.1827	2.6955E-02	1.0291E-01	ENSG00000116667
C1orf21-DT	0.1636	1.0730E-01	2.6654E-01	ENSG00000271387
C1orf210	0.0468	1.6545E-01		ENSG00000253313
C1orf216	-0.3520	3.1860E-04	3.7670E-03	ENSG00000142686
C1orf220	0.5471	1.4460E-03	1.1947E-02	ENSG00000213057
C1orf226	-0.0929	2.9116E-01	5.0148E-01	ENSG00000239887
C1orf232	0.5810	5.6009E-03	3.2773E-02	ENSG00000282872
C1orf35	0.3595	8.5878E-07	3.2922E-05	ENSG00000143793
C1orf43	-0.0433	4.6016E-01	6.5789E-01	ENSG00000143612
C1orf50	-0.2649	3.5246E-03	2.3175E-02	ENSG00000164008
C1orf52	0.1846	1.7304E-03	1.3531E-02	ENSG00000162642
C1orf53	0.0567	4.3228E-01	6.3368E-01	ENSG00000203724
C1orf54	-0.1057	2.2300E-01	4.2660E-01	ENSG00000118292
C1orf56	-0.2268	1.3026E-02	6.1389E-02	ENSG00000143443
C1orf74	0.0195	6.9205E-01		ENSG00000162757
C1orf87	-0.0247	4.8854E-01		ENSG00000162598
C1QBP	-0.2671	6.3659E-07	2.5762E-05	ENSG00000108561
C1QL1	-0.3736	9.3579E-03	4.8301E-02	ENSG00000131094
C1QL2	-0.0603	3.7358E-01	5.8210E-01	ENSG00000144119
C1QL3	0.1162	1.7159E-01	3.6079E-01	ENSG00000165985
C1QL4	-0.2972	1.2950E-02	6.1134E-02	ENSG00000186897
C1QTNF1	-0.1250	1.8489E-01	3.7842E-01	ENSG00000173918
C1QTNF12	0.5295	7.9855E-03	4.2807E-02	ENSG00000184163
C1QTNF2	-0.3292	9.2598E-03	4.7935E-02	ENSG00000145861
C1QTNF3	0.1023	2.3201E-01	4.3693E-01	ENSG00000082196
C1QTNF3-AMACR	-0.0163	6.8279E-01		ENSG00000273294
C1QTNF4	-0.1004	2.4376E-01	4.5150E-01	ENSG00000172247
C1QTNF5	0.0238	4.5653E-01		ENSG00000223953
C1QTNF6	0.3955	1.4028E-04	1.9752E-03	ENSG00000133466
C1QTNF7	0.0305	5.2841E-01	7.1307E-01	ENSG00000163145
C1QTNF7-AS1	0.0348	6.4105E-01	7.9464E-01	ENSG00000249252
C1QTNF8	-0.0084	7.4900E-01		ENSG00000184471
C1QTNF9	0.1913	1.9316E-02	8.0957E-02	ENSG00000240654
C1R	0.3235	8.1419E-05	1.3111E-03	ENSG00000159403
C1RL	-0.0216	7.9528E-01	8.9183E-01	ENSG00000139178
C1RL-AS1	0.0455	5.8039E-01	7.5184E-01	ENSG00000205885
C1S	0.4461	1.6404E-03	1.3033E-02	ENSG00000182326
C2	0.1064	2.0094E-01	3.9943E-01	ENSG00000166278
C2-AS1	0.0559	1.3092E-01		ENSG00000281756
C2-AS1	0.0770	1.8648E-01	3.8082E-01	ENSG00000229776
C20orf144	-0.0332	6.4324E-01	7.9622E-01	ENSG00000149609
C20orf173	-0.0031	6.2198E-01		ENSG00000125975
C20orf202	0.0899	1.6152E-01	3.4742E-01	ENSG00000215595
C20orf203	0.0514	3.3045E-01	5.4072E-01	ENSG00000198547
C20orf204	0.0460	5.4131E-01	7.2323E-01	ENSG00000196421
C20orf96	0.0504	4.8642E-01	6.8010E-01	ENSG00000196476

C21orf140	-0.0333	4.9782E-01	6.8911E-01	ENSG00000222018
C21orf58	0.0982	2.6832E-01	4.7778E-01	ENSG00000160298
C21orf91	0.2214	1.3017E-02	6.1358E-02	ENSG00000154642
C22orf15	0.0695	1.2432E-01		ENSG00000169314
C22orf23	0.2958	1.8697E-02	7.9124E-02	ENSG00000128346
C22orf31	-0.0034	9.5900E-01	9.7954E-01	ENSG00000100249
C22orf39	0.1378	8.1487E-02	2.2208E-01	ENSG00000242259
C22orf46P	0.1363	1.0319E-01	2.6007E-01	ENSG00000184208
C2CD2	-0.1778	8.2472E-02	2.2373E-01	ENSG00000157617
C2CD2L	-0.0331	6.2031E-01	7.8055E-01	ENSG00000172375
C2CD3	0.0263	6.8511E-01	8.2451E-01	ENSG00000168014
C2CD4A	0.1053	1.8526E-01	3.7894E-01	ENSG00000198535
C2CD4B	0.0416	5.7705E-01	7.4906E-01	ENSG00000205502
C2CD4C	-0.1568	8.3460E-02	2.2563E-01	ENSG00000183186
C2CD4D	-0.2126	5.6080E-02	1.7302E-01	ENSG00000225556
C2CD5	0.1994	1.0432E-02	5.2266E-02	ENSG00000111731
C2CD6	0.1924	4.7060E-02	1.5328E-01	ENSG00000155754
C2orf15	0.5741	1.7641E-04	2.3539E-03	ENSG00000273045
C2orf27A	0.0631	4.2332E-01	6.2590E-01	ENSG00000287151
C2orf42	0.0420	5.8681E-01	7.5618E-01	ENSG00000115998
C2orf49	0.0605	3.8330E-01	5.9154E-01	ENSG00000135974
C2orf50	0.0837	3.3007E-01	5.4037E-01	ENSG00000150873
C2orf66	0.1205	1.9505E-01	3.9190E-01	ENSG00000187944
C2orf68	0.1439	6.6501E-02	1.9403E-01	ENSG00000168887
C2orf69	-0.0609	2.8608E-01	4.9679E-01	ENSG00000178074
C2orf72	-0.2672	1.3894E-02	6.4428E-02	ENSG00000204128
C2orf73	0.0622	3.9151E-01	5.9859E-01	ENSG00000177994
C2orf74	-0.0539	4.3019E-01	6.3186E-01	ENSG00000237651
C2orf74-AS1	0.0031	9.2549E-01		ENSG00000271889
C2orf74-DT	0.0851	3.1982E-01	5.2998E-01	ENSG00000212978
C2orf76	-0.1895	1.1086E-02	5.4621E-02	ENSG00000186132
C2orf80	-0.0960	1.1109E-01	2.7290E-01	ENSG00000188674
C2orf81	0.0343	4.4209E-01	6.4149E-01	ENSG00000284308
C2orf88	-0.0116	8.8467E-01	9.4230E-01	ENSG00000187699
C2orf92	0.0621	4.5960E-01	6.5736E-01	ENSG00000228486
C3	0.0235	6.2985E-01		ENSG00000125730
C3AR1	0.0023	9.6370E-01		ENSG00000171860
C3orf18	-0.0147	8.4325E-01	9.1965E-01	ENSG00000088543
C3orf20	-0.0054	8.2181E-01	9.0773E-01	ENSG00000131379
C3orf33	-0.2593	2.3098E-03	1.6783E-02	ENSG00000174928
C3orf38	-0.0287	6.3333E-01	7.8970E-01	ENSG00000179021
C3orf49	0.0437	4.9140E-01	6.8372E-01	ENSG00000163632
C3orf52	0.2299	5.1714E-02	1.6309E-01	ENSG00000114529
C3orf62	0.2698	4.3058E-03	2.6931E-02	ENSG00000188315
C3orf70	-0.0759	3.5906E-01	5.6858E-01	ENSG00000187068
C3orf80	0.4289	9.7918E-03	4.9943E-02	ENSG00000180044
C3orf84	-0.0138	4.9498E-01		ENSG00000236980
C3orf86P	0.0697	8.1437E-02		ENSG00000225873
C4orf17	0.0312	1.1929E-01		ENSG00000138813

C4orf19	0.3503	6.6865E-03	3.7501E-02	ENSG00000154274
C4orf3	0.0868	8.2834E-02	2.2449E-01	ENSG00000164096
C4orf33	-0.0417	5.0945E-01	6.9831E-01	ENSG00000151470
C4orf36	0.0313	6.8261E-01		ENSG00000285458
C4orf36	-0.0221	6.2732E-01		ENSG00000163633
C4orf46	-0.0702	2.3250E-01	4.3750E-01	ENSG00000205208
C4orf50	0.0307	6.6449E-01	8.1125E-01	ENSG00000181215
C4orf51	0.0250	2.1481E-01		ENSG00000237136
C4orf54	0.0226	5.1106E-01		ENSG00000248713
C5	0.1292	1.6955E-01	3.5798E-01	ENSG00000106804
C5AR1	0.1125	1.6251E-01	3.4861E-01	ENSG00000197405
C5AR2	-0.0214	7.1326E-01		ENSG00000134830
C5orf15	0.1800	1.1120E-03	9.8069E-03	ENSG00000113583
C5orf22	0.1310	3.7493E-02	1.3054E-01	ENSG00000082213
C5orf24	-0.2046	1.4618E-05	3.3089E-04	ENSG00000181904
C5orf34	0.1428	1.0616E-01	2.6496E-01	ENSG00000172244
C5orf46	0.0208	2.0081E-01		ENSG00000178776
C5orf47	0.1903	5.3328E-02	1.6668E-01	ENSG00000185056
C5orf52	0.0026	9.3839E-01		ENSG00000187658
C5orf58	0.0071	4.1637E-01		ENSG00000234511
C5orf60	-0.0140	5.6556E-01		ENSG00000204661
C5orf63	0.0435	5.6376E-01	7.3998E-01	ENSG00000164241
C5orf64	0.1258	1.3980E-01	3.1622E-01	ENSG00000178722
C6orf118	0.1668	7.5284E-02	2.1097E-01	ENSG00000112539
C6orf120	0.0660	2.7474E-01	4.8458E-01	ENSG00000185127
C6orf132	-0.0784	2.9391E-01	5.0393E-01	ENSG00000188112
C6orf136	0.0083	8.9126E-01	9.4593E-01	ENSG00000204564
C6orf141	0.0474	5.0727E-01	6.9631E-01	ENSG00000197261
C6orf163	-0.0018	9.8338E-01	9.9111E-01	ENSG00000203872
C6orf226	0.0748	2.7344E-01	4.8317E-01	ENSG00000221821
C6orf47	-0.1171	1.1785E-01	2.8369E-01	ENSG00000204439
C6orf58	0.0303	7.1827E-01	8.4512E-01	ENSG00000184530
C6orf62	0.0133	8.6949E-01	9.3437E-01	ENSG00000112308
C6orf89	0.1075	3.9877E-02	1.3648E-01	ENSG00000198663
C7	0.0694	2.7968E-01	4.8972E-01	ENSG00000112936
C7orf25	0.0023	9.3540E-01		ENSG00000136197
C7orf33	0.0234	5.7612E-01		ENSG00000170279
C7orf50	-0.4019	1.0158E-06	3.7699E-05	ENSG00000146540
C7orf57	0.0652	6.2542E-02		ENSG00000164746
C8A	0.0008	9.3590E-01		ENSG00000157131
C8B	0.0076	3.5482E-01		ENSG00000021852
C8G	0.0994	1.6159E-01	3.4750E-01	ENSG00000176919
C8orf17	0.0141	5.4852E-01		ENSG00000250733
C8orf33	0.2032	6.9080E-03	3.8438E-02	ENSG00000182307
C8orf34	0.0132	8.6350E-01	9.3122E-01	ENSG00000165084
C8orf34-AS1	-0.0139	2.4463E-01		ENSG00000248801
C8orf44	0.0760	3.3769E-01	5.4765E-01	ENSG00000288596
C8orf44-SGK3	-0.0045	9.8589E-01		ENSG00000288602
C8orf48	0.1510	1.2737E-01	2.9880E-01	ENSG00000164743

C8orf58	0.0747	2.9685E-01	5.0690E-01	ENSG00000241852
C8orf76	-0.1111	2.1316E-01	4.1423E-01	ENSG00000189376
C8orf82	-0.1862	5.4640E-03	3.2194E-02	ENSG00000213563
C8orf88	-0.5434	5.6917E-05	9.8484E-04	ENSG00000253250
C8orf89	0.0242	5.2696E-01		ENSG00000274443
C8orf90	0.0183	6.3385E-01		ENSG00000226490
C9orf152	0.0140	4.8615E-01		ENSG00000188959
C9orf153	0.0779	2.9218E-01	5.0231E-01	ENSG00000187753
C9orf163	0.0166	5.4181E-01		ENSG00000196366
C9orf40	0.1528	4.4134E-02	1.4631E-01	ENSG00000135045
C9orf43	0.3085	2.5262E-02	9.8225E-02	ENSG00000157653
C9orf50	0.1148	1.8279E-01	3.7591E-01	ENSG00000179058
C9orf57	0.0175	4.7957E-01		ENSG00000204669
C9orf72	-0.1491	2.4240E-02	9.5598E-02	ENSG00000147894
C9orf78	-0.2647	8.5901E-07	3.2922E-05	ENSG00000136819
C9orf78P2	0.0030	8.4787E-01		ENSG00000224569
C9orf85	0.0327	6.6608E-01	8.1258E-01	ENSG00000155621
CA1	0.0135	6.6008E-01		ENSG00000133742
CA10	0.1950	6.4984E-02	1.9078E-01	ENSG00000154975
CA11	0.0100	8.5586E-01	9.2695E-01	ENSG00000063180
CA12	-0.0610	4.3743E-01	6.3800E-01	ENSG00000074410
CA13	-0.1228	1.9313E-01	3.8924E-01	ENSG00000185015
CA14	-0.3435	7.5797E-04	7.3636E-03	ENSG00000118298
CA15P1	0.0230	6.2568E-01		ENSG00000241527
CA2	0.1745	7.3559E-02	2.0782E-01	ENSG00000104267
CA3	0.0768	1.7767E-01	3.6887E-01	ENSG00000164879
CA4	0.2610	3.5676E-02	1.2576E-01	ENSG00000167434
CA5A	-0.0033	8.4836E-01		ENSG00000174990
CA5B	0.0005	9.9599E-01	9.9774E-01	ENSG00000169239
CA7	-0.4063	3.1338E-03	2.1201E-02	ENSG00000168748
CA8	-0.0208	7.9413E-01	8.9120E-01	ENSG00000178538
CA9	-0.0713	1.3405E-01	3.0813E-01	ENSG00000107159
CAAP1	0.0925	1.8433E-01	3.7779E-01	ENSG00000120159
CAB39	-0.0510	4.0900E-01	6.1332E-01	ENSG00000135932
CAB39L	-0.0075	8.8882E-01	9.4476E-01	ENSG00000102547
CAB39P1	0.0093	5.5028E-01		ENSG00000251303
CABIN1	0.2444	4.2064E-03	2.6489E-02	ENSG00000099991
CABLES1	0.0824	3.3425E-01	5.4440E-01	ENSG00000134508
CABLES2	-0.0676	2.2389E-01	4.2774E-01	ENSG00000149679
CABP1	0.1603	8.6563E-02	2.3112E-01	ENSG00000157782
CABP4	0.0837	2.7316E-01	4.8280E-01	ENSG00000175544
CABP5	-0.0199	5.4777E-01		ENSG00000105507
CABP7	0.0969	2.4663E-01	4.5421E-01	ENSG00000100314
CABS1	-0.0304	6.1246E-01		ENSG00000145309
CABYR	-0.0057	9.4292E-01	9.7170E-01	ENSG00000154040
CACFD1	-0.0510	4.7537E-01	6.7094E-01	ENSG00000160325
CACHD1	0.2647	1.9454E-03	1.4764E-02	ENSG00000158966
CACNA1A	0.2386	5.5400E-03	3.2525E-02	ENSG00000141837
CACNA1B	0.2324	1.7418E-02	7.5373E-02	ENSG00000148408

CACNA1C	0.2438	3.3308E-03	2.2126E-02	ENSG00000151067
CACNA1C-AS1	0.0112	9.6208E-01		ENSG00000246627
CACNA1C-AS2	0.1673	1.0215E-01	2.5831E-01	ENSG00000256271
CACNA1C-AS4	-0.0092	5.0818E-01		ENSG00000256025
CACNA1D	0.3842	4.9726E-04	5.2815E-03	ENSG00000157388
CACNA1E	0.2421	6.6284E-03	3.7253E-02	ENSG00000198216
CACNA1F	0.0494	2.1504E-01		ENSG00000102001
CACNA1G	0.0385	6.2099E-01	7.8072E-01	ENSG00000006283
CACNA1G-AS1	-0.3201	2.2979E-02	9.1866E-02	ENSG00000250107
CACNA1H	-0.1062	1.5909E-01	3.4364E-01	ENSG00000196557
CACNA1I	0.1157	2.0896E-01	4.0900E-01	ENSG00000100346
CACNA1S	0.0006	8.1456E-01	9.0338E-01	ENSG00000081248
CACNA2D1	0.1121	2.0016E-01	3.9856E-01	ENSG00000153956
CACNA2D1-AS1	0.0268	5.4453E-01		ENSG00000223770
CACNA2D2	0.1361	1.2978E-01	3.0237E-01	ENSG00000007402
CACNA2D3	-0.1679	6.7888E-02	1.9645E-01	ENSG00000157445
CACNA2D3-AS1	0.0029	8.9909E-01		ENSG00000243715
CACNA2D4	0.0531	2.3671E-01		ENSG00000151062
CACNB1	0.0990	1.3878E-01	3.1465E-01	ENSG00000067191
CACNB3	-0.0054	9.0519E-01	9.5372E-01	ENSG00000167535
CACNB4	0.1665	3.9072E-02	1.3457E-01	ENSG00000182389
CACNG1	0.0308	6.8874E-01	8.2704E-01	ENSG00000108878
CACNG2	0.0701	4.0696E-01	6.1153E-01	ENSG00000166862
CACNG3	-0.1023	2.4584E-01	4.5357E-01	ENSG00000006116
CACNG4	-0.2420	3.1943E-03	2.1496E-02	ENSG00000075461
CACNG5	0.1098	1.6629E-01	3.5400E-01	ENSG00000075429
CACNG6	-0.0145	7.7712E-01	8.8138E-01	ENSG00000130433
CACNG7	-0.0275	7.3732E-01	8.5672E-01	ENSG00000105605
CACNG8	-0.0482	5.3616E-01	7.1921E-01	ENSG00000142408
CACTIN	-0.0841	2.4454E-01	4.5213E-01	ENSG00000105298
CACTIN-AS1	0.0116	4.1165E-01		ENSG00000226800
CACUL1	-0.0363	4.9398E-01	6.8603E-01	ENSG00000151893
CACYBP	-0.3482	1.7120E-07	8.8127E-06	ENSG00000116161
CAD	0.0286	6.8587E-01	8.2498E-01	ENSG00000084774
CADM1	-0.0887	1.5319E-01	3.3540E-01	ENSG00000182985
CADM1-AS1	0.1638	1.0726E-01	2.6649E-01	ENSG00000256315
CADM2	0.2085	1.4445E-02	6.6199E-02	ENSG00000175161
CADM3	0.0359	6.7392E-01	8.1745E-01	ENSG00000162706
CADM4	-0.2086	5.6941E-03	3.3208E-02	ENSG00000105767
CADPS	0.1072	1.7009E-01	3.5870E-01	ENSG00000163618
CADPS2	-0.0107	8.9611E-01	9.4841E-01	ENSG00000081803
CAGE1	0.0144	7.3855E-01	8.5765E-01	ENSG00000164304
CAHM	-0.0414	5.5019E-01	7.2920E-01	ENSG00000270419
CALB1	0.0454	4.0213E-01	6.0669E-01	ENSG00000104327
CALB2	-0.4408	4.4899E-04	4.8883E-03	ENSG00000172137
CALCA	-0.0096	1.5167E-01	3.3330E-01	ENSG00000110680
CALCB	0.3584	3.5416E-03	2.3263E-02	ENSG00000175868
CALCOCO1	0.0143	8.4900E-01	9.2304E-01	ENSG00000012822
CALCOCO2	-0.1470	4.8231E-02	1.5562E-01	ENSG00000136436

CALCR	-0.3154	2.4261E-02	9.5633E-02	ENSG00000004948
CALD1	0.0223	7.5006E-01	8.6429E-01	ENSG00000122786
CALHM2	0.0307	7.1725E-01	8.4462E-01	ENSG00000138172
CALHM3	-0.0026	7.8827E-01		ENSG00000183128
CALHM4	0.0229	2.0350E-01		ENSG00000164451
CALHM5	0.1252	8.5270E-02	2.2852E-01	ENSG00000178033
CALHM6	0.6823	1.0882E-02	5.3898E-02	ENSG00000188820
CALM1	-0.4685	1.2344E-07	6.8562E-06	ENSG00000198668
CALM2	-0.4187	2.6184E-07	1.2682E-05	ENSG00000143933
CALM3	-0.4390	4.9908E-10	6.1708E-08	ENSG00000160014
CALML4	-0.0228	3.2795E-01		ENSG00000129007
CALN1	-0.1234	1.7916E-01	3.7069E-01	ENSG00000183166
CALR	-0.2308	2.3350E-03	1.6918E-02	ENSG00000179218
CALR3	0.0331	9.8418E-02		ENSG00000269058
CALU	-0.0524	4.1296E-01	6.1664E-01	ENSG00000128595
CALY	0.0169	8.2343E-01	9.0841E-01	ENSG00000130643
CAMK1	-0.3425	4.9653E-04	5.2801E-03	ENSG00000134072
CAMK1D	-0.0776	2.9896E-01	5.0876E-01	ENSG00000183049
CAMK1G	0.0632	4.5365E-01	6.5222E-01	ENSG00000008118
CAMK2A	0.0727	3.6841E-01	5.7759E-01	ENSG00000070808
CAMK2B	0.0000	9.9909E-01	9.9944E-01	ENSG00000058404
CAMK2D	0.0406	5.4223E-01	7.2386E-01	ENSG00000145349
CAMK2G	0.0435	5.7289E-01	7.4605E-01	ENSG00000148660
CAMK2N1	-0.1741	2.0177E-02	8.3563E-02	ENSG00000162545
CAMK2N2	0.1319	1.4489E-01	3.2360E-01	ENSG00000163888
CAMK4	-0.0596	4.0165E-01	6.0655E-01	ENSG00000152495
CAMKK1	0.2424	1.7792E-02	7.6415E-02	ENSG00000004660
CAMKK2	-0.3074	1.0654E-04	1.6161E-03	ENSG00000110931
CAMKMT	-0.0681	3.2816E-01	5.3855E-01	ENSG00000143919
CAMKV	-0.3685	4.4405E-04	4.8446E-03	ENSG00000164076
CAMLG	-0.2751	1.9233E-04	2.5238E-03	ENSG00000164615
CAMSAP1	0.1043	1.4399E-01	3.2200E-01	ENSG00000130559
CAMSAP2	0.0857	1.8098E-01	3.7329E-01	ENSG00000118200
CAMSAP3	-0.0943	1.1441E-01	2.7845E-01	ENSG00000076826
CAMTA1	-0.0667	2.7684E-01	4.8676E-01	ENSG00000171735
CAMTA2	0.0333	5.9626E-01	7.6296E-01	ENSG00000108509
CAND1	-0.0168	7.2028E-01	8.4651E-01	ENSG00000111530
CAND2	-0.1616	4.3954E-02	1.4589E-01	ENSG00000144712
CANT1	-0.2086	1.7562E-02	7.5795E-02	ENSG00000171302
CANX	-0.1729	2.1921E-02	8.8726E-02	ENSG00000127022
CAP1	-0.1566	1.1261E-03	9.9041E-03	ENSG00000131236
CAP1P1	0.1183	3.2919E-02		ENSG00000240809
CAP1P2	-0.0220	3.5709E-01		ENSG00000232004
CAP2	-0.2178	4.8467E-03	2.9375E-02	ENSG00000112186
CAP2P1	0.0178	6.4258E-01		ENSG00000259151
CAPN1	0.0541	3.6660E-01	5.7582E-01	ENSG00000014216
CAPN1-AS1	0.0001	9.3151E-01	9.6607E-01	ENSG00000254614
CAPN10	0.0064	9.2840E-01	9.6483E-01	ENSG00000142330
CAPN10-DT	0.1312	1.5660E-01	3.4030E-01	ENSG00000260942

CAPN11	0.0151	4.7567E-01		ENSG00000137225
CAPN12	0.0375	7.8432E-01	8.8610E-01	ENSG00000182472
CAPN13	-0.0433	2.5062E-01		ENSG00000162949
CAPN14	0.1504	1.2323E-01	2.9265E-01	ENSG00000214711
CAPN15	0.0308	6.9730E-01	8.3274E-01	ENSG00000103326
CAPN2	-0.0744	2.2899E-01	4.3343E-01	ENSG00000162909
CAPN3	-0.0067	9.1175E-01	9.5641E-01	ENSG00000092529
CAPN5	-0.2501	1.0007E-03	9.0624E-03	ENSG00000149260
CAPN6	0.1878	7.8275E-02	2.1640E-01	ENSG00000077274
CAPN7	-0.0382	5.5807E-01	7.3563E-01	ENSG00000131375
CAPN8	0.1377	2.1216E-02		ENSG00000203697
CAPN9	0.0271	6.9674E-01	8.3235E-01	ENSG00000135773
CAPNS1	-0.0416	5.8648E-01	7.5596E-01	ENSG00000126247
CAPNS2	0.0136	5.0942E-01		ENSG00000256812
CAPRIN1	-0.1621	1.8349E-03	1.4126E-02	ENSG00000135387
CAPRIN2	0.2079	3.3611E-03	2.2297E-02	ENSG00000110888
CAPS	0.2659	1.6656E-02	7.3000E-02	ENSG00000105519
CAPS2	0.0995	2.5254E-01	4.6058E-01	ENSG00000180881
CAPSL	0.1040	8.4461E-02	2.2718E-01	ENSG00000152611
CAPZA1	-0.1373	2.9405E-02	1.0950E-01	ENSG00000116489
CAPZA2	-0.2186	4.5041E-04	4.8950E-03	ENSG00000198898
CAPZB	-0.3330	2.7618E-09	2.6860E-07	ENSG00000077549
CARD10	-0.0752	3.8336E-01	5.9155E-01	ENSG00000100065
CARD11	0.0423	1.8902E-01		ENSG00000198286
CARD14	0.0033	9.6258E-01	9.8133E-01	ENSG00000141527
CARD18	-0.0098	5.4728E-01		ENSG00000255501
CARD19	-0.0573	4.0747E-01	6.1186E-01	ENSG00000165233
CARD6	0.0120	7.6056E-01		ENSG00000132357
CARD8	0.0224	7.4718E-01	8.6252E-01	ENSG00000105483
CARD8-AS1	-0.0399	5.2301E-01	7.0893E-01	ENSG00000268001
CARD9	0.0347	1.9912E-01		ENSG00000187796
CARF	0.2908	4.4214E-04	4.8373E-03	ENSG00000138380
CARHSP1	-0.3495	1.0831E-04	1.6372E-03	ENSG00000153048
CARM1	-0.1360	1.0054E-01	2.5564E-01	ENSG00000142453
CARMIL1	0.0230	7.8221E-01	8.8472E-01	ENSG00000079691
CARMIL2	0.0198	8.1437E-01	9.0324E-01	ENSG00000159753
CARMIL3	0.0537	4.9007E-01	6.8287E-01	ENSG00000186648
CARNMT1	-0.1629	2.2482E-02	9.0403E-02	ENSG00000156017
CARNS1	-0.0178	8.0060E-01	8.9476E-01	ENSG00000172508
CARS1	-0.0386	5.4531E-01	7.2574E-01	ENSG00000110619
CARS1-AS1	0.0333	3.3138E-01		ENSG00000247473
CARS2	-0.3529	2.9979E-04	3.5912E-03	ENSG00000134905
CARTPT	-0.0835	2.8858E-01	4.9908E-01	ENSG00000164326
CASC11	-0.0151	4.3568E-01		ENSG00000249375
CASC16	-0.0147	4.2182E-01		ENSG00000249231
CASC17	0.0351	6.6387E-01	8.1090E-01	ENSG00000260785
CASC18	-0.0009	9.9981E-01		ENSG00000257859
CASC2	0.1396	1.1399E-01	2.7781E-01	ENSG00000177640
CASC20	0.0049	7.9146E-01		ENSG00000229876

CASC23	-0.0064	7.2070E-01		ENSG00000255420
CASC3	-0.2034	1.5737E-03	1.2675E-02	ENSG00000108349
CASC8	0.0085	8.7899E-01	9.3956E-01	ENSG00000246228
CASC9	-0.0382	6.4522E-01	7.9748E-01	ENSG00000249395
CASD1	-0.0793	2.1836E-01	4.2077E-01	ENSG00000127995
CASK	-0.1589	5.4966E-03	3.2324E-02	ENSG00000147044
CASKIN1	0.0488	5.6196E-01	7.3863E-01	ENSG00000167971
CASKIN2	-0.0565	4.9521E-01	6.8703E-01	ENSG00000177303
CASP1	0.0112	9.5308E-01	9.7686E-01	ENSG00000137752
CASP10	0.0257	5.6260E-01		ENSG00000003400
CASP16P	0.0184	6.9243E-01		ENSG00000228146
CASP2	-0.1826	2.5916E-02	1.0011E-01	ENSG00000106144
CASP3	0.1057	1.7699E-01	3.6824E-01	ENSG00000164305
CASP6	-0.1033	1.5460E-01	3.3753E-01	ENSG00000138794
CASP7	-0.1312	1.6891E-01	3.5730E-01	ENSG00000165806
CASP8	0.1723	6.8662E-02	1.9802E-01	ENSG00000064012
CASP8AP2	0.1360	3.2256E-02	1.1697E-01	ENSG00000118412
CASP9	0.0447	5.6350E-01	7.3990E-01	ENSG00000132906
CASQ1	0.2936	1.3159E-02	6.1886E-02	ENSG00000143318
CASQ2	0.0213	4.9638E-01		ENSG00000118729
CASR	0.0250	3.0638E-01		ENSG00000036828
CASS4	-0.0438	2.4792E-01		ENSG00000087589
CAST	0.2582	9.6567E-04	8.8138E-03	ENSG00000153113
CASTOR1	-0.0597	2.2441E-01	4.2827E-01	ENSG00000239282
CASTOR2	-0.2054	7.0097E-03	3.8828E-02	ENSG00000274070
CASZ1	-0.1586	1.0332E-01	2.6021E-01	ENSG00000130940
CAT	0.0104	8.8011E-01	9.3996E-01	ENSG00000121691
CATIP	0.0280	3.5790E-01		ENSG00000158428
CATSPER1	0.0025	9.5203E-01		ENSG00000175294
CATSPER2	0.2744	2.0535E-02	8.4450E-02	ENSG00000166762
CATSPER3	0.0186	7.4587E-01	8.6152E-01	ENSG00000152705
CATSPERB	-0.0493	6.0771E-02		ENSG00000133962
CATSPERD	-0.0601	3.7832E-01	5.8689E-01	ENSG00000174898
CATSPERE	0.3996	1.0701E-02	5.3263E-02	ENSG00000179397
CAV1	0.0392	6.3718E-01	7.9216E-01	ENSG00000105974
CAV2	-0.3747	1.6268E-03	1.2958E-02	ENSG00000105971
CAV3	-0.0121	5.6040E-01		ENSG00000182533
CAVIN1	-0.3633	9.8598E-03	5.0175E-02	ENSG00000177469
CAVIN2	-0.0230	2.3986E-01		ENSG00000168497
CAVIN4	0.0550	5.0993E-01	6.9867E-01	ENSG00000170681
CBARP	-0.1499	3.6023E-02	1.2664E-01	ENSG00000099625
CBFA2T2	-0.0571	4.3831E-01	6.3860E-01	ENSG00000078699
CBFA2T3	0.0068	9.3191E-01	9.6632E-01	ENSG00000129993
CBFB	-0.3097	1.2914E-05	2.9892E-04	ENSG00000067955
CBL	-0.0570	4.0729E-01	6.1177E-01	ENSG00000110395
CBLB	0.3760	6.8726E-07	2.7318E-05	ENSG00000114423
CBLC	-0.0782	2.3321E-01	4.3839E-01	ENSG00000142273
CBLL1	-0.0441	5.5360E-01	7.3198E-01	ENSG00000105879
CBLL1-AS1	-0.0419	6.2760E-01	7.8553E-01	ENSG00000241764

CBLN1	-0.0611	4.6315E-01	6.6036E-01	ENSG00000102924
CBLN2	0.5661	2.2052E-03	1.6197E-02	ENSG00000141668
CBLN3	0.0222	4.3386E-01		ENSG00000139899
CBLN4	-0.0146	8.1837E-01	9.0560E-01	ENSG00000054803
CBR1	-0.2388	2.6298E-02	1.0115E-01	ENSG00000159228
CBR1-AS1	0.0322	1.9395E-01		ENSG00000230212
CBR3	-0.5754	7.4796E-04	7.3059E-03	ENSG00000159231
CBR3-AS1	0.1847	6.8036E-02	1.9676E-01	ENSG00000236830
CBR4	0.4552	2.6578E-07	1.2848E-05	ENSG00000145439
CBS	-0.0172	7.9039E-01	8.8919E-01	ENSG00000160200
CBX1	-0.1192	3.1353E-02	1.1486E-01	ENSG00000108468
CBX2	0.2412	5.0209E-03	3.0201E-02	ENSG00000173894
CBX3	-0.1052	7.4713E-02	2.0991E-01	ENSG00000122565
CBX3P2	0.0328	6.7149E-01	8.1623E-01	ENSG00000266405
CBX4	-0.4997	8.6074E-07	3.2937E-05	ENSG00000141582
CBX5	-0.0233	7.7044E-01	8.7722E-01	ENSG00000094916
CBX6	-0.0935	1.5356E-01	3.3589E-01	ENSG00000183741
CBX7	0.1972	4.1728E-02	1.4084E-01	ENSG00000100307
CBX8	0.2032	1.5427E-02	6.9370E-02	ENSG00000141570
CBY1	-0.1118	9.5281E-02	2.4735E-01	ENSG00000100211
CBY2	0.0074	7.6072E-01		ENSG00000174015
CBY3	0.0107	8.5911E-01		ENSG00000204659
CC2D1A	0.1642	3.6570E-02	1.2799E-01	ENSG00000132024
CC2D1B	0.2839	4.3724E-03	2.7223E-02	ENSG00000154222
CC2D2A	-0.1213	5.6946E-02	1.7471E-01	ENSG00000048342
CC2D2B	0.1174	1.4401E-01	3.2202E-01	ENSG00000188649
CCAR1	0.2597	5.8564E-06	1.5940E-04	ENSG00000060339
CCAR2	0.0946	1.1738E-01	2.8294E-01	ENSG00000158941
CCBE1	0.3332	2.0843E-02	8.5468E-02	ENSG00000183287
CCDC102A	-0.2264	4.7959E-02	1.5498E-01	ENSG00000135736
CCDC102B	0.0847	2.7357E-01	4.8328E-01	ENSG00000150636
CCDC103	-0.0010	8.9514E-01		ENSG00000167131
CCDC106	-0.0805	2.3796E-01	4.4400E-01	ENSG00000173581
CCDC107	0.0016	9.9158E-01	9.9535E-01	ENSG00000159884
CCDC110	0.1295	1.7433E-01	3.6462E-01	ENSG00000168491
CCDC112	-0.0359	5.8290E-01	7.5357E-01	ENSG00000164221
CCDC113	0.0381	5.6473E-01	7.4058E-01	ENSG00000103021
CCDC115	-0.0616	2.7678E-01	4.8673E-01	ENSG00000136710
CCDC116	-0.0004	9.8614E-01	9.9239E-01	ENSG00000161180
CCDC117	-0.3174	1.8021E-04	2.3929E-03	ENSG00000159873
CCDC12	-0.2886	1.1479E-07	6.4810E-06	ENSG00000160799
CCDC120	-0.0362	6.4475E-01	7.9719E-01	ENSG00000147144
CCDC121	0.0497	5.3721E-01	7.2010E-01	ENSG00000176714
CCDC122	0.1620	5.9258E-02	1.7892E-01	ENSG00000151773
CCDC124	-0.0799	1.0557E-01	2.6407E-01	ENSG00000007080
CCDC125	-0.0219	7.8668E-01	8.8730E-01	ENSG00000183323
CCDC126	-0.1180	6.2457E-02	1.8560E-01	ENSG00000169193
CCDC127	0.0843	2.2723E-01	4.3147E-01	ENSG00000164366
CCDC13-AS1	0.2040	6.6465E-02	1.9396E-01	ENSG00000173811

CCDC134	0.0098	8.8667E-01	9.4383E-01	ENSG00000100147
CCDC136	0.1693	4.1363E-02	1.4003E-01	ENSG00000128596
CCDC137	-0.0293	6.4073E-01	7.9441E-01	ENSG00000185298
CCDC138	0.3108	1.7600E-04	2.3523E-03	ENSG00000163006
CCDC14	0.0813	1.7957E-01	3.7122E-01	ENSG00000175455
CCDC140	0.0180	4.5526E-01		ENSG00000163081
CCDC141	-0.0382	6.1730E-01	7.7831E-01	ENSG00000163492
CCDC142	-0.1177	1.7709E-01	3.6829E-01	ENSG00000135637
CCDC144A	0.0847	3.2111E-01	5.3116E-01	ENSG00000170160
CCDC146	0.1009	2.6317E-01	4.7200E-01	ENSG00000135205
CCDC148	0.1015	2.0665E-01	4.0621E-01	ENSG00000153237
CCDC148-AS1	0.0008	7.7758E-01		ENSG00000227480
CCDC149	0.1700	2.0195E-02	8.3582E-02	ENSG00000181982
CCDC15	0.4787	7.4673E-05	1.2247E-03	ENSG00000149548
CCDC15-DT	0.0975	2.7035E-01	4.7989E-01	ENSG00000285825
CCDC150	0.1576	1.0140E-01	2.5712E-01	ENSG00000144395
CCDC152	0.1515	2.4890E-02	9.7318E-02	ENSG00000198865
CCDC153	0.0046	9.6337E-01	9.8180E-01	ENSG00000248712
CCDC154	0.0193	7.7758E-01	8.8152E-01	ENSG00000197599
CCDC157	0.3971	1.8010E-05	3.9381E-04	ENSG00000187860
CCDC158	0.0162	6.4772E-01		ENSG00000163749
CCDC159	-0.1259	1.2942E-01	3.0187E-01	ENSG00000183401
CCDC160	0.2580	8.4866E-03	4.4925E-02	ENSG00000203952
CCDC162P	0.0950	1.3262E-01	3.0620E-01	ENSG00000203799
CCDC163	-0.1305	1.2579E-01	2.9643E-01	ENSG00000280670
CCDC167	-0.2766	8.2482E-05	1.3221E-03	ENSG00000198937
CCDC168	-0.0109	8.2432E-01	9.0879E-01	ENSG00000175820
CCDC169	-0.2917	7.7218E-03	4.1766E-02	ENSG00000242715
CCDC169-SOHLH2	-0.1282	4.5460E-02	1.4962E-01	ENSG00000250709
CCDC17	0.0412	5.5827E-01	7.3578E-01	ENSG00000159588
CCDC170	0.0249	7.4222E-01	8.5956E-01	ENSG00000120262
CCDC171	0.2601	1.6914E-04	2.2767E-03	ENSG00000164989
CCDC174	0.3686	1.4643E-05	3.3115E-04	ENSG00000154781
CCDC175	0.4778	7.3853E-04	7.2310E-03	ENSG00000151838
CCDC177	0.1165	1.4607E-01	3.2537E-01	ENSG00000267909
CCDC178	0.0103	8.6765E-01	9.3335E-01	ENSG00000166960
CCDC18	0.1374	1.0321E-01	2.6008E-01	ENSG00000122483
CCDC180	-0.0440	5.8693E-01	7.5626E-01	ENSG00000197816
CCDC181	0.1212	1.0826E-01	2.6824E-01	ENSG00000117477
CCDC183	0.0774	3.0875E-01	5.1898E-01	ENSG00000213213
CCDC183-AS1	-0.0213	5.7644E-01	7.4871E-01	ENSG00000228544
CCDC184	0.0989	1.5092E-01	3.3220E-01	ENSG00000177875
CCDC186	0.1605	1.4877E-02	6.7560E-02	ENSG00000165813
CCDC187	0.1509	8.4605E-02	2.2731E-01	ENSG00000260220
CCDC188	0.0263	3.6009E-01		ENSG00000234409
CCDC191	0.3426	9.8341E-06	2.4173E-04	ENSG00000163617
CCDC192	0.1807	7.5477E-02	2.1127E-01	ENSG00000230561
CCDC194	-0.0341	5.5234E-01	7.3094E-01	ENSG00000269720
CCDC196	0.0843	2.5141E-01	4.5965E-01	ENSG00000196553

CCDC198	-0.0206	6.1957E-01	7.7989E-01	ENSG00000100557
CCDC200	-0.1621	8.7419E-02	2.3281E-01	ENSG00000236383
CCDC22	-0.2588	1.0480E-02	5.2473E-02	ENSG00000101997
CCDC24	-0.0828	2.0561E-01	4.0506E-01	ENSG00000159214
CCDC25	0.1571	3.6584E-02	1.2802E-01	ENSG00000147419
CCDC26	0.0552	3.8939E-01	5.9680E-01	ENSG00000229140
CCDC27	-0.0402	5.6809E-01	7.4271E-01	ENSG00000162592
CCDC28A	-0.0838	3.0057E-01	5.1035E-01	ENSG00000024862
CCDC28A-AS1	0.0249	7.6475E-01	8.7357E-01	ENSG00000279968
CCDC28B	-0.5276	7.4265E-12	1.8838E-09	ENSG00000160050
CCDC3	0.0897	3.0688E-01	5.1720E-01	ENSG00000151468
CCDC30	0.5120	2.4416E-08	1.7515E-06	ENSG00000186409
CCDC32	-0.2395	7.2082E-04	7.1004E-03	ENSG00000128891
CCDC33	0.2908	2.4420E-02	9.5954E-02	ENSG00000140481
CCDC34	0.0700	3.7203E-01	5.8075E-01	ENSG00000109881
CCDC38	0.1078	2.0778E-01	4.0744E-01	ENSG00000165972
CCDC39	0.0375	2.8010E-01		ENSG00000284862
CCDC40	0.4160	8.7958E-06	2.2016E-04	ENSG00000141519
CCDC43	-0.0381	5.6853E-01	7.4298E-01	ENSG00000180329
CCDC47	-0.0072	8.6368E-01	9.3131E-01	ENSG00000108588
CCDC50	0.1040	1.4066E-01	3.1738E-01	ENSG00000152492
CCDC51	0.1339	1.1869E-01	2.8520E-01	ENSG00000164051
CCDC54	0.0021	9.1824E-01		ENSG00000138483
CCDC57	0.2737	6.2221E-03	3.5472E-02	ENSG00000176155
CCDC59	-0.3190	1.0934E-05	2.6378E-04	ENSG00000133773
CCDC6	-0.1878	6.2724E-03	3.5675E-02	ENSG00000108091
CCDC60	0.2514	1.1664E-02	5.6831E-02	ENSG00000183273
CCDC61	-0.0606	4.8409E-01	6.7805E-01	ENSG00000104983
CCDC62	0.0497	5.7498E-01	7.4775E-01	ENSG00000130783
CCDC63	0.0776	1.6593E-01	3.5355E-01	ENSG00000173093
CCDC65	0.2008	4.4833E-02	1.4808E-01	ENSG00000139537
CCDC66	0.1624	1.4808E-02	6.7422E-02	ENSG00000180376
CCDC68	0.0422	9.9115E-01	9.9515E-01	ENSG00000166510
CCDC69	0.0418	6.1293E-01	7.7486E-01	ENSG00000198624
CCDC7	0.2875	1.0800E-03	9.6174E-03	ENSG00000216937
CCDC70	-0.0157	5.0095E-01		ENSG00000123171
CCDC71	0.0397	5.8976E-01	7.5823E-01	ENSG00000177352
CCDC71L	-0.0276	7.2700E-01	8.5014E-01	ENSG00000253276
CCDC73	0.0229	6.4211E-01	7.9538E-01	ENSG00000186714
CCDC74A	0.1802	1.2455E-03	1.0690E-02	ENSG00000163040
CCDC74B	0.0879	2.2151E-01	4.2481E-01	ENSG00000152076
CCDC77	0.2595	1.7017E-03	1.3367E-02	ENSG00000120647
CCDC78	0.1199	1.4285E-01	3.2045E-01	ENSG00000162004
CCDC8	-0.1730	7.9680E-02	2.1912E-01	ENSG00000169515
CCDC80	0.2465	3.7601E-02	1.3084E-01	ENSG00000091986
CCDC81	0.0255	7.1219E-01	8.4207E-01	ENSG00000149201
CCDC82	0.2155	5.6762E-05	9.8354E-04	ENSG00000149231
CCDC83	0.0692	8.1160E-02		ENSG00000150676
CCDC85A	0.1513	1.2424E-01	2.9402E-01	ENSG00000055813

CCDC85B	-0.0934	1.8236E-01	3.7530E-01	ENSG00000175602
CCDC85C	-0.0871	2.7611E-01	4.8589E-01	ENSG00000205476
CCDC86	-0.1151	1.4662E-01	3.2634E-01	ENSG00000110104
CCDC87	0.1709	3.2821E-02	1.1841E-01	ENSG00000182791
CCDC88A	0.0424	5.6158E-01	7.3847E-01	ENSG00000115355
CCDC88B	-0.0039	9.6097E-01	9.8050E-01	ENSG00000168071
CCDC88C	-0.1634	7.1639E-02	2.0411E-01	ENSG00000015133
CCDC89	0.0352	5.6779E-01	7.4244E-01	ENSG00000179071
CCDC9	-0.0668	4.1261E-01	6.1626E-01	ENSG00000105321
CCDC90B	-0.0609	2.3408E-01	4.3912E-01	ENSG00000137500
CCDC91	-0.1955	2.8037E-03	1.9394E-02	ENSG00000123106
CCDC92	-0.0852	1.9579E-01	3.9292E-01	ENSG00000119242
CCDC92B	-0.0244	7.6730E-01	8.7523E-01	ENSG00000277200
CCDC93	0.1450	2.9419E-02	1.0950E-01	ENSG00000125633
CCDC96	0.1222	1.9101E-01	3.8627E-01	ENSG00000173013
CCDC97	0.0786	1.9451E-01	3.9123E-01	ENSG00000142039
CCDC9B	-0.0427	5.3418E-01	7.1776E-01	ENSG00000188549
CCER2	0.1033	2.5487E-01	4.6291E-01	ENSG00000262484
CCHCR1	-0.0215	7.3115E-01	8.5293E-01	ENSG00000204536
CCIN	0.0176	8.2126E-01	9.0737E-01	ENSG00000185972
CCK	-0.0188	5.9664E-01	7.6321E-01	ENSG00000187094
CCKAR	0.0310	5.7173E-01	7.4525E-01	ENSG00000163394
CCKBR	-0.0022	9.8276E-01	9.9076E-01	ENSG00000110148
CCL13	0.0259	2.9774E-01		ENSG00000181374
CCL16	0.0156	3.0754E-01		ENSG00000275152
CCL17	0.0012	8.9873E-01		ENSG00000102970
CCL18	0.0248	5.7045E-02	1.7484E-01	ENSG00000275385
CCL19	-0.0043	7.0655E-01		ENSG00000172724
CCL2	0.1271	5.2671E-02	1.6524E-01	ENSG00000108691
CCL20	-0.0670	1.3351E-02		ENSG00000115009
CCL26	0.0410	6.7436E-02		ENSG00000006606
CCL28	-0.0426	2.5139E-01	4.5965E-01	ENSG00000151882
CCL3	0.0304	3.8366E-01		ENSG00000277632
CCL3L1	-0.0092	9.1013E-01		ENSG00000276085
CCL4	0.0376	2.8869E-01	4.9910E-01	ENSG00000275302
CCL4L2	0.0292	3.4106E-01		ENSG00000276070
CCL5	-0.0903	3.5417E-02		ENSG00000271503
CCL7	-0.0130	5.3086E-01		ENSG00000108688
CCL8	-0.0185	2.9645E-02	1.1022E-01	ENSG00000108700
CCM2	-0.0094	8.3509E-01	9.1514E-01	ENSG00000136280
CCM2L	-0.0859	2.7087E-01	4.8008E-01	ENSG00000101331
CCN1	0.0368	6.2311E-01	7.8228E-01	ENSG00000142871
CCN2	0.5039	2.4077E-05	4.9698E-04	ENSG00000118523
CCN3	0.0297	7.2130E-01	8.4722E-01	ENSG00000136999
CCN4	0.0008	9.9771E-01		ENSG00000104415
CCN6	-0.0423	4.4145E-01		ENSG00000112761
CCNA1	0.1339	1.4787E-01	3.2810E-01	ENSG00000133101
CCNA2	-0.0554	5.1384E-01	7.0166E-01	ENSG00000145386
CCNB1	0.0047	9.5500E-01	9.7732E-01	ENSG00000134057

CCNB1IP1	0.0972	1.3406E-01	3.0813E-01	ENSG00000100814
CCNB2	-0.1842	8.0663E-02	2.2077E-01	ENSG00000157456
CCNB3	0.4136	2.1021E-04	2.6981E-03	ENSG00000147082
CCNC	-0.0482	4.4160E-01	6.4110E-01	ENSG00000112237
CCND1	-0.0049	9.6910E-01	9.8480E-01	ENSG00000110092
CCND2-AS1	-0.2420	2.0578E-02	8.4586E-02	ENSG00000255920
CCND3	-0.0865	2.3491E-01	4.4031E-01	ENSG00000112576
CCNDBP1	-0.0093	8.8200E-01	9.4093E-01	ENSG00000166946
CCNE1	-0.2614	9.6823E-03	4.9560E-02	ENSG00000105173
CCNE2	0.0724	3.8574E-01	5.9356E-01	ENSG00000175305
CCNF	0.0674	3.8786E-01	5.9542E-01	ENSG00000162063
CCNG1	-0.0464	5.2972E-01	7.1425E-01	ENSG00000113328
CCNG2	-0.2195	2.1190E-03	1.5725E-02	ENSG00000138764
CCNH	0.0210	7.3561E-01	8.5581E-01	ENSG00000134480
CCNI	-0.1117	1.1336E-01	2.7671E-01	ENSG00000118816
CCNI2	0.0596	3.4075E-01	5.5047E-01	ENSG00000205089
CCNJ	0.0213	7.6281E-01	8.7229E-01	ENSG00000107443
CCNJL	-0.1163	2.0344E-01	4.0226E-01	ENSG00000135083
CCNK	-0.0730	2.5943E-01	4.6792E-01	ENSG00000090061
CCNL1	-0.4045	2.9518E-07	1.4002E-05	ENSG00000163660
CCNL2	0.1552	7.9776E-03	4.2802E-02	ENSG00000221978
CCNO	0.1878	6.7057E-02	1.9509E-01	ENSG00000152669
CCNP	-0.0525	3.9359E-01	6.0009E-01	ENSG00000105219
CCNQ	0.1445	2.8821E-02	1.0803E-01	ENSG00000262919
CCNT1	-0.1471	4.7328E-02	1.5381E-01	ENSG00000129315
CCNT2	-0.1435	6.0085E-03	3.4518E-02	ENSG00000082258
CCNY	-0.3282	6.2327E-06	1.6705E-04	ENSG00000108100
CCNYL1	0.2999	2.3538E-03	1.7004E-02	ENSG00000163249
CCNYL6	-0.0032	6.6522E-01		ENSG00000269136
CCNYL6	-0.0257	5.1527E-01		ENSG00000269845
CCNYL7	-0.0650	1.4600E-01		ENSG00000261528
CCP110	-0.1499	3.1650E-02	1.1553E-01	ENSG00000103540
CCPG1	0.0011	9.8918E-01	9.9399E-01	ENSG00000260916
CCR10	0.0545	5.0247E-01	6.9296E-01	ENSG00000184451
CCR12P	0.0077	7.2806E-01		ENSG00000238241
CCR2	0.0089	8.6086E-01		ENSG00000121807
CCR4	-0.0145	4.2830E-01		ENSG00000183813
CCR7	0.0724	1.1928E-01	2.8632E-01	ENSG00000126353
CCR9	-0.0172	2.1328E-01		ENSG00000173585
CCS	0.0402	4.6597E-01	6.6257E-01	ENSG00000173992
CCSER1	-0.1412	8.2867E-02	2.2450E-01	ENSG00000184305
CCSER2	-0.0176	7.0278E-01	8.3588E-01	ENSG00000107771
CCT2	-0.1708	1.6516E-02	7.2645E-02	ENSG00000166226
CCT3	-0.2515	1.4815E-05	3.3408E-04	ENSG00000163468
CCT4	-0.3983	2.2592E-08	1.6446E-06	ENSG00000115484
CCT4P2	-0.0126	8.4215E-01	9.1870E-01	ENSG00000225569
CCT5	-0.3744	3.6066E-07	1.6253E-05	ENSG00000150753
CCT5P1	-0.0099	6.8684E-01		ENSG00000250444
CCT6A	-0.2136	2.0196E-05	4.3118E-04	ENSG00000146731

CCT7	-0.3180	1.7180E-07	8.8250E-06	ENSG00000135624
CCT8	-0.2596	1.0397E-06	3.8470E-05	ENSG00000156261
CCT8L1P	0.0158	5.5943E-01		ENSG00000020219
CCT8P1	-0.0276	6.5335E-01	8.0375E-01	ENSG00000226015
CCZ1	-0.0794	1.0713E-01	2.6637E-01	ENSG00000122674
CCZ1B	-0.0776	1.4748E-01	3.2750E-01	ENSG00000146574
CCZ1P1	0.0920	3.0101E-01	5.1089E-01	ENSG00000243554
CD101	-0.0271	7.1058E-01	8.4116E-01	ENSG00000134256
CD101-AS1	-0.0341	3.2928E-01	5.3959E-01	ENSG00000236137
CD109-AS1	-0.0311	6.2455E-01	7.8329E-01	ENSG00000231652
CD14	0.1615	1.1248E-01	2.7519E-01	ENSG00000170458
CD151	-0.2542	1.7699E-03	1.3764E-02	ENSG00000177697
CD160	0.0630	3.1614E-01	5.2618E-01	ENSG00000117281
CD163	-0.0012	7.0491E-01		ENSG00000177575
CD164	-0.0065	9.0838E-01	9.5480E-01	ENSG00000135535
CD164L2	0.0017	8.5486E-01		ENSG00000174950
CD177	0.1206	1.1086E-01	2.7259E-01	ENSG00000204936
CD180	-0.0057	6.7193E-01		ENSG00000134061
CD19	0.0459	2.7741E-01		ENSG00000177455
CD1D	0.0664	2.9458E-01	5.0458E-01	ENSG00000158473
CD2	0.0092	4.7211E-01		ENSG00000116824
CD200	0.0139	8.2488E-01	9.0912E-01	ENSG00000091972
CD200R1	0.0532	2.1095E-01		ENSG00000163606
CD207	-0.0184	1.9643E-01		ENSG00000116031
CD22	0.0465	3.6013E-02		ENSG00000012124
CD226	0.0642	2.5719E-01	4.6568E-01	ENSG00000150637
CD24	-0.3693	9.6277E-07	3.6056E-05	ENSG00000272398
CD247	-0.0121	8.7340E-01	9.3651E-01	ENSG00000198821
CD248	-0.0478	5.7592E-01	7.4838E-01	ENSG00000174807
CD27	-0.0038	8.0113E-01		ENSG00000139193
CD27-AS1	0.0131	8.6604E-01	9.3231E-01	ENSG00000215039
CD274	-0.0559	3.3906E-01	5.4878E-01	ENSG00000120217
CD276	-0.0855	2.2822E-01	4.3292E-01	ENSG00000103855
CD2AP	0.2240	1.5706E-02	7.0224E-02	ENSG00000198087
CD2BP2	0.0849	8.5169E-02	2.2840E-01	ENSG00000169217
CD2BP2-DT	0.1451	1.3839E-01	3.1433E-01	ENSG00000260219
CD300A	0.0250	2.4101E-01		ENSG00000167851
CD300C	0.0669	1.5476E-01		ENSG00000167850
CD300LB	0.0262	5.0649E-01		ENSG00000178789
CD300LG	0.0355	4.3537E-01		ENSG00000161649
CD302	-0.2497	3.8138E-02	1.3229E-01	ENSG00000241399
CD320	-0.0483	3.9341E-01	5.9997E-01	ENSG00000167775
CD33	0.0212	2.0184E-01		ENSG00000105383
CD34	-0.0779	2.5228E-01	4.6034E-01	ENSG00000174059
CD36	0.0567	4.0030E-01	6.0563E-01	ENSG00000135218
CD37	0.0058	5.5702E-01		ENSG00000104894
CD38	0.0608	3.3931E-01	5.4900E-01	ENSG00000004468
CD3G	0.0037	8.4832E-01		ENSG00000160654
CD4	0.0390	6.0744E-01	7.7108E-01	ENSG00000010610

CD40	-0.2222	3.5299E-02	1.2477E-01	ENSG00000101017
CD44	-0.0386	5.8093E-01	7.5225E-01	ENSG00000026508
CD44-DT	0.0042	7.9386E-01		ENSG00000255521
CD46	0.0294	6.8924E-01	8.2719E-01	ENSG00000117335
CD46P1	-0.0115	4.9540E-01		ENSG00000244703
CD47	-0.3249	5.9959E-06	1.6248E-04	ENSG00000196776
CD5	0.0954	1.1241E-01	2.7505E-01	ENSG00000110448
CD52	0.0160	5.8472E-01		ENSG00000169442
CD53	0.0263	1.0166E-01		ENSG00000143119
CD55	0.0533	5.3510E-01	7.1834E-01	ENSG00000196352
CD58	-0.3637	4.8006E-04	5.1490E-03	ENSG00000116815
CD59	-0.1950	1.7621E-04	2.3527E-03	ENSG00000085063
CD6	0.0154	8.3717E-01	9.1604E-01	ENSG00000013725
CD63	-0.2317	5.2819E-03	3.1422E-02	ENSG00000135404
CD69	0.0054	4.2706E-01	6.2907E-01	ENSG00000110848
CD7	0.0563	7.4388E-02		ENSG00000173762
CD70	-0.1038	2.4529E-01	4.5305E-01	ENSG00000125726
CD72	0.4310	1.0907E-03	9.6782E-03	ENSG00000137101
CD74	0.1707	9.5451E-02	2.4764E-01	ENSG00000019582
CD79A	0.1512	3.4102E-02	1.2150E-01	ENSG00000105369
CD79B	0.0085	6.8569E-01		ENSG00000007312
CD80	-0.0029	5.7808E-01		ENSG00000121594
CD81	-0.1715	5.3305E-02	1.6663E-01	ENSG00000110651
CD81-AS1	-0.0504	4.7781E-01	6.7315E-01	ENSG00000238184
CD82	-0.4110	5.5809E-04	5.7915E-03	ENSG00000085117
CD83	-0.1734	3.9060E-02	1.3456E-01	ENSG00000112149
CD84	0.0135	3.6605E-01		ENSG00000066294
CD86	-0.0036	5.8107E-01		ENSG00000114013
CD8A	-0.0203	8.0977E-01	9.0001E-01	ENSG00000153563
CD8B	-0.0632	2.1251E-02		ENSG00000172116
CD9	-0.0160	8.3735E-01	9.1605E-01	ENSG00000010278
CD96	0.0015	8.9252E-01		ENSG00000153283
CD99	-0.0757	2.9847E-01	5.0832E-01	ENSG00000002586
CD99L2	-0.0303	6.9601E-01	8.3174E-01	ENSG00000102181
CDA	0.0264	2.9626E-01		ENSG00000158825
CDADC1	0.1419	2.0129E-02	8.3422E-02	ENSG00000102543
CDAN1	-0.1869	2.4296E-02	9.5680E-02	ENSG00000140326
CDC123	-0.1256	2.4904E-02	9.7342E-02	ENSG00000151465
CDC14A	0.0034	9.6541E-01	9.8290E-01	ENSG00000079335
CDC14B	-0.0954	2.1449E-01	4.1565E-01	ENSG00000081377
CDC16	0.0542	3.7685E-01	5.8542E-01	ENSG00000130177
CDC20	-0.0567	5.0420E-01	6.9434E-01	ENSG00000117399
CDC20-DT	-0.0584	2.5944E-01	4.6792E-01	ENSG00000234694
CDC20B	0.0735	7.6614E-02	2.1349E-01	ENSG00000164287
CDC20P1	0.0215	7.4120E-01	8.5895E-01	ENSG00000231007
CDC23	0.0077	9.1099E-01	9.5620E-01	ENSG00000094880
CDC25A	-0.1815	5.8361E-02	1.7728E-01	ENSG00000164045
CDC25B	-0.2006	1.0133E-02	5.1216E-02	ENSG00000101224
CDC25C	-0.0383	6.5922E-01	8.0777E-01	ENSG00000158402

CDC26	-0.0146	7.9973E-01	8.9430E-01	ENSG00000176386
CDC27	0.0083	9.0760E-01	9.5461E-01	ENSG00000004897
CDC34	-0.0283	5.1421E-01	7.0192E-01	ENSG00000099804
CDC37	-0.0398	4.4634E-01	6.4563E-01	ENSG00000105401
CDC37L1	0.2225	1.9413E-03	1.4742E-02	ENSG00000106993
CDC37L1-DT	0.2566	1.0079E-02	5.1038E-02	ENSG00000273061
CDC40	0.0650	2.8718E-01	4.9789E-01	ENSG00000168438
CDC42	-0.2867	3.2350E-03	2.1706E-02	ENSG00000070831
CDC42BPA	0.2121	8.2967E-04	7.8636E-03	ENSG00000143776
CDC42BPB	-0.1133	1.1351E-01	2.7699E-01	ENSG00000198752
CDC42BPG	-0.0835	3.2784E-01	5.3838E-01	ENSG00000171219
CDC42EP2	0.0043	6.2945E-01		ENSG00000149798
CDC42EP3	-0.1136	1.4029E-01	3.1686E-01	ENSG00000163171
CDC42EP4	-0.4106	1.4591E-06	5.1286E-05	ENSG00000179604
CDC42EP5	0.1082	2.3834E-01	4.4453E-01	ENSG00000167617
CDC42P1	-0.0472	1.4742E-01		ENSG00000234933
CDC42SE1	-0.4864	2.5955E-08	1.8404E-06	ENSG00000197622
CDC42SE2	-0.2070	2.2546E-03	1.6486E-02	ENSG00000158985
CDC45	-0.0911	3.0307E-01	5.1315E-01	ENSG00000093009
CDC5L	-0.2162	6.8790E-05	1.1507E-03	ENSG00000096401
CDC6	-0.1301	1.6123E-01	3.4697E-01	ENSG00000094804
CDC7	0.0266	7.1412E-01	8.4306E-01	ENSG00000097046
CDC73	0.0574	2.9144E-01	5.0175E-01	ENSG00000134371
CDCA2	-0.0581	5.0126E-01	6.9208E-01	ENSG00000184661
CDCA3	-0.1550	1.0301E-01	2.5981E-01	ENSG00000111665
CDCA4	-0.0951	2.4469E-01	4.5223E-01	ENSG00000170779
CDCA4P1	0.0025	6.7040E-01		ENSG00000229029
CDCA5	-0.0739	3.8203E-01	5.9026E-01	ENSG00000146670
CDCA7	-0.1144	1.7761E-01	3.6883E-01	ENSG00000144354
CDCA7L	-0.3716	1.1848E-03	1.0307E-02	ENSG00000164649
CDCA8	-0.0809	3.6015E-01	5.6961E-01	ENSG00000134690
CDCP1	-0.0204	5.9032E-01		ENSG00000163814
CDH1	0.1144	2.0819E-01	4.0805E-01	ENSG00000039068
CDH10	0.3892	2.6519E-03	1.8574E-02	ENSG00000040731
CDH11	0.3670	1.0048E-03	9.0858E-03	ENSG00000140937
CDH12	-0.0448	6.0545E-01	7.6942E-01	ENSG00000154162
CDH13	0.4817	1.5561E-03	1.2562E-02	ENSG00000140945
CDH13-AS2	0.0098	6.4029E-01		ENSG00000260228
CDH15	0.1034	1.4581E-01	3.2499E-01	ENSG00000129910
CDH17	0.0040	7.4108E-01		ENSG00000079112
CDH18-AS1	0.0199	2.2092E-01		ENSG00000249854
CDH19	0.0214	4.7478E-01		ENSG00000071991
CDH2	0.0523	3.4328E-01	5.5287E-01	ENSG00000170558
CDH20	0.0380	6.1812E-01	7.7886E-01	ENSG00000101542
CDH22	-0.0211	7.9860E-01	8.9385E-01	ENSG00000149654
CDH23	0.1567	6.0776E-02	1.8192E-01	ENSG00000107736
CDH24	-0.1358	8.2520E-02	2.2383E-01	ENSG00000139880
CDH26	0.0362	4.4951E-01		ENSG00000124215
CDH3	0.0263	7.2972E-01	8.5211E-01	ENSG00000062038

CDH4	0.1003	2.0520E-01	4.0446E-01	ENSG00000179242
CDH5	0.0099	8.5952E-01		ENSG00000179776
CDH7	0.0091	9.1292E-01	9.5707E-01	ENSG00000081138
CDH8	0.0911	2.8982E-01	5.0032E-01	ENSG00000150394
CDHR1	-0.2613	1.7686E-02	7.6156E-02	ENSG00000148600
CDHR2	0.0474	5.6360E-01	7.3991E-01	ENSG00000074276
CDHR3	-0.3044	1.1338E-04	1.6939E-03	ENSG00000128536
CDHR4	0.1078	4.2922E-02		ENSG00000187492
CDHR5	0.0379	3.1703E-01	5.2719E-01	ENSG00000099834
CDIN1	-0.0800	2.8849E-01	4.9900E-01	ENSG00000186073
CDIP1	-0.1959	8.7445E-03	4.5960E-02	ENSG00000089486
CDIPT	-0.1881	6.1085E-03	3.4972E-02	ENSG00000103502
CDIPTOSP	0.5462	1.1298E-05	2.6989E-04	ENSG00000214725
CDK1	-0.0807	3.3576E-01	5.4585E-01	ENSG00000170312
CDK10	0.0525	3.8359E-01	5.9178E-01	ENSG00000185324
CDK11A	-0.1476	8.6164E-02	2.3031E-01	ENSG00000008128
CDK11B	0.1196	6.1344E-02	1.8318E-01	ENSG00000248333
CDK12	-0.0167	8.0858E-01	8.9942E-01	ENSG00000167258
CDK13	0.1784	2.6847E-03	1.8766E-02	ENSG00000065883
CDK13-DT	0.2916	1.5621E-02	7.0012E-02	ENSG00000259826
CDK14	-0.1033	1.8858E-01	3.8354E-01	ENSG00000058091
CDK16	-0.3121	1.3623E-05	3.1180E-04	ENSG00000102225
CDK17	-0.0055	9.2351E-01	9.6227E-01	ENSG00000059758
CDK18	-0.1935	4.0803E-02	1.3869E-01	ENSG00000117266
CDK19	0.0902	2.3669E-01	4.4244E-01	ENSG00000155111
CDK2	-0.2855	1.4192E-03	1.1773E-02	ENSG00000123374
CDK20	0.2586	2.9282E-03	2.0103E-02	ENSG00000156345
CDK2AP1	-0.0473	4.3724E-01	6.3776E-01	ENSG00000111328
CDK2AP2	-0.0037	9.5213E-01	9.7649E-01	ENSG00000167797
CDK3	-0.0067	7.9301E-01		ENSG00000250506
CDK4	-0.2612	6.1176E-06	1.6451E-04	ENSG00000135446
CDK5	-0.1424	1.9735E-02	8.2243E-02	ENSG00000164885
CDK5R1	-0.4367	1.1946E-05	2.8235E-04	ENSG00000176749
CDK5R2	-0.1384	1.1974E-01	2.8698E-01	ENSG00000171450
CDK5RAP1	0.0609	2.7878E-01	4.8869E-01	ENSG00000101391
CDK5RAP2	-0.1018	1.1555E-01	2.8015E-01	ENSG00000136861
CDK5RAP3	-0.1472	9.9121E-02	2.5326E-01	ENSG00000108465
CDK6	-0.0694	3.6035E-01	5.6974E-01	ENSG00000105810
CDK6-AS1	0.0185	7.3564E-01	8.5581E-01	ENSG00000237819
CDK7	-0.0403	5.1886E-01	7.0611E-01	ENSG00000134058
CDK8	0.3571	3.1341E-04	3.7200E-03	ENSG00000132964
CDK9	0.1064	7.4818E-02	2.1008E-01	ENSG00000136807
CDKAL1	-0.4733	2.3340E-05	4.8422E-04	ENSG00000145996
CDKL1	0.4135	9.4509E-04	8.6671E-03	ENSG00000100490
CDKL2	-0.2229	3.8249E-03	2.4691E-02	ENSG00000138769
CDKL3	0.2071	2.6753E-02	1.0234E-01	ENSG00000006837
CDKL4	0.0086	5.8563E-01		ENSG00000205111
CDKL5	0.1113	1.7479E-01	3.6515E-01	ENSG00000008086
CDKN1A	-0.2514	3.2288E-02	1.1702E-01	ENSG00000124762

CDKN1B	-0.4430	6.6238E-04	6.6658E-03	ENSG00000111276
CDKN1C	0.1295	1.5119E-01	3.3255E-01	ENSG00000129757
CDKN2AIP	-0.2128	1.5686E-02	7.0188E-02	ENSG00000168564
CDKN2AIPNL	-0.1306	1.1874E-02	5.7579E-02	ENSG00000237190
CDKN2B	-0.1253	1.8554E-01	3.7937E-01	ENSG00000147883
CDKN2B-AS1	-0.1437	6.6414E-02	1.9387E-01	ENSG00000240498
CDKN2C	-0.4118	1.7179E-03	1.3470E-02	ENSG00000123080
CDKN2D	-0.1382	5.1315E-02	1.6229E-01	ENSG00000129355
CDKN3	-0.0060	9.1162E-01	9.5639E-01	ENSG00000100526
CDNF	0.1820	5.9563E-02	1.7934E-01	ENSG00000185267
CDO1	0.1105	2.1985E-01	4.2244E-01	ENSG00000129596
CDON	-0.1106	1.8477E-01	3.7835E-01	ENSG00000064309
CDPF1	0.1795	9.1779E-03	4.7622E-02	ENSG00000205643
CDR2	-0.0484	5.3894E-01	7.2105E-01	ENSG00000140743
CDR2-DT	0.1557	3.2110E-02	1.1655E-01	ENSG00000260790
CDR2L	0.1048	1.5380E-01	3.3614E-01	ENSG00000109089
CDRT15	-0.0038	4.7302E-01		ENSG00000223510
CDRT15L2	-0.0241	1.6170E-01		ENSG00000214819
CDRT15P3	-0.0176	7.9156E-01	8.8996E-01	ENSG00000186825
CDRT15P4	0.0133	2.9896E-01		ENSG00000287071
CDRT4	0.0235	1.5288E-01		ENSG00000239704
CDS1	0.2027	2.5523E-02	9.8989E-02	ENSG00000163624
CDS2	0.0079	9.3206E-01	9.6640E-01	ENSG00000101290
CDT1	-0.3071	6.8327E-03	3.8114E-02	ENSG00000167513
CDV3	-0.2932	1.5819E-05	3.5128E-04	ENSG00000091527
CDX2	-0.0169	3.3655E-01		ENSG00000165556
CDYL	0.0968	2.3941E-01	4.4606E-01	ENSG00000153046
CDYL2	0.2229	3.8282E-03	2.4697E-02	ENSG00000166446
CEACAM1	0.0961	1.2715E-01	2.9858E-01	ENSG00000079385
CEACAM19	0.2800	1.2647E-02	6.0107E-02	ENSG00000186567
CEACAM20	0.0070	7.5415E-01		ENSG00000273777
CEACAM21	0.1858	7.3483E-02	2.0773E-01	ENSG00000007129
CEACAM8	-0.0175	6.5740E-01		ENSG00000124469
CEBPA	-0.3952	9.9635E-04	9.0329E-03	ENSG00000245848
CEBPA-DT	-0.3442	1.5094E-02	6.8294E-02	ENSG00000267296
CEBPB	-0.4639	1.3086E-05	3.0148E-04	ENSG00000172216
CEBPB-AS1	0.2450	3.4846E-02	1.2351E-01	ENSG00000277449
CEBPD	-0.3083	2.2523E-02	9.0501E-02	ENSG00000221869
CEBPG	-0.3544	7.2534E-08	4.4176E-06	ENSG00000153879
CEBPZ	0.0655	2.0274E-01	4.0171E-01	ENSG00000115816
CEBPZOS	-0.2274	3.9625E-03	2.5409E-02	ENSG00000218739
CECR2	-0.1922	2.7426E-02	1.0431E-01	ENSG00000099954
CECR3	0.0457	3.4852E-01		ENSG00000241832
CEL	0.0005	9.3637E-01	9.6866E-01	ENSG00000170835
CELA2A	-0.0169	4.8112E-01		ENSG00000142615
CELF1	-0.0119	7.8135E-01	8.8411E-01	ENSG00000149187
CELF2	-0.0213	7.2591E-01	8.4955E-01	ENSG00000048740
CELF2-AS1	-0.0309	6.8180E-01	8.2250E-01	ENSG00000181800
CELF2-AS2	-0.1198	1.9968E-01	3.9807E-01	ENSG00000237986

CELF3	-0.0626	3.7542E-01	5.8397E-01	ENSG00000159409
CELF4	-0.0363	6.1299E-01	7.7486E-01	ENSG00000101489
CELF5	0.1268	1.0026E-01	2.5516E-01	ENSG00000161082
CELF6	0.0397	1.6566E-01		ENSG00000140488
CELP	0.0006	9.9896E-01		ENSG00000170827
CELSR1	0.0381	6.4487E-01	7.9726E-01	ENSG00000075275
CELSR2	0.0158	8.4205E-01	9.1870E-01	ENSG00000143126
CELSR3	0.1586	4.9436E-02	1.5826E-01	ENSG00000008300
CEMIP2	-0.0883	1.6221E-01	3.4823E-01	ENSG00000135048
CENATAC	0.1682	2.2438E-02	9.0256E-02	ENSG00000186166
CEND1	-0.3575	1.5217E-04	2.1046E-03	ENSG00000184524
CENPA	-0.0686	4.2917E-01	6.3077E-01	ENSG00000115163
CENPB	-0.1920	3.7310E-02	1.2999E-01	ENSG00000125817
CENPBD1P	0.0562	2.5783E-01	4.6650E-01	ENSG00000177946
CENPBD2P	-0.0199	7.2215E-01	8.4766E-01	ENSG00000213753
CENPC	0.0152	7.8644E-01	8.8723E-01	ENSG00000145241
CENPCP1	0.0204	2.7198E-01		ENSG00000226982
CENPE	0.0053	9.4894E-01	9.7468E-01	ENSG00000138778
CENPF	-0.0264	7.4401E-01	8.6086E-01	ENSG00000117724
CENPH	-0.0486	4.9525E-01	6.8703E-01	ENSG00000153044
CENPI	-0.0515	5.4729E-01	7.2703E-01	ENSG00000102384
CENPJ	0.0807	2.5605E-01	4.6434E-01	ENSG00000151849
CENPK	-0.1159	1.4190E-01	3.1905E-01	ENSG00000123219
CENPL	0.0003	9.9778E-01	9.9870E-01	ENSG00000120334
CENPM	-0.3738	7.6685E-04	7.4255E-03	ENSG00000100162
CENPN	-0.0787	3.5349E-01	5.6354E-01	ENSG00000166451
CENPNP1	0.0161	2.9100E-01		ENSG00000233671
CENPO	0.0526	5.4609E-01	7.2638E-01	ENSG00000138092
CENPP	0.0205	7.8795E-01	8.8795E-01	ENSG00000188312
CENPQ	0.1529	7.5871E-02	2.1218E-01	ENSG00000031691
CENPS	0.1324	1.5994E-01	3.4508E-01	ENSG00000175279
CENPS-CORT	-0.0559	4.4438E-01	6.4336E-01	ENSG00000251503
CENPT	0.0304	6.0767E-01	7.7108E-01	ENSG00000102901
CENPU	-0.3684	5.1826E-04	5.4527E-03	ENSG00000151725
CENPV	-0.3428	2.6284E-05	5.3535E-04	ENSG00000166582
CENPVL1	0.2218	1.5980E-02	7.1076E-02	ENSG00000223591
CENPVL2	0.2328	1.3058E-02	6.1481E-02	ENSG00000283093
CENPVL3	0.0416	6.2067E-01	7.8065E-01	ENSG00000224109
CENPW	-0.0944	2.6368E-01	4.7263E-01	ENSG00000203760
CENPX	0.0218	7.4476E-01	8.6115E-01	ENSG00000169689
CEP104	0.1910	1.7797E-03	1.3805E-02	ENSG00000116198
CEP112	0.1387	6.7604E-02	1.9609E-01	ENSG00000154240
CEP120	0.0791	2.0875E-01	4.0878E-01	ENSG00000168944
CEP126	0.4206	2.6281E-06	8.3332E-05	ENSG00000110318
CEP128	0.2343	1.0610E-02	5.2963E-02	ENSG00000100629
CEP131	-0.0047	9.4472E-01	9.7243E-01	ENSG00000141577
CEP135	0.0215	7.7954E-01	8.8300E-01	ENSG00000174799
CEP15	-0.1037	1.3504E-01	3.0956E-01	ENSG00000114405
CEP152	0.1152	1.7927E-01	3.7088E-01	ENSG00000103995

CEP162	0.1466	5.7894E-02	1.7634E-01	ENSG00000135315
CEP164	0.2219	2.6641E-04	3.2781E-03	ENSG00000110274
CEP170	-0.2569	2.5358E-04	3.1587E-03	ENSG00000143702
CEP170B	-0.0219	7.9113E-01	8.8966E-01	ENSG00000099814
CEP19	-0.0913	1.2507E-01	2.9519E-01	ENSG00000174007
CEP192	0.0329	6.3021E-01	7.8706E-01	ENSG00000101639
CEP20	-0.0609	3.4214E-01	5.5185E-01	ENSG00000133393
CEP250	0.0436	5.2215E-01	7.0863E-01	ENSG00000126001
CEP290	0.3180	7.4228E-05	1.2208E-03	ENSG00000198707
CEP295	0.1033	1.4242E-01	3.1991E-01	ENSG00000166004
CEP295NL	0.0320	6.5193E-01	8.0290E-01	ENSG00000178404
CEP350	0.2597	1.5454E-04	2.1300E-03	ENSG00000135837
CEP41	-0.0116	8.4040E-01	9.1792E-01	ENSG00000106477
CEP43	-0.0031	9.6267E-01	9.8133E-01	ENSG00000213066
CEP44	0.1079	1.7664E-01	3.6792E-01	ENSG00000164118
CEP55	-0.0098	9.0768E-01	9.5461E-01	ENSG00000138180
CEP57	0.0704	1.8861E-01	3.8354E-01	ENSG00000166037
CEP57L1	-0.0608	3.1519E-01	5.2525E-01	ENSG00000183137
CEP63	0.1268	1.1559E-01	2.8018E-01	ENSG00000182923
CEP68	0.1837	2.7571E-03	1.9137E-02	ENSG00000011523
CEP70	-0.2513	3.0166E-04	3.6118E-03	ENSG00000114107
CEP72	0.2309	2.1013E-02	8.5924E-02	ENSG00000112877
CEP76	-0.1240	1.0198E-01	2.5802E-01	ENSG00000101624
CEP78	-0.0399	5.2015E-01	7.0717E-01	ENSG00000148019
CEP83	-0.0857	2.3349E-01	4.3870E-01	ENSG00000173588
CEP83-DT	0.2719	8.9267E-03	4.6742E-02	ENSG00000278916
CEP85	0.0914	1.9605E-01	3.9329E-01	ENSG00000130695
CEP85L	-0.2260	7.4437E-03	4.0610E-02	ENSG00000111860
CEP89	-0.0550	3.9463E-01	6.0085E-01	ENSG00000121289
CEP95	0.0942	1.1335E-01	2.7671E-01	ENSG00000258890
CEP97	-0.1267	7.5085E-02	2.1063E-01	ENSG00000182504
CEPT1	0.0790	3.4653E-01	5.5673E-01	ENSG00000134255
CER1	-0.0032	6.8301E-01		ENSG00000147869
CERCAM	-0.1813	3.6860E-02	1.2884E-01	ENSG00000167123
CERK	-0.0848	1.1738E-01	2.8294E-01	ENSG00000100422
CERKL	0.0323	3.8207E-01		ENSG00000188452
CERNA3	0.0742	6.0500E-02		ENSG00000253603
CEROX1	-0.4674	1.5303E-06	5.3560E-05	ENSG00000260807
CERS1	-0.0879	3.0502E-01	5.1535E-01	ENSG00000223802
CERS2	0.3930	2.6990E-05	5.4567E-04	ENSG00000143418
CERS3	-0.0373	3.0204E-01		ENSG00000154227
CERS4	-0.3347	1.2807E-09	1.3673E-07	ENSG00000090661
CERS5	-0.1016	1.4016E-01	3.1670E-01	ENSG00000139624
CERS6-AS1	0.0272	9.8922E-01		ENSG00000227617
CERT1	-0.1757	2.8861E-03	1.9897E-02	ENSG00000113163
CES1	-0.0306	5.1255E-01	7.0051E-01	ENSG00000198848
CES2	0.0187	8.0974E-01	9.0001E-01	ENSG00000172831
CES3	0.1436	1.4096E-01	3.1764E-01	ENSG00000172828
CES4A	-0.1429	1.1682E-01	2.8203E-01	ENSG00000172824

CES5A	0.0352	6.2507E-01	7.8348E-01	ENSG00000159398
CETN2	-0.0495	3.6486E-01	5.7404E-01	ENSG00000147400
CETN3	0.0165	8.3152E-01	9.1301E-01	ENSG00000153140
CETP	0.0352	2.2159E-01		ENSG00000087237
CFAP100	0.0486	4.3621E-01	6.3708E-01	ENSG00000163885
CFAP107	0.0343	4.8298E-01		ENSG00000157330
CFAP126	-0.0414	6.2247E-01	7.8179E-01	ENSG00000188931
CFAP141	0.0333	8.0895E-01		ENSG00000163263
CFAP157	0.0392	5.4542E-01	7.2585E-01	ENSG00000160401
CFAP161	0.0160	6.4037E-01		ENSG00000156206
CFAP20	-0.0942	1.4399E-01	3.2200E-01	ENSG00000070761
CFAP206	-0.0091	8.6125E-01	9.3034E-01	ENSG00000272514
CFAP20DC	0.3520	2.2438E-03	1.6421E-02	ENSG00000163689
CFAP20DC-AS1	0.0038	9.5059E-01	9.7545E-01	ENSG00000242428
CFAP221	0.4779	9.8308E-05	1.5261E-03	ENSG00000163075
CFAP276	0.0790	3.0979E-01	5.2007E-01	ENSG00000179902
CFAP298	0.1041	2.4166E-01	4.4886E-01	ENSG00000159079
CFAP298-TCP10L	-0.1383	1.3972E-01	3.1608E-01	ENSG00000265590
CFAP299	0.0027	9.4137E-01		ENSG00000197826
CFAP300	0.2603	1.6747E-02	7.3293E-02	ENSG00000137691
CFAP36	0.0720	1.7326E-01	3.6320E-01	ENSG00000163001
CFAP410	0.3163	2.9127E-05	5.8078E-04	ENSG00000160226
CFAP418	0.1322	1.5012E-01	3.3111E-01	ENSG00000156172
CFAP418-AS1	0.1604	7.3798E-02	2.0823E-01	ENSG00000253773
CFAP43	0.0019	9.8100E-01	9.9019E-01	ENSG00000197748
CFAP44	0.2263	4.7349E-02	1.5384E-01	ENSG00000206530
CFAP44-AS1	-0.0116	5.2213E-01		ENSG00000243849
CFAP45	0.0668	2.4891E-01	4.5664E-01	ENSG00000213085
CFAP46	0.5108	3.0764E-04	3.6663E-03	ENSG00000171811
CFAP52	0.0405	4.7838E-01	6.7365E-01	ENSG00000166596
CFAP53	0.3895	1.1296E-03	9.9227E-03	ENSG00000172361
CFAP54	0.3469	1.4886E-02	6.7588E-02	ENSG00000188596
CFAP58	0.0278	6.2731E-01	7.8530E-01	ENSG00000120051
CFAP58-DT	0.0348	6.6116E-01	8.0909E-01	ENSG00000231233
CFAP61	0.0654	4.5283E-01	6.5158E-01	ENSG00000089101
CFAP65	0.3272	1.9965E-02	8.2924E-02	ENSG00000181378
CFAP68	0.1140	8.1987E-02	2.2278E-01	ENSG00000137720
CFAP73	0.1093	1.8617E-02		ENSG00000186710
CFAP74	0.1485	1.3048E-01	3.0333E-01	ENSG00000142609
CFAP77	0.1060	1.0735E-01	2.6661E-01	ENSG00000188523
CFAP90	0.4241	4.0543E-04	4.5220E-03	ENSG00000215217
CFAP91	0.5982	3.7577E-04	4.2647E-03	ENSG00000183833
CFAP92	0.1884	3.0265E-02	1.1194E-01	ENSG00000114656
CFAP97	-0.0508	3.5758E-01	5.6739E-01	ENSG00000164323
CFAP97D1	0.0579	3.7830E-01	5.8689E-01	ENSG00000231256
CFAP97D2	0.0468	4.4749E-01	6.4695E-01	ENSG00000283361
CFAP99	0.1108	6.9873E-02	2.0061E-01	ENSG00000206113
CFB	0.1356	2.3202E-02	9.2540E-02	ENSG00000243649
CFDP1	-0.2652	3.0467E-06	9.3822E-05	ENSG00000153774

CFH	0.0083	7.5045E-01		ENSG00000000971
CFHR1	0.1050	2.3080E-01	4.3564E-01	ENSG00000244414
CFHR3	0.0056	5.5570E-01		ENSG00000116785
CFHR5	-0.0144	5.7603E-01		ENSG00000134389
CFI	0.0970	1.2732E-01	2.9873E-01	ENSG00000205403
CFL1	-0.5316	2.2087E-10	3.1054E-08	ENSG00000172757
CFL2	-0.2943	1.2772E-05	2.9640E-04	ENSG00000165410
CFLAR-AS1	0.0172	5.5122E-01		ENSG00000226312
CFP	0.0191	4.2477E-01	6.2715E-01	ENSG00000126759
CFTR	0.1385	1.4570E-02		ENSG00000001626
CGA	-0.0274	4.9271E-01	6.8480E-01	ENSG00000135346
CGAS	-0.0016	9.1229E-01		ENSG00000164430
CGB1	0.0438	3.1647E-01		ENSG00000267631
CGB2	0.0550	2.6689E-01	4.7617E-01	ENSG00000104818
CGB5	0.0440	3.3354E-01		ENSG00000189052
CGB7	0.1458	1.1110E-01	2.7290E-01	ENSG00000196337
CGB8	0.0195	6.5278E-01		ENSG00000213030
CGGBP1	-0.0851	1.0358E-01	2.6065E-01	ENSG00000163320
CGN	0.1640	7.6801E-02	2.1374E-01	ENSG00000143375
CGNL1	0.0997	1.9610E-01	3.9337E-01	ENSG00000128849
CGREF1	-0.0358	6.0416E-01	7.6833E-01	ENSG00000138028
CGRRF1	0.0184	7.9992E-01	8.9440E-01	ENSG00000100532
CH25H	-0.1105	1.3500E-01	3.0954E-01	ENSG00000138135
CHAC1	-0.0532	5.2795E-01	7.1270E-01	ENSG00000128965
CHAC2	0.0988	2.5884E-01	4.6757E-01	ENSG00000143942
CHAD	0.0059	9.4005E-01		ENSG00000136457
CHADL	0.0410	6.0474E-01	7.6865E-01	ENSG00000100399
CHAF1A	-0.0892	2.7569E-01	4.8561E-01	ENSG00000167670
CHAF1B	-0.1471	5.9033E-02	1.7856E-01	ENSG00000159259
CHAMP1	-0.1195	8.0921E-02	2.2113E-01	ENSG00000198824
CHAT	-0.0282	6.7110E-01	8.1595E-01	ENSG00000070748
CHCHD1	0.0067	9.1140E-01	9.5627E-01	ENSG00000172586
CHCHD10	-0.0610	4.6917E-01	6.6566E-01	ENSG00000250479
CHCHD2	-0.3494	2.2469E-05	4.7069E-04	ENSG00000106153
CHCHD3	-0.1184	1.8297E-02	7.7887E-02	ENSG00000106554
CHCHD4	0.2110	1.5473E-03	1.2508E-02	ENSG00000163528
CHCHD5	0.0531	3.8141E-01	5.8966E-01	ENSG00000125611
CHCHD6	0.0073	9.1429E-01	9.5786E-01	ENSG00000159685
CHCHD7	0.0706	3.3019E-01	5.4046E-01	ENSG00000170791
CHCT1	0.0052	9.1748E-01		ENSG00000141371
CHD1	0.0823	2.4920E-01	4.5693E-01	ENSG00000153922
CHD1L	0.3054	9.2832E-03	4.7993E-02	ENSG00000131778
CHD2	-0.0453	3.6728E-01	5.7651E-01	ENSG00000173575
CHD3	0.0101	9.0675E-01	9.5437E-01	ENSG00000170004
CHD4	0.2383	6.9819E-03	3.8735E-02	ENSG00000111642
CHD5	0.1368	9.9041E-02	2.5313E-01	ENSG00000116254
CHD6	0.0511	4.2995E-01	6.3161E-01	ENSG00000124177
CHD7	-0.0626	3.6986E-01	5.7913E-01	ENSG00000171316
CHD8	0.0226	7.2514E-01	8.4928E-01	ENSG00000100888

CHD9	-0.0501	3.3333E-01	5.4349E-01	ENSG00000177200
CHD9NB	0.0129	8.2461E-01		ENSG00000277639
CHDH	-0.0366	6.5248E-01	8.0312E-01	ENSG00000016391
CHEK1	-0.0559	4.2093E-01	6.2377E-01	ENSG00000149554
CHEK2	-0.2254	1.0559E-02	5.2783E-02	ENSG00000183765
CHERP	-0.0934	1.4712E-01	3.2693E-01	ENSG00000085872
CHFR	0.0227	7.3510E-01	8.5555E-01	ENSG00000072609
CHFR-DT	0.0655	4.5188E-01	6.5089E-01	ENSG00000236617
CHGA	0.2913	8.0713E-03	4.3163E-02	ENSG00000100604
CHGB	0.0381	6.3046E-01	7.8714E-01	ENSG00000089199
CHI3L1	0.0009	7.2245E-01		ENSG00000133048
CHI3L2	0.0386	1.1743E-01		ENSG00000064886
CHIC1	0.4881	8.2885E-05	1.3260E-03	ENSG00000204116
CHIC2	-0.0562	3.8816E-01	5.9568E-01	ENSG00000109220
CHID1	-0.0446	4.2538E-01	6.2767E-01	ENSG00000177830
CHIT1	0.0227	2.3019E-01		ENSG00000133063
CHKA	-0.1852	8.5674E-03	4.5275E-02	ENSG00000110721
CHKB	0.0104	8.8828E-01	9.4444E-01	ENSG00000100288
CHKB-CPT1B	-0.0179	7.2221E-01		ENSG00000254413
CHKB-DT	0.4196	1.1110E-06	4.0680E-05	ENSG00000205559
CHL1	-0.5449	1.2508E-07	6.9004E-06	ENSG00000134121
CHL1-AS1	-0.0454	1.4651E-01		ENSG00000234661
CHL1-AS2	-0.3286	4.2401E-03	2.6621E-02	ENSG00000224318
CHM	0.0905	2.1868E-01	4.2115E-01	ENSG00000188419
CHML	0.2492	9.7265E-03	4.9724E-02	ENSG00000203668
CHMP1A	-0.1232	6.8776E-02	1.9826E-01	ENSG00000131165
CHMP1B	-0.2589	1.4389E-04	2.0151E-03	ENSG00000255112
CHMP1B2P	-0.0167	5.5158E-01	7.3037E-01	ENSG00000278530
CHMP2A	-0.0617	3.0820E-01	5.1844E-01	ENSG00000130724
CHMP2B	0.1072	1.4847E-01	3.2899E-01	ENSG00000083937
CHMP3	0.0244	7.1375E-01	8.4287E-01	ENSG00000115561
CHMP3-AS1	-0.0396	6.3983E-01	7.9402E-01	ENSG00000228363
CHMP4A	-0.0870	3.2313E-01	5.3309E-01	ENSG00000254505
CHMP4B	0.0707	3.1756E-01	5.2780E-01	ENSG00000101421
CHMP4C	0.0595	1.3205E-01		ENSG00000164695
CHMP5	-0.1171	5.9941E-02	1.8019E-01	ENSG00000086065
CHMP6	-0.0135	8.2868E-01	9.1131E-01	ENSG00000176108
CHMP7	0.0407	5.4813E-01	7.2768E-01	ENSG00000147457
CHN1	0.0815	2.7601E-01	4.8585E-01	ENSG00000128656
CHN2	0.5089	3.0337E-04	3.6271E-03	ENSG00000106069
CHORDC1	-0.1332	1.5698E-01	3.4064E-01	ENSG00000110172
CHP1	-0.2272	3.5198E-03	2.3150E-02	ENSG00000187446
CHP1P3	-0.0011	9.8482E-01		ENSG00000226153
CHP2	0.0450	4.3112E-01	6.3288E-01	ENSG00000166869
CHPF	0.2020	5.9267E-02	1.7893E-01	ENSG00000123989
CHPF2	0.0407	5.9579E-01	7.6268E-01	ENSG00000033100
CHPT1	-0.2444	6.3600E-05	1.0806E-03	ENSG00000111666
CHRAC1	-0.1224	7.1291E-02	2.0335E-01	ENSG00000104472
CHRD	-0.2858	7.8255E-03	4.2181E-02	ENSG00000090539

CHRDL2	-0.0112	8.4868E-01	9.2283E-01	ENSG00000054938
CHRFAM7A	-0.3070	1.4881E-04	2.0698E-03	ENSG00000166664
CHRM2	-0.0475	4.2847E-01	6.3035E-01	ENSG00000181072
CHRM3	0.0350	6.8615E-01	8.2504E-01	ENSG00000133019
CHRM3-AS2	-0.0550	2.0602E-01	4.0560E-01	ENSG00000233355
CHRM4	-0.2061	5.3609E-02	1.6736E-01	ENSG00000180720
CHRM5	0.2018	7.0186E-02	2.0111E-01	ENSG00000184984
CHRNA1	-0.0127	8.0529E-01	8.9799E-01	ENSG00000138435
CHRNA10	0.0840	1.4389E-01	3.2190E-01	ENSG00000129749
CHRNA2	0.0050	9.4527E-01	9.7278E-01	ENSG00000120903
CHRNA3	-0.2666	2.7737E-03	1.9224E-02	ENSG00000080644
CHRNA4	-0.2417	7.7675E-03	4.1958E-02	ENSG00000101204
CHRNA5	-0.4855	8.0414E-06	2.0589E-04	ENSG00000169684
CHRNA6	-0.1249	1.3261E-01	3.0620E-01	ENSG00000147434
CHRNA7	-0.2308	1.5074E-03	1.2298E-02	ENSG00000175344
CHRNB1	-0.2387	6.8916E-04	6.8603E-03	ENSG00000170175
CHRNB2	-0.0495	5.2298E-01	7.0893E-01	ENSG00000160716
CHRNB3	0.0181	4.1586E-01		ENSG00000147432
CHRNB4	-0.5727	8.7940E-07	3.3391E-05	ENSG00000117971
CHRND	0.0064	9.0248E-01		ENSG00000135902
CHRNE	0.0536	1.8337E-01		ENSG00000108556
CHRNG	0.0041	9.1218E-01		ENSG00000196811
CHROMR	-0.1099	1.5624E-01	3.4014E-01	ENSG00000223960
CHST1	0.1771	8.0733E-02	2.2088E-01	ENSG00000175264
CHST10	-0.5295	1.1507E-08	9.4377E-07	ENSG00000115526
CHST11	-0.1509	2.4027E-02	9.4969E-02	ENSG00000171310
CHST12	0.1437	4.3790E-02	1.4552E-01	ENSG00000136213
CHST13	-0.0076	6.9676E-01		ENSG00000180767
CHST14	0.0388	6.4567E-01	7.9776E-01	ENSG00000169105
CHST15	-0.3556	1.5638E-03	1.2612E-02	ENSG00000182022
CHST2	-0.2031	2.7074E-02	1.0328E-01	ENSG00000175040
CHST3	0.0337	6.7710E-01	8.1908E-01	ENSG00000122863
CHST4	0.0151	6.7411E-01		ENSG00000140835
CHST5	0.0555	2.9898E-01	5.0876E-01	ENSG00000135702
CHST6	0.1167	1.8370E-01	3.7705E-01	ENSG00000183196
CHST7	0.0461	5.8393E-01	7.5414E-01	ENSG00000147119
CHST8	0.0172	8.3561E-01	9.1524E-01	ENSG00000124302
CHST9	0.4651	2.3055E-04	2.9121E-03	ENSG00000154080
CHSY1	-0.1254	3.5138E-02	1.2440E-01	ENSG00000131873
CHSY3	0.0275	7.1613E-01	8.4408E-01	ENSG00000198108
CHTF18	0.0158	8.2852E-01	9.1123E-01	ENSG00000127586
CHTF8	0.0957	2.1903E-01	4.2144E-01	ENSG00000168802
CHTOP	-0.1198	1.4597E-02	6.6670E-02	ENSG00000160679
CHUK	0.0566	4.0028E-01	6.0563E-01	ENSG00000213341
CHURC1	-0.0462	2.6110E-01	4.6963E-01	ENSG00000258289
CIAO1	0.0168	7.7470E-01	8.8011E-01	ENSG00000144021
CIAO2A	-0.0572	4.0055E-01	6.0586E-01	ENSG00000166797
CIAO2B	-0.2183	7.2970E-03	4.0041E-02	ENSG00000166595
CIAO3	0.1819	1.1414E-02	5.5900E-02	ENSG00000103245

CIAPIN1	-0.0269	5.8806E-01	7.5699E-01	ENSG00000005194
CIB1	0.0703	3.5478E-01	5.6460E-01	ENSG00000185043
CIB2	0.0794	2.2258E-01	4.2605E-01	ENSG00000136425
CIB3	-0.0036	9.0607E-01		ENSG00000141977
CIB4	0.0394	4.6418E-01		ENSG00000157884
CIBAR1	-0.2935	7.8347E-04	7.5390E-03	ENSG00000188343
CIBAR1-DT	-0.1974	2.7727E-02	1.0502E-01	ENSG00000246662
CIBAR2	0.0052	9.1441E-01	9.5788E-01	ENSG00000153789
CIC	0.0552	4.7741E-01	6.7281E-01	ENSG00000079432
CICP21	0.0265	1.7327E-01		ENSG00000278455
CICP27	0.0486	5.4910E-01	7.2838E-01	ENSG00000233750
CIDEA	-0.0512	1.0863E-01		ENSG00000176194
CIDEC	-0.0096	5.0960E-01		ENSG00000187288
CIDECP1	-0.2514	2.9182E-06	9.1121E-05	ENSG00000186162
CIITA	-0.0048	9.4822E-01	9.7452E-01	ENSG00000179583
CILK1	0.0507	4.2686E-01	6.2892E-01	ENSG00000112144
CILP	0.0556	3.6788E-01	5.7712E-01	ENSG00000138615
CILP2	-0.5041	2.0683E-05	4.3921E-04	ENSG00000160161
CIMAP1A	0.0103	4.0987E-01		ENSG00000177947
CIMAP1B	-0.1791	8.5120E-02	2.2834E-01	ENSG00000177989
CIMAP1C	0.0640	3.4866E-01	5.5845E-01	ENSG00000182950
CIMAP1D	0.0197	4.7015E-01		ENSG00000181781
CIMAP2	0.0549	1.6238E-01		ENSG00000162398
CIMAP3	0.5419	1.2497E-04	1.8236E-03	ENSG00000173947
CIMIP1	0.0135	3.3708E-01		ENSG00000124237
CIMIP2A	0.0727	3.1646E-01	5.2650E-01	ENSG00000188163
CIMIP2B	-0.0205	3.2219E-01		ENSG00000215187
CIMIP2C	0.0216	5.2142E-01		ENSG00000173557
CINP	-0.0982	1.3552E-01	3.1028E-01	ENSG00000100865
CIP2A	0.0486	5.5589E-01	7.3389E-01	ENSG00000163507
CIPC	-0.2510	1.0819E-03	9.6241E-03	ENSG00000198894
CIR1	0.0568	4.4301E-01	6.4225E-01	ENSG00000138433
CIRBP	-0.0485	3.8836E-01	5.9587E-01	ENSG00000099622
CIRBP-AS1	-0.0192	8.1545E-01	9.0400E-01	ENSG00000267493
CISD1	-0.0991	9.7321E-02	2.5061E-01	ENSG00000122873
CISD2	0.0192	7.6782E-01	8.7557E-01	ENSG00000145354
CISD3	-0.0889	2.6153E-01	4.7022E-01	ENSG00000277972
CISH	-0.0206	8.1098E-01	9.0103E-01	ENSG00000114737
CIST1	-0.0162	7.6656E-01		ENSG00000284797
CIT	-0.0256	7.4533E-01	8.6131E-01	ENSG00000122966
CITED1	-0.1331	1.4455E-01	3.2300E-01	ENSG00000125931
CITED2	-0.5876	1.0701E-09	1.1754E-07	ENSG00000164442
CITED4	-0.1663	1.5927E-02	7.0931E-02	ENSG00000179862
CIZ1	-0.1543	1.9444E-02	8.1309E-02	ENSG00000148337
CKAP2	-0.2848	1.7523E-04	2.3457E-03	ENSG00000136108
CKAP2L	-0.2700	1.4832E-02	6.7458E-02	ENSG00000169607
CKAP2LP1	0.0099	3.9480E-01		ENSG00000271461
CKAP4	-0.1731	3.9941E-03	2.5552E-02	ENSG00000136026
CKAP5	-0.0812	1.8928E-01	3.8426E-01	ENSG00000175216

CKB	-0.3556	3.5093E-04	4.0443E-03	ENSG00000166165
CKLF	-0.0475	5.4286E-01	7.2427E-01	ENSG00000217555
CKLF-CMTM1	-0.0749	2.9010E-01	5.0049E-01	ENSG00000254788
CKM	0.1322	6.2601E-02	1.8585E-01	ENSG00000104879
CKMT1A	-0.0363	6.3972E-01	7.9402E-01	ENSG00000223572
CKMT1B	-0.0175	8.1996E-01	9.0642E-01	ENSG00000237289
CKMT2	0.0915	1.5437E-01	3.3709E-01	ENSG00000131730
CKMT2-AS1	0.2529	6.1804E-03	3.5274E-02	ENSG00000247572
CKS1B	-0.1041	1.9759E-01	3.9535E-01	ENSG00000173207
CKS2	-0.2594	1.6016E-02	7.1146E-02	ENSG00000123975
CLASP1	-0.0226	7.2606E-01	8.4961E-01	ENSG00000074054
CLASP2	-0.0591	3.1934E-01	5.2957E-01	ENSG00000163539
CLASRP	-0.2845	7.1396E-04	7.0607E-03	ENSG00000104859
CLBA1	0.1306	1.1593E-01	2.8061E-01	ENSG00000140104
CLC	0.0019	8.1642E-01		ENSG00000105205
CLCA2	0.0007	7.9346E-01		ENSG00000137975
CLCA4	0.0091	6.4208E-01		ENSG00000016602
CLCA4-AS1	-0.0060	9.5363E-01	9.7701E-01	ENSG00000236915
CLCC1	-0.2133	9.3432E-04	8.6054E-03	ENSG00000121940
CLCF1	-0.0301	7.1629E-01	8.4412E-01	ENSG00000175505
CLCN1	0.0535	5.3121E-01	7.1529E-01	ENSG00000188037
CLCN2	-0.1521	6.8092E-02	1.9690E-01	ENSG00000114859
CLCN3	-0.1492	1.3580E-02	6.3342E-02	ENSG00000109572
CLCN3P1	-0.0212	7.8520E-01	8.8651E-01	ENSG00000232000
CLCN4	0.0066	9.2811E-01	9.6469E-01	ENSG00000073464
CLCN5	0.2437	5.8991E-03	3.4120E-02	ENSG00000171365
CLCN6	0.0803	2.0633E-01	4.0603E-01	ENSG00000011021
CLCN7	-0.0551	4.2604E-01	6.2831E-01	ENSG00000103249
CLCNKA	0.0265	6.4056E-01	7.9432E-01	ENSG00000186510
CLCNKB	0.0342	6.2406E-01	7.8294E-01	ENSG00000184908
CLDN1	0.1622	1.0546E-01	2.6389E-01	ENSG00000163347
CLDN10	-0.5719	1.0569E-06	3.8928E-05	ENSG00000134873
CLDN11	0.0809	1.4329E-01		ENSG00000013297
CLDN12	-0.0282	7.0967E-01	8.4042E-01	ENSG00000157224
CLDN15	0.4898	9.9083E-04	9.0027E-03	ENSG00000106404
CLDN16	0.0540	1.1491E-01		ENSG00000113946
CLDN18	0.0044	9.8936E-01		ENSG00000066405
CLDN19	0.2661	2.1170E-02	8.6427E-02	ENSG00000164007
CLDN2	-0.0821	2.9185E-01	5.0208E-01	ENSG00000165376
CLDN20	0.0014	9.2855E-01		ENSG00000171217
CLDN23	0.0097	8.8714E-01	9.4386E-01	ENSG00000253958
CLDN3	0.1389	1.3851E-01	3.1439E-01	ENSG00000165215
CLDN34	0.0088	8.6420E-01	9.3150E-01	ENSG00000234469
CLDN5	0.0932	1.7127E-01	3.6042E-01	ENSG00000184113
CLDN6	-0.2824	2.3402E-02	9.3142E-02	ENSG00000184697
CLDN7	-0.0768	3.1005E-01	5.2031E-01	ENSG00000181885
CLDN9	0.0318	6.1576E-01	7.7708E-01	ENSG00000213937
CLDND1	-0.0215	7.5500E-01	8.6698E-01	ENSG00000080822
CLDND2	-0.0090	9.1314E-01	9.5718E-01	ENSG00000160318

CLEC11A	0.3320	7.2661E-05	1.1997E-03	ENSG00000105472
CLEC14A	0.0246	6.1503E-01		ENSG00000176435
CLEC16A	-0.0598	4.0284E-01	6.0735E-01	ENSG00000038532
CLEC17A	0.0143	6.1874E-01		ENSG00000187912
CLEC19A	-0.0022	5.7730E-01	7.4914E-01	ENSG00000261210
CLEC2A	0.0507	2.1010E-01		ENSG00000188393
CLEC2B	0.0374	1.5812E-01		ENSG00000110852
CLEC2D	0.0887	3.2394E-01	5.3401E-01	ENSG00000069493
CLEC2L	-0.0553	5.1181E-01	7.0026E-01	ENSG00000236279
CLEC3A	-0.1005	1.1950E-01	2.8664E-01	ENSG00000166509
CLEC3B	0.0180	3.1703E-01		ENSG00000163815
CLEC4C	0.0401	2.5151E-02		ENSG00000198178
CLEC4D	0.0082	4.5875E-01		ENSG00000166527
CLEC4F	0.0093	7.1707E-01		ENSG00000152672
CLEC7A	0.0325	1.8355E-01		ENSG00000172243
CLGN	0.0969	1.7718E-01	3.6833E-01	ENSG00000153132
CLHC1	-0.1214	7.7770E-02	2.1555E-01	ENSG00000162994
CLIC1	-0.1408	4.3861E-02	1.4568E-01	ENSG00000213719
CLIC2	0.3164	1.8640E-02	7.8951E-02	ENSG00000155962
CLIC3	-0.0018	8.1317E-01		ENSG00000169583
CLIC4	-0.2667	1.4736E-03	1.2126E-02	ENSG00000169504
CLIC5	0.0444	2.9256E-01	5.0262E-01	ENSG00000112782
CLIC6	-0.0179	7.5875E-01	8.6962E-01	ENSG00000159212
CLINT1	0.0286	5.9236E-01	7.6007E-01	ENSG00000113282
CLIP1	0.1195	5.1726E-02	1.6309E-01	ENSG00000130779
CLIP2	-0.1061	1.2411E-01	2.9387E-01	ENSG00000106665
CLIP3	-0.2217	1.2123E-04	1.7808E-03	ENSG00000105270
CLIP4	0.4439	8.5598E-05	1.3641E-03	ENSG00000115295
CLK1	-0.3931	3.4190E-05	6.6135E-04	ENSG00000013441
CLK2	0.0356	6.3982E-01	7.9402E-01	ENSG00000176444
CLK3	-0.1875	2.5736E-03	1.8159E-02	ENSG00000179335
CLK4	0.0290	6.8561E-01	8.2489E-01	ENSG00000113240
CLMAT3	-0.0245	7.1140E-01	8.4162E-01	ENSG00000249035
CLMN	0.1187	1.7369E-01	3.6387E-01	ENSG00000165959
CLMP	0.1082	2.2893E-01	4.3343E-01	ENSG00000166250
CLN3	0.1753	8.7782E-02	2.3355E-01	ENSG00000188603
CLN5	0.0399	5.6726E-01	7.4199E-01	ENSG00000102805
CLN6	0.0537	5.3348E-01	7.1725E-01	ENSG00000128973
CLN8	0.1607	1.9244E-02	8.0760E-02	ENSG00000182372
CLN8-AS1	-0.2965	2.7088E-03	1.8902E-02	ENSG00000253982
CLNK	0.0461	3.1958E-01		ENSG00000109684
CLNS1A	-0.0732	2.5027E-01	4.5823E-01	ENSG00000074201
CLNS1AP1	0.0542	2.2163E-01		ENSG00000213335
CLOCK	0.2227	4.9538E-03	2.9848E-02	ENSG00000134852
CLP1	0.0063	9.3101E-01	9.6587E-01	ENSG00000172409
CLPB	-0.2033	5.9619E-03	3.4322E-02	ENSG00000162129
CLPP	-0.1589	1.0652E-02	5.3090E-02	ENSG00000125656
CLPSL1	0.0185	6.1541E-01		ENSG00000204140
CLPSL2	0.1295	7.9374E-02	2.1853E-01	ENSG00000196748

CLPTM1	-0.3190	2.1448E-06	7.0645E-05	ENSG00000104853
CLPTM1L	-0.1668	9.5343E-03	4.8996E-02	ENSG00000049656
CLPX	-0.0797	1.4523E-01	3.2405E-01	ENSG00000166855
CLRN1	-0.0083	9.2173E-01	9.6140E-01	ENSG00000163646
CLRN2	0.0076	6.6920E-01		ENSG00000249581
CLSPN	-0.0765	3.3090E-01	5.4112E-01	ENSG00000092853
CLSTN1	-0.1027	1.5762E-01	3.4149E-01	ENSG00000171603
CLSTN2	0.4572	7.6395E-05	1.2468E-03	ENSG00000158258
CLSTN2-AS1	0.0983	7.1832E-02		ENSG00000250433
CLSTN3	-0.1684	4.8469E-02	1.5620E-01	ENSG00000139182
CLTA	-0.2313	6.2640E-05	1.0703E-03	ENSG00000122705
CLTB	-0.0893	9.8123E-02	2.5173E-01	ENSG00000175416
CLTC	-0.0026	9.6355E-01	9.8180E-01	ENSG00000141367
CLTCL1	0.0654	4.3492E-01	6.3598E-01	ENSG00000070371
CLTRN	0.0119	8.7153E-01	9.3531E-01	ENSG00000147003
CLU	-0.2266	2.3102E-02	9.2217E-02	ENSG00000120885
CLUAP1	-0.0227	7.2181E-01	8.4753E-01	ENSG00000103351
CLUH	0.0296	6.5335E-01	8.0375E-01	ENSG00000132361
CLUL1	0.0571	4.9721E-01	6.8849E-01	ENSG00000079101
CLVS1	-0.1388	1.0991E-01	2.7090E-01	ENSG00000177182
CLVS2	0.1479	3.3554E-02	1.2032E-01	ENSG00000146352
CLXN	-0.0338	6.9013E-01	8.2779E-01	ENSG00000034239
CLYBL	-0.0066	9.2227E-01	9.6166E-01	ENSG00000125246
CLYBL-AS1	0.0020	9.1898E-01		ENSG00000234303
CLYBL-AS2	-0.0147	3.2585E-01		ENSG00000227659
CMAHP	0.1096	8.2891E-02	2.2453E-01	ENSG00000168405
CMAS	0.0381	5.9607E-01	7.6280E-01	ENSG00000111726
CMBL	-0.1843	1.6626E-02	7.2944E-02	ENSG00000164237
CMC1	-0.0918	1.9180E-01	3.8713E-01	ENSG00000187118
CMC2	-0.1822	8.6676E-04	8.1426E-03	ENSG00000103121
CMC4	-0.0726	3.6721E-01	5.7649E-01	ENSG00000182712
CMIP	0.3635	2.1830E-06	7.1713E-05	ENSG00000153815
CMKLR1	-0.0089	5.8634E-01		ENSG00000174600
CMKLR2	0.0934	2.8571E-01	4.9643E-01	ENSG00000183671
CMPK1	-0.0512	3.2479E-01	5.3483E-01	ENSG00000162368
CMPK2	0.1701	7.5136E-02	2.1073E-01	ENSG00000134326
CMSS1	0.0813	2.0498E-01	4.0427E-01	ENSG00000184220
CMTM1	-0.1174	2.0641E-01	4.0606E-01	ENSG00000089505
CMTM2	0.0377	3.1085E-01	5.2105E-01	ENSG00000140932
CMTM3	-0.3988	3.4943E-05	6.7276E-04	ENSG00000140931
CMTM4	0.2273	3.4861E-03	2.2971E-02	ENSG00000183723
CMTM5	0.0941	1.6611E-01	3.5384E-01	ENSG00000166091
CMTM6	0.0568	3.8854E-01	5.9594E-01	ENSG00000091317
CMTM7	0.0412	6.1820E-01	7.7892E-01	ENSG00000153551
CMTM8	0.0139	8.6384E-01	9.3141E-01	ENSG00000170293
CMTR1	-0.1691	2.8590E-02	1.0745E-01	ENSG00000137200
CMTR2	0.1512	8.1869E-02	2.2263E-01	ENSG00000180917
CMYA5	0.0913	3.1422E-01	5.2452E-01	ENSG00000164309
CNBD1	0.0028	6.8678E-01		ENSG00000176571

CNBD2	-0.0272	6.8602E-01	8.2504E-01	ENSG00000149646
CNBP	-0.3540	7.3877E-09	6.4005E-07	ENSG00000169714
CNDP1	0.1123	1.4663E-01	3.2634E-01	ENSG00000150656
CNDP2	-0.1190	1.0264E-01	2.5918E-01	ENSG00000133313
CNEP1R1	-0.0849	1.9630E-01	3.9365E-01	ENSG00000205423
CNEP1R1P1	-0.0143	3.2763E-01		ENSG00000180284
CNFN	0.1252	1.3896E-01	3.1494E-01	ENSG00000105427
CNGA1	0.0025	9.7573E-01	9.8791E-01	ENSG00000198515
CNGA3	0.1787	7.7358E-02	2.1486E-01	ENSG00000144191
CNGA4	0.0345	2.2928E-01		ENSG00000132259
CNGB1	0.2700	2.8776E-02	1.0788E-01	ENSG00000070729
CNGB3	0.0350	5.5233E-01	7.3094E-01	ENSG00000170289
CNIH1	-0.0442	4.3390E-01	6.3507E-01	ENSG00000100528
CNIH2	-0.4408	3.8716E-04	4.3597E-03	ENSG00000174871
CNIH3	0.4033	4.0866E-05	7.6174E-04	ENSG00000143786
CNIH3-AS2	-0.0746	2.6019E-01	4.6872E-01	ENSG00000233384
CNIH4	0.0505	4.4314E-01	6.4240E-01	ENSG00000143771
CNKSR1	-0.0971	2.8439E-01	4.9499E-01	ENSG00000142675
CNKSR2	-0.2195	4.2672E-03	2.6750E-02	ENSG00000149970
CNKSR3	0.2468	2.5602E-02	9.9233E-02	ENSG00000153721
CNMD	0.0743	3.7849E-01	5.8703E-01	ENSG00000136110
CNN1	-0.1313	1.3637E-01	3.1154E-01	ENSG00000130176
CNN2	-0.4704	7.1041E-08	4.3374E-06	ENSG00000064666
CNN3	-0.3632	7.7392E-05	1.2602E-03	ENSG00000117519
CNNM1	-0.1627	4.5988E-02	1.5083E-01	ENSG00000119946
CNNM2	-0.1754	1.6934E-02	7.3843E-02	ENSG00000148842
CNNM3	0.0141	8.3079E-01	9.1253E-01	ENSG00000168763
CNNM3-DT	0.0740	2.2553E-01	4.2927E-01	ENSG00000273265
CNNM4	0.4065	5.5796E-07	2.3388E-05	ENSG00000158158
CNOT1	-0.0008	9.5723E-01	9.7887E-01	ENSG00000125107
CNOT10	-0.0256	6.4921E-01	8.0061E-01	ENSG00000182973
CNOT11	0.0064	9.4600E-01	9.7333E-01	ENSG00000158435
CNOT2	-0.0337	4.6067E-01	6.5823E-01	ENSG00000111596
CNOT3	-0.2024	8.2193E-03	4.3755E-02	ENSG00000088038
CNOT4	0.0509	4.2808E-01	6.3000E-01	ENSG00000080802
CNOT6	0.1096	9.8729E-02	2.5262E-01	ENSG00000113300
CNOT6L	0.1031	2.1558E-01	4.1691E-01	ENSG00000138767
CNOT7	-0.1451	2.1276E-03	1.5759E-02	ENSG00000198791
CNOT8	-0.2365	2.0706E-05	4.3921E-04	ENSG00000155508
CNOT9	-0.1366	1.7548E-02	7.5747E-02	ENSG00000144580
CNP	-0.1796	1.3623E-03	1.1429E-02	ENSG00000173786
CNPPD1	-0.0779	2.0991E-01	4.1017E-01	ENSG00000115649
CNPY1	0.0204	4.0690E-01		ENSG00000146910
CNPY2	-0.0110	8.8402E-01	9.4185E-01	ENSG00000257727
CNPY3	-0.1548	3.8830E-02	1.3396E-01	ENSG00000137161
CNPY4	-0.2529	1.7752E-03	1.3783E-02	ENSG00000166997
CNR1	0.0038	9.5781E-01	9.7922E-01	ENSG00000118432
CNR2	0.0112	6.2061E-01		ENSG00000188822
CNRIP1	-0.0421	5.8207E-01	7.5309E-01	ENSG00000119865

CNST	0.0534	3.9502E-01	6.0127E-01	ENSG00000162852
CNTD1	0.2449	3.0796E-02	1.1337E-01	ENSG00000176563
CNTF	0.0161	8.6232E-01	9.3070E-01	ENSG00000242689
CNTFR	-0.2527	1.4074E-04	1.9787E-03	ENSG00000122756
CNTFR-AS1	-0.2758	3.1246E-02	1.1456E-01	ENSG00000237159
CNTLN	-0.1276	7.4561E-02	2.0962E-01	ENSG00000044459
CNTN1	-0.1774	1.5276E-02	6.8851E-02	ENSG00000018236
CNTN2	-0.0666	4.3402E-01	6.3509E-01	ENSG00000184144
CNTN3	-0.2889	3.1138E-03	2.1094E-02	ENSG00000113805
CNTN4	-0.0648	4.5138E-01	6.5033E-01	ENSG00000144619
CNTN6	-0.0052	5.0837E-01		ENSG00000134115
CNTNAP1	0.1195	1.9285E-01	3.8885E-01	ENSG00000108797
CNTNAP2	0.0265	6.9595E-01	8.3174E-01	ENSG00000174469
CNTNAP2-AS1	0.0156	2.0547E-01		ENSG00000236795
CNTNAP3	0.5476	2.3085E-06	7.5030E-05	ENSG00000106714
CNTNAP3	0.4136	1.0024E-03	9.0713E-03	ENSG00000283378
CNTNAP3B	0.3722	2.1583E-03	1.5914E-02	ENSG00000154529
CNTNAP4	-0.0362	6.5883E-01	8.0745E-01	ENSG00000152910
CNTRL	0.1349	5.4351E-02	1.6905E-01	ENSG00000119397
CNTROB	0.0981	1.4987E-01	3.3072E-01	ENSG00000170037
COA1	0.0118	8.3521E-01	9.1518E-01	ENSG00000106603
COA3	-0.2587	1.2321E-05	2.8762E-04	ENSG00000183978
COA4	-0.2218	1.7118E-03	1.3435E-02	ENSG00000181924
COA5	0.0077	9.0274E-01	9.5245E-01	ENSG00000183513
COA6	0.1103	1.5366E-01	3.3601E-01	ENSG00000168275
COA6-AS1	0.2645	1.3460E-02	6.2998E-02	ENSG00000231663
COA7	-0.1370	5.0703E-02	1.6105E-01	ENSG00000162377
COA8	-0.3913	9.9331E-06	2.4392E-04	ENSG00000256053
COASY	-0.1488	5.7800E-02	1.7621E-01	ENSG00000068120
COBL	-0.5278	1.0630E-03	9.5128E-03	ENSG00000106078
COBLL1	0.2054	5.6201E-02	1.7321E-01	ENSG00000082438
COCH	-0.0935	2.5614E-01	4.6444E-01	ENSG00000100473
COG1	0.1122	1.5396E-01	3.3643E-01	ENSG00000166685
COG2	0.1920	1.9559E-02	8.1690E-02	ENSG00000135775
COG3	-0.0099	8.6780E-01	9.3343E-01	ENSG00000136152
COG4	-0.2654	1.3004E-03	1.1064E-02	ENSG00000103051
COG5	-0.0222	7.2703E-01	8.5014E-01	ENSG00000164597
COG6	0.2161	3.7234E-02	1.2978E-01	ENSG00000133103
COG7	0.0510	4.9626E-01	6.8781E-01	ENSG00000168434
COG8	-0.1687	2.3664E-02	9.3912E-02	ENSG00000213380
COIL	-0.1221	3.9450E-02	1.3562E-01	ENSG00000121058
COL10A1	0.0186	1.5257E-01		ENSG00000123500
COL11A1	0.1065	2.2363E-01	4.2744E-01	ENSG00000060718
COL11A2	0.2371	3.5413E-02	1.2506E-01	ENSG00000204248
COL12A1	0.1961	6.8190E-02	1.9711E-01	ENSG00000111799
COL14A1	0.0765	2.1295E-01	4.1398E-01	ENSG00000187955
COL15A1	0.1543	7.4234E-02	2.0908E-01	ENSG00000204291
COL16A1	-0.2139	2.4049E-02	9.5025E-02	ENSG00000084636
COL17A1	0.0221	2.5774E-01		ENSG00000065618

COL18A1	0.0884	3.0148E-01	5.1154E-01	ENSG00000182871
COL18A1-AS1	0.0465	3.7959E-01	5.8808E-01	ENSG00000183535
COL18A1-AS2	0.0009	8.6715E-01		ENSG00000224574
COL1A1	0.2722	2.1124E-02	8.6294E-02	ENSG00000108821
COL1A2	0.2131	4.5755E-02	1.5037E-01	ENSG00000164692
COL20A1	0.0998	2.6442E-01	4.7345E-01	ENSG00000101203
COL21A1	0.0018	9.7362E-01	9.8715E-01	ENSG00000124749
COL22A1	-0.0281	7.1281E-01	8.4243E-01	ENSG00000169436
COL23A1	0.0739	3.2624E-01	5.3640E-01	ENSG00000050767
COL24A1	-0.5708	2.2863E-03	1.6648E-02	ENSG00000171502
COL25A1	0.0634	4.0973E-01	6.1397E-01	ENSG00000188517
COL25A1-DT	0.0368	1.2636E-01		ENSG00000246774
COL26A1	-0.0932	2.4986E-01	4.5777E-01	ENSG00000160963
COL27A1	0.0867	3.1568E-01	5.2581E-01	ENSG00000196739
COL2A1	0.4746	7.2687E-04	7.1510E-03	ENSG00000139219
COL3A1	0.0309	3.8240E-01		ENSG00000168542
COL4A1	0.0674	4.2883E-01	6.3061E-01	ENSG00000187498
COL4A2	0.0207	7.9484E-01	8.9159E-01	ENSG00000134871
COL4A2-AS2	-0.0053	6.5926E-01		ENSG00000224821
COL4A3	0.0743	3.1239E-01	5.2253E-01	ENSG00000169031
COL4A4	0.2040	5.9244E-02	1.7892E-01	ENSG00000081052
COL4A5	0.0794	2.5907E-01	4.6768E-01	ENSG00000188153
COL4A6	0.0169	8.0012E-01	8.9450E-01	ENSG00000197565
COL5A1	0.0143	8.4025E-01	9.1780E-01	ENSG00000130635
COL5A2	0.2579	3.2555E-02	1.1771E-01	ENSG00000204262
COL5A3	0.0442	4.1831E-01	6.2150E-01	ENSG00000080573
COL6A1	0.0165	7.7813E-01	8.8190E-01	ENSG00000142156
COL6A2	-0.4555	5.5215E-05	9.6148E-04	ENSG00000142173
COL6A3	0.0137	7.2047E-01		ENSG00000163359
COL6A5	0.0044	8.8313E-01		ENSG00000172752
COL6A6	-0.0131	3.3547E-01		ENSG00000206384
COL7A1	0.0292	7.2310E-01	8.4820E-01	ENSG00000114270
COL8A1	-0.0827	1.1253E-01	2.7524E-01	ENSG00000144810
COL8A2	0.0208	7.8195E-01	8.8447E-01	ENSG00000171812
COL9A1	-0.3967	2.7135E-03	1.8905E-02	ENSG00000112280
COL9A2	0.0176	8.3459E-01	9.1499E-01	ENSG00000049089
COL9A3	-0.3788	4.0100E-06	1.1760E-04	ENSG00000092758
COLCA1	0.0155	6.1895E-01		ENSG00000196167
COLEC10	0.0184	6.9858E-01		ENSG00000184374
COLEC11	0.0370	5.0693E-01	6.9624E-01	ENSG00000118004
COLEC12	0.0063	9.0774E-01	9.5461E-01	ENSG00000158270
COLGALT1	-0.1013	1.2999E-01	3.0267E-01	ENSG00000130309
COLGALT2	0.0411	6.1757E-01	7.7853E-01	ENSG00000198756
COLQ	0.0002	9.9727E-01	9.9837E-01	ENSG00000206561
COMMD1	0.0413	5.4251E-01	7.2404E-01	ENSG00000173163
COMMD10	-0.0988	1.4307E-01	3.2081E-01	ENSG00000145781
COMMD2	-0.0751	1.6508E-01	3.5226E-01	ENSG00000114744
COMMD3	-0.1508	3.7219E-02	1.2977E-01	ENSG00000148444
COMMD4	-0.0626	2.6666E-01	4.7595E-01	ENSG00000140365

COMMD5	-0.1416	2.0973E-02	8.5848E-02	ENSG00000170619
COMMD6	-0.0448	4.9699E-01	6.8827E-01	ENSG00000188243
COMMD7	-0.2842	8.8852E-04	8.2936E-03	ENSG00000149600
COMMD8	0.0141	8.4073E-01	9.1800E-01	ENSG00000169019
COMMD9	0.1243	4.2484E-02	1.4257E-01	ENSG00000110442
COMP	-0.0375	4.9977E-01	6.9083E-01	ENSG00000105664
COMT	-0.3053	5.4955E-06	1.5125E-04	ENSG00000093010
COMTD1	-0.1145	1.8002E-01	3.7188E-01	ENSG00000165644
COP1	0.0091	9.0794E-01	9.5461E-01	ENSG00000143207
COPA	-0.0058	9.1625E-01	9.5863E-01	ENSG00000122218
COPB1	0.0275	6.7147E-01	8.1623E-01	ENSG00000129083
COPB2	0.1196	1.0672E-01	2.6571E-01	ENSG00000184432
COPB2-DT	0.3307	1.2043E-05	2.8356E-04	ENSG00000248932
COPE	-0.1770	5.1438E-03	3.0749E-02	ENSG00000105669
COPG1	0.0431	5.8451E-01	7.5451E-01	ENSG00000181789
COPG2	0.4173	1.6610E-05	3.6753E-04	ENSG00000158623
COPRS	-0.0561	3.6840E-01	5.7759E-01	ENSG00000172301
COPS2	-0.0771	1.1600E-01	2.8071E-01	ENSG00000166200
COPS3	-0.2635	1.0411E-04	1.5892E-03	ENSG00000141030
COPS4	-0.0568	2.6841E-01	4.7785E-01	ENSG00000138663
COPS5	-0.2211	7.5527E-04	7.3510E-03	ENSG00000121022
COPS6	-0.1986	6.1272E-05	1.0513E-03	ENSG00000168090
COPS7A	-0.0203	7.4760E-01	8.6279E-01	ENSG00000111652
COPS7B	-0.1218	8.6582E-02	2.3113E-01	ENSG00000144524
COPS8	-0.1129	2.8976E-02	1.0834E-01	ENSG00000198612
COPS8-DT	0.2860	1.6852E-03	1.3286E-02	ENSG00000227252
COPS9	-0.3730	1.7964E-05	3.9325E-04	ENSG00000172428
COPZ1	-0.1782	3.1159E-03	2.1098E-02	ENSG00000111481
COPZ2	0.0715	4.1849E-01	6.2165E-01	ENSG00000005243
COQ10A	0.1679	3.4361E-02	1.2217E-01	ENSG00000135469
COQ10B	-0.1095	1.2730E-01	2.9873E-01	ENSG00000115520
COQ2	-0.0035	9.4836E-01	9.7458E-01	ENSG00000173085
COQ3	-0.0764	1.8337E-01	3.7665E-01	ENSG00000132423
COQ4	0.1383	1.4687E-02	6.7032E-02	ENSG00000167113
COQ5	-0.0481	4.2616E-01	6.2837E-01	ENSG00000110871
COQ6	0.0405	5.8292E-01	7.5357E-01	ENSG00000119723
COQ7	0.0416	4.5720E-01	6.5545E-01	ENSG00000167186
COQ7-DT	0.0200	7.1147E-01		ENSG00000261465
COQ8A	-0.0127	8.5402E-01	9.2585E-01	ENSG00000163050
COQ8B	0.0496	4.5682E-01	6.5502E-01	ENSG00000123815
COQ9	0.0130	8.5861E-01	9.2866E-01	ENSG00000088682
CORIN	0.0171	2.4711E-01	4.5483E-01	ENSG00000145244
CORO1A	0.0084	9.0790E-01	9.5461E-01	ENSG00000102879
CORO1B	-0.2395	2.0615E-03	1.5394E-02	ENSG00000172725
CORO1C	-0.4544	2.8914E-09	2.8009E-07	ENSG00000110880
CORO2A	-0.0549	4.9239E-01	6.8459E-01	ENSG00000106789
CORO2B	-0.2226	3.6066E-03	2.3595E-02	ENSG00000103647
CORO6	0.1594	8.3465E-02	2.2563E-01	ENSG00000167549
CORO7	-0.0327	6.1709E-01	7.7812E-01	ENSG00000262246

CORT	-0.0100	6.1756E-01	7.7853E-01	ENSG00000241563
COSMOC	-0.0059	7.9749E-01		ENSG00000260552
COTL1	-0.1003	6.7070E-02	1.9509E-01	ENSG00000103187
COX1	0.0292	7.2510E-01	8.4928E-01	ENSG00000198804
COX10	-0.1319	4.1745E-02	1.4086E-01	ENSG00000006695
COX10-DT	0.2647	5.9965E-04	6.1324E-03	ENSG00000236088
COX11	-0.0406	3.4883E-01	5.5864E-01	ENSG00000166260
COX14	0.0128	8.9088E-01	9.4581E-01	ENSG00000178449
COX15	0.0094	8.9920E-01	9.5042E-01	ENSG00000014919
COX16	-0.0558	3.7856E-01	5.8704E-01	ENSG00000133983
COX17	0.0685	3.2029E-01	5.3036E-01	ENSG00000138495
COX18	0.1648	1.6426E-02	7.2339E-02	ENSG00000163626
COX19	0.2173	4.4019E-03	2.7351E-02	ENSG00000240230
COX2	-0.0865	2.7020E-01	4.7977E-01	ENSG00000198712
COX20	-0.0288	7.3286E-01	8.5407E-01	ENSG00000203667
COX3	0.0668	4.0510E-01	6.0955E-01	ENSG00000198938
COX4I1	-0.2202	1.7613E-03	1.3721E-02	ENSG00000131143
COX4I2	0.0459	2.8080E-01		ENSG00000131055
COX5A	-0.1956	1.7923E-03	1.3876E-02	ENSG00000178741
COX5B	-0.2609	2.3106E-04	2.9170E-03	ENSG00000135940
COX6A1	-0.2542	1.6195E-03	1.2912E-02	ENSG00000111775
COX6B1	-0.2691	9.7136E-05	1.5106E-03	ENSG00000126267
COX6B2	-0.2549	1.4244E-02	6.5522E-02	ENSG00000160471
COX6C	-0.2039	9.4437E-03	4.8621E-02	ENSG00000164919
COX6CP13	0.0338	5.6623E-01		ENSG00000234144
COX6CP14	-0.0160	1.5602E-01		ENSG00000239576
COX7A1	-0.0618	1.7864E-01		ENSG00000161281
COX7A2	-0.2201	8.4935E-03	4.4952E-02	ENSG00000112695
COX7A2L	-0.1006	1.2540E-01	2.9561E-01	ENSG00000115944
COX7B	-0.1713	8.6329E-03	4.5535E-02	ENSG00000131174
COX7B2	0.0312	1.8565E-01		ENSG00000170516
COX7C	-0.2660	3.7796E-04	4.2771E-03	ENSG00000127184
COX8A	-0.2436	1.5942E-03	1.2773E-02	ENSG00000176340
COX8C	-0.0248	4.4953E-01		ENSG00000187581
CP	0.0148	4.9414E-01		ENSG00000047457
CPA1	0.0123	2.6168E-01		ENSG00000091704
CPA2	0.3114	1.2740E-02	6.0478E-02	ENSG00000158516
CPA4	0.5019	8.3598E-03	4.4349E-02	ENSG00000128510
CPA5	0.0362	2.9593E-01	5.0592E-01	ENSG00000158525
CPA6	-0.0134	8.0460E-01	8.9752E-01	ENSG00000165078
CPAMD8	0.1533	1.2183E-01	2.9050E-01	ENSG00000160111
CPB1	0.0830	6.7533E-01	8.1785E-01	ENSG00000153002
CPB2	0.0485	5.0723E-01	6.9631E-01	ENSG00000080618
CPB2-AS1	0.2277	3.8359E-02	1.3279E-01	ENSG00000235903
CPD	0.0568	4.0526E-01	6.0968E-01	ENSG00000108582
CPE	-0.0722	2.8444E-01	4.9499E-01	ENSG00000109472
CPEB1	-0.2901	2.0746E-03	1.5469E-02	ENSG00000214575
CPEB1-AS1	-0.0309	5.5809E-01	7.3563E-01	ENSG00000259462
CPEB2	0.0678	3.7681E-01	5.8542E-01	ENSG00000137449

CPEB2-DT	0.0204	9.9211E-01	9.9552E-01	ENSG00000247624
CPEB3	-0.1781	1.8812E-02	7.9462E-02	ENSG00000107864
CPEB4	-0.0277	6.5582E-01	8.0557E-01	ENSG00000113742
CPED1	-0.0133	5.4644E-01	7.2651E-01	ENSG00000106034
CPHXL	-0.0149	4.8186E-01		ENSG00000283755
CPHXL2	-0.0192	2.9651E-01		ENSG00000284484
CPLANE1	0.3524	2.3131E-06	7.5030E-05	ENSG00000197603
CPLANE1-AS1	0.5451	3.2176E-03	2.1607E-02	ENSG00000286193
CPLANE2	-0.0074	9.2235E-01	9.6166E-01	ENSG00000132881
CPLX1	-0.3464	8.7323E-04	8.1882E-03	ENSG00000168993
CPLX2	-0.2281	9.2616E-03	4.7935E-02	ENSG00000145920
CPLX4	-0.0149	3.8468E-01		ENSG00000166569
CPM	0.1081	2.0625E-01	4.0594E-01	ENSG00000135678
CPNE1	-0.2090	6.3039E-03	3.5821E-02	ENSG00000214078
CPNE2	-0.1733	1.6048E-03	1.2820E-02	ENSG00000140848
CPNE3	-0.0938	7.7525E-02	2.1520E-01	ENSG00000085719
CPNE4	-0.1106	2.0707E-01	4.0669E-01	ENSG00000196353
CPNE5	0.0222	7.7736E-01	8.8147E-01	ENSG00000124772
CPNE6	0.2089	5.9674E-02	1.7959E-01	ENSG00000100884
CPNE7	-0.3947	1.1392E-03	9.9893E-03	ENSG00000178773
CPNE8	-0.0470	5.5995E-01	7.3725E-01	ENSG00000139117
CPNE8-AS1	0.0345	6.8372E-01	8.2356E-01	ENSG00000257718
CPNE9	-0.0606	4.5399E-01	6.5254E-01	ENSG00000144550
CPO	0.0037	6.1954E-01		ENSG00000144410
CPOX	0.3035	6.8489E-04	6.8365E-03	ENSG00000080819
CPPED1	0.2696	6.3341E-04	6.4268E-03	ENSG00000103381
CPQ	0.0616	4.3588E-01	6.3678E-01	ENSG00000104324
CPS1	-0.0446	5.6853E-01	7.4298E-01	ENSG00000021826
CPSF1	-0.0982	1.8398E-01	3.7736E-01	ENSG00000071894
CPSF2	0.0366	5.0697E-01	6.9625E-01	ENSG00000165934
CPSF3	0.0742	2.4266E-01	4.5037E-01	ENSG00000119203
CPSF4	0.0777	2.5461E-01	4.6263E-01	ENSG00000160917
CPSF4L	0.0370	4.5618E-01		ENSG00000187959
CPSF6	0.1356	1.2601E-02	5.9925E-02	ENSG00000111605
CPSF7	-0.2050	2.9937E-03	2.0490E-02	ENSG00000149532
CPT1A	-0.5982	3.0059E-07	1.4223E-05	ENSG00000110090
CPT1B	0.0207	7.0889E-01		ENSG00000205560
CPT1C	-0.1474	2.4695E-02	9.6755E-02	ENSG00000169169
CPT2	-0.0934	2.4679E-01	4.5438E-01	ENSG00000157184
CPTP	-0.2531	1.3877E-03	1.1582E-02	ENSG00000224051
CPVL	0.2381	4.3873E-02	1.4570E-01	ENSG00000106066
CPVL-AS2	0.0559	3.1234E-01		ENSG00000272568
CPXM1	0.1203	1.6131E-01	3.4710E-01	ENSG00000088882
CPXM2	0.0055	9.3956E-01	9.7013E-01	ENSG00000121898
CR1	-0.0025	4.7516E-01		ENSG00000203710
CR1L	-0.0045	7.7081E-01		ENSG00000197721
CR2	0.0077	6.4961E-01		ENSG00000117322
CRABP2	-0.3620	1.3422E-04	1.9127E-03	ENSG00000143320
CRACD	-0.0399	5.4715E-01	7.2700E-01	ENSG00000109265

CRACDL	-0.2110	5.0175E-02	1.6004E-01	ENSG00000196872
CRACR2A	0.1951	6.7988E-02	1.9667E-01	ENSG00000130038
CRACR2B	0.1591	4.9345E-02	1.5807E-01	ENSG00000177685
CRADD	0.1048	1.2350E-01	2.9307E-01	ENSG00000169372
CRADD-AS1	0.0965	1.3083E-01	3.0373E-01	ENSG00000258274
CRAMP1	0.1471	1.0604E-01	2.6481E-01	ENSG00000007545
CRAT	-0.1468	5.4166E-02	1.6864E-01	ENSG00000095321
CRAT37	0.0255	3.0778E-01		ENSG00000258551
CRB1	-0.3108	2.5733E-04	3.1962E-03	ENSG00000134376
CRB2	0.0363	6.5851E-01	8.0731E-01	ENSG00000148204
CRB3	-0.0208	7.6559E-01	8.7404E-01	ENSG00000130545
CRBN	-0.3519	4.5195E-11	8.6204E-09	ENSG00000113851
CRCP	-0.0339	5.3134E-01	7.1538E-01	ENSG00000241258
CREB1	-0.0381	4.4215E-01	6.4153E-01	ENSG00000118260
CREB3	0.0928	1.5167E-01	3.3330E-01	ENSG00000107175
CREB3L1	-0.2358	1.9303E-02	8.0923E-02	ENSG00000157613
CREB3L2	0.1918	1.5239E-02	6.8737E-02	ENSG00000182158
CREB3L2-AS1	0.0138	6.7023E-01		ENSG00000237243
CREB3L3	0.0276	4.0416E-01		ENSG00000060566
CREB3L4	0.2536	1.3584E-02	6.3349E-02	ENSG00000143578
CREB5	-0.1186	1.4355E-01	3.2153E-01	ENSG00000146592
CREBBP	0.0424	5.6707E-01	7.4191E-01	ENSG00000005339
CREBL2	0.0314	6.8694E-01	8.2574E-01	ENSG00000111269
CREBRF	-0.2482	5.7866E-04	5.9498E-03	ENSG00000164463
CREBZF	0.2859	9.3651E-05	1.4658E-03	ENSG00000137504
CREG1	-0.2472	2.9394E-04	3.5314E-03	ENSG00000143162
CREG2	-0.2486	5.7230E-03	3.3348E-02	ENSG00000175874
CRELD1	0.1419	7.9399E-02	2.1857E-01	ENSG00000163703
CRELD2	-0.0491	3.2748E-01	5.3793E-01	ENSG00000184164
CREM	0.1968	4.4390E-03	2.7477E-02	ENSG00000095794
CRH	0.3875	1.4302E-02	6.5713E-02	ENSG00000147571
CRHBP	0.5109	7.4328E-03	4.0560E-02	ENSG00000145708
CRHR1	0.0087	8.3832E-01		ENSG00000120088
CRHR1	-0.0156	6.6840E-01		ENSG00000263715
CRHR2	-0.4485	7.9833E-04	7.6491E-03	ENSG00000106113
CRIM1	-0.0110	8.8331E-01	9.4147E-01	ENSG00000150938
CRIP1	0.0461	5.4648E-01	7.2651E-01	ENSG00000213145
CRIP2	-0.1488	4.8925E-02	1.5716E-01	ENSG00000182809
CRIP3	-0.0081	8.8817E-01	9.4440E-01	ENSG00000146215
CRIPT	-0.1270	7.9547E-02	2.1881E-01	ENSG00000119878
CRIPTO3	0.0068	5.5824E-01		ENSG00000225366
CRISPLD1	0.0724	3.5589E-01	5.6577E-01	ENSG00000121005
CRISPLD2	0.0817	3.5575E-01	5.6564E-01	ENSG00000103196
CRK	0.1186	3.1925E-02	1.1605E-01	ENSG00000167193
CRKL	-0.2831	1.0353E-04	1.5841E-03	ENSG00000099942
CRLF2	-0.0240	6.5762E-01		ENSG00000205755
CRLF3	0.0700	2.8796E-01	4.9850E-01	ENSG00000176390
CRLS1	-0.0292	7.1847E-01	8.4517E-01	ENSG00000088766
CRMP1	-0.3879	4.1623E-05	7.7061E-04	ENSG00000072832

CRNDE	-0.1242	1.2048E-01	2.8815E-01	ENSG00000245694
CRNKL1	0.0322	6.0884E-01	7.7193E-01	ENSG00000101343
CROCC	0.1369	8.1142E-02	2.2147E-01	ENSG00000058453
CROCC2	0.0428	2.7223E-01		ENSG00000226321
CROCCP1	0.0132	6.9262E-01		ENSG00000225769
CROCCP3	-0.1153	2.0574E-01	4.0523E-01	ENSG00000080947
CROCCP4	-0.0179	4.5924E-01	6.5703E-01	ENSG00000227684
CROT	0.1258	1.1593E-01	2.8061E-01	ENSG00000005469
CRP	0.0173	2.4239E-01		ENSG00000132693
CRPPA	0.0765	3.4456E-01	5.5416E-01	ENSG00000214960
CRTAC1	-0.2843	9.1278E-03	4.7442E-02	ENSG00000095713
CRTAM	0.1076	1.2585E-01	2.9650E-01	ENSG00000109943
CRTAP	-0.0392	5.2958E-01	7.1411E-01	ENSG00000170275
CRTC1	0.1858	4.9250E-02	1.5787E-01	ENSG00000105662
CRTC2	-0.0924	1.6936E-01	3.5786E-01	ENSG00000160741
CRTC3	0.0649	2.6784E-01	4.7723E-01	ENSG00000140577
CRTC3-AS1	0.0021	9.8469E-01	9.9198E-01	ENSG00000259736
CRX	0.0194	7.2225E-01	8.4773E-01	ENSG00000105392
CRY1	-0.1190	1.2449E-01	2.9425E-01	ENSG00000008405
CRY2	-0.1468	6.1386E-02	1.8325E-01	ENSG00000121671
CRYAA	0.0390	2.3905E-01	4.4553E-01	ENSG00000160202
CRYAB	0.0687	2.4570E-01	4.5347E-01	ENSG00000109846
CRYBA1	-0.0085	8.9440E-01		ENSG00000108255
CRYBA2	-0.0446	3.9037E-01	5.9766E-01	ENSG00000163499
CRYBA4	0.1614	1.3501E-02		ENSG00000196431
CRYBB1	0.1074	3.1501E-03	2.1270E-02	ENSG00000100122
CRYBB2	0.1899	3.3972E-02	1.2111E-01	ENSG00000244752
CRYBG1	0.1332	1.3659E-01	3.1194E-01	ENSG00000112297
CRYBG2	0.2751	3.1078E-02	1.1418E-01	ENSG00000176092
CRYBG3	0.1510	1.2492E-01	2.9495E-01	ENSG00000080200
CRYGA	0.0367	5.2889E-01	7.1353E-01	ENSG00000168582
CRYGC	0.3899	9.3092E-03	4.8110E-02	ENSG00000163254
CRYGD	0.0901	2.9053E-01	5.0088E-01	ENSG00000118231
CRYGN	-0.0030	6.8837E-01		ENSG00000127377
CRYL1	-0.1867	1.3740E-02	6.3912E-02	ENSG00000165475
CRYZ	0.0550	4.7335E-01	6.6931E-01	ENSG00000116791
CRYZL1	-0.0452	3.8488E-01	5.9280E-01	ENSG00000205758
CRYZL2P	0.2784	2.5108E-02	9.7814E-02	ENSG00000242193
CS	-0.1006	8.0401E-02	2.2039E-01	ENSG00000062485
CSAD	0.0244	7.1467E-01	8.4332E-01	ENSG00000139631
CSAG2	0.0350	1.8289E-01		ENSG00000268902
CSAG3	0.0411	1.2973E-01	3.0229E-01	ENSG00000268916
CSDC2	-0.4028	1.7167E-06	5.8913E-05	ENSG00000172346
CSDE1	-0.2644	4.2318E-06	1.2221E-04	ENSG00000009307
CSE1L	-0.0997	1.1363E-01	2.7718E-01	ENSG00000124207
CSE1L-DT	0.0717	1.5813E-01	3.4225E-01	ENSG00000227431
CSF1	-0.2461	5.8657E-03	3.3967E-02	ENSG00000184371
CSF1R	-0.0985	2.4990E-01	4.5778E-01	ENSG00000182578
CSF2RA	0.0219	7.2867E-01	8.5119E-01	ENSG00000198223

CSF2RBP1	0.0080	5.1736E-01		ENSG00000232254
CSF3	0.0079	7.8950E-01		ENSG00000108342
CSGALNACT1	0.0552	5.1252E-01	7.0051E-01	ENSG00000147408
CSGALNACT2	-0.3296	6.7469E-04	6.7566E-03	ENSG00000169826
CSGALNACT2P1	0.0007	4.6284E-01		ENSG00000182574
CSK	0.0384	6.1733E-01	7.7831E-01	ENSG00000103653
CSKMT	-0.1268	1.7700E-01	3.6824E-01	ENSG00000214756
CSMD1	0.1333	1.6616E-01	3.5384E-01	ENSG00000183117
CSMD2	0.3203	5.9386E-04	6.0852E-03	ENSG00000121904
CSMD2-AS1	0.0245	4.1236E-01		ENSG00000231163
CSMD3	0.3113	8.3895E-03	4.4488E-02	ENSG00000164796
CSNK1A1	-0.1296	1.9010E-02	8.0040E-02	ENSG00000113712
CSNK1A1L	-0.0682	3.2026E-01	5.3036E-01	ENSG00000180138
CSNK1D	0.0872	1.7831E-01	3.6957E-01	ENSG00000141551
CSNK1E	-0.0684	3.5633E-01	5.6619E-01	ENSG00000213923
CSNK1G1	0.0515	4.7670E-01	6.7217E-01	ENSG00000169118
CSNK1G2	0.0536	4.1240E-01	6.1608E-01	ENSG00000133275
CSNK1G2-AS1	-0.0091	8.5943E-01		ENSG00000180846
CSNK1G3	-0.0933	7.0501E-02	2.0157E-01	ENSG00000151292
CSNK2A1	-0.1282	4.5407E-03	2.7973E-02	ENSG00000101266
CSNK2A2	-0.0999	2.6129E-01	4.6984E-01	ENSG00000070770
CSNK2A3	-0.0125	8.7989E-01	9.3994E-01	ENSG00000254598
CSNK2B	-0.1403	3.3817E-02	1.2081E-01	ENSG00000204435
CSNKA2IP	0.0141	3.1582E-01		ENSG00000283434
CSP2	-0.3703	1.7608E-02	7.5914E-02	ENSG00000241218
CSPG4	0.0424	6.0669E-01	7.7068E-01	ENSG00000173546
CSPG4BP	0.0189	8.9802E-01	9.4955E-01	ENSG00000232517
CSPG4P10	0.0411	6.1660E-01	7.7770E-01	ENSG00000276710
CSPG4P12	-0.0074	9.1166E-01	9.5639E-01	ENSG00000259295
CSPG4P13	0.0582	3.3022E-02		ENSG00000260139
CSPG5	-0.3101	4.9009E-05	8.7635E-04	ENSG00000114646
CSPP1	0.1836	2.2286E-03	1.6330E-02	ENSG00000104218
CSRNP2	-0.0273	6.4694E-01	7.9897E-01	ENSG00000110925
CSRNP3	-0.0864	1.4494E-01	3.2368E-01	ENSG00000178662
CSRP1	0.0565	4.9903E-01	6.9000E-01	ENSG00000159176
CSRP2	-0.2807	2.6601E-02	1.0195E-01	ENSG00000175183
CSRP3	0.0143	5.9897E-01		ENSG00000129170
CST3	-0.1094	1.5334E-01	3.3558E-01	ENSG00000101439
CST6	-0.0245	2.4488E-01		ENSG00000175315
CSTA	0.0312	1.5465E-01		ENSG00000121552
CSTB	-0.1396	6.8254E-02	1.9718E-01	ENSG00000160213
CSTF1	-0.1293	1.3661E-02	6.3650E-02	ENSG00000101138
CSTF2	0.1397	5.3399E-02	1.6684E-01	ENSG00000101811
CSTF2T	0.0113	8.7344E-01	9.3651E-01	ENSG00000177613
CSTF3	0.0749	2.2159E-01	4.2486E-01	ENSG00000176102
CSTF3-DT	0.2276	3.1524E-02	1.1524E-01	ENSG00000247151
CSTPP1	-0.1155	4.8783E-02	1.5680E-01	ENSG00000149179
CT45A1	0.0786	2.4808E-01	4.5579E-01	ENSG00000268940
CT45A10	0.0349	6.5148E-01	8.0263E-01	ENSG00000269586

CT45A11P	-0.0248	4.5608E-01		ENSG00000230162
CT45A2	-0.0161	7.6785E-01		ENSG00000271449
CT45A3	0.0784	1.9153E-01	3.8681E-01	ENSG00000269096
CT45A5	0.0626	5.6698E-01	7.4191E-01	ENSG00000228836
CT45A6	0.0379	7.7559E-01	8.8055E-01	ENSG00000278289
CT45A7	0.0379	7.7559E-01	8.8055E-01	ENSG00000273696
CT45A8	-0.0164	7.1726E-01		ENSG00000278085
CT45A9	-0.0233	4.2872E-01		ENSG00000270946
CT55	-0.0147	7.1933E-01		ENSG00000169551
CT62	0.0119	7.1960E-01		ENSG00000225362
CT66	-0.0387	6.2948E-01	7.8664E-01	ENSG00000234215
CT69	0.0306	1.9751E-01		ENSG00000231971
CT70	-0.0125	8.1642E-01	9.0447E-01	ENSG00000230013
CTAG1A	-0.0601	4.0155E-01	6.0655E-01	ENSG00000268651
CTAG1B	-0.0347	6.0736E-01	7.7108E-01	ENSG00000184033
CTAGE14P	0.0103	7.3617E-01		ENSG00000214211
CTAGE15	0.0268	1.2109E-01		ENSG00000271079
CTAGE3P	0.0425	4.0214E-01	6.0669E-01	ENSG00000232872
CTAGE4	0.0500	8.8687E-02		ENSG00000288784
CTAGE6	0.0179	1.6937E-01		ENSG00000271321
CTAGE8	0.0524	1.0793E-01		ENSG00000289604
CTAGE9	0.0060	8.6267E-01		ENSG00000236761
CTB-1I21.1	-0.0266	9.8333E-02	2.5203E-01	ENSG00000250284
CTB-30L5.1	0.0076	8.2865E-01		ENSG00000267052
CTBP1	-0.1465	1.5406E-02	6.9338E-02	ENSG00000159692
CTBP1-AS	-0.0186	5.9800E-01		ENSG00000280927
CTBP1-DT	0.1644	1.6514E-02	7.2645E-02	ENSG00000196810
CTBP2	-0.2160	7.1608E-03	3.9496E-02	ENSG00000175029
CTBP2P4	-0.0045	8.3721E-01		ENSG00000251102
CTBP2P7	0.0153	4.9232E-01		ENSG00000267091
CTBS	-0.1392	1.1726E-01	2.8284E-01	ENSG00000117151
CTC-338M12.4	-0.0408	5.7464E-01	7.4742E-01	ENSG00000233937
CTC1	0.1014	2.0363E-01	4.0249E-01	ENSG00000178971
CTCF	0.0272	6.9608E-01	8.3174E-01	ENSG00000102974
CTCF-DT	0.0215	7.6582E-01	8.7418E-01	ENSG00000259804
CTCFL	0.0289	4.1260E-01		ENSG00000124092
CTD-2350J17.1	-0.0259	3.6592E-01		ENSG00000250250
CTDNEP1	-0.1353	1.0065E-01	2.5582E-01	ENSG00000175826
CTDP1	0.1668	2.5053E-02	9.7767E-02	ENSG00000060069
CTDP1-DT	-0.0083	5.7063E-01		ENSG00000178412
CTDSP1	-0.0778	3.3544E-01	5.4550E-01	ENSG00000144579
CTDSP2	-0.3013	7.3509E-03	4.0239E-02	ENSG00000175215
CTDSPL	0.2751	5.4029E-03	3.1956E-02	ENSG00000144677
CTDSPL2	-0.2442	3.6831E-03	2.4013E-02	ENSG00000137770
CTF1	-0.2545	7.7957E-03	4.2064E-02	ENSG00000150281
CTH	-0.1304	1.4521E-01	3.2405E-01	ENSG00000116761
CTHRC1	0.4471	1.1471E-02	5.6101E-02	ENSG00000164932
CTIF	0.0145	8.5269E-01	9.2499E-01	ENSG00000134030
CTNNA1	-0.0659	2.9293E-01	5.0300E-01	ENSG00000044115

CTNNA1-AS1	-0.0137	3.8014E-01		ENSG00000253404
CTNNA2	-0.0903	2.2361E-01	4.2744E-01	ENSG00000066032
CTNNA3	0.2598	3.7393E-02	1.3025E-01	ENSG00000183230
CTNNAL1	-0.0425	4.4380E-01	6.4290E-01	ENSG00000119326
CTNNB1	-0.3563	5.6192E-04	5.8166E-03	ENSG00000168036
CTNNBIP1	0.0446	5.3672E-01	7.1967E-01	ENSG00000178585
CTNNBL1	-0.0789	2.2341E-01	4.2718E-01	ENSG00000132792
CTNND1	-0.1989	1.4979E-02	6.7901E-02	ENSG00000198561
CTNND2	-0.0732	1.3230E-01	3.0579E-01	ENSG00000169862
CTNS	0.0862	2.8443E-01	4.9499E-01	ENSG00000040531
CTPS1	-0.0387	5.7569E-01	7.4819E-01	ENSG00000171793
CTPS2	-0.0915	1.6062E-01	3.4607E-01	ENSG00000047230
CTR9	-0.0703	2.2952E-01	4.3402E-01	ENSG00000198730
CTRB1	0.0663	2.6068E-01	4.6918E-01	ENSG00000168925
CTRB2	-0.0229	6.6785E-01	8.1403E-01	ENSG00000168928
CTRC	0.1669	7.4101E-02	2.0880E-01	ENSG00000162438
CTSA	-0.0977	1.4957E-01	3.3032E-01	ENSG00000064601
CTSB	-0.0185	7.7692E-01	8.8125E-01	ENSG00000164733
CTSC	0.0400	6.0003E-01	7.6572E-01	ENSG00000109861
CTSD	0.1253	1.3716E-01	3.1258E-01	ENSG00000117984
CTSE	0.0024	4.7651E-01		ENSG00000196188
CTSF	0.3451	1.6332E-03	1.3001E-02	ENSG00000174080
CTSH	0.0488	4.9602E-01	6.8770E-01	ENSG00000103811
CTSK	0.3506	8.2482E-04	7.8388E-03	ENSG00000143387
CTSL	-0.0538	4.2175E-01	6.2442E-01	ENSG00000135047
CTSLP3	0.0508	4.8875E-01	6.8194E-01	ENSG00000280913
CTSO	0.0509	5.3100E-01	7.1517E-01	ENSG00000256043
CTSS	-0.0216	7.6949E-01	8.7668E-01	ENSG00000163131
CTSV	0.0495	5.5322E-01	7.3163E-01	ENSG00000136943
CTSZ	-0.0139	8.8232E-01	9.4107E-01	ENSG00000101160
CTTN	0.0053	9.2189E-01	9.6143E-01	ENSG00000085733
CTTNBP2	0.5129	5.3905E-07	2.2828E-05	ENSG00000077063
CTTNBP2NL	0.0531	3.4241E-01	5.5198E-01	ENSG00000143079
CTU1	-0.0310	6.7960E-01	8.2059E-01	ENSG00000142544
CTU2	0.1523	8.2212E-02	2.2325E-01	ENSG00000174177
CTXN1	-0.1955	4.7126E-02	1.5340E-01	ENSG00000178531
CTXN2	-0.0255	6.7176E-01	8.1641E-01	ENSG00000233932
CTXN3	-0.0312	6.0118E-01	7.6642E-01	ENSG00000205279
CTXND1	-0.0060	9.3135E-01	9.6601E-01	ENSG00000259417
CUBN	0.3538	3.3048E-03	2.2024E-02	ENSG00000107611
CUBNP2	-0.0002	7.4639E-01		ENSG00000234993
CUEDC1	-0.0261	6.7170E-01	8.1641E-01	ENSG00000180891
CUEDC2	-0.1513	1.1517E-03	1.0081E-02	ENSG00000107874
CUL1	-0.1927	6.4009E-03	3.6264E-02	ENSG00000055130
CUL2	-0.0831	1.6417E-01	3.5102E-01	ENSG00000108094
CUL3	-0.2423	2.8300E-05	5.6890E-04	ENSG00000036257
CUL4A	0.1496	3.9687E-02	1.3618E-01	ENSG00000139842
CUL4B	-0.0713	1.7792E-01	3.6918E-01	ENSG00000158290
CUL5	-0.2392	1.5461E-04	2.1300E-03	ENSG00000166266

CUL7	0.0426	5.5205E-01	7.3071E-01	ENSG00000044090
CUL9	0.2427	8.6186E-04	8.1094E-03	ENSG00000112659
CUTA	-0.1362	2.5694E-02	9.9480E-02	ENSG00000112514
CUTALP	0.4106	2.5406E-06	8.1079E-05	ENSG00000226752
CUTC	0.1599	1.4359E-02	6.5879E-02	ENSG00000119929
CUX1	-0.0308	6.9608E-01	8.3174E-01	ENSG00000257923
CUX2	-0.1517	1.2125E-01	2.8952E-01	ENSG00000111249
CWC15	-0.2367	9.5215E-04	8.7222E-03	ENSG00000150316
CWC22	0.2563	1.7622E-04	2.3527E-03	ENSG00000163510
CWC25	0.0213	7.7650E-01	8.8102E-01	ENSG00000273559
CWC27	-0.1685	3.9353E-04	4.4214E-03	ENSG00000153015
CWF19L1	-0.0684	2.9999E-01	5.0968E-01	ENSG00000095485
CWF19L2	0.3308	6.3635E-05	1.0806E-03	ENSG00000152404
CWH43	-0.0140	7.5616E-01		ENSG00000109182
CX3CL1	0.0078	9.2800E-01	9.6466E-01	ENSG00000006210
CX3CR1	0.0202	7.5614E-01	8.6792E-01	ENSG00000168329
CXADR	-0.1505	4.9219E-02	1.5785E-01	ENSG00000154639
CXADRP1	-0.0101	5.4927E-01		ENSG00000214319
CXADRP3	-0.0055	7.0214E-01		ENSG00000265766
CXCL1	0.0419	2.9216E-01	5.0231E-01	ENSG00000163739
CXCL11	0.0021	9.8182E-01		ENSG00000169248
CXCL12	-0.0399	6.0762E-01	7.7108E-01	ENSG00000107562
CXCL13	0.0065	9.9531E-01		ENSG00000156234
CXCL14	0.4010	1.2956E-02	6.1154E-02	ENSG00000145824
CXCL16	-0.1561	8.1568E-02	2.2221E-01	ENSG00000161921
CXCL17	0.0204	6.6585E-01		ENSG00000189377
CXCL5	0.0139	6.0372E-01		ENSG00000163735
CXCL6	-0.0036	9.5348E-01	9.7699E-01	ENSG00000124875
CXCR3	0.0070	6.4919E-01		ENSG00000186810
CXCR6	-0.0023	9.7167E-01		ENSG00000172215
CXorf38	0.1182	6.9399E-02	1.9948E-01	ENSG00000185753
CXorf51A	0.0327	9.1300E-02		ENSG00000224440
CXorf51B	0.0327	9.1300E-02		ENSG00000235699
CXorf58	0.2246	4.3064E-02	1.4391E-01	ENSG00000165182
CXXC1	0.0260	7.0832E-01	8.3965E-01	ENSG00000154832
CXXC4	-0.0165	8.0042E-01	8.9475E-01	ENSG00000168772
CXXC4-AS1	0.0293	5.2704E-01		ENSG00000245384
CXXC5	-0.1348	1.2721E-01	2.9864E-01	ENSG00000171604
CXXC5-AS1	-0.0366	5.1488E-01	7.0256E-01	ENSG00000250635
CYB561	0.2157	2.0006E-02	8.3066E-02	ENSG00000008283
CYB561A3	0.0803	2.7004E-01	4.7972E-01	ENSG00000162144
CYB561D1	0.0562	4.5900E-01	6.5682E-01	ENSG00000174151
CYB561D2	0.1145	1.3326E-01	3.0696E-01	ENSG00000114395
CYB5A	-0.1125	1.2200E-01	2.9074E-01	ENSG00000166347
CYB5B	0.1017	8.0369E-02	2.2033E-01	ENSG00000103018
CYB5D1	0.0166	9.2409E-01		ENSG00000182224
CYB5D2	0.4283	5.9190E-11	1.0631E-08	ENSG00000167740
CYB5R1	0.0768	1.6559E-01	3.5308E-01	ENSG00000159348
CYB5R2	0.2778	3.1680E-02	1.1560E-01	ENSG00000166394

CYB5R3	-0.0501	5.1202E-01	7.0032E-01	ENSG00000100243
CYB5R4	0.1762	1.0070E-02	5.1022E-02	ENSG00000065615
CYB5RL	0.0111	8.9946E-01	9.5058E-01	ENSG00000215883
CYBA	-0.0976	2.2577E-01	4.2962E-01	ENSG00000051523
CYBB	-0.0478	8.8868E-02		ENSG00000165168
CYBC1	0.1215	6.5714E-02	1.9233E-01	ENSG00000178927
CYBRD1	0.1030	2.5510E-01	4.6319E-01	ENSG00000071967
CYC1	-0.1927	5.1198E-04	5.3975E-03	ENSG00000179091
CYCS	-0.1013	1.4342E-01	3.2139E-01	ENSG00000172115
CYCSP3	0.0427	3.9678E-01	6.0264E-01	ENSG00000188512
CYCSP52	-0.0580	2.0786E-01	4.0753E-01	ENSG00000235700
CYFIP1	-0.1984	1.9197E-02	8.0647E-02	ENSG00000273749
CYFIP2	-0.2221	1.0810E-03	9.6195E-03	ENSG00000055163
CYGB	-0.1047	2.4415E-01	4.5180E-01	ENSG00000161544
CYLC1	0.0096	6.8481E-01		ENSG00000183035
CYLC2	0.0298	2.6582E-01		ENSG00000155833
CYLD	0.0725	2.7067E-01	4.8008E-01	ENSG00000083799
CYLD-AS1	-0.0335	1.9412E-01		ENSG00000261644
CYLD-AS2	0.0069	8.9730E-01		ENSG00000260616
CYMP	0.0096	6.5753E-01		ENSG00000240194
CYP11A1	0.2123	9.8413E-03	5.0133E-02	ENSG00000140459
CYP17A1	-0.0154	2.2903E-01		ENSG00000148795
CYP19A1	0.1121	1.1085E-01	2.7259E-01	ENSG00000137869
CYP1A1	0.0549	5.1676E-01	7.0434E-01	ENSG00000140465
CYP1A2	-0.0232	3.9533E-01		ENSG00000140505
CYP1B1	0.0298	4.6112E-01	6.5849E-01	ENSG00000138061
CYP1B1-AS1	0.1274	4.9315E-02		ENSG00000232973
CYP20A1	-0.1586	1.5362E-02	6.9188E-02	ENSG00000119004
CYP21A2	0.1144	7.8019E-02	2.1606E-01	ENSG00000231852
CYP24A1	-0.0452	5.3836E-01	7.2078E-01	ENSG00000019186
CYP26A1	0.0278	5.5257E-01	7.3112E-01	ENSG00000095596
CYP26B1	-0.0953	2.7819E-01	4.8808E-01	ENSG00000003137
CYP26C1	0.0487	4.3653E-01	6.3728E-01	ENSG00000187553
CYP27A1	-0.0444	5.9187E-01	7.5967E-01	ENSG00000135929
CYP27B1	0.0195	7.9775E-01	8.9353E-01	ENSG00000111012
CYP27C1	0.1146	2.0724E-01	4.0688E-01	ENSG00000186684
CYP2A7	0.0112	8.9131E-01		ENSG00000198077
CYP2AB1P	-0.0069	6.7648E-01		ENSG00000233441
CYP2C8	0.1575	1.0508E-01	2.6327E-01	ENSG00000138115
CYP2D6	0.2824	1.7116E-02	7.4405E-02	ENSG00000100197
CYP2D8P	0.1017	1.8976E-01	3.8487E-01	ENSG00000226450
CYP2E1	-0.0828	3.5401E-01	5.6400E-01	ENSG00000130649
CYP2F1	0.0059	5.6587E-01		ENSG00000197446
CYP2J2	-0.2943	2.3047E-02	9.2056E-02	ENSG00000134716
CYP2R1	0.2840	2.4773E-04	3.0972E-03	ENSG00000186104
CYP2S1	-0.5401	2.9237E-04	3.5143E-03	ENSG00000167600
CYP2T1P	0.0655	4.2680E-01	6.2892E-01	ENSG00000233622
CYP2T3P	-0.0026	8.0189E-01		ENSG00000268529
CYP2U1	-0.2021	5.9559E-03	3.4312E-02	ENSG00000155016

CYP2U1-AS1	-0.0359	6.6528E-01	8.1193E-01	ENSG00000245293
CYP2W1	0.0765	1.1999E-01		ENSG00000073067
CYP39A1	-0.1258	1.8139E-01	3.7392E-01	ENSG00000146233
CYP3A4	-0.0023	9.5290E-01		ENSG00000160868
CYP3A43	-0.0636	1.6249E-01		ENSG00000021461
CYP3A5	-0.0161	8.4529E-01	9.2085E-01	ENSG00000106258
CYP3A7	0.0278	1.5905E-01		ENSG00000160870
CYP46A1	0.0832	2.8924E-01	4.9966E-01	ENSG00000036530
CYP4A22	0.0283	4.5387E-01		ENSG00000162365
CYP4B1	0.0019	8.7796E-01		ENSG00000142973
CYP4F11	-0.0102	1.7296E-01	3.6282E-01	ENSG00000171903
CYP4F12	-0.0047	8.6059E-01		ENSG00000186204
CYP4F2	0.0056	8.3105E-01	9.1269E-01	ENSG00000186115
CYP4F22	-0.0093	5.5262E-01		ENSG00000171954
CYP4F27P	0.0046	5.9555E-01		ENSG00000248313
CYP4F3	0.0122	9.5113E-01		ENSG00000186529
CYP4V2	0.0143	8.5800E-01	9.2834E-01	ENSG00000145476
CYP4X1	0.0354	6.6789E-01	8.1403E-01	ENSG00000186377
CYP4Z1	0.0648	1.2942E-01		ENSG00000186160
CYP51A1	-0.4214	2.8380E-06	8.8841E-05	ENSG00000001630
CYP51A1-AS1	0.1476	1.2008E-01	2.8751E-01	ENSG00000188693
CYP7B1	-0.0194	8.0937E-01	8.9973E-01	ENSG00000172817
CYREN	0.0849	2.3747E-01	4.4342E-01	ENSG00000122783
CYRIA	-0.1343	9.0763E-02	2.3940E-01	ENSG00000197872
CYRIB	-0.1628	2.9218E-03	2.0085E-02	ENSG00000153310
CYS1	-0.1764	4.2346E-02	1.4224E-01	ENSG00000205795
CYSLTR1	-0.0059	7.4518E-01		ENSG00000173198
CYSLTR2	0.0668	2.7759E-01	4.8758E-01	ENSG00000152207
CYSRT1	0.5199	1.9962E-03	1.5057E-02	ENSG00000197191
CYSTM1	-0.0156	8.3711E-01	9.1604E-01	ENSG00000120306
CYTB	0.0385	6.1902E-01	7.7960E-01	ENSG00000198727
CYTH1	-0.1744	2.3488E-02	9.3395E-02	ENSG00000108669
CYTH1P1	0.0069	4.8799E-01		ENSG00000271286
CYTH2	-0.0892	1.5271E-01	3.3479E-01	ENSG00000105443
CYTH3	0.0615	3.6430E-01	5.7342E-01	ENSG00000008256
CYTH4	0.0248	3.4082E-01		ENSG00000100055
CYTIP	-0.1512	2.3349E-02	9.3006E-02	ENSG00000115165
CYTL1	-0.0041	7.5306E-01		ENSG00000170891
CYTOR	0.4743	1.1932E-04	1.7665E-03	ENSG00000222041
CYYR1	-0.0203	7.9971E-01	8.9430E-01	ENSG00000166265
CYYR1-AS1	0.1653	9.0180E-02	2.3831E-01	ENSG00000197934
CZIB	-0.1342	3.1576E-02	1.1534E-01	ENSG00000162384
D21S2088E	0.0125	4.1430E-01		ENSG00000228592
D2HGDH	0.0389	6.0831E-01	7.7147E-01	ENSG00000180902
DAAM1	0.2104	6.9941E-03	3.8783E-02	ENSG00000100592
DAAM2	-0.0470	5.8163E-01	7.5277E-01	ENSG00000146122
DAAM2-AS1	-0.0003	6.4626E-01		ENSG00000235033
DAB1	0.1199	1.1957E-01	2.8671E-01	ENSG00000173406
DAB1-AS1	0.0541	4.0847E-01	6.1271E-01	ENSG00000226759

DAB2IP	0.1740	4.7912E-02	1.5490E-01	ENSG00000136848
DACH1	-0.0463	5.4010E-01	7.2209E-01	ENSG00000276644
DACH2	0.0504	5.3731E-01	7.2018E-01	ENSG00000126733
DACT1	-0.2174	1.2230E-02	5.8717E-02	ENSG00000165617
DACT2	0.0952	2.3160E-01	4.3644E-01	ENSG00000164488
DACT3	-0.0186	7.7506E-01	8.8028E-01	ENSG00000197380
DACT3-AS1	0.1642	9.2891E-02	2.4317E-01	ENSG00000245598
DAD1	0.0434	5.4498E-01	7.2555E-01	ENSG00000129562
DAG1	-0.1011	1.3178E-01	3.0505E-01	ENSG00000173402
DAGLA	-0.0748	3.3874E-01	5.4852E-01	ENSG00000134780
DAGLB	0.0539	4.1182E-01	6.1583E-01	ENSG00000164535
DALRD3	0.0235	7.3385E-01	8.5469E-01	ENSG00000178149
DANCR	0.0330	5.8449E-01	7.5451E-01	ENSG00000226950
DAND5	0.0301	6.2771E-01	7.8564E-01	ENSG00000179284
DANT2	0.0158	8.3269E-01	9.1367E-01	ENSG00000235244
DAO	-0.0032	9.5750E-01	9.7911E-01	ENSG00000110887
DAP	0.0343	6.0762E-01	7.7108E-01	ENSG00000112977
DAP3	-0.0291	6.0825E-01	7.7147E-01	ENSG00000132676
DAPK1	0.1450	2.8027E-02	1.0591E-01	ENSG00000196730
DAPK1-IT1	0.0807	1.7769E-01	3.6887E-01	ENSG00000236709
DAPK2	-0.1777	5.5461E-02	1.7165E-01	ENSG00000035664
DAPK3	-0.0153	8.1740E-01	9.0498E-01	ENSG00000167657
DAPL1	-0.2596	1.6004E-02	7.1117E-02	ENSG00000163331
DAPP1	0.0180	7.6954E-01	8.7668E-01	ENSG00000070190
DARS1	0.0507	3.7119E-01	5.7998E-01	ENSG00000115866
DARS2	-0.0470	4.7907E-01	6.7430E-01	ENSG00000117593
DAW1	0.0946	2.1942E-01	4.2176E-01	ENSG00000123977
DAXX	-0.2037	8.1889E-04	7.7974E-03	ENSG00000204209
DAZAP1	0.0357	6.4142E-01	7.9494E-01	ENSG00000071626
DAZAP2	-0.1727	4.5313E-03	2.7943E-02	ENSG00000183283
DAZL	-0.0108	3.6954E-01		ENSG00000092345
DBF4	-0.0515	4.8186E-01	6.7638E-01	ENSG00000006634
DBF4B	0.1156	2.0374E-01	4.0262E-01	ENSG00000161692
DBH	0.0261	2.3396E-01		ENSG00000123454
DBH-AS1	0.0737	3.5683E-01	5.6664E-01	ENSG00000225756
DBI	-0.3482	7.3403E-04	7.2067E-03	ENSG00000155368
DBIL5P	0.0343	6.7471E-01	8.1764E-01	ENSG00000231784
DBIL5P2	-0.0152	4.5584E-01		ENSG00000242412
DBN1	-0.3238	1.5281E-04	2.1124E-03	ENSG00000113758
DBNDD1	-0.1335	2.8931E-02	1.0828E-01	ENSG00000003249
DBNDD2	-0.2493	3.8278E-02	1.3254E-01	ENSG00000244274
DBNL	-0.1706	1.4732E-03	1.2126E-02	ENSG00000136279
DBP	-0.3463	3.6473E-05	6.9675E-04	ENSG00000105516
DBR1	-0.2332	2.2314E-04	2.8331E-03	ENSG00000138231
DBT	0.1745	4.8760E-02	1.5677E-01	ENSG00000137992
DBX1	-0.0044	7.8073E-01		ENSG00000109851
DBX2	0.0425	4.3856E-01	6.3861E-01	ENSG00000185610
DCAF1	0.1554	6.3996E-02	1.8878E-01	ENSG00000145041
DCAF10	-0.0797	1.2751E-01	2.9903E-01	ENSG00000122741

DCAF11	-0.0883	1.7916E-01	3.7069E-01	ENSG00000100897
DCAF12	0.0413	5.7044E-01	7.4425E-01	ENSG00000198876
DCAF12L1	0.0142	9.2746E-01		ENSG00000198889
DCAF12L2	-0.0015	9.8003E-01	9.8991E-01	ENSG00000198354
DCAF13	0.0687	3.6871E-01	5.7783E-01	ENSG00000164934
DCAF13P1	0.0340	2.9086E-01		ENSG00000242562
DCAF15	-0.1122	1.1067E-01	2.7237E-01	ENSG00000132017
DCAF16	-0.0010	9.7855E-01	9.8925E-01	ENSG00000163257
DCAF17	-0.0879	2.0528E-01	4.0456E-01	ENSG00000115827
DCAF4	-0.1112	1.5103E-01	3.3236E-01	ENSG00000119599
DCAF4L1	0.1715	9.6021E-02	2.4868E-01	ENSG00000182308
DCAF4L2	0.0128	2.1850E-01		ENSG00000176566
DCAF5	-0.0804	1.2050E-01	2.8815E-01	ENSG00000139990
DCAF6	-0.1655	2.9146E-02	1.0889E-01	ENSG00000143164
DCAF7	-0.0623	2.0652E-01	4.0618E-01	ENSG00000136485
DCAF8	0.1216	1.0948E-01	2.7024E-01	ENSG00000132716
DCAF8-DT	-0.0863	3.2840E-01	5.3886E-01	ENSG00000228606
DCAF8L1	0.0324	1.7953E-01	3.7118E-01	ENSG00000226372
DCAF8L2	0.0699	7.1562E-03	3.9479E-02	ENSG00000189186
DCAKD	0.0076	9.0406E-01	9.5319E-01	ENSG00000172992
DCBLD1	-0.0373	6.4742E-01	7.9949E-01	ENSG00000164465
DCBLD2	0.1433	2.5628E-02	9.9294E-02	ENSG00000057019
DCC	0.1949	3.5928E-02	1.2639E-01	ENSG00000187323
DCDC1	0.2915	1.4570E-02	6.6610E-02	ENSG00000170959
DCDC2B	0.0788	2.5430E-01	4.6234E-01	ENSG00000222046
DCDC2C	0.0460	1.4223E-01		ENSG00000214866
DCHS1	0.0374	6.4394E-01	7.9679E-01	ENSG00000166341
DCHS2	0.1112	2.1779E-01	4.1991E-01	ENSG00000197410
DCK	-0.1209	4.3610E-02	1.4520E-01	ENSG00000156136
DCLK1	-0.0593	4.1752E-01	6.2089E-01	ENSG00000133083
DCLK2	-0.2060	3.8928E-03	2.5041E-02	ENSG00000170390
DCLK3	-0.0192	8.3740E-01	9.1605E-01	ENSG00000163673
DCLRE1A	-0.0240	7.6943E-01	8.7668E-01	ENSG00000198924
DCLRE1B	-0.0186	7.7441E-01	8.8002E-01	ENSG00000118655
DCLRE1C	0.1316	8.1515E-02	2.2211E-01	ENSG00000152457
DCN	-0.1560	5.6283E-02	1.7336E-01	ENSG00000011465
DCP1A	-0.2089	1.9104E-05	4.1306E-04	ENSG00000272886
DCP1B	0.3479	4.8264E-06	1.3745E-04	ENSG00000151065
DCP2	-0.4957	1.2848E-09	1.3673E-07	ENSG00000172795
DCPS	-0.3637	1.8074E-06	6.1281E-05	ENSG00000110063
DCST1	0.0427	5.5970E-01	7.3712E-01	ENSG00000163357
DCST2	0.2130	5.4045E-02	1.6845E-01	ENSG00000163354
DCSTAMP	0.0190	3.8339E-01		ENSG00000164935
DCT	-0.0058	9.3252E-01	9.6658E-01	ENSG00000080166
DCTD	-0.0107	8.5721E-01	9.2780E-01	ENSG00000129187
DCTN1	-0.1422	1.1210E-01	2.7453E-01	ENSG00000204843
DCTN1-AS1	0.5127	5.1968E-03	3.1023E-02	ENSG00000237737
DCTN2	-0.3009	1.7544E-08	1.3368E-06	ENSG00000175203
DCTN3	-0.0831	2.2383E-01	4.2766E-01	ENSG00000137100

DCTN4	-0.0440	3.9349E-01	6.0001E-01	ENSG00000132912
DCTN5	0.1149	2.0518E-02	8.4430E-02	ENSG00000166847
DCTN6	-0.0982	9.8527E-02	2.5234E-01	ENSG00000104671
DCTPP1	-0.1970	1.0809E-03	9.6195E-03	ENSG00000179958
DCUN1D1	-0.2203	6.8147E-04	6.8161E-03	ENSG00000043093
DCUN1D2	0.3790	2.9187E-05	5.8150E-04	ENSG00000150401
DCUN1D3	0.1177	1.5241E-01	3.3443E-01	ENSG00000188215
DCUN1D4	-0.1032	1.0795E-01	2.6776E-01	ENSG00000109184
DCUN1D5	0.0017	9.6342E-01	9.8180E-01	ENSG00000137692
DCX	-0.1480	6.9547E-02	1.9983E-01	ENSG00000077279
DCXR	-0.2820	3.2748E-04	3.8406E-03	ENSG00000169738
DCXR-DT	-0.0205	7.9177E-01	8.9001E-01	ENSG00000264569
DDA1	-0.1488	2.5816E-02	9.9857E-02	ENSG00000130311
DDAH1	-0.0033	9.4387E-01	9.7218E-01	ENSG00000153904
DDAH2	-0.4208	8.1508E-08	4.8559E-06	ENSG00000213722
DDB1	0.0232	7.2506E-01	8.4928E-01	ENSG00000167986
DDB2	-0.0512	4.0891E-01	6.1323E-01	ENSG00000134574
DDC	0.1989	5.9294E-02	1.7897E-01	ENSG00000132437
DDC-AS1	0.0209	3.4782E-01		ENSG00000226122
DDHD1	0.1475	2.8362E-02	1.0685E-01	ENSG00000100523
DDHD1-DT	0.0818	8.1229E-02		ENSG00000258731
DDHD2	0.0608	2.3692E-01	4.4260E-01	ENSG00000085788
DDI2	0.1481	6.1903E-02	1.8446E-01	ENSG00000197312
DDIAS	0.0634	4.4450E-01	6.4339E-01	ENSG00000165490
DDIT3	-0.5464	1.1058E-07	6.2834E-06	ENSG00000175197
DDIT4	-0.4192	2.2641E-06	7.3785E-05	ENSG00000168209
DDIT4L	0.1513	3.6043E-02	1.2669E-01	ENSG00000145358
DDN	-0.1065	2.2419E-01	4.2808E-01	ENSG00000181418
DDN-AS1	0.0272	7.3944E-01	8.5776E-01	ENSG00000257913
DDO	0.0729	2.1931E-01	4.2176E-01	ENSG00000203797
DDOST	-0.0050	9.0009E-01	9.5105E-01	ENSG00000244038
DDR1	-0.0767	2.5028E-01	4.5823E-01	ENSG00000204580
DDR2	0.5316	7.4842E-06	1.9384E-04	ENSG00000162733
DDRGK1	-0.1519	1.7146E-02	7.4470E-02	ENSG00000198171
DDT	0.0577	4.8622E-01	6.7994E-01	ENSG00000099977
DDTL	0.0776	2.9927E-01	5.0897E-01	ENSG00000099974
DDX1	-0.0855	1.2300E-01	2.9222E-01	ENSG00000079785
DDX10	0.0880	2.0219E-01	4.0097E-01	ENSG00000178105
DDX10P1	-0.0197	5.0639E-01		ENSG00000237135
DDX11	0.0595	3.9669E-01	6.0261E-01	ENSG00000013573
DDX11-AS1	-0.2424	3.7562E-02	1.3076E-01	ENSG00000245614
DDX17	0.3653	8.4771E-05	1.3535E-03	ENSG00000100201
DDX18	0.2482	3.3157E-04	3.8756E-03	ENSG00000088205
DDX18P1	0.0264	7.3991E-01		ENSG00000259165
DDX19A	-0.1765	5.0556E-03	3.0347E-02	ENSG00000168872
DDX19A-DT	0.0932	2.9108E-01	5.0140E-01	ENSG00000261777
DDX19B	-0.0347	5.6537E-01	7.4090E-01	ENSG00000157349
DDX20	0.0312	6.0967E-01	7.7240E-01	ENSG00000064703
DDX20P1	-0.0343	2.9198E-01		ENSG00000276662

DDX21	0.0899	1.7507E-01	3.6558E-01	ENSG00000165732
DDX23	-0.0223	7.0035E-01	8.3440E-01	ENSG00000174243
DDX24	0.0119	8.5796E-01	9.2834E-01	ENSG00000089737
DDX25	-0.0316	6.4531E-01	7.9755E-01	ENSG00000109832
DDX27	0.0675	1.9317E-01	3.8924E-01	ENSG00000124228
DDX28	-0.5378	3.4844E-12	1.0797E-09	ENSG00000182810
DDX31	0.2088	2.7452E-02	1.0440E-01	ENSG00000125485
DDX39A	-0.2208	1.4906E-03	1.2205E-02	ENSG00000123136
DDX39B	-0.1508	5.7001E-02	1.7477E-01	ENSG00000198563
DDX39B-AS1	-0.0097	9.3563E-01	9.6833E-01	ENSG00000234006
DDX3P2	0.0226	6.9731E-01		ENSG00000230986
DDX3X	-0.0868	2.5663E-01	4.6502E-01	ENSG00000215301
DDX4	0.0750	9.6929E-02		ENSG00000152670
DDX41	0.1534	2.9198E-02	1.0905E-01	ENSG00000183258
DDX42	0.1373	5.0072E-02	1.5988E-01	ENSG00000198231
DDX43	0.0796	5.2970E-02		ENSG00000080007
DDX46	0.1729	4.7518E-03	2.8955E-02	ENSG00000145833
DDX47	-0.4008	2.9967E-04	3.5912E-03	ENSG00000213782
DDX49	-0.0225	7.0547E-01	8.3771E-01	ENSG00000105671
DDX5	-0.1397	1.7285E-02	7.4910E-02	ENSG00000108654
DDX50	0.0489	3.4330E-01	5.5287E-01	ENSG00000107625
DDX51	-0.0103	8.6180E-01	9.3057E-01	ENSG00000185163
DDX52	0.0103	8.7672E-01	9.3851E-01	ENSG00000278053
DDX53	0.0003	8.7908E-01		ENSG00000184735
DDX54	-0.0711	2.5198E-01	4.6024E-01	ENSG00000123064
DDX55	0.0407	5.4292E-01	7.2427E-01	ENSG00000111364
DDX55P1	0.0269	3.4203E-01		ENSG00000270863
DDX56	0.1151	8.1250E-02	2.2166E-01	ENSG00000136271
DDX59	0.0157	8.1641E-01	9.0447E-01	ENSG00000118197
DDX59-AS1	-0.0263	7.4544E-01	8.6131E-01	ENSG00000260088
DDX6	-0.4106	1.1087E-10	1.8432E-08	ENSG00000110367
DDX60	-0.0024	9.7606E-01	9.8803E-01	ENSG00000137628
DDX60L	0.2516	3.8479E-02	1.3310E-01	ENSG00000181381
DEAF1	0.1209	9.3668E-02	2.4443E-01	ENSG00000177030
DECR1	-0.1750	5.1281E-03	3.0685E-02	ENSG00000104325
DECR2	0.1104	9.7157E-02	2.5043E-01	ENSG00000242612
DEDD	-0.0686	2.3480E-01	4.4019E-01	ENSG00000158796
DEDD2	0.0215	7.3833E-01	8.5753E-01	ENSG00000160570
DEF6	0.1065	1.8681E-01	3.8112E-01	ENSG00000023892
DEF8	-0.0708	1.8554E-01	3.7937E-01	ENSG00000140995
DEFA5	-0.0271	2.0535E-01		ENSG00000164816
DEFB1	-0.0051	7.4515E-01		ENSG00000164825
DEFB109D	0.0782	5.5522E-02		ENSG00000254866
DEFB125	0.0047	8.8265E-01		ENSG00000178591
DEFB131C	-0.0048	7.9649E-01		ENSG00000254700
DEFB131E	-0.0231	6.5753E-01	8.0662E-01	ENSG00000254507
DEFB135	0.0140	6.6015E-01		ENSG00000205883
DEGS1	-0.2828	3.4360E-06	1.0380E-04	ENSG00000143753
DEGS2	-0.4924	2.5753E-03	1.8161E-02	ENSG00000168350

DEK	-0.2548	1.8666E-03	1.4299E-02	ENSG00000124795
DELE1	0.0484	4.7255E-01	6.6877E-01	ENSG00000081791
DELEC1	0.3038	4.2942E-02	1.4364E-01	ENSG00000173077
DENND10	-0.0515	3.1809E-01	5.2832E-01	ENSG00000119979
DENND11	0.0562	4.6935E-01	6.6580E-01	ENSG00000257093
DENND1A	-0.0226	7.5255E-01	8.6574E-01	ENSG00000119522
DENND1B	0.2840	4.8658E-03	2.9445E-02	ENSG00000213047
DENND1C	0.1856	5.3897E-02	1.6814E-01	ENSG00000205744
DENND2A	-0.2839	4.2234E-03	2.6537E-02	ENSG00000146966
DENND2B	0.0839	2.2549E-01	4.2926E-01	ENSG00000166444
DENND2C	0.1350	1.3574E-01	3.1055E-01	ENSG00000175984
DENND2D	0.0302	6.4383E-01	7.9674E-01	ENSG00000162777
DENND3	0.2756	2.3930E-03	1.7228E-02	ENSG00000105339
DENND3-AS1	-0.1920	7.2143E-02	2.0512E-01	ENSG00000253210
DENND4A	0.1509	3.8876E-02	1.3410E-01	ENSG00000174485
DENND4B	0.2447	1.5541E-03	1.2549E-02	ENSG00000198837
DENND4C	-0.1118	1.3945E-01	3.1568E-01	ENSG00000137145
DENND5A	-0.1599	1.1496E-02	5.6199E-02	ENSG00000184014
DENND5B	0.0311	5.5837E-01	7.3579E-01	ENSG00000170456
DENND5B-AS1	0.0301	7.1121E-01	8.4147E-01	ENSG00000255867
DENND6A	0.0291	6.7001E-01	8.1498E-01	ENSG00000174839
DENND6A-DT	0.0054	8.3905E-01	9.1723E-01	ENSG00000241933
DENND6B	0.0772	2.4884E-01	4.5654E-01	ENSG00000205593
DENR	-0.1822	5.6625E-04	5.8418E-03	ENSG00000139726
DEPDC1	-0.0525	5.2583E-01	7.1107E-01	ENSG00000024526
DEPDC1-AS1	-0.0152	9.4727E-01		ENSG00000234264
DEPDC1B	0.1099	2.1204E-01	4.1294E-01	ENSG00000035499
DEPDC4	0.2143	3.1086E-02	1.1419E-01	ENSG00000166153
DEPDC5	0.1746	3.1866E-02	1.1598E-01	ENSG00000100150
DEPDC7	-0.4945	4.7916E-05	8.5869E-04	ENSG00000121690
DEPP1	-0.4458	2.1000E-05	4.4390E-04	ENSG00000165507
DEPTOR	0.0184	7.7597E-01	8.8074E-01	ENSG00000155792
DERA	-0.0500	4.6482E-01	6.6150E-01	ENSG00000023697
DERL1	0.0642	3.6515E-01	5.7429E-01	ENSG00000136986
DERL2	-0.1327	2.1046E-02	8.6006E-02	ENSG00000072849
DERL3	0.0038	9.3625E-01	9.6866E-01	ENSG00000099958
DES	0.0691	1.9257E-01	3.8842E-01	ENSG00000175084
DESI1	-0.0436	4.9794E-01	6.8915E-01	ENSG00000100418
DESI2	-0.0986	7.6028E-02	2.1243E-01	ENSG00000121644
DET1	0.4375	7.1462E-03	3.9442E-02	ENSG00000140543
DEUP1	0.1762	6.9001E-02	1.9862E-01	ENSG00000165325
DEXI	-0.5033	3.9692E-03	2.5433E-02	ENSG00000182108
DFFA	-0.3898	1.8618E-05	4.0417E-04	ENSG00000160049
DFFB	-0.1712	3.3276E-02	1.1967E-01	ENSG00000169598
DFFBP1	-0.0087	8.6339E-01	9.3121E-01	ENSG00000232303
DGAT1	-0.1032	8.3929E-02	2.2638E-01	ENSG00000185000
DGAT2	-0.0304	7.0407E-01	8.3679E-01	ENSG00000062282
DGAT2L6	-0.0062	7.4114E-01		ENSG00000184210
DGAT2L7P	0.0153	5.4651E-01		ENSG00000205267

DGCR11	0.0146	8.6738E-01	9.3318E-01	ENSG00000273311
DGCR2	0.1307	4.2388E-02	1.4234E-01	ENSG00000070413
DGCR5	0.1081	2.0880E-01	4.0884E-01	ENSG00000273032
DGCR6	0.1784	2.5023E-03	1.7787E-02	ENSG00000278817
DGCR6	0.1458	3.2619E-02	1.1785E-01	ENSG00000183628
DGCR6L	0.1799	3.9916E-03	2.5550E-02	ENSG00000128185
DGCR8	0.0723	3.6237E-01	5.7174E-01	ENSG00000128191
DGKA	-0.0082	9.1667E-01	9.5870E-01	ENSG00000065357
DGKB	-0.0943	2.6648E-01	4.7581E-01	ENSG00000136267
DGKD	-0.0068	9.2050E-01	9.6092E-01	ENSG00000077044
DGKE	0.0544	3.9299E-01	5.9976E-01	ENSG00000153933
DGKG	0.2773	1.4477E-02	6.6285E-02	ENSG00000058866
DGKH	0.1841	5.4645E-02	1.6977E-01	ENSG00000102780
DGKI	-0.0209	7.8056E-01	8.8359E-01	ENSG00000157680
DGKQ	0.0430	5.1164E-01	7.0009E-01	ENSG00000145214
DGKZ	-0.1523	1.8134E-02	7.7411E-02	ENSG00000149091
DGKZP1	-0.0352	4.8443E-01	6.7822E-01	ENSG00000179611
DGLUCY	-0.1910	1.2651E-02	6.0114E-02	ENSG00000133943
DGUOK	0.0782	2.4763E-01	4.5544E-01	ENSG00000114956
DHCR24	-0.1550	6.1958E-02	1.8458E-01	ENSG00000116133
DHCR7	-0.1396	1.3041E-01	3.0320E-01	ENSG00000172893
DHDDS	0.0426	5.0323E-01	6.9370E-01	ENSG00000117682
DHDH	0.0813	2.9678E-01	5.0683E-01	ENSG00000104808
DHFR	-0.0805	3.2352E-01	5.3349E-01	ENSG00000228716
DHFR2	0.1640	5.9972E-02	1.8026E-01	ENSG00000178700
DHH	0.0399	5.9140E-01	7.5922E-01	ENSG00000139549
DHODH	0.1260	9.2013E-02	2.4154E-01	ENSG00000102967
DHPS	0.2955	1.6852E-02	7.3636E-02	ENSG00000095059
DHRS1	0.0455	5.3247E-01	7.1608E-01	ENSG00000157379
DHRS11	0.2705	9.6234E-03	4.9335E-02	ENSG00000278535
DHRS12	-0.0368	6.5519E-01	8.0518E-01	ENSG00000102796
DHRS13	0.0410	6.2704E-01	7.8504E-01	ENSG00000167536
DHRS3	-0.1061	1.8234E-01	3.7528E-01	ENSG00000162496
DHRS4	-0.1346	7.7905E-02	2.1584E-01	ENSG00000157326
DHRS4-AS1	0.0585	3.6512E-01	5.7429E-01	ENSG00000215256
DHRS4L1	-0.0227	6.9247E-01	8.2944E-01	ENSG00000285467
DHRS4L2	-0.0735	3.3807E-01	5.4799E-01	ENSG00000187630
DHRS7	-0.1021	1.2909E-01	3.0136E-01	ENSG00000100612
DHRS7B	0.1350	4.7793E-02	1.5473E-01	ENSG00000109016
DHRS9	0.0909	6.9195E-02		ENSG00000073737
DHRSX	-0.1405	3.5663E-02	1.2573E-01	ENSG00000169084
DHTKD1	-0.0869	1.4925E-01	3.2992E-01	ENSG00000181192
DHX15	-0.0105	7.9948E-01	8.9428E-01	ENSG00000109606
DHX16	0.1205	5.5396E-02	1.7149E-01	ENSG00000204560
DHX29	-0.0004	9.6888E-01	9.8475E-01	ENSG00000067248
DHX30	0.1302	1.1960E-02	5.7837E-02	ENSG00000132153
DHX32	0.0838	2.2585E-01	4.2972E-01	ENSG00000089876
DHX33	0.0390	6.2238E-01	7.8178E-01	ENSG00000005100
DHX33-DT	-0.0081	5.2849E-01		ENSG00000262099

DHX34	-0.1665	7.7729E-02	2.1550E-01	ENSG00000134815
DHX35	-0.0695	3.0475E-01	5.1500E-01	ENSG00000101452
DHX36	0.2663	2.3309E-05	4.8397E-04	ENSG00000174953
DHX37	-0.1242	1.7959E-01	3.7124E-01	ENSG00000150990
DHX38	-0.1001	7.7359E-02	2.1486E-01	ENSG00000140829
DHX40	0.1922	2.6906E-03	1.8791E-02	ENSG00000108406
DHX40P1	0.0051	8.9991E-01		ENSG00000266992
DHX57	-0.0336	5.7312E-01	7.4620E-01	ENSG00000163214
DHX58	0.0263	7.4702E-01	8.6252E-01	ENSG00000108771
DHX8	0.0503	3.9991E-01	6.0530E-01	ENSG00000067596
DHX9	-0.1774	5.5127E-03	3.2388E-02	ENSG00000135829
DIABLO	-0.0086	9.1043E-01	9.5580E-01	ENSG00000184047
DIAPH1	0.2112	1.0187E-02	5.1404E-02	ENSG00000131504
DIAPH1-AS1	0.0261	6.7926E-01	8.2053E-01	ENSG00000246422
DIAPH2	-0.2477	2.4730E-03	1.7670E-02	ENSG00000147202
DIAPH2-AS1	0.0439	3.4710E-01		ENSG00000236256
DIAPH3	-0.1841	4.8233E-02	1.5562E-01	ENSG00000139734
DIAPH3-AS1	-0.0310	3.0787E-01		ENSG00000227528
DICER1	0.2184	5.5017E-04	5.7335E-03	ENSG00000100697
DICER1-AS1	0.1739	3.3654E-03	2.2320E-02	ENSG00000235706
DIDO1	0.4500	2.6730E-11	5.7183E-09	ENSG00000101191
DIMT1	-0.1712	1.2861E-02	6.0852E-02	ENSG00000086189
DINOL	0.1095	1.9680E-01	3.9431E-01	ENSG00000285244
DIO1	0.0141	7.0345E-01		ENSG00000211452
DIO2	0.5197	5.8885E-03	3.4075E-02	ENSG00000211448
DIO2-AS1	0.0765	2.7026E-01	4.7981E-01	ENSG00000258766
DIO3	0.1150	1.1703E-01	2.8246E-01	ENSG00000197406
DIO3OS	0.4685	1.0690E-03	9.5505E-03	ENSG00000258498
DIP2A	0.2140	2.5742E-03	1.8159E-02	ENSG00000160305
DIP2A-IT1	-0.0343	4.7497E-01	6.7061E-01	ENSG00000223692
DIP2B	0.0006	9.9700E-01	9.9831E-01	ENSG00000066084
DIP2C	0.1441	3.1774E-02	1.1580E-01	ENSG00000151240
DIP2C-AS1	-0.0541	5.0639E-01	6.9604E-01	ENSG00000180525
DIPK1A	-0.0894	2.4211E-01	4.4953E-01	ENSG00000154511
DIPK1B	-0.1617	6.1042E-03	3.4967E-02	ENSG00000165716
DIPK1C	-0.0468	4.6476E-01	6.6150E-01	ENSG00000187773
DIPK2A	0.1671	5.9058E-02	1.7856E-01	ENSG00000181744
DIPK2B	0.0078	8.4481E-01		ENSG00000147113
DIRAS1	-0.2547	5.5917E-03	3.2743E-02	ENSG00000176490
DIRAS2	0.2977	3.7246E-03	2.4224E-02	ENSG00000165023
DIRAS3	0.4297	2.2876E-04	2.8939E-03	ENSG00000162595
DIS3	0.0505	4.5080E-01	6.4991E-01	ENSG00000083520
DIS3L	-0.0690	3.7938E-01	5.8784E-01	ENSG00000166938
DIS3L2	0.0050	9.2938E-01	9.6527E-01	ENSG00000144535
DISC1	-0.0113	8.9747E-01	9.4936E-01	ENSG00000162946
DISC1-IT1	0.0336	5.1480E-01		ENSG00000226758
DISC1FP1	0.0662	3.6763E-01	5.7688E-01	ENSG00000261645
DISP1	0.1712	5.8250E-02	1.7708E-01	ENSG00000154309
DISP2	-0.1521	4.9218E-02	1.5785E-01	ENSG00000140323

DISP3	-0.0238	7.7822E-01	8.8195E-01	ENSG00000204624
DIXDC1	0.0587	2.7715E-01	4.8719E-01	ENSG00000150764
DKC1	0.0698	2.1655E-01	4.1821E-01	ENSG00000130826
DKFZP434A062	-0.0181	5.7949E-01	7.5104E-01	ENSG00000262075
DKFZp451B082	0.1654	8.6567E-02	2.3112E-01	ENSG00000285564
DKK1	-0.1454	1.0648E-01	2.6528E-01	ENSG00000107984
DKK2	0.0245	5.4542E-01		ENSG00000155011
DKK3	-0.1079	1.4869E-01	3.2923E-01	ENSG00000050165
DKK4	0.0473	3.7585E-01	5.8443E-01	ENSG00000104371
DKKL1	-0.0593	4.8848E-01	6.8170E-01	ENSG00000104901
DLAT	0.0261	7.2255E-01	8.4792E-01	ENSG00000150768
DLC1	-0.0422	6.1946E-01	7.7989E-01	ENSG00000164741
DLD	0.0079	8.9928E-01	9.5046E-01	ENSG00000091140
DLEC1	0.0997	2.3062E-01	4.3539E-01	ENSG00000008226
DLEU1	-0.1870	1.6166E-02	7.1536E-02	ENSG00000176124
DLEU2	-0.2587	1.6572E-03	1.3128E-02	ENSG00000231607
DLEU7	-0.1189	1.9813E-01	3.9605E-01	ENSG00000186047
DLG1	-0.1098	9.6796E-02	2.4993E-01	ENSG00000075711
DLG1-AS1	-0.0011	7.6408E-01		ENSG00000227375
DLG2	0.0258	7.2251E-01	8.4792E-01	ENSG00000150672
DLG3	0.1240	6.9392E-02	1.9948E-01	ENSG00000082458
DLG3-AS1	0.0068	5.6356E-01		ENSG00000231651
DLG4	-0.0777	2.6848E-01	4.7790E-01	ENSG00000132535
DLG5	0.0351	6.2847E-01	7.8609E-01	ENSG00000151208
DLGAP1	-0.2443	4.2558E-03	2.6701E-02	ENSG00000170579
DLGAP1-AS3	-0.0097	7.3529E-01	8.5570E-01	ENSG00000263724
DLGAP1-AS5	0.0153	8.4595E-01	9.2100E-01	ENSG00000261520
DLGAP2	0.1613	8.3556E-02	2.2574E-01	ENSG00000198010
DLGAP2-AS1	0.0037	9.3102E-01		ENSG00000253267
DLGAP3	-0.1955	3.3150E-02	1.1933E-01	ENSG00000116544
DLGAP4	-0.3942	2.0967E-13	1.0318E-10	ENSG00000080845
DLGAP4-AS1	0.2323	3.6303E-02	1.2726E-01	ENSG00000232907
DLGAP5	0.0240	7.6341E-01	8.7268E-01	ENSG00000126787
DLK1	-0.2178	5.2501E-02	1.6488E-01	ENSG00000185559
DLK2	-0.0912	2.9193E-01	5.0210E-01	ENSG00000171462
DLL1	-0.2307	5.4191E-03	3.2029E-02	ENSG00000198719
DLL3	-0.1069	9.6595E-02	2.4966E-01	ENSG00000090932
DLL4	0.0100	9.2193E-01	9.6143E-01	ENSG00000128917
DLST	-0.4050	2.9590E-06	9.1695E-05	ENSG00000119689
DLX1	-0.3261	1.8232E-03	1.4046E-02	ENSG00000144355
DLX2	-0.2006	3.1781E-02	1.1581E-01	ENSG00000115844
DLX2-DT	0.0363	5.1599E-01	7.0353E-01	ENSG00000236651
DLX3	-0.0735	2.5916E-01	4.6768E-01	ENSG00000064195
DLX4	-0.1060	1.7247E-01	3.6214E-01	ENSG00000108813
DLX5	0.0538	5.3077E-01	7.1508E-01	ENSG00000105880
DLX6	-0.2954	1.4843E-02	6.7461E-02	ENSG00000006377
DM1-AS	0.0038	9.7975E-01		ENSG00000267395
DMAC1	0.0018	9.7706E-01	9.8847E-01	ENSG00000137038
DMAC2	-0.1948	2.1252E-03	1.5750E-02	ENSG00000105341

DMAC2L	0.1116	1.6965E-01	3.5816E-01	ENSG00000125375
DMAP1	-0.1218	4.4434E-02	1.4715E-01	ENSG00000178028
DMBT1L1	0.0110	5.3056E-01		ENSG00000176584
DMBX1	0.1255	4.7938E-02	1.5493E-01	ENSG00000197587
DMC1	0.0601	4.7801E-01	6.7338E-01	ENSG00000100206
DMD	0.0890	2.2136E-01	4.2457E-01	ENSG00000198947
DMGDH	0.2433	2.1292E-02	8.6839E-02	ENSG00000132837
DMKN	0.2189	5.1736E-02	1.6310E-01	ENSG00000161249
DMP1	0.0119	4.2748E-01		ENSG00000152592
DMPK	0.1058	2.0556E-01	4.0501E-01	ENSG00000104936
DMRT2	-0.1166	1.7554E-01	3.6638E-01	ENSG00000173253
DMRT3	-0.0567	5.0442E-01	6.9438E-01	ENSG00000064218
DMRTA2	0.1354	1.6020E-01	3.4542E-01	ENSG00000142700
DMRTB1	0.0096	6.3157E-01		ENSG00000143006
DMRTC1	0.0706	3.1824E-01	5.2846E-01	ENSG00000269502
DMRTC1B	0.0676	2.9960E-01	5.0917E-01	ENSG00000184911
DMTF1	-0.1550	8.7456E-03	4.5960E-02	ENSG00000135164
DMTF1-AS1	0.1841	3.1445E-02	1.1506E-01	ENSG00000224046
DMTN	-0.1129	1.6540E-01	3.5282E-01	ENSG00000158856
DMWD	-0.1009	1.0479E-01	2.6267E-01	ENSG00000185800
DMXL1	-0.0297	6.2310E-01	7.8228E-01	ENSG00000172869
DMXL2	0.2640	5.6137E-04	5.8158E-03	ENSG00000104093
DNA2	0.0269	7.3640E-01	8.5612E-01	ENSG00000138346
DNAAF10	0.0123	8.8956E-01	9.4523E-01	ENSG00000243667
DNAAF11	-0.1691	7.7732E-02	2.1550E-01	ENSG00000129295
DNAAF2	0.0629	3.0534E-01	5.1560E-01	ENSG00000165506
DNAAF3	0.0235	7.7700E-01	8.8130E-01	ENSG00000167646
DNAAF4	0.1374	1.4765E-01	3.2780E-01	ENSG00000256061
DNAAF5	0.1487	1.1086E-01	2.7259E-01	ENSG00000164818
DNAAF6	0.0233	5.9531E-01		ENSG00000080572
DNAAF8	0.2102	5.9801E-02	1.7986E-01	ENSG00000166246
DNAAF9	0.0725	3.3227E-01	5.4247E-01	ENSG00000088854
DNAH1	0.3717	8.8647E-03	4.6467E-02	ENSG00000114841
DNAH10	0.2766	2.7117E-03	1.8905E-02	ENSG00000197653
DNAH11	0.1152	2.0321E-01	4.0209E-01	ENSG00000105877
DNAH12	0.1545	1.2105E-01	2.8920E-01	ENSG00000174844
DNAH14	-0.0865	1.5566E-01	3.3933E-01	ENSG00000185842
DNAH17	0.0429	6.1199E-01	7.7417E-01	ENSG00000187775
DNAH2	0.3151	1.3393E-02	6.2757E-02	ENSG00000183914
DNAH3	0.3924	1.6001E-02	7.1117E-02	ENSG00000158486
DNAH5	0.4782	1.4934E-03	1.2224E-02	ENSG00000039139
DNAH7	0.1396	1.5020E-01	3.3125E-01	ENSG00000118997
DNAH9	-0.0093	9.0851E-01	9.5481E-01	ENSG00000007174
DNAI1	0.0950	2.8006E-01	4.9007E-01	ENSG00000122735
DNAI2	0.0999	6.9060E-02	1.9876E-01	ENSG00000171595
DNAI3	0.0662	2.7221E-01	4.8147E-01	ENSG00000162643
DNAI4	-0.0279	7.3698E-01	8.5644E-01	ENSG00000152763
DNAJA1	-0.2074	7.1949E-03	3.9625E-02	ENSG00000086061
DNAJA2	-0.0955	1.1755E-01	2.8313E-01	ENSG00000069345

DNAJA3	-0.0312	6.6230E-01	8.0981E-01	ENSG00000103423
DNAJA4	-0.1327	1.2384E-01	2.9362E-01	ENSG00000140403
DNAJB1	-0.1118	2.2112E-01	4.2423E-01	ENSG00000132002
DNAJB11	-0.0926	1.4368E-01	3.2175E-01	ENSG00000090520
DNAJB12	-0.1051	1.3125E-01	3.0423E-01	ENSG00000148719
DNAJB13	-0.0242	5.7104E-01		ENSG00000187726
DNAJB14	-0.0697	1.4793E-01	3.2816E-01	ENSG00000164031
DNAJB2	0.0108	8.8687E-01	9.4386E-01	ENSG00000135924
DNAJB4	-0.1091	2.0946E-01	4.0953E-01	ENSG00000162616
DNAJB5	-0.1060	1.1712E-01	2.8255E-01	ENSG00000137094
DNAJB5-DT	0.0138	8.9600E-01		ENSG00000281491
DNAJB6	-0.2767	5.2155E-06	1.4462E-04	ENSG00000105993
DNAJB6P1	-0.2089	8.4599E-04	7.9753E-03	ENSG00000254612
DNAJB6P8	0.0055	5.5836E-01		ENSG00000264529
DNAJB7	0.0397	5.3539E-01	7.1856E-01	ENSG00000172404
DNAJB9	-0.1705	1.9793E-02	8.2403E-02	ENSG00000128590
DNAJC1	0.2362	2.2698E-03	1.6567E-02	ENSG00000136770
DNAJC10	0.2840	6.4897E-06	1.7281E-04	ENSG00000077232
DNAJC11	0.0754	3.3792E-01	5.4787E-01	ENSG00000007923
DNAJC12	-0.1748	1.7875E-02	7.6688E-02	ENSG00000108176
DNAJC13	0.1261	1.3055E-01	3.0344E-01	ENSG00000138246
DNAJC14	-0.1163	1.8358E-01	3.7692E-01	ENSG00000135392
DNAJC16	0.0038	9.6537E-01	9.8290E-01	ENSG00000116138
DNAJC17	0.0954	1.4973E-01	3.3059E-01	ENSG00000104129
DNAJC18	-0.0264	5.8273E-01	7.5352E-01	ENSG00000170464
DNAJC19	0.1498	2.0821E-02	8.5413E-02	ENSG00000205981
DNAJC2	-0.0202	7.1373E-01	8.4287E-01	ENSG00000105821
DNAJC21	0.0919	1.2777E-01	2.9956E-01	ENSG00000168724
DNAJC22	-0.0601	4.0150E-01	6.0651E-01	ENSG00000178401
DNAJC24	0.2134	4.1287E-03	2.6142E-02	ENSG00000170946
DNAJC25	0.2046	2.3642E-02	9.3839E-02	ENSG00000059769
DNAJC27	-0.1918	1.9810E-02	8.2434E-02	ENSG00000115137
DNAJC27-AS1	0.0309	6.8434E-01	8.2402E-01	ENSG00000224165
DNAJC28	0.0481	5.7590E-01	7.4838E-01	ENSG00000177692
DNAJC3	-0.0019	9.8058E-01	9.9002E-01	ENSG00000102580
DNAJC3-DT	0.2122	1.0383E-02	5.2080E-02	ENSG00000247400
DNAJC30	-0.0503	4.8117E-01	6.7583E-01	ENSG00000176410
DNAJC4	0.1026	1.0803E-01	2.6786E-01	ENSG00000110011
DNAJC5	0.2339	5.1266E-03	3.0684E-02	ENSG00000101152
DNAJC5B	0.1444	5.2865E-02	1.6562E-01	ENSG00000147570
DNAJC5G	0.0402	2.0660E-01	4.0618E-01	ENSG00000163793
DNAJC6	-0.1821	1.3514E-02	6.3142E-02	ENSG00000116675
DNAJC7	-0.0511	4.0593E-01	6.1041E-01	ENSG00000168259
DNAJC8	-0.2063	9.3292E-05	1.4611E-03	ENSG00000126698
DNAJC9	-0.0363	5.7079E-01	7.4447E-01	ENSG00000213551
DNAJC9-AS1	0.2672	1.1604E-02	5.6560E-02	ENSG00000227540
DNAJC9-AS1	0.0748	2.6172E-01	4.7042E-01	ENSG00000236756
DNAL1	-0.0294	6.3490E-01	7.9057E-01	ENSG00000119661
DNAL4	-0.0391	4.6094E-01	6.5837E-01	ENSG00000100246

DNALI1	-0.0816	2.1942E-01	4.2176E-01	ENSG00000163879
DNASE1	0.2465	5.0549E-03	3.0347E-02	ENSG00000213918
DNASE1L1	-0.1563	9.8998E-02	2.5306E-01	ENSG00000013563
DNASE1L2	0.0239	6.1929E-01		ENSG00000167968
DNASE1L3	-0.0070	6.3309E-01		ENSG00000163687
DNASE2	-0.1252	7.1846E-02	2.0452E-01	ENSG00000105612
DNASE2B	0.1194	1.5096E-01	3.3226E-01	ENSG00000137976
DNER	-0.1111	1.5987E-01	3.4499E-01	ENSG00000187957
DNHD1	0.1531	8.3622E-02	2.2585E-01	ENSG00000179532
DNLZ	0.0442	3.7318E-01	5.8178E-01	ENSG00000213221
DNM1	0.0986	1.5630E-01	3.4022E-01	ENSG00000106976
DNM1L	-0.1142	1.2758E-02	6.0495E-02	ENSG00000087470
DNM1P29	0.0185	3.2531E-01		ENSG00000274966
DNM2	-0.1091	9.2119E-02	2.4167E-01	ENSG00000079805
DNM3	0.1514	5.3914E-02	1.6815E-01	ENSG00000197959
DNM3-IT1	-0.0556	5.1119E-01	6.9964E-01	ENSG00000233540
DNMBP	0.0715	3.6850E-01	5.7769E-01	ENSG00000107554
DNMBP-AS1	-0.0427	2.6427E-01		ENSG00000227695
DNMT1	-0.0504	4.5225E-01	6.5119E-01	ENSG00000130816
DNMT3A	-0.0475	4.3456E-01	6.3569E-01	ENSG00000119772
DNMT3B	0.0576	3.9533E-01	6.0161E-01	ENSG00000088305
DNPEP	0.0925	1.7664E-01	3.6792E-01	ENSG00000123992
DNPH1	0.0145	8.4895E-01	9.2304E-01	ENSG00000112667
DNTT	0.0008	9.8516E-01		ENSG00000107447
DNTTIP1	0.0678	3.1452E-01	5.2456E-01	ENSG00000101457
DNTTIP2	-0.4129	4.2083E-09	3.9205E-07	ENSG00000067334
DOC2A	-0.1048	2.0885E-01	4.0887E-01	ENSG00000149927
DOC2B	-0.3170	1.9038E-04	2.5036E-03	ENSG00000272636
DOCK1	-0.0037	9.5876E-01	9.7939E-01	ENSG00000150760
DOCK10	0.0531	5.2624E-01	7.1144E-01	ENSG00000135905
DOCK11	0.0339	6.5826E-01	8.0708E-01	ENSG00000147251
DOCK2	1.2547	3.0944E-03		ENSG00000134516
DOCK3	0.2201	7.8804E-03	4.2374E-02	ENSG00000088538
DOCK4	0.2262	2.8705E-03	1.9812E-02	ENSG00000128512
DOCK4-AS1	0.0477	4.1945E-01	6.2219E-01	ENSG00000225572
DOCK5	0.2088	4.9376E-02	1.5811E-01	ENSG00000147459
DOCK6	-0.1353	1.0204E-01	2.5809E-01	ENSG00000130158
DOCK6-AS1	-0.0192	7.6905E-01	8.7636E-01	ENSG00000267082
DOCK7	-0.0684	2.4634E-01	4.5414E-01	ENSG00000116641
DOCK8	0.0644	1.3250E-02		ENSG00000107099
DOCK8-AS1	0.0344	2.5591E-01		ENSG00000183784
DOCK9	-0.0104	9.0959E-01	9.5525E-01	ENSG00000088387
DOHH	-0.0038	9.3961E-01	9.7013E-01	ENSG00000129932
DOK1	-0.0058	9.4219E-01	9.7141E-01	ENSG00000115325
DOK2	-0.0063	6.9879E-01		ENSG00000147443
DOK3	0.0218	7.3875E-01	8.5776E-01	ENSG00000146094
DOK4	-0.3122	2.1703E-03	1.5975E-02	ENSG00000125170
DOK5	-0.2722	1.4905E-03	1.2205E-02	ENSG00000101134
DOK6	-0.3322	9.9448E-04	9.0211E-03	ENSG00000206052

DOK7	0.1875	3.9708E-02	1.3618E-01	ENSG00000175920
DOLK	-0.0571	3.6797E-01	5.7713E-01	ENSG00000175283
DOLPP1	-0.0051	9.3226E-01	9.6651E-01	ENSG00000167130
DONSON	0.0746	2.6928E-01	4.7870E-01	ENSG00000159147
DONSONP1	0.0279	5.3285E-01		ENSG00000227267
DOP1B	0.1143	1.0640E-01	2.6523E-01	ENSG00000142197
DOT1L	0.0580	4.6883E-01	6.6553E-01	ENSG00000104885
DPAGT1	-0.0999	1.9312E-01	3.8924E-01	ENSG00000172269
DPCD	0.3487	4.6789E-07	2.0304E-05	ENSG00000166171
DPEP1	0.0295	1.7728E-01		ENSG00000015413
DPEP2	0.0138	5.0154E-01		ENSG00000167261
DPF1	-0.0829	2.9100E-01	5.0136E-01	ENSG00000011332
DPF2	-0.0856	1.7660E-01	3.6789E-01	ENSG00000133884
DPF3	-0.1967	6.5499E-02	1.9186E-01	ENSG00000205683
DPH1	-0.2514	4.0893E-03	2.5991E-02	ENSG00000108963
DPH2	0.0334	6.3869E-01	7.9344E-01	ENSG00000132768
DPH3	0.1045	8.7850E-02	2.3371E-01	ENSG00000154813
DPH5	-0.1387	4.4992E-02	1.4840E-01	ENSG00000117543
DPH5-DT	-0.3320	5.4714E-05	9.5411E-04	ENSG00000233184
DPH6	0.2432	8.8633E-03	4.6467E-02	ENSG00000134146
DPH7	0.0128	8.6208E-01	9.3066E-01	ENSG00000148399
DPM1	-0.1285	6.7891E-02	1.9645E-01	ENSG00000000419
DPM2	0.0188	7.4495E-01	8.6115E-01	ENSG00000136908
DPM3	0.0900	2.0739E-01	4.0704E-01	ENSG00000179085
DPP10	0.1298	1.3319E-01	3.0687E-01	ENSG00000175497
DPP10-AS1	0.2051	6.4748E-02	1.9040E-01	ENSG00000235026
DPP3	0.0268	7.2616E-01	8.4964E-01	ENSG00000254986
DPP3-DT	0.3008	5.1461E-03	3.0756E-02	ENSG00000255517
DPP4	-0.0700	4.1776E-01	6.2109E-01	ENSG00000197635
DPP6	-0.1204	1.1078E-01	2.7252E-01	ENSG00000130226
DPP7	-0.0636	2.4302E-01	4.5075E-01	ENSG00000176978
DPP8	-0.0043	8.9459E-01	9.4750E-01	ENSG00000074603
DPP9	-0.0096	8.8650E-01	9.4368E-01	ENSG00000142002
DPP9-AS1	0.0252	4.5575E-01		ENSG00000205790
DPPA2	-0.0143	3.0543E-01		ENSG00000163530
DPPA3P6	-0.0184	7.9031E-01	8.8919E-01	ENSG00000262516
DPPA4	-0.0339	6.7832E-01	8.1971E-01	ENSG00000121570
DPPA4P2	-0.0102	5.8013E-01		ENSG00000249691
DPPA5	0.0084	4.8954E-01		ENSG00000203909
DPRX	0.0116	6.7655E-01		ENSG00000204595
DPRXP3	0.0000	7.5658E-01		ENSG00000282308
DPRXP4	-0.0053	7.9199E-01		ENSG00000264743
DPY19L1	-0.1769	6.7767E-02	1.9628E-01	ENSG00000173852
DPY19L2	0.3034	4.6200E-04	4.9968E-03	ENSG00000177990
DPY19L2P5	0.0042	6.7989E-01		ENSG00000225100
DPY19L3	-0.1078	1.6155E-01	3.4747E-01	ENSG00000178904
DPY19L3-DT	0.0263	7.0179E-01	8.3529E-01	ENSG00000267213
DPY19L4	0.0700	2.6020E-01	4.6872E-01	ENSG00000156162
DPY19L4P1	0.0066	7.7209E-01		ENSG00000229663

DPY30	-0.1502	2.1016E-02	8.5924E-02	ENSG00000162961
DPYD	0.5047	3.1125E-06	9.5489E-05	ENSG00000188641
DPYD-AS1	0.0255	3.2646E-01		ENSG00000232878
DPYD-AS2	0.0033	8.6532E-01		ENSG00000235777
DPYS	0.1685	5.7770E-02	1.7617E-01	ENSG00000147647
DPYSL2	-0.1663	2.2002E-02	8.8980E-02	ENSG00000092964
DPYSL3	-0.5472	1.9492E-10	2.8212E-08	ENSG00000113657
DPYSL4	0.1267	1.1662E-01	2.8182E-01	ENSG00000151640
DPYSL5	-0.1367	5.6946E-02	1.7471E-01	ENSG00000157851
DQX1	0.0704	1.6758E-01		ENSG00000144045
DR1	-0.2880	2.8347E-08	1.9648E-06	ENSG00000117505
DRAIC	0.0333	6.7332E-01	8.1724E-01	ENSG00000245750
DRAM1	0.0578	4.9699E-01	6.8827E-01	ENSG00000136048
DRAM2	-0.2040	3.6866E-04	4.2014E-03	ENSG00000156171
DRAP1	-0.0615	3.0775E-01	5.1804E-01	ENSG00000175550
DRAXIN	-0.5063	1.2438E-08	1.0034E-06	ENSG00000162490
DRC1	0.4970	6.8100E-03	3.8038E-02	ENSG00000157856
DRC3	0.3283	3.8511E-03	2.4825E-02	ENSG00000171962
DRC7	0.0471	4.6542E-01	6.6199E-01	ENSG00000159625
DRD1	-0.0033	8.0167E-01	8.9537E-01	ENSG00000184845
DRD2	-0.0032	9.7782E-01	9.8899E-01	ENSG00000149295
DRD3	0.0673	3.4089E-01	5.5066E-01	ENSG00000151577
DRD4	0.1046	2.2461E-01	4.2852E-01	ENSG00000069696
DRD5	-0.1154	6.4367E-02	1.8966E-01	ENSG00000169676
DRG1	-0.1128	1.4663E-02	6.6934E-02	ENSG00000185721
DRG1P2	-0.1203	1.7973E-01	3.7144E-01	ENSG00000240393
DRG2	-0.1093	6.9923E-02	2.0071E-01	ENSG00000108591
DRGX	0.0139	4.7327E-01		ENSG00000165606
DRICH1	-0.0293	2.0344E-01		ENSG00000189269
DROSHA	-0.0212	7.3178E-01	8.5327E-01	ENSG00000113360
DRP2	0.2031	5.2333E-02	1.6441E-01	ENSG00000102385
DSC1	0.0479	3.5369E-01	5.6374E-01	ENSG00000134765
DSC2	0.2599	3.4315E-03	2.2667E-02	ENSG00000134755
DSC3	-0.0454	5.4475E-01	7.2546E-01	ENSG00000134762
DSCAM	0.1396	1.4988E-01	3.3072E-01	ENSG00000171587
DSCAM-IT1	0.0032	8.0709E-01		ENSG00000233756
DSCAML1	-0.2771	5.2786E-03	3.1410E-02	ENSG00000177103
DSCC1	-0.2589	2.1455E-02	8.7265E-02	ENSG00000136982
DSCR8	0.0464	1.5406E-01		ENSG00000198054
DSCR9	-0.0399	5.1775E-01	7.0518E-01	ENSG00000230366
DSE	0.0154	8.3218E-01	9.1345E-01	ENSG00000111817
DSEL	0.3635	2.3144E-03	1.6803E-02	ENSG00000171451
DSEL-AS1	0.0064	9.4697E-01		ENSG00000265533
DSG1	0.0145	6.1621E-01		ENSG00000134760
DSG2	0.0848	2.9659E-01	5.0660E-01	ENSG00000046604
DSG2-AS1	-0.0130	5.6328E-01		ENSG00000264859
DSG3	0.0256	2.9703E-01		ENSG00000134757
DSN1	-0.1209	1.3818E-01	3.1413E-01	ENSG00000149636
DSP	-0.0078	9.2417E-01	9.6252E-01	ENSG00000096696

DSP-AS1	0.1134	7.8456E-02	2.1678E-01	ENSG00000261189
DSPP	0.0055	7.8768E-01		ENSG00000152591
DST	0.2936	1.7003E-04	2.2862E-03	ENSG00000151914
DSTN	-0.3315	2.5612E-07	1.2479E-05	ENSG00000125868
DSTNP3	0.0020	9.3910E-01	9.7000E-01	ENSG00000253833
DSTYK	0.2136	7.3842E-04	7.2310E-03	ENSG00000133059
DTD1	-0.2011	7.0282E-04	6.9757E-03	ENSG00000125821
DTD1-AS1	0.0089	8.4796E-01		ENSG00000233993
DTD2	-0.1585	8.5256E-02	2.2851E-01	ENSG00000129480
DTHD1	0.0938	8.4287E-02	2.2690E-01	ENSG00000197057
DTL	-0.1241	1.3319E-01	3.0687E-01	ENSG00000143476
DTNA	0.0490	4.1222E-01	6.1603E-01	ENSG00000134769
DTNB	0.0102	8.9022E-01	9.4564E-01	ENSG00000138101
DTNBP1	-0.0348	5.4879E-01	7.2820E-01	ENSG00000047579
DTWD1	0.0308	6.7897E-01	8.2033E-01	ENSG00000104047
DTWD2	0.2908	1.4984E-02	6.7908E-02	ENSG00000169570
DTX1	-0.0400	5.5837E-01	7.3579E-01	ENSG00000135144
DTX2	0.1083	2.2430E-01	4.2812E-01	ENSG00000091073
DTX2P1-UPK3BP1-PMS2F	0.0296	7.1581E-01	8.4379E-01	ENSG00000265479
DTX3	-0.4404	8.7740E-07	3.3367E-05	ENSG00000178498
DTX3L	-0.0116	8.6845E-01	9.3380E-01	ENSG00000163840
DTX4	-0.0643	3.5960E-01	5.6890E-01	ENSG00000110042
DTYMK	-0.0427	5.2199E-01	7.0854E-01	ENSG00000168393
DUBR	-0.0122	8.6874E-01	9.3400E-01	ENSG00000243701
DUOX1	0.0041	9.4948E-01	9.7495E-01	ENSG00000137857
DUOX2	0.0857	2.1201E-01	4.1293E-01	ENSG00000140279
DUOXA1	-0.0473	4.0841E-01	6.1270E-01	ENSG00000140254
DUOXA2	-0.0007	9.8588E-01		ENSG00000140274
DUS1L	0.1659	1.9896E-02	8.2692E-02	ENSG00000169718
DUS2	0.0407	6.2069E-01	7.8065E-01	ENSG00000167264
DUS3L	0.1328	6.7845E-02	1.9644E-01	ENSG00000141994
DUS4L	0.1039	2.0089E-01	3.9940E-01	ENSG00000105865
DUS4L-BCAP29	-0.0009	9.8867E-01	9.9379E-01	ENSG00000288558
DUSP1	0.0731	3.8476E-01	5.9268E-01	ENSG00000120129
DUSP11	0.0048	9.4091E-01	9.7074E-01	ENSG00000144048
DUSP12	-0.2015	6.8912E-04	6.8603E-03	ENSG00000081721
DUSP13B	0.0806	1.3017E-01	3.0295E-01	ENSG00000079393
DUSP14	0.0261	6.9766E-01	8.3292E-01	ENSG00000276023
DUSP15	0.2905	1.1486E-03	1.0061E-02	ENSG00000149599
DUSP18	-0.1567	3.6196E-02	1.2696E-01	ENSG00000167065
DUSP19	-0.1287	1.6929E-01	3.5780E-01	ENSG00000162999
DUSP2	-0.2912	1.0750E-02	5.3436E-02	ENSG00000158050
DUSP22	-0.0481	5.2747E-01	7.1240E-01	ENSG00000112679
DUSP23	0.0059	9.2465E-01	9.6292E-01	ENSG00000158716
DUSP26	-0.1581	6.9553E-02	1.9983E-01	ENSG00000133878
DUSP28	-0.0441	4.9284E-01	6.8483E-01	ENSG00000188542
DUSP3	-0.1669	9.5811E-04	8.7604E-03	ENSG00000108861
DUSP4	-0.0256	7.4486E-01	8.6115E-01	ENSG00000120875
DUSP5	-0.5269	3.8526E-06	1.1339E-04	ENSG00000138166

DUSP6	-0.1756	3.2822E-02	1.1841E-01	ENSG00000139318
DUSP7	0.0520	5.3191E-01	7.1594E-01	ENSG00000164086
DUSP8	-0.2152	1.0087E-02	5.1059E-02	ENSG00000184545
DUSP9	-0.0684	4.2497E-01	6.2734E-01	ENSG00000130829
DUT	-0.2267	1.3781E-03	1.1514E-02	ENSG00000128951
DUTP6	0.0315	6.9346E-01	8.3001E-01	ENSG00000225171
DUTP7	-0.0387	4.0994E-01	6.1422E-01	ENSG00000250473
DUX4	0.0134	7.6222E-01		ENSG00000260596
DUX4L26	0.0045	5.4436E-01		ENSG00000236138
DUX4L34	-0.0050	6.3193E-01	7.8843E-01	ENSG00000282935
DUX4L9	-0.0241	4.2822E-01	6.3016E-01	ENSG00000224807
DUXAP10	0.5179	9.1919E-03	4.7664E-02	ENSG00000244306
DUXAP8	0.2779	1.5754E-02	7.0376E-02	ENSG00000206195
DUXAP9	0.2891	1.9802E-02	8.2418E-02	ENSG00000225210
DUXB	0.0442	2.6904E-01		ENSG00000282757
DVL1	-0.1733	1.4440E-02	6.6188E-02	ENSG00000107404
DVL2	-0.1321	5.3042E-02	1.6606E-01	ENSG00000004975
DVL3	0.0746	2.1149E-01	4.1231E-01	ENSG00000161202
DXO	0.0322	5.8731E-01	7.5642E-01	ENSG00000204348
DYDC1	0.0040	6.9813E-01		ENSG00000170788
DYDC2	0.0569	4.9518E-01	6.8703E-01	ENSG00000133665
DYM	-0.1273	1.2361E-02	5.9137E-02	ENSG00000141627
DYNAP	0.0070	6.3146E-01		ENSG00000178690
DYNC1H1	0.0001	9.8882E-01	9.9379E-01	ENSG00000197102
DYNC1I1	0.0692	3.7168E-01	5.8043E-01	ENSG00000158560
DYNC1I2	-0.0143	7.9902E-01	8.9407E-01	ENSG00000077380
DYNC1I2P1	0.0125	8.7248E-01	9.3588E-01	ENSG00000225137
DYNC1LI1	-0.1453	1.5301E-02	6.8954E-02	ENSG00000144635
DYNC1LI2	-0.0161	7.6255E-01	8.7218E-01	ENSG00000135720
DYNC1LI2-DT	-0.0397	4.9652E-01	6.8801E-01	ENSG00000246777
DYNC2H1	0.4775	8.4790E-08	5.0271E-06	ENSG00000187240
DYNC2I1	0.4918	6.4045E-13	2.6264E-10	ENSG00000126870
DYNC2I2	-0.0814	2.9350E-01	5.0357E-01	ENSG00000119333
DYNC2LI1	0.0629	3.0274E-01	5.1286E-01	ENSG00000138036
DYNLL1	-0.4626	4.6451E-08	3.0156E-06	ENSG00000088986
DYNLL2	-0.0710	3.7837E-01	5.8692E-01	ENSG00000264364
DYNLL2-DT	0.0000	8.8995E-01		ENSG00000266290
DYNLRB1	-0.1845	7.1211E-02	2.0325E-01	ENSG00000125971
DYNLT1	-0.1756	5.3317E-03	3.1663E-02	ENSG00000146425
DYNLT2	0.4135	1.2149E-04	1.7836E-03	ENSG00000184786
DYNLT2B	-0.0520	4.4554E-01	6.4463E-01	ENSG00000213123
DYNLT3	-0.1661	2.8913E-02	1.0823E-01	ENSG00000165169
DYNLT4	0.0242	2.6050E-01		ENSG00000188396
DYNLT5	-0.0158	6.4416E-01	7.9692E-01	ENSG00000152760
DYRK1A	-0.0591	4.4126E-01	6.4093E-01	ENSG00000157540
DYRK1B	0.3970	1.0438E-04	1.5923E-03	ENSG00000105204
DYRK2	-0.3986	1.5294E-09	1.5945E-07	ENSG00000127334
DYRK3	0.0996	2.2847E-01	4.3305E-01	ENSG00000143479
DYRK3-AS1	0.1768	7.0355E-02	2.0131E-01	ENSG00000237605

DYRK4	0.0137	8.4084E-01	9.1807E-01	ENSG00000010219
DYSF	-0.0128	9.3692E-01		ENSG00000135636
DYTN	0.0117	6.9108E-01		ENSG00000232125
DZANK1	0.2396	1.6367E-03	1.3016E-02	ENSG00000089091
DZIP1	0.0066	9.2587E-01	9.6379E-01	ENSG00000134874
DZIP1L	0.3563	5.2725E-04	5.5298E-03	ENSG00000158163
DZIP3	0.1756	9.5889E-03	4.9200E-02	ENSG00000198919
E2F1	-0.1182	1.5756E-01	3.4138E-01	ENSG00000101412
E2F2	-0.0656	4.4842E-01	6.4759E-01	ENSG00000007968
E2F3	0.0306	6.9803E-01	8.3309E-01	ENSG00000112242
E2F3-IT1	-0.0283	4.3344E-01		ENSG00000224707
E2F4	0.0224	7.1333E-01	8.4277E-01	ENSG00000205250
E2F5	0.3512	2.0691E-05	4.3921E-04	ENSG00000133740
E2F5-DT	0.0249	5.8371E-01	7.5412E-01	ENSG00000260493
E2F6	-0.0371	5.7987E-01	7.5133E-01	ENSG00000169016
E2F7	-0.0288	7.2394E-01	8.4859E-01	ENSG00000165891
E2F8	-0.1011	2.0513E-01	4.0445E-01	ENSG00000129173
E4F1	0.0714	2.7570E-01	4.8561E-01	ENSG00000167967
EAF1	-0.1365	4.7933E-02	1.5493E-01	ENSG00000144597
EAF2	0.0828	3.2311E-01	5.3309E-01	ENSG00000145088
EAPP	-0.1849	2.3325E-03	1.6909E-02	ENSG00000129518
EARS2	0.1000	1.7382E-01	3.6401E-01	ENSG00000103356
EBAG9	0.0859	1.1805E-01	2.8409E-01	ENSG00000147654
EBAG9P1	0.0525	1.9100E-01	3.8627E-01	ENSG00000233690
EBF2	0.2051	2.9351E-02	1.0944E-01	ENSG00000221818
EBF3	-0.1743	8.4564E-02	2.2730E-01	ENSG00000108001
EBF4	0.0186	8.2467E-01	9.0906E-01	ENSG00000088881
EBI3	0.0359	1.5325E-01		ENSG00000105246
EBLN1	0.0164	3.8070E-01		ENSG00000223601
EBLN2	-0.0068	9.3768E-01	9.6919E-01	ENSG00000255423
EBLN3P	0.1280	8.7857E-03	4.6112E-02	ENSG00000281649
EBNA1BP2	0.0081	9.1084E-01	9.5615E-01	ENSG00000117395
EBP	-0.2926	7.3835E-07	2.9067E-05	ENSG00000147155
EBPL	-0.1202	5.2326E-02	1.6441E-01	ENSG00000123179
ECD	-0.0570	3.0999E-01	5.2025E-01	ENSG00000122882
ECE1	-0.2760	1.2858E-03	1.0965E-02	ENSG00000117298
ECE1-AS1	-0.0606	3.3828E-01	5.4817E-01	ENSG00000231105
ECE2	0.0487	3.0924E-01		ENSG00000145194
ECEL1	-0.0926	3.1483E-01	5.2486E-01	ENSG00000171551
ECH1	-0.1973	2.1558E-03	1.5900E-02	ENSG00000104823
ECHDC1	-0.0503	4.2347E-01	6.2594E-01	ENSG00000093144
ECHDC3	-0.0791	3.4794E-01	5.5811E-01	ENSG00000134463
ECHS1	-0.1691	5.2250E-03	3.1151E-02	ENSG00000127884
ECI1	-0.1503	5.7782E-02	1.7617E-01	ENSG00000167969
ECI2	0.0160	8.2403E-01	9.0862E-01	ENSG00000198721
ECI2-DT	-0.0045	8.3434E-01		ENSG00000234817
ECM1	0.5189	3.7006E-04	4.2095E-03	ENSG00000143369
ECM2	0.0599	4.0928E-01	6.1356E-01	ENSG00000106823
ECPAS	0.0632	3.2349E-01	5.3348E-01	ENSG00000136813

ECRG4	-0.1960	4.7206E-02	1.5355E-01	ENSG00000119147
ECSIT	-0.1527	1.3775E-02	6.4020E-02	ENSG00000130159
ECT2	-0.1478	7.8753E-02	2.1735E-01	ENSG00000114346
ECT2L	-0.0316	6.8293E-01	8.2313E-01	ENSG00000203734
EDA	-0.2679	3.3992E-02	1.2114E-01	ENSG00000158813
EDA2R	-0.0810	3.5165E-01	5.6191E-01	ENSG00000131080
EDAR	-0.0032	7.3454E-01		ENSG00000135960
EDARADD	-0.0185	7.8259E-01	8.8495E-01	ENSG00000186197
EDC3	0.2526	2.9520E-04	3.5448E-03	ENSG00000179151
EDC4	-0.0494	5.7215E-01	7.4555E-01	ENSG00000038358
EDDM13	-0.0163	8.4576E-01	9.2100E-01	ENSG00000267710
EDEM1	-0.1003	1.6099E-01	3.4652E-01	ENSG00000134109
EDEM2	0.0783	2.0467E-01	4.0388E-01	ENSG00000088298
EDEM3	0.1153	1.2789E-01	2.9962E-01	ENSG00000116406
EDF1	-0.1813	3.2453E-03	2.1734E-02	ENSG00000107223
EDIL3	-0.0395	6.2204E-01	7.8152E-01	ENSG00000164176
EDIL3-DT	0.1036	2.1283E-01	4.1384E-01	ENSG00000250320
EDN1	0.0878	1.8398E-01	3.7736E-01	ENSG00000078401
EDN2	-0.0842	3.1068E-01	5.2094E-01	ENSG00000127129
EDN3	-0.0041	9.2826E-01	9.6476E-01	ENSG00000124205
EDNRA	0.0151	7.3625E-01	8.5604E-01	ENSG00000151617
EDNRB	0.0035	9.6663E-01	9.8340E-01	ENSG00000136160
EDRF1	0.0743	2.0741E-01	4.0704E-01	ENSG00000107938
EDRF1-AS1	-0.0482	4.8885E-01	6.8202E-01	ENSG00000236991
EDRF1-DT	-0.0148	8.7853E-01		ENSG00000224023
EEA1	0.3223	1.5020E-06	5.2646E-05	ENSG00000102189
EED	0.1652	3.2175E-02	1.1675E-01	ENSG00000074266
EEF1A1	-0.4320	4.0621E-07	1.8041E-05	ENSG00000156508
EEF1A1P10	-0.0657	2.9715E-01	5.0716E-01	ENSG00000243746
EEF1A1P14	-0.0414	5.5673E-01	7.3461E-01	ENSG00000233057
EEF1A1P16	-0.0475	5.1936E-02		ENSG00000213235
EEF1A1P17	-0.0084	9.8520E-01		ENSG00000257907
EEF1A1P19	-0.4005	1.5848E-03	1.2730E-02	ENSG00000249855
EEF1A1P20	-0.0054	6.7990E-01		ENSG00000249963
EEF1A1P22	-0.2206	1.3295E-02	6.2392E-02	ENSG00000259612
EEF1A1P23	-0.0706	7.0092E-02		ENSG00000236297
EEF1A1P24	-0.0422	2.1342E-01		ENSG00000223668
EEF1A1P25	-0.3438	7.0771E-03	3.9122E-02	ENSG00000241429
EEF1A1P3	-0.0884	3.1566E-01	5.2581E-01	ENSG00000232587
EEF1A1P31	0.0159	3.3524E-01		ENSG00000237859
EEF1A1P34	0.0054	9.3902E-01		ENSG00000233324
EEF1A1P35	-0.0128	5.2956E-01		ENSG00000250144
EEF1A1P39	-0.0083	6.8298E-01		ENSG00000230125
EEF1A1P4	-0.3174	1.8590E-02	7.8782E-02	ENSG00000245205
EEF1A1P8	-0.0009	9.3879E-01	9.6983E-01	ENSG00000223529
EEF1AKMT1	-0.1152	8.6791E-02	2.3164E-01	ENSG00000150456
EEF1AKMT2	0.0225	6.6796E-01	8.1403E-01	ENSG00000203791
EEF1AKMT3	0.0514	5.3973E-01	7.2183E-01	ENSG00000123427
EEF1B2	-0.0361	5.6317E-01	7.3970E-01	ENSG00000114942

EEF1B2P2	-0.0150	7.4345E-01	8.6042E-01	ENSG00000213864
EEF1B2P3	0.1198	1.9709E-01	3.9473E-01	ENSG00000232472
EEF1B2P6	-0.0083	9.2987E-01	9.6538E-01	ENSG00000213261
EEF1B2P7	-0.0407	3.8851E-01	5.9594E-01	ENSG00000213055
EEF1D	-0.0487	4.7084E-01	6.6711E-01	ENSG00000104529
EEF1DP1	-0.0924	1.3197E-01	3.0529E-01	ENSG00000228887
EEF1DP4	0.3734	1.3407E-02	6.2809E-02	ENSG00000213640
EEF1DP7	-0.0217	7.7339E-01	8.7911E-01	ENSG00000263883
EEF1E1	0.0110	8.8906E-01	9.4486E-01	ENSG00000124802
EEF1GP3	0.0199	3.4540E-01		ENSG00000226845
EEF1GP5	-0.0539	1.4383E-01		ENSG00000234785
EEF1GP7	-0.0434	4.6704E-01	6.6364E-01	ENSG00000236290
EEF2	-0.2036	1.5694E-02	7.0188E-02	ENSG00000167658
EEF2K	0.0338	6.4264E-01	7.9583E-01	ENSG00000103319
EEF2KMT	0.1591	5.9345E-02	1.7907E-01	ENSG00000118894
EEFSEC	-0.1012	1.3913E-01	3.1521E-01	ENSG00000132394
EEIG1	0.0117	8.6014E-01	9.2958E-01	ENSG00000167106
EEIG2	-0.1620	1.8433E-02	7.8319E-02	ENSG00000162636
EEPD1	-0.1746	7.0381E-02	2.0134E-01	ENSG00000122547
EFCAB10	0.0307	6.9196E-01	8.2910E-01	ENSG00000185055
EFCAB10-AS1	-0.0040	9.5411E-01	9.7701E-01	ENSG00000272604
EFCAB11	-0.0752	3.0604E-01	5.1615E-01	ENSG00000140025
EFCAB12	0.3681	1.2985E-02	6.1274E-02	ENSG00000172771
EFCAB13	-0.0572	5.1060E-01	6.9914E-01	ENSG00000178852
EFCAB13-DT	0.0921	1.2268E-01	2.9161E-01	ENSG00000263293
EFCAB14	0.1000	6.6917E-02	1.9483E-01	ENSG00000159658
EFCAB14-AS1	-0.0060	8.2489E-01		ENSG00000228237
EFCAB15P	-0.0697	4.2143E-01	6.2428E-01	ENSG00000233483
EFCAB2	-0.0139	8.6422E-01	9.3150E-01	ENSG00000203666
EFCAB5	0.0381	6.5254E-01	8.0314E-01	ENSG00000176927
EFCAB6	0.0863	3.3323E-01	5.4349E-01	ENSG00000186976
EFCAB7	0.1262	7.3312E-02	2.0744E-01	ENSG00000203965
EFCAB8	0.0082	7.7363E-01		ENSG00000215529
EFCAB9	0.0402	4.7456E-01	6.7038E-01	ENSG00000214360
EFCC1	-0.1260	1.7879E-01	3.7024E-01	ENSG00000114654
EFEMP1	0.1392	1.0111E-01	2.5672E-01	ENSG00000115380
EFEMP2	-0.0921	2.4042E-01	4.4722E-01	ENSG00000172638
EFHC1	0.4182	1.4256E-09	1.5055E-07	ENSG00000096093
EFHC2	-0.0099	9.1487E-01	9.5806E-01	ENSG00000183690
EFHD1	-0.2426	3.0369E-03	2.0676E-02	ENSG00000115468
EFHD2	-0.1028	1.2581E-01	2.9644E-01	ENSG00000142634
EFHD2-AS1	0.0353	5.5699E-01	7.3477E-01	ENSG00000228140
EFL1	-0.1854	2.0070E-02	8.3229E-02	ENSG00000140598
EFNA1	-0.4802	6.1021E-06	1.6429E-04	ENSG00000169242
EFNA2	0.3665	1.5354E-05	3.4344E-04	ENSG00000099617
EFNA3	-0.2716	5.1383E-04	5.4145E-03	ENSG00000143590
EFNA4	0.3923	3.1949E-03	2.1496E-02	ENSG00000243364
EFNA5	0.4108	4.9625E-04	5.2801E-03	ENSG00000184349
EFNB1	-0.3321	6.9135E-06	1.8252E-04	ENSG00000090776

EFNB2	0.0054	9.4441E-01	9.7218E-01	ENSG00000125266
EFNB3	0.1821	4.4622E-02	1.4763E-01	ENSG00000108947
EFR3A	0.0028	9.6930E-01	9.8487E-01	ENSG00000132294
EFR3B	0.0994	1.4376E-01	3.2181E-01	ENSG00000084710
EFTUD2	-0.0228	7.3872E-01	8.5776E-01	ENSG00000108883
EGF	0.1867	8.2351E-02	2.2355E-01	ENSG00000138798
EGFL6	0.2529	1.7044E-02	7.4172E-02	ENSG00000198759
EGFL7	-0.0772	3.4152E-01	5.5117E-01	ENSG00000172889
EGFL8	0.0528	3.7214E-01	5.8078E-01	ENSG00000241404
EGFLAM	0.0857	2.6834E-01	4.7778E-01	ENSG00000164318
EGFLAM-AS4	-0.0025	9.7794E-01		ENSG00000248730
EGFR	-0.1398	1.3447E-01	3.0881E-01	ENSG00000146648
EGILA	0.0023	8.3343E-01		ENSG00000258451
EGLN1	-0.1743	7.1631E-02	2.0411E-01	ENSG00000135766
EGLN2	0.0964	1.9404E-01	3.9061E-01	ENSG00000269858
EGLN3	0.1147	1.7968E-01	3.7140E-01	ENSG00000129521
EGOT	0.0450	5.0666E-01	6.9620E-01	ENSG00000235947
EGR1	-0.0450	5.0118E-01	6.9204E-01	ENSG00000120738
EGR2	-0.0299	6.2390E-01	7.8285E-01	ENSG00000122877
EGR3	0.0654	4.2543E-01	6.2768E-01	ENSG00000179388
EGR4	-0.1600	8.2686E-02	2.2421E-01	ENSG00000135625
EHBP1	-0.3180	2.4095E-03	1.7333E-02	ENSG00000115504
EHBP1-AS1	-0.0145	8.6248E-01	9.3070E-01	ENSG00000231609
EHBP1L1	-0.2794	5.9063E-03	3.4138E-02	ENSG00000173442
EHD1	0.1379	1.4326E-01	3.2115E-01	ENSG00000110047
EHD2	-0.3815	1.8137E-03	1.4002E-02	ENSG00000024422
EHD3	-0.0914	2.4679E-01	4.5438E-01	ENSG00000013016
EHD4	0.1737	9.7561E-02	2.5097E-01	ENSG00000103966
EHD4-AS1	0.0078	6.7423E-01		ENSG00000259883
EHF	0.0411	7.0038E-02		ENSG00000135373
EHHADH	-0.0159	8.4389E-01	9.1997E-01	ENSG00000113790
EHMT1	0.0360	5.5500E-01	7.3317E-01	ENSG00000181090
EHMT2	-0.1070	7.4345E-02	2.0922E-01	ENSG00000204371
EHMT2-AS1	-0.0085	8.3332E-01		ENSG00000237080
EI24	-0.1741	1.4952E-03	1.2231E-02	ENSG00000149547
EID1	-0.1957	5.7719E-04	5.9376E-03	ENSG00000255302
EID2	-0.1868	2.0909E-04	2.6893E-03	ENSG00000176396
EID2B	0.0398	5.5262E-01	7.3115E-01	ENSG00000176401
EID3	-0.1408	9.2075E-02	2.4167E-01	ENSG00000255150
EIF1	-0.4338	5.1917E-09	4.6621E-07	ENSG00000173812
EIF1AD	-0.1556	5.2877E-02	1.6563E-01	ENSG00000175376
EIF1AX	-0.0772	1.7769E-01	3.6887E-01	ENSG00000173674
EIF1AX-AS1	-0.0311	5.7978E-01	7.5125E-01	ENSG00000225037
EIF1AY	-0.0350	6.6896E-02		ENSG00000198692
EIF1B	-0.0574	3.6285E-01	5.7197E-01	ENSG00000114784
EIF1B-AS1	0.1435	1.3781E-01	3.1354E-01	ENSG00000280739
EIF1P5	-0.0379	7.4337E-01	8.6037E-01	ENSG00000266563
EIF2A	-0.0869	8.5874E-02	2.2976E-01	ENSG00000144895
EIF2AK1	0.0226	7.2169E-01	8.4747E-01	ENSG00000086232

EIF2AK2	-0.1649	8.7170E-03	4.5862E-02	ENSG00000055332
EIF2AK3	-0.1470	8.6472E-02	2.3099E-01	ENSG00000172071
EIF2AK3-DT	0.1830	5.6985E-02	1.7476E-01	ENSG00000234028
EIF2AK4	-0.0957	1.4884E-01	3.2942E-01	ENSG00000128829
EIF2B1	0.0916	1.2389E-01	2.9362E-01	ENSG00000111361
EIF2B2	0.0003	9.9908E-01	9.9944E-01	ENSG00000119718
EIF2B3	-0.0696	1.8882E-01	3.8374E-01	ENSG00000070785
EIF2B4	-0.0925	2.0466E-01	4.0388E-01	ENSG00000115211
EIF2B5	0.2011	3.6992E-03	2.4092E-02	ENSG00000145191
EIF2D	0.1983	1.3557E-02	6.3264E-02	ENSG00000143486
EIF2S1	-0.1515	1.1065E-02	5.4557E-02	ENSG00000134001
EIF2S2	-0.1110	5.0900E-02	1.6139E-01	ENSG00000125977
EIF2S3	-0.4094	3.6033E-10	4.7159E-08	ENSG00000130741
EIF2S3B	-0.0746	2.7393E-01	4.8372E-01	ENSG00000180574
EIF3A	0.0183	8.0545E-01	8.9800E-01	ENSG00000107581
EIF3B	0.0244	6.8359E-01	8.2353E-01	ENSG00000106263
EIF3C	-0.0003	9.9527E-01	9.9741E-01	ENSG00000184110
EIF3CL	0.0085	9.0737E-01	9.5461E-01	ENSG00000205609
EIF3D	-0.0012	9.8306E-01	9.9100E-01	ENSG00000100353
EIF3E	-0.0988	1.1978E-01	2.8698E-01	ENSG00000104408
EIF3EP3	-0.0650	3.3013E-01	5.4040E-01	ENSG00000277998
EIF3F	-0.0475	4.1787E-01	6.2117E-01	ENSG00000175390
EIF3FP2	-0.0237	4.9941E-01		ENSG00000279081
EIF3FP3	0.0209	7.9585E-01	8.9207E-01	ENSG00000233426
EIF3G	-0.1301	1.1094E-02	5.4647E-02	ENSG00000130811
EIF3H	-0.2833	7.3040E-09	6.3503E-07	ENSG00000147677
EIF3I	-0.4138	2.6429E-11	5.7183E-09	ENSG00000084623
EIF3J	0.0018	9.7840E-01	9.8925E-01	ENSG00000104131
EIF3J-DT	0.1632	3.3381E-02	1.1988E-01	ENSG00000179523
EIF3K	0.0100	8.8388E-01	9.4179E-01	ENSG00000178982
EIF3KP1	0.0065	6.0323E-01		ENSG00000175749
EIF3L	-0.1406	2.5765E-02	9.9724E-02	ENSG00000100129
EIF3LP2	-0.0405	4.5294E-01		ENSG00000233837
EIF3LP3	-0.0289	1.4100E-01		ENSG00000226086
EIF3M	-0.0595	1.6918E-01	3.5763E-01	ENSG00000149100
EIF4A1	-0.1188	9.3818E-02	2.4467E-01	ENSG00000161960
EIF4A1P10	-0.1181	1.8493E-01	3.7845E-01	ENSG00000229132
EIF4A1P2	-0.1267	2.4140E-02	9.5323E-02	ENSG00000235001
EIF4A1P4	-0.0371	3.6135E-01		ENSG00000257790
EIF4A1P7	-0.0387	3.9193E-01		ENSG00000235472
EIF4A2	-0.5235	3.2904E-18	5.7829E-15	ENSG00000156976
EIF4A3	-0.0875	2.5718E-01	4.6568E-01	ENSG00000141543
EIF4B	-0.1518	3.9850E-02	1.3641E-01	ENSG00000063046
EIF4BP2	0.0301	2.6466E-01		ENSG00000228753
EIF4BP3	-0.0785	3.3209E-01	5.4247E-01	ENSG00000224546
EIF4BP5	-0.0054	7.2696E-01		ENSG00000261336
EIF4E	-0.1861	1.2709E-03	1.0858E-02	ENSG00000151247
EIF4E1B	0.0726	3.8010E-01	5.8854E-01	ENSG00000175766
EIF4E2	-0.3032	2.6830E-11	5.7183E-09	ENSG00000135930

EIF4E3	-0.0670	4.0680E-01	6.1141E-01	ENSG00000163412
EIF4EBP1	-0.4528	2.3145E-06	7.5030E-05	ENSG00000187840
EIF4EBP2	-0.0368	4.2398E-01	6.2629E-01	ENSG00000148730
EIF4EBP3	-0.0129	4.6866E-01	6.6532E-01	ENSG00000243056
EIF4ENIF1	0.0661	2.7195E-01	4.8125E-01	ENSG00000184708
EIF4G1	-0.0952	8.1658E-02	2.2231E-01	ENSG00000114867
EIF4G2	-0.3120	1.2926E-04	1.8719E-03	ENSG00000110321
EIF4G3	0.0481	3.8838E-01	5.9587E-01	ENSG00000075151
EIF4H	-0.2160	7.9351E-04	7.6178E-03	ENSG00000106682
EIF5	0.0926	1.3383E-01	3.0777E-01	ENSG00000100664
EIF5A	-0.3317	2.0382E-10	2.9328E-08	ENSG00000132507
EIF5A2	-0.0693	3.3433E-01	5.4449E-01	ENSG00000163577
EIF5AL1	-0.1755	7.1467E-02	2.0378E-01	ENSG00000253626
EIF5AP2	-0.0070	6.1180E-01		ENSG00000267679
EIF5B	0.1926	5.3264E-03	3.1648E-02	ENSG00000158417
EIF6	-0.0563	2.5218E-01	4.6034E-01	ENSG00000242372
EIPR1	-0.2473	4.5985E-04	4.9816E-03	ENSG00000032389
ELAC1	0.1277	3.0014E-02	1.1127E-01	ENSG00000141642
ELAC2	-0.0299	7.0395E-01	8.3671E-01	ENSG00000006744
ELANE	0.0072	7.6556E-01		ENSG00000197561
ELAPOR1	0.1163	1.2273E-01	2.9171E-01	ENSG00000116299
ELAPOR2	0.1182	1.1597E-01	2.8065E-01	ENSG00000164659
ELAVL1	-0.0187	7.7341E-01	8.7911E-01	ENSG00000066044
ELAVL2	-0.1075	1.0121E-01	2.5683E-01	ENSG00000107105
ELAVL3	-0.1268	1.4366E-01	3.2175E-01	ENSG00000196361
ELAVL4	-0.0213	7.1430E-01	8.4315E-01	ENSG00000162374
ELF1	0.1077	1.9883E-01	3.9705E-01	ENSG00000120690
ELF2	-0.1202	4.4991E-02	1.4840E-01	ENSG00000109381
ELF3	0.2367	4.6603E-02	1.5218E-01	ENSG00000163435
ELF4	-0.0378	6.4272E-01	7.9583E-01	ENSG00000102034
ELFN1	-0.2708	8.7822E-03	4.6103E-02	ENSG00000225968
ELFN2	-0.1302	1.6295E-01	3.4925E-01	ENSG00000166897
ELK1	-0.2971	1.5021E-04	2.0857E-03	ENSG00000126767
ELK3	0.0689	4.2941E-01	6.3101E-01	ENSG00000111145
ELK4	0.1703	1.8296E-02	7.7887E-02	ENSG00000158711
ELL	0.0838	3.0921E-01	5.1942E-01	ENSG00000105656
ELL2	-0.1088	2.1673E-01	4.1845E-01	ENSG00000118985
ELL3	-0.0303	2.6687E-01		ENSG00000128886
ELMO1	-0.1461	4.1600E-02	1.4059E-01	ENSG00000155849
ELMO2	-0.0763	2.6226E-01	4.7110E-01	ENSG00000062598
ELMOD1	-0.0160	8.4399E-01	9.1997E-01	ENSG00000110675
ELMOD2	0.1097	7.3927E-02	2.0841E-01	ENSG00000179387
ELMOD3	0.1319	6.7224E-02	1.9543E-01	ENSG00000115459
ELN	0.5146	6.3895E-07	2.5815E-05	ENSG00000049540
ELN-AS1	0.0027	8.6543E-01		ENSG00000232415
ELOA	-0.1124	1.5920E-01	3.4378E-01	ENSG00000011007
ELOA-AS1	0.1432	8.7007E-02	2.3194E-01	ENSG00000236810
ELOA2	0.0270	3.5415E-01		ENSG00000206181
ELOA3BP	-0.0037	6.9601E-01		ENSG00000288607

ELOA3DP	-0.0079	6.1003E-01		ENSG00000288616
ELOB	-0.2473	2.8568E-03	1.9734E-02	ENSG00000103363
ELOBP1	-0.0090	9.9470E-01		ENSG00000234152
ELOBP2	0.0017	9.7468E-01		ENSG00000255262
ELOBP4	-0.0699	1.3255E-01	3.0614E-01	ENSG00000234167
ELOC	-0.0522	4.2795E-01	6.2993E-01	ENSG00000154582
ELOCP22	-0.0086	5.7449E-01		ENSG00000255006
ELOCP31	0.0476	2.2161E-01		ENSG00000256021
ELOF1	-0.0651	2.0675E-01	4.0622E-01	ENSG00000130165
ELOVL1	-0.0792	3.2938E-01	5.3973E-01	ENSG00000066322
ELOVL2	-0.1620	1.1883E-02	5.7591E-02	ENSG00000197977
ELOVL2-AS1	0.0405	6.3640E-01	7.9156E-01	ENSG00000230314
ELOVL3	0.0467	5.7142E-01	7.4493E-01	ENSG00000119915
ELOVL4	0.0890	2.5764E-01	4.6618E-01	ENSG00000118402
ELOVL5	-0.0838	2.5860E-01	4.6733E-01	ENSG00000012660
ELOVL6	-0.1963	9.7411E-03	4.9778E-02	ENSG00000170522
ELOVL7	-0.0116	8.8836E-01	9.4448E-01	ENSG00000164181
ELP1	0.0096	8.8676E-01	9.4386E-01	ENSG00000070061
ELP2	0.0422	4.4785E-01	6.4717E-01	ENSG00000134759
ELP3	-0.0890	2.3187E-01	4.3680E-01	ENSG00000134014
ELP4	-0.0252	7.2777E-01	8.5064E-01	ENSG00000109911
ELP5	-0.0373	6.1191E-01	7.7413E-01	ENSG00000170291
ELP6	0.2068	3.5923E-04	4.1186E-03	ENSG00000163832
EMC1	-0.0254	6.7149E-01	8.1623E-01	ENSG00000127463
EMC1-AS1	-0.1209	2.0057E-01	3.9896E-01	ENSG00000230424
EMC10	0.1416	4.0596E-02	1.3820E-01	ENSG00000161671
EMC2	0.1780	5.2250E-02	1.6425E-01	ENSG00000104412
EMC3	-0.1620	1.8966E-03	1.4475E-02	ENSG00000125037
EMC4	-0.0781	1.6429E-01	3.5111E-01	ENSG00000128463
EMC6	-0.3704	1.1923E-03	1.0348E-02	ENSG00000127774
EMC7	-0.0019	9.7101E-01	9.8543E-01	ENSG00000134153
EMC8	-0.0720	2.4970E-01	4.5758E-01	ENSG00000131148
EMC9	-0.1277	1.1976E-01	2.8698E-01	ENSG00000100908
EMCN	0.0848	5.7970E-02	1.7651E-01	ENSG00000164035
EMD	-0.2261	1.7773E-02	7.6347E-02	ENSG00000102119
EME1	0.1294	1.3837E-01	3.1433E-01	ENSG00000154920
EME2	0.1419	1.0301E-01	2.5981E-01	ENSG00000197774
EMG1	0.0426	5.9279E-01	7.6028E-01	ENSG00000126749
EMID1	-0.1081	9.6253E-02	2.4906E-01	ENSG00000186998
EMILIN1	0.1797	7.9709E-02	2.1914E-01	ENSG00000138080
EMILIN2	0.2112	5.4190E-02	1.6867E-01	ENSG00000132205
EMILIN3	-0.4851	1.6578E-04	2.2425E-03	ENSG00000183798
EML1	-0.1961	3.2911E-02	1.1867E-01	ENSG00000066629
EML2	-0.1678	3.2368E-02	1.1721E-01	ENSG00000125746
EML3	-0.0330	6.8276E-01	8.2313E-01	ENSG00000149499
EML4	0.0438	5.0905E-01	6.9801E-01	ENSG00000143924
EML5	0.1629	1.0412E-01	2.6155E-01	ENSG00000165521
EML6	0.2402	1.1058E-02	5.4541E-02	ENSG00000214595
EMP1	0.0162	8.3229E-01	9.1349E-01	ENSG00000134531

EMP2	-0.1466	4.6390E-02	1.5173E-01	ENSG00000213853
EMP3	-0.2504	1.9367E-02	8.1065E-02	ENSG00000142227
EMSY	-0.0325	5.9089E-01	7.5893E-01	ENSG00000158636
EMSY-DT	0.1762	1.4826E-02	6.7452E-02	ENSG00000255135
EMX1	0.0085	8.8171E-01	9.4078E-01	ENSG00000135638
EMX2	0.3613	3.9886E-03	2.5537E-02	ENSG00000170370
EMX2OS	0.0610	4.4819E-01	6.4746E-01	ENSG00000229847
EN1	0.0323	5.8356E-01		ENSG00000163064
EN2	0.0683	4.2292E-01	6.2562E-01	ENSG00000164778
ENAH	-0.1359	6.3809E-02	1.8837E-01	ENSG00000154380
ENAM	0.0130	6.4002E-01	7.9402E-01	ENSG00000132464
ENC1	-0.4065	1.1165E-02	5.4910E-02	ENSG00000171617
ENDOD1	-0.1210	9.4689E-02	2.4626E-01	ENSG00000149218
ENDOG	-0.0873	1.8745E-01	3.8205E-01	ENSG00000167136
ENDOU	-0.0492	4.0968E-01	6.1393E-01	ENSG00000111405
ENDOV	0.2277	8.0919E-05	1.3039E-03	ENSG00000173818
ENG	-0.0356	6.5316E-01	8.0373E-01	ENSG00000106991
ENGASE	-0.0465	5.4809E-01	7.2768E-01	ENSG00000167280
ENHO	-0.1948	1.1396E-02	5.5833E-02	ENSG00000168913
ENKD1	-0.2095	4.4383E-03	2.7477E-02	ENSG00000124074
ENKUR	0.0856	3.4542E-01	5.5520E-01	ENSG00000151023
ENO1	0.1269	1.8484E-01	3.7842E-01	ENSG00000074800
ENO1-AS1	0.2065	4.8523E-02	1.5634E-01	ENSG00000230679
ENO1P3	-0.0079	4.9087E-01		ENSG00000243986
ENO2	-0.0477	5.6479E-01	7.4058E-01	ENSG00000111674
ENO3	-0.1435	7.6607E-02	2.1349E-01	ENSG00000108515
ENO4	0.1694	9.3101E-02	2.4346E-01	ENSG00000188316
ENOPH1	-0.1881	6.5815E-03	3.7065E-02	ENSG00000145293
ENOSF1	0.0840	3.1339E-01	5.2363E-01	ENSG00000132199
ENOX1	0.1514	4.1798E-02	1.4096E-01	ENSG00000120658
ENOX2	0.2064	1.4159E-02	6.5311E-02	ENSG00000165675
ENPP1	0.0074	9.2614E-01	9.6390E-01	ENSG00000197594
ENPP2	-0.0220	7.9406E-01	8.9120E-01	ENSG00000136960
ENPP3	0.1362	1.4262E-01	3.2015E-01	ENSG00000154269
ENPP4	0.1948	4.6885E-02	1.5290E-01	ENSG00000001561
ENPP5	-0.0382	6.5674E-01	8.0622E-01	ENSG00000112796
ENPP6	0.0036	9.6995E-01	9.8504E-01	ENSG00000164303
ENPP7P1	0.0485	5.4176E-01	7.2348E-01	ENSG00000249188
ENPP7P10	0.0170	5.0408E-01		ENSG00000249767
ENPP7P11	-0.0171	6.0338E-01		ENSG00000250942
ENPP7P12	0.0380	4.2959E-01		ENSG00000254527
ENPP7P15	0.0355	3.4331E-01		ENSG00000284605
ENPP7P2	-0.0134	7.1717E-01		ENSG00000239959
ENPP7P4	0.0355	6.1068E-01	7.7309E-01	ENSG00000241278
ENPP7P6	-0.0005	9.4299E-01		ENSG00000255549
ENPP7P7	-0.0506	4.6538E-01	6.6197E-01	ENSG00000273819
ENPP7P8	0.0027	9.6900E-01		ENSG00000255319
ENPP7P9	-0.0031	8.2432E-01		ENSG00000250476
ENSA	-0.1901	7.6516E-04	7.4150E-03	ENSG00000143420

ENTHD1	0.0155	2.7720E-01		ENSG00000176177
ENTPD1	0.0793	3.1802E-01	5.2826E-01	ENSG00000138185
ENTPD1-AS1	0.2700	7.2885E-03	4.0012E-02	ENSG00000226688
ENTPD2	-0.0117	8.8981E-01	9.4541E-01	ENSG00000054179
ENTPD3-AS1	-0.1001	2.2053E-01	4.2341E-01	ENSG00000223797
ENTPD4	0.1390	3.8692E-02	1.3367E-01	ENSG00000197217
ENTPD4-DT	0.0351	3.1865E-01		ENSG00000287166
ENTPD5	-0.0465	5.1648E-01	7.0415E-01	ENSG00000187097
ENTPD6	0.1350	7.7109E-02	2.1434E-01	ENSG00000197586
ENTPD7	0.4908	6.9910E-07	2.7744E-05	ENSG00000198018
ENTPD8	0.0084	9.1500E-01	9.5811E-01	ENSG00000188833
ENTR1	-0.0059	9.3078E-01	9.6586E-01	ENSG00000165689
ENTREP1	0.4850	3.9636E-03	2.5409E-02	ENSG00000135063
ENTREP2	0.0015	9.8480E-01	9.9202E-01	ENSG00000104059
ENTREP3	-0.0374	6.2176E-01	7.8129E-01	ENSG00000160767
ENY2	-0.1238	3.3116E-02	1.1927E-01	ENSG00000120533
EOGT	0.1464	1.3113E-01	3.0419E-01	ENSG00000163378
EOLA1	0.2169	1.3298E-02	6.2393E-02	ENSG00000197620
EOLA1-DT	0.1204	1.7857E-01	3.6986E-01	ENSG00000241769
EOLA2	0.1171	1.0468E-01	2.6252E-01	ENSG00000197021
EOLA2-DT	0.0545	5.2270E-01	7.0893E-01	ENSG00000235703
EOMES	-0.0801	2.9625E-01	5.0624E-01	ENSG00000163508
EP300	-0.0030	9.4887E-01	9.7468E-01	ENSG00000100393
EP400	0.1888	2.4215E-02	9.5527E-02	ENSG00000183495
EPAS1	0.1253	1.2147E-01	2.8993E-01	ENSG00000116016
EPB41	-0.1186	4.5801E-02	1.5048E-01	ENSG00000159023
EPB41L1	0.2844	4.3910E-03	2.7304E-02	ENSG00000088367
EPB41L2	-0.0570	4.6406E-01	6.6105E-01	ENSG00000079819
EPB41L3	-0.1196	1.5308E-01	3.3527E-01	ENSG00000082397
EPB41L4A	-0.0829	3.4177E-01	5.5139E-01	ENSG00000129595
EPB41L4A-DT	-0.0209	7.9580E-01	8.9207E-01	ENSG00000278921
EPB41L4B	0.3769	1.2256E-03	1.0562E-02	ENSG00000095203
EPB41L5	-0.1945	1.2184E-03	1.0523E-02	ENSG00000115109
EPC1	-0.4248	1.9204E-06	6.4287E-05	ENSG00000120616
EPC1-AS1	-0.1691	2.6595E-02	1.0195E-01	ENSG00000229327
EPC2	-0.1327	1.0771E-01	2.6724E-01	ENSG00000135999
EPCAM	-0.3602	2.4445E-03	1.7510E-02	ENSG00000119888
EPCAM-DT	-0.1459	1.3700E-01	3.1238E-01	ENSG00000234690
EPDR1	-0.0089	9.0534E-01	9.5372E-01	ENSG00000086289
EPG5	0.3289	2.5962E-04	3.2182E-03	ENSG00000152223
EPGN	0.0058	6.8763E-01		ENSG00000182585
EPHA1	-0.1068	1.4229E-01	3.1965E-01	ENSG00000146904
EPHA1-AS1	0.1098	1.6249E-01	3.4860E-01	ENSG00000229153
EPHA10	-0.0003	9.7227E-01	9.8618E-01	ENSG00000183317
EPHA2	-0.1179	1.4674E-01	3.2636E-01	ENSG00000142627
EPHA3	0.2762	2.5078E-02	9.7781E-02	ENSG00000044524
EPHA4	0.1686	2.5078E-02	9.7781E-02	ENSG00000116106
EPHA5	-0.3178	4.4555E-03	2.7558E-02	ENSG00000145242
EPHA5-AS1	0.1210	1.7980E-01	3.7154E-01	ENSG00000250846

EPHA7	-0.0313	6.8735E-01	8.2611E-01	ENSG00000135333
EPHA8	-0.0288	7.1706E-01	8.4451E-01	ENSG00000070886
EPHB1	-0.0751	3.5035E-01	5.6056E-01	ENSG00000154928
EPHB2	-0.1138	8.4592E-02	2.2730E-01	ENSG00000133216
EPHB3	0.0063	9.4337E-01	9.7195E-01	ENSG00000182580
EPHB4	0.0236	7.8554E-01	8.8661E-01	ENSG00000196411
EPHB6	0.1327	9.4343E-02	2.4565E-01	ENSG00000106123
EPHX1	-0.5210	3.8727E-05	7.3242E-04	ENSG00000143819
EPHX2	-0.2874	1.2758E-02	6.0495E-02	ENSG00000120915
EPHX3	0.0387	4.9171E-01	6.8388E-01	ENSG00000105131
EPHX4	0.1831	3.1154E-02	1.1433E-01	ENSG00000172031
EPIC1	0.0052	9.7593E-01		ENSG00000224271
EPM2A	0.1062	1.6229E-01	3.4829E-01	ENSG00000112425
EPM2A-DT	0.0426	5.1999E-01	7.0703E-01	ENSG00000235652
EPM2AIP1	0.1453	2.1452E-02	8.7265E-02	ENSG00000178567
EPN1	-0.0874	1.8722E-01	3.8177E-01	ENSG00000063245
EPN2	-0.2610	1.8142E-04	2.4038E-03	ENSG00000072134
EPN2-AS1	0.0073	9.8286E-01		ENSG00000235397
EPN3	-0.0108	8.5095E-01	9.2415E-01	ENSG00000049283
EPO	0.0094	7.6999E-01		ENSG00000130427
EPOP	0.1723	6.7856E-02	1.9645E-01	ENSG00000273604
EPOR	0.1253	8.3455E-02	2.2563E-01	ENSG00000187266
EPPK1	0.0211	7.9670E-01	8.9266E-01	ENSG00000261150
EPRS1	-0.0040	9.0454E-01	9.5332E-01	ENSG00000136628
EPS15	-0.0899	1.8326E-01	3.7649E-01	ENSG00000085832
EPS15-AS1	0.0190	5.8498E-01		ENSG00000227070
EPS15L1	-0.0565	3.6659E-01	5.7582E-01	ENSG00000127527
EPS8	-0.0700	3.4274E-01	5.5221E-01	ENSG00000151491
EPS8L1	0.2615	5.8124E-04	5.9714E-03	ENSG00000131037
EPS8L2	-0.0132	8.7075E-01	9.3476E-01	ENSG00000177106
EPSTI1	0.0681	1.3246E-01	3.0599E-01	ENSG00000133106
EPX	-0.0386	2.8635E-01		ENSG00000121053
EPYC	-0.0021	8.8821E-01		ENSG00000083782
EQTN	-0.0934	4.7367E-02	1.5388E-01	ENSG00000120160
ERAL1	-0.1779	2.6403E-03	1.8514E-02	ENSG00000132591
ERAP1	0.0418	5.9088E-01	7.5893E-01	ENSG00000164307
ERAP2	0.0148	8.9313E-01	9.4686E-01	ENSG00000164308
ERAS	-0.0273	7.3427E-01	8.5491E-01	ENSG00000187682
ERBB2	0.0774	3.4446E-01	5.5407E-01	ENSG00000141736
ERBB3	0.2705	3.0274E-02	1.1195E-01	ENSG00000065361
ERBB4	-0.0801	3.4458E-01	5.5416E-01	ENSG00000178568
ERBIN	0.1467	5.1906E-02	1.6344E-01	ENSG00000112851
ERC1	-0.1202	6.8323E-02	1.9733E-01	ENSG00000082805
ERC2	-0.0159	8.1429E-01	9.0320E-01	ENSG00000187672
ERC2-IT1	0.0974	2.0175E-01	4.0032E-01	ENSG00000281708
ERCC1	0.0927	6.0809E-02	1.8195E-01	ENSG00000012061
ERCC2	-0.0142	8.1800E-01	9.0539E-01	ENSG00000104884
ERCC3	-0.0020	9.7433E-01	9.8741E-01	ENSG00000163161
ERCC4	0.1678	7.7954E-03	4.2064E-02	ENSG00000175595

ERCC5	0.1891	3.9142E-02	1.3471E-01	ENSG00000134899
ERCC6	-0.1286	4.4458E-02	1.4721E-01	ENSG00000225830
ERCC6L	-0.0501	5.5072E-01	7.2951E-01	ENSG00000186871
ERCC6L2	0.0400	4.7122E-01	6.6746E-01	ENSG00000182150
ERCC6L2-AS1	0.1375	9.3177E-02	2.4361E-01	ENSG00000175611
ERCC8	-0.0842	1.8787E-01	3.8264E-01	ENSG00000049167
EREG	0.0227	4.1413E-01		ENSG00000124882
ERF	0.2742	3.3876E-03	2.2413E-02	ENSG00000105722
ERFE	-0.1237	1.7345E-01	3.6349E-01	ENSG00000178752
ERFL	-0.0407	3.4746E-01	5.5771E-01	ENSG00000268041
ERG	0.0105	7.7396E-01		ENSG00000157554
ERG28	-0.0975	1.2252E-01	2.9143E-01	ENSG00000133935
ERGIC1	0.0181	7.3326E-01	8.5427E-01	ENSG00000113719
ERGIC2	-0.0735	3.2044E-01	5.3047E-01	ENSG00000087502
ERGIC3	0.0409	4.2450E-01	6.2694E-01	ENSG00000125991
ERH	-0.3366	2.2437E-04	2.8427E-03	ENSG00000100632
ERI1	0.0876	2.3127E-01	4.3620E-01	ENSG00000104626
ERI2	-0.0066	9.2760E-01	9.6455E-01	ENSG00000196678
ERI3	-0.0169	7.2646E-01	8.4992E-01	ENSG00000117419
ERICH1	0.1157	4.1245E-02	1.3982E-01	ENSG00000104714
ERICH2-DT	0.1969	6.8161E-02	1.9707E-01	ENSG00000234350
ERICH3	0.0104	8.7696E-01	9.3873E-01	ENSG00000178965
ERICH4	-0.0208	8.5814E-01		ENSG00000204978
ERICH5	-0.0105	8.9201E-01	9.4631E-01	ENSG00000177459
ERICH6	1.4835	4.5329E-03		ENSG00000163645
ERICH6-AS1	0.1548	1.0674E-01	2.6574E-01	ENSG00000240137
ERICH6B	0.3842	1.6406E-02	7.2275E-02	ENSG00000165837
ERLEC1	0.0260	6.4381E-01	7.9674E-01	ENSG00000068912
ERLIN1	-0.2128	1.2475E-02	5.9563E-02	ENSG00000107566
ERLIN2	0.1267	1.1158E-01	2.7356E-01	ENSG00000147475
ERMAP	0.1127	2.1754E-01	4.1954E-01	ENSG00000164010
ERMARD	0.2043	5.9558E-03	3.4312E-02	ENSG00000130023
ERMN	-0.2297	3.3947E-02	1.2107E-01	ENSG00000136541
ERMP1	-0.2259	1.8871E-02	7.9645E-02	ENSG00000099219
ERN1	0.0404	5.2787E-01	7.1270E-01	ENSG00000178607
ERN2	0.4811	1.1542E-02	5.6328E-02	ENSG00000134398
ERO1A	0.0758	3.3028E-01	5.4056E-01	ENSG00000197930
ERO1B	0.3713	3.7365E-06	1.1050E-04	ENSG00000086619
ERP27	-0.0085	4.3630E-01		ENSG00000139055
ERP29	-0.2639	7.1525E-05	1.1835E-03	ENSG00000089248
ERP44	-0.0337	5.2444E-01	7.0997E-01	ENSG00000023318
ERRFI1	-0.0877	2.4772E-01	4.5550E-01	ENSG00000116285
ERV3-1	-0.2121	2.7982E-02	1.0578E-01	ENSG00000213462
ERVFRD-1	-0.0829	1.8002E-01	3.7188E-01	ENSG00000244476
ERVH48-1	0.1192	1.3454E-01	3.0891E-01	ENSG00000233056
ERVK3-1	-0.2706	2.1711E-04	2.7765E-03	ENSG00000142396
ERVMER34-1	0.0484	6.3227E-01		ENSG00000226887
ERVV-1	-0.0279	9.2976E-02		ENSG00000269526
ERVV-2	0.0134	6.0619E-01		ENSG00000268964

ERVW-1	0.0757	1.8193E-01		ENSG00000242950
ESAM	0.1628	1.0999E-01	2.7106E-01	ENSG00000149564
ESAM-AS1	0.0801	1.7329E-01		ENSG00000250073
ESCO1	-0.0202	7.3462E-01	8.5520E-01	ENSG00000141446
ESCO2	-0.2440	2.6383E-02	1.0133E-01	ENSG00000171320
ESD	-0.1572	1.2466E-02	5.9537E-02	ENSG00000139684
ESF1	0.2448	2.0205E-05	4.3118E-04	ENSG00000089048
ESM1	0.0434	3.0183E-01		ENSG00000164283
ESPL1	0.0942	2.9779E-01	5.0763E-01	ENSG00000135476
ESPN	0.0383	5.3714E-01	7.2010E-01	ENSG00000187017
ESR1	-0.0397	2.1421E-01	4.1533E-01	ENSG00000091831
ESR2	0.0764	3.4326E-01	5.5287E-01	ENSG00000140009
ESRG	0.0394	4.5719E-02		ENSG00000265992
ESRP1	0.0253	6.7578E-01	8.1820E-01	ENSG00000104413
ESRP2	0.0958	1.7387E-01	3.6405E-01	ENSG00000103067
ESRRA	0.0917	2.3040E-01	4.3514E-01	ENSG00000173153
ESRRB	0.0912	2.6353E-01	4.7243E-01	ENSG00000119715
ESRRG	-0.1022	2.2802E-01	4.3267E-01	ENSG00000196482
ESS2	0.1099	1.2939E-01	3.0183E-01	ENSG00000100056
ESX1	0.0661	3.2231E-01	5.3228E-01	ENSG00000123576
ESYT1	0.1377	4.2737E-02	1.4314E-01	ENSG00000139641
ESYT2	0.0409	5.2931E-01	7.1387E-01	ENSG00000117868
ESYT3	-0.3027	2.2666E-02	9.0928E-02	ENSG00000158220
ETAA1	0.0045	9.5628E-01	9.7830E-01	ENSG00000143971
ETDA	0.0161	5.8558E-01		ENSG00000238210
ETDB	0.0094	8.6914E-01		ENSG00000224107
ETF1	-0.0952	1.2399E-01	2.9372E-01	ENSG00000120705
ETFA	-0.2263	1.3144E-04	1.8912E-03	ENSG00000140374
ETFB	0.1321	5.2762E-02	1.6546E-01	ENSG00000105379
ETFBKMT	0.0252	7.0712E-01	8.3893E-01	ENSG00000139160
ETFDH	0.3653	1.4658E-07	7.8233E-06	ENSG00000171503
ETFRF1	-0.0023	9.9137E-01	9.9518E-01	ENSG00000205707
ETHE1	-0.3613	3.3665E-06	1.0226E-04	ENSG00000105755
ETNK1	0.0499	4.7287E-01	6.6894E-01	ENSG00000139163
ETNK2	-0.0533	4.3057E-01	6.3225E-01	ENSG00000143845
ETNPPL	-0.0350	3.0322E-01	5.1333E-01	ENSG00000164089
ETS1	0.0502	2.1267E-01	4.1365E-01	ENSG00000134954
ETS2	0.1163	1.9454E-01	3.9125E-01	ENSG00000157557
ETV1	-0.0395	6.2463E-01	7.8330E-01	ENSG00000006468
ETV2	0.0818	3.5755E-01	5.6739E-01	ENSG00000105672
ETV3	-0.2516	1.8679E-03	1.4304E-02	ENSG00000117036
ETV3L	0.0240	5.4972E-01	7.2897E-01	ENSG00000253831
ETV4	0.0885	3.1201E-01	5.2208E-01	ENSG00000175832
ETV5	0.0566	4.2624E-01	6.2845E-01	ENSG00000244405
ETV6	0.0711	3.9613E-01	6.0213E-01	ENSG00000139083
EVA1A	0.0960	1.7460E-01	3.6494E-01	ENSG00000115363
EVA1C	-0.0885	2.9188E-01	5.0208E-01	ENSG00000166979
EVA1CP5	-0.0097	7.4421E-01		ENSG00000244699
EVC	-0.0744	3.3227E-01	5.4247E-01	ENSG00000072840

EVC2	-0.1237	1.8054E-01	3.7279E-01	ENSG00000173040
EVI2A	0.2013	6.3256E-02	1.8730E-01	ENSG00000126860
EVI2B	0.0115	4.0109E-01		ENSG00000185862
EVI5	0.1201	4.7474E-02	1.5414E-01	ENSG00000067208
EVI5L	-0.0160	8.0678E-01	8.9843E-01	ENSG00000142459
EVL	0.0284	7.0618E-01	8.3814E-01	ENSG00000196405
EVPL	0.0094	9.5228E-01		ENSG00000167880
EVX1	0.0255	3.5252E-01		ENSG00000106038
EVX1-AS	0.0010	9.4551E-01		ENSG00000253405
EVX2	-0.0368	2.0396E-01		ENSG00000174279
EWSAT1	-0.1503	1.2242E-01	2.9129E-01	ENSG00000212766
EWSR1	-0.1892	4.1171E-03	2.6095E-02	ENSG00000182944
EXD1	0.0596	3.8783E-01	5.9542E-01	ENSG00000178997
EXD2	-0.0358	5.0675E-01	6.9620E-01	ENSG00000081177
EXD3	-0.1673	5.2551E-02	1.6496E-01	ENSG00000187609
EXO1	-0.0417	6.3083E-01	7.8742E-01	ENSG00000174371
EXO5	-0.1835	2.1839E-02	8.8477E-02	ENSG00000164002
EXOC1	0.0997	9.1770E-02	2.4116E-01	ENSG00000090989
EXOC2	-0.1736	1.1232E-02	5.5220E-02	ENSG00000112685
EXOC3	0.1146	1.8571E-01	3.7967E-01	ENSG00000180104
EXOC3L1	0.1210	1.9578E-01	3.9292E-01	ENSG00000179044
EXOC3L2	0.0737	1.2345E-01	2.9305E-01	ENSG00000283632
EXOC3L4	0.0009	9.4365E-01		ENSG00000205436
EXOC4	-0.1086	9.8792E-02	2.5276E-01	ENSG00000131558
EXOC5	0.0113	8.3551E-01	9.1524E-01	ENSG00000070367
EXOC5P1	0.0160	5.9077E-01		ENSG00000180673
EXOC6	0.3436	3.1198E-05	6.1213E-04	ENSG00000138190
EXOC6B	-0.1190	6.1102E-02	1.8259E-01	ENSG00000144036
EXOC7	-0.0074	9.1381E-01	9.5762E-01	ENSG00000182473
EXOC8	-0.1913	3.5461E-02	1.2518E-01	ENSG00000116903
EXOG	0.0421	4.5458E-01	6.5306E-01	ENSG00000157036
EXOSC1	-0.0815	1.2804E-01	2.9973E-01	ENSG00000171311
EXOSC10	-0.0619	3.5828E-01	5.6777E-01	ENSG00000171824
EXOSC10-AS1	0.0570	3.9307E-01	5.9980E-01	ENSG00000230337
EXOSC2	-0.3705	6.4772E-08	4.0245E-06	ENSG00000130713
EXOSC3	0.1662	4.1240E-02	1.3982E-01	ENSG00000107371
EXOSC4	0.0809	2.1443E-01	4.1559E-01	ENSG00000178896
EXOSC5	-0.1435	5.8809E-02	1.7816E-01	ENSG00000077348
EXOSC6	-0.2587	8.5861E-07	3.2922E-05	ENSG00000223496
EXOSC7	-0.0835	1.3813E-01	3.1409E-01	ENSG00000075914
EXOSC8	-0.1203	4.1689E-02	1.4077E-01	ENSG00000120699
EXOSC9	-0.0704	1.3532E-01	3.1006E-01	ENSG00000123737
EXPH5	-0.0501	5.3075E-01	7.1508E-01	ENSG00000110723
EXT1	-0.0091	9.0405E-01	9.5319E-01	ENSG00000182197
EXT2	-0.0733	2.9596E-01	5.0595E-01	ENSG00000151348
EXTL1	-0.0167	8.4587E-01	9.2100E-01	ENSG00000158008
EXTL2	0.0784	1.4096E-01	3.1764E-01	ENSG00000162694
EXTL3	-0.1289	1.8707E-02	7.9143E-02	ENSG00000012232
EXTL3-AS1	0.0619	4.5966E-01	6.5736E-01	ENSG00000246339

EYA1	0.1561	1.0848E-01	2.6863E-01	ENSG00000104313
EYA2	0.1300	1.5730E-01	3.4109E-01	ENSG00000064655
EYA3	0.3042	3.1450E-04	3.7302E-03	ENSG00000158161
EYA4	0.0853	3.2345E-01	5.3344E-01	ENSG00000112319
EYS	0.0669	4.1595E-01	6.1934E-01	ENSG00000188107
EZH1	0.1073	6.8574E-02	1.9790E-01	ENSG00000108799
EZH2	0.0225	7.6100E-01	8.7155E-01	ENSG00000106462
EZHIP	0.0027	8.3591E-01		ENSG00000187690
EZR	-0.4980	4.3528E-07	1.9130E-05	ENSG00000092820
EZR-AS1	-0.0196	7.2045E-01	8.4663E-01	ENSG00000233893
F10	0.0410	6.2676E-01	7.8481E-01	ENSG00000126218
F10-AS1	-0.0299	6.2090E-01	7.8072E-01	ENSG00000231882
F11-AS1	0.0608	5.9613E-02		ENSG00000251165
F11R	-0.0017	9.8264E-01	9.9071E-01	ENSG00000158769
F12	0.1401	9.4665E-02	2.4622E-01	ENSG00000131187
F13A1	0.0065	6.6921E-01		ENSG00000124491
F2	-0.0169	5.5404E-01		ENSG00000180210
F2R	-0.2244	2.3850E-03	1.7179E-02	ENSG00000181104
F2RL1	-0.4059	5.7236E-03	3.3348E-02	ENSG00000164251
F2RL2	0.0324	9.6440E-01	9.8237E-01	ENSG00000164220
F2RL3	0.0079	6.3938E-01		ENSG00000127533
F3	-0.0489	5.4294E-01	7.2427E-01	ENSG00000117525
F7	-0.0226	7.6959E-01	8.7669E-01	ENSG00000057593
F8	0.4063	1.7199E-03	1.3477E-02	ENSG00000185010
F8A1	-0.2161	6.9165E-05	1.1546E-03	ENSG00000288722
F8A2	-0.2030	1.2292E-03	1.0582E-02	ENSG00000288709
F8A3	-0.2114	4.1178E-04	4.5721E-03	ENSG00000277150
FA2H	0.0011	6.5742E-01		ENSG00000103089
FAAH	0.0811	2.8589E-01	4.9663E-01	ENSG00000117480
FAAH2	0.0339	6.9059E-01	8.2817E-01	ENSG00000165591
FAAP100	0.0455	5.3241E-01	7.1608E-01	ENSG00000185504
FAAP20	0.0221	7.6195E-01	8.7200E-01	ENSG00000162585
FAAP24	0.3758	3.1790E-04	3.7623E-03	ENSG00000131944
FABP1	0.0939	3.6716E-02		ENSG00000163586
FABP12	0.0043	8.8354E-01		ENSG00000197416
FABP3	-0.0221	7.6383E-01	8.7292E-01	ENSG00000121769
FABP5	-0.5398	4.0015E-06	1.1749E-04	ENSG00000164687
FABP5P3	-0.1226	1.7643E-01	3.6758E-01	ENSG00000241735
FABP6	0.1748	9.2330E-02	2.4204E-01	ENSG00000170231
FABP7	0.0113	8.8310E-01	9.4146E-01	ENSG00000164434
FADD	0.2722	1.4167E-02	6.5312E-02	ENSG00000168040
FADS1	-0.1798	4.1394E-03	2.6178E-02	ENSG00000149485
FADS2	-0.3994	1.2689E-07	6.9691E-06	ENSG00000134824
FADS3	0.1130	9.7292E-02	2.5056E-01	ENSG00000221968
FADS6	-0.0390	6.1651E-01	7.7763E-01	ENSG00000172782
FAF1	-0.1696	1.6887E-02	7.3738E-02	ENSG00000185104
FAF2	0.0060	9.6689E-01	9.8344E-01	ENSG00000113194
FAH	-0.2013	3.0469E-02	1.1246E-01	ENSG00000103876
FAHD1	-0.2400	7.4712E-05	1.2247E-03	ENSG00000180185

FAHD2A	-0.0713	2.9424E-01	5.0413E-01	ENSG00000115042
FAHD2B	-0.0870	2.8660E-01	4.9746E-01	ENSG00000144199
FAIM	-0.0336	6.4082E-01	7.9444E-01	ENSG00000158234
FAIM2	-0.2041	4.2568E-03	2.6701E-02	ENSG00000135472
FALEC	0.2826	7.0017E-03	3.8794E-02	ENSG00000228126
FAM106A	0.1492	2.4296E-02	9.5680E-02	ENSG00000273018
FAM106C	0.0454	2.7539E-01		ENSG00000288235
FAM107A	0.1170	2.1291E-01	4.1396E-01	ENSG00000168309
FAM107B	-0.1012	1.7514E-01	3.6568E-01	ENSG00000065809
FAM110A	-0.1039	1.8229E-01	3.7524E-01	ENSG00000125898
FAM110B	-0.0649	3.1596E-01	5.2600E-01	ENSG00000169122
FAM110C	-0.0303	6.6783E-01	8.1403E-01	ENSG00000184731
FAM110D	-0.0452	5.9660E-01	7.6320E-01	ENSG00000197245
FAM111A	-0.2759	1.4820E-02	6.7448E-02	ENSG00000166801
FAM111A-DT	-0.0216	7.7556E-01	8.8055E-01	ENSG00000245571
FAM111B	-0.1031	2.1345E-01	4.1439E-01	ENSG00000189057
FAM114A1	-0.1652	4.0065E-02	1.3690E-01	ENSG00000197712
FAM114A2	0.0375	5.4008E-01	7.2209E-01	ENSG00000055147
FAM117A	0.2677	3.1375E-02	1.1490E-01	ENSG00000121104
FAM117B	-0.2647	3.1028E-04	3.6900E-03	ENSG00000138439
FAM118A	0.1120	1.4772E-01	3.2790E-01	ENSG00000100376
FAM118B	-0.1232	7.3045E-02	2.0692E-01	ENSG00000197798
FAM120A	0.1513	4.3440E-02	1.4486E-01	ENSG00000048828
FAM120AOS	-0.0657	2.2842E-01	4.3305E-01	ENSG00000188938
FAM120B	-0.0118	8.7029E-01	9.3460E-01	ENSG00000112584
FAM120C	0.0030	9.7046E-01	9.8515E-01	ENSG00000184083
FAM124A	-0.4013	8.8358E-07	3.3447E-05	ENSG00000150510
FAM124B	0.4749	9.4115E-03	4.8527E-02	ENSG00000124019
FAM131A	-0.1076	1.2419E-01	2.9398E-01	ENSG00000175182
FAM131B	-0.1397	3.7927E-02	1.3175E-01	ENSG00000159784
FAM131C	-0.1159	1.2157E-01	2.9011E-01	ENSG00000185519
FAM131C2P	-0.1862	6.6952E-02	1.9488E-01	ENSG00000232456
FAM133A	0.3433	5.7224E-04	5.8961E-03	ENSG00000179083
FAM133B	0.0084	9.0950E-01	9.5525E-01	ENSG00000234545
FAM133FP	-0.0231	4.2060E-01		ENSG00000225934
FAM135A	0.3298	1.0318E-05	2.5187E-04	ENSG00000082269
FAM135A-AS1	0.1008	7.6681E-02		ENSG00000224349
FAM135B	-0.0048	9.3657E-01	9.6868E-01	ENSG00000147724
FAM136A	0.0156	8.0227E-01	8.9572E-01	ENSG00000035141
FAM138C	0.1100	6.3301E-02	1.8736E-01	ENSG00000218839
FAM138D	0.0878	4.8331E-02		ENSG00000249054
FAM138E	1.4845	1.7352E-03		ENSG00000248893
FAM13A	-0.3557	8.9076E-08	5.2433E-06	ENSG00000138640
FAM13A-AS1	0.2035	6.2866E-02	1.8636E-01	ENSG00000248019
FAM13B	-0.0008	9.5905E-01	9.7955E-01	ENSG00000031003
FAM13C	-0.1340	6.3986E-02	1.8877E-01	ENSG00000148541
FAM149A	-0.1885	1.3697E-02	6.3792E-02	ENSG00000109794
FAM149B1	-0.1276	9.0776E-02	2.3940E-01	ENSG00000138286
FAM151A	0.0132	6.0137E-01		ENSG00000162391

FAM151B	0.0042	9.6030E-01	9.8016E-01	ENSG00000152380
FAM153A	0.1514	9.3311E-02	2.4380E-01	ENSG00000170074
FAM153CP	0.1556	8.0910E-02	2.2113E-01	ENSG00000204677
FAM156A	0.3370	4.4946E-05	8.1676E-04	ENSG00000268350
FAM156B	0.3349	4.6298E-05	8.3331E-04	ENSG00000179304
FAM161A	0.0064	9.5381E-01	9.7701E-01	ENSG00000170264
FAM161B	0.0931	2.9049E-01	5.0083E-01	ENSG00000156050
FAM162A	0.3916	4.8422E-03	2.9360E-02	ENSG00000114023
FAM162B	-0.0197	8.0462E-01	8.9752E-01	ENSG00000183807
FAM163A	-0.0195	7.8494E-01	8.8631E-01	ENSG00000143340
FAM163B	-0.2229	3.9998E-02	1.3674E-01	ENSG00000196990
FAM167A	-0.2188	3.7112E-02	1.2956E-01	ENSG00000154319
FAM167A-AS1	-0.0243	3.9744E-01		ENSG00000184608
FAM167B	0.0328	6.2513E-01	7.8348E-01	ENSG00000183615
FAM168A	-0.0224	7.6132E-01	8.7163E-01	ENSG00000054965
FAM168B	-0.0117	7.9776E-01	8.9353E-01	ENSG00000152102
FAM169A	0.0654	4.2372E-01	6.2604E-01	ENSG00000198780
FAM169BP	0.0402	8.8639E-02		ENSG00000283597
FAM171A1	0.0999	1.0330E-01	2.6021E-01	ENSG00000148468
FAM171A2	-0.3430	6.1984E-07	2.5292E-05	ENSG00000161682
FAM171B	-0.2568	2.8944E-04	3.4876E-03	ENSG00000144369
FAM174A	0.4127	7.4488E-07	2.9277E-05	ENSG00000174132
FAM174A-DT	0.4724	9.6574E-04	8.8138E-03	ENSG00000247877
FAM174B	-0.1827	8.9404E-03	4.6784E-02	ENSG00000185442
FAM174C	0.1921	1.3347E-02	6.2578E-02	ENSG00000228300
FAM177A1	0.0249	6.7721E-01	8.1917E-01	ENSG00000151327
FAM177B	0.0850	2.5235E-02		ENSG00000197520
FAM180B	0.0531	2.9300E-01	5.0301E-01	ENSG00000196666
FAM181A	0.2551	2.5840E-02	9.9904E-02	ENSG00000140067
FAM181A-AS1	0.0513	2.9074E-01	5.0116E-01	ENSG00000258584
FAM181B	-0.0710	3.5270E-01	5.6293E-01	ENSG00000182103
FAM182A	0.1724	9.9616E-02	2.5400E-01	ENSG00000125804
FAM183BP	0.0009	9.9045E-01	9.9461E-01	ENSG00000164556
FAM184A	-0.0229	7.1294E-01	8.4243E-01	ENSG00000111879
FAM184B	-0.0646	4.3294E-01	6.3423E-01	ENSG00000047662
FAM185A	0.0209	7.9312E-01	8.9076E-01	ENSG00000222011
FAM186A	0.0223	2.3819E-01		ENSG00000185958
FAM186B	0.0494	4.0093E-01	6.0619E-01	ENSG00000135436
FAM187B2P	0.0335	7.1922E-01		ENSG00000262497
FAM193A	-0.0616	3.6183E-01	5.7107E-01	ENSG00000125386
FAM193B	-0.0217	7.6197E-01	8.7200E-01	ENSG00000146067
FAM199X	-0.0729	2.1439E-01	4.1556E-01	ENSG00000123575
FAM200A	0.2238	2.5828E-02	9.9888E-02	ENSG00000221909
FAM200B	0.1045	1.4524E-01	3.2405E-01	ENSG00000237765
FAM200C	0.0443	6.0465E-01	7.6862E-01	ENSG00000221886
FAM204A	-0.0110	7.9329E-01	8.9078E-01	ENSG00000165669
FAM209A	-0.1243	6.6329E-02	1.9369E-01	ENSG00000124103
FAM209B	-0.0636	4.1025E-01	6.1437E-01	ENSG00000213714
FAM20A	0.0576	3.9324E-01	5.9985E-01	ENSG00000108950

FAM20B	-0.0734	1.9879E-01	3.9702E-01	ENSG00000116199
FAM20C	-0.0019	9.7883E-01	9.8938E-01	ENSG00000177706
FAM210A	0.2084	2.9988E-02	1.1122E-01	ENSG00000177150
FAM210B	-0.1870	2.0799E-03	1.5494E-02	ENSG00000124098
FAM216A	0.0729	3.1308E-01	5.2339E-01	ENSG00000204856
FAM216B	-0.0743	1.8527E-01	3.7894E-01	ENSG00000179813
FAM217A	-0.0015	9.6645E-01		ENSG00000145975
FAM217B	-0.0388	4.9858E-01	6.8959E-01	ENSG00000196227
FAM218A	0.3792	9.4009E-05	1.4686E-03	ENSG00000250486
FAM219A	-0.0461	4.8696E-01	6.8028E-01	ENSG00000164970
FAM219B	-0.0746	3.2681E-01	5.3704E-01	ENSG00000178761
FAM21FP	0.3934	1.3532E-02	6.3181E-02	ENSG00000237840
FAM220A	-0.0259	2.2774E-01		ENSG00000178397
FAM221A	-0.0121	8.6611E-01	9.3231E-01	ENSG00000188732
FAM221B	0.0506	4.4292E-01	6.4220E-01	ENSG00000204930
FAM222A	0.0516	5.3742E-01	7.2025E-01	ENSG00000139438
FAM222A-AS1	-0.0100	8.9476E-01	9.4750E-01	ENSG00000255650
FAM222B	0.3824	5.6793E-07	2.3684E-05	ENSG00000173065
FAM223A	0.0152	5.6169E-01		ENSG00000279245
FAM223B	0.0152	5.6169E-01		ENSG00000272681
FAM225A	0.0595	1.8229E-01		ENSG00000231528
FAM225B	0.0999	3.8398E-02		ENSG00000225684
FAM227A	0.4992	3.2289E-06	9.8691E-05	ENSG00000184949
FAM227B	-0.0077	9.2241E-01	9.6166E-01	ENSG00000166262
FAM228A	0.1191	1.3203E-01	3.0536E-01	ENSG00000186453
FAM228B	-0.0137	7.9158E-01	8.8996E-01	ENSG00000219626
FAM229A	0.1678	7.9504E-02	2.1879E-01	ENSG00000225828
FAM229B	0.0802	2.0844E-01	4.0847E-01	ENSG00000203778
FAM230A	0.0068	8.2906E-01		ENSG00000277870
FAM230B	0.0364	1.2150E-01		ENSG00000215498
FAM230E	0.0183	5.3537E-01		ENSG00000182824
FAM230H	0.0478	8.3945E-02		ENSG00000206142
FAM230I	0.2403	3.8944E-02	1.3426E-01	ENSG00000178248
FAM230J	0.0380	2.8077E-01		ENSG00000274044
FAM234A	0.0816	2.3795E-01	4.4400E-01	ENSG00000167930
FAM234B	-0.1773	2.7386E-03	1.9030E-02	ENSG00000084444
FAM236A	0.0368	4.0767E-01		ENSG00000275520
FAM236B	0.0368	4.0767E-01		ENSG00000268994
FAM237A	0.0460	3.9480E-01		ENSG00000235118
FAM237B	-0.0002	8.6859E-01		ENSG00000283267
FAM240C	0.0334	6.7810E-01	8.1969E-01	ENSG00000216921
FAM241A	0.0720	3.5510E-01	5.6476E-01	ENSG00000174749
FAM241B	0.1082	1.4688E-01	3.2662E-01	ENSG00000171224
FAM242F	0.0271	1.5361E-01		ENSG00000275239
FAM245A	-0.0396	2.5007E-02		ENSG00000228055
FAM246A	0.0095	8.3909E-01		ENSG00000286102
FAM246B	-0.0051	6.9334E-01		ENSG00000286175
FAM246C	-0.0186	7.3883E-01	8.5776E-01	ENSG00000286025
FAM247A	-0.0339	6.2331E-01	7.8239E-01	ENSG00000288811

FAM24B	-0.1431	9.8814E-02	2.5279E-01	ENSG00000213185
FAM27C	0.0770	3.2085E-01	5.3094E-01	ENSG00000231527
FAM32A	-0.1592	9.9687E-03	5.0594E-02	ENSG00000105058
FAM3A	-0.0236	7.1707E-01	8.4451E-01	ENSG00000071889
FAM3B	0.0409	4.4240E-01	6.4174E-01	ENSG00000183844
FAM3C	-0.0634	2.8747E-01	4.9814E-01	ENSG00000196937
FAM3D	0.0443	1.1440E-01		ENSG00000198643
FAM3D-AS1	0.0486	3.7120E-01	5.7998E-01	ENSG00000244383
FAM41C	0.0736	2.9157E-01	5.0185E-01	ENSG00000230368
FAM43A	-0.2323	7.4764E-03	4.0743E-02	ENSG00000185112
FAM43B	-0.1835	2.2805E-02	9.1358E-02	ENSG00000183114
FAM47E-STBD1	0.0231	2.0047E-01		ENSG00000272414
FAM50A	-0.2387	2.3123E-05	4.8142E-04	ENSG00000071859
FAM50B	0.1623	9.7202E-02	2.5043E-01	ENSG00000145945
FAM53A	0.0079	9.2856E-01	9.6486E-01	ENSG00000174137
FAM53B	-0.2034	4.2291E-02	1.4216E-01	ENSG00000189319
FAM53C	-0.3571	1.3058E-05	3.0112E-04	ENSG00000120709
FAM66B	0.2445	1.4163E-02	6.5311E-02	ENSG00000215374
FAM66C	0.3320	8.7706E-04	8.2107E-03	ENSG00000226711
FAM66E	0.0633	4.6333E-01	6.6050E-01	ENSG00000225725
FAM72A	-0.0508	5.4896E-01	7.2827E-01	ENSG00000196550
FAM72B	-0.0936	2.9779E-01	5.0763E-01	ENSG00000188610
FAM72C	-0.1658	9.2111E-02	2.4167E-01	ENSG00000263513
FAM72D	-0.1567	1.0308E-01	2.5994E-01	ENSG00000215784
FAM76A	-0.1480	7.5422E-02	2.1119E-01	ENSG00000009780
FAM76B	0.0122	8.6974E-01	9.3437E-01	ENSG00000077458
FAM78A	-0.0509	4.5090E-01	6.4997E-01	ENSG00000126882
FAM78B	0.0647	4.4184E-01	6.4129E-01	ENSG00000188859
FAM81A	0.0101	8.9058E-01	9.4581E-01	ENSG00000157470
FAM83A	0.0062	8.3896E-01		ENSG00000147689
FAM83C	-0.0228	1.7219E-01		ENSG00000125998
FAM83C-AS1	-0.0153	3.1397E-01		ENSG00000235214
FAM83D	-0.1500	1.2013E-01	2.8754E-01	ENSG00000101447
FAM83E	-0.1376	8.4371E-02	2.2703E-01	ENSG00000105523
FAM83F	0.0546	4.2896E-01	6.3073E-01	ENSG00000133477
FAM83G	-0.0652	3.8602E-01	5.9380E-01	ENSG00000188522
FAM83H	0.2186	5.0241E-02	1.6013E-01	ENSG00000180921
FAM86B1	-0.0451	5.6451E-01	7.4050E-01	ENSG00000186523
FAM86B2	-0.0258	7.5018E-01	8.6435E-01	ENSG00000145002
FAM86B3P	0.3626	1.0117E-02	5.1185E-02	ENSG00000173295
FAM86C1P	-0.0154	8.5110E-01	9.2415E-01	ENSG00000158483
FAM86DP	0.1196	1.3806E-01	3.1401E-01	ENSG00000244026
FAM86GP	-0.0097	9.0124E-01	9.5157E-01	ENSG00000166492
FAM86KP	-0.0049	7.6543E-01		ENSG00000163612
FAM87A	0.0599	4.1526E-01	6.1867E-01	ENSG00000182366
FAM87B	0.1109	2.3549E-01	4.4097E-01	ENSG00000177757
FAM88B	0.1052	2.0673E-01	4.0621E-01	ENSG00000170161
FAM88C	0.0663	4.2604E-01	6.2831E-01	ENSG00000204802
FAM89A	0.2522	9.8155E-03	5.0044E-02	ENSG00000182118

FAM89B	-0.1975	2.8133E-02	1.0617E-01	ENSG00000176973
FAM8A1	0.1165	9.7409E-02	2.5074E-01	ENSG00000137414
FAM90A1	0.0070	8.6959E-01	9.3437E-01	ENSG00000171847
FAM90A11	-0.0126	4.9989E-01		ENSG00000233115
FAM91A1	0.2471	7.5760E-04	7.3636E-03	ENSG00000176853
FAM98A	0.0221	7.3957E-01	8.5776E-01	ENSG00000119812
FAM98B	0.0850	2.3992E-01	4.4664E-01	ENSG00000171262
FAM98C	0.0111	8.7909E-01	9.3957E-01	ENSG00000130244
FAM9B	0.0162	4.7737E-01		ENSG00000177138
FAM9C	0.0511	2.2021E-01		ENSG00000187268
FAN1	-0.0755	3.0839E-01	5.1859E-01	ENSG00000198690
FANCA	-0.1180	1.2212E-01	2.9093E-01	ENSG00000187741
FANCB	-0.0132	8.7500E-01	9.3736E-01	ENSG00000181544
FANCC	0.1403	7.7338E-02	2.1485E-01	ENSG00000158169
FANCD2	-0.0890	3.0244E-01	5.1253E-01	ENSG00000144554
FANCD2OS	0.0037	8.9726E-01		ENSG00000163705
FANCD2P2	-0.0030	9.6612E-01		ENSG00000230342
FANCE	-0.2905	1.7675E-05	3.8863E-04	ENSG00000112039
FANCF	0.2532	1.2692E-03	1.0847E-02	ENSG00000183161
FANCG	-0.2134	6.6627E-03	3.7403E-02	ENSG00000221829
FANCI	-0.0715	3.5954E-01	5.6890E-01	ENSG00000140525
FANCL	-0.0871	2.8255E-01	4.9303E-01	ENSG00000115392
FANCM	0.3246	1.0994E-03	9.7355E-03	ENSG00000187790
FANK1	0.4205	1.5263E-03	1.2386E-02	ENSG00000203780
FAP	0.0077	8.4813E-01		ENSG00000078098
FAR1	0.1785	1.5845E-02	7.0677E-02	ENSG00000197601
FAR2	-0.1010	2.6028E-01	4.6876E-01	ENSG00000064763
FAR2P1	0.0520	5.4288E-01	7.2427E-01	ENSG00000286058
FAR2P3	-0.2765	2.2672E-02	9.0928E-02	ENSG00000240253
FAR2P4	-0.0084	8.8560E-01	9.4289E-01	ENSG00000231431
FARP1	-0.1918	5.3343E-03	3.1665E-02	ENSG00000152767
FARP2	-0.1349	1.0177E-01	2.5767E-01	ENSG00000006607
FARS2	-0.1110	1.0604E-01	2.6481E-01	ENSG00000145982
FARSA	-0.1864	7.2423E-03	3.9829E-02	ENSG00000179115
FARSA-AS1	0.0249	7.6070E-01	8.7130E-01	ENSG00000266975
FARSB	-0.1030	8.9716E-02	2.3731E-01	ENSG00000116120
FAS	-0.0460	5.7125E-01	7.4485E-01	ENSG00000026103
FASN	-0.1008	2.2628E-01	4.3026E-01	ENSG00000169710
FASTK	0.0198	7.1510E-01	8.4343E-01	ENSG00000164896
FASTKD1	0.0962	2.1185E-01	4.1282E-01	ENSG00000138399
FASTKD2	0.1985	1.0874E-03	9.6591E-03	ENSG00000118246
FASTKD3	0.1749	2.2462E-02	9.0338E-02	ENSG00000124279
FASTKD5	-0.1344	3.5770E-02	1.2600E-01	ENSG00000215251
FAT1	0.3260	1.4173E-04	1.9871E-03	ENSG00000083857
FAT2	0.0817	2.2679E-01	4.3096E-01	ENSG00000086570
FAT3	0.3096	3.1013E-03	2.1039E-02	ENSG00000165323
FAT4	0.0127	8.7295E-01	9.3623E-01	ENSG00000196159
FATE1	-0.0008	7.2517E-01		ENSG00000147378
FAU	-0.1351	6.9184E-02	1.9898E-01	ENSG00000149806

FAXC	0.0827	2.1968E-01	4.2215E-01	ENSG00000146267
FAXDC2	-0.0201	7.9793E-01	8.9360E-01	ENSG00000170271
FBF1	0.0317	6.5265E-01	8.0324E-01	ENSG00000188878
FBH1	-0.1944	2.1452E-02	8.7265E-02	ENSG00000134452
FBL	-0.2443	4.2202E-03	2.6530E-02	ENSG00000105202
FBLIM1	-0.0373	6.1642E-01	7.7758E-01	ENSG00000162458
FBLL1	-0.0290	7.2104E-01	8.4711E-01	ENSG00000188573
FBLN1	-0.0093	9.0691E-01	9.5442E-01	ENSG00000077942
FBLN2	0.0361	6.5026E-01	8.0162E-01	ENSG00000163520
FBLN5	0.0733	3.7444E-01	5.8308E-01	ENSG00000140092
FBLN7	0.0150	8.3637E-01	9.1564E-01	ENSG00000144152
FBN1	0.3669	1.1882E-03	1.0327E-02	ENSG00000166147
FBN1-DT	0.1708	9.1822E-02	2.4125E-01	ENSG00000259705
FBN2	0.5470	1.2781E-05	2.9640E-04	ENSG00000138829
FBN3	0.1579	5.6670E-02	1.7408E-01	ENSG00000142449
FBP1	0.0291	7.2854E-01	8.5112E-01	ENSG00000165140
FBP2	0.0664	2.0011E-01		ENSG00000130957
FBRS	0.0142	8.6107E-01	9.3034E-01	ENSG00000156860
FBRSL1	-0.0244	7.4798E-01	8.6302E-01	ENSG00000112787
FBXL12	-0.2244	4.4102E-03	2.7376E-02	ENSG00000127452
FBXL13	0.4337	4.3533E-04	4.7690E-03	ENSG00000161040
FBXL14	-0.1962	2.4609E-02	9.6493E-02	ENSG00000171823
FBXL15	0.0273	6.7833E-01	8.1971E-01	ENSG00000107872
FBXL16	-0.0491	5.6364E-01	7.3991E-01	ENSG00000127585
FBXL17	-0.0623	3.4669E-01	5.5695E-01	ENSG00000145743
FBXL18	0.0833	2.4859E-01	4.5643E-01	ENSG00000155034
FBXL19	-0.0038	9.6361E-01	9.8181E-01	ENSG00000099364
FBXL19-AS1	0.0129	8.6602E-01	9.3231E-01	ENSG00000260852
FBXL2	0.0737	3.4029E-01	5.5002E-01	ENSG00000153558
FBXL20	0.0626	3.9310E-01	5.9980E-01	ENSG00000108306
FBXL22	0.0119	8.8403E-01	9.4185E-01	ENSG00000197361
FBXL3	-0.1230	6.0716E-02	1.8185E-01	ENSG00000005812
FBXL4	0.3161	7.5526E-04	7.3510E-03	ENSG00000112234
FBXL5	-0.2380	5.0913E-04	5.3834E-03	ENSG00000118564
FBXL6	0.1725	5.0221E-02	1.6011E-01	ENSG00000182325
FBXL7	-0.0142	8.5953E-01	9.2921E-01	ENSG00000183580
FBXL8	-0.0253	7.6138E-01	8.7163E-01	ENSG00000135722
FBXL9P	0.0155	8.5375E-01	9.2560E-01	ENSG00000125122
FBXO10	0.0917	2.9250E-01	5.0254E-01	ENSG00000147912
FBXO11	-0.2304	8.2647E-04	7.8499E-03	ENSG00000138081
FBXO16	0.5706	1.4827E-03	1.2177E-02	ENSG00000214050
FBXO17	-0.3977	7.7164E-07	3.0233E-05	ENSG00000269190
FBXO2	-0.2725	2.4118E-03	1.7333E-02	ENSG00000116661
FBXO21	-0.0005	9.8821E-01	9.9354E-01	ENSG00000135108
FBXO22	0.2599	1.2244E-03	1.0559E-02	ENSG00000167196
FBXO24	0.0873	2.7406E-01	4.8382E-01	ENSG00000106336
FBXO25	-0.0740	3.5265E-01	5.6290E-01	ENSG00000147364
FBXO27	-0.2220	3.1730E-02	1.1574E-01	ENSG00000161243
FBXO28	-0.2224	6.7941E-04	6.8010E-03	ENSG00000143756

FBXO3	-0.0962	8.3216E-02	2.2510E-01	ENSG00000110429
FBXO3-DT	-0.0132	6.7133E-01		ENSG00000254508
FBXO30	0.3506	1.1656E-05	2.7674E-04	ENSG00000118496
FBXO31	0.1309	6.0808E-02	1.8195E-01	ENSG00000103264
FBXO33	-0.3747	5.8951E-05	1.0186E-03	ENSG00000165355
FBXO34	0.2157	1.3392E-03	1.1304E-02	ENSG00000178974
FBXO34-AS1	0.0067	9.2710E-01	9.6425E-01	ENSG00000258413
FBXO36	0.2694	4.2704E-03	2.6763E-02	ENSG00000153832
FBXO38	0.4121	1.0603E-06	3.8997E-05	ENSG00000145868
FBXO38-DT	0.1018	1.9898E-01	3.9719E-01	ENSG00000247199
FBXO4	0.5316	1.4452E-05	3.2825E-04	ENSG00000151876
FBXO40	-0.0113	4.7130E-01		ENSG00000163833
FBXO41	0.2112	7.8575E-03	4.2305E-02	ENSG00000163013
FBXO42	0.0148	8.3919E-01	9.1734E-01	ENSG00000037637
FBXO43	0.0109	8.8187E-01	9.4091E-01	ENSG00000156509
FBXO44	-0.2242	1.1684E-03	1.0195E-02	ENSG00000132879
FBXO45	-0.2258	7.9522E-04	7.6282E-03	ENSG00000174013
FBXO46	-0.0725	3.0830E-01	5.1854E-01	ENSG00000177051
FBXO48	0.0845	3.4726E-01	5.5760E-01	ENSG00000204923
FBXO5	-0.1326	1.3874E-01	3.1465E-01	ENSG00000112029
FBXO6	-0.1093	2.2951E-01	4.3402E-01	ENSG00000116663
FBXO7	-0.1014	1.0031E-01	2.5521E-01	ENSG00000100225
FBXO8	-0.0299	6.9379E-01	8.3033E-01	ENSG00000164117
FBXO9	0.0121	8.5450E-01	9.2596E-01	ENSG00000112146
FBXW10	-0.0507	3.6189E-01	5.7107E-01	ENSG00000171931
FBXW10B	0.0892	1.9174E-01	3.8705E-01	ENSG00000241322
FBXW11	-0.0844	1.2805E-01	2.9973E-01	ENSG00000072803
FBXW11P1	0.0749	2.3664E-01	4.4242E-01	ENSG00000230870
FBXW12	0.0044	9.4672E-01	9.7359E-01	ENSG00000164049
FBXW2	0.0177	7.7511E-01	8.8029E-01	ENSG00000119402
FBXW4	0.0413	6.3396E-01	7.9012E-01	ENSG00000107829
FBXW4P1	0.0216	7.8360E-01	8.8552E-01	ENSG00000230701
FBXW5	0.0191	7.7717E-01	8.8138E-01	ENSG00000159069
FBXW7	-0.1334	3.6531E-02	1.2788E-01	ENSG00000109670
FBXW7-AS1	-0.0575	8.3490E-02		ENSG00000270751
FBXW8	-0.1016	1.6393E-01	3.5067E-01	ENSG00000174989
FBXW9	0.3058	1.7955E-03	1.3897E-02	ENSG00000132004
FCAR	0.0158	6.2585E-01		ENSG00000186431
FCER1A	0.0243	3.2884E-01		ENSG00000179639
FCER2	-0.0235	4.1747E-01		ENSG00000104921
FCF1	-0.2182	8.7095E-03	4.5839E-02	ENSG00000119616
FCF1P4	0.0219	1.4640E-01		ENSG00000226112
FCF1P5	0.0196	5.5531E-01		ENSG00000220392
FCGR1A	0.0110	4.7054E-01		ENSG00000150337
FCGR1CP	-0.0081	6.8089E-01		ENSG00000265531
FCGR2A	0.2511	3.8141E-02	1.3229E-01	ENSG00000143226
FCGR2B	-0.0011	8.9560E-01		ENSG00000072694
FCGRT	-0.1411	3.8900E-02	1.3413E-01	ENSG00000104870
FCHO1	-0.1445	6.1094E-02	1.8259E-01	ENSG00000130475

FCHO2	-0.1225	8.2308E-02	2.2346E-01	ENSG00000157107
FCHSD1	0.0097	9.0882E-01	9.5493E-01	ENSG00000197948
FCHSD2	-0.1339	2.0032E-02	8.3131E-02	ENSG00000137478
FCMR	0.0906	2.3350E-01	4.3870E-01	ENSG00000162894
FCN1	-0.0009	8.5844E-01		ENSG00000085265
FCN2	-0.0062	7.2366E-01		ENSG00000160339
FCN3	0.0786	5.8189E-02	1.7701E-01	ENSG00000142748
FCRL3	2.8948	1.0384E-02	5.2080E-02	ENSG00000160856
FCRL6	0.0336	8.8028E-02		ENSG00000181036
FCRLA	0.0126	5.2656E-01		ENSG00000132185
FCRLB	0.1532	1.1952E-01	2.8664E-01	ENSG00000162746
FCSK	0.0593	3.9052E-01	5.9774E-01	ENSG00000157353
FDFT1	-0.4338	4.8567E-04	5.1933E-03	ENSG00000079459
FDPS	-0.1752	1.6278E-02	7.1855E-02	ENSG00000160752
FDPSP3	-0.0029	4.1510E-01		ENSG00000258872
FDPSP4	0.0110	6.0356E-01		ENSG00000254793
FDPSP7	-0.0078	9.5875E-01		ENSG00000231087
FDX1	0.0610	3.2874E-01	5.3913E-01	ENSG00000137714
FDX2	-0.0105	8.5696E-01	9.2766E-01	ENSG00000267673
FDXACB1	-0.0986	2.3356E-01	4.3874E-01	ENSG00000255561
FDXR	-0.1030	2.5297E-01	4.6090E-01	ENSG00000161513
FECH	-0.0996	1.4129E-01	3.1815E-01	ENSG00000066926
FEM1A	-0.2582	8.0625E-03	4.3125E-02	ENSG00000141965
FEM1AP2	0.0003	9.5338E-01		ENSG00000265296
FEM1B	-0.3659	5.6004E-10	6.8217E-08	ENSG00000169018
FEM1C	-0.1641	3.2972E-02	1.1887E-01	ENSG00000145780
FEN1	0.0301	6.7961E-01	8.2059E-01	ENSG00000168496
FENDRR	0.0495	1.1652E-01		ENSG00000268388
FER	-0.0496	3.0576E-01	5.1592E-01	ENSG00000151422
FER1L4	0.4653	1.7338E-05	3.8276E-04	ENSG00000088340
FER1L6	0.0391	3.7561E-01		ENSG00000214814
FERMT1	-0.0198	6.3762E-01	7.9240E-01	ENSG00000101311
FERMT2	-0.2215	9.0115E-03	4.7036E-02	ENSG00000073712
FERMT3	-0.1049	2.1844E-01	4.2088E-01	ENSG00000149781
FES	0.1559	6.4010E-02	1.8880E-01	ENSG00000182511
FETUB	0.1103	1.8397E-02		ENSG00000090512
FEV	-0.0001	9.9516E-01	9.9739E-01	ENSG00000163497
FEZ1	-0.3184	1.1775E-06	4.2731E-05	ENSG00000149557
FEZ2	0.0629	3.9279E-01	5.9950E-01	ENSG00000171055
FEZF1	0.0466	4.9033E-01	6.8300E-01	ENSG00000128610
FEZF1-AS1	0.2118	5.1867E-02	1.6336E-01	ENSG00000230316
FEZF2	-0.1152	2.0238E-01	4.0123E-01	ENSG00000153266
FFAR1	0.0247	3.5583E-01		ENSG00000126266
FFAR3	0.0109	4.3358E-01		ENSG00000185897
FFAR4	0.0990	2.3597E-01	4.4162E-01	ENSG00000186188
FGD1	-0.3717	4.9037E-06	1.3916E-04	ENSG00000102302
FGD2	0.0117	5.6307E-01		ENSG00000146192
FGD3	-0.0145	8.6456E-01	9.3172E-01	ENSG00000127084
FGD4	-0.2506	8.4688E-06	2.1426E-04	ENSG00000139132

FGD5	-0.0343	6.8634E-01	8.2522E-01	ENSG00000154783
FGD5-AS1	-0.1251	1.1694E-02	5.6908E-02	ENSG00000225733
FGD6	0.3006	3.6922E-04	4.2023E-03	ENSG00000180263
FGF1	0.1824	6.5339E-02	1.9159E-01	ENSG00000113578
FGF10	-0.0044	9.4360E-01		ENSG00000070193
FGF11	0.0593	4.9341E-01	6.8544E-01	ENSG00000161958
FGF12	-0.0491	5.6089E-01	7.3793E-01	ENSG00000114279
FGF12-AS1	-0.0259	1.2962E-01		ENSG00000231383
FGF12-AS2	0.0252	6.1736E-01		ENSG00000230126
FGF13	-0.1691	2.3264E-02	9.2729E-02	ENSG00000129682
FGF13-AS1	-0.0570	3.2213E-01	5.3212E-01	ENSG00000226031
FGF14	0.2831	9.5649E-04	8.7522E-03	ENSG00000102466
FGF14-AS2	0.0875	2.2206E-01	4.2545E-01	ENSG00000272143
FGF14-IT1	-0.0294	7.0106E-01	8.3485E-01	ENSG00000243319
FGF16	0.0386	7.5301E-01	8.6595E-01	ENSG00000196468
FGF17	-0.0287	6.4669E-01	7.9882E-01	ENSG00000158815
FGF18	-0.1733	7.8300E-02	2.1642E-01	ENSG00000156427
FGF19	-0.0215	4.5476E-01		ENSG00000162344
FGF2	0.1520	5.3485E-02	1.6707E-01	ENSG00000138685
FGF20	0.0349	6.0907E-01	7.7208E-01	ENSG00000078579
FGF21	0.0054	8.8694E-01		ENSG00000105550
FGF22	0.1197	1.6944E-01	3.5792E-01	ENSG00000070388
FGF23	-0.0006	6.8072E-01		ENSG00000118972
FGF3	0.0174	7.9227E-01		ENSG00000186895
FGF5	0.0796	2.0395E-01	4.0298E-01	ENSG00000138675
FGF7	0.0723	4.1195E-01	6.1595E-01	ENSG00000140285
FGF8	-0.0012	6.0554E-01		ENSG00000107831
FGF9	0.2086	1.5944E-02	7.0967E-02	ENSG00000102678
FGFBP1	-0.0053	5.1451E-01		ENSG00000137440
FGFBP3	0.2789	7.1955E-03	3.9625E-02	ENSG00000174721
FGFR1	0.0368	5.7073E-01	7.4443E-01	ENSG00000077782
FGFR1OP2	-0.1427	2.1971E-02	8.8870E-02	ENSG00000111790
FGFR2	-0.0974	2.5391E-01	4.6192E-01	ENSG00000066468
FGFR3	-0.1712	9.1011E-02	2.3973E-01	ENSG00000068078
FGFR4	-0.0459	5.8720E-01	7.5641E-01	ENSG00000160867
FGFRL1	-0.1106	2.2137E-01	4.2457E-01	ENSG00000127418
FGGY	0.1508	7.5061E-02	2.1061E-01	ENSG00000172456
FGL1	0.0025	9.5182E-01		ENSG00000104760
FGL2	0.0108	8.6497E-01	9.3177E-01	ENSG00000127951
FGR	0.0369	4.4554E-01		ENSG00000000938
FH	0.0108	8.3952E-01	9.1741E-01	ENSG00000091483
FHAD1	0.1470	1.3719E-01	3.1258E-01	ENSG00000142621
FHAD1-AS1	-0.0003	8.1232E-01		ENSG00000233485
FHDC1	-0.0329	6.8155E-01	8.2228E-01	ENSG00000137460
FHIP1A	0.0711	2.5505E-01	4.6314E-01	ENSG00000164142
FHIP1B	0.0851	2.1926E-01	4.2175E-01	ENSG00000051009
FHIP2A	0.0313	6.2961E-01	7.8675E-01	ENSG00000151553
FHIP2B	-0.0293	6.1386E-01	7.7548E-01	ENSG00000158863
FHIT	0.1011	2.2168E-01	4.2496E-01	ENSG00000189283

FHL1	-0.2441	1.9977E-03	1.5064E-02	ENSG00000022267
FHL1P1	0.0835	1.7702E-01	3.6824E-01	ENSG00000239219
FHL2	-0.3672	6.5470E-06	1.7359E-04	ENSG00000115641
FHL3	-0.2609	3.2021E-03	2.1515E-02	ENSG00000183386
FHL5	0.0049	8.7171E-01		ENSG00000112214
FHOD1	0.0310	7.2295E-01	8.4819E-01	ENSG00000135723
FHOD3	-0.1845	2.1351E-02	8.6997E-02	ENSG00000134775
FIBCD1	0.0088	9.2027E-01	9.6091E-01	ENSG00000130720
FIBIN	0.1731	9.6269E-02	2.4907E-01	ENSG00000176971
FIBP	0.0015	9.7947E-01	9.8975E-01	ENSG00000172500
FICD	0.4068	2.9419E-06	9.1557E-05	ENSG00000198855
FIG4	-0.2648	9.1252E-04	8.4599E-03	ENSG00000112367
FIGN	0.4137	7.3456E-06	1.9105E-04	ENSG00000182263
FIGNL1	-0.0613	3.9471E-01	6.0091E-01	ENSG00000132436
FIGNL2	0.0171	7.4707E-01		ENSG00000261308
FIGNL2-DT	0.0642	1.3434E-01		ENSG00000259887
FILIP1	-0.1148	2.0426E-01	4.0342E-01	ENSG00000118407
FILIP1L	0.0364	6.6323E-01	8.1050E-01	ENSG00000168386
FILNC1	0.0181	3.4957E-01		ENSG00000231426
FIP1L1	-0.1368	6.2750E-02	1.8613E-01	ENSG00000145216
FIRRE	0.5234	1.3743E-06	4.8656E-05	ENSG00000213468
FIRRM	0.1771	4.9963E-02	1.5965E-01	ENSG00000000460
FIS1	-0.0229	6.5542E-01	8.0537E-01	ENSG00000214253
FITM1	-0.0078	8.0743E-01		ENSG00000139914
FITM2	0.1824	2.1457E-02	8.7265E-02	ENSG00000197296
FIZ1	0.0209	7.8665E-01	8.8730E-01	ENSG00000179943
FJX1	-0.4744	8.1543E-07	3.1646E-05	ENSG00000179431
FKBP10	0.0747	3.7120E-01	5.7998E-01	ENSG00000141756
FKBP11	0.0084	9.1357E-01	9.5757E-01	ENSG00000134285
FKBP14	0.0504	5.1222E-01	7.0032E-01	ENSG00000106080
FKBP15	0.1960	6.7195E-03	3.7644E-02	ENSG00000119321
FKBP1A	-0.3877	3.2176E-07	1.4938E-05	ENSG00000088832
FKBP1B	-0.1395	6.3449E-02	1.8771E-01	ENSG00000119782
FKBP1C	0.0637	3.8059E-01	5.8896E-01	ENSG00000198225
FKBP2	-0.0429	4.7380E-01	6.6967E-01	ENSG00000173486
FKBP3	-0.2057	4.2288E-03	2.6563E-02	ENSG00000100442
FKBP5	0.2793	7.8946E-03	4.2440E-02	ENSG00000096060
FKBP6	-0.0106	8.5339E-01	9.2550E-01	ENSG00000077800
FKBP7	0.1697	1.9896E-02	8.2692E-02	ENSG00000079150
FKBP8	-0.3581	1.1198E-06	4.0891E-05	ENSG00000105701
FKBP9	-0.0759	3.0567E-01	5.1581E-01	ENSG00000122642
FKBPL	0.1575	8.8401E-02	2.3471E-01	ENSG00000204315
FKRP	0.0660	3.9309E-01	5.9980E-01	ENSG00000181027
FKTN	0.2465	2.3808E-04	2.9979E-03	ENSG00000106692
FLACC1	0.0603	3.7741E-01	5.8591E-01	ENSG00000155749
FLAD1	-0.0240	6.7725E-01	8.1917E-01	ENSG00000160688
FLCN	0.0117	8.6971E-01	9.3437E-01	ENSG00000154803
FLG	-0.0060	7.8091E-01	8.8382E-01	ENSG00000143631
FLG-AS1	0.0263	6.5986E-01	8.0831E-01	ENSG00000237975

FLI1	-0.0105	4.8076E-01		ENSG00000151702
FLII	0.0073	9.1183E-01	9.5646E-01	ENSG00000177731
FLJ12825	0.0148	5.5242E-01		ENSG00000248265
FLJ13224	0.0449	5.7010E-01	7.4394E-01	ENSG00000177340
FLJ16779	0.0954	2.2262E-01	4.2605E-01	ENSG00000275620
FLJ20021	0.0699	4.0845E-01	6.1271E-01	ENSG00000254531
FLJ30679	0.0104	9.0011E-01	9.5105E-01	ENSG00000280278
FLJ32255	0.0705	4.1137E-01	6.1553E-01	ENSG00000287263
FLJ33534	0.0709	1.8827E-01	3.8314E-01	ENSG00000145063
FLJ38576	-0.0381	6.0941E-01	7.7221E-01	ENSG00000279943
FLJ40194	0.0289	5.5605E-01	7.3399E-01	ENSG00000177369
FLJ42393	0.1768	6.6827E-02	1.9464E-01	ENSG00000279891
FLJ42969	-0.0043	9.8691E-01		ENSG00000248599
FLJ46284	0.2153	4.6818E-02	1.5272E-01	ENSG00000248858
FLNA	0.0548	4.1339E-01	6.1699E-01	ENSG00000196924
FLNB	0.2048	2.5597E-03	1.8108E-02	ENSG00000136068
FLNB-AS1	-0.0017	9.6920E-01		ENSG00000244161
FLNC	-0.0867	3.3581E-01	5.4586E-01	ENSG00000128591
FLOT1	-0.3153	1.7010E-06	5.8617E-05	ENSG00000137312
FLOT2	-0.1960	3.0261E-03	2.0625E-02	ENSG00000132589
FLRT1	-0.2305	1.1059E-02	5.4541E-02	ENSG00000126500
FLRT2	0.1127	2.1405E-01	4.1517E-01	ENSG00000185070
FLRT2-AS1	-0.0746	3.8059E-01	5.8896E-01	ENSG00000205562
FLRT3	-0.5324	3.7440E-06	1.1059E-04	ENSG00000125848
FLT1	0.0491	2.9503E-01		ENSG00000102755
FLT3	-0.0062	9.2110E-01	9.6136E-01	ENSG00000122025
FLT3LG	0.0026	9.8478E-01		ENSG00000090554
FLT4	-0.1965	5.7036E-02	1.7484E-01	ENSG00000037280
FLVCR1	0.1233	7.0204E-02	2.0114E-01	ENSG00000162769
FLVCR1-DT	0.1274	1.2254E-01	2.9145E-01	ENSG00000198468
FLVCR2	0.0160	8.1771E-01	9.0519E-01	ENSG00000119686
FLYWCH1	-0.0012	9.7885E-01	9.8938E-01	ENSG00000059122
FLYWCH2	0.1122	5.1683E-02	1.6302E-01	ENSG00000162076
FMC1	0.1217	1.7784E-01	3.6908E-01	ENSG00000164898
FMN1	0.0089	9.1613E-01	9.5863E-01	ENSG00000248905
FMN2	-0.0995	1.8502E-01	3.7854E-01	ENSG00000155816
FMNL1	0.0102	9.0427E-01	9.5324E-01	ENSG00000184922
FMNL1-DT	-0.1625	7.5250E-02	2.1096E-01	ENSG00000267121
FMNL2	0.1115	1.7685E-01	3.6810E-01	ENSG00000157827
FMNL3	-0.0030	9.7869E-01	9.8935E-01	ENSG00000161791
FMO1	0.0120	9.8989E-01		ENSG00000010932
FMO3	-0.0010	7.9394E-01		ENSG00000007933
FMO4	-0.0537	4.9789E-01	6.8912E-01	ENSG00000076258
FMO5	0.0409	6.1087E-01	7.7325E-01	ENSG00000131781
FMOD	0.0346	4.7151E-01	6.6767E-01	ENSG00000122176
FMR1	-0.0801	1.0188E-01	2.5786E-01	ENSG00000102081
FMR1-AS1	0.0374	4.8440E-01	6.7822E-01	ENSG00000268066
FN3K	-0.2744	6.9852E-05	1.1629E-03	ENSG00000167363
FN3KRP	-0.1990	1.0220E-02	5.1478E-02	ENSG00000141560

FNBP1	-0.0284	5.9252E-01	7.6020E-01	ENSG00000187239
FNBP1L	-0.1563	1.6963E-02	7.3899E-02	ENSG00000137942
FNBP4	-0.1201	1.4419E-02	6.6118E-02	ENSG00000109920
FNDC1	-0.0344	3.0846E-01	5.1866E-01	ENSG00000164694
FNDC10	-0.0828	3.0236E-01	5.1243E-01	ENSG00000228594
FNDC11	0.0101	9.0336E-01	9.5277E-01	ENSG00000125531
FNDC3A	0.2117	1.2571E-03	1.0764E-02	ENSG00000102531
FNDC3B	0.0365	5.4554E-01	7.2596E-01	ENSG00000075420
FNDC4	-0.0426	4.9021E-01	6.8299E-01	ENSG00000115226
FNDC5	-0.1317	1.3820E-01	3.1413E-01	ENSG00000160097
FNDC7	0.0242	6.5098E-01	8.0211E-01	ENSG00000143107
FNDC8	0.0188	3.3545E-01		ENSG00000073598
FNDC9	0.0062	7.1843E-01		ENSG00000172568
FNIP1	-0.0586	3.6185E-01	5.7107E-01	ENSG00000217128
FNIP2	0.0657	2.8368E-01	4.9423E-01	ENSG00000052795
FNTA	-0.0758	1.0525E-01	2.6353E-01	ENSG00000168522
FNTB	0.0107	8.9383E-01	9.4735E-01	ENSG00000257365
FOCAD	-0.0130	8.5351E-01	9.2556E-01	ENSG00000188352
FOCAD-AS1	-0.1064	1.7389E-01	3.6405E-01	ENSG00000227071
FOLH1	-0.0472	4.6433E-01	6.6131E-01	ENSG00000086205
FOLR1	-0.0050	9.1647E-01	9.5863E-01	ENSG00000110195
FOLR2	0.0219	1.3551E-01		ENSG00000165457
FOLR3	0.0633	2.2630E-01		ENSG00000110203
FOS	-0.0877	3.1548E-01	5.2555E-01	ENSG00000170345
FOSB	0.0621	3.5394E-01	5.6393E-01	ENSG00000125740
FOSL1	-0.4734	6.5194E-04	6.5823E-03	ENSG00000175592
FOSL2	0.0010	9.9077E-01	9.9485E-01	ENSG00000075426
FOSL2-AS1	0.0378	2.1505E-01		ENSG00000229951
FOXA1	-0.1562	4.0291E-02	1.3750E-01	ENSG00000129514
FOXA2	0.0108	5.7702E-01		ENSG00000125798
FOXA3	0.0080	8.8342E-01	9.4147E-01	ENSG00000170608
FOXB1	0.1315	9.3711E-02	2.4449E-01	ENSG00000171956
FOXC1	0.0263	6.3166E-01	7.8818E-01	ENSG00000054598
FOXC2	0.2855	2.6171E-02	1.0081E-01	ENSG00000176692
FOXD2	-0.0670	3.1940E-01	5.2958E-01	ENSG00000186564
FOXD2-AS1	-0.5501	4.0504E-03	2.5812E-02	ENSG00000237424
FOXD3	-0.0286	5.9133E-01	7.5918E-01	ENSG00000187140
FOXD3-AS1	-0.0033	9.5858E-01	9.7936E-01	ENSG00000230798
FOXD4	0.0375	5.3227E-01	7.1608E-01	ENSG00000170122
FOXD4L1	-0.0014	8.7414E-01		ENSG00000184492
FOXD4L3	-0.0083	5.8421E-01		ENSG00000187559
FOXD4L4	-0.0019	9.0091E-01		ENSG00000184659
FOXD4L5	0.0208	4.9724E-01		ENSG00000204779
FOXD4L6	-0.0129	4.6720E-01		ENSG00000273514
FOXE1	-0.0189	5.7220E-01		ENSG00000178919
FOXE3	0.0171	7.3619E-01	8.5602E-01	ENSG00000186790
FOXF1	0.0564	3.6017E-01	5.6961E-01	ENSG00000103241
FOXF2	-0.0382	4.7241E-01	6.6862E-01	ENSG00000137273
FOXG1	-0.4149	1.7263E-04	2.3148E-03	ENSG00000176165

FOXJ1	0.0754	3.7268E-01	5.8143E-01	ENSG00000129654
FOXJ2	-0.1800	1.2316E-02	5.9002E-02	ENSG00000065970
FOXJ3	0.0132	8.2836E-01	9.1112E-01	ENSG00000198815
FOXK1	-0.0471	4.5750E-01	6.5561E-01	ENSG00000164916
FOXK2	-0.3140	4.9119E-05	8.7768E-04	ENSG00000141568
FOXL1	0.0397	4.4107E-01	6.4089E-01	ENSG00000176678
FOXL2	-0.0110	6.5494E-01		ENSG00000183770
FOXL2NB	-0.0003	9.2786E-01		ENSG00000206262
FOXM1	-0.2345	3.5229E-02	1.2459E-01	ENSG00000111206
FOXN3	-0.3970	7.5978E-10	8.7296E-08	ENSG00000053254
FOXN3-AS1	-0.0180	8.3783E-01	9.1629E-01	ENSG00000258920
FOXN3-AS2	0.0004	8.5438E-01		ENSG00000259073
FOXN4	-0.0828	3.2922E-01	5.3953E-01	ENSG00000139445
FOXO1	0.0150	8.6314E-01	9.3102E-01	ENSG00000150907
FOXO1B	-0.0124	5.2223E-01		ENSG00000214295
FOXO3	-0.1207	8.5688E-02	2.2934E-01	ENSG00000118689
FOXO3B	-0.0930	1.5716E-01	3.4093E-01	ENSG00000240445
FOXO4	-0.2848	1.7735E-02	7.6285E-02	ENSG00000184481
FOXO6	-0.4090	3.5752E-03	2.3433E-02	ENSG00000204060
FOXO6-AS1	0.0527	2.8183E-01		ENSG00000229901
FOXP1	0.0354	6.7390E-01	8.1745E-01	ENSG00000114861
FOXP1-AS1	0.0035	9.5433E-01	9.7705E-01	ENSG00000244203
FOXP1-DT	0.0448	3.2090E-01		ENSG00000270562
FOXP2	0.3624	2.9140E-03	2.0053E-02	ENSG00000128573
FOXP3	0.0014	9.3132E-01	9.6601E-01	ENSG00000049768
FOXP4	-0.2893	6.1432E-03	3.5103E-02	ENSG00000137166
FOXP4-AS1	-0.2646	3.0586E-02	1.1276E-01	ENSG00000234753
FOXQ1	0.0235	8.4229E-01		ENSG00000164379
FOXR1	-0.0283	6.0771E-01	7.7108E-01	ENSG00000176302
FOXRED1	0.0980	1.0373E-01	2.6089E-01	ENSG00000110074
FOXRED2	0.0004	9.7475E-01	9.8760E-01	ENSG00000100350
FOXS1	0.0747	3.1546E-01	5.2555E-01	ENSG00000179772
FPGS	-0.0415	4.8238E-01	6.7672E-01	ENSG00000136877
FPGT	0.1309	1.6733E-01	3.5550E-01	ENSG00000254685
FPGT-TNNI3K	0.2743	3.2294E-02	1.1702E-01	ENSG00000259030
FPR1	-0.0223	3.0729E-01		ENSG00000171051
FPR2	-0.0004	9.8324E-01		ENSG00000171049
FPR3	0.0107	5.9174E-01		ENSG00000187474
FRA10AC1	-0.1052	1.1100E-01	2.7281E-01	ENSG00000148690
FRAS1	0.4154	2.7009E-04	3.3161E-03	ENSG00000138759
FRAT1	-0.1241	1.7222E-01	3.6170E-01	ENSG00000165879
FRAT2	0.2112	1.8186E-02	7.7589E-02	ENSG00000181274
FREM1	-0.0229	7.7641E-01	8.8102E-01	ENSG00000164946
FREM2	0.2033	6.2986E-02	1.8661E-01	ENSG00000150893
FREM3	-0.0248	5.2520E-01	7.1061E-01	ENSG00000183090
FREY1	-0.0418	4.4928E-01	6.4836E-01	ENSG00000234776
FRG1	-0.1131	7.3124E-02	2.0707E-01	ENSG00000109536
FRG1-DT	0.3862	1.1016E-03	9.7433E-03	ENSG00000245685
FRG1CP	0.0488	4.1588E-01	6.1934E-01	ENSG00000282826

FRG2	0.0944	2.6829E-02	1.0253E-01	ENSG00000205097
FRG2B	0.1041	1.9474E-02	8.1413E-02	ENSG00000225899
FRG2C	0.0772	9.0544E-02	2.3904E-01	ENSG00000172969
FRK	0.0831	1.1843E-01	2.8483E-01	ENSG00000111816
FRMD1	0.0248	7.3938E-01	8.5776E-01	ENSG00000153303
FRMD3	0.1431	1.3273E-01	3.0631E-01	ENSG00000172159
FRMD4A	-0.1203	7.7953E-02	2.1592E-01	ENSG00000151474
FRMD4B	0.0656	3.1452E-01	5.2456E-01	ENSG00000114541
FRMD5	-0.0278	7.1806E-01	8.4504E-01	ENSG00000171877
FRMD6	-0.0933	2.9928E-01	5.0897E-01	ENSG00000139926
FRMD6-AS1	0.1165	1.6436E-01	3.5113E-01	ENSG00000258537
FRMD7	0.0553	5.7720E-01	7.4911E-01	ENSG00000165694
FRMD8	0.2004	2.0384E-02	8.4053E-02	ENSG00000126391
FRMPD1	-0.3219	1.1804E-02	5.7330E-02	ENSG00000070601
FRMPD2B	0.1407	1.1969E-01	2.8694E-01	ENSG00000150175
FRMPD3	0.3262	5.4369E-03	3.2088E-02	ENSG00000147234
FRMPD3-AS1	-0.0454	2.8051E-01		ENSG00000227610
FRMPD4	0.0242	7.6539E-01	8.7393E-01	ENSG00000169933
FRRS1	0.1062	1.5264E-01	3.3479E-01	ENSG00000156869
FRRS1L	0.0792	2.0820E-01	4.0805E-01	ENSG00000260230
FRS2	-0.0936	2.1428E-01	4.1543E-01	ENSG00000166225
FRS3	0.0284	6.6628E-01	8.1274E-01	ENSG00000137218
FRY	0.0301	6.7952E-01	8.2059E-01	ENSG00000073910
FRYL	0.1194	1.0825E-01	2.6824E-01	ENSG00000075539
FSBP	-0.0360	6.3903E-01	7.9370E-01	ENSG00000265817
FSCN1	-0.2138	9.0775E-04	8.4348E-03	ENSG00000075618
FSCN2	-0.0178	4.2418E-01		ENSG00000186765
FSCN3	-0.1900	6.0202E-02	1.8077E-01	ENSG00000106328
FSD1	-0.1375	2.6346E-02	1.0124E-01	ENSG00000105255
FSD1L	0.1287	3.1912E-02	1.1605E-01	ENSG00000106701
FSD2	0.2446	4.1039E-02	1.3927E-01	ENSG00000186628
FSHR	0.0140	6.9711E-01	8.3255E-01	ENSG00000170820
FSIP1	-0.0047	9.5416E-01	9.7701E-01	ENSG00000150667
FSIP2	0.3906	2.8530E-07	1.3684E-05	ENSG00000188738
FSIP2-AS1	0.5653	4.6066E-03	2.8301E-02	ENSG00000231646
FSIP2-AS2	0.0157	7.8535E-01	8.8655E-01	ENSG00000226747
FST	-0.0469	5.8658E-01	7.5597E-01	ENSG00000134363
FSTL1	0.0325	6.4962E-01	8.0100E-01	ENSG00000163430
FSTL3	0.0040	9.6228E-01	9.8123E-01	ENSG00000070404
FSTL4	0.0239	7.7943E-01	8.8292E-01	ENSG00000053108
FSTL5	0.0191	8.1268E-01	9.0226E-01	ENSG00000168843
FTCDNL1	-0.1075	2.3291E-01	4.3806E-01	ENSG00000226124
FTH1P22	-0.0041	6.0608E-01		ENSG00000225079
FTH1P25	0.0047	6.3471E-01		ENSG00000232792
FTL	-0.0895	1.8321E-01	3.7641E-01	ENSG00000087086
FTLP12	-0.1128	3.6385E-02		ENSG00000265095
FTO	-0.3614	1.5532E-04	2.1357E-03	ENSG00000140718
FTSJ1	0.0699	2.5585E-01	4.6408E-01	ENSG00000068438
FTSJ3	0.1943	1.1464E-02	5.6076E-02	ENSG00000108592

FTX	0.3209	7.1654E-04	7.0720E-03	ENSG00000230590
FUBP1	0.0509	3.7475E-01	5.8334E-01	ENSG00000162613
FUBP3	0.0387	5.2076E-01	7.0760E-01	ENSG00000107164
FUCA1	0.1197	1.0332E-01	2.6021E-01	ENSG00000179163
FUCA2	0.0438	5.9579E-01	7.6268E-01	ENSG00000001036
FUNDC1	-0.0876	1.6055E-01	3.4595E-01	ENSG00000069509
FUNDC2	-0.3162	7.5718E-06	1.9525E-04	ENSG00000165775
FUOM	0.0078	9.1890E-01	9.5996E-01	ENSG00000148803
FURIN	-0.0245	7.1627E-01	8.4412E-01	ENSG00000140564
FUS	-0.3557	3.0707E-07	1.4446E-05	ENSG00000089280
FUT1	-0.3027	2.0944E-02	8.5759E-02	ENSG00000174951
FUT10	-0.0309	7.1191E-01	8.4206E-01	ENSG00000172728
FUT11	0.1288	1.4506E-01	3.2385E-01	ENSG00000196968
FUT2	-0.2622	3.6179E-02	1.2695E-01	ENSG00000176920
FUT4	-0.3355	2.2027E-03	1.6193E-02	ENSG00000196371
FUT5	0.0242	4.2945E-01		ENSG00000130383
FUT6	-0.0399	5.3206E-01	7.1596E-01	ENSG00000156413
FUT7	0.0512	3.9100E-01	5.9804E-01	ENSG00000180549
FUT8	0.1469	3.0719E-02	1.1315E-01	ENSG00000033170
FUT9	-0.0348	6.6196E-01	8.0976E-01	ENSG00000172461
FUZ	-0.3391	2.9119E-03	2.0047E-02	ENSG00000010361
FXN	0.0602	4.4904E-01	6.4817E-01	ENSG00000165060
FXNP2	0.0067	5.5130E-01		ENSG00000177736
FXR1	-0.1990	2.4385E-03	1.7492E-02	ENSG00000114416
FXR2	0.0050	9.3650E-01	9.6866E-01	ENSG00000129245
FXYD1	-0.0284	3.2123E-01		ENSG00000266964
FXYD2	0.2160	5.8637E-02	1.7786E-01	ENSG00000137731
FXYD3	0.0576	9.8232E-02		ENSG00000089356
FXYD5	-0.1348	1.5178E-01	3.3343E-01	ENSG00000089327
FXYD6	-0.4585	9.4456E-13	3.4178E-10	ENSG00000137726
FXYD6-FXYD2	0.0195	6.1388E-01		ENSG00000255245
FXYD7	0.0535	5.1026E-01	6.9892E-01	ENSG00000221946
FYB1	0.0085	8.0044E-01		ENSG00000082074
FYCO1	0.0563	5.1016E-01	6.9888E-01	ENSG00000163820
FYN	-0.1870	6.2894E-03	3.5764E-02	ENSG00000010810
FYTTD1	-0.2045	1.6421E-03	1.3038E-02	ENSG00000122068
FZD1	-0.0849	3.3123E-01	5.4148E-01	ENSG00000157240
FZD10	-0.0159	7.8410E-01	8.8596E-01	ENSG00000111432
FZD10-AS1	-0.0920	1.6734E-01	3.5550E-01	ENSG00000250208
FZD2	-0.2413	3.0066E-03	2.0549E-02	ENSG00000180340
FZD3	0.0336	6.6680E-01	8.1315E-01	ENSG00000104290
FZD4	0.0643	4.5262E-01	6.5145E-01	ENSG00000174804
FZD4-DT	0.0402	5.2727E-01	7.1232E-01	ENSG00000246523
FZD5	0.3807	7.2787E-03	3.9967E-02	ENSG00000163251
FZD6	0.0991	2.0448E-01	4.0369E-01	ENSG00000164930
FZD7	-0.0361	6.4435E-01	7.9692E-01	ENSG00000155760
FZD8	-0.0771	3.5836E-01	5.6777E-01	ENSG00000177283
FZD9	0.0586	5.0315E-01	6.9363E-01	ENSG00000188763
FZR1	0.0444	5.6722E-01	7.4199E-01	ENSG00000105325

G0S2	-0.0395	5.8097E-01	7.5225E-01	ENSG00000123689
G2E3	0.3232	1.4005E-05	3.2026E-04	ENSG00000092140
G2E3-AS1	0.0101	3.7468E-01		ENSG00000257636
G3BP1	-0.2175	6.9170E-05	1.1546E-03	ENSG00000145907
G3BP2	-0.0407	4.2529E-01	6.2767E-01	ENSG00000138757
G6PC1	-0.0107	4.5596E-01		ENSG00000131482
G6PC2	-0.0176	7.6115E-01	8.7162E-01	ENSG00000152254
G6PC3	0.1810	2.6791E-02	1.0244E-01	ENSG00000141349
G6PD	-0.3982	2.1605E-03	1.5926E-02	ENSG00000160211
GAA	0.1937	7.2006E-03	3.9644E-02	ENSG00000171298
GAB1	-0.1191	1.6607E-01	3.5378E-01	ENSG00000109458
GAB2	0.1013	1.1447E-01	2.7857E-01	ENSG00000033327
GAB3	0.0068	8.5580E-01		ENSG00000160219
GABARAP	-0.5139	6.1092E-07	2.5067E-05	ENSG00000170296
GABARAPL1	-0.2918	4.7867E-06	1.3647E-04	ENSG00000139112
GABARAPL2	-0.1567	5.2662E-03	3.1359E-02	ENSG00000034713
GABARAPL3	0.0189	3.1933E-01		ENSG00000279980
GABBR1	-0.0515	4.7295E-01	6.6895E-01	ENSG00000204681
GABBR2	0.0561	4.7806E-01	6.7338E-01	ENSG00000136928
GABPA	-0.0562	2.9231E-01	5.0240E-01	ENSG00000154727
GABPB1	-0.1906	2.0242E-02	8.3626E-02	ENSG00000104064
GABPB1-IT1	0.0097	8.8170E-01	9.4078E-01	ENSG00000285410
GABPB2	-0.2742	2.2137E-04	2.8178E-03	ENSG00000143458
GABRA1	0.0953	2.8740E-01	4.9810E-01	ENSG00000022355
GABRA2	0.2206	8.9937E-03	4.6963E-02	ENSG00000151834
GABRA3	-0.2767	3.3734E-03	2.2361E-02	ENSG00000011677
GABRA4	-0.0789	3.6034E-01	5.6974E-01	ENSG00000109158
GABRA5	-0.4338	1.0325E-04	1.5841E-03	ENSG00000186297
GABRA6	0.0431	1.1149E-01		ENSG00000145863
GABRB1	0.0293	7.3947E-01	8.5776E-01	ENSG00000163288
GABRB2	0.1992	2.6589E-02	1.0195E-01	ENSG00000145864
GABRB3	-0.3035	2.2477E-05	4.7069E-04	ENSG00000166206
GABRD	-0.0459	5.8868E-01	7.5740E-01	ENSG00000187730
GABRE	0.2792	2.2077E-02	8.9225E-02	ENSG00000102287
GABRG1	-0.2188	3.8181E-02	1.3237E-01	ENSG00000163285
GABRG2	0.0505	5.0637E-01	6.9604E-01	ENSG00000113327
GABRG3	-0.1419	1.2413E-01	2.9390E-01	ENSG00000182256
GABRG3-AS1	0.0388	5.6833E-01	7.4283E-01	ENSG00000228740
GABRP	0.0285	4.3053E-01		ENSG00000094755
GABRQ	0.0160	8.3565E-01	9.1524E-01	ENSG00000268089
GABRR1	0.0960	1.0607E-01	2.6484E-01	ENSG00000146276
GABRR2	0.0149	7.5611E-01		ENSG00000111886
GACAT2	0.3659	1.5926E-02	7.0931E-02	ENSG00000265962
GACAT3	-0.0020	9.0440E-01		ENSG00000236289
GAD1	0.1011	1.3562E-01	3.1034E-01	ENSG00000128683
GAD2	0.3181	6.2114E-04	6.3258E-03	ENSG00000136750
GADD45A	-0.5348	1.4096E-07	7.5729E-06	ENSG00000116717
GADD45B	-0.1314	1.3082E-01	3.0373E-01	ENSG00000099860
GADD45GIP1	-0.2187	7.9651E-05	1.2893E-03	ENSG00000179271

GADL1	0.1288	1.5779E-02	7.0474E-02	ENSG00000144644
GAGE1	0.0485	5.2040E-02		ENSG00000205777
GAGE10	0.0258	3.8443E-01		ENSG00000215274
GAGE12B	0.0063	4.7843E-01		ENSG00000275113
GAGE12J	0.0201	1.5641E-01		ENSG00000224659
GAGE13	0.0288	4.2711E-02		ENSG00000274274
GAGE2C	-0.0021	9.1027E-01		ENSG00000236362
GAGE2E	0.0126	4.6949E-01		ENSG00000189064
GAK	0.0617	2.7041E-01	4.7990E-01	ENSG00000178950
GAL	-0.1787	6.8991E-02	1.9861E-01	ENSG00000069482
GAL3ST1	0.0027	9.6467E-01	9.8256E-01	ENSG00000128242
GAL3ST2	0.0709	6.9651E-02		ENSG00000154252
GAL3ST3	-0.2986	2.5367E-04	3.1587E-03	ENSG00000175229
GAL3ST4	-0.0504	4.9859E-01	6.8959E-01	ENSG00000197093
GALC	0.2992	7.2936E-04	7.1669E-03	ENSG00000054983
GALE	0.0933	1.8610E-01	3.8025E-01	ENSG00000117308
GALK1	-0.2046	1.8881E-03	1.4428E-02	ENSG00000108479
GALK2	-0.0077	9.3564E-01	9.6833E-01	ENSG00000156958
GALM	-0.0802	3.5819E-01	5.6777E-01	ENSG00000143891
GALNS	-0.1809	3.6303E-02	1.2726E-01	ENSG00000141012
GALNT1	0.0519	3.0421E-01	5.1436E-01	ENSG00000141429
GALNT10	0.2017	3.9322E-02	1.3526E-01	ENSG00000164574
GALNT11	-0.1350	1.1393E-02	5.5831E-02	ENSG00000178234
GALNT12	0.4251	8.6526E-04	8.1321E-03	ENSG00000119514
GALNT13	-0.2613	6.5723E-03	3.7039E-02	ENSG00000144278
GALNT13-AS1	-0.0016	9.4122E-01		ENSG00000224675
GALNT15	-0.5849	2.4910E-04	3.1112E-03	ENSG00000131386
GALNT16	-0.0213	7.7475E-01	8.8013E-01	ENSG00000100626
GALNT16-AS1	0.2283	5.1921E-02	1.6347E-01	ENSG00000258957
GALNT17	0.0305	6.9817E-01	8.3318E-01	ENSG00000185274
GALNT18	0.0204	8.0465E-01	8.9752E-01	ENSG00000110328
GALNT2	-0.0325	5.6555E-01	7.4090E-01	ENSG00000143641
GALNT3	0.0460	5.3285E-01	7.1651E-01	ENSG00000115339
GALNT4	0.0059	8.5693E-01		ENSG00000257594
GALNT5	-0.0997	2.2520E-01	4.2897E-01	ENSG00000136542
GALNT6	-0.3114	2.1628E-02	8.7757E-02	ENSG00000139629
GALNT7	-0.1085	1.4432E-01	3.2256E-01	ENSG00000109586
GALNT7-DT	-0.0376	5.9703E-01	7.6354E-01	ENSG00000245213
GALNT8	0.1371	1.3863E-01	3.1449E-01	ENSG00000130035
GALNT9	-0.0052	9.4462E-01	9.7237E-01	ENSG00000182870
GALNTL5	0.0178	7.0032E-01	8.3440E-01	ENSG00000106648
GALNTL6	0.2440	2.5281E-02	9.8268E-02	ENSG00000174473
GALR2	-0.2022	3.3957E-02	1.2107E-01	ENSG00000182687
GALR3	-0.0207	6.7414E-01	8.1746E-01	ENSG00000128310
GALT	0.3934	8.5834E-05	1.3661E-03	ENSG00000213930
GAMT	-0.0880	1.3652E-01	3.1183E-01	ENSG00000130005
GAN	0.3119	2.9268E-06	9.1271E-05	ENSG00000261609
GANAB	0.0021	9.8567E-01	9.9239E-01	ENSG00000089597
GANC	0.2081	2.5500E-02	9.8916E-02	ENSG00000214013

GAP43	-0.1117	1.4533E-01	3.2410E-01	ENSG00000172020
GAPDH	-0.1746	2.3943E-02	9.4683E-02	ENSG00000111640
GAPDHP16	0.0036	8.8444E-01		ENSG00000225502
GAPDHP22	0.0164	8.2816E-01		ENSG00000234005
GAPDHP36	0.0222	4.0112E-01		ENSG00000213158
GAPDHP55	-0.0220	7.4470E-01		ENSG00000224055
GAPDHP62	0.0178	7.2312E-01	8.4820E-01	ENSG00000251013
GAPDHS	-0.0105	5.5513E-01		ENSG00000105679
GAPLINC	0.0224	5.2423E-01		ENSG00000266835
GAPVD1	0.0428	4.1557E-01	6.1899E-01	ENSG00000165219
GAR1	-0.1130	6.0580E-02	1.8160E-01	ENSG00000109534
GAREM1	0.0794	2.8782E-01	4.9847E-01	ENSG00000141441
GAREM2	-0.1710	4.3389E-03	2.7075E-02	ENSG00000157833
GARIN1A	-0.0354	6.0343E-01	7.6793E-01	ENSG00000205085
GARIN1B	0.1388	1.5059E-01	3.3175E-01	ENSG00000135248
GARIN2	0.1795	4.8554E-02	1.5637E-01	ENSG00000172717
GARIN5A	0.2239	1.7451E-02	7.5462E-02	ENSG00000142530
GARIN5B	0.0055	8.7296E-01		ENSG00000180043
GARIN6	-0.0131	8.3784E-01		ENSG00000180219
GARNL3	-0.0350	5.8790E-01	7.5699E-01	ENSG00000136895
GARRE1	0.0930	2.2590E-01	4.2977E-01	ENSG00000166398
GARS1	-0.1041	9.2954E-02	2.4323E-01	ENSG00000106105
GART	0.0484	4.7513E-01	6.7068E-01	ENSG00000159131
GAS1	0.1907	4.2866E-02	1.4348E-01	ENSG00000180447
GAS1RR	-0.0149	8.4536E-01	9.2085E-01	ENSG00000226237
GAS2	0.4362	1.5282E-03	1.2389E-02	ENSG00000148935
GAS2L1	-0.4970	2.2030E-06	7.2272E-05	ENSG00000185340
GAS2L2	0.1004	1.7707E-01	3.6829E-01	ENSG00000270765
GAS2L3	-0.1554	6.5583E-02	1.9207E-01	ENSG00000139354
GAS5	-0.1087	1.2946E-01	3.0194E-01	ENSG00000234741
GAS6	-0.1850	4.1194E-02	1.3971E-01	ENSG00000183087
GAS6-DT	0.0952	2.8758E-01	4.9827E-01	ENSG00000272695
GAS7	-0.0051	9.4194E-01	9.7127E-01	ENSG00000007237
GAS8	0.2384	9.4368E-03	4.8618E-02	ENSG00000141013
GAS8-AS1	-0.0106	7.0434E-01		ENSG00000221819
GASK1A	0.3696	1.6248E-02	7.1751E-02	ENSG00000144649
GASK1B	0.0945	2.7676E-01	4.8673E-01	ENSG00000164125
GASK1B-AS1	0.0061	9.3135E-01		ENSG00000248429
GAST	0.0597	2.6370E-01	4.7263E-01	ENSG00000184502
GATA1	0.1453	1.5897E-02		ENSG00000102145
GATA2	0.0320	6.0594E-01	7.6990E-01	ENSG00000179348
GATA3	0.2349	4.6412E-02	1.5176E-01	ENSG00000107485
GATA3-AS1	0.0101	5.5749E-01		ENSG00000197308
GATA4	-0.0544	2.8787E-01	4.9850E-01	ENSG00000136574
GATA6	-0.0178	6.9074E-01		ENSG00000141448
GATA6-AS1	0.0088	7.3890E-01		ENSG00000266010
GATAD1	0.0108	8.8079E-01	9.4037E-01	ENSG00000157259
GATAD2A	-0.0911	1.0428E-01	2.6187E-01	ENSG00000167491
GATAD2B	-0.0414	6.0483E-01	7.6873E-01	ENSG00000143614

GATB	-0.0270	6.6856E-01	8.1427E-01	ENSG00000059691
GATC	-0.1295	6.9145E-02	1.9894E-01	ENSG00000257218
GATD1	-0.3425	9.9263E-05	1.5361E-03	ENSG00000177225
GATD1-DT	0.0098	8.5162E-01	9.2443E-01	ENSG00000255284
GATD3	-0.2098	8.5260E-04	8.0315E-03	ENSG00000160221
GATM	-0.2133	5.8409E-02	1.7734E-01	ENSG00000171766
GAU1	-0.0390	5.0437E-01	6.9435E-01	ENSG00000255474
GBA1	-0.1136	9.7943E-02	2.5136E-01	ENSG00000177628
GBA2	-0.0442	5.0712E-01	6.9630E-01	ENSG00000070610
GBA3	0.0078	5.9621E-01		ENSG00000249948
GBE1	0.4446	1.0997E-04	1.6570E-03	ENSG00000114480
GBF1	0.0654	2.2226E-01	4.2562E-01	ENSG00000107862
GBGT1	-0.0326	1.1160E-01		ENSG00000148288
GBP1	-0.0209	2.8866E-01	4.9908E-01	ENSG00000117228
GBP2	0.1330	3.9931E-02	1.3655E-01	ENSG00000162645
GBP3	-0.0143	4.3346E-01		ENSG00000117226
GBP4	-0.0117	4.4377E-01		ENSG00000162654
GBP5	-0.0032	9.6313E-01		ENSG00000154451
GBP6	0.0418	1.8874E-01		ENSG00000183347
GBX1	-0.0145	3.9367E-01		ENSG00000164900
GBX2	0.0163	8.2227E-01	9.0802E-01	ENSG00000168505
GCA	-0.0357	6.2245E-01	7.8179E-01	ENSG00000115271
GCAT	-0.0440	5.7660E-01	7.4871E-01	ENSG00000100116
GCATP1	0.0024	7.6109E-01		ENSG00000270878
GCAWKR	-0.0076	5.1213E-01		ENSG00000272808
GCC1	-0.0663	3.9729E-01	6.0308E-01	ENSG00000179562
GCC2	0.3719	6.2759E-12	1.6427E-09	ENSG00000135968
GCC2-AS1	0.0652	4.2153E-01	6.2430E-01	ENSG00000214184
GCDH	-0.2256	7.3925E-03	4.0386E-02	ENSG00000105607
GCFC2	0.0297	6.2517E-01	7.8349E-01	ENSG00000005436
GCG	0.1678	9.6448E-03	4.9403E-02	ENSG00000115263
GCGR	-0.1900	7.5383E-02	2.1114E-01	ENSG00000215644
GCH1	-0.0139	8.6387E-01	9.3141E-01	ENSG00000131979
GCHFR	-0.1931	3.9568E-02	1.3596E-01	ENSG00000137880
GCK	-0.1157	1.2208E-01	2.9086E-01	ENSG00000106633
GCKR	0.0124	8.6583E-01	9.3228E-01	ENSG00000084734
GCLC	-0.1633	6.6510E-02	1.9403E-01	ENSG00000001084
GCLM	0.0140	8.5176E-01	9.2443E-01	ENSG00000023909
GCM2	0.0181	3.7925E-01		ENSG00000124827
GCN1	0.0384	5.4376E-01	7.2472E-01	ENSG00000089154
GCNA	0.0501	4.5239E-01	6.5124E-01	ENSG00000147174
GCNT1	0.4520	6.2024E-04	6.3193E-03	ENSG00000187210
GCNT1P3	0.0007	8.5998E-01		ENSG00000231047
GCNT2	-0.1816	3.0182E-02	1.1174E-01	ENSG00000111846
GCNT3	-0.4403	9.8177E-03	5.0044E-02	ENSG00000140297
GCNT4	-0.0527	4.8732E-01	6.8062E-01	ENSG00000176928
GCNT7	-0.0368	6.3549E-01	7.9092E-01	ENSG00000124091
GCOM1	-0.0219	6.4624E-01		ENSG00000137878
GCSAM	-0.0049	6.7067E-01		ENSG00000174500

GCSH	-0.0636	3.6108E-01	5.7065E-01	ENSG00000140905
GCSIR	0.4123	1.1321E-03	9.9375E-03	ENSG00000232520
GDA	0.1321	1.1396E-01	2.7778E-01	ENSG00000119125
GDAP1	0.0293	6.5659E-01	8.0614E-01	ENSG00000104381
GDAP1L1	-0.1482	2.0716E-02	8.5038E-02	ENSG00000124194
GDAP2	0.0507	4.5656E-01	6.5483E-01	ENSG00000196505
GDE1	-0.0934	4.6005E-02	1.5087E-01	ENSG00000006007
GDF10	0.0795	3.1378E-01	5.2406E-01	ENSG00000266524
GDF11	-0.0093	9.0513E-01	9.5372E-01	ENSG00000135414
GDF15	-0.0562	3.6912E-01	5.7837E-01	ENSG00000130513
GDF2	0.0141	3.5024E-01		ENSG00000263761
GDF3	0.1133	7.9141E-03	4.2517E-02	ENSG00000184344
GDF5	0.3281	2.2866E-02	9.1557E-02	ENSG00000125965
GDF6	0.0290	7.1839E-01	8.4517E-01	ENSG00000156466
GDF7	0.1352	1.0870E-01	2.6899E-01	ENSG00000143869
GDF9	0.2569	1.2435E-02	5.9445E-02	ENSG00000164404
GDI1	-0.2639	1.0281E-03	9.2662E-03	ENSG00000203879
GDI2	-0.1799	1.3048E-02	6.1469E-02	ENSG00000057608
GDI2P2	0.0413	6.0090E-01	7.6623E-01	ENSG00000233994
GDNF	0.0064	9.1758E-01	9.5907E-01	ENSG00000168621
GDPD1	0.0569	4.0144E-01	6.0651E-01	ENSG00000153982
GDPD2	0.0158	8.6403E-01	9.3150E-01	ENSG00000130055
GDPD3	0.0792	2.2960E-01	4.3409E-01	ENSG00000102886
GDPD5	0.1303	5.7146E-02	1.7506E-01	ENSG00000158555
GDPGP1	0.0059	9.3273E-01	9.6664E-01	ENSG00000183208
GEM	-0.0554	4.9024E-01	6.8299E-01	ENSG00000164949
GEMIN2	0.0044	9.5442E-01	9.7705E-01	ENSG00000092208
GEMIN4	-0.1093	1.2645E-01	2.9761E-01	ENSG00000179409
GEMIN5	0.1547	4.0606E-02	1.3821E-01	ENSG00000082516
GEMIN6	0.2086	1.8764E-02	7.9313E-02	ENSG00000152147
GEMIN7	0.0338	6.7687E-01	8.1900E-01	ENSG00000142252
GEMIN7-AS1	0.1003	2.2511E-01	4.2887E-01	ENSG00000267348
GEMIN8	-0.2095	9.3846E-04	8.6246E-03	ENSG00000046647
GEMIN8P3	-0.0172	2.7656E-01		ENSG00000223772
GEN1	0.2648	1.4580E-02	6.6631E-02	ENSG00000178295
GET1	0.0513	3.2870E-01	5.3911E-01	ENSG00000182093
GET1-SH3BGR	-0.0134	4.1331E-01		ENSG00000285815
GET1P1	0.0181	3.3623E-01		ENSG00000248633
GET3	-0.1899	1.3602E-03	1.1419E-02	ENSG00000198356
GET4	0.1040	2.3025E-01	4.3489E-01	ENSG00000239857
GFAP	0.1633	9.6139E-02	2.4892E-01	ENSG00000131095
GFER	-0.0899	2.8693E-01	4.9773E-01	ENSG00000127554
GFI1	0.0435	2.2231E-01		ENSG00000162676
GFI1B	0.0020	9.8617E-01	9.9239E-01	ENSG00000165702
GFM1	-0.1038	6.8610E-02	1.9795E-01	ENSG00000168827
GFM2	0.0261	7.2327E-01	8.4820E-01	ENSG00000164347
GFOD1	0.0657	3.2297E-01	5.3298E-01	ENSG00000145990
GFOD2	0.0683	2.6506E-01	4.7425E-01	ENSG00000141098
GFOD3P	-0.0652	3.1907E-01	5.2935E-01	ENSG00000227372

GFPT1	-0.0442	5.1813E-01	7.0547E-01	ENSG00000198380
GFPT2	0.0193	7.9927E-01	8.9419E-01	ENSG00000131459
GFRA1	-0.0022	9.7484E-01	9.8765E-01	ENSG00000151892
GFRA2	0.0929	2.3905E-01	4.4553E-01	ENSG00000168546
GFRA3	-0.0133	8.6926E-01	9.3433E-01	ENSG00000146013
GFRA4	-0.0092	8.6451E-01		ENSG00000125861
GFRAL	0.0025	8.3399E-01		ENSG00000187871
GFUS	-0.3672	3.7739E-06	1.1134E-04	ENSG00000104522
GFY	-0.1142	1.5527E-01	3.3867E-01	ENSG00000261949
GGA1	-0.0891	1.8791E-01	3.8267E-01	ENSG00000100083
GGA2	-0.1458	4.2066E-02	1.4153E-01	ENSG00000103365
GGA3	0.0470	4.8244E-01	6.7676E-01	ENSG00000125447
GGACT	0.3684	3.2250E-04	3.7949E-03	ENSG00000134864
GGCT	0.0209	7.0008E-01	8.3420E-01	ENSG00000006625
GGCX	-0.0217	7.5445E-01	8.6686E-01	ENSG00000115486
GGH	-0.2708	6.5375E-05	1.1061E-03	ENSG00000137563
GGN	0.0001	9.9680E-01	9.9822E-01	ENSG00000179168
GGNBP1	0.0050	9.0795E-01		ENSG00000204188
GGNBP2	-0.0047	9.2630E-01	9.6393E-01	ENSG00000278311
GGPS1	0.1506	5.1111E-02	1.6177E-01	ENSG00000152904
GGT1	0.0212	4.5579E-01		ENSG00000100031
GGT3P	0.0268	5.9377E-01		ENSG00000197421
GGT5	0.1525	7.1610E-02	2.0407E-01	ENSG00000099998
GGT6	0.0101	6.6126E-01		ENSG00000167741
GGT7	0.0067	9.1463E-01	9.5792E-01	ENSG00000131067
GGTA1	-0.0858	1.3218E-01	3.0558E-01	ENSG00000204136
GGTA2P	0.0423	4.9144E-01	6.8374E-01	ENSG00000237766
GGTLC2	0.0578	1.9575E-01		ENSG00000100121
GGTLC3	0.0100	7.1516E-01		ENSG00000274252
GGTLC5P	0.0236	2.8119E-01		ENSG00000276160
GHDC	-0.0087	8.9412E-01	9.4738E-01	ENSG00000167925
GHET1	0.0331	6.7355E-01	8.1736E-01	ENSG00000281189
GHITM	-0.1603	8.0583E-03	4.3112E-02	ENSG00000165678
GHR	0.1178	1.0261E-01	2.5913E-01	ENSG00000112964
GHRHR	0.0196	7.8806E-01	8.8795E-01	ENSG00000106128
GHRL	0.0362	6.1106E-01	7.7343E-01	ENSG00000157017
GHRLOS	-0.1698	6.8186E-02	1.9711E-01	ENSG00000240288
GHSR	-0.0012	8.2142E-01		ENSG00000121853
GID4	0.0203	7.8970E-01	8.8891E-01	ENSG00000141034
GID8	-0.0432	5.2979E-01	7.1431E-01	ENSG00000101193
GIGYF1	-0.0341	6.7955E-01	8.2059E-01	ENSG00000146830
GIGYF2	0.1906	2.7563E-04	3.3674E-03	ENSG00000204120
GIHCG	0.1455	2.4639E-02	9.6568E-02	ENSG00000257698
GIMAP8	-0.0045	7.3158E-01		ENSG00000171115
GIN1	0.0686	3.7481E-01	5.8335E-01	ENSG00000145723
GINM1	0.0615	3.5899E-01	5.6851E-01	ENSG00000055211
GINS1	-0.0745	3.3332E-01	5.4349E-01	ENSG00000101003
GINS2	-0.1181	1.4673E-01	3.2636E-01	ENSG00000131153
GINS3	-0.0625	4.0750E-01	6.1186E-01	ENSG00000181938

GINS4	-0.0516	5.4358E-01	7.2458E-01	ENSG00000147536
GIP	0.0201	6.4166E-01		ENSG00000159224
GIPC1	-0.1222	3.5859E-02	1.2622E-01	ENSG00000123159
GIPC2	0.0331	6.5078E-01	8.0209E-01	ENSG00000137960
GIPC3	-0.1543	1.9154E-02	8.0537E-02	ENSG00000179855
GIPR	0.1137	1.5798E-01	3.4203E-01	ENSG00000010310
GIRGL	-0.0636	2.6250E-01	4.7137E-01	ENSG00000233834
GIT1	-0.0522	3.7517E-01	5.8373E-01	ENSG00000108262
GIT2	-0.0867	1.9671E-01	3.9421E-01	ENSG00000139436
GJA1	0.0488	5.1325E-01	7.0112E-01	ENSG00000152661
GJA3	-0.0288	1.7298E-01		ENSG00000121743
GJA5	-0.0086	3.4242E-01		ENSG00000265107
GJA9	0.0085	7.6489E-01		ENSG00000131233
GJB1	0.0905	6.3579E-02		ENSG00000169562
GJB2	-0.0419	3.9457E-01	6.0080E-01	ENSG00000165474
GJB3	-0.0093	7.0687E-01		ENSG00000188910
GJB5	-0.0014	9.5799E-01		ENSG00000189280
GJB6	-0.0102	5.3454E-01		ENSG00000121742
GJB7	-0.0342	6.2846E-01	7.8609E-01	ENSG00000164411
GJC1	-0.2248	7.9948E-04	7.6571E-03	ENSG00000182963
GJC2	0.0619	3.0193E-01	5.1188E-01	ENSG00000198835
GJC3	0.0184	7.3778E-01		ENSG00000176402
GJD3	-0.0067	8.9640E-01	9.4857E-01	ENSG00000183153
GK	0.1461	8.6382E-02	2.3080E-01	ENSG00000198814
GK3	0.0026	9.8629E-01		ENSG00000229894
GK5	0.3547	2.9562E-05	5.8706E-04	ENSG00000175066
GKAP1	-0.1133	1.3194E-01	3.0528E-01	ENSG00000165113
GLA	-0.1572	1.2198E-02	5.8619E-02	ENSG00000102393
GLB1	-0.0273	6.5710E-01	8.0650E-01	ENSG00000170266
GLB1L	0.1849	7.3365E-02	2.0749E-01	ENSG00000163521
GLB1L2	0.1438	8.5345E-02	2.2863E-01	ENSG00000149328
GLB1L3	-0.0658	4.4742E-01	6.4693E-01	ENSG00000166105
GLCCI1	-0.4377	2.0023E-08	1.4795E-06	ENSG00000106415
GLDC	-0.1463	3.3792E-02	1.2075E-01	ENSG00000178445
GLDN	0.0828	2.8820E-01	4.9873E-01	ENSG00000186417
GLE1	0.0236	7.0732E-01	8.3906E-01	ENSG00000119392
GLG1	0.1666	1.9227E-03	1.4632E-02	ENSG00000090863
GLI1	0.0000	9.9665E-01		ENSG00000111087
GLI2	-0.3051	7.7043E-03	4.1681E-02	ENSG00000074047
GLI3	-0.0342	6.6814E-01	8.1415E-01	ENSG00000106571
GLI4	-0.0308	6.9104E-01	8.2844E-01	ENSG00000250571
GLIDR	0.0437	5.8237E-01	7.5322E-01	ENSG00000278175
GLIPR1	0.0033	9.6622E-01	9.8327E-01	ENSG00000139278
GLIPR1L1	0.1972	5.9124E-02	1.7871E-01	ENSG00000173401
GLIPR1L2	-0.4528	9.2384E-03	4.7838E-02	ENSG00000180481
GLIPR2	-0.4613	9.7203E-08	5.6143E-06	ENSG00000122694
GLIS1	-0.0647	3.9305E-01	5.9980E-01	ENSG00000174332
GLIS2	-0.2415	1.0704E-02	5.3263E-02	ENSG00000126603
GLIS2-AS1	0.0030	9.6747E-01	9.8383E-01	ENSG00000262686

GLIS3	0.1879	7.1318E-02	2.0338E-01	ENSG00000107249
GLIS3-AS1	0.0236	6.8499E-01		ENSG00000237009
GLMN	0.1058	1.2708E-01	2.9844E-01	ENSG00000174842
GLMP	0.0914	2.4992E-01	4.5778E-01	ENSG00000198715
GLO1	-0.1767	8.2723E-03	4.3971E-02	ENSG00000124767
GLOD4	-0.0501	3.8351E-01	5.9173E-01	ENSG00000167699
GLP1R	0.1713	5.7871E-02	1.7631E-01	ENSG00000112164
GLRA2	-0.0665	4.3540E-01	6.3646E-01	ENSG00000101958
GLRA3	-0.0272	5.1916E-01	7.0638E-01	ENSG00000145451
GLRB	-0.0624	4.0925E-01	6.1355E-01	ENSG00000109738
GLRX	-0.2348	4.1269E-03	2.6137E-02	ENSG00000173221
GLRX2	-0.0387	6.3586E-01	7.9121E-01	ENSG00000023572
GLRX3	-0.1418	1.9592E-02	8.1759E-02	ENSG00000108010
GLRX5	-0.0608	3.4317E-01	5.5281E-01	ENSG00000182512
GLS	0.0985	2.0057E-01	3.9896E-01	ENSG00000115419
GLS2	0.0050	9.3143E-01	9.6603E-01	ENSG00000135423
GLT1D1	0.0472	5.1570E-01	7.0329E-01	ENSG00000151948
GLT6D1	0.0143	6.3676E-01		ENSG00000204007
GLT8D1	0.1040	8.1652E-02	2.2231E-01	ENSG00000016864
GLT8D2	0.0734	3.8638E-01	5.9406E-01	ENSG00000120820
GLTP	-0.3812	2.9711E-10	3.9516E-08	ENSG00000139433
GLTPD2	0.1555	1.0563E-01	2.6413E-01	ENSG00000182327
GLUD1	-0.1212	1.1579E-01	2.8040E-01	ENSG00000148672
GLUD2	-0.0759	3.6200E-01	5.7123E-01	ENSG00000182890
GLUL	-0.2337	8.2166E-03	4.3750E-02	ENSG00000135821
GLULP4	0.0058	9.0534E-01		ENSG00000178723
GLYATL1	-0.1107	2.2466E-01	4.2858E-01	ENSG00000166840
GLYATL1B	0.0089	6.3522E-01		ENSG00000255151
GLYATL1P1	-0.0029	9.4268E-01	9.7154E-01	ENSG00000255189
GLYATL1P2	0.0866	3.3049E-01	5.4075E-01	ENSG00000254717
GLYATL2	0.0062	9.4252E-01	9.7152E-01	ENSG00000156689
GLYCTK	0.5531	1.9974E-04	2.5883E-03	ENSG00000168237
GLYR1	0.0150	7.6783E-01	8.7557E-01	ENSG00000140632
GM2A	-0.1930	5.0963E-02	1.6153E-01	ENSG00000196743
GMCL1	0.1223	1.1674E-01	2.8187E-01	ENSG00000087338
GMCL2	0.0159	4.6440E-01		ENSG00000244234
GMDS	-0.2594	2.9934E-05	5.9302E-04	ENSG00000112699
GMDS-DT	0.1172	1.4205E-01	3.1927E-01	ENSG00000250903
GMEB1	-0.0706	2.4079E-01	4.4772E-01	ENSG00000162419
GMEB2	-0.0182	8.0928E-01	8.9970E-01	ENSG00000101216
GMFB	-0.3120	3.4094E-04	3.9588E-03	ENSG00000197045
GMFG	0.3257	2.2251E-02	8.9766E-02	ENSG00000130755
GMIP	-0.0830	2.6841E-01	4.7785E-01	ENSG00000089639
GMNC	0.0131	6.1695E-01		ENSG00000205835
GMNN	-0.0411	5.6009E-01	7.3729E-01	ENSG00000112312
GMPPA	0.0075	9.4397E-01	9.7218E-01	ENSG00000144591
GMPPB	0.0418	5.7253E-01	7.4582E-01	ENSG00000173540
GMPR	0.2892	9.1723E-03	4.7613E-02	ENSG00000137198
GMPR2	-0.0707	2.9706E-01	5.0712E-01	ENSG00000100938

GMPS	-0.0680	3.4219E-01	5.5185E-01	ENSG00000163655
GMPSP1	0.0153	6.9027E-01	8.2789E-01	ENSG00000250471
GNA11	-0.2194	5.8540E-03	3.3919E-02	ENSG00000088256
GNA12	0.0375	5.5503E-01	7.3317E-01	ENSG00000146535
GNA13	-0.1707	1.1655E-02	5.6799E-02	ENSG00000120063
GNA14	0.0361	5.1841E-01	7.0566E-01	ENSG00000156049
GNA14-AS1	-0.0108	6.2179E-01		ENSG00000231373
GNA15	0.0151	3.1986E-01		ENSG00000060558
GNA15-DT	0.0010	8.8908E-01		ENSG00000267551
GNAI1	-0.0707	3.2163E-01	5.3172E-01	ENSG00000127955
GNAI2	-0.1849	4.6457E-03	2.8484E-02	ENSG00000114353
GNAI3	-0.0496	4.5446E-01	6.5293E-01	ENSG00000065135
GNAL	0.0475	5.3424E-01	7.1776E-01	ENSG00000141404
GNAO1	-0.2050	7.1670E-03	3.9521E-02	ENSG00000087258
GNAO1-AS1	0.0936	2.8695E-01	4.9773E-01	ENSG00000261439
GNAO1-DT	0.4595	3.0222E-05	5.9680E-04	ENSG00000246379
GNAQ	-0.2310	1.7616E-03	1.3721E-02	ENSG00000156052
GNAS	-0.3555	6.7068E-06	1.7744E-04	ENSG00000087460
GNAS-AS1	0.1820	4.6984E-02	1.5313E-01	ENSG00000235590
GNAT1	0.1075	8.1932E-02	2.2268E-01	ENSG00000114349
GNAT2	0.0212	6.5908E-01		ENSG00000134183
GNAT3	-0.0130	3.5420E-01		ENSG00000214415
GNAZ	-0.1360	6.8206E-02	1.9714E-01	ENSG00000128266
GNB1	-0.2384	2.1705E-03	1.5975E-02	ENSG00000078369
GNB1-DT	0.4278	2.4900E-03	1.7738E-02	ENSG00000231050
GNB1L	-0.2072	7.8100E-03	4.2132E-02	ENSG00000185838
GNB2	-0.1503	8.1703E-02	2.2238E-01	ENSG00000172354
GNB3	0.3321	3.8765E-03	2.4963E-02	ENSG00000111664
GNB4	-0.0851	1.7318E-01	3.6315E-01	ENSG00000114450
GNB5	0.0334	5.2730E-01	7.1232E-01	ENSG00000069966
GNE	-0.2772	1.1503E-04	1.7112E-03	ENSG00000159921
GNG10	0.1265	1.7495E-01	3.6539E-01	ENSG00000242616
GNG11	0.0105	9.4069E-01	9.7067E-01	ENSG00000127920
GNG12	-0.2036	9.0611E-03	4.7175E-02	ENSG00000172380
GNG12-AS1	0.0210	8.0248E-01	8.9586E-01	ENSG00000232284
GNG13	0.0230	7.1271E-01	8.4237E-01	ENSG00000127588
GNG2	-0.1636	3.8396E-02	1.3287E-01	ENSG00000186469
GNG3	-0.0846	2.5841E-01	4.6721E-01	ENSG00000162188
GNG4	-0.4388	3.4672E-06	1.0442E-04	ENSG00000168243
GNG5	-0.1291	1.4489E-01	3.2360E-01	ENSG00000174021
GNG5B	-0.0225	7.2558E-01	8.4945E-01	ENSG00000133136
GNG7	-0.2569	1.2793E-03	1.0918E-02	ENSG00000176533
GNG8	-0.3795	3.8896E-03	2.5027E-02	ENSG00000167414
GNGT2	0.0416	4.0814E-01		ENSG00000167083
GNL1	-0.0874	1.3429E-01	3.0848E-01	ENSG00000204590
GNL2	0.1065	1.7930E-01	3.7092E-01	ENSG00000134697
GNL3	0.1529	2.2326E-02	8.9963E-02	ENSG00000163938
GNL3L	0.0007	9.9585E-01	9.9767E-01	ENSG00000130119
GNLY	0.0112	9.0671E-01		ENSG00000115523

GNMT	0.0410	6.2966E-01	7.8675E-01	ENSG00000124713
GNPAT	-0.0742	1.7746E-01	3.6864E-01	ENSG00000116906
GNPDA1	-0.2804	2.7308E-04	3.3479E-03	ENSG00000113552
GNPDA2	0.0070	9.3037E-01	9.6564E-01	ENSG00000163281
GNPNAT1	-0.0538	4.6787E-01	6.6442E-01	ENSG00000100522
GNPTAB	-0.2376	2.8264E-04	3.4323E-03	ENSG00000111670
GNPTG	-0.0670	2.2883E-01	4.3334E-01	ENSG00000090581
GNRH1	0.2248	4.7001E-03	2.8740E-02	ENSG00000147437
GNRH2	0.0138	6.7342E-01		ENSG00000125787
GNRHR	0.1910	4.9190E-02	1.5780E-01	ENSG00000109163
GNRHR2	-0.1026	2.0564E-01	4.0506E-01	ENSG00000211451
GNRHR2P1	-0.3983	2.3492E-03	1.7001E-02	ENSG00000259169
GNS	0.1823	2.3300E-03	1.6901E-02	ENSG00000135677
GOLGA1	0.4120	3.3291E-07	1.5368E-05	ENSG00000136935
GOLGA2	0.2467	1.5308E-03	1.2402E-02	ENSG00000167110
GOLGA3	0.2490	2.0285E-03	1.5218E-02	ENSG00000090615
GOLGA4	0.4443	6.1423E-11	1.0873E-08	ENSG00000144674
GOLGA4-AS1	-0.0422	6.0744E-01	7.7108E-01	ENSG00000270194
GOLGA5	0.1880	2.4875E-02	9.7288E-02	ENSG00000066455
GOLGA6A	-0.0022	8.6427E-01	9.3150E-01	ENSG00000159289
GOLGA6B	0.0871	2.5263E-01	4.6058E-01	ENSG00000215186
GOLGA6C	0.0660	3.8246E-01	5.9066E-01	ENSG00000167195
GOLGA6D	0.0263	6.2212E-01		ENSG00000140478
GOLGA6L1	0.0127	5.5403E-01		ENSG00000273976
GOLGA6L17P	0.1169	1.2706E-01	2.9844E-01	ENSG00000230373
GOLGA6L2	0.0117	7.7210E-01	8.7846E-01	ENSG00000174450
GOLGA6L22	0.0349	1.4563E-01		ENSG00000277865
GOLGA6L22	0.0278	1.6549E-01		ENSG00000237850
GOLGA6L25	0.0250	1.8541E-01		ENSG00000227717
GOLGA6L26	0.0087	9.2585E-01		ENSG00000273756
GOLGA6L3P	-0.0269	7.4138E-01	8.5899E-01	ENSG00000188388
GOLGA6L4	0.2186	6.9413E-03	3.8579E-02	ENSG00000184206
GOLGA6L4	0.1231	1.4081E-01	3.1753E-01	ENSG00000278662
GOLGA6L6	0.0051	7.7768E-01		ENSG00000277322
GOLGA6L7	0.0925	1.6857E-01	3.5695E-01	ENSG00000261649
GOLGA6L9	0.0167	8.2485E-01	9.0912E-01	ENSG00000197978
GOLGA7	-0.2186	5.3726E-04	5.6205E-03	ENSG00000147533
GOLGA7B	-0.2256	3.4636E-02	1.2299E-01	ENSG00000155265
GOLGA7B-DT	-0.0243	6.1907E-01	7.7962E-01	ENSG00000227356
GOLGA8A	0.3885	2.8403E-05	5.7049E-04	ENSG00000175265
GOLGA8B	0.4342	5.3142E-08	3.3928E-06	ENSG00000215252
GOLGA8CP	-0.0030	9.0037E-01		ENSG00000181984
GOLGA8DP	0.0114	4.0079E-01		ENSG00000175676
GOLGA8F	0.0218	4.0014E-01		ENSG00000153684
GOLGA8G	0.0253	2.8843E-01		ENSG00000183629
GOLGA8H	0.0767	3.8390E-01	5.9203E-01	ENSG00000261794
GOLGA8J	0.0640	4.5342E-01	6.5211E-01	ENSG00000179938
GOLGA8K	0.1255	1.8041E-01	3.7258E-01	ENSG00000249931
GOLGA8M	0.1244	1.0256E-01	2.5907E-01	ENSG00000188626

GOLGA8M	-0.0032	7.7350E-01	8.7915E-01	ENSG00000261480
GOLGA8N	0.0075	9.3759E-01	9.6914E-01	ENSG00000232653
GOLGA8O	0.0154	8.5435E-01	9.2588E-01	ENSG00000206127
GOLGA8Q	0.0308	6.9598E-01	8.3174E-01	ENSG00000178115
GOLGA8R	0.0696	4.2301E-01	6.2571E-01	ENSG00000186399
GOLGA8S	-0.0230	5.9611E-01		ENSG00000261739
GOLGA8T	0.0417	6.3152E-01	7.8804E-01	ENSG00000261247
GOLGB1	0.4010	9.9474E-17	1.2831E-13	ENSG00000173230
GOLIM4	-0.0455	4.6761E-01	6.6422E-01	ENSG00000173905
GOLM1	-0.0384	5.9951E-01	7.6548E-01	ENSG00000135052
GOLM2	-0.0617	3.1665E-01	5.2674E-01	ENSG00000166734
GOLPH3	0.1105	6.6324E-02	1.9369E-01	ENSG00000113384
GOLPH3L	0.2426	2.0989E-02	8.5886E-02	ENSG00000143457
GOLT1A	-0.0791	3.6144E-01	5.7095E-01	ENSG00000174567
GOLT1B	0.2030	1.0326E-02	5.1873E-02	ENSG00000111711
GON4L	0.2586	5.4103E-05	9.4730E-04	ENSG00000116580
GON7	0.2883	1.0311E-03	9.2791E-03	ENSG00000170270
GOPC	0.0480	4.3583E-01	6.3678E-01	ENSG00000047932
GORAB	0.1268	1.4348E-01	3.2145E-01	ENSG00000120370
GORASP1	-0.0844	2.7116E-01	4.8044E-01	ENSG00000114745
GORASP2	-0.0180	7.6528E-01	8.7387E-01	ENSG00000115806
GOSR1	0.1006	9.3504E-02	2.4410E-01	ENSG00000108587
GOSR2	0.0987	1.1296E-01	2.7595E-01	ENSG00000108433
GOT1	0.0524	4.6557E-01	6.6215E-01	ENSG00000120053
GOT1-DT	0.3146	4.3173E-03	2.6961E-02	ENSG00000224934
GOT2	-0.1895	1.3160E-03	1.1159E-02	ENSG00000125166
GOT2P6	0.0675	7.3421E-02		ENSG00000214745
GP1BA	0.0229	6.5564E-01	8.0547E-01	ENSG00000185245
GP5	0.0231	7.7741E-01		ENSG00000178732
GP6	-0.0935	2.7243E-01	4.8172E-01	ENSG00000088053
GPA33	0.0224	3.4940E-01		ENSG00000143167
GPAA1	-0.0617	3.6522E-01	5.7435E-01	ENSG00000197858
GPALPP1	0.1958	5.1340E-03	3.0698E-02	ENSG00000133114
GPAM	-0.0458	5.1345E-01	7.0124E-01	ENSG00000119927
GPANK1	-0.0238	7.0330E-01	8.3621E-01	ENSG00000204438
GPAT2	0.5159	2.7709E-04	3.3752E-03	ENSG00000186281
GPAT2P1	0.0174	5.2549E-01		ENSG00000237510
GPAT3	0.5370	1.0124E-02	5.1188E-02	ENSG00000138678
GPAT4	-0.1780	8.8236E-03	4.6291E-02	ENSG00000158669
GPATCH1	0.1933	1.5733E-02	7.0320E-02	ENSG00000076650
GPATCH11	0.0718	2.9599E-01	5.0595E-01	ENSG00000152133
GPATCH2	0.5231	1.4055E-11	3.4241E-09	ENSG00000092978
GPATCH2L	0.0466	3.4717E-01	5.5760E-01	ENSG00000089916
GPATCH3	-0.0300	6.8428E-01	8.2402E-01	ENSG00000198746
GPATCH4	0.0254	6.6162E-01	8.0942E-01	ENSG00000160818
GPATCH8	0.0664	2.7084E-01	4.8008E-01	ENSG00000186566
GPBAR1	0.0742	3.1114E-01	5.2118E-01	ENSG00000179921
GPBP1	-0.1804	2.6058E-03	1.8324E-02	ENSG00000062194
GPBP1L1	-0.0440	5.1232E-01	7.0032E-01	ENSG00000159592

GPC1	-0.0619	3.4117E-01	5.5100E-01	ENSG00000063660
GPC1-AS1	-0.3151	8.2516E-03	4.3870E-02	ENSG00000218416
GPC2	-0.2589	2.6531E-04	3.2673E-03	ENSG00000213420
GPC3	-0.0805	3.2439E-01	5.3438E-01	ENSG00000147257
GPC3-AS1	-0.0071	6.5415E-01		ENSG00000286096
GPC4	-0.3044	1.7717E-03	1.3771E-02	ENSG00000076716
GPC5	-0.0262	7.6034E-01	8.7100E-01	ENSG00000179399
GPC5-AS1	0.0870	1.6903E-01	3.5752E-01	ENSG00000235984
GPC6	-0.0718	4.0772E-01	6.1205E-01	ENSG00000183098
GPC6-AS1	-0.0567	1.7449E-01		ENSG00000236520
GPCPD1	-0.0451	4.4157E-01	6.4110E-01	ENSG00000125772
GPD1L	-0.3150	1.5297E-04	2.1134E-03	ENSG00000152642
GPD2	-0.3456	2.7073E-04	3.3223E-03	ENSG00000115159
GPER1	0.1439	1.1551E-01	2.8013E-01	ENSG00000164850
GPHA2	-0.0044	9.1901E-01		ENSG00000149735
GPHN	-0.0657	2.6201E-01	4.7077E-01	ENSG00000171723
GPI	0.0410	6.2691E-01	7.8491E-01	ENSG00000105220
GPKOW	0.0276	6.7119E-01	8.1598E-01	ENSG00000068394
GPLD1	0.4442	6.3570E-05	1.0806E-03	ENSG00000112293
GPM6A	-0.2156	1.2719E-03	1.0863E-02	ENSG00000150625
GPM6A-DT	-0.0418	6.0126E-01	7.6642E-01	ENSG00000249106
GPM6B	-0.1646	4.1956E-02	1.4136E-01	ENSG00000046653
GPN1	0.0177	7.9880E-01	8.9391E-01	ENSG00000198522
GPN2	-0.1023	6.8242E-02	1.9717E-01	ENSG00000142751
GPN3	0.1013	1.9557E-01	3.9266E-01	ENSG00000111231
GPNMB	0.2312	4.5482E-02	1.4967E-01	ENSG00000136235
GPR101	-0.0848	2.9637E-01	5.0633E-01	ENSG00000165370
GPR107	0.1947	2.1073E-03	1.5669E-02	ENSG00000148358
GPR108	0.3102	8.5858E-07	3.2922E-05	ENSG00000125734
GPR12	-0.1387	1.1225E-01	2.7478E-01	ENSG00000132975
GPR132	0.0055	9.4023E-01	9.7052E-01	ENSG00000183484
GPR135	0.0848	3.5255E-01	5.6288E-01	ENSG00000181619
GPR137	-0.0681	3.8021E-01	5.8862E-01	ENSG00000173264
GPR137B	0.0036	9.7967E-01	9.8977E-01	ENSG00000077585
GPR137C	0.3651	7.7482E-05	1.2609E-03	ENSG00000180998
GPR139	0.0304	6.7759E-01	8.1935E-01	ENSG00000180269
GPR142	0.0297	5.3452E-01		ENSG00000257008
GPR143	0.0949	2.5714E-01	4.6567E-01	ENSG00000101850
GPR146	-0.0737	3.4856E-01	5.5845E-01	ENSG00000164849
GPR148	-0.0036	7.0647E-01		ENSG00000173302
GPR149	0.1116	1.7163E-01	3.6079E-01	ENSG00000174948
GPR15	0.0190	5.5049E-01		ENSG00000154165
GPR150	0.0747	3.5759E-01	5.6739E-01	ENSG00000178015
GPR151	0.0983	8.5508E-02	2.2891E-01	ENSG00000173250
GPR152	0.0188	2.9641E-01		ENSG00000175514
GPR153	-0.2041	1.6778E-02	7.3417E-02	ENSG00000158292
GPR155	0.0420	5.0133E-01	6.9213E-01	ENSG00000163328
GPR156	-0.1958	6.7708E-02	1.9622E-01	ENSG00000175697
GPR157	-0.2015	3.7024E-02	1.2931E-01	ENSG00000180758

GPR158	0.1495	1.2831E-01	2.9995E-01	ENSG00000151025
GPR158-AS1	-0.0747	8.3674E-02	2.2594E-01	ENSG00000233642
GPR160	-0.2914	2.2844E-02	9.1482E-02	ENSG00000173890
GPR161	-0.0448	4.8289E-01	6.7716E-01	ENSG00000143147
GPR162	-0.2194	7.8762E-03	4.2374E-02	ENSG00000250510
GPR17	0.0069	8.6348E-01		ENSG00000144230
GPR171	0.1124	1.1008E-01	2.7125E-01	ENSG00000174946
GPR173	0.1986	3.5248E-02	1.2465E-01	ENSG00000184194
GPR176	0.0175	8.2488E-01	9.0912E-01	ENSG00000166073
GPR176-DT	0.0690	3.1438E-01	5.2456E-01	ENSG00000246863
GPR179	-0.0592	4.1933E-01	6.2209E-01	ENSG00000277399
GPR180	-0.2107	6.2109E-03	3.5416E-02	ENSG00000152749
GPR182	-0.0163	4.0707E-01		ENSG00000166856
GPR183	0.0773	2.0009E-01	3.9851E-01	ENSG00000169508
GPR19	0.2291	9.1767E-03	4.7622E-02	ENSG00000183150
GPR20	0.0190	6.7754E-01		ENSG00000204882
GPR22	0.1119	2.2511E-01	4.2887E-01	ENSG00000172209
GPR25	0.0063	8.4633E-01		ENSG00000170128
GPR26	0.4361	1.3018E-03	1.1072E-02	ENSG00000154478
GPR27	-0.4528	4.1654E-06	1.2083E-04	ENSG00000170837
GPR3	-0.3499	1.2247E-03	1.0559E-02	ENSG00000181773
GPR32	-0.0073	8.2015E-01		ENSG00000142511
GPR34	0.3491	1.1265E-02	5.5333E-02	ENSG00000171659
GPR35	-0.0150	8.2339E-01	9.0841E-01	ENSG00000178623
GPR37	0.2050	4.1302E-02	1.3995E-01	ENSG00000170775
GPR37L1	0.0651	3.6670E-01	5.7590E-01	ENSG00000170075
GPR39	-0.0469	3.8910E-01	5.9646E-01	ENSG00000183840
GPR4	-0.0182	6.4929E-01	8.0066E-01	ENSG00000177464
GPR45	0.0163	8.2682E-01	9.1032E-01	ENSG00000135973
GPR50	-0.4593	1.6980E-03	1.3348E-02	ENSG00000102195
GPR50-AS1	-0.0015	9.3049E-01		ENSG00000234696
GPR55	0.0166	7.2445E-01		ENSG00000135898
GPR6	-0.0169	8.4590E-01	9.2100E-01	ENSG00000146360
GPR61	-0.0520	5.4213E-01	7.2377E-01	ENSG00000156097
GPR62	-0.0595	4.0903E-01	6.1334E-01	ENSG00000180929
GPR63	0.1643	9.5551E-02	2.4782E-01	ENSG00000112218
GPR68	0.0179	8.0871E-01	8.9949E-01	ENSG00000119714
GPR75	-0.0337	2.5130E-01	4.5955E-01	ENSG00000119737
GPR82	0.0090	9.0797E-01	9.5461E-01	ENSG00000171657
GPR83	0.1489	1.2446E-01	2.9424E-01	ENSG00000123901
GPR84	0.0032	7.2899E-01		ENSG00000139572
GPR84-AS1	0.0465	8.5467E-02		ENSG00000258137
GPR85	0.0039	9.5455E-01	9.7709E-01	ENSG00000164604
GPR88	0.0248	7.5671E-01	8.6825E-01	ENSG00000181656
GPR89A	0.0347	5.8365E-01	7.5412E-01	ENSG00000117262
GPR89B	0.0504	4.1395E-01	6.1758E-01	ENSG00000188092
GPRACR	0.0078	9.1572E-01		ENSG00000196979
GPRASP1	-0.0672	3.4373E-01	5.5332E-01	ENSG00000198932
GPRASP2	-0.0152	7.9440E-01	8.9134E-01	ENSG00000158301

GPRASP3	-0.0022	9.1100E-01	9.5620E-01	ENSG00000198908
GPRC5A	0.0662	4.7749E-01	6.7285E-01	ENSG00000013588
GPRC5B	-0.2822	4.1409E-03	2.6178E-02	ENSG00000167191
GPRC5C	-0.5338	3.6594E-08	2.4702E-06	ENSG00000170412
GPRC5D-AS1	-0.0377	3.7913E-01		ENSG00000247498
GPRC6A	-0.0110	3.7577E-01		ENSG00000173612
GPRIN1	-0.1098	1.4388E-01	3.2190E-01	ENSG00000169258
GPRIN2	0.1037	2.5185E-01	4.6018E-01	ENSG00000204175
GPRIN3	-0.0140	8.6565E-01	9.3217E-01	ENSG00000185477
GPS1	-0.1316	2.8159E-02	1.0621E-01	ENSG00000169727
GPS2	0.1604	5.9516E-02	1.7934E-01	ENSG00000132522
GPS2P1	-0.0014	5.6148E-01		ENSG00000236496
GPSM1	-0.0670	2.9832E-01	5.0815E-01	ENSG00000160360
GPSM2	-0.0716	3.6008E-01	5.6955E-01	ENSG00000121957
GPSM3	0.1118	2.2827E-01	4.3299E-01	ENSG00000213654
GPT2	0.0694	3.2452E-01	5.3457E-01	ENSG00000166123
GPX1	-0.2200	5.6078E-03	3.2798E-02	ENSG00000233276
GPX2	0.1008	1.5666E-01	3.4031E-01	ENSG00000176153
GPX3	0.0666	4.3665E-01	6.3732E-01	ENSG00000211445
GPX4	-0.1195	6.3283E-02	1.8734E-01	ENSG00000167468
GPX7	0.0874	3.1583E-01	5.2595E-01	ENSG00000116157
GPX8	0.0243	7.4975E-01	8.6410E-01	ENSG00000164294
GRAMD1A	-0.1580	1.6179E-02	7.1559E-02	ENSG00000089351
GRAMD1B	0.0333	6.2374E-01	7.8273E-01	ENSG00000023171
GRAMD1C	0.2241	1.5665E-02	7.0156E-02	ENSG00000178075
GRAMD2A	0.0301	6.6364E-01	8.1086E-01	ENSG00000175318
GRAMD2B	-0.3258	1.5949E-03	1.2774E-02	ENSG00000155324
GRAMD4	-0.3108	2.3739E-05	4.9167E-04	ENSG00000075240
GRAP	0.0546	1.1825E-01		ENSG00000154016
GRAP2	0.0367	1.8210E-01		ENSG00000100351
GRAPL	0.0452	1.8160E-01		ENSG00000189152
GRASLND	-0.0457	5.6770E-01	7.4237E-01	ENSG00000228203
GRB10	0.1775	2.1377E-02	8.7056E-02	ENSG00000106070
GRB14	-0.1329	1.3553E-01	3.1028E-01	ENSG00000115290
GRB2	-0.2784	2.5422E-08	1.8184E-06	ENSG00000177885
GRB7	-0.5747	1.5958E-03	1.2775E-02	ENSG00000141738
GREB1	0.4603	3.1850E-04	3.7670E-03	ENSG00000196208
GREB1L-DT	0.1358	1.7622E-01	3.6726E-01	ENSG00000265984
GREM1	-0.2310	3.0100E-02	1.1152E-01	ENSG00000166923
GREM1-AS1	-0.0404	1.8312E-01		ENSG00000259721
GREM2	0.3183	1.8049E-02	7.7125E-02	ENSG00000180875
GREP1	-0.0479	3.9517E-01	6.0143E-01	ENSG00000262152
GRHL1	0.0157	8.4587E-01	9.2100E-01	ENSG00000134317
GRHL2	0.0031	5.9944E-01		ENSG00000083307
GRHL3	0.1260	7.8112E-02	2.1624E-01	ENSG00000158055
GRHPR	-0.2424	8.3584E-04	7.9008E-03	ENSG00000137106
GRIA1	-0.3910	3.4895E-04	4.0290E-03	ENSG00000155511
GRIA2	0.2291	1.3971E-02	6.4713E-02	ENSG00000120251
GRIA3	-0.2975	1.7185E-03	1.3470E-02	ENSG00000125675

GRIA4	-0.4307	1.0681E-06	3.9225E-05	ENSG00000152578
GRID1	-0.1253	1.7615E-01	3.6722E-01	ENSG00000182771
GRID1-AS1	-0.0100	6.0895E-01		ENSG00000234942
GRID2	0.0026	9.6945E-01	9.8499E-01	ENSG00000152208
GRID2IP	-0.0456	5.7752E-01	7.4938E-01	ENSG00000215045
GRIFIN	-0.0074	6.5445E-01		ENSG00000275572
GRIK1	0.0395	5.1561E-01	7.0325E-01	ENSG00000171189
GRIK2	0.0680	4.3258E-01	6.3388E-01	ENSG00000164418
GRIK3	0.1010	1.9026E-01	3.8539E-01	ENSG00000163873
GRIK4	0.3846	3.2707E-03	2.1863E-02	ENSG00000149403
GRIK5	0.0149	8.5680E-01	9.2762E-01	ENSG00000105737
GRIN1	0.0444	6.0221E-01	7.6698E-01	ENSG00000176884
GRIN2A	0.0674	4.1022E-01	6.1437E-01	ENSG00000183454
GRIN2B	-0.3231	4.7323E-03	2.8870E-02	ENSG00000273079
GRIN2C	0.1509	1.0565E-01	2.6414E-01	ENSG00000161509
GRIN2D	0.0839	2.5801E-01	4.6670E-01	ENSG00000105464
GRIN3A	0.0329	6.8615E-01	8.2504E-01	ENSG00000198785
GRIN3B	-0.0791	3.5284E-01	5.6302E-01	ENSG00000116032
GRINA	-0.2413	4.8175E-03	2.9239E-02	ENSG00000178719
GRIP1	0.0179	8.1570E-01	9.0403E-01	ENSG00000155974
GRIP2	0.1285	1.6277E-01	3.4895E-01	ENSG00000144596
GRIPAP1	0.0129	8.7933E-01	9.3971E-01	ENSG00000068400
GRK1	0.0089	3.7481E-01	5.8335E-01	ENSG00000185974
GRK2	-0.1111	1.4477E-01	3.2340E-01	ENSG00000173020
GRK3	0.1565	6.3637E-02	1.8810E-01	ENSG00000100077
GRK3-AS1	-0.0076	9.7041E-01		ENSG00000234884
GRK4	0.1056	9.9062E-02	2.5316E-01	ENSG00000125388
GRK5	-0.0844	2.8443E-01	4.9499E-01	ENSG00000198873
GRK6	-0.1596	5.6437E-02	1.7364E-01	ENSG00000198055
GRK7	0.0917	1.9501E-01	3.9185E-01	ENSG00000114124
GRM2	-0.0912	3.1203E-01	5.2208E-01	ENSG00000164082
GRM3	-0.2735	7.9417E-03	4.2637E-02	ENSG00000198822
GRM4	0.1249	1.8880E-01	3.8373E-01	ENSG00000124493
GRM5	-0.5063	3.7106E-03	2.4154E-02	ENSG00000168959
GRM5-AS1	0.0023	7.8491E-01		ENSG00000255082
GRM6	0.0615	3.4485E-01	5.5446E-01	ENSG00000113262
GRM7	0.0776	3.5499E-01	5.6473E-01	ENSG00000196277
GRM7-AS1	-0.1258	5.8971E-02	1.7845E-01	ENSG00000236202
GRM7-AS2	-0.0027	7.0024E-01		ENSG00000237665
GRM7-AS3	0.0172	7.0372E-01		ENSG00000226258
GRM8	0.5254	6.3299E-04	6.4252E-03	ENSG00000179603
GRN	0.1538	2.2556E-02	9.0580E-02	ENSG00000030582
GRP	-0.0351	4.2172E-01	6.2442E-01	ENSG00000134443
GRPEL1	-0.0981	8.5643E-02	2.2925E-01	ENSG00000109519
GRPEL2	-0.1347	7.9518E-02	2.1880E-01	ENSG00000164284
GRPEL2-AS1	-0.0079	8.5157E-01		ENSG00000253618
GRPR	0.2739	2.9145E-02	1.0889E-01	ENSG00000126010
GRSF1	-0.1882	6.7451E-04	6.7566E-03	ENSG00000132463
GRTP1	-0.0626	4.1829E-01	6.2150E-01	ENSG00000139835

GRWD1	0.1341	9.2453E-02	2.4223E-01	ENSG00000105447
GRXCR1	0.0289	5.3085E-01		ENSG00000215203
GS1-204I12.4	0.3598	1.5960E-02	7.1026E-02	ENSG00000261729
GS1-24F4.2	-0.0376	5.6765E-01	7.4237E-01	ENSG00000245857
GS1-279B7.1	0.4276	1.2105E-02	5.8321E-02	ENSG00000261024
GSAP	0.0059	9.4681E-01	9.7364E-01	ENSG00000186088
GSC	-0.0011	9.4706E-01		ENSG00000133937
GSC2	-0.3100	2.2886E-02	9.1606E-02	ENSG00000063515
GSDMA	0.0006	8.5060E-01		ENSG00000167914
GSDMB	0.0336	6.9095E-01	8.2842E-01	ENSG00000073605
GSDMC	0.0027	9.4626E-01		ENSG00000147697
GSDMD	0.0669	3.4736E-01	5.5760E-01	ENSG00000104518
GSDME	-0.1219	7.7947E-02	2.1592E-01	ENSG00000105928
GSE1	-0.3212	1.7719E-03	1.3771E-02	ENSG00000131149
GSG1	0.1033	2.4032E-01	4.4717E-01	ENSG00000111305
GSG1L	0.1652	1.0096E-01	2.5638E-01	ENSG00000169181
GSK3A	-0.1299	2.6743E-02	1.0232E-01	ENSG00000105723
GSK3B	-0.1262	1.2114E-02	5.8351E-02	ENSG00000082701
GSKIP	0.0744	2.8123E-01	4.9157E-01	ENSG00000100744
GSN	-0.0412	5.7639E-01	7.4871E-01	ENSG00000148180
GSN-AS1	-0.1316	1.5144E-01	3.3296E-01	ENSG00000235865
GSPT1	-0.2617	1.4543E-06	5.1191E-05	ENSG00000103342
GSPT2	-0.0761	1.5661E-01	3.4030E-01	ENSG00000189369
GSR	-0.2993	8.1630E-03	4.3531E-02	ENSG00000104687
GSS	0.0101	8.3806E-01	9.1647E-01	ENSG00000100983
GSTA3	0.0648	5.4820E-02		ENSG00000174156
GSTA4	-0.3416	6.9491E-09	6.0848E-07	ENSG00000170899
GSTA9P	0.0532	6.9734E-02		ENSG00000243236
GSTCD	0.3509	4.5624E-04	4.9496E-03	ENSG00000138780
GSTCD-AS1	0.1071	8.1893E-02	2.2263E-01	ENSG00000251175
GSTK1	-0.0482	4.8435E-01	6.7822E-01	ENSG00000197448
GSTM1	0.0129	8.7736E-01	9.3895E-01	ENSG00000134184
GSTM2	-0.1794	1.5126E-02	6.8388E-02	ENSG00000213366
GSTM3	-0.2010	2.9164E-03	2.0061E-02	ENSG00000134202
GSTM4	0.0213	7.7624E-01	8.8092E-01	ENSG00000168765
GSTM5	-0.1077	1.5675E-01	3.4042E-01	ENSG00000134201
GSTO1	-0.1520	4.2128E-02	1.4170E-01	ENSG00000148834
GSTO2	0.1709	5.9563E-02	1.7934E-01	ENSG00000065621
GSTP1	-0.2190	2.5106E-02	9.7814E-02	ENSG00000084207
GSTT2	-0.0619	4.5414E-01	6.5260E-01	ENSG00000099984
GSTT2B	-0.1305	1.6915E-01	3.5762E-01	ENSG00000133433
GSTT4	-0.0305	1.7043E-01		ENSG00000276950
GSTZ1	0.1720	4.2833E-02	1.4341E-01	ENSG00000100577
GSX1	0.0018	7.6740E-01		ENSG00000169840
GSX2	-0.0474	5.7090E-01	7.4457E-01	ENSG00000180613
GTDC1	-0.1326	6.2313E-02	1.8544E-01	ENSG00000121964
GTF2A1	0.0185	8.0223E-01	8.9571E-01	ENSG00000165417
GTF2A2	-0.1026	5.9676E-02	1.7959E-01	ENSG00000140307
GTF2B	-0.0631	3.1160E-01	5.2157E-01	ENSG00000137947

GTF2E1	0.0374	6.4290E-01	7.9598E-01	ENSG00000153767
GTF2E2	-0.1663	1.8566E-02	7.8730E-02	ENSG00000197265
GTF2F1	0.0311	6.0969E-01	7.7240E-01	ENSG00000125651
GTF2F2	0.1311	2.0845E-02	8.5468E-02	ENSG00000188342
GTF2F2P2	-0.0084	5.6794E-01		ENSG00000233910
GTF2H1	0.1315	7.0343E-02	2.0131E-01	ENSG00000110768
GTF2H2	0.0140	8.0009E-01	8.9450E-01	ENSG00000145736
GTF2H2C	0.0131	8.0285E-01	8.9609E-01	ENSG00000183474
GTF2H3	-0.1149	4.7761E-02	1.5467E-01	ENSG00000111358
GTF2H4	0.0133	6.9846E-01		ENSG00000213780
GTF2H5	-0.0315	5.8935E-01	7.5786E-01	ENSG00000272047
GTF2I	-0.1028	6.9885E-02	2.0062E-01	ENSG00000263001
GTF2I-AS1	-0.0652	4.4411E-01	6.4312E-01	ENSG00000232729
GTF2IP4	-0.1102	6.5076E-02	1.9091E-01	ENSG00000233369
GTF2IP5	0.0530	4.5106E-01	6.5005E-01	ENSG00000224316
GTF2IP9	0.0000	7.7234E-01		ENSG00000251451
GTF2IRD1	0.0543	4.1793E-01	6.2123E-01	ENSG00000006704
GTF2IRD2	-0.0027	9.7011E-01	9.8507E-01	ENSG00000196275
GTF2IRD2B	0.0818	2.0575E-01	4.0523E-01	ENSG00000174428
GTF2IRD2P1	0.2106	1.2172E-02	5.8540E-02	ENSG00000214544
GTF3A	-0.2015	4.1429E-04	4.5917E-03	ENSG00000122034
GTF3C1	-0.0477	4.1625E-01	6.1970E-01	ENSG00000077235
GTF3C2	0.0889	1.3851E-01	3.1439E-01	ENSG00000115207
GTF3C2-AS1	-0.3763	1.7483E-02	7.5560E-02	ENSG00000234945
GTF3C3	0.0312	6.6660E-01	8.1302E-01	ENSG00000119041
GTF3C4	-0.1693	1.7085E-02	7.4303E-02	ENSG00000125484
GTF3C5	-0.0245	6.7224E-01	8.1669E-01	ENSG00000148308
GTF3C6	-0.0868	1.5648E-01	3.4030E-01	ENSG00000155115
GTPBP1	-0.0569	3.3302E-01	5.4329E-01	ENSG00000100226
GTPBP10	0.1635	2.7128E-02	1.0341E-01	ENSG00000105793
GTPBP2	0.3840	3.8665E-05	7.3228E-04	ENSG00000172432
GTPBP3	-0.0083	9.0558E-01	9.5378E-01	ENSG00000130299
GTPBP4	0.1022	1.0084E-01	2.5622E-01	ENSG00000107937
GTPBP6	-0.1613	2.6979E-02	1.0294E-01	ENSG00000178605
GTPBP8	-0.0301	7.1313E-01	8.4257E-01	ENSG00000163607
GTSE1	-0.0227	7.8498E-01	8.8631E-01	ENSG00000075218
GTSE1-DT	0.0147	8.6120E-01	9.3034E-01	ENSG00000277232
GTSF1	-0.0280	3.1404E-01	5.2432E-01	ENSG00000170627
GTSF1L	-0.0012	9.8237E-01		ENSG00000124196
GUCA1A	0.0387	5.6703E-01	7.4191E-01	ENSG00000048545
GUCA1B	0.2059	5.4963E-02	1.7054E-01	ENSG00000112599
GUCD1	-0.1399	6.9326E-02	1.9936E-01	ENSG00000138867
GUCY1A1	-0.1019	2.5451E-01	4.6257E-01	ENSG00000164116
GUCY1A2	0.0829	3.3894E-01	5.4866E-01	ENSG00000152402
GUCY1B1	0.0644	3.8526E-01	5.9312E-01	ENSG00000061918
GUCY1B2	-0.0450	5.4381E-01	7.2476E-01	ENSG00000123201
GUCY2C	0.0274	1.0119E-01		ENSG00000070019
GUCY2D	0.0023	8.8902E-01		ENSG00000132518
GUCY2F	0.0496	1.5104E-01		ENSG00000101890

GUCY2GP	0.0055	9.9327E-01		ENSG00000243316
GUF1	0.0654	3.6509E-01	5.7429E-01	ENSG00000151806
GUK1	-0.0227	7.6137E-01	8.7163E-01	ENSG00000143774
GULP1	-0.0063	9.3326E-01	9.6701E-01	ENSG00000144366
GUSB	0.0751	2.5898E-01	4.6768E-01	ENSG00000169919
GUSBP11	0.0045	9.5805E-01	9.7936E-01	ENSG00000228315
GUSBP5	0.0672	4.2799E-01	6.2993E-01	ENSG00000236296
GUSBP9	0.3383	1.7277E-03	1.3521E-02	ENSG00000215630
GVINP1	0.0701	9.0644E-02		ENSG00000254838
GVQW3	0.2847	7.8207E-04	7.5390E-03	ENSG00000179240
GXYLT1	0.2992	3.2763E-03	2.1894E-02	ENSG00000151233
GXYLT1P4	0.0142	7.5631E-01		ENSG00000275026
GXYLT1P5	0.0836	1.3106E-01	3.0410E-01	ENSG00000276128
GXYLT1P6	-0.0106	6.0377E-01		ENSG00000235310
GXYLT1P7	-0.0037	7.6763E-01		ENSG00000227265
GXYLT2	-0.1119	2.0267E-01	4.0161E-01	ENSG00000172986
GYG1	0.0077	9.1475E-01	9.5797E-01	ENSG00000163754
GYG2	-0.1036	1.2721E-01	2.9864E-01	ENSG00000056998
GYG2P2	0.0032	9.7176E-01		ENSG00000271580
GYPA	-0.0080	7.4881E-01		ENSG00000170180
GYPB	-0.0070	5.5924E-01		ENSG00000250361
GYPC	-0.1494	1.0827E-01	2.6824E-01	ENSG00000136732
GYPE	0.0162	2.4032E-01		ENSG00000197465
GYS1	0.0581	4.6351E-01	6.6060E-01	ENSG00000104812
GZF1	-0.0797	2.9831E-01	5.0815E-01	ENSG00000125812
GZMM	0.1064	1.9460E-01	3.9132E-01	ENSG00000197540
H1-1	-0.2960	1.6366E-03	1.3016E-02	ENSG00000124610
H1-5	-0.3877	4.7879E-03	2.9095E-02	ENSG00000184357
H1-6	0.0150	9.1125E-01		ENSG00000187475
H1-7	0.0312	1.4713E-01		ENSG00000187166
H19	0.0504	4.9489E-01	6.8685E-01	ENSG00000130600
H2AB1	0.0059	5.5369E-01		ENSG00000274183
H2AB2	0.0117	5.3441E-01		ENSG00000277858
H2AB3	0.0333	4.5162E-01		ENSG00000277745
H2AC10P	-0.4277	1.0827E-02	5.3719E-02	ENSG00000218690
H2AC11	0.1162	2.0075E-01	3.9924E-01	ENSG00000196787
H2AC12	-0.2694	2.9741E-02	1.1046E-01	ENSG00000274997
H2AC15	-0.0077	9.2756E-01	9.6455E-01	ENSG00000275221
H2AC16	0.0054	9.4413E-01	9.7218E-01	ENSG00000276903
H2AC17	-0.1095	1.3736E-01	3.1279E-01	ENSG00000278677
H2AC18	-0.0335	6.8900E-01	8.2709E-01	ENSG00000288825
H2AC19	-0.0338	6.8642E-01	8.2528E-01	ENSG00000288859
H2AC20	-0.4586	1.9419E-04	2.5361E-03	ENSG00000184260
H2AC21	-0.0089	3.7287E-01		ENSG00000184270
H2AC25	-0.3580	4.8540E-04	5.1933E-03	ENSG00000181218
H2AC4	0.0784	2.7650E-01	4.8646E-01	ENSG00000278463
H2AC6	-0.0064	9.3474E-01	9.6786E-01	ENSG00000180573
H2AC8	0.1891	3.0000E-02	1.1125E-01	ENSG00000277075
H2AC9P	-0.0223	1.8361E-01		ENSG00000218281

H2AJ	-0.0467	4.6125E-01	6.5852E-01	ENSG00000246705
H2AP	-0.0131	9.4731E-01		ENSG00000187516
H2AX	-0.0386	6.3948E-01	7.9390E-01	ENSG00000188486
H2AZ1	-0.3725	1.1755E-04	1.7444E-03	ENSG00000164032
H2AZ2	-0.2710	4.2274E-05	7.7913E-04	ENSG00000105968
H2BC10	-0.0242	4.6798E-01		ENSG00000278588
H2BC11	-0.0452	5.8561E-01	7.5543E-01	ENSG00000124635
H2BC12	-0.1477	1.1397E-01	2.7779E-01	ENSG00000197903
H2BC12L	0.0159	4.9042E-01		ENSG00000234289
H2BC13	0.0128	9.4280E-01		ENSG00000185130
H2BC14	0.0392	7.0545E-01	8.3771E-01	ENSG00000273703
H2BC15	0.2144	2.0503E-02	8.4404E-02	ENSG00000233822
H2BC16P	0.0019	9.6307E-01		ENSG00000217646
H2BC17	0.0941	4.3509E-01	6.3609E-01	ENSG00000274641
H2BC18	-0.0114	8.4596E-01	9.2100E-01	ENSG00000203814
H2BC19P	-0.0903	3.1633E-01	5.2637E-01	ENSG00000220323
H2BC20P	-0.1931	6.7333E-02	1.9558E-01	ENSG00000261716
H2BC21	-0.2543	5.2113E-03	3.1085E-02	ENSG00000184678
H2BC26	-0.2761	8.6626E-03	4.5641E-02	ENSG00000196890
H2BC27P	-0.0591	5.0375E-02		ENSG00000181201
H2BC3	0.2074	6.0478E-02	1.8136E-01	ENSG00000276410
H2BC5	-0.3830	5.1028E-04	5.3888E-03	ENSG00000158373
H2BC6	0.0582	4.5602E-01	6.5433E-01	ENSG00000274290
H2BC7	0.1021	2.5971E-01	4.6811E-01	ENSG00000277224
H2BC8	0.2275	3.3853E-02	1.2085E-01	ENSG00000273802
H2BC9	-0.1474	8.4805E-02	2.2775E-01	ENSG00000275713
H2BP9	0.0241	5.0539E-01		ENSG00000231787
H2BW1	0.0516	1.0514E-01		ENSG00000123569
H2BW2	-0.0525	2.8029E-01	4.9027E-01	ENSG00000101812
H3-3A	-0.4488	1.0946E-05	2.6378E-04	ENSG00000163041
H3-7	-0.2086	5.7470E-02	1.7574E-01	ENSG00000273213
H3C1	-0.0020	9.8014E-01	9.8991E-01	ENSG00000275714
H3C10	0.0052	9.4789E-01	9.7436E-01	ENSG00000278828
H3C11	0.0779	3.1830E-01	5.2853E-01	ENSG00000275379
H3C12	-0.0510	2.2172E-01	4.2497E-01	ENSG00000197153
H3C13	0.0244	8.2650E-01	9.1009E-01	ENSG00000183598
H3C14	0.0244	7.6896E-01	8.7634E-01	ENSG00000203811
H3C15	0.0370	6.4435E-01	7.9692E-01	ENSG00000203852
H3C2	0.0797	3.4723E-01	5.5760E-01	ENSG00000286522
H3C3	0.0087	9.0213E-01	9.5204E-01	ENSG00000287080
H3C5P	0.0120	3.7590E-01		ENSG00000277720
H3C6	0.0034	9.6482E-01	9.8267E-01	ENSG00000274750
H3C7	0.1064	1.9147E-01	3.8677E-01	ENSG00000277775
H3C8	0.0479	4.7344E-01	6.6941E-01	ENSG00000273983
H3C9P	-0.2970	2.2952E-02	9.1783E-02	ENSG00000220875
H3P1	-0.5829	3.7806E-03	2.4467E-02	ENSG00000230228
H3P13	-0.0636	1.8688E-01		ENSG00000243977
H3P25	-0.0330	5.5612E-01	7.3405E-01	ENSG00000275506
H3P31	-0.0052	9.8884E-01		ENSG00000232466

H3P33	-0.0122	5.9201E-01		ENSG00000255492
H3P36	-0.4813	9.3022E-05	1.4588E-03	ENSG00000236534
H3P43	-0.5387	1.8997E-03	1.4489E-02	ENSG00000226436
H3P45	0.0024	9.8001E-01		ENSG00000227211
H4C1	0.1441	1.0587E-01	2.6453E-01	ENSG00000278637
H4C13	0.0114	6.4237E-01		ENSG00000275126
H4C14	-0.2071	1.8291E-02	7.7887E-02	ENSG00000270882
H4C15	-0.2045	1.9176E-02	8.0612E-02	ENSG00000270276
H4C16	0.1099	2.2423E-01	4.2810E-01	ENSG00000197837
H4C2	0.0159	8.8325E-01	9.4147E-01	ENSG00000278705
H4C4	-0.0273	4.5688E-01	6.5507E-01	ENSG00000277157
H4C5	-0.0244	7.4312E-01	8.6031E-01	ENSG00000276966
H4C6	0.0423	3.0348E-01		ENSG00000274618
H4C8	0.1027	2.4913E-01	4.5683E-01	ENSG00000158406
H4C9	-0.0504	4.7021E-01	6.6649E-01	ENSG00000276180
H6PD	0.3628	1.0374E-04	1.5865E-03	ENSG00000049239
HAAO	0.0605	3.2485E-01		ENSG00000162882
HABP4	0.0294	7.3929E-01	8.5776E-01	ENSG00000130956
HACD1	-0.1067	1.6693E-01	3.5494E-01	ENSG00000165996
HACD2	-0.2442	3.0145E-03	2.0578E-02	ENSG00000206527
HACD3	-0.1445	4.4677E-03	2.7627E-02	ENSG00000074696
HACD4	0.0856	3.1935E-01	5.2957E-01	ENSG00000188921
HACE1	-0.0992	1.3038E-01	3.0318E-01	ENSG00000085382
HACL1	0.1189	5.9418E-02	1.7919E-01	ENSG00000131373
HADH	-0.2043	1.6586E-03	1.3134E-02	ENSG00000138796
HADHA	-0.0062	8.8118E-01	9.4050E-01	ENSG00000084754
HADHAP1	0.0342	6.0449E-01	7.6849E-01	ENSG00000251596
HADHB	0.0167	8.3162E-01	9.1304E-01	ENSG00000138029
HADHBP1	0.1217	5.5881E-02	1.7256E-01	ENSG00000238193
HAGH	-0.0931	1.0907E-01	2.6957E-01	ENSG00000063854
HAGHL	-0.1185	1.3938E-01	3.1559E-01	ENSG00000103253
HAGLR	0.0071	9.6393E-01		ENSG00000224189
HAL	-0.0045	9.4812E-01		ENSG00000084110
HAMP	-0.0228	9.3487E-01		ENSG00000105697
HAND1	-0.0569	3.0922E-01	5.1942E-01	ENSG00000113196
HAND2	-0.2450	3.7829E-02	1.3149E-01	ENSG00000164107
HAND2-AS1	0.0833	4.0016E-02		ENSG00000237125
HAP1	0.0874	3.3645E-01	5.4649E-01	ENSG00000173805
HAPLN1	0.3090	2.1573E-02	8.7639E-02	ENSG00000145681
HAPLN2	0.3229	1.9070E-03	1.4528E-02	ENSG00000132702
HAPLN3	-0.2811	1.7119E-02	7.4406E-02	ENSG00000140511
HAPLN4	-0.0068	7.1031E-01		ENSG00000187664
HAPSTR1	-0.3529	3.2177E-06	9.8471E-05	ENSG00000182831
HAPSTR2	-0.1098	2.1217E-01	4.1310E-01	ENSG00000230707
HAR1B	0.0717	3.3331E-01	5.4349E-01	ENSG00000231133
HARBI1	-0.0279	7.0187E-01	8.3533E-01	ENSG00000180423
HARS1	-0.0363	5.4163E-01	7.2342E-01	ENSG00000170445
HARS2	0.1199	1.1540E-01	2.7998E-01	ENSG00000112855
HAS1	0.0185	8.1420E-01	9.0317E-01	ENSG00000105509

HAS2	0.0574	3.0391E-01	5.1410E-01	ENSG00000170961
HAS3	0.0489	5.6051E-01	7.3766E-01	ENSG00000103044
HASPIN	-0.3383	9.9313E-03	5.0456E-02	ENSG00000177602
HAT1	-0.1227	4.1762E-02	1.4087E-01	ENSG00000128708
HAUS1	-0.0805	2.0045E-01	3.9891E-01	ENSG00000152240
HAUS2	-0.3137	3.2896E-07	1.5243E-05	ENSG00000137814
HAUS3	0.0296	6.7607E-01	8.1840E-01	ENSG00000214367
HAUS4	-0.2366	1.1008E-02	5.4368E-02	ENSG00000092036
HAUS5	-0.0153	8.2291E-01	9.0841E-01	ENSG00000249115
HAUS6	0.1999	2.3611E-03	1.7031E-02	ENSG00000147874
HAUS6P1	0.1229	1.6767E-01	3.5598E-01	ENSG00000227344
HAUS7	-0.0265	6.0942E-01	7.7221E-01	ENSG00000213397
HAUS8	-0.1422	6.1212E-02	1.8287E-01	ENSG00000131351
HAVCR1	0.0433	1.8635E-01		ENSG00000113249
HAVCR1P1	0.0219	6.1144E-01		ENSG00000268442
HAVCR1P2	0.0052	8.1366E-01		ENSG00000271053
HAVCR2	0.0884	2.4399E-01	4.5165E-01	ENSG00000135077
HAX1	-0.1058	5.4124E-02	1.6857E-01	ENSG00000143575
HBA1	0.0334	5.5246E-01	7.3102E-01	ENSG00000206172
HBA2	0.0357	5.0588E-01	6.9561E-01	ENSG00000188536
HBD	-0.0033	6.3512E-01	7.9073E-01	ENSG00000223609
HBEGF	0.0571	4.0997E-01	6.1422E-01	ENSG00000113070
HBM	0.0220	3.4396E-01		ENSG00000206177
HBP1	-0.3450	2.1296E-04	2.7320E-03	ENSG00000105856
HBQ1	-0.0668	4.0940E-01	6.1369E-01	ENSG00000086506
HBS1L	0.0300	5.6726E-01	7.4199E-01	ENSG00000112339
HBZ	0.0528	4.4916E-01	6.4827E-01	ENSG00000130656
HCAR1	0.0014	3.9847E-01		ENSG00000196917
HCAR2	0.0105	9.0611E-01		ENSG00000182782
HCAR3	-0.0258	1.6873E-01		ENSG00000255398
HCCAT5	0.0195	1.8175E-01		ENSG00000260880
HCCS	-0.0086	8.7466E-01	9.3712E-01	ENSG00000004961
HCFC1	-0.1851	1.6946E-02	7.3876E-02	ENSG00000172534
HCFC1R1	-0.4344	7.0204E-07	2.7816E-05	ENSG00000103145
HCFC2	-0.1270	8.6130E-02	2.3030E-01	ENSG00000111727
HCG11	-0.0664	3.8101E-01	5.8923E-01	ENSG00000228223
HCG14	0.0128	7.9804E-01		ENSG00000224157
HCG17	-0.0691	3.8622E-01	5.9394E-01	ENSG00000270604
HCG18	0.1688	2.6421E-03	1.8521E-02	ENSG00000231074
HCG20	0.0584	4.0066E-01	6.0598E-01	ENSG00000228022
HCG22	-0.1375	1.4992E-01	3.3078E-01	ENSG00000228789
HCG23	0.1049	2.0495E-01	4.0424E-01	ENSG00000225914
HCG25	0.0980	2.9235E-01	5.0242E-01	ENSG00000232940
HCG27	0.0277	6.2433E-01	7.8313E-01	ENSG00000206344
HCG9	-0.0279	6.3123E-01	7.8773E-01	ENSG00000204625
HCK	0.0236	6.6677E-01	8.1315E-01	ENSG00000101336
HCLS1	0.0035	8.7595E-01		ENSG00000180353
HCN1	-0.2412	2.7936E-02	1.0565E-01	ENSG00000164588
HCN2	-0.0476	4.4895E-01	6.4817E-01	ENSG00000099822

HCN3	-0.0844	2.3397E-01	4.3905E-01	ENSG00000143630
HCN4	0.1175	1.6721E-01	3.5533E-01	ENSG00000138622
HCP5	0.1473	1.2227E-01	2.9109E-01	ENSG00000206337
HCRT	0.1517	7.9759E-02	2.1918E-01	ENSG00000161610
HCRTR1	0.2412	4.6254E-02	1.5144E-01	ENSG00000121764
HCRTR2	0.0003	9.9403E-01	9.9671E-01	ENSG00000137252
HCST	-0.0057	7.6120E-01	8.7162E-01	ENSG00000126264
HDAC1	-0.2876	4.3408E-04	4.7638E-03	ENSG00000116478
HDAC10	0.1058	1.1189E-01	2.7417E-01	ENSG00000100429
HDAC11	0.1076	1.4017E-01	3.1670E-01	ENSG00000163517
HDAC11-AS1	0.0423	3.1009E-01		ENSG00000244502
HDAC2	-0.2892	3.8147E-07	1.7066E-05	ENSG00000196591
HDAC2-AS2	0.0383	6.5333E-01	8.0375E-01	ENSG00000228624
HDAC3	0.0867	9.6842E-02	2.4997E-01	ENSG00000171720
HDAC4	0.2219	1.4155E-02	6.5307E-02	ENSG00000068024
HDAC4-AS1	0.4232	8.1130E-04	7.7432E-03	ENSG00000222020
HDAC5	-0.3633	1.4441E-05	3.2825E-04	ENSG00000108840
HDAC6	-0.0322	6.6360E-01	8.1086E-01	ENSG00000094631
HDAC7	-0.1325	9.4545E-02	2.4604E-01	ENSG00000061273
HDAC8	0.1999	4.4159E-03	2.7403E-02	ENSG00000147099
HDAC9	0.0523	4.4736E-01	6.4688E-01	ENSG00000048052
HDC	0.0938	8.1267E-02	2.2168E-01	ENSG00000140287
HDDC2	-0.0451	4.6105E-01	6.5843E-01	ENSG00000111906
HDDC3	0.1333	5.5391E-02	1.7149E-01	ENSG00000184508
HDGF	-0.1407	2.5103E-02	9.7814E-02	ENSG00000143321
HDGFL1	0.0182	2.7078E-01		ENSG00000112273
HDGFL2	-0.0562	3.2019E-01	5.3035E-01	ENSG00000167674
HDGFL3	-0.1306	2.4177E-02	9.5410E-02	ENSG00000166503
HDHD2	0.0165	8.2368E-01	9.0841E-01	ENSG00000167220
HDHD3	0.0012	9.8954E-01	9.9419E-01	ENSG00000119431
HDHD5	0.0636	3.2332E-01	5.3334E-01	ENSG00000069998
HDHD5-AS1	0.1207	1.9339E-01	3.8954E-01	ENSG00000185837
HDLBP	-0.0605	2.9376E-01	5.0389E-01	ENSG00000115677
HDX	0.0886	2.2379E-01	4.2766E-01	ENSG00000165259
HEATR1	-0.0631	4.0246E-01	6.0700E-01	ENSG00000119285
HEATR3	-0.2423	4.4249E-03	2.7424E-02	ENSG00000155393
HEATR4	-0.0559	4.9988E-01	6.9086E-01	ENSG00000187105
HEATR5A	0.0781	3.7257E-01	5.8132E-01	ENSG00000129493
HEATR5A-DT	0.1945	7.2335E-02	2.0543E-01	ENSG00000250365
HEATR5B	0.3363	1.6519E-04	2.2370E-03	ENSG00000008869
HEATR6	0.1188	3.8889E-02	1.3413E-01	ENSG00000068097
HEATR6-DT	0.0515	4.7098E-01	6.6722E-01	ENSG00000267416
HEBP1	-0.1234	6.7920E-02	1.9649E-01	ENSG00000013583
HEBP2	-0.1216	5.6677E-02	1.7408E-01	ENSG00000051620
HECA	-0.2390	1.9233E-03	1.4632E-02	ENSG00000112406
HECTD1	-0.0562	3.5418E-01	5.6416E-01	ENSG00000092148
HECTD2	0.1079	1.2650E-01	2.9765E-01	ENSG00000165338
HECTD3	0.0932	2.2846E-01	4.3305E-01	ENSG00000126107
HECTD4	0.0500	4.7587E-01	6.7134E-01	ENSG00000173064

HECW1	-0.3565	3.1140E-04	3.7014E-03	ENSG00000002746
HECW1-IT1	-0.2231	4.7622E-02	1.5446E-01	ENSG00000181211
HECW2	0.4194	1.7377E-03	1.3569E-02	ENSG00000138411
HECW2-AS1	0.1663	9.3378E-03		ENSG00000229056
HEG1	0.1706	8.6155E-02	2.3031E-01	ENSG00000173706
HELB	-0.0607	4.7587E-01	6.7134E-01	ENSG00000127311
HELLS	0.1811	4.6129E-02	1.5117E-01	ENSG00000119969
HELQ	0.1579	5.8802E-02	1.7816E-01	ENSG00000163312
HELT	-0.0684	1.1803E-02	5.7330E-02	ENSG00000187821
HELZ	-0.1185	4.8723E-02	1.5671E-01	ENSG00000198265
HELZ2	0.0803	3.5278E-01	5.6299E-01	ENSG00000130589
HEMGN	-0.0373	1.6991E-01		ENSG00000136929
HEMK1	-0.1290	8.4555E-02	2.2730E-01	ENSG00000114735
HENMT1	0.2453	2.7951E-02	1.0568E-01	ENSG00000162639
HEPACAM	-0.0047	9.4182E-01	9.7118E-01	ENSG00000165478
HEPACAM2	-0.0199	7.1383E-01	8.4287E-01	ENSG00000188175
HEPH	-0.1873	4.5282E-02	1.4909E-01	ENSG00000089472
HEPHL1	-0.0001	7.0743E-01		ENSG00000181333
HERC1	0.1188	7.6070E-02	2.1250E-01	ENSG00000103657
HERC2	0.2701	1.7190E-04	2.3062E-03	ENSG00000128731
HERC2P6	0.0130	8.4069E-01	9.1800E-01	ENSG00000261418
HERC3	0.0692	4.2905E-01	6.3074E-01	ENSG00000138641
HERC3	-0.0110	5.3227E-01		ENSG00000287542
HERC4	-0.0558	5.0278E-01	6.9328E-01	ENSG00000148634
HERC5	-0.2625	2.6564E-02	1.0190E-01	ENSG00000138646
HERC6	0.0283	7.3061E-01	8.5270E-01	ENSG00000138642
HERPUD1	-0.2882	9.2744E-03	4.7971E-02	ENSG00000051108
HERPUD2	-0.3487	2.2080E-07	1.1065E-05	ENSG00000122557
HERPUD2-AS1	0.0642	3.8691E-01	5.9466E-01	ENSG00000271122
HES4	0.0400	6.0370E-01	7.6810E-01	ENSG00000188290
HES5	0.0712	4.0500E-01	6.0944E-01	ENSG00000197921
HES6	-0.3019	2.5876E-03	1.8211E-02	ENSG00000144485
HES7	0.1122	2.8747E-01	4.9814E-01	ENSG00000179111
HESX1	0.0528	5.2672E-01	7.1185E-01	ENSG00000163666
HEXA	0.0055	9.3112E-01	9.6590E-01	ENSG00000213614
HEXA-AS1	0.0394	5.2909E-01	7.1372E-01	ENSG00000260339
HEXB	0.0628	3.8024E-01	5.8864E-01	ENSG00000049860
HEXD	0.0680	2.7476E-01	4.8458E-01	ENSG00000169660
HEXIM1	-0.2073	4.7746E-03	2.9036E-02	ENSG00000186834
HEXIM2	-0.0008	9.9320E-01	9.9620E-01	ENSG00000168517
HEY1	-0.1649	6.8598E-02	1.9794E-01	ENSG00000164683
HEY2	-0.1460	1.3620E-01	3.1126E-01	ENSG00000135547
HEY2-AS1	-0.0137	9.6976E-01	9.8504E-01	ENSG00000237742
HEYL	-0.0813	2.4774E-01	4.5550E-01	ENSG00000163909
HFE	-0.0385	5.6886E-01	7.4328E-01	ENSG00000010704
HFM1	0.3251	1.3616E-03	1.1427E-02	ENSG00000162669
HGD	-0.0664	3.2089E-01	5.3094E-01	ENSG00000113924
HGF	0.0978	1.5735E-01	3.4109E-01	ENSG00000019991
HGFAC	-0.0238	7.6863E-01	8.7612E-01	ENSG00000109758

HGH1	-0.1333	7.8799E-02	2.1743E-01	ENSG00000235173
HGS	-0.0203	7.7025E-01	8.7704E-01	ENSG00000185359
HGSNAT	0.2423	6.0776E-06	1.6397E-04	ENSG00000165102
HHAT	0.1280	1.5474E-01	3.3775E-01	ENSG00000054392
HHATL	0.0389	3.9650E-02		ENSG00000010282
HHEX	-0.1873	4.1914E-02	1.4123E-01	ENSG00000152804
HHIP	0.0603	3.8260E-01	5.9085E-01	ENSG00000164161
HHIP-AS1	-0.0689	4.2755E-01	6.2952E-01	ENSG00000248890
HHIPL1	0.0045	9.7514E-01	9.8778E-01	ENSG00000182218
HHIPL2	0.0474	6.3770E-02		ENSG00000143512
HHLA1	0.0090	8.3103E-01		ENSG00000132297
HHLA2	0.0220	6.4432E-01		ENSG00000114455
HIBADH	-0.0133	8.5055E-01	9.2400E-01	ENSG00000106049
HIBCH	-0.2265	2.3258E-03	1.6876E-02	ENSG00000198130
HIC1	-0.0276	6.8606E-01	8.2504E-01	ENSG00000177374
HIC2	0.3961	1.0445E-05	2.5420E-04	ENSG00000169635
HID1	-0.0754	2.3535E-01	4.4083E-01	ENSG00000167861
HID1-AS1	-0.0460	2.4760E-01		ENSG00000263586
HIF1A	-0.2020	1.3347E-03	1.1281E-02	ENSG00000100644
HIF1A-AS3	0.1090	8.2855E-02	2.2450E-01	ENSG00000258667
HIF1AN	-0.2447	3.4317E-05	6.6278E-04	ENSG00000166135
HIF3A	-0.1158	1.6784E-01	3.5612E-01	ENSG00000124440
HIGD1A	-0.2579	5.5274E-04	5.7543E-03	ENSG00000181061
HIGD1B	-0.0052	9.1289E-01		ENSG00000131097
HIGD2A	-0.1248	2.7233E-02	1.0369E-01	ENSG00000146066
HIGD2B	0.0235	6.3383E-01		ENSG00000175202
HIKESHI	-0.0878	1.9000E-01	3.8506E-01	ENSG00000149196
HIKESHIP2	0.0280	5.5478E-01		ENSG00000267544
HILPDA	0.1186	1.4948E-01	3.3020E-01	ENSG00000135245
HINFP	0.0558	4.1835E-01	6.2151E-01	ENSG00000172273
HINT1	-0.0779	3.0650E-01	5.1674E-01	ENSG00000169567
HINT2	-0.0480	5.7514E-01	7.4788E-01	ENSG00000137133
HINT3	-0.1169	1.2742E-01	2.9887E-01	ENSG00000111911
HIP1	-0.3173	1.1436E-05	2.7240E-04	ENSG00000127946
HIP1R	0.1241	2.5893E-02	1.0005E-01	ENSG00000130787
HIPK1	-0.0322	6.0979E-01	7.7246E-01	ENSG00000163349
HIPK1-AS1	0.0934	2.9524E-01	5.0535E-01	ENSG00000235527
HIPK2	0.1546	6.6713E-02	1.9442E-01	ENSG00000064393
HIPK3	0.0488	4.8724E-01	6.8055E-01	ENSG00000110422
HIPK4	-0.0294	4.6424E-01		ENSG00000160396
HIRA	-0.0641	4.3135E-01	6.3292E-01	ENSG00000100084
HIRIP3	-0.1005	1.2829E-01	2.9994E-01	ENSG00000149929
HIVEP1	0.0804	2.5264E-01	4.6058E-01	ENSG00000095951
HIVEP2	0.3908	8.5767E-05	1.3659E-03	ENSG00000010818
HIVEP3	-0.0497	5.4499E-01	7.2555E-01	ENSG00000127124
HJURP	-0.1621	8.4993E-02	2.2815E-01	ENSG00000123485
HJV	0.0431	1.1882E-01		ENSG00000168509
HK1	0.0546	4.8666E-01	6.8026E-01	ENSG00000156515
HK3	-0.0133	5.2482E-01		ENSG00000160883

HKDC1	-0.0053	9.4795E-01	9.7436E-01	ENSG00000156510
HLA-A	-0.1944	4.0423E-02	1.3779E-01	ENSG00000206503
HLA-B	-0.1143	1.8356E-01	3.7690E-01	ENSG00000234745
HLA-C	-0.1114	1.8446E-01	3.7795E-01	ENSG00000204525
HLA-DMA	0.1991	4.7082E-02	1.5328E-01	ENSG00000204257
HLA-DMB	0.0095	9.0203E-01	9.5204E-01	ENSG00000242574
HLA-DOA	0.1054	1.2512E-01	2.9524E-01	ENSG00000204252
HLA-DOB	0.1189	7.3397E-02	2.0755E-01	ENSG00000241106
HLA-DPA1	0.0319	6.1595E-01	7.7721E-01	ENSG00000231389
HLA-DPB1	0.0597	4.8876E-01	6.8194E-01	ENSG00000223865
HLA-DPB2	0.4127	1.6121E-02	7.1455E-02	ENSG00000224557
HLA-DQA1	0.0215	3.6158E-01		ENSG00000237541
HLA-DQB1	0.0483	2.5952E-01	4.6792E-01	ENSG00000179344
HLA-DQB2	0.0194	7.0090E-01		ENSG00000232629
HLA-DRA	0.0458	4.1829E-01	6.2150E-01	ENSG00000204287
HLA-DRB1	0.0340	6.9281E-01	8.2964E-01	ENSG00000196126
HLA-DRB5	0.0021	3.0096E-01	5.1085E-01	ENSG00000198502
HLA-E	0.0391	6.3916E-01	7.9381E-01	ENSG00000204592
HLA-F	-0.0970	2.5197E-01	4.6024E-01	ENSG00000204642
HLA-F-AS1	0.0005	9.9765E-01	9.9862E-01	ENSG00000214922
HLA-G	0.0064	9.2664E-01	9.6410E-01	ENSG00000204632
HLA-K	-0.0355	6.4237E-01	7.9561E-01	ENSG00000230795
HLA-Z	0.0085	8.3018E-01		ENSG00000235301
HLCS	0.1230	9.2114E-02	2.4167E-01	ENSG00000159267
HLF	-0.0622	4.2594E-01	6.2823E-01	ENSG00000108924
HLTF	-0.0030	9.4403E-01	9.7218E-01	ENSG00000071794
HLX	-0.0909	7.2459E-02	2.0568E-01	ENSG00000136630
HLX-AS1	-0.0186	2.6461E-01		ENSG00000257551
HM13	0.0731	2.5089E-01	4.5914E-01	ENSG00000101294
HM13-AS1	0.0492	2.5470E-01		ENSG00000230613
HMBOX1	-0.0061	9.3270E-01	9.6664E-01	ENSG00000147421
HMBS	-0.0115	7.8537E-01	8.8655E-01	ENSG00000256269
HMCES	-0.1469	8.7535E-03	4.5992E-02	ENSG00000183624
HMCN1	-0.0853	3.1862E-01	5.2888E-01	ENSG00000143341
HMCN2	0.1523	1.1031E-01	2.7171E-01	ENSG00000148357
HMG20A	-0.0532	4.6224E-01	6.5926E-01	ENSG00000140382
HMG20B	-0.2089	5.3281E-03	3.1651E-02	ENSG00000064961
HMGA1	-0.3188	2.5330E-05	5.1764E-04	ENSG00000137309
HMGA2	-0.1756	9.0260E-02	2.3846E-01	ENSG00000149948
HMGA2-AS1	-0.0040	9.5664E-01	9.7852E-01	ENSG00000197301
HMGB1	-0.4042	5.7071E-06	1.5603E-04	ENSG00000189403
HMGB1P11	0.0090	9.9247E-01		ENSG00000230547
HMGB1P13	0.0036	9.2211E-01		ENSG00000220557
HMGB1P19	0.0469	1.7267E-01		ENSG00000253463
HMGB1P31	0.0949	2.8275E-01	4.9317E-01	ENSG00000233266
HMGB1P33	0.0004	8.8043E-01		ENSG00000261174
HMGB1P39	0.0477	1.7924E-01		ENSG00000203489
HMGB1P49	0.0084	8.1863E-01		ENSG00000230519
HMGB1P5	-0.3515	3.0545E-04	3.6449E-03	ENSG00000132967

HMGB1P7	-0.0233	4.1326E-01		ENSG00000231148
HMGB2	-0.3683	8.1015E-04	7.7382E-03	ENSG00000164104
HMGB3	-0.2557	4.1790E-04	4.6213E-03	ENSG00000029993
HMGB3P10	0.0052	9.3836E-01		ENSG00000223656
HMGB3P18	0.0178	3.8042E-01		ENSG00000220184
HMGB3P29	-0.0320	3.7316E-01		ENSG00000254503
HMGB3P3	0.0341	4.9890E-01		ENSG00000250730
HMGB3P4	0.0115	5.0956E-01		ENSG00000228808
HMGB3P6	-0.0056	6.9054E-01		ENSG00000213070
HMGCL	0.2175	1.0377E-02	5.2074E-02	ENSG00000117305
HMGCLL1	0.0138	8.6361E-01	9.3129E-01	ENSG00000146151
HMGCR	0.0312	6.4149E-01	7.9496E-01	ENSG00000113161
HMGCS1	-0.3870	1.4644E-03	1.2075E-02	ENSG00000112972
HMGCS2	0.0574	4.2057E-01	6.2354E-01	ENSG00000134240
HMGN1	-0.4681	2.1964E-13	1.0596E-10	ENSG00000205581
HMGN1P10	0.0026	5.5256E-01		ENSG00000244101
HMGN1P13	0.0098	8.1218E-01		ENSG00000249619
HMGN1P15	0.0101	8.4946E-01	9.2331E-01	ENSG00000250197
HMGN1P8	-0.0073	8.7421E-01		ENSG00000241120
HMGN3	-0.3307	1.3596E-04	1.9282E-03	ENSG00000118418
HMGN3-AS1	-0.3955	8.8972E-04	8.3017E-03	ENSG00000270362
HMGN4	-0.1454	1.0625E-02	5.2987E-02	ENSG00000182952
HMGN5	0.1159	1.5717E-01	3.4094E-01	ENSG00000198157
HMGXB3	0.2707	1.2222E-04	1.7931E-03	ENSG00000113716
HMGXB4	0.0007	9.5457E-01	9.7709E-01	ENSG00000100281
HMMR	-0.1438	1.3766E-01	3.1335E-01	ENSG00000072571
HMOX1	-0.0508	1.8639E-01	3.8066E-01	ENSG00000100292
HMOX2	-0.0238	6.8909E-01	8.2715E-01	ENSG00000103415
HMSD	-0.0911	2.4943E-01	4.5726E-01	ENSG00000221887
HMX2	-0.0027	6.9688E-01		ENSG00000188816
HMX3	-0.0270	4.0277E-01		ENSG00000188620
HNF1A	0.0025	8.5314E-01	9.2531E-01	ENSG00000135100
HNF1A-AS1	0.0035	9.7668E-01		ENSG00000241388
HNF1B	0.1021	1.9495E-01	3.9177E-01	ENSG00000275410
HNF4A	0.0173	5.9651E-01		ENSG00000101076
HNF4G	0.0212	8.4493E-01		ENSG00000164749
HNMT	0.1539	1.0144E-01	2.5718E-01	ENSG00000150540
HNRNPA0	-0.3919	3.6048E-07	1.6253E-05	ENSG00000177733
HNRNPA1	-0.3748	1.1423E-06	4.1574E-05	ENSG00000135486
HNRNPA1L3	-0.3083	1.1878E-03	1.0327E-02	ENSG00000224578
HNRNPA1P21	-0.0110	4.5272E-01		ENSG00000228168
HNRNPA1P27	-0.0593	2.0420E-01		ENSG00000233680
HNRNPA1P33	-0.0002	9.0748E-01		ENSG00000213412
HNRNPA1P49	0.0117	4.9420E-01		ENSG00000233231
HNRNPA2B1	-0.5004	1.5989E-10	2.3988E-08	ENSG00000122566
HNRNPA3	-0.0935	1.3030E-01	3.0308E-01	ENSG00000170144
HNRNPA3P10	0.0187	5.6253E-01		ENSG00000257851
HNRNPA3P11	0.0147	7.2543E-01		ENSG00000260689
HNRNPA3P12	0.0000	9.4060E-01		ENSG00000219102

HNRNPA3P3	-0.0132	7.3679E-01	8.5634E-01	ENSG00000214653
HNRNPA3P5	-0.0202	5.4529E-01		ENSG00000236565
HNRNPA3P7	-0.0175	7.9410E-01	8.9120E-01	ENSG00000253119
HNRNPA3P9	-0.0463	3.9370E-01	6.0014E-01	ENSG00000270903
HNRNPAB	-0.1219	5.7591E-02	1.7596E-01	ENSG00000197451
HNRNPCP3	-0.0121	4.6627E-01		ENSG00000259419
HNRNPCP6	-0.0464	4.2123E-01	6.2402E-01	ENSG00000213305
HNRNPCP7	-0.0240	5.7738E-01		ENSG00000228653
HNRNPCP8	-0.0077	7.4919E-01		ENSG00000254748
HNRNPD	-0.3212	7.5275E-07	2.9540E-05	ENSG00000138668
HNRNPD-DT	0.1198	1.4677E-01	3.2639E-01	ENSG00000272677
HNRNPDL	-0.2826	1.6811E-04	2.2676E-03	ENSG00000152795
HNRNPF	-0.1689	8.9013E-03	4.6639E-02	ENSG00000169813
HNRNPH1	-0.3203	9.1282E-09	7.6917E-07	ENSG00000169045
HNRNPH2	-0.3208	1.8388E-06	6.1978E-05	ENSG00000126945
HNRNPH3	-0.5102	2.9667E-08	2.0390E-06	ENSG00000096746
HNRNPK	-0.4776	1.3693E-10	2.1597E-08	ENSG00000165119
HNRNPKP1	-0.0340	3.7781E-01		ENSG00000250859
HNRNPKP2	-0.0454	4.5546E-01	6.5383E-01	ENSG00000227347
HNRNPKP3	0.0600	2.6689E-01	4.7617E-01	ENSG00000251557
HNRNPKP4	-0.2158	4.7590E-02	1.5442E-01	ENSG00000243547
HNRNPL	-0.3399	8.5776E-06	2.1624E-04	ENSG00000104824
HNRNPLL	0.0468	4.2900E-01	6.3074E-01	ENSG00000143889
HNRNPM	-0.2821	6.7182E-05	1.1307E-03	ENSG00000099783
HNRNPMP1	-0.0228	6.2414E-01	7.8300E-01	ENSG00000259335
HNRNPR	-0.3413	2.1338E-08	1.5672E-06	ENSG00000125944
HNRNPRP2	0.0004	9.3733E-01		ENSG00000255047
HNRNPU	-0.2517	2.6125E-04	3.2293E-03	ENSG00000153187
HNRNPUL1	-0.1239	6.3098E-02	1.8689E-01	ENSG00000105323
HNRNPUL2	0.1730	6.6910E-02	1.9483E-01	ENSG00000214753
HOATZ	0.0622	2.1486E-02		ENSG00000183644
HOGA1	0.0180	9.7790E-01	9.8899E-01	ENSG00000241935
HOMER1	0.2788	5.4284E-05	9.4862E-04	ENSG00000152413
HOMER2	-0.0185	7.9355E-01	8.9083E-01	ENSG00000103942
HOMER3	-0.1143	1.0716E-01	2.6640E-01	ENSG00000051128
HOOK1	0.1382	8.1847E-02	2.2263E-01	ENSG00000134709
HOOK2	-0.0272	6.9698E-01	8.3245E-01	ENSG00000095066
HOOK3	0.0371	5.4769E-01	7.2733E-01	ENSG00000168172
HOPX	0.0330	6.5779E-01	8.0675E-01	ENSG00000171476
HORMAD1	0.0129	4.8713E-01		ENSG00000143452
HORMAD2	0.0534	3.5778E-01	5.6754E-01	ENSG00000176635
HORMAD2-AS1	-0.0073	8.7983E-01	9.3992E-01	ENSG00000227117
HOTAIRM1	-0.0510	4.2727E-01	6.2921E-01	ENSG00000233429
HOXA-AS3	-0.0245	5.7039E-01	7.4422E-01	ENSG00000254369
HOXA1	-0.0178	3.3541E-01		ENSG00000105991
HOXA10	-0.0279	3.8081E-01		ENSG00000253293
HOXA13	0.0044	8.7933E-01		ENSG00000106031
HOXA2	-0.0124	2.2669E-01		ENSG00000105996
HOXA3	0.0007	9.1460E-01		ENSG00000105997

HOXA4	-0.0092	8.0990E-01		ENSG00000197576
HOXA5	-0.0280	6.6826E-01	8.1415E-01	ENSG00000106004
HOXA6	-0.0989	5.9988E-02	1.8029E-01	ENSG00000106006
HOXA7	-0.4962	3.1113E-03	2.1086E-02	ENSG00000122592
HOXA9	-0.0093	6.7006E-01		ENSG00000078399
HOXB-AS1	-0.0204	4.6635E-01		ENSG00000230148
HOXB13	-0.0695	1.9992E-01	3.9831E-01	ENSG00000159184
HOXB2	-0.2678	2.5917E-02	1.0011E-01	ENSG00000173917
HOXB3	0.0010	7.4654E-01		ENSG00000120093
HOXB7	0.0558	6.2062E-01	7.8065E-01	ENSG00000260027
HOXB9	0.0143	4.6658E-01		ENSG00000170689
HOXC-AS1	-0.0418	1.7000E-01		ENSG00000250451
HOXC10	-0.2496	2.7125E-02	1.0341E-01	ENSG00000180818
HOXC13	-0.1072	2.1266E-01	4.1365E-01	ENSG00000123364
HOXC13-AS	-0.0102	5.8303E-01		ENSG00000249641
HOXC4	-0.0120	4.8514E-01		ENSG00000198353
HOXC6	-0.0057	4.7517E-01		ENSG00000197757
HOXC8	0.0436	2.2120E-01		ENSG00000037965
HOXC9	-0.4880	8.6296E-03	4.5535E-02	ENSG00000180806
HOXD10	-0.0004	8.5546E-01		ENSG00000128710
HOXD13	0.0291	6.2105E-01		ENSG00000128714
HOXD3	-0.0424	2.6347E-01		ENSG00000128652
HOXD4	-0.0008	6.4286E-01		ENSG00000170166
HOXD8	-0.0291	6.8562E-01	8.2489E-01	ENSG00000175879
HOXD9	-0.2037	3.5194E-02	1.2454E-01	ENSG00000128709
HP	-0.0228	7.9706E-01	8.9291E-01	ENSG00000257017
HP1BP3	-0.1865	1.0948E-03	9.7036E-03	ENSG00000127483
HPAT5	0.1557	2.1006E-02	8.5924E-02	ENSG00000280707
HPCA	-0.4458	1.3021E-04	1.8824E-03	ENSG00000121905
HPCAL1	-0.5108	1.0605E-10	1.7751E-08	ENSG00000115756
HPCAL4	0.0478	5.4822E-01	7.2775E-01	ENSG00000116983
HPD	0.0832	2.1215E-01	4.1310E-01	ENSG00000158104
HPDL	0.2162	5.4926E-02	1.7046E-01	ENSG00000186603
HPF1	0.0011	9.8261E-01	9.9071E-01	ENSG00000056050
HPGD	0.2592	3.2518E-02	1.1761E-01	ENSG00000164120
HPN	0.0709	8.3975E-02		ENSG00000105707
HPN-AS1	0.0002	8.9700E-01		ENSG00000227392
HPR	0.1111	1.1131E-01	2.7320E-01	ENSG00000261701
HPRT1	-0.0718	2.3663E-01	4.4242E-01	ENSG00000165704
HPS1	0.0044	9.5297E-01	9.7681E-01	ENSG00000107521
HPS1-AS1	0.0695	4.0373E-01	6.0824E-01	ENSG00000287261
HPS3	-0.0225	7.0425E-01	8.3684E-01	ENSG00000163755
HPS4	0.0622	2.7363E-01	4.8336E-01	ENSG00000100099
HPS5	0.0457	5.8113E-01	7.5236E-01	ENSG00000110756
HPS6	-0.1623	7.9208E-02	2.1820E-01	ENSG00000166189
HPSE	-0.0716	1.6699E-01	3.5498E-01	ENSG00000173083
HPX	0.0537	5.1803E-01	7.0542E-01	ENSG00000110169
HR	0.0919	2.6766E-01	4.7706E-01	ENSG00000168453
HRAS	-0.0518	4.7888E-01	6.7416E-01	ENSG00000174775

HRAT17	-0.0048	8.1917E-01		ENSG00000234520
HRC	0.3459	2.0144E-02	8.3469E-02	ENSG00000130528
HRCT1	0.0050	7.5982E-01		ENSG00000196196
HRG	0.0153	5.6650E-01		ENSG00000113905
HRH2	-0.0534	5.1819E-01	7.0548E-01	ENSG00000113749
HRH3	-0.2471	1.1424E-02	5.5933E-02	ENSG00000101180
HRH4	-0.0175	3.8173E-01		ENSG00000134489
HRK	-0.5833	6.8587E-08	4.2084E-06	ENSG00000135116
HRNR	0.0081	9.4264E-01		ENSG00000197915
HROB	-0.0787	3.0504E-01	5.1535E-01	ENSG00000125319
HS1BP3	-0.0921	1.8974E-01	3.8487E-01	ENSG00000118960
HS2ST1	-0.0384	4.9452E-01	6.8662E-01	ENSG00000153936
HS3ST1	0.0418	6.2221E-01	7.8162E-01	ENSG00000002587
HS3ST2	-0.0010	9.8777E-01	9.9334E-01	ENSG00000122254
HS3ST3A1	0.1008	2.2707E-01	4.3130E-01	ENSG00000153976
HS3ST3B1	0.4354	6.5311E-03	3.6840E-02	ENSG00000125430
HS3ST4	-0.1532	1.2302E-01	2.9223E-01	ENSG00000182601
HS3ST6	0.0738	2.6266E-01	4.7152E-01	ENSG00000162040
HS6ST1	-0.1163	1.4377E-01	3.2181E-01	ENSG00000136720
HS6ST2	-0.0472	5.2793E-01	7.1270E-01	ENSG00000171004
HS6ST2-AS1	0.0039	9.3040E-01		ENSG00000235849
HS6ST3	0.0683	3.9650E-01	6.0248E-01	ENSG00000185352
HSBP1	-0.2264	1.2026E-05	2.8343E-04	ENSG00000230989
HSBP1L1	-0.1827	2.8374E-02	1.0687E-01	ENSG00000226742
HSCB	-0.1456	5.4596E-02	1.6966E-01	ENSG00000100209
HSD11B1	-0.0180	6.9404E-01	8.3042E-01	ENSG00000117594
HSD11B1-AS1	0.0461	2.9158E-01	5.0185E-01	ENSG00000227591
HSD11B1L	0.0594	3.0421E-01	5.1436E-01	ENSG00000167733
HSD11B2	-0.0416	6.1284E-01	7.7479E-01	ENSG00000176387
HSD17B1	0.0492	5.1076E-01	6.9928E-01	ENSG00000108786
HSD17B1-AS1	0.0967	2.1903E-01	4.2144E-01	ENSG00000266962
HSD17B10	-0.1106	6.9789E-02	2.0044E-01	ENSG00000072506
HSD17B11	-0.0445	5.6458E-01	7.4050E-01	ENSG00000198189
HSD17B12	0.0167	8.0137E-01	8.9522E-01	ENSG00000149084
HSD17B13	-0.0174	7.3496E-01		ENSG00000170509
HSD17B14	-0.1686	7.1525E-02	2.0388E-01	ENSG00000087076
HSD17B2	0.0406	3.3448E-01	5.4460E-01	ENSG00000086696
HSD17B4	-0.1933	2.5769E-03	1.8163E-02	ENSG00000133835
HSD17B6	0.0041	9.6757E-01	9.8389E-01	ENSG00000025423
HSD17B7	0.0202	8.3059E-01	9.1239E-01	ENSG00000132196
HSD17B8	0.1623	4.3485E-02	1.4492E-01	ENSG00000204228
HSD3B7	0.2085	5.0642E-02	1.6097E-01	ENSG00000099377
HSDL1	-0.0398	5.9652E-01	7.6315E-01	ENSG00000103160
HSDL2	-0.1708	2.9421E-02	1.0950E-01	ENSG00000119471
HSF1	-0.4272	9.2730E-10	1.0418E-07	ENSG00000185122
HSF2	-0.0724	1.7212E-01	3.6157E-01	ENSG00000025156
HSF2BP	0.3185	1.5841E-02	7.0676E-02	ENSG00000160207
HSF4	-0.0815	3.4129E-01	5.5109E-01	ENSG00000102878
HSF5	0.0361	1.1549E-01		ENSG00000176160

HSFX1	0.1801	8.0471E-02	2.2055E-01	ENSG00000171116
HSFX2	0.2491	4.2505E-02	1.4258E-01	ENSG00000268738
HSFX3	0.0131	6.3079E-01		ENSG00000283697
HSFX4	0.0274	3.3132E-01		ENSG00000283463
HSH2D	0.0261	4.2193E-01		ENSG00000196684
HSP90AA1	-0.3587	2.8151E-04	3.4239E-03	ENSG00000080824
HSP90AA6P	0.0330	4.1092E-01		ENSG00000181359
HSP90AB1	-0.4828	5.1882E-12	1.5000E-09	ENSG00000096384
HSP90AB3P	-0.1291	1.6983E-01	3.5838E-01	ENSG00000183199
HSP90B1	-0.3928	4.1177E-07	1.8255E-05	ENSG00000166598
HSP90B2P	-0.0064	9.1647E-01	9.5863E-01	ENSG00000259706
HSPA12A	-0.0195	7.3401E-01	8.5476E-01	ENSG00000165868
HSPA12B	0.0911	2.8821E-01	4.9873E-01	ENSG00000132622
HSPA13	-0.0377	5.9624E-01	7.6296E-01	ENSG00000155304
HSPA14	-0.3448	1.0310E-04	1.5841E-03	ENSG00000187522
HSPA1A	-0.0452	4.0030E-01	6.0563E-01	ENSG00000204389
HSPA1B	-0.0516	3.5806E-01	5.6773E-01	ENSG00000204388
HSPA1L	0.0637	4.4819E-01	6.4746E-01	ENSG00000204390
HSPA2	-0.0949	2.3270E-01	4.3780E-01	ENSG00000126803
HSPA2-AS1	0.0199	5.9477E-01		ENSG00000259116
HSPA4	-0.1243	4.3646E-02	1.4524E-01	ENSG00000170606
HSPA4L	-0.0054	9.4226E-01	9.7144E-01	ENSG00000164070
HSPA5P1	-0.0614	2.8939E-01	4.9983E-01	ENSG00000215895
HSPA6	-0.0192	5.7692E-01	7.4898E-01	ENSG00000173110
HSPA7	0.0118	7.5090E-01	8.6466E-01	ENSG00000225217
HSPA8	-0.4490	1.4691E-07	7.8238E-06	ENSG00000109971
HSPA8P1	-0.0390	2.8023E-01	4.9022E-01	ENSG00000234176
HSPA8P15	0.0580	2.5235E-01	4.6045E-01	ENSG00000219395
HSPA8P3	-0.0063	7.0981E-01	8.4055E-01	ENSG00000234788
HSPA8P4	-0.0096	5.7122E-01		ENSG00000248610
HSPA8P7	-0.0157	2.5215E-01		ENSG00000224773
HSPA9	-0.1337	5.0975E-02	1.6153E-01	ENSG00000113013
HSPB3	0.0543	5.2705E-01	7.1214E-01	ENSG00000169271
HSPB6	0.0623	4.4010E-01	6.4009E-01	ENSG00000004776
HSPB7	1.8409	1.4173E-03		ENSG00000173641
HSPB8	0.1223	1.7840E-01	3.6962E-01	ENSG00000152137
HSPB9	0.0030	7.3452E-01		ENSG00000260325
HSPBAP1	0.0513	5.2787E-01	7.1270E-01	ENSG00000169087
HSPBP1	-0.0210	6.9452E-01	8.3067E-01	ENSG00000133265
HSPC324	0.0100	7.0833E-01		ENSG00000228401
HSPD1	-0.2863	5.9744E-04	6.1148E-03	ENSG00000144381
HSPD1P10	-0.0182	7.2292E-01		ENSG00000216990
HSPD1P12	-0.0231	6.9292E-01		ENSG00000255964
HSPD1P5	-0.0119	6.3132E-01		ENSG00000249193
HSPD1P7	-0.0028	7.2350E-01		ENSG00000215005
HSPE1	-0.3460	4.3940E-05	8.0323E-04	ENSG00000115541
HSPE1-MOB4	-0.0001	9.1004E-01		ENSG00000270757
HSPE1P26	0.0120	7.5064E-01		ENSG00000220867
HSPE1P28	-0.0232	4.0865E-01		ENSG00000230081

HSPG2	0.3736	6.2437E-03	3.5554E-02	ENSG00000142798
HSPH1	-0.2111	5.9253E-02	1.7892E-01	ENSG00000120694
HTATIP2	-0.1026	2.5743E-01	4.6592E-01	ENSG00000109854
HTATSF1	-0.0276	6.1201E-01	7.7417E-01	ENSG00000102241
HTD2	-0.0238	6.3418E-01		ENSG00000255154
HTN1	0.1201	1.9873E-03	1.5004E-02	ENSG00000126550
HTN3	0.0267	1.3246E-01		ENSG00000205649
HTR1A	0.0576	1.4768E-01		ENSG00000178394
HTR1B	0.0002	7.7797E-01		ENSG00000135312
HTR1D	-0.1366	1.1543E-02	5.6328E-02	ENSG00000179546
HTR1F	0.0186	9.6890E-01	9.8475E-01	ENSG00000179097
HTR2A	-0.0726	4.0947E-01	6.1373E-01	ENSG00000102468
HTR2B	-0.0359	5.9821E-01	7.6422E-01	ENSG00000135914
HTR2C	0.4996	8.8514E-03	4.6417E-02	ENSG00000147246
HTR3A	0.1355	5.1518E-02	1.6271E-01	ENSG00000166736
HTR4	-0.0031	5.2842E-01		ENSG00000164270
HTR5A	0.0200	7.3915E-01	8.5776E-01	ENSG00000157219
HTR5A-AS1	0.0608	2.7126E-01	4.8048E-01	ENSG00000220575
HTR5BP	0.0302	5.9933E-02		ENSG00000125631
HTR6	-0.0797	3.5246E-01	5.6281E-01	ENSG00000158748
HTR7	-0.0486	3.9454E-01	6.0080E-01	ENSG00000148680
HTR7P1	0.0924	2.9459E-01	5.0458E-01	ENSG00000183935
HTRA1	0.0951	2.6486E-01	4.7403E-01	ENSG00000166033
HTRA2	-0.3259	2.4161E-05	4.9832E-04	ENSG00000115317
HTRA3	0.0093	8.7158E-01	9.3533E-01	ENSG00000170801
HTRA4	-0.0061	5.3673E-01		ENSG00000169495
HTT	0.2137	5.4430E-03	3.2109E-02	ENSG00000197386
HUNK	0.0176	8.2233E-01	9.0802E-01	ENSG00000142149
HUS1	-0.0846	1.5030E-01	3.3137E-01	ENSG00000136273
HUS1B	-0.0963	2.7913E-01	4.8918E-01	ENSG00000188996
HUWE1	0.1132	1.2039E-01	2.8803E-01	ENSG00000086758
HVCN1	-0.1835	8.0003E-02	2.1959E-01	ENSG00000122986
HYAL1	0.0256	7.5386E-01	8.6630E-01	ENSG00000114378
HYAL2	0.0643	2.7014E-01	4.7977E-01	ENSG00000068001
HYAL3	-0.0958	2.3751E-01	4.4347E-01	ENSG00000186792
HYAL4	0.0205	5.6089E-01		ENSG00000106302
HYCC1	-0.1105	1.3841E-01	3.1433E-01	ENSG00000122591
HYCC2	0.0485	4.7277E-01	6.6887E-01	ENSG00000155744
HYDIN	0.1369	1.2651E-01	2.9765E-01	ENSG00000157423
HYDINP1	0.0037	9.9911E-01		ENSG00000242586
HYI-AS1	0.1232	1.7947E-02	7.6946E-02	ENSG00000229348
HYKK	-0.1336	1.6360E-01	3.5018E-01	ENSG00000188266
HYLS1	0.0614	3.6298E-01	5.7206E-01	ENSG00000198331
HYMAI	0.3690	9.0170E-03	4.7055E-02	ENSG00000283122
HYOU1	-0.1872	2.8392E-02	1.0687E-01	ENSG00000149428
HYPK	0.0125	8.6455E-01	9.3172E-01	ENSG00000242028
IAH1	-0.0520	4.7274E-01	6.6887E-01	ENSG00000134330
IAPP	0.1733	8.3944E-02	2.2638E-01	ENSG00000121351
IARS1	0.0901	1.2536E-01	2.9555E-01	ENSG00000196305

IARS2	0.0640	2.9414E-01	5.0400E-01	ENSG00000067704
IBA57	0.2634	6.5178E-03	3.6790E-02	ENSG00000181873
IBA57-DT	0.0362	6.5586E-01	8.0558E-01	ENSG00000203684
IBSP	-0.0687	5.4408E-02	1.6916E-01	ENSG00000029559
IBTK	0.0744	1.5063E-01	3.3180E-01	ENSG00000005700
ICA1	0.0775	2.9563E-01	5.0570E-01	ENSG00000003147
ICA1L	0.1899	9.5886E-03	4.9200E-02	ENSG00000163596
ICAM1	-0.0118	8.3933E-01	9.1737E-01	ENSG00000090339
ICAM3	0.0488	3.5433E-02		ENSG00000076662
ICAM5	-0.0023	9.8014E-01	9.8991E-01	ENSG00000105376
ICE1	0.1929	5.8434E-03	3.3870E-02	ENSG00000164151
ICE2	0.0146	7.9589E-01	8.9208E-01	ENSG00000128915
ICMT	-0.0542	4.2590E-01	6.2822E-01	ENSG00000116237
ICOS	-0.0026	7.2281E-01		ENSG00000163600
ICOSLG	-0.1589	1.0810E-01	2.6798E-01	ENSG00000160223
ID1	0.2341	4.3076E-02	1.4393E-01	ENSG00000125968
ID2	-0.1501	1.1885E-01	2.8546E-01	ENSG00000115738
ID4	0.0811	3.4895E-01	5.5875E-01	ENSG00000172201
IDE	0.2827	1.5167E-03	1.2337E-02	ENSG00000119912
IDH1	-0.0723	3.2277E-01	5.3288E-01	ENSG00000138413
IDH1-AS1	0.0468	5.8200E-01	7.5306E-01	ENSG00000231908
IDH2	-0.1184	6.6557E-02	1.9413E-01	ENSG00000182054
IDH3A	-0.0163	7.9853E-01	8.9385E-01	ENSG00000166411
IDH3B	-0.0904	1.0775E-01	2.6732E-01	ENSG00000101365
IDH3G	-0.2537	6.4581E-05	1.0944E-03	ENSG00000067829
IDI1	-0.0027	9.6056E-01	9.8030E-01	ENSG00000067064
IDI2	0.0062	9.4899E-01		ENSG00000148377
IDI2-AS1	0.0155	8.4545E-01	9.2087E-01	ENSG00000232656
IDNK	0.3654	1.5816E-03	1.2722E-02	ENSG00000148057
IDS	0.0863	2.1902E-01	4.2144E-01	ENSG00000010404
IDUA	-0.0158	8.3561E-01	9.1524E-01	ENSG00000127415
IER2	-0.0474	5.7306E-01	7.4620E-01	ENSG00000160888
IER3	-0.3389	3.0448E-03	2.0708E-02	ENSG00000137331
IER3-AS1	0.0686	3.9561E-01	6.0182E-01	ENSG00000272273
IER3IP1	-0.1726	2.2756E-03	1.6590E-02	ENSG00000134049
IER5	-0.5115	3.4838E-06	1.0479E-04	ENSG00000162783
IER5L	-0.5042	5.7895E-07	2.3941E-05	ENSG00000188483
IFFO1	0.0348	6.6303E-01	8.1040E-01	ENSG00000010295
IFFO2	-0.4782	7.5659E-06	1.9525E-04	ENSG00000169991
IFI16	-0.0123	8.7885E-01	9.3956E-01	ENSG00000163565
IFI27	0.3398	1.0993E-03	9.7355E-03	ENSG00000165949
IFI27L1	0.2105	2.3057E-03	1.6775E-02	ENSG00000165948
IFI27L2	-0.1264	5.8866E-02	1.7822E-01	ENSG00000119632
IFI30	-0.1139	9.8646E-02	2.5252E-01	ENSG00000216490
IFI35	-0.3009	1.9783E-02	8.2377E-02	ENSG00000068079
IFI44	-0.3874	3.9927E-04	4.4716E-03	ENSG00000137965
IFI44L	0.0044	9.5851E-01	9.7936E-01	ENSG00000137959
IFI6	-0.1359	2.7600E-02	1.0470E-01	ENSG00000126709
IFIH1	0.0253	7.4683E-01	8.6239E-01	ENSG00000115267

IFIT1	-0.0539	5.2945E-01	7.1398E-01	ENSG00000185745
IFIT1B	-0.0060	7.3922E-01		ENSG00000204010
IFIT2	0.0907	3.0048E-01	5.1024E-01	ENSG00000119922
IFIT3	0.0564	4.9073E-01	6.8337E-01	ENSG00000119917
IFIT5	-0.1390	3.7798E-02	1.3141E-01	ENSG00000152778
IFITM10	0.3222	1.9867E-02	8.2630E-02	ENSG00000244242
IFITM4P	-0.0120	5.5264E-01		ENSG00000235821
IFNA21	0.0131	4.3075E-01		ENSG00000137080
IFNAR1	-0.2012	2.1403E-04	2.7443E-03	ENSG00000142166
IFNAR2	-0.1960	4.9101E-02	1.5760E-01	ENSG00000159110
IFNG	0.0004	9.6781E-01		ENSG00000111537
IFNG-AS1	-0.0241	2.5336E-01		ENSG00000255733
IFNGR1	0.2751	2.7661E-04	3.3743E-03	ENSG00000027697
IFNGR2	-0.2910	2.5710E-07	1.2502E-05	ENSG00000159128
IFNK	-0.0180	1.7341E-01		ENSG00000147896
IFNL1	-0.0043	7.7846E-01		ENSG00000182393
IFNL2	-0.0026	5.1073E-01		ENSG00000183709
IFNL3	0.0181	5.8938E-01		ENSG00000197110
IFNL4	-0.0052	8.3651E-01		ENSG00000272395
IFNLR1	-0.0278	7.3934E-01	8.5776E-01	ENSG00000185436
IFRD1	-0.4615	9.1432E-06	2.2770E-04	ENSG00000006652
IFRD2	-0.0043	9.4339E-01	9.7195E-01	ENSG00000214706
IFT122	0.2789	1.6127E-04	2.1933E-03	ENSG00000163913
IFT140	0.0926	2.5100E-01	4.5927E-01	ENSG00000187535
IFT172	0.3029	7.5789E-04	7.3636E-03	ENSG00000138002
IFT20	0.1313	3.0487E-02	1.1250E-01	ENSG00000109083
IFT22	0.0854	1.1541E-01	2.7998E-01	ENSG00000128581
IFT25	0.0555	3.8501E-01	5.9292E-01	ENSG00000081870
IFT27	0.0430	5.2059E-01	7.0753E-01	ENSG00000100360
IFT43	0.0840	1.3056E-01	3.0344E-01	ENSG00000119650
IFT46	-0.2024	1.5006E-03	1.2263E-02	ENSG00000118096
IFT52	0.0440	3.4728E-01	5.5760E-01	ENSG00000101052
IFT56	0.3009	5.8439E-04	5.9987E-03	ENSG00000105948
IFT57	0.2127	1.0679E-03	9.5442E-03	ENSG00000114446
IFT70A	0.2768	1.2093E-02	5.8285E-02	ENSG00000197557
IFT70B	0.1827	6.7520E-02	1.9593E-01	ENSG00000196659
IFT74	0.2608	3.1616E-04	3.7454E-03	ENSG00000096872
IFT74-AS1	0.0913	1.0551E-01		ENSG00000234676
IFT80	0.0774	3.3334E-01	5.4349E-01	ENSG00000068885
IFT81	-0.0155	7.5215E-01	8.6560E-01	ENSG00000122970
IFT88	0.0656	2.8332E-01	4.9374E-01	ENSG00000032742
IFTAP	0.0460	4.6345E-01	6.6059E-01	ENSG00000166352
IGBP1	-0.1497	3.2107E-03	2.1567E-02	ENSG00000089289
IGDCC3	-0.0364	6.4157E-01	7.9501E-01	ENSG00000174498
IGDCC4	0.1416	1.2518E-01	2.9533E-01	ENSG00000103742
IGF1	0.1173	1.0055E-01	2.5564E-01	ENSG00000017427
IGF1R	0.1555	5.0218E-02	1.6011E-01	ENSG00000140443
IGF2	0.0940	2.8991E-01	5.0035E-01	ENSG00000167244
IGF2-AS	-0.0203	4.8347E-01	6.7759E-01	ENSG00000099869

IGF2BP1	-0.1200	1.0476E-01	2.6264E-01	ENSG00000159217
IGF2BP2	0.2195	9.1873E-03	4.7654E-02	ENSG00000073792
IGF2BP2-AS1	-0.2568	2.4385E-02	9.5910E-02	ENSG00000163915
IGF2BP3	-0.0307	6.8025E-01	8.2115E-01	ENSG00000136231
IGF2R	0.0055	9.4658E-01	9.7352E-01	ENSG00000197081
IGFALS	0.1816	6.6437E-02	1.9391E-01	ENSG00000099769
IGFBP1	0.0175	3.3851E-01		ENSG00000146678
IGFBP2	0.1828	7.8153E-02	2.1624E-01	ENSG00000115457
IGFBP3	-0.0179	8.2428E-01	9.0879E-01	ENSG00000146674
IGFBP4	-0.1346	1.2176E-01	2.9041E-01	ENSG00000141753
IGFBP5	0.2025	6.4832E-02	1.9054E-01	ENSG00000115461
IGFBP6	-0.0196	8.0556E-01	8.9800E-01	ENSG00000167779
IGFBPL1	-0.1389	1.5137E-01	3.3285E-01	ENSG00000137142
IGFL2	-0.0011	9.8901E-01	9.9393E-01	ENSG00000204866
IGFL3	0.0178	7.0708E-01		ENSG00000188624
IGFL4	0.1945	6.7067E-02	1.9509E-01	ENSG00000204869
IGFLR1	0.0511	4.9998E-01	6.9089E-01	ENSG00000126246
IGFN1	0.0132	5.0935E-01		ENSG00000163395
IGHEP2	0.0042	8.5384E-01		ENSG00000254017
IGHG4	0.0176	4.5655E-01		ENSG00000211892
IGHMBP2	0.0202	7.9135E-01	8.8983E-01	ENSG00000132740
IGKV1OR-2	0.0037	8.5775E-01		ENSG00000156755
IGKV1OR2-1	0.0050	7.9602E-01		ENSG00000235235
IGLCOR22-2	0.0011	9.3536E-01		ENSG00000243519
IGLL1	0.0157	4.4713E-01		ENSG00000128322
IGLON5	-0.0503	5.3927E-01	7.2136E-01	ENSG00000142549
IGSF1	-0.0132	8.5127E-01	9.2425E-01	ENSG00000147255
IGSF10	-0.0046	5.9241E-01		ENSG00000152580
IGSF11	-0.1765	7.6738E-02	2.1367E-01	ENSG00000144847
IGSF21	-0.5123	4.0383E-07	1.7968E-05	ENSG00000117154
IGSF22	0.1331	1.7052E-01	3.5938E-01	ENSG00000179057
IGSF3	-0.2419	1.5338E-03	1.2418E-02	ENSG00000143061
IGSF3P1	0.0082	8.0928E-01		ENSG00000279124
IGSF3P2	-0.0130	4.2011E-01		ENSG00000223703
IGSF5	-0.0695	4.1875E-01	6.2187E-01	ENSG00000183067
IGSF6	-0.0879	2.7941E-01	4.8945E-01	ENSG00000140749
IGSF8	-0.0093	8.8095E-01	9.4042E-01	ENSG00000162729
IGSF9	-0.1143	1.1736E-01	2.8293E-01	ENSG00000085552
IGSF9B	-0.0565	4.2280E-01	6.2551E-01	ENSG00000080854
IHH	0.0286	1.5470E-01		ENSG00000163501
IK	-0.0805	1.4943E-01	3.3020E-01	ENSG00000113141
IKBIP	0.1404	3.7031E-02	1.2931E-01	ENSG00000166130
IKBKB	0.0599	4.6489E-01	6.6153E-01	ENSG00000104365
IKBKE	-0.0919	3.0248E-01	5.1254E-01	ENSG00000263528
IKBKG	-0.0211	7.4063E-01	8.5871E-01	ENSG00000269335
IKBKGP1	-0.0295	6.8925E-01	8.2719E-01	ENSG00000275882
IKZF2	0.1284	1.7356E-01	3.6369E-01	ENSG00000030419
IKZF3	-0.0040	9.3950E-01		ENSG00000161405
IKZF4	0.0850	2.6364E-01	4.7260E-01	ENSG00000123411

IKZF5	0.0214	7.5766E-01	8.6886E-01	ENSG00000095574
IL10	0.0103	8.3934E-01		ENSG00000136634
IL10RA	-0.3140	1.2486E-04	1.8232E-03	ENSG00000110324
IL10RB	0.3586	3.0278E-03	2.0628E-02	ENSG00000243646
IL11	-0.0536	4.8157E-01	6.7608E-01	ENSG00000095752
IL11RA	0.1432	1.4220E-01	3.1948E-01	ENSG00000137070
IL12A	0.1796	8.1122E-02	2.2144E-01	ENSG00000168811
IL12A-AS1	0.0524	1.8788E-01		ENSG00000244040
IL12B	0.0130	1.3011E-01		ENSG00000113302
IL12RB1	0.0077	5.1992E-01		ENSG00000096996
IL12RB2	0.0492	6.2855E-01	7.8609E-01	ENSG00000081985
IL13	0.0092	8.6555E-01	9.3216E-01	ENSG00000169194
IL13RA1	0.1704	7.0117E-02	2.0106E-01	ENSG00000131724
IL13RA2	0.0684	3.5463E-01	5.6459E-01	ENSG00000123496
IL15RA	0.0279	6.3506E-01	7.9069E-01	ENSG00000134470
IL16	0.0265	8.3697E-01	9.1604E-01	ENSG00000172349
IL17B	0.0005	9.5038E-01		ENSG00000127743
IL17C	0.0019	7.7324E-01		ENSG00000124391
IL17D	-0.2276	6.4503E-03	3.6494E-02	ENSG00000172458
IL17RA	-0.2663	4.0458E-03	2.5796E-02	ENSG00000177663
IL17RB	-0.1827	8.3019E-02	2.2471E-01	ENSG00000056736
IL17RC	-0.0724	3.6962E-01	5.7890E-01	ENSG00000163702
IL17RD	0.0033	9.7119E-01	9.8549E-01	ENSG00000144730
IL17RE	0.0198	7.5236E-01	8.6560E-01	ENSG00000163701
IL17REL	-0.0079	8.1869E-01		ENSG00000188263
IL18	0.0470	2.7942E-01		ENSG00000150782
IL18BP	0.1559	1.2016E-01	2.8754E-01	ENSG00000137496
IL18R1	0.0598	3.6598E-01	5.7511E-01	ENSG00000115604
IL1A	0.0481	3.0089E-01		ENSG00000115008
IL1B	0.0012	8.7080E-01		ENSG00000125538
IL1R1	-0.0206	6.1799E-01	7.7878E-01	ENSG00000115594
IL1R2	0.0463	1.5949E-01		ENSG00000115590
IL1RAPL1	-0.1609	4.8395E-02	1.5602E-01	ENSG00000169306
IL1RAPL2	-0.0385	5.2856E-01	7.1322E-01	ENSG00000189108
IL1RN	0.0838	3.2692E-02		ENSG00000136689
IL20RA	-0.0402	2.2710E-01		ENSG00000016402
IL20RB	-0.0316	6.9207E-01	8.2919E-01	ENSG00000174564
IL21-AS1	0.0247	6.1130E-01	7.7360E-01	ENSG00000227145
IL21R	0.0344	3.4327E-01		ENSG00000103522
IL21R-AS1	0.0265	6.1503E-01	7.7664E-01	ENSG00000259954
IL22	0.0027	6.9629E-01		ENSG00000127318
IL22RA1	-0.0077	7.7446E-01		ENSG00000142677
IL23A	0.0673	4.3938E-01	6.3944E-01	ENSG00000110944
IL26	0.0321	4.8673E-01		ENSG00000111536
IL27RA	0.1173	1.1873E-01	2.8525E-01	ENSG00000104998
IL2RB	0.0014	8.0109E-01		ENSG00000100385
IL2RG	0.0033	8.7866E-01		ENSG00000147168
IL31	0.0383	2.8909E-01		ENSG00000204671
IL31RA	0.0732	2.2908E-01	4.3343E-01	ENSG00000164509

IL32	0.0080	7.3166E-01	8.5327E-01	ENSG00000008517
IL33	-0.1923	5.7659E-02	1.7608E-01	ENSG00000137033
IL34	0.1589	1.0890E-01	2.6933E-01	ENSG00000157368
IL36G	0.0218	4.7674E-01		ENSG00000136688
IL3RA	-0.1191	1.5362E-01	3.3598E-01	ENSG00000185291
IL4	0.0175	2.1717E-01		ENSG00000113520
IL4I1	0.0357	4.1318E-01		ENSG00000104951
IL4R	0.0217	7.0050E-01	8.3446E-01	ENSG00000077238
IL5	0.0326	4.0109E-01		ENSG00000113525
IL5RA	0.2591	1.7529E-02	7.5708E-02	ENSG00000091181
IL6	0.0140	5.4714E-01		ENSG00000136244
IL6R	0.0541	4.0373E-01	6.0824E-01	ENSG00000160712
IL6R-AS1	-0.0069	7.2548E-01		ENSG00000228013
IL6ST	-0.0316	5.9959E-01	7.6551E-01	ENSG00000134352
IL6ST-DT	0.4247	5.9430E-04	6.0852E-03	ENSG00000227908
IL7	0.0047	9.4214E-01	9.7141E-01	ENSG00000104432
IL7R	0.0177	6.3529E-01		ENSG00000168685
ILDR2	-0.0006	9.9119E-01	9.9515E-01	ENSG00000143195
ILF2	-0.3075	3.0790E-06	9.4697E-05	ENSG00000143621
ILF2P1	0.0058	7.2816E-01		ENSG00000244226
ILF3	-0.1191	9.1782E-02	2.4117E-01	ENSG00000129351
ILF3-DT	-0.0009	9.8797E-01	9.9346E-01	ENSG00000267100
ILK	0.0754	3.8402E-01	5.9207E-01	ENSG00000166333
ILKAP	-0.3378	4.9481E-07	2.1322E-05	ENSG00000132323
ILRUN	0.0430	5.4883E-01	7.2822E-01	ENSG00000196821
ILRUN-AS1	0.1140	1.8867E-01	3.8354E-01	ENSG00000272288
ILVBL	0.0261	7.3841E-01	8.5757E-01	ENSG00000105135
IMMP1L	-0.0433	4.4592E-01	6.4511E-01	ENSG00000148950
IMMP2L	-0.1607	1.2188E-02	5.8595E-02	ENSG00000184903
IMMT	-0.1162	1.8200E-02	7.7638E-02	ENSG00000132305
IMMTP1	0.0040	9.3335E-01		ENSG00000229880
IMP3	-0.1764	1.1513E-03	1.0081E-02	ENSG00000177971
IMP4	-0.0761	2.4905E-01	4.5678E-01	ENSG00000136718
IMPA1	0.1471	5.0110E-02	1.5994E-01	ENSG00000133731
IMPA2	-0.0479	4.9680E-01	6.8816E-01	ENSG00000141401
IMPACT	0.0903	1.4037E-01	3.1691E-01	ENSG00000154059
IMPDH1	-0.2341	2.6815E-03	1.8749E-02	ENSG00000106348
IMPDH2	0.0781	1.9063E-01	3.8582E-01	ENSG00000178035
IMPG1	0.0320	3.6792E-01		ENSG00000112706
IMPG2	0.0161	8.7453E-01	9.3710E-01	ENSG00000081148
INA	-0.1957	2.2133E-02	8.9392E-02	ENSG00000148798
INAFM1	0.2088	7.9758E-03	4.2802E-02	ENSG00000257704
INAFM2	-0.1876	2.8568E-02	1.0738E-01	ENSG00000259330
INAVA	-0.1327	1.6422E-01	3.5102E-01	ENSG00000163362
INCA1	0.2248	1.7837E-02	7.6568E-02	ENSG00000196388
INCENP	0.0350	6.7243E-01	8.1680E-01	ENSG00000149503
INE1	0.2251	4.2122E-02	1.4170E-01	ENSG00000224975
INF2	-0.3202	1.9861E-03	1.4999E-02	ENSG00000203485
ING1	-0.1723	1.3469E-02	6.3026E-02	ENSG00000153487

ING2	-0.2737	3.0263E-04	3.6216E-03	ENSG00000168556
ING3	-0.0514	4.7951E-01	6.7454E-01	ENSG00000071243
ING4	0.0504	3.5839E-01	5.6777E-01	ENSG00000111653
ING5	0.1398	1.0620E-02	5.2982E-02	ENSG00000168395
INGX	-0.0020	9.6261E-01	9.8133E-01	ENSG00000243468
INHBA	0.1225	1.6682E-01	3.5477E-01	ENSG00000122641
INHBA-AS1	-0.0841	3.1036E-01	5.2066E-01	ENSG00000224116
INHBB	-0.4833	3.7461E-04	4.2535E-03	ENSG00000163083
INHBC	0.5553	6.5772E-04	6.6270E-03	ENSG00000175189
INHBE	0.0042	5.9356E-01		ENSG00000139269
INIP	0.1275	3.4747E-02	1.2327E-01	ENSG00000148153
INKA1	0.2048	4.4384E-02	1.4704E-01	ENSG00000185614
INKA2	-0.1178	1.4860E-01	3.2909E-01	ENSG00000197852
INKA2-AS1	-0.0622	4.5271E-01	6.5149E-01	ENSG00000227811
INMT	0.0163	7.6638E-01		ENSG00000241644
INO80	0.0222	7.5873E-01	8.6962E-01	ENSG00000128908
INO80B	0.1990	5.6972E-02	1.7476E-01	ENSG00000115274
INO80C	0.0204	7.7470E-01	8.8011E-01	ENSG00000153391
INO80D	0.0678	2.9155E-01	5.0185E-01	ENSG00000114933
INO80E	-0.2512	5.4239E-03	3.2044E-02	ENSG00000169592
INPP1	-0.1352	9.5187E-02	2.4724E-01	ENSG00000151689
INPP4A	0.0652	2.7006E-01	4.7972E-01	ENSG00000040933
INPP4B	0.1012	2.5640E-01	4.6484E-01	ENSG00000109452
INPP5A	-0.0323	6.1859E-01	7.7925E-01	ENSG00000068383
INPP5B	-0.1467	7.3332E-02	2.0744E-01	ENSG00000204084
INPP5D	0.0430	5.3414E-01	7.1776E-01	ENSG00000168918
INPP5E	-0.0423	5.1467E-01	7.0235E-01	ENSG00000148384
INPP5F	0.3191	1.2423E-04	1.8151E-03	ENSG00000198825
INPP5J	0.1271	1.1426E-01	2.7821E-01	ENSG00000185133
INPP5K	0.0515	4.4431E-01	6.4334E-01	ENSG00000132376
INPPL1	-0.1090	1.7983E-01	3.7154E-01	ENSG00000165458
INS	0.0064	9.7761E-01		ENSG00000254647
INSC	0.0165	7.2390E-01		ENSG00000188487
INSIG1	0.0080	9.0498E-01	9.5366E-01	ENSG00000186480
INSIG2	0.2968	7.0531E-04	6.9892E-03	ENSG00000125629
INSL3	0.0511	5.4947E-01	7.2875E-01	ENSG00000248099
INSL5	0.0275	2.3713E-01		ENSG00000172410
INSL6	0.0643	4.7068E-03		ENSG00000120210
INSM2	0.1402	2.6141E-02	1.0075E-01	ENSG00000168348
INSR	0.0405	5.4980E-01	7.2903E-01	ENSG00000171105
INSRR	-0.0246	6.3695E-01	7.9200E-01	ENSG00000027644
INSYN1	-0.3778	1.1819E-04	1.7518E-03	ENSG00000205363
INSYN2A	0.1825	5.9546E-02	1.7934E-01	ENSG00000188916
INSYN2B	0.0441	5.6382E-01	7.3998E-01	ENSG00000204767
INTS1	0.0217	7.6206E-01	8.7200E-01	ENSG00000164880
INTS10	0.0929	1.1229E-01	2.7484E-01	ENSG00000104613
INTS11	-0.2130	1.0264E-02	5.1655E-02	ENSG00000127054
INTS12	-0.1695	1.6024E-03	1.2813E-02	ENSG00000138785
INTS13	-0.2526	1.6915E-03	1.3311E-02	ENSG00000064102

INTS14	-0.0459	5.1154E-01	7.0002E-01	ENSG00000138614
INTS15	-0.1252	9.1199E-02	2.4010E-01	ENSG00000146576
INTS2	0.0846	3.1332E-01	5.2358E-01	ENSG00000108506
INTS3	-0.0180	7.7285E-01	8.7899E-01	ENSG00000143624
INTS4	0.2773	1.0565E-03	9.4664E-03	ENSG00000149262
INTS4P2	-0.0092	9.0533E-01	9.5372E-01	ENSG00000273024
INTS5	-0.0698	3.8143E-01	5.8966E-01	ENSG00000185085
INTS6	0.0408	4.9058E-01	6.8328E-01	ENSG00000102786
INTS6-AS1	0.1756	4.8550E-02	1.5637E-01	ENSG00000236778
INTS6L	0.1753	2.7242E-02	1.0371E-01	ENSG00000165359
INTS6L-AS1	-0.0277	6.7011E-02		ENSG00000225235
INTS7	0.0154	8.6124E-01	9.3034E-01	ENSG00000143493
INTS8	-0.0856	1.8814E-01	3.8295E-01	ENSG00000164941
INTS9	-0.0075	9.1655E-01	9.5866E-01	ENSG00000104299
INTU	0.1511	6.8359E-02	1.9739E-01	ENSG00000164066
INVS	-0.0173	8.0102E-01	8.9502E-01	ENSG00000119509
IP6K1	0.1561	2.7740E-02	1.0506E-01	ENSG00000176095
IP6K2	-0.2979	7.9091E-07	3.0934E-05	ENSG00000068745
IP6K3	0.0757	1.1507E-01		ENSG00000161896
IPCEF1	0.3871	4.5213E-03	2.7895E-02	ENSG00000074706
IPMK	0.1553	5.2801E-02	1.6550E-01	ENSG00000151151
IPO11	-0.0829	2.6057E-01	4.6904E-01	ENSG00000086200
IPO13	0.0403	5.9677E-01	7.6334E-01	ENSG00000117408
IPO4	0.0303	5.8793E-01	7.5699E-01	ENSG00000196497
IPO5	-0.1223	3.8275E-02	1.3254E-01	ENSG00000065150
IPO7	-0.2244	7.6493E-03	4.1420E-02	ENSG00000205339
IPO8	-0.2003	1.8213E-02	7.7638E-02	ENSG00000133704
IPO9	0.1196	4.2964E-02	1.4367E-01	ENSG00000198700
IPO9-AS1	-0.0478	3.9894E-01	6.0448E-01	ENSG00000231871
IPP	-0.2815	1.7834E-04	2.3719E-03	ENSG00000197429
IPPK	0.1111	2.2116E-01	4.2427E-01	ENSG00000127080
IQANK1	0.3411	1.1860E-02	5.7534E-02	ENSG00000203499
IQCA1	0.4118	3.2944E-03	2.1979E-02	ENSG00000132321
IQCA1-AS1	0.0080	7.8424E-01		ENSG00000232893
IQCA1L	0.0095	7.9468E-01		ENSG00000278685
IQCB1	-0.0663	3.3361E-01	5.4371E-01	ENSG00000173226
IQCC	0.0385	6.4912E-01	8.0054E-01	ENSG00000160051
IQCD	0.0552	4.1924E-01	6.2205E-01	ENSG00000166578
IQCE	0.0788	2.8824E-01	4.9873E-01	ENSG00000106012
IQCF1	0.0065	9.6172E-01		ENSG00000173389
IQCF4P	0.0218	6.6500E-01		ENSG00000224792
IQCG	-0.0674	3.5508E-01	5.6476E-01	ENSG00000114473
IQCH	0.2320	2.5559E-02	9.9098E-02	ENSG00000103599
IQCH-AS1	0.0631	4.5840E-01	6.5626E-01	ENSG00000259673
IQCJ	0.0602	1.6620E-01	3.5384E-01	ENSG00000214216
IQCK	0.4493	1.2586E-06	4.5210E-05	ENSG00000174628
IQCN	-0.0044	9.6186E-01	9.8091E-01	ENSG00000130518
IQGAP1	-0.1690	8.2361E-03	4.3835E-02	ENSG00000140575
IQGAP2	0.0272	7.6519E-01	8.7387E-01	ENSG00000145703

IQGAP3	-0.1205	1.9528E-01	3.9217E-01	ENSG00000183856
IQSEC1	0.0865	1.7823E-01	3.6944E-01	ENSG00000144711
IQSEC2	-0.0075	9.1308E-01	9.5717E-01	ENSG00000124313
IQSEC3	0.2999	2.1797E-03	1.6038E-02	ENSG00000120645
IQSEC3P2	0.0118	9.5113E-01		ENSG00000278654
IQSEC3P3	0.5365	2.2408E-03	1.6404E-02	ENSG00000274727
IQUB	0.1096	1.8243E-01	3.7537E-01	ENSG00000164675
IRAG1	0.0915	3.0412E-01	5.1431E-01	ENSG00000072952
IRAG1-AS1	-0.0616	4.4368E-01	6.4284E-01	ENSG00000177112
IRAG2	0.4631	1.1602E-02	5.6560E-02	ENSG00000118308
IRAK1	0.1659	4.5866E-02	1.5065E-01	ENSG00000184216
IRAK1BP1	0.1584	3.3860E-02	1.2085E-01	ENSG00000146243
IRAK2	-0.4476	9.6323E-04	8.8007E-03	ENSG00000134070
IRAK3	0.0176	9.8548E-01	9.9235E-01	ENSG00000090376
IRAK4	0.0838	2.9449E-01	5.0449E-01	ENSG00000198001
IREB2	0.0952	1.2449E-01	2.9425E-01	ENSG00000136381
IRF1	0.0095	9.2387E-01	9.6243E-01	ENSG00000125347
IRF2	0.2385	1.6936E-02	7.3843E-02	ENSG00000168310
IRF2BP1	-0.0665	3.0838E-01	5.1859E-01	ENSG00000170604
IRF2BP2	-0.4126	1.2211E-07	6.7978E-06	ENSG00000168264
IRF2BPL	-0.5211	2.9536E-07	1.4002E-05	ENSG00000119669
IRF3	-0.1031	1.0117E-01	2.5681E-01	ENSG00000126456
IRF4	0.1137	2.6312E-01	4.7196E-01	ENSG00000137265
IRF5	-0.1782	7.7753E-02	2.1554E-01	ENSG00000128604
IRF6	-0.1928	5.7041E-02	1.7484E-01	ENSG00000117595
IRF7	-0.1127	1.8280E-01	3.7591E-01	ENSG00000185507
IRF8	-0.0493	5.0510E-01	6.9504E-01	ENSG00000140968
IRF9	0.2059	5.5062E-02	1.7071E-01	ENSG00000213928
IRGC	0.0177	4.6417E-01		ENSG00000124449
IRGQ	-0.0006	9.8640E-01	9.9243E-01	ENSG00000167378
IRS1	-0.0746	3.5825E-01	5.6777E-01	ENSG00000169047
IRS2	-0.1941	4.1563E-03	2.6249E-02	ENSG00000185950
IRX1	-0.0001	9.8594E-01	9.9239E-01	ENSG00000170549
IRX2	0.4200	6.5529E-03	3.6947E-02	ENSG00000170561
IRX3	-0.0387	5.4970E-01	7.2897E-01	ENSG00000177508
IRX4	-0.0442	1.4434E-01	3.2257E-01	ENSG00000113430
IRX5	-0.1990	6.7430E-02	1.9574E-01	ENSG00000176842
IRX6	-0.0435	2.3305E-01		ENSG00000159387
ISCA1	-0.1028	4.1704E-02	1.4080E-01	ENSG00000135070
ISCA2	0.0444	5.3875E-01	7.2098E-01	ENSG00000165898
ISCU	-0.1760	2.2680E-02	9.0945E-02	ENSG00000136003
ISG15	0.0222	7.6976E-01	8.7673E-01	ENSG00000187608
ISG20	0.0844	3.4653E-01	5.5673E-01	ENSG00000172183
ISG20L2	0.0834	2.2247E-01	4.2588E-01	ENSG00000143319
ISL1	-0.0276	7.3324E-01	8.5427E-01	ENSG00000016082
ISL1-DT	-0.0760	3.5912E-02	1.2636E-01	ENSG00000259663
ISL2	-0.0823	2.0660E-01	4.0618E-01	ENSG00000159556
ISLR	0.2422	4.2570E-02	1.4270E-01	ENSG00000129009
ISLR2	-0.1059	1.8898E-01	3.8390E-01	ENSG00000167178

ISM1	0.0009	8.4215E-01	9.1870E-01	ENSG00000101230
ISM2	-0.0334	6.9214E-01	8.2924E-01	ENSG00000100593
ISOC1	-0.0028	9.4599E-01	9.7333E-01	ENSG00000066583
ISOC2	-0.0969	1.0388E-01	2.6120E-01	ENSG00000063241
IST1	-0.0859	7.3519E-02	2.0773E-01	ENSG00000182149
ISY1	-0.0977	6.4944E-02	1.9073E-01	ENSG00000240682
ISYNA1	-0.2920	4.0926E-06	1.1933E-04	ENSG00000105655
ITCH	-0.0192	7.8488E-01	8.8631E-01	ENSG00000078747
ITCH	-0.1489	3.5642E-02	1.2570E-01	ENSG00000289720
ITFG1	-0.0960	1.2134E-01	2.8965E-01	ENSG00000129636
ITFG2	-0.0458	5.3770E-01	7.2039E-01	ENSG00000111203
ITFG2-AS1	0.0462	4.6894E-01	6.6553E-01	ENSG00000258325
ITFG2-AS1	0.0148	6.4426E-01		ENSG00000256150
ITGA1	0.0233	8.0453E-01		ENSG00000213949
ITGA10	0.0021	9.7525E-01	9.8783E-01	ENSG00000143127
ITGA11	0.0159	8.6289E-01	9.3083E-01	ENSG00000137809
ITGA2	0.1465	1.2791E-01	2.9962E-01	ENSG00000164171
ITGA2B	-0.0186	8.1887E-01	9.0583E-01	ENSG00000005961
ITGA3	0.1189	1.7447E-01	3.6485E-01	ENSG00000005884
ITGA4	-0.0001	9.8379E-01	9.9133E-01	ENSG00000115232
ITGA5	-0.2323	1.1752E-02	5.7158E-02	ENSG00000161638
ITGA6	0.2081	1.4777E-02	6.7344E-02	ENSG00000091409
ITGA6-AS1	0.0119	8.4025E-01	9.1780E-01	ENSG00000232788
ITGA7	-0.0677	4.1872E-01	6.2187E-01	ENSG00000135424
ITGA8	0.0010	9.8351E-01	9.9117E-01	ENSG00000077943
ITGA9	-0.1540	1.2067E-01	2.8850E-01	ENSG00000144668
ITGA9-AS1	-0.0569	5.0986E-01	6.9865E-01	ENSG00000235257
ITGAE	0.1027	1.9013E-01	3.8519E-01	ENSG00000083457
ITGAL	0.1694	2.8636E-02	1.0752E-01	ENSG00000005844
ITGAM	0.0246	6.6620E-01		ENSG00000169896
ITGAV	0.0361	6.2735E-01	7.8531E-01	ENSG00000138448
ITGAX	0.0193	8.1447E-01		ENSG00000140678
ITGB1	0.1674	2.3511E-02	9.3469E-02	ENSG00000150093
ITGB1-DT	0.0170	8.1592E-01	9.0423E-01	ENSG00000229656
ITGB1BP1	-0.0884	1.0713E-01	2.6637E-01	ENSG00000119185
ITGB1BP2	0.1591	9.3111E-02	2.4346E-01	ENSG00000147166
ITGB2	0.0385	4.3158E-01	6.3305E-01	ENSG00000160255
ITGB2-AS1	0.0085	6.2625E-01		ENSG00000227039
ITGB3	-0.0414	4.4339E-01	6.4265E-01	ENSG00000259207
ITGB3BP	-0.0156	7.9332E-01	8.9078E-01	ENSG00000142856
ITGB4	-0.0814	3.6028E-01	5.6971E-01	ENSG00000132470
ITGB5	0.2859	2.1335E-02	8.6970E-02	ENSG00000082781
ITGB6	0.1301	8.0963E-02	2.2117E-01	ENSG00000115221
ITGB7	0.0672	1.4067E-01		ENSG00000139626
ITGB8	-0.0238	7.3177E-01	8.5327E-01	ENSG00000105855
ITGB8-AS1	0.2667	1.0791E-02	5.3572E-02	ENSG00000271133
ITGBL1	0.0305	5.2352E-01		ENSG00000198542
ITIH1	0.0115	6.6743E-01		ENSG00000055957
ITIH2	0.0780	2.4585E-01	4.5357E-01	ENSG00000151655

ITIH3	-0.0156	7.3725E-01		ENSG00000162267
ITIH4	0.0790	1.9475E-01	3.9149E-01	ENSG00000055955
ITIH5	-0.0352	5.5529E-01	7.3334E-01	ENSG00000123243
ITIH6	0.0264	1.2740E-01		ENSG00000102313
ITK	0.0385	4.6873E-01		ENSG00000113263
ITM2A	0.0846	3.3869E-01	5.4851E-01	ENSG00000078596
ITM2B	-0.2537	3.4434E-07	1.5777E-05	ENSG00000136156
ITM2C	-0.0886	1.8142E-01	3.7392E-01	ENSG00000135916
ITPA	-0.0377	4.8219E-01	6.7666E-01	ENSG00000125877
ITPK1	-0.1027	9.9419E-02	2.5368E-01	ENSG00000100605
ITPK1-AS1	-0.0172	8.3585E-01	9.1537E-01	ENSG00000258730
ITPKA	-0.0757	3.8896E-01	5.9628E-01	ENSG00000137825
ITPKB	-0.0110	8.9516E-01	9.4778E-01	ENSG00000143772
ITPKB-IT1	0.0115	4.4889E-01		ENSG00000228382
ITPKC	0.0133	8.5715E-01	9.2777E-01	ENSG00000086544
ITPR1	0.2519	2.1842E-02	8.8477E-02	ENSG00000150995
ITPR1-DT	0.1481	1.3362E-01	3.0745E-01	ENSG00000231249
ITPR2	0.0587	4.9183E-01	6.8401E-01	ENSG00000123104
ITPR3	0.1631	9.1451E-02	2.4061E-01	ENSG00000096433
ITPRID1	-0.0242	6.5050E-01	8.0185E-01	ENSG00000180347
ITPRID2	0.2322	2.8163E-03	1.9471E-02	ENSG00000138434
ITPRIP	-0.4476	2.4891E-03	1.7738E-02	ENSG00000148841
ITPRIPL2	0.0671	4.2461E-01	6.2706E-01	ENSG00000205730
ITSN1	0.0781	1.8631E-01	3.8058E-01	ENSG00000205726
ITSN2	0.2857	2.7677E-04	3.3746E-03	ENSG00000198399
IVD	-0.1522	5.5234E-02	1.7115E-01	ENSG00000128928
IVNS1ABP	-0.0773	3.5941E-01	5.6889E-01	ENSG00000116679
IWS1	0.0462	4.2076E-01	6.2366E-01	ENSG00000163166
IYD	-0.0122	7.1845E-01		ENSG00000009765
IZUMO1	0.0200	8.1935E-01	9.0615E-01	ENSG00000182264
IZUMO2	0.0123	5.3513E-01		ENSG00000161652
IZUMO4	0.0165	7.7011E-01	8.7696E-01	ENSG00000099840
JADE1	0.0812	3.1320E-01	5.2349E-01	ENSG00000077684
JADE2	0.2308	8.4232E-03	4.4638E-02	ENSG00000043143
JADE3	0.0147	8.5812E-01	9.2834E-01	ENSG00000102221
JAG1	0.2628	1.2569E-02	5.9843E-02	ENSG00000101384
JAG2	0.1363	1.1731E-01	2.8288E-01	ENSG00000184916
JAGN1	0.1239	3.9802E-02	1.3639E-01	ENSG00000171135
JAK1	-0.1064	9.7089E-02	2.5035E-01	ENSG00000162434
JAK2	0.2637	3.5968E-04	4.1201E-03	ENSG00000096968
JAK3	0.1600	6.4509E-02	1.9000E-01	ENSG00000105639
JAKMIP1	-0.1225	1.9018E-01	3.8527E-01	ENSG00000152969
JAKMIP2	-0.2057	9.6378E-05	1.5023E-03	ENSG00000176049
JAKMIP2-AS1	0.4909	2.2321E-03	1.6350E-02	ENSG00000280780
JAKMIP3	0.2754	1.5811E-02	7.0568E-02	ENSG00000188385
JAM2	-0.1179	1.3180E-01	3.0507E-01	ENSG00000154721
JAM3	-0.0686	4.2650E-01	6.2869E-01	ENSG00000166086
JAML	0.0341	4.5264E-01		ENSG00000160593
JARID2	0.0258	7.1906E-01	8.4556E-01	ENSG00000008083

JARID2-AS1	0.4911	8.7314E-03	4.5905E-02	ENSG00000235488
JAZF1	-0.0531	4.6336E-01	6.6050E-01	ENSG00000153814
JCAD	-0.1423	1.3620E-01	3.1126E-01	ENSG00000165757
JCHAIN	0.0387	4.1885E-01	6.2193E-01	ENSG00000132465
JDP2	-0.3109	1.2861E-03	1.0965E-02	ENSG00000140044
JHY	-0.0467	5.6921E-01	7.4342E-01	ENSG00000109944
JKAMP	0.0112	8.4577E-01	9.2100E-01	ENSG00000050130
JMJD1C	-0.1280	5.0384E-02	1.6042E-01	ENSG00000171988
JMJD1C-AS1	-0.0661	4.3851E-01	6.3860E-01	ENSG00000272767
JMJD4	0.0896	2.9519E-01	5.0530E-01	ENSG00000081692
JMJD6	-0.1226	1.5656E-01	3.4030E-01	ENSG00000070495
JMJD7	0.0266	4.1892E-01		ENSG00000243789
JMJD7-PLA2G4B	0.0208	8.2367E-01	9.0841E-01	ENSG00000168970
JMJD8	0.1761	4.2263E-02	1.4208E-01	ENSG00000161999
JMY	-0.1756	1.2764E-02	6.0511E-02	ENSG00000152409
JOSD1	-0.2978	2.3083E-03	1.6783E-02	ENSG00000100221
JOSD2	-0.0684	4.1094E-01	6.1507E-01	ENSG00000161677
JPH1	0.0722	3.9030E-01	5.9763E-01	ENSG00000104369
JPH2	-0.0085	6.7166E-01		ENSG00000149596
JPH3	-0.0837	2.7223E-01	4.8148E-01	ENSG00000154118
JPH4	-0.2327	2.1134E-03	1.5701E-02	ENSG00000092051
JPT1	-0.5973	2.0335E-16	2.0848E-13	ENSG00000189159
JPT2	-0.1140	1.3204E-01	3.0536E-01	ENSG00000206053
JPX	-0.0783	2.7833E-01	4.8822E-01	ENSG00000225470
JRK	0.3999	4.6701E-05	8.3874E-04	ENSG00000234616
JRKL	0.0396	5.9586E-01	7.6268E-01	ENSG00000183340
JSRP1	0.0388	5.5995E-01	7.3725E-01	ENSG00000167476
JTB	-0.3109	8.2674E-05	1.3235E-03	ENSG00000143543
JUN	-0.5984	2.4538E-07	1.2027E-05	ENSG00000177606
JUNB	-0.0378	7.0565E-01	8.3776E-01	ENSG00000171223
JUP	0.0002	9.9853E-01	9.9913E-01	ENSG00000173801
KAAG1	0.0122	4.2767E-01		ENSG00000146049
KALRN	-0.1337	6.2547E-02	1.8578E-01	ENSG00000160145
KANK1	0.2985	2.1250E-03	1.5750E-02	ENSG00000107104
KANK2	-0.1914	3.8597E-02	1.3342E-01	ENSG00000197256
KANK3	-0.1228	1.6837E-01	3.5676E-01	ENSG00000186994
KANK4	0.0794	3.6761E-01	5.7688E-01	ENSG00000132854
KANSL1	-0.1504	3.4763E-02	1.2329E-01	ENSG00000120071
KANSL1-AS1	0.2205	1.8844E-02	7.9570E-02	ENSG00000214401
KANSL1L	0.0081	9.2197E-01	9.6143E-01	ENSG00000144445
KANSL2	-0.4552	3.5350E-07	1.6081E-05	ENSG00000139620
KANSL3	-0.0012	9.8017E-01	9.8991E-01	ENSG00000114982
KANTR	0.1483	3.2668E-02	1.1796E-01	ENSG00000232593
KARS1	-0.1187	7.8129E-02	2.1624E-01	ENSG00000065427
KARS1P1	0.0167	2.9446E-01		ENSG00000229696
KASH5	0.0304	2.4289E-01		ENSG00000161609
KAT14	0.0359	5.7651E-01	7.4871E-01	ENSG00000149474
KAT2A	-0.0612	4.2533E-01	6.2767E-01	ENSG00000108773
KAT2B	-0.0674	3.7332E-01	5.8192E-01	ENSG00000114166

KAT5	0.1657	5.7565E-02	1.7595E-01	ENSG00000172977
KAT6A	-0.0599	3.5545E-01	5.6523E-01	ENSG00000083168
KAT6B	-0.1540	5.5232E-03	3.2441E-02	ENSG00000156650
KAT7	-0.2056	8.3669E-04	7.9059E-03	ENSG00000136504
KAT8	-0.0269	5.7553E-01	7.4808E-01	ENSG00000103510
KATNA1	0.0766	2.3396E-01	4.3905E-01	ENSG00000186625
KATNAL1	0.0843	1.6680E-01	3.5475E-01	ENSG00000102781
KATNAL2	0.3554	5.5513E-04	5.7682E-03	ENSG00000167216
KATNB1	0.2268	9.7432E-04	8.8691E-03	ENSG00000140854
KATNBL1	0.1349	3.0325E-02	1.1207E-01	ENSG00000134152
KATNIP	0.0885	2.7220E-01	4.8147E-01	ENSG00000047578
KAZALD1	0.1341	1.0301E-01	2.5981E-01	ENSG00000107821
KAZN	0.0199	7.7164E-01	8.7818E-01	ENSG00000189337
KAZN-AS1	0.0342	3.7745E-01		ENSG00000234593
KBTBD11	-0.2027	1.5310E-02	6.8980E-02	ENSG00000176595
KBTBD11-AS1	-0.1800	8.8486E-02	2.3489E-01	ENSG00000253764
KBTBD11-OT1	0.0123	8.6506E-01	9.3183E-01	ENSG00000253696
KBTBD12	0.0232	9.6591E-01	9.8323E-01	ENSG00000187715
KBTBD2	-0.1165	1.0251E-01	2.5904E-01	ENSG00000170852
KBTBD3	0.1474	7.7433E-02	2.1499E-01	ENSG00000182359
KBTBD4	0.0377	5.8614E-01	7.5569E-01	ENSG00000123444
KBTBD6	-0.2152	1.3502E-02	6.3124E-02	ENSG00000165572
KBTBD6-DT	0.0047	9.5509E-01	9.7733E-01	ENSG00000278390
KBTBD7	0.0106	8.7459E-01	9.3712E-01	ENSG00000120696
KBTBD8	0.2133	4.7177E-02	1.5350E-01	ENSG00000163376
KC6	0.2458	3.0809E-03	2.0917E-02	ENSG00000267313
KCMF1	-0.2089	2.1811E-04	2.7850E-03	ENSG00000176407
KCNA1	-0.5412	1.3153E-04	1.8915E-03	ENSG00000111262
KCNA2	-0.2478	2.5165E-03	1.7844E-02	ENSG00000177301
KCNA3	-0.2066	6.8989E-03	3.8404E-02	ENSG00000177272
KCNA4	0.5286	7.1588E-04	7.0720E-03	ENSG00000182255
KCNA5	0.4442	4.4195E-04	4.8373E-03	ENSG00000130037
KCNA6	-0.1722	9.8883E-02	2.5291E-01	ENSG00000151079
KCNA7	0.0268	6.1696E-01		ENSG00000104848
KCNAB1	0.0377	6.5938E-01	8.0785E-01	ENSG00000169282
KCNAB1-AS1	-0.0033	7.7322E-01		ENSG00000242370
KCNAB2	0.2558	2.9172E-03	2.0061E-02	ENSG00000069424
KCNAB3	0.0374	6.4305E-01	7.9609E-01	ENSG00000170049
KCNB1	0.0899	1.8769E-01	3.8236E-01	ENSG00000158445
KCNB2	-0.1087	2.2551E-01	4.2927E-01	ENSG00000182674
KCNC1	0.2660	6.9277E-03	3.8530E-02	ENSG00000129159
KCNC2	0.0670	4.4476E-01	6.4368E-01	ENSG00000166006
KCNC3	0.0014	9.8837E-01	9.9358E-01	ENSG00000131398
KCNC4	-0.1312	1.0585E-01	2.6451E-01	ENSG00000116396
KCND1	-0.0790	3.6188E-01	5.7107E-01	ENSG00000102057
KCND2	0.0077	9.1234E-01	9.5678E-01	ENSG00000184408
KCND3	-0.1276	1.5379E-01	3.3614E-01	ENSG00000171385
KCND3-AS1	0.3979	1.5174E-02	6.8508E-02	ENSG00000237556
KCND3-IT1	0.0089	2.9231E-01		ENSG00000232558

KCNE1	0.2460	3.8817E-02	1.3395E-01	ENSG00000180509
KCNE2	0.0043	9.9607E-01		ENSG00000159197
KCNE4	-0.2306	4.5620E-02	1.5002E-01	ENSG00000152049
KCNE5	0.0969	1.0123E-01	2.5686E-01	ENSG00000176076
KCNF1	-0.1089	2.0894E-01	4.0899E-01	ENSG00000162975
KCNG1	-0.3476	2.6009E-04	3.2206E-03	ENSG00000026559
KCNG2	-0.3653	1.4231E-02	6.5498E-02	ENSG00000178342
KCNG3	0.1258	1.2527E-01	2.9542E-01	ENSG00000171126
KCNH1	-0.3694	1.8515E-02	7.8572E-02	ENSG00000143473
KCNH2	0.0676	3.6669E-01	5.7590E-01	ENSG00000055118
KCNH3	-0.5376	1.9259E-04	2.5247E-03	ENSG00000135519
KCNH4	-0.0305	7.0392E-01	8.3671E-01	ENSG00000089558
KCNH6	0.0376	6.0766E-01	7.7108E-01	ENSG00000173826
KCNH7	0.0591	4.6053E-01	6.5807E-01	ENSG00000184611
KCNH7-AS1	0.4997	1.7736E-03	1.3778E-02	ENSG00000237750
KCNIP1	-0.0825	2.7404E-01	4.8382E-01	ENSG00000182132
KCNIP1-AS1	0.0558	2.5165E-01		ENSG00000253591
KCNIP2	-0.4551	1.3374E-04	1.9099E-03	ENSG00000120049
KCNIP4	-0.0048	9.4802E-01	9.7439E-01	ENSG00000185774
KCNIP4-IT1	0.0518	3.8866E-01		ENSG00000280650
KCNJ1	-0.0090	6.8469E-01		ENSG00000151704
KCNJ10	0.0319	6.9865E-01	8.3342E-01	ENSG00000177807
KCNJ11	-0.0850	3.0802E-01	5.1835E-01	ENSG00000187486
KCNJ12	0.0967	2.7485E-01	4.8461E-01	ENSG00000184185
KCNJ13	-0.0048	9.3702E-01	9.6903E-01	ENSG00000115474
KCNJ14	0.0581	5.0156E-01	6.9215E-01	ENSG00000182324
KCNJ16	0.0577	2.2556E-01		ENSG00000153822
KCNJ18	-0.0059	8.6052E-01		ENSG00000260458
KCNJ2	0.0071	9.2177E-01	9.6140E-01	ENSG00000123700
KCNJ2-AS1	0.0138	8.6671E-01	9.3275E-01	ENSG00000267365
KCNJ3	0.0037	9.7060E-01	9.8521E-01	ENSG00000162989
KCNJ4	-0.3380	8.6731E-03	4.5686E-02	ENSG00000168135
KCNJ5	0.5457	1.3029E-03	1.1078E-02	ENSG00000120457
KCNJ5-AS1	-0.0106	8.7451E-01	9.3710E-01	ENSG00000174370
KCNJ6	-0.0728	3.8785E-01	5.9542E-01	ENSG00000157542
KCNJ8	0.0017	9.8595E-01	9.9239E-01	ENSG00000121361
KCNJ9	0.1238	1.8792E-01	3.8267E-01	ENSG00000162728
KCNK1	-0.0325	6.9926E-01	8.3370E-01	ENSG00000135750
KCNK10	0.3261	3.1988E-04	3.7745E-03	ENSG00000100433
KCNK13	0.0010	9.9496E-01		ENSG00000152315
KCNK15	0.1106	1.2791E-01	2.9962E-01	ENSG00000124249
KCNK17	0.0527	8.3302E-02		ENSG00000124780
KCNK18	0.0117	4.3898E-01		ENSG00000186795
KCNK3	-0.1639	5.6781E-02	1.7433E-01	ENSG00000171303
KCNK4	0.0139	7.6097E-01		ENSG00000182450
KCNK4-CATSPERZ	-0.0160	4.4643E-01		ENSG00000257069
KCNK5	0.0790	5.3380E-02		ENSG00000164626
KCNK7	-0.0634	2.6902E-01	4.7837E-01	ENSG00000173338
KCNK9	-0.1286	1.6699E-01	3.5498E-01	ENSG00000169427

KCNMA1	0.0581	4.6897E-01	6.6553E-01	ENSG00000156113
KCNMA1-AS1	0.0807	1.7734E-01	3.6856E-01	ENSG00000236467
KCNMB1	0.0298	4.6331E-01		ENSG00000145936
KCNMB2	-0.0017	9.7832E-01	9.8921E-01	ENSG00000197584
KCNMB3	0.1568	7.6460E-02	2.1326E-01	ENSG00000171121
KCNMB4	0.0099	9.0604E-01	9.5400E-01	ENSG00000135643
KCNN1	-0.0977	2.1131E-01	4.1209E-01	ENSG00000105642
KCNN2	-0.0192	8.1175E-01	9.0159E-01	ENSG00000080709
KCNN3	0.0263	7.3949E-01	8.5776E-01	ENSG00000143603
KCNN4	-0.0023	9.6921E-01	9.8482E-01	ENSG00000104783
KCNQ1	-0.1525	1.2010E-01	2.8753E-01	ENSG00000053918
KCNQ1-AS1	0.0841	2.8377E-02		ENSG00000229414
KCNQ1DN	-0.0019	9.2399E-01		ENSG00000237941
KCNQ1OT1	0.5332	2.4987E-10	3.4156E-08	ENSG00000269821
KCNQ2	-0.1630	1.1794E-02	5.7304E-02	ENSG00000075043
KCNQ3	0.0087	9.1523E-01	9.5818E-01	ENSG00000184156
KCNQ4	-0.1547	1.0258E-01	2.5909E-01	ENSG00000117013
KCNQ5	-0.1430	7.8907E-02	2.1761E-01	ENSG00000185760
KCNQ5-AS1	0.0176	5.6015E-01		ENSG00000229154
KCNRG	0.0960	2.8633E-01	4.9712E-01	ENSG00000198553
KCNS1	0.0438	6.6390E-01	8.1090E-01	ENSG00000124134
KCNS2	-0.0134	8.2256E-01	9.0819E-01	ENSG00000156486
KCNS3	-0.0646	4.5182E-01	6.5084E-01	ENSG00000170745
KCNT1	0.0781	3.5232E-01	5.6269E-01	ENSG00000107147
KCNT2	0.0770	3.6263E-01	5.7192E-01	ENSG00000162687
KCNU1	-0.0048	7.7017E-01		ENSG00000215262
KCNV1	0.0150	7.6576E-01	8.7418E-01	ENSG00000164794
KCNV2	0.0163	6.8559E-01		ENSG00000168263
KCP	0.2333	2.2566E-02	9.0592E-02	ENSG00000135253
KCTD1	0.0981	2.4421E-01	4.5180E-01	ENSG00000134504
KCTD10	-0.1007	1.4896E-01	3.2951E-01	ENSG00000110906
KCTD11	0.1609	1.0510E-01	2.6327E-01	ENSG00000213859
KCTD12	0.2617	1.6032E-02	7.1165E-02	ENSG00000178695
KCTD13	0.0044	9.5028E-01	9.7529E-01	ENSG00000174943
KCTD14	-0.0018	9.7551E-01	9.8789E-01	ENSG00000151364
KCTD15	0.1869	3.8716E-02	1.3370E-01	ENSG00000153885
KCTD16	0.2437	4.4842E-03	2.7709E-02	ENSG00000183775
KCTD17	0.0036	9.6375E-01	9.8190E-01	ENSG00000100379
KCTD18	0.0827	1.8601E-01	3.8015E-01	ENSG00000155729
KCTD19	0.0967	2.7659E-01	4.8653E-01	ENSG00000168676
KCTD2	-0.1783	1.9274E-02	8.0844E-02	ENSG00000180901
KCTD20	-0.1151	3.5836E-02	1.2620E-01	ENSG00000112078
KCTD21	-0.0444	6.0307E-01	7.6768E-01	ENSG00000188997
KCTD21-AS1	-0.0453	5.4676E-01	7.2672E-01	ENSG00000246174
KCTD3	0.0331	5.9898E-01	7.6497E-01	ENSG00000136636
KCTD4	-0.0107	7.4863E-01	8.6354E-01	ENSG00000180332
KCTD5	-0.0097	8.9481E-01	9.4750E-01	ENSG00000167977
KCTD6	-0.1943	8.9606E-05	1.4169E-03	ENSG00000168301
KCTD7	-0.0505	4.9653E-01	6.8801E-01	ENSG00000243335

KCTD9	0.1106	1.8294E-01	3.7607E-01	ENSG00000104756
KDELR1	-0.0632	3.5053E-01	5.6067E-01	ENSG00000105438
KDELR2	0.1201	6.2896E-02	1.8642E-01	ENSG00000136240
KDELR3	0.2298	2.4162E-02	9.5366E-02	ENSG00000100196
KDF1	-0.0782	2.3916E-01	4.4565E-01	ENSG00000175707
KDM1A	-0.1443	2.3806E-02	9.4305E-02	ENSG00000004487
KDM1B	-0.3043	1.0945E-03	9.7036E-03	ENSG00000165097
KDM2A	-0.0296	6.6000E-01	8.0833E-01	ENSG00000173120
KDM2B	-0.1081	1.4145E-01	3.1833E-01	ENSG00000089094
KDM3A	-0.1411	2.4464E-02	9.6096E-02	ENSG00000115548
KDM3B	0.0288	6.3994E-01	7.9402E-01	ENSG00000120733
KDM4A	-0.1849	3.5267E-02	1.2469E-01	ENSG00000066135
KDM4B	0.1781	4.8687E-02	1.5665E-01	ENSG00000127663
KDM4C	0.0919	2.1415E-01	4.1527E-01	ENSG00000107077
KDM4D	0.0569	4.5825E-01	6.5619E-01	ENSG00000186280
KDM5A	0.1869	3.3960E-04	3.9489E-03	ENSG00000073614
KDM5B	-0.0559	3.0630E-01	5.1649E-01	ENSG00000117139
KDM5C	0.0302	6.7312E-01	8.1708E-01	ENSG00000126012
KDM6A	-0.0005	9.6684E-01	9.8344E-01	ENSG00000147050
KDM6B	0.0125	8.9036E-01	9.4567E-01	ENSG00000132510
KDM7A	-0.0985	2.0671E-01	4.0621E-01	ENSG00000006459
KDM7A-DT	0.5698	3.5049E-06	1.0517E-04	ENSG00000260231
KDM8	0.2473	3.4726E-02	1.2325E-01	ENSG00000155666
KDR	0.0152	9.5669E-01		ENSG00000128052
KDSR	-0.1323	2.4289E-02	9.5680E-02	ENSG00000119537
KEAP1	-0.1255	1.2445E-01	2.9424E-01	ENSG00000079999
KEL	0.0290	4.0193E-01	6.0655E-01	ENSG00000197993
KHDC1	-0.1348	8.3703E-02	2.2597E-01	ENSG00000135314
KHDC1-AS1	0.0913	1.9944E-01	3.9778E-01	ENSG00000229852
KHDC1L	0.0081	7.8408E-01		ENSG00000256980
KHDC4	0.1235	1.0007E-01	2.5481E-01	ENSG00000132680
KHDRBS1	-0.3058	1.3477E-08	1.0801E-06	ENSG00000121774
KHDRBS2	0.0558	5.1009E-01	6.9882E-01	ENSG00000112232
KHDRBS3	-0.2176	1.2123E-02	5.8361E-02	ENSG00000131773
KHK	-0.1706	1.5009E-02	6.7973E-02	ENSG00000138030
KHNYN	-0.0996	2.1468E-01	4.1593E-01	ENSG00000100441
KHSRP	-0.2983	2.8545E-03	1.9729E-02	ENSG00000088247
KIAA0040	0.0918	1.8252E-01	3.7552E-01	ENSG00000235750
KIAA0087	0.0666	1.4979E-02		ENSG00000122548
KIAA0232	0.1457	5.7469E-02	1.7574E-01	ENSG00000170871
KIAA0319	0.2603	1.4238E-02	6.5505E-02	ENSG00000137261
KIAA0319L	-0.0102	8.5943E-01	9.2918E-01	ENSG00000142687
KIAA0408	0.0386	4.2313E-01		ENSG00000189367
KIAA0513	-0.0242	7.6135E-01	8.7163E-01	ENSG00000135709
KIAA0586	-0.0145	8.1386E-01	9.0305E-01	ENSG00000100578
KIAA0753	0.0863	2.9123E-01	5.0155E-01	ENSG00000198920
KIAA0825	0.1716	8.2467E-02	2.2373E-01	ENSG00000185261
KIAA0930	-0.2646	1.3641E-03	1.1440E-02	ENSG00000100364
KIAA1143	-0.0627	3.0180E-01	5.1180E-01	ENSG00000163807

KIAA1191	-0.2243	1.0406E-05	2.5350E-04	ENSG00000122203
KIAA1191P1	0.0075	6.0918E-01		ENSG00000213480
KIAA1210	0.0077	8.5685E-01		ENSG00000250423
KIAA1217	0.0453	5.8961E-01	7.5812E-01	ENSG00000120549
KIAA1328	0.1844	4.8774E-03	2.9493E-02	ENSG00000150477
KIAA1549	-0.0200	7.9236E-01	8.9031E-01	ENSG00000122778
KIAA1549L	0.0064	9.3718E-01	9.6912E-01	ENSG00000110427
KIAA1586	0.1992	2.5641E-03	1.8124E-02	ENSG00000168116
KIAA1614	0.0174	7.9573E-01	8.9205E-01	ENSG00000135835
KIAA1614-AS1	-0.0062	8.3681E-01		ENSG00000232586
KIAA1671	0.0785	2.7972E-01	4.8975E-01	ENSG00000197077
KIAA1671-AS1	0.0821	3.5896E-01	5.6850E-01	ENSG00000203280
KIAA1755	-0.0369	5.4505E-01	7.2558E-01	ENSG00000149633
KIAA1958	-0.0297	6.8358E-01	8.2353E-01	ENSG00000165185
KIAA2012	0.3742	1.0936E-02	5.4118E-02	ENSG00000182329
KIAA2012-AS1	0.0385	3.1654E-01		ENSG00000222035
KIAA2013	-0.0288	7.1118E-01	8.4147E-01	ENSG00000116685
KIAA2026	0.2186	3.7279E-03	2.4234E-02	ENSG00000183354
KICS2	-0.0423	6.0198E-01	7.6698E-01	ENSG00000174206
KIDINS220	-0.0308	5.7220E-01	7.4555E-01	ENSG00000134313
KIF11	-0.1220	1.8981E-01	3.8487E-01	ENSG00000138160
KIF12	-0.2261	4.9756E-02	1.5912E-01	ENSG00000136883
KIF13A	-0.0180	8.0758E-01	8.9892E-01	ENSG00000137177
KIF13B	0.2734	4.0773E-03	2.5943E-02	ENSG00000197892
KIF14	-0.2846	1.6698E-02	7.3094E-02	ENSG00000118193
KIF15	-0.1161	2.0293E-01	4.0190E-01	ENSG00000163808
KIF16B	0.1438	1.0197E-01	2.5802E-01	ENSG00000089177
KIF17	0.2140	3.8760E-02	1.3378E-01	ENSG00000117245
KIF18A	-0.1094	2.2716E-01	4.3138E-01	ENSG00000121621
KIF18B	-0.0608	4.7933E-01	6.7448E-01	ENSG00000186185
KIF19	-0.4896	2.0275E-03	1.5218E-02	ENSG00000196169
KIF1A	-0.3154	1.9766E-05	4.2401E-04	ENSG00000130294
KIF1B	-0.2029	2.4133E-04	3.0264E-03	ENSG00000054523
KIF1C	-0.0733	3.4805E-01	5.5816E-01	ENSG00000129250
KIF1C-AS1	0.0079	6.2701E-01		ENSG00000227495
KIF20A	-0.1289	1.6828E-01	3.5661E-01	ENSG00000112984
KIF20B	0.0330	6.5844E-01	8.0726E-01	ENSG00000138182
KIF21A	0.1217	4.9009E-02	1.5738E-01	ENSG00000139116
KIF21B	0.0504	4.7710E-01	6.7249E-01	ENSG00000116852
KIF22	-0.0839	3.2810E-01	5.3848E-01	ENSG00000079616
KIF23	-0.0902	3.0047E-01	5.1024E-01	ENSG00000137807
KIF23-AS1	0.0398	5.5979E-01	7.3719E-01	ENSG00000259426
KIF24	0.0798	3.6355E-01	5.7268E-01	ENSG00000186638
KIF25	-0.0050	8.5368E-01	9.2560E-01	ENSG00000125337
KIF25-AS1	0.1808	1.0700E-02	5.3263E-02	ENSG00000229921
KIF26A	-0.4471	7.7702E-04	7.5004E-03	ENSG00000066735
KIF26B	0.0796	3.5057E-01	5.6067E-01	ENSG00000162849
KIF26B-AS1	-0.0196	3.7601E-01		ENSG00000232192
KIF27	0.1291	8.1611E-02	2.2227E-01	ENSG00000165115

KIF2A	-0.0454	4.6970E-01	6.6607E-01	ENSG00000068796
KIF2C	-0.1931	6.2503E-02	1.8571E-01	ENSG00000142945
KIF3A	-0.0017	9.7604E-01	9.8803E-01	ENSG00000131437
KIF3B	0.1322	5.6129E-02	1.7309E-01	ENSG00000101350
KIF3C	-0.1219	9.9407E-02	2.5367E-01	ENSG00000084731
KIF4A	-0.1222	1.8818E-01	3.8300E-01	ENSG00000090889
KIF4B	0.0220	3.0202E-01		ENSG00000226650
KIF5A	-0.1535	3.2645E-02	1.1793E-01	ENSG00000155980
KIF5B	-0.3148	4.2563E-07	1.8836E-05	ENSG00000170759
KIF5C	-0.0834	2.5485E-01	4.6291E-01	ENSG00000168280
KIF7	-0.1008	2.3725E-01	4.4308E-01	ENSG00000166813
KIF9	0.5075	6.0773E-07	2.5005E-05	ENSG00000088727
KIFAP3	-0.2560	6.0560E-05	1.0420E-03	ENSG00000075945
KIFBP	0.0451	5.2278E-01	7.0893E-01	ENSG00000198954
KIFC1	-0.2562	2.1352E-02	8.6997E-02	ENSG00000237649
KIFC2	-0.1086	2.1516E-01	4.1642E-01	ENSG00000167702
KIFC3	0.0819	2.8669E-01	4.9755E-01	ENSG00000140859
KIN	0.3156	2.8305E-04	3.4341E-03	ENSG00000151657
KIRREL1	-0.0179	7.8803E-01	8.8795E-01	ENSG00000183853
KIRREL2	-0.0925	2.7850E-01	4.8835E-01	ENSG00000126259
KIRREL3	-0.2677	9.1491E-03	4.7522E-02	ENSG00000149571
KIRREL3-AS2	-0.0030	9.6670E-01	9.8340E-01	ENSG00000254960
KIRREL3-AS3	0.0349	5.8079E-01	7.5220E-01	ENSG00000218109
KISS1	-0.0183	6.1451E-01		ENSG00000170498
KISS1R	0.0681	4.1496E-01	6.1852E-01	ENSG00000116014
KIT	-0.4204	2.1174E-03	1.5721E-02	ENSG00000157404
KITLG	-0.0465	5.6366E-01	7.3991E-01	ENSG00000049130
KIZ	-0.1789	3.0294E-02	1.1199E-01	ENSG00000088970
KIZ-AS1	0.0114	5.3096E-01		ENSG00000232712
KL	0.0026	9.7049E-01	9.8515E-01	ENSG00000133116
KLB	-0.0492	4.6889E-01	6.6553E-01	ENSG00000134962
KLC1	-0.1122	7.8977E-02	2.1773E-01	ENSG00000126214
KLC2	-0.1170	1.0473E-01	2.6263E-01	ENSG00000174996
KLC3	-0.2910	1.2638E-02	6.0078E-02	ENSG00000104892
KLC4	-0.0529	4.3701E-01	6.3765E-01	ENSG00000137171
KLF1	-0.0507	4.0478E-01	6.0933E-01	ENSG00000105610
KLF10	-0.5804	8.3548E-04	7.9005E-03	ENSG00000155090
KLF11	-0.5664	4.0635E-06	1.1874E-04	ENSG00000172059
KLF12	0.0204	8.1304E-01	9.0250E-01	ENSG00000118922
KLF13	-0.0707	4.0827E-01	6.1253E-01	ENSG00000169926
KLF14	0.0248	4.5062E-01	6.4981E-01	ENSG00000266265
KLF15	-0.0557	2.7041E-01	4.7990E-01	ENSG00000163884
KLF16	-0.1610	1.0335E-01	2.6024E-01	ENSG00000129911
KLF2	-0.4427	3.0963E-03	2.1011E-02	ENSG00000127528
KLF2P2	-0.0278	4.4187E-01	6.4129E-01	ENSG00000230646
KLF3	-0.1493	1.9941E-02	8.2836E-02	ENSG00000109787
KLF4	-0.0245	7.4594E-01	8.6157E-01	ENSG00000136826
KLF4P1	0.0180	8.4749E-01		ENSG00000233186
KLF5	0.0984	2.2918E-01	4.3354E-01	ENSG00000102554

KLF6	-0.2195	1.8007E-02	7.7000E-02	ENSG00000067082
KLF7	-0.3330	1.0839E-05	2.6223E-04	ENSG00000118263
KLF8	-0.1924	6.2192E-02	1.8513E-01	ENSG00000102349
KLHDC1	0.1697	7.6108E-02	2.1252E-01	ENSG00000197776
KLHDC10	0.1438	6.9169E-02	1.9896E-01	ENSG00000128607
KLHDC2	-0.1394	2.5159E-02	9.7964E-02	ENSG00000165516
KLHDC3	-0.1441	1.0622E-02	5.2983E-02	ENSG00000124702
KLHDC4	0.0252	6.8970E-01	8.2764E-01	ENSG00000104731
KLHDC7B	-0.0285	2.5701E-01		ENSG00000130487
KLHDC8A	-0.5602	1.2890E-10	2.0730E-08	ENSG00000162873
KLHDC8B	0.0587	4.3660E-01	6.3728E-01	ENSG00000185909
KLHDC9	0.1421	4.4828E-02	1.4808E-01	ENSG00000162755
KLHL1	0.0360	5.9586E-01	7.6268E-01	ENSG00000150361
KLHL10	-0.0106	8.2483E-01		ENSG00000161594
KLHL11	-0.5441	1.8975E-03	1.4477E-02	ENSG00000178502
KLHL12	-0.0709	3.3828E-01	5.4817E-01	ENSG00000117153
KLHL13	-0.2954	1.0204E-02	5.1436E-02	ENSG00000003096
KLHL14	-0.0553	5.1507E-01	7.0274E-01	ENSG00000197705
KLHL15	-0.4585	6.6870E-08	4.1329E-06	ENSG00000174010
KLHL17	-0.0859	3.0248E-01	5.1254E-01	ENSG00000187961
KLHL18	0.2143	1.6548E-03	1.3114E-02	ENSG00000114648
KLHL2	0.0270	7.2471E-01	8.4905E-01	ENSG00000109466
KLHL20	0.1132	7.7450E-02	2.1501E-01	ENSG00000076321
KLHL21	0.3536	1.2456E-03	1.0690E-02	ENSG00000162413
KLHL22	-0.2398	4.5693E-03	2.8121E-02	ENSG00000099910
KLHL23	-0.2764	3.4286E-04	3.9773E-03	ENSG00000213160
KLHL24	-0.0216	7.1757E-01	8.4473E-01	ENSG00000114796
KLHL25	0.0770	3.0443E-01	5.1463E-01	ENSG00000183655
KLHL26	0.2545	9.2391E-03	4.7838E-02	ENSG00000167487
KLHL28	-0.0565	3.7315E-01	5.8178E-01	ENSG00000179454
KLHL29	0.1228	1.3342E-01	3.0717E-01	ENSG00000119771
KLHL2P1	0.2220	2.3419E-02	9.3181E-02	ENSG00000250412
KLHL3	0.0969	2.7725E-01	4.8730E-01	ENSG00000146021
KLHL31	0.0685	3.4147E-01	5.5116E-01	ENSG00000124743
KLHL32	-0.0137	8.6495E-01	9.3177E-01	ENSG00000186231
KLHL33	0.0016	9.1053E-01		ENSG00000185271
KLHL34	-0.0262	4.4466E-01		ENSG00000185915
KLHL35	-0.0391	6.0108E-01	7.6637E-01	ENSG00000149243
KLHL36	-0.0550	3.8327E-01	5.9154E-01	ENSG00000135686
KLHL38	-0.0202	4.6092E-01		ENSG00000175946
KLHL4	0.5711	4.7904E-04	5.1403E-03	ENSG00000102271
KLHL41	-0.0123	7.7125E-01	8.7789E-01	ENSG00000239474
KLHL42	0.0465	3.3352E-01	5.4365E-01	ENSG00000087448
KLHL5	-0.0986	1.3751E-01	3.1310E-01	ENSG00000109790
KLHL6	0.0344	3.0731E-01		ENSG00000172578
KLHL6-AS1	0.0192	3.5247E-01		ENSG00000242522
KLHL7	0.0169	7.8179E-01	8.8438E-01	ENSG00000122550
KLHL8	0.1539	1.5416E-02	6.9358E-02	ENSG00000145332
KLHL9	-0.0687	1.9174E-01	3.8705E-01	ENSG00000198642

KLK1	0.0179	2.9667E-01		ENSG00000167748
KLK10	-0.0257	7.0563E-01	8.3776E-01	ENSG00000129451
KLK13	-0.0086	5.8670E-01		ENSG00000167759
KLK14	-1.7060	5.1836E-04		ENSG00000129437
KLK15	0.0223	4.3679E-01		ENSG00000174562
KLK2	-0.0043	7.8865E-01		ENSG00000167751
KLK4	0.3249	1.8339E-02	7.7988E-02	ENSG00000167749
KLK5	0.0112	4.9999E-01		ENSG00000167754
KLK6	0.0745	1.7918E-02		ENSG00000167755
KLK7	0.0164	6.1364E-01		ENSG00000169035
KLK8	0.0031	8.4552E-01		ENSG00000129455
KLKB1	0.1095	6.2364E-02		ENSG00000164344
KLLN	0.0902	2.5213E-01	4.6034E-01	ENSG00000227268
KLRB1	0.0222	4.1383E-01		ENSG00000111796
KLRC1	0.0338	1.2003E-01		ENSG00000134545
KLRC2	0.0481	2.1144E-01		ENSG00000205809
KLRC3	0.0179	3.7793E-01		ENSG00000205810
KLRC4	-0.0109	4.4951E-01		ENSG00000183542
KLRD1	-0.0706	1.2932E-01		ENSG00000134539
KLRF2	0.0021	9.1082E-01		ENSG00000256797
KLRG1	-0.4404	2.6675E-03	1.8672E-02	ENSG00000139187
KLRG2	0.0100	7.6107E-01		ENSG00000188883
KLRK1-AS1	0.1377	3.1406E-02		ENSG00000245648
KMO	-0.0413	3.7207E-01	5.8075E-01	ENSG00000117009
KMT2A	0.0937	2.0719E-01	4.0685E-01	ENSG00000118058
KMT2B	0.0866	2.5948E-01	4.6792E-01	ENSG00000272333
KMT2C	0.1342	4.2509E-02	1.4258E-01	ENSG00000055609
KMT2CP3	-0.0119	8.2294E-01		ENSG00000274927
KMT2D	0.1558	4.7076E-02	1.5328E-01	ENSG00000167548
KMT2E	-0.1378	1.0549E-02	5.2744E-02	ENSG00000005483
KMT5A	-0.3389	2.5146E-05	5.1517E-04	ENSG00000183955
KMT5AP2	0.0105	5.9387E-01		ENSG00000232531
KMT5B	-0.0835	1.4585E-01	3.2506E-01	ENSG00000110066
KMT5C	0.1250	1.1578E-01	2.8040E-01	ENSG00000133247
KNCN	0.0197	9.0437E-01	9.5326E-01	ENSG00000162456
KNDC1	-0.1458	7.4895E-02	2.1027E-01	ENSG00000171798
KNG1	0.0012	9.9863E-01		ENSG00000113889
KNL1	-0.1317	1.6584E-01	3.5341E-01	ENSG00000137812
KNOP1	-0.0061	9.3109E-01	9.6590E-01	ENSG00000103550
KNOP1P2	-0.0071	6.7594E-01		ENSG00000270429
KNSTRN	-0.0387	5.4302E-01	7.2429E-01	ENSG00000128944
KNTC1	-0.0245	7.5232E-01	8.6560E-01	ENSG00000184445
KPNA1	0.0555	3.7437E-01	5.8300E-01	ENSG00000114030
KPNA2	-0.2384	2.9106E-04	3.5020E-03	ENSG00000182481
KPNA2P1	0.0069	8.2064E-01		ENSG00000236530
KPNA3	-0.1983	8.8059E-05	1.3970E-03	ENSG00000102753
KPNA4	-0.1093	8.1870E-02	2.2263E-01	ENSG00000186432
KPNA4P1	0.0676	9.0845E-02		ENSG00000213754
KPNA5	0.0624	2.7753E-01	4.8754E-01	ENSG00000196911

KPNA6	-0.0367	4.8744E-01	6.8071E-01	ENSG00000025800
KPNA7	0.0833	8.2363E-02		ENSG00000185467
KPNB1	0.1469	4.2469E-02	1.4254E-01	ENSG00000108424
KPNB1-DT	0.0749	3.8917E-01	5.9653E-01	ENSG00000263766
KPTN	-0.1727	1.3441E-02	6.2944E-02	ENSG00000118162
KRAS	0.0325	6.0221E-01	7.6698E-01	ENSG00000133703
KRBA1	-0.0333	6.7953E-01	8.2059E-01	ENSG00000133619
KRBA2	0.0896	1.8745E-01	3.8205E-01	ENSG00000184619
KRBOX1	0.0085	8.1373E-01		ENSG00000240747
KRBOX4	-0.0429	4.7427E-01	6.7008E-01	ENSG00000147121
KRBOX5	0.4110	5.2455E-07	2.2368E-05	ENSG00000197302
KRCC1	0.0514	5.1215E-01	7.0032E-01	ENSG00000172086
KREMEN1	-0.1354	5.3528E-02	1.6716E-01	ENSG00000183762
KREMEN2	-0.2928	4.1977E-03	2.6463E-02	ENSG00000131650
KRI1	-0.4217	1.8568E-05	4.0387E-04	ENSG00000129347
KRIT1	0.4364	6.3949E-05	1.0851E-03	ENSG00000001631
KRR1	-0.0924	1.2284E-01	2.9189E-01	ENSG00000111615
KRT10	0.0222	8.1117E-01	9.0114E-01	ENSG00000186395
KRT10-AS1	0.2552	1.3484E-02	6.3074E-02	ENSG00000167920
KRT12	-0.0093	7.1815E-01		ENSG00000187242
KRT14	-0.0051	9.3834E-01		ENSG00000186847
KRT15	0.3504	2.0228E-02	8.3609E-02	ENSG00000171346
KRT16	0.0229	1.9889E-01		ENSG00000186832
KRT17	0.0424	3.0855E-01	5.1879E-01	ENSG00000128422
KRT18	0.0020	9.8109E-01	9.9019E-01	ENSG00000111057
KRT18P11	0.0033	6.2811E-01		ENSG00000215089
KRT18P14	-0.0136	5.0450E-01		ENSG00000186082
KRT18P15	-0.0193	5.7151E-01		ENSG00000234737
KRT18P16	0.0114	5.2172E-01		ENSG00000235275
KRT18P17	0.0258	3.2730E-01		ENSG00000213943
KRT18P18	-0.0054	6.5652E-01		ENSG00000261764
KRT18P20	0.0223	2.0829E-01		ENSG00000257758
KRT18P26	0.1241	2.2936E-02		ENSG00000229798
KRT18P31	-0.0517	2.5148E-01	4.5974E-01	ENSG00000249850
KRT18P34	-0.0332	2.7206E-01		ENSG00000244515
KRT18P38	0.0192	1.8353E-01		ENSG00000214012
KRT18P4	-0.0043	9.0673E-01		ENSG00000229222
KRT18P46	0.0105	5.1306E-01		ENSG00000235716
KRT18P48	0.0768	2.6671E-01	4.7598E-01	ENSG00000217889
KRT18P5	0.0859	2.8577E-01	4.9650E-01	ENSG00000236670
KRT18P52	0.0017	9.4354E-01		ENSG00000237007
KRT18P55	0.3103	2.4305E-02	9.5699E-02	ENSG00000265480
KRT18P57	-0.0105	5.2965E-01		ENSG00000215867
KRT18P62	-0.0267	4.1945E-01		ENSG00000233471
KRT18P63	0.0347	4.3171E-01		ENSG00000235413
KRT18P7	-0.3081	2.5923E-02	1.0011E-01	ENSG00000258951
KRT19	-0.1120	1.3482E-01	3.0926E-01	ENSG00000171345
KRT19P2	-0.0015	7.2869E-01		ENSG00000216306
KRT20	0.0079	8.5208E-01		ENSG00000171431

KRT222	-0.0981	2.6712E-01	4.7633E-01	ENSG00000213424
KRT23	0.0425	1.7843E-01		ENSG00000108244
KRT24	0.0177	2.7103E-01		ENSG00000167916
KRT25	0.0330	1.4189E-01		ENSG00000204897
KRT31	0.0091	6.3224E-01		ENSG00000094796
KRT39	0.0069	5.6705E-01		ENSG00000196859
KRT40	0.0197	4.3413E-01		ENSG00000204889
KRT5	0.0608	4.7870E-02		ENSG00000186081
KRT7	-0.0349	3.7757E-01		ENSG00000135480
KRT8	0.2397	4.1671E-02	1.4074E-01	ENSG00000170421
KRT80	0.0325	3.0934E-01		ENSG00000167767
KRT81	0.0134	8.0130E-01		ENSG00000205426
KRT86	0.0974	2.1373E-01	4.1477E-01	ENSG00000170442
KRT8P12	-0.1129	2.2511E-01	4.2887E-01	ENSG00000229320
KRT8P14	0.0100	7.4872E-01		ENSG00000214282
KRT8P15	0.5048	8.2400E-03	4.3843E-02	ENSG00000233579
KRT8P3	0.5706	8.9393E-03	4.6784E-02	ENSG00000254285
KRT8P31	-0.0170	4.7759E-01		ENSG00000250148
KRT8P32	0.0007	9.9988E-01		ENSG00000250221
KRT8P33	-0.0318	5.4858E-01	7.2804E-01	ENSG00000250539
KRT8P35	0.0275	3.2229E-01		ENSG00000242841
KRT8P36	-0.0703	2.7747E-01	4.8754E-01	ENSG00000240668
KRT8P39	0.1937	7.4553E-02	2.0962E-01	ENSG00000233560
KRT8P43	-0.0041	6.9529E-01		ENSG00000218186
KRT8P46	0.0255	6.6317E-01	8.1047E-01	ENSG00000248971
KRT8P50	0.0081	8.7554E-01		ENSG00000260799
KRT8P9	-0.0390	4.0289E-01		ENSG00000259470
KRTAP10-10	0.0164	6.9298E-01		ENSG00000221859
KRTAP10-4	-0.0066	5.2897E-01		ENSG00000215454
KRTAP19-1	0.0376	8.9214E-02		ENSG00000184351
KRTAP19-5	0.0426	3.9920E-01		ENSG00000186977
KRTAP4-2	-0.0012	8.2896E-01		ENSG00000244537
KRTAP4-6	0.0031	8.8264E-01		ENSG00000198090
KRTAP5-1	-0.0026	9.2793E-01	9.6462E-01	ENSG00000205869
KRTAP5-10	0.0375	1.5469E-01		ENSG00000204572
KRTAP5-11	-0.0014	9.7530E-01		ENSG00000204571
KRTAP5-5	0.0544	4.8623E-02		ENSG00000185940
KRTAP5-7	0.0309	2.5757E-01		ENSG00000244411
KRTAP5-8	0.0369	3.0207E-02		ENSG00000241233
KRTAP5-9	0.0117	5.8266E-01		ENSG00000254997
KRTAP5-AS1	-0.0243	7.6721E-01	8.7517E-01	ENSG00000233930
KRTAP8-1	0.0038	6.4908E-01		ENSG00000183640
KRTCAP2	0.0675	4.2541E-01	6.2768E-01	ENSG00000163463
KRTDAP	0.0175	5.0367E-01		ENSG00000188508
KSR1	0.0133	8.7731E-01	9.3894E-01	ENSG00000141068
KSR2	0.0995	1.3554E-01	3.1028E-01	ENSG00000171435
KTI12	-0.2030	3.7953E-02	1.3180E-01	ENSG00000198841
KTN1	-0.0444	5.0432E-01	6.9434E-01	ENSG00000126777
KTN1-AS1	0.0159	8.4065E-01	9.1799E-01	ENSG00000186615

KXD1	-0.0627	2.3413E-01	4.3919E-01	ENSG00000105700
KY	0.1103	1.2820E-01	2.9990E-01	ENSG00000174611
KYAT1	0.0294	5.6459E-01	7.4050E-01	ENSG00000286112
KYAT1	-0.0706	3.7346E-01	5.8205E-01	ENSG00000171097
KYAT3	0.2027	1.7743E-02	7.6285E-02	ENSG00000137944
KYNU	0.0568	1.3189E-01	3.0523E-01	ENSG00000115919
L1CAM	-0.1498	5.6672E-02	1.7408E-01	ENSG00000198910
L1CAM-AS1	0.0437	5.8123E-01	7.5242E-01	ENSG00000273769
L1TD1	0.0234	7.2421E-01	8.4866E-01	ENSG00000240563
L2HGDH	0.0659	3.3376E-01	5.4389E-01	ENSG00000087299
L3HYPDH	0.1123	1.9665E-01	3.9414E-01	ENSG00000126790
L3MBTL1	0.1143	1.0538E-01	2.6377E-01	ENSG00000185513
L3MBTL2	-0.1831	1.7743E-02	7.6285E-02	ENSG00000100395
L3MBTL3	-0.0496	5.1446E-01	7.0210E-01	ENSG00000198945
L3MBTL4	-0.3940	4.5287E-03	2.7934E-02	ENSG00000154655
L3MBTL4-AS1	-0.0343	2.3535E-01		ENSG00000264707
LACC1	0.1807	8.4468E-02	2.2718E-01	ENSG00000179630
LACTB	0.0378	6.1127E-01	7.7359E-01	ENSG00000103642
LACTB2	0.1462	1.3061E-01	3.0351E-01	ENSG00000147592
LACTB2-AS1	0.1242	1.6491E-01	3.5204E-01	ENSG00000246366
LAD1	0.0150	7.3111E-01		ENSG00000159166
LAG3	0.0111	8.9121E-01	9.4593E-01	ENSG00000089692
LAGE3	-0.0696	2.8965E-01	5.0006E-01	ENSG00000196976
LAIR1	-0.0083	4.4260E-01		ENSG00000167613
LAIR2	0.0168	3.7699E-01		ENSG00000167618
LALBA	0.0145	4.9856E-01		ENSG00000167531
LAMA1	0.0625	4.3845E-01	6.3860E-01	ENSG00000101680
LAMA3	0.0514	5.4199E-01	7.2366E-01	ENSG00000053747
LAMA4	0.4442	3.5304E-03	2.3201E-02	ENSG00000112769
LAMA5	-0.0063	9.1728E-01	9.5890E-01	ENSG00000130702
LAMA5-AS1	0.0540	3.2099E-01	5.3103E-01	ENSG00000228812
LAMB1	0.3854	2.0693E-04	2.6644E-03	ENSG00000091136
LAMB2	0.1226	9.6738E-02	2.4984E-01	ENSG00000172037
LAMB3	0.4765	5.2638E-03	3.1352E-02	ENSG00000196878
LAMB4	0.0470	1.0156E-01	2.5742E-01	ENSG00000091128
LAMC1	0.0961	2.0868E-01	4.0877E-01	ENSG00000135862
LAMC1-AS1	0.0523	1.5882E-01		ENSG00000224468
LAMC2	0.0935	2.7546E-01	4.8533E-01	ENSG00000058085
LAMC3	0.0805	3.0980E-01	5.2007E-01	ENSG00000050555
LAMP1	-0.1587	4.6670E-02	1.5236E-01	ENSG00000185896
LAMP2	-0.2870	1.5601E-04	2.1430E-03	ENSG00000005893
LAMP3	-0.2642	2.9344E-02	1.0943E-01	ENSG00000078081
LAMP5-AS1	0.0025	9.4198E-01		ENSG00000225988
LAMTOR1	-0.1878	2.6373E-04	3.2510E-03	ENSG00000149357
LAMTOR2	-0.1663	6.4081E-03	3.6297E-02	ENSG00000116586
LAMTOR3	-0.0412	4.8757E-01	6.8078E-01	ENSG00000109270
LAMTOR4	-0.0396	4.7713E-01	6.7250E-01	ENSG00000188186
LAMTOR5	-0.1435	4.2326E-02	1.4221E-01	ENSG00000134248
LAMTOR5-AS1	0.3673	1.9449E-03	1.4764E-02	ENSG00000224699

LANCL1	-0.0517	3.9984E-01	6.0523E-01	ENSG00000115365
LANCL2	0.0611	3.3214E-01	5.4247E-01	ENSG00000132434
LANCL3	-0.0862	3.2644E-01	5.3655E-01	ENSG00000147036
LAP3	0.0216	7.2541E-01	8.4935E-01	ENSG00000002549
LAPTM4A	-0.1964	2.5623E-03	1.8116E-02	ENSG00000068697
LAPTM4B	-0.0448	4.8445E-01	6.7822E-01	ENSG00000104341
LAPTM5	0.0256	5.5288E-01	7.3138E-01	ENSG00000162511
LARGE-AS1	0.0982	1.2779E-01	2.9958E-01	ENSG00000224973
LARGE1	-0.0692	3.5167E-01	5.6191E-01	ENSG00000133424
LARGE2	0.0403	6.2481E-01	7.8333E-01	ENSG00000165905
LARP1	0.0638	3.8169E-01	5.8992E-01	ENSG00000155506
LARP1B	0.1460	5.9817E-03	3.4388E-02	ENSG00000138709
LARP4	-0.0552	3.6268E-01	5.7192E-01	ENSG00000161813
LARP4B	0.1873	1.4315E-02	6.5737E-02	ENSG00000107929
LARP6	-0.0859	2.0053E-01	3.9896E-01	ENSG00000166173
LARP7	-0.0412	5.2182E-01	7.0850E-01	ENSG00000174720
LARP7P2	0.0205	4.6002E-01		ENSG00000261939
LARS1	-0.0961	5.5503E-02	1.7176E-01	ENSG00000133706
LARS2	-0.2700	4.9930E-04	5.2941E-03	ENSG00000011376
LAS1L	0.1410	5.2100E-02	1.6391E-01	ENSG00000001497
LASP1	-0.2381	2.1262E-03	1.5753E-02	ENSG00000002834
LASP1NB	-0.1389	1.3119E-01	3.0420E-01	ENSG00000263874
LAT	0.0051	9.6532E-01		ENSG00000213658
LAT2	0.1163	1.9896E-01	3.9719E-01	ENSG00000086730
LATS1	-0.2460	1.3310E-04	1.9063E-03	ENSG00000131023
LATS2	-0.0129	8.8704E-01	9.4386E-01	ENSG00000150457
LAX1	0.0029	9.9465E-01		ENSG00000122188
LAYN	-0.1707	8.6902E-02	2.3180E-01	ENSG00000204381
LBH	-0.0936	1.1818E-01	2.8436E-01	ENSG00000213626
LBHD1	-0.0653	4.4054E-01	6.4044E-01	ENSG00000162194
LBHD2	0.0277	6.2805E-01		ENSG00000283071
LBP	-0.0136	6.7567E-01		ENSG00000129988
LBR	-0.1933	2.1126E-03	1.5700E-02	ENSG00000143815
LBX1	0.0325	4.4999E-01		ENSG00000138136
LBX2	-0.0712	1.9908E-01	3.9734E-01	ENSG00000179528
LBX2-AS1	-0.0111	3.5956E-01	5.6890E-01	ENSG00000257702
LCA5	-0.0341	6.2785E-01	7.8572E-01	ENSG00000135338
LCA5L	0.5744	2.7589E-04	3.3688E-03	ENSG00000157578
LCAL1	0.2423	1.0990E-01	2.7090E-01	ENSG00000286042
LCAT	0.2204	5.0439E-02	1.6049E-01	ENSG00000213398
LCDR	0.1347	9.9443E-02	2.5371E-01	ENSG00000273148
LCE1D	-0.0034	8.1829E-01		ENSG00000172155
LCE1E	0.0037	8.1856E-01		ENSG00000186226
LCIIAR	0.0596	4.1899E-01	6.2195E-01	ENSG00000256802
LCK	0.0861	2.2555E-01	4.2928E-01	ENSG00000182866
LCLAT1	-0.0185	7.2600E-01	8.4958E-01	ENSG00000172954
LCMT1	-0.0235	6.5189E-01	8.0290E-01	ENSG00000205629
LCMT1-AS2	-0.0558	4.7706E-01	6.7249E-01	ENSG00000260034
LCMT2	0.1760	3.1923E-02	1.1605E-01	ENSG00000168806

LCN1	-0.0301	9.2263E-01	9.6176E-01	ENSG00000160349
LCN12	-0.0321	5.2783E-01	7.1270E-01	ENSG00000184925
LCN15	0.0219	3.4261E-01		ENSG00000177984
LCN1P1	0.0039	9.9265E-01		ENSG00000119440
LCN2	0.0249	2.5216E-01		ENSG00000148346
LCN9	0.0449	1.6570E-01		ENSG00000148386
LCNL1	0.0238	7.5626E-01	8.6801E-01	ENSG00000214402
LCOR	-0.0946	5.9517E-02	1.7934E-01	ENSG00000196233
LCORL	0.0050	9.5393E-01	9.7701E-01	ENSG00000178177
LCP2	0.0157	8.6295E-01		ENSG00000043462
LCTL	0.1219	1.3191E-01	3.0523E-01	ENSG00000188501
LDAF1	-0.3007	4.1294E-04	4.5789E-03	ENSG00000011638
LDAH	0.1259	9.2974E-02	2.4323E-01	ENSG00000118961
LDB1	-0.1632	3.3858E-02	1.2085E-01	ENSG00000198728
LDB2	0.0627	4.0401E-01	6.0858E-01	ENSG00000169744
LDHA	0.3309	1.8854E-02	7.9599E-02	ENSG00000134333
LDHAL6A	0.0122	5.8396E-01	7.5414E-01	ENSG00000166800
LDHAL6B	0.0110	5.2895E-01		ENSG00000171989
LDHB	-0.3799	1.9018E-07	9.7083E-06	ENSG00000111716
LDHC	-0.0027	6.2004E-01	7.8028E-01	ENSG00000166796
LDHD	0.0577	4.8660E-01	6.8025E-01	ENSG00000166816
LDLR	-0.2323	1.1819E-02	5.7381E-02	ENSG00000130164
LDLRAD2	0.0213	3.4556E-01		ENSG00000187942
LDLRAD3	-0.2556	2.9245E-03	2.0094E-02	ENSG00000179241
LDLRAD4	0.1637	6.0399E-02	1.8120E-01	ENSG00000168675
LDLRAD4-AS1	0.0647	2.3054E-01	4.3531E-01	ENSG00000267690
LDLRAP1	0.1578	1.1553E-01	2.8013E-01	ENSG00000157978
LDOC1	-0.1211	4.1317E-02	1.3997E-01	ENSG00000182195
LEAP2	0.0909	2.9866E-01	5.0843E-01	ENSG00000164406
LECT2	0.0295	8.9695E-02		ENSG00000145826
LEF1	-0.2316	4.4675E-02	1.4775E-01	ENSG00000138795
LEF1-AS1	0.0503	5.2557E-01	7.1097E-01	ENSG00000232021
LEFTY1	0.0302	6.3780E-01	7.9255E-01	ENSG00000243709
LEFTY2	0.0492	2.3502E-01	4.4039E-01	ENSG00000143768
LEFTY3P	-0.0026	8.6284E-01		ENSG00000234112
LEMD1	-0.0042	9.5367E-01	9.7701E-01	ENSG00000186007
LEMD1-AS1	0.0533	4.8086E-01	6.7551E-01	ENSG00000226235
LEMD1-DT	-0.0268	7.4774E-01	8.6287E-01	ENSG00000224717
LEMD2	-0.0503	4.0429E-01	6.0890E-01	ENSG00000161904
LEMD3	-0.1970	2.8850E-03	1.9895E-02	ENSG00000174106
LENEP	0.0348	5.5497E-02		ENSG00000163352
LENG1	-0.0253	7.4424E-01	8.6086E-01	ENSG00000105617
LENG8	-0.0338	6.1952E-01	7.7989E-01	ENSG00000167615
LENG8-AS1	0.0322	6.8490E-01	8.2442E-01	ENSG00000226696
LENG9	-0.0661	3.5234E-01	5.6269E-01	ENSG00000275183
LEO1	0.0824	2.5126E-01	4.5951E-01	ENSG00000166477
LEPROT	-0.0007	9.9038E-01	9.9461E-01	ENSG00000213625
LEPROTL1	0.0081	9.0360E-01	9.5296E-01	ENSG00000104660
LETM1	-0.1902	4.3803E-03	2.7258E-02	ENSG00000168924

LETM2	0.2343	3.3560E-03	2.2269E-02	ENSG00000165046
LETMD1	0.0123	8.2139E-01	9.0747E-01	ENSG00000050426
LETR1	-0.0125	8.6004E-01	9.2954E-01	ENSG00000248441
LFNG	0.0113	8.9245E-01	9.4647E-01	ENSG00000106003
LGALS1	0.0566	5.0231E-01	6.9282E-01	ENSG00000100097
LGALS12	-0.0161	3.8790E-01		ENSG00000133317
LGALS13	0.0150	2.9472E-01		ENSG00000105198
LGALS14	-0.0112	5.7275E-01	7.4605E-01	ENSG00000006659
LGALS2	0.1313	6.0904E-02	1.8217E-01	ENSG00000100079
LGALS3	0.1345	1.5137E-01	3.3285E-01	ENSG00000131981
LGALS3BP	0.0242	7.4639E-01	8.6200E-01	ENSG00000108679
LGALS4	-0.0296	6.8192E-01	8.2253E-01	ENSG00000171747
LGALS7	0.1599	4.1509E-02	1.4039E-01	ENSG00000205076
LGALS8	0.1232	1.2746E-01	2.9893E-01	ENSG00000116977
LGALS8-AS1	0.0216	5.2867E-01		ENSG00000223776
LGALS9	0.0089	8.7407E-01		ENSG00000168961
LGALSL	-0.2857	5.3142E-05	9.3405E-04	ENSG00000119862
LGI1	0.1022	2.3122E-01	4.3616E-01	ENSG00000108231
LGI2	0.4634	5.1840E-03	3.0959E-02	ENSG00000153012
LGI3	0.0149	8.6204E-01	9.3066E-01	ENSG00000168481
LGMN	0.0447	4.8941E-01	6.8252E-01	ENSG00000100600
LGR4	0.2392	2.6577E-02	1.0192E-01	ENSG00000205213
LGR4-AS1	0.0458	8.9870E-02		ENSG00000254862
LGR5	0.2015	3.3666E-02	1.2054E-01	ENSG00000139292
LGSN	0.0572	9.4421E-02		ENSG00000146166
LHB	0.0742	9.8710E-02		ENSG00000104826
LHCGR	0.0025	9.9166E-01		ENSG00000138039
LHFPL1	-0.2041	6.2355E-02	1.8545E-01	ENSG00000182508
LHFPL2	-0.2209	4.1565E-02	1.4052E-01	ENSG00000145685
LHFPL3	0.0858	3.2339E-01	5.3341E-01	ENSG00000187416
LHFPL3-AS1	0.0278	5.9271E-01		ENSG00000226869
LHFPL3-AS2	0.0229	2.7575E-01		ENSG00000225329
LHFPL4	-0.1502	4.9545E-02	1.5855E-01	ENSG00000156959
LHFPL5	-0.0164	8.4201E-01	9.1870E-01	ENSG00000197753
LHFPL6	0.5996	2.4238E-07	1.1904E-05	ENSG00000183722
LHFPL7	0.0167	2.3504E-01		ENSG00000206069
LHPP	-0.2435	3.0249E-03	2.0623E-02	ENSG00000107902
LHX1	0.1942	6.4831E-03	3.6629E-02	ENSG00000273706
LHX2	-0.2764	1.8538E-03	1.4223E-02	ENSG00000106689
LHX2-AS1	-0.1003	2.4658E-01	4.5421E-01	ENSG00000236668
LHX3	0.0359	2.3567E-01		ENSG00000107187
LHX4	0.0507	4.9483E-01	6.8683E-01	ENSG00000121454
LHX6	0.2954	2.8343E-02	1.0680E-01	ENSG00000106852
LHX8	0.0042	9.6182E-01		ENSG00000162624
LHX9	0.0362	6.6728E-01	8.1362E-01	ENSG00000143355
LIAS	-0.0431	4.8906E-01	6.8220E-01	ENSG00000121897
LIAT1	0.3957	5.6377E-03	3.2926E-02	ENSG00000187624
LIF	-0.0822	2.4385E-01	4.5154E-01	ENSG00000128342
LIFR	0.2877	1.0705E-03	9.5572E-03	ENSG00000113594

LIFR-AS1	0.3441	1.0546E-04	1.6038E-03	ENSG00000244968
LIG1	-0.0720	3.4421E-01	5.5391E-01	ENSG00000105486
LIG3	-0.0430	5.1812E-01	7.0547E-01	ENSG00000005156
LIG4	0.0746	2.9484E-01	5.0483E-01	ENSG00000174405
LILRA4	0.0159	5.6738E-01		ENSG00000239961
LILRA6	0.0243	6.9768E-01	8.3292E-01	ENSG00000244482
LILRB3	0.0142	8.6080E-01	9.3017E-01	ENSG00000204577
LILRB5	0.1134	7.2956E-03	4.0041E-02	ENSG00000105609
LIM2	0.0994	1.1928E-02		ENSG00000105370
LIMA1	-0.1412	3.0146E-02	1.1166E-01	ENSG00000050405
LIMCH1	-0.0508	4.5738E-01	6.5555E-01	ENSG00000064042
LIMD1	0.0324	6.9604E-01	8.3174E-01	ENSG00000144791
LIMD2	0.0688	2.9559E-01	5.0567E-01	ENSG00000136490
LIME1	0.0782	2.6241E-01	4.7129E-01	ENSG00000203896
LIMK1	-0.1241	8.7440E-02	2.3284E-01	ENSG00000106683
LIMK2	0.1332	7.1849E-02	2.0452E-01	ENSG00000182541
LIMS1	0.0585	4.4389E-01	6.4294E-01	ENSG00000169756
LIMS1-AS1	0.0155	6.0642E-01		ENSG00000228763
LIMS3	0.4608	7.3826E-03	4.0386E-02	ENSG00000256977
LIMS4	0.4675	7.2242E-03	3.9739E-02	ENSG00000256671
LIN28A	0.0509	3.5004E-01	5.6017E-01	ENSG00000131914
LIN28B	0.2452	1.8413E-02	7.8247E-02	ENSG00000187772
LIN28B-AS1	0.2882	9.3526E-03	4.8284E-02	ENSG00000203809
LIN37	0.0738	3.9250E-01	5.9937E-01	ENSG00000267796
LIN52	-0.1081	1.0522E-01	2.6353E-01	ENSG00000205659
LIN54	-0.0592	4.1139E-01	6.1553E-01	ENSG00000189308
LIN7A	-0.1812	3.8714E-02	1.3370E-01	ENSG00000111052
LIN7B	0.0988	1.9713E-01	3.9475E-01	ENSG00000104863
LIN7C	-0.1630	4.2449E-02	1.4251E-01	ENSG00000148943
LIN9	-0.0165	8.3245E-01	9.1358E-01	ENSG00000183814
LINC-PINT	-0.0682	3.2973E-01	5.4006E-01	ENSG00000231721
LINC00028	0.0062	9.2726E-01		ENSG00000233354
LINC00051	-0.0159	3.5457E-01		ENSG00000254008
LINC00052	0.1510	5.0807E-02	1.6130E-01	ENSG00000259527
LINC00092	-0.0034	8.6102E-01		ENSG00000225194
LINC00102	-0.0346	4.7946E-01	6.7454E-01	ENSG00000230542
LINC00106	0.1583	8.8797E-02	2.3533E-01	ENSG00000236871
LINC00158	0.0887	1.3340E-01	3.0717E-01	ENSG00000185433
LINC00173	0.1060	2.3898E-01	4.4550E-01	ENSG00000196668
LINC00174	0.0180	8.2709E-01	9.1051E-01	ENSG00000179406
LINC00184	0.0212	6.6254E-01		ENSG00000224939
LINC00189	0.0110	6.9085E-01		ENSG00000215533
LINC00200	0.0506	4.6045E-01	6.5803E-01	ENSG00000229205
LINC00205	0.0694	3.2280E-01	5.3288E-01	ENSG00000223768
LINC00216	0.0467	4.9097E-01	6.8345E-01	ENSG00000279636
LINC00222	0.0271	1.9026E-01		ENSG00000203801
LINC00237	-0.1487	6.7883E-02	1.9645E-01	ENSG00000225127
LINC00239	0.0340	4.0671E-01		ENSG00000258512
LINC00240	0.1493	1.2503E-01	2.9512E-01	ENSG00000224843

LINC00242	-0.0017	9.7916E-01	9.8958E-01	ENSG00000229214
LINC00243	-0.0001	7.4414E-01		ENSG00000214894
LINC00244	0.0277	2.0736E-01		ENSG00000279418
LINC00251	0.0009	8.4714E-01		ENSG00000280725
LINC00269	0.1164	1.0285E-01	2.5959E-01	ENSG00000215162
LINC00276	0.0097	4.4309E-01		ENSG00000230448
LINC00290	0.0163	3.4280E-01		ENSG00000248197
LINC00294	0.0644	4.4345E-01	6.4269E-01	ENSG00000280798
LINC00299	0.0880	1.5119E-01	3.3255E-01	ENSG00000236790
LINC00303	0.0381	6.4420E-02		ENSG00000176754
LINC00304	0.0433	4.0042E-01	6.0570E-01	ENSG00000180422
LINC00305	0.0041	8.3979E-01		ENSG00000179676
LINC00309	-0.0346	4.5809E-01	6.5611E-01	ENSG00000230923
LINC00310	0.1631	5.7391E-02	1.7561E-01	ENSG00000227456
LINC00311	0.2591	3.1510E-02	1.1520E-01	ENSG00000179219
LINC00313	0.0472	1.4334E-01		ENSG00000185186
LINC00316	0.0052	8.1868E-01		ENSG00000237664
LINC00320	-0.0472	3.5929E-02		ENSG00000224924
LINC00323	-0.0070	5.8338E-01		ENSG00000226496
LINC00324	0.2667	1.9111E-02	8.0379E-02	ENSG00000178977
LINC00326	0.1149	8.6502E-03	4.5585E-02	ENSG00000231023
LINC00327	0.0146	8.4527E-01	9.2085E-01	ENSG00000232977
LINC00330	0.0079	7.0496E-01		ENSG00000235097
LINC00334	0.0623	3.3758E-01	5.4765E-01	ENSG00000182586
LINC00339	-0.0278	7.3611E-01	8.5602E-01	ENSG00000218510
LINC00343	-0.0179	2.9379E-01		ENSG00000226620
LINC00347	0.0119	2.9350E-01		ENSG00000236678
LINC00348	0.0026	8.4544E-01		ENSG00000226846
LINC00351	2.3387	1.4032E-03		ENSG00000226317
LINC00354	0.3075	4.8903E-03	2.9557E-02	ENSG00000226903
LINC00355	0.0165	5.3155E-01		ENSG00000227674
LINC00364	-0.0056	8.1572E-01		ENSG00000230040
LINC00365	0.0258	4.6931E-01		ENSG00000224511
LINC00375	0.0027	4.0880E-01		ENSG00000226370
LINC00381	0.0399	2.9163E-01		ENSG00000226240
LINC00383	0.0307	1.1176E-01		ENSG00000235221
LINC00391	0.0521	5.3235E-01	7.1608E-01	ENSG00000238230
LINC00393	0.0260	2.8781E-01		ENSG00000224853
LINC00402	0.0008	9.5461E-01		ENSG00000235532
LINC00408	0.0508	1.4546E-01		ENSG00000226250
LINC00426	0.0904	3.3655E-02		ENSG00000238121
LINC00443	-0.0008	7.0502E-01		ENSG00000230156
LINC00449	0.0030	9.6921E-01	9.8482E-01	ENSG00000203441
LINC00456	0.0121	6.7590E-01		ENSG00000233124
LINC00457	0.0208	1.9926E-01		ENSG00000225179
LINC00458	0.0910	3.0204E-02		ENSG00000234787
LINC00460	0.0076	7.0946E-01		ENSG00000233532
LINC00463	-0.0241	7.7194E-01	8.7837E-01	ENSG00000234056
LINC00466	0.0039	9.7502E-01		ENSG00000224209

LINC00467	0.4947	5.8937E-07	2.4291E-05	ENSG00000153363
LINC00471	0.0295	7.2276E-01	8.4805E-01	ENSG00000181798
LINC00472	0.2288	3.1810E-02	1.1587E-01	ENSG00000233237
LINC00477	-0.0067	7.2988E-01		ENSG00000197503
LINC00484	0.0234	5.9231E-01		ENSG00000229694
LINC00485	-0.0049	6.0970E-01		ENSG00000258169
LINC00486	0.0858	1.4897E-01	3.2951E-01	ENSG00000230876
LINC00488	0.1426	8.8199E-02	2.3435E-01	ENSG00000214381
LINC00491	0.1569	5.1823E-02	1.6331E-01	ENSG00000250682
LINC00494	0.0011	9.7004E-01		ENSG00000235621
LINC00499	0.0108	4.5784E-01		ENSG00000251372
LINC00501	0.0113	5.9797E-01		ENSG00000203645
LINC00506	0.0020	8.5822E-01		ENSG00000281392
LINC00511	-0.0992	2.4642E-01	4.5414E-01	ENSG00000227036
LINC00515	0.0463	5.0528E-01	6.9517E-01	ENSG00000260583
LINC00519	0.0362	5.6003E-01		ENSG00000258955
LINC00520	0.0167	3.4831E-01		ENSG00000258791
LINC00523	0.0187	7.2825E-01		ENSG00000196273
LINC00528	-0.0529	1.0868E-01		ENSG00000269220
LINC00540	0.0221	1.9548E-01		ENSG00000276476
LINC00545	0.0290	1.3437E-01		ENSG00000236094
LINC00548	0.2042	1.8149E-02	7.7460E-02	ENSG00000215483
LINC00554	0.0157	4.3781E-01		ENSG00000260738
LINC00561	-0.0159	2.9067E-01		ENSG00000261206
LINC00562	0.0272	6.2280E-01	7.8203E-01	ENSG00000260388
LINC00570	0.0199	6.7830E-01		ENSG00000224177
LINC00571	0.0829	1.8067E-01	3.7287E-01	ENSG00000223685
LINC00574	0.3619	1.2634E-02	6.0068E-02	ENSG00000231690
LINC00575	-0.0027	8.0884E-01		ENSG00000231782
LINC00578	0.0668	2.0025E-01	3.9864E-01	ENSG00000228221
LINC00581	-0.0234	7.7434E-01	8.8002E-01	ENSG00000280989
LINC00589	0.1499	9.9182E-02	2.5334E-01	ENSG00000251191
LINC00592	0.0109	4.4985E-01		ENSG00000258279
LINC00595	0.0618	4.7497E-01	6.7061E-01	ENSG00000230417
LINC00602	0.0986	1.7188E-02	7.4590E-02	ENSG00000281832
LINC00605	-0.0325	5.8618E-01	7.5569E-01	ENSG00000251533
LINC00606	-0.0092	6.4859E-01		ENSG00000226567
LINC00607	0.0292	3.5692E-01		ENSG00000235770
LINC00609	0.0060	9.8853E-01		ENSG00000257585
LINC00612	0.0714	3.3300E-01	5.4329E-01	ENSG00000214851
LINC00613	0.0078	9.6417E-01		ENSG00000248330
LINC00620	0.0561	2.9571E-01	5.0576E-01	ENSG00000224514
LINC00622	0.0073	9.2571E-01	9.6366E-01	ENSG00000260941
LINC00624	0.5182	1.9693E-03	1.4897E-02	ENSG00000278811
LINC00629	-0.0560	4.1502E-01	6.1859E-01	ENSG00000227060
LINC00632	0.1482	2.6149E-02	1.0077E-01	ENSG00000203930
LINC00635	0.0514	3.3772E-01	5.4765E-01	ENSG00000241469
LINC00636	0.0802	8.3969E-02	2.2640E-01	ENSG00000240423
LINC00638	-0.0074	8.8086E-01	9.4037E-01	ENSG00000258701

LINC00639	0.1092	1.0151E-01	2.5731E-01	ENSG00000259070
LINC00641	-0.0375	5.9789E-01	7.6415E-01	ENSG00000258441
LINC00645	0.4190	6.0603E-04	6.1899E-03	ENSG00000258548
LINC00648	-0.0174	8.3440E-01	9.1498E-01	ENSG00000259129
LINC00649	0.2206	3.2323E-02	1.1706E-01	ENSG00000237945
LINC00652	-0.0453	5.8098E-01	7.5225E-01	ENSG00000179935
LINC00654	-0.1219	3.7140E-02	1.2962E-01	ENSG00000205181
LINC00658	0.0101	8.2424E-01		ENSG00000226995
LINC00659	0.0909	3.3888E-02		ENSG00000228705
LINC00662	0.0885	1.2558E-01	2.9598E-01	ENSG00000261824
LINC00664	0.0616	4.1171E-01	6.1570E-01	ENSG00000268658
LINC00665	0.0620	3.1663E-01	5.2674E-01	ENSG00000232677
LINC00667	0.0567	2.8964E-01	5.0006E-01	ENSG00000263753
LINC00671	-0.0015	9.5358E-01		ENSG00000213373
LINC00676	0.0126	8.0251E-01	8.9586E-01	ENSG00000234854
LINC00678	0.0035	8.5287E-01		ENSG00000254934
LINC00680-GUSBP4	0.0081	8.5315E-01		ENSG00000283352
LINC00681	0.0343	9.3878E-01	9.6983E-01	ENSG00000255494
LINC00682	0.0195	5.2076E-01		ENSG00000245870
LINC00689	-0.0527	2.2268E-01		ENSG00000231419
LINC00691	-0.0275	2.9909E-01		ENSG00000224074
LINC00703	0.0413	5.5477E-01	7.3302E-01	ENSG00000224382
LINC00707	0.0586	1.4328E-01		ENSG00000238266
LINC00710	-0.0016	9.4867E-01	9.7460E-01	ENSG00000229240
LINC00836	0.1010	4.1214E-01	6.1603E-01	ENSG00000280809
LINC00839	-0.1653	7.0084E-02	2.0103E-01	ENSG00000185904
LINC00842	-0.0725	5.9357E-02	1.7909E-01	ENSG00000285294
LINC00845	0.0100	8.8813E-01		ENSG00000227244
LINC00847	-0.0087	9.1120E-01	9.5625E-01	ENSG00000245060
LINC00852	0.1397	1.3141E-01	3.0449E-01	ENSG00000231177
LINC00853	0.1242	1.7815E-01	3.6944E-01	ENSG00000224805
LINC00858	0.2410	4.1871E-02	1.4113E-01	ENSG00000229404
LINC00861	0.0078	7.4443E-01		ENSG00000245164
LINC00862	0.0050	9.2697E-01		ENSG00000203721
LINC00863	0.1573	2.0844E-02	8.5468E-02	ENSG00000224914
LINC00865	-0.0270	6.0413E-01	7.6833E-01	ENSG00000232229
LINC00870	0.0053	8.9297E-01		ENSG00000243083
LINC00871	0.0538	2.8379E-01	4.9425E-01	ENSG00000258700
LINC00877	-0.0489	5.4341E-01	7.2458E-01	ENSG00000241163
LINC00885	0.0007	9.4378E-01		ENSG00000224652
LINC00886	0.1308	1.5234E-01	3.3433E-01	ENSG00000240875
LINC00887	0.0639	2.6298E-01	4.7182E-01	ENSG00000214145
LINC00901	-0.1396	1.3859E-01	3.1446E-01	ENSG00000242385
LINC00904	0.0330	5.8654E-01	7.5596E-01	ENSG00000271171
LINC00906	-0.0530	2.4207E-01		ENSG00000267339
LINC00907	0.1116	1.2685E-01	2.9819E-01	ENSG00000267586
LINC00908	0.1686	9.5844E-02	2.4840E-01	ENSG00000266256
LINC00910	0.3342	5.1648E-03	3.0860E-02	ENSG00000188825
LINC00921	0.1002	2.4647E-01	4.5415E-01	ENSG00000281005

LINC00922	-0.0035	6.2094E-01		ENSG00000261742
LINC00923	0.0649	2.7613E-01	4.8589E-01	ENSG00000251209
LINC00924	0.0117	7.4308E-01		ENSG00000259134
LINC00926	0.0192	8.0849E-01	8.9939E-01	ENSG00000247982
LINC00927	0.0247	4.5717E-01		ENSG00000259361
LINC00928	-0.0380	2.4614E-01		ENSG00000259218
LINC00934	0.0554	1.2843E-01		ENSG00000281196
LINC00937	-0.1934	7.0532E-02	2.0162E-01	ENSG00000226091
LINC00939	-0.0048	8.0663E-01		ENSG00000249267
LINC00940	0.0203	3.1080E-01		ENSG00000235049
LINC00942	0.0553	2.2363E-01	4.2744E-01	ENSG00000249628
LINC00943	-0.0243	3.6352E-01		ENSG00000189238
LINC00954	0.0691	1.0974E-01		ENSG00000228784
LINC00958	0.2390	2.6801E-02	1.0244E-01	ENSG00000251381
LINC00960	-0.3255	1.0960E-02	5.4173E-02	ENSG00000242516
LINC00964	0.2712	1.8810E-02	7.9462E-02	ENSG00000249816
LINC00967	0.1213	6.4754E-02	1.9040E-01	ENSG00000253138
LINC00970	0.0052	8.0411E-01		ENSG00000203601
LINC00971	0.0410	2.6052E-01	4.6899E-01	ENSG00000242641
LINC00973	0.1854	3.9808E-02	1.3639E-01	ENSG00000240476
LINC00987	-0.0049	7.4883E-01		ENSG00000237248
LINC00996	-0.0006	9.9593E-01		ENSG00000242258
LINC01003	0.0237	7.5158E-01	8.6517E-01	ENSG00000261455
LINC01007	-0.0153	3.8559E-01		ENSG00000233123
LINC01010	0.0115	8.3762E-01		ENSG00000236700
LINC01011	-0.0021	9.7879E-01	9.8938E-01	ENSG00000244041
LINC01012	-0.0928	2.4331E-01	4.5111E-01	ENSG00000281706
LINC01013	0.0189	9.6689E-01	9.8344E-01	ENSG00000228495
LINC01018	0.0593	4.8762E-01	6.8078E-01	ENSG00000250056
LINC01029	-0.0009	7.1213E-01		ENSG00000265843
LINC01030	0.0108	5.5896E-01		ENSG00000253799
LINC01031	0.0142	7.5059E-01		ENSG00000232077
LINC01033	-0.0048	9.3803E-01	9.6932E-01	ENSG00000249069
LINC01036	0.0607	3.6571E-01	5.7483E-01	ENSG00000230426
LINC01044	0.0125	9.0992E-01		ENSG00000223626
LINC01053	-0.0059	9.9586E-01		ENSG00000238169
LINC01060	0.1068	2.3564E-01	4.4122E-01	ENSG00000249378
LINC01068	0.0155	8.8937E-01		ENSG00000227676
LINC01085	0.0145	4.6500E-01		ENSG00000248698
LINC01087	0.0089	8.0594E-01		ENSG00000224559
LINC01088	0.0625	4.6269E-01	6.5982E-01	ENSG00000249307
LINC01089	-0.2129	5.0280E-02	1.6017E-01	ENSG00000212694
LINC01090	-0.0156	5.9479E-01		ENSG00000231689
LINC01095	0.0038	8.2519E-01		ENSG00000248809
LINC01098	0.0740	1.5571E-01		ENSG00000231171
LINC01099	0.0174	7.3090E-01		ENSG00000251504
LINC01101	0.0134	6.8876E-01		ENSG00000280409
LINC01102	0.1849	5.0313E-02	1.6023E-01	ENSG00000235597
LINC01103	0.0038	9.9678E-01		ENSG00000234781

LINC01104	0.0050	8.1606E-01		ENSG00000232084
LINC01107	0.0073	7.1627E-01		ENSG00000225493
LINC01108	0.0733	1.8731E-01	3.8184E-01	ENSG00000226673
LINC01114	0.4244	4.0478E-03	2.5802E-02	ENSG00000234177
LINC01115	-0.0074	5.9567E-01		ENSG00000272342
LINC01116	0.0175	7.2803E-01	8.5083E-01	ENSG00000163364
LINC01117	0.0173	7.0074E-01	8.3466E-01	ENSG00000224577
LINC01118	0.0569	6.5681E-02		ENSG00000222005
LINC01119	0.1184	1.2423E-01	2.9402E-01	ENSG00000239332
LINC01121	0.0009	9.1872E-01		ENSG00000205054
LINC01122	-0.0162	8.4666E-01	9.2147E-01	ENSG00000233723
LINC01123	-0.1651	1.0894E-01	2.6938E-01	ENSG00000204588
LINC01126	-0.0004	9.7668E-01	9.8837E-01	ENSG00000279873
LINC01128	0.3005	7.3816E-04	7.2310E-03	ENSG00000228794
LINC01132	0.1488	9.1885E-02	2.4133E-01	ENSG00000227630
LINC01133	-0.0091	6.7978E-01		ENSG00000224259
LINC01134	0.0242	1.9999E-01		ENSG00000236423
LINC01138	0.1121	2.0467E-01	4.0388E-01	ENSG00000274020
LINC01139	0.2566	3.3361E-02	1.1987E-01	ENSG00000215808
LINC01140	0.0691	2.1015E-01	4.1056E-01	ENSG00000267272
LINC01142	0.0158	4.8444E-01		ENSG00000224286
LINC01143	0.0093	6.6985E-01		ENSG00000237751
LINC01144	0.2599	2.2216E-02	8.9655E-02	ENSG00000281912
LINC01152	0.0228	7.6973E-01	8.7673E-01	ENSG00000256124
LINC01159	-0.2274	3.0373E-02	1.1223E-01	ENSG00000229743
LINC01162	0.0415	8.2885E-02		ENSG00000232790
LINC01163	0.0049	8.7600E-01		ENSG00000280953
LINC01164	0.0295	4.9774E-02		ENSG00000189275
LINC01166	0.0476	5.4162E-01	7.2342E-01	ENSG00000232903
LINC01167	0.0021	9.5195E-01		ENSG00000224758
LINC01168	0.0091	4.1391E-01		ENSG00000240707
LINC01173	0.0571	3.7210E-01	5.8075E-01	ENSG00000280744
LINC01176	0.2606	4.4633E-02	1.4765E-01	ENSG00000281404
LINC01179	0.0400	3.2707E-01		ENSG00000249500
LINC01182	0.0106	8.7810E-01	9.3917E-01	ENSG00000250634
LINC01191	-0.0027	5.6463E-01		ENSG00000234199
LINC01192	-0.0068	6.2525E-01		ENSG00000241369
LINC01193	0.0391	1.8390E-01		ENSG00000258710
LINC01198	0.1217	1.0725E-01	2.6649E-01	ENSG00000231817
LINC01203	-0.0088	8.9030E-01	9.4564E-01	ENSG00000226985
LINC01204	0.0414	1.7317E-01	3.6315E-01	ENSG00000229563
LINC01205	0.0024	8.0466E-01		ENSG00000228980
LINC01206	-0.0141	9.3002E-01	9.6545E-01	ENSG00000242512
LINC01209	-0.0091	4.1246E-01		ENSG00000228308
LINC01213	0.0104	7.7490E-01		ENSG00000244541
LINC01215	0.0254	1.6203E-01		ENSG00000271856
LINC01216	-0.0086	7.7486E-01		ENSG00000250223
LINC01217	0.0095	9.3387E-01		ENSG00000251219
LINC01224	0.1702	7.1974E-02	2.0480E-01	ENSG00000269416

LINC01229	0.0293	5.2968E-01		ENSG00000260876
LINC01231	-0.0053	7.9619E-01		ENSG00000236511
LINC01234	0.5899	1.7877E-03	1.3851E-02	ENSG00000249550
LINC01237	-0.0681	3.7276E-01	5.8149E-01	ENSG00000233806
LINC01238	0.1947	4.8729E-02	1.5671E-01	ENSG00000261186
LINC01238	0.0120	8.7223E-01	9.3583E-01	ENSG00000237940
LINC01239	0.0980	1.1553E-01	2.8013E-01	ENSG00000234840
LINC01249	-0.0103	9.3751E-01		ENSG00000231532
LINC01250	-0.0195	5.8023E-01		ENSG00000234423
LINC01252	0.1195	1.3675E-01	3.1213E-01	ENSG00000247157
LINC01254	-0.0347	2.8490E-01		ENSG00000260913
LINC01257	0.2479	1.1877E-02	5.7583E-02	ENSG00000204603
LINC01258	-0.0110	6.3467E-01		ENSG00000249534
LINC01265	0.5089	5.2047E-03	3.1053E-02	ENSG00000249740
LINC01266	0.0070	6.6558E-01		ENSG00000224957
LINC01269	0.0090	6.7964E-01		ENSG00000258689
LINC01270	0.0115	6.5501E-01		ENSG00000203999
LINC01273	0.0804	3.4220E-01	5.5185E-01	ENSG00000231742
LINC01275	0.1222	3.1174E-02	1.1435E-01	ENSG00000237595
LINC01278	-0.2558	1.1196E-04	1.6767E-03	ENSG00000235437
LINC01284	-0.0023	8.7847E-01		ENSG00000230317
LINC01285	0.4978	6.5410E-03	3.6888E-02	ENSG00000203650
LINC01287	0.0097	8.0292E-01		ENSG00000234722
LINC01289	0.3914	1.9476E-02	8.1413E-02	ENSG00000253734
LINC01290	0.0089	8.7943E-01		ENSG00000260468
LINC01291	0.1328	1.6636E-01	3.5404E-01	ENSG00000204792
LINC01299	0.0022	9.2567E-01		ENSG00000254081
LINC01300	-0.0420	8.9395E-02		ENSG00000253595
LINC01303	0.0115	5.9521E-01		ENSG00000250548
LINC01305	0.1842	7.2200E-02	2.0524E-01	ENSG00000231453
LINC01307	0.0761	1.5649E-01	3.4030E-01	ENSG00000231671
LINC01310	-0.0081	7.6203E-01		ENSG00000205632
LINC01315	-0.1019	1.6243E-01	3.4852E-01	ENSG00000229891
LINC01317	-0.0187	8.1762E-01	9.0514E-01	ENSG00000228262
LINC01318	0.0022	9.7123E-01		ENSG00000237790
LINC01322	0.0105	8.9796E-01	9.4955E-01	ENSG00000244128
LINC01324	0.0082	5.6623E-01		ENSG00000241767
LINC01331	0.0583	4.8599E-01	6.7981E-01	ENSG00000248673
LINC01333	0.0275	2.2766E-01		ENSG00000249343
LINC01337	-0.0015	9.1517E-01		ENSG00000251221
LINC01339	0.0006	9.7007E-01		ENSG00000248555
LINC01340	0.0788	1.1342E-01		ENSG00000250331
LINC01343	-0.0037	7.2946E-01		ENSG00000237290
LINC01348	0.0053	7.2221E-01		ENSG00000280587
LINC01350	0.0187	6.7540E-01		ENSG00000228309
LINC01352	0.0020	9.5638E-01		ENSG00000238078
LINC01353	-0.0024	9.7390E-01	9.8718E-01	ENSG00000231507
LINC01354	0.0920	2.0905E-01	4.0907E-01	ENSG00000231768
LINC01356	0.1259	1.6931E-01	3.5781E-01	ENSG00000215866

LINC01359	-0.0757	2.0489E-01		ENSG00000226891
LINC01361	-0.0325	6.5617E-01		ENSG00000236268
LINC01362	0.0156	3.7860E-01		ENSG00000230817
LINC01363	-0.0566	9.4330E-02		ENSG00000231605
LINC01364	-0.0080	7.6267E-01		ENSG00000227290
LINC01374	0.0209	5.1833E-01		ENSG00000280560
LINC01376	0.2290	2.8622E-02	1.0751E-01	ENSG00000236204
LINC01393	0.0063	7.8728E-01		ENSG00000225535
LINC01396	0.0149	2.6898E-01		ENSG00000273396
LINC01397	0.0119	6.2813E-01		ENSG00000258673
LINC01399	0.0973	2.5265E-01	4.6058E-01	ENSG00000233080
LINC01404	-0.0350	2.3686E-01	4.4251E-01	ENSG00000258240
LINC01405	-0.0209	5.2747E-01		ENSG00000185847
LINC01410	0.0982	2.6832E-01	4.7778E-01	ENSG00000238113
LINC01411	0.0806	1.4843E-01	3.2895E-01	ENSG00000249306
LINC01412	-0.0511	2.0198E-01	4.0073E-01	ENSG00000232606
LINC01414	0.2105	5.4317E-02	1.6898E-01	ENSG00000253554
LINC01416	0.0782	9.9160E-02		ENSG00000260930
LINC01426	0.8997	1.3720E-02	6.3886E-02	ENSG00000234380
LINC01431	0.0888	3.1255E-01	5.2276E-01	ENSG00000232645
LINC01433	-0.0179	5.7157E-01		ENSG00000230176
LINC01435	0.0322	1.9515E-01		ENSG00000229981
LINC01445	0.0009	8.2759E-01		ENSG00000231427
LINC01446	-0.0067	7.4033E-01		ENSG00000205628
LINC01447	-0.0008	7.3412E-01		ENSG00000236078
LINC01448	0.0043	9.2644E-01		ENSG00000238284
LINC01449	-0.0192	3.5874E-01		ENSG00000224017
LINC01451	0.0574	3.8067E-01	5.8902E-01	ENSG00000279141
LINC01456	0.2004	4.9052E-02	1.5748E-01	ENSG00000225882
LINC01460	0.0406	4.8735E-01	6.8062E-01	ENSG00000205334
LINC01465	0.0082	9.2165E-01	9.6140E-01	ENSG00000221949
LINC01470	-0.0577	3.8531E-01	5.9315E-01	ENSG00000249484
LINC01477	-0.0034	8.3210E-01		ENSG00000261715
LINC01478	0.0496	3.1112E-01		ENSG00000267337
LINC01482	-0.0086	4.5264E-01		ENSG00000267659
LINC01483	0.0084	8.3286E-01		ENSG00000227517
LINC01484	-0.0566	1.0742E-01	2.6675E-01	ENSG00000253686
LINC01485	0.0401	2.2418E-01		ENSG00000254211
LINC01493	-0.0021	9.4931E-01		ENSG00000254562
LINC01494	0.0145	8.0193E-01		ENSG00000228135
LINC01495	0.0481	1.3841E-01		ENSG00000255323
LINC01497	0.0249	7.2208E-01	8.4766E-01	ENSG00000237560
LINC01498	0.0047	8.9194E-01		ENSG00000247213
LINC01500	0.6515	9.8772E-03	5.0242E-02	ENSG00000258583
LINC01501	0.0236	3.7871E-01		ENSG00000229613
LINC01503	-0.2144	1.4847E-02	6.7461E-02	ENSG00000233901
LINC01504	0.1214	1.0274E-01	2.5937E-01	ENSG00000225434
LINC01505	0.0487	4.8253E-01	6.7686E-01	ENSG00000234323
LINC01508	0.1323	1.5540E-01	3.3890E-01	ENSG00000231107

LINC01512	0.0411	5.1686E-01	7.0434E-01	ENSG00000289313
LINC01516	0.0143	6.7000E-01		ENSG00000231422
LINC01518	0.0904	6.5689E-02		ENSG00000233515
LINC01524	0.1629	7.1812E-02	2.0448E-01	ENSG00000234948
LINC01529	-0.0519	5.0680E-01	6.9620E-01	ENSG00000225872
LINC01531	0.0129	4.0272E-01		ENSG00000205786
LINC01535	0.0517	5.4408E-01	7.2492E-01	ENSG00000226686
LINC01537	-0.0030	9.5833E-01	9.7936E-01	ENSG00000227467
LINC01539	0.0286	4.9673E-02		ENSG00000267712
LINC01541	0.0187	3.0446E-01		ENSG00000260676
LINC01545	0.0053	9.0423E-01		ENSG00000204904
LINC01547	0.2825	1.7796E-03	1.3805E-02	ENSG00000183250
LINC01550	-0.2476	1.2885E-02	6.0931E-02	ENSG00000246223
LINC01551	-0.2997	3.1793E-03	2.1438E-02	ENSG00000186960
LINC01554	0.0185	7.5477E-01	8.6696E-01	ENSG00000236882
LINC01558	-0.0406	2.1136E-01		ENSG00000146521
LINC01561	0.0005	9.9362E-01	9.9645E-01	ENSG00000177234
LINC01562	0.0024	9.6985E-01	9.8504E-01	ENSG00000203356
LINC01563	-0.2992	1.6421E-03	1.3038E-02	ENSG00000236819
LINC01564	0.0321	1.1117E-01		ENSG00000235899
LINC01565	-0.0282	6.7767E-01	8.1941E-01	ENSG00000198685
LINC01567	-0.0099	9.0416E-01	9.5319E-01	ENSG00000224310
LINC01572	0.4560	2.1404E-05	4.5206E-04	ENSG00000261008
LINC01574	0.0703	1.4948E-01	3.3020E-01	ENSG00000248859
LINC01582	0.0032	7.1566E-01		ENSG00000259611
LINC01583	-0.0024	8.9718E-01		ENSG00000259518
LINC01585	-0.0008	8.8758E-01		ENSG00000245479
LINC01586	0.3694	3.9329E-03	2.5242E-02	ENSG00000249487
LINC01587	0.0487	3.0817E-01	5.1843E-01	ENSG00000082929
LINC01592	0.0122	3.9225E-01		ENSG00000253658
LINC01599	0.0295	6.7699E-01	8.1907E-01	ENSG00000214900
LINC01600	-0.1257	6.5333E-02	1.9159E-01	ENSG00000164385
LINC01602	-0.0198	3.5649E-01		ENSG00000205293
LINC01605	0.0689	9.5196E-02		ENSG00000253414
LINC01606	-0.0167	3.7382E-01		ENSG00000253301
LINC01607	0.4191	1.5785E-03	1.2701E-02	ENSG00000272138
LINC01608	0.0072	8.8942E-01		ENSG00000253877
LINC01609	0.0060	5.2212E-01		ENSG00000253103
LINC01611	0.0455	1.9700E-01		ENSG00000231776
LINC01612	-0.0010	9.0603E-01		ENSG00000250266
LINC01616	0.0274	3.5461E-01	5.6459E-01	ENSG00000261340
LINC01618	-0.0931	1.1412E-01	2.7800E-01	ENSG00000250302
LINC01619	0.1362	1.5672E-01	3.4040E-01	ENSG00000257242
LINC01623	-0.0144	4.6558E-01		ENSG00000225595
LINC01625	0.1928	3.9951E-02	1.3660E-01	ENSG00000238099
LINC01629	0.0261	5.8913E-02		ENSG00000258602
LINC01635	0.0057	9.3647E-01	9.6866E-01	ENSG00000228397
LINC01637	0.0473	3.4860E-01		ENSG00000237476
LINC01638	0.0546	1.7852E-01		ENSG00000233521

LINC01640	0.0352	2.7055E-01		ENSG00000231253
LINC01643	0.0095	7.7595E-01		ENSG00000236052
LINC01647	0.0772	1.6587E-01	3.5345E-01	ENSG00000235643
LINC01649	0.1171	1.9892E-01	3.9715E-01	ENSG00000228127
LINC01659	0.0755	8.1010E-02	2.2123E-01	ENSG00000234928
LINC01664	0.0970	2.7207E-01	4.8134E-01	ENSG00000235478
LINC01668	-0.0540	8.9794E-02		ENSG00000283051
LINC01670	0.1174	1.6672E-01	3.5469E-01	ENSG00000279094
LINC01671	0.0232	2.6761E-01		ENSG00000225431
LINC01679	0.0356	6.8215E-02		ENSG00000237989
LINC01681	-0.0175	6.2857E-01		ENSG00000233985
LINC01686	-0.4128	1.2365E-02	5.9146E-02	ENSG00000261504
LINC01687	-0.0022	9.7806E-01		ENSG00000233215
LINC01690	-0.0025	9.6441E-01		ENSG00000228961
LINC01694	-0.0344	6.7299E-01	8.1708E-01	ENSG00000233922
LINC01695	0.0117	3.9362E-01		ENSG00000236532
LINC01696	0.0019	8.7528E-01		ENSG00000227940
LINC01697	-0.0008	9.6478E-01		ENSG00000232079
LINC01699	0.0205	2.5434E-01		ENSG00000179452
LINC01704	0.0068	8.8435E-01		ENSG00000231666
LINC01707	0.0600	1.1050E-01		ENSG00000223883
LINC01708	0.0333	4.6794E-02		ENSG00000224445
LINC01709	-0.0001	9.8737E-01		ENSG00000226715
LINC01711	0.0314	4.0201E-01		ENSG00000268941
LINC01714	0.0021	9.9657E-01		ENSG00000227634
LINC01717	0.0412	2.4385E-01	4.5154E-01	ENSG00000232812
LINC01721	0.0106	6.7542E-01		ENSG00000230133
LINC01730	0.1021	1.6952E-01	3.5798E-01	ENSG00000275491
LINC01732	0.0100	7.3620E-01	8.5602E-01	ENSG00000237292
LINC01738	-0.0283	7.9925E-01		ENSG00000227947
LINC01739	0.0140	4.9962E-01		ENSG00000229537
LINC01740	0.0143	8.2816E-01		ENSG00000228067
LINC01748	-0.0653	4.0338E-01	6.0785E-01	ENSG00000226476
LINC01749	0.0042	8.1775E-01		ENSG00000272259
LINC01750	-0.0551	5.1982E-01	7.0692E-01	ENSG00000231437
LINC01756	0.0000	8.8088E-01		ENSG00000230523
LINC01758	0.0047	6.8596E-01		ENSG00000229639
LINC01762	-0.0071	7.8515E-01		ENSG00000233154
LINC01765	0.0471	5.3181E-02		ENSG00000233730
LINC01766	0.0003	7.9285E-01		ENSG00000224910
LINC01772	0.1168	1.3667E-01	3.1206E-01	ENSG00000226029
LINC01775	-0.0657	8.0344E-02		ENSG00000267201
LINC01776	0.0427	5.6592E-01	7.4117E-01	ENSG00000226053
LINC01778	0.0243	7.5297E-01	8.6594E-01	ENSG00000223382
LINC01780	0.0155	3.5320E-01		ENSG00000232650
LINC01782	0.0103	2.2853E-01		ENSG00000267204
LINC01790	-0.0074	3.9327E-01		ENSG00000230173
LINC01791	-0.0089	5.2990E-01		ENSG00000267777
LINC01793	-0.0096	5.3239E-01	7.1608E-01	ENSG00000222030

LINC01797	0.0009	9.7354E-01		ENSG00000237179
LINC01798	0.0129	9.0819E-01	9.5476E-01	ENSG00000232046
LINC01799	0.1064	4.2441E-02		ENSG00000230525
LINC01800	0.0121	7.2203E-01		ENSG00000234572
LINC01801	-0.1663	9.6874E-02	2.4998E-01	ENSG00000267767
LINC01805	0.0549	5.5073E-02		ENSG00000223863
LINC01806	0.0108	8.1330E-01		ENSG00000227403
LINC01807	0.0944	1.0729E-02		ENSG00000232023
LINC01811	0.0531	2.9466E-01	5.0467E-01	ENSG00000226320
LINC01812	-0.0045	7.9163E-01		ENSG00000237013
LINC01814	0.0090	4.9361E-01		ENSG00000236008
LINC01816	-0.0065	9.2294E-01	9.6186E-01	ENSG00000231327
LINC01817	0.0110	6.7573E-01		ENSG00000231420
LINC01819	0.0083	3.4210E-01		ENSG00000231826
LINC01821	0.1521	4.4808E-02	1.4805E-01	ENSG00000225539
LINC01822	0.0045	6.1556E-01		ENSG00000229621
LINC01823	0.0385	3.5298E-01		ENSG00000224655
LINC01824	0.0038	9.9116E-01		ENSG00000226488
LINC01831	0.0690	1.4453E-01		ENSG00000225765
LINC01833	-0.0396	5.9063E-01	7.5880E-01	ENSG00000259439
LINC01837	0.0037	9.0320E-01		ENSG00000267489
LINC01838	0.0248	5.7232E-01		ENSG00000269037
LINC01841	0.0299	4.8159E-01		ENSG00000266913
LINC01842	-0.0006	8.4965E-01		ENSG00000267147
LINC01844	0.3761	1.6814E-02	7.3482E-02	ENSG00000236714
LINC01845	0.0095	6.8859E-01		ENSG00000253298
LINC01846	0.0203	4.2756E-01		ENSG00000248901
LINC01847	0.0506	1.1930E-01		ENSG00000253311
LINC01849	0.0438	5.2633E-01	7.1152E-01	ENSG00000234988
LINC01852	-0.0517	4.6479E-01	6.6150E-01	ENSG00000236914
LINC01854	0.0446	1.0303E-01		ENSG00000204460
LINC01856	0.0531	4.8036E-02		ENSG00000237574
LINC01858	-0.0009	8.4385E-01		ENSG00000261615
LINC01859	-0.0173	3.3383E-01		ENSG00000261558
LINC01864	0.5377	8.4495E-03	4.4739E-02	ENSG00000267522
LINC01869	0.0858	1.0277E-01	2.5941E-01	ENSG00000180279
LINC01873	-0.0039	8.4302E-01		ENSG00000232164
LINC01876	0.0789	7.7046E-02	2.1420E-01	ENSG00000226383
LINC01877	0.0103	6.6539E-01		ENSG00000238217
LINC01879	-0.0030	8.1297E-01	9.0248E-01	ENSG00000276397
LINC01880	-0.0043	6.9045E-01		ENSG00000232002
LINC01884	-0.0280	1.4835E-01		ENSG00000233587
LINC01885	0.0622	9.0706E-02		ENSG00000237880
LINC01893	-0.0050	8.1706E-01		ENSG00000264260
LINC01894	-0.0344	1.5074E-01		ENSG00000264345
LINC01895	0.0027	9.2331E-01		ENSG00000259256
LINC01903	0.0055	8.9344E-01		ENSG00000265555
LINC01905	0.1627	1.8005E-02	7.7000E-02	ENSG00000267057
LINC01907	1.0731	6.0002E-03		ENSG00000226125

LINC01909	0.0942	8.7933E-02		ENSG00000266258
LINC01910	0.0595	1.4573E-01		ENSG00000266278
LINC01914	0.0044	6.3681E-01		ENSG00000234362
LINC01915	0.0070	9.3581E-01	9.6833E-01	ENSG00000265485
LINC01917	0.0238	3.5603E-01		ENSG00000260433
LINC01918	-0.1061	2.3139E-01	4.3622E-01	ENSG00000226508
LINC01931	0.0072	7.9571E-01		ENSG00000162947
LINC01932	-0.0113	6.4632E-01		ENSG00000253315
LINC01933	0.0462	1.3840E-01		ENSG00000254226
LINC01942	0.0091	4.1287E-01		ENSG00000253428
LINC01948	0.1804	4.7231E-02	1.5358E-01	ENSG00000248727
LINC01951	-0.0056	6.5435E-01		ENSG00000204754
LINC01954	0.0354	4.9990E-01	6.9086E-01	ENSG00000271952
LINC01960	0.0084	4.9989E-01		ENSG00000260868
LINC01962	0.0170	8.3550E-01	9.1524E-01	ENSG00000248473
LINC01963	0.0331	6.9418E-01	8.3055E-01	ENSG00000260804
LINC01968	0.0740	2.0091E-01	3.9940E-01	ENSG00000237222
LINC01970	0.0103	9.0158E-01	9.5183E-01	ENSG00000265692
LINC01972	-0.0011	8.7768E-01		ENSG00000230401
LINC01973	0.1998	1.8998E-02	8.0013E-02	ENSG00000204283
LINC01976	0.0353	5.0055E-01	6.9141E-01	ENSG00000261514
LINC01979	-0.0098	6.6537E-01		ENSG00000262585
LINC01980	-0.0380	6.5685E-01	8.0624E-01	ENSG00000225548
LINC01986	0.0065	7.2104E-01		ENSG00000223587
LINC01988	0.0255	2.2252E-01		ENSG00000283036
LINC01989	-0.0216	5.6229E-01		ENSG00000261156
LINC01990	-0.0610	3.4438E-01	5.5407E-01	ENSG00000273125
LINC01993	0.0255	7.5163E-01	8.6517E-01	ENSG00000204277
LINC01994	-0.0191	6.7251E-01		ENSG00000241098
LINC01995	-0.0570	4.0717E-01	6.1166E-01	ENSG00000244247
LINC01998	0.1792	1.5447E-02	6.9443E-02	ENSG00000243321
LINC01999	-0.0053	8.0762E-01		ENSG00000267772
LINC02003	0.0109	8.9199E-01	9.4631E-01	ENSG00000264026
LINC02008	0.0831	2.0643E-01	4.0609E-01	ENSG00000239440
LINC02016	-0.0196	5.7281E-01		ENSG00000244215
LINC02018	-0.0831	3.5045E-01	5.6065E-01	ENSG00000272690
LINC02021	0.2035	6.3861E-02	1.8847E-01	ENSG00000249846
LINC02022	0.0091	9.8060E-01		ENSG00000232746
LINC02024	-0.0099	8.8491E-01	9.4244E-01	ENSG00000241213
LINC02026	0.0409	2.7043E-01		ENSG00000214146
LINC02028	-0.0054	9.0569E-01	9.5379E-01	ENSG00000230102
LINC02029	0.0356	3.5907E-01		ENSG00000241544
LINC02032	0.0960	6.3785E-02	1.8832E-01	ENSG00000241131
LINC02035	0.0364	6.4474E-01	7.9719E-01	ENSG00000273033
LINC02037	-0.0488	1.1349E-01		ENSG00000238097
LINC02043	0.0791	1.2110E-01	2.8926E-01	ENSG00000232233
LINC02044	-0.1063	2.4264E-01	4.5037E-01	ENSG00000243795
LINC02048	0.0029	7.9086E-01		ENSG00000228271
LINC02050	0.0058	9.8285E-01		ENSG00000242781

LINC02052	-0.0100	5.0269E-01		ENSG00000224406
LINC02054	-0.0097	6.3114E-01		ENSG00000224153
LINC02055	0.1143	1.1849E-01	2.8489E-01	ENSG00000254101
LINC02057	0.0007	9.8773E-01		ENSG00000249279
LINC02060	-0.0396	5.7787E-01	7.4955E-01	ENSG00000250156
LINC02067	-0.0071	6.6297E-01		ENSG00000240567
LINC02069	0.0194	1.3959E-01		ENSG00000229433
LINC02070	0.0203	4.5178E-01	6.5082E-01	ENSG00000241328
LINC02072	0.1149	6.6602E-02	1.9421E-01	ENSG00000267452
LINC02074	0.0242	5.8374E-01		ENSG00000266357
LINC02076	-0.0124	8.7840E-01	9.3937E-01	ENSG00000220161
LINC02078	0.0029	9.9435E-01		ENSG00000267719
LINC02080	-0.0179	4.2837E-01		ENSG00000267065
LINC02082	-0.0047	9.7478E-01		ENSG00000242268
LINC02089	0.0205	3.5685E-01		ENSG00000226364
LINC02092	0.0510	2.6348E-01		ENSG00000234721
LINC02097	-0.0084	5.7297E-01		ENSG00000226101
LINC02099	0.0030	9.0062E-01		ENSG00000253490
LINC02101	0.0060	8.7326E-01		ENSG00000248132
LINC02102	-0.0113	7.2136E-01		ENSG00000248677
LINC02104	0.0110	7.6210E-01		ENSG00000271334
LINC02105	0.0635	5.2796E-02	1.6550E-01	ENSG00000250447
LINC02106	-0.0037	7.6037E-01		ENSG00000251573
LINC02111	0.0037	9.6057E-01		ENSG00000250822
LINC02115	0.0948	1.0339E-02		ENSG00000248757
LINC02123	-0.0331	5.8123E-01	7.5242E-01	ENSG00000250668
LINC02128	0.0106	8.3653E-01		ENSG00000261241
LINC02133	0.0657	1.8978E-01	3.8487E-01	ENSG00000261231
LINC02138	0.0067	7.9033E-01		ENSG00000205015
LINC02139	0.0064	7.5993E-01		ENSG00000278214
LINC02142	0.0017	8.0242E-01		ENSG00000223597
LINC02145	-0.0414	4.2704E-01	6.2907E-01	ENSG00000250490
LINC02150	0.0100	9.9593E-01		ENSG00000248150
LINC02159	0.0036	9.2199E-01		ENSG00000253417
LINC02160	-0.0003	6.9779E-01		ENSG00000251443
LINC02172	-0.0039	9.1887E-01		ENSG00000251632
LINC02175	0.1328	1.6078E-01	3.4630E-01	ENSG00000262155
LINC02177	-0.0210	5.6776E-01		ENSG00000261617
LINC02181	-0.0156	6.6701E-01		ENSG00000232190
LINC02182	0.5791	3.0054E-03	2.0546E-02	ENSG00000260420
LINC02188	0.0386	4.2418E-01		ENSG00000261175
LINC02191	-0.0084	7.2347E-01		ENSG00000261636
LINC02192	0.0243	1.9332E-01	3.8947E-01	ENSG00000261325
LINC02200	0.4301	3.1641E-03	2.1347E-02	ENSG00000250358
LINC02201	-0.0157	2.9871E-01		ENSG00000251538
LINC02207	0.0320	3.2153E-01		ENSG00000258476
LINC02210	-0.0999	7.7838E-02	2.1570E-01	ENSG00000204650
LINC02217	-0.0301	5.4864E-01	7.2808E-01	ENSG00000248455
LINC02218	-0.0096	9.1664E-01		ENSG00000249662

LINC02226	0.0675	1.8901E-01		ENSG00000245729
LINC02231	0.0421	2.4765E-01		ENSG00000248995
LINC02232	0.1041	2.2684E-01	4.3100E-01	ENSG00000250125
LINC02234	-0.0265	5.9527E-01	7.6221E-01	ENSG00000248202
LINC02235	0.0847	2.6778E-01	4.7718E-01	ENSG00000254689
LINC02236	0.0885	1.7541E-01	3.6620E-01	ENSG00000251365
LINC02237	0.0215	3.3422E-01		ENSG00000253434
LINC02240	0.1162	1.9416E-01	3.9079E-01	ENSG00000260192
LINC02241	0.1491	1.8302E-02		ENSG00000251629
LINC02242	0.0076	9.5263E-01		ENSG00000249736
LINC02243	0.0187	2.6089E-01		ENSG00000236091
LINC02245	0.3353	7.9927E-05	1.2922E-03	ENSG00000237638
LINC02247	0.0100	7.5319E-01		ENSG00000223729
LINC02248	0.0181	5.7702E-01		ENSG00000235160
LINC02249	-0.1552	1.2346E-01	2.9305E-01	ENSG00000225930
LINC02253	0.4479	2.1416E-03	1.5824E-02	ENSG00000259485
LINC02254	0.0659	3.1208E-01	5.2211E-01	ENSG00000259664
LINC02256	-0.1921	2.1757E-02	8.8209E-02	ENSG00000289083
LINC02259	0.1083	1.2260E-01	2.9153E-01	ENSG00000259783
LINC02260	-0.0088	3.9939E-01		ENSG00000250456
LINC02263	0.0255	7.2591E-01	8.4955E-01	ENSG00000228358
LINC02265	0.5018	2.7336E-04	3.3480E-03	ENSG00000249241
LINC02268	0.0302	7.1883E-01	8.4541E-01	ENSG00000248174
LINC02269	0.0065	7.6047E-01		ENSG00000250708
LINC02273	0.0122	4.8812E-01		ENSG00000245954
LINC02274	-0.0452	4.6496E-01	6.6155E-01	ENSG00000258586
LINC02280	-0.0065	7.4982E-01		ENSG00000260792
LINC02281	-0.0028	7.2084E-01		ENSG00000257748
LINC02282	0.0848	3.1761E-01	5.2785E-01	ENSG00000257056
LINC02287	0.0077	4.2181E-01		ENSG00000258499
LINC02288	0.0002	9.9923E-01	9.9948E-01	ENSG00000246548
LINC02289	-0.0040	9.5909E-01	9.7955E-01	ENSG00000258819
LINC02290	0.0959	6.5601E-02		ENSG00000258502
LINC02294	0.0137	3.7760E-01		ENSG00000257845
LINC02299	-0.0017	8.8792E-01	9.4427E-01	ENSG00000258979
LINC02301	-0.0840	2.7743E-01	4.8751E-01	ENSG00000258743
LINC02303	0.0307	9.6271E-02		ENSG00000258616
LINC02313	0.0103	4.1352E-01		ENSG00000258474
LINC02316	-0.0037	6.9667E-01		ENSG00000258912
LINC02317	0.0102	4.3659E-01		ENSG00000258678
LINC02318	0.0192	3.7138E-01		ENSG00000258390
LINC02323	0.1752	3.7290E-02	1.2994E-01	ENSG00000259230
LINC02324	-0.0095	9.7488E-01		ENSG00000255002
LINC02325	0.0408	3.3128E-01	5.4153E-01	ENSG00000246084
LINC02326	0.0018	4.3999E-01		ENSG00000257523
LINC02327	-0.0536	8.9695E-02		ENSG00000258038
LINC02328	0.0277	6.9680E-01	8.3239E-01	ENSG00000258733
LINC02330	-0.0086	8.4278E-01		ENSG00000258770
LINC02331	-0.0010	8.9263E-01		ENSG00000235269

LINC02333	0.0227	3.3723E-01		ENSG00000271564
LINC02338	0.0258	4.8105E-01		ENSG00000277448
LINC02339	0.0083	8.5539E-01		ENSG00000279797
LINC02341	0.0327	2.6404E-01		ENSG00000283554
LINC02343	0.0080	7.3529E-01		ENSG00000271850
LINC02346	0.0309	7.7621E-01	8.8092E-01	ENSG00000206187
LINC02348	0.0274	6.0123E-01		ENSG00000286733
LINC02355	0.0050	9.5476E-01		ENSG00000248210
LINC02356	0.0582	4.2537E-01	6.2767E-01	ENSG00000257595
LINC02357	-0.0008	8.8711E-01		ENSG00000283314
LINC02359	0.0251	2.4146E-01		ENSG00000284848
LINC02360	0.0170	4.6302E-01		ENSG00000248300
LINC02361	0.1742	7.2502E-02	2.0576E-01	ENSG00000256576
LINC02365	0.0103	6.0734E-01		ENSG00000254233
LINC02367	-0.0296	6.9872E-01	8.3347E-01	ENSG00000260423
LINC02380	0.0082	7.2472E-01		ENSG00000248505
LINC02381	-0.5071	1.1394E-04	1.6984E-03	ENSG00000250742
LINC02387	0.0675	1.5024E-01		ENSG00000256232
LINC02389	0.0785	9.8595E-02		ENSG00000255693
LINC02395	0.0295	5.2098E-01		ENSG00000257771
LINC02398	0.0157	3.6272E-01		ENSG00000256287
LINC02399	0.0048	6.5233E-01		ENSG00000257725
LINC02404	0.0122	4.1179E-01		ENSG00000257893
LINC02405	0.0272	3.7869E-01		ENSG00000249345
LINC02408	-0.0825	3.0307E-01	5.1315E-01	ENSG00000203585
LINC02410	0.0025	7.1987E-01		ENSG00000258292
LINC02421	-0.0001	8.5335E-01		ENSG00000255970
LINC02422	0.0951	2.9015E-01	5.0050E-01	ENSG00000255760
LINC02426	-0.0153	3.8203E-01		ENSG00000257747
LINC02432	0.0969	3.2781E-02		ENSG00000248810
LINC02436	-0.0006	9.2856E-01		ENSG00000250754
LINC02440	0.5264	2.0770E-06	6.8626E-05	ENSG00000255618
LINC02443	0.0288	1.2895E-01		ENSG00000256115
LINC02444	0.0216	2.5477E-01		ENSG00000258123
LINC02449	0.0233	7.1804E-01	8.4504E-01	ENSG00000215241
LINC02453	0.4360	3.9475E-03	2.5327E-02	ENSG00000245017
LINC02465	0.0418	2.1049E-01		ENSG00000249618
LINC02466	0.0193	4.1605E-01		ENSG00000246876
LINC02470	0.0223	7.1944E-01	8.4576E-01	ENSG00000225231
LINC02472	0.0093	6.5218E-01		ENSG00000248338
LINC02478	0.1168	1.3969E-01	3.1606E-01	ENSG00000285373
LINC02481	0.0681	2.0269E-01		ENSG00000246526
LINC02482	-0.0102	8.8527E-01	9.4266E-01	ENSG00000251580
LINC02487	-0.0630	4.3803E-01	6.3845E-01	ENSG00000203688
LINC02488	0.1968	6.5375E-02	1.9165E-01	ENSG00000249362
LINC02489	0.0138	7.6665E-01	8.7481E-01	ENSG00000255007
LINC02498	0.0303	9.5342E-02		ENSG00000283083
LINC02502	0.1184	3.5187E-02	1.2454E-01	ENSG00000250392
LINC02508	0.0008	6.4057E-01		ENSG00000251619

LINC02511	0.0003	8.2806E-01		ENSG00000248869
LINC02513	-0.0041	6.9352E-01		ENSG00000248227
LINC02525	0.0367	6.6374E-01	8.1089E-01	ENSG00000230269
LINC02526	0.0344	5.7359E-01	7.4656E-01	ENSG00000229654
LINC02532	-0.0174	2.1809E-01		ENSG00000235142
LINC02538	0.0901	1.9342E-01	3.8956E-01	ENSG00000272549
LINC02541	0.0456	5.2225E-01	7.0868E-01	ENSG00000230943
LINC02546	0.0028	9.2190E-01		ENSG00000255117
LINC02547	-0.0050	6.9859E-01		ENSG00000254486
LINC02549	0.0112	5.2695E-01		ENSG00000226497
LINC02552	0.1242	1.4730E-01	3.2725E-01	ENSG00000256422
LINC02554	0.0752	1.0030E-01		ENSG00000226741
LINC02560	0.0876	1.9597E-01	3.9319E-01	ENSG00000268307
LINC02570	0.0091	4.1131E-01		ENSG00000237923
LINC02572	0.1723	9.6242E-02	2.4906E-01	ENSG00000229536
LINC02574	0.0033	9.6528E-01	9.8289E-01	ENSG00000233975
LINC02576	-0.0123	8.3014E-01	9.1211E-01	ENSG00000232613
LINC02585	0.2665	3.5075E-02	1.2421E-01	ENSG00000228350
LINC02593	0.2394	3.1506E-02	1.1520E-01	ENSG00000223764
LINC02594	0.2323	4.0953E-02	1.3904E-01	ENSG00000267440
LINC02596	0.0241	2.7186E-01		ENSG00000233431
LINC02601	0.3433	1.9484E-02	8.1434E-02	ENSG00000223714
LINC02603	0.0281	2.8887E-01		ENSG00000230262
LINC02606	0.1595	9.8395E-02	2.5214E-01	ENSG00000284693
LINC02607	0.0187	6.6215E-01	8.0977E-01	ENSG00000228971
LINC02608	0.0631	9.8782E-02		ENSG00000226251
LINC02609	0.2326	1.0203E-02	5.1436E-02	ENSG00000233593
LINC02610	0.2261	2.6296E-02	1.0115E-01	ENSG00000186235
LINC02611	0.1042	1.2444E-01	2.9424E-01	ENSG00000226791
LINC02613	0.0185	6.8523E-01		ENSG00000231367
LINC02614	0.1524	1.1449E-01	2.7857E-01	ENSG00000241288
LINC02615	0.5393	3.4140E-03	2.2557E-02	ENSG00000251432
LINC02617	0.0176	5.2170E-01		ENSG00000256288
LINC02620	0.0088	6.1165E-01		ENSG00000225768
LINC02626	-0.0113	5.8430E-01		ENSG00000236799
LINC02631	-0.0171	7.8429E-01		ENSG00000237772
LINC02642	-0.0035	9.1833E-01		ENSG00000232591
LINC02649	-0.1227	5.9094E-02	1.7865E-01	ENSG00000215244
LINC02653	-0.0062	9.3421E-01		ENSG00000236373
LINC02655	0.0074	7.9991E-01		ENSG00000231082
LINC02668	0.0613	1.5075E-01	3.3195E-01	ENSG00000226762
LINC02670	0.0313	3.2551E-01		ENSG00000224788
LINC02671	0.0088	5.8131E-01		ENSG00000224714
LINC02679	0.0085	7.9857E-01		ENSG00000226676
LINC02681	0.0113	9.6206E-01		ENSG00000229649
LINC02683	0.0260	1.8949E-01		ENSG00000254438
LINC02685	0.0330	1.2399E-01		ENSG00000254654
LINC02687	0.0180	5.8125E-01		ENSG00000255267
LINC02688	-0.0078	9.0043E-01	9.5114E-01	ENSG00000254872

LINC02691	0.0450	1.2652E-01		ENSG00000258913
LINC02693	0.1904	7.2735E-03	3.9956E-02	ENSG00000212719
LINC02694	0.1668	8.8222E-02	2.3437E-01	ENSG00000175779
LINC02696	0.0424	5.9939E-01	7.6537E-01	ENSG00000254427
LINC02702	0.0065	6.2459E-01		ENSG00000237937
LINC02709	-0.0176	7.0834E-01	8.3965E-01	ENSG00000245522
LINC02710	0.0248	5.5804E-01		ENSG00000255269
LINC02712	0.0523	2.0903E-01	4.0907E-01	ENSG00000273409
LINC02714	0.0522	2.3078E-01		ENSG00000251226
LINC02717	0.0276	5.9273E-01		ENSG00000285735
LINC02720	-0.0029	9.8770E-01		ENSG00000255178
LINC02721	-0.0052	8.8924E-01		ENSG00000255133
LINC02723	0.0525	1.8772E-01		ENSG00000231680
LINC02724	0.2304	4.8203E-02	1.5557E-01	ENSG00000181908
LINC02728	-0.0363	6.0071E-01	7.6611E-01	ENSG00000251323
LINC02731	0.0478	5.7668E-01	7.4877E-01	ENSG00000204241
LINC02733	0.0044	7.0775E-01		ENSG00000255553
LINC02734	0.0044	8.7083E-01		ENSG00000255382
LINC02743	-0.0378	5.2594E-01	7.1110E-01	ENSG00000255258
LINC02744	0.0024	9.7924E-01		ENSG00000254863
LINC02749	0.0664	2.1723E-01	4.1907E-01	ENSG00000254480
LINC02755	-0.0114	8.9211E-01		ENSG00000254530
LINC02761	-0.0329	4.8875E-01		ENSG00000255362
LINC02762	-0.0078	9.1687E-01	9.5877E-01	ENSG00000250303
LINC02763	-0.0089	8.7669E-01	9.3851E-01	ENSG00000254968
LINC02764	0.0180	8.5375E-01		ENSG00000255484
LINC02774	0.0279	7.0244E-01	8.3561E-01	ENSG00000226828
LINC02777	0.0732	3.1838E-01	5.2860E-01	ENSG00000232453
LINC02798	-0.0856	2.7103E-01	4.8029E-01	ENSG00000227082
LINC02800	0.0029	9.1241E-01		ENSG00000230023
LINC02801	0.0744	1.0698E-01	2.6609E-01	ENSG00000284240
LINC02802	-0.1989	1.8800E-03	1.4379E-02	ENSG00000232527
LINC02803	-0.0877	2.1158E-01	4.1238E-01	ENSG00000227740
LINC02805	0.0348	3.4548E-01		ENSG00000232265
LINC02817	0.0018	9.0331E-01		ENSG00000234754
LINC02826	-0.0028	7.7367E-01		ENSG00000275212
LINC02828	0.0430	4.6529E-01	6.6191E-01	ENSG00000224164
LINC02831	0.0043	5.4500E-01		ENSG00000224173
LINC02833	-0.0047	6.4211E-01		ENSG00000258742
LINC02840	0.0079	5.2957E-01		ENSG00000224893
LINC02844	-0.0220	6.6973E-01	8.1484E-01	ENSG00000253679
LINC02851	0.3120	2.1942E-02	8.8783E-02	ENSG00000229611
LINC02856	0.0984	6.1670E-02	1.8393E-01	ENSG00000285594
LINC02857	0.0078	7.5078E-01		ENSG00000230852
LINC02860	0.0257	4.4188E-01		ENSG00000222004
LINC02861	0.0425	5.6280E-01	7.3940E-01	ENSG00000262185
LINC02864	-0.0024	7.2811E-01		ENSG00000263711
LINC02868	-0.0082	4.2348E-01		ENSG00000203864
LINC02869	0.1643	3.9553E-02	1.3592E-01	ENSG00000228208

LINC02875	-0.0080	3.6530E-01		ENSG00000187013
LINC02877	0.0185	4.5027E-01		ENSG00000237787
LINC02880	0.0217	4.2849E-01		ENSG00000175967
LINC02882	0.0584	4.1952E-01	6.2224E-01	ENSG00000251138
LINC02884	-0.0035	3.9679E-01		ENSG00000231246
LINC02889	0.1184	5.9005E-02	1.7851E-01	ENSG00000236039
LINC02891	0.0176	7.1984E-01		ENSG00000249923
LINC02893	0.0837	2.7214E-01	4.8142E-01	ENSG00000269994
LINC02894	-0.0112	7.9931E-01		ENSG00000261437
LINC02897	0.4556	2.3382E-03	1.6936E-02	ENSG00000221953
LINC02898	-0.0114	5.8945E-01		ENSG00000205086
LINC02899	0.0411	6.0271E-01	7.6738E-01	ENSG00000248874
LINC02901	0.5490	2.4544E-03	1.7560E-02	ENSG00000203711
LINC02903	0.0167	4.6969E-01		ENSG00000205174
LINC02908	-0.0244	7.6488E-01	8.7362E-01	ENSG00000180539
LINC02909	0.0185	5.8384E-01		ENSG00000226397
LINC02910	0.0520	1.0673E-01		ENSG00000176659
LINC02912	-0.0017	9.5274E-01		ENSG00000280055
LINC02932	0.0187	7.4172E-01	8.5915E-01	ENSG00000243144
LINC02937	-0.0207	1.8126E-01		ENSG00000237422
LINC02944	0.0381	6.2078E-01	7.8065E-01	ENSG00000238276
LINC02947	0.0103	6.7373E-01		ENSG00000253140
LINC02955	0.0156	4.1786E-01		ENSG00000256321
LINC02981	0.4201	7.2700E-05	1.1997E-03	ENSG00000214870
LINC02982	0.0065	6.8198E-01		ENSG00000215246
LINC02983	0.0474	4.1168E-01	6.1570E-01	ENSG00000234432
LINC02984	0.0956	1.3884E-01	3.1473E-01	ENSG00000237807
LINC02987	0.0369	6.5743E-01	8.0659E-01	ENSG00000267575
LINC02994	0.0274	5.3752E-01		ENSG00000250546
LINC02995	0.0160	5.4315E-01		ENSG00000253955
LINC02997	0.0300	5.6946E-01	7.4363E-01	ENSG00000249364
LINC02999	0.0481	2.2879E-01		ENSG00000250564
LINC03002	0.1273	3.9446E-02	1.3562E-01	ENSG00000232310
LINC03004	-0.0124	5.6155E-01	7.3847E-01	ENSG00000230533
LINC03007	0.2124	5.4243E-02	1.6878E-01	ENSG00000223561
LINC03008	0.1076	6.2613E-02	1.8586E-01	ENSG00000229196
LINC03011	0.1198	9.0832E-02	2.3949E-01	ENSG00000237310
LINC03013	0.3430	1.0714E-02	5.3300E-02	ENSG00000227544
LINC03014	0.0477	3.2201E-01		ENSG00000240859
LINC03016	0.0630	3.4772E-01	5.5796E-01	ENSG00000230825
LINC03018	-0.0250	1.2701E-01	2.9843E-01	ENSG00000254139
LINC03019	0.0018	8.0997E-01		ENSG00000254813
LINC03021	0.0622	2.4761E-01		ENSG00000254319
LINC03021	-0.0178	1.8037E-01		ENSG00000277526
LINC03022	-0.0476	3.3882E-01	5.4858E-01	ENSG00000253641
LINC03025	0.0878	1.4347E-01	3.2145E-01	ENSG00000234394
LINC03025	0.1125	1.6983E-01	3.5838E-01	ENSG00000276462
LINC03026	0.1402	1.5051E-01	3.3173E-01	ENSG00000287750
LINC03033	-0.0171	3.4424E-01		ENSG00000232774

LINC03034	0.0061	3.7578E-01		ENSG00000259772
LINC03041	0.0250	4.7116E-01		ENSG00000205549
LINC03042	0.0251	7.1188E-01	8.4206E-01	ENSG00000196166
LINC03043	0.0046	5.9337E-01		ENSG00000275356
LINC03047	0.0622	2.8771E-01	4.9832E-01	ENSG00000253633
LINC03048	0.0090	8.7209E-01	9.3576E-01	ENSG00000262223
LINC03049	-0.2822	5.9310E-03	3.4216E-02	ENSG00000271109
LINC03050	-0.0125	4.1699E-01		ENSG00000235725
LINC03051	-0.1094	2.2642E-01	4.3041E-01	ENSG00000239268
LINC03053	0.0090	9.1563E-01	9.5841E-01	ENSG00000223486
LINC03060	0.1208	1.0016E-01	2.5496E-01	ENSG00000273297
LINC03062	-0.0005	9.9450E-01	9.9689E-01	ENSG00000237372
LINC03065	-0.0748	3.1645E-01	5.2650E-01	ENSG00000276524
LINC03066	0.0489	1.5944E-01		ENSG00000263667
LINC03069	0.0210	3.4686E-01		ENSG00000206129
LINC03070	0.0743	2.4908E-01	4.5678E-01	ENSG00000236120
LINC03088	0.2177	4.6151E-02	1.5120E-01	ENSG00000257883
LINC03095	0.0415	2.0082E-01		ENSG00000233760
LINC03098	0.0449	1.7746E-01		ENSG00000230392
LINC03099	0.0210	2.8072E-01		ENSG00000236393
LINC03102	0.3013	1.9738E-02	8.2243E-02	ENSG00000226088
LINC03104	0.0615	3.6350E-01	5.7262E-01	ENSG00000274333
LINC03105	0.0478	4.8950E-01	6.8252E-01	ENSG00000276077
LINC03106	0.0173	4.1546E-01		ENSG00000276759
LINC03112	-0.3926	1.9932E-06	6.6274E-05	ENSG00000289007
LINGO1	-0.3446	6.7459E-05	1.1340E-03	ENSG00000169783
LINGO1-AS1	-0.0751	5.6139E-02		ENSG00000259666
LINGO2	0.5074	7.8349E-06	2.0122E-04	ENSG00000174482
LINGO3	-0.0819	2.7518E-01	4.8509E-01	ENSG00000220008
LINGO4	-0.0075	5.3956E-01		ENSG00000213171
LINS1	0.2150	3.8277E-03	2.4697E-02	ENSG00000140471
LIPA	0.1779	1.5607E-02	6.9983E-02	ENSG00000107798
LIPC	0.0171	3.6466E-01		ENSG00000166035
LIPE	-0.1189	1.9992E-01	3.9831E-01	ENSG00000079435
LIPE-AS1	0.0230	7.8557E-01	8.8661E-01	ENSG00000213904
LIPF	0.0122	3.8320E-01		ENSG00000182333
LIPG	-0.0414	6.3462E-01	7.9048E-01	ENSG00000101670
LIPH	0.2219	5.2258E-02	1.6426E-01	ENSG00000163898
LIPI	0.1203	3.8045E-02	1.3206E-01	ENSG00000188992
LIPJ	-0.0260	3.8683E-01		ENSG00000204022
LIPK	0.0133	4.6128E-01		ENSG00000204021
LIPT1	0.0644	4.2497E-01	6.2734E-01	ENSG00000144182
LIPT2	-0.1183	1.4832E-01	3.2883E-01	ENSG00000175536
LIPT2-AS1	0.1413	7.2019E-02	2.0486E-01	ENSG00000254837
LITAF	-0.0458	5.0719E-01	6.9631E-01	ENSG00000189067
LITAFD	0.0176	6.0854E-01		ENSG00000283516
LITATS1	0.0678	4.3576E-01	6.3673E-01	ENSG00000233621
LIX1	0.3952	4.4763E-04	4.8778E-03	ENSG00000145721
LIX1L	-0.1679	3.9695E-02	1.3618E-01	ENSG00000271601

LIX1L-AS1	0.0531	5.2371E-01	7.0933E-01	ENSG00000234222
LKAAEAR1	-0.0490	5.5725E-01	7.3499E-01	ENSG00000171695
LL22NC01-81G9.3	0.0086	6.7763E-01		ENSG00000215403
LLGL1	-0.2927	3.2793E-03	2.1902E-02	ENSG00000131899
LLGL2	-0.0945	2.9976E-01	5.0941E-01	ENSG00000073350
LLPH	-0.2072	3.1160E-03	2.1098E-02	ENSG00000139233
LLPH-DT	0.0243	7.0546E-01	8.3771E-01	ENSG00000239335
LMAN1	-0.0597	2.6686E-01	4.7617E-01	ENSG00000074695
LMAN2	-0.0195	6.7370E-01	8.1742E-01	ENSG00000169223
LMAN2L	-0.1665	1.9775E-02	8.2369E-02	ENSG00000114988
LMBR1	-0.1553	2.9407E-02	1.0950E-01	ENSG00000105983
LMBR1L	0.1185	5.3950E-02	1.6822E-01	ENSG00000139636
LMBRD1	0.0464	4.5230E-01	6.5120E-01	ENSG00000168216
LMBRD2	0.0035	9.4622E-01	9.7335E-01	ENSG00000164187
LMCD1	0.2333	4.4077E-02	1.4616E-01	ENSG00000071282
LMCD1-AS1	-0.1330	1.4902E-01	3.2951E-01	ENSG00000227110
LMF1	-0.0365	5.9333E-01	7.6055E-01	ENSG00000103227
LMF1-AS1	0.0203	6.1654E-01		ENSG00000260439
LMF2	0.0524	4.0624E-01	6.1075E-01	ENSG00000100258
LMLN	0.4449	3.6934E-06	1.0970E-04	ENSG00000185621
LMNA	0.0367	5.6614E-01	7.4130E-01	ENSG00000160789
LMNB1-DT	0.0952	1.8964E-01	3.8474E-01	ENSG00000251072
LMNB2	-0.1085	1.6858E-01	3.5695E-01	ENSG00000176619
LMNTD1	0.0238	5.4879E-01		ENSG00000152936
LMNTD2	0.0602	4.8009E-01	6.7512E-01	ENSG00000185522
LMNTD2-AS1	0.1296	1.5886E-01	3.4333E-01	ENSG00000254815
LMO1	-0.0955	1.5736E-01	3.4109E-01	ENSG00000166407
LMO2	-0.1139	1.8059E-01	3.7286E-01	ENSG00000135363
LMO3	0.0576	5.0978E-01	6.9859E-01	ENSG00000048540
LMO4	-0.4590	1.6348E-07	8.5220E-06	ENSG00000143013
LMO7	-0.0631	4.5984E-01	6.5758E-01	ENSG00000136153
LMO7-AS1	0.0244	6.7383E-01	8.1745E-01	ENSG00000261105
LMOD1	-0.1360	8.9979E-02	2.3784E-01	ENSG00000163431
LMOD2	-0.0157	4.1073E-01		ENSG00000170807
LMOD3	-0.0457	3.8858E-01	5.9594E-01	ENSG00000163380
LMTK2	-0.0472	5.0465E-01	6.9457E-01	ENSG00000164715
LMTK3	-0.1730	8.2917E-02	2.2454E-01	ENSG00000142235
LMX1A	0.0655	2.6806E-01	4.7750E-01	ENSG00000162761
LMX1B	-0.0114	8.4350E-01	9.1973E-01	ENSG00000136944
LNC-LBCS	0.5362	1.4815E-04	2.0617E-03	ENSG00000228412
LNCARSR	0.0059	7.8593E-01		ENSG00000233086
LNCATV	0.0349	8.9553E-02		ENSG00000238005
LNCBRM	0.0010	8.6905E-01		ENSG00000249436
LNCOC1	0.2564	3.4293E-02	1.2195E-01	ENSG00000253741
LNCOG	0.0174	5.2549E-01		ENSG00000257219
LNCSRLR	-0.0104	5.7234E-01		ENSG00000240032
LNCTAM34A	-0.0935	2.9764E-01	5.0756E-01	ENSG00000234546
LNP1	0.0443	5.3784E-01	7.2047E-01	ENSG00000206535
LNPEP	0.2664	2.8357E-04	3.4360E-03	ENSG00000113441

LNPK	0.0934	1.7042E-01	3.5925E-01	ENSG00000144320
LNX1	0.2912	8.7207E-03	4.5869E-02	ENSG00000072201
LNX1-AS1	0.0369	4.8076E-01	6.7548E-01	ENSG00000250930
LNX2	0.0134	8.6932E-01	9.3433E-01	ENSG00000139517
LOC100128310	-0.0090	9.1389E-01	9.5764E-01	ENSG00000179253
LOC100128317	0.0405	4.3921E-01		ENSG00000233491
LOC100128334	-0.0151	6.6360E-01		ENSG00000235713
LOC100128494	0.0212	6.4849E-01		ENSG00000251143
LOC100128548	-0.0267	3.0802E-01		ENSG00000243979
LOC100128601	-0.0013	8.9208E-01	9.4631E-01	ENSG00000226241
LOC100128770	0.0517	1.2013E-01		ENSG00000205890
LOC100128906	-0.0134	7.2520E-01		ENSG00000203396
LOC100129203	-0.0709	4.0768E-01	6.1202E-01	ENSG00000245552
LOC100129215	-0.0432	5.0431E-01	6.9434E-01	ENSG00000226180
LOC100129434	0.2279	3.9825E-02	1.3639E-01	ENSG00000233251
LOC100129457	0.0141	5.5036E-01		ENSG00000253725
LOC100129534	0.0013	9.0621E-01		ENSG00000269896
LOC100129540	-0.0134	4.6029E-01		ENSG00000177699
LOC100129573	0.0146	4.4818E-01		ENSG00000232036
LOC100129734	-0.0278	1.5112E-01		ENSG00000227815
LOC100129774	-0.0248	2.0883E-01		ENSG00000265417
LOC100129844	-0.0130	4.4317E-01		ENSG00000218565
LOC100129931	-0.0801	2.8829E-01	4.9873E-01	ENSG00000245748
LOC100130018	0.0042	8.7239E-01		ENSG00000213226
LOC100130331	0.0243	7.5986E-01		ENSG00000237250
LOC100130357	-0.0124	8.7386E-01	9.3676E-01	ENSG00000215022
LOC100130548	-0.0148	7.7986E-01		ENSG00000235138
LOC100130587	-0.0124	5.6418E-01		ENSG00000203900
LOC100130620	-0.0062	9.5493E-01		ENSG00000235975
LOC100130664	0.0418	3.0754E-01		ENSG00000270832
LOC100130691	0.0833	3.3339E-01	5.4349E-01	ENSG00000213963
LOC100130714	-0.0154	3.2365E-01		ENSG00000271329
LOC100130881	0.0397	3.3856E-01		ENSG00000236114
LOC100131264	-0.0110	3.0778E-01		ENSG00000231357
LOC100131465	0.0178	7.4556E-01	8.6131E-01	ENSG00000215795
LOC100131496	0.0357	5.3117E-01	7.1528E-01	ENSG00000267882
LOC100131536	-0.0001	8.6493E-01		ENSG00000233388
LOC100131736	0.0105	7.1808E-01	8.4504E-01	ENSG00000232337
LOC100131785	0.0534	5.1475E-01	7.0242E-01	ENSG00000223886
LOC100132249	0.3922	1.8625E-04	2.4572E-03	ENSG00000279561
LOC100132686	-0.0150	4.6019E-01	6.5790E-01	ENSG00000268472
LOC100133077	-0.0337	4.8230E-01	6.7666E-01	ENSG00000203987
LOC100133102	-0.0034	8.2732E-01		ENSG00000218766
LOC100134391	0.0121	7.4026E-01		ENSG00000263574
LOC100192426	-0.0007	8.0076E-01		ENSG00000266149
LOC100271832	-0.0019	9.7701E-01	9.8846E-01	ENSG00000236854
LOC100286962	-0.0164	2.5145E-01		ENSG00000230495
LOC100287243	-0.0001	8.4227E-01		ENSG00000259394
LOC100287792	0.0190	4.1728E-01		ENSG00000204117

LOC100287808	-0.1182	2.0051E-01	3.9896E-01	ENSG00000277182
LOC100287944	0.0661	4.4228E-01	6.4169E-01	ENSG00000257545
LOC100287966	-0.0395	3.8010E-01		ENSG00000237472
LOC100288073	-0.5989	2.2584E-03	1.6504E-02	ENSG00000250746
LOC100288097	0.0435	7.1788E-02	2.0444E-01	ENSG00000255107
LOC100288123	0.0095	7.6739E-01		ENSG00000267244
LOC100288208	0.0343	1.1194E-01		ENSG00000177596
LOC100288683	0.0048	7.7299E-01		ENSG00000249148
LOC100288728	0.0221	7.7907E-01		ENSG00000261848
LOC100289206	0.0279	6.4426E-01		ENSG00000283178
LOC100289230	0.2110	3.9931E-02	1.3655E-01	ENSG00000248489
LOC100289361	0.0515	3.0260E-01	5.1270E-01	ENSG00000268129
LOC100289473	0.0641	3.4580E-01	5.5574E-01	ENSG00000232528
LOC100289495	0.2600	1.6814E-02	7.3482E-02	ENSG00000286760
LOC100289518	-0.0364	5.7048E-01	7.4426E-01	ENSG00000254783
LOC100294145	0.1021	1.6564E-01	3.5314E-01	ENSG00000289047
LOC100310782	0.0046	7.0743E-01		ENSG00000214278
LOC100418874	0.0145	7.7933E-01	8.8284E-01	ENSG00000228729
LOC100418965	0.0033	9.2984E-01		ENSG00000223905
LOC100419046	-0.0076	8.1193E-01		ENSG00000270292
LOC100419058	0.0078	7.1503E-01		ENSG00000271624
LOC100419073	-0.0007	9.0606E-01		ENSG00000217512
LOC100419436	0.0175	8.1925E-01	9.0612E-01	ENSG00000232344
LOC100419515	0.0612	3.7511E-01	5.8368E-01	ENSG00000275318
LOC100419570	0.0119	8.1222E-01		ENSG00000215444
LOC100419574	0.1032	6.4688E-02	1.9032E-01	ENSG00000254398
LOC100419668	0.0163	9.3946E-01		ENSG00000259502
LOC100419679	0.0209	5.3505E-01		ENSG00000228513
LOC100419700	-0.0032	9.0857E-01		ENSG00000256399
LOC100419713	-0.0127	3.1725E-01		ENSG00000270323
LOC100419755	-0.0209	5.8391E-01		ENSG00000243813
LOC100419783	-0.0139	4.4791E-01		ENSG00000276467
LOC100419806	0.0220	1.3943E-01		ENSG00000236775
LOC100419830	0.0073	7.5589E-01		ENSG00000275488
LOC100419851	-0.0192	4.3008E-01		ENSG00000271410
LOC100419913	-0.0126	5.8860E-01		ENSG00000273675
LOC100419917	0.0068	7.3523E-01		ENSG00000288437
LOC100419923	0.0067	7.0110E-01		ENSG00000229369
LOC100420011	0.0103	4.0523E-01		ENSG00000257752
LOC100420020	0.0076	8.6697E-01		ENSG00000254867
LOC100420052	0.0111	6.5996E-01		ENSG00000219384
LOC100420053	-0.0445	2.2603E-01		ENSG00000249848
LOC100420057	0.0058	5.7793E-01		ENSG00000225017
LOC100420114	-0.1637	4.8801E-02	1.5684E-01	ENSG00000273597
LOC100420186	-0.0008	9.2394E-01		ENSG00000258134
LOC100420247	-0.0158	4.1266E-01		ENSG00000223753
LOC100420250	1.0042	4.7249E-03		ENSG00000269475
LOC100420423	0.0261	6.8012E-01	8.2108E-01	ENSG00000232626
LOC100420580	0.0071	7.3571E-01		ENSG00000256651

LOC100420583	0.0011	9.0426E-01		ENSG00000256657
LOC100420587	0.0070	8.0860E-01		ENSG00000283403
LOC100420642	0.0053	8.3659E-01		ENSG00000259866
LOC100420800	0.0033	9.6389E-01		ENSG00000254629
LOC100420801	0.0326	2.4742E-01		ENSG00000255653
LOC100420839	0.0135	8.4485E-01		ENSG00000218631
LOC100420879	-0.0071	6.9590E-01		ENSG00000277704
LOC100420880	0.0114	2.2796E-01		ENSG00000215796
LOC100420899	-0.0831	1.1180E-01	2.7400E-01	ENSG00000255566
LOC100420981	-0.0687	4.0706E-01	6.1153E-01	ENSG00000257511
LOC100421023	0.0209	3.1764E-01		ENSG00000254491
LOC100421091	-0.0700	2.1883E-01	4.2134E-01	ENSG00000217488
LOC100421094	0.0016	7.4450E-01		ENSG00000254423
LOC100421100	0.2489	3.4220E-02	1.2177E-01	ENSG00000265713
LOC100421122	-0.0060	9.9721E-01		ENSG00000229695
LOC100421173	0.1296	3.5221E-02	1.2459E-01	ENSG00000262700
LOC100421273	0.0089	8.8748E-01		ENSG00000229635
LOC100421330	-0.0132	3.0281E-01		ENSG00000274451
LOC100421336	-0.0192	3.0328E-01		ENSG00000238090
LOC100421347	0.0136	3.5168E-01		ENSG00000271597
LOC100421402	0.0026	7.0834E-01	8.3965E-01	ENSG00000232499
LOC100421465	0.0071	4.3212E-01		ENSG00000235251
LOC100421569	0.1028	1.2343E-01	2.9304E-01	ENSG00000237788
LOC100421646	-0.0040	5.6147E-01		ENSG00000258823
LOC100421667	-0.0088	5.8584E-01		ENSG00000278552
LOC100421674	0.0071	7.2805E-01		ENSG00000278346
LOC100421692	-0.0338	5.0932E-01	6.9819E-01	ENSG00000224245
LOC100421775	0.0296	1.5284E-01		ENSG00000237115
LOC100422204	0.1202	1.4336E-01	3.2131E-01	ENSG00000251468
LOC100422225	0.0467	1.6928E-01		ENSG00000258510
LOC100422287	0.0183	3.3416E-01		ENSG00000235907
LOC100422300	-0.0085	8.6993E-01		ENSG00000213252
LOC100422317	0.0221	6.5387E-01		ENSG00000267430
LOC100422382	0.0038	9.0832E-01		ENSG00000256533
LOC100422398	0.0082	6.6434E-01		ENSG00000254877
LOC100422441	0.0263	5.4768E-01		ENSG00000253558
LOC100422491	0.0014	9.8631E-01		ENSG00000261081
LOC100422492	0.0069	8.6461E-01		ENSG00000259892
LOC100422493	0.0281	6.3463E-01	7.9048E-01	ENSG00000258448
LOC100422497	0.0399	4.9882E-01	6.8979E-01	ENSG00000267293
LOC100422614	0.0376	1.7624E-01		ENSG00000253283
LOC100422622	0.0190	2.6553E-01		ENSG00000203437
LOC100422687	0.0154	7.0220E-01		ENSG00000248791
LOC100505502	-0.0325	3.9822E-01		ENSG00000285781
LOC100505715	0.1565	4.3646E-02	1.4524E-01	ENSG00000267058
LOC100505716	0.1245	1.0088E-01	2.5625E-01	ENSG00000223522
LOC100505774	0.4043	4.1080E-04	4.5654E-03	ENSG00000226833
LOC100505851	0.0042	8.0902E-01		ENSG00000269289
LOC100505915	0.0337	5.8375E-01	7.5412E-01	ENSG00000260735

LOC100505985	1.4781	5.5113E-03		ENSG00000226733
LOC100506071	0.0598	3.3393E-01	5.4405E-01	ENSG00000258525
LOC100506207	0.4921	3.3126E-07	1.5321E-05	ENSG00000285219
LOC100506235	-0.1701	9.8229E-02	2.5191E-01	ENSG00000233230
LOC100506274	0.3903	1.4592E-02	6.6661E-02	ENSG00000229727
LOC100506281	0.0590	4.9683E-02		ENSG00000240338
LOC100506321	-0.0299	5.9557E-01	7.6247E-01	ENSG00000259118
LOC100506474	0.1185	2.0331E-01	4.0212E-01	ENSG00000225649
LOC100506489	0.0102	7.8412E-01		ENSG00000240973
LOC100506869	0.0978	1.2515E-01	2.9530E-01	ENSG00000258231
LOC100507053	0.0921	2.9736E-01	5.0728E-01	ENSG00000246090
LOC100507250	0.0710	3.9895E-01	6.0448E-01	ENSG00000247363
LOC100507384	0.0042	9.4479E-01		ENSG00000254519
LOC100507403	-0.0248	2.8433E-01		ENSG00000253123
LOC100507547	-0.0258	7.2595E-01	8.4955E-01	ENSG00000284954
LOC100533628	-0.0029	6.0793E-01	7.7116E-01	ENSG00000258385
LOC100533634	0.0098	5.8708E-01		ENSG00000232906
LOC100533679	0.0090	5.8815E-01		ENSG00000234726
LOC100631242	-0.0279	8.0515E-01		ENSG00000236576
LOC100652758	0.3941	9.0625E-05	1.4275E-03	ENSG00000275765
LOC100652833	0.0177	2.6192E-01		ENSG00000206356
LOC100887080	-0.0177	4.6234E-01		ENSG00000283399
LOC100996318	0.0173	4.4142E-01		ENSG00000233029
LOC100996379	0.1709	3.9782E-02	1.3635E-01	ENSG00000258488
LOC100996385	0.0443	6.2595E-01	7.8423E-01	ENSG00000248469
LOC100996437	0.3337	4.2054E-03	2.6489E-02	ENSG00000226824
LOC100996660	-0.1181	2.0327E-01	4.0212E-01	ENSG00000267248
LOC101059915	0.0012	9.5929E-01		ENSG00000283599
LOC101926907	0.0931	2.8502E-01	4.9564E-01	ENSG00000234807
LOC101926908	0.0041	9.5396E-01		ENSG00000253726
LOC101926953	0.0131	8.2624E-01	9.0997E-01	ENSG00000242816
LOC101926964	-0.4056	2.2594E-03	1.6506E-02	ENSG00000231252
LOC101927018	0.0155	6.1356E-01		ENSG00000265845
LOC101927023	0.0716	8.1751E-02		ENSG00000251076
LOC101927026	0.1666	1.1354E-01	2.7704E-01	ENSG00000260071
LOC101927040	0.0706	3.3345E-01	5.4357E-01	ENSG00000249328
LOC101927042	0.0248	8.9087E-01	9.4581E-01	ENSG00000287838
LOC101927141	0.0202	4.1070E-01		ENSG00000254394
LOC101927143	-0.0067	6.3829E-01		ENSG00000232628
LOC101927159	0.0138	7.8657E-01		ENSG00000229042
LOC101927164	-0.0300	4.7716E-01		ENSG00000237101
LOC101927179	0.1018	1.9725E-01	3.9490E-01	ENSG00000282917
LOC101927237	-0.0044	7.0578E-01		ENSG00000250075
LOC101927293	0.3349	2.3069E-02	9.2098E-02	ENSG00000225096
LOC101927377	-0.0196	9.2034E-01	9.6091E-01	ENSG00000231119
LOC101927383	0.0306	7.1741E-01	8.4471E-01	ENSG00000228528
LOC101927401	-0.0337	6.6170E-01	8.0949E-01	ENSG00000287315
LOC101927418	0.0360	9.4572E-02		ENSG00000258949
LOC101927468	0.0899	2.1237E-01	4.1337E-01	ENSG00000227733

LOC101927495	0.0022	5.7919E-01		ENSG00000250230
LOC101927539	0.0117	8.2764E-01		ENSG00000263499
LOC101927543	0.0087	5.9930E-01		ENSG00000248318
LOC101927551	-0.0802	3.0329E-01	5.1341E-01	ENSG00000230922
LOC101927557	0.0114	8.2263E-01		ENSG00000266100
LOC101927560	0.0059	8.6436E-01		ENSG00000233008
LOC101927571	-0.0208	3.9837E-01		ENSG00000266850
LOC101927575	0.0301	3.9252E-01		ENSG00000227463
LOC101927588	0.0093	6.8778E-01		ENSG00000214803
LOC101927605	0.0310	1.5142E-01		ENSG00000261436
LOC101927608	-0.0049	9.4448E-01		ENSG00000273805
LOC101927609	0.0185	8.0831E-01	8.9936E-01	ENSG00000237773
LOC101927636	0.0920	2.4632E-01	4.5414E-01	ENSG00000251600
LOC101927661	0.0964	2.0107E-01	3.9955E-01	ENSG00000235885
LOC101927690	-0.0007	9.2448E-01		ENSG00000258803
LOC101927692	0.0553	1.7266E-01		ENSG00000228484
LOC101927702	-0.0171	4.8034E-01	6.7525E-01	ENSG00000254718
LOC101927708	0.0831	1.3333E-01	3.0708E-01	ENSG00000255367
LOC101927727	0.0519	3.8190E-01	5.9013E-01	ENSG00000262003
LOC101927745	0.1199	2.6095E-02	1.0062E-01	ENSG00000229425
LOC101927769	0.0801	2.2320E-01	4.2686E-01	ENSG00000226097
LOC101927787	0.0577	3.5033E-02	1.2412E-01	ENSG00000228044
LOC101927793	0.0008	8.1071E-01		ENSG00000259881
LOC101927822	-0.0011	9.8250E-01		ENSG00000253593
LOC101927825	-0.0076	5.6983E-01		ENSG00000282556
LOC101927839	0.0031	8.8500E-01		ENSG00000225084
LOC101927857	0.0100	4.7185E-01		ENSG00000286559
LOC101927879	0.0148	3.4651E-01		ENSG00000283458
LOC101927888	0.0172	3.1611E-01		ENSG00000231811
LOC101927914	0.1218	4.4851E-02	1.4809E-01	ENSG00000234210
LOC101927993	-0.0036	5.6800E-01		ENSG00000223446
LOC101928002	0.0203	7.1243E-01	8.4224E-01	ENSG00000247131
LOC101928008	0.0179	2.7727E-01		ENSG00000255476
LOC101928012	0.0867	2.4389E-01	4.5158E-01	ENSG00000223646
LOC101928016	0.0519	2.0139E-01		ENSG00000253550
LOC101928052	0.0087	8.4099E-01		ENSG00000249419
LOC101928053	-0.0460	5.9256E-01	7.6021E-01	ENSG00000246308
LOC101928059	0.0915	2.7590E-01	4.8576E-01	ENSG00000284738
LOC101928087	0.1160	5.8657E-03		ENSG00000277332
LOC101928093	0.0315	5.4602E-01		ENSG00000253295
LOC101928143	-0.0211	1.6383E-01		ENSG00000284930
LOC101928251	0.0047	9.9824E-01		ENSG00000264985
LOC101928254	0.0116	6.2596E-01		ENSG00000219445
LOC101928266	0.0067	8.9582E-01		ENSG00000262966
LOC101928295	0.0850	2.3945E-01	4.4611E-01	ENSG00000268686
LOC101928334	0.0507	4.1822E-01	6.2148E-01	ENSG00000289355
LOC101928371	-0.0178	7.4512E-01	8.6120E-01	ENSG00000225420
LOC101928373	0.0095	8.7187E-01		ENSG00000286313
LOC101928380	0.0261	7.3647E-01	8.5613E-01	ENSG00000231963

LOC101928381	-0.0149	8.2339E-01	9.0841E-01	ENSG00000236252
LOC101928386	0.0049	7.2975E-01		ENSG00000232377
LOC101928389	0.0023	7.9538E-01		ENSG00000235834
LOC101928392	-0.0098	4.8119E-01		ENSG00000261285
LOC101928416	0.0601	3.5688E-01	5.6667E-01	ENSG00000255916
LOC101928438	0.1869	7.2834E-02	2.0656E-01	ENSG00000237461
LOC101928463	-0.0065	7.4631E-01		ENSG00000287359
LOC101928495	-0.0015	8.3879E-01		ENSG00000237208
LOC101928499	0.0353	5.4230E-01	7.2388E-01	ENSG00000259203
LOC101928516	0.0430	1.3545E-01		ENSG00000223786
LOC101928525	0.0007	8.5315E-01		ENSG00000226706
LOC101928557	-0.0580	2.6391E-01	4.7283E-01	ENSG00000270159
LOC101928565	0.0145	3.3434E-01		ENSG00000228697
LOC101928596	-0.0342	1.9227E-01	3.8794E-01	ENSG00000237707
LOC101928651	-0.0053	9.4376E-01		ENSG00000248529
LOC101928718	0.1756	9.0371E-02	2.3868E-01	ENSG00000284830
LOC101928728	0.0128	8.4332E-01	9.1966E-01	ENSG00000260063
LOC101928733	0.0128	3.9049E-01		ENSG00000228151
LOC101928748	-0.0165	3.0864E-01		ENSG00000236461
LOC101928834	0.1444	8.8151E-02	2.3428E-01	ENSG00000233968
LOC101928844	0.0295	3.1649E-01		ENSG00000267709
LOC101928847	-0.0455	5.8442E-01	7.5448E-01	ENSG00000247416
LOC101928855	0.0570	4.4407E-01	6.4311E-01	ENSG00000262833
LOC101928868	-0.0124	8.8086E-01	9.4037E-01	ENSG00000287260
LOC101928894	0.0276	3.8474E-01		ENSG00000255314
LOC101928896	0.0086	8.5721E-01		ENSG00000255084
LOC101928937	-0.0040	7.2554E-01		ENSG00000258039
LOC101928940	0.0232	6.4188E-01		ENSG00000256195
LOC101928952	0.0028	7.4905E-01		ENSG00000271143
LOC101928965	0.0245	5.7442E-01		ENSG00000271858
LOC101928988	-0.0585	2.0416E-01		ENSG00000259351
LOC101928994	0.0447	2.1839E-01		ENSG00000229261
LOC101929004	-0.1271	1.3147E-01	3.0457E-01	ENSG00000288009
LOC101929021	0.0692	2.2838E-01	4.3305E-01	ENSG00000236154
LOC101929053	0.0161	5.7325E-01	7.4625E-01	ENSG00000270857
LOC101929073	0.0126	3.2857E-01		ENSG00000226304
LOC101929106	0.0675	1.4050E-01		ENSG00000198491
LOC101929109	0.0721	2.7952E-01	4.8957E-01	ENSG00000249713
LOC101929130	0.0003	9.9738E-01	9.9843E-01	ENSG00000283175
LOC101929141	-0.0291	4.3832E-01	6.3860E-01	ENSG00000250111
LOC101929174	-0.4201	9.4373E-03	4.8618E-02	ENSG00000288018
LOC101929200	0.1392	1.5272E-01	3.3479E-01	ENSG00000286753
LOC101929227	0.0107	3.7625E-01		ENSG00000250493
LOC101929237	0.0053	9.7065E-01		ENSG00000248738
LOC101929269	0.0325	5.2221E-01	7.0867E-01	ENSG00000254839
LOC101929341	0.0105	3.2674E-01		ENSG00000253551
LOC101929372	-0.0416	1.3725E-01		ENSG00000236754
LOC101929408	0.0195	7.6696E-01	8.7499E-01	ENSG00000260288
LOC101929427	-0.0170	7.9035E-01	8.8919E-01	ENSG00000254607

LOC101929439	0.0227	3.3827E-01		ENSG00000259422
LOC101929452	-0.0185	7.9170E-01	8.9001E-01	ENSG00000223884
LOC101929457	0.0050	8.8274E-01		ENSG00000259362
LOC101929460	0.0028	8.4305E-01		ENSG00000229720
LOC101929470	0.0702	4.6029E-02		ENSG00000248964
LOC101929494	0.0032	8.9278E-01		ENSG00000276170
LOC101929528	-0.0015	9.9678E-01		ENSG00000253281
LOC101929536	-0.0366	6.4727E-02		ENSG00000261798
LOC101929552	-0.0224	7.7986E-01	8.8321E-01	ENSG00000263063
LOC101929563	0.1834	8.4071E-02	2.2651E-01	ENSG00000283982
LOC101929566	0.2331	4.0569E-02	1.3814E-01	ENSG00000263280
LOC101929577	0.0072	8.2210E-01		ENSG00000249635
LOC101929586	0.0023	9.6564E-01		ENSG00000259175
LOC101929609	0.3182	1.2598E-02	5.9921E-02	ENSG00000288573
LOC101929626	0.3742	3.5930E-03	2.3525E-02	ENSG00000227857
LOC101929653	0.4401	3.2866E-03	2.1939E-02	ENSG00000237654
LOC101929657	0.0248	7.3103E-01		ENSG00000273523
LOC101929667	0.0067	6.1747E-01		ENSG00000287255
LOC101929691	0.0114	8.3123E-01		ENSG00000285872
LOC101929698	0.0114	6.5299E-01		ENSG00000277301
LOC101929710	0.0181	7.4272E-01		ENSG00000251314
LOC101929718	-0.0109	8.1135E-01		ENSG00000258471
LOC101929719	-0.0350	5.3315E-01	7.1684E-01	ENSG00000254363
LOC101929748	0.4254	1.2129E-02	5.8378E-02	ENSG00000288062
LOC101929918	0.0675	3.8466E-01	5.9262E-01	ENSG00000286626
LOC101930090	0.0130	8.0460E-01	8.9752E-01	ENSG00000283541
LOC101930276	0.1016	3.7220E-02		ENSG00000283462
LOC101930420	0.0291	6.9243E-01		ENSG00000283544
LOC101930421	0.0378	4.2824E-01	6.3016E-01	ENSG00000225140
LOC102723313	-0.0260	5.9645E-01	7.6313E-01	ENSG00000248896
LOC102723323	-0.0607	4.2893E-01	6.3071E-01	ENSG00000260850
LOC102723335	0.0865	7.3804E-02		ENSG00000259540
LOC102723341	0.0115	3.7053E-01		ENSG00000226409
LOC102723360	0.0418	5.5015E-01	7.2920E-01	ENSG00000275496
LOC102723451	-0.0158	8.1487E-01	9.0367E-01	ENSG00000277277
LOC102723475	-0.0087	9.6901E-01	9.8478E-01	ENSG00000276289
LOC102723536	0.0007	9.1490E-01		ENSG00000231876
LOC102723553	-0.1733	8.6405E-03	4.5554E-02	ENSG00000273590
LOC102723566	0.0029	9.5980E-01	9.8006E-01	ENSG00000225032
LOC102723604	0.0486	5.0434E-01	6.9434E-01	ENSG00000258928
LOC102723663	-0.0043	8.6057E-01		ENSG00000285906
LOC102723692	-0.0144	3.4852E-01		ENSG00000261448
LOC102723701	-0.0915	2.3134E-01	4.3622E-01	ENSG00000183154
LOC102723878	-0.0033	6.0534E-01		ENSG00000236935
LOC102723996	-0.1052	1.0643E-01	2.6523E-01	ENSG00000277117
LOC102724019	0.1619	1.0906E-01	2.6957E-01	ENSG00000240086
LOC102724023	-0.0670	4.3125E-01	6.3290E-01	ENSG00000280071
LOC102724104	-0.0062	7.3616E-01		ENSG00000287958
LOC102724159	0.2274	1.4275E-02	6.5638E-02	ENSG00000275464

LOC102724200	0.1592	7.5001E-02	2.1054E-01	ENSG00000280433
LOC102724250	0.1608	2.8068E-02	1.0602E-01	ENSG00000271254
LOC102724289	-0.0166	5.5894E-01		ENSG00000287706
LOC102724334	0.0178	4.1899E-01		ENSG00000274559
LOC102724354	0.0173	2.8273E-01		ENSG00000280191
LOC102724421	-0.0383	4.2339E-01	6.2590E-01	ENSG00000257083
LOC102724428	-0.3704	1.2247E-03	1.0559E-02	ENSG00000275993
LOC102724474	0.2772	5.0032E-03	3.0114E-02	ENSG00000283828
LOC102724560	-0.0683	3.6306E-01	5.7216E-01	ENSG00000274276
LOC102724593	-0.4107	7.6097E-03	4.1260E-02	ENSG00000231187
LOC102724594	-0.0681	2.7739E-01	4.8747E-01	ENSG00000275895
LOC102724602	0.0064	7.6948E-01		ENSG00000277882
LOC102724612	0.0961	8.1758E-02	2.2248E-01	ENSG00000253894
LOC102724637	-0.0018	9.4267E-01		ENSG00000214942
LOC102724642	0.0018	9.9330E-01	9.9625E-01	ENSG00000204745
LOC102724646	0.0369	6.6069E-01	8.0883E-01	ENSG00000289256
LOC102724652	0.0623	1.3705E-01	3.1240E-01	ENSG00000276076
LOC102724680	0.3388	1.8944E-03	1.4466E-02	ENSG00000289218
LOC102724701	0.0072	9.2699E-01	9.6425E-01	ENSG00000280145
LOC102724710	0.0236	4.2381E-01		ENSG00000253634
LOC102724719	0.0438	5.1905E-01	7.0634E-01	ENSG00000223502
LOC102724737	-0.0314	6.4399E-01		ENSG00000281087
LOC102724768	0.0483	4.9083E-01	6.8345E-01	ENSG00000237949
LOC102724843	0.0448	5.1835E-01	7.0562E-01	ENSG00000277067
LOC102724859	0.2577	2.3707E-02	9.4020E-02	ENSG00000279249
LOC102724900	0.0492	6.6684E-02	1.9437E-01	ENSG00000230051
LOC102724934	-0.1506	1.3158E-01	3.0473E-01	ENSG00000257522
LOC102725254	0.1073	2.0762E-01	4.0732E-01	ENSG00000268650
LOC103171574	0.0756	2.8151E-01	4.9191E-01	ENSG00000259774
LOC103344931	0.1297	1.5378E-01	3.3614E-01	ENSG00000260917
LOC105274304	0.0256	7.1573E-01	8.4374E-01	ENSG00000261335
LOC105369165	0.0128	7.8953E-01		ENSG00000228033
LOC105369187	0.0018	7.4377E-01		ENSG00000250748
LOC105369306	-0.0169	7.5092E-01	8.6466E-01	ENSG00000233393
LOC105369329	0.2054	6.2759E-02	1.8614E-01	ENSG00000255931
LOC105369344	-0.0213	7.1428E-01	8.4315E-01	ENSG00000287917
LOC105369363	0.0632	4.4755E-01	6.4697E-01	ENSG00000286369
LOC105369373	0.0098	8.8493E-01		ENSG00000255539
LOC105369391	-0.0029	9.0802E-01		ENSG00000255326
LOC105369496	0.0653	2.0331E-01	4.0212E-01	ENSG00000287006
LOC105369576	0.2219	2.8708E-02	1.0773E-01	ENSG00000288013
LOC105369685	0.0050	9.3480E-01	9.6788E-01	ENSG00000287075
LOC105369728	0.0360	3.9146E-02		ENSG00000256442
LOC105369850	0.0956	2.2700E-01	4.3120E-01	ENSG00000257526
LOC105369907	-0.0081	6.6157E-01		ENSG00000257746
LOC105369911	0.0033	9.7824E-01		ENSG00000257283
LOC105370024	0.0204	2.0663E-01		ENSG00000256884
LOC105370027	0.0976	6.4654E-02		ENSG00000248636
LOC105370047	-0.0283	6.7434E-01	8.1751E-01	ENSG00000214650

LOC105370061	0.0268	3.1010E-01		ENSG00000256286
LOC105370105	-0.0010	7.8425E-01		ENSG00000235438
LOC105370152	0.0307	3.1209E-01		ENSG00000289381
LOC105370174	0.0450	7.3314E-01	8.5427E-01	ENSG00000288598
LOC105370203	0.1853	5.7327E-02	1.7546E-01	ENSG00000288743
LOC105370259	0.0495	5.3405E-01	7.1773E-01	ENSG00000286330
LOC105370295	0.0545	1.3160E-01		ENSG00000287376
LOC105370489	-0.0383	6.2062E-01	7.8065E-01	ENSG00000258843
LOC105370500	0.0718	3.9898E-01	6.0449E-01	ENSG00000285664
LOC105370525	0.0103	8.6980E-01		ENSG00000258926
LOC105370532	0.0254	7.5343E-01	8.6615E-01	ENSG00000274015
LOC105370586	-0.0187	5.6378E-01		ENSG00000258419
LOC105370616	-0.0040	8.7768E-01		ENSG00000258792
LOC105370622	0.0249	4.1916E-01		ENSG00000258716
LOC105370645	0.0028	8.9036E-01		ENSG00000287350
LOC105370650	0.0658	7.8954E-03		ENSG00000287501
LOC105370689	0.0500	7.9917E-02		ENSG00000287346
LOC105370705	0.0702	2.9887E-01	5.0867E-01	ENSG00000288795
LOC105370740	0.0168	5.0687E-01		ENSG00000287280
LOC105370783	-0.0107	5.5129E-01		ENSG00000259269
LOC105370791	0.1210	1.3345E-01	3.0720E-01	ENSG00000260926
LOC105370802	0.1618	2.7212E-02	1.0366E-01	ENSG00000259200
LOC105370804	0.1412	7.5353E-03		ENSG00000259588
LOC105370890	-0.0103	7.2410E-01		ENSG00000259650
LOC105370906	0.0772	2.5283E-02		ENSG00000259420
LOC105370941	0.1404	9.1011E-02	2.3973E-01	ENSG00000261136
LOC105370954	0.0065	5.7869E-01		ENSG00000259636
LOC105370969	0.0731	2.7399E-01	4.8378E-01	ENSG00000259704
LOC105371022	0.0033	6.1662E-01		ENSG00000259219
LOC105371046	0.0262	1.8525E-01		ENSG00000260989
LOC105371083	0.0070	8.8940E-01		ENSG00000263080
LOC105371090	0.0109	7.9497E-01		ENSG00000260378
LOC105371115	0.0260	4.5422E-01		ENSG00000261195
LOC105371335	0.0047	7.8649E-01		ENSG00000260520
LOC105371354	0.0053	9.4452E-01		ENSG00000288821
LOC105371361	0.0619	1.0977E-01		ENSG00000261838
LOC105371366	0.0124	7.4414E-01		ENSG00000260788
LOC105371414	0.2885	1.2219E-02	5.8707E-02	ENSG00000260279
LOC105371430	-0.0237	6.8069E-01	8.2147E-01	ENSG00000241525
LOC105371485	-0.0150	7.4416E-01		ENSG00000228133
LOC105371672	0.0073	6.8577E-01		ENSG00000287364
LOC105371689	-0.0471	4.7888E-01	6.7416E-01	ENSG00000286383
LOC105371729	-0.1082	1.7430E-01	3.6462E-01	ENSG00000285677
LOC105371730	-0.4358	3.2541E-03	2.1775E-02	ENSG00000214708
LOC105371734	0.0129	5.4236E-01		ENSG00000265222
LOC105371745	0.0083	9.6475E-01		ENSG00000270240
LOC105371749	0.0164	6.5411E-01	8.0456E-01	ENSG00000289011
LOC105371789	-0.1269	1.3885E-01	3.1473E-01	ENSG00000267638
LOC105371795	0.0398	3.7562E-01		ENSG00000267288

LOC105371814	-0.2110	2.8936E-02	1.0829E-01	ENSG00000248278
LOC105371824	0.0128	3.9983E-01		ENSG00000250286
LOC105371841	0.2806	2.3065E-02	9.2098E-02	ENSG00000287337
LOC105371855	0.0586	1.3164E-01		ENSG00000265702
LOC105371899	-0.0468	5.0745E-01	6.9644E-01	ENSG00000267568
LOC105371956	0.0042	6.4034E-01		ENSG00000263745
LOC105371967	0.3310	2.3411E-02	9.3163E-02	ENSG00000266401
LOC105372066	0.0089	6.7496E-01		ENSG00000267627
LOC105372069	0.1024	1.4977E-01	3.3063E-01	ENSG00000267039
LOC105372143	0.0025	9.3651E-01		ENSG00000267743
LOC105372165	0.1254	1.8681E-01	3.8112E-01	ENSG00000283667
LOC105372179	0.0088	4.5197E-01		ENSG00000264845
LOC105372180	0.1735	3.3095E-02	1.1922E-01	ENSG00000287646
LOC105372202	0.0216	2.9023E-01		ENSG00000287295
LOC105372268	-0.0153	7.0966E-01		ENSG00000287960
LOC105372316	-0.0405	1.3494E-01		ENSG00000269043
LOC105372321	-0.1022	2.5243E-01	4.6050E-01	ENSG00000268119
LOC105372401	0.0444	5.8285E-01	7.5356E-01	ENSG00000286177
LOC105372421	0.0835	1.9616E-01	3.9346E-01	ENSG00000268401
LOC105372432	0.0229	4.9970E-01		ENSG00000286024
LOC105372435	0.0251	1.8259E-01		ENSG00000269194
LOC105372558	0.0034	8.4572E-01		ENSG00000225280
LOC105372710	0.0049	7.8669E-01		ENSG00000233017
LOC105372832	0.0008	8.2910E-01		ENSG00000232124
LOC105372839	0.6691	1.0482E-02	5.2473E-02	ENSG00000289448
LOC105372971	0.2310	4.7266E-02	1.5364E-01	ENSG00000229770
LOC105372990	0.0408	2.8287E-01		ENSG00000273428
LOC105373044	-0.1742	9.2381E-02	2.4212E-01	ENSG00000286491
LOC105373100	0.0237	5.4422E-01		ENSG00000225929
LOC105373170	0.0555	2.6252E-01	4.7139E-01	ENSG00000286071
LOC105373244	0.0544	3.3274E-01	5.4302E-01	ENSG00000284391
LOC105373273	-0.0641	1.0397E-01	2.6131E-01	ENSG00000289566
LOC105373289	0.4796	4.8197E-03	2.9242E-02	ENSG00000286389
LOC105373299	0.0716	3.2343E-01	5.3344E-01	ENSG00000286794
LOC105373335	-0.0125	6.7556E-01	8.1798E-01	ENSG00000235189
LOC105373346	0.0274	5.8930E-01	7.5784E-01	ENSG00000235779
LOC105373373	0.0534	2.0149E-01		ENSG00000273877
LOC105373383	0.1945	1.8041E-02	7.7115E-02	ENSG00000260081
LOC105373390	-0.0232	5.3646E-01		ENSG00000237720
LOC105373422	-0.0008	9.5933E-01		ENSG00000287305
LOC105373429	0.1461	1.0731E-01	2.6654E-01	ENSG00000285569
LOC105373436	-0.0091	8.9350E-01	9.4712E-01	ENSG00000285876
LOC105373496	0.0166	2.6743E-01	4.7676E-01	ENSG00000235480
LOC105373508	-0.0080	4.8730E-01		ENSG00000286737
LOC105373526	0.0018	9.0212E-01		ENSG00000230690
LOC105373553	0.0336	6.6987E-01	8.1494E-01	ENSG00000282033
LOC105373682	0.0052	8.5706E-01		ENSG00000286081
LOC105373696	-0.0380	3.9045E-01	5.9771E-01	ENSG00000230991
LOC105373742	0.0132	4.7188E-01		ENSG00000286557

LOC105373780	0.3984	3.7912E-03	2.4511E-02	ENSG00000283839
LOC105373845	0.0246	2.3382E-01		ENSG00000237843
LOC105373973	-0.1043	1.3860E-01	3.1446E-01	ENSG00000288080
LOC105374069	0.0262	3.7329E-01		ENSG00000286827
LOC105374085	0.1600	9.4588E-02	2.4610E-01	ENSG00000250012
LOC105374101	-0.2183	4.4992E-02	1.4840E-01	ENSG00000289469
LOC105374114	0.0167	7.9863E-01	8.9385E-01	ENSG00000248468
LOC105374174	-0.0581	4.9441E-01	6.8651E-01	ENSG00000286585
LOC105374191	0.0306	2.6265E-01		ENSG00000289884
LOC105374254	0.2157	5.3963E-02	1.6824E-01	ENSG00000286086
LOC105374312	-0.0154	5.2114E-01		ENSG00000272840
LOC105374328	0.0184	3.1472E-01		ENSG00000232642
LOC105374338	-0.0535	4.1066E-01	6.1488E-01	ENSG00000283183
LOC105374344	0.0595	9.6531E-02		ENSG00000251652
LOC105374428	0.0017	9.2917E-01	9.6510E-01	ENSG00000250781
LOC105374438	0.0162	3.5343E-01		ENSG00000250863
LOC105374516	0.0019	8.9307E-01		ENSG00000250092
LOC105374524	-0.0007	9.1729E-01		ENSG00000248837
LOC105374625	0.0091	7.3004E-01		ENSG00000288978
LOC105374647	0.0029	9.2859E-01		ENSG00000249782
LOC105374736	0.0676	1.5610E-01	3.4000E-01	ENSG00000287597
LOC105374780	0.0423	3.6272E-01	5.7192E-01	ENSG00000234255
LOC105374802	0.0249	4.6058E-01		ENSG00000287435
LOC105374811	0.0179	6.9041E-01		ENSG00000270571
LOC105374981	0.1303	9.3650E-02	2.4441E-01	ENSG00000288887
LOC105374988	0.0675	2.0896E-01		ENSG00000284607
LOC105374989	0.0639	1.2072E-01		ENSG00000275846
LOC105375050	-0.0016	7.7529E-01		ENSG00000227131
LOC105375115	-0.0059	8.1081E-01		ENSG00000239715
LOC105375146	0.0012	9.8580E-01	9.9239E-01	ENSG00000235431
LOC105375170	0.0013	7.7232E-01		ENSG00000289189
LOC105375216	0.0479	4.7993E-01	6.7493E-01	ENSG00000286847
LOC105375224	0.0002	9.3042E-01		ENSG00000231418
LOC105375387	0.0533	8.6187E-02		ENSG00000227863
LOC105375421	0.0868	2.9868E-01	5.0843E-01	ENSG00000284523
LOC105375483	0.0313	1.5564E-01		ENSG00000241345
LOC105375484	0.0050	9.7365E-01		ENSG00000243574
LOC105375501	0.0103	6.2148E-01		ENSG00000286380
LOC105375523	-0.0076	7.6251E-01		ENSG00000230649
LOC105375556	0.0039	6.9624E-01		ENSG00000230190
LOC105375614	0.1862	5.4067E-02	1.6850E-01	ENSG00000222012
LOC105375638	0.0068	9.9429E-01		ENSG00000253682
LOC105375709	0.0158	2.9503E-01		ENSG00000287819
LOC105375713	-0.0062	4.9018E-01		ENSG00000289382
LOC105375743	0.0142	7.2524E-01		ENSG00000255491
LOC105375759	-0.0007	8.3030E-01		ENSG00000253407
LOC105375844	-0.0133	7.1425E-01	8.4315E-01	ENSG00000253857
LOC105375861	-0.0032	8.8065E-01		ENSG00000254775
LOC105375903	-0.0196	3.2294E-01		ENSG00000253983

LOC105375924	0.1829	7.5613E-02	2.1163E-01	ENSG00000253214
LOC105375976	-0.0120	5.5123E-01		ENSG00000224935
LOC105376030	0.0064	9.3827E-01		ENSG00000287514
LOC105376070	0.1156	1.3728E-01	3.1267E-01	ENSG00000233178
LOC105376121	0.0293	1.4337E-01		ENSG00000285634
LOC105376244	0.0526	1.3522E-01		ENSG00000284977
LOC105376291	0.0759	3.6029E-02		ENSG00000226355
LOC105376306	0.1967	5.9158E-02	1.7875E-01	ENSG00000288989
LOC105376520	0.1146	1.0027E-01	2.5517E-01	ENSG00000290098
LOC105376588	0.0667	1.6882E-01	3.5726E-01	ENSG00000255357
LOC105376650	-0.0007	8.2470E-01		ENSG00000287984
LOC105376713	-0.0247	6.7939E-01	8.2059E-01	ENSG00000259617
LOC105376805	-0.0215	7.6339E-01	8.7268E-01	ENSG00000238142
LOC105376860	-0.0171	6.5315E-01		ENSG00000289835
LOC105376975	0.0023	6.6585E-01		ENSG00000286352
LOC105377139	0.1173	2.4613E-02		ENSG00000285413
LOC105377146	-0.0186	6.5042E-01		ENSG00000286967
LOC105377188	0.0020	8.8351E-01		ENSG00000287158
LOC105377205	0.0077	9.1495E-01	9.5810E-01	ENSG00000260613
LOC105377209	0.5242	4.8956E-04	5.2293E-03	ENSG00000287215
LOC105377213	-0.0075	8.2710E-01		ENSG00000214915
LOC105377294	-0.0880	2.9093E-01	5.0136E-01	ENSG00000289586
LOC105377378	0.0556	3.6441E-02		ENSG00000288781
LOC105377448	-0.0083	7.2636E-01		ENSG00000250195
LOC105377451	-0.0382	3.4160E-01		ENSG00000286896
LOC105377503	0.0259	2.7078E-01		ENSG00000287730
LOC105377603	0.0143	7.2299E-01		ENSG00000249642
LOC105377724	0.0119	6.3720E-01		ENSG00000285914
LOC105377727	-0.0520	5.0082E-01	6.9169E-01	ENSG00000287814
LOC105377848	-0.0059	8.9599E-01		ENSG00000287300
LOC105377862	0.0074	6.9476E-01		ENSG00000271945
LOC105378044	0.0455	3.2830E-01		ENSG00000226249
LOC105378052	0.0294	3.0719E-01		ENSG00000216621
LOC105378137	0.0051	8.9119E-01		ENSG00000237987
LOC105378231	-0.0232	5.0065E-01		ENSG00000260581
LOC105378268	0.0858	3.2001E-01	5.3019E-01	ENSG00000285884
LOC105378311	-0.0090	6.6026E-01		ENSG00000234173
LOC105378318	0.0477	2.6760E-01		ENSG00000235140
LOC105378355	0.0501	4.5849E-01	6.5631E-01	ENSG00000289362
LOC105378453	0.0143	7.4429E-01		ENSG00000289441
LOC105378473	0.0562	3.5958E-01	5.6890E-01	ENSG00000287047
LOC105378714	0.0455	4.6015E-01	6.5789E-01	ENSG00000284700
LOC105378736	-0.0038	9.1866E-01		ENSG00000284601
LOC105378798	0.0347	2.6381E-01		ENSG00000225087
LOC105378976	0.0624	3.0395E-01	5.1410E-01	ENSG00000249236
LOC105378998	0.0232	3.1762E-01		ENSG00000289916
LOC105379100	0.0147	2.9902E-01		ENSG00000249787
LOC105379130	0.0233	4.5139E-01		ENSG00000289497
LOC105379199	-0.0625	4.1258E-01	6.1625E-01	ENSG00000286615

LOC105379230	0.0404	4.5227E-01	6.5119E-01	ENSG00000254237
LOC105379242	0.0475	4.8327E-01	6.7743E-01	ENSG00000286985
LOC105379301	-0.0086	6.9027E-01		ENSG00000254242
LOC105379340	0.0614	2.6024E-01		ENSG00000253888
LOC105379362	0.0723	3.2701E-01	5.3734E-01	ENSG00000247134
LOC105379428	0.1507	9.9360E-02	2.5363E-01	ENSG00000278932
LOC105379499	0.1032	2.0304E-01	4.0199E-01	ENSG00000280018
LOC105379839	-0.0528	5.3764E-01	7.2037E-01	ENSG00000286718
LOC107983987	0.0755	1.7844E-01	3.6963E-01	ENSG00000278961
LOC107984172	0.0216	8.1555E-01		ENSG00000289386
LOC107984195	-0.0011	8.6859E-01		ENSG00000227101
LOC107984203	0.0684	2.9742E-02	1.1046E-01	ENSG00000287277
LOC107984236	0.0013	8.1003E-01	9.0022E-01	ENSG00000289989
LOC107984265	-0.0384	3.0123E-01		ENSG00000286575
LOC107984322	0.0235	1.4200E-01		ENSG00000255375
LOC107984399	0.0078	6.9859E-01		ENSG00000176984
LOC107984450	0.0775	3.6234E-01	5.7171E-01	ENSG00000256001
LOC107984548	0.0082	8.2420E-01		ENSG00000258308
LOC107984685	0.0004	9.9009E-01	9.9452E-01	ENSG00000258028
LOC107984761	0.0063	8.1001E-01		ENSG00000259392
LOC107984784	0.0006	6.2465E-01		ENSG00000259697
LOC107984827	-0.0107	7.3553E-01		ENSG00000261170
LOC107984850	-0.0078	6.9747E-01		ENSG00000288531
LOC107984948	-0.0122	8.8422E-01	9.4199E-01	ENSG00000230615
LOC107984970	0.0273	5.1420E-01		ENSG00000270553
LOC107985049	-0.0064	9.5210E-01		ENSG00000271268
LOC107985074	0.0180	4.3389E-01		ENSG00000289070
LOC107985126	0.0705	8.9618E-02		ENSG00000264151
LOC107985177	0.1230	4.8074E-02	1.5529E-01	ENSG00000287281
LOC107985206	-0.1108	2.2235E-01	4.2571E-01	ENSG00000286391
LOC107985216	0.0105	8.6285E-01		ENSG00000272668
LOC107985265	-0.0195	3.5278E-01		ENSG00000268747
LOC107985380	0.0208	6.5777E-01	8.0675E-01	ENSG00000234271
LOC107985563	0.0022	9.7657E-01		ENSG00000289963
LOC107985579	-0.0099	7.9357E-01		ENSG00000181123
LOC107985629	-0.0066	8.1606E-01		ENSG00000232042
LOC107985645	0.0109	8.9744E-01		ENSG00000229733
LOC107985688	-0.0220	8.6698E-01		ENSG00000228427
LOC107985892	0.0266	6.2040E-01	7.8055E-01	ENSG00000289156
LOC107985953	-0.0807	2.8186E-01	4.9228E-01	ENSG00000286679
LOC107986163	0.0246	7.6490E-01	8.7362E-01	ENSG00000272970
LOC107986178	0.0486	6.4540E-02		ENSG00000249631
LOC107986277	0.0744	4.3634E-02	1.4524E-01	ENSG00000282904
LOC107986374	0.0035	9.2752E-01	9.6455E-01	ENSG00000248648
LOC107986524	0.0465	5.2276E-01	7.0893E-01	ENSG00000286339
LOC107986550	0.0463	1.7196E-01		ENSG00000289090
LOC107986626	0.0711	3.7981E-01	5.8830E-01	ENSG00000287578
LOC107986669	-0.0073	8.9231E-01	9.4643E-01	ENSG00000287189
LOC107986760	0.0663	5.4528E-01	7.2573E-01	ENSG00000289352

LOC107986821	0.0359	4.1911E-01	6.2200E-01	ENSG00000285964
LOC107986837	0.1504	1.4083E-01	3.1753E-01	ENSG00000225457
LOC107986951	0.0050	7.4306E-01		ENSG00000254337
LOC107987014	0.0177	6.7365E-01		ENSG00000230894
LOC107987423	0.0426	6.9338E-01	8.3001E-01	ENSG00000228695
LOC112267859	0.0354	1.3784E-01		ENSG00000279688
LOC112267871	0.0589	4.6507E-01	6.6166E-01	ENSG00000228549
LOC112267968	-0.0086	6.6350E-01		ENSG00000285492
LOC112267986	-0.0098	8.5030E-01		ENSG00000287672
LOC112268065	-0.0019	9.7332E-01		ENSG00000286683
LOC112268073	-0.0281	1.8410E-01		ENSG00000256282
LOC112268165	0.0507	3.5287E-01	5.6303E-01	ENSG00000286894
LOC112268198	-0.0105	8.8879E-01	9.4476E-01	ENSG00000279801
LOC112268208	-0.0626	3.2507E-01	5.3511E-01	ENSG00000285095
LOC112268239	0.2123	1.2591E-02	5.9905E-02	ENSG00000288880
LOC112268269	0.0905	3.0950E-01	5.1975E-01	ENSG00000268858
LOC112268276	0.0188	7.5756E-01	8.6879E-01	ENSG00000229588
LOC112268408	-0.0017	8.5851E-01		ENSG00000266988
LOC112268411	0.0860	3.2867E-01	5.3911E-01	ENSG00000236106
LOC112268419	0.0175	7.8314E-01	8.8525E-01	ENSG00000286244
LOC112694756	-0.0021	9.7438E-01	9.8743E-01	ENSG00000285043
LOC114841035	0.0373	6.5216E-01	8.0300E-01	ENSG00000286264
LOC115308161	-0.0577	3.1216E-01	5.2222E-01	ENSG00000271551
LOC116435278	0.0204	2.9208E-01		ENSG00000286710
LOC119746555	0.1955	4.4492E-02	1.4728E-01	ENSG00000260257
LOC121725015	-0.0861	3.1838E-01	5.2860E-01	ENSG00000264449
LOC122319436	0.2268	3.1758E-02	1.1578E-01	ENSG00000288658
LOC122455342	0.0051	9.1219E-01	9.5669E-01	ENSG00000284526
LOC122526776	0.0901	1.0644E-01	2.6523E-01	ENSG00000285938
LOC122526780	0.0089	8.0093E-01		ENSG00000282936
LOC122526782	-0.0546	4.8301E-01	6.7726E-01	ENSG00000233461
LOC122539214	-0.0049	9.5294E-01	9.7681E-01	ENSG00000269825
LOC124900179	0.0072	7.7239E-01		ENSG00000200999
LOC124900180	-0.0086	9.5743E-01		ENSG00000221639
LOC124900183	-0.0914	2.0398E-01	4.0300E-01	ENSG00000201863
LOC124900184	0.0376	2.6437E-01		ENSG00000212458
LOC124900187	0.0150	4.2945E-01		ENSG00000221245
LOC124900188	0.1254	2.2885E-02		ENSG00000239005
LOC124900193	0.3937	1.9658E-03	1.4882E-02	ENSG00000245146
LOC124900202	0.0055	9.5796E-01		ENSG00000212567
LOC124900205	0.0018	9.9383E-01		ENSG00000206592
LOC124900215	-0.0379	6.6293E-01	8.1040E-01	ENSG00000290049
LOC124900217	-0.1527	9.7168E-02	2.5043E-01	ENSG00000225177
LOC124900226	-0.0209	4.3715E-01		ENSG00000201807
LOC124900235	0.0118	9.5868E-01		ENSG00000202023
LOC124900239	0.0164	2.1489E-01		ENSG00000200753
LOC124900242	0.0464	4.3597E-02		ENSG00000200113
LOC124900243	0.0150	5.3977E-01		ENSG00000201465
LOC124900256	-0.0039	8.8630E-01		ENSG00000201157

LOC124900261	-0.0102	8.2040E-01		ENSG00000252505
LOC124900265	-0.0452	2.6927E-01		ENSG00000200075
LOC124900269	-0.0030	8.4107E-01		ENSG00000212342
LOC124900271	-0.0033	6.9960E-01		ENSG00000238966
LOC124900275	0.0414	6.1516E-01	7.7668E-01	ENSG00000204054
LOC124900277	0.0030	8.1018E-01		ENSG00000200026
LOC124900296	-0.0146	7.2807E-01	8.5083E-01	ENSG00000222588
LOC124900314	0.0027	7.3553E-01		ENSG00000252427
LOC124900321	-0.0060	7.6258E-01		ENSG00000251893
LOC124900332	-0.0630	4.2544E-02		ENSG00000212461
LOC124900339	0.0973	2.5243E-01	4.6050E-01	ENSG00000252128
LOC124900341	-0.0028	7.8921E-01		ENSG00000200072
LOC124900349	0.0208	6.4111E-01		ENSG00000251858
LOC124900356	0.0161	7.6251E-01		ENSG00000207430
LOC124900356	-0.0020	9.0610E-01		ENSG00000206987
LOC124900357	0.0033	9.7947E-01		ENSG00000238519
LOC124900359	0.0186	3.3094E-01		ENSG00000207432
LOC124900372	0.3810	7.4581E-04	7.2936E-03	ENSG00000261971
LOC124900379	0.0066	7.4900E-01		ENSG00000212445
LOC124900382	-0.0162	7.5411E-01		ENSG00000252122
LOC124900394	0.0077	5.2954E-01		ENSG00000252657
LOC124900399	-0.0005	9.0724E-01		ENSG00000200063
LOC124900423	-0.1147	1.8016E-01	3.7212E-01	ENSG00000201944
LOC124900426	0.0220	4.4931E-01		ENSG00000253042
LOC124900429	-0.0039	7.9222E-01		ENSG00000201619
LOC124900430	0.0340	6.5684E-01	8.0624E-01	ENSG00000200237
LOC124900432	-0.0549	1.0196E-02		ENSG00000212338
LOC124900442	0.1281	8.8714E-02	2.3519E-01	ENSG00000206878
LOC124900443	0.0041	9.4866E-01		ENSG00000207022
LOC124900447	-0.0101	8.0951E-01		ENSG00000212624
LOC124900452	0.0281	5.4036E-01		ENSG00000221083
LOC124900457	-0.0086	5.5851E-01		ENSG00000238934
LOC124900464	-0.0095	8.5934E-01		ENSG00000212517
LOC124900469	-0.0417	3.5885E-01		ENSG00000238390
LOC124900476	0.0234	2.6145E-01		ENSG00000280623
LOC124900488	0.0153	3.1259E-01		ENSG00000199226
LOC124900492	0.0611	5.6314E-02		ENSG00000201407
LOC124900498	0.0876	1.7123E-01	3.6042E-01	ENSG00000202231
LOC124900516	0.0204	6.7038E-01		ENSG00000201806
LOC124900517	0.0255	4.9542E-01		ENSG00000202537
LOC124900537	-0.0137	7.3330E-01		ENSG00000212175
LOC124900542	0.0171	2.3060E-01		ENSG00000221300
LOC124900553	-0.0098	7.3940E-01		ENSG00000212145
LOC124900556	0.0041	5.1934E-01		ENSG00000202379
LOC124900560	-0.0321	1.1216E-01		ENSG00000201957
LOC124900564	0.0552	3.3166E-01	5.4200E-01	ENSG00000253092
LOC124900607	0.0710	3.7662E-01	5.8522E-01	ENSG00000223734
LOC124900656	0.0046	9.1318E-01		ENSG00000287164
LOC124900694	0.0146	3.0357E-01		ENSG00000287659

LOC124900697	0.0145	7.7157E-01	8.7814E-01	ENSG00000289643
LOC124900711	0.2669	9.5559E-03	4.9076E-02	ENSG00000289019
LOC124900774	0.0403	3.0562E-01		ENSG00000287951
LOC124900778	0.0185	3.1970E-01		ENSG00000248187
LOC124900787	-0.0344	1.0899E-01		ENSG00000248335
LOC124900842	0.0065	9.1145E-01		ENSG00000251325
LOC124900848	0.0906	9.0349E-02		ENSG00000286891
LOC124900957	-0.0570	2.7125E-01	4.8048E-01	ENSG00000286543
LOC124900989	0.0123	7.1341E-01		ENSG00000286647
LOC124901067	0.0020	9.0479E-01		ENSG00000250244
LOC124901076	-0.1062	2.4403E-01	4.5166E-01	ENSG00000249803
LOC124901107	-0.0018	8.1457E-01		ENSG00000286657
LOC124901122	-0.0018	7.9672E-01		ENSG00000254135
LOC124901131	0.0144	6.5441E-01		ENSG00000288912
LOC124901151	0.0125	8.6987E-01	9.3441E-01	ENSG00000248367
LOC124901189	-0.0044	7.2219E-01		ENSG00000202082
LOC124901197	0.0088	5.6555E-01		ENSG00000238326
LOC124901220	0.0020	9.7060E-01		ENSG00000201368
LOC124901261	-0.0166	6.2896E-01		ENSG00000289257
LOC124901296	-0.0065	9.3785E-01	9.6928E-01	ENSG00000225173
LOC124901310	-0.0087	8.7487E-01		ENSG00000289456
LOC124901333	0.2998	8.9960E-03	4.6965E-02	ENSG00000227885
LOC124901351	0.0122	8.7863E-01		ENSG00000287816
LOC124901355	0.1534	5.9396E-02	1.7918E-01	ENSG00000272008
LOC124901358	-0.0195	4.2503E-01		ENSG00000286871
LOC124901391	-0.0173	5.6514E-01		ENSG00000287258
LOC124901407	0.0057	7.5529E-01		ENSG00000287094
LOC124901408	-0.2236	4.7915E-02	1.5490E-01	ENSG00000289312
LOC124901427	-0.0633	5.8957E-02		ENSG00000273132
LOC124901447	-0.0511	1.4540E-01		ENSG00000287591
LOC124901457	0.0085	3.1983E-01		ENSG00000286498
LOC124901521	-0.0089	9.9593E-01		ENSG00000222145
LOC124901525	-0.0080	5.9397E-01		ENSG00000200706
LOC124901526	0.0046	9.5093E-01		ENSG00000202343
LOC124901604	-0.0201	7.8293E-01		ENSG00000260951
LOC124901612	0.0045	9.7126E-01		ENSG00000236494
LOC124901628	0.0244	5.5079E-01		ENSG00000286315
LOC124901635	-0.0085	7.5700E-01		ENSG00000228735
LOC124901656	0.0043	9.8924E-01		ENSG00000287317
LOC124901661	-0.0050	9.4176E-01	9.7117E-01	ENSG00000289108
LOC124901671	-0.0145	8.5162E-01	9.2443E-01	ENSG00000285886
LOC124901813	0.0110	6.7324E-01		ENSG00000287592
LOC124901827	0.0326	1.0940E-01		ENSG00000238906
LOC124901856	0.0086	7.8275E-01		ENSG00000238832
LOC124901859	0.0132	8.0251E-01		ENSG00000199370
LOC124901861	0.0036	9.2219E-01		ENSG00000238297
LOC124901872	0.0090	9.1612E-01		ENSG00000287747
LOC124901882	-0.0142	4.5536E-01		ENSG00000254340
LOC124901944	-0.0007	9.1406E-01		ENSG00000253844

LOC124901966	0.0403	5.6085E-01	7.3792E-01	ENSG00000285758
LOC124901990	0.0964	7.8923E-02		ENSG00000253842
LOC124902013	0.0065	6.2197E-01		ENSG00000253607
LOC124902026	-0.0992	2.7534E-01	4.8520E-01	ENSG00000286535
LOC124902027	-0.0013	8.2170E-01		ENSG00000287201
LOC124902031	-0.0363	3.2301E-01		ENSG00000287325
LOC124902033	0.0142	4.8165E-01		ENSG00000253196
LOC124902062	-0.0120	6.3041E-01		ENSG00000253452
LOC124902108	0.0094	6.5214E-01		ENSG00000227155
LOC124902114	0.0260	7.3170E-01	8.5327E-01	ENSG00000286162
LOC124902128	-0.0153	7.9060E-01	8.8927E-01	ENSG00000286685
LOC124902204	0.2955	2.4110E-03	1.7333E-02	ENSG00000287769
LOC124902229	-0.0387	5.9903E-01	7.6499E-01	ENSG00000285706
LOC124902280	0.0232	7.7545E-01	8.8055E-01	ENSG00000227218
LOC124902301	0.0334	2.7346E-01		ENSG00000286502
LOC124902337	-0.0104	6.5847E-01		ENSG00000252985
LOC124902371	0.0444	5.2468E-01	7.1015E-01	ENSG00000213994
LOC124902388	0.1385	1.4897E-01	3.2951E-01	ENSG00000285852
LOC124902392	0.1495	1.0119E-01	2.5683E-01	ENSG00000285254
LOC124902477	0.5810	1.1467E-03	1.0048E-02	ENSG00000232110
LOC124902537	0.0927	2.7817E-01	4.8808E-01	ENSG00000288657
LOC124902542	0.0015	4.9893E-01		ENSG00000228636
LOC124902546	-0.1595	6.6250E-02	1.9357E-01	ENSG00000287016
LOC124902571	-0.0017	9.3845E-01		ENSG00000212411
LOC124902606	-0.0334	6.0686E-01	7.7080E-01	ENSG00000287935
LOC124902641	-0.0177	5.7083E-01		ENSG00000287898
LOC124902694	0.1668	1.0106E-01	2.5662E-01	ENSG00000255320
LOC124902771	0.0301	2.5470E-01		ENSG00000287545
LOC124902809	0.0204	4.8300E-01		ENSG00000255167
LOC124902819	0.0116	4.8787E-01		ENSG00000283489
LOC124902824	0.0012	9.7097E-01		ENSG00000206913
LOC124902860	-0.0524	5.0514E-01	6.9505E-01	ENSG00000278356
LOC124902904	0.0396	4.2381E-01		ENSG00000256625
LOC124902947	-0.0688	3.9964E-01	6.0504E-01	ENSG00000287200
LOC124902964	0.0678	2.7919E-02		ENSG00000257761
LOC124902988	-0.0096	9.0256E-01	9.5233E-01	ENSG00000258035
LOC124902995	-0.0076	4.3327E-01		ENSG00000258204
LOC124903002	-0.0116	7.8475E-01		ENSG00000257732
LOC124903055	0.0319	1.4936E-01		ENSG00000286462
LOC124903167	0.0336	2.8242E-01		ENSG00000225727
LOC124903233	-0.0093	8.2930E-01		ENSG00000277047
LOC124903236	0.0114	6.4060E-01		ENSG00000287996
LOC124903342	-0.0153	6.1008E-01		ENSG00000285518
LOC124903356	-0.0367	3.3801E-01		ENSG00000283627
LOC124903416	-0.0339	2.7473E-01		ENSG00000212615
LOC124903417	0.0087	7.6820E-01		ENSG00000201036
LOC124903448	0.0203	6.9084E-01		ENSG00000289522
LOC124903525	0.0136	7.6413E-01	8.7310E-01	ENSG00000277749
LOC124903590	-0.0012	8.1143E-01		ENSG00000200677

LOC124903592	0.0095	6.0775E-01		ENSG00000212428
LOC124903615	-0.0159	8.1843E-01	9.0561E-01	ENSG00000239197
LOC124903619	0.1846	4.4646E-03		ENSG00000260316
LOC124903640	-0.0034	8.5238E-01		ENSG00000287340
LOC124903658	-0.0047	4.8373E-01		ENSG00000286790
LOC124903659	0.0033	8.3439E-01		ENSG00000260592
LOC124903670	-0.3339	8.3314E-03	4.4228E-02	ENSG00000246465
LOC124903729	0.0009	9.5992E-01		ENSG00000261472
LOC124903776	-0.0155	8.8840E-01		ENSG00000260115
LOC124903825	0.0224	1.9943E-01		ENSG00000287396
LOC124903940	-0.0480	4.2720E-01	6.2915E-01	ENSG00000286743
LOC124904009	0.0154	8.1098E-01	9.0103E-01	ENSG00000288961
LOC124904017	0.5493	8.6340E-03	4.5535E-02	ENSG00000289599
LOC124904027	0.0657	3.3988E-01	5.4964E-01	ENSG00000284650
LOC124904048	0.1096	1.4724E-01	3.2715E-01	ENSG00000265055
LOC124904072	-0.0027	8.6785E-01		ENSG00000261978
LOC124904076	0.0517	1.9060E-01		ENSG00000287403
LOC124904117	-0.0314	5.4260E-01	7.2409E-01	ENSG00000239129
LOC124904122	0.5045	1.3363E-03	1.1287E-02	ENSG00000290039
LOC124904135	-0.0257	8.3485E-01		ENSG00000275616
LOC124904136	-0.0352	7.3793E-01		ENSG00000273709
LOC124904137	-0.0265	6.8730E-01		ENSG00000274432
LOC124904138	0.0156	1.7536E-01		ENSG00000274862
LOC124904138	0.0024	5.3512E-01		ENSG00000277903
LOC124904138	-0.0164	8.6178E-01		ENSG00000274062
LOC124904138	-0.0256	4.4458E-01		ENSG00000278591
LOC124904138	-0.0489	1.0195E-01		ENSG00000275219
LOC124904145	0.0046	6.3876E-01		ENSG00000274452
LOC124904146	0.0156	1.7536E-01		ENSG00000278774
LOC124904152	-0.0211	3.8131E-01		ENSG00000221044
LOC124904154	0.4004	1.4517E-02	6.6430E-02	ENSG00000212195
LOC124904219	-0.0031	9.6545E-01		ENSG00000287797
LOC124904288	0.0314	6.9555E-01	8.3149E-01	ENSG00000285940
LOC124904332	0.0111	6.4342E-01		ENSG00000267015
LOC124904362	0.0895	8.3079E-02		ENSG00000199977
LOC124904381	0.0040	4.9165E-01		ENSG00000199856
LOC124904382	0.0116	3.7761E-01		ENSG00000212539
LOC124904403	0.0023	5.6142E-01		ENSG00000224481
LOC124904411	-0.0210	7.3947E-01	8.5776E-01	ENSG00000233030
LOC124904434	0.0239	1.4888E-01		ENSG00000290105
LOC124904535	0.0209	7.7151E-01	8.7814E-01	ENSG00000269934
LOC124904542	0.0195	6.3248E-01		ENSG00000227006
LOC124904553	0.0411	4.0340E-01	6.0785E-01	ENSG00000233332
LOC124904559	0.0800	1.2932E-01		ENSG00000285177
LOC124904601	0.1837	8.1629E-02	2.2227E-01	ENSG00000286496
LOC124904611	-0.0150	1.6343E-01		ENSG00000267122
LOC124904613	0.2813	1.8238E-02	7.7705E-02	ENSG00000275405
LOC124904613	0.2570	4.1858E-02	1.4110E-01	ENSG00000273768
LOC124904616	0.1292	1.2188E-01	2.9058E-01	ENSG00000277918

LOC124904619	-0.0095	7.5519E-01	8.6699E-01	ENSG00000270722
LOC124904620	0.0703	4.3840E-01	6.3860E-01	ENSG00000268536
LOC124904621	0.2957	1.4458E-02	6.6231E-02	ENSG00000274210
LOC124904624	0.4162	3.5203E-04	4.0533E-03	ENSG00000275291
LOC124904627	0.4843	4.4088E-03	2.7376E-02	ENSG00000206828
LOC124904631	0.4596	1.5622E-03	1.2602E-02	ENSG00000273727
LOC124904634	0.4574	1.1851E-03	1.0307E-02	ENSG00000274428
LOC124904641	0.0071	7.7169E-01		ENSG00000273694
LOC124904686	-0.1453	1.1460E-02		ENSG00000283442
LOC124904687	-0.0218	6.4318E-01	7.9621E-01	ENSG00000283575
LOC124904695	0.3357	1.4580E-02	6.6631E-02	ENSG00000267024
LOC124904710	-0.0036	6.6339E-01		ENSG00000268055
LOC124904721	0.0061	8.0671E-01		ENSG00000283414
LOC124904795	0.0075	6.9111E-01		ENSG00000239137
LOC124904917	0.0106	8.7899E-01	9.3956E-01	ENSG00000273828
LOC124904972	0.0062	6.4435E-01		ENSG00000283527
LOC124904983	-0.0119	7.1325E-01		ENSG00000201346
LOC124905013	-0.0068	4.5691E-01		ENSG00000273102
LOC124905119	0.0158	3.1049E-01		ENSG00000224794
LOC124905134	-0.0525	5.3906E-01	7.2116E-01	ENSG00000273145
LOC124905142	0.0152	3.2789E-01	5.3838E-01	ENSG00000287225
LOC124905143	-0.0005	9.4063E-01		ENSG00000285722
LOC124905171	0.0046	7.0736E-01		ENSG00000201209
LOC124905202	-0.0676	4.2533E-01	6.2767E-01	ENSG00000289132
LOC124905218	0.1686	6.7697E-02	1.9622E-01	ENSG00000286060
LOC124905233	0.0046	8.8318E-01		ENSG00000289575
LOC124905237	0.0385	5.1353E-01	7.0128E-01	ENSG00000286939
LOC124905265	0.0092	4.1917E-01		ENSG00000275268
LOC124905312	0.2148	1.4108E-02	6.5149E-02	ENSG00000278903
LOC124905349	0.0863	3.1120E-02		ENSG00000224272
LOC124905442	0.0450	3.8867E-01	5.9595E-01	ENSG00000242296
LOC124906119	-0.0281	6.1692E-01	7.7795E-01	ENSG00000288898
LOC124906135	0.0104	3.3138E-01		ENSG00000238782
LOC124906143	0.0029	7.8377E-01		ENSG00000206731
LOC124906209	-0.0035	7.5051E-01	8.6450E-01	ENSG00000235978
LOC124906237	0.0205	4.6896E-01		ENSG00000288063
LOC124906280	0.0318	1.9419E-01		ENSG00000279328
LOC124906284	0.0419	5.3309E-01	7.1679E-01	ENSG00000284731
LOC124906285	0.0359	3.5487E-01		ENSG00000288111
LOC124906319	0.0114	6.5786E-01		ENSG00000289165
LOC124906339	-0.0058	7.3163E-01		ENSG00000283418
LOC124906347	0.0054	6.5426E-01		ENSG00000238902
LOC124906471	0.0133	8.7311E-01	9.3632E-01	ENSG00000288930
LOC124906529	-0.0018	9.7678E-01	9.8837E-01	ENSG00000249109
LOC124906608	0.1343	1.5364E-01	3.3600E-01	ENSG00000223969
LOC124906685	-0.0206	2.1264E-01		ENSG00000253363
LOC124906839	0.1920	3.9388E-02	1.3545E-01	ENSG00000276422
LOC124906859	0.0126	8.0613E-01		ENSG00000225655
LOC124906938	0.0000	7.6475E-01		ENSG00000289997

LOC124906976	0.0290	5.2397E-01		ENSG00000274659
LOC124907114	0.3112	9.4010E-04	8.6246E-03	ENSG00000275757
LOC124907114	0.2131	1.5546E-02	6.9771E-02	ENSG00000273730
LOC124907392	0.0940	2.8800E-01	4.9854E-01	ENSG00000258636
LOC124907726	0.3539	3.5908E-04	4.1186E-03	ENSG00000287126
LOC124907760	0.0261	2.4231E-01		ENSG00000231848
LOC124907763	-0.0692	3.2011E-01	5.3028E-01	ENSG00000226087
LOC124907878	0.1257	1.6383E-01	3.5056E-01	ENSG00000238207
LOC124907880	0.0056	7.9672E-01		ENSG00000271709
LOC124907890	-0.0424	3.1097E-01	5.2107E-01	ENSG00000229797
LOC124908011	0.0063	7.7400E-01		ENSG00000288082
LOC124908056	0.0646	4.4280E-01	6.4210E-01	ENSG00000228999
LOC124908074	0.0111	3.9510E-01		ENSG00000267308
LOC124909384	-0.0202	7.3750E-01	8.5688E-01	ENSG00000286952
LOC124909396	-0.0153	7.2847E-01	8.5110E-01	ENSG00000287595
LOC124909397	-0.1154	1.6525E-01	3.5254E-01	ENSG00000286447
LOC124909412	0.0234	4.1059E-01		ENSG00000289153
LOC124909423	0.0088	6.8065E-01		ENSG00000288022
LOC124909426	-0.0066	8.8441E-01	9.4208E-01	ENSG00000287232
LOC124909451	-0.0145	5.5313E-01		ENSG00000288787
LOC124909475	-0.0132	8.6964E-01	9.3437E-01	ENSG00000287005
LOC124909494	0.0711	2.4608E-01	4.5391E-01	ENSG00000243620
LOC127814295	0.1760	8.9299E-02	2.3639E-01	ENSG00000232995
LOC128125816	-0.0541	3.5008E-01	5.6020E-01	ENSG00000284934
LOC128125822	0.0284	4.6939E-01		ENSG00000285976
LOC149844	0.0298	4.3733E-01		ENSG00000234241
LOC149935	-0.0200	3.8496E-01		ENSG00000224628
LOC153910	0.0229	4.0259E-01		ENSG00000236366
LOC154761	0.1532	8.9634E-02	2.3714E-01	ENSG00000253882
LOC157273	0.3954	1.0281E-02	5.1698E-02	ENSG00000248538
LOC254896	0.0330	6.5897E-01		ENSG00000284948
LOC283038	0.0009	8.2559E-01		ENSG00000228021
LOC283045	-0.1095	2.1602E-01	4.1750E-01	ENSG00000288011
LOC283194	-0.0086	9.1703E-01	9.5883E-01	ENSG00000255240
LOC283299	-0.0008	9.6805E-01		ENSG00000254951
LOC283387	-0.0466	5.8520E-01	7.5511E-01	ENSG00000245651
LOC283683	0.3622	7.5927E-05	1.2413E-03	ENSG00000274253
LOC283731	0.0102	9.0705E-01	9.5449E-01	ENSG00000248540
LOC284685	-0.0712	2.8424E-01	4.9492E-01	ENSG00000215835
LOC284798	-0.0191	6.8948E-01		ENSG00000230725
LOC284933	0.0073	8.6285E-01		ENSG00000281732
LOC285097	0.1487	1.3007E-01	3.0283E-01	ENSG00000280119
LOC285453	-0.0085	6.9494E-01		ENSG00000227040
LOC285626	0.2074	4.5672E-02	1.5016E-01	ENSG00000249738
LOC285638	0.0227	7.6842E-01		ENSG00000249476
LOC285804	0.0040	5.3179E-01		ENSG00000271820
LOC286059	-0.0035	9.4809E-01	9.7443E-01	ENSG00000246130
LOC286083	0.5565	2.0621E-03	1.5394E-02	ENSG00000275427
LOC317727	0.0076	7.7985E-01		ENSG00000224280

LOC338963	0.0309	5.5113E-01	7.2989E-01	ENSG00000228141
LOC339059	0.0164	4.7639E-01		ENSG00000182376
LOC339166	0.5657	2.6986E-03	1.8838E-02	ENSG00000284837
LOC339260	-0.1665	2.7590E-02	1.0468E-01	ENSG00000233098
LOC339666	0.0205	7.7304E-01	8.7903E-01	ENSG00000230736
LOC339966	0.0351	9.0745E-02		ENSG00000271676
LOC344967	-0.0347	1.4786E-01		ENSG00000205794
LOC349160	-0.0199	2.9113E-01		ENSG00000234352
LOC375196	0.0140	5.4903E-01		ENSG00000269210
LOC388248	0.0066	7.4596E-01		ENSG00000260628
LOC388282	0.0307	2.3742E-01		ENSG00000187185
LOC388996	0.0420	5.2568E-01	7.1100E-01	ENSG00000278131
LOC389199	-0.0817	3.5790E-01	5.6756E-01	ENSG00000228919
LOC389831	0.1454	3.8759E-02	1.3378E-01	ENSG00000276256
LOC389906	-0.3422	5.4714E-10	6.6978E-08	ENSG00000285756
LOC390332	-0.0024	6.8061E-01		ENSG00000278399
LOC390877	0.0141	7.8836E-01		ENSG00000214347
LOC391741	-0.2600	8.9439E-03	4.6786E-02	ENSG00000248699
LOC392196	0.0158	5.1546E-01		ENSG00000254923
LOC392266	-0.0301	4.6288E-01		ENSG00000253336
LOC392787	-0.0750	1.4958E-01		ENSG00000224163
LOC400499	0.0268	6.6733E-01	8.1362E-01	ENSG00000188897
LOC400622	0.0686	4.0920E-01	6.1354E-01	ENSG00000267665
LOC400627	0.0597	3.3220E-01	5.4247E-01	ENSG00000261924
LOC401127	0.0472	4.3475E-01	6.3581E-01	ENSG00000224097
LOC401442	0.0095	8.5855E-01		ENSG00000282375
LOC401589	0.0469	5.6076E-01	7.3783E-01	ENSG00000182776
LOC401703	-0.0474	3.3840E-01		ENSG00000213365
LOC401913	0.0085	4.9068E-01		ENSG00000270624
LOC401957	-0.0063	6.2346E-01		ENSG00000232952
LOC403312	-0.1016	2.6281E-01	4.7165E-01	ENSG00000255537
LOC439933	0.0040	5.6554E-01	7.4090E-01	ENSG00000247193
LOC440700	0.0516	5.0348E-01	6.9392E-01	ENSG00000215838
LOC440742	-0.0215	6.3194E-01		ENSG00000279774
LOC441179	-0.0054	9.4670E-01		ENSG00000269155
LOC441239	0.0165	4.3114E-01		ENSG00000189316
LOC441711	-0.0023	7.5069E-01		ENSG00000273679
LOC442155	-0.0123	6.4650E-01		ENSG00000220685
LOC574538	0.2184	2.0788E-02	8.5293E-02	ENSG00000249695
LOC613206	-0.0400	5.6315E-01	7.3970E-01	ENSG00000235204
LOC613266	0.0172	4.3867E-01		ENSG00000286546
LOC642361	0.1088	1.9804E-01	3.9590E-01	ENSG00000272447
LOC643015	0.0332	9.4268E-02		ENSG00000183171
LOC643307	0.0053	8.3268E-01		ENSG00000249878
LOC643342	0.0013	8.9584E-01		ENSG00000230221
LOC644169	-0.1153	2.2857E-01	4.3311E-01	ENSG00000254595
LOC644456	-0.0014	9.7975E-01	9.8977E-01	ENSG00000231043
LOC644584	0.0258	3.3541E-01		ENSG00000257307
LOC644656	0.3294	6.1044E-03	3.4967E-02	ENSG00000268403

LOC645433	0.0440	1.1826E-01		ENSG00000248608
LOC645485	0.1060	3.2258E-02		ENSG00000246331
LOC645853	0.0230	5.0165E-01		ENSG00000251211
LOC645967	-0.0117	8.9255E-01	9.4648E-01	ENSG00000278899
LOC646665	0.0728	1.3972E-01		ENSG00000260144
LOC646828	0.0136	6.8727E-01		ENSG00000257639
LOC646870	-0.0028	6.4353E-01		ENSG00000231615
LOC651337	0.0410	5.5314E-01	7.3158E-01	ENSG00000255585
LOC653653	-0.0173	7.8228E-01	8.8472E-01	ENSG00000280852
LOC728138	0.0691	4.1673E-01	6.2023E-01	ENSG00000261819
LOC728307	0.0376	1.0685E-01		ENSG00000224931
LOC728485	0.0137	8.7422E-01	9.3709E-01	ENSG00000267260
LOC728488	0.3349	6.8406E-03	3.8140E-02	ENSG00000250138
LOC728506	0.2561	1.3893E-02	6.4428E-02	ENSG00000251158
LOC728743	-0.1808	3.4581E-02	1.2283E-01	ENSG00000284691
LOC728877	0.0288	7.0415E-01	8.3682E-01	ENSG00000204790
LOC729558	-0.0039	7.8391E-01		ENSG00000249690
LOC729707	0.0110	5.4787E-01		ENSG00000248560
LOC729732	-0.4196	2.9147E-04	3.5052E-03	ENSG00000283674
LOC729867	-0.1129	2.1207E-01	4.1298E-01	ENSG00000227741
LOC729870	-0.0946	2.9407E-01	5.0394E-01	ENSG00000287642
LOC729998	-0.2232	1.3491E-02	6.3094E-02	ENSG00000283041
LOC730098	-0.0726	3.9387E-01	6.0037E-01	ENSG00000187186
LOC730183	0.3437	4.1820E-03	2.6391E-02	ENSG00000261840
LOC730668	-0.0041	8.8799E-01		ENSG00000280424
LOC731157	0.0130	8.7750E-01	9.3898E-01	ENSG00000255647
LOC732229	-0.0059	4.7487E-01		ENSG00000269374
LOC780529	0.0114	6.7919E-01		ENSG00000271776
LOC84214	0.0348	2.9672E-01		ENSG00000236529
LOC93429	0.1368	8.6815E-02	2.3165E-01	ENSG00000268460
LOC93463	0.0130	6.2083E-01		ENSG00000124835
LOH12CR2	0.0557	4.7057E-01	6.6681E-01	ENSG00000205791
LONP1	0.0383	5.9707E-01	7.6356E-01	ENSG00000196365
LONP2	-0.1809	4.9316E-03	2.9770E-02	ENSG00000102910
LONRF1	-0.0618	2.7791E-01	4.8800E-01	ENSG00000154359
LONRF2	-0.0392	6.0328E-01	7.6791E-01	ENSG00000170500
LONRF2P1	0.0000	7.9807E-01		ENSG00000266605
LONRF3	-0.2898	2.3446E-02	9.3270E-02	ENSG00000175556
LORICRIN	0.1061	1.6352E-01	3.5010E-01	ENSG00000203782
LOX	-0.0165	8.3499E-01	9.1514E-01	ENSG00000113083
LOXHD1	0.0385	3.4806E-01		ENSG00000167210
LOXL1	0.0351	6.4655E-01	7.9869E-01	ENSG00000129038
LOXL1-AS1	-0.1420	4.5191E-02	1.4881E-01	ENSG00000261801
LOXL2	-0.4127	1.4312E-03	1.1852E-02	ENSG00000134013
LOXL2-AS1	0.0147	9.0953E-01	9.5525E-01	ENSG00000253837
LOXL3	0.0035	9.8161E-01	9.9039E-01	ENSG00000115318
LOXL4	0.1022	1.7149E-01	3.6067E-01	ENSG00000138131
LPA	-0.0434	3.0507E-01	5.1535E-01	ENSG00000198670
LPAR1	0.0457	5.6193E-01	7.3863E-01	ENSG00000198121

LPAR2	-0.1564	1.0810E-02	5.3656E-02	ENSG00000064547
LPAR3	-0.0007	9.5385E-01		ENSG00000171517
LPAR4	0.0407	6.3988E-01	7.9402E-01	ENSG00000147145
LPAR5	0.0165	6.6794E-01		ENSG00000184574
LPAR6	-0.0569	4.9813E-01	6.8926E-01	ENSG00000139679
LPCAT1	-0.2431	5.8592E-03	3.3937E-02	ENSG00000153395
LPCAT2	0.0098	8.7898E-01	9.3956E-01	ENSG00000087253
LPCAT3	0.0123	8.8078E-01	9.4037E-01	ENSG00000111684
LPCAT4	0.1616	4.8410E-02	1.5605E-01	ENSG00000176454
LPGAT1	-0.2011	2.5993E-03	1.8283E-02	ENSG00000123684
LPGAT1-AS1	0.0048	6.9490E-01		ENSG00000229258
LPIN1	0.2755	3.0319E-06	9.3602E-05	ENSG00000134324
LPIN2	0.0506	3.8505E-01	5.9292E-01	ENSG00000101577
LPIN3	0.2067	5.4154E-02	1.6864E-01	ENSG00000132793
LPL	-0.3953	1.4081E-02	6.5065E-02	ENSG00000175445
LPO	0.0662	1.1572E-01		ENSG00000167419
LPP	0.2369	6.6451E-03	3.7338E-02	ENSG00000145012
LPP-AS2	0.0400	5.6163E-01	7.3847E-01	ENSG00000270959
LPXN	0.2481	5.9226E-03	3.4184E-02	ENSG00000110031
LRAT	-0.0460	5.7818E-01	7.4978E-01	ENSG00000121207
LRATD1	0.0770	3.0168E-01	5.1175E-01	ENSG00000162981
LRATD2	-0.3460	9.2093E-04	8.5154E-03	ENSG00000168672
LRBA	0.0253	7.1066E-01	8.4116E-01	ENSG00000198589
LRCH1	-0.1508	9.0930E-02	2.3967E-01	ENSG00000136141
LRCH2	0.0500	4.9755E-01	6.8889E-01	ENSG00000130224
LRCH3	0.2538	4.2971E-04	4.7243E-03	ENSG00000186001
LRCH4	0.0134	8.4696E-01	9.2161E-01	ENSG00000077454
LRCOL1	0.0563	9.7736E-02		ENSG00000204583
LRFN1	-0.1067	1.3686E-01	3.1228E-01	ENSG00000128011
LRFN2	0.1854	8.4075E-02	2.2651E-01	ENSG00000156564
LRFN2	0.0519	2.2640E-01		ENSG00000226454
LRFN3	-0.0786	3.5710E-01	5.6690E-01	ENSG00000126243
LRFN4	-0.1039	1.5436E-01	3.3709E-01	ENSG00000173621
LRFN5	0.0988	2.1684E-01	4.1855E-01	ENSG00000165379
LRG1	-0.0108	9.6002E-01	9.8006E-01	ENSG00000171236
LRIF1	0.2928	2.0560E-03	1.5368E-02	ENSG00000121931
LRIG1	-0.0515	5.4295E-01	7.2427E-01	ENSG00000144749
LRIG2	0.1539	2.6301E-02	1.0115E-01	ENSG00000198799
LRIG2-DT	-0.0466	5.8730E-01	7.5642E-01	ENSG00000238198
LRIG3	-0.1120	1.8141E-01	3.7392E-01	ENSG00000139263
LRIT2	-0.0079	8.3294E-01		ENSG00000204033
LRIT3	0.1027	6.3486E-02		ENSG00000183423
LRMDA	0.5288	3.3799E-04	3.9376E-03	ENSG00000148655
LRP1	0.0459	5.7490E-01	7.4772E-01	ENSG00000123384
LRP1-AS	0.0140	4.4675E-01		ENSG00000259125
LRP10	-0.1562	6.2566E-02	1.8579E-01	ENSG00000197324
LRP11	-0.0535	5.0623E-01	6.9598E-01	ENSG00000120256
LRP12	0.0188	8.0160E-01	8.9534E-01	ENSG00000147650
LRP1B	0.1597	1.0168E-01	2.5762E-01	ENSG00000168702

LRP2BP	0.2039	2.7520E-02	1.0457E-01	ENSG00000109771
LRP3	-0.3783	1.7510E-05	3.8605E-04	ENSG00000130881
LRP4	0.0458	5.8850E-01	7.5729E-01	ENSG00000134569
LRP4-AS1	0.2228	4.2536E-02	1.4263E-01	ENSG00000247675
LRP5	-0.1875	5.6898E-02	1.7463E-01	ENSG00000162337
LRP6	0.1365	6.2576E-02	1.8580E-01	ENSG00000070018
LRP8	0.0229	7.3934E-01	8.5776E-01	ENSG00000157193
LRP8-DT	0.0041	8.9170E-01	9.4623E-01	ENSG00000225675
LRPAP1	-0.1535	9.2055E-03	4.7724E-02	ENSG00000163956
LRPPRC	0.0723	2.0818E-01	4.0805E-01	ENSG00000138095
LRR1	0.2255	1.4021E-02	6.4858E-02	ENSG00000165501
LRRC1	-0.0836	2.8185E-01	4.9228E-01	ENSG00000137269
LRRC10B	-0.1471	1.2229E-01	2.9111E-01	ENSG00000204950
LRRC14	-0.0720	3.9915E-01	6.0463E-01	ENSG00000160959
LRRC14B	0.0446	3.0193E-01		ENSG00000185028
LRRC15	0.0248	4.4248E-01		ENSG00000172061
LRRC17	0.0957	2.8309E-01	4.9349E-01	ENSG00000128606
LRRC18	0.0563	2.2520E-01		ENSG00000165383
LRRC19	-0.0350	6.0748E-01	7.7108E-01	ENSG00000184434
LRRC2	0.0470	2.9027E-01		ENSG00000163827
LRRC20	0.0336	5.6545E-01	7.4090E-01	ENSG00000172731
LRRC23	0.0233	7.0495E-01	8.3733E-01	ENSG00000010626
LRRC24	-0.0029	8.9030E-01		ENSG00000254402
LRRC25	-0.0042	5.2696E-01	7.1211E-01	ENSG00000175489
LRRC26	-0.0947	2.6936E-01	4.7880E-01	ENSG00000184709
LRRC27	-0.0330	6.4797E-01	7.9987E-01	ENSG00000148814
LRRC28	0.1493	1.3544E-02	6.3224E-02	ENSG00000168904
LRRC3	0.1222	1.4051E-01	3.1718E-01	ENSG00000160233
LRRC3-DT	0.4336	3.5713E-03	2.3420E-02	ENSG00000229356
LRRC32	0.0008	9.8609E-01	9.9239E-01	ENSG00000137507
LRRC34	0.0956	2.8752E-01	4.9820E-01	ENSG00000171757
LRRC36	0.0337	6.7790E-01	8.1961E-01	ENSG00000159708
LRRC37A	0.0181	8.1799E-01	9.0539E-01	ENSG00000176681
LRRC37A14P	-0.1348	1.6082E-02	7.1336E-02	ENSG00000215771
LRRC37A15P	-0.0149	7.7655E-01	8.8102E-01	ENSG00000230069
LRRC37A17P	-0.0254	5.3956E-01		ENSG00000263142
LRRC37A2	0.0206	7.9612E-01	8.9226E-01	ENSG00000238083
LRRC37A2	-0.0230	7.8586E-01	8.8674E-01	ENSG00000260075
LRRC37A3	0.0657	3.9920E-01	6.0467E-01	ENSG00000176809
LRRC37A6P	0.0378	5.6326E-01	7.3974E-01	ENSG00000230445
LRRC37B	-0.1434	4.4843E-02	1.4808E-01	ENSG00000185158
LRRC39	0.1584	1.0462E-01	2.6244E-01	ENSG00000122477
LRRC3B	0.0809	3.2437E-01	5.3438E-01	ENSG00000179796
LRRC3B-AS1	0.0933	2.8696E-01	4.9773E-01	ENSG00000225386
LRRC3C	0.0339	2.9829E-01		ENSG00000204913
LRRC4	0.0214	7.8043E-01	8.8353E-01	ENSG00000128594
LRRC40	0.0728	2.8005E-01	4.9007E-01	ENSG00000066557
LRRC41	-0.1012	1.1076E-01	2.7252E-01	ENSG00000132128
LRRC42	-0.0544	3.6071E-01	5.7021E-01	ENSG00000116212

LRRC43	0.1075	2.3999E-01	4.4674E-01	ENSG00000158113
LRRC45	0.0396	6.1567E-01	7.7701E-01	ENSG00000169683
LRRC47	-0.1186	7.4675E-02	2.0987E-01	ENSG00000130764
LRRC49	0.0589	3.0291E-01	5.1302E-01	ENSG00000137821
LRRC4B	-0.4382	4.5992E-03	2.8270E-02	ENSG00000131409
LRRC4C	0.3872	1.1961E-03	1.0373E-02	ENSG00000148948
LRRC51	-0.1877	2.5354E-02	9.8458E-02	ENSG00000184154
LRRC52	0.0599	3.2101E-01		ENSG00000162763
LRRC52-AS1	0.0095	8.4591E-01	9.2100E-01	ENSG00000237463
LRRC53	0.0228	6.2314E-01		ENSG00000162621
LRRC55	-0.0530	5.3765E-01	7.2037E-01	ENSG00000183908
LRRC56	0.0996	2.6923E-01	4.7868E-01	ENSG00000161328
LRRC57	-0.1795	6.0729E-03	3.4820E-02	ENSG00000180979
LRRC58	0.0211	7.5010E-01	8.6430E-01	ENSG00000163428
LRRC59	-0.2348	8.7147E-04	8.1748E-03	ENSG00000108829
LRRC61	-0.3132	8.0475E-04	7.7000E-03	ENSG00000127399
LRRC63	-0.1391	1.2708E-01	2.9844E-01	ENSG00000173988
LRRC66	-0.0317	6.0723E-01	7.7108E-01	ENSG00000188993
LRRC69	0.0187	7.2449E-01		ENSG00000214954
LRRC7	-0.0331	6.7311E-01	8.1708E-01	ENSG00000033122
LRRC70	0.0656	1.9236E-01	3.8808E-01	ENSG00000186105
LRRC71	0.1820	4.4082E-02	1.4616E-01	ENSG00000160838
LRRC72	-0.0064	7.7880E-01		ENSG00000205858
LRRC73	0.1324	1.6442E-01	3.5117E-01	ENSG00000204052
LRRC74A	-0.0093	5.4586E-01		ENSG00000100565
LRRC74B	0.1207	8.4586E-02	2.2730E-01	ENSG00000187905
LRRC75A	0.0326	7.0341E-01	8.3626E-01	ENSG00000181350
LRRC75B	0.0143	8.3521E-01	9.1518E-01	ENSG00000178026
LRRC77P	0.0152	7.1931E-01		ENSG00000244227
LRRC8A	-0.1631	9.4606E-03	4.8688E-02	ENSG00000136802
LRRC8B	0.0917	1.9373E-01	3.9008E-01	ENSG00000197147
LRRC8C	-0.2444	4.0968E-02	1.3907E-01	ENSG00000171488
LRRC8C-DT	0.0230	7.9329E-01	8.9078E-01	ENSG00000231999
LRRC8D	0.1955	3.5650E-02	1.2570E-01	ENSG00000171492
LRRC8E	0.0018	6.0426E-01	7.6833E-01	ENSG00000171017
LRRC9	0.4253	2.3028E-03	1.6759E-02	ENSG00000131951
LRRCC1	0.0350	5.8654E-01	7.5596E-01	ENSG00000133739
LRRD1	0.0179	7.4369E-02		ENSG00000240720
LRRFIP1	-0.0104	8.0564E-01	8.9800E-01	ENSG00000124831
LRRFIP2	0.0269	6.5887E-01	8.0746E-01	ENSG00000093167
LRRIQ3	0.2682	6.0386E-02	1.8119E-01	ENSG00000162620
LRRIQ4	0.0171	4.3734E-01		ENSG00000188306
LRRK1	0.0488	5.3514E-01	7.1834E-01	ENSG00000154237
LRRK2	0.0630	2.3013E-01	4.3474E-01	ENSG00000188906
LRRN1	0.2644	9.1040E-03	4.7338E-02	ENSG00000175928
LRRN2	-0.2279	1.7031E-02	7.4130E-02	ENSG00000170382
LRRN3	0.2207	1.0003E-02	5.0745E-02	ENSG00000173114
LRRN4	0.0079	9.0374E-01	9.5304E-01	ENSG00000125872
LRRN4CL	0.0440	5.3875E-01	7.2098E-01	ENSG00000177363

LRRTM1	0.0606	4.5764E-01	6.5569E-01	ENSG00000162951
LRRTM2	0.0906	2.1389E-01	4.1505E-01	ENSG00000146006
LRRTM3	0.0018	9.8018E-01	9.8991E-01	ENSG00000198739
LRRTM4	0.0405	5.8619E-01	7.5569E-01	ENSG00000176204
LRRTM4-AS1	-0.0016	8.2564E-01		ENSG00000234653
LRSAM1	-0.0519	4.3026E-01	6.3189E-01	ENSG00000148356
LRTM1	0.0377	4.0823E-01		ENSG00000144771
LRTM2	-0.0610	4.0453E-01	6.0914E-01	ENSG00000166159
LRTOMT	0.0275	6.7538E-01		ENSG00000284922
LRWD1	-0.0525	4.3322E-01	6.3436E-01	ENSG00000161036
LSAMP	-0.0012	9.8035E-01	9.8996E-01	ENSG00000185565
LSAMP-AS1	0.0273	6.7185E-01	8.1641E-01	ENSG00000240922
LSG1	0.1975	4.9542E-03	2.9848E-02	ENSG00000041802
LSM1	-0.0870	1.2418E-01	2.9398E-01	ENSG00000175324
LSM10	-0.1290	2.2894E-02	9.1624E-02	ENSG00000181817
LSM11	0.2468	2.2026E-02	8.9035E-02	ENSG00000155858
LSM12	-0.2154	3.6907E-06	1.0970E-04	ENSG00000161654
LSM14A	-0.3119	4.2016E-09	3.9205E-07	ENSG00000257103
LSM14B	0.0017	9.8204E-01	9.9056E-01	ENSG00000149657
LSM2	-0.1670	1.4973E-02	6.7887E-02	ENSG00000204392
LSM3	-0.1931	6.6829E-03	3.7501E-02	ENSG00000170860
LSM4	-0.2755	5.0943E-06	1.4292E-04	ENSG00000130520
LSM5	-0.0168	7.6857E-01	8.7610E-01	ENSG00000106355
LSM6	0.4558	4.2387E-08	2.7784E-06	ENSG00000164167
LSM7	-0.1630	1.7862E-02	7.6646E-02	ENSG00000130332
LSM8	0.2839	1.4751E-05	3.3297E-04	ENSG00000128534
LSMEM1	-0.0029	8.7959E-01		ENSG00000181016
LSMEM2	-0.0396	4.4719E-01	6.4667E-01	ENSG00000179564
LSP1	-0.0451	3.5079E-01	5.6094E-01	ENSG00000130592
LSP1P4	-0.0776	2.0852E-01	4.0859E-01	ENSG00000143429
LSP1P5	-0.2352	1.0577E-04	1.6055E-03	ENSG00000288905
LSR	0.2304	4.1190E-02	1.3971E-01	ENSG00000105699
LSS	-0.0256	7.3119E-01	8.5293E-01	ENSG00000160285
LST1	0.0485	3.9915E-01	6.0463E-01	ENSG00000204482
LTA	-0.0545	3.7511E-01	5.8368E-01	ENSG00000226979
LTA4H	-0.1105	1.0683E-01	2.6592E-01	ENSG00000111144
LTB	0.1109	1.8624E-01	3.8047E-01	ENSG00000227507
LTB4R	0.1958	6.0140E-02	1.8064E-01	ENSG00000213903
LTB4R2	0.0269	3.7295E-01		ENSG00000213906
LTBP1	-0.2073	3.1470E-02	1.1511E-01	ENSG00000049323
LTBP2	0.0542	5.2761E-01	7.1251E-01	ENSG00000119681
LTBP3	-0.0890	2.1938E-01	4.2176E-01	ENSG00000168056
LTBP4	0.1138	9.2941E-02	2.4323E-01	ENSG00000090006
LTBR	-0.0280	2.6323E-01		ENSG00000111321
LTC4S	0.0273	6.6879E-01	8.1435E-01	ENSG00000213316
LTF	0.0650	2.2494E-01	4.2887E-01	ENSG00000012223
LTK	-0.0517	3.7663E-01	5.8522E-01	ENSG00000062524
LTN1	0.0151	8.0843E-01	8.9937E-01	ENSG00000198862
LTO1	0.1205	1.4056E-01	3.1724E-01	ENSG00000149716

LTO1P1	-0.0034	7.4799E-01		ENSG00000251008
LTV1	-0.0342	6.2605E-01	7.8431E-01	ENSG00000135521
LUARIS	0.0045	9.3375E-01	9.6721E-01	ENSG00000231638
LUC7L	0.0003	9.8561E-01	9.9239E-01	ENSG00000007392
LUC7L2	-0.0213	6.6449E-01	8.1125E-01	ENSG00000146963
LUC7L3	0.4345	9.1532E-08	5.3495E-06	ENSG00000108848
LUCAT1	0.0230	7.5585E-01	8.6763E-01	ENSG00000248323
LUM	-0.0324	6.7000E-01	8.1498E-01	ENSG00000139329
LURAP1	0.1307	1.7251E-01	3.6221E-01	ENSG00000171357
LURAP1L	0.5176	2.2451E-04	2.8430E-03	ENSG00000153714
LUZP1	-0.2758	5.2621E-06	1.4564E-04	ENSG00000169641
LUZP2	0.0120	8.8439E-01	9.4208E-01	ENSG00000187398
LVRN	0.0479	4.5334E-01	6.5203E-01	ENSG00000172901
LXN	-0.3283	7.5645E-03	4.1123E-02	ENSG00000079257
LY6D	-0.0155	7.0849E-01	8.3971E-01	ENSG00000167656
LY6E	-0.3338	7.5372E-06	1.9501E-04	ENSG00000160932
LY6G5B	0.2422	4.0851E-02	1.3877E-01	ENSG00000240053
LY6G5C	0.0899	3.0614E-01	5.1626E-01	ENSG00000204428
LY6G6C	0.0339	6.0127E-01	7.6642E-01	ENSG00000204421
LY6G6D	0.0089	5.8925E-01		ENSG00000244355
LY6H	-0.3021	1.6010E-03	1.2806E-02	ENSG00000176956
LY6K	0.0051	9.7479E-01		ENSG00000160886
LY6L	-0.0069	9.1203E-01	9.5659E-01	ENSG00000261667
LY6S-AS1	0.0038	9.5991E-01	9.8006E-01	ENSG00000177335
LY75	0.0362	2.6223E-01		ENSG00000054219
LY86	0.0133	4.8700E-01		ENSG00000112799
LY86-AS1	-0.0414	8.0288E-02		ENSG00000216863
LY96	0.0278	9.3369E-01	9.6721E-01	ENSG00000154589
LYAR	0.1242	1.0579E-01	2.6445E-01	ENSG00000145220
LYG1	0.0359	6.6731E-01	8.1362E-01	ENSG00000144214
LYG2	0.1946	4.5103E-02	1.4863E-01	ENSG00000185674
LYL1	0.0294	4.8573E-01		ENSG00000104903
LYN	0.0411	6.2824E-01	7.8605E-01	ENSG00000254087
LYNX1	0.1454	9.5805E-02	2.4834E-01	ENSG00000180155
LYNX1-SLURP2	0.0044	9.4145E-01	9.7101E-01	ENSG00000284505
LYPD1	-0.0851	3.2127E-01	5.3125E-01	ENSG00000150551
LYPD3	-0.2103	6.5883E-03	3.7095E-02	ENSG00000124466
LYPD5	-0.4376	7.5494E-03	4.1067E-02	ENSG00000159871
LYPD6	-0.1045	2.4545E-01	4.5323E-01	ENSG00000187123
LYPD6B	-0.2227	4.8897E-02	1.5708E-01	ENSG00000150556
LYPLA1	-0.1342	5.3143E-02	1.6627E-01	ENSG00000120992
LYPLA2	-0.0896	8.3414E-02	2.2556E-01	ENSG00000011009
LYPLAL1	0.1884	3.0046E-02	1.1135E-01	ENSG00000143353
LYPLAL1-DT	0.0822	3.0706E-01	5.1735E-01	ENSG00000228063
LYRM1	-0.0942	8.2139E-02	2.2307E-01	ENSG00000102897
LYRM2	0.0188	7.5577E-01	8.6758E-01	ENSG00000083099
LYRM4	-0.2611	4.4016E-05	8.0403E-04	ENSG00000214113
LYRM4-AS1	-0.1177	1.8904E-01	3.8397E-01	ENSG00000272142
LYRM7	-0.0881	8.5297E-02	2.2857E-01	ENSG00000186687

LYRM9	0.1836	2.0198E-02	8.3583E-02	ENSG00000232859
LYSET	0.0598	3.9067E-01	5.9782E-01	ENSG00000153485
LYSMD1	-0.0639	4.3970E-01	6.3971E-01	ENSG00000163155
LYSMD2	-0.2022	3.3607E-02	1.2040E-01	ENSG00000140280
LYSMD3	0.2418	3.2602E-03	2.1804E-02	ENSG00000176018
LYSMD4	0.2174	1.0891E-02	5.3919E-02	ENSG00000183060
LYST	0.0586	3.1852E-01	5.2878E-01	ENSG00000143669
LYVE1	-0.0060	5.2463E-01		ENSG00000133800
LYZ	0.0201	5.2307E-01		ENSG00000090382
LYZL4	0.0076	7.1090E-01		ENSG00000157093
LZIC	-0.2208	4.1861E-05	7.7427E-04	ENSG00000162441
LZTFL1	0.1925	2.1004E-02	8.5924E-02	ENSG00000163818
LZTR1	0.0107	8.8541E-01	9.4277E-01	ENSG00000099949
LZTS1	-0.0936	2.7073E-01	4.8008E-01	ENSG00000061337
LZTS2	-0.0658	2.4719E-01	4.5495E-01	ENSG00000107816
LZTS3	-0.0501	4.7882E-01	6.7416E-01	ENSG00000088899
M1AP	0.0260	6.5981E-01		ENSG00000159374
M6PR	0.1754	6.3593E-03	3.6062E-02	ENSG00000003056
MAB21L1	0.2721	2.3766E-02	9.4219E-02	ENSG00000180660
MAB21L2	0.0481	3.3623E-01	5.4628E-01	ENSG00000181541
MAB21L3	0.0087	7.4103E-01		ENSG00000173212
MACC1	0.0275	4.2149E-01	6.2429E-01	ENSG00000183742
MACC1-AS1	0.0203	5.0174E-01		ENSG00000228598
MACF1	0.1047	1.0767E-01	2.6716E-01	ENSG00000127603
MACIR	-0.0424	4.8661E-01	6.8025E-01	ENSG00000181751
MACO1	0.0288	6.7485E-01	8.1764E-01	ENSG00000204178
MACORIS	0.0393	3.1429E-01		ENSG00000237797
MACROD1	-0.2578	2.0652E-03	1.5413E-02	ENSG00000133315
MACROD2	0.0808	3.0383E-01	5.1409E-01	ENSG00000172264
MACROD2-IT1	0.0216	3.3950E-01		ENSG00000227927
MACROH2A1	-0.3147	1.4983E-08	1.1776E-06	ENSG00000113648
MACROH2A2	-0.0997	1.1283E-01	2.7574E-01	ENSG00000099284
MAD1L1	0.0079	9.0556E-01	9.5378E-01	ENSG00000002822
MAD2L1	-0.0952	2.5886E-01	4.6757E-01	ENSG00000164109
MAD2L1BP	-0.1546	4.9231E-02	1.5785E-01	ENSG00000124688
MAD2L2	-0.1382	8.4343E-03	4.4677E-02	ENSG00000116670
MADCAM1	0.0366	6.5815E-01	8.0707E-01	ENSG00000099866
MADD	0.0790	2.4774E-01	4.5550E-01	ENSG00000110514
MADD-AS1	0.0061	9.1487E-01		ENSG00000256746
MAEA	0.2479	1.6747E-05	3.7023E-04	ENSG00000090316
MAEL	0.4290	5.4793E-03	3.2238E-02	ENSG00000143194
MAF	0.3388	7.2689E-03	3.9940E-02	ENSG00000178573
MAF1	-0.1802	6.8108E-03	3.8038E-02	ENSG00000179632
MAFA	-0.5968	7.2132E-03	3.9705E-02	ENSG00000182759
MAFA-AS1	-0.0106	8.3100E-01	9.1267E-01	ENSG00000254338
MAFB	-0.3010	2.3516E-03	1.7004E-02	ENSG00000204103
MAFF	-0.4674	1.3624E-06	4.8302E-05	ENSG00000185022
MAFG	0.1370	7.6949E-02	2.1399E-01	ENSG00000197063
MAFIP	0.0029	9.5156E-01		ENSG00000277400

MAFK	0.0038	9.8356E-01	9.9117E-01	ENSG00000198517
MAFTRR	0.3186	2.6312E-02	1.0117E-01	ENSG00000261390
MAGEA12	0.0245	2.0340E-01		ENSG00000213401
MAGEA2	0.0160	8.4475E-01	9.2042E-01	ENSG00000268606
MAGEA2	0.0155	8.5416E-01	9.2588E-01	ENSG00000183305
MAGEA3	0.0145	3.8892E-01		ENSG00000221867
MAGEA6	0.0416	7.0390E-02		ENSG00000197172
MAGEA9	-0.0009	8.8226E-01		ENSG00000123584
MAGEA9B	0.0110	6.6684E-01		ENSG00000267978
MAGEB6	0.0666	2.3106E-02		ENSG00000176746
MAGEC2	-0.0043	7.3603E-01		ENSG00000046774
MAGEC3	0.0624	6.1325E-02		ENSG00000165509
MAGED1	-0.0488	5.1156E-01	7.0002E-01	ENSG00000179222
MAGED2	-0.2321	1.3467E-03	1.1340E-02	ENSG00000102316
MAGED4	-0.0730	2.2026E-01	4.2304E-01	ENSG00000154545
MAGED4B	-0.0719	2.2294E-01	4.2653E-01	ENSG00000187243
MAGEE1	-0.1956	1.9665E-02	8.2008E-02	ENSG00000198934
MAGEE2	0.4022	1.3236E-02	6.2186E-02	ENSG00000186675
MAGEF1	0.1051	1.0007E-01	2.5481E-01	ENSG00000177383
MAGEH1	-0.0533	4.8313E-01	6.7731E-01	ENSG00000187601
MAGEL2	0.1802	5.4161E-02	1.6864E-01	ENSG00000254585
MAGI1	0.0630	3.6649E-01	5.7576E-01	ENSG00000151276
MAGI1-AS1	-0.0114	6.1255E-01		ENSG00000240175
MAGI1-IT1	-0.0156	2.6074E-01		ENSG00000272610
MAGI2	0.0105	8.9422E-01	9.4739E-01	ENSG00000187391
MAGI2-AS3	0.0559	2.5346E-01	4.6143E-01	ENSG00000234456
MAGI3	-0.0830	2.0666E-01	4.0621E-01	ENSG00000081026
MAGIX	0.1352	1.3608E-01	3.1110E-01	ENSG00000269313
MAGOH	0.0124	8.8029E-01	9.4001E-01	ENSG00000162385
MAGOH-DT	0.0694	3.9964E-01	6.0504E-01	ENSG00000226754
MAGOHB	-0.0614	2.4647E-01	4.5415E-01	ENSG00000111196
MAGT1	-0.0838	2.1199E-01	4.1293E-01	ENSG00000102158
MAIP1	-0.1060	1.4474E-01	3.2338E-01	ENSG00000162972
MAJIN	0.0303	5.5209E-01	7.3073E-01	ENSG00000168070
MAK	0.2285	4.6507E-02	1.5201E-01	ENSG00000111837
MAK16	0.1318	6.4821E-02	1.9054E-01	ENSG00000198042
MAL	-0.0757	3.7592E-01	5.8449E-01	ENSG00000172005
MAL2	-0.2191	3.0134E-02	1.1163E-01	ENSG00000147676
MALAT1	-0.1758	2.5418E-02	9.8661E-02	ENSG00000251562
MALL	-0.0308	5.9927E-01	7.6525E-01	ENSG00000144063
MALRD1	-0.0288	7.4575E-01	8.6142E-01	ENSG00000204740
MALSU1	0.0102	8.5793E-01	9.2834E-01	ENSG00000156928
MALT1	0.0672	2.7328E-01	4.8295E-01	ENSG00000172175
MALT1-AS1	-0.0326	6.9190E-01	8.2907E-01	ENSG00000267226
MAMDC2	0.0612	4.7581E-01	6.7134E-01	ENSG00000165072
MAMDC2-AS1	0.0286	7.2574E-01	8.4951E-01	ENSG00000204706
MAMDC4	-0.1161	2.0679E-01	4.0627E-01	ENSG00000177943
MAML1	-0.0379	5.8491E-01	7.5488E-01	ENSG00000161021
MAML2	0.0075	9.2124E-01	9.6140E-01	ENSG00000184384

MAML3	0.0545	4.6410E-01	6.6107E-01	ENSG00000196782
MAMLD1	-0.0655	3.8675E-01	5.9452E-01	ENSG00000013619
MAMSTR	0.0021	9.8080E-01	9.9009E-01	ENSG00000176909
MAN1A1	0.0618	4.5504E-01	6.5341E-01	ENSG00000111885
MAN1A2	0.1034	1.5696E-01	3.4064E-01	ENSG00000198162
MAN1B1	0.1273	9.5337E-02	2.4737E-01	ENSG00000177239
MAN1B1-DT	0.1923	2.8212E-02	1.0637E-01	ENSG00000268996
MAN1C1	-0.1703	5.9508E-02	1.7934E-01	ENSG00000117643
MAN2A1	0.1710	6.3966E-02	1.8874E-01	ENSG00000112893
MAN2A2	-0.0810	2.4701E-01	4.5470E-01	ENSG00000196547
MAN2B1	0.2383	6.6150E-03	3.7203E-02	ENSG00000104774
MAN2B2	0.0553	4.6984E-01	6.6616E-01	ENSG00000013288
MAN2C1	-0.2542	1.0603E-03	9.4938E-03	ENSG00000140400
MANBA	0.1012	1.1146E-01	2.7338E-01	ENSG00000109323
MANBAL	0.0745	1.6722E-01	3.5533E-01	ENSG00000101363
MANCR	0.0133	4.8900E-01		ENSG00000231298
MANEA	0.1125	1.1295E-01	2.7595E-01	ENSG00000172469
MANEA-DT	-0.0177	8.4898E-01	9.2304E-01	ENSG00000261366
MANEAL	-0.1300	7.0348E-02	2.0131E-01	ENSG00000185090
MANF	-0.1516	6.7988E-03	3.8028E-02	ENSG00000145050
MANSC1	-0.4832	4.8929E-11	9.1900E-09	ENSG00000111261
MANSC4	-0.0545	3.0726E-01	5.1752E-01	ENSG00000205693
MAOA	-0.2471	2.7216E-03	1.8954E-02	ENSG00000189221
MAOB	0.0090	8.7582E-01	9.3799E-01	ENSG00000069535
MAP10	-0.0035	9.6608E-01	9.8326E-01	ENSG00000212916
MAP1A	-0.0544	4.6397E-01	6.6099E-01	ENSG00000166963
MAP1B	-0.2471	2.9050E-04	3.4986E-03	ENSG00000131711
MAP1LC3A	-0.2819	1.1354E-03	9.9599E-03	ENSG00000101460
MAP1LC3B	-0.2310	7.8144E-04	7.5372E-03	ENSG00000140941
MAP1LC3B2	-0.0016	9.8108E-01	9.9019E-01	ENSG00000258102
MAP1LC3C	0.1120	2.8002E-01	4.9006E-01	ENSG00000197769
MAP1S	-0.2030	3.4096E-03	2.2540E-02	ENSG00000130479
MAP2	-0.1617	4.1390E-03	2.6178E-02	ENSG00000078018
MAP2K1	-0.0010	9.9645E-01	9.9803E-01	ENSG00000169032
MAP2K2	-0.0980	9.3234E-02	2.4371E-01	ENSG00000126934
MAP2K3	-0.0475	5.2463E-01	7.1012E-01	ENSG00000034152
MAP2K4	-0.0103	8.6782E-01	9.3343E-01	ENSG00000065559
MAP2K4P1	0.0259	7.0899E-01	8.4006E-01	ENSG00000269904
MAP2K5	-0.0818	2.3121E-01	4.3616E-01	ENSG00000137764
MAP2K6	0.1156	6.3757E-02	1.8826E-01	ENSG00000108984
MAP2K7	0.0076	9.2650E-01	9.6404E-01	ENSG00000076984
MAP3K1	0.0556	4.9322E-01	6.8521E-01	ENSG00000095015
MAP3K10	0.0201	8.0546E-01	8.9800E-01	ENSG00000130758
MAP3K11	0.1868	1.6951E-02	7.3877E-02	ENSG00000173327
MAP3K12	-0.0453	5.3391E-01	7.1766E-01	ENSG00000139625
MAP3K13	0.0920	9.9121E-02	2.5326E-01	ENSG00000073803
MAP3K14	-0.2510	1.8897E-02	7.9716E-02	ENSG00000006062
MAP3K15	-0.3213	2.0072E-02	8.3229E-02	ENSG00000180815
MAP3K19	1.1569	6.5334E-03		ENSG00000176601

MAP3K2	0.2556	1.4584E-04	2.0377E-03	ENSG00000169967
MAP3K20	-0.3073	5.5717E-04	5.7869E-03	ENSG00000091436
MAP3K21	-0.0840	2.7076E-01	4.8008E-01	ENSG00000143674
MAP3K3	0.1887	2.6704E-02	1.0223E-01	ENSG00000198909
MAP3K4	0.1610	3.8188E-02	1.3238E-01	ENSG00000085511
MAP3K4-AS1	-0.2516	9.4866E-03	4.8806E-02	ENSG00000272841
MAP3K5	0.0460	5.6016E-01	7.3732E-01	ENSG00000197442
MAP3K6	-0.0508	5.4356E-01	7.2458E-01	ENSG00000142733
MAP3K7	0.1449	1.4002E-02	6.4822E-02	ENSG00000135341
MAP3K7CL	0.1300	9.1544E-02	2.4075E-01	ENSG00000156265
MAP3K8	-0.1092	1.8083E-01	3.7311E-01	ENSG00000107968
MAP3K9	0.1231	1.0554E-01	2.6405E-01	ENSG00000006432
MAP3K9-DT	0.0916	1.3438E-01	3.0864E-01	ENSG00000259153
MAP4	-0.1212	5.0271E-02	1.6016E-01	ENSG00000047849
MAP4K1	-0.0308	7.1067E-01	8.4116E-01	ENSG00000104814
MAP4K1-AS1	-0.0334	1.8806E-01	3.8289E-01	ENSG00000267291
MAP4K2	0.0052	9.4122E-01	9.7089E-01	ENSG00000168067
MAP4K3	-0.0006	9.6592E-01	9.8323E-01	ENSG00000011566
MAP4K3-DT	0.1810	2.7708E-03	1.9216E-02	ENSG00000231312
MAP4K4	-0.3245	9.7248E-04	8.8556E-03	ENSG00000071054
MAP4K5	0.0288	5.5825E-01	7.3578E-01	ENSG00000012983
MAP6	0.1414	4.8144E-02	1.5542E-01	ENSG00000171533
MAP6D1	-0.2885	2.1509E-02	8.7434E-02	ENSG00000180834
MAP7	0.4441	5.3192E-07	2.2604E-05	ENSG00000135525
MAP7D1	-0.1565	2.0028E-02	8.3131E-02	ENSG00000116871
MAP7D2	0.2516	9.0564E-03	4.7160E-02	ENSG00000184368
MAP7D3	0.0686	3.5452E-01	5.6452E-01	ENSG00000129680
MAP9	0.4095	1.4435E-07	7.7339E-06	ENSG00000164114
MAP9-AS1	0.0375	4.2786E-01		ENSG00000250910
MAPK1	0.0187	7.4718E-01	8.6252E-01	ENSG00000100030
MAPK10	0.1775	3.7455E-03	2.4322E-02	ENSG00000109339
MAPK10-AS1	0.0841	3.4281E-02		ENSG00000250062
MAPK11	0.0437	5.9820E-01	7.6422E-01	ENSG00000185386
MAPK12	-0.1768	2.2083E-02	8.9228E-02	ENSG00000188130
MAPK13	0.0087	9.1577E-01	9.5846E-01	ENSG00000156711
MAPK14	0.1111	1.3121E-01	3.0423E-01	ENSG00000112062
MAPK15	-0.0618	4.7427E-01	6.7008E-01	ENSG00000181085
MAPK1IP1L	-0.1658	1.7574E-04	2.3500E-03	ENSG00000168175
MAPK3	-0.2041	1.0368E-02	5.2043E-02	ENSG00000102882
MAPK4	-0.1759	3.5516E-02	1.2534E-01	ENSG00000141639
MAPK6	-0.1827	7.9562E-04	7.6291E-03	ENSG00000069956
MAPK6-DT	-0.0129	7.4495E-01	8.6115E-01	ENSG00000259438
MAPK7	-0.1156	1.4131E-01	3.1817E-01	ENSG00000166484
MAPK8	-0.0085	8.9806E-01	9.4955E-01	ENSG00000107643
MAPK8IP1	-0.2652	1.6780E-03	1.3246E-02	ENSG00000121653
MAPK8IP1P1	0.0203	2.2422E-01		ENSG00000262500
MAPK8IP2	-0.0854	2.4777E-01	4.5553E-01	ENSG00000008735
MAPK8IP3	0.1541	1.6129E-02	7.1455E-02	ENSG00000138834
MAPK8IP3-AS1	-0.1421	1.1235E-02	5.5222E-02	ENSG00000261399

MAPK9	0.0813	2.0059E-01	3.9896E-01	ENSG00000050748
MAPKAP1	-0.0743	2.0765E-01	4.0732E-01	ENSG00000119487
MAPKAPK2	0.0094	9.0956E-01	9.5525E-01	ENSG00000162889
MAPKAPK3	0.1178	1.2708E-01	2.9844E-01	ENSG00000114738
MAPKAPK5	0.1120	6.0491E-02	1.8138E-01	ENSG00000089022
MAPKAPK5-AS1	-0.1891	3.1979E-03	2.1504E-02	ENSG00000234608
MAPKBP1	0.0118	8.7067E-01	9.3476E-01	ENSG00000137802
MAPRE1	-0.2334	5.7843E-07	2.3941E-05	ENSG00000101367
MAPRE2	-0.1753	1.8651E-02	7.8987E-02	ENSG00000166974
MAPRE3	-0.1345	4.4015E-02	1.4601E-01	ENSG00000084764
MAPRE3-AS1	-0.0760	3.8668E-01	5.9449E-01	ENSG00000205500
MAPT	-0.1110	1.3694E-01	3.1236E-01	ENSG00000186868
MAPT-IT1	-0.2179	2.5195E-02	9.8074E-02	ENSG00000279685
MARCHF1	0.2335	1.2057E-02	5.8214E-02	ENSG00000145416
MARCHF10-DT	0.0265	2.9771E-01		ENSG00000265000
MARCHF11	0.2147	5.4732E-02	1.6999E-01	ENSG00000183654
MARCHF11-AS1	0.0512	2.0932E-01		ENSG00000250981
MARCHF2	0.0216	6.7515E-01	8.1780E-01	ENSG00000099785
MARCHF3	-0.1486	7.0165E-02	2.0107E-01	ENSG00000173926
MARCHF4	-0.0743	3.9216E-01	5.9911E-01	ENSG00000144583
MARCHF5	-0.3028	7.3678E-08	4.4762E-06	ENSG00000198060
MARCHF6	-0.0786	2.0372E-01	4.0261E-01	ENSG00000145495
MARCHF6-DT	0.3686	4.0079E-05	7.5394E-04	ENSG00000259802
MARCHF7	-0.0543	3.7111E-01	5.7998E-01	ENSG00000136536
MARCHF8	0.0726	3.3836E-01	5.4823E-01	ENSG00000165406
MARCHF9	0.1717	3.9881E-02	1.3648E-01	ENSG00000139266
MARCKS	-0.0615	3.5473E-01	5.6460E-01	ENSG00000277443
MARCKSL1	-0.4819	1.4128E-10	2.2001E-08	ENSG00000175130
MARCOL	0.0117	6.2117E-01		ENSG00000248109
MARF1	0.2618	7.1599E-04	7.0720E-03	ENSG00000166783
MARK1	0.0664	3.0397E-01	5.1410E-01	ENSG00000116141
MARK2	-0.1336	5.6438E-02	1.7364E-01	ENSG00000072518
MARK2P10	-0.0050	7.1538E-01		ENSG00000227284
MARK2P18	0.0931	1.4851E-01	3.2905E-01	ENSG00000219712
MARK2P19	-0.0068	8.7949E-01	9.3972E-01	ENSG00000270773
MARK3	-0.0822	1.6434E-01	3.5113E-01	ENSG00000075413
MARK4	-0.0056	9.3572E-01	9.6833E-01	ENSG00000007047
MARS1	-0.0644	3.9113E-01	5.9811E-01	ENSG00000166986
MARS2	0.1207	1.9150E-01	3.8679E-01	ENSG00000247626
MARVELD1	-0.2528	2.0077E-03	1.5130E-02	ENSG00000155254
MARVELD2	-0.0696	3.3725E-01	5.4734E-01	ENSG00000152939
MARVELD3	0.1379	9.9659E-02	2.5408E-01	ENSG00000140832
MAS1	-0.0474	4.3858E-01	6.3861E-01	ENSG00000130368
MASP1	0.5761	1.3872E-06	4.9039E-05	ENSG00000127241
MASP2	0.1897	7.8800E-02	2.1743E-01	ENSG00000009724
MAST1	-0.0473	4.7894E-01	6.7419E-01	ENSG00000105613
MAST2	-0.0189	7.4881E-01	8.6354E-01	ENSG00000086015
MAST3	-0.3810	7.9348E-05	1.2876E-03	ENSG00000099308
MAST4	0.2855	8.4597E-04	7.9753E-03	ENSG00000069020

MASTL	-0.2478	1.9999E-03	1.5076E-02	ENSG00000120539
MAT1A	0.1153	1.8449E-01	3.7796E-01	ENSG00000151224
MAT2A	0.0614	4.5402E-01	6.5254E-01	ENSG00000168906
MAT2B	-0.0076	9.1563E-01	9.5841E-01	ENSG00000038274
MATCAP1	0.0594	4.2174E-01	6.2442E-01	ENSG00000196123
MATCAP2	-0.2040	4.9904E-03	3.0044E-02	ENSG00000164542
MATK	-0.3648	1.6659E-03	1.3172E-02	ENSG00000007264
MATN1	0.0963	1.8148E-01	3.7398E-01	ENSG00000162510
MATN1-AS1	0.0521	4.9611E-01	6.8774E-01	ENSG00000186056
MATN2	0.0002	9.9314E-01	9.9618E-01	ENSG00000132561
MATN3	0.0808	2.4396E-01	4.5165E-01	ENSG00000132031
MATN4	0.1029	2.5916E-01	4.6768E-01	ENSG00000124159
MATR3	0.0278	5.1063E-01		ENSG00000280987
MATR3	-0.2365	2.4428E-02	9.5968E-02	ENSG00000015479
MAU2	0.0625	2.6204E-01	4.7080E-01	ENSG00000129933
MAVS	-0.1610	1.8332E-02	7.7978E-02	ENSG00000088888
MAX	0.0187	7.6746E-01	8.7535E-01	ENSG00000125952
MAZ	-0.1744	5.5167E-02	1.7100E-01	ENSG00000103495
MB	0.1365	8.4319E-02	2.2696E-01	ENSG00000198125
MB21D2	0.1482	1.1541E-01	2.7998E-01	ENSG00000180611
MBD1	-0.0020	9.7181E-01	9.8589E-01	ENSG00000141644
MBD2	-0.1573	2.8738E-02	1.0779E-01	ENSG00000134046
MBD3	0.1694	2.8667E-02	1.0762E-01	ENSG00000071655
MBD3L3	0.0289	4.9779E-01		ENSG00000182315
MBD4	0.2545	6.5243E-04	6.5845E-03	ENSG00000129071
MBD5	0.1377	1.6104E-02	7.1409E-02	ENSG00000204406
MBD6	-0.0647	4.2025E-01	6.2321E-01	ENSG00000166987
MBIP	-0.1755	5.1250E-03	3.0681E-02	ENSG00000151332
MBL1P	-0.0070	8.2455E-01		ENSG00000242600
MBL3P	0.0080	6.8642E-01		ENSG00000219430
MBLAC1	-0.0213	8.0837E-01	8.9937E-01	ENSG00000214309
MBLAC2	0.2873	2.3572E-03	1.7013E-02	ENSG00000176055
MBNL1	-0.2415	3.1908E-03	2.1486E-02	ENSG00000152601
MBNL1-AS1	-0.0101	8.6217E-01	9.3070E-01	ENSG00000229619
MBNL2	0.0077	9.0126E-01	9.5157E-01	ENSG00000139793
MBNL3	0.0757	3.9084E-01	5.9793E-01	ENSG00000076770
MBOAT1	-0.0972	2.1017E-01	4.1058E-01	ENSG00000172197
MBOAT2	-0.0777	1.3493E-01	3.0945E-01	ENSG00000143797
MBOAT4	-0.5951	7.6018E-03	4.1226E-02	ENSG00000177669
MBOAT7	-0.0482	4.1457E-01	6.1810E-01	ENSG00000125505
MBP	-0.4060	2.2673E-03	1.6554E-02	ENSG00000197971
MBTD1	-0.0774	2.9230E-01	5.0240E-01	ENSG00000011258
MBTPS1	0.0773	2.3622E-01	4.4196E-01	ENSG00000140943
MBTPS2	-0.0050	9.3578E-01	9.6833E-01	ENSG00000012174
MC1R	0.0432	5.9999E-01	7.6571E-01	ENSG00000258839
MC4R	0.0982	1.3553E-01	3.1028E-01	ENSG00000166603
MC5R	0.0824	1.7692E-01	3.6815E-01	ENSG00000176136
MCAM	-0.0578	3.5052E-01	5.6067E-01	ENSG00000076706
MCAT	0.1446	3.7482E-02	1.3052E-01	ENSG00000100294

MCC	-0.1308	1.5568E-01	3.3933E-01	ENSG00000171444
MCCC1	-0.1510	3.1552E-02	1.1530E-01	ENSG00000078070
MCCC2	0.0651	3.5110E-01	5.6122E-01	ENSG00000131844
MCEE	0.3299	2.8684E-04	3.4699E-03	ENSG00000124370
MCEMP1	0.4053	1.3951E-02	6.4638E-02	ENSG00000183019
MCF2	0.3009	1.0211E-02	5.1450E-02	ENSG00000101977
MCF2L	0.0428	4.7645E-01	6.7189E-01	ENSG00000126217
MCF2L2	0.0126	8.7803E-01	9.3917E-01	ENSG00000053524
MCFD2	0.0992	7.1607E-02	2.0407E-01	ENSG00000180398
MCHR1	-0.0705	4.1622E-01	6.1970E-01	ENSG00000128285
MCHR2	0.0223	8.4346E-01		ENSG00000152034
MCHR2-AS1	0.0175	1.7405E-01		ENSG00000229315
MCIDAS	-0.0075	7.1125E-01		ENSG00000234602
MCL1	-0.1615	3.8532E-02	1.3327E-01	ENSG00000143384
MCM10	-0.2918	1.0125E-02	5.1188E-02	ENSG00000065328
MCM2	-0.1131	2.0986E-01	4.1013E-01	ENSG00000073111
MCM3	-0.0050	9.4526E-01	9.7278E-01	ENSG00000112118
MCM3AP	-0.0725	2.9207E-01	5.0222E-01	ENSG00000160294
MCM3AP-AS1	-0.0348	6.6420E-01	8.1109E-01	ENSG00000215424
MCM4	-0.3271	2.1333E-03	1.5782E-02	ENSG00000104738
MCM5	-0.0800	3.2286E-01	5.3288E-01	ENSG00000100297
MCM6	-0.0988	1.6567E-01	3.5317E-01	ENSG00000076003
MCM7	-0.1230	1.7000E-01	3.5861E-01	ENSG00000166508
MCM8	0.3635	1.0497E-03	9.4190E-03	ENSG00000125885
MCM8-AS1	0.0585	2.6239E-01		ENSG00000278719
MCM9	-0.1681	8.2100E-02	2.2302E-01	ENSG00000111877
MCMBP	-0.0316	6.3956E-01	7.9393E-01	ENSG00000197771
MCMDC2	0.0308	6.6124E-01	8.0912E-01	ENSG00000178460
MCOLN1	-0.1072	1.0802E-01	2.6786E-01	ENSG00000090674
MCOLN2	-0.0222	5.9779E-01		ENSG00000153898
MCOLN3	0.0005	9.8817E-01	9.9354E-01	ENSG00000055732
MCPH1	-0.1512	3.3797E-03	2.2372E-02	ENSG00000147316
MCPH1-DT	-0.0519	4.6093E-01	6.5837E-01	ENSG00000246089
MCRIP1	-0.1452	1.6959E-02	7.3893E-02	ENSG00000225663
MCRIP2	-0.1575	2.3772E-02	9.4219E-02	ENSG00000172366
MCRS1	-0.0849	1.5696E-01	3.4064E-01	ENSG00000187778
MCTP1	0.3093	1.6900E-03	1.3311E-02	ENSG00000175471
MCTP2	0.0373	5.0662E-01	6.9620E-01	ENSG00000140563
MCTS1	-0.2035	4.3045E-04	4.7303E-03	ENSG00000232119
MCU	-0.1890	1.9091E-02	8.0309E-02	ENSG00000156026
MCUB	-0.1019	1.4596E-01	3.2516E-01	ENSG00000005059
MCUR1	-0.1501	3.1411E-02	1.1496E-01	ENSG00000050393
MDC1	0.1164	1.0352E-01	2.6056E-01	ENSG00000137337
MDFIC	-0.1843	5.8295E-02	1.7717E-01	ENSG00000135272
MDGA1	-0.1436	1.2782E-01	2.9960E-01	ENSG00000112139
MDGA2	0.1418	1.1251E-01	2.7524E-01	ENSG00000139915
MDH1	-0.1992	4.3116E-03	2.6946E-02	ENSG00000014641
MDH1B	0.2312	1.7200E-02	7.4628E-02	ENSG00000138400
MDH2	-0.2986	5.7299E-08	3.5965E-06	ENSG00000146701

MDK	-0.3058	7.2973E-04	7.1677E-03	ENSG00000110492
MDM1	-0.1101	1.9074E-01	3.8595E-01	ENSG00000111554
MDM2	0.0286	6.3672E-01	7.9185E-01	ENSG00000135679
MDM4	-0.1181	7.9211E-02	2.1820E-01	ENSG00000198625
MDN1	0.3180	2.0162E-04	2.6065E-03	ENSG00000112159
MDP1	-0.0061	8.8560E-01		ENSG00000213920
MDS2	0.0024	8.7244E-01		ENSG00000197880
ME1	-0.0376	5.5012E-01	7.2920E-01	ENSG00000065833
ME2	-0.3415	5.7164E-08	3.5965E-06	ENSG00000082212
ME3	0.0141	8.6718E-01	9.3301E-01	ENSG00000151376
MEA1	-0.1898	8.5360E-04	8.0348E-03	ENSG00000124733
MEAF6	-0.1851	3.6054E-04	4.1263E-03	ENSG00000163875
MEAK7	0.0603	3.5697E-01	5.6676E-01	ENSG00000140950
MECOM	0.0897	1.5311E-01	3.3528E-01	ENSG00000085276
MECP2	0.0995	1.4671E-01	3.2636E-01	ENSG00000169057
MECR	0.0626	3.5754E-01	5.6739E-01	ENSG00000116353
MED1	-0.1586	1.7994E-02	7.6998E-02	ENSG00000125686
MED10	-0.2121	1.7335E-03	1.3545E-02	ENSG00000133398
MED11	-0.2168	5.9606E-03	3.4322E-02	ENSG00000161920
MED12	0.0073	9.1670E-01	9.5870E-01	ENSG00000184634
MED12L	0.0446	5.2805E-01	7.1271E-01	ENSG00000144893
MED13	0.0139	8.6675E-01	9.3275E-01	ENSG00000108510
MED13L	-0.1002	1.7396E-01	3.6410E-01	ENSG00000123066
MED14	0.0764	3.2062E-01	5.3067E-01	ENSG00000180182
MED15	-0.0578	3.2116E-01	5.3120E-01	ENSG00000099917
MED15P5	0.0222	4.6665E-01		ENSG00000236595
MED16	-0.1781	2.0220E-02	8.3609E-02	ENSG00000175221
MED17	-0.4607	4.4686E-07	1.9495E-05	ENSG00000042429
MED18	0.2715	7.3831E-03	4.0386E-02	ENSG00000130772
MED19	0.0737	2.4751E-01	4.5528E-01	ENSG00000156603
MED20	0.1839	1.4512E-02	6.6420E-02	ENSG00000124641
MED21	-0.0222	7.1250E-01	8.4225E-01	ENSG00000152944
MED22	-0.0087	8.8491E-01	9.4244E-01	ENSG00000148297
MED23	0.1016	1.4260E-01	3.2015E-01	ENSG00000112282
MED24	-0.0196	7.9327E-01	8.9078E-01	ENSG00000008838
MED25	-0.1868	9.1395E-03	4.7493E-02	ENSG00000104973
MED26	0.0097	9.0901E-01	9.5505E-01	ENSG00000105085
MED27	-0.0437	3.6890E-01	5.7811E-01	ENSG00000160563
MED28	0.1784	1.7381E-02	7.5251E-02	ENSG00000118579
MED28P3	0.0109	5.9740E-01		ENSG00000227692
MED29	-0.1031	9.3808E-02	2.4467E-01	ENSG00000063322
MED30	-0.1777	1.3512E-02	6.3142E-02	ENSG00000164758
MED31	0.0555	4.5363E-01	6.5222E-01	ENSG00000108590
MED4	0.0099	8.6561E-01	9.3217E-01	ENSG00000136146
MED4-AS1	-0.0722	1.5652E-01	3.4030E-01	ENSG00000229111
MED6	0.0825	1.8292E-01	3.7607E-01	ENSG00000133997
MED7	-0.0479	4.4236E-01	6.4173E-01	ENSG00000155868
MED8	0.2011	9.7712E-03	4.9869E-02	ENSG00000159479
MED9	-0.0147	7.9141E-01	8.8985E-01	ENSG00000141026

MEDAG	0.0366	8.2068E-02		ENSG00000102802
MEF2A	-0.1870	6.1089E-03	3.4972E-02	ENSG00000068305
MEF2C	-0.2033	1.3498E-02	6.3116E-02	ENSG00000081189
MEF2C-AS2	0.1199	1.8439E-01	3.7783E-01	ENSG00000245864
MEF2D	-0.2813	4.0168E-05	7.5467E-04	ENSG00000116604
MEFV	0.0727	7.7231E-02		ENSG00000103313
MEG3	0.1583	3.6839E-02	1.2881E-01	ENSG00000214548
MEGF10	0.0492	5.6480E-01	7.4058E-01	ENSG00000145794
MEGF11	0.0645	4.4057E-01	6.4044E-01	ENSG00000157890
MEGF6	0.1760	3.7144E-02	1.2962E-01	ENSG00000162591
MEGF8	0.0422	5.3519E-01	7.1834E-01	ENSG00000105429
MEGF9	-0.0510	3.2637E-01	5.3649E-01	ENSG00000106780
MEI4	0.3302	1.3560E-02	6.3264E-02	ENSG00000269964
MEIG1	0.0848	3.2328E-01	5.3330E-01	ENSG00000197889
MEIOB	-0.0084	8.8558E-01	9.4289E-01	ENSG00000162039
MEIOC	-0.3400	1.2944E-02	6.1129E-02	ENSG00000180336
MEIOSIN	-0.0075	6.0330E-01		ENSG00000237452
MEIS1	0.3022	5.4227E-04	5.6585E-03	ENSG00000143995
MEIS1-AS2	0.0481	4.3234E-01	6.3368E-01	ENSG00000230749
MEIS2	-0.1282	1.2921E-01	3.0154E-01	ENSG00000134138
MEIS3	-0.2153	1.7887E-04	2.3764E-03	ENSG00000105419
MELK	-0.0969	2.5865E-01	4.6737E-01	ENSG00000165304
MELTF	-0.2091	5.8572E-02	1.7772E-01	ENSG00000163975
MELTF-AS1	-0.0950	2.8457E-01	4.9518E-01	ENSG00000228109
MEMO1	0.3038	5.4292E-03	3.2065E-02	ENSG00000162959
MEMO1P4	0.0358	1.0381E-01		ENSG00000230376
MEN1	0.0065	9.2292E-01	9.6186E-01	ENSG00000133895
MEOX1	-0.0105	8.7860E-01		ENSG00000005102
MEP1B	0.0515	5.8558E-02	1.7770E-01	ENSG00000141434
MEPCE	-0.1035	2.0242E-01	4.0127E-01	ENSG00000146834
MERTK	0.0237	7.8523E-01	8.8651E-01	ENSG00000153208
MESD	0.0117	8.4355E-01	9.1973E-01	ENSG00000117899
MESDP1	-0.0129	4.1502E-01		ENSG00000257368
MESP1	-0.3577	1.8639E-05	4.0417E-04	ENSG00000166823
MESP2	0.0526	4.6401E-01	6.6102E-01	ENSG00000188095
MEST	-0.0049	9.3752E-01	9.6914E-01	ENSG00000106484
MESTIT1	0.0568	5.1206E-01	7.0032E-01	ENSG00000272701
MET	0.0811	1.6758E-01	3.5589E-01	ENSG00000105976
METAP1	0.0120	8.7808E-01	9.3917E-01	ENSG00000164024
METAP1D	-0.0274	7.1915E-01	8.4556E-01	ENSG00000172878
METAP2	0.0229	6.7058E-01	8.1544E-01	ENSG00000111142
METRN	-0.0732	3.8856E-01	5.9594E-01	ENSG00000103260
METRNL	-0.0084	9.2394E-01	9.6247E-01	ENSG00000176845
METTL1	-0.0787	3.1366E-01	5.2393E-01	ENSG00000037897
METTL13	0.1809	1.1163E-02	5.4910E-02	ENSG00000010165
METTL14	0.1142	5.8321E-02	1.7722E-01	ENSG00000145388
METTL14-DT	0.5123	3.7463E-03	2.4322E-02	ENSG00000281731
METTL15	0.1019	1.8892E-01	3.8389E-01	ENSG00000169519
METTL16	-0.0127	8.0727E-01	8.9873E-01	ENSG00000127804

METTL17	0.0516	4.1350E-01	6.1711E-01	ENSG00000165792
METTL18	-0.0032	9.5428E-01	9.7703E-01	ENSG00000171806
METTL21A	0.1319	6.2438E-02	1.8559E-01	ENSG00000144401
METTL21C	-0.0293	3.5203E-01		ENSG00000139780
METTL21EP	0.0263	4.8138E-01		ENSG00000250878
METTL22	0.2626	5.5359E-04	5.7570E-03	ENSG00000067365
METTL23	-0.0499	4.6178E-01	6.5898E-01	ENSG00000181038
METTL24	0.1044	9.2463E-02	2.4223E-01	ENSG00000053328
METTL25	0.1562	1.0661E-01	2.6556E-01	ENSG00000127720
METTL25B	-0.1301	6.0050E-02	1.8045E-01	ENSG00000143303
METTL26	-0.0968	1.6447E-01	3.5126E-01	ENSG00000130731
METTL27	-0.1044	2.3892E-01	4.4542E-01	ENSG00000165171
METTL2A	-0.0735	1.3028E-01	3.0306E-01	ENSG00000087995
METTL2B	-0.1248	3.2141E-02	1.1664E-01	ENSG00000165055
METTL3	0.4556	4.1631E-08	2.7610E-06	ENSG00000165819
METTL4	0.0498	4.9671E-01	6.8812E-01	ENSG00000101574
METTL5	-0.2157	3.2000E-04	3.7745E-03	ENSG00000138382
METTL5P2	0.0241	4.0713E-01		ENSG00000248557
METTL6	0.0067	9.0671E-01	9.5437E-01	ENSG00000206562
METTL8	-0.0707	2.3853E-01	4.4486E-01	ENSG00000123600
METTL9	-0.1483	6.4805E-03	3.6622E-02	ENSG00000197006
MEX3A	0.0871	2.9952E-01	5.0913E-01	ENSG00000254726
MEX3B	-0.0803	3.5314E-01	5.6336E-01	ENSG00000183496
MEX3C	-0.3581	3.5939E-06	1.0731E-04	ENSG00000176624
MEX3D	-0.1002	2.3810E-01	4.4418E-01	ENSG00000181588
MFAP1	0.2007	3.2589E-03	2.1802E-02	ENSG00000140259
MFAP2	-0.0566	4.5893E-01	6.5677E-01	ENSG00000117122
MFAP3	0.1341	4.3915E-02	1.4580E-01	ENSG00000037749
MFAP3L	0.0507	5.0002E-01	6.9089E-01	ENSG00000198948
MFAP4	0.0046	9.4313E-01	9.7180E-01	ENSG00000166482
MFF	-0.0942	6.2921E-02	1.8646E-01	ENSG00000168958
MFF-DT	0.0773	3.6990E-01	5.7915E-01	ENSG00000236432
MFGE8	-0.1621	9.1523E-02	2.4072E-01	ENSG00000140545
MFHAS1	-0.2821	6.8118E-03	3.8038E-02	ENSG00000147324
MFN1	0.1825	1.3045E-02	6.1466E-02	ENSG00000171109
MFN2	0.0301	6.5247E-01	8.0312E-01	ENSG00000116688
MFNG	-0.4298	4.8225E-05	8.6297E-04	ENSG00000100060
MFRP	0.0231	3.9391E-01		ENSG00000235718
MFSD1	-0.1762	6.5780E-03	3.7055E-02	ENSG00000118855
MFSD10	-0.0454	4.9491E-01	6.8685E-01	ENSG00000109736
MFSD11	0.0773	2.5082E-01	4.5906E-01	ENSG00000092931
MFSD12	0.0673	2.9916E-01	5.0892E-01	ENSG00000161091
MFSD13A	-0.1697	1.6862E-02	7.3640E-02	ENSG00000138111
MFSD13B	-0.0189	7.3983E-01	8.5797E-01	ENSG00000230872
MFSD14A	-0.1717	8.2753E-02	2.2432E-01	ENSG00000156875
MFSD14B	0.2097	9.7137E-03	4.9679E-02	ENSG00000148110
MFSD2A	0.0888	2.7036E-01	4.7989E-01	ENSG00000168389
MFSD2B	-0.0233	7.5994E-01	8.7070E-01	ENSG00000205639
MFSD3	-0.0916	1.7083E-01	3.5979E-01	ENSG00000167700

MFSD4A	0.4934	3.3272E-05	6.4664E-04	ENSG00000174514
MFSD4B	0.1814	4.2929E-02	1.4361E-01	ENSG00000173214
MFSD5	-0.2554	3.2291E-04	3.7976E-03	ENSG00000182544
MFSD6	-0.0378	6.5399E-01	8.0444E-01	ENSG00000151690
MFSD8	0.0682	2.6227E-01	4.7110E-01	ENSG00000164073
MFSD9	0.0436	5.2322E-01	7.0909E-01	ENSG00000135953
MGA	0.1740	1.9196E-02	8.0647E-02	ENSG00000174197
MGAM	0.0028	9.3897E-01		ENSG00000257335
MGAM2	0.0024	9.4901E-01		ENSG00000257743
MGAT1	0.1773	1.1129E-02	5.4778E-02	ENSG00000131446
MGAT2	-0.0279	6.7742E-01	8.1922E-01	ENSG00000168282
MGAT3	-0.1010	2.4401E-01	4.5165E-01	ENSG00000128268
MGAT3-AS1	-0.0114	8.4541E-01	9.2086E-01	ENSG00000227188
MGAT4A	-0.1541	2.1834E-02	8.8476E-02	ENSG00000071073
MGAT4B	-0.1000	1.5596E-01	3.3975E-01	ENSG00000161013
MGAT4C	-0.0454	5.8601E-01	7.5563E-01	ENSG00000182050
MGAT4D	0.0114	4.7126E-01		ENSG00000205301
MGAT4EP	-0.0022	8.9596E-01		ENSG00000184774
MGAT5	-0.0118	8.4111E-01	9.1820E-01	ENSG00000152127
MGAT5B	-0.0057	9.2150E-01	9.6140E-01	ENSG00000167889
MGC12916	0.0529	1.0181E-01	2.5771E-01	ENSG00000266709
MGC15885	-0.0007	9.2151E-01		ENSG00000259458
MGC16275	-0.0687	3.8995E-01	5.9733E-01	ENSG00000246731
MGC32805	0.0137	7.5412E-01		ENSG00000250328
MGLL	-0.3318	1.7979E-03	1.3909E-02	ENSG00000074416
MGME1	-0.2664	1.8766E-03	1.4358E-02	ENSG00000125871
MGMT	-0.1256	4.6685E-02	1.5239E-01	ENSG00000170430
MGP	0.0237	6.8988E-01	8.2772E-01	ENSG00000111341
MGRN1	-0.0117	8.3899E-01	9.1719E-01	ENSG00000102858
MGST1	-0.0773	3.1577E-01	5.2593E-01	ENSG00000008394
MGST2	0.1869	6.4599E-02	1.9015E-01	ENSG00000085871
MGST3	-0.1282	7.2286E-02	2.0539E-01	ENSG00000143198
MHENCR	0.3552	5.6473E-04	5.8375E-03	ENSG00000232442
MIA	-0.0207	2.5582E-01		ENSG00000261857
MIA2	-0.0971	1.6915E-01	3.5762E-01	ENSG00000150527
MIA2-AS1	0.0430	5.2496E-01	7.1041E-01	ENSG00000258940
MIA3	-0.0163	7.2562E-01	8.4946E-01	ENSG00000154305
MIAT	0.0318	6.3369E-01	7.8999E-01	ENSG00000225783
MIB1	-0.0102	8.5850E-01	9.2863E-01	ENSG00000101752
MIB2	0.0025	9.7575E-01	9.8791E-01	ENSG00000197530
MICA	0.0581	5.0356E-01	6.9395E-01	ENSG00000204520
MICAL1	-0.2495	8.2764E-04	7.8534E-03	ENSG00000135596
MICAL2	-0.2505	2.4390E-02	9.5910E-02	ENSG00000133816
MICAL3	0.2086	3.6883E-03	2.4034E-02	ENSG00000243156
MICALL1	-0.1636	5.9802E-02	1.7986E-01	ENSG00000100139
MICALL2	0.0037	9.6793E-01	9.8416E-01	ENSG00000164877
MICALL2-DT	-0.0229	5.4508E-01		ENSG00000225981
MICB-DT	-0.0718	1.8287E-01	3.7602E-01	ENSG00000286940
MICOS10	-0.1382	4.0626E-02	1.3823E-01	ENSG00000173436

MICOS10-DT	0.0168	6.6514E-01		ENSG00000235185
MICOS13	0.0675	2.8723E-01	4.9794E-01	ENSG00000174917
MICU1	-0.1025	2.4954E-02	9.7476E-02	ENSG00000107745
MICU2	0.0231	7.1487E-01	8.4338E-01	ENSG00000165487
MICU3	0.5018	3.8981E-12	1.1418E-09	ENSG00000155970
MID1	-0.0693	2.5731E-01	4.6580E-01	ENSG00000101871
MID1IP1	-0.4923	6.5662E-07	2.6313E-05	ENSG00000165175
MID2	0.0020	9.8066E-01	9.9004E-01	ENSG00000080561
MIDEAS	-0.2751	1.0728E-05	2.5982E-04	ENSG00000156030
MIDN	-0.0985	1.8868E-01	3.8354E-01	ENSG00000167470
MIEF1	0.0167	7.9546E-01	8.9200E-01	ENSG00000100335
MIEF2	0.0909	2.4090E-01	4.4785E-01	ENSG00000177427
MIEN1	-0.3086	2.7944E-06	8.7923E-05	ENSG00000141741
MIER1	-0.0453	4.8062E-01	6.7548E-01	ENSG00000198160
MIER2	-0.0254	7.5804E-01	8.6905E-01	ENSG00000105556
MIER3	-0.1502	2.8516E-02	1.0722E-01	ENSG00000155545
MIF	-0.1738	5.4002E-02	1.6834E-01	ENSG00000240972
MIF4GD	0.0556	3.9751E-01	6.0337E-01	ENSG00000125457
MIF4GD-DT	0.1654	9.2960E-02	2.4323E-01	ENSG00000263843
MIGA1	0.1382	4.2338E-02	1.4224E-01	ENSG00000180488
MIGA2	0.1723	2.8904E-02	1.0821E-01	ENSG00000148343
MIIP	-0.0111	7.9914E-01	8.9413E-01	ENSG00000116691
MILIP	0.0279	7.3066E-01	8.5271E-01	ENSG00000265688
MILR1	-0.0179	5.1349E-01		ENSG00000271605
MIMT1	-0.0932	1.0802E-01	2.6786E-01	ENSG00000268654
MINAR1	0.0938	2.9676E-01	5.0682E-01	ENSG00000169330
MINAR2	0.1036	5.2388E-02	1.6456E-01	ENSG00000186367
MINCR	0.2834	2.6367E-04	3.2510E-03	ENSG00000253716
MINDY1	-0.0341	6.5498E-01	8.0515E-01	ENSG00000143409
MINDY2	0.0413	4.7092E-01	6.6718E-01	ENSG00000128923
MINDY2-DT	0.0680	4.2109E-01	6.2389E-01	ENSG00000245975
MINDY3	0.0570	3.5244E-01	5.6281E-01	ENSG00000148481
MINDY4	0.0182	8.1405E-01	9.0309E-01	ENSG00000106125
MINK1	0.0412	4.6789E-01	6.6442E-01	ENSG00000141503
MINPP1	0.0255	7.3027E-01	8.5247E-01	ENSG00000107789
MIOS	0.0103	8.8368E-01	9.4162E-01	ENSG00000164654
MIOX	-0.0565	1.0793E-01		ENSG00000100253
MIP	-0.0486	3.5376E-01	5.6375E-01	ENSG00000135517
MIPEP	0.0503	4.7382E-01	6.6967E-01	ENSG00000027001
MIPOL1	-0.0200	7.9816E-01	8.9364E-01	ENSG00000151338
MIR1-1HG	0.0187	4.8620E-01		ENSG00000174407
MIR100HG	0.0803	3.2598E-01	5.3618E-01	ENSG00000255248
MIR101-1	0.0058	6.7595E-01		ENSG00000199135
MIR103A2	-0.0443	4.1237E-01	6.1608E-01	ENSG00000199024
MIR106AHG	0.0299	2.0466E-01		ENSG00000283638
MIR1179	0.0112	5.9340E-01		ENSG00000221630
MIR12136	0.0530	5.1724E-01	7.0464E-01	ENSG00000210151
MIR122HG	-0.0773	2.3226E-01	4.3724E-01	ENSG00000267391
MIR124-1HG	-0.0480	5.6891E-01	7.4328E-01	ENSG00000253230

MIR124-2HG	0.3141	1.9486E-03	1.4775E-02	ENSG00000254377
MIR1244-2	-0.2871	2.1366E-03	1.5802E-02	ENSG00000283498
MIR1244-3	-0.0516	5.4849E-01	7.2795E-01	ENSG00000283429
MIR1244-4	-0.0445	6.0301E-01	7.6765E-01	ENSG00000283475
MIR1252	-0.0337	1.8742E-01		ENSG00000221788
MIR125B1	0.1017	5.3473E-02	1.6705E-01	ENSG00000207971
MIR1268B	0.0058	7.2420E-01		ENSG00000265561
MIR1271	-0.0283	1.8224E-01		ENSG00000221464
MIR1276	0.0045	8.6960E-01		ENSG00000221634
MIR1277	0.0125	5.3833E-01		ENSG00000221463
MIR128-1	0.0002	8.8986E-01		ENSG00000207654
MIR128-2	-0.0137	6.7658E-01		ENSG00000207625
MIR1281	-0.0371	4.4504E-01		ENSG00000284015
MIR1285-1	0.0108	5.2274E-01		ENSG00000221520
MIR1289-1	-0.0303	1.1494E-01		ENSG00000221763
MIR1290	-0.0133	5.6864E-01		ENSG00000221662
MIR1296	-0.0683	2.5291E-01	4.6087E-01	ENSG00000221063
MIR130A	-0.0216	5.4556E-01		ENSG00000208009
MIR132	0.0129	7.4229E-01		ENSG00000267200
MIR1322	0.0073	7.5588E-01		ENSG00000283210
MIR133A1HG	0.5389	1.1830E-03	1.0296E-02	ENSG00000265142
MIR135B	0.0057	8.8104E-01		ENSG00000199059
MIR138-2	0.0030	8.4515E-01		ENSG00000207649
MIR149	0.0240	3.5386E-01		ENSG00000207611
MIR153-1	0.0184	3.8083E-01		ENSG00000207647
MIR155HG	0.0676	8.7888E-02		ENSG00000234883
MIR15B	0.0034	8.6459E-01		ENSG00000207779
MIR16-2	-0.0066	8.5156E-01		ENSG00000198987
MIR17HG	0.1276	8.9290E-02	2.3639E-01	ENSG00000215417
MIR181A2	0.0009	9.4661E-01		ENSG00000207595
MIR181A2HG	0.0509	5.5599E-01	7.3396E-01	ENSG00000224020
MIR181B1	0.0294	1.1853E-01		ENSG00000207975
MIR186	0.0018	8.0031E-01		ENSG00000207721
MIR191	-0.0192	4.9173E-01		ENSG00000207605
MIR1913	0.0020	9.8682E-01		ENSG00000222958
MIR1915	0.0166	3.9714E-01		ENSG00000222071
MIR1915HG	0.1656	6.2824E-02	1.8626E-01	ENSG00000204682
MIR193BHG	-0.2583	3.8231E-02	1.3245E-01	ENSG00000262454
MIR194-2HG	-0.0337	2.8192E-01		ENSG00000229719
MIR1972-1	-0.0023	9.6040E-01		ENSG00000238728
MIR1972-2	0.0045	9.2070E-01		ENSG00000239118
MIR202HG	0.0119	4.0514E-01		ENSG00000166917
MIR2052HG	-0.0306	4.5782E-01		ENSG00000254349
MIR205HG	0.0044	9.0517E-01		ENSG00000230937
MIR210HG	0.0791	2.4874E-01	4.5649E-01	ENSG00000247095
MIR2115	-0.0104	7.4283E-01		ENSG00000252466
MIR221	0.0138	4.5704E-01		ENSG00000207870
MIR2276	-0.4465	4.1149E-03	2.6088E-02	ENSG00000252695
MIR22HG	-0.0775	3.6050E-01	5.6993E-01	ENSG00000186594

MIR2467	-0.0002	9.1689E-01		ENSG00000264292
MIR25	0.0161	2.7906E-01		ENSG00000207547
MIR26A2	0.0003	7.2525E-01		ENSG00000207789
MIR301B	0.0011	8.9897E-01		ENSG00000212102
MIR302CHG	-0.0167	7.9625E-01		ENSG00000249532
MIR3115	-0.0151	5.4604E-01		ENSG00000263793
MIR3117	0.1339	2.0525E-02		ENSG00000264720
MIR3124	-0.0007	8.7102E-01		ENSG00000264500
MIR3125	0.0117	4.2023E-01		ENSG00000264370
MIR3128	-0.0064	7.6162E-01		ENSG00000265396
MIR3134	0.0072	7.3177E-01		ENSG00000264354
MIR3143	-0.0109	4.7757E-01		ENSG00000265565
MIR3146	-0.0091	7.3812E-01		ENSG00000265932
MIR3150BHG	0.0155	4.3095E-01		ENSG00000245080
MIR3153	0.0773	7.5818E-02		ENSG00000265112
MIR3167	0.0098	7.7128E-01		ENSG00000266215
MIR3171HG	0.1673	5.0695E-02	1.6105E-01	ENSG00000258932
MIR3174	-0.0135	7.2685E-01		ENSG00000265871
MIR3175	-0.0192	3.4578E-01		ENSG00000284324
MIR3178	-0.0130	4.8131E-01		ENSG00000266232
MIR3179-3	0.1279	2.8013E-02		ENSG00000266454
MIR3180-5	0.0182	4.0404E-01		ENSG00000264397
MIR3188	0.4519	1.2359E-02	5.9137E-02	ENSG00000267959
MIR3192	-0.0114	5.0239E-01		ENSG00000265137
MIR3198-1	0.0277	3.8691E-01		ENSG00000264757
MIR31HG	-0.0474	4.9466E-01	6.8670E-01	ENSG00000171889
MIR320A	-0.0252	3.4617E-01		ENSG00000208037
MIR320C1	-0.0031	8.7171E-01		ENSG00000221493
MIR324	0.0106	5.6889E-01		ENSG00000199053
MIR325	0.0000	8.7282E-01		ENSG00000207995
MIR325HG	0.2302	1.0222E-02	5.1478E-02	ENSG00000280870
MIR339	0.0176	8.3595E-01	9.1539E-01	ENSG00000199023
MIR345	0.0260	6.2560E-01		ENSG00000198984
MIR34AHG	-0.1979	6.0546E-02	1.8152E-01	ENSG00000228526
MIR3605	-0.0072	7.1724E-01		ENSG00000284154
MIR3609	0.0051	5.1768E-01		ENSG00000266019
MIR3648-1	0.0183	8.2815E-01	9.1103E-01	ENSG00000275708
MIR3648-2	-0.0063	9.2971E-01	9.6530E-01	ENSG00000264462
MIR3651	-0.0061	8.0082E-01		ENSG00000281156
MIR3660	0.0220	1.6536E-01		ENSG00000264342
MIR3662	0.0134	3.5216E-01		ENSG00000283409
MIR3663	0.0099	9.2217E-01		ENSG00000266782
MIR3663HG	0.0062	9.8015E-01		ENSG00000234474
MIR3667HG	-0.0017	8.8408E-01		ENSG00000188511
MIR3670-1	0.0078	5.1683E-01		ENSG00000263918
MIR3670-2	0.0078	5.1683E-01		ENSG00000264722
MIR3670-3	0.0078	5.1683E-01		ENSG00000265776
MIR3670-4	0.0078	5.1683E-01		ENSG00000274025
MIR3671	0.0251	7.6556E-01		ENSG00000265996

MIR3677	0.0239	2.9298E-01		ENSG00000266643
MIR3677HG	0.1650	8.4938E-02	2.2806E-01	ENSG00000260778
MIR3679	-0.0843	1.9718E-01	3.9479E-01	ENSG00000263813
MIR3680-1	-0.1852	1.6559E-02	7.2759E-02	ENSG00000265462
MIR3680-2	0.0156	6.5050E-01		ENSG00000266758
MIR3681HG	0.0284	7.0962E-01	8.4042E-01	ENSG00000224184
MIR3682	0.0965	1.5678E-01	3.4045E-01	ENSG00000265452
MIR3685	0.0577	1.8832E-01		ENSG00000265917
MIR378A	0.0569	1.0657E-01		ENSG00000199047
MIR378B	-0.0068	7.1047E-01		ENSG00000264534
MIR378D2HG	0.4236	5.3935E-03	3.1908E-02	ENSG00000264448
MIR378H	-0.0186	5.5090E-01		ENSG00000263361
MIR378J	0.0000	9.5349E-01		ENSG00000274620
MIR381HG	0.0372	8.9243E-02		ENSG00000223403
MIR3909	-0.0134	2.9908E-01		ENSG00000266320
MIR3922	-0.0068	8.8300E-01		ENSG00000264295
MIR3923	0.0052	6.6634E-01		ENSG00000265193
MIR3928	-0.0020	8.9052E-01		ENSG00000264141
MIR3929	0.0076	6.5159E-01		ENSG00000265641
MIR3935	-0.0050	8.5997E-01		ENSG00000265281
MIR3936HG	-0.2511	6.1103E-03	3.4972E-02	ENSG00000233006
MIR3939	0.0143	6.5360E-01		ENSG00000265828
MIR3943	0.0620	2.8223E-01	4.9265E-01	ENSG00000264069
MIR3944	-0.0200	5.3590E-01		ENSG00000265395
MIR3945HG	0.0447	2.3408E-01		ENSG00000251230
MIR3975	-0.0029	8.3999E-01		ENSG00000283514
MIR3976HG	0.0147	5.9067E-01		ENSG00000261738
MIR425	0.0194	6.7100E-01	8.1591E-01	ENSG00000199032
MIR4256	0.0172	4.4159E-01		ENSG00000283296
MIR4258	0.0329	5.2081E-01	7.0760E-01	ENSG00000264349
MIR4259	0.0179	7.3938E-01		ENSG00000266458
MIR4263	0.0279	5.2267E-01	7.0893E-01	ENSG00000265321
MIR4265	-0.0162	4.8958E-01		ENSG00000264934
MIR4284	-0.0397	2.1359E-01		ENSG00000265724
MIR4288	-0.0207	3.8170E-01		ENSG00000265251
MIR4292	-0.0123	6.4265E-01		ENSG00000265806
MIR4300HG	-0.0608	2.5763E-01	4.6618E-01	ENSG00000245832
MIR4307HG	0.0071	7.3021E-01		ENSG00000257612
MIR4319	-0.0049	8.4395E-01		ENSG00000265957
MIR4322	0.0194	6.0745E-01		ENSG00000264266
MIR4424	0.0050	9.8632E-01		ENSG00000266417
MIR4432HG	0.0122	4.5354E-01		ENSG00000228590
MIR4434	0.0360	5.6610E-01	7.4130E-01	ENSG00000283204
MIR4435-2HG	0.3166	2.9388E-03	2.0159E-02	ENSG00000172965
MIR4453HG	0.3087	3.5381E-03	2.3246E-02	ENSG00000268471
MIR4458HG	0.1625	3.9007E-02	1.3442E-01	ENSG00000247516
MIR4469	0.0101	5.0115E-01		ENSG00000284062
MIR4477A	0.0000	9.9850E-01		ENSG00000276029
MIR4477B	-0.0214	6.8990E-01	8.2772E-01	ENSG00000266017

MIR4482	0.1027	2.6813E-01	4.7755E-01	ENSG00000266852
MIR4484	0.0234	1.6371E-01		ENSG00000265092
MIR4485	-0.0481	2.1776E-01	4.1987E-01	ENSG00000283813
MIR4489	-0.0076	7.7535E-01		ENSG00000265874
MIR4500HG	0.0576	5.0687E-01	6.9620E-01	ENSG00000228824
MIR4505	-0.3735	1.0199E-02	5.1436E-02	ENSG00000264741
MIR4512	0.0164	8.4817E-01	9.2260E-01	ENSG00000266589
MIR4515	-0.0314	2.0044E-01		ENSG00000263643
MIR4522	0.0082	4.5861E-01		ENSG00000263583
MIR4527HG	-0.0116	3.3548E-01		ENSG00000267761
MIR4530	-0.0203	6.3242E-02		ENSG00000266559
MIR4633	-0.0233	2.3366E-01		ENSG00000264563
MIR4634	0.0107	5.3418E-01		ENSG00000266890
MIR4637	-0.0062	9.0517E-01		ENSG00000264792
MIR4641	-0.0031	6.2169E-01		ENSG00000266494
MIR4644	0.0004	9.5121E-01		ENSG00000266245
MIR4646	-0.0048	8.1436E-01		ENSG00000266776
MIR4663	-0.0063	8.2706E-01		ENSG00000266324
MIR4664	-0.0086	7.3953E-01		ENSG00000265660
MIR4670	-0.0211	4.2473E-01		ENSG00000264158
MIR4676	-0.0093	8.1933E-01		ENSG00000266719
MIR4677	-0.0035	9.4609E-01		ENSG00000265201
MIR4678	0.0520	7.6257E-02		ENSG00000283672
MIR4681	0.0035	8.6706E-01		ENSG00000265719
MIR4713HG	0.1483	6.1024E-02	1.8244E-01	ENSG00000259240
MIR4727	0.0641	2.7132E-01	4.8052E-01	ENSG00000274054
MIR4734	-0.0297	9.8841E-02		ENSG00000275238
MIR4740	0.0207	6.0089E-01		ENSG00000266392
MIR4748	-0.0063	8.1097E-01		ENSG00000265879
MIR4755	-0.5068	5.6064E-03	3.2797E-02	ENSG00000264616
MIR4760	-0.0027	9.5031E-01		ENSG00000263973
MIR4766	0.0065	6.4863E-01		ENSG00000266594
MIR4771-1	-0.0001	8.0895E-01		ENSG00000264793
MIR4771-2	0.0089	8.5669E-01		ENSG00000266063
MIR4777	0.0009	9.9201E-01		ENSG00000263641
MIR4786	-0.0015	8.6749E-01		ENSG00000264279
MIR4793	0.0228	2.0482E-01		ENSG00000284575
MIR4794	0.0175	5.2880E-01		ENSG00000264470
MIR4803	-0.0327	4.2716E-01		ENSG00000264099
MIR4804	0.0021	7.8446E-01		ENSG00000263593
MIR491	0.0338	6.2511E-01	7.8348E-01	ENSG00000207609
MIR497HG	-0.0148	5.1151E-01		ENSG00000267532
MIR4999	0.0339	4.8426E-01		ENSG00000265390
MIR5000	0.0050	9.6700E-01		ENSG00000263909
MIR5002	0.0067	9.7751E-01		ENSG00000266383
MIR5009	-0.0383	4.2098E-01		ENSG00000264796
MIR5010	0.0129	7.2411E-01		ENSG00000283929
MIR505	-0.0088	8.7448E-01		ENSG00000207633
MIR5087	0.0286	7.5714E-02		ENSG00000283676

MIR5089	0.0310	6.3213E-02		ENSG00000264999
MIR5091	0.0393	3.8973E-01		ENSG00000266240
MIR5094	-0.0008	9.9508E-01		ENSG00000264966
MIR5188	0.0119	5.0848E-01		ENSG00000265345
MIR5191	-0.0027	7.1111E-01		ENSG00000264802
MIR5194	0.0153	4.7762E-01		ENSG00000264653
MIR544B	0.0000	8.6686E-01		ENSG00000265981
MIR548AH	-0.0036	8.8645E-01		ENSG00000283682
MIR548AK	-0.0614	2.7866E-01	4.8852E-01	ENSG00000265653
MIR548AN	0.0097	6.1773E-01		ENSG00000263515
MIR548AR	0.0023	8.6002E-01		ENSG00000264539
MIR548AT	0.0058	9.3629E-01		ENSG00000264314
MIR548AV	-0.0140	5.0717E-01		ENSG00000263750
MIR548E	-0.0173	3.5578E-01		ENSG00000221214
MIR548F2	0.0004	7.9288E-01		ENSG00000221782
MIR548K	0.0118	5.2867E-01		ENSG00000221333
MIR548L	-0.0127	6.4016E-01		ENSG00000221230
MIR548O	-0.0342	5.2363E-01	7.0931E-01	ENSG00000221510
MIR548S	0.0133	6.5544E-01		ENSG00000265056
MIR548V	-0.0080	5.2645E-01		ENSG00000265520
MIR548XHG	0.0563	2.7673E-01	4.8673E-01	ENSG00000224141
MIR548Y	0.0025	9.6760E-01		ENSG00000263945
MIR553	-0.0126	8.4487E-01		ENSG00000207750
MIR556	-0.0010	9.8166E-01		ENSG00000207729
MIR5579	0.0014	9.5626E-01		ENSG00000266570
MIR558	0.0031	6.9577E-01		ENSG00000207653
MIR5581	-0.0176	5.5184E-01		ENSG00000263675
MIR5585	0.0480	3.1972E-01		ENSG00000266203
MIR5687	-0.0010	8.5896E-01		ENSG00000265135
MIR5695	0.0578	2.3870E-01		ENSG00000266721
MIR570	0.0145	7.6086E-01		ENSG00000207650
MIR573	0.0702	9.3235E-02		ENSG00000207697
MIR576	-0.0026	8.5820E-01		ENSG00000207988
MIR578	0.0389	3.8480E-01		ENSG00000207559
MIR5787	-0.0975	2.2462E-02		ENSG00000275334
MIR579	-0.0390	3.1089E-01		ENSG00000207956
MIR589	0.0026	9.7541E-01		ENSG00000207973
MIR590	-0.0739	1.1892E-01		ENSG00000207741
MIR593	-0.0260	2.1241E-01		ENSG00000207588
MIR600HG	0.2902	5.8237E-03	3.3787E-02	ENSG00000236901
MIR604	-0.0130	4.8994E-01		ENSG00000207612
MIR6077	0.0120	6.6836E-01		ENSG00000278596
MIR608	0.0165	4.7652E-01		ENSG00000207551
MIR6124	-0.0074	6.9757E-01		ENSG00000275373
MIR616	-0.0852	3.3097E-01	5.4112E-01	ENSG00000208028
MIR6165	0.0068	8.6392E-01		ENSG00000277478
MIR618	-0.0417	2.3790E-01		ENSG00000208022
MIR624	-0.0060	9.0510E-01		ENSG00000207952
MIR628	-0.0101	6.5039E-01		ENSG00000283891

MIR632	-0.0024	6.9589E-01		ENSG00000283774
MIR635	0.0134	7.6612E-01		ENSG00000207561
MIR644A	-0.0607	3.2404E-01	5.3405E-01	ENSG00000207997
MIR645	-0.0125	5.6314E-01		ENSG00000208018
MIR646HG	0.3942	1.4137E-02	6.5247E-02	ENSG00000228340
MIR647	0.0528	4.3592E-01	6.3680E-01	ENSG00000207554
MIR6512	-0.0027	7.4710E-01		ENSG00000278418
MIR6514	-0.0063	7.6254E-01		ENSG00000274066
MIR659	0.0589	2.7375E-01		ENSG00000207696
MIR663AHG	0.0362	4.4198E-01	6.4136E-01	ENSG00000227195
MIR670	0.0077	9.0486E-01		ENSG00000211568
MIR670HG	-0.0203	7.1893E-01	8.4549E-01	ENSG00000235661
MIR6724-1	-0.0021	7.2405E-01		ENSG00000275950
MIR6724-2	0.0190	3.5787E-01		ENSG00000274060
MIR6724-3	-0.0021	7.2405E-01		ENSG00000277379
MIR6724-4	-0.0006	9.9595E-01		ENSG00000275692
MIR6739	-0.0115	6.3920E-01		ENSG00000277681
MIR6740	0.0109	4.9725E-01		ENSG00000275207
MIR6746	-0.0006	6.7829E-01		ENSG00000277892
MIR6747	-0.0020	8.3276E-01		ENSG00000276102
MIR6772	-0.0137	4.0143E-01		ENSG00000274816
MIR6775	-0.0073	7.3677E-01		ENSG00000278598
MIR6781	-0.0298	4.6150E-01		ENSG00000278447
MIR6796	0.0005	6.2810E-01		ENSG00000275652
MIR6797	0.0067	9.3638E-01		ENSG00000276926
MIR6801	0.0138	6.7302E-01		ENSG00000274380
MIR6841	0.0081	6.1073E-01		ENSG00000283327
MIR6861	0.0079	7.6053E-01		ENSG00000283793
MIR7-3	0.0473	5.1264E-01	7.0055E-01	ENSG00000207630
MIR7-3HG	-0.0904	2.7060E-01	4.8008E-01	ENSG00000176840
MIR708	0.0565	3.6893E-01	5.7811E-01	ENSG00000211997
MIR7111	-0.0172	6.1655E-01		ENSG00000276712
MIR744	-0.0193	6.2920E-01	7.8650E-01	ENSG00000266297
MIR7515HG	-0.0061	8.3843E-01		ENSG00000236172
MIR760	0.0416	2.7266E-01		ENSG00000211575
MIR762HG	0.1947	1.8443E-02	7.8321E-02	ENSG00000260083
MIR766	0.0203	4.1224E-01		ENSG00000211578
MIR7845	0.0319	6.1617E-01	7.7736E-01	ENSG00000277590
MIR7856	0.1039	1.6524E-02		ENSG00000278281
MIR7974	0.0038	7.8470E-01		ENSG00000274713
MIR7978	0.0177	6.3220E-01		ENSG00000283281
MIR8063	-0.0413	5.6676E-01		ENSG00000277202
MIR8066	0.0058	8.4319E-01		ENSG00000273882
MIR8075	0.0024	9.8647E-01		ENSG00000277942
MIR8485	0.0655	1.4149E-01		ENSG00000216191
MIR9-1	0.0183	7.5649E-01		ENSG00000207933
MIR9-1HG	-0.4975	7.0221E-05	1.1678E-03	ENSG00000125462
MIR9-2HG	-0.0476	4.9803E-01	6.8916E-01	ENSG00000245526
MIR9-3	0.0229	1.7632E-01		ENSG00000284329

MIR9-3HG	0.0670	4.3792E-01	6.3837E-01	ENSG00000255571
MIR920	-0.0385	8.3635E-02		ENSG00000216192
MIR924HG	-0.0094	9.0784E-01	9.5461E-01	ENSG00000267374
MIR933	-0.0790	1.5763E-01		ENSG00000215973
MIR98	-0.0086	8.4221E-01	9.1872E-01	ENSG00000271886
MIR99AHG	0.0829	2.8704E-01	4.9775E-01	ENSG00000215386
MIRLET7A1HG	0.2275	4.6737E-02	1.5252E-01	ENSG00000269929
MIRLET7BHG	-0.1918	7.5022E-02	2.1055E-01	ENSG00000197182
MIRLET7G	-0.0042	5.6257E-01		ENSG00000199150
MIRLET7IHG	0.4383	9.2498E-04	8.5497E-03	ENSG00000257354
MIS12	0.1047	8.2046E-02	2.2289E-01	ENSG00000167842
MIS18A	0.1142	1.5496E-01	3.3810E-01	ENSG00000159055
MIS18BP1	-0.0376	6.4601E-01	7.9814E-01	ENSG00000129534
MISP	-0.1488	1.2828E-01	2.9994E-01	ENSG00000099812
MISP3	0.0499	5.1565E-01	7.0326E-01	ENSG00000141854
MITD1	0.2697	2.4127E-03	1.7333E-02	ENSG00000158411
MITF	-0.0458	5.7011E-01	7.4394E-01	ENSG00000187098
MIX23	0.0954	2.6109E-01	4.6963E-01	ENSG00000160124
MIXL1	-0.1694	9.1854E-02	2.4131E-01	ENSG00000185155
MKI67	-0.0349	6.4681E-01	7.9886E-01	ENSG00000148773
MKKS	-0.0410	5.2793E-01	7.1270E-01	ENSG00000125863
MKLN1	0.1570	1.2192E-02	5.8603E-02	ENSG00000128585
MKLN1-AS	0.0621	3.9238E-01	5.9933E-01	ENSG00000236753
MKNK1	0.0530	4.6143E-01	6.5872E-01	ENSG00000079277
MKNK1-AS1	0.0072	9.9280E-01		ENSG00000269956
MKNK2	-0.3458	3.6177E-04	4.1382E-03	ENSG00000099875
MKRN1	-0.2469	4.6230E-04	4.9978E-03	ENSG00000133606
MKRN2	-0.0681	3.3099E-01	5.4112E-01	ENSG00000075975
MKRN2OS	0.1722	8.6397E-02	2.3081E-01	ENSG00000225526
MKRN3	0.1936	3.3775E-02	1.2075E-01	ENSG00000179455
MKRN7P	0.0122	1.1787E-01		ENSG00000225849
MKS1	0.1348	1.0006E-01	2.5481E-01	ENSG00000011143
MKX	0.0751	3.5057E-01	5.6067E-01	ENSG00000150051
MLANA	0.0085	6.7608E-01		ENSG00000120215
MLC1	-0.3379	6.1225E-03	3.5018E-02	ENSG00000100427
MLEC	0.0651	3.3891E-01	5.4865E-01	ENSG00000110917
MLF1	0.0479	4.6045E-01	6.5803E-01	ENSG00000178053
MLF1-DT	0.5317	4.2385E-03	2.6618E-02	ENSG00000243150
MLF2	-0.1451	2.8669E-03	1.9792E-02	ENSG00000089693
MLH1	0.0236	6.8889E-01	8.2709E-01	ENSG00000076242
MLH3	0.2317	1.1037E-02	5.4454E-02	ENSG00000119684
MLIP	0.3277	1.6911E-02	7.3775E-02	ENSG00000146147
MLKL	0.0214	3.4338E-01		ENSG00000168404
MLLT1	0.1509	5.2192E-02	1.6409E-01	ENSG00000130382
MLLT10	-0.1201	1.3678E-01	3.1218E-01	ENSG00000078403
MLLT11	-0.3941	3.3205E-06	1.0099E-04	ENSG00000213190
MLLT3	-0.0136	8.3895E-01	9.1719E-01	ENSG00000171843
MLLT6	0.0787	3.4263E-01	5.5221E-01	ENSG00000275023
MLN	0.0035	8.3554E-01		ENSG00000096395

MLNR	0.0126	8.8443E-01		ENSG00000102539
MLPH	-0.0185	2.6821E-01		ENSG00000115648
MLST8	-0.0324	6.2151E-01	7.8105E-01	ENSG00000167965
MLX	0.1332	6.1681E-02	1.8394E-01	ENSG00000108788
MLXIP	0.0267	6.9342E-01	8.3001E-01	ENSG00000175727
MLXIPL	-0.0910	3.0946E-01	5.1971E-01	ENSG00000009950
MLYCD	0.2296	1.8310E-02	7.7917E-02	ENSG00000103150
MMAA	0.1193	1.3009E-01	3.0283E-01	ENSG00000151611
MMAB	-0.2181	8.1750E-04	7.7903E-03	ENSG00000139428
MMACHC	0.0168	8.5209E-01	9.2462E-01	ENSG00000132763
MMADHC	-0.2118	5.2370E-05	9.2371E-04	ENSG00000168288
MMADHC-DT	0.3704	1.1889E-02	5.7607E-02	ENSG00000231969
MMD	-0.1882	1.7227E-03	1.3491E-02	ENSG00000108960
MMD2	-0.2256	4.4839E-02	1.4808E-01	ENSG00000136297
MME	-0.0232	6.1811E-01		ENSG00000196549
MMEL1	-0.0953	2.8917E-01	4.9962E-01	ENSG00000142606
MMGT1	-0.2223	2.5211E-03	1.7861E-02	ENSG00000169446
MMP10	0.0303	6.4484E-01	7.9726E-01	ENSG00000166670
MMP11	-0.4941	6.8750E-09	6.0414E-07	ENSG00000099953
MMP12	0.0027	7.5190E-01		ENSG00000262406
MMP13	0.0023	8.7578E-01		ENSG00000137745
MMP14	-0.0368	6.5732E-01	8.0659E-01	ENSG00000157227
MMP15	0.0169	8.2934E-01	9.1170E-01	ENSG00000102996
MMP16	0.1026	1.9133E-01	3.8669E-01	ENSG00000156103
MMP17	-0.2135	2.4387E-02	9.5910E-02	ENSG00000198598
MMP19	0.0341	4.7819E-01	6.7349E-01	ENSG00000123342
MMP2	-0.2293	4.7731E-02	1.5464E-01	ENSG00000087245
MMP21	-0.0036	9.4729E-01	9.7396E-01	ENSG00000154485
MMP23A	0.1606	7.4001E-02	2.0859E-01	ENSG00000215914
MMP23B	0.2160	4.7810E-02	1.5473E-01	ENSG00000189409
MMP24	-0.0985	2.5661E-01	4.6501E-01	ENSG00000125966
MMP24OS	0.1829	5.0714E-02	1.6105E-01	ENSG00000126005
MMP25	0.0706	2.4960E-01	4.5742E-01	ENSG00000008516
MMP28	-0.0069	9.3394E-01	9.6728E-01	ENSG00000271447
MMP7	0.0272	1.8065E-01		ENSG00000137673
MMP9	0.0660	3.1088E-01	5.2105E-01	ENSG00000100985
MMRN2	0.0981	8.5478E-02	2.2886E-01	ENSG00000173269
MMS19	0.0644	3.7139E-01	5.8008E-01	ENSG00000155229
MMS22L	0.0793	3.1596E-01	5.2600E-01	ENSG00000146263
MMUT	0.0719	3.3791E-01	5.4787E-01	ENSG00000146085
MN1	-0.0758	3.4610E-01	5.5612E-01	ENSG00000169184
MNAT1	0.1705	4.7333E-03	2.8870E-02	ENSG00000020426
MND1	0.0291	6.0458E-01	7.6858E-01	ENSG00000121211
MNS1	-0.0953	2.6237E-01	4.7124E-01	ENSG00000138587
MNT	0.1301	1.3025E-01	3.0303E-01	ENSG00000070444
MNX1	-0.2709	2.8730E-02	1.0778E-01	ENSG00000130675
MNX1-AS1	0.0500	6.8054E-01	8.2142E-01	ENSG00000243479
MNX1-AS2	-0.0150	4.3044E-01		ENSG00000235029
MOAP1	-0.2110	2.3933E-03	1.7228E-02	ENSG00000165943

MOB1A	-0.1033	4.5026E-02	1.4845E-01	ENSG00000114978
MOB1B	0.1593	1.7918E-02	7.6848E-02	ENSG00000173542
MOB2	-0.1269	7.6918E-02	2.1395E-01	ENSG00000182208
MOB3A	-0.0064	9.1284E-01	9.5707E-01	ENSG00000172081
MOB3B	-0.5526	1.6020E-07	8.3865E-06	ENSG00000120162
MOB3C	0.0404	5.7554E-01	7.4808E-01	ENSG00000142961
MOB4	-0.2880	8.4816E-06	2.1426E-04	ENSG00000115540
MOBP	0.0844	1.7853E-02		ENSG00000168314
MOCOS	-0.0263	7.4114E-01	8.5894E-01	ENSG00000075643
MOCS1	-0.0423	6.0885E-01	7.7193E-01	ENSG00000124615
MOCS2	-0.0907	6.4028E-02	1.8883E-01	ENSG00000164172
MOCS2-DT	-0.1014	2.4573E-01	4.5348E-01	ENSG00000247796
MOCS3	-0.1293	9.0978E-02	2.3969E-01	ENSG00000124217
MOG	0.0024	9.6102E-01		ENSG00000204655
MOGAT1	0.0334	5.1089E-01		ENSG00000124003
MOGAT3	-0.0123	3.2563E-01		ENSG00000106384
MOGS	0.2393	5.5064E-04	5.7360E-03	ENSG00000115275
MON1A	-0.0207	7.4093E-01	8.5887E-01	ENSG00000164077
MON1B	0.1343	5.7758E-02	1.7617E-01	ENSG00000103111
MON2	0.1854	3.4807E-03	2.2949E-02	ENSG00000061987
MORC1	0.0015	9.6114E-01		ENSG00000114487
MORC2	0.0713	2.8030E-01	4.9027E-01	ENSG00000133422
MORC2-AS1	-0.0203	7.7446E-01	8.8003E-01	ENSG00000235989
MORC3	-0.1872	1.5181E-03	1.2344E-02	ENSG00000159256
MORC4	-0.0688	3.2405E-01	5.3405E-01	ENSG00000133131
MORF4L1	-0.2690	9.7486E-07	3.6398E-05	ENSG00000185787
MORF4L1P4	-0.0057	6.5809E-01		ENSG00000225133
MORF4L2	-0.1642	2.1214E-02	8.6590E-02	ENSG00000123562
MORN1	0.0527	5.3047E-01	7.1490E-01	ENSG00000116151
MORN2	0.0277	5.8886E-01	7.5749E-01	ENSG00000188010
MORN3	0.2545	4.6217E-03	2.8375E-02	ENSG00000139714
MORN4	0.2151	3.5446E-03	2.3276E-02	ENSG00000171160
MORN5	0.0696	4.2030E-01	6.2325E-01	ENSG00000185681
MOS	0.0573	2.1349E-01		ENSG00000172680
MOSMO	0.4083	8.3087E-04	7.8660E-03	ENSG00000185716
MOSPD1	-0.1601	1.7429E-02	7.5407E-02	ENSG00000101928
MOSPD2	0.2232	1.0158E-02	5.1297E-02	ENSG00000130150
MOSPD3	0.1610	2.4604E-02	9.6489E-02	ENSG00000106330
MOV10	-0.0290	7.1542E-01	8.4351E-01	ENSG00000155363
MOXD1	-0.1555	1.1229E-01	2.7484E-01	ENSG00000079931
MPC1	0.1914	2.7315E-02	1.0396E-01	ENSG00000060762
MPC2	-0.3276	1.3207E-05	3.0399E-04	ENSG00000143158
MPDU1	-0.0893	1.1196E-01	2.7428E-01	ENSG00000129255
MPDU1-AS1	0.2343	2.9687E-02	1.1034E-01	ENSG00000233223
MPDZ	-0.1520	5.1370E-02	1.6240E-01	ENSG00000107186
MPEG1	0.0117	5.5033E-01		ENSG00000197629
MPG	-0.1052	1.0629E-01	2.6508E-01	ENSG00000103152
MPHOSPH10	-0.1292	4.0824E-02	1.3872E-01	ENSG00000124383
MPHOSPH10P3	-0.0097	8.2980E-01	9.1190E-01	ENSG00000283345

MPHOSPH6	0.0068	9.2654E-01	9.6404E-01	ENSG00000135698
MPHOSPH8	0.1004	9.0537E-02	2.3904E-01	ENSG00000196199
MPHOSPH9	-0.1159	1.2738E-01	2.9880E-01	ENSG00000051825
MPI	0.0438	5.1031E-01	6.9892E-01	ENSG00000178802
MPIG6B	-0.2494	3.9821E-02	1.3639E-01	ENSG00000204420
MPL	-0.0282	5.1797E-01		ENSG00000117400
MPLKIP	0.1186	1.3692E-01	3.1235E-01	ENSG00000168303
MPND	-0.3384	1.4877E-03	1.2194E-02	ENSG00000008382
MPO	0.0531	6.9101E-02		ENSG00000005381
MPP1	-0.1722	1.4586E-02	6.6645E-02	ENSG00000130830
MPP2	0.0471	4.9876E-01	6.8975E-01	ENSG00000108852
MPP3	-0.1348	4.1531E-02	1.4044E-01	ENSG00000161647
MPP4	-0.0169	7.3732E-01		ENSG00000082126
MPP7	0.4955	1.2070E-05	2.8391E-04	ENSG00000150054
MPPE1	0.0746	1.8314E-01	3.7635E-01	ENSG00000154889
MPPED1	-0.4171	1.4137E-04	1.9832E-03	ENSG00000186732
MPPED2	-0.0014	9.8549E-01	9.9235E-01	ENSG00000066382
MPPED2-AS1	-0.0380	6.5049E-01	8.0185E-01	ENSG00000254489
MPRIP	-0.1000	1.7822E-01	3.6944E-01	ENSG00000133030
MPST	-0.0685	1.8271E-01	3.7583E-01	ENSG00000128309
MPV17	-0.1289	2.4416E-02	9.5950E-02	ENSG00000115204
MPV17L	-0.0196	8.0604E-01	8.9800E-01	ENSG00000156968
MPV17L	-0.0126	4.5144E-01		ENSG00000261130
MPV17L2	0.1641	5.9512E-02	1.7934E-01	ENSG00000254858
MPZ	-0.0641	3.3504E-01	5.4508E-01	ENSG00000158887
MPZL1	-0.0299	6.3428E-01	7.9032E-01	ENSG00000197965
MPZL2	0.2352	1.3453E-02	6.2978E-02	ENSG00000149573
MPZL3	0.0822	3.5716E-01	5.6697E-01	ENSG00000160588
MR1	0.1394	4.4021E-02	1.4601E-01	ENSG00000153029
MRAP2	0.0212	7.8541E-01	8.8655E-01	ENSG00000135324
MRAS	-0.1358	5.3129E-02	1.6625E-01	ENSG00000158186
MRC1	0.0426	1.3922E-01		ENSG00000260314
MRC2	0.0943	2.7893E-01	4.8885E-01	ENSG00000011028
MRE11	-0.0919	2.1265E-01	4.1365E-01	ENSG00000020922
MREG	-0.0239	7.7342E-01	8.7911E-01	ENSG00000118242
MRFAP1	-0.1861	4.1410E-05	7.6897E-04	ENSG00000179010
MRFAP1L1	0.0678	2.1306E-01	4.1411E-01	ENSG00000178988
MRFAP1L2	0.2605	9.6645E-04	8.8170E-03	ENSG00000170846
MRGBP	0.0786	2.3610E-01	4.4183E-01	ENSG00000101189
MRGPRE	-0.0043	8.9158E-01		ENSG00000184350
MRGPRF	0.0267	5.9311E-01	7.6039E-01	ENSG00000172935
MRI1	-0.2441	7.3330E-03	4.0188E-02	ENSG00000037757
MRLN	0.0007	5.7353E-01		ENSG00000227877
MRM1	-0.0278	6.7310E-01	8.1708E-01	ENSG00000278619
MRM2	0.0729	3.9112E-01	5.9811E-01	ENSG00000122687
MRM3	-0.0662	3.0084E-01	5.1071E-01	ENSG00000171861
MRNIP	0.1334	9.0655E-02	2.3923E-01	ENSG00000161010
MRNIP-DT	0.1328	1.1929E-01	2.8632E-01	ENSG00000245317
MRO	-0.2474	3.3953E-02	1.2107E-01	ENSG00000134042

MROCKI	0.0165	7.6147E-01		ENSG00000227502
MROH1	0.1544	8.3485E-02	2.2566E-01	ENSG00000179832
MROH2A	0.0012	6.9757E-01		ENSG00000185038
MROH2B	0.0013	9.8261E-01		ENSG00000171495
MROH3P	0.0407	8.7813E-02		ENSG00000233217
MROH5	-0.0239	1.3643E-01		ENSG00000226807
MROH6	0.0143	8.7604E-01	9.3803E-01	ENSG00000204839
MROH7	0.0318	1.4783E-01		ENSG00000184313
MROH8	0.2403	1.9639E-02	8.1930E-02	ENSG00000101353
MRPL1	-0.3481	9.1073E-06	2.2727E-04	ENSG00000169288
MRPL10	0.0471	4.5303E-01	6.5178E-01	ENSG00000159111
MRPL11	-0.1535	1.3766E-02	6.4003E-02	ENSG00000174547
MRPL12	-0.2900	1.5181E-04	2.1032E-03	ENSG00000262814
MRPL13	0.0611	3.5672E-01	5.6652E-01	ENSG00000172172
MRPL14	0.0253	5.9473E-01	7.6176E-01	ENSG00000180992
MRPL15	0.0408	4.7595E-01	6.7141E-01	ENSG00000137547
MRPL16	-0.1066	7.1871E-02	2.0453E-01	ENSG00000166902
MRPL17	0.1591	3.0623E-02	1.1288E-01	ENSG00000158042
MRPL18	-0.0462	4.6538E-01	6.6197E-01	ENSG00000112110
MRPL19	-0.0246	6.5522E-01	8.0518E-01	ENSG00000115364
MRPL2	-0.0135	8.3011E-01	9.1211E-01	ENSG00000112651
MRPL20	0.0833	2.0306E-01	4.0199E-01	ENSG00000242485
MRPL20-AS1	0.0335	6.6134E-01	8.0921E-01	ENSG00000224870
MRPL21	-0.0813	1.8939E-01	3.8445E-01	ENSG00000197345
MRPL22	-0.0142	8.0916E-01	8.9970E-01	ENSG00000082515
MRPL23	0.0948	1.2194E-01	2.9069E-01	ENSG00000276345
MRPL23	0.0963	1.4672E-01	3.2636E-01	ENSG00000214026
MRPL23-AS1	-0.0305	1.4477E-01		ENSG00000226416
MRPL24	-0.2227	1.0572E-04	1.6055E-03	ENSG00000143314
MRPL27	0.2308	1.4338E-03	1.1870E-02	ENSG00000108826
MRPL28	-0.1037	6.0748E-02	1.8187E-01	ENSG00000086504
MRPL3	0.0745	2.7270E-01	4.8213E-01	ENSG00000114686
MRPL30	-0.0405	5.4718E-01	7.2700E-01	ENSG00000185414
MRPL32	-0.1361	2.0676E-02	8.4929E-02	ENSG00000106591
MRPL33	0.0063	9.1892E-01	9.5996E-01	ENSG00000243147
MRPL34	-0.1512	1.1980E-02	5.7902E-02	ENSG00000130312
MRPL35	0.1053	1.2909E-01	3.0136E-01	ENSG00000132313
MRPL35P2	0.0077	9.1819E-01		ENSG00000232075
MRPL36	-0.1198	6.6827E-02	1.9464E-01	ENSG00000171421
MRPL37	-0.1437	1.1595E-02	5.6538E-02	ENSG00000116221
MRPL38	0.0266	7.4819E-01	8.6319E-01	ENSG00000204316
MRPL39	0.1638	2.8962E-02	1.0833E-01	ENSG00000154719
MRPL4	-0.0108	8.3431E-01	9.1493E-01	ENSG00000105364
MRPL40	-0.0917	1.5684E-01	3.4055E-01	ENSG00000185608
MRPL40P1	0.0232	5.8523E-01		ENSG00000256037
MRPL41	-0.2115	7.3304E-03	4.0188E-02	ENSG00000182154
MRPL42	-0.0459	3.9465E-01	6.0085E-01	ENSG00000198015
MRPL43	-0.1314	3.3786E-02	1.2075E-01	ENSG00000055950
MRPL44	-0.4816	2.1233E-14	1.2742E-11	ENSG00000135900

MRPL45	-0.0765	2.5441E-01	4.6245E-01	ENSG00000278845
MRPL46	0.0786	3.3173E-01	5.4202E-01	ENSG00000259494
MRPL47	-0.0543	3.1466E-01	5.2468E-01	ENSG00000136522
MRPL48	-0.0940	1.0170E-01	2.5762E-01	ENSG00000175581
MRPL49	-0.1450	2.4123E-02	9.5272E-02	ENSG00000149792
MRPL50	0.0302	6.7587E-01	8.1828E-01	ENSG00000136897
MRPL51	-0.0292	6.5183E-01	8.0290E-01	ENSG00000111639
MRPL52	-0.1776	3.4546E-03	2.2794E-02	ENSG00000172590
MRPL53P1	-0.1097	2.1330E-01	4.1434E-01	ENSG00000235299
MRPL54	-0.0803	2.0474E-01	4.0395E-01	ENSG00000183617
MRPL55	-0.0805	2.2872E-01	4.3327E-01	ENSG00000162910
MRPL57	-0.0001	9.7204E-01	9.8602E-01	ENSG00000173141
MRPL57P3	0.0139	4.3499E-01		ENSG00000275940
MRPL58	0.0058	9.3460E-01	9.6780E-01	ENSG00000167862
MRPL9	-0.3152	1.3437E-05	3.0813E-04	ENSG00000143436
MRPS10	-0.1231	6.0926E-02	1.8222E-01	ENSG00000048544
MRPS10P1	0.0097	8.8587E-01		ENSG00000235378
MRPS11	0.0576	2.5074E-01	4.5893E-01	ENSG00000181991
MRPS12	-0.0530	4.0997E-01	6.1422E-01	ENSG00000128626
MRPS14	-0.0657	2.8893E-01	4.9937E-01	ENSG00000120333
MRPS15	-0.1097	6.2095E-02	1.8495E-01	ENSG00000116898
MRPS16	-0.1686	1.6902E-03	1.3311E-02	ENSG00000182180
MRPS17	-0.1415	2.0312E-02	8.3870E-02	ENSG00000239789
MRPS18A	-0.1367	6.4152E-02	1.8910E-01	ENSG00000096080
MRPS18B	0.1002	1.5577E-01	3.3944E-01	ENSG00000204568
MRPS18C	0.0871	2.1337E-01	4.1436E-01	ENSG00000163319
MRPS18CP7	0.0092	4.2031E-01		ENSG00000235916
MRPS2	-0.0907	1.4066E-01	3.1738E-01	ENSG00000122140
MRPS21	0.0051	9.1400E-01	9.5772E-01	ENSG00000266472
MRPS22	0.0017	9.9520E-01	9.9739E-01	ENSG00000175110
MRPS23	-0.0092	8.6903E-01	9.3422E-01	ENSG00000181610
MRPS24	-0.1061	2.4768E-01	4.5550E-01	ENSG00000062582
MRPS25	0.0262	7.0018E-01	8.3428E-01	ENSG00000131368
MRPS26	-0.0230	6.1045E-01	7.7295E-01	ENSG00000125901
MRPS27	0.0293	6.2545E-01	7.8374E-01	ENSG00000113048
MRPS28	0.0350	6.0784E-01	7.7116E-01	ENSG00000147586
MRPS30	-0.3511	1.4884E-10	2.2746E-08	ENSG00000112996
MRPS31	0.0330	6.3473E-01	7.9048E-01	ENSG00000102738
MRPS31P1	-0.0057	4.8267E-01		ENSG00000275256
MRPS33	0.0642	3.1871E-01	5.2899E-01	ENSG00000090263
MRPS34	-0.2195	3.2854E-04	3.8476E-03	ENSG00000074071
MRPS35	-0.1915	8.6404E-04	8.1268E-03	ENSG00000061794
MRPS36	-0.0694	2.5682E-01	4.6532E-01	ENSG00000134056
MRPS5	-0.0233	7.7721E-01	8.8138E-01	ENSG00000144029
MRPS6	-0.1066	1.3298E-01	3.0663E-01	ENSG00000243927
MRPS6P1	-0.0457	4.2881E-01	6.3061E-01	ENSG00000270733
MRPS7	-0.0717	2.1646E-01	4.1812E-01	ENSG00000125445
MRPS9	0.1848	3.3536E-02	1.2027E-01	ENSG00000135972
MRPS9-AS1	0.0615	2.8995E-01	5.0035E-01	ENSG00000231851

MRPS9-AS2	-0.0633	3.8390E-01	5.9203E-01	ENSG00000224509
MRRF	0.2226	7.2727E-04	7.1515E-03	ENSG00000148187
MRS2	0.1075	8.5147E-02	2.2837E-01	ENSG00000124532
MRTFA	-0.0111	8.6351E-01	9.3122E-01	ENSG00000196588
MRTFA-AS1	-0.1179	1.4533E-01	3.2410E-01	ENSG00000232564
MRTFB	0.2305	9.0918E-04	8.4417E-03	ENSG00000186260
MRTO4	-0.1174	1.4839E-02	6.7461E-02	ENSG00000053372
MS4A10	-0.0043	6.9084E-01		ENSG00000172689
MS4A12	-0.0018	7.1268E-01		ENSG00000071203
MS4A2	-0.0175	3.1357E-01		ENSG00000149534
MS4A3	0.0832	2.1274E-02		ENSG00000149516
MS4A4A	0.0103	4.6753E-01		ENSG00000110079
MS4A4E	-0.0065	6.3226E-01		ENSG00000214787
MS4A6A	3.8051	2.6870E-04		ENSG00000110077
MS4A6E	0.0339	4.0786E-01		ENSG00000166926
MS4A7	0.0965	5.5625E-02	1.7201E-01	ENSG00000166927
MS4A8	0.0981	1.3587E-01	3.1072E-01	ENSG00000166959
MSANTD2	0.3994	1.9958E-04	2.5883E-03	ENSG00000120458
MSANTD2-AS1	-0.1105	1.7683E-01	3.6810E-01	ENSG00000245498
MSANTD3	-0.0294	5.6364E-01	7.3991E-01	ENSG00000066697
MSANTD3-TMEFF1	-0.0293	2.5087E-01		ENSG00000251349
MSANTD4	0.2484	2.6084E-04	3.2267E-03	ENSG00000170903
MSANTD7	-0.1907	4.8477E-03	2.9375E-02	ENSG00000284024
MSC	-0.1323	1.3107E-01	3.0410E-01	ENSG00000178860
MSC-AS1	-0.0706	2.6640E-01	4.7571E-01	ENSG00000235531
MSGN1	0.0087	6.1812E-01		ENSG00000151379
MSH2	-0.0511	4.9066E-01	6.8334E-01	ENSG00000095002
MSH2-OT1	-0.0165	6.4075E-01		ENSG00000235760
MSH3	-0.0564	3.9593E-01	6.0197E-01	ENSG00000113318
MSH4	-0.0343	3.9998E-01		ENSG00000057468
MSH5	-0.0476	5.7253E-01	7.4582E-01	ENSG00000204410
MSH5-SAPCD1	-0.0283	8.6852E-02		ENSG00000255152
MSH6	-0.1970	6.0452E-03	3.4696E-02	ENSG00000116062
MSI1	-0.1009	2.3502E-01	4.4039E-01	ENSG00000135097
MSI2	0.1630	5.8731E-02	1.7803E-01	ENSG00000153944
MSL1	0.0062	9.4253E-01	9.7152E-01	ENSG00000188895
MSL2	-0.3324	9.6629E-05	1.5038E-03	ENSG00000174579
MSL3	0.2831	1.6743E-04	2.2610E-03	ENSG00000005302
MSL3P1	0.0626	4.6016E-01	6.5789E-01	ENSG00000224287
MSLNL	0.0463	2.0945E-01		ENSG00000162006
MSMB	-0.0164	1.7557E-01		ENSG00000263639
MSMO1	-0.1639	7.9548E-02	2.1881E-01	ENSG00000052802
MSN	-0.2647	1.1035E-03	9.7525E-03	ENSG00000147065
MSR1	0.0005	6.9567E-01		ENSG00000038945
MSRB1	-0.0592	3.8213E-01	5.9030E-01	ENSG00000198736
MSRB2	-0.2607	1.1073E-04	1.6653E-03	ENSG00000148450
MSRB3	-0.1326	1.4424E-01	3.2242E-01	ENSG00000174099
MSS51	0.1394	1.1104E-01	2.7289E-01	ENSG00000166343
MST1	0.5848	5.1205E-08	3.2982E-06	ENSG00000173531

MST1L	0.3994	1.1289E-02	5.5408E-02	ENSG00000186715
MST1R	0.0136	6.6422E-01		ENSG00000164078
MSTN	-0.0276	6.9084E-01	8.2835E-01	ENSG00000138379
MSTO1	0.1966	1.0845E-02	5.3765E-02	ENSG00000125459
MSX1	-0.1259	1.7177E-01	3.6095E-01	ENSG00000163132
MSX2	-0.0405	5.9113E-01	7.5904E-01	ENSG00000120149
MT1A	0.0397	5.6543E-01	7.4090E-01	ENSG00000205362
MT1E	-0.0387	6.3603E-01	7.9127E-01	ENSG00000169715
MT1F	-0.1574	3.0034E-02	1.1133E-01	ENSG00000198417
MT1G	0.0382	5.6456E-01	7.4050E-01	ENSG00000125144
MT1H	0.0589	4.5727E-01	6.5547E-01	ENSG00000205358
MT1M	-0.0930	2.9110E-01	5.0140E-01	ENSG00000205364
MT1P3	-0.0123	4.9678E-01		ENSG00000229230
MT1X	0.0723	4.0121E-01	6.0633E-01	ENSG00000187193
MT2A	-0.0659	4.2705E-01	6.2907E-01	ENSG00000125148
MT3	-0.2096	1.7885E-02	7.6717E-02	ENSG00000087250
MTA1	-0.3021	1.7659E-03	1.3741E-02	ENSG00000182979
MTA1-DT	-0.1058	2.4327E-01	4.5110E-01	ENSG00000251602
MTA2	-0.1075	1.6909E-01	3.5760E-01	ENSG00000149480
MTA3	-0.0184	8.0106E-01	8.9502E-01	ENSG00000057935
MTAP	0.1269	1.5077E-01	3.3195E-01	ENSG00000099810
MTARC1	0.2113	3.6356E-02	1.2741E-01	ENSG00000186205
MTARC2	-0.0575	5.0233E-01	6.9282E-01	ENSG00000117791
MTATP6P1	0.0606	4.2374E-01	6.2605E-01	ENSG00000248527
MTATP6P19	0.0040	8.0590E-01		ENSG00000228316
MTATP6P2	0.0041	9.6267E-01		ENSG00000270307
MTATP6P27	0.0507	5.3118E-01	7.1528E-01	ENSG00000234003
MTATP8P1	0.2443	4.5788E-02	1.5046E-01	ENSG00000240409
MTATP8P2	0.0194	5.8689E-01		ENSG00000229604
MTBP	0.1692	4.1256E-02	1.3982E-01	ENSG00000172167
MTCH1	-0.2970	1.1114E-05	2.6699E-04	ENSG00000137409
MTCH2	-0.1619	9.2589E-04	8.5539E-03	ENSG00000109919
MTCL1	0.3176	1.3056E-04	1.8853E-03	ENSG00000168502
MTCL2	0.0601	4.0522E-01	6.0966E-01	ENSG00000149639
MTCL3	-0.0178	7.8824E-01	8.8801E-01	ENSG00000214338
MTCO1P11	0.0131	6.6442E-01		ENSG00000224083
MTCO1P12	0.0684	4.2081E-01	6.2369E-01	ENSG00000237973
MTCO1P19	0.0377	1.7655E-01		ENSG00000237300
MTCO1P22	0.0210	1.2663E-01		ENSG00000271207
MTCO1P27	0.0122	3.6375E-01		ENSG00000232579
MTCO1P28	0.0015	9.9311E-01		ENSG00000262118
MTCO1P30	-0.0086	5.6316E-01		ENSG00000248729
MTCO1P40	0.1310	1.2929E-01	3.0167E-01	ENSG00000262902
MTCO1P42	0.0049	2.4102E-01		ENSG00000230327
MTCO1P53	0.0133	4.1024E-01		ENSG00000230916
MTCO1P54	0.0044	6.5231E-01		ENSG00000233311
MTCO2P11	0.0865	9.7187E-02		ENSG00000235917
MTCO2P12	-0.0819	2.7528E-01	4.8516E-01	ENSG00000229344
MTCO2P17	-0.0105	7.7990E-01		ENSG00000233888

MTCO2P19	-0.0020	8.1678E-01		ENSG00000237628
MTCO2P2	-0.0621	4.0109E-01	6.0633E-01	ENSG00000267541
MTCO2P22	-0.0158	7.5566E-01		ENSG00000270225
MTCO2P27	-0.0164	8.9075E-01		ENSG00000230321
MTCO3P11	0.0129	8.0853E-01		ENSG00000237711
MTCO3P12	0.0751	3.5348E-01	5.6354E-01	ENSG00000198744
MTCO3P29	-0.0164	4.5049E-01		ENSG00000228069
MTCP1	-0.0153	8.1553E-01	9.0400E-01	ENSG00000214827
MTCYBP18	0.0591	3.4009E-01	5.4984E-01	ENSG00000244921
MTCYBP21	-0.0054	8.8880E-01		ENSG00000224747
MTCYBP28	0.0374	3.3320E-01		ENSG00000260161
MTCYBP3	-0.0480	3.8730E-01		ENSG00000232373
MTCYBP32	-0.0658	2.0313E-01	4.0206E-01	ENSG00000229794
MTCYBP39	0.0184	2.6312E-01		ENSG00000226341
MTCYBP43	0.0106	3.4769E-01		ENSG00000250624
MTDH	-0.0314	5.9290E-01	7.6032E-01	ENSG00000147649
MTDHP1	-0.0043	9.3100E-01	9.6587E-01	ENSG00000270948
MTERF1	0.0707	3.0034E-01	5.1017E-01	ENSG00000127989
MTERF2	-0.1204	1.1401E-01	2.7782E-01	ENSG00000120832
MTERF3	-0.1138	1.5652E-01	3.4030E-01	ENSG00000156469
MTERF4	0.1149	3.8993E-02	1.3439E-01	ENSG00000122085
MTF1	0.0628	3.6086E-01	5.7037E-01	ENSG00000188786
MTF2	-0.0345	5.6157E-01	7.3847E-01	ENSG00000143033
MTFMT	0.0377	5.6545E-01	7.4090E-01	ENSG00000103707
MTFP1	-0.0371	6.5942E-01	8.0786E-01	ENSG00000242114
MTFR1	0.0674	4.2312E-01	6.2580E-01	ENSG00000066855
MTFR1L	-0.0279	6.4457E-01	7.9709E-01	ENSG00000117640
MTFR2	-0.0325	7.0461E-01	8.3708E-01	ENSG00000146410
MTG1	0.1610	6.7868E-02	1.9645E-01	ENSG00000148824
MTG2	0.1923	9.5739E-03	4.9158E-02	ENSG00000101181
MTHFD1	-0.2156	8.1435E-03	4.3465E-02	ENSG00000100714
MTHFD1L	0.1933	1.1157E-02	5.4902E-02	ENSG00000120254
MTHFD1P1	0.0110	9.1420E-01		ENSG00000231831
MTHFD2	-0.1079	1.3470E-01	3.0916E-01	ENSG00000065911
MTHFD2L	0.1432	6.6469E-02	1.9396E-01	ENSG00000163738
MTHFR	0.2419	2.3598E-03	1.7027E-02	ENSG00000177000
MTHFS	-0.0505	5.1586E-01	7.0345E-01	ENSG00000136371
MTHFSD	-0.1799	4.6049E-02	1.5097E-01	ENSG00000103248
MTIF2	-0.0017	9.6511E-01	9.8281E-01	ENSG00000085760
MTIF3	0.0678	2.9828E-01	5.0815E-01	ENSG00000122033
MTLN	0.1911	2.0122E-02	8.3406E-02	ENSG00000175701
MTM1	-0.1157	1.4042E-01	3.1701E-01	ENSG00000171100
MTMR1	-0.2149	7.8644E-03	4.2333E-02	ENSG00000063601
MTMR10	0.3434	4.9657E-04	5.2801E-03	ENSG00000166912
MTMR11	-0.0560	4.8431E-01	6.7822E-01	ENSG00000014914
MTMR12	0.0540	4.3594E-01	6.3680E-01	ENSG00000150712
MTMR14	0.0079	9.0213E-01	9.5204E-01	ENSG00000163719
MTMR2	0.0726	2.5455E-01	4.6260E-01	ENSG00000087053
MTMR3	0.1204	9.7168E-02	2.5043E-01	ENSG00000100330

MTMR4	-0.0215	6.4393E-01	7.9679E-01	ENSG00000108389
MTMR6	0.2249	2.0434E-03	1.5310E-02	ENSG00000139505
MTMR7	0.4737	1.1817E-10	1.9514E-08	ENSG00000003987
MTMR8	-0.1432	1.3298E-01	3.0663E-01	ENSG00000102043
MTMR9	0.0329	5.0155E-01	6.9215E-01	ENSG00000104643
MTND1P11	0.0228	4.3573E-01		ENSG00000228166
MTND1P23	0.1644	2.9621E-02	1.1016E-01	ENSG00000225972
MTND1P32	-0.0271	4.0070E-01		ENSG00000232282
MTND1P36	0.0015	9.3210E-01		ENSG00000253245
MTND1P37	0.0156	8.9164E-01		ENSG00000283456
MTND1P6	0.0014	5.2067E-01		ENSG00000254346
MTND1P8	0.0182	5.8792E-01		ENSG00000263177
MTND1P9	-0.0347	2.2647E-01		ENSG00000228995
MTND2P11	-0.0064	7.6533E-01		ENSG00000237124
MTND2P12	-0.0091	4.2026E-01		ENSG00000228725
MTND2P2	0.0634	2.1791E-01	4.2005E-01	ENSG00000229954
MTND2P28	0.2213	3.7611E-03	2.4372E-02	ENSG00000225630
MTND2P32	-0.0011	7.1159E-01		ENSG00000254090
MTND2P40	-0.0505	3.3188E-01	5.4223E-01	ENSG00000236483
MTND3P23	-0.0521	1.1509E-01		ENSG00000238288
MTND3P25	-0.0082	5.1788E-01		ENSG00000249192
MTND4LP1	-0.0255	1.2587E-01		ENSG00000231501
MTND4LP30	-0.0589	1.9626E-01	3.9361E-01	ENSG00000198868
MTND4LP7	-1.1365	7.8591E-03		ENSG00000240791
MTND4P10	0.0033	9.8585E-01		ENSG00000226663
MTND4P12	0.1772	8.3027E-02	2.2471E-01	ENSG00000247627
MTND4P14	0.0058	7.5250E-01		ENSG00000236254
MTND4P15	-0.1011	1.7234E-01	3.6190E-01	ENSG00000227321
MTND4P24	-0.0088	7.8206E-01		ENSG00000232177
MTND4P35	0.0290	8.8512E-01		ENSG00000270906
MTND4P9	-0.0362	3.7347E-01		ENSG00000250050
MTND5P1	-0.0102	8.8133E-01	9.4058E-01	ENSG00000227999
MTND5P11	0.0163	9.6117E-01	9.8058E-01	ENSG00000248923
MTND5P16	0.0095	6.7883E-01		ENSG00000243658
MTND5P19	0.0277	2.9284E-01		ENSG00000231199
MTND5P2	-0.0595	1.5057E-01	3.3174E-01	ENSG00000229622
MTND5P28	-0.0080	6.2554E-01		ENSG00000223549
MTND5P33	-0.0073	4.7530E-01		ENSG00000261904
MTND6P14	0.0267	3.2402E-01		ENSG00000235638
MTND6P18	-0.0558	1.3419E-01		ENSG00000227714
MTND6P21	-0.0285	7.6715E-02		ENSG00000223431
MTND6P22	0.0132	6.3714E-01		ENSG00000270230
MTND6P3	-0.0412	4.1372E-01	6.1731E-01	ENSG00000254132
MTND6P32	0.0163	4.0728E-01		ENSG00000227649
MTND6P4	0.1507	1.3130E-01	3.0431E-01	ENSG00000249119
MTNR1A	0.1100	2.3194E-01	4.3688E-01	ENSG00000168412
MTNR1B	0.0128	6.5712E-01		ENSG00000134640
MTO1	-0.3443	2.9704E-05	5.8894E-04	ENSG00000135297
MTOR	-0.0281	6.5938E-01	8.0785E-01	ENSG00000198793

MTOR-AS1	0.0362	3.9931E-01		ENSG00000225602
MTPAP	-0.1228	9.7948E-02	2.5136E-01	ENSG00000107951
MTPN	-0.2126	6.9074E-04	6.8725E-03	ENSG00000105887
MTR	0.3810	4.0370E-06	1.1821E-04	ENSG00000116984
MTRES1	-0.0742	2.5152E-01	4.5978E-01	ENSG00000130349
MTREX	-0.0769	2.6125E-01	4.6982E-01	ENSG00000039123
MTRF1	-0.0661	3.7276E-01	5.8149E-01	ENSG00000120662
MTRF1L	-0.1632	1.6162E-02	7.1534E-02	ENSG00000112031
MTRF1LP1	-0.0072	6.0706E-01		ENSG00000256091
MTRFR	0.1729	3.9707E-02	1.3618E-01	ENSG00000130921
MTRR	0.2088	7.5022E-03	4.0866E-02	ENSG00000124275
MTSS1	-0.3083	1.0850E-07	6.2085E-06	ENSG00000170873
MTSS2	-0.4487	2.4071E-06	7.7522E-05	ENSG00000132613
MTTP	0.2407	3.2472E-02	1.1747E-01	ENSG00000138823
MTURN	-0.0498	4.1993E-01	6.2278E-01	ENSG00000180354
MTUS1	0.0955	2.0102E-01	3.9952E-01	ENSG00000129422
MTUS2	0.0231	7.8300E-01	8.8518E-01	ENSG00000132938
MTX1	-0.1958	1.3854E-04	1.9569E-03	ENSG00000173171
MTX1LP	-0.1435	1.2083E-02	5.8271E-02	ENSG00000236675
MTX2	0.0314	5.7997E-01	7.5142E-01	ENSG00000128654
MTX3	-0.0269	6.5235E-01	8.0312E-01	ENSG00000177034
MUC12	0.1554	1.1831E-01	2.8461E-01	ENSG00000205277
MUC12-AS1	-0.0155	1.9809E-01		ENSG00000227053
MUC13	0.0237	4.3341E-01		ENSG00000173702
MUC16	0.0121	4.3923E-01		ENSG00000181143
MUC17	-0.0136	5.4133E-01		ENSG00000169876
MUC19	0.1454	5.3519E-02	1.6715E-01	ENSG00000205592
MUC2	0.0558	1.2574E-01		ENSG00000198788
MUC3A	-0.3486	9.7564E-03	4.9836E-02	ENSG00000169894
MUC4	0.1730	5.6538E-02	1.7385E-01	ENSG00000145113
MUC5AC	0.0240	4.8664E-01		ENSG00000215182
MUC5B	0.0401	3.3384E-01		ENSG00000117983
MUC6	-0.6963	5.9200E-02	1.7883E-01	ENSG00000184956
MUCL1	0.0488	1.9774E-01		ENSG00000172551
MUCL3	0.0119	5.1101E-01		ENSG00000168631
MUL1	-0.0242	7.1396E-01	8.4294E-01	ENSG00000090432
MUS81	-0.1081	1.1584E-01	2.8048E-01	ENSG00000172732
MUSK	0.1181	1.4293E-02	6.5698E-02	ENSG00000030304
MUTYH	-0.0230	7.7860E-01	8.8223E-01	ENSG00000132781
MVB12A	0.0048	9.5474E-01	9.7718E-01	ENSG00000141971
MVB12B	0.1718	1.7955E-02	7.6954E-02	ENSG00000196814
MVD	-0.1484	2.1341E-02	8.6981E-02	ENSG00000167508
MVK	0.0425	5.8834E-01	7.5723E-01	ENSG00000110921
MVP	-0.1267	1.6205E-01	3.4802E-01	ENSG00000013364
MVP-DT	-0.0756	2.8202E-01	4.9241E-01	ENSG00000238045
MX1	0.0709	4.0304E-01	6.0753E-01	ENSG00000157601
MX2	0.0640	2.0230E-01		ENSG00000183486
MXD3	-0.0653	4.5469E-01	6.5318E-01	ENSG00000213347
MXD4	-0.0536	4.6934E-01	6.6580E-01	ENSG00000123933

MXI1	-0.4276	5.8142E-11	1.0519E-08	ENSG00000119950
MXRA5	0.4646	1.0839E-02	5.3749E-02	ENSG00000101825
MXRA7	-0.0739	2.5222E-01	4.6034E-01	ENSG00000182534
MXRA8	0.0213	7.9430E-01	8.9127E-01	ENSG00000162576
MYADM	-0.0503	4.1704E-01	6.2047E-01	ENSG00000179820
MYADML	0.0266	5.5650E-01		ENSG00000239649
MYADML2	0.0547	5.0753E-01	6.9649E-01	ENSG00000185105
MYB	0.1200	1.6203E-01	3.4802E-01	ENSG00000118513
MYBBP1A	0.1056	1.4138E-01	3.1824E-01	ENSG00000132382
MYBL1	-0.0824	3.1354E-01	5.2377E-01	ENSG00000185697
MYBL2	-0.1030	2.5106E-01	4.5932E-01	ENSG00000101057
MYBPC1	0.1959	2.3131E-02	9.2304E-02	ENSG00000196091
MYBPC2	0.1089	1.0917E-01	2.6972E-01	ENSG00000086967
MYBPC3	-0.0041	8.4857E-01		ENSG00000134571
MYBPH	0.0366	1.6422E-01		ENSG00000133055
MYBPHL	-0.0944	1.9990E-01	3.9831E-01	ENSG00000221986
MYCBP	0.2708	1.1921E-02	5.7715E-02	ENSG00000214114
MYCBP2	0.3734	1.1224E-05	2.6918E-04	ENSG00000005810
MYCBP2-AS1	0.1517	5.8200E-02	1.7701E-01	ENSG00000236051
MYCBP2-AS2	0.0092	5.3656E-01		ENSG00000229521
MYCBPAP	-0.0392	6.3801E-01	7.9276E-01	ENSG00000136449
MYCL	0.0943	2.3293E-01	4.3806E-01	ENSG00000116990
MYCL-AS1	-0.0051	7.4471E-01		ENSG00000236546
MYCN	-0.1949	4.1852E-02	1.4110E-01	ENSG00000134323
MYCNOS	-0.0900	3.0873E-01	5.1898E-01	ENSG00000233718
MYCT1	0.0350	2.6906E-01		ENSG00000120279
MYD88	-0.2604	7.4550E-03	4.0654E-02	ENSG00000172936
MYDGF	-0.1075	7.0128E-02	2.0106E-01	ENSG00000074842
MYEF2	-0.1391	1.9837E-02	8.2517E-02	ENSG00000104177
MYF6	-0.0015	5.4765E-01	7.2733E-01	ENSG00000111046
MYG1	0.0662	4.0724E-01	6.1173E-01	ENSG00000139637
MYH10	-0.0065	9.1274E-01	9.5704E-01	ENSG00000133026
MYH11	-0.0373	6.2209E-01	7.8154E-01	ENSG00000133392
MYH14	0.0501	5.1797E-01	7.0537E-01	ENSG00000105357
MYH15	0.5707	4.8037E-03	2.9172E-02	ENSG00000144821
MYH16	-0.0193	7.9875E-01	8.9389E-01	ENSG00000002079
MYH2	-0.0028	9.0111E-01		ENSG00000125414
MYH3	0.0145	8.2230E-01	9.0802E-01	ENSG00000109063
MYH6	0.1470	2.0103E-02	8.3344E-02	ENSG00000197616
MYH7B	0.3311	1.9702E-02	8.2136E-02	ENSG00000078814
MYH8	0.1186	1.3234E-01	3.0582E-01	ENSG00000133020
MYH9	-0.0523	3.5992E-01	5.6932E-01	ENSG00000100345
MYL10	0.0267	3.3642E-01		ENSG00000106436
MYL11	0.1284	1.1613E-01	2.8090E-01	ENSG00000180209
MYL12-AS1	0.2636	3.5102E-02	1.2429E-01	ENSG00000264235
MYL12A	0.1374	7.3274E-02	2.0742E-01	ENSG00000101608
MYL12B	-0.2937	2.2153E-05	4.6508E-04	ENSG00000118680
MYL3	0.0372	6.6399E-01	8.1095E-01	ENSG00000160808
MYL4	0.0601	1.9433E-01		ENSG00000198336

MYL5	0.4450	1.1289E-05	2.6989E-04	ENSG00000215375
MYL6	-0.4297	7.4579E-10	8.6558E-08	ENSG00000092841
MYL6B	-0.2482	1.3396E-05	3.0747E-04	ENSG00000196465
MYL7	0.0023	7.1466E-01	8.4332E-01	ENSG00000106631
MYL9	0.0851	3.6282E-01	5.7197E-01	ENSG00000101335
MYLIP	-0.3925	4.4405E-04	4.8446E-03	ENSG00000007944
MYLK	0.1708	8.3984E-02	2.2640E-01	ENSG00000065534
MYLK-AS1	0.2116	2.5194E-02	9.8074E-02	ENSG00000239523
MYLK-AS2	-0.0018	6.9227E-01		ENSG00000250174
MYLK2	0.0456	1.9273E-01		ENSG00000101306
MYLK3	0.0632	4.1753E-01	6.2089E-01	ENSG00000140795
MYLK4	0.0348	8.1552E-01	9.0400E-01	ENSG00000145949
MYMX	-0.0065	6.1820E-01		ENSG00000262179
MYNN	-0.0657	3.0714E-01	5.1743E-01	ENSG00000085274
MYO10	0.0102	8.9086E-01	9.4581E-01	ENSG00000145555
MYO15A	0.3030	2.7311E-03	1.8993E-02	ENSG00000091536
MYO15B	0.2922	8.2907E-03	4.4050E-02	ENSG00000266714
MYO16	0.1743	8.6908E-02	2.3180E-01	ENSG00000041515
MYO16-AS1	0.0289	2.3495E-01		ENSG00000236242
MYO18A	-0.0738	2.4878E-01	4.5649E-01	ENSG00000196535
MYO18B	0.0960	1.8531E-01	3.7899E-01	ENSG00000133454
MYO19	0.2689	7.5416E-04	7.3489E-03	ENSG00000278259
MYO1A	0.0013	9.4377E-01		ENSG00000166866
MYO1B	-0.0100	8.6519E-01	9.3189E-01	ENSG00000128641
MYO1C	0.4085	2.4590E-05	5.0545E-04	ENSG00000197879
MYO1D	-0.0501	5.4199E-01	7.2366E-01	ENSG00000176658
MYO1D-DT	0.0163	7.6815E-01		ENSG00000236377
MYO1E	-0.0699	4.2190E-01	6.2453E-01	ENSG00000157483
MYO1F	0.2115	6.1276E-02	1.8304E-01	ENSG00000142347
MYO1G	0.0226	7.0939E-01		ENSG00000136286
MYO1H	0.1174	1.7389E-01	3.6405E-01	ENSG00000174527
MYO3A	-0.1069	2.4620E-01	4.5407E-01	ENSG00000095777
MYO3B	0.1531	8.8672E-02	2.3510E-01	ENSG00000071909
MYO3B-AS1	-0.0074	9.7565E-01	9.8791E-01	ENSG00000231898
MYO5A	0.0604	4.2827E-01	6.3017E-01	ENSG00000197535
MYO5B	-0.1634	9.9904E-02	2.5449E-01	ENSG00000167306
MYO5BP2	0.0060	6.2335E-01		ENSG00000238245
MYO5C	0.2344	4.4797E-02	1.4803E-01	ENSG00000128833
MYO6	0.1291	9.2010E-02	2.4154E-01	ENSG00000196586
MYO7A	0.4850	4.3900E-03	2.7304E-02	ENSG00000137474
MYO9A	0.2348	8.4018E-04	7.9297E-03	ENSG00000066933
MYO9B	-0.0046	9.3571E-01	9.6833E-01	ENSG00000099331
MYOC	-0.0151	4.8256E-01		ENSG00000034971
MYOCOS	-0.0213	3.8246E-01		ENSG00000283683
MYOD1	0.0504	1.2583E-01		ENSG00000129152
MYOF	0.0178	7.6257E-01	8.7218E-01	ENSG00000138119
MYOG	0.0570	2.3635E-01	4.4217E-01	ENSG00000122180
MYOM1	0.2232	4.8366E-02	1.5597E-01	ENSG00000101605
MYOM2	0.0098	9.1133E-01	9.5625E-01	ENSG00000036448

MYOM3	0.0077	8.0548E-01		ENSG00000142661
MYORG	0.0854	2.8865E-01	4.9908E-01	ENSG00000164976
MYOSLID	0.0310	3.8616E-01		ENSG00000229647
MYOT	-0.0046	9.3912E-01		ENSG00000120729
MYOZ1	0.4765	2.1676E-03	1.5973E-02	ENSG00000177791
MYOZ2	0.0904	1.8774E-01	3.8243E-01	ENSG00000172399
MYOZ3	-0.4961	1.5961E-03	1.2775E-02	ENSG00000164591
MYPN	-0.0074	8.7051E-01		ENSG00000138347
MYPOP	0.3866	1.5062E-02	6.8162E-02	ENSG00000176182
MYRF	-0.0653	4.5067E-01	6.4983E-01	ENSG00000124920
MYRF-AS1	0.0011	9.1471E-01		ENSG00000124915
MYRFL	0.0120	2.9043E-01	5.0077E-01	ENSG00000166268
MYRIP	0.0249	7.6154E-01	8.7173E-01	ENSG00000170011
MYSM1	-0.0416	4.7150E-01	6.6767E-01	ENSG00000162601
MYT1	-0.5223	1.8581E-05	4.0387E-04	ENSG00000196132
MYT1L	-0.0242	7.1533E-01	8.4351E-01	ENSG00000186487
MYT1L-AS1	-0.1295	1.4023E-01	3.1680E-01	ENSG00000225619
MYZAP	0.0284	8.6854E-01	9.3385E-01	ENSG00000263155
MZB1	0.0381	3.6419E-01		ENSG00000170476
MZF1	0.2301	1.1596E-03	1.0137E-02	ENSG00000099326
MZF1-AS1	0.3665	4.5100E-03	2.7839E-02	ENSG00000267858
MZT1	0.1767	7.3927E-03	4.0386E-02	ENSG00000204899
MZT2A	-0.1184	8.0619E-02	2.2072E-01	ENSG00000173272
MZT2B	-0.1975	1.6565E-02	7.2759E-02	ENSG00000152082
N4BP1	-0.3524	3.4014E-06	1.0320E-04	ENSG00000102921
N4BP2	0.0355	5.7648E-01	7.4871E-01	ENSG00000078177
N4BP2L2	0.1561	4.2546E-02	1.4264E-01	ENSG00000244754
N4BP2L2-IT2	0.1681	7.4477E-02	2.0948E-01	ENSG00000281026
N4BP3	0.1838	6.5595E-02	1.9207E-01	ENSG00000145911
N6AMT1	-0.0459	5.7279E-01	7.4605E-01	ENSG00000156239
NA	0.5793	2.7414E-11	5.7651E-09	ENSG00000185596
NA	0.5528	1.7314E-10	2.5510E-08	ENSG00000249637
NA	0.4423	1.2833E-07	7.0326E-06	ENSG00000279088
NA	0.5217	1.4868E-07	7.9011E-06	ENSG00000280234
NA	0.4053	2.8990E-07	1.3878E-05	ENSG00000246695
NA	0.3926	3.1671E-07	1.4787E-05	ENSG00000224032
NA	0.3741	4.2837E-07	1.8889E-05	ENSG00000170919
NA	0.5575	5.1133E-07	2.1880E-05	ENSG00000242474
NA	0.4175	5.5295E-07	2.3337E-05	ENSG00000268205
NA	0.5898	1.0506E-06	3.8756E-05	ENSG00000257261
NA	0.3187	1.3924E-06	4.9152E-05	ENSG00000206149
NA	0.4514	3.4356E-06	1.0380E-04	ENSG00000244119
NA	0.4860	5.1236E-06	1.4342E-04	ENSG00000282508
NA	0.5642	5.3231E-06	1.4716E-04	ENSG00000229956
NA	0.5557	5.4433E-06	1.4998E-04	ENSG00000283050
NA	0.4096	5.9914E-06	1.6248E-04	ENSG00000234062
NA	0.4675	6.9805E-06	1.8370E-04	ENSG00000271576
NA	0.5598	9.1189E-06	2.2733E-04	ENSG00000244055
NA	0.3869	9.4456E-06	2.3358E-04	ENSG00000204177

NA	0.5944	1.0485E-05	2.5492E-04	ENSG00000260806
NA	0.5554	1.4742E-05	3.3297E-04	ENSG00000263272
NA	0.5794	2.3127E-05	4.8142E-04	ENSG00000270953
NA	0.4390	2.4334E-05	5.0061E-04	ENSG00000233325
NA	0.4628	2.8877E-05	5.7769E-04	ENSG00000272525
NA	0.3849	2.9622E-05	5.8778E-04	ENSG00000223546
NA	0.3794	3.4163E-05	6.6135E-04	ENSG00000223820
NA	0.3405	3.8534E-05	7.3102E-04	ENSG00000227518
NA	0.4034	4.0383E-05	7.5604E-04	ENSG00000253596
NA	0.3106	4.3307E-05	7.9402E-04	ENSG00000185684
NA	0.4252	4.5163E-05	8.1949E-04	ENSG00000260920
NA	0.4903	6.5411E-05	1.1061E-03	ENSG00000272702
NA	0.4937	6.7734E-05	1.1376E-03	ENSG00000261420
NA	0.5000	6.8866E-05	1.1511E-03	ENSG00000289207
NA	0.3073	7.5878E-05	1.2413E-03	ENSG00000259943
NA	0.5719	7.6991E-05	1.2554E-03	ENSG00000270012
NA	0.4969	8.8789E-05	1.4049E-03	ENSG00000288752
NA	0.5030	9.7189E-05	1.5106E-03	ENSG00000269911
NA	0.5381	9.9135E-05	1.5351E-03	ENSG00000231769
NA	0.4064	1.0005E-04	1.5473E-03	ENSG00000228393
NA	0.4638	1.1768E-04	1.7453E-03	ENSG00000227855
NA	0.4001	1.2348E-04	1.8085E-03	ENSG00000172014
NA	0.5014	1.3122E-04	1.8903E-03	ENSG00000286408
NA	0.3669	1.3533E-04	1.9224E-03	ENSG00000288764
NA	0.3286	1.4033E-04	1.9752E-03	ENSG00000245910
NA	0.3602	1.5477E-04	2.1301E-03	ENSG00000236682
NA	0.5595	1.5922E-04	2.1717E-03	ENSG00000272335
NA	0.5461	1.6819E-04	2.2676E-03	ENSG00000258001
NA	0.2506	1.7718E-04	2.3604E-03	ENSG00000215908
NA	0.3833	1.8048E-04	2.3952E-03	ENSG00000176912
NA	0.4792	1.8125E-04	2.4029E-03	ENSG00000289055
NA	0.3845	1.8836E-04	2.4811E-03	ENSG00000258704
NA	0.3780	1.9402E-04	2.5361E-03	ENSG00000253320
NA	0.5941	1.9976E-04	2.5883E-03	ENSG00000273108
NA	0.3834	2.1005E-04	2.6974E-03	ENSG00000230606
NA	0.3671	2.5992E-04	3.2202E-03	ENSG00000240024
NA	0.5802	2.6288E-04	3.2471E-03	ENSG00000279456
NA	2.0276	2.7284E-04		ENSG00000258590
NA	0.3676	2.8339E-04	3.4360E-03	ENSG00000230091
NA	0.2509	2.8362E-04	3.4360E-03	ENSG00000146556
NA	0.3105	2.9065E-04	3.4987E-03	ENSG00000236438
NA	0.4514	2.9835E-04	3.5794E-03	ENSG00000184441
NA	0.5525	3.1906E-04	3.7706E-03	ENSG00000267778
NA	0.5565	3.2235E-04	3.7949E-03	ENSG00000236021
NA	0.4841	3.2592E-04	3.8260E-03	ENSG00000289045
NA	0.5791	3.4240E-04	3.9739E-03	ENSG00000206120
NA	0.4732	3.4355E-04	3.9836E-03	ENSG00000284052
NA	0.3801	3.4436E-04	3.9910E-03	ENSG00000284707
NA	0.4104	3.8638E-04	4.3529E-03	ENSG00000287918

NA	0.4020	4.0213E-04	4.4995E-03	ENSG00000277701
NA	0.4435	4.1026E-04	4.5635E-03	ENSG00000288586
NA	0.4998	4.1482E-04	4.5955E-03	ENSG00000272795
NA	0.2734	4.3328E-04	4.7572E-03	ENSG00000267002
NA	0.3517	4.5999E-04	4.9816E-03	ENSG00000276517
NA	0.4493	4.6083E-04	4.9863E-03	ENSG00000239922
NA	0.5667	4.6639E-04	5.0353E-03	ENSG00000273486
NA	0.5511	4.6873E-04	5.0561E-03	ENSG00000280660
NA	0.5298	4.7031E-04	5.0710E-03	ENSG00000232093
NA	0.5274	4.7505E-04	5.1064E-03	ENSG00000289858
NA	0.5246	4.7623E-04	5.1168E-03	ENSG00000254536
NA	0.3345	4.8054E-04	5.1519E-03	ENSG00000289021
NA	0.4180	4.9607E-04	5.2801E-03	ENSG00000284642
NA	0.3584	5.1122E-04	5.3962E-03	ENSG00000278600
NA	0.3550	5.1796E-04	5.4527E-03	ENSG00000235944
NA	0.5271	5.2620E-04	5.5235E-03	ENSG00000289043
NA	0.4359	5.6407E-04	5.8339E-03	ENSG00000290110
NA	0.5139	5.6576E-04	5.8416E-03	ENSG00000275294
NA	2.8460	5.9563E-04		ENSG00000288783
NA	0.5971	6.8538E-04	6.8365E-03	ENSG00000251095
NA	0.3226	6.8546E-04	6.8365E-03	ENSG00000220804
NA	0.2749	7.1747E-04	7.0783E-03	ENSG00000244879
NA	0.3300	7.2115E-04	7.1004E-03	ENSG00000226287
NA	0.2569	7.7271E-04	7.4705E-03	ENSG00000196295
NA	2.4916	8.0049E-04		ENSG00000230226
NA	0.4541	8.2153E-04	7.8147E-03	ENSG00000264204
NA	0.3113	8.2165E-04	7.8147E-03	ENSG00000289370
NA	0.4261	8.3011E-04	7.8648E-03	ENSG00000274667
NA	0.3862	8.8608E-04	8.2772E-03	ENSG00000273008
NA	0.5701	9.6716E-04	8.8202E-03	ENSG00000270761
NA	0.5389	1.0158E-03	9.1688E-03	ENSG00000232692
NA	0.4179	1.0419E-03	9.3599E-03	ENSG00000272631
NA	0.2332	1.0757E-03	9.5916E-03	ENSG00000279457
NA	0.4793	1.0869E-03	9.6579E-03	ENSG00000260296
NA	0.5830	1.1004E-03	9.7355E-03	ENSG00000261120
NA	0.2874	1.1031E-03	9.7525E-03	ENSG00000260000
NA	0.3054	1.1109E-03	9.8043E-03	ENSG00000272163
NA	0.1804	1.1633E-03	1.0157E-02	ENSG00000215105
NA	0.3636	1.1824E-03	1.0295E-02	ENSG00000288926
NA	0.3494	1.1955E-03	1.0372E-02	ENSG00000272829
NA	0.4071	1.2429E-03	1.0678E-02	ENSG00000276203
NA	0.5144	1.2979E-03	1.1050E-02	ENSG00000242880
NA	0.4849	1.3308E-03	1.1253E-02	ENSG00000253736
NA	0.3024	1.3660E-03	1.1452E-02	ENSG00000268584
NA	0.3747	1.3972E-03	1.1639E-02	ENSG00000287190
NA	0.3039	1.4216E-03	1.1785E-02	ENSG00000217801
NA	0.4866	1.4414E-03	1.1921E-02	ENSG00000270361
NA	0.4225	1.4448E-03	1.1941E-02	ENSG00000246477
NA	0.3468	1.4544E-03	1.2013E-02	ENSG00000223751

NA	0.2942	1.4611E-03	1.2051E-02	ENSG00000235859
NA	0.4022	1.4784E-03	1.2148E-02	ENSG00000248769
NA	0.5276	1.5091E-03	1.2304E-02	ENSG00000283128
NA	0.3089	1.5092E-03	1.2304E-02	ENSG00000235501
NA	0.3670	1.5604E-03	1.2592E-02	ENSG00000245904
NA	0.3255	1.5893E-03	1.2742E-02	ENSG00000205212
NA	0.4147	1.6075E-03	1.2838E-02	ENSG00000261220
NA	0.3377	1.6431E-03	1.3042E-02	ENSG00000261799
NA	0.3275	1.6808E-03	1.3259E-02	ENSG00000239569
NA	0.1375	1.7152E-03		ENSG00000244157
NA	0.3491	1.7307E-03	1.3531E-02	ENSG00000250575
NA	0.2452	1.7364E-03	1.3563E-02	ENSG00000264575
NA	0.3379	1.7452E-03	1.3606E-02	ENSG00000259577
NA	0.3611	1.7478E-03	1.3622E-02	ENSG00000267939
NA	0.4622	1.7739E-03	1.3778E-02	ENSG00000250420
NA	1.6590	1.7819E-03		ENSG00000234949
NA	0.3646	1.8168E-03	1.4009E-02	ENSG00000120555
NA	0.2736	1.8757E-03	1.4355E-02	ENSG00000289901
NA	0.4951	1.9072E-03	1.4528E-02	ENSG00000238105
NA	0.4197	1.9255E-03	1.4637E-02	ENSG00000269918
NA	0.4886	1.9311E-03	1.4674E-02	ENSG00000213543
NA	0.3874	2.0120E-03	1.5153E-02	ENSG00000278766
NA	0.4352	2.0131E-03	1.5157E-02	ENSG00000249492
NA	0.2938	2.0775E-03	1.5480E-02	ENSG00000287222
NA	0.3820	2.1021E-03	1.5642E-02	ENSG00000289537
NA	0.4510	2.1223E-03	1.5738E-02	ENSG00000286681
NA	1.7914	2.1337E-03		ENSG00000272085
NA	0.3875	2.1696E-03	1.5975E-02	ENSG00000290085
NA	0.5720	2.2039E-03	1.6197E-02	ENSG00000287650
NA	0.5721	2.2058E-03	1.6197E-02	ENSG00000273142
NA	0.4485	2.2261E-03	1.6326E-02	ENSG00000166763
NA	0.5186	2.2285E-03	1.6330E-02	ENSG00000239827
NA	0.3668	2.2668E-03	1.6554E-02	ENSG00000237436
NA	0.3855	2.4119E-03	1.7333E-02	ENSG00000276975
NA	0.3064	2.4607E-03	1.7596E-02	ENSG00000185495
NA	0.5150	2.5672E-03	1.8137E-02	ENSG00000228084
NA	0.4007	2.5818E-03	1.8186E-02	ENSG00000254208
NA	0.4721	2.7101E-03	1.8905E-02	ENSG00000289347
NA	0.4041	2.7296E-03	1.8993E-02	ENSG00000289190
NA	0.4060	2.7317E-03	1.8993E-02	ENSG00000255008
NA	1.2542	2.7325E-03		ENSG00000227748
NA	0.4247	2.8939E-03	1.9940E-02	ENSG00000283930
NA	0.2616	2.9031E-03	1.9998E-02	ENSG00000226007
NA	0.5036	2.9282E-03	2.0103E-02	ENSG00000287575
NA	0.2442	2.9591E-03	2.0281E-02	ENSG00000251661
NA	0.3430	2.9717E-03	2.0356E-02	ENSG00000260196
NA	0.4465	2.9945E-03	2.0490E-02	ENSG00000289446
NA	0.5688	3.0916E-03	2.0984E-02	ENSG00000279253
NA	0.3964	3.1033E-03	2.1046E-02	ENSG00000289929

NA	0.3588	3.1354E-03	2.1204E-02	ENSG00000286787
NA	0.3988	3.1360E-03	2.1204E-02	ENSG00000246250
NA	0.3156	3.1445E-03	2.1244E-02	ENSG00000263004
NA	0.5467	3.2482E-03	2.1744E-02	ENSG00000261441
NA	0.4207	3.3191E-03	2.2088E-02	ENSG00000234206
NA	0.2517	3.3353E-03	2.2146E-02	ENSG00000211459
NA	0.2124	3.4131E-03	2.2557E-02	ENSG00000205763
NA	0.3566	3.4468E-03	2.2749E-02	ENSG00000250461
NA	0.2214	3.4849E-03	2.2969E-02	ENSG00000225889
NA	0.3488	3.6244E-03	2.3686E-02	ENSG00000279791
NA	0.4646	3.6289E-03	2.3709E-02	ENSG00000273284
NA	0.3679	3.7496E-03	2.4328E-02	ENSG00000289577
NA	0.2786	3.7713E-03	2.4426E-02	ENSG00000249679
NA	0.5405	3.8439E-03	2.4785E-02	ENSG00000260273
NA	0.3811	3.8765E-03	2.4963E-02	ENSG00000262583
NA	0.2456	3.9068E-03	2.5092E-02	ENSG00000228327
NA	0.3480	3.9333E-03	2.5242E-02	ENSG00000276791
NA	0.3047	4.0007E-03	2.5562E-02	ENSG00000269044
NA	0.3755	4.0029E-03	2.5569E-02	ENSG00000259556
NA	0.5850	4.0546E-03	2.5830E-02	ENSG00000277235
NA	0.2653	4.0656E-03	2.5875E-02	ENSG00000215158
NA	1.3117	4.0814E-03		ENSG00000230964
NA	0.4892	4.2146E-03	2.6504E-02	ENSG00000272977
NA	0.4903	4.3037E-03	2.6931E-02	ENSG00000287851
NA	0.2762	4.3491E-03	2.7111E-02	ENSG00000243289
NA	0.4224	4.4013E-03	2.7351E-02	ENSG00000261324
NA	0.3006	4.4220E-03	2.7413E-02	ENSG00000234665
NA	0.4702	4.6199E-03	2.8375E-02	ENSG00000272420
NA	0.4384	4.6249E-03	2.8385E-02	ENSG00000188801
NA	0.3342	4.6761E-03	2.8621E-02	ENSG00000282416
NA	0.4122	4.7100E-03	2.8785E-02	ENSG00000242375
NA	0.3525	4.7220E-03	2.8844E-02	ENSG00000273382
NA	1.2969	4.7431E-03		ENSG00000285703
NA	0.3161	4.7730E-03	2.9033E-02	ENSG00000288826
NA	0.2127	4.8041E-03	2.9172E-02	ENSG00000229018
NA	0.4433	4.8772E-03	2.9493E-02	ENSG00000237886
NA	0.1450	4.9104E-03		ENSG00000272170
NA	0.4626	4.9506E-03	2.9848E-02	ENSG00000272247
NA	0.5044	4.9535E-03	2.9848E-02	ENSG00000287234
NA	0.4624	5.0622E-03	3.0372E-02	ENSG00000260306
NA	0.4678	5.0873E-03	3.0493E-02	ENSG00000279667
NA	0.1037	5.1205E-03		ENSG00000288938
NA	0.3761	5.1228E-03	3.0676E-02	ENSG00000259660
NA	0.3521	5.1323E-03	3.0696E-02	ENSG00000242686
NA	0.4987	5.3167E-03	3.1602E-02	ENSG00000116652
NA	0.2661	5.3808E-03	3.1871E-02	ENSG00000215014
NA	0.2412	5.4497E-03	3.2133E-02	ENSG00000215237
NA	0.1238	5.4688E-03	3.2207E-02	ENSG00000287690
NA	0.1136	5.4691E-03		ENSG00000287821

NA	0.4034	5.6156E-03	3.2836E-02	ENSG00000225938
NA	0.1000	5.7094E-03		ENSG00000250775
NA	0.3974	5.7671E-03	3.3526E-02	ENSG00000189229
NA	0.4525	5.8705E-03	3.3987E-02	ENSG00000286810
NA	0.3503	5.8984E-03	3.4120E-02	ENSG00000289410
NA	0.1408	5.9343E-03	3.4227E-02	ENSG00000279798
NA	0.4731	5.9525E-03	3.4312E-02	ENSG00000286703
NA	0.3505	5.9744E-03	3.4362E-02	ENSG00000240005
NA	1.1796	5.9747E-03		ENSG00000223724
NA	0.4780	5.9785E-03	3.4377E-02	ENSG00000289117
NA	0.2552	6.0664E-03	3.4810E-02	ENSG00000205578
NA	0.4265	6.0738E-03	3.4820E-02	ENSG00000271966
NA	0.3120	6.0768E-03	3.4828E-02	ENSG00000272195
NA	0.3704	6.1256E-03	3.5027E-02	ENSG00000256340
NA	0.4498	6.2008E-03	3.5375E-02	ENSG00000229017
NA	0.3746	6.2240E-03	3.5474E-02	ENSG00000240291
NA	1.2524	6.2603E-03		ENSG00000225192
NA	0.4896	6.4353E-03	3.6417E-02	ENSG00000242628
NA	0.1753	6.4376E-03		ENSG00000275676
NA	0.3321	6.4581E-03	3.6529E-02	ENSG00000274460
NA	0.3302	6.4664E-03	3.6559E-02	ENSG00000225746
NA	0.0859	6.5016E-03		ENSG00000224442
NA	0.3710	6.5049E-03	3.6743E-02	ENSG00000225449
NA	0.3363	6.5079E-03	3.6752E-02	ENSG00000290112
NA	0.2148	6.6840E-03	3.7501E-02	ENSG00000100058
NA	0.5213	6.6878E-03	3.7501E-02	ENSG00000276728
NA	0.3698	6.7760E-03	3.7926E-02	ENSG00000289340
NA	0.3133	6.8131E-03	3.8038E-02	ENSG00000286659
NA	0.4663	6.8174E-03	3.8045E-02	ENSG00000235436
NA	0.3240	6.8925E-03	3.8378E-02	ENSG00000280164
NA	0.3143	6.9388E-03	3.8574E-02	ENSG00000233264
NA	0.1853	6.9463E-03	3.8585E-02	ENSG00000221990
NA	0.0663	7.1201E-03		ENSG00000231346
NA	1.3235	7.1384E-03		ENSG00000275191
NA	0.0958	7.1452E-03	3.9442E-02	ENSG00000231748
NA	0.2856	7.2189E-03	3.9728E-02	ENSG00000272426
NA	0.3655	7.2496E-03	3.9856E-02	ENSG00000279750
NA	0.5274	7.2752E-03	3.9957E-02	ENSG00000287756
NA	0.4468	7.3101E-03	4.0086E-02	ENSG00000286267
NA	0.2455	7.3352E-03	4.0188E-02	ENSG00000276550
NA	0.3370	7.3441E-03	4.0223E-02	ENSG00000288075
NA	0.0522	7.3544E-03		ENSG00000257294
NA	0.5594	7.3860E-03	4.0386E-02	ENSG00000187686
NA	0.2683	7.3915E-03	4.0386E-02	ENSG00000288756
NA	0.2405	7.4107E-03	4.0475E-02	ENSG00000263072
NA	0.1355	7.4151E-03	4.0481E-02	ENSG00000272650
NA	0.4667	7.4199E-03	4.0498E-02	ENSG00000261512
NA	0.4825	7.5463E-03	4.1061E-02	ENSG00000265298
NA	0.1099	7.6002E-03		ENSG00000213386

NA	0.1745	7.6270E-03	4.1326E-02	ENSG00000205583
NA	0.3520	7.6365E-03	4.1369E-02	ENSG00000214439
NA	0.2456	7.7257E-03	4.1778E-02	ENSG00000279602
NA	0.5055	7.7453E-03	4.1866E-02	ENSG00000149656
NA	0.0594	7.7517E-03		ENSG00000277797
NA	0.4195	7.7835E-03	4.2035E-02	ENSG00000279474
NA	0.4322	7.8553E-03	4.2302E-02	ENSG00000228857
NA	0.1305	7.8633E-03		ENSG00000287268
NA	0.3400	7.8669E-03	4.2337E-02	ENSG00000235934
NA	0.2910	7.8806E-03	4.2374E-02	ENSG00000237094
NA	0.2393	7.9799E-03	4.2805E-02	ENSG00000234630
NA	0.5956	8.0775E-03	4.3178E-02	ENSG00000261243
NA	0.0803	8.1183E-03		ENSG00000254873
NA	0.4331	8.1924E-03	4.3653E-02	ENSG00000286155
NA	0.4304	8.1930E-03	4.3653E-02	ENSG00000250045
NA	0.2834	8.2080E-03	4.3714E-02	ENSG00000288349
NA	0.4551	8.2412E-03	4.3843E-02	ENSG00000172971
NA	0.4052	8.3138E-03	4.4162E-02	ENSG00000214837
NA	0.4771	8.3866E-03	4.4482E-02	ENSG00000251680
NA	0.1708	8.6012E-03		ENSG00000279085
NA	0.3897	8.6089E-03	4.5455E-02	ENSG00000272157
NA	0.3885	8.6177E-03	4.5492E-02	ENSG00000270638
NA	0.4681	8.6439E-03	4.5562E-02	ENSG00000288858
NA	0.3678	8.7259E-03	4.5886E-02	ENSG00000213433
NA	0.3462	8.8081E-03	4.6219E-02	ENSG00000260253
NA	0.1746	8.9569E-03	4.6830E-02	ENSG00000259366
NA	0.3644	9.0282E-03	4.7093E-02	ENSG00000289979
NA	0.5616	9.0337E-03	4.7102E-02	ENSG00000254352
NA	0.4308	9.0435E-03	4.7113E-02	ENSG00000258490
NA	0.2936	9.0988E-03	4.7321E-02	ENSG00000258526
NA	0.4783	9.1585E-03	4.7555E-02	ENSG00000276116
NA	0.1701	9.2678E-03		ENSG00000271440
NA	0.4959	9.2735E-03	4.7971E-02	ENSG00000261758
NA	0.2448	9.2793E-03	4.7986E-02	ENSG00000260261
NA	0.3895	9.4037E-03	4.8507E-02	ENSG00000288750
NA	0.4559	9.4519E-03	4.8654E-02	ENSG00000250933
NA	0.2270	9.4875E-03	4.8806E-02	ENSG00000287613
NA	0.0964	9.4957E-03		ENSG00000285473
NA	0.3215	9.5098E-03	4.8900E-02	ENSG00000230658
NA	0.4535	9.5966E-03	4.9223E-02	ENSG00000267506
NA	0.2007	9.6244E-03	4.9335E-02	ENSG00000289406
NA	0.3842	9.6927E-03	4.9592E-02	ENSG00000276337
NA	0.3832	9.7631E-03	4.9852E-02	ENSG00000273204
NA	0.2037	9.7879E-03	4.9940E-02	ENSG00000255052
NA	0.5186	9.8197E-03	5.0044E-02	ENSG00000228061
NA	0.4932	9.8335E-03	5.0104E-02	ENSG00000289505
NA	0.6007	9.8498E-03	5.0156E-02	ENSG00000260422
NA	0.4839	9.8564E-03	5.0175E-02	ENSG00000231829
NA	0.0820	9.8899E-03		ENSG00000235888

NA	0.1063	9.9116E-03	5.0387E-02	ENSG00000231412
NA	0.1053	9.9226E-03		ENSG00000234156
NA	0.1178	1.0060E-02		ENSG00000214533
NA	0.4033	1.0067E-02	5.1022E-02	ENSG00000232529
NA	0.6737	1.0159E-02	5.1297E-02	ENSG00000232827
NA	0.7642	1.0188E-02	5.1404E-02	ENSG00000279675
NA	0.5311	1.0276E-02	5.1694E-02	ENSG00000285812
NA	0.2668	1.0281E-02	5.1698E-02	ENSG00000225138
NA	0.2048	1.0320E-02	5.1864E-02	ENSG00000227232
NA	0.4549	1.0348E-02	5.1949E-02	ENSG00000278206
NA	0.2813	1.0413E-02	5.2190E-02	ENSG00000267011
NA	0.1859	1.0428E-02		ENSG00000285898
NA	0.4893	1.0444E-02	5.2315E-02	ENSG00000258162
NA	0.3008	1.0481E-02	5.2473E-02	ENSG00000238035
NA	0.0650	1.0493E-02		ENSG00000259907
NA	0.5164	1.0496E-02	5.2521E-02	ENSG00000283491
NA	0.4696	1.0506E-02	5.2563E-02	ENSG00000286622
NA	0.5110	1.0549E-02	5.2744E-02	ENSG00000284846
NA	0.2724	1.0596E-02	5.2927E-02	ENSG00000272894
NA	0.4245	1.0609E-02	5.2963E-02	ENSG00000286931
NA	0.3728	1.0639E-02	5.3032E-02	ENSG00000261098
NA	0.1187	1.0686E-02	5.3234E-02	ENSG00000274602
NA	0.3991	1.0768E-02	5.3498E-02	ENSG00000256925
NA	0.5059	1.0771E-02	5.3498E-02	ENSG00000260443
NA	0.1262	1.0830E-02		ENSG00000289069
NA	0.3938	1.0838E-02	5.3749E-02	ENSG00000246898
NA	0.5221	1.0876E-02	5.3887E-02	ENSG00000286638
NA	0.3252	1.0879E-02	5.3893E-02	ENSG00000279692
NA	0.1217	1.0895E-02		ENSG00000263846
NA	0.1308	1.0901E-02		ENSG00000228280
NA	0.2741	1.1082E-02	5.4609E-02	ENSG00000235092
NA	0.5644	1.1111E-02	5.4711E-02	ENSG00000228252
NA	0.2458	1.1277E-02	5.5373E-02	ENSG00000277969
NA	0.0731	1.1281E-02		ENSG00000259807
NA	0.1269	1.1307E-02		ENSG00000260036
NA	0.0659	1.1339E-02		ENSG00000265460
NA	0.3075	1.1376E-02	5.5767E-02	ENSG00000229178
NA	0.4542	1.1384E-02	5.5799E-02	ENSG00000278931
NA	0.4793	1.1430E-02	5.5933E-02	ENSG00000263982
NA	0.3483	1.1518E-02	5.6265E-02	ENSG00000225880
NA	0.1329	1.1530E-02	5.6305E-02	ENSG00000289002
NA	0.3048	1.1531E-02	5.6305E-02	ENSG00000232648
NA	0.4364	1.1552E-02	5.6363E-02	ENSG00000280426
NA	0.4682	1.1591E-02	5.6532E-02	ENSG00000261369
NA	0.2672	1.1736E-02	5.7089E-02	ENSG00000288818
NA	0.4255	1.1849E-02	5.7514E-02	ENSG00000272103
NA	0.1194	1.1852E-02		ENSG00000249111
NA	0.4535	1.1859E-02	5.7534E-02	ENSG00000272192
NA	0.2961	1.1922E-02	5.7715E-02	ENSG00000271009

NA	0.2221	1.1941E-02	5.7778E-02	ENSG00000286604
NA	0.3208	1.2074E-02	5.8251E-02	ENSG00000270022
NA	0.2099	1.2146E-02	5.8451E-02	ENSG00000203761
NA	0.5081	1.2253E-02	5.8781E-02	ENSG00000179082
NA	0.3097	1.2285E-02	5.8901E-02	ENSG00000277715
NA	0.2935	1.2309E-02	5.8978E-02	ENSG00000280073
NA	0.0854	1.2356E-02	5.9136E-02	ENSG00000260618
NA	0.3055	1.2490E-02	5.9581E-02	ENSG00000289225
NA	0.3618	1.2515E-02	5.9681E-02	ENSG00000226578
NA	0.5347	1.2555E-02	5.9810E-02	ENSG00000274341
NA	0.3556	1.2559E-02	5.9818E-02	ENSG00000286214
NA	0.2502	1.2592E-02	5.9905E-02	ENSG00000289008
NA	0.5003	1.2694E-02	6.0295E-02	ENSG00000274628
NA	0.4197	1.2796E-02	6.0619E-02	ENSG00000279726
NA	0.5902	1.2802E-02	6.0634E-02	ENSG00000250994
NA	0.2911	1.2813E-02	6.0676E-02	ENSG00000262267
NA	0.0527	1.2964E-02		ENSG00000262777
NA	0.3052	1.3057E-02	6.1481E-02	ENSG00000244471
NA	0.3857	1.3073E-02	6.1523E-02	ENSG00000161149
NA	0.3484	1.3073E-02	6.1523E-02	ENSG00000225647
NA	0.4017	1.3089E-02	6.1579E-02	ENSG00000226239
NA	0.3027	1.3165E-02	6.1899E-02	ENSG00000237337
NA	0.2519	1.3168E-02	6.1902E-02	ENSG00000272931
NA	0.4301	1.3426E-02	6.2886E-02	ENSG00000273375
NA	0.4312	1.3443E-02	6.2944E-02	ENSG00000275601
NA	0.2688	1.3529E-02	6.3181E-02	ENSG00000274422
NA	0.4069	1.3741E-02	6.3912E-02	ENSG00000227914
NA	0.3238	1.3772E-02	6.4019E-02	ENSG00000286872
NA	0.4686	1.3786E-02	6.4048E-02	ENSG00000288762
NA	0.4349	1.3874E-02	6.4371E-02	ENSG00000243491
NA	0.3803	1.3920E-02	6.4524E-02	ENSG00000272541
NA	0.2580	1.3952E-02	6.4638E-02	ENSG00000257563
NA	0.3459	1.3983E-02	6.4744E-02	ENSG00000285091
NA	0.2395	1.4110E-02	6.5149E-02	ENSG00000257543
NA	0.4044	1.4195E-02	6.5407E-02	ENSG00000272948
NA	0.1225	1.4264E-02		ENSG00000287379
NA	0.1317	1.4304E-02		ENSG00000233891
NA	0.3559	1.4320E-02	6.5746E-02	ENSG00000251279
NA	0.1004	1.4400E-02		ENSG00000251412
NA	0.3610	1.4438E-02	6.6188E-02	ENSG00000269958
NA	0.3417	1.4537E-02	6.6496E-02	ENSG00000285979
NA	0.4006	1.4608E-02	6.6711E-02	ENSG00000249772
NA	0.3948	1.4705E-02	6.7069E-02	ENSG00000286288
NA	0.1931	1.4706E-02	6.7069E-02	ENSG00000261377
NA	0.3316	1.4861E-02	6.7499E-02	ENSG00000228423
NA	0.1320	1.4878E-02		ENSG00000285622
NA	0.3365	1.4896E-02	6.7600E-02	ENSG00000267852
NA	0.3060	1.4959E-02	6.7833E-02	ENSG00000289687
NA	0.2948	1.5151E-02	6.8438E-02	ENSG00000268364

NA	0.4030	1.5171E-02	6.8507E-02	ENSG00000289875
NA	0.1869	1.5189E-02	6.8546E-02	ENSG00000286862
NA	0.0720	1.5217E-02		ENSG00000254001
NA	0.2538	1.5246E-02	6.8755E-02	ENSG00000282798
NA	0.3484	1.5256E-02	6.8787E-02	ENSG00000266777
NA	0.0780	1.5393E-02	6.9293E-02	ENSG00000272128
NA	0.0941	1.5407E-02		ENSG00000227083
NA	0.2906	1.5412E-02	6.9350E-02	ENSG00000251867
NA	0.0713	1.5447E-02		ENSG00000272002
NA	0.3689	1.5536E-02	6.9748E-02	ENSG00000258847
NA	0.3837	1.5548E-02	6.9771E-02	ENSG00000280078
NA	0.3148	1.5611E-02	6.9983E-02	ENSG00000289342
NA	0.0742	1.5629E-02		ENSG00000258500
NA	0.1656	1.5636E-02		ENSG00000253194
NA	0.3179	1.5732E-02	7.0320E-02	ENSG00000246203
NA	0.3520	1.5806E-02	7.0568E-02	ENSG00000231441
NA	0.2959	1.5832E-02	7.0647E-02	ENSG00000288998
NA	0.3734	1.5862E-02	7.0717E-02	ENSG00000231711
NA	0.3131	1.5969E-02	7.1040E-02	ENSG00000285966
NA	0.2668	1.5986E-02	7.1088E-02	ENSG00000289585
NA	0.0455	1.6093E-02		ENSG00000285532
NA	0.2728	1.6102E-02	7.1409E-02	ENSG00000272556
NA	0.4041	1.6136E-02	7.1472E-02	ENSG00000261401
NA	0.3530	1.6141E-02	7.1472E-02	ENSG00000272986
NA	0.3519	1.6156E-02	7.1523E-02	ENSG00000231752
NA	0.2089	1.6171E-02	7.1538E-02	ENSG00000272150
NA	0.1581	1.6188E-02	7.1582E-02	ENSG00000174353
NA	0.3743	1.6211E-02	7.1663E-02	ENSG00000286977
NA	0.3563	1.6235E-02	7.1712E-02	ENSG00000279880
NA	0.3215	1.6436E-02	7.2371E-02	ENSG00000288043
NA	0.2526	1.6498E-02	7.2593E-02	ENSG00000257366
NA	0.2541	1.6566E-02	7.2759E-02	ENSG00000250166
NA	0.0860	1.6598E-02		ENSG00000250501
NA	0.2281	1.6633E-02	7.2947E-02	ENSG00000275494
NA	0.2037	1.6685E-02	7.3060E-02	ENSG00000267278
NA	0.3154	1.6693E-02	7.3083E-02	ENSG00000250053
NA	0.3608	1.6789E-02	7.3426E-02	ENSG00000285923
NA	0.2158	1.6796E-02	7.3442E-02	ENSG00000225446
NA	0.2238	1.6900E-02	7.3754E-02	ENSG00000286947
NA	0.0445	1.6942E-02		ENSG00000278001
NA	0.0641	1.7009E-02		ENSG00000232451
NA	0.0594	1.7192E-02		ENSG00000224330
NA	0.2021	1.7227E-02	7.4704E-02	ENSG00000263657
NA	0.3702	1.7399E-02	7.5304E-02	ENSG00000282807
NA	0.3051	1.7652E-02	7.6051E-02	ENSG00000250519
NA	0.2835	1.7669E-02	7.6097E-02	ENSG00000288904
NA	0.1597	1.7736E-02	7.6285E-02	ENSG00000227527
NA	0.3684	1.7837E-02	7.6568E-02	ENSG00000279058
NA	0.1208	1.7906E-02		ENSG00000228444

NA	0.5513	1.7943E-02	7.6939E-02	ENSG00000279668
NA	0.3501	1.7997E-02	7.6998E-02	ENSG00000287299
NA	0.2571	1.7999E-02	7.6998E-02	ENSG00000279565
NA	0.1352	1.8005E-02		ENSG00000213076
NA	0.3060	1.8055E-02	7.7128E-02	ENSG00000287516
NA	0.1007	1.8062E-02		ENSG00000273980
NA	0.3265	1.8236E-02	7.7705E-02	ENSG00000276213
NA	0.0522	1.8252E-02		ENSG00000234277
NA	0.3338	1.8267E-02	7.7813E-02	ENSG00000289701
NA	0.1160	1.8369E-02		ENSG00000258216
NA	0.3452	1.8608E-02	7.8832E-02	ENSG00000200502
NA	0.2619	1.8772E-02	7.9335E-02	ENSG00000268903
NA	0.0839	1.8785E-02		ENSG00000227038
NA	0.2006	1.8820E-02	7.9481E-02	ENSG00000289620
NA	0.2722	1.8916E-02	7.9767E-02	ENSG00000277310
NA	0.0409	1.9192E-02		ENSG00000273027
NA	0.2127	1.9210E-02	8.0676E-02	ENSG00000237877
NA	0.3370	1.9290E-02	8.0900E-02	ENSG00000272754
NA	0.2578	1.9320E-02	8.0957E-02	ENSG00000268949
NA	0.3076	1.9364E-02	8.1065E-02	ENSG00000279544
NA	0.2902	1.9366E-02	8.1065E-02	ENSG00000258752
NA	0.0641	1.9367E-02		ENSG00000229312
NA	0.2343	1.9369E-02	8.1065E-02	ENSG00000183148
NA	0.1409	1.9403E-02		ENSG00000276030
NA	0.3589	1.9436E-02	8.1288E-02	ENSG00000259209
NA	0.1992	1.9562E-02	8.1690E-02	ENSG00000238043
NA	0.1719	1.9668E-02	8.2008E-02	ENSG00000277147
NA	0.0503	1.9743E-02		ENSG00000286741
NA	0.3531	1.9915E-02	8.2754E-02	ENSG00000279332
NA	0.2787	1.9997E-02	8.3042E-02	ENSG00000184906
NA	0.1256	2.0032E-02	8.3131E-02	ENSG00000288015
NA	0.0563	2.0112E-02		ENSG00000247011
NA	0.3054	2.0189E-02	8.3570E-02	ENSG00000267519
NA	0.3400	2.0220E-02	8.3609E-02	ENSG00000267565
NA	0.3294	2.0227E-02	8.3609E-02	ENSG00000286149
NA	0.1774	2.0229E-02	8.3609E-02	ENSG00000276649
NA	0.3154	2.0233E-02	8.3615E-02	ENSG00000270996
NA	0.1330	2.0331E-02	8.3890E-02	ENSG00000279864
NA	0.2858	2.0339E-02	8.3910E-02	ENSG00000268573
NA	0.2865	2.0347E-02	8.3925E-02	ENSG00000258311
NA	0.3448	2.0435E-02	8.4179E-02	ENSG00000286443
NA	0.3789	2.0450E-02	8.4212E-02	ENSG00000289308
NA	0.3021	2.0576E-02	8.4586E-02	ENSG00000260947
NA	0.0449	2.0694E-02		ENSG00000275091
NA	0.2370	2.0789E-02	8.5293E-02	ENSG00000258354
NA	0.0791	2.0825E-02		ENSG00000260981
NA	0.2753	2.0874E-02	8.5548E-02	ENSG00000259891
NA	0.3494	2.0875E-02	8.5548E-02	ENSG00000285651
NA	0.3288	2.0882E-02	8.5553E-02	ENSG00000228050

NA	0.2598	2.0883E-02	8.5553E-02	ENSG00000251034
NA	0.2889	2.0914E-02	8.5665E-02	ENSG00000255410
NA	0.2971	2.1369E-02	8.7035E-02	ENSG00000222000
NA	0.3285	2.1488E-02	8.7361E-02	ENSG00000233554
NA	0.3371	2.1568E-02	8.7639E-02	ENSG00000272862
NA	0.3346	2.1574E-02	8.7639E-02	ENSG00000287264
NA	0.3077	2.1601E-02	8.7691E-02	ENSG00000280279
NA	0.0614	2.1633E-02		ENSG00000279488
NA	0.1988	2.1692E-02	8.7986E-02	ENSG00000285593
NA	0.0783	2.1841E-02		ENSG00000287473
NA	0.0371	2.1929E-02		ENSG00000231534
NA	0.1791	2.1965E-02	8.8861E-02	ENSG00000274996
NA	0.1345	2.2008E-02		ENSG00000279415
NA	0.1439	2.2163E-02		ENSG00000278979
NA	0.1900	2.2185E-02	8.9560E-02	ENSG00000242588
NA	0.2879	2.2198E-02	8.9598E-02	ENSG00000196096
NA	0.0723	2.2215E-02		ENSG00000224888
NA	0.2602	2.2251E-02	8.9766E-02	ENSG00000276386
NA	0.3252	2.2304E-02	8.9934E-02	ENSG00000253944
NA	0.0675	2.2309E-02		ENSG00000258594
NA	0.2781	2.2327E-02	8.9963E-02	ENSG00000288095
NA	0.1241	2.2328E-02	8.9963E-02	ENSG00000287335
NA	0.5598	2.2348E-02	9.0010E-02	ENSG00000265799
NA	0.1495	2.2671E-02	9.0928E-02	ENSG00000275540
NA	0.2391	2.2912E-02	9.1666E-02	ENSG00000288093
NA	0.1360	2.2928E-02	9.1714E-02	ENSG00000287929
NA	0.2969	2.2981E-02	9.1866E-02	ENSG00000261572
NA	0.0909	2.3188E-02		ENSG00000286805
NA	0.1504	2.3250E-02	9.2686E-02	ENSG00000233708
NA	0.0851	2.3275E-02		ENSG00000249199
NA	0.0636	2.3326E-02		ENSG00000260585
NA	0.1084	2.3356E-02		ENSG00000254092
NA	0.3166	2.3575E-02	9.3678E-02	ENSG00000288850
NA	0.2007	2.3594E-02	9.3725E-02	ENSG00000268756
NA	0.0402	2.3649E-02		ENSG00000244310
NA	0.3108	2.3693E-02	9.3981E-02	ENSG00000254910
NA	0.1521	2.3760E-02	9.4215E-02	ENSG00000188002
NA	0.2051	2.3847E-02	9.4388E-02	ENSG00000203706
NA	0.1711	2.3849E-02	9.4388E-02	ENSG00000289871
NA	0.1824	2.3940E-02	9.4683E-02	ENSG00000288695
NA	0.3027	2.4058E-02	9.5031E-02	ENSG00000272750
NA	0.2194	2.4220E-02	9.5531E-02	ENSG00000280184
NA	0.2268	2.4250E-02	9.5606E-02	ENSG00000272645
NA	0.2362	2.4286E-02	9.5680E-02	ENSG00000261522
NA	0.2708	2.4290E-02	9.5680E-02	ENSG00000273783
NA	0.2315	2.4294E-02	9.5680E-02	ENSG00000286620
NA	0.3011	2.4311E-02	9.5709E-02	ENSG00000234281
NA	0.1081	2.4340E-02		ENSG00000270988
NA	0.2858	2.4341E-02	9.5780E-02	ENSG00000289255

NA	0.0881	2.4452E-02		ENSG00000217746
NA	0.2818	2.4499E-02	9.6201E-02	ENSG00000237672
NA	0.2071	2.4543E-02	9.6328E-02	ENSG00000289827
NA	0.1112	2.4728E-02		ENSG00000286191
NA	0.3139	2.4814E-02	9.7144E-02	ENSG00000265888
NA	0.2906	2.4841E-02	9.7233E-02	ENSG00000286416
NA	0.1021	2.4904E-02	9.7342E-02	ENSG00000287923
NA	0.1006	2.4945E-02	9.7458E-02	ENSG00000287841
NA	0.2498	2.4946E-02	9.7458E-02	ENSG00000232862
NA	0.1834	2.5057E-02	9.7767E-02	ENSG00000231160
NA	0.2573	2.5105E-02	9.7814E-02	ENSG00000260361
NA	0.1207	2.5256E-02	9.8216E-02	ENSG00000218676
NA	0.2084	2.5279E-02	9.8268E-02	ENSG00000259865
NA	0.1687	2.5298E-02	9.8316E-02	ENSG00000250132
NA	0.2878	2.5312E-02	9.8316E-02	ENSG00000273026
NA	0.0884	2.5462E-02		ENSG00000269019
NA	0.1421	2.5589E-02		ENSG00000225218
NA	0.2930	2.5630E-02	9.9294E-02	ENSG00000271888
NA	0.2735	2.5652E-02	9.9365E-02	ENSG00000226981
NA	0.2317	2.5659E-02	9.9379E-02	ENSG00000233716
NA	0.5745	2.5747E-02	9.9672E-02	ENSG00000278897
NA	0.0675	2.5776E-02	9.9752E-02	ENSG00000287880
NA	0.2822	2.5781E-02	9.9757E-02	ENSG00000276529
NA	0.2856	2.5865E-02	9.9971E-02	ENSG00000269973
NA	0.1223	2.5928E-02		ENSG00000256972
NA	0.0627	2.6025E-02		ENSG00000287272
NA	0.3169	2.6138E-02	1.0075E-01	ENSG00000282393
NA	0.1777	2.6172E-02	1.0081E-01	ENSG00000255910
NA	0.2547	2.6192E-02	1.0085E-01	ENSG00000276840
NA	0.2194	2.6244E-02	1.0101E-01	ENSG00000287966
NA	0.1406	2.6303E-02	1.0115E-01	ENSG00000256101
NA	0.2899	2.6400E-02	1.0138E-01	ENSG00000280067
NA	0.2205	2.6554E-02	1.0188E-01	ENSG00000280327
NA	0.0232	2.6644E-02		ENSG00000248546
NA	0.2777	2.6646E-02	1.0208E-01	ENSG00000258983
NA	0.2546	2.6686E-02	1.0218E-01	ENSG00000226519
NA	0.3114	2.6738E-02	1.0232E-01	ENSG00000272186
NA	0.1546	2.6950E-02	1.0290E-01	ENSG00000149531
NA	0.1554	2.6982E-02	1.0294E-01	ENSG00000255556
NA	0.2902	2.7090E-02	1.0331E-01	ENSG00000267651
NA	0.0828	2.7190E-02		ENSG00000231512
NA	0.2090	2.7347E-02	1.0406E-01	ENSG00000273117
NA	0.2546	2.7464E-02	1.0443E-01	ENSG00000272068
NA	0.2938	2.7477E-02	1.0446E-01	ENSG00000287170
NA	0.0685	2.7510E-02		ENSG00000287088
NA	0.2400	2.7578E-02	1.0467E-01	ENSG00000223930
NA	0.2699	2.7585E-02	1.0468E-01	ENSG00000267505
NA	0.0945	2.7618E-02		ENSG00000276845
NA	0.2344	2.7640E-02	1.0478E-01	ENSG00000288982

NA	0.2094	2.7642E-02	1.0478E-01	ENSG00000289122
NA	0.0635	2.7654E-02		ENSG00000286984
NA	0.2905	2.7666E-02	1.0484E-01	ENSG00000182912
NA	0.2964	2.7900E-02	1.0556E-01	ENSG00000272115
NA	0.2863	2.8153E-02	1.0621E-01	ENSG00000289511
NA	0.2072	2.8169E-02	1.0624E-01	ENSG00000235558
NA	0.2370	2.8223E-02	1.0639E-01	ENSG00000269176
NA	0.0395	2.8314E-02		ENSG00000265425
NA	0.2088	2.8335E-02	1.0678E-01	ENSG00000223715
NA	0.1216	2.8340E-02		ENSG00000184617
NA	0.2835	2.8386E-02	1.0687E-01	ENSG00000240591
NA	0.0970	2.8463E-02	1.0709E-01	ENSG00000225964
NA	0.1595	2.8482E-02	1.0713E-01	ENSG00000278472
NA	0.1616	2.8627E-02	1.0751E-01	ENSG00000279568
NA	0.1007	2.8770E-02		ENSG00000278908
NA	0.2862	2.8841E-02	1.0805E-01	ENSG00000261172
NA	0.1078	2.8845E-02		ENSG00000267316
NA	0.2701	2.8867E-02	1.0813E-01	ENSG00000260475
NA	0.1551	2.8949E-02	1.0830E-01	ENSG00000223745
NA	0.2725	2.8967E-02	1.0834E-01	ENSG00000234772
NA	0.0877	2.8974E-02		ENSG00000217314
NA	0.2841	2.9229E-02	1.0912E-01	ENSG00000289222
NA	0.2747	2.9288E-02	1.0927E-01	ENSG00000287064
NA	0.2279	2.9431E-02	1.0950E-01	ENSG00000278920
NA	0.1362	2.9599E-02	1.1009E-01	ENSG00000259709
NA	0.2055	2.9779E-02	1.1055E-01	ENSG00000272777
NA	0.3647	2.9876E-02	1.1087E-01	ENSG00000237950
NA	0.2450	2.9884E-02	1.1089E-01	ENSG00000225259
NA	0.2920	2.9979E-02	1.1121E-01	ENSG00000286736
NA	0.0860	3.0015E-02		ENSG00000287466
NA	0.1212	3.0055E-02		ENSG00000257095
NA	0.1770	3.0161E-02	1.1170E-01	ENSG00000273080
NA	0.2591	3.0189E-02	1.1175E-01	ENSG00000286372
NA	0.2611	3.0214E-02	1.1182E-01	ENSG00000276571
NA	0.1707	3.0247E-02	1.1190E-01	ENSG00000201217
NA	0.1926	3.0318E-02	1.1206E-01	ENSG00000260708
NA	0.2929	3.0423E-02	1.1233E-01	ENSG00000269981
NA	0.1818	3.0442E-02	1.1238E-01	ENSG00000249353
NA	0.1542	3.0527E-02	1.1260E-01	ENSG00000280212
NA	0.2878	3.0528E-02	1.1260E-01	ENSG00000236514
NA	0.0715	3.0626E-02		ENSG00000255363
NA	0.2791	3.0667E-02	1.1299E-01	ENSG00000287828
NA	0.2794	3.0698E-02	1.1309E-01	ENSG00000287221
NA	0.2601	3.0779E-02	1.1332E-01	ENSG00000261251
NA	0.1052	3.0786E-02		ENSG00000287349
NA	0.1413	3.0894E-02	1.1365E-01	ENSG00000223914
NA	0.1436	3.0913E-02		ENSG00000289379
NA	0.2808	3.0941E-02	1.1377E-01	ENSG00000235357
NA	0.1259	3.0943E-02	1.1377E-01	ENSG00000273091

NA	0.0536	3.0952E-02		ENSG00000287024
NA	0.0660	3.1096E-02		ENSG00000286506
NA	0.2671	3.1145E-02	1.1433E-01	ENSG00000263826
NA	0.2778	3.1173E-02	1.1435E-01	ENSG00000256667
NA	0.0837	3.1208E-02		ENSG00000226965
NA	0.0981	3.1242E-02		ENSG00000267838
NA	0.2795	3.1314E-02	1.1474E-01	ENSG00000267327
NA	0.1351	3.1388E-02	1.1491E-01	ENSG00000256637
NA	0.1463	3.1402E-02	1.1494E-01	ENSG00000272692
NA	0.1372	3.1558E-02	1.1531E-01	ENSG00000272374
NA	0.1000	3.1562E-02		ENSG00000288970
NA	0.2187	3.1811E-02	1.1587E-01	ENSG00000257246
NA	0.0778	3.1821E-02		ENSG00000236975
NA	0.2728	3.1846E-02	1.1597E-01	ENSG00000259775
NA	0.2536	3.1870E-02	1.1598E-01	ENSG00000236540
NA	0.1820	3.1906E-02	1.1605E-01	ENSG00000289031
NA	0.1905	3.1975E-02	1.1614E-01	ENSG00000275216
NA	0.1136	3.2146E-02		ENSG00000276118
NA	0.2213	3.2261E-02	1.1697E-01	ENSG00000255198
NA	0.0887	3.2266E-02		ENSG00000226928
NA	0.2828	3.2279E-02	1.1702E-01	ENSG00000273062
NA	0.1896	3.2373E-02	1.1721E-01	ENSG00000287070
NA	0.2097	3.2447E-02	1.1740E-01	ENSG00000214796
NA	0.1209	3.2496E-02		ENSG00000230067
NA	0.2511	3.2728E-02	1.1816E-01	ENSG00000280057
NA	0.2753	3.2820E-02	1.1841E-01	ENSG00000286677
NA	0.2741	3.3071E-02	1.1919E-01	ENSG00000210195
NA	0.2717	3.3121E-02	1.1927E-01	ENSG00000289492
NA	0.1088	3.3189E-02	1.1944E-01	ENSG00000256995
NA	0.2500	3.3345E-02	1.1985E-01	ENSG00000228305
NA	0.2557	3.3414E-02	1.1993E-01	ENSG00000257252
NA	0.1750	3.3428E-02	1.1995E-01	ENSG00000230510
NA	0.2052	3.3529E-02	1.2026E-01	ENSG00000278002
NA	0.0668	3.3563E-02		ENSG00000225399
NA	0.0548	3.3582E-02		ENSG00000289518
NA	0.2647	3.3596E-02	1.2040E-01	ENSG00000287737
NA	0.2338	3.3734E-02	1.2068E-01	ENSG00000276449
NA	0.1219	3.3799E-02		ENSG00000108785
NA	0.2320	3.3839E-02	1.2084E-01	ENSG00000284735
NA	0.2720	3.3855E-02	1.2085E-01	ENSG00000261570
NA	0.0358	3.3861E-02		ENSG00000252436
NA	0.0614	3.3894E-02		ENSG00000201451
NA	0.2359	3.3934E-02	1.2106E-01	ENSG00000230869
NA	0.2050	3.3945E-02	1.2107E-01	ENSG00000288823
NA	0.2414	3.3980E-02	1.2112E-01	ENSG00000237357
NA	0.2667	3.4090E-02	1.2148E-01	ENSG00000282564
NA	0.1873	3.4176E-02	1.2169E-01	ENSG00000226779
NA	0.2146	3.4193E-02	1.2174E-01	ENSG00000279584
NA	0.1955	3.4206E-02	1.2175E-01	ENSG00000259726

NA	0.2283	3.4508E-02	1.2261E-01	ENSG00000228680
NA	0.1374	3.4578E-02		ENSG00000253161
NA	0.0790	3.4603E-02		ENSG00000287174
NA	0.0682	3.4721E-02		ENSG00000235042
NA	0.0842	3.5274E-02		ENSG00000277831
NA	0.1309	3.5322E-02	1.2484E-01	ENSG00000275228
NA	0.1819	3.5340E-02	1.2488E-01	ENSG00000176933
NA	0.2540	3.5381E-02	1.2497E-01	ENSG00000262619
NA	0.2634	3.5704E-02	1.2584E-01	ENSG00000279549
NA	0.0807	3.5722E-02		ENSG00000258868
NA	0.0642	3.5750E-02	1.2595E-01	ENSG00000235993
NA	0.2491	3.5983E-02	1.2657E-01	ENSG00000263011
NA	0.0519	3.5996E-02		ENSG00000273971
NA	0.2125	3.6015E-02	1.2663E-01	ENSG00000289041
NA	0.2536	3.6015E-02	1.2663E-01	ENSG00000246334
NA	0.2495	3.6090E-02	1.2675E-01	ENSG00000286830
NA	0.2528	3.6125E-02	1.2680E-01	ENSG00000286198
NA	0.0487	3.6126E-02		ENSG00000261728
NA	0.0877	3.6405E-02		ENSG00000261632
NA	0.0633	3.6548E-02		ENSG00000206781
NA	0.2504	3.6894E-02	1.2895E-01	ENSG00000285980
NA	0.1961	3.7008E-02	1.2927E-01	ENSG00000224391
NA	0.0956	3.7102E-02		ENSG00000286121
NA	0.2466	3.7182E-02	1.2971E-01	ENSG00000270095
NA	0.2253	3.7217E-02	1.2977E-01	ENSG00000280310
NA	0.0640	3.7536E-02		ENSG00000205976
NA	0.0512	3.7730E-02		ENSG00000277350
NA	0.1928	3.7774E-02	1.3137E-01	ENSG00000251188
NA	0.2098	3.7839E-02	1.3149E-01	ENSG00000224903
NA	0.0349	3.7843E-02		ENSG00000218682
NA	0.0869	3.7873E-02		ENSG00000266282
NA	0.2399	3.7926E-02	1.3175E-01	ENSG00000286406
NA	0.2364	3.7932E-02	1.3175E-01	ENSG00000258776
NA	0.0569	3.7954E-02		ENSG00000267733
NA	0.2517	3.8016E-02	1.3199E-01	ENSG00000230699
NA	0.0539	3.8089E-02		ENSG00000272094
NA	0.0763	3.8342E-02		ENSG00000254042
NA	0.2553	3.8418E-02	1.3293E-01	ENSG00000278743
NA	0.2369	3.8593E-02	1.3342E-01	ENSG00000285601
NA	0.1844	3.8597E-02	1.3342E-01	ENSG00000279595
NA	0.1255	3.8721E-02	1.3370E-01	ENSG00000259038
NA	0.1805	3.8822E-02	1.3395E-01	ENSG00000251003
NA	0.2478	3.8898E-02	1.3413E-01	ENSG00000256705
NA	0.0958	3.9136E-02		ENSG00000159904
NA	0.2532	3.9228E-02	1.3497E-01	ENSG00000288002
NA	0.0978	3.9509E-02		ENSG00000286100
NA	0.2468	3.9511E-02	1.3581E-01	ENSG00000270127
NA	0.1247	3.9575E-02	1.3596E-01	ENSG00000289361
NA	0.1491	3.9607E-02	1.3601E-01	ENSG00000253203

NA	0.1229	3.9611E-02	1.3601E-01	ENSG00000288925
NA	0.0361	3.9698E-02		ENSG00000260194
NA	0.2500	3.9827E-02	1.3639E-01	ENSG00000225507
NA	0.1360	3.9918E-02	1.3655E-01	ENSG00000270147
NA	0.2321	3.9920E-02	1.3655E-01	ENSG00000287262
NA	0.1146	3.9970E-02		ENSG00000275328
NA	0.1564	4.0036E-02	1.3686E-01	ENSG00000272667
NA	0.2074	4.0053E-02	1.3688E-01	ENSG00000202415
NA	0.2105	4.0122E-02	1.3705E-01	ENSG00000240429
NA	0.1187	4.0180E-02		ENSG00000229385
NA	0.0426	4.0259E-02		ENSG00000286824
NA	0.1597	4.0284E-02	1.3749E-01	ENSG00000260193
NA	0.2432	4.0336E-02	1.3763E-01	ENSG00000260919
NA	0.2442	4.0363E-02	1.3771E-01	ENSG00000250699
NA	0.2052	4.0423E-02	1.3779E-01	ENSG00000244625
NA	0.0761	4.0472E-02	1.3791E-01	ENSG00000280850
NA	0.1586	4.0630E-02	1.3823E-01	ENSG00000285619
NA	0.1692	4.0796E-02	1.3869E-01	ENSG00000273306
NA	0.0395	4.0975E-02		ENSG00000254066
NA	0.2494	4.1016E-02	1.3922E-01	ENSG00000269054
NA	0.1005	4.1128E-02		ENSG00000289058
NA	0.0877	4.1241E-02		ENSG00000288794
NA	0.2417	4.1329E-02	1.3999E-01	ENSG00000257557
NA	0.0800	4.1341E-02	1.3999E-01	ENSG00000227887
NA	0.2477	4.1354E-02	1.4002E-01	ENSG00000279342
NA	0.1469	4.1492E-02	1.4037E-01	ENSG00000283913
NA	0.0489	4.1510E-02		ENSG00000259396
NA	0.0426	4.1537E-02		ENSG00000286109
NA	0.1907	4.1610E-02	1.4060E-01	ENSG00000285667
NA	0.2445	4.1765E-02	1.4087E-01	ENSG00000274929
NA	0.0703	4.1888E-02		ENSG00000257270
NA	0.2426	4.1996E-02	1.4143E-01	ENSG00000283696
NA	0.2288	4.2043E-02	1.4150E-01	ENSG00000251129
NA	0.1714	4.2137E-02	1.4171E-01	ENSG00000288065
NA	0.2475	4.2233E-02	1.4200E-01	ENSG00000286437
NA	0.2356	4.2435E-02	1.4248E-01	ENSG00000287957
NA	0.1915	4.2515E-02	1.4258E-01	ENSG00000278935
NA	0.1999	4.2678E-02	1.4301E-01	ENSG00000288770
NA	0.1903	4.2685E-02	1.4301E-01	ENSG00000146722
NA	0.0994	4.2702E-02		ENSG00000230323
NA	0.1127	4.2766E-02		ENSG00000268051
NA	0.0677	4.2859E-02		ENSG00000289477
NA	0.0702	4.2883E-02		ENSG00000271590
NA	0.2441	4.3042E-02	1.4387E-01	ENSG00000250938
NA	0.1555	4.3106E-02	1.4399E-01	ENSG00000227486
NA	0.0502	4.3311E-02		ENSG00000222024
NA	0.1596	4.3370E-02	1.4469E-01	ENSG00000287561
NA	0.2416	4.3443E-02	1.4486E-01	ENSG00000285697
NA	0.1362	4.3461E-02	1.4486E-01	ENSG00000280190

NA	0.2212	4.3516E-02	1.4498E-01	ENSG00000253671
NA	0.1353	4.3568E-02	1.4514E-01	ENSG00000273013
NA	0.0520	4.3588E-02		ENSG00000253673
NA	0.2076	4.3597E-02	1.4518E-01	ENSG00000218996
NA	0.2308	4.3688E-02	1.4532E-01	ENSG00000263006
NA	0.2293	4.3790E-02	1.4552E-01	ENSG00000288896
NA	0.1287	4.3811E-02	1.4555E-01	ENSG00000261019
NA	0.1660	4.3824E-02	1.4558E-01	ENSG00000267284
NA	0.1678	4.4186E-02	1.4646E-01	ENSG00000255237
NA	0.2276	4.4405E-02	1.4709E-01	ENSG00000231240
NA	0.1328	4.4564E-02	1.4748E-01	ENSG00000242072
NA	0.0507	4.4833E-02		ENSG00000231604
NA	0.2043	4.4977E-02	1.4840E-01	ENSG00000249572
NA	0.2182	4.5026E-02	1.4845E-01	ENSG00000289238
NA	0.1492	4.5037E-02	1.4846E-01	ENSG00000290095
NA	0.2112	4.5106E-02	1.4863E-01	ENSG00000259088
NA	0.0864	4.5326E-02		ENSG00000272980
NA	0.0337	4.5328E-02		ENSG00000267443
NA	0.2361	4.5537E-02	1.4981E-01	ENSG00000227896
NA	0.1902	4.5730E-02	1.5030E-01	ENSG00000270964
NA	0.0906	4.5732E-02		ENSG00000259932
NA	0.1509	4.5839E-02	1.5058E-01	ENSG00000281920
NA	0.2270	4.5877E-02	1.5067E-01	ENSG00000255310
NA	0.2075	4.5912E-02	1.5068E-01	ENSG00000231154
NA	0.0821	4.5912E-02		ENSG00000213121
NA	0.2203	4.5936E-02	1.5074E-01	ENSG00000259994
NA	0.1969	4.5972E-02	1.5082E-01	ENSG00000289970
NA	0.1757	4.6183E-02	1.5129E-01	ENSG00000289476
NA	0.0478	4.6234E-02	1.5143E-01	ENSG00000229988
NA	0.1834	4.6367E-02	1.5169E-01	ENSG00000283162
NA	0.1166	4.6434E-02		ENSG00000258698
NA	0.1709	4.6588E-02	1.5215E-01	ENSG00000188185
NA	0.2336	4.6791E-02	1.5265E-01	ENSG00000236816
NA	0.2052	4.6921E-02	1.5297E-01	ENSG00000278917
NA	0.1914	4.7053E-02	1.5328E-01	ENSG00000236939
NA	0.1783	4.7168E-02	1.5349E-01	ENSG00000225080
NA	0.2191	4.7230E-02	1.5358E-01	ENSG00000274400
NA	0.0925	4.7305E-02		ENSG00000275070
NA	0.1007	4.7326E-02		ENSG00000238290
NA	0.0788	4.7440E-02		ENSG00000287988
NA	0.0738	4.7479E-02		ENSG00000258857
NA	0.1374	4.7584E-02	1.5442E-01	ENSG00000228547
NA	0.1995	4.7694E-02	1.5461E-01	ENSG00000277579
NA	0.1442	4.7709E-02	1.5462E-01	ENSG00000283058
NA	0.2050	4.7725E-02	1.5464E-01	ENSG00000287808
NA	0.2316	4.7740E-02	1.5464E-01	ENSG00000182021
NA	0.0649	4.7851E-02		ENSG00000242140
NA	0.1788	4.7874E-02	1.5483E-01	ENSG00000181097
NA	0.1493	4.8087E-02	1.5531E-01	ENSG00000229487

NA	0.0539	4.8105E-02		ENSG00000256943
NA	0.2273	4.8616E-02	1.5653E-01	ENSG00000289496
NA	0.2194	4.8648E-02	1.5658E-01	ENSG00000277245
NA	0.1856	4.8730E-02	1.5671E-01	ENSG00000281909
NA	0.1418	4.8986E-02	1.5733E-01	ENSG00000261131
NA	0.0428	4.9095E-02		ENSG00000279304
NA	0.0492	4.9215E-02		ENSG00000236900
NA	0.2281	4.9224E-02	1.5785E-01	ENSG00000262352
NA	0.2268	4.9264E-02	1.5789E-01	ENSG00000276071
NA	0.2028	4.9337E-02	1.5807E-01	ENSG00000279706
NA	0.2223	4.9376E-02	1.5811E-01	ENSG00000260450
NA	0.1979	4.9495E-02	1.5843E-01	ENSG00000285741
NA	0.1148	4.9633E-02	1.5880E-01	ENSG00000255489
NA	0.2189	4.9722E-02	1.5903E-01	ENSG00000230303
NA	0.1259	4.9781E-02	1.5918E-01	ENSG00000285592
NA	0.0712	4.9822E-02		ENSG00000279839
NA	0.2252	5.0116E-02	1.5994E-01	ENSG00000286997
NA	0.1111	5.0120E-02		ENSG00000289247
NA	0.2160	5.0123E-02	1.5994E-01	ENSG00000259818
NA	0.1045	5.0219E-02	1.6011E-01	ENSG00000253535
NA	0.1368	5.0503E-02	1.6063E-01	ENSG00000273308
NA	0.1908	5.0672E-02	1.6104E-01	ENSG00000286816
NA	0.1934	5.0780E-02	1.6124E-01	ENSG00000259488
NA	0.2114	5.0835E-02	1.6134E-01	ENSG00000261669
NA	0.1326	5.0839E-02	1.6134E-01	ENSG00000272049
NA	0.1654	5.0845E-02	1.6134E-01	ENSG00000272023
NA	0.2202	5.0957E-02	1.6153E-01	ENSG00000236255
NA	0.1194	5.1013E-02	1.6156E-01	ENSG00000272994
NA	0.1422	5.1323E-02	1.6229E-01	ENSG00000285850
NA	0.2194	5.1836E-02	1.6331E-01	ENSG00000289155
NA	0.2255	5.1893E-02	1.6342E-01	ENSG00000289855
NA	0.1224	5.1926E-02	1.6347E-01	ENSG00000259113
NA	0.2050	5.2005E-02	1.6367E-01	ENSG00000289486
NA	0.0344	5.2031E-02		ENSG00000232057
NA	0.0554	5.2057E-02		ENSG00000269256
NA	0.1150	5.2132E-02	1.6397E-01	ENSG00000279985
NA	0.0483	5.2419E-02		ENSG00000266893
NA	0.2169	5.2517E-02	1.6489E-01	ENSG00000177822
NA	0.1828	5.2662E-02	1.6523E-01	ENSG00000276805
NA	0.0984	5.2816E-02	1.6552E-01	ENSG00000277621
NA	0.1339	5.2866E-02	1.6562E-01	ENSG00000289173
NA	0.0782	5.2960E-02		ENSG00000259360
NA	0.1091	5.3078E-02	1.6613E-01	ENSG00000279361
NA	0.0609	5.3207E-02		ENSG00000276436
NA	0.0486	5.3220E-02		ENSG00000254874
NA	0.2167	5.3227E-02	1.6643E-01	ENSG00000276334
NA	0.0998	5.3543E-02		ENSG00000289027
NA	0.1542	5.3611E-02	1.6736E-01	ENSG00000251363
NA	0.0299	5.3675E-02		ENSG00000248975

NA	0.1033	5.3744E-02		ENSG00000290012
NA	0.0633	5.3840E-02		ENSG00000289107
NA	0.0506	5.3904E-02		ENSG00000226340
NA	0.1431	5.3937E-02	1.6820E-01	ENSG00000279026
NA	0.1006	5.3951E-02		ENSG00000249096
NA	0.1587	5.4089E-02	1.6851E-01	ENSG00000227220
NA	0.0943	5.4212E-02		ENSG00000264083
NA	0.0766	5.4379E-02		ENSG00000253456
NA	0.0594	5.4726E-02		ENSG00000213735
NA	0.1140	5.4752E-02		ENSG00000273017
NA	0.0464	5.4892E-02		ENSG00000167798
NA	0.0458	5.4996E-02		ENSG00000271795
NA	0.1831	5.5036E-02	1.7066E-01	ENSG00000262587
NA	0.0884	5.5178E-02		ENSG00000286349
NA	0.2115	5.5240E-02	1.7115E-01	ENSG00000235410
NA	0.1427	5.5299E-02	1.7128E-01	ENSG00000269139
NA	0.1791	5.5425E-02	1.7156E-01	ENSG00000250535
NA	0.0496	5.5513E-02		ENSG00000255477
NA	0.1333	5.5573E-02	1.7187E-01	ENSG00000257769
NA	0.1929	5.5736E-02	1.7226E-01	ENSG00000230641
NA	0.1622	5.5780E-02	1.7237E-01	ENSG00000180229
NA	0.1104	5.6098E-02	1.7305E-01	ENSG00000276291
NA	0.2058	5.6266E-02	1.7334E-01	ENSG00000243433
NA	0.1073	5.6296E-02	1.7337E-01	ENSG00000290165
NA	0.1871	5.6300E-02	1.7337E-01	ENSG00000271830
NA	0.2126	5.6334E-02	1.7341E-01	ENSG00000250220
NA	0.1262	5.6404E-02	1.7361E-01	ENSG00000185986
NA	0.2085	5.6514E-02	1.7382E-01	ENSG00000289272
NA	0.1044	5.6531E-02	1.7385E-01	ENSG00000287154
NA	0.0459	5.6568E-02		ENSG00000280435
NA	0.2119	5.6637E-02	1.7404E-01	ENSG00000273619
NA	0.2175	5.6677E-02	1.7408E-01	ENSG00000255062
NA	0.0397	5.6761E-02		ENSG00000257964
NA	0.1298	5.6775E-02	1.7433E-01	ENSG00000238260
NA	0.1287	5.6879E-02	1.7459E-01	ENSG00000226886
NA	0.1579	5.6994E-02	1.7477E-01	ENSG00000279575
NA	0.1649	5.7075E-02	1.7491E-01	ENSG00000233614
NA	0.2136	5.7339E-02	1.7547E-01	ENSG00000225285
NA	0.1367	5.7427E-02	1.7570E-01	ENSG00000287332
NA	0.1203	5.7508E-02	1.7584E-01	ENSG00000202190
NA	0.1652	5.7597E-02	1.7596E-01	ENSG00000257456
NA	0.1388	5.7702E-02	1.7617E-01	ENSG00000254701
NA	0.2149	5.7752E-02	1.7617E-01	ENSG00000288852
NA	0.0647	5.7767E-02		ENSG00000289068
NA	0.0459	5.7842E-02		ENSG00000261816
NA	0.1537	5.7891E-02	1.7634E-01	ENSG00000288105
NA	0.2126	5.7951E-02	1.7647E-01	ENSG00000277602
NA	0.1389	5.8252E-02	1.7708E-01	ENSG00000267731
NA	0.0714	5.8278E-02		ENSG00000244063

NA	0.0410	5.8975E-02		ENSG00000286384
NA	0.0294	5.9004E-02		ENSG00000237525
NA	0.1822	5.9130E-02	1.7871E-01	ENSG00000227953
NA	0.3080	5.9200E-02	1.7883E-01	ENSG00000287778
NA	0.1281	5.9293E-02	1.7897E-01	ENSG00000273432
NA	0.1693	5.9463E-02	1.7930E-01	ENSG00000260018
NA	0.2103	5.9535E-02	1.7934E-01	ENSG00000231890
NA	0.0265	5.9664E-02		ENSG00000289851
NA	0.0611	5.9714E-02		ENSG00000224289
NA	0.0528	5.9832E-02		ENSG00000231040
NA	0.0596	5.9847E-02		ENSG00000285201
NA	0.1404	5.9931E-02	1.8018E-01	ENSG00000267249
NA	0.1471	6.0097E-02	1.8055E-01	ENSG00000214546
NA	0.0861	6.0224E-02		ENSG00000231255
NA	0.1842	6.0260E-02	1.8093E-01	ENSG00000278133
NA	0.1876	6.0286E-02	1.8098E-01	ENSG00000259959
NA	0.1535	6.0306E-02	1.8100E-01	ENSG00000269892
NA	0.2074	6.0320E-02	1.8102E-01	ENSG00000250770
NA	0.2002	6.0469E-02	1.8136E-01	ENSG00000288885
NA	0.0836	6.0473E-02		ENSG00000260368
NA	0.0570	6.0578E-02		ENSG00000257398
NA	0.1878	6.0588E-02	1.8160E-01	ENSG00000253116
NA	0.2130	6.0622E-02	1.8168E-01	ENSG00000239462
NA	0.0758	6.0690E-02	1.8182E-01	ENSG00000273853
NA	0.0332	6.1037E-02	1.8246E-01	ENSG00000243368
NA	0.0918	6.1321E-02	1.8315E-01	ENSG00000236740
NA	0.1440	6.1386E-02	1.8325E-01	ENSG00000273270
NA	0.1351	6.1460E-02	1.8341E-01	ENSG00000267741
NA	0.0996	6.1498E-02		ENSG00000260658
NA	0.1559	6.1710E-02	1.8400E-01	ENSG00000260922
NA	0.1843	6.2140E-02	1.8506E-01	ENSG00000266718
NA	0.2095	6.2180E-02	1.8513E-01	ENSG00000257556
NA	0.1738	6.2328E-02	1.8544E-01	ENSG00000225544
NA	0.1310	6.2333E-02	1.8544E-01	ENSG00000250415
NA	0.1957	6.2335E-02	1.8544E-01	ENSG00000289092
NA	0.1451	6.2384E-02	1.8549E-01	ENSG00000233586
NA	0.2055	6.2634E-02	1.8590E-01	ENSG00000261167
NA	0.1294	6.2671E-02	1.8599E-01	ENSG00000231443
NA	0.1788	6.2822E-02	1.8626E-01	ENSG00000245025
NA	0.1016	6.2853E-02		ENSG00000227200
NA	0.0491	6.2902E-02		ENSG00000287972
NA	0.1908	6.2980E-02	1.8661E-01	ENSG00000289438
NA	0.0829	6.3032E-02		ENSG00000229953
NA	0.0573	6.3083E-02		ENSG00000280025
NA	0.0697	6.3097E-02		ENSG00000213355
NA	0.0999	6.3113E-02		ENSG00000289891
NA	0.1839	6.3288E-02	1.8734E-01	ENSG00000176134
NA	0.1982	6.3370E-02	1.8752E-01	ENSG00000280047
NA	0.2093	6.3418E-02	1.8764E-01	ENSG00000260278

NA	0.0264	6.3420E-02		ENSG00000276916
NA	0.1551	6.3584E-02	1.8799E-01	ENSG00000271398
NA	0.1188	6.3702E-02	1.8821E-01	ENSG00000262879
NA	0.1133	6.3830E-02	1.8840E-01	ENSG00000272263
NA	0.0686	6.3988E-02		ENSG00000285715
NA	0.0192	6.4044E-02		ENSG00000254712
NA	0.1284	6.4053E-02	1.8886E-01	ENSG00000229458
NA	0.1386	6.4372E-02	1.8966E-01	ENSG00000203593
NA	0.0433	6.4488E-02		ENSG00000289134
NA	0.0326	6.4517E-02		ENSG00000232271
NA	0.1273	6.4689E-02	1.9032E-01	ENSG00000273402
NA	0.1435	6.4736E-02	1.9039E-01	ENSG00000233766
NA	0.0636	6.4826E-02	1.9054E-01	ENSG00000287679
NA	0.0966	6.4896E-02	1.9061E-01	ENSG00000285627
NA	0.0946	6.5519E-02		ENSG00000197099
NA	0.0349	6.5543E-02		ENSG00000283973
NA	0.1374	6.5705E-02	1.9232E-01	ENSG00000273035
NA	0.1713	6.5820E-02	1.9255E-01	ENSG00000286449
NA	0.1408	6.5922E-02	1.9282E-01	ENSG00000231584
NA	0.1623	6.6126E-02	1.9326E-01	ENSG00000271369
NA	0.0709	6.6177E-02		ENSG00000280099
NA	0.0559	6.6272E-02	1.9359E-01	ENSG00000278022
NA	0.1372	6.6273E-02	1.9359E-01	ENSG00000224418
NA	0.1704	6.6313E-02	1.9369E-01	ENSG00000264304
NA	0.2003	6.6358E-02	1.9373E-01	ENSG00000287643
NA	0.1206	6.6536E-02	1.9409E-01	ENSG00000288156
NA	0.0582	6.6601E-02		ENSG00000244723
NA	0.0979	6.6686E-02		ENSG00000231841
NA	0.1119	6.6780E-02	1.9457E-01	ENSG00000272922
NA	0.1724	6.6791E-02	1.9458E-01	ENSG00000271780
NA	0.0347	6.6990E-02		ENSG00000259935
NA	0.1038	6.7114E-02	1.9517E-01	ENSG00000279409
NA	0.2017	6.7290E-02	1.9552E-01	ENSG00000279317
NA	0.1430	6.7335E-02	1.9558E-01	ENSG00000259692
NA	0.1952	6.7392E-02	1.9567E-01	ENSG00000272070
NA	0.1029	6.7397E-02	1.9567E-01	ENSG00000288745
NA	0.0440	6.7412E-02		ENSG00000229173
NA	0.1742	6.7628E-02	1.9609E-01	ENSG00000277369
NA	0.0401	6.7735E-02		ENSG00000260578
NA	0.1879	6.7750E-02	1.9628E-01	ENSG00000279227
NA	0.1737	6.7771E-02	1.9628E-01	ENSG00000227598
NA	0.1770	6.7836E-02	1.9643E-01	ENSG00000277186
NA	0.0344	6.7873E-02		ENSG00000287732
NA	0.0376	6.7881E-02		ENSG00000259999
NA	0.0562	6.8120E-02		ENSG00000282041
NA	0.0409	6.8237E-02		ENSG00000285579
NA	0.0715	6.8290E-02		ENSG00000289573
NA	0.1635	6.8293E-02	1.9727E-01	ENSG00000264112
NA	0.0640	6.8339E-02		ENSG00000285834

NA	0.0692	6.8452E-02		ENSG00000289986
NA	0.0590	6.8531E-02		ENSG00000259442
NA	0.1449	6.8538E-02	1.9784E-01	ENSG00000287869
NA	0.1095	6.8562E-02	1.9789E-01	ENSG00000252569
NA	0.1203	6.8639E-02	1.9801E-01	ENSG00000282160
NA	0.1923	6.8665E-02	1.9802E-01	ENSG00000269559
NA	0.1770	6.8780E-02	1.9826E-01	ENSG00000267264
NA	0.1210	6.8904E-02	1.9847E-01	ENSG00000280105
NA	0.1752	6.8920E-02	1.9850E-01	ENSG00000285090
NA	0.0615	6.8946E-02		ENSG00000287645
NA	0.0912	6.8967E-02	1.9859E-01	ENSG00000287529
NA	0.0291	6.9073E-02		ENSG00000222386
NA	0.0607	6.9251E-02		ENSG00000278250
NA	0.1848	6.9541E-02	1.9983E-01	ENSG00000286000
NA	0.0706	6.9610E-02		ENSG00000259058
NA	0.0871	6.9636E-02		ENSG00000249478
NA	0.1815	6.9809E-02	2.0047E-01	ENSG00000261173
NA	0.1368	6.9849E-02	2.0056E-01	ENSG00000231764
NA	0.1321	6.9947E-02	2.0071E-01	ENSG00000288557
NA	0.1889	7.0061E-02	2.0098E-01	ENSG00000226900
NA	0.0851	7.0208E-02		ENSG00000228648
NA	0.1960	7.0252E-02	2.0120E-01	ENSG00000286084
NA	0.1204	7.0266E-02	2.0122E-01	ENSG00000253112
NA	0.1797	7.0282E-02	2.0125E-01	ENSG00000256673
NA	0.1713	7.0320E-02	2.0131E-01	ENSG00000272356
NA	0.1958	7.0345E-02	2.0131E-01	ENSG00000278576
NA	0.0504	7.0429E-02		ENSG00000249815
NA	0.0490	7.0830E-02		ENSG00000263567
NA	0.1715	7.0965E-02	2.0263E-01	ENSG00000255355
NA	0.0369	7.1137E-02		ENSG00000231390
NA	0.1403	7.1303E-02	2.0336E-01	ENSG00000280248
NA	0.0415	7.1343E-02		ENSG00000287831
NA	0.1848	7.1671E-02	2.0415E-01	ENSG00000272791
NA	0.0546	7.1811E-02		ENSG00000228527
NA	0.1420	7.2004E-02	2.0486E-01	ENSG00000235296
NA	0.1847	7.2543E-02	2.0585E-01	ENSG00000287574
NA	0.1768	7.2693E-02	2.0618E-01	ENSG00000260118
NA	0.1941	7.2845E-02	2.0656E-01	ENSG00000265625
NA	0.1293	7.2917E-02	2.0672E-01	ENSG00000255406
NA	0.1678	7.2940E-02	2.0672E-01	ENSG00000231258
NA	0.1855	7.2955E-02	2.0672E-01	ENSG00000176700
NA	0.1901	7.3313E-02	2.0744E-01	ENSG00000270557
NA	0.1769	7.3325E-02	2.0744E-01	ENSG00000238287
NA	0.0704	7.3432E-02		ENSG00000283648
NA	0.1916	7.3455E-02	2.0769E-01	ENSG00000187984
NA	0.1745	7.3483E-02	2.0773E-01	ENSG00000277270
NA	0.0513	7.3626E-02		ENSG00000200488
NA	0.1547	7.3634E-02	2.0789E-01	ENSG00000237423
NA	0.1419	7.3759E-02	2.0817E-01	ENSG00000254604

NA	0.1285	7.3906E-02	2.0837E-01	ENSG00000255639
NA	0.0545	7.3908E-02		ENSG00000224132
NA	0.0536	7.4052E-02		ENSG00000279029
NA	0.1761	7.4264E-02	2.0909E-01	ENSG00000279412
NA	0.1757	7.4294E-02	2.0911E-01	ENSG00000289396
NA	0.0777	7.4349E-02		ENSG00000241532
NA	0.1842	7.4391E-02	2.0931E-01	ENSG00000272755
NA	0.1670	7.4611E-02	2.0973E-01	ENSG00000257918
NA	0.1642	7.4647E-02	2.0981E-01	ENSG00000247679
NA	0.0876	7.4937E-02		ENSG00000288016
NA	0.1925	7.5012E-02	2.1055E-01	ENSG00000261474
NA	0.1396	7.5280E-02	2.1097E-01	ENSG00000287617
NA	0.0296	7.5403E-02		ENSG00000250697
NA	0.0508	7.5596E-02		ENSG00000274492
NA	0.1295	7.5767E-02	2.1199E-01	ENSG00000237057
NA	0.0864	7.5769E-02		ENSG00000237418
NA	0.1907	7.5952E-02	2.1231E-01	ENSG00000260641
NA	0.1148	7.6095E-02	2.1252E-01	ENSG00000225300
NA	0.1870	7.6101E-02	2.1252E-01	ENSG00000279278
NA	0.1051	7.6111E-02	2.1252E-01	ENSG00000233799
NA	0.0274	7.6307E-02		ENSG00000272109
NA	0.1834	7.6403E-02	2.1319E-01	ENSG00000228031
NA	0.0974	7.6463E-02	2.1326E-01	ENSG00000228293
NA	0.0766	7.6471E-02		ENSG00000228877
NA	0.0111	7.6544E-02	2.1337E-01	ENSG00000267333
NA	0.0723	7.6635E-02	2.1349E-01	ENSG00000250421
NA	0.0619	7.6700E-02		ENSG00000285822
NA	0.1822	7.6742E-02	2.1367E-01	ENSG00000226530
NA	0.1382	7.6916E-02	2.1395E-01	ENSG00000288891
NA	0.0347	7.7105E-02		ENSG00000228683
NA	0.1752	7.7393E-02	2.1493E-01	ENSG00000258741
NA	0.1703	7.7546E-02	2.1521E-01	ENSG00000229839
NA	0.0340	7.7562E-02		ENSG00000286318
NA	0.0681	7.7677E-02		ENSG00000248676
NA	0.1524	7.7732E-02	2.1550E-01	ENSG00000273598
NA	0.0290	7.7927E-02		ENSG00000214822
NA	0.1048	7.8140E-02	2.1624E-01	ENSG00000233862
NA	0.0522	7.8181E-02		ENSG00000253480
NA	0.0555	7.8245E-02	2.1639E-01	ENSG00000276445
NA	0.1730	7.8254E-02	2.1639E-01	ENSG00000164669
NA	0.1058	7.8440E-02	2.1676E-01	ENSG00000249255
NA	0.0354	7.8450E-02		ENSG00000229146
NA	0.1864	7.8502E-02	2.1686E-01	ENSG00000267886
NA	0.1840	7.8572E-02	2.1703E-01	ENSG00000233220
NA	0.0704	7.8693E-02		ENSG00000243915
NA	0.0530	7.8827E-02	2.1746E-01	ENSG00000286044
NA	0.1791	7.8889E-02	2.1758E-01	ENSG00000272009
NA	0.0664	7.9065E-02	2.1794E-01	ENSG00000247699
NA	0.1805	7.9072E-02	2.1794E-01	ENSG00000261449

NA	0.1859	7.9436E-02	2.1863E-01	ENSG00000236333
NA	0.0870	7.9532E-02		ENSG00000258539
NA	0.1762	7.9874E-02	2.1932E-01	ENSG00000273014
NA	0.1754	7.9958E-02	2.1950E-01	ENSG00000259562
NA	0.1803	8.0008E-02	2.1959E-01	ENSG00000254929
NA	0.0153	8.0100E-02		ENSG00000277308
NA	0.1243	8.0298E-02	2.2020E-01	ENSG00000288897
NA	0.1657	8.0304E-02	2.2020E-01	ENSG00000270074
NA	0.1844	8.0499E-02	2.2056E-01	ENSG00000260634
NA	0.0691	8.0566E-02		ENSG00000254460
NA	0.0955	8.0686E-02	2.2080E-01	ENSG00000196312
NA	0.0959	8.0835E-02	2.2104E-01	ENSG00000258998
NA	0.1256	8.0847E-02	2.2105E-01	ENSG00000269365
NA	0.0813	8.0849E-02		ENSG00000250448
NA	0.1876	8.0915E-02	2.2113E-01	ENSG00000279384
NA	0.1531	8.0926E-02	2.2113E-01	ENSG00000257470
NA	0.0439	8.0985E-02	2.2121E-01	ENSG00000230140
NA	0.0545	8.1103E-02		ENSG00000280062
NA	0.1715	8.1288E-02	2.2169E-01	ENSG00000276672
NA	0.1837	8.1328E-02	2.2177E-01	ENSG00000232063
NA	0.1372	8.1377E-02	2.2186E-01	ENSG00000261168
NA	0.0349	8.1568E-02		ENSG00000243307
NA	0.0887	8.1745E-02		ENSG00000267446
NA	0.0749	8.1749E-02		ENSG00000257155
NA	0.1799	8.2265E-02	2.2336E-01	ENSG00000279491
NA	0.1832	8.2375E-02	2.2359E-01	ENSG00000270019
NA	0.0657	8.2391E-02		ENSG00000278797
NA	0.1530	8.2657E-02	2.2416E-01	ENSG00000273329
NA	0.1377	8.3076E-02	2.2480E-01	ENSG00000240219
NA	0.0983	8.3176E-02	2.2502E-01	ENSG00000260095
NA	0.0933	8.3528E-02	2.2572E-01	ENSG00000230836
NA	0.1642	8.3860E-02	2.2635E-01	ENSG00000232537
NA	0.0883	8.3875E-02		ENSG00000286511
NA	0.1071	8.3888E-02	2.2635E-01	ENSG00000262482
NA	0.1525	8.3939E-02	2.2638E-01	ENSG00000259475
NA	0.1901	8.4027E-02	2.2647E-01	ENSG00000290058
NA	0.0314	8.4029E-02		ENSG00000201343
NA	0.0848	8.4058E-02	2.2651E-01	ENSG00000287401
NA	0.0369	8.4128E-02		ENSG00000289991
NA	0.1706	8.4169E-02	2.2670E-01	ENSG00000249494
NA	0.0648	8.4308E-02		ENSG00000253811
NA	0.0356	8.4309E-02		ENSG00000219159
NA	0.0703	8.4395E-02		ENSG00000253383
NA	0.0626	8.4476E-02		ENSG00000271427
NA	0.1641	8.4487E-02	2.2719E-01	ENSG00000249947
NA	0.1031	8.4644E-02	2.2739E-01	ENSG00000256552
NA	0.0374	8.4686E-02		ENSG00000286489
NA	0.0504	8.4855E-02		ENSG00000228215
NA	0.0310	8.4855E-02		ENSG00000236562

NA	0.1796	8.4875E-02	2.2791E-01	ENSG00000286599
NA	0.1801	8.4957E-02	2.2808E-01	ENSG00000226797
NA	0.1309	8.5050E-02		ENSG00000285081
NA	0.1219	8.5356E-02	2.2863E-01	ENSG00000280035
NA	0.1469	8.5468E-02	2.2885E-01	ENSG00000259592
NA	0.1705	8.5895E-02	2.2980E-01	ENSG00000286327
NA	0.1266	8.6157E-02	2.3031E-01	ENSG00000260084
NA	0.0238	8.6371E-02		ENSG00000285633
NA	0.1708	8.6510E-02	2.3104E-01	ENSG00000234911
NA	0.0336	8.7046E-02		ENSG00000289118
NA	0.1271	8.7061E-02	2.3206E-01	ENSG00000286850
NA	0.0989	8.7281E-02	2.3257E-01	ENSG00000227959
NA	0.1510	8.7379E-02	2.3273E-01	ENSG00000261770
NA	0.0550	8.7546E-02		ENSG00000270090
NA	0.1423	8.7617E-02	2.3321E-01	ENSG00000287202
NA	0.1705	8.7680E-02	2.3333E-01	ENSG00000289050
NA	0.0711	8.7690E-02		ENSG00000226453
NA	0.1721	8.7748E-02	2.3349E-01	ENSG00000269921
NA	0.0867	8.7898E-02		ENSG00000257513
NA	0.1709	8.7963E-02	2.3396E-01	ENSG00000275367
NA	0.1630	8.8112E-02	2.3423E-01	ENSG00000230630
NA	0.0166	8.8193E-02		ENSG00000257885
NA	0.0575	8.8198E-02		ENSG00000227987
NA	0.1432	8.8276E-02	2.3449E-01	ENSG00000275155
NA	0.0950	8.8299E-02	2.3452E-01	ENSG00000285749
NA	0.0549	8.8404E-02		ENSG00000265394
NA	0.0297	8.8411E-02		ENSG00000271401
NA	0.0721	8.8494E-02		ENSG00000278040
NA	0.1330	8.8498E-02	2.3490E-01	ENSG00000284116
NA	0.1760	8.8530E-02	2.3493E-01	ENSG00000203325
NA	0.1622	8.8637E-02	2.3510E-01	ENSG00000287245
NA	0.1505	8.8666E-02	2.3510E-01	ENSG00000228506
NA	0.0423	8.8720E-02		ENSG00000283360
NA	0.0661	8.8764E-02		ENSG00000226709
NA	0.0577	8.8875E-02		ENSG00000178636
NA	0.1265	8.9060E-02	2.3591E-01	ENSG00000237301
NA	0.1711	8.9154E-02	2.3613E-01	ENSG00000275734
NA	0.1640	8.9211E-02	2.3625E-01	ENSG00000227172
NA	0.0367	8.9274E-02		ENSG00000273957
NA	0.1378	8.9532E-02	2.3692E-01	ENSG00000276809
NA	0.1405	9.0265E-02	2.3846E-01	ENSG00000263938
NA	0.1389	9.0331E-02	2.3860E-01	ENSG00000237491
NA	0.1040	9.0488E-02		ENSG00000255162
NA	0.1740	9.0594E-02	2.3914E-01	ENSG00000274220
NA	0.0891	9.0619E-02	2.3916E-01	ENSG00000269053
NA	0.1618	9.0684E-02	2.3928E-01	ENSG00000272583
NA	0.1670	9.0779E-02	2.3940E-01	ENSG00000230454
NA	0.1767	9.0851E-02	2.3949E-01	ENSG00000271380
NA	0.0359	9.0905E-02		ENSG00000214855

NA	0.1397	9.1068E-02	2.3985E-01	ENSG00000213062
NA	0.0311	9.1594E-02		ENSG00000272161
NA	0.0561	9.1615E-02		ENSG00000287029
NA	0.0541	9.1621E-02	2.4090E-01	ENSG00000232835
NA	0.0647	9.1851E-02		ENSG00000287835
NA	0.0670	9.1892E-02		ENSG00000251614
NA	0.1743	9.1894E-02	2.4133E-01	ENSG00000277778
NA	0.1635	9.2120E-02	2.4167E-01	ENSG00000288919
NA	0.0295	9.2177E-02		ENSG00000235079
NA	0.1512	9.2622E-02	2.4260E-01	ENSG00000255236
NA	0.0258	9.2789E-02		ENSG00000271137
NA	0.1166	9.2793E-02	2.4297E-01	ENSG00000276251
NA	0.0252	9.2944E-02		ENSG00000230428
NA	0.1538	9.3023E-02	2.4334E-01	ENSG00000214273
NA	0.1396	9.3033E-02	2.4334E-01	ENSG00000273654
NA	0.1391	9.3243E-02	2.4371E-01	ENSG00000228330
NA	0.1352	9.3376E-02	2.4392E-01	ENSG00000225762
NA	0.0959	9.3412E-02		ENSG00000260830
NA	0.0825	9.3443E-02	2.4407E-01	ENSG00000286302
NA	0.0992	9.3465E-02	2.4408E-01	ENSG00000286563
NA	0.1683	9.3476E-02	2.4408E-01	ENSG00000214810
NA	0.1324	9.3494E-02	2.4410E-01	ENSG00000228956
NA	0.0276	9.3560E-02		ENSG00000267503
NA	0.0655	9.3570E-02		ENSG00000260392
NA	0.1480	9.3742E-02	2.4454E-01	ENSG00000283208
NA	0.0837	9.3807E-02		ENSG00000279972
NA	0.0937	9.3931E-02		ENSG00000263503
NA	0.1714	9.4243E-02	2.4546E-01	ENSG00000289059
NA	0.1489	9.4359E-02	2.4566E-01	ENSG00000272983
NA	0.0253	9.4472E-02		ENSG00000260672
NA	0.1370	9.4476E-02	2.4591E-01	ENSG00000229321
NA	0.1496	9.4731E-02	2.4629E-01	ENSG00000277534
NA	0.1618	9.4858E-02	2.4654E-01	ENSG00000287811
NA	0.0632	9.5092E-02		ENSG00000265262
NA	0.0918	9.5175E-02	2.4723E-01	ENSG00000279113
NA	0.1327	9.5217E-02	2.4725E-01	ENSG00000233435
NA	0.0677	9.5255E-02		ENSG00000286970
NA	0.1475	9.5294E-02	2.4736E-01	ENSG00000274184
NA	0.0269	9.5320E-02		ENSG00000277475
NA	0.1431	9.5326E-02	2.4736E-01	ENSG00000188707
NA	0.1741	9.5591E-02	2.4789E-01	ENSG00000267092
NA	0.1202	9.5715E-02	2.4816E-01	ENSG00000261003
NA	0.1677	9.5745E-02	2.4821E-01	ENSG00000287310
NA	0.0478	9.5886E-02	2.4846E-01	ENSG00000267287
NA	0.1725	9.5889E-02	2.4846E-01	ENSG00000272172
NA	0.1625	9.5964E-02	2.4857E-01	ENSG00000260436
NA	0.0338	9.5974E-02		ENSG00000254119
NA	0.1444	9.6164E-02	2.4896E-01	ENSG00000260625
NA	0.1540	9.6369E-02	2.4923E-01	ENSG00000236206

NA	0.1253	9.6420E-02	2.4933E-01	ENSG00000231697
NA	0.1627	9.6548E-02	2.4956E-01	ENSG00000272316
NA	0.0358	9.6617E-02		ENSG00000268583
NA	0.0949	9.6690E-02	2.4982E-01	ENSG00000272158
NA	0.1284	9.6724E-02	2.4983E-01	ENSG00000288899
NA	0.1468	9.6804E-02	2.4993E-01	ENSG00000226200
NA	0.1405	9.6891E-02	2.5000E-01	ENSG00000234026
NA	0.1059	9.6997E-02	2.5020E-01	ENSG00000251298
NA	0.1055	9.7042E-02	2.5029E-01	ENSG00000276110
NA	0.1615	9.7082E-02	2.5035E-01	ENSG00000260855
NA	0.1638	9.7187E-02	2.5043E-01	ENSG00000266473
NA	0.0860	9.7190E-02	2.5043E-01	ENSG00000271937
NA	0.0903	9.7244E-02	2.5049E-01	ENSG00000269843
NA	0.0798	9.7261E-02		ENSG00000262052
NA	0.1166	9.7261E-02	2.5051E-01	ENSG00000279243
NA	0.1011	9.7351E-02	2.5066E-01	ENSG00000247903
NA	0.1011	9.7424E-02	2.5074E-01	ENSG00000279711
NA	0.1425	9.7448E-02	2.5078E-01	ENSG00000225511
NA	0.1595	9.7740E-02	2.5114E-01	ENSG00000278971
NA	0.1638	9.7926E-02	2.5136E-01	ENSG00000286707
NA	0.1019	9.7943E-02	2.5136E-01	ENSG00000261207
NA	0.1217	9.8264E-02	2.5193E-01	ENSG00000267085
NA	0.1648	9.8514E-02	2.5234E-01	ENSG00000246982
NA	0.1149	9.8544E-02	2.5236E-01	ENSG00000253549
NA	0.0302	9.8856E-02		ENSG00000235035
NA	0.1481	9.9001E-02	2.5306E-01	ENSG00000251666
NA	0.0559	9.9072E-02		ENSG00000235848
NA	0.1483	9.9256E-02	2.5344E-01	ENSG00000273156
NA	0.1060	9.9559E-02	2.5393E-01	ENSG00000226571
NA	0.1645	9.9682E-02	2.5411E-01	ENSG00000248290
NA	0.1672	9.9757E-02	2.5425E-01	ENSG00000214832
NA	0.1470	9.9806E-02	2.5432E-01	ENSG00000272501
NA	0.1402	9.9859E-02	2.5440E-01	ENSG00000241549
NA	0.1095	1.0008E-01	2.5482E-01	ENSG00000214106
NA	0.0316	1.0012E-01		ENSG00000223393
NA	0.0324	1.0025E-01		ENSG00000250662
NA	0.1377	1.0042E-01	2.5537E-01	ENSG00000270069
NA	0.1628	1.0085E-01	2.5622E-01	ENSG00000285871
NA	0.0243	1.0095E-01		ENSG00000286171
NA	0.0556	1.0104E-01		ENSG00000275329
NA	0.0370	1.0111E-01		ENSG00000213548
NA	0.1079	1.0114E-01	2.5677E-01	ENSG00000254057
NA	0.0255	1.0127E-01		ENSG00000254533
NA	0.0771	1.0133E-01		ENSG00000264736
NA	0.1682	1.0138E-01	2.5708E-01	ENSG00000255277
NA	0.0334	1.0151E-01		ENSG00000227834
NA	0.0943	1.0151E-01		ENSG00000271874
NA	0.0324	1.0168E-01		ENSG00000289131
NA	0.1413	1.0172E-01	2.5762E-01	ENSG00000259740

NA	0.1560	1.0177E-01	2.5767E-01	ENSG00000255139
NA	0.1494	1.0198E-01	2.5802E-01	ENSG00000247970
NA	0.1644	1.0218E-01	2.5836E-01	ENSG00000289631
NA	0.0602	1.0228E-01		ENSG00000224430
NA	0.1678	1.0248E-01	2.5903E-01	ENSG00000270344
NA	0.1664	1.0248E-01	2.5903E-01	ENSG00000279789
NA	0.1634	1.0254E-01	2.5907E-01	ENSG00000225689
NA	0.0959	1.0299E-01	2.5981E-01	ENSG00000260100
NA	0.0293	1.0303E-01		ENSG00000257735
NA	0.1218	1.0315E-01	2.6003E-01	ENSG00000267707
NA	0.0271	1.0320E-01		ENSG00000235126
NA	0.1469	1.0342E-01	2.6035E-01	ENSG00000257433
NA	0.0719	1.0344E-01		ENSG00000273551
NA	0.0690	1.0345E-01		ENSG00000261051
NA	0.1279	1.0367E-01	2.6086E-01	ENSG00000185485
NA	0.1336	1.0371E-01	2.6089E-01	ENSG00000261630
NA	0.1169	1.0411E-01	2.6155E-01	ENSG00000286592
NA	0.1613	1.0426E-01	2.6186E-01	ENSG00000285805
NA	0.1635	1.0442E-01	2.6205E-01	ENSG00000234129
NA	0.1172	1.0442E-01	2.6205E-01	ENSG00000287698
NA	0.0174	1.0469E-01		ENSG00000279940
NA	0.0710	1.0493E-01		ENSG00000278901
NA	0.0233	1.0501E-01		ENSG00000200170
NA	0.0534	1.0501E-01		ENSG00000286103
NA	0.0552	1.0521E-01		ENSG00000257941
NA	0.0445	1.0523E-01		ENSG00000228604
NA	0.0650	1.0557E-01	2.6407E-01	ENSG00000235965
NA	0.0899	1.0562E-01	2.6413E-01	ENSG00000268743
NA	0.0424	1.0587E-01		ENSG00000236194
NA	0.1587	1.0594E-01	2.6467E-01	ENSG00000289592
NA	0.0256	1.0607E-01		ENSG00000284825
NA	0.1389	1.0620E-01	2.6496E-01	ENSG00000289147
NA	0.1580	1.0625E-01	2.6503E-01	ENSG00000267696
NA	0.1571	1.0626E-01	2.6504E-01	ENSG00000248925
NA	0.1281	1.0631E-01	2.6509E-01	ENSG00000254682
NA	0.0659	1.0632E-01		ENSG00000225144
NA	0.1519	1.0638E-01	2.6523E-01	ENSG00000256967
NA	0.0361	1.0676E-01		ENSG00000267713
NA	0.0413	1.0692E-01		ENSG00000289369
NA	0.1407	1.0697E-01	2.6609E-01	ENSG00000257074
NA	0.0547	1.0700E-01		ENSG00000286982
NA	0.1608	1.0726E-01	2.6649E-01	ENSG00000243926
NA	0.1647	1.0727E-01	2.6649E-01	ENSG00000289443
NA	0.0195	1.0732E-01		ENSG00000259397
NA	0.1121	1.0754E-01	2.6693E-01	ENSG00000286804
NA	0.0412	1.0754E-01		ENSG00000253519
NA	0.0457	1.0757E-01		ENSG00000273920
NA	0.1628	1.0765E-01	2.6713E-01	ENSG00000197815
NA	0.0202	1.0773E-01		ENSG00000167046

NA	0.0600	1.0778E-01		ENSG00000253523
NA	0.1250	1.0790E-01	2.6765E-01	ENSG00000248544
NA	0.0575	1.0793E-01		ENSG00000259762
NA	0.0563	1.0799E-01		ENSG00000273198
NA	0.0516	1.0803E-01		ENSG00000233290
NA	0.0639	1.0812E-01		ENSG00000248994
NA	0.0263	1.0828E-01		ENSG00000267642
NA	0.0430	1.0832E-01		ENSG00000225450
NA	0.1373	1.0852E-01	2.6869E-01	ENSG00000273055
NA	0.1153	1.0858E-01	2.6876E-01	ENSG00000227782
NA	0.0907	1.0858E-01	2.6876E-01	ENSG00000288889
NA	0.0239	1.0862E-01		ENSG00000273165
NA	0.1629	1.0863E-01	2.6884E-01	ENSG00000289038
NA	0.0314	1.0863E-01		ENSG00000276188
NA	0.0837	1.0880E-01	2.6917E-01	ENSG00000286460
NA	0.1610	1.0890E-01	2.6933E-01	ENSG00000280088
NA	0.0779	1.0896E-01	2.6939E-01	ENSG00000289725
NA	0.0732	1.0901E-01		ENSG00000287467
NA	0.1586	1.0908E-01	2.6957E-01	ENSG00000254528
NA	0.1592	1.0909E-01	2.6957E-01	ENSG00000205959
NA	0.0311	1.0916E-01		ENSG00000259306
NA	0.0629	1.0920E-01	2.6977E-01	ENSG00000272807
NA	0.1627	1.0926E-01	2.6987E-01	ENSG00000245322
NA	0.1331	1.0938E-01	2.7006E-01	ENSG00000230896
NA	0.0882	1.0947E-01	2.7024E-01	ENSG00000254538
NA	0.0286	1.0954E-01		ENSG00000272072
NA	0.1574	1.0971E-01	2.7073E-01	ENSG00000267890
NA	0.1143	1.0979E-01	2.7076E-01	ENSG00000275005
NA	0.0234	1.0981E-01		ENSG00000286796
NA	0.0513	1.1010E-01		ENSG00000229087
NA	0.0658	1.1024E-01		ENSG00000257986
NA	0.1560	1.1025E-01	2.7159E-01	ENSG00000288088
NA	0.0852	1.1038E-01	2.7187E-01	ENSG00000274987
NA	0.0889	1.1046E-01		ENSG00000273403
NA	0.1253	1.1050E-01	2.7207E-01	ENSG00000276900
NA	0.0962	1.1062E-01	2.7230E-01	ENSG00000248112
NA	0.0254	1.1081E-01		ENSG00000269189
NA	0.0776	1.1091E-01	2.7269E-01	ENSG00000201428
NA	0.0284	1.1096E-01		ENSG00000261029
NA	0.1592	1.1109E-01	2.7290E-01	ENSG00000231305
NA	0.0937	1.1109E-01	2.7290E-01	ENSG00000250764
NA	0.0961	1.1131E-01		ENSG00000285361
NA	0.1101	1.1135E-01	2.7325E-01	ENSG00000257838
NA	0.0416	1.1141E-01		ENSG00000288806
NA	0.0717	1.1149E-01		ENSG00000251402
NA	0.1508	1.1156E-01	2.7353E-01	ENSG00000276603
NA	0.0226	1.1158E-01		ENSG00000258759
NA	0.1089	1.1165E-01	2.7370E-01	ENSG00000213871
NA	0.1412	1.1195E-01	2.7428E-01	ENSG00000289440

NA	0.0339	1.1199E-01		ENSG00000260385
NA	0.1430	1.1208E-01	2.7453E-01	ENSG00000284128
NA	0.0977	1.1211E-01	2.7453E-01	ENSG00000259977
NA	0.1139	1.1212E-01	2.7453E-01	ENSG00000205971
NA	0.1431	1.1233E-01	2.7489E-01	ENSG00000289119
NA	0.0770	1.1294E-01		ENSG00000253376
NA	0.1511	1.1332E-01	2.7669E-01	ENSG00000287165
NA	0.0239	1.1342E-01		ENSG00000275582
NA	0.1426	1.1351E-01	2.7699E-01	ENSG00000280294
NA	0.0046	1.1376E-01		ENSG00000232874
NA	0.1282	1.1387E-01	2.7764E-01	ENSG00000215347
NA	0.1513	1.1402E-01	2.7783E-01	ENSG00000242593
NA	0.1552	1.1413E-01	2.7800E-01	ENSG00000281344
NA	0.1443	1.1427E-01	2.7822E-01	ENSG00000288721
NA	0.1022	1.1429E-01	2.7823E-01	ENSG00000274080
NA	0.1579	1.1451E-01	2.7857E-01	ENSG00000219881
NA	0.0411	1.1453E-01		ENSG00000267097
NA	0.0825	1.1453E-01	2.7860E-01	ENSG00000227512
NA	0.1023	1.1466E-01	2.7878E-01	ENSG00000287539
NA	0.0301	1.1470E-01		ENSG00000289409
NA	0.0290	1.1473E-01		ENSG00000264057
NA	0.0570	1.1478E-01		ENSG00000274373
NA	0.1330	1.1491E-01	2.7927E-01	ENSG00000196114
NA	0.1522	1.1515E-01	2.7963E-01	ENSG00000289123
NA	0.1194	1.1525E-01	2.7983E-01	ENSG00000276898
NA	0.1057	1.1541E-01	2.7998E-01	ENSG00000233967
NA	0.0453	1.1559E-01		ENSG00000289531
NA	0.1565	1.1573E-01	2.8040E-01	ENSG00000236501
NA	0.0577	1.1578E-01	2.8040E-01	ENSG00000261797
NA	0.1197	1.1591E-01	2.8061E-01	ENSG00000259424
NA	0.1556	1.1611E-01	2.8090E-01	ENSG00000289860
NA	0.1589	1.1621E-01	2.8107E-01	ENSG00000210144
NA	0.0370	1.1639E-01		ENSG00000274893
NA	0.1095	1.1647E-01	2.8154E-01	ENSG00000270558
NA	0.0335	1.1669E-01		ENSG00000254094
NA	0.1088	1.1670E-01	2.8187E-01	ENSG00000232725
NA	0.1563	1.1672E-01	2.8187E-01	ENSG00000226756
NA	0.1425	1.1677E-01	2.8194E-01	ENSG00000251011
NA	0.0741	1.1696E-01	2.8231E-01	ENSG00000279083
NA	0.1337	1.1709E-01	2.8253E-01	ENSG00000289559
NA	0.1545	1.1736E-01	2.8293E-01	ENSG00000260132
NA	0.1525	1.1747E-01	2.8301E-01	ENSG00000205702
NA	0.1454	1.1756E-01	2.8313E-01	ENSG00000276248
NA	0.0539	1.1767E-01		ENSG00000227069
NA	0.1215	1.1799E-01	2.8399E-01	ENSG00000229520
NA	0.0681	1.1811E-01		ENSG00000265752
NA	0.1409	1.1815E-01	2.8431E-01	ENSG00000253515
NA	0.1561	1.1826E-01	2.8452E-01	ENSG00000282022
NA	0.1576	1.1850E-01	2.8489E-01	ENSG00000290015

NA	0.1120	1.1864E-01	2.8512E-01	ENSG00000255366
NA	0.0325	1.1867E-01		ENSG00000275194
NA	0.0573	1.1874E-01		ENSG00000279162
NA	0.0687	1.1887E-01	2.8549E-01	ENSG00000231585
NA	0.1182	1.1900E-01	2.8576E-01	ENSG00000274444
NA	0.0236	1.1913E-01		ENSG00000226771
NA	0.0551	1.1914E-01		ENSG00000227237
NA	0.1107	1.1923E-01	2.8626E-01	ENSG00000262943
NA	0.0668	1.1925E-01		ENSG00000272733
NA	0.0764	1.1933E-01	2.8639E-01	ENSG00000261668
NA	0.0923	1.1939E-01	2.8650E-01	ENSG00000269889
NA	0.1553	1.1941E-01	2.8650E-01	ENSG00000229917
NA	0.1054	1.1946E-01	2.8660E-01	ENSG00000285730
NA	0.1366	1.1952E-01	2.8664E-01	ENSG00000279196
NA	0.0795	1.1969E-01		ENSG00000257654
NA	0.0882	1.1974E-01	2.8698E-01	ENSG00000258675
NA	0.0404	1.1982E-01		ENSG00000287806
NA	0.1576	1.1994E-01	2.8727E-01	ENSG00000248050
NA	0.1567	1.2000E-01	2.8739E-01	ENSG00000286909
NA	0.1186	1.2004E-01	2.8746E-01	ENSG00000280800
NA	0.1563	1.2012E-01	2.8754E-01	ENSG00000230333
NA	0.0835	1.2015E-01	2.8754E-01	ENSG00000286062
NA	0.0424	1.2037E-01		ENSG00000285698
NA	0.0497	1.2045E-01		ENSG00000240399
NA	0.1085	1.2049E-01	2.8815E-01	ENSG00000289433
NA	0.0414	1.2053E-01		ENSG00000234300
NA	0.1183	1.2075E-01	2.8864E-01	ENSG00000280614
NA	0.1400	1.2090E-01	2.8892E-01	ENSG00000287244
NA	0.1387	1.2124E-01	2.8951E-01	ENSG00000254577
NA	0.0796	1.2125E-01		ENSG00000267360
NA	0.1451	1.2159E-01	2.9011E-01	ENSG00000253540
NA	0.1555	1.2162E-01	2.9014E-01	ENSG00000230648
NA	0.0938	1.2169E-01	2.9027E-01	ENSG00000271392
NA	0.0376	1.2182E-01	2.9049E-01	ENSG00000268279
NA	0.0488	1.2192E-01	2.9066E-01	ENSG00000270210
NA	0.0463	1.2195E-01		ENSG00000271228
NA	0.0967	1.2200E-01	2.9074E-01	ENSG00000236491
NA	0.1481	1.2223E-01	2.9109E-01	ENSG00000289200
NA	0.1528	1.2230E-01	2.9111E-01	ENSG00000271938
NA	0.1448	1.2231E-01	2.9111E-01	ENSG00000227236
NA	0.0544	1.2235E-01		ENSG00000260954
NA	0.1472	1.2261E-01	2.9153E-01	ENSG00000235313
NA	0.0337	1.2282E-01		ENSG00000237682
NA	0.0430	1.2315E-01		ENSG00000233818
NA	0.0932	1.2322E-01	2.9264E-01	ENSG00000290000
NA	0.0653	1.2322E-01		ENSG00000251374
NA	0.1078	1.2326E-01	2.9269E-01	ENSG00000242190
NA	0.0627	1.2327E-01		ENSG00000237234
NA	0.0942	1.2334E-01	2.9285E-01	ENSG00000228933

NA	0.0412	1.2344E-01		ENSG00000273771
NA	0.1174	1.2372E-01	2.9349E-01	ENSG00000281181
NA	0.0934	1.2383E-01	2.9362E-01	ENSG00000265413
NA	0.1522	1.2392E-01	2.9362E-01	ENSG00000288935
NA	0.1347	1.2407E-01	2.9385E-01	ENSG00000276500
NA	0.0481	1.2421E-01	2.9401E-01	ENSG00000279437
NA	0.0893	1.2437E-01	2.9420E-01	ENSG00000231550
NA	0.0571	1.2444E-01		ENSG00000289877
NA	0.0546	1.2447E-01		ENSG00000288749
NA	0.0772	1.2463E-01	2.9449E-01	ENSG00000251445
NA	0.0372	1.2464E-01		ENSG00000285821
NA	0.1419	1.2481E-01	2.9482E-01	ENSG00000260271
NA	0.0548	1.2492E-01		ENSG00000279681
NA	0.1536	1.2492E-01	2.9495E-01	ENSG00000207751
NA	0.1376	1.2494E-01	2.9497E-01	ENSG00000289259
NA	0.0665	1.2500E-01		ENSG00000231193
NA	0.1507	1.2519E-01	2.9534E-01	ENSG00000280120
NA	0.0791	1.2537E-01		ENSG00000260021
NA	0.0663	1.2556E-01		ENSG00000284461
NA	0.0772	1.2580E-01		ENSG00000256312
NA	0.1255	1.2588E-01	2.9650E-01	ENSG00000289910
NA	0.1403	1.2588E-01	2.9650E-01	ENSG00000278416
NA	0.1533	1.2610E-01	2.9692E-01	ENSG00000289154
NA	0.0196	1.2622E-01		ENSG00000235994
NA	0.1497	1.2623E-01	2.9720E-01	ENSG00000280649
NA	0.0504	1.2649E-01		ENSG00000254675
NA	0.1429	1.2665E-01	2.9792E-01	ENSG00000290117
NA	0.1117	1.2671E-01	2.9799E-01	ENSG00000230011
NA	0.0545	1.2694E-01		ENSG00000289889
NA	0.0966	1.2698E-01	2.9839E-01	ENSG00000276115
NA	0.0844	1.2703E-01	2.9843E-01	ENSG00000225406
NA	0.0731	1.2730E-01	2.9873E-01	ENSG00000269387
NA	0.0326	1.2778E-01		ENSG00000282998
NA	0.1531	1.2790E-01	2.9962E-01	ENSG00000270300
NA	0.0279	1.2800E-01		ENSG00000228352
NA	0.1299	1.2803E-01	2.9973E-01	ENSG00000277453
NA	0.0313	1.2804E-01		ENSG00000258859
NA	0.1355	1.2832E-01	2.9995E-01	ENSG00000289552
NA	0.0573	1.2849E-01		ENSG00000222511
NA	0.1451	1.2864E-01	3.0050E-01	ENSG00000289297
NA	0.0685	1.2892E-01		ENSG00000287063
NA	0.0941	1.2911E-01	3.0137E-01	ENSG00000225850
NA	0.1085	1.2930E-01	3.0168E-01	ENSG00000254370
NA	0.0276	1.2943E-01		ENSG00000248802
NA	0.1224	1.2951E-01	3.0199E-01	ENSG00000274471
NA	0.0987	1.2952E-01	3.0199E-01	ENSG00000285416
NA	0.1445	1.2961E-01	3.0214E-01	ENSG00000272054
NA	0.0577	1.2997E-01		ENSG00000269489
NA	0.0426	1.2998E-01		ENSG00000258798

NA	0.0807	1.3000E-01		ENSG00000287185
NA	0.0776	1.3008E-01		ENSG00000279148
NA	0.0279	1.3010E-01		ENSG00000255555
NA	0.0943	1.3024E-01	3.0303E-01	ENSG00000272844
NA	0.1424	1.3036E-01	3.0316E-01	ENSG00000282876
NA	0.0744	1.3049E-01		ENSG00000288929
NA	0.0714	1.3061E-01		ENSG00000287922
NA	0.1173	1.3066E-01	3.0359E-01	ENSG00000253965
NA	0.0215	1.3069E-01		ENSG00000279128
NA	0.0952	1.3080E-01	3.0373E-01	ENSG00000258820
NA	0.0919	1.3084E-01	3.0373E-01	ENSG00000273702
NA	0.0626	1.3089E-01		ENSG00000287754
NA	0.1001	1.3104E-01	3.0408E-01	ENSG00000189136
NA	0.0457	1.3110E-01		ENSG00000286712
NA	0.1472	1.3117E-01	3.0420E-01	ENSG00000259867
NA	0.0286	1.3125E-01		ENSG00000210100
NA	0.0437	1.3131E-01		ENSG00000285881
NA	0.0377	1.3145E-01		ENSG00000236018
NA	0.0224	1.3145E-01		ENSG00000225790
NA	0.0334	1.3159E-01		ENSG00000286413
NA	0.1174	1.3160E-01	3.0473E-01	ENSG00000278276
NA	0.0702	1.3174E-01	3.0499E-01	ENSG00000244332
NA	0.0473	1.3197E-01		ENSG00000289839
NA	0.0248	1.3200E-01		ENSG00000226429
NA	0.0250	1.3223E-01		ENSG00000286648
NA	0.1097	1.3234E-01	3.0582E-01	ENSG00000174384
NA	0.0457	1.3249E-01	3.0603E-01	ENSG00000286800
NA	0.0859	1.3270E-01	3.0630E-01	ENSG00000272842
NA	0.1309	1.3293E-01	3.0662E-01	ENSG00000237529
NA	0.1501	1.3298E-01	3.0663E-01	ENSG00000275445
NA	0.0686	1.3314E-01	3.0684E-01	ENSG00000270460
NA	0.1446	1.3315E-01	3.0684E-01	ENSG00000272489
NA	0.0755	1.3320E-01	3.0687E-01	ENSG00000280890
NA	0.1465	1.3324E-01	3.0694E-01	ENSG00000289054
NA	0.1206	1.3339E-01	3.0717E-01	ENSG00000242094
NA	0.0221	1.3379E-01		ENSG00000286241
NA	0.0969	1.3388E-01	3.0787E-01	ENSG00000272444
NA	0.0943	1.3399E-01	3.0805E-01	ENSG00000280402
NA	0.0534	1.3404E-01		ENSG00000229400
NA	0.1345	1.3409E-01	3.0818E-01	ENSG00000287760
NA	0.0635	1.3440E-01		ENSG00000222448
NA	0.0394	1.3459E-01		ENSG00000213232
NA	0.1426	1.3483E-01	3.0926E-01	ENSG00000262877
NA	0.0628	1.3501E-01		ENSG00000286913
NA	0.0439	1.3505E-01		ENSG00000229957
NA	0.1355	1.3510E-01	3.0963E-01	ENSG00000289436
NA	0.0439	1.3513E-01		ENSG00000242142
NA	0.1396	1.3548E-01	3.1028E-01	ENSG00000278949
NA	0.0182	1.3550E-01		ENSG00000260302

NA	0.1393	1.3555E-01	3.1028E-01	ENSG00000288833
NA	0.0269	1.3567E-01		ENSG00000279852
NA	0.0870	1.3569E-01	3.1046E-01	ENSG00000232560
NA	0.0434	1.3572E-01		ENSG00000255429
NA	0.0886	1.3578E-01	3.1060E-01	ENSG00000279382
NA	0.0910	1.3604E-01		ENSG00000273367
NA	0.1036	1.3616E-01	3.1124E-01	ENSG00000287894
NA	0.1312	1.3659E-01	3.1194E-01	ENSG00000289852
NA	0.0665	1.3672E-01		ENSG00000229276
NA	0.0462	1.3700E-01		ENSG00000273106
NA	0.1368	1.3719E-01	3.1258E-01	ENSG00000275709
NA	0.0853	1.3759E-01	3.1326E-01	ENSG00000260990
NA	0.1187	1.3763E-01	3.1332E-01	ENSG00000277597
NA	0.0341	1.3765E-01		ENSG00000259564
NA	0.1362	1.3779E-01	3.1354E-01	ENSG00000267251
NA	0.1251	1.3780E-01	3.1354E-01	ENSG00000173727
NA	0.0482	1.3815E-01		ENSG00000273747
NA	0.1446	1.3818E-01	3.1413E-01	ENSG00000278983
NA	0.0956	1.3832E-01	3.1425E-01	ENSG00000286949
NA	0.0998	1.3849E-01	3.1439E-01	ENSG00000271892
NA	0.1451	1.3849E-01	3.1439E-01	ENSG00000279532
NA	0.1454	1.3860E-01	3.1446E-01	ENSG00000215156
NA	0.0716	1.3861E-01		ENSG00000287727
NA	0.0963	1.3870E-01	3.1462E-01	ENSG00000234692
NA	0.0645	1.3885E-01		ENSG00000260647
NA	0.1433	1.3909E-01	3.1516E-01	ENSG00000279863
NA	0.1420	1.3942E-01	3.1564E-01	ENSG00000284773
NA	0.0235	1.3950E-01		ENSG00000213014
NA	0.0308	1.4003E-01		ENSG00000256810
NA	0.1368	1.4006E-01	3.1661E-01	ENSG00000220848
NA	0.0554	1.4007E-01		ENSG00000280432
NA	0.0732	1.4022E-01		ENSG00000203286
NA	0.1391	1.4029E-01	3.1686E-01	ENSG00000215859
NA	0.1327	1.4036E-01	3.1691E-01	ENSG00000282386
NA	0.1461	1.4065E-01	3.1738E-01	ENSG00000262873
NA	0.0261	1.4070E-01		ENSG00000287175
NA	0.0282	1.4079E-01		ENSG00000280425
NA	0.0988	1.4084E-01	3.1753E-01	ENSG00000288939
NA	0.1080	1.4093E-01	3.1764E-01	ENSG00000227718
NA	0.0335	1.4109E-01		ENSG00000266786
NA	0.0683	1.4113E-01		ENSG00000238749
NA	0.0501	1.4160E-01		ENSG00000272911
NA	0.0371	1.4165E-01		ENSG00000215007
NA	0.0739	1.4171E-01	3.1877E-01	ENSG00000286819
NA	0.0500	1.4172E-01		ENSG00000284800
NA	0.1211	1.4179E-01	3.1887E-01	ENSG00000286504
NA	0.0708	1.4195E-01		ENSG00000279954
NA	0.0199	1.4247E-01		ENSG00000177338
NA	0.0746	1.4266E-01	3.2021E-01	ENSG00000280423

NA	0.1433	1.4288E-01	3.2049E-01	ENSG00000257060
NA	0.0307	1.4291E-01		ENSG00000237166
NA	0.0966	1.4291E-01	3.2054E-01	ENSG00000258175
NA	0.1369	1.4296E-01	3.2061E-01	ENSG00000237883
NA	0.0348	1.4321E-01		ENSG00000223875
NA	0.0225	1.4347E-01		ENSG00000227301
NA	0.0312	1.4367E-01		ENSG00000255270
NA	0.1200	1.4379E-01	3.2183E-01	ENSG00000228302
NA	0.1257	1.4414E-01	3.2228E-01	ENSG00000232618
NA	0.0251	1.4418E-01		ENSG00000289057
NA	0.0667	1.4425E-01	3.2242E-01	ENSG00000285774
NA	0.0452	1.4485E-01		ENSG00000264116
NA	0.0306	1.4494E-01		ENSG00000287549
NA	0.0662	1.4524E-01		ENSG00000274723
NA	0.1220	1.4527E-01	3.2405E-01	ENSG00000245468
NA	0.0283	1.4540E-01		ENSG00000286633
NA	0.0556	1.4562E-01	3.2462E-01	ENSG00000287101
NA	0.0240	1.4575E-01		ENSG00000273866
NA	0.0688	1.4609E-01		ENSG00000257195
NA	0.1237	1.4610E-01	3.2540E-01	ENSG00000262050
NA	0.1345	1.4618E-01	3.2556E-01	ENSG00000278017
NA	0.0162	1.4665E-01		ENSG00000237741
NA	0.1404	1.4665E-01	3.2636E-01	ENSG00000230732
NA	0.1178	1.4666E-01	3.2636E-01	ENSG00000237719
NA	0.0384	1.4694E-01		ENSG00000244050
NA	0.1172	1.4699E-01	3.2681E-01	ENSG00000229021
NA	0.0198	1.4700E-01		ENSG00000234626
NA	0.0865	1.4721E-01	3.2711E-01	ENSG00000286234
NA	0.0640	1.4739E-01		ENSG00000226862
NA	0.0841	1.4762E-01	3.2776E-01	ENSG00000279108
NA	0.0438	1.4780E-01		ENSG00000242104
NA	0.1096	1.4786E-01	3.2810E-01	ENSG00000266441
NA	0.1172	1.4787E-01	3.2810E-01	ENSG00000278952
NA	0.1023	1.4793E-01	3.2816E-01	ENSG00000289498
NA	0.0443	1.4800E-01		ENSG00000284418
NA	0.0761	1.4807E-01	3.2841E-01	ENSG00000225969
NA	0.0224	1.4810E-01		ENSG00000258657
NA	0.0338	1.4823E-01		ENSG00000270048
NA	0.1127	1.4836E-01	3.2886E-01	ENSG00000273456
NA	0.0674	1.4838E-01		ENSG00000219186
NA	0.1196	1.4846E-01	3.2899E-01	ENSG00000286835
NA	0.1036	1.4870E-01	3.2923E-01	ENSG00000275263
NA	0.0936	1.4896E-01	3.2951E-01	ENSG00000282863
NA	0.1407	1.4902E-01	3.2951E-01	ENSG00000272572
NA	0.0393	1.4906E-01		ENSG00000249094
NA	0.0914	1.4909E-01	3.2959E-01	ENSG00000223635
NA	0.0779	1.4921E-01		ENSG00000288751
NA	0.0998	1.4929E-01	3.2998E-01	ENSG00000269737
NA	0.0223	1.4947E-01		ENSG00000237756

NA	0.0256	1.4949E-01		ENSG00000287108
NA	0.0584	1.4953E-01		ENSG00000256069
NA	0.0518	1.4969E-01		ENSG00000228536
NA	0.1095	1.4988E-01	3.3072E-01	ENSG00000273628
NA	0.0770	1.4995E-01		ENSG00000287683
NA	0.0426	1.4996E-01		ENSG00000231405
NA	0.1356	1.5028E-01	3.3137E-01	ENSG00000226054
NA	0.0383	1.5035E-01		ENSG00000246016
NA	0.0582	1.5040E-01		ENSG00000272529
NA	0.0270	1.5055E-01		ENSG00000255396
NA	0.0578	1.5065E-01		ENSG00000251408
NA	0.0985	1.5099E-01	3.3231E-01	ENSG00000235669
NA	0.0529	1.5100E-01		ENSG00000205622
NA	0.0252	1.5103E-01		ENSG00000232884
NA	0.1130	1.5113E-01	3.3252E-01	ENSG00000287434
NA	0.0215	1.5127E-01		ENSG00000282535
NA	0.1188	1.5174E-01	3.3338E-01	ENSG00000280367
NA	0.0615	1.5213E-01		ENSG00000275576
NA	0.1084	1.5216E-01	3.3403E-01	ENSG00000268201
NA	0.0522	1.5217E-01		ENSG00000289886
NA	0.0403	1.5233E-01		ENSG00000278740
NA	0.0298	1.5263E-01		ENSG00000286886
NA	0.1520	1.5268E-01	3.3479E-01	ENSG00000286472
NA	0.0663	1.5271E-01		ENSG00000289338
NA	0.1085	1.5282E-01	3.3487E-01	ENSG00000287384
NA	0.1315	1.5282E-01	3.3487E-01	ENSG00000288066
NA	0.1114	1.5307E-01	3.3527E-01	ENSG00000278996
NA	0.0762	1.5352E-01	3.3582E-01	ENSG00000287078
NA	0.1379	1.5370E-01	3.3604E-01	ENSG00000264775
NA	0.0207	1.5376E-01		ENSG00000233358
NA	0.0207	1.5388E-01		ENSG00000284699
NA	0.1063	1.5392E-01	3.3637E-01	ENSG00000289435
NA	0.1318	1.5463E-01	3.3756E-01	ENSG00000289034
NA	0.0785	1.5469E-01	3.3767E-01	ENSG00000223536
NA	0.1235	1.5484E-01	3.3791E-01	ENSG00000253762
NA	0.0315	1.5499E-01		ENSG00000225693
NA	0.1091	1.5500E-01	3.3815E-01	ENSG00000260973
NA	0.0443	1.5504E-01		ENSG00000230444
NA	0.1276	1.5508E-01	3.3831E-01	ENSG00000289444
NA	0.0634	1.5559E-01		ENSG00000269161
NA	0.0820	1.5577E-01	3.3944E-01	ENSG00000278071
NA	0.1183	1.5599E-01	3.3977E-01	ENSG00000233110
NA	0.0225	1.5609E-01		ENSG00000289026
NA	0.1372	1.5613E-01	3.4003E-01	ENSG00000286834
NA	0.0675	1.5640E-01	3.4030E-01	ENSG00000288945
NA	0.0334	1.5642E-01		ENSG00000264617
NA	0.1265	1.5644E-01	3.4030E-01	ENSG00000283635
NA	0.1341	1.5650E-01	3.4030E-01	ENSG00000260469
NA	0.1308	1.5654E-01	3.4030E-01	ENSG00000225518

NA	0.1159	1.5660E-01	3.4030E-01	ENSG00000280285
NA	0.1253	1.5663E-01	3.4030E-01	ENSG00000127957
NA	0.0453	1.5668E-01		ENSG00000260466
NA	0.0755	1.5673E-01		ENSG00000289928
NA	0.1274	1.5699E-01	3.4064E-01	ENSG00000253642
NA	0.0502	1.5704E-01		ENSG00000272515
NA	0.1346	1.5714E-01	3.4092E-01	ENSG00000272953
NA	0.0955	1.5731E-01	3.4109E-01	ENSG00000223825
NA	0.0672	1.5731E-01		ENSG00000267319
NA	0.0808	1.5745E-01	3.4118E-01	ENSG00000197644
NA	0.1017	1.5785E-01	3.4184E-01	ENSG00000278058
NA	0.0745	1.5786E-01	3.4184E-01	ENSG00000233854
NA	0.0199	1.5793E-01		ENSG00000287845
NA	0.0252	1.5801E-01		ENSG00000205682
NA	0.0867	1.5812E-01	3.4225E-01	ENSG00000259314
NA	0.0573	1.5822E-01		ENSG00000205584
NA	0.0345	1.5825E-01		ENSG00000217159
NA	0.0347	1.5845E-01		ENSG00000285911
NA	0.0192	1.5855E-01		ENSG00000250642
NA	0.0832	1.5875E-01	3.4315E-01	ENSG00000236031
NA	0.1253	1.5896E-01	3.4350E-01	ENSG00000269069
NA	0.0203	1.5897E-01		ENSG00000263369
NA	0.0782	1.5897E-01	3.4350E-01	ENSG00000278385
NA	0.0987	1.5915E-01	3.4371E-01	ENSG00000289421
NA	0.0299	1.5934E-01		ENSG00000289545
NA	0.0590	1.5937E-01		ENSG00000229591
NA	0.0123	1.5952E-01		ENSG00000237324
NA	0.0539	1.5975E-01		ENSG00000290164
NA	0.0300	1.5987E-01		ENSG00000224933
NA	0.0724	1.6005E-01	3.4522E-01	ENSG00000270589
NA	0.0449	1.6011E-01		ENSG00000286180
NA	0.1025	1.6013E-01	3.4535E-01	ENSG00000286118
NA	0.0932	1.6017E-01	3.4541E-01	ENSG00000280163
NA	0.1343	1.6021E-01	3.4542E-01	ENSG00000243004
NA	0.0646	1.6023E-01		ENSG00000250167
NA	0.0135	1.6028E-01		ENSG00000231619
NA	0.0679	1.6031E-01	3.4556E-01	ENSG00000287229
NA	0.1128	1.6040E-01	3.4571E-01	ENSG00000289532
NA	0.1202	1.6067E-01	3.4613E-01	ENSG00000287439
NA	0.1295	1.6079E-01	3.4630E-01	ENSG00000204685
NA	0.1278	1.6094E-01	3.4648E-01	ENSG00000289520
NA	0.0288	1.6112E-01		ENSG00000284675
NA	0.0690	1.6113E-01	3.4679E-01	ENSG00000230783
NA	0.0628	1.6144E-01		ENSG00000279149
NA	0.0548	1.6146E-01		ENSG00000233870
NA	0.1158	1.6151E-01	3.4742E-01	ENSG00000213089
NA	0.0503	1.6159E-01	3.4750E-01	ENSG00000267529
NA	0.0393	1.6162E-01		ENSG00000260721
NA	0.1106	1.6163E-01	3.4754E-01	ENSG00000225077

NA	0.0517	1.6175E-01		ENSG00000238232
NA	0.1026	1.6188E-01	3.4792E-01	ENSG00000274455
NA	0.1324	1.6189E-01	3.4792E-01	ENSG00000274114
NA	0.0688	1.6203E-01	3.4802E-01	ENSG00000271746
NA	0.1165	1.6224E-01	3.4826E-01	ENSG00000175741
NA	0.0464	1.6229E-01	3.4829E-01	ENSG00000267051
NA	0.1263	1.6244E-01	3.4852E-01	ENSG00000269246
NA	0.0634	1.6246E-01		ENSG00000284070
NA	0.0862	1.6254E-01	3.4864E-01	ENSG00000287023
NA	0.0928	1.6274E-01	3.4895E-01	ENSG00000285632
NA	0.0894	1.6276E-01	3.4895E-01	ENSG00000200164
NA	0.0654	1.6298E-01		ENSG00000275904
NA	0.1337	1.6305E-01	3.4940E-01	ENSG00000279267
NA	0.0491	1.6312E-01		ENSG00000287084
NA	0.0669	1.6324E-01		ENSG00000253690
NA	0.0593	1.6335E-01		ENSG00000257199
NA	0.0705	1.6344E-01	3.5003E-01	ENSG00000288865
NA	0.0670	1.6350E-01	3.5010E-01	ENSG00000251621
NA	0.0195	1.6350E-01		ENSG00000260710
NA	0.0849	1.6371E-01	3.5034E-01	ENSG00000289691
NA	0.0394	1.6375E-01		ENSG00000275850
NA	0.0385	1.6409E-01		ENSG00000224063
NA	0.1047	1.6411E-01	3.5096E-01	ENSG00000240063
NA	0.0831	1.6421E-01	3.5102E-01	ENSG00000224192
NA	0.1338	1.6430E-01	3.5111E-01	ENSG00000258515
NA	0.0352	1.6433E-01		ENSG00000283457
NA	0.0410	1.6442E-01		ENSG00000235522
NA	0.1324	1.6460E-01	3.5150E-01	ENSG00000234405
NA	0.0380	1.6466E-01		ENSG00000288056
NA	0.1328	1.6481E-01	3.5188E-01	ENSG00000289602
NA	0.0101	1.6489E-01		ENSG00000253576
NA	0.0220	1.6500E-01		ENSG00000287833
NA	0.0711	1.6504E-01	3.5223E-01	ENSG00000242797
NA	0.0299	1.6512E-01		ENSG00000236118
NA	0.0505	1.6551E-01		ENSG00000282951
NA	0.0136	1.6554E-01		ENSG00000223516
NA	0.0604	1.6556E-01		ENSG00000274501
NA	0.0524	1.6560E-01		ENSG00000278962
NA	0.0688	1.6569E-01		ENSG00000261348
NA	0.0331	1.6571E-01		ENSG00000233920
NA	0.1099	1.6576E-01	3.5330E-01	ENSG00000286757
NA	0.0175	1.6600E-01		ENSG00000230552
NA	0.0187	1.6614E-01		ENSG00000254844
NA	0.1196	1.6618E-01	3.5384E-01	ENSG00000205056
NA	0.1291	1.6630E-01	3.5400E-01	ENSG00000263590
NA	0.1038	1.6693E-01	3.5494E-01	ENSG00000271964
NA	0.0384	1.6719E-01		ENSG00000285584
NA	0.0760	1.6754E-01	3.5586E-01	ENSG00000274340
NA	0.0204	1.6755E-01		ENSG00000287776

NA	0.0895	1.6758E-01	3.5589E-01	ENSG00000272338
NA	0.0158	1.6769E-01	3.5599E-01	ENSG00000288760
NA	0.0582	1.6796E-01	3.5623E-01	ENSG00000251361
NA	0.0207	1.6801E-01		ENSG00000237990
NA	0.1282	1.6806E-01	3.5638E-01	ENSG00000258302
NA	0.1093	1.6818E-01	3.5647E-01	ENSG00000286797
NA	0.0693	1.6819E-01	3.5647E-01	ENSG00000258646
NA	0.0812	1.6851E-01	3.5691E-01	ENSG00000261471
NA	0.0972	1.6855E-01	3.5695E-01	ENSG00000285778
NA	0.0832	1.6862E-01	3.5701E-01	ENSG00000238250
NA	0.1031	1.6873E-01	3.5716E-01	ENSG00000239519
NA	0.0901	1.6882E-01	3.5726E-01	ENSG00000282057
NA	0.0319	1.6884E-01		ENSG00000287904
NA	0.0186	1.6884E-01		ENSG00000283417
NA	0.0529	1.6889E-01		ENSG00000237154
NA	0.0457	1.6896E-01		ENSG00000242078
NA	0.0523	1.6909E-01		ENSG00000241362
NA	0.0227	1.6916E-01		ENSG00000252202
NA	0.0778	1.6939E-01	3.5786E-01	ENSG00000288866
NA	0.0546	1.7031E-01	3.5912E-01	ENSG00000249244
NA	0.0945	1.7037E-01	3.5921E-01	ENSG00000228409
NA	0.1202	1.7057E-01	3.5942E-01	ENSG00000250069
NA	0.1090	1.7057E-01	3.5942E-01	ENSG00000273374
NA	0.1052	1.7060E-01	3.5945E-01	ENSG00000275374
NA	0.0752	1.7065E-01	3.5951E-01	ENSG00000289392
NA	0.0443	1.7070E-01		ENSG00000289276
NA	0.0486	1.7101E-01		ENSG00000251459
NA	0.0256	1.7128E-01		ENSG00000259241
NA	0.1141	1.7135E-01	3.6050E-01	ENSG00000257489
NA	0.0527	1.7141E-01		ENSG00000236888
NA	0.0116	1.7148E-01		ENSG00000228799
NA	0.0783	1.7155E-01	3.6075E-01	ENSG00000287330
NA	0.0198	1.7162E-01		ENSG00000223436
NA	0.0871	1.7167E-01	3.6083E-01	ENSG00000254687
NA	0.0989	1.7172E-01	3.6089E-01	ENSG00000233293
NA	0.0286	1.7184E-01		ENSG00000279841
NA	0.0203	1.7191E-01		ENSG00000234170
NA	0.0414	1.7202E-01		ENSG00000287383
NA	0.0594	1.7203E-01		ENSG00000256064
NA	0.0249	1.7209E-01		ENSG00000261693
NA	0.0718	1.7275E-01	3.6252E-01	ENSG00000278212
NA	0.0332	1.7286E-01		ENSG00000271811
NA	0.1027	1.7308E-01	3.6305E-01	ENSG00000271978
NA	0.0757	1.7310E-01	3.6306E-01	ENSG00000236209
NA	0.0473	1.7348E-01		ENSG00000290056
NA	0.0299	1.7349E-01		ENSG00000262332
NA	0.1103	1.7367E-01	3.6387E-01	ENSG00000287625
NA	0.0765	1.7369E-01		ENSG00000287919
NA	0.1077	1.7378E-01	3.6398E-01	ENSG00000266573

NA	0.1261	1.7387E-01	3.6405E-01	ENSG00000273001
NA	0.1073	1.7431E-01	3.6462E-01	ENSG00000229109
NA	0.0766	1.7432E-01	3.6462E-01	ENSG00000277692
NA	0.1144	1.7438E-01	3.6469E-01	ENSG00000264660
NA	0.0909	1.7450E-01	3.6486E-01	ENSG00000226472
NA	0.1129	1.7451E-01	3.6486E-01	ENSG00000280254
NA	0.1274	1.7457E-01	3.6490E-01	ENSG00000229152
NA	0.1275	1.7477E-01	3.6515E-01	ENSG00000250130
NA	0.1142	1.7484E-01	3.6522E-01	ENSG00000182722
NA	0.0342	1.7493E-01		ENSG00000262319
NA	0.0398	1.7498E-01		ENSG00000289023
NA	0.0138	1.7508E-01		ENSG00000260777
NA	0.0356	1.7516E-01		ENSG00000289967
NA	0.0709	1.7553E-01	3.6638E-01	ENSG00000272209
NA	0.0932	1.7578E-01	3.6676E-01	ENSG00000262848
NA	0.0588	1.7583E-01		ENSG00000225555
NA	0.0216	1.7593E-01		ENSG00000289049
NA	0.1118	1.7594E-01	3.6699E-01	ENSG00000250091
NA	0.1111	1.7597E-01	3.6699E-01	ENSG00000272696
NA	0.0467	1.7599E-01		ENSG00000230107
NA	0.0427	1.7605E-01		ENSG00000274554
NA	0.0788	1.7612E-01	3.6720E-01	ENSG00000233627
NA	0.0686	1.7631E-01		ENSG00000288526
NA	0.0893	1.7710E-01	3.6829E-01	ENSG00000254556
NA	0.0876	1.7723E-01	3.6840E-01	ENSG00000256955
NA	0.1284	1.7744E-01	3.6864E-01	ENSG00000272145
NA	0.0738	1.7746E-01	3.6864E-01	ENSG00000249776
NA	0.1269	1.7762E-01	3.6883E-01	ENSG00000237082
NA	0.0277	1.7777E-01		ENSG00000280397
NA	0.0353	1.7783E-01		ENSG00000287820
NA	0.0231	1.7783E-01		ENSG00000260776
NA	0.0238	1.7795E-01		ENSG00000284702
NA	0.1225	1.7796E-01	3.6922E-01	ENSG00000272619
NA	0.0326	1.7798E-01		ENSG00000228686
NA	0.0599	1.7815E-01		ENSG00000235736
NA	0.0592	1.7869E-01		ENSG00000248115
NA	0.0745	1.7885E-01	3.7029E-01	ENSG00000286540
NA	0.0202	1.7894E-01		ENSG00000257345
NA	0.0371	1.7913E-01		ENSG00000236409
NA	0.0520	1.7917E-01		ENSG00000260851
NA	0.0357	1.7922E-01		ENSG00000250614
NA	0.0500	1.7925E-01		ENSG00000273998
NA	0.0513	1.7938E-01	3.7104E-01	ENSG00000286974
NA	0.1095	1.7943E-01	3.7107E-01	ENSG00000260528
NA	0.0322	1.7988E-01		ENSG00000200419
NA	0.0416	1.8014E-01		ENSG00000237646
NA	0.1121	1.8042E-01	3.7258E-01	ENSG00000224592
NA	0.0457	1.8051E-01		ENSG00000200421
NA	0.0264	1.8057E-01		ENSG00000259201

NA	0.1270	1.8067E-01	3.7287E-01	ENSG00000289177
NA	0.0673	1.8079E-01	3.7309E-01	ENSG00000122043
NA	0.0498	1.8088E-01		ENSG00000287130
NA	0.0242	1.8098E-01		ENSG00000231772
NA	0.0553	1.8111E-01		ENSG00000282742
NA	0.1027	1.8117E-01	3.7362E-01	ENSG00000274447
NA	0.0855	1.8120E-01	3.7365E-01	ENSG00000283031
NA	0.0700	1.8124E-01		ENSG00000273063
NA	0.0839	1.8138E-01	3.7392E-01	ENSG00000234139
NA	0.0332	1.8153E-01		ENSG00000258732
NA	0.0387	1.8175E-01		ENSG00000235813
NA	0.0520	1.8195E-01		ENSG00000225169
NA	0.0213	1.8197E-01		ENSG00000154198
NA	0.0243	1.8202E-01		ENSG00000276002
NA	0.0191	1.8206E-01		ENSG00000260944
NA	0.0139	1.8216E-01		ENSG00000229012
NA	0.0572	1.8220E-01	3.7508E-01	ENSG00000287129
NA	0.0355	1.8224E-01		ENSG00000262171
NA	0.0918	1.8231E-01	3.7526E-01	ENSG00000263887
NA	0.0132	1.8263E-01		ENSG00000273710
NA	0.0365	1.8289E-01		ENSG00000285961
NA	0.0625	1.8296E-01	3.7608E-01	ENSG00000288921
NA	0.0555	1.8335E-01		ENSG00000280118
NA	0.0695	1.8375E-01	3.7707E-01	ENSG00000260500
NA	0.1255	1.8382E-01	3.7717E-01	ENSG00000266947
NA	0.0739	1.8397E-01	3.7736E-01	ENSG00000254238
NA	0.1161	1.8428E-01	3.7773E-01	ENSG00000233143
NA	0.0466	1.8461E-01		ENSG00000285581
NA	0.0527	1.8461E-01		ENSG00000236015
NA	0.1149	1.8466E-01	3.7818E-01	ENSG00000235724
NA	0.0622	1.8473E-01		ENSG00000141028
NA	0.0877	1.8480E-01	3.7837E-01	ENSG00000271350
NA	0.0206	1.8483E-01		ENSG00000236325
NA	0.0295	1.8503E-01		ENSG00000243007
NA	0.0596	1.8527E-01		ENSG00000271151
NA	0.0742	1.8596E-01		ENSG00000251189
NA	0.1219	1.8598E-01	3.8013E-01	ENSG00000266313
NA	0.1091	1.8603E-01	3.8015E-01	ENSG00000272630
NA	0.0923	1.8621E-01	3.8045E-01	ENSG00000248240
NA	0.0750	1.8632E-01	3.8058E-01	ENSG00000215184
NA	0.1234	1.8638E-01	3.8066E-01	ENSG00000235319
NA	0.0939	1.8666E-01	3.8104E-01	ENSG00000285041
NA	0.1142	1.8667E-01	3.8104E-01	ENSG00000269514
NA	0.1243	1.8671E-01	3.8106E-01	ENSG00000285728
NA	0.1054	1.8683E-01	3.8112E-01	ENSG00000271933
NA	0.0329	1.8693E-01		ENSG00000278603
NA	0.1256	1.8724E-01	3.8177E-01	ENSG00000270071
NA	0.0950	1.8726E-01	3.8177E-01	ENSG00000276832
NA	0.1022	1.8747E-01	3.8206E-01	ENSG00000287343

NA	0.0588	1.8766E-01		ENSG00000262031
NA	0.0695	1.8794E-01		ENSG00000225213
NA	0.0305	1.8814E-01		ENSG00000288990
NA	0.1249	1.8838E-01	3.8331E-01	ENSG00000289993
NA	0.1015	1.8866E-01	3.8354E-01	ENSG00000261159
NA	0.0513	1.8867E-01		ENSG00000256944
NA	0.1203	1.8896E-01	3.8390E-01	ENSG00000226055
NA	0.1229	1.8911E-01	3.8404E-01	ENSG00000248740
NA	0.0437	1.8919E-01		ENSG00000259302
NA	0.0645	1.8921E-01		ENSG00000228283
NA	0.0739	1.8922E-01	3.8420E-01	ENSG00000254246
NA	0.0686	1.8926E-01	3.8425E-01	ENSG00000254343
NA	0.0109	1.8943E-01		ENSG00000269540
NA	0.0621	1.8945E-01		ENSG00000272858
NA	0.1211	1.8977E-01	3.8487E-01	ENSG00000227848
NA	0.1214	1.8989E-01	3.8493E-01	ENSG00000254740
NA	0.0381	1.8991E-01		ENSG00000276754
NA	0.0915	1.9000E-01	3.8506E-01	ENSG00000235020
NA	0.0369	1.9002E-01	3.8506E-01	ENSG00000266968
NA	0.1151	1.9048E-01	3.8568E-01	ENSG00000286358
NA	0.0196	1.9054E-01		ENSG00000201555
NA	0.0497	1.9069E-01		ENSG00000285769
NA	0.0329	1.9099E-01		ENSG00000286572
NA	0.0849	1.9103E-01	3.8628E-01	ENSG00000214313
NA	0.1219	1.9108E-01	3.8632E-01	ENSG00000284879
NA	0.0253	1.9108E-01		ENSG00000256226
NA	0.0208	1.9133E-01		ENSG00000260550
NA	0.0264	1.9140E-01		ENSG00000227813
NA	0.1180	1.9167E-01	3.8701E-01	ENSG00000164845
NA	0.0529	1.9170E-01		ENSG00000286017
NA	0.1200	1.9171E-01	3.8705E-01	ENSG00000255136
NA	0.1177	1.9183E-01	3.8717E-01	ENSG00000279283
NA	0.1214	1.9193E-01	3.8731E-01	ENSG00000239407
NA	0.0346	1.9246E-01		ENSG00000250258
NA	0.0500	1.9255E-01		ENSG00000186481
NA	0.0195	1.9284E-01		ENSG00000200065
NA	0.0350	1.9286E-01		ENSG00000277233
NA	0.0477	1.9296E-01		ENSG00000226774
NA	0.1148	1.9317E-01	3.8924E-01	ENSG00000260025
NA	0.1228	1.9330E-01	3.8947E-01	ENSG00000289375
NA	0.0683	1.9337E-01	3.8954E-01	ENSG00000244480
NA	0.0244	1.9361E-01		ENSG00000286048
NA	0.0191	1.9388E-01		ENSG00000289966
NA	0.0511	1.9394E-01		ENSG00000232969
NA	0.0418	1.9409E-01		ENSG00000287495
NA	0.0401	1.9415E-01		ENSG00000271002
NA	0.0695	1.9419E-01		ENSG00000253832
NA	0.0361	1.9425E-01		ENSG00000263904
NA	0.0488	1.9427E-01		ENSG00000270987

NA	0.0668	1.9430E-01	3.9100E-01	ENSG00000222044
NA	0.0814	1.9438E-01	3.9111E-01	ENSG00000210164
NA	0.0726	1.9460E-01	3.9132E-01	ENSG00000279463
NA	0.1188	1.9482E-01	3.9160E-01	ENSG00000279059
NA	0.0686	1.9495E-01	3.9177E-01	ENSG00000176349
NA	0.0654	1.9510E-01	3.9194E-01	ENSG00000280157
NA	0.0405	1.9519E-01		ENSG00000273582
NA	0.0281	1.9523E-01	3.9213E-01	ENSG00000241462
NA	0.0371	1.9538E-01		ENSG00000289426
NA	0.0685	1.9547E-01		ENSG00000258919
NA	0.0968	1.9567E-01	3.9282E-01	ENSG00000289883
NA	0.0801	1.9580E-01	3.9292E-01	ENSG00000229751
NA	0.0458	1.9581E-01		ENSG00000259255
NA	0.0205	1.9598E-01		ENSG00000258210
NA	0.0501	1.9615E-01		ENSG00000277010
NA	0.1103	1.9630E-01	3.9365E-01	ENSG00000290047
NA	0.1158	1.9640E-01	3.9375E-01	ENSG00000271714
NA	0.1045	1.9643E-01	3.9378E-01	ENSG00000226281
NA	0.0428	1.9653E-01		ENSG00000282059
NA	0.0355	1.9658E-01		ENSG00000268889
NA	0.0278	1.9666E-01		ENSG00000233427
NA	0.0619	1.9684E-01		ENSG00000267717
NA	0.0866	1.9686E-01	3.9438E-01	ENSG00000274441
NA	0.0581	1.9688E-01		ENSG00000286147
NA	0.0210	1.9692E-01		ENSG00000286848
NA	0.1165	1.9694E-01	3.9451E-01	ENSG00000278879
NA	0.0708	1.9702E-01		ENSG00000289553
NA	0.0276	1.9703E-01		ENSG00000286429
NA	0.0432	1.9733E-01		ENSG00000261821
NA	0.0818	1.9735E-01	3.9499E-01	ENSG00000277767
NA	0.0327	1.9737E-01		ENSG00000251488
NA	0.0824	1.9743E-01	3.9510E-01	ENSG00000289983
NA	0.1016	1.9749E-01	3.9520E-01	ENSG00000267474
NA	0.0532	1.9752E-01		ENSG00000287990
NA	0.0313	1.9756E-01		ENSG00000229670
NA	0.1004	1.9771E-01	3.9554E-01	ENSG00000281383
NA	0.0179	1.9773E-01		ENSG00000289885
NA	0.1025	1.9774E-01	3.9556E-01	ENSG00000184414
NA	0.1109	1.9798E-01	3.9582E-01	ENSG00000232320
NA	0.0976	1.9820E-01	3.9609E-01	ENSG00000241596
NA	0.0612	1.9827E-01		ENSG00000235023
NA	0.0233	1.9926E-01		ENSG00000264125
NA	0.0629	1.9931E-01		ENSG00000202514
NA	0.0703	1.9964E-01	3.9804E-01	ENSG00000254459
NA	0.0914	1.9971E-01	3.9807E-01	ENSG00000248863
NA	0.0458	1.9981E-01		ENSG00000287738
NA	0.0262	1.9985E-01		ENSG00000224142
NA	0.0879	1.9986E-01	3.9827E-01	ENSG00000182397
NA	0.0235	1.9987E-01		ENSG00000279951

NA	0.0485	1.9989E-01		ENSG00000258536
NA	0.0539	1.9993E-01		ENSG00000247324
NA	0.0484	2.0012E-01		ENSG00000279541
NA	0.1066	2.0014E-01	3.9854E-01	ENSG00000233559
NA	0.0260	2.0039E-01		ENSG00000237027
NA	0.0329	2.0040E-01		ENSG00000272078
NA	0.0648	2.0048E-01	3.9894E-01	ENSG00000231084
NA	0.0343	2.0063E-01		ENSG00000286998
NA	0.0058	2.0079E-01		ENSG00000237836
NA	0.0906	2.0082E-01	3.9935E-01	ENSG00000223711
NA	0.0640	2.0106E-01	3.9955E-01	ENSG00000238009
NA	0.0299	2.0114E-01		ENSG00000214041
NA	0.0206	2.0118E-01		ENSG00000278893
NA	0.1194	2.0125E-01	3.9979E-01	ENSG00000243024
NA	0.0856	2.0127E-01	3.9979E-01	ENSG00000266896
NA	0.0147	2.0129E-01		ENSG00000214144
NA	0.1078	2.0145E-01	4.0006E-01	ENSG00000271040
NA	0.0759	2.0151E-01	4.0010E-01	ENSG00000270457
NA	0.1079	2.0155E-01	4.0015E-01	ENSG00000285582
NA	0.0479	2.0202E-01		ENSG00000277695
NA	0.0223	2.0228E-01		ENSG00000286870
NA	0.0766	2.0228E-01	4.0113E-01	ENSG00000274248
NA	0.0411	2.0248E-01		ENSG00000283752
NA	0.0493	2.0252E-01		ENSG00000279605
NA	0.1073	2.0258E-01	4.0148E-01	ENSG00000200090
NA	0.1196	2.0263E-01	4.0155E-01	ENSG00000267904
NA	0.0891	2.0280E-01	4.0176E-01	ENSG00000250982
NA	0.0615	2.0295E-01		ENSG00000286439
NA	0.0764	2.0321E-01	4.0209E-01	ENSG00000254305
NA	0.0554	2.0335E-01		ENSG00000268707
NA	0.0657	2.0363E-01	4.0249E-01	ENSG00000279135
NA	0.0396	2.0364E-01	4.0249E-01	ENSG00000289741
NA	0.0299	2.0378E-01		ENSG00000270492
NA	0.0593	2.0382E-01		ENSG00000219993
NA	0.0388	2.0392E-01		ENSG00000210154
NA	0.1170	2.0409E-01	4.0315E-01	ENSG00000288881
NA	0.0178	2.0410E-01		ENSG00000226447
NA	0.0374	2.0425E-01		ENSG00000235008
NA	0.1151	2.0461E-01	4.0385E-01	ENSG00000232098
NA	0.0555	2.0462E-01		ENSG00000250538
NA	0.0582	2.0464E-01		ENSG00000228153
NA	0.0887	2.0471E-01	4.0392E-01	ENSG00000259920
NA	0.0747	2.0485E-01	4.0410E-01	ENSG00000289528
NA	0.0700	2.0495E-01		ENSG00000240520
NA	0.0883	2.0505E-01	4.0436E-01	ENSG00000269621
NA	0.0215	2.0509E-01		ENSG00000286485
NA	0.0833	2.0518E-01	4.0446E-01	ENSG00000287146
NA	0.0875	2.0538E-01	4.0473E-01	ENSG00000198237
NA	0.0257	2.0554E-01		ENSG00000252230

NA	0.0250	2.0562E-01		ENSG00000287850
NA	0.1135	2.0582E-01	4.0530E-01	ENSG00000274565
NA	0.0147	2.0587E-01		ENSG00000225718
NA	0.0226	2.0592E-01		ENSG00000256146
NA	0.0155	2.0598E-01		ENSG00000229569
NA	0.1130	2.0605E-01	4.0562E-01	ENSG00000257497
NA	0.0664	2.0638E-01	4.0606E-01	ENSG00000286509
NA	0.0527	2.0653E-01		ENSG00000273137
NA	0.0859	2.0653E-01	4.0618E-01	ENSG00000253620
NA	0.1113	2.0655E-01	4.0618E-01	ENSG00000289015
NA	0.0869	2.0657E-01	4.0618E-01	ENSG00000275964
NA	0.0219	2.0666E-01		ENSG00000290119
NA	0.0153	2.0667E-01		ENSG00000266527
NA	0.0999	2.0670E-01	4.0621E-01	ENSG00000259554
NA	0.1030	2.0685E-01	4.0635E-01	ENSG00000206417
NA	0.0218	2.0719E-01		ENSG00000269933
NA	0.1044	2.0740E-01	4.0704E-01	ENSG00000272933
NA	0.0750	2.0744E-01	4.0706E-01	ENSG00000286251
NA	0.0458	2.0777E-01	4.0744E-01	ENSG00000270822
NA	0.0139	2.0819E-01		ENSG00000283636
NA	0.0597	2.0832E-01		ENSG00000279181
NA	0.0674	2.0855E-01	4.0861E-01	ENSG00000262823
NA	0.0327	2.0856E-01		ENSG00000260005
NA	0.0774	2.0858E-01	4.0861E-01	ENSG00000286593
NA	0.0689	2.0900E-01	4.0904E-01	ENSG00000241535
NA	0.1155	2.0915E-01	4.0916E-01	ENSG00000270876
NA	0.0315	2.0919E-01	4.0916E-01	ENSG00000259505
NA	0.0168	2.0921E-01		ENSG00000229345
NA	0.0429	2.0921E-01		ENSG00000283383
NA	0.0415	2.0940E-01	4.0946E-01	ENSG00000288720
NA	0.0634	2.0944E-01	4.0952E-01	ENSG00000286645
NA	0.0470	2.0948E-01		ENSG00000287338
NA	0.0693	2.0968E-01		ENSG00000224342
NA	0.0394	2.1022E-01		ENSG00000226089
NA	0.0557	2.1024E-01		ENSG00000275805
NA	0.0296	2.1056E-01		ENSG00000248510
NA	0.1028	2.1068E-01	4.1129E-01	ENSG00000238278
NA	0.0176	2.1080E-01		ENSG00000290067
NA	0.0308	2.1085E-01		ENSG00000275139
NA	0.0436	2.1089E-01		ENSG00000256148
NA	0.0836	2.1131E-01	4.1209E-01	ENSG00000226580
NA	0.0664	2.1145E-01	4.1230E-01	ENSG00000244675
NA	0.0965	2.1161E-01	4.1242E-01	ENSG00000232811
NA	0.0493	2.1192E-01		ENSG00000268744
NA	0.0234	2.1206E-01		ENSG00000254298
NA	0.0189	2.1207E-01		ENSG00000273069
NA	0.0658	2.1261E-01	4.1365E-01	ENSG00000274624
NA	0.1169	2.1263E-01	4.1365E-01	ENSG00000258150
NA	0.0194	2.1272E-01		ENSG00000201207

NA	0.1087	2.1276E-01	4.1379E-01	ENSG00000270441
NA	0.1142	2.1281E-01	4.1383E-01	ENSG00000260918
NA	0.1111	2.1281E-01	4.1383E-01	ENSG00000203321
NA	0.1162	2.1295E-01	4.1398E-01	ENSG00000259349
NA	0.0663	2.1303E-01	4.1409E-01	ENSG00000283692
NA	0.0410	2.1311E-01		ENSG00000236773
NA	0.1137	2.1332E-01	4.1434E-01	ENSG00000160172
NA	0.0758	2.1338E-01	4.1436E-01	ENSG00000288890
NA	0.0369	2.1351E-01		ENSG00000228585
NA	0.0333	2.1391E-01		ENSG00000278607
NA	0.0609	2.1405E-01		ENSG00000287830
NA	0.0397	2.1409E-01		ENSG00000234962
NA	0.0280	2.1436E-01	4.1553E-01	ENSG00000260351
NA	0.0461	2.1451E-01		ENSG00000259665
NA	0.0625	2.1464E-01	4.1588E-01	ENSG00000286666
NA	0.0356	2.1466E-01		ENSG00000220695
NA	0.0741	2.1496E-01	4.1628E-01	ENSG00000249335
NA	0.0815	2.1497E-01	4.1628E-01	ENSG00000287601
NA	0.0426	2.1510E-01	4.1635E-01	ENSG00000260035
NA	0.0181	2.1513E-01		ENSG00000215734
NA	0.0743	2.1527E-01	4.1657E-01	ENSG00000251131
NA	0.0784	2.1544E-01	4.1673E-01	ENSG00000234917
NA	0.0264	2.1550E-01		ENSG00000255239
NA	0.0156	2.1556E-01		ENSG00000234273
NA	0.0870	2.1575E-01	4.1713E-01	ENSG00000249593
NA	0.0608	2.1598E-01	4.1749E-01	ENSG00000231760
NA	0.0457	2.1599E-01		ENSG00000287993
NA	0.0519	2.1600E-01		ENSG00000270909
NA	0.0010	2.1601E-01		ENSG00000277958
NA	0.0241	2.1611E-01		ENSG00000234182
NA	0.1133	2.1624E-01	4.1784E-01	ENSG00000259404
NA	0.0207	2.1628E-01		ENSG00000237262
NA	0.0197	2.1656E-01	4.1821E-01	ENSG00000287217
NA	0.0850	2.1690E-01	4.1862E-01	ENSG00000284664
NA	0.0980	2.1726E-01	4.1910E-01	ENSG00000285018
NA	0.0493	2.1736E-01		ENSG00000228748
NA	0.0545	2.1755E-01		ENSG00000269873
NA	0.0712	2.1760E-01	4.1963E-01	ENSG00000232934
NA	0.0364	2.1761E-01		ENSG00000260905
NA	0.1133	2.1768E-01	4.1974E-01	ENSG00000280206
NA	0.1076	2.1787E-01	4.2002E-01	ENSG00000249621
NA	0.0202	2.1789E-01		ENSG00000214534
NA	0.1106	2.1795E-01	4.2005E-01	ENSG00000280020
NA	0.0287	2.1818E-01		ENSG00000272417
NA	0.0451	2.1821E-01		ENSG00000231606
NA	0.0276	2.1830E-01		ENSG00000286806
NA	0.0189	2.1836E-01		ENSG00000269842
NA	0.0683	2.1889E-01	4.2137E-01	ENSG00000279019
NA	0.0628	2.1890E-01	4.2137E-01	ENSG00000260179

NA	0.1063	2.1895E-01	4.2144E-01	ENSG00000234509
NA	0.1134	2.1897E-01	4.2144E-01	ENSG00000226179
NA	0.0426	2.1905E-01		ENSG00000258658
NA	0.0222	2.1918E-01		ENSG00000229052
NA	0.0489	2.1922E-01		ENSG00000239351
NA	0.1050	2.1940E-01	4.2176E-01	ENSG00000235381
NA	0.0530	2.2016E-01		ENSG00000258897
NA	0.0594	2.2044E-01		ENSG00000283438
NA	0.0461	2.2052E-01		ENSG00000263300
NA	0.0625	2.2052E-01		ENSG00000278330
NA	0.0226	2.2064E-01		ENSG00000223962
NA	0.0998	2.2071E-01	4.2373E-01	ENSG00000289212
NA	0.1022	2.2094E-01	4.2412E-01	ENSG00000289121
NA	0.0207	2.2105E-01		ENSG00000236173
NA	0.0191	2.2142E-01		ENSG00000275799
NA	0.0724	2.2156E-01	4.2486E-01	ENSG00000279199
NA	0.0522	2.2164E-01		ENSG00000206168
NA	0.1055	2.2183E-01	4.2508E-01	ENSG00000262089
NA	0.0435	2.2191E-01		ENSG00000256694
NA	0.0430	2.2193E-01		ENSG00000225226
NA	0.0163	2.2198E-01	4.2534E-01	ENSG00000275927
NA	0.0955	2.2207E-01	4.2545E-01	ENSG00000272973
NA	0.0633	2.2224E-01	4.2560E-01	ENSG00000272831
NA	0.0339	2.2231E-01		ENSG00000286501
NA	0.0135	2.2240E-01		ENSG00000241048
NA	0.0502	2.2243E-01		ENSG00000248744
NA	0.0174	2.2243E-01		ENSG00000276868
NA	0.0335	2.2254E-01		ENSG00000259130
NA	0.0350	2.2255E-01		ENSG00000223503
NA	0.0369	2.2256E-01		ENSG00000269153
NA	0.0906	2.2260E-01	4.2605E-01	ENSG00000270059
NA	0.0792	2.2282E-01	4.2635E-01	ENSG00000234902
NA	0.0161	2.2299E-01		ENSG00000229203
NA	0.0208	2.2341E-01		ENSG00000232184
NA	0.0444	2.2369E-01		ENSG00000187979
NA	0.0406	2.2378E-01		ENSG00000093100
NA	0.0340	2.2387E-01		ENSG00000278112
NA	0.1045	2.2395E-01	4.2780E-01	ENSG00000178631
NA	0.1114	2.2398E-01	4.2780E-01	ENSG00000271538
NA	0.1051	2.2401E-01	4.2780E-01	ENSG00000289152
NA	0.0767	2.2410E-01	4.2795E-01	ENSG00000285680
NA	0.1088	2.2430E-01	4.2812E-01	ENSG00000241269
NA	0.0242	2.2431E-01		ENSG00000289178
NA	0.0938	2.2459E-01	4.2850E-01	ENSG00000287897
NA	0.1004	2.2481E-01	4.2882E-01	ENSG00000289063
NA	0.0579	2.2489E-01		ENSG00000230623
NA	0.0202	2.2492E-01	4.2887E-01	ENSG00000289529
NA	0.0517	2.2493E-01		ENSG00000286249
NA	0.0247	2.2499E-01		ENSG00000249316

NA	0.0758	2.2502E-01	4.2887E-01	ENSG00000287477
NA	0.0623	2.2512E-01		ENSG00000259609
NA	0.0789	2.2535E-01	4.2912E-01	ENSG00000262481
NA	0.0242	2.2540E-01		ENSG00000227257
NA	0.0242	2.2540E-01		ENSG00000224815
NA	0.0160	2.2564E-01		ENSG00000289996
NA	0.0718	2.2565E-01	4.2943E-01	ENSG00000276934
NA	0.0880	2.2579E-01	4.2964E-01	ENSG00000228043
NA	0.0427	2.2614E-01		ENSG00000284651
NA	0.0568	2.2623E-01		ENSG00000278493
NA	0.0161	2.2639E-01		ENSG00000232633
NA	0.0148	2.2645E-01		ENSG00000226669
NA	0.0105	2.2657E-01		ENSG00000236180
NA	0.0243	2.2661E-01		ENSG00000230694
NA	0.0557	2.2673E-01	4.3088E-01	ENSG00000255141
NA	0.0164	2.2756E-01		ENSG00000287989
NA	0.0207	2.2758E-01		ENSG00000257534
NA	0.1050	2.2764E-01	4.3212E-01	ENSG00000289354
NA	0.0313	2.2796E-01		ENSG00000264655
NA	0.0396	2.2814E-01		ENSG00000279904
NA	0.1124	2.2841E-01	4.3305E-01	ENSG00000257391
NA	0.1111	2.2879E-01	4.3332E-01	ENSG00000246596
NA	0.0181	2.2885E-01		ENSG00000233574
NA	0.1069	2.2892E-01	4.3343E-01	ENSG00000289046
NA	0.0572	2.2902E-01	4.3343E-01	ENSG00000281772
NA	0.0616	2.2903E-01	4.3343E-01	ENSG00000231212
NA	0.0178	2.2908E-01		ENSG00000284430
NA	0.0173	2.2913E-01		ENSG00000227589
NA	0.0110	2.2924E-01		ENSG00000287324
NA	0.0434	2.2966E-01		ENSG00000251224
NA	0.0958	2.2997E-01	4.3461E-01	ENSG00000287766
NA	0.1007	2.3004E-01	4.3466E-01	ENSG00000231991
NA	0.1071	2.3008E-01	4.3466E-01	ENSG00000198416
NA	0.0344	2.3013E-01		ENSG00000280604
NA	0.1109	2.3056E-01	4.3531E-01	ENSG00000273320
NA	0.0814	2.3074E-01	4.3557E-01	ENSG00000284677
NA	0.0956	2.3075E-01	4.3557E-01	ENSG00000288103
NA	0.0871	2.3086E-01	4.3567E-01	ENSG00000235790
NA	0.0215	2.3109E-01		ENSG00000277532
NA	0.0296	2.3125E-01		ENSG00000287795
NA	0.0933	2.3215E-01	4.3709E-01	ENSG00000271474
NA	0.0241	2.3258E-01		ENSG00000289287
NA	0.0743	2.3275E-01	4.3783E-01	ENSG00000285080
NA	0.0800	2.3328E-01	4.3846E-01	ENSG00000250899
NA	0.0205	2.3355E-01		ENSG00000289850
NA	0.0724	2.3361E-01	4.3877E-01	ENSG00000288046
NA	0.0274	2.3374E-01		ENSG00000224467
NA	0.0353	2.3379E-01		ENSG00000260823
NA	0.0667	2.3386E-01	4.3905E-01	ENSG00000258088

NA	0.0656	2.3389E-01	4.3905E-01	ENSG00000267382
NA	0.1047	2.3391E-01	4.3905E-01	ENSG00000260398
NA	0.0805	2.3396E-01	4.3905E-01	ENSG00000287009
NA	0.0653	2.3408E-01		ENSG00000261886
NA	0.0392	2.3408E-01		ENSG00000287751
NA	0.0918	2.3432E-01	4.3945E-01	ENSG00000260455
NA	0.0919	2.3442E-01	4.3956E-01	ENSG00000289100
NA	0.0865	2.3481E-01	4.4019E-01	ENSG00000289401
NA	0.0819	2.3486E-01	4.4026E-01	ENSG00000255229
NA	0.0314	2.3495E-01		ENSG00000225948
NA	0.0736	2.3524E-01	4.4067E-01	ENSG00000285676
NA	0.0257	2.3541E-01		ENSG00000289286
NA	0.0289	2.3547E-01		ENSG00000260671
NA	0.0181	2.3559E-01		ENSG00000275860
NA	0.0223	2.3562E-01		ENSG00000267006
NA	0.0448	2.3582E-01		ENSG00000214514
NA	0.0906	2.3584E-01	4.4149E-01	ENSG00000231863
NA	0.0962	2.3612E-01	4.4183E-01	ENSG00000264458
NA	0.0733	2.3613E-01	4.4183E-01	ENSG00000289935
NA	0.0497	2.3658E-01		ENSG00000278770
NA	0.0522	2.3659E-01	4.4242E-01	ENSG00000234136
NA	0.0385	2.3694E-01		ENSG00000260410
NA	0.0355	2.3706E-01		ENSG00000286209
NA	0.0773	2.3717E-01	4.4295E-01	ENSG00000265943
NA	0.0861	2.3758E-01	4.4354E-01	ENSG00000261537
NA	0.0315	2.3767E-01		ENSG00000279730
NA	0.0847	2.3777E-01	4.4384E-01	ENSG00000225559
NA	0.1049	2.3787E-01	4.4396E-01	ENSG00000288976
NA	0.0794	2.3791E-01	4.4398E-01	ENSG00000197585
NA	0.0858	2.3814E-01	4.4421E-01	ENSG00000284543
NA	0.0563	2.3824E-01		ENSG00000277013
NA	0.0223	2.3832E-01		ENSG00000236075
NA	0.0490	2.3875E-01		ENSG00000259585
NA	0.0913	2.3875E-01	4.4524E-01	ENSG00000227001
NA	0.0284	2.3906E-01		ENSG00000231140
NA	0.0499	2.3911E-01		ENSG00000286890
NA	0.0532	2.3920E-01		ENSG00000233304
NA	0.0707	2.3949E-01	4.4614E-01	ENSG00000263366
NA	0.0281	2.3981E-01		ENSG00000249513
NA	0.0864	2.3987E-01	4.4661E-01	ENSG00000285669
NA	0.0369	2.4002E-01		ENSG00000281469
NA	0.0386	2.4010E-01		ENSG00000272866
NA	0.0732	2.4019E-01	4.4701E-01	ENSG00000259370
NA	0.0972	2.4023E-01	4.4705E-01	ENSG00000204789
NA	0.0890	2.4033E-01	4.4717E-01	ENSG00000182487
NA	0.0840	2.4037E-01	4.4720E-01	ENSG00000287378
NA	0.0461	2.4077E-01	4.4771E-01	ENSG00000279225
NA	0.0568	2.4088E-01		ENSG00000287913
NA	0.0832	2.4088E-01	4.4785E-01	ENSG00000234936

NA	0.1030	2.4106E-01	4.4809E-01	ENSG00000233783
NA	0.0780	2.4122E-01	4.4826E-01	ENSG00000289324
NA	0.0184	2.4123E-01		ENSG00000286649
NA	0.0273	2.4123E-01		ENSG00000266869
NA	0.0303	2.4124E-01		ENSG00000287424
NA	0.0827	2.4144E-01	4.4853E-01	ENSG00000241489
NA	0.0208	2.4148E-01		ENSG00000249028
NA	0.0708	2.4157E-01	4.4873E-01	ENSG00000248268
NA	0.0191	2.4158E-01		ENSG00000280916
NA	0.0310	2.4180E-01		ENSG00000250243
NA	0.0308	2.4182E-01		ENSG00000233942
NA	0.0153	2.4185E-01		ENSG00000289732
NA	0.0374	2.4203E-01		ENSG00000286326
NA	0.0375	2.4206E-01		ENSG00000286889
NA	0.0187	2.4213E-01		ENSG00000195401
NA	0.0260	2.4235E-01		ENSG00000251152
NA	0.0361	2.4238E-01		ENSG00000255669
NA	0.0904	2.4276E-01	4.5048E-01	ENSG00000249937
NA	0.0348	2.4314E-01		ENSG00000280295
NA	0.0892	2.4346E-01	4.5132E-01	ENSG00000276136
NA	0.0820	2.4348E-01	4.5132E-01	ENSG00000279601
NA	0.0910	2.4362E-01	4.5142E-01	ENSG00000268412
NA	0.0293	2.4368E-01		ENSG00000283769
NA	0.0457	2.4402E-01		ENSG00000269997
NA	0.0248	2.4410E-01		ENSG00000287134
NA	0.0726	2.4416E-01	4.5180E-01	ENSG00000234832
NA	0.0526	2.4426E-01		ENSG00000276963
NA	0.0417	2.4436E-01		ENSG00000236274
NA	0.0970	2.4470E-01	4.5223E-01	ENSG00000250731
NA	0.1052	2.4476E-01	4.5231E-01	ENSG00000219928
NA	0.0760	2.4488E-01	4.5249E-01	ENSG00000261535
NA	0.0642	2.4509E-01	4.5280E-01	ENSG00000280399
NA	0.0587	2.4512E-01		ENSG00000287607
NA	0.0411	2.4541E-01		ENSG00000261898
NA	0.0147	2.4564E-01		ENSG00000260303
NA	0.0688	2.4571E-01	4.5347E-01	ENSG00000287587
NA	0.0566	2.4630E-01		ENSG00000234476
NA	0.0234	2.4636E-01		ENSG00000251552
NA	0.0954	2.4637E-01	4.5414E-01	ENSG00000227248
NA	0.0554	2.4646E-01		ENSG00000234383
NA	0.0878	2.4677E-01	4.5438E-01	ENSG00000245059
NA	0.0202	2.4706E-01		ENSG00000260277
NA	0.1009	2.4747E-01	4.5528E-01	ENSG00000226823
NA	0.0698	2.4750E-01	4.5528E-01	ENSG00000215154
NA	0.0583	2.4780E-01		ENSG00000236095
NA	0.0667	2.4789E-01	4.5568E-01	ENSG00000270050
NA	0.1035	2.4807E-01	4.5579E-01	ENSG00000289555
NA	0.1045	2.4811E-01	4.5580E-01	ENSG00000281091
NA	0.1049	2.4812E-01	4.5580E-01	ENSG00000278840

NA	0.0856	2.4824E-01	4.5595E-01	ENSG00000273176
NA	0.0514	2.4825E-01		ENSG00000280309
NA	0.0048	2.4840E-01		ENSG00000285666
NA	0.0426	2.4857E-01		ENSG00000287722
NA	0.0733	2.4858E-01	4.5643E-01	ENSG00000283384
NA	0.0692	2.4870E-01	4.5649E-01	ENSG00000286093
NA	0.0266	2.4884E-01		ENSG00000269637
NA	0.0184	2.4888E-01		ENSG00000258837
NA	0.0683	2.4904E-01	4.5678E-01	ENSG00000230438
NA	0.0191	2.4943E-01		ENSG00000226438
NA	0.0181	2.4950E-01		ENSG00000206249
NA	0.1057	2.4951E-01	4.5733E-01	ENSG00000288840
NA	0.0453	2.4968E-01		ENSG00000259876
NA	0.0325	2.4977E-01		ENSG00000248830
NA	0.1061	2.4981E-01	4.5773E-01	ENSG00000258130
NA	0.1028	2.4982E-01	4.5773E-01	ENSG00000280038
NA	0.0592	2.5005E-01	4.5794E-01	ENSG00000249849
NA	0.0161	2.5009E-01		ENSG00000234459
NA	0.0403	2.5027E-01		ENSG00000244300
NA	0.0130	2.5032E-01		ENSG00000226694
NA	0.1045	2.5061E-01	4.5874E-01	ENSG00000287862
NA	0.0550	2.5067E-01		ENSG00000236283
NA	0.0203	2.5086E-01		ENSG00000279620
NA	0.0371	2.5087E-01		ENSG00000213642
NA	0.0252	2.5125E-01		ENSG00000271963
NA	0.0236	2.5136E-01		ENSG00000207417
NA	0.0199	2.5149E-01		ENSG00000285610
NA	0.0642	2.5158E-01	4.5986E-01	ENSG00000238242
NA	0.0432	2.5181E-01		ENSG00000285939
NA	0.0350	2.5188E-01		ENSG00000279732
NA	0.0995	2.5195E-01	4.6024E-01	ENSG00000269038
NA	0.0868	2.5201E-01	4.6024E-01	ENSG00000203709
NA	0.0750	2.5244E-01	4.6050E-01	ENSG00000231793
NA	0.0237	2.5244E-01		ENSG00000286290
NA	0.0790	2.5251E-01	4.6058E-01	ENSG00000253366
NA	0.0934	2.5259E-01	4.6058E-01	ENSG00000285813
NA	0.0508	2.5265E-01		ENSG00000175730
NA	0.0568	2.5268E-01		ENSG00000258631
NA	0.0567	2.5271E-01	4.6066E-01	ENSG00000226390
NA	0.0180	2.5283E-01		ENSG00000286858
NA	0.0304	2.5286E-01		ENSG00000210112
NA	0.0850	2.5286E-01	4.6083E-01	ENSG00000286791
NA	0.0543	2.5289E-01		ENSG00000289159
NA	0.0190	2.5320E-01		ENSG00000287451
NA	0.0235	2.5325E-01		ENSG00000278877
NA	0.0174	2.5335E-01		ENSG00000255440
NA	0.0705	2.5338E-01	4.6137E-01	ENSG00000286294
NA	0.0553	2.5351E-01	4.6146E-01	ENSG00000254366
NA	0.0214	2.5353E-01		ENSG00000201600

NA	0.1020	2.5356E-01	4.6149E-01	ENSG00000204352
NA	0.0183	2.5420E-01		ENSG00000216412
NA	0.0222	2.5440E-01		ENSG00000241754
NA	0.1043	2.5462E-01	4.6263E-01	ENSG00000171084
NA	0.0210	2.5467E-01		ENSG00000260072
NA	0.0203	2.5469E-01		ENSG00000232252
NA	0.0809	2.5470E-01	4.6270E-01	ENSG00000260369
NA	0.0502	2.5535E-01	4.6351E-01	ENSG00000286973
NA	0.0964	2.5544E-01	4.6357E-01	ENSG00000268266
NA	0.0537	2.5557E-01		ENSG00000224251
NA	0.0279	2.5580E-01		ENSG00000285929
NA	0.0207	2.5608E-01		ENSG00000219404
NA	0.0999	2.5615E-01	4.6444E-01	ENSG00000232300
NA	0.1002	2.5627E-01	4.6463E-01	ENSG00000281571
NA	0.0911	2.5643E-01	4.6485E-01	ENSG00000224093
NA	0.0353	2.5646E-01		ENSG00000232358
NA	0.0682	2.5657E-01	4.6497E-01	ENSG00000290045
NA	0.0534	2.5677E-01		ENSG00000224761
NA	0.0628	2.5691E-01	4.6542E-01	ENSG00000227743
NA	0.0871	2.5694E-01	4.6543E-01	ENSG00000235036
NA	0.0557	2.5710E-01	4.6566E-01	ENSG00000231227
NA	0.0889	2.5723E-01	4.6570E-01	ENSG00000260816
NA	0.0432	2.5747E-01		ENSG00000227193
NA	0.0416	2.5750E-01		ENSG00000275812
NA	0.0430	2.5820E-01		ENSG00000253377
NA	0.0572	2.5838E-01	4.6721E-01	ENSG00000200091
NA	0.0926	2.5850E-01	4.6729E-01	ENSG00000224647
NA	0.0201	2.5853E-01		ENSG00000254302
NA	0.1036	2.5853E-01	4.6729E-01	ENSG00000288771
NA	0.0266	2.5855E-01		ENSG00000287079
NA	0.0870	2.5861E-01	4.6733E-01	ENSG00000254510
NA	0.0163	2.5873E-01		ENSG00000267610
NA	0.0553	2.5899E-01		ENSG00000283371
NA	0.0397	2.5904E-01		ENSG00000253567
NA	0.0185	2.5911E-01		ENSG00000249956
NA	0.1026	2.5913E-01	4.6768E-01	ENSG00000286427
NA	0.0338	2.5922E-01		ENSG00000287076
NA	0.1028	2.5926E-01	4.6782E-01	ENSG00000261519
NA	0.0071	2.5939E-01	4.6791E-01	ENSG00000279814
NA	0.0514	2.5944E-01		ENSG00000287135
NA	0.0351	2.5961E-01		ENSG00000259792
NA	0.0418	2.5971E-01		ENSG00000269938
NA	0.0768	2.5986E-01	4.6829E-01	ENSG00000226840
NA	0.0990	2.6008E-01	4.6856E-01	ENSG00000279048
NA	0.0986	2.6027E-01	4.6876E-01	ENSG00000279616
NA	0.0420	2.6058E-01		ENSG00000260037
NA	0.0844	2.6096E-01	4.6950E-01	ENSG00000229127
NA	0.0438	2.6117E-01	4.6970E-01	ENSG00000231927
NA	0.0178	2.6125E-01		ENSG00000250407

NA	0.0306	2.6143E-01		ENSG00000287218
NA	0.0623	2.6161E-01	4.7033E-01	ENSG00000278231
NA	0.1023	2.6163E-01	4.7033E-01	ENSG00000289611
NA	0.0743	2.6182E-01	4.7058E-01	ENSG00000285737
NA	0.0691	2.6185E-01	4.7058E-01	ENSG00000276412
NA	0.0612	2.6190E-01	4.7062E-01	ENSG00000259463
NA	0.0639	2.6208E-01	4.7082E-01	ENSG00000288085
NA	0.0528	2.6216E-01		ENSG00000235843
NA	0.0217	2.6243E-01		ENSG00000234502
NA	0.0420	2.6258E-01		ENSG00000269303
NA	0.0452	2.6265E-01		ENSG00000279993
NA	0.0913	2.6269E-01	4.7155E-01	ENSG00000247121
NA	0.0129	2.6272E-01		ENSG00000258168
NA	0.0157	2.6273E-01		ENSG00000255817
NA	0.0135	2.6282E-01	4.7165E-01	ENSG00000287713
NA	0.0567	2.6291E-01	4.7173E-01	ENSG00000286525
NA	0.0508	2.6321E-01		ENSG00000272836
NA	0.0826	2.6334E-01	4.7220E-01	ENSG00000270068
NA	0.0213	2.6340E-01		ENSG00000259100
NA	0.0256	2.6357E-01		ENSG00000267992
NA	0.0598	2.6371E-01	4.7263E-01	ENSG00000267740
NA	0.0559	2.6383E-01		ENSG00000275075
NA	0.0302	2.6385E-01		ENSG00000267577
NA	0.0268	2.6387E-01		ENSG00000259096
NA	0.1009	2.6391E-01	4.7283E-01	ENSG00000261033
NA	0.0979	2.6394E-01	4.7283E-01	ENSG00000275764
NA	0.0515	2.6396E-01	4.7283E-01	ENSG00000275997
NA	0.0250	2.6411E-01		ENSG00000226945
NA	0.0918	2.6426E-01	4.7321E-01	ENSG00000279879
NA	0.0478	2.6483E-01		ENSG00000273261
NA	0.0967	2.6490E-01	4.7406E-01	ENSG00000279233
NA	0.0794	2.6503E-01	4.7425E-01	ENSG00000240225
NA	0.0191	2.6504E-01		ENSG00000224324
NA	0.0083	2.6508E-01	4.7425E-01	ENSG00000286672
NA	0.0456	2.6513E-01		ENSG00000233478
NA	0.0555	2.6526E-01	4.7436E-01	ENSG00000288079
NA	0.0976	2.6552E-01	4.7474E-01	ENSG00000272077
NA	0.0552	2.6567E-01	4.7492E-01	ENSG00000232504
NA	0.0196	2.6577E-01		ENSG00000288964
NA	0.0607	2.6578E-01	4.7504E-01	ENSG00000289971
NA	0.0935	2.6582E-01	4.7504E-01	ENSG00000224905
NA	0.0714	2.6585E-01	4.7504E-01	ENSG00000256481
NA	0.0152	2.6595E-01		ENSG00000287311
NA	0.0840	2.6597E-01	4.7515E-01	ENSG00000259172
NA	0.0559	2.6599E-01		ENSG00000232682
NA	0.0454	2.6608E-01		ENSG00000286418
NA	0.0296	2.6619E-01		ENSG00000269068
NA	0.0992	2.6640E-01	4.7571E-01	ENSG00000237402
NA	0.0194	2.6649E-01		ENSG00000280189

NA	0.0665	2.6655E-01	4.7587E-01	ENSG00000260608
NA	0.0231	2.6663E-01		ENSG00000254362
NA	0.0234	2.6675E-01		ENSG00000255226
NA	0.0686	2.6689E-01	4.7617E-01	ENSG00000261315
NA	0.0420	2.6697E-01		ENSG00000225506
NA	0.0988	2.6700E-01	4.7620E-01	ENSG00000273363
NA	0.0632	2.6716E-01	4.7637E-01	ENSG00000286990
NA	0.0238	2.6728E-01		ENSG00000268458
NA	0.0997	2.6745E-01	4.7676E-01	ENSG00000286527
NA	0.0396	2.6760E-01		ENSG00000240571
NA	0.0277	2.6772E-01		ENSG00000255299
NA	0.0981	2.6797E-01	4.7741E-01	ENSG00000213025
NA	0.0969	2.6811E-01	4.7755E-01	ENSG00000219392
NA	0.0437	2.6811E-01		ENSG00000271993
NA	0.1002	2.6848E-01	4.7790E-01	ENSG00000234636
NA	0.0799	2.6869E-01	4.7802E-01	ENSG00000225339
NA	0.0956	2.6873E-01	4.7807E-01	ENSG00000275180
NA	0.0389	2.6894E-01		ENSG00000271955
NA	0.0946	2.6895E-01	4.7836E-01	ENSG00000289084
NA	0.0986	2.6899E-01	4.7837E-01	ENSG00000289731
NA	0.0112	2.6900E-01		ENSG00000287621
NA	0.0658	2.6901E-01	4.7837E-01	ENSG00000272574
NA	0.0495	2.6906E-01		ENSG00000287214
NA	0.0774	2.6926E-01	4.7870E-01	ENSG00000073905
NA	0.0259	2.6931E-01		ENSG00000279043
NA	0.0828	2.6942E-01	4.7888E-01	ENSG00000280216
NA	0.0222	2.6943E-01		ENSG00000286481
NA	0.0418	2.6963E-01		ENSG00000277327
NA	0.0651	2.6972E-01	4.7928E-01	ENSG00000285803
NA	0.0564	2.6974E-01	4.7928E-01	ENSG00000236861
NA	0.0195	2.6983E-01		ENSG00000234977
NA	0.0369	2.6996E-01		ENSG00000275024
NA	0.0304	2.6999E-01		ENSG00000251461
NA	0.0474	2.7017E-01		ENSG00000225181
NA	0.0668	2.7069E-01	4.8008E-01	ENSG00000229618
NA	0.0950	2.7085E-01	4.8008E-01	ENSG00000248664
NA	0.0218	2.7090E-01		ENSG00000250129
NA	0.0640	2.7118E-01	4.8045E-01	ENSG00000280107
NA	0.0308	2.7135E-01		ENSG00000286032
NA	0.0187	2.7146E-01		ENSG00000253613
NA	0.0541	2.7159E-01		ENSG00000237757
NA	0.0282	2.7198E-01		ENSG00000270281
NA	0.0945	2.7208E-01	4.8134E-01	ENSG00000224786
NA	0.0278	2.7213E-01		ENSG00000257530
NA	0.0206	2.7221E-01		ENSG00000286590
NA	0.0707	2.7237E-01	4.8164E-01	ENSG00000179342
NA	0.0315	2.7239E-01		ENSG00000280326
NA	0.0465	2.7274E-01		ENSG00000253796
NA	0.0994	2.7290E-01	4.8241E-01	ENSG00000111788

NA	0.0155	2.7300E-01		ENSG00000207955
NA	0.0514	2.7361E-01		ENSG00000289560
NA	0.0151	2.7391E-01		ENSG00000273913
NA	0.0248	2.7403E-01		ENSG00000273172
NA	0.0951	2.7411E-01	4.8385E-01	ENSG00000272990
NA	0.0433	2.7419E-01		ENSG00000287507
NA	0.0233	2.7427E-01		ENSG00000256424
NA	0.0594	2.7434E-01	4.8416E-01	ENSG00000289689
NA	0.0234	2.7439E-01		ENSG00000199444
NA	0.0635	2.7454E-01	4.8445E-01	ENSG00000207195
NA	0.0380	2.7456E-01		ENSG00000232433
NA	0.0736	2.7466E-01	4.8451E-01	ENSG00000268107
NA	0.0303	2.7479E-01		ENSG00000262339
NA	0.0468	2.7486E-01		ENSG00000261056
NA	0.0974	2.7579E-01	4.8571E-01	ENSG00000282740
NA	0.0761	2.7589E-01	4.8576E-01	ENSG00000286168
NA	0.0924	2.7592E-01	4.8576E-01	ENSG00000260948
NA	0.0406	2.7593E-01		ENSG00000254563
NA	0.0638	2.7612E-01	4.8589E-01	ENSG00000256443
NA	0.0192	2.7650E-01		ENSG00000255418
NA	0.0630	2.7654E-01	4.8647E-01	ENSG00000274775
NA	0.0297	2.7661E-01		ENSG00000287025
NA	0.0190	2.7696E-01		ENSG00000288032
NA	0.0331	2.7698E-01	4.8693E-01	ENSG00000272223
NA	0.0321	2.7700E-01		ENSG00000210174
NA	0.0146	2.7705E-01		ENSG00000224594
NA	0.1080	2.7771E-01	4.8774E-01	ENSG00000260093
NA	0.0443	2.7774E-01		ENSG00000258199
NA	0.0188	2.7778E-01		ENSG00000235575
NA	0.0371	2.7780E-01		ENSG00000271367
NA	0.0901	2.7798E-01	4.8808E-01	ENSG00000273576
NA	0.0619	2.7802E-01	4.8808E-01	ENSG00000261226
NA	0.0100	2.7806E-01		ENSG00000287007
NA	0.0138	2.7812E-01		ENSG00000280232
NA	0.0640	2.7812E-01	4.8808E-01	ENSG00000236364
NA	0.0827	2.7815E-01	4.8808E-01	ENSG00000274993
NA	0.0672	2.7824E-01	4.8813E-01	ENSG00000279103
NA	0.0185	2.7827E-01		ENSG00000235347
NA	0.0754	2.7829E-01	4.8819E-01	ENSG00000287868
NA	0.0917	2.7919E-01	4.8920E-01	ENSG00000289042
NA	0.0934	2.7930E-01	4.8934E-01	ENSG00000285184
NA	0.0967	2.7936E-01	4.8940E-01	ENSG00000289201
NA	0.0933	2.7958E-01	4.8962E-01	ENSG00000261351
NA	0.0851	2.7964E-01	4.8968E-01	ENSG00000289414
NA	0.0190	2.7981E-01		ENSG00000237861
NA	0.0925	2.7984E-01	4.8989E-01	ENSG00000258592
NA	0.0205	2.8002E-01		ENSG00000272958
NA	0.0416	2.8004E-01		ENSG00000268873
NA	0.0687	2.8020E-01	4.9022E-01	ENSG00000269815

NA	0.0506	2.8029E-01		ENSG00000270497
NA	0.0940	2.8067E-01	4.9082E-01	ENSG00000285679
NA	0.0934	2.8089E-01	4.9106E-01	ENSG00000267383
NA	0.0375	2.8094E-01		ENSG00000273243
NA	0.0951	2.8103E-01	4.9125E-01	ENSG00000266538
NA	0.0350	2.8113E-01		ENSG00000272040
NA	0.0141	2.8118E-01		ENSG00000271901
NA	0.0152	2.8128E-01		ENSG00000236056
NA	0.0928	2.8138E-01	4.9174E-01	ENSG00000232891
NA	0.0226	2.8152E-01		ENSG00000236544
NA	0.0221	2.8159E-01		ENSG00000225813
NA	0.0105	2.8180E-01		ENSG00000261692
NA	0.0788	2.8182E-01	4.9227E-01	ENSG00000285555
NA	0.0111	2.8206E-01		ENSG00000232347
NA	0.1084	2.8211E-01	4.9254E-01	ENSG00000231549
NA	0.0149	2.8211E-01		ENSG00000236744
NA	0.0390	2.8213E-01		ENSG00000279942
NA	0.0691	2.8216E-01	4.9259E-01	ENSG00000273319
NA	0.0945	2.8251E-01	4.9299E-01	ENSG00000258768
NA	0.0955	2.8260E-01	4.9308E-01	ENSG00000235618
NA	0.0223	2.8264E-01		ENSG00000286212
NA	0.0596	2.8274E-01	4.9317E-01	ENSG00000253583
NA	0.0690	2.8288E-01	4.9332E-01	ENSG00000287054
NA	0.0691	2.8294E-01	4.9336E-01	ENSG00000279443
NA	0.0401	2.8308E-01		ENSG00000286562
NA	0.0508	2.8314E-01	4.9352E-01	ENSG00000289974
NA	0.0185	2.8322E-01		ENSG00000229336
NA	0.0798	2.8331E-01	4.9374E-01	ENSG00000266680
NA	0.0500	2.8349E-01	4.9394E-01	ENSG00000233203
NA	0.0811	2.8349E-01	4.9394E-01	ENSG00000280401
NA	0.0306	2.8353E-01		ENSG00000227214
NA	0.0146	2.8378E-01		ENSG00000286248
NA	0.0894	2.8378E-01	4.9425E-01	ENSG00000276390
NA	0.0277	2.8402E-01		ENSG00000230498
NA	0.0143	2.8407E-01		ENSG00000241529
NA	0.0512	2.8422E-01		ENSG00000289981
NA	0.0285	2.8425E-01		ENSG00000257180
NA	0.0520	2.8429E-01	4.9494E-01	ENSG00000236627
NA	0.0259	2.8436E-01		ENSG00000258722
NA	0.0331	2.8451E-01		ENSG00000258053
NA	0.0488	2.8464E-01		ENSG00000271730
NA	0.0933	2.8469E-01	4.9526E-01	ENSG00000253553
NA	0.0968	2.8470E-01	4.9526E-01	ENSG00000289285
NA	0.0737	2.8480E-01	4.9536E-01	ENSG00000259236
NA	0.0067	2.8530E-01		ENSG00000232615
NA	0.0759	2.8539E-01	4.9612E-01	ENSG00000229766
NA	0.0693	2.8545E-01	4.9615E-01	ENSG00000287283
NA	0.0792	2.8559E-01	4.9632E-01	ENSG00000253816
NA	0.0156	2.8568E-01		ENSG00000276241

NA	0.0227	2.8575E-01		ENSG00000251182
NA	0.0727	2.8588E-01	4.9663E-01	ENSG00000272010
NA	0.0286	2.8590E-01		ENSG00000235497
NA	0.0477	2.8596E-01		ENSG00000280000
NA	0.0966	2.8661E-01	4.9746E-01	ENSG00000242338
NA	0.0741	2.8672E-01	4.9755E-01	ENSG00000287004
NA	0.0941	2.8672E-01	4.9755E-01	ENSG00000229587
NA	0.0954	2.8686E-01	4.9772E-01	ENSG00000286885
NA	0.0679	2.8689E-01	4.9773E-01	ENSG00000271967
NA	0.0477	2.8699E-01	4.9773E-01	ENSG00000256420
NA	0.0628	2.8712E-01	4.9785E-01	ENSG00000279175
NA	0.0228	2.8727E-01		ENSG00000271587
NA	0.0253	2.8759E-01		ENSG00000277223
NA	0.0354	2.8784E-01		ENSG00000233894
NA	0.0872	2.8792E-01	4.9850E-01	ENSG00000289328
NA	0.0621	2.8829E-01	4.9873E-01	ENSG00000248564
NA	0.0444	2.8878E-01		ENSG00000225022
NA	0.0906	2.8905E-01	4.9952E-01	ENSG00000264070
NA	0.0783	2.8918E-01	4.9962E-01	ENSG00000289301
NA	0.0473	2.8959E-01		ENSG00000231086
NA	0.0354	2.8968E-01		ENSG00000285868
NA	0.0786	2.8997E-01	5.0035E-01	ENSG00000234283
NA	0.0376	2.9025E-01		ENSG00000263916
NA	0.0881	2.9039E-01	5.0077E-01	ENSG00000287513
NA	0.0234	2.9056E-01		ENSG00000213197
NA	0.0828	2.9062E-01	5.0099E-01	ENSG00000285780
NA	0.0441	2.9076E-01		ENSG00000254416
NA	0.0944	2.9097E-01	5.0136E-01	ENSG00000289018
NA	0.0578	2.9107E-01		ENSG00000236958
NA	0.0833	2.9144E-01	5.0175E-01	ENSG00000278013
NA	0.0261	2.9152E-01		ENSG00000272516
NA	0.0705	2.9157E-01	5.0185E-01	ENSG00000288674
NA	0.0103	2.9165E-01		ENSG00000279378
NA	0.0477	2.9172E-01		ENSG00000256612
NA	0.0517	2.9181E-01	5.0208E-01	ENSG00000260051
NA	0.0578	2.9193E-01	5.0210E-01	ENSG00000287787
NA	0.0811	2.9216E-01	5.0231E-01	ENSG00000273321
NA	0.0584	2.9228E-01	5.0240E-01	ENSG00000223519
NA	0.0369	2.9232E-01		ENSG00000181741
NA	0.0493	2.9240E-01		ENSG00000259212
NA	0.0758	2.9242E-01	5.0249E-01	ENSG00000214176
NA	0.0638	2.9270E-01	5.0275E-01	ENSG00000266824
NA	0.0859	2.9282E-01	5.0287E-01	ENSG00000272812
NA	0.0780	2.9300E-01	5.0301E-01	ENSG00000271855
NA	0.0205	2.9320E-01		ENSG00000281780
NA	0.0208	2.9323E-01		ENSG00000272425
NA	0.0703	2.9328E-01	5.0333E-01	ENSG00000067601
NA	0.0075	2.9330E-01		ENSG00000238008
NA	0.0204	2.9336E-01		ENSG00000260997

NA	0.0900	2.9344E-01	5.0353E-01	ENSG00000233871
NA	0.0801	2.9373E-01	5.0389E-01	ENSG00000231104
NA	0.0232	2.9384E-01		ENSG00000220924
NA	0.0569	2.9403E-01	5.0394E-01	ENSG00000235659
NA	0.0561	2.9407E-01	5.0394E-01	ENSG00000226599
NA	0.0773	2.9407E-01	5.0394E-01	ENSG00000260186
NA	0.0244	2.9408E-01		ENSG00000235349
NA	0.0487	2.9421E-01		ENSG00000265656
NA	0.0240	2.9451E-01		ENSG00000256897
NA	0.0149	2.9460E-01		ENSG00000234168
NA	0.0146	2.9463E-01		ENSG00000254222
NA	0.0584	2.9479E-01	5.0482E-01	ENSG00000255966
NA	0.0442	2.9500E-01		ENSG00000286904
NA	0.0926	2.9503E-01	5.0511E-01	ENSG00000228629
NA	0.0902	2.9541E-01	5.0554E-01	ENSG00000205930
NA	0.0274	2.9544E-01		ENSG00000271156
NA	0.0937	2.9547E-01	5.0561E-01	ENSG00000227704
NA	0.0468	2.9554E-01		ENSG00000263698
NA	0.0443	2.9556E-01	5.0565E-01	ENSG00000279254
NA	0.0424	2.9569E-01		ENSG00000289391
NA	0.0246	2.9581E-01		ENSG00000248696
NA	0.0187	2.9585E-01		ENSG00000271555
NA	0.0848	2.9591E-01	5.0592E-01	ENSG00000273893
NA	0.0887	2.9603E-01	5.0596E-01	ENSG00000233396
NA	0.0784	2.9652E-01	5.0651E-01	ENSG00000189366
NA	0.0540	2.9670E-01		ENSG00000267637
NA	0.0157	2.9692E-01		ENSG00000286755
NA	0.0189	2.9696E-01		ENSG00000259828
NA	0.0527	2.9704E-01	5.0712E-01	ENSG00000233334
NA	0.0505	2.9710E-01	5.0715E-01	ENSG00000290076
NA	0.0630	2.9712E-01	5.0716E-01	ENSG00000264491
NA	0.0921	2.9719E-01	5.0720E-01	ENSG00000282143
NA	0.0160	2.9720E-01		ENSG00000255968
NA	0.0528	2.9736E-01		ENSG00000279469
NA	0.0697	2.9756E-01	5.0752E-01	ENSG00000223537
NA	0.0622	2.9766E-01	5.0756E-01	ENSG00000229828
NA	0.0214	2.9792E-01		ENSG00000257228
NA	0.0286	2.9808E-01		ENSG00000285848
NA	0.0318	2.9811E-01		ENSG00000259407
NA	0.0611	2.9814E-01		ENSG00000287742
NA	0.0279	2.9836E-01		ENSG00000267203
NA	0.0600	2.9853E-01	5.0832E-01	ENSG00000225766
NA	0.0718	2.9864E-01	5.0843E-01	ENSG00000289040
NA	0.0198	2.9871E-01		ENSG00000260764
NA	0.0216	2.9884E-01		ENSG00000171483
NA	0.0362	2.9896E-01		ENSG00000250261
NA	0.0283	2.9921E-01		ENSG00000266111
NA	0.0758	2.9927E-01	5.0897E-01	ENSG00000272662
NA	0.0303	2.9934E-01		ENSG00000265775

NA	0.0350	2.9936E-01		ENSG00000236928
NA	0.0795	2.9942E-01	5.0907E-01	ENSG00000271119
NA	0.0188	2.9956E-01		ENSG00000237689
NA	0.0457	2.9957E-01		ENSG00000287908
NA	0.0225	3.0004E-01		ENSG00000288050
NA	0.0582	3.0011E-01	5.0986E-01	ENSG00000215464
NA	0.0921	3.0049E-01	5.1024E-01	ENSG00000214770
NA	0.0120	3.0055E-01		ENSG00000267749
NA	0.0227	3.0059E-01		ENSG00000264404
NA	0.0923	3.0087E-01	5.1073E-01	ENSG00000215146
NA	0.0462	3.0088E-01		ENSG00000287249
NA	0.0308	3.0112E-01		ENSG00000277840
NA	0.0143	3.0133E-01		ENSG00000202255
NA	0.0239	3.0152E-01		ENSG00000259294
NA	0.0641	3.0169E-01	5.1175E-01	ENSG00000237854
NA	0.0436	3.0180E-01		ENSG00000290163
NA	0.0365	3.0181E-01		ENSG00000248794
NA	0.0794	3.0185E-01	5.1182E-01	ENSG00000281741
NA	0.0649	3.0187E-01	5.1182E-01	ENSG00000273369
NA	0.0235	3.0217E-01		ENSG00000235420
NA	0.0198	3.0238E-01		ENSG00000287748
NA	0.0319	3.0256E-01		ENSG00000254695
NA	0.0437	3.0268E-01		ENSG00000266602
NA	0.0411	3.0271E-01		ENSG00000272627
NA	0.0260	3.0274E-01		ENSG00000258357
NA	0.0918	3.0275E-01	5.1286E-01	ENSG00000289334
NA	0.0412	3.0283E-01		ENSG00000251185
NA	0.0199	3.0286E-01		ENSG00000202382
NA	0.0868	3.0289E-01	5.1302E-01	ENSG00000272654
NA	0.0356	3.0299E-01		ENSG00000235689
NA	0.0143	3.0318E-01		ENSG00000205266
NA	0.0434	3.0321E-01	5.1333E-01	ENSG00000273958
NA	0.0219	3.0330E-01		ENSG00000252652
NA	0.0217	3.0365E-01		ENSG00000238291
NA	0.0207	3.0385E-01		ENSG00000259633
NA	0.0266	3.0387E-01		ENSG00000250891
NA	0.0906	3.0389E-01	5.1410E-01	ENSG00000289000
NA	0.0556	3.0402E-01		ENSG00000237832
NA	0.0638	3.0421E-01	5.1436E-01	ENSG00000285725
NA	0.0730	3.0429E-01	5.1447E-01	ENSG00000258891
NA	0.0258	3.0447E-01		ENSG00000269400
NA	0.0131	3.0453E-01		ENSG00000271732
NA	0.0342	3.0490E-01		ENSG00000237398
NA	0.0205	3.0495E-01		ENSG00000288972
NA	0.0507	3.0509E-01		ENSG00000255872
NA	0.0403	3.0527E-01		ENSG00000286591
NA	0.0523	3.0527E-01		ENSG00000286507
NA	0.0641	3.0528E-01	5.1557E-01	ENSG00000267940
NA	0.0123	3.0529E-01		ENSG00000227407

NA	0.0468	3.0554E-01		ENSG00000287066
NA	0.0214	3.0566E-01		ENSG00000279490
NA	0.0133	3.0590E-01		ENSG00000249920
NA	0.0169	3.0595E-01		ENSG00000234756
NA	0.0359	3.0604E-01		ENSG00000273816
NA	0.0263	3.0605E-01		ENSG00000224844
NA	0.0872	3.0652E-01	5.1675E-01	ENSG00000260669
NA	0.0155	3.0669E-01		ENSG00000258896
NA	0.0452	3.0674E-01		ENSG00000266288
NA	0.0253	3.0674E-01		ENSG00000269779
NA	0.0222	3.0702E-01		ENSG00000236141
NA	0.0313	3.0705E-01		ENSG00000249216
NA	0.0713	3.0723E-01	5.1752E-01	ENSG00000257835
NA	0.0185	3.0724E-01		ENSG00000285945
NA	0.0496	3.0727E-01	5.1752E-01	ENSG00000260701
NA	0.0295	3.0769E-01		ENSG00000284626
NA	0.0767	3.0806E-01	5.1836E-01	ENSG00000286864
NA	0.0259	3.0838E-01		ENSG00000257350
NA	0.0639	3.0840E-01	5.1859E-01	ENSG00000268555
NA	0.0226	3.0875E-01		ENSG00000285688
NA	0.0895	3.0900E-01	5.1924E-01	ENSG00000229337
NA	0.0138	3.0909E-01		ENSG00000289264
NA	0.0121	3.0924E-01		ENSG00000186543
NA	0.0884	3.0930E-01	5.1948E-01	ENSG00000289878
NA	0.0459	3.0966E-01		ENSG00000284071
NA	0.0459	3.0966E-01		ENSG00000284380
NA	0.0155	3.1020E-01		ENSG00000213630
NA	0.0245	3.1033E-01		ENSG00000213046
NA	0.0542	3.1046E-01	5.2074E-01	ENSG00000227836
NA	0.0713	3.1060E-01	5.2087E-01	ENSG00000272512
NA	0.0357	3.1070E-01		ENSG00000266805
NA	0.0567	3.1090E-01	5.2105E-01	ENSG00000266385
NA	0.0724	3.1093E-01	5.2107E-01	ENSG00000278266
NA	0.0865	3.1123E-01	5.2129E-01	ENSG00000280046
NA	0.0908	3.1141E-01	5.2142E-01	ENSG00000283537
NA	0.0625	3.1146E-01	5.2147E-01	ENSG00000183666
NA	0.0727	3.1160E-01	5.2157E-01	ENSG00000286577
NA	0.0753	3.1183E-01	5.2187E-01	ENSG00000272438
NA	0.0438	3.1185E-01		ENSG00000228829
NA	0.0204	3.1197E-01		ENSG00000250306
NA	0.0856	3.1237E-01	5.2253E-01	ENSG00000270402
NA	0.0103	3.1239E-01		ENSG00000261368
NA	0.0350	3.1243E-01		ENSG00000251410
NA	0.0725	3.1264E-01	5.2285E-01	ENSG00000278926
NA	0.0355	3.1277E-01		ENSG00000243501
NA	0.0832	3.1283E-01	5.2308E-01	ENSG00000250387
NA	0.0413	3.1295E-01	5.2321E-01	ENSG00000284902
NA	0.0667	3.1346E-01	5.2369E-01	ENSG00000128262
NA	0.0899	3.1347E-01	5.2369E-01	ENSG00000276216

NA	0.0552	3.1377E-01	5.2406E-01	ENSG00000253372
NA	0.0397	3.1388E-01		ENSG00000250961
NA	0.0283	3.1395E-01		ENSG00000244461
NA	0.0210	3.1396E-01		ENSG00000255219
NA	0.0120	3.1411E-01		ENSG00000280356
NA	0.0416	3.1438E-01		ENSG00000250325
NA	0.0911	3.1440E-01	5.2456E-01	ENSG00000289277
NA	0.0630	3.1470E-01	5.2472E-01	ENSG00000260744
NA	0.0304	3.1475E-01		ENSG00000254739
NA	0.0330	3.1490E-01		ENSG00000233420
NA	0.0217	3.1494E-01		ENSG00000288029
NA	0.0188	3.1498E-01		ENSG00000228625
NA	0.0901	3.1504E-01	5.2514E-01	ENSG00000258789
NA	0.0614	3.1528E-01	5.2535E-01	ENSG00000285777
NA	0.0292	3.1540E-01		ENSG00000196534
NA	0.0517	3.1541E-01	5.2554E-01	ENSG00000289052
NA	0.0260	3.1549E-01		ENSG00000286347
NA	0.0295	3.1580E-01	5.2594E-01	ENSG00000289214
NA	0.0122	3.1591E-01		ENSG00000289072
NA	0.0684	3.1592E-01	5.2600E-01	ENSG00000254907
NA	0.0771	3.1617E-01	5.2621E-01	ENSG00000260923
NA	0.0115	3.1623E-01		ENSG00000253476
NA	0.0252	3.1644E-01		ENSG00000287307
NA	0.0144	3.1714E-01		ENSG00000242640
NA	0.0140	3.1734E-01		ENSG00000279521
NA	0.0438	3.1739E-01		ENSG00000278737
NA	0.0150	3.1740E-01		ENSG00000225264
NA	0.0202	3.1746E-01		ENSG00000224648
NA	0.0655	3.1747E-01	5.2775E-01	ENSG00000287564
NA	0.0790	3.1751E-01	5.2775E-01	ENSG00000259146
NA	0.0588	3.1767E-01	5.2790E-01	ENSG00000290086
NA	0.0336	3.1775E-01		ENSG00000278028
NA	0.0360	3.1791E-01		ENSG00000287685
NA	0.0387	3.1792E-01		ENSG00000288560
NA	0.0197	3.1797E-01		ENSG00000236528
NA	0.0176	3.1822E-01		ENSG00000224969
NA	0.0892	3.1854E-01	5.2879E-01	ENSG00000288908
NA	0.0210	3.1879E-01		ENSG00000288854
NA	0.0545	3.1891E-01	5.2919E-01	ENSG00000235038
NA	0.0168	3.1901E-01		ENSG00000228384
NA	0.0128	3.1903E-01		ENSG00000285300
NA	0.0889	3.1912E-01	5.2939E-01	ENSG00000279722
NA	0.0810	3.1925E-01	5.2957E-01	ENSG00000269352
NA	0.0176	3.1928E-01		ENSG00000231162
NA	0.0882	3.1946E-01	5.2964E-01	ENSG00000239881
NA	0.0359	3.1948E-01		ENSG00000289368
NA	0.0660	3.1959E-01	5.2970E-01	ENSG00000289844
NA	0.0886	3.1960E-01	5.2970E-01	ENSG00000290051
NA	0.0510	3.1966E-01	5.2976E-01	ENSG00000249125

NA	0.0879	3.1990E-01	5.3006E-01	ENSG00000272735
NA	0.0627	3.1991E-01	5.3006E-01	ENSG00000284705
NA	0.0224	3.1993E-01		ENSG00000274769
NA	0.0567	3.2001E-01		ENSG00000289639
NA	0.0817	3.2022E-01	5.3035E-01	ENSG00000287055
NA	0.0217	3.2022E-01		ENSG00000233448
NA	0.0696	3.2030E-01	5.3036E-01	ENSG00000260167
NA	0.0561	3.2037E-01	5.3039E-01	ENSG00000279971
NA	0.0856	3.2083E-01	5.3094E-01	ENSG00000286220
NA	0.0770	3.2089E-01	5.3094E-01	ENSG00000180178
NA	0.0774	3.2092E-01	5.3094E-01	ENSG00000270039
NA	0.0172	3.2097E-01		ENSG00000232202
NA	0.0443	3.2109E-01		ENSG00000225767
NA	0.0231	3.2117E-01		ENSG00000230918
NA	0.0573	3.2125E-01	5.3125E-01	ENSG00000277151
NA	0.0616	3.2160E-01	5.3171E-01	ENSG00000270035
NA	0.0570	3.2170E-01	5.3176E-01	ENSG00000261211
NA	0.0883	3.2176E-01	5.3176E-01	ENSG00000272734
NA	0.0670	3.2177E-01	5.3176E-01	ENSG00000205534
NA	0.0145	3.2179E-01		ENSG00000176115
NA	0.0634	3.2189E-01	5.3183E-01	ENSG00000260498
NA	0.0825	3.2191E-01	5.3184E-01	ENSG00000278730
NA	0.0072	3.2194E-01		ENSG00000238168
NA	0.0753	3.2205E-01	5.3202E-01	ENSG00000250848
NA	0.0857	3.2220E-01	5.3220E-01	ENSG00000288736
NA	0.0188	3.2231E-01		ENSG00000277950
NA	0.0294	3.2245E-01		ENSG00000289473
NA	0.0142	3.2264E-01		ENSG00000234267
NA	0.0445	3.2282E-01	5.3288E-01	ENSG00000159860
NA	0.0231	3.2290E-01		ENSG00000272848
NA	0.0479	3.2300E-01	5.3300E-01	ENSG00000213033
NA	0.0836	3.2306E-01	5.3305E-01	ENSG00000123965
NA	0.0398	3.2320E-01		ENSG00000290017
NA	0.0432	3.2370E-01		ENSG00000288588
NA	0.0206	3.2378E-01		ENSG00000270321
NA	0.0667	3.2395E-01	5.3401E-01	ENSG00000214077
NA	0.0860	3.2409E-01	5.3407E-01	ENSG00000237058
NA	0.0462	3.2411E-01	5.3407E-01	ENSG00000279796
NA	0.0146	3.2413E-01		ENSG00000234148
NA	0.0857	3.2426E-01	5.3428E-01	ENSG00000099251
NA	0.0218	3.2430E-01		ENSG00000249465
NA	0.0482	3.2434E-01	5.3437E-01	ENSG00000231881
NA	0.0521	3.2477E-01		ENSG00000274292
NA	0.0265	3.2482E-01		ENSG00000273104
NA	0.0599	3.2491E-01	5.3492E-01	ENSG00000227542
NA	0.0640	3.2505E-01	5.3511E-01	ENSG00000224078
NA	0.0274	3.2508E-01		ENSG00000243230
NA	0.0210	3.2537E-01		ENSG00000267136
NA	0.0443	3.2542E-01		ENSG00000257322

NA	0.0176	3.2548E-01		ENSG00000279660
NA	0.0538	3.2565E-01		ENSG00000267295
NA	0.0739	3.2576E-01	5.3592E-01	ENSG00000260796
NA	0.0843	3.2592E-01	5.3611E-01	ENSG00000258645
NA	0.0187	3.2599E-01		ENSG00000268758
NA	0.0880	3.2606E-01	5.3624E-01	ENSG00000279529
NA	0.0572	3.2609E-01	5.3625E-01	ENSG00000263345
NA	0.0435	3.2649E-01		ENSG00000262810
NA	0.0055	3.2654E-01		ENSG00000286849
NA	0.0160	3.2668E-01		ENSG00000286442
NA	0.0297	3.2708E-01		ENSG00000226352
NA	0.0168	3.2726E-01		ENSG00000251527
NA	0.0869	3.2741E-01	5.3784E-01	ENSG00000278784
NA	0.0231	3.2747E-01		ENSG00000256923
NA	0.0405	3.2758E-01		ENSG00000233609
NA	0.0120	3.2758E-01		ENSG00000287556
NA	0.0159	3.2759E-01		ENSG00000270575
NA	0.0877	3.2777E-01	5.3837E-01	ENSG00000280136
NA	0.0546	3.2800E-01	5.3839E-01	ENSG00000260370
NA	0.0150	3.2823E-01		ENSG00000214772
NA	0.0704	3.2837E-01	5.3885E-01	ENSG00000286732
NA	0.0422	3.2855E-01		ENSG00000278370
NA	0.0677	3.2870E-01	5.3911E-01	ENSG00000231977
NA	0.0124	3.2875E-01		ENSG00000272797
NA	0.0169	3.2881E-01		ENSG00000230184
NA	0.0573	3.2888E-01		ENSG00000259126
NA	0.0860	3.2890E-01	5.3925E-01	ENSG00000289810
NA	0.0568	3.2909E-01	5.3942E-01	ENSG00000228157
NA	0.0730	3.2963E-01	5.4001E-01	ENSG00000270681
NA	0.0467	3.2968E-01	5.4003E-01	ENSG00000286688
NA	0.0549	3.2976E-01	5.4006E-01	ENSG00000260518
NA	0.0290	3.2978E-01		ENSG00000286122
NA	0.0348	3.3037E-01		ENSG00000258620
NA	0.0867	3.3058E-01	5.4085E-01	ENSG00000286340
NA	0.0347	3.3073E-01		ENSG00000232027
NA	0.0249	3.3078E-01		ENSG00000225574
NA	0.0568	3.3086E-01	5.4112E-01	ENSG00000286938
NA	0.0318	3.3095E-01		ENSG00000290043
NA	0.0762	3.3098E-01	5.4112E-01	ENSG00000253361
NA	0.0870	3.3098E-01	5.4112E-01	ENSG00000290057
NA	0.0209	3.3117E-01		ENSG00000260619
NA	0.0182	3.3120E-01		ENSG00000275665
NA	0.0575	3.3147E-01	5.4173E-01	ENSG00000240499
NA	0.0318	3.3148E-01		ENSG00000273302
NA	0.0215	3.3154E-01		ENSG00000259192
NA	0.0214	3.3160E-01		ENSG00000269425
NA	0.0257	3.3223E-01		ENSG00000249309
NA	0.0215	3.3264E-01		ENSG00000277566
NA	0.0182	3.3290E-01		ENSG00000257677

NA	0.0470	3.3317E-01		ENSG00000286586
NA	0.0800	3.3387E-01	5.4400E-01	ENSG00000259969
NA	0.0063	3.3414E-01		ENSG00000254458
NA	0.0214	3.3417E-01		ENSG00000278954
NA	0.0463	3.3437E-01	5.4451E-01	ENSG00000225422
NA	0.0246	3.3446E-01		ENSG00000232742
NA	0.0836	3.3465E-01	5.4471E-01	ENSG00000133519
NA	0.0097	3.3467E-01		ENSG00000254620
NA	0.0353	3.3482E-01		ENSG00000285642
NA	0.0239	3.3496E-01		ENSG00000286717
NA	0.0646	3.3497E-01	5.4507E-01	ENSG00000225643
NA	0.0299	3.3517E-01		ENSG00000286134
NA	0.0866	3.3541E-01	5.4550E-01	ENSG00000289393
NA	0.0094	3.3549E-01		ENSG00000253656
NA	0.0795	3.3563E-01	5.4574E-01	ENSG00000179028
NA	0.0367	3.3570E-01		ENSG00000232807
NA	0.0857	3.3571E-01	5.4583E-01	ENSG00000286619
NA	0.0774	3.3578E-01	5.4585E-01	ENSG00000273064
NA	0.0334	3.3586E-01		ENSG00000239263
NA	0.0532	3.3613E-01	5.4615E-01	ENSG00000289204
NA	0.0307	3.3620E-01		ENSG00000216676
NA	0.0189	3.3645E-01		ENSG00000265948
NA	0.0258	3.3678E-01		ENSG00000255434
NA	0.0332	3.3678E-01		ENSG00000284820
NA	0.0840	3.3679E-01	5.4686E-01	ENSG00000283443
NA	0.0675	3.3687E-01	5.4696E-01	ENSG00000259322
NA	0.0165	3.3704E-01		ENSG00000229116
NA	0.0612	3.3727E-01	5.4734E-01	ENSG00000248479
NA	0.0178	3.3731E-01		ENSG00000279201
NA	0.0394	3.3739E-01		ENSG00000275897
NA	0.0831	3.3750E-01	5.4755E-01	ENSG00000260747
NA	0.0710	3.3769E-01	5.4765E-01	ENSG00000272092
NA	0.0339	3.3775E-01		ENSG00000272189
NA	0.0693	3.3786E-01	5.4785E-01	ENSG00000272509
NA	0.0576	3.3808E-01	5.4799E-01	ENSG00000279696
NA	0.0146	3.3827E-01		ENSG00000234675
NA	0.0143	3.3857E-01		ENSG00000223479
NA	0.0846	3.3868E-01	5.4851E-01	ENSG00000273355
NA	0.0284	3.3869E-01		ENSG00000182048
NA	0.0053	3.3879E-01		ENSG00000278831
NA	0.0478	3.3880E-01		ENSG00000225579
NA	0.0155	3.3899E-01		ENSG00000286450
NA	0.0242	3.3903E-01		ENSG00000286072
NA	0.0767	3.3911E-01	5.4880E-01	ENSG00000279434
NA	0.0792	3.3925E-01	5.4898E-01	ENSG00000176826
NA	0.0374	3.3926E-01		ENSG00000287964
NA	0.0559	3.3941E-01		ENSG00000258531
NA	0.0561	3.3944E-01	5.4911E-01	ENSG00000248840
NA	0.0047	3.3954E-01		ENSG00000232335

NA	0.0191	3.3964E-01		ENSG00000277575
NA	0.0973	3.3969E-01	5.4940E-01	ENSG00000237749
NA	0.0211	3.4000E-01		ENSG00000249678
NA	0.0478	3.4001E-01		ENSG00000274001
NA	0.0202	3.4019E-01		ENSG00000228108
NA	0.0618	3.4027E-01		ENSG00000285996
NA	0.0619	3.4033E-01	5.5005E-01	ENSG00000224883
NA	0.0125	3.4050E-01		ENSG00000237138
NA	0.0227	3.4086E-01		ENSG00000251435
NA	0.0836	3.4113E-01	5.5097E-01	ENSG00000228172
NA	0.0170	3.4153E-01		ENSG00000287895
NA	0.0181	3.4175E-01		ENSG00000250945
NA	0.0170	3.4181E-01		ENSG00000255753
NA	0.0451	3.4188E-01	5.5153E-01	ENSG00000288565
NA	0.0087	3.4201E-01		ENSG00000256739
NA	0.0788	3.4203E-01	5.5174E-01	ENSG00000288856
NA	0.0193	3.4210E-01		ENSG00000276292
NA	0.0819	3.4220E-01	5.5185E-01	ENSG00000279673
NA	0.0119	3.4228E-01		ENSG00000238279
NA	0.0478	3.4230E-01		ENSG00000257264
NA	0.0767	3.4231E-01	5.5193E-01	ENSG00000286584
NA	0.0671	3.4235E-01	5.5197E-01	ENSG00000254454
NA	0.0315	3.4245E-01		ENSG00000213985
NA	0.0109	3.4260E-01		ENSG00000283095
NA	0.0636	3.4267E-01	5.5221E-01	ENSG00000249673
NA	0.0607	3.4273E-01	5.5221E-01	ENSG00000279413
NA	0.0180	3.4275E-01		ENSG00000250516
NA	0.0175	3.4278E-01		ENSG00000226468
NA	0.0190	3.4280E-01		ENSG00000260078
NA	0.0110	3.4287E-01		ENSG00000256006
NA	0.0531	3.4291E-01		ENSG00000254467
NA	0.0198	3.4319E-01		ENSG00000279769
NA	0.0499	3.4321E-01	5.5284E-01	ENSG00000235060
NA	0.0577	3.4336E-01	5.5294E-01	ENSG00000227487
NA	0.0447	3.4365E-01	5.5323E-01	ENSG00000279250
NA	0.0152	3.4386E-01		ENSG00000254631
NA	0.0566	3.4411E-01	5.5382E-01	ENSG00000259345
NA	0.0535	3.4424E-01		ENSG00000270031
NA	0.0582	3.4444E-01	5.5407E-01	ENSG00000275888
NA	0.0835	3.4445E-01	5.5407E-01	ENSG00000286588
NA	0.0495	3.4446E-01	5.5407E-01	ENSG00000261453
NA	0.0807	3.4459E-01	5.5416E-01	ENSG00000273437
NA	0.0072	3.4488E-01		ENSG00000279394
NA	0.0690	3.4514E-01	5.5490E-01	ENSG00000224038
NA	0.0721	3.4530E-01	5.5508E-01	ENSG00000289474
NA	0.0320	3.4676E-01		ENSG00000224417
NA	0.0169	3.4707E-01		ENSG00000178503
NA	0.0801	3.4725E-01	5.5760E-01	ENSG00000210196
NA	0.0359	3.4727E-01	5.5760E-01	ENSG00000286282

NA	0.0811	3.4730E-01	5.5760E-01	ENSG00000236519
NA	0.0175	3.4744E-01		ENSG00000275332
NA	0.0361	3.4747E-01		ENSG00000260017
NA	0.0384	3.4756E-01		ENSG00000220256
NA	0.0412	3.4770E-01		ENSG00000230435
NA	0.0689	3.4803E-01	5.5816E-01	ENSG00000257086
NA	0.0209	3.4821E-01		ENSG00000251054
NA	0.0176	3.4822E-01		ENSG00000287242
NA	0.0180	3.4824E-01		ENSG00000226441
NA	0.0787	3.4833E-01	5.5839E-01	ENSG00000229666
NA	0.0377	3.4836E-01		ENSG00000257897
NA	0.0741	3.4840E-01	5.5839E-01	ENSG00000232713
NA	0.0410	3.4849E-01		ENSG00000243694
NA	0.0713	3.4862E-01	5.5845E-01	ENSG00000273199
NA	0.0359	3.4863E-01		ENSG00000251636
NA	0.0404	3.4902E-01		ENSG00000235454
NA	0.0413	3.4904E-01		ENSG00000279678
NA	0.0572	3.4940E-01	5.5930E-01	ENSG00000285644
NA	0.0347	3.4944E-01		ENSG00000235829
NA	0.0344	3.4956E-01		ENSG00000278892
NA	0.0260	3.4956E-01		ENSG00000289628
NA	0.0513	3.4964E-01	5.5961E-01	ENSG00000227535
NA	0.0727	3.4966E-01	5.5961E-01	ENSG00000261526
NA	0.0734	3.5018E-01	5.6033E-01	ENSG00000285770
NA	0.0184	3.5063E-01		ENSG00000257514
NA	0.0186	3.5077E-01		ENSG00000187472
NA	0.0177	3.5078E-01		ENSG00000285586
NA	0.0302	3.5110E-01		ENSG00000285519
NA	0.0054	3.5151E-01		ENSG00000224256
NA	0.0597	3.5180E-01	5.6208E-01	ENSG00000288991
NA	0.0206	3.5203E-01		ENSG00000279875
NA	0.0178	3.5232E-01		ENSG00000254330
NA	0.0342	3.5261E-01		ENSG00000286642
NA	0.0220	3.5277E-01		ENSG00000267472
NA	0.0115	3.5298E-01		ENSG00000286749
NA	0.0381	3.5302E-01		ENSG00000280135
NA	0.0509	3.5306E-01	5.6328E-01	ENSG00000260495
NA	0.0096	3.5315E-01		ENSG00000274841
NA	0.0090	3.5341E-01	5.6352E-01	ENSG00000232985
NA	0.0277	3.5362E-01		ENSG00000218748
NA	0.0597	3.5369E-01	5.6374E-01	ENSG00000207497
NA	0.0746	3.5405E-01	5.6402E-01	ENSG00000242242
NA	0.0263	3.5410E-01		ENSG00000280042
NA	0.0546	3.5425E-01	5.6418E-01	ENSG00000246100
NA	0.0349	3.5440E-01		ENSG00000286838
NA	0.0109	3.5440E-01		ENSG00000287087
NA	0.0219	3.5456E-01		ENSG00000250979
NA	0.0163	3.5458E-01		ENSG00000274310
NA	0.0248	3.5468E-01		ENSG00000260657

NA	0.0585	3.5473E-01	5.6460E-01	ENSG00000238061
NA	0.0817	3.5478E-01	5.6460E-01	ENSG00000289268
NA	0.0725	3.5479E-01	5.6460E-01	ENSG00000269486
NA	0.0820	3.5480E-01	5.6460E-01	ENSG00000223653
NA	0.0165	3.5488E-01		ENSG00000281365
NA	0.0261	3.5490E-01		ENSG00000266335
NA	0.0615	3.5509E-01	5.6476E-01	ENSG00000262663
NA	0.0292	3.5536E-01		ENSG00000259843
NA	0.0163	3.5574E-01		ENSG00000234763
NA	0.0313	3.5589E-01		ENSG00000287411
NA	0.0677	3.5592E-01		ENSG00000270720
NA	0.0500	3.5593E-01	5.6577E-01	ENSG00000280286
NA	0.0515	3.5609E-01	5.6595E-01	ENSG00000287092
NA	0.0367	3.5613E-01		ENSG00000277087
NA	0.0471	3.5626E-01		ENSG00000286596
NA	0.0846	3.5626E-01	5.6615E-01	ENSG00000261754
NA	0.0624	3.5628E-01	5.6615E-01	ENSG00000284633
NA	0.0160	3.5632E-01		ENSG00000260725
NA	0.0187	3.5635E-01		ENSG00000254406
NA	0.0248	3.5655E-01		ENSG00000119660
NA	0.0405	3.5665E-01		ENSG00000288845
NA	0.0445	3.5684E-01	5.6664E-01	ENSG00000272979
NA	0.0162	3.5703E-01		ENSG00000227087
NA	0.0136	3.5738E-01		ENSG00000228876
NA	0.0121	3.5758E-01		ENSG00000288615
NA	0.0231	3.5784E-01		ENSG00000289606
NA	0.0307	3.5788E-01	5.6756E-01	ENSG00000284513
NA	0.0809	3.5790E-01	5.6756E-01	ENSG00000251161
NA	0.0800	3.5815E-01	5.6777E-01	ENSG00000283757
NA	0.0648	3.5834E-01	5.6777E-01	ENSG00000286215
NA	0.0387	3.5845E-01		ENSG00000258753
NA	0.0400	3.5845E-01		ENSG00000271761
NA	0.0158	3.5852E-01		ENSG00000237130
NA	0.0750	3.5870E-01	5.6817E-01	ENSG00000225292
NA	0.0318	3.5884E-01		ENSG00000217455
NA	0.0346	3.5897E-01		ENSG00000232627
NA	0.0191	3.5915E-01		ENSG00000203266
NA	0.0327	3.5925E-01		ENSG00000207317
NA	0.0100	3.5931E-01		ENSG00000271065
NA	0.0717	3.5934E-01	5.6885E-01	ENSG00000277954
NA	0.0752	3.5938E-01	5.6887E-01	ENSG00000275202
NA	0.0536	3.5944E-01		ENSG00000238189
NA	0.0793	3.5960E-01	5.6890E-01	ENSG00000100068
NA	0.0415	3.5975E-01		ENSG00000267142
NA	0.0128	3.6029E-01		ENSG00000238285
NA	0.0106	3.6088E-01		ENSG00000254185
NA	0.0156	3.6095E-01		ENSG00000271958
NA	0.0310	3.6098E-01		ENSG00000261611
NA	0.0398	3.6112E-01		ENSG00000229498

NA	0.0177	3.6117E-01		ENSG00000237167
NA	0.0611	3.6121E-01	5.7076E-01	ENSG00000254275
NA	0.0251	3.6129E-01		ENSG00000258284
NA	0.0199	3.6132E-01		ENSG00000267784
NA	0.0293	3.6133E-01		ENSG00000187589
NA	0.0802	3.6151E-01	5.7097E-01	ENSG00000230583
NA	0.0467	3.6171E-01	5.7107E-01	ENSG00000206682
NA	0.0132	3.6183E-01		ENSG00000213816
NA	0.0365	3.6186E-01	5.7107E-01	ENSG00000274776
NA	0.0197	3.6192E-01		ENSG00000225458
NA	0.0310	3.6215E-01		ENSG00000278182
NA	0.0513	3.6219E-01		ENSG00000273181
NA	0.0140	3.6223E-01		ENSG00000289544
NA	0.0539	3.6247E-01	5.7182E-01	ENSG00000285973
NA	0.0187	3.6256E-01		ENSG00000281613
NA	0.0096	3.6275E-01		ENSG00000255160
NA	0.0543	3.6329E-01	5.7241E-01	ENSG00000228196
NA	0.0148	3.6334E-01		ENSG00000248138
NA	0.0391	3.6334E-01		ENSG00000276790
NA	0.0105	3.6340E-01		ENSG00000253676
NA	0.0591	3.6373E-01	5.7288E-01	ENSG00000261462
NA	0.0558	3.6377E-01	5.7291E-01	ENSG00000272800
NA	0.0229	3.6416E-01		ENSG00000225931
NA	0.0744	3.6419E-01	5.7328E-01	ENSG00000261447
NA	0.0223	3.6447E-01		ENSG00000228275
NA	0.0479	3.6460E-01	5.7374E-01	ENSG00000228998
NA	0.0363	3.6470E-01		ENSG00000271996
NA	0.0312	3.6498E-01		ENSG00000285849
NA	0.0264	3.6548E-01		ENSG00000287182
NA	0.0517	3.6553E-01	5.7467E-01	ENSG00000287142
NA	0.0303	3.6554E-01		ENSG00000257951
NA	0.0572	3.6565E-01	5.7478E-01	ENSG00000275025
NA	0.0837	3.6566E-01	5.7478E-01	ENSG00000285454
NA	0.0644	3.6588E-01	5.7501E-01	ENSG00000279897
NA	0.0639	3.6595E-01	5.7509E-01	ENSG00000258230
NA	0.0704	3.6610E-01	5.7525E-01	ENSG00000240207
NA	0.0459	3.6620E-01		ENSG00000260492
NA	0.0534	3.6654E-01	5.7580E-01	ENSG00000241738
NA	0.0227	3.6658E-01		ENSG00000233996
NA	0.0526	3.6660E-01		ENSG00000230967
NA	0.0199	3.6689E-01		ENSG00000280002
NA	0.0447	3.6715E-01	5.7643E-01	ENSG00000263219
NA	0.0791	3.6727E-01	5.7651E-01	ENSG00000269983
NA	0.0505	3.6743E-01	5.7668E-01	ENSG00000271344
NA	0.0467	3.6769E-01		ENSG00000267413
NA	0.0520	3.6779E-01	5.7706E-01	ENSG00000287620
NA	0.0086	3.6791E-01		ENSG00000215905
NA	0.0674	3.6797E-01	5.7713E-01	ENSG00000235105
NA	0.0189	3.6804E-01		ENSG00000286654

NA	0.0410	3.6805E-01		ENSG00000248734
NA	0.0111	3.6835E-01		ENSG00000287739
NA	0.0312	3.6841E-01		ENSG00000237481
NA	0.0215	3.6842E-01		ENSG00000261313
NA	0.0056	3.6851E-01		ENSG00000239453
NA	0.0608	3.6854E-01	5.7769E-01	ENSG00000277459
NA	0.0216	3.6854E-01	5.7769E-01	ENSG00000286723
NA	0.0790	3.6861E-01	5.7776E-01	ENSG00000243176
NA	0.0246	3.6877E-01		ENSG00000248399
NA	0.0712	3.6920E-01	5.7842E-01	ENSG00000279495
NA	0.0336	3.6941E-01		ENSG00000269846
NA	0.0621	3.6942E-01	5.7865E-01	ENSG00000287373
NA	0.0547	3.6944E-01	5.7866E-01	ENSG00000228467
NA	0.0492	3.6950E-01		ENSG00000249725
NA	0.0216	3.6979E-01		ENSG00000266696
NA	0.0156	3.6995E-01		ENSG00000276851
NA	0.0298	3.7021E-01		ENSG00000266654
NA	0.0571	3.7047E-01	5.7965E-01	ENSG00000248184
NA	0.0479	3.7056E-01	5.7969E-01	ENSG00000235833
NA	0.0790	3.7060E-01	5.7969E-01	ENSG00000219891
NA	0.0665	3.7061E-01	5.7969E-01	ENSG00000248554
NA	0.0458	3.7082E-01		ENSG00000287048
NA	0.0635	3.7083E-01	5.7981E-01	ENSG00000262147
NA	0.0169	3.7088E-01		ENSG00000282048
NA	0.0673	3.7129E-01	5.8001E-01	ENSG00000233672
NA	0.0609	3.7139E-01	5.8008E-01	ENSG00000288934
NA	0.0235	3.7149E-01		ENSG00000258653
NA	0.0207	3.7182E-01		ENSG00000235832
NA	0.0372	3.7190E-01		ENSG00000286568
NA	0.0538	3.7190E-01	5.8063E-01	ENSG00000273419
NA	0.0131	3.7196E-01		ENSG00000274685
NA	0.0068	3.7200E-01		ENSG00000258454
NA	0.0065	3.7214E-01		ENSG00000281974
NA	0.0143	3.7262E-01		ENSG00000259639
NA	0.0452	3.7264E-01		ENSG00000285709
NA	0.0739	3.7316E-01	5.8178E-01	ENSG00000287979
NA	0.0138	3.7326E-01		ENSG00000272748
NA	0.0323	3.7332E-01		ENSG00000269980
NA	0.0326	3.7343E-01		ENSG00000288579
NA	0.0311	3.7344E-01		ENSG00000276627
NA	0.0035	3.7345E-01		ENSG00000230433
NA	0.0237	3.7353E-01		ENSG00000235881
NA	0.0551	3.7355E-01	5.8209E-01	ENSG00000214776
NA	0.0771	3.7355E-01	5.8209E-01	ENSG00000279198
NA	0.0351	3.7360E-01		ENSG00000250974
NA	0.0143	3.7365E-01		ENSG00000226942
NA	0.0785	3.7385E-01	5.8245E-01	ENSG00000268628
NA	0.0182	3.7392E-01		ENSG00000266989
NA	0.0097	3.7412E-01		ENSG00000258294

NA	0.0172	3.7427E-01		ENSG00000288040
NA	0.0237	3.7459E-01		ENSG00000231529
NA	0.0638	3.7464E-01	5.8325E-01	ENSG00000231770
NA	0.0467	3.7468E-01	5.8325E-01	ENSG00000255122
NA	0.0777	3.7491E-01	5.8347E-01	ENSG00000259479
NA	0.0294	3.7543E-01		ENSG00000268879
NA	0.0736	3.7548E-01	5.8402E-01	ENSG00000287900
NA	0.0519	3.7572E-01	5.8429E-01	ENSG00000200788
NA	0.0114	3.7572E-01		ENSG00000254978
NA	0.0245	3.7575E-01		ENSG00000228577
NA	0.0321	3.7577E-01		ENSG00000231612
NA	0.0389	3.7592E-01		ENSG00000254060
NA	0.0574	3.7605E-01	5.8458E-01	ENSG00000277324
NA	0.0052	3.7654E-01		ENSG00000227712
NA	0.0217	3.7655E-01	5.8517E-01	ENSG00000235837
NA	0.0250	3.7663E-01		ENSG00000267207
NA	0.0405	3.7670E-01		ENSG00000180610
NA	0.0166	3.7681E-01		ENSG00000252290
NA	0.0189	3.7686E-01		ENSG00000275322
NA	0.0312	3.7703E-01	5.8556E-01	ENSG00000163016
NA	0.0477	3.7714E-01	5.8569E-01	ENSG00000277978
NA	0.0676	3.7720E-01	5.8571E-01	ENSG00000266910
NA	0.0307	3.7726E-01	5.8572E-01	ENSG00000224185
NA	0.0647	3.7785E-01	5.8637E-01	ENSG00000196922
NA	0.0616	3.7801E-01	5.8655E-01	ENSG00000253659
NA	0.0693	3.7819E-01	5.8680E-01	ENSG00000285971
NA	0.0079	3.7823E-01		ENSG00000228019
NA	0.0182	3.7833E-01		ENSG00000253180
NA	0.0302	3.7849E-01		ENSG00000230695
NA	0.0133	3.7850E-01		ENSG00000276063
NA	0.0390	3.7855E-01		ENSG00000213108
NA	0.0493	3.7860E-01		ENSG00000280011
NA	0.0277	3.7868E-01		ENSG00000274370
NA	0.0771	3.7898E-01	5.8752E-01	ENSG00000267419
NA	0.0025	3.7902E-01		ENSG00000289314
NA	0.0501	3.7904E-01	5.8752E-01	ENSG00000288979
NA	0.0139	3.7910E-01		ENSG00000230730
NA	0.0504	3.7915E-01	5.8762E-01	ENSG00000278668
NA	0.0125	3.7917E-01	5.8762E-01	ENSG00000271185
NA	0.0747	3.7924E-01	5.8768E-01	ENSG00000228242
NA	0.0092	3.7930E-01		ENSG00000290096
NA	0.0532	3.7939E-01	5.8784E-01	ENSG00000288574
NA	0.0090	3.7949E-01		ENSG00000287095
NA	0.0157	3.7975E-01		ENSG00000202523
NA	0.0071	3.7977E-01		ENSG00000243276
NA	0.0709	3.7978E-01	5.8830E-01	ENSG00000242622
NA	0.0221	3.7983E-01		ENSG00000234814
NA	0.0126	3.7986E-01		ENSG00000197376
NA	0.0138	3.7999E-01		ENSG00000230790

NA	0.0425	3.8002E-01	5.8851E-01	ENSG00000255200
NA	0.0042	3.8003E-01		ENSG00000239254
NA	0.0444	3.8015E-01		ENSG00000288769
NA	0.0331	3.8061E-01		ENSG00000275710
NA	0.0421	3.8087E-01	5.8916E-01	ENSG00000289864
NA	0.0513	3.8094E-01	5.8923E-01	ENSG00000227398
NA	0.0390	3.8103E-01	5.8923E-01	ENSG00000263089
NA	0.0200	3.8106E-01		ENSG00000199466
NA	0.0382	3.8117E-01		ENSG00000232555
NA	0.0296	3.8133E-01		ENSG00000258376
NA	0.0245	3.8137E-01		ENSG00000284823
NA	0.0362	3.8137E-01		ENSG00000199331
NA	0.0359	3.8144E-01		ENSG00000224595
NA	0.0528	3.8162E-01	5.8984E-01	ENSG00000253106
NA	0.0735	3.8175E-01	5.8997E-01	ENSG00000274828
NA	0.0233	3.8187E-01		ENSG00000250202
NA	0.0029	3.8187E-01		ENSG00000288083
NA	0.0220	3.8190E-01		ENSG00000271825
NA	0.0172	3.8202E-01		ENSG00000267607
NA	0.0347	3.8216E-01		ENSG00000277095
NA	0.0141	3.8229E-01		ENSG00000260008
NA	0.0338	3.8242E-01		ENSG00000199567
NA	0.0656	3.8245E-01	5.9066E-01	ENSG00000279476
NA	0.0324	3.8261E-01		ENSG00000259890
NA	0.0311	3.8275E-01		ENSG00000244378
NA	0.0540	3.8286E-01	5.9103E-01	ENSG00000277561
NA	0.0144	3.8317E-01		ENSG00000288690
NA	0.0579	3.8331E-01	5.9154E-01	ENSG00000234650
NA	0.0231	3.8339E-01		ENSG00000228328
NA	0.0167	3.8339E-01		ENSG00000254688
NA	0.0212	3.8361E-01		ENSG00000207021
NA	0.0499	3.8366E-01	5.9181E-01	ENSG00000246792
NA	0.0164	3.8389E-01		ENSG00000270469
NA	0.0439	3.8392E-01	5.9203E-01	ENSG00000287636
NA	0.0687	3.8414E-01	5.9211E-01	ENSG00000226308
NA	0.0483	3.8433E-01	5.9229E-01	ENSG00000261123
NA	0.0545	3.8456E-01	5.9252E-01	ENSG00000288983
NA	0.0636	3.8465E-01	5.9262E-01	ENSG00000256417
NA	0.0326	3.8467E-01		ENSG00000253666
NA	0.0296	3.8470E-01		ENSG00000261795
NA	0.0162	3.8472E-01		ENSG00000280711
NA	0.0173	3.8481E-01		ENSG00000266340
NA	0.0141	3.8498E-01		ENSG00000244076
NA	0.0533	3.8533E-01	5.9315E-01	ENSG00000284968
NA	0.0274	3.8535E-01		ENSG00000250106
NA	0.0450	3.8583E-01	5.9366E-01	ENSG00000244159
NA	0.0372	3.8584E-01		ENSG00000223838
NA	0.0496	3.8588E-01	5.9366E-01	ENSG00000285739
NA	0.0287	3.8589E-01		ENSG00000260661

NA	0.0592	3.8601E-01	5.9380E-01	ENSG00000288719
NA	0.0216	3.8606E-01		ENSG00000253925
NA	0.0153	3.8621E-01		ENSG00000275438
NA	0.0202	3.8631E-01		ENSG00000289302
NA	0.0274	3.8632E-01		ENSG00000227088
NA	0.0675	3.8633E-01	5.9403E-01	ENSG00000266933
NA	0.0213	3.8644E-01		ENSG00000249526
NA	0.0144	3.8676E-01		ENSG00000285205
NA	0.0505	3.8677E-01	5.9452E-01	ENSG00000255438
NA	0.0143	3.8683E-01		ENSG00000269927
NA	0.0285	3.8683E-01		ENSG00000286012
NA	0.0756	3.8702E-01	5.9475E-01	ENSG00000258682
NA	0.0515	3.8715E-01	5.9488E-01	ENSG00000255506
NA	0.0747	3.8719E-01	5.9491E-01	ENSG00000225945
NA	0.0253	3.8721E-01		ENSG00000287056
NA	0.0173	3.8722E-01		ENSG00000287905
NA	0.0774	3.8744E-01	5.9508E-01	ENSG00000289506
NA	0.0670	3.8747E-01	5.9508E-01	ENSG00000272732
NA	0.0141	3.8749E-01		ENSG00000268926
NA	0.0512	3.8808E-01	5.9565E-01	ENSG00000260148
NA	0.0646	3.8818E-01	5.9568E-01	ENSG00000256742
NA	0.0149	3.8833E-01		ENSG00000243744
NA	0.0077	3.8840E-01		ENSG00000207032
NA	0.0501	3.8850E-01	5.9594E-01	ENSG00000239791
NA	0.0278	3.8852E-01		ENSG00000276384
NA	0.0199	3.8856E-01		ENSG00000287226
NA	0.0480	3.8880E-01	5.9607E-01	ENSG00000253819
NA	0.0169	3.8921E-01		ENSG00000231654
NA	0.0683	3.8965E-01	5.9714E-01	ENSG00000229048
NA	0.0613	3.8974E-01	5.9721E-01	ENSG00000279908
NA	0.0399	3.8978E-01		ENSG00000286722
NA	0.0663	3.8995E-01	5.9733E-01	ENSG00000286320
NA	0.0733	3.8999E-01	5.9733E-01	ENSG00000154874
NA	0.0253	3.9001E-01		ENSG00000237065
NA	0.0582	3.9002E-01	5.9733E-01	ENSG00000230612
NA	0.0481	3.9020E-01	5.9751E-01	ENSG00000261707
NA	0.0165	3.9030E-01		ENSG00000277144
NA	0.0462	3.9032E-01	5.9763E-01	ENSG00000272024
NA	0.0157	3.9051E-01		ENSG00000285654
NA	0.0629	3.9069E-01	5.9782E-01	ENSG00000226432
NA	0.0705	3.9073E-01	5.9784E-01	ENSG00000248559
NA	0.0118	3.9075E-01		ENSG00000251513
NA	0.0193	3.9078E-01		ENSG00000279040
NA	0.0184	3.9124E-01		ENSG00000218048
NA	0.0492	3.9157E-01	5.9864E-01	ENSG00000289095
NA	0.0356	3.9173E-01	5.9877E-01	ENSG00000251294
NA	0.0476	3.9179E-01	5.9880E-01	ENSG00000213204
NA	0.0190	3.9223E-01		ENSG00000287611
NA	0.0161	3.9267E-01		ENSG00000261025

NA	0.0622	3.9278E-01	5.9950E-01	ENSG00000266918
NA	0.0583	3.9315E-01	5.9983E-01	ENSG00000287759
NA	0.0697	3.9322E-01	5.9985E-01	ENSG00000286989
NA	0.0117	3.9342E-01		ENSG00000282728
NA	0.0133	3.9352E-01		ENSG00000288036
NA	0.0410	3.9379E-01		ENSG00000206739
NA	0.0265	3.9393E-01		ENSG00000225963
NA	0.0648	3.9399E-01	6.0048E-01	ENSG00000264290
NA	0.0548	3.9411E-01	6.0049E-01	ENSG00000263571
NA	0.0438	3.9418E-01	6.0050E-01	ENSG00000223704
NA	0.0753	3.9427E-01	6.0060E-01	ENSG00000279674
NA	0.0146	3.9456E-01		ENSG00000258670
NA	0.0248	3.9461E-01		ENSG00000276542
NA	0.0352	3.9471E-01		ENSG00000285509
NA	0.0533	3.9485E-01	6.0108E-01	ENSG00000233765
NA	0.0139	3.9490E-01		ENSG00000242741
NA	0.0608	3.9491E-01	6.0113E-01	ENSG00000215790
NA	0.0183	3.9499E-01		ENSG00000285210
NA	0.0328	3.9535E-01		ENSG00000251523
NA	0.0316	3.9543E-01		ENSG00000286310
NA	0.0659	3.9550E-01	6.0177E-01	ENSG00000236017
NA	0.0533	3.9585E-01	6.0197E-01	ENSG00000275479
NA	0.0700	3.9585E-01	6.0197E-01	ENSG00000287839
NA	0.0744	3.9609E-01	6.0212E-01	ENSG00000260233
NA	0.0575	3.9652E-01	6.0248E-01	ENSG00000228522
NA	0.0208	3.9678E-01		ENSG00000267423
NA	0.0370	3.9754E-01		ENSG00000254691
NA	0.0728	3.9758E-01	6.0337E-01	ENSG00000232611
NA	0.0484	3.9759E-01	6.0337E-01	ENSG00000287356
NA	0.0465	3.9777E-01	6.0353E-01	ENSG00000228028
NA	0.0742	3.9779E-01	6.0353E-01	ENSG00000230090
NA	0.0315	3.9790E-01		ENSG00000258072
NA	0.0726	3.9794E-01	6.0359E-01	ENSG00000289483
NA	0.0288	3.9797E-01		ENSG00000280036
NA	0.0191	3.9825E-01		ENSG00000225098
NA	0.0208	3.9836E-01		ENSG00000279104
NA	0.0490	3.9837E-01	6.0394E-01	ENSG00000236998
NA	0.0232	3.9844E-01		ENSG00000252993
NA	0.0667	3.9850E-01	6.0410E-01	ENSG00000228878
NA	0.0146	3.9853E-01		ENSG00000267283
NA	0.0166	3.9859E-01		ENSG00000277825
NA	0.0294	3.9861E-01		ENSG00000278396
NA	0.0600	3.9869E-01	6.0424E-01	ENSG00000274002
NA	0.0448	3.9870E-01	6.0424E-01	ENSG00000274315
NA	0.0436	3.9925E-01	6.0472E-01	ENSG00000281903
NA	0.0392	3.9932E-01		ENSG00000288805
NA	0.0249	3.9934E-01		ENSG00000202279
NA	0.0255	3.9946E-01		ENSG00000289953
NA	0.0483	3.9977E-01	6.0517E-01	ENSG00000256427

NA	0.0134	3.9997E-01		ENSG00000248601
NA	0.0355	4.0060E-01		ENSG00000206976
NA	0.0446	4.0081E-01	6.0606E-01	ENSG00000225400
NA	0.0270	4.0102E-01		ENSG00000235819
NA	0.0161	4.0106E-01		ENSG00000287483
NA	0.0173	4.0109E-01		ENSG00000289862
NA	0.0186	4.0114E-01		ENSG00000287334
NA	0.0424	4.0114E-01	6.0633E-01	ENSG00000215481
NA	0.0291	4.0119E-01	6.0633E-01	ENSG00000244009
NA	0.0339	4.0127E-01		ENSG00000226939
NA	0.0301	4.0162E-01		ENSG00000236166
NA	0.0724	4.0170E-01	6.0655E-01	ENSG00000189212
NA	0.0652	4.0173E-01	6.0655E-01	ENSG00000260404
NA	0.0558	4.0180E-01	6.0655E-01	ENSG00000280063
NA	0.0458	4.0180E-01	6.0655E-01	ENSG00000281721
NA	0.0155	4.0182E-01		ENSG00000250656
NA	0.0328	4.0185E-01	6.0655E-01	ENSG00000234513
NA	0.0090	4.0192E-01		ENSG00000232081
NA	0.0094	4.0196E-01		ENSG00000256379
NA	0.0093	4.0200E-01		ENSG00000289098
NA	0.0446	4.0217E-01		ENSG00000285525
NA	0.0719	4.0218E-01	6.0672E-01	ENSG00000271737
NA	0.0714	4.0226E-01	6.0681E-01	ENSG00000230185
NA	0.0208	4.0279E-01		ENSG00000276831
NA	0.0403	4.0283E-01		ENSG00000285570
NA	0.0097	4.0298E-01		ENSG00000236471
NA	0.0452	4.0318E-01	6.0771E-01	ENSG00000233868
NA	0.0367	4.0337E-01		ENSG00000278987
NA	0.0212	4.0347E-01		ENSG00000286205
NA	0.0698	4.0357E-01	6.0808E-01	ENSG00000243753
NA	0.0293	4.0359E-01		ENSG00000266783
NA	0.0241	4.0365E-01		ENSG00000248122
NA	0.0736	4.0385E-01	6.0839E-01	ENSG00000273391
NA	0.0204	4.0387E-01		ENSG00000269373
NA	0.0331	4.0399E-01		ENSG00000270139
NA	0.0108	4.0411E-01		ENSG00000237617
NA	0.0369	4.0412E-01		ENSG00000289343
NA	0.0709	4.0420E-01	6.0879E-01	ENSG00000277741
NA	0.0128	4.0431E-01		ENSG00000228700
NA	0.0638	4.0434E-01	6.0894E-01	ENSG00000204556
NA	0.0175	4.0443E-01		ENSG00000286607
NA	0.0670	4.0474E-01	6.0931E-01	ENSG00000224593
NA	0.0406	4.0494E-01		ENSG00000230304
NA	0.0200	4.0504E-01		ENSG00000279042
NA	0.0369	4.0512E-01		ENSG00000274677
NA	0.0441	4.0530E-01	6.0970E-01	ENSG00000234500
NA	0.0262	4.0539E-01	6.0976E-01	ENSG00000239402
NA	0.0174	4.0545E-01		ENSG00000231794
NA	0.0247	4.0556E-01		ENSG00000188850

NA	0.0177	4.0603E-01		ENSG00000279572
NA	0.0574	4.0611E-01	6.1059E-01	ENSG00000255946
NA	0.0232	4.0656E-01		ENSG00000237645
NA	0.0711	4.0690E-01	6.1152E-01	ENSG00000284624
NA	0.0238	4.0703E-01		ENSG00000227230
NA	0.0198	4.0709E-01		ENSG00000213579
NA	0.0321	4.0710E-01		ENSG00000259798
NA	0.0028	4.0719E-01		ENSG00000289526
NA	0.0417	4.0770E-01		ENSG00000236654
NA	0.0116	4.0772E-01		ENSG00000285982
NA	0.0231	4.0778E-01		ENSG00000254579
NA	0.0169	4.0779E-01		ENSG00000287316
NA	0.0713	4.0812E-01	6.1237E-01	ENSG00000249042
NA	0.0688	4.0813E-01	6.1237E-01	ENSG00000290124
NA	0.0233	4.0863E-01		ENSG00000234021
NA	0.0229	4.0875E-01	6.1306E-01	ENSG00000269903
NA	0.0102	4.0911E-01		ENSG00000270710
NA	0.0138	4.0911E-01		ENSG00000279190
NA	0.0209	4.0924E-01		ENSG00000233229
NA	0.0510	4.0925E-01	6.1355E-01	ENSG00000287879
NA	0.0205	4.0943E-01		ENSG00000248243
NA	0.0363	4.0950E-01		ENSG00000232709
NA	0.0575	4.0967E-01	6.1393E-01	ENSG00000229107
NA	0.0352	4.1003E-01		ENSG00000233535
NA	0.0618	4.1006E-01	6.1426E-01	ENSG00000285637
NA	0.0316	4.1010E-01		ENSG00000232352
NA	0.0345	4.1011E-01		ENSG00000286644
NA	0.0141	4.1026E-01		ENSG00000228918
NA	0.0662	4.1032E-01	6.1444E-01	ENSG00000254186
NA	0.0216	4.1047E-01		ENSG00000243025
NA	0.0417	4.1077E-01		ENSG00000217261
NA	0.0240	4.1080E-01		ENSG00000289014
NA	0.0147	4.1083E-01		ENSG00000226022
NA	0.0229	4.1114E-01		ENSG00000271778
NA	0.0152	4.1161E-01		ENSG00000230537
NA	0.0569	4.1161E-01	6.1567E-01	ENSG00000229948
NA	0.0092	4.1163E-01		ENSG00000285660
NA	0.0173	4.1166E-01		ENSG00000253746
NA	0.0423	4.1168E-01	6.1570E-01	ENSG00000286020
NA	0.0128	4.1192E-01		ENSG00000279562
NA	0.0559	4.1203E-01	6.1601E-01	ENSG00000282542
NA	0.0384	4.1222E-01		ENSG00000285969
NA	0.0250	4.1286E-01		ENSG00000215399
NA	0.0164	4.1292E-01		ENSG00000261546
NA	0.0083	4.1296E-01		ENSG00000227563
NA	0.0101	4.1305E-01		ENSG00000275881
NA	0.0275	4.1335E-01		ENSG00000289321
NA	0.0158	4.1336E-01		ENSG00000184566
NA	0.0574	4.1366E-01	6.1726E-01	ENSG00000260269

NA	0.0248	4.1387E-01		ENSG00000233511
NA	0.0165	4.1401E-01		ENSG00000287586
NA	0.0472	4.1404E-01	6.1769E-01	ENSG00000213713
NA	0.0174	4.1422E-01		ENSG00000248692
NA	0.0824	4.1423E-01	6.1789E-01	ENSG00000287502
NA	0.0498	4.1444E-01	6.1802E-01	ENSG00000286115
NA	0.0129	4.1449E-01		ENSG00000253899
NA	0.0264	4.1457E-01	6.1810E-01	ENSG00000273825
NA	0.0649	4.1470E-01	6.1825E-01	ENSG00000260949
NA	0.0546	4.1479E-01	6.1835E-01	ENSG00000290044
NA	0.0676	4.1485E-01	6.1840E-01	ENSG00000287400
NA	0.0313	4.1499E-01		ENSG00000255057
NA	0.0425	4.1509E-01	6.1862E-01	ENSG00000280013
NA	0.0245	4.1515E-01	6.1862E-01	ENSG00000272825
NA	0.0406	4.1515E-01		ENSG00000265574
NA	0.0706	4.1519E-01	6.1865E-01	ENSG00000262728
NA	0.0125	4.1520E-01		ENSG00000285802
NA	0.0502	4.1592E-01	6.1934E-01	ENSG00000241599
NA	0.0036	4.1625E-01		ENSG00000270002
NA	0.0688	4.1643E-01	6.1990E-01	ENSG00000285702
NA	0.0521	4.1646E-01	6.1990E-01	ENSG00000288804
NA	0.0152	4.1686E-01	6.2031E-01	ENSG00000287858
NA	0.0441	4.1710E-01		ENSG00000229399
NA	0.0262	4.1718E-01		ENSG00000267783
NA	0.0156	4.1734E-01		ENSG00000230233
NA	0.0272	4.1741E-01		ENSG00000258469
NA	0.0171	4.1744E-01		ENSG00000227262
NA	0.0323	4.1746E-01		ENSG00000267764
NA	0.0637	4.1752E-01	6.2089E-01	ENSG00000289013
NA	0.0345	4.1766E-01		ENSG00000288761
NA	0.0825	4.1782E-01	6.2114E-01	ENSG00000254539
NA	0.0640	4.1816E-01	6.2141E-01	ENSG00000283236
NA	0.0386	4.1825E-01		ENSG00000287171
NA	0.0274	4.1833E-01		ENSG00000265115
NA	0.0655	4.1841E-01	6.2157E-01	ENSG00000253559
NA	0.0366	4.1867E-01		ENSG00000235224
NA	0.0716	4.1886E-01	6.2193E-01	ENSG00000272944
NA	0.0707	4.1893E-01	6.2195E-01	ENSG00000289077
NA	0.0268	4.1976E-01		ENSG00000255146
NA	0.0239	4.2008E-01		ENSG00000284309
NA	0.0080	4.2025E-01		ENSG00000223727
NA	0.0131	4.2032E-01		ENSG00000280836
NA	0.0351	4.2037E-01		ENSG00000267098
NA	0.0181	4.2041E-01		ENSG00000253347
NA	0.0668	4.2041E-01	6.2337E-01	ENSG00000258458
NA	0.0122	4.2077E-01		ENSG00000225402
NA	0.0287	4.2089E-01		ENSG00000258068
NA	0.0136	4.2117E-01		ENSG00000249623
NA	0.0636	4.2145E-01	6.2428E-01	ENSG00000288892

NA	0.0378	4.2158E-01		ENSG00000225106
NA	0.0509	4.2162E-01	6.2437E-01	ENSG00000254468
NA	0.0394	4.2182E-01	6.2448E-01	ENSG00000285930
NA	0.0670	4.2189E-01	6.2453E-01	ENSG00000230992
NA	0.0354	4.2203E-01		ENSG00000288581
NA	0.0222	4.2217E-01		ENSG00000275345
NA	0.0258	4.2229E-01		ENSG00000268970
NA	0.0653	4.2236E-01	6.2509E-01	ENSG00000279186
NA	0.0688	4.2246E-01	6.2521E-01	ENSG00000290127
NA	0.0190	4.2247E-01		ENSG00000264520
NA	0.0646	4.2265E-01	6.2536E-01	ENSG00000230092
NA	0.0413	4.2321E-01	6.2585E-01	ENSG00000203307
NA	0.0250	4.2327E-01		ENSG00000272235
NA	0.0229	4.2328E-01		ENSG00000259605
NA	0.0662	4.2338E-01	6.2590E-01	ENSG00000187229
NA	0.0613	4.2358E-01	6.2602E-01	ENSG00000268047
NA	0.0246	4.2358E-01		ENSG00000242488
NA	0.0477	4.2359E-01	6.2602E-01	ENSG00000289065
NA	0.0698	4.2366E-01	6.2604E-01	ENSG00000290121
NA	0.0678	4.2370E-01	6.2604E-01	ENSG00000224908
NA	0.0658	4.2370E-01	6.2604E-01	ENSG00000146001
NA	0.0550	4.2378E-01	6.2606E-01	ENSG00000287069
NA	0.0485	4.2422E-01	6.2661E-01	ENSG00000251637
NA	0.0575	4.2433E-01	6.2673E-01	ENSG00000278434
NA	0.0176	4.2453E-01		ENSG00000225216
NA	0.0189	4.2460E-01		ENSG00000259720
NA	0.0209	4.2475E-01		ENSG00000254080
NA	0.0263	4.2497E-01	6.2734E-01	ENSG00000272434
NA	0.0689	4.2554E-01	6.2779E-01	ENSG00000274712
NA	0.0119	4.2604E-01		ENSG00000227068
NA	0.0289	4.2612E-01		ENSG00000176654
NA	0.0290	4.2619E-01		ENSG00000262313
NA	0.0625	4.2647E-01	6.2869E-01	ENSG00000227370
NA	0.0369	4.2674E-01		ENSG00000282989
NA	0.0712	4.2685E-01	6.2892E-01	ENSG00000289558
NA	0.0195	4.2698E-01		ENSG00000235840
NA	0.0195	4.2741E-01		ENSG00000280037
NA	0.0125	4.2767E-01		ENSG00000271742
NA	0.0127	4.2768E-01		ENSG00000287792
NA	0.0390	4.2839E-01		ENSG00000214992
NA	0.0377	4.2880E-01		ENSG00000261357
NA	0.0181	4.2882E-01	6.3061E-01	ENSG00000272647
NA	0.0442	4.2883E-01		ENSG00000289175
NA	0.0256	4.2893E-01		ENSG00000226141
NA	0.0419	4.2913E-01	6.3074E-01	ENSG00000289385
NA	0.0116	4.2922E-01		ENSG00000259022
NA	0.0548	4.2949E-01	6.3109E-01	ENSG00000278974
NA	0.0357	4.3018E-01	6.3186E-01	ENSG00000266588
NA	0.0197	4.3020E-01		ENSG00000249008

NA	0.0415	4.3025E-01		ENSG00000233785
NA	0.0651	4.3036E-01	6.3199E-01	ENSG00000272768
NA	0.0216	4.3044E-01		ENSG00000235271
NA	0.0038	4.3088E-01		ENSG00000281852
NA	0.0300	4.3090E-01		ENSG00000284299
NA	0.0114	4.3103E-01		ENSG00000258740
NA	0.0486	4.3127E-01	6.3290E-01	ENSG00000258131
NA	0.0654	4.3162E-01	6.3305E-01	ENSG00000289146
NA	0.0152	4.3194E-01		ENSG00000286671
NA	0.0384	4.3206E-01		ENSG00000230310
NA	0.0103	4.3208E-01		ENSG00000264769
NA	0.0308	4.3209E-01		ENSG00000237844
NA	0.0166	4.3217E-01		ENSG00000289142
NA	0.0188	4.3231E-01		ENSG00000273212
NA	0.0669	4.3272E-01	6.3402E-01	ENSG00000280255
NA	0.0284	4.3274E-01		ENSG00000262445
NA	0.0780	4.3276E-01	6.3404E-01	ENSG00000280734
NA	0.0336	4.3289E-01		ENSG00000273591
NA	0.0244	4.3308E-01	6.3436E-01	ENSG00000259690
NA	0.0356	4.3314E-01		ENSG00000271973
NA	0.0176	4.3316E-01		ENSG00000237903
NA	0.0206	4.3319E-01		ENSG00000224851
NA	0.0444	4.3324E-01	6.3436E-01	ENSG00000273372
NA	0.0498	4.3324E-01	6.3436E-01	ENSG00000262312
NA	0.0238	4.3345E-01		ENSG00000287998
NA	0.0144	4.3348E-01		ENSG00000259584
NA	0.0168	4.3391E-01		ENSG00000256159
NA	0.0498	4.3398E-01	6.3509E-01	ENSG00000244490
NA	0.0065	4.3404E-01		ENSG00000144188
NA	0.0093	4.3410E-01		ENSG00000232894
NA	0.0100	4.3413E-01		ENSG00000199691
NA	0.0212	4.3467E-01		ENSG00000286846
NA	0.0143	4.3476E-01		ENSG00000259003
NA	0.0177	4.3486E-01		ENSG00000273786
NA	0.0431	4.3494E-01	6.3598E-01	ENSG00000266236
NA	0.0278	4.3498E-01		ENSG00000233242
NA	0.0238	4.3500E-01		ENSG00000223475
NA	0.0643	4.3500E-01	6.3600E-01	ENSG00000240401
NA	0.0092	4.3516E-01		ENSG00000225498
NA	0.0157	4.3517E-01		ENSG00000223379
NA	0.0136	4.3529E-01		ENSG00000228380
NA	0.0204	4.3536E-01		ENSG00000260536
NA	0.0186	4.3568E-01		ENSG00000251537
NA	0.0109	4.3584E-01		ENSG00000177418
NA	0.0162	4.3618E-01		ENSG00000227702
NA	0.0139	4.3630E-01		ENSG00000255456
NA	0.0102	4.3644E-01		ENSG00000227033
NA	0.0129	4.3644E-01	6.3728E-01	ENSG00000279414
NA	0.0392	4.3660E-01	6.3728E-01	ENSG00000249020

NA	0.0210	4.3661E-01		ENSG00000287446
NA	0.0297	4.3703E-01		ENSG00000287026
NA	0.0213	4.3707E-01		ENSG00000259121
NA	0.0578	4.3716E-01	6.3776E-01	ENSG00000232234
NA	0.0483	4.3752E-01	6.3808E-01	ENSG00000286167
NA	0.0663	4.3763E-01	6.3812E-01	ENSG00000205740
NA	0.0399	4.3785E-01	6.3830E-01	ENSG00000257222
NA	0.0467	4.3796E-01	6.3839E-01	ENSG00000280776
NA	0.0015	4.3823E-01		ENSG00000228005
NA	0.0261	4.3837E-01		ENSG00000183674
NA	0.0167	4.3882E-01		ENSG00000287195
NA	0.0183	4.3887E-01		ENSG00000249737
NA	0.0141	4.3902E-01		ENSG00000231394
NA	0.0194	4.3931E-01		ENSG00000235494
NA	0.0315	4.3952E-01		ENSG00000258232
NA	0.0334	4.3958E-01		ENSG00000271267
NA	0.0227	4.3981E-01		ENSG00000236885
NA	0.0107	4.4011E-01		ENSG00000254757
NA	0.0665	4.4019E-01	6.4011E-01	ENSG00000214248
NA	0.0215	4.4023E-01		ENSG00000286915
NA	0.0379	4.4029E-01	6.4019E-01	ENSG00000241641
NA	0.0416	4.4112E-01		ENSG00000264016
NA	0.0243	4.4121E-01	6.4092E-01	ENSG00000254557
NA	0.0585	4.4188E-01	6.4129E-01	ENSG00000267199
NA	0.0556	4.4194E-01	6.4134E-01	ENSG00000257023
NA	0.0159	4.4234E-01		ENSG00000253207
NA	0.0319	4.4244E-01	6.4177E-01	ENSG00000230587
NA	0.0105	4.4249E-01		ENSG00000227080
NA	0.0380	4.4276E-01	6.4208E-01	ENSG00000280211
NA	0.0116	4.4288E-01		ENSG00000253853
NA	0.0172	4.4293E-01		ENSG00000286142
NA	0.0134	4.4294E-01		ENSG00000225752
NA	0.0186	4.4297E-01		ENSG00000266744
NA	0.0130	4.4306E-01		ENSG00000279246
NA	0.0358	4.4337E-01		ENSG00000259648
NA	0.0397	4.4363E-01		ENSG00000231741
NA	0.0641	4.4374E-01	6.4286E-01	ENSG00000289574
NA	0.0329	4.4391E-01	6.4294E-01	ENSG00000259840
NA	0.0305	4.4403E-01		ENSG00000236062
NA	0.0321	4.4418E-01		ENSG00000275481
NA	0.0235	4.4435E-01		ENSG00000258571
NA	0.0049	4.4438E-01		ENSG00000286283
NA	0.0669	4.4445E-01	6.4339E-01	ENSG00000185065
NA	0.0630	4.4474E-01	6.4368E-01	ENSG00000283045
NA	0.0103	4.4494E-01		ENSG00000212743
NA	0.0136	4.4495E-01		ENSG00000254391
NA	0.0140	4.4515E-01		ENSG00000286981
NA	0.0526	4.4523E-01	6.4422E-01	ENSG00000276282
NA	0.0190	4.4608E-01		ENSG00000255104

NA	0.0009	4.4614E-01		ENSG00000223878
NA	0.0241	4.4655E-01		ENSG00000231344
NA	0.0336	4.4676E-01		ENSG00000267594
NA	0.0376	4.4689E-01	6.4639E-01	ENSG00000270522
NA	0.0526	4.4699E-01	6.4645E-01	ENSG00000230450
NA	0.0290	4.4723E-01		ENSG00000278068
NA	0.0447	4.4729E-01		ENSG00000217929
NA	0.0135	4.4730E-01		ENSG00000260338
NA	0.0579	4.4759E-01	6.4698E-01	ENSG00000289133
NA	0.0368	4.4769E-01	6.4701E-01	ENSG00000271857
NA	0.0111	4.4769E-01		ENSG00000286661
NA	0.0207	4.4814E-01		ENSG00000279721
NA	0.0150	4.4820E-01		ENSG00000267405
NA	0.0381	4.4823E-01	6.4746E-01	ENSG00000285904
NA	0.0157	4.4833E-01		ENSG00000219565
NA	0.0073	4.4836E-01		ENSG00000236028
NA	0.0190	4.4888E-01		ENSG00000280169
NA	0.0333	4.4919E-01		ENSG00000225886
NA	0.0505	4.4926E-01	6.4836E-01	ENSG00000286045
NA	0.0195	4.4933E-01		ENSG00000235546
NA	0.0650	4.4973E-01	6.4885E-01	ENSG00000269378
NA	0.0127	4.4973E-01		ENSG00000232783
NA	0.0491	4.5002E-01	6.4924E-01	ENSG00000259158
NA	0.0534	4.5050E-01	6.4974E-01	ENSG00000227279
NA	0.0082	4.5065E-01		ENSG00000286414
NA	0.0503	4.5123E-01	6.5022E-01	ENSG00000133475
NA	0.0157	4.5124E-01		ENSG00000287141
NA	0.0566	4.5147E-01	6.5041E-01	ENSG00000280193
NA	0.0162	4.5168E-01		ENSG00000267341
NA	0.0179	4.5213E-01		ENSG00000228539
NA	0.0161	4.5233E-01		ENSG00000224713
NA	0.0656	4.5237E-01	6.5124E-01	ENSG00000272663
NA	0.0280	4.5247E-01		ENSG00000237212
NA	0.0540	4.5272E-01	6.5149E-01	ENSG00000271971
NA	0.0328	4.5282E-01		ENSG00000272506
NA	0.0381	4.5304E-01		ENSG00000285560
NA	0.0283	4.5319E-01		ENSG00000272832
NA	0.0284	4.5335E-01		ENSG00000236452
NA	0.0136	4.5337E-01		ENSG00000255361
NA	0.0140	4.5365E-01		ENSG00000279958
NA	0.0110	4.5383E-01		ENSG00000236601
NA	0.0646	4.5388E-01	6.5247E-01	ENSG00000234019
NA	0.0341	4.5420E-01	6.5263E-01	ENSG00000276968
NA	0.0373	4.5439E-01	6.5287E-01	ENSG00000230223
NA	0.0049	4.5441E-01		ENSG00000288007
NA	0.0164	4.5451E-01		ENSG00000238224
NA	0.0334	4.5465E-01		ENSG00000233090
NA	0.0611	4.5475E-01	6.5323E-01	ENSG00000277476
NA	0.0627	4.5481E-01	6.5327E-01	ENSG00000260464

NA	0.0424	4.5489E-01	6.5332E-01	ENSG00000285550
NA	0.0176	4.5493E-01		ENSG00000290100
NA	0.0350	4.5507E-01		ENSG00000239767
NA	0.0410	4.5524E-01	6.5359E-01	ENSG00000279995
NA	0.0096	4.5538E-01		ENSG00000289601
NA	0.0133	4.5583E-01		ENSG00000287503
NA	0.0276	4.5590E-01		ENSG00000288101
NA	0.0421	4.5602E-01	6.5433E-01	ENSG00000170089
NA	0.0620	4.5613E-01	6.5440E-01	ENSG00000184068
NA	0.0401	4.5631E-01	6.5458E-01	ENSG00000240996
NA	0.0191	4.5639E-01		ENSG00000237373
NA	0.0150	4.5658E-01		ENSG00000206846
NA	0.0239	4.5660E-01		ENSG00000262529
NA	0.0385	4.5671E-01	6.5493E-01	ENSG00000273007
NA	0.0192	4.5714E-01		ENSG00000249425
NA	0.0038	4.5715E-01		ENSG00000220614
NA	0.0114	4.5716E-01		ENSG00000286040
NA	0.0233	4.5725E-01		ENSG00000228817
NA	0.0372	4.5726E-01	6.5547E-01	ENSG00000288958
NA	0.0450	4.5743E-01	6.5558E-01	ENSG00000233200
NA	0.0200	4.5745E-01		ENSG00000231297
NA	0.0128	4.5765E-01		ENSG00000228620
NA	0.0502	4.5773E-01	6.5578E-01	ENSG00000267372
NA	0.0086	4.5780E-01		ENSG00000290089
NA	0.0420	4.5785E-01	6.5591E-01	ENSG00000287265
NA	0.0490	4.5790E-01	6.5591E-01	ENSG00000224356
NA	0.0251	4.5804E-01		ENSG00000234577
NA	0.0103	4.5823E-01		ENSG00000284623
NA	0.0355	4.5854E-01		ENSG00000286011
NA	0.0104	4.5876E-01		ENSG00000236510
NA	0.0133	4.5891E-01		ENSG00000236184
NA	0.0222	4.5921E-01		ENSG00000279982
NA	0.0108	4.5932E-01		ENSG00000250064
NA	0.0624	4.5951E-01	6.5726E-01	ENSG00000287110
NA	0.0329	4.5962E-01		ENSG00000259707
NA	0.0176	4.5980E-01		ENSG00000272564
NA	0.0237	4.5984E-01		ENSG00000258312
NA	0.0630	4.6037E-01	6.5803E-01	ENSG00000259683
NA	0.0327	4.6038E-01	6.5803E-01	ENSG00000253394
NA	0.0189	4.6053E-01		ENSG00000278344
NA	0.0520	4.6071E-01	6.5825E-01	ENSG00000176289
NA	0.0463	4.6087E-01	6.5837E-01	ENSG00000271167
NA	0.0260	4.6087E-01	6.5837E-01	ENSG00000214100
NA	0.0110	4.6087E-01		ENSG00000288853
NA	0.0284	4.6103E-01		ENSG00000287551
NA	0.0630	4.6119E-01	6.5852E-01	ENSG00000227775
NA	0.0062	4.6131E-01		ENSG00000286999
NA	0.0494	4.6162E-01	6.5882E-01	ENSG00000278383
NA	0.0304	4.6176E-01		ENSG00000230863

NA	0.0397	4.6188E-01	6.5900E-01	ENSG00000279028
NA	0.0359	4.6191E-01	6.5900E-01	ENSG00000248456
NA	0.0280	4.6191E-01		ENSG00000258215
NA	0.0077	4.6229E-01		ENSG00000267088
NA	0.0135	4.6269E-01		ENSG00000256633
NA	0.0229	4.6281E-01		ENSG00000224121
NA	0.0266	4.6297E-01		ENSG00000174325
NA	0.0026	4.6304E-01		ENSG00000260382
NA	0.0613	4.6319E-01	6.6037E-01	ENSG00000271904
NA	0.0238	4.6338E-01		ENSG00000261687
NA	0.0504	4.6344E-01		ENSG00000288700
NA	0.0188	4.6347E-01		ENSG00000236453
NA	0.0187	4.6352E-01		ENSG00000187999
NA	0.0522	4.6355E-01	6.6061E-01	ENSG00000267737
NA	0.0292	4.6355E-01		ENSG00000270689
NA	0.0488	4.6362E-01	6.6064E-01	ENSG00000286808
NA	0.0568	4.6386E-01	6.6087E-01	ENSG00000261790
NA	0.0197	4.6412E-01		ENSG00000231662
NA	0.0131	4.6457E-01		ENSG00000287030
NA	0.0373	4.6475E-01	6.6150E-01	ENSG00000268231
NA	0.0247	4.6480E-01		ENSG00000286686
NA	0.0267	4.6513E-01		ENSG00000267108
NA	0.0358	4.6583E-01	6.6245E-01	ENSG00000276740
NA	0.0619	4.6593E-01	6.6256E-01	ENSG00000250159
NA	0.0628	4.6602E-01	6.6257E-01	ENSG00000288839
NA	0.0253	4.6612E-01		ENSG00000249131
NA	0.0618	4.6612E-01	6.6268E-01	ENSG00000273271
NA	0.0306	4.6614E-01		ENSG00000289896
NA	0.0213	4.6631E-01		ENSG00000286828
NA	0.0590	4.6660E-01	6.6325E-01	ENSG00000278238
NA	0.0478	4.6694E-01	6.6358E-01	ENSG00000265136
NA	0.0080	4.6702E-01		ENSG00000236823
NA	0.0347	4.6751E-01	6.6415E-01	ENSG00000250600
NA	0.0356	4.6764E-01		ENSG00000260366
NA	0.0297	4.6766E-01		ENSG00000289376
NA	0.0160	4.6781E-01	6.6442E-01	ENSG00000260041
NA	0.0364	4.6786E-01	6.6442E-01	ENSG00000268375
NA	0.0124	4.6814E-01		ENSG00000248958
NA	0.0228	4.6818E-01		ENSG00000229161
NA	0.0459	4.6820E-01	6.6478E-01	ENSG00000235493
NA	0.0567	4.6831E-01	6.6490E-01	ENSG00000198155
NA	0.0110	4.6833E-01		ENSG00000277883
NA	0.0104	4.6862E-01		ENSG00000287098
NA	0.0345	4.6864E-01		ENSG00000232912
NA	0.0174	4.6873E-01		ENSG00000229853
NA	0.0193	4.6883E-01		ENSG00000234311
NA	0.0274	4.6923E-01		ENSG00000286742
NA	0.0513	4.6944E-01	6.6582E-01	ENSG00000275632
NA	0.0535	4.6945E-01	6.6582E-01	ENSG00000218226

NA	0.0538	4.6945E-01	6.6582E-01	ENSG00000261117
NA	0.0324	4.6974E-01		ENSG00000284636
NA	0.0832	4.7010E-01	6.6645E-01	ENSG00000261235
NA	0.0538	4.7013E-01	6.6645E-01	ENSG00000279489
NA	0.0518	4.7026E-01	6.6652E-01	ENSG00000287878
NA	0.0152	4.7034E-01	6.6655E-01	ENSG00000228886
NA	0.0366	4.7034E-01		ENSG00000200579
NA	0.0179	4.7042E-01		ENSG00000274695
NA	0.0176	4.7045E-01		ENSG00000230280
NA	0.0118	4.7079E-01		ENSG00000227078
NA	0.0383	4.7110E-01		ENSG00000224077
NA	0.0167	4.7110E-01		ENSG00000289241
NA	0.0456	4.7111E-01	6.6737E-01	ENSG00000257027
NA	0.0183	4.7125E-01		ENSG00000259771
NA	0.0447	4.7134E-01		ENSG00000239040
NA	0.0196	4.7154E-01		ENSG00000213514
NA	0.0284	4.7168E-01		ENSG00000253784
NA	0.0294	4.7174E-01		ENSG00000234493
NA	0.0186	4.7196E-01		ENSG00000229497
NA	0.0079	4.7215E-01		ENSG00000287426
NA	0.0115	4.7236E-01		ENSG00000231867
NA	0.0289	4.7242E-01		ENSG00000285791
NA	0.0606	4.7242E-01	6.6862E-01	ENSG00000260086
NA	0.0518	4.7270E-01	6.6887E-01	ENSG00000280379
NA	0.0519	4.7286E-01	6.6894E-01	ENSG00000286961
NA	0.0159	4.7303E-01		ENSG00000270789
NA	0.0121	4.7308E-01		ENSG00000199732
NA	0.0155	4.7318E-01		ENSG00000276691
NA	0.0198	4.7318E-01		ENSG00000235052
NA	0.0449	4.7326E-01	6.6929E-01	ENSG00000260103
NA	0.0405	4.7330E-01	6.6929E-01	ENSG00000237821
NA	0.0346	4.7359E-01		ENSG00000234699
NA	0.0560	4.7412E-01	6.7003E-01	ENSG00000275645
NA	0.0600	4.7421E-01	6.7008E-01	ENSG00000287763
NA	0.0360	4.7431E-01	6.7008E-01	ENSG00000213540
NA	0.0109	4.7432E-01		ENSG00000237325
NA	0.0127	4.7457E-01		ENSG00000287649
NA	0.0651	4.7469E-01	6.7052E-01	ENSG00000279799
NA	0.0434	4.7484E-01	6.7059E-01	ENSG00000265008
NA	0.0616	4.7501E-01	6.7062E-01	ENSG00000266904
NA	0.0218	4.7513E-01		ENSG00000230432
NA	0.0070	4.7535E-01		ENSG00000258871
NA	0.0090	4.7539E-01		ENSG00000263717
NA	0.0166	4.7559E-01		ENSG00000260668
NA	0.0427	4.7588E-01	6.7134E-01	ENSG00000273557
NA	0.0614	4.7598E-01	6.7141E-01	ENSG00000290094
NA	0.0083	4.7660E-01		ENSG00000187762
NA	0.0128	4.7689E-01		ENSG00000233754
NA	0.0063	4.7701E-01		ENSG00000254913

NA	0.0612	4.7710E-01	6.7249E-01	ENSG00000286667
NA	0.0095	4.7731E-01		ENSG00000287224
NA	0.0094	4.7742E-01		ENSG00000221042
NA	0.0157	4.7761E-01		ENSG00000289848
NA	0.0397	4.7773E-01	6.7308E-01	ENSG00000272711
NA	0.0138	4.7786E-01		ENSG00000288685
NA	0.0297	4.7804E-01	6.7338E-01	ENSG00000230490
NA	0.0041	4.7825E-01		ENSG00000278819
NA	0.0231	4.7828E-01		ENSG00000231128
NA	0.0109	4.7828E-01		ENSG00000237586
NA	0.0113	4.7845E-01		ENSG00000228655
NA	0.0166	4.7848E-01		ENSG00000254258
NA	0.0113	4.7854E-01		ENSG00000266957
NA	0.0168	4.7855E-01		ENSG00000224689
NA	0.0157	4.7855E-01		ENSG00000253483
NA	0.0351	4.7874E-01		ENSG00000286271
NA	0.0423	4.7876E-01		ENSG00000254401
NA	0.0185	4.7892E-01		ENSG00000243831
NA	0.0138	4.7906E-01		ENSG00000288800
NA	0.0326	4.7934E-01		ENSG00000273724
NA	0.0053	4.7964E-01		ENSG00000287062
NA	0.0208	4.7968E-01		ENSG00000279930
NA	0.0179	4.7974E-01		ENSG00000234084
NA	0.0608	4.8029E-01	6.7525E-01	ENSG00000272277
NA	0.0035	4.8061E-01		ENSG00000271882
NA	0.0411	4.8070E-01	6.7548E-01	ENSG00000289736
NA	0.0007	4.8072E-01		ENSG00000269729
NA	0.0234	4.8098E-01		ENSG00000280431
NA	0.0152	4.8109E-01		ENSG00000233589
NA	0.0139	4.8114E-01		ENSG00000255109
NA	0.0196	4.8119E-01		ENSG00000230848
NA	0.0601	4.8120E-01	6.7583E-01	ENSG00000225742
NA	0.0153	4.8152E-01		ENSG00000256433
NA	0.0236	4.8153E-01		ENSG00000286272
NA	0.0317	4.8162E-01		ENSG00000232295
NA	0.0435	4.8174E-01	6.7624E-01	ENSG00000272384
NA	0.0124	4.8179E-01		ENSG00000248283
NA	0.0065	4.8190E-01		ENSG00000240535
NA	0.0249	4.8197E-01		ENSG00000224029
NA	0.0458	4.8216E-01	6.7666E-01	ENSG00000248475
NA	0.0089	4.8242E-01		ENSG00000233571
NA	0.0031	4.8243E-01		ENSG00000266599
NA	0.0298	4.8255E-01		ENSG00000231703
NA	0.0200	4.8268E-01		ENSG00000289690
NA	0.0401	4.8284E-01	6.7716E-01	ENSG00000263624
NA	0.0181	4.8290E-01		ENSG00000229533
NA	0.0143	4.8299E-01		ENSG00000259529
NA	0.0244	4.8304E-01		ENSG00000258225
NA	0.0353	4.8310E-01	6.7731E-01	ENSG00000280269

NA	0.0383	4.8312E-01	6.7731E-01	ENSG00000268496
NA	0.0212	4.8325E-01		ENSG00000249084
NA	0.0304	4.8331E-01		ENSG00000227067
NA	0.0121	4.8361E-01		ENSG00000226790
NA	0.0329	4.8365E-01	6.7777E-01	ENSG00000287715
NA	0.0153	4.8372E-01		ENSG00000231758
NA	0.0165	4.8398E-01		ENSG00000230833
NA	0.0457	4.8407E-01	6.7805E-01	ENSG00000267927
NA	0.0476	4.8410E-01	6.7805E-01	ENSG00000267582
NA	0.0106	4.8416E-01		ENSG00000286746
NA	0.0246	4.8457E-01	6.7829E-01	ENSG00000259937
NA	0.0542	4.8458E-01	6.7829E-01	ENSG00000287276
NA	0.0128	4.8461E-01		ENSG00000289076
NA	0.0400	4.8503E-01	6.7885E-01	ENSG00000234367
NA	0.0208	4.8508E-01		ENSG00000289377
NA	0.0083	4.8576E-01		ENSG00000267246
NA	0.0288	4.8587E-01		ENSG00000285692
NA	0.0554	4.8605E-01	6.7981E-01	ENSG00000217027
NA	0.0056	4.8609E-01		ENSG00000289418
NA	0.0162	4.8615E-01		ENSG00000280205
NA	0.0083	4.8678E-01		ENSG00000234860
NA	0.0398	4.8682E-01	6.8026E-01	ENSG00000273448
NA	0.0469	4.8691E-01	6.8026E-01	ENSG00000289267
NA	0.0517	4.8700E-01	6.8030E-01	ENSG00000289126
NA	0.0139	4.8706E-01		ENSG00000289550
NA	0.0144	4.8710E-01		ENSG00000261395
NA	0.0084	4.8758E-01		ENSG00000267412
NA	0.0197	4.8791E-01		ENSG00000278876
NA	0.0449	4.8806E-01	6.8127E-01	ENSG00000287460
NA	0.0206	4.8828E-01		ENSG00000274833
NA	0.0230	4.8864E-01		ENSG00000271259
NA	0.0090	4.8889E-01		ENSG00000267507
NA	0.0172	4.8904E-01		ENSG00000242411
NA	0.0384	4.8959E-01	6.8252E-01	ENSG00000283317
NA	0.0398	4.8969E-01	6.8252E-01	ENSG00000275956
NA	0.0470	4.8974E-01	6.8252E-01	ENSG00000273812
NA	0.0153	4.9025E-01	6.8299E-01	ENSG00000285605
NA	0.0516	4.9056E-01	6.8328E-01	ENSG00000221184
NA	0.0536	4.9087E-01	6.8345E-01	ENSG00000205485
NA	0.0571	4.9098E-01	6.8345E-01	ENSG00000225868
NA	0.0429	4.9109E-01	6.8345E-01	ENSG00000269397
NA	0.0363	4.9118E-01	6.8348E-01	ENSG00000274678
NA	0.0477	4.9186E-01	6.8401E-01	ENSG00000279277
NA	0.0059	4.9203E-01		ENSG00000286807
NA	0.0342	4.9210E-01		ENSG00000259528
NA	0.0204	4.9214E-01		ENSG00000240015
NA	0.0273	4.9228E-01		ENSG00000257830
NA	0.0114	4.9247E-01		ENSG00000227094
NA	0.0105	4.9275E-01		ENSG00000232208

NA	0.0403	4.9275E-01	6.8482E-01	ENSG00000267192
NA	0.0049	4.9280E-01		ENSG00000252975
NA	0.0395	4.9291E-01	6.8488E-01	ENSG00000287209
NA	0.0101	4.9296E-01		ENSG00000268100
NA	0.0493	4.9313E-01	6.8512E-01	ENSG00000240661
NA	0.0135	4.9366E-01		ENSG00000289479
NA	0.0388	4.9387E-01	6.8595E-01	ENSG00000261407
NA	0.0137	4.9393E-01		ENSG00000267691
NA	0.0559	4.9422E-01	6.8632E-01	ENSG00000106133
NA	0.0280	4.9437E-01		ENSG00000270020
NA	0.0052	4.9443E-01		ENSG00000260070
NA	0.0401	4.9455E-01	6.8663E-01	ENSG00000273004
NA	0.0609	4.9498E-01	6.8685E-01	ENSG00000234134
NA	0.0638	4.9499E-01	6.8685E-01	ENSG00000233189
NA	0.0397	4.9526E-01		ENSG00000267004
NA	0.0167	4.9534E-01		ENSG00000279567
NA	0.0266	4.9536E-01		ENSG00000227128
NA	0.0492	4.9544E-01	6.8717E-01	ENSG00000287279
NA	0.0103	4.9563E-01		ENSG00000241350
NA	0.0424	4.9582E-01	6.8758E-01	ENSG00000267299
NA	0.0232	4.9584E-01		ENSG00000239465
NA	0.0091	4.9611E-01		ENSG00000257880
NA	0.0301	4.9626E-01		ENSG00000230506
NA	0.0524	4.9630E-01	6.8781E-01	ENSG00000258903
NA	0.0211	4.9637E-01		ENSG00000248343
NA	0.0219	4.9646E-01		ENSG00000240793
NA	0.0477	4.9667E-01	6.8809E-01	ENSG00000237343
NA	0.0145	4.9667E-01		ENSG00000272688
NA	0.0570	4.9678E-01	6.8816E-01	ENSG00000250986
NA	0.0067	4.9683E-01		ENSG00000231482
NA	0.0442	4.9685E-01	6.8819E-01	ENSG00000283709
NA	0.0145	4.9739E-01		ENSG00000251685
NA	0.0339	4.9748E-01		ENSG00000285696
NA	0.0152	4.9749E-01		ENSG00000204894
NA	0.0133	4.9777E-01		ENSG00000289513
NA	0.0151	4.9782E-01		ENSG00000222788
NA	0.0573	4.9889E-01	6.8985E-01	ENSG00000205464
NA	0.0068	4.9905E-01		ENSG00000200610
NA	0.0157	4.9918E-01		ENSG00000225172
NA	0.0137	4.9927E-01		ENSG00000286085
NA	0.0032	4.9949E-01		ENSG00000231189
NA	0.0220	4.9955E-01		ENSG00000281162
NA	0.0369	4.9967E-01		ENSG00000227338
NA	0.0279	4.9990E-01		ENSG00000277313
NA	0.0114	5.0004E-01	6.9089E-01	ENSG00000289408
NA	0.0157	5.0009E-01		ENSG00000229278
NA	0.0543	5.0012E-01	6.9092E-01	ENSG00000274213
NA	0.0127	5.0078E-01		ENSG00000271916
NA	0.0439	5.0085E-01	6.9169E-01	ENSG00000233221

NA	0.0093	5.0096E-01	6.9178E-01	ENSG00000280334
NA	0.0357	5.0155E-01	6.9215E-01	ENSG00000289210
NA	0.0132	5.0158E-01		ENSG00000225513
NA	0.0379	5.0159E-01	6.9215E-01	ENSG00000214018
NA	0.0559	5.0169E-01	6.9225E-01	ENSG00000288663
NA	0.0337	5.0176E-01		ENSG00000287903
NA	0.0375	5.0189E-01	6.9240E-01	ENSG00000268520
NA	0.0287	5.0190E-01		ENSG00000258603
NA	0.0303	5.0218E-01		ENSG00000188459
NA	0.0499	5.0229E-01	6.9282E-01	ENSG00000286373
NA	0.0216	5.0232E-01		ENSG00000242737
NA	0.0427	5.0249E-01	6.9296E-01	ENSG00000268836
NA	0.0135	5.0260E-01		ENSG00000224295
NA	0.0184	5.0262E-01		ENSG00000237417
NA	0.0237	5.0269E-01		ENSG00000258399
NA	0.0185	5.0297E-01		ENSG00000273123
NA	0.0446	5.0301E-01	6.9351E-01	ENSG00000241772
NA	0.0058	5.0301E-01		ENSG00000277829
NA	0.0229	5.0314E-01		ENSG00000289346
NA	0.0338	5.0328E-01	6.9370E-01	ENSG00000234418
NA	0.0638	5.0382E-01	6.9420E-01	ENSG00000223675
NA	0.0165	5.0389E-01		ENSG00000287413
NA	0.0208	5.0414E-01		ENSG00000270983
NA	0.0575	5.0418E-01	6.9434E-01	ENSG00000259685
NA	0.0098	5.0440E-01		ENSG00000243415
NA	0.0113	5.0444E-01		ENSG00000256748
NA	0.0128	5.0488E-01		ENSG00000230479
NA	0.0189	5.0513E-01		ENSG00000254447
NA	0.0241	5.0548E-01		ENSG00000253500
NA	0.0348	5.0550E-01	6.9527E-01	ENSG00000280485
NA	0.0293	5.0554E-01		ENSG00000261419
NA	0.0366	5.0572E-01	6.9547E-01	ENSG00000278864
NA	0.0152	5.0574E-01		ENSG00000260514
NA	0.0095	5.0575E-01		ENSG00000249614
NA	0.0286	5.0580E-01		ENSG00000224751
NA	0.0135	5.0581E-01		ENSG00000286735
NA	0.0053	5.0585E-01		ENSG00000207091
NA	0.0268	5.0587E-01		ENSG00000273257
NA	0.0487	5.0588E-01	6.9561E-01	ENSG00000287250
NA	0.0231	5.0590E-01		ENSG00000205879
NA	0.0214	5.0605E-01		ENSG00000282944
NA	0.0115	5.0605E-01		ENSG00000244062
NA	0.0434	5.0624E-01	6.9598E-01	ENSG00000288648
NA	0.0551	5.0637E-01	6.9604E-01	ENSG00000258634
NA	0.0055	5.0670E-01		ENSG00000274017
NA	0.0418	5.0688E-01	6.9620E-01	ENSG00000252473
NA	0.0103	5.0718E-01		ENSG00000216560
NA	0.0468	5.0737E-01	6.9641E-01	ENSG00000268670
NA	0.0293	5.0767E-01		ENSG00000285919

NA	0.0101	5.0767E-01		ENSG00000267179
NA	0.0561	5.0777E-01	6.9672E-01	ENSG00000276298
NA	0.0379	5.0800E-01	6.9692E-01	ENSG00000285672
NA	0.0345	5.0817E-01		ENSG00000223947
NA	0.0150	5.0837E-01		ENSG00000267373
NA	0.0350	5.0853E-01	6.9754E-01	ENSG00000206145
NA	0.0169	5.0861E-01		ENSG00000280104
NA	0.0316	5.0871E-01		ENSG00000225342
NA	0.0202	5.0875E-01		ENSG00000218834
NA	0.0354	5.0912E-01		ENSG00000243187
NA	0.0077	5.0917E-01	6.9806E-01	ENSG00000287011
NA	0.0132	5.0944E-01		ENSG00000229912
NA	0.0040	5.0950E-01		ENSG00000235572
NA	0.0519	5.0955E-01	6.9834E-01	ENSG00000278909
NA	0.0333	5.0968E-01	6.9849E-01	ENSG00000259459
NA	0.0282	5.0978E-01		ENSG00000273179
NA	0.0286	5.0982E-01		ENSG00000285934
NA	0.0094	5.1014E-01		ENSG00000272443
NA	0.0263	5.1017E-01		ENSG00000265511
NA	0.0095	5.1018E-01		ENSG00000279220
NA	0.0202	5.1038E-01		ENSG00000232448
NA	0.0126	5.1056E-01		ENSG00000251026
NA	0.0081	5.1071E-01		ENSG00000258854
NA	0.0106	5.1077E-01	6.9928E-01	ENSG00000273687
NA	0.0118	5.1086E-01		ENSG00000227179
NA	0.0381	5.1089E-01	6.9940E-01	ENSG00000260891
NA	0.0321	5.1099E-01	6.9947E-01	ENSG00000289365
NA	0.0173	5.1124E-01		ENSG00000286425
NA	0.0174	5.1131E-01		ENSG00000213608
NA	0.0171	5.1162E-01		ENSG00000231188
NA	0.0673	5.1194E-01	7.0030E-01	ENSG00000261140
NA	0.0328	5.1201E-01		ENSG00000258881
NA	0.0132	5.1208E-01		ENSG00000250978
NA	0.0312	5.1208E-01		ENSG00000267275
NA	0.0146	5.1229E-01		ENSG00000289975
NA	0.0343	5.1232E-01	7.0032E-01	ENSG00000287133
NA	0.0268	5.1235E-01		ENSG00000219992
NA	0.0126	5.1244E-01		ENSG00000256403
NA	0.0299	5.1307E-01	7.0099E-01	ENSG00000270462
NA	0.0352	5.1311E-01	7.0100E-01	ENSG00000215304
NA	0.0394	5.1314E-01	7.0100E-01	ENSG00000226008
NA	0.0202	5.1318E-01		ENSG00000254776
NA	0.0461	5.1328E-01	7.0113E-01	ENSG00000272033
NA	0.0190	5.1342E-01		ENSG00000285736
NA	0.0058	5.1378E-01		ENSG00000272945
NA	0.0102	5.1396E-01		ENSG00000259514
NA	0.0365	5.1404E-01	7.0181E-01	ENSG00000251079
NA	0.0133	5.1409E-01		ENSG00000231802
NA	0.0269	5.1410E-01		ENSG00000239899

NA	0.0340	5.1415E-01	7.0191E-01	ENSG00000266202
NA	0.0412	5.1434E-01	7.0198E-01	ENSG00000263041
NA	0.0131	5.1446E-01		ENSG00000289987
NA	0.0195	5.1487E-01		ENSG00000258661
NA	0.0214	5.1504E-01		ENSG00000268754
NA	0.0486	5.1517E-01	7.0284E-01	ENSG00000286545
NA	0.0095	5.1525E-01		ENSG00000286417
NA	0.0336	5.1542E-01	7.0307E-01	ENSG00000272137
NA	0.0229	5.1572E-01		ENSG00000267692
NA	0.0529	5.1587E-01	7.0345E-01	ENSG00000267750
NA	0.0038	5.1632E-01		ENSG00000153923
NA	0.0255	5.1658E-01		ENSG00000205822
NA	0.0091	5.1669E-01		ENSG00000226708
NA	0.0274	5.1679E-01		ENSG00000236485
NA	0.0492	5.1681E-01	7.0434E-01	ENSG00000283103
NA	0.0106	5.1683E-01		ENSG00000277199
NA	0.0125	5.1709E-01		ENSG00000287067
NA	0.0458	5.1712E-01	7.0459E-01	ENSG00000259002
NA	0.0325	5.1764E-01		ENSG00000255260
NA	0.0316	5.1777E-01	7.0518E-01	ENSG00000248626
NA	0.0173	5.1788E-01		ENSG00000285684
NA	0.0317	5.1800E-01		ENSG00000202310
NA	0.0464	5.1835E-01	7.0562E-01	ENSG00000264365
NA	0.0077	5.1868E-01		ENSG00000256542
NA	0.0429	5.1868E-01	7.0591E-01	ENSG00000279786
NA	0.0257	5.1888E-01		ENSG00000259521
NA	0.0247	5.1899E-01		ENSG00000254431
NA	0.0114	5.1914E-01		ENSG00000275875
NA	0.0525	5.1917E-01	7.0638E-01	ENSG00000272853
NA	0.0468	5.1920E-01	7.0638E-01	ENSG00000232876
NA	0.0246	5.1930E-01		ENSG00000268938
NA	0.0204	5.1953E-01		ENSG00000249959
NA	0.0113	5.1953E-01		ENSG00000230898
NA	0.0247	5.1955E-01		ENSG00000203496
NA	0.0101	5.1996E-01		ENSG00000254114
NA	0.0269	5.2001E-01	7.0703E-01	ENSG00000275409
NA	0.0546	5.2002E-01	7.0703E-01	ENSG00000253408
NA	0.0266	5.2052E-01		ENSG00000272745
NA	0.0107	5.2054E-01		ENSG00000255605
NA	0.0537	5.2059E-01	7.0753E-01	ENSG00000264107
NA	0.0364	5.2067E-01		ENSG00000286136
NA	0.0337	5.2074E-01	7.0760E-01	ENSG00000256984
NA	0.0506	5.2078E-01	7.0760E-01	ENSG00000288868
NA	0.0095	5.2085E-01		ENSG00000250509
NA	0.0381	5.2091E-01	7.0761E-01	ENSG00000274038
NA	0.0408	5.2102E-01	7.0772E-01	ENSG00000273287
NA	0.0172	5.2113E-01		ENSG00000271623
NA	0.0135	5.2131E-01		ENSG00000239736
NA	0.0540	5.2144E-01	7.0814E-01	ENSG00000226067

NA	0.0382	5.2153E-01	7.0823E-01	ENSG00000236963
NA	0.0251	5.2209E-01		ENSG00000201297
NA	0.0164	5.2222E-01		ENSG00000234709
NA	0.0164	5.2226E-01		ENSG00000278464
NA	0.0505	5.2244E-01	7.0883E-01	ENSG00000271848
NA	0.0399	5.2254E-01	7.0893E-01	ENSG00000236417
NA	0.0358	5.2256E-01		ENSG00000222007
NA	0.0547	5.2261E-01	7.0893E-01	ENSG00000180458
NA	0.0229	5.2289E-01		ENSG00000250027
NA	0.0364	5.2306E-01		ENSG00000286812
NA	0.0077	5.2322E-01		ENSG00000204933
NA	0.0335	5.2326E-01	7.0909E-01	ENSG00000267606
NA	0.0539	5.2377E-01	7.0938E-01	ENSG00000282988
NA	0.0246	5.2381E-01	7.0938E-01	ENSG00000283517
NA	0.0533	5.2383E-01	7.0938E-01	ENSG00000258917
NA	0.0292	5.2401E-01		ENSG00000272140
NA	0.0253	5.2401E-01	7.0955E-01	ENSG00000262119
NA	0.0202	5.2406E-01		ENSG00000231705
NA	0.0236	5.2409E-01		ENSG00000248373
NA	0.0244	5.2473E-01		ENSG00000289569
NA	0.0219	5.2503E-01		ENSG00000287389
NA	0.0411	5.2522E-01	7.1061E-01	ENSG00000279923
NA	0.0067	5.2531E-01		ENSG00000242017
NA	0.0303	5.2532E-01	7.1070E-01	ENSG00000254315
NA	0.0136	5.2537E-01		ENSG00000273183
NA	0.0443	5.2558E-01	7.1097E-01	ENSG00000289294
NA	0.0061	5.2568E-01		ENSG00000270304
NA	0.0381	5.2570E-01	7.1100E-01	ENSG00000232608
NA	0.0424	5.2572E-01	7.1100E-01	ENSG00000250900
NA	0.0093	5.2586E-01		ENSG00000236213
NA	0.0452	5.2590E-01	7.1110E-01	ENSG00000236993
NA	0.0486	5.2591E-01	7.1110E-01	ENSG00000288937
NA	0.0092	5.2597E-01		ENSG00000224816
NA	0.0319	5.2602E-01	7.1118E-01	ENSG00000265478
NA	0.0090	5.2609E-01		ENSG00000280009
NA	0.0044	5.2644E-01		ENSG00000232455
NA	0.0087	5.2661E-01		ENSG00000269392
NA	0.0163	5.2697E-01	7.1211E-01	ENSG00000270823
NA	0.0396	5.2727E-01	7.1232E-01	ENSG00000255234
NA	0.0471	5.2805E-01	7.1271E-01	ENSG00000260281
NA	0.0158	5.2846E-01		ENSG00000223754
NA	0.0112	5.2850E-01		ENSG00000290063
NA	0.0526	5.2858E-01	7.1322E-01	ENSG00000289378
NA	0.0177	5.2905E-01		ENSG00000254991
NA	0.0073	5.2933E-01		ENSG00000267212
NA	0.0030	5.2935E-01		ENSG00000229241
NA	0.0304	5.2943E-01		ENSG00000256400
NA	0.0339	5.2950E-01		ENSG00000214702
NA	0.0092	5.2993E-01		ENSG00000285935

NA	0.0232	5.2997E-01		ENSG00000266950
NA	0.0355	5.3003E-01		ENSG00000279835
NA	0.0535	5.3006E-01	7.1460E-01	ENSG00000264808
NA	0.0322	5.3009E-01	7.1460E-01	ENSG00000228137
NA	0.0166	5.3031E-01		ENSG00000260159
NA	0.0532	5.3038E-01	7.1488E-01	ENSG00000262265
NA	0.0403	5.3052E-01	7.1491E-01	ENSG00000280381
NA	0.0051	5.3053E-01		ENSG00000233661
NA	0.0470	5.3081E-01	7.1508E-01	ENSG00000224541
NA	0.0244	5.3084E-01		ENSG00000266373
NA	0.0270	5.3092E-01		ENSG00000197665
NA	0.0080	5.3098E-01		ENSG00000255552
NA	0.0465	5.3099E-01		ENSG00000288723
NA	0.0259	5.3124E-01		ENSG00000279557
NA	0.0149	5.3153E-01		ENSG00000250140
NA	0.0280	5.3160E-01	7.1570E-01	ENSG00000262693
NA	0.0701	5.3206E-01	7.1596E-01	ENSG00000270299
NA	0.0148	5.3216E-01		ENSG00000259354
NA	0.0155	5.3223E-01		ENSG00000232959
NA	0.0508	5.3228E-01	7.1608E-01	ENSG00000248752
NA	0.0377	5.3234E-01	7.1608E-01	ENSG00000276570
NA	0.0077	5.3235E-01		ENSG00000230866
NA	0.0128	5.3267E-01		ENSG00000214856
NA	0.0316	5.3270E-01		ENSG00000253282
NA	0.0088	5.3282E-01		ENSG00000229992
NA	0.0135	5.3300E-01		ENSG00000228507
NA	0.0080	5.3315E-01		ENSG00000227400
NA	0.0145	5.3334E-01		ENSG00000198022
NA	0.0060	5.3334E-01		ENSG00000250523
NA	0.0305	5.3377E-01	7.1751E-01	ENSG00000285909
NA	0.0150	5.3385E-01		ENSG00000267375
NA	0.0475	5.3397E-01	7.1770E-01	ENSG00000286129
NA	0.0307	5.3401E-01	7.1772E-01	ENSG00000260213
NA	0.0268	5.3412E-01		ENSG00000235674
NA	0.0070	5.3421E-01		ENSG00000287647
NA	0.0488	5.3431E-01	7.1780E-01	ENSG00000287127
NA	0.0621	5.3438E-01	7.1782E-01	ENSG00000282886
NA	0.0109	5.3442E-01		ENSG00000249876
NA	0.0269	5.3457E-01		ENSG00000277250
NA	0.0362	5.3463E-01		ENSG00000262539
NA	0.0157	5.3463E-01		ENSG00000271252
NA	0.0333	5.3465E-01	7.1807E-01	ENSG00000279212
NA	0.0364	5.3473E-01	7.1813E-01	ENSG00000287550
NA	0.0147	5.3482E-01		ENSG00000258479
NA	0.0533	5.3485E-01	7.1818E-01	ENSG00000261227
NA	0.0065	5.3510E-01		ENSG00000278967
NA	0.0536	5.3520E-01	7.1834E-01	ENSG00000230006
NA	0.0177	5.3530E-01		ENSG00000250260
NA	0.0101	5.3558E-01		ENSG00000258909

NA	0.0509	5.3560E-01	7.1877E-01	ENSG00000253475
NA	0.0173	5.3564E-01		ENSG00000227359
NA	0.0112	5.3655E-01		ENSG00000223816
NA	0.0258	5.3662E-01	7.1963E-01	ENSG00000249755
NA	0.0334	5.3670E-01	7.1967E-01	ENSG00000265179
NA	0.0252	5.3710E-01		ENSG00000245667
NA	0.0507	5.3716E-01	7.2010E-01	ENSG00000249790
NA	0.0038	5.3720E-01		ENSG00000226056
NA	0.0065	5.3728E-01		ENSG00000278683
NA	0.0413	5.3751E-01	7.2034E-01	ENSG00000276302
NA	0.0490	5.3764E-01	7.2037E-01	ENSG00000279089
NA	0.0145	5.3764E-01		ENSG00000197882
NA	0.0226	5.3773E-01		ENSG00000286153
NA	0.0259	5.3774E-01		ENSG00000169662
NA	0.0177	5.3796E-01		ENSG00000287252
NA	0.0213	5.3796E-01		ENSG00000210191
NA	0.0182	5.3801E-01		ENSG00000206995
NA	0.0135	5.3818E-01		ENSG00000205414
NA	0.0370	5.3835E-01	7.2078E-01	ENSG00000250536
NA	0.0471	5.3836E-01	7.2078E-01	ENSG00000273230
NA	0.0178	5.3845E-01	7.2086E-01	ENSG00000286817
NA	0.0503	5.3854E-01	7.2091E-01	ENSG00000280161
NA	0.0316	5.3870E-01	7.2098E-01	ENSG00000271283
NA	0.0050	5.3908E-01		ENSG00000287981
NA	0.0239	5.3980E-01		ENSG00000264714
NA	0.0268	5.3991E-01		ENSG00000251812
NA	0.0326	5.3996E-01	7.2201E-01	ENSG00000261326
NA	0.0207	5.4001E-01		ENSG00000201668
NA	0.0330	5.4009E-01		ENSG00000105694
NA	0.0171	5.4031E-01		ENSG00000251455
NA	0.0223	5.4058E-01		ENSG00000229072
NA	0.0425	5.4068E-01	7.2262E-01	ENSG00000259926
NA	0.0149	5.4080E-01		ENSG00000244657
NA	0.0298	5.4087E-01		ENSG00000280353
NA	0.0458	5.4092E-01	7.2282E-01	ENSG00000247728
NA	0.0243	5.4103E-01		ENSG00000259931
NA	0.0542	5.4112E-01	7.2305E-01	ENSG00000210140
NA	0.0048	5.4113E-01		ENSG00000259686
NA	0.0186	5.4114E-01		ENSG00000275467
NA	0.0451	5.4144E-01	7.2329E-01	ENSG00000237938
NA	0.0134	5.4157E-01		ENSG00000251535
NA	0.0183	5.4184E-01		ENSG00000278927
NA	0.0475	5.4191E-01	7.2364E-01	ENSG00000285701
NA	0.0263	5.4197E-01		ENSG00000286811
NA	0.0249	5.4210E-01		ENSG00000253693
NA	0.0176	5.4219E-01		ENSG00000270421
NA	0.0464	5.4230E-01	7.2388E-01	ENSG00000216775
NA	0.0117	5.4236E-01		ENSG00000275210
NA	0.0309	5.4242E-01		ENSG00000272217

NA	0.0337	5.4243E-01	7.2397E-01	ENSG00000229886
NA	0.0176	5.4283E-01		ENSG00000207009
NA	0.0048	5.4295E-01		ENSG00000286960
NA	0.0377	5.4344E-01	7.2458E-01	ENSG00000286223
NA	0.0098	5.4349E-01		ENSG00000273973
NA	0.0504	5.4374E-01	7.2472E-01	ENSG00000289135
NA	0.0155	5.4385E-01		ENSG00000227719
NA	0.0455	5.4393E-01	7.2484E-01	ENSG00000287425
NA	0.0486	5.4406E-01	7.2492E-01	ENSG00000267475
NA	0.0063	5.4427E-01		ENSG00000279208
NA	0.0277	5.4444E-01		ENSG00000241135
NA	0.0096	5.4451E-01		ENSG00000279300
NA	0.0051	5.4459E-01		ENSG00000251411
NA	0.0054	5.4459E-01		ENSG00000284600
NA	0.0500	5.4459E-01	7.2532E-01	ENSG00000268030
NA	0.0305	5.4462E-01		ENSG00000260750
NA	0.0190	5.4483E-01		ENSG00000288835
NA	0.0455	5.4489E-01	7.2555E-01	ENSG00000233613
NA	0.0113	5.4516E-01		ENSG00000286514
NA	0.0107	5.4618E-01	7.2638E-01	ENSG00000259817
NA	0.0088	5.4629E-01		ENSG00000260209
NA	0.0255	5.4644E-01		ENSG00000237251
NA	0.0264	5.4652E-01	7.2651E-01	ENSG00000259223
NA	0.0304	5.4663E-01	7.2658E-01	ENSG00000279439
NA	0.0509	5.4683E-01	7.2674E-01	ENSG00000272716
NA	0.0457	5.4684E-01	7.2674E-01	ENSG00000267500
NA	0.0070	5.4686E-01		ENSG00000238713
NA	0.0008	5.4717E-01		ENSG00000176320
NA	0.0399	5.4727E-01	7.2703E-01	ENSG00000236526
NA	0.0321	5.4727E-01		ENSG00000213376
NA	0.0388	5.4750E-01	7.2719E-01	ENSG00000267321
NA	0.0092	5.4773E-01		ENSG00000250946
NA	0.0456	5.4799E-01	7.2760E-01	ENSG00000280152
NA	0.0067	5.4806E-01		ENSG00000249514
NA	0.0383	5.4812E-01	7.2768E-01	ENSG00000226332
NA	0.0290	5.4824E-01	7.2775E-01	ENSG00000259081
NA	0.0316	5.4826E-01		ENSG00000239705
NA	0.0339	5.4891E-01	7.2827E-01	ENSG00000278816
NA	0.0362	5.4911E-01	7.2838E-01	ENSG00000259434
NA	0.0503	5.4942E-01	7.2872E-01	ENSG00000174403
NA	0.0226	5.4993E-01		ENSG00000235748
NA	0.0204	5.4996E-01	7.2915E-01	ENSG00000264659
NA	0.0082	5.5004E-01		ENSG00000279442
NA	0.0455	5.5020E-01	7.2920E-01	ENSG00000279066
NA	0.0225	5.5037E-01		ENSG00000258445
NA	0.0259	5.5038E-01		ENSG00000260690
NA	0.0272	5.5066E-01		ENSG00000280440
NA	0.0130	5.5104E-01		ENSG00000255035
NA	0.0319	5.5116E-01	7.2989E-01	ENSG00000279294

NA	0.0163	5.5126E-01		ENSG00000286653
NA	0.0112	5.5134E-01		ENSG00000232241
NA	0.0041	5.5135E-01		ENSG00000271554
NA	0.0340	5.5200E-01	7.3071E-01	ENSG00000226259
NA	0.0044	5.5269E-01		ENSG00000283005
NA	0.0081	5.5318E-01		ENSG00000285661
NA	0.0097	5.5324E-01		ENSG00000225580
NA	0.0067	5.5330E-01		ENSG00000235806
NA	0.0430	5.5358E-01	7.3198E-01	ENSG00000224616
NA	0.0383	5.5372E-01	7.3210E-01	ENSG00000236140
NA	0.0409	5.5386E-01	7.3224E-01	ENSG00000269947
NA	0.0060	5.5393E-01		ENSG00000287319
NA	0.0246	5.5396E-01		ENSG00000237015
NA	0.0086	5.5414E-01		ENSG00000267211
NA	0.0276	5.5427E-01	7.3257E-01	ENSG00000243696
NA	0.0069	5.5478E-01		ENSG00000288963
NA	0.0126	5.5484E-01		ENSG00000273712
NA	0.0239	5.5491E-01		ENSG00000224928
NA	0.0340	5.5507E-01	7.3317E-01	ENSG00000260160
NA	0.0168	5.5508E-01		ENSG00000289405
NA	0.0117	5.5518E-01		ENSG00000282980
NA	0.0138	5.5521E-01		ENSG00000254295
NA	0.0040	5.5538E-01		ENSG00000248458
NA	0.0278	5.5542E-01		ENSG00000227240
NA	0.0155	5.5544E-01		ENSG00000285549
NA	0.0505	5.5564E-01	7.3361E-01	ENSG00000197536
NA	0.0204	5.5566E-01		ENSG00000268087
NA	0.0416	5.5596E-01	7.3395E-01	ENSG00000288060
NA	0.0499	5.5634E-01	7.3423E-01	ENSG00000289074
NA	0.0081	5.5640E-01		ENSG00000285836
NA	0.0172	5.5668E-01		ENSG00000286370
NA	0.0104	5.5723E-01		ENSG00000206721
NA	0.0181	5.5725E-01		ENSG00000247853
NA	0.0500	5.5731E-01	7.3503E-01	ENSG00000286067
NA	0.0294	5.5734E-01	7.3503E-01	ENSG00000267560
NA	0.0213	5.5753E-01		ENSG00000269516
NA	0.0277	5.5775E-01	7.3536E-01	ENSG00000265443
NA	0.0156	5.5780E-01		ENSG00000235288
NA	0.0290	5.5790E-01		ENSG00000268896
NA	0.0146	5.5798E-01	7.3555E-01	ENSG00000275767
NA	0.0105	5.5807E-01		ENSG00000249379
NA	0.0110	5.5837E-01		ENSG00000254573
NA	0.0333	5.5846E-01	7.3587E-01	ENSG00000287884
NA	0.0067	5.5859E-01		ENSG00000286309
NA	0.0010	5.5885E-01		ENSG00000259543
NA	0.0282	5.5892E-01		ENSG00000279255
NA	0.0104	5.5910E-01		ENSG00000250603
NA	0.0057	5.5937E-01		ENSG00000229303
NA	0.0178	5.5944E-01		ENSG00000237939

NA	0.0107	5.5963E-01		ENSG00000213026
NA	0.0395	5.5971E-01	7.3712E-01	ENSG00000124593
NA	0.0130	5.6003E-01		ENSG00000289557
NA	0.0422	5.6011E-01	7.3729E-01	ENSG00000286650
NA	0.0148	5.6014E-01		ENSG00000290020
NA	0.0143	5.6046E-01		ENSG00000262402
NA	0.0235	5.6057E-01	7.3770E-01	ENSG00000289332
NA	0.0102	5.6080E-01		ENSG00000279174
NA	0.0095	5.6082E-01		ENSG00000226096
NA	0.0239	5.6122E-01		ENSG00000282787
NA	0.0113	5.6152E-01		ENSG00000232032
NA	0.0494	5.6162E-01	7.3847E-01	ENSG00000270504
NA	0.0306	5.6171E-01	7.3848E-01	ENSG00000228366
NA	0.0135	5.6177E-01		ENSG00000269012
NA	0.0044	5.6177E-01		ENSG00000267898
NA	0.0305	5.6178E-01	7.3850E-01	ENSG00000260483
NA	0.0204	5.6184E-01		ENSG00000258699
NA	0.0043	5.6185E-01		ENSG00000261646
NA	0.0095	5.6201E-01		ENSG00000289195
NA	0.0474	5.6205E-01	7.3870E-01	ENSG00000279259
NA	0.0170	5.6209E-01		ENSG00000206660
NA	0.0128	5.6233E-01		ENSG00000206914
NA	0.0458	5.6241E-01	7.3913E-01	ENSG00000235078
NA	0.0299	5.6244E-01	7.3914E-01	ENSG00000175604
NA	0.0160	5.6278E-01	7.3940E-01	ENSG00000237153
NA	0.0237	5.6302E-01		ENSG00000271412
NA	0.0104	5.6368E-01		ENSG00000223575
NA	0.0314	5.6388E-01	7.3998E-01	ENSG00000257058
NA	0.0097	5.6443E-01		ENSG00000236797
NA	0.0123	5.6487E-01		ENSG00000254088
NA	0.0236	5.6499E-01		ENSG00000237868
NA	0.0359	5.6511E-01	7.4080E-01	ENSG00000254659
NA	0.0175	5.6520E-01		ENSG00000118976
NA	0.0095	5.6531E-01		ENSG00000225315
NA	0.0114	5.6533E-01		ENSG00000287153
NA	0.0045	5.6535E-01		ENSG00000287144
NA	0.0224	5.6541E-01		ENSG00000229282
NA	0.0231	5.6542E-01		ENSG00000238221
NA	0.0454	5.6543E-01	7.4090E-01	ENSG00000287783
NA	0.0127	5.6543E-01		ENSG00000228351
NA	0.0316	5.6550E-01	7.4090E-01	ENSG00000277482
NA	0.0360	5.6556E-01	7.4090E-01	ENSG00000277879
NA	0.0369	5.6563E-01	7.4095E-01	ENSG00000259017
NA	0.0115	5.6573E-01		ENSG00000215720
NA	0.0493	5.6590E-01	7.4117E-01	ENSG00000270659
NA	0.0185	5.6591E-01		ENSG00000275936
NA	0.0486	5.6636E-01	7.4146E-01	ENSG00000277368
NA	0.0464	5.6693E-01	7.4191E-01	ENSG00000283390
NA	0.0488	5.6707E-01	7.4191E-01	ENSG00000275769

NA	0.0107	5.6757E-01		ENSG00000240935
NA	0.0175	5.6767E-01		ENSG00000240652
NA	0.0482	5.6771E-01	7.4237E-01	ENSG00000260360
NA	0.0064	5.6791E-01		ENSG00000260479
NA	0.0199	5.6791E-01		ENSG00000277198
NA	0.0441	5.6793E-01	7.4259E-01	ENSG00000216285
NA	0.0392	5.6816E-01	7.4273E-01	ENSG00000255118
NA	0.0145	5.6824E-01		ENSG00000289579
NA	0.0414	5.6826E-01	7.4278E-01	ENSG00000259877
NA	0.0457	5.6844E-01	7.4294E-01	ENSG00000287600
NA	0.0328	5.6890E-01	7.4328E-01	ENSG00000225398
NA	0.0474	5.6891E-01	7.4328E-01	ENSG00000283156
NA	0.0472	5.6893E-01	7.4328E-01	ENSG00000168852
NA	0.0195	5.6895E-01		ENSG00000226426
NA	0.0251	5.6900E-01		ENSG00000288828
NA	0.0495	5.6914E-01	7.4337E-01	ENSG00000289128
NA	0.0068	5.6920E-01		ENSG00000260765
NA	0.0472	5.6928E-01	7.4346E-01	ENSG00000226970
NA	0.0206	5.6939E-01		ENSG00000287525
NA	0.0174	5.6953E-01		ENSG00000268157
NA	0.0406	5.6961E-01	7.4363E-01	ENSG00000273565
NA	0.0344	5.6973E-01		ENSG00000285588
NA	0.0104	5.7032E-01		ENSG00000207293
NA	0.0194	5.7038E-01		ENSG00000253778
NA	0.0136	5.7071E-01		ENSG00000270614
NA	0.0258	5.7109E-01		ENSG00000260600
NA	0.0265	5.7112E-01		ENSG00000229403
NA	0.0347	5.7124E-01	7.4485E-01	ENSG00000215097
NA	0.0224	5.7126E-01		ENSG00000287192
NA	0.0423	5.7130E-01	7.4485E-01	ENSG00000273226
NA	0.0110	5.7189E-01		ENSG00000230425
NA	0.0089	5.7203E-01		ENSG00000200874
NA	0.0230	5.7213E-01		ENSG00000234952
NA	0.0185	5.7281E-01		ENSG00000288098
NA	0.0031	5.7307E-01		ENSG00000263893
NA	0.0078	5.7318E-01		ENSG00000254593
NA	0.0441	5.7321E-01	7.4624E-01	ENSG00000287431
NA	0.0365	5.7321E-01	7.4624E-01	ENSG00000276408
NA	0.0042	5.7324E-01		ENSG00000270846
NA	0.0019	5.7324E-01		ENSG00000253105
NA	0.0118	5.7331E-01		ENSG00000250914
NA	0.0078	5.7344E-01		ENSG00000279962
NA	0.0252	5.7351E-01		ENSG00000279946
NA	0.0189	5.7370E-01		ENSG00000249664
NA	0.0273	5.7370E-01		ENSG00000272669
NA	0.0149	5.7379E-01		ENSG00000236936
NA	0.0334	5.7398E-01	7.4685E-01	ENSG00000231952
NA	0.0109	5.7435E-01		ENSG00000270711
NA	0.0110	5.7439E-01		ENSG00000278982

NA	0.0084	5.7441E-01		ENSG00000234020
NA	0.0089	5.7455E-01		ENSG00000254865
NA	0.0175	5.7456E-01		ENSG00000256538
NA	0.0371	5.7469E-01		ENSG00000254730
NA	0.0124	5.7477E-01		ENSG00000253263
NA	0.0096	5.7500E-01		ENSG00000277463
NA	0.0232	5.7506E-01		ENSG00000261310
NA	0.0150	5.7506E-01		ENSG00000284490
NA	0.0254	5.7507E-01	7.4782E-01	ENSG00000264475
NA	0.0209	5.7507E-01		ENSG00000225933
NA	0.0309	5.7507E-01		ENSG00000226040
NA	0.0007	5.7520E-01		ENSG00000253619
NA	0.0225	5.7534E-01		ENSG00000240350
NA	0.0454	5.7539E-01	7.4808E-01	ENSG00000279865
NA	0.0198	5.7571E-01		ENSG00000187904
NA	0.0299	5.7600E-01		ENSG00000259856
NA	0.0425	5.7616E-01	7.4860E-01	ENSG00000265519
NA	0.0214	5.7616E-01		ENSG00000228952
NA	0.0021	5.7628E-01		ENSG00000257943
NA	0.0332	5.7654E-01	7.4871E-01	ENSG00000278900
NA	0.0183	5.7654E-01		ENSG00000253539
NA	0.0454	5.7658E-01	7.4871E-01	ENSG00000272518
NA	0.0195	5.7671E-01		ENSG00000289876
NA	0.0118	5.7678E-01		ENSG00000286146
NA	0.0429	5.7698E-01	7.4900E-01	ENSG00000285740
NA	0.0446	5.7721E-01	7.4911E-01	ENSG00000273066
NA	0.0451	5.7722E-01	7.4911E-01	ENSG00000290008
NA	0.0419	5.7729E-01	7.4914E-01	ENSG00000286388
NA	0.0151	5.7750E-01		ENSG00000266289
NA	0.0143	5.7755E-01		ENSG00000285838
NA	0.0334	5.7760E-01	7.4945E-01	ENSG00000255928
NA	0.0383	5.7776E-01	7.4955E-01	ENSG00000277991
NA	0.0289	5.7778E-01	7.4955E-01	ENSG00000272240
NA	0.0361	5.7780E-01	7.4955E-01	ENSG00000214243
NA	0.0470	5.7795E-01	7.4959E-01	ENSG00000288911
NA	0.0277	5.7818E-01	7.4978E-01	ENSG00000214955
NA	0.0155	5.7838E-01	7.4995E-01	ENSG00000287100
NA	0.0361	5.7841E-01	7.4995E-01	ENSG00000254099
NA	0.0271	5.7845E-01		ENSG00000230205
NA	0.0183	5.7846E-01		ENSG00000274124
NA	0.0121	5.7860E-01		ENSG00000203397
NA	0.0154	5.7860E-01		ENSG00000251577
NA	0.0416	5.7861E-01	7.5008E-01	ENSG00000286387
NA	0.0002	5.7885E-01		ENSG00000232457
NA	0.0133	5.7929E-01		ENSG00000258734
NA	0.0187	5.7934E-01		ENSG00000226535
NA	0.0473	5.8002E-01	7.5144E-01	ENSG00000289280
NA	0.0061	5.8025E-01		ENSG00000237528
NA	0.0140	5.8045E-01		ENSG00000226440

NA	0.0267	5.8054E-01		ENSG00000260871
NA	0.0124	5.8071E-01		ENSG00000279146
NA	0.0170	5.8102E-01		ENSG00000217527
NA	0.0044	5.8130E-01		ENSG00000200742
NA	0.0182	5.8135E-01		ENSG00000271049
NA	0.0038	5.8140E-01		ENSG00000177197
NA	0.0258	5.8142E-01		ENSG00000235070
NA	0.0408	5.8146E-01	7.5263E-01	ENSG00000257568
NA	0.0028	5.8158E-01		ENSG00000229915
NA	0.0095	5.8162E-01		ENSG00000255079
NA	0.0357	5.8173E-01	7.5286E-01	ENSG00000275759
NA	0.0141	5.8173E-01		ENSG00000211491
NA	0.0049	5.8180E-01		ENSG00000223774
NA	0.0020	5.8197E-01		ENSG00000253976
NA	0.0066	5.8197E-01		ENSG00000255327
NA	0.0102	5.8198E-01		ENSG00000200135
NA	0.0144	5.8202E-01		ENSG00000250214
NA	0.0106	5.8218E-01		ENSG00000126952
NA	0.0096	5.8219E-01		ENSG00000273904
NA	0.0152	5.8241E-01		ENSG00000241054
NA	0.0123	5.8251E-01		ENSG00000274584
NA	0.0448	5.8270E-01	7.5352E-01	ENSG00000278765
NA	0.0131	5.8289E-01		ENSG00000229289
NA	0.0167	5.8293E-01		ENSG00000281530
NA	0.0069	5.8305E-01		ENSG00000261775
NA	0.0488	5.8314E-01	7.5372E-01	ENSG00000231806
NA	0.0126	5.8376E-01		ENSG00000287040
NA	0.0454	5.8379E-01	7.5412E-01	ENSG00000273897
NA	0.0396	5.8380E-01	7.5412E-01	ENSG00000282051
NA	0.0354	5.8388E-01	7.5414E-01	ENSG00000241170
NA	0.0206	5.8412E-01	7.5417E-01	ENSG00000254180
NA	0.0126	5.8437E-01		ENSG00000234232
NA	0.0351	5.8470E-01	7.5468E-01	ENSG00000277020
NA	0.0010	5.8481E-01	7.5478E-01	ENSG00000289837
NA	0.0432	5.8528E-01	7.5516E-01	ENSG00000260588
NA	0.0122	5.8536E-01		ENSG00000232470
NA	0.0215	5.8555E-01		ENSG00000279989
NA	0.0284	5.8558E-01	7.5543E-01	ENSG00000288779
NA	0.0175	5.8569E-01	7.5545E-01	ENSG00000249049
NA	0.0232	5.8573E-01	7.5546E-01	ENSG00000283528
NA	0.0363	5.8578E-01	7.5549E-01	ENSG00000272129
NA	0.0097	5.8582E-01		ENSG00000235292
NA	0.0289	5.8600E-01	7.5563E-01	ENSG00000267698
NA	0.0430	5.8623E-01	7.5570E-01	ENSG00000272108
NA	0.0235	5.8625E-01		ENSG00000266379
NA	0.0116	5.8642E-01		ENSG00000235361
NA	0.0262	5.8649E-01		ENSG00000289508
NA	0.0128	5.8675E-01		ENSG00000259162
NA	0.0192	5.8695E-01		ENSG00000260742

NA	0.0102	5.8697E-01		ENSG00000256356
NA	0.0443	5.8707E-01	7.5636E-01	ENSG00000243970
NA	0.0193	5.8720E-01		ENSG00000279887
NA	0.0204	5.8720E-01		ENSG00000285573
NA	0.0170	5.8729E-01		ENSG00000279886
NA	0.0215	5.8730E-01		ENSG00000271452
NA	0.0280	5.8770E-01		ENSG00000247925
NA	0.0237	5.8776E-01		ENSG00000240959
NA	0.0160	5.8781E-01		ENSG00000270917
NA	0.0228	5.8782E-01		ENSG00000251423
NA	0.0503	5.8809E-01	7.5699E-01	ENSG00000270367
NA	0.0116	5.8828E-01		ENSG00000255187
NA	0.0137	5.8833E-01		ENSG00000289952
NA	0.0021	5.8837E-01		ENSG00000231401
NA	0.0084	5.8852E-01		ENSG00000258624
NA	0.0453	5.8881E-01	7.5748E-01	ENSG00000233013
NA	0.0239	5.8885E-01		ENSG00000254705
NA	0.0452	5.8888E-01	7.5749E-01	ENSG00000253653
NA	0.0221	5.8889E-01		ENSG00000272159
NA	0.0308	5.8897E-01	7.5757E-01	ENSG00000285816
NA	0.0184	5.8939E-01		ENSG00000256721
NA	0.0268	5.8984E-01	7.5829E-01	ENSG00000289688
NA	0.0203	5.8994E-01		ENSG00000259575
NA	0.0140	5.9002E-01		ENSG00000203520
NA	0.0371	5.9015E-01	7.5856E-01	ENSG00000289603
NA	0.0279	5.9016E-01		ENSG00000275830
NA	0.0337	5.9018E-01	7.5856E-01	ENSG00000261101
NA	0.0166	5.9027E-01		ENSG00000182632
NA	0.0320	5.9051E-01	7.5874E-01	ENSG00000279653
NA	0.0196	5.9054E-01		ENSG00000272130
NA	0.0375	5.9060E-01		ENSG00000260645
NA	0.0330	5.9102E-01	7.5902E-01	ENSG00000261329
NA	0.0222	5.9158E-01	7.5942E-01	ENSG00000271959
NA	0.0008	5.9171E-01	7.5952E-01	ENSG00000229905
NA	0.0394	5.9172E-01	7.5952E-01	ENSG00000286675
NA	0.0097	5.9173E-01		ENSG00000251391
NA	0.0424	5.9203E-01	7.5981E-01	ENSG00000286196
NA	0.0249	5.9219E-01		ENSG00000261253
NA	0.0096	5.9233E-01		ENSG00000239641
NA	0.0216	5.9235E-01		ENSG00000246323
NA	0.0075	5.9236E-01		ENSG00000286401
NA	0.0265	5.9240E-01		ENSG00000285918
NA	0.0505	5.9274E-01	7.6028E-01	ENSG00000261431
NA	0.0250	5.9280E-01	7.6028E-01	ENSG00000253503
NA	0.0290	5.9294E-01	7.6034E-01	ENSG00000254288
NA	0.0359	5.9303E-01	7.6037E-01	ENSG00000287045
NA	0.0146	5.9344E-01		ENSG00000228120
NA	0.0450	5.9359E-01	7.6081E-01	ENSG00000226310
NA	0.0130	5.9360E-01		ENSG00000272265

NA	0.0428	5.9365E-01	7.6085E-01	ENSG00000289872
NA	0.0343	5.9388E-01	7.6102E-01	ENSG00000250765
NA	0.0132	5.9411E-01		ENSG00000234466
NA	0.0415	5.9417E-01	7.6126E-01	ENSG00000288048
NA	0.0466	5.9422E-01	7.6126E-01	ENSG00000279099
NA	0.0129	5.9450E-01		ENSG00000279883
NA	0.0227	5.9466E-01		ENSG00000174912
NA	0.0387	5.9479E-01	7.6177E-01	ENSG00000277382
NA	0.0445	5.9481E-01	7.6177E-01	ENSG00000272419
NA	0.0188	5.9489E-01		ENSG00000180019
NA	0.0353	5.9509E-01	7.6206E-01	ENSG00000290023
NA	0.0329	5.9519E-01		ENSG00000290061
NA	0.0204	5.9528E-01		ENSG00000261430
NA	0.0084	5.9532E-01		ENSG00000267220
NA	0.0316	5.9537E-01	7.6230E-01	ENSG00000285578
NA	0.0099	5.9554E-01		ENSG00000277191
NA	0.0232	5.9559E-01		ENSG00000287291
NA	0.0041	5.9588E-01	7.6268E-01	ENSG00000270532
NA	0.0193	5.9600E-01	7.6275E-01	ENSG00000232211
NA	0.0006	5.9605E-01		ENSG00000234162
NA	0.0385	5.9634E-01	7.6302E-01	ENSG00000238251
NA	0.0126	5.9655E-01		ENSG00000239628
NA	0.0070	5.9680E-01		ENSG00000253557
NA	0.0518	5.9698E-01		ENSG00000282602
NA	0.0442	5.9727E-01	7.6374E-01	ENSG00000290048
NA	0.0239	5.9785E-01		ENSG00000250409
NA	0.0051	5.9787E-01		ENSG00000224387
NA	0.0390	5.9791E-01	7.6415E-01	ENSG00000261386
NA	0.0169	5.9801E-01	7.6415E-01	ENSG00000279440
NA	0.0172	5.9802E-01		ENSG00000286581
NA	0.0431	5.9812E-01	7.6419E-01	ENSG00000288025
NA	0.0102	5.9848E-01		ENSG00000282142
NA	0.0408	5.9861E-01	7.6462E-01	ENSG00000214999
NA	0.0139	5.9875E-01		ENSG00000250726
NA	0.0020	5.9879E-01		ENSG00000239207
NA	0.0153	5.9908E-01		ENSG00000219150
NA	0.0059	5.9925E-01		ENSG00000271547
NA	0.0006	5.9931E-01		ENSG00000234261
NA	0.0032	5.9942E-01		ENSG00000250980
NA	0.0271	5.9945E-01		ENSG00000214820
NA	0.0189	5.9969E-01		ENSG00000272431
NA	0.0098	5.9976E-01		ENSG00000237389
NA	0.0160	6.0021E-01		ENSG00000223822
NA	0.0258	6.0024E-01	7.6590E-01	ENSG00000228779
NA	0.0444	6.0085E-01	7.6620E-01	ENSG00000272918
NA	0.0182	6.0093E-01		ENSG00000233845
NA	0.0141	6.0106E-01		ENSG00000228561
NA	0.0267	6.0141E-01		ENSG00000282996
NA	0.0444	6.0156E-01	7.6664E-01	ENSG00000214425

NA	0.0118	6.0198E-01		ENSG00000219797
NA	0.0080	6.0210E-01		ENSG00000274680
NA	0.0317	6.0213E-01	7.6698E-01	ENSG00000289912
NA	0.0445	6.0214E-01	7.6698E-01	ENSG00000289437
NA	0.0133	6.0222E-01	7.6698E-01	ENSG00000269887
NA	0.0349	6.0224E-01	7.6698E-01	ENSG00000289635
NA	0.0133	6.0231E-01		ENSG00000265964
NA	0.0453	6.0231E-01		ENSG00000290071
NA	0.0269	6.0231E-01	7.6703E-01	ENSG00000228459
NA	0.0267	6.0241E-01	7.6712E-01	ENSG00000287422
NA	0.0180	6.0243E-01		ENSG00000286178
NA	0.0112	6.0276E-01		ENSG00000279237
NA	0.0099	6.0289E-01		ENSG00000244676
NA	0.0090	6.0292E-01		ENSG00000270975
NA	0.0401	6.0299E-01	7.6765E-01	ENSG00000215478
NA	0.0117	6.0301E-01		ENSG00000188662
NA	0.0002	6.0317E-01		ENSG00000286801
NA	0.0164	6.0318E-01		ENSG00000285659
NA	0.0077	6.0330E-01		ENSG00000285894
NA	0.0048	6.0337E-01		ENSG00000236686
NA	0.0315	6.0343E-01		ENSG00000289073
NA	0.0441	6.0349E-01	7.6794E-01	ENSG00000273729
NA	0.0413	6.0371E-01	7.6810E-01	ENSG00000279967
NA	0.0431	6.0380E-01	7.6817E-01	ENSG00000289213
NA	0.0376	6.0388E-01	7.6824E-01	ENSG00000244513
NA	0.0437	6.0401E-01	7.6833E-01	ENSG00000289304
NA	0.0405	6.0409E-01	7.6833E-01	ENSG00000255725
NA	0.0015	6.0423E-01		ENSG00000275418
NA	0.0162	6.0426E-01		ENSG00000226542
NA	0.0143	6.0447E-01		ENSG00000270874
NA	0.0098	6.0448E-01		ENSG00000254526
NA	0.0257	6.0488E-01		ENSG00000241975
NA	0.0125	6.0494E-01		ENSG00000254932
NA	0.0028	6.0530E-01		ENSG00000275348
NA	0.0332	6.0574E-01	7.6972E-01	ENSG00000214867
NA	0.0170	6.0580E-01		ENSG00000226539
NA	0.0300	6.0597E-01		ENSG00000286210
NA	0.0191	6.0599E-01		ENSG00000224669
NA	0.0112	6.0601E-01		ENSG00000243038
NA	0.0203	6.0616E-01		ENSG00000281769
NA	0.0087	6.0617E-01		ENSG00000272416
NA	0.0336	6.0648E-01		ENSG00000253286
NA	0.0005	6.0659E-01		ENSG00000250030
NA	0.0096	6.0662E-01		ENSG00000251244
NA	0.0070	6.0691E-01		ENSG00000289116
NA	0.0406	6.0701E-01	7.7090E-01	ENSG00000288988
NA	0.0353	6.0723E-01	7.7108E-01	ENSG00000225916
NA	0.0006	6.0781E-01		ENSG00000259735
NA	0.0412	6.0818E-01	7.7143E-01	ENSG00000272588

NA	0.0056	6.0833E-01	7.7147E-01	ENSG00000269318
NA	0.0193	6.0848E-01		ENSG00000236480
NA	0.0089	6.0856E-01		ENSG00000280384
NA	0.0007	6.0864E-01		ENSG00000279965
NA	0.0244	6.0883E-01		ENSG00000235698
NA	0.0325	6.0898E-01	7.7201E-01	ENSG00000286482
NA	0.0321	6.0913E-01	7.7208E-01	ENSG00000280022
NA	0.0245	6.0923E-01	7.7217E-01	ENSG00000284292
NA	0.0126	6.0969E-01		ENSG00000253100
NA	0.0065	6.0975E-01		ENSG00000225299
NA	0.0307	6.0980E-01	7.7246E-01	ENSG00000215493
NA	0.0223	6.0986E-01		ENSG00000236848
NA	0.0262	6.0991E-01		ENSG00000261481
NA	0.0414	6.1002E-01	7.7257E-01	ENSG00000260924
NA	0.0099	6.1056E-01		ENSG00000262098
NA	0.0103	6.1067E-01		ENSG00000257803
NA	0.0102	6.1090E-01		ENSG00000274937
NA	0.0257	6.1129E-01		ENSG00000254842
NA	0.0080	6.1144E-01		ENSG00000279090
NA	0.0291	6.1152E-01	7.7380E-01	ENSG00000283235
NA	0.0134	6.1154E-01		ENSG00000286505
NA	0.0419	6.1161E-01	7.7383E-01	ENSG00000286682
NA	0.0152	6.1171E-01		ENSG00000249863
NA	0.0158	6.1201E-01		ENSG00000204529
NA	0.0304	6.1232E-01	7.7442E-01	ENSG00000278861
NA	0.0122	6.1291E-01		ENSG00000234426
NA	0.0182	6.1293E-01		ENSG00000238117
NA	0.0278	6.1298E-01	7.7486E-01	ENSG00000268081
NA	0.0244	6.1310E-01	7.7496E-01	ENSG00000285943
NA	0.0192	6.1326E-01	7.7508E-01	ENSG00000268635
NA	0.0353	6.1332E-01	7.7508E-01	ENSG00000260335
NA	0.0082	6.1339E-01		ENSG00000262488
NA	0.0244	6.1380E-01		ENSG00000270190
NA	0.0232	6.1434E-01		ENSG00000237271
NA	0.0388	6.1440E-01		ENSG00000238039
NA	0.0395	6.1443E-01	7.7604E-01	ENSG00000280069
NA	0.0285	6.1453E-01	7.7612E-01	ENSG00000259712
NA	0.0122	6.1470E-01		ENSG00000264217
NA	0.0138	6.1473E-01		ENSG00000249840
NA	0.0115	6.1477E-01		ENSG00000272123
NA	0.0067	6.1480E-01		ENSG00000238001
NA	0.0328	6.1493E-01	7.7655E-01	ENSG00000261663
NA	0.0112	6.1538E-01		ENSG00000259565
NA	0.0264	6.1547E-01		ENSG00000254050
NA	0.0367	6.1552E-01	7.7690E-01	ENSG00000287527
NA	0.0231	6.1566E-01		ENSG00000225146
NA	0.0420	6.1591E-01	7.7721E-01	ENSG00000288793
NA	0.0253	6.1613E-01	7.7735E-01	ENSG00000240915
NA	0.0123	6.1636E-01		ENSG00000278456

NA	0.0041	6.1648E-01		ENSG00000223476
NA	0.0277	6.1654E-01		ENSG00000229642
NA	0.0317	6.1669E-01	7.7778E-01	ENSG00000286207
NA	0.0203	6.1684E-01		ENSG00000258485
NA	0.0120	6.1709E-01		ENSG00000213484
NA	0.0131	6.1712E-01		ENSG00000259598
NA	0.0247	6.1724E-01		ENSG00000257954
NA	0.0177	6.1741E-01		ENSG00000267595
NA	0.0227	6.1790E-01		ENSG00000278075
NA	0.0080	6.1820E-01		ENSG00000289911
NA	0.0363	6.1894E-01	7.7960E-01	ENSG00000283341
NA	0.0037	6.1898E-01	7.7960E-01	ENSG00000213478
NA	0.0384	6.1899E-01	7.7960E-01	ENSG00000274525
NA	0.0356	6.1921E-01	7.7976E-01	ENSG00000259414
NA	0.0244	6.1939E-01		ENSG00000258844
NA	0.0290	6.1971E-01	7.7995E-01	ENSG00000280129
NA	0.0073	6.1972E-01		ENSG00000254187
NA	0.0107	6.2022E-01		ENSG00000263305
NA	0.0048	6.2034E-01		ENSG00000259196
NA	0.0072	6.2074E-01		ENSG00000267160
NA	0.0350	6.2074E-01	7.8065E-01	ENSG00000274372
NA	0.0282	6.2103E-01	7.8072E-01	ENSG00000264630
NA	0.0338	6.2104E-01	7.8072E-01	ENSG00000274105
NA	0.0050	6.2119E-01		ENSG00000273328
NA	0.0377	6.2123E-01	7.8089E-01	ENSG00000205041
NA	0.0419	6.2125E-01	7.8089E-01	ENSG00000257839
NA	0.0042	6.2129E-01		ENSG00000279757
NA	0.0397	6.2139E-01	7.8098E-01	ENSG00000240057
NA	0.0396	6.2150E-01	7.8105E-01	ENSG00000250068
NA	0.0055	6.2159E-01		ENSG00000277171
NA	0.0161	6.2169E-01		ENSG00000232194
NA	0.0257	6.2169E-01		ENSG00000253171
NA	0.0097	6.2171E-01		ENSG00000224629
NA	0.0130	6.2172E-01		ENSG00000287872
NA	0.0288	6.2196E-01		ENSG00000226163
NA	0.0101	6.2210E-01		ENSG00000263326
NA	0.0100	6.2214E-01		ENSG00000283689
NA	0.0126	6.2275E-01	7.8201E-01	ENSG00000286269
NA	0.0099	6.2323E-01		ENSG00000287282
NA	0.0075	6.2337E-01		ENSG00000259072
NA	0.0368	6.2338E-01	7.8240E-01	ENSG00000279861
NA	0.0203	6.2366E-01		ENSG00000273979
NA	0.0243	6.2381E-01		ENSG00000288809
NA	0.0410	6.2383E-01	7.8281E-01	ENSG00000270021
NA	0.0193	6.2403E-01	7.8294E-01	ENSG00000277693
NA	0.0022	6.2408E-01		ENSG00000290122
NA	0.0091	6.2436E-01		ENSG00000259199
NA	0.0102	6.2450E-01		ENSG00000287702
NA	0.0132	6.2485E-01		ENSG00000288692

NA	0.0415	6.2491E-01	7.8340E-01	ENSG00000278291
NA	0.0114	6.2522E-01		ENSG00000238259
NA	0.0108	6.2524E-01		ENSG00000287784
NA	0.0064	6.2535E-01		ENSG00000263154
NA	0.0175	6.2577E-01		ENSG00000227945
NA	0.0072	6.2594E-01		ENSG00000216657
NA	0.0099	6.2650E-01		ENSG00000226291
NA	0.0109	6.2674E-01		ENSG00000231272
NA	0.0244	6.2675E-01	7.8481E-01	ENSG00000229673
NA	0.0054	6.2703E-01		ENSG00000201885
NA	0.0054	6.2703E-01		ENSG00000201959
NA	0.0054	6.2703E-01		ENSG00000201282
NA	0.0054	6.2703E-01		ENSG00000273927
NA	0.0054	6.2703E-01		ENSG00000274984
NA	0.0054	6.2703E-01		ENSG00000199913
NA	0.0054	6.2703E-01		ENSG00000267828
NA	0.0054	6.2703E-01		ENSG00000202021
NA	0.0054	6.2703E-01		ENSG00000199870
NA	0.0026	6.2714E-01		ENSG00000280143
NA	0.0407	6.2829E-01	7.8608E-01	ENSG00000247373
NA	0.0410	6.2840E-01	7.8609E-01	ENSG00000242540
NA	0.0415	6.2842E-01	7.8609E-01	ENSG00000261485
NA	0.0153	6.2852E-01		ENSG00000259533
NA	0.0311	6.2858E-01	7.8609E-01	ENSG00000289141
NA	0.0070	6.2891E-01		ENSG00000286779
NA	0.0044	6.2904E-01		ENSG00000237170
NA	0.0244	6.2932E-01		ENSG00000287248
NA	0.0179	6.2933E-01		ENSG00000287695
NA	0.0215	6.2948E-01		ENSG00000284237
NA	0.0042	6.2950E-01		ENSG00000232342
NA	0.0395	6.3009E-01	7.8705E-01	ENSG00000259658
NA	0.0127	6.3017E-01		ENSG00000231830
NA	0.0367	6.3026E-01	7.8706E-01	ENSG00000261584
NA	0.0081	6.3029E-01		ENSG00000256306
NA	0.0408	6.3035E-01	7.8710E-01	ENSG00000275557
NA	0.0136	6.3051E-01		ENSG00000264421
NA	0.0046	6.3065E-01		ENSG00000204709
NA	0.0211	6.3065E-01	7.8728E-01	ENSG00000255445
NA	0.0268	6.3077E-01	7.8738E-01	ENSG00000263033
NA	0.0177	6.3122E-01		ENSG00000280302
NA	0.0116	6.3168E-01		ENSG00000274275
NA	0.0177	6.3211E-01		ENSG00000287788
NA	0.0007	6.3223E-01		ENSG00000288788
NA	0.0033	6.3242E-01		ENSG00000260030
NA	0.0410	6.3286E-01	7.8928E-01	ENSG00000289594
NA	0.0150	6.3294E-01	7.8929E-01	ENSG00000285819
NA	0.0165	6.3295E-01		ENSG00000235619
NA	0.0166	6.3300E-01		ENSG00000207342
NA	0.0287	6.3309E-01	7.8944E-01	ENSG00000225082

NA	0.0018	6.3326E-01		ENSG00000253908
NA	0.0045	6.3328E-01		ENSG00000289454
NA	0.0147	6.3329E-01		ENSG00000261469
NA	0.0064	6.3333E-01		ENSG00000189002
NA	0.0092	6.3366E-01		ENSG00000286844
NA	0.0157	6.3367E-01		ENSG00000280040
NA	0.0454	6.3369E-01	7.8999E-01	ENSG00000269635
NA	0.0290	6.3385E-01	7.9007E-01	ENSG00000289990
NA	0.0344	6.3393E-01	7.9012E-01	ENSG00000234869
NA	0.0047	6.3414E-01		ENSG00000286423
NA	0.0225	6.3425E-01		ENSG00000266922
NA	0.0383	6.3426E-01	7.9032E-01	ENSG00000185834
NA	0.0089	6.3436E-01		ENSG00000200241
NA	0.0141	6.3437E-01		ENSG00000256268
NA	0.0345	6.3449E-01	7.9046E-01	ENSG00000287506
NA	0.0084	6.3460E-01		ENSG00000229612
NA	0.0233	6.3466E-01		ENSG00000267048
NA	0.0365	6.3469E-01	7.9048E-01	ENSG00000280128
NA	0.0344	6.3480E-01	7.9053E-01	ENSG00000221930
NA	0.0011	6.3489E-01		ENSG00000251250
NA	0.0137	6.3504E-01		ENSG00000276631
NA	0.0138	6.3507E-01		ENSG00000227933
NA	0.0087	6.3523E-01		ENSG00000236445
NA	0.0112	6.3523E-01		ENSG00000225818
NA	0.0140	6.3561E-01		ENSG00000179420
NA	0.0236	6.3570E-01		ENSG00000265982
NA	0.0243	6.3579E-01		ENSG00000258647
NA	0.0067	6.3592E-01		ENSG00000266801
NA	0.0165	6.3596E-01		ENSG00000290002
NA	0.0055	6.3600E-01	7.9127E-01	ENSG00000252690
NA	0.0013	6.3604E-01	7.9127E-01	ENSG00000257494
NA	0.0225	6.3604E-01		ENSG00000233133
NA	0.0054	6.3610E-01		ENSG00000234428
NA	0.0074	6.3621E-01		ENSG00000270933
NA	0.0229	6.3622E-01		ENSG00000289202
NA	0.0102	6.3644E-01		ENSG00000254485
NA	0.0204	6.3688E-01		ENSG00000255216
NA	0.0137	6.3693E-01		ENSG00000289570
NA	0.0374	6.3735E-01	7.9226E-01	ENSG00000260136
NA	0.0332	6.3747E-01	7.9228E-01	ENSG00000283662
NA	0.0202	6.3752E-01		ENSG00000268379
NA	0.0147	6.3765E-01		ENSG00000286023
NA	0.0078	6.3784E-01		ENSG00000234826
NA	0.0125	6.3785E-01		ENSG00000286784
NA	0.0114	6.3792E-01		ENSG00000288737
NA	0.0065	6.3799E-01		ENSG00000241220
NA	0.0074	6.3808E-01		ENSG00000251668
NA	0.0254	6.3839E-01	7.9319E-01	ENSG00000224128
NA	0.0187	6.3851E-01		ENSG00000288734

NA	0.0219	6.3857E-01		ENSG00000224746
NA	0.0217	6.3865E-01		ENSG00000285931
NA	0.0332	6.3886E-01	7.9356E-01	ENSG00000232909
NA	0.0111	6.3904E-01		ENSG00000215887
NA	0.0393	6.3925E-01	7.9381E-01	ENSG00000269102
NA	0.0379	6.3943E-01	7.9390E-01	ENSG00000196302
NA	0.0315	6.3973E-01	7.9402E-01	ENSG00000258511
NA	0.0177	6.3990E-01		ENSG00000256564
NA	0.0403	6.4003E-01	7.9402E-01	ENSG00000175170
NA	0.0203	6.4010E-01		ENSG00000288605
NA	0.0054	6.4024E-01		ENSG00000244061
NA	0.0120	6.4026E-01		ENSG00000279794
NA	0.0398	6.4028E-01	7.9415E-01	ENSG00000270605
NA	0.0015	6.4071E-01		ENSG00000250762
NA	0.0191	6.4107E-01	7.9464E-01	ENSG00000289752
NA	0.0386	6.4165E-01	7.9504E-01	ENSG00000218596
NA	0.0307	6.4178E-01	7.9511E-01	ENSG00000273041
NA	0.0277	6.4193E-01	7.9526E-01	ENSG00000266171
NA	0.0354	6.4196E-01	7.9526E-01	ENSG00000250568
NA	0.0084	6.4202E-01		ENSG00000237781
NA	0.0344	6.4212E-01	7.9538E-01	ENSG00000249509
NA	0.0089	6.4225E-01		ENSG00000271803
NA	0.0035	6.4225E-01		ENSG00000227096
NA	0.0129	6.4261E-01		ENSG00000226191
NA	0.0091	6.4321E-01		ENSG00000259590
NA	0.0277	6.4332E-01		ENSG00000289637
NA	0.0152	6.4353E-01		ENSG00000279758
NA	0.0076	6.4376E-01		ENSG00000286629
NA	0.0082	6.4398E-01		ENSG00000231362
NA	0.0029	6.4405E-01		ENSG00000255642
NA	0.0052	6.4417E-01		ENSG00000207484
NA	0.0227	6.4428E-01		ENSG00000278891
NA	0.0257	6.4431E-01	7.9692E-01	ENSG00000289067
NA	0.0279	6.4437E-01	7.9692E-01	ENSG00000289417
NA	0.0157	6.4438E-01		ENSG00000249373
NA	0.0056	6.4485E-01		ENSG00000260031
NA	0.0181	6.4491E-01		ENSG00000262097
NA	0.0105	6.4497E-01		ENSG00000231942
NA	0.0130	6.4499E-01		ENSG00000227653
NA	0.0082	6.4520E-01		ENSG00000224100
NA	0.0062	6.4530E-01		ENSG00000269118
NA	0.0259	6.4549E-01	7.9767E-01	ENSG00000287717
NA	0.0072	6.4578E-01		ENSG00000241667
NA	0.0165	6.4587E-01		ENSG00000267735
NA	0.0259	6.4602E-01		ENSG00000273989
NA	0.0053	6.4638E-01		ENSG00000289033
NA	0.0185	6.4665E-01		ENSG00000236723
NA	0.0207	6.4667E-01		ENSG00000260773
NA	0.0088	6.4681E-01		ENSG00000278147

NA	0.0102	6.4688E-01		ENSG00000275654
NA	0.0157	6.4689E-01		ENSG00000224404
NA	0.0160	6.4700E-01		ENSG00000237076
NA	0.0136	6.4714E-01		ENSG00000289481
NA	0.0138	6.4715E-01		ENSG00000271099
NA	0.0255	6.4729E-01		ENSG00000226956
NA	0.0112	6.4737E-01		ENSG00000215065
NA	0.0087	6.4761E-01		ENSG00000224167
NA	0.0338	6.4763E-01	7.9966E-01	ENSG00000224680
NA	0.0175	6.4769E-01		ENSG00000285752
NA	0.0136	6.4770E-01		ENSG00000279659
NA	0.0107	6.4777E-01		ENSG00000228721
NA	0.0370	6.4840E-01	8.0021E-01	ENSG00000223773
NA	0.0166	6.4850E-01		ENSG00000279833
NA	0.0348	6.4880E-01	8.0035E-01	ENSG00000256988
NA	0.0053	6.4897E-01	8.0041E-01	ENSG00000261670
NA	0.0021	6.4909E-01		ENSG00000277127
NA	0.0054	6.4968E-01		ENSG00000254900
NA	0.0100	6.4991E-01		ENSG00000186831
NA	0.0167	6.4994E-01	8.0135E-01	ENSG00000275476
NA	0.0078	6.5001E-01		ENSG00000290055
NA	0.0092	6.5006E-01		ENSG00000225328
NA	0.0247	6.5011E-01	8.0152E-01	ENSG00000274682
NA	0.0102	6.5062E-01		ENSG00000232118
NA	0.0020	6.5071E-01		ENSG00000284748
NA	0.0068	6.5081E-01		ENSG00000229272
NA	0.0388	6.5093E-01	8.0210E-01	ENSG00000289890
NA	0.0078	6.5101E-01		ENSG00000261058
NA	0.0176	6.5111E-01		ENSG00000230830
NA	0.0107	6.5133E-01		ENSG00000230170
NA	0.0046	6.5149E-01		ENSG00000213144
NA	0.0334	6.5150E-01	8.0263E-01	ENSG00000288538
NA	0.0049	6.5173E-01		ENSG00000253536
NA	0.0117	6.5180E-01		ENSG00000286066
NA	0.0092	6.5196E-01		ENSG00000286538
NA	0.0098	6.5206E-01		ENSG00000242675
NA	0.0099	6.5208E-01		ENSG00000286882
NA	0.0079	6.5228E-01		ENSG00000281856
NA	0.0006	6.5275E-01		ENSG00000257376
NA	0.0241	6.5277E-01		ENSG00000266717
NA	0.0337	6.5281E-01	8.0340E-01	ENSG00000289499
NA	0.0098	6.5290E-01		ENSG00000286285
NA	0.0151	6.5311E-01		ENSG00000235267
NA	0.0191	6.5342E-01		ENSG00000278829
NA	0.0181	6.5382E-01		ENSG00000266969
NA	0.0269	6.5417E-01	8.0459E-01	ENSG00000289109
NA	0.0124	6.5422E-01		ENSG00000229357
NA	0.0043	6.5464E-01		ENSG00000226622
NA	0.0204	6.5478E-01		ENSG00000259035

NA	0.0253	6.5482E-01	8.0512E-01	ENSG00000286943
NA	0.0137	6.5484E-01		ENSG00000224992
NA	0.0096	6.5500E-01		ENSG00000257809
NA	0.0263	6.5516E-01	8.0518E-01	ENSG00000277007
NA	0.0204	6.5521E-01		ENSG00000279479
NA	0.0311	6.5549E-01		ENSG00000272205
NA	0.0112	6.5563E-01		ENSG00000259589
NA	0.0114	6.5579E-01		ENSG00000253256
NA	0.0116	6.5595E-01		ENSG00000259846
NA	0.0159	6.5599E-01		ENSG00000202078
NA	0.0045	6.5614E-01		ENSG00000230267
NA	0.0074	6.5649E-01		ENSG00000290115
NA	0.0221	6.5661E-01	8.0614E-01	ENSG00000271788
NA	0.0184	6.5664E-01		ENSG00000272457
NA	0.0184	6.5679E-01		ENSG00000232699
NA	0.0305	6.5683E-01	8.0624E-01	ENSG00000253168
NA	0.0121	6.5685E-01		ENSG00000253896
NA	0.0073	6.5695E-01		ENSG00000274979
NA	0.0382	6.5714E-01	8.0651E-01	ENSG00000285847
NA	0.0215	6.5747E-01	8.0660E-01	ENSG00000256682
NA	0.0107	6.5752E-01		ENSG00000281008
NA	0.0090	6.5764E-01		ENSG00000233519
NA	0.0360	6.5776E-01	8.0675E-01	ENSG00000230551
NA	0.0121	6.5812E-01		ENSG00000236474
NA	0.0117	6.5827E-01		ENSG00000279611
NA	0.0033	6.5896E-01		ENSG00000234775
NA	0.0200	6.5920E-01		ENSG00000277595
NA	0.0236	6.5922E-01	8.0777E-01	ENSG00000279138
NA	0.0135	6.5931E-01		ENSG00000242828
NA	0.0299	6.5933E-01	8.0785E-01	ENSG00000259985
NA	0.0035	6.5977E-01		ENSG00000213133
NA	0.0140	6.6002E-01		ENSG00000289584
NA	0.0064	6.6020E-01		ENSG00000236493
NA	0.0002	6.6056E-01	8.0881E-01	ENSG00000261411
NA	0.0203	6.6063E-01		ENSG00000287676
NA	0.0367	6.6064E-01	8.0883E-01	ENSG00000289183
NA	0.0056	6.6072E-01		ENSG00000279960
NA	0.0352	6.6083E-01	8.0888E-01	ENSG00000260274
NA	0.0240	6.6095E-01	8.0897E-01	ENSG00000288739
NA	0.0271	6.6098E-01	8.0897E-01	ENSG00000261766
NA	0.0178	6.6176E-01		ENSG00000213234
NA	0.0098	6.6198E-01		ENSG00000280265
NA	0.0182	6.6205E-01		ENSG00000244125
NA	0.0374	6.6208E-01	8.0977E-01	ENSG00000276564
NA	0.0253	6.6220E-01	8.0977E-01	ENSG00000282100
NA	0.0121	6.6258E-01		ENSG00000227141
NA	0.0035	6.6269E-01		ENSG00000278727
NA	0.0300	6.6304E-01	8.1040E-01	ENSG00000268945
NA	0.0266	6.6316E-01	8.1047E-01	ENSG00000280420

NA	0.0077	6.6353E-01		ENSG00000259079
NA	0.0094	6.6365E-01		ENSG00000235028
NA	0.0210	6.6416E-01	8.1108E-01	ENSG00000255449
NA	0.0095	6.6426E-01		ENSG00000259228
NA	0.0370	6.6435E-01	8.1119E-01	ENSG00000273987
NA	0.0032	6.6438E-01		ENSG00000261239
NA	0.0406	6.6444E-01	8.1125E-01	ENSG00000261141
NA	0.0087	6.6446E-01		ENSG00000287962
NA	0.0317	6.6453E-01		ENSG00000233695
NA	0.0024	6.6469E-01		ENSG00000288871
NA	0.0113	6.6487E-01		ENSG00000229723
NA	0.0144	6.6493E-01		ENSG00000272912
NA	0.0015	6.6501E-01		ENSG00000286957
NA	0.0145	6.6502E-01	8.1177E-01	ENSG00000231393
NA	0.0267	6.6509E-01	8.1182E-01	ENSG00000229122
NA	0.0188	6.6524E-01		ENSG00000255557
NA	0.0100	6.6532E-01		ENSG00000199797
NA	0.0282	6.6545E-01	8.1205E-01	ENSG00000259135
NA	0.0122	6.6553E-01		ENSG00000286875
NA	0.0068	6.6560E-01		ENSG00000290111
NA	0.0165	6.6562E-01		ENSG00000257262
NA	0.0128	6.6571E-01	8.1229E-01	ENSG00000250343
NA	0.0194	6.6578E-01		ENSG00000261722
NA	0.0189	6.6613E-01		ENSG00000286976
NA	0.0224	6.6622E-01	8.1271E-01	ENSG00000214433
NA	0.0172	6.6652E-01		ENSG00000236940
NA	0.0084	6.6681E-01		ENSG00000277299
NA	0.0124	6.6685E-01		ENSG00000284703
NA	0.0131	6.6710E-01	8.1346E-01	ENSG00000257458
NA	0.0270	6.6750E-01	8.1378E-01	ENSG00000272950
NA	0.0076	6.6754E-01		ENSG00000259296
NA	0.0360	6.6782E-01	8.1403E-01	ENSG00000289149
NA	0.0272	6.6827E-01	8.1415E-01	ENSG00000233778
NA	0.0328	6.6828E-01	8.1415E-01	ENSG00000259921
NA	0.0207	6.6834E-01	8.1415E-01	ENSG00000263276
NA	0.0196	6.6868E-01		ENSG00000224066
NA	0.0119	6.6871E-01		ENSG00000283097
NA	0.0067	6.6933E-01		ENSG00000228312
NA	0.0055	6.6950E-01		ENSG00000276620
NA	0.0313	6.6951E-01	8.1482E-01	ENSG00000286415
NA	0.0257	6.6976E-01	8.1484E-01	ENSG00000225806
NA	0.0091	6.6991E-01		ENSG00000271208
NA	0.0159	6.6991E-01		ENSG00000289865
NA	0.0111	6.7027E-01		ENSG00000229848
NA	0.0232	6.7042E-01		ENSG00000273711
NA	0.0305	6.7056E-01	8.1544E-01	ENSG00000267868
NA	0.0294	6.7058E-01	8.1544E-01	ENSG00000289909
NA	0.0142	6.7062E-01		ENSG00000263321
NA	0.0042	6.7083E-01		ENSG00000226324

NA	0.0059	6.7087E-01		ENSG00000230154
NA	0.0105	6.7091E-01		ENSG00000284646
NA	0.0111	6.7104E-01		ENSG00000224273
NA	0.0081	6.7104E-01		ENSG00000285082
NA	0.0331	6.7114E-01	8.1596E-01	ENSG00000278993
NA	0.0347	6.7179E-01	8.1641E-01	ENSG00000229728
NA	0.0145	6.7183E-01		ENSG00000272167
NA	0.0104	6.7206E-01		ENSG00000241985
NA	0.0097	6.7213E-01		ENSG00000240758
NA	0.0193	6.7231E-01		ENSG00000199906
NA	0.0063	6.7263E-01		ENSG00000285016
NA	0.0353	6.7265E-01	8.1695E-01	ENSG00000272969
NA	0.0361	6.7273E-01	8.1700E-01	ENSG00000271918
NA	0.0423	6.7284E-01	8.1702E-01	ENSG00000271200
NA	0.0272	6.7308E-01	8.1708E-01	ENSG00000259250
NA	0.0083	6.7322E-01		ENSG00000283304
NA	0.0359	6.7339E-01	8.1729E-01	ENSG00000290072
NA	0.0139	6.7352E-01		ENSG00000285932
NA	0.0043	6.7417E-01		ENSG00000234663
NA	0.0311	6.7418E-01	8.1747E-01	ENSG00000289727
NA	0.0098	6.7418E-01		ENSG00000222352
NA	0.0350	6.7444E-01	8.1755E-01	ENSG00000234292
NA	0.0085	6.7444E-01		ENSG00000271454
NA	0.0328	6.7450E-01	8.1758E-01	ENSG00000283886
NA	0.0149	6.7466E-01		ENSG00000286880
NA	0.0304	6.7470E-01	8.1764E-01	ENSG00000215452
NA	0.0085	6.7480E-01		ENSG00000223783
NA	0.0356	6.7485E-01	8.1764E-01	ENSG00000249896
NA	0.0292	6.7485E-01	8.1764E-01	ENSG00000213338
NA	0.0436	6.7488E-01	8.1764E-01	ENSG00000267113
NA	0.0052	6.7512E-01		ENSG00000271018
NA	0.0356	6.7513E-01	8.1780E-01	ENSG00000289102
NA	0.0583	6.7515E-01	8.1780E-01	ENSG00000231992
NA	0.0135	6.7522E-01		ENSG00000285162
NA	0.0353	6.7534E-01	8.1785E-01	ENSG00000289341
NA	0.0142	6.7547E-01		ENSG00000237899
NA	0.0070	6.7552E-01		ENSG00000287987
NA	0.0093	6.7573E-01		ENSG00000288536
NA	0.0016	6.7608E-01		ENSG00000224265
NA	0.0029	6.7609E-01		ENSG00000265490
NA	0.0180	6.7614E-01		ENSG00000277948
NA	0.0011	6.7656E-01		ENSG00000256473
NA	0.0240	6.7664E-01	8.1885E-01	ENSG00000272721
NA	0.0187	6.7670E-01		ENSG00000237321
NA	0.0181	6.7678E-01		ENSG00000287985
NA	0.0131	6.7689E-01		ENSG00000257256
NA	0.0121	6.7705E-01		ENSG00000255478
NA	0.0239	6.7705E-01	8.1908E-01	ENSG00000285952
NA	0.0073	6.7716E-01		ENSG00000289726

NA	0.0272	6.7740E-01	8.1922E-01	ENSG00000258608
NA	0.0170	6.7769E-01		ENSG00000230186
NA	0.0039	6.7779E-01		ENSG00000288755
NA	0.0181	6.7782E-01		ENSG00000223732
NA	0.0352	6.7814E-01	8.1969E-01	ENSG00000239415
NA	0.0023	6.7817E-01	8.1969E-01	ENSG00000271917
NA	0.0168	6.7818E-01		ENSG00000279289
NA	0.0241	6.7830E-01	8.1971E-01	ENSG00000228944
NA	0.0089	6.7835E-01		ENSG00000249228
NA	0.0105	6.7837E-01		ENSG00000253858
NA	0.0212	6.7856E-01		ENSG00000218350
NA	0.0144	6.7862E-01		ENSG00000288051
NA	0.0296	6.7892E-01	8.2031E-01	ENSG00000285991
NA	0.0304	6.7922E-01	8.2051E-01	ENSG00000272717
NA	0.0199	6.7925E-01		ENSG00000237390
NA	0.0236	6.7943E-01		ENSG00000286478
NA	0.0063	6.7946E-01		ENSG00000254154
NA	0.0032	6.7949E-01		ENSG00000289562
NA	0.0178	6.7970E-01		ENSG00000227242
NA	0.0004	6.7982E-01		ENSG00000277548
NA	0.0273	6.7985E-01	8.2083E-01	ENSG00000213071
NA	0.0166	6.7994E-01		ENSG00000287626
NA	0.0352	6.8040E-01	8.2129E-01	ENSG00000205236
NA	0.0418	6.8063E-01	8.2146E-01	ENSG00000223349
NA	0.0144	6.8069E-01		ENSG00000254552
NA	0.0174	6.8088E-01		ENSG00000257464
NA	0.0091	6.8113E-01		ENSG00000266932
NA	0.0272	6.8128E-01	8.2207E-01	ENSG00000257953
NA	0.0166	6.8136E-01		ENSG00000287381
NA	0.0240	6.8143E-01	8.2221E-01	ENSG00000262712
NA	0.0258	6.8150E-01		ENSG00000263105
NA	0.0146	6.8155E-01		ENSG00000247570
NA	0.0120	6.8201E-01		ENSG00000262652
NA	0.0122	6.8209E-01		ENSG00000181355
NA	0.0257	6.8250E-01	8.2310E-01	ENSG00000280274
NA	0.0201	6.8261E-01	8.2313E-01	ENSG00000286314
NA	0.0055	6.8263E-01		ENSG00000257169
NA	0.0135	6.8269E-01		ENSG00000289847
NA	0.0068	6.8272E-01		ENSG00000286284
NA	0.0051	6.8288E-01		ENSG00000289206
NA	0.0273	6.8296E-01	8.2313E-01	ENSG00000287941
NA	0.0034	6.8298E-01		ENSG00000286468
NA	0.0322	6.8305E-01	8.2313E-01	ENSG00000279792
NA	0.0102	6.8312E-01		ENSG00000267521
NA	0.0126	6.8318E-01		ENSG00000277587
NA	0.0100	6.8326E-01		ENSG00000248131
NA	0.0172	6.8331E-01		ENSG00000287907
NA	0.0064	6.8342E-01		ENSG00000287536
NA	0.0146	6.8392E-01		ENSG00000267036

NA	0.0064	6.8440E-01		ENSG00000286767
NA	0.0214	6.8443E-01		ENSG00000277589
NA	0.0089	6.8447E-01	8.2406E-01	ENSG00000261402
NA	0.0334	6.8471E-01	8.2427E-01	ENSG00000279021
NA	0.0023	6.8480E-01		ENSG00000248200
NA	0.0189	6.8483E-01		ENSG00000235151
NA	0.0088	6.8484E-01		ENSG00000258943
NA	0.0322	6.8496E-01	8.2445E-01	ENSG00000288023
NA	0.0196	6.8547E-01		ENSG00000244036
NA	0.0121	6.8565E-01		ENSG00000275995
NA	0.0270	6.8582E-01	8.2498E-01	ENSG00000286903
NA	0.0261	6.8583E-01	8.2498E-01	ENSG00000263126
NA	0.0035	6.8588E-01		ENSG00000250488
NA	0.0330	6.8590E-01	8.2498E-01	ENSG00000270171
NA	0.0302	6.8590E-01	8.2498E-01	ENSG00000280355
NA	0.0206	6.8591E-01		ENSG00000256824
NA	0.0035	6.8596E-01		ENSG00000270702
NA	0.0095	6.8598E-01		ENSG00000254213
NA	0.0237	6.8611E-01	8.2504E-01	ENSG00000226772
NA	0.0102	6.8632E-01		ENSG00000240163
NA	0.0174	6.8643E-01		ENSG00000264693
NA	0.0074	6.8653E-01		ENSG00000227658
NA	0.0055	6.8667E-01		ENSG00000273972
NA	0.0085	6.8689E-01		ENSG00000188755
NA	0.0089	6.8699E-01		ENSG00000287458
NA	0.0095	6.8707E-01		ENSG00000227602
NA	0.0179	6.8708E-01		ENSG00000280060
NA	0.0064	6.8728E-01		ENSG00000236230
NA	0.0037	6.8755E-01		ENSG00000166104
NA	0.0174	6.8820E-01	8.2684E-01	ENSG00000250934
NA	0.0280	6.8840E-01	8.2685E-01	ENSG00000281195
NA	0.0143	6.8850E-01		ENSG00000278966
NA	0.0021	6.8863E-01		ENSG00000287834
NA	0.0049	6.8867E-01		ENSG00000278408
NA	0.0300	6.8875E-01	8.2704E-01	ENSG00000229939
NA	0.0111	6.8878E-01		ENSG00000258626
NA	0.0246	6.8890E-01	8.2709E-01	ENSG00000254635
NA	0.0179	6.8892E-01		ENSG00000219693
NA	0.0168	6.8934E-01		ENSG00000225703
NA	0.0224	6.8961E-01		ENSG00000229227
NA	0.0070	6.8990E-01		ENSG00000215812
NA	0.0227	6.9061E-01	8.2817E-01	ENSG00000229816
NA	0.0078	6.9088E-01		ENSG00000249129
NA	0.0001	6.9106E-01		ENSG00000261629
NA	0.0242	6.9122E-01	8.2862E-01	ENSG00000286319
NA	0.0063	6.9162E-01		ENSG00000225249
NA	0.0206	6.9176E-01		ENSG00000240107
NA	0.0294	6.9181E-01	8.2904E-01	ENSG00000278922
NA	0.0141	6.9194E-01		ENSG00000248254

NA	0.0083	6.9194E-01		ENSG00000278638
NA	0.0112	6.9232E-01		ENSG00000201308
NA	0.0309	6.9286E-01	8.2966E-01	ENSG00000289216
NA	0.0070	6.9295E-01		ENSG00000216475
NA	0.0262	6.9333E-01	8.3001E-01	ENSG00000286895
NA	0.0342	6.9336E-01	8.3001E-01	ENSG00000230530
NA	0.0029	6.9337E-01		ENSG00000235358
NA	0.0275	6.9339E-01	8.3001E-01	ENSG00000260249
NA	0.0393	6.9395E-01	8.3042E-01	ENSG00000286721
NA	0.0312	6.9400E-01	8.3042E-01	ENSG00000258813
NA	0.0299	6.9438E-01	8.3064E-01	ENSG00000259642
NA	0.0139	6.9461E-01		ENSG00000266279
NA	0.0083	6.9485E-01		ENSG00000213155
NA	0.0063	6.9489E-01		ENSG00000224750
NA	0.0109	6.9501E-01	8.3113E-01	ENSG00000289367
NA	0.0108	6.9539E-01		ENSG00000226859
NA	0.0141	6.9566E-01		ENSG00000228667
NA	0.0064	6.9571E-01		ENSG00000248356
NA	0.0227	6.9580E-01		ENSG00000255886
NA	0.0322	6.9586E-01	8.3174E-01	ENSG00000269837
NA	0.0075	6.9604E-01		ENSG00000286965
NA	0.0062	6.9606E-01		ENSG00000267439
NA	0.0107	6.9615E-01		ENSG00000286855
NA	0.0107	6.9625E-01		ENSG00000279041
NA	0.0197	6.9638E-01		ENSG00000286788
NA	0.0247	6.9645E-01	8.3210E-01	ENSG00000243167
NA	0.0128	6.9649E-01		ENSG00000285727
NA	0.0185	6.9664E-01		ENSG00000267919
NA	0.0103	6.9677E-01		ENSG00000217950
NA	0.0333	6.9687E-01	8.3240E-01	ENSG00000247934
NA	0.0263	6.9688E-01	8.3240E-01	ENSG00000289425
NA	0.0133	6.9694E-01		ENSG00000233674
NA	0.0035	6.9703E-01		ENSG00000235337
NA	0.0054	6.9740E-01		ENSG00000234943
NA	0.0108	6.9752E-01		ENSG00000267150
NA	0.0045	6.9763E-01		ENSG00000286343
NA	0.0175	6.9768E-01	8.3292E-01	ENSG00000248966
NA	0.0158	6.9774E-01		ENSG00000223528
NA	0.0062	6.9779E-01		ENSG00000213087
NA	0.0301	6.9784E-01	8.3296E-01	ENSG00000237977
NA	0.0312	6.9792E-01	8.3301E-01	ENSG00000279425
NA	0.0205	6.9811E-01	8.3315E-01	ENSG00000277425
NA	0.0082	6.9820E-01		ENSG00000259155
NA	0.0111	6.9822E-01		ENSG00000215692
NA	0.0038	6.9875E-01		ENSG00000254632
NA	0.0098	6.9890E-01		ENSG00000225770
NA	0.0233	6.9892E-01	8.3364E-01	ENSG00000269993
NA	0.0123	6.9903E-01		ENSG00000280231
NA	0.0246	6.9922E-01	8.3370E-01	ENSG00000224016

NA	0.0168	6.9941E-01		ENSG00000260405
NA	0.0105	6.9944E-01		ENSG00000277373
NA	0.0080	6.9946E-01		ENSG00000289539
NA	0.0069	6.9951E-01		ENSG00000273325
NA	0.0326	6.9971E-01	8.3388E-01	ENSG00000170629
NA	0.0116	6.9978E-01		ENSG00000261158
NA	0.0061	6.9978E-01		ENSG00000259871
NA	0.0080	6.9994E-01		ENSG00000276097
NA	0.0157	7.0003E-01		ENSG00000275741
NA	0.0170	7.0008E-01		ENSG00000223799
NA	0.0323	7.0077E-01	8.3466E-01	ENSG00000214919
NA	0.0338	7.0085E-01	8.3468E-01	ENSG00000223345
NA	0.0154	7.0095E-01	8.3476E-01	ENSG00000267117
NA	0.0295	7.0120E-01	8.3489E-01	ENSG00000235072
NA	0.0115	7.0134E-01		ENSG00000233842
NA	0.0264	7.0144E-01	8.3509E-01	ENSG00000261659
NA	0.0134	7.0165E-01		ENSG00000260345
NA	0.0083	7.0167E-01		ENSG00000282924
NA	0.0136	7.0170E-01		ENSG00000269910
NA	0.0117	7.0183E-01		ENSG00000285789
NA	0.0208	7.0196E-01	8.3536E-01	ENSG00000262248
NA	0.0103	7.0201E-01		ENSG00000243081
NA	0.0217	7.0203E-01	8.3540E-01	ENSG00000270091
NA	0.0159	7.0204E-01		ENSG00000280202
NA	0.0168	7.0229E-01	8.3561E-01	ENSG00000254694
NA	0.0058	7.0231E-01		ENSG00000268333
NA	0.0317	7.0245E-01	8.3561E-01	ENSG00000286123
NA	0.0378	7.0275E-01	8.3588E-01	ENSG00000262801
NA	0.0059	7.0278E-01		ENSG00000228222
NA	0.0197	7.0297E-01	8.3603E-01	ENSG00000226937
NA	0.0269	7.0304E-01	8.3607E-01	ENSG00000144158
NA	0.0046	7.0334E-01		ENSG00000279628
NA	0.0239	7.0409E-01	8.3679E-01	ENSG00000289402
NA	0.0105	7.0442E-01		ENSG00000287873
NA	0.0071	7.0445E-01		ENSG00000289488
NA	0.0088	7.0454E-01		ENSG00000273443
NA	0.0116	7.0464E-01		ENSG00000224677
NA	0.0282	7.0481E-01	8.3721E-01	ENSG00000259086
NA	0.0124	7.0492E-01		ENSG00000246095
NA	0.0088	7.0508E-01		ENSG00000254921
NA	0.0296	7.0514E-01	8.3752E-01	ENSG00000280417
NA	0.0138	7.0526E-01		ENSG00000256973
NA	0.0036	7.0545E-01		ENSG00000255727
NA	0.0301	7.0554E-01	8.3775E-01	ENSG00000290114
NA	0.0162	7.0570E-01		ENSG00000287535
NA	0.0214	7.0576E-01	8.3785E-01	ENSG00000220157
NA	0.0267	7.0583E-01	8.3788E-01	ENSG00000279035
NA	0.0323	7.0594E-01	8.3798E-01	ENSG00000285856
NA	0.0183	7.0606E-01	8.3808E-01	ENSG00000273058

NA	0.0144	7.0649E-01		ENSG00000287799
NA	0.0004	7.0650E-01		ENSG00000271234
NA	0.0020	7.0687E-01		ENSG00000271919
NA	0.0052	7.0704E-01		ENSG00000287417
NA	0.0049	7.0712E-01		ENSG00000220745
NA	0.0150	7.0717E-01		ENSG00000260177
NA	0.0121	7.0721E-01		ENSG00000273609
NA	0.0085	7.0730E-01	8.3906E-01	ENSG00000249359
NA	0.0019	7.0735E-01		ENSG00000205745
NA	0.0080	7.0751E-01		ENSG00000263781
NA	0.0080	7.0764E-01		ENSG00000227705
NA	0.0185	7.0767E-01	8.3931E-01	ENSG00000248459
NA	0.0057	7.0771E-01		ENSG00000287731
NA	0.0273	7.0788E-01	8.3952E-01	ENSG00000230393
NA	0.0058	7.0795E-01		ENSG00000249605
NA	0.0063	7.0803E-01		ENSG00000200344
NA	0.0373	7.0810E-01	8.3965E-01	ENSG00000286132
NA	0.0358	7.0827E-01	8.3965E-01	ENSG00000289519
NA	0.0006	7.0842E-01		ENSG00000277544
NA	0.0087	7.0870E-01		ENSG00000289957
NA	0.0192	7.0906E-01	8.4006E-01	ENSG00000263923
NA	0.0167	7.0928E-01		ENSG00000270060
NA	0.0093	7.0932E-01		ENSG00000244327
NA	0.0170	7.0953E-01		ENSG00000256616
NA	0.0258	7.0964E-01	8.4042E-01	ENSG00000244604
NA	0.0098	7.0969E-01		ENSG00000251148
NA	0.0022	7.0974E-01		ENSG00000225726
NA	0.0073	7.0988E-01		ENSG00000241907
NA	0.0062	7.0994E-01		ENSG00000282859
NA	0.0080	7.1017E-01	8.4083E-01	ENSG00000269600
NA	0.0008	7.1043E-01		ENSG00000215102
NA	0.0018	7.1044E-01		ENSG00000288776
NA	0.0147	7.1079E-01		ENSG00000285830
NA	0.0200	7.1087E-01		ENSG00000271717
NA	0.0065	7.1093E-01		ENSG00000285117
NA	0.0192	7.1114E-01	8.4147E-01	ENSG00000267549
NA	0.0179	7.1161E-01		ENSG00000258593
NA	0.0095	7.1182E-01		ENSG00000234622
NA	0.0053	7.1200E-01		ENSG00000285598
NA	0.0032	7.1208E-01		ENSG00000286385
NA	0.0108	7.1217E-01		ENSG00000230404
NA	0.0154	7.1221E-01		ENSG00000274813
NA	0.0193	7.1239E-01		ENSG00000286576
NA	0.0295	7.1252E-01	8.4225E-01	ENSG00000227888
NA	0.0036	7.1346E-01		ENSG00000231207
NA	0.0139	7.1363E-01		ENSG00000274868
NA	0.0139	7.1363E-01		ENSG00000275664
NA	0.0107	7.1366E-01		ENSG00000254024
NA	0.0264	7.1377E-01		ENSG00000275703

NA	0.0254	7.1379E-01	8.4287E-01	ENSG00000286692
NA	0.0189	7.1380E-01		ENSG00000260157
NA	0.0150	7.1386E-01		ENSG00000258875
NA	0.0063	7.1400E-01		ENSG00000287796
NA	0.0014	7.1406E-01		ENSG00000261736
NA	0.0156	7.1416E-01		ENSG00000287661
NA	0.0028	7.1446E-01		ENSG00000280095
NA	0.0212	7.1472E-01	8.4332E-01	ENSG00000272211
NA	0.0316	7.1474E-01	8.4332E-01	ENSG00000267632
NA	0.0262	7.1476E-01	8.4332E-01	ENSG00000281379
NA	0.0260	7.1479E-01	8.4332E-01	ENSG00000272360
NA	0.0257	7.1501E-01	8.4343E-01	ENSG00000286277
NA	0.0084	7.1504E-01		ENSG00000260310
NA	0.0295	7.1514E-01	8.4343E-01	ENSG00000270062
NA	0.0068	7.1516E-01	8.4343E-01	ENSG00000228499
NA	0.0220	7.1542E-01	8.4351E-01	ENSG00000233885
NA	0.0006	7.1560E-01		ENSG00000288969
NA	0.0060	7.1564E-01		ENSG00000131982
NA	0.0075	7.1590E-01		ENSG00000282834
NA	0.0003	7.1615E-01		ENSG00000258117
NA	0.0175	7.1621E-01		ENSG00000289427
NA	0.0154	7.1635E-01		ENSG00000220913
NA	0.0121	7.1637E-01		ENSG00000253406
NA	0.0240	7.1639E-01	8.4413E-01	ENSG00000229582
NA	0.0261	7.1646E-01	8.4415E-01	ENSG00000233817
NA	0.0163	7.1686E-01		ENSG00000276742
NA	0.0175	7.1693E-01		ENSG00000272182
NA	0.0145	7.1699E-01		ENSG00000278083
NA	0.0012	7.1728E-01		ENSG00000270118
NA	0.0050	7.1738E-01		ENSG00000257904
NA	0.0074	7.1797E-01		ENSG00000230138
NA	0.0034	7.1823E-01		ENSG00000287798
NA	0.0087	7.1827E-01		ENSG00000229820
NA	0.0148	7.1832E-01		ENSG00000283525
NA	0.0202	7.1846E-01	8.4517E-01	ENSG00000273167
NA	0.0051	7.1847E-01		ENSG00000237169
NA	0.0153	7.1911E-01		ENSG00000224003
NA	0.0014	7.1916E-01		ENSG00000270277
NA	0.0137	7.1919E-01		ENSG00000249245
NA	0.0180	7.1949E-01	8.4578E-01	ENSG00000258066
NA	0.0125	7.1952E-01		ENSG00000254575
NA	0.0063	7.1967E-01		ENSG00000287176
NA	0.0049	7.2007E-01		ENSG00000201581
NA	0.0286	7.2012E-01	8.4640E-01	ENSG00000279303
NA	0.0256	7.2019E-01		ENSG00000224988
NA	0.0060	7.2024E-01		ENSG00000286694
NA	0.0223	7.2032E-01	8.4651E-01	ENSG00000236833
NA	0.0073	7.2090E-01		ENSG00000224555
NA	0.0282	7.2120E-01	8.4718E-01	ENSG00000287630

NA	0.0039	7.2156E-01		ENSG00000255050
NA	0.0051	7.2157E-01		ENSG00000289071
NA	0.0098	7.2159E-01		ENSG00000288055
NA	0.0257	7.2159E-01	8.4743E-01	ENSG00000280106
NA	0.0293	7.2162E-01	8.4743E-01	ENSG00000179277
NA	0.0061	7.2302E-01		ENSG00000279391
NA	0.0204	7.2309E-01	8.4820E-01	ENSG00000288848
NA	0.0067	7.2328E-01		ENSG00000222439
NA	0.0261	7.2329E-01	8.4820E-01	ENSG00000228192
NA	0.0212	7.2341E-01	8.4824E-01	ENSG00000270955
NA	0.0032	7.2356E-01		ENSG00000276863
NA	0.0246	7.2357E-01	8.4829E-01	ENSG00000274021
NA	0.0224	7.2384E-01	8.4854E-01	ENSG00000260907
NA	0.0038	7.2388E-01		ENSG00000226161
NA	0.0030	7.2394E-01		ENSG00000258122
NA	0.0267	7.2413E-01	8.4864E-01	ENSG00000276266
NA	0.0057	7.2425E-01		ENSG00000275016
NA	0.0097	7.2451E-01		ENSG00000226819
NA	0.0007	7.2465E-01		ENSG00000257680
NA	0.0165	7.2479E-01		ENSG00000286299
NA	0.0200	7.2484E-01		ENSG00000224725
NA	0.0179	7.2496E-01		ENSG00000223665
NA	0.0029	7.2539E-01		ENSG00000290022
NA	0.0047	7.2567E-01		ENSG00000214027
NA	0.0000	7.2567E-01	8.4947E-01	ENSG00000285745
NA	0.0015	7.2589E-01		ENSG00000257507
NA	0.0169	7.2593E-01		ENSG00000237415
NA	0.0208	7.2602E-01		ENSG00000284659
NA	0.0515	7.2614E-01	8.4964E-01	ENSG00000262136
NA	0.0104	7.2622E-01		ENSG00000265678
NA	0.0129	7.2650E-01		ENSG00000282842
NA	0.0293	7.2661E-01	8.4993E-01	ENSG00000287387
NA	0.0119	7.2678E-01		ENSG00000236676
NA	0.0095	7.2682E-01		ENSG00000203258
NA	0.0139	7.2711E-01		ENSG00000198857
NA	0.0011	7.2722E-01		ENSG00000214195
NA	0.0262	7.2739E-01	8.5039E-01	ENSG00000237126
NA	0.0187	7.2742E-01		ENSG00000281468
NA	0.0277	7.2767E-01	8.5058E-01	ENSG00000286758
NA	0.0077	7.2768E-01		ENSG00000230882
NA	0.0063	7.2814E-01		ENSG00000213761
NA	0.0072	7.2833E-01		ENSG00000213731
NA	0.0071	7.2901E-01		ENSG00000225715
NA	0.0081	7.2902E-01		ENSG00000275515
NA	0.0267	7.2903E-01		ENSG00000223571
NA	0.0066	7.2905E-01		ENSG00000258633
NA	0.0003	7.2928E-01		ENSG00000289103
NA	0.0090	7.2947E-01		ENSG00000271609
NA	0.0166	7.2965E-01	8.5207E-01	ENSG00000279110

NA	0.0278	7.2982E-01	8.5219E-01	ENSG00000273084
NA	0.0197	7.3000E-01	8.5231E-01	ENSG00000254532
NA	0.0106	7.3017E-01		ENSG00000250969
NA	0.0172	7.3042E-01		ENSG00000285215
NA	0.0042	7.3055E-01		ENSG00000254826
NA	0.0023	7.3067E-01		ENSG00000224371
NA	0.0165	7.3075E-01		ENSG00000260064
NA	0.0081	7.3097E-01		ENSG00000289938
NA	0.0296	7.3097E-01		ENSG00000236212
NA	0.0083	7.3099E-01		ENSG00000256898
NA	0.0308	7.3107E-01	8.5293E-01	ENSG00000267231
NA	0.0060	7.3110E-01		ENSG00000237947
NA	0.0089	7.3112E-01		ENSG00000279375
NA	0.0097	7.3149E-01		ENSG00000287051
NA	0.0072	7.3172E-01		ENSG00000271715
NA	0.0141	7.3174E-01		ENSG00000274227
NA	0.0233	7.3179E-01	8.5327E-01	ENSG00000259232
NA	0.0200	7.3189E-01	8.5335E-01	ENSG00000183604
NA	0.0006	7.3192E-01		ENSG00000234714
NA	0.0175	7.3206E-01		ENSG00000274514
NA	0.0255	7.3214E-01		ENSG00000273056
NA	0.0287	7.3259E-01	8.5396E-01	ENSG00000284602
NA	0.0253	7.3273E-01	8.5404E-01	ENSG00000231205
NA	0.0286	7.3281E-01	8.5405E-01	ENSG00000235513
NA	0.0004	7.3306E-01		ENSG00000242943
NA	0.0018	7.3307E-01	8.5423E-01	ENSG00000289330
NA	0.0074	7.3320E-01		ENSG00000249934
NA	0.0204	7.3327E-01	8.5427E-01	ENSG00000284698
NA	0.0075	7.3330E-01		ENSG00000237063
NA	0.0064	7.3368E-01		ENSG00000273986
NA	0.0178	7.3395E-01	8.5476E-01	ENSG00000247311
NA	0.0047	7.3408E-01		ENSG00000225336
NA	0.0197	7.3415E-01	8.5488E-01	ENSG00000271727
NA	0.0051	7.3455E-01		ENSG00000274135
NA	0.0161	7.3459E-01	8.5520E-01	ENSG00000238783
NA	0.0225	7.3464E-01		ENSG00000289632
NA	0.0108	7.3506E-01	8.5554E-01	ENSG00000264914
NA	0.0034	7.3506E-01		ENSG00000228968
NA	0.0035	7.3508E-01		ENSG00000286936
NA	0.0035	7.3518E-01		ENSG00000270491
NA	0.0200	7.3536E-01	8.5571E-01	ENSG00000283312
NA	0.0286	7.3538E-01	8.5571E-01	ENSG00000273763
NA	0.0224	7.3541E-01	8.5571E-01	ENSG00000287374
NA	0.0206	7.3592E-01	8.5597E-01	ENSG00000250210
NA	0.0151	7.3594E-01	8.5597E-01	ENSG00000248719
NA	0.0274	7.3598E-01	8.5597E-01	ENSG00000260236
NA	0.0043	7.3640E-01		ENSG00000224658
NA	0.0226	7.3642E-01	8.5612E-01	ENSG00000285763
NA	0.0100	7.3644E-01		ENSG00000248909

NA	0.0226	7.3644E-01		ENSG00000225527
NA	0.0011	7.3690E-01		ENSG00000276058
NA	0.0227	7.3696E-01	8.5644E-01	ENSG00000258739
NA	0.0352	7.3698E-01		ENSG00000285545
NA	0.0161	7.3737E-01		ENSG00000283023
NA	0.0116	7.3754E-01		ENSG00000255308
NA	0.0082	7.3762E-01		ENSG00000276391
NA	0.0085	7.3794E-01		ENSG00000265015
NA	0.0015	7.3802E-01		ENSG00000242756
NA	0.0140	7.3831E-01		ENSG00000265018
NA	0.0276	7.3881E-01	8.5776E-01	ENSG00000184809
NA	0.0280	7.3903E-01	8.5776E-01	ENSG00000255145
NA	0.0245	7.3906E-01	8.5776E-01	ENSG00000268362
NA	0.0215	7.3906E-01	8.5776E-01	ENSG00000246350
NA	0.0097	7.3916E-01		ENSG00000256280
NA	0.0255	7.3958E-01	8.5776E-01	ENSG00000288815
NA	0.0075	7.3990E-01		ENSG00000229976
NA	0.0149	7.4020E-01		ENSG00000189372
NA	0.0079	7.4037E-01		ENSG00000228979
NA	0.0091	7.4042E-01		ENSG00000274127
NA	0.0050	7.4075E-01		ENSG00000270499
NA	0.0188	7.4075E-01	8.5875E-01	ENSG00000281961
NA	0.0057	7.4093E-01		ENSG00000231901
NA	0.0181	7.4104E-01		ENSG00000225678
NA	0.0122	7.4126E-01		ENSG00000274553
NA	0.0020	7.4146E-01		ENSG00000214354
NA	0.0275	7.4149E-01	8.5907E-01	ENSG00000224614
NA	0.0019	7.4158E-01		ENSG00000230823
NA	0.0320	7.4161E-01	8.5907E-01	ENSG00000213411
NA	0.0171	7.4178E-01		ENSG00000285873
NA	0.0031	7.4191E-01		ENSG00000251309
NA	0.0213	7.4196E-01	8.5939E-01	ENSG00000279129
NA	0.0053	7.4219E-01		ENSG00000250098
NA	0.0094	7.4224E-01		ENSG00000261222
NA	0.0161	7.4225E-01		ENSG00000254777
NA	0.0126	7.4231E-01		ENSG00000286375
NA	0.0154	7.4262E-01		ENSG00000285557
NA	0.0157	7.4280E-01		ENSG00000261789
NA	0.0079	7.4307E-01		ENSG00000230990
NA	0.0080	7.4310E-01		ENSG00000230365
NA	0.0028	7.4312E-01		ENSG00000279834
NA	0.0021	7.4316E-01		ENSG00000285402
NA	0.0222	7.4316E-01	8.6031E-01	ENSG00000235363
NA	0.0238	7.4323E-01	8.6033E-01	ENSG00000238151
NA	0.0286	7.4326E-01	8.6033E-01	ENSG00000229422
NA	0.0075	7.4336E-01		ENSG00000279743
NA	0.0036	7.4363E-01		ENSG00000249293
NA	0.0141	7.4388E-01		ENSG00000267432
NA	0.0217	7.4389E-01	8.6081E-01	ENSG00000273209

NA	0.0136	7.4394E-01		ENSG00000254244
NA	0.0204	7.4398E-01	8.6086E-01	ENSG00000280434
NA	0.0275	7.4422E-01	8.6086E-01	ENSG00000272562
NA	0.0239	7.4423E-01	8.6086E-01	ENSG00000265218
NA	0.0224	7.4425E-01	8.6086E-01	ENSG00000260317
NA	0.0279	7.4428E-01	8.6086E-01	ENSG00000288973
NA	0.0064	7.4432E-01		ENSG00000270343
NA	0.0275	7.4463E-01	8.6114E-01	ENSG00000253300
NA	0.0096	7.4470E-01		ENSG00000289290
NA	0.0159	7.4482E-01	8.6115E-01	ENSG00000278009
NA	0.0090	7.4508E-01		ENSG00000287329
NA	0.0217	7.4514E-01	8.6120E-01	ENSG00000270133
NA	0.0055	7.4515E-01		ENSG00000277290
NA	0.0142	7.4539E-01		ENSG00000273240
NA	0.0175	7.4552E-01	8.6131E-01	ENSG00000250046
NA	0.0306	7.4556E-01	8.6131E-01	ENSG00000251682
NA	0.0138	7.4600E-01		ENSG00000286799
NA	0.0061	7.4616E-01		ENSG00000284986
NA	0.0199	7.4620E-01		ENSG00000166984
NA	0.0183	7.4628E-01		ENSG00000213569
NA	0.0158	7.4657E-01	8.6217E-01	ENSG00000259512
NA	0.0198	7.4735E-01	8.6259E-01	ENSG00000268056
NA	0.0241	7.4765E-01	8.6281E-01	ENSG00000234764
NA	0.0124	7.4844E-01		ENSG00000248242
NA	0.0031	7.4858E-01		ENSG00000103832
NA	0.0235	7.4862E-01	8.6354E-01	ENSG00000289841
NA	0.0193	7.4866E-01	8.6354E-01	ENSG00000267786
NA	0.0038	7.4873E-01		ENSG00000224839
NA	0.0271	7.4874E-01	8.6354E-01	ENSG00000237846
NA	0.0237	7.4876E-01	8.6354E-01	ENSG00000288748
NA	0.0105	7.4881E-01		ENSG00000266891
NA	0.0223	7.4891E-01	8.6357E-01	ENSG00000288877
NA	0.0091	7.4897E-01		ENSG00000224232
NA	0.0279	7.4897E-01	8.6360E-01	ENSG00000232815
NA	0.0065	7.4923E-01		ENSG00000249099
NA	0.0201	7.4957E-01	8.6410E-01	ENSG00000265630
NA	0.0216	7.4975E-01	8.6410E-01	ENSG00000254639
NA	0.0162	7.4976E-01		ENSG00000239774
NA	0.0011	7.4984E-01		ENSG00000285521
NA	0.0081	7.4985E-01		ENSG00000228573
NA	0.0132	7.4985E-01	8.6417E-01	ENSG00000253720
NA	0.0020	7.4988E-01		ENSG00000228195
NA	0.0161	7.4999E-01		ENSG00000259797
NA	0.0184	7.5002E-01	8.6428E-01	ENSG00000235997
NA	0.0045	7.5026E-01		ENSG00000273177
NA	0.0085	7.5030E-01		ENSG00000229887
NA	0.0183	7.5035E-01		ENSG00000287406
NA	0.0158	7.5046E-01	8.6450E-01	ENSG00000272849
NA	0.0213	7.5055E-01	8.6450E-01	ENSG00000255471

NA	0.0016	7.5056E-01	8.6450E-01	ENSG00000288575
NA	0.0253	7.5064E-01	8.6456E-01	ENSG00000287160
NA	0.0095	7.5116E-01		ENSG00000280182
NA	0.0048	7.5154E-01		ENSG00000224795
NA	0.0114	7.5158E-01		ENSG00000285030
NA	0.0220	7.5160E-01	8.6517E-01	ENSG00000286549
NA	0.0147	7.5165E-01		ENSG00000279410
NA	0.0341	7.5180E-01	8.6533E-01	ENSG00000259378
NA	0.0052	7.5184E-01		ENSG00000228051
NA	0.0113	7.5218E-01		ENSG00000273474
NA	0.0217	7.5232E-01	8.6560E-01	ENSG00000270087
NA	0.0021	7.5242E-01		ENSG00000249234
NA	0.0206	7.5277E-01	8.6591E-01	ENSG00000259275
NA	0.0252	7.5283E-01	8.6591E-01	ENSG00000289854
NA	0.0233	7.5289E-01	8.6591E-01	ENSG00000223343
NA	0.0048	7.5313E-01		ENSG00000237478
NA	0.0250	7.5318E-01	8.6598E-01	ENSG00000231856
NA	0.0194	7.5326E-01	8.6603E-01	ENSG00000285799
NA	0.0226	7.5355E-01	8.6617E-01	ENSG00000258384
NA	0.0226	7.5361E-01	8.6620E-01	ENSG00000286669
NA	0.0029	7.5364E-01		ENSG00000286355
NA	0.0266	7.5388E-01	8.6630E-01	ENSG00000232519
NA	0.0046	7.5390E-01		ENSG00000278236
NA	0.0147	7.5392E-01		ENSG00000265194
NA	0.0093	7.5411E-01		ENSG00000240244
NA	0.0210	7.5457E-01	8.6686E-01	ENSG00000234740
NA	0.0112	7.5483E-01		ENSG00000260743
NA	0.0115	7.5498E-01		ENSG00000234504
NA	0.0247	7.5503E-01	8.6698E-01	ENSG00000250734
NA	0.0204	7.5514E-01	8.6698E-01	ENSG00000237118
NA	0.0140	7.5549E-01		ENSG00000260487
NA	0.0075	7.5597E-01		ENSG00000248394
NA	0.0068	7.5666E-01		ENSG00000180172
NA	0.0029	7.5681E-01		ENSG00000224842
NA	0.0044	7.5685E-01		ENSG00000286637
NA	0.0258	7.5691E-01	8.6841E-01	ENSG00000233175
NA	0.0224	7.5699E-01	8.6843E-01	ENSG00000287844
NA	0.0247	7.5704E-01	8.6843E-01	ENSG00000284727
NA	0.0266	7.5713E-01	8.6849E-01	ENSG00000286747
NA	0.0022	7.5724E-01		ENSG00000237828
NA	0.0092	7.5737E-01		ENSG00000225521
NA	0.0507	7.5755E-01	8.6879E-01	ENSG00000248431
NA	0.0262	7.5775E-01	8.6888E-01	ENSG00000206567
NA	0.0046	7.5811E-01		ENSG00000289836
NA	0.0181	7.5814E-01		ENSG00000242861
NA	0.0115	7.5831E-01		ENSG00000272375
NA	0.0079	7.5838E-01		ENSG00000207105
NA	0.0242	7.5871E-01	8.6962E-01	ENSG00000198580
NA	0.0160	7.5879E-01		ENSG00000266994

NA	0.0128	7.5881E-01		ENSG00000249237
NA	0.0125	7.5887E-01		ENSG00000277911
NA	0.0151	7.5926E-01		ENSG00000238164
NA	0.0118	7.5929E-01		ENSG00000286148
NA	0.0133	7.5930E-01		ENSG00000264078
NA	0.0017	7.5935E-01		ENSG00000238246
NA	0.0109	7.5955E-01		ENSG00000223513
NA	0.0126	7.5998E-01		ENSG00000244556
NA	0.0185	7.6009E-01	8.7079E-01	ENSG00000249779
NA	0.0032	7.6015E-01		ENSG00000283408
NA	0.0058	7.6031E-01		ENSG00000260996
NA	0.0029	7.6070E-01		ENSG00000267274
NA	0.0154	7.6074E-01		ENSG00000223642
NA	0.0061	7.6078E-01		ENSG00000242683
NA	0.0030	7.6102E-01		ENSG00000200888
NA	0.0077	7.6102E-01		ENSG00000256902
NA	0.0045	7.6145E-01		ENSG00000225839
NA	0.0150	7.6173E-01	8.7190E-01	ENSG00000258443
NA	0.0215	7.6180E-01		ENSG00000273335
NA	0.0059	7.6184E-01		ENSG00000271725
NA	0.0227	7.6226E-01	8.7214E-01	ENSG00000278066
NA	0.0027	7.6240E-01		ENSG00000270427
NA	0.0198	7.6241E-01	8.7215E-01	ENSG00000287678
NA	0.0093	7.6247E-01		ENSG00000261637
NA	0.0114	7.6251E-01		ENSG00000207305
NA	0.0153	7.6264E-01		ENSG00000289874
NA	0.0069	7.6267E-01		ENSG00000261673
NA	0.0137	7.6275E-01		ENSG00000270894
NA	0.0119	7.6284E-01		ENSG00000287043
NA	0.0282	7.6289E-01	8.7229E-01	ENSG00000289151
NA	0.0226	7.6300E-01	8.7238E-01	ENSG00000280187
NA	0.0187	7.6313E-01		ENSG00000219163
NA	0.0069	7.6317E-01		ENSG00000235933
NA	0.0020	7.6331E-01		ENSG00000251506
NA	0.0016	7.6354E-01		ENSG00000286411
NA	0.0024	7.6359E-01		ENSG00000249240
NA	0.0142	7.6367E-01		ENSG00000267769
NA	0.0029	7.6455E-01		ENSG00000229672
NA	0.0076	7.6465E-01		ENSG00000267780
NA	0.0265	7.6476E-01		ENSG00000236508
NA	0.0067	7.6477E-01		ENSG00000228115
NA	0.0086	7.6480E-01		ENSG00000278594
NA	0.0044	7.6499E-01		ENSG00000273289
NA	0.0084	7.6526E-01		ENSG00000281641
NA	0.0202	7.6542E-01	8.7393E-01	ENSG00000261839
NA	0.0069	7.6560E-01		ENSG00000273368
NA	0.0122	7.6583E-01		ENSG00000176268
NA	0.0016	7.6591E-01		ENSG00000289331
NA	0.0047	7.6600E-01		ENSG00000227962

NA	0.0088	7.6635E-01		ENSG00000231509
NA	0.0032	7.6674E-01		ENSG00000279882
NA	0.0084	7.6744E-01		ENSG00000228718
NA	0.0142	7.6752E-01	8.7535E-01	ENSG00000257624
NA	0.0009	7.6753E-01		ENSG00000257268
NA	0.0216	7.6772E-01	8.7554E-01	ENSG00000234477
NA	0.0045	7.6787E-01		ENSG00000270110
NA	0.0006	7.6804E-01		ENSG00000237031
NA	0.0172	7.6807E-01	8.7573E-01	ENSG00000276814
NA	0.0044	7.6811E-01		ENSG00000274099
NA	0.0026	7.6824E-01		ENSG00000261502
NA	0.0145	7.6868E-01		ENSG00000289982
NA	0.0033	7.6897E-01		ENSG00000255395
NA	0.0103	7.6911E-01		ENSG00000279926
NA	0.0118	7.6915E-01		ENSG00000226131
NA	0.0072	7.6953E-01		ENSG00000278878
NA	0.0070	7.6953E-01		ENSG00000229224
NA	0.0016	7.6984E-01		ENSG00000280003
NA	0.0105	7.7025E-01		ENSG00000268870
NA	0.0110	7.7070E-01		ENSG00000279315
NA	0.0112	7.7076E-01		ENSG00000272248
NA	0.0115	7.7100E-01		ENSG00000261476
NA	0.0123	7.7110E-01		ENSG00000249055
NA	0.0107	7.7117E-01		ENSG00000225913
NA	0.0121	7.7132E-01		ENSG00000230131
NA	0.0150	7.7153E-01		ENSG00000227034
NA	0.0112	7.7160E-01		ENSG00000248498
NA	0.0171	7.7185E-01	8.7833E-01	ENSG00000258007
NA	0.0023	7.7222E-01		ENSG00000278818
NA	0.0078	7.7239E-01		ENSG00000236772
NA	0.0227	7.7256E-01	8.7893E-01	ENSG00000223039
NA	0.0066	7.7274E-01	8.7896E-01	ENSG00000285595
NA	0.0127	7.7279E-01		ENSG00000241661
NA	0.0282	7.7306E-01	8.7903E-01	ENSG00000260693
NA	0.0006	7.7356E-01		ENSG00000275720
NA	0.0092	7.7405E-01		ENSG00000289465
NA	0.0350	7.7407E-01		ENSG00000283141
NA	0.0017	7.7412E-01		ENSG00000244280
NA	0.0099	7.7421E-01		ENSG00000286199
NA	0.0202	7.7433E-01	8.8002E-01	ENSG00000184844
NA	0.0228	7.7440E-01	8.8002E-01	ENSG00000258598
NA	0.0221	7.7454E-01	8.8003E-01	ENSG00000289412
NA	0.0092	7.7461E-01		ENSG00000263677
NA	0.0080	7.7475E-01		ENSG00000267583
NA	0.0029	7.7531E-01		ENSG00000275129
NA	0.0114	7.7545E-01		ENSG00000276488
NA	0.0152	7.7546E-01		ENSG00000285731
NA	0.0027	7.7565E-01		ENSG00000240375
NA	0.0222	7.7588E-01	8.8072E-01	ENSG00000236308

NA	0.0161	7.7610E-01	8.8085E-01	ENSG00000259536
NA	0.0179	7.7637E-01	8.8102E-01	ENSG00000259909
NA	0.0229	7.7646E-01		ENSG00000230524
NA	0.0063	7.7666E-01		ENSG00000285901
NA	0.0237	7.7668E-01	8.8109E-01	ENSG00000251615
NA	0.0168	7.7676E-01	8.8111E-01	ENSG00000289615
NA	0.0358	7.7716E-01	8.8138E-01	ENSG00000287475
NA	0.0241	7.7739E-01		ENSG00000227835
NA	0.0222	7.7741E-01	8.8148E-01	ENSG00000237276
NA	0.0046	7.7753E-01		ENSG00000285662
NA	0.0276	7.7753E-01	8.8152E-01	ENSG00000289349
NA	0.0006	7.7779E-01		ENSG00000234055
NA	0.0147	7.7781E-01		ENSG00000251405
NA	0.0095	7.7799E-01		ENSG00000259032
NA	0.0085	7.7830E-01		ENSG00000280375
NA	0.0016	7.7836E-01		ENSG00000274346
NA	0.0045	7.7853E-01		ENSG00000201365
NA	0.0000	7.7854E-01		ENSG00000284722
NA	0.0078	7.7891E-01		ENSG00000286410
NA	0.0091	7.7924E-01		ENSG00000253772
NA	0.0138	7.7930E-01	8.8284E-01	ENSG00000236643
NA	0.0131	7.7948E-01		ENSG00000285844
NA	0.0045	7.7997E-01		ENSG00000228156
NA	0.0087	7.8013E-01		ENSG00000248641
NA	0.0224	7.8024E-01	8.8343E-01	ENSG00000228903
NA	0.0238	7.8058E-01	8.8359E-01	ENSG00000273674
NA	0.0045	7.8069E-01		ENSG00000224525
NA	0.0029	7.8075E-01		ENSG00000225760
NA	0.0173	7.8078E-01	8.8371E-01	ENSG00000255464
NA	0.0047	7.8085E-01		ENSG00000203435
NA	0.0021	7.8104E-01		ENSG00000253512
NA	0.0026	7.8142E-01		ENSG00000279910
NA	0.0230	7.8159E-01	8.8427E-01	ENSG00000237732
NA	0.0064	7.8179E-01		ENSG00000223602
NA	0.0011	7.8197E-01		ENSG00000279630
NA	0.0044	7.8221E-01		ENSG00000281904
NA	0.0103	7.8223E-01		ENSG00000277708
NA	0.0134	7.8273E-01	8.8502E-01	ENSG00000259591
NA	0.0140	7.8287E-01		ENSG00000287721
NA	0.0098	7.8298E-01		ENSG00000270792
NA	0.0149	7.8301E-01	8.8518E-01	ENSG00000238262
NA	0.0082	7.8311E-01		ENSG00000261204
NA	0.0055	7.8327E-01		ENSG00000237263
NA	0.0229	7.8333E-01	8.8530E-01	ENSG00000280273
NA	0.0139	7.8357E-01		ENSG00000259177
NA	0.0091	7.8409E-01		ENSG00000225078
NA	0.0014	7.8411E-01		ENSG00000270574
NA	0.0134	7.8418E-01		ENSG00000234207
NA	0.0231	7.8455E-01	8.8623E-01	ENSG00000241860

NA	0.0120	7.8471E-01		ENSG00000226780
NA	0.0197	7.8479E-01	8.8630E-01	ENSG00000230409
NA	0.0046	7.8488E-01		ENSG00000229237
NA	0.0006	7.8491E-01		ENSG00000232875
NA	0.0039	7.8524E-01		ENSG00000288810
NA	0.0044	7.8527E-01		ENSG00000228295
NA	0.0126	7.8540E-01		ENSG00000235660
NA	0.0078	7.8556E-01		ENSG00000229207
NA	0.0218	7.8566E-01	8.8667E-01	ENSG00000270218
NA	0.0010	7.8579E-01	8.8674E-01	ENSG00000236953
NA	0.0077	7.8580E-01		ENSG00000233278
NA	0.0137	7.8604E-01		ENSG00000223452
NA	0.0064	7.8605E-01		ENSG00000227076
NA	0.0189	7.8629E-01	8.8713E-01	ENSG00000260633
NA	0.0049	7.8634E-01		ENSG00000213194
NA	0.0096	7.8643E-01		ENSG00000280140
NA	0.0092	7.8661E-01		ENSG00000260012
NA	0.0168	7.8686E-01	8.8742E-01	ENSG00000251474
NA	0.0035	7.8694E-01		ENSG00000259198
NA	0.0212	7.8720E-01	8.8755E-01	ENSG00000239280
NA	0.0216	7.8758E-01	8.8782E-01	ENSG00000260448
NA	0.0180	7.8776E-01	8.8790E-01	ENSG00000259918
NA	0.0228	7.8788E-01	8.8795E-01	ENSG00000283355
NA	0.0223	7.8815E-01	8.8798E-01	ENSG00000272267
NA	0.0056	7.8819E-01		ENSG00000286021
NA	0.0188	7.8850E-01	8.8805E-01	ENSG00000236397
NA	0.0089	7.8868E-01	8.8806E-01	ENSG00000272689
NA	0.0181	7.8873E-01	8.8806E-01	ENSG00000255542
NA	0.0083	7.8884E-01		ENSG00000282692
NA	0.0080	7.8906E-01		ENSG00000269148
NA	0.0131	7.8925E-01		ENSG00000289388
NA	0.0091	7.8940E-01		ENSG00000243014
NA	0.0035	7.8960E-01		ENSG00000259711
NA	0.0097	7.8970E-01		ENSG00000277447
NA	0.0233	7.8994E-01	8.8896E-01	ENSG00000233058
NA	0.0019	7.8997E-01		ENSG00000236229
NA	0.0082	7.9002E-01		ENSG00000263501
NA	0.0045	7.9016E-01		ENSG00000227541
NA	0.0084	7.9037E-01		ENSG00000260272
NA	0.0226	7.9043E-01	8.8920E-01	ENSG00000287168
NA	0.0121	7.9047E-01		ENSG00000261305
NA	0.0149	7.9048E-01		ENSG00000225108
NA	0.0026	7.9138E-01		ENSG00000287583
NA	0.0007	7.9143E-01		ENSG00000286424
NA	0.0050	7.9162E-01		ENSG00000225928
NA	0.0225	7.9164E-01	8.8999E-01	ENSG00000272455
NA	0.0007	7.9169E-01		ENSG00000290093
NA	0.0063	7.9178E-01		ENSG00000275340
NA	0.0206	7.9188E-01	8.9001E-01	ENSG00000290032

NA	0.0058	7.9191E-01		ENSG00000243960
NA	0.0101	7.9232E-01		ENSG00000272324
NA	0.0236	7.9253E-01		ENSG00000222343
NA	0.0004	7.9259E-01		ENSG00000270665
NA	0.0062	7.9273E-01		ENSG00000260593
NA	0.0070	7.9294E-01		ENSG00000278887
NA	0.0180	7.9297E-01	8.9075E-01	ENSG00000235555
NA	0.0048	7.9299E-01		ENSG00000287367
NA	0.0172	7.9300E-01		ENSG00000237705
NA	0.0039	7.9302E-01		ENSG00000259432
NA	0.0046	7.9304E-01		ENSG00000236989
NA	0.0011	7.9321E-01		ENSG00000285695
NA	0.0128	7.9338E-01	8.9078E-01	ENSG00000274949
NA	0.0197	7.9347E-01	8.9083E-01	ENSG00000287336
NA	0.0186	7.9354E-01	8.9083E-01	ENSG00000278768
NA	0.0207	7.9362E-01	8.9087E-01	ENSG00000271239
NA	0.0064	7.9368E-01		ENSG00000258090
NA	0.0084	7.9396E-01		ENSG00000261915
NA	0.0045	7.9453E-01		ENSG00000224375
NA	0.0077	7.9454E-01		ENSG00000287231
NA	0.0141	7.9471E-01	8.9153E-01	ENSG00000227755
NA	0.0077	7.9474E-01		ENSG00000269504
NA	0.0124	7.9474E-01		ENSG00000288271
NA	0.0038	7.9499E-01		ENSG00000228950
NA	0.0042	7.9529E-01		ENSG00000237540
NA	0.0068	7.9537E-01		ENSG00000288081
NA	0.0190	7.9566E-01	8.9205E-01	ENSG00000253771
NA	0.0174	7.9593E-01	8.9209E-01	ENSG00000272154
NA	0.0179	7.9619E-01	8.9230E-01	ENSG00000267342
NA	0.0002	7.9621E-01		ENSG00000249352
NA	0.0176	7.9637E-01		ENSG00000200922
NA	0.0365	7.9647E-01	8.9252E-01	ENSG00000265313
NA	0.0042	7.9670E-01		ENSG00000277349
NA	0.0101	7.9672E-01		ENSG00000257194
NA	0.0092	7.9707E-01		ENSG00000233117
NA	0.0053	7.9719E-01		ENSG00000279609
NA	0.0017	7.9750E-01		ENSG00000287528
NA	0.0099	7.9752E-01		ENSG00000267634
NA	0.0206	7.9768E-01	8.9352E-01	ENSG00000287807
NA	0.0200	7.9797E-01	8.9360E-01	ENSG00000290126
NA	0.0060	7.9821E-01		ENSG00000233081
NA	0.0137	7.9824E-01	8.9370E-01	ENSG00000230457
NA	0.0241	7.9886E-01	8.9394E-01	ENSG00000227579
NA	0.0126	7.9906E-01	8.9408E-01	ENSG00000271138
NA	0.0104	7.9932E-01		ENSG00000206645
NA	0.0190	7.9953E-01	8.9428E-01	ENSG00000260519
NA	0.0192	7.9962E-01	8.9430E-01	ENSG00000279500
NA	0.0155	7.9981E-01		ENSG00000288879
NA	0.0112	8.0033E-01		ENSG00000224228

NA	0.0124	8.0036E-01		ENSG00000229023
NA	0.0028	8.0046E-01		ENSG00000232917
NA	0.0047	8.0056E-01	8.9476E-01	ENSG00000288714
NA	0.0176	8.0061E-01	8.9476E-01	ENSG00000286362
NA	0.0012	8.0073E-01	8.9485E-01	ENSG00000289003
NA	0.0151	8.0129E-01		ENSG00000234427
NA	0.0062	8.0149E-01		ENSG00000272203
NA	0.0104	8.0150E-01		ENSG00000289902
NA	0.0122	8.0153E-01	8.9530E-01	ENSG00000259788
NA	0.0051	8.0161E-01		ENSG00000261457
NA	0.0075	8.0161E-01		ENSG00000278171
NA	0.0190	8.0183E-01	8.9537E-01	ENSG00000247317
NA	0.0117	8.0236E-01		ENSG00000286365
NA	0.0060	8.0271E-01		ENSG00000258455
NA	0.0172	8.0286E-01	8.9609E-01	ENSG00000272347
NA	0.0209	8.0294E-01	8.9614E-01	ENSG00000228065
NA	0.0079	8.0328E-01		ENSG00000233936
NA	0.0112	8.0330E-01		ENSG00000265091
NA	0.0077	8.0336E-01		ENSG00000289610
NA	0.0076	8.0360E-01		ENSG00000273965
NA	0.0019	8.0383E-01		ENSG00000231933
NA	0.0027	8.0386E-01	8.9696E-01	ENSG00000205105
NA	0.0127	8.0411E-01		ENSG00000287352
NA	0.0062	8.0421E-01		ENSG00000287333
NA	0.0143	8.0439E-01		ENSG00000286954
NA	0.0122	8.0444E-01		ENSG00000287239
NA	0.0190	8.0453E-01	8.9752E-01	ENSG00000253447
NA	0.0179	8.0505E-01	8.9784E-01	ENSG00000236814
NA	0.0163	8.0507E-01		ENSG00000212163
NA	0.0048	8.0527E-01		ENSG00000255710
NA	0.0280	8.0551E-01	8.9800E-01	ENSG00000289339
NA	0.0207	8.0556E-01	8.9800E-01	ENSG00000270728
NA	0.0031	8.0563E-01		ENSG00000287840
NA	0.0124	8.0579E-01	8.9800E-01	ENSG00000259682
NA	0.0200	8.0601E-01	8.9800E-01	ENSG00000197550
NA	0.0054	8.0605E-01		ENSG00000254495
NA	0.0145	8.0625E-01		ENSG00000276523
NA	0.0104	8.0627E-01		ENSG00000229601
NA	0.0021	8.0628E-01		ENSG00000266466
NA	0.0130	8.0670E-01	8.9839E-01	ENSG00000249085
NA	0.0087	8.0688E-01		ENSG00000272941
NA	0.0098	8.0723E-01		ENSG00000232768
NA	0.0099	8.0758E-01		ENSG00000260459
NA	0.0083	8.0763E-01		ENSG00000248159
NA	0.0026	8.0764E-01		ENSG00000220267
NA	0.0142	8.0770E-01	8.9894E-01	ENSG00000270108
NA	0.0206	8.0788E-01	8.9902E-01	ENSG00000288765
NA	0.0197	8.0805E-01	8.9912E-01	ENSG00000235121
NA	0.0026	8.0841E-01		ENSG00000242507

NA	0.0126	8.0846E-01		ENSG00000259347
NA	0.0166	8.0862E-01	8.9942E-01	ENSG00000255580
NA	0.0080	8.0869E-01		ENSG00000203395
NA	0.0189	8.0878E-01	8.9952E-01	ENSG00000226465
NA	0.0007	8.0932E-01		ENSG00000227176
NA	0.0009	8.0958E-01		ENSG00000228289
NA	0.0122	8.0960E-01	8.9994E-01	ENSG00000286004
NA	0.0080	8.0960E-01		ENSG00000243295
NA	0.0085	8.0981E-01		ENSG00000285809
NA	0.0047	8.0985E-01		ENSG00000201384
NA	0.0152	8.0993E-01	9.0014E-01	ENSG00000272106
NA	0.0198	8.1019E-01	9.0032E-01	ENSG00000288559
NA	0.0053	8.1039E-01		ENSG00000286518
NA	0.0024	8.1047E-01		ENSG00000286523
NA	0.0074	8.1054E-01		ENSG00000288005
NA	0.0060	8.1079E-01		ENSG00000259359
NA	0.0198	8.1102E-01	9.0103E-01	ENSG00000246889
NA	0.0106	8.1104E-01		ENSG00000235781
NA	0.0271	8.1175E-01		ENSG00000225787
NA	0.0147	8.1178E-01	9.0159E-01	ENSG00000271078
NA	0.0173	8.1207E-01	9.0183E-01	ENSG00000285287
NA	0.0128	8.1218E-01		ENSG00000215002
NA	0.0163	8.1236E-01	9.0205E-01	ENSG00000240497
NA	0.0164	8.1238E-01	9.0205E-01	ENSG00000248569
NA	0.0080	8.1248E-01		ENSG00000285853
NA	0.0086	8.1254E-01		ENSG00000173231
NA	0.0106	8.1286E-01		ENSG00000229376
NA	0.0079	8.1303E-01		ENSG00000244349
NA	0.0105	8.1316E-01		ENSG00000270228
NA	0.0157	8.1322E-01	9.0266E-01	ENSG00000279329
NA	0.0135	8.1356E-01		ENSG00000287121
NA	0.0035	8.1360E-01		ENSG00000225066
NA	0.0149	8.1379E-01	9.0304E-01	ENSG00000285517
NA	0.0007	8.1430E-01		ENSG00000272321
NA	0.0003	8.1470E-01		ENSG00000259222
NA	0.0088	8.1498E-01		ENSG00000277873
NA	0.0175	8.1509E-01	9.0384E-01	ENSG00000225393
NA	0.0176	8.1533E-01	9.0399E-01	ENSG00000262678
NA	0.0036	8.1546E-01		ENSG00000223598
NA	0.0186	8.1567E-01	9.0403E-01	ENSG00000253807
NA	0.0016	8.1571E-01		ENSG00000286656
NA	0.0016	8.1587E-01		ENSG00000225794
NA	0.0027	8.1606E-01		ENSG00000231461
NA	0.0055	8.1607E-01		ENSG00000231808
NA	0.0038	8.1612E-01		ENSG00000273011
NA	0.0019	8.1623E-01		ENSG00000257097
NA	0.0183	8.1625E-01	9.0439E-01	ENSG00000273599
NA	0.0059	8.1627E-01		ENSG00000285329
NA	0.0114	8.1647E-01	9.0447E-01	ENSG00000226361

NA	0.0191	8.1663E-01	9.0452E-01	ENSG00000226963
NA	0.0071	8.1664E-01		ENSG00000279924
NA	0.0104	8.1682E-01		ENSG00000234848
NA	0.0105	8.1687E-01		ENSG00000283422
NA	0.0191	8.1695E-01	9.0480E-01	ENSG00000257122
NA	0.0204	8.1713E-01		ENSG00000236391
NA	0.0138	8.1717E-01	9.0492E-01	ENSG00000257449
NA	0.0044	8.1733E-01		ENSG00000229593
NA	0.0077	8.1743E-01		ENSG00000254115
NA	0.0065	8.1755E-01		ENSG00000277672
NA	0.0209	8.1764E-01		ENSG00000267589
NA	0.0055	8.1795E-01		ENSG00000259481
NA	0.0056	8.1833E-01		ENSG00000272824
NA	0.0024	8.1833E-01		ENSG00000267550
NA	0.0028	8.1845E-01		ENSG00000254271
NA	0.0038	8.1846E-01		ENSG00000268603
NA	0.0037	8.1849E-01		ENSG00000257302
NA	0.0019	8.1858E-01		ENSG00000279953
NA	0.0003	8.1873E-01		ENSG00000232494
NA	0.0177	8.1879E-01	9.0582E-01	ENSG00000279086
NA	0.0051	8.1898E-01		ENSG00000213078
NA	0.0028	8.1931E-01		ENSG00000279251
NA	0.0070	8.1945E-01		ENSG00000276095
NA	0.0027	8.1956E-01		ENSG00000271618
NA	0.0124	8.1958E-01	9.0633E-01	ENSG00000228169
NA	0.0026	8.1967E-01		ENSG00000254034
NA	0.0144	8.1977E-01	9.0638E-01	ENSG00000236711
NA	0.0069	8.1989E-01	9.0638E-01	ENSG00000259563
NA	0.0243	8.1989E-01	9.0638E-01	ENSG00000273295
NA	0.0166	8.2038E-01	9.0672E-01	ENSG00000250170
NA	0.0054	8.2063E-01		ENSG00000232396
NA	0.0038	8.2080E-01	9.0703E-01	ENSG00000260604
NA	0.0037	8.2102E-01		ENSG00000224014
NA	0.0097	8.2111E-01		ENSG00000233971
NA	0.0176	8.2157E-01	9.0762E-01	ENSG00000277342
NA	0.0073	8.2163E-01		ENSG00000287292
NA	0.0170	8.2164E-01	9.0762E-01	ENSG00000268049
NA	0.0187	8.2226E-01	9.0802E-01	ENSG00000285879
NA	0.0055	8.2229E-01		ENSG00000225092
NA	0.0179	8.2285E-01	9.0841E-01	ENSG00000266371
NA	0.0056	8.2288E-01		ENSG00000228986
NA	0.0058	8.2289E-01		ENSG00000253326
NA	0.0076	8.2290E-01		ENSG00000232793
NA	0.0174	8.2311E-01	9.0841E-01	ENSG00000256745
NA	0.0099	8.2314E-01		ENSG00000279433
NA	0.0162	8.2332E-01	9.0841E-01	ENSG00000267388
NA	0.0174	8.2334E-01		ENSG00000280219
NA	0.0009	8.2351E-01		ENSG00000223866
NA	0.0022	8.2368E-01		ENSG00000285994

NA	0.0072	8.2371E-01		ENSG00000278630
NA	0.0004	8.2379E-01		ENSG00000232794
NA	0.0064	8.2421E-01		ENSG00000279130
NA	0.0151	8.2429E-01	9.0879E-01	ENSG00000216624
NA	0.0199	8.2458E-01	9.0903E-01	ENSG00000279494
NA	0.0261	8.2461E-01		ENSG00000236451
NA	0.0081	8.2469E-01		ENSG00000235782
NA	0.0048	8.2477E-01		ENSG00000236531
NA	0.0064	8.2477E-01		ENSG00000201573
NA	0.0141	8.2478E-01	9.0912E-01	ENSG00000182257
NA	0.0020	8.2482E-01		ENSG00000235610
NA	0.0183	8.2495E-01	9.0915E-01	ENSG00000271335
NA	0.0174	8.2499E-01	9.0916E-01	ENSG00000287837
NA	0.0164	8.2505E-01	9.0918E-01	ENSG00000263878
NA	0.0179	8.2523E-01	9.0931E-01	ENSG00000288820
NA	0.0124	8.2532E-01	9.0932E-01	ENSG00000285888
NA	0.0055	8.2545E-01		ENSG00000269403
NA	0.0057	8.2563E-01		ENSG00000231894
NA	0.0121	8.2585E-01		ENSG00000274378
NA	0.0087	8.2598E-01		ENSG00000262815
NA	0.0197	8.2600E-01	9.0987E-01	ENSG00000248780
NA	0.0169	8.2621E-01	9.0997E-01	ENSG00000273253
NA	0.0135	8.2650E-01		ENSG00000276403
NA	0.0009	8.2665E-01		ENSG00000233919
NA	0.0064	8.2697E-01		ENSG00000134612
NA	0.0076	8.2715E-01		ENSG00000189089
NA	0.0113	8.2726E-01	9.1051E-01	ENSG00000244371
NA	0.0166	8.2732E-01	9.1051E-01	ENSG00000288045
NA	0.0044	8.2761E-01		ENSG00000248249
NA	0.0091	8.2764E-01		ENSG00000287293
NA	0.0122	8.2771E-01	9.1079E-01	ENSG00000219608
NA	0.0055	8.2773E-01		ENSG00000274892
NA	0.0089	8.2774E-01		ENSG00000279330
NA	0.0118	8.2779E-01	9.1081E-01	ENSG00000270533
NA	0.0107	8.2782E-01		ENSG00000255092
NA	0.0078	8.2806E-01	9.1103E-01	ENSG00000287220
NA	0.0142	8.2810E-01	9.1103E-01	ENSG00000224363
NA	0.0166	8.2825E-01		ENSG00000278969
NA	0.0180	8.2853E-01	9.1123E-01	ENSG00000226803
NA	0.0106	8.2857E-01	9.1123E-01	ENSG00000263680
NA	0.0106	8.2860E-01		ENSG00000257475
NA	0.0032	8.2907E-01		ENSG00000286897
NA	0.0040	8.2914E-01		ENSG00000237527
NA	0.0078	8.2964E-01		ENSG00000235518
NA	0.0007	8.2971E-01		ENSG00000259122
NA	0.0050	8.2981E-01		ENSG00000226486
NA	0.0003	8.2989E-01		ENSG00000279499
NA	0.0036	8.3031E-01		ENSG00000288985
NA	0.0053	8.3033E-01		ENSG00000275232

NA	0.0176	8.3051E-01	9.1239E-01	ENSG00000250999
NA	0.0115	8.3067E-01		ENSG00000204622
NA	0.0048	8.3097E-01		ENSG00000287348
NA	0.0089	8.3128E-01		ENSG00000233010
NA	0.0037	8.3130E-01		ENSG00000217612
NA	0.0046	8.3152E-01		ENSG00000255572
NA	0.0080	8.3183E-01		ENSG00000289567
NA	0.0078	8.3198E-01		ENSG00000213724
NA	0.0058	8.3201E-01		ENSG00000223427
NA	0.0075	8.3215E-01		ENSG00000248176
NA	0.0005	8.3232E-01		ENSG00000220378
NA	0.0016	8.3261E-01		ENSG00000278341
NA	0.0016	8.3267E-01		ENSG00000244706
NA	0.0061	8.3274E-01		ENSG00000235795
NA	0.0091	8.3288E-01		ENSG00000286524
NA	0.0085	8.3325E-01		ENSG00000277671
NA	0.0085	8.3325E-01		ENSG00000278775
NA	0.0085	8.3325E-01		ENSG00000273739
NA	0.0085	8.3325E-01		ENSG00000276197
NA	0.0060	8.3331E-01		ENSG00000276578
NA	0.0019	8.3348E-01		ENSG00000271199
NA	0.0062	8.3350E-01		ENSG00000200591
NA	0.0039	8.3351E-01	9.1436E-01	ENSG00000272056
NA	0.0050	8.3431E-01		ENSG00000280242
NA	0.0069	8.3443E-01		ENSG00000222031
NA	0.0021	8.3453E-01	9.1499E-01	ENSG00000250360
NA	0.0118	8.3460E-01		ENSG00000223800
NA	0.0028	8.3487E-01		ENSG00000264853
NA	0.0501	8.3510E-01	9.1514E-01	ENSG00000286582
NA	0.0030	8.3511E-01		ENSG00000249460
NA	0.0066	8.3525E-01		ENSG00000241886
NA	0.0004	8.3526E-01		ENSG00000254964
NA	0.0093	8.3531E-01	9.1524E-01	ENSG00000238755
NA	0.0114	8.3595E-01		ENSG00000229424
NA	0.0039	8.3602E-01		ENSG00000241324
NA	0.0011	8.3606E-01		ENSG00000286338
NA	0.0170	8.3638E-01	9.1564E-01	ENSG00000254348
NA	0.0066	8.3641E-01		ENSG00000248545
NA	0.0170	8.3652E-01	9.1576E-01	ENSG00000271755
NA	0.0028	8.3671E-01		ENSG00000226954
NA	0.0111	8.3713E-01	9.1604E-01	ENSG00000105988
NA	0.0069	8.3719E-01		ENSG00000253172
NA	0.0097	8.3722E-01		ENSG00000273248
NA	0.0081	8.3726E-01		ENSG00000132204
NA	0.0069	8.3777E-01	9.1627E-01	ENSG00000275910
NA	0.0056	8.3781E-01		ENSG00000258958
NA	0.0092	8.3788E-01		ENSG00000269534
NA	0.0004	8.3791E-01		ENSG00000278275
NA	0.0118	8.3802E-01		ENSG00000215559

NA	0.0001	8.3830E-01		ENSG00000259668
NA	0.0162	8.3863E-01	9.1701E-01	ENSG00000240356
NA	0.0089	8.3875E-01	9.1705E-01	ENSG00000267543
NA	0.0162	8.3897E-01	9.1719E-01	ENSG00000288996
NA	0.0071	8.3897E-01		ENSG00000254347
NA	0.0070	8.3957E-01		ENSG00000275038
NA	0.0028	8.3961E-01		ENSG00000233635
NA	0.0145	8.3973E-01		ENSG00000231464
NA	0.0060	8.3993E-01		ENSG00000257052
NA	0.0111	8.4001E-01	9.1766E-01	ENSG00000230910
NA	0.0018	8.4004E-01		ENSG00000225558
NA	0.0053	8.4024E-01		ENSG00000289630
NA	0.0062	8.4036E-01		ENSG00000282418
NA	0.0031	8.4069E-01		ENSG00000282849
NA	0.0038	8.4070E-01		ENSG00000285783
NA	0.0091	8.4087E-01		ENSG00000223650
NA	0.0042	8.4098E-01		ENSG00000251526
NA	0.0113	8.4102E-01	9.1819E-01	ENSG00000251379
NA	0.0014	8.4105E-01		ENSG00000213558
NA	0.0160	8.4110E-01	9.1820E-01	ENSG00000271500
NA	0.0131	8.4114E-01	9.1820E-01	ENSG00000233912
NA	0.0031	8.4164E-01		ENSG00000225492
NA	0.0087	8.4213E-01		ENSG00000233191
NA	0.0117	8.4236E-01		ENSG00000222017
NA	0.0029	8.4250E-01		ENSG00000248978
NA	0.0013	8.4251E-01		ENSG00000197210
NA	0.0150	8.4279E-01		ENSG00000279756
NA	0.0036	8.4291E-01		ENSG00000214761
NA	0.0200	8.4325E-01		ENSG00000261595
NA	0.0119	8.4358E-01	9.1973E-01	ENSG00000213830
NA	0.0022	8.4369E-01		ENSG00000236427
NA	0.0078	8.4371E-01		ENSG00000286583
NA	0.0013	8.4386E-01		ENSG00000290077
NA	0.0147	8.4399E-01	9.1997E-01	ENSG00000277450
NA	0.0018	8.4408E-01		ENSG00000229020
NA	0.0158	8.4414E-01	9.2003E-01	ENSG00000268912
NA	0.0011	8.4428E-01		ENSG00000280362
NA	0.0087	8.4431E-01		ENSG00000285887
NA	0.0148	8.4462E-01	9.2037E-01	ENSG00000274487
NA	0.0043	8.4470E-01		ENSG00000229065
NA	0.0023	8.4478E-01		ENSG00000223492
NA	0.0007	8.4497E-01		ENSG00000218586
NA	0.0013	8.4516E-01		ENSG00000266907
NA	0.0165	8.4533E-01	9.2085E-01	ENSG00000235946
NA	0.0004	8.4551E-01		ENSG00000234022
NA	0.0154	8.4631E-01	9.2119E-01	ENSG00000280287
NA	0.0023	8.4635E-01		ENSG00000279662
NA	0.0089	8.4646E-01		ENSG00000237885
NA	0.0163	8.4653E-01		ENSG00000250869

NA	0.0077	8.4654E-01		ENSG00000233653
NA	0.0005	8.4661E-01		ENSG00000272915
NA	0.0038	8.4675E-01		ENSG00000213036
NA	0.0144	8.4713E-01	9.2176E-01	ENSG00000287031
NA	0.0017	8.4759E-01		ENSG00000249012
NA	0.0017	8.4786E-01		ENSG00000243023
NA	0.0011	8.4786E-01		ENSG00000287357
NA	0.0061	8.4788E-01		ENSG00000225510
NA	0.0165	8.4791E-01		ENSG00000287616
NA	0.0011	8.4816E-01		ENSG00000289384
NA	0.0056	8.4835E-01		ENSG00000259181
NA	0.0016	8.4836E-01		ENSG00000266601
NA	0.0041	8.4838E-01		ENSG00000213574
NA	0.0143	8.4843E-01	9.2267E-01	ENSG00000203279
NA	0.0156	8.4857E-01	9.2275E-01	ENSG00000226445
NA	0.0025	8.4877E-01		ENSG00000287362
NA	0.0032	8.4899E-01		ENSG00000288837
NA	0.0118	8.4912E-01		ENSG00000262296
NA	0.0162	8.4932E-01	9.2328E-01	ENSG00000288924
NA	0.0075	8.4934E-01		ENSG00000214552
NA	0.0028	8.4942E-01		ENSG00000231102
NA	0.0054	8.4947E-01		ENSG00000271443
NA	0.0121	8.4987E-01		ENSG00000289988
NA	0.0147	8.4989E-01	9.2365E-01	ENSG00000289415
NA	0.0067	8.4989E-01		ENSG00000287877
NA	0.0055	8.4994E-01		ENSG00000286980
NA	0.0169	8.5030E-01	9.2385E-01	ENSG00000233340
NA	0.0144	8.5068E-01	9.2404E-01	ENSG00000287523
NA	0.0105	8.5070E-01	9.2404E-01	ENSG00000230286
NA	0.0083	8.5076E-01		ENSG00000259993
NA	0.0109	8.5082E-01		ENSG00000214549
NA	0.0154	8.5088E-01	9.2412E-01	ENSG00000273492
NA	0.0128	8.5103E-01		ENSG00000266217
NA	0.0096	8.5112E-01		ENSG00000289060
NA	0.0159	8.5133E-01	9.2425E-01	ENSG00000260267
NA	0.0101	8.5144E-01		ENSG00000259152
NA	0.0093	8.5169E-01	9.2443E-01	ENSG00000280010
NA	0.0078	8.5173E-01		ENSG00000285955
NA	0.0107	8.5178E-01	9.2443E-01	ENSG00000280339
NA	0.0087	8.5187E-01		ENSG00000273449
NA	0.0094	8.5201E-01	9.2461E-01	ENSG00000278177
NA	0.0152	8.5221E-01	9.2466E-01	ENSG00000274922
NA	0.0142	8.5230E-01	9.2466E-01	ENSG00000289209
NA	0.0156	8.5270E-01	9.2499E-01	ENSG00000285796
NA	0.0064	8.5282E-01		ENSG00000251170
NA	0.0155	8.5298E-01	9.2525E-01	ENSG00000273010
NA	0.0029	8.5338E-01		ENSG00000251360
NA	0.0057	8.5350E-01		ENSG00000233003
NA	0.0122	8.5356E-01	9.2556E-01	ENSG00000235576

NA	0.0068	8.5361E-01		ENSG00000286640
NA	0.0035	8.5382E-01		ENSG00000225779
NA	0.0003	8.5409E-01		ENSG00000287912
NA	0.0110	8.5414E-01	9.2588E-01	ENSG00000286409
NA	0.0077	8.5436E-01		ENSG00000258591
NA	0.0112	8.5457E-01	9.2596E-01	ENSG00000286910
NA	0.0006	8.5479E-01		ENSG00000186676
NA	0.0100	8.5485E-01		ENSG00000229368
NA	0.0040	8.5503E-01		ENSG00000285653
NA	0.0060	8.5506E-01		ENSG00000228054
NA	0.0106	8.5508E-01	9.2626E-01	ENSG00000289543
NA	0.0090	8.5530E-01		ENSG00000261218
NA	0.0105	8.5530E-01	9.2647E-01	ENSG00000259746
NA	0.0107	8.5537E-01		ENSG00000213104
NA	0.0031	8.5545E-01		ENSG00000237979
NA	0.0053	8.5550E-01		ENSG00000236457
NA	0.0001	8.5565E-01		ENSG00000279739
NA	0.0139	8.5621E-01		ENSG00000276312
NA	0.0082	8.5666E-01		ENSG00000253356
NA	0.0057	8.5671E-01		ENSG00000254325
NA	0.0121	8.5674E-01		ENSG00000230912
NA	0.0249	8.5697E-01	9.2766E-01	ENSG00000273139
NA	0.0048	8.5718E-01		ENSG00000289424
NA	0.0045	8.5720E-01		ENSG00000269752
NA	0.0048	8.5737E-01		ENSG00000224159
NA	0.0025	8.5743E-01		ENSG00000234698
NA	0.0029	8.5753E-01		ENSG00000253013
NA	0.0107	8.5762E-01		ENSG00000256084
NA	0.0112	8.5812E-01	9.2834E-01	ENSG00000272305
NA	0.0016	8.5821E-01		ENSG00000272719
NA	0.0007	8.5826E-01		ENSG00000238160
NA	0.0029	8.5832E-01		ENSG00000227352
NA	0.0035	8.5891E-01		ENSG00000258234
NA	0.0117	8.5892E-01	9.2883E-01	ENSG00000269907
NA	0.0186	8.5898E-01		ENSG00000289270
NA	0.0058	8.5914E-01		ENSG00000226798
NA	0.0139	8.5917E-01	9.2898E-01	ENSG00000249661
NA	0.0098	8.5938E-01		ENSG00000286493
NA	0.0153	8.5940E-01		ENSG00000287059
NA	0.0155	8.5956E-01		ENSG00000279872
NA	0.0115	8.6002E-01		ENSG00000286773
NA	0.0154	8.6006E-01	9.2954E-01	ENSG00000248206
NA	0.0061	8.6011E-01		ENSG00000234558
NA	0.0062	8.6028E-01		ENSG00000265206
NA	0.0002	8.6030E-01		ENSG00000287836
NA	0.0047	8.6032E-01		ENSG00000234338
NA	0.0005	8.6092E-01		ENSG00000230320
NA	0.0132	8.6117E-01	9.3034E-01	ENSG00000279623
NA	0.0134	8.6132E-01	9.3034E-01	ENSG00000246263

NA	0.0067	8.6136E-01		ENSG00000257159
NA	0.0008	8.6149E-01		ENSG00000243423
NA	0.0027	8.6175E-01		ENSG00000213063
NA	0.0053	8.6183E-01	9.3057E-01	ENSG00000236354
NA	0.0149	8.6188E-01	9.3057E-01	ENSG00000289960
NA	0.0130	8.6195E-01	9.3059E-01	ENSG00000287038
NA	0.0003	8.6235E-01		ENSG00000280828
NA	0.0026	8.6243E-01		ENSG00000228255
NA	0.0088	8.6257E-01		ENSG00000257818
NA	0.0091	8.6258E-01		ENSG00000248378
NA	0.0007	8.6259E-01		ENSG00000203363
NA	0.0110	8.6269E-01		ENSG00000290016
NA	0.0149	8.6273E-01	9.3079E-01	ENSG00000242154
NA	0.0115	8.6321E-01		ENSG00000257191
NA	0.0083	8.6327E-01		ENSG00000243384
NA	0.0004	8.6331E-01		ENSG00000279613
NA	0.0126	8.6331E-01	9.3117E-01	ENSG00000288728
NA	0.0004	8.6375E-01		ENSG00000271394
NA	0.0019	8.6405E-01		ENSG00000229466
NA	0.0075	8.6411E-01		ENSG00000225125
NA	0.0065	8.6434E-01		ENSG00000249806
NA	0.0030	8.6445E-01		ENSG00000275719
NA	0.0008	8.6448E-01		ENSG00000237875
NA	0.0134	8.6469E-01	9.3172E-01	ENSG00000262691
NA	0.0016	8.6472E-01		ENSG00000259874
NA	0.0134	8.6473E-01	9.3172E-01	ENSG00000289086
NA	0.0138	8.6484E-01	9.3175E-01	ENSG00000279144
NA	0.0000	8.6495E-01		ENSG00000219027
NA	0.0022	8.6508E-01		ENSG00000250015
NA	0.0038	8.6584E-01		ENSG00000280312
NA	0.0027	8.6588E-01		ENSG00000259545
NA	0.0140	8.6610E-01	9.3231E-01	ENSG00000202399
NA	0.0011	8.6634E-01		ENSG00000275223
NA	0.0112	8.6667E-01		ENSG00000289950
NA	0.0131	8.6762E-01		ENSG00000258933
NA	0.0048	8.6819E-01		ENSG00000235559
NA	0.0189	8.6825E-01	9.3371E-01	ENSG00000201501
NA	0.0049	8.6833E-01		ENSG00000228285
NA	0.0137	8.6871E-01	9.3400E-01	ENSG00000280195
NA	0.0006	8.6874E-01		ENSG00000271148
NA	0.0040	8.6875E-01		ENSG00000276174
NA	0.0009	8.6877E-01		ENSG00000287219
NA	0.0078	8.6932E-01		ENSG00000213082
NA	0.0097	8.6957E-01		ENSG00000235537
NA	0.0069	8.6959E-01		ENSG00000151631
NA	0.0034	8.6985E-01		ENSG00000284095
NA	0.0104	8.6989E-01	9.3441E-01	ENSG00000281332
NA	0.0119	8.6989E-01		ENSG00000236516
NA	0.0002	8.7011E-01		ENSG00000258034

NA	0.0064	8.7013E-01		ENSG00000183022
NA	0.0043	8.7023E-01		ENSG00000289262
NA	0.0036	8.7029E-01		ENSG00000232332
NA	0.0132	8.7038E-01	9.3461E-01	ENSG00000265366
NA	0.0107	8.7104E-01	9.3503E-01	ENSG00000282021
NA	0.0012	8.7111E-01		ENSG00000223356
NA	0.0091	8.7128E-01	9.3517E-01	ENSG00000243155
NA	0.0124	8.7168E-01	9.3540E-01	ENSG00000254602
NA	0.0001	8.7189E-01		ENSG00000265316
NA	0.0028	8.7191E-01		ENSG00000287876
NA	0.0102	8.7191E-01	9.3561E-01	ENSG00000254973
NA	0.0068	8.7201E-01		ENSG00000256209
NA	0.0121	8.7284E-01	9.3615E-01	ENSG00000269935
NA	0.0113	8.7288E-01		ENSG00000234322
NA	0.0027	8.7320E-01	9.3637E-01	ENSG00000223298
NA	0.0060	8.7334E-01		ENSG00000224857
NA	0.0120	8.7363E-01	9.3663E-01	ENSG00000214719
NA	0.0065	8.7381E-01		ENSG00000275343
NA	0.0115	8.7430E-01	9.3710E-01	ENSG00000260418
NA	0.0124	8.7438E-01	9.3710E-01	ENSG00000159712
NA	0.0071	8.7469E-01		ENSG00000287404
NA	0.0111	8.7475E-01	9.3717E-01	ENSG00000288993
NA	0.0017	8.7488E-01		ENSG00000271163
NA	0.0108	8.7491E-01		ENSG00000259845
NA	0.0036	8.7508E-01		ENSG00000230696
NA	0.0058	8.7550E-01		ENSG00000258446
NA	0.0090	8.7551E-01	9.3775E-01	ENSG00000286900
NA	0.0109	8.7551E-01		ENSG00000250540
NA	0.0129	8.7559E-01		ENSG00000230911
NA	0.0064	8.7576E-01		ENSG00000236922
NA	0.0020	8.7579E-01		ENSG00000254331
NA	0.0080	8.7580E-01		ENSG00000228487
NA	0.0011	8.7584E-01		ENSG00000272183
NA	0.0101	8.7585E-01	9.3799E-01	ENSG00000259594
NA	0.0177	8.7600E-01	9.3803E-01	ENSG00000249609
NA	0.0125	8.7604E-01	9.3803E-01	ENSG00000185607
NA	0.0096	8.7608E-01	9.3803E-01	ENSG00000269924
NA	0.0038	8.7610E-01		ENSG00000286937
NA	0.0021	8.7616E-01		ENSG00000285552
NA	0.0118	8.7716E-01	9.3886E-01	ENSG00000239801
NA	0.0007	8.7738E-01		ENSG00000237584
NA	0.0007	8.7740E-01		ENSG00000266908
NA	0.0014	8.7744E-01	9.3895E-01	ENSG00000256843
NA	0.0141	8.7767E-01	9.3908E-01	ENSG00000259065
NA	0.0106	8.7771E-01	9.3908E-01	ENSG00000264666
NA	0.0038	8.7775E-01		ENSG00000232482
NA	0.0201	8.7781E-01	9.3909E-01	ENSG00000280414
NA	0.0117	8.7785E-01	9.3909E-01	ENSG00000161912
NA	0.0045	8.7804E-01		ENSG00000223220

NA	0.0087	8.7827E-01		ENSG00000228983
NA	0.0030	8.7898E-01		ENSG00000265380
NA	0.0033	8.7900E-01		ENSG00000259104
NA	0.0150	8.7928E-01	9.3970E-01	ENSG00000260978
NA	0.0107	8.7938E-01	9.3972E-01	ENSG00000288829
NA	0.0022	8.7952E-01		ENSG00000225105
NA	0.0119	8.7977E-01	9.3992E-01	ENSG00000272971
NA	0.0081	8.7998E-01	9.3996E-01	ENSG00000265791
NA	0.0127	8.7999E-01	9.3996E-01	ENSG00000257135
NA	0.0296	8.8017E-01	9.3996E-01	ENSG00000237438
NA	0.0069	8.8017E-01	9.3996E-01	ENSG00000280347
NA	0.0122	8.8018E-01	9.3996E-01	ENSG00000180015
NA	0.0008	8.8019E-01		ENSG00000279599
NA	0.0065	8.8029E-01		ENSG00000223831
NA	0.0079	8.8035E-01	9.4003E-01	ENSG00000272081
NA	0.0057	8.8052E-01		ENSG00000264187
NA	0.0098	8.8090E-01		ENSG00000272854
NA	0.0384	8.8113E-01	9.4050E-01	ENSG00000287644
NA	0.0055	8.8130E-01		ENSG00000226455
NA	0.0173	8.8141E-01	9.4059E-01	ENSG00000214857
NA	0.0021	8.8159E-01		ENSG00000289904
NA	0.0012	8.8164E-01		ENSG00000216753
NA	0.0075	8.8197E-01	9.4093E-01	ENSG00000262133
NA	0.0036	8.8206E-01		ENSG00000258970
NA	0.0010	8.8228E-01		ENSG00000238092
NA	0.0023	8.8234E-01		ENSG00000285647
NA	0.0077	8.8247E-01		ENSG00000277541
NA	0.0056	8.8253E-01		ENSG00000287427
NA	0.0059	8.8256E-01	9.4121E-01	ENSG00000233611
NA	0.0019	8.8257E-01	9.4121E-01	ENSG00000267353
NA	0.0118	8.8261E-01	9.4121E-01	ENSG00000234571
NA	0.0003	8.8268E-01		ENSG00000258344
NA	0.0139	8.8311E-01		ENSG00000241493
NA	0.0027	8.8370E-01		ENSG00000237174
NA	0.0067	8.8384E-01		ENSG00000218475
NA	0.0042	8.8423E-01		ENSG00000253362
NA	0.0054	8.8499E-01		ENSG00000253667
NA	0.0050	8.8501E-01		ENSG00000241679
NA	0.0092	8.8511E-01		ENSG00000289540
NA	0.0239	8.8535E-01		ENSG00000274093
NA	0.0016	8.8536E-01		ENSG00000235763
NA	0.0091	8.8545E-01		ENSG00000232305
NA	0.0033	8.8548E-01		ENSG00000201134
NA	0.0075	8.8555E-01		ENSG00000277504
NA	0.0007	8.8562E-01		ENSG00000255487
NA	0.0026	8.8630E-01		ENSG00000267905
NA	0.0070	8.8632E-01		ENSG00000234451
NA	0.0087	8.8637E-01		ENSG00000228791
NA	0.0036	8.8673E-01		ENSG00000225900

NA	0.0022	8.8701E-01		ENSG00000267699
NA	0.0036	8.8717E-01		ENSG00000253369
NA	0.0133	8.8719E-01		ENSG00000286163
NA	0.0307	8.8721E-01	9.4386E-01	ENSG00000225920
NA	0.0008	8.8742E-01		ENSG00000287447
NA	0.0075	8.8764E-01	9.4425E-01	ENSG00000279785
NA	0.0030	8.8768E-01		ENSG00000289284
NA	0.0010	8.8783E-01		ENSG00000280348
NA	0.0020	8.8805E-01		ENSG00000200814
NA	0.0067	8.8833E-01		ENSG00000253355
NA	0.0058	8.8837E-01		ENSG00000263612
NA	0.0070	8.8880E-01	9.4476E-01	ENSG00000258569
NA	0.0019	8.8911E-01		ENSG00000287963
NA	0.0073	8.8927E-01		ENSG00000261653
NA	0.0083	8.8932E-01		ENSG00000239511
NA	0.0030	8.8933E-01		ENSG00000223944
NA	0.0051	8.8933E-01		ENSG00000232736
NA	0.0093	8.8947E-01	9.4517E-01	ENSG00000260992
NA	0.0096	8.8965E-01		ENSG00000269800
NA	0.0033	8.8978E-01		ENSG00000272473
NA	0.0024	8.8978E-01		ENSG00000255851
NA	0.0093	8.8985E-01	9.4541E-01	ENSG00000231533
NA	0.0001	8.9000E-01	9.4552E-01	ENSG00000287891
NA	0.0015	8.9011E-01		ENSG00000270815
NA	0.0013	8.9013E-01		ENSG00000258422
NA	0.0098	8.9018E-01	9.4564E-01	ENSG00000223742
NA	0.0027	8.9021E-01		ENSG00000254270
NA	0.0024	8.9034E-01		ENSG00000254587
NA	0.0030	8.9040E-01		ENSG00000287368
NA	0.0029	8.9073E-01		ENSG00000262623
NA	0.0003	8.9074E-01		ENSG00000275088
NA	0.0084	8.9082E-01		ENSG00000289245
NA	0.0060	8.9083E-01		ENSG00000279340
NA	0.0097	8.9119E-01	9.4593E-01	ENSG00000269481
NA	0.0062	8.9127E-01		ENSG00000258473
NA	0.0056	8.9164E-01		ENSG00000231345
NA	0.0089	8.9174E-01	9.4623E-01	ENSG00000261560
NA	0.0045	8.9182E-01		ENSG00000240869
NA	0.0012	8.9203E-01		ENSG00000289298
NA	0.0077	8.9224E-01	9.4642E-01	ENSG00000283633
NA	0.0019	8.9238E-01		ENSG00000243107
NA	0.0025	8.9256E-01		ENSG00000280336
NA	0.0001	8.9257E-01		ENSG00000254909
NA	0.0015	8.9277E-01		ENSG00000285597
NA	0.0012	8.9307E-01		ENSG00000268533
NA	0.0110	8.9314E-01	9.4686E-01	ENSG00000286833
NA	0.0086	8.9315E-01		ENSG00000287020
NA	0.0035	8.9316E-01		ENSG00000267054
NA	0.0028	8.9326E-01		ENSG00000258177

NA	0.0185	8.9378E-01		ENSG00000255201
NA	0.0008	8.9385E-01		ENSG00000236352
NA	0.0064	8.9405E-01		ENSG00000227992
NA	0.0095	8.9406E-01		ENSG00000189149
NA	0.0035	8.9415E-01	9.4738E-01	ENSG00000280092
NA	0.0041	8.9424E-01		ENSG00000250761
NA	0.0127	8.9439E-01		ENSG00000226647
NA	0.0054	8.9444E-01		ENSG00000274019
NA	0.0143	8.9513E-01		ENSG00000259103
NA	0.0086	8.9513E-01		ENSG00000199319
NA	0.0088	8.9554E-01	9.4810E-01	ENSG00000227159
NA	0.0013	8.9568E-01		ENSG00000263684
NA	0.0006	8.9569E-01		ENSG00000275485
NA	0.0073	8.9606E-01		ENSG00000278633
NA	0.0114	8.9607E-01	9.4841E-01	ENSG00000279453
NA	0.0119	8.9608E-01	9.4841E-01	ENSG00000247345
NA	0.0068	8.9613E-01		ENSG00000229854
NA	0.0004	8.9646E-01		ENSG00000235615
NA	0.0032	8.9657E-01		ENSG00000241475
NA	0.0054	8.9676E-01	9.4874E-01	ENSG00000237980
NA	0.0024	8.9685E-01		ENSG00000273554
NA	0.0027	8.9721E-01		ENSG00000255272
NA	0.0013	8.9736E-01		ENSG00000232097
NA	0.0070	8.9766E-01	9.4953E-01	ENSG00000251288
NA	0.0041	8.9790E-01		ENSG00000260910
NA	0.0034	8.9824E-01		ENSG00000225330
NA	0.0107	8.9832E-01	9.4974E-01	ENSG00000223508
NA	0.0046	8.9849E-01		ENSG00000254606
NA	0.0039	8.9852E-01		ENSG00000277677
NA	0.0075	8.9863E-01	9.4994E-01	ENSG00000276744
NA	0.0011	8.9864E-01		ENSG00000289517
NA	0.0000	8.9872E-01		ENSG00000258736
NA	0.0070	8.9887E-01		ENSG00000289861
NA	0.0093	8.9932E-01	9.5047E-01	ENSG00000287408
NA	0.0022	8.9933E-01		ENSG00000213755
NA	0.0006	8.9945E-01		ENSG00000248165
NA	0.0081	8.9960E-01	9.5068E-01	ENSG00000279955
NA	0.0045	8.9970E-01		ENSG00000286036
NA	0.0096	8.9993E-01	9.5094E-01	ENSG00000267655
NA	0.0047	8.9995E-01		ENSG00000250039
NA	0.0048	9.0018E-01		ENSG00000267224
NA	0.0074	9.0019E-01	9.5105E-01	ENSG00000268947
NA	0.0020	9.0025E-01		ENSG00000231515
NA	0.0036	9.0026E-01		ENSG00000266036
NA	0.0086	9.0036E-01		ENSG00000287532
NA	0.0011	9.0058E-01		ENSG00000241211
NA	0.0060	9.0075E-01	9.5132E-01	ENSG00000201084
NA	0.0146	9.0083E-01		ENSG00000254551
NA	0.0018	9.0086E-01		ENSG00000251010

NA	0.0104	9.0093E-01	9.5147E-01	ENSG00000247287
NA	0.0105	9.0100E-01	9.5150E-01	ENSG00000287670
NA	0.0108	9.0113E-01	9.5152E-01	ENSG00000244560
NA	0.0013	9.0115E-01		ENSG00000183911
NA	0.0025	9.0116E-01		ENSG00000280159
NA	0.0040	9.0126E-01		ENSG00000232053
NA	0.0122	9.0173E-01		ENSG00000280330
NA	0.0122	9.0173E-01		ENSG00000279177
NA	0.0037	9.0187E-01		ENSG00000254497
NA	0.0022	9.0191E-01		ENSG00000252211
NA	0.0093	9.0201E-01	9.5204E-01	ENSG00000271851
NA	0.0105	9.0207E-01	9.5204E-01	ENSG00000273951
NA	0.0048	9.0218E-01	9.5206E-01	ENSG00000271204
NA	0.0102	9.0234E-01	9.5215E-01	ENSG00000239213
NA	0.0089	9.0276E-01		ENSG00000230831
NA	0.0075	9.0294E-01	9.5253E-01	ENSG00000279345
NA	0.0003	9.0348E-01		ENSG00000279600
NA	0.0061	9.0362E-01	9.5296E-01	ENSG00000260874
NA	0.0129	9.0375E-01		ENSG00000227666
NA	0.0056	9.0399E-01		ENSG00000267922
NA	0.0006	9.0400E-01		ENSG00000268027
NA	0.0083	9.0402E-01	9.5319E-01	ENSG00000249112
NA	0.0131	9.0416E-01		ENSG00000214081
NA	0.0008	9.0436E-01		ENSG00000286865
NA	0.0009	9.0467E-01		ENSG00000288786
NA	0.0032	9.0515E-01		ENSG00000277340
NA	0.0065	9.0529E-01		ENSG00000286295
NA	0.0105	9.0530E-01	9.5372E-01	ENSG00000204437
NA	0.0084	9.0540E-01		ENSG00000251613
NA	0.0085	9.0559E-01	9.5378E-01	ENSG00000287726
NA	0.0083	9.0567E-01	9.5379E-01	ENSG00000223834
NA	0.0072	9.0576E-01	9.5383E-01	ENSG00000234518
NA	0.0045	9.0587E-01		ENSG00000287191
NA	0.0102	9.0590E-01	9.5389E-01	ENSG00000187952
NA	0.0113	9.0664E-01		ENSG00000254028
NA	0.0099	9.0682E-01	9.5437E-01	ENSG00000273493
NA	0.0051	9.0738E-01		ENSG00000251357
NA	0.0090	9.0766E-01	9.5461E-01	ENSG00000270804
NA	0.0143	9.0782E-01	9.5461E-01	ENSG00000236708
NA	0.0052	9.0811E-01		ENSG00000286074
NA	0.0217	9.0812E-01	9.5472E-01	ENSG00000235538
NA	0.0038	9.0825E-01		ENSG00000260797
NA	0.0040	9.0828E-01		ENSG00000229367
NA	0.0065	9.0839E-01	9.5480E-01	ENSG00000232486
NA	0.0062	9.0844E-01	9.5481E-01	ENSG00000264548
NA	0.0055	9.0851E-01		ENSG00000288717
NA	0.0099	9.0856E-01	9.5481E-01	ENSG00000262049
NA	0.0068	9.0869E-01	9.5487E-01	ENSG00000234685
NA	0.0007	9.0881E-01		ENSG00000231434

NA	0.0069	9.0907E-01	9.5507E-01	ENSG00000249212
NA	0.0178	9.0930E-01		ENSG00000234496
NA	0.0149	9.0931E-01	9.5524E-01	ENSG00000237748
NA	0.0025	9.0935E-01		ENSG00000274653
NA	0.0115	9.0942E-01		ENSG00000229204
NA	0.0048	9.0974E-01		ENSG00000224885
NA	0.0021	9.0977E-01		ENSG00000280049
NA	0.0075	9.0979E-01	9.5529E-01	ENSG00000281566
NA	0.0080	9.0979E-01	9.5529E-01	ENSG00000289613
NA	0.0068	9.0979E-01		ENSG00000266598
NA	0.0100	9.1010E-01		ENSG00000266501
NA	0.0043	9.1012E-01		ENSG00000267920
NA	0.0107	9.1035E-01		ENSG00000268951
NA	0.0055	9.1040E-01		ENSG00000228626
NA	0.0087	9.1047E-01	9.5580E-01	ENSG00000276509
NA	0.0049	9.1048E-01		ENSG00000280311
NA	0.0025	9.1051E-01		ENSG00000223589
NA	0.0028	9.1052E-01		ENSG00000226977
NA	0.0033	9.1055E-01		ENSG00000259915
NA	0.0039	9.1058E-01		ENSG00000230849
NA	0.0015	9.1066E-01		ENSG00000252824
NA	0.0002	9.1088E-01		ENSG00000271598
NA	0.0012	9.1111E-01		ENSG00000285878
NA	0.0062	9.1132E-01	9.5625E-01	ENSG00000271781
NA	0.0046	9.1142E-01	9.5627E-01	ENSG00000231905
NA	0.0022	9.1143E-01		ENSG00000258051
NA	0.0023	9.1155E-01		ENSG00000288553
NA	0.0044	9.1160E-01		ENSG00000199472
NA	0.0060	9.1169E-01	9.5639E-01	ENSG00000255330
NA	0.0005	9.1206E-01		ENSG00000289452
NA	0.0010	9.1208E-01		ENSG00000286565
NA	0.0070	9.1220E-01	9.5669E-01	ENSG00000250643
NA	0.0020	9.1225E-01		ENSG00000286333
NA	0.0070	9.1236E-01		ENSG00000270813
NA	0.0089	9.1242E-01	9.5679E-01	ENSG00000228150
NA	0.0083	9.1246E-01	9.5679E-01	ENSG00000225891
NA	0.0010	9.1264E-01		ENSG00000261792
NA	0.0078	9.1265E-01		ENSG00000267537
NA	0.0030	9.1267E-01		ENSG00000229291
NA	0.0003	9.1329E-01		ENSG00000202071
NA	0.0023	9.1368E-01		ENSG00000270149
NA	0.0014	9.1415E-01		ENSG00000237676
NA	0.0059	9.1420E-01	9.5780E-01	ENSG00000234104
NA	0.0048	9.1430E-01		ENSG00000237331
NA	0.0063	9.1443E-01	9.5788E-01	ENSG00000280186
NA	0.0060	9.1448E-01	9.5789E-01	ENSG00000274425
NA	0.0082	9.1459E-01	9.5792E-01	ENSG00000260038
NA	0.0040	9.1516E-01		ENSG00000267152
NA	0.0088	9.1523E-01		ENSG00000286519

NA	0.0196	9.1548E-01		ENSG00000255021
NA	0.0125	9.1564E-01		ENSG00000279927
NA	0.0078	9.1564E-01	9.5841E-01	ENSG00000239381
NA	0.0106	9.1583E-01		ENSG00000227098
NA	0.0097	9.1589E-01	9.5855E-01	ENSG00000282772
NA	0.0016	9.1595E-01		ENSG00000259631
NA	0.0069	9.1600E-01		ENSG00000280283
NA	0.0078	9.1605E-01	9.5863E-01	ENSG00000286360
NA	0.0012	9.1613E-01		ENSG00000255231
NA	0.0089	9.1623E-01	9.5863E-01	ENSG00000274561
NA	0.0103	9.1625E-01	9.5863E-01	ENSG00000286729
NA	0.0025	9.1655E-01		ENSG00000271936
NA	0.0040	9.1658E-01		ENSG00000234724
NA	0.0072	9.1658E-01	9.5866E-01	ENSG00000279057
NA	0.0039	9.1658E-01		ENSG00000287906
NA	0.0078	9.1673E-01	9.5870E-01	ENSG00000279970
NA	0.0048	9.1680E-01		ENSG00000225836
NA	0.0117	9.1703E-01	9.5883E-01	ENSG00000215006
NA	0.0089	9.1763E-01	9.5907E-01	ENSG00000224846
NA	0.0001	9.1764E-01	9.5907E-01	ENSG00000287777
NA	0.0042	9.1786E-01		ENSG00000279853
NA	0.0032	9.1802E-01		ENSG00000288900
NA	0.0002	9.1813E-01		ENSG00000249797
NA	0.0050	9.1822E-01		ENSG00000261442
NA	0.0136	9.1840E-01		ENSG00000274849
NA	0.0078	9.1853E-01	9.5976E-01	ENSG00000235316
NA	0.0033	9.1864E-01		ENSG00000273901
NA	0.0039	9.1870E-01		ENSG00000196274
NA	0.0031	9.1896E-01		ENSG00000221406
NA	0.0036	9.1910E-01		ENSG00000271806
NA	0.0079	9.1911E-01	9.6004E-01	ENSG00000259687
NA	0.0044	9.1917E-01		ENSG00000280029
NA	0.0061	9.1926E-01	9.6011E-01	ENSG00000272764
NA	0.0066	9.1926E-01		ENSG00000260988
NA	0.0054	9.1931E-01		ENSG00000233359
NA	0.0065	9.1934E-01		ENSG00000286912
NA	0.0172	9.1935E-01	9.6016E-01	ENSG00000229585
NA	0.0095	9.1937E-01		ENSG00000236440
NA	0.0036	9.1945E-01		ENSG00000254202
NA	0.0012	9.1976E-01	9.6048E-01	ENSG00000240970
NA	0.0068	9.1979E-01		ENSG00000232597
NA	0.0011	9.2021E-01		ENSG00000286490
NA	0.0060	9.2029E-01	9.6091E-01	ENSG00000237493
NA	0.0061	9.2044E-01		ENSG00000277558
NA	0.0066	9.2049E-01	9.6092E-01	ENSG00000253695
NA	0.0068	9.2049E-01	9.6092E-01	ENSG00000286874
NA	0.0056	9.2113E-01		ENSG00000231699
NA	0.0049	9.2123E-01		ENSG00000287790
NA	0.0060	9.2139E-01	9.6140E-01	ENSG00000234043

NA	0.0054	9.2161E-01		ENSG00000269952
NA	0.0083	9.2178E-01	9.6140E-01	ENSG00000289236
NA	0.0030	9.2184E-01		ENSG00000225008
NA	0.0072	9.2198E-01		ENSG00000278862
NA	0.0038	9.2198E-01		ENSG00000277545
NA	0.0029	9.2201E-01		ENSG00000196564
NA	0.0007	9.2226E-01		ENSG00000187893
NA	0.0025	9.2231E-01	9.6166E-01	ENSG00000286348
NA	0.0071	9.2244E-01	9.6166E-01	ENSG00000285877
NA	0.0061	9.2247E-01	9.6166E-01	ENSG00000279207
NA	0.0107	9.2272E-01		ENSG00000255306
NA	0.0028	9.2298E-01		ENSG00000258696
NA	0.0035	9.2298E-01		ENSG00000238166
NA	0.0129	9.2338E-01		ENSG00000255358
NA	0.0041	9.2341E-01		ENSG00000268742
NA	0.0075	9.2343E-01	9.6226E-01	ENSG00000227627
NA	0.0029	9.2347E-01		ENSG00000203387
NA	0.0075	9.2348E-01		ENSG00000239912
NA	0.0082	9.2385E-01		ENSG00000226549
NA	0.0006	9.2395E-01		ENSG00000249116
NA	0.0062	9.2412E-01	9.6252E-01	ENSG00000264148
NA	0.0002	9.2417E-01		ENSG00000261610
NA	0.0030	9.2424E-01		ENSG00000251485
NA	0.0034	9.2430E-01		ENSG00000279147
NA	0.0016	9.2442E-01		ENSG00000198106
NA	0.0109	9.2465E-01		ENSG00000270403
NA	0.0049	9.2483E-01		ENSG00000278572
NA	0.0023	9.2497E-01		ENSG00000279205
NA	0.0080	9.2509E-01	9.6322E-01	ENSG00000287594
NA	0.0115	9.2510E-01		ENSG00000248359
NA	0.0028	9.2517E-01		ENSG00000225940
NA	0.0014	9.2543E-01	9.6345E-01	ENSG00000266541
NA	0.0075	9.2549E-01	9.6347E-01	ENSG00000287780
NA	0.0069	9.2574E-01		ENSG00000216723
NA	0.0072	9.2619E-01	9.6392E-01	ENSG00000272079
NA	0.0092	9.2649E-01		ENSG00000261248
NA	0.0043	9.2650E-01		ENSG00000264324
NA	0.0212	9.2652E-01	9.6404E-01	ENSG00000244538
NA	0.0013	9.2652E-01		ENSG00000270679
NA	0.0032	9.2676E-01		ENSG00000232491
NA	0.0007	9.2687E-01		ENSG00000214089
NA	0.0113	9.2688E-01	9.6421E-01	ENSG00000272086
NA	0.0023	9.2700E-01		ENSG00000232738
NA	0.0066	9.2702E-01	9.6425E-01	ENSG00000228657
NA	0.0121	9.2715E-01		ENSG00000267149
NA	0.0008	9.2730E-01		ENSG00000270893
NA	0.0079	9.2748E-01	9.6455E-01	ENSG00000260853
NA	0.0058	9.2751E-01		ENSG00000279551
NA	0.0031	9.2769E-01		ENSG00000229930

NA	0.0001	9.2781E-01		ENSG00000237281
NA	0.0116	9.2800E-01		ENSG00000273267
NA	0.0050	9.2802E-01		ENSG00000229550
NA	0.0034	9.2805E-01		ENSG00000237273
NA	0.0076	9.2896E-01	9.6500E-01	ENSG00000228107
NA	0.0055	9.2957E-01	9.6530E-01	ENSG00000229325
NA	0.0113	9.2966E-01	9.6530E-01	ENSG00000232546
NA	0.0050	9.2972E-01	9.6530E-01	ENSG00000235159
NA	0.0064	9.3001E-01	9.6545E-01	ENSG00000253893
NA	0.0004	9.3003E-01		ENSG00000286050
NA	0.0037	9.3043E-01		ENSG00000230563
NA	0.0044	9.3050E-01		ENSG00000286378
NA	0.0068	9.3054E-01	9.6574E-01	ENSG00000289913
NA	0.0000	9.3058E-01		ENSG00000206028
NA	0.0016	9.3060E-01		ENSG00000235024
NA	0.0020	9.3084E-01		ENSG00000286734
NA	0.0045	9.3105E-01		ENSG00000279366
NA	0.0006	9.3109E-01		ENSG00000241449
NA	0.0073	9.3126E-01	9.6600E-01	ENSG00000214826
NA	0.0023	9.3131E-01		ENSG00000204183
NA	0.0047	9.3137E-01		ENSG00000268892
NA	0.0130	9.3144E-01	9.6603E-01	ENSG00000257298
NA	0.0066	9.3144E-01		ENSG00000249888
NA	0.0069	9.3151E-01		ENSG00000260601
NA	0.0051	9.3156E-01	9.6607E-01	ENSG00000267742
NA	0.0019	9.3182E-01		ENSG00000283084
NA	0.0011	9.3185E-01		ENSG00000275248
NA	0.0075	9.3210E-01	9.6640E-01	ENSG00000224550
NA	0.0121	9.3212E-01		ENSG00000229332
NA	0.0041	9.3213E-01		ENSG00000269570
NA	0.0103	9.3224E-01		ENSG00000258465
NA	0.0005	9.3229E-01		ENSG00000262304
NA	0.0010	9.3234E-01		ENSG00000290068
NA	0.0022	9.3241E-01		ENSG00000259106
NA	0.0071	9.3255E-01	9.6658E-01	ENSG00000233270
NA	0.0023	9.3276E-01		ENSG00000250422
NA	0.0015	9.3379E-01	9.6721E-01	ENSG00000284471
NA	0.0271	9.3385E-01	9.6722E-01	ENSG00000233952
NA	0.0105	9.3399E-01		ENSG00000227203
NA	0.0006	9.3416E-01		ENSG00000230865
NA	0.0026	9.3452E-01		ENSG00000268790
NA	0.0036	9.3458E-01		ENSG00000264188
NA	0.0012	9.3468E-01		ENSG00000217447
NA	0.0030	9.3477E-01		ENSG00000264212
NA	0.0064	9.3502E-01	9.6803E-01	ENSG00000250838
NA	0.0058	9.3503E-01		ENSG00000227042
NA	0.0015	9.3566E-01		ENSG00000289618
NA	0.0017	9.3568E-01		ENSG00000250371
NA	0.0048	9.3596E-01		ENSG00000259649

NA	0.0006	9.3607E-01		ENSG00000199476
NA	0.0033	9.3614E-01		ENSG00000272940
NA	0.0093	9.3639E-01	9.6866E-01	ENSG00000250698
NA	0.0045	9.3659E-01	9.6868E-01	ENSG00000232855
NA	0.0049	9.3662E-01		ENSG00000258380
NA	0.0054	9.3663E-01		ENSG00000272366
NA	0.0016	9.3672E-01		ENSG00000286781
NA	0.0057	9.3705E-01	9.6903E-01	ENSG00000290146
NA	0.0048	9.3738E-01	9.6914E-01	ENSG00000254561
NA	0.0047	9.3742E-01	9.6914E-01	ENSG00000285611
NA	0.0052	9.3742E-01		ENSG00000224238
NA	0.0027	9.3787E-01		ENSG00000259048
NA	0.0068	9.3794E-01	9.6929E-01	ENSG00000272523
NA	0.0046	9.3813E-01		ENSG00000186466
NA	0.0013	9.3829E-01		ENSG00000271615
NA	0.0049	9.3845E-01		ENSG00000250796
NA	0.0067	9.3849E-01		ENSG00000287663
NA	0.0021	9.3853E-01		ENSG00000201724
NA	0.0027	9.3877E-01		ENSG00000243792
NA	0.0007	9.3911E-01		ENSG00000270104
NA	0.0044	9.3950E-01	9.7013E-01	ENSG00000237429
NA	0.0052	9.3953E-01	9.7013E-01	ENSG00000230751
NA	0.0069	9.3961E-01	9.7013E-01	ENSG00000260329
NA	0.0045	9.3991E-01	9.7035E-01	ENSG00000289208
NA	0.0059	9.3994E-01	9.7035E-01	ENSG00000258938
NA	0.0077	9.4022E-01		ENSG00000287544
NA	0.0073	9.4024E-01		ENSG00000275468
NA	0.0146	9.4025E-01		ENSG00000275963
NA	0.0056	9.4039E-01	9.7056E-01	ENSG00000230311
NA	0.0137	9.4050E-01		ENSG00000287852
NA	0.0052	9.4052E-01	9.7063E-01	ENSG00000215840
NA	0.0059	9.4053E-01		ENSG00000225463
NA	0.0070	9.4053E-01	9.7063E-01	ENSG00000229119
NA	0.0031	9.4112E-01		ENSG00000283057
NA	0.0030	9.4142E-01	9.7101E-01	ENSG00000288172
NA	0.0051	9.4192E-01		ENSG00000239528
NA	0.0015	9.4209E-01		ENSG00000224416
NA	0.0062	9.4219E-01	9.7141E-01	ENSG00000231125
NA	0.0046	9.4231E-01	9.7145E-01	ENSG00000288187
NA	0.0085	9.4232E-01		ENSG00000236165
NA	0.0050	9.4281E-01		ENSG00000260460
NA	0.0021	9.4317E-01		ENSG00000237346
NA	0.0002	9.4317E-01		ENSG00000226302
NA	0.0091	9.4319E-01	9.7182E-01	ENSG00000259205
NA	0.0128	9.4328E-01		ENSG00000236852
NA	0.0057	9.4331E-01		ENSG00000254006
NA	0.0013	9.4338E-01		ENSG00000230046
NA	0.0030	9.4385E-01		ENSG00000274918
NA	0.0021	9.4388E-01		ENSG00000271911

NA	0.0001	9.4411E-01		ENSG00000199204
NA	0.0123	9.4418E-01		ENSG00000233876
NA	0.0007	9.4422E-01		ENSG00000174930
NA	0.0023	9.4436E-01		ENSG00000275011
NA	0.0021	9.4438E-01	9.7218E-01	ENSG00000259425
NA	0.0039	9.4439E-01		ENSG00000277022
NA	0.0000	9.4440E-01	9.7218E-01	ENSG00000254162
NA	0.0052	9.4452E-01		ENSG00000266959
NA	0.0064	9.4467E-01		ENSG00000279586
NA	0.0022	9.4486E-01		ENSG00000231206
NA	0.0066	9.4503E-01	9.7267E-01	ENSG00000284625
NA	0.0033	9.4524E-01	9.7278E-01	ENSG00000239556
NA	0.0348	9.4549E-01	9.7290E-01	ENSG00000267571
NA	0.0140	9.4611E-01		ENSG00000289320
NA	0.0030	9.4630E-01	9.7340E-01	ENSG00000237194
NA	0.0022	9.4651E-01		ENSG00000279405
NA	0.0058	9.4685E-01		ENSG00000259728
NA	0.0049	9.4758E-01		ENSG00000279202
NA	0.0076	9.4763E-01	9.7425E-01	ENSG00000289915
NA	0.0048	9.4767E-01	9.7425E-01	ENSG00000285103
NA	0.0052	9.4782E-01		ENSG00000231046
NA	0.0043	9.4793E-01	9.7436E-01	ENSG00000259124
NA	0.0118	9.4843E-01		ENSG00000265334
NA	0.0011	9.4844E-01		ENSG00000229700
NA	0.0050	9.4867E-01	9.7460E-01	ENSG00000250397
NA	0.0031	9.4884E-01		ENSG00000215296
NA	0.0012	9.4913E-01		ENSG00000233721
NA	0.0069	9.4946E-01		ENSG00000285640
NA	0.0032	9.4952E-01		ENSG00000253859
NA	0.0060	9.4957E-01		ENSG00000287438
NA	0.0043	9.4964E-01	9.7496E-01	ENSG00000289194
NA	0.0047	9.4987E-01		ENSG00000228292
NA	0.0014	9.5001E-01		ENSG00000269976
NA	0.0054	9.5006E-01	9.7521E-01	ENSG00000188383
NA	0.0019	9.5012E-01		ENSG00000255866
NA	0.0036	9.5018E-01	9.7527E-01	ENSG00000204110
NA	0.0102	9.5032E-01		ENSG00000285663
NA	0.0052	9.5044E-01	9.7537E-01	ENSG00000260643
NA	0.0225	9.5056E-01	9.7545E-01	ENSG00000042304
NA	0.0033	9.5070E-01		ENSG00000286124
NA	0.0076	9.5088E-01		ENSG00000236054
NA	0.0025	9.5091E-01		ENSG00000262558
NA	0.0234	9.5098E-01		ENSG00000275636
NA	0.0138	9.5100E-01		ENSG00000288729
NA	0.0034	9.5107E-01	9.7582E-01	ENSG00000253766
NA	0.0043	9.5108E-01		ENSG00000234782
NA	0.0011	9.5143E-01		ENSG00000258394
NA	0.0244	9.5169E-01	9.7623E-01	ENSG00000231884
NA	0.0024	9.5195E-01		ENSG00000224875

NA	0.0005	9.5197E-01		ENSG00000229455
NA	0.0100	9.5209E-01		ENSG00000286307
NA	0.0030	9.5221E-01		ENSG00000258086
NA	0.0029	9.5222E-01		ENSG00000227678
NA	0.0044	9.5233E-01	9.7666E-01	ENSG00000286138
NA	0.0228	9.5240E-01	9.7668E-01	ENSG00000230847
NA	0.0097	9.5257E-01	9.7678E-01	ENSG00000254787
NA	0.0040	9.5275E-01	9.7681E-01	ENSG00000245958
NA	0.0054	9.5286E-01	9.7681E-01	ENSG00000253570
NA	0.0001	9.5358E-01		ENSG00000270249
NA	0.0020	9.5358E-01		ENSG00000255091
NA	0.0017	9.5375E-01		ENSG00000265010
NA	0.0049	9.5382E-01	9.7701E-01	ENSG00000285636
NA	0.0001	9.5393E-01		ENSG00000258853
NA	0.0169	9.5411E-01	9.7701E-01	ENSG00000231167
NA	0.0012	9.5413E-01		ENSG00000257870
NA	0.0042	9.5414E-01		ENSG00000275691
NA	0.0002	9.5431E-01		ENSG00000243810
NA	0.0005	9.5445E-01		ENSG00000225563
NA	0.0036	9.5453E-01		ENSG00000279949
NA	0.0033	9.5461E-01		ENSG00000184795
NA	0.0033	9.5462E-01	9.7710E-01	ENSG00000274414
NA	0.0035	9.5480E-01		ENSG00000228750
NA	0.0050	9.5489E-01	9.7729E-01	ENSG00000289428
NA	0.0049	9.5503E-01	9.7732E-01	ENSG00000246877
NA	0.0167	9.5517E-01		ENSG00000289158
NA	0.0074	9.5540E-01		ENSG00000260685
NA	0.0001	9.5569E-01		ENSG00000229437
NA	0.0079	9.5579E-01		ENSG00000272293
NA	0.0046	9.5582E-01		ENSG00000267426
NA	0.0071	9.5593E-01		ENSG00000228201
NA	0.0007	9.5604E-01		ENSG00000285561
NA	0.0013	9.5617E-01		ENSG00000236234
NA	0.0046	9.5627E-01	9.7830E-01	ENSG00000264015
NA	0.0035	9.5631E-01	9.7830E-01	ENSG00000272817
NA	0.0021	9.5638E-01		ENSG00000286700
NA	0.0045	9.5647E-01	9.7842E-01	ENSG00000250222
NA	0.0060	9.5648E-01		ENSG00000279895
NA	0.0060	9.5648E-01		ENSG00000280019
NA	0.0060	9.5648E-01		ENSG00000279728
NA	0.0043	9.5664E-01		ENSG00000273445
NA	0.0001	9.5679E-01		ENSG00000254506
NA	0.0033	9.5710E-01	9.7880E-01	ENSG00000229431
NA	0.0226	9.5713E-01		ENSG00000224700
NA	0.0031	9.5717E-01		ENSG00000225674
NA	0.0027	9.5756E-01	9.7913E-01	ENSG00000272156
NA	0.0010	9.5766E-01		ENSG00000228368
NA	0.0041	9.5771E-01	9.7921E-01	ENSG00000231340
NA	0.0010	9.5784E-01		ENSG00000289351

NA	0.0011	9.5791E-01		ENSG00000281420
NA	0.0012	9.5797E-01		ENSG00000226677
NA	0.0072	9.5797E-01		ENSG00000231907
NA	0.0049	9.5808E-01		ENSG00000278925
NA	0.0160	9.5808E-01		ENSG00000258981
NA	0.0024	9.5854E-01	9.7936E-01	ENSG00000227113
NA	0.0045	9.5868E-01	9.7939E-01	ENSG00000279319
NA	0.0000	9.5877E-01		ENSG00000261049
NA	0.0037	9.5881E-01	9.7939E-01	ENSG00000272720
NA	0.0039	9.5931E-01	9.7970E-01	ENSG00000275140
NA	0.0029	9.5948E-01		ENSG00000288927
NA	0.0090	9.5960E-01		ENSG00000189348
NA	0.0005	9.5965E-01		ENSG00000286612
NA	0.0021	9.6007E-01		ENSG00000280387
NA	0.0113	9.6011E-01		ENSG00000272482
NA	0.0034	9.6032E-01		ENSG00000244585
NA	0.0037	9.6034E-01		ENSG00000287724
NA	0.0032	9.6071E-01	9.8038E-01	ENSG00000123870
NA	0.0042	9.6072E-01		ENSG00000278601
NA	0.0061	9.6072E-01		ENSG00000269444
NA	0.0043	9.6077E-01	9.8038E-01	ENSG00000235636
NA	0.0028	9.6083E-01	9.8040E-01	ENSG00000223901
NA	0.0056	9.6104E-01	9.8053E-01	ENSG00000273096
NA	0.0064	9.6114E-01		ENSG00000266505
NA	0.0028	9.6114E-01		ENSG00000179141
NA	0.0107	9.6126E-01		ENSG00000251330
NA	0.0043	9.6143E-01	9.8076E-01	ENSG00000288741
NA	0.0040	9.6170E-01	9.8083E-01	ENSG00000128692
NA	0.0032	9.6183E-01	9.8091E-01	ENSG00000280407
NA	0.0010	9.6189E-01		ENSG00000254987
NA	0.0062	9.6201E-01		ENSG00000265547
NA	0.0034	9.6237E-01		ENSG00000270837
NA	0.0070	9.6238E-01		ENSG00000237522
NA	0.0043	9.6257E-01		ENSG00000261187
NA	0.0013	9.6258E-01		ENSG00000287675
NA	0.0175	9.6263E-01	9.8133E-01	ENSG00000234705
NA	0.0040	9.6270E-01	9.8133E-01	ENSG00000278867
NA	0.0107	9.6272E-01		ENSG00000228481
NA	0.0032	9.6278E-01		ENSG00000217718
NA	0.0014	9.6310E-01		ENSG00000288089
NA	0.0039	9.6330E-01	9.8180E-01	ENSG00000234072
NA	0.0161	9.6349E-01	9.8180E-01	ENSG00000237351
NA	0.0015	9.6365E-01		ENSG00000287284
NA	0.0027	9.6370E-01		ENSG00000221571
NA	0.0143	9.6380E-01		ENSG00000288768
NA	0.0036	9.6398E-01		ENSG00000259278
NA	0.0006	9.6413E-01		ENSG00000259215
NA	0.0030	9.6427E-01		ENSG00000241912
NA	0.0019	9.6433E-01		ENSG00000279862

NA	0.0079	9.6437E-01		ENSG00000239572
NA	0.0029	9.6457E-01		ENSG00000289600
NA	0.0009	9.6463E-01		ENSG00000251598
NA	0.0028	9.6471E-01		ENSG00000214305
NA	0.0026	9.6491E-01	9.8273E-01	ENSG00000235236
NA	0.0028	9.6526E-01		ENSG00000273937
NA	0.0020	9.6539E-01		ENSG00000224452
NA	0.0091	9.6586E-01		ENSG00000288696
NA	0.0039	9.6586E-01		ENSG00000270052
NA	0.0048	9.6590E-01		ENSG00000284697
NA	0.0025	9.6595E-01		ENSG00000249786
NA	0.0008	9.6608E-01		ENSG00000274216
NA	0.0021	9.6623E-01	9.8327E-01	ENSG00000260646
NA	0.0023	9.6627E-01	9.8327E-01	ENSG00000278627
NA	0.0025	9.6630E-01	9.8327E-01	ENSG00000256464
NA	0.0093	9.6632E-01	9.8327E-01	ENSG00000255503
NA	0.0110	9.6648E-01		ENSG00000262020
NA	0.0012	9.6650E-01		ENSG00000240435
NA	0.0178	9.6659E-01		ENSG00000253824
NA	0.0028	9.6662E-01	9.8340E-01	ENSG00000234925
NA	0.0011	9.6682E-01		ENSG00000263167
NA	0.0013	9.6689E-01		ENSG00000258011
NA	0.0047	9.6690E-01		ENSG00000258378
NA	0.0047	9.6708E-01		ENSG00000272799
NA	0.0000	9.6723E-01		ENSG00000223634
NA	0.0018	9.6771E-01		ENSG00000235081
NA	0.0020	9.6785E-01		ENSG00000288915
NA	0.0042	9.6785E-01		ENSG00000237679
NA	0.0031	9.6789E-01		ENSG00000267143
NA	0.0022	9.6816E-01		ENSG00000287986
NA	0.0111	9.6819E-01		ENSG00000270705
NA	0.0021	9.6857E-01		ENSG00000255608
NA	0.0048	9.6870E-01		ENSG00000231837
NA	0.0173	9.6879E-01	9.8475E-01	ENSG00000188013
NA	0.0003	9.6893E-01		ENSG00000257289
NA	0.0023	9.6911E-01	9.8480E-01	ENSG00000286412
NA	0.0017	9.6922E-01		ENSG00000286573
NA	0.0004	9.6929E-01		ENSG00000267014
NA	0.0065	9.6968E-01		ENSG00000256589
NA	0.0140	9.6979E-01	9.8504E-01	ENSG00000179766
NA	0.0005	9.6979E-01		ENSG00000286186
NA	0.0046	9.6993E-01		ENSG00000255403
NA	0.0022	9.6995E-01	9.8504E-01	ENSG00000251550
NA	0.0138	9.7006E-01		ENSG00000287939
NA	0.0039	9.7017E-01	9.8507E-01	ENSG00000226065
NA	0.0028	9.7017E-01	9.8507E-01	ENSG00000289589
NA	0.0017	9.7020E-01		ENSG00000249741
NA	0.0031	9.7027E-01	9.8508E-01	ENSG00000285652
NA	0.0018	9.7028E-01		ENSG00000259258

NA	0.0051	9.7033E-01		ENSG00000233055
NA	0.0002	9.7034E-01		ENSG00000267315
NA	0.0030	9.7055E-01		ENSG00000229919
NA	0.0184	9.7070E-01	9.8528E-01	ENSG00000283118
NA	0.0013	9.7086E-01	9.8536E-01	ENSG00000231503
NA	0.0023	9.7093E-01		ENSG00000271264
NA	0.0303	9.7096E-01		ENSG00000230729
NA	0.0038	9.7145E-01		ENSG00000269463
NA	0.0048	9.7186E-01		ENSG00000255328
NA	0.0029	9.7191E-01		ENSG00000223430
NA	0.0025	9.7228E-01		ENSG00000257962
NA	0.0127	9.7237E-01		ENSG00000213667
NA	0.0027	9.7252E-01		ENSG00000254397
NA	0.0030	9.7252E-01	9.8635E-01	ENSG00000237356
NA	0.0031	9.7258E-01		ENSG00000288777
NA	0.0056	9.7282E-01	9.8661E-01	ENSG00000287548
NA	0.0124	9.7283E-01		ENSG00000235908
NA	0.0016	9.7287E-01	9.8662E-01	ENSG00000240373
NA	0.0097	9.7290E-01		ENSG00000272908
NA	0.0004	9.7310E-01		ENSG00000288563
NA	0.0016	9.7316E-01		ENSG00000225401
NA	0.0030	9.7318E-01	9.8689E-01	ENSG00000280007
NA	0.0010	9.7327E-01		ENSG00000288940
NA	0.0017	9.7331E-01		ENSG00000251611
NA	0.0026	9.7337E-01		ENSG00000236938
NA	0.0016	9.7345E-01		ENSG00000266961
NA	0.0041	9.7353E-01		ENSG00000284654
NA	0.0047	9.7353E-01	9.8713E-01	ENSG00000241622
NA	0.0139	9.7367E-01		ENSG00000257894
NA	0.0004	9.7371E-01		ENSG00000263829
NA	0.0023	9.7385E-01	9.8718E-01	ENSG00000258365
NA	0.0121	9.7389E-01	9.8718E-01	ENSG00000224183
NA	0.0010	9.7413E-01		ENSG00000276092
NA	0.0070	9.7414E-01		ENSG00000223726
NA	0.0012	9.7419E-01		ENSG00000227382
NA	0.0001	9.7421E-01		ENSG00000225378
NA	0.0025	9.7425E-01	9.8741E-01	ENSG00000225489
NA	0.0023	9.7431E-01	9.8741E-01	ENSG00000259635
NA	0.0094	9.7446E-01		ENSG00000207223
NA	0.0026	9.7463E-01	9.8758E-01	ENSG00000279140
NA	0.0008	9.7491E-01		ENSG00000280396
NA	0.0074	9.7501E-01		ENSG00000258807
NA	0.0054	9.7504E-01		ENSG00000289228
NA	0.0007	9.7511E-01		ENSG00000207142
NA	0.0013	9.7519E-01		ENSG00000235616
NA	0.0030	9.7524E-01		ENSG00000278595
NA	0.0002	9.7553E-01		ENSG00000288084
NA	0.0012	9.7578E-01		ENSG00000279699
NA	0.0013	9.7579E-01		ENSG00000271525

NA	0.0078	9.7580E-01		ENSG00000264000
NA	0.0104	9.7585E-01		ENSG00000280116
NA	0.0025	9.7590E-01		ENSG00000277304
NA	0.0055	9.7591E-01		ENSG00000279176
NA	0.0028	9.7601E-01		ENSG00000284686
NA	0.0003	9.7639E-01		ENSG00000267449
NA	0.0018	9.7654E-01		ENSG00000215515
NA	0.0028	9.7664E-01		ENSG00000258018
NA	0.0019	9.7665E-01	9.8837E-01	ENSG00000267481
NA	0.0022	9.7671E-01	9.8837E-01	ENSG00000131401
NA	0.0057	9.7673E-01		ENSG00000278518
NA	0.0095	9.7701E-01		ENSG00000229407
NA	0.0145	9.7710E-01		ENSG00000278573
NA	0.0024	9.7712E-01	9.8848E-01	ENSG00000276952
NA	0.0014	9.7731E-01	9.8860E-01	ENSG00000233064
NA	0.0033	9.7747E-01		ENSG00000249610
NA	0.0117	9.7750E-01		ENSG00000249771
NA	0.0028	9.7756E-01		ENSG00000279497
NA	0.0051	9.7762E-01		ENSG00000262362
NA	0.0041	9.7773E-01		ENSG00000266818
NA	0.0039	9.7774E-01		ENSG00000271253
NA	0.0008	9.7785E-01		ENSG00000236896
NA	0.0011	9.7786E-01	9.8899E-01	ENSG00000238199
NA	0.0182	9.7796E-01		ENSG00000283213
NA	0.0005	9.7838E-01		ENSG00000231173
NA	0.0013	9.7847E-01	9.8925E-01	ENSG00000289265
NA	0.0185	9.7849E-01		ENSG00000287901
NA	0.0002	9.7850E-01		ENSG00000270179
NA	0.0256	9.7852E-01	9.8925E-01	ENSG00000268543
NA	0.0025	9.7854E-01		ENSG00000274654
NA	0.0058	9.7891E-01		ENSG00000236681
NA	0.0023	9.7906E-01		ENSG00000232568
NA	0.0014	9.7908E-01		ENSG00000272905
NA	0.0017	9.7909E-01		ENSG00000255028
NA	0.0020	9.7914E-01		ENSG00000244757
NA	0.0016	9.7923E-01		ENSG00000253330
NA	0.0115	9.7944E-01		ENSG00000213856
NA	0.0026	9.7957E-01	9.8977E-01	ENSG00000234624
NA	0.0042	9.7972E-01	9.8977E-01	ENSG00000106610
NA	0.0011	9.7972E-01	9.8977E-01	ENSG00000273141
NA	0.0024	9.7999E-01		ENSG00000289510
NA	0.0062	9.8003E-01		ENSG00000236662
NA	0.0018	9.8022E-01	9.8991E-01	ENSG00000235044
NA	0.0004	9.8038E-01		ENSG00000258723
NA	0.0014	9.8050E-01	9.9000E-01	ENSG00000277957
NA	0.0156	9.8051E-01		ENSG00000259793
NA	0.0031	9.8060E-01		ENSG00000270919
NA	0.0150	9.8067E-01		ENSG00000228124
NA	0.0009	9.8070E-01		ENSG00000224490

NA	0.0030	9.8087E-01		ENSG00000229269
NA	0.0033	9.8107E-01		ENSG00000286602
NA	0.0002	9.8111E-01		ENSG00000286015
NA	0.0052	9.8133E-01		ENSG00000231827
NA	0.0001	9.8158E-01		ENSG00000255342
NA	0.0026	9.8161E-01	9.9039E-01	ENSG00000262333
NA	0.0024	9.8162E-01		ENSG00000227154
NA	0.0026	9.8166E-01		ENSG00000227673
NA	0.0023	9.8168E-01		ENSG00000270799
NA	0.0008	9.8189E-01		ENSG00000279465
NA	0.0073	9.8202E-01		ENSG00000284612
NA	0.0052	9.8216E-01		ENSG00000286836
NA	0.0032	9.8227E-01		ENSG00000285541
NA	0.0013	9.8243E-01	9.9071E-01	ENSG00000205424
NA	0.0019	9.8247E-01	9.9071E-01	ENSG00000254428
NA	0.0013	9.8261E-01	9.9071E-01	ENSG00000230804
NA	0.0011	9.8265E-01	9.9071E-01	ENSG00000260577
NA	0.0005	9.8285E-01		ENSG00000230661
NA	0.0043	9.8285E-01		ENSG00000279200
NA	0.0044	9.8289E-01		ENSG00000223390
NA	0.0107	9.8298E-01		ENSG00000287089
NA	0.0014	9.8328E-01	9.9105E-01	ENSG00000279092
NA	0.0001	9.8333E-01		ENSG00000253911
NA	0.0019	9.8366E-01	9.9123E-01	ENSG00000273151
NA	0.0018	9.8386E-01		ENSG00000234818
NA	0.0154	9.8435E-01	9.9172E-01	ENSG00000279543
NA	0.0017	9.8439E-01		ENSG00000267079
NA	0.0011	9.8440E-01		ENSG00000176970
NA	0.0101	9.8449E-01		ENSG00000288849
NA	0.0102	9.8455E-01		ENSG00000213703
NA	0.0027	9.8490E-01		ENSG00000261487
NA	0.0021	9.8512E-01		ENSG00000200332
NA	0.0014	9.8514E-01	9.9215E-01	ENSG00000274265
NA	0.0086	9.8548E-01		ENSG00000216809
NA	0.0027	9.8556E-01		ENSG00000260123
NA	0.0041	9.8556E-01		ENSG00000289371
NA	0.0015	9.8568E-01	9.9239E-01	ENSG00000236035
NA	0.0048	9.8579E-01		ENSG00000260142
NA	0.0074	9.8599E-01	9.9239E-01	ENSG00000231536
NA	0.0032	9.8599E-01	9.9239E-01	ENSG00000289482
NA	0.0032	9.8606E-01		ENSG00000248790
NA	0.0013	9.8611E-01		ENSG00000278384
NA	0.0014	9.8633E-01	9.9243E-01	ENSG00000253636
NA	0.0012	9.8639E-01	9.9243E-01	ENSG00000258077
NA	0.0014	9.8642E-01	9.9243E-01	ENSG00000248514
NA	0.0008	9.8655E-01		ENSG00000254662
NA	0.0071	9.8666E-01		ENSG00000280171
NA	0.0015	9.8677E-01	9.9262E-01	ENSG00000289115
NA	0.0006	9.8701E-01		ENSG00000225811

NA	0.0058	9.8707E-01		ENSG00000272088
NA	0.0086	9.8717E-01		ENSG00000260430
NA	0.0037	9.8743E-01	9.9312E-01	ENSG00000285827
NA	0.0056	9.8760E-01		ENSG00000236464
NA	0.0074	9.8761E-01	9.9322E-01	ENSG00000279537
NA	0.0032	9.8783E-01		ENSG00000234645
NA	0.0031	9.8806E-01		ENSG00000259637
NA	0.0024	9.8820E-01		ENSG00000233664
NA	0.0046	9.8825E-01	9.9354E-01	ENSG00000253190
NA	0.0040	9.8868E-01		ENSG00000254596
NA	0.0015	9.8878E-01	9.9379E-01	ENSG00000281538
NA	0.0063	9.8881E-01		ENSG00000287496
NA	0.0001	9.8886E-01		ENSG00000206738
NA	0.0045	9.8890E-01		ENSG00000224479
NA	0.0011	9.8928E-01	9.9405E-01	ENSG00000273175
NA	0.0045	9.8940E-01	9.9411E-01	ENSG00000278703
NA	0.0027	9.8943E-01	9.9411E-01	ENSG00000287287
NA	0.0015	9.8991E-01		ENSG00000234929
NA	0.0063	9.8998E-01		ENSG00000267587
NA	0.0001	9.9003E-01		ENSG00000257681
NA	0.0037	9.9011E-01		ENSG00000230781
NA	0.0024	9.9021E-01		ENSG00000286878
NA	0.0022	9.9026E-01		ENSG00000228702
NA	0.0092	9.9033E-01	9.9461E-01	ENSG00000267986
NA	0.0117	9.9037E-01	9.9461E-01	ENSG00000228027
NA	0.0132	9.9065E-01	9.9477E-01	ENSG00000225193
NA	0.0031	9.9066E-01		ENSG00000253170
NA	0.0020	9.9073E-01		ENSG00000253749
NA	0.0021	9.9101E-01		ENSG00000215223
NA	0.0022	9.9114E-01		ENSG00000227779
NA	0.0065	9.9151E-01		ENSG00000273568
NA	0.0038	9.9172E-01		ENSG00000285685
NA	0.0062	9.9221E-01		ENSG00000277764
NA	0.0011	9.9232E-01		ENSG00000282852
NA	0.0019	9.9239E-01		ENSG00000271181
NA	0.0057	9.9263E-01		ENSG00000259161
NA	0.0007	9.9302E-01	9.9612E-01	ENSG00000287576
NA	0.0019	9.9310E-01		ENSG00000282390
NA	0.0032	9.9313E-01		ENSG00000279098
NA	0.0005	9.9313E-01		ENSG00000228322
NA	0.0007	9.9336E-01		ENSG00000213594
NA	0.0046	9.9341E-01		ENSG00000274560
NA	0.0005	9.9342E-01	9.9629E-01	ENSG00000288863
NA	0.0015	9.9374E-01		ENSG00000287302
NA	0.0012	9.9379E-01		ENSG00000144785
NA	0.0007	9.9398E-01	9.9671E-01	ENSG00000234648
NA	0.0166	9.9409E-01	9.9671E-01	ENSG00000278543
NA	0.0013	9.9439E-01	9.9686E-01	ENSG00000268034
NA	0.0000	9.9458E-01	9.9689E-01	ENSG00000266368

NA	0.0034	9.9478E-01		ENSG00000227308
NA	0.0010	9.9497E-01		ENSG00000254644
NA	0.0030	9.9508E-01		ENSG00000234141
NA	0.0051	9.9515E-01		ENSG00000279633
NA	0.0021	9.9518E-01		ENSG00000273584
NA	0.0092	9.9535E-01		ENSG00000285530
NA	0.0070	9.9568E-01		ENSG00000253582
NA	0.0017	9.9575E-01		ENSG00000227406
NA	0.0036	9.9579E-01		ENSG00000251216
NA	0.0030	9.9588E-01	9.9767E-01	ENSG00000241954
NA	0.0021	9.9592E-01		ENSG00000272787
NA	0.0065	9.9594E-01		ENSG00000287818
NA	0.0097	9.9609E-01		ENSG00000258303
NA	0.0030	9.9615E-01		ENSG00000260711
NA	0.0113	9.9635E-01		ENSG00000260112
NA	0.0002	9.9639E-01	9.9802E-01	ENSG00000254786
NA	0.0011	9.9706E-01	9.9831E-01	ENSG00000272906
NA	0.0186	9.9707E-01	9.9831E-01	ENSG00000253236
NA	0.0026	9.9719E-01		ENSG00000250948
NA	0.0014	9.9720E-01		ENSG00000233005
NA	0.0034	9.9726E-01		ENSG00000229393
NA	0.0141	9.9728E-01		ENSG00000267480
NA	0.0021	9.9734E-01		ENSG00000268288
NA	0.0022	9.9738E-01		ENSG00000286635
NA	0.0007	9.9756E-01		ENSG00000206699
NA	0.0056	9.9768E-01		ENSG00000223620
NA	0.0022	9.9786E-01		ENSG00000237217
NA	0.0063	9.9799E-01		ENSG00000232715
NA	0.0006	9.9801E-01	9.9882E-01	ENSG00000213683
NA	0.0022	9.9858E-01		ENSG00000258565
NA	0.0165	9.9859E-01	9.9913E-01	ENSG00000275898
NA	0.0029	9.9860E-01		ENSG00000263331
NA	0.0000	9.9872E-01		ENSG00000233820
NA	0.0032	9.9885E-01		ENSG00000273233
NA	0.0002	9.9909E-01	9.9944E-01	ENSG00000289453
NA	0.0008	9.9917E-01	9.9946E-01	ENSG00000266578
NA	0.0050	9.9924E-01		ENSG00000287289
NA	0.0018	9.9926E-01		ENSG00000265378
NA	0.0074	9.9933E-01		ENSG00000266521
NA	0.0013	9.9939E-01		ENSG00000253125
NA	0.0003	9.9944E-01		ENSG00000236811
NA	0.0001	9.9964E-01	9.9976E-01	ENSG00000289943
NA	0.0120	9.9970E-01	9.9976E-01	ENSG00000229370
NA	0.0010	9.9979E-01		ENSG00000288109
NA	0.0032	9.9988E-01		ENSG00000289061
NA	0.0009	9.9999E-01		ENSG00000228408
NA	-0.0025	9.9886E-01		ENSG00000283518
NA	-0.0022	9.9849E-01		ENSG00000236505
NA	-0.0007	9.9844E-01		ENSG00000237670

NA	-0.0002	9.9785E-01	9.9871E-01	ENSG00000273691
NA	-0.0016	9.9694E-01		ENSG00000280767
NA	-0.0010	9.9675E-01		ENSG00000231720
NA	-0.0009	9.9674E-01		ENSG00000235904
NA	-0.0046	9.9654E-01	9.9804E-01	ENSG00000235198
NA	-0.0101	9.9633E-01		ENSG00000276756
NA	-0.0005	9.9625E-01		ENSG00000251342
NA	-0.0046	9.9606E-01		ENSG00000283096
NA	-0.0116	9.9544E-01		ENSG00000255741
NA	-0.0001	9.9530E-01	9.9741E-01	ENSG00000274883
NA	-0.0004	9.9494E-01	9.9721E-01	ENSG00000180385
NA	-0.0139	9.9464E-01		ENSG00000271716
NA	-0.0308	9.9430E-01		ENSG00000287032
NA	-0.0005	9.9411E-01	9.9671E-01	ENSG00000289030
NA	-0.0005	9.9409E-01	9.9671E-01	ENSG00000255182
NA	-0.0010	9.9369E-01		ENSG00000286579
NA	-0.0010	9.9314E-01		ENSG00000279137
NA	-0.0018	9.9259E-01		ENSG00000241651
NA	-0.0052	9.9255E-01	9.9579E-01	ENSG00000280194
NA	-0.0033	9.9217E-01		ENSG00000274515
NA	-0.0024	9.9206E-01		ENSG00000267626
NA	-0.0010	9.9190E-01		ENSG00000287925
NA	-0.0005	9.9188E-01	9.9540E-01	ENSG00000287691
NA	-0.0004	9.9182E-01		ENSG00000254287
NA	-0.0005	9.9176E-01	9.9540E-01	ENSG00000256894
NA	-0.0009	9.9167E-01	9.9535E-01	ENSG00000248472
NA	-0.0231	9.9135E-01		ENSG00000257509
NA	-0.0006	9.9128E-01		ENSG00000279650
NA	-0.0017	9.8993E-01	9.9442E-01	ENSG00000259363
NA	-0.0007	9.8992E-01		ENSG00000268278
NA	-0.0089	9.8985E-01		ENSG00000213253
NA	-0.0007	9.8983E-01	9.9435E-01	ENSG00000230202
NA	-0.0005	9.8962E-01	9.9422E-01	ENSG00000237457
NA	-0.0009	9.8945E-01		ENSG00000213560
NA	0.0000	9.8930E-01		ENSG00000236257
NA	-0.0028	9.8854E-01		ENSG00000227189
NA	-0.0002	9.8811E-01		ENSG00000269944
NA	-0.0068	9.8781E-01		ENSG00000276434
NA	-0.0104	9.8748E-01		ENSG00000219529
NA	-0.0007	9.8742E-01	9.9312E-01	ENSG00000200737
NA	-0.0069	9.8701E-01		ENSG00000253399
NA	-0.0035	9.8692E-01		ENSG00000230828
NA	-0.0061	9.8685E-01		ENSG00000258154
NA	-0.0008	9.8668E-01	9.9257E-01	ENSG00000204801
NA	-0.0057	9.8656E-01		ENSG00000254449
NA	-0.0039	9.8654E-01	9.9247E-01	ENSG00000285536
NA	-0.0003	9.8644E-01		ENSG00000286853
NA	-0.0027	9.8604E-01	9.9239E-01	ENSG00000269755
NA	-0.0035	9.8597E-01		ENSG00000285650

NA	-0.0007	9.8538E-01	9.9231E-01	ENSG00000260259
NA	-0.0009	9.8529E-01		ENSG00000215270
NA	-0.0036	9.8527E-01		ENSG00000287394
NA	-0.0057	9.8510E-01	9.9215E-01	ENSG00000227973
NA	-0.0011	9.8506E-01	9.9215E-01	ENSG00000269086
NA	-0.0039	9.8501E-01		ENSG00000274403
NA	-0.0009	9.8421E-01		ENSG00000231395
NA	-0.0043	9.8411E-01		ENSG00000271275
NA	-0.0019	9.8326E-01		ENSG00000285933
NA	-0.0101	9.8325E-01		ENSG00000228323
NA	-0.0040	9.8323E-01		ENSG00000235749
NA	-0.0060	9.8267E-01		ENSG00000225000
NA	-0.0017	9.8262E-01	9.9071E-01	ENSG00000260163
NA	-0.0020	9.8242E-01		ENSG00000225815
NA	-0.0009	9.8233E-01	9.9070E-01	ENSG00000287306
NA	0.0000	9.8208E-01		ENSG00000258796
NA	-0.0014	9.8207E-01	9.9056E-01	ENSG00000259587
NA	-0.0022	9.8200E-01		ENSG00000287983
NA	-0.0083	9.8181E-01		ENSG00000288707
NA	-0.0027	9.8160E-01	9.9039E-01	ENSG00000270175
NA	-0.0023	9.8155E-01	9.9039E-01	ENSG00000289114
NA	-0.0003	9.8143E-01		ENSG00000266389
NA	-0.0090	9.8139E-01		ENSG00000251484
NA	-0.0017	9.8133E-01	9.9030E-01	ENSG00000227698
NA	-0.0056	9.8063E-01		ENSG00000225670
NA	-0.0005	9.7995E-01		ENSG00000289568
NA	-0.0032	9.7985E-01		ENSG00000244712
NA	-0.0042	9.7984E-01		ENSG00000288659
NA	-0.0013	9.7949E-01	9.8975E-01	ENSG00000257539
NA	-0.0014	9.7920E-01	9.8958E-01	ENSG00000267672
NA	-0.0105	9.7846E-01		ENSG00000256681
NA	-0.0030	9.7842E-01		ENSG00000286991
NA	-0.0044	9.7830E-01		ENSG00000286316
NA	-0.0022	9.7820E-01	9.8916E-01	ENSG00000259474
NA	-0.0023	9.7795E-01	9.8900E-01	ENSG00000251417
NA	-0.0018	9.7792E-01		ENSG00000238258
NA	-0.0040	9.7729E-01		ENSG00000279189
NA	-0.0027	9.7685E-01	9.8837E-01	ENSG00000289306
NA	-0.0019	9.7642E-01	9.8835E-01	ENSG00000286457
NA	-0.0076	9.7628E-01		ENSG00000256789
NA	-0.0016	9.7596E-01		ENSG00000257966
NA	-0.0018	9.7582E-01		ENSG00000279815
NA	-0.0033	9.7578E-01		ENSG00000286775
NA	-0.0003	9.7560E-01		ENSG00000254790
NA	-0.0002	9.7557E-01	9.8789E-01	ENSG00000235051
NA	-0.0015	9.7556E-01	9.8789E-01	ENSG00000286181
NA	-0.0009	9.7529E-01	9.8783E-01	ENSG00000232485
NA	-0.0018	9.7502E-01	9.8770E-01	ENSG00000224220
NA	-0.0017	9.7498E-01		ENSG00000237249

NA	-0.0070	9.7494E-01		ENSG00000268955
NA	-0.0032	9.7465E-01		ENSG00000224224
NA	-0.0018	9.7442E-01		ENSG00000232524
NA	-0.0016	9.7410E-01		ENSG00000234315
NA	0.0000	9.7381E-01		ENSG00000286500
NA	-0.0097	9.7353E-01		ENSG00000206582
NA	-0.0004	9.7300E-01		ENSG00000287688
NA	-0.0019	9.7131E-01		ENSG00000287322
NA	-0.0013	9.7128E-01		ENSG00000253555
NA	-0.0080	9.7122E-01		ENSG00000240108
NA	-0.0047	9.7095E-01		ENSG00000278492
NA	-0.0052	9.7092E-01		ENSG00000280215
NA	-0.0007	9.7049E-01		ENSG00000223784
NA	-0.0021	9.6994E-01	9.8504E-01	ENSG00000262766
NA	-0.0010	9.6994E-01		ENSG00000252305
NA	-0.0029	9.6957E-01		ENSG00000250657
NA	-0.0021	9.6949E-01		ENSG00000279656
NA	-0.0034	9.6919E-01		ENSG00000280173
NA	-0.0090	9.6880E-01		ENSG00000286992
NA	-0.0096	9.6872E-01		ENSG00000199523
NA	-0.0010	9.6852E-01		ENSG00000278215
NA	-0.0023	9.6839E-01	9.8444E-01	ENSG00000288888
NA	-0.0035	9.6825E-01		ENSG00000271198
NA	-0.0003	9.6821E-01		ENSG00000206756
NA	-0.0042	9.6807E-01	9.8421E-01	ENSG00000226415
NA	-0.0028	9.6804E-01		ENSG00000237470
NA	-0.0074	9.6802E-01		ENSG00000272707
NA	-0.0033	9.6791E-01		ENSG00000263338
NA	-0.0014	9.6779E-01		ENSG00000235847
NA	-0.0026	9.6770E-01		ENSG00000240490
NA	-0.0046	9.6760E-01		ENSG00000276786
NA	-0.0011	9.6751E-01		ENSG00000260648
NA	-0.0034	9.6715E-01	9.8362E-01	ENSG00000224358
NA	-0.0005	9.6709E-01		ENSG00000287926
NA	-0.0021	9.6666E-01	9.8340E-01	ENSG00000254272
NA	-0.0010	9.6620E-01		ENSG00000234235
NA	-0.0082	9.6618E-01		ENSG00000232630
NA	-0.0037	9.6614E-01		ENSG00000260815
NA	-0.0034	9.6489E-01		ENSG00000234060
NA	-0.0004	9.6440E-01	9.8237E-01	ENSG00000259342
NA	-0.0002	9.6426E-01		ENSG00000261429
NA	-0.0010	9.6404E-01		ENSG00000224967
NA	-0.0012	9.6402E-01		ENSG00000254756
NA	-0.0036	9.6392E-01	9.8204E-01	ENSG00000267023
NA	-0.0013	9.6334E-01	9.8180E-01	ENSG00000279957
NA	-0.0029	9.6332E-01	9.8180E-01	ENSG00000274423
NA	-0.0008	9.6309E-01		ENSG00000253142
NA	-0.0025	9.6294E-01	9.8153E-01	ENSG00000100181
NA	-0.0094	9.6271E-01		ENSG00000286274

NA	-0.0098	9.6267E-01	9.8133E-01	ENSG00000253357
NA	-0.0008	9.6267E-01		ENSG00000287762
NA	-0.0034	9.6253E-01	9.8133E-01	ENSG00000278126
NA	-0.0003	9.6249E-01		ENSG00000227758
NA	-0.0047	9.6246E-01		ENSG00000271278
NA	-0.0046	9.6222E-01		ENSG00000275371
NA	-0.0036	9.6220E-01		ENSG00000232028
NA	-0.0018	9.6213E-01		ENSG00000267546
NA	-0.0026	9.6161E-01	9.8083E-01	ENSG00000286259
NA	-0.0079	9.6097E-01		ENSG00000269274
NA	-0.0024	9.6074E-01	9.8038E-01	ENSG00000271789
NA	-0.0003	9.6057E-01	9.8030E-01	ENSG00000261757
NA	-0.0122	9.6056E-01		ENSG00000266973
NA	-0.0089	9.6049E-01		ENSG00000276704
NA	-0.0054	9.6041E-01		ENSG00000250348
NA	-0.0132	9.6041E-01		ENSG00000279619
NA	-0.0015	9.6036E-01		ENSG00000276054
NA	-0.0019	9.6032E-01	9.8016E-01	ENSG00000285115
NA	-0.0022	9.6018E-01		ENSG00000236908
NA	-0.0038	9.6009E-01	9.8008E-01	ENSG00000229036
NA	-0.0025	9.5996E-01	9.8006E-01	ENSG00000273759
NA	-0.0050	9.5936E-01		ENSG00000289130
NA	-0.0008	9.5915E-01		ENSG00000254678
NA	-0.0017	9.5860E-01		ENSG00000251009
NA	-0.0205	9.5822E-01	9.7936E-01	ENSG00000273890
NA	-0.0030	9.5818E-01		ENSG00000278462
NA	-0.0015	9.5818E-01		ENSG00000272834
NA	-0.0027	9.5811E-01		ENSG00000273792
NA	-0.0006	9.5795E-01		ENSG00000224739
NA	-0.0095	9.5791E-01		ENSG00000229287
NA	-0.0112	9.5739E-01		ENSG00000277411
NA	-0.0058	9.5716E-01		ENSG00000257940
NA	-0.0063	9.5686E-01		ENSG00000279807
NA	-0.0066	9.5650E-01		ENSG00000259290
NA	-0.0011	9.5645E-01		ENSG00000273341
NA	-0.0014	9.5623E-01		ENSG00000252719
NA	-0.0033	9.5590E-01		ENSG00000224817
NA	-0.0026	9.5585E-01		ENSG00000254021
NA	-0.0192	9.5579E-01		ENSG00000267762
NA	-0.0052	9.5578E-01		ENSG00000285948
NA	-0.0011	9.5471E-01		ENSG00000226825
NA	-0.0029	9.5429E-01		ENSG00000250686
NA	-0.0048	9.5427E-01	9.7703E-01	ENSG00000285693
NA	-0.0033	9.5418E-01	9.7701E-01	ENSG00000259555
NA	-0.0051	9.5388E-01	9.7701E-01	ENSG00000277959
NA	-0.0001	9.5383E-01		ENSG00000267156
NA	-0.0047	9.5352E-01	9.7699E-01	ENSG00000261654
NA	-0.0045	9.5351E-01	9.7699E-01	ENSG00000287493
NA	-0.0066	9.5326E-01		ENSG00000218502

NA	-0.0035	9.5319E-01		ENSG00000219375
NA	-0.0041	9.5299E-01	9.7681E-01	ENSG00000289985
NA	-0.0009	9.5291E-01		ENSG00000272254
NA	-0.0086	9.5270E-01		ENSG00000260908
NA	-0.0148	9.5243E-01	9.7668E-01	ENSG00000233073
NA	-0.0056	9.5219E-01		ENSG00000267708
NA	-0.0008	9.5217E-01		ENSG00000230313
NA	-0.0037	9.5182E-01	9.7623E-01	ENSG00000268189
NA	-0.0093	9.5180E-01		ENSG00000225728
NA	-0.0015	9.5120E-01		ENSG00000287896
NA	-0.0037	9.5106E-01	9.7582E-01	ENSG00000224579
NA	-0.0022	9.5100E-01		ENSG00000216915
NA	-0.0039	9.5030E-01	9.7529E-01	ENSG00000287624
NA	-0.0011	9.5018E-01		ENSG00000229190
NA	-0.0002	9.5016E-01		ENSG00000224219
NA	-0.0016	9.4951E-01	9.7495E-01	ENSG00000286503
NA	-0.0094	9.4944E-01		ENSG00000207922
NA	-0.0030	9.4925E-01		ENSG00000289538
NA	-0.0005	9.4916E-01		ENSG00000241073
NA	-0.0005	9.4911E-01	9.7478E-01	ENSG00000267334
NA	-0.0042	9.4902E-01	9.7473E-01	ENSG00000286366
NA	-0.0016	9.4891E-01		ENSG00000236116
NA	-0.0026	9.4869E-01	9.7460E-01	ENSG00000287534
NA	-0.0070	9.4865E-01		ENSG00000250500
NA	-0.0019	9.4859E-01		ENSG00000215199
NA	0.0000	9.4858E-01		ENSG00000230027
NA	-0.0019	9.4803E-01		ENSG00000284721
NA	-0.0014	9.4739E-01		ENSG00000285576
NA	-0.0021	9.4735E-01		ENSG00000268105
NA	-0.0022	9.4729E-01		ENSG00000287052
NA	-0.0098	9.4704E-01		ENSG00000226986
NA	-0.0061	9.4691E-01	9.7366E-01	ENSG00000186076
NA	-0.0023	9.4686E-01	9.7365E-01	ENSG00000266644
NA	-0.0155	9.4658E-01		ENSG00000260545
NA	-0.0054	9.4616E-01	9.7334E-01	ENSG00000283108
NA	-0.0025	9.4599E-01		ENSG00000207450
NA	-0.0032	9.4546E-01		ENSG00000286708
NA	-0.0005	9.4527E-01		ENSG00000264924
NA	-0.0005	9.4512E-01		ENSG00000270775
NA	-0.0004	9.4497E-01		ENSG00000286553
NA	-0.0052	9.4482E-01	9.7249E-01	ENSG00000273143
NA	-0.0036	9.4451E-01		ENSG00000225486
NA	-0.0005	9.4419E-01	9.7218E-01	ENSG00000286099
NA	-0.0053	9.4407E-01	9.7218E-01	ENSG00000273748
NA	-0.0107	9.4375E-01		ENSG00000237963
NA	-0.0018	9.4342E-01		ENSG00000248245
NA	-0.0023	9.4270E-01		ENSG00000223313
NA	-0.0011	9.4260E-01		ENSG00000217835
NA	-0.0050	9.4240E-01		ENSG00000289829

NA	-0.0088	9.4209E-01		ENSG00000242163
NA	-0.0011	9.4142E-01		ENSG00000206964
NA	-0.0038	9.4126E-01	9.7089E-01	ENSG00000253981
NA	-0.0011	9.4122E-01		ENSG00000285565
NA	-0.0019	9.4112E-01		ENSG00000200201
NA	-0.0041	9.4111E-01	9.7086E-01	ENSG00000275426
NA	-0.0034	9.4084E-01	9.7074E-01	ENSG00000274799
NA	-0.0017	9.4013E-01	9.7046E-01	ENSG00000225111
NA	-0.0064	9.3977E-01		ENSG00000177776
NA	-0.0054	9.3968E-01	9.7016E-01	ENSG00000274421
NA	-0.0063	9.3956E-01	9.7013E-01	ENSG00000275198
NA	-0.0009	9.3940E-01		ENSG00000273464
NA	-0.0045	9.3930E-01	9.7006E-01	ENSG00000228395
NA	-0.0065	9.3923E-01		ENSG00000286966
NA	-0.0434	9.3893E-01	9.6988E-01	ENSG00000257139
NA	-0.0125	9.3892E-01		ENSG00000285734
NA	-0.0013	9.3866E-01		ENSG00000275678
NA	-0.0062	9.3863E-01		ENSG00000230149
NA	-0.0029	9.3858E-01		ENSG00000232987
NA	-0.0031	9.3849E-01		ENSG00000271490
NA	-0.0006	9.3825E-01		ENSG00000251107
NA	-0.0041	9.3817E-01	9.6941E-01	ENSG00000259551
NA	-0.0014	9.3788E-01		ENSG00000232474
NA	-0.0017	9.3767E-01		ENSG00000231496
NA	-0.0002	9.3741E-01		ENSG00000229722
NA	-0.0039	9.3727E-01	9.6914E-01	ENSG00000229509
NA	-0.0170	9.3715E-01		ENSG00000225411
NA	-0.0009	9.3704E-01		ENSG00000276533
NA	-0.0168	9.3679E-01		ENSG00000233044
NA	-0.0063	9.3669E-01	9.6874E-01	ENSG00000267265
NA	-0.0145	9.3649E-01		ENSG00000279206
NA	-0.0002	9.3643E-01	9.6866E-01	ENSG00000248787
NA	-0.0027	9.3640E-01		ENSG00000275084
NA	-0.0047	9.3639E-01	9.6866E-01	ENSG00000259654
NA	-0.0053	9.3630E-01		ENSG00000283765
NA	-0.0017	9.3627E-01		ENSG00000226356
NA	-0.0006	9.3560E-01		ENSG00000260635
NA	-0.0003	9.3538E-01		ENSG00000279420
NA	-0.0005	9.3537E-01		ENSG00000232422
NA	-0.0048	9.3476E-01		ENSG00000285520
NA	-0.0050	9.3472E-01	9.6786E-01	ENSG00000218189
NA	-0.0042	9.3459E-01		ENSG00000254630
NA	-0.0051	9.3445E-01	9.6769E-01	ENSG00000248121
NA	-0.0067	9.3415E-01	9.6741E-01	ENSG00000227543
NA	-0.0118	9.3406E-01		ENSG00000229970
NA	-0.0015	9.3399E-01		ENSG00000270282
NA	-0.0046	9.3361E-01	9.6718E-01	ENSG00000233860
NA	-0.0045	9.3342E-01		ENSG00000230946
NA	-0.0015	9.3339E-01		ENSG00000254609

NA	-0.0066	9.3338E-01	9.6701E-01	ENSG00000230637
NA	-0.0019	9.3321E-01	9.6701E-01	ENSG00000289831
NA	-0.0034	9.3315E-01		ENSG00000218358
NA	-0.0193	9.3313E-01		ENSG00000250282
NA	-0.0022	9.3258E-01		ENSG00000256407
NA	-0.0079	9.3251E-01	9.6658E-01	ENSG00000268798
NA	-0.0188	9.3233E-01		ENSG00000232056
NA	-0.0003	9.3192E-01		ENSG00000167774
NA	-0.0116	9.3174E-01	9.6618E-01	ENSG00000279641
NA	-0.0125	9.3124E-01		ENSG00000234419
NA	-0.0005	9.3111E-01		ENSG00000258521
NA	-0.0001	9.3096E-01	9.6587E-01	ENSG00000255311
NA	-0.0044	9.3071E-01		ENSG00000231724
NA	-0.0057	9.3029E-01	9.6563E-01	ENSG00000175658
NA	-0.0021	9.3022E-01		ENSG00000241067
NA	-0.0040	9.2961E-01	9.6530E-01	ENSG00000264007
NA	-0.0010	9.2954E-01		ENSG00000183909
NA	-0.0001	9.2917E-01		ENSG00000199781
NA	-0.0039	9.2913E-01		ENSG00000286174
NA	-0.0006	9.2906E-01		ENSG00000287520
NA	-0.0038	9.2896E-01		ENSG00000267070
NA	-0.0031	9.2831E-01		ENSG00000233979
NA	-0.0003	9.2827E-01		ENSG00000269940
NA	-0.0370	9.2789E-01	9.6462E-01	ENSG00000259358
NA	-0.0072	9.2787E-01	9.6462E-01	ENSG00000205771
NA	-0.0066	9.2782E-01	9.6462E-01	ENSG00000262227
NA	-0.0002	9.2755E-01		ENSG00000250543
NA	-0.0042	9.2743E-01		ENSG00000203327
NA	-0.0061	9.2725E-01		ENSG00000233272
NA	-0.0050	9.2722E-01		ENSG00000261588
NA	-0.0032	9.2720E-01		ENSG00000203648
NA	-0.0012	9.2686E-01		ENSG00000258179
NA	-0.0008	9.2668E-01		ENSG00000221857
NA	-0.0030	9.2655E-01		ENSG00000228039
NA	-0.0129	9.2637E-01		ENSG00000235672
NA	-0.0039	9.2606E-01	9.6390E-01	ENSG00000275833
NA	-0.0007	9.2595E-01		ENSG00000267653
NA	-0.0011	9.2583E-01		ENSG00000262903
NA	-0.0015	9.2571E-01		ENSG00000253321
NA	-0.0017	9.2568E-01		ENSG00000229922
NA	-0.0022	9.2558E-01		ENSG00000236472
NA	-0.0012	9.2513E-01		ENSG00000207189
NA	-0.0168	9.2484E-01	9.6304E-01	ENSG00000200397
NA	-0.0057	9.2418E-01	9.6252E-01	ENSG00000278700
NA	-0.0059	9.2408E-01	9.6252E-01	ENSG00000260490
NA	-0.0047	9.2397E-01		ENSG00000212663
NA	-0.0014	9.2365E-01		ENSG00000273407
NA	-0.0077	9.2359E-01	9.6227E-01	ENSG00000288851
NA	-0.0074	9.2317E-01	9.6203E-01	ENSG00000183929

NA	-0.0183	9.2309E-01		ENSG00000286763
NA	-0.0047	9.2287E-01	9.6186E-01	ENSG00000223188
NA	-0.0052	9.2277E-01	9.6182E-01	ENSG00000272995
NA	-0.0067	9.2268E-01	9.6177E-01	ENSG00000225447
NA	-0.0081	9.2209E-01		ENSG00000289289
NA	0.0000	9.2200E-01	9.6143E-01	ENSG00000287405
NA	-0.0422	9.2175E-01	9.6140E-01	ENSG00000232545
NA	-0.0029	9.2163E-01		ENSG00000236542
NA	-0.0053	9.2158E-01	9.6140E-01	ENSG00000260541
NA	-0.0031	9.2153E-01		ENSG00000262011
NA	-0.0020	9.2150E-01		ENSG00000275064
NA	-0.0056	9.2150E-01	9.6140E-01	ENSG00000289879
NA	-0.0010	9.2143E-01		ENSG00000256581
NA	-0.0311	9.2131E-01	9.6140E-01	ENSG00000230470
NA	-0.0081	9.2115E-01	9.6136E-01	ENSG00000262668
NA	-0.0046	9.2114E-01	9.6136E-01	ENSG00000256278
NA	-0.0017	9.2093E-01		ENSG00000265125
NA	-0.0006	9.2080E-01	9.6115E-01	ENSG00000226532
NA	-0.0010	9.2076E-01		ENSG00000275674
NA	-0.0002	9.2070E-01		ENSG00000248113
NA	-0.0106	9.2060E-01		ENSG00000274507
NA	-0.0066	9.2055E-01	9.6093E-01	ENSG00000237588
NA	-0.0073	9.2048E-01	9.6092E-01	ENSG00000289056
NA	-0.0023	9.2029E-01		ENSG00000255520
NA	-0.0071	9.1998E-01		ENSG00000253121
NA	-0.0001	9.1992E-01		ENSG00000225183
NA	-0.0029	9.1963E-01		ENSG00000290019
NA	-0.0061	9.1941E-01	9.6019E-01	ENSG00000223812
NA	-0.0021	9.1939E-01		ENSG00000270921
NA	-0.0010	9.1922E-01		ENSG00000228417
NA	-0.0012	9.1899E-01		ENSG00000249753
NA	-0.0149	9.1898E-01		ENSG00000289411
NA	-0.0243	9.1895E-01	9.5996E-01	ENSG00000289521
NA	-0.0166	9.1870E-01	9.5989E-01	ENSG00000258111
NA	-0.0160	9.1843E-01		ENSG00000272682
NA	-0.0073	9.1822E-01		ENSG00000225311
NA	-0.0002	9.1818E-01		ENSG00000233851
NA	-0.0016	9.1800E-01		ENSG00000238132
NA	-0.0008	9.1783E-01		ENSG00000259028
NA	-0.0073	9.1773E-01	9.5913E-01	ENSG00000214773
NA	-0.0111	9.1756E-01		ENSG00000280183
NA	-0.0073	9.1713E-01	9.5883E-01	ENSG00000287450
NA	-0.0041	9.1694E-01		ENSG00000273450
NA	-0.0068	9.1675E-01		ENSG00000237806
NA	-0.0007	9.1661E-01		ENSG00000264596
NA	-0.0407	9.1635E-01	9.5863E-01	ENSG00000231628
NA	-0.0066	9.1629E-01		ENSG00000222421
NA	-0.0019	9.1602E-01		ENSG00000254456
NA	-0.0016	9.1546E-01		ENSG00000271420

NA	-0.0065	9.1529E-01	9.5821E-01	ENSG00000167807
NA	-0.0074	9.1521E-01		ENSG00000213239
NA	-0.0419	9.1499E-01		ENSG00000267834
NA	-0.0079	9.1476E-01	9.5797E-01	ENSG00000256196
NA	-0.0019	9.1450E-01		ENSG00000236430
NA	-0.0005	9.1427E-01		ENSG00000233021
NA	-0.0066	9.1371E-01	9.5757E-01	ENSG00000258748
NA	-0.0010	9.1357E-01		ENSG00000250081
NA	-0.0065	9.1351E-01		ENSG00000225187
NA	-0.0236	9.1317E-01		ENSG00000249311
NA	-0.0069	9.1309E-01	9.5717E-01	ENSG00000279452
NA	0.0000	9.1306E-01		ENSG00000232294
NA	-0.0056	9.1290E-01	9.5707E-01	ENSG00000213997
NA	-0.0029	9.1230E-01		ENSG00000261103
NA	-0.0221	9.1203E-01	9.5659E-01	ENSG00000276178
NA	-0.0186	9.1201E-01		ENSG00000285606
NA	-0.0003	9.1168E-01		ENSG00000249238
NA	-0.0051	9.1122E-01	9.5625E-01	ENSG00000226149
NA	-0.0122	9.1114E-01	9.5625E-01	ENSG00000289581
NA	-0.0031	9.1100E-01		ENSG00000248927
NA	-0.0014	9.1026E-01		ENSG00000238222
NA	-0.0034	9.1026E-01		ENSG00000259995
NA	-0.0089	9.0984E-01	9.5530E-01	ENSG00000235314
NA	-0.0048	9.0943E-01		ENSG00000230947
NA	-0.0051	9.0940E-01	9.5525E-01	ENSG00000233460
NA	-0.0007	9.0919E-01		ENSG00000278090
NA	-0.0077	9.0880E-01	9.5493E-01	ENSG00000258581
NA	-0.0004	9.0877E-01		ENSG00000288153
NA	-0.0092	9.0859E-01	9.5481E-01	ENSG00000285571
NA	-0.0003	9.0856E-01		ENSG00000214369
NA	-0.0037	9.0828E-01		ENSG00000213149
NA	-0.0083	9.0823E-01		ENSG00000239455
NA	-0.0007	9.0807E-01		ENSG00000286933
NA	-0.0015	9.0756E-01		ENSG00000267298
NA	-0.0017	9.0686E-01		ENSG00000286446
NA	-0.0098	9.0676E-01		ENSG00000279047
NA	-0.0028	9.0671E-01		ENSG00000227017
NA	-0.0058	9.0639E-01	9.5421E-01	ENSG00000231704
NA	-0.0030	9.0624E-01		ENSG00000241984
NA	-0.0006	9.0607E-01		ENSG00000264196
NA	-0.0096	9.0533E-01	9.5372E-01	ENSG00000272173
NA	-0.0025	9.0527E-01		ENSG00000250602
NA	-0.0044	9.0525E-01		ENSG00000226522
NA	-0.0095	9.0511E-01	9.5372E-01	ENSG00000259869
NA	-0.0072	9.0500E-01		ENSG00000240454
NA	-0.0098	9.0482E-01	9.5357E-01	ENSG00000264964
NA	-0.0168	9.0472E-01		ENSG00000253869
NA	-0.0043	9.0448E-01		ENSG00000265728
NA	-0.0010	9.0443E-01		ENSG00000289832

NA	-0.0085	9.0436E-01	9.5326E-01	ENSG00000237575
NA	-0.0083	9.0400E-01	9.5319E-01	ENSG00000236312
NA	-0.0005	9.0398E-01		ENSG00000248125
NA	-0.0047	9.0393E-01		ENSG00000243730
NA	-0.0014	9.0378E-01		ENSG00000285159
NA	-0.0094	9.0369E-01		ENSG00000254054
NA	-0.0022	9.0333E-01		ENSG00000273394
NA	-0.0055	9.0328E-01		ENSG00000272910
NA	-0.0094	9.0311E-01		ENSG00000253785
NA	-0.0098	9.0292E-01	9.5253E-01	ENSG00000234688
NA	-0.0020	9.0273E-01		ENSG00000228372
NA	-0.0041	9.0272E-01		ENSG00000228848
NA	-0.0032	9.0265E-01		ENSG00000234881
NA	-0.0066	9.0255E-01		ENSG00000251000
NA	-0.0056	9.0206E-01	9.5204E-01	ENSG00000260198
NA	-0.0069	9.0192E-01	9.5204E-01	ENSG00000259499
NA	-0.0121	9.0113E-01	9.5152E-01	ENSG00000253706
NA	-0.0045	9.0112E-01		ENSG00000228852
NA	-0.0054	9.0075E-01		ENSG00000284652
NA	-0.0054	9.0039E-01	9.5114E-01	ENSG00000234332
NA	-0.0032	9.0030E-01	9.5109E-01	ENSG00000279344
NA	-0.0071	9.0018E-01	9.5105E-01	ENSG00000286337
NA	-0.0102	8.9976E-01	9.5081E-01	ENSG00000261884
NA	-0.0026	8.9927E-01		ENSG00000256827
NA	-0.0016	8.9916E-01		ENSG00000253204
NA	-0.0046	8.9891E-01		ENSG00000279981
NA	-0.0022	8.9876E-01		ENSG00000285556
NA	-0.0010	8.9864E-01		ENSG00000287938
NA	-0.0104	8.9858E-01	9.4993E-01	ENSG00000230832
NA	-0.0013	8.9825E-01		ENSG00000232604
NA	-0.0104	8.9796E-01	9.4955E-01	ENSG00000227014
NA	-0.0065	8.9792E-01	9.4955E-01	ENSG00000184319
NA	-0.0002	8.9784E-01		ENSG00000257512
NA	-0.0076	8.9777E-01	9.4954E-01	ENSG00000229525
NA	-0.0024	8.9768E-01		ENSG00000255105
NA	-0.0014	8.9684E-01		ENSG00000286426
NA	-0.0042	8.9648E-01		ENSG00000272100
NA	-0.0019	8.9635E-01	9.4857E-01	ENSG00000226207
NA	-0.0013	8.9617E-01		ENSG00000235141
NA	-0.0025	8.9610E-01		ENSG00000272864
NA	-0.0071	8.9610E-01	9.4841E-01	ENSG00000275854
NA	-0.0159	8.9602E-01		ENSG00000278635
NA	-0.0074	8.9584E-01	9.4837E-01	ENSG00000258559
NA	-0.0012	8.9580E-01		ENSG00000255528
NA	-0.0090	8.9572E-01		ENSG00000288803
NA	-0.0042	8.9569E-01		ENSG00000277855
NA	-0.0026	8.9568E-01		ENSG00000267463
NA	-0.0024	8.9529E-01		ENSG00000289833
NA	-0.0102	8.9468E-01	9.4750E-01	ENSG00000231466

NA	-0.0182	8.9442E-01	9.4747E-01	ENSG00000262768
NA	-0.0145	8.9411E-01	9.4738E-01	ENSG00000227304
NA	-0.0106	8.9410E-01	9.4738E-01	ENSG00000227671
NA	-0.0074	8.9385E-01		ENSG00000258553
NA	-0.0050	8.9364E-01	9.4723E-01	ENSG00000265451
NA	-0.0003	8.9338E-01		ENSG00000205215
NA	-0.0022	8.9337E-01	9.4702E-01	ENSG00000232928
NA	-0.0338	8.9301E-01	9.4680E-01	ENSG00000261997
NA	-0.0016	8.9283E-01		ENSG00000225333
NA	-0.0051	8.9270E-01		ENSG00000286281
NA	-0.0101	8.9265E-01	9.4653E-01	ENSG00000214135
NA	-0.0019	8.9235E-01		ENSG00000261303
NA	-0.0141	8.9202E-01		ENSG00000243498
NA	-0.0050	8.9197E-01		ENSG00000257643
NA	-0.0075	8.9181E-01	9.4623E-01	ENSG00000231414
NA	-0.0102	8.9178E-01	9.4623E-01	ENSG00000256083
NA	-0.0172	8.9169E-01	9.4623E-01	ENSG00000199415
NA	-0.0086	8.9166E-01		ENSG00000286738
NA	-0.0046	8.9164E-01		ENSG00000283269
NA	-0.0062	8.9139E-01		ENSG00000231978
NA	-0.0083	8.9128E-01		ENSG00000256343
NA	-0.0082	8.9126E-01	9.4593E-01	ENSG00000248774
NA	-0.0001	8.9108E-01		ENSG00000279744
NA	-0.0059	8.9104E-01		ENSG00000276223
NA	-0.0121	8.9098E-01	9.4587E-01	ENSG00000233978
NA	-0.0038	8.9089E-01		ENSG00000229256
NA	-0.0081	8.9086E-01	9.4581E-01	ENSG00000261557
NA	-0.0066	8.9071E-01		ENSG00000235013
NA	-0.0080	8.9065E-01	9.4581E-01	ENSG00000253697
NA	-0.0101	8.9030E-01	9.4564E-01	ENSG00000273162
NA	-0.0027	8.9027E-01		ENSG00000255027
NA	-0.0063	8.8924E-01		ENSG00000286444
NA	-0.0038	8.8885E-01	9.4476E-01	ENSG00000231903
NA	-0.0086	8.8884E-01	9.4476E-01	ENSG00000229502
NA	-0.0027	8.8872E-01		ENSG00000231883
NA	0.0000	8.8864E-01		ENSG00000229808
NA	-0.0024	8.8855E-01	9.4464E-01	ENSG00000278997
NA	-0.0077	8.8833E-01		ENSG00000258404
NA	-0.0025	8.8786E-01		ENSG00000289383
NA	-0.0033	8.8775E-01		ENSG00000268282
NA	-0.0113	8.8761E-01		ENSG00000279461
NA	-0.0032	8.8742E-01		ENSG00000287286
NA	-0.0055	8.8723E-01		ENSG00000276918
NA	-0.0102	8.8719E-01	9.4386E-01	ENSG00000267676
NA	-0.0118	8.8707E-01	9.4386E-01	ENSG00000285600
NA	-0.0087	8.8704E-01	9.4386E-01	ENSG00000242531
NA	-0.0028	8.8702E-01	9.4386E-01	ENSG00000219747
NA	-0.0019	8.8667E-01		ENSG00000239544
NA	-0.0073	8.8661E-01		ENSG00000266479

NA	-0.0079	8.8598E-01	9.4322E-01	ENSG00000254035
NA	-0.0009	8.8592E-01		ENSG00000259910
NA	-0.0069	8.8592E-01		ENSG00000285559
NA	-0.0019	8.8585E-01		ENSG00000237842
NA	-0.0080	8.8523E-01	9.4266E-01	ENSG00000286652
NA	-0.0037	8.8480E-01	9.4240E-01	ENSG00000278905
NA	-0.0050	8.8430E-01		ENSG00000234107
NA	-0.0066	8.8405E-01	9.4185E-01	ENSG00000283897
NA	-0.0043	8.8404E-01		ENSG00000235279
NA	-0.0009	8.8388E-01		ENSG00000255031
NA	-0.0119	8.8333E-01	9.4147E-01	ENSG00000276663
NA	-0.0090	8.8333E-01	9.4147E-01	ENSG00000257729
NA	-0.0119	8.8302E-01	9.4141E-01	ENSG00000213777
NA	-0.0123	8.8292E-01	9.4134E-01	ENSG00000286445
NA	-0.0058	8.8287E-01		ENSG00000255672
NA	-0.0065	8.8242E-01		ENSG00000274191
NA	-0.0132	8.8227E-01	9.4106E-01	ENSG00000265907
NA	-0.0076	8.8201E-01		ENSG00000225230
NA	-0.0159	8.8175E-01		ENSG00000274064
NA	-0.0046	8.8174E-01		ENSG00000279036
NA	-0.0004	8.8167E-01		ENSG00000279006
NA	-0.0082	8.8159E-01	9.4073E-01	ENSG00000261505
NA	-0.0195	8.8138E-01	9.4059E-01	ENSG00000206676
NA	-0.0011	8.8119E-01		ENSG00000273189
NA	-0.0119	8.8116E-01	9.4050E-01	ENSG00000249839
NA	-0.0060	8.8078E-01		ENSG00000229832
NA	-0.0030	8.8030E-01		ENSG00000177173
NA	-0.0007	8.8020E-01		ENSG00000261501
NA	-0.0032	8.8016E-01		ENSG00000266196
NA	-0.0007	8.7983E-01		ENSG00000273312
NA	-0.0043	8.7964E-01		ENSG00000289703
NA	-0.0126	8.7947E-01	9.3972E-01	ENSG00000225279
NA	-0.0181	8.7942E-01	9.3972E-01	ENSG00000286043
NA	-0.0112	8.7915E-01	9.3960E-01	ENSG00000232536
NA	-0.0111	8.7899E-01	9.3956E-01	ENSG00000273891
NA	-0.0253	8.7873E-01		ENSG00000270140
NA	-0.0020	8.7839E-01		ENSG00000284431
NA	-0.0076	8.7815E-01		ENSG00000280377
NA	-0.0061	8.7811E-01		ENSG00000236281
NA	-0.0010	8.7808E-01	9.3917E-01	ENSG00000288909
NA	-0.0106	8.7777E-01	9.3909E-01	ENSG00000260077
NA	-0.0129	8.7756E-01	9.3900E-01	ENSG00000210077
NA	-0.0025	8.7745E-01		ENSG00000287991
NA	-0.0010	8.7738E-01		ENSG00000251609
NA	-0.0088	8.7724E-01	9.3890E-01	ENSG00000224415
NA	-0.0019	8.7659E-01		ENSG00000249338
NA	-0.0125	8.7652E-01	9.3837E-01	ENSG00000279133
NA	-0.0045	8.7647E-01		ENSG00000233040
NA	-0.0033	8.7618E-01		ENSG00000272030

NA	-0.0129	8.7603E-01	9.3803E-01	ENSG00000289507
NA	-0.0031	8.7600E-01		ENSG00000287943
NA	-0.0003	8.7454E-01		ENSG00000285618
NA	-0.0085	8.7395E-01		ENSG00000250383
NA	-0.0073	8.7394E-01		ENSG00000239435
NA	-0.0004	8.7374E-01		ENSG00000250906
NA	-0.0113	8.7351E-01	9.3654E-01	ENSG00000269906
NA	-0.0123	8.7342E-01	9.3651E-01	ENSG00000229990
NA	-0.0021	8.7277E-01		ENSG00000259286
NA	-0.0135	8.7254E-01	9.3592E-01	ENSG00000234771
NA	-0.0124	8.7247E-01		ENSG00000278487
NA	-0.0106	8.7223E-01	9.3583E-01	ENSG00000234183
NA	-0.0002	8.7174E-01		ENSG00000271218
NA	-0.0030	8.7158E-01		ENSG00000257900
NA	-0.0110	8.7139E-01	9.3520E-01	ENSG00000290074
NA	-0.0349	8.7134E-01	9.3519E-01	ENSG00000288753
NA	-0.0078	8.7124E-01	9.3517E-01	ENSG00000266708
NA	-0.0068	8.7122E-01		ENSG00000207368
NA	-0.0022	8.7118E-01		ENSG00000289972
NA	-0.0006	8.7088E-01		ENSG00000253616
NA	-0.0032	8.7051E-01		ENSG00000272966
NA	-0.0002	8.7037E-01		ENSG00000260362
NA	-0.0026	8.7030E-01		ENSG00000234306
NA	-0.0125	8.7001E-01	9.3446E-01	ENSG00000239883
NA	-0.0134	8.6952E-01	9.3437E-01	ENSG00000260537
NA	-0.0104	8.6939E-01	9.3437E-01	ENSG00000289187
NA	-0.0066	8.6938E-01		ENSG00000261695
NA	-0.0039	8.6907E-01		ENSG00000251446
NA	-0.0063	8.6901E-01		ENSG00000287936
NA	-0.0123	8.6885E-01	9.3407E-01	ENSG00000272148
NA	-0.0109	8.6879E-01		ENSG00000224447
NA	-0.0052	8.6834E-01		ENSG00000256571
NA	-0.0068	8.6823E-01		ENSG00000201565
NA	-0.0013	8.6814E-01		ENSG00000251187
NA	-0.0027	8.6803E-01		ENSG00000286587
NA	-0.0126	8.6795E-01	9.3343E-01	ENSG00000232044
NA	-0.0010	8.6769E-01		ENSG00000279231
NA	-0.0049	8.6651E-01	9.3265E-01	ENSG00000243486
NA	-0.0010	8.6645E-01		ENSG00000249022
NA	0.0000	8.6643E-01		ENSG00000250997
NA	-0.0083	8.6634E-01	9.3252E-01	ENSG00000288855
NA	-0.0121	8.6611E-01		ENSG00000259984
NA	-0.0030	8.6599E-01		ENSG00000258930
NA	-0.0002	8.6577E-01		ENSG00000253154
NA	-0.0035	8.6574E-01		ENSG00000279781
NA	-0.0006	8.6572E-01		ENSG00000287860
NA	-0.0020	8.6552E-01		ENSG00000232328
NA	-0.0117	8.6548E-01	9.3212E-01	ENSG00000259367
NA	-0.0016	8.6536E-01		ENSG00000232983

NA	-0.0007	8.6494E-01		ENSG00000288569
NA	-0.0030	8.6477E-01		ENSG00000218965
NA	-0.0142	8.6473E-01	9.3172E-01	ENSG00000289372
NA	-0.0010	8.6435E-01		ENSG00000236889
NA	-0.0039	8.6419E-01		ENSG00000232259
NA	-0.0089	8.6340E-01		ENSG00000244586
NA	-0.0068	8.6291E-01		ENSG00000255014
NA	-0.0109	8.6280E-01	9.3082E-01	ENSG00000289941
NA	-0.0010	8.6260E-01		ENSG00000289695
NA	-0.0137	8.6249E-01	9.3070E-01	ENSG00000218175
NA	-0.0141	8.6236E-01	9.3070E-01	ENSG00000262468
NA	-0.0089	8.6230E-01		ENSG00000271396
NA	-0.0036	8.6214E-01		ENSG00000268153
NA	-0.0111	8.6187E-01	9.3057E-01	ENSG00000226160
NA	-0.0001	8.6181E-01		ENSG00000259447
NA	-0.0103	8.6170E-01	9.3057E-01	ENSG00000285865
NA	-0.0287	8.6165E-01		ENSG00000287044
NA	-0.0032	8.6139E-01		ENSG00000234997
NA	-0.0119	8.6113E-01	9.3034E-01	ENSG00000279145
NA	-0.0011	8.6092E-01		ENSG00000225739
NA	-0.0034	8.6039E-01		ENSG00000226904
NA	-0.0050	8.6028E-01		ENSG00000207069
NA	-0.0043	8.6000E-01	9.2954E-01	ENSG00000229873
NA	-0.0008	8.5989E-01		ENSG00000235389
NA	-0.0006	8.5974E-01		ENSG00000260217
NA	-0.0038	8.5962E-01		ENSG00000254988
NA	-0.0025	8.5954E-01		ENSG00000280392
NA	-0.0027	8.5952E-01		ENSG00000267146
NA	-0.0047	8.5939E-01		ENSG00000283392
NA	-0.0111	8.5910E-01	9.2898E-01	ENSG00000230312
NA	-0.0043	8.5901E-01		ENSG00000286613
NA	-0.0135	8.5866E-01	9.2868E-01	ENSG00000238165
NA	-0.0071	8.5863E-01		ENSG00000259441
NA	-0.0127	8.5855E-01	9.2863E-01	ENSG00000219433
NA	-0.0100	8.5830E-01	9.2849E-01	ENSG00000273416
NA	-0.0110	8.5812E-01	9.2834E-01	ENSG00000229598
NA	-0.0035	8.5807E-01		ENSG00000286286
NA	-0.0071	8.5778E-01		ENSG00000270412
NA	-0.0007	8.5777E-01		ENSG00000267033
NA	-0.0114	8.5744E-01	9.2791E-01	ENSG00000249278
NA	-0.0087	8.5744E-01	9.2791E-01	ENSG00000269694
NA	-0.0109	8.5700E-01	9.2766E-01	ENSG00000233705
NA	-0.0108	8.5685E-01	9.2762E-01	ENSG00000213352
NA	-0.0085	8.5656E-01		ENSG00000279936
NA	-0.0124	8.5622E-01	9.2714E-01	ENSG00000238286
NA	-0.0053	8.5607E-01	9.2707E-01	ENSG00000275389
NA	-0.0019	8.5605E-01		ENSG00000257346
NA	-0.0123	8.5596E-01	9.2701E-01	ENSG00000186493
NA	-0.0095	8.5567E-01		ENSG00000254248

NA	-0.0082	8.5508E-01		ENSG00000260440
NA	-0.0186	8.5505E-01		ENSG00000225761
NA	-0.0105	8.5482E-01	9.2614E-01	ENSG00000234575
NA	-0.0073	8.5465E-01		ENSG00000211829
NA	-0.0013	8.5461E-01		ENSG00000251448
NA	-0.0031	8.5402E-01		ENSG00000289980
NA	-0.0066	8.5384E-01		ENSG00000289039
NA	-0.0133	8.5375E-01	9.2560E-01	ENSG00000215190
NA	-0.0053	8.5365E-01		ENSG00000271989
NA	-0.0124	8.5359E-01	9.2556E-01	ENSG00000283078
NA	-0.0026	8.5269E-01		ENSG00000266117
NA	-0.0090	8.5256E-01		ENSG00000286823
NA	-0.0029	8.5246E-01		ENSG00000274944
NA	-0.0122	8.5180E-01	9.2443E-01	ENSG00000220925
NA	-0.0017	8.5179E-01		ENSG00000287740
NA	-0.0065	8.5167E-01		ENSG00000270612
NA	-0.0034	8.5134E-01	9.2425E-01	ENSG00000259519
NA	-0.0112	8.5115E-01		ENSG00000278052
NA	-0.0138	8.5109E-01	9.2415E-01	ENSG00000268678
NA	-0.0159	8.5104E-01	9.2415E-01	ENSG00000289005
NA	-0.0119	8.5091E-01		ENSG00000270739
NA	-0.0152	8.5080E-01	9.2411E-01	ENSG00000231728
NA	-0.0015	8.5072E-01		ENSG00000256029
NA	-0.0034	8.5058E-01	9.2400E-01	ENSG00000249413
NA	-0.0145	8.5013E-01	9.2375E-01	ENSG00000255158
NA	-0.0293	8.5009E-01	9.2374E-01	ENSG00000186743
NA	-0.0080	8.5005E-01	9.2374E-01	ENSG00000270760
NA	-0.0044	8.4980E-01		ENSG00000279878
NA	-0.0046	8.4946E-01		ENSG00000285524
NA	-0.0049	8.4941E-01		ENSG00000279708
NA	-0.0006	8.4911E-01		ENSG00000289467
NA	-0.0007	8.4885E-01		ENSG00000264699
NA	-0.0254	8.4837E-01	9.2265E-01	ENSG00000227638
NA	-0.0089	8.4816E-01	9.2260E-01	ENSG00000225112
NA	-0.0070	8.4813E-01	9.2260E-01	ENSG00000210107
NA	-0.0037	8.4796E-01		ENSG00000235390
NA	-0.0033	8.4742E-01		ENSG00000256341
NA	-0.0033	8.4735E-01		ENSG00000285974
NA	-0.0125	8.4721E-01	9.2180E-01	ENSG00000283504
NA	-0.0034	8.4715E-01		ENSG00000231378
NA	-0.0069	8.4685E-01		ENSG00000285553
NA	-0.0109	8.4676E-01	9.2147E-01	ENSG00000251348
NA	-0.0137	8.4673E-01	9.2147E-01	ENSG00000232006
NA	-0.0037	8.4665E-01		ENSG00000182000
NA	-0.0139	8.4663E-01	9.2147E-01	ENSG00000244733
NA	-0.0034	8.4646E-01		ENSG00000287793
NA	-0.0100	8.4621E-01		ENSG00000226330
NA	-0.0041	8.4620E-01		ENSG00000275401
NA	-0.0094	8.4606E-01	9.2104E-01	ENSG00000255256

NA	-0.0120	8.4564E-01	9.2099E-01	ENSG00000279689
NA	-0.0032	8.4556E-01		ENSG00000203573
NA	-0.0104	8.4529E-01		ENSG00000199875
NA	-0.0160	8.4515E-01	9.2082E-01	ENSG00000251669
NA	-0.0058	8.4512E-01		ENSG00000270690
NA	-0.0035	8.4490E-01		ENSG00000258693
NA	-0.0039	8.4486E-01		ENSG00000215589
NA	-0.0153	8.4467E-01	9.2038E-01	ENSG00000226009
NA	-0.0067	8.4433E-01		ENSG00000225415
NA	-0.0050	8.4423E-01		ENSG00000269792
NA	-0.0020	8.4414E-01		ENSG00000286843
NA	-0.0125	8.4394E-01	9.1997E-01	ENSG00000283529
NA	-0.0159	8.4370E-01	9.1982E-01	ENSG00000213300
NA	-0.0125	8.4361E-01		ENSG00000273387
NA	-0.0046	8.4332E-01		ENSG00000264765
NA	-0.0101	8.4311E-01		ENSG00000248785
NA	-0.0152	8.4305E-01		ENSG00000286627
NA	-0.0001	8.4295E-01		ENSG00000235679
NA	-0.0074	8.4284E-01		ENSG00000267682
NA	-0.0056	8.4274E-01		ENSG00000285895
NA	-0.0059	8.4229E-01		ENSG00000236388
NA	-0.0018	8.4216E-01		ENSG00000287385
NA	-0.0132	8.4215E-01	9.1870E-01	ENSG00000179994
NA	-0.0075	8.4200E-01		ENSG00000264243
NA	-0.0001	8.4195E-01		ENSG00000274603
NA	-0.0168	8.4194E-01		ENSG00000252254
NA	-0.0032	8.4194E-01		ENSG00000261060
NA	-0.0019	8.4193E-01		ENSG00000263394
NA	-0.0025	8.4180E-01		ENSG00000268184
NA	-0.0075	8.4170E-01		ENSG00000231840
NA	-0.0093	8.4142E-01	9.1838E-01	ENSG00000233928
NA	-0.0023	8.4138E-01		ENSG00000217769
NA	-0.0013	8.4134E-01		ENSG00000279889
NA	-0.0009	8.4107E-01		ENSG00000286941
NA	-0.0034	8.4059E-01		ENSG00000232757
NA	-0.0159	8.4049E-01	9.1794E-01	ENSG00000223804
NA	-0.0002	8.4034E-01		ENSG00000276718
NA	-0.0096	8.4031E-01		ENSG00000240093
NA	-0.0121	8.4006E-01	9.1767E-01	ENSG00000269793
NA	-0.0159	8.3982E-01	9.1755E-01	ENSG00000251634
NA	-0.0102	8.3976E-01		ENSG00000287714
NA	-0.0029	8.3962E-01	9.1747E-01	ENSG00000213406
NA	-0.0022	8.3937E-01		ENSG00000277747
NA	-0.0156	8.3926E-01	9.1737E-01	ENSG00000242337
NA	-0.0012	8.3923E-01		ENSG00000286090
NA	-0.0058	8.3916E-01		ENSG00000248476
NA	-0.0026	8.3888E-01		ENSG00000287061
NA	-0.0200	8.3821E-01	9.1658E-01	ENSG00000279598
NA	-0.0042	8.3819E-01		ENSG00000263278

NA	-0.0110	8.3790E-01	9.1633E-01	ENSG00000288918
NA	-0.0093	8.3788E-01		ENSG00000278011
NA	-0.0130	8.3723E-01	9.1604E-01	ENSG00000260331
NA	-0.0280	8.3718E-01	9.1604E-01	ENSG00000286463
NA	-0.0013	8.3700E-01		ENSG00000275162
NA	-0.0093	8.3688E-01		ENSG00000279044
NA	-0.0120	8.3673E-01	9.1587E-01	ENSG00000230777
NA	-0.0056	8.3672E-01		ENSG00000267178
NA	-0.0118	8.3661E-01	9.1578E-01	ENSG00000289081
NA	-0.0026	8.3654E-01		ENSG00000285671
NA	-0.0006	8.3629E-01		ENSG00000229151
NA	-0.0020	8.3591E-01		ENSG00000254847
NA	-0.0038	8.3568E-01		ENSG00000287652
NA	-0.0170	8.3509E-01	9.1514E-01	ENSG00000263823
NA	-0.0154	8.3473E-01	9.1500E-01	ENSG00000279591
NA	-0.0120	8.3439E-01		ENSG00000259755
NA	-0.0093	8.3434E-01		ENSG00000280089
NA	-0.0091	8.3431E-01		ENSG00000201786
NA	-0.0114	8.3424E-01	9.1489E-01	ENSG00000213250
NA	-0.0030	8.3409E-01		ENSG00000276505
NA	-0.0132	8.3405E-01		ENSG00000251730
NA	-0.0170	8.3400E-01	9.1470E-01	ENSG00000268575
NA	-0.0013	8.3384E-01		ENSG00000262094
NA	-0.0087	8.3367E-01	9.1446E-01	ENSG00000243404
NA	-0.0181	8.3353E-01	9.1436E-01	ENSG00000262001
NA	-0.0094	8.3345E-01	9.1435E-01	ENSG00000240731
NA	-0.0027	8.3337E-01		ENSG00000255447
NA	-0.0093	8.3259E-01		ENSG00000259782
NA	-0.0140	8.3233E-01	9.1349E-01	ENSG00000274528
NA	-0.0001	8.3158E-01		ENSG00000219410
NA	-0.0168	8.3152E-01	9.1301E-01	ENSG00000234233
NA	-0.0095	8.3077E-01		ENSG00000249685
NA	-0.0016	8.3077E-01		ENSG00000250751
NA	-0.0034	8.3074E-01		ENSG00000207117
NA	-0.0022	8.3054E-01	9.1239E-01	ENSG00000253341
NA	-0.0107	8.2998E-01	9.1205E-01	ENSG00000257325
NA	-0.0073	8.2995E-01		ENSG00000279822
NA	-0.0171	8.2977E-01	9.1190E-01	ENSG00000187534
NA	-0.0154	8.2941E-01	9.1170E-01	ENSG00000255621
NA	-0.0115	8.2928E-01	9.1169E-01	ENSG00000278213
NA	-0.0098	8.2921E-01		ENSG00000286470
NA	-0.0055	8.2883E-01		ENSG00000229016
NA	-0.0101	8.2883E-01		ENSG00000233565
NA	-0.0121	8.2863E-01		ENSG00000249986
NA	-0.0060	8.2860E-01		ENSG00000270426
NA	-0.0005	8.2860E-01		ENSG00000258074
NA	-0.0069	8.2840E-01		ENSG00000256588
NA	-0.0050	8.2807E-01		ENSG00000290053
NA	-0.0139	8.2773E-01	9.1079E-01	ENSG00000244932

NA	-0.0010	8.2747E-01		ENSG00000253507
NA	-0.0148	8.2744E-01	9.1060E-01	ENSG00000215548
NA	-0.0048	8.2741E-01		ENSG00000285826
NA	-0.0096	8.2718E-01	9.1051E-01	ENSG00000289554
NA	-0.0014	8.2717E-01		ENSG00000199398
NA	-0.0110	8.2689E-01		ENSG00000258827
NA	-0.0048	8.2668E-01		ENSG00000229009
NA	-0.0073	8.2662E-01		ENSG00000237919
NA	-0.0039	8.2659E-01		ENSG00000287961
NA	-0.0145	8.2653E-01	9.1009E-01	ENSG00000260615
NA	-0.0056	8.2650E-01	9.1009E-01	ENSG00000224810
NA	-0.0033	8.2642E-01		ENSG00000289605
NA	-0.0036	8.2608E-01		ENSG00000276131
NA	-0.0031	8.2605E-01		ENSG00000229846
NA	-0.0119	8.2597E-01		ENSG00000280113
NA	-0.0146	8.2592E-01	9.0982E-01	ENSG00000224478
NA	-0.0035	8.2528E-01		ENSG00000261166
NA	-0.0101	8.2517E-01		ENSG00000230684
NA	-0.0175	8.2404E-01	9.0862E-01	ENSG00000274840
NA	-0.0115	8.2387E-01		ENSG00000236411
NA	-0.0065	8.2386E-01		ENSG00000254974
NA	-0.0040	8.2379E-01		ENSG00000230993
NA	-0.0172	8.2364E-01	9.0841E-01	ENSG00000237004
NA	-0.0068	8.2362E-01		ENSG00000259821
NA	-0.0161	8.2358E-01	9.0841E-01	ENSG00000230084
NA	-0.0031	8.2358E-01		ENSG00000288533
NA	-0.0028	8.2347E-01		ENSG00000196970
NA	-0.0178	8.2332E-01	9.0841E-01	ENSG00000244257
NA	-0.0007	8.2329E-01		ENSG00000253939
NA	-0.0001	8.2328E-01	9.0841E-01	ENSG00000273716
NA	-0.0104	8.2324E-01	9.0841E-01	ENSG00000260971
NA	-0.0049	8.2297E-01		ENSG00000210176
NA	-0.0060	8.2295E-01		ENSG00000266469
NA	-0.0021	8.2290E-01		ENSG00000288812
NA	-0.0063	8.2226E-01		ENSG00000197670
NA	-0.0036	8.2202E-01		ENSG00000238231
NA	-0.0042	8.2192E-01		ENSG00000223271
NA	-0.0074	8.2172E-01		ENSG00000260252
NA	-0.0123	8.2170E-01	9.0765E-01	ENSG00000286751
NA	-0.0064	8.2168E-01		ENSG00000255856
NA	-0.0052	8.2149E-01		ENSG00000228643
NA	-0.0112	8.2097E-01		ENSG00000261296
NA	-0.0036	8.2085E-01	9.0704E-01	ENSG00000272219
NA	-0.0077	8.2075E-01		ENSG00000289293
NA	-0.0163	8.2075E-01	9.0701E-01	ENSG00000275630
NA	-0.0115	8.2054E-01	9.0686E-01	ENSG00000285649
NA	-0.0081	8.2040E-01		ENSG00000259416
NA	-0.0130	8.2005E-01		ENSG00000279464
NA	-0.0012	8.1985E-01		ENSG00000234460

NA	-0.0171	8.1977E-01	9.0638E-01	ENSG00000278012
NA	-0.0087	8.1963E-01		ENSG00000268818
NA	-0.0075	8.1961E-01		ENSG00000250909
NA	-0.0044	8.1941E-01	9.0618E-01	ENSG00000275265
NA	-0.0191	8.1932E-01	9.0615E-01	ENSG00000248714
NA	-0.0047	8.1928E-01		ENSG00000260678
NA	-0.0107	8.1904E-01	9.0597E-01	ENSG00000276867
NA	-0.0048	8.1899E-01		ENSG00000274297
NA	-0.0178	8.1861E-01	9.0574E-01	ENSG00000229358
NA	-0.0111	8.1832E-01		ENSG00000264937
NA	-0.0014	8.1815E-01		ENSG00000273186
NA	-0.0009	8.1780E-01		ENSG00000258373
NA	-0.0159	8.1760E-01	9.0514E-01	ENSG00000233170
NA	-0.0011	8.1752E-01		ENSG00000254744
NA	-0.0091	8.1746E-01		ENSG00000289859
NA	-0.0086	8.1739E-01		ENSG00000249031
NA	-0.0005	8.1717E-01	9.0492E-01	ENSG00000277118
NA	-0.0138	8.1713E-01	9.0492E-01	ENSG00000265496
NA	-0.0071	8.1692E-01		ENSG00000202512
NA	-0.0007	8.1674E-01		ENSG00000219487
NA	-0.0114	8.1645E-01	9.0447E-01	ENSG00000287789
NA	-0.0162	8.1618E-01	9.0439E-01	ENSG00000225156
NA	-0.0134	8.1612E-01	9.0437E-01	ENSG00000233846
NA	-0.0078	8.1592E-01		ENSG00000254297
NA	-0.0043	8.1581E-01		ENSG00000253992
NA	-0.0183	8.1568E-01	9.0403E-01	ENSG00000287254
NA	-0.0168	8.1563E-01	9.0403E-01	ENSG00000165121
NA	-0.0095	8.1553E-01	9.0400E-01	ENSG00000256167
NA	-0.0006	8.1542E-01		ENSG00000267141
NA	-0.0159	8.1540E-01	9.0400E-01	ENSG00000248587
NA	-0.0006	8.1471E-01		ENSG00000251536
NA	-0.0014	8.1454E-01		ENSG00000224727
NA	-0.0118	8.1449E-01		ENSG00000254433
NA	-0.0196	8.1444E-01	9.0328E-01	ENSG00000269001
NA	-0.0030	8.1424E-01		ENSG00000228002
NA	-0.0163	8.1382E-01	9.0304E-01	ENSG00000289462
NA	-0.0073	8.1359E-01		ENSG00000285675
NA	-0.0015	8.1331E-01	9.0268E-01	ENSG00000258342
NA	-0.0077	8.1329E-01		ENSG00000202019
NA	-0.0066	8.1325E-01		ENSG00000287934
NA	-0.0111	8.1298E-01	9.0248E-01	ENSG00000284655
NA	-0.0167	8.1292E-01		ENSG00000276988
NA	-0.0035	8.1284E-01		ENSG00000234393
NA	-0.0087	8.1220E-01		ENSG00000247872
NA	-0.0032	8.1205E-01		ENSG00000260441
NA	-0.0029	8.1197E-01		ENSG00000272914
NA	-0.0046	8.1171E-01		ENSG00000256385
NA	-0.0139	8.1163E-01	9.0156E-01	ENSG00000262410
NA	-0.0212	8.1091E-01	9.0103E-01	ENSG00000287104

NA	-0.0020	8.1072E-01		ENSG00000250264
NA	-0.0172	8.1037E-01	9.0047E-01	ENSG00000276148
NA	-0.0070	8.1010E-01		ENSG00000276763
NA	-0.0050	8.1007E-01		ENSG00000261633
NA	-0.0016	8.1004E-01		ENSG00000258856
NA	-0.0001	8.0987E-01		ENSG00000230756
NA	-0.0079	8.0971E-01		ENSG00000226767
NA	-0.0097	8.0918E-01	8.9970E-01	ENSG00000286305
NA	-0.0227	8.0916E-01		ENSG00000254165
NA	-0.0200	8.0914E-01	8.9970E-01	ENSG00000277782
NA	-0.0009	8.0904E-01		ENSG00000236838
NA	-0.0100	8.0903E-01		ENSG00000248511
NA	-0.0173	8.0897E-01	8.9965E-01	ENSG00000223803
NA	-0.0058	8.0891E-01		ENSG00000229893
NA	-0.0140	8.0891E-01	8.9962E-01	ENSG00000280214
NA	-0.0070	8.0833E-01		ENSG00000286829
NA	-0.0066	8.0815E-01		ENSG00000278896
NA	-0.0133	8.0771E-01	8.9894E-01	ENSG00000223935
NA	-0.0194	8.0747E-01	8.9887E-01	ENSG00000210135
NA	-0.0004	8.0705E-01		ENSG00000253227
NA	-0.0119	8.0698E-01		ENSG00000267530
NA	-0.0152	8.0697E-01	8.9856E-01	ENSG00000255883
NA	-0.0026	8.0693E-01		ENSG00000239323
NA	-0.0064	8.0690E-01		ENSG00000240673
NA	-0.0023	8.0619E-01		ENSG00000229559
NA	-0.0163	8.0606E-01	8.9800E-01	ENSG00000289182
NA	-0.0148	8.0604E-01	8.9800E-01	ENSG00000259015
NA	-0.0165	8.0603E-01	8.9800E-01	ENSG00000273343
NA	-0.0152	8.0586E-01	8.9800E-01	ENSG00000260751
NA	-0.0183	8.0585E-01		ENSG00000249773
NA	-0.0137	8.0567E-01	8.9800E-01	ENSG00000227492
NA	-0.0116	8.0540E-01	8.9800E-01	ENSG00000288862
NA	-0.0021	8.0530E-01		ENSG00000287167
NA	-0.0104	8.0525E-01		ENSG00000289223
NA	-0.0121	8.0508E-01	8.9784E-01	ENSG00000289373
NA	-0.0033	8.0469E-01		ENSG00000279932
NA	-0.0013	8.0426E-01		ENSG00000269419
NA	-0.0020	8.0404E-01		ENSG00000275287
NA	-0.0269	8.0397E-01	8.9701E-01	ENSG00000261970
NA	-0.0161	8.0393E-01	8.9700E-01	ENSG00000272927
NA	-0.0058	8.0374E-01		ENSG00000259672
NA	-0.0227	8.0372E-01		ENSG00000286252
NA	-0.0221	8.0337E-01		ENSG00000225905
NA	-0.0049	8.0298E-01		ENSG00000260532
NA	-0.0044	8.0296E-01		ENSG00000249945
NA	-0.0075	8.0256E-01		ENSG00000279548
NA	-0.0022	8.0254E-01		ENSG00000259341
NA	-0.0003	8.0237E-01		ENSG00000241397
NA	-0.0045	8.0234E-01		ENSG00000260473

NA	-0.0141	8.0174E-01	8.9537E-01	ENSG00000272459
NA	-0.0030	8.0169E-01		ENSG00000283400
NA	-0.0024	8.0153E-01		ENSG00000251276
NA	-0.0036	8.0076E-01		ENSG00000270591
NA	-0.0034	8.0075E-01		ENSG00000213590
NA	-0.0007	8.0068E-01		ENSG00000277647
NA	-0.0032	8.0049E-01	8.9476E-01	ENSG00000240231
NA	-0.0159	8.0004E-01	8.9449E-01	ENSG00000289788
NA	-0.0013	7.9966E-01		ENSG00000278794
NA	-0.0111	7.9953E-01	8.9428E-01	ENSG00000270640
NA	-0.0062	7.9952E-01		ENSG00000287390
NA	-0.0153	7.9923E-01	8.9419E-01	ENSG00000277945
NA	-0.0110	7.9895E-01		ENSG00000236086
NA	-0.0168	7.9867E-01		ENSG00000253385
NA	-0.0194	7.9861E-01	8.9385E-01	ENSG00000202260
NA	-0.0020	7.9842E-01		ENSG00000287216
NA	-0.0022	7.9773E-01		ENSG00000287824
NA	-0.0014	7.9755E-01		ENSG00000251075
NA	-0.0050	7.9727E-01		ENSG00000288893
NA	-0.0086	7.9705E-01		ENSG00000262732
NA	-0.0054	7.9638E-01		ENSG00000279325
NA	-0.0217	7.9636E-01	8.9244E-01	ENSG00000289088
NA	-0.0082	7.9632E-01		ENSG00000232369
NA	-0.0083	7.9616E-01		ENSG00000272479
NA	-0.0021	7.9607E-01		ENSG00000285782
NA	-0.0076	7.9607E-01		ENSG00000260723
NA	-0.0163	7.9570E-01	8.9205E-01	ENSG00000287569
NA	-0.0204	7.9503E-01	8.9166E-01	ENSG00000243403
NA	-0.0136	7.9501E-01	8.9166E-01	ENSG00000258096
NA	-0.0203	7.9466E-01	8.9153E-01	ENSG00000196933
NA	-0.0196	7.9458E-01	8.9151E-01	ENSG00000272345
NA	-0.0103	7.9435E-01		ENSG00000274825
NA	-0.0062	7.9433E-01		ENSG00000252874
NA	-0.0124	7.9425E-01		ENSG00000265750
NA	-0.0217	7.9418E-01	8.9122E-01	ENSG00000226889
NA	-0.0217	7.9407E-01	8.9120E-01	ENSG00000234618
NA	-0.0028	7.9389E-01		ENSG00000219951
NA	-0.0079	7.9359E-01		ENSG00000289188
NA	-0.0083	7.9332E-01		ENSG00000285840
NA	-0.0080	7.9332E-01		ENSG00000258886
NA	-0.0058	7.9321E-01		ENSG00000277130
NA	-0.0124	7.9315E-01		ENSG00000230295
NA	-0.0083	7.9311E-01		ENSG00000237387
NA	-0.0056	7.9255E-01		ENSG00000254273
NA	-0.0217	7.9254E-01	8.9043E-01	ENSG00000264207
NA	-0.0183	7.9240E-01	8.9031E-01	ENSG00000276007
NA	-0.0210	7.9236E-01	8.9031E-01	ENSG00000255495
NA	-0.0051	7.9220E-01		ENSG00000276179
NA	-0.0114	7.9219E-01		ENSG00000270236

NA	-0.0037	7.9217E-01		ENSG00000231976
NA	-0.0213	7.9205E-01	8.9013E-01	ENSG00000253730
NA	-0.0042	7.9204E-01		ENSG00000279376
NA	-0.0202	7.9187E-01	8.9001E-01	ENSG00000255581
NA	-0.0138	7.9187E-01	8.9001E-01	ENSG00000259066
NA	-0.0060	7.9174E-01		ENSG00000254537
NA	-0.0003	7.9160E-01		ENSG00000206728
NA	-0.0022	7.9107E-01		ENSG00000212533
NA	-0.0029	7.9100E-01		ENSG00000230952
NA	-0.0106	7.9091E-01		ENSG00000224412
NA	-0.0190	7.9085E-01	8.8943E-01	ENSG00000275930
NA	-0.0138	7.9081E-01	8.8942E-01	ENSG00000225465
NA	-0.0041	7.9073E-01		ENSG00000279903
NA	-0.0060	7.9055E-01		ENSG00000258181
NA	-0.0141	7.9049E-01	8.8923E-01	ENSG00000271833
NA	-0.0063	7.9016E-01		ENSG00000279314
NA	-0.0258	7.8997E-01	8.8896E-01	ENSG00000287109
NA	-0.0223	7.8993E-01	8.8896E-01	ENSG00000256008
NA	-0.0221	7.8986E-01	8.8896E-01	ENSG00000231566
NA	-0.0197	7.8971E-01	8.8891E-01	ENSG00000277159
NA	-0.0226	7.8957E-01	8.8884E-01	ENSG00000271991
NA	-0.0048	7.8938E-01		ENSG00000249453
NA	-0.0052	7.8931E-01		ENSG00000250794
NA	-0.0189	7.8893E-01	8.8820E-01	ENSG00000249459
NA	-0.0111	7.8873E-01		ENSG00000235728
NA	-0.0087	7.8862E-01		ENSG00000225620
NA	-0.0021	7.8858E-01	8.8805E-01	ENSG00000258711
NA	-0.0205	7.8833E-01	8.8801E-01	ENSG00000271869
NA	-0.0155	7.8800E-01	8.8795E-01	ENSG00000260052
NA	-0.0114	7.8771E-01	8.8790E-01	ENSG00000271862
NA	-0.0059	7.8755E-01		ENSG00000230958
NA	-0.0038	7.8735E-01		ENSG00000231985
NA	-0.0122	7.8727E-01		ENSG00000270433
NA	-0.0022	7.8726E-01		ENSG00000264958
NA	-0.0053	7.8720E-01		ENSG00000223911
NA	-0.0151	7.8713E-01		ENSG00000287046
NA	-0.0201	7.8698E-01	8.8747E-01	ENSG00000278991
NA	-0.0094	7.8649E-01		ENSG00000255176
NA	-0.0025	7.8621E-01		ENSG00000231261
NA	-0.0008	7.8504E-01		ENSG00000248528
NA	-0.0218	7.8497E-01	8.8631E-01	ENSG00000234589
NA	-0.0054	7.8488E-01		ENSG00000282440
NA	-0.0052	7.8484E-01		ENSG00000202461
NA	-0.0082	7.8473E-01		ENSG00000261523
NA	-0.0017	7.8464E-01		ENSG00000240687
NA	0.0000	7.8394E-01		ENSG00000290003
NA	-0.0135	7.8343E-01	8.8537E-01	ENSG00000262772
NA	-0.0056	7.8317E-01		ENSG00000258858
NA	-0.0066	7.8297E-01	8.8518E-01	ENSG00000287579

NA	-0.0046	7.8286E-01		ENSG00000233099
NA	-0.0053	7.8253E-01		ENSG00000287274
NA	-0.0204	7.8234E-01	8.8475E-01	ENSG00000268883
NA	-0.0177	7.8213E-01		ENSG00000227161
NA	-0.0023	7.8177E-01		ENSG00000286825
NA	-0.0183	7.8170E-01	8.8435E-01	ENSG00000288033
NA	-0.0066	7.8164E-01		ENSG00000251062
NA	-0.0078	7.8137E-01		ENSG00000282879
NA	-0.0142	7.8127E-01	8.8407E-01	ENSG00000273365
NA	-0.0096	7.8077E-01		ENSG00000223374
NA	-0.0036	7.8071E-01		ENSG00000232489
NA	-0.0216	7.8041E-01		ENSG00000243280
NA	-0.0214	7.8030E-01	8.8346E-01	ENSG00000280321
NA	-0.0025	7.8011E-01		ENSG00000285486
NA	-0.0153	7.8005E-01	8.8338E-01	ENSG00000256658
NA	-0.0176	7.7985E-01	8.8321E-01	ENSG00000253390
NA	-0.0029	7.7985E-01		ENSG00000254317
NA	-0.0153	7.7958E-01		ENSG00000254420
NA	-0.0077	7.7910E-01		ENSG00000284776
NA	-0.0165	7.7862E-01		ENSG00000263235
NA	-0.0097	7.7851E-01		ENSG00000262492
NA	-0.0099	7.7807E-01		ENSG00000259287
NA	-0.0196	7.7806E-01	8.8190E-01	ENSG00000267526
NA	-0.0189	7.7804E-01	8.8190E-01	ENSG00000261096
NA	-0.0104	7.7790E-01		ENSG00000278514
NA	-0.0195	7.7781E-01		ENSG00000241720
NA	-0.0223	7.7742E-01		ENSG00000272181
NA	-0.0129	7.7657E-01	8.8102E-01	ENSG00000200309
NA	-0.0049	7.7651E-01	8.8102E-01	ENSG00000267723
NA	-0.0098	7.7570E-01		ENSG00000248416
NA	-0.0081	7.7570E-01	8.8060E-01	ENSG00000254263
NA	-0.0069	7.7564E-01		ENSG00000199975
NA	-0.0067	7.7552E-01		ENSG00000270947
NA	-0.0236	7.7523E-01	8.8039E-01	ENSG00000288670
NA	-0.0010	7.7498E-01		ENSG00000287432
NA	-0.0112	7.7488E-01		ENSG00000289326
NA	-0.0033	7.7482E-01		ENSG00000259137
NA	-0.0030	7.7440E-01		ENSG00000261147
NA	-0.0074	7.7409E-01		ENSG00000286104
NA	-0.0019	7.7367E-01		ENSG00000256994
NA	-0.0084	7.7359E-01		ENSG00000258050
NA	-0.0045	7.7340E-01		ENSG00000288819
NA	-0.0197	7.7335E-01	8.7911E-01	ENSG00000269883
NA	-0.0233	7.7320E-01	8.7905E-01	ENSG00000289419
NA	-0.0081	7.7317E-01		ENSG00000262408
NA	-0.0408	7.7282E-01	8.7899E-01	ENSG00000254936
NA	-0.0013	7.7276E-01		ENSG00000214268
NA	-0.0047	7.7275E-01		ENSG00000211683
NA	-0.0043	7.7242E-01		ENSG00000212432

NA	-0.0041	7.7226E-01		ENSG00000232493
NA	-0.0053	7.7210E-01		ENSG00000287015
NA	-0.0209	7.7206E-01	8.7846E-01	ENSG00000288021
NA	-0.0020	7.7179E-01		ENSG00000256913
NA	-0.0040	7.7167E-01		ENSG00000254780
NA	-0.0013	7.7152E-01		ENSG00000274397
NA	-0.0001	7.7136E-01		ENSG00000258428
NA	-0.0013	7.7111E-01		ENSG00000224402
NA	-0.0095	7.7111E-01		ENSG00000232173
NA	-0.0143	7.7107E-01		ENSG00000223813
NA	-0.0064	7.7091E-01		ENSG00000280134
NA	-0.0017	7.7081E-01		ENSG00000286046
NA	-0.0096	7.7056E-01		ENSG00000271428
NA	-0.0069	7.7025E-01		ENSG00000261293
NA	-0.0076	7.7024E-01		ENSG00000203588
NA	-0.0154	7.7017E-01		ENSG00000243680
NA	-0.0006	7.6993E-01		ENSG00000265912
NA	-0.0033	7.6980E-01		ENSG00000276406
NA	-0.0075	7.6974E-01		ENSG00000233990
NA	-0.0061	7.6972E-01	8.7673E-01	ENSG00000286145
NA	-0.0082	7.6972E-01		ENSG00000261145
NA	-0.0133	7.6967E-01	8.7673E-01	ENSG00000279917
NA	-0.0093	7.6914E-01		ENSG00000226253
NA	-0.0247	7.6901E-01	8.7636E-01	ENSG00000229931
NA	-0.0059	7.6896E-01		ENSG00000227934
NA	-0.0171	7.6894E-01	8.7634E-01	ENSG00000270154
NA	-0.0022	7.6884E-01		ENSG00000248750
NA	-0.0036	7.6817E-01		ENSG00000232998
NA	-0.0153	7.6815E-01	8.7579E-01	ENSG00000267262
NA	-0.0203	7.6797E-01	8.7566E-01	ENSG00000230982
NA	-0.0161	7.6752E-01	8.7535E-01	ENSG00000277763
NA	-0.0047	7.6736E-01		ENSG00000253737
NA	-0.0126	7.6726E-01		ENSG00000246820
NA	-0.0200	7.6722E-01	8.7517E-01	ENSG00000254938
NA	-0.0079	7.6719E-01		ENSG00000272180
NA	-0.0012	7.6669E-01		ENSG00000168967
NA	-0.0241	7.6643E-01	8.7471E-01	ENSG00000204652
NA	-0.0030	7.6591E-01		ENSG00000237307
NA	-0.0124	7.6581E-01	8.7418E-01	ENSG00000236129
NA	-0.0081	7.6545E-01		ENSG00000274303
NA	-0.0168	7.6522E-01	8.7387E-01	ENSG00000267219
NA	-0.0046	7.6504E-01		ENSG00000232953
NA	-0.0025	7.6444E-01		ENSG00000267282
NA	-0.0016	7.6419E-01		ENSG00000286561
NA	-0.0163	7.6404E-01	8.7310E-01	ENSG00000251307
NA	-0.0202	7.6382E-01	8.7292E-01	ENSG00000283303
NA	-0.0200	7.6366E-01	8.7285E-01	ENSG00000253829
NA	-0.0047	7.6344E-01		ENSG00000238085
NA	-0.0159	7.6319E-01	8.7252E-01	ENSG00000244124

NA	-0.0103	7.6281E-01	8.7229E-01	ENSG00000267980
NA	-0.0149	7.6270E-01		ENSG00000255081
NA	-0.0182	7.6249E-01	8.7216E-01	ENSG00000231010
NA	-0.0244	7.6245E-01	8.7215E-01	ENSG00000267198
NA	-0.0170	7.6230E-01	8.7214E-01	ENSG00000288910
NA	-0.0138	7.6207E-01		ENSG00000225387
NA	-0.0237	7.6202E-01	8.7200E-01	ENSG00000235450
NA	-0.0163	7.6194E-01	8.7200E-01	ENSG00000265554
NA	-0.0082	7.6143E-01		ENSG00000263729
NA	-0.0113	7.6126E-01		ENSG00000253273
NA	-0.0032	7.6124E-01		ENSG00000257277
NA	-0.0212	7.6120E-01	8.7162E-01	ENSG00000229447
NA	-0.0034	7.6071E-01		ENSG00000257948
NA	-0.0035	7.6063E-01		ENSG00000265751
NA	-0.0053	7.6031E-01		ENSG00000285771
NA	-0.0076	7.6024E-01	8.7093E-01	ENSG00000289269
NA	-0.0076	7.5992E-01		ENSG00000287027
NA	-0.0085	7.5963E-01	8.7043E-01	ENSG00000259408
NA	-0.0001	7.5963E-01		ENSG00000253813
NA	-0.0083	7.5962E-01		ENSG00000279365
NA	-0.0225	7.5940E-01	8.7029E-01	ENSG00000242759
NA	-0.0043	7.5926E-01		ENSG00000231755
NA	-0.0006	7.5892E-01		ENSG00000210184
NA	-0.0197	7.5889E-01		ENSG00000266445
NA	-0.0172	7.5872E-01	8.6962E-01	ENSG00000288044
NA	-0.0052	7.5816E-01		ENSG00000228923
NA	-0.0230	7.5799E-01	8.6905E-01	ENSG00000257800
NA	-0.0175	7.5797E-01		ENSG00000275393
NA	-0.0050	7.5791E-01		ENSG00000241169
NA	-0.0133	7.5759E-01		ENSG00000232218
NA	-0.0170	7.5736E-01		ENSG00000285608
NA	-0.0228	7.5729E-01	8.6856E-01	ENSG00000264968
NA	-0.0011	7.5681E-01		ENSG00000235802
NA	-0.0002	7.5679E-01		ENSG00000255689
NA	-0.0133	7.5672E-01		ENSG00000267765
NA	-0.0020	7.5657E-01		ENSG00000225603
NA	-0.0009	7.5656E-01		ENSG00000214646
NA	-0.0062	7.5650E-01		ENSG00000255142
NA	-0.0023	7.5616E-01		ENSG00000288631
NA	-0.0011	7.5542E-01		ENSG00000188933
NA	-0.0006	7.5542E-01		ENSG00000258580
NA	-0.0263	7.5506E-01	8.6698E-01	ENSG00000286092
NA	-0.0105	7.5481E-01		ENSG00000290009
NA	-0.0070	7.5459E-01		ENSG00000278041
NA	-0.0022	7.5436E-01		ENSG00000286756
NA	-0.0017	7.5395E-01		ENSG00000261200
NA	-0.0158	7.5374E-01	8.6622E-01	ENSG00000223624
NA	-0.0193	7.5350E-01	8.6617E-01	ENSG00000260206
NA	-0.0078	7.5329E-01		ENSG00000226816

NA	-0.0015	7.5328E-01		ENSG00000278239
NA	-0.0008	7.5300E-01		ENSG00000254177
NA	-0.0086	7.5266E-01		ENSG00000286233
NA	-0.0061	7.5258E-01		ENSG00000251521
NA	-0.0097	7.5253E-01		ENSG00000260733
NA	-0.0085	7.5238E-01		ENSG00000257658
NA	-0.0261	7.5206E-01	8.6554E-01	ENSG00000203546
NA	-0.0192	7.5185E-01	8.6535E-01	ENSG00000279502
NA	-0.0057	7.5126E-01	8.6491E-01	ENSG00000205325
NA	-0.0052	7.5098E-01		ENSG00000276673
NA	-0.0107	7.5088E-01		ENSG00000271644
NA	-0.0142	7.5085E-01	8.6466E-01	ENSG00000267714
NA	-0.0099	7.5075E-01		ENSG00000253334
NA	-0.0052	7.5070E-01		ENSG00000270781
NA	-0.0045	7.5054E-01		ENSG00000286778
NA	-0.0046	7.5032E-01		ENSG00000289977
NA	-0.0112	7.5023E-01	8.6435E-01	ENSG00000283010
NA	-0.0087	7.4986E-01		ENSG00000281133
NA	-0.0047	7.4985E-01		ENSG00000213740
NA	-0.0099	7.4974E-01	8.6410E-01	ENSG00000236779
NA	-0.0208	7.4964E-01	8.6410E-01	ENSG00000261349
NA	-0.0256	7.4950E-01	8.6409E-01	ENSG00000288799
NA	-0.0145	7.4918E-01		ENSG00000288067
NA	-0.0048	7.4879E-01		ENSG00000264772
NA	-0.0255	7.4869E-01	8.6354E-01	ENSG00000209082
NA	-0.0052	7.4852E-01		ENSG00000236545
NA	-0.0097	7.4841E-01		ENSG00000284621
NA	-0.0065	7.4840E-01		ENSG00000279665
NA	-0.0019	7.4825E-01		ENSG00000204031
NA	-0.0089	7.4810E-01		ENSG00000214460
NA	-0.0090	7.4793E-01		ENSG00000231156
NA	-0.0232	7.4787E-01	8.6299E-01	ENSG00000289295
NA	-0.0011	7.4756E-01		ENSG00000230647
NA	-0.0239	7.4753E-01	8.6275E-01	ENSG00000243406
NA	-0.0267	7.4732E-01	8.6259E-01	ENSG00000289253
NA	-0.0032	7.4724E-01		ENSG00000287767
NA	-0.0017	7.4711E-01		ENSG00000286433
NA	-0.0086	7.4700E-01		ENSG00000279421
NA	-0.0242	7.4692E-01	8.6245E-01	ENSG00000231731
NA	-0.0072	7.4675E-01		ENSG00000241295
NA	-0.0092	7.4660E-01		ENSG00000261219
NA	-0.0025	7.4647E-01		ENSG00000225979
NA	-0.0139	7.4635E-01		ENSG00000252915
NA	-0.0187	7.4558E-01	8.6131E-01	ENSG00000255992
NA	-0.0233	7.4545E-01	8.6131E-01	ENSG00000267645
NA	-0.0032	7.4541E-01		ENSG00000267808
NA	-0.0219	7.4520E-01	8.6123E-01	ENSG00000278763
NA	-0.0094	7.4517E-01		ENSG00000224040
NA	-0.0109	7.4510E-01		ENSG00000272699

NA	-0.0181	7.4495E-01	8.6115E-01	ENSG00000271797
NA	0.0000	7.4473E-01		ENSG00000231698
NA	-0.0149	7.4463E-01		ENSG00000184303
NA	-0.0144	7.4445E-01		ENSG00000257773
NA	-0.0099	7.4445E-01		ENSG00000207512
NA	-0.0064	7.4441E-01		ENSG00000255340
NA	-0.0137	7.4391E-01		ENSG00000287608
NA	-0.0051	7.4388E-01		ENSG00000274281
NA	-0.0060	7.4379E-01		ENSG00000214190
NA	-0.0271	7.4352E-01	8.6047E-01	ENSG00000251259
NA	-0.0047	7.4340E-01		ENSG00000224207
NA	-0.0185	7.4333E-01	8.6037E-01	ENSG00000289317
NA	-0.0045	7.4321E-01		ENSG00000271101
NA	-0.0109	7.4318E-01		ENSG00000259045
NA	-0.0273	7.4317E-01	8.6031E-01	ENSG00000289184
NA	-0.0153	7.4257E-01	8.5985E-01	ENSG00000286632
NA	-0.0120	7.4255E-01		ENSG00000235887
NA	-0.0071	7.4249E-01		ENSG00000233601
NA	-0.0230	7.4249E-01	8.5979E-01	ENSG00000287969
NA	-0.0108	7.4228E-01		ENSG00000287920
NA	-0.0130	7.4180E-01		ENSG00000231561
NA	-0.0080	7.4174E-01		ENSG00000248583
NA	-0.0094	7.4139E-01		ENSG00000214286
NA	-0.0118	7.4133E-01		ENSG00000258115
NA	-0.0012	7.4123E-01		ENSG00000215086
NA	-0.0200	7.4103E-01	8.5887E-01	ENSG00000279894
NA	-0.0029	7.4093E-01		ENSG00000232130
NA	-0.0133	7.4058E-01		ENSG00000255983
NA	-0.0247	7.4032E-01	8.5846E-01	ENSG00000256594
NA	-0.0064	7.4029E-01		ENSG00000280054
NA	-0.0017	7.3988E-01		ENSG00000276087
NA	-0.0053	7.3979E-01		ENSG00000227505
NA	-0.0041	7.3978E-01		ENSG00000259513
NA	-0.0028	7.3918E-01		ENSG00000237851
NA	-0.0077	7.3837E-01		ENSG00000255045
NA	-0.0278	7.3834E-01	8.5753E-01	ENSG00000287358
NA	-0.0068	7.3810E-01		ENSG00000263096
NA	-0.0284	7.3784E-01	8.5716E-01	ENSG00000224126
NA	-0.0115	7.3775E-01	8.5710E-01	ENSG00000225808
NA	-0.0259	7.3759E-01	8.5695E-01	ENSG00000260729
NA	-0.0031	7.3703E-01		ENSG00000290107
NA	-0.0068	7.3649E-01		ENSG00000237514
NA	-0.0038	7.3605E-01		ENSG00000249006
NA	-0.0361	7.3595E-01	8.5597E-01	ENSG00000226499
NA	-0.0022	7.3589E-01		ENSG00000267016
NA	-0.0261	7.3563E-01	8.5581E-01	ENSG00000282381
NA	-0.0022	7.3537E-01		ENSG00000277039
NA	-0.0277	7.3530E-01	8.5570E-01	ENSG00000281021
NA	-0.0012	7.3479E-01		ENSG00000257239

NA	-0.0288	7.3477E-01	8.5528E-01	ENSG00000240449
NA	-0.0006	7.3471E-01		ENSG00000268847
NA	-0.0101	7.3457E-01		ENSG00000201034
NA	-0.0050	7.3457E-01		ENSG00000264472
NA	-0.0030	7.3418E-01		ENSG00000268173
NA	-0.0070	7.3388E-01		ENSG00000255213
NA	-0.0044	7.3377E-01		ENSG00000259773
NA	-0.0077	7.3373E-01		ENSG00000254013
NA	-0.0124	7.3278E-01	8.5405E-01	ENSG00000273133
NA	-0.0277	7.3266E-01	8.5400E-01	ENSG00000250790
NA	-0.0121	7.3225E-01		ENSG00000234091
NA	-0.0280	7.3218E-01	8.5365E-01	ENSG00000254409
NA	-0.0142	7.3194E-01		ENSG00000262470
NA	-0.0107	7.3108E-01	8.5293E-01	ENSG00000213721
NA	-0.0237	7.3106E-01	8.5293E-01	ENSG00000286458
NA	-0.0189	7.3058E-01		ENSG00000260350
NA	-0.0038	7.3037E-01		ENSG00000228941
NA	-0.0264	7.3019E-01	8.5241E-01	ENSG00000275672
NA	-0.0277	7.3011E-01	8.5236E-01	ENSG00000288597
NA	-0.0142	7.3004E-01	8.5232E-01	ENSG00000230482
NA	-0.0141	7.2985E-01		ENSG00000256769
NA	-0.0056	7.2974E-01		ENSG00000256293
NA	-0.0145	7.2969E-01		ENSG00000287241
NA	-0.0157	7.2951E-01		ENSG00000289937
NA	-0.0020	7.2946E-01		ENSG00000251293
NA	-0.0020	7.2888E-01		ENSG00000267170
NA	-0.0182	7.2886E-01	8.5131E-01	ENSG00000287563
NA	-0.0076	7.2885E-01		ENSG00000266311
NA	-0.0239	7.2848E-01	8.5110E-01	ENSG00000230183
NA	-0.0107	7.2838E-01		ENSG00000289887
NA	-0.0218	7.2829E-01	8.5096E-01	ENSG00000226632
NA	-0.0145	7.2822E-01		ENSG00000230666
NA	-0.0044	7.2807E-01		ENSG00000264812
NA	-0.0232	7.2806E-01		ENSG00000286606
NA	-0.0056	7.2796E-01		ENSG00000241721
NA	-0.0292	7.2782E-01	8.5066E-01	ENSG00000197332
NA	-0.0286	7.2756E-01	8.5055E-01	ENSG00000278107
NA	-0.0102	7.2754E-01		ENSG00000222649
NA	-0.0116	7.2734E-01		ENSG00000231665
NA	-0.0092	7.2711E-01		ENSG00000275978
NA	-0.0094	7.2698E-01		ENSG00000258107
NA	-0.0064	7.2685E-01		ENSG00000234181
NA	-0.0148	7.2685E-01	8.5004E-01	ENSG00000254879
NA	-0.0050	7.2672E-01		ENSG00000242951
NA	-0.0288	7.2668E-01	8.4997E-01	ENSG00000258427
NA	-0.0050	7.2659E-01		ENSG00000286893
NA	-0.0261	7.2657E-01	8.4993E-01	ENSG00000229251
NA	-0.0012	7.2642E-01		ENSG00000241438
NA	-0.0050	7.2639E-01	8.4988E-01	ENSG00000286932

NA	-0.0087	7.2617E-01		ENSG00000260342
NA	-0.0107	7.2583E-01		ENSG00000258461
NA	-0.0040	7.2568E-01		ENSG00000233069
NA	-0.0229	7.2546E-01	8.4935E-01	ENSG00000266677
NA	-0.0101	7.2538E-01		ENSG00000225063
NA	-0.0113	7.2522E-01		ENSG00000257043
NA	-0.0261	7.2519E-01	8.4928E-01	ENSG00000275390
NA	-0.0158	7.2500E-01		ENSG00000260594
NA	-0.0002	7.2495E-01		ENSG00000285859
NA	-0.0074	7.2470E-01		ENSG00000215057
NA	-0.0091	7.2470E-01		ENSG00000286364
NA	-0.0042	7.2425E-01		ENSG00000249617
NA	-0.0264	7.2422E-01	8.4866E-01	ENSG00000274798
NA	-0.0100	7.2410E-01		ENSG00000259276
NA	-0.0287	7.2399E-01	8.4859E-01	ENSG00000269473
NA	-0.0064	7.2390E-01		ENSG00000199676
NA	-0.0023	7.2373E-01		ENSG00000233451
NA	-0.0031	7.2370E-01		ENSG00000253760
NA	-0.0183	7.2341E-01		ENSG00000248155
NA	-0.0253	7.2331E-01	8.4820E-01	ENSG00000267811
NA	-0.0024	7.2319E-01		ENSG00000225233
NA	-0.0077	7.2312E-01		ENSG00000104970
NA	-0.0098	7.2291E-01		ENSG00000226194
NA	-0.0231	7.2261E-01	8.4795E-01	ENSG00000223896
NA	-0.0222	7.2200E-01	8.4764E-01	ENSG00000277977
NA	-0.0209	7.2199E-01		ENSG00000280936
NA	-0.0176	7.2185E-01		ENSG00000268222
NA	-0.0054	7.2172E-01		ENSG00000278351
NA	-0.0072	7.2159E-01		ENSG00000287533
NA	-0.0050	7.2149E-01		ENSG00000255334
NA	-0.0186	7.2135E-01	8.4724E-01	ENSG00000231799
NA	-0.0178	7.2107E-01	8.4711E-01	ENSG00000269836
NA	-0.0025	7.2095E-01		ENSG00000233776
NA	-0.0186	7.2074E-01	8.4688E-01	ENSG00000228404
NA	-0.0095	7.2040E-01		ENSG00000250267
NA	-0.0111	7.2032E-01		ENSG00000225857
NA	-0.0267	7.2030E-01	8.4651E-01	ENSG00000179978
NA	-0.0234	7.1927E-01		ENSG00000287631
NA	-0.0212	7.1927E-01	8.4564E-01	ENSG00000251171
NA	-0.0307	7.1916E-01	8.4556E-01	ENSG00000259343
NA	-0.0095	7.1913E-01		ENSG00000253961
NA	-0.0081	7.1876E-01		ENSG00000235096
NA	-0.0109	7.1872E-01	8.4531E-01	ENSG00000226363
NA	-0.0118	7.1867E-01		ENSG00000259254
NA	-0.0254	7.1858E-01	8.4519E-01	ENSG00000286064
NA	-0.0108	7.1832E-01		ENSG00000231398
NA	-0.0066	7.1786E-01		ENSG00000285750
NA	-0.0086	7.1758E-01		ENSG00000231896
NA	-0.0176	7.1736E-01	8.4469E-01	ENSG00000277662

NA	-0.0052	7.1720E-01		ENSG00000279373
NA	-0.0046	7.1697E-01		ENSG00000254855
NA	-0.0054	7.1686E-01		ENSG00000277001
NA	-0.0297	7.1680E-01	8.4433E-01	ENSG00000235545
NA	-0.0109	7.1671E-01		ENSG00000275649
NA	-0.0041	7.1660E-01		ENSG00000176857
NA	-0.0257	7.1658E-01	8.4418E-01	ENSG00000258824
NA	-0.0103	7.1654E-01		ENSG00000234527
NA	-0.0088	7.1647E-01		ENSG00000285732
NA	-0.0076	7.1641E-01		ENSG00000254044
NA	-0.0302	7.1640E-01	8.4413E-01	ENSG00000234286
NA	-0.0052	7.1630E-01		ENSG00000269888
NA	-0.0094	7.1625E-01	8.4412E-01	ENSG00000288583
NA	-0.0034	7.1610E-01		ENSG00000250853
NA	-0.0308	7.1606E-01	8.4404E-01	ENSG00000235370
NA	-0.0024	7.1562E-01		ENSG00000279265
NA	-0.0003	7.1555E-01		ENSG00000234274
NA	-0.0183	7.1534E-01	8.4351E-01	ENSG00000260927
NA	-0.0111	7.1530E-01		ENSG00000233884
NA	-0.0050	7.1514E-01	8.4343E-01	ENSG00000253395
NA	-0.0137	7.1512E-01	8.4343E-01	ENSG00000253477
NA	-0.0063	7.1502E-01		ENSG00000238007
NA	-0.0297	7.1474E-01	8.4332E-01	ENSG00000229539
NA	-0.0067	7.1437E-01		ENSG00000279998
NA	-0.0188	7.1391E-01	8.4293E-01	ENSG00000284645
NA	-0.0247	7.1379E-01	8.4287E-01	ENSG00000222019
NA	-0.0183	7.1374E-01		ENSG00000258153
NA	-0.0187	7.1370E-01	8.4287E-01	ENSG00000262096
NA	-0.0264	7.1367E-01		ENSG00000236478
NA	-0.0283	7.1361E-01	8.4286E-01	ENSG00000248103
NA	-0.0278	7.1353E-01	8.4284E-01	ENSG00000286611
NA	-0.0300	7.1352E-01	8.4284E-01	ENSG00000218418
NA	-0.0221	7.1351E-01		ENSG00000241095
NA	-0.0123	7.1302E-01		ENSG00000272461
NA	-0.0088	7.1301E-01		ENSG00000259616
NA	-0.0092	7.1265E-01	8.4237E-01	ENSG00000184523
NA	-0.0086	7.1263E-01		ENSG00000255042
NA	-0.0108	7.1222E-01		ENSG00000224553
NA	-0.0088	7.1215E-01		ENSG00000232015
NA	-0.0202	7.1209E-01		ENSG00000279074
NA	-0.0109	7.1204E-01	8.4207E-01	ENSG00000288785
NA	-0.0307	7.1194E-01	8.4206E-01	ENSG00000225313
NA	-0.0239	7.1182E-01	8.4204E-01	ENSG00000269427
NA	-0.0092	7.1174E-01		ENSG00000265542
NA	-0.0251	7.1163E-01	8.4185E-01	ENSG00000284512
NA	-0.0143	7.1129E-01		ENSG00000273765
NA	-0.0042	7.1127E-01		ENSG00000277152
NA	-0.0117	7.1087E-01		ENSG00000289237
NA	-0.0155	7.1085E-01		ENSG00000274884

NA	-0.0055	7.1075E-01		ENSG00000278255
NA	-0.0100	7.1063E-01		ENSG00000270381
NA	-0.0135	7.1032E-01		ENSG00000259727
NA	-0.0105	7.1020E-01		ENSG00000285471
NA	-0.0172	7.1019E-01	8.4083E-01	ENSG00000272566
NA	-0.0090	7.1009E-01		ENSG00000264246
NA	-0.0179	7.0966E-01	8.4042E-01	ENSG00000287704
NA	-0.0248	7.0950E-01	8.4040E-01	ENSG00000213018
NA	-0.0251	7.0937E-01	8.4031E-01	ENSG00000286035
NA	-0.0048	7.0911E-01		ENSG00000244641
NA	-0.0140	7.0899E-01		ENSG00000230665
NA	-0.0099	7.0892E-01		ENSG00000276807
NA	-0.0017	7.0875E-01		ENSG00000232009
NA	-0.0053	7.0873E-01		ENSG00000226666
NA	-0.0265	7.0862E-01	8.3974E-01	ENSG00000219274
NA	-0.0037	7.0860E-01	8.3974E-01	ENSG00000272006
NA	-0.0283	7.0831E-01	8.3965E-01	ENSG00000289235
NA	-0.0178	7.0813E-01	8.3965E-01	ENSG00000289196
NA	-0.0132	7.0770E-01		ENSG00000225588
NA	-0.0308	7.0767E-01	8.3931E-01	ENSG00000225442
NA	-0.0066	7.0756E-01		ENSG00000162699
NA	-0.0097	7.0736E-01		ENSG00000264215
NA	-0.0262	7.0727E-01	8.3906E-01	ENSG00000258611
NA	-0.0090	7.0714E-01		ENSG00000285705
NA	-0.0064	7.0686E-01		ENSG00000189166
NA	-0.0056	7.0684E-01		ENSG00000217330
NA	-0.0076	7.0660E-01		ENSG00000236792
NA	-0.0047	7.0654E-01		ENSG00000233733
NA	-0.0034	7.0652E-01		ENSG00000238516
NA	-0.0079	7.0652E-01		ENSG00000229661
NA	-0.0023	7.0648E-01		ENSG00000279221
NA	-0.0004	7.0644E-01	8.3829E-01	ENSG00000280096
NA	-0.0117	7.0642E-01		ENSG00000258915
NA	-0.0066	7.0641E-01		ENSG00000280181
NA	-0.0023	7.0611E-01		ENSG00000225639
NA	-0.0245	7.0590E-01		ENSG00000243797
NA	-0.0029	7.0563E-01		ENSG00000236695
NA	-0.0320	7.0535E-01	8.3769E-01	ENSG00000275297
NA	-0.0040	7.0527E-01		ENSG00000272463
NA	-0.0144	7.0513E-01		ENSG00000234515
NA	-0.0091	7.0513E-01		ENSG00000227313
NA	-0.0051	7.0492E-01		ENSG00000288843
NA	-0.0251	7.0478E-01	8.3721E-01	ENSG00000254919
NA	-0.0004	7.0458E-01		ENSG00000227757
NA	-0.0143	7.0447E-01		ENSG00000264647
NA	-0.0157	7.0443E-01		ENSG00000255909
NA	-0.0119	7.0436E-01		ENSG00000259277
NA	-0.0142	7.0417E-01		ENSG00000248213
NA	-0.0323	7.0390E-01	8.3671E-01	ENSG00000262198

NA	-0.0321	7.0387E-01	8.3671E-01	ENSG00000267605
NA	-0.0041	7.0367E-01		ENSG00000183239
NA	-0.0183	7.0330E-01	8.3621E-01	ENSG00000242791
NA	-0.0162	7.0297E-01		ENSG00000240048
NA	-0.0105	7.0288E-01		ENSG00000261433
NA	-0.0065	7.0285E-01		ENSG00000243402
NA	-0.0239	7.0245E-01	8.3561E-01	ENSG00000249485
NA	-0.0063	7.0207E-01		ENSG00000249319
NA	-0.0074	7.0188E-01		ENSG00000260255
NA	-0.0067	7.0184E-01		ENSG00000279752
NA	-0.0213	7.0180E-01	8.3529E-01	ENSG00000267476
NA	-0.0296	7.0171E-01	8.3529E-01	ENSG00000287697
NA	-0.0068	7.0159E-01	8.3522E-01	ENSG00000210127
NA	-0.0310	7.0130E-01	8.3497E-01	ENSG00000271646
NA	-0.0213	7.0129E-01		ENSG00000225807
NA	-0.0047	7.0111E-01		ENSG00000287459
NA	-0.0091	7.0105E-01		ENSG00000289945
NA	-0.0084	7.0081E-01		ENSG00000231878
NA	-0.0274	7.0043E-01	8.3446E-01	ENSG00000272622
NA	-0.0081	7.0010E-01		ENSG00000269091
NA	-0.0316	6.9994E-01	8.3412E-01	ENSG00000260176
NA	-0.0214	6.9968E-01	8.3388E-01	ENSG00000272281
NA	-0.0144	6.9961E-01		ENSG00000279387
NA	-0.0171	6.9948E-01		ENSG00000287041
NA	-0.0227	6.9906E-01	8.3370E-01	ENSG00000215283
NA	-0.0001	6.9864E-01	8.3342E-01	ENSG00000263990
NA	-0.0074	6.9858E-01	8.3342E-01	ENSG00000279812
NA	-0.0184	6.9844E-01		ENSG00000229758
NA	-0.0192	6.9826E-01		ENSG00000231468
NA	-0.0146	6.9822E-01		ENSG00000250338
NA	-0.0038	6.9813E-01		ENSG00000250292
NA	-0.0024	6.9812E-01		ENSG00000289169
NA	-0.0046	6.9810E-01		ENSG00000229928
NA	-0.0049	6.9804E-01		ENSG00000287022
NA	-0.0321	6.9785E-01	8.3296E-01	ENSG00000288710
NA	-0.0266	6.9757E-01	8.3290E-01	ENSG00000271553
NA	-0.0065	6.9754E-01		ENSG00000286720
NA	-0.0221	6.9746E-01		ENSG00000278965
NA	-0.0290	6.9744E-01	8.3283E-01	ENSG00000280122
NA	-0.0140	6.9728E-01		ENSG00000234702
NA	-0.0085	6.9647E-01		ENSG00000224237
NA	-0.0095	6.9630E-01		ENSG00000232848
NA	-0.0065	6.9615E-01		ENSG00000279640
NA	-0.0052	6.9598E-01		ENSG00000226919
NA	-0.0023	6.9560E-01		ENSG00000258648
NA	-0.0081	6.9540E-01		ENSG00000255843
NA	-0.0065	6.9468E-01		ENSG00000218069
NA	-0.0045	6.9461E-01		ENSG00000268108
NA	-0.0076	6.9456E-01		ENSG00000270842

NA	-0.0050	6.9448E-01		ENSG00000250071
NA	-0.0116	6.9425E-01	8.3059E-01	ENSG00000250031
NA	-0.0093	6.9413E-01		ENSG00000261616
NA	0.0000	6.9404E-01		ENSG00000265743
NA	-0.0118	6.9395E-01		ENSG00000218347
NA	-0.0317	6.9392E-01		ENSG00000258904
NA	-0.0330	6.9374E-01	8.3031E-01	ENSG00000224858
NA	-0.0097	6.9367E-01		ENSG00000235064
NA	-0.0001	6.9351E-01		ENSG00000280280
NA	-0.0303	6.9334E-01	8.3001E-01	ENSG00000228801
NA	-0.0053	6.9318E-01		ENSG00000262079
NA	-0.0060	6.9291E-01		ENSG00000249047
NA	-0.0060	6.9276E-01		ENSG00000236115
NA	-0.0290	6.9257E-01	8.2947E-01	ENSG00000218018
NA	-0.0214	6.9240E-01	8.2939E-01	ENSG00000286341
NA	-0.0173	6.9232E-01	8.2933E-01	ENSG00000228663
NA	-0.0304	6.9227E-01	8.2932E-01	ENSG00000272343
NA	-0.0299	6.9226E-01	8.2932E-01	ENSG00000279649
NA	-0.0061	6.9201E-01		ENSG00000258558
NA	-0.0142	6.9171E-01	8.2897E-01	ENSG00000132832
NA	-0.0267	6.9167E-01	8.2897E-01	ENSG00000289629
NA	-0.0294	6.9159E-01	8.2896E-01	ENSG00000232480
NA	-0.0092	6.9147E-01		ENSG00000277867
NA	-0.0249	6.9086E-01	8.2835E-01	ENSG00000233542
NA	-0.0056	6.8919E-01	8.2719E-01	ENSG00000199366
NA	-0.0336	6.8897E-01	8.2709E-01	ENSG00000235100
NA	-0.0064	6.8894E-01		ENSG00000259244
NA	-0.0061	6.8885E-01		ENSG00000279607
NA	-0.0282	6.8876E-01	8.2704E-01	ENSG00000232721
NA	-0.0114	6.8874E-01		ENSG00000253181
NA	-0.0231	6.8851E-01	8.2690E-01	ENSG00000219201
NA	-0.0265	6.8846E-01	8.2689E-01	ENSG00000259986
NA	-0.0145	6.8835E-01	8.2684E-01	ENSG00000225475
NA	-0.0198	6.8833E-01	8.2684E-01	ENSG00000290021
NA	-0.0322	6.8831E-01	8.2684E-01	ENSG00000278943
NA	-0.0076	6.8830E-01		ENSG00000260254
NA	-0.0078	6.8818E-01		ENSG00000225407
NA	-0.0113	6.8813E-01		ENSG00000260417
NA	-0.0080	6.8803E-01		ENSG00000207326
NA	-0.0072	6.8781E-01		ENSG00000286780
NA	-0.0011	6.8756E-01		ENSG00000225170
NA	-0.0302	6.8716E-01	8.2593E-01	ENSG00000249159
NA	-0.0263	6.8706E-01	8.2585E-01	ENSG00000245330
NA	-0.0142	6.8692E-01		ENSG00000226957
NA	-0.0219	6.8683E-01	8.2570E-01	ENSG00000261375
NA	-0.0230	6.8682E-01		ENSG00000288936
NA	-0.0092	6.8680E-01		ENSG00000219682
NA	-0.0297	6.8679E-01	8.2569E-01	ENSG00000285639
NA	-0.0062	6.8641E-01		ENSG00000223518

NA	-0.0078	6.8607E-01		ENSG00000275406
NA	-0.0106	6.8600E-01		ENSG00000237633
NA	-0.0320	6.8563E-01	8.2489E-01	ENSG00000289626
NA	-0.0243	6.8550E-01	8.2489E-01	ENSG00000278985
NA	-0.0202	6.8503E-01	8.2448E-01	ENSG00000254038
NA	-0.0052	6.8458E-01		ENSG00000228471
NA	-0.0081	6.8456E-01		ENSG00000259138
NA	-0.0079	6.8442E-01		ENSG00000287377
NA	-0.0066	6.8419E-01		ENSG00000227255
NA	-0.0008	6.8399E-01		ENSG00000286273
NA	-0.0172	6.8384E-01		ENSG00000286979
NA	-0.0255	6.8369E-01	8.2356E-01	ENSG00000254649
NA	-0.0326	6.8363E-01	8.2353E-01	ENSG00000286242
NA	-0.0066	6.8352E-01		ENSG00000229083
NA	-0.0121	6.8327E-01		ENSG00000287857
NA	-0.0241	6.8299E-01	8.2313E-01	ENSG00000280422
NA	-0.0117	6.8293E-01		ENSG00000214366
NA	-0.0331	6.8285E-01	8.2313E-01	ENSG00000288814
NA	-0.0195	6.8265E-01	8.2313E-01	ENSG00000271029
NA	-0.0340	6.8261E-01	8.2313E-01	ENSG00000223416
NA	-0.0038	6.8248E-01		ENSG00000231069
NA	-0.0048	6.8247E-01		ENSG00000227288
NA	-0.0151	6.8234E-01		ENSG00000254898
NA	-0.0144	6.8189E-01		ENSG00000270977
NA	-0.0051	6.8156E-01		ENSG00000224819
NA	-0.0197	6.8124E-01		ENSG00000228485
NA	-0.0125	6.8095E-01		ENSG00000286292
NA	-0.0107	6.8068E-01		ENSG00000274272
NA	-0.0211	6.8053E-01		ENSG00000232946
NA	-0.0072	6.8033E-01		ENSG00000268764
NA	-0.0102	6.8024E-01		ENSG00000287512
NA	-0.0319	6.8017E-01	8.2110E-01	ENSG00000280087
NA	-0.0122	6.7933E-01		ENSG00000239969
NA	-0.0228	6.7911E-01	8.2046E-01	ENSG00000246575
NA	-0.0204	6.7846E-01	8.1981E-01	ENSG00000257657
NA	-0.0001	6.7781E-01		ENSG00000229530
NA	-0.0093	6.7773E-01		ENSG00000281849
NA	-0.0070	6.7764E-01		ENSG00000182814
NA	-0.0194	6.7756E-01	8.1935E-01	ENSG00000279530
NA	-0.0042	6.7712E-01		ENSG00000289828
NA	-0.0248	6.7698E-01	8.1907E-01	ENSG00000279390
NA	-0.0039	6.7677E-01		ENSG00000204121
NA	-0.0158	6.7659E-01		ENSG00000289704
NA	-0.0169	6.7624E-01		ENSG00000286108
NA	-0.0083	6.7611E-01	8.1840E-01	ENSG00000286594
NA	-0.0134	6.7592E-01		ENSG00000255780
NA	-0.0043	6.7590E-01		ENSG00000222432
NA	-0.0063	6.7581E-01		ENSG00000271849
NA	-0.0049	6.7575E-01		ENSG00000225751

NA	-0.0057	6.7565E-01		ENSG00000276067
NA	-0.0110	6.7562E-01		ENSG00000286063
NA	-0.0062	6.7550E-01		ENSG00000231072
NA	-0.0121	6.7547E-01		ENSG00000273381
NA	-0.0333	6.7536E-01	8.1785E-01	ENSG00000288612
NA	-0.0050	6.7535E-01		ENSG00000226994
NA	-0.0105	6.7523E-01		ENSG00000255663
NA	-0.0222	6.7518E-01	8.1780E-01	ENSG00000260029
NA	-0.0288	6.7499E-01	8.1774E-01	ENSG00000234585
NA	-0.0262	6.7495E-01		ENSG00000214875
NA	-0.0090	6.7489E-01		ENSG00000276359
NA	-0.0364	6.7483E-01	8.1764E-01	ENSG00000279203
NA	-0.0282	6.7464E-01	8.1764E-01	ENSG00000274367
NA	-0.0089	6.7417E-01		ENSG00000273744
NA	-0.0322	6.7402E-01	8.1745E-01	ENSG00000289111
NA	-0.0358	6.7400E-01	8.1745E-01	ENSG00000280077
NA	-0.0185	6.7388E-01	8.1745E-01	ENSG00000220908
NA	-0.0091	6.7377E-01		ENSG00000280115
NA	-0.0356	6.7364E-01	8.1742E-01	ENSG00000274204
NA	-0.0344	6.7355E-01	8.1736E-01	ENSG00000189014
NA	-0.0260	6.7346E-01		ENSG00000271265
NA	-0.0136	6.7331E-01		ENSG00000233682
NA	-0.0055	6.7279E-01		ENSG00000237713
NA	-0.0244	6.7255E-01	8.1686E-01	ENSG00000271662
NA	-0.0087	6.7242E-01		ENSG00000226206
NA	-0.0244	6.7237E-01	8.1677E-01	ENSG00000259405
NA	-0.0138	6.7230E-01		ENSG00000237640
NA	-0.0254	6.7228E-01	8.1670E-01	ENSG00000270755
NA	-0.0285	6.7226E-01		ENSG00000279803
NA	-0.0152	6.7221E-01		ENSG00000272379
NA	-0.0347	6.7189E-01	8.1641E-01	ENSG00000259972
NA	-0.0141	6.7160E-01		ENSG00000260211
NA	-0.0093	6.7136E-01		ENSG00000258888
NA	-0.0010	6.7083E-01		ENSG00000279347
NA	-0.0085	6.7068E-01		ENSG00000229759
NA	-0.0038	6.7063E-01		ENSG00000275933
NA	-0.0140	6.7013E-01		ENSG00000226138
NA	-0.0210	6.6989E-01		ENSG00000253678
NA	-0.0099	6.6983E-01		ENSG00000261367
NA	-0.0248	6.6967E-01		ENSG00000286643
NA	-0.0252	6.6954E-01	8.1482E-01	ENSG00000224321
NA	-0.0086	6.6952E-01		ENSG00000257829
NA	-0.0292	6.6867E-01	8.1432E-01	ENSG00000264546
NA	-0.0321	6.6848E-01		ENSG00000231530
NA	-0.0341	6.6844E-01	8.1417E-01	ENSG00000272368
NA	-0.0142	6.6827E-01		ENSG00000261215
NA	-0.0025	6.6814E-01		ENSG00000275381
NA	-0.0188	6.6796E-01	8.1403E-01	ENSG00000287033
NA	-0.0323	6.6793E-01	8.1403E-01	ENSG00000267787

NA	-0.0095	6.6744E-01		ENSG00000231208
NA	-0.0041	6.6737E-01		ENSG00000266304
NA	-0.0086	6.6631E-01		ENSG00000270966
NA	-0.0132	6.6625E-01		ENSG00000276931
NA	-0.0068	6.6611E-01		ENSG00000227413
NA	-0.0309	6.6576E-01	8.1231E-01	ENSG00000213881
NA	-0.0118	6.6566E-01		ENSG00000263370
NA	-0.0236	6.6565E-01		ENSG00000232162
NA	-0.0267	6.6544E-01		ENSG00000267096
NA	-0.0017	6.6543E-01		ENSG00000280257
NA	-0.0357	6.6512E-01	8.1182E-01	ENSG00000261135
NA	-0.0044	6.6428E-01		ENSG00000261205
NA	-0.0026	6.6413E-01	8.1108E-01	ENSG00000285744
NA	-0.0156	6.6402E-01		ENSG00000273473
NA	-0.0155	6.6399E-01		ENSG00000229386
NA	-0.0265	6.6391E-01	8.1090E-01	ENSG00000249207
NA	-0.0011	6.6390E-01		ENSG00000226822
NA	-0.0070	6.6372E-01		ENSG00000267005
NA	-0.0339	6.6368E-01	8.1086E-01	ENSG00000279159
NA	-0.0351	6.6366E-01	8.1086E-01	ENSG00000275441
NA	-0.0301	6.6358E-01	8.1086E-01	ENSG00000289288
NA	-0.0287	6.6339E-01		ENSG00000230668
NA	-0.0296	6.6297E-01	8.1040E-01	ENSG00000249565
NA	-0.0188	6.6255E-01	8.1004E-01	ENSG00000228960
NA	-0.0019	6.6230E-01		ENSG00000278698
NA	-0.0214	6.6224E-01	8.0978E-01	ENSG00000229999
NA	-0.0357	6.6220E-01	8.0977E-01	ENSG00000289614
NA	-0.0107	6.6169E-01		ENSG00000286944
NA	-0.0073	6.6165E-01		ENSG00000234457
NA	-0.0135	6.6152E-01		ENSG00000185390
NA	-0.0168	6.6150E-01	8.0931E-01	ENSG00000287270
NA	-0.0186	6.6127E-01		ENSG00000278273
NA	-0.0339	6.6118E-01	8.0909E-01	ENSG00000284648
NA	-0.0245	6.6105E-01	8.0901E-01	ENSG00000123009
NA	-0.0250	6.6085E-01		ENSG00000253915
NA	-0.0349	6.6071E-01	8.0883E-01	ENSG00000254851
NA	-0.0130	6.6067E-01		ENSG00000270296
NA	-0.0074	6.6040E-01		ENSG00000267387
NA	-0.0014	6.6033E-01	8.0861E-01	ENSG00000263220
NA	-0.0040	6.6020E-01		ENSG00000242267
NA	-0.0176	6.6016E-01		ENSG00000227769
NA	-0.0099	6.5995E-01	8.0833E-01	ENSG00000255348
NA	-0.0347	6.5988E-01	8.0831E-01	ENSG00000227663
NA	-0.0124	6.5982E-01		ENSG00000264300
NA	-0.0108	6.5966E-01		ENSG00000251393
NA	-0.0105	6.5966E-01		ENSG00000224688
NA	-0.0049	6.5939E-01		ENSG00000237025
NA	-0.0383	6.5912E-01	8.0773E-01	ENSG00000177337
NA	-0.0260	6.5879E-01	8.0745E-01	ENSG00000289546

NA	-0.0114	6.5825E-01		ENSG00000235996
NA	-0.0085	6.5818E-01		ENSG00000285921
NA	-0.0210	6.5812E-01		ENSG00000259929
NA	-0.0288	6.5808E-01	8.0702E-01	ENSG00000217653
NA	-0.0044	6.5782E-01		ENSG00000267501
NA	-0.0007	6.5769E-01		ENSG00000215467
NA	-0.0277	6.5743E-01	8.0659E-01	ENSG00000280369
NA	-0.0106	6.5728E-01		ENSG00000253924
NA	-0.0102	6.5684E-01		ENSG00000270632
NA	-0.0063	6.5662E-01		ENSG00000279625
NA	-0.0079	6.5648E-01		ENSG00000242352
NA	-0.0183	6.5632E-01	8.0591E-01	ENSG00000284985
NA	-0.0046	6.5625E-01		ENSG00000272381
NA	-0.0234	6.5612E-01	8.0570E-01	ENSG00000200834
NA	-0.0017	6.5602E-01		ENSG00000239498
NA	-0.0109	6.5592E-01		ENSG00000269839
NA	-0.0053	6.5577E-01		ENSG00000230676
NA	-0.0115	6.5567E-01		ENSG00000254048
NA	-0.0064	6.5566E-01		ENSG00000290001
NA	-0.0047	6.5565E-01		ENSG00000289873
NA	-0.0074	6.5531E-01		ENSG00000244345
NA	-0.0051	6.5522E-01		ENSG00000158482
NA	-0.0272	6.5481E-01	8.0512E-01	ENSG00000273424
NA	-0.0227	6.5452E-01	8.0490E-01	ENSG00000272153
NA	-0.0141	6.5429E-01		ENSG00000233859
NA	-0.0142	6.5372E-01		ENSG00000233038
NA	-0.0148	6.5368E-01		ENSG00000266076
NA	-0.0179	6.5353E-01		ENSG00000224031
NA	-0.0221	6.5336E-01	8.0375E-01	ENSG00000287445
NA	-0.0079	6.5295E-01		ENSG00000256500
NA	-0.0067	6.5290E-01		ENSG00000284744
NA	-0.0125	6.5269E-01		ENSG00000230010
NA	-0.0059	6.5264E-01		ENSG00000240156
NA	-0.0132	6.5258E-01		ENSG00000228639
NA	-0.0259	6.5247E-01	8.0312E-01	ENSG00000224765
NA	-0.0167	6.5247E-01	8.0312E-01	ENSG00000213131
NA	-0.0312	6.5240E-01	8.0312E-01	ENSG00000254477
NA	-0.0061	6.5232E-01		ENSG00000232734
NA	-0.0260	6.5225E-01	8.0307E-01	ENSG00000235979
NA	-0.0014	6.5211E-01		ENSG00000276715
NA	-0.0230	6.5201E-01	8.0290E-01	ENSG00000268746
NA	-0.0170	6.5198E-01	8.0290E-01	ENSG00000253708
NA	-0.0095	6.5181E-01		ENSG00000286987
NA	-0.0044	6.5159E-01		ENSG00000267130
NA	-0.0209	6.5140E-01		ENSG00000223576
NA	-0.0106	6.5126E-01		ENSG00000287035
NA	-0.0075	6.5120E-01		ENSG00000259982
NA	-0.0144	6.5092E-01		ENSG00000248629
NA	-0.0072	6.5086E-01		ENSG00000286479

NA	-0.0092	6.5072E-01		ENSG00000264176
NA	-0.0202	6.5055E-01		ENSG00000286771
NA	-0.0106	6.5035E-01		ENSG00000276417
NA	-0.0338	6.5020E-01	8.0159E-01	ENSG00000267277
NA	-0.0138	6.5003E-01		ENSG00000287915
NA	-0.0101	6.4929E-01		ENSG00000228622
NA	-0.0404	6.4883E-01	8.0035E-01	ENSG00000270820
NA	-0.0310	6.4868E-01	8.0033E-01	ENSG00000275493
NA	-0.0355	6.4868E-01	8.0033E-01	ENSG00000272954
NA	-0.0136	6.4867E-01		ENSG00000243256
NA	-0.0373	6.4865E-01	8.0033E-01	ENSG00000235254
NA	-0.0116	6.4818E-01		ENSG00000232354
NA	-0.0229	6.4795E-01	7.9987E-01	ENSG00000223908
NA	-0.0359	6.4771E-01	7.9972E-01	ENSG00000265100
NA	-0.0040	6.4739E-01		ENSG00000227453
NA	-0.0093	6.4737E-01		ENSG00000241825
NA	-0.0373	6.4713E-01	7.9917E-01	ENSG00000279168
NA	-0.0291	6.4648E-01	7.9865E-01	ENSG00000279166
NA	-0.0002	6.4647E-01		ENSG00000267552
NA	-0.0104	6.4619E-01		ENSG00000278941
NA	-0.0046	6.4591E-01		ENSG00000224061
NA	-0.0191	6.4579E-01		ENSG00000232228
NA	-0.0175	6.4576E-01		ENSG00000287508
NA	-0.0296	6.4564E-01	7.9776E-01	ENSG00000239503
NA	-0.0099	6.4554E-01		ENSG00000229962
NA	-0.0180	6.4554E-01		ENSG00000259767
NA	-0.0169	6.4517E-01	7.9748E-01	ENSG00000271314
NA	-0.0133	6.4478E-01		ENSG00000288886
NA	-0.0225	6.4477E-01		ENSG00000213003
NA	-0.0219	6.4453E-01	7.9707E-01	ENSG00000274383
NA	-0.0259	6.4378E-01		ENSG00000260082
NA	-0.0304	6.4357E-01	7.9650E-01	ENSG00000205106
NA	-0.0110	6.4275E-01		ENSG00000287997
NA	-0.0051	6.4275E-01		ENSG00000290106
NA	-0.0374	6.4275E-01	7.9583E-01	ENSG00000279337
NA	-0.0133	6.4269E-01		ENSG00000258576
NA	-0.0388	6.4262E-01	7.9583E-01	ENSG00000237863
NA	-0.0284	6.4247E-01	7.9569E-01	ENSG00000261543
NA	-0.0095	6.4204E-01		ENSG00000241520
NA	-0.0025	6.4125E-01		ENSG00000171658
NA	-0.0122	6.4118E-01		ENSG00000276593
NA	-0.0126	6.4112E-01		ENSG00000264145
NA	-0.0257	6.4093E-01		ENSG00000225076
NA	-0.0388	6.4072E-01	7.9441E-01	ENSG00000279161
NA	-0.0379	6.4020E-01	7.9415E-01	ENSG00000287665
NA	-0.0151	6.4020E-01		ENSG00000258672
NA	-0.0366	6.4002E-01	7.9402E-01	ENSG00000241255
NA	-0.0138	6.3992E-01	7.9402E-01	ENSG00000179038
NA	-0.0127	6.3990E-01		ENSG00000283991

NA	-0.0128	6.3982E-01		ENSG00000279569
NA	-0.0101	6.3958E-01		ENSG00000259557
NA	-0.0366	6.3947E-01	7.9390E-01	ENSG00000274898
NA	-0.0149	6.3896E-01		ENSG00000237626
NA	-0.0321	6.3877E-01		ENSG00000277930
NA	-0.0153	6.3871E-01		ENSG00000225165
NA	-0.0074	6.3827E-01		ENSG00000257119
NA	-0.0129	6.3809E-01		ENSG00000234175
NA	-0.0027	6.3805E-01		ENSG00000254455
NA	-0.0067	6.3761E-01		ENSG00000223691
NA	-0.0037	6.3749E-01		ENSG00000201554
NA	-0.0341	6.3739E-01	7.9226E-01	ENSG00000248568
NA	-0.0068	6.3710E-01		ENSG00000227773
NA	-0.0362	6.3707E-01	7.9207E-01	ENSG00000241411
NA	-0.0297	6.3700E-01	7.9202E-01	ENSG00000172186
NA	-0.0196	6.3695E-01		ENSG00000275437
NA	-0.0193	6.3687E-01		ENSG00000237668
NA	-0.0360	6.3665E-01	7.9183E-01	ENSG00000282855
NA	-0.0168	6.3650E-01		ENSG00000223725
NA	-0.0222	6.3633E-01	7.9151E-01	ENSG00000241627
NA	-0.0224	6.3630E-01		ENSG00000281016
NA	-0.0286	6.3627E-01	7.9148E-01	ENSG00000265261
NA	-0.0209	6.3575E-01	7.9111E-01	ENSG00000277112
NA	-0.0238	6.3573E-01		ENSG00000265798
NA	-0.0243	6.3553E-01	7.9092E-01	ENSG00000227355
NA	-0.0357	6.3550E-01	7.9092E-01	ENSG00000228634
NA	-0.0066	6.3547E-01		ENSG00000267340
NA	-0.0291	6.3516E-01	7.9073E-01	ENSG00000259326
NA	-0.0039	6.3512E-01		ENSG00000287197
NA	-0.0138	6.3468E-01	7.9048E-01	ENSG00000284708
NA	-0.0077	6.3430E-01		ENSG00000232852
NA	-0.0441	6.3417E-01	7.9030E-01	ENSG00000252965
NA	-0.0324	6.3383E-01	7.9007E-01	ENSG00000262580
NA	-0.0045	6.3375E-01		ENSG00000285820
NA	-0.0124	6.3355E-01		ENSG00000286713
NA	-0.0104	6.3329E-01		ENSG00000228432
NA	-0.0200	6.3287E-01	7.8928E-01	ENSG00000272865
NA	-0.0131	6.3265E-01		ENSG00000244701
NA	-0.0131	6.3257E-01		ENSG00000266667
NA	-0.0254	6.3255E-01		ENSG00000259723
NA	-0.0042	6.3214E-01		ENSG00000227920
NA	-0.0030	6.3213E-01		ENSG00000199357
NA	-0.0102	6.3208E-01		ENSG00000257759
NA	-0.0194	6.3200E-01		ENSG00000273821
NA	-0.0114	6.3174E-01		ENSG00000228261
NA	-0.0176	6.3149E-01		ENSG00000230585
NA	-0.0150	6.3125E-01		ENSG00000254522
NA	-0.0337	6.3106E-01	7.8762E-01	ENSG00000245768
NA	-0.0224	6.3100E-01	7.8758E-01	ENSG00000232901

NA	-0.0117	6.3096E-01		ENSG00000246225
NA	-0.0059	6.3050E-01		ENSG00000286639
NA	-0.0203	6.3041E-01	7.8713E-01	ENSG00000225334
NA	-0.0049	6.3024E-01		ENSG00000281100
NA	-0.0101	6.3018E-01		ENSG00000254197
NA	-0.0236	6.2996E-01		ENSG00000254732
NA	-0.0094	6.2988E-01		ENSG00000283480
NA	-0.0098	6.2981E-01		ENSG00000262920
NA	-0.0389	6.2970E-01	7.8675E-01	ENSG00000288953
NA	-0.0134	6.2968E-01		ENSG00000268931
NA	-0.0086	6.2941E-01		ENSG00000227519
NA	-0.0182	6.2939E-01		ENSG00000286328
NA	-0.0011	6.2931E-01		ENSG00000285591
NA	-0.0145	6.2912E-01		ENSG00000273406
NA	-0.0076	6.2896E-01	7.8647E-01	ENSG00000229596
NA	-0.0096	6.2892E-01		ENSG00000229855
NA	-0.0118	6.2830E-01		ENSG00000202470
NA	-0.0272	6.2816E-01	7.8599E-01	ENSG00000241057
NA	-0.0221	6.2787E-01	7.8572E-01	ENSG00000257910
NA	-0.0142	6.2757E-01		ENSG00000272477
NA	-0.0079	6.2740E-01		ENSG00000289965
NA	-0.0074	6.2737E-01		ENSG00000273362
NA	-0.0060	6.2728E-01		ENSG00000234562
NA	-0.0123	6.2722E-01		ENSG00000286216
NA	-0.0101	6.2702E-01		ENSG00000272144
NA	-0.0245	6.2697E-01		ENSG00000279509
NA	-0.0107	6.2672E-01		ENSG00000235495
NA	-0.0169	6.2661E-01		ENSG00000225297
NA	-0.0344	6.2657E-01	7.8473E-01	ENSG00000272037
NA	-0.0319	6.2652E-01	7.8473E-01	ENSG00000269954
NA	-0.0167	6.2592E-01		ENSG00000213237
NA	-0.0219	6.2577E-01	7.8404E-01	ENSG00000279349
NA	-0.0064	6.2555E-01		ENSG00000289091
NA	-0.0309	6.2553E-01	7.8379E-01	ENSG00000287619
NA	-0.0038	6.2494E-01		ENSG00000286144
NA	-0.0187	6.2467E-01		ENSG00000218902
NA	-0.0400	6.2467E-01	7.8330E-01	ENSG00000237039
NA	-0.0368	6.2437E-01	7.8313E-01	ENSG00000286158
NA	-0.0317	6.2433E-01	7.8313E-01	ENSG00000246851
NA	-0.0099	6.2395E-01		ENSG00000270098
NA	-0.0137	6.2377E-01		ENSG00000254924
NA	-0.0387	6.2373E-01	7.8273E-01	ENSG00000258702
NA	-0.0108	6.2338E-01		ENSG00000266126
NA	-0.0353	6.2327E-01	7.8239E-01	ENSG00000288584
NA	-0.0237	6.2323E-01	7.8238E-01	ENSG00000261842
NA	-0.0059	6.2221E-01		ENSG00000237410
NA	-0.0231	6.2184E-01	7.8135E-01	ENSG00000170409
NA	-0.0164	6.2145E-01		ENSG00000204398
NA	-0.0050	6.2133E-01		ENSG00000288059

NA	-0.0062	6.2129E-01		ENSG00000228973
NA	-0.0027	6.2111E-01		ENSG00000282206
NA	-0.0081	6.2084E-01		ENSG00000286351
NA	-0.0085	6.2081E-01		ENSG00000255202
NA	-0.0111	6.2027E-01		ENSG00000155070
NA	-0.0187	6.2019E-01		ENSG00000286194
NA	-0.0112	6.2015E-01		ENSG00000227073
NA	-0.0063	6.1980E-01		ENSG00000279631
NA	-0.0145	6.1960E-01		ENSG00000225505
NA	-0.0306	6.1956E-01	7.7989E-01	ENSG00000183055
NA	-0.0252	6.1956E-01	7.7989E-01	ENSG00000279526
NA	-0.0367	6.1953E-01	7.7989E-01	ENSG00000289480
NA	-0.0221	6.1936E-01	7.7989E-01	ENSG00000265282
NA	-0.0164	6.1912E-01		ENSG00000225039
NA	-0.0060	6.1841E-01		ENSG00000241357
NA	-0.0297	6.1828E-01	7.7892E-01	ENSG00000198221
NA	-0.0185	6.1812E-01		ENSG00000228113
NA	-0.0296	6.1785E-01	7.7877E-01	ENSG00000256654
NA	-0.0341	6.1769E-01	7.7864E-01	ENSG00000272372
NA	-0.0036	6.1737E-01		ENSG00000286130
NA	-0.0165	6.1678E-01		ENSG00000237077
NA	-0.0018	6.1672E-01		ENSG00000282304
NA	-0.0069	6.1670E-01		ENSG00000289593
NA	-0.0024	6.1660E-01		ENSG00000237864
NA	-0.0416	6.1638E-01	7.7758E-01	ENSG00000133624
NA	-0.0119	6.1636E-01		ENSG00000249791
NA	-0.0126	6.1622E-01		ENSG00000240710
NA	-0.0054	6.1597E-01		ENSG00000287093
NA	-0.0114	6.1574E-01		ENSG00000287537
NA	-0.0153	6.1562E-01		ENSG00000255148
NA	-0.0265	6.1556E-01	7.7691E-01	ENSG00000258323
NA	-0.0142	6.1548E-01	7.7689E-01	ENSG00000261451
NA	-0.0073	6.1490E-01		ENSG00000272382
NA	-0.0155	6.1392E-01		ENSG00000257359
NA	-0.0151	6.1384E-01	7.7548E-01	ENSG00000237380
NA	-0.0091	6.1372E-01		ENSG00000256377
NA	-0.0076	6.1334E-01		ENSG00000141979
NA	-0.0058	6.1322E-01		ENSG00000237971
NA	-0.0087	6.1317E-01		ENSG00000273203
NA	-0.0215	6.1278E-01	7.7475E-01	ENSG00000273245
NA	-0.0136	6.1240E-01	7.7442E-01	ENSG00000286866
NA	-0.0083	6.1236E-01		ENSG00000226972
NA	-0.0419	6.1218E-01	7.7431E-01	ENSG00000224738
NA	-0.0130	6.1209E-01		ENSG00000259656
NA	-0.0117	6.1198E-01		ENSG00000236012
NA	-0.0024	6.1172E-01		ENSG00000287169
NA	-0.0125	6.1161E-01		ENSG00000239413
NA	-0.0122	6.1160E-01		ENSG00000269588
NA	-0.0092	6.1153E-01		ENSG00000279482

NA	-0.0078	6.1134E-01		ENSG00000232059
NA	-0.0137	6.1122E-01		ENSG00000254205
NA	-0.0082	6.1108E-01		ENSG00000220937
NA	-0.0059	6.1087E-01		ENSG00000242390
NA	-0.0078	6.1039E-01		ENSG00000258534
NA	-0.0028	6.1035E-01		ENSG00000287183
NA	-0.0117	6.1032E-01		ENSG00000223563
NA	-0.0430	6.1031E-01	7.7286E-01	ENSG00000265749
NA	-0.0317	6.1030E-01	7.7286E-01	ENSG00000265801
NA	-0.0080	6.1015E-01		ENSG00000236437
NA	-0.0090	6.1000E-01		ENSG00000237612
NA	-0.0167	6.0981E-01		ENSG00000249577
NA	-0.0381	6.0945E-01	7.7221E-01	ENSG00000286813
NA	-0.0435	6.0929E-01	7.7220E-01	ENSG00000244086
NA	-0.0052	6.0851E-01		ENSG00000281825
NA	-0.0115	6.0814E-01		ENSG00000218233
NA	-0.0351	6.0790E-01	7.7116E-01	ENSG00000236986
NA	-0.0082	6.0787E-01		ENSG00000271232
NA	-0.0335	6.0780E-01	7.7116E-01	ENSG00000227212
NA	-0.0424	6.0770E-01	7.7108E-01	ENSG00000272449
NA	-0.0420	6.0770E-01	7.7108E-01	ENSG00000264063
NA	-0.0129	6.0733E-01		ENSG00000248578
NA	-0.0072	6.0730E-01		ENSG00000236559
NA	-0.0200	6.0721E-01		ENSG00000268750
NA	-0.0196	6.0699E-01		ENSG00000286693
NA	-0.0426	6.0670E-01	7.7068E-01	ENSG00000264895
NA	-0.0046	6.0663E-01		ENSG00000283582
NA	-0.0063	6.0652E-01		ENSG00000251624
NA	-0.0017	6.0620E-01		ENSG00000273669
NA	-0.0204	6.0592E-01		ENSG00000269138
NA	-0.0064	6.0582E-01		ENSG00000279334
NA	-0.0141	6.0552E-01		ENSG00000226197
NA	-0.0115	6.0541E-01		ENSG00000267437
NA	-0.0234	6.0535E-01		ENSG00000257515
NA	-0.0128	6.0476E-01		ENSG00000257120
NA	-0.0384	6.0443E-01	7.6845E-01	ENSG00000289395
NA	-0.0178	6.0372E-01		ENSG00000225083
NA	-0.0457	6.0370E-01	7.6810E-01	ENSG00000241231
NA	-0.0021	6.0346E-01		ENSG00000232605
NA	-0.0124	6.0305E-01		ENSG00000235371
NA	-0.0201	6.0281E-01	7.6747E-01	ENSG00000287077
NA	-0.0424	6.0246E-01	7.6715E-01	ENSG00000289390
NA	-0.0080	6.0236E-01		ENSG00000248222
NA	-0.0076	6.0212E-01		ENSG00000249206
NA	-0.0373	6.0211E-01	7.6698E-01	ENSG00000217416
NA	-0.0050	6.0197E-01		ENSG00000255444
NA	-0.0153	6.0132E-01		ENSG00000248984
NA	-0.0305	6.0121E-01	7.6642E-01	ENSG00000287083
NA	-0.0060	6.0112E-01		ENSG00000233351

NA	-0.0089	6.0085E-01		ENSG00000279512
NA	-0.0431	6.0077E-01	7.6615E-01	ENSG00000196741
NA	-0.0242	6.0066E-01	7.6611E-01	ENSG00000279764
NA	-0.0231	6.0052E-01		ENSG00000269533
NA	-0.0017	6.0034E-01		ENSG00000225721
NA	-0.0202	6.0015E-01		ENSG00000260318
NA	-0.0101	5.9932E-01		ENSG00000288110
NA	-0.0079	5.9900E-01		ENSG00000228121
NA	-0.0138	5.9896E-01		ENSG00000269902
NA	-0.0137	5.9884E-01		ENSG00000278989
NA	-0.0122	5.9869E-01		ENSG00000249318
NA	-0.0338	5.9863E-01		ENSG00000287735
NA	-0.0240	5.9863E-01		ENSG00000271751
NA	-0.0164	5.9853E-01		ENSG00000267455
NA	-0.0312	5.9848E-01	7.6453E-01	ENSG00000228335
NA	-0.0105	5.9844E-01		ENSG00000253420
NA	-0.0208	5.9816E-01		ENSG00000287453
NA	-0.0241	5.9807E-01		ENSG00000284719
NA	-0.0157	5.9798E-01		ENSG00000244151
NA	-0.0206	5.9764E-01		ENSG00000228076
NA	-0.0119	5.9763E-01		ENSG00000266490
NA	-0.0425	5.9761E-01	7.6387E-01	ENSG00000267222
NA	-0.0438	5.9753E-01	7.6387E-01	ENSG00000213430
NA	-0.0444	5.9747E-01	7.6387E-01	ENSG00000229334
NA	-0.0091	5.9736E-01		ENSG00000182347
NA	-0.0310	5.9710E-01	7.6356E-01	ENSG00000261542
NA	-0.0084	5.9668E-01		ENSG00000223429
NA	-0.0444	5.9653E-01	7.6315E-01	ENSG00000244558
NA	-0.0109	5.9647E-01		ENSG00000266998
NA	-0.0067	5.9607E-01		ENSG00000255522
NA	-0.0091	5.9577E-01		ENSG00000274895
NA	-0.0242	5.9543E-01		ENSG00000272576
NA	-0.0148	5.9528E-01		ENSG00000265908
NA	-0.0097	5.9519E-01		ENSG00000226652
NA	-0.0437	5.9515E-01	7.6210E-01	ENSG00000108448
NA	-0.0148	5.9505E-01		ENSG00000255480
NA	-0.0082	5.9430E-01		ENSG00000289888
NA	-0.0257	5.9400E-01		ENSG00000287290
NA	-0.0248	5.9387E-01		ENSG00000283977
NA	-0.0223	5.9355E-01		ENSG00000288956
NA	-0.0379	5.9349E-01	7.6072E-01	ENSG00000288746
NA	-0.0125	5.9349E-01		ENSG00000236152
NA	-0.0155	5.9341E-01		ENSG00000263316
NA	-0.0166	5.9309E-01		ENSG00000225971
NA	-0.0224	5.9306E-01		ENSG00000264829
NA	0.0000	5.9301E-01		ENSG00000225380
NA	-0.0113	5.9297E-01		ENSG00000235772
NA	-0.0229	5.9273E-01	7.6028E-01	ENSG00000203334
NA	-0.0452	5.9267E-01	7.6028E-01	ENSG00000287853

NA	-0.0165	5.9260E-01		ENSG00000236132
NA	-0.0223	5.9217E-01	7.5986E-01	ENSG00000231073
NA	-0.0282	5.9215E-01	7.5986E-01	ENSG00000286550
NA	-0.0016	5.9164E-01		ENSG00000227475
NA	-0.0093	5.9122E-01		ENSG00000228719
NA	-0.0345	5.9087E-01	7.5893E-01	ENSG00000286724
NA	-0.0454	5.9076E-01	7.5892E-01	ENSG00000225978
NA	-0.0257	5.9040E-01		ENSG00000254710
NA	-0.0077	5.9034E-01	7.5862E-01	ENSG00000181126
NA	-0.0355	5.9034E-01	7.5862E-01	ENSG00000227160
NA	-0.0360	5.9018E-01	7.5856E-01	ENSG00000269859
NA	-0.0083	5.9017E-01		ENSG00000178107
NA	-0.0151	5.9013E-01		ENSG00000278733
NA	-0.0236	5.9008E-01		ENSG00000253205
NA	-0.0271	5.8998E-01		ENSG00000203635
NA	-0.0158	5.8975E-01		ENSG00000215198
NA	-0.0103	5.8949E-01		ENSG00000259632
NA	-0.0413	5.8941E-01	7.5790E-01	ENSG00000242553
NA	-0.0053	5.8908E-01		ENSG00000251126
NA	-0.0095	5.8901E-01		ENSG00000230104
NA	-0.0040	5.8889E-01		ENSG00000253307
NA	-0.0444	5.8879E-01	7.5748E-01	ENSG00000217643
NA	-0.0423	5.8843E-01	7.5725E-01	ENSG00000243538
NA	-0.0216	5.8842E-01		ENSG00000254990
NA	-0.0347	5.8828E-01	7.5720E-01	ENSG00000272004
NA	-0.0095	5.8805E-01		ENSG00000224906
NA	-0.0125	5.8749E-01		ENSG00000234361
NA	-0.0053	5.8747E-01		ENSG00000235432
NA	-0.0297	5.8734E-01	7.5642E-01	ENSG00000285747
NA	-0.0156	5.8704E-01		ENSG00000265094
NA	-0.0105	5.8673E-01		ENSG00000254387
NA	-0.0424	5.8668E-01	7.5605E-01	ENSG00000259319
NA	-0.0093	5.8584E-01		ENSG00000228060
NA	-0.0051	5.8555E-01		ENSG00000231937
NA	-0.0382	5.8521E-01	7.5511E-01	ENSG00000248124
NA	-0.0221	5.8517E-01		ENSG00000272472
NA	-0.0460	5.8512E-01	7.5511E-01	ENSG00000285804
NA	-0.0075	5.8492E-01		ENSG00000215311
NA	-0.0384	5.8459E-01	7.5459E-01	ENSG00000259429
NA	-0.0272	5.8409E-01		ENSG00000287843
NA	-0.0107	5.8394E-01		ENSG00000287105
NA	-0.0400	5.8381E-01	7.5412E-01	ENSG00000279339
NA	-0.0285	5.8375E-01	7.5412E-01	ENSG00000266897
NA	-0.0405	5.8318E-01	7.5372E-01	ENSG00000244730
NA	-0.0076	5.8303E-01		ENSG00000279274
NA	-0.0076	5.8303E-01		ENSG00000279115
NA	-0.0346	5.8297E-01	7.5360E-01	ENSG00000280079
NA	-0.0174	5.8258E-01		ENSG00000270001
NA	-0.0350	5.8254E-01	7.5336E-01	ENSG00000280378

NA	-0.0419	5.8237E-01	7.5322E-01	ENSG00000228519
NA	-0.0431	5.8221E-01	7.5317E-01	ENSG00000226478
NA	-0.0095	5.8190E-01		ENSG00000286950
NA	-0.0156	5.8189E-01	7.5302E-01	ENSG00000250410
NA	-0.0197	5.8164E-01		ENSG00000282915
NA	-0.0238	5.8152E-01		ENSG00000272328
NA	-0.0006	5.8145E-01		ENSG00000226491
NA	-0.0097	5.8114E-01		ENSG00000282097
NA	-0.0465	5.8083E-01	7.5220E-01	ENSG00000224043
NA	-0.0085	5.8058E-01		ENSG00000236658
NA	-0.0172	5.8022E-01		ENSG00000225099
NA	-0.0137	5.8020E-01		ENSG00000234946
NA	-0.0100	5.7996E-01		ENSG00000289931
NA	-0.0374	5.7960E-01	7.5109E-01	ENSG00000288041
NA	-0.0342	5.7950E-01	7.5104E-01	ENSG00000232150
NA	-0.0139	5.7884E-01		ENSG00000231961
NA	-0.0110	5.7855E-01		ENSG00000271992
NA	-0.0177	5.7837E-01		ENSG00000289914
NA	-0.0131	5.7832E-01		ENSG00000212541
NA	-0.0117	5.7822E-01	7.4978E-01	ENSG00000224971
NA	-0.0386	5.7816E-01	7.4978E-01	ENSG00000258033
NA	-0.0072	5.7764E-01		ENSG00000273870
NA	-0.0307	5.7693E-01	7.4898E-01	ENSG00000279031
NA	-0.0242	5.7684E-01	7.4894E-01	ENSG00000286924
NA	-0.0002	5.7650E-01		ENSG00000226220
NA	-0.0473	5.7646E-01	7.4871E-01	ENSG00000278376
NA	-0.0108	5.7616E-01		ENSG00000188223
NA	-0.0036	5.7603E-01		ENSG00000261723
NA	-0.0155	5.7563E-01		ENSG00000276651
NA	-0.0465	5.7563E-01	7.4816E-01	ENSG00000258982
NA	-0.0091	5.7562E-01		ENSG00000287085
NA	-0.0434	5.7553E-01	7.4808E-01	ENSG00000204816
NA	-0.0309	5.7553E-01	7.4808E-01	ENSG00000231369
NA	-0.0200	5.7483E-01		ENSG00000271075
NA	0.0000	5.7475E-01		ENSG00000259950
NA	-0.0189	5.7466E-01		ENSG00000287599
NA	-0.0111	5.7425E-01		ENSG00000263494
NA	-0.0462	5.7419E-01	7.4700E-01	ENSG00000233974
NA	-0.0062	5.7412E-01		ENSG00000203392
NA	-0.0131	5.7401E-01		ENSG00000257272
NA	-0.0416	5.7378E-01	7.4670E-01	ENSG00000188365
NA	-0.0118	5.7306E-01		ENSG00000232136
NA	-0.0103	5.7271E-01		ENSG00000200714
NA	-0.0417	5.7131E-01	7.4485E-01	ENSG00000258457
NA	-0.0437	5.7061E-01	7.4430E-01	ENSG00000255026
NA	-0.0178	5.7018E-01		ENSG00000272885
NA	-0.0217	5.7009E-01		ENSG00000290082
NA	-0.0477	5.7004E-01	7.4394E-01	ENSG00000228709
NA	-0.0485	5.6970E-01	7.4363E-01	ENSG00000280222

NA	-0.0253	5.6960E-01	7.4363E-01	ENSG00000258520
NA	-0.0451	5.6957E-01	7.4363E-01	ENSG00000198658
NA	-0.0080	5.6899E-01	7.4329E-01	ENSG00000288031
NA	-0.0089	5.6850E-01		ENSG00000289470
NA	-0.0088	5.6848E-01		ENSG00000274469
NA	-0.0066	5.6825E-01	7.4278E-01	ENSG00000233067
NA	-0.0107	5.6822E-01		ENSG00000269476
NA	-0.0200	5.6821E-01		ENSG00000285984
NA	-0.0432	5.6812E-01	7.4272E-01	ENSG00000261342
NA	-0.0150	5.6800E-01		ENSG00000286068
NA	-0.0030	5.6769E-01		ENSG00000261426
NA	-0.0349	5.6762E-01	7.4237E-01	ENSG00000287584
NA	-0.0270	5.6740E-01		ENSG00000227765
NA	-0.0052	5.6721E-01		ENSG00000203804
NA	-0.0078	5.6708E-01		ENSG00000253865
NA	-0.0207	5.6703E-01	7.4191E-01	ENSG00000272202
NA	-0.0337	5.6696E-01	7.4191E-01	ENSG00000224367
NA	-0.0033	5.6639E-01		ENSG00000284906
NA	-0.0272	5.6628E-01	7.4141E-01	ENSG00000287705
NA	-0.0096	5.6618E-01		ENSG00000229010
NA	-0.0111	5.6591E-01		ENSG00000263393
NA	-0.0449	5.6591E-01	7.4117E-01	ENSG00000288949
NA	-0.0232	5.6589E-01		ENSG00000232727
NA	-0.0181	5.6564E-01		ENSG00000287490
NA	-0.0087	5.6549E-01		ENSG00000280244
NA	-0.0241	5.6549E-01	7.4090E-01	ENSG00000287430
NA	-0.0111	5.6533E-01		ENSG00000231424
NA	-0.0145	5.6530E-01		ENSG00000213244
NA	-0.0177	5.6524E-01		ENSG00000255329
NA	-0.0392	5.6502E-01	7.4079E-01	ENSG00000257524
NA	-0.0018	5.6476E-01		ENSG00000236920
NA	-0.0182	5.6446E-01		ENSG00000273509
NA	-0.0159	5.6427E-01		ENSG00000216802
NA	-0.0303	5.6389E-01	7.3998E-01	ENSG00000239920
NA	-0.0043	5.6369E-01		ENSG00000259602
NA	-0.0323	5.6341E-01	7.3982E-01	ENSG00000267044
NA	-0.0177	5.6313E-01		ENSG00000267533
NA	-0.0054	5.6311E-01		ENSG00000269124
NA	-0.0299	5.6300E-01	7.3959E-01	ENSG00000229786
NA	-0.0141	5.6278E-01		ENSG00000262038
NA	-0.0119	5.6258E-01		ENSG00000267209
NA	-0.0151	5.6223E-01		ENSG00000231877
NA	-0.0154	5.6223E-01		ENSG00000262292
NA	-0.0071	5.6142E-01		ENSG00000229068
NA	-0.0080	5.6120E-01		ENSG00000285761
NA	-0.0157	5.6119E-01		ENSG00000223843
NA	-0.0248	5.6101E-01		ENSG00000178146
NA	-0.0077	5.6074E-01		ENSG00000279724
NA	-0.0122	5.6058E-01		ENSG00000227253

NA	-0.0068	5.6053E-01		ENSG00000285960
NA	-0.0124	5.6046E-01		ENSG00000260059
NA	-0.0226	5.6032E-01		ENSG00000239377
NA	-0.0138	5.6029E-01		ENSG00000251155
NA	-0.0163	5.6017E-01		ENSG00000168122
NA	-0.0412	5.6000E-01	7.3725E-01	ENSG00000235720
NA	-0.0281	5.5944E-01	7.3684E-01	ENSG00000285567
NA	-0.0073	5.5943E-01		ENSG00000279422
NA	-0.0180	5.5938E-01		ENSG00000215388
NA	-0.0009	5.5937E-01	7.3679E-01	ENSG00000288942
NA	-0.0132	5.5909E-01		ENSG00000254897
NA	-0.0210	5.5852E-01		ENSG00000232887
NA	-0.0117	5.5793E-01		ENSG00000286530
NA	-0.0093	5.5790E-01		ENSG00000232467
NA	-0.0219	5.5762E-01		ENSG00000287433
NA	-0.0078	5.5747E-01		ENSG00000223559
NA	-0.0188	5.5716E-01		ENSG00000288878
NA	-0.0369	5.5716E-01		ENSG00000226205
NA	-0.0081	5.5713E-01		ENSG00000274717
NA	-0.0339	5.5680E-01	7.3467E-01	ENSG00000232978
NA	-0.0114	5.5638E-01		ENSG00000238344
NA	-0.0107	5.5619E-01		ENSG00000278732
NA	-0.0125	5.5555E-01		ENSG00000260836
NA	-0.0274	5.5553E-01		ENSG00000286487
NA	-0.0152	5.5548E-01		ENSG00000287414
NA	-0.0089	5.5533E-01		ENSG00000257696
NA	-0.0081	5.5513E-01		ENSG00000229209
NA	-0.0139	5.5498E-01		ENSG00000273174
NA	-0.0298	5.5453E-01	7.3281E-01	ENSG00000230773
NA	-0.0353	5.5394E-01	7.3231E-01	ENSG00000280351
NA	-0.0117	5.5390E-01		ENSG00000283737
NA	-0.0088	5.5386E-01		ENSG00000254758
NA	-0.0388	5.5335E-01	7.3172E-01	ENSG00000282870
NA	-0.0178	5.5320E-01		ENSG00000255292
NA	0.0000	5.5281E-01		ENSG00000244060
NA	-0.0187	5.5244E-01		ENSG00000257210
NA	-0.0417	5.5186E-01	7.3062E-01	ENSG00000176761
NA	-0.0122	5.5141E-01		ENSG00000229044
NA	-0.0133	5.5122E-01		ENSG00000250310
NA	-0.0139	5.5092E-01		ENSG00000275532
NA	-0.0222	5.5082E-01		ENSG00000279123
NA	-0.0148	5.5066E-01		ENSG00000286279
NA	-0.0482	5.5053E-01	7.2945E-01	ENSG00000257303
NA	-0.0125	5.5044E-01		ENSG00000249996
NA	-0.0117	5.5036E-01		ENSG00000224972
NA	-0.0280	5.5026E-01		ENSG00000279554
NA	-0.0069	5.5022E-01		ENSG00000289857
NA	-0.0384	5.5018E-01	7.2920E-01	ENSG00000261996
NA	-0.0312	5.5010E-01		ENSG00000255074

NA	-0.0135	5.4959E-01		ENSG00000261435
NA	-0.0051	5.4927E-01		ENSG00000287582
NA	-0.0103	5.4871E-01		ENSG00000284603
NA	-0.0199	5.4857E-01		ENSG00000275945
NA	-0.0313	5.4848E-01	7.2795E-01	ENSG00000287921
NA	-0.0385	5.4832E-01	7.2781E-01	ENSG00000276674
NA	-0.0124	5.4823E-01		ENSG00000200685
NA	-0.0122	5.4792E-01		ENSG00000288684
NA	-0.0091	5.4710E-01		ENSG00000229484
NA	-0.0465	5.4695E-01	7.2682E-01	ENSG00000223396
NA	-0.0489	5.4686E-01	7.2674E-01	ENSG00000261338
NA	-0.0063	5.4674E-01		ENSG00000288539
NA	-0.0201	5.4629E-01	7.2645E-01	ENSG00000226038
NA	-0.0151	5.4610E-01		ENSG00000283148
NA	-0.0163	5.4600E-01		ENSG00000233144
NA	-0.0464	5.4600E-01	7.2629E-01	ENSG00000254893
NA	-0.0207	5.4596E-01		ENSG00000279228
NA	-0.0126	5.4548E-01		ENSG00000286888
NA	-0.0096	5.4540E-01		ENSG00000272164
NA	-0.0269	5.4524E-01		ENSG00000212961
NA	-0.0286	5.4516E-01	7.2565E-01	ENSG00000232750
NA	-0.0242	5.4506E-01		ENSG00000268204
NA	-0.0153	5.4469E-01		ENSG00000270409
NA	-0.0076	5.4467E-01		ENSG00000284772
NA	-0.0117	5.4467E-01		ENSG00000250455
NA	-0.0418	5.4443E-01	7.2523E-01	ENSG00000230423
NA	-0.0183	5.4425E-01		ENSG00000225345
NA	-0.0432	5.4423E-01	7.2500E-01	ENSG00000232022
NA	-0.0131	5.4407E-01		ENSG00000276768
NA	-0.0507	5.4359E-01	7.2458E-01	ENSG00000258744
NA	-0.0500	5.4335E-01	7.2458E-01	ENSG00000254966
NA	-0.0482	5.4325E-01	7.2452E-01	ENSG00000229335
NA	-0.0437	5.4316E-01	7.2443E-01	ENSG00000245662
NA	-0.0234	5.4314E-01		ENSG00000285800
NA	-0.0189	5.4310E-01		ENSG00000199530
NA	-0.0146	5.4282E-01		ENSG00000236426
NA	-0.0285	5.4259E-01		ENSG00000259109
NA	-0.0292	5.4246E-01		ENSG00000267658
NA	-0.0503	5.4118E-01	7.2309E-01	ENSG00000237753
NA	-0.0530	5.4048E-01	7.2247E-01	ENSG00000228232
NA	-0.0089	5.4006E-01		ENSG00000270259
NA	-0.0112	5.3999E-01		ENSG00000290123
NA	-0.0264	5.3984E-01		ENSG00000287971
NA	-0.0363	5.3980E-01	7.2187E-01	ENSG00000228034
NA	-0.0323	5.3944E-01	7.2155E-01	ENSG00000197083
NA	-0.0391	5.3913E-01	7.2121E-01	ENSG00000212789
NA	-0.0105	5.3907E-01		ENSG00000255397
NA	-0.0088	5.3892E-01		ENSG00000237606
NA	-0.0129	5.3882E-01		ENSG00000285641

NA	-0.0208	5.3875E-01		ENSG00000206679
NA	-0.0444	5.3832E-01	7.2078E-01	ENSG00000286548
NA	-0.0175	5.3820E-01		ENSG00000231158
NA	-0.0061	5.3815E-01		ENSG00000285977
NA	-0.0326	5.3784E-01	7.2047E-01	ENSG00000287193
NA	-0.0037	5.3777E-01		ENSG00000262519
NA	-0.0496	5.3716E-01	7.2010E-01	ENSG00000279070
NA	-0.0492	5.3659E-01	7.1963E-01	ENSG00000227632
NA	-0.0217	5.3635E-01	7.1941E-01	ENSG00000286715
NA	-0.0371	5.3617E-01	7.1921E-01	ENSG00000230805
NA	-0.0489	5.3603E-01	7.1910E-01	ENSG00000226396
NA	-0.0240	5.3590E-01		ENSG00000288010
NA	-0.0127	5.3572E-01		ENSG00000289281
NA	-0.0282	5.3519E-01	7.1834E-01	ENSG00000240132
NA	-0.0256	5.3494E-01		ENSG00000234937
NA	-0.0215	5.3488E-01		ENSG00000230216
NA	-0.0119	5.3449E-01		ENSG00000266963
NA	-0.0530	5.3434E-01	7.1782E-01	ENSG00000288398
NA	-0.0076	5.3367E-01		ENSG00000286690
NA	-0.0145	5.3329E-01		ENSG00000273489
NA	-0.0233	5.3315E-01		ENSG00000207416
NA	-0.0467	5.3247E-01	7.1608E-01	ENSG00000266651
NA	-0.0091	5.3233E-01		ENSG00000241059
NA	-0.0122	5.3227E-01		ENSG00000204776
NA	-0.0453	5.3193E-01	7.1594E-01	ENSG00000232702
NA	-0.0195	5.3182E-01		ENSG00000244479
NA	-0.0077	5.3178E-01		ENSG00000272076
NA	-0.0096	5.3132E-01		ENSG00000271659
NA	-0.0333	5.3124E-01	7.1529E-01	ENSG00000242307
NA	-0.0140	5.3119E-01		ENSG00000289730
NA	-0.0071	5.3114E-01		ENSG00000253173
NA	-0.0451	5.3111E-01	7.1526E-01	ENSG00000258815
NA	-0.0491	5.3089E-01	7.1511E-01	ENSG00000257004
NA	-0.0374	5.3058E-01	7.1495E-01	ENSG00000224731
NA	-0.0505	5.3033E-01	7.1484E-01	ENSG00000239445
NA	-0.0077	5.3024E-01		ENSG00000221879
NA	-0.0181	5.3022E-01		ENSG00000258045
NA	-0.0518	5.3009E-01	7.1460E-01	ENSG00000276075
NA	-0.0075	5.2977E-01		ENSG00000257137
NA	-0.0294	5.2970E-01		ENSG00000255317
NA	-0.0086	5.2956E-01		ENSG00000260187
NA	-0.0105	5.2952E-01		ENSG00000255475
NA	-0.0131	5.2937E-01		ENSG00000286395
NA	-0.0331	5.2893E-01	7.1354E-01	ENSG00000253392
NA	-0.0142	5.2883E-01		ENSG00000228360
NA	-0.0363	5.2862E-01	7.1322E-01	ENSG00000261238
NA	-0.0008	5.2862E-01		ENSG00000201228
NA	-0.0248	5.2857E-01		ENSG00000279845
NA	-0.0081	5.2826E-01		ENSG00000285538

NA	-0.0027	5.2778E-01		ENSG00000287297
NA	-0.0111	5.2760E-01		ENSG00000267234
NA	-0.0528	5.2733E-01	7.1233E-01	ENSG00000282572
NA	-0.0309	5.2705E-01	7.1214E-01	ENSG00000289094
NA	-0.0144	5.2702E-01		ENSG00000235286
NA	-0.0067	5.2698E-01		ENSG00000224733
NA	-0.0100	5.2673E-01		ENSG00000229950
NA	-0.0162	5.2645E-01		ENSG00000237280
NA	-0.0425	5.2575E-01	7.1100E-01	ENSG00000249916
NA	-0.0311	5.2516E-01		ENSG00000289357
NA	-0.0523	5.2507E-01	7.1052E-01	ENSG00000270111
NA	-0.0077	5.2484E-01		ENSG00000199471
NA	-0.0535	5.2446E-01	7.0997E-01	ENSG00000290073
NA	-0.0176	5.2434E-01		ENSG00000201800
NA	-0.0301	5.2428E-01		ENSG00000267612
NA	-0.0330	5.2424E-01	7.0982E-01	ENSG00000225057
NA	-0.0190	5.2416E-01		ENSG00000286887
NA	-0.0078	5.2381E-01		ENSG00000253166
NA	-0.0274	5.2370E-01		ENSG00000234534
NA	-0.0318	5.2346E-01		ENSG00000228802
NA	-0.0544	5.2319E-01	7.0909E-01	ENSG00000279348
NA	-0.0496	5.2309E-01	7.0901E-01	ENSG00000214189
NA	-0.0006	5.2298E-01		ENSG00000282527
NA	-0.0149	5.2297E-01	7.0893E-01	ENSG00000273487
NA	-0.0263	5.2291E-01	7.0893E-01	ENSG00000269151
NA	-0.0188	5.2288E-01		ENSG00000228540
NA	-0.0501	5.2282E-01	7.0893E-01	ENSG00000288928
NA	-0.0175	5.2268E-01		ENSG00000258870
NA	-0.0129	5.2251E-01		ENSG00000235461
NA	-0.0172	5.2209E-01		ENSG00000226425
NA	-0.0422	5.2194E-01	7.0854E-01	ENSG00000228988
NA	-0.0211	5.2193E-01		ENSG00000233771
NA	-0.0147	5.2173E-01		ENSG00000272567
NA	-0.0304	5.2136E-01	7.0806E-01	ENSG00000237934
NA	-0.0194	5.2127E-01		ENSG00000261837
NA	-0.0234	5.2050E-01	7.0748E-01	ENSG00000265936
NA	-0.0550	5.2049E-01	7.0748E-01	ENSG00000279691
NA	-0.0119	5.2029E-01		ENSG00000237285
NA	-0.0213	5.2021E-01		ENSG00000250234
NA	-0.0080	5.1990E-01		ENSG00000242198
NA	-0.0227	5.1972E-01		ENSG00000229046
NA	-0.0532	5.1958E-01	7.0675E-01	ENSG00000227077
NA	-0.0107	5.1903E-01		ENSG00000259531
NA	-0.0251	5.1863E-01	7.0588E-01	ENSG00000254554
NA	-0.0246	5.1830E-01		ENSG00000278886
NA	-0.0356	5.1818E-01	7.0548E-01	ENSG00000289472
NA	-0.0442	5.1748E-01	7.0490E-01	ENSG00000261916
NA	-0.0176	5.1723E-01		ENSG00000284618
NA	-0.0066	5.1720E-01		ENSG00000285707

NA	-0.0435	5.1720E-01	7.0464E-01	ENSG00000232739
NA	-0.0298	5.1714E-01	7.0459E-01	ENSG00000230199
NA	-0.0079	5.1689E-01		ENSG00000214659
NA	-0.0110	5.1612E-01		ENSG00000270666
NA	-0.0419	5.1558E-01		ENSG00000270313
NA	-0.0540	5.1539E-01	7.0307E-01	ENSG00000269388
NA	-0.0110	5.1518E-01		ENSG00000223669
NA	-0.0110	5.1498E-01		ENSG00000279022
NA	-0.0299	5.1491E-01	7.0256E-01	ENSG00000262521
NA	-0.0259	5.1475E-01		ENSG00000248873
NA	-0.0198	5.1446E-01		ENSG00000228737
NA	-0.0477	5.1393E-01	7.0170E-01	ENSG00000268218
NA	-0.0051	5.1383E-01		ENSG00000279121
NA	-0.0423	5.1343E-01	7.0124E-01	ENSG00000286684
NA	-0.0107	5.1342E-01		ENSG00000267943
NA	-0.0453	5.1334E-01	7.0116E-01	ENSG00000260496
NA	-0.0553	5.1273E-01	7.0056E-01	ENSG00000289407
NA	-0.0401	5.1272E-01	7.0056E-01	ENSG00000263648
NA	-0.0195	5.1252E-01		ENSG00000261461
NA	-0.0279	5.1244E-01		ENSG00000266903
NA	-0.0248	5.1197E-01	7.0030E-01	ENSG00000286631
NA	-0.0087	5.1195E-01		ENSG00000286076
NA	-0.0376	5.1183E-01	7.0026E-01	ENSG00000282080
NA	-0.0295	5.1171E-01		ENSG00000226661
NA	-0.0140	5.1169E-01		ENSG00000227455
NA	-0.0218	5.1164E-01		ENSG00000234766
NA	-0.0216	5.1120E-01	6.9964E-01	ENSG00000289254
NA	-0.0004	5.1107E-01	6.9953E-01	ENSG00000285928
NA	-0.0225	5.1027E-01		ENSG00000283405
NA	-0.0077	5.1005E-01		ENSG00000287444
NA	-0.0404	5.0998E-01	6.9871E-01	ENSG00000259560
NA	-0.0339	5.0992E-01	6.9867E-01	ENSG00000218890
NA	-0.0124	5.0984E-01		ENSG00000250831
NA	-0.0153	5.0949E-01		ENSG00000227619
NA	-0.0204	5.0945E-01		ENSG00000277895
NA	-0.0542	5.0907E-01	6.9801E-01	ENSG00000245248
NA	-0.0436	5.0905E-01	6.9801E-01	ENSG00000272168
NA	-0.0178	5.0858E-01		ENSG00000254500
NA	-0.0431	5.0824E-01	6.9718E-01	ENSG00000260232
NA	-0.0323	5.0819E-01		ENSG00000261270
NA	-0.0112	5.0790E-01		ENSG00000231713
NA	-0.0520	5.0711E-01	6.9630E-01	ENSG00000270084
NA	-0.0382	5.0710E-01	6.9630E-01	ENSG00000253954
NA	-0.0292	5.0672E-01		ENSG00000250101
NA	-0.0229	5.0672E-01	6.9620E-01	ENSG00000204959
NA	-0.0136	5.0653E-01		ENSG00000287746
NA	-0.0209	5.0613E-01		ENSG00000286716
NA	-0.0050	5.0604E-01		ENSG00000235486
NA	-0.0149	5.0575E-01		ENSG00000253945

NA	-0.0227	5.0564E-01		ENSG00000233828
NA	-0.0270	5.0563E-01		ENSG00000268438
NA	-0.0443	5.0549E-01	6.9527E-01	ENSG00000235010
NA	-0.0123	5.0534E-01	6.9521E-01	ENSG00000289868
NA	-0.0149	5.0532E-01		ENSG00000282865
NA	-0.0286	5.0519E-01		ENSG00000237740
NA	-0.0129	5.0491E-01		ENSG00000287303
NA	-0.0193	5.0464E-01		ENSG00000275236
NA	-0.0366	5.0394E-01		ENSG00000234710
NA	-0.0080	5.0389E-01		ENSG00000213205
NA	-0.0501	5.0358E-01	6.9395E-01	ENSG00000286069
NA	-0.0332	5.0327E-01	6.9370E-01	ENSG00000180189
NA	-0.0203	5.0193E-01		ENSG00000279091
NA	-0.0510	5.0182E-01	6.9238E-01	ENSG00000275580
NA	-0.0091	5.0172E-01		ENSG00000278785
NA	-0.0066	5.0146E-01	6.9215E-01	ENSG00000235510
NA	-0.0463	5.0087E-01	6.9169E-01	ENSG00000279912
NA	-0.0071	5.0084E-01		ENSG00000236646
NA	-0.0135	5.0083E-01		ENSG00000267215
NA	-0.0093	4.9998E-01		ENSG00000273804
NA	-0.0531	4.9971E-01	6.9078E-01	ENSG00000272769
NA	-0.0260	4.9872E-01		ENSG00000231934
NA	-0.0254	4.9847E-01	6.8954E-01	ENSG00000277968
NA	-0.0530	4.9803E-01	6.8916E-01	ENSG00000260782
NA	-0.0387	4.9777E-01	6.8908E-01	ENSG00000285841
NA	-0.0240	4.9747E-01		ENSG00000286951
NA	-0.0231	4.9739E-01	6.8871E-01	ENSG00000259694
NA	-0.0201	4.9728E-01		ENSG00000290108
NA	-0.0155	4.9722E-01		ENSG00000284969
NA	-0.0347	4.9690E-01	6.8822E-01	ENSG00000279996
NA	-0.0333	4.9665E-01	6.8809E-01	ENSG00000287887
NA	-0.0248	4.9616E-01	6.8778E-01	ENSG00000234886
NA	-0.0423	4.9552E-01	6.8724E-01	ENSG00000223525
NA	-0.0128	4.9551E-01		ENSG00000287003
NA	-0.0073	4.9537E-01		ENSG00000285681
NA	-0.0607	4.9526E-01	6.8703E-01	ENSG00000233797
NA	-0.0147	4.9498E-01		ENSG00000230166
NA	-0.0582	4.9498E-01	6.8685E-01	ENSG00000273472
NA	-0.0148	4.9492E-01		ENSG00000226801
NA	-0.0323	4.9430E-01	6.8640E-01	ENSG00000234743
NA	-0.0136	4.9398E-01		ENSG00000278475
NA	-0.0598	4.9393E-01	6.8600E-01	ENSG00000258017
NA	-0.0107	4.9392E-01		ENSG00000235077
NA	-0.0119	4.9350E-01		ENSG00000228421
NA	-0.0169	4.9336E-01		ENSG00000267581
NA	-0.0468	4.9258E-01	6.8474E-01	ENSG00000279672
NA	-0.0563	4.9250E-01	6.8470E-01	ENSG00000234345
NA	-0.0585	4.9238E-01	6.8459E-01	ENSG00000260400
NA	-0.0437	4.9216E-01	6.8438E-01	ENSG00000259321

NA	-0.0285	4.9207E-01		ENSG00000177803
NA	-0.0155	4.9186E-01		ENSG00000289278
NA	-0.0205	4.9175E-01		ENSG00000274322
NA	-0.0581	4.9165E-01	6.8387E-01	ENSG00000250896
NA	-0.0415	4.9151E-01	6.8375E-01	ENSG00000266268
NA	-0.0553	4.9147E-01	6.8374E-01	ENSG00000231754
NA	-0.0147	4.9132E-01		ENSG00000286127
NA	-0.0393	4.9120E-01	6.8348E-01	ENSG00000287671
NA	-0.0138	4.9110E-01		ENSG00000234491
NA	-0.0074	4.9104E-01		ENSG00000249669
NA	-0.0590	4.9095E-01	6.8345E-01	ENSG00000124549
NA	-0.0115	4.9079E-01		ENSG00000255790
NA	-0.0173	4.9043E-01		ENSG00000169075
NA	-0.0304	4.9021E-01		ENSG00000237734
NA	-0.0235	4.9008E-01		ENSG00000279406
NA	-0.0362	4.8981E-01	6.8255E-01	ENSG00000290083
NA	-0.0107	4.8974E-01		ENSG00000228162
NA	-0.0574	4.8972E-01	6.8252E-01	ENSG00000254884
NA	-0.0592	4.8951E-01	6.8252E-01	ENSG00000236264
NA	-0.0585	4.8946E-01	6.8252E-01	ENSG00000283064
NA	-0.0112	4.8942E-01		ENSG00000200325
NA	-0.0573	4.8933E-01	6.8246E-01	ENSG00000256176
NA	-0.0063	4.8930E-01		ENSG00000249984
NA	-0.0169	4.8922E-01		ENSG00000249894
NA	-0.0332	4.8858E-01	6.8181E-01	ENSG00000256030
NA	-0.0248	4.8813E-01		ENSG00000218428
NA	-0.0591	4.8809E-01	6.8127E-01	ENSG00000235045
NA	-0.0195	4.8806E-01		ENSG00000268140
NA	-0.0483	4.8787E-01	6.8109E-01	ENSG00000197813
NA	-0.0127	4.8769E-01		ENSG00000287637
NA	-0.0141	4.8767E-01		ENSG00000272642
NA	-0.0147	4.8716E-01		ENSG00000184084
NA	-0.0230	4.8706E-01		ENSG00000229491
NA	-0.0435	4.8688E-01	6.8026E-01	ENSG00000273650
NA	-0.0474	4.8688E-01	6.8026E-01	ENSG00000258637
NA	-0.0580	4.8678E-01	6.8026E-01	ENSG00000235386
NA	-0.0360	4.8678E-01		ENSG00000279039
NA	-0.0230	4.8667E-01	6.8026E-01	ENSG00000224856
NA	-0.0380	4.8638E-01	6.8008E-01	ENSG00000286113
NA	-0.0280	4.8622E-01		ENSG00000206844
NA	-0.0138	4.8610E-01		ENSG00000230614
NA	-0.0063	4.8586E-01		ENSG00000224172
NA	-0.0552	4.8541E-01	6.7910E-01	ENSG00000216829
NA	-0.0535	4.8516E-01	6.7891E-01	ENSG00000237758
NA	-0.0124	4.8441E-01		ENSG00000284540
NA	-0.0426	4.8436E-01	6.7822E-01	ENSG00000226851
NA	-0.0608	4.8408E-01	6.7805E-01	ENSG00000242294
NA	-0.0281	4.8394E-01		ENSG00000288234
NA	-0.0158	4.8394E-01		ENSG00000237848

NA	-0.0126	4.8386E-01		ENSG00000230886
NA	-0.0372	4.8377E-01	6.7782E-01	ENSG00000257443
NA	-0.0600	4.8369E-01	6.7778E-01	ENSG00000286456
NA	-0.0264	4.8365E-01		ENSG00000267114
NA	-0.0405	4.8354E-01	6.7765E-01	ENSG00000289171
NA	-0.0606	4.8336E-01	6.7747E-01	ENSG00000261268
NA	-0.0449	4.8334E-01	6.7747E-01	ENSG00000260290
NA	-0.0102	4.8331E-01		ENSG00000279235
NA	-0.0139	4.8306E-01		ENSG00000287309
NA	-0.0551	4.8278E-01	6.7712E-01	ENSG00000249626
NA	-0.0311	4.8273E-01		ENSG00000287916
NA	-0.0092	4.8268E-01		ENSG00000289964
NA	-0.0481	4.8231E-01	6.7666E-01	ENSG00000258944
NA	-0.0573	4.8228E-01	6.7666E-01	ENSG00000285531
NA	-0.0098	4.8190E-01		ENSG00000218521
NA	-0.0574	4.8170E-01	6.7623E-01	ENSG00000263847
NA	-0.0033	4.8156E-01		ENSG00000272823
NA	-0.0161	4.8150E-01		ENSG00000260240
NA	-0.0250	4.8142E-01	6.7598E-01	ENSG00000249412
NA	-0.0252	4.8126E-01	6.7584E-01	ENSG00000280303
NA	-0.0092	4.8099E-01		ENSG00000204780
NA	-0.0186	4.8081E-01		ENSG00000275894
NA	-0.0172	4.8068E-01		ENSG00000279590
NA	-0.0498	4.8014E-01	6.7515E-01	ENSG00000232043
NA	-0.0130	4.8006E-01		ENSG00000277740
NA	-0.0094	4.7985E-01		ENSG00000207092
NA	-0.0398	4.7983E-01	6.7483E-01	ENSG00000280266
NA	-0.0147	4.7975E-01		ENSG00000224427
NA	-0.0079	4.7968E-01		ENSG00000228139
NA	-0.0126	4.7961E-01		ENSG00000285602
NA	-0.0509	4.7943E-01	6.7454E-01	ENSG00000253284
NA	-0.0108	4.7929E-01		ENSG00000286879
NA	-0.0359	4.7918E-01	6.7438E-01	ENSG00000289447
NA	-0.0067	4.7905E-01		ENSG00000228236
NA	-0.0539	4.7887E-01	6.7416E-01	ENSG00000280365
NA	-0.0555	4.7836E-01	6.7365E-01	ENSG00000267491
NA	-0.0229	4.7800E-01		ENSG00000271361
NA	-0.0600	4.7769E-01	6.7305E-01	ENSG00000233830
NA	-0.0608	4.7729E-01	6.7269E-01	ENSG00000289353
NA	-0.0072	4.7726E-01		ENSG00000288986
NA	-0.0254	4.7692E-01		ENSG00000260425
NA	-0.0012	4.7678E-01	6.7224E-01	ENSG00000258605
NA	-0.0297	4.7676E-01		ENSG00000234076
NA	-0.0584	4.7617E-01	6.7157E-01	ENSG00000235587
NA	-0.0365	4.7609E-01	6.7153E-01	ENSG00000229782
NA	-0.0552	4.7580E-01	6.7134E-01	ENSG00000213178
NA	-0.0242	4.7579E-01		ENSG00000289180
NA	-0.0126	4.7543E-01		ENSG00000260185
NA	-0.0159	4.7541E-01		ENSG00000271627

NA	-0.0133	4.7539E-01		ENSG00000265096
NA	-0.0081	4.7491E-01		ENSG00000287196
NA	-0.0356	4.7433E-01	6.7008E-01	ENSG00000269296
NA	-0.0475	4.7431E-01	6.7008E-01	ENSG00000254141
NA	-0.0292	4.7386E-01		ENSG00000266993
NA	-0.0406	4.7359E-01	6.6951E-01	ENSG00000243659
NA	-0.0619	4.7329E-01	6.6929E-01	ENSG00000207425
NA	-0.0173	4.7263E-01		ENSG00000279273
NA	-0.0160	4.7262E-01		ENSG00000243008
NA	-0.0103	4.7185E-01		ENSG00000287494
NA	-0.0246	4.7117E-01		ENSG00000284196
NA	-0.0414	4.7048E-01	6.6672E-01	ENSG00000269961
NA	-0.0329	4.7040E-01		ENSG00000236456
NA	-0.0069	4.7026E-01		ENSG00000184423
NA	-0.0156	4.7018E-01		ENSG00000242156
NA	-0.0179	4.7013E-01		ENSG00000261810
NA	-0.0477	4.7009E-01	6.6645E-01	ENSG00000267799
NA	-0.0257	4.6978E-01		ENSG00000278918
NA	-0.0612	4.6974E-01	6.6609E-01	ENSG00000189423
NA	-0.0100	4.6968E-01		ENSG00000261826
NA	-0.0581	4.6965E-01	6.6603E-01	ENSG00000254719
NA	-0.0122	4.6950E-01		ENSG00000274902
NA	-0.0329	4.6926E-01		ENSG00000290005
NA	-0.0243	4.6900E-01		ENSG00000237158
NA	-0.0102	4.6826E-01		ENSG00000204584
NA	-0.0505	4.6820E-01	6.6478E-01	ENSG00000238072
NA	-0.0245	4.6815E-01		ENSG00000224829
NA	-0.0119	4.6794E-01		ENSG00000224834
NA	-0.0183	4.6776E-01		ENSG00000261072
NA	-0.0569	4.6776E-01	6.6439E-01	ENSG00000284630
NA	-0.0477	4.6743E-01	6.6413E-01	ENSG00000253454
NA	-0.0100	4.6732E-01		ENSG00000275106
NA	-0.0253	4.6650E-01		ENSG00000258026
NA	-0.0189	4.6625E-01		ENSG00000280023
NA	-0.0346	4.6624E-01	6.6276E-01	ENSG00000286634
NA	-0.0633	4.6622E-01	6.6276E-01	ENSG00000224505
NA	-0.0137	4.6609E-01		ENSG00000277651
NA	-0.0090	4.6597E-01		ENSG00000283123
NA	-0.0162	4.6582E-01		ENSG00000289082
NA	-0.0350	4.6582E-01	6.6245E-01	ENSG00000234604
NA	-0.0204	4.6550E-01		ENSG00000279925
NA	-0.0140	4.6500E-01		ENSG00000289292
NA	-0.0781	4.6494E-01	6.6155E-01	ENSG00000259570
NA	-0.0377	4.6469E-01	6.6150E-01	ENSG00000224635
NA	-0.0389	4.6461E-01	6.6148E-01	ENSG00000284747
NA	-0.0491	4.6370E-01	6.6072E-01	ENSG00000269243
NA	-0.0604	4.6357E-01	6.6061E-01	ENSG00000018607
NA	-0.0194	4.6325E-01		ENSG00000270993
NA	-0.0462	4.6322E-01	6.6039E-01	ENSG00000283427

NA	-0.0431	4.6308E-01	6.6033E-01	ENSG00000236390
NA	-0.0457	4.6249E-01	6.5956E-01	ENSG00000286110
NA	-0.0552	4.6195E-01	6.5902E-01	ENSG00000231341
NA	-0.0201	4.6172E-01		ENSG00000223815
NA	-0.0180	4.6170E-01		ENSG00000202479
NA	-0.0079	4.6161E-01		ENSG00000270986
NA	-0.0168	4.6159E-01		ENSG00000259415
NA	-0.0618	4.6155E-01	6.5878E-01	ENSG00000277437
NA	-0.0163	4.6151E-01		ENSG00000223849
NA	-0.0571	4.6146E-01	6.5872E-01	ENSG00000288997
NA	-0.0449	4.6145E-01	6.5872E-01	ENSG00000284728
NA	-0.0099	4.6131E-01		ENSG00000243349
NA	-0.0468	4.6121E-01	6.5852E-01	ENSG00000287125
NA	-0.0127	4.6112E-01		ENSG00000215284
NA	-0.0200	4.6041E-01		ENSG00000256211
NA	-0.0629	4.6040E-01	6.5803E-01	ENSG00000286907
NA	-0.0014	4.6036E-01		ENSG00000286089
NA	-0.0219	4.6006E-01		ENSG00000219930
NA	-0.0069	4.5986E-01		ENSG00000287959
NA	-0.0072	4.5966E-01		ENSG00000215811
NA	-0.0596	4.5928E-01	6.5705E-01	ENSG00000226121
NA	-0.0642	4.5885E-01	6.5669E-01	ENSG00000287829
NA	-0.0399	4.5884E-01	6.5669E-01	ENSG00000233299
NA	-0.0181	4.5868E-01		ENSG00000232756
NA	-0.0438	4.5859E-01	6.5641E-01	ENSG00000278000
NA	-0.0131	4.5836E-01		ENSG00000253122
NA	-0.0256	4.5825E-01		ENSG00000285870
NA	-0.0161	4.5769E-01		ENSG00000259380
NA	-0.0541	4.5753E-01	6.5561E-01	ENSG00000223660
NA	-0.0130	4.5737E-01		ENSG00000290066
NA	-0.0311	4.5724E-01		ENSG00000258171
NA	-0.0113	4.5683E-01		ENSG00000217897
NA	-0.0093	4.5666E-01		ENSG00000228938
NA	-0.0532	4.5629E-01	6.5458E-01	ENSG00000288772
NA	-0.0308	4.5628E-01		ENSG00000280233
NA	-0.0150	4.5588E-01		ENSG00000214244
NA	-0.0193	4.5539E-01		ENSG00000267646
NA	-0.0147	4.5514E-01		ENSG00000233025
NA	-0.0567	4.5497E-01	6.5339E-01	ENSG00000255085
NA	-0.0641	4.5490E-01	6.5332E-01	ENSG00000259448
NA	-0.0601	4.5403E-01	6.5254E-01	ENSG00000288831
NA	-0.0140	4.5353E-01		ENSG00000249169
NA	-0.0233	4.5347E-01		ENSG00000202417
NA	-0.0127	4.5315E-01		ENSG00000264956
NA	-0.0091	4.5311E-01		ENSG00000277690
NA	-0.0383	4.5311E-01	6.5178E-01	ENSG00000289468
NA	-0.0475	4.5311E-01	6.5178E-01	ENSG00000174715
NA	-0.0417	4.5293E-01	6.5168E-01	ENSG00000254450
NA	-0.0619	4.5227E-01	6.5119E-01	ENSG00000212994

NA	-0.0312	4.5179E-01		ENSG00000250238
NA	-0.0181	4.5164E-01		ENSG00000288933
NA	-0.0166	4.5159E-01		ENSG00000243974
NA	-0.0650	4.5130E-01	6.5024E-01	ENSG00000248503
NA	-0.0130	4.5103E-01		ENSG00000261393
NA	-0.0485	4.5094E-01	6.4998E-01	ENSG00000287757
NA	-0.0480	4.5088E-01	6.4997E-01	ENSG00000279748
NA	-0.0134	4.5081E-01		ENSG00000286393
NA	-0.0402	4.5077E-01	6.4991E-01	ENSG00000277491
NA	-0.0213	4.5069E-01		ENSG00000236324
NA	-0.0635	4.5063E-01	6.4981E-01	ENSG00000184612
NA	-0.0460	4.5042E-01	6.4967E-01	ENSG00000288096
NA	-0.0406	4.5038E-01	6.4965E-01	ENSG00000261124
NA	-0.0076	4.4881E-01		ENSG00000271947
NA	-0.0622	4.4852E-01	6.4766E-01	ENSG00000267809
NA	-0.0102	4.4815E-01		ENSG00000242440
NA	-0.0265	4.4799E-01		ENSG00000212440
NA	-0.0651	4.4767E-01	6.4701E-01	ENSG00000237729
NA	-0.0220	4.4762E-01		ENSG00000223749
NA	-0.0523	4.4695E-01	6.4644E-01	ENSG00000224216
NA	-0.0208	4.4639E-01		ENSG00000278673
NA	-0.0639	4.4518E-01	6.4418E-01	ENSG00000262420
NA	-0.0207	4.4479E-01		ENSG00000269543
NA	-0.0445	4.4449E-01	6.4339E-01	ENSG00000239917
NA	-0.0353	4.4444E-01	6.4339E-01	ENSG00000228251
NA	-0.0632	4.4435E-01	6.4336E-01	ENSG00000258044
NA	-0.0614	4.4414E-01	6.4312E-01	ENSG00000284657
NA	-0.0458	4.4390E-01	6.4294E-01	ENSG00000258256
NA	-0.0083	4.4363E-01		ENSG00000285128
NA	-0.0109	4.4331E-01		ENSG00000259676
NA	-0.0139	4.4186E-01		ENSG00000250332
NA	-0.0594	4.4184E-01	6.4129E-01	ENSG00000289311
NA	-0.0249	4.4167E-01		ENSG00000232085
NA	-0.0466	4.4162E-01	6.4110E-01	ENSG00000282995
NA	-0.0661	4.4129E-01	6.4093E-01	ENSG00000275454
NA	-0.0566	4.4119E-01	6.4092E-01	ENSG00000259238
NA	-0.0585	4.4114E-01	6.4089E-01	ENSG00000267390
NA	-0.0668	4.4109E-01	6.4089E-01	ENSG00000285130
NA	-0.0150	4.4103E-01		ENSG00000256646
NA	-0.0201	4.4098E-01		ENSG00000271192
NA	-0.0534	4.4079E-01	6.4069E-01	ENSG00000275131
NA	-0.0230	4.4055E-01		ENSG00000272334
NA	-0.0167	4.4042E-01		ENSG00000271707
NA	-0.0078	4.4017E-01		ENSG00000229019
NA	-0.0397	4.4014E-01	6.4009E-01	ENSG00000259644
NA	-0.0476	4.3975E-01	6.3975E-01	ENSG00000218537
NA	-0.0090	4.3970E-01		ENSG00000262140
NA	-0.0462	4.3951E-01	6.3954E-01	ENSG00000268015
NA	-0.0236	4.3943E-01		ENSG00000273154

NA	-0.0659	4.3923E-01	6.3934E-01	ENSG00000227615
NA	-0.0545	4.3917E-01	6.3928E-01	ENSG00000289956
NA	-0.0478	4.3913E-01	6.3927E-01	ENSG00000286018
NA	-0.0405	4.3880E-01	6.3889E-01	ENSG00000256116
NA	-0.0627	4.3845E-01	6.3860E-01	ENSG00000275807
NA	-0.0133	4.3827E-01		ENSG00000230618
NA	-0.0387	4.3820E-01	6.3860E-01	ENSG00000289303
NA	-0.0337	4.3774E-01		ENSG00000236762
NA	-0.0326	4.3762E-01		ENSG00000289164
NA	-0.0417	4.3724E-01	6.3776E-01	ENSG00000223723
NA	-0.0147	4.3709E-01		ENSG00000256826
NA	-0.0303	4.3698E-01		ENSG00000232437
NA	-0.0501	4.3692E-01	6.3756E-01	ENSG00000268163
NA	-0.0269	4.3691E-01		ENSG00000279817
NA	-0.0552	4.3685E-01	6.3748E-01	ENSG00000259659
NA	-0.0350	4.3679E-01	6.3744E-01	ENSG00000225984
NA	-0.0690	4.3673E-01	6.3739E-01	ENSG00000244245
NA	-0.0578	4.3620E-01	6.3708E-01	ENSG00000287275
NA	-0.0437	4.3556E-01	6.3656E-01	ENSG00000270184
NA	-0.0188	4.3504E-01		ENSG00000214976
NA	-0.0055	4.3494E-01		ENSG00000249258
NA	-0.0481	4.3435E-01	6.3543E-01	ENSG00000276573
NA	-0.0380	4.3435E-01		ENSG00000289969
NA	-0.0117	4.3428E-01		ENSG00000238018
NA	-0.0177	4.3424E-01		ENSG00000268992
NA	-0.0196	4.3376E-01		ENSG00000261582
NA	-0.0229	4.3375E-01		ENSG00000271980
NA	-0.0249	4.3320E-01		ENSG00000260509
NA	-0.0117	4.3307E-01		ENSG00000229120
NA	-0.0167	4.3306E-01		ENSG00000226971
NA	-0.0191	4.3285E-01		ENSG00000286881
NA	-0.0412	4.3284E-01	6.3411E-01	ENSG00000256263
NA	-0.0551	4.3258E-01	6.3388E-01	ENSG00000281327
NA	-0.0666	4.3236E-01	6.3368E-01	ENSG00000228925
NA	-0.0391	4.3216E-01	6.3357E-01	ENSG00000278532
NA	-0.0188	4.3194E-01		ENSG00000228838
NA	-0.0131	4.3188E-01	6.3331E-01	ENSG00000234617
NA	-0.0191	4.3160E-01		ENSG00000275512
NA	-0.0319	4.3152E-01		ENSG00000233980
NA	-0.0240	4.3138E-01	6.3292E-01	ENSG00000286883
NA	-0.0155	4.3123E-01		ENSG00000266086
NA	-0.0151	4.3119E-01		ENSG00000235119
NA	-0.0049	4.3060E-01		ENSG00000260042
NA	-0.0411	4.3055E-01		ENSG00000262039
NA	-0.0103	4.3051E-01		ENSG00000256034
NA	-0.0159	4.3035E-01		ENSG00000256149
NA	-0.0613	4.3027E-01	6.3189E-01	ENSG00000235893
NA	-0.0236	4.3006E-01		ENSG00000260282
NA	-0.0627	4.2991E-01	6.3159E-01	ENSG00000124224

NA	-0.0135	4.2981E-01		ENSG00000234061
NA	-0.0206	4.2967E-01		ENSG00000235209
NA	-0.0539	4.2966E-01	6.3126E-01	ENSG00000278934
NA	-0.0691	4.2903E-01	6.3074E-01	ENSG00000288632
NA	-0.0135	4.2892E-01		ENSG00000262959
NA	-0.0073	4.2880E-01		ENSG00000287143
NA	-0.0068	4.2873E-01		ENSG00000230287
NA	-0.0116	4.2867E-01		ENSG00000260337
NA	-0.0311	4.2811E-01		ENSG00000222881
NA	-0.0262	4.2810E-01		ENSG00000227726
NA	-0.0062	4.2781E-01		ENSG00000279914
NA	-0.0701	4.2772E-01	6.2965E-01	ENSG00000222043
NA	-0.0572	4.2738E-01	6.2930E-01	ENSG00000283217
NA	-0.0087	4.2719E-01		ENSG00000267402
NA	-0.0420	4.2707E-01	6.2907E-01	ENSG00000244198
NA	-0.0221	4.2682E-01	6.2892E-01	ENSG00000270808
NA	-0.0165	4.2673E-01		ENSG00000237666
NA	-0.0585	4.2673E-01	6.2887E-01	ENSG00000286618
NA	-0.0156	4.2629E-01		ENSG00000290024
NA	-0.0693	4.2611E-01	6.2833E-01	ENSG00000256968
NA	-0.0617	4.2607E-01	6.2831E-01	ENSG00000277287
NA	-0.0628	4.2562E-01	6.2788E-01	ENSG00000213189
NA	-0.0087	4.2522E-01		ENSG00000232542
NA	-0.0440	4.2474E-01	6.2715E-01	ENSG00000275811
NA	-0.0190	4.2469E-01		ENSG00000276166
NA	-0.0198	4.2448E-01		ENSG00000254143
NA	-0.0098	4.2420E-01		ENSG00000285897
NA	-0.0154	4.2404E-01		ENSG00000261555
NA	-0.0488	4.2330E-01	6.2590E-01	ENSG00000254912
NA	-0.0433	4.2249E-01	6.2521E-01	ENSG00000269535
NA	-0.0358	4.2197E-01	6.2460E-01	ENSG00000211513
NA	-0.0145	4.2180E-01		ENSG00000270115
NA	-0.0151	4.2169E-01		ENSG00000199890
NA	-0.0164	4.2112E-01		ENSG00000287741
NA	-0.0633	4.2100E-01	6.2383E-01	ENSG00000248127
NA	-0.0459	4.2050E-01	6.2346E-01	ENSG00000214765
NA	-0.0712	4.1961E-01	6.2234E-01	ENSG00000230562
NA	-0.0133	4.1926E-01		ENSG00000227742
NA	-0.0214	4.1926E-01		ENSG00000244196
NA	-0.0315	4.1919E-01		ENSG00000264834
NA	-0.0693	4.1877E-01	6.2187E-01	ENSG00000246451
NA	-0.0210	4.1872E-01		ENSG00000187172
NA	-0.0165	4.1867E-01		ENSG00000262703
NA	-0.0103	4.1848E-01		ENSG00000224936
NA	-0.0556	4.1815E-01	6.2141E-01	ENSG00000261002
NA	-0.0124	4.1741E-01		ENSG00000266397
NA	-0.0157	4.1728E-01		ENSG00000270482
NA	-0.0667	4.1714E-01	6.2053E-01	ENSG00000228415
NA	-0.0174	4.1692E-01		ENSG00000280277

NA	-0.0095	4.1682E-01		ENSG00000254949
NA	-0.0660	4.1677E-01	6.2024E-01	ENSG00000217702
NA	-0.0330	4.1626E-01		ENSG00000232667
NA	-0.0133	4.1609E-01		ENSG00000285614
NA	-0.0465	4.1576E-01	6.1924E-01	ENSG00000244380
NA	-0.0174	4.1574E-01		ENSG00000224685
NA	-0.0417	4.1551E-01		ENSG00000289229
NA	-0.0140	4.1508E-01		ENSG00000275834
NA	-0.0103	4.1488E-01		ENSG00000236187
NA	-0.0144	4.1447E-01	6.1802E-01	ENSG00000232710
NA	-0.0297	4.1373E-01		ENSG00000188828
NA	-0.0232	4.1357E-01		ENSG00000272657
NA	-0.0605	4.1317E-01	6.1676E-01	ENSG00000270177
NA	-0.0637	4.1313E-01	6.1674E-01	ENSG00000261118
NA	-0.0373	4.1305E-01		ENSG00000266839
NA	-0.0173	4.1292E-01		ENSG00000260810
NA	-0.0693	4.1268E-01	6.1633E-01	ENSG00000279693
NA	-0.0546	4.1226E-01	6.1603E-01	ENSG00000273254
NA	-0.0472	4.1222E-01	6.1603E-01	ENSG00000280149
NA	-0.0285	4.1210E-01		ENSG00000226849
NA	-0.0741	4.1149E-01	6.1553E-01	ENSG00000265342
NA	-0.0414	4.1110E-01	6.1524E-01	ENSG00000279513
NA	-0.0524	4.1101E-01	6.1514E-01	ENSG00000279286
NA	-0.0712	4.1082E-01	6.1496E-01	ENSG00000285755
NA	-0.0160	4.1054E-01		ENSG00000285882
NA	-0.0111	4.1050E-01		ENSG00000272703
NA	-0.0390	4.1032E-01		ENSG00000250541
NA	-0.0294	4.1016E-01		ENSG00000261765
NA	-0.0159	4.0963E-01		ENSG00000228901
NA	-0.0215	4.0957E-01		ENSG00000230500
NA	-0.0268	4.0942E-01		ENSG00000234801
NA	-0.0301	4.0917E-01		ENSG00000237456
NA	-0.0232	4.0882E-01		ENSG00000287712
NA	-0.0151	4.0862E-01		ENSG00000287370
NA	-0.0308	4.0814E-01	6.1237E-01	ENSG00000234297
NA	-0.0639	4.0806E-01	6.1236E-01	ENSG00000243802
NA	-0.0474	4.0793E-01	6.1225E-01	ENSG00000260784
NA	-0.0623	4.0787E-01	6.1222E-01	ENSG00000275120
NA	-0.0097	4.0773E-01		ENSG00000228711
NA	-0.0550	4.0763E-01	6.1198E-01	ENSG00000285257
NA	-0.0210	4.0759E-01		ENSG00000254236
NA	-0.0255	4.0749E-01		ENSG00000206702
NA	-0.0716	4.0745E-01	6.1186E-01	ENSG00000231686
NA	-0.0318	4.0719E-01		ENSG00000286877
NA	-0.0587	4.0705E-01	6.1153E-01	ENSG00000205579
NA	-0.0191	4.0673E-01		ENSG00000260621
NA	-0.0197	4.0661E-01		ENSG00000270405
NA	-0.0665	4.0644E-01	6.1102E-01	ENSG00000229206
NA	-0.0195	4.0599E-01		ENSG00000254170

NA	-0.0237	4.0590E-01		ENSG00000271215
NA	-0.0294	4.0579E-01		ENSG00000286676
NA	-0.0067	4.0538E-01		ENSG00000286962
NA	-0.0166	4.0504E-01		ENSG00000285978
NA	-0.0113	4.0419E-01		ENSG00000236665
NA	-0.0727	4.0403E-01	6.0858E-01	ENSG00000241556
NA	-0.0161	4.0397E-01		ENSG00000270385
NA	-0.0191	4.0379E-01		ENSG00000277572
NA	-0.0064	4.0371E-01		ENSG00000233951
NA	-0.0056	4.0370E-01		ENSG00000266378
NA	-0.0098	4.0331E-01		ENSG00000274686
NA	-0.0714	4.0245E-01	6.0700E-01	ENSG00000236200
NA	-0.0329	4.0243E-01		ENSG00000287666
NA	-0.0207	4.0220E-01		ENSG00000260011
NA	-0.0343	4.0195E-01		ENSG00000267197
NA	-0.0386	4.0191E-01	6.0655E-01	ENSG00000214593
NA	-0.0359	4.0166E-01		ENSG00000271214
NA	-0.0654	4.0160E-01	6.0655E-01	ENSG00000267734
NA	-0.0492	4.0146E-01	6.0651E-01	ENSG00000226699
NA	-0.0211	4.0139E-01	6.0651E-01	ENSG00000230626
NA	-0.0140	4.0130E-01		ENSG00000215319
NA	-0.0752	4.0125E-01	6.0635E-01	ENSG00000274225
NA	-0.0174	4.0109E-01		ENSG00000255296
NA	-0.0123	4.0091E-01		ENSG00000280395
NA	-0.0164	4.0087E-01		ENSG00000226548
NA	-0.0619	4.0070E-01	6.0601E-01	ENSG00000273733
NA	-0.0273	4.0038E-01		ENSG00000282024
NA	-0.0401	4.0021E-01		ENSG00000269950
NA	-0.0252	4.0006E-01		ENSG00000250280
NA	-0.0233	3.9933E-01		ENSG00000229057
NA	-0.0596	3.9860E-01	6.0421E-01	ENSG00000228551
NA	-0.0183	3.9846E-01		ENSG00000271266
NA	-0.0076	3.9844E-01		ENSG00000233330
NA	-0.0165	3.9835E-01		ENSG00000252822
NA	-0.0217	3.9824E-01		ENSG00000254314
NA	-0.0569	3.9802E-01	6.0363E-01	ENSG00000253586
NA	-0.0503	3.9793E-01	6.0359E-01	ENSG00000278999
NA	-0.0406	3.9773E-01	6.0352E-01	ENSG00000287120
NA	-0.0268	3.9772E-01		ENSG00000266936
NA	-0.0733	3.9705E-01	6.0286E-01	ENSG00000224861
NA	-0.0721	3.9702E-01	6.0286E-01	ENSG00000236556
NA	-0.0244	3.9696E-01		ENSG00000227417
NA	-0.0154	3.9691E-01		ENSG00000268510
NA	-0.0232	3.9674E-01	6.0261E-01	ENSG00000235297
NA	-0.0283	3.9662E-01		ENSG00000287866
NA	-0.0497	3.9592E-01	6.0197E-01	ENSG00000232823
NA	-0.0530	3.9590E-01	6.0197E-01	ENSG00000229294
NA	-0.0170	3.9547E-01		ENSG00000234660
NA	-0.0361	3.9545E-01		ENSG00000287729

NA	-0.0223	3.9489E-01		ENSG00000223828
NA	-0.0184	3.9479E-01		ENSG00000277135
NA	-0.0596	3.9448E-01	6.0076E-01	ENSG00000224208
NA	-0.0305	3.9435E-01		ENSG00000231967
NA	-0.0344	3.9403E-01	6.0049E-01	ENSG00000213866
NA	-0.0577	3.9399E-01	6.0048E-01	ENSG00000251141
NA	-0.0272	3.9386E-01		ENSG00000249286
NA	-0.0124	3.9379E-01		ENSG00000227590
NA	-0.0390	3.9366E-01	6.0013E-01	ENSG00000289307
NA	-0.0302	3.9342E-01	5.9997E-01	ENSG00000232949
NA	-0.0456	3.9334E-01	5.9997E-01	ENSG00000259732
NA	-0.0129	3.9328E-01		ENSG00000287114
NA	-0.0154	3.9288E-01		ENSG00000279536
NA	-0.0202	3.9276E-01		ENSG00000286868
NA	-0.0587	3.9270E-01	5.9947E-01	ENSG00000279762
NA	-0.0304	3.9269E-01	5.9947E-01	ENSG00000214067
NA	-0.0305	3.9223E-01		ENSG00000286869
NA	-0.0502	3.9217E-01	5.9911E-01	ENSG00000272330
NA	-0.0371	3.9214E-01	5.9911E-01	ENSG00000268987
NA	-0.0455	3.9196E-01	5.9898E-01	ENSG00000249650
NA	-0.0734	3.9175E-01	5.9877E-01	ENSG00000223760
NA	-0.0154	3.9126E-01		ENSG00000256720
NA	-0.0169	3.9106E-01		ENSG00000260395
NA	-0.0173	3.9085E-01		ENSG00000253688
NA	-0.0660	3.9058E-01	5.9778E-01	ENSG00000264513
NA	-0.0176	3.9040E-01		ENSG00000177725
NA	-0.0159	3.9034E-01		ENSG00000203411
NA	-0.0155	3.9031E-01		ENSG00000279606
NA	-0.0248	3.9031E-01		ENSG00000219553
NA	-0.0059	3.9013E-01		ENSG00000280177
NA	-0.0556	3.8976E-01	5.9722E-01	ENSG00000279423
NA	-0.0171	3.8892E-01		ENSG00000224830
NA	-0.0753	3.8876E-01	5.9604E-01	ENSG00000260488
NA	-0.0725	3.8859E-01	5.9594E-01	ENSG00000271943
NA	-0.0498	3.8854E-01	5.9594E-01	ENSG00000228674
NA	-0.0306	3.8830E-01		ENSG00000232828
NA	-0.0160	3.8828E-01		ENSG00000256904
NA	-0.0508	3.8825E-01	5.9576E-01	ENSG00000231201
NA	-0.0155	3.8819E-01		ENSG00000269898
NA	-0.0756	3.8793E-01	5.9549E-01	ENSG00000231079
NA	-0.0378	3.8789E-01		ENSG00000232460
NA	-0.0072	3.8787E-01		ENSG00000251127
NA	-0.0179	3.8737E-01		ENSG00000276633
NA	-0.0622	3.8695E-01	5.9468E-01	ENSG00000285867
NA	-0.0259	3.8641E-01		ENSG00000261832
NA	-0.0165	3.8610E-01		ENSG00000285668
NA	-0.0546	3.8608E-01	5.9380E-01	ENSG00000230068
NA	-0.0618	3.8438E-01	5.9232E-01	ENSG00000204072
NA	-0.0737	3.8423E-01	5.9219E-01	ENSG00000249249

NA	-0.0743	3.8414E-01	5.9211E-01	ENSG00000248334
NA	-0.0536	3.8410E-01	5.9211E-01	ENSG00000237001
NA	-0.0734	3.8395E-01	5.9203E-01	ENSG00000271976
NA	-0.0512	3.8389E-01	5.9203E-01	ENSG00000275413
NA	-0.0685	3.8382E-01	5.9201E-01	ENSG00000224086
NA	-0.0210	3.8378E-01		ENSG00000256218
NA	-0.0100	3.8322E-01		ENSG00000225606
NA	-0.0212	3.8293E-01		ENSG00000225711
NA	-0.0453	3.8242E-01	5.9066E-01	ENSG00000200059
NA	-0.0586	3.8217E-01	5.9032E-01	ENSG00000250299
NA	-0.0146	3.8215E-01		ENSG00000232413
NA	-0.0266	3.8197E-01		ENSG00000272282
NA	-0.0263	3.8140E-01		ENSG00000267385
NA	-0.0697	3.8111E-01	5.8931E-01	ENSG00000255933
NA	-0.0225	3.8058E-01		ENSG00000261067
NA	-0.0168	3.8034E-01		ENSG00000283445
NA	-0.0325	3.8033E-01		ENSG00000258623
NA	-0.0638	3.8009E-01	5.8854E-01	ENSG00000248015
NA	-0.0206	3.7991E-01		ENSG00000283914
NA	-0.0107	3.7960E-01		ENSG00000254590
NA	-0.0332	3.7931E-01		ENSG00000254887
NA	-0.0328	3.7928E-01		ENSG00000225282
NA	-0.0247	3.7916E-01		ENSG00000250329
NA	-0.0200	3.7881E-01		ENSG00000241228
NA	-0.0185	3.7867E-01		ENSG00000275286
NA	-0.0153	3.7864E-01		ENSG00000273512
NA	-0.0175	3.7855E-01		ENSG00000248634
NA	-0.0283	3.7841E-01		ENSG00000231531
NA	-0.0336	3.7812E-01		ENSG00000228510
NA	-0.0291	3.7799E-01		ENSG00000254373
NA	-0.0390	3.7797E-01		ENSG00000256325
NA	-0.0312	3.7784E-01		ENSG00000261018
NA	-0.0621	3.7780E-01	5.8634E-01	ENSG00000272405
NA	-0.0151	3.7762E-01		ENSG00000253384
NA	-0.0561	3.7724E-01	5.8572E-01	ENSG00000260651
NA	-0.0135	3.7710E-01		ENSG00000227747
NA	-0.0223	3.7674E-01		ENSG00000288106
NA	-0.0158	3.7661E-01		ENSG00000213060
NA	-0.0082	3.7627E-01	5.8481E-01	ENSG00000228118
NA	-0.0598	3.7621E-01	5.8475E-01	ENSG00000268070
NA	-0.0202	3.7612E-01		ENSG00000287238
NA	-0.0274	3.7604E-01		ENSG00000233998
NA	-0.0508	3.7597E-01	5.8453E-01	ENSG00000274578
NA	-0.0197	3.7587E-01		ENSG00000259402
NA	-0.0561	3.7563E-01	5.8419E-01	ENSG00000251139
NA	-0.0461	3.7556E-01	5.8411E-01	ENSG00000223890
NA	-0.0287	3.7514E-01		ENSG00000236567
NA	-0.0712	3.7499E-01	5.8356E-01	ENSG00000219085
NA	-0.0273	3.7493E-01		ENSG00000289617

NA	-0.0764	3.7467E-01	5.8325E-01	ENSG00000169668
NA	-0.0315	3.7464E-01		ENSG00000240143
NA	-0.0688	3.7452E-01	5.8316E-01	ENSG00000290033
NA	-0.0431	3.7435E-01	5.8300E-01	ENSG00000272402
NA	-0.0528	3.7412E-01	5.8276E-01	ENSG00000272989
NA	-0.0260	3.7373E-01		ENSG00000285577
NA	-0.0402	3.7350E-01		ENSG00000201071
NA	-0.0526	3.7327E-01	5.8187E-01	ENSG00000268564
NA	-0.0719	3.7318E-01	5.8178E-01	ENSG00000284610
NA	-0.0757	3.7317E-01	5.8178E-01	ENSG00000275457
NA	-0.0397	3.7309E-01	5.8178E-01	ENSG00000254388
NA	-0.0785	3.7258E-01	5.8132E-01	ENSG00000288872
NA	-0.0296	3.7246E-01		ENSG00000226526
NA	-0.0044	3.7229E-01		ENSG00000130612
NA	-0.0787	3.7210E-01	5.8075E-01	ENSG00000234921
NA	-0.0631	3.7185E-01	5.8058E-01	ENSG00000236698
NA	-0.0373	3.7137E-01		ENSG00000286293
NA	-0.0121	3.7113E-01	5.7998E-01	ENSG00000162840
NA	-0.0165	3.7103E-01		ENSG00000230118
NA	-0.0368	3.7098E-01		ENSG00000249330
NA	-0.0696	3.7031E-01	5.7942E-01	ENSG00000231563
NA	-0.0782	3.7018E-01	5.7931E-01	ENSG00000259744
NA	-0.0344	3.7011E-01		ENSG00000286916
NA	-0.0571	3.7001E-01	5.7929E-01	ENSG00000225031
NA	-0.0194	3.6979E-01		ENSG00000287437
NA	-0.0115	3.6966E-01		ENSG00000259133
NA	-0.0502	3.6964E-01	5.7890E-01	ENSG00000260190
NA	-0.0235	3.6929E-01		ENSG00000242021
NA	-0.0167	3.6929E-01		ENSG00000213440
NA	-0.0197	3.6925E-01		ENSG00000288757
NA	-0.0345	3.6875E-01		ENSG00000277795
NA	-0.0345	3.6797E-01	5.7713E-01	ENSG00000274598
NA	-0.0574	3.6779E-01	5.7706E-01	ENSG00000269653
NA	-0.0714	3.6737E-01	5.7663E-01	ENSG00000233247
NA	-0.0738	3.6705E-01	5.7634E-01	ENSG00000239524
NA	-0.0382	3.6704E-01		ENSG00000283698
NA	-0.0207	3.6704E-01		ENSG00000236153
NA	-0.0279	3.6686E-01		ENSG00000233583
NA	-0.0538	3.6677E-01	5.7597E-01	ENSG00000234789
NA	-0.0257	3.6655E-01		ENSG00000258260
NA	-0.0234	3.6652E-01		ENSG00000254843
NA	-0.0707	3.6634E-01	5.7559E-01	ENSG00000232034
NA	-0.0080	3.6592E-01		ENSG00000224307
NA	-0.0649	3.6554E-01	5.7467E-01	ENSG00000289291
NA	-0.0475	3.6534E-01	5.7450E-01	ENSG00000289004
NA	-0.0519	3.6462E-01	5.7374E-01	ENSG00000233514
NA	-0.0159	3.6461E-01		ENSG00000243018
NA	-0.0280	3.6445E-01	5.7358E-01	ENSG00000274967
NA	-0.0184	3.6436E-01		ENSG00000259701

NA	-0.0800	3.6382E-01	5.7295E-01	ENSG00000260293
NA	-0.0287	3.6374E-01		ENSG00000260612
NA	-0.0171	3.6367E-01		ENSG00000273113
NA	-0.0237	3.6354E-01		ENSG00000267729
NA	-0.0219	3.6337E-01		ENSG00000271032
NA	-0.0494	3.6332E-01	5.7242E-01	ENSG00000272744
NA	-0.0249	3.6331E-01		ENSG00000260120
NA	-0.0175	3.6320E-01		ENSG00000275839
NA	-0.0304	3.6306E-01		ENSG00000251271
NA	-0.0428	3.6288E-01	5.7197E-01	ENSG00000224993
NA	-0.0323	3.6288E-01		ENSG00000233673
NA	-0.0136	3.6276E-01		ENSG00000253114
NA	-0.0397	3.6275E-01		ENSG00000287149
NA	-0.0424	3.6213E-01		ENSG00000267042
NA	-0.0692	3.6152E-01	5.7097E-01	ENSG00000272540
NA	-0.0369	3.6150E-01		ENSG00000279570
NA	-0.0367	3.6120E-01		ENSG00000285710
NA	-0.0179	3.6087E-01		ENSG00000237921
NA	-0.0190	3.6057E-01		ENSG00000244381
NA	-0.0446	3.5996E-01		ENSG00000269949
NA	-0.0782	3.5945E-01	5.6889E-01	ENSG00000249456
NA	-0.0101	3.5934E-01		ENSG00000250290
NA	-0.0639	3.5920E-01	5.6866E-01	ENSG00000213839
NA	-0.0644	3.5910E-01	5.6860E-01	ENSG00000257740
NA	-0.0240	3.5910E-01		ENSG00000213981
NA	-0.0592	3.5834E-01	5.6777E-01	ENSG00000235912
NA	-0.0135	3.5823E-01		ENSG00000287657
NA	-0.0221	3.5816E-01		ENSG00000237953
NA	-0.0123	3.5812E-01	5.6775E-01	ENSG00000237352
NA	-0.0304	3.5800E-01		ENSG00000276846
NA	-0.0631	3.5784E-01	5.6756E-01	ENSG00000223599
NA	-0.0529	3.5772E-01	5.6749E-01	ENSG00000278948
NA	-0.0803	3.5764E-01	5.6743E-01	ENSG00000223972
NA	-0.0199	3.5755E-01		ENSG00000274737
NA	-0.0144	3.5721E-01		ENSG00000232362
NA	-0.0190	3.5714E-01		ENSG00000259316
NA	-0.0226	3.5702E-01	5.6682E-01	ENSG00000213201
NA	-0.0137	3.5633E-01		ENSG00000284716
NA	-0.0269	3.5626E-01		ENSG00000261559
NA	-0.0812	3.5615E-01	5.6602E-01	ENSG00000258101
NA	-0.0175	3.5594E-01		ENSG00000238137
NA	-0.0457	3.5583E-01	5.6573E-01	ENSG00000263050
NA	-0.0641	3.5530E-01	5.6504E-01	ENSG00000270165
NA	-0.0704	3.5502E-01	5.6473E-01	ENSG00000261026
NA	-0.0806	3.5495E-01	5.6473E-01	ENSG00000289494
NA	-0.0816	3.5480E-01	5.6460E-01	ENSG00000287509
NA	-0.0521	3.5409E-01		ENSG00000260911
NA	-0.0784	3.5407E-01	5.6402E-01	ENSG00000284966
NA	-0.0509	3.5382E-01	5.6380E-01	ENSG00000268683

NA	-0.0453	3.5376E-01	5.6375E-01	ENSG00000283285
NA	-0.0173	3.5375E-01		ENSG00000228392
NA	-0.0808	3.5317E-01	5.6336E-01	ENSG00000217624
NA	-0.0217	3.5310E-01		ENSG00000224431
NA	-0.0296	3.5302E-01		ENSG00000226427
NA	-0.0312	3.5302E-01		ENSG00000286245
NA	-0.0826	3.5266E-01	5.6290E-01	ENSG00000280160
NA	-0.0721	3.5263E-01	5.6290E-01	ENSG00000235609
NA	-0.0179	3.5243E-01		ENSG00000266312
NA	-0.0159	3.5208E-01		ENSG00000259515
NA	-0.0306	3.5190E-01		ENSG00000280217
NA	-0.0182	3.5175E-01		ENSG00000230980
NA	-0.0094	3.5132E-01		ENSG00000236244
NA	-0.0555	3.5123E-01	5.6135E-01	ENSG00000238084
NA	-0.0128	3.5113E-01		ENSG00000254619
NA	-0.0197	3.5110E-01		ENSG00000232882
NA	-0.0344	3.5059E-01		ENSG00000274499
NA	-0.0119	3.5045E-01		ENSG00000218757
NA	-0.0585	3.5045E-01	5.6065E-01	ENSG00000201882
NA	-0.0229	3.4956E-01		ENSG00000279197
NA	-0.0306	3.4936E-01		ENSG00000266846
NA	-0.0396	3.4923E-01		ENSG00000254676
NA	-0.0310	3.4898E-01		ENSG00000268660
NA	-0.0115	3.4884E-01		ENSG00000279485
NA	-0.0635	3.4861E-01	5.5845E-01	ENSG00000242660
NA	-0.0414	3.4829E-01	5.5839E-01	ENSG00000256603
NA	-0.0164	3.4811E-01		ENSG00000225918
NA	-0.0479	3.4780E-01	5.5805E-01	ENSG00000259376
NA	-0.0624	3.4766E-01	5.5789E-01	ENSG00000274591
NA	-0.0733	3.4753E-01	5.5776E-01	ENSG00000288744
NA	-0.0350	3.4742E-01		ENSG00000289075
NA	-0.0199	3.4701E-01		ENSG00000213058
NA	-0.0632	3.4693E-01	5.5731E-01	ENSG00000254027
NA	-0.0387	3.4686E-01		ENSG00000233403
NA	-0.0333	3.4616E-01		ENSG00000232696
NA	-0.0136	3.4529E-01		ENSG00000279404
NA	-0.0154	3.4459E-01		ENSG00000204990
NA	-0.0322	3.4453E-01		ENSG00000267223
NA	-0.0454	3.4439E-01		ENSG00000225632
NA	-0.0185	3.4432E-01		ENSG00000266944
NA	-0.0100	3.4403E-01		ENSG00000264456
NA	-0.0174	3.4381E-01		ENSG00000249406
NA	-0.0581	3.4361E-01	5.5321E-01	ENSG00000255409
NA	-0.0121	3.4337E-01		ENSG00000176043
NA	-0.0752	3.4242E-01	5.5198E-01	ENSG00000203362
NA	-0.0208	3.4179E-01		ENSG00000258524
NA	-0.0669	3.4157E-01	5.5118E-01	ENSG00000227064
NA	-0.0193	3.4150E-01		ENSG00000255422
NA	-0.0461	3.4147E-01	5.5116E-01	ENSG00000078319

NA	-0.0082	3.4144E-01		ENSG00000232391
NA	-0.0721	3.4044E-01	5.5015E-01	ENSG00000266946
NA	-0.0752	3.4038E-01	5.5009E-01	ENSG00000259251
NA	-0.0156	3.4028E-01		ENSG00000267652
NA	-0.0574	3.4016E-01	5.4987E-01	ENSG00000213212
NA	-0.0697	3.4009E-01	5.4984E-01	ENSG00000264164
NA	-0.0750	3.3975E-01	5.4947E-01	ENSG00000286837
NA	-0.0614	3.3965E-01	5.4937E-01	ENSG00000231169
NA	-0.0520	3.3944E-01	5.4911E-01	ENSG00000280053
NA	-0.0580	3.3928E-01	5.4899E-01	ENSG00000274023
NA	-0.0766	3.3912E-01	5.4880E-01	ENSG00000290091
NA	-0.0837	3.3885E-01	5.4859E-01	ENSG00000261613
NA	-0.0799	3.3856E-01	5.4841E-01	ENSG00000250462
NA	-0.0438	3.3851E-01		ENSG00000260105
NA	-0.0157	3.3784E-01		ENSG00000217824
NA	-0.0587	3.3772E-01	5.4765E-01	ENSG00000233225
NA	-0.0740	3.3765E-01	5.4765E-01	ENSG00000278986
NA	-0.0287	3.3758E-01		ENSG00000282840
NA	-0.0824	3.3734E-01	5.4741E-01	ENSG00000157306
NA	-0.0220	3.3731E-01		ENSG00000280227
NA	-0.0515	3.3724E-01	5.4734E-01	ENSG00000279187
NA	-0.0841	3.3698E-01	5.4707E-01	ENSG00000263307
NA	-0.0086	3.3696E-01	5.4707E-01	ENSG00000289572
NA	-0.0464	3.3671E-01	5.4678E-01	ENSG00000286268
NA	-0.0515	3.3671E-01	5.4678E-01	ENSG00000286994
NA	-0.0732	3.3649E-01	5.4652E-01	ENSG00000235688
NA	-0.0148	3.3633E-01		ENSG00000286080
NA	-0.0224	3.3627E-01		ENSG00000253711
NA	-0.0813	3.3627E-01	5.4631E-01	ENSG00000279467
NA	-0.0351	3.3618E-01		ENSG00000237451
NA	-0.0278	3.3609E-01		ENSG00000278153
NA	-0.0580	3.3598E-01	5.4599E-01	ENSG00000237347
NA	-0.0499	3.3589E-01	5.4588E-01	ENSG00000207370
NA	-0.0555	3.3587E-01	5.4588E-01	ENSG00000215878
NA	-0.0134	3.3585E-01		ENSG00000259274
NA	-0.0604	3.3584E-01	5.4587E-01	ENSG00000273015
NA	-0.0137	3.3539E-01		ENSG00000286440
NA	-0.0181	3.3535E-01		ENSG00000227107
NA	-0.0261	3.3516E-01		ENSG00000225358
NA	-0.0673	3.3505E-01	5.4508E-01	ENSG00000238021
NA	-0.0134	3.3494E-01		ENSG00000258073
NA	-0.0248	3.3481E-01		ENSG00000285908
NA	-0.0639	3.3469E-01	5.4471E-01	ENSG00000279288
NA	-0.0324	3.3460E-01		ENSG00000248968
NA	-0.0822	3.3448E-01	5.4460E-01	ENSG00000239218
NA	-0.0409	3.3410E-01		ENSG00000183562
NA	-0.0288	3.3404E-01		ENSG00000230260
NA	-0.0264	3.3351E-01		ENSG00000258437
NA	-0.0452	3.3342E-01		ENSG00000239480

NA	-0.0864	3.3335E-01	5.4349E-01	ENSG00000226853
NA	-0.0626	3.3321E-01	5.4349E-01	ENSG00000280198
NA	-0.0161	3.3321E-01		ENSG00000287500
NA	-0.0324	3.3298E-01	5.4329E-01	ENSG00000219491
NA	-0.0132	3.3275E-01		ENSG00000214265
NA	-0.0298	3.3263E-01		ENSG00000290166
NA	-0.0856	3.3254E-01	5.4281E-01	ENSG00000289112
NA	-0.0368	3.3215E-01		ENSG00000286803
NA	-0.0843	3.3215E-01	5.4247E-01	ENSG00000237976
NA	-0.0808	3.3194E-01	5.4229E-01	ENSG00000289136
NA	-0.0131	3.3193E-01		ENSG00000279742
NA	-0.0848	3.3143E-01	5.4171E-01	ENSG00000272843
NA	-0.0115	3.3133E-01		ENSG00000251031
NA	-0.0738	3.3095E-01	5.4112E-01	ENSG00000279416
NA	-0.0210	3.3062E-01		ENSG00000258450
NA	-0.0717	3.3045E-01	5.4072E-01	ENSG00000227123
NA	-0.0643	3.3029E-01	5.4056E-01	ENSG00000235932
NA	-0.0327	3.2982E-01		ENSG00000232389
NA	-0.0661	3.2946E-01	5.3978E-01	ENSG00000264943
NA	-0.0610	3.2916E-01	5.3946E-01	ENSG00000289840
NA	-0.0379	3.2792E-01	5.3839E-01	ENSG00000214881
NA	-0.0374	3.2758E-01		ENSG00000261544
NA	-0.0376	3.2720E-01	5.3762E-01	ENSG00000214559
NA	-0.0792	3.2630E-01	5.3646E-01	ENSG00000258317
NA	-0.0566	3.2624E-01		ENSG00000288543
NA	-0.0139	3.2616E-01		ENSG00000253754
NA	-0.0878	3.2587E-01	5.3607E-01	ENSG00000288759
NA	-0.0163	3.2581E-01		ENSG00000271013
NA	-0.0833	3.2525E-01	5.3531E-01	ENSG00000271743
NA	-0.0159	3.2524E-01		ENSG00000225417
NA	-0.0703	3.2481E-01	5.3484E-01	ENSG00000255384
NA	-0.0154	3.2460E-01		ENSG00000287469
NA	-0.0157	3.2458E-01		ENSG00000269191
NA	-0.0144	3.2403E-01		ENSG00000227432
NA	-0.0166	3.2363E-01		ENSG00000233852
NA	-0.0687	3.2295E-01	5.3298E-01	ENSG00000226540
NA	-0.0206	3.2291E-01		ENSG00000280758
NA	-0.0337	3.2291E-01		ENSG00000261635
NA	-0.0248	3.2185E-01		ENSG00000268499
NA	-0.0148	3.2153E-01		ENSG00000287856
NA	-0.0160	3.2141E-01		ENSG00000277851
NA	-0.0191	3.2121E-01		ENSG00000258527
NA	-0.0512	3.2111E-01		ENSG00000279352
NA	-0.0117	3.2111E-01	5.3116E-01	ENSG00000238063
NA	-0.0087	3.2111E-01		ENSG00000249283
NA	-0.0298	3.2088E-01		ENSG00000285999
NA	-0.0474	3.2063E-01		ENSG00000227516
NA	-0.0214	3.2059E-01		ENSG00000223631
NA	-0.0669	3.2054E-01	5.3056E-01	ENSG00000258662

NA	-0.0320	3.2051E-01		ENSG00000269480
NA	-0.0165	3.2034E-01		ENSG00000229002
NA	-0.0845	3.2019E-01	5.3035E-01	ENSG00000285190
NA	-0.0403	3.1949E-01		ENSG00000251689
NA	-0.0679	3.1936E-01	5.2957E-01	ENSG00000286230
NA	-0.0404	3.1901E-01		ENSG00000266803
NA	-0.0523	3.1878E-01	5.2903E-01	ENSG00000283047
NA	-0.0868	3.1812E-01	5.2833E-01	ENSG00000269399
NA	-0.0711	3.1676E-01	5.2679E-01	ENSG00000244627
NA	-0.0580	3.1668E-01	5.2676E-01	ENSG00000290040
NA	-0.0115	3.1662E-01		ENSG00000244260
NA	-0.0304	3.1639E-01		ENSG00000287975
NA	-0.0207	3.1606E-01		ENSG00000262151
NA	-0.0098	3.1600E-01		ENSG00000277628
NA	-0.0401	3.1557E-01		ENSG00000279118
NA	-0.0269	3.1547E-01		ENSG00000256757
NA	-0.0138	3.1545E-01		ENSG00000250403
NA	-0.0636	3.1517E-01	5.2525E-01	ENSG00000213260
NA	-0.0616	3.1502E-01	5.2514E-01	ENSG00000285417
NA	-0.0417	3.1498E-01		ENSG00000226885
NA	-0.0665	3.1440E-01	5.2456E-01	ENSG00000259318
NA	-0.0308	3.1431E-01	5.2456E-01	ENSG00000259838
NA	-0.0797	3.1396E-01	5.2427E-01	ENSG00000215093
NA	-0.0317	3.1343E-01		ENSG00000222604
NA	-0.0387	3.1287E-01		ENSG00000231355
NA	-0.0272	3.1247E-01		ENSG00000224323
NA	-0.0121	3.1218E-01		ENSG00000212241
NA	-0.0135	3.1180E-01		ENSG00000259195
NA	-0.0429	3.1162E-01		ENSG00000279594
NA	-0.0204	3.1144E-01		ENSG00000200953
NA	-0.0909	3.1132E-01	5.2136E-01	ENSG00000287463
NA	-0.0419	3.1130E-01		ENSG00000287870
NA	-0.0164	3.1120E-01		ENSG00000271522
NA	-0.0806	3.1051E-01	5.2077E-01	ENSG00000182841
NA	-0.0162	3.1043E-01		ENSG00000231092
NA	-0.0623	3.1032E-01	5.2065E-01	ENSG00000239650
NA	-0.0346	3.0999E-01		ENSG00000272660
NA	-0.0288	3.0988E-01		ENSG00000225871
NA	-0.0574	3.0985E-01	5.2011E-01	ENSG00000289501
NA	-0.0893	3.0953E-01	5.1976E-01	ENSG00000188985
NA	-0.0141	3.0915E-01		ENSG00000226186
NA	-0.0708	3.0906E-01	5.1924E-01	ENSG00000225051
NA	-0.0669	3.0867E-01	5.1891E-01	ENSG00000261868
NA	-0.0327	3.0835E-01		ENSG00000270714
NA	-0.0769	3.0788E-01	5.1816E-01	ENSG00000261578
NA	-0.0130	3.0788E-01		ENSG00000289954
NA	-0.0443	3.0765E-01		ENSG00000283132
NA	-0.0426	3.0755E-01		ENSG00000287454
NA	-0.0223	3.0754E-01		ENSG00000286218

NA	-0.0294	3.0742E-01		ENSG00000227201
NA	-0.0656	3.0735E-01	5.1754E-01	ENSG00000286459
NA	-0.0138	3.0728E-01		ENSG00000254127
NA	-0.0429	3.0707E-01		ENSG00000271155
NA	-0.0188	3.0693E-01		ENSG00000285851
NA	-0.0295	3.0661E-01		ENSG00000254419
NA	-0.0562	3.0659E-01	5.1680E-01	ENSG00000289305
NA	-0.0672	3.0553E-01	5.1574E-01	ENSG00000227586
NA	-0.0558	3.0506E-01	5.1535E-01	ENSG00000239804
NA	-0.0373	3.0503E-01	5.1535E-01	ENSG00000226243
NA	-0.0243	3.0487E-01		ENSG00000213684
NA	-0.0363	3.0475E-01		ENSG00000235149
NA	-0.0896	3.0459E-01	5.1481E-01	ENSG00000287978
NA	-0.0294	3.0432E-01		ENSG00000218459
NA	-0.0566	3.0397E-01	5.1410E-01	ENSG00000233577
NA	-0.0441	3.0390E-01		ENSG00000273481
NA	-0.0377	3.0377E-01		ENSG00000255808
NA	-0.0275	3.0362E-01		ENSG00000254972
NA	-0.0223	3.0362E-01		ENSG00000269421
NA	-0.0241	3.0359E-01		ENSG00000263708
NA	-0.0904	3.0359E-01	5.1381E-01	ENSG00000289745
NA	-0.0486	3.0321E-01		ENSG00000277581
NA	-0.0205	3.0298E-01		ENSG00000236355
NA	-0.0742	3.0179E-01	5.1180E-01	ENSG00000233747
NA	-0.0578	3.0174E-01	5.1180E-01	ENSG00000273447
NA	-0.0901	3.0147E-01	5.1154E-01	ENSG00000272432
NA	-0.0124	3.0129E-01		ENSG00000249114
NA	-0.0128	3.0121E-01		ENSG00000251987
NA	-0.0295	3.0120E-01		ENSG00000234397
NA	-0.0248	3.0109E-01		ENSG00000273483
NA	-0.0927	3.0040E-01	5.1021E-01	ENSG00000274642
NA	-0.0222	3.0022E-01		ENSG00000271653
NA	-0.0146	2.9998E-01		ENSG00000257258
NA	-0.0325	2.9974E-01		ENSG00000277744
NA	-0.0404	2.9974E-01		ENSG00000267346
NA	-0.0467	2.9966E-01		ENSG00000230299
NA	-0.0784	2.9944E-01	5.0907E-01	ENSG00000244346
NA	-0.0718	2.9935E-01	5.0903E-01	ENSG00000241409
NA	-0.0552	2.9917E-01	5.0892E-01	ENSG00000260602
NA	-0.0085	2.9894E-01		ENSG00000242790
NA	-0.0769	2.9782E-01	5.0763E-01	ENSG00000286195
NA	-0.0908	2.9779E-01	5.0763E-01	ENSG00000273218
NA	-0.0246	2.9778E-01		ENSG00000169253
NA	-0.0234	2.9737E-01		ENSG00000269806
NA	-0.0771	2.9688E-01	5.0692E-01	ENSG00000227449
NA	-0.0245	2.9669E-01		ENSG00000285535
NA	-0.0386	2.9652E-01		ENSG00000237329
NA	-0.0949	2.9645E-01	5.0644E-01	ENSG00000279928
NA	-0.0859	2.9616E-01	5.0611E-01	ENSG00000288830

NA	-0.0923	2.9600E-01	5.0595E-01	ENSG00000267325
NA	-0.0922	2.9573E-01	5.0576E-01	ENSG00000273314
NA	-0.0602	2.9571E-01	5.0576E-01	ENSG00000285900
NA	-0.0936	2.9555E-01	5.0565E-01	ENSG00000269978
NA	-0.0368	2.9546E-01		ENSG00000264750
NA	-0.0154	2.9480E-01		ENSG00000231296
NA	-0.0484	2.9462E-01		ENSG00000237188
NA	-0.0431	2.9417E-01		ENSG00000250644
NA	-0.0678	2.9390E-01	5.0393E-01	ENSG00000287559
NA	-0.0541	2.9381E-01	5.0390E-01	ENSG00000279354
NA	-0.0720	2.9301E-01	5.0301E-01	ENSG00000263301
NA	-0.0708	2.9268E-01	5.0274E-01	ENSG00000277728
NA	-0.0783	2.9266E-01	5.0274E-01	ENSG00000251586
NA	-0.0277	2.9242E-01		ENSG00000261334
NA	-0.0790	2.9199E-01	5.0217E-01	ENSG00000261499
NA	-0.0275	2.9179E-01		ENSG00000288637
NA	-0.0180	2.9139E-01		ENSG00000285754
NA	-0.0910	2.9128E-01	5.0161E-01	ENSG00000234268
NA	-0.0350	2.9103E-01		ENSG00000258960
NA	-0.0935	2.9101E-01	5.0136E-01	ENSG00000196381
NA	-0.0736	2.9096E-01	5.0136E-01	ENSG00000260565
NA	-0.0856	2.9096E-01	5.0136E-01	ENSG00000231521
NA	-0.0293	2.9088E-01		ENSG00000237775
NA	-0.0205	2.9027E-01		ENSG00000272909
NA	-0.0694	2.8989E-01	5.0035E-01	ENSG00000267345
NA	-0.0434	2.8956E-01		ENSG00000256361
NA	-0.0781	2.8882E-01	4.9926E-01	ENSG00000264538
NA	-0.0295	2.8875E-01		ENSG00000289523
NA	-0.0088	2.8863E-01		ENSG00000249926
NA	-0.0330	2.8834E-01		ENSG00000277385
NA	-0.0366	2.8760E-01		ENSG00000271828
NA	-0.0957	2.8714E-01	4.9785E-01	ENSG00000288551
NA	-0.0350	2.8706E-01		ENSG00000259805
NA	-0.0332	2.8622E-01		ENSG00000269304
NA	-0.0739	2.8601E-01	4.9670E-01	ENSG00000274721
NA	-0.0244	2.8599E-01		ENSG00000172912
NA	-0.0589	2.8542E-01	4.9614E-01	ENSG00000258568
NA	-0.0316	2.8541E-01		ENSG00000203620
NA	-0.0666	2.8533E-01	4.9604E-01	ENSG00000258285
NA	-0.0625	2.8532E-01	4.9604E-01	ENSG00000270255
NA	-0.0371	2.8510E-01		ENSG00000279663
NA	-0.0155	2.8495E-01		ENSG00000257715
NA	-0.0926	2.8478E-01	4.9536E-01	ENSG00000230555
NA	-0.0243	2.8462E-01		ENSG00000248936
NA	-0.0948	2.8460E-01	4.9520E-01	ENSG00000235354
NA	-0.0323	2.8401E-01		ENSG00000213297
NA	-0.0356	2.8397E-01		ENSG00000287124
NA	-0.0899	2.8375E-01	4.9425E-01	ENSG00000286334
NA	-0.0841	2.8344E-01	4.9392E-01	ENSG00000203644

NA	-0.0902	2.8282E-01	4.9325E-01	ENSG00000261872
NA	-0.0204	2.8164E-01		ENSG00000215049
NA	-0.0231	2.8097E-01		ENSG00000267408
NA	-0.0540	2.8065E-01	4.9082E-01	ENSG00000236262
NA	-0.0924	2.7999E-01	4.9005E-01	ENSG00000256139
NA	-0.0734	2.7987E-01	4.8991E-01	ENSG00000224837
NA	-0.0311	2.7883E-01		ENSG00000287744
NA	-0.0950	2.7858E-01	4.8842E-01	ENSG00000250377
NA	-0.0419	2.7838E-01		ENSG00000280486
NA	-0.0431	2.7755E-01		ENSG00000230807
NA	-0.0371	2.7743E-01		ENSG00000229316
NA	-0.0290	2.7738E-01		ENSG00000236942
NA	-0.0276	2.7731E-01		ENSG00000266179
NA	-0.0347	2.7697E-01		ENSG00000261114
NA	-0.0825	2.7682E-01	4.8676E-01	ENSG00000277072
NA	-0.0361	2.7634E-01		ENSG00000226987
NA	-0.0894	2.7569E-01	4.8561E-01	ENSG00000257126
NA	-0.0955	2.7528E-01	4.8516E-01	ENSG00000275029
NA	-0.0962	2.7482E-01	4.8461E-01	ENSG00000272655
NA	-0.0161	2.7476E-01		ENSG00000255680
NA	-0.0981	2.7475E-01	4.8458E-01	ENSG00000288948
NA	-0.0219	2.7461E-01		ENSG00000258751
NA	-0.0801	2.7454E-01	4.8445E-01	ENSG00000253712
NA	-0.0340	2.7437E-01		ENSG00000224621
NA	-0.0679	2.7394E-01	4.8372E-01	ENSG00000267394
NA	-0.0118	2.7388E-01		ENSG00000256103
NA	-0.0440	2.7384E-01		ENSG00000262921
NA	-0.0707	2.7353E-01	4.8325E-01	ENSG00000260352
NA	-0.0616	2.7345E-01	4.8317E-01	ENSG00000238082
NA	-0.0141	2.7344E-01		ENSG00000225359
NA	-0.0123	2.7290E-01		ENSG00000233987
NA	-0.0561	2.7207E-01		ENSG00000237382
NA	-0.0756	2.7185E-01	4.8111E-01	ENSG00000204173
NA	-0.0477	2.7181E-01		ENSG00000259364
NA	-0.0389	2.7169E-01		ENSG00000288030
NA	-0.0453	2.7166E-01	4.8089E-01	ENSG00000249061
NA	-0.0522	2.7156E-01	4.8080E-01	ENSG00000249602
NA	-0.0213	2.7153E-01		ENSG00000285724
NA	-0.0658	2.7125E-01	4.8048E-01	ENSG00000274767
NA	-0.0548	2.7061E-01	4.8008E-01	ENSG00000231132
NA	-0.0223	2.7031E-01		ENSG00000270016
NA	-0.0424	2.7021E-01		ENSG00000267892
NA	-0.0998	2.7019E-01	4.7977E-01	ENSG00000248593
NA	-0.0279	2.7001E-01		ENSG00000287091
NA	-0.0842	2.6988E-01	4.7949E-01	ENSG00000241962
NA	-0.0489	2.6928E-01		ENSG00000278972
NA	-0.0990	2.6921E-01	4.7868E-01	ENSG00000279608
NA	-0.0255	2.6905E-01		ENSG00000261647
NA	-0.1009	2.6883E-01	4.7820E-01	ENSG00000223695

NA	-0.0555	2.6700E-01	4.7620E-01	ENSG00000232762
NA	-0.0625	2.6661E-01	4.7592E-01	ENSG00000260884
NA	-0.0531	2.6639E-01	4.7571E-01	ENSG00000228810
NA	-0.0528	2.6633E-01		ENSG00000269652
NA	-0.0778	2.6597E-01	4.7515E-01	ENSG00000288792
NA	-0.0147	2.6548E-01		ENSG00000269583
NA	-0.0507	2.6506E-01	4.7425E-01	ENSG00000257271
NA	-0.0139	2.6497E-01		ENSG00000231726
NA	-0.0257	2.6483E-01		ENSG00000236796
NA	-0.0194	2.6481E-01		ENSG00000272221
NA	-0.0235	2.6448E-01		ENSG00000235205
NA	-0.0960	2.6427E-01	4.7321E-01	ENSG00000273356
NA	-0.0339	2.6426E-01		ENSG00000255339
NA	-0.0619	2.6394E-01	4.7283E-01	ENSG00000251648
NA	-0.0931	2.6330E-01	4.7216E-01	ENSG00000289085
NA	-0.0279	2.6321E-01		ENSG00000214121
NA	-0.0656	2.6305E-01	4.7189E-01	ENSG00000234275
NA	-0.0997	2.6301E-01	4.7184E-01	ENSG00000286873
NA	-0.0831	2.6276E-01	4.7160E-01	ENSG00000160766
NA	-0.0280	2.6217E-01		ENSG00000198019
NA	-0.0193	2.6201E-01		ENSG00000240634
NA	-0.0753	2.6191E-01	4.7062E-01	ENSG00000225026
NA	-0.0224	2.6145E-01		ENSG00000224808
NA	-0.0808	2.6130E-01	4.6984E-01	ENSG00000287479
NA	-0.0132	2.6085E-01		ENSG00000235742
NA	-0.0579	2.6083E-01	4.6937E-01	ENSG00000228835
NA	-0.1009	2.6069E-01	4.6918E-01	ENSG00000287558
NA	-0.0608	2.6028E-01		ENSG00000231822
NA	-0.0929	2.5995E-01	4.6837E-01	ENSG00000259511
NA	-0.1046	2.5945E-01	4.6792E-01	ENSG00000289296
NA	-0.0744	2.5852E-01	4.6729E-01	ENSG00000276984
NA	-0.1005	2.5849E-01	4.6729E-01	ENSG00000277383
NA	-0.0453	2.5846E-01		ENSG00000241219
NA	-0.1009	2.5835E-01	4.6721E-01	ENSG00000283849
NA	-0.0310	2.5812E-01		ENSG00000279771
NA	-0.0148	2.5735E-01		ENSG00000229271
NA	-0.0357	2.5720E-01		ENSG00000228466
NA	-0.0940	2.5714E-01	4.6567E-01	ENSG00000248180
NA	-0.0729	2.5696E-01	4.6544E-01	ENSG00000259020
NA	-0.1005	2.5690E-01	4.6542E-01	ENSG00000286232
NA	-0.1008	2.5652E-01	4.6496E-01	ENSG00000267009
NA	-0.0232	2.5611E-01		ENSG00000263017
NA	-0.0882	2.5584E-01	4.6408E-01	ENSG00000280376
NA	-0.0536	2.5580E-01		ENSG00000257225
NA	-0.0655	2.5576E-01	4.6401E-01	ENSG00000256747
NA	-0.0334	2.5571E-01		ENSG00000287456
NA	-0.0517	2.5539E-01	4.6351E-01	ENSG00000226881
NA	-0.0190	2.5440E-01		ENSG00000258282
NA	-0.0557	2.5431E-01	4.6234E-01	ENSG00000253374

NA	-0.0007	2.5416E-01	4.6213E-01	ENSG00000287842
NA	-0.0861	2.5414E-01	4.6213E-01	ENSG00000235411
NA	-0.0817	2.5398E-01	4.6198E-01	ENSG00000246067
NA	-0.0183	2.5388E-01		ENSG00000287278
NA	-0.1032	2.5364E-01	4.6157E-01	ENSG00000279759
NA	-0.0943	2.5346E-01	4.6143E-01	ENSG00000284614
NA	-0.0175	2.5335E-01		ENSG00000271150
NA	-0.0783	2.5318E-01	4.6111E-01	ENSG00000244081
NA	-0.0783	2.5318E-01	4.6111E-01	ENSG00000237904
NA	-0.0577	2.5317E-01	4.6111E-01	ENSG00000227854
NA	-0.0721	2.5277E-01	4.6074E-01	ENSG00000253919
NA	-0.0256	2.5164E-01		ENSG00000213303
NA	-0.0340	2.5127E-01		ENSG00000258712
NA	-0.0394	2.5112E-01		ENSG00000267185
NA	-0.1032	2.5094E-01	4.5920E-01	ENSG00000272430
NA	-0.0699	2.5059E-01	4.5872E-01	ENSG00000273618
NA	-0.0266	2.5027E-01		ENSG00000228056
NA	-0.0163	2.5016E-01		ENSG00000268601
NA	-0.1042	2.5009E-01	4.5798E-01	ENSG00000255121
NA	-0.0387	2.4963E-01		ENSG00000232060
NA	-0.0904	2.4908E-01	4.5678E-01	ENSG00000259467
NA	-0.0537	2.4879E-01	4.5649E-01	ENSG00000253985
NA	-0.0870	2.4870E-01	4.5649E-01	ENSG00000274270
NA	-0.0905	2.4848E-01	4.5629E-01	ENSG00000284959
NA	-0.0697	2.4839E-01	4.5619E-01	ENSG00000279821
NA	-0.0785	2.4823E-01	4.5595E-01	ENSG00000256056
NA	-0.1037	2.4798E-01	4.5577E-01	ENSG00000285367
NA	-0.0231	2.4762E-01		ENSG00000289587
NA	-0.1035	2.4749E-01	4.5528E-01	ENSG00000263146
NA	-0.0305	2.4715E-01		ENSG00000222515
NA	-0.0973	2.4706E-01	4.5476E-01	ENSG00000253333
NA	-0.0241	2.4680E-01		ENSG00000215268
NA	-0.0768	2.4648E-01	4.5415E-01	ENSG00000286001
NA	-0.0606	2.4642E-01	4.5414E-01	ENSG00000270116
NA	-0.0816	2.4634E-01	4.5414E-01	ENSG00000260060
NA	-0.0415	2.4622E-01		ENSG00000288107
NA	-0.0596	2.4602E-01		ENSG00000228816
NA	-0.0191	2.4578E-01		ENSG00000289089
NA	-0.0232	2.4573E-01		ENSG00000267417
NA	-0.0399	2.4566E-01		ENSG00000257666
NA	-0.0706	2.4561E-01	4.5339E-01	ENSG00000264270
NA	-0.0707	2.4529E-01	4.5305E-01	ENSG00000263466
NA	-0.1100	2.4471E-01	4.5223E-01	ENSG00000287809
NA	-0.0262	2.4466E-01	4.5223E-01	ENSG00000243304
NA	-0.0641	2.4462E-01	4.5220E-01	ENSG00000259523
NA	-0.0281	2.4443E-01		ENSG00000230731
NA	-0.0833	2.4443E-01	4.5199E-01	ENSG00000279392
NA	-0.0455	2.4440E-01		ENSG00000254332
NA	-0.0969	2.4428E-01	4.5185E-01	ENSG00000284634

NA	-0.0631	2.4420E-01	4.5180E-01	ENSG00000269560
NA	-0.1061	2.4400E-01	4.5165E-01	ENSG00000242970
NA	-0.0636	2.4378E-01	4.5150E-01	ENSG00000280176
NA	-0.0568	2.4376E-01	4.5150E-01	ENSG00000225067
NA	-0.0804	2.4370E-01	4.5146E-01	ENSG00000272942
NA	-0.0859	2.4367E-01	4.5143E-01	ENSG00000205955
NA	-0.0593	2.4349E-01		ENSG00000273350
NA	-0.0914	2.4346E-01	4.5132E-01	ENSG00000236266
NA	-0.1053	2.4303E-01	4.5075E-01	ENSG00000261596
NA	-0.0649	2.4277E-01	4.5048E-01	ENSG00000233588
NA	-0.1030	2.4217E-01	4.4961E-01	ENSG00000279179
NA	-0.0645	2.4211E-01	4.4953E-01	ENSG00000286480
NA	-0.0633	2.4205E-01	4.4951E-01	ENSG00000266248
NA	-0.0217	2.4156E-01		ENSG00000253923
NA	-0.0219	2.4156E-01		ENSG00000285527
NA	-0.1061	2.4137E-01	4.4842E-01	ENSG00000256663
NA	-0.0979	2.4123E-01	4.4826E-01	ENSG00000239763
NA	-0.1019	2.4093E-01	4.4787E-01	ENSG00000265205
NA	-0.0366	2.4072E-01		ENSG00000234120
NA	-0.1046	2.4046E-01	4.4727E-01	ENSG00000255129
NA	-0.0176	2.4043E-01		ENSG00000239405
NA	-0.0306	2.3997E-01		ENSG00000230022
NA	-0.0837	2.3961E-01	4.4626E-01	ENSG00000251442
NA	-0.1057	2.3960E-01	4.4626E-01	ENSG00000267649
NA	-0.0334	2.3939E-01		ENSG00000285704
NA	-0.0269	2.3877E-01		ENSG00000228834
NA	-0.0454	2.3809E-01		ENSG00000254321
NA	-0.0453	2.3728E-01		ENSG00000286488
NA	-0.0236	2.3722E-01		ENSG00000276121
NA	-0.1086	2.3685E-01	4.4251E-01	ENSG00000284669
NA	-0.1089	2.3679E-01	4.4251E-01	ENSG00000215271
NA	-0.0640	2.3642E-01	4.4222E-01	ENSG00000240766
NA	-0.0472	2.3612E-01		ENSG00000213872
NA	-0.0232	2.3578E-01		ENSG00000259820
NA	-0.0682	2.3499E-01	4.4039E-01	ENSG00000226989
NA	-0.0224	2.3458E-01		ENSG00000236285
NA	-0.0273	2.3426E-01		ENSG00000264705
NA	-0.0987	2.3419E-01	4.3927E-01	ENSG00000253669
NA	-0.0791	2.3407E-01	4.3912E-01	ENSG00000228217
NA	-0.0969	2.3380E-01	4.3899E-01	ENSG00000279080
NA	-0.0708	2.3314E-01	4.3830E-01	ENSG00000273073
NA	-0.0266	2.3305E-01		ENSG00000286240
NA	-0.0372	2.3304E-01	4.3820E-01	ENSG00000267424
NA	-0.0620	2.3269E-01	4.3780E-01	ENSG00000235489
NA	-0.1117	2.3212E-01	4.3709E-01	ENSG00000178660
NA	-0.0475	2.3182E-01		ENSG00000275339
NA	-0.1062	2.3160E-01	4.3644E-01	ENSG00000233929
NA	-0.1075	2.3159E-01	4.3644E-01	ENSG00000264589
NA	-0.0900	2.3056E-01	4.3531E-01	ENSG00000289534

NA	-0.0189	2.3040E-01		ENSG00000258925
NA	-0.0971	2.3023E-01	4.3488E-01	ENSG00000266865
NA	-0.0352	2.2978E-01		ENSG00000225331
NA	-0.0188	2.2974E-01		ENSG00000223787
NA	-0.0612	2.2958E-01	4.3409E-01	ENSG00000277498
NA	-0.1092	2.2906E-01	4.3343E-01	ENSG00000238186
NA	-0.0784	2.2906E-01	4.3343E-01	ENSG00000267504
NA	-0.0622	2.2883E-01	4.3334E-01	ENSG00000239828
NA	-0.0691	2.2842E-01	4.3305E-01	ENSG00000228862
NA	-0.0297	2.2802E-01	4.3267E-01	ENSG00000234816
NA	-0.0292	2.2719E-01		ENSG00000236217
NA	-0.0449	2.2706E-01		ENSG00000286934
NA	-0.0257	2.2705E-01		ENSG00000258172
NA	-0.0915	2.2656E-01	4.3059E-01	ENSG00000266456
NA	-0.1100	2.2643E-01	4.3041E-01	ENSG00000228274
NA	-0.0625	2.2625E-01		ENSG00000285621
NA	-0.0774	2.2610E-01	4.2996E-01	ENSG00000213216
NA	-0.0553	2.2601E-01	4.2988E-01	ENSG00000269984
NA	-0.1116	2.2600E-01	4.2988E-01	ENSG00000224094
NA	-0.0929	2.2549E-01	4.2926E-01	ENSG00000185275
NA	-0.0276	2.2531E-01		ENSG00000234584
NA	-0.0278	2.2515E-01		ENSG00000250659
NA	-0.0880	2.2509E-01	4.2887E-01	ENSG00000234160
NA	-0.0764	2.2496E-01	4.2887E-01	ENSG00000254802
NA	-0.1004	2.2448E-01	4.2834E-01	ENSG00000280132
NA	-0.0265	2.2411E-01		ENSG00000234551
NA	-0.0355	2.2319E-01		ENSG00000290088
NA	-0.0780	2.2316E-01	4.2680E-01	ENSG00000249930
NA	-0.0293	2.2303E-01		ENSG00000263924
NA	-0.0796	2.2271E-01	4.2618E-01	ENSG00000257531
NA	-0.0841	2.2244E-01	4.2586E-01	ENSG00000255321
NA	-0.0814	2.2231E-01	4.2568E-01	ENSG00000231329
NA	-0.0949	2.2215E-01	4.2551E-01	ENSG00000264932
NA	-0.0427	2.2210E-01	4.2547E-01	ENSG00000214821
NA	-0.1126	2.2171E-01	4.2497E-01	ENSG00000245970
NA	-0.0168	2.2156E-01		ENSG00000266648
NA	-0.1075	2.2134E-01	4.2457E-01	ENSG00000178082
NA	-0.0325	2.2117E-01		ENSG00000288971
NA	-0.1114	2.2101E-01	4.2417E-01	ENSG00000233381
NA	-0.0260	2.1999E-01		ENSG00000258717
NA	-0.0567	2.1963E-01		ENSG00000254336
NA	-0.0818	2.1942E-01	4.2176E-01	ENSG00000229180
NA	-0.0324	2.1892E-01		ENSG00000268366
NA	-0.0276	2.1883E-01		ENSG00000225094
NA	-0.0864	2.1879E-01	4.2129E-01	ENSG00000226377
NA	-0.0222	2.1875E-01		ENSG00000283402
NA	-0.0843	2.1850E-01	4.2088E-01	ENSG00000267397
NA	-0.0396	2.1772E-01		ENSG00000262681
NA	-0.0555	2.1722E-01		ENSG00000226284

NA	-0.1039	2.1688E-01	4.1860E-01	ENSG00000225071
NA	-0.0259	2.1674E-01		ENSG00000213383
NA	-0.0239	2.1574E-01		ENSG00000224566
NA	-0.1153	2.1572E-01	4.1711E-01	ENSG00000231993
NA	-0.0676	2.1531E-01	4.1659E-01	ENSG00000287449
NA	-0.0387	2.1523E-01		ENSG00000233578
NA	-0.1151	2.1486E-01	4.1618E-01	ENSG00000227008
NA	-0.0172	2.1451E-01		ENSG00000226789
NA	-0.1044	2.1405E-01	4.1517E-01	ENSG00000273784
NA	-0.1168	2.1355E-01	4.1451E-01	ENSG00000227388
NA	-0.0941	2.1347E-01	4.1439E-01	ENSG00000108958
NA	-0.0183	2.1298E-01		ENSG00000182625
NA	-0.0708	2.1257E-01	4.1364E-01	ENSG00000233877
NA	-0.0256	2.1245E-01		ENSG00000287581
NA	-0.0925	2.1233E-01	4.1331E-01	ENSG00000264273
NA	-0.0175	2.1229E-01		ENSG00000286841
NA	-0.0866	2.1200E-01	4.1293E-01	ENSG00000259270
NA	-0.0438	2.1145E-01	4.1230E-01	ENSG00000236022
NA	-0.0206	2.1133E-01		ENSG00000243944
NA	-0.1159	2.1090E-01	4.1160E-01	ENSG00000280229
NA	-0.1024	2.1083E-01	4.1151E-01	ENSG00000288061
NA	-0.0231	2.1065E-01		ENSG00000279713
NA	-0.0856	2.1064E-01	4.1124E-01	ENSG00000289028
NA	-0.0833	2.1039E-01	4.1081E-01	ENSG00000260714
NA	-0.0270	2.1025E-01		ENSG00000268731
NA	-0.0878	2.0960E-01	4.0975E-01	ENSG00000284946
NA	-0.0615	2.0908E-01	4.0909E-01	ENSG00000259483
NA	-0.0367	2.0903E-01		ENSG00000223505
NA	-0.0326	2.0874E-01		ENSG00000237629
NA	-0.0952	2.0857E-01	4.0861E-01	ENSG00000235421
NA	-0.0226	2.0821E-01		ENSG00000214973
NA	-0.0253	2.0802E-01		ENSG00000258412
NA	-0.0316	2.0801E-01		ENSG00000231334
NA	-0.1030	2.0766E-01	4.0732E-01	ENSG00000233297
NA	-0.1079	2.0742E-01	4.0704E-01	ENSG00000272892
NA	-0.0898	2.0735E-01	4.0704E-01	ENSG00000250090
NA	-0.0272	2.0723E-01		ENSG00000251687
NA	-0.0478	2.0704E-01		ENSG00000240298
NA	-0.0207	2.0702E-01		ENSG00000279298
NA	-0.0397	2.0695E-01		ENSG00000241282
NA	-0.0487	2.0689E-01	4.0640E-01	ENSG00000242088
NA	-0.0756	2.0666E-01	4.0621E-01	ENSG00000262881
NA	-0.1043	2.0617E-01	4.0583E-01	ENSG00000260805
NA	-0.1037	2.0598E-01	4.0556E-01	ENSG00000288999
NA	-0.0316	2.0590E-01		ENSG00000280117
NA	-0.0290	2.0572E-01		ENSG00000228554
NA	-0.0532	2.0536E-01		ENSG00000263477
NA	-0.0172	2.0534E-01		ENSG00000271395
NA	-0.0897	2.0491E-01	4.0419E-01	ENSG00000273797

NA	-0.1147	2.0481E-01	4.0405E-01	ENSG00000235957
NA	-0.0998	2.0459E-01	4.0384E-01	ENSG00000275552
NA	-0.0680	2.0442E-01	4.0362E-01	ENSG00000271754
NA	-0.0695	2.0440E-01	4.0362E-01	ENSG00000234036
NA	-0.0432	2.0397E-01		ENSG00000220643
NA	-0.0195	2.0387E-01		ENSG00000287118
NA	-0.0891	2.0354E-01	4.0238E-01	ENSG00000288965
NA	-0.0944	2.0351E-01	4.0236E-01	ENSG00000271664
NA	-0.0898	2.0335E-01	4.0214E-01	ENSG00000236875
NA	-0.0570	2.0307E-01		ENSG00000286536
NA	-0.1191	2.0288E-01	4.0186E-01	ENSG00000272476
NA	-0.1180	2.0206E-01	4.0082E-01	ENSG00000269707
NA	-0.1169	2.0202E-01	4.0077E-01	ENSG00000288827
NA	-0.0152	2.0202E-01		ENSG00000225355
NA	-0.1199	2.0170E-01	4.0028E-01	ENSG00000260367
NA	-0.0309	2.0149E-01		ENSG00000287119
NA	-0.0291	2.0130E-01		ENSG00000264254
NA	-0.0400	2.0104E-01		ENSG00000255040
NA	-0.0373	2.0094E-01		ENSG00000254207
NA	-0.0181	2.0066E-01		ENSG00000257175
NA	-0.1154	2.0032E-01	3.9874E-01	ENSG00000215414
NA	-0.0296	1.9960E-01		ENSG00000274284
NA	-0.1098	1.9930E-01	3.9765E-01	ENSG00000270157
NA	-0.0127	1.9918E-01		ENSG00000258829
NA	-0.1067	1.9818E-01	3.9609E-01	ENSG00000248415
NA	-0.1110	1.9779E-01	3.9560E-01	ENSG00000232065
NA	-0.0375	1.9763E-01		ENSG00000271711
NA	-0.0566	1.9753E-01		ENSG00000238140
NA	-0.0278	1.9728E-01		ENSG00000237458
NA	-0.0146	1.9686E-01		ENSG00000278881
NA	-0.0267	1.9604E-01		ENSG00000269172
NA	-0.1207	1.9597E-01	3.9319E-01	ENSG00000227157
NA	-0.0710	1.9572E-01	3.9287E-01	ENSG00000263020
NA	-0.1185	1.9569E-01	3.9283E-01	ENSG00000231551
NA	-0.0557	1.9552E-01	3.9259E-01	ENSG00000218313
NA	-0.0227	1.9541E-01		ENSG00000279714
NA	-0.0249	1.9540E-01		ENSG00000289503
NA	-0.1217	1.9436E-01	3.9109E-01	ENSG00000288913
NA	-0.1043	1.9407E-01	3.9063E-01	ENSG00000286070
NA	-0.0386	1.9368E-01		ENSG00000237631
NA	-0.1169	1.9302E-01	3.8909E-01	ENSG00000235449
NA	-0.0311	1.9300E-01		ENSG00000275006
NA	-0.0311	1.9300E-01		ENSG00000265889
NA	-0.1041	1.9299E-01	3.8909E-01	ENSG00000206659
NA	-0.1139	1.9188E-01	3.8724E-01	ENSG00000260526
NA	-0.0276	1.9147E-01		ENSG00000223254
NA	-0.0193	1.9134E-01		ENSG00000273044
NA	-0.0993	1.9120E-01	3.8654E-01	ENSG00000201592
NA	-0.1137	1.9093E-01	3.8622E-01	ENSG00000230322

NA	-0.0848	1.9060E-01	3.8582E-01	ENSG00000269925
NA	-0.0596	1.9057E-01	3.8580E-01	ENSG00000261783
NA	-0.0460	1.9036E-01	3.8549E-01	ENSG00000213956
NA	-0.1231	1.9010E-01	3.8516E-01	ENSG00000197258
NA	-0.0305	1.9010E-01		ENSG00000286975
NA	-0.1116	1.9004E-01	3.8507E-01	ENSG00000277142
NA	-0.0279	1.8989E-01		ENSG00000263606
NA	-0.0184	1.8970E-01		ENSG00000224079
NA	-0.0749	1.8953E-01	3.8462E-01	ENSG00000174680
NA	-0.1232	1.8913E-01	3.8404E-01	ENSG00000157021
NA	-0.0207	1.8911E-01		ENSG00000258777
NA	-0.0273	1.8887E-01		ENSG00000253213
NA	-0.0711	1.8878E-01	3.8371E-01	ENSG00000285970
NA	-0.0531	1.8864E-01	3.8354E-01	ENSG00000289564
NA	-0.0752	1.8761E-01	3.8227E-01	ENSG00000287155
NA	-0.0373	1.8756E-01		ENSG00000266821
NA	-0.0571	1.8751E-01		ENSG00000231114
NA	-0.0545	1.8742E-01		ENSG00000235656
NA	-0.0254	1.8669E-01		ENSG00000255097
NA	-0.0421	1.8615E-01		ENSG00000249400
NA	-0.0642	1.8554E-01		ENSG00000267062
NA	-0.1247	1.8539E-01	3.7913E-01	ENSG00000280385
NA	-0.1219	1.8436E-01	3.7782E-01	ENSG00000268093
NA	-0.0387	1.8384E-01		ENSG00000266802
NA	-0.1103	1.8373E-01	3.7707E-01	ENSG00000251396
NA	-0.0568	1.8360E-01	3.7694E-01	ENSG00000226609
NA	-0.0432	1.8351E-01		ENSG00000271784
NA	-0.1170	1.8294E-01	3.7607E-01	ENSG00000263164
NA	-0.1199	1.8276E-01	3.7589E-01	ENSG00000234354
NA	-0.0867	1.8243E-01	3.7537E-01	ENSG00000244265
NA	-0.0293	1.8200E-01		ENSG00000228692
NA	-0.0504	1.8178E-01		ENSG00000271064
NA	-0.0456	1.8174E-01		ENSG00000286534
NA	-0.0360	1.8107E-01		ENSG00000275017
NA	-0.0215	1.8075E-01		ENSG00000234336
NA	-0.0348	1.8073E-01		ENSG00000229595
NA	-0.0800	1.8066E-01	3.7287E-01	ENSG00000254481
NA	-0.0413	1.8025E-01		ENSG00000257548
NA	-0.0406	1.7964E-01		ENSG00000259815
NA	-0.0669	1.7951E-01	3.7117E-01	ENSG00000231460
NA	-0.0208	1.7932E-01		ENSG00000258811
NA	-0.0204	1.7914E-01		ENSG00000234998
NA	-0.0215	1.7905E-01		ENSG00000226068
NA	-0.0839	1.7895E-01	3.7045E-01	ENSG00000251639
NA	-0.1254	1.7885E-01	3.7029E-01	ENSG00000230578
NA	-0.0492	1.7885E-01		ENSG00000279107
NA	-0.0339	1.7881E-01		ENSG00000267590
NA	-0.0866	1.7842E-01	3.6962E-01	ENSG00000289231
NA	-0.1071	1.7841E-01	3.6962E-01	ENSG00000264860

NA	-0.0795	1.7834E-01	3.6959E-01	ENSG00000261513
NA	-0.1264	1.7786E-01	3.6908E-01	ENSG00000287419
NA	-0.1027	1.7753E-01	3.6874E-01	ENSG00000213790
NA	-0.0301	1.7685E-01		ENSG00000255893
NA	-0.1261	1.7671E-01	3.6798E-01	ENSG00000246560
NA	-0.0285	1.7670E-01		ENSG00000257921
NA	-0.1024	1.7620E-01	3.6726E-01	ENSG00000278467
NA	-0.0413	1.7574E-01		ENSG00000235115
NA	-0.0259	1.7519E-01		ENSG00000260033
NA	-0.0734	1.7478E-01	3.6515E-01	ENSG00000202079
NA	-0.1038	1.7476E-01	3.6515E-01	ENSG00000253200
NA	-0.0868	1.7455E-01	3.6489E-01	ENSG00000255291
NA	-0.0613	1.7416E-01		ENSG00000235872
NA	-0.0306	1.7413E-01		ENSG00000225303
NA	-0.1019	1.7405E-01	3.6421E-01	ENSG00000267640
NA	-0.0594	1.7377E-01		ENSG00000284726
NA	-0.1293	1.7368E-01	3.6387E-01	ENSG00000285589
NA	-0.0359	1.7289E-01		ENSG00000289893
NA	-0.0515	1.7276E-01		ENSG00000287519
NA	-0.1183	1.7274E-01	3.6252E-01	ENSG00000234185
NA	-0.1084	1.7264E-01	3.6240E-01	ENSG00000225284
NA	-0.1113	1.7256E-01	3.6227E-01	ENSG00000232533
NA	-0.0109	1.7151E-01		ENSG00000289274
NA	-0.1151	1.7136E-01	3.6050E-01	ENSG00000288838
NA	-0.1242	1.7130E-01	3.6047E-01	ENSG00000268942
NA	-0.0866	1.7097E-01	3.6005E-01	ENSG00000275092
NA	-0.0341	1.7061E-01		ENSG00000261089
NA	-0.1300	1.6967E-01	3.5817E-01	ENSG00000131484
NA	-0.0450	1.6965E-01		ENSG00000229015
NA	-0.0978	1.6952E-01	3.5798E-01	ENSG00000237994
NA	-0.1021	1.6949E-01	3.5798E-01	ENSG00000246790
NA	-0.0682	1.6938E-01	3.5786E-01	ENSG00000273076
NA	-0.0305	1.6919E-01		ENSG00000236948
NA	-0.1072	1.6910E-01	3.5760E-01	ENSG00000272693
NA	-0.0423	1.6843E-01		ENSG00000240759
NA	-0.1300	1.6842E-01	3.5682E-01	ENSG00000287886
NA	-0.1041	1.6813E-01	3.5641E-01	ENSG00000225591
NA	-0.0230	1.6776E-01		ENSG00000278469
NA	-0.0307	1.6760E-01	3.5589E-01	ENSG00000285366
NA	-0.1168	1.6730E-01	3.5548E-01	ENSG00000250321
NA	-0.0523	1.6704E-01		ENSG00000232260
NA	-0.0320	1.6683E-01		ENSG00000225642
NA	-0.0857	1.6602E-01	3.5370E-01	ENSG00000259280
NA	-0.0291	1.6515E-01		ENSG00000256381
NA	-0.1192	1.6489E-01	3.5203E-01	ENSG00000289037
NA	-0.0383	1.6469E-01		ENSG00000200741
NA	-0.1294	1.6424E-01	3.5104E-01	ENSG00000272256
NA	-0.0231	1.6399E-01		ENSG00000276823
NA	-0.1340	1.6394E-01	3.5067E-01	ENSG00000237350

NA	-0.0281	1.6393E-01		ENSG00000250257
NA	-0.0435	1.6388E-01		ENSG00000265739
NA	-0.0344	1.6384E-01		ENSG00000290069
NA	-0.1139	1.6325E-01	3.4968E-01	ENSG00000279539
NA	-0.0534	1.6321E-01		ENSG00000274943
NA	-0.0477	1.6311E-01		ENSG00000259013
NA	-0.0274	1.6303E-01		ENSG00000282943
NA	-0.0443	1.6284E-01		ENSG00000256351
NA	-0.0234	1.6281E-01		ENSG00000260555
NA	-0.0611	1.6259E-01	3.4866E-01	ENSG00000259719
NA	-0.0275	1.6230E-01		ENSG00000275234
NA	-0.0206	1.6210E-01		ENSG00000217862
NA	-0.0332	1.6188E-01		ENSG00000230572
NA	-0.0633	1.6163E-01		ENSG00000288393
NA	-0.0338	1.6154E-01		ENSG00000264635
NA	-0.0766	1.6143E-01	3.4733E-01	ENSG00000249604
NA	-0.0764	1.6088E-01	3.4645E-01	ENSG00000285925
NA	-0.1337	1.6075E-01	3.4629E-01	ENSG00000248708
NA	-0.0394	1.6069E-01		ENSG00000248773
NA	-0.1250	1.6049E-01	3.4583E-01	ENSG00000280247
NA	-0.0248	1.6034E-01		ENSG00000254219
NA	-0.0526	1.6020E-01		ENSG00000230953
NA	-0.1330	1.6011E-01	3.4533E-01	ENSG00000285554
NA	-0.0336	1.5987E-01		ENSG00000285656
NA	-0.0146	1.5902E-01		ENSG00000274751
NA	-0.1207	1.5856E-01	3.4279E-01	ENSG00000230747
NA	-0.1280	1.5830E-01	3.4246E-01	ENSG00000235082
NA	-0.0359	1.5821E-01		ENSG00000232387
NA	-0.1348	1.5819E-01	3.4236E-01	ENSG00000261762
NA	-0.0194	1.5811E-01		ENSG00000272896
NA	-0.1233	1.5769E-01	3.4158E-01	ENSG00000233848
NA	-0.1164	1.5766E-01	3.4155E-01	ENSG00000286311
NA	-0.1341	1.5743E-01	3.4117E-01	ENSG00000281501
NA	-0.0780	1.5742E-01	3.4117E-01	ENSG00000235066
NA	-0.0649	1.5737E-01	3.4109E-01	ENSG00000262434
NA	-0.1368	1.5736E-01	3.4109E-01	ENSG00000259377
NA	-0.0248	1.5721E-01		ENSG00000200688
NA	-0.0640	1.5689E-01		ENSG00000213177
NA	-0.1210	1.5689E-01	3.4056E-01	ENSG00000186940
NA	-0.0481	1.5684E-01		ENSG00000275569
NA	-0.0288	1.5679E-01		ENSG00000228286
NA	-0.1267	1.5663E-01	3.4030E-01	ENSG00000228158
NA	-0.1044	1.5630E-01	3.4022E-01	ENSG00000236337
NA	-0.0614	1.5620E-01		ENSG00000278616
NA	-0.0325	1.5609E-01		ENSG00000235298
NA	-0.0404	1.5578E-01		ENSG00000250990
NA	-0.0938	1.5575E-01	3.3944E-01	ENSG00000289463
NA	-0.0545	1.5537E-01		ENSG00000232754
NA	-0.1049	1.5413E-01	3.3675E-01	ENSG00000224965

NA	-0.0403	1.5379E-01		ENSG00000238034
NA	-0.0330	1.5378E-01		ENSG00000226890
NA	-0.0527	1.5344E-01		ENSG00000242602
NA	-0.1168	1.5335E-01	3.3558E-01	ENSG00000268297
NA	-0.0162	1.5319E-01		ENSG00000241030
NA	-0.1099	1.5294E-01	3.3510E-01	ENSG00000231351
NA	-0.0926	1.5277E-01	3.3481E-01	ENSG00000276103
NA	-0.1362	1.5273E-01	3.3479E-01	ENSG00000279738
NA	-0.0768	1.5263E-01	3.3479E-01	ENSG00000283196
NA	-0.0554	1.5239E-01		ENSG00000288698
NA	-0.1356	1.5224E-01	3.3416E-01	ENSG00000223756
NA	-0.1367	1.5207E-01	3.3390E-01	ENSG00000258655
NA	-0.1336	1.5161E-01	3.3322E-01	ENSG00000275055
NA	-0.0584	1.5144E-01		ENSG00000215480
NA	-0.1237	1.5118E-01	3.3255E-01	ENSG00000227946
NA	-0.0841	1.5078E-01	3.3195E-01	ENSG00000277053
NA	-0.0929	1.5067E-01	3.3187E-01	ENSG00000286856
NA	-0.1252	1.5038E-01	3.3146E-01	ENSG00000257390
NA	-0.0600	1.4999E-01	3.3091E-01	ENSG00000257703
NA	-0.0817	1.4983E-01	3.3070E-01	ENSG00000250472
NA	-0.1356	1.4981E-01	3.3067E-01	ENSG00000257802
NA	-0.0256	1.4980E-01		ENSG00000213673
NA	-0.1351	1.4978E-01	3.3063E-01	ENSG00000289283
NA	-0.0898	1.4956E-01	3.3032E-01	ENSG00000267592
NA	-0.0317	1.4949E-01		ENSG00000227607
NA	-0.1355	1.4948E-01	3.3020E-01	ENSG00000285907
NA	-0.0349	1.4935E-01		ENSG00000267703
NA	-0.1302	1.4903E-01	3.2951E-01	ENSG00000231747
NA	-0.1400	1.4887E-01	3.2945E-01	ENSG00000279069
NA	-0.1342	1.4879E-01	3.2934E-01	ENSG00000286786
NA	-0.1350	1.4867E-01	3.2923E-01	ENSG00000224599
NA	-0.0948	1.4856E-01	3.2908E-01	ENSG00000259677
NA	-0.1049	1.4842E-01	3.2895E-01	ENSG00000210156
NA	-0.1324	1.4822E-01	3.2866E-01	ENSG00000234004
NA	-0.0239	1.4822E-01		ENSG00000270839
NA	-0.0520	1.4710E-01	3.2693E-01	ENSG00000280406
NA	-0.1028	1.4707E-01	3.2692E-01	ENSG00000226608
NA	-0.0722	1.4669E-01	3.2636E-01	ENSG00000233644
NA	-0.0732	1.4646E-01	3.2610E-01	ENSG00000254670
NA	-0.1441	1.4593E-01	3.2514E-01	ENSG00000263535
NA	-0.0509	1.4561E-01		ENSG00000259669
NA	-0.0232	1.4537E-01		ENSG00000279386
NA	-0.1293	1.4526E-01	3.2405E-01	ENSG00000231170
NA	-0.1371	1.4526E-01	3.2405E-01	ENSG00000228218
NA	-0.1362	1.4478E-01	3.2340E-01	ENSG00000269688
NA	-0.0756	1.4437E-01		ENSG00000255159
NA	-0.0978	1.4380E-01	3.2183E-01	ENSG00000214553
NA	-0.1220	1.4369E-01	3.2175E-01	ENSG00000287355
NA	-0.0498	1.4324E-01		ENSG00000274356

NA	-0.0296	1.4295E-01		ENSG00000259581
NA	-0.1381	1.4275E-01	3.2039E-01	ENSG00000204620
NA	-0.0201	1.4274E-01		ENSG00000231544
NA	-0.0732	1.4262E-01	3.2015E-01	ENSG00000279217
NA	-0.0465	1.4254E-01		ENSG00000213050
NA	-0.0181	1.4219E-01	3.1947E-01	ENSG00000234446
NA	-0.1159	1.4216E-01	3.1944E-01	ENSG00000273249
NA	-0.0642	1.4120E-01	3.1804E-01	ENSG00000234197
NA	-0.1440	1.4089E-01	3.1760E-01	ENSG00000289455
NA	-0.0285	1.4017E-01		ENSG00000201114
NA	-0.0967	1.3983E-01	3.1625E-01	ENSG00000220130
NA	-0.0348	1.3957E-01		ENSG00000237852
NA	-0.0648	1.3917E-01	3.1529E-01	ENSG00000224099
NA	-0.0743	1.3847E-01	3.1439E-01	ENSG00000273107
NA	-0.1110	1.3831E-01	3.1425E-01	ENSG00000266990
NA	-0.1422	1.3787E-01	3.1363E-01	ENSG00000224790
NA	-0.1365	1.3775E-01	3.1347E-01	ENSG00000289394
NA	-0.0871	1.3767E-01		ENSG00000271983
NA	-0.0396	1.3759E-01		ENSG00000241656
NA	-0.1272	1.3712E-01	3.1250E-01	ENSG00000240695
NA	-0.1194	1.3701E-01	3.1238E-01	ENSG00000233108
NA	-0.0759	1.3602E-01	3.1098E-01	ENSG00000259006
NA	-0.1451	1.3600E-01	3.1097E-01	ENSG00000267053
NA	-0.1308	1.3596E-01	3.1091E-01	ENSG00000228386
NA	-0.1444	1.3545E-01	3.1026E-01	ENSG00000272483
NA	-0.0555	1.3540E-01		ENSG00000281112
NA	-0.1443	1.3535E-01	3.1009E-01	ENSG00000236498
NA	-0.0225	1.3514E-01		ENSG00000203402
NA	-0.1425	1.3505E-01	3.0956E-01	ENSG00000235785
NA	-0.1448	1.3451E-01	3.0888E-01	ENSG00000251495
NA	-0.0698	1.3435E-01		ENSG00000253721
NA	-0.1378	1.3418E-01	3.0831E-01	ENSG00000254610
NA	-0.1396	1.3412E-01	3.0821E-01	ENSG00000272710
NA	-0.1325	1.3397E-01	3.0803E-01	ENSG00000279369
NA	-0.0602	1.3380E-01		ENSG00000235085
NA	-0.0372	1.3377E-01		ENSG00000286918
NA	-0.1414	1.3355E-01	3.0730E-01	ENSG00000267422
NA	-0.1324	1.3355E-01	3.0730E-01	ENSG00000260007
NA	-0.1461	1.3341E-01	3.0717E-01	ENSG00000213885
NA	-0.1360	1.3303E-01	3.0671E-01	ENSG00000289001
NA	-0.1066	1.3272E-01	3.0630E-01	ENSG00000255286
NA	-0.1342	1.3262E-01	3.0620E-01	ENSG00000204791
NA	-0.0307	1.3204E-01		ENSG00000232970
NA	-0.1170	1.3195E-01	3.0528E-01	ENSG00000285204
NA	-0.0711	1.3170E-01		ENSG00000253439
NA	-0.0971	1.3159E-01	3.0473E-01	ENSG00000274594
NA	-0.0685	1.3114E-01		ENSG00000280281
NA	-0.0676	1.3110E-01		ENSG00000278202
NA	-0.1418	1.3079E-01	3.0373E-01	ENSG00000272822

NA	-0.1329	1.3076E-01	3.0370E-01	ENSG00000285053
NA	-0.1202	1.3073E-01	3.0365E-01	ENSG00000289044
NA	-0.0425	1.3057E-01		ENSG00000236073
NA	-0.1348	1.3024E-01	3.0303E-01	ENSG00000213888
NA	-0.0978	1.3019E-01	3.0297E-01	ENSG00000224985
NA	-0.1490	1.3011E-01	3.0284E-01	ENSG00000287623
NA	-0.0223	1.2965E-01		ENSG00000265975
NA	-0.1348	1.2959E-01	3.0212E-01	ENSG00000285382
NA	-0.0525	1.2946E-01		ENSG00000227911
NA	-0.0845	1.2939E-01	3.0183E-01	ENSG00000224825
NA	-0.0251	1.2909E-01	3.0136E-01	ENSG00000228347
NA	-0.0552	1.2856E-01	3.0033E-01	ENSG00000259699
NA	-0.1336	1.2849E-01	3.0021E-01	ENSG00000227097
NA	-0.0757	1.2845E-01	3.0018E-01	ENSG00000266900
NA	-0.1440	1.2824E-01	2.9991E-01	ENSG00000272871
NA	-0.1229	1.2808E-01	2.9974E-01	ENSG00000286451
NA	-0.1475	1.2801E-01	2.9973E-01	ENSG00000290090
NA	-0.0348	1.2748E-01		ENSG00000253104
NA	-0.0233	1.2701E-01		ENSG00000286185
NA	-0.1420	1.2691E-01	2.9827E-01	ENSG00000265800
NA	-0.0706	1.2676E-01	2.9805E-01	ENSG00000234819
NA	-0.0782	1.2651E-01		ENSG00000250240
NA	-0.0354	1.2573E-01		ENSG00000258788
NA	-0.1475	1.2534E-01	2.9553E-01	ENSG00000287117
NA	-0.1416	1.2527E-01	2.9542E-01	ENSG00000260558
NA	-0.0548	1.2489E-01		ENSG00000269119
NA	-0.0291	1.2471E-01		ENSG00000270682
NA	-0.0437	1.2450E-01		ENSG00000287931
NA	-0.1431	1.2406E-01	2.9384E-01	ENSG00000238058
NA	-0.1492	1.2388E-01	2.9362E-01	ENSG00000228175
NA	-0.0497	1.2299E-01		ENSG00000262380
NA	-0.0926	1.2264E-01	2.9159E-01	ENSG00000260447
NA	-0.1383	1.2250E-01	2.9141E-01	ENSG00000272715
NA	-0.1142	1.2236E-01	2.9116E-01	ENSG00000286616
NA	-0.1498	1.2227E-01	2.9109E-01	ENSG00000258424
NA	-0.0586	1.2171E-01		ENSG00000254648
NA	-0.0182	1.2112E-01		ENSG00000252192
NA	-0.0434	1.2110E-01		ENSG00000227852
NA	-0.0355	1.2095E-01		ENSG00000206797
NA	-0.0354	1.2072E-01		ENSG00000224394
NA	-0.0645	1.2040E-01		ENSG00000236327
NA	-0.0316	1.2032E-01		ENSG00000206734
NA	-0.1530	1.1986E-01	2.8710E-01	ENSG00000235084
NA	-0.1506	1.1905E-01	2.8587E-01	ENSG00000212829
NA	-0.0643	1.1864E-01		ENSG00000230197
NA	-0.0939	1.1861E-01	2.8509E-01	ENSG00000249149
NA	-0.1045	1.1755E-01	2.8313E-01	ENSG00000284661
NA	-0.1435	1.1741E-01	2.8294E-01	ENSG00000253931
NA	-0.0873	1.1729E-01	2.8288E-01	ENSG00000283945

NA	-0.0782	1.1714E-01	2.8258E-01	ENSG00000288984
NA	-0.0821	1.1666E-01	2.8186E-01	ENSG00000286331
NA	-0.0805	1.1649E-01	2.8156E-01	ENSG00000288548
NA	-0.0674	1.1648E-01		ENSG00000180389
NA	-0.1591	1.1551E-01	2.8013E-01	ENSG00000270083
NA	-0.0261	1.1511E-01		ENSG00000285807
NA	-0.0733	1.1501E-01		ENSG00000276250
NA	-0.0879	1.1498E-01	2.7940E-01	ENSG00000258604
NA	-0.0349	1.1389E-01		ENSG00000227383
NA	-0.1056	1.1376E-01	2.7743E-01	ENSG00000283972
NA	-0.0702	1.1373E-01		ENSG00000225416
NA	-0.0930	1.1366E-01	2.7722E-01	ENSG00000252096
NA	-0.0501	1.1316E-01		ENSG00000230534
NA	-0.2355	1.1296E-01	2.7595E-01	ENSG00000288808
NA	-0.0229	1.1273E-01		ENSG00000230757
NA	-0.1285	1.1269E-01	2.7548E-01	ENSG00000285151
NA	-0.0903	1.1264E-01	2.7543E-01	ENSG00000224376
NA	-0.0889	1.1236E-01	2.7495E-01	ENSG00000259308
NA	-0.0571	1.1233E-01		ENSG00000176183
NA	-0.0936	1.1221E-01	2.7471E-01	ENSG00000236549
NA	-0.1488	1.1176E-01	2.7391E-01	ENSG00000241112
NA	-0.1414	1.1144E-01	2.7336E-01	ENSG00000287555
NA	-0.0986	1.1121E-01	2.7302E-01	ENSG00000230536
NA	-0.0257	1.1093E-01		ENSG00000285835
NA	-0.0250	1.1075E-01		ENSG00000237327
NA	-0.1580	1.1070E-01	2.7241E-01	ENSG00000270124
NA	-0.1479	1.1049E-01	2.7207E-01	ENSG00000248161
NA	-0.1203	1.0988E-01	2.7090E-01	ENSG00000271843
NA	-0.1385	1.0973E-01	2.7073E-01	ENSG00000258337
NA	-0.0239	1.0944E-01		ENSG00000224222
NA	-0.0431	1.0927E-01		ENSG00000231625
NA	-0.1317	1.0824E-01	2.6824E-01	ENSG00000288817
NA	-0.0761	1.0787E-01		ENSG00000279481
NA	-0.0914	1.0754E-01	2.6693E-01	ENSG00000282885
NA	-0.1440	1.0754E-01	2.6693E-01	ENSG00000275383
NA	-0.1635	1.0698E-01	2.6609E-01	ENSG00000279306
NA	-0.1449	1.0683E-01	2.6592E-01	ENSG00000254653
NA	-0.1371	1.0671E-01	2.6571E-01	ENSG00000232406
NA	-0.1569	1.0618E-01	2.6496E-01	ENSG00000263551
NA	-0.0596	1.0596E-01		ENSG00000253238
NA	-0.1025	1.0575E-01	2.6438E-01	ENSG00000253516
NA	-0.1577	1.0539E-01	2.6378E-01	ENSG00000231519
NA	-0.1460	1.0522E-01	2.6353E-01	ENSG00000269584
NA	-0.0308	1.0487E-01		ENSG00000280166
NA	-0.0351	1.0485E-01		ENSG00000250882
NA	-0.0441	1.0471E-01		ENSG00000248590
NA	-0.0849	1.0467E-01	2.6252E-01	ENSG00000251467
NA	-0.1527	1.0449E-01	2.6219E-01	ENSG00000263179
NA	-0.1469	1.0372E-01	2.6089E-01	ENSG00000206768

NA	-0.0745	1.0321E-01		ENSG00000260467
NA	-0.1658	1.0310E-01	2.5994E-01	ENSG00000288957
NA	-0.0253	1.0309E-01		ENSG00000287511
NA	-0.1451	1.0309E-01	2.5994E-01	ENSG00000225335
NA	-0.1179	1.0295E-01	2.5978E-01	ENSG00000249210
NA	-0.0933	1.0255E-01	2.5907E-01	ENSG00000258466
NA	-0.1594	1.0171E-01	2.5762E-01	ENSG00000230306
NA	-0.1562	1.0169E-01	2.5762E-01	ENSG00000253683
NA	-0.1596	1.0138E-01	2.5708E-01	ENSG00000258728
NA	-0.1362	1.0128E-01	2.5694E-01	ENSG00000239486
NA	-0.0462	1.0125E-01		ENSG00000259691
NA	-0.0294	1.0125E-01		ENSG00000259356
NA	-0.0916	1.0061E-01	2.5575E-01	ENSG00000269886
NA	-0.0522	1.0046E-01		ENSG00000215210
NA	-0.1680	9.9508E-02	2.5382E-01	ENSG00000289032
NA	-0.0752	9.9207E-02		ENSG00000253047
NA	-0.0368	9.8925E-02		ENSG00000289478
NA	-0.1567	9.8692E-02	2.5261E-01	ENSG00000206706
NA	-0.1090	9.8641E-02	2.5252E-01	ENSG00000268154
NA	-0.1683	9.8225E-02	2.5191E-01	ENSG00000256712
NA	-0.0364	9.8141E-02		ENSG00000261612
NA	-0.0493	9.7930E-02	2.5136E-01	ENSG00000255986
NA	-0.1616	9.7819E-02	2.5121E-01	ENSG00000232445
NA	-0.1276	9.7724E-02	2.5114E-01	ENSG00000286899
NA	-0.1694	9.7662E-02	2.5109E-01	ENSG00000228434
NA	-0.1458	9.7498E-02	2.5086E-01	ENSG00000231707
NA	-0.1426	9.7483E-02	2.5084E-01	ENSG00000255670
NA	-0.1514	9.7415E-02	2.5074E-01	ENSG00000274818
NA	-0.0556	9.7388E-02		ENSG00000213409
NA	-0.0762	9.6986E-02	2.5019E-01	ENSG00000234607
NA	-0.1596	9.6849E-02	2.4997E-01	ENSG00000226702
NA	-0.1728	9.6715E-02	2.4983E-01	ENSG00000277938
NA	-0.0244	9.6562E-02		ENSG00000224007
NA	-0.0648	9.6462E-02		ENSG00000238273
NA	-0.0245	9.5881E-02		ENSG00000228541
NA	-0.1305	9.5505E-02	2.4775E-01	ENSG00000251186
NA	-0.0425	9.5372E-02		ENSG00000241478
NA	-0.1220	9.5305E-02	2.4736E-01	ENSG00000228782
NA	-0.1716	9.4917E-02	2.4664E-01	ENSG00000286431
NA	-0.1721	9.4868E-02	2.4654E-01	ENSG00000229927
NA	-0.1174	9.4235E-02	2.4546E-01	ENSG00000274364
NA	-0.1677	9.4133E-02	2.4528E-01	ENSG00000234678
NA	-0.1352	9.4013E-02	2.4504E-01	ENSG00000286164
NA	-0.0771	9.3918E-02	2.4487E-01	ENSG00000261553
NA	-0.1348	9.3822E-02	2.4467E-01	ENSG00000286608
NA	-0.0442	9.3769E-02		ENSG00000232600
NA	-0.0707	9.3470E-02		ENSG00000269506
NA	-0.0575	9.3240E-02		ENSG00000236527
NA	-0.0691	9.3039E-02		ENSG00000259055

NA	-0.1371	9.2886E-02	2.4317E-01	ENSG00000154608
NA	-0.1450	9.2670E-02	2.4270E-01	ENSG00000234042
NA	-0.0307	9.2550E-02		ENSG00000289275
NA	-0.1060	9.2350E-02	2.4207E-01	ENSG00000267801
NA	-0.0931	9.2302E-02	2.4202E-01	ENSG00000278044
NA	-0.1170	9.2113E-02	2.4167E-01	ENSG00000224728
NA	-0.0449	9.1883E-02		ENSG00000257754
NA	-0.0513	9.1753E-02		ENSG00000236813
NA	-0.0406	9.1320E-02		ENSG00000235816
NA	-0.1731	9.0852E-02	2.3949E-01	ENSG00000223864
NA	-0.0274	9.0824E-02		ENSG00000199751
NA	-0.0826	9.0762E-02	2.3940E-01	ENSG00000267144
NA	-0.0410	9.0706E-02		ENSG00000213650
NA	-0.0745	9.0668E-02		ENSG00000232454
NA	-0.1719	8.9734E-02	2.3733E-01	ENSG00000280162
NA	-0.0656	8.9531E-02		ENSG00000201916
NA	-0.1784	8.9388E-02	2.3660E-01	ENSG00000243193
NA	-0.1653	8.8724E-02	2.3519E-01	ENSG00000236710
NA	-0.0438	8.8114E-02		ENSG00000271868
NA	-0.0458	8.7676E-02		ENSG00000264727
NA	-0.1561	8.7448E-02	2.3284E-01	ENSG00000226268
NA	-0.1433	8.7337E-02	2.3264E-01	ENSG00000256458
NA	-0.1781	8.7323E-02	2.3263E-01	ENSG00000280061
NA	-0.0881	8.6589E-02	2.3113E-01	ENSG00000265174
NA	-0.1753	8.5003E-02	2.2815E-01	ENSG00000240622
NA	-0.0657	8.4797E-02		ENSG00000287068
NA	-0.1754	8.4530E-02	2.2728E-01	ENSG00000272275
NA	-0.1761	8.4418E-02	2.2711E-01	ENSG00000213228
NA	-0.1804	8.4179E-02	2.2670E-01	ENSG00000227227
NA	-0.0601	8.3920E-02		ENSG00000263368
NA	-0.0576	8.2938E-02	2.2455E-01	ENSG00000258016
NA	-0.1606	8.2905E-02	2.2453E-01	ENSG00000233205
NA	-0.1455	8.2903E-02	2.2453E-01	ENSG00000174028
NA	-0.0174	8.2865E-02	2.2450E-01	ENSG00000232368
NA	-0.0639	8.2510E-02		ENSG00000240211
NA	-0.0573	8.2111E-02	2.2302E-01	ENSG00000259338
NA	-0.0583	8.2001E-02		ENSG00000261478
NA	-0.1780	8.1963E-02	2.2274E-01	ENSG00000178162
NA	-0.0991	8.1872E-02	2.2263E-01	ENSG00000234208
NA	-0.1886	8.1673E-02	2.2232E-01	ENSG00000289515
NA	-0.1865	8.1495E-02	2.2208E-01	ENSG00000260838
NA	-0.0815	8.1045E-02		ENSG00000262228
NA	-0.1819	8.0996E-02	2.2121E-01	ENSG00000289198
NA	-0.1472	8.0940E-02	2.2113E-01	ENSG00000224597
NA	-0.0696	8.0766E-02		ENSG00000267523
NA	-0.1617	8.0537E-02	2.2059E-01	ENSG00000268516
NA	-0.1628	8.0228E-02	2.2009E-01	ENSG00000271025
NA	-0.1085	8.0147E-02	2.1994E-01	ENSG00000277440
NA	-0.1682	7.9687E-02	2.1912E-01	ENSG00000251050

NA	-0.1339	7.9288E-02	2.1836E-01	ENSG00000198406
NA	-0.0810	7.8748E-02		ENSG00000269487
NA	-0.0259	7.8672E-02		ENSG00000253605
NA	-0.0107	7.8154E-02	2.1624E-01	ENSG00000289413
NA	-0.0542	7.7917E-02		ENSG00000279356
NA	-0.1796	7.7704E-02	2.1550E-01	ENSG00000235253
NA	-0.0249	7.7658E-02		ENSG00000280028
NA	-0.0814	7.7365E-02		ENSG00000233367
NA	-0.0614	7.7278E-02		ENSG00000279589
NA	-0.1603	7.7147E-02	2.1441E-01	ENSG00000179899
NA	-0.1884	7.7111E-02	2.1434E-01	ENSG00000278963
NA	-0.0584	7.7056E-02		ENSG00000271382
NA	-0.1722	7.6845E-02	2.1384E-01	ENSG00000265293
NA	-0.1334	7.6677E-02	2.1357E-01	ENSG00000218582
NA	-0.0806	7.6579E-02		ENSG00000261987
NA	-0.1812	7.6492E-02	2.1332E-01	ENSG00000210049
NA	-0.1147	7.6419E-02	2.1321E-01	ENSG00000273247
NA	-0.1043	7.6395E-02	2.1319E-01	ENSG00000205940
NA	-0.0892	7.6145E-02	2.1256E-01	ENSG00000286782
NA	-0.0949	7.5970E-02	2.1232E-01	ENSG00000213509
NA	-0.0409	7.5797E-02		ENSG00000231953
NA	-0.1153	7.5438E-02	2.1119E-01	ENSG00000283515
NA	-0.1003	7.5438E-02	2.1119E-01	ENSG00000277634
NA	-0.0664	7.5292E-02		ENSG00000272468
NA	-0.0308	7.5032E-02		ENSG00000231120
NA	-0.1776	7.4492E-02	2.0950E-01	ENSG00000233405
NA	-0.1966	7.4446E-02	2.0941E-01	ENSG00000268230
NA	-0.1869	7.4263E-02	2.0909E-01	ENSG00000279417
NA	-0.0964	7.3744E-02	2.0815E-01	ENSG00000256982
NA	-0.1807	7.3620E-02	2.0789E-01	ENSG00000251301
NA	-0.1914	7.3616E-02	2.0789E-01	ENSG00000264577
NA	-0.1855	7.3511E-02	2.0773E-01	ENSG00000198134
NA	-0.1799	7.3351E-02	2.0747E-01	ENSG00000235280
NA	-0.1819	7.3293E-02	2.0744E-01	ENSG00000230593
NA	-0.1910	7.3062E-02	2.0692E-01	ENSG00000225695
NA	-0.0239	7.2885E-02		ENSG00000285708
NA	-0.1837	7.2260E-02	2.0538E-01	ENSG00000228010
NA	-0.1777	7.2019E-02	2.0486E-01	ENSG00000164616
NA	-0.1908	7.1871E-02	2.0453E-01	ENSG00000287817
NA	-0.0383	7.1643E-02		ENSG00000272279
NA	-0.1909	7.1505E-02	2.0386E-01	ENSG00000254461
NA	-0.0694	7.1088E-02		ENSG00000231564
NA	-0.0840	7.0665E-02	2.0187E-01	ENSG00000241473
NA	-0.0847	7.0468E-02		ENSG00000283907
NA	-0.1823	7.0425E-02	2.0140E-01	ENSG00000228007
NA	-0.0961	7.0148E-02	2.0106E-01	ENSG00000279372
NA	-0.0999	6.9980E-02	2.0077E-01	ENSG00000289110
NA	-0.1013	6.9729E-02		ENSG00000214289
NA	-0.1735	6.9595E-02	1.9993E-01	ENSG00000213467

NA	-0.1946	6.9169E-02	1.9896E-01	ENSG00000203497
NA	-0.1796	6.9079E-02	1.9879E-01	ENSG00000289504
NA	-0.0656	6.8894E-02		ENSG00000229032
NA	-0.1134	6.8749E-02	1.9821E-01	ENSG00000236671
NA	-0.0824	6.8026E-02	1.9675E-01	ENSG00000226899
NA	-0.0548	6.7841E-02		ENSG00000213522
NA	-0.1554	6.7761E-02	1.9628E-01	ENSG00000285766
NA	-0.1322	6.7551E-02	1.9600E-01	ENSG00000261037
NA	-0.0398	6.7547E-02		ENSG00000287347
NA	-0.1337	6.7475E-02	1.9583E-01	ENSG00000262692
NA	-0.1606	6.7255E-02	1.9547E-01	ENSG00000244313
NA	-0.1274	6.7066E-02	1.9509E-01	ENSG00000230305
NA	-0.1194	6.6740E-02	1.9447E-01	ENSG00000258851
NA	-0.0513	6.6657E-02		ENSG00000270487
NA	-0.0608	6.6638E-02		ENSG00000288556
NA	-0.1552	6.5787E-02	1.9251E-01	ENSG00000270072
NA	-0.1002	6.5436E-02	1.9170E-01	ENSG00000251118
NA	-0.0841	6.5105E-02		ENSG00000235554
NA	-0.1896	6.4998E-02	1.9079E-01	ENSG00000239559
NA	-0.1019	6.4973E-02	1.9077E-01	ENSG00000269901
NA	-0.2019	6.4879E-02	1.9061E-01	ENSG00000284606
NA	-0.1135	6.4793E-02	1.9049E-01	ENSG00000269066
NA	-0.2005	6.4708E-02	1.9033E-01	ENSG00000213236
NA	-0.1651	6.4387E-02	1.8969E-01	ENSG00000234287
NA	-0.2038	6.4141E-02	1.8909E-01	ENSG00000261094
NA	-0.0327	6.3948E-02		ENSG00000278446
NA	-0.1001	6.3745E-02	1.8824E-01	ENSG00000230415
NA	-0.0886	6.3736E-02	1.8824E-01	ENSG00000237446
NA	-0.2030	6.3736E-02	1.8824E-01	ENSG00000288253
NA	-0.1198	6.3530E-02	1.8788E-01	ENSG00000235090
NA	-0.0683	6.3364E-02		ENSG00000269967
NA	-0.0555	6.3249E-02		ENSG00000236535
NA	-0.0586	6.3046E-02		ENSG00000235852
NA	-0.1757	6.2717E-02	1.8608E-01	ENSG00000265916
NA	-0.1152	6.1955E-02	1.8458E-01	ENSG00000259917
NA	-0.0732	6.1075E-02		ENSG00000203647
NA	-0.1320	6.0667E-02	1.8177E-01	ENSG00000235262
NA	-0.1040	6.0619E-02		ENSG00000253335
NA	-0.0975	6.0202E-02		ENSG00000217241
NA	-0.0886	6.0159E-02		ENSG00000263206
NA	-0.0788	5.9986E-02		ENSG00000253509
NA	-0.1538	5.9799E-02	1.7986E-01	ENSG00000287825
NA	-0.1304	5.9683E-02	1.7959E-01	ENSG00000235174
NA	-0.2077	5.9552E-02	1.7934E-01	ENSG00000276698
NA	-0.1720	5.9420E-02	1.7919E-01	ENSG00000239911
NA	-0.2021	5.9337E-02	1.7907E-01	ENSG00000279766
NA	-0.1950	5.8846E-02	1.7820E-01	ENSG00000207294
NA	-0.2133	5.8623E-02	1.7786E-01	ENSG00000278834
NA	-0.1952	5.8448E-02	1.7741E-01	ENSG00000257576

NA	-0.1789	5.8376E-02	1.7729E-01	ENSG00000270587
NA	-0.2127	5.8244E-02	1.7708E-01	ENSG00000255389
NA	-0.2121	5.8241E-02	1.7708E-01	ENSG00000279281
NA	-0.0350	5.7951E-02		ENSG00000220660
NA	-0.1406	5.7846E-02	1.7630E-01	ENSG00000262298
NA	-0.1916	5.7777E-02	1.7617E-01	ENSG00000279827
NA	-0.2106	5.7733E-02	1.7617E-01	ENSG00000288577
NA	-0.1311	5.7593E-02	1.7596E-01	ENSG00000244573
NA	-0.0485	5.7557E-02	1.7594E-01	ENSG00000219149
NA	-0.1112	5.7556E-02	1.7594E-01	ENSG00000279459
NA	-0.1465	5.7305E-02	1.7542E-01	ENSG00000289698
NA	-0.2207	5.6444E-02	1.7364E-01	ENSG00000284620
NA	-0.0573	5.6261E-02		ENSG00000213370
NA	-0.0987	5.6183E-02	1.7321E-01	ENSG00000273855
NA	-0.1640	5.5991E-02	1.7281E-01	ENSG00000254708
NA	-0.2021	5.5974E-02	1.7278E-01	ENSG00000250950
NA	-0.0923	5.5906E-02		ENSG00000258846
NA	-0.1173	5.5873E-02	1.7256E-01	ENSG00000266941
NA	-0.2079	5.5784E-02	1.7237E-01	ENSG00000259083
NA	-0.0384	5.5663E-02		ENSG00000286942
NA	-0.1999	5.5649E-02	1.7204E-01	ENSG00000270503
NA	-0.0303	5.5575E-02		ENSG00000223795
NA	-0.2147	5.5249E-02	1.7115E-01	ENSG00000236603
NA	-0.1149	5.4425E-02		ENSG00000253875
NA	-0.1488	5.4390E-02	1.6912E-01	ENSG00000249870
NA	-0.1503	5.4088E-02	1.6851E-01	ENSG00000263120
NA	-0.0702	5.4023E-02		ENSG00000214192
NA	-0.2137	5.3867E-02	1.6807E-01	ENSG00000279838
NA	-0.0264	5.3652E-02		ENSG00000228976
NA	-0.1854	5.3553E-02	1.6722E-01	ENSG00000260664
NA	-0.2114	5.3194E-02	1.6639E-01	ENSG00000247735
NA	-0.0390	5.2979E-02		ENSG00000233581
NA	-0.2082	5.2946E-02	1.6578E-01	ENSG00000277511
NA	-0.2207	5.2939E-02	1.6578E-01	ENSG00000287804
NA	-0.1653	5.2569E-02	1.6498E-01	ENSG00000287180
NA	-0.0689	5.2190E-02	1.6409E-01	ENSG00000287707
NA	-0.2267	5.2057E-02	1.6379E-01	ENSG00000280033
NA	-0.1037	5.1784E-02		ENSG00000267255
NA	-0.1823	5.1687E-02	1.6302E-01	ENSG00000236924
NA	-0.1833	5.1650E-02	1.6299E-01	ENSG00000287855
NA	-0.1060	5.1399E-02	1.6245E-01	ENSG00000286263
NA	-0.2213	5.0982E-02	1.6153E-01	ENSG00000253966
NA	-0.1834	5.0843E-02	1.6134E-01	ENSG00000223984
NA	-0.0296	5.0728E-02		ENSG00000279587
NA	-0.1739	5.0711E-02	1.6105E-01	ENSG00000226964
NA	-0.0737	5.0320E-02		ENSG00000276746
NA	-0.1068	5.0250E-02	1.6013E-01	ENSG00000240122
NA	-0.2069	5.0152E-02	1.5999E-01	ENSG00000233966
NA	-0.1683	5.0006E-02	1.5975E-01	ENSG00000206927

NA	-0.1670	4.9855E-02	1.5935E-01	ENSG00000288704
NA	-0.1183	4.9680E-02	1.5892E-01	ENSG00000276724
NA	-0.1022	4.9637E-02	1.5880E-01	ENSG00000249203
NA	-0.1223	4.8713E-02	1.5671E-01	ENSG00000287351
NA	-0.0738	4.8469E-02		ENSG00000267557
NA	-0.0369	4.8384E-02		ENSG00000257884
NA	-0.1807	4.8383E-02	1.5600E-01	ENSG00000285596
NA	-0.2348	4.8291E-02	1.5577E-01	ENSG00000261079
NA	-0.0941	4.8241E-02	1.5563E-01	ENSG00000236434
NA	-0.2059	4.8108E-02	1.5536E-01	ENSG00000177855
NA	-0.0780	4.7942E-02		ENSG00000280443
NA	-0.1355	4.7889E-02	1.5486E-01	ENSG00000239300
NA	-0.1819	4.7809E-02	1.5473E-01	ENSG00000248079
NA	-0.1622	4.7753E-02	1.5466E-01	ENSG00000267174
NA	-0.0913	4.7622E-02		ENSG00000272582
NA	-0.2300	4.7523E-02	1.5428E-01	ENSG00000287743
NA	-0.1291	4.7333E-02	1.5381E-01	ENSG00000254328
NA	-0.1381	4.7070E-02	1.5328E-01	ENSG00000289551
NA	-0.1781	4.6976E-02	1.5313E-01	ENSG00000272597
NA	-0.2214	4.6753E-02	1.5255E-01	ENSG00000282933
NA	-0.0893	4.6690E-02		ENSG00000229750
NA	-0.2111	4.6535E-02	1.5206E-01	ENSG00000288766
NA	-0.2183	4.6349E-02	1.5165E-01	ENSG00000242615
NA	-0.1418	4.6126E-02	1.5117E-01	ENSG00000232693
NA	-0.2039	4.5902E-02	1.5067E-01	ENSG00000234769
NA	-0.2100	4.5452E-02	1.4961E-01	ENSG00000226701
NA	-0.0750	4.5229E-02		ENSG00000227192
NA	-0.2209	4.4990E-02	1.4840E-01	ENSG00000154898
NA	-0.0913	4.4615E-02		ENSG00000279071
NA	-0.1282	4.4076E-02	1.4616E-01	ENSG00000279358
NA	-0.1218	4.3969E-02	1.4592E-01	ENSG00000267662
NA	-0.2334	4.3772E-02	1.4550E-01	ENSG00000159247
NA	-0.1623	4.3274E-02	1.4444E-01	ENSG00000255224
NA	-0.1843	4.3047E-02	1.4387E-01	ENSG00000273521
NA	-0.1354	4.3016E-02	1.4380E-01	ENSG00000240919
NA	-0.2316	4.2959E-02	1.4367E-01	ENSG00000284617
NA	-0.1380	4.2877E-02	1.4350E-01	ENSG00000242992
NA	-0.2290	4.2460E-02	1.4253E-01	ENSG00000212769
NA	-0.2020	4.2051E-02	1.4150E-01	ENSG00000271437
NA	-0.1960	4.2032E-02	1.4150E-01	ENSG00000280383
NA	-0.1071	4.1839E-02		ENSG00000226658
NA	-0.1180	4.1837E-02	1.4107E-01	ENSG00000261216
NA	-0.2385	4.1723E-02	1.4084E-01	ENSG00000210082
NA	-0.0687	4.1237E-02		ENSG00000277358
NA	-0.2189	4.1205E-02	1.3972E-01	ENSG00000197582
NA	-0.0992	4.0971E-02		ENSG00000265746
NA	-0.1707	4.0826E-02	1.3872E-01	ENSG00000264281
NA	-0.2320	4.0696E-02	1.3842E-01	ENSG00000254846
NA	-0.1775	4.0535E-02	1.3806E-01	ENSG00000251414

NA	-0.2476	4.0258E-02	1.3742E-01	ENSG00000263531
NA	-0.2167	4.0140E-02	1.3710E-01	ENSG00000279583
NA	-0.2269	4.0086E-02	1.3695E-01	ENSG00000249502
NA	-0.2509	4.0044E-02	1.3686E-01	ENSG00000214578
NA	-0.0447	3.9992E-02		ENSG00000243494
NA	-0.2359	3.9838E-02	1.3639E-01	ENSG00000175772
NA	-0.2344	3.9736E-02	1.3625E-01	ENSG00000283761
NA	-0.1535	3.9649E-02	1.3612E-01	ENSG00000289644
NA	-0.2000	3.9600E-02	1.3601E-01	ENSG00000236439
NA	-0.2367	3.8986E-02	1.3438E-01	ENSG00000231245
NA	-0.0647	3.8798E-02		ENSG00000205100
NA	-0.0576	3.8724E-02		ENSG00000287859
NA	-0.1706	3.8646E-02	1.3353E-01	ENSG00000234219
NA	-0.0612	3.8069E-02		ENSG00000278959
NA	-0.2487	3.7798E-02	1.3141E-01	ENSG00000241990
NA	-0.0379	3.7283E-02		ENSG00000254251
NA	-0.2052	3.6530E-02	1.2788E-01	ENSG00000241547
NA	-0.2593	3.6523E-02	1.2788E-01	ENSG00000236539
NA	-0.0428	3.6380E-02		ENSG00000250508
NA	-0.1527	3.6114E-02	1.2679E-01	ENSG00000267466
NA	-0.2156	3.6101E-02	1.2677E-01	ENSG00000288091
NA	-0.2325	3.5612E-02	1.2563E-01	ENSG00000233328
NA	-0.2067	3.5214E-02	1.2459E-01	ENSG00000228502
NA	-0.0778	3.5200E-02		ENSG00000277423
NA	-0.0637	3.4827E-02		ENSG00000251532
NA	-0.2611	3.4542E-02	1.2271E-01	ENSG00000263786
NA	-0.1859	3.4433E-02	1.2238E-01	ENSG00000241464
NA	-0.1719	3.3900E-02	1.2096E-01	ENSG00000289627
NA	-0.1974	3.3824E-02	1.2081E-01	ENSG00000258477
NA	-0.1732	3.3819E-02	1.2081E-01	ENSG00000264350
NA	-0.2019	3.3670E-02	1.2054E-01	ENSG00000214331
NA	-0.1130	3.3440E-02		ENSG00000272371
NA	-0.2020	3.3374E-02	1.1988E-01	ENSG00000241185
NA	-0.1537	3.3023E-02	1.1903E-01	ENSG00000287875
NA	-0.1598	3.2858E-02	1.1851E-01	ENSG00000269867
NA	-0.1289	3.2831E-02	1.1843E-01	ENSG00000225648
NA	-0.2314	3.2770E-02	1.1830E-01	ENSG00000260774
NA	-0.2594	3.2231E-02	1.1690E-01	ENSG00000255565
NA	-0.2701	3.2207E-02	1.1683E-01	ENSG00000240463
NA	-0.2226	3.2022E-02	1.1628E-01	ENSG00000187653
NA	-0.1659	3.1881E-02	1.1598E-01	ENSG00000226148
NA	-0.1451	3.1874E-02	1.1598E-01	ENSG00000249072
NA	-0.2257	3.1754E-02	1.1578E-01	ENSG00000278254
NA	-0.1993	3.1693E-02	1.1563E-01	ENSG00000286705
NA	-0.1404	3.1667E-02	1.1557E-01	ENSG00000234337
NA	-0.0627	3.1427E-02		ENSG00000254698
NA	-0.2759	3.1377E-02	1.1490E-01	ENSG00000225234
NA	-0.2652	3.1175E-02	1.1435E-01	ENSG00000242353
NA	-0.2175	3.1096E-02	1.1419E-01	ENSG00000227081

NA	-0.1609	3.0967E-02	1.1384E-01	ENSG00000287965
NA	-0.1963	3.0943E-02	1.1377E-01	ENSG00000261064
NA	-0.1290	3.0400E-02	1.1228E-01	ENSG00000273950
NA	-0.2235	3.0279E-02	1.1195E-01	ENSG00000226471
NA	-0.1346	3.0241E-02	1.1189E-01	ENSG00000264881
NA	-0.2662	3.0089E-02	1.1150E-01	ENSG00000225976
NA	-0.2838	2.9760E-02	1.1050E-01	ENSG00000256262
NA	-0.0476	2.9760E-02		ENSG00000220343
NA	-0.0724	2.9456E-02		ENSG00000225885
NA	-0.1344	2.9428E-02	1.0950E-01	ENSG00000250959
NA	-0.2720	2.9394E-02	1.0950E-01	ENSG00000228665
NA	-0.2692	2.9371E-02	1.0946E-01	ENSG00000279694
NA	-0.2865	2.8978E-02	1.0834E-01	ENSG00000259242
NA	-0.1227	2.8826E-02	1.0804E-01	ENSG00000248585
NA	-0.0930	2.8655E-02		ENSG00000213587
NA	-0.2026	2.8453E-02	1.0706E-01	ENSG00000224543
NA	-0.1745	2.8451E-02	1.0706E-01	ENSG00000227063
NA	-0.2806	2.8376E-02	1.0687E-01	ENSG00000280239
NA	-0.2343	2.8301E-02	1.0667E-01	ENSG00000285892
NA	-0.2680	2.8121E-02	1.0617E-01	ENSG00000279632
NA	-0.2681	2.8039E-02	1.0594E-01	ENSG00000277687
NA	-0.2720	2.7916E-02	1.0560E-01	ENSG00000220506
NA	-0.2489	2.7650E-02	1.0480E-01	ENSG00000206341
NA	-0.2765	2.7574E-02	1.0467E-01	ENSG00000286971
NA	-0.2038	2.7533E-02	1.0457E-01	ENSG00000236307
NA	-0.2079	2.7483E-02	1.0447E-01	ENSG00000253133
NA	-0.2200	2.7232E-02	1.0369E-01	ENSG00000276538
NA	-0.2422	2.7167E-02	1.0352E-01	ENSG00000287910
NA	-0.2860	2.6884E-02	1.0271E-01	ENSG00000288802
NA	-0.2709	2.6780E-02	1.0243E-01	ENSG00000249684
NA	-0.3017	2.6534E-02	1.0184E-01	ENSG00000268061
NA	-0.3038	2.6318E-02	1.0118E-01	ENSG00000287115
NA	-0.1511	2.6155E-02	1.0077E-01	ENSG00000272599
NA	-0.2422	2.5528E-02	9.8993E-02	ENSG00000288917
NA	-0.1814	2.5308E-02	9.8316E-02	ENSG00000279483
NA	-0.2962	2.5239E-02	9.8197E-02	ENSG00000237892
NA	-0.2566	2.5073E-02	9.7781E-02	ENSG00000220785
NA	-0.2599	2.5024E-02	9.7719E-02	ENSG00000268751
NA	-0.3076	2.4560E-02	9.6355E-02	ENSG00000263884
NA	-0.3064	2.4513E-02	9.6242E-02	ENSG00000273451
NA	-0.3046	2.4416E-02	9.5950E-02	ENSG00000289380
NA	-0.1089	2.4249E-02	9.5606E-02	ENSG00000218980
NA	-0.2556	2.4203E-02	9.5497E-02	ENSG00000229567
NA	-0.0929	2.4200E-02		ENSG00000285748
NA	-0.1619	2.3674E-02	9.3937E-02	ENSG00000228446
NA	-0.0675	2.3646E-02		ENSG00000253244
NA	-0.1763	2.3399E-02	9.3142E-02	ENSG00000277806
NA	-0.3104	2.2884E-02	9.1606E-02	ENSG00000202222
NA	-0.2567	2.2822E-02	9.1412E-02	ENSG00000287708

NA	-0.1123	2.2712E-02		ENSG00000275586
NA	-0.2055	2.2415E-02	9.0209E-02	ENSG00000214223
NA	-0.0579	2.1772E-02		ENSG00000235445
NA	-0.2092	2.1463E-02	8.7276E-02	ENSG00000249936
NA	-0.1333	2.1423E-02		ENSG00000235677
NA	-0.2293	2.1230E-02	8.6627E-02	ENSG00000269982
NA	-0.3006	2.0926E-02	8.5702E-02	ENSG00000285548
NA	-0.3067	2.0670E-02	8.4918E-02	ENSG00000229519
NA	-0.1997	2.0665E-02	8.4914E-02	ENSG00000231579
NA	-0.3242	2.0606E-02	8.4686E-02	ENSG00000226520
NA	-0.1769	2.0435E-02	8.4179E-02	ENSG00000277702
NA	-0.2943	2.0401E-02	8.4083E-02	ENSG00000243389
NA	-0.0976	2.0327E-02	8.3890E-02	ENSG00000252759
NA	-0.1399	2.0248E-02		ENSG00000258359
NA	-0.3248	2.0043E-02	8.3163E-02	ENSG00000279432
NA	-0.3432	1.9824E-02	8.2475E-02	ENSG00000242659
NA	-0.0358	1.9651E-02	8.1965E-02	ENSG00000288035
NA	-0.2231	1.9341E-02	8.1017E-02	ENSG00000228532
NA	-0.2625	1.9317E-02	8.0957E-02	ENSG00000231113
NA	-0.3609	1.9271E-02	8.0844E-02	ENSG00000289260
NA	-0.3432	1.9262E-02	8.0821E-02	ENSG00000282978
NA	-0.0544	1.9240E-02		ENSG00000267063
NA	-0.2242	1.9236E-02	8.0741E-02	ENSG00000272462
NA	-0.2996	1.9224E-02	8.0710E-02	ENSG00000261575
NA	-0.3448	1.9061E-02	8.0227E-02	ENSG00000261460
NA	-0.3292	1.8208E-02	7.7638E-02	ENSG00000225822
NA	-0.1359	1.8132E-02	7.7411E-02	ENSG00000214174
NA	-0.3323	1.7738E-02	7.6285E-02	ENSG00000281333
NA	-0.3506	1.7701E-02	7.6194E-02	ENSG00000243094
NA	-0.3454	1.7694E-02	7.6179E-02	ENSG00000289106
NA	-0.3178	1.7369E-02	7.5216E-02	ENSG00000268869
NA	-0.3209	1.7287E-02	7.4910E-02	ENSG00000272779
NA	-0.3376	1.7249E-02	7.4786E-02	ENSG00000288995
NA	-0.1114	1.7154E-02		ENSG00000261592
NA	-0.2655	1.7086E-02	7.4303E-02	ENSG00000279114
NA	-0.3524	1.6905E-02	7.3764E-02	ENSG00000236060
NA	-0.3307	1.6860E-02	7.3640E-02	ENSG00000257179
NA	-0.3248	1.6789E-02	7.3426E-02	ENSG00000279652
NA	-0.3791	1.6677E-02	7.3040E-02	ENSG00000257438
NA	-0.2620	1.6674E-02	7.3040E-02	ENSG00000179967
NA	-0.2957	1.6635E-02	7.2947E-02	ENSG00000229932
NA	-0.3121	1.6532E-02	7.2676E-02	ENSG00000280474
NA	-0.1091	1.6517E-02		ENSG00000235408
NA	-0.2904	1.6492E-02	7.2578E-02	ENSG00000280388
NA	-0.0742	1.6438E-02		ENSG00000266079
NA	-0.3661	1.6362E-02	7.2147E-02	ENSG00000271100
NA	-0.3877	1.6270E-02	7.1834E-02	ENSG00000289397
NA	-0.3553	1.6168E-02	7.1536E-02	ENSG00000277501
NA	-0.2867	1.6142E-02	7.1472E-02	ENSG00000234773

NA	-0.3551	1.6016E-02	7.1146E-02	ENSG00000251333
NA	-0.1863	1.5850E-02	7.0677E-02	ENSG00000226334
NA	-0.3924	1.5686E-02	7.0188E-02	ENSG00000224080
NA	-0.3677	1.5644E-02	7.0087E-02	ENSG00000206228
NA	-0.0821	1.5557E-02		ENSG00000230515
NA	-0.8597	1.5500E-02	6.9646E-02	ENSG00000254985
NA	-0.3905	1.5149E-02	6.8438E-02	ENSG00000251456
NA	-0.3315	1.4958E-02	6.7833E-02	ENSG00000236044
NA	-0.3301	1.4838E-02	6.7461E-02	ENSG00000233045
NA	-0.3156	1.4822E-02	6.7448E-02	ENSG00000214199
NA	-0.4132	1.4798E-02	6.7403E-02	ENSG00000235586
NA	-0.2919	1.4223E-02	6.5476E-02	ENSG00000215482
NA	-0.3861	1.4024E-02	6.4860E-02	ENSG00000243609
NA	-0.1145	1.3942E-02	6.4614E-02	ENSG00000237882
NA	-0.3274	1.3758E-02	6.3979E-02	ENSG00000213080
NA	-0.3067	1.3738E-02	6.3912E-02	ENSG00000239719
NA	-0.4138	1.3377E-02	6.2696E-02	ENSG00000286342
NA	-0.4195	1.3209E-02	6.2072E-02	ENSG00000239677
NA	-0.3626	1.3111E-02	6.1672E-02	ENSG00000287655
NA	-0.3166	1.3009E-02	6.1336E-02	ENSG00000288992
NA	-0.1785	1.3006E-02		ENSG00000267317
NA	-0.3628	1.2949E-02	6.1134E-02	ENSG00000245156
NA	-0.2387	1.2780E-02	6.0567E-02	ENSG00000226976
NA	-0.2950	1.2477E-02	5.9563E-02	ENSG00000277675
NA	-0.1966	1.2393E-02	5.9254E-02	ENSG00000248092
NA	-0.3066	1.2320E-02	5.9003E-02	ENSG00000263731
NA	-0.3251	1.2090E-02	5.8282E-02	ENSG00000226781
NA	-0.3238	1.1966E-02	5.7856E-02	ENSG00000249014
NA	-0.4052	1.1879E-02	5.7583E-02	ENSG00000237017
NA	-0.4962	1.1766E-02	5.7213E-02	ENSG00000287236
NA	-0.4441	1.1681E-02	5.6868E-02	ENSG00000248100
NA	-0.2843	1.1372E-02	5.5759E-02	ENSG00000217094
NA	-0.4704	1.1256E-02	5.5303E-02	ENSG00000287391
NA	-0.1637	1.1013E-02	5.4380E-02	ENSG00000250705
NA	-0.1926	1.0780E-02	5.3532E-02	ENSG00000279236
NA	-0.2987	1.0747E-02	5.3436E-02	ENSG00000279064
NA	-0.4461	1.0561E-02	5.2783E-02	ENSG00000234614
NA	-0.3043	1.0420E-02	5.2217E-02	ENSG00000276141
NA	-0.3035	1.0187E-02	5.1404E-02	ENSG00000182057
NA	-0.3327	1.0125E-02	5.1188E-02	ENSG00000224945
NA	-0.2962	1.0072E-02	5.1023E-02	ENSG00000223612
NA	-0.2011	1.0046E-02	5.0942E-02	ENSG00000251229
NA	-0.5377	9.7655E-03	4.9852E-02	ENSG00000213592
NA	-0.2936	9.6478E-03	4.9403E-02	ENSG00000287042
NA	-0.3361	9.6155E-03	4.9310E-02	ENSG00000258674
NA	-0.2384	9.5776E-03	4.9167E-02	ENSG00000260549
NA	-0.2789	9.3156E-03	4.8123E-02	ENSG00000227799
NA	-0.3634	9.3150E-03	4.8123E-02	ENSG00000267127
NA	-0.3919	9.1881E-03	4.7654E-02	ENSG00000227725

NA	-0.1917	9.0504E-03		ENSG00000267938
NA	-0.5116	9.0487E-03	4.7130E-02	ENSG00000270207
NA	-0.3594	9.0409E-03	4.7113E-02	ENSG00000213600
NA	-0.1624	8.7626E-03	4.6020E-02	ENSG00000232273
NA	-0.3346	8.6350E-03	4.5535E-02	ENSG00000248126
NA	-0.4373	8.5900E-03	4.5375E-02	ENSG00000272505
NA	-0.5535	8.2513E-03	4.3870E-02	ENSG00000270802
NA	-0.4660	8.1698E-03	4.3558E-02	ENSG00000262412
NA	-0.4203	8.0445E-03	4.3076E-02	ENSG00000289466
NA	-0.1461	8.0297E-03	4.3017E-02	ENSG00000184188
NA	-0.4831	7.9834E-03	4.2807E-02	ENSG00000261620
NA	-0.5561	7.6206E-03	4.1301E-02	ENSG00000238249
NA	-0.4300	7.6164E-03	4.1287E-02	ENSG00000277170
NA	-0.3332	7.6014E-03	4.1226E-02	ENSG00000256591
NA	-0.3822	7.5508E-03	4.1067E-02	ENSG00000228477
NA	-0.1252	7.5323E-03		ENSG00000276396
NA	-0.2293	7.3450E-03	4.0223E-02	ENSG00000196656
NA	-0.5350	7.1947E-03	3.9625E-02	ENSG00000259398
NA	-0.5021	7.1435E-03	3.9442E-02	ENSG00000223461
NA	-0.4896	7.0519E-03	3.9018E-02	ENSG00000233538
NA	-0.3525	6.6988E-03	3.7546E-02	ENSG00000242262
NA	-0.2274	6.6935E-03	3.7524E-02	ENSG00000249898
NA	-0.5028	6.6576E-03	3.7391E-02	ENSG00000214222
NA	-0.4630	6.2392E-03	3.5536E-02	ENSG00000279555
NA	-0.1741	6.1950E-03	3.5349E-02	ENSG00000255059
NA	-0.2133	6.1519E-03	3.5145E-02	ENSG00000229944
NA	-0.1833	6.1178E-03	3.4998E-02	ENSG00000279336
NA	-0.2869	5.9843E-03	3.4395E-02	ENSG00000214975
NA	-0.4746	5.7308E-03	3.3374E-02	ENSG00000260057
NA	-0.4903	5.7274E-03	3.3362E-02	ENSG00000286353
NA	-0.2275	5.5725E-03	3.2657E-02	ENSG00000213453
NA	-0.3169	5.4049E-03	3.1961E-02	ENSG00000266920
NA	-0.4196	5.1978E-03	3.1023E-02	ENSG00000270607
NA	-0.4014	4.8308E-03	2.9298E-02	ENSG00000249264
NA	-0.5536	4.7433E-03	2.8917E-02	ENSG00000290046
NA	-0.2226	4.7265E-03	2.8850E-02	ENSG00000229129
NA	-0.2226	4.7265E-03	2.8850E-02	ENSG00000229465
NA	-0.2354	4.6702E-03	2.8598E-02	ENSG00000270055
NA	-0.5213	4.3438E-03	2.7096E-02	ENSG00000287271
NA	-0.5851	4.0315E-03	2.5732E-02	ENSG00000254791
NA	-0.2568	3.8157E-03	2.4656E-02	ENSG00000256393
NA	-0.3568	3.7825E-03	2.4472E-02	ENSG00000224848
NA	-0.2775	3.6213E-03	2.3679E-02	ENSG00000230074
NA	-0.2056	3.6202E-03	2.3678E-02	ENSG00000221883
NA	-0.1902	3.5872E-03	2.3505E-02	ENSG00000180581
NA	-0.3601	3.3309E-03	2.2126E-02	ENSG00000124097
NA	-0.4918	3.3268E-03	2.2117E-02	ENSG00000259953
NA	-0.3998	3.3196E-03	2.2088E-02	ENSG00000227256
NA	-0.3397	3.1098E-03	2.1085E-02	ENSG00000249019

NA	-0.4285	3.0582E-03	2.0776E-02	ENSG00000261242
NA	-0.5373	3.0158E-03	2.0578E-02	ENSG00000210194
NA	-0.3597	2.8552E-03	1.9729E-02	ENSG00000271121
NA	-0.3710	2.7285E-03	1.8993E-02	ENSG00000197744
NA	-0.3012	2.6249E-03	1.8422E-02	ENSG00000290018
NA	-0.2953	2.4961E-03	1.7759E-02	ENSG00000277406
NA	-0.3200	2.3533E-03	1.7004E-02	ENSG00000287021
NA	-0.2133	2.3475E-03	1.6993E-02	ENSG00000112096
NA	-0.2105	2.3103E-03	1.6783E-02	ENSG00000232024
NA	-0.3544	2.2719E-03	1.6577E-02	ENSG00000235238
NA	-0.4543	2.2272E-03	1.6329E-02	ENSG00000204860
NA	-0.2748	1.8059E-03	1.3960E-02	ENSG00000235043
NA	-0.2194	1.5891E-03	1.2742E-02	ENSG00000239521
NA	-0.5271	1.5888E-03	1.2742E-02	ENSG00000249302
NA	-0.4716	1.5281E-03	1.2389E-02	ENSG00000267191
NA	-0.3343	1.5060E-03	1.2292E-02	ENSG00000224411
NA	-0.2431	1.4582E-03	1.2032E-02	ENSG00000225151
NA	-0.5381	1.3114E-03	1.1134E-02	ENSG00000287967
NA	-0.3552	1.2094E-03	1.0459E-02	ENSG00000250182
NA	-0.2856	1.1180E-03	9.8524E-03	ENSG00000226085
NA	-0.3800	1.0775E-03	9.5992E-03	ENSG00000289017
NA	-0.4078	9.8863E-04	8.9860E-03	ENSG00000229212
NA	-0.3250	9.5298E-04	8.7265E-03	ENSG00000180867
NA	-0.2951	9.2817E-04	8.5663E-03	ENSG00000186312
NA	-0.5900	8.9022E-04	8.3032E-03	ENSG00000286039
NA	-0.5777	8.8347E-04	8.2590E-03	ENSG00000232748
NA	-0.4544	8.8081E-04	8.2373E-03	ENSG00000186369
NA	-0.2314	8.7089E-04	8.1725E-03	ENSG00000218283
NA	-0.3088	8.3050E-04	7.8655E-03	ENSG00000228589
NA	-0.2783	7.5140E-04	7.3299E-03	ENSG00000274104
NA	-0.4447	7.2750E-04	7.1515E-03	ENSG00000188765
NA	-0.4164	6.6283E-04	6.6676E-03	ENSG00000263081
NA	-0.3275	5.7723E-04	5.9376E-03	ENSG00000196205
NA	-0.4267	5.0317E-04	5.3227E-03	ENSG00000269951
NA	-0.3615	4.3107E-04	4.7350E-03	ENSG00000289843
NA	-0.4289	3.9306E-04	4.4201E-03	ENSG00000283538
NA	-0.4622	3.5149E-04	4.0489E-03	ENSG00000231409
NA	-0.4186	3.4500E-04	3.9947E-03	ENSG00000240036
NA	-0.5473	3.0434E-04	3.6368E-03	ENSG00000228809
NA	-0.4632	2.8445E-04	3.4444E-03	ENSG00000279573
NA	-0.2558	2.3505E-04	2.9613E-03	ENSG00000174977
NA	-0.4837	2.2030E-04	2.8071E-03	ENSG00000240914
NA	-0.5214	1.9198E-04	2.5220E-03	ENSG00000236876
NA	-0.4369	1.6849E-04	2.2691E-03	ENSG00000255769
NA	-0.5842	1.3441E-04	1.9127E-03	ENSG00000179362
NA	-0.4725	1.3204E-04	1.8954E-03	ENSG00000289138
NA	-0.3227	1.3180E-04	1.8931E-03	ENSG00000215492
NA	-0.3559	1.2290E-04	1.8021E-03	ENSG00000259781
NA	-0.3541	1.1294E-04	1.6883E-03	ENSG00000215302

NA	-0.3673	1.1128E-04	1.6695E-03	ENSG00000185641
NA	-0.4608	1.0236E-04	1.5751E-03	ENSG00000213362
NA	-0.3327	9.3157E-05	1.4599E-03	ENSG00000286156
NA	-0.4629	8.4643E-05	1.3529E-03	ENSG00000279901
NA	-0.2609	7.9534E-05	1.2883E-03	ENSG00000172974
NA	-0.5312	6.3171E-05	1.0779E-03	ENSG00000273345
NA	-0.3285	5.9131E-05	1.0196E-03	ENSG00000261279
NA	-0.4424	5.6496E-05	9.7962E-04	ENSG00000289318
NA	-0.5133	4.6019E-05	8.3075E-04	ENSG00000230204
NA	-0.5677	3.8468E-05	7.3033E-04	ENSG00000229097
NA	-0.3333	2.7417E-05	5.5339E-04	ENSG00000215861
NA	-0.4836	2.0780E-05	4.4038E-04	ENSG00000289516
NA	-0.4345	1.0691E-05	2.5915E-04	ENSG00000233476
NA	-0.4812	9.5467E-06	2.3560E-04	ENSG00000234664
NA	-0.4391	7.0842E-06	1.8563E-04	ENSG00000255046
NA	-0.3552	5.8034E-06	1.5831E-04	ENSG00000223509
NA	-0.5656	5.1588E-06	1.4365E-04	ENSG00000234964
NA	-0.4935	1.2288E-06	4.4398E-05	ENSG00000284428
NA	-0.4646	9.4767E-07	3.5545E-05	ENSG00000280138
NA	-0.5219	8.7237E-07	3.3227E-05	ENSG00000235655
NA	-0.3725	1.5234E-07	8.0609E-06	ENSG00000198618
NA	-0.5658	1.2994E-07	7.0893E-06	ENSG00000178458
NA	-0.5685	5.8511E-08	3.6633E-06	ENSG00000229989
NA	-0.4709	2.6564E-08	1.8675E-06	ENSG00000263563
NA	-0.4642	2.7299E-09	2.6654E-07	ENSG00000235734
NA	-0.4731	1.4244E-10	2.2043E-08	ENSG00000230037
NA	-0.5830	6.5622E-14	3.5101E-11	ENSG00000228407
NAA10	-0.0490	3.4892E-01	5.5875E-01	ENSG00000102030
NAA15	0.0387	5.5767E-01	7.3530E-01	ENSG00000164134
NAA16	-0.0586	3.7420E-01	5.8281E-01	ENSG00000172766
NAA20	0.0264	6.6948E-01	8.1482E-01	ENSG00000173418
NAA25	-0.0356	4.7484E-01	6.7059E-01	ENSG00000111300
NAA30	-0.1514	2.3125E-02	9.2294E-02	ENSG00000139977
NAA35	0.2227	5.1158E-04	5.3975E-03	ENSG00000135040
NAA38	0.0941	1.0833E-01	2.6835E-01	ENSG00000183011
NAA40	0.0950	1.5562E-01	3.3928E-01	ENSG00000110583
NAA50	-0.0566	4.1009E-01	6.1426E-01	ENSG00000121579
NAA60	-0.0089	8.6838E-01	9.3379E-01	ENSG00000122390
NAA80	0.2135	4.6876E-03	2.8684E-02	ENSG00000243477
NAAA	-0.2740	1.0695E-03	9.5516E-03	ENSG00000138744
NAALAD2	0.3172	2.2016E-02	8.9023E-02	ENSG00000077616
NAALADL1	-0.0300	5.9759E-01	7.6387E-01	ENSG00000168060
NAALADL2	0.1280	1.6081E-01	3.4632E-01	ENSG00000177694
NAB1	-0.0979	1.3137E-01	3.0443E-01	ENSG00000138386
NAB2	-0.1226	1.3876E-01	3.1465E-01	ENSG00000166886
NABP2	-0.0448	5.4134E-01	7.2323E-01	ENSG00000139579
NACA	-0.3443	4.8707E-06	1.3839E-04	ENSG00000196531
NACA2	-0.0072	6.6389E-01	8.1090E-01	ENSG00000253506
NACA3P	-0.0436	3.9557E-01	6.0182E-01	ENSG00000121089

NACA4P	-0.0991	2.0303E-01	4.0199E-01	ENSG00000228224
NACAD	0.0685	3.5974E-01	5.6908E-01	ENSG00000136274
NACAP2	-0.0156	4.2026E-01		ENSG00000180230
NACC1	-0.0346	6.5562E-01	8.0547E-01	ENSG00000160877
NACC2	0.0799	3.5333E-01	5.6347E-01	ENSG00000148411
NADK	-0.1645	2.7847E-02	1.0541E-01	ENSG00000008130
NADK2	-0.2053	1.9488E-02	8.1436E-02	ENSG00000152620
NADSYN1	0.0638	2.8768E-01	4.9832E-01	ENSG00000172890
NAE1	-0.0109	8.1724E-01	9.0494E-01	ENSG00000159593
NAF1	-0.0001	9.9676E-01	9.9822E-01	ENSG00000145414
NAGA	-0.0041	9.4933E-01	9.7491E-01	ENSG00000198951
NAGK	-0.0337	6.3918E-01	7.9381E-01	ENSG00000124357
NAGLU	0.0160	9.0418E-01	9.5319E-01	ENSG00000108784
NAGPA	-0.1176	1.8405E-01	3.7741E-01	ENSG00000103174
NAGS	0.0612	4.8053E-01	6.7541E-01	ENSG00000161653
NAIF1	-0.0354	6.5723E-01	8.0655E-01	ENSG00000171169
NAIP	0.0728	3.3492E-01	5.4501E-01	ENSG00000249437
NAIPP1	0.1609	1.0616E-01	2.6496E-01	ENSG00000250687
NAIPP3	0.0267	7.5332E-01	8.6607E-01	ENSG00000248477
NALCN	0.4325	1.2280E-05	2.8721E-04	ENSG00000102452
NALCN-AS1	0.0545	3.3529E-01	5.4533E-01	ENSG00000233009
NALF1	0.5348	6.0698E-06	1.6394E-04	ENSG00000204442
NALF2	-0.3602	1.1583E-04	1.7210E-03	ENSG00000130054
NAMA	0.0517	2.1031E-01	4.1073E-01	ENSG00000271086
NAMPT	0.0575	4.1338E-01	6.1699E-01	ENSG00000105835
NAMPTP1	0.4108	3.3761E-03	2.2369E-02	ENSG00000229644
NANOG	0.1078	2.8441E-02		ENSG00000111704
NANOS1	-0.0814	2.9505E-01	5.0511E-01	ENSG00000188613
NANOS2	0.0230	5.1512E-01		ENSG00000188425
NANOS3	0.0235	7.6454E-01	8.7345E-01	ENSG00000187556
NANP	-0.2212	1.0215E-02	5.1461E-02	ENSG00000170191
NANS	-0.1655	1.5426E-02	6.9370E-02	ENSG00000095380
NAP1L1	0.0009	9.9711E-01	9.9831E-01	ENSG00000187109
NAP1L1P1	-0.0135	8.3718E-01	9.1604E-01	ENSG00000254759
NAP1L2	-0.0873	2.9827E-01	5.0815E-01	ENSG00000186462
NAP1L3	-0.2995	1.8157E-03	1.4007E-02	ENSG00000186310
NAP1L4	-0.1601	1.3191E-03	1.1177E-02	ENSG00000205531
NAP1L5	-0.3653	5.1109E-06	1.4323E-04	ENSG00000177432
NAP1L6P	-0.0149	7.3903E-01	8.5776E-01	ENSG00000204118
NAPA	-0.1720	7.8468E-03	4.2266E-02	ENSG00000105402
NAPB	-0.0057	9.5555E-01	9.7768E-01	ENSG00000125814
NAPEPLD	0.0884	2.3664E-01	4.4242E-01	ENSG00000161048
NAPG	0.0086	9.0332E-01	9.5277E-01	ENSG00000134265
NAPRT	-0.3003	2.3821E-05	4.9294E-04	ENSG00000147813
NAPSA	0.0751	2.2799E-01	4.3267E-01	ENSG00000131400
NARF	0.0972	1.9947E-01	3.9778E-01	ENSG00000141562
NARF-AS2	-0.0296	7.1827E-01	8.4512E-01	ENSG00000265458
NARS1	-0.0587	3.2632E-01	5.3646E-01	ENSG00000134440
NARS2	0.0711	2.8740E-01	4.9810E-01	ENSG00000137513

NASP	-0.3324	1.0348E-04	1.5841E-03	ENSG00000132780
NAT10	0.1369	4.0379E-02	1.3772E-01	ENSG00000135372
NAT14	-0.0440	5.2931E-01	7.1387E-01	ENSG00000090971
NAT16	0.1517	6.7359E-02	1.9562E-01	ENSG00000167011
NAT2	0.0624	5.0398E-01	6.9429E-01	ENSG00000156006
NAT8L	0.1850	5.3645E-02	1.6744E-01	ENSG00000185818
NAT9	0.2600	1.3450E-03	1.1329E-02	ENSG00000109065
NATD1	0.1056	1.6955E-01	3.5798E-01	ENSG00000274180
NAV1	-0.0195	7.8987E-01	8.8896E-01	ENSG00000134369
NAV2	0.1495	5.6590E-02	1.7392E-01	ENSG00000166833
NAV2-AS2	0.0187	4.7147E-01		ENSG00000254453
NAV2-AS3	-0.0198	5.4654E-01		ENSG00000254542
NAV2-AS4	-0.0004	9.0157E-01		ENSG00000254622
NAV2-AS5	-0.0162	9.9087E-01		ENSG00000255043
NAV3	0.0321	6.5057E-01	8.0189E-01	ENSG00000067798
NAXD	0.0323	6.8812E-01	8.2680E-01	ENSG00000213995
NAXD-AS1	0.0729	3.4475E-01	5.5435E-01	ENSG00000275880
NAXE	0.0587	3.3517E-01	5.4517E-01	ENSG00000163382
NBAS	0.0433	4.0499E-01	6.0944E-01	ENSG00000151779
NBDY	-0.2029	8.0710E-05	1.3021E-03	ENSG00000204272
NBEA	0.3191	2.0255E-04	2.6148E-03	ENSG00000172915
NBEAL1	0.1675	9.5901E-03	4.9200E-02	ENSG00000144426
NBEAL2	0.0208	7.9496E-01	8.9166E-01	ENSG00000160796
NBEAP2	0.0785	1.8983E-01	3.8487E-01	ENSG00000197927
NBN	-0.0147	7.9867E-01	8.9385E-01	ENSG00000104320
NBPF1	0.1287	7.3897E-02	2.0837E-01	ENSG00000219481
NBPF10	0.0794	3.4899E-01	5.5875E-01	ENSG00000271425
NBPF11	0.1923	4.3136E-03	2.6952E-02	ENSG00000263956
NBPF12	0.2190	6.0708E-03	3.4818E-02	ENSG00000268043
NBPF14	0.2824	9.0898E-03	4.7284E-02	ENSG00000270629
NBPF15	0.0723	2.3971E-01	4.4641E-01	ENSG00000266338
NBPF19	0.0174	4.7362E-01	6.6951E-01	ENSG00000271383
NBPF20	0.1939	2.7940E-02	1.0565E-01	ENSG00000162825
NBPF26	0.1145	1.4966E-01	3.3050E-01	ENSG00000273136
NBPF3	0.1429	9.7413E-02	2.5074E-01	ENSG00000142794
NBPF4	0.0142	5.5150E-01		ENSG00000196427
NBPF8	0.0886	1.7326E-01	3.6320E-01	ENSG00000270231
NBPF9	0.1360	3.6649E-02	1.2822E-01	ENSG00000269713
NBR1	-0.1175	1.0477E-01	2.6264E-01	ENSG00000188554
NBR2	-0.1028	2.1873E-01	4.2120E-01	ENSG00000198496
NCALD	-0.1449	3.9699E-02	1.3618E-01	ENSG00000104490
NCAM1	-0.3260	1.6797E-06	5.8210E-05	ENSG00000149294
NCAM2	0.3130	9.7140E-04	8.8524E-03	ENSG00000154654
NCAN	-0.2841	1.7723E-05	3.8935E-04	ENSG00000130287
NCAPD2	-0.0172	8.0751E-01	8.9887E-01	ENSG00000010292
NCAPD3	-0.2397	7.5877E-03	4.1186E-02	ENSG00000151503
NCAPG	-0.3301	3.6491E-03	2.3822E-02	ENSG00000109805
NCAPG2	-0.0314	6.9953E-01	8.3383E-01	ENSG00000146918
NCAPH	-0.2218	3.2474E-02	1.1747E-01	ENSG00000121152

NCAPH2	-0.0837	2.4016E-01	4.4698E-01	ENSG00000025770
NCBP1	-0.0579	3.0898E-01	5.1924E-01	ENSG00000136937
NCBP2	-0.0320	5.8191E-01	7.5302E-01	ENSG00000114503
NCBP2-AS1	-0.0048	8.7110E-01		ENSG00000225578
NCBP2AS2	-0.2104	2.9884E-03	2.0463E-02	ENSG00000270170
NCBP2L	0.0114	5.4797E-01		ENSG00000170935
NCBP3	0.0140	8.3114E-01	9.1275E-01	ENSG00000074356
NCCRP1	-0.0728	2.4041E-01	4.4722E-01	ENSG00000188505
NCDN	0.0154	8.2287E-01	9.0841E-01	ENSG00000020129
NCEH1	0.1041	2.4847E-01	4.5629E-01	ENSG00000144959
NCF1	0.0274	7.2826E-01	8.5096E-01	ENSG00000158517
NCF1C	-0.0512	4.9294E-01	6.8489E-01	ENSG00000165178
NCF2	-0.0639	3.8768E-01	5.9532E-01	ENSG00000116701
NCF4	0.0049	4.9763E-01		ENSG00000100365
NCK1	-0.1528	2.6758E-03	1.8714E-02	ENSG00000158092
NCK2	-0.1383	1.1358E-02	5.5704E-02	ENSG00000071051
NCKAP1	-0.0417	4.2912E-01	6.3074E-01	ENSG00000061676
NCKAP5	-0.1140	1.7144E-01	3.6060E-01	ENSG00000176771
NCKAP5-AS1	-0.0202	5.1402E-01		ENSG00000233729
NCKAP5-AS2	-0.0200	7.8257E-01	8.8495E-01	ENSG00000226953
NCKAP5L	0.0287	6.4325E-01	7.9622E-01	ENSG00000167566
NCKIPSD	0.1585	4.9344E-02	1.5807E-01	ENSG00000213672
NCL	-0.2487	4.1623E-05	7.7061E-04	ENSG00000115053
NCLN	0.0317	6.1164E-01	7.7383E-01	ENSG00000125912
NCMAP	-0.0174	6.4668E-01		ENSG00000184454
NCOA1	0.0619	3.2946E-01	5.3978E-01	ENSG00000084676
NCOA2	0.1207	1.2138E-01	2.8973E-01	ENSG00000140396
NCOA3	0.1164	6.7018E-02	1.9505E-01	ENSG00000124151
NCOA4	-0.1109	6.6596E-02	1.9421E-01	ENSG00000266412
NCOA5	0.2166	8.9404E-03	4.6784E-02	ENSG00000124160
NCOA6	-0.1932	2.6897E-03	1.8790E-02	ENSG00000198646
NCOA7	0.1096	1.0029E-01	2.5517E-01	ENSG00000111912
NCOA7-AS1	-0.0075	7.1253E-01		ENSG00000232131
NCOR1	0.0264	5.5422E-01	7.3256E-01	ENSG00000141027
NCOR2	-0.1716	5.4324E-03	3.2077E-02	ENSG00000196498
NCR3	-0.0122	4.2174E-01		ENSG00000204475
NCR3LG1	0.0669	4.1905E-01	6.2195E-01	ENSG00000188211
NCRNA00250	0.0202	2.3097E-01		ENSG00000253433
NCS1	-0.3050	3.3533E-04	3.9104E-03	ENSG00000107130
NCSTN	0.0008	9.8501E-01	9.9215E-01	ENSG00000162736
ND1	0.1505	2.4547E-02	9.6328E-02	ENSG00000198888
ND2	0.1161	9.6689E-02	2.4982E-01	ENSG00000198763
ND3	0.2884	1.2471E-03	1.0695E-02	ENSG00000198840
ND4	0.0526	5.0809E-01	6.9701E-01	ENSG00000198886
ND4L	0.0723	3.4839E-01	5.5839E-01	ENSG00000212907
ND5	0.0668	4.0076E-01	6.0602E-01	ENSG00000198786
ND6	-0.1089	2.3139E-01	4.3622E-01	ENSG00000198695
NDC1	-0.0246	7.2484E-01	8.4915E-01	ENSG00000058804
NDC80	-0.2785	1.1907E-02	5.7670E-02	ENSG00000080986

NDE1	-0.2794	1.1296E-03	9.9227E-03	ENSG00000072864
NDEL1	-0.0008	9.7187E-01	9.8589E-01	ENSG00000166579
NDFIP1	-0.3330	7.1176E-07	2.8156E-05	ENSG00000131507
NDFIP2	0.0440	5.0704E-01	6.9630E-01	ENSG00000102471
NDN	-0.0569	3.4239E-01	5.5198E-01	ENSG00000182636
NDNF	0.5865	5.4132E-05	9.4730E-04	ENSG00000173376
NDOR1	-0.0685	3.9130E-01	5.9830E-01	ENSG00000188566
NDP-AS1	0.5168	3.2449E-03	2.1734E-02	ENSG00000236276
NDRG1	0.0437	5.8070E-01	7.5212E-01	ENSG00000104419
NDRG2	-0.0968	8.9812E-02	2.3749E-01	ENSG00000165795
NDRG3	-0.1841	2.6752E-03	1.8714E-02	ENSG00000101079
NDRG4	0.1487	3.5531E-02	1.2537E-01	ENSG00000103034
NDST1	-0.0758	2.5908E-01	4.6768E-01	ENSG00000070614
NDST1-AS1	0.1815	6.8970E-02	1.9859E-01	ENSG00000254333
NDST2	0.0401	5.4172E-01	7.2346E-01	ENSG00000166507
NDST3	0.3440	1.6912E-03	1.3311E-02	ENSG00000164100
NDST4	-0.2306	1.6648E-02	7.2977E-02	ENSG00000138653
NDUFA1	-0.1904	4.3082E-03	2.6938E-02	ENSG00000125356
NDUFA10	-0.1496	8.7821E-03	4.6103E-02	ENSG00000130414
NDUFA11	-0.3892	3.4761E-05	6.6977E-04	ENSG00000174886
NDUFA12	-0.2112	1.2303E-03	1.0588E-02	ENSG00000184752
NDUFA13	-0.0664	4.0850E-01	6.1272E-01	ENSG00000186010
NDUFA2	-0.1254	6.1744E-02	1.8408E-01	ENSG00000131495
NDUFA3	-0.2336	6.6592E-04	6.6878E-03	ENSG00000170906
NDUFA3P2	-0.0136	6.2005E-01		ENSG00000254856
NDUFA3P4	-0.0378	4.7616E-01	6.7157E-01	ENSG00000259262
NDUFA4	-0.2069	1.0240E-02	5.1543E-02	ENSG00000189043
NDUFA4L2	0.0881	1.2806E-01	2.9973E-01	ENSG00000185633
NDUFA5	-0.2095	1.8265E-04	2.4162E-03	ENSG00000128609
NDUFA5P3	0.0065	7.3023E-01		ENSG00000234853
NDUFA6	-0.2060	2.0668E-03	1.5420E-02	ENSG00000184983
NDUFA6-DT	0.0217	7.6656E-01	8.7479E-01	ENSG00000237037
NDUFA7	-0.0099	9.0746E-01	9.5461E-01	ENSG00000267855
NDUFA8	-0.0602	2.2698E-01	4.3120E-01	ENSG00000119421
NDUFA9	-0.0866	1.3502E-01	3.0956E-01	ENSG00000139180
NDUFA9P1	0.0227	7.8073E-01	8.8370E-01	ENSG00000237406
NDUFAB1	-0.1213	8.3318E-02	2.2535E-01	ENSG00000004779
NDUFAB1P1	0.1167	2.6856E-02	1.0262E-01	ENSG00000217767
NDUFAF1	-0.0437	4.8900E-01	6.8220E-01	ENSG00000137806
NDUFAF2	0.0586	3.9812E-01	6.0371E-01	ENSG00000164182
NDUFAF2P1	0.0164	8.4668E-01	9.2147E-01	ENSG00000279307
NDUFAF3	-0.3010	8.7694E-06	2.1976E-04	ENSG00000178057
NDUFAF4	-0.0509	4.3393E-01	6.3507E-01	ENSG00000123545
NDUFAF5	-0.0437	4.4264E-01	6.4194E-01	ENSG00000101247
NDUFAF6	0.0474	3.9115E-01	5.9811E-01	ENSG00000156170
NDUFAF7	0.2497	1.5869E-03	1.2739E-02	ENSG00000003509
NDUFAF8	0.0192	7.3816E-01	8.5741E-01	ENSG00000224877
NDUFB1	-0.0997	1.8862E-01	3.8354E-01	ENSG00000183648
NDUFB10	-0.0963	1.5323E-01	3.3544E-01	ENSG00000140990

NDUFB10P2	0.0144	6.1135E-01		ENSG00000261624
NDUFB11	-0.4157	1.0673E-08	8.8417E-07	ENSG00000147123
NDUFB1P2	0.0145	2.6967E-01		ENSG00000234523
NDUFB2	-0.2605	2.8042E-04	3.4123E-03	ENSG00000090266
NDUFB2-AS1	0.0761	3.7586E-01	5.8443E-01	ENSG00000240889
NDUFB3	0.0043	9.5397E-01	9.7701E-01	ENSG00000119013
NDUFB4	-0.0709	3.0226E-01	5.1234E-01	ENSG00000065518
NDUFB4P11	0.0289	1.9817E-01		ENSG00000259374
NDUFB4P12	0.0277	7.4922E-01	8.6385E-01	ENSG00000124399
NDUFB4P2	0.0028	7.5290E-01		ENSG00000251306
NDUFB4P6	0.0213	3.2922E-01		ENSG00000235258
NDUFB4P8	0.0300	4.9418E-01		ENSG00000223663
NDUFB5	-0.1111	1.3382E-01	3.0777E-01	ENSG00000136521
NDUFB6	-0.1128	7.5923E-02	2.1226E-01	ENSG00000165264
NDUFB7	-0.0217	7.5982E-01	8.7060E-01	ENSG00000099795
NDUFB8	-0.2197	1.1633E-03	1.0157E-02	ENSG00000166136
NDUFB8P2	0.0375	6.3118E-01		ENSG00000270264
NDUFB9	-0.1589	1.4110E-02	6.5149E-02	ENSG00000147684
NDUFB9P1	-0.0032	9.2237E-01		ENSG00000271045
NDUFC1	-0.0747	1.9451E-01	3.9123E-01	ENSG00000109390
NDUFC2	-0.3189	4.7498E-06	1.3589E-04	ENSG00000151366
NDUFC2-KCTD14	-0.0031	6.8726E-01		ENSG00000259112
NDUFS1	0.0173	7.5508E-01	8.6698E-01	ENSG00000023228
NDUFS2	0.0151	7.6265E-01	8.7222E-01	ENSG00000158864
NDUFS3	-0.1248	2.2532E-02	9.0501E-02	ENSG00000213619
NDUFS4	-0.0796	2.2864E-01	4.3321E-01	ENSG00000164258
NDUFS5	-0.3010	1.1842E-05	2.8044E-04	ENSG00000168653
NDUFS5P1	0.0012	8.7690E-01		ENSG00000218424
NDUFS6	-0.1485	1.7631E-02	7.5989E-02	ENSG00000145494
NDUFS7	-0.0689	2.6517E-01	4.7431E-01	ENSG00000115286
NDUFS8	-0.0305	5.8212E-01	7.5309E-01	ENSG00000110717
NDUFV1	0.0058	9.2472E-01	9.6296E-01	ENSG00000167792
NDUFV1-DT	0.0971	1.0525E-01	2.6353E-01	ENSG00000184224
NDUFV2	-0.1265	1.2206E-01	2.9085E-01	ENSG00000178127
NDUFV2-AS1	-0.0822	2.4324E-01	4.5107E-01	ENSG00000266053
NDUFV3	0.0164	7.9798E-01	8.9360E-01	ENSG00000160194
NEAT1	0.1204	1.8152E-01	3.7399E-01	ENSG00000245532
NEB	0.4591	6.1341E-03	3.5067E-02	ENSG00000183091
NEBL	-0.0217	7.5808E-01	8.6906E-01	ENSG00000078114
NEBL-AS1	-0.0022	9.7688E-01	9.8837E-01	ENSG00000231920
NECAB1	-0.1257	1.1507E-01	2.7956E-01	ENSG00000123119
NECAB2	0.0072	9.4267E-01	9.7154E-01	ENSG00000103154
NECAB3	-0.1464	6.6950E-02	1.9488E-01	ENSG00000125967
NECAP1	-0.1542	1.9383E-02	8.1095E-02	ENSG00000089818
NECAP1P2	0.0321	4.3720E-01		ENSG00000234632
NECAP2	0.1106	7.2966E-02	2.0672E-01	ENSG00000157191
NECTIN1	0.1237	1.3688E-01	3.1231E-01	ENSG00000110400
NECTIN1-AS1	0.0069	9.8829E-01		ENSG00000255247
NECTIN1-DT	0.0586	4.6953E-01	6.6590E-01	ENSG00000254854

NECTIN2	-0.1738	5.8815E-03	3.4043E-02	ENSG00000130202
NECTIN3	-0.0987	2.5869E-01	4.6739E-01	ENSG00000177707
NECTIN4	0.0452	2.3482E-01		ENSG00000143217
NEDD1	-0.1149	1.8393E-01	3.7736E-01	ENSG00000139350
NEDD4	-0.1728	6.0402E-02	1.8120E-01	ENSG00000069869
NEDD4L	-0.3126	3.2335E-04	3.7976E-03	ENSG00000049759
NEDD8	-0.1462	2.6652E-02	1.0208E-01	ENSG00000129559
NEDD8-MDP1	-0.0176	5.0886E-01		ENSG00000255526
NEDD9	-0.4054	3.9079E-05	7.3795E-04	ENSG00000111859
NEFH	0.0195	7.8744E-01	8.8772E-01	ENSG00000100285
NEFL	0.1219	1.6866E-01	3.5706E-01	ENSG00000277586
NEFM	0.5878	5.8371E-06	1.5905E-04	ENSG00000104722
NEGR1	0.1245	1.3427E-01	3.0848E-01	ENSG00000172260
NEIL1	0.0900	2.0003E-01	3.9843E-01	ENSG00000140398
NEIL2	0.0729	2.2358E-01	4.2744E-01	ENSG00000154328
NEIL3	-0.0245	7.7744E-01	8.8148E-01	ENSG00000109674
NEK1	-0.0515	3.6642E-01	5.7569E-01	ENSG00000137601
NEK11	-0.0089	9.1438E-01	9.5788E-01	ENSG00000114670
NEK2	-0.1592	9.5321E-02	2.4736E-01	ENSG00000117650
NEK2-DT	0.0627	2.5347E-01	4.6143E-01	ENSG00000231057
NEK3	0.1533	2.6551E-02	1.0188E-01	ENSG00000136098
NEK4	0.1683	3.6116E-02	1.2679E-01	ENSG00000114904
NEK4P2	0.0215	3.9883E-01		ENSG00000264359
NEK5	0.0666	4.1773E-01	6.2109E-01	ENSG00000197168
NEK6	0.0122	8.7980E-01	9.3992E-01	ENSG00000119408
NEK7	-0.1287	1.4783E-01	3.2810E-01	ENSG00000151414
NEK8	0.0946	2.9636E-01	5.0633E-01	ENSG00000160602
NEK9	-0.0099	8.7027E-01	9.3460E-01	ENSG00000119638
NELFA	-0.1240	2.7528E-02	1.0457E-01	ENSG00000185049
NELFB	-0.1856	8.2462E-03	4.3860E-02	ENSG00000188986
NELFCD	0.0280	2.9197E-01		ENSG00000101158
NELFE	-0.0658	2.7171E-01	4.8089E-01	ENSG00000204356
NELL1	0.1239	1.7397E-01	3.6410E-01	ENSG00000165973
NELL2	-0.2437	9.3264E-03	4.8169E-02	ENSG00000184613
NEMF	0.0227	7.0856E-01	8.3974E-01	ENSG00000165525
NEMP1	0.0379	6.6961E-01	8.1482E-01	ENSG00000166881
NEMP2	-0.0290	7.2857E-01	8.5112E-01	ENSG00000189362
NEMP2-DT	-0.4483	9.1483E-04	8.4781E-03	ENSG00000233654
NENF	-0.1848	4.8164E-03	2.9239E-02	ENSG00000117691
NENFP1	-0.0350	2.1675E-01		ENSG00000233647
NEO1	0.0068	9.2876E-01	9.6491E-01	ENSG00000067141
NEPRO	0.1185	7.8206E-02	2.1633E-01	ENSG00000163608
NES	0.0664	3.4475E-01	5.5435E-01	ENSG00000132688
NETO1	-0.0807	3.6278E-01	5.7197E-01	ENSG00000166342
NETO1-DT	-0.0415	6.1786E-01	7.7877E-01	ENSG00000263958
NETO2	-0.2704	1.2269E-03	1.0566E-02	ENSG00000171208
NEU1	0.0025	9.8049E-01	9.9000E-01	ENSG00000204386
NEU3	0.1581	4.0491E-02	1.3795E-01	ENSG00000162139
NEU4	-0.0071	9.6584E-01		ENSG00000204099

NEURL1	0.3247	9.6072E-04	8.7811E-03	ENSG00000107954
NEURL1-AS1	0.0172	7.2661E-01	8.4993E-01	ENSG00000235470
NEURL1B	-0.1708	8.5236E-02	2.2848E-01	ENSG00000214357
NEURL2	0.0242	6.4001E-01	7.9402E-01	ENSG00000124257
NEURL3	-0.0474	4.3236E-01	6.3368E-01	ENSG00000163121
NEURL4	0.1795	4.3635E-02	1.4524E-01	ENSG00000215041
NEUROD1	-0.0709	2.7386E-01	4.8366E-01	ENSG00000162992
NEUROD2	-0.3300	2.0188E-02	8.3570E-02	ENSG00000171532
NEUROD4	0.0106	8.4281E-01	9.1921E-01	ENSG00000123307
NEUROD6	-0.0461	4.0583E-01	6.1032E-01	ENSG00000164600
NEUROG1	0.0062	7.1685E-01	8.4433E-01	ENSG00000181965
NEUROG2	0.0068	9.0520E-01	9.5372E-01	ENSG00000178403
NEUROG3	0.0038	6.3876E-01		ENSG00000122859
NEXMIF	0.1156	1.1626E-01	2.8115E-01	ENSG00000050030
NEXN	0.0560	5.1249E-01	7.0051E-01	ENSG00000162614
NEXN-AS1	0.0318	6.3423E-01	7.9032E-01	ENSG00000235927
NF1	0.1884	1.8982E-02	7.9992E-02	ENSG00000196712
NF1P1	0.0227	4.1053E-01		ENSG00000270831
NF1P8	-0.0104	3.6089E-01		ENSG00000236956
NF2	-0.0986	5.3120E-02	1.6624E-01	ENSG00000186575
NFAM1	0.0329	5.1685E-01	7.0434E-01	ENSG00000235568
NFASC	0.1865	6.5714E-03	3.7039E-02	ENSG00000163531
NFAT5	-0.1461	1.2307E-02	5.8978E-02	ENSG00000102908
NFATC1	-0.2739	1.9427E-02	8.1264E-02	ENSG00000131196
NFATC2	-0.0017	9.8872E-01	9.9379E-01	ENSG00000101096
NFATC2IP	-0.0543	4.1435E-01	6.1800E-01	ENSG00000176953
NFATC3	-0.0816	3.0659E-01	5.1680E-01	ENSG00000072736
NFATC4	-0.0416	5.9193E-01	7.5972E-01	ENSG00000100968
NFE2	0.0617	3.3466E-01	5.4471E-01	ENSG00000123405
NFE2L1	0.0101	8.4802E-01	9.2256E-01	ENSG00000082641
NFE2L1-DT	-0.0158	8.4062E-01	9.1799E-01	ENSG00000263412
NFE2L2	-0.1521	3.0241E-02	1.1189E-01	ENSG00000116044
NFE2L3	0.0015	9.8618E-01	9.9239E-01	ENSG00000050344
NFIA	-0.3925	3.3492E-04	3.9074E-03	ENSG00000162599
NFIA-AS1	0.0190	7.7018E-01		ENSG00000237853
NFIA-AS2	-0.1086	2.3513E-01	4.4052E-01	ENSG00000237928
NFIB	-0.5028	3.4421E-05	6.6425E-04	ENSG00000147862
NFIC	-0.2973	1.1950E-04	1.7672E-03	ENSG00000141905
NFIL3	-0.5549	3.4481E-11	6.8924E-09	ENSG00000165030
NFILZ	0.0015	9.1370E-01		ENSG00000268480
NFKB1	-0.3322	1.0826E-03	9.6269E-03	ENSG00000109320
NFKB2	-0.0866	3.2004E-01	5.3020E-01	ENSG00000077150
NFKBIB	-0.1338	3.3754E-02	1.2073E-01	ENSG00000104825
NFKBID	0.3906	5.0930E-03	3.0520E-02	ENSG00000167604
NFKBIE	-0.1556	6.4546E-02	1.9006E-01	ENSG00000146232
NFKBIL1	-0.0227	6.9553E-01	8.3149E-01	ENSG00000204498
NFKBIZ	-0.3502	1.3269E-02	6.2295E-02	ENSG00000144802
NFRKB	-0.0278	6.4931E-01	8.0066E-01	ENSG00000170322
NFS1	-0.0242	7.2397E-01	8.4859E-01	ENSG00000244005

NFU1	0.0025	9.7049E-01	9.8515E-01	ENSG00000169599
NFX1	0.2907	2.8737E-05	5.7580E-04	ENSG00000086102
NFXL1	0.1381	9.7818E-02	2.5121E-01	ENSG00000170448
NFYA	0.0599	3.9354E-01	6.0005E-01	ENSG00000001167
NFYB	-0.1043	6.7155E-02	1.9527E-01	ENSG00000120837
NFYC	-0.0411	4.7505E-01	6.7064E-01	ENSG00000066136
NGB	-0.0443	5.3811E-01	7.2071E-01	ENSG00000165553
NGDN	-0.2780	1.2994E-07	7.0893E-06	ENSG00000129460
NGEF	-0.3796	7.8232E-04	7.5390E-03	ENSG00000066248
NGF	0.3351	1.8795E-02	7.9417E-02	ENSG00000134259
NGFR	-0.5335	1.8400E-05	4.0069E-04	ENSG00000064300
NGLY1	0.0537	4.0158E-01	6.0655E-01	ENSG00000151092
NGRN	-0.3574	1.5172E-05	3.3989E-04	ENSG00000182768
NHEJ1	0.1920	7.2125E-02	2.0509E-01	ENSG00000187736
NHERF1	-0.0738	4.0150E-01	6.0651E-01	ENSG00000109062
NHERF2	-0.1732	5.8958E-02	1.7843E-01	ENSG00000065054
NHERF4	-0.0185	4.6371E-01		ENSG00000172367
NHLH1	-0.1662	9.6348E-02	2.4920E-01	ENSG00000171786
NHLH2	0.1400	1.4641E-01	3.2603E-01	ENSG00000177551
NHLRC1	0.3376	1.3276E-03	1.1237E-02	ENSG00000187566
NHLRC2	0.1814	2.1155E-02	8.6381E-02	ENSG00000196865
NHLRC4	0.0123	7.0228E-01		ENSG00000257108
NHP2	-0.2497	2.1969E-05	4.6241E-04	ENSG00000145912
NHS	-0.0668	3.5428E-01	5.6418E-01	ENSG00000188158
NHS-AS1	-0.0070	6.8301E-01		ENSG00000230020
NHSL1	-0.4255	4.2399E-05	7.8027E-04	ENSG00000135540
NHSL2	-0.1452	9.8907E-02	2.5295E-01	ENSG00000204131
NHSL3	0.1912	1.6784E-02	7.3426E-02	ENSG00000162522
NIBAN1	0.1890	7.6624E-02	2.1349E-01	ENSG00000135842
NIBAN2	-0.1917	6.2404E-02	1.8551E-01	ENSG00000136830
NIBAN3	0.0238	7.4083E-01	8.5880E-01	ENSG00000167483
NICOL1	-0.1483	5.5036E-02	1.7066E-01	ENSG00000243449
NID1	0.0275	7.3994E-01	8.5805E-01	ENSG00000116962
NID2	-0.0101	9.0307E-01	9.5263E-01	ENSG00000087303
NIF3L1	-0.0361	4.5901E-01	6.5682E-01	ENSG00000196290
NIFK	0.0289	6.4534E-01	7.9755E-01	ENSG00000155438
NIFK-AS1	0.0033	9.6671E-01	9.8340E-01	ENSG00000236859
NIFKP8	0.0269	2.8022E-01		ENSG00000259449
NIM1K	0.0117	8.8808E-01	9.4438E-01	ENSG00000177453
NIN	-0.0145	8.2559E-01	9.0954E-01	ENSG00000100503
NINJ1	-0.0729	2.6313E-01	4.7196E-01	ENSG00000131669
NINJ2	0.1159	2.1947E-03		ENSG00000171840
NINJ2-AS1	0.0281	6.7799E-01	8.1967E-01	ENSG00000177406
NINL	0.2675	8.9503E-04	8.3449E-03	ENSG00000101004
NIP7	-0.0559	3.9447E-01	6.0076E-01	ENSG00000132603
NIPA1	0.0473	4.3557E-01	6.3656E-01	ENSG00000170113
NIPA2	0.0317	6.3231E-01	7.8883E-01	ENSG00000140157
NIPAL1	0.0092	8.9207E-01	9.4631E-01	ENSG00000163293
NIPAL2	0.0358	5.4520E-01	7.2567E-01	ENSG00000104361

NIPAL3	-0.0069	9.1873E-01	9.5989E-01	ENSG00000001461
NIPAL4	0.0672	2.4801E-01	4.5577E-01	ENSG00000172548
NIPBL	-0.0279	6.8505E-01	8.2448E-01	ENSG00000164190
NIPBL-DT	0.2068	1.8537E-03	1.4223E-02	ENSG00000285967
NIPSNAP1	-0.2582	5.0552E-06	1.4235E-04	ENSG00000184117
NIPSNAP2	-0.1297	3.8254E-03	2.4691E-02	ENSG00000146729
NIPSNAP3A	-0.2478	6.4063E-04	6.4840E-03	ENSG00000136783
NIPSNAP3B	0.0016	9.8918E-01	9.9399E-01	ENSG00000165028
NISCH	-0.1485	1.4923E-02	6.7707E-02	ENSG00000010322
NIT1	-0.1269	5.8833E-02	1.7820E-01	ENSG00000158793
NIT2	0.1064	1.5325E-01	3.3544E-01	ENSG00000114021
NKAIN1	0.0471	5.2392E-01	7.0947E-01	ENSG00000084628
NKAIN2	-0.4448	1.6357E-03	1.3016E-02	ENSG00000188580
NKAIN3	0.0761	3.5766E-01	5.6743E-01	ENSG00000185942
NKAIN4	-0.3309	1.4782E-03	1.2148E-02	ENSG00000101198
NKAP	0.2563	1.9961E-03	1.5057E-02	ENSG00000101882
NKAPD1	-0.0489	4.1514E-01	6.1862E-01	ENSG00000150776
NKAPL	0.5888	1.1428E-02	5.5933E-02	ENSG00000189134
NKAPP1	-0.0263	7.5277E-01	8.6591E-01	ENSG00000233382
NKD1	0.1020	2.2424E-01	4.2810E-01	ENSG00000140807
NKD2	-0.3007	2.4542E-03	1.7560E-02	ENSG00000145506
NKG7	0.0159	6.6304E-01		ENSG00000105374
NKILA	0.0341	6.7807E-01	8.1969E-01	ENSG00000278709
NKIRAS1	0.0772	2.0304E-01	4.0199E-01	ENSG00000197885
NKIRAS2	-0.0862	1.3635E-01	3.1153E-01	ENSG00000168256
NKPD1	0.2234	5.4445E-02	1.6925E-01	ENSG00000179846
NKRF	-0.0078	9.1620E-01	9.5863E-01	ENSG00000186416
NKTR	0.1068	1.3264E-01	3.0622E-01	ENSG00000114857
NKX1-1	-0.0008	8.8078E-01		ENSG00000235608
NKX1-2	-0.0545	1.8082E-01		ENSG00000229544
NKX2-3	0.0616	3.6314E-01	5.7225E-01	ENSG00000119919
NKX2-4	-0.0130	5.9119E-01		ENSG00000125816
NKX2-5	0.0028	9.4996E-01	9.7516E-01	ENSG00000183072
NKX2-6	0.0319	3.4976E-01		ENSG00000180053
NKX3-1	-0.0229	5.8805E-01	7.5699E-01	ENSG00000167034
NKX3-2	-0.0269	6.6039E-01	8.0864E-01	ENSG00000109705
NKX6-1	0.0140	7.3716E-01		ENSG00000163623
NKX6-2	0.1228	2.6035E-02		ENSG00000148826
NKX6-3	0.1029	7.7614E-02	2.1532E-01	ENSG00000165066
NLE1	0.1626	7.0313E-02	2.0131E-01	ENSG00000073536
NLGN1	0.0576	4.5569E-01	6.5397E-01	ENSG00000169760
NLGN1-AS1	-0.0204	5.7512E-01		ENSG00000228213
NLGN2	0.0059	9.3519E-01	9.6814E-01	ENSG00000169992
NLGN3	-0.0051	9.3341E-01	9.6701E-01	ENSG00000196338
NLGN4X	0.0564	4.0241E-01	6.0699E-01	ENSG00000146938
NLGN4Y	0.0002	9.9860E-01	9.9913E-01	ENSG00000165246
NLK	0.2990	1.2520E-03	1.0734E-02	ENSG00000087095
NLN	-0.0056	9.1640E-01	9.5863E-01	ENSG00000123213
NLRC3	-0.0019	9.8076E-01	9.9009E-01	ENSG00000167984

NLRC4	0.0027	8.4321E-01		ENSG00000091106
NLRC5	0.0524	5.2494E-01	7.1041E-01	ENSG00000140853
NLRP1	0.1809	7.2648E-02	2.0610E-01	ENSG00000091592
NLRP11	0.0073	9.0331E-01	9.5277E-01	ENSG00000179873
NLRP12	-0.0247	2.7545E-01		ENSG00000142405
NLRP14	0.3550	1.0581E-02	5.2864E-02	ENSG00000158077
NLRP2	0.1911	2.9719E-02	1.1044E-01	ENSG00000022556
NLRP3	0.0257	5.7318E-01		ENSG00000162711
NLRP6	-0.0053	6.5104E-01		ENSG00000174885
NLRP9	-0.0360	4.1759E-01		ENSG00000185792
NLRX1	0.0497	5.5640E-01	7.3425E-01	ENSG00000160703
NMB	0.2004	2.1148E-02	8.6366E-02	ENSG00000197696
NMBR	0.0366	7.3921E-01	8.5776E-01	ENSG00000135577
NMD3	-0.1439	3.2305E-02	1.1705E-01	ENSG00000169251
NME1	0.0341	5.8803E-01	7.5699E-01	ENSG00000239672
NME2	-0.3430	2.1924E-03	1.6127E-02	ENSG00000243678
NME3	-0.5964	8.1436E-13	3.0360E-10	ENSG00000103024
NME4	-0.2953	5.2327E-05	9.2361E-04	ENSG00000103202
NME5	0.1163	1.3497E-01	3.0951E-01	ENSG00000112981
NME6	-0.0294	6.2969E-01	7.8675E-01	ENSG00000172113
NME7	0.1603	2.0531E-02	8.4449E-02	ENSG00000143156
NME8	0.0118	5.7166E-01		ENSG00000086288
NME9	0.3118	6.2711E-03	3.5675E-02	ENSG00000181322
NMI	-0.0348	6.2035E-01	7.8055E-01	ENSG00000123609
NMNAT1	-0.0852	2.4632E-01	4.5414E-01	ENSG00000173614
NMNAT2	-0.1694	1.4251E-02	6.5539E-02	ENSG00000157064
NMNAT3	-0.3025	1.2161E-05	2.8496E-04	ENSG00000163864
NMRAL1	-0.1991	2.5161E-03	1.7844E-02	ENSG00000153406
NMRK1	-0.1170	8.5904E-02	2.2980E-01	ENSG00000106733
NMRK2	-0.0830	3.2405E-01	5.3405E-01	ENSG00000077009
NMS	0.0027	9.0951E-01		ENSG00000204640
NMT1	-0.1250	1.8316E-02	7.7929E-02	ENSG00000136448
NMT2	-0.0605	2.9704E-01	5.0712E-01	ENSG00000152465
NMU	-0.2376	2.1052E-02	8.6017E-02	ENSG00000109255
NMUR1	0.0290	7.6131E-01	8.7163E-01	ENSG00000171596
NMUR2	0.0685	1.8852E-01	3.8349E-01	ENSG00000132911
NNAT	0.1502	1.8577E-02	7.8743E-02	ENSG00000053438
NNMT	-0.0629	3.7030E-01	5.7942E-01	ENSG00000166741
NNT	-0.2606	2.6358E-05	5.3599E-04	ENSG00000112992
NOA1	-0.2856	3.2048E-04	3.7783E-03	ENSG00000084092
NOB1	-0.1931	1.9803E-02	8.2418E-02	ENSG00000141101
NOC2L	-0.1184	1.4117E-01	3.1799E-01	ENSG00000188976
NOC2LP1	0.1636	8.3918E-02	2.2638E-01	ENSG00000213225
NOC3L	0.0860	2.8269E-01	4.9317E-01	ENSG00000173145
NOC4L	0.0473	4.5821E-01	6.5617E-01	ENSG00000184967
NOCT	-0.5498	3.0349E-05	5.9787E-04	ENSG00000151014
NOD1	-0.0459	5.8521E-01	7.5511E-01	ENSG00000106100
NOD2	0.0267	7.8539E-01	8.8655E-01	ENSG00000167207
NODAL	-0.0063	8.3499E-01	9.1514E-01	ENSG00000156574

NOG	-0.0717	4.0038E-01	6.0570E-01	ENSG00000183691
NOL10	0.0019	9.8236E-01	9.9070E-01	ENSG00000115761
NOL11	-0.0672	3.1907E-01	5.2935E-01	ENSG00000130935
NOL12	0.0758	3.8310E-01	5.9131E-01	ENSG00000273899
NOL3	-0.1531	5.9217E-02	1.7886E-01	ENSG00000140939
NOL4	-0.0899	2.1602E-01	4.1750E-01	ENSG00000101746
NOL4L	-0.0664	2.3645E-01	4.4223E-01	ENSG00000197183
NOL4L-DT	0.0114	5.6853E-01		ENSG00000204393
NOL6	0.2672	3.2358E-03	2.1706E-02	ENSG00000165271
NOL7	-0.0258	6.6836E-01	8.1415E-01	ENSG00000225921
NOL8	0.0292	6.3017E-01	7.8706E-01	ENSG00000198000
NOL9	-0.2913	4.0385E-05	7.5604E-04	ENSG00000162408
NOLC1	0.1130	1.8424E-01	3.7770E-01	ENSG00000166197
NOLC1P1	0.0192	2.4702E-01		ENSG00000225271
NOM1	-0.0295	6.7547E-01	8.1791E-01	ENSG00000146909
NOMO1	0.0656	3.8571E-01	5.9356E-01	ENSG00000103512
NOMO2	0.1288	5.1099E-02	1.6175E-01	ENSG00000185164
NOMO3	0.1242	8.3658E-02	2.2593E-01	ENSG00000103226
NONO	-0.1652	2.7355E-04	3.3485E-03	ENSG00000147140
NOP10	-0.2966	2.5612E-04	3.1860E-03	ENSG00000182117
NOP14	0.0286	6.7947E-01	8.2059E-01	ENSG00000087269
NOP16	0.0645	3.4007E-01	5.4984E-01	ENSG00000048162
NOP2	-0.0009	9.9041E-01	9.9461E-01	ENSG00000111641
NOP53	-0.0449	4.5788E-01	6.5591E-01	ENSG00000105373
NOP53-AS1	-0.0210	6.8243E-01		ENSG00000269656
NOP56	-0.0788	8.8423E-02	2.3475E-01	ENSG00000101361
NOP58	-0.1808	2.3537E-03	1.7004E-02	ENSG00000055044
NOP9	-0.1166	2.0309E-01	4.0202E-01	ENSG00000196943
NOPCHAP1	-0.2360	1.6222E-04	2.2027E-03	ENSG00000151131
NORAD	-0.0134	8.1167E-01	9.0156E-01	ENSG00000260032
NOS1	-0.5995	1.0782E-04	1.6326E-03	ENSG00000089250
NOS1AP	-0.1500	5.8782E-02	1.7814E-01	ENSG00000198929
NOS2	0.2242	5.0870E-02	1.6134E-01	ENSG00000007171
NOS3	0.0493	5.4266E-01	7.2413E-01	ENSG00000164867
NOSIP	-0.0270	5.5115E-01	7.2989E-01	ENSG00000142546
NOSTRIN	-0.0342	6.2942E-01	7.8664E-01	ENSG00000163072
NOTCH1	0.0249	7.5628E-01	8.6801E-01	ENSG00000148400
NOTCH2	0.0627	4.1231E-01	6.1603E-01	ENSG00000134250
NOTCH2NLA	0.1785	6.7572E-02	1.9602E-01	ENSG00000264343
NOTCH2NLB	0.2067	4.1665E-02	1.4074E-01	ENSG00000286019
NOTCH2NLC	0.2610	1.1815E-02	5.7372E-02	ENSG00000286219
NOTCH2NLR	-0.0023	9.7421E-01	9.8741E-01	ENSG00000286106
NOTCH3	0.1257	1.2093E-01	2.8897E-01	ENSG00000074181
NOTCH4	0.3206	6.1069E-04	6.2349E-03	ENSG00000204301
NOTO	0.0066	8.3269E-01		ENSG00000214513
NOTUM	-0.4114	2.1193E-03	1.5725E-02	ENSG00000185269
NOVA1	0.3662	1.0492E-07	6.0174E-06	ENSG00000139910
NOVA2	0.0774	2.9448E-01	5.0449E-01	ENSG00000104967
NOX1	0.0729	2.4535E-01	4.5312E-01	ENSG00000007952

NOX3	0.0342	7.3110E-01	8.5293E-01	ENSG00000074771
NOX4P1	0.0036	8.3738E-01		ENSG00000255532
NOX5	0.0234	3.1030E-01		ENSG00000255346
NOXA1	0.1118	1.9387E-01	3.9029E-01	ENSG00000188747
NOXO1	-0.0045	6.6183E-01		ENSG00000196408
NOXRED1	0.0101	2.8791E-01		ENSG00000165555
NPAP1	0.0304	1.7950E-01		ENSG00000185823
NPAS1	-0.4164	1.3252E-04	1.8997E-03	ENSG00000130751
NPAS2	-0.0767	2.8896E-01	4.9940E-01	ENSG00000170485
NPAS3	0.0887	2.6583E-01	4.7504E-01	ENSG00000151322
NPAS4	-0.2024	5.5328E-02	1.7134E-01	ENSG00000174576
NPAT	0.2295	3.9644E-03	2.5409E-02	ENSG00000149308
NPBWR1	0.0965	1.9094E-01	3.8622E-01	ENSG00000288611
NPBWR2	0.0596	6.3552E-02		ENSG00000125522
NPC1	0.0718	3.4122E-01	5.5104E-01	ENSG00000141458
NPC1L1	0.0726	2.8220E-01	4.9262E-01	ENSG00000015520
NPC2	0.0806	3.1939E-01	5.2958E-01	ENSG00000119655
NPDC1	-0.3788	2.3860E-08	1.7166E-06	ENSG00000107281
NPEPL1	0.3637	1.0218E-04	1.5733E-03	ENSG00000215440
NPEPPS	-0.1023	8.1487E-02	2.2208E-01	ENSG00000141279
NPEPPSP1	-0.0412	5.6477E-01	7.4058E-01	ENSG00000274615
NPFF	0.0858	1.0201E-01	2.5807E-01	ENSG00000139574
NPFFR1	-0.1472	8.5039E-02	2.2818E-01	ENSG00000148734
NPFFR2	0.0929	1.1985E-01	2.8710E-01	ENSG00000056291
NPHP1	0.5039	1.5566E-07	8.1835E-06	ENSG00000144061
NPHP3	0.1692	7.9773E-02	2.1918E-01	ENSG00000113971
NPHP3-AS1	-0.0347	1.8361E-01		ENSG00000248724
NPHP4	0.2782	4.9635E-03	2.9896E-02	ENSG00000131697
NPHS1	0.3478	1.7811E-02	7.6483E-02	ENSG00000161270
NPIPA1	0.2093	2.0391E-02	8.4068E-02	ENSG00000183426
NPIPA2	0.2263	1.9081E-02	8.0296E-02	ENSG00000254852
NPIPA3	0.1326	1.4009E-01	3.1664E-01	ENSG00000224712
NPIPA5	0.1583	7.8288E-02	2.1641E-01	ENSG00000183793
NPIPA7	0.1293	1.1672E-01	2.8187E-01	ENSG00000214967
NPIPA8	0.2752	2.7539E-02	1.0457E-01	ENSG00000214940
NPIPA9	0.1244	1.4101E-01	3.1769E-01	ENSG00000233024
NPIPA9	0.1126	1.7604E-01	3.6711E-01	ENSG00000183889
NPIPB11	0.5763	1.1439E-06	4.1574E-05	ENSG00000254206
NPIPB12	0.4843	2.6052E-06	8.2816E-05	ENSG00000169203
NPIPB13	0.4948	1.2366E-06	4.4550E-05	ENSG00000198064
NPIPB15	0.5086	4.2697E-04	4.7068E-03	ENSG00000196436
NPIPB2	0.0816	2.8808E-01	4.9865E-01	ENSG00000234719
NPIPB3	0.4353	7.2664E-06	1.8960E-04	ENSG00000169246
NPIPB4	0.4115	1.0579E-05	2.5671E-04	ENSG00000185864
NPIPB5	0.3737	4.9417E-05	8.8173E-04	ENSG00000243716
NPIPB6	0.5162	1.0832E-04	1.6372E-03	ENSG00000198156
NPIPB7	0.0620	3.3077E-01	5.4110E-01	ENSG00000233232
NPIPB8	0.2287	4.9369E-02	1.5811E-01	ENSG00000255524
NPIPB9	0.4397	4.2927E-04	4.7216E-03	ENSG00000196993

NPIPP1	0.1103	2.3117E-01	4.3613E-01	ENSG00000188599
NPL	0.0669	3.9974E-01	6.0515E-01	ENSG00000135838
NPLOC4	0.0337	5.0121E-01	6.9204E-01	ENSG00000182446
NPM1	-0.2382	1.9471E-04	2.5402E-03	ENSG00000181163
NPM1P30	0.0084	4.3081E-01		ENSG00000228219
NPM1P38	-0.0143	4.6958E-01		ENSG00000219257
NPM1P39	-0.0376	5.9311E-01	7.6039E-01	ENSG00000225159
NPM1P40	-0.0096	5.2732E-01		ENSG00000236523
NPM1P48	-0.0187	3.1476E-01		ENSG00000231821
NPM1P49	0.0105	8.0716E-01		ENSG00000226556
NPM2	-0.4634	4.2881E-04	4.7187E-03	ENSG00000158806
NPM3	-0.4385	2.0127E-04	2.6050E-03	ENSG00000107833
NPNT	-0.2465	3.2446E-02	1.1740E-01	ENSG00000168743
NPPA	-0.0204	7.6155E-01	8.7173E-01	ENSG00000175206
NPPB	0.0488	5.3410E-01	7.1776E-01	ENSG00000120937
NPPC	-0.2499	8.0911E-03	4.3241E-02	ENSG00000163273
NPR1	-0.0130	8.6516E-01	9.3189E-01	ENSG00000169418
NPR2	-0.0349	6.7675E-01	8.1893E-01	ENSG00000159899
NPR3	0.1204	9.4511E-02	2.4597E-01	ENSG00000113389
NPRL2	0.2049	2.3292E-02	9.2811E-02	ENSG00000114388
NPRL3	-0.1275	1.0194E-01	2.5800E-01	ENSG00000103148
NPSR1	0.0436	3.0752E-01	5.1769E-01	ENSG00000187258
NPSR1-AS1	-0.0071	9.5921E-01		ENSG00000197085
NPTN	-0.0300	6.6827E-01	8.1415E-01	ENSG00000156642
NPTX1	0.0611	4.8217E-01	6.7666E-01	ENSG00000171246
NPTX2	0.0859	2.8023E-01	4.9022E-01	ENSG00000106236
NPTXR	-0.5831	1.5472E-07	8.1520E-06	ENSG00000221890
NPW	-0.2293	3.2883E-02	1.1858E-01	ENSG00000183971
NPY	0.0181	7.6042E-01	8.7105E-01	ENSG00000122585
NPY2R	-0.0047	5.5701E-01	7.3477E-01	ENSG00000185149
NPY4R	-0.0291	3.8016E-01		ENSG00000204174
NPY4R2	-0.0105	5.8293E-01		ENSG00000264717
NPY5R	0.1107	2.0232E-01	4.0117E-01	ENSG00000164129
NQO1	-0.0550	2.9980E-01	5.0942E-01	ENSG00000181019
NQO2	0.0288	6.0547E-01	7.6942E-01	ENSG00000124588
NR0B1	0.0019	5.2320E-01		ENSG00000169297
NR0B2	0.0358	3.1648E-01		ENSG00000131910
NR1D2	-0.4502	2.1728E-10	3.0725E-08	ENSG00000174738
NR1H2	-0.0660	3.0041E-01	5.1021E-01	ENSG00000131408
NR1H3	-0.2535	6.4338E-03	3.6417E-02	ENSG00000025434
NR1H4	0.0095	8.0289E-01		ENSG00000012504
NR1I2	0.0354	3.1667E-01		ENSG00000144852
NR1I3	0.0172	7.9812E-01	8.9364E-01	ENSG00000143257
NR2C1	0.2264	4.4482E-03	2.7521E-02	ENSG00000120798
NR2C2	0.1004	2.3006E-01	4.3466E-01	ENSG00000177463
NR2C2AP	-0.1427	9.3460E-02	2.4408E-01	ENSG00000184162
NR2E1	-0.4289	6.1171E-05	1.0505E-03	ENSG00000112333
NR2E3	-0.0201	4.5832E-01	6.5625E-01	ENSG00000278570
NR2F1	-0.2172	1.7988E-02	7.6998E-02	ENSG00000175745

NR2F1-AS1	0.0316	6.6424E-01	8.1110E-01	ENSG00000237187
NR2F2	-0.0098	9.0935E-01	9.5524E-01	ENSG00000185551
NR2F2-AS1	0.1032	2.3355E-01	4.3874E-01	ENSG00000247809
NR2F6	-0.2997	1.3721E-03	1.1476E-02	ENSG00000160113
NR3C1	-0.1335	1.1743E-01	2.8297E-01	ENSG00000113580
NR3C1P1	0.0925	5.8052E-02		ENSG00000276393
NR3C2	-0.1077	2.1325E-01	4.1434E-01	ENSG00000151623
NR4A1	0.0427	6.1313E-01	7.7496E-01	ENSG00000123358
NR4A1AS	0.0124	5.1054E-01		ENSG00000259884
NR4A3	-0.0980	2.8231E-01	4.9274E-01	ENSG00000119508
NR5A1	-0.0160	3.2133E-01		ENSG00000136931
NR5A2	0.0312	3.0994E-01		ENSG00000116833
NR6A1	0.0517	5.3962E-01	7.2175E-01	ENSG00000148200
NRAD1	0.0125	8.1822E-01		ENSG00000233725
NRAP	0.0513	1.7152E-01		ENSG00000197893
NRARP	-0.0846	3.2725E-01	5.3766E-01	ENSG00000198435
NRAS	-0.2707	2.2929E-05	4.7852E-04	ENSG00000213281
NRAV	0.0511	5.1926E-01	7.0638E-01	ENSG00000248008
NRBF2	0.0138	7.8095E-01	8.8383E-01	ENSG00000148572
NRBP1	-0.0079	8.9242E-01	9.4647E-01	ENSG00000115216
NRBP2	0.2553	1.7125E-03	1.3436E-02	ENSG00000185189
NRCAM	0.0221	7.4550E-01	8.6131E-01	ENSG00000091129
NRDC	-0.0896	8.9890E-02	2.3764E-01	ENSG00000078618
NRDE2	0.3302	7.4526E-05	1.2241E-03	ENSG00000119720
NREP	-0.1087	1.5934E-01	3.4399E-01	ENSG00000134986
NRF1	-0.0400	6.0871E-01	7.7186E-01	ENSG00000106459
NRG1	0.2078	2.7374E-02	1.0415E-01	ENSG00000157168
NRG1-IT1	0.0963	1.1077E-01	2.7252E-01	ENSG00000253974
NRG1-IT3	0.0044	8.9634E-01		ENSG00000254049
NRG2	0.0176	8.2876E-01	9.1134E-01	ENSG00000158458
NRG3	-0.2029	1.2452E-02	5.9505E-02	ENSG00000185737
NRG3-AS1	0.1073	5.8832E-02		ENSG00000225738
NRG4	0.0374	5.2185E-01	7.0850E-01	ENSG00000169752
NRGN	-0.5489	4.1128E-05	7.6548E-04	ENSG00000154146
NRIP1	-0.0681	3.1751E-01	5.2775E-01	ENSG00000180530
NRIP2	0.0161	8.3015E-01	9.1211E-01	ENSG00000053702
NRIP3	-0.1911	2.5219E-02	9.8136E-02	ENSG00000175352
NRIP3-DT	0.1808	8.3991E-02	2.2640E-01	ENSG00000253973
NRK	0.0106	7.6224E-01		ENSG00000123572
NRL	0.0520	5.4297E-01	7.2427E-01	ENSG00000129535
NRM	-0.0231	7.3548E-01	8.5574E-01	ENSG00000137404
NRN1	0.3203	1.0734E-02	5.3390E-02	ENSG00000124785
NRP1	0.1284	1.2812E-01	2.9979E-01	ENSG00000099250
NRP2	-0.2818	9.1459E-05	1.4379E-03	ENSG00000118257
NRROS	-0.0122	4.5803E-01		ENSG00000174004
NRSN1	-0.0409	5.9695E-01	7.6349E-01	ENSG00000152954
NRSN2	0.1071	1.6953E-01	3.5798E-01	ENSG00000125841
NRSN2-AS1	-0.2999	6.3190E-04	6.4195E-03	ENSG00000225377
NRTN	-0.1529	7.9710E-02	2.1914E-01	ENSG00000171119

NRXN1	-0.3060	7.6144E-05	1.2440E-03	ENSG00000179915
NRXN1-DT	-0.0363	3.8852E-01		ENSG00000231918
NRXN2	-0.0505	5.1124E-01	6.9966E-01	ENSG00000110076
NRXN3	-0.1427	5.6206E-02	1.7321E-01	ENSG00000021645
NSA2	0.0950	1.7114E-01	3.6028E-01	ENSG00000164346
NSD1	-0.0428	4.7743E-01	6.7281E-01	ENSG00000165671
NSD2	-0.0744	2.5481E-01	4.6287E-01	ENSG00000109685
NSD3	-0.2151	3.6700E-04	4.1844E-03	ENSG00000147548
NSDHL	-0.1154	6.1455E-02	1.8341E-01	ENSG00000147383
NSF	-0.0333	6.6830E-01	8.1415E-01	ENSG00000073969
NSFL1C	-0.0842	9.9451E-02	2.5371E-01	ENSG00000088833
NSG1	-0.2444	2.0157E-03	1.5172E-02	ENSG00000168824
NSG2	-0.1084	1.4856E-01	3.2908E-01	ENSG00000170091
NSL1	-0.0852	2.1149E-01	4.1231E-01	ENSG00000117697
NSMAF	0.2681	1.8089E-03	1.3979E-02	ENSG00000035681
NSMCE1	-0.3151	5.1097E-07	2.1880E-05	ENSG00000169189
NSMCE1-DT	0.1655	5.4354E-02	1.6905E-01	ENSG00000245888
NSMCE2	0.0707	3.8068E-01	5.8902E-01	ENSG00000156831
NSMCE3	-0.2970	3.3205E-09	3.1790E-07	ENSG00000185115
NSMCE4A	-0.1346	1.3586E-02	6.3349E-02	ENSG00000107672
NSMF	-0.1861	9.4339E-03	4.8618E-02	ENSG00000165802
NSRP1	0.2468	1.0076E-05	2.4668E-04	ENSG00000126653
NSRP1P1	0.5588	1.2466E-03	1.0695E-02	ENSG00000235613
NSUN2	0.1582	4.0710E-02	1.3845E-01	ENSG00000037474
NSUN3	-0.0052	9.3555E-01	9.6833E-01	ENSG00000178694
NSUN4	-0.0206	7.5372E-01	8.6622E-01	ENSG00000117481
NSUN5	0.1018	1.1836E-01	2.8470E-01	ENSG00000130305
NSUN5P1	0.2127	5.7349E-03	3.3390E-02	ENSG00000223705
NSUN6	0.3611	1.3642E-07	7.3611E-06	ENSG00000241058
NSUN7	-0.0536	4.7080E-01	6.6709E-01	ENSG00000179299
NT5C	-0.0640	3.4441E-01	5.5407E-01	ENSG00000125458
NT5C1A	0.0025	9.7683E-01	9.8837E-01	ENSG00000116981
NT5C1B	-0.0035	9.0857E-01		ENSG00000185013
NT5C2	-0.0809	3.3137E-01	5.4165E-01	ENSG00000076685
NT5C3A	-0.1132	1.1673E-02	5.6852E-02	ENSG00000122643
NT5C3AP1	-0.0095	8.6537E-01	9.3204E-01	ENSG00000213492
NT5C3B	-0.1105	7.4791E-02	2.1005E-01	ENSG00000141698
NT5DC1	0.0654	3.8475E-01	5.9268E-01	ENSG00000178425
NT5DC2	-0.2213	4.2808E-03	2.6815E-02	ENSG00000168268
NT5DC3	-0.3065	1.4027E-04	1.9752E-03	ENSG00000111696
NT5DC4	0.0099	3.3840E-01		ENSG00000144130
NT5E	0.0622	3.9629E-01	6.0227E-01	ENSG00000135318
NT5M	-0.0703	2.9827E-01	5.0815E-01	ENSG00000205309
NTAN1	-0.2645	8.2042E-05	1.3177E-03	ENSG00000157045
NTAN1P1	0.0048	7.4518E-01		ENSG00000270965
NTAN1P2	0.0252	6.1519E-01		ENSG00000250569
NTAQ1	-0.1222	4.9016E-02	1.5738E-01	ENSG00000156795
NTF3	0.0899	2.8823E-01	4.9873E-01	ENSG00000185652
NTF4	-0.2666	3.3734E-02	1.2068E-01	ENSG00000225950

NTHL1	-0.1989	1.3162E-03	1.1159E-02	ENSG00000065057
NTM	0.1294	1.2654E-01	2.9770E-01	ENSG00000182667
NTMT1	0.0413	5.1675E-01	7.0434E-01	ENSG00000148335
NTMT2	0.3723	1.4013E-02	6.4849E-02	ENSG00000203740
NTN1	-0.0895	3.2124E-01	5.3125E-01	ENSG00000065320
NTN3	0.0256	7.4728E-01	8.6259E-01	ENSG00000162068
NTN4	-0.0584	4.8973E-01	6.8252E-01	ENSG00000074527
NTN5	0.0345	6.3456E-01	7.9048E-01	ENSG00000142233
NTNG1	0.2388	1.3876E-02	6.4371E-02	ENSG00000162631
NTNG2	-0.2693	9.7285E-03	4.9724E-02	ENSG00000196358
NTPCR	-0.1111	4.4583E-02	1.4752E-01	ENSG00000135778
NTRK1	-0.1960	7.0648E-02	2.0185E-01	ENSG00000198400
NTRK2	0.0002	9.9993E-01	9.9993E-01	ENSG00000148053
NTRK3	-0.0417	6.3016E-01	7.8706E-01	ENSG00000140538
NTRK3-AS1	-0.0003	7.2034E-01		ENSG00000260305
NTSR1	-0.5157	4.2520E-05	7.8193E-04	ENSG00000101188
NTSR2	0.0428	2.8833E-01		ENSG00000169006
NUAK1	-0.2169	4.5746E-03	2.8146E-02	ENSG00000074590
NUAK2	-0.1579	7.2360E-02	2.0547E-01	ENSG00000163545
NUB1	0.1247	4.7735E-02	1.5464E-01	ENSG00000013374
NUBP1	0.1527	3.3616E-02	1.2040E-01	ENSG00000103274
NUBP2	0.1402	2.4912E-02	9.7355E-02	ENSG00000095906
NUBPL	0.1329	7.0240E-02	2.0120E-01	ENSG00000151413
NUCB1	-0.1870	1.6664E-02	7.3021E-02	ENSG00000104805
NUCB2	-0.0669	1.5340E-01	3.3565E-01	ENSG00000070081
NUCKS1	-0.1064	1.8589E-01	3.7999E-01	ENSG00000069275
NUDC	-0.2941	7.5134E-08	4.5272E-06	ENSG00000090273
NUDCD1	0.1129	1.1419E-01	2.7809E-01	ENSG00000120526
NUDCD2	0.1260	6.7082E-02	1.9510E-01	ENSG00000170584
NUDCD3	-0.0528	3.4127E-01	5.5109E-01	ENSG00000015676
NUDT1	-0.1759	1.4505E-02	6.6397E-02	ENSG00000106268
NUDT10	-0.0445	4.3327E-01	6.3436E-01	ENSG00000122824
NUDT11	-0.2096	5.0941E-04	5.3840E-03	ENSG00000196368
NUDT12	0.5398	1.6986E-10	2.5177E-08	ENSG00000112874
NUDT13	0.0191	8.1730E-01	9.0494E-01	ENSG00000166321
NUDT14	-0.1588	1.7167E-02	7.4548E-02	ENSG00000183828
NUDT15	0.1117	1.6973E-01	3.5826E-01	ENSG00000136159
NUDT16	0.1426	2.9229E-02	1.0912E-01	ENSG00000198585
NUDT16-DT	0.2473	4.1604E-02	1.4059E-01	ENSG00000250608
NUDT16L1	-0.0962	8.3562E-02	2.2574E-01	ENSG00000168101
NUDT16L2P	-0.0353	3.0671E-01		ENSG00000246082
NUDT17	-0.1900	2.4055E-02	9.5031E-02	ENSG00000186364
NUDT18	0.4961	1.3169E-04	1.8926E-03	ENSG00000275074
NUDT19	-0.0722	3.8786E-01	5.9542E-01	ENSG00000213965
NUDT2	0.0033	9.9340E-01	9.9629E-01	ENSG00000164978
NUDT21	-0.1648	3.7752E-03	2.4438E-02	ENSG00000167005
NUDT22	0.0684	3.5184E-01	5.6211E-01	ENSG00000149761
NUDT4	-0.4311	3.9205E-07	1.7475E-05	ENSG00000173598
NUDT4B	-0.3685	3.2994E-06	1.0047E-04	ENSG00000177144

NUDT5	-0.2764	4.6127E-05	8.3207E-04	ENSG00000165609
NUDT6	0.1161	1.9314E-01	3.8924E-01	ENSG00000170917
NUDT7	0.2155	2.5925E-02	1.0011E-01	ENSG00000140876
NUDT8	0.0507	5.1712E-01	7.0459E-01	ENSG00000167799
NUDT9	-0.2295	1.5996E-04	2.1780E-03	ENSG00000170502
NUF2	0.0083	9.1458E-01	9.5792E-01	ENSG00000143228
NUFIP1	-0.1122	5.2596E-02	1.6504E-01	ENSG00000083635
NUFIP2	-0.0766	1.9816E-01	3.9607E-01	ENSG00000108256
NUGGC	-0.0041	8.5835E-01		ENSG00000189233
NUMA1	-0.1081	9.7773E-02	2.5117E-01	ENSG00000137497
NUMB	0.0086	9.0775E-01	9.5461E-01	ENSG00000133961
NUMBL	-0.1919	1.5167E-03	1.2337E-02	ENSG00000105245
NUP107	0.2008	9.9418E-03	5.0488E-02	ENSG00000111581
NUP133	0.1525	5.7095E-03	3.3289E-02	ENSG00000069248
NUP153	-0.1017	2.1242E-01	4.1343E-01	ENSG00000124789
NUP153-AS1	-0.0232	6.2946E-01	7.8664E-01	ENSG00000272269
NUP155	0.0469	4.8071E-01	6.7548E-01	ENSG00000113569
NUP160	0.4537	6.7060E-11	1.1702E-08	ENSG00000030066
NUP188	0.0230	7.2523E-01	8.4928E-01	ENSG00000095319
NUP205	-0.0514	4.5534E-01	6.5369E-01	ENSG00000155561
NUP210	0.0424	5.5884E-01	7.3617E-01	ENSG00000132182
NUP210L	0.0112	8.6840E-01	9.3379E-01	ENSG00000143552
NUP214	-0.0459	4.5503E-01	6.5341E-01	ENSG00000126883
NUP35	0.2611	4.8549E-04	5.1933E-03	ENSG00000163002
NUP37	0.1852	9.8475E-03	5.0155E-02	ENSG00000075188
NUP42	-0.0459	4.4748E-01	6.4695E-01	ENSG00000136243
NUP43	-0.0948	2.2602E-01	4.2988E-01	ENSG00000120253
NUP50	0.0134	8.5318E-01	9.2531E-01	ENSG00000093000
NUP50-DT	-0.1287	9.7717E-02	2.5114E-01	ENSG00000226328
NUP54	0.0492	4.1060E-01	6.1483E-01	ENSG00000138750
NUP58	0.1207	5.3215E-02	1.6643E-01	ENSG00000139496
NUP62	-0.1642	6.6874E-03	3.7501E-02	ENSG00000213024
NUP62CL	0.0655	4.4326E-01	6.4250E-01	ENSG00000198088
NUP85	0.3532	2.8676E-08	1.9819E-06	ENSG00000125450
NUP88	-0.1148	8.1218E-02	2.2165E-01	ENSG00000108559
NUP93	-0.0421	5.7331E-01	7.4628E-01	ENSG00000102900
NUP93-DT	-0.1390	1.3035E-01	3.0316E-01	ENSG00000261302
NUP98	-0.0503	4.1584E-01	6.1932E-01	ENSG00000110713
NUPR1	0.0210	7.5106E-01	8.6472E-01	ENSG00000176046
NUPR2	-0.0239	1.6840E-01		ENSG00000185290
NUS1	-0.0166	8.1809E-01	9.0544E-01	ENSG00000153989
NUSAP1	-0.1604	9.4100E-02	2.4522E-01	ENSG00000137804
NUTF2	-0.3885	2.3543E-11	5.2188E-09	ENSG00000102898
NUTF2P5	-0.0025	9.3021E-01		ENSG00000215227
NUTF2P6	0.0105	7.7496E-01		ENSG00000259710
NUTM1	0.0215	4.6474E-01		ENSG00000184507
NUTM2A	-0.0184	7.8486E-01	8.8631E-01	ENSG00000184923
NUTM2A-AS1	-0.0538	2.8087E-01	4.9106E-01	ENSG00000223482
NUTM2B	0.0179	7.9301E-01	8.9075E-01	ENSG00000188199

NUTM2B-AS1	0.0624	3.0908E-01	5.1924E-01	ENSG00000225484
NUTM2D	0.0281	7.3957E-01	8.5776E-01	ENSG00000214562
NUTM2E	-0.0095	8.9650E-01	9.4857E-01	ENSG00000228570
NUTM2F	0.2195	3.1191E-02	1.1439E-01	ENSG00000130950
NUTM2G	0.1576	1.1535E-01	2.7998E-01	ENSG00000188152
NVL	0.0431	4.6600E-01	6.6257E-01	ENSG00000143748
NWD1	0.0931	2.1502E-01	4.1628E-01	ENSG00000188039
NWD2	0.0014	9.6627E-01	9.8327E-01	ENSG00000174145
NXF1	-0.2063	6.7405E-03	3.7745E-02	ENSG00000162231
NXF3	-0.0015	9.8778E-01		ENSG00000147206
NXN	-0.2668	4.9491E-05	8.8178E-04	ENSG00000167693
NXNL2	0.1157	1.9995E-01	3.9834E-01	ENSG00000130045
NXPE2	-0.0112	2.6806E-01		ENSG00000204361
NXPE3	-0.0589	3.5275E-01	5.6298E-01	ENSG00000144815
NXPH1	0.0024	9.7678E-01	9.8837E-01	ENSG00000122584
NXPH2	0.1006	7.8586E-02	2.1704E-01	ENSG00000144227
NXPH3	-0.1079	2.3681E-01	4.4251E-01	ENSG00000182575
NXPH4	-0.0185	8.1252E-01	9.0217E-01	ENSG00000182379
NXT1	-0.1489	7.9538E-02	2.1881E-01	ENSG00000132661
NXT2	-0.0139	8.4249E-01	9.1898E-01	ENSG00000101888
NYAP1	-0.6000	1.8487E-03	1.4211E-02	ENSG00000166924
NYAP2	-0.1297	1.4594E-01	3.2514E-01	ENSG00000144460
NYNRIN	0.0696	3.8495E-01	5.9287E-01	ENSG00000205978
NYX	0.0299	1.1103E-01		ENSG00000188937
OAF	-0.1128	2.2548E-01	4.2926E-01	ENSG00000184232
OARD1	-0.0668	2.5400E-01	4.6198E-01	ENSG00000124596
OAS1	0.0025	7.9124E-01		ENSG00000089127
OAS2	-0.0241	3.2149E-01		ENSG00000111335
OAS3	0.0600	2.7816E-01	4.8808E-01	ENSG00000111331
OASL	-0.0173	5.6662E-01		ENSG00000135114
OAT	-0.1879	8.1819E-03	4.3613E-02	ENSG00000065154
OAZ1	-0.4185	8.5182E-13	3.1282E-10	ENSG00000104904
OAZ2	-0.1615	2.3943E-02	9.4683E-02	ENSG00000180304
OAZ3	-0.0453	5.6618E-01	7.4132E-01	ENSG00000143450
OBI1	0.4673	8.0742E-08	4.8220E-06	ENSG00000152193
OBI1-AS1	0.0132	6.1505E-01		ENSG00000234377
OBSCN	0.1262	1.1595E-01	2.8063E-01	ENSG00000154358
OBSCN-AS1	0.2269	4.0419E-02	1.3779E-01	ENSG00000162913
OBSL1	0.2276	1.4719E-02	6.7106E-02	ENSG00000124006
OCA2	-0.1234	1.5852E-01	3.4276E-01	ENSG00000104044
OCEL1	-0.1417	5.9868E-02	1.8004E-01	ENSG00000099330
OCIAD1	-0.1006	6.4043E-02	1.8885E-01	ENSG00000109180
OCIAD1-AS1	-0.0355	6.0937E-01	7.7221E-01	ENSG00000248256
OCIAD2	-0.3386	1.2095E-04	1.7805E-03	ENSG00000145247
OCLN	0.0118	8.8792E-01	9.4427E-01	ENSG00000197822
OCM	0.0984	2.3214E-01	4.3709E-01	ENSG00000122543
OCM2	0.0125	6.7510E-01		ENSG00000135175
OCRL	0.0219	7.1679E-01	8.4433E-01	ENSG00000122126
OCSTAMP	0.0582	1.0985E-01		ENSG00000149635

ODAD1	-0.0152	8.3314E-01	9.1405E-01	ENSG00000105479
ODAD2	0.2197	5.5551E-02	1.7182E-01	ENSG00000169126
ODAD3	0.1731	8.5349E-02	2.2863E-01	ENSG00000198003
ODAD4	0.0952	2.3113E-01	4.3609E-01	ENSG00000204815
ODAM	0.0103	6.9141E-01		ENSG00000109205
ODAPH	-0.0532	4.8383E-01	6.7787E-01	ENSG00000174792
ODC1	-0.5571	5.2555E-12	1.5000E-09	ENSG00000115758
ODF1	-0.0580	2.1565E-01		ENSG00000155087
ODF2	0.1908	2.4403E-03	1.7495E-02	ENSG00000136811
ODF2-AS1	0.0234	5.2758E-01		ENSG00000225951
ODF2L	0.3110	2.6741E-08	1.8745E-06	ENSG00000122417
ODF4	-0.0144	5.0858E-01		ENSG00000184650
ODR4	0.0285	6.3683E-01	7.9193E-01	ENSG00000157181
OFD1	0.1400	3.7733E-02	1.3124E-01	ENSG00000046651
OFD1P17	0.0266	6.9659E-01	8.3222E-01	ENSG00000228212
OGA	-0.1161	7.7773E-02	2.1555E-01	ENSG00000198408
OGDH	-0.1575	7.8799E-03	4.2374E-02	ENSG00000105953
OGDHL	0.2089	3.9093E-02	1.3462E-01	ENSG00000197444
OGFOD1	0.0253	7.2580E-01	8.4951E-01	ENSG00000087263
OGFOD2	0.2695	1.1679E-02	5.6868E-02	ENSG00000111325
OGFOD3	0.0554	3.9599E-01	6.0204E-01	ENSG00000181396
OGFR	-0.1968	8.5284E-03	4.5098E-02	ENSG00000060491
OGFRL1	-0.1240	9.2241E-02	2.4191E-01	ENSG00000119900
OGG1	-0.2299	2.4396E-03	1.7495E-02	ENSG00000114026
OGN	-0.0713	3.3225E-01	5.4247E-01	ENSG00000106809
OGT	0.1711	4.0200E-02	1.3726E-01	ENSG00000147162
OIP5	-0.2272	3.0014E-02	1.1127E-01	ENSG00000104147
OIP5-AS1	-0.1028	7.2474E-02	2.0570E-01	ENSG00000247556
OLA1	-0.2360	4.2321E-05	7.7941E-04	ENSG00000138430
OLA1P2	0.0006	6.4628E-01		ENSG00000213671
OLAH	-0.0234	3.7898E-01		ENSG00000152463
OLFM1	-0.2328	1.0341E-02	5.1926E-02	ENSG00000130558
OLFM2	-0.3200	6.3323E-05	1.0790E-03	ENSG00000105088
OLFM3	0.5444	4.1257E-03	2.6136E-02	ENSG00000118733
OLFM4	0.0586	3.0229E-01	5.1236E-01	ENSG00000102837
OLFML1	0.0113	8.9142E-01	9.4602E-01	ENSG00000183801
OLFML2A	0.0513	5.4740E-01	7.2713E-01	ENSG00000185585
OLFML2B	0.0699	2.7537E-01	4.8521E-01	ENSG00000162745
OLFML3	0.0549	4.9967E-01	6.9077E-01	ENSG00000116774
OLIG1	-0.0209	7.5219E-01	8.6560E-01	ENSG00000184221
OLIG2	-0.1221	5.7650E-02	1.7608E-01	ENSG00000205927
OLIG3	-0.0555	2.0930E-01	4.0931E-01	ENSG00000177468
OLMALINC	-0.2490	8.9198E-03	4.6716E-02	ENSG00000235823
OMA1	-0.3953	2.3455E-03	1.6984E-02	ENSG00000162600
OMD	0.0169	9.4399E-01	9.7218E-01	ENSG00000127083
OMG	-0.0206	8.0514E-01	8.9786E-01	ENSG00000126861
ONECUT3	-0.0787	3.3808E-01	5.4799E-01	ENSG00000205922
OOSP1	0.0305	2.0938E-01		ENSG00000284873
OOSP3	0.0190	7.4841E-01		ENSG00000285231

OPA1	-0.0103	8.6006E-01	9.2954E-01	ENSG00000198836
OPA1-AS1	-0.0310	4.9259E-01		ENSG00000224855
OPA3	-0.0019	9.7534E-01	9.8783E-01	ENSG00000125741
OPCML	0.3027	1.7883E-03	1.3851E-02	ENSG00000183715
OPCML-IT1	0.0388	3.8119E-01		ENSG00000254896
OPHN1	-0.0605	3.0817E-01	5.1843E-01	ENSG00000079482
OPLAH	-0.5064	7.6303E-04	7.4031E-03	ENSG00000178814
OPN1SW	-0.0432	4.6917E-01	6.6566E-01	ENSG00000128617
OPN5	0.0478	2.3572E-01		ENSG00000124818
OPRD1	-0.1762	7.4196E-02	2.0900E-01	ENSG00000116329
OPRK1	-0.0217	7.8882E-01	8.8812E-01	ENSG00000082556
OPRL1	0.2849	3.0884E-02	1.1364E-01	ENSG00000125510
OPTC	-0.0145	9.1722E-01		ENSG00000188770
OPTN	-0.1398	3.8390E-02	1.3287E-01	ENSG00000123240
OR10A4	0.0219	1.3321E-01		ENSG00000170782
OR10A5	0.0180	3.9869E-01		ENSG00000166363
OR10AD1	0.0117	3.5241E-01		ENSG00000172640
OR10AH1P	-0.0079	7.9171E-01		ENSG00000272595
OR10D1P	0.0262	1.9638E-01		ENSG00000196403
OR10H1	-0.0110	6.2455E-01		ENSG00000186723
OR10H5	-0.0177	2.3307E-01		ENSG00000172519
OR10Q1	-0.0264	3.4629E-01		ENSG00000180475
OR11G2	-0.0045	9.5658E-01		ENSG00000196832
OR11H7	0.0070	7.1788E-01		ENSG00000258806
OR11M1P	0.1122	4.4936E-02	1.4833E-01	ENSG00000224709
OR11N1P	0.0021	9.4247E-01		ENSG00000185903
OR13A1	0.0686	1.3361E-01		ENSG00000256574
OR13J1	-0.0013	9.2115E-01		ENSG00000168828
OR1F1	-0.2675	3.3566E-02	1.2034E-01	ENSG00000168124
OR1F2P	-0.0266	3.5669E-01		ENSG00000203581
OR1I1	0.0045	6.2965E-01		ENSG00000094661
OR1L8	0.0334	3.8705E-01		ENSG00000171496
OR1N2	0.0055	8.5305E-01		ENSG00000171501
OR1R1P	0.0050	9.3147E-01		ENSG00000180042
OR2A20P	0.0135	4.7246E-01		ENSG00000170356
OR2A4	0.0011	7.8144E-01		ENSG00000180658
OR2A7	0.0859	1.6639E-02		ENSG00000243896
OR2AG1	2.8246	2.0455E-05		ENSG00000279486
OR2AT4	-0.0055	9.0561E-01		ENSG00000171561
OR2B11	0.0020	8.7208E-01		ENSG00000177535
OR2C1	0.0231	3.2081E-01		ENSG00000168158
OR2D2	0.0602	1.1881E-01		ENSG00000166368
OR2D3	0.0298	2.9587E-01		ENSG00000178358
OR2F1	0.0303	4.0419E-01		ENSG00000213215
OR2H2	0.0116	4.6693E-01	6.6358E-01	ENSG00000204657
OR2I1P	-0.0004	8.7214E-01		ENSG00000237988
OR2K2	0.0051	7.4805E-01		ENSG00000171133
OR2L13	-0.0031	7.7450E-01	8.8003E-01	ENSG00000196071
OR2L1P	-0.0038	7.7720E-01		ENSG00000224227

OR2L3	0.0158	4.6012E-01		ENSG00000198128
OR2M4	0.0016	8.7463E-01		ENSG00000171180
OR2S1P	0.0060	5.9433E-01		ENSG00000228307
OR2T8	-0.0118	3.5281E-01		ENSG00000177462
OR2V2	0.0406	1.6534E-01		ENSG00000182613
OR2W3	-0.0276	5.6173E-01		ENSG00000238243
OR2W6P	-0.0161	5.2418E-01		ENSG00000168126
OR3A1	-0.0031	9.8706E-01		ENSG00000180090
OR3A2	-0.0332	4.7853E-01	6.7381E-01	ENSG00000221882
OR3A3	0.0062	7.1535E-01		ENSG00000159961
OR4D1	0.0151	6.4080E-01	7.9444E-01	ENSG00000141194
OR4G1P	0.0154	4.9612E-01		ENSG00000267310
OR4G3P	-0.0005	9.0463E-01		ENSG00000282137
OR4G6P	0.0138	5.5981E-01		ENSG00000275771
OR51B5	0.0171	5.9938E-01		ENSG00000167355
OR51E2	-0.0021	7.4423E-01		ENSG00000167332
OR51M1	0.0266	4.6109E-01		ENSG00000184698
OR52B5P	-0.0007	7.6236E-01		ENSG00000231049
OR52H2P	0.0092	4.4660E-01		ENSG00000248553
OR52I2	-0.0083	8.5083E-01		ENSG00000226288
OR52K3P	0.4292	1.3807E-02	6.4120E-02	ENSG00000225101
OR52N2	0.0186	5.2537E-01		ENSG00000180988
OR52N4	-0.0031	9.2736E-01		ENSG00000181074
OR52T1P	0.0122	6.4773E-01		ENSG00000233646
OR56A1	-0.0088	4.9872E-01		ENSG00000180934
OR5A1	-0.0090	9.8890E-01		ENSG00000172320
OR5AU1	0.0179	3.0591E-01		ENSG00000169327
OR5BA1P	0.0199	3.3825E-01		ENSG00000255303
OR5BT1P	0.0106	5.6384E-01		ENSG00000258024
OR5K2	0.0163	2.7238E-01		ENSG00000231861
OR5M2P	0.0469	2.2433E-02		ENSG00000254752
OR5M8	0.0928	1.5261E-02	6.8799E-02	ENSG00000181371
OR6A2	0.0057	7.6126E-01		ENSG00000184933
OR6V1	-0.0235	3.0880E-01		ENSG00000225781
OR7A17	-0.0325	1.8170E-01		ENSG00000185385
OR7C1	-0.0194	7.4473E-01		ENSG00000127530
OR7D2	0.0378	4.5004E-01		ENSG00000188000
OR7E110P	0.0022	9.3596E-01		ENSG00000235623
OR7E121P	-0.0450	2.3952E-01		ENSG00000244222
OR7E122P	-0.0316	3.5341E-01	5.6352E-01	ENSG00000215160
OR7E125P	0.0132	9.7374E-01		ENSG00000177306
OR7E128P	0.0141	4.8044E-01		ENSG00000228915
OR7E12P	0.0881	2.5176E-01	4.6008E-01	ENSG00000189398
OR7E14P	0.1013	2.6563E-01	4.7488E-01	ENSG00000184669
OR7E154P	0.0100	8.9086E-01	9.4581E-01	ENSG00000254715
OR7E162P	-0.0010	9.4412E-01		ENSG00000234386
OR7E22P	0.0919	8.5080E-02		ENSG00000179799
OR7E28P	-0.0171	7.7118E-01	8.7786E-01	ENSG00000251491
OR7E29P	-0.1133	3.2057E-02	1.1639E-01	ENSG00000243429

OR7E36P	-0.0643	1.2849E-01	3.0021E-01	ENSG00000205240
OR7E7P	-0.1157	1.9129E-01	3.8665E-01	ENSG00000238228
OR7E90P	0.0193	6.5609E-01		ENSG00000188668
OR7M1P	0.0005	2.4861E-01		ENSG00000273336
OR8A1	0.0044	6.9995E-01		ENSG00000196119
OR8D1	0.0710	8.6544E-03		ENSG00000196341
OR8G1	0.0215	3.6041E-01		ENSG00000197849
OR8G3P	0.0306	1.4539E-01		ENSG00000255425
OR8G5	0.0553	1.0586E-01		ENSG00000255298
OR8T1P	-0.0154	3.3529E-01		ENSG00000226413
ORAI1	-0.1106	1.2043E-01	2.8807E-01	ENSG00000276045
ORAI2	0.0914	9.2285E-02	2.4200E-01	ENSG00000160991
ORAI3	0.0961	2.5820E-01	4.6700E-01	ENSG00000175938
ORC1	-0.1252	1.6673E-01	3.5469E-01	ENSG00000085840
ORC2	0.4707	2.2097E-06	7.2397E-05	ENSG00000115942
ORC3	0.0390	5.7415E-01	7.4700E-01	ENSG00000135336
ORC4	0.0142	8.2894E-01	9.1144E-01	ENSG00000115947
ORC5	0.1618	1.7012E-02	7.4060E-02	ENSG00000164815
ORC6	0.1151	1.2084E-01	2.8882E-01	ENSG00000091651
ORM1	-0.0074	7.1182E-01		ENSG00000229314
ORM2	-0.0695	2.2713E-01	4.3138E-01	ENSG00000228278
ORMDL1	0.0555	1.8211E-01	3.7499E-01	ENSG00000128699
ORMDL2	-0.0241	7.2520E-01	8.4928E-01	ENSG00000123353
ORMDL3	-0.1162	1.1416E-01	2.7806E-01	ENSG00000172057
OS9	-0.2142	4.4193E-03	2.7410E-02	ENSG00000135506
OSBP	-0.0218	7.1470E-01	8.4332E-01	ENSG00000110048
OSBP2	0.1074	1.4828E-01	3.2877E-01	ENSG00000184792
OSBPL10	-0.0350	6.8415E-01	8.2396E-01	ENSG00000144645
OSBPL10-AS1	0.0123	6.5194E-01		ENSG00000232490
OSBPL11	-0.0415	5.5012E-01	7.2920E-01	ENSG00000144909
OSBPL1A	0.0210	7.3345E-01	8.5431E-01	ENSG00000141447
OSBPL2	-0.0141	8.0648E-01	8.9825E-01	ENSG00000130703
OSBPL3	0.1680	8.7652E-02	2.3328E-01	ENSG00000070882
OSBPL5	-0.0544	4.1801E-01	6.2128E-01	ENSG00000021762
OSBPL6	-0.0286	7.0177E-01	8.3529E-01	ENSG00000079156
OSBPL7	-0.1496	7.2318E-02	2.0540E-01	ENSG00000006025
OSBPL8	-0.0132	8.1349E-01	9.0281E-01	ENSG00000091039
OSBPL9	0.0734	2.1094E-01	4.1166E-01	ENSG00000117859
OSCAR	-0.0400	6.2918E-01	7.8650E-01	ENSG00000170909
OSCP1	0.3289	9.1788E-06	2.2825E-04	ENSG00000116885
OSER1	-0.3129	1.1697E-05	2.7728E-04	ENSG00000132823
OSER1-DT	-0.0392	5.0522E-01	6.9513E-01	ENSG00000223891
OSGEP	-0.0093	8.4522E-01	9.2085E-01	ENSG00000092094
OSGEPL1	0.1252	7.0602E-02	2.0174E-01	ENSG00000128694
OSGIN1	-0.0557	1.7395E-01	3.6410E-01	ENSG00000140961
OSGIN2	-0.0252	6.8919E-01	8.2719E-01	ENSG00000164823
OSM	-0.0214	4.4738E-01		ENSG00000099985
OSMR	-0.0426	5.5024E-01	7.2922E-01	ENSG00000145623
OSR1	0.0278	2.9635E-01		ENSG00000143867

OSR2	0.0243	5.2800E-01		ENSG00000164920
OST4	-0.1684	2.0407E-02	8.4094E-02	ENSG00000228474
OSTC	0.0722	3.2782E-01	5.3838E-01	ENSG00000198856
OSTF1	-0.2044	1.4950E-02	6.7818E-02	ENSG00000134996
OSTM1	-0.0112	8.7124E-01	9.3517E-01	ENSG00000081087
OSTM1-AS1	-0.0355	6.5582E-01	8.0557E-01	ENSG00000225174
OTC	0.2047	4.2513E-02	1.4258E-01	ENSG00000036473
OTOA	0.0459	4.1000E-01		ENSG00000155719
OTOG	0.0800	2.2429E-01	4.2812E-01	ENSG00000188162
OTOGL	0.0395	4.7184E-01	6.6806E-01	ENSG00000165899
OTOP3	0.0070	8.1224E-01		ENSG00000182938
OTOR	0.0081	8.1609E-01		ENSG00000125879
OTOS	0.1863	6.3538E-02	1.8788E-01	ENSG00000178602
OTP	0.1311	2.9223E-02	1.0912E-01	ENSG00000171540
OTUB1	-0.1731	4.7893E-04	5.1403E-03	ENSG00000167770
OTUB2	0.1242	1.4757E-01	3.2767E-01	ENSG00000089723
OTUD1	-0.2471	8.0515E-03	4.3086E-02	ENSG00000165312
OTUD3	-0.0500	4.4362E-01	6.4284E-01	ENSG00000169914
OTUD4	0.0077	9.2357E-01	9.6227E-01	ENSG00000164164
OTUD5	-0.0300	6.5089E-01	8.0209E-01	ENSG00000068308
OTUD6A	-0.0184	4.2645E-01		ENSG00000189401
OTUD6B	-0.0524	4.6048E-01	6.5803E-01	ENSG00000155100
OTUD6B-AS1	0.0542	3.7907E-01	5.8753E-01	ENSG00000253738
OTUD7A	0.0166	8.3180E-01	9.1311E-01	ENSG00000169918
OTUD7B	0.2501	5.4495E-03	3.2133E-02	ENSG00000264522
OTULIN	0.0479	4.8604E-01	6.7981E-01	ENSG00000154124
OTULIN-DT	0.1296	1.5947E-01	3.4422E-01	ENSG00000261360
OTULINL	-0.0031	9.6738E-01	9.8378E-01	ENSG00000145569
OTX1	-0.0302	7.9849E-01	8.9385E-01	ENSG00000115507
OVCH1	-0.0423	5.1029E-01	6.9892E-01	ENSG00000187950
OVCH1-AS1	0.0138	8.7035E-01	9.3461E-01	ENSG00000257599
OVGP1	0.5686	1.9169E-05	4.1410E-04	ENSG00000085465
OVOL1	0.0778	1.9911E-01	3.9736E-01	ENSG00000172818
OVOL2	-0.1875	3.2528E-02	1.1763E-01	ENSG00000125850
OVOL3	-0.0024	8.5431E-01		ENSG00000105261
OVOS2	-0.1091	1.6029E-01	3.4556E-01	ENSG00000177359
OXA1L	0.1915	1.3505E-02	6.3127E-02	ENSG00000155463
OXCT1	-0.1108	7.0377E-02	2.0134E-01	ENSG00000083720
OXCT1-AS1	0.2580	2.2331E-02	8.9963E-02	ENSG00000248668
OXCT2	0.0305	7.5459E-01	8.6686E-01	ENSG00000198754
OXCT2P1	0.0350	5.3119E-01		ENSG00000237624
OXGR1	-0.0018	9.7137E-01	9.8559E-01	ENSG00000165621
OXLD1	0.1558	8.9895E-03	4.6955E-02	ENSG00000204237
OXNAD1	0.0165	8.2878E-01	9.1134E-01	ENSG00000154814
OXR1	-0.2701	3.8195E-05	7.2571E-04	ENSG00000164830
OXSM	0.3596	4.0240E-04	4.5005E-03	ENSG00000151093
OXSR1	-0.0550	3.2889E-01	5.3925E-01	ENSG00000172939
OXT	-0.0390	4.2830E-01	6.3017E-01	ENSG00000101405
OXTR	0.0388	5.6954E-01	7.4363E-01	ENSG00000180914

P2RX1	0.0063	6.3394E-01		ENSG00000108405
P2RX2	-0.1609	1.1120E-01	2.7302E-01	ENSG00000187848
P2RX3	-0.0694	3.9671E-01	6.0261E-01	ENSG00000109991
P2RX4	-0.0119	8.5652E-01	9.2741E-01	ENSG00000135124
P2RX5	0.0206	7.9930E-01	8.9419E-01	ENSG00000083454
P2RX5-TAX1BP3	-0.3099	5.1182E-03	3.0656E-02	ENSG00000257950
P2RX6	0.0834	2.9106E-01	5.0140E-01	ENSG00000099957
P2RX7	0.0876	3.2856E-01	5.3903E-01	ENSG00000089041
P2RY1	-0.1208	1.7103E-01	3.6012E-01	ENSG00000169860
P2RY11	0.2091	5.0041E-02	1.5984E-01	ENSG00000244165
P2RY12	0.1602	3.5180E-02	1.2453E-01	ENSG00000169313
P2RY13	0.0884	1.0298E-01		ENSG00000181631
P2RY14	0.0239	8.1392E-01	9.0305E-01	ENSG00000174944
P2RY2	-0.0040	8.1012E-01	9.0028E-01	ENSG00000175591
P2RY4	0.0222	2.6721E-01		ENSG00000186912
P2RY6	0.0674	1.5907E-01	3.4363E-01	ENSG00000171631
P2RY8	0.0436	2.0651E-01		ENSG00000182162
P3H1	0.1508	3.3714E-02	1.2066E-01	ENSG00000117385
P3H2	0.2810	2.4766E-02	9.6987E-02	ENSG00000090530
P3H3	0.1187	1.5266E-01	3.3479E-01	ENSG00000110811
P3H4	-0.0208	7.8111E-01	8.8392E-01	ENSG00000141696
P4HA1	0.0903	2.5303E-01	4.6096E-01	ENSG00000122884
P4HA2	-0.0864	3.0556E-01	5.1576E-01	ENSG00000072682
P4HA3	-0.4208	1.2824E-02	6.0713E-02	ENSG00000149380
P4HB	0.1551	1.0991E-01	2.7090E-01	ENSG00000185624
P4HTM	-0.0110	8.6482E-01	9.3175E-01	ENSG00000178467
PA2G4	-0.2081	8.7706E-06	2.1976E-04	ENSG00000170515
PAAF1	0.1064	8.1603E-02	2.2227E-01	ENSG00000175575
PABIR1	-0.0025	9.6109E-01	9.8054E-01	ENSG00000187866
PABIR2	-0.1593	1.1542E-01	2.7998E-01	ENSG00000156504
PABIR3	0.0918	1.9684E-01	3.9437E-01	ENSG00000156500
PABPC1	-0.0844	2.9592E-01	5.0592E-01	ENSG00000070756
PABPC1L	-0.0720	3.9864E-01	6.0424E-01	ENSG00000101104
PABPC1L2A	0.1620	1.0839E-01	2.6847E-01	ENSG00000186288
PABPC1L2B	0.0307	7.2354E-01	8.4829E-01	ENSG00000184388
PABPC1L2B-AS1	0.0177	8.0766E-01	8.9894E-01	ENSG00000226725
PABPC4	-0.0128	8.6284E-01	9.3083E-01	ENSG00000090621
PABPC4L	-0.0038	9.4842E-01	9.7460E-01	ENSG00000254535
PABPC5	0.1631	1.0746E-01	2.6680E-01	ENSG00000174740
PABPC5-AS1	0.0635	4.2659E-01	6.2878E-01	ENSG00000234161
PABPN1	-0.0123	8.5360E-01	9.2556E-01	ENSG00000100836
PABPN1L	-0.0018	7.3400E-01	8.5476E-01	ENSG00000205022
PACC1	-0.1513	2.7407E-02	1.0426E-01	ENSG00000065600
PACRG	0.4461	4.1693E-06	1.2083E-04	ENSG00000112530
PACRG-AS1	-0.0070	8.0613E-01	8.9800E-01	ENSG00000281692
PACRG-AS3	0.0833	2.1579E-01	4.1719E-01	ENSG00000225683
PACRGL	0.2448	4.3638E-03	2.7182E-02	ENSG00000163138
PACS1	-0.0214	7.3569E-01	8.5583E-01	ENSG00000175115
PACS2	-0.0436	4.4114E-01	6.4089E-01	ENSG00000179364

PACSIN1	0.0949	2.1684E-01	4.1855E-01	ENSG00000124507
PACSIN2	-0.0535	4.5245E-01	6.5125E-01	ENSG00000100266
PACSIN3	-0.3670	1.1221E-04	1.6794E-03	ENSG00000165912
PADI1	0.0754	1.3147E-01		ENSG00000142623
PADI2	0.0175	8.3586E-01	9.1537E-01	ENSG00000117115
PADI3	0.0052	6.6925E-01		ENSG00000142619
PADI4	0.0010	9.2022E-01		ENSG00000159339
PAEPP1	-0.0014	7.3656E-01		ENSG00000224045
PAF1	-0.1407	3.8211E-02	1.3240E-01	ENSG00000006712
PAFAH1B1	-0.0595	3.2551E-01	5.3559E-01	ENSG00000007168
PAFAH1B2	-0.1263	7.1272E-02	2.0333E-01	ENSG00000168092
PAFAH1B2P2	0.0528	3.8101E-01	5.8923E-01	ENSG00000257501
PAFAH1B3	-0.1359	2.5990E-02	1.0030E-01	ENSG00000079462
PAFAH2	-0.1109	1.8403E-01	3.7741E-01	ENSG00000158006
PAG1	0.1587	9.7218E-02	2.5045E-01	ENSG00000076641
PAGE2	0.0241	1.2841E-01		ENSG00000234068
PAGE2B	0.0274	5.6770E-01		ENSG00000238269
PAGE4	0.0217	6.5460E-01		ENSG00000101951
PAGE5	0.0064	7.1533E-01		ENSG00000158639
PAGR1	-0.0995	2.0729E-01	4.0695E-01	ENSG00000280789
PAH	0.0864	1.3934E-01	3.1555E-01	ENSG00000171759
PAICS	-0.0899	1.9527E-01	3.9217E-01	ENSG00000128050
PAIP1	-0.2248	1.5451E-05	3.4499E-04	ENSG00000172239
PAIP1P1	-0.0266	4.4359E-01		ENSG00000233892
PAIP2	-0.1888	2.1689E-04	2.7752E-03	ENSG00000120727
PAIP2B	-0.1610	2.8089E-02	1.0608E-01	ENSG00000124374
PAK1	-0.0627	3.4146E-01	5.5116E-01	ENSG00000149269
PAK1IP1	0.0611	3.7163E-01	5.8038E-01	ENSG00000111845
PAK2	-0.1599	1.2578E-02	5.9874E-02	ENSG00000180370
PAK3	-0.0812	2.3138E-01	4.3622E-01	ENSG00000077264
PAK4	-0.0854	2.9853E-01	5.0832E-01	ENSG00000130669
PAK5	-0.1602	3.3793E-02	1.2075E-01	ENSG00000101349
PAK6	0.0304	6.8300E-01	8.2313E-01	ENSG00000137843
PAK6-AS1	-0.0192	2.7350E-01		ENSG00000176753
PALB2	0.0180	8.0183E-01	8.9537E-01	ENSG00000083093
PALD1	-0.2610	1.4364E-03	1.1884E-02	ENSG00000107719
PALLD	0.1020	2.1633E-01	4.1793E-01	ENSG00000129116
PALM	-0.0934	2.5124E-01	4.5951E-01	ENSG00000099864
PALM2AKAP2	-0.2467	1.2119E-02	5.8361E-02	ENSG00000157654
PALM3	-0.2919	9.8935E-03	5.0306E-02	ENSG00000187867
PALMD	0.3010	1.2236E-02	5.8736E-02	ENSG00000099260
PALS1	-0.0663	3.1159E-01	5.2157E-01	ENSG00000072415
PALS2	0.3856	2.3428E-09	2.3625E-07	ENSG00000105926
PAM	0.0327	6.8306E-01	8.2313E-01	ENSG00000145730
PAM16	-0.0021	9.7855E-01	9.8925E-01	ENSG00000217930
PAMR1	0.1397	1.1624E-01	2.8112E-01	ENSG00000149090
PAN2	0.0430	5.4773E-01	7.2734E-01	ENSG00000135473
PAN3	0.0828	3.2889E-01	5.3925E-01	ENSG00000152520
PANK1	-0.0913	2.3250E-01	4.3750E-01	ENSG00000152782

PANK1-AS1	0.0071	9.8435E-01	9.9172E-01	ENSG00000232936
PANK2	-0.4240	1.0137E-09	1.1235E-07	ENSG00000125779
PANK3	0.1116	1.2824E-01	2.9991E-01	ENSG00000120137
PANK4	0.2442	4.5586E-03	2.8062E-02	ENSG00000157881
PANO1	0.1661	5.7255E-02	1.7531E-01	ENSG00000288675
PANTR1	-0.3216	1.4453E-02	6.6225E-02	ENSG00000233639
PANX1	-0.0876	2.3500E-01	4.4039E-01	ENSG00000110218
PANX2	0.2917	6.8527E-03	3.8191E-02	ENSG00000073150
PANX3	0.0231	4.6643E-01		ENSG00000154143
PAOX	0.0677	3.6840E-01	5.7759E-01	ENSG00000148832
PAPLN	-0.4427	3.2251E-03	2.1652E-02	ENSG00000100767
PAPOLA	-0.2961	8.9316E-07	3.3706E-05	ENSG00000090060
PAPOLB	0.0193	6.9857E-01	8.3342E-01	ENSG00000218823
PAPOLG	-0.0830	3.0593E-01	5.1604E-01	ENSG00000115421
PAPPA	0.0860	3.1265E-01	5.2285E-01	ENSG00000182752
PAPPA2	0.1788	8.8072E-02	2.3415E-01	ENSG00000116183
PAPSS1	-0.0797	1.7269E-01	3.6248E-01	ENSG00000138801
PAPSS2	0.2533	3.3085E-02	1.1922E-01	ENSG00000198682
PAQR3	0.0590	3.5083E-01	5.6096E-01	ENSG00000163291
PAQR4	-0.1645	4.8527E-02	1.5634E-01	ENSG00000162073
PAQR5	-0.5704	9.4005E-04	8.6246E-03	ENSG00000137819
PAQR5-DT	0.0147	6.0819E-01		ENSG00000261634
PAQR6	0.0161	8.5045E-01	9.2398E-01	ENSG00000160781
PAQR7	-0.0302	7.0905E-01	8.4006E-01	ENSG00000182749
PAQR8	-0.2160	4.3597E-02	1.4518E-01	ENSG00000170915
PAQR9	0.1859	7.5377E-02	2.1114E-01	ENSG00000188582
PAQR9-AS1	0.0439	5.8145E-01	7.5263E-01	ENSG00000241570
PARAIL	0.0586	3.8686E-01	5.9462E-01	ENSG00000251136
PARD3	-0.0661	3.5725E-01	5.6707E-01	ENSG00000148498
PARD3-DT	0.1201	9.7611E-02	2.5103E-01	ENSG00000226386
PARD3B	-0.1069	1.6364E-01	3.5025E-01	ENSG00000116117
PARD6A	0.0497	5.2494E-01	7.1041E-01	ENSG00000102981
PARD6B	0.0505	4.5682E-01	6.5502E-01	ENSG00000124171
PARD6G	0.0662	4.0537E-01	6.0976E-01	ENSG00000178184
PARD6G-AS1	0.2277	3.8278E-02	1.3254E-01	ENSG00000267270
PARG	0.1208	6.5938E-02	1.9284E-01	ENSG00000227345
PARK7	-0.2322	1.0939E-05	2.6378E-04	ENSG00000116288
PARK7P1	0.0441	3.3728E-01		ENSG00000257243
PARL	0.0938	1.8084E-01	3.7311E-01	ENSG00000175193
PARLP1	-0.0089	7.1557E-01		ENSG00000239835
PARLP2	-0.0213	7.7000E-01	8.7692E-01	ENSG00000217648
PARM1	0.0430	5.9997E-01	7.6571E-01	ENSG00000169116
PARM1-AS1	0.0189	3.7538E-01		ENSG00000249717
PARN	-0.1499	1.8738E-02	7.9218E-02	ENSG00000140694
PARP1	-0.1230	1.7597E-02	7.5892E-02	ENSG00000143799
PARP10	0.2268	4.3754E-02	1.4546E-01	ENSG00000178685
PARP11	0.1039	1.7743E-01	3.6864E-01	ENSG00000111224
PARP11-AS1	0.0532	4.0291E-01	6.0738E-01	ENSG00000256862
PARP12	0.0864	3.3226E-01	5.4247E-01	ENSG00000059378

PARP14	0.0589	4.7242E-01	6.6862E-01	ENSG00000173193
PARP15	0.0088	7.1278E-01		ENSG00000173200
PARP16	0.2984	3.9970E-05	7.5246E-04	ENSG00000138617
PARP2	-0.0472	4.1532E-01	6.1874E-01	ENSG00000129484
PARP3	0.4063	1.2868E-03	1.0967E-02	ENSG00000041880
PARP4	-0.1483	6.0815E-02	1.8195E-01	ENSG00000102699
PARP4P2	0.0072	9.4318E-01		ENSG00000224976
PARP6	-0.0550	4.8861E-01	6.8181E-01	ENSG00000137817
PARP8	0.0622	4.3765E-01	6.3812E-01	ENSG00000151883
PARP9	-0.0152	8.5429E-01	9.2588E-01	ENSG00000138496
PARPBP	-0.0200	7.8785E-01	8.8795E-01	ENSG00000185480
PARS2	0.3650	4.0907E-03	2.5991E-02	ENSG00000162396
PART1	0.1021	1.0737E-01	2.6665E-01	ENSG00000152931
PARTICL	-0.0167	7.9267E-01	8.9050E-01	ENSG00000286532
PARVA	-0.0831	2.2051E-01	4.2341E-01	ENSG00000197702
PARVB	0.3493	1.3784E-02	6.4048E-02	ENSG00000188677
PARVG	0.0213	3.0646E-01		ENSG00000138964
PASD1	0.0048	7.4138E-01		ENSG00000166049
PASK	0.0146	8.5427E-01	9.2588E-01	ENSG00000115687
PATE2	0.0579	1.7829E-01		ENSG00000196844
PATJ	0.1297	6.6852E-02	1.9469E-01	ENSG00000132849
PATL1	0.0318	6.2655E-01	7.8473E-01	ENSG00000166889
PATL2	0.0684	2.9954E-01	5.0913E-01	ENSG00000229474
PATZ1	-0.1549	1.2160E-02	5.8496E-02	ENSG00000100105
PAWR	-0.3209	3.2853E-04	3.8476E-03	ENSG00000177425
PAWRP2	0.0104	8.2609E-01	9.0992E-01	ENSG00000270424
PAX1	0.0673	3.8282E-01	5.9099E-01	ENSG00000125813
PAX2	-0.0395	3.9567E-01	6.0188E-01	ENSG00000075891
PAX3	0.4141	1.4064E-02	6.4999E-02	ENSG00000135903
PAX5	-0.0021	9.5858E-01	9.7936E-01	ENSG00000196092
PAX6	-0.3482	9.1130E-05	1.4346E-03	ENSG00000007372
PAX6-AS1	-0.1725	9.6025E-02	2.4868E-01	ENSG00000281880
PAX7	-0.0165	6.0580E-01		ENSG00000009709
PAX8	-0.0159	8.1168E-01	9.0156E-01	ENSG00000125618
PAX8-AS1	-0.0306	7.1639E-01	8.4413E-01	ENSG00000189223
PAX9	0.0283	2.2342E-01		ENSG00000198807
PAXBP1	-0.0078	8.9461E-01	9.4750E-01	ENSG00000159086
PAXBP1-AS1	-0.0826	3.0071E-01	5.1052E-01	ENSG00000238197
PAXIP1	-0.0462	5.7788E-01	7.4955E-01	ENSG00000157212
PAXIP1-DT	0.1554	3.7447E-02	1.3041E-01	ENSG00000273344
PAXX	-0.1102	9.2724E-02	2.4281E-01	ENSG00000148362
PBDC1	-0.0638	2.5787E-01	4.6653E-01	ENSG00000102390
PBK	-0.0416	6.0164E-01	7.6665E-01	ENSG00000168078
PBLD	0.1235	9.4205E-02	2.4541E-01	ENSG00000108187
PBOV1	0.0060	9.1084E-01		ENSG00000254440
PBRM1	-0.0614	2.5603E-01	4.6433E-01	ENSG00000163939
PBX1	0.1705	2.5432E-02	9.8682E-02	ENSG00000185630
PBX1-AS1	0.1240	5.5952E-02	1.7273E-01	ENSG00000233693
PBX2	-0.3036	1.9211E-02	8.0676E-02	ENSG00000204304

PBX3	0.2456	3.4483E-02	1.2254E-01	ENSG00000167081
PBX4	-0.0083	8.9594E-01	9.4840E-01	ENSG00000105717
PBXIP1	-0.0599	4.8510E-01	6.7890E-01	ENSG00000163346
PC	-0.1112	1.4398E-01	3.2200E-01	ENSG00000173599
PCA3	0.0396	6.2673E-01	7.8481E-01	ENSG00000225937
PCARE	0.0676	2.2232E-01		ENSG00000179270
PCAT18	-0.0134	8.4922E-01	9.2321E-01	ENSG00000265369
PCAT19	0.0814	2.9729E-01	5.0724E-01	ENSG00000267107
PCAT2	0.0343	7.2411E-01	8.4864E-01	ENSG00000254166
PCAT5	0.0064	9.9261E-01		ENSG00000280719
PCAT6	0.3836	1.8036E-05	3.9381E-04	ENSG00000228288
PCBD1	-0.3336	2.5116E-05	5.1498E-04	ENSG00000166228
PCBD2	0.0805	2.5256E-01	4.6058E-01	ENSG00000132570
PCBP1	-0.5484	2.1443E-04	2.7461E-03	ENSG00000169564
PCBP1-AS1	0.1034	1.0527E-01	2.6356E-01	ENSG00000179818
PCBP2	-0.0653	4.1595E-01	6.1934E-01	ENSG00000197111
PCBP2-OT1	-0.0506	1.5914E-01		ENSG00000282977
PCBP3	-0.1612	5.1466E-02	1.6260E-01	ENSG00000183570
PCBP4	-0.0803	2.7999E-01	4.9005E-01	ENSG00000090097
PCCA	0.0186	7.8710E-01	8.8748E-01	ENSG00000175198
PCCA-DT	-0.0193	8.1418E-01	9.0317E-01	ENSG00000274605
PCCB	-0.2058	7.8699E-04	7.5670E-03	ENSG00000114054
PCDH1	0.0433	6.1797E-01	7.7878E-01	ENSG00000156453
PCDH10	0.1566	8.3086E-02	2.2480E-01	ENSG00000138650
PCDH11X	0.1858	7.8931E-02	2.1765E-01	ENSG00000102290
PCDH11Y	0.1302	1.6843E-01	3.5682E-01	ENSG00000099715
PCDH12	0.1011	2.5911E-01	4.6768E-01	ENSG00000113555
PCDH15	0.1940	7.0141E-02	2.0106E-01	ENSG00000150275
PCDH17	0.3047	5.5731E-03	3.2657E-02	ENSG00000118946
PCDH18	0.2226	3.8244E-02	1.3248E-01	ENSG00000189184
PCDH19	-0.1794	1.8735E-02	7.9217E-02	ENSG00000165194
PCDH20	0.0085	7.2825E-01		ENSG00000280165
PCDH7	0.2792	2.4067E-03	1.7320E-02	ENSG00000169851
PCDH8	-0.1375	1.3298E-01	3.0663E-01	ENSG00000136099
PCDH9	0.3069	1.2217E-03	1.0544E-02	ENSG00000184226
PCDH9-AS2	0.0012	7.9060E-01		ENSG00000228842
PCDHA10	0.0055	9.4058E-01	9.7064E-01	ENSG00000250120
PCDHA11	-0.1133	2.2854E-01	4.3311E-01	ENSG00000249158
PCDHA12	-0.0747	3.9797E-01	6.0359E-01	ENSG00000251664
PCDHA13	0.0485	5.2745E-01	7.1240E-01	ENSG00000239389
PCDHA2	-0.5986	1.7478E-06	5.9730E-05	ENSG00000204969
PCDHA3	0.0433	6.0593E-01	7.6990E-01	ENSG00000255408
PCDHA4	-0.0881	2.6262E-01	4.7149E-01	ENSG00000204967
PCDHA5	0.2079	6.1800E-02	1.8420E-01	ENSG00000204965
PCDHA6	-0.3176	8.7564E-03	4.5997E-02	ENSG00000081842
PCDHA7	0.0722	4.0671E-01	6.1130E-01	ENSG00000204963
PCDHA8	-0.0283	6.3878E-01	7.9351E-01	ENSG00000204962
PCDHA9	0.1441	1.3690E-01	3.1232E-01	ENSG00000204961
PCDHAC1	0.0575	4.9819E-01	6.8929E-01	ENSG00000248383

PCDHB1	-0.0146	6.3390E-01		ENSG00000171815
PCDHB10	-0.2390	7.4882E-04	7.3085E-03	ENSG00000120324
PCDHB11	-0.2054	2.9419E-02	1.0950E-01	ENSG00000197479
PCDHB12	0.2564	3.1049E-02	1.1413E-01	ENSG00000120328
PCDHB13	0.0275	7.3671E-01	8.5629E-01	ENSG00000187372
PCDHB14	-0.2217	5.0403E-03	3.0300E-02	ENSG00000120327
PCDHB15	-0.0697	3.9112E-01	5.9811E-01	ENSG00000113248
PCDHB16	0.1720	6.2726E-02	1.8608E-01	ENSG00000272674
PCDHB17P	-0.0284	6.2290E-01	7.8212E-01	ENSG00000255622
PCDHB2	-0.3709	9.3083E-08	5.4145E-06	ENSG00000112852
PCDHB3	-0.0371	6.5743E-01	8.0659E-01	ENSG00000113205
PCDHB4	0.0926	3.0543E-01	5.1565E-01	ENSG00000081818
PCDHB5	-0.1954	2.8118E-02	1.0617E-01	ENSG00000113209
PCDHB6	-0.0019	9.8237E-01	9.9070E-01	ENSG00000113211
PCDHB7	0.4586	3.4903E-03	2.2993E-02	ENSG00000113212
PCDHB8	0.0728	3.5495E-01	5.6473E-01	ENSG00000120322
PCDHB9	-0.2265	4.9848E-03	3.0017E-02	ENSG00000177839
PCDHGA1	0.1095	2.0519E-01	4.0446E-01	ENSG00000204956
PCDHGA10	-0.0950	2.3433E-01	4.3945E-01	ENSG00000253846
PCDHGA11	-0.1077	2.3669E-01	4.4244E-01	ENSG00000253873
PCDHGA12	0.0918	3.0389E-01	5.1410E-01	ENSG00000253159
PCDHGA2	0.0135	9.5990E-01	9.8006E-01	ENSG00000081853
PCDHGA3	-0.0249	7.3418E-01	8.5488E-01	ENSG00000254245
PCDHGA4	0.0384	5.9795E-01	7.6415E-01	ENSG00000262576
PCDHGA5	0.0557	4.2087E-01	6.2371E-01	ENSG00000253485
PCDHGA6	-0.1102	2.1546E-01	4.1675E-01	ENSG00000253731
PCDHGA7	-0.1350	1.0326E-01	2.6018E-01	ENSG00000253537
PCDHGA8	-0.0643	4.6509E-01	6.6166E-01	ENSG00000253767
PCDHGA9	-0.0243	7.1787E-01	8.4501E-01	ENSG00000261934
PCDHGB1	-0.1024	2.4790E-01	4.5568E-01	ENSG00000254221
PCDHGB2	-0.0794	3.2370E-01	5.3376E-01	ENSG00000253910
PCDHGB3	0.0259	2.3571E-01		ENSG00000262209
PCDHGB4	-0.2342	3.2783E-02	1.1833E-01	ENSG00000253953
PCDHGB5	-0.0899	3.1454E-01	5.2456E-01	ENSG00000276547
PCDHGB6	-0.4221	2.2028E-05	4.6286E-04	ENSG00000253305
PCDHGB7	-0.1687	7.7696E-02	2.1550E-01	ENSG00000254122
PCDHGB8P	-0.1176	1.9522E-01	3.9213E-01	ENSG00000248449
PCDHGB9P	-0.0565	5.0433E-01	6.9434E-01	ENSG00000276545
PCDHGC3	-0.3279	7.4622E-03	4.0684E-02	ENSG00000240184
PCDHGC4	-0.0738	3.6539E-01	5.7453E-01	ENSG00000242419
PCDHGC5	-0.0199	6.1542E-01		ENSG00000240764
PCED1A	-0.1002	1.5149E-01	3.3303E-01	ENSG00000132635
PCED1B	-0.0689	2.3912E-01	4.4562E-01	ENSG00000179715
PCF11	-0.0390	5.6597E-01	7.4117E-01	ENSG00000165494
PCGF1	-0.1070	1.2392E-01	2.9362E-01	ENSG00000115289
PCGF2	-0.0561	5.1216E-01	7.0032E-01	ENSG00000277258
PCGF3	0.1849	5.9178E-03	3.4172E-02	ENSG00000185619
PCGF3-AS1	-0.2472	2.0332E-03	1.5243E-02	ENSG00000249592
PCGF5	0.0314	6.7424E-01	8.1750E-01	ENSG00000180628

PCGF6	-0.1923	4.4387E-03	2.7477E-02	ENSG00000156374
PCID2	-0.0987	8.2796E-02	2.2441E-01	ENSG00000126226
PCIF1	-0.2526	1.6483E-04	2.2346E-03	ENSG00000100982
PCK1	0.0904	2.3324E-01	4.3842E-01	ENSG00000124253
PCK2	-0.0209	7.8323E-01	8.8527E-01	ENSG00000100889
PCLAF	-0.3148	4.8902E-03	2.9557E-02	ENSG00000166803
PCLO	0.2714	2.1451E-04	2.7461E-03	ENSG00000186472
PCM1	-0.0176	7.1810E-01	8.4504E-01	ENSG00000078674
PCMT1	-0.0569	2.8739E-01	4.9810E-01	ENSG00000120265
PCMTD1	0.0061	9.3747E-01	9.6914E-01	ENSG00000168300
PCMTD2	0.0011	9.8458E-01	9.9192E-01	ENSG00000203880
PCNA	-0.2237	5.5693E-03	3.2650E-02	ENSG00000132646
PCNP	-0.2396	2.0457E-05	4.3580E-04	ENSG00000081154
PCNT	0.1267	1.0670E-01	2.6571E-01	ENSG00000160299
PCNX1	0.3036	8.9724E-05	1.4176E-03	ENSG00000100731
PCNX2	0.2146	7.2504E-03	3.9856E-02	ENSG00000135749
PCNX3	0.1007	2.0170E-01	4.0028E-01	ENSG00000197136
PCNX4	0.1424	1.9722E-02	8.2207E-02	ENSG00000126773
PCOLCE	-0.0055	9.4009E-01	9.7046E-01	ENSG00000106333
PCOLCE-AS1	0.0445	5.6322E-01	7.3973E-01	ENSG00000224729
PCOLCE2	-0.0142	8.5165E-01	9.2443E-01	ENSG00000163710
PCOTH	-0.0797	9.9542E-02		ENSG00000205861
PCP2	-0.0350	4.7497E-01	6.7061E-01	ENSG00000174788
PCP4	0.0513	4.9999E-01	6.9089E-01	ENSG00000183036
PCP4L1	0.4328	4.5337E-03	2.7944E-02	ENSG00000248485
PCSK1N	0.0392	6.2750E-01	7.8545E-01	ENSG00000102109
PCSK2	0.1156	1.7679E-01	3.6808E-01	ENSG00000125851
PCSK4	0.1953	5.8339E-02	1.7725E-01	ENSG00000115257
PCSK5	-0.1441	1.4282E-01	3.2043E-01	ENSG00000099139
PCSK6	-0.0270	7.4423E-01	8.6086E-01	ENSG00000140479
PCSK6-AS1	0.0336	2.7592E-01		ENSG00000259764
PCSK7	0.1806	6.6589E-03	3.7391E-02	ENSG00000160613
PCSK9	-0.0034	9.5497E-01	9.7732E-01	ENSG00000169174
PCTP	0.2165	2.3288E-02	9.2810E-02	ENSG00000141179
PCYOX1	-0.0615	2.2217E-01	4.2551E-01	ENSG00000116005
PCYOX1L	0.0594	3.7743E-01	5.8591E-01	ENSG00000145882
PCYT1A	0.0101	8.9417E-01	9.4738E-01	ENSG00000161217
PCYT1B	-0.2303	1.8107E-03	1.3984E-02	ENSG00000102230
PCYT1B-AS1	-0.0106	5.0634E-01		ENSG00000236836
PCYT2	-0.2043	1.5379E-02	6.9250E-02	ENSG00000185813
PDAP1	-0.1732	1.2323E-02	5.9003E-02	ENSG00000106244
PDCD1	0.0086	8.4417E-01		ENSG00000188389
PDCD10	0.1231	7.0128E-02	2.0106E-01	ENSG00000114209
PDCD11	0.2988	1.7857E-05	3.9159E-04	ENSG00000148843
PDCD1LG2	0.1571	1.2350E-02	5.9121E-02	ENSG00000197646
PDCD2	-0.0142	7.5944E-01	8.7029E-01	ENSG00000071994
PDCD2L	0.0383	5.6267E-01	7.3936E-01	ENSG00000126249
PDCD4	-0.0951	2.5292E-01	4.6087E-01	ENSG00000150593
PDCD5	-0.0881	1.7163E-01	3.6079E-01	ENSG00000105185

PDCD6	0.0296	7.2180E-01	8.4753E-01	ENSG00000249915
PDCD6IP	0.0150	7.9981E-01	8.9433E-01	ENSG00000170248
PDCD6IP-DT	-0.0282	7.1309E-01	8.4256E-01	ENSG00000271643
PDCD6IPP1	-0.0416	5.8250E-01	7.5335E-01	ENSG00000275325
PDCD7	0.0513	3.9537E-01	6.0161E-01	ENSG00000090470
PDCL	-0.1382	3.0755E-02	1.1325E-01	ENSG00000136940
PDCL2	0.0105	7.2826E-01		ENSG00000163440
PDCL3	-0.1176	5.9037E-02	1.7856E-01	ENSG00000115539
PDCL3P6	0.0145	7.2966E-01		ENSG00000224255
PDE10A	-0.0277	7.0914E-01	8.4012E-01	ENSG00000112541
PDE11A	0.2393	4.4694E-02	1.4779E-01	ENSG00000128655
PDE11A-AS1	0.1066	5.2844E-02		ENSG00000229941
PDE12	0.0102	8.7385E-01	9.3676E-01	ENSG00000174840
PDE1A	-0.2497	1.5527E-02	6.9741E-02	ENSG00000115252
PDE1B	0.2461	1.0266E-02	5.1655E-02	ENSG00000123360
PDE1C	-0.1589	7.2202E-02	2.0524E-01	ENSG00000154678
PDE3A	0.1670	8.0216E-02	2.2008E-01	ENSG00000172572
PDE3B	0.3437	3.5063E-03	2.3074E-02	ENSG00000152270
PDE4A	-0.3198	1.4333E-05	3.2683E-04	ENSG00000065989
PDE4B	-0.1154	6.8216E-02	1.9714E-01	ENSG00000184588
PDE4B-AS1	-0.0682	2.3339E-01	4.3863E-01	ENSG00000227466
PDE4C	0.0728	2.3789E-01	4.4398E-01	ENSG00000105650
PDE4C	-0.0004	9.2468E-01		ENSG00000285188
PDE4D	-0.1355	3.1970E-02	1.1614E-01	ENSG00000113448
PDE4DIP	-0.0955	1.7327E-01	3.6320E-01	ENSG00000178104
PDE5A	0.1297	1.3347E-01	3.0720E-01	ENSG00000138735
PDE6A	0.0618	1.1119E-01		ENSG00000132915
PDE6B	-0.0819	2.8016E-01	4.9021E-01	ENSG00000133256
PDE6C	0.1388	5.6243E-02	1.7329E-01	ENSG00000095464
PDE6D	-0.1682	3.9782E-03	2.5484E-02	ENSG00000156973
PDE6G	-0.0355	5.3658E-01	7.1963E-01	ENSG00000185527
PDE6H	0.2876	1.5537E-02	6.9748E-02	ENSG00000139053
PDE7A	0.1476	5.2556E-02	1.6496E-01	ENSG00000205268
PDE7B	0.1698	9.8459E-02	2.5225E-01	ENSG00000171408
PDE8A	0.1094	1.6407E-01	3.5091E-01	ENSG00000073417
PDE8B	-0.2016	6.2712E-02	1.8608E-01	ENSG00000113231
PDE9A	-0.1711	2.3540E-02	9.3570E-02	ENSG00000160191
PDE9A-AS1	0.0514	1.5902E-01		ENSG00000225731
PDF	0.0078	9.1509E-01	9.5813E-01	ENSG00000258429
PDGFA	-0.1912	5.4084E-02	1.6851E-01	ENSG00000197461
PDGFA-DT	-0.0481	3.9268E-01	5.9947E-01	ENSG00000223855
PDGFB	-0.2397	1.8334E-02	7.7978E-02	ENSG00000100311
PDGFC	0.0523	5.3475E-01	7.1813E-01	ENSG00000145431
PDGFD	-0.0639	4.7236E-01	6.6862E-01	ENSG00000170962
PDGFRB	0.1110	2.3244E-01	4.3747E-01	ENSG00000113721
PDGFRL	0.1174	2.0112E-01	3.9959E-01	ENSG00000104213
PDHA1	-0.2055	5.5745E-04	5.7874E-03	ENSG00000131828
PDHA1P1	-0.0159	4.5122E-01		ENSG00000270772
PDHB	0.0079	8.8678E-01	9.4386E-01	ENSG00000168291

PDHX	0.1583	3.1252E-02	1.1457E-01	ENSG00000110435
PDIA2	0.0677	4.1649E-01	6.1990E-01	ENSG00000185615
PDIA3	-0.2314	1.2000E-04	1.7701E-03	ENSG00000167004
PDIA4	-0.2383	8.5140E-03	4.5041E-02	ENSG00000155660
PDIA5	0.1332	1.0584E-01	2.6451E-01	ENSG00000065485
PDIA6	-0.3493	1.4414E-05	3.2808E-04	ENSG00000143870
PDIK1L	-0.0969	1.5240E-01	3.3443E-01	ENSG00000175087
PDK1	0.4849	1.7629E-03	1.3722E-02	ENSG00000152256
PDK1-AS1	0.0040	9.4858E-01	9.7460E-01	ENSG00000225205
PDK2	-0.1066	1.7106E-01	3.6014E-01	ENSG00000005882
PDK3	0.2536	2.3193E-04	2.9264E-03	ENSG00000067992
PDK4	-0.1004	2.1116E-01	4.1199E-01	ENSG00000004799
PDLIM1P4	0.0115	2.9512E-01		ENSG00000249274
PDLIM2	-0.0542	4.7310E-01	6.6911E-01	ENSG00000120913
PDLIM3	-0.1104	2.2806E-01	4.3272E-01	ENSG00000154553
PDLIM4	-0.1287	3.4825E-02	1.2345E-01	ENSG00000131435
PDLIM5	0.0850	2.6553E-01	4.7474E-01	ENSG00000163110
PDLIM7	-0.0084	8.8266E-01	9.4123E-01	ENSG00000196923
PDP1	-0.3207	3.7667E-04	4.2709E-03	ENSG00000164951
PDP2	0.1852	7.5314E-02	2.1101E-01	ENSG00000172840
PDPK1	0.0713	1.9143E-01	3.8676E-01	ENSG00000140992
PDPK2P	0.0087	9.1362E-01	9.5757E-01	ENSG00000205918
PDPN	-0.2439	2.6600E-02	1.0195E-01	ENSG00000162493
PDPR	0.0388	5.8062E-01	7.5207E-01	ENSG00000090857
PDRG1	-0.1465	2.1878E-02	8.8582E-02	ENSG00000088356
PDS5A	-0.1527	2.0311E-03	1.5232E-02	ENSG00000121892
PDS5B	-0.0894	1.8670E-01	3.8106E-01	ENSG00000083642
PDSS1	0.1739	1.8168E-02	7.7526E-02	ENSG00000148459
PDSS2	-0.0250	7.1800E-01	8.4504E-01	ENSG00000164494
PDX1	0.0249	5.0662E-01		ENSG00000139515
PDXDC1	0.2035	2.0166E-03	1.5174E-02	ENSG00000179889
PDXDC2P	0.0175	7.8447E-01	8.8622E-01	ENSG00000255185
PDXDC2P-NPIPB14P	0.2518	2.5303E-02	9.8316E-02	ENSG00000196696
PDXK	-0.0796	1.4123E-01	3.1807E-01	ENSG00000160209
PDXP	0.0519	4.7683E-01	6.7226E-01	ENSG00000241360
PDXP-DT	0.0208	7.3688E-01	8.5640E-01	ENSG00000233360
PDYN	-0.0154	7.5787E-01	8.6898E-01	ENSG00000101327
PDYN-AS1	0.0128	4.2498E-01		ENSG00000233896
PDZD11	-0.1891	6.3298E-05	1.0790E-03	ENSG00000120509
PDZD2	-0.0150	8.6298E-01	9.3089E-01	ENSG00000133401
PDZD4	-0.0518	4.8755E-01	6.8078E-01	ENSG00000067840
PDZD7	0.1601	7.1755E-02	2.0437E-01	ENSG00000186862
PDZD8	-0.0620	3.9338E-01	5.9997E-01	ENSG00000165650
PDZD9	-0.0147	8.2232E-01	9.0802E-01	ENSG00000155714
PDZK1	0.4034	1.2680E-02	6.0240E-02	ENSG00000174827
PDZK1IP1	0.0010	7.8702E-01		ENSG00000162366
PDZPH1P	-0.0006	8.7914E-01		ENSG00000226926
PDZRN3	-0.3703	1.5774E-04	2.1587E-03	ENSG00000121440
PDZRN4	0.0251	7.5636E-01	8.6801E-01	ENSG00000165966

PEA15	-0.2510	8.1508E-03	4.3494E-02	ENSG00000162734
PEAK1	0.1800	1.5141E-02	6.8423E-02	ENSG00000173517
PEAK3	-0.3714	1.4205E-02	6.5415E-02	ENSG00000188305
PEAR1	0.1257	1.7813E-01	3.6944E-01	ENSG00000187800
PEBP1	-0.3303	6.2820E-07	2.5464E-05	ENSG00000089220
PECAM1	0.0435	2.0873E-01		ENSG00000261371
PECR	-0.0649	3.3575E-01	5.4585E-01	ENSG00000115425
PEDS1	-0.0682	2.1586E-01	4.1728E-01	ENSG00000240849
PEF1	0.0265	6.8287E-01	8.2313E-01	ENSG00000162517
PEG10	0.0367	6.4844E-01	8.0022E-01	ENSG00000242265
PEG13	0.0479	5.6173E-01	7.3848E-01	ENSG00000282164
PEG3	-0.0409	5.6383E-01	7.3998E-01	ENSG00000198300
PELATON	0.0009	9.5392E-01		ENSG00000224397
PELI1	-0.3457	7.4877E-04	7.3085E-03	ENSG00000197329
PELI2	0.0608	4.0177E-01	6.0655E-01	ENSG00000139946
PELI3	-0.2269	5.9090E-03	3.4145E-02	ENSG00000174516
PELO	0.1278	7.2962E-02	2.0672E-01	ENSG00000152684
PELP1	-0.0290	6.8295E-01	8.2313E-01	ENSG00000141456
PELP1-DT	0.1204	1.7104E-01	3.6012E-01	ENSG00000244184
PEMT	0.1822	6.4104E-03	3.6301E-02	ENSG00000133027
PENK	-0.4120	7.8199E-03	4.2176E-02	ENSG00000181195
PENK-AS1	-0.0551	2.3575E-01	4.4139E-01	ENSG00000254254
PEPD	-0.2122	1.5652E-03	1.2617E-02	ENSG00000124299
PER1	-0.3192	4.2142E-06	1.2184E-04	ENSG00000179094
PER2	-0.1544	2.4474E-02	9.6117E-02	ENSG00000132326
PER3	-0.2221	1.5612E-02	6.9983E-02	ENSG00000049246
PERP	0.0472	5.7219E-01	7.4555E-01	ENSG00000112378
PES1	0.0902	1.8764E-01	3.8229E-01	ENSG00000100029
PET100	-0.1514	7.1177E-02	2.0319E-01	ENSG00000229833
PET117	-0.2813	8.5920E-03	4.5376E-02	ENSG00000232838
PEX1	0.1082	6.3458E-02	1.8771E-01	ENSG00000127980
PEX10	-0.1149	1.6285E-01	3.4909E-01	ENSG00000157911
PEX11A	-0.1047	1.4316E-01	3.2097E-01	ENSG00000166821
PEX11B	0.0803	2.4330E-01	4.5111E-01	ENSG00000131779
PEX11G	0.5064	1.4854E-05	3.3408E-04	ENSG00000104883
PEX12	-0.0550	4.5669E-01	6.5493E-01	ENSG00000108733
PEX13	-0.2469	3.4663E-04	4.0079E-03	ENSG00000162928
PEX14	0.0215	7.7481E-01	8.8015E-01	ENSG00000142655
PEX16	0.1455	1.5142E-02	6.8423E-02	ENSG00000121680
PEX19	-0.0207	7.5675E-01	8.6825E-01	ENSG00000162735
PEX2	0.1520	5.1544E-02	1.6274E-01	ENSG00000164751
PEX26	-0.2437	8.5378E-03	4.5128E-02	ENSG00000215193
PEX3	0.1861	9.2149E-03	4.7753E-02	ENSG00000034693
PEX5	-0.0133	8.3124E-01	9.1283E-01	ENSG00000139197
PEX6	-0.1843	1.5687E-02	7.0188E-02	ENSG00000124587
PEX7	-0.0022	9.8123E-01	9.9024E-01	ENSG00000112357
PF4V1	0.0074	8.6022E-01		ENSG00000109272
PFAS	-0.0111	8.6415E-01	9.3150E-01	ENSG00000178921
PFDN1	-0.2636	5.0480E-06	1.4235E-04	ENSG00000113068

PFDN1P2	0.0067	5.3060E-01		ENSG00000248188
PFDN2	-0.2019	4.3093E-03	2.6938E-02	ENSG00000143256
PFDN4	-0.2138	1.6190E-02	7.1582E-02	ENSG00000101132
PFDN5	-0.0005	9.9843E-01	9.9913E-01	ENSG00000123349
PFDN6	-0.2181	2.9466E-03	2.0206E-02	ENSG00000204220
PFKFB1	0.0413	5.5174E-01	7.3050E-01	ENSG00000158571
PFKFB2	0.2126	2.3627E-02	9.3824E-02	ENSG00000123836
PFKFB3	0.0758	3.8010E-01	5.8854E-01	ENSG00000170525
PFKFB4	0.4993	4.1599E-05	7.7061E-04	ENSG00000114268
PFKL	-0.0254	7.3573E-01	8.5584E-01	ENSG00000141959
PFKM	-0.1456	1.7140E-02	7.4458E-02	ENSG00000152556
PFKP	0.0514	5.3895E-01	7.2105E-01	ENSG00000067057
PFN1	-0.4445	2.1591E-08	1.5811E-06	ENSG00000108518
PFN1P2	0.0503	4.9269E-01	6.8480E-01	ENSG00000270392
PFN2	-0.1834	5.9732E-03	3.4362E-02	ENSG00000070087
PFN3	0.0234	6.7815E-01	8.1969E-01	ENSG00000196570
PFN4	0.0914	3.0262E-01	5.1271E-01	ENSG00000176732
PGA3	0.0544	3.1190E-01	5.2195E-01	ENSG00000229859
PGA4	0.0600	2.1511E-01	4.1635E-01	ENSG00000229183
PGA5	0.0657	1.7701E-01	3.6824E-01	ENSG00000256713
PGAM1	0.0124	8.6467E-01	9.3172E-01	ENSG00000171314
PGAM1P11	-0.0105	6.9214E-01		ENSG00000233623
PGAM1P5	-0.0005	6.0753E-01		ENSG00000257150
PGAM1P6	0.0080	3.5043E-01		ENSG00000224464
PGAM4	0.1233	1.8083E-01	3.7311E-01	ENSG00000226784
PGAM5	-0.2356	1.1012E-04	1.6582E-03	ENSG00000247077
PGAP1	0.2133	2.1079E-03	1.5669E-02	ENSG00000197121
PGAP2	-0.0250	6.5090E-01	8.0209E-01	ENSG00000148985
PGAP3	0.3280	8.0755E-05	1.3021E-03	ENSG00000161395
PGAP4	-0.1254	8.8658E-02	2.3510E-01	ENSG00000165152
PGAP6	-0.0382	6.0340E-01	7.6793E-01	ENSG00000129925
PGBD1	-0.1718	1.0118E-02	5.1185E-02	ENSG00000137338
PGBD2	0.4675	2.0592E-03	1.5386E-02	ENSG00000185220
PGBD4	-0.0480	5.4587E-01	7.2621E-01	ENSG00000182405
PGBD5	-0.0654	3.7286E-01	5.8156E-01	ENSG00000177614
PGC	0.0193	6.6540E-01	8.1203E-01	ENSG00000096088
PGD	-0.2489	1.5381E-02	6.9250E-02	ENSG00000142657
PGDP1	-0.0066	6.7523E-01		ENSG00000265273
PGF	-0.0185	8.2403E-01	9.0862E-01	ENSG00000119630
PGGHG	0.0335	6.8388E-01	8.2367E-01	ENSG00000142102
PGGT1B	0.0155	7.9026E-01	8.8919E-01	ENSG00000164219
PGK1	0.0843	3.1742E-01	5.2771E-01	ENSG00000102144
PGK1P2	0.0180	1.9913E-01	3.9737E-01	ENSG00000213290
PGLS	-0.0554	3.9249E-01	5.9937E-01	ENSG00000130313
PGLS-DT	0.1353	1.1952E-01	2.8664E-01	ENSG00000269439
PGLYRP2	0.0092	8.1226E-01		ENSG00000161031
PGLYRP4	0.0233	2.0930E-01		ENSG00000163218
PGM1	-0.0443	5.5721E-01	7.3497E-01	ENSG00000079739
PGM2	0.0289	6.8315E-01	8.2320E-01	ENSG00000169299

PGM2L1	0.0115	8.8786E-01	9.4427E-01	ENSG00000165434
PGM3	0.2922	7.0337E-05	1.1678E-03	ENSG00000013375
PGM5	-0.0535	5.1096E-01	6.9947E-01	ENSG00000154330
PGM5-AS1	0.0523	3.7065E-01	5.7969E-01	ENSG00000224958
PGM5P3-AS1	0.0761	1.5845E-01	3.4272E-01	ENSG00000277631
PGM5P4-AS1	0.0184	7.5465E-01	8.6686E-01	ENSG00000231943
PGP	0.0861	2.0780E-01	4.0745E-01	ENSG00000184207
PGPEP1	0.0177	8.2981E-01	9.1190E-01	ENSG00000130517
PGPEP1L	0.0000	7.7301E-01		ENSG00000183571
PGR	0.0402	4.7030E-01		ENSG00000082175
PGRMC1	-0.1120	9.8451E-02	2.5225E-01	ENSG00000101856
PGRMC2	-0.1147	7.6369E-02	2.1314E-01	ENSG00000164040
PGS1	-0.1938	4.2147E-03	2.6504E-02	ENSG00000087157
PHACTR1	0.0618	4.3131E-01	6.3290E-01	ENSG00000112137
PHACTR2	0.2127	1.2715E-02	6.0372E-02	ENSG00000112419
PHACTR2-AS1	0.0378	2.2186E-01		ENSG00000235740
PHACTR3	-0.0570	4.8035E-01	6.7525E-01	ENSG00000087495
PHACTR3-AS1	0.0322	4.7279E-01		ENSG00000238194
PHACTR4	0.0529	3.4156E-01	5.5118E-01	ENSG00000204138
PHAF1	0.0769	3.4272E-01	5.5221E-01	ENSG00000125149
PHAX	-0.3120	2.7034E-09	2.6636E-07	ENSG00000164902
PHB1	-0.2428	1.7223E-06	5.9023E-05	ENSG00000167085
PHB1P15	0.0180	7.7116E-01	8.7786E-01	ENSG00000262648
PHB1P4	0.0041	9.2345E-01		ENSG00000230251
PHB1P5	0.0306	6.3623E-01	7.9146E-01	ENSG00000233523
PHB1P6	0.0451	4.8490E-01	6.7871E-01	ENSG00000276592
PHB1P9	-0.3409	1.7282E-02	7.4910E-02	ENSG00000230224
PHB2	-0.0723	9.7917E-02	2.5136E-01	ENSG00000215021
PHC1	-0.0794	1.0588E-01	2.6453E-01	ENSG00000111752
PHC2	-0.2181	2.1555E-02	8.7603E-02	ENSG00000134686
PHC2-AS1	0.1665	4.3991E-02	1.4597E-01	ENSG00000233246
PHC3	-0.0049	9.0665E-01	9.5437E-01	ENSG00000173889
PHETA1	0.0419	5.9746E-01	7.6387E-01	ENSG00000198324
PHETA2	0.1083	2.3198E-01	4.3691E-01	ENSG00000177096
PHF1	-0.0117	8.4752E-01	9.2210E-01	ENSG00000112511
PHF10	0.1476	4.6146E-02	1.5120E-01	ENSG00000130024
PHF11	0.0787	3.7242E-01	5.8118E-01	ENSG00000136147
PHF12	-0.1560	3.6958E-02	1.2913E-01	ENSG00000109118
PHF13	-0.1194	1.3987E-01	3.1630E-01	ENSG00000116273
PHF14	0.3143	5.3149E-07	2.2604E-05	ENSG00000106443
PHF19	-0.3182	4.0989E-03	2.6013E-02	ENSG00000119403
PHF2	-0.0294	6.8615E-01	8.2504E-01	ENSG00000197724
PHF20	-0.0444	3.5097E-01	5.6112E-01	ENSG00000025293
PHF20L1	0.0934	4.3277E-02	1.4444E-01	ENSG00000129292
PHF21A	-0.0106	8.5218E-01	9.2466E-01	ENSG00000135365
PHF21B	-0.0343	6.8836E-01	8.2684E-01	ENSG00000056487
PHF23	0.0064	9.1779E-01	9.5915E-01	ENSG00000040633
PHF24	0.1203	1.4550E-01	3.2443E-01	ENSG00000122733
PHF3	0.0291	6.6901E-01	8.1453E-01	ENSG00000118482

PHF5A	-0.0536	3.9535E-01	6.0161E-01	ENSG00000100410
PHF6	-0.1043	2.6431E-02	1.0145E-01	ENSG00000156531
PHF7	0.1835	3.1381E-02	1.1490E-01	ENSG00000010318
PHF8	-0.0244	7.2237E-01	8.4783E-01	ENSG00000172943
PHGDH	-0.3084	1.2688E-04	1.8429E-03	ENSG00000092621
PHIP	0.2065	2.9144E-03	2.0053E-02	ENSG00000146247
PHKA1	0.0674	4.0458E-01	6.0918E-01	ENSG00000067177
PHKA1-AS1	0.0275	5.3750E-01		ENSG00000231944
PHKA2	0.1010	2.1885E-01	4.2135E-01	ENSG00000044446
PHKB	-0.1205	4.4964E-02	1.4840E-01	ENSG00000102893
PHKG1	0.0847	3.0365E-01	5.1388E-01	ENSG00000164776
PHKG2	-0.0223	6.9949E-01	8.3382E-01	ENSG00000156873
PHLDA1	-0.2994	2.8025E-03	1.9391E-02	ENSG00000139289
PHLDA2	0.1070	2.2875E-01	4.3329E-01	ENSG00000181649
PHLDA3	-0.0473	4.5058E-01	6.4981E-01	ENSG00000174307
PHLDB1	0.1279	5.6110E-02	1.7305E-01	ENSG00000019144
PHLDB2	0.1224	1.9097E-01	3.8625E-01	ENSG00000144824
PHLDB3	-0.2055	3.0384E-02	1.1225E-01	ENSG00000176531
PHLPP1	-0.0846	3.0977E-01	5.2007E-01	ENSG00000081913
PHLPP2	0.1494	4.2037E-02	1.4150E-01	ENSG00000040199
PHOSPHO1	0.0272	6.0790E-01	7.7116E-01	ENSG00000173868
PHOSPHO2	0.3460	3.8523E-03	2.4826E-02	ENSG00000144362
PHOX2A	-0.0277	6.1215E-01	7.7431E-01	ENSG00000165462
PHPT1	-0.2129	3.4031E-03	2.2503E-02	ENSG00000054148
PHRF1	0.2534	1.7624E-03	1.3722E-02	ENSG00000070047
PHTF1	0.1268	2.6982E-02	1.0294E-01	ENSG00000116793
PHTF2	0.0248	6.8194E-01	8.2253E-01	ENSG00000006576
PHYH	-0.1800	9.0361E-03	4.7105E-02	ENSG00000107537
PHYHD1	0.0471	5.4990E-01	7.2912E-01	ENSG00000175287
PHYHIP	-0.0545	4.9622E-01	6.8781E-01	ENSG00000168490
PHYHIPL	0.1398	3.2593E-02	1.1779E-01	ENSG00000165443
PHYKPL	-0.1737	1.3184E-02	6.1964E-02	ENSG00000175309
PI15	-0.3794	1.3637E-02	6.3558E-02	ENSG00000137558
PI16	0.0306	6.2972E-01	7.8675E-01	ENSG00000164530
PI4K2A	0.0297	6.7398E-01	8.1745E-01	ENSG00000155252
PI4K2B	0.5615	4.2458E-08	2.7784E-06	ENSG00000038210
PI4KA	-0.0369	5.2091E-01	7.0761E-01	ENSG00000241973
PI4KAP2	0.0941	2.0349E-02	8.3925E-02	ENSG00000183506
PI4KB	-0.0637	3.0739E-01	5.1754E-01	ENSG00000143393
PIANP	0.0254	7.3254E-01	8.5394E-01	ENSG00000139200
PIAS1	0.0169	7.7306E-01	8.7903E-01	ENSG00000033800
PIAS2	-0.0671	1.6783E-01	3.5612E-01	ENSG00000078043
PIAS3	0.0767	2.5167E-01	4.5999E-01	ENSG00000131788
PIAS4	0.0582	4.0199E-01	6.0655E-01	ENSG00000105229
PIBF1	0.1826	4.3706E-03	2.7218E-02	ENSG00000083535
PICALM	-0.0565	4.0263E-01	6.0710E-01	ENSG00000073921
PICART1	0.0389	4.5098E-01	6.4998E-01	ENSG00000246640
PICK1	0.0724	3.3368E-01	5.4379E-01	ENSG00000100151
PID1	-0.5029	1.7089E-16	1.8281E-13	ENSG00000153823

PIDD1	0.4240	4.2224E-09	3.9205E-07	ENSG00000177595
PIERCE2	0.0852	3.0615E-01	5.1626E-01	ENSG00000261652
PIEZO1	-0.1435	1.3232E-01	3.0582E-01	ENSG00000103335
PIEZO2	0.0508	5.5832E-01	7.3579E-01	ENSG00000154864
PIF1	-0.0783	3.4163E-01	5.5123E-01	ENSG00000140451
PIGA	-0.0355	6.5580E-01	8.0557E-01	ENSG00000165195
PIGB	0.1671	6.3689E-02	1.8819E-01	ENSG00000069943
PIGBOS1	0.0004	9.9565E-01	9.9755E-01	ENSG00000225973
PIGC	-0.0377	4.5656E-01	6.5483E-01	ENSG00000135845
PIGF	-0.0935	1.2277E-01	2.9177E-01	ENSG00000151665
PIGG	0.0147	8.4799E-01	9.2256E-01	ENSG00000174227
PIGH	0.0559	3.8208E-01	5.9030E-01	ENSG00000100564
PIGK	-0.0849	1.6680E-01	3.5475E-01	ENSG00000142892
PIGL	0.3048	3.0913E-04	3.6798E-03	ENSG00000108474
PIGM	0.3206	3.8220E-04	4.3153E-03	ENSG00000143315
PIGN	0.0462	5.2258E-01	7.0893E-01	ENSG00000197563
PIGO	0.0525	4.8957E-01	6.8252E-01	ENSG00000165282
PIGP	0.1846	2.4630E-02	9.6546E-02	ENSG00000185808
PIGQ	-0.0412	5.4416E-01	7.2495E-01	ENSG00000007541
PIGR	-0.0008	9.5155E-01		ENSG00000162896
PIGS	0.0257	6.2936E-01	7.8663E-01	ENSG00000087111
PIGT	0.0826	2.0199E-01	4.0073E-01	ENSG00000124155
PIGU	-0.0810	2.0722E-01	4.0688E-01	ENSG00000101464
PIGV	0.0921	2.6289E-01	4.7173E-01	ENSG00000060642
PIGW	0.2409	9.9150E-03	5.0394E-02	ENSG00000277161
PIGX	-0.0934	6.3123E-02	1.8694E-01	ENSG00000163964
PIGY-DT	0.2275	7.0912E-03	3.9191E-02	ENSG00000285122
PIGZ	-0.0040	9.5152E-01	9.7615E-01	ENSG00000119227
PIH1D1	-0.1208	2.5125E-02	9.7861E-02	ENSG00000104872
PIH1D2	0.3290	1.3354E-03	1.1283E-02	ENSG00000150773
PIK3AP1	-0.0012	9.8029E-01	9.8995E-01	ENSG00000155629
PIK3C2A	0.1248	4.9388E-02	1.5812E-01	ENSG00000011405
PIK3C2B	0.1209	8.5355E-02	2.2863E-01	ENSG00000133056
PIK3C2G	0.0080	8.3959E-01		ENSG00000139144
PIK3C3	0.2012	9.7238E-04	8.8556E-03	ENSG00000078142
PIK3CA	0.0420	5.5295E-01	7.3140E-01	ENSG00000121879
PIK3CA-DT	0.0105	8.5112E-01		ENSG00000229102
PIK3CB	-0.0467	4.7142E-01	6.6766E-01	ENSG00000051382
PIK3CD	0.0219	7.7004E-01	8.7693E-01	ENSG00000171608
PIK3CD-AS1	-0.0080	3.4243E-01		ENSG00000179840
PIK3CD-AS2	0.0634	3.9276E-01	5.9950E-01	ENSG00000231789
PIK3CG	0.0179	5.0505E-01		ENSG00000105851
PIK3IP1	-0.5282	3.7857E-05	7.2040E-04	ENSG00000100100
PIK3IP1-DT	-0.0708	3.0526E-01	5.1557E-01	ENSG00000228839
PIK3R1	-0.0735	2.4744E-01	4.5528E-01	ENSG00000145675
PIK3R2	-0.0628	3.4736E-01	5.5760E-01	ENSG00000105647
PIK3R3	-0.2179	3.9989E-03	2.5562E-02	ENSG00000117461
PIK3R4	0.2789	2.6902E-04	3.3047E-03	ENSG00000196455
PIK3R5	0.0174	9.9515E-01		ENSG00000141506

PIKFYVE	0.0326	6.4780E-01	7.9979E-01	ENSG00000115020
PILRA	0.1640	9.7605E-02	2.5103E-01	ENSG00000085514
PILRB	0.1596	1.0584E-01	2.6451E-01	ENSG00000121716
PIM2	0.0278	7.3633E-01	8.5608E-01	ENSG00000102096
PIM3	0.0179	7.9403E-01	8.9120E-01	ENSG00000198355
PIMREG	-0.0823	3.4427E-01	5.5397E-01	ENSG00000129195
PIN1	-0.1268	4.3588E-02	1.4518E-01	ENSG00000127445
PIN1-DT	-0.0153	7.4266E-01	8.5987E-01	ENSG00000267289
PIN1P1	0.1209	1.8547E-02		ENSG00000229359
PIN4	-0.0861	9.7703E-02	2.5114E-01	ENSG00000102309
PINCR	0.0191	6.4086E-01		ENSG00000224294
PINK1	0.0052	9.5446E-01	9.7705E-01	ENSG00000158828
PINK1-AS	0.1140	2.1866E-01	4.2114E-01	ENSG00000117242
PINLYP	-0.0326	6.9585E-01	8.3174E-01	ENSG00000234465
PINX1	-0.1544	4.6994E-02	1.5313E-01	ENSG00000254093
PIP4K2A	-0.3665	5.7692E-07	2.3938E-05	ENSG00000150867
PIP4K2B	-0.0999	1.4642E-01	3.2604E-01	ENSG00000276293
PIP4K2C	0.0417	5.8155E-01	7.5271E-01	ENSG00000166908
PIP4P1	-0.0431	4.6177E-01	6.5898E-01	ENSG00000165782
PIP4P2	-0.0574	3.9406E-01	6.0049E-01	ENSG00000155099
PIP5K1A	0.0999	1.5484E-01	3.3791E-01	ENSG00000143398
PIP5K1B	0.3849	9.1796E-05	1.4408E-03	ENSG00000107242
PIP5K1C	-0.0714	2.8557E-01	4.9632E-01	ENSG00000186111
PIP5KL1	-0.1664	8.3080E-02	2.2480E-01	ENSG00000167103
PIPOX	0.0860	3.0827E-01	5.1852E-01	ENSG00000179761
PIPSL	0.0018	9.8047E-01	9.9000E-01	ENSG00000180764
PIR	-0.2711	3.1762E-02	1.1578E-01	ENSG00000087842
PIRAT1	0.0059	8.9633E-01		ENSG00000237803
PIRT	0.0277	2.9145E-01		ENSG00000233670
PISD	0.0929	1.6785E-01	3.5612E-01	ENSG00000241878
PITHD1	0.0025	9.7424E-01	9.8741E-01	ENSG00000057757
PITPNA	0.0043	9.4779E-01	9.7432E-01	ENSG00000174238
PITPNA-AS1	-0.2605	2.5139E-02	9.7900E-02	ENSG00000236618
PITPNB	-0.0747	2.5841E-01	4.6721E-01	ENSG00000180957
PITPNC1	-0.0434	5.0404E-01	6.9432E-01	ENSG00000154217
PITPNM1	-0.0312	6.9505E-01	8.3115E-01	ENSG00000110697
PITPNM2	0.0753	3.8567E-01	5.9356E-01	ENSG00000090975
PITPNM2-AS1	-0.0044	7.7355E-01		ENSG00000251497
PITPNM3	-0.0403	6.3124E-01	7.8773E-01	ENSG00000091622
PITRM1	0.0404	5.5789E-01	7.3551E-01	ENSG00000107959
PITRM1-AS1	-0.0376	4.5333E-01	6.5203E-01	ENSG00000237399
PITX1	-0.1451	5.7756E-02	1.7617E-01	ENSG00000069011
PITX1-AS1	0.0193	7.7878E-01	8.8230E-01	ENSG00000224186
PITX2	0.0677	3.6502E-01	5.7426E-01	ENSG00000164093
PITX3	-0.0013	8.8942E-01	9.4516E-01	ENSG00000107859
PIWIL1	0.0054	9.5348E-01		ENSG00000125207
PIWIL2	0.2784	2.9363E-02	1.0945E-01	ENSG00000197181
PIWIL2-DT	-0.0010	9.8605E-01	9.9239E-01	ENSG00000254064
PIWIL3	-0.0060	8.2276E-01		ENSG00000184571

PIWIL4	-0.5104	5.3074E-03	3.1558E-02	ENSG00000134627
PIWIL4-AS1	0.0251	4.4191E-01		ENSG00000255929
PJA1	-0.3357	2.3707E-05	4.9141E-04	ENSG00000181191
PJA2	-0.0951	1.6096E-01	3.4648E-01	ENSG00000198961
PJVK	0.2790	1.3450E-03	1.1329E-02	ENSG00000204311
PKD1	0.1025	1.8652E-01	3.8084E-01	ENSG00000008710
PKD1-AS1	0.0128	7.9395E-01	8.9120E-01	ENSG00000259933
PKD1L1	0.0219	7.7272E-01	8.7896E-01	ENSG00000158683
PKD1L2	0.1126	1.4589E-01	3.2509E-01	ENSG00000166473
PKD1L3	0.0939	1.9076E-01	3.8596E-01	ENSG00000277481
PKD1P2	0.0054	9.4828E-01	9.7454E-01	ENSG00000227827
PKD1P3	0.0271	7.1782E-01	8.4499E-01	ENSG00000183458
PKD1P4	0.0208	8.0117E-01	8.9510E-01	ENSG00000205746
PKD1P5	-0.0163	8.2720E-01	9.1051E-01	ENSG00000254681
PKD1P6	-0.1849	1.7485E-02	7.5560E-02	ENSG00000250251
PKD1P6-NPIPP1	0.1485	1.3471E-01	3.0916E-01	ENSG00000270580
PKD2	0.0199	8.0061E-01	8.9476E-01	ENSG00000118762
PKD2L1	0.0646	2.2513E-01	4.2887E-01	ENSG00000107593
PKD2L2	0.0658	3.9003E-01	5.9733E-01	ENSG00000078795
PKDCC	-0.3229	9.7891E-03	4.9940E-02	ENSG00000162878
PKDREJ	0.0919	1.3820E-01	3.1413E-01	ENSG00000130943
PKHD1	0.0047	7.9415E-01		ENSG00000170927
PKHD1L1	0.2341	2.0984E-02	8.5879E-02	ENSG00000205038
PKIA	-0.0451	5.3042E-01	7.1489E-01	ENSG00000171033
PKIA-AS1	0.0371	6.5826E-01	8.0708E-01	ENSG00000254266
PKIB	0.0948	1.4804E-01	3.2836E-01	ENSG00000135549
PKIG	0.1511	1.6129E-02	7.1455E-02	ENSG00000168734
PKLR	0.0054	9.3333E-01	9.6701E-01	ENSG00000143627
PKM	0.0820	3.5356E-01	5.6361E-01	ENSG00000067225
PKMYT1	-0.0918	2.8235E-01	4.9278E-01	ENSG00000127564
PKN1	-0.3079	3.3245E-08	2.2597E-06	ENSG00000123143
PKN2	0.0873	2.0961E-01	4.0975E-01	ENSG00000065243
PKN2-AS1	0.0041	9.5414E-01	9.7701E-01	ENSG00000237505
PKN3	-0.1677	7.3603E-02	2.0789E-01	ENSG00000160447
PKNOX1	-0.0782	2.2507E-01	4.2887E-01	ENSG00000160199
PKNOX2	0.0471	5.4742E-01	7.2713E-01	ENSG00000165495
PKNOX2-DT	0.0013	9.2605E-01	9.6390E-01	ENSG00000254880
PKP1	-0.0107	6.2085E-01		ENSG00000081277
PKP2	-0.2303	3.2432E-02	1.1740E-01	ENSG00000057294
PKP3	0.0146	4.9687E-01		ENSG00000184363
PKP4	-0.1843	1.8488E-02	7.8487E-02	ENSG00000144283
PKP4-AS1	-0.0224	3.5479E-01		ENSG00000204380
PLA1A	0.0298	3.6090E-01		ENSG00000144837
PLA2G10	-0.0197	6.0516E-01		ENSG00000069764
PLA2G10EP	0.0011	7.3263E-01		ENSG00000255037
PLA2G10IP	0.0029	9.1841E-01		ENSG00000276484
PLA2G10KP	0.0258	2.6068E-01		ENSG00000278528
PLA2G12A	-0.0382	5.5399E-01	7.3233E-01	ENSG00000123739
PLA2G12B	0.0346	1.2380E-01		ENSG00000138308

PLA2G15	-0.0871	2.7577E-01	4.8571E-01	ENSG00000103066
PLA2G1B	-0.0449	2.7789E-01		ENSG00000170890
PLA2G2A	0.0004	8.0483E-01		ENSG00000188257
PLA2G2C	0.0120	6.4758E-01	7.9964E-01	ENSG00000187980
PLA2G2D	0.0126	4.0686E-01		ENSG00000117215
PLA2G3	0.1774	4.3419E-02	1.4482E-01	ENSG00000100078
PLA2G4A	0.0219	8.7538E-01	9.3768E-01	ENSG00000116711
PLA2G4B	-0.0614	1.6273E-01		ENSG00000243708
PLA2G4C	-0.0017	9.7907E-01	9.8952E-01	ENSG00000105499
PLA2G4D	0.0045	8.8704E-01		ENSG00000159337
PLA2G4E-AS1	-0.0068	5.7730E-01		ENSG00000246740
PLA2G5	0.0303	4.7479E-01	6.7059E-01	ENSG00000127472
PLA2G6	-0.2102	1.2003E-02	5.8002E-02	ENSG00000184381
PLA2G7	0.0031	9.5008E-01	9.7521E-01	ENSG00000146070
PLA2R1	0.0665	3.4866E-01	5.5845E-01	ENSG00000153246
PLAA	-0.1421	1.8368E-02	7.8085E-02	ENSG00000137055
PLAAT1	0.3832	6.3638E-05	1.0806E-03	ENSG00000127252
PLAAT2	-0.0032	8.5660E-01		ENSG00000133328
PLAAT3	0.0638	4.6453E-01	6.6144E-01	ENSG00000176485
PLAAT4	0.0593	5.4897E-01	7.2827E-01	ENSG00000133321
PLAAT5	-0.0142	7.9093E-01		ENSG00000168004
PLAC1	-0.0001	7.8479E-01	8.8630E-01	ENSG00000170965
PLAC4	0.0094	5.1406E-01		ENSG00000280109
PLAC8	0.0042	9.1400E-01		ENSG00000145287
PLAC8L1	0.0432	4.0335E-01	6.0785E-01	ENSG00000173261
PLAC9	-0.5595	2.2118E-03	1.6235E-02	ENSG00000189129
PLAG1	0.2816	7.4724E-03	4.0730E-02	ENSG00000181690
PLAGL1	0.1303	9.1386E-02	2.4046E-01	ENSG00000118495
PLAGL2	0.1326	4.2887E-02	1.4351E-01	ENSG00000126003
PLAT	0.0987	2.0709E-01	4.0669E-01	ENSG00000104368
PLAU	-0.1644	1.1131E-01	2.7320E-01	ENSG00000122861
PLAUR	-0.1144	1.9971E-01	3.9807E-01	ENSG00000011422
PLB1	0.1780	9.6861E-02	2.4997E-01	ENSG00000163803
PLBD1	0.0454	5.1038E-01	6.9899E-01	ENSG00000121316
PLBD1-AS1	0.0088	9.0981E-01		ENSG00000256751
PLBD2	0.3544	8.4155E-06	2.1347E-04	ENSG00000151176
PLCB1	-0.1869	7.5290E-03	4.0985E-02	ENSG00000182621
PLCB1-IT1	-0.0096	8.3202E-01	9.1332E-01	ENSG00000225479
PLCB2	0.0388	6.4029E-01	7.9415E-01	ENSG00000137841
PLCB3	-0.2524	6.7021E-03	3.7555E-02	ENSG00000149782
PLCB4	0.3364	5.1674E-06	1.4367E-04	ENSG00000101333
PLCD1	-0.1171	1.6209E-01	3.4805E-01	ENSG00000187091
PLCD3	0.0200	8.0780E-01	8.9899E-01	ENSG00000161714
PLCD4	0.0062	9.3774E-01	9.6922E-01	ENSG00000115556
PLCE1	0.1137	1.7066E-01	3.5952E-01	ENSG00000138193
PLCE1-AS1	-0.0010	7.4986E-01		ENSG00000268894
PLCE1-AS2	-0.0129	7.3777E-01		ENSG00000232913
PLCG1	-0.0444	4.9863E-01	6.8961E-01	ENSG00000124181
PLCG1-AS1	0.5635	2.0655E-05	4.3921E-04	ENSG00000226648

PLCG2	0.0071	9.1846E-01	9.5972E-01	ENSG00000197943
PLCH1	-0.0236	7.6605E-01	8.7440E-01	ENSG00000114805
PLCH2	-0.3257	3.2982E-04	3.8595E-03	ENSG00000149527
PLCL1	-0.0757	3.3847E-01	5.4833E-01	ENSG00000115896
PLCL2	0.0418	5.0482E-01	6.9477E-01	ENSG00000154822
PLCXD1	-0.2292	7.6015E-03	4.1226E-02	ENSG00000182378
PLCXD2	-0.3499	1.3390E-05	3.0747E-04	ENSG00000240891
PLCZ1	0.0092	9.8511E-01		ENSG00000139151
PLD1	0.4226	5.4761E-03	3.2235E-02	ENSG00000075651
PLD2	-0.0335	6.3689E-01	7.9196E-01	ENSG00000129219
PLD3	0.0405	5.6467E-01	7.4056E-01	ENSG00000105223
PLD4	0.0050	4.7254E-01		ENSG00000166428
PLD5	0.3499	1.3252E-03	1.1224E-02	ENSG00000180287
PLD6	-0.0856	3.1395E-01	5.2427E-01	ENSG00000179598
PLEC	0.1924	1.9589E-02	8.1759E-02	ENSG00000178209
PLEK	0.0436	1.6718E-01		ENSG00000115956
PLEK2	0.0080	8.8062E-01	9.4028E-01	ENSG00000100558
PLEKHA1	0.2552	4.9564E-05	8.8243E-04	ENSG00000107679
PLEKHA2	-0.2613	1.7061E-02	7.4231E-02	ENSG00000169499
PLEKHA3	-0.1649	4.2151E-03	2.6504E-02	ENSG00000116095
PLEKHA4	-0.0256	7.5460E-01	8.6686E-01	ENSG00000105559
PLEKHA5	0.2138	1.9731E-05	4.2363E-04	ENSG00000052126
PLEKHA6	0.3395	3.8493E-04	4.3406E-03	ENSG00000143850
PLEKHA7	-0.0789	3.2896E-01	5.3931E-01	ENSG00000166689
PLEKHA8	0.1214	5.0311E-02	1.6023E-01	ENSG00000106086
PLEKHA8P1	0.1197	2.0166E-01	4.0028E-01	ENSG00000134297
PLEKHB1	-0.3754	2.0228E-03	1.5202E-02	ENSG00000021300
PLEKHB2	-0.2492	7.4369E-05	1.2223E-03	ENSG00000115762
PLEKHD1	0.0236	7.3241E-01	8.5387E-01	ENSG00000175985
PLEKHF1	-0.1194	2.0141E-01	4.0004E-01	ENSG00000166289
PLEKHF2	-0.3710	1.3897E-03	1.1591E-02	ENSG00000175895
PLEKHG1	0.1202	1.3460E-01	3.0904E-01	ENSG00000120278
PLEKHG2	-0.0962	1.7573E-01	3.6668E-01	ENSG00000090924
PLEKHG3	0.1120	1.9726E-01	3.9490E-01	ENSG00000126822
PLEKHG4	-0.0173	8.2062E-01	9.0691E-01	ENSG00000196155
PLEKHG4B	0.1200	1.0033E-01	2.5521E-01	ENSG00000153404
PLEKHG5	-0.3314	2.9407E-05	5.8539E-04	ENSG00000171680
PLEKHG6	-0.0270	6.7327E-01	8.1722E-01	ENSG00000008323
PLEKHG7	-0.0035	8.7829E-01		ENSG00000187510
PLEKHH1	0.1500	8.0749E-02	2.2088E-01	ENSG00000054690
PLEKHH2	-0.0831	2.9733E-01	5.0727E-01	ENSG00000152527
PLEKHH3	-0.2853	3.2987E-04	3.8595E-03	ENSG00000068137
PLEKHJ1	0.0598	3.1204E-01	5.2208E-01	ENSG00000104886
PLEKHM1	-0.1631	2.9338E-02	1.0942E-01	ENSG00000225190
PLEKHM2	-0.0796	1.3144E-01	3.0452E-01	ENSG00000116786
PLEKHM3	-0.0314	6.4432E-01	7.9692E-01	ENSG00000178385
PLEKHN1	0.0069	7.6982E-01		ENSG00000187583
PLEKHO1	-0.1234	5.8722E-02	1.7802E-01	ENSG00000023902
PLEKHO2	0.2374	3.3613E-02	1.2040E-01	ENSG00000241839

PLET1	0.2101	4.0933E-02	1.3900E-01	ENSG00000188771
PLG	0.0147	8.7768E-01		ENSG00000122194
PLGLB1	0.0626	4.4782E-01	6.4716E-01	ENSG00000183281
PLGLB2	0.0321	6.6944E-01	8.1482E-01	ENSG00000125551
PLGRKT	-0.1446	4.6271E-02	1.5146E-01	ENSG00000107020
PLIN1	0.1052	8.4195E-02	2.2670E-01	ENSG00000166819
PLIN2	-0.1045	1.8858E-01	3.8354E-01	ENSG00000147872
PLIN3	-0.2477	1.3091E-03	1.1122E-02	ENSG00000105355
PLIN4	0.0683	4.1935E-01	6.2209E-01	ENSG00000167676
PLIN5	0.0095	8.7787E-01	9.3909E-01	ENSG00000214456
PLK1	-0.0471	5.7228E-01	7.4561E-01	ENSG00000166851
PLK2	-0.0956	2.5741E-01	4.6591E-01	ENSG00000145632
PLK3	0.0915	1.6215E-01	3.4814E-01	ENSG00000173846
PLK4	-0.1212	1.7613E-01	3.6720E-01	ENSG00000142731
PLK5	-0.0211	7.9847E-01	8.9385E-01	ENSG00000185988
PLLP	-0.4465	5.0113E-04	5.3076E-03	ENSG00000102934
PLN	-0.0914	1.6044E-01	3.4578E-01	ENSG00000198523
PLOD1	-0.0280	7.3138E-01	8.5304E-01	ENSG00000083444
PLOD2	0.2894	7.5597E-03	4.1106E-02	ENSG00000152952
PLOD3	0.0194	7.5997E-01	8.7070E-01	ENSG00000106397
PLP1	-0.0984	2.4870E-01	4.5649E-01	ENSG00000123560
PLP2	-0.4356	1.4621E-06	5.1321E-05	ENSG00000102007
PLPBP	-0.0031	9.4964E-01	9.7496E-01	ENSG00000147471
PLPP1	0.0421	5.2451E-01	7.0999E-01	ENSG00000067113
PLPP3	-0.0822	3.4069E-01	5.5044E-01	ENSG00000162407
PLPP4	-0.1282	1.7171E-01	3.6089E-01	ENSG00000203805
PLPP5	-0.1591	5.7990E-02	1.7655E-01	ENSG00000147535
PLPP6	0.2705	6.1052E-03	3.4967E-02	ENSG00000205808
PLPP7	0.5648	4.8099E-05	8.6134E-04	ENSG00000160539
PLPPR1	-0.5210	3.6159E-06	1.0771E-04	ENSG00000148123
PLPPR2	-0.0307	6.6551E-01	8.1209E-01	ENSG00000105520
PLPPR3	-0.2328	1.3716E-03	1.1475E-02	ENSG00000129951
PLPPR4	-0.0848	3.0298E-01	5.1306E-01	ENSG00000117600
PLPPR5	-0.0563	4.3755E-01	6.3810E-01	ENSG00000117598
PLPPR5-AS1	-0.0261	5.6416E-01	7.4015E-01	ENSG00000232825
PLRG1	0.0916	2.0330E-01	4.0212E-01	ENSG00000171566
PLS1	-0.0651	4.3116E-01	6.3289E-01	ENSG00000120756
PLS3	-0.0219	7.6481E-01	8.7359E-01	ENSG00000102024
PLS3-AS1	0.0070	9.2090E-01	9.6121E-01	ENSG00000271826
PLSCR1	0.0421	5.9994E-01	7.6571E-01	ENSG00000188313
PLSCR2	0.0056	6.4294E-01		ENSG00000163746
PLSCR3	0.0058	9.0600E-01		ENSG00000187838
PLSCR4	-0.0039	9.6512E-01	9.8281E-01	ENSG00000114698
PLSCR5	-0.0044	5.9300E-01		ENSG00000231213
PLTP	0.1338	1.3125E-01	3.0423E-01	ENSG00000100979
PLVAP	-0.0054	4.1233E-01		ENSG00000130300
PLXDC1	-0.1458	8.2383E-02	2.2359E-01	ENSG00000161381
PLXDC2	0.1132	1.3975E-01	3.1613E-01	ENSG00000120594
PLXNA1	-0.0144	8.4832E-01	9.2265E-01	ENSG00000114554

PLXNA2	0.1472	7.5981E-02	2.1232E-01	ENSG00000076356
PLXNA3	-0.0169	7.8383E-01	8.8574E-01	ENSG00000130827
PLXNA4	0.0660	4.1439E-01	6.1800E-01	ENSG00000221866
PLXNB1	0.3795	1.8644E-05	4.0417E-04	ENSG00000164050
PLXNB2	0.1020	2.0433E-01	4.0353E-01	ENSG00000196576
PLXNB3	0.1230	1.9072E-01	3.8595E-01	ENSG00000198753
PLXNC1	-0.0783	3.1435E-01	5.2456E-01	ENSG00000136040
PLXND1	-0.4158	1.0385E-05	2.5325E-04	ENSG00000004399
PM20D1	0.0172	9.1638E-01		ENSG00000162877
PM20D2	-0.3005	1.1030E-04	1.6598E-03	ENSG00000146281
PMAIP1	-0.2187	3.7631E-03	2.4379E-02	ENSG00000141682
PMCH	-0.0403	1.8438E-01	3.7783E-01	ENSG00000183395
PMCHL2	0.0284	3.1431E-01		ENSG00000169040
PMEL	0.3089	1.9557E-02	8.1690E-02	ENSG00000185664
PMEPA1	0.0494	5.4169E-01	7.2346E-01	ENSG00000124225
PMF1	-0.0703	3.0989E-01	5.2012E-01	ENSG00000160783
PMFBP1	0.5116	2.1441E-03	1.5833E-02	ENSG00000118557
PML	-0.0667	3.5482E-01	5.6460E-01	ENSG00000140464
PMM1	-0.0613	2.6574E-01	4.7501E-01	ENSG00000100417
PMM2	0.0050	9.2520E-01	9.6329E-01	ENSG00000140650
PMP2	-0.5414	5.4210E-05	9.4801E-04	ENSG00000147588
PMP22	0.0664	4.1221E-01	6.1603E-01	ENSG00000109099
PMPCA	0.0555	3.6123E-01	5.7076E-01	ENSG00000165688
PMPCB	0.0251	6.1358E-01	7.7529E-01	ENSG00000105819
PMS1	-0.0475	4.7115E-01	6.6739E-01	ENSG00000064933
PMS2	-0.0341	6.0036E-01	7.6598E-01	ENSG00000122512
PMS2CL	-0.1115	5.1067E-02	1.6169E-01	ENSG00000187953
PMS2P12	0.0007	8.8663E-01		ENSG00000228546
PMS2P14	0.0682	4.2857E-01	6.3037E-01	ENSG00000277125
PMVK	-0.0394	3.8075E-01	5.8906E-01	ENSG00000163344
PNCK	-0.0732	3.7069E-01	5.7969E-01	ENSG00000130822
PNISR	-0.0711	2.4555E-01	4.5333E-01	ENSG00000132424
PNKD	0.2347	9.3722E-04	8.6207E-03	ENSG00000127838
PNKP	0.0964	1.8031E-01	3.7240E-01	ENSG00000039650
PNLDC1	-0.0081	5.2195E-01		ENSG00000146453
PNLIP	-0.0851	9.4410E-03	4.8618E-02	ENSG00000175535
PNLIPRP3	0.0045	5.4953E-01		ENSG00000203837
PNMA1	-0.2492	2.2171E-04	2.8206E-03	ENSG00000176903
PNMA2	-0.0201	7.2769E-01	8.5058E-01	ENSG00000240694
PNMA3	0.1767	5.1842E-02	1.6331E-01	ENSG00000183837
PNMA5	0.0752	3.6939E-01	5.7865E-01	ENSG00000198883
PNMA6A	-0.0189	7.8746E-01	8.8772E-01	ENSG00000235961
PNMA6E	0.0014	8.9724E-01		ENSG00000214897
PNMA6F	0.0690	4.0010E-01	6.0552E-01	ENSG00000225110
PNMA8A	-0.0593	3.2178E-01	5.3176E-01	ENSG00000182013
PNMA8B	0.4549	2.6688E-03	1.8676E-02	ENSG00000204851
PNMA8C	0.0916	3.0689E-01	5.1720E-01	ENSG00000277531
PNMT	-0.1631	8.8379E-02	2.3468E-01	ENSG00000141744
PNN	0.3308	9.3862E-06	2.3258E-04	ENSG00000100941

PNO1	0.2066	6.7797E-03	3.7938E-02	ENSG00000115946
PNP	-0.0073	8.8342E-01	9.4147E-01	ENSG00000198805
PNPLA1	0.1033	3.0190E-02		ENSG00000180316
PNPLA2	0.0377	5.8056E-01	7.5202E-01	ENSG00000177666
PNPLA3	-0.2148	6.9346E-03	3.8560E-02	ENSG00000100344
PNPLA4	0.0560	3.8760E-01	5.9524E-01	ENSG00000006757
PNPLA5	0.0487	3.4820E-01	5.5833E-01	ENSG00000100341
PNPLA6	-0.0134	8.6932E-01	9.3433E-01	ENSG00000032444
PNPLA7	0.3081	1.4164E-02	6.5311E-02	ENSG00000130653
PNPLA8	-0.1181	9.0140E-02	2.3823E-01	ENSG00000135241
PNPO	-0.2723	6.9464E-03	3.8585E-02	ENSG00000108439
PNPT1	0.1280	7.2408E-02	2.0558E-01	ENSG00000138035
PNRC1	-0.4824	1.5285E-08	1.1939E-06	ENSG00000146278
PNRC2	0.0422	4.8156E-01	6.7608E-01	ENSG00000189266
POC1A	-0.1842	6.8241E-02	1.9717E-01	ENSG00000164087
POC1B	-0.0418	5.9102E-01	7.5902E-01	ENSG00000139323
POC1B-GALNT4	0.0811	1.7819E-01	3.6944E-01	ENSG00000259075
POC5	0.1459	5.6324E-02	1.7340E-01	ENSG00000152359
PODN	0.0152	8.0857E-01	8.9942E-01	ENSG00000174348
PODNL1	0.1333	1.1257E-01	2.7530E-01	ENSG00000132000
PODXL	-0.0589	4.9645E-01	6.8798E-01	ENSG00000128567
PODXL2	-0.0255	6.8885E-01	8.2709E-01	ENSG00000114631
POF1B	-0.0137	8.1158E-01		ENSG00000124429
POFUT1	0.0956	1.6417E-01	3.5102E-01	ENSG00000101346
POFUT2	0.2872	1.3583E-03	1.1410E-02	ENSG00000186866
POGK	-0.1707	2.0550E-03	1.5368E-02	ENSG00000143157
POGLUT1	-0.0107	8.6138E-01	9.3034E-01	ENSG00000163389
POGLUT2	0.0494	5.2362E-01	7.0931E-01	ENSG00000134901
POGLUT3	-0.1201	1.5832E-01	3.4246E-01	ENSG00000178202
POGZ	0.0103	8.8923E-01	9.4500E-01	ENSG00000143442
POLA1	-0.1718	5.0197E-02	1.6009E-01	ENSG00000101868
POLA2	-0.0239	7.7306E-01	8.7903E-01	ENSG00000014138
POLB	-0.1169	3.4263E-02	1.2188E-01	ENSG00000070501
POLD1	-0.1826	6.4625E-02	1.9018E-01	ENSG00000062822
POLD2	-0.1735	5.4781E-03	3.2238E-02	ENSG00000106628
POLD3	0.0206	7.7276E-01	8.7896E-01	ENSG00000077514
POLD4	0.1477	1.2918E-01	3.0151E-01	ENSG00000175482
POLDIP2	-0.0697	2.3594E-01	4.4161E-01	ENSG00000004142
POLDIP3	-0.0384	5.0553E-01	6.9528E-01	ENSG00000100227
POLE	0.1272	1.1335E-01	2.7671E-01	ENSG00000177084
POLE2	-0.1549	3.7588E-02	1.3082E-01	ENSG00000100479
POLE3	-0.1223	3.8619E-02	1.3348E-01	ENSG00000148229
POLE4	0.0049	9.2949E-01	9.6530E-01	ENSG00000115350
POLG	-0.0834	2.3882E-01	4.4530E-01	ENSG00000140521
POLG2	0.0301	7.0332E-01	8.3621E-01	ENSG00000256525
POLH	0.0729	2.9530E-01	5.0541E-01	ENSG00000170734
POLI	0.2374	1.2776E-04	1.8524E-03	ENSG00000101751
POLK	0.0341	6.1680E-01	7.7788E-01	ENSG00000122008
POLL	0.2311	6.9018E-03	3.8412E-02	ENSG00000166169

POLM	0.0929	2.4441E-01	4.5199E-01	ENSG00000122678
POLN	0.0994	1.1941E-01	2.8650E-01	ENSG00000130997
POLQ	-0.0441	6.0162E-01	7.6665E-01	ENSG00000051341
POLR1A	0.0790	2.4501E-01	4.5268E-01	ENSG00000068654
POLR1B	0.1743	4.6704E-02	1.5243E-01	ENSG00000125630
POLR1C	0.3605	1.7581E-06	5.9998E-05	ENSG00000171453
POLR1D	-0.2297	1.0714E-04	1.6243E-03	ENSG00000186184
POLR1E	-0.1532	2.2743E-02	9.1155E-02	ENSG00000137054
POLR1F	-0.2655	1.0469E-04	1.5940E-03	ENSG00000105849
POLR1G	-0.0110	8.9207E-01	9.4631E-01	ENSG00000117877
POLR1H	0.2593	3.8158E-03	2.4656E-02	ENSG00000066379
POLR1HASP	0.1860	5.8476E-02	1.7748E-01	ENSG00000204623
POLR2A	0.0095	8.9652E-01	9.4857E-01	ENSG00000181222
POLR2B	-0.0185	7.3971E-01	8.5787E-01	ENSG00000047315
POLR2C	-0.0186	7.5487E-01	8.6698E-01	ENSG00000102978
POLR2D	-0.1283	2.9048E-02	1.0857E-01	ENSG00000144231
POLR2E	-0.1726	4.7558E-03	2.8956E-02	ENSG00000099817
POLR2F	-0.1419	3.5063E-02	1.2419E-01	ENSG00000100142
POLR2G	-0.1238	1.1266E-01	2.7543E-01	ENSG00000168002
POLR2H	0.1111	5.1015E-02	1.6156E-01	ENSG00000163882
POLR2I	0.0016	9.8189E-01	9.9046E-01	ENSG00000105258
POLR2J	0.0559	3.0903E-01	5.1924E-01	ENSG00000005075
POLR2J2	-0.1436	7.7590E-02	2.1530E-01	ENSG00000228049
POLR2J3	-0.1143	7.9314E-02	2.1841E-01	ENSG00000285437
POLR2J3-UPK3BL2	0.0614	4.5965E-01	6.5736E-01	ENSG00000168255
POLR2J4	-0.0867	3.2989E-01	5.4011E-01	ENSG00000214783
POLR2K	0.0558	4.4132E-01	6.4093E-01	ENSG00000147669
POLR2L	-0.1153	8.6537E-02	2.3109E-01	ENSG00000177700
POLR2M	-0.2383	1.0077E-04	1.5536E-03	ENSG00000255529
POLR3A	0.0826	1.2984E-01	3.0243E-01	ENSG00000148606
POLR3B	-0.1978	2.4340E-02	9.5780E-02	ENSG00000013503
POLR3C	0.0583	3.2800E-01	5.3839E-01	ENSG00000186141
POLR3D	-0.1180	1.1958E-01	2.8672E-01	ENSG00000168495
POLR3DP1	-0.0079	5.2872E-01		ENSG00000214626
POLR3E	0.1023	9.6634E-02	2.4973E-01	ENSG00000058600
POLR3F	0.1018	1.4074E-01	3.1746E-01	ENSG00000132664
POLR3G	0.1775	3.8204E-02	1.3239E-01	ENSG00000113356
POLR3GL	-0.0498	4.2859E-01	6.3037E-01	ENSG00000121851
POLR3H	-0.0237	7.2079E-01	8.4690E-01	ENSG00000100413
POLR3K	-0.0395	4.8961E-01	6.8252E-01	ENSG00000161980
POLRMT	-0.0126	8.5017E-01	9.2376E-01	ENSG00000099821
POLRMTP1	0.1205	1.4655E-01	3.2626E-01	ENSG00000266066
POM121	0.1516	2.5073E-02	9.7781E-02	ENSG00000196313
POM121C	0.3239	7.2481E-06	1.8932E-04	ENSG00000272391
POM121L15P	0.0317	7.0238E-01	8.3561E-01	ENSG00000161103
POMC	-0.0276	2.7581E-01	4.8571E-01	ENSG00000115138
POMGNT1	-0.0154	7.9983E-01	8.9433E-01	ENSG00000085998
POMGNT2	-0.0649	2.9767E-01	5.0756E-01	ENSG00000144647
POMK	-0.0950	1.6503E-01	3.5223E-01	ENSG00000185900

POMP	-0.1824	1.2996E-02	6.1303E-02	ENSG00000132963
POMT1	0.1856	6.5047E-04	6.5729E-03	ENSG00000130714
POMT2	0.0675	3.4340E-01	5.5297E-01	ENSG00000009830
POMZP3	-0.0280	7.2322E-01	8.4820E-01	ENSG00000146707
PON1	0.2800	2.5872E-02	9.9980E-02	ENSG00000005421
PON2	-0.0343	6.7384E-01	8.1745E-01	ENSG00000105854
PON3	0.0154	3.3680E-01		ENSG00000105852
POP1	0.3369	2.6988E-03	1.8838E-02	ENSG00000104356
POP4	-0.0618	3.3043E-01	5.4072E-01	ENSG00000105171
POP5	-0.0708	1.8580E-01	3.7983E-01	ENSG00000167272
POP7	-0.2293	1.0543E-03	9.4506E-03	ENSG00000172336
POPDC2	0.2612	8.0763E-03	4.3178E-02	ENSG00000121577
POPDC3	0.3251	8.3315E-03	4.4228E-02	ENSG00000132429
POR	-0.0329	6.5589E-01	8.0558E-01	ENSG00000127948
PORCN	0.0193	7.2536E-01	8.4935E-01	ENSG00000102312
PORCN-DT	0.2119	5.9673E-02	1.7959E-01	ENSG00000224292
POT1	0.3944	8.1146E-07	3.1542E-05	ENSG00000128513
POT1-AS1	0.4776	1.9417E-04	2.5361E-03	ENSG00000224897
POTED	0.0199	2.5879E-01		ENSG00000166351
POTEE	-0.4997	4.7652E-06	1.3602E-04	ENSG00000188219
POTEF	-0.4004	1.5659E-04	2.1470E-03	ENSG00000196604
POTEH	0.0072	5.9542E-01		ENSG00000198062
POTEI	-0.4850	1.4781E-04	2.0589E-03	ENSG00000196834
POTEJ	-0.4749	1.4135E-04	1.9832E-03	ENSG00000222038
POU1F1	0.0065	7.6575E-01		ENSG00000064835
POU2AF1	-0.0108	5.5956E-01		ENSG00000110777
POU2AF3	0.1543	1.2041E-01	2.8806E-01	ENSG00000214290
POU2F1	0.0701	3.7341E-01	5.8202E-01	ENSG00000143190
POU2F3	0.0306	4.8645E-01		ENSG00000137709
POU3F1	-0.0267	7.0747E-01	8.3915E-01	ENSG00000185668
POU3F2	0.2930	4.5807E-03	2.8177E-02	ENSG00000184486
POU3F3	-0.0784	2.6739E-01	4.7672E-01	ENSG00000198914
POU3F4	-0.0003	9.9547E-01	9.9750E-01	ENSG00000196767
POU4F1	0.1022	2.0450E-01	4.0370E-01	ENSG00000152192
POU4F2	-0.0303	6.8799E-01	8.2673E-01	ENSG00000151615
POU4F3	0.0074	9.0651E-01	9.5429E-01	ENSG00000091010
POU5F1	-0.0374	4.9866E-01		ENSG00000204531
POU5F1B	-0.0074	3.4665E-01		ENSG00000212993
POU5F1P3	0.0170	6.6668E-01		ENSG00000235602
POU5F1P4	0.0216	5.0410E-01		ENSG00000237872
POU5F1P6	0.0113	2.7984E-01	4.8989E-01	ENSG00000242551
POU5F2	0.0941	2.6975E-01	4.7928E-01	ENSG00000248483
POU6F1	0.0920	2.3242E-01	4.3747E-01	ENSG00000184271
POU6F2	0.2868	1.2924E-02	6.1070E-02	ENSG00000106536
PP12613	0.0285	2.1716E-01		ENSG00000226757
PP2D1	-0.5643	1.4045E-03	1.1675E-02	ENSG00000183977
PPA1	-0.2503	8.6573E-07	3.3025E-05	ENSG00000180817
PPA2	-0.1752	2.7817E-03	1.9269E-02	ENSG00000138777
PPAN	0.0827	3.2517E-01	5.3521E-01	ENSG00000130810

PPARA	0.1574	6.5438E-02	1.9170E-01	ENSG00000186951
PPARD	0.0449	5.4459E-01	7.2532E-01	ENSG00000112033
PPARG	-0.1760	8.8906E-02	2.3560E-01	ENSG00000132170
PPARGC1A	-0.2130	1.3240E-02	6.2192E-02	ENSG00000109819
PPARGC1B	0.0718	3.7248E-01	5.8123E-01	ENSG00000155846
PPAT	-0.0332	6.3673E-01	7.9185E-01	ENSG00000128059
PPATP1	-0.0016	9.6492E-01		ENSG00000241293
PPCDC	0.1302	1.3114E-01	3.0419E-01	ENSG00000138621
PPCS	-0.0178	7.2761E-01	8.5057E-01	ENSG00000127125
PPDPF	-0.4667	5.8205E-07	2.4029E-05	ENSG00000125534
PPDPFL	0.0194	3.9563E-01		ENSG00000168333
PPEF1	-0.0434	5.8842E-01	7.5725E-01	ENSG00000086717
PPEF2	-0.0018	9.3362E-01		ENSG00000156194
PPFIA1	0.1641	1.1541E-02	5.6328E-02	ENSG00000131626
PPFIA2	0.0757	3.4027E-01	5.5001E-01	ENSG00000139220
PPFIA3	0.0618	3.4132E-01	5.5110E-01	ENSG00000177380
PPFIA4	0.5155	1.4196E-05	3.2432E-04	ENSG00000143847
PPFIBP1	-0.1439	5.4813E-02	1.7020E-01	ENSG00000110841
PPFIBP2	0.0630	4.6314E-01	6.6036E-01	ENSG00000166387
PPHLN1	-0.0333	5.8411E-01	7.5417E-01	ENSG00000134283
PPIA	-0.3653	1.1896E-07	6.6373E-06	ENSG00000196262
PPIAL4C	-0.0526	1.4554E-01	3.2449E-01	ENSG00000288867
PPIAL4G	-0.0784	9.9832E-02	2.5436E-01	ENSG00000236334
PPIAL4H	0.0085	5.7843E-01		ENSG00000270339
PPIAP20	-0.0124	7.2274E-01		ENSG00000235686
PPIAP30	-0.5724	7.3345E-03	4.0188E-02	ENSG00000206448
PPIAP36	0.0051	9.7516E-01		ENSG00000236872
PPIAP41	0.1546	2.8843E-02	1.0805E-01	ENSG00000254463
PPIAP46	0.0076	8.2028E-01		ENSG00000260266
PPIAP49	-0.0134	3.1672E-01		ENSG00000260471
PPIAP50	0.0175	9.9111E-01		ENSG00000261330
PPIAP51	-0.0437	4.9069E-01	6.8335E-01	ENSG00000260229
PPIAP52	-0.0133	7.9064E-01	8.8927E-01	ENSG00000270606
PPIAP64	0.0082	6.0178E-01		ENSG00000236259
PPIAP68	-0.0165	4.9770E-01		ENSG00000213739
PPIAP69	-0.0493	1.7857E-01		ENSG00000228337
PPIB	-0.2611	2.6582E-06	8.3851E-05	ENSG00000166794
PPIC	-0.0487	5.6543E-01	7.4090E-01	ENSG00000168938
PPID	-0.3831	6.6476E-06	1.7606E-04	ENSG00000171497
PPIE	-0.0728	1.9080E-01	3.8601E-01	ENSG00000084072
PPIF	-0.1251	2.5053E-02	9.7767E-02	ENSG00000108179
PPIG	-0.0705	1.9946E-01	3.9778E-01	ENSG00000138398
PPIH	-0.0468	3.2037E-01	5.3039E-01	ENSG00000171960
PPIL1	0.0129	8.5484E-01	9.2614E-01	ENSG00000137168
PPIL2	0.1449	1.8441E-02	7.8321E-02	ENSG00000100023
PPIL3	0.1396	3.2617E-02	1.1785E-01	ENSG00000240344
PPIL4	0.1838	8.9557E-03	4.6830E-02	ENSG00000131013
PPIL6	-0.0555	4.3143E-01	6.3296E-01	ENSG00000185250
PPIP5K1	0.0150	8.4267E-01	9.1913E-01	ENSG00000168781

PPIP5K2	0.0101	8.9324E-01	9.4692E-01	ENSG00000145725
PPL	-0.0567	5.0744E-01	6.9644E-01	ENSG00000118898
PPM1A	-0.0508	4.1293E-01	6.1662E-01	ENSG00000100614
PPM1AP1	-0.0105	8.3260E-01	9.1366E-01	ENSG00000250483
PPM1B	0.2245	1.9125E-04	2.5137E-03	ENSG00000138032
PPM1D	-0.3520	6.2170E-05	1.0652E-03	ENSG00000170836
PPM1E	-0.2845	3.0450E-05	5.9937E-04	ENSG00000175175
PPM1F	0.1509	5.6036E-02	1.7291E-01	ENSG00000100034
PPM1G	0.0167	7.7497E-01	8.8026E-01	ENSG00000115241
PPM1H	0.1162	2.0325E-01	4.0212E-01	ENSG00000111110
PPM1J	0.0748	3.7420E-01	5.8281E-01	ENSG00000155367
PPM1K	0.1086	8.4412E-02	2.2711E-01	ENSG00000163644
PPM1K-DT	0.0709	6.2101E-01	7.8072E-01	ENSG00000246375
PPM1L	-0.1996	5.7584E-03	3.3503E-02	ENSG00000163590
PPM1M	-0.3762	9.3855E-04	8.6246E-03	ENSG00000164088
PPM1N	-0.3899	7.9362E-03	4.2621E-02	ENSG00000213889
PPME1	-0.0151	7.7644E-01	8.8102E-01	ENSG00000214517
PPOX	0.1164	9.2970E-02	2.4323E-01	ENSG00000143224
PPP1CA	-0.1414	1.7355E-02	7.5179E-02	ENSG00000172531
PPP1CB	-0.1286	1.6370E-02	7.2170E-02	ENSG00000213639
PPP1CC	-0.1185	1.4389E-02	6.5991E-02	ENSG00000186298
PPP1R10	0.1223	7.9321E-02	2.1841E-01	ENSG00000204569
PPP1R11	-0.0536	3.2170E-01	5.3176E-01	ENSG00000204619
PPP1R12A	0.1286	1.4337E-02	6.5788E-02	ENSG00000058272
PPP1R12B	-0.0176	7.8023E-01	8.8343E-01	ENSG00000077157
PPP1R12C	0.2510	2.2531E-03	1.6480E-02	ENSG00000125503
PPP1R13B	0.0444	5.2745E-01	7.1240E-01	ENSG00000088808
PPP1R13B-DT	0.0152	6.8299E-01		ENSG00000258735
PPP1R13L	0.0097	9.0764E-01	9.5461E-01	ENSG00000104881
PPP1R14B	-0.5094	6.1370E-07	2.5125E-05	ENSG00000173457
PPP1R14B-AS1	0.0013	8.6693E-01		ENSG00000256940
PPP1R14C	-0.1546	1.0131E-01	2.5697E-01	ENSG00000198729
PPP1R14D	-0.0100	6.3022E-01		ENSG00000166143
PPP1R15A	-0.5090	5.6733E-10	6.8765E-08	ENSG00000087074
PPP1R15B	-0.1822	9.7182E-03	4.9692E-02	ENSG00000158615
PPP1R16A	0.0943	1.9385E-01	3.9029E-01	ENSG00000160972
PPP1R16B	-0.2957	3.2900E-03	2.1955E-02	ENSG00000101445
PPP1R17	0.1903	7.1838E-02	2.0452E-01	ENSG00000106341
PPP1R18	-0.0159	8.2958E-01	9.1184E-01	ENSG00000146112
PPP1R1A	-0.0306	6.2615E-01	7.8440E-01	ENSG00000135447
PPP1R1B	-0.4625	3.5263E-05	6.7731E-04	ENSG00000131771
PPP1R1C	0.3117	6.3183E-03	3.5878E-02	ENSG00000150722
PPP1R2	-0.2308	2.8276E-04	3.4323E-03	ENSG00000184203
PPP1R21	-0.2191	6.0238E-04	6.1552E-03	ENSG00000162869
PPP1R26	0.0034	9.7663E-01	9.8837E-01	ENSG00000196422
PPP1R26-AS1	-0.0331	6.6910E-01	8.1461E-01	ENSG00000225361
PPP1R26P1	0.1199	6.5944E-02	1.9284E-01	ENSG00000238086
PPP1R27	-0.0622	2.1310E-01	4.1417E-01	ENSG00000182676
PPP1R2B	0.0073	8.9448E-01	9.4747E-01	ENSG00000231989

PPP1R35	0.0220	7.6181E-01	8.7195E-01	ENSG00000160813
PPP1R36	-0.0024	6.7258E-01		ENSG00000165807
PPP1R37	-0.1419	1.0239E-01	2.5885E-01	ENSG00000104866
PPP1R3A	0.1373	2.1031E-02	8.5960E-02	ENSG00000154415
PPP1R3B	0.0585	4.7950E-01	6.7454E-01	ENSG00000173281
PPP1R3C	-0.0781	3.7378E-01	5.8237E-01	ENSG00000119938
PPP1R3D	-0.0374	6.3257E-01	7.8899E-01	ENSG00000132825
PPP1R3E	0.1393	5.4861E-02	1.7032E-01	ENSG00000235194
PPP1R3F	-0.0700	3.5325E-01	5.6341E-01	ENSG00000049769
PPP1R3G	-0.0304	6.5555E-01	8.0547E-01	ENSG00000219607
PPP1R7	-0.2583	3.2627E-05	6.3612E-04	ENSG00000115685
PPP1R8	-0.3676	9.4642E-09	7.9477E-07	ENSG00000117751
PPP1R8P1	0.5738	9.5437E-03	4.9024E-02	ENSG00000224986
PPP1R9A	0.1082	1.4810E-01	3.2844E-01	ENSG00000158528
PPP1R9A-AS1	0.0914	2.7259E-01	4.8197E-01	ENSG00000236197
PPP1R9B	-0.1098	2.2632E-01	4.3032E-01	ENSG00000108819
PPP2CA	-0.2300	1.2599E-03	1.0775E-02	ENSG00000113575
PPP2CB	-0.0435	5.6277E-01	7.3940E-01	ENSG00000104695
PPP2R1A	-0.1630	2.0527E-02	8.4446E-02	ENSG00000105568
PPP2R1B	-0.1253	8.9822E-02	2.3749E-01	ENSG00000137713
PPP2R2A	0.0591	3.7004E-01	5.7930E-01	ENSG00000221914
PPP2R2B	-0.2092	8.5076E-04	8.0172E-03	ENSG00000156475
PPP2R2C	-0.3407	3.3187E-04	3.8774E-03	ENSG00000074211
PPP2R2D	-0.1632	6.2921E-03	3.5771E-02	ENSG00000175470
PPP2R3A	-0.0158	7.8852E-01	8.8805E-01	ENSG00000073711
PPP2R3B	-0.2418	3.7162E-04	4.2216E-03	ENSG00000167393
PPP2R3C	-0.1283	2.4408E-02	9.5950E-02	ENSG00000092020
PPP2R5A	-0.1078	1.9960E-01	3.9799E-01	ENSG00000066027
PPP2R5B	-0.0581	4.6156E-01	6.5878E-01	ENSG00000068971
PPP2R5C	0.0984	7.3880E-02	2.0835E-01	ENSG00000078304
PPP2R5CP	0.0210	7.2678E-01		ENSG00000239557
PPP2R5D	-0.1886	3.1453E-02	1.1506E-01	ENSG00000112640
PPP2R5E	-0.1006	4.8811E-02	1.5685E-01	ENSG00000154001
PPP3CA	0.0843	2.8990E-01	5.0035E-01	ENSG00000138814
PPP3CB	0.0656	3.8572E-01	5.9356E-01	ENSG00000107758
PPP3CB-AS1	0.0038	9.5876E-01	9.7939E-01	ENSG00000221817
PPP3CC	0.0027	9.5712E-01	9.7880E-01	ENSG00000120910
PPP3R1	-0.1833	4.6459E-02	1.5189E-01	ENSG00000221823
PPP4C	-0.1059	7.7597E-02	2.1530E-01	ENSG00000149923
PPP4R1	0.0861	1.4216E-01	3.1944E-01	ENSG00000154845
PPP4R1-AS1	0.1141	1.3160E-01	3.0473E-01	ENSG00000263627
PPP4R2	-0.1892	1.5139E-02	6.8423E-02	ENSG00000163605
PPP4R3A	-0.1951	1.5123E-03	1.2317E-02	ENSG00000100796
PPP4R3B	0.0337	4.5850E-01	6.5631E-01	ENSG00000275052
PPP4R4	0.1863	5.1430E-02	1.6253E-01	ENSG00000119698
PPP5C	-0.1656	6.8351E-03	3.8118E-02	ENSG00000011485
PPP6C	-0.0239	6.4814E-01	7.9997E-01	ENSG00000119414
PPP6R1	-0.0297	6.9256E-01	8.2947E-01	ENSG00000105063
PPP6R2	-0.0085	8.7307E-01	9.3632E-01	ENSG00000100239

PPP6R3	0.0912	1.3771E-01	3.1342E-01	ENSG00000110075
PPRC1	0.1399	7.5833E-02	2.1215E-01	ENSG00000148840
PPT1	-0.0076	9.0417E-01	9.5319E-01	ENSG00000131238
PPT2	-0.0053	9.2771E-01	9.6456E-01	ENSG00000221988
PPT2-EGFL8	0.0238	6.7195E-01	8.1642E-01	ENSG00000258388
PPTC7	-0.4443	1.1994E-10	1.9585E-08	ENSG00000196850
PPWD1	0.1046	1.5852E-01	3.4276E-01	ENSG00000113593
PPY	0.0755	8.1079E-02	2.2134E-01	ENSG00000108849
PQBP1	-0.1381	1.0411E-02	5.2190E-02	ENSG00000102103
PRADC1	0.1880	1.1794E-02	5.7304E-02	ENSG00000135617
PRAF2	0.0435	5.1056E-01	6.9914E-01	ENSG00000243279
PRAG1	-0.1078	1.8340E-01	3.7665E-01	ENSG00000275342
PRAM1	0.0380	4.5819E-01	6.5617E-01	ENSG00000133246
PRAME	0.0197	5.5769E-01		ENSG00000185686
PRANCR	0.2129	1.6952E-02	7.3877E-02	ENSG00000257815
PRAP1	0.0402	3.6867E-01		ENSG00000165828
PRB1	0.0018	7.7054E-01		ENSG00000251655
PRB2	-0.0035	6.2735E-01		ENSG00000121335
PRB3	0.0638	2.7385E-01	4.8366E-01	ENSG00000197870
PRC1	-0.0181	8.4160E-01	9.1850E-01	ENSG00000198901
PRC1-AS1	-0.0749	3.8722E-01	5.9491E-01	ENSG00000258725
PRCC	-0.1950	1.9826E-03	1.4978E-02	ENSG00000143294
PRCD	-0.1096	2.2999E-01	4.3461E-01	ENSG00000214140
PRCP	0.0434	5.1659E-01	7.0423E-01	ENSG00000137509
PRDM1	-0.0067	8.6787E-01	9.3343E-01	ENSG00000057657
PRDM10	0.3490	1.4284E-03	1.1834E-02	ENSG00000170325
PRDM11	0.1828	5.7214E-02	1.7522E-01	ENSG00000019485
PRDM12	0.0964	2.5211E-01	4.6034E-01	ENSG00000130711
PRDM13	0.1184	1.4948E-01	3.3020E-01	ENSG00000112238
PRDM14	0.0089	6.7431E-01		ENSG00000147596
PRDM15	0.1025	2.3520E-01	4.4062E-01	ENSG00000141956
PRDM16	0.0979	2.6514E-01	4.7431E-01	ENSG00000142611
PRDM2	0.2025	5.4068E-04	5.6442E-03	ENSG00000116731
PRDM4	-0.0139	8.0093E-01	8.9499E-01	ENSG00000110851
PRDM4-AS1	-0.3417	4.0392E-03	2.5767E-02	ENSG00000258136
PRDM5	0.2882	1.2903E-02	6.0984E-02	ENSG00000138738
PRDM6	-0.0039	9.9458E-01		ENSG00000061455
PRDM7	-0.0223	4.0207E-01		ENSG00000126856
PRDM8	-0.0431	5.7847E-01	7.4999E-01	ENSG00000152784
PRDX1	-0.1087	2.2094E-01	4.2412E-01	ENSG00000117450
PRDX2	-0.3105	1.6012E-06	5.5723E-05	ENSG00000167815
PRDX3	-0.1266	5.9056E-02	1.7856E-01	ENSG00000165672
PRDX4	0.1008	1.0694E-01	2.6609E-01	ENSG00000123131
PRDX5	-0.1802	1.3259E-03	1.1226E-02	ENSG00000126432
PRDX6	0.0042	9.9882E-01	9.9931E-01	ENSG00000117592
PRDX6-AS1	0.1877	2.7775E-02	1.0515E-01	ENSG00000203739
PREB	0.0389	5.8390E-01	7.5414E-01	ENSG00000138073
PRECSIT	0.1646	4.2730E-02	1.4314E-01	ENSG00000255874
PRELID1	-0.0333	6.1541E-01	7.7684E-01	ENSG00000169230

PRELID1P1	0.0406	4.6484E-01	6.6150E-01	ENSG00000217325
PRELID2	0.1589	3.8111E-02	1.3222E-01	ENSG00000186314
PRELID3A	-0.1258	1.7810E-01	3.6944E-01	ENSG00000141391
PRELID3B	-0.1673	1.7317E-02	7.5030E-02	ENSG00000101166
PRELID3BP5	0.0061	9.1285E-01		ENSG00000251656
PRELID3BP8	0.0107	5.5309E-01		ENSG00000270741
PRELP	-0.1933	6.7752E-02	1.9628E-01	ENSG00000188783
PREP	0.0546	3.8585E-01	5.9366E-01	ENSG00000085377
PREPL	-0.0086	8.9137E-01	9.4601E-01	ENSG00000138078
PREX1	-0.1267	1.6771E-01	3.5599E-01	ENSG00000124126
PREX2	-0.0604	4.8044E-01	6.7535E-01	ENSG00000046889
PRF1	0.0087	8.0298E-01		ENSG00000180644
PRG2	-0.0010	8.4780E-01		ENSG00000186652
PRG3	-0.0300	2.8402E-01		ENSG00000156575
PRG4	0.0226	7.0427E-01	8.3684E-01	ENSG00000116690
PRH1	0.4039	5.6139E-05	9.7550E-04	ENSG00000231887
PRH1	0.0270	3.4407E-01		ENSG00000111215
PRH1-PRR4	-0.0050	9.3645E-01	9.6866E-01	ENSG00000275778
PRH2	0.1008	1.8960E-01	3.8470E-01	ENSG00000134551
PRICKLE1	0.0819	3.2386E-01	5.3397E-01	ENSG00000139174
PRICKLE2	-0.0306	6.7278E-01	8.1702E-01	ENSG00000163637
PRICKLE2-AS1	0.0178	7.3852E-01		ENSG00000241111
PRICKLE2-AS3	0.0154	9.3922E-01	9.7005E-01	ENSG00000226017
PRICKLE2-DT	0.0124	5.3622E-01		ENSG00000244564
PRICKLE3	-0.0292	6.7189E-01	8.1641E-01	ENSG00000012211
PRICKLE4	0.0167	4.2247E-01		ENSG00000278224
PRIM1	-0.3881	2.2008E-05	4.6283E-04	ENSG00000198056
PRIM2	0.0921	1.8167E-01	3.7421E-01	ENSG00000146143
PRIM2BP	0.0902	1.9714E-01	3.9475E-01	ENSG00000283453
PRIMA1	0.1412	7.6232E-02	2.1278E-01	ENSG00000175785
PRIMPOL	-0.3119	4.9149E-04	5.2419E-03	ENSG00000164306
PRKAA1	0.0327	6.1108E-01	7.7343E-01	ENSG00000132356
PRKAA2	0.0569	4.3656E-01	6.3728E-01	ENSG00000162409
PRKAB1	0.0066	9.3077E-01	9.6586E-01	ENSG00000111725
PRKAB2	-0.1076	9.7178E-02	2.5043E-01	ENSG00000131791
PRKACA	0.1167	1.7939E-01	3.7104E-01	ENSG00000072062
PRKACB	-0.0474	4.3088E-01	6.3264E-01	ENSG00000142875
PRKAG1	0.1018	2.4292E-01	4.5067E-01	ENSG00000181929
PRKAG2	0.0812	2.2002E-01	4.2267E-01	ENSG00000106617
PRKAR1A	-0.0299	6.4853E-01	8.0026E-01	ENSG00000108946
PRKAR1B	-0.1425	6.1393E-02	1.8325E-01	ENSG00000188191
PRKAR2A	0.0186	7.9272E-01	8.9051E-01	ENSG00000114302
PRKAR2A-AS1	0.3012	1.2749E-02	6.0489E-02	ENSG00000224424
PRKAR2B	-0.0172	7.8743E-01	8.8772E-01	ENSG00000005249
PRKCA	-0.1293	1.2845E-01	3.0018E-01	ENSG00000154229
PRKCB	-0.3452	9.1736E-04	8.4919E-03	ENSG00000166501
PRKCD	-0.1597	4.5669E-02	1.5016E-01	ENSG00000163932
PRKCE	-0.2178	9.9897E-03	5.0690E-02	ENSG00000171132
PRKCG	-0.0829	2.8914E-01	4.9962E-01	ENSG00000126583

PRKCH	-0.2295	4.7575E-02	1.5442E-01	ENSG00000027075
PRKCI	-0.3130	2.2168E-07	1.1086E-05	ENSG00000163558
PRKCQ	-0.0893	3.1450E-01	5.2456E-01	ENSG00000065675
PRKCQ-AS1	-0.1275	1.3052E-01	3.0339E-01	ENSG00000237943
PRKCSH	-0.0504	4.3927E-01	6.3935E-01	ENSG00000130175
PRKCZ	-0.1524	7.6750E-02	2.1367E-01	ENSG00000067606
PRKCZ-AS1	-0.0903	3.1454E-01	5.2456E-01	ENSG00000182873
PRKD1	-0.2252	1.7739E-02	7.6285E-02	ENSG00000184304
PRKD2	0.0022	9.7388E-01	9.8718E-01	ENSG00000105287
PRKD3	-0.1259	1.2215E-01	2.9098E-01	ENSG00000115825
PRKDC	0.0611	4.1769E-01	6.2109E-01	ENSG00000253729
PRKG1	-0.0556	5.1427E-01	7.0197E-01	ENSG00000185532
PRKG2	0.1512	1.0438E-01	2.6202E-01	ENSG00000138669
PRKN	0.1274	7.3150E-02	2.0712E-01	ENSG00000185345
PRKRA	-0.0565	2.7143E-01	4.8061E-01	ENSG00000180228
PRKRIP1	0.3010	8.5032E-07	3.2793E-05	ENSG00000128563
PRKX	-0.2307	1.4770E-04	2.0589E-03	ENSG00000183943
PRKX-AS1	0.0131	8.6085E-01		ENSG00000236188
PRKY	-0.0187	6.3743E-01		ENSG00000099725
PRL	0.0917	2.2818E-01	4.3287E-01	ENSG00000172179
PRLHR	0.0299	5.7108E-01	7.4476E-01	ENSG00000119973
PRLR	-0.0444	6.0214E-01	7.6698E-01	ENSG00000113494
PRMT1	-0.2149	6.9657E-05	1.1604E-03	ENSG00000126457
PRMT2	-0.0037	9.3575E-01	9.6833E-01	ENSG00000160310
PRMT3	0.0010	9.9037E-01	9.9461E-01	ENSG00000185238
PRMT5	-0.0872	1.7941E-01	3.7104E-01	ENSG00000100462
PRMT5-AS1	0.0895	3.1709E-01	5.2724E-01	ENSG00000237054
PRMT5-DT	0.0317	6.9054E-01	8.2817E-01	ENSG00000257285
PRMT6	0.1100	1.2966E-01	3.0219E-01	ENSG00000198890
PRMT7	0.0414	5.5293E-01	7.3140E-01	ENSG00000132600
PRMT8	0.2577	1.7590E-02	7.5877E-02	ENSG00000111218
PRMT9	0.0652	3.0788E-01	5.1816E-01	ENSG00000164169
PRNCR1	0.1928	7.5159E-02	2.1075E-01	ENSG00000282961
PRND	0.0055	9.4768E-01	9.7425E-01	ENSG00000171864
PRNP	-0.3428	5.3193E-05	9.3405E-04	ENSG00000171867
PRO1804	0.0090	8.8462E-01		ENSG00000278873
PROB1	-0.0088	9.2347E-01	9.6226E-01	ENSG00000228672
PROC	-0.0240	7.1727E-01	8.4462E-01	ENSG00000115718
PROCA1	0.2088	4.0160E-02	1.3715E-01	ENSG00000167525
PROCR	-0.0529	5.2081E-01	7.0760E-01	ENSG00000101000
PRODH	0.2037	2.6943E-02	1.0289E-01	ENSG00000277196
PRODH	0.1696	7.3811E-02	2.0823E-01	ENSG00000100033
PRODH2	0.0180	4.7992E-01		ENSG00000250799
PRODHLP	0.0309	6.9893E-01	8.3364E-01	ENSG00000161132
PROK1	0.0694	8.5472E-02		ENSG00000143125
PROK2	-0.3072	1.6237E-02	7.1712E-02	ENSG00000163421
PROKR1	-0.1326	9.7678E-02	2.5111E-01	ENSG00000169618
PROM1	-0.1227	1.6394E-01	3.5067E-01	ENSG00000007062
PROM2	-0.0550	4.4822E-01	6.4746E-01	ENSG00000155066

PRORP	-0.0182	4.7555E-01		ENSG00000258790
PRORP	-0.0730	4.0118E-01	6.0633E-01	ENSG00000100890
PRORSD1P	0.1528	1.2510E-01	2.9524E-01	ENSG00000162997
PROS1	-0.0177	8.2960E-01	9.1184E-01	ENSG00000184500
PROSER1	0.0128	8.4099E-01	9.1819E-01	ENSG00000120685
PROSER2	-0.1759	5.7726E-02	1.7617E-01	ENSG00000148426
PROSER2-AS1	-0.0732	1.6776E-01	3.5602E-01	ENSG00000225778
PROSER3	-0.0120	8.5086E-01	9.2412E-01	ENSG00000167595
PROX1	-0.4927	1.2316E-05	2.8762E-04	ENSG00000117707
PROX1-AS1	0.0023	9.8321E-01	9.9105E-01	ENSG00000230461
PROX2	-0.0660	3.4585E-02		ENSG00000119608
PROZ	0.0095	5.6525E-01		ENSG00000126231
PRPF18	0.0883	1.2227E-01	2.9109E-01	ENSG00000165630
PRPF19	-0.2402	3.2692E-03	2.1858E-02	ENSG00000110107
PRPF3	0.0807	2.5657E-01	4.6497E-01	ENSG00000117360
PRPF31	-0.2088	1.5345E-04	2.1175E-03	ENSG00000105618
PRPF38A	0.1367	3.6312E-02	1.2727E-01	ENSG00000134748
PRPF38B	-0.0353	5.6984E-01	7.4374E-01	ENSG00000134186
PRPF39	-0.2251	2.6437E-03	1.8527E-02	ENSG00000185246
PRPF39-DT	0.0238	7.4703E-02		ENSG00000249163
PRPF4	0.2355	1.9643E-03	1.4876E-02	ENSG00000136875
PRPF40A	-0.0166	6.4519E-01	7.9748E-01	ENSG00000196504
PRPF40B	-0.3330	6.7460E-04	6.7566E-03	ENSG00000110844
PRPF4B	0.1603	7.2602E-03	3.9901E-02	ENSG00000112739
PRPF6	0.1320	3.1882E-02	1.1598E-01	ENSG00000101161
PRPF8	0.1536	4.6404E-03	2.8470E-02	ENSG00000174231
PRPH	-0.0224	7.5674E-01	8.6825E-01	ENSG00000135406
PRPH2	0.0051	8.5318E-01		ENSG00000112619
PRPS1	-0.2380	7.5513E-04	7.3510E-03	ENSG00000147224
PRPS1L1	0.0041	7.3515E-01		ENSG00000229937
PRPS2	-0.1376	1.0461E-01	2.6242E-01	ENSG00000101911
PRPSAP1	-0.0013	9.8060E-01	9.9002E-01	ENSG00000161542
PRPSAP2	-0.0931	8.1237E-02	2.2165E-01	ENSG00000141127
PRR11	-0.1985	6.6128E-02	1.9326E-01	ENSG00000068489
PRR12	-0.0596	4.8682E-01	6.8026E-01	ENSG00000126464
PRR13	-0.0457	5.0930E-01	6.9819E-01	ENSG00000205352
PRR14	-0.1921	1.3804E-02	6.4120E-02	ENSG00000156858
PRR14L	0.1935	1.9256E-03	1.4637E-02	ENSG00000183530
PRR15	0.0475	2.7228E-01		ENSG00000176532
PRR15L	-0.0234	2.2465E-01		ENSG00000167183
PRR16	0.5920	4.0182E-05	7.5467E-04	ENSG00000184838
PRR18	0.1217	1.8339E-01	3.7665E-01	ENSG00000176381
PRR20G	0.0264	1.4364E-01		ENSG00000239620
PRR22	0.0222	6.9928E-01	8.3370E-01	ENSG00000212123
PRR23D1	0.0475	5.2084E-01	7.0760E-01	ENSG00000255251
PRR23D2	0.0766	2.7478E-01	4.8458E-01	ENSG00000255378
PRR29	0.0636	3.9090E-01	5.9796E-01	ENSG00000224383
PRR29-AS1	0.0469	3.0977E-01		ENSG00000264954
PRR3	0.1018	9.0978E-02	2.3969E-01	ENSG00000204576

PRR32	0.0359	3.8678E-01		ENSG00000183631
PRR35	-0.0575	3.4288E-01	5.5238E-01	ENSG00000161992
PRR36	-0.0588	3.4227E-01	5.5190E-01	ENSG00000183248
PRR5	-0.1758	7.6703E-02	2.1361E-01	ENSG00000186654
PRR5-ARHGAP8	-0.0205	4.0740E-01		ENSG00000248405
PRR5L	-0.1618	1.0965E-01	2.7064E-01	ENSG00000135362
PRR7	-0.0068	8.9804E-01	9.4955E-01	ENSG00000131188
PRRC1	-0.1047	1.1569E-01	2.8037E-01	ENSG00000164244
PRRC2A	-0.2644	6.5782E-03	3.7055E-02	ENSG00000204469
PRRC2B	-0.1156	9.9292E-02	2.5351E-01	ENSG00000288701
PRRC2C	0.2093	3.2224E-05	6.2877E-04	ENSG00000117523
PRRG1	-0.0231	7.6928E-01	8.7659E-01	ENSG00000130962
PRRG2	0.0299	6.7488E-01	8.1764E-01	ENSG00000126460
PRRG3	-0.1859	6.5702E-02	1.9232E-01	ENSG00000130032
PRRG4	0.0492	5.4393E-01	7.2484E-01	ENSG00000135378
PRRT1	-0.1325	1.1152E-01	2.7348E-01	ENSG00000204314
PRRT1B	0.0095	9.7944E-01		ENSG00000283526
PRRT2	-0.1764	1.6401E-02	7.2275E-02	ENSG00000167371
PRRT3	-0.0988	1.3262E-01	3.0620E-01	ENSG00000163704
PRRT3-AS1	0.0879	7.5966E-02	2.1232E-01	ENSG00000230082
PRRT4	0.0217	7.9519E-01	8.9178E-01	ENSG00000224940
PRRX1	-0.0771	2.1946E-01	4.2177E-01	ENSG00000116132
PRRX2	-0.5803	9.7543E-05	1.5152E-03	ENSG00000167157
PRSS12	-0.0509	5.1976E-01	7.0687E-01	ENSG00000164099
PRSS16	-0.0562	4.8562E-01	6.7932E-01	ENSG00000112812
PRSS22	0.0095	3.9756E-01		ENSG00000005001
PRSS27	0.4488	7.3310E-05	1.2082E-03	ENSG00000172382
PRSS3	0.0002	9.9911E-01	9.9944E-01	ENSG00000010438
PRSS30P	0.0407	2.1781E-01		ENSG00000172460
PRSS33	0.1099	1.8400E-01	3.7736E-01	ENSG00000103355
PRSS35	0.0064	9.3256E-01	9.6658E-01	ENSG00000146250
PRSS36	0.4409	7.4479E-03	4.0624E-02	ENSG00000178226
PRSS37	0.0092	8.6746E-01		ENSG00000165076
PRSS48	0.0284	4.3469E-01		ENSG00000189099
PRSS51	-0.0368	2.5547E-01	4.6360E-01	ENSG00000253649
PRSS52P	0.0108	4.6484E-01		ENSG00000285237
PRSS53	0.0336	5.6171E-01	7.3848E-01	ENSG00000151006
PRSS54	0.0326	5.2854E-01		ENSG00000103023
PRSS55	-0.0641	2.0096E-01	3.9944E-01	ENSG00000184647
PRSS56	-0.0650	3.4853E-01	5.5845E-01	ENSG00000237412
PRSS8	0.1300	1.3674E-01	3.1213E-01	ENSG00000052344
PRTFDC1	0.0854	1.4835E-01	3.2886E-01	ENSG00000099256
PRTN3	0.0127	8.8970E-01	9.4533E-01	ENSG00000196415
PRUNE1	0.0141	8.4334E-01	9.1966E-01	ENSG00000143363
PRUNE2	0.1294	7.6640E-02	2.1349E-01	ENSG00000106772
PRX	-0.0265	7.4225E-01	8.5956E-01	ENSG00000105227
PRXL2A	-0.2235	1.2113E-04	1.7805E-03	ENSG00000122378
PRXL2B	-0.0245	7.2966E-01	8.5207E-01	ENSG00000157870
PRXL2C	-0.0296	7.0869E-01	8.3974E-01	ENSG00000158122

PSAP	-0.0459	5.5068E-01	7.2951E-01	ENSG00000197746
PSAT1	-0.0558	4.8540E-01	6.7910E-01	ENSG00000135069
PSAT1P3	-0.0007	9.5746E-01		ENSG00000230787
PSCA	0.0192	5.3656E-01		ENSG00000167653
PSD	-0.0491	4.4897E-01	6.4817E-01	ENSG00000059915
PSD2	0.0648	3.7776E-01	5.8634E-01	ENSG00000146005
PSD3	-0.0202	7.5801E-01	8.6905E-01	ENSG00000156011
PSD4	0.0338	6.4174E-01	7.9510E-01	ENSG00000125637
PSEN1	0.0454	4.2291E-01	6.2562E-01	ENSG00000080815
PSEN2	0.0865	2.9469E-01	5.0468E-01	ENSG00000143801
PSENEN	0.0442	5.1029E-01	6.9892E-01	ENSG00000205155
PSG1	0.0014	9.8408E-01	9.9153E-01	ENSG00000231924
PSG3	0.0139	3.4439E-01		ENSG00000221826
PSG4	-0.0058	9.6314E-01		ENSG00000243137
PSG8	-0.0106	5.1541E-01		ENSG00000124467
PSG8-AS1	0.0036	4.7140E-01		ENSG00000225877
PSG9	-0.0014	7.2626E-01		ENSG00000183668
PSIP1	-0.2534	7.7372E-05	1.2602E-03	ENSG00000164985
PSKH1	0.1472	5.2733E-02	1.6539E-01	ENSG00000159792
PSKH2	-0.0003	5.9079E-01		ENSG00000147613
PSMA1	0.0212	7.1075E-01	8.4121E-01	ENSG00000129084
PSMA2	-0.1036	2.4736E-01	4.5519E-01	ENSG00000106588
PSMA3	-0.1078	6.7459E-02	1.9580E-01	ENSG00000100567
PSMA3-AS1	-0.1835	3.9042E-03	2.5082E-02	ENSG00000257621
PSMA4	-0.1855	2.4202E-03	1.7377E-02	ENSG00000041357
PSMA5	-0.0313	5.8724E-01	7.5641E-01	ENSG00000143106
PSMA6	-0.3601	1.3502E-06	4.8009E-05	ENSG00000100902
PSMA7	-0.1557	9.5140E-03	4.8912E-02	ENSG00000101182
PSMB1	-0.1554	3.7211E-02	1.2977E-01	ENSG00000008018
PSMB10	0.0107	8.8496E-01	9.4245E-01	ENSG00000205220
PSMB2	-0.2178	8.6556E-06	2.1764E-04	ENSG00000126067
PSMB3	-0.1483	2.2025E-02	8.9035E-02	ENSG00000277791
PSMB4	-0.0121	8.3941E-01	9.1739E-01	ENSG00000159377
PSMB5	-0.0870	1.3879E-01	3.1465E-01	ENSG00000100804
PSMB6	-0.2730	2.4705E-04	3.0918E-03	ENSG00000142507
PSMB7	-0.1379	6.4003E-03	3.6264E-02	ENSG00000136930
PSMB8	-0.0886	3.0116E-01	5.1111E-01	ENSG00000204264
PSMB8-AS1	-0.0326	6.6681E-01	8.1315E-01	ENSG00000204261
PSMB9	-0.0263	7.5096E-01	8.6466E-01	ENSG00000240065
PSMC1	-0.0939	1.2410E-01	2.9387E-01	ENSG00000100764
PSMC1P1	-0.0695	3.0463E-01	5.1483E-01	ENSG00000241506
PSMC1P10	0.0026	7.5499E-01		ENSG00000236348
PSMC1P11	0.0108	6.4481E-01		ENSG00000217385
PSMC1P12	0.0205	4.0404E-01		ENSG00000226126
PSMC1P3	-0.0291	3.5289E-01		ENSG00000231244
PSMC1P4	-0.0230	3.2923E-01		ENSG00000248781
PSMC1P5	0.0580	3.8054E-01	5.8896E-01	ENSG00000250273
PSMC1P9	-0.0382	2.1479E-01	4.1610E-01	ENSG00000255993
PSMC2	-0.1277	2.2382E-02	9.0134E-02	ENSG00000161057

PSMC2P1	-0.0047	9.7927E-01		ENSG00000240854
PSMC3	-0.2579	6.3769E-04	6.4600E-03	ENSG00000165916
PSMC3IP	-0.0984	1.9445E-01	3.9116E-01	ENSG00000131470
PSMC3P1	0.0166	3.9798E-01		ENSG00000236680
PSMC4	-0.0880	1.5911E-01	3.4365E-01	ENSG00000013275
PSMC5	-0.1263	7.9853E-03	4.2807E-02	ENSG00000087191
PSMC6	-0.1436	3.2650E-02	1.1793E-01	ENSG00000100519
PSMD1	0.0082	8.7384E-01	9.3676E-01	ENSG00000173692
PSMD10	-0.2987	6.0861E-10	7.2693E-08	ENSG00000101843
PSMD10P3	0.0069	9.5471E-01		ENSG00000232041
PSMD11	-0.1191	1.3275E-02	6.2309E-02	ENSG00000108671
PSMD12	0.1069	9.9602E-02	2.5400E-01	ENSG00000197170
PSMD13	-0.0971	1.1108E-01	2.7290E-01	ENSG00000185627
PSMD14	0.0100	8.6344E-01	9.3122E-01	ENSG00000115233
PSMD2	0.0051	9.3881E-01	9.6983E-01	ENSG00000175166
PSMD2P1	-0.0034	9.7289E-01		ENSG00000228818
PSMD3	-0.2125	3.3958E-04	3.9489E-03	ENSG00000108344
PSMD4	-0.1746	1.1305E-03	9.9272E-03	ENSG00000159352
PSMD5	-0.0269	6.5155E-01	8.0266E-01	ENSG00000095261
PSMD6	-0.0874	5.5006E-02	1.7061E-01	ENSG00000163636
PSMD6-AS2	-0.0578	4.8082E-01	6.7550E-01	ENSG00000239653
PSMD7	-0.2382	1.9227E-04	2.5238E-03	ENSG00000103035
PSMD7-DT	0.3233	1.8706E-02	7.9143E-02	ENSG00000261404
PSMD8	-0.1412	3.4108E-02	1.2150E-01	ENSG00000099341
PSMD9	-0.0222	7.5730E-01	8.6856E-01	ENSG00000110801
PSME1	-0.2413	7.1280E-04	7.0521E-03	ENSG00000092010
PSME2	-0.0768	2.3507E-01	4.4044E-01	ENSG00000100911
PSME2P1	0.0308	4.1211E-01		ENSG00000238000
PSME2P2	0.0041	9.6208E-01		ENSG00000225131
PSME2P3	-0.0016	9.7477E-01		ENSG00000248988
PSME2P4	0.0083	7.0403E-01		ENSG00000251332
PSME2P5	0.0027	9.1843E-01	9.5972E-01	ENSG00000253208
PSME3	-0.0389	4.5605E-01	6.5434E-01	ENSG00000131467
PSME3IP1	-0.0444	2.8504E-01	4.9564E-01	ENSG00000172775
PSME4	0.0780	2.3891E-01	4.4542E-01	ENSG00000068878
PSMF1	-0.0547	2.6828E-01	4.7778E-01	ENSG00000125818
PSMG1	-0.0746	2.9036E-01	5.0077E-01	ENSG00000183527
PSMG2	-0.0699	2.8617E-01	4.9691E-01	ENSG00000128789
PSMG3	-0.0587	3.3255E-01	5.4281E-01	ENSG00000157778
PSMG3-AS1	0.0909	2.2930E-01	4.3370E-01	ENSG00000230487
PSMG3P2	0.0093	9.1334E-01		ENSG00000239805
PSMG4	0.5381	2.8096E-10	3.7776E-08	ENSG00000180822
PSORS1C1	0.0362	4.7352E-01	6.6947E-01	ENSG00000204540
PSPC1	0.0631	3.1096E-01	5.2107E-01	ENSG00000121390
PSPC1P1	0.1182	2.0011E-01	3.9852E-01	ENSG00000227879
PSPH	-0.2659	8.6705E-04	8.1426E-03	ENSG00000146733
PSPHP1	-0.1951	6.3049E-02	1.8677E-01	ENSG00000226278
PSPN	-0.2076	5.8197E-02	1.7701E-01	ENSG00000125650
PSRC1	-0.0461	5.5065E-01	7.2951E-01	ENSG00000134222

PSTK	-0.0523	3.6828E-01	5.7750E-01	ENSG00000179988
PSTPIP1	0.0594	4.4853E-01	6.4766E-01	ENSG00000140368
PSTPIP2	-0.1782	4.2746E-02	1.4316E-01	ENSG00000152229
PTAFR	0.0833	1.1356E-01	2.7705E-01	ENSG00000169403
PTAR1	-0.0732	3.3336E-01	5.4349E-01	ENSG00000188647
PTBP1	0.0703	2.6786E-01	4.7723E-01	ENSG00000011304
PTBP2	0.2081	1.4423E-03	1.1925E-02	ENSG00000117569
PTBP3	-0.0802	2.0921E-01	4.0916E-01	ENSG00000119314
PTCD1	-0.1650	6.7227E-02	1.9543E-01	ENSG00000106246
PTCD2	0.0970	1.8986E-01	3.8491E-01	ENSG00000049883
PTCD3	-0.1109	8.9031E-02	2.3585E-01	ENSG00000132300
PTCH1	0.1214	9.4384E-02	2.4570E-01	ENSG00000185920
PTCH2	0.4034	5.1835E-04	5.4527E-03	ENSG00000117425
PTCHD1	0.5363	7.5471E-06	1.9506E-04	ENSG00000165186
PTCHD4	0.3488	1.4861E-03	1.2192E-02	ENSG00000244694
PTCSC1	0.0144	6.5152E-01		ENSG00000287736
PTCSC2	0.0483	1.1704E-01		ENSG00000236130
PTDSS1	-0.0829	2.4456E-01	4.5214E-01	ENSG00000156471
PTDSS2	-0.0022	9.5180E-01	9.7623E-01	ENSG00000174915
PTEN	-0.1017	5.6581E-02	1.7392E-01	ENSG00000171862
PTENP1	-0.0387	6.3175E-01	7.8825E-01	ENSG00000237984
PTENP1-AS	0.1154	2.1124E-01	4.1206E-01	ENSG00000281128
PTER	0.3112	7.3927E-03	4.0386E-02	ENSG00000165983
PTF1A	-0.0450	5.5698E-01	7.3477E-01	ENSG00000168267
PTGDR	0.0008	8.9840E-01		ENSG00000168229
PTGDR2	0.0395	3.7270E-01		ENSG00000183134
PTGDS	-0.0802	3.2287E-01	5.3288E-01	ENSG00000107317
PTGER1	0.0579	4.3416E-01	6.3526E-01	ENSG00000160951
PTGER2	0.0621	2.5295E-01	4.6088E-01	ENSG00000125384
PTGER3	0.0254	6.7683E-01	8.1899E-01	ENSG00000050628
PTGER4	0.0687	1.8065E-01	3.7287E-01	ENSG00000171522
PTGER4P2-CDK2AP2P2	0.0404	4.5683E-01		ENSG00000275450
PTGES	-0.0807	2.7380E-01	4.8363E-01	ENSG00000148344
PTGES2	0.0225	7.3721E-01	8.5666E-01	ENSG00000148334
PTGES2-AS1	0.0425	5.0669E-01	6.9620E-01	ENSG00000232850
PTGES3	-0.3623	2.7630E-13	1.2590E-10	ENSG00000110958
PTGES3L	0.2288	3.6807E-02	1.2873E-01	ENSG00000267060
PTGES3L-AARSD1	0.0042	7.1283E-01		ENSG00000108825
PTGFR	-0.0534	3.4862E-01	5.5845E-01	ENSG00000122420
PTGFRN	-0.1534	2.7615E-02	1.0474E-01	ENSG00000134247
PTGIR	0.0270	6.2457E-01	7.8329E-01	ENSG00000160013
PTGIS	0.0017	9.8730E-01	9.9311E-01	ENSG00000124212
PTGR1	-0.0724	3.1731E-01	5.2757E-01	ENSG00000106853
PTGR2	-0.1111	2.1407E-01	4.1517E-01	ENSG00000140043
PTGR3	-0.1461	3.5417E-02	1.2506E-01	ENSG00000180011
PTGS1	0.0465	4.9785E-01	6.8911E-01	ENSG00000095303
PTGS2	-0.0159	7.4428E-01	8.6086E-01	ENSG00000073756
PTH	0.0100	4.7033E-01		ENSG00000152266
PTH1R	-0.0046	9.5032E-01	9.7529E-01	ENSG00000160801

PTH2R	-0.1211	2.1031E-01	4.1073E-01	ENSG00000144407
PTHLH	0.0822	2.0697E-01	4.0652E-01	ENSG00000087494
PTK2	-0.0574	3.3088E-01	5.4112E-01	ENSG00000169398
PTK2B	0.0041	9.7186E-01	9.8589E-01	ENSG00000120899
PTK6	-0.1823	5.4978E-02	1.7054E-01	ENSG00000101213
PTK7	-0.1195	9.3055E-02	2.4337E-01	ENSG00000112655
PTMA	-0.4366	1.8402E-05	4.0069E-04	ENSG00000187514
PTMAP5	-0.5191	2.6596E-05	5.3904E-04	ENSG00000214182
PTMS	-0.3441	1.6116E-03	1.2859E-02	ENSG00000159335
PTN	-0.0002	9.9686E-01	9.9824E-01	ENSG00000105894
PTOV1	-0.3968	2.4205E-09	2.4230E-07	ENSG00000104960
PTOV1-AS1	-0.0127	8.7425E-01	9.3709E-01	ENSG00000268006
PTOV1P1	0.0173	7.1247E-01		ENSG00000240882
PTP4A1	-0.0676	2.9142E-01	5.0175E-01	ENSG00000112245
PTP4A1P3	0.0007	9.9417E-01		ENSG00000226254
PTP4A2	-0.2618	1.0997E-07	6.2636E-06	ENSG00000184007
PTP4A3	0.0929	1.6124E-01	3.4697E-01	ENSG00000184489
PTPA	-0.1006	1.5822E-01	3.4237E-01	ENSG00000119383
PTPDC1	0.1539	3.2319E-02	1.1706E-01	ENSG00000158079
PTPMT1	-0.0430	5.4875E-01	7.2818E-01	ENSG00000110536
PTPN1	-0.0541	3.8155E-01	5.8981E-01	ENSG00000196396
PTPN11	-0.0181	6.8280E-01	8.2313E-01	ENSG00000179295
PTPN12	0.0847	3.1417E-01	5.2447E-01	ENSG00000127947
PTPN13	0.1572	5.2700E-02	1.6531E-01	ENSG00000163629
PTPN14	-0.3195	2.0217E-02	8.3609E-02	ENSG00000152104
PTPN18	-0.0424	4.5373E-01	6.5229E-01	ENSG00000072135
PTPN2	-0.0499	4.1917E-01	6.2202E-01	ENSG00000175354
PTPN20	0.0978	1.1501E-01	2.7947E-01	ENSG00000204179
PTPN21	0.2162	1.8568E-02	7.8730E-02	ENSG00000070778
PTPN22	0.1060	7.8634E-02	2.1708E-01	ENSG00000134242
PTPN23	0.0015	9.8279E-01	9.9076E-01	ENSG00000076201
PTPN3	-0.2336	2.6325E-02	1.0119E-01	ENSG00000070159
PTPN4	0.2746	3.0312E-04	3.6258E-03	ENSG00000088179
PTPN5	-0.1009	2.5224E-01	4.6034E-01	ENSG00000110786
PTPN6	0.3578	1.1067E-02	5.4557E-02	ENSG00000111679
PTPN7	0.1230	1.3171E-02		ENSG00000143851
PTPN9	0.0142	7.9804E-01	8.9363E-01	ENSG00000169410
PTPRA	-0.1572	1.7175E-02	7.4569E-02	ENSG00000132670
PTPRB	0.0825	7.8633E-02	2.1708E-01	ENSG00000127329
PTPRC	0.0183	7.5617E-01		ENSG00000081237
PTPRD	0.5705	2.7172E-09	2.6636E-07	ENSG00000153707
PTPRD-AS1	-0.2541	9.9235E-03	5.0427E-02	ENSG00000225706
PTPRE	0.2935	2.1024E-03	1.5642E-02	ENSG00000132334
PTPRF	0.0559	4.4021E-01	6.4011E-01	ENSG00000142949
PTPRG	0.2645	2.9498E-03	2.0223E-02	ENSG00000144724
PTPRG-AS1	0.1124	1.4245E-01	3.1992E-01	ENSG00000241472
PTPRJ	-0.0027	9.6604E-01	9.8326E-01	ENSG00000149177
PTPRK	-0.0643	3.5659E-01	5.6643E-01	ENSG00000152894
PTPRM	0.1651	4.1750E-02	1.4086E-01	ENSG00000173482

PTPRN	0.3609	6.6990E-04	6.7250E-03	ENSG00000054356
PTPRN2	-0.1435	1.1196E-01	2.7428E-01	ENSG00000155093
PTPRO	-0.0635	4.4575E-01	6.4489E-01	ENSG00000151490
PTPRQ	0.1026	4.2409E-02		ENSG00000139304
PTPRR	0.4128	6.0221E-03	3.4588E-02	ENSG00000153233
PTPRS	-0.1876	1.4818E-02	6.7448E-02	ENSG00000105426
PTPRT	0.4804	3.4052E-04	3.9559E-03	ENSG00000196090
PTPRT-DT	0.0200	8.1423E-01	9.0317E-01	ENSG00000233508
PTPRU	0.1602	7.5038E-02	2.1057E-01	ENSG00000060656
PTPRVP	0.0438	3.2261E-02		ENSG00000243323
PTPRZ1	-0.0427	5.9020E-01	7.5856E-01	ENSG00000106278
PTRH1	0.0310	7.8829E-01	8.8801E-01	ENSG00000187024
PTRH2	0.0544	4.9029E-01	6.8299E-01	ENSG00000141378
PTRHD1	0.0106	8.9417E-01	9.4738E-01	ENSG00000184924
PTS	-0.0343	5.7361E-01	7.4656E-01	ENSG00000150787
PTTG1	-0.0936	3.1034E-01	5.2065E-01	ENSG00000164611
PTTG1IP	-0.0501	4.5841E-01	6.5626E-01	ENSG00000183255
PTTG2	0.0125	6.4716E-01		ENSG00000250254
PTX3	-0.3316	1.7752E-02	7.6299E-02	ENSG00000163661
PUDP	0.0992	1.3095E-01	3.0393E-01	ENSG00000130021
PUF60	-0.0584	3.3463E-01	5.4471E-01	ENSG00000179950
PUM1	-0.0359	4.4139E-01	6.4096E-01	ENSG00000134644
PUM2	0.1406	4.3289E-02	1.4446E-01	ENSG00000055917
PUM3	0.3723	8.3334E-07	3.2290E-05	ENSG00000080608
PURA	0.3007	3.4995E-04	4.0368E-03	ENSG00000185129
PURB	-0.1400	5.6438E-02	1.7364E-01	ENSG00000146676
PURG	-0.1527	7.9790E-02	2.1918E-01	ENSG00000172733
PURPL	0.1798	8.3553E-02	2.2574E-01	ENSG00000250337
PUS1	0.0561	5.0387E-01	6.9420E-01	ENSG00000177192
PUS10	-0.1188	1.4002E-01	3.1655E-01	ENSG00000162927
PUS3	0.3553	1.2668E-05	2.9460E-04	ENSG00000110060
PUS7	0.0714	2.7719E-01	4.8723E-01	ENSG00000091127
PUS7L	0.1417	2.7537E-02	1.0457E-01	ENSG00000129317
PUSL1	0.2746	3.7608E-03	2.4372E-02	ENSG00000169972
PVALB	0.0930	2.9224E-01	5.0238E-01	ENSG00000100362
PVALEF	0.0131	6.3150E-01		ENSG00000225180
PVR	0.0058	9.4408E-01	9.7218E-01	ENSG00000073008
PVRIG	-0.0326	3.8861E-01		ENSG00000213413
PVT1	-0.4474	1.7440E-08	1.3368E-06	ENSG00000249859
PWP1	-0.0878	8.0363E-02	2.2033E-01	ENSG00000136045
PWP2	0.2026	2.0700E-02	8.4988E-02	ENSG00000241945
PWRN1	0.5388	9.8617E-03	5.0175E-02	ENSG00000259905
PWWP2A	-0.4037	1.7409E-08	1.3368E-06	ENSG00000170234
PWWP2B	-0.0834	3.5384E-01	5.6380E-01	ENSG00000171813
PWWP3A	0.2764	2.5401E-05	5.1866E-04	ENSG00000160953
PWWP3B	0.1571	1.1393E-01	2.7774E-01	ENSG00000157502
PWWP4	0.0511	1.1095E-01	2.7275E-01	ENSG00000278803
PXDC1	-0.1718	3.8707E-02	1.3370E-01	ENSG00000168994
PXDN	0.0920	1.9949E-01	3.9780E-01	ENSG00000130508

PXDNL	0.0441	5.9303E-01	7.6037E-01	ENSG00000147485
PXK	0.0142	8.3756E-01	9.1612E-01	ENSG00000168297
PXMP2	-0.3706	6.8250E-04	6.8208E-03	ENSG00000176894
PXMP4	0.0813	2.7159E-01	4.8082E-01	ENSG00000101417
PXN	0.0690	3.5091E-01	5.6105E-01	ENSG00000089159
PXN-AS1	0.2442	1.4032E-02	6.4885E-02	ENSG00000255857
PXT1	0.4286	1.4853E-02	6.7476E-02	ENSG00000179165
PXYLP1	-0.1432	5.1221E-02	1.6203E-01	ENSG00000155893
PYCARD	-0.2978	1.9337E-02	8.1015E-02	ENSG00000103490
PYCR1	-0.1190	5.5250E-02	1.7115E-01	ENSG00000183010
PYCR2	-0.0097	8.9512E-01	9.4777E-01	ENSG00000143811
PYCR3	-0.0213	7.9782E-01	8.9354E-01	ENSG00000104524
PYDC1	-0.0014	9.8749E-01	9.9314E-01	ENSG00000169900
PYGB	0.0483	4.9467E-01	6.8670E-01	ENSG00000100994
PYGL	0.0333	6.7791E-01	8.1961E-01	ENSG00000100504
PYGM	0.0603	4.2474E-01	6.2715E-01	ENSG00000068976
PYGO1	-0.1326	8.1461E-02	2.2206E-01	ENSG00000171016
PYGO2	0.1061	1.6698E-01	3.5498E-01	ENSG00000163348
PYM1	0.2092	6.6395E-04	6.6719E-03	ENSG00000170473
PYROXD1	0.0614	3.7704E-01	5.8556E-01	ENSG00000121350
PYROXD2	-0.0336	6.9100E-01	8.2844E-01	ENSG00000119943
PYY	0.0115	9.8211E-01		ENSG00000131096
PZP	0.0512	4.5905E-01	6.5683E-01	ENSG00000126838
QARS1	0.1829	4.0379E-03	2.5766E-02	ENSG00000172053
QDPR	-0.2942	6.2456E-07	2.5359E-05	ENSG00000151552
QKI	-0.2955	4.8211E-04	5.1643E-03	ENSG00000112531
QNG1	0.1122	2.2310E-01	4.2672E-01	ENSG00000165118
QPCT	-0.3225	5.7173E-03	3.3327E-02	ENSG00000115828
QPCTL	0.2708	3.7056E-03	2.4127E-02	ENSG00000011478
QPRT	-0.1719	5.0995E-02	1.6155E-01	ENSG00000103485
QRFP	-0.0201	4.5489E-01		ENSG00000188710
QRFPR	0.0216	7.4223E-01	8.5956E-01	ENSG00000186867
QRICH1	-0.1085	3.3889E-02	1.2093E-01	ENSG00000198218
QRICH2	-0.1212	1.9164E-01	3.8698E-01	ENSG00000129646
QRSL1	0.0728	2.2525E-01	4.2900E-01	ENSG00000130348
QRSL1P3	0.0695	2.2780E-01	4.3238E-01	ENSG00000257957
QSER1	0.1346	1.2482E-01	2.9482E-01	ENSG00000060749
QSOX1	0.2132	3.7119E-03	2.4156E-02	ENSG00000116260
QSOX2	0.1550	8.7303E-02	2.3260E-01	ENSG00000165661
QTRT1	-0.0455	4.7488E-01	6.7059E-01	ENSG00000213339
QTRT2	0.0902	2.1360E-01	4.1459E-01	ENSG00000151576
R3HCC1	0.0052	9.5182E-01	9.7623E-01	ENSG00000104679
R3HCC1L	0.0967	1.6293E-01	3.4924E-01	ENSG00000166024
R3HDM1	0.0384	5.9761E-01	7.6387E-01	ENSG00000048991
R3HDM2	-0.1499	4.7536E-03	2.8956E-02	ENSG00000179912
R3HDM4	0.0664	2.9350E-01	5.0357E-01	ENSG00000198858
R3HDML	0.0372	1.3049E-01		ENSG00000101074
RAB10	-0.4739	2.9106E-10	3.8922E-08	ENSG00000084733
RAB11A	-0.3753	1.6270E-10	2.4262E-08	ENSG00000103769

RAB11B	-0.1159	1.0851E-01	2.6867E-01	ENSG00000185236
RAB11B-AS1	-0.4044	7.8005E-05	1.2677E-03	ENSG00000269386
RAB11FIP1	-0.1179	1.4895E-01	3.2951E-01	ENSG00000156675
RAB11FIP2	0.0323	6.3502E-01	7.9068E-01	ENSG00000107560
RAB11FIP3	0.1737	2.6609E-02	1.0196E-01	ENSG00000090565
RAB11FIP4	-0.1043	1.1450E-01	2.7857E-01	ENSG00000131242
RAB11FIP5	0.0484	4.5360E-01	6.5222E-01	ENSG00000135631
RAB12	-0.2023	3.0267E-02	1.1194E-01	ENSG00000206418
RAB13	0.0622	4.2570E-01	6.2796E-01	ENSG00000143545
RAB14	-0.0989	1.4103E-02	6.5149E-02	ENSG00000119396
RAB15	-0.2481	3.2004E-03	2.1510E-02	ENSG00000139998
RAB17	0.0685	1.0855E-01		ENSG00000124839
RAB18	-0.2021	1.0189E-02	5.1404E-02	ENSG00000099246
RAB19	0.0269	4.8081E-01		ENSG00000146955
RAB1A	-0.1410	1.1355E-02	5.5698E-02	ENSG00000138069
RAB1B	-0.0392	6.4162E-01	7.9504E-01	ENSG00000174903
RAB20	0.1690	9.7940E-02	2.5136E-01	ENSG00000139832
RAB21	-0.2428	7.3799E-05	1.2154E-03	ENSG00000080371
RAB22A	-0.0594	2.0915E-01	4.0916E-01	ENSG00000124209
RAB23	-0.0749	3.7603E-01	5.8458E-01	ENSG00000112210
RAB24	0.1073	2.1191E-01	4.1290E-01	ENSG00000169228
RAB25	-0.0521	5.1218E-01	7.0032E-01	ENSG00000132698
RAB26	0.0143	8.4149E-01	9.1842E-01	ENSG00000167964
RAB27A	0.1824	7.9722E-02	2.1914E-01	ENSG00000069974
RAB27B	0.0435	5.9544E-01	7.6234E-01	ENSG00000041353
RAB28	-0.0410	5.2563E-01	7.1100E-01	ENSG00000157869
RAB29	-0.1828	2.0439E-02	8.4182E-02	ENSG00000117280
RAB2A	-0.2429	7.0494E-06	1.8492E-04	ENSG00000104388
RAB2B	-0.3219	2.3278E-07	1.1524E-05	ENSG00000129472
RAB30	-0.2920	3.6056E-04	4.1263E-03	ENSG00000137502
RAB31	0.0203	7.8710E-01	8.8748E-01	ENSG00000168461
RAB32	-0.0092	9.0414E-01	9.5319E-01	ENSG00000118508
RAB33A	-0.1983	1.1027E-02	5.4437E-02	ENSG00000134594
RAB33B	-0.5550	1.3375E-07	7.2810E-06	ENSG00000172007
RAB34	-0.1635	3.8625E-02	1.3348E-01	ENSG00000109113
RAB35	-0.1433	8.6810E-02	2.3165E-01	ENSG00000111737
RAB35-AS1	0.5109	7.1436E-07	2.8213E-05	ENSG00000277283
RAB36	0.2084	1.2245E-02	5.8765E-02	ENSG00000100228
RAB37	0.0242	7.6950E-01	8.7668E-01	ENSG00000172794
RAB38	0.0922	2.9801E-01	5.0787E-01	ENSG00000123892
RAB39A	-0.2717	1.3736E-02	6.3912E-02	ENSG00000179331
RAB39B	0.0901	2.2609E-01	4.2996E-01	ENSG00000155961
RAB3A	-0.2336	3.9333E-04	4.4212E-03	ENSG00000105649
RAB3B	-0.2869	2.5699E-04	3.1951E-03	ENSG00000169213
RAB3C	-0.1771	7.7626E-03	4.1941E-02	ENSG00000152932
RAB3D	-0.4142	7.8373E-05	1.2729E-03	ENSG00000105514
RAB3GAP1	-0.0429	4.2503E-01	6.2738E-01	ENSG00000115839
RAB3GAP2	0.2792	1.7849E-03	1.3841E-02	ENSG00000118873
RAB3IL1	-0.1983	2.8551E-02	1.0733E-01	ENSG00000167994

RAB3IP	-0.0139	7.7978E-01	8.8319E-01	ENSG00000127328
RAB40A	0.3783	1.0939E-02	5.4119E-02	ENSG00000172476
RAB40AL	0.0190	6.6810E-01		ENSG00000102128
RAB40B	0.1106	1.1784E-01	2.8369E-01	ENSG00000141542
RAB40C	0.1118	1.5688E-01	3.4056E-01	ENSG00000197562
RAB41	-0.0039	8.8311E-01		ENSG00000147127
RAB42	-0.0108	8.9798E-01	9.4955E-01	ENSG00000188060
RAB43	-0.0441	3.1461E-01		ENSG00000172780
RAB44	-0.0094	4.7594E-01		ENSG00000255587
RAB4A	-0.1771	1.5908E-04	2.1710E-03	ENSG00000168118
RAB4A-AS1	0.0964	2.8170E-01	4.9214E-01	ENSG00000177788
RAB4B	0.0871	3.0457E-01	5.1481E-01	ENSG00000167578
RAB5A	-0.1189	8.7280E-02	2.3257E-01	ENSG00000144566
RAB5B	-0.1536	4.6703E-03	2.8598E-02	ENSG00000111540
RAB5C	-0.2632	6.6124E-03	3.7203E-02	ENSG00000108774
RAB5IF	-0.3236	1.5043E-05	3.3803E-04	ENSG00000101084
RAB6A	-0.0314	6.1789E-01	7.7877E-01	ENSG00000175582
RAB6B	-0.1142	7.4752E-02	2.0999E-01	ENSG00000154917
RAB6C	0.1344	1.6787E-01	3.5613E-01	ENSG00000222014
RAB6D	0.1527	1.0927E-01	2.6987E-01	ENSG00000233087
RAB7A	-0.1238	3.8459E-02	1.3305E-01	ENSG00000075785
RAB7B	-0.0407	5.6659E-01	7.4166E-01	ENSG00000276600
RAB8A	-0.1376	4.2666E-02	1.4299E-01	ENSG00000167461
RAB8B	-0.2322	3.4846E-04	4.0272E-03	ENSG00000166128
RAB9A	-0.0624	3.8117E-01	5.8937E-01	ENSG00000123595
RAB9B	0.3412	7.0275E-05	1.1678E-03	ENSG00000123570
RABAC1	-0.1537	3.7317E-02	1.3000E-01	ENSG00000105404
RABEP1	0.0133	8.3055E-01	9.1239E-01	ENSG00000029725
RABEP2	-0.0158	8.3568E-01	9.1524E-01	ENSG00000177548
RABEPK	-0.1875	1.5608E-04	2.1430E-03	ENSG00000136933
RABGAP1	-0.0623	2.2400E-01	4.2780E-01	ENSG00000011454
RABGAP1L	-0.1023	1.4192E-01	3.1906E-01	ENSG00000152061
RABGAP1L-AS1	-0.0029	9.6048E-01	9.8028E-01	ENSG00000229531
RABGAP1L-DT	0.0658	4.0750E-01	6.1186E-01	ENSG00000227373
RABGEF1	0.0455	5.9124E-01	7.5910E-01	ENSG00000154710
RABGEF1P2	0.0361	3.9338E-01		ENSG00000230189
RABGGTA	0.1055	1.0081E-01	2.5619E-01	ENSG00000100949
RABGGTB	0.0133	8.3931E-01	9.1737E-01	ENSG00000137955
RABIF	0.1256	6.0144E-02	1.8064E-01	ENSG00000183155
RABL2A	0.0250	7.4318E-01	8.6031E-01	ENSG00000144134
RABL2B	0.0078	9.3167E-01	9.6615E-01	ENSG00000079974
RABL3	-0.0109	8.3746E-01	9.1605E-01	ENSG00000144840
RABL6	-0.0287	6.9927E-01	8.3370E-01	ENSG00000196642
RAC1	-0.2810	5.5236E-06	1.5168E-04	ENSG00000136238
RAC2	0.2329	4.2517E-02	1.4258E-01	ENSG00000128340
RACGAP1	-0.2538	2.2611E-02	9.0744E-02	ENSG00000161800
RACK1	-0.2226	1.9543E-04	2.5471E-03	ENSG00000204628
RAD1	0.1658	1.0612E-02	5.2963E-02	ENSG00000113456
RAD17	0.0059	9.1089E-01	9.5616E-01	ENSG00000152942

RAD18	0.1231	1.0120E-01	2.5683E-01	ENSG00000070950
RAD21	-0.0682	3.0707E-01	5.1735E-01	ENSG00000164754
RAD21-AS1	0.1018	1.5898E-01	3.4350E-01	ENSG00000253327
RAD21L1	0.0162	4.0393E-01		ENSG00000244588
RAD23A	-0.2210	1.5479E-04	2.1301E-03	ENSG00000179262
RAD23B	-0.3512	3.4912E-06	1.0488E-04	ENSG00000119318
RAD50	-0.0112	8.8128E-01	9.4057E-01	ENSG00000113522
RAD51	-0.0119	8.9478E-01	9.4750E-01	ENSG00000051180
RAD51-AS1	-0.0347	6.2919E-01	7.8650E-01	ENSG00000245849
RAD51AP1	-0.0791	3.3406E-01	5.4418E-01	ENSG00000111247
RAD51AP2	-0.0075	5.9380E-01	7.6096E-01	ENSG00000214842
RAD51B	-0.1022	2.2818E-01	4.3287E-01	ENSG00000182185
RAD51C	-0.0643	3.2226E-01	5.3227E-01	ENSG00000108384
RAD51D	-0.0062	9.4430E-01	9.7218E-01	ENSG00000185379
RAD52	0.0897	2.6864E-01	4.7801E-01	ENSG00000002016
RAD54B	-0.0048	9.5337E-01	9.7697E-01	ENSG00000197275
RAD54L	-0.0155	8.4682E-01	9.2150E-01	ENSG00000085999
RAD54L2	0.0350	5.0036E-01	6.9121E-01	ENSG00000164080
RAD9A	0.0114	8.7881E-01	9.3956E-01	ENSG00000172613
RADIL	0.1190	1.2438E-01	2.9422E-01	ENSG00000157927
RADX	0.0628	3.8804E-01	5.9562E-01	ENSG00000147231
RAE1	-0.1829	2.1415E-03	1.5824E-02	ENSG00000101146
RAET1E	0.0093	2.5948E-01		ENSG00000164520
RAET1E-AS1	-0.0039	8.1238E-01		ENSG00000268592
RAET1G	0.0132	7.3028E-01		ENSG00000203722
RAET1L	0.0446	2.9192E-01		ENSG00000155918
RAF1	-0.1078	1.2429E-01	2.9406E-01	ENSG00000132155
RAG1	0.0747	2.9919E-01	5.0892E-01	ENSG00000166349
RAG2	0.0138	5.5530E-01		ENSG00000175097
RAI1	0.0257	7.0995E-01	8.4067E-01	ENSG00000108557
RAI1-AS1	0.0081	8.9123E-01	9.4593E-01	ENSG00000237328
RAI14	-0.2395	9.3993E-03	4.8495E-02	ENSG00000039560
RAI2	-0.1958	3.3521E-02	1.2025E-01	ENSG00000131831
RALA	-0.0549	3.4140E-01	5.5115E-01	ENSG00000006451
RALB	0.0059	9.2918E-01	9.6510E-01	ENSG00000144118
RALBP1	0.1757	5.4762E-03	3.2235E-02	ENSG00000017797
RALGAPA1	-0.0124	8.2573E-01	9.0965E-01	ENSG00000174373
RALGAPA1P1	0.1502	9.2156E-02	2.4174E-01	ENSG00000229419
RALGAPA2	-0.0715	3.7090E-01	5.7984E-01	ENSG00000188559
RALGAPB	0.1031	5.6587E-02	1.7392E-01	ENSG00000170471
RALGDS	-0.1275	8.1739E-02	2.2245E-01	ENSG00000160271
RALGPS1	-0.0264	6.8967E-01	8.2764E-01	ENSG00000136828
RALGPS2	-0.0310	6.3843E-01	7.9320E-01	ENSG00000116191
RALY	-0.0611	2.9826E-01	5.0815E-01	ENSG00000125970
RALY-AS1	-0.0124	8.9267E-01	9.4653E-01	ENSG00000285230
RALYL	0.0683	3.9835E-01	6.0394E-01	ENSG00000184672
RAMAC	-0.1038	5.0415E-02	1.6045E-01	ENSG00000169612
RAMACL	-0.0441	4.3181E-01	6.3324E-01	ENSG00000235272
RAMP1	-0.1246	1.5793E-01	3.4194E-01	ENSG00000132329

RAMP2	-0.2342	2.7532E-03	1.9115E-02	ENSG00000131477
RAMP2-AS1	-0.1556	7.2931E-02	2.0672E-01	ENSG00000197291
RAMP3	0.0082	8.4938E-01	9.2330E-01	ENSG00000122679
RAN	-0.3971	4.3651E-15	3.1589E-12	ENSG00000132341
RANBP1	-0.1476	2.5564E-02	9.9101E-02	ENSG00000099901
RANBP10	-0.0107	8.7005E-01	9.3446E-01	ENSG00000141084
RANBP17	0.2550	5.5286E-04	5.7543E-03	ENSG00000204764
RANBP2	-0.1667	1.0886E-02	5.3907E-02	ENSG00000153201
RANBP3	-0.1276	5.8715E-02	1.7802E-01	ENSG00000031823
RANBP3-DT	0.1155	2.0110E-01	3.9959E-01	ENSG00000266983
RANBP3L	-0.0148	8.3739E-01	9.1605E-01	ENSG00000164188
RANBP6	-0.3996	8.9777E-07	3.3807E-05	ENSG00000137040
RANBP9	-0.1848	1.7388E-02	7.5271E-02	ENSG00000010017
RANGAP1	-0.0618	3.6254E-01	5.7188E-01	ENSG00000100401
RANGRF	0.0908	3.0897E-01	5.1924E-01	ENSG00000108961
RAP1A	-0.0516	3.5808E-01	5.6773E-01	ENSG00000116473
RAP1B	-0.0637	2.6023E-01	4.6873E-01	ENSG00000127314
RAP1GAP	-0.1922	4.7859E-03	2.9090E-02	ENSG00000076864
RAP1GAP2	0.0764	3.8738E-01	5.9508E-01	ENSG00000132359
RAP1GDS1	-0.1641	3.1182E-03	2.1107E-02	ENSG00000138698
RAP2A	-0.0138	8.1525E-01	9.0394E-01	ENSG00000125249
RAP2B	-0.0604	4.0702E-01	6.1153E-01	ENSG00000181467
RAP2C	-0.1642	1.8457E-02	7.8368E-02	ENSG00000123728
RAP2C-AS1	0.1095	1.2224E-01	2.9109E-01	ENSG00000232160
RAP2CP1	0.0044	9.1140E-01		ENSG00000270938
RAPGEF1	-0.0821	1.5055E-01	3.3174E-01	ENSG00000107263
RAPGEF2	0.0167	8.3746E-01	9.1605E-01	ENSG00000109756
RAPGEF3	-0.1607	1.0559E-01	2.6409E-01	ENSG00000079337
RAPGEF4	0.0681	4.2999E-01	6.3164E-01	ENSG00000091428
RAPGEF4-AS1	-0.0991	1.5199E-01	3.3377E-01	ENSG00000228016
RAPGEF5	0.1385	1.4080E-01	3.1753E-01	ENSG00000136237
RAPGEFL1	-0.0382	6.1242E-01	7.7442E-01	ENSG00000108352
RAPH1	-0.5611	1.2315E-06	4.4431E-05	ENSG00000173166
RARA	-0.2683	4.4930E-03	2.7748E-02	ENSG00000131759
RARA-AS1	-0.0661	4.3326E-01	6.3436E-01	ENSG00000265666
RARB	-0.2633	6.3455E-03	3.6000E-02	ENSG00000077092
RARG	0.0200	8.0793E-01	8.9902E-01	ENSG00000172819
RARRES1	-0.0597	4.7933E-01	6.7448E-01	ENSG00000118849
RARRES2	-0.3439	3.0330E-05	5.9787E-04	ENSG00000106538
RARRES2P3	0.0008	5.9220E-01		ENSG00000231913
RARS1	0.3073	1.3096E-04	1.8889E-03	ENSG00000113643
RARS2	0.0001	9.9966E-01	9.9976E-01	ENSG00000146282
RASA1	0.1794	2.4800E-02	9.7105E-02	ENSG00000145715
RASA2	-0.0697	3.0741E-01	5.1754E-01	ENSG00000155903
RASA3	-0.0069	9.2296E-01	9.6186E-01	ENSG00000185989
RASA4	-0.0861	2.7407E-01	4.8382E-01	ENSG00000105808
RASA4	-0.1584	2.6801E-02	1.0244E-01	ENSG00000170667
RASAL1	-0.2464	4.0508E-02	1.3799E-01	ENSG00000111344
RASAL2	0.0968	1.6193E-01	3.4796E-01	ENSG00000075391

RASAL2-AS1	0.0321	6.3599E-01	7.9127E-01	ENSG00000224687
RASAL3	0.3018	2.9439E-02	1.0952E-01	ENSG00000105122
RASD1	-0.2482	1.1453E-02	5.6035E-02	ENSG00000108551
RASD2	-0.4966	2.6328E-06	8.3365E-05	ENSG00000100302
RASEF	-0.0918	3.0639E-01	5.1660E-01	ENSG00000165105
RASGEF1A	-0.1770	4.7657E-02	1.5453E-01	ENSG00000198915
RASGEF1B	-0.1810	8.5958E-02	2.2989E-01	ENSG00000138670
RASGEF1C	-0.0863	3.1889E-01	5.2919E-01	ENSG00000146090
RASGRF1	-0.0125	8.6788E-01	9.3343E-01	ENSG00000058335
RASGRF2	0.5935	1.5287E-03	1.2389E-02	ENSG00000113319
RASGRF2-AS1	-0.0305	1.1731E-01		ENSG00000251450
RASGRP1	-0.2224	1.4144E-02	6.5271E-02	ENSG00000172575
RASGRP2	-0.5798	7.4686E-06	1.9384E-04	ENSG00000068831
RASGRP4	0.0116	8.0931E-01	8.9970E-01	ENSG00000171777
RASIP1	-0.0751	3.7523E-01	5.8374E-01	ENSG00000105538
RASL10A	-0.3293	1.3915E-02	6.4516E-02	ENSG00000100276
RASL10B	-0.0869	2.7010E-01	4.7974E-01	ENSG00000270885
RASL11A	0.0187	8.1872E-01	9.0582E-01	ENSG00000122035
RASL11B	-0.0118	8.7843E-01	9.3937E-01	ENSG00000128045
RASL12	-0.0922	1.4012E-01	3.1665E-01	ENSG00000103710
RASSF1	0.0720	3.4139E-01	5.5115E-01	ENSG00000068028
RASSF10	-0.0352	6.1370E-01	7.7536E-01	ENSG00000189431
RASSF2	-0.3969	9.9611E-06	2.4436E-04	ENSG00000101265
RASSF3	0.0548	4.3767E-01	6.3812E-01	ENSG00000153179
RASSF4	-0.0538	3.9940E-01	6.0486E-01	ENSG00000107551
RASSF5	0.0903	2.9806E-01	5.0791E-01	ENSG00000266094
RASSF6	-0.0063	9.8251E-01		ENSG00000169435
RASSF7	0.2523	1.4036E-03	1.1671E-02	ENSG00000099849
RASSF8	0.2231	2.8705E-02	1.0773E-01	ENSG00000123094
RASSF9	0.1106	9.9335E-02	2.5359E-01	ENSG00000198774
RAVER1	-0.3594	3.4602E-05	6.6722E-04	ENSG00000161847
RAVER2	-0.2150	1.9225E-02	8.0710E-02	ENSG00000162437
RAX	0.0569	9.9718E-02		ENSG00000134438
RB1	-0.0621	3.1105E-01	5.2110E-01	ENSG00000139687
RB1-DT	0.5069	6.2946E-04	6.3999E-03	ENSG00000231473
RB1CC1	0.0614	2.3978E-01	4.4651E-01	ENSG00000023287
RBAK	0.2816	3.1310E-04	3.7181E-03	ENSG00000146587
RBAK-RBAKDN	-0.0584	2.1848E-01		ENSG00000272968
RBBP4	-0.2470	4.3076E-06	1.2425E-04	ENSG00000162521
RBBP4P2	-0.0133	7.6866E-01	8.7612E-01	ENSG00000242457
RBBP5	-0.1504	3.1211E-02	1.1445E-01	ENSG00000117222
RBBP6	-0.1203	3.4824E-02	1.2345E-01	ENSG00000122257
RBBP7	-0.1956	9.5667E-05	1.4926E-03	ENSG00000102054
RBBP8	-0.0481	4.4098E-01	6.4089E-01	ENSG00000101773
RBBP9	0.0546	4.5226E-01	6.5119E-01	ENSG00000089050
RBCK1	0.1867	9.3962E-04	8.6246E-03	ENSG00000125826
RBFA	-0.0776	2.4869E-01	4.5649E-01	ENSG00000101546
RBFADN	-0.1453	1.0819E-01	2.6815E-01	ENSG00000261126
RBFOX1	-0.0216	7.9484E-01	8.9159E-01	ENSG00000078328

RBFOX2	-0.2620	1.0288E-03	9.2690E-03	ENSG00000100320
RBFOX3	-0.5413	4.4646E-05	8.1312E-04	ENSG00000167281
RBIS	0.0496	4.8034E-01	6.7525E-01	ENSG00000176731
RBISP5	-0.0061	5.7554E-01		ENSG00000224058
RBL1	-0.0857	3.1044E-01	5.2074E-01	ENSG00000080839
RBL2	-0.0382	5.7461E-01	7.4742E-01	ENSG00000103479
RBM10	-0.4425	4.4644E-07	1.9495E-05	ENSG00000182872
RBM11	0.2145	1.7962E-02	7.6956E-02	ENSG00000185272
RBM12	-0.0211	6.9148E-01	8.2889E-01	ENSG00000244462
RBM12B	0.1882	4.3166E-02	1.4417E-01	ENSG00000183808
RBM12B-AS1	0.0290	7.4541E-01	8.6131E-01	ENSG00000279331
RBM12B-DT	-0.0816	1.7039E-01	3.5921E-01	ENSG00000253848
RBM14	-0.1007	9.9404E-02	2.5367E-01	ENSG00000239306
RBM14-RBM4	0.0273	7.0457E-01	8.3708E-01	ENSG00000248643
RBM15	-0.1579	4.6550E-02	1.5208E-01	ENSG00000162775
RBM15-AS1	0.1099	1.6620E-01	3.5384E-01	ENSG00000227963
RBM15B	-0.1411	3.9287E-02	1.3516E-01	ENSG00000259956
RBM17	-0.1134	5.2149E-02	1.6400E-01	ENSG00000134453
RBM18	-0.0491	4.3347E-01	6.3463E-01	ENSG00000119446
RBM19	0.2768	1.3698E-03	1.1472E-02	ENSG00000122965
RBM20	0.0848	2.7918E-01	4.8920E-01	ENSG00000203867
RBM22	-0.1065	5.2795E-02	1.6550E-01	ENSG00000086589
RBM23	0.2530	1.5194E-04	2.1038E-03	ENSG00000100461
RBM24	-0.4885	5.1598E-06	1.4365E-04	ENSG00000112183
RBM25	0.1096	5.3379E-02	1.6680E-01	ENSG00000119707
RBM26	0.1555	3.3164E-02	1.1937E-01	ENSG00000139746
RBM26-AS1	0.4533	4.9486E-05	8.8178E-04	ENSG00000227354
RBM27	0.1181	1.5827E-01	3.4244E-01	ENSG00000091009
RBM28	0.2610	5.1585E-05	9.1379E-04	ENSG00000106344
RBM3	0.1674	3.4752E-02	1.2327E-01	ENSG00000102317
RBM33	0.1444	6.1837E-02	1.8429E-01	ENSG00000184863
RBM33-DT	-0.0015	9.8814E-01	9.9354E-01	ENSG00000216895
RBM34	-0.2609	5.3874E-03	3.1895E-02	ENSG00000188739
RBM38	-0.3020	2.6575E-05	5.3904E-04	ENSG00000132819
RBM39	-0.3618	4.2094E-06	1.2184E-04	ENSG00000131051
RBM39P1	-0.0026	7.8441E-01		ENSG00000214019
RBM4	0.0289	5.7629E-01	7.4871E-01	ENSG00000173933
RBM41	0.0279	6.2808E-01	7.8594E-01	ENSG00000089682
RBM42	-0.0619	2.8467E-01	4.9526E-01	ENSG00000126254
RBM43	0.0827	3.4713E-01	5.5758E-01	ENSG00000184898
RBM44	0.1109	2.2166E-01	4.2495E-01	ENSG00000177483
RBM45	-0.1004	1.1097E-01	2.7278E-01	ENSG00000155636
RBM46	-0.0200	6.0460E-01		ENSG00000151962
RBM47	0.0309	7.1139E-01	8.4162E-01	ENSG00000163694
RBM48	-0.3089	2.0946E-04	2.6920E-03	ENSG00000127993
RBM4B	0.0250	7.1528E-01	8.4351E-01	ENSG00000173914
RBM5	0.2481	6.9427E-04	6.9048E-03	ENSG00000003756
RBM5-AS1	0.1001	1.5805E-01	3.4214E-01	ENSG00000281691
RBM6	-0.1152	5.8856E-02	1.7821E-01	ENSG00000004534

RBM7	-0.0850	1.8690E-01	3.8119E-01	ENSG00000076053
RBM8A	-0.2966	1.4843E-05	3.3408E-04	ENSG00000265241
RBMS1	0.2207	2.1130E-02	8.6306E-02	ENSG00000153250
RBMS2	-0.1475	5.4588E-02	1.6965E-01	ENSG00000076067
RBMS3	-0.3358	5.9543E-03	3.4312E-02	ENSG00000144642
RBMS3-AS2	0.0001	9.6480E-01		ENSG00000203506
RBMX	-0.2999	2.4225E-05	4.9910E-04	ENSG00000147274
RBMX2	0.1178	8.4793E-02	2.2774E-01	ENSG00000134597
RBMXL1	-0.1409	1.1692E-02	5.6908E-02	ENSG00000213516
RBP1	-0.2858	4.2964E-03	2.6899E-02	ENSG00000114115
RBP2	0.0120	8.1508E-01		ENSG00000114113
RBP3	-0.0110	8.0188E-01		ENSG00000265203
RBP4	-0.2621	2.9197E-02	1.0905E-01	ENSG00000138207
RBP5	0.0457	5.4728E-01	7.2703E-01	ENSG00000139194
RBP7	-0.1082	2.3150E-01	4.3635E-01	ENSG00000162444
RBPJ	-0.0720	2.5585E-01	4.6408E-01	ENSG00000168214
RBPMS	0.3264	1.0690E-02	5.3242E-02	ENSG00000157110
RBPMS-AS1	0.0150	5.5800E-01		ENSG00000254109
RBPMS2	0.2849	2.1601E-02	8.7691E-02	ENSG00000166831
RBSN	-0.1053	9.2007E-02	2.4154E-01	ENSG00000131381
RBX1	-0.2386	2.5674E-03	1.8137E-02	ENSG00000100387
RC3H1	-0.0853	1.6553E-01	3.5300E-01	ENSG00000135870
RC3H1-DT	-0.0291	7.3933E-01	8.5776E-01	ENSG00000224977
RC3H2	-0.1008	3.8096E-02	1.3219E-01	ENSG00000056586
RCAN1	0.0981	1.8470E-01	3.7824E-01	ENSG00000159200
RCAN2	0.2861	8.1097E-03	4.3312E-02	ENSG00000172348
RCAN3	-0.1774	3.0825E-02	1.1345E-01	ENSG00000117602
RCAN3AS	0.0026	8.5908E-01		ENSG00000286061
RCBTB1	0.0510	4.2765E-01	6.2958E-01	ENSG00000136144
RCBTB2	-0.3786	4.3469E-04	4.7650E-03	ENSG00000136161
RCC1	0.0030	9.6771E-01	9.8398E-01	ENSG00000180198
RCC1L	-0.0082	8.9852E-01	9.4990E-01	ENSG00000274523
RCC2	-0.1196	1.4084E-01	3.1753E-01	ENSG00000179051
RCCD1	-0.0326	6.3923E-01	7.9381E-01	ENSG00000166965
RCE1	-0.1233	1.4278E-01	3.2041E-01	ENSG00000173653
RCHY1	0.0363	5.2799E-01	7.1271E-01	ENSG00000163743
RCL1	-0.0970	1.5188E-01	3.3359E-01	ENSG00000120158
RCN1	-0.0074	9.0615E-01	9.5408E-01	ENSG00000049449
RCN2	-0.0232	7.0050E-01	8.3446E-01	ENSG00000117906
RCOR1	0.0302	7.0746E-01	8.3915E-01	ENSG00000089902
RCOR2	-0.4537	3.6050E-05	6.8968E-04	ENSG00000167771
RCOR3	-0.0860	1.0032E-01	2.5521E-01	ENSG00000117625
RCSD1	-0.0114	4.9964E-01		ENSG00000198771
RCVRN	0.0191	3.3116E-01		ENSG00000109047
RD3	0.0631	3.3255E-01	5.4281E-01	ENSG00000198570
RD3L	0.0395	5.7558E-01		ENSG00000227729
RDH10	0.3944	7.6415E-05	1.2468E-03	ENSG00000121039
RDH10-AS1	0.0165	3.9872E-01		ENSG00000250295
RDH11	-0.2188	9.5439E-06	2.3560E-04	ENSG00000072042

RDH12	-0.0109	8.8899E-01	9.4486E-01	ENSG00000139988
RDH13	0.1033	1.3798E-01	3.1385E-01	ENSG00000160439
RDH14	-0.0529	3.8161E-01	5.8984E-01	ENSG00000240857
RDH16	0.0383	6.1799E-01	7.7878E-01	ENSG00000139547
RDH5	0.0299	6.9000E-01	8.2779E-01	ENSG00000135437
RDH8	0.0173	3.5977E-01		ENSG00000080511
RDM1	-0.3920	2.8048E-03	1.9397E-02	ENSG00000278023
RDM1P1	0.0831	2.0842E-01	4.0846E-01	ENSG00000266504
RDM1P2	0.0322	6.8799E-01	8.2673E-01	ENSG00000266497
RDM1P4	0.0673	2.2999E-01	4.3461E-01	ENSG00000271974
RDM1P5	-0.0089	9.0584E-01	9.5387E-01	ENSG00000263818
RDX	-0.3176	4.8540E-06	1.3807E-04	ENSG00000137710
RDXP1	0.0087	5.8673E-01		ENSG00000255387
RDXP3	0.0126	6.6648E-01		ENSG00000238247
REC114	-0.0244	3.5298E-01		ENSG00000183324
REC8	-0.2196	1.2868E-02	6.0864E-02	ENSG00000100918
RECK	0.1521	5.8018E-02	1.7661E-01	ENSG00000122707
RECQL	0.1413	5.0607E-02	1.6090E-01	ENSG00000004700
RECQL4	0.0242	7.6446E-01	8.7340E-01	ENSG00000160957
RECQL5	0.0563	4.9770E-01	6.8902E-01	ENSG00000108469
REELD1	0.3520	1.3516E-02	6.3142E-02	ENSG00000250673
REEP1	-0.1181	1.4165E-01	3.1868E-01	ENSG00000068615
REEP2	0.0045	9.9137E-01	9.9518E-01	ENSG00000132563
REEP3	-0.0127	8.3605E-01	9.1544E-01	ENSG00000165476
REEP4	0.1673	5.0629E-02	1.6095E-01	ENSG00000168476
REEP5	-0.0230	5.8583E-01	7.5551E-01	ENSG00000129625
REEP6	0.2497	2.2206E-03	1.6295E-02	ENSG00000115255
REG3G	0.0001	7.4493E-01		ENSG00000143954
REG4	0.0077	8.4831E-01		ENSG00000134193
REL	-0.0264	7.0634E-01	8.3829E-01	ENSG00000162924
REL-DT	0.1047	1.8145E-01	3.7395E-01	ENSG00000228414
RELA	-0.1094	4.1662E-02	1.4074E-01	ENSG00000173039
RELA-DT	0.1997	1.2174E-02	5.8540E-02	ENSG00000285533
RELB	-0.0566	4.9280E-01	6.8483E-01	ENSG00000104856
RELCH	0.1456	1.5994E-02	7.1110E-02	ENSG00000134444
RELL1	-0.0763	3.1595E-01	5.2600E-01	ENSG00000181826
RELL2	0.0224	7.7562E-01	8.8055E-01	ENSG00000164620
RELT	0.1165	1.5105E-01	3.3236E-01	ENSG00000054967
REM1	0.0267	4.0602E-01		ENSG00000088320
REM2	-0.3585	1.6768E-04	2.2631E-03	ENSG00000139890
REN	-0.0158	8.6663E-01	9.3274E-01	ENSG00000143839
RENBP	-0.0013	9.8878E-01	9.9379E-01	ENSG00000102032
REPIN1	-0.0081	8.7743E-01	9.3895E-01	ENSG00000214022
REPS1	-0.1737	5.5504E-03	3.2555E-02	ENSG00000135597
REPS2	-0.0168	8.3453E-01	9.1499E-01	ENSG00000169891
RER1	0.1984	1.7189E-02	7.4590E-02	ENSG00000157916
RERE	0.1280	5.6578E-02	1.7392E-01	ENSG00000142599
RERG	0.0498	5.4411E-01	7.2492E-01	ENSG00000134533
RERGL	0.0755	1.5624E-01	3.4014E-01	ENSG00000111404

RESF1	-0.0249	7.1685E-01	8.4433E-01	ENSG00000174718
RESP18	0.5277	1.6742E-03	1.3224E-02	ENSG00000182698
REST	-0.0314	6.3739E-01	7.9226E-01	ENSG00000084093
RET	0.2089	5.3224E-02	1.6643E-01	ENSG00000165731
RETREG1	-0.0723	3.2050E-01	5.3053E-01	ENSG00000154153
RETREG1-AS1	-0.0324	6.1644E-01	7.7758E-01	ENSG00000246214
RETREG2	-0.0777	2.0521E-01	4.0446E-01	ENSG00000144567
RETREG3	0.0324	6.2680E-01	7.8481E-01	ENSG00000141699
RETSAT	0.0788	2.8999E-01	5.0035E-01	ENSG00000042445
REV1	-0.0441	4.2937E-01	6.3099E-01	ENSG00000135945
REV3L	0.0742	3.1113E-01	5.2118E-01	ENSG00000009413
REX1BD	0.1333	1.1923E-02	5.7715E-02	ENSG00000006015
REXO1	-0.0657	3.5320E-01	5.6337E-01	ENSG00000079313
REXO2	-0.0348	6.4144E-01	7.9494E-01	ENSG00000076043
REXO4	-0.1988	1.2745E-02	6.0489E-02	ENSG00000148300
REXO5	0.0526	4.7030E-01	6.6653E-01	ENSG00000005189
RFC1	0.0638	3.4926E-01	5.5912E-01	ENSG00000035928
RFC2	-0.2554	1.1120E-03	9.8069E-03	ENSG00000049541
RFC3	0.3694	6.3422E-06	1.6925E-04	ENSG00000133119
RFC4	-0.0328	5.5193E-01	7.3067E-01	ENSG00000163918
RFC5	-0.0526	4.3624E-01	6.3708E-01	ENSG00000111445
RFESD	0.1136	2.1396E-01	4.1512E-01	ENSG00000175449
RFFL	-0.1088	1.8498E-01	3.7851E-01	ENSG00000092871
RFK	-0.0567	3.8570E-01	5.9356E-01	ENSG00000135002
RFLNA	-0.0200	8.0232E-01	8.9573E-01	ENSG00000178882
RFLNB	-0.2012	2.8196E-02	1.0632E-01	ENSG00000183688
RFNG	-0.3035	2.1433E-04	2.7461E-03	ENSG00000169733
RFPL2	0.2272	4.6344E-02	1.5165E-01	ENSG00000128253
RFPL3	0.0002	9.5677E-01		ENSG00000128276
RFPL3S	-0.1711	9.3328E-02	2.4382E-01	ENSG00000205853
RFPL4A	0.0059	6.5339E-01	8.0375E-01	ENSG00000223638
RFPL4AL1	0.0458	8.4044E-02		ENSG00000229292
RFT1	0.1992	2.8842E-03	1.9895E-02	ENSG00000163933
RFTN1	0.2464	2.5422E-02	9.8661E-02	ENSG00000131378
RFTN2	-0.1898	3.8360E-02	1.3279E-01	ENSG00000162944
RFWD3	-0.3078	8.8703E-04	8.2829E-03	ENSG00000168411
RFX1	-0.0004	9.9435E-01	9.9686E-01	ENSG00000132005
RFX2	0.0760	3.2904E-01	5.3937E-01	ENSG00000087903
RFX3	-0.0726	2.8243E-01	4.9289E-01	ENSG00000080298
RFX4	0.1068	2.4872E-01	4.5649E-01	ENSG00000111783
RFX5	0.0528	4.4367E-01	6.4284E-01	ENSG00000143390
RFX6	0.0183	3.3786E-01		ENSG00000185002
RFX7	0.2402	1.6044E-03	1.2820E-02	ENSG00000181827
RFX8	0.0410	5.6333E-01	7.3975E-01	ENSG00000196460
RFXANK	-0.4946	6.8474E-12	1.7550E-09	ENSG00000064490
RFXAP	-0.1343	5.7853E-02	1.7630E-01	ENSG00000133111
RGCC	-0.0842	2.9785E-01	5.0763E-01	ENSG00000102760
RGL1	0.0751	2.4992E-01	4.5778E-01	ENSG00000143344
RGL2	-0.0063	9.2829E-01	9.6476E-01	ENSG00000237441

RGL3	0.0454	4.6420E-01	6.6117E-01	ENSG00000205517
RGL4	-0.0155	6.1463E-01		ENSG00000159496
RGMA	0.0234	7.7456E-01	8.8003E-01	ENSG00000182175
RGMB	-0.0533	5.2227E-01	7.0868E-01	ENSG00000174136
RGMB-AS1	0.0132	8.7448E-01	9.3710E-01	ENSG00000246763
RGN	0.2363	4.9539E-02	1.5855E-01	ENSG00000130988
RGP1	-0.0075	9.0784E-01	9.5461E-01	ENSG00000107185
RGPD1	-0.0367	6.6960E-01	8.1482E-01	ENSG00000187627
RGPD2	-0.0214	7.9657E-01	8.9256E-01	ENSG00000185304
RGPD3	0.0140	8.6408E-01	9.3150E-01	ENSG00000153165
RGPD4	0.0557	5.2116E-01	7.0783E-01	ENSG00000196862
RGPD5	0.0176	7.5512E-01	8.6698E-01	ENSG00000015568
RGPD6	0.0236	7.1912E-01	8.4556E-01	ENSG00000183054
RGPD8	0.0740	2.2835E-01	4.3305E-01	ENSG00000169629
RGR	-0.5881	2.4866E-03	1.7729E-02	ENSG00000148604
RGS1	0.0222	3.6262E-01		ENSG00000090104
RGS11	-0.5489	3.5011E-04	4.0368E-03	ENSG00000076344
RGS12	-0.0665	3.1513E-01	5.2522E-01	ENSG00000159788
RGS13	0.0368	4.3709E-01	6.3773E-01	ENSG00000127074
RGS14	-0.1700	3.1586E-02	1.1536E-01	ENSG00000169220
RGS16	-0.3643	2.7699E-03	1.9214E-02	ENSG00000143333
RGS17	0.2761	4.0514E-04	4.5218E-03	ENSG00000091844
RGS19	-0.4453	2.8268E-04	3.4323E-03	ENSG00000171700
RGS2	-0.1329	8.2024E-02	2.2286E-01	ENSG00000116741
RGS20	0.2797	9.0259E-04	8.3963E-03	ENSG00000147509
RGS21	0.0218	2.0053E-01		ENSG00000253148
RGS22	0.1918	2.3024E-02	9.2009E-02	ENSG00000132554
RGS3	-0.0382	6.1513E-01	7.7668E-01	ENSG00000138835
RGS4	0.5930	2.4787E-03	1.7693E-02	ENSG00000117152
RGS5	0.1916	5.4866E-02	1.7032E-01	ENSG00000143248
RGS6	0.1412	1.4127E-01	3.1814E-01	ENSG00000182732
RGS7	0.0540	4.9966E-01	6.9077E-01	ENSG00000182901
RGS7BP	-0.1288	1.3289E-01	3.0662E-01	ENSG00000186479
RGS8	0.0889	3.0561E-01	5.1579E-01	ENSG00000135824
RGS9	-0.1219	1.5248E-01	3.3455E-01	ENSG00000108370
RGS9BP	-0.0784	2.7754E-01	4.8754E-01	ENSG00000186326
RGSL1	-0.0009	7.6105E-01		ENSG00000121446
RHAG	-0.0092	4.0645E-01		ENSG00000112077
RHBDD1	-0.0464	5.3203E-01	7.1596E-01	ENSG00000144468
RHBDD2	-0.0233	6.8583E-01	8.2498E-01	ENSG00000005486
RHBDD3	-0.0271	5.7398E-01	7.4685E-01	ENSG00000100263
RHBDF1	0.0122	8.8356E-01	9.4153E-01	ENSG00000007384
RHBDF1P1	0.0188	6.3499E-01		ENSG00000234123
RHBDF2	-0.2724	2.9246E-02	1.0915E-01	ENSG00000129667
RHBDL1	-0.0414	6.0027E-01	7.6590E-01	ENSG00000103269
RHBDL2	0.0062	9.4063E-01	9.7065E-01	ENSG00000158315
RHBDL3	-0.0399	6.0473E-01	7.6865E-01	ENSG00000141314
RHBG	-0.1384	1.2585E-01	2.9650E-01	ENSG00000132677
RHCE	0.4600	4.4585E-05	8.1260E-04	ENSG00000188672

RHCG	0.0229	7.3886E-01	8.5776E-01	ENSG00000140519
RHD	0.1253	1.6988E-01	3.5845E-01	ENSG00000187010
RHEB	-0.3449	3.1752E-06	9.7294E-05	ENSG00000106615
RHEBL1	0.2234	2.7265E-02	1.0378E-01	ENSG00000167550
RHEX	0.0058	8.5749E-01		ENSG00000263961
RHNO1	-0.1255	3.0388E-02	1.1225E-01	ENSG00000171792
RHO	-0.0010	9.4771E-01		ENSG00000163914
RHOA	-0.1833	3.6640E-04	4.1832E-03	ENSG00000067560
RHOB	-0.2974	4.2040E-03	2.6489E-02	ENSG00000143878
RHOBTB1	0.2506	1.9238E-03	1.4632E-02	ENSG00000072422
RHOBTB2	0.3019	3.8620E-04	4.3529E-03	ENSG00000008853
RHOBTB3	-0.1927	1.6324E-02	7.1992E-02	ENSG00000164292
RHOC	-0.1109	1.0601E-01	2.6477E-01	ENSG00000155366
RHOD	-0.0131	8.5730E-01	9.2786E-01	ENSG00000173156
RHOF	-0.1290	1.7583E-01	3.6681E-01	ENSG00000139725
RHOG	0.1103	1.7342E-01	3.6346E-01	ENSG00000177105
RHOG2P	-0.0080	6.6261E-01		ENSG00000213706
RHOH	0.0009	9.9135E-01	9.9518E-01	ENSG00000168421
RHOJ	0.1259	1.6644E-01	3.5416E-01	ENSG00000126785
RHOQ	0.1934	1.0581E-02	5.2864E-02	ENSG00000119729
RHOQ-AS1	0.2336	2.1791E-02	8.8329E-02	ENSG00000250116
RHOT1	-0.0722	2.5916E-01	4.6768E-01	ENSG00000126858
RHOT2	0.0361	5.4472E-01	7.2546E-01	ENSG00000140983
RHOU	-0.0296	7.1217E-01	8.4207E-01	ENSG00000116574
RHOV	-0.5655	2.3961E-07	1.1815E-05	ENSG00000104140
RHOXF1	-0.0344	5.3193E-01	7.1594E-01	ENSG00000101883
RHOXF1-AS1	0.0010	9.2311E-01		ENSG00000258545
RHOXF2	0.1677	2.3971E-02	9.4777E-02	ENSG00000131721
RHOXF2B	0.0127	4.2360E-01		ENSG00000203989
RHPN1	-0.0178	7.9313E-01	8.9076E-01	ENSG00000158106
RHPN1-AS1	0.0385	6.5558E-01	8.0547E-01	ENSG00000254389
RHPN2	-0.0052	9.4079E-01	9.7073E-01	ENSG00000131941
RIBC1	0.2389	3.4717E-02	1.2324E-01	ENSG00000158423
RIBC2	0.0708	3.5911E-01	5.6860E-01	ENSG00000128408
RIC1	-0.1024	1.3704E-01	3.1240E-01	ENSG00000107036
RIC3	0.2135	8.2576E-05	1.3228E-03	ENSG00000166405
RIC8A	-0.0524	4.1946E-01	6.2219E-01	ENSG00000177963
RIC8B	0.0501	4.3607E-01	6.3696E-01	ENSG00000111785
RICTOR	0.1633	2.8626E-02	1.0751E-01	ENSG00000164327
RIDA	-0.2143	7.0405E-03	3.8972E-02	ENSG00000132541
RIF1	0.1260	3.9711E-02	1.3618E-01	ENSG00000080345
RIGI	0.0800	3.3843E-01	5.4830E-01	ENSG00000107201
RIIAD1	0.4359	1.1404E-05	2.7190E-04	ENSG00000178796
RILP	0.0338	6.5318E-01	8.0373E-01	ENSG00000167705
RILPL1	-0.0886	2.2909E-01	4.3343E-01	ENSG00000188026
RILPL2	0.0057	9.3240E-01	9.6657E-01	ENSG00000150977
RIMBP2	0.1775	1.9195E-02	8.0647E-02	ENSG00000060709
RIMBP3	0.0189	8.2816E-01	9.1103E-01	ENSG00000275793
RIMBP3B	0.0380	6.4472E-01	7.9719E-01	ENSG00000274600

RIMBP3C	0.0337	6.7109E-01	8.1595E-01	ENSG00000183246
RIMKLA	-0.0131	8.0336E-01	8.9653E-01	ENSG00000177181
RIMKLB	-0.3530	8.7387E-09	7.4689E-07	ENSG00000166532
RIMOC1	-0.1001	1.4736E-01	3.2733E-01	ENSG00000205765
RIMS1	0.3362	2.7347E-03	1.9008E-02	ENSG00000079841
RIMS2	0.4284	1.4384E-05	3.2770E-04	ENSG00000176406
RIMS3	0.0723	3.1085E-01	5.2105E-01	ENSG00000117016
RIMS4	-0.2316	1.1969E-02	5.7858E-02	ENSG00000101098
RIN1	0.0518	5.4616E-01	7.2638E-01	ENSG00000174791
RIN2	0.2253	1.6860E-02	7.3640E-02	ENSG00000132669
RIN3	0.0403	5.9737E-01	7.6382E-01	ENSG00000100599
RING1	0.0870	1.8653E-01	3.8084E-01	ENSG00000204227
RINL	-0.0080	9.0929E-01	9.5524E-01	ENSG00000187994
RINT1	0.0117	8.7509E-01	9.3742E-01	ENSG00000135249
RIOK1	0.1209	8.6289E-02	2.3060E-01	ENSG00000124784
RIOK2	0.0649	3.3806E-01	5.4799E-01	ENSG00000058729
RIOK3	0.0041	9.6500E-01	9.8277E-01	ENSG00000101782
RIOX1	-0.0724	2.6727E-01	4.7654E-01	ENSG00000170468
RIOX2	-0.0756	3.5655E-01	5.6643E-01	ENSG00000170854
RIPK1	0.0388	6.0891E-01	7.7196E-01	ENSG00000137275
RIPK2	-0.2542	7.1811E-04	7.0818E-03	ENSG00000104312
RIPK3	0.0269	3.6655E-01		ENSG00000129465
RIPK4	0.0523	4.7512E-01	6.7068E-01	ENSG00000183421
RIPOR1	-0.2123	9.1064E-04	8.4520E-03	ENSG00000039523
RIPOR2	0.0350	6.7217E-01	8.1665E-01	ENSG00000111913
RIPOR3	0.0038	9.6440E-01	9.8237E-01	ENSG00000042062
RIPPLY1	-0.0007	9.1106E-01		ENSG00000147223
RIPPLY2	0.0758	3.5282E-01	5.6301E-01	ENSG00000203877
RIPPLY3	0.3044	1.8726E-02	7.9193E-02	ENSG00000183145
RIT1	-0.2324	1.0079E-02	5.1038E-02	ENSG00000143622
RIT2	-0.2909	2.1389E-02	8.7073E-02	ENSG00000152214
RITA1	-0.0914	2.1157E-01	4.1238E-01	ENSG00000139405
RLBP1	0.0611	2.9392E-01	5.0393E-01	ENSG00000140522
RLF	-0.0469	4.4321E-01	6.4246E-01	ENSG00000117000
RLIG1	0.0418	5.5155E-01	7.3036E-01	ENSG00000133641
RLIM	-0.0715	1.7126E-01	3.6042E-01	ENSG00000131263
RLIMP1	0.0064	9.1955E-01		ENSG00000229456
RLIMP3	0.0266	2.2170E-01		ENSG00000259388
RLN1	-0.0546	5.1966E-01	7.0681E-01	ENSG00000107018
RLN2	-0.0374	6.5866E-01	8.0739E-01	ENSG00000107014
RLN3	0.0007	8.1591E-01		ENSG00000171136
RMC1	-0.1377	5.5692E-02	1.7215E-01	ENSG00000141452
RMDN1	0.1076	9.3281E-02	2.4378E-01	ENSG00000176623
RMDN2	-0.0131	8.4599E-01	9.2100E-01	ENSG00000115841
RMDN3	-0.0816	2.0113E-01	3.9959E-01	ENSG00000137824
RMI1	0.2000	8.8346E-03	4.6339E-02	ENSG00000178966
RMI2	0.0076	9.2139E-01	9.6140E-01	ENSG00000175643
RMND1	-0.0376	5.0673E-01	6.9620E-01	ENSG00000155906
RMND5A	-0.1616	1.6973E-02	7.3927E-02	ENSG00000153561

RMND5B	-0.0840	2.1249E-01	4.1353E-01	ENSG00000145916
RN7SKP106	-0.0024	9.9500E-01		ENSG00000222764
RN7SKP11	0.0066	6.1367E-01		ENSG00000252827
RN7SKP110	0.0074	9.4313E-01	9.7180E-01	ENSG00000222078
RN7SKP112	0.0853	1.0312E-01	2.5996E-01	ENSG00000223145
RN7SKP114	-0.0653	1.5140E-01		ENSG00000222259
RN7SKP151	-0.0307	5.8758E-01	7.5670E-01	ENSG00000222162
RN7SKP16	0.0371	4.7141E-01	6.6766E-01	ENSG00000222112
RN7SKP160	0.0032	4.3842E-01		ENSG00000200674
RN7SKP225	-0.0312	3.5182E-01		ENSG00000222337
RN7SKP23	0.0076	9.0638E-01	9.5421E-01	ENSG00000280039
RN7SKP248	-0.0016	9.2477E-01		ENSG00000252087
RN7SKP253	0.0164	4.6885E-01		ENSG00000252233
RN7SKP266	-0.0117	9.8506E-01		ENSG00000222164
RN7SKP272	0.0213	4.6903E-01		ENSG00000252797
RN7SKP276	-0.0251	7.2184E-01	8.4753E-01	ENSG00000252051
RN7SKP283	-0.0071	6.7658E-01		ENSG00000253006
RN7SKP287	-0.0149	5.2570E-01		ENSG00000252355
RN7SKP292	-0.1445	7.9416E-02	2.1860E-01	ENSG00000202392
RN7SKP30	-0.0448	2.0188E-01		ENSG00000223305
RN7SKP38	-0.0202	4.2001E-01		ENSG00000222714
RN7SKP4	0.0369	3.9187E-01		ENSG00000271818
RN7SKP40	-0.0050	7.2927E-01		ENSG00000253020
RN7SKP55	-0.0236	2.1556E-01		ENSG00000201758
RN7SKP56	0.0611	2.2338E-01		ENSG00000222445
RN7SKP57	-0.0224	5.3373E-01		ENSG00000253057
RN7SKP64	0.0027	9.6791E-01		ENSG00000253015
RN7SKP76	0.0251	3.3309E-01		ENSG00000201289
RN7SKP80	0.1829	7.2278E-02	2.0539E-01	ENSG00000202058
RN7SKP83	-0.0279	2.4052E-01		ENSG00000252321
RN7SKP88	0.0336	4.3289E-01		ENSG00000252656
RN7SKP96	0.0219	3.5213E-01		ENSG00000252757
RN7SKP97	-0.0068	7.2714E-01		ENSG00000222898
RN7SL105P	0.0062	6.9744E-01		ENSG00000243704
RN7SL113P	0.0167	9.1360E-01		ENSG00000278590
RN7SL11P	0.0142	4.2809E-01		ENSG00000239316
RN7SL12P	-0.0071	6.1093E-01		ENSG00000239553
RN7SL130P	-0.0097	6.7618E-01		ENSG00000244256
RN7SL138P	0.0000	8.3027E-01		ENSG00000266274
RN7SL141P	-0.0064	8.4096E-01		ENSG00000243398
RN7SL144P	0.0074	9.7457E-01		ENSG00000242559
RN7SL145P	0.0001	8.3819E-01		ENSG00000244357
RN7SL146P	-0.0361	4.1059E-01		ENSG00000240106
RN7SL149P	0.0072	6.8227E-01		ENSG00000241834
RN7SL162P	-0.0264	3.0733E-01		ENSG00000266503
RN7SL163P	0.0149	2.1869E-01		ENSG00000266195
RN7SL166P	-0.0160	4.2082E-01		ENSG00000275014
RN7SL177P	-0.0127	4.1738E-01		ENSG00000242976
RN7SL182P	-0.0157	3.5241E-01		ENSG00000241461

RN7SL189P	0.0221	1.9459E-01		ENSG00000243817
RN7SL192P	-0.0322	4.9468E-01	6.8670E-01	ENSG00000276757
RN7SL196P	-0.0012	6.3962E-01		ENSG00000274424
RN7SL199P	0.1778	8.4701E-02	2.2752E-01	ENSG00000265315
RN7SL200P	-0.0187	4.5925E-01		ENSG00000265123
RN7SL202P	-0.0286	1.4266E-01		ENSG00000266627
RN7SL20P	0.0066	8.9810E-01		ENSG00000242251
RN7SL217P	-0.0252	3.3722E-01	5.4734E-01	ENSG00000264706
RN7SL220P	0.0043	9.5581E-01		ENSG00000266467
RN7SL230P	-0.0179	6.7200E-01		ENSG00000264916
RN7SL233P	0.0498	3.4946E-01	5.5936E-01	ENSG00000242971
RN7SL239P	0.0101	5.6989E-01		ENSG00000242999
RN7SL23P	-0.0208	2.9053E-01		ENSG00000240823
RN7SL242P	-0.0154	9.1409E-01		ENSG00000244389
RN7SL246P	0.0602	1.5398E-01		ENSG00000265093
RN7SL258P	0.0301	3.3961E-01		ENSG00000240589
RN7SL262P	0.0110	6.0925E-01		ENSG00000265033
RN7SL263P	-0.0167	2.9584E-01		ENSG00000240160
RN7SL265P	-0.0030	9.1638E-01		ENSG00000241709
RN7SL268P	0.0521	4.4145E-01	6.4100E-01	ENSG00000244425
RN7SL269P	0.0555	4.2691E-02		ENSG00000243051
RN7SL273P	-0.0823	2.0237E-01	4.0122E-01	ENSG00000239953
RN7SL274P	0.0066	9.5900E-01		ENSG00000242430
RN7SL280P	0.0100	9.6519E-01		ENSG00000244671
RN7SL285P	-0.1271	1.1543E-01	2.7998E-01	ENSG00000243313
RN7SL286P	-0.0018	7.1309E-01		ENSG00000277464
RN7SL297P	0.0038	5.4851E-01		ENSG00000240183
RN7SL3	-0.0658	7.7144E-02		ENSG00000278771
RN7SL306P	-0.0008	8.5744E-01		ENSG00000242241
RN7SL308P	0.0035	3.3440E-01		ENSG00000243951
RN7SL30P	-0.0092	6.3166E-01		ENSG00000243845
RN7SL313P	0.0098	4.8753E-01		ENSG00000275655
RN7SL328P	0.0245	2.6159E-01		ENSG00000240853
RN7SL329P	0.0776	2.0150E-01	4.0010E-01	ENSG00000242170
RN7SL331P	-0.0126	4.4262E-01		ENSG00000278422
RN7SL336P	-0.0685	3.6167E-01	5.7107E-01	ENSG00000264017
RN7SL338P	-0.0380	2.3706E-01		ENSG00000241568
RN7SL34P	0.0159	6.1224E-01		ENSG00000239868
RN7SL350P	0.0098	5.4344E-01		ENSG00000243254
RN7SL351P	0.0106	3.5263E-01		ENSG00000240098
RN7SL359P	-0.0143	4.0890E-01		ENSG00000244384
RN7SL364P	-0.0433	4.3652E-01		ENSG00000243560
RN7SL370P	-0.0329	3.2593E-01		ENSG00000243437
RN7SL378P	0.0282	9.4276E-02		ENSG00000265684
RN7SL37P	0.0185	7.8463E-01	8.8624E-01	ENSG00000242493
RN7SL381P	-0.0252	3.9526E-01		ENSG00000263968
RN7SL382P	0.1044	1.7817E-01	3.6944E-01	ENSG00000240723
RN7SL384P	0.0035	9.2823E-01		ENSG00000242912
RN7SL385P	0.0382	2.7069E-01		ENSG00000241333

RN7SL388P	0.1860	3.3336E-02	1.1983E-01	ENSG00000239577
RN7SL394P	0.0152	6.2975E-01		ENSG00000239942
RN7SL395P	0.0014	9.2546E-01		ENSG00000244307
RN7SL399P	-0.0048	5.7659E-01		ENSG00000239542
RN7SL39P	-0.0120	3.1302E-01		ENSG00000241223
RN7SL402P	-0.0606	6.9917E-02		ENSG00000243968
RN7SL405P	-0.0305	1.2152E-01		ENSG00000264540
RN7SL40P	0.0080	6.0741E-01		ENSG00000242696
RN7SL413P	-0.0568	4.9133E-02		ENSG00000242893
RN7SL417P	0.0088	9.0335E-01	9.5277E-01	ENSG00000244056
RN7SL423P	-0.0008	9.3190E-01		ENSG00000244372
RN7SL430P	0.0195	4.4307E-01		ENSG00000243911
RN7SL434P	-0.0130	3.6920E-01		ENSG00000241868
RN7SL441P	-0.0921	1.3725E-01	3.1266E-01	ENSG00000241413
RN7SL443P	0.1088	2.3245E-01	4.3747E-01	ENSG00000241229
RN7SL444P	-0.0549	4.0610E-01	6.1059E-01	ENSG00000265753
RN7SL448P	0.0380	2.9443E-02		ENSG00000264322
RN7SL449P	-0.0164	7.8841E-01		ENSG00000242889
RN7SL452P	-0.0187	7.4303E-01		ENSG00000243103
RN7SL459P	0.0067	9.3723E-01		ENSG00000240993
RN7SL470P	-0.0174	5.0689E-01		ENSG00000263669
RN7SL472P	-0.0115	5.0194E-01		ENSG00000240457
RN7SL473P	0.0174	7.9062E-01	8.8927E-01	ENSG00000277452
RN7SL477P	-0.0172	3.4356E-01		ENSG00000239367
RN7SL47P	-0.0122	6.0045E-01		ENSG00000244710
RN7SL481P	0.0254	2.0643E-01		ENSG00000240322
RN7SL488P	-0.0239	1.5532E-01		ENSG00000264271
RN7SL498P	0.0068	4.3200E-01		ENSG00000263755
RN7SL499P	0.0147	4.3234E-01		ENSG00000239202
RN7SL49P	0.0207	6.3380E-01		ENSG00000265802
RN7SL502P	0.0197	6.3819E-01		ENSG00000240733
RN7SL505P	-0.0215	6.1464E-01		ENSG00000241420
RN7SL508P	0.0116	8.6709E-01	9.3299E-01	ENSG00000244112
RN7SL510P	-0.0112	4.8423E-01		ENSG00000266308
RN7SL516P	0.0364	2.9563E-01		ENSG00000242699
RN7SL521P	0.0129	8.6952E-01	9.3437E-01	ENSG00000240877
RN7SL524P	-0.4178	4.9277E-03	2.9754E-02	ENSG00000266754
RN7SL525P	-0.0069	6.0019E-01		ENSG00000264910
RN7SL526P	-0.0322	4.9338E-01		ENSG00000243642
RN7SL530P	0.0293	2.5714E-01		ENSG00000239910
RN7SL531P	0.0032	9.9175E-01		ENSG00000264071
RN7SL538P	0.0043	9.0696E-01		ENSG00000240692
RN7SL539P	0.0004	6.1515E-01		ENSG00000274076
RN7SL544P	0.0134	1.9292E-01		ENSG00000275230
RN7SL547P	0.0182	6.4368E-01		ENSG00000240584
RN7SL558P	-0.0147	7.2859E-01		ENSG00000243260
RN7SL559P	0.0717	3.0864E-02		ENSG00000240750
RN7SL55P	-0.0413	1.6614E-01		ENSG00000243227
RN7SL565P	0.0070	7.6271E-01		ENSG00000276562

RN7SL566P	0.0985	9.1339E-02	2.4038E-01	ENSG00000241983
RN7SL569P	-0.0233	2.5531E-01		ENSG00000239468
RN7SL574P	0.0426	3.4348E-01		ENSG00000266075
RN7SL577P	-0.0045	9.3537E-01	9.6827E-01	ENSG00000265942
RN7SL57P	0.0027	5.6384E-01		ENSG00000242256
RN7SL582P	-0.0142	6.5580E-01		ENSG00000241156
RN7SL587P	-0.2342	3.1936E-02	1.1607E-01	ENSG00000240233
RN7SL589P	0.0324	2.3944E-01		ENSG00000239247
RN7SL600P	0.1136	6.0495E-02		ENSG00000274963
RN7SL605P	-0.0033	8.3636E-01		ENSG00000265566
RN7SL606P	-0.0008	9.1319E-01		ENSG00000239932
RN7SL608P	-0.0213	3.4953E-01		ENSG00000239884
RN7SL610P	0.0177	4.1451E-01		ENSG00000243847
RN7SL612P	0.0155	2.8066E-01		ENSG00000266160
RN7SL614P	0.0103	5.5617E-01		ENSG00000243267
RN7SL615P	-0.1161	7.2025E-03		ENSG00000263989
RN7SL621P	-0.0165	4.2868E-01		ENSG00000240189
RN7SL622P	0.0028	8.5873E-01		ENSG00000265052
RN7SL623P	-0.0185	3.1315E-01		ENSG00000244501
RN7SL628P	0.0110	1.7627E-01		ENSG00000277467
RN7SL635P	0.0071	7.4643E-01		ENSG00000243029
RN7SL638P	0.0013	9.9247E-01		ENSG00000243702
RN7SL648P	-0.0022	8.9060E-01		ENSG00000265727
RN7SL649P	0.0333	3.3922E-01		ENSG00000243539
RN7SL652P	-0.0075	5.6077E-01		ENSG00000265559
RN7SL655P	0.0259	1.1731E-01		ENSG00000241963
RN7SL656P	0.1198	1.8169E-01	3.7422E-01	ENSG00000265411
RN7SL65P	0.0004	7.2842E-01		ENSG00000243770
RN7SL660P	0.1401	3.3758E-02	1.2073E-01	ENSG00000278746
RN7SL663P	-0.0267	4.4863E-01		ENSG00000275132
RN7SL665P	0.0098	5.0745E-01		ENSG00000264169
RN7SL670P	-0.0025	7.0848E-01		ENSG00000264041
RN7SL672P	0.0041	9.4119E-01	9.7089E-01	ENSG00000239742
RN7SL678P	0.0148	5.1787E-01		ENSG00000263969
RN7SL67P	0.0409	2.7205E-01		ENSG00000243854
RN7SL683P	-0.0131	4.6817E-01		ENSG00000242330
RN7SL687P	-0.0023	8.7526E-01		ENSG00000244335
RN7SL688P	0.0014	9.4814E-01		ENSG00000239726
RN7SL689P	-0.0346	5.4411E-01	7.2492E-01	ENSG00000263432
RN7SL68P	-0.0587	4.3106E-01	6.3286E-01	ENSG00000242020
RN7SL693P	0.0084	7.8219E-01		ENSG00000265272
RN7SL697P	-0.0009	9.1648E-01		ENSG00000264484
RN7SL698P	0.0120	5.1124E-01		ENSG00000244232
RN7SL716P	-0.0211	3.1418E-01		ENSG00000263558
RN7SL718P	0.0045	4.1345E-01		ENSG00000264423
RN7SL722P	0.0084	7.2467E-01		ENSG00000273940
RN7SL724P	0.0100	6.6693E-01		ENSG00000244692
RN7SL735P	-0.0242	1.4023E-01		ENSG00000244044
RN7SL738P	-0.4060	1.4891E-02	6.7600E-02	ENSG00000243339

RN7SL73P	0.0060	7.2397E-01		ENSG00000265882
RN7SL743P	0.0563	4.5218E-01	6.5119E-01	ENSG00000243954
RN7SL744P	-0.0271	7.5141E-02		ENSG00000264028
RN7SL749P	-0.0377	5.5464E-01	7.3288E-01	ENSG00000242853
RN7SL757P	-0.0074	8.4102E-01		ENSG00000239249
RN7SL75P	-0.0086	7.4253E-01		ENSG00000239908
RN7SL760P	0.0079	5.9223E-01		ENSG00000272469
RN7SL763P	0.0118	3.2152E-01		ENSG00000277774
RN7SL76P	0.0279	4.3271E-01		ENSG00000241959
RN7SL771P	0.0025	7.1203E-01		ENSG00000240173
RN7SL774P	0.0008	9.9112E-01		ENSG00000263882
RN7SL775P	-0.0185	6.1320E-01		ENSG00000243370
RN7SL778P	-0.0039	8.7263E-01		ENSG00000272412
RN7SL787P	0.0259	7.1836E-02		ENSG00000274098
RN7SL788P	0.0284	1.7394E-01		ENSG00000241745
RN7SL789P	-0.0391	3.1829E-01		ENSG00000242436
RN7SL791P	0.0331	5.1008E-01		ENSG00000241291
RN7SL794P	0.0320	2.6211E-01		ENSG00000240235
RN7SL79P	-0.0039	8.8267E-01		ENSG00000239595
RN7SL803P	0.0065	8.5438E-01		ENSG00000272197
RN7SL806P	0.0087	4.5422E-01		ENSG00000242719
RN7SL809P	-0.0731	1.5297E-01	3.3512E-01	ENSG00000241217
RN7SL812P	-0.0600	2.5370E-01		ENSG00000242876
RN7SL815P	0.0292	1.9976E-01		ENSG00000243359
RN7SL81P	-0.0125	8.0780E-01		ENSG00000244218
RN7SL827P	-0.0217	5.3126E-01		ENSG00000271986
RN7SL82P	-0.0076	7.6917E-01		ENSG00000278696
RN7SL832P	0.3764	1.3309E-03	1.1253E-02	ENSG00000243819
RN7SL833P	-0.0336	2.9501E-01		ENSG00000244080
RN7SL83P	0.0171	5.0059E-01		ENSG00000241756
RN7SL846P	-0.0019	8.9664E-01		ENSG00000242818
RN7SL849P	0.0040	8.8030E-01		ENSG00000243115
RN7SL850P	0.0025	8.7550E-01		ENSG00000263955
RN7SL851P	-0.0142	7.6638E-01	8.7469E-01	ENSG00000240718
RN7SL856P	0.0449	3.5195E-01	5.6220E-01	ENSG00000276653
RNA5-8SN1	0.2102	1.4472E-02	6.6272E-02	ENSG00000278189
RNA5-8SN2	0.1810	3.7610E-02	1.3085E-01	ENSG00000278233
RNA5-8SN3	0.1872	3.0531E-02	1.1260E-01	ENSG00000275215
RNA5-8SN4	0.3217	9.0375E-04	8.4009E-03	ENSG00000276700
RNA5-8SN5	0.2111	1.6471E-02	7.2500E-02	ENSG00000274917
RNA5-8SP10	0.2416	6.9223E-03	3.8508E-02	ENSG00000277739
RNA5-8SP6	0.0128	6.4180E-01		ENSG00000251705
RNA5SP118	0.0356	1.3706E-01	3.1240E-01	ENSG00000222317
RNA5SP122	-0.0015	8.7257E-01		ENSG00000207186
RNA5SP123	0.0200	2.7717E-01		ENSG00000200114
RNA5SP130	0.0030	8.3258E-01		ENSG00000201301
RNA5SP131	-0.0151	6.5962E-01		ENSG00000202322
RNA5SP132	-0.0045	7.6483E-01		ENSG00000201595
RNA5SP141	0.0028	8.6948E-01		ENSG00000201413

RNA5SP149	-0.0034	8.2958E-01		ENSG00000201822
RNA5SP151	0.0123	6.4836E-01		ENSG00000202502
RNA5SP159	0.0431	1.4671E-01		ENSG00000252970
RNA5SP174	-0.0019	7.9171E-01		ENSG00000199572
RNA5SP18	0.0758	2.7749E-01	4.8754E-01	ENSG00000212237
RNA5SP180	0.0135	3.8592E-01		ENSG00000251990
RNA5SP191	-0.0240	6.0272E-01		ENSG00000199455
RNA5SP194	0.0252	6.0739E-01		ENSG00000201532
RNA5SP195	-0.0394	2.8552E-01		ENSG00000199545
RNA5SP212	0.0228	3.4078E-01		ENSG00000212525
RNA5SP217	0.0264	2.4248E-01		ENSG00000251768
RNA5SP218	0.0354	4.6288E-01		ENSG00000200058
RNA5SP221	-0.0321	5.6118E-01	7.3819E-01	ENSG00000223203
RNA5SP233	-0.0041	9.9195E-01		ENSG00000238391
RNA5SP235	0.0187	4.8140E-01		ENSG00000202281
RNA5SP246	0.0010	8.9229E-01		ENSG00000239021
RNA5SP247	-0.0575	1.1393E-01		ENSG00000223113
RNA5SP255	0.0364	1.0989E-01		ENSG00000252179
RNA5SP259	-0.0055	7.0061E-01		ENSG00000202411
RNA5SP278	-0.0189	5.8540E-01		ENSG00000252864
RNA5SP282	-0.0435	3.3363E-01		ENSG00000252164
RNA5SP289	0.0007	9.2705E-01		ENSG00000199202
RNA5SP295	0.0064	8.2415E-01		ENSG00000199525
RNA5SP298	0.0146	5.7631E-01		ENSG00000201861
RNA5SP301	0.0034	9.4437E-01	9.7218E-01	ENSG00000199407
RNA5SP304	0.0415	2.3359E-01	4.3877E-01	ENSG00000223131
RNA5SP305	-0.0071	9.5807E-01		ENSG00000199535
RNA5SP306	0.0081	4.2658E-01		ENSG00000223019
RNA5SP310	0.0276	4.6212E-01		ENSG00000239152
RNA5SP311	-0.0204	6.9821E-01		ENSG00000238405
RNA5SP312	-0.0246	6.7254E-01	8.1686E-01	ENSG00000252877
RNA5SP315	-0.0294	5.8697E-01		ENSG00000252149
RNA5SP317	0.0360	3.2492E-01		ENSG00000222108
RNA5SP319	-0.0292	5.7024E-01		ENSG00000199638
RNA5SP33	0.0053	9.7073E-01		ENSG00000252496
RNA5SP330	-0.0221	1.8203E-01		ENSG00000252905
RNA5SP337	0.0042	8.8555E-01		ENSG00000252816
RNA5SP341	0.0465	1.6826E-01		ENSG00000252070
RNA5SP343	0.0037	7.3375E-01		ENSG00000284736
RNA5SP351	0.0068	6.6328E-01		ENSG00000238965
RNA5SP363	-0.0136	7.0477E-01	8.3721E-01	ENSG00000201942
RNA5SP364	0.0209	2.0481E-01		ENSG00000252747
RNA5SP366	0.0293	3.0704E-01		ENSG00000202324
RNA5SP37	-0.0025	9.6809E-01	9.8421E-01	ENSG00000202290
RNA5SP372	-0.0347	2.1622E-01		ENSG00000212138
RNA5SP382	-0.0095	6.3804E-01		ENSG00000200225
RNA5SP388	0.0056	9.6112E-01		ENSG00000199454
RNA5SP395	-0.0068	7.8854E-01		ENSG00000252376
RNA5SP437	0.1598	4.1337E-02	1.3999E-01	ENSG00000253058

RNA5SP441	0.0194	1.9479E-01		ENSG00000252618
RNA5SP442	0.0083	7.8162E-01		ENSG00000201920
RNA5SP443	0.0156	3.4704E-01		ENSG00000199953
RNA5SP450	-0.0246	5.8851E-01		ENSG00000223138
RNA5SP452	0.0057	6.6255E-01		ENSG00000199874
RNA5SP46	0.0101	6.7771E-01		ENSG00000199240
RNA5SP462	-0.0004	9.5199E-01		ENSG00000252539
RNA5SP463	0.0090	4.3448E-01		ENSG00000285674
RNA5SP464	-0.0052	8.0246E-01		ENSG00000252060
RNA5SP465	-0.0391	4.2909E-01	6.3074E-01	ENSG00000212497
RNA5SP466	-0.0226	2.4799E-01		ENSG00000252546
RNA5SP469	0.0233	2.2356E-01		ENSG00000201035
RNA5SP474	0.0237	5.7216E-01		ENSG00000212536
RNA5SP477	-0.0262	5.9289E-01	7.6032E-01	ENSG00000199509
RNA5SP479	-0.0066	6.8520E-01		ENSG00000201728
RNA5SP48	-0.0231	7.3614E-01	8.5602E-01	ENSG00000200839
RNA5SP481	0.0089	4.8572E-01		ENSG00000252623
RNA5SP488	-0.0037	9.7252E-01		ENSG00000201812
RNA5SP492	-0.0816	2.9981E-01	5.0942E-01	ENSG00000223262
RNA5SP493	0.0007	9.0021E-01		ENSG00000200985
RNA5SP494	0.0107	5.7258E-01		ENSG00000222585
RNA5SP495	0.0109	4.0371E-01		ENSG00000252267
RNA5SP497	0.0098	7.5452E-01		ENSG00000251890
RNA5SP508	0.0231	3.9550E-01		ENSG00000223259
RNA5SP511	-0.0197	2.5132E-01		ENSG00000222419
RNA5SP514	0.0052	5.3746E-01		ENSG00000200587
RNA5SP515	0.0245	2.3791E-01		ENSG00000201440
RNA5SP525	0.0266	2.4122E-01		ENSG00000212595
RNA5SP53	-0.0207	2.9964E-01		ENSG00000212601
RNA5SP530	0.0092	7.3436E-01		ENSG00000277049
RNA5SP60	-0.0029	7.3341E-01		ENSG00000222552
RNA5SP75	-0.0039	6.5042E-01		ENSG00000252650
RNA5SP78	0.0043	9.4662E-01	9.7352E-01	ENSG00000201492
RNA5SP85	0.0074	3.1641E-01		ENSG00000285626
RNA5SP88	0.0408	2.4336E-01		ENSG00000202430
RNA5SP92	-0.0027	8.4209E-01		ENSG00000252246
RNA5SP93	0.0102	5.8285E-01		ENSG00000199395
RNASE1	0.1788	1.4771E-02	6.7330E-02	ENSG00000129538
RNASE10	-0.0172	4.8916E-01		ENSG00000182545
RNASE4	0.0162	8.0594E-01	8.9800E-01	ENSG00000258818
RNASE7	0.0015	9.2126E-01		ENSG00000165799
RNASEH1	0.0206	7.2442E-01	8.4882E-01	ENSG00000171865
RNASEH1-DT	0.0587	3.7720E-01	5.8571E-01	ENSG00000234171
RNASEH1P1	0.0220	9.3080E-01	9.6586E-01	ENSG00000265790
RNASEH2A	0.0319	7.0228E-01	8.3561E-01	ENSG00000104889
RNASEH2B	-0.3781	1.9363E-06	6.4643E-05	ENSG00000136104
RNASEH2C	-0.1201	9.3565E-02	2.4421E-01	ENSG00000172922
RNASEK	-0.0363	6.4040E-01	7.9424E-01	ENSG00000219200
RNASEK-C17orf49	-0.0164	7.0965E-01		ENSG00000161939

RNASEL	-0.0305	7.1358E-01	8.4286E-01	ENSG00000135828
RNASET2	0.0578	3.6143E-01	5.7095E-01	ENSG00000026297
RND2	-0.1263	6.2771E-02	1.8615E-01	ENSG00000108830
RND3	-0.3080	3.0165E-05	5.9616E-04	ENSG00000115963
RNF10	-0.1820	6.9537E-03	3.8605E-02	ENSG00000022840
RNF103	0.1094	7.4788E-02	2.1005E-01	ENSG00000239305
RNF103-CHMP3	-0.1184	1.3244E-01	3.0597E-01	ENSG00000249884
RNF11	-0.2585	1.2998E-05	3.0029E-04	ENSG00000123091
RNF111	0.1253	6.6336E-02	1.9369E-01	ENSG00000157450
RNF112	0.0192	8.2032E-01	9.0672E-01	ENSG00000128482
RNF113A	-0.1573	2.8950E-02	1.0830E-01	ENSG00000125352
RNF113B	-0.0101	8.1761E-01		ENSG00000139797
RNF114	-0.1941	9.5770E-04	8.7600E-03	ENSG00000124226
RNF115	0.0344	6.1441E-01	7.7604E-01	ENSG00000265491
RNF121	0.1679	6.1486E-02	1.8347E-01	ENSG00000137522
RNF122	-0.3828	1.2179E-03	1.0522E-02	ENSG00000133874
RNF123	-0.0179	8.2306E-01	9.0841E-01	ENSG00000164068
RNF125	-0.0728	3.3474E-01	5.4477E-01	ENSG00000101695
RNF126	-0.0507	3.1704E-01	5.2719E-01	ENSG00000070423
RNF128	-0.0470	5.4931E-01	7.2861E-01	ENSG00000133135
RNF13	0.1898	1.0155E-02	5.1296E-02	ENSG00000082996
RNF130	0.0740	1.7819E-01	3.6944E-01	ENSG00000113269
RNF135	0.5141	2.5466E-03	1.8026E-02	ENSG00000181481
RNF138	-0.2718	2.3989E-04	3.0145E-03	ENSG00000134758
RNF139	-0.5998	3.6451E-12	1.0937E-09	ENSG00000170881
RNF139-DT	0.2199	1.7977E-02	7.6978E-02	ENSG00000245149
RNF14	0.1118	1.0501E-01	2.6311E-01	ENSG00000013561
RNF141	-0.0542	3.7129E-01	5.8001E-01	ENSG00000110315
RNF144A	0.0008	9.9855E-01	9.9913E-01	ENSG00000151692
RNF144B	0.0383	3.9938E-01		ENSG00000137393
RNF145	-0.2490	5.6221E-03	3.2866E-02	ENSG00000145860
RNF146	0.2220	3.7475E-03	2.4323E-02	ENSG00000118518
RNF148	-0.0088	4.4719E-01		ENSG00000235631
RNF149	0.1616	8.6814E-03	4.5711E-02	ENSG00000163162
RNF14P3	0.0270	5.6339E-01		ENSG00000238109
RNF150	0.1210	1.4853E-01	3.2906E-01	ENSG00000170153
RNF151	0.0300	3.3099E-01		ENSG00000179580
RNF152	0.2602	2.0274E-02	8.3728E-02	ENSG00000176641
RNF157	-0.0846	1.5419E-01	3.3678E-01	ENSG00000141576
RNF157-AS1	0.1083	1.7772E-01	3.6889E-01	ENSG00000267128
RNF166	0.0128	8.5968E-01	9.2928E-01	ENSG00000158717
RNF167	0.0908	2.0171E-01	4.0028E-01	ENSG00000108523
RNF168	-0.1002	1.2266E-01	2.9160E-01	ENSG00000163961
RNF169	-0.1179	1.4174E-01	3.1881E-01	ENSG00000166439
RNF17	0.0052	8.2293E-01		ENSG00000132972
RNF170	0.1430	4.8128E-02	1.5540E-01	ENSG00000120925
RNF175	-0.2514	5.1022E-03	3.0567E-02	ENSG00000145428
RNF180	-0.0348	6.4115E-01	7.9470E-01	ENSG00000164197
RNF181	-0.0237	7.1516E-01	8.4343E-01	ENSG00000168894

RNF182	-0.0868	3.1088E-01	5.2105E-01	ENSG00000180537
RNF183	0.1010	8.5142E-02	2.2837E-01	ENSG00000165188
RNF185	0.0588	3.8084E-01	5.8916E-01	ENSG00000138942
RNF187	-0.4842	1.1416E-12	3.9561E-10	ENSG00000168159
RNF19A	-0.2199	1.4846E-03	1.2184E-02	ENSG00000034677
RNF19B	-0.2793	1.4945E-03	1.2229E-02	ENSG00000116514
RNF2	-0.1099	1.0060E-01	2.5573E-01	ENSG00000121481
RNF20	-0.0189	7.5492E-01	8.6698E-01	ENSG00000155827
RNF207	-0.0218	8.6752E-01	9.3325E-01	ENSG00000158286
RNF207-AS1	0.0948	1.5210E-01	3.3395E-01	ENSG00000226944
RNF208	0.1020	2.5551E-01	4.6363E-01	ENSG00000212864
RNF212	0.3031	8.9178E-03	4.6715E-02	ENSG00000178222
RNF212B	0.0590	2.5126E-01	4.5951E-01	ENSG00000215277
RNF213	0.1455	5.8792E-02	1.7815E-01	ENSG00000173821
RNF213-AS1	0.5062	4.0930E-04	4.5549E-03	ENSG00000263069
RNF214	-0.1714	1.4802E-02	6.7410E-02	ENSG00000167257
RNF215	0.0058	9.3092E-01	9.6587E-01	ENSG00000099999
RNF216	0.0765	1.6663E-01	3.5455E-01	ENSG00000011275
RNF216-IT1	-0.0331	3.6894E-01		ENSG00000237738
RNF216P1	0.1243	1.8107E-02	7.7320E-02	ENSG00000196204
RNF217	-0.0511	4.7555E-01	6.7112E-01	ENSG00000146373
RNF217-AS1	0.1452	1.1709E-01	2.8253E-01	ENSG00000236548
RNF220	-0.2319	2.7761E-03	1.9235E-02	ENSG00000187147
RNF222	-0.0142	9.3998E-01		ENSG00000189051
RNF223	0.0010	8.8619E-01		ENSG00000237330
RNF224	0.0404	9.2690E-02		ENSG00000233198
RNF225	-0.0233	2.0184E-01		ENSG00000269855
RNF227	0.0380	5.7534E-01	7.4806E-01	ENSG00000179859
RNF24	-0.0331	5.7281E-01	7.4605E-01	ENSG00000101236
RNF25	0.5159	5.3340E-09	4.7552E-07	ENSG00000163481
RNF26	-0.2877	8.2961E-04	7.8636E-03	ENSG00000173456
RNF31	-0.1890	5.0894E-02	1.6139E-01	ENSG00000092098
RNF32	0.1618	7.6120E-02	2.1252E-01	ENSG00000105982
RNF32-DT	0.0970	1.0500E-01	2.6311E-01	ENSG00000182648
RNF34	0.2169	1.5881E-03	1.2742E-02	ENSG00000170633
RNF38	-0.1885	9.2614E-04	8.5539E-03	ENSG00000137075
RNF39	-0.0895	3.1799E-01	5.2826E-01	ENSG00000204618
RNF4	0.1132	7.7406E-02	2.1494E-01	ENSG00000063978
RNF40	-0.0549	5.1763E-01	7.0506E-01	ENSG00000103549
RNF41	-0.1216	3.6900E-02	1.2895E-01	ENSG00000181852
RNF43	0.0611	4.0577E-01	6.1027E-01	ENSG00000108375
RNF44	0.2305	1.0379E-02	5.2076E-02	ENSG00000146083
RNF5	-0.1175	5.7580E-02	1.7596E-01	ENSG00000204308
RNF6	-0.1904	2.0697E-02	8.4988E-02	ENSG00000127870
RNF7	-0.0245	5.9422E-01	7.6126E-01	ENSG00000114125
RNF8	0.0476	4.1544E-01	6.1887E-01	ENSG00000112130
RNFT1	-0.0137	8.5226E-01	9.2466E-01	ENSG00000189050
RNFT1-DT	-0.0917	2.6595E-01	4.7515E-01	ENSG00000267302
RNFT2	-0.0438	4.9281E-01	6.8483E-01	ENSG00000135119

RNGTT	0.2004	1.4374E-02	6.5937E-02	ENSG00000111880
RNH1	-0.0902	1.9508E-01	3.9193E-01	ENSG00000023191
RNLS	0.1525	1.2110E-01	2.8926E-01	ENSG00000184719
RNMT	0.1006	1.4133E-01	3.1818E-01	ENSG00000101654
RNPC3	0.5795	4.6958E-13	2.0270E-10	ENSG00000185946
RNPC3-DT	0.0064	9.2525E-01	9.6331E-01	ENSG00000224613
RNPEP	-0.0267	6.9964E-01	8.3388E-01	ENSG00000176393
RNPEPL1	0.0678	3.1073E-01	5.2095E-01	ENSG00000142327
RNPS1	-0.2380	1.0853E-04	1.6393E-03	ENSG00000205937
RNU1-1	0.4937	1.6399E-03	1.3033E-02	ENSG00000206652
RNU1-100P	-0.0550	1.1876E-01		ENSG00000202125
RNU1-103P	-0.0336	6.2928E-01	7.8656E-01	ENSG00000252311
RNU1-124P	0.0259	4.7898E-01		ENSG00000200731
RNU1-129P	0.0091	8.8128E-01		ENSG00000206791
RNU1-134P	-0.0115	8.5187E-01		ENSG00000199805
RNU1-154P	-0.0080	8.4907E-01		ENSG00000270384
RNU1-2	0.2754	2.4877E-02	9.7288E-02	ENSG00000207005
RNU1-27P	0.4307	7.1622E-04	7.0720E-03	ENSG00000206596
RNU1-28P	0.4313	7.3661E-04	7.2208E-03	ENSG00000206588
RNU1-3	0.2244	5.2041E-02	1.6376E-01	ENSG00000207513
RNU1-30P	0.0110	5.5755E-01		ENSG00000272020
RNU1-36P	0.0030	9.7890E-01		ENSG00000202000
RNU1-4	0.1632	1.0163E-01	2.5755E-01	ENSG00000207389
RNU1-47P	-0.0076	8.4997E-01		ENSG00000199836
RNU1-8P	0.0162	5.0859E-01		ENSG00000207056
RNU1-94P	0.0221	3.4646E-01		ENSG00000199497
RNU105B	-0.0243	3.1488E-01		ENSG00000201348
RNU11	-0.0085	8.5089E-01		ENSG00000274978
RNU11-2P	-0.0109	4.3449E-01		ENSG00000252707
RNU11-6P	0.0108	5.2110E-01		ENSG00000212429
RNU12-2P	0.0005	8.0085E-01		ENSG00000201659
RNU2-1	-0.0140	5.4798E-01		ENSG00000274585
RNU2-10P	0.0006	7.7514E-01		ENSG00000252184
RNU2-11P	-0.0231	1.7089E-01		ENSG00000239122
RNU2-17P	0.0103	5.6796E-01	7.4259E-01	ENSG00000222222
RNU2-22P	0.0605	1.7532E-01		ENSG00000223198
RNU2-25P	-0.0013	7.7469E-01		ENSG00000222973
RNU2-26P	-0.0034	6.2899E-01		ENSG00000222440
RNU2-33P	-0.0040	9.6372E-01		ENSG00000222276
RNU2-39P	-0.0179	8.0167E-01		ENSG00000222536
RNU2-59P	-0.0033	7.5045E-01		ENSG00000222414
RNU2-5P	0.0098	7.9629E-01		ENSG00000222465
RNU2-63P	0.2554	2.8045E-02	1.0595E-01	ENSG00000222724
RNU2-65P	0.0035	9.0155E-01		ENSG00000222094
RNU2-68P	0.0229	1.8787E-01		ENSG00000222810
RNU2-7P	0.0315	5.5765E-01	7.3530E-01	ENSG00000222726
RNU4-1	0.0935	2.9416E-02	1.0950E-01	ENSG00000200795
RNU4-14P	0.0136	4.4846E-01		ENSG00000222790
RNU4-16P	0.0061	9.3765E-01		ENSG00000201607

RNU4-22P	0.0064	9.7560E-01		ENSG00000222990
RNU4-24P	-0.0107	5.6324E-01		ENSG00000201435
RNU4-36P	-0.0157	8.3471E-01	9.1500E-01	ENSG00000201164
RNU4-39P	-0.0255	7.4097E-01	8.5887E-01	ENSG00000199325
RNU4-40P	0.0211	7.2682E-01	8.5004E-01	ENSG00000201221
RNU4-47P	0.0161	4.0894E-01		ENSG00000222808
RNU4-51P	-0.0013	8.7726E-01		ENSG00000201076
RNU4-68P	-0.0146	4.6412E-01		ENSG00000201184
RNU4-78P	-0.0123	6.1014E-01		ENSG00000222872
RNU4-86P	0.0062	7.9163E-01		ENSG00000222067
RNU4ATAC11P	0.0075	7.2172E-01		ENSG00000251748
RNU4ATAC16P	-0.0138	5.8318E-01		ENSG00000221439
RNU4ATAC18P	0.0042	9.2497E-01		ENSG00000251988
RNU5A-1	0.4979	2.0910E-03	1.5567E-02	ENSG00000199568
RNU5A-3P	0.0141	4.3931E-01		ENSG00000254172
RNU5A-8P	0.1916	2.6416E-02	1.0141E-01	ENSG00000200972
RNU5D-1	0.3654	1.5811E-02	7.0568E-02	ENSG00000200169
RNU5E-10P	-0.0211	3.4515E-01		ENSG00000200376
RNU5E-4P	0.0456	3.9376E-01		ENSG00000201801
RNU5E-8P	-0.0507	3.8488E-01	5.9280E-01	ENSG00000200372
RNU5F-1	0.1311	6.6117E-02	1.9326E-01	ENSG00000199377
RNU6-1	0.0145	4.9397E-01		ENSG00000206625
RNU6-1003P	0.0265	4.1486E-01		ENSG00000252908
RNU6-1004P	-0.0002	8.8129E-01		ENSG00000252393
RNU6-1005P	-0.0198	3.6679E-01		ENSG00000207248
RNU6-1011P	-0.0035	8.6346E-01		ENSG00000207399
RNU6-1016P	0.0461	5.3461E-01	7.1806E-01	ENSG00000252498
RNU6-1019P	0.0100	7.0451E-01		ENSG00000201294
RNU6-101P	0.0598	3.3768E-01	5.4765E-01	ENSG00000222255
RNU6-1023P	0.0171	3.8265E-01		ENSG00000251946
RNU6-1024P	-0.0485	2.3773E-01		ENSG00000206926
RNU6-1025P	-0.0916	5.6368E-02		ENSG00000222561
RNU6-1028P	-0.0032	8.3610E-01		ENSG00000252973
RNU6-103P	0.0449	5.7880E-01	7.5030E-01	ENSG00000200556
RNU6-1043P	0.0024	8.6129E-01		ENSG00000222287
RNU6-1046P	0.0199	2.7749E-01		ENSG00000252347
RNU6-1053P	0.1042	1.1794E-01	2.8389E-01	ENSG00000251739
RNU6-1061P	0.0189	6.1420E-01		ENSG00000252339
RNU6-106P	0.0334	9.6977E-02		ENSG00000207134
RNU6-1076P	0.0152	7.0895E-01		ENSG00000222329
RNU6-1079P	0.0068	7.7167E-01		ENSG00000199731
RNU6-1086P	-0.0061	9.5735E-01		ENSG00000222862
RNU6-1093P	0.0127	6.2244E-01		ENSG00000212496
RNU6-1099P	0.1497	1.0691E-01	2.6603E-01	ENSG00000200403
RNU6-10P	0.0025	9.9541E-01		ENSG00000206763
RNU6-1100P	0.0275	5.2811E-01		ENSG00000222623
RNU6-1107P	-0.0062	9.3989E-01		ENSG00000201687
RNU6-1111P	0.0355	1.7970E-01		ENSG00000212420
RNU6-1123P	0.0153	6.5742E-01		ENSG00000199217

RNU6-1138P	-0.0242	2.4227E-01		ENSG00000202229
RNU6-1141P	0.0084	5.7851E-01		ENSG00000201390
RNU6-1154P	-0.0057	6.2102E-01		ENSG00000212303
RNU6-1157P	-0.0033	9.9094E-01		ENSG00000207185
RNU6-1158P	-0.0309	1.6052E-01		ENSG00000212469
RNU6-1162P	-0.0006	9.3123E-01		ENSG00000207172
RNU6-1165P	0.0134	5.8449E-01		ENSG00000222051
RNU6-116P	0.0159	6.0270E-01		ENSG00000206769
RNU6-1178P	-0.5647	8.3243E-03	4.4209E-02	ENSG00000252483
RNU6-1187P	0.0046	8.7121E-01		ENSG00000201641
RNU6-1188P	0.0244	2.3267E-01		ENSG00000200665
RNU6-118P	-0.0207	8.2116E-01	9.0730E-01	ENSG00000252361
RNU6-1196P	-0.0067	6.6426E-01		ENSG00000252030
RNU6-1199P	-0.0077	6.5666E-01		ENSG00000223181
RNU6-1200P	0.0283	5.1698E-01		ENSG00000206889
RNU6-1208P	-0.0063	6.0622E-01		ENSG00000238482
RNU6-1209P	-0.0066	6.6003E-01		ENSG00000200525
RNU6-1214P	-0.0538	1.8697E-01		ENSG00000200520
RNU6-1216P	-0.0052	6.6233E-01		ENSG00000199460
RNU6-1223P	0.0062	7.7241E-01		ENSG00000252933
RNU6-122P	0.0136	4.2845E-01		ENSG00000252627
RNU6-1254P	0.0086	6.8469E-01		ENSG00000212568
RNU6-1258P	-0.0056	7.0607E-01		ENSG00000252720
RNU6-125P	0.1045	1.7712E-01	3.6830E-01	ENSG00000207234
RNU6-1262P	-0.0028	8.6697E-01		ENSG00000252026
RNU6-1263P	0.0713	2.1012E-01	4.1054E-01	ENSG00000207331
RNU6-1267P	-0.0115	7.6060E-01		ENSG00000222679
RNU6-1272P	-0.0056	7.0768E-01		ENSG00000199646
RNU6-1275P	-0.0173	5.1827E-01		ENSG00000212389
RNU6-1284P	0.0042	7.1393E-01		ENSG00000200571
RNU6-1285P	0.0705	2.8885E-01	4.9927E-01	ENSG00000200350
RNU6-1294P	-0.0094	6.9630E-01		ENSG00000251804
RNU6-1301P	0.0181	1.5576E-01		ENSG00000199594
RNU6-130P	-0.0035	9.5171E-01		ENSG00000223044
RNU6-1316P	-0.0062	7.8212E-01		ENSG00000206969
RNU6-131P	0.0076	9.5745E-01		ENSG00000212446
RNU6-1321P	0.0102	5.9615E-01		ENSG00000212207
RNU6-1327P	-0.0074	7.1330E-01		ENSG00000202172
RNU6-1331P	0.0045	7.8649E-01		ENSG00000200528
RNU6-1333P	-0.0170	5.8785E-01		ENSG00000251971
RNU6-1337P	0.0561	2.5715E-01		ENSG00000252334
RNU6-135P	-0.0229	5.3177E-01		ENSG00000252578
RNU6-137P	-0.0340	3.8403E-01		ENSG00000200550
RNU6-143P	0.0018	5.5399E-01		ENSG00000252751
RNU6-151P	-0.0815	6.2411E-02		ENSG00000201028
RNU6-155P	0.0116	4.7377E-01		ENSG00000252156
RNU6-157P	0.0028	8.7677E-01		ENSG00000206684
RNU6-171P	-0.0075	6.8471E-01	8.2427E-01	ENSG00000207082
RNU6-177P	-0.0070	6.4329E-01		ENSG00000223189

RNU6-179P	0.0227	2.0631E-01		ENSG00000200220
RNU6-188P	-0.0147	5.4403E-01		ENSG00000201077
RNU6-190P	0.0103	7.3785E-01		ENSG00000206881
RNU6-197P	0.0144	4.3956E-01		ENSG00000252489
RNU6-198P	-0.0061	6.3174E-01		ENSG00000206985
RNU6-2	0.0235	3.0539E-01	5.1563E-01	ENSG00000207357
RNU6-204P	-0.0004	9.1141E-01		ENSG00000199335
RNU6-213P	-0.0161	4.0970E-01		ENSG00000206778
RNU6-218P	-0.0166	5.0395E-01		ENSG00000252929
RNU6-223P	0.0347	2.9216E-01		ENSG00000199700
RNU6-228P	0.0144	6.6256E-01		ENSG00000199570
RNU6-230P	0.0192	5.4028E-01		ENSG00000199327
RNU6-233P	-0.0317	4.1462E-02		ENSG00000222777
RNU6-236P	0.0283	4.5291E-01		ENSG00000200756
RNU6-238P	0.0818	6.7093E-02		ENSG00000200183
RNU6-247P	0.0146	1.9081E-01		ENSG00000199506
RNU6-254P	0.0112	7.5242E-01		ENSG00000200247
RNU6-262P	-0.0072	5.7829E-01		ENSG00000222249
RNU6-268P	-0.0075	7.4251E-01		ENSG00000201044
RNU6-26P	1.8142	2.6101E-03		ENSG00000206712
RNU6-288P	0.0511	2.0734E-01		ENSG00000200560
RNU6-28P	0.0285	1.8043E-01		ENSG00000199248
RNU6-294P	0.0072	6.7071E-01		ENSG00000201869
RNU6-298P	-0.0069	6.5111E-01		ENSG00000212190
RNU6-307P	-0.0131	5.1712E-01		ENSG00000252552
RNU6-30P	0.0328	6.0699E-01	7.7090E-01	ENSG00000207291
RNU6-310P	0.0131	3.8292E-01		ENSG00000207385
RNU6-312P	-0.0029	6.7399E-01		ENSG00000201499
RNU6-313P	-0.0189	3.3032E-01		ENSG00000252126
RNU6-315P	-0.0024	8.7267E-01		ENSG00000199536
RNU6-319P	0.0049	6.3028E-01		ENSG00000251834
RNU6-320P	0.0218	2.1687E-01		ENSG00000252320
RNU6-321P	0.0626	1.5572E-01		ENSG00000222844
RNU6-322P	0.0070	9.1414E-01		ENSG00000251819
RNU6-32P	-0.1239	6.7620E-02	1.9609E-01	ENSG00000206675
RNU6-335P	0.0377	3.6454E-01		ENSG00000201433
RNU6-353P	0.0301	1.3382E-01		ENSG00000201136
RNU6-354P	0.0369	6.7315E-02		ENSG00000206589
RNU6-355P	0.0204	6.3071E-01		ENSG00000222359
RNU6-361P	0.0606	4.5431E-02		ENSG00000201324
RNU6-375P	-0.0143	4.5536E-01		ENSG00000252859
RNU6-379P	0.0080	6.2657E-01		ENSG00000200683
RNU6-37P	0.0110	8.8904E-01	9.4486E-01	ENSG00000199562
RNU6-387P	0.0480	2.4169E-01		ENSG00000223263
RNU6-388P	0.0540	7.3759E-02		ENSG00000252821
RNU6-396P	0.0485	2.8443E-01		ENSG00000202239
RNU6-405P	-0.0074	8.3134E-01		ENSG00000252446
RNU6-407P	0.0807	6.4781E-02		ENSG00000202150
RNU6-418P	0.0189	2.7742E-01		ENSG00000206762

RNU6-422P	-0.0068	5.6955E-01		ENSG00000207362
RNU6-429P	0.0064	8.2281E-01		ENSG00000207312
RNU6-430P	0.0115	6.9513E-01		ENSG00000252887
RNU6-431P	-0.0433	2.3572E-01		ENSG00000206601
RNU6-433P	-0.0188	9.0050E-01		ENSG00000200086
RNU6-446P	-0.0017	9.9066E-01		ENSG00000199697
RNU6-447P	-0.0153	3.9311E-01		ENSG00000222225
RNU6-455P	-0.0026	8.4507E-01		ENSG00000207412
RNU6-469P	0.1462	2.3360E-02	9.3022E-02	ENSG00000252062
RNU6-476P	-0.1932	7.1258E-02	2.0333E-01	ENSG00000207457
RNU6-479P	0.0001	9.2290E-01		ENSG00000252157
RNU6-482P	0.1643	5.5749E-03		ENSG00000212370
RNU6-48P	-0.0381	5.2156E-01	7.0823E-01	ENSG00000206888
RNU6-495P	-0.0026	8.5420E-01		ENSG00000199865
RNU6-501P	-0.0559	2.2997E-01		ENSG00000200942
RNU6-503P	-0.0183	3.2075E-01		ENSG00000252338
RNU6-504P	-0.0069	5.5164E-01		ENSG00000252377
RNU6-514P	-0.0294	2.8876E-01		ENSG00000206935
RNU6-518P	0.0116	5.2639E-01		ENSG00000251865
RNU6-519P	-0.0017	6.0658E-01		ENSG00000222522
RNU6-522P	0.0016	8.1910E-01		ENSG00000201367
RNU6-527P	-0.0079	9.0173E-01		ENSG00000200295
RNU6-531P	0.0035	9.8716E-01		ENSG00000252503
RNU6-539P	-0.0112	6.9817E-01		ENSG00000252474
RNU6-540P	0.0140	6.3724E-01		ENSG00000207352
RNU6-541P	0.0144	5.6754E-01		ENSG00000207440
RNU6-554P	-0.0012	8.4235E-01		ENSG00000222398
RNU6-558P	-0.0069	9.5848E-01		ENSG00000201382
RNU6-564P	0.0043	8.1917E-01		ENSG00000222915
RNU6-574P	-0.1265	1.8065E-01	3.7287E-01	ENSG00000206992
RNU6-577P	-0.0081	8.0853E-01		ENSG00000252756
RNU6-593P	0.0006	8.0893E-01		ENSG00000201586
RNU6-598P	-0.0194	6.0697E-01		ENSG00000200887
RNU6-603P	-0.0077	7.4531E-01		ENSG00000223335
RNU6-606P	-0.0162	9.7907E-01		ENSG00000207452
RNU6-60P	0.0644	2.6630E-01	4.7564E-01	ENSG00000201662
RNU6-610P	0.0655	1.2442E-01		ENSG00000206991
RNU6-611P	-0.0462	4.9537E-01	6.8710E-01	ENSG00000207003
RNU6-613P	-0.0116	7.5280E-01		ENSG00000222344
RNU6-618P	-0.0028	8.1141E-01		ENSG00000200388
RNU6-625P	-0.0111	7.1978E-01		ENSG00000238658
RNU6-628P	-0.3174	2.2993E-02	9.1903E-02	ENSG00000222607
RNU6-638P	-0.0037	9.1318E-01		ENSG00000252391
RNU6-644P	0.0296	5.7856E-01	7.5006E-01	ENSG00000212457
RNU6-645P	-0.0339	3.7984E-01	5.8831E-01	ENSG00000201519
RNU6-652P	0.0425	4.9165E-01	6.8387E-01	ENSG00000202358
RNU6-661P	-0.0230	6.3614E-01		ENSG00000199279
RNU6-662P	-0.0071	9.4723E-01		ENSG00000206980
RNU6-674P	0.0073	9.5103E-01		ENSG00000200701

RNU6-678P	-0.0038	8.6478E-01		ENSG00000252641
RNU6-681P	0.0059	7.9090E-01		ENSG00000200882
RNU6-689P	0.0048	7.5893E-01		ENSG00000206924
RNU6-697P	0.0248	4.2765E-01		ENSG00000200218
RNU6-7	0.0065	5.2124E-01		ENSG00000201654
RNU6-701P	-0.0053	9.6698E-01		ENSG00000206786
RNU6-703P	0.0350	1.5940E-01		ENSG00000252755
RNU6-705P	-0.0327	4.4444E-01		ENSG00000222533
RNU6-711P	-0.0024	8.3704E-01		ENSG00000253064
RNU6-723P	-0.0033	7.0919E-01		ENSG00000206700
RNU6-729P	-0.0324	2.6464E-01	4.7380E-01	ENSG00000207128
RNU6-731P	-0.0152	6.9315E-01		ENSG00000253022
RNU6-757P	-0.0425	6.1377E-01		ENSG00000222266
RNU6-758P	0.0173	5.8922E-01		ENSG00000252101
RNU6-762P	0.0245	3.2777E-01		ENSG00000252916
RNU6-781P	0.1428	8.5312E-03	4.5103E-02	ENSG00000252186
RNU6-784P	0.0012	4.6236E-01		ENSG00000207058
RNU6-785P	0.0198	5.8198E-01		ENSG00000223256
RNU6-786P	0.0150	8.3471E-01		ENSG00000252658
RNU6-789P	-0.0301	2.5166E-01		ENSG00000252914
RNU6-790P	0.0797	4.5180E-02	1.4880E-01	ENSG00000207208
RNU6-79P	0.0084	4.3092E-01		ENSG00000199381
RNU6-8	0.0251	3.0312E-01		ENSG00000202337
RNU6-807P	-0.0044	9.4034E-01	9.7056E-01	ENSG00000252614
RNU6-808P	-0.0214	9.7939E-02		ENSG00000212535
RNU6-824P	0.0099	4.3813E-01		ENSG00000200594
RNU6-826P	0.0172	3.9435E-01		ENSG00000252636
RNU6-828P	0.0286	2.2425E-01		ENSG00000201746
RNU6-82P	0.0294	2.2049E-01		ENSG00000200840
RNU6-834P	0.0107	7.9869E-01		ENSG00000199237
RNU6-850P	-0.0309	5.6655E-01	7.4164E-01	ENSG00000252743
RNU6-85P	-0.0047	8.0599E-01		ENSG00000271932
RNU6-860P	-0.0227	3.2577E-01		ENSG00000222792
RNU6-862P	0.0032	9.0438E-01		ENSG00000199674
RNU6-875P	-0.0154	3.5270E-01		ENSG00000252297
RNU6-881P	-0.0040	9.6936E-01		ENSG00000252622
RNU6-883P	0.1110	3.6651E-02		ENSG00000207327
RNU6-886P	0.0172	3.8618E-01		ENSG00000207046
RNU6-890P	-0.0078	7.6679E-01		ENSG00000206848
RNU6-892P	0.1121	5.5223E-02	1.7115E-01	ENSG00000222267
RNU6-895P	-0.0185	3.8522E-01		ENSG00000206852
RNU6-904P	0.0063	8.7280E-01		ENSG00000252015
RNU6-905P	0.0110	4.0948E-01		ENSG00000207163
RNU6-91P	-0.0040	7.6028E-01		ENSG00000272439
RNU6-920P	0.0001	8.7149E-01		ENSG00000222858
RNU6-924P	-0.0007	7.6474E-01		ENSG00000199796
RNU6-925P	0.0516	2.9944E-01		ENSG00000207359
RNU6-928P	-0.0072	6.5062E-01		ENSG00000252685
RNU6-934P	0.0090	7.7964E-01		ENSG00000202024

RNU6-936P	0.0462	1.8325E-01		ENSG00000206732
RNU6-942P	-0.0044	8.5380E-01		ENSG00000199872
RNU6-945P	-0.0123	8.8801E-01		ENSG00000206674
RNU6-946P	0.0083	6.9953E-01		ENSG00000206605
RNU6-94P	-0.0759	3.0593E-01	5.1604E-01	ENSG00000271819
RNU6-95P	0.0147	4.6858E-01		ENSG00000207428
RNU6-969P	-0.0135	4.2537E-01		ENSG00000206627
RNU6-975P	0.0006	8.6230E-01		ENSG00000207023
RNU6-97P	-0.0361	2.5198E-01		ENSG00000200257
RNU6-998P	-0.0139	8.7699E-01		ENSG00000251703
RNU6ATAC	0.0934	1.7998E-02	7.6998E-02	ENSG00000221676
RNU6ATAC12P	0.0332	1.3763E-01		ENSG00000252351
RNU6ATAC16P	0.0115	5.9206E-01		ENSG00000221518
RNU6ATAC24P	-0.0202	5.0389E-01		ENSG00000252620
RNU6ATAC26P	-0.0609	6.2797E-02		ENSG00000210841
RNU6ATAC27P	0.0183	6.6810E-01		ENSG00000221216
RNU6ATAC39P	0.0253	9.7151E-01		ENSG00000252118
RNU6ATAC42P	-0.0016	8.3465E-01		ENSG00000221564
RNU7-107P	-0.0007	9.3092E-01		ENSG00000238523
RNU7-110P	-0.0074	8.0061E-01		ENSG00000252700
RNU7-115P	0.0018	8.9210E-01		ENSG00000252242
RNU7-123P	-0.0058	8.9547E-01	9.4806E-01	ENSG00000251720
RNU7-133P	0.0001	7.4900E-01		ENSG00000238987
RNU7-140P	-0.3903	1.7188E-02	7.4590E-02	ENSG00000238364
RNU7-141P	-0.0344	3.1868E-01		ENSG00000238777
RNU7-143P	-0.0003	9.9617E-01		ENSG00000252590
RNU7-151P	0.0108	6.1422E-01		ENSG00000252739
RNU7-169P	-0.0206	3.4312E-01		ENSG00000238457
RNU7-171P	0.0079	7.3837E-01		ENSG00000238406
RNU7-181P	0.0051	6.6994E-01	8.1498E-01	ENSG00000253043
RNU7-185P	0.0008	9.6442E-01		ENSG00000239102
RNU7-187P	-0.0567	1.3318E-01		ENSG00000238998
RNU7-194P	-0.0018	9.7046E-01		ENSG00000238721
RNU7-195P	-0.0393	3.9001E-01		ENSG00000239151
RNU7-35P	0.0344	3.4911E-01		ENSG00000252983
RNU7-38P	-0.0069	6.7721E-01		ENSG00000238446
RNU7-3P	0.0274	6.7061E-01		ENSG00000252244
RNU7-40P	0.0089	3.7144E-01		ENSG00000252206
RNU7-43P	-0.0069	7.3432E-01		ENSG00000252363
RNU7-49P	0.0029	7.6218E-01		ENSG00000251991
RNU7-56P	-0.0103	5.1490E-01		ENSG00000238709
RNU7-57P	0.0118	2.8485E-01		ENSG00000238365
RNU7-59P	0.0587	1.5170E-01		ENSG00000238880
RNU7-63P	-0.0343	3.1601E-01		ENSG00000238417
RNU7-75P	-0.0428	3.5211E-01		ENSG00000251880
RNU7-77P	0.0537	3.2982E-01	5.4006E-01	ENSG00000253054
RNU7-80P	-0.0139	9.2633E-01		ENSG00000238778
RNU7-81P	-0.0263	6.1226E-01		ENSG00000252507
RNVU1-15	0.3414	1.1280E-02	5.5376E-02	ENSG00000207205

RNVU1-18	0.1079	2.1537E-01	4.1665E-01	ENSG00000206737
RNVU1-2	0.0299	1.0807E-01		ENSG00000238825
RNVU1-22	0.0960	1.7861E-01	3.6993E-01	ENSG00000199879
RNVU1-23	0.0106	7.5624E-01		ENSG00000252826
RNVU1-24	0.5493	4.5608E-04	4.9496E-03	ENSG00000201699
RNVU1-2A	0.2166	5.7141E-02	1.7506E-01	ENSG00000278099
RNVU1-3	0.0737	2.0919E-01	4.0916E-01	ENSG00000201183
RNVU1-33	-0.0193	1.4196E-01		ENSG00000200597
RNVU1-34	0.1131	6.6688E-02	1.9437E-01	ENSG00000200997
RNVU1-4	0.3692	1.8966E-02	7.9960E-02	ENSG00000277610
RNVU1-7	0.1124	1.2639E-01	2.9750E-01	ENSG00000206585
RNVU1-8	0.0124	9.8184E-01	9.9046E-01	ENSG00000286172
RNY1	-0.0073	7.1957E-01		ENSG00000201098
RNY1P12	-0.0836	9.7023E-02		ENSG00000201121
RNY1P14	-0.0332	2.0533E-01		ENSG00000207155
RNY1P16	-0.0839	9.0027E-03		ENSG00000199933
RNY1P2	0.0167	3.1408E-01		ENSG00000201690
RNY1P4	-0.0031	8.7692E-01		ENSG00000207325
RNY1P5	0.1052	1.8981E-01	3.8487E-01	ENSG00000206617
RNY1P9	-0.0079	6.6929E-01		ENSG00000255156
RNY3P14	-0.0009	8.2295E-01		ENSG00000207036
RNY3P16	0.0090	7.7726E-01		ENSG00000252147
RNY4P10	0.0622	6.9436E-02		ENSG00000202441
RNY4P17	0.0079	5.3451E-01		ENSG00000201818
RNY4P19	-0.0127	7.1163E-01		ENSG00000199400
RNY4P25	-0.0517	2.4437E-01	4.5199E-01	ENSG00000238711
RNY4P34	0.0221	2.0556E-01		ENSG00000201649
RNY4P36	-0.0013	9.9278E-01		ENSG00000212418
RNY4P37	0.0205	4.4668E-01		ENSG00000252034
RNY4P6	0.0029	6.9368E-01		ENSG00000200537
RO60	-0.0252	5.8655E-01	7.5596E-01	ENSG00000116747
ROBO1	0.0118	8.6715E-01	9.3301E-01	ENSG00000169855
ROBO2	0.1144	1.5998E-01	3.4514E-01	ENSG00000185008
ROBO3	0.0656	3.0215E-01	5.1218E-01	ENSG00000154134
ROBO4	-0.0317	2.6208E-01		ENSG00000154133
ROCK1	0.0864	1.1298E-01	2.7597E-01	ENSG00000067900
ROCK2	0.2173	1.7340E-04	2.3226E-03	ENSG00000134318
ROGDI	-0.4553	2.5159E-10	3.4201E-08	ENSG00000067836
ROM1	0.0353	6.6640E-01	8.1284E-01	ENSG00000149489
ROMO1	0.0690	3.6180E-01	5.7107E-01	ENSG00000125995
ROPN1	0.0817	1.5724E-01	3.4106E-01	ENSG00000065371
ROPN1B	-0.0606	4.6084E-01	6.5837E-01	ENSG00000114547
ROR1	-0.0891	2.7199E-01	4.8126E-01	ENSG00000185483
ROR1-AS1	-0.0467	4.2764E-01	6.2958E-01	ENSG00000223949
ROR2	0.0102	8.8793E-01	9.4427E-01	ENSG00000169071
RORA	0.0795	2.8824E-01	4.9873E-01	ENSG00000069667
RORA-AS1	-0.0306	6.6832E-01	8.1415E-01	ENSG00000245534
RORA-AS2	-0.0029	5.8758E-01		ENSG00000259482
RORB	0.0179	8.1523E-01	9.0394E-01	ENSG00000198963

RORC	-0.1253	9.1196E-02	2.4010E-01	ENSG00000143365
ROS1	-0.0116	5.9674E-01		ENSG00000047936
RP1	-0.0003	8.3625E-01		ENSG00000104237
RP1L1	0.0052	9.2798E-01		ENSG00000183638
RP2	0.0431	5.7789E-01	7.4955E-01	ENSG00000102218
RP9	-0.0633	3.2665E-01	5.3682E-01	ENSG00000164610
RPA1	-0.1213	6.4951E-02	1.9073E-01	ENSG00000132383
RPA2	-0.0621	1.9828E-01	3.9618E-01	ENSG00000117748
RPA3	0.0392	5.3485E-01	7.1818E-01	ENSG00000106399
RPA4	0.0010	9.9345E-01		ENSG00000204086
RPAIN	-0.0319	6.9269E-01	8.2954E-01	ENSG00000129197
RPAP1	0.1084	9.2527E-02	2.4238E-01	ENSG00000103932
RPAP2	-0.0109	8.1981E-01	9.0638E-01	ENSG00000122484
RPAP3	0.2405	9.3451E-04	8.6054E-03	ENSG00000005175
RPE	-0.1205	1.6000E-01	3.4514E-01	ENSG00000197713
RPEL1	0.0222	2.0909E-01		ENSG00000235376
RPF1	-0.1288	4.0894E-02	1.3890E-01	ENSG00000117133
RPF2	0.0997	1.7684E-01	3.6810E-01	ENSG00000197498
RPF2P1	-0.0143	4.2604E-01		ENSG00000225357
RPGR	0.4105	1.5496E-05	3.4536E-04	ENSG00000156313
RPGRIP1	-0.0530	4.8621E-01	6.7994E-01	ENSG00000092200
RPGRIP1L	0.0898	2.2303E-01	4.2662E-01	ENSG00000103494
RPH3A	0.2032	1.2290E-02	5.8912E-02	ENSG00000089169
RPH3AL	0.1039	2.5512E-01	4.6319E-01	ENSG00000181031
RPH3AL-AS1	0.0142	6.6551E-01		ENSG00000262061
RPIA	-0.1724	2.7879E-02	1.0550E-01	ENSG00000153574
RPL10	-0.0986	1.8346E-01	3.7674E-01	ENSG00000147403
RPL10A	-0.1060	1.1140E-01	2.7329E-01	ENSG00000198755
RPL10AP1	-0.0958	3.3721E-02		ENSG00000244691
RPL10AP11	0.0123	6.3407E-01		ENSG00000244593
RPL10AP2	0.0404	6.1537E-01	7.7684E-01	ENSG00000188873
RPL10AP6	0.0378	6.6139E-01	8.0923E-01	ENSG00000226360
RPL10AP7	0.0080	5.0621E-01		ENSG00000214846
RPL10AP9	0.0423	5.2029E-01	7.0731E-01	ENSG00000242477
RPL10P1	0.0061	5.6425E-01		ENSG00000217026
RPL10P12	0.0156	8.3152E-01	9.1301E-01	ENSG00000234040
RPL10P13	0.0214	6.4594E-01		ENSG00000258245
RPL10P15	-0.1653	1.0642E-01	2.6523E-01	ENSG00000267119
RPL10P16	-0.0625	4.4250E-01	6.4177E-01	ENSG00000178464
RPL10P18	-0.0201	2.9663E-01		ENSG00000253995
RPL10P3	-0.0421	5.6512E-01	7.4080E-01	ENSG00000230734
RPL10P4	-0.0186	5.3960E-01		ENSG00000238003
RPL10P5	0.0058	6.8071E-01		ENSG00000235644
RPL10P6	0.0050	9.5265E-01	9.7678E-01	ENSG00000230076
RPL10P8	0.0064	7.6784E-01		ENSG00000254012
RPL10P9	-0.0724	4.0198E-01	6.0655E-01	ENSG00000233913
RPL11	-0.2367	2.1330E-03	1.5782E-02	ENSG00000142676
RPL11P1	-0.0352	7.9444E-02		ENSG00000227394
RPL12	-0.0642	3.2566E-01	5.3580E-01	ENSG00000197958

RPL12P10	0.0200	6.5875E-01		ENSG00000226279
RPL12P12	-0.0006	7.4468E-01		ENSG00000236992
RPL12P20	-0.0233	2.8504E-01		ENSG00000239627
RPL12P24	0.0165	5.2543E-01		ENSG00000243601
RPL12P25	-0.0253	2.5356E-01		ENSG00000235330
RPL12P37	0.0383	3.6625E-01		ENSG00000240270
RPL12P43	-0.0253	3.1144E-01		ENSG00000234524
RPL12P6	0.0051	6.7407E-01	8.1746E-01	ENSG00000243824
RPL12P8	0.0080	5.5107E-01		ENSG00000219932
RPL13	-0.3864	1.6380E-06	5.6924E-05	ENSG00000167526
RPL13A	-0.1845	2.5379E-02	9.8539E-02	ENSG00000142541
RPL13AP11	0.0636	9.9778E-02		ENSG00000229657
RPL13AP12	0.0485	5.6492E-01	7.4069E-01	ENSG00000236047
RPL13AP14	-0.0180	7.2219E-01		ENSG00000242858
RPL13AP16	-0.0093	4.0843E-01		ENSG00000226624
RPL13AP17	0.0200	2.7695E-01		ENSG00000231322
RPL13AP2	0.0119	5.9640E-01		ENSG00000244053
RPL13AP20	-0.2601	3.6131E-02	1.2680E-01	ENSG00000234498
RPL13AP22	-0.0350	5.3855E-01	7.2091E-01	ENSG00000243517
RPL13AP25	-0.1495	1.2667E-01	2.9794E-01	ENSG00000136149
RPL13AP26	-0.1258	1.6520E-01	3.5247E-01	ENSG00000250318
RPL13AP3	-0.0782	2.5221E-01	4.6034E-01	ENSG00000177350
RPL13AP5	-0.2314	2.3594E-02	9.3725E-02	ENSG00000236552
RPL13AP6	-0.2450	4.5169E-02	1.4880E-01	ENSG00000234118
RPL13P12	-0.3703	4.6048E-04	4.9846E-03	ENSG00000215030
RPL13P4	-0.0285	7.4406E-02		ENSG00000241890
RPL13P5	-0.0692	4.1513E-01	6.1862E-01	ENSG00000240370
RPL13P6	-0.0249	1.3586E-01		ENSG00000213176
RPL13P7	0.0146	4.9102E-01		ENSG00000232463
RPL13P8	0.5774	6.9485E-03	3.8585E-02	ENSG00000241634
RPL14	-0.2222	3.1874E-03	2.1475E-02	ENSG00000188846
RPL14P1	-0.1082	2.2923E-01	4.3360E-01	ENSG00000139239
RPL14P2	-0.0495	1.2942E-01		ENSG00000225662
RPL14P3	0.0528	2.1461E-01		ENSG00000241923
RPL14P4	0.0180	6.6317E-01		ENSG00000237774
RPL15	-0.4276	4.5867E-11	8.6812E-09	ENSG00000174748
RPL15P11	-0.0480	1.4927E-01		ENSG00000223718
RPL15P16	-0.0188	6.4076E-01		ENSG00000234751
RPL15P17	-0.0635	2.8079E-01	4.9100E-01	ENSG00000243830
RPL15P18	-0.1111	1.2823E-01	2.9991E-01	ENSG00000228501
RPL15P20	-0.0076	8.3694E-01	9.1604E-01	ENSG00000215003
RPL15P3	-0.5873	4.4646E-10	5.5788E-08	ENSG00000212802
RPL17	-0.4970	9.4581E-08	5.4886E-06	ENSG00000265681
RPL17-C18orf32	-0.0747	3.5569E-02		ENSG00000215472
RPL17P15	0.0050	8.2219E-01		ENSG00000229326
RPL17P16	-0.0062	7.3813E-01		ENSG00000236768
RPL17P18	0.0157	8.3554E-01	9.1524E-01	ENSG00000234742
RPL17P19	-0.0235	3.6592E-01		ENSG00000241261
RPL17P22	-0.0386	3.2703E-01		ENSG00000243592

RPL17P23	0.0941	4.5571E-02		ENSG00000220694
RPL17P25	-0.0079	5.1023E-01		ENSG00000219547
RPL17P26	-0.0578	1.1274E-01	2.7554E-01	ENSG00000216854
RPL17P27	-0.0017	9.1406E-01		ENSG00000243099
RPL17P29	-0.0087	7.1217E-01		ENSG00000231965
RPL17P34	0.0672	8.5091E-02	2.2829E-01	ENSG00000213432
RPL17P36	0.0067	7.7808E-01	8.8190E-01	ENSG00000236058
RPL17P38	-0.0163	5.1301E-01		ENSG00000240441
RPL17P39	-0.0780	3.7399E-01	5.8263E-01	ENSG00000212664
RPL17P43	-0.0205	2.6245E-01	4.7133E-01	ENSG00000228331
RPL17P44	-0.0219	1.9434E-01		ENSG00000241088
RPL17P5	0.0119	4.9436E-01		ENSG00000230721
RPL17P50	0.0264	6.4793E-01	7.9987E-01	ENSG00000213700
RPL17P6	-0.0846	2.9899E-01	5.0876E-01	ENSG00000226084
RPL17P7	-0.0886	3.0582E-01	5.1598E-01	ENSG00000244716
RPL17P8	-0.0676	1.9031E-01	3.8544E-01	ENSG00000213041
RPL17P9	-0.0010	8.1402E-01		ENSG00000223583
RPL18	-0.2011	5.8393E-03	3.3854E-02	ENSG00000063177
RPL18A	-0.3738	5.2194E-06	1.4462E-04	ENSG00000105640
RPL18AP1	0.0353	8.3773E-02		ENSG00000240096
RPL18AP10	-0.0617	2.9550E-01	5.0562E-01	ENSG00000213717
RPL18AP11	-0.0244	6.1627E-01		ENSG00000212695
RPL18AP12	-0.0624	1.1010E-01		ENSG00000276810
RPL18AP13	-0.0395	2.7471E-01		ENSG00000213304
RPL18AP15	0.0003	7.6095E-01		ENSG00000236189
RPL18AP3	-0.4403	2.9412E-07	1.3998E-05	ENSG00000213442
RPL18AP6	-0.3830	1.5501E-03	1.2526E-02	ENSG00000230979
RPL18AP7	-0.0946	9.9728E-02	2.5420E-01	ENSG00000232439
RPL18AP9	-0.0301	5.8687E-01		ENSG00000223916
RPL18P10	0.0027	9.4264E-01		ENSG00000214359
RPL18P13	-0.0149	9.6533E-01		ENSG00000244485
RPL19	-0.2894	2.5083E-04	3.1281E-03	ENSG00000108298
RPL19P14	-0.0467	2.5461E-01		ENSG00000240870
RPL19P19	0.0034	5.6416E-01		ENSG00000243234
RPL19P2	-0.0001	8.8097E-01		ENSG00000229863
RPL19P20	-0.0023	6.7470E-01	8.1764E-01	ENSG00000229168
RPL19P21	-0.0060	9.2690E-01	9.6421E-01	ENSG00000230508
RPL19P5	-0.0092	8.2848E-01		ENSG00000230580
RPL19P6	-0.1437	7.3634E-02	2.0789E-01	ENSG00000233597
RPL19P8	0.0027	9.0832E-01		ENSG00000241877
RPL21	-0.1580	4.2575E-02	1.4270E-01	ENSG00000122026
RPL21P1	-0.0679	4.0541E-01	6.0977E-01	ENSG00000214760
RPL21P10	0.0529	5.1550E-01	7.0314E-01	ENSG00000239272
RPL21P100	0.0224	7.3320E-01	8.5427E-01	ENSG00000242405
RPL21P105	-0.0687	2.3345E-01	4.3868E-01	ENSG00000239374
RPL21P11	-0.1199	7.2943E-02	2.0672E-01	ENSG00000242571
RPL21P116	0.0305	3.9019E-01		ENSG00000243711
RPL21P119	-0.1346	1.2256E-01	2.9148E-01	ENSG00000220793
RPL21P120	-0.1162	1.0619E-01	2.6496E-01	ENSG00000244582

RPL21P123	0.0617	1.1168E-01		ENSG00000240531
RPL21P125	-0.0267	4.5353E-01	6.5220E-01	ENSG00000240898
RPL21P128	-0.0507	1.0831E-01		ENSG00000244527
RPL21P130	-0.0128	5.0572E-01		ENSG00000243771
RPL21P134	-0.3346	1.7443E-02	7.5451E-02	ENSG00000233254
RPL21P137	0.0182	6.6540E-01		ENSG00000236732
RPL21P14	-0.0513	4.1707E-01	6.2047E-01	ENSG00000242747
RPL21P16	-0.1499	8.4573E-02	2.2730E-01	ENSG00000220842
RPL21P18	-0.0591	2.4706E-01		ENSG00000213343
RPL21P19	-0.1105	2.2748E-01	4.3189E-01	ENSG00000219133
RPL21P2	0.0031	9.2830E-01		ENSG00000215063
RPL21P20	0.0069	6.4432E-01		ENSG00000227311
RPL21P21	-0.0164	2.5881E-01		ENSG00000224340
RPL21P23	-0.0234	2.1501E-01		ENSG00000236776
RPL21P27	0.0171	3.0812E-01		ENSG00000225272
RPL21P28	-0.2956	4.1862E-03	2.6404E-02	ENSG00000220749
RPL21P29	-0.0105	6.1312E-01		ENSG00000232037
RPL21P3	-0.0271	4.4326E-01		ENSG00000225419
RPL21P32	0.1933	1.2273E-02	5.8863E-02	ENSG00000224019
RPL21P34	-0.0027	7.4899E-01		ENSG00000238180
RPL21P37	-0.0902	3.1458E-01	5.2458E-01	ENSG00000229503
RPL21P39	-0.0513	5.4650E-01	7.2651E-01	ENSG00000239797
RPL21P40	0.0096	4.5224E-01		ENSG00000235670
RPL21P45	0.0057	5.3480E-01		ENSG00000239532
RPL21P46	-0.0151	5.8905E-01	7.5759E-01	ENSG00000241612
RPL21P48	0.0266	4.6328E-01		ENSG00000242318
RPL21P5	-0.0295	3.5776E-01		ENSG00000258640
RPL21P51	-0.0088	9.4806E-01		ENSG00000240674
RPL21P52	-0.0262	4.4244E-01		ENSG00000242431
RPL21P53	-0.1535	9.6453E-02	2.4939E-01	ENSG00000244021
RPL21P54	0.0259	4.4249E-01		ENSG00000241829
RPL21P57	-0.0130	6.0647E-01		ENSG00000241187
RPL21P59	0.0085	4.7061E-01		ENSG00000253946
RPL21P60	0.0067	9.4939E-01	9.7491E-01	ENSG00000240729
RPL21P61	-0.0094	6.0246E-01		ENSG00000217379
RPL21P63	0.0054	9.6192E-01		ENSG00000217181
RPL21P65	-0.0211	2.0918E-01		ENSG00000216977
RPL21P69	-0.0353	2.5578E-01		ENSG00000216480
RPL21P7	-0.0299	2.8761E-01		ENSG00000258613
RPL21P75	-0.1912	2.8684E-02	1.0767E-01	ENSG00000213860
RPL21P80	-0.0792	3.6715E-01	5.7643E-01	ENSG00000243181
RPL21P82	0.0022	9.3215E-01		ENSG00000227399
RPL21P83	-0.0310	3.2360E-01		ENSG00000230188
RPL21P86	-0.0231	5.4857E-01		ENSG00000213669
RPL21P87	-0.0845	2.7947E-01	4.8952E-01	ENSG00000228053
RPL21P89	0.1574	4.5487E-02	1.4967E-01	ENSG00000229870
RPL21P90	0.0148	4.0051E-01		ENSG00000227805
RPL21P93	-0.0996	2.7168E-01	4.8089E-01	ENSG00000229605
RPL21P94	0.0148	1.7760E-01		ENSG00000241678

RPL21P95	0.2824	2.3848E-02	9.4388E-02	ENSG00000241782
RPL21P97	-0.0754	3.6281E-01	5.7197E-01	ENSG00000239470
RPL21P98	-0.2302	4.8612E-02	1.5653E-01	ENSG00000243071
RPL21P99	0.0403	1.3678E-01		ENSG00000242986
RPL22	-0.1126	1.2630E-01	2.9732E-01	ENSG00000116251
RPL22L1	-0.0841	2.9301E-01	5.0301E-01	ENSG00000163584
RPL22P16	0.0201	4.3876E-01		ENSG00000213935
RPL22P19	0.0164	1.8564E-01		ENSG00000241129
RPL22P2	0.0022	9.4381E-01		ENSG00000241081
RPL22P3	-0.3222	2.0327E-02	8.3890E-02	ENSG00000226457
RPL23	-0.0990	1.8111E-01	3.7353E-01	ENSG00000125691
RPL23A	-0.3317	6.5072E-07	2.6204E-05	ENSG00000198242
RPL23AP12	-0.0421	7.2064E-02		ENSG00000228861
RPL23AP15	-0.0073	7.9853E-01		ENSG00000230841
RPL23AP18	-0.3782	1.1959E-02	5.7837E-02	ENSG00000225338
RPL23AP21	0.2257	2.2422E-02	9.0209E-02	ENSG00000228463
RPL23AP23	-0.0295	1.3667E-01		ENSG00000236863
RPL23AP24	0.1291	1.6891E-01	3.5730E-01	ENSG00000236679
RPL23AP25	-0.1124	2.1909E-01	4.2147E-01	ENSG00000233084
RPL23AP3	-0.0367	1.4167E-01		ENSG00000214914
RPL23AP36	0.0196	6.3837E-01		ENSG00000225124
RPL23AP4	0.0141	8.6497E-01	9.3177E-01	ENSG00000212932
RPL23AP41	0.0153	5.0415E-01		ENSG00000224085
RPL23AP42	-0.5158	3.4382E-06	1.0380E-04	ENSG00000234851
RPL23AP43	-0.1496	4.5704E-02	1.5024E-01	ENSG00000227337
RPL23AP45	0.1813	8.9687E-02	2.3726E-01	ENSG00000213285
RPL23AP47	0.1023	2.4686E-01	4.5446E-01	ENSG00000219814
RPL23AP49	-0.1593	9.3244E-02	2.4371E-01	ENSG00000243422
RPL23AP5	-0.1827	7.8489E-02	2.1685E-01	ENSG00000236829
RPL23AP50	0.0029	8.2708E-01		ENSG00000232778
RPL23AP51	0.0132	7.0669E-01		ENSG00000234718
RPL23AP52	0.0103	6.5389E-01		ENSG00000230370
RPL23AP57	-0.0111	7.9195E-01		ENSG00000206147
RPL23AP60	0.1854	5.4199E-02	1.6868E-01	ENSG00000213147
RPL23AP61	0.0098	8.5886E-01		ENSG00000280012
RPL23AP65	-0.1961	7.0247E-02	2.0120E-01	ENSG00000243964
RPL23AP67	0.0243	5.2438E-01		ENSG00000240991
RPL23AP69	-0.0130	3.6889E-01		ENSG00000235315
RPL23AP74	-0.0192	2.3332E-01		ENSG00000227694
RPL23AP75	0.0341	2.5706E-01		ENSG00000240125
RPL23AP78	-0.0223	1.6464E-01		ENSG00000269244
RPL23AP80	0.0175	3.4907E-01		ENSG00000213035
RPL23AP81	-0.0183	7.1794E-01		ENSG00000242748
RPL23AP84	0.1484	8.5009E-02	2.2815E-01	ENSG00000261914
RPL23AP87	-0.0558	5.0254E-01	6.9299E-01	ENSG00000232938
RPL23AP92	0.0000	9.5076E-01		ENSG00000270723
RPL23P2	0.0119	8.8118E-01	9.4050E-01	ENSG00000176054
RPL23P6	-0.0116	7.7503E-01		ENSG00000180211
RPL23P7	0.0113	7.8576E-01		ENSG00000232383

RPL23P8	-0.0521	4.9591E-01	6.8762E-01	ENSG00000219451
RPL24	-0.2725	2.4311E-03	1.7444E-02	ENSG00000114391
RPL24P2	-0.0915	8.0484E-02	2.2055E-01	ENSG00000235065
RPL24P3	-0.0165	3.9874E-01		ENSG00000244237
RPL24P4	-0.1028	2.0285E-01	4.0183E-01	ENSG00000181524
RPL24P5	-0.0143	6.7606E-01		ENSG00000235582
RPL24P6	0.0036	8.1708E-01		ENSG00000236844
RPL24P8	0.0195	9.3756E-01	9.6914E-01	ENSG00000236801
RPL26	-0.0907	1.8425E-01	3.7770E-01	ENSG00000161970
RPL26L1	-0.2191	1.3794E-03	1.1521E-02	ENSG00000037241
RPL26L1-AS1	0.2333	4.3347E-02	1.4464E-01	ENSG00000204758
RPL26P10	0.0018	9.8283E-01		ENSG00000226984
RPL26P19	-0.1396	1.2182E-01	2.9049E-01	ENSG00000226221
RPL26P22	-0.0145	3.9604E-01		ENSG00000229977
RPL26P23	-0.0008	7.0802E-01		ENSG00000234450
RPL26P27	0.0130	5.8551E-01		ENSG00000226493
RPL26P29	-0.0226	3.1014E-01		ENSG00000234806
RPL26P32	0.0058	7.5057E-01		ENSG00000230291
RPL26P35	-0.0081	3.2994E-01		ENSG00000244229
RPL26P36	0.0247	7.3048E-01	8.5263E-01	ENSG00000225912
RPL26P37	0.0140	8.6109E-01		ENSG00000235175
RPL26P4	-0.0600	2.1654E-01	4.1821E-01	ENSG00000241494
RPL26P6	0.0784	1.4454E-01		ENSG00000229659
RPL27	-0.1430	5.1132E-02	1.6179E-01	ENSG00000131469
RPL27A	-0.2758	1.3553E-03	1.1401E-02	ENSG00000166441
RPL27AP5	-0.0365	1.0811E-01		ENSG00000182383
RPL27AP6	-0.0956	2.8598E-01	4.9669E-01	ENSG00000218426
RPL27AP8	0.0239	1.2935E-01		ENSG00000229423
RPL27P1	0.0107	2.4102E-01		ENSG00000213867
RPL27P11	0.0005	9.3368E-01		ENSG00000233287
RPL27P3	-0.0237	5.2435E-01		ENSG00000226545
RPL27P4	-0.0099	6.2738E-01		ENSG00000225616
RPL27P5	-0.0075	6.6134E-01		ENSG00000232403
RPL27P9	0.0226	2.4925E-01		ENSG00000236722
RPL28	-0.0079	9.0695E-01	9.5442E-01	ENSG00000108107
RPL28P2	-0.0463	4.7955E-01	6.7455E-01	ENSG00000237641
RPL28P5	0.0189	7.7861E-01	8.8223E-01	ENSG00000213293
RPL29	-0.1967	3.7443E-03	2.4322E-02	ENSG00000162244
RPL29P2	0.0190	5.4587E-01		ENSG00000240480
RPL29P21	-0.0094	4.1738E-01		ENSG00000213779
RPL29P34	-0.0242	2.9924E-01		ENSG00000239255
RPL3	-0.2105	2.9281E-03	2.0103E-02	ENSG00000100316
RPL30	-0.1483	3.3384E-02	1.1988E-01	ENSG00000156482
RPL30P13	-0.0025	9.4443E-01		ENSG00000229014
RPL31	-0.1773	2.6118E-02	1.0069E-01	ENSG00000071082
RPL31P10	0.0307	2.3137E-01		ENSG00000213942
RPL31P12	0.0265	4.2760E-01		ENSG00000227207
RPL31P14	0.0070	5.8598E-01		ENSG00000237930
RPL31P17	0.0541	3.5455E-01		ENSG00000235225

RPL31P18	0.0422	2.3198E-01		ENSG00000213849
RPL31P19	0.0038	9.0997E-01		ENSG00000227309
RPL31P2	0.0058	8.7825E-01		ENSG00000230837
RPL31P21	0.0043	9.6553E-01		ENSG00000241400
RPL31P22	0.0274	1.6338E-01		ENSG00000227433
RPL31P28	-0.0444	1.5849E-01		ENSG00000219863
RPL31P30	0.0133	8.1608E-01		ENSG00000230702
RPL31P31	-0.0079	7.4083E-01		ENSG00000239983
RPL31P34	-0.0047	9.1276E-01		ENSG00000231359
RPL31P38	0.0004	9.4909E-01		ENSG00000179131
RPL31P4	-0.0721	4.1248E-01	6.1614E-01	ENSG00000213598
RPL31P49	-0.0447	1.4227E-01		ENSG00000241680
RPL31P52	0.1200	1.2347E-01	2.9305E-01	ENSG00000219355
RPL31P58	-0.0252	6.4942E-01		ENSG00000243053
RPL31P61	0.0594	2.0443E-01	4.0362E-01	ENSG00000243297
RPL31P63	0.0406	5.5744E-01	7.3508E-01	ENSG00000236735
RPL31P7	-0.0304	2.8218E-01		ENSG00000232083
RPL32	-0.1994	9.3786E-03	4.8398E-02	ENSG00000144713
RPL32P13	-0.0154	7.0420E-01		ENSG00000241002
RPL32P18	-0.3001	1.4303E-02	6.5713E-02	ENSG00000146677
RPL32P22	-0.0054	9.1313E-01		ENSG00000213529
RPL32P27	-0.0779	3.3357E-01	5.4368E-01	ENSG00000239617
RPL32P32	0.0273	2.5059E-01		ENSG00000241157
RPL34	-0.1691	2.9416E-02	1.0950E-01	ENSG00000109475
RPL34-DT	0.0817	3.5191E-01	5.6219E-01	ENSG00000234492
RPL34P1	0.0052	6.9569E-01		ENSG00000237049
RPL34P10	-0.0337	1.7217E-01		ENSG00000236489
RPL34P16	-0.0119	9.2133E-01		ENSG00000217239
RPL34P18	-0.0464	5.5742E-01	7.3508E-01	ENSG00000240509
RPL34P20	-0.0446	1.3999E-01		ENSG00000231508
RPL34P21	-0.0095	3.8804E-01		ENSG00000244451
RPL34P22	-0.0058	8.3131E-01		ENSG00000244267
RPL34P24	-0.0529	3.7075E-01	5.7974E-01	ENSG00000240808
RPL34P26	0.0064	7.9898E-01		ENSG00000223676
RPL34P27	-0.1024	2.9288E-02	1.0927E-01	ENSG00000232858
RPL34P28	-0.0060	4.7338E-01		ENSG00000239686
RPL34P31	-0.0051	7.7833E-01		ENSG00000239223
RPL34P4	0.0227	2.2695E-01		ENSG00000225854
RPL34P5	0.0490	4.5541E-01		ENSG00000231416
RPL34P6	0.0210	7.7671E-01	8.8109E-01	ENSG00000231333
RPL35	-0.1875	1.3256E-02	6.2246E-02	ENSG00000136942
RPL35A	-0.1257	8.0594E-02	2.2069E-01	ENSG00000182899
RPL35AP14	0.0226	2.7421E-01		ENSG00000240436
RPL35AP6	0.0495	3.6781E-01	5.7706E-01	ENSG00000228879
RPL35P1	-0.0083	7.7814E-01	8.8190E-01	ENSG00000237991
RPL35P5	-0.0346	4.5194E-01	6.5094E-01	ENSG00000225573
RPL35P6	-0.0339	6.1161E-01	7.7383E-01	ENSG00000244018
RPL36	-0.2131	1.2887E-02	6.0931E-02	ENSG00000130255
RPL36A	0.1271	1.4349E-01	3.2145E-01	ENSG00000241343

RPL36AL	-0.2748	2.6646E-04	3.2781E-03	ENSG00000165502
RPL36AP11	-0.0097	6.2124E-01		ENSG00000237003
RPL36AP21	-0.0376	2.1449E-01	4.1565E-01	ENSG00000240785
RPL36AP23	-0.0121	4.9116E-01		ENSG00000241722
RPL36AP32	0.0149	5.2220E-01		ENSG00000242958
RPL36AP37	-0.4231	3.2673E-05	6.3652E-04	ENSG00000244398
RPL36AP41	-0.1098	9.2744E-03		ENSG00000242087
RPL36AP45	-0.1352	1.2701E-02	6.0320E-02	ENSG00000240366
RPL36AP46	-0.1964	4.3450E-02	1.4486E-01	ENSG00000224011
RPL36AP48	-0.0249	3.1113E-01		ENSG00000213180
RPL36AP49	-0.0628	1.5653E-01	3.4030E-01	ENSG00000243779
RPL36AP51	-0.0005	8.8218E-01		ENSG00000242291
RPL36AP52	-0.0656	5.7070E-02		ENSG00000214925
RPL36AP54	-0.0296	3.7661E-01		ENSG00000226107
RPL36P12	-0.0053	5.0034E-01		ENSG00000213361
RPL36P15	0.0251	2.4933E-01		ENSG00000240100
RPL36P19	0.0016	9.5978E-01		ENSG00000232540
RPL36P20	0.0038	8.5396E-01		ENSG00000234270
RPL36P6	0.1196	9.5237E-02	2.4726E-01	ENSG00000224348
RPL36P7	-0.0088	8.5129E-01	9.2425E-01	ENSG00000213519
RPL37	-0.0780	2.5459E-01	4.6263E-01	ENSG00000145592
RPL37A	-0.0848	2.3128E-01	4.3620E-01	ENSG00000197756
RPL37AP6	0.0121	6.9017E-01		ENSG00000228341
RPL37P10	-0.0060	6.6988E-01		ENSG00000224627
RPL37P18	-0.0127	3.9715E-01		ENSG00000229235
RPL37P20	0.0095	6.8245E-01		ENSG00000239701
RPL37P25	-0.0005	8.9514E-01		ENSG00000251597
RPL37P6	-0.0226	4.7380E-01		ENSG00000241431
RPL37P9	0.0026	8.8333E-01		ENSG00000227950
RPL38	-0.0438	5.3791E-01	7.2053E-01	ENSG00000172809
RPL39	-0.1439	3.0896E-02	1.1365E-01	ENSG00000198918
RPL39L	-0.0549	4.5345E-01	6.5212E-01	ENSG00000163923
RPL39P16	0.0096	8.2260E-01		ENSG00000227470
RPL3L	0.0037	9.8473E-01	9.9198E-01	ENSG00000140986
RPL3P1	-0.0626	2.4111E-01	4.4812E-01	ENSG00000228149
RPL3P11	0.0058	5.9184E-01		ENSG00000223568
RPL3P2	0.0052	9.4867E-01	9.7460E-01	ENSG00000227939
RPL3P4	-0.1703	6.9350E-02	1.9939E-01	ENSG00000232573
RPL3P6	0.0320	3.3485E-01		ENSG00000213891
RPL3P7	0.0169	7.5951E-01	8.7034E-01	ENSG00000225093
RPL3P9	-0.0334	4.2352E-01		ENSG00000240540
RPL4	-0.1481	2.9026E-02	1.0851E-01	ENSG00000174444
RPL41	-0.1674	3.4198E-02	1.2174E-01	ENSG00000229117
RPL41P2	-0.1375	1.3948E-01	3.1569E-01	ENSG00000256338
RPL4P2	0.0117	3.4372E-01		ENSG00000232341
RPL4P3	-0.0235	8.7440E-01	9.3710E-01	ENSG00000230364
RPL4P4	-0.0784	3.6316E-01	5.7225E-01	ENSG00000229638
RPL4P5	-0.0860	2.9000E-01	5.0035E-01	ENSG00000230207
RPL4P6	-0.0828	3.2719E-02		ENSG00000230071

RPL5	-0.1473	3.3388E-02	1.1988E-01	ENSG00000122406
RPL5P1	0.0323	6.7649E-01	8.1874E-01	ENSG00000241061
RPL5P11	0.0423	3.0257E-01		ENSG00000242882
RPL5P12	0.0135	5.1978E-01		ENSG00000242697
RPL5P14	-0.0042	7.2658E-01		ENSG00000243914
RPL5P18	0.0545	1.7974E-01		ENSG00000220744
RPL5P22	-0.0337	4.5416E-01	6.5260E-01	ENSG00000239198
RPL5P23	0.0094	9.0778E-01	9.5461E-01	ENSG00000240395
RPL5P24	0.0210	4.4564E-01		ENSG00000244052
RPL5P28	0.0180	8.0283E-01		ENSG00000235264
RPL5P29	0.0111	7.7209E-01		ENSG00000242208
RPL5P3	-0.0129	7.1511E-01		ENSG00000242276
RPL5P30	-0.0298	6.2841E-01	7.8609E-01	ENSG00000243431
RPL5P34	-0.1431	1.2826E-01	2.9993E-01	ENSG00000234009
RPL5P4	0.0101	9.1040E-01	9.5580E-01	ENSG00000229994
RPL5P5	0.0546	2.9211E-01		ENSG00000213051
RPL5P6	0.0238	3.9433E-01		ENSG00000232393
RPL5P8	-0.0104	4.5513E-01		ENSG00000213917
RPL5P9	-0.0368	3.9168E-01		ENSG00000236330
RPL6	-0.1045	1.5877E-01	3.4315E-01	ENSG00000089009
RPL6P1	0.0033	9.7865E-01		ENSG00000236111
RPL6P10	0.0824	3.4150E-01	5.5117E-01	ENSG00000243199
RPL6P13	-0.0140	4.3381E-01		ENSG00000243312
RPL6P16	0.0030	7.1631E-01		ENSG00000240159
RPL6P19	-0.0171	8.3662E-01	9.1578E-01	ENSG00000230383
RPL6P20	0.0070	7.2944E-01		ENSG00000236976
RPL6P24	-0.0892	6.9538E-02		ENSG00000227474
RPL6P26	-0.0422	1.9673E-01		ENSG00000240305
RPL6P27	-0.0535	5.0627E-01	6.9599E-01	ENSG00000235552
RPL6P28	0.0723	2.9319E-01	5.0323E-01	ENSG00000223730
RPL6P31	0.0275	5.0714E-01		ENSG00000223945
RPL6P6	0.0052	8.5444E-01		ENSG00000231063
RPL7	-0.2723	4.4831E-04	4.8830E-03	ENSG00000147604
RPL7A	-0.1660	2.8023E-02	1.0591E-01	ENSG00000148303
RPL7AP10	-0.1106	1.2554E-02		ENSG00000240522
RPL7AP11	-0.1078	1.8486E-01	3.7842E-01	ENSG00000242445
RPL7AP14	-0.1238	5.1018E-02	1.6156E-01	ENSG00000213979
RPL7AP15	0.0119	6.7610E-01		ENSG00000236806
RPL7AP16	-0.0726	2.6948E-01	4.7896E-01	ENSG00000234329
RPL7AP20	0.0051	8.8607E-01		ENSG00000234981
RPL7AP21	0.0025	7.1970E-01		ENSG00000229757
RPL7AP22	0.0087	5.9872E-01		ENSG00000227331
RPL7AP24	-0.0171	5.4368E-01		ENSG00000241307
RPL7AP25	-0.0109	4.3266E-01		ENSG00000235294
RPL7AP26	-0.0180	6.8364E-01		ENSG00000214988
RPL7AP27	0.0008	7.2464E-01		ENSG00000242169
RPL7AP29	0.0037	8.2296E-01		ENSG00000248843
RPL7AP3	0.0045	6.4983E-01		ENSG00000256243
RPL7AP30	-0.0024	7.1406E-01	8.4302E-01	ENSG00000241741

RPL7AP31	0.0067	8.8349E-01		ENSG00000242083
RPL7AP35	-0.0281	4.6117E-01		ENSG00000216639
RPL7AP39	0.4316	1.8612E-03	1.4267E-02	ENSG00000213385
RPL7AP42	0.0053	8.7477E-01		ENSG00000234358
RPL7AP43	0.2673	2.8510E-02	1.0721E-01	ENSG00000213542
RPL7AP45	0.0860	2.9089E-01	5.0136E-01	ENSG00000275160
RPL7AP46	0.0123	5.7971E-01		ENSG00000233555
RPL7AP47	0.0075	8.1468E-01		ENSG00000234982
RPL7AP48	-0.0014	8.5787E-01		ENSG00000237088
RPL7AP50	0.0292	6.9160E-01	8.2896E-01	ENSG00000213609
RPL7AP53	0.0026	7.2418E-01		ENSG00000235962
RPL7AP55	-0.0016	7.8730E-01		ENSG00000241008
RPL7AP58	0.0126	2.6875E-01		ENSG00000240554
RPL7AP6	-0.1777	5.8385E-02	1.7729E-01	ENSG00000242071
RPL7AP60	-0.1942	6.7774E-02	1.9628E-01	ENSG00000213152
RPL7AP62	-0.0118	8.5873E-01	9.2870E-01	ENSG00000213862
RPL7AP63	0.0021	9.7254E-01		ENSG00000243697
RPL7AP65	-0.0579	2.6485E-01	4.7403E-01	ENSG00000228000
RPL7AP66	-0.0822	3.3881E-01	5.4858E-01	ENSG00000175886
RPL7AP70	0.0366	6.4024E-01	7.9415E-01	ENSG00000235776
RPL7AP71	0.1127	2.1833E-02		ENSG00000234566
RPL7AP73	-0.0025	9.8140E-01		ENSG00000237952
RPL7AP76	0.3043	1.5848E-02	7.0677E-02	ENSG00000231995
RPL7AP77	0.0010	6.8159E-01		ENSG00000225005
RPL7AP79	-0.0088	4.9103E-01		ENSG00000255106
RPL7AP82	0.2615	2.2668E-02	9.0928E-02	ENSG00000228955
RPL7AP83	-0.0530	2.3639E-02		ENSG00000229339
RPL7AP9	0.0507	7.8493E-02		ENSG00000213272
RPL7L1	-0.2430	1.8682E-06	6.2811E-05	ENSG00000146223
RPL7L1P16	-0.0115	6.6252E-01		ENSG00000260494
RPL7L1P8	-0.0010	9.1266E-01		ENSG00000228766
RPL7P1	-0.2098	1.4310E-02	6.5727E-02	ENSG00000214485
RPL7P10	-0.0235	3.9101E-01		ENSG00000229752
RPL7P11	-0.0097	8.4244E-01		ENSG00000234619
RPL7P12	-0.0212	4.9446E-01		ENSG00000227770
RPL7P13	-0.0645	2.3423E-01		ENSG00000234253
RPL7P16	0.0063	8.0998E-01		ENSG00000242899
RPL7P18	0.0125	4.5450E-01		ENSG00000243806
RPL7P19	-0.4399	5.2043E-04	5.4723E-03	ENSG00000241458
RPL7P20	0.0085	9.7586E-01	9.8791E-01	ENSG00000254252
RPL7P22	0.0088	9.2437E-01		ENSG00000226544
RPL7P23	-0.2884	3.3059E-03	2.2026E-02	ENSG00000244363
RPL7P24	0.0716	3.6404E-01	5.7312E-01	ENSG00000240003
RPL7P28	-0.0092	4.3868E-01		ENSG00000218632
RPL7P32	-0.0957	2.6477E-01	4.7397E-01	ENSG00000231006
RPL7P38	-0.0505	1.8510E-01		ENSG00000239473
RPL7P4	0.0147	7.7978E-01	8.8319E-01	ENSG00000230449
RPL7P43	-0.0203	1.2242E-01		ENSG00000241045
RPL7P44	-0.0286	3.9929E-01	6.0473E-01	ENSG00000233873

RPL7P47	-0.0647	2.6255E-01	4.7140E-01	ENSG00000228071
RPL7P48	-0.0355	4.4209E-01		ENSG00000187504
RPL7P49	-0.1237	1.4069E-01	3.1741E-01	ENSG00000242931
RPL7P50	-0.0098	4.8871E-01		ENSG00000268785
RPL7P52	0.0088	5.8573E-01		ENSG00000228599
RPL7P56	0.0617	2.8096E-01	4.9116E-01	ENSG00000231164
RPL7P57	0.0018	9.3913E-01	9.7000E-01	ENSG00000224401
RPL7P6	-0.0535	5.0066E-01	6.9151E-01	ENSG00000227525
RPL7P60	0.0447	3.9558E-01	6.0182E-01	ENSG00000214142
RPL7P7	-0.0130	6.9784E-01		ENSG00000224315
RPL7P9	-0.2436	5.7602E-03	3.3506E-02	ENSG00000137970
RPL8	-0.3982	6.1312E-10	7.2878E-08	ENSG00000161016
RPL8P3	0.0119	8.0632E-01		ENSG00000240950
RPL8P4	-0.1128	1.1063E-01	2.7230E-01	ENSG00000213169
RPL9	-0.1822	2.6921E-02	1.0282E-01	ENSG00000163682
RPL9P10	-0.0199	2.3277E-01		ENSG00000232166
RPL9P11	-0.0391	5.8212E-01	7.5309E-01	ENSG00000231181
RPL9P14	-0.0113	5.3612E-01		ENSG00000185078
RPL9P17	-0.0116	7.5648E-01		ENSG00000224282
RPL9P18	-0.0194	5.2542E-01		ENSG00000213309
RPL9P2	0.0366	4.0075E-01	6.0602E-01	ENSG00000234324
RPL9P20	-0.0072	5.9411E-01		ENSG00000244113
RPL9P22	0.0109	8.9780E-01		ENSG00000240174
RPL9P27	0.0195	4.2440E-01		ENSG00000244251
RPL9P28	-0.0265	1.3883E-01		ENSG00000242067
RPL9P3	-0.0090	8.6578E-01	9.3227E-01	ENSG00000240392
RPL9P30	-0.0637	3.7863E-01	5.8711E-01	ENSG00000240074
RPL9P32	-0.0820	3.3210E-01	5.4247E-01	ENSG00000242100
RPL9P7	-0.2471	5.7513E-03	3.3470E-02	ENSG00000238103
RPL9P8	-0.2954	5.1030E-04	5.3888E-03	ENSG00000237550
RPLP0	-0.1348	6.6172E-02	1.9337E-01	ENSG00000089157
RPLP0P2	0.0332	6.6973E-01	8.1484E-01	ENSG00000243742
RPLP0P3	-0.0013	9.8337E-01		ENSG00000240023
RPLP0P5	0.0023	9.8885E-01		ENSG00000232883
RPLP0P6	-0.0673	3.8992E-01	5.9733E-01	ENSG00000213553
RPLP1	-0.1243	8.2735E-02	2.2432E-01	ENSG00000137818
RPLP1P13	-0.0062	4.7774E-01		ENSG00000232163
RPLP1P3	-0.0171	3.6020E-01		ENSG00000224600
RPLP2	-0.0396	5.3865E-01	7.2098E-01	ENSG00000177600
RPN1	-0.0575	3.5371E-01	5.6374E-01	ENSG00000163902
RPN2	-0.0291	5.9086E-01	7.5893E-01	ENSG00000118705
RPP14	0.1572	9.9998E-02	2.5470E-01	ENSG00000163684
RPP21	-0.0683	4.1075E-01	6.1494E-01	ENSG00000241370
RPP25	-0.3992	2.8291E-06	8.8676E-05	ENSG00000178718
RPP25L	-0.0959	2.1222E-01	4.1317E-01	ENSG00000164967
RPP30	0.0207	7.6221E-01	8.7213E-01	ENSG00000148688
RPP38	0.1792	1.1776E-02	5.7253E-02	ENSG00000152464
RPP38-DT	0.1421	1.3533E-01	3.1006E-01	ENSG00000176236
RPP40	-0.1978	2.8393E-02	1.0687E-01	ENSG00000124787

RPRD1A	0.0916	3.7267E-02	1.2988E-01	ENSG00000141425
RPRD1B	0.1634	1.2795E-02	6.0619E-02	ENSG00000101413
RPRD2	-0.1127	6.0712E-02	1.8185E-01	ENSG00000163125
RPRM	-0.1094	1.8129E-01	3.7381E-01	ENSG00000177519
RPRML	0.0339	6.7709E-01	8.1908E-01	ENSG00000179673
RPS10	-0.2953	9.1426E-03	4.7499E-02	ENSG00000124614
RPS10P1	-0.0323	6.0832E-01	7.7147E-01	ENSG00000217275
RPS10P10	0.0107	6.4646E-01		ENSG00000226082
RPS10P11	0.0082	9.7776E-01	9.8898E-01	ENSG00000243648
RPS10P12	-0.0207	1.9884E-01		ENSG00000240627
RPS10P13	-0.0384	4.2384E-01	6.2611E-01	ENSG00000217130
RPS10P15	-0.5307	5.7974E-03	3.3659E-02	ENSG00000213280
RPS10P2	-0.0511	4.7977E-01	6.7479E-01	ENSG00000213950
RPS10P22	-0.0728	7.6437E-02		ENSG00000242667
RPS10P23	0.0637	2.6089E-01	4.6941E-01	ENSG00000240861
RPS10P24	0.0033	9.8152E-01		ENSG00000241183
RPS10P26	-0.2775	2.2418E-02	9.0209E-02	ENSG00000239246
RPS10P27	-0.0252	2.4696E-01		ENSG00000267752
RPS10P3	-0.1869	7.4715E-02	2.0991E-01	ENSG00000217716
RPS10P4	-0.0344	3.6270E-01	5.7192E-01	ENSG00000240776
RPS10P5	-0.0463	2.5514E-01	4.6319E-01	ENSG00000101278
RPS10P6	-0.1364	1.4742E-01	3.2743E-01	ENSG00000223861
RPS11	-0.0978	1.8787E-01	3.8264E-01	ENSG00000142534
RPS11P7	0.0003	9.7447E-01		ENSG00000214047
RPS12	0.0395	6.0049E-01	7.6602E-01	ENSG00000112306
RPS12P12	-0.0125	4.7986E-01		ENSG00000220920
RPS12P29	0.0550	5.0140E-01	6.9215E-01	ENSG00000213939
RPS12P31	-0.0181	5.1107E-01		ENSG00000242325
RPS12P7	0.0044	8.5711E-01		ENSG00000243314
RPS12P9	0.0434	1.7158E-01		ENSG00000226439
RPS13	-0.1252	1.1357E-01	2.7707E-01	ENSG00000110700
RPS13P1	0.0035	9.0744E-01		ENSG00000230175
RPS13P2	-0.1495	1.3041E-01	3.0320E-01	ENSG00000228929
RPS13P4	-0.0548	3.1174E-01	5.2176E-01	ENSG00000238111
RPS13P6	-0.0044	7.5119E-01		ENSG00000239615
RPS14	-0.2175	1.3009E-02	6.1336E-02	ENSG00000164587
RPS14P1	-0.5737	1.9609E-03	1.4855E-02	ENSG00000224114
RPS15	-0.3357	3.6621E-05	6.9904E-04	ENSG00000115268
RPS15A	-0.1549	5.0700E-02	1.6105E-01	ENSG00000134419
RPS15AP1	-0.0602	4.7521E-01	6.7076E-01	ENSG00000214535
RPS15AP11	0.0160	6.7256E-01		ENSG00000234093
RPS15AP12	-0.0781	1.9472E-01	3.9147E-01	ENSG00000232134
RPS15AP13	-0.0013	9.7813E-01		ENSG00000228568
RPS15AP14	0.0356	1.4506E-01		ENSG00000233131
RPS15AP16	-0.0035	9.2440E-01		ENSG00000239483
RPS15AP17	0.0055	6.3048E-01	7.8714E-01	ENSG00000225405
RPS15AP18	0.0172	6.3457E-01		ENSG00000242979
RPS15AP19	-0.0854	3.2272E-01	5.3285E-01	ENSG00000219023
RPS15AP24	0.0096	9.7520E-01		ENSG00000241511

RPS15AP25	-0.0363	2.2564E-01		ENSG00000236191
RPS15AP29	0.0221	4.2826E-01		ENSG00000237827
RPS15AP30	0.0142	7.7576E-01		ENSG00000227560
RPS15AP35	-0.0116	5.3078E-01		ENSG00000214917
RPS15AP36	-0.0062	5.9436E-01		ENSG00000213013
RPS15AP37	0.0087	5.1485E-01		ENSG00000235573
RPS15AP40	-0.0132	5.5751E-01		ENSG00000233921
RPS15AP5	0.0136	5.6819E-01		ENSG00000223540
RPS15AP6	0.0678	3.5665E-01	5.6644E-01	ENSG00000233072
RPS15AP8	-0.0126	6.1801E-01		ENSG00000235002
RPS15P1	-0.0056	6.7816E-01		ENSG00000236887
RPS15P2	-0.0189	5.9251E-01	7.6020E-01	ENSG00000235605
RPS15P4	-0.5733	2.5587E-07	1.2479E-05	ENSG00000233762
RPS15P5	-0.0411	6.0885E-01	7.7193E-01	ENSG00000229806
RPS15P7	-0.0411	5.6511E-01	7.4080E-01	ENSG00000213970
RPS15P8	0.0090	6.3309E-01		ENSG00000243122
RPS16	-0.1195	1.0407E-01	2.6151E-01	ENSG00000105193
RPS16P1	-0.0149	6.7598E-01	8.1837E-01	ENSG00000178715
RPS16P4	0.0196	3.8802E-01		ENSG00000231449
RPS16P7	0.0379	5.6709E-01	7.4191E-01	ENSG00000243445
RPS17	-0.1083	2.1558E-01	4.1691E-01	ENSG00000182774
RPS17P11	0.0100	5.2875E-01		ENSG00000244621
RPS17P16	-0.1658	8.5808E-02	2.2962E-01	ENSG00000232346
RPS17P2	-0.0060	8.1364E-01		ENSG00000197575
RPS17P3	0.0819	1.6504E-01	3.5223E-01	ENSG00000242439
RPS18	-0.1616	4.0614E-02	1.3822E-01	ENSG00000231500
RPS18P12	-0.0215	7.0233E-01	8.3561E-01	ENSG00000230897
RPS18P14	-0.0716	1.8317E-01	3.7637E-01	ENSG00000214093
RPS18P2	-0.1159	2.0894E-01	4.0899E-01	ENSG00000213315
RPS18P5	-0.0838	3.2850E-01	5.3895E-01	ENSG00000242299
RPS18P6	-0.0064	8.0047E-01		ENSG00000244048
RPS18P7	0.0202	4.0701E-01		ENSG00000240388
RPS19	-0.3791	1.1954E-04	1.7672E-03	ENSG00000105372
RPS19BP1	0.1374	1.6408E-02	7.2275E-02	ENSG00000187051
RPS19P1	-0.0395	4.9473E-01	6.8672E-01	ENSG00000214612
RPS19P5	-0.0378	2.8095E-01		ENSG00000219559
RPS19P7	-0.0947	1.2802E-01	2.9973E-01	ENSG00000228462
RPS2	-0.3194	4.1573E-04	4.6035E-03	ENSG00000140988
RPS20	-0.1121	1.5120E-01	3.3255E-01	ENSG00000008988
RPS20P15	-0.0083	8.6700E-01	9.3294E-01	ENSG00000227523
RPS20P17	-0.0243	5.1207E-01		ENSG00000239393
RPS20P28	0.0474	4.6415E-01		ENSG00000240739
RPS20P31	-0.0244	3.5948E-01		ENSG00000240925
RPS20P33	0.0651	2.9396E-01	5.0394E-01	ENSG00000242085
RPS20P6	-0.0102	4.8922E-01		ENSG00000228399
RPS21	0.0982	1.7558E-01	3.6643E-01	ENSG00000171858
RPS23	-0.1976	5.5976E-03	3.2767E-02	ENSG00000186468
RPS23P1	-0.0428	2.8874E-01	4.9916E-01	ENSG00000243504
RPS23P2	-0.0569	1.6523E-01		ENSG00000213448

RPS23P8	-0.1675	7.3053E-02	2.0692E-01	ENSG00000230629
RPS24	-0.2926	6.7470E-05	1.1340E-03	ENSG00000138326
RPS25	-0.2455	1.0939E-03	9.7026E-03	ENSG00000118181
RPS25P10	0.1056	1.3069E-01	3.0363E-01	ENSG00000234735
RPS25P3	0.0523	1.7673E-01		ENSG00000233416
RPS25P7	0.0113	5.8905E-01		ENSG00000218472
RPS26	-0.1207	1.3008E-01	3.0283E-01	ENSG00000197728
RPS26P12	-0.0304	3.2931E-01		ENSG00000235869
RPS26P21	0.0556	6.5898E-02		ENSG00000242829
RPS26P22	-0.0375	2.0977E-01		ENSG00000242199
RPS26P23	0.0234	6.2423E-01		ENSG00000240775
RPS26P31	-0.0441	6.0423E-01	7.6833E-01	ENSG00000235459
RPS26P48	0.0121	7.4589E-01		ENSG00000239438
RPS26P57	0.0301	4.3893E-01		ENSG00000236880
RPS27	-0.0609	4.1079E-01	6.1496E-01	ENSG00000177954
RPS27A	-0.2648	2.2059E-03	1.6197E-02	ENSG00000143947
RPS27AP1	-0.0781	2.5983E-01	4.6829E-01	ENSG00000242692
RPS27AP11	-0.1501	1.2805E-01	2.9973E-01	ENSG00000218208
RPS27AP16	-0.2439	1.7004E-02	7.4035E-02	ENSG00000224631
RPS27AP18	-0.0025	7.4465E-01		ENSG00000249851
RPS27AP19	-0.0106	3.6898E-01		ENSG00000269228
RPS27AP2	0.0292	3.3767E-01		ENSG00000232333
RPS27AP20	-0.0028	6.1486E-01		ENSG00000182888
RPS27AP5	-0.4497	5.2755E-03	3.1407E-02	ENSG00000231767
RPS27AP6	0.1227	1.0613E-01	2.6496E-01	ENSG00000224800
RPS27AP8	0.0289	4.0239E-01		ENSG00000237483
RPS27AP9	0.0490	1.0501E-01		ENSG00000242706
RPS27L	-0.0484	5.3580E-01	7.1899E-01	ENSG00000185088
RPS27P30	-0.0266	3.4619E-01		ENSG00000270285
RPS28	-0.0414	5.1535E-01	7.0305E-01	ENSG00000233927
RPS28P2	-0.0110	5.8090E-01		ENSG00000236317
RPS29	-0.0343	6.4072E-01	7.9441E-01	ENSG00000213741
RPS29P14	-0.0165	6.7043E-01		ENSG00000229054
RPS29P19	0.0294	3.1339E-01		ENSG00000243353
RPS2P1	0.1002	1.3671E-01	3.1209E-01	ENSG00000225246
RPS2P11	-0.1169	1.7972E-01	3.7144E-01	ENSG00000234080
RPS2P14	0.0481	2.3746E-01		ENSG00000232450
RPS2P15	-0.0002	9.0521E-01		ENSG00000235440
RPS2P17	-0.1090	2.1030E-01	4.1073E-01	ENSG00000213399
RPS2P19	0.0136	7.4902E-01		ENSG00000244249
RPS2P20	-0.2535	1.6403E-02	7.2275E-02	ENSG00000228981
RPS2P21	0.0005	7.0522E-01		ENSG00000213851
RPS2P23	-0.0234	2.4714E-01		ENSG00000205644
RPS2P24	-0.0342	1.0516E-01		ENSG00000244019
RPS2P28	-0.0561	1.3345E-01		ENSG00000179157
RPS2P29	-0.0923	2.6697E-01	4.7620E-01	ENSG00000219470
RPS2P3	-0.0039	9.2155E-01	9.6140E-01	ENSG00000242888
RPS2P31	-0.0247	1.9092E-01		ENSG00000213296
RPS2P32	0.0229	7.6203E-01	8.7200E-01	ENSG00000232818

RPS2P33	-0.3030	5.1263E-02	1.6215E-01	ENSG00000213750
RPS2P34	0.0027	9.0560E-01		ENSG00000235377
RPS2P35	-0.0678	3.0990E-01	5.2012E-01	ENSG00000238172
RPS2P4	-0.0636	3.9064E-01	5.9782E-01	ENSG00000196183
RPS2P40	-0.0391	5.3996E-01	7.2201E-01	ENSG00000197149
RPS2P42	0.1084	1.5953E-01	3.4431E-01	ENSG00000243916
RPS2P46	-0.2967	1.3511E-03	1.1370E-02	ENSG00000189343
RPS2P47	-0.0627	4.4813E-01	6.4746E-01	ENSG00000239291
RPS2P48	-0.0930	1.2160E-01	2.9011E-01	ENSG00000233380
RPS2P49	0.0351	5.7303E-01	7.4620E-01	ENSG00000274308
RPS2P5	-0.3098	4.6366E-04	5.0103E-03	ENSG00000240342
RPS2P50	-0.3857	1.3477E-02	6.3054E-02	ENSG00000274756
RPS2P51	0.0195	7.6856E-01	8.7610E-01	ENSG00000240418
RPS2P53	0.0737	2.5170E-01	4.6001E-01	ENSG00000234750
RPS2P54	-0.0466	2.1404E-01	4.1517E-01	ENSG00000215450
RPS2P55	-0.1623	8.2929E-02	2.2455E-01	ENSG00000216866
RPS2P7	-0.3502	5.4654E-03	3.2194E-02	ENSG00000235508
RPS3	-0.3265	3.0200E-07	1.4235E-05	ENSG00000149273
RPS3A	-0.3608	2.2277E-06	7.2722E-05	ENSG00000145425
RPS3AP12	0.0700	2.9875E-01	5.0853E-01	ENSG00000236804
RPS3AP14	-0.0192	8.4239E-01		ENSG00000242229
RPS3AP15	-0.0025	8.2783E-01		ENSG00000244674
RPS3AP16	-0.0500	2.3608E-01		ENSG00000243894
RPS3AP18	-0.0047	7.3728E-01		ENSG00000243417
RPS3AP19	-0.0105	9.7873E-01		ENSG00000244004
RPS3AP20	-0.1711	7.2099E-02	2.0506E-01	ENSG00000213757
RPS3AP21	-0.2692	2.0952E-02	8.5777E-02	ENSG00000214784
RPS3AP22	-0.0067	7.6983E-01		ENSG00000240083
RPS3AP25	-0.1912	7.5893E-02	2.1220E-01	ENSG00000232385
RPS3AP26	-0.2499	6.6044E-03	3.7169E-02	ENSG00000214389
RPS3AP28	0.0010	9.8392E-01		ENSG00000234995
RPS3AP29	-0.0397	4.4843E-01	6.4759E-01	ENSG00000237818
RPS3AP3	0.0538	1.5959E-01	3.4442E-01	ENSG00000231241
RPS3AP30	-0.0199	5.0313E-01		ENSG00000228744
RPS3AP31	-0.0124	9.4405E-01		ENSG00000244427
RPS3AP34	-0.0821	3.3338E-01	5.4349E-01	ENSG00000242607
RPS3AP35	-0.0506	2.3961E-01	4.4626E-01	ENSG00000244289
RPS3AP36	-0.0189	2.6600E-01		ENSG00000214362
RPS3AP39	0.0020	5.5283E-01		ENSG00000237226
RPS3AP40	0.0003	9.5910E-01		ENSG00000243672
RPS3AP43	-0.1184	1.2061E-01	2.8839E-01	ENSG00000242661
RPS3AP44	-0.4122	1.2122E-02	5.8361E-02	ENSG00000234031
RPS3AP47	-0.0491	5.6134E-01	7.3832E-01	ENSG00000205871
RPS3AP48	-0.0141	3.0846E-01		ENSG00000242278
RPS3AP49	-0.0628	4.2667E-01	6.2882E-01	ENSG00000242060
RPS3AP5	-0.2323	4.2013E-02	1.4147E-01	ENSG00000178429
RPS3AP6	-0.2429	2.6485E-03	1.8555E-02	ENSG00000234797
RPS3AP7	0.0249	6.1462E-01		ENSG00000229965
RPS3AP8	0.0262	2.7870E-01		ENSG00000233562

RPS3P1	0.0059	7.9534E-01		ENSG00000230935
RPS3P2	-0.0615	2.3229E-01	4.3726E-01	ENSG00000231307
RPS3P3	-0.0511	5.3204E-01	7.1596E-01	ENSG00000228205
RPS3P4	-0.0944	1.6436E-01	3.5113E-01	ENSG00000220472
RPS3P6	0.0014	9.1610E-01		ENSG00000213363
RPS3P7	-0.0554	1.4684E-01		ENSG00000243101
RPS4X	-0.2569	4.0007E-04	4.4785E-03	ENSG00000198034
RPS4XP1	0.0292	5.6671E-01		ENSG00000214203
RPS4XP11	-0.0232	5.8245E-01		ENSG00000234335
RPS4XP13	0.0095	9.5184E-01		ENSG00000240371
RPS4XP14	0.0112	4.6976E-01		ENSG00000243663
RPS4XP2	0.1227	4.5942E-02	1.5074E-01	ENSG00000226948
RPS4XP20	0.0098	7.8260E-01		ENSG00000249987
RPS4XP21	0.0110	5.0352E-01		ENSG00000186008
RPS4XP22	-0.0312	2.5275E-01		ENSG00000239830
RPS4XP3	0.0321	4.9291E-01		ENSG00000214541
RPS4XP4	0.0274	4.8685E-01		ENSG00000215520
RPS4XP5	0.0638	1.3094E-01		ENSG00000229920
RPS4XP6	0.0176	6.8647E-01		ENSG00000244073
RPS4XP7	-0.0110	6.8024E-01		ENSG00000218265
RPS4Y1P1	0.0224	5.5382E-01		ENSG00000213318
RPS5	-0.0581	3.8075E-01	5.8906E-01	ENSG00000083845
RPS5P3	0.0049	8.1577E-01		ENSG00000215088
RPS5P4	0.0002	9.5695E-01		ENSG00000236905
RPS5P5	-0.0019	7.4348E-01		ENSG00000213791
RPS6	-0.3545	1.6197E-05	3.5935E-04	ENSG00000137154
RPS6KA1	-0.0325	6.4055E-01	7.9432E-01	ENSG00000117676
RPS6KA2	0.4958	5.2161E-10	6.4171E-08	ENSG00000071242
RPS6KA2-IT1	-0.0081	8.5039E-01		ENSG00000232082
RPS6KA3	0.2497	2.3760E-03	1.7124E-02	ENSG00000177189
RPS6KA4	-0.1368	1.5405E-01	3.3660E-01	ENSG00000162302
RPS6KA5	0.1694	2.4736E-02	9.6885E-02	ENSG00000100784
RPS6KA6	0.0313	6.6875E-01	8.1434E-01	ENSG00000072133
RPS6KB1	-0.0056	9.0979E-01	9.5529E-01	ENSG00000108443
RPS6KB2	0.0724	3.0683E-01	5.1716E-01	ENSG00000175634
RPS6KC1	-0.0483	4.6199E-01	6.5902E-01	ENSG00000136643
RPS6KL1	-0.1202	5.4117E-02	1.6857E-01	ENSG00000198208
RPS6P1	-0.1796	8.9447E-03	4.6786E-02	ENSG00000241352
RPS6P10	0.0381	5.1354E-01	7.0128E-01	ENSG00000232176
RPS6P12	0.0584	3.9753E-01	6.0337E-01	ENSG00000233309
RPS6P16	0.0399	3.4876E-01		ENSG00000243250
RPS6P21	0.0194	3.8434E-01		ENSG00000243499
RPS6P25	-0.0451	5.9996E-01	7.6571E-01	ENSG00000240616
RPS6P9	-0.1351	1.6899E-02	7.3754E-02	ENSG00000239344
RPS7	-0.2345	2.3516E-03	1.7004E-02	ENSG00000171863
RPS7P1	-0.1604	8.5200E-02	2.2843E-01	ENSG00000263266
RPS7P10	-0.0021	9.8167E-01	9.9040E-01	ENSG00000226525
RPS7P11	-0.0090	9.1241E-01	9.5679E-01	ENSG00000213326
RPS7P14	0.0075	7.6419E-01		ENSG00000213695

RPS7P3	0.0023	9.9239E-01	9.9571E-01	ENSG00000231940
RPS7P4	-0.0116	4.4780E-01		ENSG00000229133
RPS7P8	-0.0075	7.7261E-01		ENSG00000213183
RPS8	-0.1893	1.5470E-02	6.9525E-02	ENSG00000142937
RPS8P1	-0.0640	4.3830E-01	6.3860E-01	ENSG00000225200
RPS8P10	-0.0411	3.6981E-01		ENSG00000223877
RPS8P4	-0.0026	8.6412E-01		ENSG00000227437
RPS8P8	-0.0104	8.8284E-01	9.4130E-01	ENSG00000240376
RPS8P9	-0.0640	3.0583E-01	5.1598E-01	ENSG00000233668
RPS9	-0.0909	2.1326E-01	4.1434E-01	ENSG00000170889
RPS9P2	-0.0322	5.9467E-01	7.6172E-01	ENSG00000237214
RPS9P4	0.0237	6.8278E-01	8.2313E-01	ENSG00000243829
RPSA	-0.1971	4.1327E-03	2.6160E-02	ENSG00000168028
RPSA2	-0.1000	1.7676E-01	3.6805E-01	ENSG00000288920
RPSAP12	-0.0588	4.8527E-01	6.7900E-01	ENSG00000240087
RPSAP13	0.0160	7.0642E-01		ENSG00000233924
RPSAP15	-0.0351	6.6200E-01	8.0977E-01	ENSG00000237506
RPSAP16	-0.0367	4.6994E-01		ENSG00000235145
RPSAP17	-0.0485	2.9206E-01	5.0222E-01	ENSG00000226855
RPSAP18	-0.0481	4.6897E-01	6.6553E-01	ENSG00000224261
RPSAP19	-0.0286	7.3130E-01	8.5302E-01	ENSG00000183298
RPSAP21	0.0018	9.7223E-01	9.8618E-01	ENSG00000226498
RPSAP23	0.0167	4.4043E-01		ENSG00000230391
RPSAP29	0.0005	3.0824E-01		ENSG00000244722
RPSAP3	0.0019	5.1142E-01		ENSG00000242952
RPSAP31	0.0173	7.5643E-01		ENSG00000234371
RPSAP36	0.0959	1.4112E-01	3.1791E-01	ENSG00000243175
RPSAP4	-0.0489	2.2042E-01	4.2327E-01	ENSG00000239269
RPSAP43	0.0073	6.0605E-01		ENSG00000216471
RPSAP44	0.0705	3.3443E-01	5.4458E-01	ENSG00000220773
RPSAP45	-0.0269	2.0662E-01		ENSG00000219773
RPSAP46	0.0326	1.4900E-01		ENSG00000213394
RPSAP47	-0.0578	4.9823E-01	6.8929E-01	ENSG00000188856
RPSAP49	0.0015	7.0401E-01		ENSG00000226327
RPSAP5	0.0011	9.1180E-01		ENSG00000243904
RPSAP51	-0.0346	6.0706E-01		ENSG00000244131
RPSAP52	-0.0686	3.9644E-01	6.0245E-01	ENSG00000241749
RPSAP53	-0.0014	6.7518E-01		ENSG00000214263
RPSAP54	-0.0510	5.4514E-01	7.2565E-01	ENSG00000213621
RPSAP55	0.0016	8.9241E-01		ENSG00000214896
RPSAP56	0.0412	5.5431E-01	7.3257E-01	ENSG00000284670
RPSAP57	-0.0228	5.0503E-01		ENSG00000267524
RPSAP6	-0.0129	6.4513E-01		ENSG00000214629
RPSAP61	-0.0339	5.8769E-01	7.5676E-01	ENSG00000214016
RPSAP70	0.0080	9.1829E-01	9.5963E-01	ENSG00000250327
RPSAP74	0.0071	3.5055E-01		ENSG00000253621
RPSAP8	-0.0149	3.4935E-01		ENSG00000230592
RPTOR	0.0025	9.7775E-01	9.8898E-01	ENSG00000141564
RPUSD1	0.2072	3.9979E-03	2.5562E-02	ENSG00000007376

RPUSD2	-0.2418	1.1186E-03	9.8545E-03	ENSG00000166133
RPUSD3	-0.1330	2.9731E-02	1.1046E-01	ENSG00000156990
RPUSD4	0.0729	3.1327E-01	5.2355E-01	ENSG00000165526
RRAD	-0.1688	7.3579E-02	2.0786E-01	ENSG00000166592
RRAGA	-0.2248	9.4577E-05	1.4766E-03	ENSG00000155876
RRAGB	-0.1946	5.3476E-03	3.1705E-02	ENSG00000083750
RRAGC	0.2910	2.2793E-03	1.6607E-02	ENSG00000116954
RRAGC-DT	0.0934	2.5917E-01	4.6768E-01	ENSG00000228436
RRAGD	-0.4132	5.9422E-12	1.5892E-09	ENSG00000025039
RRAS	-0.3084	5.1984E-03	3.1023E-02	ENSG00000126458
RRAS2	-0.0225	6.5520E-01	8.0518E-01	ENSG00000133818
RRBP1	0.2798	1.1186E-04	1.6762E-03	ENSG00000125844
RREB1	-0.4287	1.2124E-05	2.8464E-04	ENSG00000124782
RRH	0.1160	1.3997E-01	3.1645E-01	ENSG00000180245
RRM1	-0.0621	3.1445E-01	5.2456E-01	ENSG00000167325
RRM2	-0.1225	1.8150E-01	3.7399E-01	ENSG00000171848
RRM2B	-0.1286	1.0497E-01	2.6306E-01	ENSG00000048392
RRM2P2	0.0219	3.6759E-01		ENSG00000236484
RRN3	-0.1713	7.5718E-03	4.1138E-02	ENSG00000085721
RRN3P2	0.0388	7.5864E-02		ENSG00000103472
RRP1	-0.0057	9.2879E-01	9.6491E-01	ENSG00000160214
RRP12	0.0919	2.1754E-01	4.1954E-01	ENSG00000052749
RRP15	0.1856	1.6897E-02	7.3754E-02	ENSG00000067533
RRP1B	0.0849	1.4280E-01	3.2041E-01	ENSG00000160208
RRP36	-0.0701	1.2675E-01	2.9805E-01	ENSG00000124541
RRP7A	-0.1259	4.8132E-02	1.5540E-01	ENSG00000189306
RRP8	0.1780	7.7907E-03	4.2056E-02	ENSG00000132275
RRP9	-0.0951	2.1197E-01	4.1293E-01	ENSG00000114767
RRS1	0.1814	6.5073E-02	1.9091E-01	ENSG00000179041
RRS1-DT	0.0401	1.8095E-01	3.7325E-01	ENSG00000246145
RS1	0.0356	1.8647E-01		ENSG00000102104
RSAD1	0.0387	5.0947E-01	6.9831E-01	ENSG00000136444
RSAD2	-0.0148	8.3984E-01	9.1755E-01	ENSG00000134321
RSBN1	0.1247	5.5820E-02	1.7244E-01	ENSG00000081019
RSBN1L	0.1193	7.4296E-02	2.0911E-01	ENSG00000187257
RSF1	0.1318	2.6575E-02	1.0192E-01	ENSG00000048649
RSKR	0.1584	9.9233E-02	2.5341E-01	ENSG00000167524
RSL1D1	-0.1789	2.4919E-03	1.7746E-02	ENSG00000171490
RSL24D1	-0.2190	9.3323E-04	8.6033E-03	ENSG00000137876
RSPH1	0.1876	6.2528E-02	1.8574E-01	ENSG00000160188
RSPH1-DT	0.0117	7.8814E-01	8.8798E-01	ENSG00000239930
RSPH10B	0.0643	3.3272E-01	5.4302E-01	ENSG00000155026
RSPH10B2	0.0515	4.7554E-01	6.7112E-01	ENSG00000169402
RSPH14	0.5319	9.8087E-06	2.4134E-04	ENSG00000100218
RSPH3	0.2682	5.5036E-03	3.2350E-02	ENSG00000130363
RSPH4A	0.2387	3.5221E-02	1.2459E-01	ENSG00000111834
RSPH6A	0.0073	8.3023E-01		ENSG00000104941
RSPH9	0.1369	1.3096E-01	3.0393E-01	ENSG00000172426
RSPO3	0.0226	6.9011E-01	8.2779E-01	ENSG00000146374

RSPO4	0.0693	3.1952E-01	5.2966E-01	ENSG00000101282
RSPRY1	0.3741	8.2978E-10	9.4087E-08	ENSG00000159579
RSRC1	-0.0511	3.5105E-01	5.6121E-01	ENSG00000174891
RSRC2	-0.1530	6.7670E-03	3.7884E-02	ENSG00000111011
RSRP1	0.1670	1.5005E-02	6.7966E-02	ENSG00000117616
RSU1	-0.1363	2.0063E-02	8.3219E-02	ENSG00000148484
RTBDN	0.0490	5.5881E-01	7.3617E-01	ENSG00000132026
RTCA	-0.0303	6.3123E-01	7.8773E-01	ENSG00000137996
RTCB	0.1975	9.3979E-04	8.6246E-03	ENSG00000100220
RTEL1	0.2686	3.0654E-02	1.1296E-01	ENSG00000258366
RTEL1-TNFRSF6B	-0.0152	8.2631E-01	9.1001E-01	ENSG00000026036
RTF1	0.0507	3.3851E-01	5.4835E-01	ENSG00000137815
RTF2	-0.0912	1.7597E-01	3.6699E-01	ENSG00000022277
RTKN	-0.1769	6.6013E-02	1.9302E-01	ENSG00000114993
RTKN2	0.0585	4.7216E-01	6.6840E-01	ENSG00000182010
RTL1	0.1411	1.2115E-01	2.8934E-01	ENSG00000254656
RTL10	0.0770	2.8199E-01	4.9240E-01	ENSG00000215012
RTL4	0.0231	4.1579E-01		ENSG00000187823
RTL5	-0.0854	2.3272E-01	4.3781E-01	ENSG00000242732
RTL6	0.0131	8.5499E-01	9.2621E-01	ENSG00000188636
RTL8A	-0.1156	5.6191E-02	1.7321E-01	ENSG00000203950
RTL8B	-0.1690	1.0197E-02	5.1434E-02	ENSG00000212747
RTL8C	-0.1750	1.1116E-02	5.4723E-02	ENSG00000134590
RTL9	0.1671	8.8073E-02	2.3415E-01	ENSG00000243978
RTN1	-0.3090	1.1854E-06	4.2954E-05	ENSG00000139970
RTN2	-0.2354	1.7982E-03	1.3909E-02	ENSG00000125744
RTN3	-0.4564	2.7182E-13	1.2590E-10	ENSG00000133318
RTN4	-0.0839	2.1102E-01	4.1179E-01	ENSG00000115310
RTN4IP1	-0.0074	9.1713E-01	9.5883E-01	ENSG00000130347
RTN4R	-0.0215	7.4970E-01	8.6410E-01	ENSG00000040608
RTN4RL1	-0.3183	4.3525E-03	2.7119E-02	ENSG00000185924
RTN4RL2	-0.3411	6.9965E-04	6.9499E-03	ENSG00000186907
RTP5	0.0259	1.3420E-01		ENSG00000188011
RTRAF	-0.1092	7.3497E-02	2.0773E-01	ENSG00000087302
RTTN	0.3462	7.5161E-04	7.3299E-03	ENSG00000176225
RUBCN	-0.0367	5.8868E-01	7.5740E-01	ENSG00000145016
RUBCNL	0.2244	4.5179E-02	1.4880E-01	ENSG00000102445
RUFY1	0.0354	5.8109E-01	7.5235E-01	ENSG00000176783
RUFY1-AS1	-0.0067	8.4213E-01		ENSG00000244945
RUFY2	-0.0321	4.3243E-01	6.3374E-01	ENSG00000204130
RUFY3	-0.1531	1.4050E-02	6.4947E-02	ENSG00000018189
RUFY4	-0.0224	6.0460E-01		ENSG00000188282
RUNDC1	0.0164	8.2406E-01	9.0862E-01	ENSG00000198863
RUNDC3A	-0.2510	2.7494E-03	1.9093E-02	ENSG00000108309
RUNDC3B	0.0726	3.4378E-01	5.5337E-01	ENSG00000105784
RUNX1	0.1014	1.5620E-01	3.4011E-01	ENSG00000159216
RUNX1T1	0.1941	1.4465E-02	6.6253E-02	ENSG00000079102
RUNX2	0.0254	7.5133E-01	8.6494E-01	ENSG00000124813
RUNX3	0.0089	8.8219E-01	9.4106E-01	ENSG00000020633

RUSC1	-0.0496	4.7359E-01	6.6951E-01	ENSG00000160753
RUSC1-AS1	0.1931	7.3876E-02	2.0835E-01	ENSG00000225855
RUSC2	0.0496	4.5838E-01	6.5626E-01	ENSG00000198853
RUSF1	0.1472	1.1466E-01	2.7878E-01	ENSG00000140688
RUVBL1	-0.0010	9.6985E-01	9.8504E-01	ENSG00000175792
RUVBL1-AS1	0.0139	5.5291E-01		ENSG00000239608
RUVBL2	0.0601	3.0738E-01	5.1754E-01	ENSG00000183207
RWDD1	-0.0643	2.8596E-01	4.9669E-01	ENSG00000111832
RWDD2A	-0.0631	3.3009E-01	5.4037E-01	ENSG00000013392
RWDD2B	0.1308	1.0410E-01	2.6155E-01	ENSG00000156253
RWDD3	0.0194	7.9706E-01	8.9291E-01	ENSG00000122481
RWDD3-DT	0.1004	2.2856E-01	4.3311E-01	ENSG00000226026
RWDD4	0.1703	7.1835E-03	3.9603E-02	ENSG00000182552
RXFP1	0.0062	8.6214E-01		ENSG00000171509
RXFP2	-0.0949	4.3805E-02	1.4555E-01	ENSG00000133105
RXFP3	-0.0001	7.0228E-01		ENSG00000182631
RXFP4	0.0332	5.9016E-01	7.5856E-01	ENSG00000173080
RXRA	-0.0224	7.5550E-01	8.6731E-01	ENSG00000186350
RXRB	0.0301	6.9772E-01	8.3293E-01	ENSG00000204231
RXRG	-0.1148	1.6877E-01	3.5722E-01	ENSG00000143171
RXYLT1	0.1927	5.5544E-03	3.2570E-02	ENSG00000118600
RYBP	0.0290	7.0114E-01	8.3486E-01	ENSG00000163602
RYK	-0.1329	3.6821E-02	1.2876E-01	ENSG00000163785
RYR1	0.4322	5.5496E-03	3.2555E-02	ENSG00000196218
RYR2	0.3719	4.1361E-03	2.6175E-02	ENSG00000198626
RYR3	0.1840	5.8957E-02	1.7843E-01	ENSG00000198838
RYR3-DT	0.0211	6.4894E-01	8.0041E-01	ENSG00000259446
S100A10	0.1325	1.1867E-01	2.8516E-01	ENSG00000197747
S100A11	0.5958	1.1603E-04	1.7229E-03	ENSG00000163191
S100A13	0.3934	4.2035E-05	7.7626E-04	ENSG00000189171
S100A14	-0.0320	2.2755E-01		ENSG00000189334
S100A16	-0.0060	9.2762E-01	9.6455E-01	ENSG00000188643
S100A2	0.0684	3.1627E-01	5.2634E-01	ENSG00000196754
S100A3	0.0172	5.6793E-01		ENSG00000188015
S100A6	-0.0179	8.3447E-01	9.1499E-01	ENSG00000197956
S100A8	0.0048	4.6991E-01		ENSG00000143546
S100A9	0.0065	5.3999E-01		ENSG00000163220
S100B	0.3515	3.9010E-03	2.5071E-02	ENSG00000160307
S100G	-0.0025	9.8520E-01		ENSG00000169906
S100P	-0.0127	5.8289E-01		ENSG00000163993
S100PBP	-0.1109	1.4532E-01	3.2410E-01	ENSG00000116497
S1PR1	0.1178	1.0332E-01	2.6021E-01	ENSG00000170989
S1PR2	0.0879	2.8960E-01	5.0003E-01	ENSG00000267534
S1PR3	-0.1358	1.1458E-01	2.7870E-01	ENSG00000213694
S1PR5	-0.1474	1.3367E-01	3.0751E-01	ENSG00000180739
SAA1	0.0618	1.0199E-01		ENSG00000173432
SAAL1	-0.0132	8.1820E-01	9.0545E-01	ENSG00000166788
SAC3D1	-0.1606	2.4380E-02	9.5910E-02	ENSG00000168061
SACM1L	0.3121	9.0584E-05	1.4275E-03	ENSG00000211456

SACS	-0.0194	7.6819E-01	8.7579E-01	ENSG00000151835
SACS-AS1	0.0188	3.8635E-01		ENSG00000229558
SAE1	-0.1700	1.2449E-02	5.9500E-02	ENSG00000142230
SAFB	-0.1730	1.8043E-02	7.7115E-02	ENSG00000160633
SAFB2	-0.0391	5.7230E-01	7.4561E-01	ENSG00000130254
SAG	-0.0005	8.9134E-01		ENSG00000130561
SAGE1	0.0033	6.0838E-01		ENSG00000181433
SALL1	-0.2082	7.7319E-03	4.1802E-02	ENSG00000103449
SALL2	-0.1381	7.6953E-02	2.1399E-01	ENSG00000165821
SALL3	0.0646	4.5361E-01	6.5222E-01	ENSG00000256463
SALL4	0.1782	6.4570E-02	1.9011E-01	ENSG00000101115
SAMD1	-0.3984	5.4025E-05	9.4679E-04	ENSG00000141858
SAMD10	0.1049	2.0317E-01	4.0209E-01	ENSG00000130590
SAMD11	0.0310	7.2327E-01	8.4820E-01	ENSG00000187634
SAMD11P1	-0.0192	3.5953E-01		ENSG00000262480
SAMD12	-0.2913	4.7003E-03	2.8740E-02	ENSG00000177570
SAMD13	-0.0189	8.1285E-01	9.0242E-01	ENSG00000203943
SAMD14	-0.0460	5.2325E-01	7.0909E-01	ENSG00000167100
SAMD15	0.1636	1.0806E-01	2.6792E-01	ENSG00000100583
SAMD4A	0.1884	6.2458E-02	1.8560E-01	ENSG00000020577
SAMD4B	-0.0117	8.7865E-01	9.3956E-01	ENSG00000179134
SAMD5	0.3939	1.9762E-04	2.5686E-03	ENSG00000203727
SAMD7	0.0050	6.5111E-01		ENSG00000187033
SAMD8	-0.2439	6.7932E-06	1.7954E-04	ENSG00000156671
SAMD9	-0.0228	6.7742E-01	8.1922E-01	ENSG00000205413
SAMD9L	0.0966	1.5201E-01	3.3381E-01	ENSG00000177409
SAMHD1	-0.4624	2.1270E-08	1.5669E-06	ENSG00000101347
SAMM50	-0.0421	5.2925E-01	7.1386E-01	ENSG00000100347
SAMSN1	0.1443	7.5723E-02	2.1192E-01	ENSG00000155307
SANBR	0.0419	5.0416E-01	6.9434E-01	ENSG00000162929
SAP130	-0.0526	5.1432E-01	7.0198E-01	ENSG00000136715
SAP18	-0.2594	8.0278E-05	1.2969E-03	ENSG00000150459
SAP30	-0.3143	6.1423E-04	6.2632E-03	ENSG00000164105
SAP30-DT	0.1481	5.5118E-02	1.7087E-01	ENSG00000272870
SAP30BP	-0.0164	7.8855E-01	8.8805E-01	ENSG00000161526
SAP30L	0.0201	7.5308E-01	8.6598E-01	ENSG00000164576
SAP30L-AS1	0.4329	1.5022E-03	1.2268E-02	ENSG00000245275
SAPCD1	0.0403	4.8617E-01	6.7994E-01	ENSG00000228727
SAPCD2	-0.2119	4.9249E-02	1.5787E-01	ENSG00000186193
SAR1A	-0.1511	4.1539E-03	2.6241E-02	ENSG00000079332
SAR1B	-0.0323	5.0449E-01	6.9443E-01	ENSG00000152700
SARAF	-0.3420	2.0762E-06	6.8626E-05	ENSG00000133872
SARDH	-0.0319	6.9517E-01	8.3125E-01	ENSG00000123453
SARM1	0.0113	8.7869E-01	9.3956E-01	ENSG00000004139
SARNP	-0.3884	2.0547E-03	1.5368E-02	ENSG00000205323
SARS1	-0.1997	2.1297E-03	1.5765E-02	ENSG00000031698
SARS2	0.2063	5.4172E-02	1.6864E-01	ENSG00000104835
SART1	0.1133	1.0290E-01	2.5968E-01	ENSG00000175467
SART3	0.2294	3.5313E-04	4.0620E-03	ENSG00000075856

SASH1	-0.0537	5.0496E-01	6.9492E-01	ENSG00000111961
SASH3	0.0060	7.9544E-01		ENSG00000122122
SASS6	0.0946	2.3732E-01	4.4318E-01	ENSG00000156876
SAT1	0.0546	5.1394E-01	7.0170E-01	ENSG00000130066
SAT2	-0.2169	1.1922E-03	1.0348E-02	ENSG00000141504
SATB1	-0.1875	5.8633E-02	1.7786E-01	ENSG00000182568
SATB2	-0.0816	3.5425E-01	5.6418E-01	ENSG00000119042
SATB2-AS1	-0.0940	2.3140E-01	4.3622E-01	ENSG00000225953
SATL1	-0.0157	8.2529E-01	9.0932E-01	ENSG00000184788
SAV1	-0.0356	6.2393E-01	7.8285E-01	ENSG00000151748
SAXO1	0.5395	1.0947E-02	5.4140E-02	ENSG00000155875
SAXO2	0.4799	3.2080E-04	3.7803E-03	ENSG00000188659
SAXO3	-0.0034	6.6725E-01		ENSG00000268655
SAXO4	-0.0065	9.3211E-01	9.6640E-01	ENSG00000162148
SAXO5	-0.0106	8.9061E-01	9.4581E-01	ENSG00000198723
SAYSD1	0.0826	3.0068E-01	5.1051E-01	ENSG00000112167
SBDS	-0.3371	1.9125E-07	9.7357E-06	ENSG00000126524
SBF1	-0.0049	9.2172E-01	9.6140E-01	ENSG00000100241
SBF1P1	-0.0077	5.9305E-01		ENSG00000248522
SBF2	0.1601	1.0307E-02	5.1807E-02	ENSG00000133812
SBF2-AS1	-0.0023	9.8018E-01	9.8991E-01	ENSG00000246273
SBK1	0.0314	6.3369E-01	7.8999E-01	ENSG00000188322
SBK2	0.1298	9.6211E-02	2.4902E-01	ENSG00000187550
SBK3	0.0208	5.2945E-01		ENSG00000231274
SBNO1	0.0350	5.0212E-01	6.9269E-01	ENSG00000139697
SBNO1-AS1	0.1655	1.0272E-01	2.5935E-01	ENSG00000256092
SBNO2	-0.0357	6.6304E-01	8.1040E-01	ENSG00000064932
SBSN	-0.0081	9.0198E-01		ENSG00000189001
SBSPON	0.0792	2.4678E-01	4.5438E-01	ENSG00000164764
SC5D	0.0542	3.7407E-01	5.8271E-01	ENSG00000109929
SCAANT1	0.0073	6.4788E-01		ENSG00000280620
SCAF1	-0.0637	3.9696E-01	6.0282E-01	ENSG00000126461
SCAF11	0.1327	2.5810E-03	1.8186E-02	ENSG00000139218
SCAF4	-0.0240	6.8174E-01	8.2246E-01	ENSG00000156304
SCAF8	-0.0935	1.5031E-01	3.3137E-01	ENSG00000213079
SCAI	-0.0663	2.8417E-01	4.9483E-01	ENSG00000173611
SCAMP1	-0.1224	2.8148E-02	1.0621E-01	ENSG00000085365
SCAMP1-AS1	-0.0621	3.5789E-01	5.6756E-01	ENSG00000245556
SCAMP2	-0.0148	7.9203E-01	8.9013E-01	ENSG00000140497
SCAMP3	-0.1431	1.8383E-02	7.8133E-02	ENSG00000116521
SCAMP4	-0.2903	6.6052E-05	1.1141E-03	ENSG00000227500
SCAMP5	0.0608	3.7177E-01	5.8049E-01	ENSG00000198794
SCAND1	-0.1832	7.1904E-03	3.9625E-02	ENSG00000171222
SCAND3	0.1791	5.4364E-02	1.6906E-01	ENSG00000232040
SCAND3P1	0.0090	7.0923E-01		ENSG00000232795
SCAP	-0.0202	7.2940E-01	8.5185E-01	ENSG00000114650
SCAPER	-0.2003	1.4673E-03	1.2095E-02	ENSG00000140386
SCARA3	-0.1534	6.8685E-02	1.9805E-01	ENSG00000168077
SCARA5	0.0022	9.0470E-01		ENSG00000168079

SCARB1	-0.0863	1.7140E-01	3.6054E-01	ENSG00000073060
SCARB2	-0.2556	6.4858E-07	2.6161E-05	ENSG00000138760
SCARF1	0.4054	6.3284E-03	3.5911E-02	ENSG00000074660
SCARF2	-0.0637	4.5671E-01	6.5493E-01	ENSG00000244486
SCARNA1	-0.2624	3.4424E-02	1.2236E-01	ENSG00000252947
SCARNA14	-0.0596	2.9419E-01		ENSG00000252712
SCARNA17	0.0015	9.8120E-01	9.9024E-01	ENSG00000267322
SCARNA18	0.0223	6.6659E-01		ENSG00000238835
SCARNA18B	-0.1324	1.1712E-01	2.8255E-01	ENSG00000238754
SCARNA21	0.0024	5.7654E-01	7.4871E-01	ENSG00000252835
SCARNA23	-0.0263	6.3865E-01	7.9343E-01	ENSG00000251869
SCARNA3	0.0768	1.3714E-01		ENSG00000252906
SCARNA5	0.0101	8.6054E-01		ENSG00000252010
SCARNA6	0.0237	2.9713E-01		ENSG00000251791
SCARNA7	0.0706	1.5548E-01	3.3903E-01	ENSG00000238741
SCARNA8	0.0695	1.8535E-01		ENSG00000251733
SCARNA9	0.0129	7.6015E-01		ENSG00000254911
SCART1	0.0764	2.8760E-01	4.9827E-01	ENSG00000214279
SCAT1	0.0014	7.6426E-01		ENSG00000267123
SCAT2	0.0224	4.6550E-01		ENSG00000257596
SCAT8	0.1641	1.7477E-02	7.5560E-02	ENSG00000236345
SCCPDH	-0.1135	3.3402E-02	1.1991E-01	ENSG00000143653
SCD	-0.2931	5.4982E-03	3.2326E-02	ENSG00000099194
SCD5	-0.4698	4.1453E-06	1.2067E-04	ENSG00000145284
SCEL	0.1019	2.0976E-01	4.0999E-01	ENSG00000136155
SCFD1	0.0548	3.7176E-01	5.8049E-01	ENSG00000092108
SCFD2	-0.0068	9.2630E-01	9.6393E-01	ENSG00000184178
SCG2	-0.2062	1.2323E-02	5.9003E-02	ENSG00000171951
SCG3	-0.1198	5.7761E-02	1.7617E-01	ENSG00000104112
SCG5	0.0787	3.7057E-01	5.7969E-01	ENSG00000166922
SCG5-AS1	0.0184	7.6883E-01	8.7627E-01	ENSG00000241818
SCGB1A1	-0.0159	6.2107E-01		ENSG00000149021
SCGB1D2	-0.0015	8.3071E-01		ENSG00000124935
SCGB1D4	-0.0070	6.9494E-01		ENSG00000197745
SCGB2A1	0.0582	8.7153E-02	2.3228E-01	ENSG00000124939
SCGB2B2	0.0613	4.6450E-01	6.6144E-01	ENSG00000205209
SCGB3A1	0.0167	9.4893E-01	9.7468E-01	ENSG00000161055
SCGB3A2	-0.0099	5.1154E-01		ENSG00000164265
SCHIP1	0.0338	6.8433E-01	8.2402E-01	ENSG00000283154
SCHIP1	-0.0955	2.5202E-01	4.6024E-01	ENSG00000151967
SCHLAP1	0.0029	9.0305E-01		ENSG00000281131
SCIMP	0.0452	5.1061E-01	6.9914E-01	ENSG00000161929
SCIN	0.0039	9.5398E-01	9.7701E-01	ENSG00000006747
SCIRT	0.3827	1.2581E-03	1.0767E-02	ENSG00000237686
SCLT1	-0.0758	1.9777E-01	3.9559E-01	ENSG00000151466
SCLY	0.3745	2.8577E-03	1.9734E-02	ENSG00000132330
SCMH1	-0.0904	1.5518E-01	3.3849E-01	ENSG00000010803
SCML1	-0.1947	4.0904E-03	2.5991E-02	ENSG00000047634
SCML2	-0.1754	1.9087E-02	8.0309E-02	ENSG00000102098

SCN11A	0.2029	6.0833E-02	1.8198E-01	ENSG00000168356
SCN1A	0.3953	1.0970E-05	2.6411E-04	ENSG00000144285
SCN1A-AS1	0.1014	1.6172E-01	3.4763E-01	ENSG00000236107
SCN1B	0.0160	8.5876E-01	9.2870E-01	ENSG00000105711
SCN2B	-0.1076	2.3685E-01	4.4251E-01	ENSG00000149575
SCN3A	0.5198	7.5255E-08	4.5272E-06	ENSG00000153253
SCN3B	-0.1813	2.3641E-02	9.3839E-02	ENSG00000166257
SCN4A	0.0935	2.0025E-01	3.9864E-01	ENSG00000007314
SCN4B	0.3027	1.2061E-02	5.8221E-02	ENSG00000177098
SCN5A	-0.2390	4.6012E-03	2.8275E-02	ENSG00000183873
SCN7A	0.1515	1.6813E-01	3.5641E-01	ENSG00000136546
SCN8A	0.1185	1.1062E-01	2.7230E-01	ENSG00000196876
SCN9A	0.3749	4.3150E-03	2.6954E-02	ENSG00000169432
SCNM1	-0.0560	3.5825E-01	5.6777E-01	ENSG00000163156
SCNN1A	0.0285	4.5539E-01		ENSG00000111319
SCNN1B	-0.0784	2.1258E-01	4.1364E-01	ENSG00000168447
SCNN1D	0.0126	8.7234E-01	9.3586E-01	ENSG00000162572
SCNN1G	-0.0016	7.7890E-01		ENSG00000166828
SCO1	0.0781	2.1794E-01	4.2005E-01	ENSG00000133028
SCO2	-0.0043	9.2865E-01	9.6489E-01	ENSG00000284194
SCOC	-0.0497	4.9798E-01	6.8916E-01	ENSG00000153130
SCOC-AS1	0.4169	7.2840E-06	1.8976E-04	ENSG00000196951
SCP2	-0.0731	2.2506E-01	4.2887E-01	ENSG00000116171
SCPEP1	-0.2383	1.9693E-03	1.4897E-02	ENSG00000121064
SCRG1	-0.2210	4.9952E-02	1.5964E-01	ENSG00000164106
SCRIB	-0.1372	1.1666E-01	2.8186E-01	ENSG00000180900
SCRN1	-0.1687	5.2168E-03	3.1110E-02	ENSG00000136193
SCRN2	-0.0294	6.5607E-01	8.0568E-01	ENSG00000141295
SCRN3	0.0937	1.8488E-01	3.7842E-01	ENSG00000144306
SCRT1	-0.1502	1.2429E-01	2.9406E-01	ENSG00000261678
SCRT2	-0.2830	2.6177E-03	1.8387E-02	ENSG00000215397
SCT	-0.0009	9.7491E-01		ENSG00000070031
SCTR	-0.0077	7.1860E-01		ENSG00000080293
SCTR-AS1	-0.0026	9.7187E-01		ENSG00000231013
SCUBE1	-0.2581	3.0494E-03	2.0726E-02	ENSG00000159307
SCUBE1-AS1	0.0284	6.9910E-01	8.3370E-01	ENSG00000203527
SCUBE2	-0.0328	6.8352E-01	8.2352E-01	ENSG00000175356
SCUBE3	0.3987	3.5938E-04	4.1186E-03	ENSG00000146197
SCX	0.1025	1.0718E-01	2.6644E-01	ENSG00000260428
SCYGR3	0.0031	7.1259E-01		ENSG00000284704
SCYL1	-0.0450	4.8141E-01	6.7598E-01	ENSG00000142186
SCYL2	-0.1640	1.0292E-02	5.1746E-02	ENSG00000136021
SCYL2P1	-0.0372	5.9762E-01	7.6387E-01	ENSG00000213090
SCYL3	0.1457	6.4304E-02	1.8953E-01	ENSG00000000457
SDAD1	-0.2465	3.7753E-04	4.2749E-03	ENSG00000198301
SDAD1-AS1	0.0931	1.1654E-01	2.8164E-01	ENSG00000245928
SDAD1P1	0.5274	1.2283E-08	9.9415E-07	ENSG00000228451
SDC1	-0.1207	1.3648E-01	3.1177E-01	ENSG00000115884
SDC2	0.2161	1.3726E-02	6.3904E-02	ENSG00000169439

SDC3	-0.0643	3.5958E-01	5.6890E-01	ENSG00000162512
SDC4	-0.3738	1.5917E-03	1.2757E-02	ENSG00000124145
SDCBP	-0.2895	1.8014E-03	1.3929E-02	ENSG00000137575
SDCBP2	0.2573	4.9447E-03	2.9832E-02	ENSG00000125775
SDCBP2-AS1	-0.1500	7.4554E-02	2.0962E-01	ENSG00000234684
SDCBP2P1	0.0112	7.6143E-01		ENSG00000223389
SDCBPP3	-0.0432	1.0452E-01		ENSG00000232801
SDCCAG8	-0.0064	9.0232E-01	9.5215E-01	ENSG00000054282
SDE2	-0.3378	8.5183E-06	2.1497E-04	ENSG00000143751
SDF2	-0.0550	3.2517E-01	5.3521E-01	ENSG00000132581
SDF2L1	-0.1049	1.3617E-01	3.1124E-01	ENSG00000128228
SDF4	-0.0002	9.8806E-01	9.9352E-01	ENSG00000078808
SDHA	-0.1758	1.8972E-02	7.9972E-02	ENSG00000073578
SDHAF1	0.3790	5.1740E-05	9.1521E-04	ENSG00000205138
SDHAF2	0.0794	2.7088E-01	4.8008E-01	ENSG00000167985
SDHAF3	0.2343	1.9925E-03	1.5038E-02	ENSG00000196636
SDHAF4	0.0778	3.0530E-01	5.1557E-01	ENSG00000154079
SDHAP2	0.4583	3.7313E-08	2.5056E-06	ENSG00000242086
SDHB	-0.1988	2.0607E-03	1.5393E-02	ENSG00000117118
SDHC	-0.2248	4.7098E-04	5.0738E-03	ENSG00000143252
SDHD	-0.0732	2.6051E-01	4.6899E-01	ENSG00000204370
SDK1	0.0988	2.0872E-01	4.0877E-01	ENSG00000146555
SDK2	0.0341	6.4430E-01	7.9692E-01	ENSG00000069188
SDR16C5	0.0867	6.6744E-02		ENSG00000170786
SDR39U1	0.0718	3.3070E-01	5.4101E-01	ENSG00000100445
SDR42E1	0.1966	3.6452E-02	1.2769E-01	ENSG00000184860
SDR42E2	0.5737	3.9016E-03	2.5071E-02	ENSG00000183921
SDR9C7	0.0133	4.2526E-01		ENSG00000170426
SDS	0.1106	2.3543E-01	4.4096E-01	ENSG00000135094
SDSL	0.0502	5.4142E-01	7.2329E-01	ENSG00000139410
SEBOX	-0.0146	4.7795E-01		ENSG00000274529
SEC11A	-0.0329	5.6001E-01	7.3725E-01	ENSG00000140612
SEC11C	-0.1465	4.1506E-02	1.4039E-01	ENSG00000166562
SEC13	0.0158	8.2726E-01	9.1051E-01	ENSG00000157020
SEC14L1	-0.2291	1.5765E-03	1.2693E-02	ENSG00000129657
SEC14L1P1	-0.0741	3.9268E-01	5.9947E-01	ENSG00000213693
SEC14L2	0.1370	1.3466E-01	3.0914E-01	ENSG00000100003
SEC14L3	0.0303	1.9497E-01		ENSG00000100012
SEC14L4	0.0197	2.9022E-01		ENSG00000133488
SEC14L5	0.4141	4.6411E-03	2.8470E-02	ENSG00000103184
SEC14L6	0.0529	3.9450E-01	6.0076E-01	ENSG00000214491
SEC16A	0.1706	5.0702E-03	3.0412E-02	ENSG00000148396
SEC16B	0.0982	1.9912E-02		ENSG00000120341
SEC1P	0.0193	7.0651E-01	8.3833E-01	ENSG00000232871
SEC22A	0.0989	1.4888E-01	3.2945E-01	ENSG00000121542
SEC22B	-0.1477	4.0375E-02	1.3772E-01	ENSG00000265808
SEC22C	-0.0536	3.3169E-01	5.4202E-01	ENSG00000093183
SEC23A	-0.0640	3.6547E-01	5.7463E-01	ENSG00000100934
SEC23A-AS1	-0.0189	5.3121E-01		ENSG00000258651

SEC23B	-0.0024	9.5330E-01	9.7696E-01	ENSG00000101310
SEC23IP	0.1490	2.9823E-02	1.1070E-01	ENSG00000107651
SEC24A	0.1491	4.6987E-02	1.5313E-01	ENSG00000113615
SEC24B	-0.0189	7.6320E-01	8.7252E-01	ENSG00000138802
SEC24B-AS1	0.1312	1.0978E-01	2.7076E-01	ENSG00000247950
SEC24C	-0.0320	6.9699E-01	8.3245E-01	ENSG00000176986
SEC24D	0.0763	3.2186E-01	5.3182E-01	ENSG00000150961
SEC31A	0.1137	1.0890E-01	2.6933E-01	ENSG00000138674
SEC31B	0.1036	1.1128E-01	2.7318E-01	ENSG00000075826
SEC61A1	0.0300	6.9594E-01	8.3174E-01	ENSG00000058262
SEC61A2	0.0686	2.6032E-01	4.6879E-01	ENSG00000065665
SEC61B	-0.1468	4.7150E-02	1.5346E-01	ENSG00000106803
SEC61G	0.1322	1.2295E-01	2.9211E-01	ENSG00000132432
SEC61G-DT	0.2790	3.1916E-02	1.1605E-01	ENSG00000234707
SEC62	0.0880	1.2446E-01	2.9424E-01	ENSG00000008952
SEC63	-0.0603	1.7186E-01	3.6109E-01	ENSG00000025796
SEC63P1	0.0660	9.3121E-02		ENSG00000228057
SECISBP2	0.0112	8.7071E-01	9.3476E-01	ENSG00000187742
SECISBP2L	-0.0433	3.9432E-01	6.0063E-01	ENSG00000138593
SECTM1	-0.4439	1.1249E-02	5.5279E-02	ENSG00000141574
SEH1L	-0.0695	2.3400E-01	4.3906E-01	ENSG00000085415
SEL1L	0.0465	3.8397E-01	5.9203E-01	ENSG00000071537
SEL1L2	0.0214	7.8584E-01	8.8674E-01	ENSG00000101251
SEL1L3	-0.3019	7.9598E-06	2.0401E-04	ENSG00000091490
SELE	0.0089	5.4230E-01		ENSG00000007908
SELENBP1	-0.0365	6.3778E-01	7.9255E-01	ENSG00000143416
SELENOF	-0.0941	9.1292E-02	2.4032E-01	ENSG00000183291
SELENOH	-0.1198	8.2628E-02	2.2410E-01	ENSG00000211450
SELENOI	0.2541	5.4411E-04	5.6728E-03	ENSG00000138018
SELENOK	-0.0305	6.3416E-01	7.9030E-01	ENSG00000113811
SELENOM	0.1408	5.3156E-02	1.6629E-01	ENSG00000198832
SELENON	-0.0605	3.2978E-01	5.4006E-01	ENSG00000162430
SELENOO	0.0790	2.6044E-01	4.6896E-01	ENSG00000073169
SELENOP	0.0708	4.1219E-01	6.1603E-01	ENSG00000250722
SELENOS	0.1131	1.2818E-01	2.9988E-01	ENSG00000131871
SELENOT	-0.1733	4.2585E-04	4.6966E-03	ENSG00000198843
SELENOV	-0.2803	2.3816E-02	9.4319E-02	ENSG00000186838
SELENOW	-0.4536	3.7684E-11	7.3589E-09	ENSG00000178980
SELL	0.0722	1.4379E-02		ENSG00000188404
SELP	-0.0058	8.5455E-01	9.2596E-01	ENSG00000174175
SELPLG	-0.0162	7.7851E-01	8.8220E-01	ENSG00000110876
SEM1	-0.0463	4.6750E-01	6.6415E-01	ENSG00000127922
SEMA3A	-0.0237	7.2657E-01	8.4993E-01	ENSG00000075213
SEMA3C	-0.1347	1.1645E-01	2.8152E-01	ENSG00000075223
SEMA3D	0.0500	4.6756E-01	6.6419E-01	ENSG00000153993
SEMA3F	-0.1199	1.8658E-01	3.8091E-01	ENSG00000001617
SEMA3F-AS1	-0.0681	4.3327E-01	6.3436E-01	ENSG00000235016
SEMA3G	0.0279	4.2289E-01		ENSG00000010319
SEMA4A	-0.1170	1.0037E-01	2.5529E-01	ENSG00000196189

SEMA4B	0.1136	1.5857E-01	3.4279E-01	ENSG00000185033
SEMA4C	-0.0308	6.7962E-01	8.2059E-01	ENSG00000168758
SEMA4D	0.0919	2.0819E-01	4.0805E-01	ENSG00000187764
SEMA4F	0.2604	1.5110E-04	2.0945E-03	ENSG00000135622
SEMA4G	0.1076	1.5832E-01	3.4246E-01	ENSG00000095539
SEMA5A	-0.1705	7.8811E-02	2.1744E-01	ENSG00000112902
SEMA5A-AS1	0.0474	4.5520E-01	6.5356E-01	ENSG00000251370
SEMA5B	-0.0358	6.7396E-01	8.1745E-01	ENSG00000082684
SEMA6A	0.1784	1.3980E-02	6.4744E-02	ENSG00000092421
SEMA6A-AS2	0.0170	8.1119E-01	9.0114E-01	ENSG00000249167
SEMA6B	-0.4472	6.6848E-08	4.1329E-06	ENSG00000167680
SEMA6C	-0.0980	1.3852E-01	3.1439E-01	ENSG00000143434
SEMA6D	0.3040	5.3060E-04	5.5579E-03	ENSG00000137872
SEMA7A	0.0407	6.3291E-01	7.8929E-01	ENSG00000138623
SENP1	-0.0308	6.0767E-01	7.7108E-01	ENSG00000079387
SENP2	0.1489	1.7773E-02	7.6347E-02	ENSG00000163904
SENP3	-0.1265	1.3720E-01	3.1258E-01	ENSG00000161956
SENP5	0.0457	4.9821E-01	6.8929E-01	ENSG00000119231
SENP6	-0.0431	3.8271E-01	5.9091E-01	ENSG00000112701
SENP7	0.2719	5.6389E-04	5.8339E-03	ENSG00000138468
SENP8	0.3531	2.7134E-03	1.8905E-02	ENSG00000166192
SEPHS1	0.0513	4.0492E-01	6.0944E-01	ENSG00000086475
SEPHS2	-0.2400	5.8194E-05	1.0062E-03	ENSG00000179918
SEPT5-GP1BB	0.0768	1.7479E-01	3.6515E-01	ENSG00000284874
SEPTIN1	0.0858	3.3453E-01	5.4464E-01	ENSG00000180096
SEPTIN10	0.0571	4.2798E-01	6.2993E-01	ENSG00000186522
SEPTIN11	-0.2197	1.5056E-02	6.8149E-02	ENSG00000138758
SEPTIN12	0.0619	3.5875E-01	5.6820E-01	ENSG00000140623
SEPTIN2	-0.2295	4.1404E-03	2.6178E-02	ENSG00000168385
SEPTIN3	-0.3097	1.0962E-04	1.6533E-03	ENSG00000100167
SEPTIN4	-0.2978	3.3369E-04	3.8949E-03	ENSG00000108387
SEPTIN4-AS1	-0.0474	1.2937E-01		ENSG00000264672
SEPTIN5	-0.1277	1.7816E-01	3.6944E-01	ENSG00000184702
SEPTIN6	0.0359	5.6981E-01	7.4373E-01	ENSG00000125354
SEPTIN7	-0.2731	6.1556E-06	1.6535E-04	ENSG00000122545
SEPTIN7P1	0.0502	3.6132E-01	5.7085E-01	ENSG00000259090
SEPTIN7P10	-0.0411	1.0697E-01		ENSG00000248885
SEPTIN7P12	-0.0045	5.9398E-01		ENSG00000275562
SEPTIN7P15	-0.0154	3.1954E-01		ENSG00000275061
SEPTIN7P3	-0.0409	7.9644E-02		ENSG00000226102
SEPTIN7P4	-0.0154	3.1954E-01		ENSG00000240651
SEPTIN7P5	-0.0154	3.1954E-01		ENSG00000164740
SEPTIN7P6	-0.0177	5.4125E-01		ENSG00000241007
SEPTIN7P7	-0.3397	1.8056E-02	7.7128E-02	ENSG00000229897
SEPTIN8	-0.2266	1.2863E-02	6.0852E-02	ENSG00000164402
SEPTIN9	-0.1192	1.1692E-01	2.8223E-01	ENSG00000184640
SERAC1	0.0143	8.3259E-01	9.1366E-01	ENSG00000122335
SERBP1	-0.1138	2.8611E-02	1.0749E-01	ENSG00000142864
SERF1A	-0.0558	3.0588E-01	5.1602E-01	ENSG00000172058

SERF1A	-0.0716	2.2900E-01	4.3343E-01	ENSG00000205572
SERF2	-0.4460	8.2656E-08	4.9124E-06	ENSG00000140264
SERGEF	-0.0058	9.2670E-01	9.6412E-01	ENSG00000129158
SERHL	-0.0459	5.4079E-01	7.2272E-01	ENSG00000172250
SERHL2	-0.0167	8.4058E-01	9.1799E-01	ENSG00000183569
SERINC1	-0.2649	8.1740E-04	7.7903E-03	ENSG00000111897
SERINC2	0.0448	5.8714E-01	7.5640E-01	ENSG00000168528
SERINC3	0.1403	1.7955E-02	7.6954E-02	ENSG00000132824
SERINC5	0.0100	9.0283E-01	9.5250E-01	ENSG00000164300
SERP1	-0.1236	7.7210E-02	2.1456E-01	ENSG00000120742
SERP2	-0.0820	3.2185E-01	5.3182E-01	ENSG00000151778
SERPINA1	0.0725	1.5446E-01		ENSG00000197249
SERPINA10	0.0817	9.0525E-02	2.3904E-01	ENSG00000140093
SERPINA3	0.0399	3.3412E-01		ENSG00000196136
SERPINA5	0.0541	1.2796E-01	2.9971E-01	ENSG00000188488
SERPINB1	0.1399	1.4702E-01	3.2685E-01	ENSG00000021355
SERPINB6	0.0344	6.7046E-01	8.1542E-01	ENSG00000124570
SERPINB7	0.0049	7.8227E-01		ENSG00000166396
SERPINB8	0.1099	1.8424E-01	3.7770E-01	ENSG00000166401
SERPINB9	-0.1643	1.0145E-01	2.5718E-01	ENSG00000170542
SERPINC1	-0.0126	7.9207E-01		ENSG00000117601
SERPIND1	-0.1223	1.3486E-01	3.0930E-01	ENSG00000099937
SERPINE1	0.0347	6.7343E-01	8.1729E-01	ENSG00000106366
SERPINE2	0.0276	7.2241E-01	8.4784E-01	ENSG00000135919
SERPINE3	0.0169	5.3702E-01	7.2003E-01	ENSG00000253309
SERPINF1	0.2280	4.8768E-02	1.5677E-01	ENSG00000132386
SERPINF2	0.0453	3.9841E-01		ENSG00000167711
SERPING1	-0.2159	1.2588E-02	5.9905E-02	ENSG00000149131
SERPINH1	-0.1522	1.0882E-01	2.6921E-01	ENSG00000149257
SERPINI1	-0.2272	4.4006E-02	1.4600E-01	ENSG00000163536
SERPINI2	0.0500	4.3126E-01	6.3290E-01	ENSG00000114204
SERTAD1	-0.1930	2.9126E-02	1.0885E-01	ENSG00000197019
SERTAD2	-0.0087	8.8781E-01	9.4427E-01	ENSG00000179833
SERTAD3	-0.1058	1.1535E-01	2.7998E-01	ENSG00000167565
SERTAD4	-0.2576	7.1650E-04	7.0720E-03	ENSG00000082497
SERTM1	0.0702	3.4577E-01	5.5572E-01	ENSG00000180440
SERTM2	0.1032	1.0614E-01	2.6496E-01	ENSG00000260802
SESN1	-0.1814	1.4716E-02	6.7104E-02	ENSG00000080546
SESN2	-0.1989	6.3908E-03	3.6232E-02	ENSG00000130766
SESN3	-0.0223	7.4891E-01	8.6357E-01	ENSG00000149212
SESTD1	0.2314	6.8640E-04	6.8404E-03	ENSG00000187231
SET	-0.4482	2.6403E-09	2.6195E-07	ENSG00000119335
SETBP1	-0.0063	9.4639E-01	9.7346E-01	ENSG00000152217
SETBP1-DT	0.0364	6.6587E-01	8.1241E-01	ENSG00000267414
SETD1A	-0.0110	8.9251E-01	9.4648E-01	ENSG00000099381
SETD1B	0.0258	7.5394E-01	8.6633E-01	ENSG00000139718
SETD2	-0.1304	3.6792E-02	1.2870E-01	ENSG00000181555
SETD3	0.0325	6.1687E-01	7.7793E-01	ENSG00000183576
SETD4	0.4477	2.7071E-09	2.6636E-07	ENSG00000185917

SETD5	0.0763	2.2936E-01	4.3377E-01	ENSG00000168137
SETD6	0.0074	9.0203E-01	9.5204E-01	ENSG00000103037
SETD7	-0.5191	3.5106E-12	1.0797E-09	ENSG00000145391
SETD9	0.0632	4.4607E-01	6.4528E-01	ENSG00000155542
SETDB1	-0.1562	1.6984E-02	7.3964E-02	ENSG00000143379
SETDB2	0.0581	4.3950E-01	6.3954E-01	ENSG00000136169
SETMAR	0.0247	7.2061E-01	8.4677E-01	ENSG00000170364
SETP11	0.0196	6.8931E-01		ENSG00000239570
SETSIP	-0.0467	6.3691E-02		ENSG00000230667
SETX	0.1271	8.8130E-02	2.3425E-01	ENSG00000107290
SEZ6L	-0.0126	8.7028E-01	9.3460E-01	ENSG00000100095
SEZ6L2	-0.1201	6.0303E-02	1.8100E-01	ENSG00000174938
SF1	-0.0891	1.7812E-01	3.6944E-01	ENSG00000168066
SF3A1	-0.0664	2.4453E-01	4.5213E-01	ENSG00000099995
SF3A2	-0.1552	1.6065E-02	7.1274E-02	ENSG00000104897
SF3A3	-0.0899	1.5373E-01	3.3607E-01	ENSG00000183431
SF3A3P1	-0.0018	7.7968E-01		ENSG00000230568
SF3B1	-0.3142	2.4225E-09	2.4230E-07	ENSG00000115524
SF3B2	-0.1158	4.2306E-02	1.4218E-01	ENSG00000087365
SF3B3	-0.0686	2.0660E-01	4.0618E-01	ENSG00000189091
SF3B4	-0.1149	4.0720E-02	1.3846E-01	ENSG00000143368
SF3B4P1	0.0234	1.7129E-01		ENSG00000258988
SF3B5	-0.1881	6.4642E-03	3.6555E-02	ENSG00000169976
SF3B6	-0.1972	5.6336E-03	3.2918E-02	ENSG00000115128
SFI1	0.0310	5.9954E-01	7.6548E-01	ENSG00000198089
SFMBT1	0.2823	1.5392E-04	2.1229E-03	ENSG00000163935
SFMBT2	0.2892	4.0912E-03	2.5991E-02	ENSG00000198879
SFN	0.0101	9.1553E-01		ENSG00000175793
SFPQ	-0.0151	7.7296E-01	8.7903E-01	ENSG00000116560
SFR1	-0.1870	2.6888E-02	1.0271E-01	ENSG00000156384
SFRP2	0.3873	1.2771E-03	1.0903E-02	ENSG00000145423
SFRP4	-0.1821	8.6929E-02	2.3183E-01	ENSG00000106483
SFRP5	0.0859	2.6379E-01	4.7273E-01	ENSG00000120057
SFSWAP	-0.0693	3.1048E-01	5.2074E-01	ENSG00000061936
SFT2D1	0.1873	4.3506E-03	2.7114E-02	ENSG00000198818
SFT2D2	-0.1728	6.0285E-03	3.4616E-02	ENSG00000213064
SFTA1P	-0.0261	5.2198E-01	7.0854E-01	ENSG00000225383
SFTA2	0.0896	2.1907E-01	4.2147E-01	ENSG00000196260
SFTPA1	0.0242	3.7140E-01		ENSG00000122852
SFTPA2	-0.0318	5.0493E-01		ENSG00000185303
SFTPB	-0.0190	6.1584E-01		ENSG00000168878
SFTPC	-0.0734	2.2509E-01	4.2887E-01	ENSG00000168484
SFTPD	-0.0694	3.4751E-01	5.5776E-01	ENSG00000133661
SFXN1	-0.1610	1.1853E-02	5.7522E-02	ENSG00000164466
SFXN2	0.1051	2.4544E-01	4.5323E-01	ENSG00000156398
SFXN3	-0.1146	1.1172E-01	2.7384E-01	ENSG00000107819
SFXN4	-0.0944	1.2651E-01	2.9765E-01	ENSG00000183605
SFXN5	-0.0208	7.8421E-01	8.8605E-01	ENSG00000144040
SGCA	0.1187	4.8536E-02	1.5635E-01	ENSG00000108823

SGCB	-0.2006	9.9468E-04	9.0211E-03	ENSG00000163069
SGCD	0.4372	1.5972E-03	1.2780E-02	ENSG00000170624
SGCE	0.0532	3.2965E-01	5.4001E-01	ENSG00000127990
SGCG	-0.1004	2.5384E-01	4.6187E-01	ENSG00000102683
SGCZ	0.0197	7.2216E-01	8.4766E-01	ENSG00000185053
SGF29	0.1338	5.1670E-02	1.6301E-01	ENSG00000176476
SGIP1	0.0283	6.0418E-01	7.6833E-01	ENSG00000118473
SGK1	-0.3967	2.5815E-04	3.2032E-03	ENSG00000118515
SGK2	0.0470	4.6225E-01	6.5926E-01	ENSG00000101049
SGK3	0.0623	3.6920E-01	5.7842E-01	ENSG00000104205
SGMS1	-0.1829	3.9698E-02	1.3618E-01	ENSG00000198964
SGMS2	0.3418	1.8270E-02	7.7813E-02	ENSG00000164023
SGO1	-0.0790	3.5558E-01	5.6541E-01	ENSG00000129810
SGO1-AS1	-0.0508	3.4734E-01	5.5760E-01	ENSG00000231304
SGO1P1	-0.1245	5.9473E-03		ENSG00000270615
SGO2	-0.0166	8.2721E-01	9.1051E-01	ENSG00000163535
SGPL1	-0.0443	4.9171E-01	6.8388E-01	ENSG00000166224
SGPP1	-0.1330	8.3882E-02	2.2635E-01	ENSG00000126821
SGPP2	0.0698	4.3131E-01	6.3290E-01	ENSG00000163082
SGSH	0.4261	6.5205E-07	2.6215E-05	ENSG00000181523
SGSM1	0.1041	2.0626E-01	4.0594E-01	ENSG00000167037
SGSM2	0.0413	5.5432E-01	7.3257E-01	ENSG00000141258
SGSM3	-0.0327	6.8002E-01	8.2100E-01	ENSG00000100359
SGTA	-0.0120	9.1763E-01	9.5907E-01	ENSG00000104969
SGTB	-0.2143	1.8843E-03	1.4407E-02	ENSG00000197860
SH2B1	-0.0147	8.4411E-01	9.2003E-01	ENSG00000178188
SH2B2	-0.1671	4.6266E-02	1.5146E-01	ENSG00000160999
SH2B3	-0.1450	7.5261E-02	2.1096E-01	ENSG00000111252
SH2D1A	0.0020	9.7972E-01		ENSG00000183918
SH2D2A	0.0025	9.7136E-01	9.8559E-01	ENSG00000027869
SH2D3A	-0.3636	1.3639E-02	6.3558E-02	ENSG00000125731
SH2D3C	-0.3492	1.6721E-04	2.2593E-03	ENSG00000095370
SH2D4A	0.0566	3.4012E-01	5.4985E-01	ENSG00000104611
SH2D4B	-0.1031	2.1941E-01	4.2176E-01	ENSG00000178217
SH2D5	0.0435	6.0041E-01	7.6598E-01	ENSG00000189410
SH2D6	0.0251	7.4509E-01	8.6120E-01	ENSG00000152292
SH2D7	-0.2250	4.5898E-02	1.5067E-01	ENSG00000183476
SH3BGR	-0.0717	3.8424E-01	5.9219E-01	ENSG00000185437
SH3BGRL	-0.0916	1.2244E-01	2.9129E-01	ENSG00000131171
SH3BGRL2	0.1428	1.2386E-01	2.9362E-01	ENSG00000198478
SH3BGRL3	-0.2780	3.9403E-04	4.4245E-03	ENSG00000142669
SH3BP1	-0.0498	5.0885E-01	6.9786E-01	ENSG00000100092
SH3BP2	0.0923	1.9368E-01	3.9002E-01	ENSG00000087266
SH3BP4	-0.0280	6.8689E-01	8.2573E-01	ENSG00000130147
SH3BP5	-0.3183	3.0450E-03	2.0708E-02	ENSG00000131370
SH3BP5-AS1	0.1357	1.3875E-01	3.1465E-01	ENSG00000224660
SH3BP5L	-0.0433	6.0764E-01	7.7108E-01	ENSG00000175137
SH3D19	-0.0654	3.9169E-01	5.9876E-01	ENSG00000109686
SH3GL1	-0.0708	2.5254E-01	4.6058E-01	ENSG00000141985

SH3GL2	-0.4119	4.1540E-06	1.2067E-04	ENSG00000107295
SH3GL3	0.0408	5.5635E-01	7.3423E-01	ENSG00000140600
SH3GLB1	-0.0773	9.3855E-02	2.4473E-01	ENSG00000097033
SH3GLB2	-0.0688	2.7053E-01	4.8008E-01	ENSG00000148341
SH3KBP1	0.1929	1.5184E-02	6.8538E-02	ENSG00000147010
SH3PXD2A	-0.0016	9.7501E-01	9.8770E-01	ENSG00000107957
SH3PXD2A-AS1	-0.0452	5.5204E-01	7.3071E-01	ENSG00000280693
SH3PXD2B	0.1789	8.9733E-03	4.6899E-02	ENSG00000174705
SH3RF1	-0.1728	1.2522E-02	5.9696E-02	ENSG00000154447
SH3RF2	0.2030	3.4730E-02	1.2325E-01	ENSG00000156463
SH3RF3	-0.4169	4.5668E-05	8.2622E-04	ENSG00000172985
SH3TC1	0.0363	3.6502E-01		ENSG00000125089
SH3TC2	0.1477	1.0616E-02	5.2974E-02	ENSG00000169247
SH3TC2-DT	0.0223	5.2762E-01		ENSG00000250072
SH3YL1	-0.0398	5.0410E-01	6.9433E-01	ENSG00000035115
SHANK1	0.1066	2.2985E-01	4.3450E-01	ENSG00000161681
SHANK2	-0.1575	2.0748E-02	8.5155E-02	ENSG00000162105
SHANK2-AS1	-0.1966	4.2919E-02	1.4360E-01	ENSG00000226627
SHANK3	0.2295	2.4879E-02	9.7288E-02	ENSG00000251322
SHARPIN	-0.0832	1.5988E-01	3.4499E-01	ENSG00000179526
SHB	-0.0683	3.8050E-01	5.8893E-01	ENSG00000107338
SHBG	0.0677	4.3492E-01	6.3598E-01	ENSG00000129214
SHC1	-0.1002	1.9247E-01	3.8824E-01	ENSG00000160691
SHC2	-0.3133	3.9265E-06	1.1543E-04	ENSG00000129946
SHC3	0.1896	7.5348E-03	4.1007E-02	ENSG00000148082
SHC4	0.1403	1.1741E-01	2.8294E-01	ENSG00000185634
SHCBP1	-0.0502	5.4651E-01	7.2651E-01	ENSG00000171241
SHD	-0.1817	2.1490E-03	1.5860E-02	ENSG00000105251
SHE	-0.2268	4.6898E-02	1.5292E-01	ENSG00000169291
SHF	-0.2585	3.1460E-05	6.1617E-04	ENSG00000138606
SHFL	-0.1318	9.7509E-02	2.5086E-01	ENSG00000130813
SHH	0.0058	9.0063E-01	9.5128E-01	ENSG00000164690
SHISA2	0.0032	9.7094E-01	9.8540E-01	ENSG00000180730
SHISA3	-0.0575	4.0702E-01	6.1153E-01	ENSG00000178343
SHISA4	-0.0184	7.7268E-01	8.7896E-01	ENSG00000198892
SHISA5	-0.1847	5.2536E-04	5.5170E-03	ENSG00000164054
SHISA6	-0.5336	3.5916E-04	4.1186E-03	ENSG00000188803
SHISA7	-0.1048	1.6094E-01	3.4648E-01	ENSG00000187902
SHISA8	-0.4912	2.6014E-06	8.2803E-05	ENSG00000234965
SHISA9	-0.0713	3.9653E-01	6.0248E-01	ENSG00000237515
SHISAL1	0.5331	1.0412E-04	1.5892E-03	ENSG00000138944
SHISAL2A	0.3042	2.3153E-02	9.2375E-02	ENSG00000182183
SHISAL2B	0.0005	9.7492E-01	9.8769E-01	ENSG00000145642
SHKBP1	-0.0565	3.7019E-01	5.7931E-01	ENSG00000160410
SHLD1	0.4222	8.8294E-05	1.3998E-03	ENSG00000171984
SHLD2	0.1834	2.6742E-02	1.0232E-01	ENSG00000122376
SHLD2P1	0.0481	5.6598E-01	7.4117E-01	ENSG00000165874
SHLD3	-0.2949	3.0931E-04	3.6802E-03	ENSG00000253251
SHMT1	0.2353	3.9502E-03	2.5338E-02	ENSG00000176974

SHMT2	-0.0813	3.6978E-01	5.7903E-01	ENSG00000182199
SHOC1	-0.0555	2.1998E-01		ENSG00000165181
SHOC2	-0.0099	8.2333E-01	9.0841E-01	ENSG00000108061
SHOX	-0.0167	8.4101E-01		ENSG00000185960
SHOX2	0.0851	2.6104E-01	4.6958E-01	ENSG00000168779
SHPK	-0.0732	3.9781E-01	6.0353E-01	ENSG00000197417
SHPRH	0.4035	9.8432E-08	5.6719E-06	ENSG00000146414
SHQ1	0.4976	4.5275E-05	8.2092E-04	ENSG00000144736
SHROOM1	0.1296	1.6198E-01	3.4802E-01	ENSG00000164403
SHROOM2	-0.0489	5.2671E-01	7.1185E-01	ENSG00000146950
SHROOM3	0.0564	4.4810E-01	6.4745E-01	ENSG00000138771
SHTN1	-0.2852	1.4125E-04	1.9832E-03	ENSG00000187164
SIAE	0.1626	4.1051E-02	1.3928E-01	ENSG00000110013
SIAH1	0.0888	1.7203E-01	3.6142E-01	ENSG00000196470
SIAH2	-0.2287	3.1917E-03	2.1486E-02	ENSG00000181788
SIDT1	-0.0720	3.6154E-01	5.7097E-01	ENSG00000072858
SIDT2	0.1654	4.8469E-02	1.5620E-01	ENSG00000149577
SIGIRR	-0.1233	1.2390E-01	2.9362E-01	ENSG00000185187
SIGLEC1	0.0301	6.7847E-01	8.1981E-01	ENSG00000088827
SIGLEC10	0.0187	3.3687E-01		ENSG00000142512
SIGLEC11	-0.0216	2.7765E-01		ENSG00000161640
SIGLEC15	0.0046	6.5392E-01		ENSG00000197046
SIGLEC16	0.0221	3.7772E-01		ENSG00000161643
SIGLEC5	0.0157	4.5250E-01		ENSG00000268500
SIGLEC6	-0.0093	5.0231E-01		ENSG00000105492
SIGMAR1	-0.0519	3.0548E-01	5.1571E-01	ENSG00000147955
SIK1	-0.3250	2.5759E-03	1.8161E-02	ENSG00000142178
SIK2	0.4001	1.4786E-04	2.0589E-03	ENSG00000170145
SIK3	-0.0009	9.9248E-01	9.9576E-01	ENSG00000160584
SIKE1	0.1234	8.3497E-02	2.2566E-01	ENSG00000052723
SIL1	0.2685	5.8955E-04	6.0441E-03	ENSG00000120725
SIM1	0.0032	6.4632E-01		ENSG00000112246
SIM2	0.0113	7.5828E-01		ENSG00000159263
SIMC1	-0.0108	8.5913E-01	9.2898E-01	ENSG00000170085
SIN3A	0.0077	9.2500E-01	9.6316E-01	ENSG00000169375
SIN3B	-0.0911	1.6312E-01	3.4953E-01	ENSG00000127511
SINHCAF	-0.2557	4.2736E-04	4.7090E-03	ENSG00000139146
SIPA1	-0.4436	2.2885E-05	4.7800E-04	ENSG00000213445
SIPA1L1	0.1747	3.1144E-02	1.1433E-01	ENSG00000197555
SIPA1L2	0.0990	1.6359E-01	3.5018E-01	ENSG00000116991
SIPA1L3	0.0242	7.2320E-01	8.4820E-01	ENSG00000105738
SIRPA	-0.1156	1.5190E-01	3.3362E-01	ENSG00000198053
SIRPB1	-0.0003	8.6408E-01		ENSG00000101307
SIRT1	-0.1416	1.9569E-02	8.1706E-02	ENSG00000096717
SIRT2	-0.2102	1.4964E-03	1.2233E-02	ENSG00000068903
SIRT3	0.2046	6.5871E-04	6.6316E-03	ENSG00000142082
SIRT4	0.1109	2.0775E-01	4.0744E-01	ENSG00000089163
SIRT5	-0.1833	9.2846E-03	4.7993E-02	ENSG00000124523
SIRT6	-0.0482	4.8126E-01	6.7584E-01	ENSG00000077463

SIRT7	-0.0598	3.6098E-01	5.7052E-01	ENSG00000187531
SIT1	0.0313	4.3421E-01		ENSG00000137078
SIVA1	0.0266	7.0840E-01	8.3966E-01	ENSG00000184990
SIX1	-0.0034	7.8355E-01		ENSG00000126778
SIX2	-0.0466	3.2602E-01	5.3620E-01	ENSG00000170577
SIX3	0.2505	5.8263E-03	3.3788E-02	ENSG00000138083
SIX3-AS1	0.1015	2.1795E-01	4.2005E-01	ENSG00000236502
SIX4	-0.2203	4.7704E-02	1.5462E-01	ENSG00000100625
SIX5	0.0497	3.9412E-01	6.0049E-01	ENSG00000177045
SIX6	-0.0050	9.2807E-01	9.6469E-01	ENSG00000184302
SKA1	-0.3096	4.3863E-03	2.7288E-02	ENSG00000154839
SKA2	-0.1244	4.6376E-02	1.5170E-01	ENSG00000182628
SKA3	-0.1898	5.6103E-02	1.7305E-01	ENSG00000165480
SKAP1	-0.0084	8.0647E-01	8.9825E-01	ENSG00000141293
SKAP1-AS2	-0.0095	2.4078E-01		ENSG00000235300
SKAP2	-0.0078	9.2182E-01	9.6140E-01	ENSG00000005020
SKI	-0.1329	1.2501E-01	2.9510E-01	ENSG00000157933
SKIC2	-0.1594	5.0270E-02	1.6016E-01	ENSG00000204351
SKIC3	0.2281	4.3039E-05	7.9027E-04	ENSG00000198677
SKIC8	0.1374	4.1481E-02	1.4035E-01	ENSG00000140395
SKIDA1	-0.1413	9.2211E-02	2.4186E-01	ENSG00000180592
SKIL	-0.0859	1.3210E-01	3.0546E-01	ENSG00000136603
SKOR1	0.1121	1.8159E-01	3.7411E-01	ENSG00000188779
SKOR1-AS1	0.0138	9.9926E-01		ENSG00000245719
SKOR2	-0.0079	7.9749E-01	8.9334E-01	ENSG00000215474
SKP1	-0.1030	4.1574E-02	1.4053E-01	ENSG00000113558
SKP2	-0.0807	2.3369E-01	4.3890E-01	ENSG00000145604
SLA	0.0803	3.5260E-01	5.6290E-01	ENSG00000155926
SLA2	-0.0159	9.8010E-01		ENSG00000101082
SLAIN1	-0.2094	4.0421E-03	2.5779E-02	ENSG00000139737
SLAIN2	-0.1342	2.8744E-02	1.0780E-01	ENSG00000109171
SLAMF6	0.0132	7.6420E-01		ENSG00000162739
SLAMF7	0.0124	6.6617E-01		ENSG00000026751
SLAMF9	0.0185	3.2752E-01		ENSG00000162723
SLBP	-0.0859	1.5308E-01	3.3527E-01	ENSG00000163950
SLC10A1	-0.0102	7.2430E-01		ENSG00000100652
SLC10A2	0.0170	3.6907E-01		ENSG00000125255
SLC10A3	-0.0123	8.7711E-01	9.3885E-01	ENSG00000126903
SLC10A4	-0.1837	7.3327E-02	2.0744E-01	ENSG00000145248
SLC10A5	0.0657	2.5038E-01	4.5838E-01	ENSG00000253598
SLC10A7	0.0363	6.4873E-01	8.0034E-01	ENSG00000120519
SLC11A2	0.1249	6.5004E-02	1.9079E-01	ENSG00000110911
SLC12A2	0.1401	8.0764E-02	2.2090E-01	ENSG00000064651
SLC12A2-DT	0.1246	8.0158E-02	2.1995E-01	ENSG00000245937
SLC12A3	-0.0047	9.4787E-01		ENSG00000070915
SLC12A4	-0.2441	1.8101E-02	7.7308E-02	ENSG00000124067
SLC12A5	-0.0498	5.2838E-01	7.1307E-01	ENSG00000124140
SLC12A5-AS1	0.4082	1.6295E-02	7.1918E-02	ENSG00000204044
SLC12A6	-0.1944	1.4215E-02	6.5448E-02	ENSG00000140199

SLC12A7	-0.0707	4.0464E-01	6.0923E-01	ENSG00000113504
SLC12A8	-0.0122	8.9482E-01	9.4750E-01	ENSG00000221955
SLC12A9	-0.1060	1.9140E-01	3.8676E-01	ENSG00000146828
SLC12A9-AS1	-0.0165	6.6578E-01		ENSG00000236305
SLC13A3	0.2759	3.3665E-03	2.2321E-02	ENSG00000158296
SLC13A5	0.0184	8.1606E-01	9.0435E-01	ENSG00000141485
SLC14A1	0.0025	9.6905E-01		ENSG00000141469
SLC14A2	0.1105	2.4258E-01	4.5029E-01	ENSG00000132874
SLC15A1	0.0226	8.7230E-01	9.3585E-01	ENSG00000088386
SLC15A2	-0.0002	9.9448E-01	9.9689E-01	ENSG00000163406
SLC15A3	0.1924	7.2124E-02	2.0509E-01	ENSG00000110446
SLC15A4	-0.0910	2.4630E-01	4.5414E-01	ENSG00000139370
SLC16A1	-0.0491	4.8515E-01	6.7891E-01	ENSG00000155380
SLC16A1-AS1	-0.0474	5.2750E-01	7.1240E-01	ENSG00000226419
SLC16A10	0.3506	6.8599E-04	6.8390E-03	ENSG00000112394
SLC16A11	-0.1544	1.1884E-01	2.8546E-01	ENSG00000174326
SLC16A12	0.0818	1.1312E-01		ENSG00000152779
SLC16A13	0.0950	2.9327E-01	5.0333E-01	ENSG00000174327
SLC16A14	-0.1136	6.9090E-02	1.9880E-01	ENSG00000163053
SLC16A2	-0.1232	1.6121E-02	7.1455E-02	ENSG00000147100
SLC16A3	-0.0704	4.2204E-01	6.2466E-01	ENSG00000141526
SLC16A4	-0.0190	8.1798E-01	9.0539E-01	ENSG00000168679
SLC16A5	0.0654	3.9872E-01	6.0424E-01	ENSG00000170190
SLC16A6	-0.3301	3.6037E-03	2.3582E-02	ENSG00000108932
SLC16A7	0.0796	2.7464E-01	4.8451E-01	ENSG00000118596
SLC16A8	0.0014	9.7376E-01	9.8718E-01	ENSG00000100156
SLC16A9	-0.0773	2.8085E-01	4.9106E-01	ENSG00000165449
SLC17A4	0.0053	9.9767E-01		ENSG00000146039
SLC17A5	-0.0594	3.7771E-01	5.8631E-01	ENSG00000119899
SLC17A6	0.3005	2.2282E-02	8.9861E-02	ENSG00000091664
SLC17A7	0.1504	1.2597E-01	2.9667E-01	ENSG00000104888
SLC17A8	0.1363	6.5795E-02	1.9251E-01	ENSG00000179520
SLC17A9	0.0094	8.3758E-01		ENSG00000101194
SLC18A1	-0.0340	6.6033E-01	8.0861E-01	ENSG00000036565
SLC18A2-AS1	0.0043	9.9473E-01		ENSG00000225936
SLC18A3	0.0202	8.0793E-01	8.9902E-01	ENSG00000187714
SLC18B1	-0.3572	3.1459E-05	6.1617E-04	ENSG00000146409
SLC19A1	-0.1495	8.1063E-02	2.2132E-01	ENSG00000173638
SLC19A2	0.0538	4.5557E-01	6.5386E-01	ENSG00000117479
SLC19A3	-0.0198	7.9559E-01	8.9205E-01	ENSG00000135917
SLC1A1	-0.1187	1.4417E-01	3.2232E-01	ENSG00000106688
SLC1A2	-0.0879	3.2861E-01	5.3907E-01	ENSG00000110436
SLC1A2-AS1	0.0080	6.3045E-01		ENSG00000255004
SLC1A3	-0.0794	3.1382E-01	5.2410E-01	ENSG00000079215
SLC1A3-AS1	0.0299	4.3902E-01	6.3918E-01	ENSG00000250155
SLC1A4	0.0124	8.8227E-01	9.4106E-01	ENSG00000115902
SLC1A5	-0.3579	6.1179E-05	1.0505E-03	ENSG00000105281
SLC1A6	0.0158	8.3952E-01	9.1741E-01	ENSG00000105143
SLC1A7	0.0199	4.9138E-01		ENSG00000162383

SLC20A1	-0.0127	8.3475E-01	9.1500E-01	ENSG00000144136
SLC20A2	0.1245	1.0856E-01	2.6875E-01	ENSG00000168575
SLC22A1	-0.0396	4.6046E-01	6.5803E-01	ENSG00000175003
SLC22A10	0.0040	7.6393E-01		ENSG00000184999
SLC22A11	0.1218	9.4300E-02	2.4558E-01	ENSG00000168065
SLC22A12	0.0165	8.0091E-01	8.9499E-01	ENSG00000197891
SLC22A13	0.0257	2.1344E-01		ENSG00000172940
SLC22A14	0.0042	8.3193E-01		ENSG00000144671
SLC22A15	-0.0112	8.8705E-01	9.4386E-01	ENSG00000163393
SLC22A17	-0.0389	4.3991E-01	6.3991E-01	ENSG00000092096
SLC22A18	-0.0198	8.0303E-01	8.9620E-01	ENSG00000110628
SLC22A18AS	0.0379	5.2181E-01		ENSG00000254827
SLC22A2	-0.0265	6.1594E-01		ENSG00000112499
SLC22A20P	0.0116	8.7002E-01	9.3446E-01	ENSG00000197847
SLC22A23	-0.0794	2.3811E-01	4.4418E-01	ENSG00000137266
SLC22A3	-0.0738	2.4570E-01	4.5347E-01	ENSG00000146477
SLC22A31	0.1930	6.5416E-02	1.9169E-01	ENSG00000259803
SLC22A4	0.5223	1.1601E-03	1.0137E-02	ENSG00000197208
SLC22A5	-0.0748	3.3900E-01	5.4872E-01	ENSG00000197375
SLC22A6	-0.0024	8.6063E-01		ENSG00000197901
SLC22A7	-0.0283	4.9982E-01	6.9086E-01	ENSG00000137204
SLC23A1	-0.0703	3.7790E-01	5.8642E-01	ENSG00000170482
SLC23A2	0.0613	3.7121E-01	5.7998E-01	ENSG00000089057
SLC24A1	0.3406	8.0326E-03	4.3022E-02	ENSG00000074621
SLC24A2	-0.2107	4.1457E-02	1.4030E-01	ENSG00000155886
SLC24A3	-0.0837	3.4787E-01	5.5811E-01	ENSG00000185052
SLC24A3-AS1	0.0183	5.1546E-01		ENSG00000179447
SLC24A4	-0.1188	1.1577E-01	2.8040E-01	ENSG00000140090
SLC24A5	0.0834	1.9912E-01		ENSG00000188467
SLC25A1	-0.2055	4.6950E-03	2.8722E-02	ENSG00000100075
SLC25A10	0.1017	2.5519E-01	4.6325E-01	ENSG00000183048
SLC25A11	-0.1995	4.2415E-04	4.6842E-03	ENSG00000108528
SLC25A12	-0.0463	4.3932E-01	6.3938E-01	ENSG00000115840
SLC25A13	0.3065	3.0502E-04	3.6414E-03	ENSG00000004864
SLC25A14	0.0468	4.6459E-01	6.6148E-01	ENSG00000102078
SLC25A15	0.0261	7.3102E-01	8.5293E-01	ENSG00000102743
SLC25A16	0.0562	4.3852E-01	6.3860E-01	ENSG00000122912
SLC25A17	0.0473	5.0881E-01	6.9784E-01	ENSG00000100372
SLC25A18	0.2465	2.6404E-02	1.0138E-01	ENSG00000182902
SLC25A19	-0.1431	1.2360E-01	2.9328E-01	ENSG00000125454
SLC25A2	-0.0308	1.4717E-01		ENSG00000120329
SLC25A20	-0.0579	3.8276E-01	5.9094E-01	ENSG00000178537
SLC25A21	0.5438	5.7758E-03	3.3565E-02	ENSG00000183032
SLC25A21-AS1	0.0224	7.9677E-01	8.9270E-01	ENSG00000258708
SLC25A22	-0.0895	1.9423E-01	3.9089E-01	ENSG00000177542
SLC25A23	-0.1084	4.3747E-02	1.4546E-01	ENSG00000125648
SLC25A24	-0.0398	5.5239E-01	7.3097E-01	ENSG00000085491
SLC25A26	-0.1767	1.5941E-02	7.0967E-02	ENSG00000144741
SLC25A27	0.0197	7.7758E-01	8.8152E-01	ENSG00000153291

SLC25A28	-0.0455	5.0650E-01	6.9611E-01	ENSG00000155287
SLC25A29	-0.1222	1.1645E-01	2.8152E-01	ENSG00000197119
SLC25A3	-0.1686	2.5509E-03	1.8051E-02	ENSG00000075415
SLC25A30	0.1534	1.2375E-01	2.9354E-01	ENSG00000174032
SLC25A30-AS1	-0.0134	8.6250E-01	9.3070E-01	ENSG00000251015
SLC25A31	0.0306	2.9523E-01		ENSG00000151475
SLC25A32	0.0019	9.8390E-01	9.9139E-01	ENSG00000164933
SLC25A33	-0.2466	1.4718E-04	2.0541E-03	ENSG00000171612
SLC25A34	-0.1273	1.7745E-01	3.6864E-01	ENSG00000162461
SLC25A35	0.0458	5.9061E-01	7.5880E-01	ENSG00000125434
SLC25A36	-0.0297	5.4632E-01	7.2645E-01	ENSG00000114120
SLC25A36P1	-0.0502	7.6649E-02		ENSG00000267781
SLC25A37	0.0158	8.1913E-01	9.0603E-01	ENSG00000147454
SLC25A38	-0.2204	1.5326E-03	1.2413E-02	ENSG00000144659
SLC25A39	-0.1558	1.6220E-02	7.1689E-02	ENSG00000013306
SLC25A3P1	0.1388	5.1511E-02	1.6271E-01	ENSG00000236253
SLC25A4	-0.2811	7.1961E-05	1.1899E-03	ENSG00000151729
SLC25A40	0.4075	1.1347E-04	1.6941E-03	ENSG00000075303
SLC25A41	0.1152	1.8904E-01	3.8397E-01	ENSG00000181240
SLC25A42	0.0390	5.6333E-01	7.3975E-01	ENSG00000181035
SLC25A43	-0.0980	2.7484E-01	4.8461E-01	ENSG00000077713
SLC25A44	0.0338	6.2547E-01	7.8374E-01	ENSG00000160785
SLC25A45	0.2751	2.3399E-02	9.3142E-02	ENSG00000162241
SLC25A46	-0.0333	5.3095E-01	7.1513E-01	ENSG00000164209
SLC25A47	0.0343	2.6686E-01		ENSG00000140107
SLC25A48	0.0663	4.2717E-01	6.2915E-01	ENSG00000145832
SLC25A5	-0.3272	1.6917E-06	5.8378E-05	ENSG00000005022
SLC25A5-AS1	0.2195	7.5758E-03	4.1145E-02	ENSG00000224281
SLC25A51	-0.2159	1.0387E-02	5.2084E-02	ENSG00000122696
SLC25A52	-0.0184	2.2987E-01		ENSG00000141437
SLC25A53	0.1172	1.6369E-01	3.5031E-01	ENSG00000269743
SLC25A5P5	-0.0107	7.1724E-01		ENSG00000226421
SLC25A6	-0.0590	3.2268E-01	5.3285E-01	ENSG00000169100
SLC26A1	0.0276	7.2153E-01	8.4741E-01	ENSG00000145217
SLC26A10P	-0.0014	9.3506E-01		ENSG00000135502
SLC26A11	0.0525	4.6985E-01	6.6616E-01	ENSG00000181045
SLC26A3	0.0139	7.4921E-01		ENSG00000091138
SLC26A4	-0.0199	7.8142E-01	8.8416E-01	ENSG00000091137
SLC26A5	0.0230	5.7811E-01		ENSG00000170615
SLC26A5-AS1	0.0089	8.3840E-01		ENSG00000234715
SLC26A6	0.0451	5.7184E-01	7.4532E-01	ENSG00000225697
SLC26A7	0.1910	1.1212E-01	2.7453E-01	ENSG00000147606
SLC26A8	0.0352	5.9995E-01	7.6571E-01	ENSG00000112053
SLC26A9	0.0133	8.7479E-01		ENSG00000174502
SLC27A1	-0.1652	4.4217E-02	1.4655E-01	ENSG00000130304
SLC27A2	0.0553	5.1060E-01	6.9914E-01	ENSG00000140284
SLC27A3	-0.1390	1.2234E-01	2.9114E-01	ENSG00000143554
SLC27A4	0.0104	8.9047E-01	9.4574E-01	ENSG00000167114
SLC27A5	-0.0196	7.7015E-01	8.7696E-01	ENSG00000083807

SLC27A6	-0.0323	4.0333E-01	6.0785E-01	ENSG00000113396
SLC28A1	0.0539	1.4450E-01		ENSG00000156222
SLC28A2	-0.0035	9.5795E-01	9.7933E-01	ENSG00000137860
SLC28A2-AS1	-0.0126	8.7627E-01	9.3820E-01	ENSG00000259520
SLC28A3	0.0672	3.3716E-02		ENSG00000197506
SLC28A3-AS1	0.0041	2.7836E-01		ENSG00000233262
SLC29A1	-0.0339	6.2333E-01	7.8239E-01	ENSG00000112759
SLC29A2	-0.3749	1.5060E-04	2.0888E-03	ENSG00000174669
SLC29A3	0.1620	6.1520E-02	1.8355E-01	ENSG00000198246
SLC29A4	-0.0631	2.5392E-01	4.6192E-01	ENSG00000164638
SLC2A1	0.1513	9.5124E-02	2.4712E-01	ENSG00000117394
SLC2A10	-0.0174	8.2757E-01	9.1071E-01	ENSG00000197496
SLC2A11	0.2870	1.1349E-03	9.9590E-03	ENSG00000133460
SLC2A12	-0.0798	3.6433E-01	5.7342E-01	ENSG00000146411
SLC2A13	0.1124	1.6868E-01	3.5707E-01	ENSG00000151229
SLC2A14	0.1558	1.0618E-01	2.6496E-01	ENSG00000173262
SLC2A3	-0.0916	2.5900E-01	4.6768E-01	ENSG00000059804
SLC2A4	-0.0210	7.5025E-01	8.6435E-01	ENSG00000181856
SLC2A4RG	-0.4492	1.1993E-03	1.0394E-02	ENSG00000125520
SLC2A5	0.2827	2.9964E-02	1.1117E-01	ENSG00000142583
SLC2A6	-0.2241	5.5505E-03	3.2555E-02	ENSG00000160326
SLC2A8	0.1767	1.5998E-02	7.1117E-02	ENSG00000136856
SLC2A9	0.0235	6.2206E-01		ENSG00000109667
SLC30A1	0.0584	4.5275E-01	6.5149E-01	ENSG00000170385
SLC30A10	0.1769	6.5360E-02	1.9163E-01	ENSG00000196660
SLC30A2	0.0019	5.7283E-01	7.4605E-01	ENSG00000158014
SLC30A4	-0.1901	3.1807E-02	1.1587E-01	ENSG00000104154
SLC30A5	0.0579	3.8337E-01	5.9155E-01	ENSG00000145740
SLC30A6	0.2999	2.0437E-05	4.3575E-04	ENSG00000152683
SLC30A7	0.1641	2.1405E-02	8.7109E-02	ENSG00000162695
SLC30A8	0.1456	9.6323E-02	2.4916E-01	ENSG00000164756
SLC30A9	-0.0785	2.5736E-01	4.6585E-01	ENSG00000014824
SLC31A1	0.0876	2.4383E-01	4.5154E-01	ENSG00000136868
SLC31A2	-0.0368	5.5171E-01	7.3049E-01	ENSG00000136867
SLC32A1	-0.5331	1.1270E-05	2.6973E-04	ENSG00000101438
SLC33A1	0.1734	6.8661E-02	1.9802E-01	ENSG00000169359
SLC34A2	0.0928	1.6636E-01	3.5404E-01	ENSG00000157765
SLC34A3	-0.0060	7.4491E-01		ENSG00000198569
SLC35A1	-0.2277	1.5834E-03	1.2728E-02	ENSG00000164414
SLC35A2	0.3681	2.3971E-05	4.9521E-04	ENSG00000102100
SLC35A3	0.0732	2.8316E-01	4.9352E-01	ENSG00000117620
SLC35A4	0.0269	7.1004E-01	8.4074E-01	ENSG00000176087
SLC35A5	-0.0088	8.9624E-01	9.4851E-01	ENSG00000138459
SLC35B1	0.1510	1.0694E-02	5.3253E-02	ENSG00000121073
SLC35B2	0.0580	3.6051E-01	5.6993E-01	ENSG00000157593
SLC35B3	0.1690	2.1222E-02	8.6610E-02	ENSG00000124786
SLC35B4	0.2172	1.1546E-03	1.0103E-02	ENSG00000205060
SLC35C1	-0.1236	8.4341E-02	2.2697E-01	ENSG00000181830
SLC35C2	0.0022	9.7980E-01	9.8977E-01	ENSG00000080189

SLC35D1	0.1896	2.7540E-02	1.0457E-01	ENSG00000116704
SLC35D2	0.0937	2.7530E-01	4.8516E-01	ENSG00000130958
SLC35D3	-0.0346	3.4752E-01		ENSG00000182747
SLC35E1	0.0401	5.3589E-01	7.1900E-01	ENSG00000127526
SLC35E2B	0.0747	2.1394E-01	4.1511E-01	ENSG00000189339
SLC35E3	0.0721	2.5957E-01	4.6796E-01	ENSG00000175782
SLC35E4	-0.0193	7.2402E-01	8.4860E-01	ENSG00000100036
SLC35F1	-0.3484	2.0779E-06	6.8626E-05	ENSG00000196376
SLC35F2	-0.1894	7.4289E-02	2.0911E-01	ENSG00000110660
SLC35F3	0.0310	7.1479E-01	8.4332E-01	ENSG00000183780
SLC35F4	0.3742	8.4281E-03	4.4654E-02	ENSG00000151812
SLC35F5	0.0411	5.4028E-01	7.2224E-01	ENSG00000115084
SLC35F6	-0.1286	9.8941E-02	2.5298E-01	ENSG00000213699
SLC35G1	0.2468	1.0854E-03	9.6487E-03	ENSG00000176273
SLC35G2	-0.0615	3.7899E-01	5.8752E-01	ENSG00000168917
SLC35G5	0.0487	2.5291E-01		ENSG00000177710
SLC35G6	0.0398	3.8559E-01		ENSG00000259224
SLC36A1	-0.0621	4.2155E-01	6.2430E-01	ENSG00000123643
SLC36A2	-0.0081	6.0214E-01		ENSG00000186335
SLC36A3	0.0046	9.5375E-01		ENSG00000186334
SLC36A4	0.3784	1.3570E-06	4.8180E-05	ENSG00000180773
SLC37A1	0.1249	8.8003E-02	2.3401E-01	ENSG00000160190
SLC37A2	0.2102	5.1607E-02	1.6290E-01	ENSG00000134955
SLC37A3	0.0597	3.2649E-01	5.3660E-01	ENSG00000157800
SLC37A4	0.1838	1.2516E-02	5.9681E-02	ENSG00000137700
SLC38A1	0.1071	1.0534E-01	2.6369E-01	ENSG00000111371
SLC38A10	-0.0305	6.9012E-01	8.2779E-01	ENSG00000157637
SLC38A11	0.0238	2.0882E-01		ENSG00000169507
SLC38A2	0.0350	6.2221E-01	7.8162E-01	ENSG00000134294
SLC38A3	-0.0214	7.8226E-01	8.8472E-01	ENSG00000188338
SLC38A4	0.0358	2.4628E-01		ENSG00000139209
SLC38A5	0.1716	8.8947E-02	2.3568E-01	ENSG00000017483
SLC38A6	0.0594	4.2635E-01	6.2858E-01	ENSG00000139974
SLC38A7	0.0690	3.3399E-01	5.4412E-01	ENSG00000103042
SLC38A8	0.0050	8.3332E-01		ENSG00000166558
SLC38A9	0.0463	5.5316E-01	7.3158E-01	ENSG00000177058
SLC39A1	0.0837	2.9406E-01	5.0394E-01	ENSG00000143570
SLC39A10	-0.0453	5.3974E-01	7.2183E-01	ENSG00000196950
SLC39A11	-0.0941	2.0379E-01	4.0269E-01	ENSG00000133195
SLC39A12	0.0303	5.0399E-01	6.9429E-01	ENSG00000148482
SLC39A13	0.1670	3.0214E-03	2.0605E-02	ENSG00000165915
SLC39A13-AS1	0.0523	3.5591E-01	5.6577E-01	ENSG00000255197
SLC39A14	0.1848	2.8840E-02	1.0805E-01	ENSG00000104635
SLC39A2	-0.0145	4.6440E-01		ENSG00000165794
SLC39A3	0.1491	1.3244E-02	6.2200E-02	ENSG00000141873
SLC39A4	0.1312	1.6572E-01	3.5325E-01	ENSG00000147804
SLC39A5	0.0657	1.4936E-01	3.3008E-01	ENSG00000139540
SLC39A6	0.0411	4.3202E-01	6.3340E-01	ENSG00000141424
SLC39A7	0.0165	8.3719E-01	9.1604E-01	ENSG00000112473

SLC39A8	0.3119	7.3021E-03	4.0060E-02	ENSG00000138821
SLC39A9	-0.0577	2.5588E-01	4.6409E-01	ENSG00000029364
SLC3A1	0.4402	5.0152E-03	3.0178E-02	ENSG00000138079
SLC3A2	-0.3832	1.2801E-10	2.0721E-08	ENSG00000168003
SLC40A1	-0.2452	1.4327E-02	6.5760E-02	ENSG00000138449
SLC41A1	-0.1187	1.0432E-01	2.6193E-01	ENSG00000133065
SLC41A2	0.2013	4.0830E-02	1.3872E-01	ENSG00000136052
SLC41A3	-0.0870	1.3161E-01	3.0473E-01	ENSG00000114544
SLC43A1	-0.0275	7.2323E-01	8.4820E-01	ENSG00000149150
SLC43A2	-0.1942	2.2771E-03	1.6596E-02	ENSG00000167703
SLC43A3	-0.1869	5.5815E-02	1.7244E-01	ENSG00000134802
SLC44A1	-0.0375	6.0188E-01	7.6692E-01	ENSG00000070214
SLC44A2	-0.0765	3.2402E-01	5.3405E-01	ENSG00000129353
SLC44A3	0.0744	3.9580E-01	6.0197E-01	ENSG00000143036
SLC44A3-AS1	-0.0135	8.6172E-01	9.3057E-01	ENSG00000224081
SLC44A4	0.1468	1.3347E-01	3.0720E-01	ENSG00000204385
SLC44A5	0.1427	1.1049E-01	2.7207E-01	ENSG00000137968
SLC45A1	-0.0911	2.3546E-01	4.4097E-01	ENSG00000162426
SLC45A2	0.0582	2.5379E-01	4.6181E-01	ENSG00000164175
SLC45A3	-0.0055	9.4417E-01	9.7218E-01	ENSG00000158715
SLC45A4	0.1166	1.6810E-01	3.5641E-01	ENSG00000022567
SLC46A1	0.0531	5.3561E-01	7.1877E-01	ENSG00000076351
SLC46A3	0.0362	6.6211E-01	8.0977E-01	ENSG00000139508
SLC47A1	-0.4598	3.7414E-03	2.4315E-02	ENSG00000142494
SLC47A2	-0.0845	1.2468E-01	2.9458E-01	ENSG00000180638
SLC48A1	-0.2155	2.2125E-04	2.8177E-03	ENSG00000211584
SLC49A4	0.0802	2.2855E-01	4.3311E-01	ENSG00000138463
SLC4A1	0.0097	4.9165E-01		ENSG00000004939
SLC4A10	-0.1176	1.2799E-01	2.9973E-01	ENSG00000144290
SLC4A11	-0.0115	8.8346E-01	9.4147E-01	ENSG00000088836
SLC4A1AP	-0.0588	2.8683E-01	4.9770E-01	ENSG00000163798
SLC4A2	-0.0117	8.8028E-01	9.4001E-01	ENSG00000164889
SLC4A3	0.0961	2.1847E-01	4.2088E-01	ENSG00000114923
SLC4A4	-0.0174	8.1812E-01	9.0544E-01	ENSG00000080493
SLC4A5	0.5401	4.6430E-03	2.8475E-02	ENSG00000188687
SLC4A7	0.1124	1.7913E-01	3.7069E-01	ENSG00000033867
SLC4A8	-0.0215	7.3032E-01	8.5248E-01	ENSG00000050438
SLC4A9	0.0607	3.3713E-01	5.4728E-01	ENSG00000113073
SLC50A1	0.0002	9.9167E-01	9.9535E-01	ENSG00000169241
SLC51A	0.0835	3.2796E-01	5.3839E-01	ENSG00000163959
SLC51B	0.1018	4.7421E-02		ENSG00000186198
SLC52A1	0.0593	2.1657E-01		ENSG00000132517
SLC52A2	-0.0463	4.6386E-01	6.6087E-01	ENSG00000185803
SLC52A3	0.0470	9.2796E-02		ENSG00000101276
SLC5A1	0.0621	1.1676E-01		ENSG00000100170
SLC5A10	-0.0018	7.6284E-01		ENSG00000154025
SLC5A11	-0.0029	8.3629E-01		ENSG00000158865
SLC5A12	0.0432	3.9824E-01	6.0384E-01	ENSG00000148942
SLC5A2	0.0146	7.8840E-01	8.8805E-01	ENSG00000140675

SLC5A3	0.1435	4.7615E-02	1.5446E-01	ENSG00000198743
SLC5A4	0.5309	1.0951E-02	5.4149E-02	ENSG00000100191
SLC5A4-AS1	0.1742	8.3681E-02	2.2594E-01	ENSG00000242082
SLC5A5	0.0181	7.7195E-01	8.7837E-01	ENSG00000105641
SLC5A6	0.0646	4.1746E-01	6.2089E-01	ENSG00000138074
SLC5A7	-0.0148	4.1232E-01		ENSG00000115665
SLC5A8	0.0017	9.6674E-01	9.8340E-01	ENSG00000256870
SLC5A9	0.0161	5.3079E-01		ENSG00000117834
SLC66A1	0.0504	4.4955E-01	6.4863E-01	ENSG00000040487
SLC66A1L	0.0507	2.9373E-01	5.0389E-01	ENSG00000174899
SLC66A2	-0.0616	4.1075E-01	6.1494E-01	ENSG00000122490
SLC66A2P2	-0.0347	5.7352E-01	7.4651E-01	ENSG00000270480
SLC66A3	0.0051	9.5879E-01	9.7939E-01	ENSG00000162976
SLC6A1	-0.1324	7.8938E-02	2.1765E-01	ENSG00000157103
SLC6A1-AS1	-0.0272	4.6058E-01		ENSG00000232287
SLC6A11	-0.3276	1.7400E-03	1.3578E-02	ENSG00000132164
SLC6A12	0.1605	1.1331E-01	2.7669E-01	ENSG00000111181
SLC6A12-AS1	-0.0914	2.4357E-01	4.5142E-01	ENSG00000256577
SLC6A13	0.0414	2.2673E-01		ENSG00000010379
SLC6A15	-0.0856	1.7084E-01	3.5979E-01	ENSG00000072041
SLC6A16	0.2768	3.1856E-02	1.1598E-01	ENSG00000063127
SLC6A17	0.0129	8.7642E-01	9.3831E-01	ENSG00000197106
SLC6A17-AS1	0.0093	6.9026E-01		ENSG00000227091
SLC6A2	0.0063	7.2024E-01		ENSG00000103546
SLC6A20	0.0769	3.2786E-02		ENSG00000163817
SLC6A3	-0.1427	3.7163E-02	1.2966E-01	ENSG00000142319
SLC6A4	0.0074	8.9430E-01	9.4740E-01	ENSG00000108576
SLC6A5	0.0218	4.4398E-01		ENSG00000165970
SLC6A6	0.4989	7.8031E-08	4.6784E-06	ENSG00000131389
SLC6A7	0.0689	3.6174E-01	5.7107E-01	ENSG00000011083
SLC6A8	0.0990	2.1851E-01	4.2088E-01	ENSG00000130821
SLC6A9	0.0724	3.7143E-01	5.8011E-01	ENSG00000196517
SLC7A1	0.0826	3.1017E-01	5.2048E-01	ENSG00000139514
SLC7A11	0.0683	3.5946E-01	5.6889E-01	ENSG00000151012
SLC7A11-AS1	0.0648	4.5895E-02		ENSG00000250033
SLC7A14	-0.1300	1.4325E-01	3.2115E-01	ENSG00000013293
SLC7A14-AS1	0.0045	7.1328E-01		ENSG00000285051
SLC7A2	-0.0769	3.6759E-01	5.7688E-01	ENSG00000003989
SLC7A3	-0.1116	1.7178E-01	3.6095E-01	ENSG00000165349
SLC7A4	-0.0013	9.9651E-01	9.9804E-01	ENSG00000099960
SLC7A5	-0.2757	3.1382E-03	2.1213E-02	ENSG00000103257
SLC7A6	0.0579	4.3988E-01	6.3989E-01	ENSG00000103064
SLC7A6OS	-0.1271	8.1237E-02	2.2165E-01	ENSG00000103061
SLC7A7	0.0804	3.3285E-01	5.4313E-01	ENSG00000155465
SLC7A8	0.1949	5.5519E-02	1.7179E-01	ENSG00000092068
SLC7A9	-0.0603	3.3407E-01	5.4418E-01	ENSG00000021488
SLC8A1	0.2338	7.6512E-03	4.1421E-02	ENSG00000183023
SLC8A1-AS1	0.3954	1.6911E-03	1.3311E-02	ENSG00000227028
SLC8A2	-0.1629	8.4153E-02	2.2669E-01	ENSG00000118160

SLC8A3	0.1025	2.6046E-01	4.6896E-01	ENSG00000100678
SLC8B1	0.0265	7.4714E-01	8.6252E-01	ENSG00000089060
SLC9A1	0.0168	8.2308E-01	9.0841E-01	ENSG00000090020
SLC9A2	-0.0019	9.7890E-01	9.8939E-01	ENSG00000115616
SLC9A3-OT1	-0.0344	2.0142E-01		ENSG00000188242
SLC9A3P2	0.0115	5.1461E-01		ENSG00000238125
SLC9A4	0.0020	5.2350E-01	7.0922E-01	ENSG00000180251
SLC9A5	-0.0644	4.0466E-01	6.0923E-01	ENSG00000135740
SLC9A6	-0.0683	2.7131E-01	4.8052E-01	ENSG00000198689
SLC9A7	-0.1072	1.5675E-01	3.4042E-01	ENSG00000065923
SLC9A7P1	0.0180	4.1937E-01		ENSG00000227825
SLC9A8	0.0942	1.2904E-01	3.0131E-01	ENSG00000197818
SLC9A9	0.0026	9.7803E-01	9.8904E-01	ENSG00000181804
SLC9B1	0.1110	2.1344E-01	4.1439E-01	ENSG00000164037
SLC9B2	-0.2626	4.9058E-03	2.9643E-02	ENSG00000164038
SLC9C1	0.0128	6.6904E-01		ENSG00000172139
SLC9C2	0.0590	2.8795E-01	4.9850E-01	ENSG00000162753
SLCO1A2	0.2441	3.3583E-02	1.2038E-01	ENSG00000084453
SLCO1B1	0.1129	1.5451E-01	3.3735E-01	ENSG00000134538
SLCO1B3	0.0188	6.7173E-01		ENSG00000111700
SLCO1C1	0.0506	4.7408E-01	6.7000E-01	ENSG00000139155
SLCO2A1	0.0187	8.0745E-01	8.9887E-01	ENSG00000174640
SLCO2B1	0.1026	6.2947E-03	3.5778E-02	ENSG00000137491
SLCO3A1	0.2828	2.7602E-04	3.3688E-03	ENSG00000176463
SLCO4A1	-0.0450	5.9483E-01	7.6177E-01	ENSG00000101187
SLCO4A1-AS1	0.0030	7.7038E-01		ENSG00000232803
SLCO4C1	-0.1414	7.9819E-02	2.1921E-01	ENSG00000173930
SLCO5A1	-0.2358	4.7361E-03	2.8881E-02	ENSG00000137571
SLCO5A1-AS1	-0.2502	1.8717E-02	7.9170E-02	ENSG00000246528
SLCO6A1	0.0745	8.9918E-02		ENSG00000205359
SLED1	0.0254	6.1023E-01		ENSG00000249138
SLF1	0.3487	2.6469E-05	5.3778E-04	ENSG00000133302
SLF2	0.0285	6.7962E-01	8.2059E-01	ENSG00000119906
SLFN11	0.1127	1.6321E-01	3.4962E-01	ENSG00000172716
SLFN12	0.1070	1.0457E-01	2.6235E-01	ENSG00000172123
SLFN12L	-0.0129	6.8214E-01		ENSG00000205045
SLFN13	0.0840	2.5361E-01	4.6154E-01	ENSG00000154760
SLFN14	-0.0093	9.3237E-01		ENSG00000236320
SLFN5	0.1493	1.2814E-01	2.9982E-01	ENSG00000166750
SLFNL1	-0.0267	4.2752E-01		ENSG00000171790
SLFNL1-AS1	0.0568	3.2472E-01	5.3479E-01	ENSG00000281207
SLIRP	-0.1087	1.2537E-01	2.9556E-01	ENSG00000119705
SLIT1	-0.1152	2.1185E-01	4.1282E-01	ENSG00000187122
SLIT1-AS1	-0.0128	3.9869E-01		ENSG00000234855
SLIT2	0.4053	3.5644E-06	1.0656E-04	ENSG00000145147
SLIT2-IT1	-0.0113	2.8005E-01		ENSG00000248228
SLIT3	0.0504	5.8402E-01	7.5414E-01	ENSG00000184347
SLITRK1	0.2656	2.1383E-02	8.7064E-02	ENSG00000178235
SLITRK2	-0.2478	9.1592E-03	4.7555E-02	ENSG00000185985

SLITRK3	0.0565	5.1672E-01	7.0434E-01	ENSG00000121871
SLITRK4	0.3463	7.6506E-04	7.4150E-03	ENSG00000179542
SLITRK5	0.3288	4.0691E-05	7.5963E-04	ENSG00000165300
SLK	0.0818	1.0977E-01	2.7076E-01	ENSG00000065613
SLMAP	0.0047	9.4253E-01	9.7152E-01	ENSG00000163681
SLN	-0.0165	6.9455E-01	8.3067E-01	ENSG00000170290
SLPI	0.0569	2.5436E-01		ENSG00000124107
SLTM	0.0377	5.1220E-01	7.0032E-01	ENSG00000137776
SLU7	-0.2167	2.6085E-03	1.8333E-02	ENSG00000164609
SLURP2	-0.0027	8.0107E-01		ENSG00000283992
SLX1A	0.0098	8.9779E-01	9.4954E-01	ENSG00000132207
SLX1A-SULT1A3	0.0056	9.4616E-01	9.7334E-01	ENSG00000213599
SLX1B	0.1033	1.4341E-01	3.2139E-01	ENSG00000181625
SLX1B-SULT1A4	0.0016	9.8624E-01	9.9241E-01	ENSG00000260280
SLX4	0.2059	3.5856E-02	1.2622E-01	ENSG00000188827
SLX4IP	-0.0569	4.3158E-01	6.3305E-01	ENSG00000149346
SLX9	-0.0963	2.7234E-01	4.8163E-01	ENSG00000160256
SMAD1	-0.0022	9.5610E-01	9.7820E-01	ENSG00000170365
SMAD1-AS1	0.0376	3.5491E-01		ENSG00000250902
SMAD1-AS2	-0.0255	1.6017E-01		ENSG00000250582
SMAD2	-0.1783	1.0340E-04	1.5841E-03	ENSG00000175387
SMAD3	0.0130	8.6241E-01	9.3070E-01	ENSG00000166949
SMAD4	-0.0325	5.5217E-01	7.3079E-01	ENSG00000141646
SMAD5	0.1095	6.4527E-02	1.9003E-01	ENSG00000113658
SMAD5-AS1	-0.0891	6.8937E-02		ENSG00000164621
SMAD6	0.0807	2.6471E-01	4.7389E-01	ENSG00000137834
SMAD7	-0.3835	3.8842E-04	4.3720E-03	ENSG00000101665
SMAD9	-0.1931	2.2715E-02	9.1057E-02	ENSG00000120693
SMAGP	-0.5608	8.7274E-06	2.1912E-04	ENSG00000170545
SMANTIS	0.0837	6.3249E-02		ENSG00000288869
SMAP1	0.0375	5.7207E-01	7.4550E-01	ENSG00000112305
SMAP2	0.0403	5.5851E-01	7.3590E-01	ENSG00000084070
SMARCA1	-0.0851	1.3966E-01	3.1605E-01	ENSG00000102038
SMARCA2	-0.1139	4.3720E-02	1.4541E-01	ENSG00000080503
SMARCA4	-0.0680	2.7171E-01	4.8089E-01	ENSG00000127616
SMARCA5	-0.5345	3.7588E-15	2.8026E-12	ENSG00000153147
SMARCA5-AS1	-0.1473	1.3348E-01	3.0720E-01	ENSG00000245112
SMARCAD1	0.0536	3.5221E-01	5.6256E-01	ENSG00000163104
SMARCAL1	-0.0304	6.9959E-01	8.3386E-01	ENSG00000138375
SMARCB1	-0.2879	1.9325E-04	2.5292E-03	ENSG00000099956
SMARCC1	-0.0939	9.1706E-02	2.4104E-01	ENSG00000173473
SMARCC2	-0.0519	3.4275E-01	5.5221E-01	ENSG00000139613
SMARCD1	-0.1136	9.6548E-02	2.4956E-01	ENSG00000066117
SMARCD2	0.0930	2.2917E-01	4.3354E-01	ENSG00000108604
SMARCD3	-0.1187	5.1459E-02	1.6260E-01	ENSG00000082014
SMARCE1	-0.1449	3.9218E-02	1.3496E-01	ENSG00000073584
SMARCE1P1	0.0274	4.8378E-01		ENSG00000225215
SMARCE1P5	-0.0193	2.4625E-01		ENSG00000230793
SMC1A	-0.0783	3.2531E-01	5.3536E-01	ENSG00000072501

SMC1B	0.1148	1.6467E-01	3.5161E-01	ENSG00000077935
SMC2	-0.0087	8.9429E-01	9.4740E-01	ENSG00000136824
SMC2-DT	0.1248	1.2723E-01	2.9865E-01	ENSG00000270332
SMC3	-0.0282	6.0725E-01	7.7108E-01	ENSG00000108055
SMC4	-0.1168	1.8674E-01	3.8108E-01	ENSG00000113810
SMC5	-0.0934	8.9986E-02	2.3784E-01	ENSG00000198887
SMC6	0.2259	1.3418E-03	1.1318E-02	ENSG00000163029
SMCHD1	-0.0729	1.6807E-01	3.5638E-01	ENSG00000101596
SMCO1	-0.0033	5.7573E-01		ENSG00000214097
SMCO2	-0.0339	1.6054E-01		ENSG00000165935
SMCO3	0.0555	3.6411E-01	5.7320E-01	ENSG00000179256
SMCO4	0.4832	1.0477E-06	3.8708E-05	ENSG00000166002
SMCR2	-0.0108	6.2797E-01		ENSG00000223979
SMCR5	-0.0843	2.3991E-01	4.4664E-01	ENSG00000226746
SMCR8	-0.0622	4.6095E-01	6.5837E-01	ENSG00000176994
SMDT1	-0.2355	1.0150E-03	9.1681E-03	ENSG00000183172
SMG1	0.0125	8.6983E-01	9.3441E-01	ENSG00000157106
SMG1P1	-0.1710	2.5617E-02	9.9278E-02	ENSG00000237296
SMG1P3	-0.1747	7.8326E-03	4.2208E-02	ENSG00000180747
SMG1P4	-0.1235	9.1721E-02	2.4106E-01	ENSG00000185710
SMG1P6	0.1617	1.0014E-01	2.5493E-01	ENSG00000254634
SMG1P7	0.0752	3.8865E-01	5.9595E-01	ENSG00000261556
SMG5	-0.0506	4.2258E-01	6.2531E-01	ENSG00000198952
SMG6	-0.0646	3.0508E-01	5.1535E-01	ENSG00000070366
SMG7	-0.0988	1.0979E-01	2.7076E-01	ENSG00000116698
SMG7-AS1	-0.0090	9.0901E-01	9.5505E-01	ENSG00000232860
SMG8	-0.4168	1.8744E-06	6.2920E-05	ENSG00000167447
SMG9	0.0265	7.0806E-01	8.3965E-01	ENSG00000105771
SMIM1	-0.0084	9.5298E-01	9.7681E-01	ENSG00000235169
SMIM10	0.0985	2.0293E-01	4.0190E-01	ENSG00000184785
SMIM10L1	-0.0673	1.9932E-01	3.9765E-01	ENSG00000256537
SMIM10L2A	-0.0149	8.5108E-01	9.2415E-01	ENSG00000178947
SMIM10L2B	-0.0014	9.8348E-01	9.9117E-01	ENSG00000196972
SMIM11	-0.1148	4.5131E-02	1.4870E-01	ENSG00000205670
SMIM12	0.0814	1.6225E-01	3.4826E-01	ENSG00000163866
SMIM13	-0.1966	2.3568E-03	1.7013E-02	ENSG00000224531
SMIM14	-0.0942	1.5249E-01	3.3456E-01	ENSG00000163683
SMIM14-DT	0.0649	4.0743E-01	6.1186E-01	ENSG00000255458
SMIM15	-0.0346	5.4500E-01	7.2555E-01	ENSG00000188725
SMIM17	0.0194	7.9652E-01	8.9254E-01	ENSG00000268182
SMIM18	-0.1383	1.2841E-01	3.0015E-01	ENSG00000253457
SMIM19	0.0516	3.7010E-01	5.7931E-01	ENSG00000176209
SMIM2-AS1	0.0535	4.8790E-01	6.8109E-01	ENSG00000227258
SMIM20	-0.0956	9.8600E-02	2.5248E-01	ENSG00000250317
SMIM22	0.0782	1.4872E-01	3.2923E-01	ENSG00000267795
SMIM24	0.0043	9.4935E-01	9.7491E-01	ENSG00000095932
SMIM26	0.1106	9.6186E-02	2.4899E-01	ENSG00000232388
SMIM27	0.1540	3.9359E-02	1.3537E-01	ENSG00000235453
SMIM28	0.0044	6.4254E-01		ENSG00000262543

SMIM29	-0.0971	1.4054E-01	3.1722E-01	ENSG00000186577
SMIM3	-0.1291	7.0138E-02	2.0106E-01	ENSG00000256235
SMIM30	-0.0403	5.6409E-01	7.4015E-01	ENSG00000214194
SMIM32	0.0992	2.1028E-01	4.1073E-01	ENSG00000271824
SMIM34	0.0813	1.4096E-01		ENSG00000243627
SMIM35	-0.0459	7.5572E-02		ENSG00000255274
SMIM38	0.0033	5.5434E-01		ENSG00000284713
SMIM40	0.0696	1.7779E-01	3.6901E-01	ENSG00000286920
SMIM41	0.0020	9.8108E-01		ENSG00000284791
SMIM42	-0.0086	4.8714E-01		ENSG00000288460
SMIM43	0.0746	1.9782E-01	3.9563E-01	ENSG00000164112
SMIM45	-0.1531	1.1671E-01	2.8187E-01	ENSG00000205704
SMIM47	-0.0323	6.7414E-01	8.1746E-01	ENSG00000261341
SMIM5	-0.0150	7.6666E-01	8.7481E-01	ENSG00000204323
SMIM7	-0.0430	4.3657E-01	6.3728E-01	ENSG00000214046
SMIM8	0.0440	5.3493E-01	7.1822E-01	ENSG00000111850
SMKR1	-0.1002	2.1332E-01	4.1434E-01	ENSG00000240204
SMN1	-0.2652	4.8921E-07	2.1155E-05	ENSG00000172062
SMN1	-0.2721	2.4144E-07	1.1881E-05	ENSG00000205571
SMNDC1	-0.1848	1.4176E-02	6.5329E-02	ENSG00000119953
SMO	-0.0493	5.5265E-01	7.3115E-01	ENSG00000128602
SMOC1	-0.2392	2.1918E-02	8.8726E-02	ENSG00000198732
SMOC2	-0.1410	1.5075E-01	3.3195E-01	ENSG00000112562
SMOX	-0.0994	1.7567E-01	3.6659E-01	ENSG00000088826
SMPD1	0.0889	2.6421E-01	4.7321E-01	ENSG00000166311
SMPD2	-0.0251	7.6413E-01	8.7310E-01	ENSG00000135587
SMPD3	0.0154	8.4903E-01	9.2304E-01	ENSG00000103056
SMPD4	0.0564	3.5635E-01	5.6619E-01	ENSG00000136699
SMPD4BP	0.0652	4.0750E-01	6.1186E-01	ENSG00000152117
SMPD4P1	0.2655	2.4565E-02	9.6355E-02	ENSG00000223553
SMPDL3A	-0.1118	2.1850E-01	4.2088E-01	ENSG00000172594
SMPDL3B	-0.0688	3.6173E-01	5.7107E-01	ENSG00000130768
SMPX	0.0564	2.3378E-01		ENSG00000091482
SMS	-0.3111	4.4395E-05	8.1034E-04	ENSG00000102172
SMTN	-0.2336	1.5737E-02	7.0320E-02	ENSG00000183963
SMTNL1	-0.0633	4.4904E-01	6.4817E-01	ENSG00000214872
SMTNL2	-0.1752	5.0230E-02	1.6011E-01	ENSG00000188176
SMU1	-0.1623	2.5850E-03	1.8199E-02	ENSG00000122692
SMUG1	0.0974	6.7909E-02	1.9648E-01	ENSG00000123415
SMURF1	0.1392	5.8721E-02	1.7802E-01	ENSG00000198742
SMURF2	0.1356	9.1346E-02	2.4038E-01	ENSG00000108854
SMYD1	-0.0086	9.8060E-01		ENSG00000115593
SMYD2	-0.0650	3.4792E-01	5.5811E-01	ENSG00000143499
SMYD3	-0.0022	9.3727E-01	9.6914E-01	ENSG00000185420
SMYD3-AS1	-0.0524	4.0272E-01	6.0721E-01	ENSG00000226876
SMYD4	0.1455	5.4925E-02	1.7046E-01	ENSG00000186532
SMYD5	0.0209	7.4486E-01	8.6115E-01	ENSG00000135632
SNAI1	-0.0908	2.9774E-01	5.0763E-01	ENSG00000124216
SNAI2	-0.0628	4.8091E-01	6.7554E-01	ENSG00000019549

SNAI3	0.0026	9.7447E-01	9.8747E-01	ENSG00000185669
SNAI3-AS1	-0.0136	8.5803E-01	9.2834E-01	ENSG00000260630
SNAP23	-0.2715	8.5271E-03	4.5098E-02	ENSG00000092531
SNAP25	0.0472	5.1228E-01	7.0032E-01	ENSG00000132639
SNAP25-AS1	-0.0135	8.6602E-01	9.3231E-01	ENSG00000227906
SNAP29	-0.0538	2.7347E-01	4.8317E-01	ENSG00000099940
SNAP47	-0.0119	8.7969E-01	9.3989E-01	ENSG00000143740
SNAP91	-0.1141	1.1425E-01	2.7821E-01	ENSG00000065609
SNAPC1	0.1432	1.4371E-01	3.2175E-01	ENSG00000023608
SNAPC2	-0.2058	3.7754E-04	4.2749E-03	ENSG00000104976
SNAPC3	-0.0518	4.5079E-01	6.4991E-01	ENSG00000164975
SNAPC4	0.2841	1.0513E-03	9.4304E-03	ENSG00000165684
SNAPC5	0.0635	3.9875E-01	6.0426E-01	ENSG00000174446
SNAPIN	-0.0511	4.2083E-01	6.2369E-01	ENSG00000143553
SNCA	-0.1339	1.0275E-01	2.5937E-01	ENSG00000145335
SNCA-AS1	0.0392	3.9192E-01		ENSG00000247775
SNCAIP	-0.1961	3.1628E-02	1.1548E-01	ENSG00000064692
SNCB	-0.3812	1.5663E-04	2.1470E-03	ENSG00000074317
SNCG	0.2009	4.4658E-02	1.4771E-01	ENSG00000173267
SND1	0.0667	3.2915E-01	5.3946E-01	ENSG00000197157
SND1-IT1	0.1394	1.3852E-01	3.1439E-01	ENSG00000279078
SNED1	0.3176	1.2096E-02	5.8287E-02	ENSG00000162804
SNF8	0.0650	2.7143E-01	4.8061E-01	ENSG00000159210
SNHG1	0.0198	7.7779E-01	8.8171E-01	ENSG00000255717
SNHG10	0.4864	4.1887E-08	2.7705E-06	ENSG00000247092
SNHG11	0.1922	3.8789E-03	2.4968E-02	ENSG00000174365
SNHG12	0.1405	1.0699E-01	2.6609E-01	ENSG00000197989
SNHG15	-0.0388	5.7545E-01	7.4808E-01	ENSG00000232956
SNHG16	-0.0348	5.4766E-01	7.2733E-01	ENSG00000163597
SNHG17	-0.1444	4.8190E-02	1.5554E-01	ENSG00000196756
SNHG18	-0.3241	9.9398E-03	5.0488E-02	ENSG00000250786
SNHG19	0.0510	5.5071E-01	7.2951E-01	ENSG00000260260
SNHG20	0.1630	4.4732E-02	1.4786E-01	ENSG00000234912
SNHG21	0.0226	7.8332E-01	8.8530E-01	ENSG00000250988
SNHG25	-0.0109	6.1218E-01		ENSG00000266402
SNHG26	0.0189	7.7592E-01	8.8072E-01	ENSG00000228649
SNHG27	0.0107	5.1393E-01		ENSG00000251676
SNHG28	-0.0230	9.8002E-02		ENSG00000188004
SNHG29	-0.1798	9.2354E-03	4.7838E-02	ENSG00000175061
SNHG3	-0.1613	2.6217E-02	1.0092E-01	ENSG00000242125
SNHG31	0.0634	4.4130E-01	6.4093E-01	ENSG00000229267
SNHG32	-0.0770	2.8919E-01	4.9962E-01	ENSG00000204387
SNHG4	0.1930	7.4433E-02	2.0940E-01	ENSG00000281398
SNHG5	0.0802	2.9849E-01	5.0832E-01	ENSG00000203875
SNHG7	0.2578	6.5869E-04	6.6316E-03	ENSG00000233016
SNHG8	0.0927	1.8328E-01	3.7649E-01	ENSG00000269893
SNIP1	-0.0395	5.5512E-01	7.3319E-01	ENSG00000163877
SNN	-0.2375	7.8966E-03	4.2441E-02	ENSG00000184602
SNORA11	0.0301	6.3542E-01	7.9090E-01	ENSG00000221716

SNORA11F	-0.0531	3.7125E-01	5.8001E-01	ENSG00000221164
SNORA12	-0.0188	8.9701E-01	9.4896E-01	ENSG00000212464
SNORA14A	0.0400	8.2836E-02		ENSG00000201643
SNORA14B	-0.0089	8.4618E-01	9.2112E-01	ENSG00000207181
SNORA15B-1	-0.0124	6.9583E-01		ENSG00000207062
SNORA16B	0.0039	7.5952E-01		ENSG00000201544
SNORA19	0.0111	6.5430E-01		ENSG00000207468
SNORA20	-0.0152	8.5187E-01		ENSG00000207392
SNORA22	-0.0424	3.0824E-01		ENSG00000206634
SNORA22B	-0.0126	6.0265E-01		ENSG00000206603
SNORA23	-0.0184	2.2446E-01		ENSG00000201998
SNORA24B	-0.0524	4.3368E-01	6.3487E-01	ENSG00000206903
SNORA28	-0.1409	8.8161E-02	2.3428E-01	ENSG00000272533
SNORA2B	-0.0620	2.4195E-01	4.4937E-01	ENSG00000207313
SNORA2C	-0.0110	5.3645E-01		ENSG00000221491
SNORA31	-0.0039	8.3174E-01		ENSG00000199477
SNORA33	0.0531	3.8027E-01	5.8865E-01	ENSG00000200534
SNORA35	0.0015	8.6248E-01		ENSG00000208839
SNORA37	-0.0150	2.9145E-01		ENSG00000207233
SNORA38	-0.0222	4.9251E-01		ENSG00000200816
SNORA38B	0.0042	8.2040E-01		ENSG00000200394
SNORA3B	-0.0538	4.3906E-01	6.3920E-01	ENSG00000212607
SNORA40B	0.0031	5.9999E-01		ENSG00000208308
SNORA47	-0.0125	2.9241E-01		ENSG00000238961
SNORA53	0.0854	7.0589E-02	2.0172E-01	ENSG00000212443
SNORA54	0.0205	7.2982E-01		ENSG00000207008
SNORA59B	0.0395	4.0471E-01		ENSG00000239149
SNORA5C	0.0350	2.5355E-01		ENSG00000201772
SNORA63C	0.0153	6.5639E-01		ENSG00000201448
SNORA65	-0.0401	2.5219E-01		ENSG00000201302
SNORA66	0.0229	6.8446E-01	8.2406E-01	ENSG00000207523
SNORA68B	-0.0042	8.4242E-01		ENSG00000201388
SNORA70B	-0.0332	4.0069E-01		ENSG00000206937
SNORA70D	-0.0305	1.4727E-01		ENSG00000263666
SNORA70G	0.4623	1.0826E-02	5.3719E-02	ENSG00000206650
SNORA73B	-0.0028	9.5184E-01		ENSG00000200087
SNORA74C-1	0.0006	7.1881E-01		ENSG00000265733
SNORA74C-2	-0.0107	6.9497E-01		ENSG00000288603
SNORA74D	0.0117	8.4994E-01		ENSG00000252213
SNORA77	-0.0109	6.7011E-01	8.1507E-01	ENSG00000221643
SNORA79B	0.0057	7.0831E-01		ENSG00000222489
SNORA7B	-0.0202	7.3666E-01	8.5627E-01	ENSG00000207088
SNORA80A	0.0035	7.7570E-01		ENSG00000200792
SNORA80B	0.2121	6.0067E-02	1.8048E-01	ENSG00000206633
SNORA80C	0.0292	1.2316E-01		ENSG00000199787
SNORA80D	-0.0889	1.1140E-01	2.7329E-01	ENSG00000207217
SNORA80E	-0.1870	3.2189E-02	1.1678E-01	ENSG00000207475
SNORA84	-0.1040	1.9884E-01	3.9705E-01	ENSG00000239183
SNORA9B	-0.0121	8.1370E-01		ENSG00000206897

SNORC	-0.0329	6.8797E-01	8.2673E-01	ENSG00000182600
SNORD100	-0.0024	8.3652E-01		ENSG00000221500
SNORD101	0.0212	2.7895E-01		ENSG00000206754
SNORD104	0.0130	7.1081E-01	8.4121E-01	ENSG00000199753
SNORD11	-0.0066	9.3095E-01	9.6587E-01	ENSG00000238317
SNORD113-3	-0.0015	8.6147E-01		ENSG00000201700
SNORD114-2	-0.0007	3.8737E-01		ENSG00000200823
SNORD114-20	0.0084	5.6046E-01		ENSG00000202048
SNORD114-3	0.0150	3.7293E-01		ENSG00000201839
SNORD117	-0.0805	5.4151E-02		ENSG00000201785
SNORD11B	-0.0138	7.3761E-01		ENSG00000271852
SNORD124	-0.0285	2.5675E-01		ENSG00000238793
SNORD12C	0.1123	2.2503E-01	4.2887E-01	ENSG00000209042
SNORD13	-0.0104	8.5434E-01	9.2588E-01	ENSG00000239039
SNORD13E	0.1458	2.7144E-02	1.0345E-01	ENSG00000238311
SNORD13P1	-0.0011	9.4473E-01		ENSG00000238498
SNORD14A	-0.0133	8.5489E-01	9.2614E-01	ENSG00000272034
SNORD15B	0.0182	6.6275E-01		ENSG00000207445
SNORD17	-0.0308	8.7842E-01	9.3937E-01	ENSG00000212232
SNORD19	-0.0463	5.8558E-01	7.5543E-01	ENSG00000212493
SNORD37	-0.0020	8.3242E-01		ENSG00000206775
SNORD38C	-0.0198	4.8974E-01		ENSG00000207109
SNORD38D	-0.0037	5.2010E-01		ENSG00000207199
SNORD3G	-0.0071	6.8223E-01		ENSG00000199666
SNORD46	-0.0152	8.2826E-01	9.1108E-01	ENSG00000200913
SNORD59A	-0.1189	1.9364E-01	3.8996E-01	ENSG00000207031
SNORD6	-0.0025	7.8211E-01		ENSG00000202314
SNORD62A	0.0050	5.4699E-01		ENSG00000235284
SNORD62B	-0.0430	2.9913E-01	5.0892E-01	ENSG00000231587
SNORD65C	0.0212	6.9634E-01	8.3201E-01	ENSG00000212264
SNORD69	-0.0039	9.4856E-01		ENSG00000212452
SNORD70	-0.0377	3.9589E-01	6.0197E-01	ENSG00000212534
SNORD71	0.0065	6.4921E-01		ENSG00000223224
SNORD72	-0.0074	6.4680E-01		ENSG00000212296
SNORD83A	-0.0076	8.0973E-01		ENSG00000209482
SNORD89	0.0054	7.6849E-01		ENSG00000212283
SNORD94	0.0548	3.7780E-01	5.8634E-01	ENSG00000208772
SNORD96B	0.0091	3.1414E-01		ENSG00000208883
SNORD99	0.0396	3.9291E-01		ENSG00000221539
SNPH	-0.1021	1.7322E-01	3.6319E-01	ENSG00000101298
SNRK	-0.0110	9.2899E-01	9.6500E-01	ENSG00000163788
SNRNP200	0.1167	4.7857E-02	1.5481E-01	ENSG00000144028
SNRNP25	-0.0154	7.1724E-01	8.4462E-01	ENSG00000161981
SNRNP27	-0.0768	2.0751E-01	4.0716E-01	ENSG00000124380
SNRNP35	-0.1194	5.7091E-02	1.7493E-01	ENSG00000184209
SNRNP40	0.0971	6.0811E-02	1.8195E-01	ENSG00000060688
SNRNP48	-0.0668	3.7282E-01	5.8153E-01	ENSG00000168566
SNRNP70	-0.2611	1.4052E-04	1.9769E-03	ENSG00000104852
SNRPA	-0.1051	4.4430E-02	1.4715E-01	ENSG00000077312

SNRPA1	0.0201	6.9432E-01	8.3061E-01	ENSG00000131876
SNRPB	-0.1701	4.6221E-03	2.8375E-02	ENSG00000125835
SNRPB2	-0.0600	3.5210E-01	5.6241E-01	ENSG00000125870
SNRPC	-0.2577	1.0465E-04	1.5940E-03	ENSG00000124562
SNRPCP10	-0.0175	3.3506E-01		ENSG00000243794
SNRPCP11	0.0268	2.0691E-01		ENSG00000241346
SNRPCP14	-0.0118	8.1724E-01		ENSG00000254805
SNRPCP15	-0.0193	2.4093E-01		ENSG00000230106
SNRPCP17	-0.0213	1.5429E-01		ENSG00000254311
SNRPCP6	-0.0105	8.2504E-01		ENSG00000254683
SNRPCP7	-0.0171	9.0508E-01		ENSG00000277952
SNRPD1	-0.0312	5.8923E-01	7.5779E-01	ENSG00000167088
SNRPD2	-0.1964	9.6311E-03	4.9359E-02	ENSG00000125743
SNRPD3	-0.2315	8.9996E-04	8.3750E-03	ENSG00000100028
SNRPE	-0.1626	2.5080E-02	9.7781E-02	ENSG00000182004
SNRPF	-0.0497	4.3193E-01	6.3331E-01	ENSG00000139343
SNRPF-DT	0.0869	9.5742E-02		ENSG00000258343
SNRPG	-0.0669	3.1149E-01	5.2149E-01	ENSG00000143977
SNRPGP14	-0.0095	5.7155E-01		ENSG00000236577
SNRPGP18	-0.0811	1.5274E-01	3.3479E-01	ENSG00000257979
SNRPN	-0.2664	5.0231E-04	5.3158E-03	ENSG00000128739
SNTA1	-0.0395	5.1941E-01	7.0655E-01	ENSG00000101400
SNTB1	0.1974	7.0835E-02	2.0233E-01	ENSG00000172164
SNTB2	0.4111	1.2984E-06	4.6446E-05	ENSG00000168807
SNTG1	0.2891	2.2940E-03	1.6699E-02	ENSG00000147481
SNTG2	-0.1401	1.4500E-01	3.2376E-01	ENSG00000172554
SNTN	0.0123	9.3407E-01	9.6737E-01	ENSG00000188817
SNU13	-0.1493	3.1977E-03	2.1504E-02	ENSG00000100138
SNUPN	-0.0686	2.7953E-01	4.8957E-01	ENSG00000169371
SNURF	0.0935	2.8948E-01	4.9988E-01	ENSG00000273173
SNW1	-0.1729	7.8315E-04	7.5390E-03	ENSG00000100603
SNX1	0.0044	9.5852E-01	9.7936E-01	ENSG00000028528
SNX10	0.0213	7.0616E-01	8.3814E-01	ENSG00000086300
SNX10-AS1	0.3963	6.2495E-05	1.0699E-03	ENSG00000225792
SNX11	0.0387	6.0992E-01	7.7253E-01	ENSG00000002919
SNX12	0.0702	4.0291E-01	6.0738E-01	ENSG00000147164
SNX13	0.2212	5.7176E-04	5.8936E-03	ENSG00000071189
SNX14	0.2201	2.6081E-03	1.8333E-02	ENSG00000135317
SNX15	-0.0330	4.2613E-01		ENSG00000110025
SNX16	-0.0237	7.6286E-01	8.7229E-01	ENSG00000104497
SNX17	0.0503	3.8269E-01	5.9090E-01	ENSG00000115234
SNX18	-0.0789	2.4416E-01	4.5180E-01	ENSG00000178996
SNX19	0.0884	1.0828E-01	2.6824E-01	ENSG00000120451
SNX19P4	0.0068	6.5680E-01		ENSG00000276531
SNX2	-0.1733	1.0143E-02	5.1248E-02	ENSG00000205302
SNX20	0.0004	5.4913E-01		ENSG00000167208
SNX21	-0.2707	2.1042E-03	1.5651E-02	ENSG00000124104
SNX22	-0.3572	3.9731E-04	4.4536E-03	ENSG00000157734
SNX24	-0.1435	2.4159E-02	9.5366E-02	ENSG00000064652

SNX25	0.1566	4.8678E-02	1.5665E-01	ENSG00000109762
SNX25P1	-0.1562	3.2023E-02	1.1628E-01	ENSG00000236809
SNX27	-0.1198	1.1515E-01	2.7963E-01	ENSG00000143376
SNX29	0.0999	1.2689E-01	2.9824E-01	ENSG00000048471
SNX2P1	0.0091	5.6856E-01		ENSG00000270585
SNX3	-0.4100	5.1634E-13	2.1904E-10	ENSG00000112335
SNX30	0.1375	4.7805E-02	1.5473E-01	ENSG00000148158
SNX31	-0.0054	7.7449E-01		ENSG00000174226
SNX32	-0.1881	1.1427E-02	5.5933E-02	ENSG00000172803
SNX33	-0.0122	8.8724E-01	9.4386E-01	ENSG00000173548
SNX4	0.1205	7.6788E-02	2.1373E-01	ENSG00000114520
SNX5	0.0223	7.1479E-01	8.4332E-01	ENSG00000089006
SNX6	-0.1642	3.8234E-04	4.3153E-03	ENSG00000129515
SNX7	0.2167	1.9155E-02	8.0537E-02	ENSG00000162627
SNX8	0.2025	5.5392E-03	3.2525E-02	ENSG00000106266
SNX9	0.0393	5.3049E-01	7.1490E-01	ENSG00000130340
SOAT1	-0.5979	1.2883E-11	3.1698E-09	ENSG00000057252
SOAT2	0.0223	4.1355E-01		ENSG00000167780
SOBP	0.0019	9.9115E-01	9.9515E-01	ENSG00000112320
SOCS1	0.1781	4.8747E-02	1.5675E-01	ENSG00000185338
SOCS2	-0.2562	4.6485E-04	5.0209E-03	ENSG00000120833
SOCS2-AS1	-0.0390	6.2001E-01	7.8028E-01	ENSG00000246985
SOCS3	-0.1387	1.4769E-01	3.2785E-01	ENSG00000184557
SOCS3-DT	0.1038	1.2925E-01	3.0162E-01	ENSG00000266970
SOCS4	0.1657	3.6237E-03	2.3686E-02	ENSG00000180008
SOCS5	-0.2210	2.5061E-03	1.7800E-02	ENSG00000171150
SOCS5P2	-0.0253	5.2509E-01		ENSG00000224682
SOCS5P4	0.0040	6.3012E-01		ENSG00000227536
SOCS6	-0.0254	6.9748E-01	8.3284E-01	ENSG00000170677
SOCS7	-0.0031	9.6549E-01	9.8291E-01	ENSG00000274211
SOD1	-0.3999	4.4433E-09	4.0794E-07	ENSG00000142168
SOD2-OT1	-0.0474	5.4714E-01	7.2700E-01	ENSG00000285427
SOD3	0.1265	1.0169E-01	2.5762E-01	ENSG00000109610
SOHLH1	0.0203	4.4520E-01		ENSG00000165643
SOHLH2	-0.3387	5.7659E-03	3.3526E-02	ENSG00000120669
SON	0.0118	8.0617E-01	8.9800E-01	ENSG00000159140
SORBS1	-0.1189	6.3679E-02	1.8818E-01	ENSG00000095637
SORBS2	0.1698	2.2695E-02	9.0992E-02	ENSG00000154556
SORBS3	-0.0491	4.1242E-01	6.1608E-01	ENSG00000120896
SORCS1	-0.0233	7.4134E-01	8.5899E-01	ENSG00000108018
SORCS2	0.1032	2.4932E-01	4.5709E-01	ENSG00000184985
SORCS3	-0.0588	4.7755E-01	6.7289E-01	ENSG00000156395
SORCS3-AS1	0.0080	5.1982E-01		ENSG00000226387
SORD	0.0104	8.8005E-01	9.3996E-01	ENSG00000140263
SORL1	0.2755	3.9602E-04	4.4412E-03	ENSG00000137642
SORT1	-0.0054	9.0261E-01	9.5235E-01	ENSG00000134243
SOS1	-0.0264	6.3248E-01	7.8893E-01	ENSG00000115904
SOS2	-0.0116	8.6265E-01	9.3079E-01	ENSG00000100485
SOST	0.0641	8.2747E-02	2.2432E-01	ENSG00000167941

SOSTDC1	-0.3442	6.2526E-03	3.5588E-02	ENSG00000171243
SOWAHB	-0.1476	1.1341E-01	2.7679E-01	ENSG00000186212
SOWAHC	-0.1654	1.4285E-02	6.5672E-02	ENSG00000198142
SOWAHD	-0.1959	6.8870E-02	1.9842E-01	ENSG00000187808
SOX1	0.0715	4.1857E-01	6.2173E-01	ENSG00000182968
SOX1-OT	0.0419	6.2345E-01	7.8246E-01	ENSG00000224243
SOX10	0.0248	1.3210E-01		ENSG00000100146
SOX11	0.0672	4.0023E-01	6.0563E-01	ENSG00000176887
SOX12	-0.2963	5.9094E-05	1.0196E-03	ENSG00000177732
SOX13	0.0349	6.8208E-01	8.2263E-01	ENSG00000143842
SOX14	-0.0253	5.5531E-01	7.3334E-01	ENSG00000168875
SOX15	0.0698	4.0118E-01	6.0633E-01	ENSG00000129194
SOX17	0.0038	8.9697E-01		ENSG00000164736
SOX18	-0.2331	1.4790E-02	6.7389E-02	ENSG00000203883
SOX2	-0.4063	1.1327E-05	2.7033E-04	ENSG00000181449
SOX2-OT	0.0220	7.8190E-01	8.8446E-01	ENSG00000242808
SOX21	0.0970	2.7414E-01	4.8388E-01	ENSG00000125285
SOX21-AS1	0.2018	3.5047E-02	1.2415E-01	ENSG00000227640
SOX3	-0.2849	5.9169E-03	3.4172E-02	ENSG00000134595
SOX30	0.0484	3.7867E-01	5.8713E-01	ENSG00000039600
SOX5	-0.0449	5.0545E-01	6.9527E-01	ENSG00000134532
SOX5-AS1	-0.0976	1.2363E-01	2.9330E-01	ENSG00000256120
SOX6	0.1406	8.0304E-02	2.2020E-01	ENSG00000110693
SOX7	0.0682	2.6516E-01	4.7431E-01	ENSG00000171056
SOX8	-0.2663	3.8794E-03	2.4968E-02	ENSG00000005513
SOX9	-0.2362	1.7602E-02	7.5901E-02	ENSG00000125398
SOX9-AS1	-0.0853	3.3224E-01	5.4247E-01	ENSG00000234899
SP1	0.0439	5.8796E-01	7.5699E-01	ENSG00000185591
SP100	0.0876	2.6486E-01	4.7403E-01	ENSG00000067066
SP110	-0.0816	3.5824E-01	5.6777E-01	ENSG00000135899
SP140	0.1239	1.0391E-01	2.6123E-01	ENSG00000079263
SP140L	3.1217	3.4854E-05		ENSG00000185404
SP2	0.1772	2.7218E-02	1.0367E-01	ENSG00000167182
SP2-AS1	-0.1443	1.4082E-01	3.1753E-01	ENSG00000234494
SP2-DT	0.1281	1.3475E-01	3.0923E-01	ENSG00000264920
SP3	-0.2043	1.8099E-03	1.3982E-02	ENSG00000172845
SP4	0.0555	3.9433E-01	6.0063E-01	ENSG00000105866
SP5	0.5880	6.3997E-03	3.6264E-02	ENSG00000204335
SP7	0.0266	5.6181E-01		ENSG00000170374
SP8	-0.0383	6.0941E-01	7.7221E-01	ENSG00000164651
SP9	-0.2278	4.7852E-02	1.5481E-01	ENSG00000217236
SPA17	-0.0617	3.9792E-01	6.0359E-01	ENSG00000064199
SPAAR	-0.0218	5.8752E-01		ENSG00000235387
SPACA3	-0.0158	6.3497E-01		ENSG00000141316
SPACA4	0.0162	2.5634E-01		ENSG00000177202
SPACA5	0.0289	5.6172E-01		ENSG00000171489
SPACA5B	0.0522	1.2124E-01		ENSG00000171478
SPACA6	0.0984	2.7616E-01	4.8590E-01	ENSG00000182310
SPACA6-AS1	0.0161	7.9180E-01	8.9001E-01	ENSG00000269959

SPACA7	0.0186	5.1275E-01		ENSG00000153498
SPACA9	-0.1083	1.0688E-01	2.6602E-01	ENSG00000165698
SPACDR	-0.0779	2.2867E-01	4.3323E-01	ENSG00000185955
SPAG1	0.1642	9.1876E-02	2.4133E-01	ENSG00000104450
SPAG11A	-0.0027	7.3054E-01	8.5266E-01	ENSG00000178287
SPAG11B	0.0216	8.3051E-01	9.1239E-01	ENSG00000164871
SPAG16	0.0375	5.3504E-01	7.1832E-01	ENSG00000144451
SPAG4	0.4786	1.5682E-04	2.1479E-03	ENSG00000061656
SPAG5	-0.0316	6.8297E-01	8.2313E-01	ENSG00000076382
SPAG6	0.1379	1.3628E-01	3.1140E-01	ENSG00000077327
SPAG7	-0.1972	2.2394E-04	2.8402E-03	ENSG00000091640
SPAG8	0.3763	1.1271E-02	5.5354E-02	ENSG00000137098
SPAG9	-0.0106	8.5232E-01	9.2466E-01	ENSG00000008294
SPANXA2-OT1	-0.0421	6.2436E-01	7.8313E-01	ENSG00000277215
SPANXN3	0.0124	4.7858E-01		ENSG00000189252
SPARC	0.0216	7.9240E-01	8.9031E-01	ENSG00000113140
SPARCL1	-0.0044	9.4425E-01	9.7218E-01	ENSG00000152583
SPART	-0.1488	2.7918E-02	1.0560E-01	ENSG00000133104
SPART-AS1	0.0950	1.5161E-01	3.3322E-01	ENSG00000120664
SPAST	-0.1348	3.6601E-02	1.2807E-01	ENSG00000021574
SPATA1	0.1805	5.9515E-02	1.7934E-01	ENSG00000122432
SPATA12	0.1852	5.0497E-02	1.6063E-01	ENSG00000186451
SPATA13	0.0227	3.0964E-01		ENSG00000228741
SPATA13	-0.1509	6.1076E-02	1.8255E-01	ENSG00000182957
SPATA13-AS1	-0.0227	2.9977E-01		ENSG00000227213
SPATA17	0.3846	3.1742E-05	6.2034E-04	ENSG00000162814
SPATA18	-0.0237	7.5774E-01	8.6888E-01	ENSG00000163071
SPATA19	0.0011	9.7116E-01		ENSG00000166118
SPATA2	-0.1781	2.0871E-02	8.5548E-02	ENSG00000158480
SPATA20	0.1462	4.0801E-02	1.3869E-01	ENSG00000006282
SPATA21	-0.0514	5.0385E-01	6.9420E-01	ENSG00000187144
SPATA22	0.0139	5.8756E-01		ENSG00000141255
SPATA24	0.0332	6.6605E-01	8.1258E-01	ENSG00000170469
SPATA25	0.0583	2.3586E-01		ENSG00000149634
SPATA2L	0.0336	6.8196E-01	8.2253E-01	ENSG00000158792
SPATA3	0.0028	9.5168E-01		ENSG00000173699
SPATA3-AS1	-0.0023	9.4834E-01		ENSG00000238062
SPATA31C1	0.0028	2.3349E-01		ENSG00000230246
SPATA31G1	-0.0946	3.8970E-02		ENSG00000174038
SPATA31H1	0.1619	1.0982E-01	2.7080E-01	ENSG00000221843
SPATA32	0.0171	7.2830E-01		ENSG00000184361
SPATA33	0.2263	4.3786E-03	2.7254E-02	ENSG00000167523
SPATA4	0.1519	1.2072E-01	2.8861E-01	ENSG00000150628
SPATA41	-0.0160	4.7236E-01		ENSG00000189419
SPATA42	-0.0148	5.1890E-01		ENSG00000203897
SPATA45	-0.0211	5.2568E-01		ENSG00000185523
SPATA46	-0.0230	4.4936E-01		ENSG00000171722
SPATA6	0.1698	7.0587E-02	2.0172E-01	ENSG00000132122
SPATA6L	0.0602	4.6902E-01	6.6556E-01	ENSG00000106686

SPATA7	0.3490	8.2767E-06	2.1082E-04	ENSG00000042317
SPATA8	0.0223	9.5834E-01	9.7936E-01	ENSG00000185594
SPATA9	0.0442	4.6689E-01	6.6358E-01	ENSG00000145757
SPATC1	-0.0261	2.1412E-01		ENSG00000186583
SPATC1L	0.1119	4.9796E-02	1.5920E-01	ENSG00000160284
SPATS1	0.0121	9.4217E-01		ENSG00000249481
SPATS2	-0.1792	5.9219E-03	3.4184E-02	ENSG00000123352
SPATS2L	0.4620	6.0270E-06	1.6296E-04	ENSG00000196141
SPC24	-0.1912	7.2448E-02	2.0568E-01	ENSG00000161888
SPC25	-0.4105	9.1175E-04	8.4559E-03	ENSG00000152253
SPCS1	0.0139	8.1728E-01	9.0494E-01	ENSG00000114902
SPCS2	-0.2188	6.2529E-05	1.0699E-03	ENSG00000118363
SPCS2P1	-0.0054	7.8191E-01		ENSG00000260928
SPCS3	-0.1887	1.5229E-03	1.2375E-02	ENSG00000129128
SPDEF	0.0115	8.7281E-01	9.3615E-01	ENSG00000124664
SPDL1	0.1751	1.2897E-02	6.0968E-02	ENSG00000040275
SPDYA	0.2144	5.2119E-02	1.6395E-01	ENSG00000163806
SPDYE1	-0.1127	1.8947E-01	3.8459E-01	ENSG00000136206
SPDYE10	0.0887	2.6885E-01	4.7820E-01	ENSG00000274570
SPDYE11	0.0668	4.1689E-01	6.2032E-01	ENSG00000275976
SPDYE12	-0.0132	8.6181E-01	9.3057E-01	ENSG00000184616
SPDYE13	0.0227	7.8643E-01	8.8723E-01	ENSG00000286038
SPDYE14	-0.0425	6.0671E-01	7.7068E-01	ENSG00000286137
SPDYE15	0.0089	9.1920E-01	9.6009E-01	ENSG00000286014
SPDYE16	-0.0524	5.0891E-01	6.9791E-01	ENSG00000185040
SPDYE17	0.1664	1.0135E-01	2.5705E-01	ENSG00000186645
SPDYE18	0.0448	5.8844E-01	7.5725E-01	ENSG00000205482
SPDYE2	-0.2172	7.8365E-03	4.2220E-02	ENSG00000205238
SPDYE21	0.0432	6.0989E-01	7.7252E-01	ENSG00000230358
SPDYE2B	-0.2795	1.3417E-03	1.1318E-02	ENSG00000173678
SPDYE3	-0.0931	2.9181E-01	5.0208E-01	ENSG00000214300
SPDYE5	-0.0447	6.0408E-01	7.6833E-01	ENSG00000170092
SPDYE6	-0.2624	1.9760E-03	1.4937E-02	ENSG00000260097
SPDYE8	0.0448	5.8226E-01	7.5320E-01	ENSG00000273520
SPDYE9	0.0354	6.5216E-01	8.0300E-01	ENSG00000262461
SPECC1	-0.1283	9.4588E-02	2.4610E-01	ENSG00000128487
SPECC1L	0.1070	2.2740E-01	4.3176E-01	ENSG00000100014
SPECC1L-ADORA2A	-0.0020	9.6173E-01		ENSG00000258555
SPECC1P2	0.0046	9.6879E-01		ENSG00000244456
SPEF1	0.1870	7.8685E-02	2.1719E-01	ENSG00000101222
SPEG	-0.1547	6.4896E-02	1.9061E-01	ENSG00000072195
SPEM2	0.0209	2.1621E-01		ENSG00000184560
SPEM3	-0.0002	6.1832E-01		ENSG00000283439
SPEN	-0.0789	2.2872E-01	4.3327E-01	ENSG00000065526
SPEN-AS1	0.0829	2.1317E-01	4.1423E-01	ENSG00000179743
SPESP1	0.0362	5.4629E-01	7.2645E-01	ENSG00000258484
SPG11	-0.0233	7.0057E-01	8.3451E-01	ENSG00000104133
SPG21	-0.2314	1.6837E-04	2.2688E-03	ENSG00000090487
SPG7	0.1793	2.5067E-03	1.7800E-02	ENSG00000197912

SPHK1	0.2454	3.5992E-02	1.2659E-01	ENSG00000176170
SPHK2	-0.0267	7.0866E-01	8.3974E-01	ENSG00000063176
SPHKAP	0.0876	3.3832E-01	5.4820E-01	ENSG00000153820
SPI1	0.0600	1.9747E-01		ENSG00000066336
SPIB	-0.0942	6.5741E-02		ENSG00000269404
SPICE1	0.4805	1.3781E-03	1.1514E-02	ENSG00000163611
SPICP4	0.0105	4.4256E-01		ENSG00000250020
SPIDR	-0.2434	5.4543E-03	3.2144E-02	ENSG00000164808
SPIN1	-0.1421	2.1596E-02	8.7691E-02	ENSG00000106723
SPIN2A	0.1744	2.7760E-02	1.0512E-01	ENSG00000147059
SPIN2B	0.0135	8.3413E-01	9.1481E-01	ENSG00000186787
SPIN2P1	0.0180	1.0583E-01		ENSG00000237708
SPIN3	0.2685	2.5402E-03	1.7986E-02	ENSG00000204271
SPIN4	-0.1979	1.7445E-02	7.5451E-02	ENSG00000186767
SPINDOC	-0.2985	6.7874E-03	3.7973E-02	ENSG00000168005
SPINK1	-0.0059	7.1398E-01		ENSG00000164266
SPINK13	0.0095	5.4566E-01		ENSG00000214510
SPINK2	0.0469	5.3177E-01	7.1589E-01	ENSG00000128040
SPINK4	-0.0215	5.8838E-01		ENSG00000122711
SPINK5	0.2589	2.3988E-02	9.4829E-02	ENSG00000133710
SPINK6	-0.0329	2.1153E-01		ENSG00000178172
SPINK7	0.0084	9.2399E-01		ENSG00000145879
SPINK8	-0.0067	5.8211E-01		ENSG00000229453
SPINK9	0.0054	9.1991E-01		ENSG00000204909
SPINT1	-0.1761	4.3454E-02	1.4486E-01	ENSG00000166145
SPINT1-AS1	0.0070	9.1633E-01	9.5863E-01	ENSG00000261183
SPINT2	0.0504	4.8223E-01	6.7666E-01	ENSG00000167642
SPIRE1	-0.2228	7.6120E-04	7.3912E-03	ENSG00000134278
SPIRE2	0.0160	8.2161E-01	9.0762E-01	ENSG00000204991
SPMAP1	0.0045	8.8119E-01		ENSG00000275489
SPMAP2	-0.0033	6.8677E-01		ENSG00000105549
SPMAP2L	0.0206	7.5855E-01	8.6956E-01	ENSG00000249693
SPMIP1	-0.0393	6.4120E-01	7.9471E-01	ENSG00000272899
SPMIP10	0.0159	6.3080E-01		ENSG00000196900
SPMIP2	0.4827	3.7880E-03	2.4502E-02	ENSG00000164123
SPMIP3	-0.0239	1.7928E-01		ENSG00000173728
SPMIP3P1	-0.0173	3.3299E-01		ENSG00000276925
SPMIP4	-0.0701	4.4063E-01	6.4050E-01	ENSG00000153790
SPMIP5	-0.2172	1.8208E-02	7.7638E-02	ENSG00000165863
SPMIP6	0.1665	4.8826E-02	1.5688E-01	ENSG00000164972
SPMIP7	-0.0088	3.9517E-01		ENSG00000164500
SPMIP8	0.0993	2.7115E-01	4.8044E-01	ENSG00000159648
SPMIP9	0.0061	7.2952E-01		ENSG00000172073
SPN	0.1108	1.8853E-01	3.8349E-01	ENSG00000197471
SPNS1	0.0236	7.8549E-01	8.8660E-01	ENSG00000169682
SPNS2	-0.0343	6.5720E-01	8.0654E-01	ENSG00000183018
SPOCD1	0.1798	4.1305E-02	1.3995E-01	ENSG00000134668
SPOCK2	0.0082	9.3491E-01	9.6796E-01	ENSG00000107742
SPOCK3	-0.0144	8.6229E-01	9.3070E-01	ENSG00000196104

SPON1	-0.1468	6.0150E-02	1.8064E-01	ENSG00000262655
SPON2	-0.1006	2.6609E-01	4.7533E-01	ENSG00000159674
SPOP	-0.0235	6.2857E-01	7.8609E-01	ENSG00000121067
SPOPL	-0.0071	9.2946E-01	9.6530E-01	ENSG00000144228
SPOUT1	0.0055	9.2690E-01	9.6421E-01	ENSG00000198917
SPP1	0.0939	2.6662E-01	4.7592E-01	ENSG00000118785
SPPL2A	-0.0494	4.4111E-01	6.4089E-01	ENSG00000138600
SPPL2B	0.0868	2.1452E-01	4.1568E-01	ENSG00000005206
SPPL2C	0.0049	5.9065E-01		ENSG00000185294
SPPL3	-0.0016	9.4382E-01	9.7218E-01	ENSG00000157837
SPRED1	0.0788	2.7805E-01	4.8808E-01	ENSG00000166068
SPRED2	-0.1142	1.2455E-01	2.9433E-01	ENSG00000198369
SPRED3	0.0649	4.1010E-01	6.1426E-01	ENSG00000188766
SPRING1	-0.1349	1.5965E-02	7.1035E-02	ENSG00000111412
SPRR1A	0.0014	7.7694E-01		ENSG00000169474
SPRR2F	0.0077	7.0953E-01		ENSG00000244094
SPRTN	0.0343	5.8616E-01	7.5569E-01	ENSG00000010072
SPRY1	0.5431	1.6433E-04	2.2300E-03	ENSG00000164056
SPRY2	-0.3382	1.4003E-03	1.1656E-02	ENSG00000136158
SPRY3	0.1061	2.4784E-01	4.5562E-01	ENSG00000168939
SPRY4	-0.1245	1.5324E-01	3.3544E-01	ENSG00000187678
SPRY4-AS1	0.3901	1.8486E-03	1.4211E-02	ENSG00000231185
SPRYD3	0.1594	7.5748E-02	2.1196E-01	ENSG00000167778
SPRYD4	-0.0754	2.8998E-01	5.0035E-01	ENSG00000176422
SPRYD7	0.0278	7.1094E-01	8.4128E-01	ENSG00000123178
SPSB1	0.1556	8.0742E-02	2.2088E-01	ENSG00000171621
SPSB2	0.0015	9.7822E-01	9.8916E-01	ENSG00000111671
SPSB3	0.0316	5.7440E-01	7.4719E-01	ENSG00000162032
SPSB4	-0.0038	9.6154E-01	9.8079E-01	ENSG00000175093
SPTAN1	0.1005	8.6899E-02	2.3180E-01	ENSG00000197694
SPTB	0.1119	2.2348E-01	4.2729E-01	ENSG00000070182
SPTBN1	0.0939	2.0763E-01	4.0732E-01	ENSG00000115306
SPTBN2	0.0796	3.3728E-01	5.4734E-01	ENSG00000173898
SPTBN4	-0.0778	2.7813E-01	4.8808E-01	ENSG00000160460
SPTBN5	0.5013	1.0210E-02	5.1450E-02	ENSG00000137877
SPTLC1	-0.0506	3.6933E-01	5.7860E-01	ENSG00000090054
SPTLC1P1	0.0028	6.9285E-01		ENSG00000230397
SPTLC2	0.0525	4.3835E-01	6.3860E-01	ENSG00000100596
SPTLC3	0.1063	1.7046E-01	3.5931E-01	ENSG00000172296
SPTSSA	-0.0798	1.7983E-01	3.7154E-01	ENSG00000165389
SPTSSB	0.0762	2.9188E-01	5.0208E-01	ENSG00000196542
SPTY2D1	-0.0777	3.4820E-01	5.5833E-01	ENSG00000179119
SPX	0.1521	8.0301E-02	2.2020E-01	ENSG00000134548
SQLE	-0.1811	4.3242E-02	1.4436E-01	ENSG00000104549
SQLE-DT	-0.0270	5.4710E-01		ENSG00000253513
SQOR	0.0059	9.4173E-01	9.7117E-01	ENSG00000137767
SQSTM1	-0.1163	1.9532E-01	3.9222E-01	ENSG00000161011
SRA1	0.1751	2.3194E-02	9.2526E-02	ENSG00000213523
SRARP	0.0383	6.8643E-03		ENSG00000183888

SRBD1	0.0254	7.0841E-01	8.3966E-01	ENSG00000068784
SRC	0.0094	9.0030E-01	9.5109E-01	ENSG00000197122
SRCAP	0.1373	1.3583E-01	3.1066E-01	ENSG00000080603
SRCIN1	0.0931	2.5906E-01	4.6768E-01	ENSG00000277363
SRD5A1	-0.2444	6.0450E-03	3.4696E-02	ENSG00000145545
SRD5A3	0.0469	5.7175E-01	7.4525E-01	ENSG00000128039
SRD5A3-AS1	0.0539	4.3763E-01	6.3812E-01	ENSG00000249700
SREBF1	-0.1959	6.9720E-02	2.0027E-01	ENSG00000072310
SREBF2	-0.3632	6.1126E-07	2.5067E-05	ENSG00000198911
SREK1	-0.0215	7.1088E-01	8.4125E-01	ENSG00000153914
SREK1IP1	0.0982	1.7287E-01	3.6270E-01	ENSG00000153006
SRF	-0.1004	2.2715E-01	4.3138E-01	ENSG00000112658
SRFBP1	-0.0137	8.2937E-01	9.1170E-01	ENSG00000151304
SRGAP1	-0.0909	1.3838E-01	3.1433E-01	ENSG00000196935
SRGAP2	-0.3922	1.8093E-07	9.2745E-06	ENSG00000266028
SRGAP2-AS1	0.0946	2.3290E-01	4.3806E-01	ENSG00000230806
SRGAP2B	-0.3819	3.0869E-05	6.0617E-04	ENSG00000196369
SRGAP2C	-0.3336	1.8038E-05	3.9381E-04	ENSG00000171943
SRGAP3	-0.0819	2.2100E-01	4.2417E-01	ENSG00000196220
SRGAP3-AS2	-0.0510	2.8563E-01	4.9634E-01	ENSG00000228723
SRGAP3-AS3	-0.4988	9.1179E-03	4.7400E-02	ENSG00000227929
SRGAP3-AS4	0.0950	2.0977E-01	4.0999E-01	ENSG00000235830
SRGN	0.0647	1.1700E-01		ENSG00000122862
SRI	-0.1316	2.3623E-02	9.3823E-02	ENSG00000075142
SRI-AS1	-0.0160	7.7317E-01	8.7905E-01	ENSG00000254003
SRL	0.1498	1.3078E-02		ENSG00000185739
SRM	-0.1224	7.4054E-02	2.0870E-01	ENSG00000116649
SRMS	0.0589	4.7017E-01	6.6646E-01	ENSG00000125508
SRP14	-0.4252	1.1604E-07	6.5187E-06	ENSG00000140319
SRP14-DT	-0.0365	6.1060E-01	7.7307E-01	ENSG00000248508
SRP14P2	0.0713	4.6185E-01	6.5900E-01	ENSG00000234106
SRP14P3	-0.0998	1.1392E-01	2.7774E-01	ENSG00000235183
SRP14P4	0.0033	8.6197E-01		ENSG00000268062
SRP19	-0.1873	2.1387E-03	1.5812E-02	ENSG00000153037
SRP54	0.2177	5.0487E-03	3.0328E-02	ENSG00000100883
SRP68	0.0635	2.9283E-01	5.0287E-01	ENSG00000167881
SRP72	-0.0978	3.3110E-02	1.1926E-01	ENSG00000174780
SRP72P2	-0.2510	3.8156E-02	1.3232E-01	ENSG00000188451
SRP9	-0.2262	1.2892E-04	1.8681E-03	ENSG00000143742
SRPK1	-0.1175	5.6711E-02	1.7416E-01	ENSG00000096063
SRPK2	-0.0205	6.9919E-01	8.3370E-01	ENSG00000135250
SRPK2P	0.0035	9.6642E-01		ENSG00000253431
SRPK3	0.0633	2.8529E-01	4.9604E-01	ENSG00000184343
SRPRA	0.2572	4.7036E-03	2.8753E-02	ENSG00000182934
SRPRB	-0.0593	3.8407E-01	5.9210E-01	ENSG00000144867
SRPX	-0.1713	9.1462E-02	2.4061E-01	ENSG00000101955
SRPX2	0.2283	4.2362E-02	1.4228E-01	ENSG00000102359
SRR	-0.1349	1.5811E-02	7.0568E-02	ENSG00000167720
SRRD	0.1355	3.3708E-02	1.2066E-01	ENSG00000100104

SRRM1	-0.0548	3.0887E-01	5.1914E-01	ENSG00000133226
SRRM2	-0.1001	1.4144E-01	3.1833E-01	ENSG00000167978
SRRM2-AS1	0.2694	1.5567E-02	6.9832E-02	ENSG00000205913
SRRM3	0.1125	8.1799E-02	2.2256E-01	ENSG00000177679
SRRM4	-0.0396	5.7133E-01	7.4485E-01	ENSG00000139767
SRRM5	-0.1385	1.4249E-01	3.1997E-01	ENSG00000226763
SRRT	-0.0770	3.2089E-01	5.3094E-01	ENSG00000087087
SRSF1	-0.2885	7.0118E-04	6.9623E-03	ENSG00000136450
SRSF10	-0.1146	7.6778E-02	2.1373E-01	ENSG00000188529
SRSF11	0.0522	3.8631E-01	5.9403E-01	ENSG00000116754
SRSF12	-0.0545	3.7199E-01	5.8073E-01	ENSG00000154548
SRSF2	-0.1641	1.9295E-02	8.0904E-02	ENSG00000161547
SRSF3	-0.4353	2.9536E-06	9.1643E-05	ENSG00000112081
SRSF4	-0.1009	6.1761E-02	1.8411E-01	ENSG00000116350
SRSF5	-0.3033	5.3823E-08	3.4220E-06	ENSG00000100650
SRSF6	-0.0503	4.8925E-01	6.8239E-01	ENSG00000124193
SRSF7	-0.1750	2.5996E-02	1.0030E-01	ENSG00000115875
SRSF8	-0.1771	6.4122E-03	3.6303E-02	ENSG00000263465
SRSF9	-0.2530	3.4363E-06	1.0380E-04	ENSG00000111786
SRXN1	-0.0153	3.7891E-01	5.8747E-01	ENSG00000271303
SS18	-0.1252	8.8969E-02	2.3571E-01	ENSG00000141380
SS18L1	0.1068	1.6998E-01	3.5860E-01	ENSG00000184402
SS18L2	-0.3000	4.9604E-04	5.2801E-03	ENSG00000008324
SSB	-0.0457	3.9344E-01	5.9997E-01	ENSG00000138385
SSBL5P	0.0048	8.1665E-01		ENSG00000236048
SSBP1	-0.1135	4.6239E-02	1.5143E-01	ENSG00000106028
SSBP2	-0.3119	1.9947E-07	1.0098E-05	ENSG00000145687
SSBP3	0.0781	3.1778E-01	5.2800E-01	ENSG00000157216
SSBP3-AS1	0.5573	1.9695E-03	1.4897E-02	ENSG00000198711
SSBP4	-0.0962	1.5086E-01	3.3211E-01	ENSG00000130511
SSC4D	0.1026	2.5125E-01	4.5951E-01	ENSG00000146700
SSC5D	-0.0316	6.7368E-01	8.1742E-01	ENSG00000179954
SSH1	-0.0726	2.6352E-01	4.7243E-01	ENSG00000084112
SSH2	0.0382	5.6970E-01	7.4363E-01	ENSG00000141298
SSH3	-0.0458	5.9806E-01	7.6415E-01	ENSG00000172830
SSMEM1	0.0166	7.2991E-01		ENSG00000165120
SSNA1	-0.0993	1.2678E-01	2.9808E-01	ENSG00000176101
SSPN	0.0030	9.6994E-01	9.8504E-01	ENSG00000123096
SSPOP	-0.0669	4.4053E-01	6.4044E-01	ENSG00000197558
SSR1	-0.1077	2.2423E-02	9.0209E-02	ENSG00000124783
SSR2	-0.0021	9.5683E-01	9.7867E-01	ENSG00000163479
SSR3	-0.0958	1.3580E-01	3.1061E-01	ENSG00000114850
SSR4	0.1399	1.2026E-02	5.8100E-02	ENSG00000180879
SSR4P1	0.0789	2.3673E-01	4.4248E-01	ENSG00000235374
SSRP1	0.0077	9.3804E-01	9.6932E-01	ENSG00000149136
SST	0.0027	9.7179E-01	9.8589E-01	ENSG00000157005
SSTR1	0.4938	5.6364E-03	3.2926E-02	ENSG00000139874
SSTR2	-0.4147	3.7315E-05	7.1119E-04	ENSG00000180616
SSTR3	0.0136	8.2321E-01		ENSG00000278195

SSTR5	-0.1800	5.9899E-02	1.8011E-01	ENSG00000162009
SSTR5-AS1	0.0006	9.8800E-01		ENSG00000261713
SSU72	-0.0802	8.3905E-02	2.2637E-01	ENSG00000160075
SSU72L1	0.0046	6.4850E-01		ENSG00000284438
SSU72L2	0.0034	7.1321E-01		ENSG00000284306
SSU72L4	0.0044	6.9431E-01		ENSG00000283873
SSUH2	0.0489	3.1612E-01	5.2618E-01	ENSG00000125046
SSX2IP	0.3419	1.5159E-05	3.3989E-04	ENSG00000117155
ST13	-0.1941	2.3122E-03	1.6792E-02	ENSG00000100380
ST13P12	-0.0231	6.7993E-01		ENSG00000248400
ST13P15	-0.0333	3.0701E-01		ENSG00000243759
ST13P17	0.0473	2.2523E-01	4.2900E-01	ENSG00000253183
ST13P19	0.0069	9.0665E-01		ENSG00000228110
ST13P9	-0.0007	9.1273E-01		ENSG00000256070
ST14	-0.0132	6.5017E-01		ENSG00000149418
ST18	-0.1180	1.8824E-01	3.8310E-01	ENSG00000147488
ST20	-0.1131	2.2178E-01	4.2503E-01	ENSG00000180953
ST20-MTHFS	-0.0266	1.2171E-01		ENSG00000259332
ST3GAL1	0.1586	6.8861E-02	1.9842E-01	ENSG00000008513
ST3GAL2	0.0392	5.6071E-01	7.3781E-01	ENSG00000157350
ST3GAL3	0.0860	3.3516E-01	5.4517E-01	ENSG00000126091
ST3GAL3-AS1	0.0021	8.8503E-01		ENSG00000229444
ST3GAL4	-0.1102	1.4570E-01	3.2479E-01	ENSG00000110080
ST3GAL5	-0.0264	5.9373E-01	7.6091E-01	ENSG00000115525
ST3GAL6	0.0841	3.2231E-01	5.3228E-01	ENSG00000064225
ST6GAL1	-0.2594	2.3801E-02	9.4302E-02	ENSG00000073849
ST6GAL2	0.2930	5.3538E-03	3.1735E-02	ENSG00000144057
ST6GALNAC1	-0.0101	4.6519E-01		ENSG00000070526
ST6GALNAC2	0.0262	7.1624E-01	8.4412E-01	ENSG00000070731
ST6GALNAC3	0.0280	7.1747E-01	8.4473E-01	ENSG00000184005
ST6GALNAC4	-0.1124	1.7490E-01	3.6531E-01	ENSG00000136840
ST6GALNAC4P1	-0.0004	8.7043E-01		ENSG00000233469
ST6GALNAC5	-0.0452	5.8601E-01	7.5563E-01	ENSG00000117069
ST6GALNAC6	0.0945	9.5845E-02	2.4840E-01	ENSG00000160408
ST7	-0.1551	2.6080E-02	1.0058E-01	ENSG00000004866
ST7-AS1	-0.0571	3.8187E-01	5.9013E-01	ENSG00000227199
ST7-AS2	0.0100	9.0549E-01	9.5378E-01	ENSG00000226367
ST7-OT4	-0.0241	4.8951E-01		ENSG00000214188
ST7L	-0.0503	3.5798E-01	5.6763E-01	ENSG00000007341
ST8SIA1	0.3042	1.3540E-04	1.9224E-03	ENSG00000111728
ST8SIA2	0.0142	8.2903E-01	9.1149E-01	ENSG00000140557
ST8SIA3	0.2119	2.4564E-02	9.6355E-02	ENSG00000177511
ST8SIA4	0.1202	1.1580E-01	2.8041E-01	ENSG00000113532
ST8SIA5	-0.3388	3.8904E-04	4.3770E-03	ENSG00000101638
ST8SIA5-DT	0.1034	2.6342E-01	4.7231E-01	ENSG00000270112
ST8SIA6	0.1645	6.6614E-02	1.9422E-01	ENSG00000148488
ST8SIA6-AS1	0.0767	1.4898E-01		ENSG00000204832
STAB1	0.2146	3.1817E-02	1.1587E-01	ENSG00000010327
STAB2	-0.0268	2.2449E-01		ENSG00000136011

STAC	-0.1175	2.1337E-01	4.1436E-01	ENSG00000144681
STAC2	-0.2481	1.1927E-02	5.7721E-02	ENSG00000141750
STAC3	0.0150	8.2829E-01	9.1108E-01	ENSG00000185482
STAG1	-0.0601	3.0536E-01	5.1561E-01	ENSG00000118007
STAG2	-0.0530	3.3817E-01	5.4806E-01	ENSG00000101972
STAG2-AS1	-0.0301	6.8187E-01	8.2253E-01	ENSG00000232412
STAG3	-0.0405	5.7428E-01	7.4707E-01	ENSG00000066923
STAG3L5P-PVRIG2P-PILF	0.0031	9.1804E-01		ENSG00000272752
STAM	-0.0470	4.5244E-01	6.5125E-01	ENSG00000136738
STAM-DT	0.3698	1.6495E-04	2.2349E-03	ENSG00000260589
STAM2	0.0465	5.1595E-01	7.0352E-01	ENSG00000115145
STAMBP	0.1891	6.7428E-04	6.7566E-03	ENSG00000124356
STAMBPL1	0.0736	3.8608E-01	5.9380E-01	ENSG00000138134
STAP1	0.0010	7.1943E-01		ENSG00000035720
STAP2	0.1098	2.0873E-01	4.0877E-01	ENSG00000178078
STAR	0.1059	2.4556E-01	4.5333E-01	ENSG00000147465
STARD10	-0.1961	1.8882E-03	1.4428E-02	ENSG00000214530
STARD13	-0.0764	3.2130E-01	5.3125E-01	ENSG00000133121
STARD13-AS	-0.1616	1.0990E-01	2.7090E-01	ENSG00000236581
STARD3	-0.0697	2.5264E-01	4.6058E-01	ENSG00000131748
STARD3NL	-0.1582	6.6266E-03	3.7251E-02	ENSG00000010270
STARD4	-0.2193	7.3572E-03	4.0263E-02	ENSG00000164211
STARD4-AS1	0.2806	1.4018E-02	6.4857E-02	ENSG00000246859
STARD5	0.0354	6.2903E-01	7.8649E-01	ENSG00000172345
STARD6	0.3983	3.3127E-03	2.2065E-02	ENSG00000174448
STARD7	-0.1869	9.2684E-03	4.7960E-02	ENSG00000084090
STARD8	0.1381	6.8506E-02	1.9777E-01	ENSG00000130052
STARD9	0.1373	1.1758E-01	2.8314E-01	ENSG00000159433
STAT1	0.1027	2.3799E-01	4.4402E-01	ENSG00000115415
STAT2	-0.1401	7.6069E-02	2.1250E-01	ENSG00000170581
STAT3	0.0024	9.9133E-01	9.9518E-01	ENSG00000168610
STAT4	0.0284	7.1569E-01	8.4373E-01	ENSG00000138378
STAT4-AS1	-0.0024	8.7632E-01		ENSG00000231858
STAT5A	-0.0678	4.2067E-01	6.2362E-01	ENSG00000126561
STAT5B	0.0264	7.1654E-01	8.4417E-01	ENSG00000173757
STAT6	-0.0037	9.6198E-01	9.8100E-01	ENSG00000166888
STATH	0.0093	4.6254E-01		ENSG00000126549
STAU1	-0.1187	4.3748E-02	1.4546E-01	ENSG00000124214
STAU2	-0.1807	1.2529E-02	5.9720E-02	ENSG00000040341
STAU2-AS1	-0.0245	1.7445E-01		ENSG00000253302
STBD1	0.1239	1.0017E-01	2.5496E-01	ENSG00000118804
STC1	0.4437	2.8772E-04	3.4771E-03	ENSG00000159167
STEAP1	0.0462	5.3188E-01	7.1594E-01	ENSG00000164647
STEAP1B	0.3120	2.2513E-02	9.0484E-02	ENSG00000105889
STEAP1B-AS1	0.0536	1.9748E-01		ENSG00000232759
STEAP2	0.3061	1.6224E-02	7.1694E-02	ENSG00000157214
STEAP2-AS1	0.1074	9.0528E-02	2.3904E-01	ENSG00000227646
STEAP3	0.0813	2.8162E-01	4.9206E-01	ENSG00000115107
STEAP3-AS1	0.0204	7.4719E-01	8.6252E-01	ENSG00000229867

STEAP4	0.0061	9.2321E-01		ENSG00000127954
STEEP1	-0.0551	3.8985E-01	5.9731E-01	ENSG00000018610
STH	0.0516	4.6744E-01	6.6413E-01	ENSG00000256762
STIL	-0.0842	3.1513E-01	5.2522E-01	ENSG00000123473
STIM1	-0.0224	6.9082E-01	8.2835E-01	ENSG00000167323
STIM1-AS1	0.0589	2.7795E-01		ENSG00000228661
STIM2	0.0556	4.3956E-01	6.3954E-01	ENSG00000109689
STIMATE	0.1133	1.7766E-01	3.6887E-01	ENSG00000213533
STIMATE-MUSTN1	-0.0593	2.8332E-01		ENSG00000248592
STING1	0.2295	2.7077E-02	1.0328E-01	ENSG00000184584
STIP1	-0.2707	8.9799E-04	8.3661E-03	ENSG00000168439
STK10	-0.0282	7.1206E-01	8.4207E-01	ENSG00000072786
STK11	-0.0132	8.3377E-01	9.1449E-01	ENSG00000118046
STK11IP	0.3240	1.6134E-04	2.1933E-03	ENSG00000144589
STK16	-0.1516	3.6511E-02	1.2786E-01	ENSG00000115661
STK17A	0.0220	7.8288E-01	8.8515E-01	ENSG00000164543
STK17B	0.2533	1.7533E-02	7.5709E-02	ENSG00000081320
STK19	0.2348	1.5655E-03	1.2617E-02	ENSG00000204344
STK24	-0.2344	2.5708E-03	1.8145E-02	ENSG00000102572
STK25	-0.1074	1.4930E-01	3.2998E-01	ENSG00000115694
STK26	-0.1342	9.3710E-02	2.4449E-01	ENSG00000134602
STK3	-0.0493	5.3371E-01	7.1748E-01	ENSG00000104375
STK32A	-0.0475	5.6768E-01	7.4237E-01	ENSG00000169302
STK32B	0.1464	1.2773E-01	2.9948E-01	ENSG00000152953
STK32C	-0.1021	1.9052E-01	3.8573E-01	ENSG00000165752
STK33	0.0438	5.5504E-01	7.3317E-01	ENSG00000130413
STK35	-0.0486	4.3556E-01	6.3656E-01	ENSG00000125834
STK36	0.1750	4.7624E-02	1.5446E-01	ENSG00000163482
STK38	0.0694	3.0925E-01	5.1942E-01	ENSG00000112079
STK38L	0.0887	1.6317E-01	3.4957E-01	ENSG00000211455
STK39	0.0763	3.3330E-01	5.4349E-01	ENSG00000198648
STK4	0.0865	1.6738E-01	3.5556E-01	ENSG00000101109
STK4-DT	0.0117	8.0373E-01		ENSG00000227477
[truncated: 217,455 more chars]
