# Supplementary material for: Effects of microgravity on human iPSC-derived neural organoids on the International Space Station
Source: Stem Cells Transl Med. 2024 Oct 23;13(12):1186–97. doi: 10.1093/stcltm/szae070 (PMC11631337; doi:10.1093/stcltm/szae070)
Supplement: szae070_suppl_Supplementary_Materials [file szae070_suppl_supplementary_materials.zip › R1Table S2b Dopaminergic-List of differentially expressed transcripts.pdf]

DOPAMINE NEURON ORGANOIDS				
SYMBOL (NA: uncharacterized lncRNA or pseudogene)	log2FoldChange	pvalue	padj	ENSEMBL
HIGHER IN LEO THAN GROUND: LARGEST TO SMALLEST DIFFERENCE (BLUE)				
POMC	3.3416	5.1552E-19	3.9915E-16	ENSG00000115138
ANKRD20A19P	3.1951	1.1586E-08	6.506E-07	ENSG00000196593
NDUFA4L2	2.9286	6.6657E-09	4.0941E-07	ENSG00000185633
LINC02522	2.7645	1.6799E-10	1.8501E-08	ENSG00000231056
AVP	2.7170	4.0812E-04	0.00350048	ENSG00000101200
NA	2.5557	9.1249E-13	1.9592E-10	ENSG00000285534
LINC03099	2.5346	9.6882E-15	3.4865E-12	ENSG00000236393
AL121721.1	2.5213	5.2312E-11	6.8195E-09	ENSG00000243810
NMUR2	2.4979	4.6482E-11	6.1857E-09	ENSG00000132911
AC013269.2	2.4914	7.7957E-08	3.1819E-06	ENSG00000225819
GHSR	2.4755	3.0560E-10	3.0742E-08	ENSG00000121853
NA	2.3905	2.3487E-07	8.023E-06	ENSG00000286403
MGC12916	2.3099	4.9368E-07	1.4771E-05	ENSG00000266709
NA	2.2883	9.1129E-07	2.4744E-05	ENSG00000287590
CA6	2.2847	3.5809E-09	2.4734E-07	ENSG00000131686
RPL6P15	2.2553	8.8176E-10	7.6894E-08	ENSG00000240611
NA	2.2397	8.1528E-08	3.3013E-06	ENSG00000287360
AL121949.1	2.1622	6.0001E-07	1.7382E-05	ENSG00000218048
TBX19	2.1487	4.6635E-17	2.6479E-14	ENSG00000143178
AC025277.1	2.1284	1.4537E-08	7.7544E-07	ENSG00000261555
SAXO3	2.0873	7.1755E-05	0.00085834	ENSG00000268655
LINC00891	2.0398	1.1137E-06	2.9275E-05	ENSG00000281852
LINC00052	1.9664	3.2253E-13	7.7018E-11	ENSG00000259527
AC004593.1	1.9553	1.0393E-16	5.3109E-14	ENSG00000235669
AC036222.1	1.9117	3.1780E-06	6.9224E-05	ENSG00000264451
AC091951.1	1.9020	6.4998E-13	1.4568E-10	ENSG00000205976
AC104534.1	1.8166	1.0077E-10	1.2032E-08	ENSG00000268756
AC113137.1	1.8152	1.1097E-05	0.00019119	ENSG00000267098
AC127024.3	1.8096	6.6252E-04	0.00516572	ENSG00000265443
NA	1.8057	3.9215E-08	1.7989E-06	ENSG00000285923
LGSN	1.7734	3.4743E-08	1.6318E-06	ENSG00000146166
NA	1.7404	4.5365E-06	9.3027E-05	ENSG00000289072
GBP1	1.7393	1.5785E-13	3.9541E-11	ENSG00000117228
NA	1.7341	2.1893E-09	1.6211E-07	ENSG00000286176
SPN	1.7296	1.1890E-07	4.4675E-06	ENSG00000197471
IQCM	1.7153	6.7311E-04	0.00521958	ENSG00000234828
NA	1.6781	3.2562E-05	0.00045094	ENSG00000285896
TRG-AS1	1.6672	5.3709E-07	1.5865E-05	ENSG00000281103
AL161658.1	1.6538	7.8907E-13	1.7232E-10	ENSG00000280229
NA	1.6482	3.5016E-05	0.00047845	ENSG00000287530

CHRNE	1.6470	1.1815E-08	6.5942E-07	ENSG00000108556
OR9K1P	1.6317	2.2163E-07	7.642E-06	ENSG00000196534
MNX1	1.6167	5.1571E-12	8.6689E-10	ENSG00000130675
CD163L1	1.6119	6.6087E-15	2.5203E-12	ENSG00000177675
BX005019.1	1.6102	7.8386E-05	0.00091958	ENSG00000259946
ADARB2-AS1	1.6087	3.0913E-10	3.0975E-08	ENSG00000205696
LINC01483	1.5969	1.1504E-05	0.00019642	ENSG00000227517
NA	1.5930	1.4622E-08	7.7672E-07	ENSG00000287054
HRC	1.5911	2.1277E-08	1.0808E-06	ENSG00000130528
SLC38A5	1.5762	3.3121E-06	7.1732E-05	ENSG00000017483
CCDC13-AS2	1.5605	7.7217E-10	6.915E-08	ENSG00000225611
AC003092.2	1.5600	1.1378E-05	0.00019486	ENSG00000236938
AC018953.1	1.5557	8.2734E-07	2.2903E-05	ENSG00000253583
AC006277.1	1.5551	1.5575E-05	0.00025171	ENSG00000279452
CRYGEP	1.5481	7.5073E-15	2.78E-12	ENSG00000229150
GPC5-AS2	1.5254	2.7410E-06	6.2033E-05	ENSG00000232885
SKAP1	1.5224	1.2773E-10	1.4636E-08	ENSG00000141293
DHRS2	1.4930	2.9298E-09	2.0794E-07	ENSG00000100867
CSF3R	1.4533	1.2079E-04	0.00130111	ENSG00000119535
TRAV18	1.4248	1.9038E-06	4.525E-05	ENSG00000211798
SLC17A9	1.4101	8.2048E-11	1.0128E-08	ENSG00000101194
NA	1.3994	4.4650E-05	0.00058746	ENSG00000289288
AC040168.1	1.3960	5.1161E-05	0.00065556	ENSG00000279523
NA	1.3711	3.9141E-04	0.0033833	ENSG00000289220
LARGE-AS1	1.3709	5.1898E-04	0.00423525	ENSG00000224973
LOC100420587	1.3688	3.5030E-03	0.01901552	ENSG00000283403
LOC112267871	1.3671	1.2809E-04	0.00136086	ENSG00000228549
AVPR1B	1.3595	8.4837E-06	0.00015406	ENSG00000198049
AL732372.1	1.3563	3.8100E-07	1.1872E-05	ENSG00000236601
CXorf51B	1.3492	7.1980E-04	0.00549988	ENSG00000235699
NEUROD1	1.3463	9.4106E-30	4.0075E-26	ENSG00000162992
NA	1.3420	6.9822E-09	4.2376E-07	ENSG00000231672
DIRC3	1.3405	2.9826E-05	0.00042128	ENSG00000182866
AL121578.3	1.3331	1.7757E-06	4.2737E-05	ENSG00000259977
IL2RG	1.3266	2.4728E-07	8.3574E-06	ENSG00000147168
LINC02432	1.3222	6.4489E-12	1.0559E-09	ENSG00000248810
LINC00494	1.3117	1.3942E-06	3.5303E-05	ENSG00000235621
CXorf51A	1.3063	9.1460E-04	0.00663887	ENSG00000224440
SNORD94	1.2984	4.3632E-04	0.00369399	ENSG00000208772
NPPB	1.2976	2.1444E-07	7.4142E-06	ENSG00000120937
DLEU7	1.2973	6.8165E-10	6.1982E-08	ENSG00000186047
CRYGD	1.2968	1.0957E-09	9.2375E-08	ENSG00000118231
TIGIT	1.2961	5.9658E-05	0.00074133	ENSG00000181847
LINC03088	1.2925	7.7999E-05	0.00091587	ENSG00000257883
CBY2	1.2923	2.9185E-03	0.01649066	ENSG00000174015
AC100756.1	1.2839	9.6353E-04	0.00691552	ENSG00000260399
ONECUT3	1.2807	1.5376E-11	2.3526E-09	ENSG00000205922
AL161938.1	1.2787	1.8154E-05	0.00028339	ENSG00000261411
AC018553.1	1.2663	2.6219E-05	0.00038064	ENSG00000277559

NXPH4	1.2649	2.7808E-07	9.2155E-06	ENSG00000182379
AC076968.1	1.2637	5.6500E-05	0.00070906	ENSG00000260030
LEFTY1	1.2508	3.4457E-08	1.6214E-06	ENSG00000243709
AL354751.1	1.2438	1.8122E-03	0.01133254	ENSG00000234537
RNU6-2	1.2373	1.3198E-03	0.00886058	ENSG00000207357
PMEL	1.2311	2.1222E-08	1.0808E-06	ENSG00000185664
NA	1.2291	2.2271E-10	2.3132E-08	ENSG00000278600
AC015871.3	1.2277	7.0364E-06	0.00013249	ENSG00000199325
GJC3	1.2199	3.6152E-07	1.1362E-05	ENSG00000176402
BTG4	1.2198	7.9756E-04	0.00597782	ENSG00000137707
ISCA2P1	1.2193	2.1082E-08	1.0773E-06	ENSG00000226912
PKD2L1	1.2184	2.5948E-06	5.9249E-05	ENSG00000107593
LIM2	1.2128	4.3937E-10	4.2204E-08	ENSG00000105370
IRGM	1.2105	5.5757E-04	0.00449416	ENSG00000237693
LINC02242	1.2087	9.0792E-07	2.4732E-05	ENSG00000249736
SLC5A7	1.2074	2.3198E-08	1.1622E-06	ENSG00000115665
LINC01886	1.2043	5.5641E-06	0.00010961	ENSG00000224568
AL354855.1	1.2032	9.9830E-06	0.00017495	ENSG00000285287
AL031708.1	1.2016	2.0449E-04	0.00199283	ENSG00000261732
AC140912.1	1.1941	2.4119E-09	1.7608E-07	ENSG00000261227
P2RX2	1.1872	8.6264E-04	0.00634463	ENSG00000187848
RAB11FIP1P1	1.1814	2.9504E-05	0.00041765	ENSG00000228492
ABCA4	1.1753	4.7495E-06	9.6236E-05	ENSG00000198691
AL442647.1	1.1737	1.8040E-04	0.00180973	ENSG00000225689
NA	1.1629	2.0056E-03	0.01226545	ENSG00000288043
TNNI1	1.1614	6.4882E-12	1.0559E-09	ENSG00000159173
AC092119.2	1.1601	3.9404E-06	8.2707E-05	ENSG00000274460
GHRH	1.1542	6.7519E-10	6.1614E-08	ENSG00000118702
MIR210	1.1521	8.4941E-04	0.00627264	ENSG00000199038
FTCD	1.1519	1.2169E-10	1.4116E-08	ENSG00000160282
TSKS	1.1498	4.5896E-06	9.3741E-05	ENSG00000126467
PPY	1.1447	6.5730E-04	0.00512813	ENSG00000108849
NA	1.1410	3.1136E-05	0.00043568	ENSG00000287720
NA	1.1407	4.8914E-06	9.8564E-05	ENSG00000286507
S100A4	1.1407	6.8522E-09	4.1785E-07	ENSG00000196154
NA	1.1359	1.1339E-05	0.00019468	ENSG00000285601
LINC03062	1.1356	6.1628E-07	1.7773E-05	ENSG00000237372
AL731684.1	1.1330	1.2078E-03	0.00824057	ENSG00000225532
CAPN3	1.1319	2.5971E-07	8.7085E-06	ENSG00000092529
AC051619.8	1.1313	1.4188E-03	0.0093968	ENSG00000260035
LINC02481	1.1304	1.3798E-05	0.0002276	ENSG00000246526
AL390816.2	1.1282	1.6853E-05	0.00026795	ENSG00000258903
ZNF556	1.1164	1.4521E-03	0.00956744	ENSG00000172000
AL132709.1	1.1067	3.5216E-04	0.00312322	ENSG00000230805
AC134043.2	1.1045	2.8357E-05	0.00040545	ENSG00000279204
CPA1	1.1032	7.3344E-04	0.0055801	ENSG00000091704
HERC2P11	1.0997	3.4884E-03	0.01895602	ENSG00000261581
AC245060.6	1.0961	9.0947E-04	0.00661297	ENSG00000279278
DPYS	1.0935	9.5062E-06	0.00016833	ENSG00000147647

LINC02172	1.0922	1.2908E-04	0.00136855	ENSG00000251632
AL358933.1	1.0917	5.2478E-05	0.00066905	ENSG00000261839
TNFRSF10D	1.0891	6.7553E-08	2.8342E-06	ENSG00000173530
AJ011932.1	1.0879	3.0276E-08	1.4596E-06	ENSG00000274248
AC012512.1	1.0851	3.2811E-05	0.00045268	ENSG00000226539
KRT40	1.0792	3.5548E-06	7.6348E-05	ENSG00000204889
NA	1.0772	6.6020E-03	0.03100896	ENSG00000289107
AC024896.1	1.0762	1.2615E-03	0.00855614	ENSG00000247903
TRAV19	1.0759	7.5912E-05	0.0008988	ENSG00000211799
ITGAX	1.0749	6.7949E-03	0.03168772	ENSG00000140678
NTMT2	1.0748	6.5695E-05	0.00080099	ENSG00000203740
NA	1.0724	1.7598E-09	1.3498E-07	ENSG00000289207
RCN3	1.0671	4.8752E-18	3.278E-15	ENSG00000142552
LINC00326	1.0626	1.8433E-04	0.00184049	ENSG00000231023
MNX1-AS1	1.0616	1.0980E-04	0.00120752	ENSG00000243479
SAMSN1	1.0610	4.2618E-07	1.3041E-05	ENSG00000155307
TSPYL5	1.0595	2.0111E-27	5.7096E-24	ENSG00000180543
PLAC1	1.0563	6.9484E-08	2.8868E-06	ENSG00000170965
ACSBG1	1.0540	4.6159E-04	0.00385632	ENSG00000103740
AC106052.1	1.0517	1.6813E-04	0.00170875	ENSG00000272862
IZUMO2	1.0503	3.0219E-03	0.01692868	ENSG00000161652
AL603839.4	1.0493	1.2267E-03	0.00834696	ENSG00000279667
LOC101929653	1.0490	3.2289E-05	0.0004479	ENSG00000237654
NA	1.0489	1.5025E-03	0.00982082	ENSG00000287831
IRS3P	1.0436	2.7838E-10	2.8225E-08	ENSG00000184414
FRY-AS1	1.0422	1.2715E-08	6.9416E-07	ENSG00000237637
NEUROD4	1.0367	1.4331E-07	5.2237E-06	ENSG00000123307
AC116609.1	1.0362	6.3812E-09	3.9574E-07	ENSG00000223751
NA	1.0326	4.0073E-03	0.02114186	ENSG00000286136
VIP	1.0231	4.7137E-04	0.00391422	ENSG00000146469
ZNF670	1.0147	1.5663E-15	6.67E-13	ENSG00000277462
AADACP1	1.0140	4.4207E-03	0.02279606	ENSG00000240602
IFI16	1.0129	3.5605E-07	1.1218E-05	ENSG00000163565
AC106028.3	1.0124	7.4902E-05	0.00088808	ENSG00000260361
AC107375.1	1.0078	1.0966E-06	2.8945E-05	ENSG00000259891
RPL23AP32	1.0040	1.6590E-04	0.00169014	ENSG00000237887
PEAR1	0.9964	1.7295E-03	0.01094951	ENSG00000187800
HIF1A-AS3	0.9954	3.6376E-05	0.00049333	ENSG00000258667
MEG8	0.9952	9.5418E-08	3.7566E-06	ENSG00000225746
TMEM273	0.9948	1.6336E-03	0.01047979	ENSG00000204161
AL356804.1	0.9944	1.3998E-10	1.5967E-08	ENSG00000259033
NA	0.9932	7.9397E-05	0.00092863	ENSG00000287064
CPA5	0.9931	5.6392E-05	0.00070805	ENSG00000158525
KCNAB3	0.9914	3.1185E-07	1.0125E-05	ENSG00000170049
ALDOC	0.9872	3.0042E-12	5.5623E-10	ENSG00000109107
ARHGDIB	0.9849	2.1310E-06	4.9873E-05	ENSG00000111348
CHRNB3	0.9846	2.7629E-09	1.983E-07	ENSG00000147432
N4BP2L2-IT2	0.9816	3.2951E-03	0.01803603	ENSG00000281026
DHPS	0.9808	4.7084E-07	1.4204E-05	ENSG00000095059

HS3ST5	0.9796	6.6632E-07	1.8959E-05	ENSG00000249853
LINC01511	0.9776	4.8465E-05	0.00062987	ENSG00000250584
LINC02525	0.9752	5.9112E-17	3.2834E-14	ENSG00000230269
RHOXF1-AS1	0.9741	7.6298E-05	0.00090186	ENSG00000258545
MYOC	0.9710	8.0629E-04	0.00601326	ENSG00000034971
SRL	0.9705	2.0413E-04	0.00199179	ENSG00000185739
NPTX1	0.9699	7.7985E-06	0.00014471	ENSG00000171246
CDA	0.9681	2.9835E-04	0.0027167	ENSG00000158825
MIR210HG	0.9644	6.5284E-08	2.7617E-06	ENSG00000247095
NCCRP1	0.9608	6.3706E-06	0.00012294	ENSG00000188505
NA	0.9596	7.0547E-04	0.00541143	ENSG00000289354
NAT1	0.9569	1.9452E-09	1.4661E-07	ENSG00000171428
MSC	0.9558	1.0534E-05	0.00018298	ENSG00000178860
AC074389.1	0.9532	1.3962E-03	0.00928031	ENSG00000205971
LINC01572	0.9520	3.5593E-13	8.4208E-11	ENSG00000261008
LINC00626	0.9515	7.0360E-03	0.03251697	ENSG00000225826
NA	0.9508	4.3890E-03	0.02268401	ENSG00000286550
SLITRK6	0.9507	2.0536E-14	6.4778E-12	ENSG00000184564
AC129507.2	0.9490	4.6730E-04	0.00388672	ENSG00000262294
AC068418.2	0.9456	1.8716E-03	0.01163509	ENSG00000265043
LINC02440	0.9436	4.4227E-08	1.993E-06	ENSG00000255618
CAVIN3	0.9407	1.3042E-08	7.0751E-07	ENSG00000170955
SCIRT	0.9406	3.9867E-10	3.8585E-08	ENSG00000237686
NA	0.9406	9.6185E-04	0.00690929	ENSG00000287335
AC069133.1	0.9403	3.8571E-04	0.00334594	ENSG00000253762
TXNIP	0.9398	6.6126E-11	8.4479E-09	ENSG00000265972
PARP16	0.9395	6.8749E-20	6.0573E-17	ENSG00000138617
HSPA12B	0.9379	2.4235E-05	0.0003567	ENSG00000132622
MIP	0.9365	3.6220E-04	0.00319229	ENSG00000135517
RB1-DT	0.9343	3.8110E-08	1.7514E-06	ENSG00000231473
TEX54	0.9295	1.0042E-10	1.2032E-08	ENSG00000283268
AL590235.2	0.9274	3.1164E-03	0.01729141	ENSG00000270090
PMFBP1	0.9272	6.1437E-08	2.6339E-06	ENSG00000118557
NA	0.9256	5.8549E-12	9.7142E-10	ENSG00000289341
MIR3188	0.9250	1.0739E-04	0.00118476	ENSG00000267959
ONECUT1	0.9197	1.8370E-07	6.4921E-06	ENSG00000169856
BST1	0.9172	1.0071E-12	2.1444E-10	ENSG00000109743
AC108517.1	0.9160	1.4764E-04	0.00153344	ENSG00000249111
PPP1R36	0.9147	3.1255E-07	1.0134E-05	ENSG00000165807
LTK	0.9146	4.2558E-03	0.0221614	ENSG00000062524
AL445309.1	0.9143	1.4788E-03	0.00970588	ENSG00000272320
AC004257.1	0.9137	1.6461E-03	0.0105331	ENSG00000269635
WDCP	0.9123	8.7063E-10	7.6183E-08	ENSG00000163026
AC019322.4	0.9116	6.6199E-03	0.031047	ENSG00000279417
NA	0.9113	1.6727E-15	7.0062E-13	ENSG00000286149
LINC02072	0.9099	2.0831E-04	0.00201878	ENSG00000267452
PHETA2	0.9099	2.7541E-05	0.00039622	ENSG00000177096
NA	0.9084	2.1185E-04	0.00204341	ENSG00000285922
AC027228.2	0.9083	1.7463E-10	1.9009E-08	ENSG00000261762

AL627171.1	0.9075	7.6776E-14	2.0869E-11	ENSG00000278002
TBC1D10C	0.9054	7.0242E-03	0.03247833	ENSG00000175463
LINC00548	0.9050	4.7597E-03	0.02408685	ENSG00000215483
NA	0.9037	4.8807E-06	9.8426E-05	ENSG00000286069
LINC01275	0.9015	2.0892E-04	0.00202198	ENSG00000237595
TBX10	0.9012	3.5322E-05	0.00048109	ENSG00000167800
LOC286083	0.9003	5.5612E-04	0.00448675	ENSG00000275427
MIF4GD-DT	0.8993	2.7033E-05	0.0003898	ENSG00000263843
AC093627.5	0.8985	1.3311E-08	7.1907E-07	ENSG00000242474
NA	0.8969	1.8699E-03	0.0116279	ENSG00000287254
RAB40A	0.8949	2.8856E-04	0.00264073	ENSG00000172476
MEI1	0.8922	1.2769E-03	0.00863112	ENSG00000167077
LINC03060	0.8909	3.7084E-07	1.1598E-05	ENSG00000273297
NOS3	0.8883	9.4966E-06	0.00016833	ENSG00000164867
LINC01522	0.8873	9.0760E-13	1.9592E-10	ENSG00000237423
THRB	0.8840	5.5917E-06	0.00010999	ENSG00000151090
NA	0.8838	4.1304E-03	0.02166146	ENSG00000286292
ZNF225	0.8829	4.5158E-09	3.0126E-07	ENSG00000256294
NA	0.8818	4.4294E-03	0.02282225	ENSG00000289891
AL109840.2	0.8799	8.3011E-05	0.00096323	ENSG00000285091
MMP1	0.8789	9.3953E-04	0.00678707	ENSG00000196611
SCGN	0.8755	9.8359E-06	0.00017297	ENSG00000079689
LOC100419928	0.8733	1.0531E-05	0.00018298	ENSG00000255836
SLC2A14	0.8721	7.9027E-05	0.0009252	ENSG00000173262
NCF1B	0.8709	6.9882E-04	0.00538228	ENSG00000182487
DDC	0.8692	1.0158E-16	5.297E-14	ENSG00000132437
ZNF112	0.8682	1.2099E-06	3.1259E-05	ENSG00000062370
NA	0.8617	1.6965E-10	1.8604E-08	ENSG00000287918
NA	0.8613	2.7528E-13	6.6986E-11	ENSG00000286267
LINC00457	0.8584	3.0046E-06	6.6181E-05	ENSG00000225179
ZNF221	0.8583	1.5870E-05	0.00025583	ENSG00000159905
SAMSN1-AS1	0.8579	6.2900E-04	0.00494204	ENSG00000223662
ZNF701	0.8571	2.3430E-12	4.5011E-10	ENSG00000167562
RAX	0.8570	4.5976E-10	4.365E-08	ENSG00000134438
LINC02202	0.8522	2.0306E-04	0.00198488	ENSG00000245812
GALT	0.8483	2.9615E-17	1.8456E-14	ENSG00000213930
NA	0.8479	1.9409E-03	0.01196335	ENSG00000287548
AC242376.1	0.8471	2.7944E-03	0.01595518	ENSG00000259414
AL031595.3	0.8456	9.1846E-04	0.00666315	ENSG00000280434
AC005593.1	0.8446	7.0166E-08	2.9085E-06	ENSG00000279584
BANF1P2	0.8431	2.1799E-03	0.01313313	ENSG00000230306
AC004623.1	0.8421	8.3745E-03	0.03720351	ENSG00000279488
HAP1	0.8409	2.8824E-09	2.0515E-07	ENSG00000173805
PLAT	0.8401	1.2604E-08	6.896E-07	ENSG00000104368
LINC02182	0.8400	2.0843E-04	0.00201878	ENSG00000260420
DRD2	0.8398	2.7951E-12	5.2902E-10	ENSG00000149295
NA	0.8395	1.0778E-03	0.00752236	ENSG00000289928
MUC20P1	0.8393	4.9622E-06	9.9834E-05	ENSG00000224769
AC084262.1	0.8392	1.3493E-06	3.4372E-05	ENSG00000260253

AC124068.2	0.8386	2.6896E-04	0.00248999	ENSG00000261441
AC046136.1	0.8381	7.5124E-09	4.5165E-07	ENSG00000242880
NA	0.8356	2.8170E-18	2.0565E-15	ENSG00000289612
AL358473.1	0.8348	2.2254E-09	1.6293E-07	ENSG00000229191
C16orf82	0.8346	6.3685E-04	0.00499302	ENSG00000234186
AC093503.3	0.8321	3.0759E-05	0.00043157	ENSG00000279161
AZGP1	0.8313	2.7526E-05	0.00039622	ENSG00000160862
LINC01170	0.8307	2.4398E-05	0.00035847	ENSG00000253807
STARD4-AS1	0.8306	3.5788E-07	1.1261E-05	ENSG00000246859
MST1R	0.8301	3.0941E-03	0.01719764	ENSG00000164078
NA	0.8292	5.7179E-12	9.5489E-10	ENSG00000286155
ZNF616	0.8278	1.5102E-06	3.7464E-05	ENSG00000204611
MMRN2	0.8277	6.4244E-04	0.00502603	ENSG00000173269
AC083798.1	0.8271	3.5656E-03	0.01930989	ENSG00000242531
TMEM45B	0.8251	1.7554E-05	0.00027635	ENSG00000151715
SNORA24B	0.8241	2.5770E-03	0.01494453	ENSG00000206903
CDH10	0.8236	3.7876E-06	8.018E-05	ENSG00000040731
STX16-NPEPL1	0.8200	6.3945E-05	0.00078557	ENSG00000254995
NA	0.8196	9.2732E-05	0.00105307	ENSG00000287851
DENND1C	0.8184	2.9822E-12	5.5618E-10	ENSG00000205744
AC068790.5	0.8180	2.3363E-04	0.00221993	ENSG00000270061
NA	0.8169	4.3810E-04	0.00370416	ENSG00000288023
RAPGEF6	0.8168	2.2015E-09	1.6211E-07	ENSG00000158987
LOC101929269	0.8161	2.9776E-04	0.00271325	ENSG00000254839
AP002992.1	0.8156	5.4843E-04	0.00444013	ENSG00000255236
LINC01697	0.8146	2.3474E-03	0.01390976	ENSG00000232079
PTGES3P2	0.8119	3.2288E-07	1.0388E-05	ENSG00000217643
PTPRH	0.8106	6.8633E-06	0.00013015	ENSG00000080031
AC087742.1	0.8093	2.2010E-03	0.01323268	ENSG00000274363
LINC02669	0.8068	7.7310E-05	0.00091071	ENSG00000233321
AC092145.1	0.8064	2.2991E-03	0.01369028	ENSG00000261471
GIP	0.8051	1.3503E-03	0.00903107	ENSG00000159224
LOC124904138	0.8048	4.3822E-09	2.9466E-07	ENSG00000278048
ALK	0.8035	1.6772E-06	4.089E-05	ENSG00000171094
RBMS1P1	0.8034	4.7481E-04	0.00393123	ENSG00000225422
LINC02343	0.8022	1.2639E-05	0.00021157	ENSG00000271850
TRMT9B	0.8017	1.6133E-07	5.7894E-06	ENSG00000250305
SMIM24	0.8012	6.5501E-05	0.00080008	ENSG00000095932
NA	0.7987	6.0231E-03	0.02896055	ENSG00000287713
C2CD4B	0.7981	4.8815E-04	0.00401567	ENSG00000205502
ZNF496-DT	0.7979	1.4833E-12	2.9842E-10	ENSG00000289234
AC116407.3	0.7971	1.1887E-03	0.00813158	ENSG00000280020
ZC3H10	0.7970	1.2209E-13	3.1196E-11	ENSG00000135482
NA	0.7968	2.9311E-07	9.6635E-06	ENSG00000286329
AC242376.2	0.7958	3.1518E-07	1.0194E-05	ENSG00000274471
ACER2	0.7941	3.3310E-04	0.00297486	ENSG00000177076
SMPX	0.7934	1.1432E-03	0.00787951	ENSG00000091482
RNU5F-1	0.7932	5.1664E-05	0.0006597	ENSG00000199377
CMAHP	0.7919	6.5726E-04	0.00512813	ENSG00000168405

STEAP1B	0.7917	6.7298E-03	0.03142974	ENSG00000105889
LINC02930	0.7912	6.4260E-05	0.00078744	ENSG00000274461
CHRNA5	0.7882	9.9658E-15	3.507E-12	ENSG00000169684
CD19	0.7878	5.7261E-03	0.02782011	ENSG00000177455
WASH5P	0.7872	3.8453E-12	6.776E-10	ENSG00000282458
AL035446.1	0.7871	6.6859E-03	0.03127055	ENSG00000234147
SLC18A3	0.7848	8.4496E-07	2.329E-05	ENSG00000187714
NA	0.7846	8.3905E-04	0.0062033	ENSG00000287165
AOAH	0.7838	7.0044E-04	0.0053874	ENSG00000136250
RYR2	0.7834	2.3943E-07	8.1416E-06	ENSG00000198626
LOC102724612	0.7834	4.8808E-03	0.02453474	ENSG00000253894
AL033530.1	0.7833	9.3816E-07	2.5339E-05	ENSG00000285407
NA	0.7819	8.1336E-04	0.00604486	ENSG00000288758
HK2	0.7794	7.6791E-05	0.00090586	ENSG00000159399
LINC01471	0.7786	5.1083E-03	0.0254222	ENSG00000239921
CDH12	0.7773	1.3455E-05	0.0002228	ENSG00000154162
RPS8P7	0.7773	9.9972E-04	0.0071093	ENSG00000229721
SERPINA5	0.7765	1.1564E-03	0.00795575	ENSG00000188488
RN7SL499P	0.7760	8.5536E-04	0.0063093	ENSG00000239202
AC232271.1	0.7756	2.7832E-05	0.00039973	ENSG00000270012
CELA2B	0.7706	2.4303E-03	0.01429793	ENSG00000215704
PDE3B	0.7698	3.6798E-06	7.8352E-05	ENSG00000152270
GFRA1	0.7685	1.0841E-03	0.00755797	ENSG00000151892
LOC105375423	0.7681	2.0576E-06	4.8411E-05	ENSG00000286923
AL512791.1	0.7675	1.3449E-03	0.00900492	ENSG00000258424
LINC00589	0.7669	1.2240E-03	0.00833326	ENSG00000251191
CDH15	0.7668	5.1429E-06	0.0001025	ENSG00000129910
LOC124900989	0.7666	3.1513E-04	0.00285021	ENSG00000286647
NA	0.7666	4.1165E-06	8.5792E-05	ENSG00000288856
RNU5B-1	0.7665	3.1822E-05	0.00044358	ENSG00000200156
LINC01630	0.7665	8.1461E-06	0.00014932	ENSG00000227115
SCARNA18B	0.7664	2.8087E-03	0.01601935	ENSG00000238754
AARSD1	0.7662	1.5709E-06	3.8818E-05	ENSG00000266967
CASP3	0.7653	2.3030E-19	1.8982E-16	ENSG00000164305
AL359693.1	0.7645	4.9340E-04	0.00405393	ENSG00000277797
FOXP1-DT	0.7644	6.5016E-03	0.03066012	ENSG00000270562
DUSP8P5	0.7631	3.7495E-07	1.1698E-05	ENSG00000235316
NA	0.7624	1.0836E-07	4.148E-06	ENSG00000288752
ERP27	0.7612	1.2206E-05	0.00020553	ENSG00000139055
LINC01549	0.7597	1.0156E-04	0.00113197	ENSG00000232560
RNF139-DT	0.7593	7.1059E-13	1.5652E-10	ENSG00000245149
ZNF547	0.7585	9.1067E-05	0.00103692	ENSG00000152433
PCDH11X	0.7571	1.1218E-06	2.9427E-05	ENSG00000102290
ADCY8	0.7570	1.0332E-07	4.0131E-06	ENSG00000155897
LOC124901321	0.7569	9.2332E-05	0.00104898	ENSG00000289609
NA	0.7562	2.7113E-08	1.3322E-06	ENSG00000289009
AC007731.2	0.7560	7.0384E-03	0.03251697	ENSG00000215493
AL020997.2	0.7557	2.3761E-04	0.00225279	ENSG00000269971
PRDM12	0.7527	1.3022E-03	0.00876083	ENSG00000130711

INKA1	0.7526	2.6897E-04	0.00248999	ENSG00000185614
AC005519.1	0.7505	8.7981E-03	0.0386452	ENSG00000258559
AC079061.1	0.7484	5.5620E-05	0.00069939	ENSG00000248050
PLPP6	0.7481	1.2991E-14	4.3676E-12	ENSG00000205808
AADAC	0.7478	2.6031E-03	0.01505238	ENSG00000114771
NA	0.7457	2.8183E-03	0.01605933	ENSG00000286147
ZKSCAN4	0.7446	3.4487E-15	1.3351E-12	ENSG00000187626
NA	0.7445	5.2556E-09	3.4169E-07	ENSG00000290110
AC092666.1	0.7433	5.1982E-03	0.02580029	ENSG00000241449
HMCN2	0.7412	5.2294E-08	2.3077E-06	ENSG00000148357
FGFR3	0.7408	1.3745E-07	5.0531E-06	ENSG00000068078
MYO1F	0.7388	1.4873E-05	0.00024257	ENSG00000142347
LDHA	0.7386	2.3719E-05	0.00035153	ENSG00000134333
AC012508.2	0.7381	4.0660E-05	0.00054194	ENSG00000260634
FFAR2	0.7380	2.8481E-04	0.00261488	ENSG00000126262
GRIN2C	0.7377	1.8743E-03	0.01164656	ENSG00000161509
EPHX3	0.7376	2.2712E-04	0.0021694	ENSG00000105131
NA	0.7374	3.2576E-07	1.0457E-05	ENSG00000289222
AC010864.1	0.7363	6.4101E-08	2.7207E-06	ENSG00000273008
AC108134.2	0.7354	2.1940E-08	1.1079E-06	ENSG00000261889
CCDC15	0.7353	4.2732E-09	2.8809E-07	ENSG00000149548
AL662844.4	0.7345	4.1196E-07	1.2682E-05	ENSG00000272501
NA	0.7331	3.2616E-05	0.00045144	ENSG00000286327
LOC105375798	0.7326	1.8321E-04	0.00183356	ENSG00000254812
DLGAP2	0.7312	2.0411E-06	4.8155E-05	ENSG00000198010
RAB35-AS1	0.7273	1.6936E-14	5.4775E-12	ENSG00000277283
GHET1	0.7269	2.9469E-05	0.00041738	ENSG00000281189
ZNF181	0.7265	1.0728E-07	4.122E-06	ENSG00000197841
STRADA	0.7260	9.5602E-09	5.5391E-07	ENSG00000266173
APOD	0.7252	9.5923E-10	8.2246E-08	ENSG00000189058
AC103724.3	0.7234	7.4817E-03	0.03408179	ENSG00000261449
SORCS3	0.7218	8.9665E-06	0.00016066	ENSG00000156395
PLAG1	0.7217	3.7608E-14	1.0919E-11	ENSG00000181690
AC138646.1	0.7213	6.0761E-08	2.6093E-06	ENSG00000253659
NA	0.7212	7.7855E-05	0.00091545	ENSG00000289866
ZNF17	0.7201	5.8015E-06	0.0001135	ENSG00000186272
MLIP	0.7190	4.5725E-07	1.3843E-05	ENSG00000146147
LINC02506	0.7180	1.6689E-08	8.7559E-07	ENSG00000251129
LOC124901131	0.7178	4.5246E-03	0.023196	ENSG00000288912
AC013731.1	0.7173	2.3963E-03	0.01414683	ENSG00000270557
AC138305.1	0.7163	5.5414E-07	1.6293E-05	ENSG00000260658
HERC3	0.7158	1.6863E-05	0.00026795	ENSG00000138641
ZNF200	0.7155	8.9537E-14	2.3709E-11	ENSG00000010539
LTBP4	0.7154	1.1193E-14	3.8131E-12	ENSG00000090006
PAX3	0.7152	5.2987E-04	0.00431443	ENSG00000135903
NPPA	0.7132	6.1278E-04	0.00484438	ENSG00000175206
AC122129.1	0.7129	2.0885E-03	0.0126721	ENSG00000197815
SLC22A31	0.7120	3.5431E-05	0.00048231	ENSG00000259803
SLC17A6	0.7120	2.4449E-05	0.00035902	ENSG00000091664

AC005208.1	0.7113	1.7662E-03	0.01110146	ENSG00000230258
NA	0.7113	2.5924E-04	0.0024166	ENSG00000288997
MIR4453HG	0.7110	3.3326E-07	1.0644E-05	ENSG00000268471
CCDC144NL	0.7102	5.9339E-08	2.5654E-06	ENSG00000205212
LINC01664	0.7101	9.0479E-07	2.4705E-05	ENSG00000235478
SMC2-DT	0.7100	1.3061E-03	0.00878458	ENSG00000270332
RN7SL521P	0.7096	1.5318E-03	0.00997678	ENSG00000240877
AC009831.1	0.7077	1.7843E-03	0.01119199	ENSG00000263823
LOC107984273	0.7071	7.2823E-04	0.00554771	ENSG00000285587
HSPA1A	0.7052	5.5491E-08	2.4154E-06	ENSG00000204389
NA	0.7030	2.4241E-07	8.2147E-06	ENSG00000287070
DISC1FP1	0.7024	8.7963E-05	0.00100852	ENSG00000261645
CYP27B1	0.7017	1.8594E-04	0.00185367	ENSG00000111012
NTS	0.7006	6.8158E-14	1.8929E-11	ENSG00000133636
LOC728506	0.6975	3.9805E-08	1.8194E-06	ENSG00000251158
AC015909.2	0.6968	4.8889E-04	0.00402048	ENSG00000253730
AC245060.2	0.6963	2.6274E-06	5.9854E-05	ENSG00000234630
NA	0.6961	4.3277E-04	0.00366997	ENSG00000289446
MLF1-DT	0.6956	2.9084E-05	0.00041308	ENSG00000243150
ZNF786	0.6955	4.5436E-06	9.3059E-05	ENSG00000197362
AC021087.2	0.6953	5.6346E-03	0.02746443	ENSG00000250848
KIAA0087	0.6953	4.0847E-03	0.02147923	ENSG00000122548
NFYC-AS1	0.6945	6.6650E-05	0.00081055	ENSG00000272145
AC004012.1	0.6944	1.8063E-03	0.01130306	ENSG00000233942
COQ8A	0.6940	1.9765E-03	0.01213371	ENSG00000163050
CYP2T1P	0.6936	8.8659E-06	0.00015931	ENSG00000233622
AL731684.2	0.6934	2.6938E-03	0.01549867	ENSG00000230423
TBCE	0.6930	2.3774E-05	0.00035214	ENSG00000284770
AC092117.1	0.6913	3.4040E-07	1.0845E-05	ENSG00000276791
DOK3	0.6902	1.5241E-05	0.00024709	ENSG00000146094
LINC01579	0.6900	6.9269E-06	0.00013071	ENSG00000258754
GPC5	0.6893	3.8952E-07	1.208E-05	ENSG00000179399
MT-TY	0.6883	1.1065E-05	0.00019095	ENSG00000210144
LINC00632	0.6878	9.0922E-11	1.0958E-08	ENSG00000203930
NCF1	0.6876	1.6178E-03	0.01039928	ENSG00000158517
PTPRE	0.6870	6.2757E-08	2.6814E-06	ENSG00000132334
ZNF252P-AS1	0.6868	3.9718E-03	0.02100226	ENSG00000255559
AC005537.1	0.6861	2.3838E-05	0.00035268	ENSG00000232006
LOC124904122	0.6859	1.8589E-07	6.5331E-06	ENSG00000290039
LINC01201	0.6841	4.3418E-03	0.02247996	ENSG00000228659
LINC01902	0.6838	6.2052E-07	1.7875E-05	ENSG00000283503
AC138932.2	0.6824	2.8600E-06	6.3712E-05	ENSG00000258354
TCHH	0.6815	1.3314E-04	0.00140512	ENSG00000159450
ZNF595	0.6800	2.7234E-14	8.1866E-12	ENSG00000272602
ZNF555	0.6796	3.1213E-14	9.2365E-12	ENSG00000186300
EGFEM1P	0.6783	2.1366E-05	0.00032302	ENSG00000206120
CYB5R4	0.6777	5.4299E-08	2.3797E-06	ENSG00000065615
L1CAM-AS1	0.6774	2.8544E-03	0.01621833	ENSG00000273769
ST3GAL1	0.6774	3.3846E-10	3.365E-08	ENSG00000008513

NA	0.6768	8.2724E-03	0.03686886	ENSG00000286677
LINC02997	0.6762	1.0158E-04	0.00113197	ENSG00000249364
AC091982.1	0.6756	7.9596E-03	0.03582375	ENSG00000253921
AC069224.1	0.6749	5.4265E-07	1.5974E-05	ENSG00000260572
NA	0.6747	2.9573E-03	0.01666222	ENSG00000289913
DACT3-AS1	0.6739	2.5917E-04	0.0024166	ENSG00000245598
NA	0.6738	3.8791E-05	0.00052029	ENSG00000289285
DCUN1D2	0.6723	3.2735E-11	4.5706E-09	ENSG00000150401
NA	0.6721	1.9330E-10	2.0493E-08	ENSG00000285646
GRB14	0.6720	1.3037E-05	0.00021658	ENSG00000115290
GTSE1-DT	0.6710	1.8401E-05	0.00028633	ENSG00000277232
NA	0.6706	8.7970E-03	0.0386452	ENSG00000286715
CNBD2	0.6695	1.1076E-03	0.00769467	ENSG00000149646
AL606760.3	0.6692	2.3681E-04	0.00224596	ENSG00000259818
RPL34P20	0.6682	3.9089E-03	0.02074267	ENSG00000231508
FARSA-AS1	0.6680	3.2314E-04	0.00291021	ENSG00000266975
CNMD	0.6678	3.8582E-04	0.00334594	ENSG00000136110
PRSS27	0.6675	1.2368E-06	3.1761E-05	ENSG00000172382
RASIP1	0.6671	6.8166E-04	0.00527538	ENSG00000105538
MUC4	0.6666	1.0379E-02	0.04366034	ENSG00000145113
LINC02193	0.6663	9.5596E-05	0.00107839	ENSG00000260923
SND1-DT	0.6659	9.7844E-04	0.00699466	ENSG00000240790
WASH3P	0.6633	7.4098E-11	9.2807E-09	ENSG00000185596
AC007686.3	0.6625	3.3749E-05	0.00046336	ENSG00000273729
KRT8P33	0.6620	4.5689E-03	0.02338098	ENSG00000250539
INHA	0.6620	1.7314E-06	4.1933E-05	ENSG00000123999
LINC00992	0.6614	2.7433E-04	0.00252863	ENSG00000248663
LINC02610	0.6606	8.4637E-10	7.4571E-08	ENSG00000186235
PDK1	0.6604	9.7633E-09	5.5855E-07	ENSG00000152256
FAM226B	0.6597	1.2373E-07	4.6018E-06	ENSG00000269911
LOC100507336	0.6594	4.2389E-04	0.00360905	ENSG00000228793
ADGRE2	0.6589	2.2199E-03	0.01332434	ENSG00000127507
AC099568.2	0.6586	5.9733E-08	2.5738E-06	ENSG00000272931
CDR2-DT	0.6581	5.6304E-03	0.0274598	ENSG00000260790
NA	0.6565	4.2279E-07	1.2968E-05	ENSG00000286138
BNIP3P1	0.6560	1.2383E-05	0.00020789	ENSG00000197358
GALNTL6	0.6555	8.4923E-09	4.9882E-07	ENSG00000174473
AC096992.2	0.6548	1.0411E-04	0.00115656	ENSG00000273486
CSTF2	0.6547	1.0196E-07	3.9715E-06	ENSG00000101811
CCDC89	0.6539	9.5808E-04	0.00689187	ENSG00000179071
CAPN10-DT	0.6536	3.4285E-07	1.0909E-05	ENSG00000260942
CDKL3	0.6536	1.2097E-05	0.00020429	ENSG00000006837
MACROD2	0.6533	3.1728E-09	2.2271E-07	ENSG00000172264
ZNF230	0.6529	2.0857E-08	1.0679E-06	ENSG00000159882
GUCA1B	0.6529	6.7742E-06	0.00012879	ENSG00000112599
MOV10L1	0.6528	2.3890E-03	0.0141102	ENSG00000073146
AC012676.3	0.6526	1.6628E-03	0.01060856	ENSG00000277170
AC126755.4	0.6520	6.9135E-06	0.00013066	ENSG00000257563
LINC00235	0.6514	1.0290E-03	0.00726906	ENSG00000277142

NXF2	0.6508	3.7275E-04	0.00326841	ENSG00000269405
NUDT16-DT	0.6502	1.3752E-05	0.00022698	ENSG00000250608
NA	0.6502	6.9010E-05	0.00083135	ENSG00000289558
MST1L	0.6499	8.1179E-04	0.00603841	ENSG00000186715
LINC02361	0.6494	1.1161E-04	0.00122446	ENSG00000256576
AL023881.1	0.6491	7.4264E-03	0.03390242	ENSG00000276984
PTCH2	0.6487	1.5809E-08	8.3285E-07	ENSG00000117425
LINC02236	0.6482	5.2572E-03	0.02601204	ENSG00000251365
AGMO	0.6481	1.1294E-08	6.3564E-07	ENSG00000187546
NA	0.6476	9.0997E-03	0.03964603	ENSG00000287097
AC138969.3	0.6475	8.7286E-06	0.00015717	ENSG00000257366
TMEM143	0.6472	2.6703E-07	8.9071E-06	ENSG00000161558
NA	0.6469	2.4014E-05	0.00035447	ENSG00000288840
INTS2	0.6456	1.3265E-07	4.9051E-06	ENSG00000108506
FAM200A	0.6454	4.6791E-12	8.0239E-10	ENSG00000221909
HINFP	0.6453	8.0251E-11	9.9539E-09	ENSG00000172273
NA	0.6447	1.0474E-04	0.00116206	ENSG00000288826
RPL7AP76	0.6407	6.4154E-04	0.00502205	ENSG00000231995
GPRACR	0.6403	7.5156E-03	0.03420562	ENSG00000196979
FIGN	0.6403	1.0364E-05	0.00018075	ENSG00000182263
HORMAD2-AS1	0.6402	4.2372E-03	0.02207698	ENSG00000227117
LIPH	0.6399	3.0980E-06	6.7784E-05	ENSG00000163898
ANKRD42-DT	0.6393	2.1230E-09	1.5815E-07	ENSG00000247137
NA	0.6392	4.3090E-04	0.00365902	ENSG00000289041
NA	0.6390	2.6246E-05	0.00038081	ENSG00000287048
LOC101929473	0.6389	9.1900E-03	0.03990043	ENSG00000255087
ESPNL	0.6388	6.6278E-04	0.00516619	ENSG00000144488
RNU4ATAC	0.6386	1.6548E-08	8.7001E-07	ENSG00000264229
HAPLN2	0.6385	5.5781E-04	0.00449467	ENSG00000132702
NA	0.6380	3.4507E-07	1.0953E-05	ENSG00000289259
MALRD1	0.6366	4.3754E-08	1.9752E-06	ENSG00000204740
ATP8B3	0.6365	2.1203E-04	0.00204435	ENSG00000130270
U52111.1	0.6362	4.9716E-03	0.02489807	ENSG00000232725
ZBTB20-AS5	0.6362	7.2588E-05	0.00086627	ENSG00000242290
CLDN8	0.6359	6.6146E-03	0.03103368	ENSG00000156284
NA	0.6351	1.1156E-03	0.00773329	ENSG00000287170
LINC00685	0.6351	1.4448E-04	0.00150434	ENSG00000226179
CYP21A2	0.6325	5.6889E-03	0.02769232	ENSG00000231852
MST1P2	0.6317	7.0557E-06	0.00013276	ENSG00000186301
MTMR7	0.6312	9.5935E-09	5.5458E-07	ENSG00000003987
GLRA3	0.6312	3.1739E-05	0.00044291	ENSG00000145451
NRIP2	0.6307	1.8557E-03	0.0115563	ENSG00000053702
AL031985.3	0.6307	6.5305E-05	0.00079838	ENSG00000260920
LOC100132249	0.6306	4.7691E-06	9.6557E-05	ENSG00000279561
AC087276.1	0.6302	1.2295E-06	3.1669E-05	ENSG00000254577
ZNF248	0.6285	3.9828E-12	6.9227E-10	ENSG00000198105
ST18	0.6264	1.8271E-05	0.00028466	ENSG00000147488
GPC3	0.6262	4.0743E-06	8.4981E-05	ENSG00000147257
C11orf24	0.6252	1.5475E-09	1.2279E-07	ENSG00000171067

MPO	0.6236	3.1158E-03	0.01729141	ENSG00000005381
LINC00449	0.6234	6.7887E-04	0.00525627	ENSG00000203441
AL133299.1	0.6230	1.1854E-05	0.00020098	ENSG00000261120
OSBP2	0.6228	1.1979E-08	6.6556E-07	ENSG00000184792
AC006058.3	0.6226	4.3640E-06	9.0068E-05	ENSG00000272121
RENBP	0.6221	2.0123E-03	0.01229748	ENSG00000102032
HEYL	0.6217	2.7757E-03	0.01586625	ENSG00000163909
AL356488.3	0.6210	3.2761E-07	1.0503E-05	ENSG00000161653
NA	0.6209	2.8825E-05	0.00041076	ENSG00000289979
AC133552.2	0.6209	9.6351E-09	5.5573E-07	ENSG00000262587
NA	0.6191	4.1852E-03	0.0218908	ENSG00000289731
HSPA1B	0.6181	1.6383E-06	4.0059E-05	ENSG00000204388
NHLH2	0.6180	8.7591E-04	0.00642188	ENSG00000177551
AL354813.1	0.6175	1.9919E-03	0.01221097	ENSG00000255438
ITGA2B	0.6162	1.9701E-04	0.00193753	ENSG00000005961
AC097639.1	0.6144	6.7967E-06	0.00012911	ENSG00000261572
PCSK1	0.6122	8.8970E-07	2.4339E-05	ENSG00000175426
NA	0.6120	1.9784E-08	1.0171E-06	ENSG00000287966
LRRFIP1P1	0.6118	1.6835E-05	0.00026784	ENSG00000240429
AL451085.1	0.6118	4.2041E-05	0.00055744	ENSG00000270361
AC005363.2	0.6107	6.9921E-04	0.0053828	ENSG00000277602
LOC100289230	0.6103	2.7966E-05	0.00040099	ENSG00000248489
NRP1	0.6103	9.4954E-07	2.5592E-05	ENSG00000099250
NA	0.6103	2.9166E-04	0.00266821	ENSG00000289280
C1QL4	0.6098	1.5320E-04	0.00158163	ENSG00000186897
NHLRC3	0.6093	2.9947E-09	2.1196E-07	ENSG00000188811
NA	0.6091	7.0402E-03	0.03251697	ENSG00000290126
NIBAN3	0.6079	2.1813E-03	0.01313739	ENSG00000167483
POSTN	0.6072	8.5566E-04	0.00630969	ENSG00000133110
MRM2	0.6071	1.1568E-03	0.00795619	ENSG00000122687
LURAP1L	0.6059	2.6508E-14	8.1602E-12	ENSG00000153714
ONECUT2	0.6056	4.0374E-08	1.8388E-06	ENSG00000119547
MIR378D2HG	0.6055	4.7511E-03	0.02405319	ENSG00000264448
NA	0.6055	9.2616E-06	0.00016502	ENSG00000288349
USH1C	0.6051	5.0732E-05	0.00065138	ENSG00000006611
EGR3	0.6047	1.0715E-03	0.00748932	ENSG00000179388
NA	0.6047	2.7558E-06	6.2202E-05	ENSG00000235558
SPAG4	0.6044	1.9738E-05	0.00030253	ENSG00000061656
ABHD12B	0.6043	9.4027E-04	0.00679055	ENSG00000131969
MIR124-2HG	0.6032	4.7537E-07	1.4306E-05	ENSG00000254377
TMPRSS4	0.6030	1.4396E-04	0.00150018	ENSG00000137648
ANPEP	0.6030	8.9945E-03	0.03928529	ENSG00000166825
AC146949.1	0.6029	3.5817E-04	0.0031666	ENSG00000161149
GNAO1-AS1	0.6026	5.2398E-03	0.02596455	ENSG00000261439
NTNG1	0.6017	6.8979E-09	4.1964E-07	ENSG00000162631
IL10RB	0.6005	6.0476E-05	0.00074901	ENSG00000243646
TUBA3FP	0.6002	2.8865E-06	6.4188E-05	ENSG00000273382
ALDH1A2	0.6002	1.2277E-04	0.00131805	ENSG00000128918

LOWER IN LEO THAN GROUND: LARGEST TO SMALLEST DIFFERENCE (GREEN)				
CCL20	-2.8385	3.8471E-06	8.12E-05	ENSG00000115009
CXCL8	-2.751	1.1440E-09	9.55E-08	ENSG00000169429
CSN3	-2.3166	1.5892E-05	0.00025589	ENSG00000171209
SMR3B	-2.1816	2.0260E-04	0.00198112	ENSG00000171201
AC103770.1	-2.1476	1.1525E-07	4.36E-06	ENSG00000254251
RRAD	-2.0506	6.3403E-37	5.40E-33	ENSG00000166592
SCRG1	-1.9356	5.6482E-09	3.62E-07	ENSG00000164106
SLC39A12	-1.9083	6.5249E-06	0.00012507	ENSG00000148482
LINC02224	-1.8921	4.2539E-07	1.30E-05	ENSG00000249203
LOC339666	-1.8895	7.8686E-04	0.00591248	ENSG00000230736
RAMP3	-1.8608	2.1140E-07	7.32E-06	ENSG00000122679
AC073941.1	-1.7853	2.8326E-07	9.38E-06	ENSG00000259255
FABP12	-1.7644	1.3496E-03	0.00902987	ENSG00000197416
SLC22A3	-1.7631	1.0698E-07	4.12E-06	ENSG00000146477
KRTAP3-1	-1.7509	7.6109E-04	0.00574497	ENSG00000212901
FOSL1	-1.7034	5.4302E-23	6.31E-20	ENSG00000175592
CXCL13	-1.6806	3.0677E-05	0.00043092	ENSG00000156234
KRT75	-1.6746	2.6293E-03	0.01519928	ENSG00000170454
GZMB	-1.6622	1.5009E-05	0.00024419	ENSG00000100453
CXCL2	-1.6324	1.1820E-08	6.59E-07	ENSG00000081041
C10orf105	-1.6087	3.5893E-08	1.67E-06	ENSG00000214688
VCAM1	-1.6043	1.7976E-08	9.35E-07	ENSG00000162692
PGM5-AS1	-1.5835	3.1381E-16	1.51E-13	ENSG00000224958
MFRP	-1.5654	5.4649E-08	2.39E-06	ENSG00000235718
IBSP	-1.5572	8.6518E-05	0.00099712	ENSG00000029559
NFKBIA	-1.5436	3.1539E-27	7.33E-24	ENSG00000100906
SLCO1B3	-1.5304	2.0212E-04	0.00197721	ENSG00000111700
SMR3A	-1.5261	1.8353E-04	0.00183601	ENSG00000109208
RF00272	-1.516	7.5964E-03	0.0345244	ENSG00000252473
OASL	-1.5107	2.1567E-05	0.00032529	ENSG00000135114
GOLGA6A	-1.5079	1.0926E-07	4.17E-06	ENSG00000159289
CXCL3	-1.5072	5.2135E-06	0.00010367	ENSG00000163734
PLAAT4	-1.4982	1.0395E-07	4.02E-06	ENSG00000133321
TNFAIP3	-1.4865	2.6525E-25	4.52E-22	ENSG00000118503
H2BC9	-1.4744	4.2774E-16	1.99E-13	ENSG00000275713
AC020658.6	-1.4707	1.2131E-03	0.00827182	ENSG00000279409
AC091488.1	-1.4693	2.2838E-05	0.00034105	ENSG00000271320
C15orf48	-1.4667	3.1336E-03	0.01736402	ENSG00000166920
ECM2	-1.4663	5.5250E-09	3.56E-07	ENSG00000106823
AL136366.1	-1.4616	1.6515E-05	0.00026374	ENSG00000225472
SLC2A5	-1.451	7.7702E-05	0.00091407	ENSG00000142583
H4C11	-1.4368	1.0585E-18	7.95E-16	ENSG00000197238
MME	-1.4186	3.1937E-04	0.00287996	ENSG00000196549
AL512598.1	-1.4138	1.2226E-04	0.00131364	ENSG00000273107
SPARCL1	-1.3923	3.6310E-08	1.68E-06	ENSG00000152583
CDK6-AS1	-1.3902	1.5073E-12	2.99E-10	ENSG00000237819
RELB	-1.3882	1.5766E-17	1.01E-14	ENSG00000104856

H4C12	-1.3717	1.0753E-17	7.04E-15	ENSG00000273542
ARHGEF2-AS1	-1.3679	1.2127E-04	0.00130471	ENSG00000224276
GOLGA6D	-1.3552	1.5849E-06	3.91E-05	ENSG00000140478
AC104046.1	-1.3543	2.6970E-27	6.89E-24	ENSG00000259630
NA	-1.3516	4.3677E-04	0.00369653	ENSG00000289044
ANGPTL1	-1.34	5.7456E-24	7.73E-21	ENSG00000116194
NFIA-AS2	-1.3328	8.2990E-07	2.29E-05	ENSG00000237928
H1-2	-1.3173	1.0605E-25	2.08E-22	ENSG00000187837
PMF1-BGLAP	-1.3126	1.1460E-04	0.00124915	ENSG00000260238
ELN	-1.31	1.4577E-30	7.45E-27	ENSG00000049540
ZC3H12A	-1.3062	1.7437E-35	1.11E-31	ENSG00000163874
PTGER3	-1.3041	2.9337E-10	2.96E-08	ENSG00000050628
KCNA1	-1.2992	4.5083E-11	6.03E-09	ENSG00000111262
PLAC9	-1.2921	3.1860E-05	0.00044387	ENSG00000189129
SMANTIS	-1.2742	5.9609E-08	2.57E-06	ENSG00000288869
AL121672.2	-1.2705	3.2449E-04	0.00291837	ENSG00000235159
OGN	-1.2559	2.4505E-04	0.00230877	ENSG00000106809
C7	-1.2553	4.9805E-07	1.49E-05	ENSG00000112936
IGFBP3	-1.2458	1.8611E-05	0.00028883	ENSG00000146674
RPSAP18	-1.2417	2.6796E-03	0.01543049	ENSG00000224261
CD101-AS1	-1.2224	4.2267E-03	0.02204454	ENSG00000236137
AL356309.2	-1.2221	6.2852E-10	5.80E-08	ENSG00000233721
AL138756.1	-1.2177	9.6026E-06	0.00016972	ENSG00000259953
CSF1	-1.2102	1.4184E-25	2.59E-22	ENSG00000184371
IFIT2	-1.2095	2.6759E-09	1.93E-07	ENSG00000119922
AL356417.2	-1.206	5.9519E-06	0.00011591	ENSG00000233682
LINC01338	-1.1936	8.7851E-09	5.12E-07	ENSG00000281327
OR7E29P	-1.193	3.4239E-05	0.00046908	ENSG00000243429
MTND6P18	-1.1917	4.5152E-03	0.02315216	ENSG00000227714
TCERG1L	-1.187	2.4697E-04	0.00232252	ENSG00000176769
AC093908.1	-1.185	3.0673E-03	0.0171042	ENSG00000280219
FGL2	-1.1833	5.6599E-09	3.62E-07	ENSG00000127951
EPCAM-DT	-1.1777	5.1142E-13	1.17E-10	ENSG00000234690
DCN	-1.1732	1.3683E-05	0.00022614	ENSG00000011465
SLC1A3	-1.1715	4.5490E-06	9.31E-05	ENSG00000079215
INHBB	-1.1637	1.8864E-08	9.72E-07	ENSG00000163083
PROKR2	-1.163	5.7725E-05	0.00072124	ENSG00000101292
IFIT3	-1.1522	5.7717E-04	0.00462297	ENSG00000119917
TMEM233	-1.1509	4.3486E-07	1.33E-05	ENSG00000224982
TNFRSF11B	-1.1469	4.6125E-10	4.37E-08	ENSG00000164761
CTXND1	-1.1344	6.4035E-08	2.72E-06	ENSG00000259417
HPD	-1.1336	8.6828E-05	0.00099979	ENSG00000158104
SLC27A6	-1.1328	5.5289E-04	0.00446767	ENSG00000113396
FAM209A	-1.1245	1.5967E-03	0.01028695	ENSG00000124103
WDR38	-1.1215	9.0075E-10	7.78E-08	ENSG00000136918
AL031432.1	-1.1202	2.9283E-03	0.01653884	ENSG00000231953
FDCSP	-1.12	7.4060E-04	0.00562346	ENSG00000181617
LINC01088	-1.111	3.3729E-06	7.28E-05	ENSG00000249307
FAM167A-AS1	-1.1108	7.7366E-03	0.03500185	ENSG00000184608

IQGAP3	-1.1054	2.8685E-04	0.00263072	ENSG00000183856
LINC03013	-1.1011	1.2436E-05	0.00020863	ENSG00000227544
BCAP29	-1.0982	2.0035E-09	1.50E-07	ENSG00000075790
AC130456.1	-1.0951	1.5670E-04	0.00161313	ENSG00000259871
AQP4	-1.0946	6.0146E-06	0.00011704	ENSG00000171885
GAB1	-1.0943	8.3490E-22	8.53E-19	ENSG00000109458
BMPR1B-DT	-1.0902	6.0904E-04	0.00482383	ENSG00000249599
COLEC12	-1.0863	1.8458E-14	5.90E-12	ENSG00000158270
AL390198.2	-1.0806	2.3525E-05	0.00034947	ENSG00000250090
H2BC4	-1.0792	4.1806E-17	2.43E-14	ENSG00000180596
MGP	-1.0749	2.9113E-05	0.00041326	ENSG00000111341
GOLGA6B	-1.0723	1.1834E-06	3.08E-05	ENSG00000215186
LINC01621	-1.0705	1.6580E-06	4.05E-05	ENSG00000235357
LOC124905349	-1.0701	8.8543E-04	0.00647316	ENSG00000224272
COMMD5P1	-1.0683	1.4681E-05	0.00023985	ENSG00000173966
ZC3H12C	-1.0683	7.1933E-38	9.19E-34	ENSG00000149289
ADGRG6	-1.0668	7.3855E-09	4.45E-07	ENSG00000112414
CFAP52	-1.063	2.2894E-11	3.38E-09	ENSG00000166596
CLEC3A	-1.0628	3.3083E-04	0.00296181	ENSG00000166509
DYNLT5	-1.0627	1.8646E-06	4.45E-05	ENSG00000152760
NA	-1.0605	5.7625E-06	0.000113	ENSG00000290046
HTR1D	-1.0602	7.1727E-04	0.00548714	ENSG00000179546
DAW1	-1.0547	3.8489E-10	3.74E-08	ENSG00000123977
FNDC1	-1.0519	2.5833E-05	0.00037589	ENSG00000164694
NMI	-1.0502	2.0506E-06	4.83E-05	ENSG00000123609
TMEM171	-1.05	1.8150E-03	0.01134441	ENSG00000157111
LINC01354	-1.0499	2.4478E-05	0.00035924	ENSG00000231768
ATF3	-1.0498	9.1887E-51	2.35E-46	ENSG00000162772
IRF1	-1.0466	4.0377E-17	2.40E-14	ENSG00000125347
NKX2-1-AS1	-1.0368	4.3349E-04	0.00367488	ENSG00000253563
LOC124901261	-1.0364	1.7128E-03	0.01086476	ENSG00000289257
GLIPR1L2	-1.0309	7.7266E-06	0.00014368	ENSG00000180481
PTPRC	-1.0299	1.5264E-03	0.009949	ENSG00000081237
BTC	-1.0291	6.7722E-09	4.14E-07	ENSG00000174808
RNF175	-1.0255	7.0171E-07	1.98E-05	ENSG00000145428
MXRA5	-1.0248	3.8326E-04	0.0033342	ENSG00000101825
CXCL12	-1.0243	4.7023E-08	2.10E-06	ENSG00000107562
PRND	-1.0229	5.3944E-04	0.00437565	ENSG00000171864
STRIT1	-1.019	4.6250E-04	0.00385932	ENSG00000240045
ADGB	-1.0187	5.2824E-03	0.02612272	ENSG00000118492
NA	-1.0161	1.0686E-04	0.0011804	ENSG00000287251
CCL2	-1.0096	5.4595E-05	0.00068819	ENSG00000108691
AC004846.2	-1.0076	2.4381E-04	0.00229791	ENSG00000258944
AC125437.1	-1.0068	2.1047E-06	4.94E-05	ENSG00000267655
AL049781.1	-1.0062	2.6580E-03	0.0153373	ENSG00000280281
LOC107986951	-1.0033	3.4501E-04	0.00306513	ENSG00000254337
LRRC2	-1.0005	1.0958E-06	2.89E-05	ENSG00000163827
PLAU	-0.9991	7.2122E-04	0.00550745	ENSG00000122861
RN7SL1	-0.9984	9.7319E-07	2.61E-05	ENSG00000276168

NEDD9	-0.9968	2.8426E-11	4.12E-09	ENSG00000111859
DRC7	-0.9939	1.7358E-05	0.00027442	ENSG00000159625
LOC124908056	-0.9921	1.3078E-05	0.00021698	ENSG00000228999
DDIT3	-0.9886	2.1663E-22	2.31E-19	ENSG00000175197
CCDC190	-0.9884	2.1617E-04	0.00207715	ENSG00000185860
DCT	-0.9873	2.8958E-05	0.00041174	ENSG00000080166
IGFBP7-AS1	-0.9859	8.5600E-05	0.00098789	ENSG00000245067
SPR	-0.985	4.1226E-14	1.18E-11	ENSG00000116096
BARX2	-0.9843	1.4123E-03	0.00936384	ENSG00000043039
H3C2	-0.9801	5.1604E-04	0.00421525	ENSG00000286522
NFKB1	-0.9793	6.7559E-20	6.06E-17	ENSG00000109320
CAV3	-0.9776	8.3778E-03	0.03720351	ENSG00000182533
CD83	-0.9774	2.2215E-10	2.31E-08	ENSG00000112149
AC092675.1	-0.9767	1.0420E-03	0.00734084	ENSG00000222000
ASB12	-0.9754	4.3807E-04	0.00370416	ENSG00000198881
CDC42EP1	-0.9744	1.2871E-08	7.00E-07	ENSG00000128283
CCDC170	-0.9733	3.7936E-10	3.71E-08	ENSG00000120262
TMEM221	-0.9712	1.5796E-08	8.33E-07	ENSG00000188051
ITGB7	-0.9676	2.9494E-03	0.01663202	ENSG00000139626
PRELP	-0.9639	3.1794E-05	0.00044344	ENSG00000188783
CFH	-0.9614	9.6069E-06	0.00016972	ENSG00000000971
COL12A1	-0.9613	7.2539E-08	2.99E-06	ENSG00000111799
GJD2	-0.9582	1.1630E-04	0.00126398	ENSG00000159248
CD34	-0.9564	9.8842E-05	0.00110769	ENSG00000174059
GADD45A	-0.9539	6.9319E-29	2.53E-25	ENSG00000116717
LINC00348	-0.953	2.8479E-03	0.01619212	ENSG00000226846
OPN3	-0.9509	3.0129E-08	1.46E-06	ENSG00000054277
PTX3	-0.9502	5.1416E-05	0.00065752	ENSG00000163661
GOLGA6C	-0.9496	3.7354E-05	0.00050445	ENSG00000167195
SUSD5	-0.9482	1.8601E-05	0.00028883	ENSG00000173705
CSRNP1	-0.947	1.5861E-21	1.56E-18	ENSG00000144655
MDFIC	-0.9469	2.5348E-15	1.01E-12	ENSG00000135272
NFIX	-0.9386	4.7010E-08	2.10E-06	ENSG00000008441
ACTA2	-0.9359	4.3788E-04	0.00370416	ENSG00000107796
ZNF404	-0.9352	8.6508E-07	2.38E-05	ENSG00000176222
NA	-0.9351	2.7209E-04	0.00251439	ENSG00000288044
THEM7P	-0.9342	2.2193E-05	0.00033337	ENSG00000227160
SMAD9-IT1	-0.9308	1.0298E-02	0.0434189	ENSG00000236711
FGF1	-0.9295	1.2311E-06	3.17E-05	ENSG00000113578
SOD3	-0.9252	1.1824E-10	1.38E-08	ENSG00000109610
ANGPTL2	-0.9251	2.0635E-15	8.50E-13	ENSG00000136859
CLEC19A	-0.923	8.8832E-05	0.00101555	ENSG00000261210
PSMB9	-0.921	3.5241E-08	1.65E-06	ENSG00000240065
AC096570.1	-0.9201	2.7657E-03	0.01583377	ENSG00000234207
AC110792.2	-0.92	6.6861E-03	0.03127055	ENSG00000269506
GSC	-0.9165	1.8943E-03	0.0117277	ENSG00000133937
RND1	-0.9157	4.4497E-14	1.26E-11	ENSG00000172602
ITGA2	-0.9154	2.9711E-07	9.75E-06	ENSG00000164171
SPMIP6	-0.9153	2.1962E-09	1.62E-07	ENSG00000164972

ENKUR	-0.9148	3.5132E-12	6.32E-10	ENSG00000151023
KCNMB1	-0.9135	2.1591E-04	0.00207553	ENSG00000145936
GP9	-0.9122	4.1195E-03	0.02161772	ENSG00000169704
AC026124.2	-0.9091	1.6736E-14	5.48E-12	ENSG00000276853
LINC00113	-0.907	3.2648E-03	0.01792005	ENSG00000225298
LINC01896	-0.907	2.8443E-06	6.35E-05	ENSG00000263146
KCNIP3	-0.904	1.1398E-12	2.39E-10	ENSG00000115041
TTR	-0.903	2.3473E-06	5.41E-05	ENSG00000118271
LINC02306	-0.9022	4.6159E-04	0.00385632	ENSG00000257986
LINC00880	-0.9013	1.4400E-04	0.00150018	ENSG00000243629
TMPRSS7	-0.9004	2.0485E-04	0.00199547	ENSG00000176040
HBEGF	-0.8996	1.3818E-14	4.59E-12	ENSG00000113070
CXCL1	-0.899	3.9554E-03	0.0209373	ENSG00000163739
HCG17	-0.898	4.4842E-05	0.00058877	ENSG00000270604
NKX2-4	-0.8942	2.4502E-06	5.62E-05	ENSG00000125816
CKLF	-0.893	1.0389E-06	2.76E-05	ENSG00000217555
DAND5	-0.891	2.0816E-05	0.00031566	ENSG00000179284
LOC101926964	-0.8891	2.9408E-03	0.01660217	ENSG00000231252
RAB39A	-0.8842	2.6054E-08	1.28E-06	ENSG00000179331
LOC102723604	-0.8823	2.2065E-03	0.01325928	ENSG00000258928
SLC15A2	-0.8799	1.9779E-05	0.00030298	ENSG00000163406
SMIM5	-0.8796	1.1251E-09	9.43E-08	ENSG00000204323
FAM180B	-0.8757	1.5366E-03	0.01000276	ENSG00000196666
NA	-0.8751	2.6436E-06	6.01E-05	ENSG00000289516
C8orf88	-0.8729	1.5169E-06	3.76E-05	ENSG00000253250
COL22A1	-0.872	3.7509E-04	0.00328101	ENSG00000169436
ATP1A2	-0.87	1.1772E-03	0.00807253	ENSG00000018625
MATN2	-0.8691	1.3734E-09	1.10E-07	ENSG00000132561
MMP21	-0.8654	7.1365E-03	0.03284307	ENSG00000154485
MAFF	-0.8614	2.6482E-19	2.11E-16	ENSG00000185022
SCART1	-0.859	3.0206E-03	0.01692545	ENSG00000214279
EMCN	-0.8573	4.8678E-04	0.00400775	ENSG00000164035
ARHGEF26-AS1	-0.8561	7.9672E-08	3.24E-06	ENSG00000243069
HTR1A	-0.8558	7.4949E-04	0.00567747	ENSG00000178394
ADGRG3	-0.8538	9.6190E-07	2.58E-05	ENSG00000182885
CYP7B1	-0.8534	2.9554E-05	0.00041813	ENSG00000172817
AL161757.4	-0.8533	1.9945E-04	0.00195856	ENSG00000258776
IGFBP7	-0.8516	3.8623E-04	0.00334751	ENSG00000163453
TROAP	-0.8508	1.3824E-07	5.07E-06	ENSG00000135451
ZBTB2	-0.8503	1.2602E-15	5.55E-13	ENSG00000181472
LOC100129534	-0.8466	7.9171E-04	0.00594357	ENSG00000269896
IER3	-0.8462	9.7934E-09	5.59E-07	ENSG00000137331
UBE2FP1	-0.8458	1.0730E-04	0.00118425	ENSG00000224080
ZNF474	-0.8445	2.3642E-07	8.05E-06	ENSG00000164185
PTPRZ1	-0.8427	3.5808E-06	7.67E-05	ENSG00000106278
LPAR4	-0.8422	5.7720E-06	0.00011302	ENSG00000147145
PLP1	-0.841	1.7549E-05	0.00027635	ENSG00000123560
ACAN	-0.8394	9.3930E-03	0.04056348	ENSG00000157766
AC015845.2	-0.8391	3.9775E-03	0.02102675	ENSG00000265542

WFIKKN1	-0.8387	6.0766E-10	5.63E-08	ENSG00000127578
PLSCR4	-0.838	3.6153E-06	7.72E-05	ENSG00000114698
MAOB	-0.8354	1.7706E-05	0.00027823	ENSG00000069535
AC087741.1	-0.8354	5.5570E-05	0.0006991	ENSG00000262580
FAS	-0.8351	9.2563E-07	2.51E-05	ENSG00000026103
SPRY2	-0.8332	1.3053E-11	2.03E-09	ENSG00000136158
MTCYBP3	-0.8314	8.0773E-03	0.03623944	ENSG00000232373
BIRC3	-0.8301	7.3639E-05	0.00087677	ENSG00000023445
H1-3	-0.8271	1.1353E-05	0.00019468	ENSG00000124575
AL121895.1	-0.8269	8.8529E-04	0.00647316	ENSG00000232406
KCNQ4	-0.8267	4.1293E-04	0.00352989	ENSG00000117013
TBX2	-0.826	2.5811E-03	0.0149603	ENSG00000121068
MSX2	-0.8255	1.5054E-05	0.00024452	ENSG00000120149
PGM5P4-AS1	-0.8248	1.5598E-07	5.62E-06	ENSG00000231943
AC107959.3	-0.8247	5.3936E-03	0.0265489	ENSG00000253616
CFAP77	-0.8242	1.5146E-07	5.47E-06	ENSG00000188523
COL4A1	-0.8222	1.9480E-06	4.63E-05	ENSG00000187498
RORA-AS1	-0.8207	1.3216E-05	0.00021913	ENSG00000245534
GFAP	-0.8201	4.7830E-03	0.02418553	ENSG00000131095
AL590714.1	-0.8175	4.7947E-03	0.02424028	ENSG00000224985
C22orf31	-0.8161	1.6959E-04	0.00172153	ENSG00000100249
ZFP36L1	-0.8153	1.0312E-10	1.22E-08	ENSG00000185650
CTSV	-0.815	4.5507E-04	0.00381227	ENSG00000136943
LOC124906859	-0.8148	9.5200E-08	3.75E-06	ENSG00000225655
GHR	-0.8146	1.3362E-07	4.93E-06	ENSG00000112964
TMEM35B	-0.8123	2.2413E-03	0.0134401	ENSG00000243749
LINC01218	-0.8106	1.8070E-04	0.00181092	ENSG00000251636
C1orf226	-0.8079	2.9956E-08	1.45E-06	ENSG00000239887
NT5E	-0.8078	5.4320E-05	0.00068574	ENSG00000135318
NEUROG2	-0.8075	1.0496E-03	0.00737555	ENSG00000178403
GCNT4	-0.8072	3.7645E-06	7.98E-05	ENSG00000176928
ANKRD66	-0.8059	9.6307E-04	0.00691552	ENSG00000230062
AC005906.2	-0.8043	2.9638E-03	0.01669014	ENSG00000256654
P2RX6P	-0.8039	4.6572E-05	0.00060867	ENSG00000206145
PGM5P3-AS1	-0.8034	4.9238E-07	1.48E-05	ENSG00000277631
H1-4	-0.8033	3.3891E-12	6.14E-10	ENSG00000168298
SALL3	-0.8033	5.8709E-07	1.71E-05	ENSG00000256463
SMOC1	-0.803	5.6370E-07	1.65E-05	ENSG00000198732
LOC101927040	-0.8017	9.9566E-05	0.00111336	ENSG00000249328
MBOAT1	-0.8015	1.3791E-03	0.00918146	ENSG00000172197
LINC00327	-0.8002	4.8135E-03	0.02429184	ENSG00000232977
CFAP107	-0.7976	1.2149E-06	3.14E-05	ENSG00000157330
AC023886.1	-0.7968	1.3754E-03	0.00917336	ENSG00000249509
AL139156.2	-0.7963	2.2978E-26	4.89E-23	ENSG00000228407
LINC00622	-0.796	3.1875E-03	0.01762449	ENSG00000260941
NTRK2	-0.7957	2.8956E-11	4.16E-09	ENSG00000148053
CFAP161	-0.7952	4.0538E-04	0.0034805	ENSG00000156206
C11orf96	-0.7938	1.0684E-06	2.83E-05	ENSG00000187479
C16orf89	-0.7928	1.3992E-06	3.53E-05	ENSG00000153446

RGS10	-0.7924	7.8699E-04	0.00591248	ENSG00000148908
SCD	-0.7921	3.4845E-10	3.45E-08	ENSG00000099194
SPINK2	-0.7919	2.9819E-06	6.58E-05	ENSG00000128040
OLFML2B	-0.7913	9.3783E-07	2.53E-05	ENSG00000162745
HSD17B2	-0.7911	1.1092E-03	0.00769934	ENSG00000086696
YAP1	-0.7902	3.9485E-11	5.40E-09	ENSG00000137693
UNC5B-AS1	-0.7899	1.5933E-06	3.92E-05	ENSG00000237512
LOC105370705	-0.7885	4.0028E-04	0.00344243	ENSG00000288795
PDGFRB	-0.7868	1.2769E-05	0.00021311	ENSG00000113721
RHOD	-0.7831	9.0317E-06	0.0001616	ENSG00000173156
LCAT	-0.7818	5.0257E-08	2.23E-06	ENSG00000213398
LNCTAM34A	-0.7816	1.1982E-08	6.66E-07	ENSG00000234546
IGSF5	-0.7811	3.0746E-05	0.00043157	ENSG00000183067
KLF10	-0.7808	7.2023E-12	1.15E-09	ENSG00000155090
CALCRL	-0.7806	9.8184E-08	3.84E-06	ENSG00000064989
AEBP1	-0.7799	6.2272E-04	0.00490779	ENSG00000106624
MAP3K19	-0.7774	2.9286E-07	9.66E-06	ENSG00000176601
NUP153-AS1	-0.7748	3.5085E-03	0.01903316	ENSG00000272269
PGA5	-0.7736	6.4538E-03	0.03048635	ENSG00000256713
PTHLH	-0.7736	2.4077E-03	0.01420103	ENSG00000087494
CCDC81	-0.7735	1.2210E-05	0.00020553	ENSG00000149201
PARP14	-0.7709	1.4595E-09	1.16E-07	ENSG00000173193
AF228730.4	-0.7703	1.7910E-04	0.00180168	ENSG00000284614
IFFO2	-0.7702	5.2832E-13	1.19E-10	ENSG00000169991
LOC107985579	-0.7701	2.9745E-03	0.01671889	ENSG00000181123
MYOZ3	-0.7699	3.4358E-03	0.01870517	ENSG00000164591
RGR	-0.7678	2.0273E-03	0.01236841	ENSG00000148604
FBLN5	-0.7671	9.7580E-08	3.83E-06	ENSG00000140092
PGA3	-0.7665	5.7618E-03	0.02793751	ENSG00000229859
BARHL2	-0.7662	8.6530E-07	2.38E-05	ENSG00000143032
TRIM47	-0.7652	1.9283E-09	1.46E-07	ENSG00000132481
HERC5	-0.7647	3.8631E-07	1.20E-05	ENSG00000138646
OLMALINC	-0.7643	3.6409E-09	2.51E-07	ENSG00000235823
ID1	-0.7642	6.3500E-17	3.45E-14	ENSG00000125968
CYYR1	-0.7634	1.4029E-05	0.00023066	ENSG00000166265
CYP4F24P	-0.7617	2.4104E-03	0.01421362	ENSG00000267594
TNC	-0.7613	3.2679E-09	2.28E-07	ENSG00000041982
HSPA5	-0.7612	1.0971E-13	2.83E-11	ENSG00000044574
DLGAP1-AS1	-0.7611	1.1896E-06	3.08E-05	ENSG00000177337
MLC1	-0.76	5.1343E-05	0.00065725	ENSG00000100427
TICAM1	-0.7594	1.6972E-06	4.12E-05	ENSG00000127666
TAC4	-0.7593	3.6426E-08	1.68E-06	ENSG00000176358
CFAP144	-0.757	2.1916E-08	1.11E-06	ENSG00000186973
NA	-0.7557	3.5863E-03	0.01938426	ENSG00000286545
ERAP2	-0.7538	6.2964E-05	0.00077495	ENSG00000164308
LINC01216	-0.7514	8.1989E-03	0.03663052	ENSG00000250223
ZMYND10	-0.7505	1.6363E-07	5.84E-06	ENSG00000004838
EMC1-AS1	-0.7481	6.9080E-08	2.87E-06	ENSG00000230424
ERFE	-0.7475	3.8766E-03	0.02061408	ENSG00000178752

DUSP10	-0.7473	1.2792E-22	1.42E-19	ENSG00000143507
AC010319.3	-0.7468	1.0405E-03	0.00733399	ENSG00000269053
AC008514.2	-0.7467	4.2030E-04	0.00358205	ENSG00000253966
ANXA1	-0.746	4.8342E-07	1.45E-05	ENSG00000135046
AC090907.1	-0.746	6.8320E-03	0.03179114	ENSG00000259376
C8orf34	-0.7442	2.0845E-05	0.0003159	ENSG00000165084
C5orf56	-0.7432	6.9563E-07	1.97E-05	ENSG00000197536
SPDEF	-0.7429	6.4485E-06	0.00012407	ENSG00000124664
AL133163.3	-0.7419	3.1563E-04	0.00285372	ENSG00000259017
PTN	-0.7409	3.1473E-07	1.02E-05	ENSG00000105894
C2orf74	-0.7392	2.2458E-04	0.00214914	ENSG00000237651
AC069366.2	-0.7383	7.9630E-03	0.03582375	ENSG00000265801
ADAM33	-0.7379	1.2232E-03	0.00833189	ENSG00000149451
LRRC37A5P	-0.7357	1.7800E-05	0.00027933	ENSG00000204173
PCLAF	-0.7345	1.7257E-05	0.00027328	ENSG00000166803
TIPARP	-0.7337	8.2214E-28	2.63E-24	ENSG00000163659
OR2L13	-0.7331	2.1835E-03	0.01314566	ENSG00000196071
GJC2	-0.7322	4.6798E-03	0.02377196	ENSG00000198835
ALDH1A1	-0.7278	5.3887E-11	6.99E-09	ENSG00000165092
MECOM	-0.7275	5.5631E-07	1.63E-05	ENSG00000085276
PTAR1	-0.7266	3.7412E-10	3.68E-08	ENSG00000188647
GNG12-AS1	-0.7253	2.6036E-05	0.00037841	ENSG00000232284
PRRX1	-0.7249	4.9988E-04	0.0040977	ENSG00000116132
AC009054.2	-0.7237	5.4412E-03	0.02674145	ENSG00000261783
VGLL3	-0.7225	3.2963E-08	1.56E-06	ENSG00000206538
NEURL3	-0.7208	1.4685E-03	0.0096504	ENSG00000163121
ACSM5	-0.7203	5.4901E-04	0.00444336	ENSG00000183549
AC131571.1	-0.7203	6.1176E-04	0.00484084	ENSG00000228061
CFAP276	-0.72	1.4159E-10	1.61E-08	ENSG00000179902
GOLIM4	-0.7199	9.0007E-14	2.37E-11	ENSG00000173905
LRRC52	-0.7181	7.6565E-05	0.00090403	ENSG00000162763
DLX3	-0.7172	4.2172E-03	0.02200865	ENSG00000064195
IGF2BP2-AS1	-0.7172	7.2389E-07	2.03E-05	ENSG00000163915
ATE1-AS1	-0.7166	2.0416E-04	0.00199179	ENSG00000226864
RHOJ	-0.7155	2.5435E-10	2.62E-08	ENSG00000126785
AL162390.1	-0.7141	1.9336E-05	0.00029727	ENSG00000274421
OPN1SW	-0.7131	8.1547E-03	0.03652259	ENSG00000128617
ANXA4	-0.712	1.4412E-08	7.70E-07	ENSG00000196975
STAG2-AS1	-0.7119	3.0023E-05	0.00042359	ENSG00000232412
EFHD1	-0.7118	1.2601E-09	1.04E-07	ENSG00000115468
STEAP1	-0.7111	1.8360E-05	0.00028588	ENSG00000164647
SOSTDC1	-0.711	1.3604E-03	0.00908949	ENSG00000171243
CCND2-AS1	-0.7102	1.1429E-05	0.00019546	ENSG00000255920
CLIC6	-0.7092	3.4098E-04	0.00303609	ENSG00000159212
KLF9	-0.7087	6.6156E-09	4.07E-07	ENSG00000119138
MORN5	-0.7086	1.8521E-06	4.44E-05	ENSG00000185681
LOC102723834	-0.7078	7.6077E-03	0.03456356	ENSG00000227496
NFKBIE	-0.7065	2.0530E-24	3.09E-21	ENSG00000146232
CHAC1	-0.7061	1.7352E-13	4.30E-11	ENSG00000128965

GRIK4	-0.7052	5.4443E-10	5.11E-08	ENSG00000149403
MNS1	-0.7049	3.7264E-09	2.55E-07	ENSG00000138587
GPR35	-0.7047	3.2813E-03	0.01798358	ENSG00000178623
NEK11	-0.7043	6.3376E-08	2.70E-06	ENSG00000114670
MSTN	-0.7037	2.5254E-03	0.01471876	ENSG00000138379
SKA3	-0.7034	1.4876E-05	0.00024257	ENSG00000165480
AC008494.3	-0.703	1.1644E-03	0.0080001	ENSG00000271797
GPR87	-0.702	6.1544E-03	0.02942602	ENSG00000138271
FABP7	-0.7019	2.3268E-04	0.0022118	ENSG00000164434
NME4	-0.7016	6.2511E-11	8.03E-09	ENSG00000103202
DSCC1	-0.7	1.2647E-05	0.00021157	ENSG00000136982
MPIG6B	-0.6996	4.4261E-03	0.02281877	ENSG00000204420
HACD4	-0.6982	2.4953E-05	0.00036475	ENSG00000188921
HOATZ	-0.6969	4.8073E-06	9.73E-05	ENSG00000183644
TRPV4	-0.6961	6.6825E-04	0.00519455	ENSG00000111199
DIAPH3	-0.696	7.2650E-06	0.00013648	ENSG00000139734
SOX9	-0.6958	3.7690E-09	2.57E-07	ENSG00000125398
ITPKB	-0.6943	8.8137E-09	5.12E-07	ENSG00000143772
SERPINH1	-0.6933	1.1553E-06	3.02E-05	ENSG00000149257
ID3	-0.6925	9.0131E-10	7.78E-08	ENSG00000117318
MRPS30-DT	-0.6925	2.1804E-07	7.53E-06	ENSG00000251141
SLC24A5	-0.6904	4.8947E-03	0.02458499	ENSG00000188467
CA14	-0.6889	7.0071E-10	6.33E-08	ENSG00000118298
C1QTNF5	-0.6883	5.9815E-03	0.02878342	ENSG00000223953
SIDT1	-0.6875	5.2398E-04	0.00427189	ENSG00000072858
CFAP126	-0.6868	3.0522E-06	6.71E-05	ENSG00000188931
LINC00513	-0.6867	2.2480E-03	0.0134737	ENSG00000233559
STON1	-0.6847	4.0741E-08	1.85E-06	ENSG00000243244
ARMC3	-0.6844	8.2332E-08	3.32E-06	ENSG00000165309
NA	-0.6844	1.5907E-05	0.00025589	ENSG00000287620
IFIT1	-0.6839	5.1266E-03	0.02550431	ENSG00000185745
RHOH	-0.6838	2.7849E-05	0.00039976	ENSG00000168421
RPS20P22	-0.6834	9.8029E-04	0.00700041	ENSG00000239218
AC008443.5	-0.6825	1.1818E-05	0.00020077	ENSG00000250222
LRRC8C	-0.6821	2.5597E-07	8.63E-06	ENSG00000171488
CFAP100	-0.6813	1.0110E-04	0.00112857	ENSG00000163885
ABCA11P	-0.6807	6.0926E-03	0.02919025	ENSG00000251595
AL035443.1	-0.6805	6.1588E-03	0.02944104	ENSG00000224765
MTHFS	-0.6802	4.2370E-06	8.79E-05	ENSG00000136371
NPHP3-AS1	-0.6791	8.3201E-03	0.0370256	ENSG00000248724
AC145141.2	-0.679	1.5706E-03	0.01016194	ENSG00000253985
COL8A1	-0.6787	3.0275E-03	0.0169566	ENSG00000144810
RPL7AP60	-0.6784	2.4136E-03	0.01422077	ENSG00000213152
AAMDC	-0.6778	1.3627E-12	2.82E-10	ENSG00000087884
PRR34-AS1	-0.6773	1.6980E-03	0.01078685	ENSG00000241990
HFE	-0.6766	5.6337E-04	0.00453232	ENSG00000010704
ARMCX5-GPRASP2	-0.676	9.8431E-07	2.63E-05	ENSG00000271147
TMT1A	-0.6757	7.2267E-12	1.15E-09	ENSG00000185432
JMJD1C	-0.6751	8.3645E-25	1.34E-21	ENSG00000171988

CFAP54	-0.6749	5.8687E-07	1.71E-05	ENSG00000188596
SRGAP3-AS4	-0.6731	3.2929E-03	0.01803202	ENSG00000235830
AC026470.2	-0.6722	3.9190E-04	0.00338631	ENSG00000261267
HLA-DMA	-0.6712	2.0620E-07	7.16E-06	ENSG00000204257
GPHA2	-0.6708	1.8067E-03	0.01130306	ENSG00000149735
HOPX	-0.6705	3.2676E-08	1.55E-06	ENSG00000171476
CMKLR2	-0.67	2.1454E-05	0.00032398	ENSG00000183671
RNU1-103P	-0.6694	5.0080E-03	0.02502139	ENSG00000252311
C2orf73	-0.6692	6.9938E-05	0.00084094	ENSG00000177994
LRRC4	-0.6689	3.2321E-07	1.04E-05	ENSG00000128594
ZNF230-DT	-0.6687	9.9543E-04	0.00708669	ENSG00000266921
SLC6A20	-0.6686	7.6717E-03	0.03480689	ENSG00000163817
MGST1	-0.6683	1.5731E-06	3.88E-05	ENSG00000008394
OAS3	-0.6683	6.2212E-04	0.00490609	ENSG00000111331
LRRC77P	-0.6664	4.6627E-03	0.02373818	ENSG00000244227
SLC17A8	-0.6663	3.8359E-05	0.00051612	ENSG00000179520
KCNJ8	-0.6661	4.1633E-04	0.0035542	ENSG00000121361
SLC24A3	-0.666	5.1088E-06	0.00010198	ENSG00000185052
CTAG1A	-0.6658	5.6112E-03	0.02740787	ENSG00000268651
HSPA5P1	-0.6654	2.0750E-03	0.01261156	ENSG00000215895
IFI35	-0.6649	1.6302E-07	5.83E-06	ENSG00000068079
AC080023.1	-0.6643	6.2994E-03	0.0299061	ENSG00000254554
DDX60	-0.6641	2.7465E-04	0.00253067	ENSG00000137628
HSPB1	-0.6641	1.7264E-07	6.14E-06	ENSG00000106211
PARP9	-0.6634	4.8838E-08	2.17E-06	ENSG00000138496
HMCN1	-0.6623	3.1106E-04	0.00281638	ENSG00000143341
FBXW4P1	-0.6621	1.1903E-04	0.00128659	ENSG00000230701
PIGHP1	-0.662	4.5825E-08	2.06E-06	ENSG00000259657
ATP6V1C2	-0.661	7.4720E-05	0.00088757	ENSG00000143882
LOC105377862	-0.6607	8.2906E-08	3.34E-06	ENSG00000271945
EMP3	-0.6606	3.0368E-11	4.31E-09	ENSG00000142227
ZIC2	-0.6599	2.0438E-04	0.00199283	ENSG00000043355
CIART	-0.659	2.1837E-10	2.29E-08	ENSG00000159208
AL450124.1	-0.6586	1.0166E-03	0.00719923	ENSG00000204556
SPC25	-0.6578	2.0693E-04	0.00201037	ENSG00000152253
PTGS2	-0.6573	1.8578E-03	0.01156369	ENSG00000073756
SETD7	-0.6573	3.4575E-16	1.64E-13	ENSG00000145391
E2F2	-0.6568	1.8867E-04	0.00187298	ENSG00000007968
AL353150.1	-0.6564	8.6616E-04	0.00636505	ENSG00000224945
CD58	-0.6563	1.9669E-07	6.86E-06	ENSG00000116815
H2BC5	-0.6563	2.7783E-09	1.99E-07	ENSG00000158373
ZNF695	-0.6546	3.6537E-03	0.01965787	ENSG00000197472
SFTA3	-0.6537	1.4131E-03	0.00936384	ENSG00000229415
CFAP53	-0.6526	2.9486E-13	7.11E-11	ENSG00000172361
FILIP1	-0.6509	2.5097E-04	0.00235236	ENSG00000118407
H4C3	-0.6504	2.2193E-07	7.64E-06	ENSG00000197061
SLC12A4	-0.6501	9.6823E-06	0.00017073	ENSG00000124067
TBX2-AS1	-0.65	7.0438E-03	0.0325278	ENSG00000267280
HBA2	-0.6498	6.5588E-03	0.03086252	ENSG00000188536

TFPI	-0.6489	1.7721E-03	0.01113446	ENSG00000003436
ZNF732	-0.6481	3.0105E-03	0.01687991	ENSG00000186777
COBLL1	-0.6478	7.7131E-06	0.00014354	ENSG00000082438
TRIM17	-0.6445	1.4258E-03	0.00943439	ENSG00000162931
ADORA1	-0.6442	1.2239E-03	0.00833326	ENSG00000163485
TPX2	-0.6439	3.2319E-06	7.02E-05	ENSG00000088325
CHST14	-0.6434	4.8640E-06	9.82E-05	ENSG00000169105
SERF2	-0.6434	2.2652E-10	2.34E-08	ENSG00000140264
WNT8B	-0.6428	1.4382E-03	0.00949767	ENSG00000075290
FBXL7	-0.6425	2.3022E-08	1.16E-06	ENSG00000183580
CATIP	-0.6422	1.1537E-04	0.00125648	ENSG00000158428
TXLNB	-0.6416	2.3759E-08	1.19E-06	ENSG00000164440
PCP4	-0.6401	4.3342E-10	4.18E-08	ENSG00000183036
PLSCR5	-0.6393	8.0100E-03	0.03598182	ENSG00000231213
UCP3	-0.6385	1.0798E-02	0.04495854	ENSG00000175564
TUBB6	-0.6384	9.3876E-06	0.00016681	ENSG00000176014
FJX1	-0.6381	6.2569E-08	2.68E-06	ENSG00000179431
NA	-0.6377	7.0534E-05	0.00084612	ENSG00000289133
GREP1	-0.6372	1.0990E-03	0.00764956	ENSG00000262152
RPL23AP42	-0.637	4.2979E-12	7.42E-10	ENSG00000234851
MUC15	-0.6365	1.0159E-04	0.00113197	ENSG00000169550
BTN3A3	-0.6355	1.1409E-04	0.00124632	ENSG00000111801
CA8	-0.6353	4.5199E-06	9.28E-05	ENSG00000178538
RDM1	-0.6338	3.0144E-04	0.00273902	ENSG00000278023
SLC16A4	-0.6318	8.7980E-05	0.00100852	ENSG00000168679
SSPOP	-0.6318	4.4626E-04	0.00375943	ENSG00000197558
HNRNPCP4	-0.6304	6.5969E-03	0.03099642	ENSG00000263179
ITPRIP	-0.6294	5.1915E-10	4.89E-08	ENSG00000148841
HERPUD1	-0.6292	4.1329E-23	5.03E-20	ENSG00000051108
FOLR1	-0.6281	3.0685E-03	0.01710719	ENSG00000110195
SPC24	-0.6275	1.5297E-04	0.00158046	ENSG00000161888
C21orf62	-0.6274	2.1334E-06	4.99E-05	ENSG00000205929
DNAAF11	-0.6274	1.7369E-07	6.17E-06	ENSG00000129295
AC015983.2	-0.6258	7.1328E-03	0.03283776	ENSG00000234418
LINC02041	-0.6256	7.0953E-03	0.03271829	ENSG00000228952
CENPW	-0.6254	2.2222E-07	7.64E-06	ENSG00000203760
PAX6	-0.6252	2.1490E-03	0.01298098	ENSG00000007372
IRF2BP2	-0.6236	1.3224E-13	3.35E-11	ENSG00000168264
NFIC	-0.6231	4.5735E-10	4.36E-08	ENSG00000141905
RN7SL4P	-0.6231	1.5689E-03	0.01015395	ENSG00000263740
AC108693.1	-0.6229	4.7983E-03	0.02424857	ENSG00000239280
NEK2-DT	-0.6227	4.7348E-03	0.02400372	ENSG00000231057
C5	-0.6209	8.5633E-06	0.00015496	ENSG00000106804
USP2	-0.6206	1.4693E-10	1.64E-08	ENSG00000036672
ATOH8	-0.6203	3.1777E-07	1.03E-05	ENSG00000168874
AC012513.3	-0.6193	2.9531E-04	0.00269385	ENSG00000279348
VAMP5	-0.6176	1.7370E-05	0.00027442	ENSG00000168899
NGB	-0.6169	2.2731E-03	0.01358035	ENSG00000165553
CPS1	-0.6168	4.3600E-05	0.00057591	ENSG00000021826

AC098864.1	-0.616	1.0369E-07	4.02E-06	ENSG00000177822
KLF15	-0.6154	4.4490E-03	0.02288158	ENSG00000163884
CEP55	-0.6142	3.2982E-05	0.0004543	ENSG00000138180
ARID5B	-0.6136	1.9355E-23	2.47E-20	ENSG00000150347
SIAE	-0.6134	7.7952E-09	4.68E-07	ENSG00000110013
APOA1	-0.6117	1.7499E-03	0.01102108	ENSG00000118137
LEPR	-0.6108	2.0115E-07	6.99E-06	ENSG00000116678
B3GNT5	-0.6099	2.1298E-11	3.17E-09	ENSG00000176597
VEPH1	-0.6096	5.4909E-05	0.00069181	ENSG00000197415
RIPK2	-0.608	4.8400E-11	6.37E-09	ENSG00000104312
AC074212.1	-0.6077	5.5254E-03	0.02705632	ENSG00000259605
AL162231.2	-0.606	1.8225E-05	0.00028412	ENSG00000230074
CCDC59	-0.6049	1.1067E-19	9.43E-17	ENSG00000133773
NA	-0.6046	4.3666E-03	0.0225851	ENSG00000289578
LTBP3	-0.6041	2.5155E-08	1.24E-06	ENSG00000168056
SLC22A5	-0.6037	1.8471E-07	6.51E-06	ENSG00000197375
DYDC2	-0.6033	4.6367E-06	9.45E-05	ENSG00000133665
TUBA4B	-0.602	1.4755E-03	0.00969098	ENSG00000243910
CHST6	-0.6011	7.2239E-05	0.00086373	ENSG00000183196
**NO SIGNIFICANT DIFFERENCE BETWEEN LEO AND GROUND (Alphabetical)**				
A1BG	0.0147	8.7729E-01	9.38E-01	ENSG00000121410
A1BG-AS1	0.0210	7.9987E-01	8.93E-01	ENSG00000268895
A1CF	-0.0809	1.8457E-01		ENSG00000148584
A2M	0.3450	2.6524E-03	1.53E-02	ENSG00000175899
A2M-AS1	-0.0626	4.8659E-01	6.71E-01	ENSG00000245105
A2ML1	-0.0699	2.4073E-01		ENSG00000166535
A3GALT2	-0.0014	6.4744E-01		ENSG00000184389
A4GALT	0.0865	3.7087E-01	5.66E-01	ENSG00000128274
AAAS	0.0429	4.9130E-01	6.75E-01	ENSG00000094914
AACS	-0.0346	5.1200E-01	6.92E-01	ENSG00000081760
AADACL2	0.0123	5.6171E-01		ENSG00000197953
AADAT	0.0008	9.7773E-01	9.88E-01	ENSG00000109576
AAGAB	0.1238	1.4555E-01	3.04E-01	ENSG00000103591
AAK1	0.0411	5.8164E-01	7.48E-01	ENSG00000115977
AAMP	0.1931	1.4314E-03	9.46E-03	ENSG00000127837
AANAT	0.0828	3.9468E-01	5.89E-01	ENSG00000129673
AAR2	-0.0381	5.1164E-01	6.92E-01	ENSG00000131043
AARD	-0.0145	8.4473E-01	9.19E-01	ENSG00000205002
AARS1	-0.0028	9.6717E-01	9.83E-01	ENSG00000090861
AARS2	0.0485	5.8094E-01	7.47E-01	ENSG00000124608
AARSD1P1	0.0315	2.0335E-01	3.81E-01	ENSG00000234969
AASDH	0.0932	2.2066E-01	4.02E-01	ENSG00000157426
AASDHPPT	0.1512	3.7053E-02	1.14E-01	ENSG00000149313
AASS	-0.5688	8.6308E-08	3.46E-06	ENSG00000008311
AATBC	0.1853	1.0851E-01	2.49E-01	ENSG00000215458
AATF	-0.0780	1.8274E-01	3.55E-01	ENSG00000275700
AATK	-0.0018	9.6818E-01	9.84E-01	ENSG00000181409

ABALON	0.0167	3.5205E-01		ENSG00000281376
ABAT	0.2927	5.2699E-04	4.29E-03	ENSG00000183044
ABCA1	-0.5845	1.7435E-05	2.75E-04	ENSG00000165029
ABCA10	-0.0130	8.5499E-01	9.26E-01	ENSG00000154263
ABCA12	0.2563	3.9744E-02	1.21E-01	ENSG00000144452
ABCA13	0.0228	8.0315E-01	8.95E-01	ENSG00000179869
ABCA17P	0.0261	7.8969E-01	8.87E-01	ENSG00000238098
ABCA2	-0.1698	6.8569E-02	1.80E-01	ENSG00000107331
ABCA3	-0.0008	9.8770E-01	9.93E-01	ENSG00000167972
ABCA5	-0.3618	6.3950E-05	7.86E-04	ENSG00000154265
ABCA6	0.0601	3.9100E-01	5.85E-01	ENSG00000154262
ABCA7	0.2434	2.5397E-03	1.48E-02	ENSG00000064687
ABCA8	-0.3933	8.1651E-03	3.65E-02	ENSG00000141338
ABCA9	-0.0129	7.0226E-01		ENSG00000154258
ABCA9-AS1	0.0341	1.1181E-01		ENSG00000231749
ABCB1	0.2489	2.3043E-02	8.01E-02	ENSG00000085563
ABCB10	0.0696	3.4877E-01	5.44E-01	ENSG00000135776
ABCB10P4	-0.0029	8.1315E-01		ENSG00000260053
ABCB11	0.0500	1.1417E-01		ENSG00000073734
ABCB4	0.1146	2.6748E-01	4.57E-01	ENSG00000005471
ABCB5	0.0169	3.2123E-01		ENSG00000004846
ABCB6	0.1136	2.7257E-01	4.63E-01	ENSG00000115657
ABCB7	-0.1056	1.5748E-01	3.21E-01	ENSG00000131269
ABCB8	-0.0222	7.5589E-01	8.67E-01	ENSG00000197150
ABCB9	0.3508	2.0996E-04	2.03E-03	ENSG00000150967
ABCC1	-0.2465	1.4939E-02	5.78E-02	ENSG00000103222
ABCC10	0.5939	1.1297E-07	4.29E-06	ENSG00000124574
ABCC11	0.0116	8.9568E-01	9.48E-01	ENSG00000121270
ABCC12	0.1697	2.9505E-03	1.66E-02	ENSG00000140798
ABCC2	0.0909	2.3429E-01	4.18E-01	ENSG00000023839
ABCC3	0.0942	3.2724E-01	5.22E-01	ENSG00000108846
ABCC4	0.0362	6.9473E-01	8.29E-01	ENSG00000125257
ABCC5	0.0836	2.0735E-01	3.86E-01	ENSG00000114770
ABCC5-AS1	-0.0537	5.0316E-01	6.84E-01	ENSG00000223882
ABCC6	-0.3094	7.9014E-03	3.56E-02	ENSG00000091262
ABCC8	-0.0660	4.8009E-01	6.66E-01	ENSG00000006071
ABCC9	-0.2034	6.9778E-02	1.82E-01	ENSG00000069431
ABCD1	-0.0343	7.0281E-01	8.34E-01	ENSG00000101986
ABCD2	-0.0191	8.2360E-01	9.07E-01	ENSG00000173208
ABCD3	-0.1354	4.8494E-03	2.44E-02	ENSG00000117528
ABCD4	0.0368	5.8146E-01	7.47E-01	ENSG00000119688
ABCE1	0.2095	1.2543E-02	5.05E-02	ENSG00000164163
ABCF1	-0.1038	1.2281E-01	2.71E-01	ENSG00000204574
ABCF2	-0.1215	1.2982E-01	2.81E-01	ENSG00000033050
ABCF3	0.2366	5.8975E-04	4.70E-03	ENSG00000161204
ABCG1	-0.0135	8.0291E-01	8.95E-01	ENSG00000160179
ABCG2	0.0060	9.8851E-01		ENSG00000118777
ABCG4	0.2393	2.3843E-02	8.22E-02	ENSG00000172350
ABCG5	0.1279	1.6279E-01	3.29E-01	ENSG00000138075

ABCG8	0.0118	8.5315E-01		ENSG00000143921
ABHD1	-0.2883	9.0115E-03	3.93E-02	ENSG00000143994
ABHD10	0.0199	7.3247E-01	8.53E-01	ENSG00000144827
ABHD11	-0.1045	1.5314E-01	3.15E-01	ENSG00000106077
ABHD12	0.1165	3.3770E-02	1.07E-01	ENSG00000100997
ABHD13	0.0087	8.8769E-01	9.44E-01	ENSG00000139826
ABHD14A	-0.5428	4.7616E-24	6.76E-21	ENSG00000248487
ABHD14A-ACY1	-0.0430	7.2662E-01	8.49E-01	ENSG00000114786
ABHD14B	-0.1235	1.6526E-01	3.32E-01	ENSG00000114779
ABHD15	0.0377	6.6560E-01	8.08E-01	ENSG00000168792
ABHD16A	0.0412	6.7247E-01	8.12E-01	ENSG00000204427
ABHD17A	-0.0755	8.7552E-02	2.14E-01	ENSG00000129968
ABHD17B	-0.1710	4.0210E-02	1.22E-01	ENSG00000107362
ABHD17C	0.0891	2.9256E-01	4.84E-01	ENSG00000136379
ABHD18	0.2935	5.8022E-05	7.24E-04	ENSG00000164074
ABHD2	-0.2144	5.3340E-04	4.34E-03	ENSG00000140526
ABHD3	-0.3108	4.5855E-04	3.84E-03	ENSG00000158201
ABHD4	-0.2512	5.7404E-03	2.79E-02	ENSG00000100439
ABHD5	-0.2169	3.7452E-04	3.28E-03	ENSG00000011198
ABHD6	0.1838	1.2223E-02	4.95E-02	ENSG00000163686
ABHD8	-0.1196	5.5279E-02	1.54E-01	ENSG00000127220
ABI1	-0.2319	1.0308E-03	7.28E-03	ENSG00000136754
ABI2	-0.1330	3.0205E-02	9.80E-02	ENSG00000138443
ABI3	-0.0095	3.4944E-01		ENSG00000108798
ABI3BP	-0.5891	6.3108E-05	7.76E-04	ENSG00000154175
ABITRAM	0.4405	3.5387E-08	1.65E-06	ENSG00000119328
ABL1	-0.1755	7.2915E-03	3.34E-02	ENSG00000097007
ABL2	0.2203	1.7216E-02	6.44E-02	ENSG00000143322
ABLIM1	-0.2767	1.5442E-06	3.82E-05	ENSG00000099204
ABLIM2	-0.0585	4.4839E-01	6.38E-01	ENSG00000163995
ABLIM3	0.1809	1.0147E-01	2.37E-01	ENSG00000173210
ABO	0.1932	9.4272E-02	2.26E-01	ENSG00000175164
ABR	-0.1412	1.4826E-02	5.75E-02	ENSG00000159842
ABRA	0.0013	8.3591E-01		ENSG00000174429
ABRACL	-0.1329	5.3225E-02	1.50E-01	ENSG00000146386
ABRAXAS1	-0.0568	5.0953E-01	6.90E-01	ENSG00000163322
ABRAXAS2	0.1081	8.3391E-02	2.07E-01	ENSG00000165660
ABT1	-0.0081	8.7550E-01	9.37E-01	ENSG00000146109
ABTB1	-0.0701	4.0467E-01	5.99E-01	ENSG00000114626
ABTB2	-0.0206	8.1680E-01	9.03E-01	ENSG00000166016
ABTB3	0.0164	8.2033E-01	9.05E-01	ENSG00000151136
ACAA1	0.0526	4.0611E-01	6.00E-01	ENSG00000060971
ACAA2	-0.0730	3.6521E-01	5.61E-01	ENSG00000167315
ACAA2P1	0.0262	7.3979E-01	8.57E-01	ENSG00000233585
ACACA	-0.1953	5.0252E-03	2.51E-02	ENSG00000278540
ACACB	-0.1381	1.5028E-01	3.11E-01	ENSG00000076555
ACAD10	0.1340	6.6267E-02	1.75E-01	ENSG00000111271
ACAD11	0.0942	3.3940E-01	5.34E-01	ENSG00000240303
ACAD8	0.1749	1.4853E-02	5.76E-02	ENSG00000151498

ACAD9	0.0154	8.7306E-01	9.36E-01	ENSG00000177646
ACADL	-0.2508	5.0549E-02	1.44E-01	ENSG00000115361
ACADM	-0.1583	3.5692E-03	1.93E-02	ENSG00000117054
ACADS	0.1556	7.9396E-02	2.00E-01	ENSG00000122971
ACADSB	0.1823	1.5470E-02	5.93E-02	ENSG00000196177
ACADVL	0.0432	5.6427E-01	7.33E-01	ENSG00000072778
ACAP1	0.1039	1.2045E-01	2.68E-01	ENSG00000072818
ACAP2	0.0060	9.2834E-01	9.65E-01	ENSG00000114331
ACAP3	0.3330	9.1663E-05	1.04E-03	ENSG00000131584
ACAT1	-0.0538	4.3835E-01	6.29E-01	ENSG00000075239
ACAT2	-0.2069	4.4883E-02	1.32E-01	ENSG00000120437
ACBD3	-0.1815	7.3548E-04	5.59E-03	ENSG00000182827
ACBD3-AS1	0.0061	9.7741E-01	9.88E-01	ENSG00000234478
ACBD4	-0.0446	5.6392E-01	7.33E-01	ENSG00000181513
ACBD5	-0.0387	4.8221E-01	6.67E-01	ENSG00000107897
ACBD6	-0.0038	8.5142E-01	9.24E-01	ENSG00000230124
ACBD7	0.0711	3.8889E-01	5.83E-01	ENSG00000176244
ACCS	0.2570	2.6117E-02	8.79E-02	ENSG00000110455
ACD	0.1560	2.1925E-03	1.32E-02	ENSG00000102977
ACE	0.0183	8.2601E-01	9.09E-01	ENSG00000159640
ACE2	-0.0050	9.3948E-01		ENSG00000130234
ACER1	-0.0722	3.1090E-01	5.04E-01	ENSG00000167769
ACER3	-0.1157	1.0752E-01	2.47E-01	ENSG00000078124
ACHE	0.1808	5.7884E-02	1.59E-01	ENSG00000087085
ACIN1	-0.3103	5.2501E-05	6.69E-04	ENSG00000100813
ACKR1	0.0126	8.9136E-01	9.46E-01	ENSG00000213088
ACKR2	0.0838	4.0569E-01	5.99E-01	ENSG00000144648
ACKR3	-0.3421	7.1756E-03	3.30E-02	ENSG00000144476
ACKR4	0.0049	4.7137E-01		ENSG00000129048
ACLY	-0.0654	2.2587E-01	4.08E-01	ENSG00000131473
ACMSD	0.0005	6.1151E-01	7.70E-01	ENSG00000153086
ACO1	-0.1384	1.9579E-02	7.08E-02	ENSG00000122729
ACO2	-0.1004	2.1898E-01	4.00E-01	ENSG00000100412
ACOT1	0.0803	3.7474E-01	5.70E-01	ENSG00000184227
ACOT11	-0.1427	1.4975E-01	3.10E-01	ENSG00000162390
ACOT12	0.1453	5.4161E-02	1.52E-01	ENSG00000172497
ACOT13	-0.1498	1.1186E-01	2.54E-01	ENSG00000112304
ACOT2	0.0808	3.3068E-01	5.25E-01	ENSG00000119673
ACOT4	-0.0182	8.0415E-01	8.96E-01	ENSG00000177465
ACOT6	-0.0036	9.5970E-01	9.80E-01	ENSG00000205669
ACOT7	-0.0535	4.0112E-01	5.95E-01	ENSG00000097021
ACOT8	-0.2294	6.5887E-03	3.10E-02	ENSG00000101473
ACOT9	-0.2725	1.1806E-03	8.09E-03	ENSG00000123130
ACOX1	0.0019	9.7555E-01	9.87E-01	ENSG00000161533
ACOX2	-0.2563	5.2342E-03	2.59E-02	ENSG00000168306
ACOX3	0.2100	3.2742E-03	1.80E-02	ENSG00000087008
ACOXL	0.1341	2.0229E-01	3.80E-01	ENSG00000153093
ACOXL-AS1	-0.0283	8.9071E-01	9.45E-01	ENSG00000204581
ACP1	-0.0240	5.8896E-01	7.53E-01	ENSG00000143727

ACP2	-0.2466	6.5513E-03	3.08E-02	ENSG00000134575
ACP3	-0.1342	1.8832E-01	3.63E-01	ENSG00000014257
ACP4	0.0423	6.4484E-01	7.94E-01	ENSG00000142513
ACP5	0.4132	3.2832E-03	1.80E-02	ENSG00000102575
ACP6	0.0445	5.9574E-01	7.58E-01	ENSG00000162836
ACP7	-0.1013	2.5752E-01	4.46E-01	ENSG00000183760
ACR	0.0011	9.3642E-01		ENSG00000100312
ACRBP	0.4727	1.0442E-03	7.35E-03	ENSG00000111644
ACRV1	-0.0634	4.0327E-01	5.97E-01	ENSG00000134940
ACSBG2	-0.0026	9.6972E-01	9.84E-01	ENSG00000130377
ACSF2	-0.4181	3.5206E-05	4.80E-04	ENSG00000167107
ACSF3	0.0263	6.0700E-01	7.66E-01	ENSG00000176715
ACSL1	-0.0125	8.8013E-01	9.39E-01	ENSG00000151726
ACSL3	-0.1865	6.0643E-03	2.91E-02	ENSG00000123983
ACSL4	-0.1489	7.7073E-02	1.95E-01	ENSG00000068366
ACSL5	-0.0167	9.5472E-01		ENSG00000197142
ACSL6	-0.0227	7.8058E-01	8.82E-01	ENSG00000164398
ACSL6-AS1	-0.0143	9.0171E-01	9.51E-01	ENSG00000223548
ACSM1	-0.1147	2.6849E-01	4.58E-01	ENSG00000166743
ACSM2A	-0.0140	4.4449E-01		ENSG00000183747
ACSM2B	-0.0559	6.4951E-02	1.73E-01	ENSG00000066813
ACSM3	0.0100	8.9589E-01	9.48E-01	ENSG00000005187
ACSM4	0.0157	4.9638E-01		ENSG00000215009
ACSM6	0.0868	2.5025E-01	4.37E-01	ENSG00000173124
ACSS1	-0.5525	1.9505E-04	1.92E-03	ENSG00000154930
ACSS2	0.0410	5.5088E-01	7.22E-01	ENSG00000131069
ACSS3	-0.1092	1.2759E-01	2.78E-01	ENSG00000111058
ACTA1	0.0480	5.2617E-01	7.02E-01	ENSG00000143632
ACTA2-AS1	0.0277	2.8589E-01		ENSG00000180139
ACTB	-0.1163	1.2600E-01	2.76E-01	ENSG00000075624
ACTBL2	0.0269	3.1051E-02		ENSG00000169067
ACTBP1	-0.0788	7.3343E-01	8.54E-01	ENSG00000229145
ACTBP14	0.0164	1.1232E-01		ENSG00000229001
ACTC1	0.0212	7.8506E-01	8.85E-01	ENSG00000159251
ACTE1P	-0.0144	6.6774E-01		ENSG00000172900
ACTG1	0.0053	9.3171E-01	9.66E-01	ENSG00000184009
ACTG1P12	-0.0308	5.4331E-01		ENSG00000226642
ACTG1P14	-0.0573	5.0130E-01	6.83E-01	ENSG00000230581
ACTG1P15	-0.0108	7.7909E-01		ENSG00000259904
ACTG1P16	0.0081	6.0876E-01		ENSG00000260298
ACTG1P17	0.1119	2.8264E-01	4.74E-01	ENSG00000259315
ACTG1P19	-0.0501	6.7124E-01	8.11E-01	ENSG00000237999
ACTG1P23	-0.0490	9.8605E-01		ENSG00000230362
ACTG1P24	-0.0253	6.6507E-01		ENSG00000226359
ACTG1P25	0.3656	2.3315E-03	1.38E-02	ENSG00000234996
ACTG2	-0.1314	2.1183E-01	3.92E-01	ENSG00000163017
ACTL10	-0.0200	8.3404E-01	9.13E-01	ENSG00000288649
ACTL11P	0.0190	3.1455E-01		ENSG00000234667
ACTL6A	0.0437	4.4518E-01	6.35E-01	ENSG00000136518

ACTL6B	0.1927	3.0457E-02	9.85E-02	ENSG00000077080
ACTL7A	-0.0073	7.1119E-01		ENSG00000187003
ACTL7B	-0.0098	8.4511E-01		ENSG00000148156
ACTL8	0.0059	3.0619E-01		ENSG00000117148
ACTMAP	0.0930	2.7544E-01	4.66E-01	ENSG00000188493
ACTN1	-0.0950	7.7771E-02	1.97E-01	ENSG00000072110
ACTN1-DT	-0.0246	7.6337E-01		ENSG00000259062
ACTN2	0.0970	2.7159E-01	4.62E-01	ENSG00000077522
ACTN3	0.0034	9.5824E-01	9.79E-01	ENSG00000248746
ACTN4	-0.0803	2.6231E-01	4.51E-01	ENSG00000130402
ACTR10	-0.0671	2.0893E-01	3.88E-01	ENSG00000131966
ACTR1A	0.0303	5.9852E-01	7.60E-01	ENSG00000138107
ACTR1B	-0.2715	5.3319E-03	2.63E-02	ENSG00000115073
ACTR2	-0.1631	4.4366E-03	2.28E-02	ENSG00000138071
ACTR3	-0.3141	6.9790E-05	8.40E-04	ENSG00000115091
ACTR3B	0.2147	3.9459E-03	2.09E-02	ENSG00000133627
ACTR3C	0.0096	9.3049E-01	9.66E-01	ENSG00000106526
ACTR5	0.4235	7.1242E-05	8.53E-04	ENSG00000101442
ACTR6	-0.1470	1.2795E-02	5.13E-02	ENSG00000075089
ACTR8	0.1931	2.3474E-02	8.13E-02	ENSG00000113812
ACTRT3	0.3354	4.7400E-03	2.40E-02	ENSG00000184378
ACVR1	-0.2127	3.2716E-03	1.79E-02	ENSG00000115170
ACVR1B	0.0601	4.3675E-01	6.28E-01	ENSG00000135503
ACVR1C	-0.0388	6.8769E-01	8.24E-01	ENSG00000123612
ACVR2A	0.2268	2.1331E-02	7.56E-02	ENSG00000121989
ACVR2B	0.4821	1.1406E-04	1.25E-03	ENSG00000114739
ACVR2B-AS1	0.1080	2.9408E-01	4.86E-01	ENSG00000229589
ACVRL1	0.0881	5.5522E-02		ENSG00000139567
ACY1	-0.0524	5.4250E-01	7.15E-01	ENSG00000243989
ACYP1	-0.4443	4.4523E-11	5.99E-09	ENSG00000119640
ACYP2	-0.1127	2.2119E-02	7.77E-02	ENSG00000170634
ADA	-0.1442	1.6449E-01	3.31E-01	ENSG00000196839
ADA2	0.1098	2.1249E-01	3.92E-01	ENSG00000093072
ADAD2	0.0511	3.4212E-02	1.08E-01	ENSG00000140955
ADAL	0.0530	4.2319E-01	6.15E-01	ENSG00000168803
ADAM10	0.0736	1.2035E-01	2.68E-01	ENSG00000137845
ADAM11	0.2834	8.1641E-03	3.65E-02	ENSG00000073670
ADAM12	0.3576	4.5785E-03	2.34E-02	ENSG00000148848
ADAM15	0.0832	2.0630E-01	3.85E-01	ENSG00000143537
ADAM17	-0.1769	1.2813E-02	5.13E-02	ENSG00000151694
ADAM19	0.0507	5.0240E-01	6.84E-01	ENSG00000135074
ADAM1B	-0.0311	8.5091E-01		ENSG00000226469
ADAM20	-0.0088	7.2872E-01		ENSG00000134007
ADAM21	-0.0801	3.3753E-01	5.33E-01	ENSG00000139985
ADAM21P1	-0.0343	1.8291E-01		ENSG00000235812
ADAM22	-0.0401	6.3546E-01	7.88E-01	ENSG00000008277
ADAM23	-0.0412	5.5422E-01	7.25E-01	ENSG00000114948
ADAM28	-0.2129	6.5496E-02	1.74E-01	ENSG00000042980
ADAM29	0.5723	1.0036E-03	7.13E-03	ENSG00000168594

ADAM32	-0.1355	1.2834E-01	2.79E-01	ENSG00000197140
ADAM3B	-0.0010	9.2885E-01		ENSG00000260089
ADAM7	0.0378	5.6638E-02		ENSG00000069206
ADAM8	0.2322	5.3447E-02	1.50E-01	ENSG00000151651
ADAM9	-0.2840	1.5866E-04	1.63E-03	ENSG00000168615
ADAMTS1	0.4991	8.4114E-06	1.53E-04	ENSG00000154734
ADAMTS10	0.1876	6.1924E-02	1.67E-01	ENSG00000142303
ADAMTS12	-0.1760	6.9322E-02	1.81E-01	ENSG00000151388
ADAMTS13	-0.0293	7.2876E-01	8.51E-01	ENSG00000160323
ADAMTS14	0.0472	5.9922E-01	7.60E-01	ENSG00000138316
ADAMTS15	-0.3328	2.7432E-02	9.11E-02	ENSG00000166106
ADAMTS16	-0.0754	4.4533E-01	6.35E-01	ENSG00000145536
ADAMTS16-DT	0.3889	1.5772E-02	6.02E-02	ENSG00000250579
ADAMTS17	-0.0815	3.3925E-01	5.34E-01	ENSG00000140470
ADAMTS18	-0.0965	3.2245E-01	5.16E-01	ENSG00000140873
ADAMTS19	-0.0244	6.7607E-01	8.15E-01	ENSG00000145808
ADAMTS19-AS1	-0.0299	6.9162E-01		ENSG00000249421
ADAMTS2	0.2665	1.4153E-02	5.55E-02	ENSG00000087116
ADAMTS20	-0.0396	6.4531E-01	7.94E-01	ENSG00000173157
ADAMTS3	-0.4696	2.0088E-03	1.23E-02	ENSG00000156140
ADAMTS4	0.2515	2.8894E-02	9.48E-02	ENSG00000158859
ADAMTS5	-0.5141	5.6593E-04	4.55E-03	ENSG00000154736
ADAMTS6	0.0110	9.2473E-01	9.63E-01	ENSG00000049192
ADAMTS7	-0.0022	9.8370E-01	9.91E-01	ENSG00000136378
ADAMTS7P1	-0.0239	7.0756E-01	8.36E-01	ENSG00000274376
ADAMTS7P3	0.0137	6.0342E-01		ENSG00000261143
ADAMTS7P4	0.0359	4.3138E-02		ENSG00000218052
ADAMTS8	-0.1382	1.4201E-01	2.99E-01	ENSG00000134917
ADAMTS9	-0.0321	7.1204E-01	8.40E-01	ENSG00000163638
ADAMTS9-AS1	-0.0742	3.7484E-01	5.70E-01	ENSG00000241158
ADAMTS9-AS2	0.0843	3.9031E-01	5.85E-01	ENSG00000241684
ADAMTSL1	0.3577	1.6640E-03	1.06E-02	ENSG00000178031
ADAMTSL2	-0.5892	5.5460E-04	4.48E-03	ENSG00000197859
ADAMTSL3	0.0571	4.8321E-01	6.68E-01	ENSG00000156218
ADAMTSL4	-0.1724	1.2013E-01	2.67E-01	ENSG00000143382
ADAMTSL5	-0.0619	4.9847E-01	6.81E-01	ENSG00000185761
ADAP1	-0.0792	2.6770E-01	4.57E-01	ENSG00000105963
ADAP2	-0.0202	8.1599E-01	9.03E-01	ENSG00000184060
ADAR	-0.0954	3.6845E-02	1.14E-01	ENSG00000160710
ADARB1	0.2268	5.0776E-03	2.53E-02	ENSG00000197381
ADARB2	0.5000	2.7911E-06	6.27E-05	ENSG00000185736
ADAT1	0.1677	1.5212E-02	5.86E-02	ENSG00000065457
ADAT2	0.0952	2.3625E-01	4.20E-01	ENSG00000189007
ADAT3	0.1679	1.1465E-01	2.59E-01	ENSG00000213638
ADCK1	-0.0308	6.7299E-01	8.13E-01	ENSG00000063761
ADCK2	-0.0463	5.2346E-01	7.00E-01	ENSG00000133597
ADCK5	-0.0042	9.6118E-01	9.80E-01	ENSG00000173137
ADCY1	-0.0252	7.4849E-01	8.62E-01	ENSG00000164742
ADCY10	-0.0518	3.7549E-01		ENSG00000143199

ADCY2	-0.0410	6.2706E-01	7.82E-01	ENSG00000078295
ADCY3	0.1662	8.4872E-02	2.09E-01	ENSG00000138031
ADCY4	0.0555	4.2498E-01	6.17E-01	ENSG00000129467
ADCY5	-0.0397	6.4791E-01	7.96E-01	ENSG00000173175
ADCY6	-0.0505	4.7862E-01	6.65E-01	ENSG00000174233
ADCY6-DT	-0.2052	8.6160E-02	2.12E-01	ENSG00000257660
ADCY7	-0.0837	3.7821E-01	5.73E-01	ENSG00000121281
ADCY9	0.2198	1.7630E-02	6.55E-02	ENSG00000162104
ADCYAP1	0.5488	1.0342E-04	1.15E-03	ENSG00000141433
ADCYAP1R1	-0.0793	4.1616E-01	6.09E-01	ENSG00000078549
ADD1	-0.0466	3.8549E-01	5.80E-01	ENSG00000087274
ADD2	-0.0773	3.6521E-01	5.61E-01	ENSG00000075340
ADD3	-0.5667	1.6308E-06	3.99E-05	ENSG00000148700
ADD3-AS1	-0.0954	3.4411E-01	5.39E-01	ENSG00000203876
ADGRA1	-0.0003	9.9654E-01	9.98E-01	ENSG00000197177
ADGRA2	-0.1756	1.0917E-01	2.50E-01	ENSG00000020181
ADGRA3	-0.1344	2.8952E-02	9.50E-02	ENSG00000152990
ADGRB1	-0.5152	6.1701E-03	2.95E-02	ENSG00000181790
ADGRB2	-0.2264	1.9073E-02	6.95E-02	ENSG00000121753
ADGRB3	-0.1100	1.8387E-01	3.57E-01	ENSG00000135298
ADGRD1	0.1821	1.0921E-01	2.50E-01	ENSG00000111452
ADGRD2	-0.0224	8.6842E-01		ENSG00000180264
ADGRE5	-0.0211	8.0452E-01	8.96E-01	ENSG00000123146
ADGRF1	-0.0693	2.3690E-01	4.21E-01	ENSG00000153292
ADGRF2	0.0628	3.7154E-01	5.67E-01	ENSG00000164393
ADGRF3	-0.0264	7.6995E-01	8.75E-01	ENSG00000173567
ADGRF4	-0.0320	7.0800E-01	8.37E-01	ENSG00000153294
ADGRF5	0.0155	4.3635E-01		ENSG00000069122
ADGRF5P1	-0.0080	8.7851E-01	9.38E-01	ENSG00000227582
ADGRG1	-0.2080	1.1378E-03	7.85E-03	ENSG00000205336
ADGRG2	0.0502	5.2946E-01	7.04E-01	ENSG00000173698
ADGRG4	0.0136	4.2386E-01		ENSG00000156920
ADGRG5	-0.1694	1.2138E-01	2.69E-01	ENSG00000159618
ADGRG7	0.0388	6.3914E-01	7.90E-01	ENSG00000144820
ADGRL1	-0.1589	6.3513E-02	1.70E-01	ENSG00000072071
ADGRL1-AS1	-0.0736	4.4395E-01	6.34E-01	ENSG00000267169
ADGRL2	0.1123	4.8539E-02	1.40E-01	ENSG00000117114
ADGRL3	-0.0249	7.4937E-01	8.63E-01	ENSG00000150471
ADGRL4	0.1673	2.6195E-02	8.81E-02	ENSG00000162618
ADGRV1	-0.0683	3.1056E-01	5.03E-01	ENSG00000164199
ADH1A	0.0126	3.2670E-01		ENSG00000187758
ADH1B	-0.0269	8.0844E-01		ENSG00000196616
ADH1C	-0.0124	6.8106E-01		ENSG00000248144
ADH4	0.0110	5.6773E-01		ENSG00000198099
ADH5	-0.1555	1.5001E-02	5.80E-02	ENSG00000197894
ADH5P3	-0.0046	9.9290E-01		ENSG00000223694
ADH5P5	-0.0096	7.0376E-01		ENSG00000234865
ADH6	-0.0084	8.9397E-01	9.47E-01	ENSG00000172955
ADH7	-0.0093	7.2850E-01		ENSG00000196344

ADHFE1	-0.2514	1.8712E-02	6.86E-02	ENSG00000147576
ADI1	-0.2999	6.6304E-04	5.17E-03	ENSG00000182551
ADIPOQ	0.0037	7.6073E-01		ENSG00000181092
ADIPOR1	-0.0428	4.6417E-01	6.52E-01	ENSG00000159346
ADIPOR2	-0.2812	4.4377E-03	2.28E-02	ENSG00000006831
ADISSP	0.0879	1.4507E-01	3.03E-01	ENSG00000101220
ADK	-0.0262	6.2434E-01	7.80E-01	ENSG00000156110
ADM	0.0502	6.0426E-01	7.64E-01	ENSG00000148926
ADM2	-0.1648	1.3804E-01	2.93E-01	ENSG00000128165
ADM5	-0.0316	7.4052E-01	8.57E-01	ENSG00000224420
ADNP	-0.1539	2.5977E-02	8.76E-02	ENSG00000101126
ADNP-AS1	-0.3229	1.7131E-02	6.41E-02	ENSG00000259456
ADNP2	-0.2916	4.9071E-05	6.37E-04	ENSG00000101544
ADO	-0.0025	9.7108E-01	9.85E-01	ENSG00000181915
ADORA2A	-0.0439	6.0618E-01		ENSG00000128271
ADORA2A-AS1	-0.0400	6.0030E-01		ENSG00000178803
ADORA2B	-0.0680	4.6885E-01	6.56E-01	ENSG00000170425
ADPGK	0.0225	7.5139E-01	8.64E-01	ENSG00000159322
ADPGK-AS1	0.0058	9.8351E-01	9.91E-01	ENSG00000260898
ADPRH	-0.1460	1.7261E-01	3.42E-01	ENSG00000144843
ADPRHL1	-0.0258	7.9205E-01	8.88E-01	ENSG00000153531
ADPRM	0.1684	1.9110E-02	6.96E-02	ENSG00000170222
ADPRS	0.1128	4.5695E-02	1.34E-01	ENSG00000116863
ADRA1A	0.0051	9.4391E-01	9.72E-01	ENSG00000120907
ADRA1B	0.3163	1.6262E-02	6.16E-02	ENSG00000170214
ADRA1D	-0.0081	9.8122E-01		ENSG00000171873
ADRA2A	-0.0131	8.8978E-01	9.45E-01	ENSG00000150594
ADRA2B	-0.0110	7.7134E-01	8.76E-01	ENSG00000274286
ADRA2C	0.0543	5.7468E-01	7.42E-01	ENSG00000184160
ADRB1	0.0057	9.5249E-01	9.76E-01	ENSG00000043591
ADRB2	-0.1069	2.8487E-01	4.76E-01	ENSG00000169252
ADRM1	0.1071	5.2759E-02	1.49E-01	ENSG00000130706
ADSL	-0.1005	2.2392E-01	4.06E-01	ENSG00000239900
ADSS1	0.2202	1.9018E-04	1.88E-03	ENSG00000185100
ADSS2	-0.1299	3.4486E-02	1.08E-01	ENSG00000035687
ADTRP	0.0312	7.1804E-01	8.44E-01	ENSG00000111863
AEBP2	-0.4846	1.0028E-08	5.69E-07	ENSG00000139154
AEN	0.1159	9.6093E-02	2.28E-01	ENSG00000181026
AFAP1	0.0285	7.1528E-01	8.41E-01	ENSG00000196526
AFAP1-AS1	-0.0317	8.7096E-01	9.34E-01	ENSG00000272620
AFAP1L1	0.3227	2.4804E-02	8.47E-02	ENSG00000157510
AFAP1L2	0.1374	1.8030E-01	3.52E-01	ENSG00000169129
AFDN	-0.1193	2.5101E-02	8.54E-02	ENSG00000130396
AFF1	-0.2910	1.5820E-03	1.02E-02	ENSG00000172493
AFF2	0.1731	9.1777E-02	2.21E-01	ENSG00000155966
AFF3	-0.0627	4.6594E-01	6.54E-01	ENSG00000144218
AFF4	-0.0436	4.4459E-01	6.35E-01	ENSG00000072364
AFG1L	-0.2825	3.2524E-03	1.79E-02	ENSG00000135537
AFG2A	0.1406	1.2862E-01	2.80E-01	ENSG00000145375

AFG2B	0.0061	9.3305E-01	9.67E-01	ENSG00000171763
AFG3L1P	0.2771	2.2642E-03	1.35E-02	ENSG00000223959
AFG3L2	0.0672	2.8451E-01	4.76E-01	ENSG00000141385
AFMID	0.1510	7.3648E-02	1.89E-01	ENSG00000183077
AFP	0.1115	1.8872E-01	3.63E-01	ENSG00000081051
AFTPH	-0.1784	3.0520E-02	9.86E-02	ENSG00000119844
AFTPH-DT	0.2559	1.1941E-02	4.86E-02	ENSG00000260101
AGA	-0.0307	6.5033E-01	7.97E-01	ENSG00000038002
AGA-DT	0.1113	2.7642E-01	4.67E-01	ENSG00000250131
AGAP1	0.0810	2.5525E-01	4.43E-01	ENSG00000157985
AGAP1-IT1	-0.0123	8.9998E-01	9.50E-01	ENSG00000235529
AGAP2	0.0802	4.1185E-01	6.05E-01	ENSG00000135439
AGAP3	-0.1481	2.5392E-02	8.62E-02	ENSG00000133612
AGAP4	0.0410	6.5293E-01	7.99E-01	ENSG00000188234
AGAP5	-0.0206	4.6792E-01		ENSG00000172650
AGAP6	0.0043	9.5840E-01	9.79E-01	ENSG00000204149
AGAP9	0.1263	2.1770E-01	3.99E-01	ENSG00000204172
AGBL1	0.0015	4.0233E-01	5.96E-01	ENSG00000273540
AGBL1-AS1	0.0097	3.4635E-01		ENSG00000260125
AGBL2	-0.1424	1.5693E-01	3.20E-01	ENSG00000165923
AGBL3	0.1650	1.1689E-01	2.62E-01	ENSG00000146856
AGBL4	0.0937	2.7620E-01	4.67E-01	ENSG00000186094
AGBL4-AS1	-0.0094	6.5555E-01		ENSG00000230114
AGBL4-IT1	-0.0034	6.7344E-01		ENSG00000225623
AGBL5	0.2105	2.8782E-03	1.63E-02	ENSG00000084693
AGBL5-AS1	0.0781	1.8970E-01	3.64E-01	ENSG00000231636
AGER	-0.1384	1.9693E-01	3.73E-01	ENSG00000204305
AGFG1	-0.0300	5.8390E-01	7.49E-01	ENSG00000173744
AGFG2	0.0828	3.8031E-01	5.75E-01	ENSG00000106351
AGGF1	0.0375	5.6901E-01	7.37E-01	ENSG00000164252
AGK	0.1424	2.7992E-02	9.25E-02	ENSG00000006530
AGKP1	-0.0339	4.2199E-01		ENSG00000226555
AGL	-0.0317	6.3249E-01	7.86E-01	ENSG00000162688
AGMAT	0.1048	2.6796E-01	4.57E-01	ENSG00000116771
AGO1	-0.1105	1.6996E-01	3.38E-01	ENSG00000092847
AGO2	-0.0313	6.9298E-01	8.28E-01	ENSG00000123908
AGO3	-0.0395	5.4764E-01	7.19E-01	ENSG00000126070
AGO4	0.2386	7.9712E-03	3.58E-02	ENSG00000134698
AGPAT1	-0.2188	1.6364E-03	1.05E-02	ENSG00000204310
AGPAT2	-0.1421	9.4701E-02	2.26E-01	ENSG00000169692
AGPAT3	-0.2023	5.5073E-03	2.70E-02	ENSG00000160216
AGPAT4	0.0400	5.1820E-01	6.96E-01	ENSG00000026652
AGPAT5	-0.0269	6.0180E-01	7.62E-01	ENSG00000155189
AGPS	-0.0794	1.6051E-01	3.26E-01	ENSG00000018510
AGR2	-0.1340	1.6547E-01	3.32E-01	ENSG00000106541
AGR3	-0.1323	2.0069E-01	3.78E-01	ENSG00000173467
AGRN	-0.1017	1.9311E-01	3.69E-01	ENSG00000188157
AGRP	-0.0701	3.0818E-01	5.01E-01	ENSG00000159723
AGT	-0.5204	6.1910E-03	2.95E-02	ENSG00000135744

AGTPBP1	0.2911	2.0934E-03	1.27E-02	ENSG00000135049
AGTR1	0.0202	5.9610E-01		ENSG00000144891
AGTRAP	-0.5361	1.0122E-09	8.62E-08	ENSG00000177674
AGXT	-0.0136	9.7474E-01		ENSG00000172482
AHCTF1	-0.0079	9.1568E-01	9.58E-01	ENSG00000153207
AHCY	-0.0831	6.5220E-02	1.73E-01	ENSG00000101444
AHCYL1	-0.1147	5.7960E-02	1.59E-01	ENSG00000168710
AHCYL2	-0.0202	7.5754E-01	8.68E-01	ENSG00000158467
AHCYP3	-0.0478	4.9287E-01		ENSG00000233955
AHDC1	-0.1980	2.0595E-02	7.36E-02	ENSG00000126705
AHI1	0.4311	1.3253E-07	4.91E-06	ENSG00000135541
AHI1-DT	0.2600	2.5909E-03	1.50E-02	ENSG00000231028
AHNAK	0.0293	6.6653E-01	8.08E-01	ENSG00000124942
AHNAK2	0.1344	1.5631E-01	3.19E-01	ENSG00000185567
AHR	-0.1254	1.8134E-01	3.53E-01	ENSG00000106546
AHRR	-0.0124	8.9140E-01	9.46E-01	ENSG00000063438
AHSA1	-0.0399	3.6261E-01	5.58E-01	ENSG00000100591
AHSA2P	0.0222	7.7598E-01	8.79E-01	ENSG00000173209
AHSG	0.0244	1.2608E-01		ENSG00000145192
AHSP	0.0217	6.4184E-01	7.92E-01	ENSG00000169877
AIDA	-0.1918	4.1243E-03	2.16E-02	ENSG00000186063
AIDAP2	0.0168	2.1644E-01		ENSG00000251429
AIF1	0.1023	8.9251E-03	3.90E-02	ENSG00000204472
AIF1L	0.0069	9.5040E-01	9.75E-01	ENSG00000126878
AIFM1	-0.0129	8.4662E-01	9.21E-01	ENSG00000156709
AIFM2	-0.1914	6.2936E-02	1.69E-01	ENSG00000042286
AIFM3	-0.3850	6.4315E-03	3.04E-02	ENSG00000183773
AIG1	-0.3861	4.8331E-07	1.45E-05	ENSG00000146416
AIM2	-0.0192	8.2963E-01	9.11E-01	ENSG00000163568
AIMP1	-0.0388	5.7317E-01	7.41E-01	ENSG00000164022
AIMP1P1	0.0205	3.8628E-01		ENSG00000234187
AIMP2	-0.0039	9.3147E-01	9.66E-01	ENSG00000106305
AIP	-0.1610	3.3016E-02	1.05E-01	ENSG00000110711
AIPL1	0.0986	2.8750E-01	4.79E-01	ENSG00000129221
AIRE	-0.0020	6.6150E-01		ENSG00000160224
AIRIM	0.2991	4.4954E-04	3.78E-03	ENSG00000116922
AIRN	-0.0575	5.3321E-01	7.07E-01	ENSG00000268257
AJAP1	0.4172	1.1273E-03	7.79E-03	ENSG00000196581
AJM1	-0.1492	9.2375E-02	2.23E-01	ENSG00000232434
AJUBA	-0.5267	2.7251E-07	9.05E-06	ENSG00000129474
AK1	-0.5451	7.0235E-08	2.91E-06	ENSG00000106992
AK2	0.0626	3.8915E-01	5.84E-01	ENSG00000004455
AK2P1	0.0710	3.8216E-01	5.77E-01	ENSG00000185839
AK3	0.0018	9.6201E-01	9.81E-01	ENSG00000147853
AK3P3	0.4397	1.5481E-02	5.93E-02	ENSG00000230042
AK4	0.2245	8.9787E-03	3.92E-02	ENSG00000162433
AK5	-0.0947	3.1076E-01	5.04E-01	ENSG00000154027
AK6	-0.2826	3.0922E-06	6.78E-05	ENSG00000085231
AK7	0.0047	9.4375E-01	9.72E-01	ENSG00000140057

AK8	0.1014	1.8967E-01	3.64E-01	ENSG00000165695
AK9	0.0781	1.3006E-01	2.82E-01	ENSG00000155085
AKAIN1	-0.0518	5.8309E-01	7.49E-01	ENSG00000231824
AKAP1	0.1808	5.4889E-03	2.69E-02	ENSG00000121057
AKAP10	0.5172	4.9061E-09	3.22E-07	ENSG00000108599
AKAP11	-0.0581	4.0544E-01	5.99E-01	ENSG00000023516
AKAP12	-0.0734	1.6246E-01	3.28E-01	ENSG00000131016
AKAP13	-0.2377	4.4659E-04	3.76E-03	ENSG00000170776
AKAP14	-0.4223	6.1482E-04	4.86E-03	ENSG00000186471
AKAP17A	0.0510	4.7469E-01	6.61E-01	ENSG00000197976
AKAP3	-0.1450	1.3917E-01	2.95E-01	ENSG00000111254
AKAP4	-0.0328	3.1789E-01		ENSG00000147081
AKAP5	-0.0385	6.8301E-01	8.21E-01	ENSG00000179841
AKAP6	0.2044	6.9171E-03	3.21E-02	ENSG00000151320
AKAP7	-0.0201	7.8808E-01	8.86E-01	ENSG00000118507
AKAP8	0.0375	6.0176E-01	7.62E-01	ENSG00000105127
AKAP8L	0.2547	9.0826E-04	6.61E-03	ENSG00000011243
AKAP9	0.1527	8.0257E-03	3.60E-02	ENSG00000127914
AKIP1	-0.0683	1.2917E-01	2.80E-01	ENSG00000166452
AKIRIN1	-0.0819	1.5412E-01	3.16E-01	ENSG00000174574
AKIRIN2	-0.2589	1.6269E-05	2.60E-04	ENSG00000135334
AKNA	-0.1750	1.0614E-01	2.45E-01	ENSG00000106948
AKNAD1	0.1901	9.0527E-02	2.19E-01	ENSG00000162641
AKR1A1	-0.1216	1.0285E-01	2.39E-01	ENSG00000117448
AKR1B1	0.0377	6.8161E-01	8.20E-01	ENSG00000085662
AKR1B10	0.1540	8.6837E-03	3.83E-02	ENSG00000198074
AKR1B15	0.0214	3.6764E-01		ENSG00000227471
AKR1B1P1	0.0219	5.1022E-01		ENSG00000229991
AKR1C1	0.0064	9.3079E-01	9.66E-01	ENSG00000187134
AKR1C2	0.0185	7.8349E-01	8.83E-01	ENSG00000151632
AKR1C3	0.1268	6.6120E-02	1.75E-01	ENSG00000196139
AKR1C4	0.0073	9.7354E-01	9.86E-01	ENSG00000198610
AKR1C7P	0.0211	2.0492E-01		ENSG00000215267
AKR1D1	-0.0369	7.6181E-01		ENSG00000122787
AKR1E2	0.5729	1.5246E-03	9.94E-03	ENSG00000165568
AKR7A2	-0.2604	4.1019E-03	2.16E-02	ENSG00000053371
AKR7A3	-0.0456	6.3449E-01	7.87E-01	ENSG00000162482
AKR7L	0.4972	2.2941E-03	1.37E-02	ENSG00000211454
AKT1	0.1969	1.6930E-02	6.35E-02	ENSG00000142208
AKT1S1	0.0297	6.0840E-01	7.67E-01	ENSG00000204673
AKT2	0.0493	4.2520E-01	6.17E-01	ENSG00000105221
AKT3	-0.2990	1.0775E-03	7.52E-03	ENSG00000117020
AKT3-IT1	-0.0047	9.5242E-01		ENSG00000228939
AKTIP	0.0876	2.1413E-01	3.95E-01	ENSG00000166971
ALAD	-0.0293	5.9952E-01	7.60E-01	ENSG00000148218
ALAS1	-0.0060	9.2209E-01	9.61E-01	ENSG00000023330
ALAS2	0.0065	5.7684E-01		ENSG00000158578
ALB	0.1411	1.0256E-01	2.39E-01	ENSG00000163631
ALCAM	-0.0851	1.9847E-01	3.75E-01	ENSG00000170017

ALDH16A1	-0.0887	2.9367E-01	4.86E-01	ENSG00000161618
ALDH18A1	-0.0641	3.3302E-01	5.28E-01	ENSG00000059573
ALDH1A3	-0.0518	5.5597E-01	7.27E-01	ENSG00000184254
ALDH1A3-AS1	-0.0002	7.0903E-01		ENSG00000259583
ALDH1B1	-0.0619	4.9778E-01	6.81E-01	ENSG00000137124
ALDH1L1	0.0915	3.6208E-01	5.58E-01	ENSG00000144908
ALDH1L1-AS1	0.0017	7.2861E-01		ENSG00000250218
ALDH1L1-AS2	-0.0144	8.6945E-01		ENSG00000246022
ALDH1L2	0.0154	8.6529E-01	9.31E-01	ENSG00000136010
ALDH2	-0.1425	4.7733E-02	1.38E-01	ENSG00000111275
ALDH3A1	0.0701	3.9810E-01	5.92E-01	ENSG00000108602
ALDH3A2	-0.1116	1.0793E-01	2.48E-01	ENSG00000072210
ALDH3B1	-0.1974	4.8963E-02	1.41E-01	ENSG00000006534
ALDH3B2	0.1936	6.6858E-02	1.76E-01	ENSG00000132746
ALDH4A1	-0.4799	1.8036E-05	2.82E-04	ENSG00000159423
ALDH5A1	-0.3898	3.9774E-05	5.32E-04	ENSG00000112294
ALDH6A1	-0.4072	7.7073E-06	1.44E-04	ENSG00000119711
ALDH7A1	-0.0589	3.3605E-01	5.31E-01	ENSG00000164904
ALDH7A1P1	-0.0190	7.9867E-01	8.92E-01	ENSG00000251400
ALDH8A1	-0.0217	7.8966E-01	8.87E-01	ENSG00000118514
ALDH9A1	-0.2761	1.8337E-03	1.14E-02	ENSG00000143149
ALDOA	0.5771	3.1898E-06	6.94E-05	ENSG00000149925
ALDOB	0.0103	5.9731E-01		ENSG00000136872
ALG1	0.1602	3.4721E-02	1.09E-01	ENSG00000033011
ALG10	0.1847	9.9385E-02	2.34E-01	ENSG00000139133
ALG10B	0.0338	7.0739E-01	8.36E-01	ENSG00000175548
ALG11	-0.0383	6.3034E-01	7.84E-01	ENSG00000253710
ALG12	-0.1938	4.8875E-02	1.41E-01	ENSG00000182858
ALG13	0.0043	9.8525E-01	9.92E-01	ENSG00000101901
ALG14	-0.0686	3.4189E-01	5.37E-01	ENSG00000172339
ALG14-AS1	-0.0277	4.4140E-01		ENSG00000230427
ALG1L10P	-0.0024	2.9720E-01	4.89E-01	ENSG00000254016
ALG1L11P	-0.0601	1.0415E-01		ENSG00000249889
ALG1L2	0.0302	5.8345E-01	7.49E-01	ENSG00000251287
ALG1L5P	0.0091	6.0357E-01		ENSG00000226943
ALG1L8P	0.0357	4.8109E-01	6.66E-01	ENSG00000227620
ALG1L9P	-0.0546	5.6469E-01	7.33E-01	ENSG00000248671
ALG2	-0.0823	2.1380E-01	3.94E-01	ENSG00000119523
ALG3	0.0766	2.6488E-01	4.54E-01	ENSG00000214160
ALG5	0.0377	4.0991E-01	6.03E-01	ENSG00000120697
ALG6	-0.2208	3.0887E-03	1.72E-02	ENSG00000088035
ALG8	-0.1115	7.4710E-02	1.91E-01	ENSG00000159063
ALG9	0.3776	3.2638E-04	2.93E-03	ENSG00000086848
ALKAL1	0.0178	8.2460E-01	9.08E-01	ENSG00000196711
ALKAL2	-0.0836	3.4154E-01	5.37E-01	ENSG00000189292
ALKBH1	0.2891	3.5454E-04	3.14E-03	ENSG00000100601
ALKBH2	0.4486	1.1588E-11	1.83E-09	ENSG00000189046
ALKBH3	0.2232	5.1468E-04	4.21E-03	ENSG00000166199
ALKBH3-AS1	0.3749	1.5829E-02	6.04E-02	ENSG00000244926

ALKBH4	0.0240	7.5780E-01	8.68E-01	ENSG00000160993
ALKBH5	-0.0515	4.2130E-01	6.14E-01	ENSG00000091542
ALKBH6	0.1893	9.4233E-02	2.26E-01	ENSG00000239382
ALKBH7	-0.2981	3.0777E-07	1.00E-05	ENSG00000125652
ALKBH8	0.2191	5.1029E-02	1.45E-01	ENSG00000137760
ALLC	0.0115	5.5491E-01		ENSG00000151360
ALMS1	-0.1695	3.1065E-02	9.99E-02	ENSG00000116127
ALMS1-IT1	0.0469	6.1020E-01	7.69E-01	ENSG00000230002
ALOX12	0.3023	2.9941E-02	9.73E-02	ENSG00000108839
ALOX12-AS1	0.0966	1.6008E-01	3.25E-01	ENSG00000215067
ALOX12B	0.0011	9.9068E-01	9.95E-01	ENSG00000179477
ALOX15	-0.0375	9.6980E-01	9.84E-01	ENSG00000161905
ALOX15B	0.1665	7.9516E-03	3.58E-02	ENSG00000179593
ALOX5	0.2303	6.3737E-02	1.70E-01	ENSG00000012779
ALOX5AP	0.1148	8.7687E-02		ENSG00000132965
ALOXE3	-0.0792	3.9581E-01	5.90E-01	ENSG00000179148
ALPI	-0.0241	7.1029E-01		ENSG00000163295
ALPK1	0.1743	8.2332E-02	2.05E-01	ENSG00000073331
ALPK2	-0.0051	9.9926E-01		ENSG00000198796
ALPK3	0.4127	1.6757E-03	1.07E-02	ENSG00000136383
ALPL	-0.0905	3.5089E-01	5.46E-01	ENSG00000162551
ALS2	0.1890	1.9907E-03	1.22E-02	ENSG00000003393
ALS2CL	0.1646	1.2475E-01	2.74E-01	ENSG00000178038
ALX1	-0.0262	7.7812E-01	8.80E-01	ENSG00000180318
ALX4	0.0030	9.2330E-01	9.62E-01	ENSG00000052850
ALYREF	-0.3185	3.6322E-07	1.14E-05	ENSG00000183684
AMACR	0.0479	4.7898E-01	6.65E-01	ENSG00000242110
AMBN	-0.0158	8.5048E-01	9.23E-01	ENSG00000178522
AMBP	-0.0002	9.8871E-01		ENSG00000106927
AMBRA1	-0.0054	9.3758E-01	9.69E-01	ENSG00000110497
AMD1	-0.0814	2.6236E-01	4.51E-01	ENSG00000123505
AMD1P1	0.0209	6.9036E-01		ENSG00000228339
AMDHD1	0.0566	5.1500E-01	6.94E-01	ENSG00000139344
AMDHD2	0.2177	3.4838E-02	1.09E-01	ENSG00000162066
AMELX	0.0140	3.8105E-01		ENSG00000125363
AMELY	-0.0491	3.5554E-01	5.51E-01	ENSG00000099721
AMER1	-0.0165	8.6023E-01	9.29E-01	ENSG00000184675
AMER2	0.1529	1.0088E-01	2.36E-01	ENSG00000165566
AMER3	-0.0970	2.9037E-01	4.82E-01	ENSG00000178171
AMFR	0.0028	9.9007E-01	9.94E-01	ENSG00000159461
AMH	0.0545	5.5966E-01	7.29E-01	ENSG00000104899
AMHR2	0.0884	3.2494E-01	5.19E-01	ENSG00000135409
AMIGO1	-0.1489	3.4702E-02	1.09E-01	ENSG00000181754
AMIGO2	-0.2547	2.6741E-03	1.54E-02	ENSG00000139211
AMMECR1	0.0639	4.4076E-01	6.31E-01	ENSG00000101935
AMMECR1L	-0.2451	3.0857E-03	1.72E-02	ENSG00000144233
AMN	0.3698	2.1541E-02	7.62E-02	ENSG00000166126
AMN1	-0.0263	7.4051E-01	8.57E-01	ENSG00000151743
AMOT	-0.2645	1.7961E-04	1.81E-03	ENSG00000126016

AMOTL1	-0.0593	3.3123E-01	5.26E-01	ENSG00000166025
AMOTL2	-0.2088	1.7035E-02	6.38E-02	ENSG00000114019
AMPD1	-0.0216	3.8515E-01		ENSG00000116748
AMPD2	0.2292	8.1208E-04	6.04E-03	ENSG00000116337
AMPD3	0.0911	3.2522E-01	5.19E-01	ENSG00000133805
AMPH	0.0250	7.8734E-01	8.86E-01	ENSG00000078053
AMT	0.0524	3.3957E-01	5.35E-01	ENSG00000145020
AMTN	-0.1021	2.9175E-01	4.83E-01	ENSG00000187689
AMY1A	0.4309	4.1463E-03	2.17E-02	ENSG00000237763
AMY1B	0.4391	3.8926E-03	2.07E-02	ENSG00000174876
AMY1C	0.4102	5.9125E-03	2.85E-02	ENSG00000187733
AMY2A	0.0347	6.4694E-01	7.95E-01	ENSG00000243480
AMY2B	0.3391	3.3072E-03	1.81E-02	ENSG00000240038
AMYP1	-0.0303	7.3590E-01	8.55E-01	ENSG00000227408
AMZ1	-0.0954	3.3184E-01	5.27E-01	ENSG00000174945
AMZ2	-0.0605	3.0570E-01	4.98E-01	ENSG00000196704
ANAPC1	0.0698	2.5297E-01	4.40E-01	ENSG00000153107
ANAPC10	0.1065	1.1886E-01	2.65E-01	ENSG00000164162
ANAPC11	-0.1833	9.5646E-04	6.89E-03	ENSG00000141552
ANAPC13	-0.0376	4.2661E-01	6.18E-01	ENSG00000129055
ANAPC15	0.0289	6.9758E-01	8.31E-01	ENSG00000110200
ANAPC16	-0.2036	3.3119E-03	1.81E-02	ENSG00000166295
ANAPC1P2	0.1156	2.2189E-01	4.03E-01	ENSG00000285793
ANAPC1P3	-0.0048	5.1369E-01		ENSG00000230395
ANAPC2	0.1025	2.5763E-01	4.46E-01	ENSG00000176248
ANAPC4	-0.0819	1.3328E-01	2.86E-01	ENSG00000053900
ANAPC5	0.2619	4.6389E-04	3.87E-03	ENSG00000089053
ANAPC7	-0.1866	1.1192E-02	4.62E-02	ENSG00000196510
ANG	-0.0317	7.2012E-01	8.45E-01	ENSG00000214274
ANGEL1	0.0834	2.8874E-01	4.80E-01	ENSG00000013523
ANGEL2	-0.0664	3.1687E-01	5.10E-01	ENSG00000174606
ANGPT1	-0.3359	1.0424E-02	4.38E-02	ENSG00000154188
ANGPT2	0.1394	1.8834E-01	3.63E-01	ENSG00000091879
ANGPT4	-0.0197	4.7567E-01		ENSG00000101280
ANGPTL3	-0.0382	5.5401E-01		ENSG00000132855
ANGPTL4	0.0386	6.8200E-01	8.20E-01	ENSG00000167772
ANGPTL5	0.1667	1.3577E-01	2.90E-01	ENSG00000187151
ANGPTL6	0.4021	9.4085E-03	4.06E-02	ENSG00000130812
ANGPTL7	-0.0487	5.9561E-01	7.58E-01	ENSG00000171819
ANGPTL8	-0.0006	9.9396E-01	9.97E-01	ENSG00000130173
ANK1	0.0336	6.8959E-01	8.26E-01	ENSG00000029534
ANK2	-0.0458	5.1029E-01	6.90E-01	ENSG00000145362
ANK2-AS1	0.5139	1.7438E-03	1.10E-02	ENSG00000248152
ANK3	0.2390	9.7905E-04	6.99E-03	ENSG00000151150
ANKAR	0.1610	8.8641E-02	2.16E-01	ENSG00000151687
ANKDD1A	0.0577	4.1879E-01	6.11E-01	ENSG00000166839
ANKDD1B	0.0220	7.8700E-01	8.86E-01	ENSG00000189045
ANKEF1	0.0318	7.0511E-01	8.35E-01	ENSG00000132623
ANKFN1	-0.3427	1.5759E-02	6.02E-02	ENSG00000153930

ANKFY1	0.1010	1.6438E-01	3.31E-01	ENSG00000185722
ANKH	0.4335	2.2312E-07	7.66E-06	ENSG00000154122
ANKHD1	-0.0459	5.8180E-01	7.48E-01	ENSG00000131503
ANKHD1-EIF4EBP3	-0.0604	5.2456E-01	7.01E-01	ENSG00000254996
ANKIB1	-0.2879	1.0371E-03	7.32E-03	ENSG00000001629
ANKK1	-0.0976	1.6486E-01	3.31E-01	ENSG00000170209
ANKLE1	0.1046	2.9827E-01	4.90E-01	ENSG00000160117
ANKLE2	-0.1984	9.3499E-05	1.06E-03	ENSG00000176915
ANKMY1	0.2589	1.4758E-02	5.73E-02	ENSG00000144504
ANKMY2	0.2279	4.4629E-03	2.29E-02	ENSG00000106524
ANKRA2	0.1520	8.8419E-03	3.88E-02	ENSG00000164331
ANKRD1	-0.4864	1.6040E-03	1.03E-02	ENSG00000148677
ANKRD10	-0.1480	8.5633E-03	3.79E-02	ENSG00000088448
ANKRD11	0.0433	4.6140E-01	6.50E-01	ENSG00000167522
ANKRD12	0.1566	4.3038E-03	2.24E-02	ENSG00000101745
ANKRD13A	0.3767	2.3898E-05	3.53E-04	ENSG00000076513
ANKRD13B	-0.1630	6.5803E-02	1.74E-01	ENSG00000198720
ANKRD13C	0.1883	5.4720E-03	2.69E-02	ENSG00000118454
ANKRD13C-DT	-0.5485	5.8894E-06	1.15E-04	ENSG00000197568
ANKRD13D	-0.0049	9.2468E-01	9.63E-01	ENSG00000172932
ANKRD16	0.1860	1.6808E-02	6.32E-02	ENSG00000134461
ANKRD17	-0.0445	4.0233E-01	5.96E-01	ENSG00000132466
ANKRD18A	-0.1059	2.9557E-01	4.88E-01	ENSG00000180071
ANKRD18B	-0.2036	7.6834E-02	1.95E-01	ENSG00000230453
ANKRD18DP	-0.0287	7.7675E-01		ENSG00000226435
ANKRD18EP	0.0705	4.0617E-01	6.00E-01	ENSG00000217165
ANKRD2	0.0651	2.3985E-01	4.24E-01	ENSG00000165887
ANKRD20A1	-0.1751	1.2060E-01	2.68E-01	ENSG00000260691
ANKRD20A17P	-0.0053	8.4202E-01		ENSG00000251056
ANKRD20A18P	-0.0004	9.1705E-01		ENSG00000249493
ANKRD20A8P	-0.0656	3.4961E-01	5.45E-01	ENSG00000229089
ANKRD22	-0.4057	1.3399E-03	8.98E-03	ENSG00000152766
ANKRD23	-0.0615	9.1707E-01	9.59E-01	ENSG00000163126
ANKRD24	-0.2884	1.1431E-02	4.70E-02	ENSG00000089847
ANKRD26	-0.0133	8.1750E-01	9.03E-01	ENSG00000107890
ANKRD26P4	-0.0091	9.4949E-01		ENSG00000229427
ANKRD27	0.4895	7.4219E-05	8.83E-04	ENSG00000105186
ANKRD28	-0.0573	3.7111E-01	5.67E-01	ENSG00000206560
ANKRD29	-0.4322	1.4631E-06	3.66E-05	ENSG00000154065
ANKRD30B	-0.0036	9.8695E-01		ENSG00000180777
ANKRD30BL	0.1672	1.3446E-01	2.88E-01	ENSG00000163046
ANKRD31	0.2950	2.8040E-02	9.26E-02	ENSG00000145700
ANKRD33	0.0563	1.9766E-01	3.74E-01	ENSG00000167612
ANKRD33B	-0.4022	1.5964E-02	6.08E-02	ENSG00000164236
ANKRD33BP1	0.0093	3.2961E-01		ENSG00000229689
ANKRD33BP8	-0.0017	8.9404E-01		ENSG00000254558
ANKRD34A	-0.0586	5.4207E-01	7.15E-01	ENSG00000272031
ANKRD34B	-0.0948	3.1637E-01	5.10E-01	ENSG00000189127
ANKRD34C-AS1	0.0078	6.3806E-01		ENSG00000259234

ANKRD35	0.0270	7.5916E-01	8.69E-01	ENSG00000198483
ANKRD36	0.2612	1.0716E-03	7.49E-03	ENSG00000135976
ANKRD36B	0.2432	3.5958E-03	1.94E-02	ENSG00000196912
ANKRD36C	0.2958	7.0934E-04	5.43E-03	ENSG00000174501
ANKRD37	0.1334	1.0318E-01	2.40E-01	ENSG00000186352
ANKRD39	-0.0384	5.5332E-01	7.24E-01	ENSG00000213337
ANKRD40	0.0053	9.4601E-01	9.73E-01	ENSG00000154945
ANKRD40CL	-0.0457	4.4753E-01	6.37E-01	ENSG00000167117
ANKRD42	-0.0268	7.0286E-01	8.34E-01	ENSG00000137494
ANKRD44	0.2552	8.7593E-03	3.85E-02	ENSG00000065413
ANKRD44-IT1	0.0660	3.5928E-01	5.55E-01	ENSG00000236977
ANKRD45	-0.1908	9.7876E-02	2.31E-01	ENSG00000183831
ANKRD46	0.2556	1.1344E-03	7.83E-03	ENSG00000186106
ANKRD49	0.2056	4.4323E-04	3.74E-03	ENSG00000168876
ANKRD49P2	0.0213	3.8859E-01		ENSG00000225349
ANKRD50	0.1690	2.8168E-02	9.29E-02	ENSG00000151458
ANKRD52	0.2153	3.8008E-03	2.03E-02	ENSG00000139645
ANKRD53	-0.2179	6.9050E-02	1.81E-01	ENSG00000144031
ANKRD54	0.0213	7.7434E-01	8.78E-01	ENSG00000100124
ANKRD55	0.1642	1.3604E-01	2.90E-01	ENSG00000164512
ANKRD6	-0.1529	5.0651E-02	1.44E-01	ENSG00000135299
ANKRD61	0.0876	3.6592E-01	5.62E-01	ENSG00000157999
ANKRD63	-0.0863	1.5134E-01	3.12E-01	ENSG00000230778
ANKRD65	-0.1219	1.5804E-01	3.22E-01	ENSG00000235098
ANKRD7	-0.0580	6.5619E-01	8.02E-01	ENSG00000106013
ANKRD9	0.0673	3.2400E-01	5.18E-01	ENSG00000156381
ANKS1A	0.0661	3.6816E-01	5.64E-01	ENSG00000064999
ANKS1B	0.4031	5.6422E-03	2.75E-02	ENSG00000185046
ANKS3	0.5320	3.6401E-06	7.76E-05	ENSG00000168096
ANKS4B	-0.0183	4.0423E-01		ENSG00000175311
ANKS6	-0.2235	1.0655E-03	7.46E-03	ENSG00000165138
ANKUB1	-0.0454	6.2789E-01	7.82E-01	ENSG00000206199
ANKZF1	0.3845	1.3768E-07	5.05E-06	ENSG00000163516
ANLN	-0.2230	6.2774E-02	1.69E-01	ENSG00000011426
ANO1	0.0418	6.1646E-01	7.74E-01	ENSG00000131620
ANO10	-0.1078	1.3424E-01	2.88E-01	ENSG00000160746
ANO2	0.0354	6.7395E-01	8.13E-01	ENSG00000047617
ANO3	0.0148	8.6137E-01	9.29E-01	ENSG00000134343
ANO4	-0.2326	2.3092E-02	8.03E-02	ENSG00000151572
ANO5	0.0373	5.8835E-01	7.52E-01	ENSG00000171714
ANO6	-0.2596	3.8158E-03	2.03E-02	ENSG00000177119
ANO7	-0.1257	2.2991E-01	4.13E-01	ENSG00000146205
ANO8	-0.0605	5.1888E-01	6.96E-01	ENSG00000074855
ANO9	-0.1745	1.0092E-01	2.36E-01	ENSG00000185101
ANOS1	-0.2383	2.3507E-02	8.13E-02	ENSG00000011201
ANP32A	-0.2758	3.8664E-04	3.35E-03	ENSG00000140350
ANP32AP1	0.0085	5.6929E-01		ENSG00000259516
ANP32B	-0.5356	6.4781E-10	5.93E-08	ENSG00000136938
ANP32BP1	-0.0230	2.2437E-01		ENSG00000259790

ANP32E	-0.3299	8.8262E-07	2.42E-05	ENSG00000143401
ANTKMT	-0.0628	3.2738E-01	5.22E-01	ENSG00000103254
ANTXR1	-0.4341	3.5157E-05	4.80E-04	ENSG00000169604
ANTXR2	-0.0469	6.0638E-01	7.66E-01	ENSG00000163297
ANXA10	0.0237	2.4843E-01		ENSG00000109511
ANXA11	-0.0459	5.3480E-01	7.09E-01	ENSG00000122359
ANXA13	-0.0290	7.3857E-01	8.56E-01	ENSG00000104537
ANXA2	-0.1665	8.5532E-02	2.10E-01	ENSG00000182718
ANXA2R	-0.0860	4.2451E-01	6.16E-01	ENSG00000177721
ANXA2R-AS1	-0.1745	1.1970E-01	2.67E-01	ENSG00000215068
ANXA2R-OT1	-0.1897	4.8905E-02	1.41E-01	ENSG00000177738
ANXA3	-0.4622	4.4199E-03	2.28E-02	ENSG00000138772
ANXA5	-0.4523	1.2734E-06	3.26E-05	ENSG00000164111
ANXA6	-0.2130	3.1670E-02	1.01E-01	ENSG00000197043
ANXA7	0.0523	5.1179E-01	6.92E-01	ENSG00000138279
ANXA8	-0.0614	4.2139E-01	6.14E-01	ENSG00000265190
ANXA8	-0.0623	2.3046E-01	4.14E-01	ENSG00000276850
ANXA8L1	-0.0307	5.7004E-01	7.38E-01	ENSG00000264230
ANXA9	-0.1229	2.2312E-01	4.05E-01	ENSG00000143412
AOC1	0.1041	2.9707E-01	4.89E-01	ENSG00000002726
AOC2	0.0603	5.3552E-01	7.09E-01	ENSG00000131480
AOC3	-0.0081	9.2436E-01	9.63E-01	ENSG00000131471
AOPEP	-0.4045	3.6093E-06	7.71E-05	ENSG00000148120
AOX1	-0.0205	8.2740E-01	9.09E-01	ENSG00000138356
AOX2P	0.0673	1.0479E-01		ENSG00000243478
AP1AR	0.2224	1.7352E-02	6.47E-02	ENSG00000138660
AP1B1	0.0728	1.6623E-01	3.33E-01	ENSG00000100280
AP1G1	0.0002	9.7599E-01	9.88E-01	ENSG00000166747
AP1G2	0.3648	1.5811E-04	1.63E-03	ENSG00000213983
AP1M1	0.1158	7.5706E-02	1.93E-01	ENSG00000072958
AP1M2	0.0724	4.5418E-01	6.44E-01	ENSG00000129354
AP1M2P1	-0.0092	7.6973E-01		ENSG00000227534
AP1S1	0.1938	3.4832E-02	1.09E-01	ENSG00000106367
AP1S2	0.0927	3.2552E-01	5.20E-01	ENSG00000182287
AP1S3	0.5248	5.9476E-05	7.40E-04	ENSG00000152056
AP2A1	-0.1263	5.9676E-02	1.63E-01	ENSG00000196961
AP2A2	-0.1618	1.5411E-02	5.92E-02	ENSG00000183020
AP2B1	-0.1551	4.1115E-03	2.16E-02	ENSG00000006125
AP2B1P1	-0.0382	2.5909E-01		ENSG00000234130
AP2M1	-0.0359	5.0654E-01	6.87E-01	ENSG00000161203
AP2S1	-0.0961	9.1892E-02	2.22E-01	ENSG00000042753
AP3B1	-0.1881	8.9553E-04	6.53E-03	ENSG00000132842
AP3B2	0.0010	9.9892E-01	1.00E+00	ENSG00000103723
AP3D1	-0.0309	5.4199E-01	7.15E-01	ENSG00000065000
AP3M1	0.4288	2.7682E-06	6.24E-05	ENSG00000185009
AP3M2	0.0528	4.4809E-01	6.38E-01	ENSG00000070718
AP3S1	-0.1429	3.4194E-02	1.08E-01	ENSG00000177879
AP3S2	0.1431	5.9030E-02	1.61E-01	ENSG00000157823
AP4B1	0.2533	4.3136E-03	2.24E-02	ENSG00000134262

AP4B1-AS1	0.0051	6.5180E-01		ENSG00000226167
AP4E1	0.3560	5.3188E-04	4.33E-03	ENSG00000081014
AP4M1	-0.0032	9.3002E-01	9.66E-01	ENSG00000221838
AP4S1	-0.2534	4.0090E-03	2.11E-02	ENSG00000100478
AP5B1	-0.0062	9.2112E-01	9.61E-01	ENSG00000254470
AP5M1	-0.2162	2.7597E-03	1.58E-02	ENSG00000053770
AP5S1	-0.2865	1.0909E-03	7.60E-03	ENSG00000125843
AP5Z1	0.1489	5.1572E-02	1.46E-01	ENSG00000242802
APAF1	-0.1136	1.3443E-01	2.88E-01	ENSG00000120868
APBA1	0.0863	3.7186E-01	5.67E-01	ENSG00000107282
APBA2	0.2159	1.7590E-02	6.54E-02	ENSG00000034053
APBA3	-0.0899	2.0952E-01	3.89E-01	ENSG00000011132
APBB1	0.1747	2.5798E-02	8.72E-02	ENSG00000166313
APBB2	0.1291	6.6967E-02	1.76E-01	ENSG00000163697
APBB3	0.2364	4.4732E-04	3.77E-03	ENSG00000113108
APC	-0.0578	3.8339E-01	5.78E-01	ENSG00000134982
APC2	-0.0629	3.6848E-01	5.64E-01	ENSG00000115266
APCDD1	-0.0604	4.8749E-01	6.72E-01	ENSG00000154856
APCDD1L	0.0152	5.7068E-01		ENSG00000198768
APCDD1L-DT	0.0034	2.7872E-01		ENSG00000231290
APEH	0.1812	4.8886E-03	2.46E-02	ENSG00000164062
APELA	-0.0817	2.8810E-01	4.79E-01	ENSG00000248329
APEX1	-0.0177	6.1769E-01	7.75E-01	ENSG00000100823
APEX2	0.1630	5.6394E-02	1.56E-01	ENSG00000169188
APH1A	-0.1397	9.5982E-03	4.12E-02	ENSG00000117362
APH1B	-0.0849	2.4564E-01	4.31E-01	ENSG00000138613
API5	0.0550	3.7548E-01	5.71E-01	ENSG00000166181
APIP	0.0983	2.3411E-01	4.18E-01	ENSG00000149089
APLF	-0.0715	4.5471E-01	6.44E-01	ENSG00000169621
APLN	0.2607	1.9021E-02	6.94E-02	ENSG00000171388
APLNR	0.0468	2.1583E-01	3.97E-01	ENSG00000134817
APLP1	0.1322	8.3920E-02	2.08E-01	ENSG00000105290
APLP2	-0.1632	3.9275E-03	2.08E-02	ENSG00000084234
APMAP	0.1824	7.8669E-03	3.55E-02	ENSG00000101474
APOA1-AS	-0.0057	5.5722E-01		ENSG00000235910
APOA2	-0.0148	9.4475E-01		ENSG00000158874
APOB	-0.1194	3.6988E-02		ENSG00000084674
APOBEC1	-0.0312	9.6000E-01	9.80E-01	ENSG00000111701
APOBEC2	0.1047	2.7629E-01	4.67E-01	ENSG00000124701
APOBEC3A	-0.0415	6.5048E-01		ENSG00000128383
APOBEC3B	0.0951	3.4137E-01	5.37E-01	ENSG00000179750
APOBEC3C	-0.3081	3.0994E-03	1.72E-02	ENSG00000244509
APOBEC3D	-0.0423	7.4540E-01	8.61E-01	ENSG00000243811
APOBEC3F	-0.5284	1.2758E-05	2.13E-04	ENSG00000128394
APOBEC3G	-0.2317	5.6678E-02	1.57E-01	ENSG00000239713
APOBEC3H	-0.0459	5.1170E-01	6.92E-01	ENSG00000100298
APOBEC4	-0.1900	9.4719E-02	2.26E-01	ENSG00000173627
APOBR	-0.0269	2.3535E-01		ENSG00000184730
APOC1	-0.1581	1.3680E-01	2.91E-01	ENSG00000130208

APOE	0.0136	8.8491E-01	9.42E-01	ENSG00000130203
APOF	-0.2666	4.7332E-02	1.37E-01	ENSG00000175336
APOH	-0.1700	2.8001E-02	9.25E-02	ENSG00000091583
APOL1	0.0938	2.6589E-01	4.55E-01	ENSG00000100342
APOL2	0.0921	2.4535E-01	4.31E-01	ENSG00000128335
APOL3	0.0152	8.0187E-01	8.94E-01	ENSG00000128284
APOL4	0.0894	3.5908E-01	5.55E-01	ENSG00000100336
APOL6	-0.4161	1.5458E-03	1.00E-02	ENSG00000221963
APOLD1	0.1782	7.9423E-02	2.00E-01	ENSG00000178878
APOM	0.0107	8.9805E-01	9.49E-01	ENSG00000204444
APOO	-0.2003	1.4120E-03	9.36E-03	ENSG00000184831
APOOL	-0.4435	6.8140E-08	2.85E-06	ENSG00000155008
APOOP2	0.0250	3.3477E-01		ENSG00000240902
APP	0.0310	7.0055E-01	8.33E-01	ENSG00000142192
APPBP2	0.0015	9.7922E-01	9.89E-01	ENSG00000062725
APPL1	-0.0726	1.8931E-01	3.64E-01	ENSG00000157500
APPL2	-0.1859	2.0416E-03	1.24E-02	ENSG00000136044
APRG1	0.0905	3.6788E-01	5.64E-01	ENSG00000198590
APRT	-0.2437	5.9597E-04	4.74E-03	ENSG00000198931
APTR	0.0406	4.7725E-01	6.64E-01	ENSG00000214293
APTX	0.0450	3.8861E-01	5.83E-01	ENSG00000137074
AQP1	-0.1020	3.1111E-01	5.04E-01	ENSG00000240583
AQP10	-0.0398	6.3099E-01		ENSG00000143595
AQP11	-0.0892	2.1492E-01	3.95E-01	ENSG00000178301
AQP12A	-0.0054	8.7109E-01		ENSG00000184945
AQP12B	0.0185	6.9531E-01		ENSG00000185176
AQP3	0.0050	9.5909E-01	9.79E-01	ENSG00000165272
AQP4-AS1	0.0752	4.1259E-01	6.06E-01	ENSG00000260372
AQP5	0.0129	8.8507E-01	9.42E-01	ENSG00000161798
AQP5-AS1	-0.1316	1.9413E-01	3.70E-01	ENSG00000257588
AQP6	0.0223	8.1268E-01	9.01E-01	ENSG00000086159
AQP7	0.0263	4.2095E-01		ENSG00000165269
AQP7B	-0.0305	8.7109E-01		ENSG00000259916
AQP7P2	-0.0259	6.1445E-01	7.73E-01	ENSG00000181997
AQP8	0.2226	6.4792E-02	1.73E-01	ENSG00000103375
AQP9	-0.0006	9.0729E-01		ENSG00000103569
AQR	-0.0016	9.7382E-01	9.87E-01	ENSG00000021776
AR	0.0038	9.6772E-01	9.83E-01	ENSG00000169083
ARAF	-0.0835	2.5169E-01	4.38E-01	ENSG00000078061
ARAP1	-0.1281	8.5374E-02	2.10E-01	ENSG00000186635
ARAP1-AS2	-0.0317	6.2048E-01		ENSG00000245148
ARAP2	-0.4471	2.5628E-03	1.49E-02	ENSG00000047365
ARAP3	-0.0339	7.2175E-01	8.46E-01	ENSG00000120318
ARB2A	-0.3083	3.0621E-05	4.31E-04	ENSG00000113391
ARC	-0.0076	9.3457E-01	9.68E-01	ENSG00000198576
ARCN1	0.0863	1.4976E-01	3.10E-01	ENSG00000095139
AREG	-0.0504	6.0185E-01	7.62E-01	ENSG00000109321
AREL1	0.0696	3.5293E-01	5.48E-01	ENSG00000119682
ARF1	-0.0143	7.3660E-01	8.55E-01	ENSG00000143761

ARF3	-0.2936	4.7240E-04	3.92E-03	ENSG00000134287
ARF4	0.3916	6.0321E-03	2.90E-02	ENSG00000168374
ARF4-AS1	-0.1159	2.2930E-01	4.12E-01	ENSG00000272146
ARF5	-0.1304	1.7729E-03	1.11E-02	ENSG00000004059
ARF6	-0.2489	1.6479E-04	1.68E-03	ENSG00000165527
ARFGAP1	0.2348	6.7812E-03	3.16E-02	ENSG00000101199
ARFGAP2	0.0455	4.8021E-01	6.66E-01	ENSG00000149182
ARFGAP3	-0.1860	8.7664E-04	6.43E-03	ENSG00000242247
ARFGEF1	0.0666	3.1460E-01	5.08E-01	ENSG00000066777
ARFGEF2	-0.1660	4.2077E-02	1.26E-01	ENSG00000124198
ARFGEF3	0.0354	5.8391E-01	7.49E-01	ENSG00000112379
ARFIP1	-0.0881	1.9472E-01	3.71E-01	ENSG00000164144
ARFIP2	0.2441	9.9187E-05	1.11E-03	ENSG00000132254
ARFRP1	-0.0876	1.4269E-01	3.00E-01	ENSG00000101246
ARG1	0.0590	4.1194E-01	6.05E-01	ENSG00000118520
ARG2	0.2680	1.2697E-02	5.10E-02	ENSG00000081181
ARGLU1	-0.1519	1.9686E-02	7.11E-02	ENSG00000134884
ARGLU1-DT	-0.2590	4.4142E-02	1.30E-01	ENSG00000272274
ARHGAP1	0.0293	6.2783E-01	7.82E-01	ENSG00000175220
ARHGAP10	-0.0376	6.9046E-01	8.26E-01	ENSG00000071205
ARHGAP11A	-0.1572	1.5261E-01	3.14E-01	ENSG00000198826
ARHGAP11A-SCG5	-0.0027	6.6550E-01		ENSG00000288864
ARHGAP11B	-0.0425	6.6169E-01		ENSG00000285077
ARHGAP12	-0.2388	4.4779E-06	9.20E-05	ENSG00000165322
ARHGAP15	0.1693	1.2238E-01	2.71E-01	ENSG00000075884
ARHGAP17	0.0973	7.9190E-02	1.99E-01	ENSG00000140750
ARHGAP18	0.1555	4.0365E-02	1.22E-01	ENSG00000146376
ARHGAP19	0.0870	3.5138E-01	5.47E-01	ENSG00000213390
ARHGAP19-SLIT1	-0.1221	1.9508E-01	3.71E-01	ENSG00000269891
ARHGAP20	0.3649	1.1420E-02	4.70E-02	ENSG00000137727
ARHGAP21	-0.0450	5.4185E-01	7.15E-01	ENSG00000107863
ARHGAP22	0.5107	2.3515E-03	1.39E-02	ENSG00000128805
ARHGAP23	-0.0370	5.6528E-01	7.34E-01	ENSG00000275832
ARHGAP24	-0.2305	2.1687E-03	1.31E-02	ENSG00000138639
ARHGAP25	0.1428	1.7677E-01	3.47E-01	ENSG00000163219
ARHGAP26	0.1263	3.1546E-02	1.01E-01	ENSG00000145819
ARHGAP26-AS1	-0.1026	1.6534E-01	3.32E-01	ENSG00000226272
ARHGAP26-IT1	-0.0429	4.7784E-01		ENSG00000230789
ARHGAP27	0.0036	9.7027E-01	9.85E-01	ENSG00000159314
ARHGAP27P1-BPTFP1-K	-0.2612	4.4487E-02	1.31E-01	ENSG00000215769
ARHGAP28	0.2218	4.0105E-02	1.21E-01	ENSG00000088756
ARHGAP29	0.3382	1.8885E-03	1.17E-02	ENSG00000137962
ARHGAP29-AS1	-0.0639	3.4634E-01	5.41E-01	ENSG00000226835
ARHGAP30	0.5963	3.0413E-05	4.28E-04	ENSG00000186517
ARHGAP31	-0.4385	1.2392E-02	5.00E-02	ENSG00000031081
ARHGAP31-AS1	0.0047	9.5117E-01	9.75E-01	ENSG00000241155
ARHGAP32	0.0260	7.2979E-01	8.51E-01	ENSG00000134909
ARHGAP33	0.0267	7.5800E-01	8.69E-01	ENSG00000004777
ARHGAP35	-0.0939	2.0777E-01	3.87E-01	ENSG00000160007

ARHGAP36	0.4025	8.6718E-03	3.82E-02	ENSG00000147256
ARHGAP39	0.0359	6.6499E-01	8.07E-01	ENSG00000147799
ARHGAP4	0.4831	3.0870E-05	4.32E-04	ENSG00000089820
ARHGAP40	0.0269	2.3152E-01		ENSG00000124143
ARHGAP42	-0.4976	7.2695E-06	1.36E-04	ENSG00000165895
ARHGAP42-AS1	0.4420	1.2662E-02	5.09E-02	ENSG00000248027
ARHGAP44	0.0916	2.6089E-01	4.49E-01	ENSG00000006740
ARHGAP44-AS1	0.0153	1.9811E-01		ENSG00000265489
ARHGAP45	0.3324	1.7316E-03	1.09E-02	ENSG00000180448
ARHGAP5	-0.2064	1.4010E-03	9.30E-03	ENSG00000100852
ARHGAP6	-0.1365	1.9957E-01	3.77E-01	ENSG00000047648
ARHGAP8	0.5736	1.3614E-03	9.09E-03	ENSG00000241484
ARHGAP9	-0.2007	6.3253E-02	1.70E-01	ENSG00000123329
ARHGDIA	-0.0015	9.5493E-01	9.77E-01	ENSG00000141522
ARHGDIG	-0.2787	7.0448E-04	5.41E-03	ENSG00000242173
ARHGEF1	0.1306	9.8145E-02	2.32E-01	ENSG00000076928
ARHGEF10	0.1154	2.4702E-01	4.33E-01	ENSG00000104728
ARHGEF10L	-0.0329	5.6814E-01	7.36E-01	ENSG00000074964
ARHGEF11	0.0831	2.2529E-01	4.08E-01	ENSG00000132694
ARHGEF12	-0.0486	4.7204E-01	6.59E-01	ENSG00000196914
ARHGEF15	0.2459	2.9161E-02	9.55E-02	ENSG00000198844
ARHGEF16	0.2540	3.1337E-02	1.01E-01	ENSG00000130762
ARHGEF17	-0.3054	9.8855E-04	7.05E-03	ENSG00000110237
ARHGEF17-AS1	-0.0045	9.4577E-01		ENSG00000257038
ARHGEF18	-0.0998	1.6521E-01	3.32E-01	ENSG00000104880
ARHGEF19	0.5107	1.9330E-07	6.76E-06	ENSG00000142632
ARHGEF2	-0.0633	4.4032E-01	6.31E-01	ENSG00000116584
ARHGEF2-AS2	-0.1028	2.1206E-01	3.92E-01	ENSG00000273002
ARHGEF25	-0.0244	8.0459E-01		ENSG00000240771
ARHGEF26	-0.2790	2.0341E-02	7.29E-02	ENSG00000114790
ARHGEF28	0.1032	2.2652E-01	4.09E-01	ENSG00000214944
ARHGEF3	0.2992	6.6956E-04	5.20E-03	ENSG00000163947
ARHGEF33	0.2508	3.6851E-02	1.14E-01	ENSG00000214694
ARHGEF35	-0.2260	2.5134E-02	8.55E-02	ENSG00000213214
ARHGEF37	0.1762	5.5938E-02	1.55E-01	ENSG00000183111
ARHGEF38	0.0603	5.2751E-01	7.03E-01	ENSG00000236699
ARHGEF38-IT1	-0.0624	3.3137E-01	5.26E-01	ENSG00000249885
ARHGEF39	0.4108	1.7598E-03	1.11E-02	ENSG00000137135
ARHGEF4	-0.1804	1.6135E-02	6.13E-02	ENSG00000136002
ARHGEF40	0.0381	5.9897E-01	7.60E-01	ENSG00000165801
ARHGEF5	-0.1225	1.8973E-01	3.64E-01	ENSG00000050327
ARHGEF6	-0.1353	1.9054E-01	3.66E-01	ENSG00000129675
ARHGEF7	-0.0583	3.6379E-01	5.59E-01	ENSG00000102606
ARHGEF7-AS2	-0.0582	4.3912E-01	6.30E-01	ENSG00000235875
ARHGEF9	-0.1073	1.2054E-01	2.68E-01	ENSG00000131089
ARHGEF9-IT1	-0.0377	3.9377E-01		ENSG00000231729
ARID1A	-0.1578	3.7999E-02	1.16E-01	ENSG00000117713
ARID1B	-0.0350	5.7750E-01	7.44E-01	ENSG00000049618
ARID2	0.0194	7.7597E-01	8.79E-01	ENSG00000189079

ARID3A	0.3285	5.0299E-03	2.51E-02	ENSG00000116017
ARID3B	0.1613	5.7235E-02	1.58E-01	ENSG00000179361
ARID3C	-0.2228	1.2913E-01	2.80E-01	ENSG00000205143
ARID4A	0.0357	4.7255E-01	6.60E-01	ENSG00000032219
ARID4B	0.0293	4.7322E-01	6.60E-01	ENSG00000054267
ARID5A	-0.1920	3.8081E-02	1.17E-01	ENSG00000196843
ARIH1	0.0278	6.5162E-01	7.98E-01	ENSG00000166233
ARIH2	-0.0022	9.0657E-01	9.53E-01	ENSG00000177479
ARK2C	0.2909	2.2506E-03	1.35E-02	ENSG00000141622
ARK2N	-0.0958	1.5247E-01	3.14E-01	ENSG00000152242
ARL1	-0.0056	9.1388E-01	9.57E-01	ENSG00000120805
ARL10	-0.0433	5.3296E-01	7.07E-01	ENSG00000175414
ARL11	0.0212	7.6128E-01	8.70E-01	ENSG00000152213
ARL13A	0.0016	8.2540E-01		ENSG00000174225
ARL13B	0.1515	2.8016E-02	9.25E-02	ENSG00000169379
ARL14	-0.1304	7.8357E-02	1.98E-01	ENSG00000179674
ARL14EP	0.0389	4.8539E-01	6.70E-01	ENSG00000152219
ARL14EPL	0.0139	6.4607E-01		ENSG00000268223
ARL14EPP1	-0.0256	4.3147E-01		ENSG00000224891
ARL15	0.0221	7.9728E-01	8.92E-01	ENSG00000185305
ARL16	-0.0351	4.4443E-01	6.35E-01	ENSG00000214087
ARL17A	0.0124	8.4044E-01	9.17E-01	ENSG00000185829
ARL17A	-0.0320	5.6234E-01	7.31E-01	ENSG00000228696
ARL2	-0.1419	3.9149E-02	1.19E-01	ENSG00000213465
ARL2BP	-0.0671	4.8360E-01	6.68E-01	ENSG00000102931
ARL3	-0.0697	2.0229E-01	3.80E-01	ENSG00000138175
ARL4A	0.0567	4.5780E-01	6.47E-01	ENSG00000122644
ARL4C	-0.1843	6.7689E-04	5.24E-03	ENSG00000188042
ARL4D	0.2207	2.8169E-02	9.29E-02	ENSG00000175906
ARL5A	-0.0385	4.6974E-01	6.57E-01	ENSG00000162980
ARL5AP3	-0.0209	6.1583E-01		ENSG00000228431
ARL5B	0.0184	8.1392E-01	9.02E-01	ENSG00000165997
ARL5C	-0.0100	6.3768E-01		ENSG00000141748
ARL6	0.0940	2.0277E-01	3.80E-01	ENSG00000113966
ARL6IP1	-0.1538	7.0302E-02	1.83E-01	ENSG00000170540
ARL6IP4	0.4096	7.9461E-03	3.58E-02	ENSG00000182196
ARL6IP5	-0.3138	8.0284E-06	1.48E-04	ENSG00000144746
ARL6IP6	-0.0515	4.6186E-01	6.50E-01	ENSG00000177917
ARL8A	-0.0376	5.8619E-01	7.51E-01	ENSG00000143862
ARL8B	-0.2533	1.7031E-06	4.13E-05	ENSG00000134108
ARL9	-0.2637	4.7103E-02	1.37E-01	ENSG00000196503
ARMC1	0.2150	6.3834E-06	1.23E-04	ENSG00000104442
ARMC10	-0.1104	4.5293E-02	1.33E-01	ENSG00000170632
ARMC12	0.0368	6.9645E-01	8.30E-01	ENSG00000157343
ARMC2	-0.0761	2.7035E-01	4.60E-01	ENSG00000118690
ARMC5	0.0380	5.8779E-01	7.52E-01	ENSG00000140691
ARMC6	-0.1188	1.1041E-01	2.52E-01	ENSG00000105676
ARMC7	0.0770	4.3802E-01	6.29E-01	ENSG00000125449
ARMC8	0.0558	3.8124E-01	5.76E-01	ENSG00000114098

ARMC9	0.0559	4.1894E-01	6.11E-01	ENSG00000135931
ARMCX1	-0.0225	7.8525E-01	8.85E-01	ENSG00000126947
ARMCX2	-0.2284	1.9119E-03	1.18E-02	ENSG00000184867
ARMCX3	-0.0031	9.5298E-01	9.76E-01	ENSG00000102401
ARMCX4	0.1902	1.0586E-02	4.43E-02	ENSG00000196440
ARMCX5	0.2509	6.7076E-04	5.21E-03	ENSG00000125962
ARMCX5-GPRASP2	-0.0198	8.5285E-01		ENSG00000286237
ARMCX6	-0.1302	3.6192E-03	1.95E-02	ENSG00000198960
ARMH1	0.4292	1.8857E-06	4.49E-05	ENSG00000198520
ARMH3	-0.1088	5.5256E-02	1.54E-01	ENSG00000120029
ARMH4	0.1023	7.4016E-02	1.90E-01	ENSG00000139971
ARMS2	-0.0110	8.4506E-01		ENSG00000254636
ARMT1	0.0917	2.2559E-01	4.08E-01	ENSG00000146476
ARNT	0.0711	3.6543E-01	5.61E-01	ENSG00000143437
ARNT2	-0.0803	3.4308E-01	5.38E-01	ENSG00000172379
ARNT2-DT	0.1028	1.4629E-01	3.05E-01	ENSG00000259495
ARPC1A	-0.1998	1.5221E-03	9.93E-03	ENSG00000241685
ARPC1B	0.2931	2.9406E-02	9.61E-02	ENSG00000130429
ARPC2	-0.0759	1.0038E-01	2.35E-01	ENSG00000163466
ARPC3	-0.2490	3.9822E-05	5.32E-04	ENSG00000111229
ARPC4	-0.0628	3.1953E-01	5.13E-01	ENSG00000241553
ARPC4-TTLL3	0.0672	1.5791E-01	3.22E-01	ENSG00000250151
ARPC5	0.0575	3.6934E-01	5.65E-01	ENSG00000162704
ARPC5L	-0.0603	2.5313E-01	4.40E-01	ENSG00000136950
ARPIN	-0.4902	1.5020E-06	3.73E-05	ENSG00000242498
ARPIN-AP3S2	-0.0277	8.3762E-01		ENSG00000250021
ARPP19	-0.1407	2.4908E-03	1.46E-02	ENSG00000128989
ARPP21	0.1554	8.0271E-02	2.01E-01	ENSG00000172995
ARR3	0.0996	2.2157E-01	4.03E-01	ENSG00000120500
ARRB1	0.0281	7.4760E-01	8.62E-01	ENSG00000137486
ARRB2	-0.0063	9.2113E-01	9.61E-01	ENSG00000141480
ARRDC1	-0.0485	5.0434E-01	6.86E-01	ENSG00000197070
ARRDC1-AS1	-0.1685	6.4000E-03	3.03E-02	ENSG00000203993
ARRDC2	0.0332	7.2296E-01	8.47E-01	ENSG00000105643
ARRDC3	-0.2572	5.3489E-04	4.35E-03	ENSG00000113369
ARRDC3-AS1	0.2621	1.5277E-02	5.88E-02	ENSG00000281357
ARRDC4	0.5071	9.8153E-08	3.84E-06	ENSG00000140450
ARSA	-0.4142	2.0381E-05	3.10E-04	ENSG00000100299
ARSB	0.0721	2.8577E-01	4.77E-01	ENSG00000113273
ARSD	-0.1552	1.8658E-02	6.84E-02	ENSG00000006756
ARSF	0.0263	2.9820E-01		ENSG00000062096
ARSG	0.1096	1.7974E-01	3.51E-01	ENSG00000141337
ARSH	0.0725	1.0759E-01	2.47E-01	ENSG00000205667
ARSI	-0.0366	6.8290E-01	8.20E-01	ENSG00000183876
ARSJ	-0.0963	2.7850E-01	4.69E-01	ENSG00000180801
ARSK	-0.0206	7.1334E-01	8.40E-01	ENSG00000164291
ARSL	-0.0188	8.1445E-01	9.02E-01	ENSG00000157399
ART3	-0.0432	2.2384E-01		ENSG00000156219
ART4	0.0089	7.6194E-01		ENSG00000111339

ART5	0.2617	1.8423E-02	6.78E-02	ENSG00000167311
ARTN	-0.0726	4.3686E-01	6.28E-01	ENSG00000117407
ARV1	0.2461	2.8836E-04	2.64E-03	ENSG00000173409
ARVCF	0.0309	6.5295E-01	7.99E-01	ENSG00000099889
ARX	0.3810	6.7783E-07	1.92E-05	ENSG00000004848
AS3MT	-0.0317	7.8161E-01		ENSG00000214435
ASAH1	-0.1156	5.4576E-02	1.52E-01	ENSG00000104763
ASAH1-AS1	0.0153	8.7331E-01	9.36E-01	ENSG00000245281
ASAH2	0.3375	2.7688E-03	1.58E-02	ENSG00000188611
ASAH2B	0.2452	1.5312E-03	9.98E-03	ENSG00000204147
ASAP1	-0.0259	6.4402E-01	7.94E-01	ENSG00000153317
ASAP1-IT2	-0.0837	9.6971E-01	9.84E-01	ENSG00000280543
ASAP2	-0.0540	4.5314E-01	6.43E-01	ENSG00000151693
ASAP3	-0.0051	9.6715E-01	9.83E-01	ENSG00000088280
ASB1	-0.0396	5.4869E-01	7.20E-01	ENSG00000065802
ASB13	0.3763	1.7307E-03	1.09E-02	ENSG00000196372
ASB14	-0.1318	2.0997E-01	3.89E-01	ENSG00000239388
ASB15	0.0017	6.7961E-01		ENSG00000146809
ASB15-AS1	-0.0164	8.3309E-01		ENSG00000230442
ASB16	0.2324	6.5497E-02	1.74E-01	ENSG00000161664
ASB16-AS1	-0.2091	2.0292E-03	1.24E-02	ENSG00000267080
ASB18	0.0226	3.3725E-01		ENSG00000182177
ASB2	-0.5075	6.9982E-04	5.38E-03	ENSG00000100628
ASB3	0.0144	8.8222E-01	9.41E-01	ENSG00000115239
ASB4	0.0034	9.7740E-01	9.88E-01	ENSG00000005981
ASB5	-0.0568	5.4151E-01	7.14E-01	ENSG00000164122
ASB6	-0.1244	2.3206E-02	8.06E-02	ENSG00000148331
ASB7	0.0686	3.4782E-01	5.43E-01	ENSG00000183475
ASB8	0.1514	2.5544E-03	1.48E-02	ENSG00000177981
ASB9	0.1052	2.9637E-01	4.88E-01	ENSG00000102048
ASCC1	0.0836	1.7971E-01	3.51E-01	ENSG00000138303
ASCC2	0.1548	2.8242E-02	9.31E-02	ENSG00000100325
ASCC3	0.0634	3.8227E-01	5.77E-01	ENSG00000112249
ASCL1	-0.0909	1.5337E-01	3.15E-01	ENSG00000139352
ASCL2	0.1659	1.3860E-01	2.94E-01	ENSG00000183734
ASCL4	0.0572	3.3757E-01	5.33E-01	ENSG00000187855
ASCL5	0.0077	8.8378E-01	9.41E-01	ENSG00000232237
ASF1A	0.1156	1.2330E-01	2.72E-01	ENSG00000111875
ASF1B	-0.2118	6.1222E-02	1.66E-01	ENSG00000105011
ASGR1	0.2955	2.1217E-04	2.04E-03	ENSG00000141505
ASGR2	0.0607	5.3131E-01	7.06E-01	ENSG00000161944
ASH1L	0.0401	5.7563E-01	7.42E-01	ENSG00000116539
ASH1L-AS1	0.1485	4.2766E-02	1.27E-01	ENSG00000235919
ASH2L	0.0820	2.5652E-01	4.44E-01	ENSG00000129691
ASIC1	-0.0945	2.9541E-01	4.87E-01	ENSG00000110881
ASIC2	0.1591	1.1064E-01	2.52E-01	ENSG00000108684
ASIC3	0.3430	1.0116E-02	4.28E-02	ENSG00000213199
ASIC4	0.3981	7.6416E-04	5.76E-03	ENSG00000072182
ASIC5	-0.0054	8.5885E-01		ENSG00000256394

ASIP	-0.0894	1.4047E-01		ENSG00000101440
ASL	0.5552	7.2758E-09	4.39E-07	ENSG00000126522
ASMT	0.0464	4.5223E-01		ENSG00000196433
ASMTL	-0.0132	8.2031E-01	9.05E-01	ENSG00000169093
ASNS	0.3126	1.5271E-02	5.88E-02	ENSG00000070669
ASNSD1	-0.0338	6.3274E-01	7.86E-01	ENSG00000138381
ASNSP3	-0.0221	6.3427E-01		ENSG00000236554
ASPA	-0.0477	3.2249E-01		ENSG00000108381
ASPDH	0.0466	5.9895E-01	7.60E-01	ENSG00000204653
ASPG	0.0461	3.2115E-01	5.15E-01	ENSG00000166183
ASPH	0.1372	3.3375E-02	1.06E-01	ENSG00000198363
ASPHD1	0.1642	2.6752E-02	8.95E-02	ENSG00000174939
ASPHD2	-0.0426	5.2938E-01	7.04E-01	ENSG00000128203
ASPM	-0.2229	5.6634E-02	1.57E-01	ENSG00000066279
ASPN	0.0347	7.0036E-01	8.33E-01	ENSG00000106819
ASPSCR1	0.0548	3.0508E-01	4.98E-01	ENSG00000169696
ASRGL1	0.0197	6.9617E-01	8.30E-01	ENSG00000162174
ASS1	-0.1803	6.8542E-02	1.80E-01	ENSG00000130707
ASS1P2	-0.0651	4.5832E-01	6.47E-01	ENSG00000223922
ASTE1	0.1253	1.5900E-01	3.23E-01	ENSG00000034533
ASTILCS	0.1094	2.8738E-01	4.79E-01	ENSG00000244998
ASTL	0.0816	3.4919E-01	5.44E-01	ENSG00000188886
ASTN1	-0.1325	1.7255E-01	3.42E-01	ENSG00000152092
ASTN2	0.0648	3.9026E-01	5.85E-01	ENSG00000148219
ASTN2-AS1	-0.0121	4.7937E-01		ENSG00000229105
ASXL1	-0.0282	6.5140E-01	7.98E-01	ENSG00000171456
ASXL2	-0.0808	3.2823E-01	5.23E-01	ENSG00000143970
ASXL3	0.0399	6.2607E-01	7.81E-01	ENSG00000141431
ATAD1	0.2051	2.0405E-03	1.24E-02	ENSG00000138138
ATAD2	-0.4065	9.9951E-04	7.11E-03	ENSG00000156802
ATAD2B	0.1729	4.8324E-02	1.40E-01	ENSG00000119778
ATAD3A	0.3739	3.1104E-05	4.35E-04	ENSG00000197785
ATAD3B	0.5838	2.4433E-06	5.61E-05	ENSG00000160072
ATAD3C	0.1023	3.1680E-01	5.10E-01	ENSG00000215915
ATAD5	0.1005	2.4933E-01	4.36E-01	ENSG00000176208
ATAT1	0.0138	8.0470E-01	8.96E-01	ENSG00000137343
ATCAY	0.1118	1.6070E-01	3.26E-01	ENSG00000167654
ATE1	0.0690	1.5653E-01	3.20E-01	ENSG00000107669
ATF1	0.2234	2.8777E-03	1.63E-02	ENSG00000123268
ATF2	-0.1136	6.5841E-02	1.74E-01	ENSG00000115966
ATF4	-0.2548	1.2436E-10	1.43E-08	ENSG00000128272
ATF5	0.1050	2.6482E-01	4.54E-01	ENSG00000169136
ATF6	-0.1482	3.3140E-03	1.81E-02	ENSG00000118217
ATF6B	0.3289	2.8391E-04	2.61E-03	ENSG00000213676
ATF7	-0.1624	6.1556E-02	1.66E-01	ENSG00000170653
ATF7-NPFF	-0.0137	4.6836E-01		ENSG00000267281
ATF7IP	0.1144	8.8571E-02	2.16E-01	ENSG00000171681
ATF7IP2	-0.1536	4.4359E-02	1.31E-01	ENSG00000166669
ATG10	0.0947	1.3434E-01	2.88E-01	ENSG00000152348

ATG101	-0.0856	1.3192E-01	2.84E-01	ENSG00000123395
ATG12	0.1726	8.0338E-04	6.00E-03	ENSG00000145782
ATG13	0.0556	3.5207E-01	5.47E-01	ENSG00000175224
ATG14	-0.2912	4.2569E-06	8.81E-05	ENSG00000126775
ATG16L1	0.4013	6.5542E-06	1.25E-04	ENSG00000085978
ATG16L2	-0.1245	1.2634E-01	2.77E-01	ENSG00000168010
ATG2A	-0.0311	7.0493E-01	8.35E-01	ENSG00000110046
ATG2B	0.4445	5.2426E-06	1.04E-04	ENSG00000066739
ATG3	0.0536	4.5553E-01	6.45E-01	ENSG00000144848
ATG4A	-0.0176	7.7105E-01	8.76E-01	ENSG00000101844
ATG4B	0.1171	5.6034E-02	1.56E-01	ENSG00000168397
ATG4C	-0.0473	5.2473E-01	7.01E-01	ENSG00000125703
ATG4D	0.1186	1.1893E-01	2.65E-01	ENSG00000130734
ATG5	0.0694	3.2962E-01	5.24E-01	ENSG00000057663
ATG7	0.0973	2.1056E-01	3.90E-01	ENSG00000197548
ATG9A	0.0623	4.6644E-01	6.54E-01	ENSG00000198925
ATG9B	0.4549	6.9018E-06	1.31E-04	ENSG00000181652
ATIC	-0.0810	3.0226E-01	4.95E-01	ENSG00000138363
ATL1	-0.0930	2.6404E-01	4.53E-01	ENSG00000198513
ATL2	-0.0854	1.9476E-01	3.71E-01	ENSG00000119787
ATL3	0.0565	3.9338E-01	5.88E-01	ENSG00000184743
ATM	-0.0432	5.3312E-01	7.07E-01	ENSG00000149311
ATMIN	-0.0691	1.5913E-01	3.24E-01	ENSG00000166454
ATN1	-0.2149	1.5319E-02	5.89E-02	ENSG00000111676
ATOH1	-0.0021	8.7711E-01		ENSG00000172238
ATOH7	0.2339	2.1935E-02	7.72E-02	ENSG00000179774
ATOSA	-0.1222	5.8959E-02	1.61E-01	ENSG00000047346
ATOSB	0.4106	2.8761E-06	6.40E-05	ENSG00000005238
ATOX1	-0.1600	3.2206E-03	1.77E-02	ENSG00000177556
ATP10A	0.0084	9.1391E-01	9.57E-01	ENSG00000206190
ATP10B	0.1712	6.6079E-02	1.75E-01	ENSG00000118322
ATP10D	-0.3258	4.1838E-06	8.69E-05	ENSG00000145246
ATP11A	-0.1380	4.2660E-02	1.27E-01	ENSG00000068650
ATP11A-AS1	-0.2153	7.3293E-02	1.88E-01	ENSG00000232684
ATP11AUN	0.0077	4.6103E-01		ENSG00000197595
ATP11B	-0.0545	3.7345E-01	5.69E-01	ENSG00000058063
ATP11C	0.0528	5.2373E-01	7.00E-01	ENSG00000101974
ATP12A	-0.0796	1.7320E-01		ENSG00000075673
ATP13A1	-0.1472	1.2479E-02	5.03E-02	ENSG00000105726
ATP13A2	0.0366	5.9787E-01	7.59E-01	ENSG00000159363
ATP13A3	-0.1089	2.0059E-01	3.78E-01	ENSG00000133657
ATP13A4	-0.1619	1.4104E-01	2.98E-01	ENSG00000127249
ATP13A5	-0.0170	8.5940E-01	9.28E-01	ENSG00000187527
ATP1A1	0.0096	8.9091E-01	9.46E-01	ENSG00000163399
ATP1A1-AS1	0.1774	4.1406E-02	1.24E-01	ENSG00000203865
ATP1A3	0.2523	1.3462E-02	5.34E-02	ENSG00000105409
ATP1A4	-0.1696	5.7459E-02	1.58E-01	ENSG00000132681
ATP1B1	-0.1060	4.4718E-02	1.32E-01	ENSG00000143153
ATP1B2	-0.2854	3.2155E-05	4.47E-04	ENSG00000129244

ATP1B3	-0.2080	6.8646E-03	3.19E-02	ENSG00000069849
ATP1B4	-0.0103	8.5860E-01		ENSG00000101892
ATP23	-0.0908	2.2672E-01	4.09E-01	ENSG00000166896
ATP2A1	-0.0164	8.6112E-01	9.29E-01	ENSG00000196296
ATP2A1-AS1	-0.5062	4.3154E-04	3.66E-03	ENSG00000260442
ATP2A2	-0.2816	3.9255E-04	3.39E-03	ENSG00000174437
ATP2A3	0.1852	8.5062E-02	2.10E-01	ENSG00000074370
ATP2B1	0.0375	4.0172E-01	5.96E-01	ENSG00000070961
ATP2B1-AS1	0.4699	4.3847E-18	3.112E-15	ENSG00000271614
ATP2B1-AS1	0.4699	4.3847E-18	3.11E-15	ENSG00000271614
ATP2B2	0.0347	7.0171E-01	8.33E-01	ENSG00000157087
ATP2B3	-0.0075	9.3279E-01	9.67E-01	ENSG00000067842
ATP2B4	-0.0709	3.4762E-01	5.43E-01	ENSG00000058668
ATP2C1	-0.2554	1.3951E-04	1.46E-03	ENSG00000017260
ATP2C2	-0.1788	1.0266E-01	2.39E-01	ENSG00000064270
ATP2C2-AS1	0.0231	5.4234E-01		ENSG00000261286
ATP4A	0.1226	5.8430E-03	2.82E-02	ENSG00000105675
ATP4B	0.0048	8.3987E-01	9.16E-01	ENSG00000186009
ATP5F1A	-0.0314	6.7025E-01	8.11E-01	ENSG00000152234
ATP5F1AP1	-0.0076	9.0691E-01		ENSG00000234861
ATP5F1AP2	0.0263	2.3956E-01		ENSG00000227682
ATP5F1AP3	0.0004	8.6766E-01		ENSG00000263232
ATP5F1AP7	-0.0004	6.4619E-01		ENSG00000241571
ATP5F1AP8	-0.0013	7.3860E-01		ENSG00000234720
ATP5F1B	0.0262	7.1175E-01	8.39E-01	ENSG00000110955
ATP5F1C	-0.0823	1.4667E-01	3.06E-01	ENSG00000165629
ATP5F1CP1	-0.1023	6.6358E-01	8.07E-01	ENSG00000224004
ATP5F1D	-0.2113	1.4820E-03	9.72E-03	ENSG00000099624
ATP5F1E	-0.3071	8.5452E-06	1.55E-04	ENSG00000124172
ATP5IF1	-0.3435	3.3919E-09	2.36E-07	ENSG00000130770
ATP5MC1	-0.0576	4.1145E-01	6.05E-01	ENSG00000159199
ATP5MC2	-0.2809	1.4936E-04	1.55E-03	ENSG00000135390
ATP5MC3	-0.2035	9.9713E-03	4.24E-02	ENSG00000154518
ATP5ME	-0.5178	6.1250E-12	1.01E-09	ENSG00000169020
ATP5MF	-0.4356	6.6245E-13	1.47E-10	ENSG00000241468
ATP5MF-PTCD1	-0.0023	9.5760E-01		ENSG00000248919
ATP5MFP1	0.0189	6.3208E-01		ENSG00000224421
ATP5MG	-0.1920	8.2232E-03	3.67E-02	ENSG00000167283
ATP5MGL	0.2200	6.1336E-02	1.66E-01	ENSG00000249222
ATP5MGP3	-0.0367	8.4261E-01		ENSG00000249256
ATP5MJ	-0.2525	1.0294E-04	1.15E-03	ENSG00000156411
ATP5MK	-0.1720	6.0986E-03	2.92E-02	ENSG00000173915
ATP5PB	-0.0846	1.5267E-01	3.14E-01	ENSG00000116459
ATP5PBP1	-0.1033	5.8692E-01	7.51E-01	ENSG00000224451
ATP5PD	-0.2072	1.4354E-03	9.48E-03	ENSG00000167863
ATP5PDP2	-0.0036	9.6923E-01		ENSG00000229184
ATP5PF	-0.2987	3.0385E-05	4.28E-04	ENSG00000154723
ATP5PO	-0.1775	8.3352E-03	3.71E-02	ENSG00000241837
ATP6	-0.0374	6.7222E-01	8.12E-01	ENSG00000198899

ATP6AP1	-0.0344	6.0076E-01	7.61E-01	ENSG00000071553
ATP6AP1-DT	-0.2705	5.6760E-04	4.55E-03	ENSG00000197180
ATP6AP2	0.0560	4.7391E-01	6.61E-01	ENSG00000182220
ATP6V0A1	0.1620	3.8141E-02	1.17E-01	ENSG00000033627
ATP6V0A2	0.1980	3.1240E-02	1.00E-01	ENSG00000185344
ATP6V0A4	0.0633	2.3141E-01	4.15E-01	ENSG00000105929
ATP6V0B	-0.1164	6.1624E-02	1.67E-01	ENSG00000117410
ATP6V0C	-0.2613	3.4095E-02	1.07E-01	ENSG00000185883
ATP6V0CP4	-0.0228	6.3880E-01		ENSG00000284138
ATP6V0D1	0.0583	3.9913E-01	5.93E-01	ENSG00000159720
ATP6V0D1-DT	-0.1739	8.7254E-02	2.14E-01	ENSG00000270049
ATP6V0D2	0.0786	2.0179E-01	3.79E-01	ENSG00000147614
ATP6V0E1	-0.2624	2.5828E-04	2.41E-03	ENSG00000113732
ATP6V0E1P1	0.0395	5.9506E-01	7.57E-01	ENSG00000225364
ATP6V0E2	-0.1268	5.3736E-02	1.51E-01	ENSG00000171130
ATP6V0E2-AS1	0.0078	9.3636E-01	9.69E-01	ENSG00000204934
ATP6V1A	-0.1216	5.6042E-02	1.56E-01	ENSG00000114573
ATP6V1B1	-0.0316	6.9553E-01	8.30E-01	ENSG00000116039
ATP6V1B2	0.1272	1.1398E-01	2.58E-01	ENSG00000147416
ATP6V1C1	0.0438	5.3212E-01	7.06E-01	ENSG00000155097
ATP6V1D	-0.1749	4.7966E-04	3.96E-03	ENSG00000100554
ATP6V1E1	-0.0253	7.1206E-01	8.40E-01	ENSG00000131100
ATP6V1E2	-0.1879	2.7791E-02	9.20E-02	ENSG00000250565
ATP6V1F	-0.1893	3.0879E-03	1.72E-02	ENSG00000128524
ATP6V1G1	-0.2780	3.6666E-06	7.81E-05	ENSG00000136888
ATP6V1G1P4	0.0260	4.3070E-01		ENSG00000233343
ATP6V1G2	-0.0020	9.7885E-01	9.89E-01	ENSG00000213760
ATP6V1G3	-0.0941	9.7362E-02	2.30E-01	ENSG00000151418
ATP6V1H	-0.1127	4.7233E-02	1.37E-01	ENSG00000047249
ATP7A	0.0109	8.9149E-01	9.46E-01	ENSG00000165240
ATP7B	-0.0100	8.8921E-01	9.44E-01	ENSG00000123191
ATP8	-0.1108	1.9643E-01	3.73E-01	ENSG00000228253
ATP8A1	-0.0051	9.4685E-01	9.73E-01	ENSG00000124406
ATP8A2	-0.0800	2.1831E-01	3.99E-01	ENSG00000132932
ATP8B1	0.1372	1.9244E-01	3.68E-01	ENSG00000081923
ATP8B1-AS1	-0.0421	6.5457E-01	8.00E-01	ENSG00000267040
ATP8B2	0.3774	1.0913E-04	1.20E-03	ENSG00000143515
ATP8B4	0.0695	4.2054E-01	6.13E-01	ENSG00000104043
ATP9A	-0.0412	5.7667E-01	7.43E-01	ENSG00000054793
ATP9B	-0.2087	3.9378E-04	3.40E-03	ENSG00000166377
ATPAF1	-0.1667	2.5840E-02	8.73E-02	ENSG00000123472
ATPAF2	-0.2511	1.2812E-05	2.14E-04	ENSG00000171953
ATPSCKMT	-0.2620	4.8728E-04	4.01E-03	ENSG00000150756
ATR	0.2326	1.6827E-03	1.07E-02	ENSG00000175054
ATRAID	-0.2306	2.8874E-03	1.64E-02	ENSG00000138085
ATRIP	0.1623	4.0189E-02	1.22E-01	ENSG00000164053
ATRN	0.2602	2.4138E-03	1.42E-02	ENSG00000088812
ATRNL1	-0.0447	5.5291E-01	7.24E-01	ENSG00000107518
ATRX	-0.1211	2.9055E-02	9.52E-02	ENSG00000085224

ATXN1	-0.0948	1.2772E-01	2.79E-01	ENSG00000124788
ATXN10	-0.2025	2.2879E-04	2.18E-03	ENSG00000130638
ATXN1L	0.0822	3.1056E-01	5.03E-01	ENSG00000224470
ATXN2	-0.0049	9.1040E-01	9.55E-01	ENSG00000204842
ATXN2-AS	0.4464	3.7070E-03	1.99E-02	ENSG00000258099
ATXN2L	-0.1516	1.8192E-02	6.71E-02	ENSG00000168488
ATXN3	0.0385	5.6747E-01	7.36E-01	ENSG00000066427
ATXN7	0.0749	3.6493E-01	5.61E-01	ENSG00000163635
ATXN7L1	-0.0839	2.3561E-01	4.20E-01	ENSG00000146776
ATXN7L2	-0.0794	3.3252E-01	5.27E-01	ENSG00000162650
ATXN7L3	0.0129	8.7062E-01	9.34E-01	ENSG00000087152
ATXN7L3-AS1	-0.1440	2.7676E-02	9.17E-02	ENSG00000260793
ATXN7L3B	-0.0535	3.6982E-01	5.66E-01	ENSG00000253719
AUH	-0.0794	3.6404E-01	5.60E-01	ENSG00000148090
AUNIP	-0.1758	1.1492E-01	2.59E-01	ENSG00000127423
AUP1	-0.1366	1.1421E-02	4.70E-02	ENSG00000115307
AURKA	0.0101	9.1473E-01	9.57E-01	ENSG00000087586
AURKAIP1	-0.0985	8.7713E-02	2.14E-01	ENSG00000175756
AURKB	-0.2918	8.3653E-03	3.72E-02	ENSG00000178999
AURKC	0.1076	2.7153E-01	4.61E-01	ENSG00000105146
AUTS2	0.1301	8.6155E-02	2.12E-01	ENSG00000158321
AVEN	-0.2732	8.2016E-04	6.09E-03	ENSG00000169857
AVIL	0.0258	7.7732E-01	8.80E-01	ENSG00000135407
AVL9	0.1313	5.9131E-02	1.62E-01	ENSG00000105778
AVPI1	0.0034	9.7932E-01	9.89E-01	ENSG00000119986
AVPR1A	-0.1310	6.5218E-02	1.73E-01	ENSG00000166148
AWAT1	-0.0177	4.3289E-01		ENSG00000204195
AWAT2	0.0136	3.4263E-01		ENSG00000147160
AXDND1	0.2909	2.7776E-02	9.20E-02	ENSG00000162779
AXIN1	0.0989	2.1004E-01	3.89E-01	ENSG00000103126
AXIN2	-0.2750	5.9111E-03	2.85E-02	ENSG00000168646
AXL	-0.1530	6.5569E-02	1.74E-01	ENSG00000167601
AZI2	0.0226	7.0403E-01	8.34E-01	ENSG00000163512
AZIN1	-0.3281	5.4267E-07	1.60E-05	ENSG00000155096
AZIN2	0.1796	2.6995E-02	9.01E-02	ENSG00000142920
AZU1	-0.1826	7.4373E-02	1.90E-01	ENSG00000172232
B2M	-0.5003	1.5553E-06	3.85E-05	ENSG00000166710
B3GALNT1	-0.2507	7.7371E-03	3.50E-02	ENSG00000169255
B3GALNT2	-0.2209	4.2285E-03	2.20E-02	ENSG00000162885
B3GALT1	-0.1012	2.5565E-01	4.43E-01	ENSG00000172318
B3GALT1-AS1	0.0314	4.5002E-01		ENSG00000235335
B3GALT2	0.3065	1.1049E-02	4.58E-02	ENSG00000162630
B3GALT4	-0.0086	8.9687E-01	9.49E-01	ENSG00000235863
B3GALT5	0.1530	1.2687E-01	2.77E-01	ENSG00000183778
B3GALT6	0.0468	3.2970E-01	5.24E-01	ENSG00000176022
B3GALT9	0.0432	6.3432E-01	7.87E-01	ENSG00000214654
B3GAT1	-0.5141	3.3189E-04	2.97E-03	ENSG00000109956
B3GAT1-DT	0.1063	2.8960E-01	4.81E-01	ENSG00000255545
B3GAT2	-0.0209	8.2946E-01	9.11E-01	ENSG00000112309

B3GAT3	0.1919	3.8386E-04	3.34E-03	ENSG00000149541
B3GLCT	-0.3101	4.4672E-03	2.29E-02	ENSG00000187676
B3GNT2	-0.2873	5.1981E-03	2.58E-02	ENSG00000170340
B3GNT3	0.1012	1.3745E-01	2.92E-01	ENSG00000179913
B3GNT4	0.3735	2.5435E-03	1.48E-02	ENSG00000176383
B3GNT6	0.0684	1.8820E-01		ENSG00000198488
B3GNT7	-0.1196	2.4121E-01	4.26E-01	ENSG00000156966
B3GNT8	-0.1869	5.7642E-02	1.59E-01	ENSG00000177191
B3GNT9	-0.2993	9.4385E-03	4.07E-02	ENSG00000237172
B3GNTL1	-0.1142	2.2182E-01	4.03E-01	ENSG00000175711
B4GALNT1	0.1147	2.3586E-01	4.20E-01	ENSG00000135454
B4GALNT3	0.1119	2.7779E-01	4.69E-01	ENSG00000139044
B4GALNT4	-0.2134	4.3326E-02	1.29E-01	ENSG00000182272
B4GALT1	-0.2867	2.8985E-03	1.64E-02	ENSG00000086062
B4GALT2	-0.0632	3.9403E-01	5.89E-01	ENSG00000117411
B4GALT3	0.0210	7.5577E-01	8.67E-01	ENSG00000158850
B4GALT4	0.1050	1.0533E-01	2.44E-01	ENSG00000121578
B4GALT4-AS1	0.0024	8.0529E-01		ENSG00000240254
B4GALT5	-0.2527	1.5485E-03	1.00E-02	ENSG00000158470
B4GALT6	0.0994	2.8375E-01	4.75E-01	ENSG00000118276
B4GALT7	-0.1117	5.5142E-02	1.54E-01	ENSG00000027847
B4GAT1	-0.1255	6.9409E-02	1.81E-01	ENSG00000174684
B4GAT1-DT	-0.1483	6.3177E-02	1.70E-01	ENSG00000255468
B9D1	-0.2643	5.3671E-04	4.36E-03	ENSG00000108641
B9D2	-0.4908	3.2490E-09	2.27E-07	ENSG00000123810
BAALC	-0.3158	9.1129E-04	6.62E-03	ENSG00000164929
BAAT	-0.0261	8.8715E-01	9.43E-01	ENSG00000136881
BABAM1	-0.0389	5.7435E-01	7.41E-01	ENSG00000105393
BABAM2	-0.1360	5.1235E-02	1.45E-01	ENSG00000158019
BACE1	0.0180	7.8917E-01	8.87E-01	ENSG00000186318
BACE2	-0.2389	2.0584E-02	7.35E-02	ENSG00000182240
BACH1	-0.1429	1.5342E-02	5.90E-02	ENSG00000156273
BACH2	0.2241	1.1310E-02	4.66E-02	ENSG00000112182
BAD	-0.2220	7.4619E-07	2.09E-05	ENSG00000002330
BAG1	-0.0397	4.2533E-01	6.17E-01	ENSG00000107262
BAG2	0.0146	8.7623E-01	9.37E-01	ENSG00000112208
BAG3	-0.4988	1.2229E-08	6.76E-07	ENSG00000151929
BAG4	-0.1649	1.4428E-02	5.63E-02	ENSG00000156735
BAG5	-0.3646	8.8158E-09	5.12E-07	ENSG00000166170
BAG6	-0.1966	4.0889E-02	1.23E-01	ENSG00000204463
BAHCC1	0.3337	5.7728E-03	2.80E-02	ENSG00000266074
BAHD1	-0.1889	5.4913E-02	1.53E-01	ENSG00000140320
BAIAP2	-0.0812	1.6757E-01	3.35E-01	ENSG00000175866
BAIAP2-DT	-0.1068	2.0251E-01	3.80E-01	ENSG00000226137
BAIAP2L1	-0.0561	5.5050E-01	7.22E-01	ENSG00000006453
BAIAP2L2	0.4607	3.9299E-04	3.39E-03	ENSG00000128298
BAIAP3	0.1789	1.5249E-02	5.87E-02	ENSG00000007516
BAK1	0.0304	6.9590E-01	8.30E-01	ENSG00000030110
BAMBI	-0.4305	2.8367E-04	2.61E-03	ENSG00000095739

BANCR	-0.0519	2.2650E-01		ENSG00000278910
BANF1	-0.2903	8.4626E-06	1.54E-04	ENSG00000175334
BANK1	-0.0614	4.7185E-01	6.59E-01	ENSG00000153064
BANP	0.0104	8.8808E-01	9.44E-01	ENSG00000172530
BAP1	-0.0422	5.7188E-01	7.39E-01	ENSG00000163930
BARD1	-0.2560	1.7219E-02	6.44E-02	ENSG00000138376
BARHL1	-0.0987	1.8508E-03	1.15E-02	ENSG00000125492
BARX1	0.2112	7.8985E-02	1.99E-01	ENSG00000131668
BARX1-DT	-0.0024	9.4933E-01		ENSG00000235601
BASP1	0.0651	4.2715E-01	6.19E-01	ENSG00000176788
BASP1-AS1	0.1753	7.3684E-02	1.89E-01	ENSG00000215196
BATF	0.0174	3.7890E-01		ENSG00000156127
BATF2	-0.0194	7.9372E-01	8.89E-01	ENSG00000168062
BATF3	-0.1494	1.7176E-01	3.41E-01	ENSG00000123685
BAX	-0.3329	3.0812E-06	6.76E-05	ENSG00000087088
BAZ1A	0.0005	9.9523E-01	9.97E-01	ENSG00000198604
BAZ1A-AS1	-0.1820	1.0313E-01	2.40E-01	ENSG00000258738
BAZ1B	-0.3425	5.1000E-12	8.63E-10	ENSG00000009954
BAZ2A	0.1223	1.3271E-01	2.85E-01	ENSG00000076108
BAZ2B	0.1661	8.1270E-03	3.64E-02	ENSG00000123636
BAZ2B	0.2446	1.6132E-02	6.13E-02	ENSG00000226266
BAZ2B-AS1	-0.1277	1.2533E-01	2.75E-01	ENSG00000224152
BBC3	-0.0045	9.5547E-01	9.77E-01	ENSG00000105327
BBIP1	-0.0969	7.4043E-02	1.90E-01	ENSG00000214413
BBLN	-0.0230	6.8080E-01	8.19E-01	ENSG00000171159
BBOF1	-0.0159	8.0303E-01	8.95E-01	ENSG00000119636
BBOX1	0.0314	7.4034E-01	8.57E-01	ENSG00000129151
BBOX1-AS1	0.0222	8.0447E-01	8.96E-01	ENSG00000254560
BBS1	0.0672	4.9378E-01	6.77E-01	ENSG00000174483
BBS10	0.4814	4.3700E-07	1.33E-05	ENSG00000179941
BBS12	0.4166	1.8020E-04	1.81E-03	ENSG00000181004
BBS2	0.0842	2.8499E-01	4.76E-01	ENSG00000125124
BBS4	0.1604	1.2618E-03	8.56E-03	ENSG00000140463
BBS5	0.1690	5.9676E-02	1.63E-01	ENSG00000163093
BBS7	0.0590	3.4109E-01	5.36E-01	ENSG00000138686
BBS9	0.0489	5.2338E-01	7.00E-01	ENSG00000122507
BBX	-0.0892	1.5376E-01	3.16E-01	ENSG00000114439
BCAM	-0.0022	9.8003E-01	9.89E-01	ENSG00000187244
BCAN	-0.3841	1.5573E-03	1.01E-02	ENSG00000132692
BCAP31	-0.1073	7.4743E-03	3.41E-02	ENSG00000185825
BCAP31P1	0.0156	2.7520E-01		ENSG00000261217
BCAP31P2	0.0124	4.5781E-01		ENSG00000260047
BCAR1	-0.0139	8.0142E-01	8.94E-01	ENSG00000050820
BCAR3	-0.1075	1.8875E-01	3.63E-01	ENSG00000137936
BCAR4	-0.1174	1.4045E-01	2.97E-01	ENSG00000262117
BCAS1	0.0813	4.1235E-01	6.05E-01	ENSG00000064787
BCAS2	-0.2311	1.5668E-05	2.53E-04	ENSG00000116752
BCAS3	0.0335	6.5267E-01	7.99E-01	ENSG00000141376
BCAS4	-0.2107	7.5403E-04	5.70E-03	ENSG00000124243

BCAT1	-0.1686	6.0353E-02	1.64E-01	ENSG00000060982
BCAT2	-0.0401	5.6569E-01	7.34E-01	ENSG00000105552
BCCIP	-0.0191	7.1209E-01	8.40E-01	ENSG00000107949
BCDIN3D	0.5591	1.3406E-08	7.21E-07	ENSG00000186666
BCDIN3D-AS1	0.0473	6.2655E-01	7.81E-01	ENSG00000258057
BCHE	-0.3503	1.6802E-05	2.67E-04	ENSG00000114200
BCKDHA	-0.0234	7.8296E-01	8.83E-01	ENSG00000248098
BCKDHB	-0.0321	5.9188E-01	7.55E-01	ENSG00000083123
BCKDK	0.0240	7.7334E-01	8.77E-01	ENSG00000103507
BCL10	-0.2925	1.0918E-05	1.89E-04	ENSG00000142867
BCL11A	0.1581	1.0909E-01	2.50E-01	ENSG00000119866
BCL11B	0.3300	2.1515E-02	7.61E-02	ENSG00000127152
BCL2	-0.3591	4.8286E-04	3.98E-03	ENSG00000171791
BCL2A1	-0.0980	7.5047E-02		ENSG00000140379
BCL2L1	-0.2100	1.6330E-03	1.05E-02	ENSG00000171552
BCL2L10	-0.1313	7.7434E-02	1.96E-01	ENSG00000137875
BCL2L11	-0.2365	1.6085E-03	1.03E-02	ENSG00000153094
BCL2L12	-0.1247	1.3688E-01	2.91E-01	ENSG00000126453
BCL2L12P1	-0.0021	8.2975E-01		ENSG00000248530
BCL2L13	-0.0441	5.3247E-01	7.07E-01	ENSG00000099968
BCL2L14	0.0802	2.4760E-01	4.34E-01	ENSG00000121380
BCL2L15	0.0248	1.2331E-01	2.72E-01	ENSG00000188761
BCL2L2	0.0983	2.8241E-01	4.74E-01	ENSG00000129473
BCL2L2-PABPN1	-0.1048	2.5320E-01	4.40E-01	ENSG00000258643
BCL3	-0.5015	4.4258E-05	5.84E-04	ENSG00000069399
BCL6	0.0480	5.4202E-01	7.15E-01	ENSG00000113916
BCL6B	0.0160	5.9870E-01		ENSG00000161940
BCL7A	0.0467	3.8956E-01	5.84E-01	ENSG00000110987
BCL7B	-0.2073	2.6976E-03	1.55E-02	ENSG00000106635
BCL7C	-0.2574	1.9958E-06	4.73E-05	ENSG00000099385
BCL9	-0.0349	6.6188E-01	8.05E-01	ENSG00000116128
BCL9L	-0.3586	1.5892E-03	1.03E-02	ENSG00000186174
BCLAF1	-0.2336	1.4657E-03	9.64E-03	ENSG00000029363
BCLAF1P2	-0.1199	1.7642E-01	3.47E-01	ENSG00000279800
BCLAF3	0.4422	3.6444E-05	4.94E-04	ENSG00000173681
BCO1	-0.1666	1.3148E-01	2.84E-01	ENSG00000135697
BCO2	-0.0880	3.6606E-01	5.62E-01	ENSG00000197580
BCOR	-0.2213	2.0171E-03	1.23E-02	ENSG00000183337
BCORL1	0.1216	1.4564E-01	3.04E-01	ENSG00000085185
BCR	-0.0627	2.6133E-01	4.50E-01	ENSG00000186716
BCRP7	-0.0285	2.7949E-01		ENSG00000215544
BCRP8	0.1068	1.7102E-01	3.40E-01	ENSG00000236794
BCRP9	-0.0117	7.3824E-01		ENSG00000271287
BCS1L	-0.0710	1.5032E-01	3.11E-01	ENSG00000074582
BCYRN1	-0.0618	4.9839E-01	6.81E-01	ENSG00000236824
BDH1	-0.1383	9.6674E-02	2.29E-01	ENSG00000161267
BDH2	-0.1501	2.6743E-02	8.95E-02	ENSG00000164039
BDKRB1	-0.1436	8.8325E-03		ENSG00000100739
BDKRB2	-0.2596	4.4763E-02	1.32E-01	ENSG00000168398

BDNF	0.5049	2.0082E-03	1.23E-02	ENSG00000176697
BDNF-AS	-0.0713	3.6282E-01	5.59E-01	ENSG00000245573
BDP1	0.2369	1.4885E-03	9.75E-03	ENSG00000145734
BEAN1	-0.1380	1.9341E-01	3.69E-01	ENSG00000166546
BEAN1-AS1	-0.0223	9.2712E-01		ENSG00000261656
BECN1	0.1889	6.7968E-03	3.17E-02	ENSG00000126581
BEGAIN	0.1214	1.4450E-01	3.03E-01	ENSG00000183092
BEND3	-0.1284	1.5675E-01	3.20E-01	ENSG00000178409
BEND4	-0.0048	9.4123E-01	9.71E-01	ENSG00000188848
BEND5	-0.0946	1.2438E-01	2.74E-01	ENSG00000162373
BEND6	-0.3555	6.8175E-04	5.28E-03	ENSG00000151917
BEND7	-0.0902	1.7406E-01	3.44E-01	ENSG00000165626
BEST1	0.3095	4.3926E-03	2.27E-02	ENSG00000167995
BEST2	0.0450	5.9557E-01	7.58E-01	ENSG00000039987
BEST3	0.0263	6.9846E-01	8.31E-01	ENSG00000127325
BEST4	-0.0914	3.6253E-01	5.58E-01	ENSG00000142959
BET1	0.3444	6.2454E-06	1.21E-04	ENSG00000105829
BET1L	0.0187	7.8061E-01	8.82E-01	ENSG00000177951
BEX1	-0.1679	9.2871E-04	6.72E-03	ENSG00000133169
BEX2	-0.1619	1.7645E-03	1.11E-02	ENSG00000133134
BEX3	-0.3682	1.0844E-07	4.15E-06	ENSG00000166681
BEX4	-0.1862	3.7404E-04	3.28E-03	ENSG00000102409
BEX5	0.2823	2.5996E-03	1.50E-02	ENSG00000184515
BFAR	0.0807	1.8895E-01	3.64E-01	ENSG00000103429
BFSP1	-0.0565	5.4105E-01	7.14E-01	ENSG00000125864
BFSP2	0.0186	3.0296E-01		ENSG00000170819
BGLAP	0.0118	8.1318E-01		ENSG00000242252
BGN	0.0190	8.3591E-01	9.14E-01	ENSG00000182492
BHLHA15	0.0846	3.9407E-01	5.89E-01	ENSG00000180535
BHLHE22	-0.2447	4.6280E-02	1.35E-01	ENSG00000180828
BHLHE22-AS1	-0.0037	7.9097E-01		ENSG00000254102
BHLHE23	-0.0605	2.0417E-01		ENSG00000125533
BHLHE40	-0.2894	8.5697E-04	6.32E-03	ENSG00000134107
BHLHE40-AS1	0.2264	3.7788E-02	1.16E-01	ENSG00000235831
BHLHE41	-0.1132	1.6899E-01	3.37E-01	ENSG00000123095
BHMT	0.0747	2.1349E-01	3.94E-01	ENSG00000145692
BHMT2	0.0472	3.5359E-01		ENSG00000132840
BICC1	0.0531	5.7486E-01	7.42E-01	ENSG00000122870
BICD1	-0.0398	5.0538E-01	6.86E-01	ENSG00000151746
BICD1P1	-0.0109	9.5066E-01		ENSG00000224346
BICD2	-0.0584	4.1624E-01	6.09E-01	ENSG00000185963
BICDL1	0.3674	6.6106E-05	8.05E-04	ENSG00000135127
BICDL2	-0.0402	6.5151E-01	7.98E-01	ENSG00000162069
BICRA	-0.0897	3.5472E-01	5.50E-01	ENSG00000063169
BICRAL	-0.0961	2.4028E-01	4.25E-01	ENSG00000112624
BID	-0.0463	5.8184E-01	7.48E-01	ENSG00000015475
BIK	-0.1389	1.5807E-01	3.22E-01	ENSG00000100290
BIN1	0.1727	6.7266E-03	3.14E-02	ENSG00000136717
BIN3	0.1617	1.0043E-03	7.13E-03	ENSG00000147439

BIRC2	-0.0105	8.5983E-01	9.29E-01	ENSG00000110330
BIRC5	-0.2011	8.5454E-02	2.10E-01	ENSG00000089685
BIRC6	0.0912	1.9541E-01	3.71E-01	ENSG00000115760
BIRC7	0.0972	5.5739E-02	1.55E-01	ENSG00000101197
BISPR	0.1981	9.2597E-02	2.23E-01	ENSG00000282851
BIVM	-0.0432	5.2313E-01	7.00E-01	ENSG00000134897
BLACAT1	0.0181	8.2782E-01	9.09E-01	ENSG00000281406
BLCAP	0.2201	9.5988E-04	6.90E-03	ENSG00000166619
BLK	0.0401	1.4385E-01		ENSG00000136573
BLM	0.1457	1.7931E-01	3.51E-01	ENSG00000197299
BLMH	-0.1346	3.0595E-02	9.88E-02	ENSG00000108578
BLNK	-0.4063	1.7321E-03	1.09E-02	ENSG00000095585
BLOC1S1	-0.2753	2.4789E-03	1.45E-02	ENSG00000135441
BLOC1S2	-0.0551	2.0572E-01	3.84E-01	ENSG00000196072
BLOC1S3	0.0394	5.9386E-01	7.56E-01	ENSG00000189114
BLOC1S4	0.0308	6.5115E-01	7.98E-01	ENSG00000186222
BLOC1S5	0.0610	3.8246E-01	5.77E-01	ENSG00000188428
BLOC1S6	-0.1447	2.0326E-03	1.24E-02	ENSG00000104164
BLTP1	-0.1365	3.5614E-02	1.11E-01	ENSG00000138688
BLTP2	0.0725	2.2681E-01	4.09E-01	ENSG00000007202
BLTP3A	-0.0995	1.8667E-01	3.61E-01	ENSG00000065060
BLTP3B	-0.0617	3.5173E-01	5.47E-01	ENSG00000111647
BLVRA	-0.2373	5.4530E-04	4.42E-03	ENSG00000106605
BLVRB	-0.0413	6.4250E-01	7.93E-01	ENSG00000090013
BLVRBP1	-0.0331	5.9048E-01		ENSG00000273682
BLZF1	0.5065	1.9837E-05	3.04E-04	ENSG00000117475
BMAL1	0.1065	1.4661E-01	3.06E-01	ENSG00000133794
BMAL2	0.0801	3.1870E-01	5.12E-01	ENSG00000029153
BMAL2-AS1	-0.0155	6.3049E-01		ENSG00000245311
BMERB1	0.0267	7.0657E-01	8.36E-01	ENSG00000166780
BMF	-0.0338	7.0148E-01	8.33E-01	ENSG00000104081
BMI1	-0.1225	9.5497E-02	2.28E-01	ENSG00000168283
BMP1	-0.1342	4.6998E-02	1.37E-01	ENSG00000168487
BMP2	-0.1026	2.4367E-01	4.29E-01	ENSG00000125845
BMP2K	0.0906	3.6053E-01	5.57E-01	ENSG00000138756
BMP2KL	-0.0089	5.8615E-01		ENSG00000204113
BMP3	0.1278	1.5632E-01	3.19E-01	ENSG00000152785
BMP4	-0.0756	4.3606E-01	6.27E-01	ENSG00000125378
BMP5	-0.1243	1.9578E-01	3.72E-01	ENSG00000112175
BMP6	-0.1358	1.3181E-01	2.84E-01	ENSG00000153162
BMP7	-0.2972	9.7921E-04	6.99E-03	ENSG00000101144
BMP8A	-0.3627	2.8892E-03	1.64E-02	ENSG00000183682
BMP8B	-0.0808	4.0430E-01	5.98E-01	ENSG00000116985
BMPER	0.0344	6.7677E-01	8.15E-01	ENSG00000164619
BMPR1A	-0.0105	8.3623E-01	9.14E-01	ENSG00000107779
BMPR1AP1	0.0046	6.9355E-01		ENSG00000219642
BMPR1B	-0.5433	2.3213E-05	3.46E-04	ENSG00000138696
BMPR2	-0.2022	1.3991E-02	5.50E-02	ENSG00000204217
BMS1	0.1792	1.2754E-02	5.12E-02	ENSG00000165733

BMS1P10	-0.2349	3.9830E-03	2.10E-02	ENSG00000237238
BMS1P11	-0.1577	1.1062E-01	2.52E-01	ENSG00000225883
BMS1P13	-0.1980	5.6887E-02	1.57E-01	ENSG00000231701
BMS1P15	-0.1388	1.6634E-01	3.33E-01	ENSG00000258780
BMS1P16	0.0564	5.0938E-01	6.89E-01	ENSG00000258684
BMS1P4	0.0039	6.4646E-01		ENSG00000271816
BMS1P9	-0.1522	1.2872E-01	2.80E-01	ENSG00000198312
BMT2	-0.0382	5.6617E-01	7.35E-01	ENSG00000164603
BNC1	-0.0157	5.8702E-01		ENSG00000169594
BNC2	-0.1232	1.1880E-01	2.65E-01	ENSG00000173068
BNC2-AS1	-0.1896	9.9552E-02	2.34E-01	ENSG00000234779
BNIP1	-0.2794	5.5987E-07	1.64E-05	ENSG00000113734
BNIP2	-0.0382	5.4379E-01	7.16E-01	ENSG00000140299
BNIP3	0.4104	2.0559E-05	3.12E-04	ENSG00000176171
BNIP3L	0.2357	6.2578E-04	4.92E-03	ENSG00000104765
BNIP3P16	0.0002	8.5557E-01		ENSG00000267641
BNIP3P18	0.0265	3.0621E-01		ENSG00000269055
BNIP3P22	0.0025	5.6023E-01		ENSG00000268174
BNIP3P25	-0.0574	1.4362E-01		ENSG00000268322
BNIP3P28	-0.0058	8.7081E-01	9.34E-01	ENSG00000271095
BNIP3P30	0.0345	4.9638E-01	6.79E-01	ENSG00000268079
BNIP3P40	-0.0106	9.2153E-01		ENSG00000268058
BNIP3P47	-0.0052	8.7232E-01		ENSG00000273791
BNIP3P9	0.0130	4.5486E-01		ENSG00000270325
BNIP5	0.0186	4.4697E-01		ENSG00000189325
BNIPL	-0.0445	6.3559E-01	7.88E-01	ENSG00000163141
BOC	-0.3593	2.0254E-03	1.24E-02	ENSG00000144857
BOD1	-0.1735	7.2941E-03	3.34E-02	ENSG00000145919
BOD1L1	0.1587	1.0474E-02	4.39E-02	ENSG00000038219
BOK	-0.2114	3.0424E-02	9.84E-02	ENSG00000176720
BOLA1	-0.2154	1.2771E-02	5.12E-02	ENSG00000178096
BOLA2	0.0800	2.6399E-01	4.53E-01	ENSG00000169627
BOLA2-SMG1P6	0.0774	3.7923E-01	5.74E-01	ENSG00000261740
BOLA2P3	-0.0496	4.8796E-01	6.72E-01	ENSG00000220771
BOLA3	-0.3030	1.8367E-04	1.84E-03	ENSG00000163170
BOLA3-DT	-0.0168	7.6354E-01	8.72E-01	ENSG00000225439
BOLL	0.0055	6.9977E-01		ENSG00000152430
BOP1	0.1239	5.9672E-02	1.63E-01	ENSG00000261236
BORA	0.5734	4.3614E-05	5.76E-04	ENSG00000136122
BORCS5	0.0062	9.2351E-01	9.62E-01	ENSG00000165714
BORCS6	0.2560	3.8186E-03	2.03E-02	ENSG00000196544
BORCS7	-0.2714	8.8991E-04	6.49E-03	ENSG00000166275
BORCS8	-0.0718	3.4362E-01	5.39E-01	ENSG00000254901
BORCS8-MEF2B	-0.0131	9.8119E-01		ENSG00000213999
BPGM	-0.0834	1.4917E-01	3.09E-01	ENSG00000172331
BPHL	-0.0844	1.9497E-01	3.71E-01	ENSG00000137274
BPIFA1	-0.0602	3.0697E-03	1.71E-02	ENSG00000198183
BPIFA2	0.0043	6.8323E-01		ENSG00000131050
BPIFB1	-0.0687	2.0470E-01		ENSG00000125999

BPIFB2	-0.0211	5.8624E-01		ENSG00000078898
BPIFB9P	-0.0257	9.2750E-01		ENSG00000125997
BPIFC	-0.0095	8.4565E-01		ENSG00000184459
BPNT1	0.0097	9.4592E-01	9.73E-01	ENSG00000162813
BPNT2	-0.0109	8.5086E-01	9.23E-01	ENSG00000104331
BPTF	-0.0916	1.8951E-01	3.64E-01	ENSG00000171634
BRAF	0.0325	6.4482E-01	7.94E-01	ENSG00000157764
BRAFP1	-0.0329	5.2488E-01		ENSG00000224775
BRAP	0.1169	8.4876E-02	2.09E-01	ENSG00000089234
BRAT1	0.0993	1.5770E-01	3.21E-01	ENSG00000106009
BRCA1	-0.5584	3.8958E-07	1.21E-05	ENSG00000012048
BRCA2	-0.0850	3.9099E-01	5.85E-01	ENSG00000139618
BRCC3	-0.0203	6.8720E-01	8.24E-01	ENSG00000185515
BRCC3P1	-0.0340	5.9858E-01		ENSG00000251667
BRD1	0.2081	3.2162E-03	1.77E-02	ENSG00000100425
BRD2	-0.1635	2.8027E-03	1.60E-02	ENSG00000204256
BRD3	-0.1338	1.3047E-02	5.21E-02	ENSG00000169925
BRD3OS	-0.0214	7.4192E-01	8.58E-01	ENSG00000235106
BRD4	0.0565	2.7492E-01	4.66E-01	ENSG00000141867
BRD7	-0.2233	1.1586E-04	1.26E-03	ENSG00000166164
BRD7P2	-0.1504	1.3566E-01	2.89E-01	ENSG00000184100
BRD8	0.1043	9.9792E-02	2.34E-01	ENSG00000112983
BRD9	0.0727	2.7902E-01	4.70E-01	ENSG00000028310
BRF1	-0.3232	1.4953E-04	1.55E-03	ENSG00000185024
BRF2	0.0498	5.8680E-01	7.51E-01	ENSG00000104221
BRI3	-0.0990	1.9711E-01	3.74E-01	ENSG00000164713
BRI3BP	-0.2975	1.3980E-03	9.29E-03	ENSG00000184992
BRICD5	-0.0419	6.4700E-01	7.96E-01	ENSG00000182685
BRINP1	0.3616	7.2435E-04	5.52E-03	ENSG00000078725
BRINP2	0.1447	1.8181E-01	3.54E-01	ENSG00000198797
BRINP3	-0.0505	5.8962E-01	7.53E-01	ENSG00000162670
BRIP1	-0.1435	1.7850E-01	3.50E-01	ENSG00000136492
BRIX1	-0.0146	7.9026E-01	8.87E-01	ENSG00000113460
BRK1	-0.0890	4.7309E-02	1.37E-01	ENSG00000254999
BRME1	0.4071	5.8858E-04	4.70E-03	ENSG00000132016
BRMS1	0.0683	2.1349E-01	3.94E-01	ENSG00000174744
BRMS1L	-0.0126	8.6404E-01	9.31E-01	ENSG00000100916
BROX	-0.1159	7.5588E-02	1.93E-01	ENSG00000162819
BRPF1	0.0044	9.6615E-01	9.83E-01	ENSG00000156983
BRPF3	-0.3422	8.7086E-05	1.00E-03	ENSG00000096070
BRS3	-0.0682	5.8508E-02		ENSG00000102239
BRSK1	-0.0943	3.4563E-01	5.41E-01	ENSG00000160469
BRSK2	0.1173	1.1517E-01	2.59E-01	ENSG00000174672
BRWD1	0.1429	3.9377E-02	1.20E-01	ENSG00000185658
BRWD1-AS1	-0.0331	9.3712E-01		ENSG00000238141
BRWD1-AS2	0.2970	5.1802E-03	2.57E-02	ENSG00000255568
BRWD3	0.0446	6.0626E-01	7.66E-01	ENSG00000165288
BSCL2	0.2915	4.0355E-04	3.47E-03	ENSG00000168000
BSDC1	0.1275	3.7663E-02	1.16E-01	ENSG00000160058

BSG	-0.0190	7.3954E-01	8.57E-01	ENSG00000172270
BSG-AS1	0.0579	5.5139E-01	7.23E-01	ENSG00000267751
BSN	0.2433	1.3198E-03	8.86E-03	ENSG00000164061
BSN-DT	-0.2922	3.5422E-02	1.11E-01	ENSG00000226913
BSND	0.0160	4.6823E-01	6.56E-01	ENSG00000162399
BSPRY	0.1263	2.0564E-01	3.84E-01	ENSG00000119411
BST2	0.1462	1.6390E-01	3.30E-01	ENSG00000130303
BSX	0.0058	6.4982E-01		ENSG00000188909
BTAF1	0.5593	2.0023E-09	1.50E-07	ENSG00000095564
BTBD1	0.1098	1.2083E-01	2.68E-01	ENSG00000064726
BTBD10	0.1886	9.7376E-03	4.16E-02	ENSG00000148925
BTBD10P1	-0.0007	8.3136E-01		ENSG00000257675
BTBD16	0.0079	9.1174E-01	9.55E-01	ENSG00000138152
BTBD17	-0.0321	7.3006E-01	8.51E-01	ENSG00000204347
BTBD18	0.0946	3.2872E-01	5.23E-01	ENSG00000233436
BTBD19	-0.0962	3.1537E-01	5.09E-01	ENSG00000222009
BTBD2	-0.0566	4.0842E-01	6.02E-01	ENSG00000133243
BTBD3	-0.2437	1.7577E-03	1.11E-02	ENSG00000132640
BTBD6	0.0177	8.0985E-01	8.99E-01	ENSG00000184887
BTBD7	0.0015	9.8570E-01	9.92E-01	ENSG00000011114
BTBD8	0.1949	4.2942E-02	1.28E-01	ENSG00000189195
BTBD9	-0.0810	2.4206E-01	4.27E-01	ENSG00000183826
BTBD9-AS1	-0.1156	6.6369E-02	1.75E-01	ENSG00000226533
BTD	-0.0687	3.0542E-01	4.98E-01	ENSG00000169814
BTF3	-0.1878	4.1520E-04	3.55E-03	ENSG00000145741
BTF3L4	0.0641	1.8195E-01	3.54E-01	ENSG00000134717
BTG1	-0.0810	1.2170E-01	2.70E-01	ENSG00000133639
BTG2	0.0788	3.2781E-01	5.22E-01	ENSG00000159388
BTG2-DT	0.0820	3.9949E-01	5.94E-01	ENSG00000233791
BTG3	-0.3153	1.1965E-04	1.29E-03	ENSG00000154640
BTG3-AS1	-0.4786	3.7626E-04	3.29E-03	ENSG00000280594
BTK	0.0314	6.4890E-01	7.97E-01	ENSG00000010671
BTLA	-0.0250	2.9366E-01		ENSG00000186265
BTN1A1	-0.0174	5.1507E-01		ENSG00000124557
BTN2A1	0.0912	1.9921E-01	3.76E-01	ENSG00000112763
BTN2A2	-0.1192	1.4294E-01	3.01E-01	ENSG00000124508
BTN3A1	-0.3331	3.9709E-03	2.10E-02	ENSG00000026950
BTN3A2	-0.4746	1.1954E-03	8.17E-03	ENSG00000186470
BTNL2	0.0305	3.5016E-01		ENSG00000204290
BTNL9	-0.0104	6.3436E-01	7.87E-01	ENSG00000165810
BTRC	0.1415	1.5021E-02	5.80E-02	ENSG00000166167
BUB1	-0.2310	4.6178E-02	1.35E-01	ENSG00000169679
BUB1B	-0.0471	6.2465E-01	7.80E-01	ENSG00000156970
BUB3	0.1507	9.5505E-03	4.11E-02	ENSG00000154473
BUD13	-0.0046	9.5349E-01	9.76E-01	ENSG00000137656
BUD23	0.0609	3.0873E-01	5.01E-01	ENSG00000071462
BUD31	-0.2406	3.4993E-07	1.11E-05	ENSG00000106245
BVES	-0.1908	9.8843E-02	2.33E-01	ENSG00000112276
BVES-AS1	-0.0914	3.4241E-01	5.38E-01	ENSG00000203808

BYSL	0.4958	3.0490E-07	9.95E-06	ENSG00000112578
BZW1	0.0540	4.2861E-01	6.20E-01	ENSG00000082153
BZW1-AS1	-0.0287	2.5221E-01		ENSG00000230408
BZW2	0.1709	7.3800E-03	3.37E-02	ENSG00000136261
C10orf126	-0.0458	6.1707E-01	7.74E-01	ENSG00000232624
C10orf143	-0.0205	8.2883E-01	9.10E-01	ENSG00000237489
C10orf53	-0.0521	8.7513E-01	9.37E-01	ENSG00000178645
C10orf55	0.0580	1.4255E-01		ENSG00000222047
C10orf67	-0.4894	4.8407E-03	2.44E-02	ENSG00000179133
C10orf67-AS1	-0.1249	1.6725E-01	3.34E-01	ENSG00000224215
C10orf71	-0.0257	5.1388E-01		ENSG00000177354
C10orf88	0.2844	2.3601E-03	1.40E-02	ENSG00000119965
C10orf88B	0.0057	5.0756E-01		ENSG00000255624
C10orf90	-0.0172	7.1674E-01		ENSG00000154493
C10orf95	0.0060	5.2434E-01		ENSG00000120055
C10orf95-AS1	0.1390	5.1160E-02	1.45E-01	ENSG00000269609
C11orf16	-0.0452	6.2369E-01	7.79E-01	ENSG00000176029
C11orf21	0.1305	3.4570E-02	1.09E-01	ENSG00000110665
C11orf52	0.0612	2.4601E-01	4.32E-01	ENSG00000149300
C11orf54	0.0944	2.3798E-01	4.22E-01	ENSG00000182919
C11orf58	-0.1478	2.3057E-03	1.37E-02	ENSG00000110696
C11orf65	0.0178	8.1313E-01	9.01E-01	ENSG00000166323
C11orf68	0.0533	4.5676E-01	6.46E-01	ENSG00000175573
C11orf71	0.2854	8.0462E-06	1.48E-04	ENSG00000180425
C11orf86	-0.0117	6.3492E-01		ENSG00000173237
C11orf87	-0.1490	1.4267E-01	3.00E-01	ENSG00000185742
C11orf91	-0.0092	9.6870E-01		ENSG00000205177
C11orf97	-0.0637	5.0445E-01	6.86E-01	ENSG00000257057
C11orf98	-0.1099	1.9226E-01	3.68E-01	ENSG00000278615
C11orf98P1	-0.0139	7.1356E-01		ENSG00000232158
C11orf98P3	0.0130	7.4454E-01		ENSG00000228171
C12orf4	0.0808	2.5252E-01	4.39E-01	ENSG00000047621
C12orf40	-0.0126	8.5600E-01		ENSG00000180116
C12orf42	0.0685	4.7083E-01	6.58E-01	ENSG00000179088
C12orf43	-0.0444	5.4191E-01	7.15E-01	ENSG00000157895
C12orf50	0.0071	7.1756E-01		ENSG00000165805
C12orf54	-0.0673	2.6922E-01		ENSG00000177627
C12orf56	-0.0908	3.4065E-01	5.36E-01	ENSG00000185306
C12orf57	-0.1474	6.9147E-02	1.81E-01	ENSG00000111678
C12orf60	0.0633	4.3376E-01	6.25E-01	ENSG00000182993
C12orf71	-0.0181	6.7300E-01		ENSG00000214700
C12orf75	-0.4943	1.2446E-07	4.62E-06	ENSG00000235162
C12orf76	0.1764	8.3218E-03	3.70E-02	ENSG00000174456
C13orf42	-0.0053	7.6870E-01		ENSG00000226792
C13orf46	-0.1020	2.9357E-01	4.86E-01	ENSG00000283199
C14orf119	-0.0116	8.3636E-01	9.14E-01	ENSG00000179933
C14orf132	-0.1910	2.1490E-03	1.30E-02	ENSG00000227051
C14orf178	0.0349	6.9735E-02		ENSG00000197734
C14orf180	0.0244	5.6668E-01		ENSG00000184601

C14orf28	0.1720	7.3166E-02	1.88E-01	ENSG00000179476
C14orf39	-0.3459	5.3913E-03	2.65E-02	ENSG00000179008
C14orf93	0.0225	7.8019E-01	8.81E-01	ENSG00000100802
C15orf32	-0.0037	5.9561E-01		ENSG00000183643
C15orf39	-0.2250	4.0915E-02	1.23E-01	ENSG00000167173
C15orf40	-0.0529	3.6361E-01	5.59E-01	ENSG00000169609
C15orf61	-0.1457	9.4882E-03	4.09E-02	ENSG00000189227
C15orf62	0.1175	2.5173E-01	4.39E-01	ENSG00000188277
C16orf46	-0.1227	2.3831E-01	4.23E-01	ENSG00000166455
C16orf54	0.0140	7.1083E-01		ENSG00000185905
C16orf74	-0.0697	4.1561E-01	6.08E-01	ENSG00000154102
C16orf86	0.1475	1.1159E-01	2.54E-01	ENSG00000159761
C16orf87	-0.2311	3.7418E-05	5.05E-04	ENSG00000155330
C16orf90	0.0042	7.6040E-01		ENSG00000215131
C16orf92	-0.0388	6.1811E-01	7.75E-01	ENSG00000167194
C16orf95	0.0044	9.6399E-01	9.82E-01	ENSG00000260456
C16orf95-DT	-0.0887	3.5105E-01	5.46E-01	ENSG00000270006
C16orf96	0.0112	5.2151E-01	6.99E-01	ENSG00000205832
C17orf100	0.4471	1.8914E-06	4.50E-05	ENSG00000256806
C17orf107	0.1067	1.1242E-01	2.55E-01	ENSG00000205710
C17orf113	0.0628	5.2117E-01	6.98E-01	ENSG00000267221
C17orf114	-0.4379	2.2098E-04	2.12E-03	ENSG00000262165
C17orf49	0.0310	7.3848E-01	8.56E-01	ENSG00000258315
C17orf50	-0.0032	9.7216E-01	9.86E-01	ENSG00000270806
C17orf58	-0.0928	1.5349E-01	3.15E-01	ENSG00000186665
C17orf67	0.0573	5.2051E-01	6.98E-01	ENSG00000214226
C17orf75	-0.1046	4.6525E-02	1.36E-01	ENSG00000108666
C17orf78	0.0040	6.5042E-01		ENSG00000278505
C17orf80	0.2712	3.4807E-03	1.89E-02	ENSG00000141219
C17orf99	-0.0011	9.1146E-01		ENSG00000187997
C18orf15	-0.0096	8.3293E-01	9.13E-01	ENSG00000279020
C18orf21	0.1052	1.0385E-01	2.41E-01	ENSG00000141428
C18orf32	-0.0804	2.8076E-01	4.72E-01	ENSG00000177576
C18orf54	0.0630	4.6860E-01	6.56E-01	ENSG00000166845
C19orf12	0.0238	7.0476E-01	8.35E-01	ENSG00000131943
C19orf18	0.2443	5.7062E-02	1.58E-01	ENSG00000177025
C19orf25	0.1331	2.9046E-02	9.52E-02	ENSG00000119559
C19orf38	-0.0411	6.6411E-01	8.07E-01	ENSG00000214212
C19orf44	-0.0846	3.9920E-01	5.93E-01	ENSG00000105072
C19orf47	0.2593	1.0027E-02	4.26E-02	ENSG00000160392
C19orf48P	0.0371	5.3593E-01	7.10E-01	ENSG00000167747
C19orf53	-0.4096	1.3379E-08	7.21E-07	ENSG00000104979
C19orf67	-0.0494	4.1330E-01	6.06E-01	ENSG00000188032
C19orf73	0.0145	8.8641E-01	9.43E-01	ENSG00000221916
C19orf81	-0.1494	9.6827E-02	2.30E-01	ENSG00000235034
C19orf84	0.0428	7.1222E-02		ENSG00000262874
C19orf85	-0.0263	9.1325E-01	9.56E-01	ENSG00000283567
C1D	-0.2661	1.9107E-05	2.95E-04	ENSG00000197223
C1GALT1	0.0083	8.9931E-01	9.50E-01	ENSG00000106392

C1GALT1C1	-0.2606	8.2168E-04	6.10E-03	ENSG00000171155
C1GALT1C1L	0.1244	2.3602E-01	4.20E-01	ENSG00000223658
C1orf105	-0.0200	8.9909E-01		ENSG00000180999
C1orf115	0.0087	9.1506E-01	9.57E-01	ENSG00000162817
C1orf116	-0.0006	9.9852E-01	9.99E-01	ENSG00000182795
C1orf122	-0.2407	8.8814E-05	1.02E-03	ENSG00000197982
C1orf127	-0.2078	8.7169E-02	2.14E-01	ENSG00000175262
C1orf131	0.0590	3.8145E-01	5.76E-01	ENSG00000143633
C1orf141	-0.0800	2.5936E-01	4.48E-01	ENSG00000203963
C1orf146	-0.0403	5.3481E-01		ENSG00000203910
C1orf159	0.1513	3.4746E-02	1.09E-01	ENSG00000131591
C1orf162	0.0619	4.4277E-01	6.34E-01	ENSG00000143110
C1orf167	-0.0480	7.0054E-01		ENSG00000215910
C1orf174	-0.0649	2.4036E-01	4.25E-01	ENSG00000198912
C1orf198	0.1218	7.7478E-02	1.96E-01	ENSG00000119280
C1orf202	-0.0332	5.5145E-01	7.23E-01	ENSG00000284188
C1orf21	-0.1982	1.9246E-03	1.19E-02	ENSG00000116667
C1orf21-DT	0.2027	8.1123E-02	2.03E-01	ENSG00000271387
C1orf210	0.2339	3.7900E-02	1.16E-01	ENSG00000253313
C1orf216	0.1318	1.8112E-01	3.53E-01	ENSG00000142686
C1orf220	0.2630	4.9253E-02	1.42E-01	ENSG00000213057
C1orf232	0.0659	5.0749E-01	6.88E-01	ENSG00000282872
C1orf35	0.0467	4.8038E-01	6.66E-01	ENSG00000143793
C1orf43	0.1131	2.0249E-02	7.27E-02	ENSG00000143612
C1orf50	-0.2664	1.2636E-03	8.56E-03	ENSG00000164008
C1orf52	0.0434	4.7957E-01	6.65E-01	ENSG00000162642
C1orf53	-0.5854	3.1348E-06	6.85E-05	ENSG00000203724
C1orf54	-0.3272	5.6380E-04	4.53E-03	ENSG00000118292
C1orf56	-0.3558	2.0015E-04	1.96E-03	ENSG00000143443
C1orf74	-0.0395	5.6469E-01	7.33E-01	ENSG00000162757
C1orf87	-0.1043	1.5985E-01	3.25E-01	ENSG00000162598
C1orf94	-0.0226	7.6082E-01	8.70E-01	ENSG00000142698
C1QA	0.3015	2.3694E-02	8.17E-02	ENSG00000173372
C1QBP	-0.0572	2.4299E-01	4.28E-01	ENSG00000108561
C1QL1	0.2450	9.5977E-04	6.90E-03	ENSG00000131094
C1QL2	-0.1099	9.1451E-02	2.21E-01	ENSG00000144119
C1QL3	0.1892	1.0193E-01	2.38E-01	ENSG00000165985
C1QTNF1	-0.1058	2.9957E-01	4.92E-01	ENSG00000173918
C1QTNF12	-0.0420	6.4027E-01	7.91E-01	ENSG00000184163
C1QTNF2	-0.2079	8.3611E-02	2.07E-01	ENSG00000145861
C1QTNF3	-0.1108	1.8903E-01	3.64E-01	ENSG00000082196
C1QTNF3-AMACR	0.0036	8.4297E-01		ENSG00000273294
C1QTNF4	-0.3689	2.0690E-03	1.26E-02	ENSG00000172247
C1QTNF6	-0.0758	3.0892E-01	5.02E-01	ENSG00000133466
C1QTNF7	-0.0325	6.7334E-01	8.13E-01	ENSG00000163145
C1QTNF7-AS1	-0.0579	5.5025E-01	7.22E-01	ENSG00000249252
C1QTNF8	-0.0314	9.7947E-01		ENSG00000184471
C1QTNF9	-0.0197	6.8521E-01		ENSG00000240654
C1QTNF9B	-0.0167	6.7744E-01		ENSG00000205863

C1R	0.0065	9.5238E-01	9.76E-01	ENSG00000159403
C1RL	-0.1597	7.1192E-02	1.84E-01	ENSG00000139178
C1RL-AS1	-0.0116	9.0292E-01	9.52E-01	ENSG00000205885
C1S	0.3338	1.4336E-02	5.61E-02	ENSG00000182326
C2	0.2473	5.6230E-02	1.56E-01	ENSG00000166278
C2-AS1	0.0037	5.4687E-01		ENSG00000281756
C2-AS1	0.0106	8.9233E-01	9.46E-01	ENSG00000229776
C20orf141	0.0242	1.2460E-01		ENSG00000258713
C20orf144	0.5530	3.7821E-04	3.30E-03	ENSG00000149609
C20orf202	-0.0089	9.3731E-01	9.69E-01	ENSG00000215595
C20orf203	-0.0051	9.3567E-01	9.68E-01	ENSG00000198547
C20orf204	0.2030	8.8012E-02	2.15E-01	ENSG00000196421
C20orf96	0.1289	9.2600E-02	2.23E-01	ENSG00000196476
C21orf140	-0.0391	8.2774E-01		ENSG00000222018
C21orf58	-0.1394	1.5139E-01	3.12E-01	ENSG00000160298
C21orf91	0.1869	2.4512E-02	8.40E-02	ENSG00000154642
C21orf91-OT1	-0.0277	3.2626E-01		ENSG00000240770
C22orf15	-0.2336	5.3858E-02	1.51E-01	ENSG00000169314
C22orf23	0.3336	1.7955E-03	1.12E-02	ENSG00000128346
C22orf39	0.1469	1.0274E-02	4.33E-02	ENSG00000242259
C22orf42	0.0835	2.4089E-01	4.26E-01	ENSG00000205856
C22orf46P	-0.0876	2.3167E-01	4.15E-01	ENSG00000184208
C2CD2	-0.4713	8.5601E-05	9.88E-04	ENSG00000157617
C2CD2L	0.3163	1.7025E-04	1.73E-03	ENSG00000172375
C2CD3	0.0394	5.3213E-01	7.06E-01	ENSG00000168014
C2CD4A	0.4405	8.1668E-03	3.66E-02	ENSG00000198535
C2CD4C	-0.0547	3.9116E-01	5.85E-01	ENSG00000183186
C2CD4D	0.5409	6.2599E-03	2.98E-02	ENSG00000225556
C2CD5	0.2938	9.2149E-04	6.68E-03	ENSG00000111731
C2CD6	0.1651	9.0739E-02	2.20E-01	ENSG00000155754
C2orf15	0.3214	9.6817E-04	6.94E-03	ENSG00000273045
C2orf27A	-0.0244	7.8820E-01	8.86E-01	ENSG00000287151
C2orf42	0.1805	3.0473E-02	9.85E-02	ENSG00000115998
C2orf49	0.3000	1.7564E-05	2.76E-04	ENSG00000135974
C2orf50	-0.4254	1.6166E-06	3.97E-05	ENSG00000150873
C2orf66	0.0229	7.9367E-01	8.89E-01	ENSG00000187944
C2orf68	0.0463	5.6520E-01	7.34E-01	ENSG00000168887
C2orf69	0.1931	1.9658E-03	1.21E-02	ENSG00000178074
C2orf72	-0.4691	2.1714E-03	1.31E-02	ENSG00000204128
C2orf74-AS1	0.0111	4.7229E-01		ENSG00000271889
C2orf74-DT	-0.2606	4.2560E-03	2.22E-02	ENSG00000212978
C2orf76	-0.3502	2.0031E-05	3.06E-04	ENSG00000186132
C2orf78	-0.0064	4.9837E-01		ENSG00000187833
C2orf80	0.2136	5.3880E-02	1.51E-01	ENSG00000188674
C2orf81	-0.0822	3.1390E-01	5.07E-01	ENSG00000284308
C2orf88	-0.3080	2.4657E-02	8.44E-02	ENSG00000187699
C2orf92	-0.0479	6.0737E-01	7.67E-01	ENSG00000228486
C3	0.0020	4.4717E-01	6.37E-01	ENSG00000125730
C3AR1	-0.0070	9.4819E-01	9.74E-01	ENSG00000171860

C3orf18	0.1050	1.2707E-01	2.78E-01	ENSG00000088543
C3orf20	0.0050	9.1139E-01	9.55E-01	ENSG00000131379
C3orf22	-0.0085	5.7476E-01		ENSG00000180697
C3orf33	-0.2170	5.6902E-02	1.57E-01	ENSG00000174928
C3orf38	-0.1327	6.6401E-02	1.75E-01	ENSG00000179021
C3orf49	-0.0380	5.1598E-01	6.95E-01	ENSG00000163632
C3orf52	-0.0208	8.1850E-01	9.04E-01	ENSG00000114529
C3orf62	-0.0179	8.4411E-01	9.19E-01	ENSG00000188315
C3orf70	0.2895	5.5744E-03	2.73E-02	ENSG00000187068
C3orf80	0.2008	8.9474E-02	2.18E-01	ENSG00000180044
C3orf84	-0.0030	9.5080E-01	9.75E-01	ENSG00000236980
C3orf86P	0.0066	4.1845E-01		ENSG00000225873
C4A	-0.4678	1.4229E-03	9.42E-03	ENSG00000244731
C4B	-0.4875	1.0761E-03	7.52E-03	ENSG00000224389
C4BPA	0.0080	7.3604E-01		ENSG00000123838
C4BPB	-0.0002	8.6393E-01		ENSG00000123843
C4orf19	-0.0468	6.1668E-01	7.74E-01	ENSG00000154274
C4orf3	-0.0109	8.6540E-01	9.31E-01	ENSG00000164096
C4orf33	0.0714	1.9663E-01	3.73E-01	ENSG00000151470
C4orf36	-0.0642	5.7392E-01		ENSG00000163633
C4orf36	-0.2373	7.9969E-02	2.00E-01	ENSG00000285458
C4orf46	0.1044	2.1304E-01	3.93E-01	ENSG00000205208
C4orf50	-0.1235	2.2315E-01	4.05E-01	ENSG00000181215
C4orf51	0.0519	3.5109E-01		ENSG00000237136
C5AR1	-0.0690	4.5054E-01	6.40E-01	ENSG00000197405
C5AR2	0.0092	6.1625E-01		ENSG00000134830
C5orf15	-0.0223	7.4916E-01	8.63E-01	ENSG00000113583
C5orf22	0.2827	2.6763E-05	3.86E-04	ENSG00000082213
C5orf24	0.0171	7.7726E-01	8.80E-01	ENSG00000181904
C5orf34	0.1051	3.0582E-01	4.98E-01	ENSG00000172244
C5orf46	-0.0217	8.2371E-01		ENSG00000178776
C5orf47	-0.0231	7.9667E-01	8.91E-01	ENSG00000185056
C5orf52	0.0096	6.2228E-01		ENSG00000187658
C5orf58	0.0227	2.1668E-01		ENSG00000234511
C5orf60	-0.0031	9.6561E-01	9.83E-01	ENSG00000204661
C5orf63	-0.0110	9.0865E-01	9.54E-01	ENSG00000164241
C5orf64	-0.0376	7.3093E-01	8.52E-01	ENSG00000178722
C6	-0.0577	4.1159E-01	6.05E-01	ENSG00000039537
C6orf118	-0.3801	3.8390E-04	3.34E-03	ENSG00000112539
C6orf120	0.4123	6.0451E-08	2.60E-06	ENSG00000185127
C6orf132	-0.1341	1.8832E-01	3.63E-01	ENSG00000188112
C6orf136	0.0029	9.7925E-01	9.89E-01	ENSG00000204564
C6orf141	-0.0799	3.9662E-01	5.91E-01	ENSG00000197261
C6orf15	0.0143	5.5642E-01	7.27E-01	ENSG00000204542
C6orf163	0.2522	4.8115E-02	1.39E-01	ENSG00000203872
C6orf226	0.0089	8.7844E-01	9.38E-01	ENSG00000221821
C6orf47	0.1589	5.2043E-02	1.47E-01	ENSG00000204439
C6orf52	0.0075	9.1044E-01	9.55E-01	ENSG00000137434
C6orf58	0.1065	3.0056E-01	4.93E-01	ENSG00000184530

C6orf62	-0.2129	2.4569E-02	8.41E-02	ENSG00000112308
C6orf89	-0.0940	6.2660E-02	1.69E-01	ENSG00000198663
C7orf25	-0.0059	9.3128E-01	9.66E-01	ENSG00000136197
C7orf33	-0.0260	9.2620E-01		ENSG00000170279
C7orf50	-0.2247	1.5799E-03	1.02E-02	ENSG00000146540
C7orf57	-0.0971	3.2639E-01	5.21E-01	ENSG00000164746
C8B	-0.0235	6.9826E-01		ENSG00000021852
C8G	-0.0452	6.0900E-01	7.68E-01	ENSG00000176919
C8orf17	-0.0118	5.9870E-01		ENSG00000250733
C8orf33	0.1906	1.8994E-03	1.18E-02	ENSG00000182307
C8orf34-AS1	-0.1128	1.1920E-01	2.66E-01	ENSG00000248801
C8orf44	0.0700	3.3241E-01	5.27E-01	ENSG00000288596
C8orf44-SGK3	0.0046	7.2126E-01		ENSG00000288602
C8orf48	0.1228	2.3475E-01	4.19E-01	ENSG00000164743
C8orf58	0.0640	4.2359E-01	6.16E-01	ENSG00000241852
C8orf76	-0.4288	1.4600E-05	2.39E-04	ENSG00000189376
C8orf82	-0.0759	2.5979E-01	4.48E-01	ENSG00000213563
C8orf89	0.1381	3.4021E-02	1.07E-01	ENSG00000274443
C8orf90	-0.0001	7.6684E-01		ENSG00000226490
C9orf152	0.0349	3.8771E-01		ENSG00000188959
C9orf153	0.0313	6.0384E-01	7.64E-01	ENSG00000187753
C9orf163	0.0093	9.0427E-01	9.52E-01	ENSG00000196366
C9orf40	-0.0116	8.8572E-01	9.42E-01	ENSG00000135045
C9orf43	0.2856	1.6459E-02	6.22E-02	ENSG00000157653
C9orf50	0.1347	1.5421E-01	3.16E-01	ENSG00000179058
C9orf72	-0.0101	8.6233E-01	9.30E-01	ENSG00000147894
C9orf78	-0.0255	4.1044E-01	6.04E-01	ENSG00000136819
C9orf78P2	-0.0025	9.7910E-01		ENSG00000224569
C9orf85	0.0244	6.5925E-01	8.03E-01	ENSG00000155621
CA1	0.0112	6.8442E-01		ENSG00000133742
CA10	0.0014	9.9713E-01	9.99E-01	ENSG00000154975
CA11	0.0415	5.1421E-01	6.93E-01	ENSG00000063180
CA12	0.0153	8.5988E-01	9.29E-01	ENSG00000074410
CA13	-0.2472	7.7544E-03	3.51E-02	ENSG00000185015
CA15P1	-0.0479	5.3312E-01		ENSG00000241527
CA2	-0.5708	2.5827E-03	1.50E-02	ENSG00000104267
CA3	-0.3173	3.0175E-02	9.79E-02	ENSG00000164879
CA4	-0.1319	1.0581E-01	2.44E-01	ENSG00000167434
CA5A	-0.0629	4.9176E-01		ENSG00000174990
CA5B	0.0452	5.5738E-01	7.28E-01	ENSG00000169239
CA7	-0.1151	2.4009E-01	4.25E-01	ENSG00000168748
CA9	0.0062	9.4275E-01	9.72E-01	ENSG00000107159
CAAP1	-0.0864	1.0856E-01	2.49E-01	ENSG00000120159
CAB39	0.1467	3.7882E-02	1.16E-01	ENSG00000135932
CAB39L	0.0588	3.7795E-01	5.73E-01	ENSG00000102547
CABCOCO1	0.2924	1.3670E-02	5.40E-02	ENSG00000183346
CABIN1	0.1114	7.2997E-02	1.88E-01	ENSG00000099991
CABLES1	-0.0490	5.9530E-01	7.57E-01	ENSG00000134508
CABLES2	0.1820	1.6395E-02	6.21E-02	ENSG00000149679

CABP1	0.1040	3.0733E-01	5.00E-01	ENSG00000157782
CABP2	0.0490	4.0352E-01	5.98E-01	ENSG00000167791
CABP4	-0.0658	4.2562E-01	6.17E-01	ENSG00000175544
CABP7	-0.5252	1.7110E-03	1.09E-02	ENSG00000100314
CABS1	-0.0796	3.9968E-01	5.94E-01	ENSG00000145309
CABYR	0.1754	7.4067E-02	1.90E-01	ENSG00000154040
CACFD1	-0.0039	9.3034E-01	9.66E-01	ENSG00000160325
CACHD1	-0.1138	5.6665E-02	1.57E-01	ENSG00000158966
CACNA1A	-0.0137	8.4418E-01	9.19E-01	ENSG00000141837
CACNA1B	0.2758	1.4275E-02	5.59E-02	ENSG00000148408
CACNA1C	0.2145	1.4683E-02	5.71E-02	ENSG00000151067
CACNA1C-AS1	-0.0174	6.6633E-01		ENSG00000246627
CACNA1C-AS2	-0.0192	8.3538E-01	9.14E-01	ENSG00000256271
CACNA1C-AS4	-0.0317	8.7312E-01		ENSG00000256025
CACNA1D	0.3711	3.8591E-04	3.35E-03	ENSG00000157388
CACNA1E	0.1809	6.7466E-02	1.77E-01	ENSG00000198216
CACNA1F	0.5971	3.2384E-03	1.78E-02	ENSG00000102001
CACNA1G	-0.0044	9.5849E-01	9.79E-01	ENSG00000006283
CACNA1G-AS1	0.0469	5.2784E-01	7.03E-01	ENSG00000250107
CACNA1H	-0.0195	7.9959E-01	8.93E-01	ENSG00000196557
CACNA1I	0.1546	1.5371E-01	3.16E-01	ENSG00000100346
CACNA1S	-0.0396	9.6357E-01	9.81E-01	ENSG00000081248
CACNA2D1	-0.0691	2.5905E-01	4.47E-01	ENSG00000153956
CACNA2D1-AS1	-0.1458	9.2519E-02	2.23E-01	ENSG00000223770
CACNA2D2	-0.1617	3.1506E-02	1.01E-01	ENSG00000007402
CACNA2D3	0.5634	3.1912E-03	1.76E-02	ENSG00000157445
CACNA2D4	-0.1141	2.6044E-01	4.49E-01	ENSG00000151062
CACNB1	0.3998	4.9827E-04	4.09E-03	ENSG00000067191
CACNB2	0.1175	1.6090E-01	3.26E-01	ENSG00000165995
CACNB3	0.0306	6.6377E-01	8.07E-01	ENSG00000167535
CACNB4	0.2220	7.0551E-03	3.26E-02	ENSG00000182389
CACNG1	0.0135	8.8426E-01	9.42E-01	ENSG00000108878
CACNG2	0.2114	6.9525E-02	1.81E-01	ENSG00000166862
CACNG3	-0.1347	1.8753E-01	3.62E-01	ENSG00000006116
CACNG4	-0.0318	7.0295E-01	8.34E-01	ENSG00000075461
CACNG5	0.0057	9.5244E-01	9.76E-01	ENSG00000075429
CACNG6	0.1089	1.8615E-01	3.60E-01	ENSG00000130433
CACNG7	-0.1021	2.9415E-01	4.86E-01	ENSG00000105605
CACNG8	-0.0450	6.3415E-01	7.87E-01	ENSG00000142408
CACTIN	0.2132	2.7237E-02	9.06E-02	ENSG00000105298
CACUL1	-0.0294	5.0552E-01	6.87E-01	ENSG00000151893
CACYBP	-0.1735	5.3762E-05	6.80E-04	ENSG00000116161
CAD	-0.1114	1.3522E-01	2.89E-01	ENSG00000084774
CADM1	-0.1986	2.3562E-03	1.39E-02	ENSG00000182985
CADM1-AS1	-0.1715	1.2570E-01	2.75E-01	ENSG00000256315
CADM2	0.0733	2.4338E-01	4.29E-01	ENSG00000175161
CADM2-AS2	-0.0112	9.7319E-01		ENSG00000241648
CADM3	-0.4615	4.4837E-05	5.89E-04	ENSG00000162706
CADM4	-0.1551	5.8554E-02	1.61E-01	ENSG00000105767

CADPS	-0.0391	5.0291E-01	6.84E-01	ENSG00000163618
CADPS2	-0.1983	2.3590E-02	8.15E-02	ENSG00000081803
CAGE1	0.0439	6.0612E-01	7.66E-01	ENSG00000164304
CAHM	0.1295	7.7883E-02	1.97E-01	ENSG00000270419
CALB1	0.3159	7.1082E-03	3.28E-02	ENSG00000104327
CALB2	-0.1837	8.2848E-02	2.06E-01	ENSG00000172137
CALCA	0.2713	4.2116E-02	1.26E-01	ENSG00000110680
CALCB	0.1079	2.7686E-01	4.67E-01	ENSG00000175868
CALCOCO1	0.1253	7.5764E-02	1.93E-01	ENSG00000012822
CALCOCO2	-0.0265	6.9758E-01	8.31E-01	ENSG00000136436
CALCR	-0.0037	8.7821E-01		ENSG00000004948
CALD1	-0.3327	1.2588E-04	1.34E-03	ENSG00000122786
CALHM1	0.1157	2.1679E-01	3.97E-01	ENSG00000185933
CALHM2	0.0432	6.5029E-01	7.97E-01	ENSG00000138172
CALHM3	-0.0002	8.0813E-01		ENSG00000183128
CALHM4	-0.0230	3.3313E-01		ENSG00000164451
CALHM5	-0.0247	7.2071E-01		ENSG00000178033
CALHM6	-0.0495	7.6928E-01	8.75E-01	ENSG00000188820
CALM1	-0.1986	1.3698E-04	1.44E-03	ENSG00000198668
CALM1P1	-0.0343	6.1926E-01	7.75E-01	ENSG00000223467
CALM2	-0.0224	7.0903E-01	8.37E-01	ENSG00000143933
CALM3	-0.0015	9.2858E-01	9.65E-01	ENSG00000160014
CALML3	0.0154	3.7271E-01		ENSG00000178363
CALML6	0.0023	9.2553E-01		ENSG00000169885
CALN1	-0.0630	5.1354E-01	6.93E-01	ENSG00000183166
CALR	-0.1468	9.8195E-02	2.32E-01	ENSG00000179218
CALR3	0.2183	2.1062E-02	7.49E-02	ENSG00000269058
CALU	-0.0662	3.4961E-01	5.45E-01	ENSG00000128595
CALY	0.2494	1.7731E-03	1.11E-02	ENSG00000130643
CAMK1	-0.3395	1.3571E-05	2.24E-04	ENSG00000134072
CAMK1D	-0.1819	5.9702E-02	1.63E-01	ENSG00000183049
CAMK1G	0.5095	9.6587E-05	1.09E-03	ENSG00000008118
CAMK2A	0.3407	7.4766E-04	5.67E-03	ENSG00000070808
CAMK2B	-0.1653	6.2462E-03	2.97E-02	ENSG00000058404
CAMK2D	-0.1225	6.2851E-02	1.69E-01	ENSG00000145349
CAMK2G	-0.0026	9.7741E-01	9.88E-01	ENSG00000148660
CAMK2N1	-0.1071	1.4684E-01	3.06E-01	ENSG00000162545
CAMK2N2	0.1464	1.0509E-01	2.43E-01	ENSG00000163888
CAMK4	-0.0214	8.1518E-01	9.02E-01	ENSG00000152495
CAMKK1	0.1429	9.1224E-02	2.20E-01	ENSG00000004660
CAMKK2	0.0782	2.2013E-01	4.01E-01	ENSG00000110931
CAMKMT	0.1610	1.0226E-02	4.32E-02	ENSG00000143919
CAMKV	0.3762	3.2913E-04	2.95E-03	ENSG00000164076
CAMLG	-0.0877	1.1118E-01	2.53E-01	ENSG00000164615
CAMP	-0.0175	6.8375E-01		ENSG00000164047
CAMSAP1	-0.0865	2.9132E-01	4.83E-01	ENSG00000130559
CAMSAP2	0.0647	4.1967E-01	6.12E-01	ENSG00000118200
CAMSAP3	-0.0006	9.8911E-01	9.94E-01	ENSG00000076826
CAMTA1	-0.1622	3.7566E-03	2.01E-02	ENSG00000171735

CAMTA1-AS2	0.0358	1.6507E-01		ENSG00000237728
CAMTA2	-0.0996	1.7637E-01	3.47E-01	ENSG00000108509
CAND1	-0.0954	5.7836E-02	1.59E-01	ENSG00000111530
CAND2	-0.2237	1.0535E-02	4.41E-02	ENSG00000144712
CANT1	-0.2096	8.7566E-03	3.85E-02	ENSG00000171302
CANX	-0.2545	5.9705E-05	7.41E-04	ENSG00000127022
CAP1	0.0128	8.6019E-01	9.29E-01	ENSG00000131236
CAP1P1	-0.0034	9.6052E-01		ENSG00000240809
CAP1P2	0.0070	5.9144E-01		ENSG00000232004
CAP2	-0.1717	6.0461E-03	2.90E-02	ENSG00000112186
CAP2P1	-0.0331	4.9508E-01		ENSG00000259151
CAPG	0.0917	3.5477E-01	5.50E-01	ENSG00000042493
CAPN1	0.0851	1.5862E-01	3.23E-01	ENSG00000014216
CAPN1-AS1	-0.1092	2.4956E-01	4.36E-01	ENSG00000254614
CAPN10	0.0295	6.7394E-01	8.13E-01	ENSG00000142330
CAPN11	0.0006	2.0020E-01		ENSG00000137225
CAPN12	0.2932	3.5800E-02	1.11E-01	ENSG00000182472
CAPN13	0.4895	2.1182E-03	1.28E-02	ENSG00000162949
CAPN14	0.1275	2.1836E-01	3.99E-01	ENSG00000214711
CAPN15	0.0204	8.4108E-01	9.17E-01	ENSG00000103326
CAPN2	-0.1621	2.3482E-03	1.39E-02	ENSG00000162909
CAPN5	0.0717	2.8173E-01	4.73E-01	ENSG00000149260
CAPN6	0.1033	2.8440E-01	4.76E-01	ENSG00000077274
CAPN7	0.0224	7.3467E-01	8.54E-01	ENSG00000131375
CAPN8	-0.1412	8.2550E-02	2.05E-01	ENSG00000203697
CAPN9	0.0403	6.7732E-01	8.16E-01	ENSG00000135773
CAPNS1	-0.2504	3.2243E-03	1.78E-02	ENSG00000126247
CAPNS2	0.0053	7.3575E-01	8.55E-01	ENSG00000256812
CAPRIN1	-0.0977	8.8347E-03	3.88E-02	ENSG00000135387
CAPRIN2	-0.0117	8.5140E-01	9.24E-01	ENSG00000110888
CAPS	-0.0560	5.2188E-01	6.99E-01	ENSG00000105519
CAPS2	-0.1027	1.5997E-01	3.25E-01	ENSG00000180881
CAPSL	-0.1150	2.2910E-01	4.12E-01	ENSG00000152611
CAPZA1	-0.1014	9.4551E-02	2.26E-01	ENSG00000116489
CAPZA2	-0.0816	2.0225E-01	3.80E-01	ENSG00000198898
CAPZA3	0.0043	8.3272E-01		ENSG00000177938
CAPZB	-0.1638	5.0690E-05	6.51E-04	ENSG00000077549
CARD10	0.1795	4.9877E-02	1.43E-01	ENSG00000100065
CARD11	0.0495	1.6290E-01	3.29E-01	ENSG00000198286
CARD14	-0.0309	7.4536E-01	8.61E-01	ENSG00000141527
CARD16	0.0114	4.1206E-01	6.05E-01	ENSG00000204397
CARD18	0.0044	9.2128E-01		ENSG00000255501
CARD19	0.0093	8.5735E-01	9.27E-01	ENSG00000165233
CARD6	0.0051	6.1707E-01		ENSG00000132357
CARD8	-0.3111	1.7621E-03	1.11E-02	ENSG00000105483
CARD8-AS1	0.0002	9.9965E-01	1.00E+00	ENSG00000268001
CARD9	-0.0207	8.7051E-01		ENSG00000187796
CARF	0.2873	7.6539E-04	5.77E-03	ENSG00000138380
CARHSP1	0.0982	2.5790E-01	4.46E-01	ENSG00000153048

CARM1	0.0865	3.2842E-01	5.23E-01	ENSG00000142453
CARMIL1	0.1414	1.0129E-01	2.37E-01	ENSG00000079691
CARMIL2	-0.1109	2.1410E-01	3.94E-01	ENSG00000159753
CARMIL3	-0.0331	7.0609E-01	8.36E-01	ENSG00000186648
CARNMT1	-0.2346	9.6193E-03	4.13E-02	ENSG00000156017
CARNS1	-0.0814	4.0158E-01	5.96E-01	ENSG00000172508
CARS1	0.1350	6.1565E-02	1.66E-01	ENSG00000110619
CARS1-AS1	-0.0866	1.1827E-01		ENSG00000247473
CARS1P2	-0.0104	7.9620E-01		ENSG00000253756
CARS2	-0.3426	2.2156E-04	2.12E-03	ENSG00000134905
CARTPT	0.1484	1.3295E-01	2.86E-01	ENSG00000164326
CASC11	-0.0074	9.6495E-01		ENSG00000249375
CASC16	-0.0186	7.7704E-01	8.79E-01	ENSG00000249231
CASC18	-0.0228	5.4511E-01		ENSG00000257859
CASC2	-0.0760	2.8868E-01	4.80E-01	ENSG00000177640
CASC3	-0.0538	3.4157E-01	5.37E-01	ENSG00000108349
CASC8	0.0214	3.0508E-01		ENSG00000246228
CASC9	-0.1807	1.1108E-02	4.60E-02	ENSG00000249395
CASD1	0.0154	7.7545E-01	8.79E-01	ENSG00000127995
CASK	-0.0145	8.2510E-01	9.08E-01	ENSG00000147044
CASKIN1	0.1223	2.1838E-01	3.99E-01	ENSG00000167971
CASKIN2	-0.2897	1.1916E-03	8.15E-03	ENSG00000177303
CASP1	-0.0059	8.6280E-01		ENSG00000137752
CASP10	-0.0107	8.0203E-01		ENSG00000003400
CASP16P	0.0209	3.5510E-01		ENSG00000228146
CASP2	0.0126	8.6053E-01	9.29E-01	ENSG00000106144
CASP4	-0.0142	9.6645E-01	9.83E-01	ENSG00000196954
CASP5	0.0019	9.8113E-01		ENSG00000137757
CASP6	-0.1395	5.1387E-02	1.46E-01	ENSG00000138794
CASP7	-0.0148	8.3470E-01	9.14E-01	ENSG00000165806
CASP8	-0.0331	7.2644E-01	8.49E-01	ENSG00000064012
CASP8AP2	0.1078	1.8960E-01	3.64E-01	ENSG00000118412
CASP9	-0.0256	7.3830E-01	8.56E-01	ENSG00000132906
CASQ1	-0.0525	5.9305E-01	7.55E-01	ENSG00000143318
CASQ2	0.1432	7.0249E-02		ENSG00000118729
CAST	0.0210	7.1615E-01	8.42E-01	ENSG00000153113
CASTOR1	0.1257	2.3064E-01	4.14E-01	ENSG00000239282
CASTOR2	-0.1052	1.2267E-01	2.71E-01	ENSG00000274070
CASZ1	-0.0657	4.8490E-01	6.69E-01	ENSG00000130940
CAT	0.0134	8.9353E-01	9.47E-01	ENSG00000121691
CATSPER1	-0.0105	9.1155E-01		ENSG00000175294
CATSPER2	0.2147	3.1848E-02	1.02E-01	ENSG00000166762
CATSPER3	-0.0550	4.2507E-01	6.17E-01	ENSG00000152705
CATSPERB	-0.0796	5.7195E-01	7.39E-01	ENSG00000133962
CATSPERD	-0.2051	6.9607E-02	1.82E-01	ENSG00000174898
CATSPERE	0.1700	9.6463E-02	2.29E-01	ENSG00000179397
CATSPERG	0.1199	2.4325E-01	4.28E-01	ENSG00000099338
CATSPERZ	0.0159	5.9094E-01		ENSG00000219435
CAV1	-0.4382	5.9906E-03	2.88E-02	ENSG00000105974

CAV2	0.0253	7.7304E-01	8.77E-01	ENSG00000105971
CAVIN1	-0.5529	2.3027E-05	3.43E-04	ENSG00000177469
CAVIN2	-0.0806	3.9052E-01	5.85E-01	ENSG00000168497
CAVIN4	0.1020	3.2878E-01	5.23E-01	ENSG00000170681
CBARP	0.0217	8.2221E-01	9.06E-01	ENSG00000099625
CBFA2T2	0.1127	1.0544E-01	2.44E-01	ENSG00000078699
CBFA2T3	-0.0889	2.9960E-01	4.92E-01	ENSG00000129993
CBFB	0.0194	7.9978E-01	8.93E-01	ENSG00000067955
CBL	-0.3422	5.8857E-05	7.34E-04	ENSG00000110395
CBLB	0.1349	4.5164E-02	1.33E-01	ENSG00000114423
CBLC	0.5118	1.6899E-03	1.08E-02	ENSG00000142273
CBLL1	-0.0865	1.7823E-01	3.49E-01	ENSG00000105879
CBLL1-AS1	-0.0803	4.0217E-01	5.96E-01	ENSG00000241764
CBLN1	-0.2174	2.9507E-02	9.63E-02	ENSG00000102924
CBLN2	0.3610	7.9634E-03	3.58E-02	ENSG00000141668
CBLN3	-0.0202	8.0433E-01	8.96E-01	ENSG00000139899
CBR1	-0.1367	1.8082E-01	3.53E-01	ENSG00000159228
CBR1-AS1	0.0323	5.6184E-01	7.31E-01	ENSG00000230212
CBR3	-0.1325	1.9448E-01	3.70E-01	ENSG00000159231
CBR3-AS1	0.0614	4.6267E-01	6.51E-01	ENSG00000236830
CBR4	0.3347	1.0665E-06	2.83E-05	ENSG00000145439
CBS	0.0787	2.3218E-01	4.16E-01	ENSG00000160200
CBX1	-0.3708	1.3184E-08	7.14E-07	ENSG00000108468
CBX2	0.5518	2.7372E-10	2.79E-08	ENSG00000173894
CBX3	0.0134	8.8724E-01	9.43E-01	ENSG00000122565
CBX3P2	0.1518	1.1105E-01	2.53E-01	ENSG00000266405
CBX4	-0.0702	2.1499E-01	3.95E-01	ENSG00000141582
CBX5	-0.0388	5.7763E-01	7.44E-01	ENSG00000094916
CBX6	-0.0893	2.4910E-01	4.35E-01	ENSG00000183741
CBX7	0.5417	6.1460E-05	7.59E-04	ENSG00000100307
CBX8	0.1578	6.6373E-02	1.75E-01	ENSG00000141570
CBY1	-0.1126	6.1512E-02	1.66E-01	ENSG00000100211
CBY3	-0.0225	7.4203E-01		ENSG00000204659
CC2D1A	0.4215	4.2014E-06	8.72E-05	ENSG00000132024
CC2D1B	0.1040	2.3237E-01	4.16E-01	ENSG00000154222
CC2D2A	-0.2704	6.2072E-04	4.90E-03	ENSG00000048342
CC2D2B	-0.0906	2.5077E-01	4.37E-01	ENSG00000188649
CCAR1	0.2247	1.8741E-04	1.86E-03	ENSG00000060339
CCAR2	0.0987	1.1619E-01	2.61E-01	ENSG00000158941
CCBE1	-0.0034	9.9100E-01	9.95E-01	ENSG00000183287
CCDC102A	-0.3685	1.1227E-02	4.63E-02	ENSG00000135736
CCDC102B	0.0552	5.0274E-01	6.84E-01	ENSG00000150636
CCDC103	-0.0034	7.8188E-01		ENSG00000167131
CCDC106	0.0207	7.8333E-01	8.83E-01	ENSG00000173581
CCDC107	-0.0522	4.1685E-01	6.09E-01	ENSG00000159884
CCDC110	-0.0315	7.3996E-01	8.57E-01	ENSG00000168491
CCDC112	-0.0213	7.4619E-01	8.61E-01	ENSG00000164221
CCDC113	0.1149	5.9319E-02	1.62E-01	ENSG00000103021
CCDC115	0.1369	1.0075E-02	4.27E-02	ENSG00000136710

CCDC116	-0.0827	1.8937E-01		ENSG00000161180
CCDC117	0.0051	9.5232E-01	9.76E-01	ENSG00000159873
CCDC12	-0.2680	5.3529E-06	1.06E-04	ENSG00000160799
CCDC120	-0.0743	3.9568E-01	5.90E-01	ENSG00000147144
CCDC121	-0.0909	3.2697E-01	5.21E-01	ENSG00000176714
CCDC122	0.0486	5.0299E-01	6.84E-01	ENSG00000151773
CCDC124	-0.0695	2.0691E-01	3.86E-01	ENSG00000007080
CCDC125	-0.1988	2.5496E-02	8.65E-02	ENSG00000183323
CCDC126	-0.1523	4.4867E-02	1.32E-01	ENSG00000169193
CCDC127	0.0785	1.6660E-01	3.34E-01	ENSG00000164366
CCDC13	0.1206	2.3937E-01	4.24E-01	ENSG00000244607
CCDC13-AS1	0.2683	3.3427E-02	1.06E-01	ENSG00000173811
CCDC134	0.1337	1.3667E-01	2.91E-01	ENSG00000100147
CCDC136	0.1008	1.6496E-01	3.31E-01	ENSG00000128596
CCDC137	0.1943	1.2696E-03	8.59E-03	ENSG00000185298
CCDC138	0.3914	1.1523E-06	3.01E-05	ENSG00000163006
CCDC14	0.0850	1.6854E-01	3.36E-01	ENSG00000175455
CCDC141	-0.0573	4.8004E-01	6.66E-01	ENSG00000163492
CCDC142	0.1851	6.1050E-02	1.66E-01	ENSG00000135637
CCDC144A	0.0127	8.7641E-01	9.37E-01	ENSG00000170160
CCDC146	-0.5374	7.8821E-06	1.46E-04	ENSG00000135205
CCDC148	-0.0390	6.6079E-01	8.05E-01	ENSG00000153237
CCDC148-AS1	0.0118	4.6677E-01		ENSG00000227480
CCDC149	0.1348	1.0616E-01	2.45E-01	ENSG00000181982
CCDC15-DT	0.2590	3.3437E-02	1.06E-01	ENSG00000285825
CCDC150	0.0619	5.2512E-01	7.01E-01	ENSG00000144395
CCDC152	-0.0102	9.0931E-01	9.55E-01	ENSG00000198865
CCDC153	0.0182	8.4896E-01	9.22E-01	ENSG00000248712
CCDC154	0.1753	9.8617E-02	2.32E-01	ENSG00000197599
CCDC157	-0.1204	7.7548E-02	1.96E-01	ENSG00000187860
CCDC158	0.1341	6.0414E-02		ENSG00000163749
CCDC159	-0.4396	8.9490E-05	1.02E-03	ENSG00000183401
CCDC160	-0.0251	7.2303E-01	8.47E-01	ENSG00000203952
CCDC162P	-0.0094	9.1948E-01	9.60E-01	ENSG00000203799
CCDC163	0.2567	1.0455E-02	4.39E-02	ENSG00000280670
CCDC167	-0.2897	2.5929E-06	5.92E-05	ENSG00000198937
CCDC168	-0.0424	7.1841E-01		ENSG00000175820
CCDC169	-0.2436	3.1176E-02	1.00E-01	ENSG00000242715
CCDC169-SOHLH2	-0.0106	8.1010E-01		ENSG00000250709
CCDC17	-0.0732	4.1867E-01	6.11E-01	ENSG00000159588
CCDC171	0.1842	1.1177E-02	4.62E-02	ENSG00000164989
CCDC174	0.1813	3.6708E-02	1.13E-01	ENSG00000154781
CCDC175	-0.0185	8.1249E-01	9.01E-01	ENSG00000151838
CCDC177	0.1006	3.1534E-01	5.09E-01	ENSG00000267909
CCDC178	-0.2344	5.4188E-02	1.52E-01	ENSG00000166960
CCDC18	-0.0073	9.2114E-01	9.61E-01	ENSG00000122483
CCDC180	0.3942	1.7557E-02	6.53E-02	ENSG00000197816
CCDC181	-0.1067	1.7626E-01	3.47E-01	ENSG00000117477
CCDC183	0.1545	1.3005E-01	2.82E-01	ENSG00000213213

CCDC183-AS1	0.0373	6.6459E-01	8.07E-01	ENSG00000228544
CCDC184	0.0425	6.3873E-01	7.90E-01	ENSG00000177875
CCDC186	-0.0340	4.2289E-01	6.15E-01	ENSG00000165813
CCDC187	-0.1440	1.8239E-01	3.55E-01	ENSG00000260220
CCDC188	0.1940	9.8899E-02	2.33E-01	ENSG00000234409
CCDC191	-0.1662	1.8851E-02	6.89E-02	ENSG00000163617
CCDC192	0.2073	8.3940E-02	2.08E-01	ENSG00000230561
CCDC194	0.1526	4.9906E-02	1.43E-01	ENSG00000269720
CCDC195	-0.0926	1.2339E-01	2.72E-01	ENSG00000283428
CCDC196	-0.0522	6.9266E-01	8.28E-01	ENSG00000196553
CCDC197	0.0041	9.5984E-01		ENSG00000175699
CCDC198	-0.1253	1.5441E-01	3.17E-01	ENSG00000100557
CCDC200	0.0199	3.5589E-02	1.11E-01	ENSG00000236383
CCDC22	-0.2018	1.8130E-02	6.69E-02	ENSG00000101997
CCDC24	-0.1686	5.3317E-02	1.50E-01	ENSG00000159214
CCDC25	0.0576	3.0677E-01	4.99E-01	ENSG00000147419
CCDC26	-0.0145	8.4947E-01		ENSG00000229140
CCDC27	-0.0172	8.3576E-01	9.14E-01	ENSG00000162592
CCDC28A	-0.0391	5.4853E-01	7.20E-01	ENSG00000024862
CCDC28A-AS1	-0.1276	2.1389E-01	3.94E-01	ENSG00000279968
CCDC28B	-0.4585	4.0047E-08	1.83E-06	ENSG00000160050
CCDC3	0.0223	8.1695E-01	9.03E-01	ENSG00000151468
CCDC30	-0.1084	4.4802E-02	1.32E-01	ENSG00000186409
CCDC32	0.0010	9.9799E-01	9.99E-01	ENSG00000128891
CCDC33	-0.0187	8.4319E-01	9.18E-01	ENSG00000140481
CCDC34	-0.3780	4.3916E-07	1.34E-05	ENSG00000109881
CCDC38	0.0815	3.6811E-01	5.64E-01	ENSG00000165972
CCDC39	0.0116	8.6065E-01	9.29E-01	ENSG00000284862
CCDC40	0.1743	1.5135E-02	5.84E-02	ENSG00000141519
CCDC42	0.0231	1.6550E-01		ENSG00000161973
CCDC43	-0.0789	1.9622E-01	3.73E-01	ENSG00000180329
CCDC47	0.0495	4.6966E-01	6.57E-01	ENSG00000108588
CCDC50	-0.1234	2.1820E-02	7.69E-02	ENSG00000152492
CCDC51	0.2092	1.4976E-02	5.79E-02	ENSG00000164051
CCDC57	0.1270	8.1693E-02	2.04E-01	ENSG00000176155
CCDC6	-0.4282	4.0441E-13	9.39E-11	ENSG00000108091
CCDC60	-0.0211	9.9771E-01	9.99E-01	ENSG00000183273
CCDC61	-0.0457	6.3682E-01	7.89E-01	ENSG00000104983
CCDC62	0.1689	1.3564E-01	2.89E-01	ENSG00000130783
CCDC63	-0.0045	8.5852E-01		ENSG00000173093
CCDC65	-0.3220	2.8932E-03	1.64E-02	ENSG00000139537
CCDC66	-0.0040	9.5097E-01	9.75E-01	ENSG00000180376
CCDC68	-0.3396	1.7949E-02	6.64E-02	ENSG00000166510
CCDC69	0.5134	3.7650E-06	7.98E-05	ENSG00000198624
CCDC7	-0.0415	6.0200E-01	7.62E-01	ENSG00000216937
CCDC71	0.0113	9.0192E-01	9.51E-01	ENSG00000177352
CCDC71L	-0.1780	6.3466E-02	1.70E-01	ENSG00000253276
CCDC73	-0.0250	5.6157E-01		ENSG00000186714
CCDC74A	-0.0250	7.6259E-01	8.71E-01	ENSG00000163040

CCDC74B	-0.0967	1.9375E-01	3.69E-01	ENSG00000152076
CCDC77	0.3411	1.1166E-03	7.74E-03	ENSG00000120647
CCDC78	-0.0190	8.4125E-01	9.17E-01	ENSG00000162004
CCDC8	-0.2313	7.7455E-03	3.50E-02	ENSG00000169515
CCDC80	-0.2329	1.8346E-02	6.76E-02	ENSG00000091986
CCDC82	0.2267	2.9165E-03	1.65E-02	ENSG00000149231
CCDC83	-0.0724	5.8413E-01		ENSG00000150676
CCDC85A	0.1817	1.1258E-01	2.55E-01	ENSG00000055813
CCDC85B	-0.1155	7.0133E-02	1.83E-01	ENSG00000175602
CCDC85C	0.0808	3.0518E-01	4.98E-01	ENSG00000205476
CCDC86	-0.0754	2.4624E-01	4.32E-01	ENSG00000110104
CCDC87	0.0696	4.1284E-01	6.06E-01	ENSG00000182791
CCDC88A	0.0741	2.1660E-01	3.97E-01	ENSG00000115355
CCDC88B	0.3668	4.1712E-03	2.18E-02	ENSG00000168071
CCDC88C	-0.3474	8.4229E-04	6.22E-03	ENSG00000015133
CCDC9	-0.0340	6.3362E-01	7.86E-01	ENSG00000105321
CCDC90B	-0.0686	2.0511E-01	3.83E-01	ENSG00000137500
CCDC91	-0.1578	8.3485E-03	3.71E-02	ENSG00000123106
CCDC92	0.0511	4.8833E-01	6.72E-01	ENSG00000119242
CCDC92B	-0.0758	4.0770E-01	6.01E-01	ENSG00000277200
CCDC93	0.1588	5.6901E-02	1.57E-01	ENSG00000125633
CCDC96	-0.0225	7.7467E-01	8.78E-01	ENSG00000173013
CCDC97	0.0251	7.3440E-01	8.54E-01	ENSG00000142039
CCDC9B	-0.3120	1.8894E-02	6.90E-02	ENSG00000188549
CCER2	0.0090	9.0797E-01	9.54E-01	ENSG00000262484
CCHCR1	-0.0083	8.9874E-01	9.49E-01	ENSG00000204536
CCIN	0.0318	4.6572E-01	6.54E-01	ENSG00000185972
CCK	0.1078	1.9704E-01	3.74E-01	ENSG00000187094
CCKAR	0.0995	3.1809E-01	5.12E-01	ENSG00000163394
CCKBR	0.0334	7.2730E-01	8.50E-01	ENSG00000110148
CCL13	0.0034	6.8627E-01		ENSG00000181374
CCL16	-0.0133	6.2562E-01		ENSG00000275152
CCL17	-0.0167	8.0281E-01		ENSG00000102970
CCL18	-0.0095	6.0071E-01	7.61E-01	ENSG00000275385
CCL19	0.0060	6.0035E-01		ENSG00000172724
CCL25	0.4222	1.1784E-02	4.81E-02	ENSG00000131142
CCL26	0.0114	1.3164E-01	2.84E-01	ENSG00000006606
CCL28	-0.0657	3.7874E-01	5.74E-01	ENSG00000151882
CCL3	0.0376	2.1910E-01	4.00E-01	ENSG00000277632
CCL3L1	0.1126	1.6461E-01	3.31E-01	ENSG00000276085
CCL4	-0.0240	6.3824E-01	7.90E-01	ENSG00000275302
CCL4L2	-0.0826	5.9970E-02	1.63E-01	ENSG00000276070
CCL5	-0.1141	4.7641E-02		ENSG00000271503
CCL7	-1.8590	1.6278E-03		ENSG00000108688
CCM2	0.1650	9.8133E-03	4.19E-02	ENSG00000136280
CCM2L	-0.0061	9.5653E-01	9.78E-01	ENSG00000101331
CCN1	-0.3878	8.6592E-04	6.37E-03	ENSG00000142871
CCN2	0.0678	4.4533E-01	6.35E-01	ENSG00000118523
CCN3	-0.2667	2.9791E-02	9.70E-02	ENSG00000136999

CCN4	-0.0066	9.5945E-01		ENSG00000104415
CCN5	-0.0180	7.6157E-01	8.70E-01	ENSG00000064205
CCN6	0.0100	5.7354E-01		ENSG00000112761
CCNA1	-0.2481	1.3368E-02	5.31E-02	ENSG00000133101
CCNA2	-0.1180	2.4660E-01	4.32E-01	ENSG00000145386
CCNB1	-0.2332	3.3120E-02	1.05E-01	ENSG00000134057
CCNB1IP1	0.0359	5.5850E-01	7.29E-01	ENSG00000100814
CCNB2	-0.2922	1.7304E-02	6.46E-02	ENSG00000157456
CCNB3	0.2788	1.6661E-02	6.28E-02	ENSG00000147082
CCNC	0.0751	2.1636E-01	3.97E-01	ENSG00000112237
CCND1	-0.2816	2.4573E-03	1.44E-02	ENSG00000110092
CCND2	-0.1280	8.7946E-02	2.15E-01	ENSG00000118971
CCND3	0.2934	3.8113E-05	5.13E-04	ENSG00000112576
CCNDBP1	-0.0693	2.9380E-01	4.86E-01	ENSG00000166946
CCNE1	0.0889	3.0887E-01	5.02E-01	ENSG00000105173
CCNE2	-0.1665	1.3535E-01	2.89E-01	ENSG00000175305
CCNF	0.2373	1.3039E-02	5.20E-02	ENSG00000162063
CCNG1	-0.0205	6.1670E-01	7.74E-01	ENSG00000113328
CCNG2	0.0593	4.1486E-01	6.08E-01	ENSG00000138764
CCNH	0.1564	1.7369E-02	6.48E-02	ENSG00000134480
CCNI	-0.1202	7.5456E-02	1.92E-01	ENSG00000118816
CCNI2	-0.0797	2.4661E-01	4.32E-01	ENSG00000205089
CCNJ	0.1810	2.6752E-02	8.95E-02	ENSG00000107443
CCNJL	0.0521	5.8322E-01	7.49E-01	ENSG00000135083
CCNK	-0.2641	1.2180E-03	8.30E-03	ENSG00000090061
CCNL1	-0.5366	2.0035E-21	1.90E-18	ENSG00000163660
CCNL2	0.1260	3.0043E-02	9.76E-02	ENSG00000221978
CCNO	-0.0115	9.0118E-01	9.51E-01	ENSG00000152669
CCNP	0.1154	2.6064E-01	4.49E-01	ENSG00000105219
CCNQ	-0.0194	6.8392E-01	8.21E-01	ENSG00000262919
CCNT1	0.0546	4.0498E-01	5.99E-01	ENSG00000129315
CCNT2	0.0162	7.7755E-01	8.80E-01	ENSG00000082258
CCNY	-0.0825	6.5164E-02	1.73E-01	ENSG00000108100
CCNYL1	0.1130	2.3184E-01	4.15E-01	ENSG00000163249
CCNYL6	0.0041	7.9199E-01		ENSG00000269845
CCNYL7	0.0049	7.2164E-01		ENSG00000261528
CCP110	0.1457	3.6658E-02	1.13E-01	ENSG00000103540
CCPG1	-0.1294	1.0294E-01	2.40E-01	ENSG00000260916
CCR1	-0.0101	7.1167E-01		ENSG00000163823
CCR10	0.2140	9.9740E-03	4.24E-02	ENSG00000184451
CCR3	0.0887	1.5240E-01	3.14E-01	ENSG00000183625
CCR7	0.0088	5.7280E-01		ENSG00000126353
CCR9	-0.0252	3.6252E-01		ENSG00000173585
CCRL2	0.0148	7.6558E-01		ENSG00000121797
CCS	-0.0358	6.3668E-01	7.89E-01	ENSG00000173992
CCSAP	-0.1174	1.1039E-01	2.52E-01	ENSG00000154429
CCSER1	0.0624	5.0157E-01	6.83E-01	ENSG00000184305
CCSER2	0.1750	2.3190E-03	1.38E-02	ENSG00000107771
CCT2	0.0041	9.8602E-01	9.92E-01	ENSG00000166226

CCT3	-0.0209	7.1376E-01	8.40E-01	ENSG00000163468
CCT4	-0.2780	1.0421E-04	1.16E-03	ENSG00000115484
CCT4P2	-0.0617	3.8006E-01	5.75E-01	ENSG00000225569
CCT5	-0.1551	2.4434E-02	8.37E-02	ENSG00000150753
CCT5P1	-0.0021	9.7797E-01		ENSG00000250444
CCT6A	0.0502	3.2847E-01	5.23E-01	ENSG00000146731
CCT6B	0.4248	4.7360E-05	6.17E-04	ENSG00000132141
CCT6P2	0.0026	7.1307E-01		ENSG00000250526
CCT7	-0.1284	2.2047E-02	7.75E-02	ENSG00000135624
CCT7P1	0.0069	4.5878E-01		ENSG00000217733
CCT8	-0.0787	9.4455E-02	2.26E-01	ENSG00000156261
CCT8L1P	0.0290	1.9793E-01		ENSG00000020219
CCT8P1	0.0587	5.1504E-01	6.94E-01	ENSG00000226015
CCZ1	0.1526	2.3416E-02	8.11E-02	ENSG00000122674
CCZ1B	0.1314	6.6355E-02	1.75E-01	ENSG00000146574
CCZ1P1	0.1445	1.5977E-01	3.25E-01	ENSG00000243554
CD101	-0.1593	1.3064E-01	2.82E-01	ENSG00000134256
CD109	-0.1561	1.3010E-01	2.82E-01	ENSG00000156535
CD109-AS1	-0.0160	7.9026E-01	8.87E-01	ENSG00000231652
CD14	0.0495	5.9086E-01	7.54E-01	ENSG00000170458
CD151	-0.1211	9.2198E-02	2.22E-01	ENSG00000177697
CD160	0.0227	6.6330E-01		ENSG00000117281
CD163	-0.0146	8.1742E-01	9.03E-01	ENSG00000177575
CD164	-0.0394	6.2321E-01	7.79E-01	ENSG00000135535
CD164L2	0.0390	6.6503E-01	8.07E-01	ENSG00000174950
CD177	0.0358	2.6310E-01	4.52E-01	ENSG00000204936
CD180	-0.0034	7.8972E-01		ENSG00000134061
CD1D	0.0662	4.8102E-01	6.66E-01	ENSG00000158473
CD2	0.0464	2.8927E-01	4.81E-01	ENSG00000116824
CD200	-0.1579	4.7295E-02	1.37E-01	ENSG00000091972
CD200R1	0.1382	1.6559E-01	3.32E-01	ENSG00000163606
CD207	0.0073	8.3737E-01		ENSG00000116031
CD22	0.0254	1.4524E-01		ENSG00000012124
CD226	0.0142	8.3179E-01		ENSG00000150637
CD24	-0.0972	1.8506E-01	3.58E-01	ENSG00000272398
CD247	-0.0100	9.0820E-01	9.54E-01	ENSG00000198821
CD248	0.1926	3.6647E-02	1.13E-01	ENSG00000174807
CD27	-0.0057	9.4246E-01		ENSG00000139193
CD27-AS1	-0.2070	7.3840E-03	3.37E-02	ENSG00000215039
CD274	-0.1519	1.6612E-01	3.33E-01	ENSG00000120217
CD276	-0.2353	3.7979E-03	2.03E-02	ENSG00000103855
CD2AP	-0.3581	1.3618E-04	1.43E-03	ENSG00000198087
CD2BP2	0.1358	1.9870E-02	7.16E-02	ENSG00000169217
CD2BP2-DT	-0.1188	2.0999E-01	3.89E-01	ENSG00000260219
CD300A	0.0232	4.0245E-02		ENSG00000167851
CD300C	0.0364	5.8727E-01	7.52E-01	ENSG00000167850
CD300LG	-0.0266	8.2043E-01		ENSG00000161649
CD302	0.0806	4.1233E-01	6.05E-01	ENSG00000241399
CD320	-0.2033	4.4956E-04	3.78E-03	ENSG00000167775

CD36	-0.4342	1.1111E-03	7.71E-03	ENSG00000135218
CD37	-0.0316	6.1703E-01		ENSG00000104894
CD38	-0.0803	2.0324E-01		ENSG00000004468
CD3D	0.0717	2.5427E-01		ENSG00000167286
CD3G	0.0804	1.9870E-01	3.76E-01	ENSG00000160654
CD4	0.1340	2.0010E-01	3.77E-01	ENSG00000010610
CD40	-0.0242	7.9041E-01	8.88E-01	ENSG00000101017
CD44	-0.5205	8.9650E-10	7.78E-08	ENSG00000026508
CD44-DT	-0.0168	6.7834E-01		ENSG00000255521
CD46	0.0561	4.9535E-01	6.79E-01	ENSG00000117335
CD46P1	0.0575	1.8499E-01		ENSG00000244703
CD47	-0.2309	1.1620E-02	4.76E-02	ENSG00000196776
CD5	0.1023	2.3576E-01	4.20E-01	ENSG00000110448
CD52	-0.1088	2.5509E-01	4.42E-01	ENSG00000169442
CD53	-0.0085	8.5909E-01		ENSG00000143119
CD55	-0.0833	1.9961E-01	3.77E-01	ENSG00000196352
CD59	-0.3184	1.2571E-04	1.34E-03	ENSG00000085063
CD6	0.5289	4.6290E-03	2.36E-02	ENSG00000013725
CD63	-0.3775	1.4765E-08	7.83E-07	ENSG00000135404
CD63-AS1	-0.3782	2.3999E-03	1.42E-02	ENSG00000258056
CD68	-0.0161	7.8587E-01		ENSG00000129226
CD7	0.0267	7.7919E-01	8.81E-01	ENSG00000173762
CD70	0.0427	7.5744E-02	1.93E-01	ENSG00000125726
CD72	0.2954	1.4798E-02	5.75E-02	ENSG00000137101
CD74	0.2531	4.4586E-02	1.31E-01	ENSG00000019582
CD79A	0.2272	6.2492E-02	1.68E-01	ENSG00000105369
CD79B	0.0356	5.8817E-02		ENSG00000007312
CD80	-0.0039	1.5069E-01		ENSG00000121594
CD81	-0.4084	1.4970E-04	1.55E-03	ENSG00000110651
CD81-AS1	-0.1504	1.6071E-01	3.26E-01	ENSG00000238184
CD82	-0.0290	7.2428E-01	8.47E-01	ENSG00000085117
CD86	-0.0058	9.6454E-01	9.82E-01	ENSG00000114013
CD8A	-0.2180	3.5369E-02	1.10E-01	ENSG00000153563
CD8B	-0.0189	7.5857E-01	8.69E-01	ENSG00000172116
CD8B2	0.0152	7.6367E-01		ENSG00000254126
CD9	-0.4654	1.4282E-04	1.49E-03	ENSG00000010278
CD93	-0.0030	8.8552E-01		ENSG00000125810
CD96	0.0492	4.5108E-01	6.41E-01	ENSG00000153283
CD99	-0.3958	6.7034E-06	1.28E-04	ENSG00000002586
CD99L2	-0.0031	9.1193E-01	9.56E-01	ENSG00000102181
CDADC1	0.1226	3.5095E-02	1.10E-01	ENSG00000102543
CDAN1	0.3426	9.2051E-05	1.05E-03	ENSG00000140326
CDC123	0.0012	9.8494E-01	9.92E-01	ENSG00000151465
CDC14A	-0.0614	4.5792E-01	6.47E-01	ENSG00000079335
CDC14B	-0.1287	6.5455E-02	1.74E-01	ENSG00000081377
CDC16	-0.0691	2.3104E-01	4.14E-01	ENSG00000130177
CDC20	-0.1536	1.4298E-01	3.01E-01	ENSG00000117399
CDC20-DT	-0.0780	6.2569E-01	7.81E-01	ENSG00000234694
CDC20B	-0.2850	1.7038E-02	6.38E-02	ENSG00000164287

CDC20P1	-0.0333	4.3656E-01		ENSG00000231007
CDC23	0.1656	3.4829E-03	1.89E-02	ENSG00000094880
CDC25A	-0.0409	6.6953E-01	8.10E-01	ENSG00000164045
CDC25B	-0.3547	2.2675E-07	7.78E-06	ENSG00000101224
CDC25C	-0.4139	1.4831E-03	9.73E-03	ENSG00000158402
CDC26	-0.0765	1.6599E-01	3.33E-01	ENSG00000176386
CDC27	-0.0102	6.7547E-01	8.14E-01	ENSG00000004897
CDC34	-0.0476	2.2635E-01	4.09E-01	ENSG00000099804
CDC37	0.1270	3.9104E-02	1.19E-01	ENSG00000105401
CDC37L1	0.3023	3.7026E-08	1.70E-06	ENSG00000106993
CDC37L1-DT	-0.0835	3.6709E-01	5.63E-01	ENSG00000273061
CDC40	-0.0360	4.6659E-01	6.55E-01	ENSG00000168438
CDC42	-0.1329	9.2411E-02	2.23E-01	ENSG00000070831
CDC42BPA	0.1032	1.0334E-01	2.40E-01	ENSG00000143776
CDC42BPB	-0.1526	2.1372E-02	7.57E-02	ENSG00000198752
CDC42BPG	0.1882	8.4797E-02	2.09E-01	ENSG00000171219
CDC42EP3	-0.1272	6.7199E-02	1.77E-01	ENSG00000163171
CDC42EP4	-0.3468	1.2870E-03	8.68E-03	ENSG00000179604
CDC42EP5	0.1011	3.0373E-01	4.96E-01	ENSG00000167617
CDC42P1	0.0508	1.6825E-01		ENSG00000234933
CDC42SE1	-0.2356	1.7073E-05	2.71E-04	ENSG00000197622
CDC42SE2	-0.3201	1.8506E-05	2.88E-04	ENSG00000158985
CDC45	-0.4184	6.5499E-03	3.08E-02	ENSG00000093009
CDC5L	-0.2064	5.5364E-06	1.09E-04	ENSG00000096401
CDC6	-0.2479	3.6918E-02	1.14E-01	ENSG00000094804
CDC7	0.2186	2.4834E-02	8.48E-02	ENSG00000097046
CDC73	-0.1179	3.2910E-02	1.05E-01	ENSG00000134371
CDCA2	-0.1971	6.8875E-02	1.80E-01	ENSG00000184661
CDCA3	-0.3532	6.0244E-04	4.78E-03	ENSG00000111665
CDCA4	-0.1390	1.3339E-01	2.86E-01	ENSG00000170779
CDCA4P1	-0.0002	3.4211E-01		ENSG00000229029
CDCA5	-0.2447	2.3802E-02	8.21E-02	ENSG00000146670
CDCA7	-0.1431	1.3608E-01	2.90E-01	ENSG00000144354
CDCA7L	-0.2194	3.0336E-02	9.82E-02	ENSG00000164649
CDCA8	-0.2355	3.5556E-02	1.11E-01	ENSG00000134690
CDCP1	-0.2422	5.7331E-02	1.58E-01	ENSG00000163814
CDH1	-0.0244	7.6531E-01	8.73E-01	ENSG00000039068
CDH11	0.3049	8.7708E-04	6.43E-03	ENSG00000140937
CDH12P1	0.1979	8.8368E-02	2.16E-01	ENSG00000254335
CDH12P2	0.1910	9.6012E-02	2.28E-01	ENSG00000249230
CDH12P3	0.1850	1.0687E-01	2.46E-01	ENSG00000253492
CDH12P4	0.1413	1.6497E-01	3.31E-01	ENSG00000253900
CDH13	0.1208	2.3490E-01	4.19E-01	ENSG00000140945
CDH13-AS2	0.0254	1.2170E-01		ENSG00000260228
CDH16	-0.0078	8.2473E-01		ENSG00000166589
CDH17	0.0569	5.4396E-03		ENSG00000079112
CDH18	0.0719	4.4779E-01	6.38E-01	ENSG00000145526
CDH18-AS1	0.0195	1.9916E-01		ENSG00000249854
CDH19	0.0235	1.9100E-02		ENSG00000071991

CDH2	-0.2320	3.5593E-04	3.15E-03	ENSG00000170558
CDH20	0.4286	5.8869E-03	2.84E-02	ENSG00000101542
CDH22	0.3340	8.8682E-04	6.48E-03	ENSG00000149654
CDH23	-0.1841	6.1319E-02	1.66E-01	ENSG00000107736
CDH24	-0.0921	3.0395E-01	4.96E-01	ENSG00000139880
CDH26	0.0232	8.1534E-01	9.03E-01	ENSG00000124215
CDH3	-0.0275	7.7704E-01	8.79E-01	ENSG00000062038
CDH4	-0.0383	6.5112E-01	7.98E-01	ENSG00000179242
CDH5	-0.0721	9.7766E-02		ENSG00000179776
CDH6	-0.0998	1.0648E-01	2.45E-01	ENSG00000113361
CDH7	0.1365	1.5628E-01	3.19E-01	ENSG00000081138
CDH8	0.2877	5.9854E-03	2.88E-02	ENSG00000150394
CDH9	0.1607	1.3423E-01	2.88E-01	ENSG00000113100
CDHR1	0.0362	7.1116E-01	8.39E-01	ENSG00000148600
CDHR2	0.0389	6.5521E-01	8.01E-01	ENSG00000074276
CDHR3	0.0981	2.6757E-01	4.57E-01	ENSG00000128536
CDHR4	-0.1568	1.1814E-01	2.64E-01	ENSG00000187492
CDHR5	-0.0410	7.1012E-01	8.38E-01	ENSG00000099834
CDIN1	0.0787	3.5587E-01	5.51E-01	ENSG00000186073
CDIP1	-0.0903	1.8508E-01	3.58E-01	ENSG00000089486
CDIPT	-0.0586	1.8004E-01	3.51E-01	ENSG00000103502
CDIPTOSP	0.0312	7.5865E-01	8.69E-01	ENSG00000214725
CDK1	-0.4375	1.9531E-03	1.20E-02	ENSG00000170312
CDK10	0.0296	6.7657E-01	8.15E-01	ENSG00000185324
CDK11A	0.1914	3.2428E-02	1.03E-01	ENSG00000008128
CDK11B	-0.0408	5.2310E-01	7.00E-01	ENSG00000248333
CDK12	-0.1582	4.1701E-02	1.25E-01	ENSG00000167258
CDK13	0.0823	2.5824E-01	4.46E-01	ENSG00000065883
CDK13-DT	0.0994	3.2277E-01	5.17E-01	ENSG00000259826
CDK14	-0.2251	1.5418E-03	1.00E-02	ENSG00000058091
CDK15	-0.0590	5.1265E-01	6.92E-01	ENSG00000138395
CDK16	-0.0167	7.8353E-01	8.83E-01	ENSG00000102225
CDK17	0.0126	9.3780E-01	9.69E-01	ENSG00000059758
CDK18	0.0190	8.1908E-01	9.05E-01	ENSG00000117266
CDK19	0.0556	5.1942E-01	6.97E-01	ENSG00000155111
CDK2	-0.0913	2.4666E-01	4.33E-01	ENSG00000123374
CDK20	0.0984	2.8623E-01	4.77E-01	ENSG00000156345
CDK2AP1	-0.4480	1.3692E-06	3.47E-05	ENSG00000111328
CDK2AP2	-0.1948	1.1449E-04	1.25E-03	ENSG00000167797
CDK3	-0.0693	2.3676E-01		ENSG00000250506
CDK4	-0.2180	1.6615E-02	6.27E-02	ENSG00000135446
CDK5	0.0013	9.6968E-01	9.84E-01	ENSG00000164885
CDK5R1	0.0369	6.7130E-01	8.11E-01	ENSG00000176749
CDK5R2	-0.0639	4.3985E-01	6.31E-01	ENSG00000171450
CDK5RAP1	0.1254	8.1906E-02	2.04E-01	ENSG00000101391
CDK5RAP2	-0.0918	1.9075E-01	3.66E-01	ENSG00000136861
CDK5RAP3	0.2731	1.9587E-03	1.20E-02	ENSG00000108465
CDK6	-0.4037	1.9157E-04	1.89E-03	ENSG00000105810
CDK7	0.1617	1.2196E-02	4.95E-02	ENSG00000134058

CDK8	-0.0115	8.8615E-01	9.43E-01	ENSG00000132964
CDK9	0.0303	4.8376E-01	6.69E-01	ENSG00000136807
CDKAL1	-0.0551	4.2089E-01	6.13E-01	ENSG00000145996
CDKL1	-0.0490	5.9568E-01	7.58E-01	ENSG00000100490
CDKL2	-0.5104	5.9891E-10	5.56E-08	ENSG00000138769
CDKL4	-0.0416	4.5407E-01		ENSG00000205111
CDKL5	0.1556	9.1506E-02	2.21E-01	ENSG00000008086
CDKN1A	-0.3624	3.0217E-04	2.74E-03	ENSG00000124762
CDKN1B	-0.3028	3.6230E-03	1.95E-02	ENSG00000111276
CDKN1C	0.1460	7.4023E-02	1.90E-01	ENSG00000129757
CDKN2A	-0.2602	2.4285E-02	8.33E-02	ENSG00000147889
CDKN2AIP	-0.1576	4.0171E-02	1.21E-01	ENSG00000168564
CDKN2AIPNL	0.0365	3.9360E-01	5.88E-01	ENSG00000237190
CDKN2B	0.0487	6.1585E-01	7.74E-01	ENSG00000147883
CDKN2B-AS1	-0.0334	7.0064E-01	8.33E-01	ENSG00000240498
CDKN2C	-0.1013	2.8458E-01	4.76E-01	ENSG00000123080
CDKN2D	0.1726	2.9089E-02	9.53E-02	ENSG00000129355
CDKN3	-0.1900	4.2389E-02	1.27E-01	ENSG00000100526
CDNF	0.1734	6.4944E-02	1.73E-01	ENSG00000185267
CDO1	-0.2447	1.6961E-02	6.36E-02	ENSG00000129596
CDON	-0.3559	3.4164E-03	1.86E-02	ENSG00000064309
CDPF1	0.0053	9.2977E-01	9.66E-01	ENSG00000205643
CDR2	0.2053	4.6725E-03	2.38E-02	ENSG00000140743
CDR2L	0.0250	7.2861E-01	8.51E-01	ENSG00000109089
CDRT15	0.0166	3.5531E-01		ENSG00000223510
CDRT15P3	-0.0018	9.7995E-01	9.89E-01	ENSG00000186825
CDRT4	0.0276	1.9720E-01		ENSG00000239704
CDS1	0.0063	9.1274E-01	9.56E-01	ENSG00000163624
CDS2	-0.1300	2.9235E-02	9.57E-02	ENSG00000101290
CDT1	-0.1377	1.1796E-01	2.64E-01	ENSG00000167513
CDV3	-0.1776	2.3222E-03	1.38E-02	ENSG00000091527
CDX2	-0.0003	9.9549E-01		ENSG00000165556
CDYL	-0.1034	1.9695E-01	3.73E-01	ENSG00000153046
CDYL2	-0.2603	8.9032E-05	1.02E-03	ENSG00000166446
CEACAM1	0.1369	1.9230E-01	3.68E-01	ENSG00000079385
CEACAM19	0.0818	3.8733E-01	5.82E-01	ENSG00000186567
CEACAM20	-0.0037	8.1274E-01		ENSG00000273777
CEACAM21	-0.1122	2.4871E-01	4.35E-01	ENSG00000007129
CEACAM5	0.0516	3.0880E-01	5.02E-01	ENSG00000105388
CEACAM6	-0.0586	4.4357E-01	6.34E-01	ENSG00000086548
CEACAM7	-0.0095	6.9498E-01	8.29E-01	ENSG00000007306
CEACAM8	-0.0504	1.1529E-01		ENSG00000124469
CEBPA	0.2481	3.0297E-02	9.82E-02	ENSG00000245848
CEBPA-DT	0.1738	1.2492E-01	2.74E-01	ENSG00000267296
CEBPB	-0.3042	4.1048E-04	3.52E-03	ENSG00000172216
CEBPB-AS1	0.2784	3.9571E-02	1.20E-01	ENSG00000277449
CEBPD	-0.4097	1.7921E-03	1.12E-02	ENSG00000221869
CEBPE	-0.0102	6.6920E-01		ENSG00000092067
CEBPG	-0.3959	1.6152E-09	1.27E-07	ENSG00000153879

CEBPZ	0.1557	1.3135E-02	5.23E-02	ENSG00000115816
CEBPZOS	-0.2078	9.1877E-03	3.99E-02	ENSG00000218739
CECR2	0.0172	8.1076E-01	9.00E-01	ENSG00000099954
CECR3	0.0166	2.5468E-01	4.42E-01	ENSG00000241832
CEL	0.2249	2.1615E-02	7.64E-02	ENSG00000170835
CELA1	0.0017	9.6827E-01		ENSG00000139610
CELA2A	0.0093	3.4780E-01		ENSG00000142615
CELF1	0.2039	8.8601E-04	6.47E-03	ENSG00000149187
CELF2	0.2591	6.3037E-04	4.95E-03	ENSG00000048740
CELF2-AS1	-0.0131	8.5980E-01	9.29E-01	ENSG00000181800
CELF2-AS2	-0.0671	4.8274E-01	6.68E-01	ENSG00000237986
CELF3	0.1754	2.3929E-02	8.24E-02	ENSG00000159409
CELF4	0.1494	7.1354E-02	1.85E-01	ENSG00000101489
CELF5	0.0950	2.7579E-01	4.66E-01	ENSG00000161082
CELF6	-0.0337	4.9368E-01	6.77E-01	ENSG00000140488
CELP	0.1891	1.0120E-01	2.37E-01	ENSG00000170827
CELSR1	-0.3345	1.5798E-03	1.02E-02	ENSG00000075275
CELSR2	-0.1553	3.6219E-02	1.12E-01	ENSG00000143126
CELSR3	0.2748	5.6978E-03	2.77E-02	ENSG00000008300
CEMIP	-0.1486	1.5895E-01	3.23E-01	ENSG00000103888
CEMIP2	-0.0318	6.5392E-01	8.00E-01	ENSG00000135048
CENATAC	0.0554	4.6072E-01	6.49E-01	ENSG00000186166
CEND1	0.1003	2.6291E-01	4.52E-01	ENSG00000184524
CENPA	-0.3667	4.0982E-03	2.15E-02	ENSG00000115163
CENPB	-0.2512	1.1513E-02	4.73E-02	ENSG00000125817
CENPBD1P	0.3887	2.5002E-08	1.24E-06	ENSG00000177946
CENPBD2P	-0.2196	1.8302E-03	1.14E-02	ENSG00000213753
CENPC	-0.0956	8.1437E-02	2.03E-01	ENSG00000145241
CENPCP1	-0.0189	6.0660E-01		ENSG00000226982
CENPE	-0.2277	1.6831E-02	6.32E-02	ENSG00000138778
CENPF	-0.4174	1.8595E-03	1.16E-02	ENSG00000117724
CENPH	-0.0358	5.9796E-01	7.59E-01	ENSG00000153044
CENPI	-0.3513	1.9764E-02	7.13E-02	ENSG00000102384
CENPJ	-0.0073	9.3661E-01	9.69E-01	ENSG00000151849
CENPK	-0.1873	6.0770E-02	1.65E-01	ENSG00000123219
CENPL	0.1143	2.4029E-01	4.25E-01	ENSG00000120334
CENPM	-0.3823	1.6154E-04	1.65E-03	ENSG00000100162
CENPN	-0.4457	7.7556E-05	9.13E-04	ENSG00000166451
CENPO	-0.3291	4.4160E-03	2.28E-02	ENSG00000138092
CENPP	0.0953	3.2112E-01	5.15E-01	ENSG00000188312
CENPQ	-0.1279	1.0232E-01	2.39E-01	ENSG00000031691
CENPS	-0.3258	6.5131E-03	3.07E-02	ENSG00000175279
CENPS-CORT	-0.0283	7.3304E-01	8.53E-01	ENSG00000251503
CENPT	0.1473	2.9152E-02	9.55E-02	ENSG00000102901
CENPU	-0.2567	1.1435E-02	4.70E-02	ENSG00000151725
CENPV	-0.0044	8.9776E-01	9.49E-01	ENSG00000166582
CENPVL1	0.1255	2.0568E-01	3.84E-01	ENSG00000223591
CENPVL2	0.1318	1.9581E-01	3.72E-01	ENSG00000283093
CENPVL3	0.0278	7.3400E-01	8.54E-01	ENSG00000224109

CENPX	0.1006	1.1559E-01	2.60E-01	ENSG00000169689
CEP104	0.0427	5.2863E-01	7.04E-01	ENSG00000116198
CEP112	0.1769	1.7829E-02	6.61E-02	ENSG00000154240
CEP120	0.3605	8.5658E-05	9.88E-04	ENSG00000168944
CEP126	-0.1797	1.8123E-02	6.69E-02	ENSG00000110318
CEP128	-0.0300	7.5284E-01	8.65E-01	ENSG00000100629
CEP131	0.2243	2.4548E-02	8.41E-02	ENSG00000141577
CEP135	-0.0649	4.7394E-01	6.61E-01	ENSG00000174799
CEP15	0.0003	9.9758E-01	9.99E-01	ENSG00000114405
CEP152	0.0970	3.0817E-01	5.01E-01	ENSG00000103995
CEP162	0.0322	6.6784E-01	8.09E-01	ENSG00000135315
CEP164	-0.0321	5.8856E-01	7.52E-01	ENSG00000110274
CEP170	0.1132	2.0157E-01	3.79E-01	ENSG00000143702
CEP170B	0.1583	1.0319E-01	2.40E-01	ENSG00000099814
CEP19	-0.0524	5.4198E-01	7.15E-01	ENSG00000174007
CEP192	-0.2450	4.0199E-03	2.12E-02	ENSG00000101639
CEP20	0.1099	1.2150E-01	2.69E-01	ENSG00000133393
CEP250	0.1655	4.2848E-02	1.28E-01	ENSG00000126001
CEP290	-0.0303	6.0261E-01	7.63E-01	ENSG00000198707
CEP295	0.0299	6.6233E-01	8.06E-01	ENSG00000166004
CEP295NL	-0.0158	8.5802E-01	9.27E-01	ENSG00000178404
CEP350	0.2421	2.4913E-04	2.34E-03	ENSG00000135837
CEP41	-0.1022	1.5917E-01	3.24E-01	ENSG00000106477
CEP43	-0.0072	8.9344E-01	9.47E-01	ENSG00000213066
CEP44	0.3906	3.1119E-06	6.80E-05	ENSG00000164118
CEP57	0.0564	3.2848E-01	5.23E-01	ENSG00000166037
CEP57L1	0.0747	1.8289E-01	3.55E-01	ENSG00000183137
CEP63	0.0754	3.4057E-01	5.36E-01	ENSG00000182923
CEP68	0.2751	3.5694E-06	7.65E-05	ENSG00000011523
CEP70	-0.2354	2.0949E-04	2.03E-03	ENSG00000114107
CEP72	0.0523	5.8480E-01	7.50E-01	ENSG00000112877
CEP76	0.0875	2.7875E-01	4.70E-01	ENSG00000101624
CEP78	-0.2372	4.3236E-03	2.24E-02	ENSG00000148019
CEP83	-0.1680	1.4438E-02	5.64E-02	ENSG00000173588
CEP83-DT	0.5564	4.3164E-04	3.66E-03	ENSG00000278916
CEP85	0.5257	1.1886E-06	3.08E-05	ENSG00000130695
CEP85L	-0.0004	9.7146E-01	9.85E-01	ENSG00000111860
CEP89	-0.1186	2.7206E-02	9.06E-02	ENSG00000121289
CEP95	0.1175	1.0601E-01	2.45E-01	ENSG00000258890
CEP97	-0.2627	3.7945E-04	3.31E-03	ENSG00000182504
CEPT1	0.2323	1.4603E-02	5.69E-02	ENSG00000134255
CER1	-0.1648	1.1893E-01	2.65E-01	ENSG00000147869
CERCAM	0.0047	9.7514E-01	9.87E-01	ENSG00000167123
CERK	0.2983	1.3076E-04	1.38E-03	ENSG00000100422
CERKL	0.0556	5.5905E-01	7.29E-01	ENSG00000188452
CERNA3	-0.0180	7.4961E-01		ENSG00000253603
CEROX1	-0.1714	4.7167E-02	1.37E-01	ENSG00000260807
CERS1	-0.3399	2.2003E-04	2.11E-03	ENSG00000223802
CERS2	-0.1170	1.5976E-01	3.25E-01	ENSG00000143418

CERS3	-0.0299	6.7696E-01	8.15E-01	ENSG00000154227
CERS4	-0.1965	7.7547E-03	3.51E-02	ENSG00000090661
CERS5	-0.0561	2.6496E-01	4.54E-01	ENSG00000139624
CERS6	-0.1175	1.8872E-01	3.63E-01	ENSG00000172292
CERS6-AS1	-0.0040	7.4752E-01		ENSG00000227617
CERT1	-0.1224	4.2017E-02	1.26E-01	ENSG00000113163
CES1	0.0544	3.4776E-01	5.43E-01	ENSG00000198848
CES2	0.0557	4.0745E-01	6.01E-01	ENSG00000172831
CES3	-0.4283	1.1881E-03	8.13E-03	ENSG00000172828
CES4A	0.0478	5.7874E-01	7.45E-01	ENSG00000172824
CES5A	-0.0041	4.4038E-01		ENSG00000159398
CETN2	-0.2957	1.1144E-04	1.22E-03	ENSG00000147400
CETN3	-0.1495	5.3191E-02	1.50E-01	ENSG00000153140
CETP	0.0024	8.0513E-01		ENSG00000087237
CFAP119	0.2236	3.5577E-02	1.11E-01	ENSG00000196118
CFAP141	-0.5616	3.7711E-03	2.01E-02	ENSG00000163263
CFAP157	-0.3007	1.6962E-02	6.36E-02	ENSG00000160401
CFAP20	-0.1265	1.7042E-02	6.38E-02	ENSG00000070761
CFAP206	0.2348	6.0647E-02	1.65E-01	ENSG00000272514
CFAP20DC	0.0844	3.3913E-01	5.34E-01	ENSG00000163689
CFAP20DC-AS1	-0.0662	2.7248E-01		ENSG00000242428
CFAP210	-0.2105	2.5916E-02	8.75E-02	ENSG00000154479
CFAP221	0.0119	8.9755E-01	9.49E-01	ENSG00000163075
CFAP251	-0.0070	9.3385E-01	9.67E-01	ENSG00000158023
CFAP298	-0.1521	4.2548E-02	1.27E-01	ENSG00000159079
CFAP298-TCP10L	-0.1166	2.5340E-01	4.41E-01	ENSG00000265590
CFAP299	-0.2473	4.2984E-02	1.28E-01	ENSG00000197826
CFAP300	-0.2846	9.1149E-03	3.97E-02	ENSG00000137691
CFAP36	-0.0659	1.2065E-01	2.68E-01	ENSG00000163001
CFAP410	0.2482	8.0357E-03	3.61E-02	ENSG00000160226
CFAP418	-0.3561	4.2560E-04	3.62E-03	ENSG00000156172
CFAP418-AS1	-0.0136	8.7624E-01	9.37E-01	ENSG00000253773
CFAP43	-0.5368	1.7727E-04	1.79E-03	ENSG00000197748
CFAP44	-0.1439	1.7238E-01	3.41E-01	ENSG00000206530
CFAP44-AS1	-0.0359	3.3077E-01		ENSG00000243849
CFAP45	-0.5241	3.1660E-05	4.42E-04	ENSG00000213085
CFAP46	-0.3103	4.7985E-04	3.96E-03	ENSG00000171811
CFAP47	-0.1794	1.0917E-01	2.50E-01	ENSG00000165164
CFAP57	-0.1062	2.9533E-01	4.87E-01	ENSG00000243710
CFAP58	0.1048	2.7129E-01	4.61E-01	ENSG00000120051
CFAP58-DT	0.5268	1.8032E-04	1.81E-03	ENSG00000231233
CFAP61	-0.0887	3.6806E-01	5.64E-01	ENSG00000089101
CFAP65	-0.1011	2.2403E-01	4.06E-01	ENSG00000181378
CFAP68	-0.0546	4.2139E-01	6.14E-01	ENSG00000137720
CFAP69	0.1456	1.2259E-01	2.71E-01	ENSG00000105792
CFAP70	-0.1703	6.9962E-02	1.82E-01	ENSG00000156042
CFAP73	-0.2183	7.3361E-02	1.89E-01	ENSG00000186710
CFAP74	-0.2504	1.5969E-02	6.08E-02	ENSG00000142609
CFAP90	-0.5168	8.5517E-06	1.55E-04	ENSG00000215217

CFAP91	-0.0574	5.0578E-01	6.87E-01	ENSG00000183833
CFAP92	-0.0899	2.5536E-01	4.43E-01	ENSG00000114656
CFAP95	-0.1140	2.5397E-01	4.41E-01	ENSG00000204711
CFAP95-DT	-0.1834	7.0830E-02	1.84E-01	ENSG00000225626
CFAP96	-0.0285	7.0312E-01	8.34E-01	ENSG00000205129
CFAP97	-0.1440	2.0167E-02	7.25E-02	ENSG00000164323
CFAP97D1	0.0659	8.6744E-02	2.13E-01	ENSG00000231256
CFAP97D2	-0.1321	1.9048E-01	3.65E-01	ENSG00000283361
CFAP99	-0.0937	3.3144E-01	5.26E-01	ENSG00000206113
CFB	0.3089	1.4383E-02	5.62E-02	ENSG00000243649
CFC1	0.0342	7.1360E-01	8.40E-01	ENSG00000136698
CFC1B	0.0378	6.8515E-01	8.22E-01	ENSG00000152093
CFD	-0.1388	1.8006E-01	3.51E-01	ENSG00000197766
CFDP1	-0.2530	1.7273E-03	1.09E-02	ENSG00000153774
CFHR1	-0.0651	4.9227E-01	6.76E-01	ENSG00000244414
CFHR3	0.0714	1.4294E-01		ENSG00000116785
CFHR4	0.0005	7.3627E-01		ENSG00000134365
CFI	0.0805	3.2234E-01	5.16E-01	ENSG00000205403
CFL1	-0.0925	1.0184E-01	2.38E-01	ENSG00000172757
CFL2	-0.1303	8.3263E-02	2.06E-01	ENSG00000165410
CFLAR	-0.4364	2.7627E-05	3.97E-04	ENSG00000003402
CFLAR-AS1	0.0030	7.8934E-01		ENSG00000226312
CFP	0.0414	5.5507E-01	7.26E-01	ENSG00000126759
CFTR	0.1300	2.1408E-01	3.94E-01	ENSG00000001626
CGA	0.0941	3.1830E-01	5.12E-01	ENSG00000135346
CGAS	-0.0603	3.5109E-01	5.46E-01	ENSG00000164430
CGB1	0.0394	7.4317E-01		ENSG00000267631
CGB2	0.0278	9.6375E-01		ENSG00000104818
CGB5	0.0139	8.2918E-01		ENSG00000189052
CGB7	-0.0234	7.4882E-01	8.63E-01	ENSG00000196337
CGB8	-0.0090	7.6739E-01		ENSG00000213030
CGGBP1	-0.1235	2.1249E-02	7.54E-02	ENSG00000163320
CGN	0.2145	3.0187E-02	9.79E-02	ENSG00000143375
CGNL1	-0.0080	9.0889E-01	9.54E-01	ENSG00000128849
CGREF1	0.1264	1.2124E-01	2.69E-01	ENSG00000138028
CGRRF1	-0.1273	2.8125E-02	9.28E-02	ENSG00000100532
CH25H	0.1521	1.0671E-01	2.46E-01	ENSG00000138135
CHAC2	-0.0389	6.4483E-01	7.94E-01	ENSG00000143942
CHAD	-0.0002	7.1279E-01		ENSG00000136457
CHADL	-0.0872	3.1685E-01	5.10E-01	ENSG00000100399
CHAER1	-0.0051	8.2825E-01		ENSG00000288944
CHAF1A	-0.0879	2.5826E-01	4.46E-01	ENSG00000167670
CHAF1B	0.0387	5.9052E-01	7.54E-01	ENSG00000159259
CHAMP1	-0.3104	1.1508E-05	1.96E-04	ENSG00000198824
CHAT	0.1015	1.0051E-01	2.36E-01	ENSG00000070748
CHCHD1	-0.2290	3.6207E-04	3.19E-03	ENSG00000172586
CHCHD10	-0.0631	5.1216E-01	6.92E-01	ENSG00000250479
CHCHD2	-0.0335	7.0076E-01	8.33E-01	ENSG00000106153
CHCHD3	-0.0658	2.5585E-01	4.43E-01	ENSG00000106554

CHCHD4	0.1798	1.7046E-02	6.38E-02	ENSG00000163528
CHCHD5	-0.1654	1.7422E-02	6.49E-02	ENSG00000125611
CHCHD6	0.1864	1.1408E-02	4.70E-02	ENSG00000159685
CHCHD7	0.2134	1.3329E-04	1.41E-03	ENSG00000170791
CHCT1	0.0626	1.7912E-01		ENSG00000141371
CHD1	-0.0580	4.1720E-01	6.10E-01	ENSG00000153922
CHD1L	0.3460	8.6591E-03	3.82E-02	ENSG00000131778
CHD2	-0.0318	5.5226E-01	7.24E-01	ENSG00000173575
CHD3	-0.3198	2.8436E-03	1.62E-02	ENSG00000170004
CHD4	-0.0750	4.2206E-01	6.15E-01	ENSG00000111642
CHD5	-0.0507	5.4328E-01	7.15E-01	ENSG00000116254
CHD6	-0.2981	1.6712E-04	1.70E-03	ENSG00000124177
CHD7	0.0259	6.4187E-01	7.92E-01	ENSG00000171316
CHD8	0.0794	2.6915E-01	4.58E-01	ENSG00000100888
CHD9	-0.0788	9.0902E-02	2.20E-01	ENSG00000177200
CHD9NB	-0.2221	6.3697E-02	1.70E-01	ENSG00000277639
CHDH	0.0598	5.3540E-01	7.09E-01	ENSG00000016391
CHEK1	-0.3152	4.9321E-03	2.47E-02	ENSG00000149554
CHEK2	-0.4695	2.2459E-05	3.36E-04	ENSG00000183765
CHERP	0.1198	1.1111E-01	2.53E-01	ENSG00000085872
CHFR	0.3443	4.4710E-06	9.20E-05	ENSG00000072609
CHFR-DT	-0.0633	5.1437E-01	6.93E-01	ENSG00000236617
CHGA	0.1754	1.2156E-01	2.69E-01	ENSG00000100604
CHGB	-0.1029	2.5183E-01	4.39E-01	ENSG00000089199
CHI3L1	-0.0718	1.0142E-01	2.37E-01	ENSG00000133048
CHI3L2	-0.1497	5.5255E-03		ENSG00000064886
CHIC1	0.1065	2.5973E-01	4.48E-01	ENSG00000204116
CHIC2	-0.2726	1.0798E-05	1.87E-04	ENSG00000109220
CHID1	-0.2309	6.9839E-03	3.23E-02	ENSG00000177830
CHIT1	0.0778	3.0035E-01	4.93E-01	ENSG00000133063
CHKA	-0.0404	5.0925E-01	6.89E-01	ENSG00000110721
CHKB	0.3646	1.3823E-02	5.45E-02	ENSG00000100288
CHKB-CPT1B	0.0219	6.8402E-01		ENSG00000254413
CHKB-DT	0.4382	1.1839E-05	2.01E-04	ENSG00000205559
CHL1	-0.0868	4.7540E-01	6.62E-01	ENSG00000134121
CHL1-AS1	0.0421	1.1547E-01	2.60E-01	ENSG00000234661
CHL1-AS2	0.0690	1.9584E-01	3.72E-01	ENSG00000224318
CHM	-0.2329	4.8532E-03	2.44E-02	ENSG00000188419
CHML	-0.0871	3.4254E-01	5.38E-01	ENSG00000203668
CHMP1A	0.0255	6.5493E-01	8.01E-01	ENSG00000131165
CHMP1B	-0.1101	8.4345E-02	2.08E-01	ENSG00000255112
CHMP1B2P	-0.2779	4.1487E-02	1.25E-01	ENSG00000278530
CHMP2A	0.0136	8.0390E-01	8.96E-01	ENSG00000130724
CHMP2B	-0.1111	3.8885E-02	1.19E-01	ENSG00000083937
CHMP3	0.0004	9.6215E-01	9.81E-01	ENSG00000115561
CHMP3-AS1	0.3841	5.7234E-03	2.78E-02	ENSG00000228363
CHMP4A	0.2825	2.2061E-02	7.75E-02	ENSG00000254505
CHMP4B	-0.3336	5.2618E-05	6.70E-04	ENSG00000101421
CHMP4C	-0.0333	7.0310E-01	8.34E-01	ENSG00000164695

CHMP5	-0.0310	5.1316E-01	6.93E-01	ENSG00000086065
CHMP6	-0.0164	7.9622E-01	8.91E-01	ENSG00000176108
CHMP7	0.4067	2.3637E-05	3.51E-04	ENSG00000147457
CHN1	-0.1301	1.1915E-01	2.66E-01	ENSG00000128656
CHN2	0.4543	2.6283E-06	5.99E-05	ENSG00000106069
CHODL	0.4877	2.5827E-05	3.76E-04	ENSG00000154645
CHORDC1	-0.2014	2.8668E-02	9.42E-02	ENSG00000110172
CHP1	-0.3663	6.3123E-09	3.93E-07	ENSG00000187446
CHP1P3	0.0058	9.5595E-01	9.77E-01	ENSG00000226153
CHPF	0.2389	1.1753E-03	8.07E-03	ENSG00000123989
CHPF2	0.1257	1.1570E-01	2.60E-01	ENSG00000033100
CHPT1	-0.0307	6.2198E-01	7.78E-01	ENSG00000111666
CHRAC1	-0.1377	2.9067E-02	9.53E-02	ENSG00000104472
CHRD	0.2831	1.9659E-03	1.21E-02	ENSG00000090539
CHRDL1	-0.0155	8.6550E-01	9.31E-01	ENSG00000101938
CHRDL2	-0.1554	1.4998E-01	3.10E-01	ENSG00000054938
CHRFAM7A	-0.1747	6.1446E-02	1.66E-01	ENSG00000166664
CHRM1	-0.0053	6.8373E-01		ENSG00000168539
CHRM2	0.0152	8.7074E-01	9.34E-01	ENSG00000181072
CHRM3	-0.3918	3.2648E-04	2.93E-03	ENSG00000133019
CHRM3-AS2	-0.0323	7.0430E-01	8.34E-01	ENSG00000233355
CHRM4	-0.0961	2.7706E-01	4.68E-01	ENSG00000180720
CHRM5	-0.3467	5.2102E-04	4.25E-03	ENSG00000184984
CHRNA1	-0.0585	5.4459E-01	7.17E-01	ENSG00000138435
CHRNA10	-0.0237	7.3721E-01	8.56E-01	ENSG00000129749
CHRNA2	0.1154	1.7565E-01	3.46E-01	ENSG00000120903
CHRNA3	0.0441	6.0771E-01	7.67E-01	ENSG00000080644
CHRNA4	-0.1111	2.3250E-01	4.16E-01	ENSG00000101204
CHRNA6	0.0271	7.5938E-01	8.69E-01	ENSG00000147434
CHRNA7	-0.2211	1.1824E-02	4.82E-02	ENSG00000175344
CHRNA9	0.0263	6.6483E-01	8.07E-01	ENSG00000174343
CHRNB1	-0.1850	2.3138E-02	8.04E-02	ENSG00000170175
CHRNB2	-0.0377	6.6561E-01	8.08E-01	ENSG00000160716
CHRNB4	0.5131	8.7640E-05	1.01E-03	ENSG00000117971
CHRND	0.3644	2.1085E-02	7.49E-02	ENSG00000135902
CHRNG	0.0272	5.1116E-01	6.91E-01	ENSG00000196811
CHROMR	-0.0172	7.9086E-01	8.88E-01	ENSG00000223960
CHST1	0.2489	9.0124E-03	3.93E-02	ENSG00000175264
CHST10	-0.0802	3.5198E-01	5.47E-01	ENSG00000115526
CHST11	-0.1125	1.4343E-01	3.01E-01	ENSG00000171310
CHST12	-0.2031	4.4466E-03	2.29E-02	ENSG00000136213
CHST13	0.0057	6.6446E-01		ENSG00000180767
CHST15	-0.4963	3.2515E-04	2.92E-03	ENSG00000182022
CHST2	0.3678	3.5684E-05	4.85E-04	ENSG00000175040
CHST3	-0.5798	1.4169E-05	2.33E-04	ENSG00000122863
CHST4	-0.1311	1.3210E-01	2.85E-01	ENSG00000140835
CHST5	-0.1256	1.0210E-01	2.38E-01	ENSG00000135702
CHST7	-0.1239	1.6054E-01	3.26E-01	ENSG00000147119
CHST8	0.3103	1.9469E-02	7.05E-02	ENSG00000124302

CHST9	-0.0227	8.0503E-01	8.96E-01	ENSG00000154080
CHSY1	-0.3738	1.4290E-04	1.49E-03	ENSG00000131873
CHSY3	0.0285	6.8597E-01	8.23E-01	ENSG00000198108
CHTF18	0.4628	2.6619E-05	3.84E-04	ENSG00000127586
CHTF8	0.0560	2.6871E-01	4.58E-01	ENSG00000168802
CHTOP	-0.0378	4.2958E-01	6.21E-01	ENSG00000160679
CHUK	0.2989	4.7655E-04	3.94E-03	ENSG00000213341
CHURC1	-0.1555	2.0427E-02	7.32E-02	ENSG00000258289
CHURC1-FNTB	0.0012	7.2808E-01		ENSG00000125954
CIAO1	0.1385	1.5413E-02	5.92E-02	ENSG00000144021
CIAO2A	-0.1016	9.8088E-02	2.32E-01	ENSG00000166797
CIAO2B	-0.2205	7.2274E-05	8.64E-04	ENSG00000166595
CIAO3	0.2145	4.9374E-03	2.48E-02	ENSG00000103245
CIAPIN1	0.0727	2.4318E-01	4.28E-01	ENSG00000005194
CIB1	-0.0612	1.4537E-01	3.04E-01	ENSG00000185043
CIB2	-0.1861	9.8439E-03	4.20E-02	ENSG00000136425
CIB3	-0.0059	9.2185E-01		ENSG00000141977
CIB4	-0.0601	4.9063E-01	6.75E-01	ENSG00000157884
CIBAR1	-0.0191	7.3074E-01	8.52E-01	ENSG00000188343
CIBAR1-DT	0.4350	8.4296E-05	9.76E-04	ENSG00000246662
CIBAR2	-0.0848	3.8018E-01	5.75E-01	ENSG00000153789
CIC	-0.0976	2.0387E-01	3.82E-01	ENSG00000079432
CICP27	0.0455	6.1607E-01	7.74E-01	ENSG00000233750
CIDEC	0.0020	9.3661E-01		ENSG00000187288
CIDECP1	-0.2461	3.4421E-05	4.71E-04	ENSG00000186162
CIITA	0.2582	5.0562E-02	1.44E-01	ENSG00000179583
CILK1	0.3314	2.1076E-03	1.28E-02	ENSG00000112144
CILP	-0.4974	7.0098E-03	3.24E-02	ENSG00000138615
CILP2	0.2029	5.1551E-02	1.46E-01	ENSG00000160161
CIMAP1A	0.0212	1.9180E-01		ENSG00000177947
CIMAP1B	-0.1618	1.3137E-01	2.84E-01	ENSG00000177989
CIMAP1C	-0.1409	1.8660E-01	3.61E-01	ENSG00000182950
CIMAP1D	0.0202	4.5717E-01		ENSG00000181781
CIMAP2	-0.0309	3.2499E-01		ENSG00000162398
CIMAP3	-0.3181	1.6153E-04	1.65E-03	ENSG00000173947
CIMIP1	-0.0579	5.4493E-01	7.17E-01	ENSG00000124237
CIMIP2A	0.4088	1.6622E-02	6.27E-02	ENSG00000188163
CIMIP2B	0.0266	3.1396E-01		ENSG00000215187
CIMIP2C	-0.1598	7.9754E-02	2.00E-01	ENSG00000173557
CINP	-0.0475	4.3331E-01	6.25E-01	ENSG00000100865
CIP2A	-0.0419	6.5596E-01	8.01E-01	ENSG00000163507
CIPC	-0.0061	9.2779E-01	9.64E-01	ENSG00000198894
CIR1	-0.1072	5.2079E-02	1.47E-01	ENSG00000138433
CIRBP	0.0346	4.5505E-01	6.45E-01	ENSG00000099622
CIRBP-AS1	0.3580	6.2236E-03	2.97E-02	ENSG00000267493
CIROP	-0.0281	3.5535E-01		ENSG00000283654
CISD1	-0.1628	1.0686E-02	4.46E-02	ENSG00000122873
CISD2	-0.1934	1.3364E-03	8.96E-03	ENSG00000145354
CISD3	-0.1693	2.7598E-02	9.15E-02	ENSG00000277972

CISH	0.0992	2.4104E-01	4.26E-01	ENSG00000114737
CIST1	-0.0421	3.7796E-01		ENSG00000284797
CIT	0.3080	7.5231E-04	5.69E-03	ENSG00000122966
CITED1	-0.0377	7.1373E-01	8.40E-01	ENSG00000125931
CITED2	0.0746	3.6929E-01	5.65E-01	ENSG00000164442
CITED4	-0.2396	6.2128E-04	4.90E-03	ENSG00000179862
CIZ1	0.1331	1.0533E-01	2.44E-01	ENSG00000148337
CKAP2	-0.2369	1.8623E-04	1.86E-03	ENSG00000136108
CKAP2L	-0.1212	2.2980E-01	4.13E-01	ENSG00000169607
CKAP2LP1	0.0184	5.5271E-01		ENSG00000271461
CKAP4	-0.1195	6.2993E-02	1.69E-01	ENSG00000136026
CKAP5	-0.1425	4.1792E-03	2.19E-02	ENSG00000175216
CKB	0.0226	6.9758E-01	8.31E-01	ENSG00000166165
CKLF-CMTM1	-0.1067	3.2813E-01	5.23E-01	ENSG00000254788
CKM	0.1799	1.0675E-01	2.46E-01	ENSG00000104879
CKMT1A	-0.1316	1.1440E-01	2.58E-01	ENSG00000223572
CKMT1B	-0.1355	8.6588E-02	2.12E-01	ENSG00000237289
CKMT2	-0.0407	6.5080E-01	7.98E-01	ENSG00000131730
CKMT2-AS1	0.3698	3.1897E-06	6.94E-05	ENSG00000247572
CKS1B	-0.1486	7.3487E-02	1.89E-01	ENSG00000173207
CKS2	-0.5443	1.3483E-06	3.44E-05	ENSG00000123975
CLASP1	0.0320	6.5713E-01	8.02E-01	ENSG00000074054
CLASP2	0.0781	3.2261E-01	5.16E-01	ENSG00000163539
CLASRP	0.2371	5.5748E-03	2.73E-02	ENSG00000104859
CLBA1	-0.0036	9.3576E-01	9.68E-01	ENSG00000140104
CLC	-0.0522	3.8183E-01	5.77E-01	ENSG00000105205
CLCA1	2.0599	1.3329E-03		ENSG00000016490
CLCA2	0.0009	8.8982E-01		ENSG00000137975
CLCA4-AS1	0.1050	2.2633E-01	4.09E-01	ENSG00000236915
CLCC1	0.0522	5.0089E-01	6.83E-01	ENSG00000121940
CLCF1	-0.5372	7.0246E-05	8.44E-04	ENSG00000175505
CLCN1	-0.0726	2.6451E-01	4.53E-01	ENSG00000188037
CLCN2	0.2168	4.0533E-02	1.22E-01	ENSG00000114859
CLCN3	-0.1496	1.6678E-02	6.28E-02	ENSG00000109572
CLCN3P1	-0.2210	6.1713E-03	2.95E-02	ENSG00000232000
CLCN4	0.1197	1.1760E-01	2.63E-01	ENSG00000073464
CLCN5	-0.2004	9.3800E-04	6.78E-03	ENSG00000171365
CLCN6	0.1579	1.0728E-01	2.47E-01	ENSG00000011021
CLCN7	0.1055	2.1429E-01	3.95E-01	ENSG00000103249
CLCNKA	-0.0280	7.6577E-01	8.73E-01	ENSG00000186510
CLCNKB	0.2028	8.4491E-02	2.09E-01	ENSG00000184908
CLDN1	-0.0153	8.6937E-01	9.34E-01	ENSG00000163347
CLDN10	0.0565	5.4760E-01	7.19E-01	ENSG00000134873
CLDN11	0.2004	3.7754E-02	1.16E-01	ENSG00000013297
CLDN12	-0.0158	8.0098E-01	8.94E-01	ENSG00000157224
CLDN14	-0.0005	6.0271E-01		ENSG00000159261
CLDN15	0.0108	9.0609E-01	9.53E-01	ENSG00000106404
CLDN16	0.0073	7.8548E-01		ENSG00000113946
CLDN19	0.0590	4.5145E-01	6.41E-01	ENSG00000164007

CLDN2	-0.0470	3.3645E-01	5.32E-01	ENSG00000165376
CLDN20	-0.0613	1.4183E-01		ENSG00000171217
CLDN23	0.2355	5.7049E-02	1.58E-01	ENSG00000253958
CLDN25	-0.0034	9.3267E-01		ENSG00000228607
CLDN3	0.1355	1.5538E-01	3.18E-01	ENSG00000165215
CLDN34	-0.0608	3.8451E-01		ENSG00000234469
CLDN4	-0.2200	4.6114E-02	1.35E-01	ENSG00000189143
CLDN5	0.4019	4.0487E-04	3.48E-03	ENSG00000184113
CLDN6	-0.0850	3.8785E-01	5.83E-01	ENSG00000184697
CLDN7	0.1622	9.5533E-02	2.28E-01	ENSG00000181885
CLDN9	-0.5440	1.3008E-05	2.16E-04	ENSG00000213937
CLDND1	-0.0708	2.0017E-01	3.77E-01	ENSG00000080822
CLDND2	-0.2445	2.6217E-02	8.81E-02	ENSG00000160318
CLEC11A	0.0656	2.7210E-01	4.62E-01	ENSG00000105472
CLEC14A	0.0170	2.7639E-01		ENSG00000176435
CLEC16A	0.1722	2.8987E-03	1.64E-02	ENSG00000038532
CLEC17A	-0.0079	6.8967E-01		ENSG00000187912
CLEC18A	-0.2136	7.4630E-03	3.40E-02	ENSG00000157322
CLEC18B	-0.2122	6.3815E-03	3.03E-02	ENSG00000140839
CLEC18C	-0.1809	3.9843E-02	1.21E-01	ENSG00000157335
CLEC1A	0.0583	2.4302E-01		ENSG00000150048
CLEC20A	-0.0006	8.3499E-01		ENSG00000188585
CLEC2A	-0.0833	7.9756E-02		ENSG00000188393
CLEC2B	-0.0636	2.5647E-01		ENSG00000110852
CLEC2D	-0.1186	2.4457E-01	4.30E-01	ENSG00000069493
CLEC2L	0.0663	5.0606E-01	6.87E-01	ENSG00000236279
CLEC3B	-0.2235	7.3257E-02	1.88E-01	ENSG00000163815
CLEC4D	-0.0400	4.6892E-01		ENSG00000166527
CLEC4E	0.0025	6.9075E-01		ENSG00000166523
CLEC7A	-0.0186	8.6409E-01		ENSG00000172243
CLGN	0.2674	6.6378E-04	5.17E-03	ENSG00000153132
CLHC1	-0.2190	7.0983E-03	3.27E-02	ENSG00000162994
CLIC1	-0.1235	1.5324E-02	5.89E-02	ENSG00000213719
CLIC2	0.4048	1.3216E-02	5.26E-02	ENSG00000155962
CLIC3	-0.3400	2.5729E-02	8.70E-02	ENSG00000169583
CLIC4	-0.1980	5.5996E-04	4.51E-03	ENSG00000169504
CLIC5	0.4437	8.7510E-03	3.85E-02	ENSG00000112782
CLINT1	-0.0262	5.9244E-01	7.55E-01	ENSG00000113282
CLIP1	0.0639	1.8668E-01	3.61E-01	ENSG00000130779
CLIP2	-0.2596	1.7386E-03	1.10E-02	ENSG00000106665
CLIP3	-0.2398	1.5362E-04	1.59E-03	ENSG00000105270
CLIP4	0.1398	1.4009E-01	2.96E-01	ENSG00000115295
CLK1	-0.2670	2.5459E-03	1.48E-02	ENSG00000013441
CLK2	0.1153	9.1960E-02	2.22E-01	ENSG00000176444
CLK3	-0.1551	3.4156E-03	1.86E-02	ENSG00000179335
CLK4	-0.0439	5.9906E-01	7.60E-01	ENSG00000113240
CLMAT3	0.0310	7.3710E-01	8.56E-01	ENSG00000249035
CLMN	-0.2543	1.0163E-02	4.30E-02	ENSG00000165959
CLMP	0.0459	6.2997E-01	7.84E-01	ENSG00000166250

CLN3	-0.0338	6.5970E-01	8.04E-01	ENSG00000188603
CLN5	-0.2710	9.0879E-04	6.61E-03	ENSG00000102805
CLN6	0.1384	1.7902E-01	3.50E-01	ENSG00000128973
CLN8	0.1195	4.1347E-02	1.24E-01	ENSG00000182372
CLN8-AS1	-0.1289	6.4504E-02	1.72E-01	ENSG00000253982
CLNK	0.0511	4.2990E-01	6.22E-01	ENSG00000109684
CLNS1A	-0.2256	1.1878E-03	8.13E-03	ENSG00000074201
CLNS1AP1	-0.0051	3.2617E-01		ENSG00000213335
CLOCK	-0.0366	6.5200E-01	7.99E-01	ENSG00000134852
CLP1	0.2336	1.3781E-03	9.18E-03	ENSG00000172409
CLPB	-0.1204	9.1195E-02	2.20E-01	ENSG00000162129
CLPP	-0.0142	7.1327E-01	8.40E-01	ENSG00000125656
CLPS	0.0212	4.5060E-01		ENSG00000137392
CLPSL1	0.0080	5.9300E-01		ENSG00000204140
CLPSL2	-0.1073	1.8256E-01	3.55E-01	ENSG00000196748
CLPTM1	0.0398	5.8757E-01	7.52E-01	ENSG00000104853
CLPTM1L	-0.1493	6.4565E-03	3.05E-02	ENSG00000049656
CLPX	-0.0868	9.6815E-02	2.30E-01	ENSG00000166855
CLRN2	-0.0014	7.9996E-01		ENSG00000249581
CLRN3	0.0580	2.2844E-01		ENSG00000180745
CLSPN	-0.3732	1.2030E-04	1.30E-03	ENSG00000092853
CLSTN1	-0.3547	1.4054E-09	1.13E-07	ENSG00000171603
CLSTN2	-0.2212	2.5544E-02	8.66E-02	ENSG00000158258
CLSTN2-AS1	-0.0039	4.3896E-01		ENSG00000250433
CLSTN3	0.2183	2.4845E-02	8.48E-02	ENSG00000139182
CLTA	-0.2633	7.9781E-05	9.31E-04	ENSG00000122705
CLTB	-0.0092	8.6054E-01	9.29E-01	ENSG00000175416
CLTC	-0.0944	8.1574E-02	2.04E-01	ENSG00000141367
CLTCL1	0.0930	2.9366E-01	4.86E-01	ENSG00000070371
CLTRN	-0.0686	4.3255E-01	6.24E-01	ENSG00000147003
CLU	-0.4281	9.9344E-05	1.11E-03	ENSG00000120885
CLUAP1	-0.0926	1.6236E-01	3.28E-01	ENSG00000103351
CLUH	0.0564	4.5433E-01	6.44E-01	ENSG00000132361
CLUHP6	-0.0113	7.7350E-01		ENSG00000265279
CLUL1	-0.1958	7.4967E-02	1.91E-01	ENSG00000079101
CLVS1	-0.0734	4.4614E-01	6.36E-01	ENSG00000177182
CLVS2	0.2920	2.8875E-02	9.48E-02	ENSG00000146352
CLXN	-0.4397	1.4443E-03	9.53E-03	ENSG00000034239
CLYBL	0.0729	3.1292E-01	5.06E-01	ENSG00000125246
CMA1	0.0043	5.5305E-01		ENSG00000092009
CMAS	0.0341	6.2570E-01	7.81E-01	ENSG00000111726
CMBL	-0.5163	1.5980E-05	2.56E-04	ENSG00000164237
CMC1	-0.2312	3.0660E-03	1.71E-02	ENSG00000187118
CMC2	-0.2441	5.7702E-05	7.21E-04	ENSG00000103121
CMC4	-0.4428	5.6979E-03	2.77E-02	ENSG00000182712
CMIP	-0.1959	3.7542E-02	1.15E-01	ENSG00000153815
CMKLR1	0.0368	6.3013E-01	7.84E-01	ENSG00000174600
CMPK1	-0.0454	4.4915E-01	6.39E-01	ENSG00000162368
CMPK2	-0.1318	2.0463E-01	3.83E-01	ENSG00000134326

CMSS1	0.0488	3.6092E-01	5.57E-01	ENSG00000184220
CMTM1	-0.1165	2.5411E-01	4.41E-01	ENSG00000089505
CMTM2	-0.0041	9.7331E-01	9.86E-01	ENSG00000140932
CMTM3	-0.2269	1.0194E-02	4.31E-02	ENSG00000140931
CMTM4	-0.2052	3.2929E-02	1.05E-01	ENSG00000183723
CMTM5	-0.0915	2.7639E-01	4.67E-01	ENSG00000166091
CMTM6	-0.1935	5.7644E-03	2.79E-02	ENSG00000091317
CMTM7	-0.0312	7.3176E-01	8.53E-01	ENSG00000153551
CMTM8	-0.1322	1.2701E-01	2.78E-01	ENSG00000170293
CMTR1	0.1239	8.6256E-02	2.12E-01	ENSG00000137200
CMTR2	0.2074	2.0946E-03	1.27E-02	ENSG00000180917
CMYA5	0.2307	1.3896E-02	5.47E-02	ENSG00000164309
CNBD1	-0.0264	7.1818E-01	8.44E-01	ENSG00000176571
CNBP	-0.0411	4.5363E-01	6.43E-01	ENSG00000169714
CNDP1	-0.0279	1.0047E-01	2.35E-01	ENSG00000150656
CNDP2	0.0460	4.8871E-01	6.73E-01	ENSG00000133313
CNEP1R1	0.3528	7.0483E-07	1.99E-05	ENSG00000205423
CNFN	-0.1813	1.0219E-01	2.38E-01	ENSG00000105427
CNGA1	0.0949	3.4718E-01	5.42E-01	ENSG00000198515
CNGA3	0.2753	2.1650E-03	1.31E-02	ENSG00000144191
CNGA4	-0.0456	6.2467E-01	7.80E-01	ENSG00000132259
CNGB1	-0.0394	6.7005E-01	8.10E-01	ENSG00000070729
CNGB3	0.0833	3.4710E-01	5.42E-01	ENSG00000170289
CNIH1	-0.1757	2.4692E-03	1.45E-02	ENSG00000100528
CNIH2	0.0132	8.8009E-01	9.39E-01	ENSG00000174871
CNIH3	0.1126	2.0759E-01	3.87E-01	ENSG00000143786
CNIH3-AS2	0.0941	1.7215E-01	3.41E-01	ENSG00000233384
CNIH4	-0.0295	6.6757E-01	8.09E-01	ENSG00000143771
CNKSR1	0.2755	1.3158E-02	5.24E-02	ENSG00000142675
CNKSR2	0.2990	1.2838E-02	5.14E-02	ENSG00000149970
CNKSR3	-0.1665	9.5800E-02	2.28E-01	ENSG00000153721
CNN1	0.1046	2.6824E-01	4.57E-01	ENSG00000130176
CNN2	0.1945	1.8911E-02	6.90E-02	ENSG00000064666
CNN3	-0.4546	9.7701E-06	1.72E-04	ENSG00000117519
CNNM1	0.1990	4.9887E-02	1.43E-01	ENSG00000119946
CNNM2	-0.1998	8.7567E-03	3.85E-02	ENSG00000148842
CNNM3	-0.0329	5.5808E-01	7.28E-01	ENSG00000168763
CNNM3-DT	0.0856	2.8040E-01	4.71E-01	ENSG00000273265
CNNM4	0.2982	6.4179E-04	5.02E-03	ENSG00000158158
CNOT1	-0.1282	1.9932E-02	7.18E-02	ENSG00000125107
CNOT10	0.1205	1.3262E-01	2.85E-01	ENSG00000182973
CNOT11	0.0846	1.4925E-01	3.10E-01	ENSG00000158435
CNOT2	-0.0562	2.7714E-01	4.68E-01	ENSG00000111596
CNOT3	-0.0369	6.5956E-01	8.04E-01	ENSG00000088038
CNOT4	0.0483	4.5993E-01	6.49E-01	ENSG00000080802
CNOT6	0.0722	3.9028E-01	5.85E-01	ENSG00000113300
CNOT6L	0.1019	2.2155E-01	4.03E-01	ENSG00000138767
CNOT7	-0.1381	2.8698E-03	1.63E-02	ENSG00000198791
CNOT8	0.0428	5.1158E-01	6.92E-01	ENSG00000155508

CNOT9	-0.2414	5.1551E-05	6.59E-04	ENSG00000144580
CNP	-0.1841	2.3438E-03	1.39E-02	ENSG00000173786
CNPPD1	0.1584	9.9083E-03	4.22E-02	ENSG00000115649
CNPY1	-0.0011	9.6735E-01		ENSG00000146910
CNPY2	-0.0544	4.0449E-01	5.98E-01	ENSG00000257727
CNPY3	-0.4703	3.7406E-07	1.17E-05	ENSG00000137161
CNPY4	-0.4067	2.2711E-05	3.39E-04	ENSG00000166997
CNR1	0.2304	4.1009E-02	1.23E-01	ENSG00000118432
CNR2	0.0087	5.5044E-01		ENSG00000188822
CNRIP1	0.0257	7.6970E-01	8.75E-01	ENSG00000119865
CNST	0.1282	8.7629E-02	2.14E-01	ENSG00000162852
CNTD1	0.0877	3.7753E-01	5.73E-01	ENSG00000176563
CNTF	-0.1026	2.4620E-01	4.32E-01	ENSG00000242689
CNTFR	0.0180	8.7996E-01	9.39E-01	ENSG00000122756
CNTFR-AS1	0.0997	7.1483E-02	1.85E-01	ENSG00000237159
CNTLN	-0.1997	7.1114E-03	3.28E-02	ENSG00000044459
CNTN1	-0.1144	1.4893E-01	3.09E-01	ENSG00000018236
CNTN2	-0.3495	2.5922E-03	1.50E-02	ENSG00000184144
CNTN3	0.1486	2.9279E-02	9.58E-02	ENSG00000113805
CNTN4	0.1805	8.8545E-02	2.16E-01	ENSG00000144619
CNTN4-AS2	0.0217	3.3194E-01		ENSG00000227588
CNTN5	0.4252	1.7407E-03	1.10E-02	ENSG00000149972
CNTN6	-0.1205	1.7917E-01	3.50E-01	ENSG00000134115
CNTNAP1	0.1473	1.2392E-01	2.73E-01	ENSG00000108797
CNTNAP2	-0.2328	5.3150E-03	2.62E-02	ENSG00000174469
CNTNAP2-AS1	-0.0062	9.9045E-01		ENSG00000236795
CNTNAP3	0.3348	9.5917E-03	4.12E-02	ENSG00000283378
CNTNAP3	0.0881	3.1356E-01	5.07E-01	ENSG00000106714
CNTNAP3B	0.0470	5.8514E-01	7.50E-01	ENSG00000154529
CNTNAP4	0.0192	8.3942E-01	9.16E-01	ENSG00000152910
CNTNAP5	0.3399	1.1973E-02	4.87E-02	ENSG00000155052
CNTNAP5-DT	0.1782	1.1895E-01	2.65E-01	ENSG00000228400
CNTRL	0.1180	1.0593E-01	2.45E-01	ENSG00000119397
CNTROB	0.2911	4.0045E-03	2.11E-02	ENSG00000170037
COA1	-0.0165	7.6389E-01	8.72E-01	ENSG00000106603
COA3	-0.4236	1.7643E-09	1.35E-07	ENSG00000183978
COA4	-0.4032	6.5491E-07	1.87E-05	ENSG00000181924
COA5	-0.2096	1.2028E-05	2.04E-04	ENSG00000183513
COA6	-0.1010	1.6993E-01	3.38E-01	ENSG00000168275
COA6-AS1	0.0414	6.3086E-01	7.84E-01	ENSG00000231663
COA7	0.0462	4.6882E-01	6.56E-01	ENSG00000162377
COA8	-0.4458	2.1554E-10	2.27E-08	ENSG00000256053
COASY	-0.0019	8.8627E-01	9.43E-01	ENSG00000068120
COBL	-0.1571	6.9731E-02	1.82E-01	ENSG00000106078
COCH	0.2159	3.3250E-02	1.05E-01	ENSG00000100473
COG1	0.4149	9.3879E-06	1.67E-04	ENSG00000166685
COG2	0.2048	3.6295E-03	1.95E-02	ENSG00000135775
COG3	-0.2307	4.1450E-05	5.51E-04	ENSG00000136152
COG4	-0.1022	1.7458E-01	3.44E-01	ENSG00000103051

COG5	0.0430	6.0539E-01	7.65E-01	ENSG00000164597
COG6	0.0604	4.6754E-01	6.55E-01	ENSG00000133103
COG7	0.0608	3.3581E-01	5.31E-01	ENSG00000168434
COG8	0.1033	1.9632E-01	3.73E-01	ENSG00000213380
COIL	-0.1822	7.0209E-03	3.25E-02	ENSG00000121058
COL10A1	0.0154	3.0418E-01		ENSG00000123500
COL11A1	-0.0767	4.3253E-01	6.24E-01	ENSG00000060718
COL11A2	0.3132	7.1355E-03	3.28E-02	ENSG00000204248
COL13A1	0.0877	3.0636E-01	4.99E-01	ENSG00000197467
COL14A1	-0.4569	1.2797E-02	5.13E-02	ENSG00000187955
COL15A1	0.0146	8.5107E-01	9.23E-01	ENSG00000204291
COL16A1	0.0960	3.4029E-01	5.35E-01	ENSG00000084636
COL17A1	0.1229	1.7514E-01	3.45E-01	ENSG00000065618
COL18A1	0.0079	9.5101E-01	9.75E-01	ENSG00000182871
COL18A1-AS1	-0.3320	2.4656E-02	8.44E-02	ENSG00000183535
COL18A1-AS2	-0.0155	8.0263E-01		ENSG00000224574
COL19A1	0.0160	8.3650E-01	9.14E-01	ENSG00000082293
COL1A1	0.1405	1.3124E-01	2.83E-01	ENSG00000108821
COL1A2	0.2288	2.6371E-02	8.85E-02	ENSG00000164692
COL20A1	0.0379	6.6667E-01	8.08E-01	ENSG00000101203
COL21A1	-0.1477	1.1854E-01	2.65E-01	ENSG00000124749
COL23A1	-0.3175	2.7390E-02	9.10E-02	ENSG00000050767
COL24A1	-0.0889	3.3192E-01	5.27E-01	ENSG00000171502
COL25A1	0.0370	6.8520E-01	8.22E-01	ENSG00000188517
COL25A1-DT	-0.0643	3.3588E-01	5.31E-01	ENSG00000246774
COL26A1	-0.2889	6.2942E-05	7.75E-04	ENSG00000160963
COL27A1	-0.2696	3.5911E-02	1.12E-01	ENSG00000196739
COL28A1	0.5222	1.5758E-04	1.62E-03	ENSG00000215018
COL2A1	0.5018	7.2438E-07	2.03E-05	ENSG00000139219
COL3A1	-0.0965	2.8572E-01	4.77E-01	ENSG00000168542
COL4A2	-0.4738	4.0591E-05	5.42E-04	ENSG00000134871
COL4A3	-0.0353	7.1091E-01	8.39E-01	ENSG00000169031
COL4A4	-0.3969	8.2179E-04	6.10E-03	ENSG00000081052
COL4A5	-0.4464	3.8315E-05	5.16E-04	ENSG00000188153
COL4A6	-0.2777	4.4141E-03	2.28E-02	ENSG00000197565
COL5A1	0.0048	9.5836E-01	9.79E-01	ENSG00000130635
COL5A2	-0.1036	2.3011E-01	4.13E-01	ENSG00000204262
COL5A3	-0.1021	8.4122E-02	2.08E-01	ENSG00000080573
COL6A1	0.0619	3.1612E-01	5.10E-01	ENSG00000142156
COL6A2	-0.0383	6.7956E-01	8.18E-01	ENSG00000142173
COL6A3	0.0015	9.7311E-01	9.86E-01	ENSG00000163359
COL6A5	0.0073	5.6273E-01		ENSG00000172752
COL6A6	-0.0649	1.2087E-01		ENSG00000206384
COL7A1	-0.1482	1.4839E-01	3.09E-01	ENSG00000114270
COL8A2	-0.1162	1.5070E-01	3.12E-01	ENSG00000171812
COL9A1	-0.5423	4.6696E-03	2.37E-02	ENSG00000112280
COL9A2	-0.5070	3.7429E-04	3.28E-03	ENSG00000049089
COL9A3	-0.3859	7.0680E-03	3.26E-02	ENSG00000092758
COLCA1	-0.0401	3.8016E-01		ENSG00000196167

COLEC10	0.1856	9.7195E-02	2.30E-01	ENSG00000184374
COLEC11	0.1998	8.5757E-02	2.11E-01	ENSG00000118004
COLGALT1	0.2323	2.8554E-05	4.07E-04	ENSG00000130309
COLGALT2	-0.3149	8.5607E-03	3.79E-02	ENSG00000198756
COLQ	0.0075	9.3811E-01	9.69E-01	ENSG00000206561
COMMD1	-0.1213	7.8067E-03	3.52E-02	ENSG00000173163
COMMD10	-0.2389	5.0925E-03	2.54E-02	ENSG00000145781
COMMD2	0.0801	4.9726E-02	1.43E-01	ENSG00000114744
COMMD3	-0.1947	3.9723E-04	3.42E-03	ENSG00000148444
COMMD4	-0.2376	3.2955E-04	2.95E-03	ENSG00000140365
COMMD5	-0.3093	1.7350E-04	1.75E-03	ENSG00000170619
COMMD6	-0.2742	4.3079E-04	3.66E-03	ENSG00000188243
COMMD7	-0.3601	1.0175E-07	3.97E-06	ENSG00000149600
COMMD8	0.0018	9.8265E-01	9.91E-01	ENSG00000169019
COMMD9	0.0785	2.6345E-01	4.52E-01	ENSG00000110442
COMP	0.0820	2.1800E-01	3.99E-01	ENSG00000105664
COMT	-0.4088	1.2343E-07	4.60E-06	ENSG00000093010
COMTD1	-0.1472	5.1053E-02	1.45E-01	ENSG00000165644
COP1	0.1724	1.9673E-02	7.11E-02	ENSG00000143207
COPA	0.0628	2.5506E-01	4.42E-01	ENSG00000122218
COPB1	0.0023	9.8956E-01	9.94E-01	ENSG00000129083
COPB2	0.1014	1.8964E-01	3.64E-01	ENSG00000184432
COPB2-DT	0.2028	1.4514E-03	9.57E-03	ENSG00000248932
COPE	-0.0134	7.5209E-01	8.65E-01	ENSG00000105669
COPG1	0.0852	2.3561E-01	4.20E-01	ENSG00000181789
COPG2	0.0735	4.0667E-01	6.00E-01	ENSG00000158623
COPRS	-0.2779	1.5754E-07	5.67E-06	ENSG00000172301
COPS2	-0.2315	2.0055E-06	4.74E-05	ENSG00000166200
COPS3	0.0593	3.7151E-01	5.67E-01	ENSG00000141030
COPS4	-0.0032	9.5822E-01	9.79E-01	ENSG00000138663
COPS5	-0.3319	1.7485E-08	9.12E-07	ENSG00000121022
COPS6	-0.1125	1.0421E-02	4.37E-02	ENSG00000168090
COPS7A	0.0476	4.9716E-01	6.80E-01	ENSG00000111652
COPS7B	0.1918	3.0757E-03	1.71E-02	ENSG00000144524
COPS8	-0.0584	3.7778E-01	5.73E-01	ENSG00000198612
COPS8-DT	0.5940	3.0918E-07	1.01E-05	ENSG00000227252
COPS8P3	0.0079	5.4538E-01		ENSG00000254697
COPS9	-0.4659	1.4322E-11	2.20E-09	ENSG00000172428
COPZ1	-0.1812	1.0505E-02	4.40E-02	ENSG00000111481
COPZ2	-0.2003	4.1021E-02	1.23E-01	ENSG00000005243
COQ10A	0.2284	9.3990E-03	4.06E-02	ENSG00000135469
COQ10B	-0.1390	4.2438E-02	1.27E-01	ENSG00000115520
COQ2	-0.2100	1.8649E-02	6.84E-02	ENSG00000173085
COQ3	0.1237	9.8219E-02	2.32E-01	ENSG00000132423
COQ4	-0.1002	2.0946E-02	7.46E-02	ENSG00000167113
COQ5	-0.0164	7.6482E-01	8.72E-01	ENSG00000110871
COQ6	0.0408	5.3349E-01	7.08E-01	ENSG00000119723
COQ7	0.1694	1.3821E-03	9.20E-03	ENSG00000167186
COQ7-DT	0.0098	8.6467E-01	9.31E-01	ENSG00000261465

COQ8B	0.3433	3.2973E-04	2.96E-03	ENSG00000123815
COQ9	-0.0263	7.4022E-01	8.57E-01	ENSG00000088682
CORIN	-0.1422	1.5784E-01	3.22E-01	ENSG00000145244
CORO1A	0.2261	5.0008E-03	2.50E-02	ENSG00000102879
CORO1B	-0.1585	2.8326E-02	9.33E-02	ENSG00000172725
CORO1C	-0.0866	1.6596E-01	3.33E-01	ENSG00000110880
CORO2A	0.1131	2.3185E-01	4.15E-01	ENSG00000106789
CORO2B	0.0972	2.5847E-01	4.47E-01	ENSG00000103647
CORO6	0.3863	3.7888E-03	2.02E-02	ENSG00000167549
CORO7	0.0727	3.8286E-01	5.77E-01	ENSG00000262246
CORO7-PAM16	-0.0027	9.7644E-01		ENSG00000103426
CORT	0.1060	2.5823E-01	4.46E-01	ENSG00000241563
COSMOC	-0.1774	1.2877E-02		ENSG00000260552
COTL1	0.1104	8.0174E-02	2.01E-01	ENSG00000103187
COX1	0.0755	4.4077E-01	6.31E-01	ENSG00000198804
COX10	-0.0626	3.2574E-01	5.20E-01	ENSG00000006695
COX10-DT	0.4637	2.0060E-04	1.97E-03	ENSG00000236088
COX11	0.1009	7.6509E-02	1.94E-01	ENSG00000166260
COX14	-0.0979	1.0194E-01	2.38E-01	ENSG00000178449
COX15	-0.0043	9.1532E-01	9.57E-01	ENSG00000014919
COX16	-0.0253	7.2868E-01	8.51E-01	ENSG00000133983
COX17	-0.1347	5.4321E-02	1.52E-01	ENSG00000138495
COX18	0.2117	3.3814E-03	1.84E-02	ENSG00000163626
COX19	0.1528	1.4355E-02	5.62E-02	ENSG00000240230
COX2	-0.1065	2.9534E-01	4.87E-01	ENSG00000198712
COX20	0.2108	5.3800E-02	1.51E-01	ENSG00000203667
COX3	0.0637	4.6939E-01	6.57E-01	ENSG00000198938
COX4I1	-0.1944	1.0864E-03	7.57E-03	ENSG00000131143
COX4I2	0.0871	3.7837E-04	3.30E-03	ENSG00000131055
COX5A	-0.1430	2.4092E-02	8.28E-02	ENSG00000178741
COX5B	-0.2874	2.8232E-06	6.32E-05	ENSG00000135940
COX6A1	-0.2091	2.0397E-03	1.24E-02	ENSG00000111775
COX6A2	-0.0473	6.8201E-01		ENSG00000156885
COX6B1	-0.2971	1.0016E-05	1.75E-04	ENSG00000126267
COX6B2	0.1465	1.7943E-01	3.51E-01	ENSG00000160471
COX6C	-0.3574	1.5076E-07	5.46E-06	ENSG00000164919
COX6CP13	-0.0026	6.4723E-01	7.96E-01	ENSG00000234144
COX7A1	0.2559	4.1192E-02	1.24E-01	ENSG00000161281
COX7A2	-0.1462	3.2735E-02	1.04E-01	ENSG00000112695
COX7A2L	-0.0359	4.7333E-01	6.60E-01	ENSG00000115944
COX7B	-0.2347	1.4908E-03	9.76E-03	ENSG00000131174
COX7BP4	-0.0502	3.6910E-01		ENSG00000233114
COX7C	-0.3988	9.7830E-09	5.59E-07	ENSG00000127184
COX8A	-0.3246	8.3866E-07	2.32E-05	ENSG00000176340
COX8C	0.0295	8.0357E-01		ENSG00000187581
CP	-0.1514	9.6344E-02	2.29E-01	ENSG00000047457
CPA2	0.3270	2.3866E-02	8.22E-02	ENSG00000158516
CPA3	-0.0200	5.8066E-01		ENSG00000163751
CPA4	-0.1021	1.8518E-01	3.59E-01	ENSG00000128510

CPA6	-0.1159	1.8734E-01	3.61E-01	ENSG00000165078
CPAMD8	-0.0892	3.5394E-01	5.49E-01	ENSG00000160111
CPB1	0.1448	5.2214E-02	1.47E-01	ENSG00000153002
CPB2	0.0868	3.5015E-01	5.45E-01	ENSG00000080618
CPB2-AS1	-0.0219	8.2533E-01	9.08E-01	ENSG00000235903
CPD	-0.0230	7.5601E-01	8.67E-01	ENSG00000108582
CPE	0.1134	1.6288E-01	3.29E-01	ENSG00000109472
CPEB1	-0.2372	8.2794E-03	3.69E-02	ENSG00000214575
CPEB1-AS1	0.0741	2.4702E-01		ENSG00000259462
CPEB2	0.1487	8.1277E-02	2.03E-01	ENSG00000137449
CPEB2-DT	0.1403	6.1376E-02	1.66E-01	ENSG00000247624
CPEB3	0.0379	6.1292E-01	7.71E-01	ENSG00000107864
CPEB4	-0.1154	8.7695E-02	2.14E-01	ENSG00000113742
CPED1	0.0299	7.3357E-01	8.54E-01	ENSG00000106034
CPHXL	0.0068	9.5447E-01		ENSG00000283755
CPHXL2	-0.0235	9.0438E-01		ENSG00000284484
CPLANE1	0.0799	2.4449E-01	4.30E-01	ENSG00000197603
CPLANE1-AS1	0.0181	8.5064E-01	9.23E-01	ENSG00000286193
CPLANE2	0.0868	3.7362E-01	5.69E-01	ENSG00000132881
CPLX1	0.0252	7.8400E-01	8.84E-01	ENSG00000168993
CPLX2	-0.2559	3.1550E-02	1.01E-01	ENSG00000145920
CPLX4	0.0019	9.4285E-01		ENSG00000166569
CPM	0.1869	7.0145E-02	1.83E-01	ENSG00000135678
CPN1	0.4112	1.5967E-02	6.08E-02	ENSG00000120054
CPN2	0.0035	6.8695E-01		ENSG00000178772
CPNE1	-0.1792	2.9536E-02	9.63E-02	ENSG00000214078
CPNE2	-0.4472	1.4781E-06	3.68E-05	ENSG00000140848
CPNE3	-0.0645	1.9015E-01	3.65E-01	ENSG00000085719
CPNE4	0.1299	1.3298E-01	2.86E-01	ENSG00000196353
CPNE5	-0.1019	3.0126E-01	4.94E-01	ENSG00000124772
CPNE6	0.3593	2.3674E-02	8.17E-02	ENSG00000100884
CPNE7	0.0230	7.9378E-01	8.89E-01	ENSG00000178773
CPNE8	-0.2108	6.5095E-03	3.07E-02	ENSG00000139117
CPNE8-AS1	0.0861	4.1213E-01	6.05E-01	ENSG00000257718
CPNE9	0.0416	6.6260E-01	8.06E-01	ENSG00000144550
CPO	0.0298	4.3153E-01		ENSG00000144410
CPOX	0.0315	6.9178E-01	8.27E-01	ENSG00000080819
CPPED1	0.1933	9.6609E-03	4.14E-02	ENSG00000103381
CPQ	-0.0770	4.1312E-01	6.06E-01	ENSG00000104324
CPSF1	-0.1322	6.9535E-02	1.81E-01	ENSG00000071894
CPSF1P2	0.0088	5.9072E-01		ENSG00000220132
CPSF2	-0.0249	6.1443E-01	7.73E-01	ENSG00000165934
CPSF3	0.2573	8.6024E-04	6.33E-03	ENSG00000119203
CPSF4	0.3316	1.5598E-04	1.61E-03	ENSG00000160917
CPSF4L	-0.0468	5.7352E-01		ENSG00000187959
CPSF6	0.1319	2.0749E-02	7.40E-02	ENSG00000111605
CPSF7	-0.1004	8.8247E-02	2.15E-01	ENSG00000149532
CPT1A	-0.2479	3.4363E-02	1.08E-01	ENSG00000110090
CPT1B	-0.0119	8.7549E-01	9.37E-01	ENSG00000205560

CPT1C	-0.4260	2.9226E-08	1.42E-06	ENSG00000169169
CPT2	0.2253	1.0811E-02	4.50E-02	ENSG00000157184
CPTP	-0.0674	3.0809E-01	5.01E-01	ENSG00000224051
CPVL	0.1546	3.5422E-02	1.11E-01	ENSG00000106066
CPVL-AS2	0.0037	9.5200E-01	9.76E-01	ENSG00000272568
CPXM1	0.4840	2.4517E-06	5.62E-05	ENSG00000088882
CPXM2	-0.1387	1.8207E-01	3.54E-01	ENSG00000121898
CPZ	-0.0576	2.9565E-01	4.88E-01	ENSG00000109625
CR1	-0.0142	9.2644E-01		ENSG00000203710
CR1L	-0.0252	2.7180E-01		ENSG00000197721
CR2	0.0656	7.3195E-02		ENSG00000117322
CRABP1	0.1211	1.7366E-01	3.43E-01	ENSG00000166426
CRABP2	-0.1338	1.6284E-01	3.29E-01	ENSG00000143320
CRACD	-0.0394	5.7150E-01	7.39E-01	ENSG00000109265
CRACDL	-0.1873	8.7041E-02	2.13E-01	ENSG00000196872
CRACR2A	0.5543	1.1047E-02	4.58E-02	ENSG00000130038
CRACR2B	0.1580	3.6996E-02	1.14E-01	ENSG00000177685
CRADD	0.0064	9.6089E-01	9.80E-01	ENSG00000169372
CRADD-AS1	0.0453	4.4284E-01		ENSG00000258274
CRAMP1	0.0351	7.0732E-01	8.36E-01	ENSG00000007545
CRAT	-0.0192	7.6311E-01	8.72E-01	ENSG00000095321
CRAT37	0.0096	6.0267E-02	1.64E-01	ENSG00000258551
CRB1	-0.0639	5.0110E-01	6.83E-01	ENSG00000134376
CRB2	-0.3951	1.1516E-03	7.93E-03	ENSG00000148204
CRB3	0.0962	3.3089E-01	5.26E-01	ENSG00000130545
CRBN	-0.1625	9.2187E-03	4.00E-02	ENSG00000113851
CRCP	0.0377	5.4590E-01	7.18E-01	ENSG00000241258
CREB1	-0.0142	8.2659E-01	9.09E-01	ENSG00000118260
CREB3	0.1253	6.6596E-02	1.76E-01	ENSG00000107175
CREB3L1	-0.0462	5.2861E-01	7.04E-01	ENSG00000157613
CREB3L2	-0.1089	8.6313E-02	2.12E-01	ENSG00000182158
CREB3L2-AS1	0.0015	9.9329E-01	9.96E-01	ENSG00000237243
CREB3L3	0.0148	2.1528E-01		ENSG00000060566
CREB3L4	0.1498	2.9621E-02	9.65E-02	ENSG00000143578
CREB5	-0.2825	1.2651E-04	1.35E-03	ENSG00000146592
CREBBP	-0.3156	3.5247E-05	4.80E-04	ENSG00000005339
CREBL2	0.1437	2.6849E-02	8.97E-02	ENSG00000111269
CREBRF	-0.0487	3.1018E-01	5.03E-01	ENSG00000164463
CREBZF	0.5032	8.4040E-09	4.95E-07	ENSG00000137504
CREG1	-0.0789	2.3866E-01	4.23E-01	ENSG00000143162
CREG2	-0.0194	7.6263E-01	8.71E-01	ENSG00000175874
CRELD1	0.0833	2.7275E-01	4.63E-01	ENSG00000163703
CRELD2	-0.1198	5.9835E-02	1.63E-01	ENSG00000184164
CREM	-0.1377	5.7820E-03	2.80E-02	ENSG00000095794
CRH	0.0055	9.4609E-01	9.73E-01	ENSG00000147571
CRHBP	0.2622	4.2744E-02	1.27E-01	ENSG00000145708
CRHR1	0.0810	6.5735E-02		ENSG00000120088
CRHR1	-0.0257	9.4754E-01		ENSG00000263715
CRHR2	0.5819	1.2983E-04	1.38E-03	ENSG00000106113

CRIM1	0.1411	8.4211E-02	2.08E-01	ENSG00000150938
CRIP1	0.2876	3.5798E-02	1.11E-01	ENSG00000213145
CRIP2	-0.1906	1.5682E-03	1.02E-02	ENSG00000182809
CRIP3	-0.0143	8.7343E-01	9.36E-01	ENSG00000146215
CRIPT	-0.1606	1.9878E-02	7.16E-02	ENSG00000119878
CRISP1	0.0720	5.2472E-02		ENSG00000124812
CRISP2	-0.0052	7.0297E-01		ENSG00000124490
CRISP3	0.0204	8.2359E-01	9.07E-01	ENSG00000096006
CRISPLD1	-0.1315	1.6176E-01	3.27E-01	ENSG00000121005
CRISPLD2	-0.0289	7.4992E-01	8.63E-01	ENSG00000103196
CRK	0.0398	5.1885E-01	6.96E-01	ENSG00000167193
CRKL	-0.0934	9.9688E-02	2.34E-01	ENSG00000099942
CRLF1	-0.0605	5.2284E-01	7.00E-01	ENSG00000006016
CRLF2	0.1520	1.3719E-01	2.92E-01	ENSG00000205755
CRLF3	0.1064	1.5323E-01	3.15E-01	ENSG00000176390
CRLS1	-0.1207	1.0878E-01	2.49E-01	ENSG00000088766
CRMP1	0.1234	1.5715E-01	3.21E-01	ENSG00000072832
CRNDE	0.1829	5.0722E-02	1.44E-01	ENSG00000245694
CRNKL1	0.2722	2.1410E-06	5.00E-05	ENSG00000101343
CROCC	0.0602	4.2525E-01	6.17E-01	ENSG00000058453
CROCC2	0.0135	8.5913E-01	9.28E-01	ENSG00000226321
CROCCP1	0.0146	6.4224E-01		ENSG00000225769
CROCCP3	-0.0604	5.2790E-01	7.03E-01	ENSG00000080947
CROCCP4	0.0056	8.4280E-01		ENSG00000227684
CROT	0.2116	1.9602E-03	1.21E-02	ENSG00000005469
CRPPA	-0.1463	1.6213E-01	3.28E-01	ENSG00000214960
CRPPA-AS1	0.0025	7.4927E-01		ENSG00000229688
CRTAC1	0.0471	6.1268E-01	7.71E-01	ENSG00000095713
CRTAM	0.0078	6.3557E-01		ENSG00000109943
CRTAP	-0.0720	2.6939E-01	4.59E-01	ENSG00000170275
CRTC1	0.0430	5.8873E-01	7.53E-01	ENSG00000105662
CRTC2	0.1874	1.2270E-02	4.97E-02	ENSG00000160741
CRTC3	-0.1902	6.4451E-03	3.05E-02	ENSG00000140577
CRTC3-AS1	0.1633	1.4060E-01	2.97E-01	ENSG00000259736
CRX	-0.0385	6.7564E-01	8.15E-01	ENSG00000105392
CRY1	0.0098	9.3058E-01	9.66E-01	ENSG00000008405
CRY2	-0.1434	3.1594E-02	1.01E-01	ENSG00000121671
CRYAA	0.1631	5.7653E-02	1.59E-01	ENSG00000160202
CRYAB	-0.2777	4.0156E-02	1.21E-01	ENSG00000109846
CRYBA1	0.3783	9.3879E-03	4.06E-02	ENSG00000108255
CRYBA2	-0.0621	5.1817E-01	6.96E-01	ENSG00000163499
CRYBA4	-0.0041	9.0931E-01	9.55E-01	ENSG00000196431
CRYBB1	0.1447	1.2972E-01	2.81E-01	ENSG00000100122
CRYBB2	0.0091	9.1192E-01	9.56E-01	ENSG00000244752
CRYBG1	0.0887	3.7106E-01	5.67E-01	ENSG00000112297
CRYBG2	0.1016	2.9104E-01	4.83E-01	ENSG00000176092
CRYBG3	-0.1075	2.4178E-01	4.27E-01	ENSG00000080200
CRYGA	-0.0027	8.0315E-01		ENSG00000168582
CRYGC	0.0573	2.2683E-02	7.92E-02	ENSG00000163254

CRYGN	0.0178	6.1061E-01		ENSG00000127377
CRYGS	-0.2909	2.4186E-02	8.31E-02	ENSG00000213139
CRYL1	-0.1766	8.4733E-03	3.75E-02	ENSG00000165475
CRYM	-0.0587	4.8440E-01	6.69E-01	ENSG00000103316
CRYZ	-0.0103	8.3766E-01	9.15E-01	ENSG00000116791
CRYZL1	0.1681	4.6168E-04	3.86E-03	ENSG00000205758
CRYZL2P	-0.0898	3.6833E-01	5.64E-01	ENSG00000242193
CS	0.1500	2.2390E-02	7.84E-02	ENSG00000062485
CSAD	-0.1038	1.2062E-01	2.68E-01	ENSG00000139631
CSDC2	-0.1361	1.0659E-01	2.46E-01	ENSG00000172346
CSDE1	-0.1902	3.9577E-05	5.30E-04	ENSG00000009307
CSE1L	0.2159	1.1032E-03	7.67E-03	ENSG00000124207
CSE1L-DT	0.0034	5.5018E-01		ENSG00000227431
CSF1R	-0.3979	9.2216E-03	4.00E-02	ENSG00000182578
CSF2RA	0.0946	7.2756E-03		ENSG00000198223
CSF3	0.0266	7.0777E-01	8.37E-01	ENSG00000108342
CSGALNACT1	0.0492	6.0959E-01	7.68E-01	ENSG00000147408
CSGALNACT2	0.0328	6.5931E-01	8.03E-01	ENSG00000169826
CSK	-0.2018	3.1678E-02	1.01E-01	ENSG00000103653
CSKMT	-0.3046	1.2313E-02	4.98E-02	ENSG00000214756
CSMD1	0.0375	4.5591E-01	6.45E-01	ENSG00000183117
CSMD2	0.0200	8.0302E-01	8.95E-01	ENSG00000121904
CSMD2-AS1	-0.0077	9.4344E-01		ENSG00000231163
CSMD3	0.1401	1.7200E-01	3.41E-01	ENSG00000164796
CSN2	-0.0478	4.8364E-01	6.68E-01	ENSG00000135222
CSNK1A1	-0.1354	1.0329E-02	4.35E-02	ENSG00000113712
CSNK1A1L	-0.1516	7.5001E-02	1.92E-01	ENSG00000180138
CSNK1D	0.0744	1.9473E-01	3.71E-01	ENSG00000141551
CSNK1E	-0.3033	1.6981E-04	1.72E-03	ENSG00000213923
CSNK1G1	0.0582	3.9808E-01	5.92E-01	ENSG00000169118
CSNK1G2	0.0219	6.9368E-01	8.28E-01	ENSG00000133275
CSNK1G2-AS1	-0.0489	2.5813E-01		ENSG00000180846
CSNK1G3	0.0787	1.3401E-01	2.87E-01	ENSG00000151292
CSNK2A1	-0.0662	1.8095E-01	3.53E-01	ENSG00000101266
CSNK2A2	-0.2580	1.5723E-02	6.01E-02	ENSG00000070770
CSNK2A3	-0.0503	6.0116E-01	7.62E-01	ENSG00000254598
CSNK2B	-0.3387	4.6528E-07	1.41E-05	ENSG00000204435
CSP2	-0.0930	1.5298E-01	3.15E-01	ENSG00000241218
CSPG4	0.1077	2.9487E-01	4.87E-01	ENSG00000173546
CSPG4BP	0.0176	8.0681E-01	8.97E-01	ENSG00000232517
CSPG4P10	0.3398	1.4971E-03	9.80E-03	ENSG00000276710
CSPG4P12	0.0274	7.0554E-01	8.35E-01	ENSG00000259295
CSPG4P13	-0.0100	7.3867E-01		ENSG00000260139
CSPG5	-0.2119	4.9589E-02	1.42E-01	ENSG00000114646
CSPP1	-0.1444	3.2823E-02	1.04E-01	ENSG00000104218
CSRNP2	0.1055	1.4371E-01	3.02E-01	ENSG00000110925
CSRNP3	-0.0955	1.4347E-01	3.01E-01	ENSG00000178662
CSRP1	-0.1175	9.7034E-02	2.30E-01	ENSG00000159176
CSRP1-AS1	-0.1049	3.0092E-01	4.93E-01	ENSG00000224536

CSRP2	-0.3533	1.0916E-03	7.60E-03	ENSG00000175183
CSRP3	-0.0216	5.9378E-01		ENSG00000129170
CST1	0.2530	5.5863E-02	1.55E-01	ENSG00000170373
CST2	-0.0484	3.0373E-01		ENSG00000170369
CST3	-0.3545	1.2781E-06	3.27E-05	ENSG00000101439
CST4	0.0154	5.4066E-01		ENSG00000101441
CST6	0.0137	9.8968E-01	9.94E-01	ENSG00000175315
CSTA	-0.0043	7.2893E-01	8.51E-01	ENSG00000121552
CSTB	-0.3593	8.3903E-05	9.72E-04	ENSG00000160213
CSTF1	0.1641	2.9134E-02	9.54E-02	ENSG00000101138
CSTF2T	0.2780	5.9107E-05	7.36E-04	ENSG00000177613
CSTF3	-0.1107	7.6334E-02	1.94E-01	ENSG00000176102
CSTF3-DT	-0.1485	1.9539E-01	3.71E-01	ENSG00000247151
CSTL1	0.0014	7.0277E-01		ENSG00000125823
CSTPP1	-0.1270	2.0897E-02	7.44E-02	ENSG00000149179
CT45A1	0.0119	3.6870E-01		ENSG00000268940
CT45A3	0.0093	4.6214E-01		ENSG00000269096
CT45A5	0.0060	8.9963E-01		ENSG00000228836
CT45A6	0.0115	4.7829E-01		ENSG00000278289
CT45A7	0.0081	5.8419E-01		ENSG00000273696
CT55	0.0067	5.7691E-01		ENSG00000169551
CT62	0.0032	6.5838E-01		ENSG00000225362
CT66	0.0031	9.7148E-01	9.85E-01	ENSG00000234215
CT70	-0.0008	9.9498E-01	9.97E-01	ENSG00000230013
CTAG1B	-0.3562	2.2315E-02	7.82E-02	ENSG00000184033
CTAGE11P	-0.0010	8.9821E-01		ENSG00000214249
CTAGE12P	0.1078	4.1921E-02		ENSG00000215441
CTAGE14P	0.0134	5.8864E-01		ENSG00000214211
CTAGE15	-0.0077	9.2066E-01	9.60E-01	ENSG00000271079
CTAGE3P	-0.0155	8.2229E-01	9.06E-01	ENSG00000232872
CTAGE4	-0.2166	7.0992E-02	1.84E-01	ENSG00000288784
CTAGE6	-0.1110	1.7891E-01	3.50E-01	ENSG00000271321
CTAGE7P	0.0065	6.5691E-01		ENSG00000233122
CTAGE8	-0.2837	3.9604E-02	1.20E-01	ENSG00000289604
CTAGE9	-0.1762	7.0680E-02	1.84E-01	ENSG00000236761
CTBP1	0.0411	5.3719E-01	7.11E-01	ENSG00000159692
CTBP1-AS	-0.0646	3.4196E-01		ENSG00000280927
CTBP1-DT	0.1608	2.7081E-02	9.02E-02	ENSG00000196810
CTBP2	-0.1917	3.2653E-04	2.93E-03	ENSG00000175029
CTBP2P4	0.0061	6.5203E-01		ENSG00000251102
CTBS	-0.2868	4.5829E-03	2.34E-02	ENSG00000117151
CTC-338M12.4	0.2253	1.0909E-02	4.53E-02	ENSG00000233937
CTC1	0.1365	1.0349E-01	2.40E-01	ENSG00000178971
CTCF	-0.1430	7.4471E-03	3.40E-02	ENSG00000102974
CTCF-DT	0.2065	6.7961E-02	1.78E-01	ENSG00000259804
CTCFL	-0.0041	6.5164E-01		ENSG00000124092
CTD-2350J17.1	-0.0335	4.1149E-01		ENSG00000250250
CTDNEP1	-0.2773	3.8652E-04	3.35E-03	ENSG00000175826
CTDP1	0.3985	3.5254E-07	1.11E-05	ENSG00000060069

CTDP1-DT	0.0017	6.1176E-01		ENSG00000178412
CTDSP1	-0.3641	7.1077E-03	3.28E-02	ENSG00000144579
CTDSP2	-0.3622	5.1296E-06	1.02E-04	ENSG00000175215
CTDSPL	0.1421	8.7615E-02	2.14E-01	ENSG00000144677
CTDSPL2	0.0352	5.8798E-01	7.52E-01	ENSG00000137770
CTF1	-0.5889	6.2569E-05	7.71E-04	ENSG00000150281
CTH	-0.2371	2.7665E-03	1.58E-02	ENSG00000116761
CTHRC1	-0.0126	8.9179E-01	9.46E-01	ENSG00000164932
CTIF	-0.0357	6.4267E-01	7.93E-01	ENSG00000134030
CTNNA1	-0.1774	1.5049E-04	1.56E-03	ENSG00000044115
CTNNA1-AS1	-0.0652	3.8916E-01		ENSG00000253404
CTNNA2	0.1455	1.0715E-01	2.46E-01	ENSG00000066032
CTNNA3	0.0661	4.9203E-01	6.76E-01	ENSG00000183230
CTNNAL1	0.1188	4.7603E-02	1.38E-01	ENSG00000119326
CTNNB1	-0.1161	1.7823E-01	3.49E-01	ENSG00000168036
CTNNBIP1	0.0589	4.3561E-01	6.27E-01	ENSG00000178585
CTNNBL1	0.1746	5.6282E-03	2.75E-02	ENSG00000132792
CTNND1	-0.1142	1.6954E-01	3.38E-01	ENSG00000198561
CTNND2	-0.0131	8.7340E-01	9.36E-01	ENSG00000169862
CTNS	0.0260	7.2305E-01	8.47E-01	ENSG00000040531
CTPS1	0.1960	4.5944E-03	2.35E-02	ENSG00000171793
CTPS2	0.0148	8.4926E-01	9.22E-01	ENSG00000047230
CTR9	-0.0626	2.0082E-01	3.78E-01	ENSG00000198730
CTRB1	-0.0203	7.8652E-01	8.85E-01	ENSG00000168925
CTRB2	-0.2301	4.0003E-02	1.21E-01	ENSG00000168928
CTRC	0.0379	6.5802E-01	8.03E-01	ENSG00000162438
CTSA	-0.3601	4.5605E-05	5.97E-04	ENSG00000064601
CTSB	-0.0708	3.1870E-01	5.12E-01	ENSG00000164733
CTSC	-0.0335	7.1117E-01	8.39E-01	ENSG00000109861
CTSD	-0.0645	3.0585E-01	4.99E-01	ENSG00000117984
CTSE	0.0140	5.7307E-01		ENSG00000196188
CTSF	0.1775	6.2067E-02	1.67E-01	ENSG00000174080
CTSG	0.0043	8.4347E-01		ENSG00000100448
CTSH	-0.4326	3.6038E-06	7.71E-05	ENSG00000103811
CTSK	0.0470	6.1429E-01	7.72E-01	ENSG00000143387
CTSL	-0.1252	8.6162E-02	2.12E-01	ENSG00000135047
CTSLP3	0.1842	1.5105E-02	5.83E-02	ENSG00000280913
CTSO	-0.0892	2.8639E-01	4.77E-01	ENSG00000256043
CTSS	0.0060	9.4981E-01	9.75E-01	ENSG00000163131
CTSW	0.1410	1.6394E-01	3.30E-01	ENSG00000172543
CTSZ	-0.0175	8.1992E-01	9.05E-01	ENSG00000101160
CTTN	-0.0266	5.8763E-01	7.52E-01	ENSG00000085733
CTTNBP2	0.2567	8.8164E-03	3.87E-02	ENSG00000077063
CTTNBP2NL	0.0685	2.6359E-01	4.52E-01	ENSG00000143079
CTU1	0.0348	6.7255E-01	8.12E-01	ENSG00000142544
CTU2	0.1371	1.1635E-01	2.62E-01	ENSG00000174177
CTXN1	-0.1657	4.7194E-02	1.37E-01	ENSG00000178531
CTXN2	0.0252	7.4957E-01	8.63E-01	ENSG00000233932
CTXN3	0.5005	9.1004E-03	3.96E-02	ENSG00000205279

CTXND2	-0.0734	1.2417E-01		ENSG00000283324
CUBN	0.2082	6.8520E-02	1.80E-01	ENSG00000107611
CUEDC1	-0.0428	5.0567E-01	6.87E-01	ENSG00000180891
CUEDC2	-0.1742	1.0158E-03	7.20E-03	ENSG00000107874
CUL1	0.0493	5.0650E-01	6.87E-01	ENSG00000055130
CUL2	0.1968	1.4280E-02	5.59E-02	ENSG00000108094
CUL3	0.0083	8.9639E-01	9.48E-01	ENSG00000036257
CUL4A	-0.0590	2.0280E-01	3.80E-01	ENSG00000139842
CUL4B	-0.0494	3.8701E-01	5.82E-01	ENSG00000158290
CUL5	-0.0989	3.1795E-02	1.02E-01	ENSG00000166266
CUL7	-0.0260	7.4180E-01	8.58E-01	ENSG00000044090
CUL9	0.0500	5.0255E-01	6.84E-01	ENSG00000112659
CUTA	-0.2609	9.1062E-07	2.47E-05	ENSG00000112514
CUTALP	0.2021	3.8399E-02	1.17E-01	ENSG00000226752
CUTC	0.1807	1.6823E-03	1.07E-02	ENSG00000119929
CUX1	-0.3233	1.6351E-07	5.84E-06	ENSG00000257923
CUX2	0.0213	8.2163E-01	9.06E-01	ENSG00000111249
CWC15	-0.2111	4.0933E-05	5.45E-04	ENSG00000150316
CWC22	0.2461	8.6017E-04	6.33E-03	ENSG00000163510
CWC25	-0.3805	1.4978E-12	2.99E-10	ENSG00000273559
CWC27	-0.1853	1.1603E-04	1.26E-03	ENSG00000153015
CWF19L1	-0.0499	4.4061E-01	6.31E-01	ENSG00000095485
CWF19L2	0.1349	9.7033E-02	2.30E-01	ENSG00000152404
CX3CL1	-0.2932	1.6062E-04	1.64E-03	ENSG00000006210
CX3CR1	-0.0603	4.2016E-01	6.13E-01	ENSG00000168329
CXADR	0.0913	2.4793E-01	4.34E-01	ENSG00000154639
CXADRP1	0.0227	2.2197E-01		ENSG00000214319
CXADRP3	-0.0022	9.0837E-01		ENSG00000265766
CXCL10	-0.0243	1.4939E-01		ENSG00000169245
CXCL11	0.0588	2.7330E-01	4.64E-01	ENSG00000169248
CXCL14	-0.1328	1.3448E-01	2.88E-01	ENSG00000145824
CXCL16	-0.1515	9.4785E-02	2.26E-01	ENSG00000161921
CXCL17	0.0242	7.6607E-01	8.73E-01	ENSG00000189377
CXCL5	-0.0507	4.6904E-01	6.57E-01	ENSG00000163735
CXCL6	-0.1611	5.8832E-02	1.61E-01	ENSG00000124875
CXCR4	0.1874	7.3812E-02	1.89E-01	ENSG00000121966
CXCR6	-0.1007	6.1116E-02		ENSG00000172215
CXorf38	0.2875	1.2694E-05	2.12E-04	ENSG00000185753
CXorf49	-0.0070	7.3859E-01		ENSG00000215115
CXorf49B	-0.0079	6.7450E-01		ENSG00000215113
CXorf58	0.3263	3.0424E-02	9.84E-02	ENSG00000165182
CXXC1	0.2968	2.3362E-05	3.47E-04	ENSG00000154832
CXXC4	-0.1802	4.4932E-02	1.32E-01	ENSG00000168772
CXXC4-AS1	-0.1249	1.7457E-01	3.44E-01	ENSG00000245384
CXXC5	-0.2794	3.6276E-08	1.68E-06	ENSG00000171604
CXXC5-AS1	-0.0060	9.3747E-01	9.69E-01	ENSG00000250635
CYB561	0.0403	4.7296E-01	6.60E-01	ENSG00000008283
CYB561A3	0.1672	2.1616E-02	7.64E-02	ENSG00000162144
CYB561D1	0.4231	2.0242E-06	4.78E-05	ENSG00000174151

CYB561D2	-0.0864	2.0052E-01	3.78E-01	ENSG00000114395
CYB5A	-0.1256	1.4146E-01	2.98E-01	ENSG00000166347
CYB5B	-0.0017	9.0515E-01	9.52E-01	ENSG00000103018
CYB5D1	-0.0671	4.0777E-01	6.01E-01	ENSG00000182224
CYB5D2	0.0414	3.7778E-01	5.73E-01	ENSG00000167740
CYB5R1	0.1222	1.1696E-01	2.62E-01	ENSG00000159348
CYB5R2	0.5765	8.8968E-06	1.60E-04	ENSG00000166394
CYB5R3	-0.0962	7.2814E-02	1.88E-01	ENSG00000100243
CYB5RL	-0.0716	4.5307E-01	6.43E-01	ENSG00000215883
CYBA	-0.3684	1.3435E-09	1.09E-07	ENSG00000051523
CYBB	-0.0313	7.3544E-01	8.55E-01	ENSG00000165168
CYBC1	0.1158	1.3776E-01	2.93E-01	ENSG00000178927
CYBRD1	-0.2951	4.5546E-03	2.33E-02	ENSG00000071967
CYC1	-0.0332	6.2848E-01	7.83E-01	ENSG00000179091
CYCS	0.0058	9.3664E-01	9.69E-01	ENSG00000172115
CYCSP3	0.0350	1.3456E-01	2.88E-01	ENSG00000188512
CYCSP52	0.0466	4.9556E-01	6.79E-01	ENSG00000235700
CYFIP1	-0.4812	2.4132E-12	4.60E-10	ENSG00000273749
CYFIP2	-0.0378	4.4009E-01	6.31E-01	ENSG00000055163
CYGB	-0.3799	1.9296E-03	1.19E-02	ENSG00000161544
CYLD	-0.0076	9.0652E-01	9.53E-01	ENSG00000083799
CYLD-AS1	0.0079	5.4354E-01		ENSG00000261644
CYLD-AS2	-0.0092	9.8313E-01		ENSG00000260616
CYP11A1	-0.0384	6.3982E-01	7.91E-01	ENSG00000140459
CYP17A1	-0.0195	6.0937E-01		ENSG00000148795
CYP19A1	0.0568	1.6453E-01	3.31E-01	ENSG00000137869
CYP1A1	0.0311	7.4638E-01	8.61E-01	ENSG00000140465
CYP1A2	0.0221	7.4127E-01	8.58E-01	ENSG00000140505
CYP1B1	-0.2075	4.0908E-02	1.23E-01	ENSG00000138061
CYP1B1-AS1	0.1686	1.3124E-01	2.83E-01	ENSG00000232973
CYP20A1	-0.1594	1.0297E-02	4.34E-02	ENSG00000119004
CYP24A1	0.0311	4.2347E-01		ENSG00000019186
CYP26A1	-0.0004	8.0934E-01		ENSG00000095596
CYP26B1	0.2234	5.8102E-02	1.60E-01	ENSG00000003137
CYP27A1	-0.2836	2.2344E-02	7.83E-02	ENSG00000135929
CYP27C1	-0.1574	1.0028E-01	2.35E-01	ENSG00000186684
CYP2A13	-0.0023	8.4107E-01		ENSG00000197838
CYP2A6	-0.0492	8.8435E-01	9.42E-01	ENSG00000255974
CYP2A7	0.0186	3.3458E-01		ENSG00000198077
CYP2C18	-0.2669	1.2873E-01	2.80E-01	ENSG00000108242
CYP2C8	-0.1169	2.2862E-01	4.11E-01	ENSG00000138115
CYP2D6	0.1654	1.3921E-01	2.95E-01	ENSG00000100197
CYP2D8P	0.0742	4.1553E-01	6.08E-01	ENSG00000226450
CYP2E1	-0.2063	8.5204E-02	2.10E-01	ENSG00000130649
CYP2F1	0.0047	7.5728E-01		ENSG00000197446
CYP2J2	-0.2140	5.7511E-02	1.59E-01	ENSG00000134716
CYP2R1	0.4685	1.0893E-09	9.22E-08	ENSG00000186104
CYP2S1	-0.4332	6.4201E-03	3.04E-02	ENSG00000167600
CYP2T3P	0.0000	9.2721E-01		ENSG00000268529

CYP2U1	-0.1599	4.1065E-02	1.24E-01	ENSG00000155016
CYP2U1-AS1	0.0381	6.7859E-01	8.17E-01	ENSG00000245293
CYP2W1	0.0309	6.0835E-01	7.67E-01	ENSG00000073067
CYP39A1	0.0878	3.6076E-01	5.57E-01	ENSG00000146233
CYP3A4	-0.0089	7.4663E-01		ENSG00000160868
CYP3A43	0.0058	8.2960E-01		ENSG0000021461
CYP3A5	-0.0567	5.5693E-01	7.27E-01	ENSG00000106258
CYP3A7	0.0190	6.3055E-02		ENSG00000160870
CYP46A1	0.0214	7.9374E-01	8.89E-01	ENSG00000036530
CYP4A11	-0.0223	7.7806E-01	8.80E-01	ENSG00000187048
CYP4A22	0.0217	4.0674E-01	6.00E-01	ENSG00000162365
CYP4B1	-0.1622	2.5734E-02	8.70E-02	ENSG00000142973
CYP4F11	0.0147	6.4863E-01		ENSG00000171903
CYP4F12	-0.0339	5.3863E-01	7.12E-01	ENSG00000186204
CYP4F26P	-0.0012	9.1063E-01		ENSG00000226562
CYP4F27P	-0.0013	7.0536E-01		ENSG00000248313
CYP4F3	0.0430	6.4814E-01	7.96E-01	ENSG00000186529
CYP4V2	-0.3444	4.5164E-05	5.92E-04	ENSG00000145476
CYP4X1	0.0065	9.4321E-01	9.72E-01	ENSG00000186377
CYP4Z1	-0.0582	1.2241E-01		ENSG00000186160
CYP51A1	-0.5775	6.7088E-08	2.83E-06	ENSG00000001630
CYP51A1-AS1	0.2119	5.7454E-02	1.58E-01	ENSG00000188693
CYP8B1	-0.0028	9.5538E-01		ENSG00000180432
CYREN	0.1143	8.1252E-02	2.03E-01	ENSG00000122783
CYRIA	0.0194	8.2735E-01	9.09E-01	ENSG00000197872
CYRIB	0.0027	9.5812E-01	9.79E-01	ENSG00000153310
CYS1	-0.1083	2.5127E-01	4.38E-01	ENSG00000205795
CYSLTR1	0.0303	7.4770E-01	8.62E-01	ENSG00000173198
CYSLTR2	0.0201	2.9672E-01		ENSG00000152207
CYSRT1	0.0009	9.7483E-01	9.87E-01	ENSG00000197191
CYSTM1	-0.2538	3.5313E-04	3.13E-03	ENSG00000120306
CYTB	-0.0577	5.1383E-01	6.93E-01	ENSG00000198727
CYTH1	0.1135	1.3312E-01	2.86E-01	ENSG00000108669
CYTH1P1	-0.0286	9.7493E-01		ENSG00000271286
CYTH2	-0.0945	7.1132E-02	1.84E-01	ENSG00000105443
CYTH3	0.2808	1.5170E-03	9.90E-03	ENSG00000008256
CYTH4	0.0037	5.5117E-01		ENSG00000100055
CYTIP	0.0240	3.5797E-01		ENSG00000115165
CYTL1	-0.0406	6.6562E-01	8.08E-01	ENSG00000170891
CYTOR	0.3178	2.5501E-03	1.48E-02	ENSG00000222041
CYYR1-AS1	-0.0214	7.8760E-01	8.86E-01	ENSG00000197934
CZIB	-0.1766	1.6385E-03	1.05E-02	ENSG00000162384
D21S2088E	0.0547	1.7058E-02	6.39E-02	ENSG00000228592
D2HGDH	-0.0049	9.3591E-01	9.68E-01	ENSG00000180902
DAAM1	0.0723	2.7708E-01	4.68E-01	ENSG00000100592
DAAM2	0.4563	7.6722E-03	3.48E-02	ENSG00000146122
DAAM2-AS1	-0.0213	4.9852E-01		ENSG00000235033
DAB1	-0.0441	6.1251E-01	7.71E-01	ENSG00000173406
DAB1-AS1	0.0156	8.1782E-01	9.04E-01	ENSG00000226759

DAB2	-0.5283	1.1703E-06	3.05E-05	ENSG00000153071
DAB2IP	-0.0674	4.4030E-01	6.31E-01	ENSG00000136848
DACH1	0.0136	8.9750E-01	9.49E-01	ENSG00000276644
DACH2	-0.0441	6.3271E-01	7.86E-01	ENSG00000126733
DACT1	-0.1514	1.4054E-01	2.97E-01	ENSG00000165617
DACT2	-0.5377	5.8521E-04	4.68E-03	ENSG00000164488
DACT3	0.1942	2.2666E-02	7.91E-02	ENSG00000197380
DAD1	-0.1004	1.1489E-01	2.59E-01	ENSG00000129562
DAG1	-0.3841	1.1795E-09	9.82E-08	ENSG00000173402
DAGLA	0.5276	9.8328E-06	1.73E-04	ENSG00000134780
DAGLB	0.1566	2.9775E-02	9.69E-02	ENSG00000164535
DALRD3	0.0099	8.7639E-01	9.37E-01	ENSG00000178149
DANCR	-0.0964	1.2208E-01	2.70E-01	ENSG00000226950
DANT2	0.1138	1.5772E-01	3.21E-01	ENSG00000235244
DAO	-0.1094	7.4152E-02	1.90E-01	ENSG00000110887
DAP	-0.0012	9.2938E-01	9.65E-01	ENSG00000112977
DAP3	0.0265	6.1439E-01	7.73E-01	ENSG00000132676
DAPK1	0.1333	1.2004E-01	2.67E-01	ENSG00000196730
DAPK1-IT1	0.0095	2.2144E-01		ENSG00000236709
DAPK2	0.1872	8.9162E-02	2.17E-01	ENSG00000035664
DAPK3	0.1883	9.7060E-03	4.15E-02	ENSG00000167657
DAPL1	0.1567	1.3493E-01	2.89E-01	ENSG00000163331
DAPP1	0.0448	4.8420E-01	6.69E-01	ENSG00000070190
DARS1	0.0345	5.7894E-01	7.45E-01	ENSG00000115866
DARS2	-0.0429	5.6437E-01	7.33E-01	ENSG00000117593
DAXX	-0.0355	5.5757E-01	7.28E-01	ENSG00000204209
DAZAP1	0.1060	2.1197E-01	3.92E-01	ENSG00000071626
DAZAP2	0.0076	9.8718E-01	9.93E-01	ENSG00000183283
DAZL	-0.0075	8.3478E-01		ENSG00000092345
DBF4	0.0116	8.9114E-01	9.46E-01	ENSG00000006634
DBF4B	0.0030	9.7669E-01	9.88E-01	ENSG00000161692
DBH	-0.0354	1.9440E-01	3.70E-01	ENSG00000123454
DBH-AS1	0.0605	5.0805E-01	6.88E-01	ENSG00000225756
DBI	-0.5248	6.2388E-09	3.91E-07	ENSG00000155368
DBIL5P	0.0722	4.5355E-01	6.43E-01	ENSG00000231784
DBIL5P2	-0.0060	9.3722E-01		ENSG00000242412
DBN1	-0.0501	4.4380E-01	6.34E-01	ENSG00000113758
DBNDD1	-0.2180	6.9631E-04	5.37E-03	ENSG00000003249
DBNDD2	-0.1780	1.1652E-01	2.62E-01	ENSG00000244274
DBNL	-0.1046	5.0950E-02	1.45E-01	ENSG00000136279
DBP	0.0593	5.0362E-01	6.85E-01	ENSG00000105516
DBR1	0.0780	3.1078E-01	5.04E-01	ENSG00000138231
DBT	-0.0452	4.9776E-01	6.81E-01	ENSG00000137992
DBX1	0.3951	2.7683E-03	1.58E-02	ENSG00000109851
DBX2	0.0083	5.9058E-01		ENSG00000185610
DCAF1	0.2016	1.0666E-02	4.45E-02	ENSG00000145041
DCAF10	0.0681	1.3733E-01	2.92E-01	ENSG00000122741
DCAF11	0.1650	1.7777E-02	6.59E-02	ENSG00000100897
DCAF12	-0.0065	8.8195E-01	9.40E-01	ENSG00000198876

DCAF12L1	0.0440	4.2404E-01	6.16E-01	ENSG00000198889
DCAF12L2	-0.0762	4.2287E-01	6.15E-01	ENSG00000198354
DCAF13	0.2205	9.5070E-03	4.09E-02	ENSG00000164934
DCAF13P1	-0.0138	7.1244E-01		ENSG00000242562
DCAF15	-0.0701	2.6274E-01	4.52E-01	ENSG00000132017
DCAF16	0.0937	1.5151E-01	3.13E-01	ENSG00000163257
DCAF17	0.0226	7.4656E-01	8.61E-01	ENSG00000115827
DCAF4	0.2286	5.8788E-03	2.84E-02	ENSG00000119599
DCAF4L1	-0.0197	8.0602E-01	8.96E-01	ENSG00000182308
DCAF4L2	0.0064	7.0224E-01		ENSG00000176566
DCAF5	-0.1509	2.1333E-03	1.29E-02	ENSG00000139990
DCAF6	0.0068	9.0914E-01	9.55E-01	ENSG00000143164
DCAF7	-0.1306	8.4054E-02	2.08E-01	ENSG00000136485
DCAF8	0.1515	6.5370E-02	1.74E-01	ENSG00000132716
DCAF8-DT	0.2629	1.0876E-02	4.52E-02	ENSG00000228606
DCAF8L2	0.0240	2.8198E-02		ENSG00000189186
DCAKD	0.0419	5.2489E-01	7.01E-01	ENSG00000172992
DCBLD1	-0.2207	1.4964E-02	5.79E-02	ENSG00000164465
DCBLD2	0.0355	6.6860E-01	8.09E-01	ENSG00000057019
DCC	0.1686	1.1264E-01	2.55E-01	ENSG00000187323
DCDC1	-0.2818	4.0107E-03	2.12E-02	ENSG00000170959
DCDC2	-0.0170	8.5203E-01	9.24E-01	ENSG00000146038
DCDC2B	-0.0044	9.1609E-01	9.58E-01	ENSG00000222046
DCDC2C	-0.0073	9.8553E-01		ENSG00000214866
DCHS1	-0.0323	6.9825E-01	8.31E-01	ENSG00000166341
DCHS2	-0.1435	1.7846E-01	3.49E-01	ENSG00000197410
DCK	-0.0138	8.3426E-01	9.14E-01	ENSG00000156136
DCLK1	0.0494	5.6068E-01	7.30E-01	ENSG00000133083
DCLK2	0.0011	9.9390E-01	9.97E-01	ENSG00000170390
DCLK3	0.1539	1.5881E-01	3.23E-01	ENSG00000163673
DCLRE1A	0.0437	6.0267E-01	7.63E-01	ENSG00000198924
DCLRE1B	0.1358	9.9557E-02	2.34E-01	ENSG00000118655
DCLRE1C	-0.1236	9.9197E-02	2.33E-01	ENSG00000152457
DCP1A	-0.1795	7.7044E-05	9.08E-04	ENSG00000272886
DCP1B	0.2552	1.3031E-05	2.17E-04	ENSG00000151065
DCP2	-0.1558	2.6966E-02	9.00E-02	ENSG00000172795
DCPS	-0.2859	1.8750E-03	1.16E-02	ENSG00000110063
DCST1	0.3841	8.1922E-03	3.66E-02	ENSG00000163357
DCST2	0.1454	1.4906E-01	3.09E-01	ENSG00000163354
DCTD	-0.1733	1.6817E-02	6.32E-02	ENSG00000129187
DCTN1	0.0952	2.2979E-01	4.13E-01	ENSG00000204843
DCTN1-AS1	0.5438	1.2276E-03	8.35E-03	ENSG00000237737
DCTN2	-0.0311	3.7282E-01	5.68E-01	ENSG00000175203
DCTN3	-0.0287	5.8676E-01	7.51E-01	ENSG00000137100
DCTN4	-0.1574	1.5457E-03	1.00E-02	ENSG00000132912
DCTN5	-0.0061	8.9368E-01	9.47E-01	ENSG00000166847
DCTN6	-0.1208	3.7662E-02	1.16E-01	ENSG00000104671
DCTPP1	-0.0439	4.3137E-01	6.23E-01	ENSG00000179958
DCUN1D1	-0.1581	4.9262E-03	2.47E-02	ENSG00000043093

DCUN1D3	0.3085	8.1805E-03	3.66E-02	ENSG00000188215
DCUN1D4	-0.0101	8.8016E-01	9.39E-01	ENSG00000109184
DCUN1D5	0.0651	2.0416E-01	3.82E-01	ENSG00000137692
DCX	0.1397	9.8288E-02	2.32E-01	ENSG00000077279
DCXR	-0.1527	6.3258E-03	3.00E-02	ENSG00000169738
DCXR-DT	0.0589	4.5706E-01	6.46E-01	ENSG00000264569
DDA1	-0.0783	9.0886E-02	2.20E-01	ENSG00000130311
DDAH1	-0.4660	6.7174E-06	1.28E-04	ENSG00000153904
DDAH2	-0.0836	9.8001E-02	2.31E-01	ENSG00000213722
DDB1	0.0425	4.0518E-01	5.99E-01	ENSG00000167986
DDB2	0.0617	2.6698E-01	4.56E-01	ENSG00000134574
DDC-AS1	0.0766	3.2672E-02	1.04E-01	ENSG00000226122
DDHD1	0.3244	1.4398E-05	2.36E-04	ENSG00000100523
DDHD1-DT	-0.0449	8.1697E-01		ENSG00000258731
DDHD2	0.2034	1.9646E-04	1.93E-03	ENSG00000085788
DDI2	0.0952	2.9989E-01	4.92E-01	ENSG00000197312
DDIAS	-0.3846	1.7020E-02	6.38E-02	ENSG00000165490
DDIT4	-0.0388	4.8069E-01	6.66E-01	ENSG00000168209
DDIT4L	0.0321	7.4678E-01	8.61E-01	ENSG00000145358
DDIT4L-AS1	-0.0096	8.2736E-01		ENSG00000249710
DDN	0.2148	5.7846E-02	1.59E-01	ENSG00000181418
DDN-AS1	0.2074	3.7584E-02	1.15E-01	ENSG00000257913
DDO	-0.1247	1.9519E-01	3.71E-01	ENSG00000203797
DDOST	0.0863	1.7979E-01	3.51E-01	ENSG00000244038
DDR1	-0.3715	5.1941E-09	3.39E-07	ENSG00000204580
DDR2	-0.5942	1.9412E-05	2.98E-04	ENSG00000162733
DDRGK1	-0.1140	5.8793E-02	1.61E-01	ENSG00000198171
DDT	0.0945	3.4889E-01	5.44E-01	ENSG00000099977
DDTL	-0.0045	9.5398E-01	9.77E-01	ENSG00000099974
DDX1	0.0060	9.1668E-01	9.58E-01	ENSG00000079785
DDX10	0.0951	2.0077E-01	3.78E-01	ENSG00000178105
DDX10P1	0.0336	1.7674E-01		ENSG00000237135
DDX11	0.0402	6.6330E-01	8.07E-01	ENSG00000013573
DDX11-AS1	0.0990	3.1691E-01	5.10E-01	ENSG00000245614
DDX17	-0.0403	6.1340E-01	7.72E-01	ENSG00000100201
DDX18	0.0144	7.6420E-01	8.72E-01	ENSG00000088205
DDX18P1	-0.0569	2.3284E-01		ENSG00000259165
DDX18P2	-0.0362	7.2729E-01		ENSG00000259601
DDX19A	-0.0481	4.4155E-01	6.32E-01	ENSG00000168872
DDX19A-DT	0.0926	3.5107E-01	5.46E-01	ENSG00000261777
DDX19B	0.1587	5.1346E-04	4.20E-03	ENSG00000157349
DDX20	0.1275	1.0788E-01	2.48E-01	ENSG00000064703
DDX21	-0.0721	3.6627E-01	5.62E-01	ENSG00000165732
DDX23	0.0073	8.9375E-01	9.47E-01	ENSG00000174243
DDX24	-0.0671	1.5671E-01	3.20E-01	ENSG00000089737
DDX25	0.2567	4.4373E-04	3.74E-03	ENSG00000109832
DDX27	0.0585	3.6208E-01	5.58E-01	ENSG00000124228
DDX28	-0.0937	1.2453E-01	2.74E-01	ENSG00000182810
DDX31	-0.0353	5.9932E-01	7.60E-01	ENSG00000125485

DDX39A	-0.1006	1.2943E-01	2.81E-01	ENSG00000123136
DDX39B	-0.0629	4.1420E-01	6.07E-01	ENSG00000198563
DDX39B-AS1	-0.0789	3.1403E-01	5.07E-01	ENSG00000234006
DDX3P2	0.0009	9.0840E-01		ENSG00000230986
DDX3X	-0.0376	5.7435E-01	7.41E-01	ENSG00000215301
DDX3Y	-0.0114	8.6730E-01	9.33E-01	ENSG00000067048
DDX4	0.0182	4.7898E-01		ENSG00000152670
DDX41	0.3206	1.0939E-06	2.89E-05	ENSG00000183258
DDX42	-0.0199	7.3827E-01	8.56E-01	ENSG00000198231
DDX43	-0.0107	4.8035E-01		ENSG00000080007
DDX46	-0.0441	3.7075E-01	5.66E-01	ENSG00000145833
DDX47	-0.1904	7.8060E-02	1.97E-01	ENSG00000213782
DDX49	0.1216	8.1278E-02	2.03E-01	ENSG00000105671
DDX5	0.0824	2.7062E-01	4.60E-01	ENSG00000108654
DDX50	0.0333	4.2501E-01	6.17E-01	ENSG00000107625
DDX51	0.3803	1.6227E-04	1.66E-03	ENSG00000185163
DDX52	0.0142	8.0377E-01	8.96E-01	ENSG00000278053
DDX53	-0.0494	3.4168E-01		ENSG00000184735
DDX54	0.0559	3.8461E-01	5.79E-01	ENSG00000123064
DDX55	0.0432	5.2366E-01	7.00E-01	ENSG00000111364
DDX55P1	0.0091	7.7714E-01		ENSG00000270863
DDX56	0.1539	1.1182E-02	4.62E-02	ENSG00000136271
DDX59	0.0386	5.4059E-01	7.14E-01	ENSG00000118197
DDX59-AS1	-0.1558	1.5544E-01	3.18E-01	ENSG00000260088
DDX6	-0.3758	2.8632E-08	1.40E-06	ENSG00000110367
DDX60L	0.1116	2.4525E-01	4.31E-01	ENSG00000181381
DEAF1	0.3786	4.3228E-07	1.32E-05	ENSG00000177030
DECR1	-0.2216	7.7893E-03	3.52E-02	ENSG00000104325
DECR2	0.0853	2.6106E-01	4.50E-01	ENSG00000242612
DEDD	-0.0990	1.0061E-01	2.36E-01	ENSG00000158796
DEDD2	0.1187	1.0962E-01	2.50E-01	ENSG00000160570
DEF6	0.2822	7.5205E-03	3.42E-02	ENSG00000023892
DEF8	-0.1707	4.6694E-03	2.37E-02	ENSG00000140995
DEFA1	0.0333	1.6794E-01		ENSG00000206047
DEFA1B	0.0333	1.6794E-01		ENSG00000240247
DEFA5	-0.0903	8.9226E-01	9.46E-01	ENSG00000164816
DEFB1	0.0660	3.1976E-01	5.13E-01	ENSG00000164825
DEFB105A	0.0196	2.5615E-01		ENSG00000186562
DEFB109D	0.0012	6.8589E-01		ENSG00000254866
DEFB112	0.0691	8.4838E-02		ENSG00000180872
DEFB131C	0.0239	4.9397E-01		ENSG00000254700
DEFB131E	0.0247	7.7515E-01	8.78E-01	ENSG00000254507
DEFB4A	-0.0403	3.0623E-01	4.99E-01	ENSG00000177257
DEFB4A	-0.1583	9.6801E-03	4.14E-02	ENSG00000171711
DEGS1	-0.2055	5.6272E-03	2.75E-02	ENSG00000143753
DEGS2	-0.0249	7.9486E-01	8.90E-01	ENSG00000168350
DEK	-0.1703	4.1943E-03	2.19E-02	ENSG00000124795
DELE1	-0.0542	3.5666E-01	5.52E-01	ENSG00000081791
DELEC1	-0.0349	7.2376E-01	8.47E-01	ENSG00000173077

DENND10	-0.0365	4.2244E-01	6.15E-01	ENSG00000119979
DENND11	-0.1399	5.1738E-02	1.46E-01	ENSG00000257093
DENND1A	-0.0903	1.6165E-01	3.27E-01	ENSG00000119522
DENND1B	-0.0570	4.3890E-01	6.30E-01	ENSG00000213047
DENND2A	-0.0564	4.7772E-01	6.64E-01	ENSG00000146966
DENND2B	-0.2284	3.3431E-05	4.60E-04	ENSG00000166444
DENND2C	0.0097	9.0445E-01	9.52E-01	ENSG00000175984
DENND2D	-0.0985	3.2571E-01	5.20E-01	ENSG00000162777
DENND3	0.1944	2.1217E-02	7.53E-02	ENSG00000105339
DENND3-AS1	-0.3254	6.6608E-03	3.12E-02	ENSG00000253210
DENND4A	0.0737	3.7630E-01	5.72E-01	ENSG00000174485
DENND4B	0.0820	2.7040E-01	4.60E-01	ENSG00000198837
DENND4C	-0.0468	5.3854E-01	7.12E-01	ENSG00000137145
DENND5A	-0.2157	1.1708E-04	1.27E-03	ENSG00000184014
DENND5B	-0.0420	5.3012E-01	7.05E-01	ENSG00000170456
DENND5B-AS1	0.2335	6.3480E-02	1.70E-01	ENSG00000255867
DENND6A	0.0113	8.6310E-01	9.30E-01	ENSG00000174839
DENND6A-DT	-0.0495	5.6932E-01	7.37E-01	ENSG00000241933
DENND6B	0.0112	8.7081E-01	9.34E-01	ENSG00000205593
DENR	-0.0944	8.2605E-02	2.05E-01	ENSG00000139726
DEPDC1	-0.0958	3.2044E-01	5.14E-01	ENSG00000024526
DEPDC1-AS1	-0.0048	6.3775E-01		ENSG00000234264
DEPDC1B	-0.1333	2.0273E-01	3.80E-01	ENSG00000035499
DEPDC4	0.3379	1.0835E-03	7.56E-03	ENSG00000166153
DEPDC5	0.4148	1.7564E-07	6.22E-06	ENSG00000100150
DEPDC7	-0.1786	4.4241E-02	1.31E-01	ENSG00000121690
DEPP1	-0.4417	1.7182E-04	1.74E-03	ENSG00000165507
DEPTOR	-0.0551	5.5425E-01	7.25E-01	ENSG00000155792
DERA	-0.0289	6.1723E-01	7.74E-01	ENSG00000023697
DERL1	-0.0977	1.4898E-01	3.09E-01	ENSG00000136986
DERL2	-0.2639	6.6027E-06	1.26E-04	ENSG00000072849
DERL3	0.1465	1.7451E-01	3.44E-01	ENSG00000099958
DES	0.2066	8.1271E-02	2.03E-01	ENSG00000175084
DESI1	-0.2255	2.3544E-03	1.39E-02	ENSG00000100418
DESI2	-0.1037	1.2861E-01	2.80E-01	ENSG00000121644
DET1	0.1090	9.7806E-03	4.18E-02	ENSG00000140543
DEUP1	0.0987	3.0808E-01	5.01E-01	ENSG00000165325
DEXI	-0.0485	5.8200E-01	7.48E-01	ENSG00000182108
DFFA	-0.1813	1.4271E-02	5.59E-02	ENSG00000160049
DFFB	0.1862	3.9571E-02	1.20E-01	ENSG00000169598
DFFBP1	-0.0386	6.6234E-01		ENSG00000232303
DGAT1	-0.0466	4.0370E-01	5.98E-01	ENSG00000185000
DGAT2	0.3572	7.7869E-03	3.52E-02	ENSG00000062282
DGAT2L6	0.0310	3.8848E-01		ENSG00000184210
DGAT2L7P	0.0192	1.8594E-01		ENSG00000205267
DGCR11	0.0422	6.3176E-01	7.85E-01	ENSG00000273311
DGCR2	0.1012	1.0556E-01	2.44E-01	ENSG00000070413
DGCR5	0.1198	2.4512E-01	4.31E-01	ENSG00000273032
DGCR6	0.1269	7.8103E-02	1.97E-01	ENSG00000183628

DGCR6	0.0645	2.8557E-01	4.77E-01	ENSG00000278817
DGCR6L	0.0417	4.3067E-01	6.22E-01	ENSG00000128185
DGCR8	-0.0190	8.1678E-01	9.03E-01	ENSG00000128191
DGKA	0.0631	4.7324E-01	6.60E-01	ENSG00000065357
DGKB	0.0163	7.2804E-01	8.50E-01	ENSG00000136267
DGKD	-0.0418	5.5697E-01	7.27E-01	ENSG00000077044
DGKE	0.2381	1.1376E-02	4.69E-02	ENSG00000153933
DGKG	0.5026	3.7398E-04	3.28E-03	ENSG00000058866
DGKH	0.0155	8.6433E-01	9.31E-01	ENSG00000102780
DGKI	0.1152	1.8451E-01	3.58E-01	ENSG00000157680
DGKK	0.2700	3.3336E-02	1.06E-01	ENSG00000274588
DGKQ	0.0516	4.9319E-01	6.77E-01	ENSG00000145214
DGKZ	-0.0393	5.4189E-01	7.15E-01	ENSG00000149091
DGKZP1	-0.0293	6.8338E-01	8.21E-01	ENSG00000179611
DGLUCY	-0.0906	2.0439E-01	3.83E-01	ENSG00000133943
DGUOK	0.0474	3.7607E-01	5.71E-01	ENSG00000114956
DHCR24	-0.2515	1.8522E-02	6.81E-02	ENSG00000116133
DHCR7	-0.3827	1.0761E-04	1.19E-03	ENSG00000172893
DHDDS	0.2153	1.3167E-03	8.84E-03	ENSG00000117682
DHDH	0.3010	4.9517E-03	2.48E-02	ENSG00000104808
DHFR	-0.3326	2.9746E-03	1.67E-02	ENSG00000228716
DHFR2	-0.1191	1.9502E-01	3.71E-01	ENSG00000178700
DHH	0.4996	2.8129E-03	1.60E-02	ENSG00000139549
DHODH	0.1243	2.0215E-01	3.80E-01	ENSG00000102967
DHRS1	0.1126	8.5064E-02	2.10E-01	ENSG00000157379
DHRS11	0.1649	2.1693E-02	7.66E-02	ENSG00000278535
DHRS12	-0.0739	3.2014E-01	5.14E-01	ENSG00000102796
DHRS13	0.2752	2.3955E-03	1.41E-02	ENSG00000167536
DHRS3	-0.0944	3.4802E-01	5.43E-01	ENSG00000162496
DHRS4	-0.1123	1.4076E-01	2.97E-01	ENSG00000157326
DHRS4-AS1	-0.1806	1.5939E-03	1.03E-02	ENSG00000215256
DHRS4L1	0.0511	5.1661E-01	6.95E-01	ENSG00000285467
DHRS4L2	0.0038	9.3876E-01	9.69E-01	ENSG00000187630
DHRS7	-0.2062	2.3006E-03	1.37E-02	ENSG00000100612
DHRS7B	0.0396	5.6591E-01	7.34E-01	ENSG00000109016
DHRS7C	-0.0003	9.1886E-01		ENSG00000184544
DHRS9	0.0305	6.4838E-01	7.96E-01	ENSG00000073737
DHRSX	-0.4929	2.9695E-06	6.56E-05	ENSG00000169084
DHTKD1	-0.0446	4.2003E-01	6.12E-01	ENSG00000181192
DHX15	-0.1243	1.8749E-02	6.86E-02	ENSG00000109606
DHX16	0.0315	6.4515E-01	7.94E-01	ENSG00000204560
DHX29	-0.0046	9.2579E-01	9.63E-01	ENSG00000067248
DHX30	0.1479	7.4583E-03	3.40E-02	ENSG00000132153
DHX32	0.1241	1.2711E-01	2.78E-01	ENSG00000089876
DHX33	0.0121	8.8548E-01	9.42E-01	ENSG00000005100
DHX33-DT	0.0138	7.8834E-01		ENSG00000262099
DHX34	-0.0233	7.7036E-01	8.75E-01	ENSG00000134815
DHX35	0.0504	5.1175E-01	6.92E-01	ENSG00000101452
DHX36	0.1341	2.3556E-02	8.14E-02	ENSG00000174953

DHX37	-0.0259	7.6455E-01	8.72E-01	ENSG00000150990
DHX38	0.1198	7.7224E-02	1.96E-01	ENSG00000140829
DHX40	-0.0081	8.4404E-01	9.19E-01	ENSG00000108406
DHX40P1	0.0246	5.7512E-01		ENSG00000266992
DHX57	0.0484	4.6420E-01	6.52E-01	ENSG00000163214
DHX58	0.0321	7.3248E-01	8.53E-01	ENSG00000108771
DHX8	-0.0159	7.7251E-01	8.77E-01	ENSG00000067596
DHX9	-0.2690	3.9614E-07	1.22E-05	ENSG00000135829
DIABLO	0.2840	6.5387E-03	3.08E-02	ENSG00000184047
DIAPH1	0.0075	9.1002E-01	9.55E-01	ENSG00000131504
DIAPH1-AS1	0.0054	9.4372E-01	9.72E-01	ENSG00000246422
DIAPH2	-0.3419	2.8633E-04	2.63E-03	ENSG00000147202
DIAPH2-AS1	-0.1846	6.7542E-02	1.78E-01	ENSG00000236256
DICER1	0.0759	3.4306E-01	5.38E-01	ENSG00000100697
DICER1-AS1	0.2741	1.8992E-05	2.93E-04	ENSG00000235706
DIDO1	0.5336	1.7608E-11	2.68E-09	ENSG00000101191
DIMT1	-0.1439	5.7936E-02	1.59E-01	ENSG00000086189
DINOL	-0.1369	1.9973E-01	3.77E-01	ENSG00000285244
DIO1	-0.0606	5.1762E-01	6.96E-01	ENSG00000211452
DIO2	-0.5428	6.5103E-03	3.07E-02	ENSG00000211448
DIO2-AS1	-0.0061	8.5037E-01		ENSG00000258766
DIO3	0.1093	2.7548E-01	4.66E-01	ENSG00000197406
DIO3OS	-0.0316	7.4495E-01	8.60E-01	ENSG00000258498
DIP2A	-0.0679	3.0348E-01	4.96E-01	ENSG00000160305
DIP2A-IT1	0.0043	9.5582E-01	9.77E-01	ENSG00000223692
DIP2B	-0.2063	2.2845E-03	1.36E-02	ENSG00000066084
DIP2C	0.0852	2.1705E-01	3.98E-01	ENSG00000151240
DIP2C-AS1	0.0880	3.7592E-01	5.71E-01	ENSG00000180525
DIPK1A	-0.3570	1.3986E-04	1.46E-03	ENSG00000154511
DIPK1B	-0.1264	1.1099E-02	4.60E-02	ENSG00000165716
DIPK1C	-0.0150	4.7621E-01		ENSG00000187773
DIPK2A	-0.2333	1.3367E-02	5.31E-02	ENSG00000181744
DIRAS1	-0.2693	3.1320E-03	1.74E-02	ENSG00000176490
DIRAS2	0.1132	2.4349E-01	4.29E-01	ENSG00000165023
DIRAS3	0.0093	9.3186E-01	9.66E-01	ENSG00000162595
DIS3	0.0736	2.6989E-01	4.59E-01	ENSG00000083520
DIS3L	-0.1747	3.5690E-02	1.11E-01	ENSG00000166938
DIS3L2	0.0256	6.5125E-01	7.98E-01	ENSG00000144535
DISC1	0.5430	4.1495E-06	8.63E-05	ENSG00000162946
DISC1-IT1	0.0140	1.2578E-01		ENSG00000226758
DISP1	-0.3078	8.4577E-06	1.54E-04	ENSG00000154309
DISP2	-0.1210	1.2208E-01	2.70E-01	ENSG00000140323
DISP3	0.2347	2.9849E-02	9.71E-02	ENSG00000204624
DIXDC1	0.0764	1.9368E-01	3.69E-01	ENSG00000150764
DKC1	-0.0738	1.5094E-01	3.12E-01	ENSG00000130826
DKFZP434A062	-0.3718	2.1507E-02	7.61E-02	ENSG00000262075
DKFZp451B082	-0.0558	5.4260E-01	7.15E-01	ENSG00000285564
DKK1	-0.1887	1.0480E-01	2.43E-01	ENSG00000107984
DKK2	-0.2831	2.9430E-02	9.61E-02	ENSG00000155011

DKK3	-0.1490	4.1689E-02	1.25E-01	ENSG00000050165
DKK4	-0.2189	7.5783E-02	1.93E-01	ENSG00000104371
DKKL1	-0.3281	1.1411E-02	4.70E-02	ENSG00000104901
DLAT	0.2594	1.0575E-03	7.42E-03	ENSG00000150768
DLC1	-0.1787	9.5372E-02	2.27E-01	ENSG00000164741
DLD	0.1516	3.4869E-02	1.09E-01	ENSG00000091140
DLEC1	-0.2658	4.2590E-02	1.27E-01	ENSG00000008226
DLEU1	0.1151	4.8593E-02	1.40E-01	ENSG00000176124
DLEU2	-0.0677	4.0855E-01	6.02E-01	ENSG00000231607
DLG1	-0.1383	3.6374E-02	1.13E-01	ENSG00000075711
DLG1-AS1	-0.0004	9.3637E-01	9.69E-01	ENSG00000227375
DLG2	-0.0755	2.0076E-01	3.78E-01	ENSG00000150672
DLG3	0.0668	3.3434E-01	5.30E-01	ENSG00000082458
DLG3-AS1	-0.0095	9.0985E-01	9.55E-01	ENSG00000231651
DLG4	0.2071	6.0857E-03	2.92E-02	ENSG00000132535
DLG5	-0.0909	2.1315E-01	3.93E-01	ENSG00000151208
DLGAP1	-0.1326	1.3767E-01	2.92E-01	ENSG00000170579
DLGAP1-AS3	0.0670	3.9535E-01	5.90E-01	ENSG00000263724
DLGAP1-AS5	0.0328	6.8153E-01		ENSG00000261520
DLGAP3	0.1862	4.8834E-02	1.41E-01	ENSG00000116544
DLGAP4	-0.1966	3.8733E-04	3.35E-03	ENSG00000080845
DLGAP4-AS1	0.1600	1.3688E-01	2.91E-01	ENSG00000232907
DLGAP5	-0.3876	4.7804E-03	2.42E-02	ENSG00000126787
DLK1	-0.2356	2.6174E-02	8.80E-02	ENSG00000185559
DLK2	0.1915	1.0346E-01	2.40E-01	ENSG00000171462
DLL1	-0.1035	2.3014E-01	4.13E-01	ENSG00000198719
DLL3	0.1082	1.9314E-01	3.69E-01	ENSG00000090932
DLL4	0.0231	7.9439E-01	8.89E-01	ENSG00000128917
DLST	0.0805	3.4007E-01	5.35E-01	ENSG00000119689
DLX1	0.1175	1.9886E-01	3.76E-01	ENSG00000144355
DLX2	-0.1187	2.2335E-01	4.05E-01	ENSG00000115844
DLX2-DT	0.0094	4.5041E-01		ENSG00000236651
DLX4	0.0885	2.9920E-01	4.92E-01	ENSG00000108813
DLX5	-0.1882	2.7208E-01	4.62E-01	ENSG00000105880
DLX6	0.0145	4.9587E-01	6.79E-01	ENSG00000006377
DM1-AS	-0.0289	7.8929E-01	8.87E-01	ENSG00000267395
DMAC1	-0.1919	1.6973E-03	1.08E-02	ENSG00000137038
DMAC2	-0.1213	8.4987E-02	2.10E-01	ENSG00000105341
DMAC2L	-0.0650	3.9019E-01	5.85E-01	ENSG00000125375
DMAP1	0.1225	4.7396E-02	1.37E-01	ENSG00000178028
DMBT1	-0.0062	6.7458E-01		ENSG00000187908
DMBX1	-0.0189	8.3650E-01	9.14E-01	ENSG00000197587
DMC1	0.0582	5.5023E-01	7.22E-01	ENSG00000100206
DMD	0.0612	3.7346E-01	5.69E-01	ENSG00000198947
DMGDH	0.0740	4.3928E-01	6.30E-01	ENSG00000132837
DMKN	-0.1311	1.1376E-01	2.57E-01	ENSG00000161249
DMPK	-0.0711	3.3920E-01	5.34E-01	ENSG00000104936
DMRT1	0.0359	5.0517E-01	6.86E-01	ENSG00000137090
DMRT2	-0.0801	3.6475E-01	5.61E-01	ENSG00000173253

DMRT3	-0.4136	1.8329E-03	1.14E-02	ENSG00000064218
DMRTA1	-0.3234	1.3849E-03	9.22E-03	ENSG00000176399
DMRTA2	0.2850	1.6830E-03	1.07E-02	ENSG00000142700
DMRTB1	0.0067	1.4663E-01	3.06E-01	ENSG00000143006
DMRTC1	0.0609	3.9558E-01	5.90E-01	ENSG00000269502
DMRTC1B	0.0477	5.0207E-01	6.84E-01	ENSG00000184911
DMTF1	0.0151	8.2733E-01	9.09E-01	ENSG00000135164
DMTF1-AS1	-0.1419	9.4425E-02	2.26E-01	ENSG00000224046
DMTN	0.1084	1.4453E-01	3.03E-01	ENSG00000158856
DMWD	-0.0147	8.2103E-01	9.06E-01	ENSG00000185800
DMXL1	-0.0433	3.3394E-01	5.29E-01	ENSG00000172869
DMXL2	-0.1543	2.9875E-02	9.72E-02	ENSG00000104093
DNA2	-0.0662	4.9452E-01	6.78E-01	ENSG00000138346
DNAAF1	-0.2685	1.0360E-02	4.36E-02	ENSG00000154099
DNAAF10	-0.2253	2.6347E-02	8.84E-02	ENSG00000243667
DNAAF2	0.1156	3.3924E-02	1.07E-01	ENSG00000165506
DNAAF3	-0.1213	1.7845E-01	3.49E-01	ENSG00000167646
DNAAF4	-0.1503	1.4523E-01	3.04E-01	ENSG00000256061
DNAAF5	-0.0469	5.4793E-01	7.20E-01	ENSG00000164818
DNAAF6	-0.4269	1.4867E-02	5.76E-02	ENSG00000080572
DNAAF8	0.1774	1.1707E-01	2.63E-01	ENSG00000166246
DNAAF9	0.0896	2.9585E-01	4.88E-01	ENSG00000088854
DNAH1	0.0363	6.9737E-01	8.31E-01	ENSG00000114841
DNAH10	0.0045	9.5931E-01	9.79E-01	ENSG00000197653
DNAH11	-0.5631	1.1766E-03	8.07E-03	ENSG00000105877
DNAH12	-0.1416	1.6988E-01	3.38E-01	ENSG00000174844
DNAH14	-0.1766	3.7035E-02	1.14E-01	ENSG00000185842
DNAH17	0.3147	2.5433E-02	8.63E-02	ENSG00000187775
DNAH2	0.0118	9.1426E-01	9.57E-01	ENSG00000183914
DNAH3	-0.0998	3.2881E-01	5.23E-01	ENSG00000158486
DNAH5	0.2808	1.4975E-02	5.79E-02	ENSG00000039139
DNAH6	-0.0759	2.9816E-01	4.90E-01	ENSG00000115423
DNAH7	-0.2650	1.0772E-02	4.49E-02	ENSG00000118997
DNAH8	0.0032	4.2596E-01		ENSG00000124721
DNAH9	-0.3053	3.0334E-03	1.70E-02	ENSG00000007174
DNAI1	-0.1043	2.8690E-01	4.78E-01	ENSG00000122735
DNAI2	0.0137	8.8177E-01	9.40E-01	ENSG00000171595
DNAI3	-0.4887	1.5946E-04	1.63E-03	ENSG00000162643
DNAI4	-0.4579	3.3500E-08	1.58E-06	ENSG00000152763
DNAI7	0.2992	4.6629E-03	2.37E-02	ENSG00000118307
DNAJA1	-0.1837	3.2358E-03	1.78E-02	ENSG00000086061
DNAJA2	0.0135	8.0826E-01	8.98E-01	ENSG00000069345
DNAJA3	0.1161	1.4074E-01	2.97E-01	ENSG00000103423
DNAJA4	0.0774	3.8405E-01	5.79E-01	ENSG00000140403
DNAJB1	-0.0167	8.0504E-01	8.96E-01	ENSG00000132002
DNAJB11	-0.0758	1.9290E-01	3.68E-01	ENSG00000090520
DNAJB12	-0.2728	1.8645E-07	6.54E-06	ENSG00000148719
DNAJB13	-0.4636	1.2344E-02	4.99E-02	ENSG00000187726
DNAJB14	-0.0985	6.8731E-02	1.80E-01	ENSG00000164031

DNAJB2	-0.4337	1.8060E-04	1.81E-03	ENSG00000135924
DNAJB4	-0.3924	9.8557E-07	2.63E-05	ENSG00000162616
DNAJB5	0.2324	1.2579E-02	5.06E-02	ENSG00000137094
DNAJB5-DT	0.0020	8.3987E-01	9.16E-01	ENSG00000281491
DNAJB6	-0.0189	7.4598E-01	8.61E-01	ENSG00000105993
DNAJB6P1	0.0262	7.2391E-01	8.47E-01	ENSG00000254612
DNAJB6P8	0.0018	7.3997E-01		ENSG00000264529
DNAJB7	0.0141	1.4163E-01	2.99E-01	ENSG00000172404
DNAJB9	-0.0132	8.7647E-01	9.37E-01	ENSG00000128590
DNAJC1	-0.1094	1.1524E-01	2.60E-01	ENSG00000136770
DNAJC10	-0.0106	8.2633E-01	9.09E-01	ENSG00000077232
DNAJC11	0.3842	3.5097E-05	4.79E-04	ENSG00000007923
DNAJC12	-0.0470	4.5529E-01	6.45E-01	ENSG00000108176
DNAJC13	0.0471	5.6336E-01	7.32E-01	ENSG00000138246
DNAJC14	0.1827	6.8253E-02	1.79E-01	ENSG00000135392
DNAJC15	-0.2488	1.7951E-04	1.81E-03	ENSG00000120675
DNAJC16	0.0990	2.5337E-01	4.41E-01	ENSG00000116138
DNAJC17	0.0607	3.7671E-01	5.72E-01	ENSG00000104129
DNAJC18	-0.0209	6.9943E-01	8.32E-01	ENSG00000170464
DNAJC19	0.2247	1.9948E-02	7.18E-02	ENSG00000205981
DNAJC2	0.0674	2.1799E-01	3.99E-01	ENSG00000105821
DNAJC21	-0.1492	6.6854E-05	8.12E-04	ENSG00000168724
DNAJC22	0.1960	9.5533E-02	2.28E-01	ENSG00000178401
DNAJC24	0.0485	4.4530E-01	6.35E-01	ENSG00000170946
DNAJC25	-0.1703	1.9835E-02	7.15E-02	ENSG00000059769
DNAJC27	-0.0465	4.8616E-01	6.71E-01	ENSG00000115137
DNAJC27-AS1	-0.0398	5.6786E-01	7.36E-01	ENSG00000224165
DNAJC28	0.0377	6.5772E-01	8.02E-01	ENSG00000177692
DNAJC3	-0.1988	4.7721E-04	3.95E-03	ENSG00000102580
DNAJC3-DT	0.2690	2.9817E-05	4.21E-04	ENSG00000247400
DNAJC30	-0.2004	1.6609E-03	1.06E-02	ENSG00000176410
DNAJC4	-0.0470	4.2223E-01	6.15E-01	ENSG00000110011
DNAJC5	-0.0338	6.8356E-01	8.21E-01	ENSG00000101152
DNAJC5B	0.0093	9.0222E-01	9.51E-01	ENSG00000147570
DNAJC5G	-0.0307	3.2717E-01		ENSG00000163793
DNAJC6	0.0467	4.2908E-01	6.21E-01	ENSG00000116675
DNAJC7	-0.0287	6.6379E-01	8.07E-01	ENSG00000168259
DNAJC8	-0.2221	8.2836E-08	3.34E-06	ENSG00000126698
DNAJC9	0.0684	4.0036E-01	5.94E-01	ENSG00000213551
DNAJC9-AS1	0.3192	6.0713E-04	4.81E-03	ENSG00000227540
DNAJC9-AS1	-0.0212	8.2816E-01	9.10E-01	ENSG00000236756
DNAL1	-0.1906	2.2538E-03	1.35E-02	ENSG00000119661
DNAL4	-0.0574	3.1430E-01	5.08E-01	ENSG00000100246
DNALI1	-0.3958	9.5308E-12	1.51E-09	ENSG00000163879
DNASE1	0.1076	1.9491E-01	3.71E-01	ENSG00000213918
DNASE1L1	-0.0568	4.0751E-01	6.01E-01	ENSG00000013563
DNASE1L2	0.1601	6.8694E-02	1.80E-01	ENSG00000167968
DNASE1L3	0.0086	4.9906E-01		ENSG00000163687
DNASE2	0.0313	6.7398E-01	8.13E-01	ENSG00000105612

DNASE2B	-0.0142	7.7300E-01		ENSG00000137976
DNER	0.3677	8.6257E-04	6.34E-03	ENSG00000187957
DNHD1	-0.0601	5.1859E-01	6.96E-01	ENSG00000179532
DNLZ	0.1402	8.8465E-02	2.16E-01	ENSG00000213221
DNM1	0.1432	3.3743E-02	1.07E-01	ENSG00000106976
DNM1L	-0.0302	5.1829E-01	6.96E-01	ENSG00000087470
DNM1P29	0.0102	2.7814E-01		ENSG00000274966
DNM2	0.0591	3.9985E-01	5.94E-01	ENSG00000079805
DNM3	0.2400	9.9424E-03	4.23E-02	ENSG00000197959
DNM3-IT1	0.0560	1.7671E-01		ENSG00000233540
DNMBP	-0.2233	1.3372E-02	5.31E-02	ENSG00000107554
DNMBP-AS1	-0.0805	1.3510E-01		ENSG00000227695
DNMT1	-0.2267	6.9629E-04	5.37E-03	ENSG00000130816
DNMT3A	0.0859	2.3265E-01	4.16E-01	ENSG00000119772
DNMT3B	0.0869	3.6837E-01	5.64E-01	ENSG00000088305
DNPEP	0.0725	2.6386E-01	4.53E-01	ENSG00000123992
DNPH1	-0.3702	4.1125E-04	3.52E-03	ENSG00000112667
DNTT	0.0212	6.9941E-01		ENSG00000107447
DNTTIP1	0.2198	7.5467E-07	2.11E-05	ENSG00000101457
DNTTIP2	-0.2951	3.1726E-07	1.02E-05	ENSG00000067334
DOC2A	0.1604	8.0918E-02	2.02E-01	ENSG00000149927
DOC2B	0.1652	6.0890E-02	1.65E-01	ENSG00000272636
DOCK1	-0.3264	8.1923E-06	1.50E-04	ENSG00000150760
DOCK10	0.2723	6.0471E-03	2.90E-02	ENSG00000135905
DOCK11	0.2558	3.7332E-02	1.15E-01	ENSG00000147251
DOCK11P1	-0.0026	8.5582E-01		ENSG00000259000
DOCK2	0.1259	1.4100E-01	2.98E-01	ENSG00000134516
DOCK3	0.3748	2.3913E-04	2.26E-03	ENSG00000088538
DOCK4	0.3607	1.8896E-03	1.17E-02	ENSG00000128512
DOCK4-AS1	-0.0069	6.4228E-01		ENSG00000225572
DOCK5	0.1068	2.3017E-01	4.13E-01	ENSG00000147459
DOCK6	-0.1254	1.5990E-01	3.25E-01	ENSG00000130158
DOCK6-AS1	0.0088	9.4950E-01	9.75E-01	ENSG00000267082
DOCK7	-0.1511	3.6896E-02	1.14E-01	ENSG00000116641
DOCK8	0.0258	7.5092E-01	8.64E-01	ENSG00000107099
DOCK8-AS1	0.0474	5.6692E-01	7.35E-01	ENSG00000183784
DOCK9	0.0487	5.9082E-01	7.54E-01	ENSG00000088387
DOHH	0.1928	5.9775E-03	2.88E-02	ENSG00000129932
DOK1	0.0444	6.2938E-01	7.83E-01	ENSG00000115325
DOK4	0.0242	7.4242E-01	8.59E-01	ENSG00000125170
DOK5	-0.4233	2.8447E-06	6.35E-05	ENSG00000101134
DOK6	-0.0897	3.1684E-01	5.10E-01	ENSG00000206052
DOK7	-0.0884	3.4352E-01	5.39E-01	ENSG00000175920
DOLK	-0.0622	3.5957E-01	5.56E-01	ENSG00000175283
DOLPP1	0.0480	4.4908E-01	6.39E-01	ENSG00000167130
DONSON	0.1571	2.2824E-02	7.95E-02	ENSG00000159147
DONSONP1	0.0148	7.1183E-02		ENSG00000227267
DOP1A	0.3452	4.7785E-04	3.95E-03	ENSG00000083097
DOP1B	0.0190	8.0620E-01	8.97E-01	ENSG00000142197

DOT1L	-0.0998	2.1099E-01	3.91E-01	ENSG00000104885
DPAGT1	-0.0740	2.6415E-01	4.53E-01	ENSG00000172269
DPCD	-0.0139	7.9118E-01	8.88E-01	ENSG00000166171
DPEP1	0.2443	3.3965E-02	1.07E-01	ENSG00000015413
DPEP2	-0.0154	4.8140E-01		ENSG00000167261
DPF1	0.3635	5.2405E-03	2.60E-02	ENSG00000011332
DPF2	0.0386	5.0466E-01	6.86E-01	ENSG00000133884
DPF3	-0.1364	1.2030E-01	2.68E-01	ENSG00000205683
DPH1	0.0953	2.5343E-01	4.41E-01	ENSG00000108963
DPH2	0.4104	3.2496E-08	1.55E-06	ENSG00000132768
DPH3	0.1603	8.2363E-03	3.68E-02	ENSG00000154813
DPH5	-0.3157	6.2559E-05	7.71E-04	ENSG00000117543
DPH5-DT	-0.1819	3.1134E-02	1.00E-01	ENSG00000233184
DPH6	0.2406	5.8589E-04	4.68E-03	ENSG00000134146
DPH7	0.1581	1.3085E-02	5.22E-02	ENSG00000148399
DPM1	-0.1983	4.3010E-03	2.24E-02	ENSG00000000419
DPM2	-0.0791	1.5061E-01	3.11E-01	ENSG00000136908
DPM3	-0.4110	2.8144E-06	6.31E-05	ENSG00000179085
DPP10	-0.1040	2.1624E-01	3.97E-01	ENSG00000175497
DPP10-AS1	-0.0499	5.6387E-01	7.33E-01	ENSG00000235026
DPP3	0.1325	1.0885E-01	2.49E-01	ENSG00000254986
DPP3-DT	-0.2357	6.2243E-03	2.97E-02	ENSG00000255517
DPP4	0.0378	2.6287E-01		ENSG00000197635
DPP6	-0.2150	2.0602E-02	7.36E-02	ENSG00000130226
DPP7	-0.3204	1.3685E-03	9.13E-03	ENSG00000176978
DPP8	0.0905	1.6193E-02	6.14E-02	ENSG00000074603
DPP9	0.0460	4.7640E-01	6.63E-01	ENSG00000142002
DPP9-AS1	-0.0107	7.9545E-01		ENSG00000205790
DPPA3P6	-0.0592	6.9085E-01		ENSG00000262516
DPPA4	-0.0185	5.2414E-01		ENSG00000121570
DPRX	-0.0276	7.3019E-01		ENSG00000204595
DPRXP3	-0.1750	2.9327E-02		ENSG00000282308
DPRXP4	-0.0136	6.6420E-01		ENSG00000264743
DPY19L1	-0.2235	1.6133E-02	6.13E-02	ENSG00000173852
DPY19L2	0.1068	1.8504E-01	3.58E-01	ENSG00000177990
DPY19L3	-0.0332	6.6156E-01	8.05E-01	ENSG00000178904
DPY19L3-DT	0.0588	5.4128E-01	7.14E-01	ENSG00000267213
DPY19L4	-0.1129	8.6403E-02	2.12E-01	ENSG00000156162
DPY30	-0.2878	2.5324E-06	5.80E-05	ENSG00000162961
DPYD	0.0457	5.1995E-01	6.97E-01	ENSG00000188641
DPYD-AS1	0.0283	3.0636E-01	4.99E-01	ENSG00000232878
DPYD-AS2	0.0278	3.1747E-01		ENSG00000235777
DPYSL2	-0.1075	1.7577E-01	3.46E-01	ENSG00000092964
DPYSL3	-0.0108	8.7342E-01	9.36E-01	ENSG00000113657
DPYSL4	0.0281	7.4664E-01	8.61E-01	ENSG00000151640
DPYSL5	-0.0147	8.6397E-01	9.31E-01	ENSG00000157851
DQX1	-0.0343	2.6457E-01		ENSG00000144045
DR1	-0.1226	2.4996E-02	8.51E-02	ENSG00000117505
DRAIC	0.0094	9.2224E-01	9.61E-01	ENSG00000245750

DRAM1	-0.1134	1.4578E-01	3.04E-01	ENSG00000136048
DRAM2	-0.1657	3.1650E-02	1.01E-01	ENSG00000156171
DRAP1	0.0611	2.6414E-01	4.53E-01	ENSG00000175550
DRAXIN	-0.3212	3.8783E-03	2.06E-02	ENSG00000162490
DRC1	-0.2474	1.5089E-02	5.83E-02	ENSG00000157856
DRC3	-0.1384	1.2057E-01	2.68E-01	ENSG00000171962
DRD1	-0.1410	1.2666E-01	2.77E-01	ENSG00000184845
DRD3	0.0728	1.7677E-01	3.47E-01	ENSG00000151577
DRD4	-0.1996	9.0729E-02	2.20E-01	ENSG00000069696
DRD5	-0.0635	3.1983E-01	5.14E-01	ENSG00000169676
DRG1	-0.0020	8.9309E-01	9.47E-01	ENSG00000185721
DRG1P2	-0.0508	5.9062E-01	7.54E-01	ENSG00000240393
DRG2	-0.2438	1.3801E-04	1.45E-03	ENSG00000108591
DRGX	-0.0353	2.0347E-01		ENSG00000165606
DRICH1	-0.0592	7.2847E-01		ENSG00000189269
DROSHA	-0.0027	9.5981E-01	9.80E-01	ENSG00000113360
DRP2	0.3923	3.3555E-03	1.83E-02	ENSG00000102385
DSC1	-0.1462	1.4344E-01	3.01E-01	ENSG00000134765
DSC2	-0.1257	1.4861E-01	3.09E-01	ENSG00000134755
DSC3	-0.0386	6.4993E-01	7.97E-01	ENSG00000134762
DSCAM	0.2589	2.5445E-02	8.64E-02	ENSG00000171587
DSCAM-AS1	0.0187	5.3367E-01		ENSG00000235123
DSCAM-IT1	-0.0205	5.4327E-01		ENSG00000233756
DSCAML1	-0.1328	2.0640E-01	3.85E-01	ENSG00000177103
DSCR8	-0.2069	6.4083E-02	1.71E-01	ENSG00000198054
DSCR9	0.0869	8.3631E-02	2.07E-01	ENSG00000230366
DSE	-0.0678	4.2586E-01	6.18E-01	ENSG00000111817
DSEL	0.0816	2.6412E-01	4.53E-01	ENSG00000171451
DSEL-AS1	0.1011	1.3790E-01	2.93E-01	ENSG00000265533
DSG1	-0.0071	9.8662E-01		ENSG00000134760
DSG2	-0.0404	5.2785E-01	7.03E-01	ENSG00000046604
DSG2-AS1	-0.0294	9.0413E-01	9.52E-01	ENSG00000264859
DSG3	-0.0416	6.3989E-01	7.91E-01	ENSG00000134757
DSN1	0.0454	5.3197E-01	7.06E-01	ENSG00000149636
DSP	-0.0152	8.0501E-01	8.96E-01	ENSG00000096696
DSP-AS1	0.1163	2.2166E-01	4.03E-01	ENSG00000261189
DST	-0.1186	3.8611E-02	1.18E-01	ENSG00000151914
DSTN	-0.0341	4.9820E-01	6.81E-01	ENSG00000125868
DSTNP3	0.0217	8.1628E-01	9.03E-01	ENSG00000253833
DSTYK	-0.0551	2.6234E-01	4.51E-01	ENSG00000133059
DTD1	0.0851	1.3320E-01	2.86E-01	ENSG00000125821
DTD1-AS1	0.0251	4.2901E-01		ENSG00000233993
DTD2	-0.0645	5.0032E-01	6.83E-01	ENSG00000129480
DTHD1	0.0309	7.0350E-01	8.34E-01	ENSG00000197057
DTL	-0.3186	1.7417E-02	6.49E-02	ENSG00000143476
DTNA	-0.0396	4.3779E-01	6.29E-01	ENSG00000134769
DTNB	0.1718	1.0463E-02	4.39E-02	ENSG00000138101
DTNBP1	-0.1240	9.8784E-03	4.21E-02	ENSG00000047579
DTWD1	-0.1548	2.5451E-02	8.64E-02	ENSG00000104047

DTWD2	-0.0171	8.4628E-01	9.20E-01	ENSG00000169570
DTX1	-0.0173	7.9356E-01	8.89E-01	ENSG00000135144
DTX2	0.4061	2.0821E-04	2.02E-03	ENSG00000091073
DTX2P1-UPK3BP1-PMS2	-0.0256	7.7285E-01	8.77E-01	ENSG00000265479
DTX3	-0.3257	2.7452E-05	3.95E-04	ENSG00000178498
DTX3L	-0.1517	1.5498E-01	3.17E-01	ENSG00000163840
DTX4	-0.1726	6.8167E-02	1.79E-01	ENSG00000110042
DTYMK	-0.0545	3.5168E-01	5.47E-01	ENSG00000168393
DUBR	-0.1521	2.8777E-02	9.45E-02	ENSG00000243701
DUOX1	0.2121	7.1709E-02	1.85E-01	ENSG00000137857
DUOX2	0.0311	5.7735E-01	7.44E-01	ENSG00000140279
DUOXA1	0.1456	1.0897E-01	2.50E-01	ENSG00000140254
DUOXA2	0.0153	6.3007E-01		ENSG00000140274
DUS1L	0.2347	1.1439E-04	1.25E-03	ENSG00000169718
DUS2	0.2032	1.8928E-02	6.91E-02	ENSG00000167264
DUS3L	0.2868	9.2199E-05	1.05E-03	ENSG00000141994
DUS4L	0.0273	7.4894E-01	8.63E-01	ENSG00000105865
DUS4L-BCAP29	0.0103	9.1299E-01	9.56E-01	ENSG00000288558
DUSP1	-0.0601	4.5202E-01	6.42E-01	ENSG00000120129
DUSP11	-0.0265	6.1008E-01	7.69E-01	ENSG00000144048
DUSP12	-0.1164	6.4070E-02	1.71E-01	ENSG00000081721
DUSP13B	-0.0367	6.2500E-01	7.80E-01	ENSG00000079393
DUSP14	-0.2390	1.0646E-03	7.46E-03	ENSG00000276023
DUSP15	0.1788	4.0790E-02	1.23E-01	ENSG00000149599
DUSP16	-0.4035	3.3365E-04	2.98E-03	ENSG00000111266
DUSP18	-0.0141	8.3596E-01	9.14E-01	ENSG00000167065
DUSP19	-0.1113	2.7696E-01	4.68E-01	ENSG00000162999
DUSP2	0.0705	4.6604E-01	6.54E-01	ENSG00000158050
DUSP22	-0.1976	6.2256E-04	4.91E-03	ENSG00000112679
DUSP23	-0.0256	7.3282E-01	8.53E-01	ENSG00000158716
DUSP26	-0.0549	4.7777E-01	6.64E-01	ENSG00000133878
DUSP28	0.0752	2.2561E-01	4.08E-01	ENSG00000188542
DUSP3	-0.0900	1.1963E-01	2.67E-01	ENSG00000108861
DUSP4	0.3602	7.6311E-05	9.02E-04	ENSG00000120875
DUSP5	-0.5121	6.0723E-07	1.76E-05	ENSG00000138166
DUSP6	-0.1160	1.7716E-01	3.48E-01	ENSG00000139318
DUSP7	0.1200	1.3055E-01	2.82E-01	ENSG00000164086
DUSP8	-0.1268	6.0948E-02	1.65E-01	ENSG00000184545
DUSP9	0.1394	1.8343E-01	3.56E-01	ENSG00000130829
DUT	-0.3637	1.2698E-04	1.35E-03	ENSG00000128951
DUTP6	0.3049	3.5459E-02	1.11E-01	ENSG00000225171
DUTP7	0.0294	3.8708E-01		ENSG00000250473
DUX4	0.0066	3.2916E-01		ENSG00000260596
DUXAP1	0.0013	8.2868E-01		ENSG00000259056
DUXB	-0.0125	8.0996E-01		ENSG00000282757
DVL1	-0.1359	1.0942E-01	2.50E-01	ENSG00000107404
DVL2	-0.1574	5.7580E-03	2.79E-02	ENSG00000004975
DVL3	0.1804	1.3677E-02	5.41E-02	ENSG00000161202
DXO	-0.0443	4.6254E-01	6.51E-01	ENSG00000204348

DYDC1	-0.0313	6.0281E-01	7.63E-01	ENSG00000170788
DYM	-0.0325	4.6919E-01	6.57E-01	ENSG00000141627
DYM-AS1	-0.0185	4.2228E-01		ENSG00000264269
DYNAP	-0.0020	5.5125E-01		ENSG00000178690
DYNC1H1	-0.1206	2.7606E-02	9.15E-02	ENSG00000197102
DYNC1I1	0.0584	2.8079E-01	4.72E-01	ENSG00000158560
DYNC1I2	-0.0980	2.4092E-02	8.28E-02	ENSG00000077380
DYNC1I2P1	-0.0081	9.3002E-01	9.66E-01	ENSG00000225137
DYNC1LI1	-0.0078	8.9422E-01	9.47E-01	ENSG00000144635
DYNC1LI2	-0.1204	4.8711E-02	1.40E-01	ENSG00000135720
DYNC1LI2-DT	-0.0481	5.3429E-01	7.08E-01	ENSG00000246777
DYNC2H1	-0.2078	4.5062E-03	2.31E-02	ENSG00000187240
DYNC2I1	0.1858	4.3708E-03	2.26E-02	ENSG00000126870
DYNC2I2	-0.3571	4.9905E-07	1.49E-05	ENSG00000119333
DYNC2LI1	-0.0333	4.8428E-01	6.69E-01	ENSG00000138036
DYNLL1	-0.4732	3.4630E-11	4.78E-09	ENSG00000088986
DYNLL2	-0.2591	8.3343E-03	3.71E-02	ENSG00000264364
DYNLL2-DT	0.0251	1.2456E-01		ENSG00000266290
DYNLRB1	0.0058	9.5036E-01	9.75E-01	ENSG00000125971
DYNLRB2	-0.2854	2.3328E-03	1.38E-02	ENSG00000168589
DYNLT1	-0.1910	1.1241E-03	7.78E-03	ENSG00000146425
DYNLT2	0.1910	9.4417E-02	2.26E-01	ENSG00000184786
DYNLT2B	-0.4037	3.4726E-05	4.75E-04	ENSG00000213123
DYNLT3	0.0439	5.2464E-01	7.01E-01	ENSG00000165169
DYNLT4	-0.0902	3.3926E-01	5.34E-01	ENSG00000188396
DYRK1A	-0.3543	1.4901E-04	1.55E-03	ENSG00000157540
DYRK1B	-0.0624	3.8517E-01	5.80E-01	ENSG00000105204
DYRK2	-0.2584	1.2099E-04	1.30E-03	ENSG00000127334
DYRK3	-0.1321	1.0534E-01	2.44E-01	ENSG00000143479
DYRK3-AS1	0.0816	4.0625E-01	6.00E-01	ENSG00000237605
DYRK4	-0.3342	5.7969E-08	2.51E-06	ENSG00000010219
DYSF	-0.0652	3.2512E-01		ENSG00000135636
DYTN	-0.0063	9.2635E-01		ENSG00000232125
DZANK1	0.2080	5.9344E-03	2.86E-02	ENSG00000089091
DZIP1	-0.3281	4.8342E-04	3.99E-03	ENSG00000134874
DZIP1L	-0.3616	3.2111E-03	1.77E-02	ENSG00000158163
DZIP3	-0.1190	2.2400E-02	7.84E-02	ENSG00000198919
E2F1	-0.0052	9.5191E-01	9.76E-01	ENSG00000101412
E2F3	-0.1876	3.1009E-02	9.98E-02	ENSG00000112242
E2F3-IT1	-0.0005	8.5150E-01		ENSG00000224707
E2F4	0.1566	7.8729E-03	3.55E-02	ENSG00000205250
E2F5	0.0882	2.7980E-01	4.71E-01	ENSG00000133740
E2F5-DT	-0.0756	5.9812E-01	7.59E-01	ENSG00000260493
E2F6	0.1379	7.9183E-02	1.99E-01	ENSG00000169016
E2F7	-0.5727	6.1058E-07	1.76E-05	ENSG00000165891
E2F8	0.1703	1.2966E-01	2.81E-01	ENSG00000129173
E4F1	0.0818	2.5738E-01	4.45E-01	ENSG00000167967
EAF1	-0.1396	4.8758E-02	1.41E-01	ENSG00000144597
EAF2	-0.1315	1.0845E-01	2.49E-01	ENSG00000145088

EAPP	-0.0508	2.4308E-01	4.28E-01	ENSG00000129518
EARS2	0.3393	1.3739E-05	2.27E-04	ENSG00000103356
EBAG9	0.0523	4.0023E-01	5.94E-01	ENSG00000147654
EBAG9P1	0.0321	4.4738E-01	6.37E-01	ENSG00000233690
EBF1	0.5340	2.6523E-06	6.02E-05	ENSG00000164330
EBF2	0.0680	1.2098E-01		ENSG00000221818
EBF3	0.5116	6.8506E-05	8.26E-04	ENSG00000108001
EBF4	-0.0761	4.1287E-01	6.06E-01	ENSG00000088881
EBI3	-0.0276	8.4423E-01		ENSG00000105246
EBLN2	-0.0792	4.1955E-01	6.12E-01	ENSG00000255423
EBLN3P	0.0116	8.4477E-01	9.19E-01	ENSG00000281649
EBNA1BP2	0.0438	4.6258E-01	6.51E-01	ENSG00000117395
EBP	-0.2628	1.7930E-05	2.81E-04	ENSG00000147155
EBPL	-0.1235	1.1118E-01	2.53E-01	ENSG00000123179
ECD	0.1359	3.4546E-02	1.08E-01	ENSG00000122882
ECE1	-0.0058	9.2596E-01	9.63E-01	ENSG00000117298
ECE1-AS1	-0.1053	2.8461E-01	4.76E-01	ENSG00000231105
ECE2	0.1356	8.9611E-02	2.18E-01	ENSG00000145194
ECEL1	-0.1153	1.9041E-01	3.65E-01	ENSG00000171551
ECH1	-0.2584	2.9486E-03	1.66E-02	ENSG00000104823
ECHDC1	-0.0963	1.0286E-01	2.39E-01	ENSG00000093144
ECHDC2	-0.5732	7.7980E-05	9.16E-04	ENSG00000121310
ECHDC3	0.1624	9.6048E-02	2.28E-01	ENSG00000134463
ECHS1	-0.1758	9.0098E-04	6.56E-03	ENSG00000127884
ECI1	-0.1622	5.0453E-02	1.44E-01	ENSG00000167969
ECI2	-0.3356	2.2553E-06	5.22E-05	ENSG00000198721
ECI2-DT	0.0013	9.9463E-01		ENSG00000234817
ECM1	0.4262	6.3750E-04	4.99E-03	ENSG00000143369
ECPAS	-0.3023	3.4991E-05	4.78E-04	ENSG00000136813
ECRG4	-0.2814	3.6074E-02	1.12E-01	ENSG00000119147
ECSIT	-0.1490	1.0219E-02	4.32E-02	ENSG00000130159
ECT2	-0.3340	1.9452E-03	1.20E-02	ENSG00000114346
ECT2L	-0.0231	8.0727E-01	8.97E-01	ENSG00000203734
EDA	-0.0116	9.0240E-01	9.51E-01	ENSG00000158813
EDA2R	0.0007	9.9278E-01	9.96E-01	ENSG00000131080
EDAR	0.0174	7.5578E-01	8.67E-01	ENSG00000135960
EDARADD	-0.1082	3.4513E-01	5.40E-01	ENSG00000186197
EDC3	0.2364	1.0154E-03	7.19E-03	ENSG00000179151
EDC4	0.4145	3.2233E-03	1.78E-02	ENSG00000038358
EDDM13	0.0438	6.4526E-01	7.94E-01	ENSG00000267710
EDEM1	-0.0585	4.1480E-01	6.08E-01	ENSG00000134109
EDEM2	0.0031	9.6181E-01	9.81E-01	ENSG00000088298
EDEM3	0.3066	5.6699E-04	4.55E-03	ENSG00000116406
EDF1	-0.2191	2.6862E-05	3.88E-04	ENSG00000107223
EDIL3	0.2230	2.3064E-02	8.02E-02	ENSG00000164176
EDIL3-DT	0.5112	3.1646E-03	1.75E-02	ENSG00000250320
EDN1	-0.1704	1.2951E-01	2.81E-01	ENSG00000078401
EDN2	-0.1069	2.4694E-01	4.33E-01	ENSG00000127129
EDN3	0.0209	3.9408E-01		ENSG00000124205

EDNRA	-0.5343	2.0602E-03	1.25E-02	ENSG00000151617
EDNRB	0.0387	6.7446E-01	8.14E-01	ENSG00000136160
EDRF1	0.2409	1.1437E-03	7.88E-03	ENSG00000107938
EDRF1-AS1	-0.0434	5.5686E-01	7.27E-01	ENSG00000236991
EDRF1-DT	-0.0505	2.1315E-01		ENSG00000224023
EEA1	0.0801	2.1959E-01	4.01E-01	ENSG00000102189
EED	0.4550	2.0548E-06	4.84E-05	ENSG00000074266
EEF1A1	-0.3758	3.5203E-05	4.80E-04	ENSG00000156508
EEF1A1P10	-0.0768	3.8625E-01	5.81E-01	ENSG00000243746
EEF1A1P14	-0.0994	2.8978E-01	4.81E-01	ENSG00000233057
EEF1A1P16	-0.0213	6.1598E-01		ENSG00000213235
EEF1A1P19	-0.0634	5.1299E-01	6.92E-01	ENSG00000249855
EEF1A1P22	-0.2104	6.3659E-02	1.70E-01	ENSG00000259612
EEF1A1P23	-0.0182	6.0758E-01		ENSG00000236297
EEF1A1P24	-0.0391	7.7028E-01		ENSG00000223668
EEF1A1P25	-0.2180	5.6739E-02	1.57E-01	ENSG00000241429
EEF1A1P3	0.1579	1.4023E-01	2.96E-01	ENSG00000232587
EEF1A1P35	-0.0355	6.9867E-01	8.31E-01	ENSG00000250144
EEF1A1P39	-0.0102	7.7953E-01		ENSG00000230125
EEF1A1P4	-0.0777	4.2760E-01	6.20E-01	ENSG00000245205
EEF1A1P8	-0.1522	3.2142E-01	5.15E-01	ENSG00000223529
EEF1A2	-0.0972	2.0097E-01	3.78E-01	ENSG00000101210
EEF1AKMT1	-0.3543	1.1525E-05	1.96E-04	ENSG00000150456
EEF1AKMT2	-0.1105	6.5352E-02	1.74E-01	ENSG00000203791
EEF1AKMT3	0.1874	4.3975E-02	1.30E-01	ENSG00000123427
EEF1AKMT4	0.5051	9.8466E-05	1.10E-03	ENSG00000284753
EEF1B2	-0.0377	4.9252E-01	6.76E-01	ENSG00000114942
EEF1B2P2	-0.0866	5.4465E-01	7.17E-01	ENSG00000213864
EEF1B2P3	-0.0435	6.4420E-01	7.94E-01	ENSG00000232472
EEF1B2P6	0.0964	3.2155E-01	5.15E-01	ENSG00000213261
EEF1B2P7	0.0440	5.7669E-01	7.43E-01	ENSG00000213055
EEF1D	-0.0987	1.3738E-01	2.92E-01	ENSG00000104529
EEF1DP1	0.0502	1.3279E-01	2.86E-01	ENSG00000228887
EEF1DP4	-0.0070	5.0998E-01		ENSG00000213640
EEF1DP7	0.1328	1.4972E-01	3.10E-01	ENSG00000263883
EEF1E1	-0.0456	4.6985E-01	6.57E-01	ENSG00000124802
EEF1GP5	-0.0193	8.9162E-01		ENSG00000234785
EEF1GP7	-0.1192	8.4716E-02		ENSG00000236290
EEF2	-0.0739	3.3154E-01	5.26E-01	ENSG00000167658
EEF2K	0.2444	2.5060E-03	1.46E-02	ENSG00000103319
EEF2KMT	0.0598	4.8408E-01	6.69E-01	ENSG00000118894
EEFSEC	0.1112	1.2642E-01	2.77E-01	ENSG00000132394
EEIG1	0.1405	1.0662E-01	2.46E-01	ENSG00000167106
EEIG2	0.3280	6.5707E-05	8.01E-04	ENSG00000162636
EEPD1	-0.1090	2.3760E-01	4.22E-01	ENSG00000122547
EFCAB10	-0.3644	5.4012E-03	2.66E-02	ENSG00000185055
EFCAB10-AS1	-0.0231	7.7521E-01	8.78E-01	ENSG00000272604
EFCAB11	-0.2301	1.3443E-03	9.00E-03	ENSG00000140025
EFCAB12	-0.1230	2.2404E-01	4.06E-01	ENSG00000172771

EFCAB13	-0.0576	5.5169E-01	7.23E-01	ENSG00000178852
EFCAB13-DT	-0.0139	6.6160E-01	8.05E-01	ENSG00000263293
EFCAB14	0.0023	9.4459E-01	9.72E-01	ENSG00000159658
EFCAB15P	-0.2958	1.8901E-02	6.90E-02	ENSG00000233483
EFCAB2	-0.3963	2.3043E-06	5.32E-05	ENSG00000203666
EFCAB5	-0.1253	2.2816E-01	4.11E-01	ENSG00000176927
EFCAB6	0.0207	8.2031E-01	9.05E-01	ENSG00000186976
EFCAB7	0.0327	5.7520E-01	7.42E-01	ENSG00000203965
EFCAB8	0.0242	2.1530E-01		ENSG00000215529
EFCAB9	-0.0116	9.8449E-01		ENSG00000214360
EFCC1	-0.5328	1.3612E-04	1.43E-03	ENSG00000114654
EFEMP1	-0.4445	6.1493E-03	2.94E-02	ENSG00000115380
EFEMP2	-0.3219	5.0295E-03	2.51E-02	ENSG00000172638
EFHB	0.4731	8.0651E-06	1.48E-04	ENSG00000163576
EFHC1	0.1848	5.2566E-03	2.60E-02	ENSG00000096093
EFHC2	-0.3988	3.2306E-03	1.78E-02	ENSG00000183690
EFHD2	0.0844	1.5432E-01	3.17E-01	ENSG00000142634
EFHD2-AS1	0.0378	6.7443E-01	8.14E-01	ENSG00000228140
EFL1	0.0388	6.1510E-01	7.73E-01	ENSG00000140598
EFNA1	-0.4076	6.7345E-06	1.28E-04	ENSG00000169242
EFNA2	-0.1581	5.6293E-02	1.56E-01	ENSG00000099617
EFNA3	0.1315	1.1379E-01	2.57E-01	ENSG00000143590
EFNA4	-0.0532	4.9672E-01	6.80E-01	ENSG00000243364
EFNA5	-0.0178	8.9483E-01	9.48E-01	ENSG00000184349
EFNB1	0.0074	9.4527E-01	9.73E-01	ENSG00000090776
EFNB2	0.0114	8.9902E-01	9.49E-01	ENSG00000125266
EFNB3	-0.5310	9.6642E-06	1.71E-04	ENSG00000108947
EFR3A	-0.1706	4.2108E-02	1.26E-01	ENSG00000132294
EFR3B	0.1663	4.6296E-02	1.35E-01	ENSG00000084710
EFS	-0.4382	8.4408E-03	3.74E-02	ENSG00000100842
EFTUD2	0.3047	2.0075E-04	1.97E-03	ENSG00000108883
EGF	0.0874	3.8343E-01	5.78E-01	ENSG00000138798
EGFL6	0.4186	5.7075E-05	7.15E-04	ENSG00000198759
EGFL7	-0.1889	8.7075E-02	2.13E-01	ENSG00000172889
EGFL8	0.0025	7.4947E-01	8.63E-01	ENSG00000241404
EGFLAM	0.0654	4.2489E-01	6.17E-01	ENSG00000164318
EGFR	-0.0451	5.7852E-01	7.45E-01	ENSG00000146648
EGLN1	0.2861	1.1198E-02	4.63E-02	ENSG00000135766
EGLN2	0.0277	1.2235E-01	2.71E-01	ENSG00000269858
EGLN3	0.2171	3.6822E-02	1.14E-01	ENSG00000129521
EGOT	-0.1119	2.5416E-01	4.41E-01	ENSG00000235947
EGR1	-0.3043	1.7408E-03	1.10E-02	ENSG00000120738
EGR2	0.0755	4.3367E-01	6.25E-01	ENSG00000122877
EGR4	-0.3395	2.2929E-02	7.98E-02	ENSG00000135625
EHBP1	-0.1911	1.2610E-03	8.56E-03	ENSG00000115504
EHBP1-AS1	-0.0045	9.2145E-01	9.61E-01	ENSG00000231609
EHBP1L1	0.1009	2.9894E-01	4.91E-01	ENSG00000173442
EHD1	0.1551	9.5172E-02	2.27E-01	ENSG00000110047
EHD2	-0.2351	3.7530E-02	1.15E-01	ENSG00000024422

EHD3	0.0171	8.2425E-01	9.08E-01	ENSG00000013016
EHD4	0.1691	6.2812E-02	1.69E-01	ENSG00000103966
EHD4-AS1	-0.0335	7.3907E-01		ENSG00000259883
EHF	-0.1537	1.1500E-01	2.59E-01	ENSG00000135373
EHHADH	-0.0141	8.1453E-01	9.02E-01	ENSG00000113790
EHMT1	0.0410	4.7794E-01	6.64E-01	ENSG00000181090
EHMT2	-0.0779	1.9099E-01	3.66E-01	ENSG00000204371
EHMT2-AS1	-0.0096	8.9321E-01	9.47E-01	ENSG00000237080
EI24	0.0330	5.7216E-01	7.40E-01	ENSG00000149547
EID1	-0.1210	1.6586E-02	6.26E-02	ENSG00000255302
EID2	-0.0035	8.4131E-01	9.17E-01	ENSG00000176396
EID2B	0.1438	1.9294E-02	7.01E-02	ENSG00000176401
EID3	-0.1411	1.7958E-01	3.51E-01	ENSG00000255150
EIF1	-0.2036	4.1887E-05	5.56E-04	ENSG00000173812
EIF1AD	0.1529	1.6512E-02	6.24E-02	ENSG00000175376
EIF1AX	-0.1006	5.9733E-02	1.63E-01	ENSG00000173674
EIF1AX-AS1	-0.0371	5.1150E-01		ENSG00000225037
EIF1AY	-0.0418	4.4931E-01	6.39E-01	ENSG00000198692
EIF1B	-0.0385	3.9409E-01	5.89E-01	ENSG00000114784
EIF1B-AS1	0.0246	7.5373E-01	8.66E-01	ENSG00000280739
EIF1P5	0.3255	1.5495E-02	5.94E-02	ENSG00000266563
EIF2A	-0.0473	3.5182E-01	5.47E-01	ENSG00000144895
EIF2AK1	-0.0228	6.1768E-01	7.75E-01	ENSG00000086232
EIF2AK2	-0.2489	1.8073E-04	1.81E-03	ENSG00000055332
EIF2AK3	0.0278	7.3448E-01	8.54E-01	ENSG00000172071
EIF2AK3-DT	0.0238	7.9551E-01	8.90E-01	ENSG00000234028
EIF2AK4	-0.1530	1.4515E-02	5.66E-02	ENSG00000128829
EIF2B1	0.2781	1.8967E-06	4.51E-05	ENSG00000111361
EIF2B2	0.1091	1.4322E-01	3.01E-01	ENSG00000119718
EIF2B3	0.1845	5.1670E-03	2.57E-02	ENSG00000070785
EIF2B4	0.0882	1.2518E-01	2.75E-01	ENSG00000115211
EIF2B5	0.3769	1.3569E-06	3.45E-05	ENSG00000145191
EIF2D	-0.0355	6.5721E-01	8.02E-01	ENSG00000143486
EIF2S1	0.0850	1.0854E-01	2.49E-01	ENSG00000134001
EIF2S2	-0.1244	5.2611E-02	1.48E-01	ENSG00000125977
EIF2S3	-0.3286	3.5463E-04	3.14E-03	ENSG00000130741
EIF2S3B	0.0148	8.0922E-01	8.99E-01	ENSG00000180574
EIF3A	-0.2564	1.6717E-04	1.70E-03	ENSG00000107581
EIF3B	0.1746	6.8018E-03	3.17E-02	ENSG00000106263
EIF3C	0.0149	7.4422E-01	8.60E-01	ENSG00000184110
EIF3CL	0.0095	8.3360E-01	9.13E-01	ENSG00000205609
EIF3D	-0.0156	7.6992E-01	8.75E-01	ENSG00000100353
EIF3E	-0.2558	2.1392E-03	1.29E-02	ENSG00000104408
EIF3EP3	-0.0314	6.8947E-01	8.26E-01	ENSG00000277998
EIF3EP4	0.0014	8.5110E-01		ENSG00000270554
EIF3F	-0.1509	2.1674E-02	7.66E-02	ENSG00000175390
EIF3FP2	0.0232	1.9099E-01		ENSG00000279081
EIF3FP3	-0.0266	7.6189E-01	8.71E-01	ENSG00000233426
EIF3G	-0.0420	3.4244E-01	5.38E-01	ENSG00000130811

EIF3H	-0.2029	2.3939E-05	3.54E-04	ENSG00000147677
EIF3I	-0.1965	1.8850E-04	1.87E-03	ENSG00000084623
EIF3J	-0.0412	5.2514E-01	7.01E-01	ENSG00000104131
EIF3J-DT	0.1572	2.4371E-03	1.43E-02	ENSG00000179523
EIF3K	-0.0760	2.4251E-01	4.28E-01	ENSG00000178982
EIF3KP1	0.0247	8.5176E-02		ENSG00000175749
EIF3L	0.0175	8.1047E-01	9.00E-01	ENSG00000100129
EIF3LP2	-0.0626	3.7818E-01	5.73E-01	ENSG00000233837
EIF3LP3	-0.0262	3.2789E-01		ENSG00000226086
EIF3M	-0.0158	7.1722E-01	8.43E-01	ENSG00000149100
EIF4A1	0.0054	9.4744E-01	9.73E-01	ENSG00000161960
EIF4A1P10	0.1229	2.2743E-01	4.10E-01	ENSG00000229132
EIF4A1P12	0.0811	8.1805E-02	2.04E-01	ENSG00000257662
EIF4A1P2	0.0886	2.1448E-01	3.95E-01	ENSG00000235001
EIF4A1P4	-0.0595	4.3798E-01		ENSG00000257790
EIF4A1P7	0.0077	3.9944E-01		ENSG00000235472
EIF4A2	-0.4968	1.4911E-05	2.43E-04	ENSG00000156976
EIF4A3	-0.0116	8.4593E-01	9.20E-01	ENSG00000141543
EIF4B	-0.2784	3.6034E-03	1.94E-02	ENSG00000063046
EIF4BP2	0.0352	2.2252E-01		ENSG00000228753
EIF4BP3	-0.5986	1.2289E-03	8.36E-03	ENSG00000224546
EIF4E	-0.1163	9.6401E-02	2.29E-01	ENSG00000151247
EIF4E1B	0.5862	1.3589E-03	9.08E-03	ENSG00000175766
EIF4E2	-0.2554	5.8166E-04	4.65E-03	ENSG00000135930
EIF4E3	0.0923	2.5453E-01	4.42E-01	ENSG00000163412
EIF4EBP1	0.0607	3.7123E-01	5.67E-01	ENSG00000187840
EIF4EBP2	0.1417	2.6907E-03	1.55E-02	ENSG00000148730
EIF4EBP3	0.0552	5.7081E-01	7.39E-01	ENSG00000243056
EIF4ENIF1	0.1122	5.9113E-02	1.62E-01	ENSG00000184708
EIF4G1	-0.2667	1.5933E-06	3.92E-05	ENSG00000114867
EIF4G2	-0.2369	1.7931E-03	1.12E-02	ENSG00000110321
EIF4G3	-0.2296	7.1842E-04	5.49E-03	ENSG00000075151
EIF4H	-0.0528	2.3699E-01	4.21E-01	ENSG00000106682
EIF5	0.0104	9.1683E-01	9.58E-01	ENSG00000100664
EIF5A	-0.0417	3.7759E-01	5.73E-01	ENSG00000132507
EIF5A2	-0.0440	4.7244E-01	6.60E-01	ENSG00000163577
EIF5AL1	0.1377	1.9779E-01	3.74E-01	ENSG00000253626
EIF5AP2	-0.0244	7.2779E-01		ENSG00000267679
EIF5B	-0.0270	7.1089E-01	8.39E-01	ENSG00000158417
EIF6	-0.1045	7.3076E-02	1.88E-01	ENSG00000242372
EIPR1	-0.1341	1.8097E-02	6.68E-02	ENSG00000032389
ELAC1	0.0587	3.3641E-01	5.32E-01	ENSG00000141642
ELAC2	0.3175	1.3535E-04	1.43E-03	ENSG00000006744
ELAPOR1	-0.0479	5.8988E-01	7.53E-01	ENSG00000116299
ELAPOR2	0.4596	2.5717E-09	1.87E-07	ENSG00000164659
ELAVL1	-0.1384	3.9606E-03	2.10E-02	ENSG00000066044
ELAVL2	0.3947	8.5922E-04	6.33E-03	ENSG00000107105
ELAVL3	-0.0629	4.8319E-01	6.68E-01	ENSG00000196361
ELAVL4	0.0964	2.7561E-01	4.66E-01	ENSG00000162374

ELF1	-0.4207	1.0991E-09	9.24E-08	ENSG00000120690
ELF2	-0.0483	3.0994E-01	5.03E-01	ENSG00000109381
ELF3	-0.5686	4.4601E-09	2.99E-07	ENSG00000163435
ELF4	-0.0039	9.6687E-01	9.83E-01	ENSG00000102034
ELF5	0.1880	1.0067E-01	2.36E-01	ENSG00000135374
ELFN1	0.1962	6.1020E-02	1.66E-01	ENSG00000225968
ELFN1-AS1	-0.2394	5.3225E-02	1.50E-01	ENSG00000236081
ELFN2	0.0324	7.2358E-01	8.47E-01	ENSG00000166897
ELK1	0.0869	2.8040E-01	4.71E-01	ENSG00000126767
ELK3	-0.3649	1.4502E-05	2.38E-04	ENSG00000111145
ELK4	0.0120	8.8052E-01	9.39E-01	ENSG00000158711
ELL	0.0465	6.0974E-01	7.68E-01	ENSG00000105656
ELL2	0.0256	7.5721E-01	8.68E-01	ENSG00000118985
ELL3	-0.0844	3.7831E-01	5.73E-01	ENSG00000128886
ELMO1	0.3364	2.9639E-03	1.67E-02	ENSG00000155849
ELMO2	0.0313	6.3678E-01	7.89E-01	ENSG00000062598
ELMO3	0.1824	1.1737E-01	2.63E-01	ENSG00000102890
ELMOD1	0.0972	3.0012E-01	4.93E-01	ENSG00000110675
ELMOD2	0.0427	5.6725E-01	7.35E-01	ENSG00000179387
ELMOD3	0.2281	2.0573E-03	1.25E-02	ENSG00000115459
ELN-AS1	-0.4291	2.5800E-03	1.50E-02	ENSG00000232415
ELOA	-0.0577	3.5216E-01	5.47E-01	ENSG00000011007
ELOA-AS1	0.0328	6.7056E-01	8.11E-01	ENSG00000236810
ELOA3BP	-0.0022	6.9308E-01		ENSG00000288607
ELOA3DP	0.0157	4.7467E-01		ENSG00000288616
ELOB	-0.2188	3.0777E-05	4.32E-04	ENSG00000103363
ELOBP1	-0.0221	9.5773E-01		ENSG00000234152
ELOBP2	-0.0438	2.9573E-01		ENSG00000255262
ELOBP4	0.0073	8.8128E-01	9.40E-01	ENSG00000234167
ELOC	-0.1133	4.7346E-02	1.37E-01	ENSG00000154582
ELOCP22	-0.0284	4.2949E-01		ENSG00000255006
ELOCP31	-0.0282	6.3920E-01		ENSG00000256021
ELOF1	-0.1488	3.1074E-04	2.81E-03	ENSG00000130165
ELOVL1	0.0019	9.8644E-01	9.93E-01	ENSG00000066322
ELOVL2	-0.2273	1.0997E-02	4.57E-02	ENSG00000197977
ELOVL2-AS1	-0.1719	1.3071E-01	2.83E-01	ENSG00000230314
ELOVL3	0.1724	8.5835E-02	2.11E-01	ENSG00000119915
ELOVL4	0.1141	1.2862E-01	2.80E-01	ENSG00000118402
ELOVL5	-0.3470	1.7126E-04	1.73E-03	ENSG00000012660
ELOVL6	-0.1279	8.5804E-02	2.11E-01	ENSG00000170522
ELOVL7	-0.1116	2.5039E-01	4.37E-01	ENSG00000164181
ELP1	0.0071	9.2033E-01	9.60E-01	ENSG00000070061
ELP2	0.1528	3.8810E-03	2.06E-02	ENSG00000134759
ELP3	0.0338	5.9126E-01	7.54E-01	ENSG00000134014
ELP4	-0.2152	7.5205E-04	5.69E-03	ENSG00000109911
ELP5	0.0643	3.3667E-01	5.32E-01	ENSG00000170291
ELP6	-0.1231	9.9210E-03	4.22E-02	ENSG00000163832
EMB	-0.3120	9.7785E-05	1.10E-03	ENSG00000170571
EMC1	-0.0385	5.0993E-01	6.90E-01	ENSG00000127463

EMC10	-0.1816	7.1449E-03	3.29E-02	ENSG00000161671
EMC2	-0.0291	6.4300E-01	7.93E-01	ENSG00000104412
EMC3	-0.1089	1.4613E-02	5.69E-02	ENSG00000125037
EMC4	-0.0338	5.2734E-01	7.03E-01	ENSG00000128463
EMC6	-0.3130	8.8344E-04	6.46E-03	ENSG00000127774
EMC7	-0.0371	4.8715E-01	6.71E-01	ENSG00000134153
EMC8	-0.0214	6.6123E-01	8.05E-01	ENSG00000131148
EMC9	-0.2410	1.6637E-05	2.65E-04	ENSG00000100908
EMD	-0.0006	9.9299E-01	9.96E-01	ENSG00000102119
EME1	0.0618	5.2742E-01	7.03E-01	ENSG00000154920
EME2	0.0420	6.4961E-01	7.97E-01	ENSG00000197774
EMG1	-0.0437	5.3321E-01	7.07E-01	ENSG00000126749
EMID1	-0.1385	1.0642E-01	2.45E-01	ENSG00000186998
EMILIN1	-0.3600	1.3138E-03	8.83E-03	ENSG00000138080
EMILIN2	0.0117	9.2036E-01	9.60E-01	ENSG00000132205
EMILIN3	-0.1127	2.6494E-01	4.54E-01	ENSG00000183798
EML1	0.1963	1.0314E-02	4.35E-02	ENSG00000066629
EML2	0.1605	8.0744E-02	2.02E-01	ENSG00000125746
EML3	0.0487	5.5612E-01	7.27E-01	ENSG00000149499
EML4	-0.0955	1.6778E-01	3.35E-01	ENSG00000143924
EML5	0.0944	3.4417E-01	5.39E-01	ENSG00000165521
EML6	0.2093	3.8657E-02	1.18E-01	ENSG00000214595
EMP1	-0.5595	6.6959E-04	5.20E-03	ENSG00000134531
EMP2	-0.2079	7.7823E-03	3.52E-02	ENSG00000213853
EMSY	0.0638	3.6318E-01	5.59E-01	ENSG00000158636
EMSY-DT	-0.2370	1.0658E-03	7.46E-03	ENSG00000255135
EMX1	-0.0086	8.3438E-01		ENSG00000135638
EMX2	-0.0447	6.3415E-01	7.87E-01	ENSG00000170370
EMX2OS	-0.1253	2.3319E-01	4.17E-01	ENSG00000229847
EN1	0.0140	7.0179E-01	8.33E-01	ENSG00000163064
EN2	-0.0041	9.4872E-01	9.74E-01	ENSG00000164778
ENAH	-0.5358	4.8905E-09	3.22E-07	ENSG00000154380
ENAM	0.0733	4.3604E-01	6.27E-01	ENSG00000132464
ENC1	-0.3660	4.6364E-04	3.87E-03	ENSG00000171617
ENDOD1	0.2282	4.5082E-03	2.31E-02	ENSG00000149218
ENDOG	-0.0378	5.1966E-01	6.97E-01	ENSG00000167136
ENDOU	-0.0008	8.8556E-01		ENSG00000111405
ENDOV	0.2314	8.9546E-05	1.02E-03	ENSG00000173818
ENG	-0.1065	2.9671E-01	4.89E-01	ENSG00000106991
ENGASE	0.0761	3.5444E-01	5.50E-01	ENSG00000167280
ENHO	-0.1769	1.7636E-02	6.55E-02	ENSG00000168913
ENKD1	-0.1302	5.8424E-02	1.60E-01	ENSG00000124074
ENO1	0.4186	2.6585E-05	3.84E-04	ENSG00000074800
ENO1-AS1	0.0714	4.6355E-01	6.52E-01	ENSG00000230679
ENO1P3	-0.0083	9.7717E-01		ENSG00000243986
ENO2	0.3232	7.5751E-05	8.97E-04	ENSG00000111674
ENO3	-0.0795	4.0837E-01	6.02E-01	ENSG00000108515
ENO4	-0.2017	6.6258E-02	1.75E-01	ENSG00000188316
ENOPH1	-0.0970	8.1968E-02	2.04E-01	ENSG00000145293

ENOSF1	-0.1468	7.2799E-02	1.88E-01	ENSG00000132199
ENOX1	0.0716	3.3597E-01	5.31E-01	ENSG00000120658
ENOX2	-0.0525	3.8073E-01	5.76E-01	ENSG00000165675
ENPEP	0.0113	8.9624E-01	9.48E-01	ENSG00000138792
ENPP1	-0.1687	1.2177E-01	2.70E-01	ENSG00000197594
ENPP2	-0.4618	3.5239E-04	3.12E-03	ENSG00000136960
ENPP3	-0.0655	4.8303E-01	6.68E-01	ENSG00000154269
ENPP4	-0.0437	5.1119E-01	6.91E-01	ENSG00000001561
ENPP5	0.0370	6.6926E-01	8.10E-01	ENSG00000112796
ENPP6	-0.0363	7.0763E-01	8.36E-01	ENSG00000164303
ENPP7P1	0.0141	6.0080E-01	7.61E-01	ENSG00000249188
ENPP7P10	-0.0066	6.3036E-01		ENSG00000249767
ENPP7P11	-0.1440	1.7028E-01	3.38E-01	ENSG00000250942
ENPP7P12	0.0229	3.2220E-01		ENSG00000254527
ENPP7P15	-0.0265	4.8420E-01		ENSG00000284605
ENPP7P2	-0.0057	4.0026E-01	5.94E-01	ENSG00000239959
ENPP7P4	-0.0310	6.6073E-01	8.05E-01	ENSG00000241278
ENPP7P6	0.0067	3.6051E-01		ENSG00000255549
ENPP7P7	-0.0138	8.8527E-01	9.42E-01	ENSG00000273819
ENPP7P8	0.0236	7.6725E-01	8.74E-01	ENSG00000255319
ENPP7P9	-0.0143	6.6433E-01		ENSG00000250476
ENSA	0.0505	4.8045E-01	6.66E-01	ENSG00000143420
ENSAP1	-0.0184	3.2224E-01		ENSG00000224274
ENTPD1	0.0647	4.8285E-01	6.68E-01	ENSG00000138185
ENTPD1-AS1	0.1054	2.6956E-01	4.59E-01	ENSG00000226688
ENTPD2	0.0945	3.3581E-01	5.31E-01	ENSG00000054179
ENTPD3	0.0780	4.1451E-01	6.07E-01	ENSG00000168032
ENTPD3-AS1	-0.3057	2.6383E-04	2.45E-03	ENSG00000223797
ENTPD4	0.1285	4.3245E-02	1.28E-01	ENSG00000197217
ENTPD4-DT	-0.0666	3.6445E-01		ENSG00000287166
ENTPD5	-0.0941	1.9520E-01	3.71E-01	ENSG00000187097
ENTPD6	0.3113	1.0665E-03	7.47E-03	ENSG00000197586
ENTPD7	0.3523	3.1011E-04	2.81E-03	ENSG00000198018
ENTPD8	0.1087	2.8756E-01	4.79E-01	ENSG00000188833
ENTR1	-0.0128	8.1987E-01	9.05E-01	ENSG00000165689
ENTREP1	-0.1154	2.6196E-01	4.51E-01	ENSG00000135063
ENTREP2	0.0980	2.1339E-01	3.94E-01	ENSG00000104059
ENTREP3	-0.0634	4.5967E-01	6.48E-01	ENSG00000160767
ENY2	-0.2604	2.9663E-07	9.74E-06	ENSG00000120533
EOGT	0.0808	3.3489E-01	5.30E-01	ENSG00000163378
EOLA1	0.4525	3.3346E-08	1.58E-06	ENSG00000197620
EOLA1-DT	-0.0065	9.4319E-01	9.72E-01	ENSG00000241769
EOLA2	0.2322	3.6739E-04	3.23E-03	ENSG00000197021
EOLA2-DT	-0.2125	4.2051E-02	1.26E-01	ENSG00000235703
EOMES	-0.2709	3.8658E-02	1.18E-01	ENSG00000163508
EP300	-0.0595	3.4363E-01	5.39E-01	ENSG00000100393
EP400	-0.0966	2.4508E-01	4.31E-01	ENSG00000183495
EPAS1	-0.5283	3.3002E-07	1.06E-05	ENSG00000116016
EPB41	-0.0218	7.1786E-01	8.44E-01	ENSG00000159023

EPB41L1	-0.0556	4.9140E-01	6.75E-01	ENSG00000088367
EPB41L2	-0.3830	1.5626E-05	2.52E-04	ENSG00000079819
EPB41L3	-0.0052	8.8441E-01	9.42E-01	ENSG00000082397
EPB41L4A	-0.1058	2.2030E-01	4.01E-01	ENSG00000129595
EPB41L4A-DT	0.0292	7.5973E-01	8.69E-01	ENSG00000278921
EPB41L4B	0.4302	7.4416E-05	8.84E-04	ENSG00000095203
EPB41L5	-0.0598	3.8871E-01	5.83E-01	ENSG00000115109
EPC1	-0.5249	3.5163E-11	4.83E-09	ENSG00000120616
EPC1-AS1	-0.0341	2.4238E-01		ENSG00000229327
EPC2	-0.1340	9.0739E-02	2.20E-01	ENSG00000135999
EPCAM	0.0489	5.6557E-01	7.34E-01	ENSG00000119888
EPDR1	-0.0543	5.3160E-01	7.06E-01	ENSG00000086289
EPG5	0.1399	1.5966E-01	3.24E-01	ENSG00000152223
EPGN	0.0008	9.3726E-01		ENSG00000182585
EPHA1	0.2301	4.2142E-02	1.26E-01	ENSG00000146904
EPHA1-AS1	-0.0957	3.3643E-01	5.32E-01	ENSG00000229153
EPHA10	0.3012	3.9932E-04	3.44E-03	ENSG00000183317
EPHA2	-0.5997	1.3729E-09	1.10E-07	ENSG00000142627
EPHA3	0.0331	6.9544E-01	8.29E-01	ENSG00000044524
EPHA4	-0.0156	8.4339E-01	9.18E-01	ENSG00000116106
EPHA5	0.2176	1.1528E-02	4.73E-02	ENSG00000145242
EPHA5-AS1	0.0942	3.0009E-01	4.93E-01	ENSG00000250846
EPHA6	0.4173	1.6533E-03	1.06E-02	ENSG00000080224
EPHA7	0.1658	2.6930E-02	8.99E-02	ENSG00000135333
EPHA8	0.5848	1.0529E-04	1.17E-03	ENSG00000070886
EPHB1	-0.0843	3.0393E-01	4.96E-01	ENSG00000154928
EPHB2	-0.0109	8.4748E-01	9.21E-01	ENSG00000133216
EPHB3	-0.1582	6.6883E-03	3.13E-02	ENSG00000182580
EPHB4	-0.3126	7.0324E-04	5.40E-03	ENSG00000196411
EPHB6	0.0022	9.7585E-01	9.88E-01	ENSG00000106123
EPHX1	-0.2995	3.3162E-03	1.81E-02	ENSG00000143819
EPHX2	-0.1381	1.9120E-01	3.66E-01	ENSG00000120915
EPHX4	-0.1321	1.5441E-01	3.17E-01	ENSG00000172031
EPIC1	-0.0141	6.0699E-01		ENSG00000224271
EPM2A	-0.0044	9.5649E-01	9.78E-01	ENSG00000112425
EPM2A-DT	0.0113	9.0239E-01	9.51E-01	ENSG00000235652
EPM2AIP1	0.0750	2.0013E-01	3.77E-01	ENSG00000178567
EPN1	0.1619	2.8557E-02	9.39E-02	ENSG00000063245
EPN2	-0.4139	1.6548E-06	4.04E-05	ENSG00000072134
EPN2-AS1	-0.0067	5.0410E-01		ENSG00000235397
EPN3	-0.2404	2.4344E-02	8.35E-02	ENSG00000049283
EPO	0.0020	9.7600E-01		ENSG00000130427
EPOP	-0.1293	2.2026E-01	4.01E-01	ENSG00000273604
EPOR	0.3072	9.1095E-04	6.62E-03	ENSG00000187266
EPPIN	-0.0177	4.3249E-01		ENSG00000101448
EPPK1	0.2897	4.3258E-03	2.24E-02	ENSG00000261150
EPRS1	-0.0284	5.9593E-01	7.58E-01	ENSG00000136628
EPS15	-0.0246	6.5847E-01	8.03E-01	ENSG00000085832
EPS15-AS1	-0.0362	4.0257E-01		ENSG00000227070

EPS15L1	0.1294	1.0270E-01	2.39E-01	ENSG00000127527
EPS8	-0.1867	3.9586E-03	2.09E-02	ENSG00000151491
EPS8L1	0.3406	4.0896E-05	5.45E-04	ENSG00000131037
EPS8L2	0.0382	6.4958E-01	7.97E-01	ENSG00000177106
EPS8L3	-0.0134	5.7105E-01		ENSG00000198758
EPSTI1	-0.2107	7.2416E-02	1.87E-01	ENSG00000133106
EPX	-0.0133	5.4105E-01		ENSG00000121053
EPYC	-0.0361	3.1248E-01		ENSG00000083782
ERAL1	-0.1516	3.3458E-02	1.06E-01	ENSG00000132591
ERAP1	-0.4159	5.7153E-06	1.12E-04	ENSG00000164307
ERAS	-0.4064	4.1666E-03	2.18E-02	ENSG00000187682
ERBB2	-0.4206	1.4934E-07	5.43E-06	ENSG00000141736
ERBB3	0.1490	1.2468E-01	2.74E-01	ENSG00000065361
ERBB4	-0.1590	1.1259E-01	2.55E-01	ENSG00000178568
ERBIN	-0.3511	1.1185E-08	6.31E-07	ENSG00000112851
ERC1	-0.2101	1.7491E-03	1.10E-02	ENSG00000082805
ERC2	0.1895	3.8922E-02	1.19E-01	ENSG00000187672
ERC2-IT1	0.0119	8.5117E-01	9.24E-01	ENSG00000281708
ERCC1	-0.1120	3.9648E-02	1.20E-01	ENSG00000012061
ERCC2	0.0079	9.0958E-01	9.55E-01	ENSG00000104884
ERCC3	0.1569	1.8693E-02	6.85E-02	ENSG00000163161
ERCC4	0.1456	9.7335E-03	4.16E-02	ENSG00000175595
ERCC5	0.3251	3.0884E-03	1.72E-02	ENSG00000134899
ERCC6	-0.0833	2.0922E-01	3.89E-01	ENSG00000225830
ERCC6L	-0.1148	2.5466E-01	4.42E-01	ENSG00000186871
ERCC6L2	0.1310	3.3360E-02	1.06E-01	ENSG00000182150
ERCC6L2-AS1	-0.1182	1.3665E-01	2.91E-01	ENSG00000175611
ERCC8	0.0211	7.2281E-01	8.47E-01	ENSG00000049167
EREG	-0.0481	4.6063E-01		ENSG00000124882
ERF	-0.0299	7.0239E-01	8.33E-01	ENSG00000105722
ERFL	0.0634	4.0870E-01	6.02E-01	ENSG00000268041
ERG	0.0040	9.5272E-01	9.76E-01	ENSG00000157554
ERG28	-0.5054	4.7509E-18	3.28E-15	ENSG00000133935
ERGIC1	0.0316	5.8984E-01	7.53E-01	ENSG00000113719
ERGIC2	-0.0144	7.9420E-01	8.89E-01	ENSG00000087502
ERGIC3	-0.2863	8.2606E-06	1.51E-04	ENSG00000125991
ERH	-0.1995	1.9981E-03	1.22E-02	ENSG00000100632
ERI1	0.3083	7.4678E-04	5.66E-03	ENSG00000104626
ERI2	0.3753	8.0345E-04	6.00E-03	ENSG00000196678
ERI3	0.0848	1.2747E-01	2.78E-01	ENSG00000117419
ERICH1	-0.0621	3.2117E-01	5.15E-01	ENSG00000104714
ERICH2	0.0733	2.9691E-01	4.89E-01	ENSG00000204334
ERICH2-DT	-0.2756	2.1343E-02	7.56E-02	ENSG00000234350
ERICH3	-0.3599	1.9407E-03	1.20E-02	ENSG00000178965
ERICH3-AS1	-0.0102	8.8927E-01		ENSG00000234497
ERICH4	-0.0298	4.0535E-01		ENSG00000204978
ERICH5	-0.4020	1.0468E-03	7.37E-03	ENSG00000177459
ERICH6	-0.0814	8.7733E-01	9.38E-01	ENSG00000163645
ERICH6-AS1	-0.0305	6.7289E-01	8.13E-01	ENSG00000240137

ERICH6B	-0.1724	1.2336E-01	2.72E-01	ENSG00000165837
ERLEC1	0.0217	6.6543E-01	8.08E-01	ENSG00000068912
ERLIN1	-0.0168	7.6622E-01	8.73E-01	ENSG00000107566
ERLIN2	-0.2736	1.6532E-03	1.06E-02	ENSG00000147475
ERLNC1	0.0060	6.2704E-01		ENSG00000230550
ERMAP	-0.1337	1.5461E-01	3.17E-01	ENSG00000164010
ERMARD	0.2144	6.0837E-03	2.92E-02	ENSG00000130023
ERMN	-0.1035	2.8064E-01	4.72E-01	ENSG00000136541
ERMP1	-0.4411	2.5030E-05	3.65E-04	ENSG00000099219
ERN1	-0.0457	4.6746E-01	6.55E-01	ENSG00000178607
ERN2	0.5942	4.4630E-03	2.29E-02	ENSG00000134398
ERO1A	0.2976	7.5374E-07	2.11E-05	ENSG00000197930
ERO1B	0.1979	2.7569E-02	9.14E-02	ENSG00000086619
ERP29	-0.3936	3.3810E-07	1.08E-05	ENSG00000089248
ERP44	-0.0756	1.3300E-01	2.86E-01	ENSG00000023318
ERRFI1	-0.2421	5.8397E-03	2.82E-02	ENSG00000116285
ERV3-1	-0.5793	1.2210E-10	1.41E-08	ENSG00000213462
ERVFRD-1	-0.0283	7.5876E-01	8.69E-01	ENSG00000244476
ERVH48-1	0.0340	1.6244E-01		ENSG00000233056
ERVK3-1	-0.3180	1.2383E-04	1.33E-03	ENSG00000142396
ERVMER34-1	-0.0478	2.6353E-01		ENSG00000226887
ERVV-1	-0.0349	4.3161E-01		ENSG00000269526
ERVV-2	-0.0083	8.9916E-01	9.49E-01	ENSG00000268964
ERVW-1	0.0464	1.5139E-01		ENSG00000242950
ESAM	-0.0150	8.5588E-01	9.26E-01	ENSG00000149564
ESAM-AS1	-0.0090	8.5722E-01		ENSG00000250073
ESCO1	-0.1556	1.2944E-02	5.18E-02	ENSG00000141446
ESCO2	-0.5554	7.9266E-04	5.95E-03	ENSG00000171320
ESD	-0.1267	3.4871E-02	1.09E-01	ENSG00000139684
ESF1	0.0182	7.9618E-01	8.91E-01	ENSG00000089048
ESM1	0.1408	1.7645E-01	3.47E-01	ENSG00000164283
ESPL1	0.0238	7.7969E-01	8.81E-01	ENSG00000135476
ESPN	-0.0632	4.9449E-01	6.78E-01	ENSG00000187017
ESR1	-0.0317	6.2555E-01		ENSG00000091831
ESR2	0.0215	8.1230E-01	9.01E-01	ENSG00000140009
ESRP1	-0.0817	4.0223E-01	5.96E-01	ENSG00000104413
ESRP2	0.3112	1.5713E-02	6.01E-02	ENSG00000103067
ESRRA	0.4598	1.6612E-03	1.06E-02	ENSG00000173153
ESRRB	0.1252	8.2146E-02	2.04E-01	ENSG00000119715
ESRRG	-0.0407	6.4316E-01	7.93E-01	ENSG00000196482
ESS2	0.1264	8.7747E-02	2.14E-01	ENSG00000100056
ESYT1	-0.1415	4.8192E-02	1.39E-01	ENSG00000139641
ESYT2	0.2937	5.5691E-07	1.63E-05	ENSG00000117868
ESYT3	0.1895	1.0357E-01	2.41E-01	ENSG00000158220
ETAA1	0.2600	4.0362E-04	3.47E-03	ENSG00000143971
ETDA	0.0417	7.2004E-02		ENSG00000238210
ETDB	0.0202	2.1758E-01		ENSG00000224107
ETF1	-0.0893	6.6277E-02	1.75E-01	ENSG00000120705
ETFA	-0.0989	1.1104E-01	2.53E-01	ENSG00000140374

ETFB	-0.0308	6.0379E-01	7.64E-01	ENSG00000105379
ETFBKMT	0.2169	9.0806E-03	3.96E-02	ENSG00000139160
ETFDH	0.4362	7.1397E-10	6.42E-08	ENSG00000171503
ETFRF1	-0.0969	1.0771E-01	2.47E-01	ENSG00000205707
ETHE1	-0.2824	2.4294E-04	2.29E-03	ENSG00000105755
ETNK1	0.0184	7.4927E-01	8.63E-01	ENSG00000139163
ETNK2	0.1248	1.1884E-01	2.65E-01	ENSG00000143845
ETNPPL	0.0683	4.7781E-01	6.64E-01	ENSG00000164089
ETS1	-0.0790	3.4844E-01	5.43E-01	ENSG00000134954
ETS2	0.1054	1.8143E-01	3.53E-01	ENSG00000157557
ETV1	-0.2788	8.3793E-03	3.72E-02	ENSG00000006468
ETV2	-0.2568	1.7241E-02	6.44E-02	ENSG00000105672
ETV3	-0.0603	3.4093E-01	5.36E-01	ENSG00000117036
ETV3L	0.0589	7.0319E-01	8.34E-01	ENSG00000253831
ETV4	0.1838	6.2504E-02	1.68E-01	ENSG00000175832
ETV5	-0.1178	7.9869E-02	2.00E-01	ENSG00000244405
ETV6	-0.0257	7.5262E-01	8.65E-01	ENSG00000139083
ETV7	-0.2598	4.3633E-02	1.29E-01	ENSG00000010030
EVA1A	-0.2394	5.4737E-02	1.53E-01	ENSG00000115363
EVA1A-AS	-0.0107	9.3830E-01		ENSG00000231172
EVA1C	-0.2081	1.0064E-02	4.27E-02	ENSG00000166979
EVA1CP5	0.0414	2.4830E-01		ENSG00000244699
EVC	-0.2860	1.0417E-02	4.37E-02	ENSG00000072840
EVC2	0.1803	1.1237E-01	2.55E-01	ENSG00000173040
EVI2A	0.1283	2.0204E-01	3.80E-01	ENSG00000126860
EVI2B	0.0046	4.3759E-01		ENSG00000185862
EVI5	0.0175	7.8187E-01	8.82E-01	ENSG00000067208
EVI5L	0.3958	6.5211E-05	7.98E-04	ENSG00000142459
EVL	-0.1714	2.3190E-02	8.05E-02	ENSG00000196405
EVPL	-0.0693	4.8253E-01	6.68E-01	ENSG00000167880
EWSAT1	0.0420	3.9252E-01	5.87E-01	ENSG00000212766
EWSR1	-0.0159	8.1418E-01	9.02E-01	ENSG00000182944
EXD1	-0.0830	2.2395E-01	4.06E-01	ENSG00000178997
EXD2	-0.1120	4.8291E-02	1.40E-01	ENSG00000081177
EXD3	-0.1481	7.4462E-02	1.90E-01	ENSG00000187609
EXO1	-0.0367	6.6748E-01	8.09E-01	ENSG00000174371
EXO5	0.2758	7.7052E-03	3.49E-02	ENSG00000164002
EXOC1	0.0788	2.3119E-01	4.15E-01	ENSG00000090989
EXOC1L	-0.0378	5.3089E-01		ENSG00000250821
EXOC2	0.2148	9.5739E-03	4.11E-02	ENSG00000112685
EXOC3	0.2517	2.2616E-03	1.35E-02	ENSG00000180104
EXOC3L1	0.4486	4.6825E-04	3.89E-03	ENSG00000179044
EXOC3L2	-0.0464	4.6013E-01	6.49E-01	ENSG00000283632
EXOC3L4	-0.0099	8.2546E-01		ENSG00000205436
EXOC4	-0.0167	7.6094E-01	8.70E-01	ENSG00000131558
EXOC5	0.0206	6.5424E-01	8.00E-01	ENSG00000070367
EXOC5P1	0.0242	5.6156E-01		ENSG00000180673
EXOC6	-0.0088	8.8477E-01	9.42E-01	ENSG00000138190
EXOC6B	-0.1028	9.4557E-02	2.26E-01	ENSG00000144036

EXOC7	0.2117	1.8430E-02	6.78E-02	ENSG00000182473
EXOC8	-0.1490	7.7692E-02	1.96E-01	ENSG00000116903
EXOG	0.1817	5.4760E-03	2.69E-02	ENSG00000157036
EXOSC1	-0.2046	1.1028E-02	4.58E-02	ENSG00000171311
EXOSC10	0.2340	6.6734E-03	3.12E-02	ENSG00000171824
EXOSC10-AS1	-0.2120	8.1628E-02	2.04E-01	ENSG00000230337
EXOSC2	-0.0469	5.0591E-01	6.87E-01	ENSG00000130713
EXOSC3	0.0577	4.9859E-01	6.81E-01	ENSG00000107371
EXOSC4	-0.0287	6.1870E-01	7.75E-01	ENSG00000178896
EXOSC5	0.1481	1.3997E-02	5.50E-02	ENSG00000077348
EXOSC6	0.0724	1.3243E-01	2.85E-01	ENSG00000223496
EXOSC7	-0.0512	4.1209E-01	6.05E-01	ENSG00000075914
EXOSC8	-0.0108	7.6368E-01	8.72E-01	ENSG00000120699
EXOSC9	0.0724	2.5816E-01	4.46E-01	ENSG00000123737
EXPH5	-0.1444	1.3466E-01	2.88E-01	ENSG00000110723
EXT1	0.0049	9.3172E-01	9.66E-01	ENSG00000182197
EXT2	-0.0252	6.6787E-01	8.09E-01	ENSG00000151348
EXTL1	-0.0821	3.6877E-01	5.64E-01	ENSG00000158008
EXTL2	0.0303	5.3958E-01	7.13E-01	ENSG00000162694
EXTL3	-0.2060	5.9755E-04	4.75E-03	ENSG00000012232
EXTL3-AS1	0.0518	5.5855E-01	7.29E-01	ENSG00000246339
EYA1	0.1116	2.4711E-01	4.33E-01	ENSG00000104313
EYA2	-0.1287	2.1802E-01	3.99E-01	ENSG00000064655
EYA3	0.4859	2.1317E-11	3.17E-09	ENSG00000158161
EYA4	-0.0834	3.3514E-01	5.30E-01	ENSG00000112319
EYS	0.3494	1.7846E-02	6.61E-02	ENSG00000188107
EZH1	0.2334	8.1046E-04	6.03E-03	ENSG00000108799
EZH2	0.2702	1.1937E-03	8.16E-03	ENSG00000106462
EZHIP	0.0185	4.0433E-01		ENSG00000187690
EZR	-0.1504	2.6949E-02	8.99E-02	ENSG00000092820
EZR-AS1	-0.0376	6.4735E-01	7.96E-01	ENSG00000233893
F10	0.2646	3.3537E-02	1.06E-01	ENSG00000126218
F10-AS1	0.0357	5.5625E-01	7.27E-01	ENSG00000231882
F11-AS1	-0.0011	9.9839E-01		ENSG00000251165
F11R	-0.0637	4.3684E-01	6.28E-01	ENSG00000158769
F12	0.2915	4.7965E-03	2.42E-02	ENSG00000131187
F13A1	-0.3757	2.1814E-02	7.69E-02	ENSG00000124491
F2	-0.0258	7.8306E-01		ENSG00000180210
F2R	-0.0449	5.5790E-01	7.28E-01	ENSG00000181104
F2RL1	-0.0493	5.6674E-01	7.35E-01	ENSG00000164251
F2RL2	0.0542	4.8427E-01	6.69E-01	ENSG00000164220
F2RL3	0.0150	3.4313E-01		ENSG00000127533
F3	0.1102	2.8065E-01	4.72E-01	ENSG00000117525
F5	-0.2606	2.2555E-02	7.88E-02	ENSG00000198734
F7	-0.0079	9.2542E-01	9.63E-01	ENSG00000057593
F8	0.0781	3.6293E-01	5.59E-01	ENSG00000185010
F8A1	-0.1275	6.1452E-02	1.66E-01	ENSG00000288722
F8A2	-0.1011	1.7730E-01	3.48E-01	ENSG00000288709
F8A3	-0.0911	2.0774E-01	3.87E-01	ENSG00000277150

FA2H	0.0961	3.3319E-01	5.28E-01	ENSG00000103089
FAAH	0.0258	7.4570E-01	8.61E-01	ENSG00000117480
FAAH2	-0.0739	4.2125E-01	6.14E-01	ENSG00000165591
FAAP100	0.0109	8.8840E-01	9.44E-01	ENSG00000185504
FAAP20	-0.0964	1.4626E-01	3.05E-01	ENSG00000162585
FAAP24	0.0994	3.0335E-01	4.96E-01	ENSG00000131944
FABP1	-0.0298	2.8925E-01		ENSG00000163586
FABP3	-0.3753	4.5118E-04	3.79E-03	ENSG00000121769
FABP4	-0.1929	1.4406E-02	5.63E-02	ENSG00000170323
FABP5	-0.2662	2.6768E-03	1.54E-02	ENSG00000164687
FABP5P3	0.0725	4.2386E-01	6.16E-01	ENSG00000241735
FABP6	0.3755	3.9825E-03	2.10E-02	ENSG00000170231
FADD	0.3764	4.2916E-04	3.65E-03	ENSG00000168040
FADS1	-0.0872	3.1065E-01	5.04E-01	ENSG00000149485
FADS2	-0.2416	3.0599E-02	9.88E-02	ENSG00000134824
FADS3	0.2001	1.0535E-02	4.41E-02	ENSG00000221968
FADS6	-0.1100	2.1085E-01	3.90E-01	ENSG00000172782
FAF1	0.1236	5.4951E-02	1.53E-01	ENSG00000185104
FAF2	-0.0910	2.3963E-02	8.25E-02	ENSG00000113194
FAH	-0.2035	2.3602E-02	8.16E-02	ENSG00000103876
FAHD1	-0.0988	3.0865E-02	9.95E-02	ENSG00000180185
FAHD2A	-0.3403	3.2234E-05	4.48E-04	ENSG00000115042
FAHD2B	-0.2632	1.1295E-02	4.66E-02	ENSG00000144199
FAIM	-0.2012	6.4479E-03	3.05E-02	ENSG00000158234
FAIM2	0.0045	9.5298E-01	9.76E-01	ENSG00000135472
FALEC	0.3437	5.0477E-03	2.52E-02	ENSG00000228126
FAM106A	0.0076	6.0226E-01		ENSG00000273018
FAM107A	0.0427	6.4410E-01	7.94E-01	ENSG00000168309
FAM107B	-0.0171	7.6903E-01	8.75E-01	ENSG00000065809
FAM110A	-0.0125	8.9808E-01	9.49E-01	ENSG00000125898
FAM110B	0.1771	3.4040E-02	1.07E-01	ENSG00000169122
FAM110C	-0.0619	4.8590E-01	6.70E-01	ENSG00000184731
FAM110D	-0.0658	4.8231E-01	6.67E-01	ENSG00000197245
FAM111A	-0.5232	8.6950E-07	2.39E-05	ENSG00000166801
FAM111A-DT	-0.4224	3.3158E-04	2.97E-03	ENSG00000245571
FAM111B	-0.5360	2.2341E-03	1.34E-02	ENSG00000189057
FAM114A1	-0.2232	7.0180E-03	3.25E-02	ENSG00000197712
FAM114A2	0.0733	1.5439E-01	3.17E-01	ENSG00000055147
FAM117A	-0.0433	5.9428E-01	7.57E-01	ENSG00000121104
FAM117B	-0.1304	5.9476E-02	1.62E-01	ENSG00000138439
FAM118A	0.1183	5.5660E-02	1.55E-01	ENSG00000100376
FAM118B	-0.1018	8.3241E-02	2.06E-01	ENSG00000197798
FAM120A	-0.3418	7.3106E-04	5.57E-03	ENSG00000048828
FAM120AOS	-0.1172	6.6000E-03	3.10E-02	ENSG00000188938
FAM120B	0.0804	2.3802E-01	4.22E-01	ENSG00000112584
FAM120C	-0.1167	1.7915E-01	3.50E-01	ENSG00000184083
FAM124A	-0.3093	3.6192E-03	1.95E-02	ENSG00000150510
FAM124B	0.0277	1.2993E-01		ENSG00000124019
FAM131A	0.1382	2.8922E-02	9.49E-02	ENSG00000175182

FAM131B	0.1789	7.2452E-02	1.87E-01	ENSG00000159784
FAM131C	0.4210	1.6783E-09	1.31E-07	ENSG00000185519
FAM131C2P	0.3129	1.2649E-02	5.08E-02	ENSG00000232456
FAM133A	0.0816	3.8731E-01	5.82E-01	ENSG00000179083
FAM133B	-0.3210	1.7716E-06	4.27E-05	ENSG00000234545
FAM133FP	0.0171	2.1279E-01		ENSG00000225934
FAM135A	0.2167	6.0259E-03	2.90E-02	ENSG00000082269
FAM135A-AS1	0.0157	4.8340E-01		ENSG00000224349
FAM135B	-0.0156	9.6362E-01	9.81E-01	ENSG00000147724
FAM136A	0.0104	9.1219E-01	9.56E-01	ENSG00000035141
FAM138A	-0.0922	3.5327E-01	5.49E-01	ENSG00000237613
FAM138B	-0.0381	6.8419E-01	8.21E-01	ENSG00000226516
FAM138C	-0.0499	6.0321E-01	7.63E-01	ENSG00000218839
FAM138D	0.1040	2.1925E-01	4.00E-01	ENSG00000249054
FAM138E	-0.0571	5.4276E-01	7.15E-01	ENSG00000248893
FAM138F	-0.0899	3.6903E-01	5.65E-01	ENSG00000282591
FAM13A	-0.0984	1.3360E-01	2.87E-01	ENSG00000138640
FAM13A-AS1	-0.1095	2.8085E-01	4.72E-01	ENSG00000248019
FAM13B	0.0009	9.9022E-01	9.95E-01	ENSG00000031003
FAM13C	0.2620	9.7078E-04	6.95E-03	ENSG00000148541
FAM149A	0.0022	9.8487E-01	9.92E-01	ENSG00000109794
FAM149B1	-0.1281	4.7901E-02	1.39E-01	ENSG00000138286
FAM151A	-0.0212	9.1089E-01	9.55E-01	ENSG00000162391
FAM151B	-0.0945	3.3886E-01	5.34E-01	ENSG00000152380
FAM153A	0.1539	1.3868E-01	2.94E-01	ENSG00000170074
FAM153CP	0.0400	6.6754E-01	8.09E-01	ENSG00000204677
FAM156A	0.3226	1.8654E-04	1.86E-03	ENSG00000268350
FAM156B	0.3184	2.5303E-04	2.37E-03	ENSG00000179304
FAM161A	-0.1448	3.9564E-02	1.20E-01	ENSG00000170264
FAM161B	0.2164	2.0037E-02	7.21E-02	ENSG00000156050
FAM162A	-0.0088	9.0377E-01	9.52E-01	ENSG00000114023
FAM162B	0.0537	4.9416E-01	6.78E-01	ENSG00000183807
FAM163A	0.5582	3.7085E-05	5.01E-04	ENSG00000143340
FAM163B	0.0031	9.7404E-01	9.87E-01	ENSG00000196990
FAM167A	0.0899	1.8620E-01	3.60E-01	ENSG00000154319
FAM167B	0.1320	1.4737E-01	3.07E-01	ENSG00000183615
FAM168A	-0.0293	6.6700E-01	8.09E-01	ENSG00000054965
FAM168B	-0.0843	1.7867E-01	3.50E-01	ENSG00000152102
FAM169A	-0.0321	7.0335E-01	8.34E-01	ENSG00000198780
FAM169BP	0.0471	2.3466E-01		ENSG00000283597
FAM171A1	0.0020	9.8275E-01	9.91E-01	ENSG00000148468
FAM171A2	0.1438	7.5703E-02	1.93E-01	ENSG00000161682
FAM171B	-0.1709	9.8503E-03	4.20E-02	ENSG00000144369
FAM174A	0.0767	2.4611E-01	4.32E-01	ENSG00000174132
FAM174A-DT	0.0046	9.6246E-01	9.81E-01	ENSG00000247877
FAM174B	-0.0193	7.6104E-01	8.70E-01	ENSG00000185442
FAM174C	-0.0955	1.2054E-01	2.68E-01	ENSG00000228300
FAM177A1	0.1140	1.0962E-01	2.50E-01	ENSG00000151327
FAM177B	-0.0098	7.3659E-01		ENSG00000197520

FAM178B	0.3412	1.8065E-02	6.67E-02	ENSG00000168754
FAM180A	-0.0034	7.9682E-01		ENSG00000189320
FAM181A	-0.4148	1.2402E-02	5.01E-02	ENSG00000140067
FAM181A-AS1	-0.1145	1.9580E-01	3.72E-01	ENSG00000258584
FAM181B	-0.3698	8.9987E-04	6.55E-03	ENSG00000182103
FAM182A	-0.0558	5.6188E-01	7.31E-01	ENSG00000125804
FAM183BP	0.0473	4.9582E-01	6.79E-01	ENSG00000164556
FAM184A	-0.0542	4.0796E-01	6.01E-01	ENSG00000111879
FAM184B	-0.1210	2.4292E-01	4.28E-01	ENSG00000047662
FAM185A	0.0923	1.9236E-01	3.68E-01	ENSG00000222011
FAM186B	0.0080	9.1379E-01	9.57E-01	ENSG00000135436
FAM187B	0.0120	6.0509E-01		ENSG00000177558
FAM187B2P	0.0649	2.5117E-01	4.38E-01	ENSG00000262497
FAM193A	-0.2376	1.0490E-03	7.37E-03	ENSG00000125386
FAM193B	0.1848	1.1115E-02	4.60E-02	ENSG00000146067
FAM199X	-0.2785	1.5548E-04	1.60E-03	ENSG00000123575
FAM200B	0.1368	3.8482E-02	1.17E-01	ENSG00000237765
FAM200C	0.1195	2.4686E-01	4.33E-01	ENSG00000221886
FAM204A	-0.1047	1.5637E-02	5.98E-02	ENSG00000165669
FAM209B	0.0073	9.3763E-01	9.69E-01	ENSG00000213714
FAM20A	-0.3719	3.0845E-04	2.80E-03	ENSG00000108950
FAM20B	-0.0814	1.6740E-01	3.35E-01	ENSG00000116199
FAM20C	-0.4750	5.6775E-05	7.12E-04	ENSG00000177706
FAM210A	-0.1388	3.9052E-02	1.19E-01	ENSG00000177150
FAM210B	0.0335	4.8996E-01	6.74E-01	ENSG00000124098
FAM216A	0.1139	1.0748E-01	2.47E-01	ENSG00000204856
FAM216B	-0.0890	3.6024E-01	5.56E-01	ENSG00000179813
FAM217A	-0.0328	6.6545E-01		ENSG00000145975
FAM217B	-0.0634	2.5177E-01	4.39E-01	ENSG00000196227
FAM218A	0.3831	3.2760E-03	1.80E-02	ENSG00000250486
FAM219A	0.0975	1.9348E-01	3.69E-01	ENSG00000164970
FAM219B	-0.0268	6.0939E-01	7.68E-01	ENSG00000178761
FAM21FP	0.0530	5.8403E-01	7.49E-01	ENSG00000237840
FAM220A	0.0082	4.8674E-01		ENSG00000178397
FAM221A	0.0994	1.6169E-01	3.27E-01	ENSG00000188732
FAM221B	0.0174	8.2159E-01	9.06E-01	ENSG00000204930
FAM222A	-0.0062	9.0966E-01	9.55E-01	ENSG00000139438
FAM222A-AS1	0.0711	4.6871E-01	6.56E-01	ENSG00000255650
FAM222B	0.1902	7.9804E-03	3.59E-02	ENSG00000173065
FAM223A	-0.0124	6.4592E-01		ENSG00000279245
FAM223B	-0.0124	6.4592E-01		ENSG00000272681
FAM225A	0.0197	3.6254E-01		ENSG00000231528
FAM225B	0.0259	2.3388E-01		ENSG00000225684
FAM227A	0.1501	1.0331E-01	2.40E-01	ENSG00000184949
FAM227B	-0.0103	8.7538E-01	9.37E-01	ENSG00000166262
FAM228A	0.0706	4.0162E-01	5.96E-01	ENSG00000186453
FAM228B	-0.0172	7.5869E-01	8.69E-01	ENSG00000219626
FAM229A	0.0699	4.4205E-01	6.33E-01	ENSG00000225828
FAM229B	-0.1194	1.0122E-01	2.37E-01	ENSG00000203778

FAM230A	-0.0078	9.7810E-01		ENSG00000277870
FAM230B	0.0155	3.2594E-01		ENSG00000215498
FAM230E	0.0215	6.9423E-02		ENSG00000182824
FAM230H	0.0224	3.0056E-01		ENSG00000206142
FAM230I	-0.0079	8.8352E-01		ENSG00000178248
FAM230J	-0.0291	5.7496E-01		ENSG00000274044
FAM234A	0.0504	4.3518E-01	6.26E-01	ENSG00000167930
FAM234B	0.1265	1.1758E-01	2.63E-01	ENSG00000084444
FAM236A	0.0162	3.6659E-01		ENSG00000275520
FAM236B	0.0162	3.6659E-01		ENSG00000268994
FAM237A	-0.0291	6.8374E-01		ENSG00000235118
FAM237B	0.0204	2.6721E-01		ENSG00000283267
FAM240C	-0.0737	4.2540E-01	6.17E-01	ENSG00000216921
FAM241A	0.1034	2.1936E-01	4.00E-01	ENSG00000174749
FAM241B	-0.0683	2.8990E-01	4.81E-01	ENSG00000171224
FAM242E	0.0244	2.3533E-01		ENSG00000229697
FAM242F	-0.0134	8.0600E-01		ENSG00000275239
FAM246A	0.0280	3.1417E-01		ENSG00000286102
FAM246C	0.0273	6.9106E-01	8.27E-01	ENSG00000286025
FAM247A	-0.1567	1.3374E-01	2.87E-01	ENSG00000288811
FAM24B	-0.0805	4.1639E-01	6.09E-01	ENSG00000213185
FAM27C	0.0224	7.0065E-01	8.33E-01	ENSG00000231527
FAM27E3	0.4767	2.5727E-03	1.49E-02	ENSG00000274026
FAM32A	0.0033	9.4625E-01	9.73E-01	ENSG00000105058
FAM3A	-0.0009	9.8642E-01	9.93E-01	ENSG00000071889
FAM3B	0.1498	1.6856E-01	3.36E-01	ENSG00000183844
FAM3C	-0.1271	8.4769E-02	2.09E-01	ENSG00000196937
FAM3D	-0.0099	8.7674E-01	9.37E-01	ENSG00000198643
FAM3D-AS1	0.0732	4.0435E-01	5.98E-01	ENSG00000244383
FAM41C	-0.0420	5.4501E-01	7.17E-01	ENSG00000230368
FAM43A	0.1403	1.1001E-01	2.51E-01	ENSG00000185112
FAM43B	0.0623	4.7850E-01	6.65E-01	ENSG00000183114
FAM47C	-0.0016	8.0827E-01		ENSG00000198173
FAM47E	0.0337	5.7822E-01	7.45E-01	ENSG00000189157
FAM47E-STBD1	-0.0106	8.1424E-01		ENSG00000272414
FAM50A	-0.0599	1.9117E-01	3.66E-01	ENSG00000071859
FAM50B	0.2256	3.0317E-02	9.82E-02	ENSG00000145945
FAM53A	0.2854	3.2141E-02	1.03E-01	ENSG00000174137
FAM53B	-0.1906	7.7637E-02	1.96E-01	ENSG00000189319
FAM53C	-0.0129	8.5458E-01	9.25E-01	ENSG00000120709
FAM66B	0.2669	1.8451E-02	6.78E-02	ENSG00000215374
FAM66C	0.2389	8.2034E-03	3.66E-02	ENSG00000226711
FAM66E	0.2043	8.8329E-02	2.16E-01	ENSG00000225725
FAM72A	0.0192	8.3934E-01	9.16E-01	ENSG00000196550
FAM72B	-0.0528	5.7939E-01	7.46E-01	ENSG00000188610
FAM72C	0.0425	6.4434E-01	7.94E-01	ENSG00000263513
FAM72D	0.0318	7.3968E-01	8.57E-01	ENSG00000215784
FAM74A4	0.0061	8.0378E-01		ENSG00000274583
FAM74A6	0.0061	8.0378E-01		ENSG00000274516

FAM76A	-0.2630	8.2789E-05	9.61E-04	ENSG00000009780
FAM76B	0.2201	1.0048E-02	4.27E-02	ENSG00000077458
FAM78A	-0.0268	6.5739E-01	8.02E-01	ENSG00000126882
FAM78B	0.1368	1.9738E-01	3.74E-01	ENSG00000188859
FAM81A	0.1101	1.8235E-01	3.55E-01	ENSG00000157470
FAM81B	-0.2740	4.8940E-03	2.46E-02	ENSG00000153347
FAM83A	0.0041	9.0475E-01		ENSG00000147689
FAM83A-AS1	0.0052	5.6635E-01		ENSG00000204949
FAM83B	-0.0186	8.4956E-01	9.23E-01	ENSG00000168143
FAM83C	-0.0724	1.1141E-01		ENSG00000125998
FAM83C-AS1	0.0037	6.0719E-01		ENSG00000235214
FAM83D	0.0268	7.1421E-01	8.41E-01	ENSG00000101447
FAM83E	0.1603	1.1733E-01	2.63E-01	ENSG00000105523
FAM83F	0.5784	8.8123E-04	6.45E-03	ENSG00000133477
FAM83G	-0.3121	1.4029E-02	5.51E-02	ENSG00000188522
FAM83H	-0.2364	3.3640E-02	1.06E-01	ENSG00000180921
FAM86B1	0.0475	5.8942E-01	7.53E-01	ENSG00000186523
FAM86B2	0.1086	2.6458E-01	4.53E-01	ENSG00000145002
FAM86B3P	0.0817	3.9584E-01	5.90E-01	ENSG00000173295
FAM86C1P	0.1723	1.1928E-01	2.66E-01	ENSG00000158483
FAM86DP	0.1211	1.2172E-01	2.70E-01	ENSG00000244026
FAM86GP	-0.0233	8.0929E-01	8.99E-01	ENSG00000166492
FAM86KP	0.0074	5.9143E-01		ENSG00000163612
FAM86MP	-0.0160	5.8891E-01		ENSG00000186234
FAM87A	-0.0382	6.6977E-01	8.10E-01	ENSG00000182366
FAM87B	0.3171	1.5236E-02	5.87E-02	ENSG00000177757
FAM88B	0.0262	7.7155E-01	8.76E-01	ENSG00000170161
FAM88C	0.0564	5.3435E-01	7.08E-01	ENSG00000204802
FAM89A	-0.3175	2.6600E-05	3.84E-04	ENSG00000182118
FAM89B	-0.1053	1.9164E-01	3.67E-01	ENSG00000176973
FAM8A1	0.3061	6.2507E-04	4.92E-03	ENSG00000137414
FAM90A1	-0.1976	6.8285E-02	1.79E-01	ENSG00000171847
FAM90A11	-0.0339	5.4553E-01		ENSG00000233115
FAM90A24	0.0036	9.4785E-01		ENSG00000215354
FAM91A1	0.2173	4.3106E-03	2.24E-02	ENSG00000176853
FAM95A	0.0179	6.3885E-01		ENSG00000289811
FAM98A	0.2240	1.0634E-02	4.44E-02	ENSG00000119812
FAM98B	-0.2041	1.1679E-02	4.78E-02	ENSG00000171262
FAM98C	-0.0691	2.3892E-01	4.24E-01	ENSG00000130244
FAM9A	0.0357	2.6103E-01		ENSG00000183304
FAM9B	0.0006	8.9700E-01		ENSG00000177138
FAM9C	-0.0215	6.1355E-01		ENSG00000187268
FAN1	-0.0189	7.8981E-01	8.87E-01	ENSG00000198690
FANCA	0.3936	5.7725E-06	1.13E-04	ENSG00000187741
FANCB	-0.1611	1.3274E-01	2.85E-01	ENSG00000181544
FANCC	0.1441	8.5015E-02	2.10E-01	ENSG00000158169
FANCD2	-0.1598	1.4316E-01	3.01E-01	ENSG00000144554
FANCD2OS	-0.0045	9.4000E-01		ENSG00000163705
FANCE	0.0767	2.7927E-01	4.70E-01	ENSG00000112039

FANCF	0.3795	7.2469E-05	8.65E-04	ENSG00000183161
FANCG	0.1004	2.2847E-01	4.11E-01	ENSG00000221829
FANCI	-0.0540	5.3685E-01	7.10E-01	ENSG00000140525
FANCL	-0.1531	3.0120E-02	9.77E-02	ENSG00000115392
FANCM	0.1852	1.1027E-01	2.52E-01	ENSG00000187790
FANK1	-0.1504	1.0127E-01	2.37E-01	ENSG00000203780
FAP	0.0106	4.9925E-01		ENSG00000078098
FAR1	-0.0693	2.5683E-01	4.45E-01	ENSG00000197601
FAR2	0.0447	6.4304E-01	7.93E-01	ENSG00000064763
FAR2P1	-0.0071	9.3528E-01	9.68E-01	ENSG00000286058
FAR2P3	0.0738	4.4391E-01	6.34E-01	ENSG00000240253
FAR2P4	0.1107	1.2261E-01	2.71E-01	ENSG00000231431
FARP1	-0.2223	1.9283E-05	2.97E-04	ENSG00000152767
FARP2	-0.0106	8.6875E-01	9.34E-01	ENSG00000006607
FARS2	-0.2819	8.1422E-04	6.05E-03	ENSG00000145982
FARS2-AS1	-0.0106	9.0927E-01	9.55E-01	ENSG00000269985
FARSA	0.0209	6.9723E-01	8.31E-01	ENSG00000179115
FARSB	0.1599	2.5500E-02	8.65E-02	ENSG00000116120
FASN	-0.2537	9.7522E-03	4.17E-02	ENSG00000169710
FASTK	0.0522	4.0670E-01	6.00E-01	ENSG00000164896
FASTKD1	0.0642	4.3072E-01	6.22E-01	ENSG00000138399
FASTKD2	0.0867	1.4366E-01	3.01E-01	ENSG00000118246
FASTKD3	0.5534	6.7299E-11	8.56E-09	ENSG00000124279
FASTKD5	-0.1967	3.7947E-03	2.03E-02	ENSG00000215251
FAT1	-0.3758	1.7649E-05	2.78E-04	ENSG00000083857
FAT2	0.0100	5.3756E-01	7.11E-01	ENSG00000086570
FAT3	0.3457	2.6527E-03	1.53E-02	ENSG00000165323
FAT4	-0.1926	7.9967E-02	2.00E-01	ENSG00000196159
FAU	-0.1497	6.5397E-03	3.08E-02	ENSG00000149806
FAXC	0.1680	7.4903E-02	1.91E-01	ENSG00000146267
FAXDC2	-0.1624	8.8433E-02	2.16E-01	ENSG00000170271
FBF1	-0.0270	7.5355E-01	8.66E-01	ENSG00000188878
FBH1	-0.0103	9.0241E-01	9.51E-01	ENSG00000134452
FBL	-0.0985	1.4219E-01	2.99E-01	ENSG00000105202
FBLIM1	-0.0804	3.9943E-01	5.94E-01	ENSG00000162458
FBLL1	-0.1805	4.9811E-02	1.43E-01	ENSG00000188573
FBLN1	-0.1721	1.0759E-01	2.47E-01	ENSG00000077942
FBLN2	-0.0048	9.5582E-01	9.77E-01	ENSG00000163520
FBLN7	-0.3253	4.6297E-03	2.36E-02	ENSG00000144152
FBN1	-0.1984	2.4635E-02	8.43E-02	ENSG00000166147
FBN1-DT	0.1939	1.0093E-01	2.36E-01	ENSG00000259705
FBN2	-0.3381	7.5360E-03	3.43E-02	ENSG00000138829
FBN3	0.2266	3.0630E-02	9.89E-02	ENSG00000142449
FBP1	0.0680	4.8775E-01	6.72E-01	ENSG00000165140
FBP2	-0.0236	7.9772E-01	8.92E-01	ENSG00000130957
FBRS	-0.3044	2.4541E-03	1.44E-02	ENSG00000156860
FBRSL1	0.1295	1.0863E-01	2.49E-01	ENSG00000112787
FBXL12	-0.1918	9.5732E-03	4.11E-02	ENSG00000127452
FBXL13	0.1367	1.5310E-01	3.15E-01	ENSG00000161040

FBXL14	0.2207	1.9394E-02	7.04E-02	ENSG00000171823
FBXL15	-0.0344	5.7909E-01	7.45E-01	ENSG00000107872
FBXL16	0.0563	4.9686E-01	6.80E-01	ENSG00000127585
FBXL17	0.2561	1.1002E-03	7.65E-03	ENSG00000145743
FBXL18	0.2408	6.2445E-03	2.97E-02	ENSG00000155034
FBXL19	-0.1092	2.8505E-01	4.76E-01	ENSG00000099364
FBXL19-AS1	0.0330	7.2551E-01	8.48E-01	ENSG00000260852
FBXL2	-0.0251	7.0005E-01	8.32E-01	ENSG00000153558
FBXL20	0.2127	1.4783E-03	9.70E-03	ENSG00000108306
FBXL22	0.0568	5.3126E-01	7.06E-01	ENSG00000197361
FBXL3	-0.1162	1.8883E-01	3.63E-01	ENSG00000005812
FBXL4	-0.0856	2.1271E-01	3.93E-01	ENSG00000112234
FBXL5	-0.0065	9.2414E-01	9.62E-01	ENSG00000118564
FBXL6	0.4063	4.6782E-06	9.52E-05	ENSG00000182325
FBXL8	0.4474	3.9864E-03	2.10E-02	ENSG00000135722
FBXL9P	-0.1300	1.3191E-01	2.84E-01	ENSG00000125122
FBXO10	-0.1486	7.6960E-02	1.95E-01	ENSG00000147912
FBXO11	-0.1070	1.2329E-01	2.72E-01	ENSG00000138081
FBXO15	-0.0605	4.8726E-01	6.71E-01	ENSG00000141665
FBXO16	0.0974	9.8060E-02	2.32E-01	ENSG00000214050
FBXO17	-0.1909	7.5641E-02	1.93E-01	ENSG00000269190
FBXO2	-0.1819	6.2562E-02	1.68E-01	ENSG00000116661
FBXO21	-0.0374	4.5907E-01	6.48E-01	ENSG00000135108
FBXO22	-0.0444	5.2039E-01	6.98E-01	ENSG00000167196
FBXO24	0.0549	5.6707E-01	7.35E-01	ENSG00000106336
FBXO25	0.2356	9.7361E-03	4.16E-02	ENSG00000147364
FBXO27	-0.3305	2.3548E-04	2.24E-03	ENSG00000161243
FBXO28	-0.2261	7.3081E-05	8.71E-04	ENSG00000143756
FBXO3	0.0595	4.0657E-01	6.00E-01	ENSG00000110429
FBXO3-DT	-0.0628	5.1505E-01		ENSG00000254508
FBXO30	0.0275	6.8205E-01	8.20E-01	ENSG00000118496
FBXO31	-0.0044	9.3174E-01	9.66E-01	ENSG00000103264
FBXO32	-0.3810	1.2871E-05	2.14E-04	ENSG00000156804
FBXO33	-0.1286	6.4472E-02	1.72E-01	ENSG00000165355
FBXO34	0.0891	2.0110E-01	3.78E-01	ENSG00000178974
FBXO34-AS1	0.0324	3.4186E-01	5.37E-01	ENSG00000258413
FBXO36	0.1684	2.5640E-02	8.68E-02	ENSG00000153832
FBXO38	0.3805	1.4010E-06	3.53E-05	ENSG00000145868
FBXO38-DT	0.0216	8.1555E-01	9.03E-01	ENSG00000247199
FBXO39	-0.0492	3.7464E-01		ENSG00000177294
FBXO4	0.3875	2.0031E-04	1.96E-03	ENSG00000151876
FBXO41	-0.0950	2.4814E-01	4.34E-01	ENSG00000163013
FBXO42	0.0592	3.7088E-01	5.66E-01	ENSG00000037637
FBXO43	-0.1756	1.1838E-01	2.65E-01	ENSG00000156509
FBXO44	-0.0702	2.8139E-01	4.72E-01	ENSG00000132879
FBXO45	0.0892	1.8979E-01	3.65E-01	ENSG00000174013
FBXO46	0.0321	6.7594E-01	8.15E-01	ENSG00000177051
FBXO47	0.0263	3.0969E-01		ENSG00000204952
FBXO48	-0.1973	9.1103E-02	2.20E-01	ENSG00000204923

FBXO5	-0.3003	1.4482E-02	5.65E-02	ENSG00000112029
FBXO6	-0.2057	4.6904E-02	1.36E-01	ENSG00000116663
FBXO7	-0.0911	1.5768E-01	3.21E-01	ENSG00000100225
FBXO8	0.1237	4.1709E-02	1.25E-01	ENSG00000164117
FBXO9	0.1989	9.5639E-03	4.11E-02	ENSG00000112146
FBXW10	0.0299	5.5691E-01	7.27E-01	ENSG00000171931
FBXW10B	-0.0308	7.3636E-01	8.55E-01	ENSG00000241322
FBXW11	0.0186	7.4267E-01	8.59E-01	ENSG00000072803
FBXW11P1	0.0682	3.2385E-01	5.18E-01	ENSG00000230870
FBXW12	-0.1586	1.4968E-01	3.10E-01	ENSG00000164049
FBXW2	0.0009	9.9869E-01	1.00E+00	ENSG00000119402
FBXW4	-0.3443	3.2120E-04	2.89E-03	ENSG00000107829
FBXW5	0.1516	2.4606E-02	8.42E-02	ENSG00000159069
FBXW7	-0.0065	8.7141E-01	9.35E-01	ENSG00000109670
FBXW8	0.0892	1.9982E-01	3.77E-01	ENSG00000174989
FBXW9	0.1001	1.8374E-01	3.57E-01	ENSG00000132004
FCER1G	0.0023	1.5638E-01	3.19E-01	ENSG00000158869
FCF1	0.0043	9.6794E-01	9.84E-01	ENSG00000119616
FCF1P5	-0.0055	6.7310E-01		ENSG00000220392
FCGBP	-0.1262	2.2170E-01	4.03E-01	ENSG00000275395
FCGR1A	-0.0412	2.0055E-01		ENSG00000150337
FCGR2A	-0.1787	1.0480E-01	2.43E-01	ENSG00000143226
FCGR2B	0.0151	5.1190E-01		ENSG00000072694
FCGRT	-0.5798	2.5974E-06	5.93E-05	ENSG00000104870
FCHO1	0.4217	1.0361E-05	1.81E-04	ENSG00000130475
FCHO2	-0.0159	7.9979E-01	8.93E-01	ENSG00000157107
FCHSD1	0.1105	1.8409E-01	3.57E-01	ENSG00000197948
FCHSD2	0.0762	2.2374E-01	4.06E-01	ENSG00000137478
FCMR	-0.3553	9.9291E-03	4.22E-02	ENSG00000162894
FCN3	0.0620	2.7568E-01		ENSG00000142748
FCRL3	0.0151	1.4790E-01		ENSG00000160856
FCRL6	0.0017	8.1973E-01		ENSG00000181036
FCRLA	0.0219	1.3067E-01		ENSG00000132185
FCRLB	0.1500	1.4465E-01	3.03E-01	ENSG00000162746
FCSK	0.0304	6.6573E-01	8.08E-01	ENSG00000157353
FDFT1	-0.1327	3.6731E-02	1.13E-01	ENSG00000079459
FDPS	-0.1554	4.4545E-02	1.31E-01	ENSG00000160752
FDPSP3	-0.0171	3.1390E-01		ENSG00000258872
FDPSP7	0.0228	3.2737E-01		ENSG00000231087
FDX1	-0.2147	3.6186E-04	3.19E-03	ENSG00000137714
FDX2	0.0163	1.5694E-01	3.20E-01	ENSG00000267673
FDXACB1	0.0879	3.5988E-01	5.56E-01	ENSG00000255561
FDXR	-0.2155	8.8883E-03	3.89E-02	ENSG00000161513
FECH	0.1320	1.7780E-01	3.49E-01	ENSG00000066926
FEM1A	-0.0755	4.1935E-01	6.12E-01	ENSG00000141965
FEM1AP2	-0.0041	8.7602E-01		ENSG00000265296
FEM1B	-0.3397	1.2301E-08	6.79E-07	ENSG00000169018
FEM1C	-0.1636	1.4865E-02	5.76E-02	ENSG00000145780
FEN1	0.1359	1.0377E-01	2.41E-01	ENSG00000168496

FENDRR	-0.0139	8.6518E-01		ENSG00000268388
FER	-0.2694	5.8284E-05	7.27E-04	ENSG00000151422
FER1L4	0.5534	1.9126E-05	2.95E-04	ENSG00000088340
FER1L5	0.4772	4.4831E-04	3.77E-03	ENSG00000249715
FER1L6	-0.0543	7.6366E-01	8.72E-01	ENSG00000214814
FER1L6-AS2	-0.0005	9.2568E-01		ENSG00000253868
FERD3L	-0.0359	8.2491E-01	9.08E-01	ENSG00000146618
FERMT1	-0.4758	1.5635E-03	1.01E-02	ENSG00000101311
FERMT2	-0.2244	4.3648E-03	2.26E-02	ENSG00000073712
FERMT3	-0.0869	3.7824E-01	5.73E-01	ENSG00000149781
FES	0.5785	4.5369E-04	3.80E-03	ENSG00000182511
FEV	0.0155	8.7151E-01	9.35E-01	ENSG00000163497
FEZ1	0.0277	7.6660E-01	8.73E-01	ENSG00000149557
FEZ2	-0.1951	2.2818E-02	7.95E-02	ENSG00000171055
FEZF1	-0.0295	7.5203E-01	8.65E-01	ENSG00000128610
FEZF1-AS1	-0.0233	8.1142E-01	9.00E-01	ENSG00000230316
FEZF2	0.1683	9.7560E-02	2.31E-01	ENSG00000153266
FFAR1	-0.0390	4.2299E-01		ENSG00000126266
FFAR3	0.0262	3.1030E-01		ENSG00000185897
FFAR4	-0.0198	8.1479E-01	9.02E-01	ENSG00000186188
FGB	0.0060	7.0339E-01		ENSG00000171564
FGD1	-0.1098	1.4274E-01	3.00E-01	ENSG00000102302
FGD2	0.0068	9.2664E-01	9.64E-01	ENSG00000146192
FGD3	0.1911	4.7815E-02	1.38E-01	ENSG00000127084
FGD4	-0.2146	1.5376E-03	1.00E-02	ENSG00000139132
FGD5	-0.0587	4.9357E-01	6.77E-01	ENSG00000154783
FGD5-AS1	-0.1377	3.4482E-03	1.88E-02	ENSG00000225733
FGD6	0.3392	1.1776E-04	1.28E-03	ENSG00000180263
FGF10	0.0044	8.4458E-01		ENSG00000070193
FGF11	-0.1142	2.6336E-01	4.52E-01	ENSG00000161958
FGF12	-0.0850	1.2705E-01	2.78E-01	ENSG00000114279
FGF12-AS1	-0.0450	6.8052E-01		ENSG00000231383
FGF12-AS2	0.0022	9.7951E-01	9.89E-01	ENSG00000230126
FGF13	0.0754	4.4069E-01	6.31E-01	ENSG00000129682
FGF13-AS1	-0.1230	2.1739E-01	3.98E-01	ENSG00000226031
FGF14	0.1325	7.8744E-02	1.98E-01	ENSG00000102466
FGF14-AS2	-0.0971	2.3816E-01	4.23E-01	ENSG00000272143
FGF14-IT1	0.0253	7.7386E-01	8.78E-01	ENSG00000243319
FGF16	0.0329	6.4069E-01	7.91E-01	ENSG00000196468
FGF17	-0.0840	3.7445E-01	5.70E-01	ENSG00000158815
FGF18	-0.0681	4.7138E-01	6.59E-01	ENSG00000156427
FGF19	0.0391	5.5505E-01	7.26E-01	ENSG00000162344
FGF2	-0.1682	2.9448E-02	9.61E-02	ENSG00000138685
FGF20	-0.1942	7.8158E-02	1.97E-01	ENSG00000078579
FGF22	0.1739	8.6528E-02	2.12E-01	ENSG00000070388
FGF23	0.0037	4.2456E-01		ENSG00000118972
FGF5	0.0721	4.5662E-01	6.46E-01	ENSG00000138675
FGF7	-0.0313	7.3853E-01	8.56E-01	ENSG00000140285
FGF8	-0.0350	3.5938E-01		ENSG00000107831

FGF9	0.2695	9.3376E-03	4.04E-02	ENSG00000102678
FGFBP1	-0.0371	9.0968E-01		ENSG00000137440
FGFBP2	0.0012	7.2011E-01		ENSG00000137441
FGFBP3	-0.1247	2.1472E-01	3.95E-01	ENSG00000174721
FGFR1	-0.2291	8.1463E-06	1.49E-04	ENSG00000077782
FGFR1OP2	-0.0392	5.0837E-01	6.89E-01	ENSG00000111790
FGFR2	-0.1529	6.0056E-02	1.64E-01	ENSG00000066468
FGFR4	0.0063	9.5005E-01	9.75E-01	ENSG00000160867
FGFRL1	-0.2371	4.6738E-03	2.38E-02	ENSG00000127418
FGG	0.0094	7.3427E-01		ENSG00000171557
FGGY	0.1088	1.6165E-01	3.27E-01	ENSG00000172456
FGL1	-0.2354	5.6529E-02	1.57E-01	ENSG00000104760
FGR	0.0389	1.8647E-01	3.60E-01	ENSG00000000938
FH	0.2435	4.8368E-04	3.99E-03	ENSG00000091483
FHAD1	-0.1850	9.7527E-02	2.31E-01	ENSG00000142621
FHAD1-AS1	-0.0076	6.7115E-01		ENSG00000233485
FHDC1	0.1329	2.0625E-01	3.85E-01	ENSG00000137460
FHIP1A	-0.1531	1.5286E-01	3.14E-01	ENSG00000164142
FHIP1B	-0.1433	4.4292E-02	1.31E-01	ENSG00000051009
FHIP2A	0.0743	1.9957E-01	3.77E-01	ENSG00000151553
FHIP2B	0.0450	5.0973E-01	6.90E-01	ENSG00000158863
FHIT	-0.4182	8.5842E-04	6.33E-03	ENSG00000189283
FHL1	-0.0352	6.7968E-01	8.18E-01	ENSG00000022267
FHL1P1	0.0055	8.9237E-01		ENSG00000239219
FHL2	0.2385	5.7407E-02	1.58E-01	ENSG00000115641
FHL3	-0.1718	4.2133E-02	1.26E-01	ENSG00000183386
FHOD1	-0.0262	7.9063E-01	8.88E-01	ENSG00000135723
FHOD3	0.0476	5.4281E-01	7.15E-01	ENSG00000134775
FIBCD1	-0.1854	9.9126E-02	2.33E-01	ENSG00000130720
FIBIN	-0.0206	8.2249E-01	9.07E-01	ENSG00000176971
FIBP	-0.0108	8.5411E-01	9.25E-01	ENSG00000172500
FICD	0.5692	3.0087E-09	2.12E-07	ENSG00000198855
FIG4	-0.0254	7.2020E-01	8.45E-01	ENSG00000112367
FIGNL1	0.2118	4.2747E-02	1.27E-01	ENSG00000132436
FIGNL2	0.0339	3.2636E-01	5.21E-01	ENSG00000261308
FIGNL2-DT	0.0211	6.8766E-01	8.24E-01	ENSG00000259887
FILIP1L	-0.2285	2.7316E-02	9.08E-02	ENSG00000168386
FILNC1	0.0100	5.6076E-01		ENSG00000231426
FIP1L1	-0.1039	5.4664E-02	1.53E-01	ENSG00000145216
FIRRE	0.1168	2.4295E-01	4.28E-01	ENSG00000213468
FIRRM	0.5604	4.1484E-06	8.63E-05	ENSG00000000460
FIS1	-0.0670	1.3970E-01	2.96E-01	ENSG00000214253
FITM1	0.0380	3.7285E-01	5.68E-01	ENSG00000139914
FITM2	0.2107	1.1858E-02	4.83E-02	ENSG00000197296
FIZ1	-0.0011	9.8893E-01	9.94E-01	ENSG00000179943
FKBP10	0.1300	7.2280E-02	1.87E-01	ENSG00000141756
FKBP11	-0.1649	2.6869E-02	8.98E-02	ENSG00000134285
FKBP14	-0.0071	8.8832E-01	9.44E-01	ENSG00000106080
FKBP15	0.1539	1.4377E-02	5.62E-02	ENSG00000119321

FKBP1A	-0.0232	7.2814E-01	8.50E-01	ENSG00000088832
FKBP1B	-0.0860	1.8757E-01	3.62E-01	ENSG00000119782
FKBP1C	0.0380	6.2310E-01	7.79E-01	ENSG00000198225
FKBP2	-0.1098	2.8505E-01	4.76E-01	ENSG00000173486
FKBP3	-0.2799	6.5004E-07	1.86E-05	ENSG00000100442
FKBP4	-0.2654	1.3139E-03	8.83E-03	ENSG00000004478
FKBP5	0.4703	4.2079E-04	3.58E-03	ENSG00000096060
FKBP6	0.0567	5.2400E-01	7.01E-01	ENSG00000077800
FKBP7	0.1861	5.0507E-03	2.52E-02	ENSG00000079150
FKBP8	-0.1231	3.6756E-02	1.14E-01	ENSG00000105701
FKBP9	0.0774	2.9640E-01	4.88E-01	ENSG00000122642
FKBPL	0.1447	5.1173E-02	1.45E-01	ENSG00000204315
FKRP	-0.2729	1.4076E-03	9.34E-03	ENSG00000181027
FKTN	0.0851	2.3024E-01	4.14E-01	ENSG00000106692
FKTN-AS1	0.0147	8.3477E-01	9.14E-01	ENSG00000228317
FLACC1	-0.5694	9.9430E-04	7.08E-03	ENSG00000155749
FLAD1	0.1704	4.2867E-03	2.23E-02	ENSG00000160688
FLCN	-0.0941	2.0379E-01	3.82E-01	ENSG00000154803
FLI1	-0.0278	8.4452E-01		ENSG00000151702
FLII	0.0322	6.2695E-01	7.82E-01	ENSG00000177731
FLJ13224	0.3896	1.6430E-02	6.22E-02	ENSG00000177340
FLJ16779	-0.1628	1.3288E-01	2.86E-01	ENSG00000275620
FLJ20021	-0.0828	2.7607E-01	4.67E-01	ENSG00000254531
FLJ30679	0.0191	8.4201E-01	9.18E-01	ENSG00000280278
FLJ32255	-0.2546	4.8517E-02	1.40E-01	ENSG00000287263
FLJ33534	0.0310	6.7081E-01	8.11E-01	ENSG00000145063
FLJ38576	-0.0121	8.9139E-01	9.46E-01	ENSG00000279943
FLJ40194	0.1003	1.4567E-01	3.04E-01	ENSG00000177369
FLJ42393	-0.0650	5.0755E-01	6.88E-01	ENSG00000279891
FLJ42969	0.0202	8.2909E-01	9.10E-01	ENSG00000248599
FLJ46284	-0.0415	7.0012E-01	8.32E-01	ENSG00000248858
FLNA	-0.2883	3.8085E-04	3.32E-03	ENSG00000196924
FLNB	0.0216	7.4019E-01	8.57E-01	ENSG00000136068
FLNB-AS1	0.0499	4.3331E-01	6.25E-01	ENSG00000244161
FLNC	-0.5815	4.8010E-05	6.25E-04	ENSG00000128591
FLOT1	-0.0758	1.6492E-01	3.31E-01	ENSG00000137312
FLOT2	-0.0612	3.8227E-01	5.77E-01	ENSG00000132589
FLRT1	0.0616	5.0616E-01	6.87E-01	ENSG00000126500
FLRT2	0.2890	2.9019E-03	1.64E-02	ENSG00000185070
FLRT2-AS1	0.3333	4.3061E-03	2.24E-02	ENSG00000205562
FLRT3	0.0966	1.7362E-01	3.43E-01	ENSG00000125848
FLT1	-0.1018	7.0903E-01	8.37E-01	ENSG00000102755
FLT3	0.0027	4.8883E-01		ENSG00000122025
FLT3LG	0.0378	6.3796E-01	7.90E-01	ENSG00000090554
FLT4	0.1595	1.0522E-01	2.43E-01	ENSG00000037280
FLVCR1	0.2120	8.2504E-03	3.68E-02	ENSG00000162769
FLVCR1-DT	0.4367	6.8989E-06	1.31E-04	ENSG00000198468
FLVCR2	-0.0249	7.8670E-01	8.85E-01	ENSG00000119686
FLYWCH1	0.1204	7.8350E-02	1.98E-01	ENSG00000059122

FLYWCH2	-0.1262	9.9342E-03	4.22E-02	ENSG00000162076
FMC1	0.0624	3.9398E-01	5.89E-01	ENSG00000164898
FMN1	0.0593	5.1703E-01	6.95E-01	ENSG00000248905
FMN2	-0.0109	8.7729E-01	9.38E-01	ENSG00000155816
FMNL1	0.1430	1.1090E-01	2.53E-01	ENSG00000184922
FMNL1-DT	0.0957	3.1587E-01	5.09E-01	ENSG00000267121
FMNL2	-0.3350	5.8670E-06	1.15E-04	ENSG00000157827
FMNL3	0.0197	8.0077E-01	8.93E-01	ENSG00000161791
FMO1	-0.2175	2.2710E-02	7.92E-02	ENSG00000010932
FMO2	-0.0616	2.0720E-01		ENSG00000094963
FMO3	-0.0412	5.0638E-01		ENSG00000007933
FMO4	-0.1126	2.3395E-01	4.18E-01	ENSG00000076258
FMO5	0.0083	9.3152E-01	9.66E-01	ENSG00000131781
FMO6P	-0.0004	8.9573E-01		ENSG00000117507
FMOD	0.3791	1.4650E-02	5.70E-02	ENSG00000122176
FMR1	-0.0561	2.4621E-01	4.32E-01	ENSG00000102081
FMR1-AS1	-0.0603	4.5016E-01	6.40E-01	ENSG00000268066
FMR1NB	0.0129	4.8976E-01		ENSG00000176988
FN1	-0.1895	8.3633E-02	2.07E-01	ENSG00000115414
FN3K	-0.2172	4.6628E-04	3.88E-03	ENSG00000167363
FN3KRP	-0.0364	5.5990E-01	7.30E-01	ENSG00000141560
FNBP1	0.0730	2.5178E-01	4.39E-01	ENSG00000187239
FNBP1L	-0.1256	1.2067E-01	2.68E-01	ENSG00000137942
FNBP4	-0.1984	8.9706E-05	1.02E-03	ENSG00000109920
FNDC10	-0.1313	6.1330E-02	1.66E-01	ENSG00000228594
FNDC11	0.0257	7.8312E-01	8.83E-01	ENSG00000125531
FNDC3A	-0.0053	9.3121E-01	9.66E-01	ENSG00000102531
FNDC3B	-0.2910	3.4818E-04	3.09E-03	ENSG00000075420
FNDC4	0.1367	1.2633E-01	2.77E-01	ENSG00000115226
FNDC5	-0.4748	5.0259E-06	1.01E-04	ENSG00000160097
FNDC7	-0.0877	4.5758E-01	6.47E-01	ENSG00000143107
FNDC8	-0.0588	2.1857E-01		ENSG00000073598
FNDC9	0.0193	4.1433E-01		ENSG00000172568
FNIP1	0.0625	3.7782E-01	5.73E-01	ENSG00000217128
FNIP2	0.0905	2.1590E-01	3.97E-01	ENSG00000052795
FNTA	0.0474	4.2197E-01	6.15E-01	ENSG00000168522
FNTB	0.0994	2.4611E-01	4.32E-01	ENSG00000257365
FOCAD	-0.0489	4.3538E-01	6.27E-01	ENSG00000188352
FOCAD-AS1	-0.0455	6.2429E-01	7.80E-01	ENSG00000227071
FOLH1	-0.0563	5.5848E-01	7.29E-01	ENSG00000086205
FOLR2	0.4689	7.5495E-03	3.43E-02	ENSG00000165457
FOLR3	-0.0313	5.1577E-01		ENSG00000110203
FOS	0.0258	7.5507E-01	8.67E-01	ENSG00000170345
FOSB	0.5236	2.8191E-05	4.04E-04	ENSG00000125740
FOSL2	-0.3689	7.7031E-05	9.08E-04	ENSG00000075426
FOSL2-AS1	0.1757	9.2038E-02	2.22E-01	ENSG00000229951
FOXA1	-0.1485	3.1240E-02	1.00E-01	ENSG00000129514
FOXA2	0.0888	3.3333E-01	5.28E-01	ENSG00000125798
FOXA3	-0.1680	6.9271E-02	1.81E-01	ENSG00000170608

FOXB1	0.1365	8.9123E-02	2.17E-01	ENSG00000171956
FOXC1	-0.0349	7.0844E-01	8.37E-01	ENSG00000054598
FOXC2	-0.0034	9.6691E-01	9.83E-01	ENSG00000176692
FOXD1	0.2349	4.2011E-02	1.26E-01	ENSG00000251493
FOXD2	0.0244	8.2830E-02		ENSG00000186564
FOXD2-AS1	-0.0287	5.0127E-01		ENSG00000237424
FOXD3	0.0270	6.7535E-01	8.14E-01	ENSG00000187140
FOXD3-AS1	-0.0652	4.4511E-01	6.35E-01	ENSG00000230798
FOXD4	-0.1358	9.8427E-02	2.32E-01	ENSG00000170122
FOXD4L1	0.0030	9.9025E-01		ENSG00000184492
FOXD4L3	0.0025	4.3534E-01		ENSG00000187559
FOXD4L5	-0.0109	8.5428E-01		ENSG00000204779
FOXD4L6	-0.0015	6.1539E-01		ENSG00000273514
FOXE1	-0.0269	6.0509E-01	7.65E-01	ENSG00000178919
FOXE3	0.0105	3.8662E-01		ENSG00000186790
FOXF1	0.1191	2.0879E-01	3.88E-01	ENSG00000103241
FOXF2	-0.1690	6.2375E-02	1.68E-01	ENSG00000137273
FOXG1	0.0452	8.0071E-02		ENSG00000176165
FOXH1	0.0276	1.1774E-01		ENSG00000160973
FOXI3	0.0525	4.0962E-01	6.03E-01	ENSG00000214336
FOXJ1	-0.2991	1.5268E-03	9.95E-03	ENSG00000129654
FOXJ2	-0.0910	1.8818E-01	3.63E-01	ENSG00000065970
FOXJ3	-0.1117	9.0199E-02	2.19E-01	ENSG00000198815
FOXK1	-0.1172	1.3035E-01	2.82E-01	ENSG00000164916
FOXK2	-0.2994	7.4657E-04	5.66E-03	ENSG00000141568
FOXL1	0.0621	2.7960E-01	4.71E-01	ENSG00000176678
FOXL2	-0.0019	9.5786E-01		ENSG00000183770
FOXM1	-0.1658	1.3080E-01	2.83E-01	ENSG00000111206
FOXN1	-0.0065	9.8220E-01		ENSG00000109101
FOXN2	-0.1633	7.2872E-02	1.88E-01	ENSG00000170802
FOXN3	-0.1634	8.8497E-03	3.88E-02	ENSG00000053254
FOXN3-AS1	-0.3792	8.4540E-05	9.77E-04	ENSG00000258920
FOXN4	-0.0219	7.8696E-01	8.86E-01	ENSG00000139445
FOXO1	-0.2122	5.0792E-02	1.45E-01	ENSG00000150907
FOXO3	-0.1567	4.8159E-03	2.43E-02	ENSG00000118689
FOXO3B	0.0807	1.9088E-01	3.66E-01	ENSG00000240445
FOXO4	-0.0041	9.4582E-01	9.73E-01	ENSG00000184481
FOXO6	0.3942	3.9522E-03	2.09E-02	ENSG00000204060
FOXO6-AS1	-0.0418	8.2288E-01	9.07E-01	ENSG00000229901
FOXP1	-0.2464	5.0682E-03	2.53E-02	ENSG00000114861
FOXP1-AS1	-0.1695	1.3134E-01	2.84E-01	ENSG00000244203
FOXP2	-0.2907	4.0614E-03	2.14E-02	ENSG00000128573
FOXP3	0.0046	9.4588E-01	9.73E-01	ENSG00000049768
FOXP4	0.2330	4.6277E-03	2.36E-02	ENSG00000137166
FOXP4-AS1	-0.0106	9.0973E-01	9.55E-01	ENSG00000234753
FOXQ1	0.0948	3.4533E-01	5.40E-01	ENSG00000164379
FOXR1	0.0452	3.6799E-01	5.64E-01	ENSG00000176302
FOXRED1	0.3791	3.2703E-05	4.52E-04	ENSG00000110074
FOXRED2	-0.1868	3.9660E-04	3.42E-03	ENSG00000100350

FOXS1	-0.0424	5.7970E-01	7.46E-01	ENSG00000179772
FPGS	0.0543	4.5174E-01	6.41E-01	ENSG00000136877
FPGT	-0.0826	3.4485E-01	5.40E-01	ENSG00000254685
FPGT-TNNI3K	0.1247	1.6653E-01	3.34E-01	ENSG00000259030
FPR1	-0.0077	6.4762E-01		ENSG00000171051
FPR3	-0.0349	3.1799E-01		ENSG00000187474
FRA10AC1	-0.3029	3.0744E-03	1.71E-02	ENSG00000148690
FRAS1	-0.1920	3.3988E-02	1.07E-01	ENSG00000138759
FRAT1	0.1969	2.6349E-02	8.84E-02	ENSG00000165879
FRAT2	0.2984	3.8648E-05	5.19E-04	ENSG00000181274
FREM1	-0.3930	4.9145E-05	6.37E-04	ENSG00000164946
FREM2	-0.1802	7.1042E-02	1.84E-01	ENSG00000150893
FREM3	0.0073	3.5895E-01		ENSG00000183090
FREY1	0.1234	2.0047E-01	3.78E-01	ENSG00000234776
FRG1	0.0811	1.0476E-01	2.43E-01	ENSG00000109536
FRG1-DT	0.1803	1.0277E-01	2.39E-01	ENSG00000245685
FRG1CP	0.0842	1.2940E-01	2.81E-01	ENSG00000282826
FRG2C	-0.0665	7.3232E-02		ENSG00000172969
FRK	0.1941	9.0094E-02	2.19E-01	ENSG00000111816
FRMD1	-0.0273	6.5983E-01		ENSG00000153303
FRMD3	-0.2084	3.2029E-02	1.02E-01	ENSG00000172159
FRMD4A	-0.0105	8.8755E-01	9.43E-01	ENSG00000151474
FRMD4B	-0.0910	1.4805E-01	3.08E-01	ENSG00000114541
FRMD5	-0.0838	3.8304E-01	5.78E-01	ENSG00000171877
FRMD6	-0.4514	4.2488E-06	8.80E-05	ENSG00000139926
FRMD6-AS1	0.3424	4.1200E-04	3.52E-03	ENSG00000258537
FRMD7	-0.0282	6.2501E-01		ENSG00000165694
FRMD8	0.1382	7.3888E-02	1.89E-01	ENSG00000126391
FRMPD1	0.1083	2.8621E-01	4.77E-01	ENSG00000070601
FRMPD2	-0.2603	5.0115E-02	1.43E-01	ENSG00000170324
FRMPD2B	-0.0449	5.2175E-01	6.99E-01	ENSG00000150175
FRMPD3	0.0634	5.1240E-01	6.92E-01	ENSG00000147234
FRMPD3-AS1	0.0354	2.9263E-01		ENSG00000227610
FRMPD4	-0.1433	1.4914E-01	3.09E-01	ENSG00000169933
FRRS1	-0.0870	3.7993E-01	5.75E-01	ENSG00000156869
FRRS1L	0.2230	3.5019E-03	1.90E-02	ENSG00000260230
FRS2	-0.2912	3.8536E-06	8.12E-05	ENSG00000166225
FRS3	0.3285	8.5960E-05	9.91E-04	ENSG00000137218
FRY	0.0011	9.9080E-01	9.95E-01	ENSG00000073910
FRYL	-0.1041	1.1695E-01	2.62E-01	ENSG00000075539
FRZB	-0.5649	4.9547E-03	2.48E-02	ENSG00000162998
FSBP	-0.0520	5.2084E-01	6.98E-01	ENSG00000265817
FSCN1	0.0183	8.1667E-01	9.03E-01	ENSG00000075618
FSCN2	-0.0763	2.9690E-01	4.89E-01	ENSG00000186765
FSCN3	0.0145	8.6001E-01	9.29E-01	ENSG00000106328
FSD1	-0.0284	6.0979E-01	7.68E-01	ENSG00000105255
FSD1L	-0.0731	2.8550E-01	4.77E-01	ENSG00000106701
FSD2	0.2050	8.7191E-02	2.14E-01	ENSG00000186628
FSHB	-0.1199	1.9055E-02	6.94E-02	ENSG00000131808

FSHR	0.0099	3.1589E-01		ENSG00000170820
FSIP1	-0.0497	5.7577E-01	7.43E-01	ENSG00000150667
FSIP2	0.2818	6.9503E-03	3.22E-02	ENSG00000188738
FSIP2-AS1	0.1480	1.4458E-01	3.03E-01	ENSG00000231646
FSIP2-AS2	-0.0517	5.8330E-01	7.49E-01	ENSG00000226747
FST	0.3665	5.0293E-03	2.51E-02	ENSG00000134363
FSTL1	-0.4611	4.1895E-05	5.56E-04	ENSG00000163430
FSTL3	0.0399	6.3705E-01	7.89E-01	ENSG00000070404
FSTL4	-0.1238	2.0483E-01	3.83E-01	ENSG00000053108
FSTL5	0.1275	1.9381E-01	3.69E-01	ENSG00000168843
FTCDNL1	-0.0752	4.4691E-01	6.37E-01	ENSG00000226124
FTH1	-0.4591	6.4083E-05	7.86E-04	ENSG00000167996
FTH1P10	-0.2234	3.2564E-02	1.04E-01	ENSG00000223361
FTH1P2	-0.0478	5.9513E-01	7.57E-01	ENSG00000234975
FTL	-0.1012	2.9846E-01	4.91E-01	ENSG00000087086
FTLP12	-0.0423	4.3739E-01		ENSG00000265095
FTO	-0.0451	4.4877E-01	6.39E-01	ENSG00000140718
FTSJ1	0.1559	2.4168E-02	8.30E-02	ENSG00000068438
FTSJ3	0.1716	1.1747E-02	4.80E-02	ENSG00000108592
FTX	0.5494	2.8511E-11	4.12E-09	ENSG00000230590
FUBP1	-0.2078	4.9759E-05	6.43E-04	ENSG00000162613
FUBP3	0.0819	2.3934E-01	4.24E-01	ENSG00000107164
FUCA1	0.3395	9.5694E-05	1.08E-03	ENSG00000179163
FUCA2	-0.0583	3.8160E-01	5.76E-01	ENSG00000001036
FUNDC1	-0.1764	1.6367E-03	1.05E-02	ENSG00000069509
FUNDC2	-0.2513	1.3326E-04	1.41E-03	ENSG00000165775
FUOM	0.0159	8.6830E-01	9.33E-01	ENSG00000148803
FURIN	0.0720	2.0864E-01	3.88E-01	ENSG00000140564
FUS	0.0559	4.5133E-01	6.41E-01	ENSG00000089280
FUT1	0.2804	1.2128E-02	4.92E-02	ENSG00000174951
FUT10	-0.0530	4.8556E-01	6.70E-01	ENSG00000172728
FUT11	0.1785	3.4742E-03	1.89E-02	ENSG00000196968
FUT2	-0.0308	7.4391E-01	8.60E-01	ENSG00000176920
FUT3	0.2944	3.0832E-02	9.94E-02	ENSG00000171124
FUT4	-0.0412	6.6390E-01	8.07E-01	ENSG00000196371
FUT5	0.0286	4.1077E-01		ENSG00000130383
FUT6	0.0888	2.1534E-01	3.96E-01	ENSG00000156413
FUT7	0.1155	3.7066E-02		ENSG00000180549
FUT8	-0.0457	4.5384E-01	6.43E-01	ENSG00000033170
FUT9	0.0377	6.9293E-01	8.28E-01	ENSG00000172461
FUZ	-0.1263	1.6126E-01	3.26E-01	ENSG00000010361
FXN	0.1461	2.2204E-02	7.79E-02	ENSG00000165060
FXNP2	0.0002	8.7567E-01		ENSG00000177736
FXR1	-0.1555	7.0887E-03	3.27E-02	ENSG00000114416
FXR2	0.2006	3.2906E-03	1.80E-02	ENSG00000129245
FXYD1	0.0765	2.0283E-01	3.80E-01	ENSG00000266964
FXYD2	0.2614	4.3504E-02	1.29E-01	ENSG00000137731
FXYD3	-0.0347	6.0930E-01	7.68E-01	ENSG00000089356
FXYD4	0.1417	5.2362E-02	1.48E-01	ENSG00000150201

FXYD5	0.1847	9.7455E-02	2.31E-01	ENSG00000089327
FXYD6	-0.1158	9.9655E-02	2.34E-01	ENSG00000137726
FXYD6-FXYD2	-0.0991	3.7857E-01	5.74E-01	ENSG00000255245
FXYD7	0.2853	6.7021E-03	3.13E-02	ENSG00000221946
FYB1	0.0000	5.0453E-01		ENSG00000082074
FYB2	0.1686	1.0243E-01	2.39E-01	ENSG00000187889
FYCO1	-0.1629	6.5942E-02	1.75E-01	ENSG00000163820
FYN	0.1225	1.3405E-01	2.87E-01	ENSG00000010810
FYTTD1	-0.0907	1.5434E-01	3.17E-01	ENSG00000122068
FZD1	0.1963	4.3465E-03	2.25E-02	ENSG00000157240
FZD10	-0.0038	9.0603E-01	9.53E-01	ENSG00000111432
FZD10-AS1	-0.0186	6.4166E-01	7.92E-01	ENSG00000250208
FZD2	-0.2484	4.9786E-03	2.49E-02	ENSG00000180340
FZD3	-0.3293	3.6986E-04	3.25E-03	ENSG00000104290
FZD4	-0.2291	5.2523E-02	1.48E-01	ENSG00000174804
FZD4-DT	-0.2755	3.9548E-02	1.20E-01	ENSG00000246523
FZD5	-0.0609	5.0625E-01	6.87E-01	ENSG00000163251
FZD6	-0.2381	8.7108E-03	3.84E-02	ENSG00000164930
FZD7	-0.4855	4.9797E-06	1.00E-04	ENSG00000155760
FZD8	-0.2707	4.5865E-02	1.34E-01	ENSG00000177283
FZD9	0.2472	2.0036E-02	7.21E-02	ENSG00000188763
FZR1	0.0417	4.9454E-01	6.78E-01	ENSG00000105325
G0S2	-0.1820	1.0486E-01	2.43E-01	ENSG00000123689
G2E3	0.0865	2.3033E-01	4.14E-01	ENSG00000092140
G2E3-AS1	0.0176	5.1934E-01		ENSG00000257636
G3BP1	-0.0405	5.2106E-01	6.98E-01	ENSG00000145907
G3BP2	0.1605	1.0406E-02	4.37E-02	ENSG00000138757
G6PC3	-0.0330	5.5891E-01	7.29E-01	ENSG00000141349
G6PD	-0.0212	8.1397E-01	9.02E-01	ENSG00000160211
GAA	-0.0074	9.2535E-01	9.63E-01	ENSG00000171298
GAB2	-0.1010	1.7332E-01	3.43E-01	ENSG00000033327
GABARAP	-0.2012	2.6866E-02	8.98E-02	ENSG00000170296
GABARAPL1	0.0731	2.4779E-01	4.34E-01	ENSG00000139112
GABARAPL2	-0.0952	6.5805E-02	1.74E-01	ENSG00000034713
GABBR1	-0.0789	3.2436E-01	5.18E-01	ENSG00000204681
GABBR2	-0.4498	7.4575E-04	5.66E-03	ENSG00000136928
GABPA	-0.1643	1.8783E-02	6.87E-02	ENSG00000154727
GABPB1	-0.0665	3.6429E-01	5.60E-01	ENSG00000104064
GABPB1-IT1	0.2011	3.6945E-04	3.25E-03	ENSG00000285410
GABPB2	-0.0279	7.0945E-01	8.38E-01	ENSG00000143458
GABRA1	-0.4637	8.0331E-05	9.37E-04	ENSG00000022355
GABRA2	0.4104	1.1870E-06	3.08E-05	ENSG00000151834
GABRA3	-0.2117	5.2627E-02	1.48E-01	ENSG00000011677
GABRA4	-0.1819	1.0018E-01	2.35E-01	ENSG00000109158
GABRA5	-0.2218	2.9248E-02	9.57E-02	ENSG00000186297
GABRA6	-0.0288	3.0416E-01		ENSG00000145863
GABRB1	-0.0652	4.6503E-01	6.53E-01	ENSG00000163288
GABRB2	-0.0320	7.4301E-01	8.59E-01	ENSG00000145864
GABRB3	-0.1812	4.9951E-03	2.50E-02	ENSG00000166206

GABRD	0.0158	8.6283E-01	9.30E-01	ENSG00000187730
GABRE	0.5103	3.6052E-04	3.18E-03	ENSG00000102287
GABRG1	-0.1444	1.4560E-01	3.04E-01	ENSG00000163285
GABRG2	-0.0304	6.9013E-01	8.26E-01	ENSG00000113327
GABRG3	-0.2801	1.4189E-02	5.56E-02	ENSG00000182256
GABRG3-AS1	-0.0337	4.5344E-01		ENSG00000228740
GABRP	-0.0668	2.0867E-01	3.88E-01	ENSG00000094755
GABRQ	0.1630	1.4223E-01	2.99E-01	ENSG00000268089
GABRR1	0.4310	5.5069E-04	4.45E-03	ENSG00000146276
GABRR2	0.0039	9.6102E-01	9.80E-01	ENSG00000111886
GABRR3	-0.0230	6.3192E-01		ENSG00000183185
GACAT2	0.1149	2.1602E-01	3.97E-01	ENSG00000265962
GACAT3	0.0122	7.4821E-01		ENSG00000236289
GAD1	0.3881	2.7592E-05	3.97E-04	ENSG00000128683
GAD2	0.1372	3.5920E-02	1.12E-01	ENSG00000136750
GADD45B	-0.2584	9.3253E-05	1.06E-03	ENSG00000099860
GADD45G	-0.1057	5.9234E-02	1.62E-01	ENSG00000130222
GADD45GIP1	-0.3482	7.7510E-07	2.16E-05	ENSG00000179271
GADL1	0.1230	2.3178E-01	4.15E-01	ENSG00000144644
GAGE10	0.0257	5.7418E-01		ENSG00000215274
GAK	-0.0276	6.3292E-01	7.86E-01	ENSG00000178950
GAL	-0.1040	2.8197E-01	4.73E-01	ENSG00000069482
GAL3ST1	0.0692	4.7841E-01	6.64E-01	ENSG00000128242
GAL3ST2	0.0415	3.8379E-01		ENSG00000154252
GAL3ST3	-0.3737	2.9960E-04	2.73E-03	ENSG00000175229
GAL3ST4	0.1296	1.7319E-01	3.42E-01	ENSG00000197093
GALC	0.0293	7.3083E-01	8.52E-01	ENSG00000054983
GALE	0.2795	1.4055E-04	1.47E-03	ENSG00000117308
GALK1	-0.0286	7.0289E-01	8.34E-01	ENSG00000108479
GALK2	0.0413	5.8672E-01	7.51E-01	ENSG00000156958
GALM	-0.1093	2.6554E-01	4.54E-01	ENSG00000143891
GALNS	-0.3891	3.1699E-06	6.91E-05	ENSG00000141012
GALNT1	-0.0758	1.4799E-01	3.08E-01	ENSG00000141429
GALNT10	-0.2995	2.4124E-04	2.28E-03	ENSG00000164574
GALNT11	-0.1218	2.5236E-02	8.58E-02	ENSG00000178234
GALNT12	-0.0360	6.2122E-01	7.77E-01	ENSG00000119514
GALNT13	-0.1997	1.3991E-02	5.50E-02	ENSG00000144278
GALNT13-AS1	0.0790	3.4561E-01	5.41E-01	ENSG00000224675
GALNT14	-0.0559	5.4579E-01	7.18E-01	ENSG00000158089
GALNT15	-0.5496	2.7487E-03	1.58E-02	ENSG00000131386
GALNT16	-0.1735	8.7769E-02	2.14E-01	ENSG00000100626
GALNT16-AS1	-0.0359	6.8671E-01	8.23E-01	ENSG00000258957
GALNT17	0.0208	8.2562E-01	9.08E-01	ENSG00000185274
GALNT18	0.2824	1.1495E-03	7.92E-03	ENSG00000110328
GALNT2	-0.1516	5.7006E-02	1.58E-01	ENSG00000143641
GALNT3	-0.2872	2.0419E-02	7.31E-02	ENSG00000115339
GALNT4	-0.0827	1.6578E-01		ENSG00000257594
GALNT5	-0.3593	1.3477E-02	5.34E-02	ENSG00000136542
GALNT6	0.1773	1.1083E-01	2.52E-01	ENSG00000139629

GALNT7	-0.2952	2.8760E-04	2.63E-03	ENSG00000109586
GALNT7-DT	-0.1688	1.1998E-01	2.67E-01	ENSG00000245213
GALNT8	-0.2692	1.3700E-02	5.41E-02	ENSG00000130035
GALNT9	0.0791	3.3583E-01	5.31E-01	ENSG00000182870
GALNTL5	0.0347	1.4548E-01		ENSG00000106648
GALP	0.1473	1.1921E-01	2.66E-01	ENSG00000197487
GALR1	-0.0378	4.8028E-01	6.66E-01	ENSG00000166573
GALR2	-0.0099	9.1292E-01	9.56E-01	ENSG00000182687
GALR3	0.0776	4.3855E-01	6.29E-01	ENSG00000128310
GAMT	-0.1966	9.3799E-03	4.05E-02	ENSG00000130005
GAN	0.1831	3.2198E-02	1.03E-01	ENSG00000261609
GANAB	-0.1412	3.0930E-02	9.96E-02	ENSG00000089597
GANC	0.1067	2.2656E-01	4.09E-01	ENSG00000214013
GAP43	-0.0416	6.1810E-01	7.75E-01	ENSG00000172020
GAPDH	0.1923	1.3442E-02	5.33E-02	ENSG00000111640
GAPDHP22	-0.0978	1.1788E-01	2.64E-01	ENSG00000234005
GAPDHP47	-0.0169	6.5989E-01		ENSG00000243033
GAPDHP49	0.0053	6.1855E-01		ENSG00000234285
GAPDHP55	0.0032	8.9575E-01		ENSG00000224055
GAPDHP62	0.0089	7.5980E-01		ENSG00000251013
GAPDHS	-0.0112	5.9126E-01		ENSG00000105679
GAPLINC	0.0833	4.0605E-01	6.00E-01	ENSG00000266835
GAPVD1	0.1977	2.7943E-04	2.57E-03	ENSG00000165219
GAR1	0.0720	3.5842E-01	5.54E-01	ENSG00000109534
GAREM1	0.1932	7.3227E-03	3.35E-02	ENSG00000141441
GAREM2	0.1045	1.6971E-01	3.38E-01	ENSG00000157833
GARIN1A	-0.0317	7.3046E-01	8.52E-01	ENSG00000205085
GARIN1B	0.2462	1.5764E-02	6.02E-02	ENSG00000135248
GARIN2	0.0413	4.2440E-01	6.16E-01	ENSG00000172717
GARIN5A	-0.0839	3.4584E-01	5.41E-01	ENSG00000142530
GARIN5B	0.0368	1.7974E-01		ENSG00000180043
GARIN6	0.0061	5.3955E-01		ENSG00000180219
GARNL3	0.2033	2.1840E-02	7.70E-02	ENSG00000136895
GARRE1	-0.0919	2.6746E-01	4.57E-01	ENSG00000166398
GARS1	0.1926	9.0164E-03	3.93E-02	ENSG00000106105
GART	0.2789	1.2652E-05	2.12E-04	ENSG00000159131
GAS1	-0.3480	1.2274E-02	4.97E-02	ENSG00000180447
GAS1RR	-0.0356	5.9817E-01	7.59E-01	ENSG00000226237
GAS2	0.0764	4.1635E-01	6.09E-01	ENSG00000148935
GAS2L1	-0.5731	1.7514E-07	6.21E-06	ENSG00000185340
GAS2L2	0.0411	5.4251E-01	7.15E-01	ENSG00000270765
GAS2L3	-0.0698	4.6773E-01	6.55E-01	ENSG00000139354
GAS5	-0.3036	1.3993E-03	9.29E-03	ENSG00000234741
GAS6	-0.3342	3.3738E-03	1.84E-02	ENSG00000183087
GAS6-DT	0.0246	7.8893E-01	8.87E-01	ENSG00000272695
GAS7	-0.1920	4.3899E-02	1.30E-01	ENSG00000007237
GAS8	0.0892	2.6386E-01	4.53E-01	ENSG00000141013
GAS8-AS1	0.0248	6.3979E-02		ENSG00000221819
GASK1A	-0.2753	3.6146E-02	1.12E-01	ENSG00000144649

GASK1B	-0.5299	5.7076E-05	7.15E-04	ENSG00000164125
GASK1B-AS1	0.0212	5.4321E-01	7.15E-01	ENSG00000248429
GAST	-0.0073	6.1076E-01		ENSG00000184502
GATA1	0.0430	3.4814E-01	5.43E-01	ENSG00000102145
GATA2	-0.1016	2.8023E-01	4.71E-01	ENSG00000179348
GATA3	-0.1383	3.6938E-02	1.14E-01	ENSG00000107485
GATA4	0.0140	2.9331E-01		ENSG00000136574
GATA5	-0.0046	8.6620E-01		ENSG00000130700
GATA6	-0.4825	1.1742E-02	4.80E-02	ENSG00000141448
GATA6-AS1	-0.0364	2.4102E-01		ENSG00000266010
GATAD1	0.1324	5.8469E-02	1.60E-01	ENSG00000157259
GATAD2A	-0.0002	9.9701E-01	9.98E-01	ENSG00000167491
GATAD2B	-0.0948	2.9718E-01	4.89E-01	ENSG00000143614
GATB	-0.0480	4.3725E-01	6.28E-01	ENSG00000059691
GATC	0.2584	1.3106E-04	1.39E-03	ENSG00000257218
GATD1	-0.3724	5.1787E-06	1.03E-04	ENSG00000177225
GATD1-DT	-0.3124	6.5802E-06	1.26E-04	ENSG00000255284
GATD3	-0.1940	2.1271E-02	7.55E-02	ENSG00000160221
GATM	-0.4494	6.0728E-05	7.51E-04	ENSG00000171766
GAU1	-0.0318	7.1893E-01	8.44E-01	ENSG00000255474
GBA1	-0.0592	3.6509E-01	5.61E-01	ENSG00000177628
GBA2	0.2240	1.6452E-03	1.05E-02	ENSG00000070610
GBA3	-0.0009	8.7750E-01		ENSG00000249948
GBE1	0.2923	1.9071E-03	1.18E-02	ENSG00000114480
GBF1	0.1907	1.2748E-02	5.12E-02	ENSG00000107862
GBGT1	-0.1969	4.2722E-02	1.27E-01	ENSG00000148288
GBP2	-0.0798	3.3954E-01	5.35E-01	ENSG00000162645
GBP3	-0.0498	5.4555E-01	7.18E-01	ENSG00000117226
GBP5	-0.0130	8.3320E-01		ENSG00000154451
GBP6	0.0082	5.8588E-01	7.51E-01	ENSG00000183347
GBX1	-0.0023	9.8817E-01		ENSG00000164900
GBX2	0.0717	3.0517E-01	4.98E-01	ENSG00000168505
GC	-0.0879	8.8922E-01	9.44E-01	ENSG00000145321
GCA	0.0901	1.7644E-01	3.47E-01	ENSG00000115271
GCAT	-0.1414	4.9202E-02	1.41E-01	ENSG00000100116
GCC1	0.1607	6.4196E-02	1.71E-01	ENSG00000179562
GCC2	0.0208	6.9919E-01	8.32E-01	ENSG00000135968
GCC2-AS1	-0.0960	3.4279E-01	5.38E-01	ENSG00000214184
GCDH	0.1090	2.5455E-01	4.42E-01	ENSG00000105607
GCFC2	-0.1070	6.3690E-02	1.70E-01	ENSG00000005436
GCG	-0.0185	8.7455E-01	9.37E-01	ENSG00000115263
GCGR	0.5659	9.3431E-04	6.76E-03	ENSG00000215644
GCH1	0.0526	4.8250E-01	6.68E-01	ENSG00000131979
GCHFR	0.0014	9.8909E-01	9.94E-01	ENSG00000137880
GCK	0.4202	2.5733E-04	2.40E-03	ENSG00000106633
GCKR	0.0862	3.0086E-01	4.93E-01	ENSG00000084734
GCLC	0.0418	6.6388E-01	8.07E-01	ENSG00000001084
GCLM	0.0274	7.7468E-01	8.78E-01	ENSG00000023909
GCM2	-0.0335	3.9029E-01		ENSG00000124827

GCN1	-0.0290	6.8594E-01	8.23E-01	ENSG00000089154
GCNA	-0.0608	4.3441E-01	6.26E-01	ENSG00000147174
GCNT1	-0.0041	9.5351E-01	9.76E-01	ENSG00000187210
GCNT2	-0.0605	5.3016E-01	7.05E-01	ENSG00000111846
GCNT3	-0.0135	8.7399E-01	9.36E-01	ENSG00000140297
GCNT7	-0.0588	5.2729E-01	7.03E-01	ENSG00000124091
GCOM1	-0.1299	1.6982E-01	3.38E-01	ENSG00000137878
GCSAM	-0.0370	6.4990E-01	7.97E-01	ENSG00000174500
GCSH	-0.1784	5.6228E-03	2.74E-02	ENSG00000140905
GCSHP6	0.0108	9.0370E-01		ENSG00000270538
GCSIR	0.2333	6.0570E-02	1.65E-01	ENSG00000232520
GDA	-0.2954	2.6558E-02	8.90E-02	ENSG00000119125
GDAP1	0.2011	1.3878E-02	5.47E-02	ENSG00000104381
GDAP1L1	0.0523	4.4676E-01	6.37E-01	ENSG00000124194
GDAP2	0.2982	4.7100E-04	3.91E-03	ENSG00000196505
GDE1	0.0633	4.0492E-01	5.99E-01	ENSG00000006007
GDF10	-0.0643	5.0799E-01	6.88E-01	ENSG00000266524
GDF11	-0.3975	2.4757E-04	2.33E-03	ENSG00000135414
GDF15	-0.0102	8.9487E-01	9.48E-01	ENSG00000130513
GDF5	-0.2233	6.7365E-02	1.77E-01	ENSG00000125965
GDF6	0.0518	5.3365E-01	7.08E-01	ENSG00000156466
GDF7	0.0818	4.0122E-01	5.95E-01	ENSG00000143869
GDF9	0.3718	9.8743E-03	4.21E-02	ENSG00000164404
GDI1	0.0174	8.4791E-01	9.22E-01	ENSG00000203879
GDI2	0.0082	9.7736E-01	9.88E-01	ENSG00000057608
GDI2P2	0.0308	7.0725E-01	8.36E-01	ENSG00000233994
GDNF	-0.2080	8.1599E-02	2.04E-01	ENSG00000168621
GDPD1	-0.1712	1.8604E-02	6.83E-02	ENSG00000153982
GDPD2	-0.1237	9.9487E-02	2.34E-01	ENSG00000130055
GDPD3	-0.0593	4.9188E-01	6.75E-01	ENSG00000102886
GDPD4	0.0028	5.3054E-01		ENSG00000178795
GDPD5	0.0032	9.6702E-01	9.83E-01	ENSG00000158555
GDPGP1	-0.0149	8.4951E-01	9.23E-01	ENSG00000183208
GEM	-0.3440	5.5018E-04	4.45E-03	ENSG00000164949
GEMIN2	0.0991	1.3649E-01	2.91E-01	ENSG00000092208
GEMIN4	0.0542	4.9706E-01	6.80E-01	ENSG00000179409
GEMIN5	0.0977	2.3954E-01	4.24E-01	ENSG00000082516
GEMIN6	0.2205	1.6143E-02	6.13E-02	ENSG00000152147
GEMIN7	0.0048	9.6370E-01	9.81E-01	ENSG00000142252
GEMIN7-AS1	0.3862	2.7017E-04	2.50E-03	ENSG00000267348
GEMIN8	-0.2217	1.2306E-03	8.37E-03	ENSG00000046647
GEMIN8P3	0.0215	3.0707E-01		ENSG00000223772
GEN1	0.0080	9.2637E-01	9.64E-01	ENSG00000178295
GET1	-0.0408	5.9530E-01	7.57E-01	ENSG00000182093
GET1-SH3BGR	-0.0699	2.8132E-01	4.72E-01	ENSG00000285815
GET3	-0.1610	4.9177E-03	2.47E-02	ENSG00000198356
GET4	0.0307	7.1336E-01	8.40E-01	ENSG00000239857
GFER	-0.1649	6.0262E-02	1.64E-01	ENSG00000127554
GFI1	0.3350	2.6095E-02	8.78E-02	ENSG00000162676

GFI1B	0.0362	9.9056E-02	2.33E-01	ENSG00000165702
GFM1	0.1783	1.3496E-02	5.35E-02	ENSG00000168827
GFM2	0.1796	4.3688E-02	1.30E-01	ENSG00000164347
GFOD1	0.3266	2.5273E-04	2.37E-03	ENSG00000145990
GFOD2	0.2646	1.5008E-03	9.82E-03	ENSG00000141098
GFOD3P	-0.0193	7.7071E-01	8.76E-01	ENSG00000227372
GFPT1	0.1332	4.8225E-02	1.39E-01	ENSG00000198380
GFPT2	0.1968	7.3294E-03	3.35E-02	ENSG00000131459
GFRA2	0.1113	2.6300E-01	4.52E-01	ENSG00000168546
GFRA3	-0.2127	5.3570E-02	1.50E-01	ENSG00000146013
GFRA4	0.0245	4.9548E-01		ENSG00000125861
GFUS	-0.1401	1.1122E-02	4.60E-02	ENSG00000104522
GFY	0.4898	1.0418E-02	4.37E-02	ENSG00000261949
GGA1	-0.0413	5.5588E-01	7.27E-01	ENSG00000100083
GGA2	0.0312	6.1443E-01	7.73E-01	ENSG00000103365
GGA3	0.1180	1.7963E-01	3.51E-01	ENSG00000125447
GGACT	0.1373	7.7355E-02	1.96E-01	ENSG00000134864
GGCT	-0.0853	1.9244E-01	3.68E-01	ENSG00000006625
GGCX	-0.1945	6.4098E-03	3.04E-02	ENSG00000115486
GGH	-0.3838	1.5846E-04	1.63E-03	ENSG00000137563
GGN	-0.1873	9.7579E-02	2.31E-01	ENSG00000179168
GGNBP1	0.0333	5.7080E-01		ENSG00000204188
GGNBP2	0.0426	4.5300E-01	6.43E-01	ENSG00000278311
GGPS1	0.0127	8.2053E-01	9.05E-01	ENSG00000152904
GGT1	-0.0332	6.7368E-01	8.13E-01	ENSG00000100031
GGT3P	-0.0066	8.2052E-01		ENSG00000197421
GGT5	0.5436	2.5290E-04	2.37E-03	ENSG00000099998
GGT6	-0.0060	9.3945E-01	9.70E-01	ENSG00000167741
GGT7	0.0901	1.5619E-01	3.19E-01	ENSG00000131067
GGTA1	0.0741	3.5672E-01	5.52E-01	ENSG00000204136
GGTA2P	-0.0009	2.6231E-01		ENSG00000237766
GGTLC2	-0.0771	3.0372E-01	4.96E-01	ENSG00000100121
GGTLC5P	-0.0170	8.6345E-01		ENSG00000276160
GHDC	0.1089	1.8566E-01	3.59E-01	ENSG00000167925
GHITM	0.0535	4.4639E-01	6.36E-01	ENSG00000165678
GHRHR	-0.1110	7.2519E-02		ENSG00000106128
GHRL	0.0168	8.5969E-01	9.29E-01	ENSG00000157017
GHRLOS	-0.2059	5.4005E-02	1.51E-01	ENSG00000240288
GID4	0.2436	1.6292E-02	6.17E-02	ENSG00000141034
GID8	-0.2613	1.3776E-03	9.18E-03	ENSG00000101193
GIGYF1	0.0567	5.3773E-01	7.11E-01	ENSG00000146830
GIGYF2	0.0489	4.8635E-01	6.71E-01	ENSG00000204120
GIHCG	-0.1020	6.0318E-02	1.64E-01	ENSG00000257698
GIMAP2	-0.0197	4.6785E-01		ENSG00000106560
GIN1	0.0836	3.1627E-01	5.10E-01	ENSG00000145723
GINM1	-0.0530	3.0259E-01	4.95E-01	ENSG00000055211
GINS1	0.0344	7.0509E-01	8.35E-01	ENSG00000101003
GINS2	-0.0660	3.1558E-01	5.09E-01	ENSG00000131153
GINS3	-0.2151	5.1435E-02	1.46E-01	ENSG00000181938

GINS4	0.1059	2.4747E-01	4.34E-01	ENSG00000147536
GIPC1	-0.0444	5.1847E-01	6.96E-01	ENSG00000123159
GIPC2	-0.2383	5.9030E-02	1.61E-01	ENSG00000137960
GIPR	0.1101	1.2438E-01	2.74E-01	ENSG00000010310
GIRGL	-0.4644	2.9339E-04	2.68E-03	ENSG00000233834
GIT1	0.2173	7.0475E-03	3.25E-02	ENSG00000108262
GIT2	0.1039	2.3190E-01	4.15E-01	ENSG00000139436
GJA1	-0.3751	2.1285E-03	1.29E-02	ENSG00000152661
GJA10	-0.0181	2.4658E-01		ENSG00000135355
GJA3	-0.1445	1.4155E-01	2.98E-01	ENSG00000121743
GJA5	-0.0304	3.5696E-01		ENSG00000265107
GJA9	-0.0042	9.0749E-01		ENSG00000131233
GJB1	0.0068	9.3812E-01	9.69E-01	ENSG00000169562
GJB2	-0.2015	6.3823E-02	1.71E-01	ENSG00000165474
GJB3	-0.0939	2.2035E-01	4.01E-01	ENSG00000188910
GJB5	-0.0078	8.6097E-01		ENSG00000189280
GJB6	-0.0325	7.0615E-01		ENSG00000121742
GJB7	0.1853	9.9675E-02	2.34E-01	ENSG00000164411
GJC1	0.1257	1.5196E-01	3.13E-01	ENSG00000182963
GJD2-DT	0.0089	8.0175E-01	8.94E-01	ENSG00000250007
GJD3	-0.0036	9.6613E-01	9.83E-01	ENSG00000183153
GK	0.1755	8.1429E-02	2.03E-01	ENSG00000198814
GK3	-0.0194	8.6679E-01		ENSG00000229894
GK5	0.2816	1.8578E-03	1.16E-02	ENSG00000175066
GKAP1	-0.1889	9.0026E-03	3.93E-02	ENSG00000165113
GLA	0.1483	8.8025E-03	3.87E-02	ENSG00000102393
GLB1	-0.0234	7.6349E-01	8.72E-01	ENSG00000170266
GLB1L	-0.1003	1.7503E-01	3.45E-01	ENSG00000163521
GLB1L2	-0.0012	9.8757E-01	9.93E-01	ENSG00000149328
GLB1L3	0.0167	7.6013E-01	8.70E-01	ENSG00000166105
GLCCI1	-0.2659	2.3884E-04	2.26E-03	ENSG00000106415
GLCE	-0.1813	2.6472E-03	1.53E-02	ENSG00000138604
GLDC	-0.0195	7.9990E-01	8.93E-01	ENSG00000178445
GLDN	0.5475	3.1294E-03	1.74E-02	ENSG00000186417
GLE1	0.2064	2.9947E-03	1.68E-02	ENSG00000119392
GLG1	-0.0292	4.5860E-01	6.48E-01	ENSG00000090863
GLI1	-0.0353	5.2466E-01	7.01E-01	ENSG00000111087
GLI2	-0.3183	1.7792E-02	6.60E-02	ENSG00000074047
GLI3	-0.2203	5.3201E-03	2.63E-02	ENSG00000106571
GLI4	-0.1140	1.5517E-01	3.18E-01	ENSG00000250571
GLIDR	-0.1959	1.1031E-02	4.58E-02	ENSG00000278175
GLIPR1	-0.5262	2.8707E-05	4.09E-04	ENSG00000139278
GLIPR1L1	0.0310	7.2044E-01	8.45E-01	ENSG00000173401
GLIPR2	-0.0683	4.0670E-01	6.00E-01	ENSG00000122694
GLIS1	0.0359	7.1081E-01	8.39E-01	ENSG00000174332
GLIS2	-0.4122	1.7568E-06	4.25E-05	ENSG00000126603
GLIS2-AS1	-0.4681	7.9940E-04	5.98E-03	ENSG00000262686
GLIS3	-0.4987	9.3138E-05	1.06E-03	ENSG00000107249
GLIS3-AS1	0.0375	6.4533E-01	7.94E-01	ENSG00000237009

GLMN	0.1681	3.6020E-02	1.12E-01	ENSG00000174842
GLMP	-0.0716	2.5546E-01	4.43E-01	ENSG00000198715
GLO1	-0.1463	2.9261E-02	9.57E-02	ENSG00000124767
GLOD4	-0.0381	3.4373E-01	5.39E-01	ENSG00000167699
GLOD5	-0.0779	2.7998E-01	4.71E-01	ENSG00000171433
GLP1R	-0.3295	1.9070E-02	6.95E-02	ENSG00000112164
GLP2R	0.0155	4.9753E-01		ENSG00000065325
GLRA1	0.2704	4.0848E-02	1.23E-01	ENSG00000145888
GLRA2	0.1297	1.9303E-01	3.69E-01	ENSG00000101958
GLRB	-0.0465	5.2321E-01	7.00E-01	ENSG00000109738
GLRX	-0.0034	9.6047E-01	9.80E-01	ENSG00000173221
GLRX2	-0.1223	9.3587E-02	2.25E-01	ENSG00000023572
GLRX3	-0.1132	3.0852E-02	9.95E-02	ENSG00000108010
GLRX5	-0.1143	1.8513E-02	6.80E-02	ENSG00000182512
GLRX5P2	-0.0019	9.2561E-01		ENSG00000232879
GLS	-0.0018	9.7737E-01	9.88E-01	ENSG00000115419
GLS2	0.0344	7.1869E-01	8.44E-01	ENSG00000135423
GLT1D1	-0.0057	7.3743E-01	8.56E-01	ENSG00000151948
GLT8D1	0.0750	1.5794E-01	3.22E-01	ENSG00000016864
GLT8D2	-0.0918	3.2611E-01	5.20E-01	ENSG00000120820
GLTP	-0.2214	1.7525E-04	1.77E-03	ENSG00000139433
GLTPD2	0.1337	1.9183E-01	3.67E-01	ENSG00000182327
GLUD1	-0.0143	7.8929E-01	8.87E-01	ENSG00000148672
GLUD2	0.2822	4.1475E-02	1.25E-01	ENSG00000182890
GLUL	0.1014	2.6306E-01	4.52E-01	ENSG00000135821
GLULP1	0.0582	1.0152E-01		ENSG00000270978
GLULP4	-0.0005	4.6791E-01		ENSG00000178723
GLYATL1	-0.1855	8.0014E-02	2.01E-01	ENSG00000166840
GLYATL1B	-0.0027	9.7466E-01		ENSG00000255151
GLYATL1P1	-0.0205	7.8353E-01	8.83E-01	ENSG00000255189
GLYATL1P2	-0.1536	1.5850E-01	3.23E-01	ENSG00000254717
GLYATL2	-0.2454	4.7750E-02	1.38E-01	ENSG00000156689
GLYATL3	-0.3041	1.0320E-02	4.35E-02	ENSG00000203972
GLYCTK	0.0890	3.2265E-01	5.16E-01	ENSG00000168237
GLYR1	0.2010	1.8081E-03	1.13E-02	ENSG00000140632
GM2A	0.1170	2.3942E-01	4.24E-01	ENSG00000196743
GMCL1	0.0174	7.7844E-01	8.80E-01	ENSG00000087338
GMDS	0.0990	1.8102E-01	3.53E-01	ENSG00000112699
GMDS-DT	0.0063	9.4503E-01	9.73E-01	ENSG00000250903
GMEB1	-0.0273	6.8767E-01	8.24E-01	ENSG00000162419
GMEB2	-0.0185	8.0126E-01	8.94E-01	ENSG00000101216
GMFB	0.0304	7.1891E-01	8.44E-01	ENSG00000197045
GMFG	-0.0940	3.5071E-01	5.46E-01	ENSG00000130755
GMIP	0.2355	2.0622E-02	7.36E-02	ENSG00000089639
GMNC	-0.2096	7.8427E-02	1.98E-01	ENSG00000205835
GMNN	-0.2172	1.6492E-02	6.23E-02	ENSG00000112312
GMPPA	0.1366	7.3158E-02	1.88E-01	ENSG00000144591
GMPPB	0.2135	5.3289E-03	2.63E-02	ENSG00000173540
GMPR	0.1238	9.5285E-02	2.27E-01	ENSG00000137198

GMPR2	-0.0136	8.3712E-01	9.15E-01	ENSG00000100938
GMPS	-0.0371	5.4601E-01	7.18E-01	ENSG00000163655
GMPSP1	-0.0459	4.5164E-01		ENSG00000250471
GNA11	-0.0020	9.7161E-01	9.85E-01	ENSG00000088256
GNA12	-0.2039	3.7031E-03	1.99E-02	ENSG00000146535
GNA13	-0.3865	9.9370E-07	2.65E-05	ENSG00000120063
GNA14	0.3511	7.0329E-03	3.25E-02	ENSG00000156049
GNA14-AS1	-0.0089	8.9919E-01		ENSG00000231373
GNA15	-0.0077	7.6251E-01		ENSG00000060558
GNAI1	-0.0532	4.9030E-01	6.74E-01	ENSG00000127955
GNAI2	-0.0295	6.4059E-01	7.91E-01	ENSG00000114353
GNAI3	0.1021	1.6359E-01	3.30E-01	ENSG00000065135
GNAL	0.0613	4.7214E-01	6.60E-01	ENSG00000141404
GNAO1	-0.0646	3.0663E-01	4.99E-01	ENSG00000087258
GNAO1-DT	0.5590	2.4810E-05	3.63E-04	ENSG00000246379
GNAQ	-0.3071	1.6949E-03	1.08E-02	ENSG00000156052
GNAS	-0.1383	4.8065E-02	1.39E-01	ENSG00000087460
GNAS-AS1	0.3365	2.5108E-02	8.54E-02	ENSG00000235590
GNAT1	0.1483	4.6521E-02		ENSG00000114349
GNAT2	0.0084	8.4038E-01	9.17E-01	ENSG00000134183
GNAZ	-0.1736	3.7073E-02	1.14E-01	ENSG00000128266
GNB1	0.0395	5.2792E-01	7.03E-01	ENSG00000078369
GNB1-DT	0.1109	2.7814E-01	4.69E-01	ENSG00000231050
GNB1L	-0.3136	7.9907E-04	5.98E-03	ENSG00000185838
GNB2	-0.0411	5.5546E-01	7.26E-01	ENSG00000172354
GNB3	0.0922	3.0658E-01	4.99E-01	ENSG00000111664
GNB4	-0.2669	2.1119E-03	1.28E-02	ENSG00000114450
GNB5	0.1226	7.0806E-02	1.84E-01	ENSG00000069966
GNE	-0.2000	5.4958E-03	2.69E-02	ENSG00000159921
GNG10	0.0425	6.6425E-01	8.07E-01	ENSG00000242616
GNG11	-0.3967	2.0707E-04	2.01E-03	ENSG00000127920
GNG12	-0.3612	5.3738E-04	4.36E-03	ENSG00000172380
GNG13	0.1378	1.7648E-01	3.47E-01	ENSG00000127588
GNG14	-0.0116	7.0898E-01		ENSG00000283980
GNG2	0.0470	6.0693E-01	7.66E-01	ENSG00000186469
GNG3	0.0796	4.1433E-01	6.07E-01	ENSG00000162188
GNG4	0.1181	4.4819E-02	1.32E-01	ENSG00000168243
GNG5	-0.5445	4.6410E-16	2.12E-13	ENSG00000174021
GNG5B	-0.0257	7.7269E-01	8.77E-01	ENSG00000133136
GNG7	-0.5031	1.1116E-05	1.91E-04	ENSG00000176533
GNG8	0.0964	3.3652E-01	5.32E-01	ENSG00000167414
GNGT1	-0.2604	3.6347E-02	1.13E-01	ENSG00000127928
GNGT2	0.0096	5.1208E-01		ENSG00000167083
GNL1	-0.0971	3.3016E-02	1.05E-01	ENSG00000204590
GNL2	0.3184	1.8484E-04	1.84E-03	ENSG00000134697
GNL3	0.2204	1.5452E-03	1.00E-02	ENSG00000163938
GNL3L	-0.1749	1.2286E-02	4.97E-02	ENSG00000130119
GNMT	0.2474	5.1974E-02	1.47E-01	ENSG00000124713
GNPAT	0.0028	9.6270E-01	9.81E-01	ENSG00000116906

GNPDA1	-0.1165	4.5237E-02	1.33E-01	ENSG00000113552
GNPDA2	-0.0756	2.3704E-01	4.21E-01	ENSG00000163281
GNPNAT1	0.0866	3.2751E-01	5.22E-01	ENSG00000100522
GNPTAB	0.0256	6.9037E-01	8.26E-01	ENSG00000111670
GNPTG	-0.3282	6.1491E-09	3.88E-07	ENSG00000090581
GNRH1	0.0539	8.8761E-03	3.89E-02	ENSG00000147437
GNRH2	0.0438	4.1865E-01	6.11E-01	ENSG00000125787
GNRHR	-0.1226	1.6486E-01	3.31E-01	ENSG00000109163
GNRHR2	-0.1003	7.0233E-01	8.33E-01	ENSG00000211451
GNRHR2P1	0.0195	8.5470E-01	9.25E-01	ENSG00000259169
GNS	-0.1374	1.2803E-02	5.13E-02	ENSG00000135677
GOLGA1	0.2198	7.4590E-03	3.40E-02	ENSG00000136935
GOLGA2	0.0440	5.7018E-01	7.38E-01	ENSG00000167110
GOLGA3	0.0975	1.7541E-01	3.45E-01	ENSG00000090615
GOLGA4	0.1526	4.3922E-02	1.30E-01	ENSG00000144674
GOLGA4-AS1	0.0759	4.2442E-01	6.16E-01	ENSG00000270194
GOLGA5	0.3655	9.7743E-09	5.59E-07	ENSG00000066455
GOLGA6L1	0.0693	2.0095E-02		ENSG00000273976
GOLGA6L17P	0.0017	9.7547E-01	9.87E-01	ENSG00000230373
GOLGA6L2	-0.0105	6.4917E-01		ENSG00000174450
GOLGA6L22	0.0494	7.6701E-02		ENSG00000277865
GOLGA6L22	0.0174	9.0425E-02		ENSG00000237850
GOLGA6L25	0.0101	2.2068E-01		ENSG00000227717
GOLGA6L26	0.0560	4.0642E-02		ENSG00000273756
GOLGA6L3P	0.0288	7.6499E-01	8.72E-01	ENSG00000188388
GOLGA6L4	0.0243	7.6856E-01	8.74E-01	ENSG00000278662
GOLGA6L4	-0.0170	8.3823E-01	9.15E-01	ENSG00000184206
GOLGA6L6	0.0413	9.9228E-02		ENSG00000277322
GOLGA6L7	-0.0139	2.7596E-01	4.67E-01	ENSG00000261649
GOLGA6L9	-0.1293	1.0977E-01	2.51E-01	ENSG00000197978
GOLGA7	0.0165	8.3521E-01	9.14E-01	ENSG00000147533
GOLGA7B	-0.2740	9.2672E-03	4.01E-02	ENSG00000155265
GOLGA7B-DT	-0.1621	6.8426E-02	1.79E-01	ENSG00000227356
GOLGA8A	0.4992	1.8249E-08	9.46E-07	ENSG00000175265
GOLGA8B	0.4886	3.5751E-08	1.66E-06	ENSG00000215252
GOLGA8H	0.2929	3.5477E-02	1.11E-01	ENSG00000261794
GOLGA8J	0.0621	5.1739E-01	6.95E-01	ENSG00000179938
GOLGA8K	-0.0105	9.0988E-01	9.55E-01	ENSG00000249931
GOLGA8M	-0.0164	8.2735E-01	9.09E-01	ENSG00000188626
GOLGA8M	-0.0717	3.7518E-01	5.71E-01	ENSG00000261480
GOLGA8N	-0.0891	3.7289E-01	5.68E-01	ENSG00000232653
GOLGA8O	-0.0653	5.0197E-01	6.84E-01	ENSG00000206127
GOLGA8Q	-0.1174	2.5762E-01	4.46E-01	ENSG00000178115
GOLGA8R	0.0048	9.5808E-01	9.79E-01	ENSG00000186399
GOLGA8S	-0.0110	6.4357E-01		ENSG00000261739
GOLGA8T	-0.0630	5.1530E-01	6.94E-01	ENSG00000261247
GOLGB1	-0.0068	9.0700E-01	9.53E-01	ENSG00000173230
GOLM1	-0.0772	3.1713E-01	5.11E-01	ENSG00000135052
GOLM2	-0.0721	2.0822E-01	3.87E-01	ENSG00000166734

GOLPH3	-0.0218	6.1730E-01	7.75E-01	ENSG00000113384
GOLPH3L	0.3663	8.4039E-07	2.32E-05	ENSG00000143457
GOLT1A	0.1478	1.3071E-01	2.83E-01	ENSG00000174567
GOLT1B	0.4147	3.3637E-05	4.62E-04	ENSG00000111711
GON4L	0.1705	4.9613E-03	2.49E-02	ENSG00000116580
GON7	-0.0562	3.7352E-01	5.69E-01	ENSG00000170270
GOPC	-0.0624	3.4018E-01	5.35E-01	ENSG00000047932
GORAB	0.0845	3.3383E-01	5.29E-01	ENSG00000120370
GORASP1	0.1552	5.0541E-02	1.44E-01	ENSG00000114745
GORASP2	0.0251	6.6191E-01	8.05E-01	ENSG00000115806
GOSR1	0.0485	3.3370E-01	5.29E-01	ENSG00000108587
GOSR2	0.1592	1.0158E-02	4.30E-02	ENSG00000108433
GOT1	0.0199	8.2011E-01	9.05E-01	ENSG00000120053
GOT1-DT	0.2572	2.6784E-02	8.95E-02	ENSG00000224934
GOT1L1	-0.0110	7.8288E-01		ENSG00000169154
GOT2	-0.0405	4.1353E-01	6.06E-01	ENSG00000125166
GOT2P6	-0.0079	9.5958E-01		ENSG00000214745
GP1BA	0.0392	3.9461E-01	5.89E-01	ENSG00000185245
GP2	-0.0572	4.3930E-01	6.30E-01	ENSG00000169347
GP5	0.0232	3.8487E-01		ENSG00000178732
GP6	-0.1342	2.0291E-01	3.81E-01	ENSG00000088053
GPA33	-0.0891	1.2121E-01		ENSG00000143167
GPAA1	-0.0844	2.1920E-01	4.00E-01	ENSG00000197858
GPALPP1	0.1200	5.5559E-02	1.55E-01	ENSG00000133114
GPAM	0.2398	8.1597E-03	3.65E-02	ENSG00000119927
GPANK1	0.1098	8.1957E-02	2.04E-01	ENSG00000204438
GPAT2	-0.0574	5.5332E-01	7.24E-01	ENSG00000186281
GPAT2P1	-0.0143	9.7559E-01		ENSG00000237510
GPAT3	-0.3026	3.6145E-02	1.12E-01	ENSG00000138678
GPAT4	0.1820	1.6662E-02	6.28E-02	ENSG00000158669
GPATCH1	0.4763	1.8966E-05	2.93E-04	ENSG00000076650
GPATCH11	0.0836	2.8190E-01	4.73E-01	ENSG00000152133
GPATCH2	0.1858	6.7373E-03	3.14E-02	ENSG00000092978
GPATCH2L	-0.1394	2.2186E-02	7.79E-02	ENSG00000089916
GPATCH3	-0.0005	9.7725E-01	9.88E-01	ENSG00000198746
GPATCH4	-0.0005	7.1837E-01		ENSG00000160818
GPATCH8	0.0543	2.6038E-01	4.49E-01	ENSG00000186566
GPBAR1	0.0963	3.2293E-01	5.17E-01	ENSG00000179921
GPBP1	-0.1247	1.8908E-02	6.90E-02	ENSG00000062194
GPBP1L1	-0.0048	9.3554E-01	9.68E-01	ENSG00000159592
GPC1	0.0625	2.7736E-01	4.68E-01	ENSG00000063660
GPC1-AS1	-0.0167	8.4837E-01	9.22E-01	ENSG00000218416
GPC2	0.4920	7.1392E-06	1.34E-04	ENSG00000213420
GPC3-AS1	0.0064	6.5653E-01		ENSG00000286096
GPC4	-0.4263	1.1928E-04	1.29E-03	ENSG00000076716
GPC5-AS1	0.0154	4.9410E-01	6.78E-01	ENSG00000235984
GPC6	0.0060	9.4635E-01	9.73E-01	ENSG00000183098
GPC6-AS1	-0.0405	3.9728E-01		ENSG00000236520
GPCPD1	-0.1170	3.2061E-02	1.02E-01	ENSG00000125772

GPD1	-0.0138	8.7956E-01	9.39E-01	ENSG00000167588
GPD1L	-0.2507	8.7402E-03	3.85E-02	ENSG00000152642
GPD2	-0.2735	8.2324E-03	3.67E-02	ENSG00000115159
GPER1	-0.1206	2.3632E-01	4.20E-01	ENSG00000164850
GPHB5	-0.0327	7.8135E-01	8.82E-01	ENSG00000179600
GPHN	0.1543	2.1836E-02	7.70E-02	ENSG00000171723
GPI	0.1152	1.5046E-01	3.11E-01	ENSG00000105220
GPIHBP1	0.0046	9.3163E-01		ENSG00000277494
GPKOW	0.0792	2.2714E-01	4.10E-01	ENSG00000068394
GPLD1	0.0303	7.4323E-01	8.59E-01	ENSG00000112293
GPM6A	-0.0280	7.5079E-01	8.64E-01	ENSG00000150625
GPM6A-DT	0.5009	4.6393E-03	2.36E-02	ENSG00000249106
GPM6B	-0.5587	1.9818E-06	4.70E-05	ENSG00000046653
GPN1	0.2732	7.0683E-04	5.42E-03	ENSG00000198522
GPN2	0.0030	9.7816E-01	9.89E-01	ENSG00000142751
GPN3	0.2475	5.7903E-04	4.64E-03	ENSG00000111231
GPNMB	-0.0917	3.1998E-01	5.14E-01	ENSG00000136235
GPR101	-0.1068	2.5798E-01	4.46E-01	ENSG00000165370
GPR107	-0.0550	4.7951E-01	6.65E-01	ENSG00000148358
GPR108	-0.0677	3.0751E-01	5.00E-01	ENSG00000125734
GPR12	0.1415	1.4026E-01	2.96E-01	ENSG00000132975
GPR132	-0.0628	2.4931E-01		ENSG00000183484
GPR135	-0.1767	8.0099E-02	2.01E-01	ENSG00000181619
GPR137	-0.0463	4.9575E-01	6.79E-01	ENSG00000173264
GPR137B	-0.2750	8.6799E-04	6.38E-03	ENSG00000077585
GPR137C	0.0189	8.4011E-01	9.16E-01	ENSG00000180998
GPR139	0.2102	8.2413E-02	2.05E-01	ENSG00000180269
GPR141	-0.0141	6.9086E-01		ENSG00000187037
GPR142	0.0021	6.5425E-01		ENSG00000257008
GPR143	-0.0081	9.2024E-01	9.60E-01	ENSG00000101850
GPR146	-0.2105	8.2910E-02	2.06E-01	ENSG00000164849
GPR148	0.1646	8.5860E-02	2.11E-01	ENSG00000173302
GPR149	-0.3387	2.1933E-02	7.72E-02	ENSG00000174948
GPR150	0.2014	9.1436E-02	2.21E-01	ENSG00000178015
GPR151	0.0717	2.2929E-01	4.12E-01	ENSG00000173250
GPR152	0.0002	8.5146E-01		ENSG00000175514
GPR153	0.0199	8.0569E-01	8.96E-01	ENSG00000158292
GPR155	-0.0099	8.6285E-01	9.30E-01	ENSG00000163328
GPR156	-0.0570	5.3671E-01	7.10E-01	ENSG00000175697
GPR157	0.1115	2.5162E-01	4.38E-01	ENSG00000180758
GPR158	0.2658	4.6039E-02	1.35E-01	ENSG00000151025
GPR158-AS1	0.0176	5.5492E-01		ENSG00000233642
GPR160	-0.1178	2.3473E-01	4.19E-01	ENSG00000173890
GPR161	-0.0670	2.0451E-01	3.83E-01	ENSG00000143147
GPR162	-0.3337	2.2225E-05	3.34E-04	ENSG00000250510
GPR171	0.0199	7.4855E-01	8.63E-01	ENSG00000174946
GPR173	-0.0122	8.9625E-01	9.48E-01	ENSG00000184194
GPR176	0.1453	1.4905E-01	3.09E-01	ENSG00000166073
GPR176-DT	0.1870	5.6935E-02	1.57E-01	ENSG00000246863

GPR179	-0.2586	5.0831E-02	1.45E-01	ENSG00000277399
GPR18	-0.1688	1.3248E-01	2.85E-01	ENSG00000125245
GPR180	0.0487	4.6535E-01	6.54E-01	ENSG00000152749
GPR183	-0.2828	4.0045E-02	1.21E-01	ENSG00000169508
GPR19	0.0533	5.2872E-01	7.04E-01	ENSG00000183150
GPR20	-0.0198	5.6354E-01	7.33E-01	ENSG00000204882
GPR21	-0.0380	4.3396E-01		ENSG00000188394
GPR22	-0.0277	7.6380E-01	8.72E-01	ENSG00000172209
GPR26	-0.0766	4.3075E-01	6.22E-01	ENSG00000154478
GPR27	-0.4381	1.7009E-04	1.72E-03	ENSG00000170837
GPR3	-0.1865	4.7162E-02	1.37E-01	ENSG00000181773
GPR34	0.1742	1.0950E-01	2.50E-01	ENSG00000171659
GPR37	0.0134	8.8592E-01	9.42E-01	ENSG00000170775
GPR37L1	0.3120	9.0790E-03	3.96E-02	ENSG00000170075
GPR39	-0.0900	6.4796E-02		ENSG00000183840
GPR4	0.4964	1.3876E-03	9.23E-03	ENSG00000177464
GPR45	-0.0644	3.2948E-01		ENSG00000135973
GPR50	0.1946	3.5752E-02	1.11E-01	ENSG00000102195
GPR50-AS1	0.0477	4.9056E-01	6.75E-01	ENSG00000234696
GPR55	0.0128	8.2387E-01		ENSG00000135898
GPR6	0.0915	1.6725E-01	3.34E-01	ENSG00000146360
GPR61	-0.1470	1.7328E-01	3.43E-01	ENSG00000156097
GPR62	0.1575	9.9669E-02	2.34E-01	ENSG00000180929
GPR63	-0.0330	7.1888E-01	8.44E-01	ENSG00000112218
GPR68	-0.1212	2.3158E-01	4.15E-01	ENSG00000119714
GPR75	-0.3072	3.2896E-02	1.05E-01	ENSG00000119737
GPR78	-0.0275	5.8108E-01		ENSG00000155269
GPR82	-0.1141	5.0821E-01	6.89E-01	ENSG00000171657
GPR83	0.3548	1.9248E-02	7.00E-02	ENSG00000123901
GPR84	0.0038	6.6720E-01		ENSG00000139572
GPR84-AS1	-0.1757	4.6537E-02	1.36E-01	ENSG00000258137
GPR85	-0.0797	2.7959E-01	4.71E-01	ENSG00000164604
GPR88	-0.2461	5.2175E-02	1.47E-01	ENSG00000181656
GPR89A	0.1233	8.4617E-02	2.09E-01	ENSG00000117262
GPR89B	0.1519	3.5999E-02	1.12E-01	ENSG00000188092
GPRASP1	-0.0787	1.8694E-01	3.61E-01	ENSG00000198932
GPRASP2	-0.0248	7.2365E-01	8.47E-01	ENSG00000158301
GPRASP3	-0.0261	8.6384E-01	9.31E-01	ENSG00000198908
GPRC5A	-0.4017	5.3837E-03	2.65E-02	ENSG00000013588
GPRC5B	-0.1240	1.4053E-01	2.97E-01	ENSG00000167191
GPRC5C	-0.2519	4.0772E-05	5.43E-04	ENSG00000170412
GPRC5D	0.0001	6.8604E-01		ENSG00000111291
GPRC5D-AS1	0.0055	6.9851E-01	8.31E-01	ENSG00000247498
GPRC6A	-0.0360	2.3943E-01		ENSG00000173612
GPRIN1	0.1717	7.3012E-02	1.88E-01	ENSG00000169258
GPRIN2	-0.0963	3.3463E-01	5.30E-01	ENSG00000204175
GPRIN3	0.3091	9.5874E-04	6.89E-03	ENSG00000185477
GPS1	0.0679	2.5645E-01	4.44E-01	ENSG00000169727
GPS2	0.2595	1.0934E-02	4.54E-02	ENSG00000132522

GPS2P1	0.1111	6.3717E-02	1.70E-01	ENSG00000236496
GPSM1	0.1887	1.6685E-02	6.28E-02	ENSG00000160360
GPSM2	0.0246	7.6025E-01	8.70E-01	ENSG00000121957
GPSM3	0.5368	1.4402E-04	1.50E-03	ENSG00000213654
GPT	-0.0080	7.1163E-01		ENSG00000167701
GPT2	0.0233	7.8276E-01	8.83E-01	ENSG00000166123
GPX1	-0.4176	2.1331E-08	1.08E-06	ENSG00000233276
GPX2	0.0551	3.3173E-01	5.27E-01	ENSG00000176153
GPX3	0.3865	2.2788E-04	2.17E-03	ENSG00000211445
GPX4	-0.1193	3.6072E-02	1.12E-01	ENSG00000167468
GPX7	-0.2709	2.9340E-02	9.59E-02	ENSG00000116157
GPX8	-0.3160	2.2906E-03	1.37E-02	ENSG00000164294
GRAMD1A	0.1372	2.5419E-02	8.63E-02	ENSG00000089351
GRAMD1B	-0.1018	1.7874E-01	3.50E-01	ENSG00000023171
GRAMD1C	-0.1094	1.9864E-01	3.76E-01	ENSG00000178075
GRAMD2A	0.0053	9.5546E-01	9.77E-01	ENSG00000175318
GRAMD2B	-0.4912	7.2681E-08	2.99E-06	ENSG00000155324
GRAMD4	0.0209	7.5837E-01	8.69E-01	ENSG00000075240
GRAP	-0.0137	6.8038E-01		ENSG00000154016
GRAP2	0.0144	8.2736E-01		ENSG00000100351
GRAPL	-0.0198	4.5660E-01		ENSG00000189152
GRASLND	0.2586	2.7064E-02	9.02E-02	ENSG00000228203
GRB10	0.5987	4.0655E-09	2.75E-07	ENSG00000106070
GRB2	-0.1761	1.0184E-05	1.78E-04	ENSG00000177885
GRB7	-0.0162	8.6171E-01	9.30E-01	ENSG00000141738
GREB1	0.1110	2.4571E-01	4.32E-01	ENSG00000196208
GREB1L	0.4654	2.5084E-05	3.66E-04	ENSG00000141449
GREB1L-DT	-0.1917	7.7996E-02	1.97E-01	ENSG00000265984
GREM1	-0.1094	1.1392E-01	2.58E-01	ENSG00000166923
GREM2	0.2145	5.8838E-02	1.61E-01	ENSG00000180875
GRHL1	-0.0229	7.9751E-01	8.92E-01	ENSG00000134317
GRHL2	0.2170	3.1067E-02	9.99E-02	ENSG00000083307
GRHL3	0.0290	7.5193E-01	8.64E-01	ENSG00000158055
GRHPR	-0.3174	1.0064E-04	1.12E-03	ENSG00000137106
GRIA1	0.3501	4.4278E-03	2.28E-02	ENSG00000155511
GRIA2	-0.0634	3.4534E-01	5.40E-01	ENSG00000120251
GRIA3	0.0022	9.8139E-01	9.90E-01	ENSG00000125675
GRIA4	0.4343	5.3654E-04	4.36E-03	ENSG00000152578
GRID1	0.1314	1.8464E-01	3.58E-01	ENSG00000182771
GRID1-AS1	0.0073	4.9043E-01		ENSG00000234942
GRID2	0.0368	6.9430E-01	8.29E-01	ENSG00000152208
GRID2IP	0.0440	6.4672E-01	7.95E-01	ENSG00000215045
GRIFIN	-0.0451	4.2559E-01		ENSG00000275572
GRIK1	-0.4839	5.7324E-03	2.78E-02	ENSG00000171189
GRIK2	0.3901	6.0412E-04	4.79E-03	ENSG00000164418
GRIK3	0.2333	1.2527E-02	5.05E-02	ENSG00000163873
GRIK5	-0.2148	2.6082E-02	8.78E-02	ENSG00000105737
GRIN1	0.1227	2.3553E-01	4.20E-01	ENSG00000176884
GRIN2A	-0.1747	3.0564E-02	9.88E-02	ENSG00000183454

GRIN2B	0.1022	2.9073E-01	4.82E-01	ENSG00000273079
GRIN2D	0.1521	1.0632E-01	2.45E-01	ENSG00000105464
GRIN3A	0.1991	6.2029E-02	1.67E-01	ENSG00000198785
GRIN3B	-0.1047	2.9785E-01	4.90E-01	ENSG00000116032
GRINA	-0.0816	2.6899E-01	4.58E-01	ENSG00000178719
GRIP1	-0.0204	8.1585E-01	9.03E-01	ENSG00000155974
GRIP2	0.1047	2.6682E-01	4.56E-01	ENSG00000144596
GRIPAP1	0.0149	8.5572E-01	9.26E-01	ENSG00000068400
GRK1	0.1209	9.7672E-02	2.31E-01	ENSG00000185974
GRK2	0.1868	2.6305E-02	8.83E-02	ENSG00000173020
GRK3	0.0964	2.1686E-01	3.98E-01	ENSG00000100077
GRK3-AS1	-0.0201	7.9182E-01	8.88E-01	ENSG00000234884
GRK4	0.0414	5.2359E-01	7.00E-01	ENSG00000125388
GRK5	0.1488	9.3904E-02	2.25E-01	ENSG00000198873
GRK6	0.1058	9.8971E-02	2.33E-01	ENSG00000198055
GRK7	0.0747	2.3243E-01	4.16E-01	ENSG00000114124
GRM1	0.0067	9.4239E-01	9.72E-01	ENSG00000152822
GRM2	0.0941	3.5132E-01	5.46E-01	ENSG00000164082
GRM3	-0.5207	1.4529E-03	9.57E-03	ENSG00000198822
GRM4	-0.0835	3.7899E-01	5.74E-01	ENSG00000124493
GRM5	-0.1152	1.0500E-01	2.43E-01	ENSG00000168959
GRM5-AS1	-0.0388	3.0392E-01		ENSG00000255082
GRM5P1	-0.0111	4.5499E-01		ENSG00000205035
GRM6	0.0061	7.3176E-01		ENSG00000113262
GRM7	0.1932	4.3614E-02	1.29E-01	ENSG00000196277
GRM7-AS1	-0.0014	8.8887E-01		ENSG00000236202
GRM7-AS3	0.0194	6.0761E-01		ENSG00000226258
GRM8	-0.0862	3.5671E-01	5.52E-01	ENSG00000179603
GRM8-AS1	-0.0008	9.6812E-01		ENSG00000236340
GRN	0.0654	3.4281E-01	5.38E-01	ENSG00000030582
GRP	-0.5698	1.9577E-03	1.20E-02	ENSG00000134443
GRPEL1	-0.0613	2.6396E-01	4.53E-01	ENSG00000109519
GRPEL2	0.0249	6.5445E-01	8.00E-01	ENSG00000164284
GRPEL2-AS1	-0.0148	6.6539E-01		ENSG00000253618
GRPR	-0.0300	6.5742E-01	8.02E-01	ENSG00000126010
GRSF1	-0.2462	2.2961E-04	2.19E-03	ENSG00000132463
GRTP1	-0.2026	8.2458E-02	2.05E-01	ENSG00000139835
GRWD1	0.0974	1.7187E-01	3.41E-01	ENSG00000105447
GRXCR1	-0.0510	4.0408E-01		ENSG00000215203
GS1-204I12.4	-0.0981	1.2884E-01		ENSG00000261729
GS1-24F4.2	-0.0390	5.8235E-01	7.48E-01	ENSG00000245857
GS1-279B7.1	-0.0283	8.9774E-01	9.49E-01	ENSG00000261024
GSAP	0.0061	9.4999E-01	9.75E-01	ENSG00000186088
GSC2	0.0440	1.9367E-01		ENSG00000063515
GSDMA	-0.0015	5.1442E-01		ENSG00000167914
GSDMB	0.0214	8.2362E-01	9.07E-01	ENSG00000073605
GSDMC	-0.0847	1.0154E-01		ENSG00000147697
GSDMD	-0.1781	1.0632E-01	2.45E-01	ENSG00000104518
GSDME	-0.5455	1.8521E-07	6.52E-06	ENSG00000105928

GSE1	-0.2095	1.4806E-02	5.75E-02	ENSG00000131149
GSG1	0.0392	5.9668E-01	7.58E-01	ENSG00000111305
GSG1L	-0.0652	5.0032E-01	6.83E-01	ENSG00000169181
GSK3A	-0.1202	5.3256E-02	1.50E-01	ENSG00000105723
GSK3B	-0.0860	1.2646E-01	2.77E-01	ENSG00000082701
GSKIP	0.0903	2.5266E-01	4.40E-01	ENSG00000100744
GSN	0.2416	9.9911E-04	7.11E-03	ENSG00000148180
GSN-AS1	-0.1492	1.6654E-01	3.34E-01	ENSG00000235865
GSPT1	-0.1621	3.8552E-04	3.35E-03	ENSG00000103342
GSPT2	-0.3102	7.8746E-06	1.46E-04	ENSG00000189369
GSR	0.1349	1.7894E-01	3.50E-01	ENSG00000104687
GSS	0.0482	3.9256E-01	5.87E-01	ENSG00000100983
GSTA1	0.0012	5.5679E-01	7.27E-01	ENSG00000243955
GSTA2	0.0064	7.3493E-01		ENSG00000244067
GSTA3	0.0703	1.8982E-01	3.65E-01	ENSG00000174156
GSTA4	-0.1893	1.1574E-02	4.74E-02	ENSG00000170899
GSTA9P	-0.0109	8.6562E-01		ENSG00000243236
GSTCD	0.1548	6.5912E-02	1.75E-01	ENSG00000138780
GSTCD-AS1	-0.0004	8.2231E-01		ENSG00000251175
GSTK1	-0.3434	7.9007E-05	9.25E-04	ENSG00000197448
GSTM1	-0.0384	8.6065E-01	9.29E-01	ENSG00000134184
GSTM2	-0.1716	1.8411E-02	6.78E-02	ENSG00000213366
GSTM3	-0.1567	4.5421E-02	1.33E-01	ENSG00000134202
GSTM4	0.0235	7.3745E-01	8.56E-01	ENSG00000168765
GSTM5	-0.1488	2.8871E-02		ENSG00000134201
GSTO1	-0.1750	3.6609E-02	1.13E-01	ENSG00000148834
GSTO2	0.0753	4.1548E-01	6.08E-01	ENSG00000065621
GSTO3P	0.0134	5.2073E-01		ENSG00000232829
GSTP1	-0.2971	3.0784E-03	1.71E-02	ENSG00000084207
GSTT2	0.3243	2.7035E-03	1.55E-02	ENSG00000099984
GSTT2B	0.3308	5.9052E-04	4.71E-03	ENSG00000133433
GSTT4	-0.0090	7.4547E-01		ENSG00000276950
GSTZ1	0.0496	5.1678E-01	6.95E-01	ENSG00000100577
GSX1	-0.0196	6.8866E-01		ENSG00000169840
GSX2	-0.0577	2.1574E-01		ENSG00000180613
GTDC1	-0.0593	3.3902E-01	5.34E-01	ENSG00000121964
GTF2A1	-0.0675	3.4091E-01	5.36E-01	ENSG00000165417
GTF2A2	-0.1144	2.2352E-02	7.83E-02	ENSG00000140307
GTF2B	-0.0022	9.6695E-01	9.83E-01	ENSG00000137947
GTF2E1	0.3835	1.0047E-05	1.76E-04	ENSG00000153767
GTF2E2	-0.2272	2.5644E-03	1.49E-02	ENSG00000197265
GTF2F1	0.0246	5.7762E-01	7.44E-01	ENSG00000125651
GTF2F2	-0.1126	4.9545E-02	1.42E-01	ENSG00000188342
GTF2H1	0.3095	1.9201E-03	1.19E-02	ENSG00000110768
GTF2H2	-0.1415	1.3832E-02	5.45E-02	ENSG00000145736
GTF2H2C	-0.0302	5.5562E-01	7.27E-01	ENSG00000183474
GTF2H3	-0.0833	1.2925E-01	2.81E-01	ENSG00000111358
GTF2H4	-0.0249	6.4565E-01		ENSG00000213780
GTF2H5	-0.2711	2.5725E-04	2.40E-03	ENSG00000272047

GTF2I	-0.2191	6.5193E-05	7.98E-04	ENSG00000263001
GTF2I-AS1	-0.1162	2.4233E-01	4.27E-01	ENSG00000232729
GTF2IP14	-0.0172	7.2541E-01	8.48E-01	ENSG00000226002
GTF2IP4	-0.1310	1.5052E-02	5.81E-02	ENSG00000233369
GTF2IP5	0.0392	5.1555E-01	6.94E-01	ENSG00000224316
GTF2IRD1	-0.2057	2.5851E-04	2.41E-03	ENSG00000006704
GTF2IRD2	0.0148	8.6671E-01	9.32E-01	ENSG00000196275
GTF2IRD2B	-0.1354	4.5804E-02	1.34E-01	ENSG00000174428
GTF2IRD2P1	-0.0795	3.8961E-01	5.84E-01	ENSG00000214544
GTF3A	0.0356	5.3719E-01	7.11E-01	ENSG00000122034
GTF3C1	-0.1892	1.9841E-03	1.22E-02	ENSG00000077235
GTF3C2	0.2600	5.7534E-05	7.20E-04	ENSG00000115207
GTF3C2-AS1	-0.0412	7.9026E-01	8.87E-01	ENSG00000234945
GTF3C3	0.0104	8.7173E-01	9.35E-01	ENSG00000119041
GTF3C4	-0.3909	2.8745E-09	2.05E-07	ENSG00000125484
GTF3C5	0.0829	1.9676E-01	3.73E-01	ENSG00000148308
GTF3C6	-0.3080	3.3321E-09	2.32E-07	ENSG00000155115
GTF3C6P1	-0.0278	4.4239E-01		ENSG00000215085
GTPBP1	0.1079	1.3082E-01	2.83E-01	ENSG00000100226
GTPBP10	0.1742	1.5094E-02	5.83E-02	ENSG00000105793
GTPBP2	0.1026	1.4358E-01	3.01E-01	ENSG00000172432
GTPBP3	0.1576	4.9800E-02	1.43E-01	ENSG00000130299
GTPBP4	0.0172	7.5489E-01	8.67E-01	ENSG00000107937
GTPBP6	-0.2819	1.4935E-04	1.55E-03	ENSG00000178605
GTPBP8	-0.1277	5.4428E-02	1.52E-01	ENSG00000163607
GTSE1	-0.3705	6.7028E-03	3.13E-02	ENSG00000075218
GUCA1A	-0.0275	7.4548E-01	8.61E-01	ENSG00000048545
GUCD1	0.1366	3.6740E-02	1.13E-01	ENSG00000138867
GUCY1A1	0.0156	8.8442E-01	9.42E-01	ENSG00000164116
GUCY1A2	-0.0989	2.9728E-01	4.89E-01	ENSG00000152402
GUCY1B1	0.2738	4.2772E-03	2.23E-02	ENSG00000061918
GUCY1B2	-0.0142	7.1479E-01		ENSG00000123201
GUCY2C	0.0242	4.6615E-02		ENSG00000070019
GUCY2D	-0.1223	1.0528E-01	2.44E-01	ENSG00000132518
GUCY2F	-0.0522	3.3535E-01	5.31E-01	ENSG00000101890
GUF1	0.0028	9.7631E-01	9.88E-01	ENSG00000151806
GUK1	0.0240	6.1852E-01	7.75E-01	ENSG00000143774
GULP1	-0.1156	1.1736E-01	2.63E-01	ENSG00000144366
GUSB	0.0170	8.6329E-01	9.30E-01	ENSG00000169919
GUSBP11	0.1447	1.6322E-01	3.29E-01	ENSG00000228315
GUSBP5	0.0412	6.5660E-01	8.02E-01	ENSG00000236296
GUSBP9	0.3864	7.0186E-04	5.39E-03	ENSG00000215630
GVINP1	0.0174	3.3113E-01		ENSG00000254838
GVQW3	0.4789	5.0107E-05	6.46E-04	ENSG00000179240
GXYLT1	0.0122	8.5035E-01	9.23E-01	ENSG00000151233
GXYLT1P4	-0.0211	7.6300E-01	8.71E-01	ENSG00000275026
GXYLT1P5	0.0122	6.2367E-01		ENSG00000276128
GXYLT1P7	-0.0138	5.4635E-01		ENSG00000227265
GXYLT2	-0.0159	7.7903E-01	8.81E-01	ENSG00000172986

GYG1	-0.0375	5.4104E-01	7.14E-01	ENSG00000163754
GYG2	0.0004	9.8354E-01	9.91E-01	ENSG00000056998
GYG2P2	0.0495	2.8372E-02		ENSG00000271580
GYPC	-0.1331	2.0150E-01	3.79E-01	ENSG00000136732
GYS1	-0.0934	1.4055E-01	2.97E-01	ENSG00000104812
GZF1	-0.0582	3.0224E-01	4.95E-01	ENSG00000125812
GZMA	-0.0111	6.7452E-01		ENSG00000145649
GZMK	-0.1113	1.2436E-01	2.74E-01	ENSG00000113088
GZMM	-0.0535	4.6731E-01	6.55E-01	ENSG00000197540
H1-0	0.3126	5.7036E-03	2.77E-02	ENSG00000189060
H1-1	-0.0139	8.7101E-01	9.35E-01	ENSG00000124610
H1-10	-0.3568	1.0430E-04	1.16E-03	ENSG00000184897
H1-5	-0.5827	2.9096E-03	1.65E-02	ENSG00000184357
H1-6	-0.0803	1.7819E-02		ENSG00000187475
H1-7	-0.0096	4.0089E-01		ENSG00000187166
H1-8	-0.0670	3.1267E-01	5.06E-01	ENSG00000178804
H19	-0.0231	8.0490E-01	8.96E-01	ENSG00000130600
H2AB1	0.0082	4.8977E-01		ENSG00000274183
H2AB2	-0.0151	6.1212E-01		ENSG00000277858
H2AB3	-0.0161	6.0157E-01		ENSG00000277745
H2AC10P	-0.0879	2.0480E-01	3.83E-01	ENSG00000218690
H2AC11	-0.0706	4.7500E-01	6.62E-01	ENSG00000196787
H2AC12	0.3572	8.7634E-03	3.85E-02	ENSG00000274997
H2AC13	-0.0744	3.9910E-01	5.93E-01	ENSG00000196747
H2AC14	-0.3744	3.3180E-02	1.05E-01	ENSG00000276368
H2AC15	0.0579	5.5566E-01	7.27E-01	ENSG00000275221
H2AC16	-0.0232	7.8324E-01	8.83E-01	ENSG00000276903
H2AC17	0.2191	6.9962E-02	1.82E-01	ENSG00000278677
H2AC18	-0.4485	3.0000E-06	6.61E-05	ENSG00000288825
H2AC19	-0.4487	2.9642E-06	6.55E-05	ENSG00000288859
H2AC20	-0.4247	6.4277E-06	1.24E-04	ENSG00000184260
H2AC21	-0.0146	9.2079E-01		ENSG00000184270
H2AC25	-0.5413	3.9541E-08	1.81E-06	ENSG00000181218
H2AC4	0.4760	5.8956E-03	2.84E-02	ENSG00000278463
H2AC6	-0.3363	8.0577E-04	6.01E-03	ENSG00000180573
H2AC7	-0.4913	2.8088E-03	1.60E-02	ENSG00000196866
H2AC8	-0.0668	4.5979E-01	6.48E-01	ENSG00000277075
H2AJ	-0.1642	1.4674E-03	9.65E-03	ENSG00000246705
H2AL3	0.0113	3.6720E-01		ENSG00000229674
H2AP	0.0067	7.5442E-01		ENSG00000187516
H2AX	-0.1810	4.0177E-02	1.21E-01	ENSG00000188486
H2AZ1	-0.2465	7.9183E-04	5.94E-03	ENSG00000164032
H2AZ2	-0.3371	1.4030E-11	2.17E-09	ENSG00000105968
H2BC10	-0.0850	2.9490E-01		ENSG00000278588
H2BC11	-0.0097	9.2623E-01	9.64E-01	ENSG00000124635
H2BC12	-0.3352	2.4310E-04	2.29E-03	ENSG00000197903
H2BC12L	0.0251	5.2751E-01		ENSG00000234289
H2BC13	-0.0322	7.7343E-01		ENSG00000185130
H2BC14	0.0422	1.0580E-01		ENSG00000273703

H2BC15	0.2479	1.0584E-02	4.43E-02	ENSG00000233822
H2BC17	-0.0689	3.9358E-01	5.88E-01	ENSG00000274641
H2BC18	0.0682	4.8847E-01	6.72E-01	ENSG00000203814
H2BC19P	-0.1467	1.3824E-01	2.93E-01	ENSG00000220323
H2BC20P	-0.1787	7.9617E-02	2.00E-01	ENSG00000261716
H2BC21	-0.1746	4.8543E-02	1.40E-01	ENSG00000184678
H2BC26	0.1000	2.3898E-01	4.24E-01	ENSG00000196890
H2BC27P	-0.0095	7.9381E-01		ENSG00000181201
H2BC3	0.0503	2.5770E-01	4.46E-01	ENSG00000276410
H2BC6	-0.0186	8.4092E-01	9.17E-01	ENSG00000274290
H2BC7	-0.1920	9.4863E-02	2.27E-01	ENSG00000277224
H2BC8	0.1172	2.3171E-01	4.15E-01	ENSG00000273802
H2BW2	0.0196	5.5726E-01		ENSG00000101812
H3-3A	-0.3996	4.1421E-07	1.27E-05	ENSG00000163041
H3-3B	-0.5460	1.8703E-06	4.46E-05	ENSG00000132475
H3-5	-0.1900	9.2443E-02	2.23E-01	ENSG00000188375
H3-7	0.0243	7.7619E-01	8.79E-01	ENSG00000273213
H3C1	-0.0187	8.3867E-01	9.16E-01	ENSG00000275714
H3C10	-0.1543	1.5621E-01	3.19E-01	ENSG00000278828
H3C11	-0.0016	9.9266E-01		ENSG00000275379
H3C12	-0.0736	4.9959E-01	6.82E-01	ENSG00000197153
H3C13	0.5954	9.3274E-04	6.75E-03	ENSG00000183598
H3C14	-0.4250	1.6342E-02	6.19E-02	ENSG00000203811
H3C15	-0.4672	1.2524E-02	5.04E-02	ENSG00000203852
H3C3	-0.0252	4.7422E-01		ENSG00000287080
H3C6	-0.0810	3.7598E-01	5.71E-01	ENSG00000274750
H3C7	-0.1733	9.4876E-02	2.27E-01	ENSG00000277775
H3C8	-0.0949	1.5131E-01		ENSG00000273983
H3C9P	-0.1035	3.1006E-01	5.03E-01	ENSG00000220875
H3P1	-0.1445	1.9505E-01	3.71E-01	ENSG00000230228
H3P13	0.0185	6.7893E-01		ENSG00000243977
H3P25	0.0709	3.6605E-01	5.62E-01	ENSG00000275506
H3P31	-0.0236	5.9160E-01		ENSG00000232466
H3P36	-0.1503	8.1306E-02	2.03E-01	ENSG00000236534
H3P43	-0.2440	5.7670E-02	1.59E-01	ENSG00000226436
H4C1	0.2290	6.2008E-02	1.67E-01	ENSG00000278637
H4C14	-0.2745	1.9295E-03	1.19E-02	ENSG00000270882
H4C15	-0.2461	5.5027E-03	2.70E-02	ENSG00000270276
H4C16	0.5626	9.3154E-05	1.06E-03	ENSG00000197837
H4C2	0.0459	6.0406E-01	7.64E-01	ENSG00000278705
H4C4	0.0715	1.9572E-01	3.72E-01	ENSG00000277157
H4C5	-0.0996	2.0904E-01	3.89E-01	ENSG00000276966
H4C8	-0.2433	4.3166E-02	1.28E-01	ENSG00000158406
H4C9	-0.1268	1.6713E-01	3.34E-01	ENSG00000276180
H6PD	0.0092	9.0401E-01	9.52E-01	ENSG00000049239
HAAO	0.1795	1.4142E-02	5.55E-02	ENSG00000162882
HABP2	0.0415	1.8607E-01	3.60E-01	ENSG00000148702
HABP4	0.3113	4.4781E-05	5.89E-04	ENSG00000130956
HACD1	-0.2303	1.1462E-02	4.71E-02	ENSG00000165996

HACD2	-0.2852	9.4686E-05	1.07E-03	ENSG00000206527
HACD3	-0.2256	9.9612E-06	1.75E-04	ENSG00000074696
HACE1	0.0647	4.5711E-01	6.46E-01	ENSG00000085382
HACL1	0.1806	8.6016E-03	3.80E-02	ENSG00000131373
HADH	-0.3774	3.4571E-05	4.73E-04	ENSG00000138796
HADHA	-0.0428	5.5162E-01	7.23E-01	ENSG00000084754
HADHAP1	-0.0380	7.1732E-01	8.43E-01	ENSG00000251596
HADHB	-0.1467	6.7517E-02	1.78E-01	ENSG00000138029
HADHBP1	0.0109	5.1619E-01		ENSG00000238193
HAGH	-0.0530	2.5867E-01	4.47E-01	ENSG00000063854
HAGHL	-0.0284	7.2845E-01	8.50E-01	ENSG00000103253
HAGLR	0.0202	2.4943E-01		ENSG00000224189
HAL	0.0222	2.0098E-01		ENSG00000084110
HAMP	-0.0609	2.1512E-01		ENSG00000105697
HAND1	-0.0599	7.7568E-02		ENSG00000113196
HAND2	0.0546	5.9080E-02		ENSG00000164107
HAND2-AS1	-0.0071	9.0749E-01		ENSG00000237125
HAPLN1	-0.4910	8.4096E-03	3.73E-02	ENSG00000145681
HAPLN3	0.0640	4.9660E-01	6.80E-01	ENSG00000140511
HAPLN4	0.0015	7.7611E-01		ENSG00000187664
HAPSTR1	-0.2238	1.4613E-03	9.62E-03	ENSG00000182831
HAPSTR2	-0.0162	6.7993E-01	8.18E-01	ENSG00000230707
HAR1B	0.0305	6.4971E-01	7.97E-01	ENSG00000231133
HARBI1	0.2202	1.2047E-02	4.89E-02	ENSG00000180423
HARS1	0.2191	4.6781E-03	2.38E-02	ENSG00000170445
HARS2	0.3998	1.7965E-06	4.31E-05	ENSG00000112855
HAS1	-0.0023	9.6061E-01	9.80E-01	ENSG00000105509
HAS2	-0.2675	4.1872E-02	1.25E-01	ENSG00000170961
HAS3	-0.1091	2.0479E-01	3.83E-01	ENSG00000103044
HASPIN	-0.0364	6.3375E-01	7.86E-01	ENSG00000177602
HAT1	-0.1273	6.5260E-02	1.73E-01	ENSG00000128708
HAUS1	-0.0660	2.3945E-01	4.24E-01	ENSG00000152240
HAUS2	-0.1201	2.0786E-02	7.41E-02	ENSG00000137814
HAUS3	0.3349	2.7717E-03	1.58E-02	ENSG00000214367
HAUS4	-0.2192	4.9400E-02	1.42E-01	ENSG00000092036
HAUS5	0.2918	1.5712E-03	1.02E-02	ENSG00000249115
HAUS6	0.1508	4.4809E-02	1.32E-01	ENSG00000147874
HAUS6P1	0.0583	4.8034E-01	6.66E-01	ENSG00000227344
HAUS7	0.1554	5.1525E-02	1.46E-01	ENSG00000213397
HAUS8	0.1444	7.8879E-02	1.99E-01	ENSG00000131351
HAVCR1	0.0264	2.3934E-01		ENSG00000113249
HAVCR1P1	0.0011	6.5708E-01		ENSG00000268442
HAVCR1P2	-0.0383	5.1835E-01		ENSG00000271053
HAVCR2	0.1207	1.5226E-01	3.14E-01	ENSG00000135077
HAX1	-0.0946	6.9200E-02	1.81E-01	ENSG00000143575
HBA1	-0.4101	1.8046E-02	6.67E-02	ENSG00000206172
HBM	0.0134	4.3125E-01		ENSG00000206177
HBP1	-0.1388	1.0626E-01	2.45E-01	ENSG00000105856
HBQ1	0.2067	5.0091E-02	1.43E-01	ENSG00000086506

HBS1L	0.0538	4.2779E-01	6.20E-01	ENSG00000112339
HBZ	-0.0473	8.3309E-01	9.13E-01	ENSG00000130656
HCAR2	0.0399	4.7677E-01	6.64E-01	ENSG00000182782
HCAR3	-0.0034	9.8268E-01		ENSG00000255398
HCCAT5	0.0057	8.2127E-01		ENSG00000260880
HCCS	0.1456	3.8259E-02	1.17E-01	ENSG00000004961
HCFC1	-0.1686	3.6879E-03	1.98E-02	ENSG00000172534
HCFC1R1	-0.0699	3.0685E-01	4.99E-01	ENSG00000103145
HCFC2	0.1967	1.9061E-02	6.94E-02	ENSG00000111727
HCG11	-0.2485	4.8807E-02	1.41E-01	ENSG00000228223
HCG14	-0.1176	2.5718E-01	4.45E-01	ENSG00000224157
HCG18	0.1115	6.0902E-02	1.65E-01	ENSG00000231074
HCG20	0.4036	8.1000E-03	3.63E-02	ENSG00000228022
HCG22	0.0657	4.3649E-01	6.28E-01	ENSG00000228789
HCG23	0.4576	8.1431E-04	6.05E-03	ENSG00000225914
HCG25	0.0268	7.6621E-01	8.73E-01	ENSG00000232940
HCG27	0.0233	7.7860E-01	8.80E-01	ENSG00000206344
HCG9	0.1163	1.5635E-01	3.19E-01	ENSG00000204625
HCK	0.4610	1.1776E-02	4.81E-02	ENSG00000101336
HCLS1	-0.0081	7.9452E-01	8.90E-01	ENSG00000180353
HCN1	-0.2495	5.4098E-02	1.51E-01	ENSG00000164588
HCN2	-0.1231	2.9326E-01	4.85E-01	ENSG00000099822
HCN3	0.1991	3.3375E-02	1.06E-01	ENSG00000143630
HCN4	0.2055	3.3369E-02	1.06E-01	ENSG00000138622
HCP5	-0.0126	8.8712E-01	9.43E-01	ENSG00000206337
HCP5B	0.0038	5.3101E-01		ENSG00000281831
HCRT	-0.0894	2.3966E-01	4.24E-01	ENSG00000161610
HCRTR1	0.0842	3.5708E-01	5.53E-01	ENSG00000121764
HCRTR2	0.0358	6.9556E-01	8.30E-01	ENSG00000137252
HCST	-0.0267	7.5642E-01	8.68E-01	ENSG00000126264
HDAC1	-0.1209	1.0533E-01	2.44E-01	ENSG00000116478
HDAC10	0.3296	2.5217E-02	8.57E-02	ENSG00000100429
HDAC11	0.1383	7.6298E-02	1.94E-01	ENSG00000163517
HDAC11-AS1	0.0641	2.5886E-01	4.47E-01	ENSG00000244502
HDAC1P2	-0.0430	6.5916E-01	8.03E-01	ENSG00000233012
HDAC2	-0.0036	8.5012E-01	9.23E-01	ENSG00000196591
HDAC2-AS2	0.3651	4.6775E-03	2.38E-02	ENSG00000228624
HDAC3	0.2061	3.2597E-03	1.79E-02	ENSG00000171720
HDAC4	0.0004	9.9670E-01	9.98E-01	ENSG00000068024
HDAC4-AS1	0.0440	6.0788E-01	7.67E-01	ENSG00000222020
HDAC5	0.0384	5.7993E-01	7.46E-01	ENSG00000108840
HDAC6	0.0718	2.0933E-01	3.89E-01	ENSG00000094631
HDAC7	-0.4125	3.8399E-05	5.16E-04	ENSG00000061273
HDAC8	0.0966	1.3252E-01	2.85E-01	ENSG00000147099
HDAC9	-0.2183	6.4213E-03	3.04E-02	ENSG00000048052
HDC	-0.2577	4.6485E-02	1.36E-01	ENSG00000140287
HDDC2	-0.1904	2.5732E-03	1.49E-02	ENSG00000111906
HDDC3	0.1339	6.9618E-02	1.82E-01	ENSG00000184508
HDGF	-0.2392	9.4237E-04	6.80E-03	ENSG00000143321

HDGFL2	-0.0040	8.8185E-01	9.40E-01	ENSG00000167674
HDGFL3	-0.1123	1.6675E-02	6.28E-02	ENSG00000166503
HDHD2	-0.0552	5.3518E-01	7.09E-01	ENSG00000167220
HDHD3	-0.1314	8.2644E-02	2.05E-01	ENSG00000119431
HDHD5	0.0676	3.6219E-01	5.58E-01	ENSG00000069998
HDHD5-AS1	0.1354	1.9741E-01	3.74E-01	ENSG00000185837
HDLBP	-0.2622	2.1744E-05	3.27E-04	ENSG00000115677
HDX	0.0574	5.0786E-01	6.88E-01	ENSG00000165259
HEATR1	0.2214	3.0726E-02	9.92E-02	ENSG00000119285
HEATR3	-0.0006	9.8129E-01	9.90E-01	ENSG00000155393
HEATR4	0.1916	9.6234E-02	2.29E-01	ENSG00000187105
HEATR5A	-0.3999	1.2779E-03	8.64E-03	ENSG00000129493
HEATR5A-DT	0.3735	1.4170E-02	5.56E-02	ENSG00000250365
HEATR5B	0.4333	2.4278E-04	2.29E-03	ENSG00000008869
HEATR6	0.1905	1.9064E-03	1.18E-02	ENSG00000068097
HEATR6-DT	-0.0804	8.7647E-01	9.37E-01	ENSG00000267416
HEBP1	-0.1680	1.9256E-02	7.00E-02	ENSG00000013583
HEBP2	-0.0958	5.1041E-02	1.45E-01	ENSG00000051620
HECA	-0.0225	7.3603E-01	8.55E-01	ENSG00000112406
HECTD1	-0.0861	1.1488E-01	2.59E-01	ENSG00000092148
HECTD2	0.3556	4.3567E-04	3.69E-03	ENSG00000165338
HECTD3	0.5656	1.7570E-09	1.35E-07	ENSG00000126107
HECTD4	-0.0114	8.3766E-01	9.15E-01	ENSG00000173064
HECW1	0.1519	9.2596E-02	2.23E-01	ENSG00000002746
HECW1-IT1	0.0116	6.8370E-01		ENSG00000181211
HECW2	0.2317	1.0316E-02	4.35E-02	ENSG00000138411
HECW2-AS1	0.0021	3.7849E-01		ENSG00000229056
HEG1	-0.5027	9.3411E-05	1.06E-03	ENSG00000173706
HELB	0.1233	2.3663E-01	4.21E-01	ENSG00000127311
HELLS	-0.0743	3.5579E-01	5.51E-01	ENSG00000119969
HELQ	0.2316	3.3227E-03	1.82E-02	ENSG00000163312
HELT	-0.0450	1.1125E-01		ENSG00000187821
HELZ	-0.0371	4.7695E-01	6.64E-01	ENSG00000198265
HELZ2	0.2283	2.1507E-02	7.61E-02	ENSG00000130589
HEMGN	-0.0092	6.9585E-01		ENSG00000136929
HEMK1	-0.3289	3.8814E-05	5.20E-04	ENSG00000114735
HENMT1	0.3930	2.7407E-04	2.53E-03	ENSG00000162639
HEPACAM	0.1306	1.6319E-01	3.29E-01	ENSG00000165478
HEPACAM2	0.1112	2.8014E-01	4.71E-01	ENSG00000188175
HEPH	-0.2792	3.0338E-03	1.70E-02	ENSG00000089472
HEPHL1	0.0461	5.8770E-01	7.52E-01	ENSG00000181333
HERC1	0.0097	8.8147E-01	9.40E-01	ENSG00000103657
HERC2	0.0302	6.1761E-01	7.75E-01	ENSG00000128731
HERC2P6	0.1324	2.8712E-02		ENSG00000261418
HERC4	-0.1521	2.6031E-02	8.77E-02	ENSG00000148634
HERC6	-0.3417	7.3535E-04	5.59E-03	ENSG00000138642
HERPUD2	-0.0450	5.2230E-01	6.99E-01	ENSG00000122557
HERPUD2-AS1	0.1468	3.0876E-02	9.95E-02	ENSG00000271122
HES1	-0.3753	1.6069E-10	1.78E-08	ENSG00000114315

HES2	-0.0555	5.1393E-01	6.93E-01	ENSG00000069812
HES4	-0.1861	1.6755E-02	6.30E-02	ENSG00000188290
HES5	-0.3593	1.3994E-02	5.50E-02	ENSG00000197921
HES6	-0.0924	2.2492E-01	4.07E-01	ENSG00000144485
HES7	0.5975	2.9730E-03	1.67E-02	ENSG00000179111
HESX1	0.1131	2.5526E-01	4.43E-01	ENSG00000163666
HEXA	-0.3057	3.3835E-04	3.02E-03	ENSG00000213614
HEXA-AS1	-0.0490	5.8415E-01	7.49E-01	ENSG00000260339
HEXB	-0.1062	6.6706E-02	1.76E-01	ENSG00000049860
HEXD	0.1956	2.7914E-03	1.59E-02	ENSG00000169660
HEXIM1	-0.1394	1.1384E-02	4.69E-02	ENSG00000186834
HEXIM2	0.1100	2.7368E-01	4.64E-01	ENSG00000168517
HEY1	-0.1812	4.0831E-02	1.23E-01	ENSG00000164683
HEY2	-0.2004	8.7768E-02	2.14E-01	ENSG00000135547
HEY2-AS1	-0.0435	4.9206E-01		ENSG00000237742
HFM1	0.1067	2.3958E-01	4.24E-01	ENSG00000162669
HGD	-0.0182	9.2167E-01	9.61E-01	ENSG00000113924
HGF	0.0118	8.8177E-01	9.40E-01	ENSG00000019991
HGFAC	-0.0768	4.3661E-01	6.28E-01	ENSG00000109758
HGH1	-0.0053	9.4444E-01	9.72E-01	ENSG00000235173
HGS	0.0710	2.7884E-01	4.70E-01	ENSG00000185359
HGSNAT	0.2115	9.2937E-04	6.72E-03	ENSG00000165102
HHAT	-0.4307	4.9333E-05	6.39E-04	ENSG00000054392
HHATL	0.1490	9.6097E-02	2.28E-01	ENSG00000010282
HHATL-AS1	-0.0335	3.3408E-01		ENSG00000230970
HHEX	-0.0127	9.1963E-01	9.60E-01	ENSG00000152804
HHIP	-0.1249	1.9953E-01	3.77E-01	ENSG00000164161
HHIP-AS1	-0.1233	2.2674E-01	4.09E-01	ENSG00000248890
HHIPL1	0.0790	3.4563E-01	5.41E-01	ENSG00000182218
HHIPL2	0.0010	9.6748E-01		ENSG00000143512
HHLA1	-0.0066	9.4447E-01		ENSG00000132297
HHLA2	0.0021	8.7036E-01	9.34E-01	ENSG00000114455
HIBADH	-0.1770	6.3787E-02	1.70E-01	ENSG00000106049
HIBCH	-0.1235	1.4516E-01	3.04E-01	ENSG00000198130
HIC1	0.0263	7.5769E-01	8.68E-01	ENSG00000177374
HIC2	0.3077	4.5092E-04	3.79E-03	ENSG00000169635
HID1	0.0049	9.3155E-01	9.66E-01	ENSG00000167861
HID1-AS1	0.0532	4.3749E-01	6.28E-01	ENSG00000263586
HIF1A	-0.2969	3.1714E-06	6.91E-05	ENSG00000100644
HIF1AN	0.0758	3.7133E-01	5.67E-01	ENSG00000166135
HIF3A	0.3550	6.0836E-06	1.18E-04	ENSG00000124440
HIGD1A	-0.1551	3.4397E-02	1.08E-01	ENSG00000181061
HIGD1B	-0.0421	3.2722E-01		ENSG00000131097
HIGD2A	-0.2676	2.0038E-05	3.06E-04	ENSG00000146066
HIGD2AP1	0.0035	9.6985E-01		ENSG00000230047
HIGD2B	0.0861	2.7269E-01	4.63E-01	ENSG00000175202
HIKESHI	-0.1296	2.8291E-02	9.32E-02	ENSG00000149196
HIKESHIP2	0.0229	4.7994E-01		ENSG00000267544
HIKESHIP3	-0.0166	7.4279E-01		ENSG00000271081

HILPDA	-0.1519	2.6367E-02	8.85E-02	ENSG00000135245
HINT1	-0.1563	1.4913E-02	5.78E-02	ENSG00000169567
HINT2	-0.2497	1.1287E-02	4.66E-02	ENSG00000137133
HINT3	0.0291	7.2956E-01	8.51E-01	ENSG00000111911
HIP1	-0.2678	1.5807E-03	1.02E-02	ENSG00000127946
HIP1R	0.1771	4.8677E-03	2.45E-02	ENSG00000130787
HIPK1	-0.3280	1.5970E-05	2.56E-04	ENSG00000163349
HIPK1-AS1	0.3154	1.0244E-02	4.32E-02	ENSG00000235527
HIPK2	-0.4031	9.6268E-05	1.08E-03	ENSG00000064393
HIPK3	-0.1576	2.6780E-02	8.95E-02	ENSG00000110422
HIPK4	-0.0073	5.7452E-01		ENSG00000160396
HIRA	0.0264	7.4758E-01	8.62E-01	ENSG00000100084
HIRIP3	0.0814	1.6488E-01	3.31E-01	ENSG00000149929
HIVEP1	-0.0545	4.5345E-01	6.43E-01	ENSG00000095951
HIVEP2	0.1309	1.2028E-01	2.68E-01	ENSG00000010818
HIVEP3	-0.0832	2.0773E-01	3.87E-01	ENSG00000127124
HJURP	-0.1278	2.2318E-01	4.05E-01	ENSG00000123485
HJV	-0.0134	8.5061E-01		ENSG00000168509
HK1	0.3044	8.0151E-05	9.35E-04	ENSG00000156515
HK3	-0.0665	1.3516E-01		ENSG00000160883
HKDC1	0.4793	7.2334E-04	5.52E-03	ENSG00000156510
HLA-A	-0.3619	3.7192E-04	3.26E-03	ENSG00000206503
HLA-B	-0.4638	5.2208E-07	1.54E-05	ENSG00000234745
HLA-C	-0.3894	1.2102E-07	4.52E-06	ENSG00000204525
HLA-DMB	-0.0594	5.1222E-01	6.92E-01	ENSG00000242574
HLA-DOA	0.3006	1.9445E-02	7.05E-02	ENSG00000204252
HLA-DOB	-0.0166	7.7460E-01	8.78E-01	ENSG00000241106
HLA-DPA1	-0.1898	1.0155E-01	2.37E-01	ENSG00000231389
HLA-DPB1	-0.0328	6.5400E-01	8.00E-01	ENSG00000223865
HLA-DPB2	0.0522	5.9155E-01	7.55E-01	ENSG00000224557
HLA-DQA1	0.0024	9.6981E-01	9.84E-01	ENSG00000196735
HLA-DQB1	0.2180	1.1449E-02	4.71E-02	ENSG00000179344
HLA-DQB1-AS1	0.0195	3.2352E-01		ENSG00000223534
HLA-DQB2	0.0134	6.0099E-01		ENSG00000232629
HLA-DRA	0.0100	8.9343E-01	9.47E-01	ENSG00000204287
HLA-DRB1	0.3520	1.1406E-03	7.87E-03	ENSG00000196126
HLA-DRB5	-0.0153	8.6945E-01	9.34E-01	ENSG00000198502
HLA-E	-0.1559	7.0738E-02	1.84E-01	ENSG00000204592
HLA-F	-0.1645	1.1806E-01	2.64E-01	ENSG00000204642
HLA-F-AS1	-0.2040	8.1103E-02	2.03E-01	ENSG00000214922
HLA-G	0.0481	6.1463E-01	7.73E-01	ENSG00000204632
HLA-K	-0.1002	2.2568E-01	4.08E-01	ENSG00000230795
HLA-Z	-0.0025	3.4385E-01		ENSG00000235301
HLCS	-0.0774	2.3794E-01	4.22E-01	ENSG00000159267
HLF	0.3328	5.4892E-03	2.69E-02	ENSG00000108924
HLTF	-0.1377	3.0541E-02	9.87E-02	ENSG00000071794
HLX	-0.0433	4.0133E-01		ENSG00000136630
HM13	-0.0806	1.7264E-01	3.42E-01	ENSG00000101294
HM13-AS1	-0.0259	7.2162E-01	8.46E-01	ENSG00000230613

HMBOX1	0.2651	1.3152E-04	1.39E-03	ENSG00000147421
HMBS	0.0318	4.8427E-01	6.69E-01	ENSG00000256269
HMCES	-0.0329	5.1543E-01	6.94E-01	ENSG00000183624
HMG20A	0.2405	6.0482E-03	2.90E-02	ENSG00000140382
HMG20B	-0.1270	1.1711E-01	2.63E-01	ENSG00000064961
HMGA1	0.0360	5.7257E-01	7.40E-01	ENSG00000137309
HMGA2	-0.2138	2.2023E-02	7.74E-02	ENSG00000149948
HMGA2-AS1	0.0408	6.4898E-01	7.97E-01	ENSG00000197301
HMGB1	-0.3173	2.6410E-06	6.01E-05	ENSG00000189403
HMGB1P11	0.0133	4.9083E-01		ENSG00000230547
HMGB1P13	0.1983	2.4994E-02	8.51E-02	ENSG00000220557
HMGB1P19	0.0110	4.5458E-01		ENSG00000253463
HMGB1P31	0.1671	1.3332E-01	2.86E-01	ENSG00000233266
HMGB1P33	-0.0046	8.8833E-01		ENSG00000261174
HMGB1P39	-0.0470	1.5274E-01		ENSG00000203489
HMGB1P49	-0.0037	8.5177E-01		ENSG00000230519
HMGB1P5	-0.3080	1.5409E-05	2.50E-04	ENSG00000132967
HMGB1P7	-0.0060	6.6353E-01		ENSG00000231148
HMGB2	-0.3226	1.3097E-03	8.81E-03	ENSG00000164104
HMGB3	-0.0885	1.9513E-01	3.71E-01	ENSG00000029993
HMGB3P10	-0.0145	7.2741E-01		ENSG00000223656
HMGB3P28	0.0049	8.5955E-01		ENSG00000267515
HMGB3P3	-0.0297	6.8819E-01		ENSG00000250730
HMGB3P6	0.0307	2.1296E-01		ENSG00000213070
HMGCL	0.1066	2.0939E-01	3.89E-01	ENSG00000117305
HMGCLL1	0.2114	1.5343E-02	5.90E-02	ENSG00000146151
HMGCR	-0.0576	4.5700E-01	6.46E-01	ENSG00000113161
HMGCS1	-0.3994	5.3758E-05	6.80E-04	ENSG00000112972
HMGCS2	-0.3947	1.5540E-02	5.95E-02	ENSG00000134240
HMGN1	-0.2583	3.3127E-06	7.17E-05	ENSG00000205581
HMGN1P13	0.0025	7.8358E-01		ENSG00000249619
HMGN1P15	-0.0020	8.3267E-01		ENSG00000250197
HMGN1P8	-0.0254	8.2398E-01		ENSG00000241120
HMGN2	-0.4733	2.9412E-08	1.43E-06	ENSG00000198830
HMGN3	-0.5616	7.1971E-11	9.06E-09	ENSG00000118418
HMGN3-AS1	-0.2075	3.3611E-02	1.06E-01	ENSG00000270362
HMGN4	-0.1790	1.3236E-02	5.26E-02	ENSG00000182952
HMGN5	-0.1569	8.9774E-03	3.92E-02	ENSG00000198157
HMGXB3	0.1122	1.5096E-01	3.12E-01	ENSG00000113716
HMGXB4	0.0487	4.7480E-01	6.62E-01	ENSG00000100281
HMMR	-0.5194	2.4558E-05	3.60E-04	ENSG00000072571
HMOX1	-0.1127	2.4036E-01	4.25E-01	ENSG00000100292
HMOX2	0.0218	6.7177E-01	8.12E-01	ENSG00000103415
HMSD	-0.2192	7.2937E-02	1.88E-01	ENSG00000221887
HMX1	-0.3198	2.5589E-02	8.67E-02	ENSG00000215612
HMX2	0.0189	3.2006E-01		ENSG00000188816
HMX3	-0.0126	8.2490E-01		ENSG00000188620
HNF1A	-0.0386	6.0534E-01		ENSG00000135100
HNF1A-AS1	0.0235	2.3261E-01		ENSG00000241388

HNF1B	0.1595	1.1816E-01	2.64E-01	ENSG00000275410
HNF4A	-0.0047	9.6436E-01		ENSG00000101076
HNF4G	-0.0733	2.7510E-01	4.66E-01	ENSG00000164749
HNMT	0.0557	5.1577E-01	6.94E-01	ENSG00000150540
HNRNPA0	-0.1348	4.6016E-02	1.35E-01	ENSG00000177733
HNRNPA1	-0.2671	1.0375E-04	1.15E-03	ENSG00000135486
HNRNPA1L3	-0.1916	5.9760E-02	1.63E-01	ENSG00000224578
HNRNPA1P27	0.0156	5.4320E-01		ENSG00000233680
HNRNPA2B1	-0.1285	9.2969E-02	2.24E-01	ENSG00000122566
HNRNPA3	-0.2708	8.2141E-07	2.28E-05	ENSG00000170144
HNRNPA3P10	-0.0048	7.4932E-01		ENSG00000257851
HNRNPA3P11	-0.0462	5.0431E-01	6.86E-01	ENSG00000260689
HNRNPA3P3	0.0421	5.8543E-01	7.50E-01	ENSG00000214653
HNRNPA3P5	0.0787	1.0793E-01	2.48E-01	ENSG00000236565
HNRNPA3P7	0.0296	2.4512E-01	4.31E-01	ENSG00000253119
HNRNPA3P9	-0.0008	9.8665E-01		ENSG00000270903
HNRNPAB	0.1153	9.0260E-02	2.19E-01	ENSG00000197451
HNRNPC	-0.4087	1.5207E-05	2.47E-04	ENSG00000092199
HNRNPCL1	0.0063	5.4927E-01		ENSG00000179172
HNRNPCP3	-0.0113	7.1962E-01		ENSG00000259419
HNRNPCP6	-0.3437	2.0569E-02	7.35E-02	ENSG00000213305
HNRNPCP7	0.0023	7.1438E-01		ENSG00000228653
HNRNPCP8	0.0006	9.9220E-01		ENSG00000254748
HNRNPD	-0.0066	8.3439E-01	9.14E-01	ENSG00000138668
HNRNPD-DT	0.2628	6.6395E-03	3.11E-02	ENSG00000272677
HNRNPDL	-0.2225	5.0197E-05	6.46E-04	ENSG00000152795
HNRNPF	-0.1373	2.7486E-02	9.12E-02	ENSG00000169813
HNRNPH1	-0.1443	1.2950E-02	5.18E-02	ENSG00000169045
HNRNPH2	-0.0647	3.4386E-01	5.39E-01	ENSG00000126945
HNRNPH3	-0.3845	9.4106E-06	1.67E-04	ENSG00000096746
HNRNPK	-0.0689	3.8500E-01	5.80E-01	ENSG00000165119
HNRNPKP1	0.0213	1.4882E-01		ENSG00000250859
HNRNPKP2	0.0759	2.9566E-01	4.88E-01	ENSG00000227347
HNRNPKP3	0.0492	3.8687E-01	5.82E-01	ENSG00000251557
HNRNPKP4	-0.1320	2.0936E-01	3.89E-01	ENSG00000243547
HNRNPL	-0.0730	3.0867E-01	5.01E-01	ENSG00000104824
HNRNPLL	-0.0051	9.3748E-01	9.69E-01	ENSG00000143889
HNRNPM	-0.0261	7.1295E-01	8.40E-01	ENSG00000099783
HNRNPMP1	-0.0826	9.4977E-02	2.27E-01	ENSG00000259335
HNRNPR	-0.0943	1.1072E-01	2.52E-01	ENSG00000125944
HNRNPU	-0.1454	3.6839E-03	1.98E-02	ENSG00000153187
HNRNPUL1	-0.0408	4.5074E-01	6.40E-01	ENSG00000105323
HNRNPUL2	-0.0203	8.2656E-01	9.09E-01	ENSG00000214753
HNRNPUL2-BSCL2	-0.0032	8.8655E-01		ENSG00000234857
HOGA1	-0.0953	3.1907E-01	5.13E-01	ENSG00000241935
HOMER1	0.2937	6.1947E-04	4.89E-03	ENSG00000152413
HOMER2	-0.1165	9.8446E-02	2.32E-01	ENSG00000103942
HOMER3	-0.2407	1.9453E-04	1.92E-03	ENSG00000051128
HOOK1	0.0467	5.6458E-01	7.33E-01	ENSG00000134709

HOOK2	0.1029	1.5739E-01	3.21E-01	ENSG00000095066
HOOK3	-0.0681	2.0615E-01	3.85E-01	ENSG00000168172
HORMAD1	0.0080	4.4359E-01		ENSG00000143452
HORMAD2	-0.0525	5.6723E-01	7.35E-01	ENSG00000176635
HOTAIRM1	0.0000	9.6820E-01	9.84E-01	ENSG00000233429
HOXA-AS3	0.0202	3.2796E-01		ENSG00000254369
HOXA2	-0.0105	5.0284E-01		ENSG00000105996
HOXA5	-0.0325	3.2208E-01		ENSG00000106004
HOXB-AS1	0.0045	9.3253E-01	9.66E-01	ENSG00000230148
HOXB13	-0.0707	4.9190E-02		ENSG00000159184
HOXB2	0.0902	3.0241E-01	4.95E-01	ENSG00000173917
HOXB3	-0.0829	2.9454E-01	4.87E-01	ENSG00000120093
HOXB4	-0.0094	5.5023E-01		ENSG00000182742
HOXB7	0.0580	1.0221E-01	2.38E-01	ENSG00000260027
HOXB9	-0.0207	6.3105E-01		ENSG00000170689
HOXC-AS1	-0.0059	6.8782E-01		ENSG00000250451
HOXC10	-0.0013	8.7026E-01		ENSG00000180818
HOXC13	0.1175	3.6801E-02		ENSG00000123364
HOXC4	0.0097	7.8176E-01		ENSG00000198353
HOXC5	0.0093	8.3971E-01		ENSG00000172789
HOXC6	-0.0155	8.5505E-01	9.26E-01	ENSG00000197757
HOXC8	-0.0219	4.2354E-01		ENSG00000037965
HOXC9	-0.0394	3.2377E-01	5.18E-01	ENSG00000180806
HOXD8	0.0508	4.1698E-02	1.25E-01	ENSG00000175879
HOXD9	0.0006	9.5485E-01		ENSG00000128709
HP	0.0988	8.2032E-02	2.04E-01	ENSG00000257017
HP1BP3	-0.2351	8.1617E-06	1.49E-04	ENSG00000127483
HPAT5	0.0124	2.9405E-01		ENSG00000280707
HPCA	-0.0411	6.6871E-01	8.09E-01	ENSG00000121905
HPCAL1	0.1443	5.0169E-02	1.43E-01	ENSG00000115756
HPCAL4	0.3152	1.3123E-02	5.23E-02	ENSG00000116983
HPDL	0.4160	7.4682E-03	3.40E-02	ENSG00000186603
HPF1	-0.1563	3.3058E-03	1.81E-02	ENSG00000056050
HPGD	0.0726	4.2025E-01	6.13E-01	ENSG00000164120
HPN	0.1277	2.1071E-01	3.90E-01	ENSG00000105707
HPN-AS1	0.0697	3.5616E-01	5.52E-01	ENSG00000227392
HPR	-0.0480	6.2783E-01	7.82E-01	ENSG00000261701
HPRT1	0.2352	1.0646E-04	1.18E-03	ENSG00000165704
HPS1	-0.1307	6.5939E-02	1.75E-01	ENSG00000107521
HPS1-AS1	0.0240	8.0253E-01	8.95E-01	ENSG00000287261
HPS3	0.2204	2.2928E-03	1.37E-02	ENSG00000163755
HPS4	0.2968	2.7036E-07	9.01E-06	ENSG00000100099
HPS5	-0.0816	3.3903E-01	5.34E-01	ENSG00000110756
HPS6	0.0091	9.1649E-01	9.58E-01	ENSG00000166189
HPSE	-0.3189	3.0798E-02	9.94E-02	ENSG00000173083
HPSE2	0.0845	2.5591E-01	4.43E-01	ENSG00000172987
HPX	0.1255	2.2674E-01	4.09E-01	ENSG00000110169
HPYR1	0.0013	7.6858E-01		ENSG00000253521
HR	-0.1516	1.1531E-01	2.60E-01	ENSG00000168453

HRAS	0.0544	4.8635E-01	6.71E-01	ENSG00000174775
HRCT1	-0.0047	7.9504E-01		ENSG00000196196
HRH1	-0.2051	6.6724E-02	1.76E-01	ENSG00000196639
HRH2	-0.4241	1.2797E-02	5.13E-02	ENSG00000113749
HRH3	0.0622	5.0490E-01	6.86E-01	ENSG00000101180
HRH4	-0.0247	5.9205E-01		ENSG00000134489
HRK	-0.3409	7.6841E-04	5.79E-03	ENSG00000135116
HROB	-0.1669	9.4090E-02	2.25E-01	ENSG00000125319
HS1BP3	0.0145	8.3594E-01	9.14E-01	ENSG00000118960
HS2ST1	-0.2267	6.3195E-04	4.96E-03	ENSG00000153936
HS3ST1	-0.4919	2.3094E-03	1.37E-02	ENSG00000002587
HS3ST2	-0.1209	2.3886E-01	4.23E-01	ENSG00000122254
HS3ST3A1	-0.4608	5.3471E-03	2.64E-02	ENSG00000153976
HS3ST3B1	0.0386	6.8716E-01	8.24E-01	ENSG00000125430
HS3ST4	-0.1778	6.9379E-02	1.81E-01	ENSG00000182601
HS3ST6	0.0111	9.0766E-01	9.54E-01	ENSG00000162040
HS6ST1	0.0976	1.1011E-01	2.51E-01	ENSG00000136720
HS6ST2	0.0330	7.1220E-01	8.40E-01	ENSG00000171004
HS6ST3	-0.0815	3.7473E-01	5.70E-01	ENSG00000185352
HSBP1	-0.1258	3.7517E-02	1.15E-01	ENSG00000230989
HSBP1L1	-0.1169	6.8041E-02	1.79E-01	ENSG00000226742
HSCB	-0.2768	6.6250E-05	8.06E-04	ENSG00000100209
HSD11B1	-0.0785	9.1564E-02		ENSG00000117594
HSD11B1-AS1	-0.0081	7.7545E-01		ENSG00000227591
HSD11B1L	-0.1120	6.2366E-02	1.68E-01	ENSG00000167733
HSD11B2	-0.1288	2.2227E-01	4.04E-01	ENSG00000176387
HSD17B1	-0.2151	6.7435E-02	1.77E-01	ENSG00000108786
HSD17B1-AS1	0.2484	5.5257E-02	1.54E-01	ENSG00000266962
HSD17B10	-0.2397	1.3970E-04	1.46E-03	ENSG00000072506
HSD17B11	-0.3731	1.9190E-05	2.96E-04	ENSG00000198189
HSD17B12	-0.2461	1.6613E-03	1.06E-02	ENSG00000149084
HSD17B13	-0.0645	2.7509E-01	4.66E-01	ENSG00000170509
HSD17B14	-0.3949	2.6635E-04	2.47E-03	ENSG00000087076
HSD17B4	-0.1862	1.2336E-02	4.99E-02	ENSG00000133835
HSD17B6	0.0758	4.2044E-01	6.13E-01	ENSG00000025423
HSD17B7	-0.2153	3.3646E-03	1.84E-02	ENSG00000132196
HSD17B8	0.0130	8.8277E-01	9.41E-01	ENSG00000204228
HSD3B7	-0.1029	2.3660E-01	4.21E-01	ENSG00000099377
HSD52	0.4529	1.9789E-03	1.21E-02	ENSG00000224609
HSDL1	0.1110	2.2846E-01	4.11E-01	ENSG00000103160
HSDL2	-0.5183	2.7158E-10	2.78E-08	ENSG00000119471
HSF1	-0.1738	1.0704E-02	4.47E-02	ENSG00000185122
HSF2	0.2258	2.3324E-03	1.38E-02	ENSG00000025156
HSF2BP	0.5093	1.4844E-03	9.74E-03	ENSG00000160207
HSF4	0.5430	8.5749E-03	3.79E-02	ENSG00000102878
HSF5	0.0074	5.2790E-01		ENSG00000176160
HSFX1	-0.0591	5.4401E-01	7.16E-01	ENSG00000171116
HSFX2	-0.0785	4.1645E-01	6.09E-01	ENSG00000268738
HSFX3	0.0191	4.6735E-01		ENSG00000283697

HSFX4	0.0128	3.2379E-01		ENSG00000283463
HSFY2	-0.0059	7.5648E-01		ENSG00000169953
HSFY3P	0.0364	3.3182E-01		ENSG00000227289
HSH2D	0.1392	1.9169E-01	3.67E-01	ENSG00000196684
HSP90AA1	-0.3602	4.9088E-11	6.43E-09	ENSG00000080824
HSP90AA6P	-0.0459	2.5563E-01		ENSG00000181359
HSP90AB1	-0.2976	7.9379E-09	4.74E-07	ENSG00000096384
HSP90AB3P	-0.1937	8.3822E-02	2.07E-01	ENSG00000183199
HSP90B1	-0.3358	6.4415E-07	1.85E-05	ENSG00000166598
HSP90B2P	0.4277	2.8340E-03	1.61E-02	ENSG00000259706
HSPA12A	0.1795	9.5507E-03	4.11E-02	ENSG00000165868
HSPA13	0.1925	1.1939E-02	4.86E-02	ENSG00000155304
HSPA14	-0.2384	2.6587E-03	1.53E-02	ENSG00000187522
HSPA1L	0.1713	9.8030E-02	2.32E-01	ENSG00000204390
HSPA2	0.5045	5.0240E-05	6.47E-04	ENSG00000126803
HSPA2-AS1	-0.0106	9.1225E-01		ENSG00000259116
HSPA4	-0.0325	5.2915E-01	7.04E-01	ENSG00000170606
HSPA4L	-0.1144	7.1075E-02	1.84E-01	ENSG00000164070
HSPA6	-0.1409	3.0261E-02	9.81E-02	ENSG00000173110
HSPA7	-0.0220	2.9054E-01	4.82E-01	ENSG00000225217
HSPA8	-0.1065	9.5919E-02	2.28E-01	ENSG00000109971
HSPA8P1	-0.0014	2.1724E-01	3.98E-01	ENSG00000234176
HSPA8P15	-0.0200	9.5645E-01	9.78E-01	ENSG00000219395
HSPA8P3	0.0172	6.6364E-01		ENSG00000234788
HSPA8P4	-0.0084	7.2403E-01		ENSG00000248610
HSPA8P7	0.0021	8.8262E-01		ENSG00000224773
HSPA9	-0.1204	1.0708E-02	4.47E-02	ENSG00000113013
HSPB3	-0.0761	1.9255E-01		ENSG00000169271
HSPB6	-0.3892	1.2731E-02	5.11E-02	ENSG00000004776
HSPB7	-0.5056	1.0110E-02	4.28E-02	ENSG00000173641
HSPB8	-0.0220	8.0894E-01	8.99E-01	ENSG00000152137
HSPB9	-0.0151	8.4677E-01		ENSG00000260325
HSPBAP1	0.0953	2.8222E-01	4.73E-01	ENSG00000169087
HSPBP1	-0.0993	3.1666E-02	1.01E-01	ENSG00000133265
HSPC324	-0.0565	2.0094E-01		ENSG00000228401
HSPD1	-0.1578	6.8420E-03	3.18E-02	ENSG00000144381
HSPD1P10	-0.0116	9.2738E-01		ENSG00000216990
HSPD1P5	-0.0566	1.5012E-01		ENSG00000249193
HSPD1P7	0.0319	2.4092E-01		ENSG00000215005
HSPE1	-0.4607	4.0015E-11	5.44E-09	ENSG00000115541
HSPE1-MOB4	0.0044	8.7232E-01		ENSG00000270757
HSPE1P26	0.0043	3.7161E-01		ENSG00000220867
HSPE1P27	-0.0336	6.7230E-01	8.12E-01	ENSG00000235112
HSPG2	-0.2513	4.4596E-03	2.29E-02	ENSG00000142798
HSPH1	-0.1371	1.1062E-01	2.52E-01	ENSG00000120694
HTATIP2	0.2440	4.1777E-02	1.25E-01	ENSG00000109854
HTATSF1	-0.0971	3.9070E-02	1.19E-01	ENSG00000102241
HTD2	-0.0228	8.2067E-01		ENSG00000255154
HTN1	0.4816	1.3858E-02	5.46E-02	ENSG00000126550

HTN3	0.1406	9.2461E-02	2.23E-01	ENSG00000205649
HTR1B	0.1271	2.2489E-01	4.07E-01	ENSG00000135312
HTR1E	0.1051	2.9359E-01	4.86E-01	ENSG00000168830
HTR1F	-0.0606	3.2858E-01		ENSG00000179097
HTR2A	-0.3202	3.0392E-03	1.70E-02	ENSG00000102468
HTR2B	-0.0530	4.9164E-01	6.75E-01	ENSG00000135914
HTR2C	-0.2248	4.5469E-02	1.33E-01	ENSG00000147246
HTR3A	-0.3833	7.9006E-03	3.56E-02	ENSG00000166736
HTR3D	-0.0001	8.5305E-01		ENSG00000186090
HTR3E	0.0263	2.0261E-01	3.80E-01	ENSG00000186038
HTR4	0.0218	5.3845E-01		ENSG00000164270
HTR5A	0.0895	3.5356E-01	5.49E-01	ENSG00000157219
HTR5A-AS1	0.1240	1.2809E-01	2.79E-01	ENSG00000220575
HTR5BP	-0.0202	3.9282E-01		ENSG00000125631
HTR6	-0.0183	9.6541E-01	9.82E-01	ENSG00000158748
HTR7	0.0925	1.8872E-01	3.63E-01	ENSG00000148680
HTR7P1	0.1965	7.1603E-02	1.85E-01	ENSG00000183935
HTRA1	-0.4708	3.0859E-06	6.77E-05	ENSG00000166033
HTRA2	-0.0255	6.2782E-01	7.82E-01	ENSG00000115317
HTRA3	0.1724	1.2588E-01	2.76E-01	ENSG00000170801
HTRA4	0.0144	5.3863E-01		ENSG00000169495
HTT	0.0345	6.6629E-01	8.08E-01	ENSG00000197386
HUNK	-0.0800	3.8427E-01	5.79E-01	ENSG00000142149
HUS1	0.0562	4.0700E-01	6.00E-01	ENSG00000136273
HUS1B	-0.0109	9.1050E-01	9.55E-01	ENSG00000188996
HUWE1	-0.1511	1.9906E-02	7.17E-02	ENSG00000086758
HVCN1	-0.1025	3.0342E-01	4.96E-01	ENSG00000122986
HYAL1	0.2393	5.6206E-02	1.56E-01	ENSG00000114378
HYAL2	-0.0853	2.1438E-01	3.95E-01	ENSG00000068001
HYAL3	-0.0750	3.4050E-01	5.36E-01	ENSG00000186792
HYAL4	-0.0711	1.6652E-01		ENSG00000106302
HYCC1	-0.1151	8.9084E-02	2.17E-01	ENSG00000122591
HYCC2	0.2264	1.3068E-02	5.21E-02	ENSG00000155744
HYDIN	-0.3510	3.7911E-04	3.31E-03	ENSG00000157423
HYDINP1	-0.0161	6.2191E-01		ENSG00000242586
HYI-AS1	-0.0079	2.4294E-01	4.28E-01	ENSG00000229348
HYKK	0.0725	4.5695E-01	6.46E-01	ENSG00000188266
HYLS1	0.2656	2.8368E-03	1.61E-02	ENSG00000198331
HYMAI	-0.0856	3.7911E-01	5.74E-01	ENSG00000283122
HYOU1	0.0234	7.8720E-01	8.86E-01	ENSG00000149428
HYPK	0.4452	4.5453E-03	2.33E-02	ENSG00000242028
IAH1	-0.1260	4.8111E-02	1.39E-01	ENSG00000134330
IAPP	0.0111	9.0655E-01	9.53E-01	ENSG00000121351
IARS1	0.2154	4.8327E-03	2.44E-02	ENSG00000196305
IARS2	0.0333	5.8884E-01	7.53E-01	ENSG00000067704
IBA57	0.2713	4.1872E-03	2.19E-02	ENSG00000181873
IBA57-DT	-0.1458	1.7081E-01	3.39E-01	ENSG00000203684
IBTK	-0.1060	2.6594E-02	8.91E-02	ENSG00000005700
ICA1	0.1306	1.0355E-01	2.41E-01	ENSG00000003147

ICA1L	-0.2085	4.6151E-03	2.36E-02	ENSG00000163596
ICAM1	-0.4901	7.1052E-03	3.28E-02	ENSG00000090339
ICAM2	0.0195	1.5023E-01		ENSG00000108622
ICAM3	-0.0133	9.5101E-01		ENSG00000076662
ICAM5	0.5501	7.7371E-04	5.82E-03	ENSG00000105376
ICE1	0.0734	2.2837E-01	4.11E-01	ENSG00000164151
ICE2	-0.1506	1.9113E-02	6.96E-02	ENSG00000128915
ICMT	0.0484	3.9507E-01	5.90E-01	ENSG00000116237
ICOSLG	-0.3113	3.0459E-02	9.85E-02	ENSG00000160223
ID2	-0.5181	1.9064E-10	2.03E-08	ENSG00000115738
ID4	-0.3552	6.7262E-04	5.22E-03	ENSG00000172201
IDE	-0.0215	7.6933E-01	8.75E-01	ENSG00000119912
IDH1	0.1198	8.9297E-02	2.17E-01	ENSG00000138413
IDH1-AS1	-0.2561	1.0844E-02	4.51E-02	ENSG00000231908
IDH2	0.0043	9.5845E-01	9.79E-01	ENSG00000182054
IDH3A	0.2761	2.3410E-02	8.11E-02	ENSG00000166411
IDH3B	0.0304	6.4251E-01	7.93E-01	ENSG00000101365
IDH3G	-0.0558	3.2806E-01	5.23E-01	ENSG00000067829
IDI1	-0.3694	6.2765E-07	1.81E-05	ENSG00000067064
IDI2	-0.0377	4.7302E-01		ENSG00000148377
IDI2-AS1	-0.0450	6.4090E-01	7.92E-01	ENSG00000232656
IDNK	-0.0336	5.9936E-01	7.60E-01	ENSG00000148057
IDO1	0.0238	5.3282E-01		ENSG00000131203
IDS	0.2041	5.3448E-03	2.64E-02	ENSG00000010404
IDUA	-0.0961	2.2271E-01	4.05E-01	ENSG00000127415
IER2	-0.0106	8.9276E-01	9.47E-01	ENSG00000160888
IER3-AS1	-0.1588	1.4715E-01	3.06E-01	ENSG00000272273
IER3IP1	-0.1066	5.4315E-02	1.52E-01	ENSG00000134049
IER5	-0.2244	3.6571E-04	3.22E-03	ENSG00000162783
IER5L	-0.0711	4.0728E-01	6.01E-01	ENSG00000188483
IFFO1	0.3275	2.0581E-04	2.00E-03	ENSG00000010295
IFI27	0.0925	3.1519E-01	5.09E-01	ENSG00000165949
IFI27L1	-0.0837	6.8754E-02	1.80E-01	ENSG00000165948
IFI27L2	-0.3413	1.2478E-05	2.09E-04	ENSG00000119632
IFI30	-0.0702	4.3202E-01	6.23E-01	ENSG00000216490
IFI44	-0.2236	3.3339E-02	1.06E-01	ENSG00000137965
IFI44L	-0.3694	1.8812E-02	6.88E-02	ENSG00000137959
IFI6	-0.4245	1.9543E-04	1.92E-03	ENSG00000126709
IFIH1	-0.4563	9.6696E-04	6.94E-03	ENSG00000115267
IFIT1B	0.0061	5.8797E-01		ENSG00000204010
IFIT5	-0.2457	1.0503E-02	4.40E-02	ENSG00000152778
IFITM1	-0.1264	2.1503E-01	3.95E-01	ENSG00000185885
IFITM10	-0.0899	3.6890E-01	5.65E-01	ENSG00000244242
IFITM2	-0.3594	1.2653E-03	8.57E-03	ENSG00000185201
IFITM3	-0.5902	2.6522E-07	8.87E-06	ENSG00000142089
IFITM4P	0.0011	8.9871E-01		ENSG00000235821
IFITM9P	-0.0194	5.1327E-01		ENSG00000213275
IFNA20P	0.0065	3.3587E-01		ENSG00000226393
IFNA21	0.0020	8.0350E-01		ENSG00000137080

IFNAR1	-0.0112	8.4556E-01	9.20E-01	ENSG00000142166
IFNAR2	0.0250	7.7423E-01	8.78E-01	ENSG00000159110
IFNAR2-IL10RB	-0.0007	6.7855E-01		ENSG00000249624
IFNG-AS1	-0.0554	1.2896E-01		ENSG00000255733
IFNGR1	0.0997	1.1382E-01	2.57E-01	ENSG00000027697
IFNGR2	-0.3238	3.2527E-05	4.51E-04	ENSG00000159128
IFNL2	-0.0036	7.9317E-01		ENSG00000183709
IFNL3	-0.0039	8.9778E-01		ENSG00000197110
IFNL4	0.0041	6.9301E-01		ENSG00000272395
IFNL4P1	-0.0086	7.9971E-01		ENSG00000272311
IFNLR1	0.0949	2.7403E-01	4.65E-01	ENSG00000185436
IFRD1	-0.3732	4.2900E-06	8.87E-05	ENSG00000006652
IFRD2	-0.0031	9.4113E-01	9.71E-01	ENSG00000214706
IFT122	0.1800	1.8386E-02	6.77E-02	ENSG00000163913
IFT140	-0.0130	8.5549E-01	9.26E-01	ENSG00000187535
IFT172	0.2254	2.0270E-03	1.24E-02	ENSG00000138002
IFT20	0.1122	7.5517E-02	1.92E-01	ENSG00000109083
IFT22	-0.2302	4.8285E-04	3.98E-03	ENSG00000128581
IFT25	-0.0977	1.0661E-01	2.46E-01	ENSG00000081870
IFT27	-0.0282	6.1470E-01	7.73E-01	ENSG00000100360
IFT43	-0.1653	2.6033E-03	1.51E-02	ENSG00000119650
IFT46	-0.3876	2.9163E-06	6.47E-05	ENSG00000118096
IFT52	0.0294	5.6314E-01	7.32E-01	ENSG00000101052
IFT56	-0.1053	1.4624E-01	3.05E-01	ENSG00000105948
IFT57	-0.0070	9.1325E-01	9.56E-01	ENSG00000114446
IFT70A	0.0200	8.0517E-01	8.96E-01	ENSG00000197557
IFT70B	0.0603	5.1590E-01	6.95E-01	ENSG00000196659
IFT74	-0.0088	8.4405E-01	9.19E-01	ENSG00000096872
IFT74-AS1	-0.0693	3.3307E-01	5.28E-01	ENSG00000234676
IFT80	-0.2062	2.3551E-03	1.39E-02	ENSG00000068885
IFT81	-0.1707	3.5877E-03	1.94E-02	ENSG00000122970
IFT88	-0.1161	4.4966E-02	1.32E-01	ENSG00000032742
IFTAP	-0.2399	3.0795E-04	2.79E-03	ENSG00000166352
IGBP1	-0.1950	1.2803E-04	1.36E-03	ENSG00000089289
IGDCC3	-0.0175	8.4535E-01	9.20E-01	ENSG00000174498
IGDCC4	-0.2877	2.4365E-03	1.43E-02	ENSG00000103742
IGF1	-0.0952	3.1849E-01	5.12E-01	ENSG00000017427
IGF1R	-0.1626	8.1289E-02	2.03E-01	ENSG00000140443
IGF2	0.2548	4.9238E-02	1.42E-01	ENSG00000167244
IGF2-AS	-0.0478	3.4514E-01		ENSG00000099869
IGF2BP1	0.0905	2.4905E-01	4.35E-01	ENSG00000159217
IGF2BP2	0.1893	1.5318E-02	5.89E-02	ENSG00000073792
IGF2BP3	0.2400	4.8212E-03	2.43E-02	ENSG00000136231
IGF2R	-0.1051	1.7158E-01	3.40E-01	ENSG00000197081
IGFALS	0.0940	3.4582E-01	5.41E-01	ENSG00000099769
IGFBP1	-0.0032	9.3913E-01		ENSG00000146678
IGFBP2	0.0654	4.8194E-01	6.67E-01	ENSG00000115457
IGFBP4	0.0210	8.0970E-01	8.99E-01	ENSG00000141753
IGFBP5	-0.1362	4.7151E-02	1.37E-01	ENSG00000115461

IGFBP6	0.1721	4.7044E-02	1.37E-01	ENSG00000167779
IGFBPL1	-0.4865	6.4587E-05	7.91E-04	ENSG00000137142
IGFL2	-0.0798	2.7300E-01	4.63E-01	ENSG00000204866
IGFL2-AS1	0.0037	6.1908E-01		ENSG00000268621
IGFL3	-0.0064	8.0601E-01		ENSG00000188624
IGFL4	0.0425	6.4609E-01	7.95E-01	ENSG00000204869
IGFLR1	-0.0587	5.4132E-01	7.14E-01	ENSG00000126246
IGFN1	-0.0402	3.3622E-01		ENSG00000163395
IGHEP2	-0.0197	7.1629E-01		ENSG00000254017
IGHMBP2	-0.0525	4.3483E-01	6.26E-01	ENSG00000132740
IGIP	-0.0779	4.1668E-01	6.09E-01	ENSG00000182700
IGKV1-12	0.0219	2.7812E-01		ENSG00000243290
IGKV1-22	0.0117	3.1551E-01		ENSG00000253578
IGKV1-6	-0.0021	9.8375E-01		ENSG00000239855
IGKV1D-43	0.0513	3.3333E-02	1.06E-01	ENSG00000242580
IGKV1D-8	0.0172	9.5047E-01	9.75E-01	ENSG00000239819
IGKV1OR-2	0.0134	4.2744E-01		ENSG00000156755
IGKV3-7	0.0104	5.4352E-01		ENSG00000243063
IGKV3D-11	-0.0319	3.3201E-01	5.27E-01	ENSG00000211632
IGKV3D-7	0.0728	6.2244E-03	2.97E-02	ENSG00000228325
IGKV6D-41	0.0047	7.6622E-01		ENSG00000211626
IGLCOR22-2	-0.0064	9.2911E-01		ENSG00000243519
IGLON5	0.0542	5.7086E-01	7.39E-01	ENSG00000142549
IGLV1-47	0.0239	7.7528E-02		ENSG00000211648
IGLV1-50	0.0090	6.8372E-01		ENSG00000211645
IGLV6-57	-0.0111	5.4972E-01		ENSG00000211640
IGLV7-43	-0.0014	7.6484E-01		ENSG00000211652
IGLV7-46	-0.0311	7.3612E-01		ENSG00000211649
IGLVI-42	0.0283	2.8559E-01		ENSG00000254175
IGSF1	-0.0438	5.6416E-01	7.33E-01	ENSG00000147255
IGSF10	0.0942	3.1255E-01	5.06E-01	ENSG00000152580
IGSF11	-0.4033	9.6533E-03	4.14E-02	ENSG00000144847
IGSF21	-0.2341	5.2182E-03	2.59E-02	ENSG00000117154
IGSF22	0.1080	2.8418E-01	4.76E-01	ENSG00000179057
IGSF3	-0.4563	1.7762E-06	4.27E-05	ENSG00000143061
IGSF6	-0.0321	6.9660E-01	8.30E-01	ENSG00000140749
IGSF8	0.0784	2.9683E-01	4.89E-01	ENSG00000162729
IGSF9	0.0669	3.3034E-01	5.25E-01	ENSG00000085552
IGSF9B	0.1609	5.1821E-02	1.47E-01	ENSG00000080854
IHH	-0.0035	8.3829E-01		ENSG00000163501
IHO1	0.0862	3.9002E-01	5.84E-01	ENSG00000173421
IK	-0.1448	3.9507E-03	2.09E-02	ENSG00000113141
IKBIP	-0.0480	4.4331E-01	6.34E-01	ENSG00000166130
IKBKB	0.1165	1.5557E-01	3.18E-01	ENSG00000104365
IKBKE	-0.0242	7.7400E-01	8.78E-01	ENSG00000263528
IKBKE-AS1	0.0003	8.0301E-01		ENSG00000162888
IKBKG	0.0527	4.5574E-01	6.45E-01	ENSG00000269335
IKBKGP1	0.2215	1.7143E-02	6.41E-02	ENSG00000275882
IKZF1	-0.0764	3.8894E-01	5.83E-01	ENSG00000185811

IKZF2	-0.0874	3.7802E-01	5.73E-01	ENSG00000030419
IKZF3	-0.2200	2.2477E-02	7.87E-02	ENSG00000161405
IKZF4	-0.0715	3.6617E-01	5.62E-01	ENSG00000123411
IKZF5	0.0620	3.8557E-01	5.80E-01	ENSG00000095574
IL10RA	0.0328	7.3361E-01	8.54E-01	ENSG00000110324
IL11	-0.2977	2.4129E-02	8.29E-02	ENSG00000095752
IL11RA	0.2461	3.8624E-02	1.18E-01	ENSG00000137070
IL12A	-0.0755	3.9931E-01	5.94E-01	ENSG00000168811
IL12A-AS1	0.0321	8.1492E-02		ENSG00000244040
IL12B	-0.0220	5.0592E-01		ENSG00000113302
IL12RB1	-0.0152	5.2857E-01		ENSG00000096996
IL12RB2	0.0313	7.2923E-01	8.51E-01	ENSG00000081985
IL13	-0.0379	8.8809E-01		ENSG00000169194
IL13RA1	-0.0231	7.3564E-01	8.55E-01	ENSG00000131724
IL13RA2	-0.1573	6.2859E-02	1.69E-01	ENSG00000123496
IL15	-0.1372	1.2023E-01	2.67E-01	ENSG00000164136
IL15RA	0.0389	6.8241E-01	8.20E-01	ENSG00000134470
IL16	0.0407	6.3333E-01	7.86E-01	ENSG00000172349
IL17B	0.0651	2.4395E-01	4.29E-01	ENSG00000127743
IL17C	-0.0202	9.9419E-01		ENSG00000124391
IL17D	-0.4263	4.5466E-06	9.31E-05	ENSG00000172458
IL17RA	0.1412	1.0454E-01	2.42E-01	ENSG00000177663
IL17RB	-0.1651	1.2246E-01	2.71E-01	ENSG00000056736
IL17RC	-0.0245	7.3430E-01	8.54E-01	ENSG00000163702
IL17RD	-0.1559	9.0246E-02	2.19E-01	ENSG00000144730
IL17RE	-0.1546	1.2664E-01	2.77E-01	ENSG00000163701
IL17REL	0.1250	8.2802E-02	2.06E-01	ENSG00000188263
IL18	-0.4169	2.2709E-03	1.36E-02	ENSG00000150782
IL18BP	-0.0119	8.8067E-01	9.39E-01	ENSG00000137496
IL18R1	0.0054	9.5026E-01	9.75E-01	ENSG00000115604
IL19	-0.0025	8.8440E-01		ENSG00000142224
IL1A	-0.1569	5.8019E-02	1.59E-01	ENSG00000115008
IL1B	0.0245	3.9626E-01		ENSG00000125538
IL1R1	0.0635	4.3652E-01	6.28E-01	ENSG00000115594
IL1R2	0.0043	2.0402E-01		ENSG00000115590
IL1RAP	0.5115	8.5852E-04	6.33E-03	ENSG00000196083
IL1RAPL1	0.0314	7.0852E-01	8.37E-01	ENSG00000169306
IL1RAPL2	-0.0024	9.5372E-01	9.77E-01	ENSG00000189108
IL1RN	0.0260	7.1054E-01	8.39E-01	ENSG00000136689
IL20RA	0.0701	4.6839E-01	6.56E-01	ENSG00000016402
IL20RB	0.1213	2.4220E-01	4.27E-01	ENSG00000174564
IL21R	-0.1949	2.5102E-02		ENSG00000103522
IL21R-AS1	-0.0293	6.7876E-01	8.17E-01	ENSG00000259954
IL22	0.1014	1.0649E-01	2.45E-01	ENSG00000127318
IL22RA1	0.0705	4.0208E-01	5.96E-01	ENSG00000142677
IL23A	-0.0647	5.0306E-01	6.84E-01	ENSG00000110944
IL26	-0.0049	7.9791E-01		ENSG00000111536
IL27	0.0046	8.0523E-01		ENSG00000197272
IL27RA	-0.1600	6.2958E-02	1.69E-01	ENSG00000104998

IL2RB	-0.0128	7.2287E-01		ENSG00000100385
IL31RA	0.0718	4.4532E-01	6.35E-01	ENSG00000164509
IL32	-0.1998	9.7193E-03	4.16E-02	ENSG00000008517
IL33	-0.0541	5.6589E-01	7.34E-01	ENSG00000137033
IL34	0.1583	1.3685E-01	2.91E-01	ENSG00000157368
IL36G	0.1704	5.3664E-02	1.51E-01	ENSG00000136688
IL36RN	-0.0059	8.7505E-01		ENSG00000136695
IL37	0.0494	1.0061E-01	2.36E-01	ENSG00000125571
IL3RA	-0.0023	9.7783E-01	9.88E-01	ENSG00000185291
IL4	-0.0073	9.2933E-01		ENSG00000113520
IL4I1	-0.0527	4.4494E-01	6.35E-01	ENSG00000104951
IL4R	-0.0484	5.9005E-01	7.53E-01	ENSG00000077238
IL5	0.0284	2.6011E-01		ENSG00000113525
IL5RA	0.0156	7.3955E-02	1.90E-01	ENSG00000091181
IL6	-0.0479	3.7215E-01	5.67E-01	ENSG00000136244
IL6R	-0.0616	5.2079E-01	6.98E-01	ENSG00000160712
IL6R-AS1	-0.1070	2.7824E-01	4.69E-01	ENSG00000228013
IL6ST	-0.1848	1.4410E-02	5.63E-02	ENSG00000134352
IL6ST-DT	0.0381	6.5736E-01	8.02E-01	ENSG00000227908
IL7	-0.4653	6.0718E-03	2.91E-02	ENSG00000104432
IL7R	-0.0060	9.8781E-01		ENSG00000168685
ILDR1	0.1450	1.6513E-01	3.32E-01	ENSG00000145103
ILDR2	-0.0334	7.2924E-01	8.51E-01	ENSG00000143195
ILF2	-0.2462	5.2465E-04	4.28E-03	ENSG00000143621
ILF2P1	-0.0290	3.0412E-01		ENSG00000244226
ILF3	-0.1670	8.2516E-03	3.68E-02	ENSG00000129351
ILF3-DT	0.3873	8.0045E-10	7.13E-08	ENSG00000267100
ILK	0.2522	2.4583E-02	8.42E-02	ENSG00000166333
ILKAP	-0.2197	6.1057E-05	7.55E-04	ENSG00000132323
ILRUN	-0.1312	1.0002E-01	2.35E-01	ENSG00000196821
ILRUN-AS1	-0.3547	7.9584E-04	5.97E-03	ENSG00000272288
ILVBL	0.1795	5.3155E-03	2.62E-02	ENSG00000105135
IMMP1L	-0.2155	1.9735E-04	1.94E-03	ENSG00000148950
IMMP2L	-0.3909	8.9261E-04	6.51E-03	ENSG00000184903
IMMT	0.0237	6.5544E-01	8.01E-01	ENSG00000132305
IMMTP1	-0.0091	7.8245E-01	8.83E-01	ENSG00000229880
IMP3	-0.0295	5.1717E-01	6.95E-01	ENSG00000177971
IMP4	0.1221	1.3952E-02	5.49E-02	ENSG00000136718
IMPA1	-0.1300	4.4349E-02	1.31E-01	ENSG00000133731
IMPA2	0.1351	1.7771E-01	3.49E-01	ENSG00000141401
IMPACT	0.1379	1.4470E-02	5.65E-02	ENSG00000154059
IMPDH1	0.0062	9.6078E-01	9.80E-01	ENSG00000106348
IMPDH2	0.2325	3.3710E-05	4.63E-04	ENSG00000178035
IMPG1	0.0051	7.4548E-01		ENSG00000112706
IMPG2	-0.2549	2.9447E-02	9.61E-02	ENSG00000081148
INA	0.0294	6.9531E-01	8.29E-01	ENSG00000148798
INAFM1	0.4280	1.5030E-05	2.44E-04	ENSG00000257704
INAFM2	0.1287	4.0359E-02	1.22E-01	ENSG00000259330
INAVA	0.0802	3.8806E-01	5.83E-01	ENSG00000163362

INCA1	0.3992	9.6145E-05	1.08E-03	ENSG00000196388
INCENP	-0.0369	7.0110E-01	8.33E-01	ENSG00000149503
INE1	0.3742	1.0039E-02	4.26E-02	ENSG00000224975
INF2	-0.3578	5.4289E-03	2.67E-02	ENSG00000203485
ING1	-0.2892	2.4781E-05	3.63E-04	ENSG00000153487
ING2	0.0818	3.5721E-01	5.53E-01	ENSG00000168556
ING3	-0.0978	1.8772E-01	3.62E-01	ENSG00000071243
ING4	0.0766	1.3984E-01	2.96E-01	ENSG00000111653
ING5	-0.0187	7.2106E-01	8.45E-01	ENSG00000168395
INGX	0.1291	1.1491E-01	2.59E-01	ENSG00000243468
INHBA	-0.1095	2.7473E-01	4.65E-01	ENSG00000122641
INHBA-AS1	0.3417	1.7794E-02	6.60E-02	ENSG00000224116
INHBC	0.5551	4.3207E-03	2.24E-02	ENSG00000175189
INHBE	0.0173	7.9043E-01	8.88E-01	ENSG00000139269
INIP	0.2825	3.4716E-04	3.08E-03	ENSG00000148153
INKA2	0.1873	1.8709E-02	6.86E-02	ENSG00000197852
INKA2-AS1	0.1268	2.2783E-01	4.11E-01	ENSG00000227811
INMT	0.1958	7.5655E-02	1.93E-01	ENSG00000241644
INO80	-0.0232	7.6221E-01	8.71E-01	ENSG00000128908
INO80B	0.0917	1.4544E-01	3.04E-01	ENSG00000115274
INO80C	-0.2503	4.4689E-05	5.88E-04	ENSG00000153391
INO80D	-0.0462	5.2075E-01	6.98E-01	ENSG00000114933
INO80E	-0.1218	1.5251E-01	3.14E-01	ENSG00000169592
INPP1	0.1269	1.1735E-01	2.63E-01	ENSG00000151689
INPP4A	0.1447	6.7396E-02	1.77E-01	ENSG00000040933
INPP4B	0.0013	9.7295E-01	9.86E-01	ENSG00000109452
INPP5A	0.1721	2.8842E-03	1.64E-02	ENSG00000068383
INPP5B	0.2129	8.1967E-03	3.66E-02	ENSG00000204084
INPP5D	0.3200	1.4513E-02	5.66E-02	ENSG00000168918
INPP5E	0.1690	2.6867E-02	8.98E-02	ENSG00000148384
INPP5F	0.1496	1.0977E-01	2.51E-01	ENSG00000198825
INPP5J	0.0997	1.9057E-01	3.66E-01	ENSG00000185133
INPP5K	0.2639	9.8618E-04	7.03E-03	ENSG00000132376
INPPL1	-0.0881	1.9731E-01	3.74E-01	ENSG00000165458
INS	0.0106	4.1834E-01	6.11E-01	ENSG00000254647
INSC	-0.0418	4.7999E-01	6.66E-01	ENSG00000188487
INSIG1	-0.0512	4.5719E-01	6.46E-01	ENSG00000186480
INSIG2	0.2289	1.0411E-02	4.37E-02	ENSG00000125629
INSL3	0.0679	4.8652E-01	6.71E-01	ENSG00000248099
INSL4	-0.0094	8.6612E-01		ENSG00000120211
INSL5	0.0158	5.7740E-01		ENSG00000172410
INSL6	0.0105	5.1003E-01		ENSG00000120210
INSM1	0.2140	4.8938E-02	1.41E-01	ENSG00000173404
INSM2	0.1719	1.0452E-01	2.42E-01	ENSG00000168348
INSR	0.1996	4.6557E-03	2.37E-02	ENSG00000171105
INSRR	-0.0592	1.1336E-01		ENSG00000027644
INSYN1	-0.1979	3.1250E-02	1.00E-01	ENSG00000205363
INSYN2A	-0.0043	9.5475E-01	9.77E-01	ENSG00000188916
INSYN2B	0.1151	2.4050E-01	4.25E-01	ENSG00000204767

INTS1	-0.0344	5.9509E-01	7.57E-01	ENSG00000164880
INTS10	0.2792	5.3749E-05	6.80E-04	ENSG00000104613
INTS11	-0.0243	5.5344E-01	7.25E-01	ENSG00000127054
INTS12	0.0012	9.8349E-01	9.91E-01	ENSG00000138785
INTS13	-0.0117	8.6670E-01	9.32E-01	ENSG00000064102
INTS14	0.1117	9.6490E-02	2.29E-01	ENSG00000138614
INTS15	0.0984	1.7401E-01	3.44E-01	ENSG00000146576
INTS3	-0.1648	7.2272E-03	3.32E-02	ENSG00000143624
INTS4	0.0500	5.0589E-01	6.87E-01	ENSG00000149262
INTS4P2	0.3439	2.7647E-03	1.58E-02	ENSG00000273024
INTS5	0.1173	1.5638E-01	3.19E-01	ENSG00000185085
INTS6	0.0662	2.6177E-01	4.51E-01	ENSG00000102786
INTS6-AS1	-0.0164	8.2377E-01	9.07E-01	ENSG00000236778
INTS6L	0.1639	1.1503E-01	2.59E-01	ENSG00000165359
INTS6L-AS1	-0.0102	8.8101E-01		ENSG00000225235
INTS7	-0.0105	9.0627E-01	9.53E-01	ENSG00000143493
INTS8	0.0287	6.6936E-01	8.10E-01	ENSG00000164941
INTS9	0.1304	3.7271E-02	1.15E-01	ENSG00000104299
INTU	-0.2293	2.0705E-02	7.39E-02	ENSG00000164066
INVS	0.0319	6.6514E-01	8.08E-01	ENSG00000119509
IP6K1	0.3126	7.0788E-05	8.48E-04	ENSG00000176095
IP6K2	-0.2679	8.3749E-06	1.53E-04	ENSG00000068745
IP6K3	0.0176	8.5323E-01	9.25E-01	ENSG00000161896
IPCEF1	-0.1064	2.9284E-01	4.85E-01	ENSG00000074706
IPMK	0.1504	1.0708E-01	2.46E-01	ENSG00000151151
IPO11	0.0979	2.2231E-01	4.04E-01	ENSG00000086200
IPO13	0.0712	3.1132E-01	5.04E-01	ENSG00000117408
IPO4	-0.3244	3.2630E-02	1.04E-01	ENSG00000196497
IPO5	-0.2502	5.9399E-05	7.39E-04	ENSG00000065150
IPO7	-0.4353	9.4436E-05	1.07E-03	ENSG00000205339
IPO8	-0.3361	1.0237E-03	7.24E-03	ENSG00000133704
IPO9	0.0193	7.4664E-01	8.61E-01	ENSG00000198700
IPO9-AS1	-0.1666	9.0040E-02	2.19E-01	ENSG00000231871
IPP	0.0258	7.2008E-01	8.45E-01	ENSG00000197429
IPPK	0.3494	5.8613E-03	2.83E-02	ENSG00000127080
IQANK1	-0.0978	2.8474E-01	4.76E-01	ENSG00000203499
IQCA1	-0.0133	8.8287E-01	9.41E-01	ENSG00000132321
IQCA1-AS1	0.1298	3.9050E-03		ENSG00000232893
IQCA1L	-0.0200	3.5300E-01		ENSG00000278685
IQCB1	0.1052	1.6520E-01	3.32E-01	ENSG00000173226
IQCC	0.2230	1.9739E-02	7.13E-02	ENSG00000160051
IQCD	-0.1679	2.0538E-02	7.34E-02	ENSG00000166578
IQCE	-0.2188	2.2991E-03	1.37E-02	ENSG00000106012
IQCF1	-0.0293	8.2711E-01		ENSG00000173389
IQCF4P	0.0465	2.5259E-01		ENSG00000224792
IQCG	-0.0299	6.5336E-01	8.00E-01	ENSG00000114473
IQCH	0.0930	2.8350E-01	4.75E-01	ENSG00000103599
IQCH-AS1	-0.2986	4.1141E-03	2.16E-02	ENSG00000259673
IQCJ	-0.0300	7.0826E-01	8.37E-01	ENSG00000214216

IQCK	-0.0547	4.5255E-01	6.42E-01	ENSG00000174628
IQCN	-0.2209	2.4431E-02	8.37E-02	ENSG00000130518
IQGAP1	-0.2212	3.6847E-07	1.15E-05	ENSG00000140575
IQGAP2	-0.0634	4.7760E-01	6.64E-01	ENSG00000145703
IQSEC1	0.1977	1.2941E-03	8.72E-03	ENSG00000144711
IQSEC2	-0.0766	3.0350E-01	4.96E-01	ENSG00000124313
IQSEC3	0.3212	1.4710E-02	5.72E-02	ENSG00000120645
IQSEC3P2	0.0085	6.9240E-01		ENSG00000278654
IQSEC3P3	0.1084	2.4622E-01	4.32E-01	ENSG00000274727
IQUB	0.0134	8.7961E-01	9.39E-01	ENSG00000164675
IRAG1	-0.1516	1.6374E-01	3.30E-01	ENSG00000072952
IRAG1-AS1	-0.1737	1.1993E-01	2.67E-01	ENSG00000177112
IRAG2	-0.0849	2.7675E-01	4.67E-01	ENSG00000118308
IRAK1	0.2648	3.2727E-03	1.80E-02	ENSG00000184216
IRAK1BP1	-0.1390	2.8744E-02	9.44E-02	ENSG00000146243
IRAK2	-0.4033	1.1017E-04	1.21E-03	ENSG00000134070
IRAK3	0.2693	4.1849E-02	1.25E-01	ENSG00000090376
IRAK4	-0.1159	2.1704E-01	3.98E-01	ENSG00000198001
IREB2	0.0701	3.3192E-01	5.27E-01	ENSG00000136381
IRF2	0.2724	4.6126E-03	2.35E-02	ENSG00000168310
IRF2BP1	-0.1399	4.7189E-02	1.37E-01	ENSG00000170604
IRF2BPL	-0.3123	9.9330E-06	1.74E-04	ENSG00000119669
IRF3	-0.0137	8.0964E-01	8.99E-01	ENSG00000126456
IRF4	-0.0556	3.4781E-01		ENSG00000137265
IRF5	-0.0105	9.0971E-01	9.55E-01	ENSG00000128604
IRF6	-0.0403	6.3057E-01	7.84E-01	ENSG00000117595
IRF7	-0.0749	3.8683E-01	5.82E-01	ENSG00000185507
IRF8	-0.0121	8.9989E-01		ENSG00000140968
IRF9	0.0045	9.6311E-01	9.81E-01	ENSG00000213928
IRGC	0.0019	7.0690E-01		ENSG00000124449
IRGQ	0.0885	1.7899E-01	3.50E-01	ENSG00000167378
IRS1	-0.1192	1.9463E-01	3.71E-01	ENSG00000169047
IRS2	-0.3799	2.4689E-07	8.36E-06	ENSG00000185950
IRS4	-0.1505	1.6133E-01	3.26E-01	ENSG00000133124
IRX1	0.0764	4.2323E-01	6.15E-01	ENSG00000170549
IRX2	0.4526	5.5475E-03	2.72E-02	ENSG00000170561
IRX3	0.5047	4.6872E-05	6.12E-04	ENSG00000177508
IRX4	0.0487	5.9028E-01	7.54E-01	ENSG00000113430
IRX5	0.4271	2.9849E-03	1.68E-02	ENSG00000176842
IRX6	0.1590	1.4176E-01	2.99E-01	ENSG00000159387
ISCA1	0.0057	9.8176E-01	9.90E-01	ENSG00000135070
ISCA2	-0.0272	6.7081E-01	8.11E-01	ENSG00000165898
ISCU	-0.2082	1.5666E-03	1.01E-02	ENSG00000136003
ISG15	-0.2691	1.6760E-02	6.30E-02	ENSG00000187608
ISG20	0.0267	7.6905E-01	8.75E-01	ENSG00000172183
ISG20L2	0.1308	5.2117E-02	1.47E-01	ENSG00000143319
ISL1	0.3862	8.4591E-03	3.75E-02	ENSG00000016082
ISL1-DT	-0.0194	4.1650E-01		ENSG00000259663
ISL2	-0.0306	7.4313E-01		ENSG00000159556

ISLR	-0.3774	3.0494E-03	1.70E-02	ENSG00000129009
ISLR2	0.1393	1.5812E-01	3.22E-01	ENSG00000167178
ISM1	-0.0392	6.3488E-01	7.87E-01	ENSG00000101230
ISM2	-0.3705	7.3421E-03	3.36E-02	ENSG00000100593
ISOC1	-0.2906	7.9734E-05	9.31E-04	ENSG00000066583
ISOC2	-0.0913	9.1146E-02	2.20E-01	ENSG00000063241
IST1	-0.0348	5.1432E-01	6.93E-01	ENSG00000182149
ISX	0.0327	1.2512E-01		ENSG00000175329
ISY1	0.0700	2.1486E-01	3.95E-01	ENSG00000240682
ISYNA1	-0.2085	8.6702E-03	3.82E-02	ENSG00000105655
ITCH	0.1871	1.7442E-02	6.50E-02	ENSG00000078747
ITCH	0.0334	6.4197E-01	7.92E-01	ENSG00000289720
ITFG1	0.0861	1.4753E-01	3.07E-01	ENSG00000129636
ITFG2	0.0889	2.5257E-01	4.39E-01	ENSG00000111203
ITFG2-AS1	0.0615	4.9427E-01	6.78E-01	ENSG00000258325
ITFG2-AS1	-0.0091	7.1961E-01		ENSG00000256150
ITGA1	-0.1573	1.2630E-01	2.77E-01	ENSG00000213949
ITGA10	-0.0205	8.0478E-01	8.96E-01	ENSG00000143127
ITGA11	0.0923	2.5497E-01	4.42E-01	ENSG00000137809
ITGA3	0.0892	1.8530E-01	3.59E-01	ENSG00000005884
ITGA4	-0.1438	1.8402E-01	3.57E-01	ENSG00000115232
ITGA5	-0.1435	1.0173E-01	2.38E-01	ENSG00000161638
ITGA6	-0.1586	9.0854E-03	3.96E-02	ENSG00000091409
ITGA6-AS1	0.1296	1.1671E-01	2.62E-01	ENSG00000232788
ITGA7	-0.1790	7.3252E-02	1.88E-01	ENSG00000135424
ITGA8	-0.0064	5.5082E-01		ENSG00000077943
ITGA9	0.0110	9.1415E-01	9.57E-01	ENSG00000144668
ITGA9-AS1	0.0661	4.9995E-01	6.82E-01	ENSG00000235257
ITGAE	-0.1010	1.3296E-01	2.86E-01	ENSG00000083457
ITGAL	0.0433	4.3307E-01	6.24E-01	ENSG00000005844
ITGAM	-0.0626	2.1275E-01	3.93E-01	ENSG00000169896
ITGAV	-0.2709	4.0395E-03	2.13E-02	ENSG00000138448
ITGB1	-0.2357	1.9273E-05	2.97E-04	ENSG00000150093
ITGB1-DT	0.2907	3.5188E-02	1.10E-01	ENSG00000229656
ITGB1BP1	0.0377	5.4754E-01	7.19E-01	ENSG00000119185
ITGB1BP2	-0.0041	9.4797E-01	9.74E-01	ENSG00000147166
ITGB2	0.0089	5.6135E-01		ENSG00000160255
ITGB2-AS1	-0.0018	8.7369E-01		ENSG00000227039
ITGB3	-0.0044	6.6858E-01		ENSG00000259207
ITGB3BP	-0.1571	4.6398E-02	1.35E-01	ENSG00000142856
ITGB4	-0.5778	9.9661E-06	1.75E-04	ENSG00000132470
ITGB5	-0.3569	4.2606E-05	5.64E-04	ENSG00000082781
ITGB6	0.0335	6.9861E-01	8.31E-01	ENSG00000115221
ITGB8	-0.5283	1.1335E-06	2.97E-05	ENSG00000105855
ITGB8-AS1	-0.0760	3.6676E-01	5.63E-01	ENSG00000271133
ITGBL1	0.0268	5.3183E-02		ENSG00000198542
ITIH1	-0.0483	1.5077E-01		ENSG00000055957
ITIH2	0.0787	3.8761E-01	5.82E-01	ENSG00000151655
ITIH3	-0.1179	9.8094E-02		ENSG00000162267

ITIH4	-0.0699	5.1502E-01	6.94E-01	ENSG00000055955
ITIH5	-0.0890	3.6844E-01	5.64E-01	ENSG00000123243
ITIH6	-0.0171	6.8745E-01		ENSG00000102313
ITK	-0.0345	5.4302E-01		ENSG00000113263
ITLN1	0.0008	9.2937E-01		ENSG00000179914
ITLN2	0.2280	6.7627E-02	1.78E-01	ENSG00000158764
ITM2A	-0.2018	5.2081E-02	1.47E-01	ENSG00000078596
ITM2B	-0.2037	1.0217E-02	4.32E-02	ENSG00000136156
ITM2C	-0.0508	4.5864E-01	6.48E-01	ENSG00000135916
ITPA	-0.0573	2.9159E-01	4.83E-01	ENSG00000125877
ITPK1	0.2185	4.8750E-03	2.45E-02	ENSG00000100605
ITPK1-AS1	0.0937	3.4967E-01	5.45E-01	ENSG00000258730
ITPKA	0.2721	4.1159E-02	1.24E-01	ENSG00000137825
ITPKB-IT1	-0.0183	9.7784E-01		ENSG00000228382
ITPKC	0.1921	2.9442E-03	1.66E-02	ENSG00000086544
ITPR1	0.2425	5.8070E-03	2.81E-02	ENSG00000150995
ITPR1-DT	-0.0054	9.5074E-01	9.75E-01	ENSG00000231249
ITPR2	0.0273	7.4020E-01	8.57E-01	ENSG00000123104
ITPR3	0.1194	2.4658E-01	4.32E-01	ENSG00000096433
ITPRID2	-0.3410	2.6712E-04	2.47E-03	ENSG00000138434
ITPRIPL1	0.0582	4.2000E-01	6.12E-01	ENSG00000198885
ITPRIPL2	0.0725	4.5415E-01	6.44E-01	ENSG00000205730
ITSN1	-0.0507	4.3376E-01	6.25E-01	ENSG00000205726
ITSN2	0.2176	1.1236E-02	4.64E-02	ENSG00000198399
IVD	-0.2421	8.9534E-06	1.61E-04	ENSG00000128928
IVL	-0.0087	7.8046E-01	8.82E-01	ENSG00000163207
IVNS1ABP	0.1896	9.4756E-03	4.08E-02	ENSG00000116679
IWS1	-0.0246	7.0108E-01	8.33E-01	ENSG00000163166
IYD	-0.0747	7.3857E-01	8.56E-01	ENSG00000009765
IZUMO1	0.0367	2.2208E-01	4.04E-01	ENSG00000182264
IZUMO4	0.0213	8.0597E-01	8.96E-01	ENSG00000099840
JADE1	-0.1128	8.6299E-02	2.12E-01	ENSG00000077684
JADE2	0.0103	9.0645E-01	9.53E-01	ENSG00000043143
JADE3	0.0164	8.6197E-01	9.30E-01	ENSG00000102221
JAG1	-0.2802	6.6125E-03	3.10E-02	ENSG00000101384
JAG2	-0.0463	6.2356E-01	7.79E-01	ENSG00000184916
JAGN1	-0.0249	7.0156E-01	8.33E-01	ENSG00000171135
JAK1	-0.1310	1.9832E-02	7.15E-02	ENSG00000162434
JAK2	0.2197	3.4592E-02	1.09E-01	ENSG00000096968
JAK3	0.3629	5.8716E-04	4.69E-03	ENSG00000105639
JAKMIP1	-0.1373	1.8553E-01	3.59E-01	ENSG00000152969
JAKMIP2	0.1597	3.9786E-02	1.21E-01	ENSG00000176049
JAKMIP2-AS1	0.5880	6.9387E-04	5.35E-03	ENSG00000280780
JAKMIP3	0.2766	2.2671E-02	7.91E-02	ENSG00000188385
JAM2	-0.5427	2.1238E-08	1.08E-06	ENSG00000154721
JAM3	0.0682	4.7921E-01	6.65E-01	ENSG00000166086
JAML	0.0068	2.5161E-01		ENSG00000160593
JARID2	0.2239	1.1097E-02	4.60E-02	ENSG00000008083
JARID2-AS1	0.3198	3.0103E-02	9.77E-02	ENSG00000235488

JAZF1	-0.0616	3.9427E-01	5.89E-01	ENSG00000153814
JCAD	-0.0471	6.0745E-01	7.67E-01	ENSG00000165757
JCHAIN	-0.0617	3.3910E-01	5.34E-01	ENSG00000132465
JDP2	-0.4890	8.2410E-07	2.28E-05	ENSG00000140044
JHY	-0.4519	1.8462E-07	6.51E-06	ENSG00000109944
JKAMP	-0.0952	2.2649E-01	4.09E-01	ENSG00000050130
JMJD1C-AS1	0.0634	4.9708E-01	6.80E-01	ENSG00000272767
JMJD4	-0.0385	6.3015E-01	7.84E-01	ENSG00000081692
JMJD4P1	0.0015	7.9889E-01		ENSG00000249505
JMJD6	-0.1100	1.4280E-01	3.00E-01	ENSG00000070495
JMJD7	0.0406	2.6079E-01		ENSG00000243789
JMJD7-PLA2G4B	0.2417	4.4543E-02	1.31E-01	ENSG00000168970
JMJD8	0.1386	8.4754E-02	2.09E-01	ENSG00000161999
JMY	-0.2995	1.8919E-05	2.92E-04	ENSG00000152409
JOSD1	-0.4021	1.1363E-04	1.24E-03	ENSG00000100221
JOSD2	0.0482	5.6166E-01	7.31E-01	ENSG00000161677
JPH1	0.0991	3.2838E-01	5.23E-01	ENSG00000104369
JPH2	-0.0334	6.0541E-01		ENSG00000149596
JPH3	-0.3064	1.6230E-03	1.04E-02	ENSG00000154118
JPH4	0.0171	8.0821E-01	8.98E-01	ENSG00000092051
JPT1	-0.0906	1.4840E-01	3.09E-01	ENSG00000189159
JPT2	-0.1435	4.3537E-02	1.29E-01	ENSG00000206053
JPX	-0.3430	4.5448E-07	1.38E-05	ENSG00000225470
JRK	-0.0477	6.1481E-01	7.73E-01	ENSG00000234616
JRKL	0.1277	1.6225E-01	3.28E-01	ENSG00000183340
JSRP1	-0.0086	8.2716E-01	9.09E-01	ENSG00000167476
JTB	-0.4748	3.0994E-11	4.38E-09	ENSG00000143543
JUN	-0.2152	1.0950E-02	4.55E-02	ENSG00000177606
JUNB	-0.0659	3.9741E-01	5.92E-01	ENSG00000171223
JUND	-0.3606	2.8569E-08	1.40E-06	ENSG00000130522
JUP	0.0489	4.8043E-01	6.66E-01	ENSG00000173801
KAAG1	-0.0983	2.7196E-01	4.62E-01	ENSG00000146049
KALRN	0.1718	5.9309E-02	1.62E-01	ENSG00000160145
KANK1	-0.1930	3.7727E-02	1.16E-01	ENSG00000107104
KANK2	-0.3304	6.8507E-04	5.30E-03	ENSG00000197256
KANK3	0.4655	1.7593E-06	4.25E-05	ENSG00000186994
KANK4	-0.1723	1.2717E-01	2.78E-01	ENSG00000132854
KANSL1	-0.0809	2.5836E-01	4.46E-01	ENSG00000120071
KANSL1-AS1	-0.1323	1.3900E-01	2.95E-01	ENSG00000214401
KANSL1L	-0.2498	1.3296E-02	5.28E-02	ENSG00000144445
KANSL2	-0.2105	2.5328E-03	1.48E-02	ENSG00000139620
KANSL3	0.0284	6.5057E-01	7.98E-01	ENSG00000114982
KANTR	0.1067	1.8176E-01	3.54E-01	ENSG00000232593
KARS1	0.1050	6.8111E-02	1.79E-01	ENSG00000065427
KARS1P1	-0.0177	6.2160E-01		ENSG00000229696
KARS1P2	-0.0062	5.4664E-01		ENSG00000230371
KASH5	0.0675	5.4182E-02	1.52E-01	ENSG00000161609
KAT14	0.0815	2.3911E-01	4.24E-01	ENSG00000149474
KAT2A	-0.0581	5.1862E-01	6.96E-01	ENSG00000108773

KAT2B	-0.2088	2.1010E-02	7.47E-02	ENSG00000114166
KAT5	0.1880	1.6631E-02	6.27E-02	ENSG00000172977
KAT6A	-0.0657	2.8773E-01	4.79E-01	ENSG00000083168
KAT6B	-0.2035	8.0889E-04	6.03E-03	ENSG00000156650
KAT7	-0.1070	1.2171E-01	2.70E-01	ENSG00000136504
KAT7P1	-0.0052	9.1052E-01		ENSG00000232842
KAT8	-0.0564	2.3701E-01	4.21E-01	ENSG00000103510
KATNA1	0.0127	8.7551E-01	9.37E-01	ENSG00000186625
KATNAL1	-0.0659	3.8675E-01	5.81E-01	ENSG00000102781
KATNAL2	0.1201	1.5061E-01	3.11E-01	ENSG00000167216
KATNB1	0.4606	3.1591E-09	2.22E-07	ENSG00000140854
KATNBL1	0.1740	3.0858E-03	1.72E-02	ENSG00000134152
KATNIP	-0.0065	9.2314E-01	9.62E-01	ENSG00000047578
KAZALD1	-0.0103	9.1389E-01	9.57E-01	ENSG00000107821
KAZN	0.2209	2.5815E-03	1.50E-02	ENSG00000189337
KAZN-AS1	0.0148	3.5913E-01		ENSG00000234593
KBTBD11	0.0729	3.8807E-01	5.83E-01	ENSG00000176595
KBTBD11-AS1	0.0555	5.5596E-01	7.27E-01	ENSG00000253764
KBTBD11-OT1	-0.1354	1.7599E-01	3.46E-01	ENSG00000253696
KBTBD12	0.0436	4.8828E-01	6.72E-01	ENSG00000187715
KBTBD2	-0.0764	1.9144E-01	3.66E-01	ENSG00000170852
KBTBD3	0.1916	1.9305E-02	7.01E-02	ENSG00000182359
KBTBD4	0.0011	9.9987E-01	1.00E+00	ENSG00000123444
KBTBD6	0.0756	3.4985E-01	5.45E-01	ENSG00000165572
KBTBD6-DT	0.0829	3.9893E-01	5.93E-01	ENSG00000278390
KBTBD7	0.1417	6.0397E-02	1.64E-01	ENSG00000120696
KBTBD8	0.0581	5.0511E-01	6.86E-01	ENSG00000163376
KC6	0.1944	5.4130E-03	2.66E-02	ENSG00000267313
KCMF1	-0.0376	4.6784E-01	6.56E-01	ENSG00000176407
KCNA2	-0.1431	1.4422E-01	3.02E-01	ENSG00000177301
KCNA3	0.1637	8.8533E-02	2.16E-01	ENSG00000177272
KCNA4	0.1787	1.2120E-01	2.69E-01	ENSG00000182255
KCNA5	-0.2641	2.1811E-02	7.69E-02	ENSG00000130037
KCNA6	-0.0551	5.4642E-01	7.18E-01	ENSG00000151079
KCNA7	-0.0103	4.8820E-01		ENSG00000104848
KCNAB1	0.1593	9.1435E-02	2.21E-01	ENSG00000169282
KCNAB1-AS2	0.0260	1.1796E-01		ENSG00000240596
KCNAB2	0.3093	1.7311E-03	1.09E-02	ENSG00000069424
KCNB1	0.1516	1.1936E-01	2.66E-01	ENSG00000158445
KCNB2	0.2424	2.8672E-02	9.42E-02	ENSG00000182674
KCNC1	-0.1118	2.4240E-01	4.28E-01	ENSG00000129159
KCNC2	-0.0492	5.3949E-01	7.13E-01	ENSG00000166006
KCNC3	0.1924	9.3696E-02	2.25E-01	ENSG00000131398
KCNC4	-0.0915	3.1928E-01	5.13E-01	ENSG00000116396
KCND1	-0.3719	3.3734E-03	1.84E-02	ENSG00000102057
KCND2	-0.1005	2.5217E-01	4.39E-01	ENSG00000184408
KCND3	-0.0549	4.8959E-01	6.74E-01	ENSG00000171385
KCND3-AS1	-0.0771	9.9667E-01	9.98E-01	ENSG00000237556
KCND3-IT1	-0.0267	6.9486E-01		ENSG00000232558

KCNE1	0.1782	1.0209E-01	2.38E-01	ENSG00000180509
KCNE3	-0.0141	9.0140E-01	9.51E-01	ENSG00000175538
KCNE4	0.3425	2.2846E-02	7.96E-02	ENSG00000152049
KCNE5	-0.0175	8.4231E-01	9.18E-01	ENSG00000176076
KCNF1	-0.1966	5.0129E-02	1.43E-01	ENSG00000162975
KCNG1	-0.3335	7.5698E-03	3.44E-02	ENSG00000026559
KCNG2	0.0106	9.0478E-01	9.52E-01	ENSG00000178342
KCNG3	-0.2343	6.0335E-02	1.64E-01	ENSG00000171126
KCNG4	-0.0586	2.8571E-01		ENSG00000168418
KCNH1	-0.0230	8.8479E-01	9.42E-01	ENSG00000143473
KCNH2	-0.0105	8.8450E-01	9.42E-01	ENSG00000055118
KCNH3	-0.0271	7.7747E-01	8.80E-01	ENSG00000135519
KCNH4	0.1787	1.1370E-01	2.57E-01	ENSG00000089558
KCNH5	0.0642	5.1386E-01	6.93E-01	ENSG00000140015
KCNH6	0.1563	9.0943E-02	2.20E-01	ENSG00000173826
KCNH7	-0.1323	1.1539E-01	2.60E-01	ENSG00000184611
KCNH7-AS1	0.0934	1.2774E-01		ENSG00000237750
KCNH8	0.3180	7.4755E-03	3.41E-02	ENSG00000183960
KCNIP1	0.0204	8.1312E-01	9.01E-01	ENSG00000182132
KCNIP1-AS1	0.0316	2.0637E-01		ENSG00000253591
KCNIP2	0.0126	9.0920E-01	9.55E-01	ENSG00000120049
KCNIP4	0.1266	2.2747E-01	4.10E-01	ENSG00000185774
KCNIP4-IT1	-0.0051	9.4569E-01		ENSG00000280650
KCNJ1	-0.0110	6.2014E-01		ENSG00000151704
KCNJ10	0.0113	2.1918E-01		ENSG00000177807
KCNJ11	-0.2345	2.9330E-02	9.59E-02	ENSG00000187486
KCNJ12	-0.0863	3.8300E-01	5.78E-01	ENSG00000184185
KCNJ13	-0.2771	3.6311E-02	1.13E-01	ENSG00000115474
KCNJ14	0.0709	4.6556E-01	6.54E-01	ENSG00000182324
KCNJ15	-0.0391	7.9267E-01	8.89E-01	ENSG00000157551
KCNJ16	0.5565	6.7389E-08	2.83E-06	ENSG00000153822
KCNJ18	0.0049	8.5944E-01		ENSG00000260458
KCNJ2	0.0964	3.3688E-01	5.32E-01	ENSG00000123700
KCNJ2-AS1	0.2531	1.5399E-02	5.91E-02	ENSG00000267365
KCNJ3	-0.0828	3.9581E-01	5.90E-01	ENSG00000162989
KCNJ4	-0.2580	1.8734E-02	6.86E-02	ENSG00000168135
KCNJ5	0.4572	1.3063E-02	5.21E-02	ENSG00000120457
KCNJ5-AS1	-0.0807	2.6777E-01	4.57E-01	ENSG00000174370
KCNJ6	0.0195	7.5711E-01	8.68E-01	ENSG00000157542
KCNJ9	0.0217	8.1767E-01	9.04E-01	ENSG00000162728
KCNK1	-0.2484	1.3277E-04	1.40E-03	ENSG00000135750
KCNK10	-0.1356	1.6332E-01	3.29E-01	ENSG00000100433
KCNK12	-0.1079	2.6192E-01	4.51E-01	ENSG00000184261
KCNK13	0.0272	6.7563E-01	8.15E-01	ENSG00000152315
KCNK15	-0.0652	4.7280E-01	6.60E-01	ENSG00000124249
KCNK16	0.0191	3.6078E-01		ENSG00000095981
KCNK17	-0.0025	9.9001E-01		ENSG00000124780
KCNK2	0.0236	7.5672E-01	8.68E-01	ENSG00000082482
KCNK3	-0.0860	3.7651E-01	5.72E-01	ENSG00000171303

KCNK4	0.0235	2.4695E-01		ENSG00000182450
KCNK4-CATSPERZ	0.0093	5.2340E-01		ENSG00000257069
KCNK5	-0.0127	6.2017E-01	7.76E-01	ENSG00000164626
KCNK6	-0.2009	7.9768E-02	2.00E-01	ENSG00000099337
KCNK7	0.0121	8.7041E-01	9.34E-01	ENSG00000173338
KCNK9	-0.1162	2.3976E-01	4.24E-01	ENSG00000169427
KCNMA1	-0.1688	6.7852E-02	1.78E-01	ENSG00000156113
KCNMA1-AS1	0.0130	5.6335E-01		ENSG00000236467
KCNMB2	-0.3006	1.1997E-02	4.88E-02	ENSG00000197584
KCNMB3	0.1330	2.0075E-01	3.78E-01	ENSG00000171121
KCNMB4	-0.3151	5.0943E-06	1.02E-04	ENSG00000135643
KCNN1	0.2220	2.3660E-02	8.17E-02	ENSG00000105642
KCNN2	0.2130	6.1964E-02	1.67E-01	ENSG00000080709
KCNN3	-0.2024	1.5600E-02	5.97E-02	ENSG00000143603
KCNN4	0.0464	5.4214E-01	7.15E-01	ENSG00000104783
KCNQ1	0.1964	5.3296E-02	1.50E-01	ENSG00000053918
KCNQ1-AS1	0.0240	5.2267E-01	7.00E-01	ENSG00000229414
KCNQ1DN	0.0222	2.9833E-01		ENSG00000237941
KCNQ1OT1	0.4450	5.1186E-05	6.56E-04	ENSG00000269821
KCNQ2	-0.0137	8.6137E-01	9.29E-01	ENSG00000075043
KCNQ3	-0.0262	7.6310E-01	8.72E-01	ENSG00000184156
KCNQ5	-0.0543	5.6119E-01	7.31E-01	ENSG00000185760
KCNQ5-AS1	-0.0264	8.9877E-01	9.49E-01	ENSG00000229154
KCNRG	0.1835	1.1053E-01	2.52E-01	ENSG00000198553
KCNS1	0.0029	8.7994E-01		ENSG00000124134
KCNS2	-0.1588	1.4039E-01	2.97E-01	ENSG00000156486
KCNS3	0.4617	5.9156E-04	4.71E-03	ENSG00000170745
KCNT1	-0.0909	2.6797E-01	4.57E-01	ENSG00000107147
KCNT2	0.0429	6.3169E-01	7.85E-01	ENSG00000162687
KCNU1	-0.1447	1.6731E-01	3.35E-01	ENSG00000215262
KCNV1	-0.1362	1.4827E-01	3.08E-01	ENSG00000164794
KCNV2	-0.0440	5.0248E-01	6.84E-01	ENSG00000168263
KCP	0.0181	8.4629E-01	9.20E-01	ENSG00000135253
KCTD1	-0.2588	7.7553E-03	3.51E-02	ENSG00000134504
KCTD10	0.2958	9.4835E-07	2.56E-05	ENSG00000110906
KCTD11	0.1738	5.4510E-02	1.52E-01	ENSG00000213859
KCTD12	-0.3677	8.6417E-04	6.35E-03	ENSG00000178695
KCTD13	0.3805	1.0589E-06	2.81E-05	ENSG00000174943
KCTD14	0.0878	3.7078E-01	5.66E-01	ENSG00000151364
KCTD15	-0.2735	2.0321E-02	7.29E-02	ENSG00000153885
KCTD16	0.2118	2.2100E-03	1.33E-02	ENSG00000183775
KCTD17	0.0272	6.4248E-01	7.93E-01	ENSG00000100379
KCTD18	0.0231	6.9255E-01	8.28E-01	ENSG00000155729
KCTD19	-0.0232	8.0067E-01	8.93E-01	ENSG00000168676
KCTD2	-0.3501	3.3986E-04	3.03E-03	ENSG00000180901
KCTD20	0.1253	9.5909E-02	2.28E-01	ENSG00000112078
KCTD21	-0.2356	2.8171E-02	9.29E-02	ENSG00000188997
KCTD21-AS1	-0.4206	8.9832E-03	3.92E-02	ENSG00000246174
KCTD3	0.0300	6.5246E-01	7.99E-01	ENSG00000136636

KCTD4	-0.1867	8.5881E-02	2.11E-01	ENSG00000180332
KCTD5	0.0819	1.8175E-01	3.54E-01	ENSG00000167977
KCTD6	-0.2473	8.0476E-04	6.01E-03	ENSG00000168301
KCTD7	0.1460	1.3128E-01	2.83E-01	ENSG00000243335
KCTD8	0.0577	4.8153E-01	6.67E-01	ENSG00000183783
KCTD9	0.2175	4.1392E-02	1.24E-01	ENSG00000104756
KDELR1	-0.2593	1.2581E-04	1.34E-03	ENSG00000105438
KDELR2	0.0224	7.3401E-01	8.54E-01	ENSG00000136240
KDELR3	0.0546	4.5913E-01	6.48E-01	ENSG00000100196
KDF1	0.0583	5.3614E-01	7.10E-01	ENSG00000175707
KDM1A	0.1335	3.9489E-02	1.20E-01	ENSG00000004487
KDM1B	-0.1952	5.5852E-02	1.55E-01	ENSG00000165097
KDM2A	-0.0688	3.7609E-01	5.71E-01	ENSG00000173120
KDM2B	0.0544	4.3204E-01	6.23E-01	ENSG00000089094
KDM3A	-0.1049	4.3561E-02	1.29E-01	ENSG00000115548
KDM3B	-0.1977	4.0211E-03	2.12E-02	ENSG00000120733
KDM4A	0.0420	6.5463E-01	8.00E-01	ENSG00000066135
KDM4B	0.2280	1.8254E-03	1.14E-02	ENSG00000127663
KDM4C	0.1959	4.8436E-03	2.44E-02	ENSG00000107077
KDM4D	0.2702	6.2065E-04	4.90E-03	ENSG00000186280
KDM5A	-0.0342	6.0236E-01	7.62E-01	ENSG00000073614
KDM5B	0.0068	8.5647E-01	9.26E-01	ENSG00000117139
KDM5C	0.1786	2.3081E-02	8.02E-02	ENSG00000126012
KDM5D	0.0370	5.9877E-01	7.60E-01	ENSG00000012817
KDM6A	0.1018	1.5500E-01	3.18E-01	ENSG00000147050
KDM6B	-0.0433	6.2587E-01	7.81E-01	ENSG00000132510
KDM7A	0.1091	1.4839E-01	3.09E-01	ENSG00000006459
KDM7A-DT	0.1116	2.1687E-01	3.98E-01	ENSG00000260231
KDM8	0.4528	2.4689E-03	1.45E-02	ENSG00000155666
KDR	-0.0408	5.3984E-01	7.13E-01	ENSG00000128052
KDSR	-0.0104	8.2417E-01	9.08E-01	ENSG00000119537
KEAP1	-0.0186	7.5628E-01	8.68E-01	ENSG00000079999
KEL	0.1205	1.4759E-01	3.07E-01	ENSG00000197993
KERA	-0.0351	6.5921E-01	8.03E-01	ENSG00000139330
KHDC1	-0.2499	1.3304E-02	5.29E-02	ENSG00000135314
KHDC1-AS1	-0.0263	7.5565E-01	8.67E-01	ENSG00000229852
KHDC1L	0.0127	1.2792E-01		ENSG00000256980
KHDC3L	0.5870	2.7270E-03	1.57E-02	ENSG00000203908
KHDC4	0.1184	1.2525E-01	2.75E-01	ENSG00000132680
KHDRBS1	-0.1673	1.2701E-04	1.35E-03	ENSG00000121774
KHDRBS2	0.3682	2.3569E-04	2.24E-03	ENSG00000112232
KHDRBS3	-0.1052	1.5123E-01	3.12E-01	ENSG00000131773
KHK	0.0858	2.5249E-01	4.39E-01	ENSG00000138030
KHNYN	0.0167	8.3586E-01	9.14E-01	ENSG00000100441
KHSRP	-0.1602	6.6635E-02	1.76E-01	ENSG00000088247
KIAA0040	0.0514	6.1455E-01	7.73E-01	ENSG00000235750
KIAA0232	0.0071	9.5949E-01	9.80E-01	ENSG00000170871
KIAA0319	-0.0674	4.7955E-01	6.65E-01	ENSG00000137261
KIAA0319L	-0.0813	5.7670E-02	1.59E-01	ENSG00000142687

KIAA0408	-0.0179	9.9745E-01		ENSG00000189367
KIAA0513	0.1154	1.7985E-01	3.51E-01	ENSG00000135709
KIAA0586	0.2309	4.4356E-03	2.28E-02	ENSG00000100578
KIAA0753	-0.4376	4.5166E-06	9.28E-05	ENSG00000198920
KIAA0825	-0.1487	1.2836E-01	2.79E-01	ENSG00000185261
KIAA0930	-0.2952	1.7028E-05	2.70E-04	ENSG00000100364
KIAA1143	-0.1718	1.3624E-02	5.39E-02	ENSG00000163807
KIAA1191	0.0454	4.0498E-01	5.99E-01	ENSG00000122203
KIAA1191P1	0.0071	8.0917E-01		ENSG00000213480
KIAA1217	0.2003	1.3415E-02	5.32E-02	ENSG00000120549
KIAA1328	0.2386	1.6966E-03	1.08E-02	ENSG00000150477
KIAA1549	-0.1286	6.5207E-02	1.73E-01	ENSG00000122778
KIAA1549L	-0.2387	5.0077E-03	2.50E-02	ENSG00000110427
KIAA1586	0.5677	1.1714E-07	4.42E-06	ENSG00000168116
KIAA1614	-0.0004	9.4475E-01	9.72E-01	ENSG00000135835
KIAA1671	-0.4163	7.9046E-05	9.25E-04	ENSG00000197077
KIAA1671-AS1	0.1211	2.4424E-01	4.30E-01	ENSG00000203280
KIAA1755	-0.0125	8.9858E-01		ENSG00000149633
KIAA1958	-0.2245	9.1362E-04	6.63E-03	ENSG00000165185
KIAA2012	-0.2030	7.9817E-02	2.00E-01	ENSG00000182329
KIAA2012-AS1	-0.0146	8.3052E-01		ENSG00000222035
KIAA2013	-0.0322	5.8975E-01	7.53E-01	ENSG00000116685
KIAA2026	0.1033	1.5865E-01	3.23E-01	ENSG00000183354
KICS2	0.1977	4.1709E-02	1.25E-01	ENSG00000174206
KIDINS220	0.0808	3.0785E-01	5.00E-01	ENSG00000134313
KIF11	-0.2412	3.8128E-02	1.17E-01	ENSG00000138160
KIF12	0.0090	9.3300E-01	9.67E-01	ENSG00000136883
KIF13A	-0.1935	3.1181E-02	1.00E-01	ENSG00000137177
KIF13B	-0.0329	6.7589E-01	8.15E-01	ENSG00000197892
KIF14	-0.1308	2.0685E-01	3.86E-01	ENSG00000118193
KIF15	-0.3016	1.4137E-02	5.55E-02	ENSG00000163808
KIF16B	-0.0225	7.4777E-01	8.62E-01	ENSG00000089177
KIF17	0.1625	1.1987E-01	2.67E-01	ENSG00000117245
KIF18A	0.0303	7.5041E-01	8.63E-01	ENSG00000121621
KIF18B	-0.0664	4.8768E-01	6.72E-01	ENSG00000186185
KIF19	0.0010	9.7735E-01	9.88E-01	ENSG00000196169
KIF1A	-0.2285	5.3519E-08	2.35E-06	ENSG00000130294
KIF1B	-0.1713	5.0799E-03	2.53E-02	ENSG00000054523
KIF1C	-0.1434	5.7124E-02	1.58E-01	ENSG00000129250
KIF1C-AS1	-0.0116	9.9003E-01		ENSG00000227495
KIF20A	-0.3911	7.0034E-03	3.24E-02	ENSG00000112984
KIF20B	-0.1788	4.6900E-02	1.36E-01	ENSG00000138182
KIF21A	0.1431	6.3499E-02	1.70E-01	ENSG00000139116
KIF21B	0.0431	6.1290E-01	7.71E-01	ENSG00000116852
KIF22	0.0073	9.3039E-01	9.66E-01	ENSG00000079616
KIF23	-0.1697	1.0389E-01	2.41E-01	ENSG00000137807
KIF23-AS1	-0.3419	9.0319E-03	3.94E-02	ENSG00000259426
KIF24	0.0421	6.6009E-01	8.04E-01	ENSG00000186638
KIF25	-0.0165	8.3088E-01	9.12E-01	ENSG00000125337

KIF25-AS1	-0.0537	4.4781E-01	6.38E-01	ENSG00000229921
KIF26A	0.4674	2.3451E-03	1.39E-02	ENSG00000066735
KIF26B	-0.2665	1.9061E-02	6.94E-02	ENSG00000162849
KIF26B-AS1	-0.0120	9.5703E-01		ENSG00000232192
KIF27	-0.2986	1.7571E-06	4.25E-05	ENSG00000165115
KIF2A	-0.2455	7.0697E-04	5.42E-03	ENSG00000068796
KIF2C	-0.3792	3.6149E-03	1.95E-02	ENSG00000142945
KIF3A	0.1555	3.1686E-02	1.01E-01	ENSG00000131437
KIF3B	-0.1141	1.1312E-01	2.56E-01	ENSG00000101350
KIF3C	-0.1310	7.4489E-02	1.90E-01	ENSG00000084731
KIF4A	-0.3195	8.5346E-03	3.78E-02	ENSG00000090889
KIF4B	0.0163	1.8581E-01		ENSG00000226650
KIF5A	-0.0670	3.5573E-01	5.51E-01	ENSG00000155980
KIF5B	-0.2122	2.5707E-10	2.64E-08	ENSG00000170759
KIF5C	0.1362	7.5237E-02	1.92E-01	ENSG00000168280
KIF6	0.1383	1.9191E-01	3.67E-01	ENSG00000164627
KIF7	-0.0851	3.6242E-01	5.58E-01	ENSG00000166813
KIF9	-0.1888	3.2205E-03	1.77E-02	ENSG00000088727
KIFAP3	-0.0702	2.3036E-01	4.14E-01	ENSG00000075945
KIFBP	0.1065	1.4019E-01	2.96E-01	ENSG00000198954
KIFC1	-0.1643	1.3240E-01	2.85E-01	ENSG00000237649
KIFC2	0.1402	1.3809E-01	2.93E-01	ENSG00000167702
KIFC3	0.1081	1.6562E-01	3.32E-01	ENSG00000140859
KILH	-0.0023	9.7436E-01		ENSG00000231683
KIN	0.2429	2.2548E-03	1.35E-02	ENSG00000151657
KIRREL1	-0.0200	7.9432E-01	8.89E-01	ENSG00000183853
KIRREL2	0.2499	5.4738E-03	2.69E-02	ENSG00000126259
KIRREL3	0.0520	5.8433E-01	7.49E-01	ENSG00000149571
KIRREL3-AS2	-0.0140	8.9315E-01		ENSG00000254960
KIRREL3-AS3	0.0146	1.8984E-01		ENSG00000218109
KISS1	-0.0441	9.4005E-01	9.70E-01	ENSG00000170498
KISS1R	0.4899	1.4852E-03	9.74E-03	ENSG00000116014
KIT	-0.1073	2.8487E-01	4.76E-01	ENSG00000157404
KITLG	-0.1969	2.7502E-02	9.12E-02	ENSG00000049130
KIZ	0.2641	4.2064E-03	2.20E-02	ENSG00000088970
KIZ-AS1	0.0049	6.0690E-01		ENSG00000232712
KL	0.0099	3.9303E-01		ENSG00000133116
KLB	0.1255	2.3363E-01	4.18E-01	ENSG00000134962
KLC1	-0.1235	2.1870E-02	7.71E-02	ENSG00000126214
KLC2	0.1050	1.1941E-01	2.66E-01	ENSG00000174996
KLC3	0.2659	2.1800E-02	7.69E-02	ENSG00000104892
KLC4	0.0932	1.5179E-01	3.13E-01	ENSG00000137171
KLF1	0.0503	4.8891E-01	6.73E-01	ENSG00000105610
KLF11	-0.0959	2.5284E-01	4.40E-01	ENSG00000172059
KLF12	-0.0354	6.2467E-01	7.80E-01	ENSG00000118922
KLF13	-0.2407	1.2310E-02	4.98E-02	ENSG00000169926
KLF14	-0.0334	5.6854E-01		ENSG00000266265
KLF16	-0.0572	5.5802E-01	7.28E-01	ENSG00000129911
KLF2	-0.0200	8.3059E-01	9.11E-01	ENSG00000127528

KLF2P2	0.0255	7.8581E-01	8.85E-01	ENSG00000230646
KLF3	-0.0646	2.6807E-01	4.57E-01	ENSG00000109787
KLF4	-0.1719	9.0307E-02	2.19E-01	ENSG00000136826
KLF4P1	-0.0002	8.9180E-01		ENSG00000233186
KLF5	-0.1214	3.4280E-02	1.08E-01	ENSG00000102554
KLF6	-0.4431	1.7686E-10	1.91E-08	ENSG00000067082
KLF7	0.1045	2.1985E-01	4.01E-01	ENSG00000118263
KLF8	-0.0504	5.7874E-01	7.45E-01	ENSG00000102349
KLHDC1	0.1103	2.5780E-01	4.46E-01	ENSG00000197776
KLHDC10	0.0141	8.7456E-01	9.37E-01	ENSG00000128607
KLHDC2	0.1520	5.2015E-02	1.47E-01	ENSG00000165516
KLHDC3	0.0390	5.6589E-01	7.34E-01	ENSG00000124702
KLHDC4	0.0565	3.8459E-01	5.79E-01	ENSG00000104731
KLHDC7A	-0.0578	4.8106E-01	6.66E-01	ENSG00000179023
KLHDC7B	-0.0047	7.9159E-01		ENSG00000130487
KLHDC8A	-0.2770	9.6310E-03	4.13E-02	ENSG00000162873
KLHDC8B	-0.2592	1.0277E-03	7.26E-03	ENSG00000185909
KLHDC9	-0.1391	2.8221E-03	1.61E-02	ENSG00000162755
KLHL1	-0.0983	2.0729E-01	3.86E-01	ENSG00000150361
KLHL10	-0.0306	5.8437E-01		ENSG00000161594
KLHL11	-0.1292	1.7987E-01	3.51E-01	ENSG00000178502
KLHL12	0.0202	8.1403E-01	9.02E-01	ENSG00000117153
KLHL13	-0.1705	7.6590E-02	1.94E-01	ENSG00000003096
KLHL14	-0.3558	1.5733E-02	6.01E-02	ENSG00000197705
KLHL15	-0.5596	3.7438E-12	6.64E-10	ENSG00000174010
KLHL17	-0.1074	1.7734E-01	3.48E-01	ENSG00000187961
KLHL18	0.1291	7.4245E-02	1.90E-01	ENSG00000114648
KLHL2	0.2292	6.2374E-03	2.97E-02	ENSG00000109466
KLHL20	0.3068	3.4763E-04	3.09E-03	ENSG00000076321
KLHL21	0.2977	1.5463E-03	1.00E-02	ENSG00000162413
KLHL22	-0.0688	3.8248E-01	5.77E-01	ENSG00000099910
KLHL23	-0.2389	2.0970E-03	1.27E-02	ENSG00000213160
KLHL24	0.3798	8.5270E-06	1.55E-04	ENSG00000114796
KLHL25	0.1580	8.5281E-02	2.10E-01	ENSG00000183655
KLHL26	0.2512	1.0599E-03	7.43E-03	ENSG00000167487
KLHL28	-0.0377	6.3198E-01	7.85E-01	ENSG00000179454
KLHL29	-0.0390	6.4508E-01	7.94E-01	ENSG00000119771
KLHL2P1	0.0193	6.8985E-01	8.26E-01	ENSG00000250412
KLHL3	-0.0565	5.5111E-01	7.22E-01	ENSG00000146021
KLHL30	0.0709	2.7205E-01		ENSG00000168427
KLHL31	-0.0760	4.3423E-01	6.25E-01	ENSG00000124743
KLHL32	0.0082	9.2517E-01	9.63E-01	ENSG00000186231
KLHL33	-0.0074	7.4696E-01		ENSG00000185271
KLHL34	0.0134	4.9115E-01		ENSG00000185915
KLHL35	0.2538	1.1385E-02	4.69E-02	ENSG00000149243
KLHL36	-0.0974	1.3555E-01	2.89E-01	ENSG00000135686
KLHL38	-0.0332	5.9959E-01		ENSG00000175946
KLHL4	-0.2567	5.2298E-02	1.48E-01	ENSG00000102271
KLHL40	0.0177	5.9370E-01		ENSG00000157119

KLHL41	-0.5431	1.3787E-03	9.18E-03	ENSG00000239474
KLHL42	-0.1816	4.8409E-03	2.44E-02	ENSG00000087448
KLHL5	0.1045	1.5247E-01	3.14E-01	ENSG00000109790
KLHL6	0.2324	5.4917E-02	1.53E-01	ENSG00000172578
KLHL6-AS1	0.0036	5.6252E-01		ENSG00000242522
KLHL7	0.1216	8.7736E-02	2.14E-01	ENSG00000122550
KLHL8	0.1856	1.1511E-02	4.73E-02	ENSG00000145332
KLHL9	-0.1267	2.1368E-02	7.57E-02	ENSG00000198642
KLK1	0.0852	4.9658E-02	1.42E-01	ENSG00000167748
KLK10	0.0525	5.8457E-01	7.50E-01	ENSG00000129451
KLK11	0.2205	6.3331E-02	1.70E-01	ENSG00000167757
KLK12	-0.1670	1.3292E-01	2.86E-01	ENSG00000186474
KLK13	0.0477	3.8823E-01	5.83E-01	ENSG00000167759
KLK14	-0.0306	8.8165E-01		ENSG00000129437
KLK15	0.0522	3.8293E-01		ENSG00000174562
KLK2	-0.0146	8.0783E-01		ENSG00000167751
KLK3	0.0056	5.9752E-01		ENSG00000142515
KLK4	0.0875	3.1816E-01	5.12E-01	ENSG00000167749
KLK5	0.0385	3.6568E-01	5.62E-01	ENSG00000167754
KLK6	0.0707	2.7502E-01	4.66E-01	ENSG00000167755
KLK7	-0.0078	6.1679E-01		ENSG00000169035
KLK8	0.0581	4.3948E-02		ENSG00000129455
KLKB1	-0.0813	2.2542E-01	4.08E-01	ENSG00000164344
KLLN	-0.0884	3.7484E-01	5.70E-01	ENSG00000227268
KLRB1	0.0069	7.0378E-01		ENSG00000111796
KLRC1	0.0082	5.1910E-01		ENSG00000134545
KLRC2	0.1110	2.7949E-01	4.71E-01	ENSG00000205809
KLRC3	-0.1362	5.6624E-02	1.57E-01	ENSG00000205810
KLRC4	0.0042	7.9347E-01		ENSG00000183542
KLRD1	-0.2503	3.3461E-02	1.06E-01	ENSG00000134539
KLRG1	-0.4108	1.3456E-03	9.01E-03	ENSG00000139187
KLRG2	0.0575	2.7434E-01	4.65E-01	ENSG00000188883
KLRK1-AS1	-0.1545	1.3748E-01	2.92E-01	ENSG00000245648
KMO	-0.3255	1.9011E-02	6.93E-02	ENSG00000117009
KMT2A	0.1263	1.1744E-01	2.63E-01	ENSG00000118058
KMT2B	-0.0650	4.7338E-01	6.60E-01	ENSG00000272333
KMT2C	0.0455	5.1605E-01	6.95E-01	ENSG00000055609
KMT2CP3	-0.0101	7.6409E-01		ENSG00000274927
KMT2D	0.1496	7.9295E-02	1.99E-01	ENSG00000167548
KMT2E	0.0256	5.7916E-01	7.45E-01	ENSG00000005483
KMT5A	-0.3130	1.2076E-04	1.30E-03	ENSG00000183955
KMT5AP2	-0.0133	8.2442E-01		ENSG00000232531
KMT5B	-0.0197	8.0153E-01	8.94E-01	ENSG00000110066
KMT5C	0.3656	1.0302E-04	1.15E-03	ENSG00000133247
KNCN	-0.0208	8.1735E-01	9.03E-01	ENSG00000162456
KNDC1	-0.0228	7.8023E-01	8.81E-01	ENSG00000171798
KNL1	-0.2158	7.5841E-02	1.93E-01	ENSG00000137812
KNOP1	0.1045	9.2423E-02	2.23E-01	ENSG00000103550
KNOP1P2	0.0115	5.0690E-01		ENSG00000270429

KNSTRN	0.1233	6.5036E-02	1.73E-01	ENSG00000128944
KNTC1	0.1396	1.3564E-01	2.89E-01	ENSG00000184445
KPNA1	0.0674	3.0342E-01	4.96E-01	ENSG00000114030
KPNA2	-0.0495	5.0592E-01	6.87E-01	ENSG00000182481
KPNA3	0.0223	7.0921E-01	8.38E-01	ENSG00000102753
KPNA4	-0.1983	1.3398E-02	5.32E-02	ENSG00000186432
KPNA4P1	-0.0250	4.5802E-01	6.47E-01	ENSG00000213754
KPNA5	-0.0531	4.9781E-01	6.81E-01	ENSG00000196911
KPNA6	-0.1661	1.0743E-02	4.48E-02	ENSG00000025800
KPNA7	-0.0008	9.8943E-01	9.94E-01	ENSG00000185467
KPNB1	-0.1984	1.7990E-02	6.66E-02	ENSG00000108424
KPNB1-DT	-0.1603	1.3609E-01	2.90E-01	ENSG00000263766
KPTN	-0.1974	3.7436E-02	1.15E-01	ENSG00000118162
KRAS	0.1191	6.1976E-02	1.67E-01	ENSG00000133703
KRBA1	-0.2290	1.5104E-02	5.83E-02	ENSG00000133619
KRBA2	0.4561	1.1696E-08	6.55E-07	ENSG00000184619
KRBOX1	0.0596	3.3290E-01		ENSG00000240747
KRBOX4	-0.0556	4.5537E-01	6.45E-01	ENSG00000147121
KRBOX5	0.1917	1.7318E-03	1.09E-02	ENSG00000197302
KRCC1	-0.2862	1.6549E-05	2.64E-04	ENSG00000172086
KREMEN1	-0.2914	5.1779E-07	1.53E-05	ENSG00000183762
KREMEN2	0.5293	2.1574E-06	5.03E-05	ENSG00000131650
KRI1	-0.0359	6.0376E-01	7.64E-01	ENSG00000129347
KRIT1	0.2402	1.2718E-02	5.11E-02	ENSG00000001631
KRR1	-0.2096	4.1038E-05	5.46E-04	ENSG00000111615
KRT10	0.0275	6.5735E-01	8.02E-01	ENSG00000186395
KRT10-AS1	0.0216	8.1947E-01	9.05E-01	ENSG00000167920
KRT12	-0.0097	9.0450E-01		ENSG00000187242
KRT14	-0.0147	6.2612E-01		ENSG00000186847
KRT15	0.1878	9.1223E-02	2.20E-01	ENSG00000171346
KRT16	-0.0453	4.6032E-01	6.49E-01	ENSG00000186832
KRT17	0.0236	7.5363E-01	8.66E-01	ENSG00000128422
KRT17P3	0.0215	3.3187E-01	5.27E-01	ENSG00000231870
KRT17P6	0.0059	6.1913E-01	7.75E-01	ENSG00000231645
KRT18	-0.1014	2.5897E-01	4.47E-01	ENSG00000111057
KRT18P1	0.0077	9.1582E-01		ENSG00000228666
KRT18P10	-0.0085	8.4530E-01	9.20E-01	ENSG00000214207
KRT18P11	0.0856	1.8010E-01	3.52E-01	ENSG00000215089
KRT18P13	-0.0078	9.7931E-01		ENSG00000214417
KRT18P15	-0.0058	9.0758E-01	9.54E-01	ENSG00000234737
KRT18P16	-0.0414	1.8210E-01		ENSG00000235275
KRT18P17	-0.0306	7.8927E-01		ENSG00000213943
KRT18P18	-0.0013	6.5570E-01		ENSG00000261764
KRT18P20	0.0030	6.5325E-01		ENSG00000257758
KRT18P21	-0.0049	7.8420E-01		ENSG00000250363
KRT18P23	-0.0031	9.7011E-01		ENSG00000215333
KRT18P26	0.0145	1.8378E-01		ENSG00000229798
KRT18P28	0.0063	7.0834E-01		ENSG00000176855
KRT18P31	0.0061	3.1546E-01		ENSG00000249850

KRT18P34	0.0032	4.8939E-01		ENSG00000244515
KRT18P38	0.0103	4.1762E-01		ENSG00000214012
KRT18P39	0.0051	7.8601E-01		ENSG00000224791
KRT18P4	0.0165	5.3281E-01		ENSG00000229222
KRT18P45	0.0047	6.7129E-01		ENSG00000249438
KRT18P48	0.0106	8.2314E-01		ENSG00000217889
KRT18P5	-0.2467	5.4182E-02	1.52E-01	ENSG00000236670
KRT18P52	-0.0262	2.5872E-01		ENSG00000237007
KRT18P55	-0.0050	6.9190E-01	8.27E-01	ENSG00000265480
KRT18P57	-0.0053	9.7955E-01		ENSG00000215867
KRT18P61	-0.0283	6.7177E-01	8.12E-01	ENSG00000267083
KRT18P62	0.0122	8.1232E-01		ENSG00000233471
KRT18P63	0.0302	4.1216E-01		ENSG00000235413
KRT18P68	0.0022	8.7562E-01		ENSG00000213100
KRT18P7	-0.1277	1.8172E-01	3.54E-01	ENSG00000258951
KRT19	0.1045	3.0796E-01	5.01E-01	ENSG00000171345
KRT19P1	0.0181	1.7124E-01		ENSG00000218014
KRT19P2	0.0179	3.7734E-01		ENSG00000216306
KRT20	0.0036	7.3046E-01		ENSG00000171431
KRT222	-0.2751	2.8924E-02	9.49E-02	ENSG00000213424
KRT23	0.2162	6.7302E-02	1.77E-01	ENSG00000108244
KRT24	0.0158	5.5293E-02		ENSG00000167916
KRT32	0.0479	5.4875E-02	1.53E-01	ENSG00000108759
KRT37	0.0088	3.8678E-01		ENSG00000108417
KRT4	-0.0540	6.1900E-01	7.75E-01	ENSG00000170477
KRT41P	0.0054	3.7044E-01		ENSG00000225438
KRT5	-0.0435	3.7481E-01		ENSG00000186081
KRT6A	-0.0221	6.1099E-01	7.69E-01	ENSG00000205420
KRT6B	-0.0183	6.8415E-01	8.21E-01	ENSG00000185479
KRT6C	-0.0225	9.2718E-01	9.64E-01	ENSG00000170465
KRT7	0.0782	4.2837E-01	6.20E-01	ENSG00000135480
KRT7-AS	-0.1030	1.7705E-01	3.47E-01	ENSG00000257671
KRT78	0.0013	8.5338E-01		ENSG00000170423
KRT8	0.1285	1.9171E-01	3.67E-01	ENSG00000170421
KRT80	0.0682	4.8767E-01	6.72E-01	ENSG00000167767
KRT81	-0.0295	7.3974E-01	8.57E-01	ENSG00000205426
KRT83	0.0080	8.3039E-01		ENSG00000170523
KRT86	-0.1681	1.2447E-01	2.74E-01	ENSG00000170442
KRT87P	-0.0231	5.6771E-01		ENSG00000135477
KRT8P10	-0.0138	6.7958E-01		ENSG00000231203
KRT8P11	0.0205	2.0925E-01		ENSG00000255815
KRT8P12	-0.2990	9.4573E-03	4.08E-02	ENSG00000229320
KRT8P14	-0.0115	9.2688E-01		ENSG00000214282
KRT8P15	0.1278	2.2232E-01	4.04E-01	ENSG00000233579
KRT8P20	0.0016	5.9887E-01		ENSG00000227404
KRT8P3	0.1060	2.9747E-01	4.89E-01	ENSG00000254285
KRT8P31	-0.0144	4.0281E-01		ENSG00000250148
KRT8P32	0.0120	2.3279E-01	4.16E-01	ENSG00000250221
KRT8P36	-0.0184	8.2000E-01	9.05E-01	ENSG00000240668

KRT8P37	-0.0156	7.3526E-01		ENSG00000213771
KRT8P39	0.0651	4.3371E-01	6.25E-01	ENSG00000233560
KRT8P41	0.0067	7.0628E-01		ENSG00000213538
KRT8P43	-0.0303	5.1517E-01		ENSG00000218186
KRT8P45	-0.0181	8.9738E-01	9.49E-01	ENSG00000224520
KRT8P46	-0.0051	9.7093E-01	9.85E-01	ENSG00000248971
KRT8P49	0.0131	4.7572E-01		ENSG00000271602
KRT8P5	-0.0026	6.4281E-01		ENSG00000267573
KRT8P50	0.0074	2.5561E-01		ENSG00000260799
KRT8P52	-0.0047	7.1477E-01		ENSG00000232479
KRT8P8	-0.0006	4.8325E-01		ENSG00000223940
KRT8P9	-0.0872	1.2390E-01		ENSG00000259470
KRTAP10-11	0.0100	3.8438E-01		ENSG00000243489
KRTAP10-2	-0.0074	8.9736E-01		ENSG00000205445
KRTAP10-3	0.0236	4.1354E-01		ENSG00000212935
KRTAP10-4	-0.0286	4.9235E-01		ENSG00000215454
KRTAP10-6	0.0147	9.7191E-01		ENSG00000188155
KRTAP10-7	0.0064	7.1962E-01		ENSG00000272804
KRTAP12-1	-0.0098	5.4760E-01		ENSG00000187175
KRTAP19-5	0.0224	1.6278E-01		ENSG00000186977
KRTAP2-1	-0.0194	7.1877E-01	8.44E-01	ENSG00000212725
KRTAP2-2	-0.0093	9.1701E-01		ENSG00000214518
KRTAP2-3	-0.0240	6.3486E-01	7.87E-01	ENSG00000212724
KRTAP2-4	-0.0477	3.7776E-01	5.73E-01	ENSG00000213417
KRTAP3-3	0.0071	8.4323E-02		ENSG00000212899
KRTAP4-4	-0.0807	2.9435E-01	4.86E-01	ENSG00000171396
KRTAP4-6	-0.0014	8.8615E-01		ENSG00000198090
KRTAP5-1	0.0572	3.6956E-02	1.14E-01	ENSG00000205869
KRTAP5-10	1.2833	5.2526E-03		ENSG00000204572
KRTAP5-11	0.0174	5.5075E-01		ENSG00000204571
KRTAP5-5	0.0045	7.0172E-01		ENSG00000185940
KRTAP5-7	0.0096	5.2517E-01		ENSG00000244411
KRTAP5-8	-0.0160	4.2850E-01		ENSG00000241233
KRTAP5-9	0.0477	4.4278E-02		ENSG00000254997
KRTAP5-AS1	0.1109	2.7985E-01	4.71E-01	ENSG00000233930
KRTCAP2	-0.0349	6.6387E-01	8.07E-01	ENSG00000163463
KRTCAP3	0.4119	2.9687E-03	1.67E-02	ENSG00000157992
KRTDAP	-0.3873	1.5857E-02	6.05E-02	ENSG00000188508
KSR1	0.0213	7.8141E-01	8.82E-01	ENSG00000141068
KSR2	0.1867	4.2379E-02	1.27E-01	ENSG00000171435
KTI12	-0.1025	1.1408E-01	2.58E-01	ENSG00000198841
KTN1	-0.4143	4.5483E-07	1.38E-05	ENSG00000126777
KTN1-AS1	-0.0484	5.6193E-01	7.31E-01	ENSG00000186615
KXD1	-0.0355	5.3007E-01	7.05E-01	ENSG00000105700
KY	-0.0966	3.3474E-01	5.30E-01	ENSG00000174611
KYAT1	0.1581	1.2481E-01	2.74E-01	ENSG00000171097
KYAT1	0.0052	9.3705E-01	9.69E-01	ENSG00000286112
KYAT3	0.0718	1.9055E-01	3.66E-01	ENSG00000137944
KYNU	0.0914	2.1878E-01	4.00E-01	ENSG00000115919

L1CAM	0.1249	1.4430E-01	3.02E-01	ENSG00000198910
L1TD1	0.0222	7.6349E-01	8.72E-01	ENSG00000240563
L2HGDH	0.3257	1.5989E-04	1.64E-03	ENSG00000087299
L3HYPDH	-0.1123	1.9819E-01	3.75E-01	ENSG00000126790
L3MBTL1	0.2363	4.4926E-03	2.31E-02	ENSG00000185513
L3MBTL2	-0.2698	4.2486E-04	3.61E-03	ENSG00000100395
L3MBTL3	-0.0806	3.8182E-01	5.77E-01	ENSG00000198945
L3MBTL4	-0.5579	1.0583E-03	7.42E-03	ENSG00000154655
L3MBTL4-AS1	-0.0143	5.4647E-01		ENSG00000264707
LACC1	0.0203	8.3958E-01	9.16E-01	ENSG00000179630
LACTB	0.1104	8.4693E-02	2.09E-01	ENSG00000103642
LACTB2	-0.0377	5.7753E-01	7.44E-01	ENSG00000147592
LACTB2-AS1	0.2907	3.9354E-02	1.20E-01	ENSG00000246366
LACTBL1	0.0329	5.6191E-01	7.31E-01	ENSG00000215906
LAD1	-0.0436	5.9236E-01	7.55E-01	ENSG00000159166
LAG3	0.1867	9.0765E-02	2.20E-01	ENSG00000089692
LAGE3	-0.2569	5.1557E-06	1.03E-04	ENSG00000196976
LAIR1	0.0101	4.7463E-01		ENSG00000167613
LAIR2	0.0388	6.3128E-02		ENSG00000167618
LAMA1	-0.5074	1.1417E-04	1.25E-03	ENSG00000101680
LAMA2	-0.0995	2.8895E-01	4.80E-01	ENSG00000196569
LAMA3	-0.1607	7.7420E-02	1.96E-01	ENSG00000053747
LAMA4	-0.3485	1.9465E-02	7.05E-02	ENSG00000112769
LAMA5	-0.0062	9.2721E-01	9.64E-01	ENSG00000130702
LAMA5-AS1	-0.1726	8.1181E-02	2.03E-01	ENSG00000228812
LAMB1	-0.2356	7.2163E-03	3.31E-02	ENSG00000091136
LAMB2	-0.2305	2.4630E-03	1.45E-02	ENSG00000172037
LAMB3	-0.1707	1.2906E-01	2.80E-01	ENSG00000196878
LAMB4	0.0030	9.6583E-01	9.83E-01	ENSG00000091128
LAMC1	-0.2548	1.1980E-04	1.29E-03	ENSG00000135862
LAMC1-AS1	0.0083	4.4202E-01		ENSG00000224468
LAMC2	-0.0335	7.2059E-01	8.45E-01	ENSG00000058085
LAMC3	0.0581	3.4644E-01	5.42E-01	ENSG00000050555
LAMP1	-0.4579	1.1956E-09	9.92E-08	ENSG00000185896
LAMP2	-0.3142	2.6537E-05	3.84E-04	ENSG00000005893
LAMP3	-0.0360	6.6665E-01	8.08E-01	ENSG00000078081
LAMP5	0.0411	6.4507E-01	7.94E-01	ENSG00000125869
LAMTOR1	-0.1625	8.2805E-05	9.61E-04	ENSG00000149357
LAMTOR2	-0.3022	1.9971E-07	6.95E-06	ENSG00000116586
LAMTOR3	-0.0125	8.0581E-01	8.96E-01	ENSG00000109270
LAMTOR4	-0.2698	1.2783E-06	3.27E-05	ENSG00000188186
LAMTOR5	-0.2186	1.3021E-04	1.38E-03	ENSG00000134248
LAMTOR5-AS1	-0.0240	7.9506E-01	8.90E-01	ENSG00000224699
LANCL1	0.2406	1.3497E-03	9.03E-03	ENSG00000115365
LANCL2	-0.0856	2.1363E-01	3.94E-01	ENSG00000132434
LANCL3	0.5264	8.8296E-05	1.01E-03	ENSG00000147036
LAP3	-0.0248	6.9333E-01	8.28E-01	ENSG00000002549
LAPTM4A	-0.2864	3.2269E-05	4.48E-04	ENSG00000068697
LAPTM4B	-0.1342	4.3224E-02	1.28E-01	ENSG00000104341

LAPTM5	0.0521	2.7815E-01	4.69E-01	ENSG00000162511
LARGE1	0.0959	1.9824E-01	3.75E-01	ENSG00000133424
LARGE2	0.0678	4.5536E-01	6.45E-01	ENSG00000165905
LARP1	-0.1697	1.7473E-02	6.50E-02	ENSG00000155506
LARP1B	0.1060	1.0713E-01	2.46E-01	ENSG00000138709
LARP4	-0.0487	4.6065E-01	6.49E-01	ENSG00000161813
LARP4B	0.0400	4.9679E-01	6.80E-01	ENSG00000107929
LARP4B-DT	-0.0007	6.7199E-01		ENSG00000229869
LARP6	0.0232	7.0501E-01	8.35E-01	ENSG00000166173
LARP7	-0.1730	2.5624E-04	2.39E-03	ENSG00000174720
LARP7P2	0.0038	8.0733E-01		ENSG00000261939
LARS1	-0.3035	6.0530E-09	3.84E-07	ENSG00000133706
LARS2	0.0329	6.9396E-01	8.29E-01	ENSG00000011376
LAS1L	0.2699	1.9745E-03	1.21E-02	ENSG00000001497
LASP1	-0.1301	8.4469E-03	3.75E-02	ENSG00000002834
LASP1NB	-0.3601	1.1648E-03	8.00E-03	ENSG00000263874
LAT	-0.0054	7.7871E-01		ENSG00000213658
LAT2	-0.0093	8.1595E-01	9.03E-01	ENSG00000086730
LATS1	-0.1126	5.9055E-02	1.61E-01	ENSG00000131023
LATS2	-0.2737	1.8272E-02	6.73E-02	ENSG00000150457
LAX1	0.0073	7.9280E-01		ENSG00000122188
LAYN	-0.0684	4.7340E-01	6.60E-01	ENSG00000204381
LBH	0.1366	4.4782E-02	1.32E-01	ENSG00000213626
LBHD1	0.0714	4.6516E-01	6.53E-01	ENSG00000162194
LBHD2	-0.1492	6.1729E-02	1.67E-01	ENSG00000283071
LBP	0.0088	6.6347E-01		ENSG00000129988
LBR	-0.1092	7.9194E-02	1.99E-01	ENSG00000143815
LBX2	-0.0505	4.3947E-01	6.30E-01	ENSG00000179528
LBX2-AS1	-0.0883	3.3548E-01	5.31E-01	ENSG00000257702
LCA5	-0.0630	3.5222E-01	5.47E-01	ENSG00000135338
LCA5L	0.1337	7.6981E-02	1.95E-01	ENSG00000157578
LCAL1	0.2003	3.6316E-02	1.13E-01	ENSG00000286042
LCDR	0.4842	4.4815E-09	3.00E-07	ENSG00000273148
LCE1D	-0.0106	4.7125E-01	6.59E-01	ENSG00000172155
LCE1E	0.0119	5.3237E-01	7.07E-01	ENSG00000186226
LCEP4	0.0015	8.3002E-01		ENSG00000226947
LCIIAR	0.0383	6.8384E-01	8.21E-01	ENSG00000256802
LCLAT1	-0.1636	1.7340E-02	6.47E-02	ENSG00000172954
LCMT1	0.0551	3.0163E-01	4.94E-01	ENSG00000205629
LCMT1-AS2	-0.7230	3.1370E-02	1.01E-01	ENSG00000260034
LCMT2	0.1000	2.9698E-01	4.89E-01	ENSG00000168806
LCN1	0.3177	2.8601E-02	9.40E-02	ENSG00000160349
LCN12	-0.3703	1.3530E-02	5.36E-02	ENSG00000184925
LCN15	0.3450	5.7328E-03	2.78E-02	ENSG00000177984
LCN1P1	-0.0025	6.7601E-01		ENSG00000119440
LCN2	0.3839	1.3965E-02	5.49E-02	ENSG00000148346
LCNL1	0.3605	2.1290E-02	7.55E-02	ENSG00000214402
LCOR	0.0801	1.4133E-01	2.98E-01	ENSG00000196233
LCORL	0.1047	2.3259E-01	4.16E-01	ENSG00000178177

LCP1	0.2030	7.8922E-02	1.99E-01	ENSG00000136167
LCP2	-0.0114	9.3725E-01		ENSG00000043462
LCTL	0.0436	6.1834E-01	7.75E-01	ENSG00000188501
LDAF1	0.0629	2.4005E-01	4.25E-01	ENSG00000011638
LDAH	0.0445	5.5251E-01	7.24E-01	ENSG00000118961
LDB1	-0.1596	1.7914E-02	6.63E-02	ENSG00000198728
LDB2	0.0162	8.4340E-01	9.18E-01	ENSG00000169744
LDB3	0.1171	2.6084E-01	4.49E-01	ENSG00000122367
LDHAL6A	-0.0158	9.5372E-01		ENSG00000166800
LDHB	-0.4743	7.9767E-09	4.74E-07	ENSG00000111716
LDHBP1	0.0052	7.2883E-01		ENSG00000232662
LDHC	0.0030	6.8332E-01		ENSG00000166796
LDHD	0.0873	3.2945E-01	5.24E-01	ENSG00000166816
LDLR	-0.4218	9.7840E-08	3.83E-06	ENSG00000130164
LDLRAD1	-0.4755	1.1349E-03	7.83E-03	ENSG00000203985
LDLRAD2	-0.0230	5.6441E-01		ENSG00000187942
LDLRAD3	-0.2145	2.4955E-02	8.51E-02	ENSG00000179241
LDLRAD4	-0.1798	3.7431E-02	1.15E-01	ENSG00000168675
LDLRAD4-AS1	-0.0113	8.5084E-01		ENSG00000267690
LDLRAP1	0.0861	3.0640E-01	4.99E-01	ENSG00000157978
LDOC1	-0.2127	1.8972E-04	1.88E-03	ENSG00000182195
LEAP2	-0.0498	5.3075E-01	7.05E-01	ENSG00000164406
LEF1	-0.1649	1.0722E-01	2.47E-01	ENSG00000138795
LEF1-AS1	-0.0875	3.8167E-01	5.77E-01	ENSG00000232021
LEFTY2	-0.0771	3.7829E-01	5.73E-01	ENSG00000143768
LEFTY3P	0.0748	1.3186E-01		ENSG00000234112
LEKR1	0.5428	9.2187E-07	2.50E-05	ENSG00000197980
LEMD1	-0.3550	3.5145E-06	7.56E-05	ENSG00000186007
LEMD1-AS1	0.0218	7.1217E-01	8.40E-01	ENSG00000226235
LEMD1-DT	0.1602	1.4967E-01	3.10E-01	ENSG00000224717
LEMD2	-0.0287	6.4023E-01	7.91E-01	ENSG00000161904
LEMD3	-0.1130	9.6344E-02	2.29E-01	ENSG00000174106
LENEP	0.0094	5.6948E-01		ENSG00000163352
LENG1	-0.0892	2.5167E-01	4.38E-01	ENSG00000105617
LENG8	0.1741	3.1203E-02	1.00E-01	ENSG00000167615
LENG8-AS1	0.1478	6.0190E-02	1.64E-01	ENSG00000226696
LENG9	-0.0453	6.2856E-01	7.83E-01	ENSG00000275183
LEO1	0.2609	4.7989E-04	3.96E-03	ENSG00000166477
LEP	-0.0070	7.0582E-01		ENSG00000174697
LEPROT	0.0176	7.9855E-01	8.92E-01	ENSG00000213625
LEPROTL1	0.0329	5.3592E-01	7.10E-01	ENSG00000104660
LETM1	0.0206	7.3798E-01	8.56E-01	ENSG00000168924
LETM2	0.2738	8.3221E-03	3.70E-02	ENSG00000165046
LETMD1	-0.0317	6.1210E-01	7.70E-01	ENSG00000050426
LETR1	0.0204	8.2464E-01	9.08E-01	ENSG00000248441
LFNG	-0.1531	1.2980E-01	2.81E-01	ENSG00000106003
LGALS1	-0.3498	2.0012E-03	1.23E-02	ENSG00000100097
LGALS12	-0.0053	6.0628E-01		ENSG00000133317
LGALS14	-0.0826	3.7486E-01	5.70E-01	ENSG00000006659

LGALS2	-0.1231	7.1642E-02	1.85E-01	ENSG00000100079
LGALS3	0.0311	7.3637E-01	8.55E-01	ENSG00000131981
LGALS3BP	-0.2003	3.0988E-02	9.98E-02	ENSG00000108679
LGALS4	-0.2831	3.9911E-02	1.21E-01	ENSG00000171747
LGALS7	0.0297	3.8535E-01	5.80E-01	ENSG00000205076
LGALS7	-0.0868	2.7733E-01	4.68E-01	ENSG00000178934
LGALS8	0.0101	8.9842E-01	9.49E-01	ENSG00000116977
LGALS8-AS1	0.0294	2.3511E-01		ENSG00000223776
LGALS9	0.0301	8.5882E-01	9.28E-01	ENSG00000168961
LGALSL	-0.1355	1.5447E-02	5.92E-02	ENSG00000119862
LGI1	-0.0270	7.2197E-01	8.46E-01	ENSG00000108231
LGI2	0.2897	2.1718E-03	1.31E-02	ENSG00000153012
LGI3	-0.0502	5.8305E-01	7.49E-01	ENSG00000168481
LGI4	0.4720	5.1792E-04	4.23E-03	ENSG00000153902
LGMN	-0.0909	1.6196E-01	3.27E-01	ENSG00000100600
LGR4	0.0467	4.7646E-01	6.63E-01	ENSG00000205213
LGR4-AS1	-0.0310	6.7671E-01	8.15E-01	ENSG00000254862
LGR5	-0.1257	2.3248E-01	4.16E-01	ENSG00000139292
LGR6	0.4686	1.2028E-02	4.89E-02	ENSG00000133067
LHB	0.2000	4.0579E-02	1.22E-01	ENSG00000104826
LHCGR	-0.0086	8.8476E-01	9.42E-01	ENSG00000138039
LHFPL1	-0.4217	6.0890E-03	2.92E-02	ENSG00000182508
LHFPL2	-0.4090	4.0896E-04	3.51E-03	ENSG00000145685
LHFPL3	-0.3141	3.0900E-02	9.96E-02	ENSG00000187416
LHFPL3-AS1	-0.0149	4.0764E-01		ENSG00000226869
LHFPL3-AS2	-0.0255	4.5272E-01	6.42E-01	ENSG00000225329
LHFPL4	-0.0693	2.7629E-01	4.67E-01	ENSG00000156959
LHFPL5	0.0608	5.0294E-01	6.84E-01	ENSG00000197753
LHFPL6	0.0742	3.2190E-01	5.16E-01	ENSG00000183722
LHFPL7	-0.0399	4.7481E-01		ENSG00000206069
LHPP	-0.1194	1.3404E-01	2.87E-01	ENSG00000107902
LHX1	-0.0443	5.7451E-01	7.42E-01	ENSG00000273706
LHX1-DT	0.0152	8.6364E-01	9.31E-01	ENSG00000277268
LHX2	-0.1008	9.5111E-02	2.27E-01	ENSG00000106689
LHX3	0.0067	5.9859E-01		ENSG00000107187
LHX4	0.0669	1.9660E-01	3.73E-01	ENSG00000121454
LHX5	0.1338	2.0964E-01	3.89E-01	ENSG00000089116
LHX6	-0.0180	8.4988E-01	9.23E-01	ENSG00000106852
LHX8	0.0518	2.5398E-01		ENSG00000162624
LHX9	0.0531	5.6131E-01	7.31E-01	ENSG00000143355
LIAS	-0.0049	9.3826E-01	9.69E-01	ENSG00000121897
LIAT1	-0.0344	6.9312E-01	8.28E-01	ENSG00000187624
LIF	-0.5680	3.7040E-03	1.99E-02	ENSG00000128342
LIF-AS1	0.0170	5.7065E-01		ENSG00000232530
LIF-AS2	-0.0055	8.0850E-01		ENSG00000268812
LIFR	-0.0199	7.6518E-01	8.73E-01	ENSG00000113594
LIFR-AS1	0.3780	4.5629E-05	5.97E-04	ENSG00000244968
LIG1	0.0756	2.9047E-01	4.82E-01	ENSG00000105486
LIG3	-0.0142	8.1124E-01	9.00E-01	ENSG00000005156

LIG4	0.0417	5.5040E-01	7.22E-01	ENSG00000174405
LILRA6	0.0163	4.5739E-01		ENSG00000244482
LILRB3	0.0105	9.1661E-01	9.58E-01	ENSG00000204577
LILRB5	0.0344	1.1944E-01		ENSG00000105609
LIMA1	-0.3385	2.4631E-08	1.23E-06	ENSG00000050405
LIMCH1	0.0357	6.4248E-01	7.93E-01	ENSG00000064042
LIMD1	0.1939	5.8616E-02	1.61E-01	ENSG00000144791
LIMD2	-0.0120	8.1458E-01	9.02E-01	ENSG00000136490
LIME1	0.1019	1.7529E-01	3.45E-01	ENSG00000203896
LIMK1	0.0071	9.2791E-01	9.64E-01	ENSG00000106683
LIMK2	0.2924	2.8856E-05	4.11E-04	ENSG00000182541
LIMS1	-0.2064	2.5054E-03	1.46E-02	ENSG00000169756
LIMS1-AS1	0.0204	1.6304E-01		ENSG00000228763
LIMS2	-0.1085	2.9095E-01	4.83E-01	ENSG00000072163
LIMS3	-0.0944	3.5304E-01	5.48E-01	ENSG00000256977
LIMS4	-0.0831	4.0741E-01	6.01E-01	ENSG00000256671
LIN28A	0.4194	2.1956E-02	7.73E-02	ENSG00000131914
LIN28B	0.2975	3.7623E-02	1.15E-01	ENSG00000187772
LIN28B-AS1	0.2330	5.9387E-02	1.62E-01	ENSG00000203809
LIN37	0.3672	1.8856E-04	1.87E-03	ENSG00000267796
LIN52	0.1790	3.9230E-02	1.19E-01	ENSG00000205659
LIN54	0.0797	3.7194E-01	5.67E-01	ENSG00000189308
LIN7A	-0.2214	2.6525E-02	8.89E-02	ENSG00000111052
LIN7B	0.1317	6.4985E-02	1.73E-01	ENSG00000104863
LIN7C	-0.0607	3.1853E-01	5.12E-01	ENSG00000148943
LIN9	0.3107	5.3032E-03	2.62E-02	ENSG00000183814
LINC-PINT	0.0052	9.3613E-01	9.68E-01	ENSG00000231721
LINC-ROR	0.0009	9.6277E-01		ENSG00000258609
LINC00092	-0.0947	3.2319E-01	5.17E-01	ENSG00000225194
LINC00102	0.0277	2.8385E-01		ENSG00000230542
LINC00106	0.0784	4.3064E-01	6.22E-01	ENSG00000236871
LINC00114	0.0312	3.0649E-01	4.99E-01	ENSG00000223806
LINC00158	-0.2112	5.7664E-02	1.59E-01	ENSG00000185433
LINC00160	0.0027	9.9361E-01		ENSG00000230978
LINC00173	-0.0272	7.7393E-01	8.78E-01	ENSG00000196668
LINC00174	0.1425	5.3393E-02	1.50E-01	ENSG00000179406
LINC00184	0.0102	4.4459E-01		ENSG00000224939
LINC00200	0.1359	2.3912E-02	8.24E-02	ENSG00000229205
LINC00205	-0.1130	1.2982E-01	2.81E-01	ENSG00000223768
LINC00207	0.0496	2.6093E-02		ENSG00000187012
LINC00216	-0.0052	9.2757E-01	9.64E-01	ENSG00000279636
LINC00237	-0.0301	9.0400E-01	9.52E-01	ENSG00000225127
LINC00239	0.2439	5.3107E-02	1.49E-01	ENSG00000258512
LINC00240	0.0083	9.0318E-01	9.52E-01	ENSG00000224843
LINC00242	0.0842	2.8055E-01	4.72E-01	ENSG00000229214
LINC00243	0.0117	4.8833E-01		ENSG00000214894
LINC00261	0.1423	1.2832E-01	2.79E-01	ENSG00000259974
LINC00269	0.0252	1.3008E-01		ENSG00000215162
LINC00278	-0.0011	9.4816E-01	9.74E-01	ENSG00000231535

LINC00294	0.3210	7.6565E-03	3.47E-02	ENSG00000280798
LINC00298	0.0089	5.6890E-01		ENSG00000235665
LINC00299	-0.0724	2.6121E-01		ENSG00000236790
LINC00301	0.1333	8.6330E-02	2.12E-01	ENSG00000181995
LINC00303	0.0553	9.3336E-02		ENSG00000176754
LINC00304	0.0711	2.5917E-01	4.47E-01	ENSG00000180422
LINC00309	0.0159	8.6620E-01	9.32E-01	ENSG00000230923
LINC00310	0.0289	7.2562E-01	8.48E-01	ENSG00000227456
LINC00311	-0.0912	2.7392E-01	4.64E-01	ENSG00000179219
LINC00313	-0.0123	8.9352E-01	9.47E-01	ENSG00000185186
LINC00314	-0.0079	8.4701E-01		ENSG00000178457
LINC00316	-0.0214	5.2073E-01		ENSG00000237664
LINC00319	0.0111	2.3029E-01		ENSG00000188660
LINC00320	-0.1818	2.7484E-02	9.12E-02	ENSG00000224924
LINC00323	-0.0172	7.7562E-01	8.79E-01	ENSG00000226496
LINC00324	0.5101	4.7371E-05	6.17E-04	ENSG00000178977
LINC00330	0.0091	9.0391E-01	9.52E-01	ENSG00000235097
LINC00334	-0.5041	1.4465E-02	5.65E-02	ENSG00000182586
LINC00339	0.0501	5.9943E-01	7.60E-01	ENSG00000218510
LINC00347	0.0202	2.6765E-01		ENSG00000236678
LINC00354	-0.0145	8.6064E-01	9.29E-01	ENSG00000226903
LINC00355	0.0040	6.9330E-01		ENSG00000227674
LINC00365	0.0117	7.3310E-01		ENSG00000224511
LINC00375	0.0075	7.1153E-01		ENSG00000226370
LINC00381	-0.0711	1.6142E-01		ENSG00000226240
LINC00391	-0.0688	4.7286E-01	6.60E-01	ENSG00000238230
LINC00393	-0.0083	9.9366E-01		ENSG00000224853
LINC00395	-0.0031	9.2952E-01		ENSG00000231061
LINC00398	-0.0236	5.3381E-01		ENSG00000237879
LINC00402	0.0105	7.3256E-01		ENSG00000235532
LINC00426	-0.0563	3.1796E-01		ENSG00000238121
LINC00443	0.0040	9.4253E-01		ENSG00000230156
LINC00445	0.0035	9.0578E-01		ENSG00000236036
LINC00456	0.0037	3.3659E-01		ENSG00000233124
LINC00458	-0.0711	4.0480E-01	5.99E-01	ENSG00000234787
LINC00460	-0.0017	9.1383E-01		ENSG00000233532
LINC00463	-0.0446	5.0200E-01	6.84E-01	ENSG00000234056
LINC00466	-0.0490	3.2399E-01		ENSG00000224209
LINC00467	0.0758	3.4797E-01	5.43E-01	ENSG00000153363
LINC00471	0.2180	6.1602E-02	1.67E-01	ENSG00000181798
LINC00472	-0.1153	2.6350E-01	4.52E-01	ENSG00000233237
LINC00474	-0.0126	9.7373E-01		ENSG00000204148
LINC00479	0.0097	6.6320E-01		ENSG00000236384
LINC00484	-0.0019	9.0910E-01		ENSG00000229694
LINC00486	-0.0178	7.0190E-01	8.33E-01	ENSG00000230876
LINC00487	0.3450	1.0701E-02	4.47E-02	ENSG00000205837
LINC00488	0.5422	3.5071E-03	1.90E-02	ENSG00000214381
LINC00491	0.1442	1.2751E-01	2.78E-01	ENSG00000250682
LINC00499	-0.0314	7.7876E-01		ENSG00000251372

LINC00501	0.0945	9.6718E-02		ENSG00000203645
LINC00504	0.3624	1.9596E-02	7.09E-02	ENSG00000248360
LINC00511	0.0271	7.3072E-01	8.52E-01	ENSG00000227036
LINC00515	-0.1162	2.1852E-01	3.99E-01	ENSG00000260583
LINC00517	-0.0929	5.3050E-02		ENSG00000259091
LINC00519	-0.0319	3.7259E-01		ENSG00000258955
LINC00520	-0.0041	7.5577E-01		ENSG00000258791
LINC00523	0.0318	6.9382E-01	8.29E-01	ENSG00000196273
LINC00524	0.0040	9.7725E-01		ENSG00000259023
LINC00525	-0.0357	2.4128E-01		ENSG00000146666
LINC00528	0.0046	8.4376E-01		ENSG00000269220
LINC00539	0.1069	2.9636E-01	4.88E-01	ENSG00000224429
LINC00540	0.0276	6.9504E-01	8.29E-01	ENSG00000276476
LINC00543	-0.0285	4.4282E-01		ENSG00000260704
LINC00545	0.0384	7.0324E-02	1.83E-01	ENSG00000236094
LINC00552	-0.0222	3.9447E-01		ENSG00000279770
LINC00561	-0.0093	7.3330E-01		ENSG00000261206
LINC00562	0.0523	5.5260E-01	7.24E-01	ENSG00000260388
LINC00567	0.0273	6.6846E-02		ENSG00000259831
LINC00570	0.0162	1.9185E-01		ENSG00000224177
LINC00571	-0.0041	9.5394E-01	9.77E-01	ENSG00000223685
LINC00574	0.2162	5.8579E-02	1.61E-01	ENSG00000231690
LINC00578	0.1055	2.5915E-01	4.47E-01	ENSG00000228221
LINC00582	0.0311	2.8804E-01		ENSG00000229228
LINC00587	0.0771	1.2806E-01	2.79E-01	ENSG00000204250
LINC00592	-0.0103	8.5101E-01		ENSG00000258279
LINC00595	-0.1376	1.9713E-01	3.74E-01	ENSG00000230417
LINC00601	-0.0421	1.8231E-01		ENSG00000235180
LINC00602	-0.0262	8.7984E-01	9.39E-01	ENSG00000281832
LINC00605	0.0474	2.6231E-01		ENSG00000251533
LINC00607	-0.0035	9.8373E-01		ENSG00000235770
LINC00609	-0.0109	7.5393E-01		ENSG00000257585
LINC00612	0.2045	8.3823E-02	2.07E-01	ENSG00000214851
LINC00613	-0.0411	7.9829E-01	8.92E-01	ENSG00000248330
LINC00616	0.0825	4.0171E-01	5.96E-01	ENSG00000248307
LINC00620	-0.0484	4.1759E-01		ENSG00000224514
LINC00624	0.1891	9.5268E-02	2.27E-01	ENSG00000278811
LINC00629	0.2854	3.5547E-02	1.11E-01	ENSG00000227060
LINC00635	0.1035	4.7205E-02	1.37E-01	ENSG00000241469
LINC00636	-0.0039	6.4641E-01	7.95E-01	ENSG00000240423
LINC00638	-0.1725	8.5603E-02	2.11E-01	ENSG00000258701
LINC00639	0.0264	5.1254E-01	6.92E-01	ENSG00000259070
LINC00641	-0.1037	2.1458E-01	3.95E-01	ENSG00000258441
LINC00642	0.0282	7.3138E-01	8.52E-01	ENSG00000233208
LINC00645	0.1591	1.5310E-01	3.15E-01	ENSG00000258548
LINC00648	-0.3164	2.8565E-03	1.62E-02	ENSG00000259129
LINC00649	0.0394	6.3870E-01	7.90E-01	ENSG00000237945
LINC00652	-0.1341	1.9267E-01	3.68E-01	ENSG00000179935
LINC00654	-0.0549	3.7951E-01	5.74E-01	ENSG00000205181

LINC00658	-0.0160	7.2539E-01		ENSG00000226995
LINC00659	0.0763	2.0316E-03		ENSG00000228705
LINC00662	0.1137	1.1847E-01	2.65E-01	ENSG00000261824
LINC00664	-0.0231	8.3575E-01	9.14E-01	ENSG00000268658
LINC00665	-0.1327	5.9662E-02	1.63E-01	ENSG00000232677
LINC00667	-0.0820	1.1701E-01	2.63E-01	ENSG00000263753
LINC00668	0.0190	2.7895E-01		ENSG00000265933
LINC00676	0.0559	1.3072E-01	2.83E-01	ENSG00000234854
LINC00678	-0.0281	4.9811E-01		ENSG00000254934
LINC00680-GUSBP4	-0.0166	9.5514E-01		ENSG00000283352
LINC00682	0.0192	1.0005E-01		ENSG00000245870
LINC00689	-0.0069	9.5240E-01		ENSG00000231419
LINC00698	0.1105	6.9471E-03	3.22E-02	ENSG00000244342
LINC00707	0.0232	6.0287E-01		ENSG00000238266
LINC00710	0.2663	3.0517E-02	9.86E-02	ENSG00000229240
LINC00836	0.0457	3.7396E-02		ENSG00000280809
LINC00842	-0.0301	2.4204E-01		ENSG00000285294
LINC00845	0.0207	5.7115E-01		ENSG00000227244
LINC00847	0.0257	7.2082E-01	8.45E-01	ENSG00000245060
LINC00852	0.1307	1.5216E-01	3.14E-01	ENSG00000231177
LINC00853	-0.0504	5.4959E-01	7.21E-01	ENSG00000224805
LINC00858	-0.1120	2.7038E-01	4.60E-01	ENSG00000229404
LINC00861	-0.0432	4.0796E-01		ENSG00000245164
LINC00862	-0.0218	4.6061E-01		ENSG00000203721
LINC00863	0.1595	2.1267E-02	7.54E-02	ENSG00000224914
LINC00865	-0.0183	4.5647E-01		ENSG00000232229
LINC00867	0.1379	1.8836E-01	3.63E-01	ENSG00000232139
LINC00868	0.0144	4.0858E-01		ENSG00000267535
LINC00870	0.0476	8.7206E-02		ENSG00000243083
LINC00871	0.0637	4.2891E-01	6.21E-01	ENSG00000258700
LINC00877	-0.0162	8.6026E-01	9.29E-01	ENSG00000241163
LINC00885	0.0127	5.1483E-01		ENSG00000224652
LINC00886	0.0115	9.0339E-01	9.52E-01	ENSG00000240875
LINC00887	0.0008	9.9345E-01	9.96E-01	ENSG00000214145
LINC00896	-0.0246	2.5097E-01		ENSG00000236499
LINC00901	-0.0064	9.5142E-01	9.75E-01	ENSG00000242385
LINC00904	-0.0609	9.3700E-01	9.69E-01	ENSG00000271171
LINC00906	-0.0238	7.2074E-01		ENSG00000267339
LINC00907	0.4609	3.2109E-03	1.77E-02	ENSG00000267586
LINC00908	-0.0685	4.7363E-01	6.60E-01	ENSG00000266256
LINC00910	0.1245	1.9840E-01	3.75E-01	ENSG00000188825
LINC00921	0.0810	3.8906E-01	5.84E-01	ENSG00000281005
LINC00922	0.5376	7.8226E-03	3.53E-02	ENSG00000261742
LINC00923	0.0589	4.0054E-01	5.95E-01	ENSG00000251209
LINC00926	0.5452	1.9271E-05	2.97E-04	ENSG00000247982
LINC00927	0.0364	1.7573E-01		ENSG00000259361
LINC00929	-0.0092	8.7871E-01		ENSG00000259150
LINC00934	0.0330	3.4427E-02		ENSG00000281196
LINC00937	0.0216	8.0906E-01	8.99E-01	ENSG00000226091

LINC00939	-0.0610	3.4273E-01	5.38E-01	ENSG00000249267
LINC00940	0.0109	6.3545E-01		ENSG00000235049
LINC00942	0.0378	2.7808E-01	4.69E-01	ENSG00000249628
LINC00943	0.0563	2.8462E-01		ENSG00000189238
LINC00951	-0.0184	8.3985E-01	9.16E-01	ENSG00000226070
LINC00954	0.1239	1.5612E-01	3.19E-01	ENSG00000228784
LINC00958	0.1222	1.9277E-01	3.68E-01	ENSG00000251381
LINC00960	-0.0317	7.3619E-01	8.55E-01	ENSG00000242516
LINC00964	-0.0097	5.6742E-01		ENSG00000249816
LINC00967	0.0119	7.2402E-01		ENSG00000253138
LINC00968	0.0098	3.8007E-01		ENSG00000246430
LINC00970	0.1295	2.9136E-02	9.54E-02	ENSG00000203601
LINC00971	0.1126	6.0313E-03	2.90E-02	ENSG00000242641
LINC00973	0.0821	3.4054E-01	5.36E-01	ENSG00000240476
LINC00987	0.0340	4.0524E-02		ENSG00000237248
LINC00994	-0.0214	3.2686E-01		ENSG00000189196
LINC00996	-0.0208	5.4981E-01		ENSG00000242258
LINC01003	0.2035	4.9002E-03	2.46E-02	ENSG00000261455
LINC01007	0.0013	7.8600E-01		ENSG00000233123
LINC01010	0.0117	3.1600E-01		ENSG00000236700
LINC01011	-0.3134	1.0383E-03	7.32E-03	ENSG00000244041
LINC01012	-0.0269	7.7453E-01	8.78E-01	ENSG00000281706
LINC01013	-0.1649	1.2241E-01	2.71E-01	ENSG00000228495
LINC01014	-0.1752	1.0501E-01	2.43E-01	ENSG00000223941
LINC01018	0.5350	1.1999E-03	8.20E-03	ENSG00000250056
LINC01019	0.0653	1.3886E-02		ENSG00000248118
LINC01028	0.0300	5.8810E-01		ENSG00000267603
LINC01030	-0.0090	9.8621E-01		ENSG00000253799
LINC01031	0.0143	3.9893E-01		ENSG00000232077
LINC01033	0.0027	8.2199E-01		ENSG00000249069
LINC01036	0.0046	9.3842E-01	9.69E-01	ENSG00000230426
LINC01048	-0.0058	5.4993E-01		ENSG00000230390
LINC01053	-0.0467	2.7291E-01		ENSG00000238169
LINC01060	-0.1462	1.7530E-01	3.45E-01	ENSG00000249378
LINC01068	-0.0840	4.2824E-02		ENSG00000227676
LINC01085	0.0416	1.6528E-01		ENSG00000248698
LINC01089	-0.4690	3.8519E-06	8.12E-05	ENSG00000212694
LINC01090	0.0139	8.8189E-01	9.40E-01	ENSG00000231689
LINC01091	-0.1394	1.1076E-01	2.52E-01	ENSG00000249464
LINC01095	-0.0054	6.8131E-01		ENSG00000248809
LINC01098	-0.0149	7.3439E-01		ENSG00000231171
LINC01099	-0.0122	8.4078E-01		ENSG00000251504
LINC01101	0.0135	6.9713E-01		ENSG00000280409
LINC01102	-0.0405	5.1522E-01		ENSG00000235597
LINC01108	-0.0415	3.3580E-01	5.31E-01	ENSG00000226673
LINC01109	0.0025	8.6526E-01		ENSG00000270866
LINC01114	-0.0161	7.6598E-01		ENSG00000234177
LINC01115	0.1218	4.0698E-02	1.23E-01	ENSG00000272342
LINC01116	0.0417	5.3900E-01	7.12E-01	ENSG00000163364

LINC01117	0.0034	6.6118E-01		ENSG00000224577
LINC01118	-0.0044	8.1660E-01		ENSG00000222005
LINC01119	-0.0965	2.9911E-01	4.91E-01	ENSG00000239332
LINC01121	-0.1827	8.3622E-02	2.07E-01	ENSG00000205054
LINC01122	0.2097	7.5430E-02	1.92E-01	ENSG00000233723
LINC01123	-0.5044	1.5930E-04	1.63E-03	ENSG00000204588
LINC01124	0.0700	4.1271E-01	6.06E-01	ENSG00000222033
LINC01126	-0.0567	9.1255E-01	9.56E-01	ENSG00000279873
LINC01128	0.1494	3.8141E-02	1.17E-01	ENSG00000228794
LINC01132	0.2324	6.0178E-02	1.64E-01	ENSG00000227630
LINC01133	0.1097	1.7188E-01	3.41E-01	ENSG00000224259
LINC01134	-0.0643	1.2436E-01		ENSG00000236423
LINC01138	-0.1169	1.0359E-01	2.41E-01	ENSG00000274020
LINC01139	0.0310	7.5433E-01	8.66E-01	ENSG00000215808
LINC01140	0.3460	2.2744E-02	7.93E-02	ENSG00000267272
LINC01144	0.1569	9.2667E-02	2.23E-01	ENSG00000281912
LINC01152	-0.0509	5.8214E-01	7.48E-01	ENSG00000256124
LINC01159	-0.0193	7.7577E-01	8.79E-01	ENSG00000229743
LINC01166	0.0086	9.1467E-01	9.57E-01	ENSG00000232903
LINC01173	-0.1489	3.0803E-02		ENSG00000280744
LINC01176	0.2036	8.0544E-02	2.02E-01	ENSG00000281404
LINC01182	0.0236	7.6622E-01	8.73E-01	ENSG00000250634
LINC01191	0.0028	7.5558E-01		ENSG00000234199
LINC01198	0.0306	3.4025E-02		ENSG00000231817
LINC01203	0.5301	6.4427E-03	3.05E-02	ENSG00000226985
LINC01204	-0.0136	9.9364E-01		ENSG00000229563
LINC01206	0.0114	6.3078E-01		ENSG00000242512
LINC01213	-0.0533	5.4132E-01	7.14E-01	ENSG00000244541
LINC01217	-0.1234	9.9664E-03		ENSG00000251219
LINC01224	0.0281	7.6113E-01	8.70E-01	ENSG00000269416
LINC01227	-0.0296	2.9161E-01		ENSG00000260737
LINC01229	0.0055	9.6483E-01	9.82E-01	ENSG00000260876
LINC01231	-0.0621	3.0463E-01	4.97E-01	ENSG00000236511
LINC01233	0.0035	9.3945E-01		ENSG00000269364
LINC01234	0.5659	1.4856E-03	9.74E-03	ENSG00000249550
LINC01235	0.0260	9.5161E-02		ENSG00000270547
LINC01237	0.0552	5.2613E-01	7.02E-01	ENSG00000233806
LINC01238	0.3153	1.7874E-02	6.62E-02	ENSG00000261186
LINC01238	0.0170	8.5412E-01	9.25E-01	ENSG00000237940
LINC01239	-0.0486	5.8472E-01	7.50E-01	ENSG00000234840
LINC01241	-0.0159	7.0557E-01		ENSG00000236306
LINC01249	-0.0106	6.9440E-01		ENSG00000231532
LINC01250	0.0389	1.4678E-01	3.06E-01	ENSG00000234423
LINC01252	-0.0352	7.1877E-01	8.44E-01	ENSG00000247157
LINC01254	-0.0006	8.3301E-01		ENSG00000260913
LINC01255	-0.0084	6.6785E-01		ENSG00000267252
LINC01257	-0.0116	8.7660E-01	9.37E-01	ENSG00000204603
LINC01258	-0.0008	8.2945E-01		ENSG00000249534
LINC01264	0.0127	5.2531E-01		ENSG00000229630

LINC01265	-0.0441	5.5171E-01	7.23E-01	ENSG00000249740
LINC01266	-0.0245	4.7952E-01	6.65E-01	ENSG00000224957
LINC01267	-0.0759	1.9474E-01	3.71E-01	ENSG00000251576
LINC01269	-0.0180	7.9769E-01		ENSG00000258689
LINC01270	0.0393	6.8138E-01	8.19E-01	ENSG00000203999
LINC01271	0.0082	4.0768E-01		ENSG00000233077
LINC01273	-0.1471	1.4181E-01	2.99E-01	ENSG00000231742
LINC01276	-0.0272	6.3347E-01		ENSG00000226917
LINC01278	-0.3751	2.1329E-06	4.99E-05	ENSG00000235437
LINC01284	-0.0246	8.8400E-01		ENSG00000230317
LINC01285	0.0234	7.8618E-01	8.85E-01	ENSG00000203650
LINC01287	0.0165	2.9853E-01		ENSG00000234722
LINC01289	-0.0948	7.3461E-01	8.54E-01	ENSG00000253734
LINC01290	-0.1076	3.5993E-02		ENSG00000260468
LINC01291	-0.0300	7.4736E-01	8.62E-01	ENSG00000204792
LINC01300	-0.0222	3.9191E-01		ENSG00000253595
LINC01303	0.0259	8.7759E-01	9.38E-01	ENSG00000250548
LINC01307	0.0025	8.0201E-01		ENSG00000231671
LINC01310	0.0062	7.2804E-01		ENSG00000205632
LINC01315	0.0562	4.4357E-01	6.34E-01	ENSG00000229891
LINC01317	0.0155	2.8695E-01		ENSG00000228262
LINC01322	0.2220	3.1045E-03	1.72E-02	ENSG00000244128
LINC01324	0.0060	8.4300E-01	9.18E-01	ENSG00000241767
LINC01331	0.0407	2.5790E-01		ENSG00000248673
LINC01339	0.0498	2.1988E-01		ENSG00000248555
LINC01340	0.0050	3.9267E-01		ENSG00000250331
LINC01342	-2.6146	1.9691E-04		ENSG00000223823
LINC01348	0.0209	5.5317E-01		ENSG00000280587
LINC01350	-0.0061	7.5805E-01		ENSG00000228309
LINC01353	0.1592	1.5148E-01	3.13E-01	ENSG00000231507
LINC01356	-0.0851	3.7081E-01	5.66E-01	ENSG00000215866
LINC01359	-0.0142	8.1083E-01		ENSG00000226891
LINC01361	-0.1010	2.1123E-01	3.91E-01	ENSG00000236268
LINC01362	0.0003	8.5188E-01	9.24E-01	ENSG00000230817
LINC01363	-0.0201	5.1901E-01		ENSG00000231605
LINC01364	-0.0097	6.9475E-01		ENSG00000227290
LINC01374	-0.1384	1.9459E-01	3.71E-01	ENSG00000280560
LINC01375	0.0132	4.5858E-01		ENSG00000226159
LINC01376	0.0022	9.8005E-01	9.89E-01	ENSG00000236204
LINC01392	-0.0091	9.4421E-01		ENSG00000233607
LINC01393	0.1295	3.7274E-02	1.15E-01	ENSG00000225535
LINC01396	-0.0075	8.1274E-01		ENSG00000273396
LINC01397	0.0350	2.7606E-01		ENSG00000258673
LINC01399	0.2921	3.4214E-02	1.08E-01	ENSG00000233080
LINC01404	0.0960	1.9419E-01	3.70E-01	ENSG00000258240
LINC01405	0.0064	7.0780E-01		ENSG00000185847
LINC01410	-0.0901	3.6029E-01	5.56E-01	ENSG00000238113
LINC01411	0.0263	1.2851E-01		ENSG00000249306
LINC01412	-0.0253	6.8772E-01		ENSG00000232606

LINC01413	0.0388	1.5866E-02		ENSG00000260172
LINC01414	0.4020	6.9588E-04	5.37E-03	ENSG00000253554
LINC01416	0.0822	3.1302E-01	5.06E-01	ENSG00000260930
LINC01426	0.2261	5.8249E-02	1.60E-01	ENSG00000234380
LINC01431	0.1258	1.7545E-01	3.45E-01	ENSG00000232645
LINC01433	-0.0578	1.3516E-01		ENSG00000230176
LINC01435	-0.0010	6.1734E-01		ENSG00000229981
LINC01443	-0.0162	4.6078E-01		ENSG00000266554
LINC01446	-0.0086	8.9195E-01		ENSG00000205628
LINC01451	-0.0464	4.9660E-01	6.80E-01	ENSG00000279141
LINC01456	0.0954	3.3017E-01	5.25E-01	ENSG00000225882
LINC01460	0.0822	2.7597E-01	4.67E-01	ENSG00000205334
LINC01465	0.1688	1.2258E-01	2.71E-01	ENSG00000221949
LINC01467	-0.0195	8.6684E-01		ENSG00000258977
LINC01470	0.0152	2.8816E-01		ENSG00000249484
LINC01474	-0.1529	7.5213E-02		ENSG00000236849
LINC01477	-0.0837	2.0995E-01	3.89E-01	ENSG00000261715
LINC01479	-0.1628	8.4120E-03	3.73E-02	ENSG00000255772
LINC01480	-0.3414	1.7903E-02	6.63E-02	ENSG00000270164
LINC01482	-0.0358	5.7003E-01	7.38E-01	ENSG00000267659
LINC01484	0.0669	4.5900E-01	6.48E-01	ENSG00000253686
LINC01485	0.0277	2.9961E-01		ENSG00000254211
LINC01492	-0.0187	4.7941E-01		ENSG00000225564
LINC01494	0.0106	9.0514E-01	9.52E-01	ENSG00000228135
LINC01495	-0.0027	8.6173E-01		ENSG00000255323
LINC01497	0.2068	5.5695E-02	1.55E-01	ENSG00000237560
LINC01498	-0.0448	5.3092E-01		ENSG00000247213
LINC01500	-0.0135	9.7012E-01	9.85E-01	ENSG00000258583
LINC01503	-0.2883	2.1629E-03	1.31E-02	ENSG00000233901
LINC01504	0.2737	2.6131E-02	8.79E-02	ENSG00000225434
LINC01505	0.0322	3.8833E-01	5.83E-01	ENSG00000234323
LINC01508	0.0219	8.6189E-01	9.30E-01	ENSG00000231107
LINC01512	0.0869	2.9155E-01	4.83E-01	ENSG00000289313
LINC01524	0.0454	3.1309E-01	5.06E-01	ENSG00000234948
LINC01526	0.0205	2.2243E-01		ENSG00000224995
LINC01529	0.4840	9.2964E-03	4.02E-02	ENSG00000225872
LINC01535	-0.0370	6.9019E-01	8.26E-01	ENSG00000226686
LINC01537	-0.0233	9.6003E-01		ENSG00000227467
LINC01539	0.0005	8.5253E-01		ENSG00000267712
LINC01541	-0.0116	8.5910E-01		ENSG00000260676
LINC01545	-0.1591	9.7938E-02	2.31E-01	ENSG00000204904
LINC01547	0.0501	5.1175E-01	6.92E-01	ENSG00000183250
LINC01550	-0.1542	1.5045E-01	3.11E-01	ENSG00000246223
LINC01551	-0.0283	8.2818E-01		ENSG00000186960
LINC01553	-0.0003	9.2995E-01		ENSG00000235931
LINC01554	-0.0431	5.0905E-01	6.89E-01	ENSG00000236882
LINC01555	-0.0154	7.6695E-01		ENSG00000180869
LINC01558	0.0032	7.7893E-01		ENSG00000146521
LINC01559	-0.0055	8.6674E-01		ENSG00000180861

LINC01561	-0.0521	5.6220E-01	7.31E-01	ENSG00000177234
LINC01562	0.0969	2.8693E-01	4.78E-01	ENSG00000203356
LINC01563	-0.0756	4.4466E-01	6.35E-01	ENSG00000236819
LINC01564	-0.0015	9.9966E-01	1.00E+00	ENSG00000235899
LINC01565	0.0436	5.8319E-01	7.49E-01	ENSG00000198685
LINC01567	0.3558	2.2767E-02	7.94E-02	ENSG00000224310
LINC01574	-0.0762	4.2657E-01	6.18E-01	ENSG00000248859
LINC01586	0.5000	1.7061E-08	8.93E-07	ENSG00000249487
LINC01587	0.0425	5.4727E-01	7.19E-01	ENSG00000082929
LINC01592	0.1020	3.7239E-02		ENSG00000253658
LINC01599	-0.0313	7.1335E-01	8.40E-01	ENSG00000214900
LINC01600	0.0081	4.4009E-01		ENSG00000164385
LINC01602	0.0171	7.7787E-01	8.80E-01	ENSG00000205293
LINC01603	0.0055	7.7102E-01		ENSG00000253479
LINC01605	0.0451	6.3707E-01	7.89E-01	ENSG00000253414
LINC01607	0.0537	5.5023E-01	7.22E-01	ENSG00000272138
LINC01608	-0.0101	8.7283E-01	9.36E-01	ENSG00000253877
LINC01611	0.0011	9.8074E-01		ENSG00000231776
LINC01612	-0.3038	2.7163E-02	9.05E-02	ENSG00000250266
LINC01614	0.0078	5.4022E-01		ENSG00000230838
LINC01615	-0.0042	9.8646E-01		ENSG00000223485
LINC01616	-0.0462	9.6752E-01	9.83E-01	ENSG00000261340
LINC01618	-0.0045	8.6624E-01	9.32E-01	ENSG00000250302
LINC01619	-0.0232	8.1370E-01	9.02E-01	ENSG00000257242
LINC01622	-0.0003	8.3213E-01		ENSG00000286785
LINC01623	-0.0208	4.2594E-01		ENSG00000225595
LINC01624	-0.0072	8.0180E-01		ENSG00000227508
LINC01625	0.1605	1.0996E-01	2.51E-01	ENSG00000238099
LINC01629	0.0152	1.2979E-01		ENSG00000258602
LINC01634	-0.0539	2.8026E-01		ENSG00000235295
LINC01635	0.0513	5.4166E-01	7.15E-01	ENSG00000228397
LINC01637	-0.0258	7.8673E-01	8.85E-01	ENSG00000237476
LINC01638	0.0066	9.4092E-01	9.71E-01	ENSG00000233521
LINC01640	-0.0062	3.9740E-01		ENSG00000231253
LINC01643	0.0258	2.8860E-01		ENSG00000236052
LINC01645	0.0014	8.8941E-01		ENSG00000224968
LINC01647	0.1188	2.1710E-02		ENSG00000235643
LINC01648	0.0073	5.7138E-01		ENSG00000233399
LINC01649	-0.0974	7.3610E-01	8.55E-01	ENSG00000228127
LINC01654	0.0608	5.7562E-03	2.79E-02	ENSG00000261781
LINC01656	-0.0066	6.6590E-01		ENSG00000232655
LINC01659	0.0319	8.3326E-02		ENSG00000234928
LINC01668	-0.0039	5.9759E-01		ENSG00000283051
LINC01670	0.2011	2.5124E-02	8.55E-02	ENSG00000279094
LINC01671	-0.0003	8.3564E-01		ENSG00000225431
LINC01679	0.0977	4.3929E-02		ENSG00000237989
LINC01681	0.0118	7.3017E-01		ENSG00000233985
LINC01686	-0.1417	1.1825E-01	2.64E-01	ENSG00000261504
LINC01687	0.0081	5.0543E-01		ENSG00000233215

LINC01694	0.0986	3.0288E-01	4.95E-01	ENSG00000233922
LINC01695	-0.2963	2.4148E-02	8.30E-02	ENSG00000236532
LINC01701	-0.0072	8.7637E-01		ENSG00000232212
LINC01704	-0.0004	5.4546E-01		ENSG00000231666
LINC01707	-0.0254	6.3737E-01		ENSG00000223883
LINC01708	0.0295	8.7592E-02		ENSG00000224445
LINC01709	0.0465	2.1485E-01		ENSG00000226715
LINC01711	0.0301	2.7486E-01		ENSG00000268941
LINC01730	-0.0615	7.6658E-01	8.73E-01	ENSG00000275491
LINC01732	-0.1101	2.7150E-01	4.61E-01	ENSG00000237292
LINC01736	0.0069	5.8404E-01		ENSG00000228058
LINC01740	-0.0139	5.8433E-01		ENSG00000228067
LINC01742	-0.0249	3.5393E-01		ENSG00000275852
LINC01748	0.1303	1.5001E-01	3.10E-01	ENSG00000226476
LINC01750	-0.3668	5.9391E-04	4.73E-03	ENSG00000231437
LINC01751	0.1002	2.4844E-01	4.35E-01	ENSG00000261249
LINC01756	-0.0098	7.9609E-01		ENSG00000230523
LINC01757	0.0217	2.4535E-01		ENSG00000228105
LINC01758	-0.0113	6.5440E-01		ENSG00000229639
LINC01760	-0.0004	9.7222E-01		ENSG00000228504
LINC01762	0.1156	1.4837E-01	3.09E-01	ENSG00000233154
LINC01765	-0.1965	9.3000E-02	2.24E-01	ENSG00000233730
LINC01766	-0.0083	8.0728E-01		ENSG00000224910
LINC01767	-0.2226	2.7726E-02	9.18E-02	ENSG00000223956
LINC01772	0.1294	1.0957E-01	2.50E-01	ENSG00000226029
LINC01776	0.0676	2.5159E-01	4.38E-01	ENSG00000226053
LINC01777	-0.0044	8.0630E-01		ENSG00000235054
LINC01778	-0.0823	3.9994E-01	5.94E-01	ENSG00000223382
LINC01783	0.1477	1.2688E-02	5.10E-02	ENSG00000233421
LINC01787	0.0098	4.5066E-01		ENSG00000231987
LINC01798	-0.0359	5.0377E-01		ENSG00000232046
LINC01800	-0.0230	5.6937E-01		ENSG00000234572
LINC01801	-0.0867	3.6729E-01	5.63E-01	ENSG00000267767
LINC01805	-0.0244	7.6918E-01		ENSG00000223863
LINC01806	0.1195	1.4835E-01	3.08E-01	ENSG00000227403
LINC01807	0.1302	2.0871E-01	3.88E-01	ENSG00000232023
LINC01811	-0.0275	7.7219E-01	8.77E-01	ENSG00000226320
LINC01814	0.1548	1.4994E-01	3.10E-01	ENSG00000236008
LINC01816	0.1130	2.6947E-01	4.59E-01	ENSG00000231327
LINC01819	0.0067	3.4176E-01		ENSG00000231826
LINC01821	0.0662	4.0532E-01	5.99E-01	ENSG00000225539
LINC01823	-0.0063	6.4740E-01		ENSG00000224655
LINC01824	0.0209	2.2276E-01		ENSG00000226488
LINC01825	-0.0200	6.3731E-01		ENSG00000223466
LINC01829	-0.0208	7.7285E-01	8.77E-01	ENSG00000236780
LINC01833	-0.0751	4.1028E-01	6.03E-01	ENSG00000259439
LINC01837	0.0150	3.4110E-01		ENSG00000267489
LINC01838	0.0479	1.7055E-02		ENSG00000269037
LINC01841	0.0235	6.1270E-01	7.71E-01	ENSG00000266913

LINC01842	0.0190	2.4658E-01		ENSG00000267147
LINC01844	0.0662	4.7078E-01	6.58E-01	ENSG00000236714
LINC01846	0.0145	1.1637E-01		ENSG00000248901
LINC01847	0.0572	1.1798E-01		ENSG00000253311
LINC01849	0.0011	9.2940E-01		ENSG00000234988
LINC01852	-0.1784	9.3824E-02	2.25E-01	ENSG00000236914
LINC01854	-0.0199	6.3232E-01		ENSG00000204460
LINC01855	-0.0199	3.9958E-01		ENSG00000267517
LINC01856	0.0008	8.6027E-01		ENSG00000237574
LINC01858	-0.0084	7.9408E-01		ENSG00000261615
LINC01863	-0.0748	4.5263E-01	6.42E-01	ENSG00000253959
LINC01864	0.3806	2.0196E-02	7.26E-02	ENSG00000267522
LINC01869	0.1029	2.1971E-01	4.01E-01	ENSG00000180279
LINC01873	-0.0008	9.6502E-01	9.82E-01	ENSG00000232164
LINC01876	0.2271	4.1741E-02	1.25E-01	ENSG00000226383
LINC01879	0.0691	2.2595E-01	4.09E-01	ENSG00000276397
LINC01884	-0.0378	2.7914E-01		ENSG00000233587
LINC01885	-0.0003	7.8981E-01	8.87E-01	ENSG00000237880
LINC01894	-0.0605	5.3324E-01		ENSG00000264345
LINC01895	0.0085	7.1474E-01		ENSG00000259256
LINC01899	-0.0146	1.5526E-01	3.18E-01	ENSG00000265352
LINC01905	-0.1041	3.0231E-01	4.95E-01	ENSG00000267057
LINC01907	0.0511	4.2568E-01		ENSG00000226125
LINC01909	0.2092	3.2688E-02	1.04E-01	ENSG00000266258
LINC01913	0.0236	1.0579E-01		ENSG00000214691
LINC01914	-0.0134	9.2359E-01		ENSG00000234362
LINC01915	0.0514	7.8965E-03	3.56E-02	ENSG00000265485
LINC01916	-0.0010	7.9016E-01		ENSG00000264232
LINC01917	0.0031	5.2783E-01		ENSG00000260433
LINC01918	0.3218	1.4801E-02	5.75E-02	ENSG00000226508
LINC01919	-0.0100	6.3130E-01		ENSG00000263438
LINC01931	-0.0406	3.3106E-01		ENSG00000162947
LINC01933	0.0919	3.5599E-01	5.51E-01	ENSG00000254226
LINC01942	0.0540	2.4760E-02		ENSG00000253428
LINC01948	-0.0060	9.9700E-01	9.98E-01	ENSG00000248727
LINC01954	0.0015	8.2703E-01		ENSG00000271952
LINC01960	-0.0292	5.8298E-01		ENSG00000260868
LINC01962	0.0865	3.1132E-01	5.04E-01	ENSG00000248473
LINC01963	-0.3533	1.1974E-02	4.87E-02	ENSG00000260804
LINC01964	-0.0408	2.8444E-01		ENSG00000260840
LINC01968	-0.0340	5.2242E-01		ENSG00000237222
LINC01970	0.5902	1.1501E-03	7.92E-03	ENSG00000265692
LINC01973	0.0832	4.0318E-01	5.97E-01	ENSG00000204283
LINC01976	0.0190	5.8880E-01		ENSG00000261514
LINC01978	-0.1373	1.0060E-01	2.36E-01	ENSG00000262188
LINC01979	-0.0606	4.6772E-01		ENSG00000262585
LINC01980	-0.3681	2.3911E-05	3.53E-04	ENSG00000225548
LINC01981	0.0035	5.0335E-01		ENSG00000229243
LINC01985	-0.0487	1.3338E-01		ENSG00000227260

LINC01987	-0.0035	8.0419E-01		ENSG00000267790
LINC01988	-0.0167	6.6283E-01		ENSG00000283036
LINC01989	0.0051	5.2547E-01		ENSG00000261156
LINC01990	-0.5156	7.9619E-03	3.58E-02	ENSG00000273125
LINC01992	-0.0149	7.0537E-01		ENSG00000260019
LINC01993	-0.1020	3.6824E-01	5.64E-01	ENSG00000204277
LINC01994	0.0129	7.7434E-01	8.78E-01	ENSG00000241098
LINC01995	0.0011	2.0630E-01	3.85E-01	ENSG00000244247
LINC01998	-0.0465	5.2947E-01	7.04E-01	ENSG00000243321
LINC02003	0.0118	8.3213E-01	9.12E-01	ENSG00000264026
LINC02004	-0.0029	6.9496E-01		ENSG00000240006
LINC02008	-0.0048	6.9027E-01		ENSG00000239440
LINC02009	0.0616	2.9550E-01	4.87E-01	ENSG00000283646
LINC02010	0.0102	5.2694E-01		ENSG00000242671
LINC02015	-0.0143	6.1071E-01		ENSG00000231574
LINC02016	-0.0126	6.6132E-01		ENSG00000244215
LINC02018	-0.3781	1.7413E-03	1.10E-02	ENSG00000272690
LINC02021	-0.2807	1.2325E-02	4.98E-02	ENSG00000249846
LINC02022	0.0259	1.4165E-01		ENSG00000232746
LINC02024	0.0571	5.1739E-01	6.95E-01	ENSG00000241213
LINC02026	0.0373	5.7967E-01	7.46E-01	ENSG00000214146
LINC02028	-0.0658	4.8088E-01	6.66E-01	ENSG00000230102
LINC02029	0.0109	7.6711E-01	8.74E-01	ENSG00000241544
LINC02032	-0.1862	5.5092E-02	1.54E-01	ENSG00000241131
LINC02035	-0.0070	9.3760E-01	9.69E-01	ENSG00000273033
LINC02037	0.0093	7.1883E-01		ENSG00000238097
LINC02043	0.0200	9.1491E-02		ENSG00000232233
LINC02044	-0.3702	7.8967E-03	3.56E-02	ENSG00000243795
LINC02048	0.0289	5.0420E-02		ENSG00000228271
LINC02052	0.0014	6.3164E-01		ENSG00000224406
LINC02054	-0.0523	2.6167E-01		ENSG00000224153
LINC02055	0.1628	7.6670E-02	1.95E-01	ENSG00000254101
LINC02057	-0.0887	3.5806E-01	5.54E-01	ENSG00000249279
LINC02067	-0.0483	1.7629E-01		ENSG00000240567
LINC02069	-0.0194	4.9147E-01		ENSG00000229433
LINC02070	0.0201	6.9084E-01	8.26E-01	ENSG00000241328
LINC02074	0.0653	2.9108E-01	4.83E-01	ENSG00000266357
LINC02076	-0.0561	7.3980E-01	8.57E-01	ENSG00000220161
LINC02078	0.0349	5.3466E-01		ENSG00000267719
LINC02080	-0.0122	4.9703E-01		ENSG00000267065
LINC02082	0.0316	6.5091E-01	7.98E-01	ENSG00000242268
LINC02092	0.0770	2.7655E-02		ENSG00000234721
LINC02101	-0.1518	8.2055E-02	2.04E-01	ENSG00000248132
LINC02102	-0.0498	4.3900E-01		ENSG00000248677
LINC02104	-0.0606	2.6497E-01		ENSG00000271334
LINC02105	-0.0086	9.4526E-01		ENSG00000250447
LINC02111	0.0249	4.7325E-01		ENSG00000250822
LINC02115	-0.0016	9.1930E-01		ENSG00000248757
LINC02123	0.0292	1.9860E-01		ENSG00000250668

LINC02126	0.0351	3.3964E-02		ENSG00000259847
LINC02128	0.3091	2.8258E-02	9.31E-02	ENSG00000261241
LINC02133	-0.0341	4.1868E-01		ENSG00000261231
LINC02138	0.0520	5.1938E-02		ENSG00000205015
LINC02139	-0.0125	9.8269E-01		ENSG00000278214
LINC02142	-0.0065	8.5749E-01		ENSG00000223597
LINC02145	0.0013	7.1693E-01		ENSG00000250490
LINC02150	0.0025	6.8725E-01		ENSG00000248150
LINC02154	0.0853	1.5836E-02		ENSG00000235385
LINC02159	0.0552	1.5951E-01		ENSG00000253417
LINC02160	-0.0038	9.3620E-01		ENSG00000251443
LINC02171	0.0426	4.4163E-02		ENSG00000250632
LINC02175	0.1875	5.0804E-02	1.45E-01	ENSG00000262155
LINC02177	-0.2380	3.6669E-02	1.13E-01	ENSG00000261617
LINC02178	0.0116	4.9025E-01		ENSG00000261092
LINC02181	-0.0216	6.3439E-01		ENSG00000232190
LINC02188	0.0305	1.5707E-02	6.00E-02	ENSG00000261175
LINC02191	-0.0036	7.7959E-01		ENSG00000261636
LINC02192	-0.0235	6.5841E-01		ENSG00000261325
LINC02195	-0.0708	1.7769E-01		ENSG00000236481
LINC02200	-0.1661	9.3461E-02	2.24E-01	ENSG00000250358
LINC02207	-0.0010	8.5471E-01		ENSG00000258476
LINC02210	-0.0973	7.0894E-02	1.84E-01	ENSG00000204650
LINC02212	-0.0314	2.8476E-01		ENSG00000249396
LINC02217	0.0024	4.1119E-01		ENSG00000248455
LINC02219	-0.0070	7.3628E-01		ENSG00000253787
LINC02226	-0.0085	9.5895E-01		ENSG00000245729
LINC02227	0.0213	7.1378E-02		ENSG00000276778
LINC02231	-0.0489	1.4861E-01		ENSG00000248995
LINC02232	0.4668	1.0680E-03	7.47E-03	ENSG00000250125
LINC02234	0.0660	5.0117E-02		ENSG00000248202
LINC02235	-0.0286	6.7817E-01	8.16E-01	ENSG00000254689
LINC02240	0.2356	6.2844E-02	1.69E-01	ENSG00000260192
LINC02241	0.0567	1.7565E-01		ENSG00000251629
LINC02245	0.1380	5.9976E-02	1.63E-01	ENSG00000237638
LINC02249	-0.0839	3.8466E-01	5.79E-01	ENSG00000225930
LINC02253	0.1985	2.3637E-02	8.16E-02	ENSG00000259485
LINC02254	-0.0063	9.4314E-01	9.72E-01	ENSG00000259664
LINC02256	-0.0874	3.1749E-01	5.11E-01	ENSG00000289083
LINC02259	-0.0519	5.2613E-01	7.02E-01	ENSG00000259783
LINC02261	-0.1176	7.6365E-02		ENSG00000249699
LINC02262	0.0003	9.1139E-01		ENSG00000224932
LINC02263	0.0035	7.2205E-01		ENSG00000228358
LINC02265	0.3846	3.7160E-03	1.99E-02	ENSG00000249241
LINC02266	0.0228	2.1129E-01		ENSG00000272727
LINC02268	0.0087	9.2841E-01	9.65E-01	ENSG00000248174
LINC02269	-0.0001	9.7533E-01		ENSG00000250708
LINC02273	0.0183	2.6313E-01		ENSG00000245954
LINC02274	0.0066	6.5652E-01	8.02E-01	ENSG00000258586

LINC02280	-0.0081	9.8368E-01		ENSG00000260792
LINC02282	0.0163	6.4658E-01	7.95E-01	ENSG00000257056
LINC02287	0.1493	1.6588E-01	3.33E-01	ENSG00000258499
LINC02288	0.0729	4.2833E-01	6.20E-01	ENSG00000246548
LINC02289	-0.1938	9.4676E-02	2.26E-01	ENSG00000258819
LINC02290	-0.0432	8.1289E-01	9.01E-01	ENSG00000258502
LINC02292	0.0028	5.6230E-01		ENSG00000258630
LINC02293	0.1226	1.3284E-01	2.86E-01	ENSG00000257185
LINC02294	-0.0391	3.5429E-01		ENSG00000257845
LINC02301	-0.0717	4.5962E-01	6.48E-01	ENSG00000258743
LINC02303	0.0601	1.4111E-01		ENSG00000258616
LINC02313	0.0209	7.1115E-01	8.39E-01	ENSG00000258474
LINC02317	0.0157	2.8144E-01		ENSG00000258678
LINC02318	-0.0068	9.5149E-01		ENSG00000258390
LINC02321	-0.0693	2.9401E-01	4.86E-01	ENSG00000258884
LINC02323	0.0725	4.2067E-02		ENSG00000259230
LINC02328	-0.0231	8.0809E-01	8.98E-01	ENSG00000258733
LINC02330	0.0307	1.1950E-01		ENSG00000258770
LINC02331	-0.0043	8.7771E-01		ENSG00000235269
LINC02332	-0.1176	1.6069E-01		ENSG00000259054
LINC02333	0.0010	7.4652E-01		ENSG00000271564
LINC02338	0.0168	3.4968E-01		ENSG00000277448
LINC02345	-0.0125	3.6552E-01		ENSG00000259225
LINC02346	-0.0190	7.1504E-01		ENSG00000206187
LINC02347	0.0129	7.2573E-01		ENSG00000214043
LINC02348	0.2111	3.6590E-02	1.13E-01	ENSG00000286733
LINC02355	-0.0652	2.1076E-01		ENSG00000248210
LINC02356	0.0127	8.9137E-01	9.46E-01	ENSG00000257595
LINC02359	0.0417	4.7930E-02		ENSG00000284848
LINC02363	0.5633	4.1092E-04	3.52E-03	ENSG00000180712
LINC02365	0.0261	1.0152E-01		ENSG00000254233
LINC02367	0.0785	3.9070E-01	5.85E-01	ENSG00000260423
LINC02372	-0.0181	4.9459E-01		ENSG00000249873
LINC02380	-0.0175	6.7648E-01		ENSG00000248505
LINC02381	0.3937	1.0149E-04	1.13E-03	ENSG00000250742
LINC02387	-0.0247	9.1755E-01	9.59E-01	ENSG00000256232
LINC02388	-0.0648	4.6284E-01	6.51E-01	ENSG00000257259
LINC02389	-0.0115	9.7648E-01		ENSG00000255693
LINC02392	-0.0239	5.4340E-01		ENSG00000258183
LINC02395	0.0136	6.3417E-01	7.87E-01	ENSG00000257771
LINC02398	0.0142	1.8484E-01		ENSG00000256287
LINC02399	0.5973	1.6957E-03	1.08E-02	ENSG00000257725
LINC02400	0.0043	1.2527E-01		ENSG00000257784
LINC02404	0.0488	1.9930E-01		ENSG00000257893
LINC02405	0.0111	2.0681E-01		ENSG00000249345
LINC02408	0.0045	7.0590E-01	8.36E-01	ENSG00000203585
LINC02418	-0.0418	8.1163E-01	9.00E-01	ENSG00000214039
LINC02421	-0.0136	6.0102E-01		ENSG00000255970
LINC02422	0.1073	2.8191E-01	4.73E-01	ENSG00000255760

LINC02426	-0.0017	7.4516E-01		ENSG00000257747
LINC02443	0.0048	4.8680E-01		ENSG00000256115
LINC02444	-0.0047	7.4658E-01		ENSG00000258123
LINC02449	-0.1846	8.8909E-02	2.17E-01	ENSG00000215241
LINC02453	0.4710	4.5318E-03	2.32E-02	ENSG00000245017
LINC02458	-0.0379	6.6922E-01	8.10E-01	ENSG00000246363
LINC02465	-0.0021	8.1562E-01		ENSG00000249618
LINC02470	0.0082	4.7068E-01	6.58E-01	ENSG00000225231
LINC02473	0.1720	1.2041E-01	2.68E-01	ENSG00000261121
LINC02475	-0.0024	9.8641E-01		ENSG00000251350
LINC02478	0.0224	7.3960E-01	8.57E-01	ENSG00000285373
LINC02482	-0.3010	4.7478E-03	2.40E-02	ENSG00000251580
LINC02487	0.0963	1.2711E-01	2.78E-01	ENSG00000203688
LINC02488	0.0004	6.4714E-01		ENSG00000249362
LINC02489	-0.1935	6.2334E-02	1.68E-01	ENSG00000255007
LINC02498	0.0031	6.4983E-01		ENSG00000283083
LINC02499	0.0071	6.4722E-01		ENSG00000250436
LINC02508	0.0060	7.7864E-01		ENSG00000251619
LINC02511	-0.0835	4.0515E-01	5.99E-01	ENSG00000248869
LINC02516	-0.0130	9.4147E-01		ENSG00000261083
LINC02518	0.0455	7.1952E-02		ENSG00000232316
LINC02526	0.2230	6.8899E-02	1.80E-01	ENSG00000229654
LINC02532	-0.2005	8.6446E-02	2.12E-01	ENSG00000235142
LINC02539	0.0179	5.1776E-01	6.96E-01	ENSG00000234956
LINC02541	0.0183	8.3481E-01	9.14E-01	ENSG00000230943
LINC02549	0.0084	8.4697E-01		ENSG00000226497
LINC02550	-0.0079	6.9943E-01		ENSG00000271584
LINC02552	0.1992	9.3593E-02	2.25E-01	ENSG00000256422
LINC02560	0.0622	5.1060E-01	6.90E-01	ENSG00000268307
LINC02567	-0.0648	2.6754E-01	4.57E-01	ENSG00000237552
LINC02572	0.1116	2.7982E-01	4.71E-01	ENSG00000229536
LINC02574	-0.1801	7.4881E-02	1.91E-01	ENSG00000233975
LINC02576	0.0180	7.9615E-01	8.91E-01	ENSG00000232613
LINC02579	-0.0424	5.3295E-01		ENSG00000233694
LINC02585	-0.0566	5.2059E-01	6.98E-01	ENSG00000228350
LINC02587	-0.1909	9.4636E-02	2.26E-01	ENSG00000229108
LINC02593	0.1742	1.1329E-01	2.57E-01	ENSG00000223764
LINC02594	0.1996	9.1025E-02	2.20E-01	ENSG00000267440
LINC02596	-0.0079	9.5029E-01		ENSG00000233431
LINC02601	0.2921	2.5748E-02	8.70E-02	ENSG00000223714
LINC02603	-0.0020	9.2295E-01		ENSG00000230262
LINC02606	-0.0641	4.9838E-01	6.81E-01	ENSG00000284693
LINC02607	0.0652	2.7763E-02	9.19E-02	ENSG00000228971
LINC02608	-0.0112	8.0131E-01		ENSG00000226251
LINC02609	-0.3433	7.9500E-06	1.47E-04	ENSG00000233593
LINC02611	0.0010	6.0296E-01		ENSG00000226791
LINC02613	-0.0128	9.7707E-01		ENSG00000231367
LINC02614	-0.0113	9.0104E-01	9.51E-01	ENSG00000241288
LINC02615	0.2249	5.2093E-02	1.47E-01	ENSG00000251432

LINC02616	0.0544	2.5680E-02		ENSG00000261761
LINC02617	-0.0323	3.8249E-01		ENSG00000256288
LINC02626	-0.0126	8.0557E-01		ENSG00000236799
LINC02631	-0.0713	1.3637E-01		ENSG00000237772
LINC02642	0.0075	6.3650E-01		ENSG00000232591
LINC02649	-0.0699	1.7362E-01		ENSG00000215244
LINC02652	0.0030	8.1413E-01		ENSG00000237128
LINC02653	-0.2000	8.2566E-02	2.05E-01	ENSG00000236373
LINC02668	0.0192	2.9635E-01		ENSG00000226762
LINC02671	0.0019	7.6912E-01		ENSG00000224714
LINC02677	-0.0026	3.9814E-01		ENSG00000242147
LINC02679	-0.0288	4.3195E-01		ENSG00000226676
LINC02681	0.0211	3.1199E-01		ENSG00000229649
LINC02683	-0.0556	6.4103E-01	7.92E-01	ENSG00000254438
LINC02685	0.0263	7.1321E-01	8.40E-01	ENSG00000254654
LINC02687	0.0288	1.4419E-01	3.02E-01	ENSG00000255267
LINC02688	0.0043	9.3797E-01	9.69E-01	ENSG00000254872
LINC02691	0.0232	1.9846E-01		ENSG00000258913
LINC02693	0.3124	8.4581E-03	3.75E-02	ENSG00000212719
LINC02694	0.1051	2.3505E-01	4.19E-01	ENSG00000175779
LINC02696	-0.0340	6.9327E-01	8.28E-01	ENSG00000254427
LINC02702	-0.0024	4.9252E-01		ENSG00000237937
LINC02709	0.0354	6.3840E-01	7.90E-01	ENSG00000245522
LINC02714	0.0148	7.1207E-01	8.40E-01	ENSG00000251226
LINC02717	-0.0175	7.2238E-01		ENSG00000285735
LINC02721	-0.0147	5.7968E-01		ENSG00000255133
LINC02723	-0.0027	9.9306E-01		ENSG00000231680
LINC02724	0.0430	5.6226E-01	7.31E-01	ENSG00000181908
LINC02728	-0.1242	2.3959E-01	4.24E-01	ENSG00000251323
LINC02731	0.1325	2.1269E-01	3.93E-01	ENSG00000204241
LINC02733	-0.0298	5.2902E-01		ENSG00000255553
LINC02734	-0.0326	5.4736E-01		ENSG00000255382
LINC02742	0.0092	4.7362E-01		ENSG00000249867
LINC02744	0.0009	6.4059E-01	7.91E-01	ENSG00000254863
LINC02749	-0.0169	8.0747E-01	8.98E-01	ENSG00000254480
LINC02751	0.0150	7.6044E-01	8.70E-01	ENSG00000254946
LINC02754	0.0032	9.7624E-01	9.88E-01	ENSG00000287312
LINC02755	0.0044	9.2960E-01		ENSG00000254530
LINC02761	0.0184	5.8888E-01		ENSG00000255362
LINC02762	0.1983	2.3161E-02	8.05E-02	ENSG00000250303
LINC02763	0.0333	2.1477E-01	3.95E-01	ENSG00000254968
LINC02764	0.0117	3.5495E-01		ENSG00000255484
LINC02774	0.0236	3.1245E-01	5.06E-01	ENSG00000226828
LINC02777	0.1439	1.6383E-01	3.30E-01	ENSG00000232453
LINC02798	-0.0675	3.5912E-01	5.55E-01	ENSG00000227082
LINC02801	0.0588	3.7268E-01	5.68E-01	ENSG00000284240
LINC02802	-0.2674	2.2974E-03	1.37E-02	ENSG00000232527
LINC02803	-0.0483	4.4076E-01	6.31E-01	ENSG00000227740
LINC02805	-0.0394	1.5605E-01		ENSG00000232265

LINC02808	-0.0508	3.6025E-01		ENSG00000284696
LINC02809	0.0029	6.4189E-01		ENSG00000276255
LINC02810	0.0100	5.8087E-01		ENSG00000236648
LINC02817	-0.0132	6.3370E-01		ENSG00000234754
LINC02824	0.0313	2.6548E-02		ENSG00000255998
LINC02826	-0.0021	7.6802E-01	8.74E-01	ENSG00000275212
LINC02828	0.0469	1.1226E-01		ENSG00000224164
LINC02830	0.0038	6.1642E-01		ENSG00000224899
LINC02831	0.0132	3.9021E-01		ENSG00000224173
LINC02833	0.5817	4.2835E-04	3.64E-03	ENSG00000258742
LINC02844	0.0115	7.3202E-01		ENSG00000253679
LINC02851	0.0462	6.1897E-01	7.75E-01	ENSG00000229611
LINC02853	-0.0126	8.1401E-01		ENSG00000259245
LINC02856	0.0254	4.8314E-01		ENSG00000285594
LINC02857	0.0144	8.7306E-01	9.36E-01	ENSG00000230852
LINC02858	0.0104	4.9274E-01		ENSG00000285869
LINC02860	0.0408	4.7461E-02		ENSG00000222004
LINC02861	0.4690	1.0987E-02	4.56E-02	ENSG00000262185
LINC02864	-0.3185	1.4904E-02	5.78E-02	ENSG00000263711
LINC02868	-0.0836	1.4788E-01		ENSG00000203864
LINC02869	-0.0358	7.0759E-01		ENSG00000228208
LINC02873	0.0014	9.1777E-01		ENSG00000175728
LINC02875	-0.0813	2.2136E-01	4.03E-01	ENSG00000187013
LINC02881	-0.0019	9.8058E-01		ENSG00000277288
LINC02882	-0.2633	1.6992E-01	3.38E-01	ENSG00000251138
LINC02884	0.0043	7.6639E-02		ENSG00000231246
LINC02889	-0.0344	8.6565E-01		ENSG00000236039
LINC02891	-0.0088	9.6531E-01	9.82E-01	ENSG00000249923
LINC02893	-0.0717	3.9393E-01	5.89E-01	ENSG00000269994
LINC02894	0.1511	1.9831E-02	7.15E-02	ENSG00000261437
LINC02897	0.3470	6.6101E-03	3.10E-02	ENSG00000221953
LINC02898	-0.0222	5.4873E-01		ENSG00000205086
LINC02899	-0.0603	8.1589E-02	2.04E-01	ENSG00000248874
LINC02900	-0.0008	6.8739E-01		ENSG00000249647
LINC02901	0.1822	1.0821E-01	2.48E-01	ENSG00000203711
LINC02906	-0.0392	6.5589E-01		ENSG00000279847
LINC02908	-0.1298	1.8622E-01	3.60E-01	ENSG00000180539
LINC02910	0.3391	2.4854E-02	8.48E-02	ENSG00000176659
LINC02915	0.0276	3.0749E-01		ENSG00000175746
LINC02932	-0.0194	8.0468E-01	8.96E-01	ENSG00000243144
LINC02941	-0.0098	5.3998E-01		ENSG00000236013
LINC02944	-0.0194	9.3222E-01	9.66E-01	ENSG00000238276
LINC02947	-0.0019	8.2715E-01		ENSG00000253140
LINC02955	-0.1858	1.0246E-01	2.39E-01	ENSG00000256321
LINC02957	-0.0196	5.4970E-01		ENSG00000260454
LINC02981	0.1216	9.5236E-02	2.27E-01	ENSG00000214870
LINC02982	-0.1080	2.7652E-02		ENSG00000215246
LINC02983	0.0102	9.0046E-01	9.50E-01	ENSG00000234432
LINC02984	-0.5678	3.0353E-03	1.70E-02	ENSG00000237807

LINC02985	0.4204	2.6563E-03	1.53E-02	ENSG00000256546
LINC02987	-0.1071	2.1788E-01	3.99E-01	ENSG00000267575
LINC02994	0.0511	5.3597E-01	7.10E-01	ENSG00000250546
LINC02995	-0.0137	8.4476E-01		ENSG00000253955
LINC02999	-0.0306	6.9848E-01		ENSG00000250564
LINC03002	0.0051	8.8165E-01		ENSG00000232310
LINC03004	0.0013	2.4640E-01	4.32E-01	ENSG00000230533
LINC03007	0.0064	5.6214E-01	7.31E-01	ENSG00000223561
LINC03008	0.0205	8.2839E-01	9.10E-01	ENSG00000229196
LINC03011	-0.2190	1.0884E-02	4.53E-02	ENSG00000237310
LINC03012	0.0135	4.6636E-01	6.54E-01	ENSG00000226770
LINC03014	-0.0050	9.6320E-01	9.81E-01	ENSG00000240859
LINC03016	-0.1946	1.0917E-01	2.50E-01	ENSG00000230825
LINC03021	-0.0115	6.8066E-01		ENSG00000254319
LINC03022	-0.0296	6.0278E-01		ENSG00000253641
LINC03025	0.0000	9.9641E-01	9.98E-01	ENSG00000276462
LINC03025	-0.0472	9.1244E-01		ENSG00000234394
LINC03026	-0.2114	5.8631E-02	1.61E-01	ENSG00000287750
LINC03033	-0.0018	9.6651E-01		ENSG00000232774
LINC03034	0.2156	3.8054E-02	1.16E-01	ENSG00000259772
LINC03040	0.0405	4.8423E-01	6.69E-01	ENSG00000181577
LINC03041	-0.0208	7.7429E-01		ENSG00000205549
LINC03042	0.0023	9.8462E-01	9.92E-01	ENSG00000196166
LINC03047	0.0309	8.5998E-02		ENSG00000253633
LINC03048	0.0186	8.2923E-01	9.11E-01	ENSG00000262223
LINC03049	-0.0498	5.7707E-01	7.44E-01	ENSG00000271109
LINC03051	0.0576	5.3297E-01	7.07E-01	ENSG00000239268
LINC03053	0.3372	2.4862E-02	8.48E-02	ENSG00000223486
LINC03065	0.1231	2.3279E-01	4.16E-01	ENSG00000276524
LINC03069	-0.0521	1.6468E-01		ENSG00000206129
LINC03070	0.0035	4.9266E-01		ENSG00000236120
LINC03095	0.0129	6.8013E-01		ENSG00000233760
LINC03098	-0.0090	8.2829E-01		ENSG00000230392
LINC03102	0.0630	2.4255E-01		ENSG00000226088
LINC03104	-0.0009	9.8784E-01	9.93E-01	ENSG00000274333
LINC03105	-0.0142	8.2331E-01	9.07E-01	ENSG00000276077
LINC03112	-0.3237	3.1905E-04	2.88E-03	ENSG00000289007
LINGO1	0.1311	9.3566E-02	2.25E-01	ENSG00000169783
LINGO1-AS1	0.0146	5.8426E-01		ENSG00000259666
LINGO2	0.0913	3.0265E-01	4.95E-01	ENSG00000174482
LINGO3	0.1938	6.6672E-02	1.76E-01	ENSG00000220008
LINGO4	-0.0086	8.5442E-01	9.25E-01	ENSG00000213171
LINS1	0.5434	1.7483E-10	1.90E-08	ENSG00000140471
LIPA	0.1762	5.3135E-02	1.49E-01	ENSG00000107798
LIPC	0.0152	8.2731E-01	9.09E-01	ENSG00000166035
LIPE	0.2081	7.6978E-02	1.95E-01	ENSG00000079435
LIPE-AS1	-0.1976	4.2646E-02	1.27E-01	ENSG00000213904
LIPF	0.0013	9.8640E-01	9.93E-01	ENSG00000182333
LIPG	0.2225	2.4032E-02	8.27E-02	ENSG00000101670

LIPI	-0.0068	9.7848E-01	9.89E-01	ENSG00000188992
LIPJ	-0.0507	9.2755E-01	9.64E-01	ENSG00000204022
LIPK	-0.0219	9.3752E-01		ENSG00000204021
LIPM	-0.0876	3.1968E-01	5.13E-01	ENSG00000173239
LIPN	0.0114	8.7031E-01	9.34E-01	ENSG00000204020
LIPT1	-0.0147	8.3302E-01	9.13E-01	ENSG00000144182
LIPT2	-0.1211	1.3942E-01	2.95E-01	ENSG00000175536
LIPT2-AS1	0.1859	2.2124E-02	7.77E-02	ENSG00000254837
LITAF	-0.2757	2.2299E-03	1.34E-02	ENSG00000189067
LITAFD	-0.0916	2.4301E-01		ENSG00000283516
LITATS1	-0.1182	2.1579E-01	3.97E-01	ENSG00000233621
LIX1	-0.2644	2.3027E-02	8.01E-02	ENSG00000145721
LIX1L	-0.1798	6.2603E-03	2.98E-02	ENSG00000271601
LIX1L-AS1	-0.0418	6.6917E-01	8.10E-01	ENSG00000234222
LKAAEAR1	-0.2938	1.7507E-02	6.51E-02	ENSG00000171695
LLGL1	-0.0781	3.2174E-01	5.16E-01	ENSG00000131899
LLGL2	0.0281	7.7200E-01	8.76E-01	ENSG00000073350
LLPH	0.0145	8.0564E-01	8.96E-01	ENSG00000139233
LLPH-DT	0.0488	2.2185E-01	4.03E-01	ENSG00000239335
LMAN1	-0.0834	1.8733E-01	3.61E-01	ENSG00000074695
LMAN2	-0.1904	9.5305E-04	6.87E-03	ENSG00000169223
LMAN2L	-0.3352	2.2834E-03	1.36E-02	ENSG00000114988
LMBR1	-0.0302	6.1608E-01	7.74E-01	ENSG00000105983
LMBR1L	0.3012	1.1531E-04	1.26E-03	ENSG00000139636
LMBRD1	-0.1621	4.5773E-03	2.34E-02	ENSG00000168216
LMBRD2	-0.0805	2.4007E-01	4.25E-01	ENSG00000164187
LMCD1	-0.0170	8.2802E-01	9.10E-01	ENSG00000071282
LMCD1-AS1	0.1440	1.3607E-01	2.90E-01	ENSG00000227110
LMF1	-0.2817	1.2192E-05	2.06E-04	ENSG00000103227
LMF1-AS1	0.0357	3.6369E-01	5.59E-01	ENSG00000260439
LMF2	0.2400	1.1453E-03	7.89E-03	ENSG00000100258
LMLN	0.1367	6.4228E-02	1.71E-01	ENSG00000185621
LMNA	-0.3182	4.9284E-06	9.92E-05	ENSG00000160789
LMNB1	-0.1188	2.4694E-01	4.33E-01	ENSG00000113368
LMNB1-DT	0.0042	4.6263E-01		ENSG00000251072
LMNB2	-0.0514	4.0620E-01	6.00E-01	ENSG00000176619
LMNTD1	-0.0213	8.0543E-01	8.96E-01	ENSG00000152936
LMNTD2	0.5945	3.0501E-04	2.77E-03	ENSG00000185522
LMNTD2-AS1	0.3699	1.9421E-02	7.04E-02	ENSG00000254815
LMO1	0.0756	4.1309E-01	6.06E-01	ENSG00000166407
LMO2	-0.2392	3.4174E-02	1.07E-01	ENSG00000135363
LMO3	0.0470	5.9142E-01	7.54E-01	ENSG00000048540
LMO4	-0.0261	7.2937E-01	8.51E-01	ENSG00000143013
LMO7	-0.4523	1.2400E-04	1.33E-03	ENSG00000136153
LMO7-AS1	-0.1346	8.2807E-02	2.06E-01	ENSG00000261105
LMOD1	-0.0683	4.7471E-01	6.61E-01	ENSG00000163431
LMOD2	-0.0430	1.1707E-01		ENSG00000170807
LMOD3	-0.0914	3.3306E-01	5.28E-01	ENSG00000163380
LMTK2	0.0847	3.0940E-01	5.02E-01	ENSG00000164715

LMTK3	-0.1344	1.9010E-01	3.65E-01	ENSG00000142235
LMX1A	0.1094	1.2063E-01	2.68E-01	ENSG00000162761
LMX1B	-0.2100	3.8370E-02	1.17E-01	ENSG00000136944
LNC-LBCS	0.0973	1.6989E-01	3.38E-01	ENSG00000228412
LNCARSR	0.0048	7.3177E-01		ENSG00000233086
LNCATV	-0.0427	6.2034E-01	7.76E-01	ENSG00000238005
LNCBRM	0.0063	1.8960E-01		ENSG00000249436
LNCDAT	-0.0029	4.5771E-01		ENSG00000288638
LNCNEF	0.0001	9.6419E-01		ENSG00000237396
LNCOC1	0.0582	4.9788E-01	6.81E-01	ENSG00000253741
LNCOG	-0.0521	5.3127E-01	7.06E-01	ENSG00000257219
LNCSRLR	-0.0248	5.4916E-01		ENSG00000240032
LNP1	-0.0316	6.6753E-01	8.09E-01	ENSG00000206535
LNPEP	0.0224	7.8066E-01	8.82E-01	ENSG00000113441
LNPK	0.0771	2.4614E-01	4.32E-01	ENSG00000144320
LNX1	0.0040	9.6185E-01	9.81E-01	ENSG00000072201
LNX1-AS1	-0.1652	7.0439E-02	1.83E-01	ENSG00000250930
LNX2	0.1188	1.3497E-01	2.89E-01	ENSG00000139517
LOC100128317	-0.0012	8.4559E-01		ENSG00000233491
LOC100128334	0.0105	4.7987E-01		ENSG00000235713
LOC100128494	-0.0414	3.6145E-01		ENSG00000251143
LOC100128548	0.0030	8.4952E-01		ENSG00000243979
LOC100128601	-0.0337	9.8277E-01	9.91E-01	ENSG00000226241
LOC100128770	-0.1340	8.8790E-02	2.16E-01	ENSG00000205890
LOC100128906	-0.0244	7.2774E-01	8.50E-01	ENSG00000203396
LOC100129138	0.0203	2.7575E-01		ENSG00000215869
LOC100129175	-0.1099	1.8429E-01	3.57E-01	ENSG00000224090
LOC100129203	0.3501	6.2273E-06	1.21E-04	ENSG00000245552
LOC100129215	0.0559	4.3128E-01	6.23E-01	ENSG00000226180
LOC100129434	-0.0265	8.7535E-01	9.37E-01	ENSG00000233251
LOC100129667	0.0098	3.7518E-01		ENSG00000253668
LOC100129734	0.0381	2.7158E-01		ENSG00000227815
LOC100129774	-0.0758	1.9571E-01		ENSG00000265417
LOC100129844	-0.0388	2.7866E-01		ENSG00000218565
LOC100129931	-0.0171	8.4782E-01	9.22E-01	ENSG00000245748
LOC100130331	0.0293	9.6625E-03		ENSG00000237250
LOC100130357	-0.0528	5.1019E-01	6.90E-01	ENSG00000215022
LOC100130548	-0.0420	5.8952E-01		ENSG00000235138
LOC100130587	-0.0163	7.9738E-01		ENSG00000203900
LOC100130664	-0.0047	8.9869E-01		ENSG00000270832
LOC100130691	-0.0580	5.3091E-01	7.05E-01	ENSG00000213963
LOC100130714	-0.0012	6.8941E-01		ENSG00000271329
LOC100130881	-0.0631	4.2537E-01	6.17E-01	ENSG00000236114
LOC100131465	-0.0624	3.8060E-01	5.76E-01	ENSG00000215795
LOC100131496	0.0322	6.6939E-01	8.10E-01	ENSG00000267882
LOC100131785	0.0077	9.3228E-01	9.66E-01	ENSG00000223886
LOC100132686	0.0856	1.7680E-01	3.47E-01	ENSG00000268472
LOC100133077	0.0081	2.7046E-01		ENSG00000203987
LOC100134391	0.0746	1.4648E-02	5.70E-02	ENSG00000263574

LOC100192426	-0.0013	9.9230E-01		ENSG00000266149
LOC100271832	0.0125	8.4502E-01	9.19E-01	ENSG00000236854
LOC100286962	-0.0239	3.9924E-01		ENSG00000230495
LOC100287792	-0.0030	8.7164E-01		ENSG00000204117
LOC100287808	-0.0234	7.9063E-01	8.88E-01	ENSG00000277182
LOC100287944	0.0650	5.0074E-01	6.83E-01	ENSG00000257545
LOC100287966	-0.0256	9.5778E-01		ENSG00000237472
LOC100288001	-0.0050	9.9649E-01		ENSG00000253223
LOC100288073	-0.4515	3.2103E-03	1.77E-02	ENSG00000250746
LOC100288097	-0.0046	8.7139E-01		ENSG00000255107
LOC100288123	0.0346	1.5917E-01		ENSG00000267244
LOC100288728	0.0609	3.8273E-02		ENSG00000261848
LOC100289206	-0.0938	1.2473E-01	2.74E-01	ENSG00000283178
LOC100289361	-0.0279	8.8311E-01	9.41E-01	ENSG00000268129
LOC100289473	0.0181	6.4411E-01		ENSG00000232528
LOC100289495	0.0399	6.6083E-01	8.05E-01	ENSG00000286760
LOC100289518	-0.1066	9.6914E-02	2.30E-01	ENSG00000254783
LOC100294145	0.2846	1.5428E-03	1.00E-02	ENSG00000289047
LOC100310756	-0.0676	3.4852E-01	5.44E-01	ENSG00000289161
LOC100418723	0.0169	2.5483E-01		ENSG00000230638
LOC100418874	0.0891	2.0499E-01		ENSG00000228729
LOC100418965	0.0877	3.7107E-02	1.14E-01	ENSG00000223905
LOC100419073	-0.0592	1.6984E-01		ENSG00000217512
LOC100419436	-0.0387	6.3446E-01	7.87E-01	ENSG00000232344
LOC100419503	-0.0170	7.3364E-01		ENSG00000258439
LOC100419506	0.0020	7.6675E-01		ENSG00000233157
LOC100419515	-0.0866	3.1766E-01	5.11E-01	ENSG00000275318
LOC100419570	-0.0139	7.7904E-01		ENSG00000215444
LOC100419574	0.0110	8.3209E-01	9.12E-01	ENSG00000254398
LOC100419668	0.0116	3.2261E-01		ENSG00000259502
LOC100419679	0.0196	5.8372E-01		ENSG00000228513
LOC100419713	0.0147	2.4748E-01		ENSG00000270323
LOC100419755	0.0047	6.4365E-01		ENSG00000243813
LOC100419783	-0.0044	8.4672E-01		ENSG00000276467
LOC100419786	-0.0013	9.3522E-01		ENSG00000226010
LOC100419824	-0.0129	8.6714E-01		ENSG00000234424
LOC100419851	0.0072	6.4017E-01		ENSG00000271410
LOC100419913	0.0355	3.7410E-01		ENSG00000273675
LOC100420006	0.0075	2.7352E-01		ENSG00000284706
LOC100420020	-0.0129	9.6586E-01		ENSG00000254867
LOC100420027	0.0286	5.0380E-01	6.85E-01	ENSG00000248185
LOC100420052	-0.0158	7.9117E-01		ENSG00000219384
LOC100420114	-0.0479	4.5647E-01	6.46E-01	ENSG00000273597
LOC100420250	0.0064	8.4837E-01		ENSG00000269475
LOC100420347	-0.0853	5.7358E-01	7.41E-01	ENSG00000258836
LOC100420423	-0.0346	6.6962E-01	8.10E-01	ENSG00000232626
LOC100420580	0.0078	7.9660E-01		ENSG00000256651
LOC100420800	-0.0248	3.5157E-01		ENSG00000254629
LOC100420839	-0.0752	3.1994E-01	5.14E-01	ENSG00000218631

LOC100420845	0.0142	7.0956E-01		ENSG00000254189
LOC100420879	0.0128	6.6507E-01		ENSG00000277704
LOC100420899	0.1774	1.0810E-02		ENSG00000255566
LOC100420981	-0.1043	3.0314E-01	4.95E-01	ENSG00000257511
LOC100421091	-0.0351	5.4219E-01		ENSG00000217488
LOC100421094	0.0014	8.9064E-01		ENSG00000254423
LOC100421100	0.1288	1.4189E-01	2.99E-01	ENSG00000265713
LOC100421122	0.0067	5.4534E-01		ENSG00000229695
LOC100421173	0.0210	7.5541E-01	8.67E-01	ENSG00000262700
LOC100421273	0.0339	5.4814E-01		ENSG00000229635
LOC100421336	0.0038	2.6348E-01		ENSG00000238090
LOC100421347	-0.0004	8.7876E-01		ENSG00000271597
LOC100421402	-0.0385	2.7235E-01	4.62E-01	ENSG00000232499
LOC100421465	0.0129	4.7883E-01		ENSG00000235251
LOC100421569	-0.0152	8.1179E-01		ENSG00000237788
LOC100421622	-0.0071	9.9880E-01		ENSG00000256723
LOC100421646	-0.0254	5.3216E-01		ENSG00000258823
LOC100421667	0.0326	8.8023E-02		ENSG00000278552
LOC100421692	0.0287	3.0370E-01		ENSG00000224245
LOC100421775	0.0286	3.4869E-01		ENSG00000237115
LOC100422204	0.3139	2.6286E-02	8.83E-02	ENSG00000251468
LOC100422225	-0.0034	7.0421E-01		ENSG00000258510
LOC100422300	0.0879	1.4418E-01		ENSG00000213252
LOC100422317	-0.0484	2.9665E-01		ENSG00000267430
LOC100422382	-0.0182	4.7057E-01		ENSG00000256533
LOC100422399	-0.0057	9.4329E-01		ENSG00000255381
LOC100422441	-0.0102	8.3944E-01	9.16E-01	ENSG00000253558
LOC100422493	-0.0388	6.2529E-01	7.80E-01	ENSG00000258448
LOC100422497	-0.0331	4.6936E-01		ENSG00000267293
LOC100422622	0.0230	2.1109E-01		ENSG00000203437
LOC100422627	-0.0779	3.8937E-01	5.84E-01	ENSG00000234841
LOC100422687	-0.0082	7.9633E-01		ENSG00000248791
LOC100500719	0.0041	8.3664E-01		ENSG00000188078
LOC100505502	0.0191	4.5605E-01		ENSG00000285781
LOC100505664	-0.0151	8.2846E-01	9.10E-01	ENSG00000230387
LOC100505715	-0.2230	1.6826E-02	6.32E-02	ENSG00000267058
LOC100505716	0.0317	7.1428E-01	8.41E-01	ENSG00000223522
LOC100505774	0.0593	4.8958E-01	6.74E-01	ENSG00000226833
LOC100505782	0.0000	5.4669E-01		ENSG00000234859
LOC100505851	-0.0016	8.3550E-01		ENSG00000269289
LOC100505915	0.1839	1.0822E-01	2.48E-01	ENSG00000260735
LOC100505978	0.0385	5.6231E-01	7.31E-01	ENSG00000257711
LOC100505985	-0.0084	7.5412E-01		ENSG00000226733
LOC100506071	0.0141	7.7914E-01		ENSG00000258525
LOC100506207	0.1678	2.0586E-02	7.35E-02	ENSG00000285219
LOC100506235	-0.2735	3.3093E-02	1.05E-01	ENSG00000233230
LOC100506271	0.0045	8.6905E-01		ENSG00000237862
LOC100506274	0.3602	1.5202E-02	5.86E-02	ENSG00000229727
LOC100506281	0.0027	7.1067E-01		ENSG00000240338

LOC100506321	0.0050	9.5597E-01	9.77E-01	ENSG00000259118
LOC100506405	0.0200	3.3226E-01		ENSG00000228496
LOC100506474	0.1054	2.6692E-01	4.56E-01	ENSG00000225649
LOC100506551	0.0589	4.2249E-01	6.15E-01	ENSG00000257279
LOC100506606	0.0000	9.9883E-01	1.00E+00	ENSG00000257176
LOC100506869	-0.1266	1.6831E-01	3.36E-01	ENSG00000258231
LOC100507053	-0.1443	1.5765E-01	3.21E-01	ENSG00000246090
LOC100507250	-0.1566	1.4843E-01	3.09E-01	ENSG00000247363
LOC100507384	0.0010	9.4086E-01		ENSG00000254519
LOC100507403	0.0115	5.1162E-01		ENSG00000253123
LOC100507547	0.0343	7.2609E-01	8.49E-01	ENSG00000284954
LOC100533628	0.0716	3.7663E-01	5.72E-01	ENSG00000258385
LOC100533629	0.0140	5.9420E-01		ENSG00000232578
LOC100533679	0.0113	4.0982E-01		ENSG00000234726
LOC100533727	-0.0088	9.0638E-01		ENSG00000224529
LOC100631242	0.0161	6.7571E-01		ENSG00000236576
LOC100652758	0.1727	8.0695E-02	2.02E-01	ENSG00000275765
LOC100887080	-0.0127	8.3458E-01		ENSG00000283399
LOC100996318	-0.0324	7.2052E-01	8.45E-01	ENSG00000233029
LOC100996379	0.0527	4.1420E-01	6.07E-01	ENSG00000258488
LOC100996385	0.0019	7.9964E-01		ENSG00000248469
LOC100996437	0.0563	5.2771E-01	7.03E-01	ENSG00000226824
LOC100996583	0.0016	6.6444E-01		ENSG00000228037
LOC100996660	-0.0955	3.3119E-01	5.26E-01	ENSG00000267248
LOC100996671	0.0171	3.3514E-01		ENSG00000256732
LOC101059915	0.0093	6.5206E-01		ENSG00000283599
LOC101926907	0.0659	4.9923E-01	6.82E-01	ENSG00000234807
LOC101926908	0.0353	2.5610E-01		ENSG00000253726
LOC101926953	-0.0031	6.5272E-01		ENSG00000242816
LOC101927018	-0.0076	9.3765E-01		ENSG00000265845
LOC101927023	-0.0529	1.8625E-01		ENSG00000251076
LOC101927026	0.0218	8.2366E-01	9.07E-01	ENSG00000260071
LOC101927042	0.0427	2.7967E-01		ENSG00000287838
LOC101927131	-0.0058	7.7088E-01		ENSG00000262999
LOC101927141	-0.0111	6.6468E-01		ENSG00000254394
LOC101927159	-0.0004	9.9806E-01		ENSG00000229042
LOC101927164	-0.0415	2.0268E-01		ENSG00000237101
LOC101927179	0.0298	6.9261E-01	8.28E-01	ENSG00000282917
LOC101927237	0.0053	4.9566E-01		ENSG00000250075
LOC101927245	0.1568	1.1702E-01	2.63E-01	ENSG00000283959
LOC101927293	-0.1641	7.9613E-02	2.00E-01	ENSG00000225096
LOC101927314	0.3738	1.1553E-02	4.74E-02	ENSG00000271860
LOC101927377	-0.0766	3.0747E-01	5.00E-01	ENSG00000231119
LOC101927383	-0.1697	1.0894E-01	2.50E-01	ENSG00000228528
LOC101927388	-0.0598	4.1221E-01	6.05E-01	ENSG00000287397
LOC101927401	0.1310	2.0777E-01	3.87E-01	ENSG00000287315
LOC101927418	0.0341	1.2504E-01	2.74E-01	ENSG00000258949
LOC101927468	0.0461	6.3092E-01	7.84E-01	ENSG00000227733
LOC101927495	-0.0152	8.6539E-01	9.31E-01	ENSG00000250230

LOC101927513	0.0272	3.1311E-01		ENSG00000254278
LOC101927531	-0.0281	5.9841E-01	7.60E-01	ENSG00000255595
LOC101927539	-0.0481	4.5837E-01		ENSG00000263499
LOC101927543	-0.1284	3.0326E-02		ENSG00000248318
LOC101927551	-0.0833	3.7547E-01	5.71E-01	ENSG00000230922
LOC101927557	-0.0964	5.7537E-01	7.42E-01	ENSG00000266100
LOC101927560	0.1263	1.0236E-01	2.39E-01	ENSG00000233008
LOC101927571	0.0037	9.7958E-01	9.89E-01	ENSG00000266850
LOC101927575	0.0410	2.9898E-01		ENSG00000227463
LOC101927588	-0.0113	8.6958E-01		ENSG00000214803
LOC101927608	-0.1025	1.4703E-01	3.06E-01	ENSG00000273805
LOC101927609	-0.0572	5.2121E-01	6.98E-01	ENSG00000237773
LOC101927636	-0.0254	9.9002E-01	9.94E-01	ENSG00000251600
LOC101927657	0.0150	2.0433E-01		ENSG00000244791
LOC101927661	0.0066	3.6129E-01		ENSG00000235885
LOC101927687	-0.0159	8.4973E-01	9.23E-01	ENSG00000232732
LOC101927692	-0.0065	9.3212E-01	9.66E-01	ENSG00000228484
LOC101927702	-0.1262	2.0591E-01	3.84E-01	ENSG00000254718
LOC101927708	-0.1199	2.0389E-01	3.82E-01	ENSG00000255367
LOC101927727	-0.0353	4.7533E-01		ENSG00000262003
LOC101927745	0.0326	6.4554E-01	7.94E-01	ENSG00000229425
LOC101927769	-0.0206	5.4185E-01		ENSG00000226097
LOC101927787	0.0071	2.5029E-01		ENSG00000228044
LOC101927825	0.0007	9.4179E-01	9.71E-01	ENSG00000282556
LOC101927827	0.0648	3.4286E-01	5.38E-01	ENSG00000229311
LOC101927857	0.0466	1.5742E-01		ENSG00000286559
LOC101927879	-0.0180	5.9409E-01		ENSG00000283458
LOC101927911	0.0552	3.0502E-02		ENSG00000262884
LOC101927950	-0.0030	9.8731E-01		ENSG00000270174
LOC101927993	-0.0018	8.7501E-01		ENSG00000223446
LOC101927995	-0.0700	3.4460E-01	5.40E-01	ENSG00000289450
LOC101928002	0.1300	8.2470E-02	2.05E-01	ENSG00000247131
LOC101928012	0.0010	6.7668E-01		ENSG00000223646
LOC101928016	-0.0206	4.8530E-01		ENSG00000253550
LOC101928053	0.0647	5.0055E-01	6.83E-01	ENSG00000246308
LOC101928059	0.1447	1.7087E-01	3.39E-01	ENSG00000284738
LOC101928087	-0.0124	8.3883E-01		ENSG00000277332
LOC101928093	-0.1218	2.3280E-01	4.16E-01	ENSG00000253295
LOC101928107	-0.0225	3.1643E-01		ENSG00000226527
LOC101928251	-0.0028	8.4485E-01		ENSG00000264985
LOC101928266	-0.0450	5.7031E-01	7.38E-01	ENSG00000262966
LOC101928295	-0.0632	2.5381E-01	4.41E-01	ENSG00000268686
LOC101928335	0.1047	1.7939E-01	3.51E-01	ENSG00000236064
LOC101928357	-0.0400	2.7299E-01		ENSG00000287568
LOC101928371	-0.3831	1.3931E-02	5.48E-02	ENSG00000225420
LOC101928372	0.0304	1.7450E-01		ENSG00000198358
LOC101928373	-0.0060	6.7861E-01		ENSG00000286313
LOC101928380	0.3250	1.7333E-02	6.47E-02	ENSG00000231963
LOC101928381	-0.0086	8.4923E-01	9.22E-01	ENSG00000236252

LOC101928386	-0.0383	8.6113E-01		ENSG00000232377
LOC101928389	-0.0122	7.8677E-01		ENSG00000235834
LOC101928398	0.0392	1.1454E-01		ENSG00000287082
LOC101928416	0.0711	1.7032E-01	3.39E-01	ENSG00000255916
LOC101928438	0.4240	8.0566E-04	6.01E-03	ENSG00000237461
LOC101928463	0.0105	6.5509E-01		ENSG00000287359
LOC101928495	0.0293	2.1669E-01		ENSG00000237208
LOC101928499	-0.0061	3.5483E-01	5.50E-01	ENSG00000259203
LOC101928505	-0.0202	3.3631E-01		ENSG00000287709
LOC101928516	-0.0464	3.7863E-01		ENSG00000223786
LOC101928517	0.0123	3.2557E-01		ENSG00000269072
LOC101928525	0.0202	3.4712E-01		ENSG00000226706
LOC101928557	-0.0103	8.6198E-01		ENSG00000270159
LOC101928565	0.0019	6.5936E-01		ENSG00000228697
LOC101928596	-0.0192	5.9847E-01	7.60E-01	ENSG00000237707
LOC101928626	0.1482	1.5853E-01	3.23E-01	ENSG00000230021
LOC101928651	0.0001	6.2953E-01		ENSG00000248529
LOC101928718	0.2275	6.9640E-02	1.82E-01	ENSG00000284830
LOC101928721	-0.0089	9.7722E-01		ENSG00000247810
LOC101928728	-0.0415	6.2437E-01	7.80E-01	ENSG00000260063
LOC101928797	0.0039	8.7249E-01		ENSG00000233569
LOC101928834	0.0198	8.2264E-01	9.07E-01	ENSG00000233968
LOC101928844	0.0077	6.0351E-01	7.63E-01	ENSG00000267709
LOC101928847	0.0623	5.2731E-01	7.03E-01	ENSG00000247416
LOC101928855	-0.0046	9.9831E-01	9.99E-01	ENSG00000262833
LOC101928868	0.0389	6.9393E-01	8.29E-01	ENSG00000287260
LOC101928896	0.0013	7.7279E-01		ENSG00000255084
LOC101928906	0.1210	2.0990E-01	3.89E-01	ENSG00000205611
LOC101928940	0.0063	7.3979E-01		ENSG00000256195
LOC101928952	0.0253	2.1714E-01		ENSG00000271143
LOC101928965	-0.0134	8.6352E-01	9.30E-01	ENSG00000271858
LOC101928988	-0.0256	8.4977E-01		ENSG00000259351
LOC101928994	0.0367	2.2548E-01		ENSG00000229261
LOC101929004	-0.1848	1.0477E-01	2.43E-01	ENSG00000288009
LOC101929021	-0.3873	1.2777E-02	5.12E-02	ENSG00000236154
LOC101929053	-0.0388	8.8500E-01		ENSG00000270857
LOC101929106	0.1312	2.7354E-02	9.09E-02	ENSG00000198491
LOC101929107	-0.0481	3.5619E-01		ENSG00000258572
LOC101929109	0.0368	2.7303E-02	9.08E-02	ENSG00000249713
LOC101929124	-0.0023	9.0990E-01		ENSG00000260599
LOC101929130	-0.0614	2.6626E-01	4.55E-01	ENSG00000283175
LOC101929141	0.0029	9.9605E-01		ENSG00000250111
LOC101929174	-0.1067	1.7092E-01	3.39E-01	ENSG00000288018
LOC101929200	0.0644	5.0876E-01	6.89E-01	ENSG00000286753
LOC101929227	-0.0246	7.8667E-01		ENSG00000250493
LOC101929237	0.0153	6.0177E-01		ENSG00000248738
LOC101929341	-0.0044	7.2321E-01		ENSG00000253551
LOC101929372	-0.0345	3.3559E-01		ENSG00000236754
LOC101929408	0.1073	1.3134E-01	2.84E-01	ENSG00000260288

LOC101929420	0.0116	7.2942E-01		ENSG00000234647
LOC101929427	-0.0651	4.0195E-01		ENSG00000254607
LOC101929452	0.0115	8.7513E-01	9.37E-01	ENSG00000223884
LOC101929457	0.0112	2.4336E-01		ENSG00000259362
LOC101929460	-0.0569	1.8330E-01		ENSG00000229720
LOC101929470	0.0094	3.4905E-01		ENSG00000248964
LOC101929494	-0.0318	8.6672E-01	9.32E-01	ENSG00000276170
LOC101929536	0.0025	6.2494E-01	7.80E-01	ENSG00000261798
LOC101929552	0.1646	1.4327E-01	3.01E-01	ENSG00000263063
LOC101929563	0.3532	2.3165E-02	8.05E-02	ENSG00000283982
LOC101929566	-0.0020	9.7977E-01	9.89E-01	ENSG00000263280
LOC101929577	-0.0354	2.7593E-01		ENSG00000249635
LOC101929586	0.0044	5.6691E-01		ENSG00000259175
LOC101929609	0.0894	3.7076E-01	5.66E-01	ENSG00000288573
LOC101929614	0.0208	7.3196E-01	8.53E-01	ENSG00000289623
LOC101929626	0.4654	3.5528E-03	1.92E-02	ENSG00000227857
LOC101929657	-0.0491	4.3640E-01		ENSG00000273523
LOC101929667	0.0033	7.9610E-01		ENSG00000287255
LOC101929691	-0.3501	1.8320E-02	6.75E-02	ENSG00000285872
LOC101929698	0.0440	1.5671E-01		ENSG00000277301
LOC101929710	-0.0671	4.4498E-01	6.35E-01	ENSG00000251314
LOC101929718	-0.0652	1.9829E-01		ENSG00000258471
LOC101929719	0.1439	1.1454E-01	2.59E-01	ENSG00000254363
LOC101929748	-0.0023	2.6429E-01	4.53E-01	ENSG00000288062
LOC101929918	0.0196	8.2339E-01	9.07E-01	ENSG00000286626
LOC101930090	-0.0209	7.3078E-01		ENSG00000283541
LOC101930276	0.0267	9.6815E-02		ENSG00000283462
LOC101930420	0.0131	2.8624E-01		ENSG00000283544
LOC101930421	-0.0012	6.6617E-01	8.08E-01	ENSG00000225140
LOC102546299	-0.5862	1.2561E-03	8.52E-03	ENSG00000241956
LOC102723313	-0.0226	7.4711E-01		ENSG00000248896
LOC102723335	-0.0035	5.3865E-01		ENSG00000259540
LOC102723360	-0.0691	2.8551E-01	4.77E-01	ENSG00000275496
LOC102723451	-0.0074	8.8943E-01	9.44E-01	ENSG00000277277
LOC102723475	-0.0204	7.1331E-01	8.40E-01	ENSG00000276289
LOC102723553	-0.2492	5.9569E-04	4.74E-03	ENSG00000273590
LOC102723566	0.0393	5.6085E-01	7.30E-01	ENSG00000225032
LOC102723663	-0.0293	2.0706E-01		ENSG00000285906
LOC102723670	-0.0502	5.6105E-01	7.30E-01	ENSG00000259868
LOC102723692	0.0066	2.8307E-01		ENSG00000261448
LOC102723701	0.0951	1.3332E-01	2.86E-01	ENSG00000183154
LOC102723878	0.0746	3.1584E-01	5.09E-01	ENSG00000236935
LOC102723996	-0.1816	7.2908E-02	1.88E-01	ENSG00000277117
LOC102724019	-0.0169	8.4255E-01	9.18E-01	ENSG00000240086
LOC102724023	-0.3316	1.2981E-02	5.19E-02	ENSG00000280071
LOC102724104	0.0089	4.8910E-01		ENSG00000287958
LOC102724159	0.2709	5.0849E-03	2.53E-02	ENSG00000275464
LOC102724200	-0.0911	3.0304E-01	4.95E-01	ENSG00000280433
LOC102724250	0.0267	7.4305E-01	8.59E-01	ENSG00000271254

LOC102724289	-0.0060	6.6641E-01		ENSG00000287706
LOC102724334	0.0247	5.3560E-01		ENSG00000274559
LOC102724354	0.0060	6.7469E-01	8.14E-01	ENSG00000280191
LOC102724421	-0.0348	4.7558E-01		ENSG00000257083
LOC102724428	-0.4360	1.2636E-04	1.35E-03	ENSG00000275993
LOC102724474	0.2753	2.8655E-02	9.42E-02	ENSG00000283828
LOC102724482	0.0425	5.8263E-03	2.82E-02	ENSG00000287687
LOC102724560	0.0135	8.7931E-01	9.39E-01	ENSG00000274276
LOC102724593	0.0220	7.7260E-01	8.77E-01	ENSG00000231187
LOC102724594	0.0819	1.2835E-01	2.79E-01	ENSG00000275895
LOC102724602	-0.0086	8.4672E-01		ENSG00000277882
LOC102724637	-0.0336	3.1097E-01		ENSG00000214942
LOC102724642	0.1008	2.8813E-01	4.79E-01	ENSG00000204745
LOC102724646	-0.0406	6.4907E-01	7.97E-01	ENSG00000289256
LOC102724652	0.1843	7.3929E-02	1.90E-01	ENSG00000276076
LOC102724680	-0.0123	8.8690E-01	9.43E-01	ENSG00000289218
LOC102724701	-0.0502	5.3851E-01	7.12E-01	ENSG00000280145
LOC102724719	-0.2897	2.8375E-02	9.34E-02	ENSG00000223502
LOC102724737	-0.0578	3.6312E-01	5.59E-01	ENSG00000281087
LOC102724768	0.0160	8.3831E-01	9.15E-01	ENSG00000237949
LOC102724843	-0.0284	6.5522E-01	8.01E-01	ENSG00000277067
LOC102724859	0.0942	2.3412E-01	4.18E-01	ENSG00000279249
LOC102724900	0.0054	7.9106E-01		ENSG00000230051
LOC102724934	0.0327	6.3484E-01	7.87E-01	ENSG00000257522
LOC102725191	-0.1026	1.8956E-01	3.64E-01	ENSG00000226690
LOC102725254	-0.0727	4.5689E-01	6.46E-01	ENSG00000268650
LOC103171574	0.0390	6.1335E-01	7.72E-01	ENSG00000259774
LOC103344931	-0.0117	9.0029E-01	9.50E-01	ENSG00000260917
LOC105274304	-0.0869	3.6832E-01	5.64E-01	ENSG00000261335
LOC105369147	0.0456	5.7197E-01	7.39E-01	ENSG00000247081
LOC105369165	0.0427	4.6577E-01		ENSG00000228033
LOC105369187	-0.0192	6.9222E-01		ENSG00000250748
LOC105369306	-0.0230	7.8309E-01		ENSG00000233393
LOC105369329	0.1699	1.2377E-01	2.73E-01	ENSG00000255931
LOC105369344	0.0043	7.7640E-01	8.79E-01	ENSG00000287917
LOC105369363	-0.2271	3.2071E-02	1.02E-01	ENSG00000286369
LOC105369373	-0.0100	7.4546E-01		ENSG00000255539
LOC105369391	-0.0093	8.8345E-01	9.41E-01	ENSG00000255326
LOC105369496	0.0716	3.8159E-01	5.76E-01	ENSG00000287006
LOC105369576	0.4655	3.0093E-03	1.69E-02	ENSG00000288013
LOC105369685	-0.0315	1.5725E-01		ENSG00000287075
LOC105369728	-0.0027	9.1334E-01		ENSG00000256442
LOC105369850	0.1037	2.9455E-01	4.87E-01	ENSG00000257526
LOC105369907	-0.0362	1.8820E-01		ENSG00000257746
LOC105370024	0.1290	1.5388E-01	3.16E-01	ENSG00000256884
LOC105370027	0.0226	7.9858E-01	8.92E-01	ENSG00000248636
LOC105370047	0.0879	2.9643E-01	4.88E-01	ENSG00000214650
LOC105370061	0.0309	6.1006E-01		ENSG00000256286
LOC105370105	-0.0560	2.4178E-01		ENSG00000235438

LOC105370122	0.0101	3.1433E-01		ENSG00000289125
LOC105370174	-0.0262	6.9452E-01	8.29E-01	ENSG00000288598
LOC105370203	-0.1194	1.1106E-01	2.53E-01	ENSG00000288743
LOC105370259	-0.1913	5.7395E-02	1.58E-01	ENSG00000286330
LOC105370315	0.0171	3.9595E-01		ENSG00000287159
LOC105370489	0.0313	7.1148E-01	8.39E-01	ENSG00000258843
LOC105370500	0.1020	3.0773E-01	5.00E-01	ENSG00000285664
LOC105370525	0.0079	7.0812E-01		ENSG00000258926
LOC105370532	-0.1721	1.2880E-01	2.80E-01	ENSG00000274015
LOC105370616	-0.0233	3.4011E-01	5.35E-01	ENSG00000258792
LOC105370622	0.0100	8.0762E-01	8.98E-01	ENSG00000258716
LOC105370689	0.0210	4.5860E-01		ENSG00000287346
LOC105370740	-0.0010	9.9642E-01		ENSG00000287280
LOC105370783	-0.0004	9.9166E-01		ENSG00000259269
LOC105370791	0.1439	9.3519E-02	2.24E-01	ENSG00000260926
LOC105370792	0.2212	6.0805E-02	1.65E-01	ENSG00000174171
LOC105370802	-0.0103	5.8646E-01	7.51E-01	ENSG00000259200
LOC105370804	0.0085	3.5345E-01		ENSG00000259588
LOC105370890	-0.0165	5.3180E-01		ENSG00000259650
LOC105370906	-0.1296	1.3707E-01	2.92E-01	ENSG00000259420
LOC105370941	0.5401	4.3210E-03	2.24E-02	ENSG00000261136
LOC105370969	-0.0406	6.4179E-01	7.92E-01	ENSG00000259704
LOC105371022	0.0000	9.6497E-01		ENSG00000259219
LOC105371046	0.0039	7.3336E-01		ENSG00000260989
LOC105371083	-0.1235	1.9848E-01	3.75E-01	ENSG00000263080
LOC105371115	-0.0107	8.3093E-01	9.12E-01	ENSG00000261195
LOC105371335	-0.0085	9.0842E-01		ENSG00000260520
LOC105371354	0.0132	2.4526E-01		ENSG00000288821
LOC105371361	0.1150	1.1991E-01	2.67E-01	ENSG00000261838
LOC105371414	0.1043	3.0258E-01	4.95E-01	ENSG00000260279
LOC105371430	0.0567	9.8897E-02		ENSG00000241525
LOC105371485	0.0440	6.9370E-02		ENSG00000228133
LOC105371689	-0.0148	8.5802E-01	9.27E-01	ENSG00000286383
LOC105371729	-0.1492	9.6237E-02	2.29E-01	ENSG00000285677
LOC105371730	-0.0892	3.6351E-01	5.59E-01	ENSG00000214708
LOC105371734	0.0292	2.9488E-01		ENSG00000265222
LOC105371745	0.0136	6.4630E-01		ENSG00000270240
LOC105371749	0.0188	2.4751E-01	4.34E-01	ENSG00000289011
LOC105371789	0.0763	3.6981E-01	5.66E-01	ENSG00000267638
LOC105371795	0.0181	8.1558E-01	9.03E-01	ENSG00000267288
LOC105371814	0.0062	9.6615E-01	9.83E-01	ENSG00000248278
LOC105371824	-0.0257	6.5968E-01		ENSG00000250286
LOC105371841	0.1062	2.7112E-01	4.61E-01	ENSG00000287337
LOC105371855	-0.0239	7.3808E-01		ENSG00000265702
LOC105371899	-0.0574	4.9763E-01	6.81E-01	ENSG00000267568
LOC105371956	0.0041	9.0614E-01	9.53E-01	ENSG00000263745
LOC105371967	-0.0086	7.0882E-01		ENSG00000266401
LOC105372066	0.0022	6.5685E-01	8.02E-01	ENSG00000267627
LOC105372069	-0.0320	8.5890E-01		ENSG00000267039

LOC105372143	-0.0082	8.5064E-01		ENSG00000267743
LOC105372165	0.1358	2.0721E-01	3.86E-01	ENSG00000283667
LOC105372180	-0.0274	8.3630E-01	9.14E-01	ENSG00000287646
LOC105372268	-0.0002	4.7846E-01		ENSG00000287960
LOC105372316	-0.0091	7.6468E-01		ENSG00000269043
LOC105372321	-0.2454	3.9290E-02	1.19E-01	ENSG00000268119
LOC105372401	0.0131	8.9246E-01	9.46E-01	ENSG00000286177
LOC105372421	0.0670	4.2438E-01	6.16E-01	ENSG00000268401
LOC105372430	0.0004	8.5707E-01		ENSG00000268530
LOC105372432	0.0900	6.9724E-03		ENSG00000286024
LOC105372435	-0.0198	7.6979E-01	8.75E-01	ENSG00000269194
LOC105372436	0.0740	4.2807E-01	6.20E-01	ENSG00000287001
LOC105372441	0.0032	5.8040E-01		ENSG00000267968
LOC105372558	-0.3522	1.5987E-02	6.08E-02	ENSG00000225280
LOC105372564	0.0285	6.5143E-01	7.98E-01	ENSG00000288974
LOC105372710	0.0076	9.0101E-01	9.51E-01	ENSG00000233017
LOC105372832	0.0020	7.0283E-01		ENSG00000232124
LOC105372839	0.0025	6.6436E-01		ENSG00000289448
LOC105372971	-0.1229	2.2663E-01	4.09E-01	ENSG00000229770
LOC105372988	0.0168	3.0950E-01		ENSG00000225676
LOC105372990	-0.0157	7.1202E-01		ENSG00000273428
LOC105373044	-0.3135	9.4756E-03	4.08E-02	ENSG00000286491
LOC105373100	-0.0031	9.5160E-01		ENSG00000225929
LOC105373170	0.1813	6.3662E-02	1.70E-01	ENSG00000286071
LOC105373244	0.0992	1.9069E-01	3.66E-01	ENSG00000284391
LOC105373273	-0.1157	1.5078E-01	3.12E-01	ENSG00000289566
LOC105373289	0.4697	4.8105E-04	3.97E-03	ENSG00000286389
LOC105373299	-0.0627	5.0888E-01	6.89E-01	ENSG00000286794
LOC105373335	-0.0502	4.2835E-01	6.20E-01	ENSG00000235189
LOC105373373	0.0395	5.4638E-01	7.18E-01	ENSG00000273877
LOC105373383	0.0613	4.7429E-01	6.61E-01	ENSG00000260081
LOC105373390	0.0083	4.1809E-01		ENSG00000237720
LOC105373422	0.0071	4.1439E-01		ENSG00000287305
LOC105373429	0.0221	7.7658E-01	8.79E-01	ENSG00000285569
LOC105373436	0.0899	3.6061E-01	5.57E-01	ENSG00000285876
LOC105373496	0.0777	3.3860E-01	5.34E-01	ENSG00000235480
LOC105373553	-0.0954	1.1828E-01	2.64E-01	ENSG00000282033
LOC105373575	-0.0125	7.4952E-01		ENSG00000286776
LOC105373696	0.0070	7.4177E-01		ENSG00000230991
LOC105373780	-0.1085	2.8753E-01	4.79E-01	ENSG00000283839
LOC105373973	-0.0274	8.2353E-01	9.07E-01	ENSG00000288080
LOC105374042	0.2466	5.4616E-02	1.53E-01	ENSG00000239482
LOC105374069	-0.0160	9.7131E-01		ENSG00000286827
LOC105374085	0.0649	4.4360E-01	6.34E-01	ENSG00000250012
LOC105374101	-0.0059	9.4459E-01	9.72E-01	ENSG00000289469
LOC105374114	-0.0190	9.3895E-01	9.69E-01	ENSG00000248468
LOC105374174	0.0431	6.5261E-01	7.99E-01	ENSG00000286585
LOC105374191	-0.0259	7.7073E-01	8.76E-01	ENSG00000289884
LOC105374254	0.0091	9.2419E-01	9.62E-01	ENSG00000286086

LOC105374312	0.0454	1.9110E-01		ENSG00000272840
LOC105374328	0.0029	8.9090E-01		ENSG00000232642
LOC105374338	0.1611	5.9969E-02	1.63E-01	ENSG00000283183
LOC105374344	-0.0084	9.2176E-01	9.61E-01	ENSG00000251652
LOC105374438	0.0065	5.7020E-01		ENSG00000250863
LOC105374736	-0.0049	9.6321E-01		ENSG00000287597
LOC105374780	-0.0027	9.4125E-01		ENSG00000234255
LOC105374802	-0.0349	7.0611E-01	8.36E-01	ENSG00000287435
LOC105374811	-0.1800	1.1485E-01	2.59E-01	ENSG00000270571
LOC105374988	-0.0044	8.8697E-01		ENSG00000284607
LOC105374989	-0.0140	6.4038E-01		ENSG00000275846
LOC105375082	0.0064	7.4795E-01		ENSG00000287485
LOC105375146	-0.0201	4.8462E-01		ENSG00000235431
LOC105375166	-0.0074	8.6222E-01	9.30E-01	ENSG00000237070
LOC105375170	-0.5220	5.7385E-03	2.79E-02	ENSG00000289189
LOC105375216	0.0586	4.4989E-01	6.40E-01	ENSG00000286847
LOC105375387	0.0529	3.1657E-02		ENSG00000227863
LOC105375421	0.3330	2.8426E-02	9.36E-02	ENSG00000284523
LOC105375501	0.0125	6.9867E-01	8.31E-01	ENSG00000286380
LOC105375556	-0.0139	4.7440E-01		ENSG00000230190
LOC105375587	0.0005	8.8168E-01		ENSG00000290007
LOC105375614	0.2138	1.3699E-02	5.41E-02	ENSG00000222012
LOC105375709	0.0156	1.6883E-01		ENSG00000287819
LOC105375713	-0.0114	5.6911E-01		ENSG00000289382
LOC105375743	-0.0135	6.1530E-01		ENSG00000255491
LOC105375844	0.0482	3.7794E-01		ENSG00000253857
LOC105375924	0.1141	7.9018E-02	1.99E-01	ENSG00000253214
LOC105375976	-0.0679	2.9060E-01		ENSG00000224935
LOC105376070	-0.0625	7.0740E-01	8.36E-01	ENSG00000233178
LOC105376121	0.0378	1.6932E-01		ENSG00000285634
LOC105376244	-0.0122	6.5907E-01		ENSG00000284977
LOC105376291	0.0454	7.7502E-02		ENSG00000226355
LOC105376306	0.0091	9.2386E-01	9.62E-01	ENSG00000288989
LOC105376381	-0.0082	8.7611E-01		ENSG00000287235
LOC105376429	-0.0598	7.5081E-01	8.64E-01	ENSG00000236495
LOC105376520	-0.0109	5.2035E-01	6.98E-01	ENSG00000290098
LOC105376588	0.0586	1.7670E-01		ENSG00000255357
LOC105376654	0.2650	6.8217E-03	3.18E-02	ENSG00000254746
LOC105376713	-0.0319	6.7094E-01	8.11E-01	ENSG00000259617
LOC105376731	-0.0010	7.2924E-01		ENSG00000260660
LOC105376805	-0.0205	8.1968E-01	9.05E-01	ENSG00000238142
LOC105376860	-0.1339	1.7949E-01	3.51E-01	ENSG00000289835
LOC105376975	-0.0069	9.5639E-01	9.78E-01	ENSG00000286352
LOC105376995	-0.0182	7.6516E-01		ENSG00000289548
LOC105377139	-0.0058	8.8284E-01		ENSG00000285413
LOC105377205	0.0285	5.1795E-01		ENSG00000260613
LOC105377209	0.1569	1.2662E-01	2.77E-01	ENSG00000287215
LOC105377213	0.1541	1.8862E-02	6.89E-02	ENSG00000214915
LOC105377224	0.1582	1.4864E-01	3.09E-01	ENSG00000288049

LOC105377294	0.1181	2.6304E-01	4.52E-01	ENSG00000289586
LOC105377378	-0.0264	5.0155E-01		ENSG00000288781
LOC105377448	0.0511	4.3134E-01	6.23E-01	ENSG00000250195
LOC105377451	0.0515	3.7404E-01		ENSG00000286896
LOC105377503	0.0946	3.1819E-04		ENSG00000287730
LOC105377727	0.1288	1.6887E-01	3.37E-01	ENSG00000287814
LOC105377927	-0.0044	9.5762E-01		ENSG00000289020
LOC105378044	0.0587	5.3620E-01	7.10E-01	ENSG00000226249
LOC105378052	-0.0104	7.2539E-01		ENSG00000216621
LOC105378231	0.1268	9.5128E-03	4.10E-02	ENSG00000260581
LOC105378268	-0.1833	4.9867E-02	1.43E-01	ENSG00000285884
LOC105378311	-0.0065	8.1628E-01		ENSG00000234173
LOC105378318	0.0430	7.5206E-02		ENSG00000235140
LOC105378353	-0.1252	3.3433E-02		ENSG00000289607
LOC105378355	0.0194	4.5601E-01		ENSG00000289362
LOC105378453	0.0117	5.1293E-01		ENSG00000289441
LOC105378464	0.0032	6.5277E-01		ENSG00000237761
LOC105378473	-0.0100	7.8082E-01		ENSG00000287047
LOC105378644	-0.0329	6.5886E-01	8.03E-01	ENSG00000284640
LOC105378714	-0.0240	7.8654E-01	8.85E-01	ENSG00000284700
LOC105378736	0.0006	7.7878E-01		ENSG00000284601
LOC105378798	-0.0217	6.5440E-01	8.00E-01	ENSG00000225087
LOC105378930	0.0029	9.9718E-01		ENSG00000287980
LOC105378976	-0.0629	4.9872E-01	6.81E-01	ENSG00000249236
LOC105378998	0.0253	3.5478E-01		ENSG00000289916
LOC105379040	-0.0484	6.2803E-01		ENSG00000289535
LOC105379100	-0.0755	3.6404E-01		ENSG00000249787
LOC105379130	0.0405	3.9166E-02		ENSG00000289497
LOC105379199	-0.0126	9.2340E-01	9.62E-01	ENSG00000286615
LOC105379230	-0.0068	8.9140E-01		ENSG00000254237
LOC105379301	0.0154	6.4426E-01		ENSG00000254242
LOC105379340	-0.0360	4.5257E-01		ENSG00000253888
LOC105379362	-0.0041	9.5987E-01	9.80E-01	ENSG00000247134
LOC105379379	0.0085	6.8035E-01		ENSG00000254111
LOC105379428	0.1015	1.1668E-02	4.77E-02	ENSG00000278932
LOC105379499	0.1452	8.6494E-02	2.12E-01	ENSG00000280018
LOC105379839	0.0480	6.1553E-01	7.73E-01	ENSG00000286718
LOC107983987	0.0158	8.2572E-01	9.08E-01	ENSG00000278961
LOC107984172	0.4920	1.0338E-02	4.35E-02	ENSG00000289386
LOC107984203	0.0127	9.6933E-01		ENSG00000287277
LOC107984236	0.0084	1.2952E-01	2.81E-01	ENSG00000289989
LOC107984265	-0.0481	4.0954E-01	6.03E-01	ENSG00000286575
LOC107984322	-0.0169	9.8364E-01		ENSG00000255375
LOC107984450	0.1969	4.1916E-03		ENSG00000256001
LOC107984536	0.0301	3.3198E-02		ENSG00000289309
LOC107984548	0.0336	2.1452E-01		ENSG00000258308
LOC107984551	-0.0127	8.4336E-01		ENSG00000288767
LOC107984685	-0.0189	7.3580E-01		ENSG00000258028
LOC107984709	0.0086	8.0427E-01		ENSG00000289583

LOC107984761	0.0477	8.8477E-02		ENSG00000259392
LOC107984827	0.0395	5.8671E-01	7.51E-01	ENSG00000261170
LOC107984850	-0.0059	6.0289E-01		ENSG00000288531
LOC107984948	0.1574	8.3197E-02	2.06E-01	ENSG00000230615
LOC107984970	-0.0392	8.3214E-01		ENSG00000270553
LOC107985049	-0.0135	6.5852E-01		ENSG00000271268
LOC107985074	0.0141	4.4695E-01		ENSG00000289070
LOC107985126	0.0080	8.9807E-01		ENSG00000264151
LOC107985177	-0.0058	9.3873E-01	9.69E-01	ENSG00000287281
LOC107985206	0.0375	6.9735E-01	8.31E-01	ENSG00000286391
LOC107985216	-0.0574	5.2021E-01	6.98E-01	ENSG00000272668
LOC107985265	0.0214	5.2754E-01		ENSG00000268747
LOC107985380	0.0923	2.0130E-01	3.79E-01	ENSG00000234271
LOC107985629	-0.0090	7.6656E-01		ENSG00000232042
LOC107985645	-0.0121	6.8849E-01		ENSG00000229733
LOC107985688	0.0109	5.5045E-01		ENSG00000228427
LOC107985892	-0.0134	8.5776E-01	9.27E-01	ENSG00000289156
LOC107985953	0.0062	5.0534E-01		ENSG00000286679
LOC107986163	-0.2101	4.8469E-02	1.40E-01	ENSG00000272970
LOC107986178	-0.0103	6.9640E-01		ENSG00000249631
LOC107986277	0.0185	6.2680E-02	1.69E-01	ENSG00000282904
LOC107986374	0.0195	6.9591E-01		ENSG00000248648
LOC107986400	0.0136	5.3443E-01		ENSG00000248973
LOC107986453	-0.0162	9.5710E-01		ENSG00000250378
LOC107986524	0.1663	9.3493E-02	2.24E-01	ENSG00000286339
LOC107986547	-0.1602	3.4675E-02	1.09E-01	ENSG00000289838
LOC107986550	0.0138	4.7041E-01		ENSG00000289090
LOC107986626	-0.0166	7.9546E-01	8.90E-01	ENSG00000287578
LOC107986669	0.3270	2.6746E-02	8.95E-02	ENSG00000287189
LOC107986742	-0.1758	1.0536E-01	2.44E-01	ENSG00000287815
LOC107986760	-0.0381	8.3336E-01	9.13E-01	ENSG00000289352
LOC107986821	0.0341	3.7365E-01		ENSG00000285964
LOC107986837	-0.0151	8.2378E-01		ENSG00000225457
LOC107986954	0.0378	8.3910E-02		ENSG00000287927
LOC107987013	-0.0115	7.9307E-01		ENSG00000232939
LOC107987014	0.0181	7.3058E-01		ENSG00000230894
LOC107987423	0.0499	4.0769E-01	6.01E-01	ENSG00000228695
LOC112267859	-0.0229	6.9083E-01		ENSG00000279688
LOC112267968	0.0203	4.2913E-01		ENSG00000285492
LOC112267986	-0.0166	8.5363E-01		ENSG00000287672
LOC112268073	0.0125	3.6084E-01		ENSG00000256282
LOC112268162	-0.0158	4.3320E-01		ENSG00000259509
LOC112268165	-0.0417	5.7819E-01	7.45E-01	ENSG00000286894
LOC112268176	-0.0135	5.5162E-01		ENSG00000261090
LOC112268198	-0.0611	9.7098E-01	9.85E-01	ENSG00000279801
LOC112268208	-0.0462	5.9280E-01	7.55E-01	ENSG00000285095
LOC112268239	0.1902	6.1668E-02	1.67E-01	ENSG00000288880
LOC112268269	0.0552	5.4693E-01	7.19E-01	ENSG00000268858
LOC112268276	-0.0025	9.2787E-01		ENSG00000229588

LOC112268411	0.1050	2.7212E-01	4.62E-01	ENSG00000236106
LOC112268419	-0.0597	8.0548E-01		ENSG00000286244
LOC112694756	0.0203	8.1834E-01	9.04E-01	ENSG00000285043
LOC114841035	0.1779	9.1141E-02	2.20E-01	ENSG00000286264
LOC115308161	-0.1556	1.0009E-01	2.35E-01	ENSG00000271551
LOC116435278	-0.0634	2.0049E-01		ENSG00000286710
LOC119746555	0.0678	4.8192E-01	6.67E-01	ENSG00000260257
LOC121725015	-0.1605	1.4310E-01	3.01E-01	ENSG00000264449
LOC122319436	-0.2487	4.2625E-02	1.27E-01	ENSG00000288658
LOC122455340	0.0232	3.2276E-01		ENSG00000283536
LOC122455341	-0.0631	6.4491E-01		ENSG00000284294
LOC122455342	-0.0998	1.7958E-01		ENSG00000284526
LOC122526776	0.0717	1.8150E-01	3.53E-01	ENSG00000285938
LOC122526780	-0.0336	6.7323E-01	8.13E-01	ENSG00000282936
LOC122526782	-0.2707	8.5484E-03	3.78E-02	ENSG00000233461
LOC122539214	0.0124	8.9743E-01	9.49E-01	ENSG00000269825
LOC124900179	0.0064	5.4693E-01		ENSG00000200999
LOC124900180	-0.0034	9.1274E-01		ENSG00000221639
LOC124900183	-0.0722	2.3629E-01	4.20E-01	ENSG00000201863
LOC124900184	-0.0326	8.3448E-01		ENSG00000212458
LOC124900188	-0.0257	9.1349E-01		ENSG00000239005
LOC124900193	0.1323	1.7481E-01	3.45E-01	ENSG00000245146
LOC124900202	0.0014	9.7273E-01		ENSG00000212567
LOC124900204	0.0076	7.2200E-01		ENSG00000251828
LOC124900205	-0.0080	6.3722E-01		ENSG00000206592
LOC124900215	-0.1715	1.2705E-01	2.78E-01	ENSG00000290049
LOC124900217	-0.2775	1.5880E-02	6.05E-02	ENSG00000225177
LOC124900224	0.0128	3.5096E-01		ENSG00000252218
LOC124900226	0.0103	2.9963E-01		ENSG00000201807
LOC124900265	-0.0125	8.1541E-01		ENSG00000200075
LOC124900270	0.0164	3.5925E-01		ENSG00000238854
LOC124900271	0.0036	6.5756E-01		ENSG00000238966
LOC124900275	0.4884	7.2789E-08	2.99E-06	ENSG00000204054
LOC124900277	-0.0090	8.3145E-01		ENSG00000200026
LOC124900296	-0.0586	3.0794E-01		ENSG00000222588
LOC124900311	-0.0049	7.1140E-01		ENSG00000201733
LOC124900314	-0.0191	7.8609E-01		ENSG00000252427
LOC124900315	-0.0235	5.3737E-01		ENSG00000252778
LOC124900324	0.0022	5.2814E-01		ENSG00000200897
LOC124900332	-0.0316	3.3732E-01		ENSG00000212461
LOC124900339	0.1512	1.5078E-01	3.12E-01	ENSG00000252128
LOC124900349	0.0150	6.7176E-01	8.12E-01	ENSG00000251858
LOC124900356	0.0245	3.8768E-01		ENSG00000207430
LOC124900357	-0.0166	6.1279E-01		ENSG00000238519
LOC124900359	-0.0119	6.8893E-01		ENSG00000207432
LOC124900372	0.3637	9.8157E-05	1.10E-03	ENSG00000261971
LOC124900382	0.0353	2.8508E-01		ENSG00000252122
LOC124900383	0.0141	4.9744E-01		ENSG00000238685
LOC124900394	0.0090	6.6846E-01		ENSG00000252657

LOC124900399	-0.0289	3.3333E-01		ENSG00000200063
LOC124900423	-0.0146	9.5058E-01	9.75E-01	ENSG00000201944
LOC124900429	-0.0053	6.9670E-01		ENSG00000201619
LOC124900430	0.0381	6.7761E-01	8.16E-01	ENSG00000200237
LOC124900432	-0.0224	4.1447E-01		ENSG00000212338
LOC124900442	0.0047	5.5166E-01		ENSG00000206878
LOC124900447	-0.0009	9.1006E-01		ENSG00000212624
LOC124900452	0.0270	4.1831E-01		ENSG00000221083
LOC124900460	0.0040	6.1724E-01		ENSG00000201151
LOC124900464	-0.0024	9.1424E-01		ENSG00000212517
LOC124900469	-0.0053	9.4686E-01		ENSG00000238390
LOC124900476	0.0241	3.6786E-02		ENSG00000280623
LOC124900488	-0.0252	5.5557E-01		ENSG00000199226
LOC124900492	-0.0056	7.0843E-01		ENSG00000201407
LOC124900498	0.2135	1.4409E-02		ENSG00000202231
LOC124900516	-0.0112	8.2994E-01		ENSG00000201806
LOC124900517	-0.0198	6.8045E-01		ENSG00000202537
LOC124900537	-0.0015	9.5612E-01		ENSG00000212175
LOC124900564	0.1046	1.8692E-01	3.61E-01	ENSG00000253092
LOC124900607	0.1005	2.6546E-01	4.54E-01	ENSG00000223734
LOC124900638	0.2380	6.0678E-02	1.65E-01	ENSG00000231616
LOC124900656	-0.0309	4.1418E-01		ENSG00000287164
LOC124900694	-0.0082	9.5494E-01		ENSG00000287659
LOC124900711	0.0961	2.4053E-01	4.25E-01	ENSG00000289019
LOC124900774	0.1703	8.4045E-02	2.08E-01	ENSG00000287951
LOC124900778	0.0243	2.6516E-01		ENSG00000248187
LOC124900787	-0.0006	7.6170E-01		ENSG00000248335
LOC124900848	-0.0735	1.6384E-01	3.30E-01	ENSG00000286891
LOC124900869	-0.0017	5.7424E-01		ENSG00000286580
LOC124900957	-0.0488	4.2434E-01	6.16E-01	ENSG00000286543
LOC124900964	-0.0271	2.7484E-01		ENSG00000251257
LOC124901013	-0.0823	2.8470E-01	4.76E-01	ENSG00000288846
LOC124901048	-0.0057	6.4925E-01		ENSG00000248709
LOC124901067	-0.0868	3.7300E-01	5.68E-01	ENSG00000250244
LOC124901076	0.0116	6.0803E-01		ENSG00000249803
LOC124901107	0.0074	7.8144E-01		ENSG00000286657
LOC124901122	-0.0046	9.1552E-01		ENSG00000254135
LOC124901151	-0.0340	7.1204E-01	8.40E-01	ENSG00000248367
LOC124901168	-0.0139	4.6228E-01		ENSG00000260763
LOC124901197	-0.0218	3.2666E-01		ENSG00000238326
LOC124901296	0.1078	2.7111E-01	4.61E-01	ENSG00000225173
LOC124901310	-0.0178	8.8159E-01		ENSG00000289456
LOC124901333	0.0012	9.9209E-01	9.96E-01	ENSG00000227885
LOC124901351	-0.0508	3.0924E-01		ENSG00000287816
LOC124901355	0.2937	1.5413E-03	1.00E-02	ENSG00000272008
LOC124901391	-0.0087	9.1242E-01	9.56E-01	ENSG00000287258
LOC124901407	-0.0034	8.5443E-01		ENSG00000287094
LOC124901408	-0.0448	6.3710E-01	7.89E-01	ENSG00000289312
LOC124901409	-0.0363	2.7171E-01		ENSG00000216613

LOC124901427	0.0844	2.9558E-01	4.88E-01	ENSG00000273132
LOC124901447	-0.0393	3.8542E-01		ENSG00000287591
LOC124901457	-0.0016	9.7269E-01		ENSG00000286498
LOC124901521	-0.0042	9.4302E-01		ENSG00000222145
LOC124901526	0.0802	2.7118E-01	4.61E-01	ENSG00000202343
LOC124901604	-0.0166	5.2130E-01		ENSG00000260951
LOC124901612	-0.0064	9.0811E-01		ENSG00000236494
LOC124901628	0.0430	2.8189E-01		ENSG00000286315
LOC124901635	0.0043	7.6864E-01		ENSG00000228735
LOC124901661	0.1321	2.1415E-01	3.95E-01	ENSG00000289108
LOC124901671	0.0750	4.4935E-01	6.39E-01	ENSG00000285886
LOC124901813	0.1098	6.1670E-02	1.67E-01	ENSG00000287592
LOC124901856	0.0048	9.4753E-01		ENSG00000238832
LOC124901861	0.0175	6.8348E-01		ENSG00000238297
LOC124901864	-0.0125	6.1983E-01		ENSG00000284858
LOC124901882	0.0352	2.6897E-01		ENSG00000254340
LOC124901944	0.0420	2.1900E-01		ENSG00000253844
LOC124901966	0.4196	1.2457E-02	5.02E-02	ENSG00000285758
LOC124901990	0.0165	8.8244E-01		ENSG00000253842
LOC124902013	0.0065	8.1231E-01		ENSG00000253607
LOC124902026	-0.1667	1.3417E-01	2.87E-01	ENSG00000286535
LOC124902031	-0.0072	2.2742E-01	4.10E-01	ENSG00000287325
LOC124902062	-0.0094	7.7899E-01		ENSG00000253452
LOC124902108	-0.0118	7.3065E-01		ENSG00000227155
LOC124902114	-0.2222	1.9100E-02	6.96E-02	ENSG00000286162
LOC124902128	0.0127	8.3006E-01	9.11E-01	ENSG00000286685
LOC124902204	0.4281	8.7281E-04	6.40E-03	ENSG00000287769
LOC124902222	0.0157	3.7769E-01		ENSG00000213612
LOC124902229	0.1678	1.3545E-01	2.89E-01	ENSG00000285706
LOC124902280	0.0021	9.8804E-01	9.93E-01	ENSG00000227218
LOC124902301	-0.0217	9.7072E-01	9.85E-01	ENSG00000286502
LOC124902371	0.1102	8.6960E-02	2.13E-01	ENSG00000213994
LOC124902388	0.3231	3.0469E-03	1.70E-02	ENSG00000285852
LOC124902392	0.0873	1.4313E-01	3.01E-01	ENSG00000285254
LOC124902439	-0.0049	9.5275E-01	9.76E-01	ENSG00000228566
LOC124902473	0.0369	1.3454E-01	2.88E-01	ENSG00000286359
LOC124902477	-0.1468	1.4082E-01	2.97E-01	ENSG00000232110
LOC124902532	0.2366	9.3875E-06	1.67E-04	ENSG00000287560
LOC124902537	-0.0841	6.6570E-01	8.08E-01	ENSG00000288657
LOC124902546	-0.3227	3.0111E-02	9.77E-02	ENSG00000287016
LOC124902606	0.0401	5.6269E-01	7.32E-01	ENSG00000287935
LOC124902629	0.0378	6.0623E-01		ENSG00000250041
LOC124902641	-0.0322	4.6566E-01	6.54E-01	ENSG00000287898
LOC124902694	-0.0254	7.9091E-01	8.88E-01	ENSG00000255320
LOC124902709	-0.0056	7.9294E-01		ENSG00000215841
LOC124902771	-0.0015	9.7180E-01	9.85E-01	ENSG00000287545
LOC124902824	0.0039	6.2760E-01		ENSG00000206913
LOC124902860	-0.0092	9.2603E-01	9.63E-01	ENSG00000278356
LOC124902904	-0.0067	7.4244E-01	8.59E-01	ENSG00000256625

LOC124902947	0.0200	8.1672E-01	9.03E-01	ENSG00000287200
LOC124902964	0.0127	4.4721E-01		ENSG00000257761
LOC124902988	0.1375	1.2703E-01	2.78E-01	ENSG00000258035
LOC124903002	-0.0278	3.0822E-01		ENSG00000257732
LOC124903055	0.0437	2.2478E-02		ENSG00000286462
LOC124903167	-0.0237	9.5063E-01	9.75E-01	ENSG00000225727
LOC124903183	0.0604	9.6711E-02		ENSG00000285572
LOC124903233	0.0247	3.8679E-01	5.81E-01	ENSG00000277047
LOC124903236	0.0322	6.8142E-02		ENSG00000287996
LOC124903317	-0.1393	8.1900E-02	2.04E-01	ENSG00000287156
LOC124903324	0.2330	6.3704E-02	1.70E-01	ENSG00000286257
LOC124903342	-0.0047	9.1230E-01		ENSG00000285518
LOC124903356	0.0257	2.6519E-01		ENSG00000283627
LOC124903372	0.0148	3.7947E-01		ENSG00000187621
LOC124903416	0.0168	5.5451E-01		ENSG00000212615
LOC124903448	-0.0479	3.7175E-01	5.67E-01	ENSG00000289522
LOC124903525	0.0839	4.0018E-01	5.94E-01	ENSG00000277749
LOC124903592	-0.0008	8.7389E-01		ENSG00000212428
LOC124903594	0.0116	4.8359E-01		ENSG00000207119
LOC124903615	-0.0199	7.4415E-01	8.60E-01	ENSG00000239197
LOC124903619	-0.0395	5.5747E-01		ENSG00000260316
LOC124903640	0.0217	7.3163E-01	8.52E-01	ENSG00000287340
LOC124903658	0.0178	7.4629E-01	8.61E-01	ENSG00000286790
LOC124903659	0.0094	6.1055E-01		ENSG00000260592
LOC124903670	-0.4389	2.0716E-03	1.26E-02	ENSG00000246465
LOC124903729	0.0083	6.9713E-01		ENSG00000261472
LOC124903770	-0.0415	6.1933E-01	7.75E-01	ENSG00000260573
LOC124903776	-0.0604	4.4044E-01	6.31E-01	ENSG00000260115
LOC124903940	-0.0235	5.4449E-01	7.17E-01	ENSG00000286743
LOC124903956	0.0123	8.9469E-01	9.47E-01	ENSG00000265246
LOC124904009	-0.0096	8.9714E-01	9.49E-01	ENSG00000288961
LOC124904017	-0.0525	5.4422E-01	7.16E-01	ENSG00000289599
LOC124904027	-0.0604	1.2921E-01		ENSG00000284650
LOC124904048	-0.0402	8.5468E-01	9.25E-01	ENSG00000265055
LOC124904072	-0.5488	1.0059E-02	4.27E-02	ENSG00000261978
LOC124904076	-0.0080	9.9362E-01		ENSG00000287403
LOC124904115	-0.0055	8.5467E-01		ENSG00000252274
LOC124904117	-0.0455	5.7398E-01		ENSG00000239129
LOC124904135	0.0202	1.5414E-01	3.16E-01	ENSG00000275616
LOC124904136	0.0033	3.1639E-01		ENSG00000273709
LOC124904137	0.0285	1.1167E-01	2.54E-01	ENSG00000274432
LOC124904138	0.0021	3.6717E-01	5.63E-01	ENSG00000278591
LOC124904138	0.0085	4.0671E-01	6.00E-01	ENSG00000274062
LOC124904138	0.0116	4.2860E-01		ENSG00000274862
LOC124904138	0.0080	4.5142E-01		ENSG00000277903
LOC124904138	-0.0158	9.3957E-01	9.70E-01	ENSG00000275219
LOC124904138	-0.0269	2.6918E-01	4.58E-01	ENSG00000276596
LOC124904145	0.0172	5.4732E-01		ENSG00000274452
LOC124904146	0.0116	4.2860E-01		ENSG00000278774

LOC124904152	0.0077	3.4351E-01		ENSG00000221044
LOC124904154	-0.0274	8.8299E-01	9.41E-01	ENSG00000212195
LOC124904288	0.2805	3.8129E-02	1.17E-01	ENSG00000285940
LOC124904294	0.0292	1.7744E-02	6.58E-02	ENSG00000289168
LOC124904362	0.0548	1.0799E-01		ENSG00000199977
LOC124904381	0.0110	6.3300E-01		ENSG00000199856
LOC124904403	-0.0140	7.8202E-01		ENSG00000224481
LOC124904411	-0.1853	9.4668E-02	2.26E-01	ENSG00000233030
LOC124904434	0.0445	5.8056E-01	7.47E-01	ENSG00000290105
LOC124904530	0.0057	9.0886E-01		ENSG00000287259
LOC124904535	0.1222	2.4059E-01	4.25E-01	ENSG00000269934
LOC124904542	0.0052	7.3116E-01		ENSG00000227006
LOC124904553	0.0736	3.1321E-01	5.06E-01	ENSG00000233332
LOC124904559	0.0289	6.5427E-02	1.74E-01	ENSG00000285177
LOC124904601	-0.0478	5.9864E-01	7.60E-01	ENSG00000286496
LOC124904611	0.0445	3.2833E-01	5.23E-01	ENSG00000267122
LOC124904613	0.4722	2.0096E-04	1.97E-03	ENSG00000275405
LOC124904613	0.1636	1.4053E-01	2.97E-01	ENSG00000273768
LOC124904616	-0.0193	9.4413E-01	9.72E-01	ENSG00000277918
LOC124904619	-0.0004	5.8803E-01	7.52E-01	ENSG00000270722
LOC124904620	-0.0993	2.1656E-01	3.97E-01	ENSG00000268536
LOC124904621	-0.2862	1.9272E-02	7.00E-02	ENSG00000274210
LOC124904624	0.5221	7.8944E-05	9.25E-04	ENSG00000275291
LOC124904627	-0.1374	1.9171E-01	3.67E-01	ENSG00000206828
LOC124904631	0.4071	1.7318E-03	1.09E-02	ENSG00000273727
LOC124904634	0.1555	1.0434E-01	2.42E-01	ENSG00000274428
LOC124904671	-0.0152	6.5821E-01		ENSG00000212538
LOC124904686	0.0020	5.4079E-01		ENSG00000283442
LOC124904687	-0.0406	4.6947E-01		ENSG00000283575
LOC124904695	0.1479	1.6890E-01	3.37E-01	ENSG00000267024
LOC124904701	0.0076	8.6986E-01		ENSG00000179066
LOC124904706	-0.0145	8.3577E-01		ENSG00000278757
LOC124904795	0.0454	4.7285E-01		ENSG00000239137
LOC124904917	0.0321	6.9891E-01	8.32E-01	ENSG00000273828
LOC124904972	0.0563	2.0988E-01		ENSG00000283527
LOC124904983	0.0149	2.2588E-01		ENSG00000201346
LOC124905013	-0.0029	9.6973E-01		ENSG00000273102
LOC124905092	-0.1463	4.5307E-02	1.33E-01	ENSG00000282012
LOC124905119	-0.0432	1.8074E-01		ENSG00000224794
LOC124905134	-0.3590	8.9001E-03	3.90E-02	ENSG00000273145
LOC124905142	-0.0140	8.2037E-01		ENSG00000287225
LOC124905143	0.0387	4.2949E-01	6.21E-01	ENSG00000285722
LOC124905177	-0.0244	7.1636E-01		ENSG00000289127
LOC124905202	-0.0275	7.7476E-01	8.78E-01	ENSG00000289132
LOC124905218	-0.1164	1.6289E-01	3.29E-01	ENSG00000286060
LOC124905233	-0.0092	9.9222E-01	9.96E-01	ENSG00000289575
LOC124905237	-0.0275	5.9799E-01	7.59E-01	ENSG00000286939
LOC124905242	0.0019	6.4164E-01		ENSG00000228543
LOC124905307	-0.0184	4.5427E-01		ENSG00000252209

LOC124905312	0.1820	2.7905E-02	9.22E-02	ENSG00000278903
LOC124905442	0.0055	6.6911E-01		ENSG00000242296
LOC124906016	-0.1200	2.5134E-01	4.38E-01	ENSG00000204929
LOC124906119	-0.0200	8.0156E-01	8.94E-01	ENSG00000288898
LOC124906143	-0.0628	1.6420E-01		ENSG00000206731
LOC124906209	-0.0090	9.1907E-01	9.60E-01	ENSG00000235978
LOC124906232	0.0186	8.0308E-01	8.95E-01	ENSG00000287629
LOC124906237	0.1457	8.0268E-02	2.01E-01	ENSG00000288063
LOC124906280	-0.0249	7.8979E-01		ENSG00000279328
LOC124906284	0.0189	2.1989E-01	4.01E-01	ENSG00000284731
LOC124906285	-0.1027	2.9753E-01	4.90E-01	ENSG00000288111
LOC124906319	0.0386	6.9223E-01	8.27E-01	ENSG00000289165
LOC124906339	0.0184	3.9596E-01		ENSG00000283418
LOC124906471	-0.1175	2.0624E-01	3.85E-01	ENSG00000288930
LOC124906529	0.0042	6.9734E-01		ENSG00000249109
LOC124906608	-0.5447	3.2550E-05	4.51E-04	ENSG00000223969
LOC124906685	-0.0012	9.8294E-01		ENSG00000253363
LOC124906789	0.0985	3.0061E-01	4.93E-01	ENSG00000248690
LOC124906839	0.2335	6.5148E-02	1.73E-01	ENSG00000276422
LOC124906938	-0.0084	7.8720E-01		ENSG00000289997
LOC124906976	0.0254	5.7825E-01	7.45E-01	ENSG00000274659
LOC124907114	0.0200	7.9382E-01	8.89E-01	ENSG00000275757
LOC124907114	0.0026	9.8542E-01	9.92E-01	ENSG00000273730
LOC124907392	-0.0551	5.4964E-01	7.21E-01	ENSG00000258636
LOC124907726	0.1944	6.6121E-02	1.75E-01	ENSG00000287126
LOC124907760	-0.0586	3.6856E-01		ENSG00000231848
LOC124907763	-0.1334	2.0686E-01	3.86E-01	ENSG00000226087
LOC124907878	0.1617	1.4544E-01	3.04E-01	ENSG00000238207
LOC124907890	-0.0387	6.5722E-01	8.02E-01	ENSG00000229797
LOC124907995	-0.0016	9.9269E-01		ENSG00000287791
LOC124909362	0.4092	7.2865E-03	3.34E-02	ENSG00000289460
LOC124909384	-0.0239	8.4392E-01		ENSG00000286952
LOC124909396	0.1897	8.8304E-02	2.15E-01	ENSG00000287595
LOC124909397	0.0203	3.1835E-02	1.02E-01	ENSG00000286447
LOC124909412	-0.0318	9.3863E-01	9.69E-01	ENSG00000289153
LOC124909423	0.0061	4.8769E-01		ENSG00000288022
LOC124909426	0.0028	9.5487E-01	9.77E-01	ENSG00000287232
LOC124909451	-0.0129	7.8861E-01		ENSG00000288787
LOC124909475	0.3129	2.3576E-02	8.15E-02	ENSG00000287005
LOC124909494	-0.0849	2.2700E-01	4.10E-01	ENSG00000243620
LOC124909495	-0.0090	5.7111E-01		ENSG00000289451
LOC127814295	-0.0119	8.8530E-01	9.42E-01	ENSG00000232995
LOC128071543	0.0071	6.2834E-01		ENSG00000243062
LOC128125816	0.0837	9.8979E-02		ENSG00000284934
LOC128125822	-0.0197	7.3794E-01		ENSG00000285976
LOC149844	-0.0045	4.5495E-01		ENSG00000234241
LOC149935	0.0106	6.1585E-01		ENSG00000224628
LOC153910	-0.0224	3.4124E-01		ENSG00000236366
LOC154761	-0.1535	1.1838E-01	2.65E-01	ENSG00000253882

LOC157273	0.0743	4.4639E-01	6.36E-01	ENSG00000248538
LOC158434	0.0119	4.3423E-01		ENSG00000275465
LOC254896	-0.0026	9.7149E-01	9.85E-01	ENSG00000284948
LOC283038	-0.0039	9.4327E-01		ENSG00000228021
LOC283045	0.2619	5.0152E-02	1.43E-01	ENSG00000288011
LOC283194	-0.4504	2.5217E-03	1.47E-02	ENSG00000255240
LOC283299	-0.0080	8.7908E-01		ENSG00000254951
LOC283387	0.0451	6.3891E-01	7.90E-01	ENSG00000245651
LOC283683	0.3882	4.8943E-04	4.02E-03	ENSG00000274253
LOC283731	-0.1355	2.0115E-01	3.78E-01	ENSG00000248540
LOC284685	0.0934	2.4614E-01	4.32E-01	ENSG00000215835
LOC284788	-0.0157	9.8943E-01		ENSG00000204684
LOC284798	-0.0078	9.5134E-01		ENSG00000230725
LOC284933	0.0068	7.1160E-01		ENSG00000281732
LOC285097	0.1037	1.3590E-01	2.90E-01	ENSG00000280119
LOC285453	0.0355	2.0151E-01		ENSG00000227040
LOC285626	0.0296	3.7932E-01	5.74E-01	ENSG00000249738
LOC285638	-0.1016	1.3256E-01	2.85E-01	ENSG00000249476
LOC285804	-0.0663	1.8139E-01	3.53E-01	ENSG00000271820
LOC286059	-0.0565	4.9376E-01		ENSG00000246130
LOC338963	0.1215	2.0057E-01	3.78E-01	ENSG00000228141
LOC339059	-0.0202	8.2854E-01		ENSG00000182376
LOC339166	0.0322	9.8152E-02		ENSG00000284837
LOC339260	-0.2770	3.0418E-03	1.70E-02	ENSG00000233098
LOC339966	-0.0301	9.4513E-01		ENSG00000271676
LOC340268	-0.0016	8.3782E-01		ENSG00000197320
LOC344967	-0.0262	6.4165E-01		ENSG00000205794
LOC349160	-0.0109	9.5748E-01	9.78E-01	ENSG00000234352
LOC359819	0.1324	1.0940E-01	2.50E-01	ENSG00000250669
LOC375196	-0.0082	9.0413E-01	9.52E-01	ENSG00000269210
LOC388248	-0.0041	9.2898E-01		ENSG00000260628
LOC388282	-0.0486	5.6871E-01	7.37E-01	ENSG00000187185
LOC388996	0.0034	7.7943E-01	8.81E-01	ENSG00000278131
LOC389199	0.4900	8.0717E-03	3.62E-02	ENSG00000228919
LOC389831	-0.0838	1.7088E-01	3.39E-01	ENSG00000276256
LOC389906	0.0266	6.7391E-01	8.13E-01	ENSG00000285756
LOC390877	-0.0031	2.7645E-01		ENSG00000214347
LOC391741	-0.0325	7.9298E-01	8.89E-01	ENSG00000248699
LOC392787	-0.1090	1.5600E-01	3.19E-01	ENSG00000224163
LOC400036	0.0503	2.2385E-02		ENSG00000257616
LOC400499	-0.0803	4.0847E-01	6.02E-01	ENSG00000188897
LOC400541	0.2307	7.0200E-02	1.83E-01	ENSG00000247228
LOC400622	0.1554	1.1275E-01	2.56E-01	ENSG00000267665
LOC400627	0.2154	3.1290E-02	1.00E-01	ENSG00000261924
LOC401127	0.0776	1.4309E-01		ENSG00000224097
LOC401442	-0.0163	6.7515E-01		ENSG00000282375
LOC401589	0.0578	1.8311E-01	3.56E-01	ENSG00000182776
LOC401703	-0.0023	8.4302E-01		ENSG00000213365
LOC401957	0.0016	4.1943E-01		ENSG00000232952

LOC403312	-0.3045	2.8440E-02	9.36E-02	ENSG00000255537
LOC439933	0.0092	5.1739E-01		ENSG00000247193
LOC440311	0.0104	4.4588E-01		ENSG00000277654
LOC440700	0.1900	6.8229E-02	1.79E-01	ENSG00000215838
LOC441239	0.0003	9.2468E-01		ENSG00000189316
LOC441711	-0.0185	8.5994E-01		ENSG00000273679
LOC442155	0.0282	5.5437E-01		ENSG00000220685
LOC442497	0.0973	1.8102E-01	3.53E-01	ENSG00000249574
LOC574538	-0.0050	3.9937E-01	5.94E-01	ENSG00000249695
LOC613206	-0.0163	8.0798E-01	8.98E-01	ENSG00000235204
LOC613266	0.0006	8.3570E-01		ENSG00000286546
LOC642361	0.2396	7.6774E-03	3.48E-02	ENSG00000272447
LOC643015	0.0205	7.6049E-01		ENSG00000183171
LOC643172	-0.0163	3.7339E-01		ENSG00000223651
LOC643342	-0.0437	1.9035E-01		ENSG00000230221
LOC644169	-0.0032	3.5364E-01	5.49E-01	ENSG00000254595
LOC644456	-0.0129	8.2945E-01	9.11E-01	ENSG00000231043
LOC644584	-0.0097	8.1486E-01		ENSG00000257307
LOC644656	0.3875	1.1616E-03	7.98E-03	ENSG00000268403
LOC645433	0.0084	3.8841E-01		ENSG00000248608
LOC645485	-0.0149	9.8946E-01		ENSG00000246331
LOC645853	-0.0579	3.6035E-01	5.56E-01	ENSG00000251211
LOC645967	0.1531	1.2940E-01	2.81E-01	ENSG00000278899
LOC646665	-0.0077	8.9314E-01	9.47E-01	ENSG00000260144
LOC646828	-0.0358	2.7543E-01		ENSG00000257639
LOC646870	0.0062	8.2609E-01		ENSG00000231615
LOC651337	0.0321	6.5586E-01	8.01E-01	ENSG00000255585
LOC653653	0.1282	4.9094E-02	1.41E-01	ENSG00000280852
LOC654780	0.0202	2.0004E-01		ENSG00000250685
LOC728138	0.1430	1.8200E-01	3.54E-01	ENSG00000261819
LOC728158	-0.0155	6.1145E-01		ENSG00000287326
LOC728307	0.0665	1.6175E-02	6.14E-02	ENSG00000224931
LOC728485	0.0269	7.5769E-01	8.68E-01	ENSG00000267260
LOC728488	0.5305	1.9454E-04	1.92E-03	ENSG00000250138
LOC728743	-0.2172	2.0062E-02	7.22E-02	ENSG00000284691
LOC728877	0.0408	4.8142E-01	6.67E-01	ENSG00000204790
LOC729141	-0.0031	7.0263E-01	8.34E-01	ENSG00000213115
LOC729732	-0.3165	6.5870E-03	3.10E-02	ENSG00000283674
LOC729867	0.0326	6.9336E-01	8.28E-01	ENSG00000227741
LOC729870	0.2558	4.6680E-02	1.36E-01	ENSG00000287642
LOC729998	-0.1856	5.1283E-02	1.46E-01	ENSG00000283041
LOC730098	-0.1093	2.6707E-01	4.56E-01	ENSG00000187186
LOC730183	-0.1964	2.2339E-02	7.83E-02	ENSG00000261840
LOC730338	-0.0419	8.4593E-02		ENSG00000233539
LOC730668	-0.0117	6.6698E-01		ENSG00000280424
LOC731157	0.0067	9.3679E-01	9.69E-01	ENSG00000255647
LOC732229	0.0329	6.3244E-01	7.86E-01	ENSG00000269374
LOC780529	-0.0132	6.6463E-01		ENSG00000271776
LOC84214	0.0123	3.9385E-01		ENSG00000236529

LOC93429	0.0330	2.0270E-01		ENSG00000268460
LOC93463	-0.0426	5.6951E-01		ENSG00000124835
LOH12CR2	0.0197	8.0199E-01	8.94E-01	ENSG00000205791
LOHAN2	0.0436	3.1030E-02		ENSG00000258779
LONP1	0.0553	3.9242E-01	5.87E-01	ENSG00000196365
LONP2	-0.1684	1.5267E-02	5.88E-02	ENSG00000102910
LONRF1	0.2329	3.2915E-03	1.80E-02	ENSG00000154359
LONRF2	-0.0671	4.4484E-01	6.35E-01	ENSG00000170500
LONRF2P1	-0.0055	8.4793E-01		ENSG00000266605
LONRF3	-0.0461	6.2760E-01	7.82E-01	ENSG00000175556
LORICRIN	0.0794	4.1582E-01	6.09E-01	ENSG00000203782
LOX	-0.4118	1.7136E-04	1.73E-03	ENSG00000113083
LOXHD1	0.1079	7.1330E-02	1.85E-01	ENSG00000167210
LOXL1	-0.0504	4.7900E-01	6.65E-01	ENSG00000129038
LOXL1-AS1	-0.2680	5.2061E-03	2.58E-02	ENSG00000261801
LOXL2	0.5018	1.7880E-03	1.12E-02	ENSG00000134013
LOXL2-AS1	0.5636	5.4959E-03	2.69E-02	ENSG00000253837
LOXL3	-0.1592	8.4610E-02	2.09E-01	ENSG00000115318
LOXL4	0.0262	7.7264E-01	8.77E-01	ENSG00000138131
LPA	-0.0515	3.1569E-01		ENSG00000198670
LPAR1	-0.0780	4.2289E-01	6.15E-01	ENSG00000198121
LPAR2	-0.1466	6.0625E-02	1.65E-01	ENSG00000064547
LPAR3	-0.1155	6.7932E-02	1.78E-01	ENSG00000171517
LPAR5	0.3391	2.1949E-02	7.73E-02	ENSG00000184574
LPAR6	-0.4032	7.0202E-04	5.39E-03	ENSG00000139679
LPCAT1	-0.0880	2.2573E-01	4.08E-01	ENSG00000153395
LPCAT2	0.0561	5.4153E-01	7.14E-01	ENSG00000087253
LPCAT3	0.0982	2.8846E-01	4.80E-01	ENSG00000111684
LPCAT4	0.1236	1.0085E-01	2.36E-01	ENSG00000176454
LPGAT1	-0.1749	1.0411E-02	4.37E-02	ENSG00000123684
LPIN1	0.2176	1.1780E-03	8.08E-03	ENSG00000134324
LPIN2	-0.2143	4.4733E-03	2.30E-02	ENSG00000101577
LPIN3	-0.1303	1.6915E-01	3.37E-01	ENSG00000132793
LPL	-0.0032	9.7021E-01	9.85E-01	ENSG00000175445
LPO	-0.0387	5.1456E-01	6.94E-01	ENSG00000167419
LPP	-0.1563	4.2247E-02	1.26E-01	ENSG00000145012
LPP-AS2	0.0152	8.7507E-01	9.37E-01	ENSG00000270959
LPXN	0.1822	3.4943E-02	1.09E-01	ENSG00000110031
LRAT	0.0839	3.9588E-01	5.90E-01	ENSG00000121207
LRATD1	0.1031	1.5587E-01	3.19E-01	ENSG00000162981
LRATD2	-0.5522	1.4612E-08	7.77E-07	ENSG00000168672
LRBA	-0.1317	1.0946E-01	2.50E-01	ENSG00000198589
LRCH1	-0.0081	9.1994E-01	9.60E-01	ENSG00000136141
LRCH2	-0.0541	5.6098E-01	7.30E-01	ENSG00000130224
LRCH3	0.2500	2.6623E-04	2.47E-03	ENSG00000186001
LRCH4	0.5101	2.2285E-05	3.34E-04	ENSG00000077454
LRCOL1	0.0086	3.0052E-01		ENSG00000204583
LRFN1	-0.1079	2.2545E-01	4.08E-01	ENSG00000128011
LRFN2	0.1496	1.7025E-01	3.38E-01	ENSG00000156564

LRFN2	-0.0221	9.7745E-01		ENSG00000226454
LRFN3	-0.4798	1.4164E-04	1.48E-03	ENSG00000126243
LRFN4	-0.2635	8.7133E-04	6.39E-03	ENSG00000173621
LRFN5	0.4974	3.2642E-05	4.51E-04	ENSG00000165379
LRG1	0.0256	7.7073E-01	8.76E-01	ENSG00000171236
LRGUK	0.0206	8.3779E-01	9.15E-01	ENSG00000155530
LRIF1	0.3193	1.2722E-03	8.61E-03	ENSG00000121931
LRIG1	0.0420	6.6338E-01	8.07E-01	ENSG00000144749
LRIG2	0.3427	2.8450E-06	6.35E-05	ENSG00000198799
LRIG2-DT	0.1711	9.5984E-02	2.28E-01	ENSG00000238198
LRIG3	-0.1296	5.6349E-02	1.56E-01	ENSG00000139263
LRIT1	0.0033	8.5577E-01		ENSG00000148602
LRIT2	-0.0029	5.7789E-01		ENSG00000204033
LRIT3	0.0017	4.9325E-01		ENSG00000183423
LRMDA	0.4038	6.4792E-06	1.25E-04	ENSG00000148655
LRP1	-0.3063	5.5451E-04	4.48E-03	ENSG00000123384
LRP10	-0.3600	3.7462E-06	7.96E-05	ENSG00000197324
LRP11	0.0197	8.1116E-01	9.00E-01	ENSG00000120256
LRP12	0.1772	9.6401E-03	4.13E-02	ENSG00000147650
LRP1B	-0.1011	2.0066E-01	3.78E-01	ENSG00000168702
LRP2	0.2120	6.1424E-02	1.66E-01	ENSG00000081479
LRP2BP	0.0747	3.2408E-01	5.18E-01	ENSG00000109771
LRP3	0.0052	9.7833E-01	9.89E-01	ENSG00000130881
LRP4	-0.1305	1.2881E-01	2.80E-01	ENSG00000134569
LRP4-AS1	0.3868	1.0110E-02	4.28E-02	ENSG00000247675
LRP5	-0.0406	6.0526E-01	7.65E-01	ENSG00000162337
LRP6	0.1831	6.6182E-03	3.10E-02	ENSG00000070018
LRP8	0.2744	8.8721E-05	1.02E-03	ENSG00000157193
LRP8-DT	-0.0814	5.0275E-01	6.84E-01	ENSG00000225675
LRPAP1	-0.2118	6.5208E-04	5.10E-03	ENSG00000163956
LRPPRC	0.0402	5.1874E-01	6.96E-01	ENSG00000138095
LRR1	0.2583	6.7294E-03	3.14E-02	ENSG00000165501
LRRC1	-0.2263	2.3019E-03	1.37E-02	ENSG00000137269
LRRC10B	0.1384	8.9707E-02	2.18E-01	ENSG00000204950
LRRC14	0.1572	7.3271E-02	1.88E-01	ENSG00000160959
LRRC14B	0.0021	9.4285E-01	9.72E-01	ENSG00000185028
LRRC15	0.0017	9.0309E-01		ENSG00000172061
LRRC17	-0.4538	6.0080E-04	4.77E-03	ENSG00000128606
LRRC18	-0.2621	4.2423E-02	1.27E-01	ENSG00000165383
LRRC19	0.1653	1.2859E-01	2.80E-01	ENSG00000184434
LRRC2-AS1	-0.0142	6.2472E-01		ENSG00000268324
LRRC20	0.0938	1.8325E-01	3.56E-01	ENSG00000172731
LRRC23	-0.0493	3.4044E-01	5.36E-01	ENSG00000010626
LRRC24	-0.0248	8.7020E-01		ENSG00000254402
LRRC25	0.0720	1.9989E-01	3.77E-01	ENSG00000175489
LRRC26	-0.2503	2.6047E-02	8.77E-02	ENSG00000184709
LRRC27	0.0237	7.5035E-01	8.63E-01	ENSG00000148814
LRRC28	0.1610	1.3474E-02	5.34E-02	ENSG00000168904
LRRC3	0.1623	9.7514E-02	2.31E-01	ENSG00000160233

LRRC3-DT	0.1672	1.3935E-01	2.95E-01	ENSG00000229356
LRRC32	-0.0357	6.4817E-01	7.96E-01	ENSG00000137507
LRRC34	-0.2928	1.1836E-02	4.83E-02	ENSG00000171757
LRRC36	0.1407	1.6137E-01	3.27E-01	ENSG00000159708
LRRC37A	-0.0493	5.7876E-01	7.45E-01	ENSG00000176681
LRRC37A14P	-0.0290	5.9201E-01		ENSG00000215771
LRRC37A15P	-0.0352	9.9442E-01		ENSG00000230069
LRRC37A17P	-0.0147	7.7083E-01		ENSG00000263142
LRRC37A2	0.4128	2.4128E-03	1.42E-02	ENSG00000260075
LRRC37A2	-0.1228	1.1464E-01	2.59E-01	ENSG00000238083
LRRC37A3	-0.2119	4.8283E-03	2.43E-02	ENSG00000176809
LRRC37A6P	0.0339	4.9601E-01	6.79E-01	ENSG00000230445
LRRC37B	-0.0314	6.1288E-01	7.71E-01	ENSG00000185158
LRRC38	-0.0350	1.9145E-01		ENSG00000162494
LRRC39	-0.0919	3.4759E-01	5.43E-01	ENSG00000122477
LRRC3B	0.2980	1.8493E-02	6.80E-02	ENSG00000179796
LRRC3B-AS1	-0.0403	8.5428E-01	9.25E-01	ENSG00000225386
LRRC3C	0.0031	4.4596E-01	6.36E-01	ENSG00000204913
LRRC40	0.2607	1.1058E-03	7.68E-03	ENSG00000066557
LRRC41	-0.1037	1.4596E-01	3.05E-01	ENSG00000132128
LRRC42	-0.1487	5.7658E-02	1.59E-01	ENSG00000116212
LRRC43	0.1889	6.1939E-02	1.67E-01	ENSG00000158113
LRRC45	0.2041	3.0354E-02	9.83E-02	ENSG00000169683
LRRC46	-0.2221	3.6060E-02	1.12E-01	ENSG00000141294
LRRC47	0.1351	6.2237E-03	2.97E-02	ENSG00000130764
LRRC49	0.0502	4.7628E-01	6.63E-01	ENSG00000137821
LRRC4B	-0.3525	9.0451E-03	3.95E-02	ENSG00000131409
LRRC4C	0.1521	1.9483E-02	7.06E-02	ENSG00000148948
LRRC51	-0.3926	2.3113E-04	2.20E-03	ENSG00000184154
LRRC52-AS1	-0.0364	6.9015E-01	8.26E-01	ENSG00000237463
LRRC53	0.0038	5.6136E-01		ENSG00000162621
LRRC55	0.1198	1.7182E-01	3.41E-01	ENSG00000183908
LRRC56	0.1856	6.5599E-02	1.74E-01	ENSG00000161328
LRRC57	-0.0028	9.6469E-01	9.82E-01	ENSG00000180979
LRRC58	-0.1631	1.0916E-02	4.54E-02	ENSG00000163428
LRRC59	-0.0067	8.8509E-01	9.42E-01	ENSG00000108829
LRRC61	-0.1061	2.0691E-01	3.86E-01	ENSG00000127399
LRRC63	0.0408	6.4597E-01	7.95E-01	ENSG00000173988
LRRC66	-0.0793	2.4146E-01	4.26E-01	ENSG00000188993
LRRC69	-0.3659	2.4744E-03	1.45E-02	ENSG00000214954
LRRC7	0.2358	1.0237E-02	4.32E-02	ENSG00000033122
LRRC70	-0.0766	5.2900E-01		ENSG00000186105
LRRC71	-0.0087	9.1153E-01	9.55E-01	ENSG00000160838
LRRC72	-0.0046	9.4000E-01		ENSG00000205858
LRRC73	0.1456	1.2977E-01	2.81E-01	ENSG00000204052
LRRC74A	0.0103	4.9019E-01		ENSG00000100565
LRRC74B	0.1002	2.6667E-01	4.56E-01	ENSG00000187905
LRRC75A	0.0352	6.1437E-01	7.73E-01	ENSG00000181350
LRRC75B	-0.1476	4.3283E-02	1.29E-01	ENSG00000178026

LRRC8A	-0.1031	1.0269E-01	2.39E-01	ENSG00000136802
LRRC8B	0.3329	1.1408E-04	1.25E-03	ENSG00000197147
LRRC8C-DT	-0.2057	2.3680E-02	8.17E-02	ENSG00000231999
LRRC8D	0.0892	2.2247E-01	4.04E-01	ENSG00000171492
LRRC8E	0.0794	3.2917E-01	5.24E-01	ENSG00000171017
LRRC9	0.1159	1.5665E-01	3.20E-01	ENSG00000131951
LRRCC1	-0.2621	2.7330E-04	2.52E-03	ENSG00000133739
LRRD1	-0.0041	8.3712E-01		ENSG00000240720
LRRFIP1	-0.0598	2.5018E-01	4.37E-01	ENSG00000124831
LRRFIP2	0.1065	5.8817E-02	1.61E-01	ENSG00000093167
LRRIQ1	-0.1585	3.2078E-02	1.02E-01	ENSG00000133640
LRRIQ3	0.0176	8.5477E-01	9.25E-01	ENSG00000162620
LRRIQ4	-0.0349	2.4338E-01		ENSG00000188306
LRRK1	0.0273	7.7068E-01	8.76E-01	ENSG00000154237
LRRK2	-0.0246	8.0830E-01	8.98E-01	ENSG00000188906
LRRN1	-0.1285	1.1571E-01	2.60E-01	ENSG00000175928
LRRN2	-0.4801	1.1826E-05	2.01E-04	ENSG00000170382
LRRN3	-0.0672	4.3182E-01	6.23E-01	ENSG00000173114
LRRN4	-0.2067	5.5262E-02	1.54E-01	ENSG00000125872
LRRN4CL	0.0302	7.5275E-01	8.65E-01	ENSG00000177363
LRRTM1	0.0258	7.8496E-01	8.85E-01	ENSG00000162951
LRRTM2	0.2214	4.1011E-02	1.23E-01	ENSG00000146006
LRRTM3	0.2883	2.2028E-02	7.75E-02	ENSG00000198739
LRRTM4	0.1029	3.0246E-01	4.95E-01	ENSG00000176204
LRSAM1	0.0979	1.7689E-01	3.47E-01	ENSG00000148356
LRTM1	-0.0531	5.4536E-01	7.17E-01	ENSG00000144771
LRTM2	-0.2094	6.5135E-02	1.73E-01	ENSG00000166159
LRTOMT	-0.0918	1.2024E-01	2.67E-01	ENSG00000284922
LRWD1	0.0598	3.9044E-01	5.85E-01	ENSG00000161036
LSAMP	-0.1108	1.8097E-01	3.53E-01	ENSG00000185565
LSAMP-AS1	0.0963	2.3314E-01	4.17E-01	ENSG00000240922
LSG1	0.3068	8.3591E-05	9.69E-04	ENSG00000041802
LSM1	-0.0576	2.6956E-01	4.59E-01	ENSG00000175324
LSM10	-0.2452	9.6115E-06	1.70E-04	ENSG00000181817
LSM11	0.2211	1.3544E-02	5.36E-02	ENSG00000155858
LSM12	-0.1481	7.3516E-04	5.59E-03	ENSG00000161654
LSM14A	-0.1193	1.5171E-02	5.85E-02	ENSG00000257103
LSM14B	-0.0744	4.0345E-01	5.97E-01	ENSG00000149657
LSM2	-0.1588	4.5689E-03	2.34E-02	ENSG00000204392
LSM3	-0.1385	1.6851E-02	6.33E-02	ENSG00000170860
LSM4	-0.1903	3.2643E-03	1.79E-02	ENSG00000130520
LSM5	-0.0760	2.1825E-01	3.99E-01	ENSG00000106355
LSM6	0.0339	5.9662E-01	7.58E-01	ENSG00000164167
LSM7	-0.2730	6.9375E-07	1.97E-05	ENSG00000130332
LSM8	0.1042	1.3501E-01	2.89E-01	ENSG00000128534
LSMEM1	-0.0313	3.9994E-01		ENSG00000181016
LSMEM2	-0.1130	1.1428E-01	2.58E-01	ENSG00000179564
LSP1	0.0243	7.5881E-01	8.69E-01	ENSG00000130592
LSP1P4	-0.0285	7.2095E-01	8.45E-01	ENSG00000143429

LSP1P5	-0.1571	6.7023E-02	1.77E-01	ENSG00000288905
LSR	0.2052	9.9009E-03	4.22E-02	ENSG00000105699
LSS	-0.0642	2.7187E-01	4.62E-01	ENSG00000160285
LST1	0.0080	8.7689E-01	9.37E-01	ENSG00000204482
LTA	-0.0101	8.7546E-01	9.37E-01	ENSG00000226979
LTA4H	-0.2429	2.0777E-03	1.26E-02	ENSG00000111144
LTB	-0.0581	8.4737E-01	9.21E-01	ENSG00000227507
LTB4R	0.1105	2.7765E-01	4.68E-01	ENSG00000213903
LTB4R2	0.0086	6.1735E-01		ENSG00000213906
LTBP1	-0.5850	1.7377E-05	2.74E-04	ENSG00000049323
LTBP2	-0.4642	4.6415E-04	3.87E-03	ENSG00000119681
LTBR	0.2147	4.9146E-02	1.41E-01	ENSG00000111321
LTC4S	-0.2229	4.4511E-02	1.31E-01	ENSG00000213316
LTF	-0.0279	8.3056E-01	9.11E-01	ENSG00000012223
LTN1	0.2078	6.9619E-03	3.23E-02	ENSG00000198862
LTO1	0.0211	7.5675E-01	8.68E-01	ENSG00000149716
LTV1	-0.0597	2.9902E-01	4.91E-01	ENSG00000135521
LUADT1	0.0153	4.0205E-01		ENSG00000196634
LUARIS	0.0931	7.8243E-03		ENSG00000231638
LUC7L	0.2618	1.2817E-03	8.66E-03	ENSG00000007392
LUC7L2	0.0055	9.2661E-01	9.64E-01	ENSG00000146963
LUC7L3	0.3142	1.2159E-05	2.05E-04	ENSG00000108848
LUCAT1	0.0418	5.9808E-01	7.59E-01	ENSG00000248323
LUM	-0.4886	1.5067E-03	9.84E-03	ENSG00000139329
LURAP1	0.0614	5.1506E-01	6.94E-01	ENSG00000171357
LURAP1L-AS1	0.1177	2.4964E-01	4.36E-01	ENSG00000235448
LUZP1	-0.3838	6.1473E-07	1.77E-05	ENSG00000169641
LUZP2	0.0264	7.4706E-01	8.61E-01	ENSG00000187398
LVRN	0.0286	5.0243E-01	6.84E-01	ENSG00000172901
LXN	-0.3807	3.2980E-05	4.54E-04	ENSG00000079257
LY6D	-0.0009	9.3891E-01		ENSG00000167656
LY6E	-0.0922	2.5271E-01	4.40E-01	ENSG00000160932
LY6G5B	0.1882	1.0479E-01	2.43E-01	ENSG00000240053
LY6G5C	0.0056	9.4795E-01	9.74E-01	ENSG00000204428
LY6G6C	-0.1222	2.4251E-01	4.28E-01	ENSG00000204421
LY6G6D	-0.0226	3.9779E-01		ENSG00000244355
LY6H	-0.5746	1.4698E-10	1.64E-08	ENSG00000176956
LY6L	0.0023	8.4763E-01		ENSG00000261667
LY6S-AS1	-0.3617	8.5652E-03	3.79E-02	ENSG00000177335
LY75	0.0529	5.1777E-01	6.96E-01	ENSG00000054219
LY86-AS1	0.0140	6.8997E-01		ENSG00000216863
LY9	-0.0409	3.9653E-01		ENSG00000122224
LY96	0.0107	9.0451E-01	9.52E-01	ENSG00000154589
LYAR	0.4577	6.2708E-05	7.73E-04	ENSG00000145220
LYG1	-0.0321	7.3356E-01	8.54E-01	ENSG00000144214
LYG2	0.1331	1.4519E-01	3.04E-01	ENSG00000185674
LYL1	-0.0293	4.3846E-01		ENSG00000104903
LYN	-0.0620	4.5707E-01	6.46E-01	ENSG00000254087
LYNX1	-0.2252	3.3607E-02	1.06E-01	ENSG00000180155

LYNX1-SLURP2	-0.0972	2.9337E-01	4.85E-01	ENSG00000284505
LYPD1	-0.5201	2.4031E-05	3.55E-04	ENSG00000150551
LYPD2	-0.0594	4.5632E-01	6.46E-01	ENSG00000197353
LYPD3	0.3816	6.6915E-05	8.12E-04	ENSG00000124466
LYPD5	-0.5259	1.0758E-02	4.48E-02	ENSG00000159871
LYPD6	-0.0187	8.4441E-01	9.19E-01	ENSG00000187123
LYPD6B	-0.1010	3.1591E-01	5.09E-01	ENSG00000150556
LYPLA1	0.0490	5.1740E-01	6.95E-01	ENSG00000120992
LYPLA2	0.1484	6.5797E-03	3.09E-02	ENSG00000011009
LYPLAL1	-0.0275	6.2957E-01	7.83E-01	ENSG00000143353
LYPLAL1-DT	-0.3405	2.2015E-02	7.74E-02	ENSG00000228063
LYRM1	-0.1094	2.6920E-02	8.99E-02	ENSG00000102897
LYRM2	-0.0079	8.5864E-01	9.28E-01	ENSG00000083099
LYRM4	-0.2339	8.7557E-05	1.01E-03	ENSG00000214113
LYRM4-AS1	-0.5542	4.9717E-04	4.08E-03	ENSG00000272142
LYRM7	0.0169	7.6826E-01	8.74E-01	ENSG00000186687
LYRM9	0.3148	1.9228E-04	1.90E-03	ENSG00000232859
LYSET	0.1216	4.7381E-02	1.37E-01	ENSG00000153485
LYSMD1	-0.1056	2.5378E-01	4.41E-01	ENSG00000163155
LYSMD2	-0.0982	1.8873E-01	3.63E-01	ENSG00000140280
LYSMD3	0.2858	7.0099E-04	5.39E-03	ENSG00000176018
LYSMD4	0.3644	2.2061E-05	3.32E-04	ENSG00000183060
LYST	0.2243	3.3354E-04	2.98E-03	ENSG00000143669
LYVE1	0.0154	5.0548E-01		ENSG00000133800
LYZ	0.0268	4.3542E-01		ENSG00000090382
LYZL4	-0.0060	9.7568E-01	9.88E-01	ENSG00000157093
LZIC	-0.1413	1.1753E-02	4.80E-02	ENSG00000162441
LZTFL1	-0.0031	9.3898E-01	9.69E-01	ENSG00000163818
LZTR1	-0.0240	7.5534E-01	8.67E-01	ENSG00000099949
LZTS1	0.1291	2.0006E-01	3.77E-01	ENSG00000061337
LZTS1-AS1	0.0712	4.0712E-02		ENSG00000253733
LZTS2	-0.0093	8.5507E-01	9.26E-01	ENSG00000107816
LZTS3	-0.1289	1.9681E-02	7.11E-02	ENSG00000088899
M1AP	0.0091	9.4393E-01	9.72E-01	ENSG00000159374
M6PR	0.2372	1.5017E-03	9.82E-03	ENSG00000003056
MAB21L1	0.0888	3.2776E-01	5.22E-01	ENSG00000180660
MAB21L2	-0.0351	5.8857E-01		ENSG00000181541
MAB21L3	-0.0228	6.8277E-01	8.20E-01	ENSG00000173212
MAB21L4	-0.0136	8.6899E-01	9.34E-01	ENSG00000172478
MACC1	-0.1040	2.8799E-01	4.79E-01	ENSG00000183742
MACC1-AS1	-0.0376	5.6307E-01		ENSG00000228598
MACF1	-0.2912	2.4883E-05	3.64E-04	ENSG00000127603
MACIR	0.4433	7.5346E-04	5.70E-03	ENSG00000181751
MACO1	0.0558	4.7811E-01	6.64E-01	ENSG00000204178
MACORIS	-0.1121	4.7105E-02	1.37E-01	ENSG00000237797
MACROD1	-0.2484	7.8064E-03	3.52E-02	ENSG00000133315
MACROD2-AS1	0.0932	2.2462E-01	4.07E-01	ENSG00000235914
MACROD2-IT1	0.0060	2.7611E-01		ENSG00000227927
MACROH2A1	-0.2231	1.1424E-05	1.95E-04	ENSG00000113648

MACROH2A2	-0.2237	2.0762E-03	1.26E-02	ENSG00000099284
MAD1L1	0.1878	1.0208E-01	2.38E-01	ENSG00000002822
MAD2L1	-0.2299	2.9628E-02	9.66E-02	ENSG00000164109
MAD2L1BP	-0.2135	3.7530E-03	2.01E-02	ENSG00000124688
MAD2L2	-0.1354	5.5641E-03	2.72E-02	ENSG00000116670
MADCAM1	0.2797	9.7272E-03	4.16E-02	ENSG00000099866
MADD	0.0802	2.3596E-01	4.20E-01	ENSG00000110514
MADD-AS1	0.4535	1.7161E-02	6.42E-02	ENSG00000256746
MAEA	0.0609	2.1567E-01	3.96E-01	ENSG00000090316
MAEL	0.0157	8.6094E-01	9.29E-01	ENSG00000143194
MAF	-0.3796	9.1284E-04	6.63E-03	ENSG00000178573
MAF1	-0.0289	5.8951E-01	7.53E-01	ENSG00000179632
MAFA	-0.0436	5.1746E-01	6.95E-01	ENSG00000182759
MAFA-AS1	0.0365	6.3286E-01	7.86E-01	ENSG00000254338
MAFB	-0.0023	9.7155E-01	9.85E-01	ENSG00000204103
MAFG	0.3691	1.4695E-06	3.67E-05	ENSG00000197063
MAFIP	0.0004	9.3455E-01	9.68E-01	ENSG00000277400
MAFK	0.4521	4.5371E-03	2.32E-02	ENSG00000198517
MAFTRR	0.0605	5.3502E-01	7.09E-01	ENSG00000261390
MAGEA11	0.0055	6.0354E-01		ENSG00000185247
MAGEA4	0.0323	3.7643E-02	1.16E-01	ENSG00000147381
MAGEA9B	0.0057	6.1094E-01		ENSG00000267978
MAGEB2	-0.0036	3.9597E-01		ENSG00000099399
MAGEC3	-0.0105	6.7364E-01		ENSG00000165509
MAGED1	-0.0686	2.9396E-01	4.86E-01	ENSG00000179222
MAGED2	-0.0844	1.9127E-01	3.66E-01	ENSG00000102316
MAGED4	-0.0046	9.5496E-01	9.77E-01	ENSG00000154545
MAGED4B	-0.0049	9.5256E-01	9.76E-01	ENSG00000187243
MAGEE1	0.0837	2.8302E-01	4.74E-01	ENSG00000198934
MAGEF1	0.1138	1.0114E-02	4.28E-02	ENSG00000177383
MAGEH1	-0.0531	5.0537E-01	6.86E-01	ENSG00000187601
MAGEL2	0.1658	3.2209E-02	1.03E-01	ENSG00000254585
MAGI1	0.1077	8.7426E-02	2.14E-01	ENSG00000151276
MAGI1-AS1	-0.0145	9.3528E-01		ENSG00000240175
MAGI1-IT1	0.0035	8.2432E-01		ENSG00000272610
MAGI2	0.1592	4.0503E-02	1.22E-01	ENSG00000187391
MAGI2-AS3	-0.1044	1.7884E-01	3.50E-01	ENSG00000234456
MAGI3	-0.0954	9.3271E-02	2.24E-01	ENSG00000081026
MAGIX	-0.0559	5.2255E-01	6.99E-01	ENSG00000269313
MAGOH	-0.2067	3.0487E-07	9.95E-06	ENSG00000162385
MAGOH-DT	-0.3398	7.1478E-03	3.29E-02	ENSG00000226754
MAGOHB	-0.1726	5.4097E-03	2.66E-02	ENSG00000111196
MAGT1	-0.4167	1.2903E-05	2.15E-04	ENSG00000102158
MAIP1	-0.2538	1.1703E-04	1.27E-03	ENSG00000162972
MAJIN	-0.0912	1.5532E-01		ENSG00000168070
MAK	-0.1235	2.3127E-01	4.15E-01	ENSG00000111837
MAK16	0.2426	5.3071E-03	2.62E-02	ENSG00000198042
MAL	-0.2074	5.0713E-02	1.44E-01	ENSG00000172005
MAL2	-0.3866	1.8834E-05	2.91E-04	ENSG00000147676

MAL2-AS1	-0.0268	9.1237E-01		ENSG00000253972
MALAT1	-0.1546	5.6635E-02	1.57E-01	ENSG00000251562
MALL	0.0501	5.1797E-01	6.96E-01	ENSG00000144063
MALSU1	-0.0326	5.7736E-01	7.44E-01	ENSG00000156928
MALT1	0.1374	1.0094E-01	2.36E-01	ENSG00000172175
MALT1-AS1	0.0746	4.0884E-01	6.02E-01	ENSG00000267226
MAMDC2	0.0312	7.3501E-01	8.54E-01	ENSG00000165072
MAMDC2-AS1	-0.1058	2.9706E-01	4.89E-01	ENSG00000204706
MAMDC4	-0.1878	1.0532E-01	2.44E-01	ENSG00000177943
MAML1	0.0913	1.5651E-01	3.20E-01	ENSG00000161021
MAML2	-0.2884	8.0693E-04	6.02E-03	ENSG00000184384
MAML3	0.1078	1.5092E-01	3.12E-01	ENSG00000196782
MAMLD1	-0.0279	7.5285E-01	8.65E-01	ENSG00000013619
MAMSTR	0.5619	1.5498E-08	8.20E-07	ENSG00000176909
MAN1A1	-0.3623	1.2723E-03	8.61E-03	ENSG00000111885
MAN1A2	-0.2755	2.2548E-04	2.16E-03	ENSG00000198162
MAN1B1	0.0515	3.7748E-01	5.73E-01	ENSG00000177239
MAN1B1-DT	0.4206	1.4378E-06	3.60E-05	ENSG00000268996
MAN1C1	-0.3141	2.9563E-03	1.67E-02	ENSG00000117643
MAN2A1	-0.1722	4.9005E-02	1.41E-01	ENSG00000112893
MAN2A2	0.0936	1.8201E-01	3.54E-01	ENSG00000196547
MAN2B1	0.3784	3.8527E-04	3.34E-03	ENSG00000104774
MAN2B2	-0.1802	1.9192E-02	6.98E-02	ENSG00000013288
MAN2C1	0.0606	4.6239E-01	6.51E-01	ENSG00000140400
MANBA	-0.2134	4.0616E-03	2.14E-02	ENSG00000109323
MANBAL	0.0368	4.0107E-01	5.95E-01	ENSG00000101363
MANCR	-0.0535	3.8323E-01		ENSG00000231298
MANEA	-0.0735	3.7065E-01	5.66E-01	ENSG00000172469
MANEA-DT	0.0549	4.7284E-01	6.60E-01	ENSG00000261366
MANEAL	0.0534	5.1058E-01	6.90E-01	ENSG00000185090
MANF	-0.0601	3.7784E-01	5.73E-01	ENSG00000145050
MANSC1	-0.2899	5.3070E-04	4.32E-03	ENSG00000111261
MANSC4	-0.0145	7.4939E-01	8.63E-01	ENSG00000205693
MAOA	-0.3187	5.2293E-05	6.67E-04	ENSG00000189221
MAP10	0.2745	3.6243E-03	1.95E-02	ENSG00000212916
MAP1A	0.0029	9.6774E-01	9.83E-01	ENSG00000166963
MAP1B	0.0214	7.8868E-01	8.87E-01	ENSG00000131711
MAP1LC3A	-0.0430	3.4427E-01	5.39E-01	ENSG00000101460
MAP1LC3B	-0.0871	3.0270E-01	4.95E-01	ENSG00000140941
MAP1LC3B2	-0.1084	2.0046E-01	3.78E-01	ENSG00000258102
MAP1LC3C	-0.0882	3.1775E-01	5.11E-01	ENSG00000197769
MAP1S	-0.0332	6.1794E-01	7.75E-01	ENSG00000130479
MAP2	-0.0560	4.2464E-01	6.16E-01	ENSG00000078018
MAP2K1	-0.0539	4.1061E-01	6.04E-01	ENSG00000169032
MAP2K2	-0.0709	1.9350E-01	3.69E-01	ENSG00000126934
MAP2K3	-0.3260	8.0154E-04	6.00E-03	ENSG00000034152
MAP2K4	0.0088	8.8243E-01	9.41E-01	ENSG00000065559
MAP2K4P1	-0.1073	1.6234E-01	3.28E-01	ENSG00000269904
MAP2K5	-0.1052	9.5445E-02	2.27E-01	ENSG00000137764

MAP2K6	-0.3087	1.6037E-03	1.03E-02	ENSG00000108984
MAP2K7	0.0507	5.7178E-01	7.39E-01	ENSG00000076984
MAP3K1	0.2591	2.3758E-02	8.19E-02	ENSG00000095015
MAP3K10	0.2148	4.6199E-02	1.35E-01	ENSG00000130758
MAP3K11	0.0185	7.8445E-01	8.84E-01	ENSG00000173327
MAP3K12	0.0810	2.3724E-01	4.21E-01	ENSG00000139625
MAP3K13	-0.0057	9.0952E-01	9.55E-01	ENSG00000073803
MAP3K14	-0.1852	6.9411E-02	1.81E-01	ENSG00000006062
MAP3K15	-0.0185	7.9224E-01	8.88E-01	ENSG00000180815
MAP3K2	0.0640	3.5821E-01	5.54E-01	ENSG00000169967
MAP3K20	-0.3955	4.4906E-05	5.89E-04	ENSG00000091436
MAP3K20-AS1	-0.1078	2.6803E-01	4.57E-01	ENSG00000238133
MAP3K21	-0.2195	1.6035E-02	6.10E-02	ENSG00000143674
MAP3K3	0.3456	6.7757E-05	8.20E-04	ENSG00000198909
MAP3K4	0.0740	3.7840E-01	5.73E-01	ENSG00000085511
MAP3K4-AS1	-0.2717	3.5840E-03	1.94E-02	ENSG00000272841
MAP3K5	-0.3989	6.0634E-04	4.81E-03	ENSG00000197442
MAP3K6	0.0813	3.6538E-01	5.61E-01	ENSG00000142733
MAP3K7	0.2082	1.5797E-03	1.02E-02	ENSG00000135341
MAP3K7CL	-0.0257	3.6178E-01	5.58E-01	ENSG00000156265
MAP3K8	-0.5411	6.2279E-09	3.91E-07	ENSG00000107968
MAP3K9	0.0838	2.3828E-01	4.23E-01	ENSG00000006432
MAP3K9-DT	0.1385	3.4586E-02	1.09E-01	ENSG00000259153
MAP4	-0.0744	2.2460E-01	4.07E-01	ENSG00000047849
MAP4K1	0.2739	5.3191E-03	2.63E-02	ENSG00000104814
MAP4K1-AS1	0.0207	5.2087E-01		ENSG00000267291
MAP4K2	0.0655	4.3162E-01	6.23E-01	ENSG00000168067
MAP4K3	0.1160	3.8400E-02	1.17E-01	ENSG00000011566
MAP4K3-DT	0.1640	1.1585E-03	7.97E-03	ENSG00000231312
MAP4K4	-0.1003	9.1432E-02	2.21E-01	ENSG00000071054
MAP4K5	0.0424	4.1247E-01	6.05E-01	ENSG00000012983
MAP6	-0.0058	9.3680E-01	9.69E-01	ENSG00000171533
MAP6D1	-0.0695	4.6061E-01	6.49E-01	ENSG00000180834
MAP7	-0.1566	3.2709E-02	1.04E-01	ENSG00000135525
MAP7D1	-0.2800	2.9901E-05	4.22E-04	ENSG00000116871
MAP7D2	0.1091	1.8164E-01	3.54E-01	ENSG00000184368
MAP7D3	-0.3728	2.9348E-04	2.68E-03	ENSG00000129680
MAP9	-0.1863	8.0500E-04	6.01E-03	ENSG00000164114
MAP9-AS1	-0.0409	6.5298E-01	7.99E-01	ENSG00000250910
MAPK1	-0.1228	3.7127E-03	1.99E-02	ENSG00000100030
MAPK10	0.0553	4.4845E-01	6.38E-01	ENSG00000109339
MAPK10-AS1	-0.0024	5.1175E-01		ENSG00000250062
MAPK11	0.1295	2.1053E-01	3.90E-01	ENSG00000185386
MAPK12	-0.1790	7.3053E-02	1.88E-01	ENSG00000188130
MAPK13	0.0908	3.6731E-01	5.63E-01	ENSG00000156711
MAPK14	0.2142	9.9427E-04	7.08E-03	ENSG00000112062
MAPK15	-0.4917	2.2363E-04	2.14E-03	ENSG00000181085
MAPK1IP1L	-0.1962	4.7668E-03	2.41E-02	ENSG00000168175
MAPK3	-0.1383	6.7975E-02	1.78E-01	ENSG00000102882

MAPK4	0.0220	8.1601E-01	9.03E-01	ENSG00000141639
MAPK6	-0.3164	1.4277E-09	1.14E-07	ENSG00000069956
MAPK6-DT	-0.1183	2.0081E-01	3.78E-01	ENSG00000259438
MAPK7	0.0243	7.3912E-01	8.57E-01	ENSG00000166484
MAPK8	0.1146	1.1334E-01	2.57E-01	ENSG00000107643
MAPK8IP1	0.0867	2.7092E-01	4.61E-01	ENSG00000121653
MAPK8IP1P1	0.0060	7.7557E-01		ENSG00000262500
MAPK8IP2	-0.1976	4.5898E-03	2.35E-02	ENSG00000008735
MAPK8IP3	0.1635	1.2228E-02	4.95E-02	ENSG00000138834
MAPK8IP3-AS1	-0.0847	3.3547E-01	5.31E-01	ENSG00000261399
MAPK9	0.1872	8.5843E-03	3.79E-02	ENSG00000050748
MAPKAP1	0.0290	5.9165E-01	7.55E-01	ENSG00000119487
MAPKAPK2	-0.2200	2.7674E-02	9.17E-02	ENSG00000162889
MAPKAPK3	0.1920	1.6467E-02	6.23E-02	ENSG00000114738
MAPKAPK5	0.1976	1.1367E-03	7.84E-03	ENSG00000089022
MAPKAPK5-AS1	0.1260	4.4019E-03	2.27E-02	ENSG00000234608
MAPKBP1	0.2285	1.3985E-02	5.50E-02	ENSG00000137802
MAPRE1	-0.1862	3.6713E-03	1.97E-02	ENSG00000101367
MAPRE2	0.1719	1.5512E-02	5.94E-02	ENSG00000166974
MAPRE3	0.1647	2.1681E-02	7.66E-02	ENSG00000084764
MAPRE3-AS1	-0.4084	4.0612E-03	2.14E-02	ENSG00000205500
MAPT	-0.0335	5.5682E-01	7.27E-01	ENSG00000186868
MAPT-IT1	0.1241	2.1869E-01	4.00E-01	ENSG00000279685
MARCHF1	0.0012	9.8420E-01	9.91E-01	ENSG00000145416
MARCHF10	-0.1379	1.9678E-01	3.73E-01	ENSG00000173838
MARCHF10-DT	0.0174	1.1216E-01		ENSG00000265000
MARCHF11	0.1363	1.8147E-01	3.53E-01	ENSG00000183654
MARCHF11-AS1	0.0250	1.7161E-01		ENSG00000250981
MARCHF2	0.0946	6.5431E-02	1.74E-01	ENSG00000099785
MARCHF3	0.0782	4.0979E-01	6.03E-01	ENSG00000173926
MARCHF4	-0.0598	4.1613E-01	6.09E-01	ENSG00000144583
MARCHF5	-0.2830	3.3071E-07	1.06E-05	ENSG00000198060
MARCHF6	-0.2244	1.2894E-04	1.37E-03	ENSG00000145495
MARCHF6-DT	0.4149	1.3989E-06	3.53E-05	ENSG00000259802
MARCHF7	-0.0691	1.4220E-01	2.99E-01	ENSG00000136536
MARCHF8	-0.1847	1.2371E-02	5.00E-02	ENSG00000165406
MARCHF9	-0.1981	4.9243E-02	1.42E-01	ENSG00000139266
MARCKS	-0.3283	4.5181E-04	3.79E-03	ENSG00000277443
MARCKSL1	0.0079	9.2425E-01	9.62E-01	ENSG00000175130
MARF1	0.0687	4.4398E-01	6.34E-01	ENSG00000166783
MARK1	-0.0216	7.1084E-01	8.39E-01	ENSG00000116141
MARK2	0.1038	1.9712E-01	3.74E-01	ENSG00000072518
MARK2P18	-0.4161	1.5839E-02	6.04E-02	ENSG00000219712
MARK2P19	-0.0809	3.1174E-01	5.05E-01	ENSG00000270773
MARK3	0.0098	8.7119E-01	9.35E-01	ENSG00000075413
MARK4	-0.1559	1.0929E-01	2.50E-01	ENSG00000007047
MARS1	0.0999	1.6130E-01	3.26E-01	ENSG00000166986
MARS2	0.1665	7.1469E-02	1.85E-01	ENSG00000247626
MARVELD1	-0.2764	6.7368E-03	3.14E-02	ENSG00000155254

MARVELD2	-0.0567	5.0897E-01	6.89E-01	ENSG00000152939
MARVELD3	0.0211	8.1602E-01	9.03E-01	ENSG00000140832
MAS1	0.0048	5.7350E-01		ENSG00000130368
MASP1	0.0069	9.4485E-01	9.72E-01	ENSG00000127241
MASP2	0.0125	8.9639E-01	9.48E-01	ENSG00000009724
MAST1	0.1555	4.1157E-02	1.24E-01	ENSG00000105613
MAST2	0.1235	1.0737E-01	2.47E-01	ENSG00000086015
MAST3	-0.1628	5.8168E-02	1.60E-01	ENSG00000099308
MAST4	0.0123	8.6806E-01	9.33E-01	ENSG00000069020
MASTL	-0.3297	2.9066E-03	1.64E-02	ENSG00000120539
MAT1A	0.1005	2.2361E-01	4.06E-01	ENSG00000151224
MAT2A	0.4500	2.0441E-05	3.11E-04	ENSG00000168906
MAT2B	0.2091	2.8024E-03	1.60E-02	ENSG00000038274
MATCAP1	0.3639	5.3845E-07	1.59E-05	ENSG00000196123
MATCAP2	-0.1791	2.7587E-02	9.14E-02	ENSG00000164542
MATK	-0.1877	9.3611E-02	2.25E-01	ENSG00000007264
MATN1	0.0799	2.8624E-01	4.77E-01	ENSG00000162510
MATN1-AS1	0.2603	5.1453E-02	1.46E-01	ENSG00000186056
MATN3	-0.1046	2.7615E-01	4.67E-01	ENSG00000132031
MATN4	-0.1694	1.2768E-01	2.78E-01	ENSG00000124159
MATR3	0.0844	4.0061E-01	5.95E-01	ENSG00000015479
MATR3	0.0034	5.7719E-01		ENSG00000280987
MAU2	0.1252	4.7524E-02	1.38E-01	ENSG00000129933
MAVS	-0.1251	5.4868E-02	1.53E-01	ENSG00000088888
MAX	0.1111	8.9070E-02	2.17E-01	ENSG00000125952
MAZ	0.0552	5.3161E-01	7.06E-01	ENSG00000103495
MB	-0.0740	4.4905E-01	6.39E-01	ENSG00000198125
MB21D2	-0.2575	1.8195E-02	6.71E-02	ENSG00000180611
MBD1	0.1488	5.8115E-03	2.81E-02	ENSG00000141644
MBD2	0.0765	2.2871E-01	4.12E-01	ENSG00000134046
MBD3	-0.0561	4.2549E-01	6.17E-01	ENSG00000071655
MBD3L3	-0.0230	9.7496E-01	9.87E-01	ENSG00000182315
MBD4	0.1377	1.8607E-02	6.83E-02	ENSG00000129071
MBD5	0.0374	5.4003E-01	7.14E-01	ENSG00000204406
MBD6	-0.0003	9.9425E-01	9.97E-01	ENSG00000166987
MBIP	-0.1338	1.6870E-02	6.33E-02	ENSG00000151332
MBL1P	0.0039	4.6865E-01		ENSG00000242600
MBLAC1	0.0197	8.1768E-01	9.04E-01	ENSG00000214309
MBLAC2	0.4042	5.6330E-04	4.53E-03	ENSG00000176055
MBNL1	-0.3413	4.0672E-06	8.50E-05	ENSG00000152601
MBNL1-AS1	-0.1470	1.2108E-01	2.69E-01	ENSG00000229619
MBNL2	0.0006	9.9153E-01	9.95E-01	ENSG00000139793
MBNL3	-0.5137	3.9858E-03	2.10E-02	ENSG00000076770
MBOAT2	0.0150	8.6979E-01	9.34E-01	ENSG00000143797
MBOAT4	-0.0012	9.8484E-01	9.92E-01	ENSG00000177669
MBOAT7	-0.1163	1.8010E-02	6.66E-02	ENSG00000125505
MBP	-0.1052	2.0897E-01	3.88E-01	ENSG00000197971
MBTD1	-0.0248	7.4340E-01	8.59E-01	ENSG00000011258
MBTPS1	0.0714	1.8070E-01	3.53E-01	ENSG00000140943

MBTPS2	0.1450	1.0627E-02	4.44E-02	ENSG00000012174
MC1R	0.3029	2.3493E-02	8.13E-02	ENSG00000258839
MC4R	-0.2766	2.6833E-02	8.97E-02	ENSG00000166603
MC5R	-0.0034	9.6761E-01	9.83E-01	ENSG00000176136
MCAM	-0.1848	2.7387E-02	9.10E-02	ENSG00000076706
MCAT	0.0043	9.6454E-01	9.82E-01	ENSG00000100294
MCC	-0.2110	4.5238E-02	1.33E-01	ENSG00000171444
MCCC1	0.0627	4.3894E-01	6.30E-01	ENSG00000078070
MCCC2	0.0049	9.9398E-01	9.97E-01	ENSG00000131844
MCEE	0.3094	6.0689E-04	4.81E-03	ENSG00000124370
MCEMP1	-0.0378	3.8517E-01		ENSG00000183019
MCF2	-0.2820	2.8474E-02	9.37E-02	ENSG00000101977
MCF2L	-0.0273	6.0975E-01	7.68E-01	ENSG00000126217
MCF2L2	0.2008	4.6406E-02	1.35E-01	ENSG00000053524
MCFD2	0.0398	5.8209E-01	7.48E-01	ENSG00000180398
MCHR1	0.0949	3.0238E-01	4.95E-01	ENSG00000128285
MCHR2	-0.0262	3.6985E-01		ENSG00000152034
MCIDAS	0.0343	1.4215E-01	2.99E-01	ENSG00000234602
MCL1	-0.2004	5.7824E-03	2.80E-02	ENSG00000143384
MCM10	-0.4883	2.9732E-03	1.67E-02	ENSG00000065328
MCM2	0.0005	9.9635E-01	9.98E-01	ENSG00000073111
MCM3	-0.0468	5.1622E-01	6.95E-01	ENSG00000112118
MCM3AP	0.1755	6.3759E-02	1.70E-01	ENSG00000160294
MCM3AP-AS1	0.1482	1.0733E-01	2.47E-01	ENSG00000215424
MCM4	-0.1677	2.6678E-02	8.93E-02	ENSG00000104738
MCM5	-0.1696	6.8251E-02	1.79E-01	ENSG00000100297
MCM6	-0.2963	1.1189E-03	7.75E-03	ENSG00000076003
MCM7	-0.1686	9.0116E-02	2.19E-01	ENSG00000166508
MCM8	0.1687	1.1876E-01	2.65E-01	ENSG00000125885
MCM8-AS1	-0.0473	7.9160E-01	8.88E-01	ENSG00000278719
MCM9	0.0133	8.7444E-01	9.37E-01	ENSG00000111877
MCMBP	0.1673	1.7797E-02	6.60E-02	ENSG00000197771
MCMDC2	-0.0560	5.5164E-01	7.23E-01	ENSG00000178460
MCOLN1	0.1876	4.3141E-02	1.28E-01	ENSG00000090674
MCOLN2	0.0832	2.2895E-01	4.12E-01	ENSG00000153898
MCOLN3	-0.1445	1.5391E-01	3.16E-01	ENSG00000055732
MCPH1	0.0433	5.0564E-01	6.87E-01	ENSG00000147316
MCPH1-DT	-0.1553	1.7261E-02	6.45E-02	ENSG00000246089
MCRIP1	0.0006	9.8945E-01	9.94E-01	ENSG00000225663
MCRIP2	-0.0704	2.9686E-01	4.89E-01	ENSG00000172366
MCRS1	-0.2908	9.7523E-09	5.59E-07	ENSG00000187778
MCTP1	-0.3639	4.5452E-04	3.81E-03	ENSG00000175471
MCTP2	0.0484	6.2774E-01	7.82E-01	ENSG00000140563
MCTS1	-0.1065	1.1728E-01	2.63E-01	ENSG00000232119
MCU	-0.0061	9.3360E-01	9.67E-01	ENSG00000156026
MCUB	-0.1819	3.1458E-02	1.01E-01	ENSG00000005059
MCUR1	-0.2111	5.0378E-08	2.23E-06	ENSG00000050393
MDC1	0.0686	3.9692E-01	5.91E-01	ENSG00000137337
MDFI	-0.0410	6.6003E-01	8.04E-01	ENSG00000112559

MDGA1	-0.0323	7.1065E-01	8.39E-01	ENSG00000112139
MDGA2	0.1345	1.9118E-01	3.66E-01	ENSG00000139915
MDH1	-0.0800	1.7454E-01	3.44E-01	ENSG00000014641
MDH1B	-0.1607	3.6751E-02	1.14E-01	ENSG00000138400
MDH2	-0.0378	5.1417E-01	6.93E-01	ENSG00000146701
MDK	-0.4717	6.3674E-06	1.23E-04	ENSG00000110492
MDM1	-0.0775	2.5067E-01	4.37E-01	ENSG00000111554
MDM2	-0.0444	4.6748E-01	6.55E-01	ENSG00000135679
MDM4	-0.0963	9.6091E-02	2.28E-01	ENSG00000198625
MDN1	0.1206	1.2572E-01	2.76E-01	ENSG00000112159
MDP1	-0.0186	8.1087E-01		ENSG00000213920
MDS2	0.1297	2.0525E-01	3.84E-01	ENSG00000197880
ME1	-0.1163	2.5230E-01	4.39E-01	ENSG00000065833
ME2	-0.2188	8.4121E-04	6.22E-03	ENSG00000082212
ME3	-0.3479	4.3893E-03	2.27E-02	ENSG00000151376
MEA1	-0.1668	1.4611E-04	1.52E-03	ENSG00000124733
MEAF6	0.0453	3.5455E-01	5.50E-01	ENSG00000163875
MEAK7	0.0884	2.1256E-01	3.92E-01	ENSG00000140950
MECOM-AS1	-0.0165	6.0360E-01		ENSG00000241479
MECP2	-0.1165	9.6142E-02	2.28E-01	ENSG00000169057
MECR	0.0678	3.4196E-01	5.37E-01	ENSG00000116353
MED1	0.0022	9.2340E-01	9.62E-01	ENSG00000125686
MED10	-0.1202	1.0568E-02	4.42E-02	ENSG00000133398
MED11	-0.2531	6.8116E-03	3.17E-02	ENSG00000161920
MED12	0.0696	3.4047E-01	5.36E-01	ENSG00000184634
MED12L	0.0292	7.1083E-01	8.39E-01	ENSG00000144893
MED13	-0.1681	4.0426E-02	1.22E-01	ENSG00000108510
MED13L	-0.0557	4.0856E-01	6.02E-01	ENSG00000123066
MED14	-0.0672	3.7153E-01	5.67E-01	ENSG00000180182
MED15	0.0117	8.4738E-01	9.21E-01	ENSG00000099917
MED15P3	0.0209	1.4606E-01		ENSG00000226831
MED15P5	0.0405	4.8546E-01		ENSG00000236595
MED16	-0.0532	4.4596E-01	6.36E-01	ENSG00000175221
MED17	-0.2033	2.2992E-02	8.00E-02	ENSG00000042429
MED18	0.3616	8.8853E-04	6.49E-03	ENSG00000130772
MED19	0.0018	9.6756E-01	9.83E-01	ENSG00000156603
MED20	0.0355	6.4263E-01	7.93E-01	ENSG00000124641
MED21	-0.0985	1.2274E-01	2.71E-01	ENSG00000152944
MED22	0.0311	7.1440E-01	8.41E-01	ENSG00000148297
MED23	0.1437	4.6369E-02	1.35E-01	ENSG00000112282
MED24	0.0834	1.9974E-01	3.77E-01	ENSG00000008838
MED25	-0.0302	6.6133E-01	8.05E-01	ENSG00000104973
MED26	-0.1355	1.3621E-01	2.90E-01	ENSG00000105085
MED27	-0.0023	9.3225E-01	9.66E-01	ENSG00000160563
MED28	-0.0254	5.8336E-01	7.49E-01	ENSG00000118579
MED28P3	-0.0115	7.7008E-01		ENSG00000227692
MED29	-0.0603	1.6514E-01	3.32E-01	ENSG00000063322
MED30	-0.2277	1.3766E-03	9.18E-03	ENSG00000164758
MED31	0.0081	8.9148E-01	9.46E-01	ENSG00000108590

MED4	0.0366	4.6142E-01	6.50E-01	ENSG00000136146
MED4-AS1	-0.0669	3.0029E-01		ENSG00000229111
MED6	0.1103	1.6896E-02	6.34E-02	ENSG00000133997
MED7	-0.1014	9.0368E-02	2.19E-01	ENSG00000155868
MED8	0.1349	6.4204E-03	3.04E-02	ENSG00000159479
MED9	0.1833	2.2548E-02	7.88E-02	ENSG00000141026
MEDAG	0.5223	1.1527E-02	4.73E-02	ENSG00000102802
MEF2A	-0.0132	8.3343E-01	9.13E-01	ENSG00000068305
MEF2C	-0.1586	7.5292E-02	1.92E-01	ENSG00000081189
MEF2C-AS1	-0.0180	6.5498E-01		ENSG00000248309
MEF2C-AS2	-0.4732	1.1808E-02	4.82E-02	ENSG00000245864
MEF2D	-0.0657	3.2884E-01	5.23E-01	ENSG00000116604
MEFV	-0.0423	6.9757E-01		ENSG00000103313
MEG3	0.3346	9.2207E-03	4.00E-02	ENSG00000214548
MEGF10	-0.4063	1.4521E-04	1.51E-03	ENSG00000145794
MEGF11	-0.4926	3.3130E-04	2.96E-03	ENSG00000157890
MEGF6	-0.0280	7.6825E-01	8.74E-01	ENSG00000162591
MEGF8	-0.2818	8.3098E-04	6.16E-03	ENSG00000105429
MEGF9	-0.1730	2.1731E-02	7.67E-02	ENSG00000106780
MEI4	0.4446	1.2641E-02	5.08E-02	ENSG00000269964
MEIG1	-0.4273	2.9708E-04	2.71E-03	ENSG00000197889
MEIOB	-0.0217	9.2358E-01		ENSG00000162039
MEIOC	0.1021	2.6726E-01	4.56E-01	ENSG00000180336
MEIOSIN	-0.0632	2.6603E-01	4.55E-01	ENSG00000237452
MEIS1	0.0165	8.6385E-01	9.31E-01	ENSG00000143995
MEIS1-AS2	-0.0024	7.1405E-01		ENSG00000230749
MEIS2	0.0337	6.4319E-01	7.93E-01	ENSG00000134138
MEIS3	-0.0704	2.3139E-01	4.15E-01	ENSG00000105419
MELK	-0.2704	1.8411E-02	6.78E-02	ENSG00000165304
MELTF	-0.1418	1.8560E-01	3.59E-01	ENSG00000163975
MELTF-AS1	-0.3831	2.9957E-03	1.68E-02	ENSG00000228109
MEMO1	0.2361	3.7858E-02	1.16E-01	ENSG00000162959
MEN1	0.0605	3.8164E-01	5.77E-01	ENSG00000133895
MEOX1	-1.2603	3.7698E-03		ENSG00000005102
MEOX2	0.2026	8.5240E-02	2.10E-01	ENSG00000106511
MEP1B	0.1569	6.6624E-02	1.76E-01	ENSG00000141434
MEPCE	0.0954	3.2505E-01	5.19E-01	ENSG00000146834
MEPE	0.0099	3.7846E-01		ENSG00000152595
MERTK	-0.0548	5.6846E-01	7.37E-01	ENSG00000153208
MESD	-0.1687	5.7902E-03	2.80E-02	ENSG00000117899
MESP1	-0.1159	2.3998E-01	4.25E-01	ENSG00000166823
MESP2	0.0418	6.1785E-01	7.75E-01	ENSG00000188095
MEST	-0.2794	2.6210E-02	8.81E-02	ENSG00000106484
MESTIT1	0.0166	8.6111E-01	9.29E-01	ENSG00000272701
MESTP3	-0.0474	4.3420E-01		ENSG00000250927
MET	-0.4248	7.2748E-04	5.54E-03	ENSG00000105976
METAP1	0.0827	2.2315E-01	4.05E-01	ENSG00000164024
METAP1D	0.1650	1.0814E-01	2.48E-01	ENSG00000172878
METAP2	-0.0399	4.3385E-01	6.25E-01	ENSG00000111142

METRN	-0.2281	2.9722E-02	9.68E-02	ENSG00000103260
METRNL	-0.2772	6.6049E-03	3.10E-02	ENSG00000176845
METTL1	-0.0803	3.2370E-01	5.18E-01	ENSG00000037897
METTL13	0.3118	4.9838E-04	4.09E-03	ENSG00000010165
METTL14	0.2567	6.7522E-05	8.17E-04	ENSG00000145388
METTL14-DT	0.2417	5.0719E-02	1.44E-01	ENSG00000281731
METTL15	0.0823	2.6278E-01	4.52E-01	ENSG00000169519
METTL16	0.0213	7.0988E-01	8.38E-01	ENSG00000127804
METTL17	0.3134	1.4104E-07	5.16E-06	ENSG00000165792
METTL18	0.1103	1.1715E-01	2.63E-01	ENSG00000171806
METTL21A	0.0886	1.3882E-01	2.94E-01	ENSG00000144401
METTL21C	0.0029	9.1154E-01		ENSG00000139780
METTL21EP	-0.0262	2.8832E-01	4.79E-01	ENSG00000250878
METTL22	0.1271	9.6486E-03	4.14E-02	ENSG00000067365
METTL23	-0.1498	2.2806E-02	7.95E-02	ENSG00000181038
METTL24	0.0016	9.8827E-01	9.94E-01	ENSG00000053328
METTL25	-0.1987	5.0765E-02	1.44E-01	ENSG00000127720
METTL25B	-0.1891	4.5355E-02	1.33E-01	ENSG00000143303
METTL26	-0.1098	6.4511E-02	1.72E-01	ENSG00000130731
METTL27	-0.3380	7.3437E-03	3.36E-02	ENSG00000165171
METTL2A	0.0378	4.1646E-01	6.09E-01	ENSG00000087995
METTL2B	-0.0842	1.8683E-01	3.61E-01	ENSG00000165055
METTL3	0.3206	5.3809E-09	3.49E-07	ENSG00000165819
METTL4	-0.0779	2.9087E-01	4.83E-01	ENSG00000101574
METTL5	-0.1068	4.7849E-02	1.39E-01	ENSG00000138382
METTL5P2	0.0022	6.0378E-01		ENSG00000248557
METTL6	0.0347	5.4130E-01	7.14E-01	ENSG00000206562
METTL8	-0.1405	5.0481E-02	1.44E-01	ENSG00000123600
METTL9	-0.0547	2.2447E-01	4.07E-01	ENSG00000197006
MEX3A	0.1593	9.8286E-02	2.32E-01	ENSG00000254726
MEX3B	-0.0914	3.3609E-01	5.31E-01	ENSG00000183496
MEX3C	-0.2584	8.0519E-04	6.01E-03	ENSG00000176624
MEX3D	-0.1049	3.0281E-01	4.95E-01	ENSG00000181588
MFAP1	-0.0221	7.1207E-01	8.40E-01	ENSG00000140259
MFAP2	-0.2499	2.5202E-03	1.47E-02	ENSG00000117122
MFAP3	-0.0074	9.1867E-01	9.60E-01	ENSG00000037749
MFAP3L	-0.2643	6.8867E-03	3.20E-02	ENSG00000198948
MFAP4	-0.3808	1.1586E-02	4.75E-02	ENSG00000166482
MFAP5	-0.0690	2.8706E-01	4.78E-01	ENSG00000197614
MFF	-0.0818	7.5423E-02	1.92E-01	ENSG00000168958
MFF-DT	-0.0914	3.5749E-01	5.53E-01	ENSG00000236432
MFGE8	-0.0704	3.3830E-01	5.33E-01	ENSG00000140545
MFHAS1	-0.2970	1.4869E-03	9.74E-03	ENSG00000147324
MFN1	0.1308	6.4012E-02	1.71E-01	ENSG00000171109
MFN2	0.1271	3.6277E-02	1.12E-01	ENSG00000116688
MFNG	0.1823	5.5502E-02	1.54E-01	ENSG00000100060
MFSD1	-0.1895	2.4083E-02	8.28E-02	ENSG00000118855
MFSD10	-0.2790	2.1726E-05	3.27E-04	ENSG00000109736
MFSD11	0.2420	6.7437E-04	5.23E-03	ENSG00000092931

MFSD12	0.1125	9.3149E-02	2.24E-01	ENSG00000161091
MFSD13A	0.0484	4.9627E-01	6.79E-01	ENSG00000138111
MFSD13B	0.0451	4.1835E-01	6.11E-01	ENSG00000230872
MFSD14A	-0.0933	3.3676E-01	5.32E-01	ENSG00000156875
MFSD14B	-0.1139	6.1777E-02	1.67E-01	ENSG00000148110
MFSD2A	0.0322	6.6586E-01	8.08E-01	ENSG00000168389
MFSD2B	-0.0184	8.8332E-01	9.41E-01	ENSG00000205639
MFSD3	-0.0546	4.4942E-01	6.39E-01	ENSG00000167700
MFSD4A	0.4501	9.3916E-05	1.06E-03	ENSG00000174514
MFSD4B	0.2324	7.4253E-03	3.39E-02	ENSG00000173214
MFSD5	-0.1836	9.3908E-03	4.06E-02	ENSG00000182544
MFSD6	0.1792	1.6195E-02	6.14E-02	ENSG00000151690
MFSD6L	0.0524	4.6126E-01	6.50E-01	ENSG00000185156
MFSD8	0.2085	1.4860E-03	9.74E-03	ENSG00000164073
MFSD9	0.1492	4.2149E-02	1.26E-01	ENSG00000135953
MGA	0.2141	8.7328E-03	3.84E-02	ENSG00000174197
MGAM	-0.0413	5.7903E-01		ENSG00000257335
MGAM2	0.0010	7.6243E-01		ENSG00000257743
MGARP	-0.1804	1.1358E-01	2.57E-01	ENSG00000137463
MGAT1	0.0719	2.3168E-01	4.15E-01	ENSG00000131446
MGAT2	0.0686	4.7986E-01	6.65E-01	ENSG00000168282
MGAT3	-0.3274	4.5324E-03	2.32E-02	ENSG00000128268
MGAT3-AS1	-0.1203	1.3911E-01	2.95E-01	ENSG00000227188
MGAT4A	-0.0511	4.6286E-01	6.51E-01	ENSG00000071073
MGAT4B	-0.1039	1.6604E-01	3.33E-01	ENSG00000161013
MGAT4C	0.0434	5.5147E-01	7.23E-01	ENSG00000182050
MGAT4EP	0.1375	3.6155E-04	3.19E-03	ENSG00000184774
MGAT5	-0.2715	8.1643E-06	1.49E-04	ENSG00000152127
MGAT5B	0.3301	1.4493E-03	9.56E-03	ENSG00000167889
MGC15885	0.0216	1.5717E-01		ENSG00000259458
MGC16275	0.0198	8.2390E-01	9.07E-01	ENSG00000246731
MGC32805	-0.0153	7.5892E-01		ENSG00000250328
MGLL	0.1829	9.3467E-03	4.04E-02	ENSG00000074416
MGME1	0.0066	9.2265E-01	9.61E-01	ENSG00000125871
MGMT	0.0228	7.5844E-01	8.69E-01	ENSG00000170430
MGRN1	0.0048	9.3759E-01	9.69E-01	ENSG00000102858
MGST2	-0.4662	4.9920E-06	1.00E-04	ENSG00000085871
MGST3	-0.1479	3.0338E-02	9.82E-02	ENSG00000143198
MHENCR	0.2019	7.2943E-03	3.34E-02	ENSG00000232442
MIA	-0.0138	8.4532E-01		ENSG00000261857
MIA2	-0.0573	2.8585E-01	4.77E-01	ENSG00000150527
MIA2-AS1	0.0174	8.5222E-01	9.24E-01	ENSG00000258940
MIA3	0.0123	8.3823E-01	9.15E-01	ENSG00000154305
MIAT	0.4026	2.2361E-08	1.13E-06	ENSG00000225783
MIB1	-0.1447	3.6080E-02	1.12E-01	ENSG00000101752
MIB2	0.1140	1.5662E-01	3.20E-01	ENSG00000197530
MICA	-0.1560	1.1134E-01	2.53E-01	ENSG00000204520
MICAL1	0.0917	2.2093E-01	4.02E-01	ENSG00000135596
MICAL2	0.5266	4.9533E-07	1.48E-05	ENSG00000133816

MICAL3	0.0453	5.9679E-01	7.58E-01	ENSG00000243156
MICALL1	-0.2291	3.5407E-03	1.92E-02	ENSG00000100139
MICALL2	0.0173	8.2524E-01	9.08E-01	ENSG00000164877
MICALL2-DT	-0.0150	8.7776E-01		ENSG00000225981
MICB	0.0535	5.4469E-01	7.17E-01	ENSG00000204516
MICB-DT	0.0011	9.2255E-01		ENSG00000286940
MICOS10	-0.1608	1.0706E-02	4.47E-02	ENSG00000173436
MICOS10-DT	-0.0528	5.3683E-01	7.10E-01	ENSG00000235185
MICOS13	-0.0982	9.4240E-02	2.26E-01	ENSG00000174917
MICU1	-0.0872	1.0661E-01	2.46E-01	ENSG00000107745
MICU2	-0.1470	1.9938E-02	7.18E-02	ENSG00000165487
MICU3	0.1181	9.0906E-02	2.20E-01	ENSG00000155970
MID1	-0.0619	4.3533E-01	6.27E-01	ENSG00000101871
MID1IP1	-0.1732	3.7175E-02	1.14E-01	ENSG00000165175
MID2	-0.2028	1.5508E-02	5.94E-02	ENSG00000080561
MIDEAS	-0.5230	2.4930E-16	1.22E-13	ENSG00000156030
MIDN	-0.4767	2.4939E-09	1.82E-07	ENSG00000167470
MIEF1	0.0009	9.8911E-01	9.94E-01	ENSG00000100335
MIEF2	0.0252	7.1983E-01	8.45E-01	ENSG00000177427
MIEN1	-0.1765	6.8707E-04	5.31E-03	ENSG00000141741
MIER1	-0.2019	1.2476E-03	8.47E-03	ENSG00000198160
MIER2	-0.0023	9.9110E-01	9.95E-01	ENSG00000105556
MIER3	-0.2270	2.9126E-03	1.65E-02	ENSG00000155545
MIF	-0.0508	7.7434E-01	8.78E-01	ENSG00000240972
MIF4GD	0.1882	9.0576E-03	3.95E-02	ENSG00000125457
MIGA1	0.0975	1.5686E-01	3.20E-01	ENSG00000180488
MIGA2	0.1091	1.5751E-01	3.21E-01	ENSG00000148343
MIIP	0.0263	6.1724E-01	7.74E-01	ENSG00000116691
MILIP	-0.2037	2.8521E-02	9.39E-02	ENSG00000265688
MILR1	-0.0177	6.3048E-01		ENSG00000271605
MIMT1	0.0062	5.6060E-01		ENSG00000268654
MINAR1	0.0964	3.3973E-01	5.35E-01	ENSG00000169330
MINCR	-0.0823	2.4656E-01	4.32E-01	ENSG00000253716
MINDY1	-0.0657	3.9553E-01	5.90E-01	ENSG00000143409
MINDY2	0.0894	1.2029E-01	2.68E-01	ENSG00000128923
MINDY2-DT	0.0241	7.9510E-01	8.90E-01	ENSG00000245975
MINDY3	0.0577	3.2521E-01	5.19E-01	ENSG00000148481
MINDY4	-0.1783	1.1895E-01	2.65E-01	ENSG00000106125
MINK1	0.1951	1.7475E-02	6.50E-02	ENSG00000141503
MINPP1	0.1331	1.0371E-01	2.41E-01	ENSG00000107789
MIOS	0.2103	2.2735E-02	7.93E-02	ENSG00000164654
MIOX	-0.0732	5.6459E-02		ENSG00000100253
MIPEP	-0.0221	7.2034E-01	8.45E-01	ENSG00000027001
MIPOL1	-0.5883	8.4474E-08	3.39E-06	ENSG00000151338
MIR100HG	-0.0152	8.5754E-01	9.27E-01	ENSG00000255248
MIR101-1	0.0071	4.6720E-01		ENSG00000199135
MIR103A2	0.0442	3.8258E-01		ENSG00000199024
MIR106AHG	-0.0104	7.6085E-01		ENSG00000283638
MIR1179	0.0099	1.6633E-01	3.33E-01	ENSG00000221630

MIR12136	0.2348	5.8048E-02	1.59E-01	ENSG00000210151
MIR124-1HG	0.2410	4.6018E-02	1.35E-01	ENSG00000253230
MIR124-3	-0.0191	2.9377E-01		ENSG00000207598
MIR1244-2	0.0350	6.4171E-01	7.92E-01	ENSG00000283498
MIR1244-3	0.0664	4.9387E-01	6.77E-01	ENSG00000283429
MIR1244-4	0.0226	8.1250E-01	9.01E-01	ENSG00000283475
MIR1251	-0.0326	5.6769E-01		ENSG00000221479
MIR1252	-0.0238	3.5109E-01		ENSG00000221788
MIR125B1	-0.0036	9.3050E-01		ENSG00000207971
MIR1268B	-0.0301	6.3722E-01		ENSG00000265561
MIR1277	0.0063	8.7462E-01		ENSG00000221463
MIR1281	0.0295	4.4369E-01		ENSG00000284015
MIR1290	0.0257	3.9029E-01		ENSG00000221662
MIR1296	-0.0963	1.3431E-01		ENSG00000221063
MIR1298	-0.0034	9.9266E-01		ENSG00000221710
MIR130A	0.0180	2.1438E-01		ENSG00000208009
MIR132	-0.2571	4.4166E-02	1.31E-01	ENSG00000267200
MIR133A1HG	0.1589	8.1225E-02	2.03E-01	ENSG00000265142
MIR135B	-0.0249	7.1521E-01		ENSG00000199059
MIR137HG	0.3805	3.3226E-03	1.82E-02	ENSG00000225206
MIR149	-0.1292	3.2843E-02		ENSG00000207611
MIR153-1	0.0277	1.6197E-01		ENSG00000207647
MIR155HG	-0.0714	4.4878E-01		ENSG00000234883
MIR16-2	-0.0255	5.0415E-01		ENSG00000198987
MIR17HG	-0.0539	6.9847E-01	8.31E-01	ENSG00000215417
MIR181A2HG	-0.5388	2.2514E-06	5.22E-05	ENSG00000224020
MIR186	-0.0320	5.3820E-01		ENSG00000207721
MIR191	0.0296	1.3762E-01	2.92E-01	ENSG00000207605
MIR1913	0.0172	3.0588E-01		ENSG00000222958
MIR1915HG	-0.2585	4.6370E-03	2.36E-02	ENSG00000204682
MIR193BHG	-0.2102	6.4603E-02	1.72E-01	ENSG00000262454
MIR194-2HG	0.0759	2.3330E-01		ENSG00000229719
MIR1972-1	-0.0385	9.1125E-01		ENSG00000238728
MIR1972-2	-0.0539	2.1262E-01		ENSG00000239118
MIR200CHG	-0.0217	8.1286E-01	9.01E-01	ENSG00000257084
MIR202HG	-0.0003	8.2718E-01		ENSG00000166917
MIR2052HG	0.0482	3.9903E-01	5.93E-01	ENSG00000254349
MIR205HG	-0.0525	4.4988E-01	6.40E-01	ENSG00000230937
MIR218-1	0.0240	6.6953E-01		ENSG00000207732
MIR2276	0.1259	1.7105E-01	3.40E-01	ENSG00000252695
MIR22HG	-0.1665	1.0695E-02	4.46E-02	ENSG00000186594
MIR26A2	0.0168	7.2047E-01		ENSG00000207789
MIR26B	-0.0203	3.7093E-01		ENSG00000199121
MIR302CHG	0.0634	8.6783E-02		ENSG00000249532
MIR3115	-0.0081	8.3364E-01		ENSG00000263793
MIR3117	-0.0178	7.2307E-01		ENSG00000264720
MIR3124	-0.0032	7.8743E-01		ENSG00000264500
MIR3127	0.0136	4.6342E-01		ENSG00000264157
MIR3128	-0.0319	5.7245E-01		ENSG00000265396

MIR3132	-0.0012	7.1882E-01		ENSG00000265252
MIR3136	-0.0115	7.2887E-01		ENSG00000265355
MIR3143	-0.0004	8.5740E-01		ENSG00000265565
MIR3146	0.0072	6.3448E-01		ENSG00000265932
MIR3150BHG	0.0052	8.6084E-01		ENSG00000245080
MIR3153	0.0072	5.3655E-01		ENSG00000265112
MIR3171HG	0.0675	4.5149E-01	6.41E-01	ENSG00000258932
MIR3175	0.0040	8.0065E-01		ENSG00000284324
MIR3178	-0.0230	6.4473E-01		ENSG00000266232
MIR3179-3	-0.0426	7.6030E-01	8.70E-01	ENSG00000266454
MIR3179-4	-0.0203	2.3943E-01	4.24E-01	ENSG00000277014
MIR3181	-0.0056	9.9746E-01		ENSG00000264947
MIR3189	0.0125	5.1048E-01		ENSG00000264175
MIR3193	0.0703	6.0168E-02		ENSG00000264395
MIR31HG	-0.0521	5.0966E-01	6.90E-01	ENSG00000171889
MIR320A	-0.0424	8.0790E-01	8.98E-01	ENSG00000208037
MIR324	-0.0163	9.6151E-01		ENSG00000199053
MIR325	0.0012	6.9418E-01		ENSG00000207995
MIR325HG	0.0359	7.0123E-01	8.33E-01	ENSG00000280870
MIR339	-0.0215	8.6987E-01	9.34E-01	ENSG00000199023
MIR345	0.0557	1.0339E-01		ENSG00000198984
MIR34AHG	-0.0926	3.4594E-01	5.41E-01	ENSG00000228526
MIR34BHG	-0.0293	5.8471E-01	7.50E-01	ENSG00000286028
MIR3605	0.0070	5.3966E-01		ENSG00000284154
MIR3609	0.0018	4.8710E-01		ENSG00000266019
MIR3648-1	0.0164	8.6760E-01	9.33E-01	ENSG00000275708
MIR3648-2	-0.0044	9.6091E-01	9.80E-01	ENSG00000264462
MIR3651	0.0068	5.7140E-01		ENSG00000281156
MIR3660	-0.0136	8.6915E-01		ENSG00000264342
MIR3662	-0.0353	2.2889E-01		ENSG00000283409
MIR3670-1	0.0069	3.0367E-01		ENSG00000263918
MIR3670-2	0.0069	3.0367E-01		ENSG00000264722
MIR3670-3	0.0069	3.0367E-01		ENSG00000265776
MIR3670-4	0.0069	3.0367E-01		ENSG00000274025
MIR3671	0.0334	8.8796E-02		ENSG00000265996
MIR3677	0.0142	3.1003E-01		ENSG00000266643
MIR3677HG	-0.0041	9.5553E-01	9.77E-01	ENSG00000260778
MIR3679	-0.0061	9.2185E-01	9.61E-01	ENSG00000263813
MIR3680-1	-0.0476	4.7538E-01	6.62E-01	ENSG00000265462
MIR3680-2	-0.0247	3.4412E-01		ENSG00000266758
MIR3681HG	0.1431	1.8332E-01	3.56E-01	ENSG00000224184
MIR3682	-0.1040	2.6620E-01	4.55E-01	ENSG00000265452
MIR3685	0.0196	2.3671E-01		ENSG00000265917
MIR375	0.0100	8.0904E-01		ENSG00000198973
MIR378A	0.0008	7.9825E-01		ENSG00000199047
MIR378H	-0.0494	7.9499E-01		ENSG00000263361
MIR381HG	0.0048	1.4267E-01		ENSG00000223403
MIR3909	-0.0433	4.0454E-01		ENSG00000266320
MIR3928	0.0083	5.9506E-01		ENSG00000264141

MIR3929	-0.0832	1.9135E-01	3.66E-01	ENSG00000265641
MIR3935	0.0059	5.8548E-01		ENSG00000265281
MIR3936HG	-0.2609	1.1184E-02	4.62E-02	ENSG00000233006
MIR3939	-0.0100	7.7758E-01		ENSG00000265828
MIR3945HG	-0.0274	8.4045E-01		ENSG00000251230
MIR3975	-0.0011	9.9766E-01		ENSG00000283514
MIR3976HG	-0.0180	9.6884E-01		ENSG00000261738
MIR425	0.2466	5.2812E-02	1.49E-01	ENSG00000199032
MIR4258	-0.0181	4.0499E-01	5.99E-01	ENSG00000264349
MIR4259	0.5039	9.4047E-03	4.06E-02	ENSG00000266458
MIR4263	-0.0552	4.2525E-01	6.17E-01	ENSG00000265321
MIR4275	0.0010	8.8045E-01		ENSG00000283275
MIR4284	-0.0776	1.7493E-01	3.45E-01	ENSG00000265724
MIR4288	0.0076	6.5117E-01		ENSG00000265251
MIR4292	0.0155	2.0832E-01		ENSG00000265806
MIR4300HG	0.0223	2.8335E-01		ENSG00000245832
MIR4307HG	0.0005	8.6953E-01		ENSG00000257612
MIR4319	-0.0233	4.0128E-01		ENSG00000265957
MIR4322	0.0193	2.5817E-01		ENSG00000264266
MIR4323	0.0042	5.9436E-01		ENSG00000266226
MIR4427	-0.0132	6.4562E-01		ENSG00000265744
MIR4432HG	0.0058	4.5849E-01		ENSG00000228590
MIR4434	-0.0305	7.1017E-01	8.38E-01	ENSG00000283204
MIR4435-2HG	0.3233	1.6299E-03	1.05E-02	ENSG00000172965
MIR4458HG	0.1162	9.1094E-02	2.20E-01	ENSG00000247516
MIR4469	0.0037	8.3031E-01		ENSG00000284062
MIR4477A	0.0266	2.5049E-01		ENSG00000276029
MIR4477B	0.0256	6.0780E-01		ENSG00000266017
MIR4482	0.4930	2.3018E-04	2.19E-03	ENSG00000266852
MIR4484	0.0091	3.7012E-01		ENSG00000265092
MIR4485	-0.0649	3.8285E-01	5.77E-01	ENSG00000283813
MIR4489	-0.0769	2.5619E-01		ENSG00000265874
MIR4500HG	0.1834	6.8123E-02	1.79E-01	ENSG00000228824
MIR4505	0.0875	3.8378E-01	5.78E-01	ENSG00000264741
MIR4511	0.0097	5.4322E-01		ENSG00000264737
MIR4512	0.4938	1.9545E-03	1.20E-02	ENSG00000266589
MIR4515	-0.0021	6.8326E-01		ENSG00000263643
MIR4516	0.0019	6.9520E-01		ENSG00000265867
MIR4526	0.0029	5.9576E-01		ENSG00000263527
MIR4527HG	0.0136	5.7307E-01		ENSG00000267761
MIR4530	0.0057	1.9485E-01		ENSG00000266559
MIR4633	-0.0124	7.2610E-01		ENSG00000264563
MIR4634	0.0035	6.9621E-01		ENSG00000266890
MIR4644	-0.0179	9.2945E-01		ENSG00000266245
MIR4653	-0.0166	4.5042E-01		ENSG00000264425
MIR4663	0.0038	7.3522E-01		ENSG00000266324
MIR4664	-0.0090	8.3612E-01		ENSG00000265660
MIR4676	-0.0220	4.7673E-01		ENSG00000266719
MIR4678	0.0244	2.0980E-01	3.89E-01	ENSG00000283672

MIR4713HG	0.4020	1.8177E-02	6.70E-02	ENSG00000259240
MIR4716	0.0219	4.2264E-01		ENSG00000264210
MIR4727	0.0401	4.0343E-01		ENSG00000274054
MIR4729	-0.0018	5.4988E-01		ENSG00000263857
MIR4730	0.0036	8.1144E-01		ENSG00000264961
MIR4734	-0.0224	5.6319E-01		ENSG00000275238
MIR4740	-0.0011	3.2270E-01		ENSG00000266392
MIR4742	-0.0138	6.0888E-01		ENSG00000266618
MIR4748	-0.0095	8.2482E-01		ENSG00000265879
MIR4754	-0.0022	7.1123E-01		ENSG00000266640
MIR4755	-0.2724	4.6118E-02	1.35E-01	ENSG00000264616
MIR4760	0.0113	5.4587E-01		ENSG00000263973
MIR4771-2	0.0020	7.2068E-01		ENSG00000266063
MIR4777	0.0018	6.6257E-01		ENSG00000263641
MIR4794	-0.0012	8.1141E-01		ENSG00000264470
MIR4803	-0.0547	3.9255E-01		ENSG00000264099
MIR491	0.1249	2.3866E-02	8.22E-02	ENSG00000207609
MIR497HG	-0.0112	6.6328E-01		ENSG00000267532
MIR4999	0.1034	9.9406E-02	2.34E-01	ENSG00000265390
MIR5003	0.0021	9.0433E-01		ENSG00000265160
MIR5009	-0.0348	5.6274E-01		ENSG00000264796
MIR5010	-0.0288	4.1081E-01		ENSG00000283929
MIR5087	0.0344	1.7316E-01		ENSG00000283676
MIR5091	0.0228	1.1069E-01		ENSG00000266240
MIR5094	0.0105	2.8969E-01		ENSG00000264966
MIR5188	-0.0208	7.2389E-01	8.47E-01	ENSG00000265345
MIR5190	-0.0198	4.2670E-01		ENSG00000266146
MIR5194	0.0097	6.7332E-01		ENSG00000264653
MIR548AK	0.0652	3.3591E-01	5.31E-01	ENSG00000265653
MIR548AL	-0.0110	9.4107E-01		ENSG00000264402
MIR548AR	0.0013	4.7449E-01		ENSG00000264539
MIR548AT	0.0078	8.5410E-01		ENSG00000264314
MIR548E	-0.0340	3.7188E-01		ENSG00000221214
MIR548F2	-0.0154	5.6950E-01		ENSG00000221782
MIR548N	0.0190	3.7332E-01		ENSG00000221669
MIR548O	-0.0082	8.9996E-01	9.50E-01	ENSG00000221510
MIR548S	0.0032	6.5648E-01		ENSG00000265056
MIR548V	0.0214	4.4700E-01		ENSG00000265520
MIR548XHG	-0.9658	1.9856E-02	7.16E-02	ENSG00000224141
MIR548Y	-0.0057	7.7371E-01		ENSG00000263945
MIR553	-0.0217	7.9268E-01		ENSG00000207750
MIR556	-0.0137	8.9074E-01		ENSG00000207729
MIR558	0.0245	8.9007E-02		ENSG00000207653
MIR5581	-0.0301	4.7055E-01		ENSG00000263675
MIR5585	-0.0283	9.6922E-01		ENSG00000266203
MIR561	-0.0339	5.4493E-01	7.17E-01	ENSG00000207951
MIR567	-0.0184	8.6955E-01		ENSG00000207940
MIR5690	-0.0075	8.4264E-01		ENSG00000265527
MIR5695	-0.0192	8.3172E-01		ENSG00000266721

MIR570	0.0092	6.4090E-01		ENSG00000207650
MIR573	0.0441	4.7894E-02		ENSG00000207697
MIR578	-0.0311	9.8503E-01	9.92E-01	ENSG00000207559
MIR5787	0.0077	8.0036E-01		ENSG00000275334
MIR579	-0.0277	5.2602E-01		ENSG00000207956
MIR589	0.0123	4.8565E-01		ENSG00000207973
MIR590	-0.0347	5.4437E-01		ENSG00000207741
MIR593	-0.0498	3.9474E-01	5.89E-01	ENSG00000207588
MIR600HG	-0.0712	4.7294E-01	6.60E-01	ENSG00000236901
MIR6077	0.5447	1.2006E-02	4.88E-02	ENSG00000278596
MIR6124	-0.0129	6.3361E-01		ENSG00000275373
MIR616	0.2933	1.6713E-02	6.29E-02	ENSG00000208028
MIR618	0.0104	4.4560E-01		ENSG00000208022
MIR619	-0.0065	9.9530E-01		ENSG00000207622
MIR624	-0.0023	8.4726E-01		ENSG00000207952
MIR628	0.0040	5.1632E-01		ENSG00000283891
MIR632	-0.0193	5.1379E-01		ENSG00000283774
MIR635	-0.0801	2.6267E-01	4.52E-01	ENSG00000207561
MIR642A	0.0224	4.2486E-01		ENSG00000207773
MIR644A	-0.0041	8.9041E-01	9.45E-01	ENSG00000207997
MIR645	0.0038	8.5200E-01		ENSG00000208018
MIR646HG	-0.1665	1.2262E-01	2.71E-01	ENSG00000228340
MIR647	0.4392	8.6218E-03	3.80E-02	ENSG00000207554
MIR6514	-0.0152	6.7673E-01		ENSG00000274066
MIR658	-0.0047	9.1961E-01		ENSG00000284197
MIR659	-0.0010	9.1141E-01	9.55E-01	ENSG00000207696
MIR663AHG	0.1746	5.3761E-02	1.51E-01	ENSG00000227195
MIR670	0.0037	6.0760E-01		ENSG00000211568
MIR670HG	0.0151	2.7511E-01		ENSG00000235661
MIR6719	-0.2180	6.1125E-02	1.66E-01	ENSG00000277759
MIR6724-1	-0.0164	9.2848E-01		ENSG00000275950
MIR6724-2	-0.0467	4.4004E-01		ENSG00000274060
MIR6724-3	-0.0164	9.2848E-01		ENSG00000277379
MIR6724-4	-0.0454	6.5393E-01		ENSG00000275692
MIR6740	0.0003	7.9839E-01		ENSG00000275207
MIR6746	-0.0031	7.2046E-01		ENSG00000277892
MIR6747	-0.0133	9.7845E-01		ENSG00000276102
MIR6772	-0.0037	7.7895E-01		ENSG00000274816
MIR6775	0.0119	4.2409E-01		ENSG00000278598
MIR6781	0.0171	6.6777E-01		ENSG00000278447
MIR6796	-0.0117	8.1911E-01		ENSG00000275652
MIR6797	0.0088	6.6560E-01		ENSG00000276926
MIR6822	-0.0340	3.9938E-01		ENSG00000284158
MIR6826	-0.0157	7.1439E-01		ENSG00000278658
MIR6841	0.0057	7.6964E-01		ENSG00000283327
MIR7-3	0.4005	1.8881E-02	6.90E-02	ENSG00000207630
MIR7-3HG	0.0778	3.4214E-01	5.37E-01	ENSG00000176840
MIR708	0.0091	4.1749E-01		ENSG00000211997
MIR7111	-0.0011	7.9874E-01		ENSG00000276712

MIR744	0.0161	8.4241E-01	9.18E-01	ENSG00000266297
MIR760	0.0343	3.2843E-01		ENSG00000211575
MIR762HG	0.0735	3.3853E-01	5.34E-01	ENSG00000260083
MIR764	0.0085	5.5833E-01		ENSG00000212100
MIR7845	-0.0307	5.5027E-01	7.22E-01	ENSG00000277590
MIR7856	-0.0078	6.7559E-01		ENSG00000278281
MIR8063	-0.0033	9.9264E-01		ENSG00000277202
MIR8066	0.0052	6.1366E-01		ENSG00000273882
MIR8075	-0.0009	8.6082E-01		ENSG00000277942
MIR8086	0.0003	9.5833E-01		ENSG00000275036
MIR8485	0.0055	9.8792E-01	9.93E-01	ENSG00000216191
MIR9-1HG	0.1115	2.8152E-01	4.73E-01	ENSG00000125462
MIR9-2HG	-0.0230	7.8107E-01	8.82E-01	ENSG00000245526
MIR9-3HG	0.3414	1.8732E-02	6.86E-02	ENSG00000255571
MIR924HG	-0.0063	9.4334E-01	9.72E-01	ENSG00000267374
MIR933	0.0199	3.0358E-01		ENSG00000215973
MIR98	0.0711	3.4269E-01	5.38E-01	ENSG00000271886
MIR99AHG	-0.4105	5.3471E-05	6.79E-04	ENSG00000215386
MIRLET7A1HG	-0.1239	2.2818E-01	4.11E-01	ENSG00000269929
MIRLET7BHG	-0.0282	7.3335E-01	8.54E-01	ENSG00000197182
MIRLET7IHG	0.1204	2.4733E-01	4.33E-01	ENSG00000257354
MIS12	0.0144	8.3267E-01	9.13E-01	ENSG00000167842
MIS18A	0.1592	6.1191E-02	1.66E-01	ENSG00000159055
MIS18BP1	-0.2205	1.6313E-02	6.18E-02	ENSG00000129534
MISFA	-0.3299	1.6158E-02	6.13E-02	ENSG00000247595
MISP	-0.1932	7.4555E-02	1.91E-01	ENSG00000099812
MISP3	-0.2058	1.2767E-03	8.63E-03	ENSG00000141854
MITD1	0.1666	4.2347E-03	2.21E-02	ENSG00000158411
MITF	-0.3360	1.6559E-03	1.06E-02	ENSG00000187098
MIX23	-0.1891	3.6650E-02	1.13E-01	ENSG00000160124
MIXL1	-0.0289	7.6063E-01	8.70E-01	ENSG00000185155
MKI67	-0.3411	7.6244E-03	3.46E-02	ENSG00000148773
MKKS	-0.0408	5.7390E-01	7.41E-01	ENSG00000125863
MKLN1	-0.0533	3.4580E-01	5.41E-01	ENSG00000128585
MKLN1-AS	-0.0037	9.5216E-01	9.76E-01	ENSG00000236753
MKNK1	0.1422	6.7260E-02	1.77E-01	ENSG00000079277
MKNK1-AS1	-0.0021	6.6426E-01		ENSG00000269956
MKNK2	-0.4603	2.1863E-06	5.08E-05	ENSG00000099875
MKRN1	0.0225	7.2807E-01	8.50E-01	ENSG00000133606
MKRN2	0.2625	6.7842E-04	5.25E-03	ENSG00000075975
MKRN2OS	-0.0210	8.5007E-01	9.23E-01	ENSG00000225526
MKRN3	0.3095	8.4567E-03	3.75E-02	ENSG00000179455
MKRN7P	-0.0345	5.1399E-01	6.93E-01	ENSG00000225849
MKS1	0.2164	1.9415E-03	1.20E-02	ENSG00000011143
MKX	0.0616	5.0118E-01	6.83E-01	ENSG00000150051
MLANA	-0.0351	5.8962E-01	7.53E-01	ENSG00000120215
MLEC	-0.2634	1.2367E-05	2.08E-04	ENSG00000110917
MLF1	-0.2188	2.3035E-03	1.37E-02	ENSG00000178053
MLF2	0.0613	1.4376E-01	3.02E-01	ENSG00000089693

MLH1	0.0734	1.7567E-01	3.46E-01	ENSG00000076242
MLH3	0.0346	6.8233E-01	8.20E-01	ENSG00000119684
MLKL	0.0014	9.2781E-01		ENSG00000168404
MLLT1	-0.3833	5.0282E-06	1.01E-04	ENSG00000130382
MLLT10	-0.2262	1.0608E-02	4.43E-02	ENSG00000078403
MLLT11	0.0350	6.8816E-01	8.24E-01	ENSG00000213190
MLLT3	-0.1873	1.2978E-02	5.19E-02	ENSG00000171843
MLLT6	-0.1668	6.3540E-02	1.70E-01	ENSG00000275023
MLN	0.0088	2.1273E-01		ENSG00000096395
MLNR	-0.0062	9.8220E-01		ENSG00000102539
MLPH	0.0299	7.5528E-01	8.67E-01	ENSG00000115648
MLST8	-0.0295	5.2962E-01	7.04E-01	ENSG00000167965
MLX	0.2567	5.5731E-05	7.00E-04	ENSG00000108788
MLXIP	-0.1056	4.3975E-02	1.30E-01	ENSG00000175727
MLXIPL	-0.0428	6.5537E-01	8.01E-01	ENSG00000009950
MLYCD	-0.0680	4.6280E-01	6.51E-01	ENSG00000103150
MMAA	-0.1356	1.1209E-01	2.55E-01	ENSG00000151611
MMAB	-0.2864	6.3450E-07	1.82E-05	ENSG00000139428
MMACHC	-0.3755	4.9461E-03	2.48E-02	ENSG00000132763
MMADHC	-0.0846	8.3517E-02	2.07E-01	ENSG00000168288
MMADHC-DT	0.1458	1.6416E-01	3.31E-01	ENSG00000231969
MMD	0.0345	6.6506E-01	8.07E-01	ENSG00000108960
MMD2	-0.2303	6.6111E-02	1.75E-01	ENSG00000136297
MMEL1	0.1813	1.1420E-01	2.58E-01	ENSG00000142606
MMGT1	0.0097	9.0205E-01	9.51E-01	ENSG00000169446
MMP10	0.1885	5.8306E-02	1.60E-01	ENSG00000166670
MMP11	0.0294	7.3881E-01	8.57E-01	ENSG00000099953
MMP12	-0.0586	4.1173E-01	6.05E-01	ENSG00000262406
MMP13	0.0376	1.1411E-01	2.58E-01	ENSG00000137745
MMP14	-0.2141	3.3162E-02	1.05E-01	ENSG00000157227
MMP15	-0.0581	5.0338E-01	6.85E-01	ENSG00000102996
MMP16	0.0459	6.1470E-01	7.73E-01	ENSG00000156103
MMP17	-0.2976	7.7650E-03	3.51E-02	ENSG00000198598
MMP19	0.0574	1.7271E-01		ENSG00000123342
MMP2	0.0555	5.7822E-01	7.45E-01	ENSG00000087245
MMP23A	0.4428	3.6246E-03	1.95E-02	ENSG00000215914
MMP23B	0.4664	1.9476E-03	1.20E-02	ENSG00000189409
MMP24	0.1371	1.8218E-01	3.54E-01	ENSG00000125966
MMP24OS	-0.1986	2.2516E-02	7.88E-02	ENSG00000126005
MMP25	0.1174	2.1243E-01	3.92E-01	ENSG00000008516
MMP26	-0.0145	8.0471E-01	8.96E-01	ENSG00000167346
MMP28	-0.4885	8.1240E-03	3.64E-02	ENSG00000271447
MMP3	0.0337	5.3047E-01		ENSG00000149968
MMP7	-0.0478	7.0239E-01	8.33E-01	ENSG00000137673
MMP9	0.0416	6.3323E-01	7.86E-01	ENSG00000100985
MMRN1	-0.4629	4.2090E-04	3.58E-03	ENSG00000138722
MMS19	0.1468	2.8057E-02	9.26E-02	ENSG00000155229
MMS22L	0.4664	4.9397E-05	6.39E-04	ENSG00000146263
MMUT	0.0237	7.6635E-01	8.73E-01	ENSG00000146085

MN1	0.0921	2.7899E-01	4.70E-01	ENSG00000169184
MNAT1	-0.2119	2.4502E-03	1.44E-02	ENSG00000020426
MND1	-0.0195	8.5481E-01		ENSG00000121211
MNT	0.0531	4.8046E-01	6.66E-01	ENSG00000070444
MNX1-AS2	0.0487	4.7568E-01	6.62E-01	ENSG00000235029
MOAP1	0.2021	1.0239E-02	4.32E-02	ENSG00000165943
MOB1A	-0.1935	3.1911E-04	2.88E-03	ENSG00000114978
MOB1B	-0.2150	1.1994E-02	4.88E-02	ENSG00000173542
MOB2	0.0477	4.2614E-01	6.18E-01	ENSG00000182208
MOB3A	0.0090	8.9915E-01	9.49E-01	ENSG00000172081
MOB3B	-0.1937	3.6823E-02	1.14E-01	ENSG00000120162
MOB3C	0.0962	3.3639E-01	5.32E-01	ENSG00000142961
MOB4	-0.0506	3.6640E-01	5.62E-01	ENSG00000115540
MOBP	0.0326	8.2530E-02		ENSG00000168314
MOCOS	0.0523	5.7429E-01	7.41E-01	ENSG00000075643
MOCS1	-0.4440	2.9071E-06	6.45E-05	ENSG00000124615
MOCS2	-0.0829	1.2617E-01	2.76E-01	ENSG00000164172
MOCS2-DT	-0.1544	1.4165E-01	2.99E-01	ENSG00000247796
MOCS3	0.0426	5.2979E-01	7.05E-01	ENSG00000124217
MOG	0.0070	6.8639E-01		ENSG00000204655
MOGAT1	0.0171	4.7267E-01		ENSG00000124003
MOGAT2	-0.0340	3.6147E-01		ENSG00000166391
MOGAT3	0.0060	7.8129E-01		ENSG00000106384
MOGS	0.3307	2.6831E-14	8.16E-12	ENSG00000115275
MOK	0.0474	5.6309E-01	7.32E-01	ENSG00000080823
MON1A	-0.0189	7.8400E-01	8.84E-01	ENSG00000164077
MON1B	0.1169	1.5559E-02	5.95E-02	ENSG00000103111
MON2	0.2679	4.6026E-05	6.02E-04	ENSG00000061987
MORC2	0.0050	9.4556E-01	9.73E-01	ENSG00000133422
MORC2-AS1	-0.0392	7.5913E-01	8.69E-01	ENSG00000235989
MORC3	-0.0528	3.2294E-01	5.17E-01	ENSG00000159256
MORC4	-0.2657	1.8560E-04	1.85E-03	ENSG00000133131
MORF4L1	-0.2190	1.1085E-03	7.70E-03	ENSG00000185787
MORF4L1P4	0.0109	5.3341E-01		ENSG00000225133
MORF4L2	0.0328	6.8466E-01	8.21E-01	ENSG00000123562
MORN1	0.0376	5.9596E-01	7.58E-01	ENSG00000116151
MORN2	-0.2689	1.3838E-04	1.45E-03	ENSG00000188010
MORN3	0.0228	7.5389E-01	8.66E-01	ENSG00000139714
MORN4	0.1436	5.2467E-03	2.60E-02	ENSG00000171160
MOS	0.0026	9.1811E-01		ENSG00000172680
MOSMO	0.0542	5.7762E-01	7.44E-01	ENSG00000185716
MOSPD1	0.0708	2.6844E-01	4.58E-01	ENSG00000101928
MOSPD2	0.0765	3.6112E-01	5.57E-01	ENSG00000130150
MOSPD3	0.2419	2.8040E-03	1.60E-02	ENSG00000106330
MOV10	-0.0963	2.1119E-01	3.91E-01	ENSG00000155363
MOXD1	-0.1496	1.3365E-01	2.87E-01	ENSG00000079931
MPC1	-0.0459	5.8832E-01	7.52E-01	ENSG00000060762
MPC2	-0.2368	2.8601E-06	6.37E-05	ENSG00000143158
MPDU1	-0.1566	2.1412E-02	7.58E-02	ENSG00000129255

MPDU1-AS1	0.1549	1.5167E-01	3.13E-01	ENSG00000233223
MPDZ	-0.5999	1.0820E-12	2.28E-10	ENSG00000107186
MPEG1	-0.0223	5.5132E-01		ENSG00000197629
MPG	-0.1338	3.3117E-02	1.05E-01	ENSG00000103152
MPHOSPH10	0.0377	5.6515E-01	7.34E-01	ENSG00000124383
MPHOSPH10P3	-0.0902	1.7264E-01	3.42E-01	ENSG00000283345
MPHOSPH6	-0.2560	8.4507E-05	9.77E-04	ENSG00000135698
MPHOSPH8	-0.1308	2.9943E-02	9.73E-02	ENSG00000196199
MPHOSPH9	0.0275	6.6562E-01	8.08E-01	ENSG00000051825
MPI	-0.1194	7.3865E-02	1.89E-01	ENSG00000178802
MPL	0.0324	7.2196E-01	8.46E-01	ENSG00000117400
MPLKIP	-0.0265	6.1654E-01	7.74E-01	ENSG00000168303
MPND	-0.3021	4.3391E-03	2.25E-02	ENSG00000008382
MPP1	0.2300	4.6576E-02	1.36E-01	ENSG00000130830
MPP2	0.0772	2.3129E-01	4.15E-01	ENSG00000108852
MPP3	0.1277	1.3087E-01	2.83E-01	ENSG00000161647
MPP4	-0.0625	1.9361E-01		ENSG00000082126
MPP7	0.1431	1.1159E-01	2.54E-01	ENSG00000150054
MPPE1	-0.0961	1.2824E-01	2.79E-01	ENSG00000154889
MPPED1	0.0803	3.7214E-01	5.67E-01	ENSG00000186732
MPPED2	0.1919	3.3603E-02	1.06E-01	ENSG00000066382
MPPED2-AS1	-0.5461	5.8505E-03	2.83E-02	ENSG00000254489
MPRIP	-0.1812	3.4453E-03	1.87E-02	ENSG00000133030
MPST	-0.0104	8.1339E-01	9.01E-01	ENSG00000128309
MPV17	-0.2416	4.7364E-05	6.17E-04	ENSG00000115204
MPV17L	0.4301	1.9100E-04	1.89E-03	ENSG00000156968
MPV17L2	0.2959	8.2465E-05	9.58E-04	ENSG00000254858
MPZ	-0.0445	6.6525E-01	8.08E-01	ENSG00000158887
MPZL1	-0.0979	1.1851E-01	2.65E-01	ENSG00000197965
MPZL2	-0.1877	6.9903E-02	1.82E-01	ENSG00000149573
MPZL3	-0.0120	8.9236E-01	9.46E-01	ENSG00000160588
MR1	-0.1611	1.3373E-01	2.87E-01	ENSG00000153029
MRAP	0.0180	3.4017E-01		ENSG00000170262
MRAP2	0.1020	1.6037E-01	3.25E-01	ENSG00000135324
MRAS	-0.1291	7.1383E-02	1.85E-01	ENSG00000158186
MRC1	0.0096	2.8476E-01		ENSG00000260314
MRC2	-0.3193	5.9972E-04	4.77E-03	ENSG00000011028
MRE11	-0.0788	2.4200E-01	4.27E-01	ENSG00000020922
MREG	0.1486	1.4464E-01	3.03E-01	ENSG00000118242
MRFAP1	-0.1819	1.6326E-03	1.05E-02	ENSG00000179010
MRFAP1L1	0.1919	1.2161E-03	8.29E-03	ENSG00000178988
MRFAP1L2	0.3225	2.5933E-07	8.71E-06	ENSG00000170846
MRGBP	0.0920	9.5264E-02	2.27E-01	ENSG00000101189
MRGPRF	0.0780	3.9351E-01	5.88E-01	ENSG00000172935
MRGPRF-AS1	-0.0059	8.1860E-01		ENSG00000256508
MRGPRX3	0.0106	2.7948E-01		ENSG00000179826
MRI1	-0.0796	3.7804E-01	5.73E-01	ENSG00000037757
MRLN	0.3208	1.4952E-02	5.79E-02	ENSG00000227877
MRM1	-0.0407	6.7003E-01	8.10E-01	ENSG00000278619

MRM3	-0.0701	2.6759E-01	4.57E-01	ENSG00000171861
MRNIP	0.0254	7.0731E-01	8.36E-01	ENSG00000161010
MRNIP-DT	0.3661	3.6310E-05	4.93E-04	ENSG00000245317
MRO	-0.0171	7.6817E-01	8.74E-01	ENSG00000134042
MROCKI	-0.0003	6.4810E-01		ENSG00000227502
MROH1	-0.0655	2.3661E-01	4.21E-01	ENSG00000179832
MROH3P	-0.0149	5.3240E-01		ENSG00000233217
MROH5	0.0002	7.5515E-01		ENSG00000226807
MROH6	0.1955	7.3043E-02	1.88E-01	ENSG00000204839
MROH7	-0.0972	1.3934E-01	2.95E-01	ENSG00000184313
MROH7-TTC4	-0.0090	8.4699E-01		ENSG00000271723
MROH8	0.0965	2.7700E-01	4.68E-01	ENSG00000101353
MROH9	-0.0232	7.1419E-01		ENSG00000117501
MRPL1	-0.2951	1.0097E-03	7.16E-03	ENSG00000169288
MRPL10	0.1346	4.4339E-02	1.31E-01	ENSG00000159111
MRPL11	-0.2953	1.2154E-04	1.31E-03	ENSG00000174547
MRPL12	-0.2356	1.2702E-02	5.10E-02	ENSG00000262814
MRPL13	-0.1400	3.0283E-02	9.81E-02	ENSG00000172172
MRPL14	-0.1335	6.6578E-02	1.76E-01	ENSG00000180992
MRPL15	0.0484	5.0047E-01	6.83E-01	ENSG00000137547
MRPL16	-0.0019	9.7839E-01	9.89E-01	ENSG00000166902
MRPL17	-0.0222	7.5968E-01	8.69E-01	ENSG00000158042
MRPL18	-0.0154	7.6241E-01	8.71E-01	ENSG00000112110
MRPL19	-0.1342	4.9935E-02	1.43E-01	ENSG00000115364
MRPL2	-0.0713	2.3259E-01	4.16E-01	ENSG00000112651
MRPL20	-0.1846	4.7341E-04	3.92E-03	ENSG00000242485
MRPL20-AS1	0.5403	7.3573E-07	2.06E-05	ENSG00000224870
MRPL21	-0.1558	7.2371E-03	3.32E-02	ENSG00000197345
MRPL22	-0.1356	4.4691E-02	1.32E-01	ENSG00000082515
MRPL23	-0.0328	6.0978E-01	7.68E-01	ENSG00000276345
MRPL23	-0.0411	3.5561E-01	5.51E-01	ENSG00000214026
MRPL23-AS1	-0.0328	6.9549E-01	8.30E-01	ENSG00000226416
MRPL24	-0.1349	2.7195E-02	9.06E-02	ENSG00000143314
MRPL27	-0.0685	1.6974E-01	3.38E-01	ENSG00000108826
MRPL28	0.0062	9.4116E-01	9.71E-01	ENSG00000086504
MRPL3	0.1913	6.8383E-03	3.18E-02	ENSG00000114686
MRPL30	0.0723	2.4978E-01	4.36E-01	ENSG00000185414
MRPL32	-0.1985	2.0223E-03	1.23E-02	ENSG00000106591
MRPL33	-0.2267	3.1475E-04	2.85E-03	ENSG00000243147
MRPL34	-0.2761	6.2573E-05	7.71E-04	ENSG00000130312
MRPL35	0.0976	2.3208E-01	4.16E-01	ENSG00000132313
MRPL35P2	-0.0461	3.9483E-01		ENSG00000232075
MRPL36	-0.2163	1.4311E-03	9.46E-03	ENSG00000171421
MRPL37	-0.0870	1.4523E-01	3.04E-01	ENSG00000116221
MRPL38	0.4719	3.9864E-04	3.43E-03	ENSG00000204316
MRPL39	-0.0434	5.1106E-01	6.91E-01	ENSG00000154719
MRPL4	-0.0450	4.3405E-01	6.25E-01	ENSG00000105364
MRPL40	-0.1602	4.2800E-03	2.23E-02	ENSG00000185608
MRPL40P1	-0.0294	6.2779E-01	7.82E-01	ENSG00000256037

MRPL41	-0.2165	1.4442E-03	9.53E-03	ENSG00000182154
MRPL42	-0.0995	7.8651E-02	1.98E-01	ENSG00000198015
MRPL43	-0.0444	4.4499E-01	6.35E-01	ENSG00000055950
MRPL44	-0.3019	2.3651E-06	5.44E-05	ENSG00000135900
MRPL45	-0.1372	8.5378E-02	2.10E-01	ENSG00000278845
MRPL46	0.0013	9.9523E-01	9.97E-01	ENSG00000259494
MRPL47	-0.2107	4.5563E-04	3.82E-03	ENSG00000136522
MRPL48	-0.3315	1.4046E-06	3.54E-05	ENSG00000175581
MRPL49	-0.0023	9.6776E-01	9.83E-01	ENSG00000149792
MRPL50	0.1341	1.1166E-02	4.62E-02	ENSG00000136897
MRPL51	-0.1699	3.7775E-03	2.02E-02	ENSG00000111639
MRPL52	-0.2933	8.2888E-06	1.51E-04	ENSG00000172590
MRPL53P1	-0.0624	5.1494E-01	6.94E-01	ENSG00000235299
MRPL54	-0.1977	1.6718E-04	1.70E-03	ENSG00000183617
MRPL55	-0.0248	6.0945E-01	7.68E-01	ENSG00000162910
MRPL57	-0.2911	5.3775E-05	6.80E-04	ENSG00000173141
MRPL57P3	-0.0170	8.0887E-01		ENSG00000275940
MRPL58	-0.1486	1.3519E-02	5.35E-02	ENSG00000167862
MRPL9	-0.2295	6.0107E-04	4.77E-03	ENSG00000143436
MRPS10	0.0843	1.7743E-01	3.48E-01	ENSG00000048544
MRPS10P1	0.0124	3.3967E-01		ENSG00000235378
MRPS11	0.0554	2.1492E-01	3.95E-01	ENSG00000181991
MRPS12	-0.0115	8.7007E-01	9.34E-01	ENSG00000128626
MRPS14	-0.2789	1.9366E-04	1.91E-03	ENSG00000120333
MRPS15	-0.2834	6.4596E-07	1.85E-05	ENSG00000116898
MRPS16	-0.1799	2.6658E-03	1.54E-02	ENSG00000182180
MRPS17	0.0804	2.4945E-01	4.36E-01	ENSG00000239789
MRPS18A	-0.1244	9.8058E-02	2.32E-01	ENSG00000096080
MRPS18B	0.1933	7.9263E-04	5.95E-03	ENSG00000204568
MRPS18C	-0.1014	1.7017E-01	3.38E-01	ENSG00000163319
MRPS18CP7	-0.0042	7.7108E-01		ENSG00000235916
MRPS2	-0.2096	2.2770E-03	1.36E-02	ENSG00000122140
MRPS21	-0.1054	5.2276E-02	1.48E-01	ENSG00000266472
MRPS21P8	0.0077	8.9969E-01		ENSG00000259978
MRPS22	0.0702	1.9251E-01	3.68E-01	ENSG00000175110
MRPS23	-0.0513	2.9720E-01	4.89E-01	ENSG00000181610
MRPS24	-0.0372	6.9783E-01	8.31E-01	ENSG00000062582
MRPS25	-0.0133	8.3752E-01	9.15E-01	ENSG00000131368
MRPS26	-0.0459	3.3332E-01	5.28E-01	ENSG00000125901
MRPS27	0.0224	7.4666E-01	8.61E-01	ENSG00000113048
MRPS28	-0.2570	1.8864E-03	1.17E-02	ENSG00000147586
MRPS30	-0.1744	1.1757E-03	8.07E-03	ENSG00000112996
MRPS31	0.0572	3.1893E-01	5.13E-01	ENSG00000102738
MRPS33	-0.1653	2.0977E-03	1.27E-02	ENSG00000090263
MRPS34	-0.1209	5.5394E-02	1.54E-01	ENSG00000074071
MRPS35	-0.1319	1.5992E-02	6.08E-02	ENSG00000061794
MRPS36	-0.2058	2.7599E-03	1.58E-02	ENSG00000134056
MRPS5	-0.1043	2.6807E-01	4.57E-01	ENSG00000144029
MRPS6	-0.0089	8.7952E-01	9.39E-01	ENSG00000243927

MRPS6P1	0.0138	6.7805E-01	8.16E-01	ENSG00000270733
MRPS7	-0.0560	3.4891E-01	5.44E-01	ENSG00000125445
MRPS9	0.0119	8.8012E-01	9.39E-01	ENSG00000135972
MRPS9-AS1	0.1470	8.9827E-02	2.18E-01	ENSG00000231851
MRPS9-AS2	-0.0662	4.5945E-01	6.48E-01	ENSG00000224509
MRRF	0.2390	2.8463E-03	1.62E-02	ENSG00000148187
MRS2	0.1295	4.8703E-02	1.40E-01	ENSG00000124532
MRTFA	-0.0655	3.5807E-01	5.54E-01	ENSG00000196588
MRTFA-AS1	0.0449	2.8429E-01	4.76E-01	ENSG00000232564
MRTFB	-0.1715	2.1240E-02	7.54E-02	ENSG00000186260
MRTO4	0.0545	2.6597E-01	4.55E-01	ENSG00000053372
MS4A10	0.0118	3.0780E-01		ENSG00000172689
MS4A12	-0.0317	2.1994E-01		ENSG00000071203
MS4A15	0.0093	5.7498E-01		ENSG00000166961
MS4A19P	-0.0294	7.5174E-01		ENSG00000283601
MS4A2	0.0199	1.2039E-01		ENSG00000149534
MS4A4A	0.0107	4.8984E-01	6.74E-01	ENSG00000110079
MS4A4E	-0.0324	6.2256E-01		ENSG00000214787
MS4A6A	0.1505	1.5760E-01	3.21E-01	ENSG00000110077
MS4A6E	-0.0170	8.4945E-01	9.23E-01	ENSG00000166926
MS4A7	0.0728	1.2339E-01	2.72E-01	ENSG00000166927
MS4A8	-0.3180	2.9415E-02	9.61E-02	ENSG00000166959
MSANTD1	0.0730	4.5316E-01	6.43E-01	ENSG00000188981
MSANTD2	0.1723	8.4095E-02	2.08E-01	ENSG00000120458
MSANTD2-AS1	-0.1693	6.1930E-02	1.67E-01	ENSG00000245498
MSANTD3	0.0237	7.2850E-01	8.50E-01	ENSG00000066697
MSANTD3-TMEFF1	-0.0134	6.9063E-01		ENSG00000251349
MSANTD4	0.3161	1.4545E-06	3.64E-05	ENSG00000170903
MSANTD7	-0.0483	4.9280E-01	6.76E-01	ENSG00000284024
MSC-AS1	0.1865	7.9271E-02	1.99E-01	ENSG00000235531
MSGN1	0.0115	4.1454E-01		ENSG00000151379
MSH2	0.0866	2.6104E-01	4.50E-01	ENSG00000095002
MSH3	-0.1663	7.1663E-03	3.29E-02	ENSG00000113318
MSH4	0.1036	1.2263E-01		ENSG00000057468
MSH5	0.4430	8.1753E-03	3.66E-02	ENSG00000204410
MSH5-SAPCD1	0.0099	5.9742E-01		ENSG00000255152
MSH6	0.0044	9.4985E-01	9.75E-01	ENSG00000116062
MSI1	-0.2188	2.5927E-02	8.75E-02	ENSG00000135097
MSI2	-0.0798	3.6047E-01	5.56E-01	ENSG00000153944
MSL1	-0.2279	1.4946E-02	5.79E-02	ENSG00000188895
MSL2	-0.0495	4.9114E-01	6.75E-01	ENSG00000174579
MSL3	0.1028	2.1015E-01	3.90E-01	ENSG00000005302
MSL3P1	0.0762	4.2984E-01	6.22E-01	ENSG00000224287
MSLN	0.2471	5.0942E-02	1.45E-01	ENSG00000102854
MSLNL	-0.0063	7.0939E-01		ENSG00000162006
MSMB	-0.1040	9.2165E-02	2.22E-01	ENSG00000263639
MSMO1	-0.2224	1.1053E-02	4.58E-02	ENSG00000052802
MSN	-0.1611	4.4905E-02	1.32E-01	ENSG00000147065
MSR1	0.2426	5.8604E-02	1.61E-01	ENSG00000038945

MSRA	-0.1154	2.5654E-01	4.44E-01	ENSG00000175806
MSRB1	-0.1933	2.2348E-02	7.83E-02	ENSG00000198736
MSRB2	-0.4409	7.8208E-09	4.68E-07	ENSG00000148450
MSRB3	-0.5857	5.3085E-06	1.05E-04	ENSG00000174099
MSS51	-0.1517	1.6481E-01	3.31E-01	ENSG00000166343
MST1	0.3929	2.6086E-05	3.79E-04	ENSG00000173531
MSTO1	0.3120	1.3636E-03	9.10E-03	ENSG00000125459
MSX1	-0.3571	2.9229E-04	2.67E-03	ENSG00000163132
MT1A	0.0209	9.2970E-01	9.66E-01	ENSG00000205362
MT1E	0.0260	7.7288E-01	8.77E-01	ENSG00000169715
MT1F	-0.0346	6.8384E-01	8.21E-01	ENSG00000198417
MT1G	0.1541	1.5938E-01	3.24E-01	ENSG00000125144
MT1H	-0.0246	6.7806E-01	8.16E-01	ENSG00000205358
MT1M	-0.0088	9.4830E-01		ENSG00000205364
MT1X	0.0775	4.0699E-01	6.00E-01	ENSG00000187193
MT2A	-0.2208	2.7851E-02	9.21E-02	ENSG00000125148
MT3	-0.1027	3.1067E-01	5.04E-01	ENSG00000087250
MTA1	-0.2958	9.3755E-04	6.78E-03	ENSG00000182979
MTA1-DT	-0.4620	1.2044E-05	2.04E-04	ENSG00000251602
MTA2	0.2444	8.9257E-03	3.90E-02	ENSG00000149480
MTA3	-0.1341	4.2164E-02	1.26E-01	ENSG00000057935
MTAP	-0.2465	5.1357E-03	2.55E-02	ENSG00000099810
MTARC1	-0.1641	4.1619E-02	1.25E-01	ENSG00000186205
MTARC2	-0.4270	2.0820E-04	2.02E-03	ENSG00000117791
MTATP6P1	0.1040	2.6303E-01	4.52E-01	ENSG00000248527
MTATP6P2	-0.0607	9.9654E-02		ENSG00000270307
MTATP6P27	-0.0749	4.9427E-01	6.78E-01	ENSG00000234003
MTATP8P1	-0.0399	7.3585E-01	8.55E-01	ENSG00000240409
MTATP8P2	0.0319	3.5492E-01	5.50E-01	ENSG00000229604
MTBP	0.1760	7.9182E-02	1.99E-01	ENSG00000172167
MTCH1	-0.0625	3.1263E-01	5.06E-01	ENSG00000137409
MTCH2	-0.0454	4.5155E-01	6.41E-01	ENSG00000109919
MTCL1	0.3006	4.6648E-03	2.37E-02	ENSG00000168502
MTCL2	-0.1443	1.0008E-01	2.35E-01	ENSG00000149639
MTCL3	-0.0412	7.0739E-01	8.36E-01	ENSG00000214338
MTCO1P11	0.0073	3.3926E-01		ENSG00000224083
MTCO1P12	0.1753	1.1995E-01	2.67E-01	ENSG00000237973
MTCO1P19	0.0086	6.4222E-01		ENSG00000237300
MTCO1P22	0.0225	2.9728E-01		ENSG00000271207
MTCO1P27	-0.0129	4.7776E-01		ENSG00000232579
MTCO1P40	0.0110	9.7248E-01	9.86E-01	ENSG00000262902
MTCO1P42	-0.0051	9.1018E-01		ENSG00000230327
MTCO1P53	0.0189	4.7935E-01		ENSG00000230916
MTCO2P11	-0.0458	4.0908E-01		ENSG00000235917
MTCO2P12	-0.0496	5.7140E-01	7.39E-01	ENSG00000229344
MTCO2P19	0.0072	4.2320E-01		ENSG00000237628
MTCO2P2	0.0367	7.0904E-01	8.37E-01	ENSG00000267541
MTCO2P22	-0.0508	3.9466E-01	5.89E-01	ENSG00000270225
MTCO2P27	-0.0867	2.0215E-01		ENSG00000230321

MTCO3P12	0.3288	9.4978E-05	1.07E-03	ENSG00000198744
MTCP1	0.0053	9.5586E-01	9.77E-01	ENSG00000214827
MTCYBP18	0.0745	3.2199E-01	5.16E-01	ENSG00000244921
MTCYBP21	0.0114	2.5686E-01		ENSG00000224747
MTCYBP28	0.0298	9.0907E-02		ENSG00000260161
MTCYBP32	0.0084	4.3785E-01		ENSG00000229794
MTDH	-0.2203	2.6343E-04	2.45E-03	ENSG00000147649
MTDHP1	0.0356	2.9208E-01		ENSG00000270948
MTERF1	0.2512	5.4102E-04	4.39E-03	ENSG00000127989
MTERF2	0.1552	2.2011E-02	7.74E-02	ENSG00000120832
MTERF3	0.1460	5.3939E-02	1.51E-01	ENSG00000156469
MTERF4	0.0374	4.5084E-01	6.41E-01	ENSG00000122085
MTF1	0.0372	6.2986E-01	7.84E-01	ENSG00000188786
MTF2	0.1364	2.3845E-02	8.22E-02	ENSG00000143033
MTFMT	0.0516	4.1547E-01	6.08E-01	ENSG00000103707
MTFP1	0.0750	4.1293E-01	6.06E-01	ENSG00000242114
MTFR1	-0.1362	9.0357E-02	2.19E-01	ENSG00000066855
MTFR1L	0.0962	1.4506E-01	3.03E-01	ENSG00000117640
MTFR2	-0.1404	1.8860E-01	3.63E-01	ENSG00000146410
MTG1	-0.0161	7.8208E-01	8.83E-01	ENSG00000148824
MTG2	0.0665	3.3949E-01	5.35E-01	ENSG00000101181
MTHFD1	-0.0568	4.2315E-01	6.15E-01	ENSG00000100714
MTHFD1L	0.1629	2.6296E-02	8.83E-02	ENSG00000120254
MTHFD1P1	-0.0265	6.2968E-01		ENSG00000231831
MTHFD2	-0.0440	5.3131E-01	7.06E-01	ENSG00000065911
MTHFD2L	-0.1919	5.1377E-03	2.55E-02	ENSG00000163738
MTHFD2P1	-0.0762	3.4045E-01		ENSG00000244681
MTHFR	0.3119	7.2346E-05	8.64E-04	ENSG00000177000
MTHFSD	-0.3806	2.6112E-04	2.43E-03	ENSG00000103248
MTIF2	0.0110	8.9070E-01	9.45E-01	ENSG00000085760
MTIF2P1	-0.0006	9.9034E-01		ENSG00000233432
MTIF3	0.0351	5.8933E-01	7.53E-01	ENSG00000122033
MTLN	-0.0630	4.5425E-01	6.44E-01	ENSG00000175701
MTM1	0.0571	5.2688E-01	7.03E-01	ENSG00000171100
MTMR1	0.1216	1.2782E-01	2.79E-01	ENSG00000063601
MTMR10	-0.0228	7.8891E-01	8.87E-01	ENSG00000166912
MTMR11	0.1062	2.2733E-01	4.10E-01	ENSG00000014914
MTMR12	0.1999	2.3610E-03	1.40E-02	ENSG00000150712
MTMR14	0.2547	5.5505E-04	4.48E-03	ENSG00000163719
MTMR2	-0.0755	1.6714E-01	3.34E-01	ENSG00000087053
MTMR3	-0.0540	4.3821E-01	6.29E-01	ENSG00000100330
MTMR4	0.2880	2.1080E-04	2.03E-03	ENSG00000108389
MTMR6	0.2156	1.0131E-02	4.29E-02	ENSG00000139505
MTMR8	-0.0035	9.6859E-01	9.84E-01	ENSG00000102043
MTMR9	0.0671	3.2575E-01	5.20E-01	ENSG00000104643
MTND1P11	-0.0030	5.2140E-01		ENSG00000228166
MTND1P23	0.0882	2.2677E-01	4.09E-01	ENSG00000225972
MTND1P27	-0.0786	1.5819E-01		ENSG00000223921
MTND1P32	-0.0192	9.8300E-01		ENSG00000232282

MTND1P37	-0.0264	7.3762E-01	8.56E-01	ENSG00000283456
MTND1P6	-0.0071	9.3384E-01		ENSG00000254346
MTND1P9	0.0108	6.7425E-01		ENSG00000228995
MTND2P11	0.0239	2.7653E-01		ENSG00000237124
MTND2P2	0.0202	4.8826E-01		ENSG00000229954
MTND2P28	-0.0178	8.0001E-01	8.93E-01	ENSG00000225630
MTND2P32	-0.0401	1.4060E-01		ENSG00000254090
MTND2P40	0.0242	6.2937E-01		ENSG00000236483
MTND2P9	0.0023	6.2311E-01		ENSG00000225901
MTND3P25	0.0067	6.9939E-01		ENSG00000249192
MTND4LP1	0.0100	7.3301E-01		ENSG00000231501
MTND4LP30	-0.0253	6.4817E-01	7.96E-01	ENSG00000198868
MTND4P1	0.0062	5.7652E-01		ENSG00000228597
MTND4P12	-0.0098	9.1289E-01	9.56E-01	ENSG00000247627
MTND4P15	-0.0557	1.9129E-01		ENSG00000227321
MTND4P24	-0.0511	3.1360E-01		ENSG00000232177
MTND4P35	-0.0798	3.6268E-01	5.59E-01	ENSG00000270906
MTND4P9	0.0061	7.5986E-01		ENSG00000250050
MTND5P1	-0.0889	2.5924E-01	4.47E-01	ENSG00000227999
MTND5P10	0.0076	7.2577E-01		ENSG00000271687
MTND5P11	-0.0986	3.1566E-01	5.09E-01	ENSG00000248923
MTND5P16	-0.0216	4.0598E-01		ENSG00000243658
MTND5P2	-0.0722	4.1088E-01	6.04E-01	ENSG00000229622
MTND5P28	0.0049	6.4164E-01		ENSG00000223549
MTND5P33	0.0190	2.9320E-01		ENSG00000261904
MTND6P21	-0.0077	9.1599E-01		ENSG00000223431
MTND6P22	-0.0265	7.6876E-01	8.74E-01	ENSG00000270230
MTND6P3	-0.1302	2.3599E-01	4.20E-01	ENSG00000254132
MTND6P4	-0.3282	1.2108E-02	4.92E-02	ENSG00000249119
MTNR1A	0.0491	5.4125E-01	7.14E-01	ENSG00000168412
MTO1	0.0035	9.7309E-01	9.86E-01	ENSG00000135297
MTOR	0.0185	7.6738E-01	8.74E-01	ENSG00000198793
MTOR-AS1	-0.0694	3.2092E-01	5.15E-01	ENSG00000225602
MTPAP	-0.0936	2.1337E-01	3.94E-01	ENSG00000107951
MTPN	-0.2653	8.7893E-07	2.41E-05	ENSG00000105887
MTR	0.1460	3.4617E-02	1.09E-01	ENSG00000116984
MTRES1	-0.0321	5.1691E-01	6.95E-01	ENSG00000130349
MTREX	0.0493	5.0333E-01	6.85E-01	ENSG00000039123
MTRF1	0.0949	2.2959E-01	4.13E-01	ENSG00000120662
MTRF1L	-0.2013	1.1581E-04	1.26E-03	ENSG00000112031
MTRF1LP1	0.0285	3.6067E-01		ENSG00000256091
MTRFR	0.1736	3.1954E-02	1.02E-01	ENSG00000130921
MTRR	0.4039	3.2108E-06	6.98E-05	ENSG00000124275
MTSS1	-0.0808	2.3999E-01	4.25E-01	ENSG00000170873
MTSS2	-0.5913	4.4813E-10	4.29E-08	ENSG00000132613
MTTP	0.0625	5.2703E-01	7.03E-01	ENSG00000138823
MTURN	-0.2587	6.4972E-06	1.25E-04	ENSG00000180354
MTUS1	0.0587	4.0069E-01	5.95E-01	ENSG00000129422
MTUS2	0.1323	1.6837E-01	3.36E-01	ENSG00000132938

MTX1	-0.0884	1.8550E-01	3.59E-01	ENSG00000173171
MTX1LP	-0.0992	1.7224E-01	3.41E-01	ENSG00000236675
MTX2	0.0016	9.8158E-01	9.90E-01	ENSG00000128654
MTX3	-0.0320	6.8896E-01	8.25E-01	ENSG00000177034
MUC1	0.0290	7.6294E-01	8.71E-01	ENSG00000185499
MUC12	-0.1432	1.5596E-01	3.19E-01	ENSG00000205277
MUC12-AS1	-0.0736	3.9244E-01	5.87E-01	ENSG00000227053
MUC13	-0.0070	6.9352E-01		ENSG00000173702
MUC16	-0.0677	3.8921E-01	5.84E-01	ENSG00000181143
MUC17	0.0011	8.2549E-01		ENSG00000169876
MUC19	0.0398	3.1733E-01		ENSG00000205592
MUC2	-0.0651	1.3446E-01		ENSG00000198788
MUC20	0.5127	5.6750E-04	4.55E-03	ENSG00000176945
MUC3A	-0.0219	8.2756E-01	9.09E-01	ENSG00000169894
MUC5AC	-0.3880	1.6304E-02	6.18E-02	ENSG00000215182
MUC5B	-0.1773	9.1538E-02	2.21E-01	ENSG00000117983
MUC6	0.0147	3.7421E-01	5.70E-01	ENSG00000184956
MUC7	0.0024	8.1714E-01		ENSG00000171195
MUL1	-0.1238	1.0690E-01	2.46E-01	ENSG00000090432
MUS81	0.1368	3.1765E-02	1.02E-01	ENSG00000172732
MUSK	0.0598	5.3518E-01	7.09E-01	ENSG00000030304
MUSTN1	0.0085	6.2261E-01		ENSG00000272573
MUTYH	0.4991	3.2938E-04	2.95E-03	ENSG00000132781
MVB12A	-0.0808	2.8411E-01	4.76E-01	ENSG00000141971
MVB12B	-0.0739	2.9968E-01	4.92E-01	ENSG00000196814
MVD	0.0197	7.8286E-01	8.83E-01	ENSG00000167508
MVK	0.0762	3.9504E-01	5.90E-01	ENSG00000110921
MVP	0.3722	2.1624E-04	2.08E-03	ENSG00000013364
MVP-DT	-0.0208	8.1858E-01	9.04E-01	ENSG00000238045
MX1	-0.3090	1.8132E-02	6.69E-02	ENSG00000157601
MX2	0.0331	1.8419E-01		ENSG00000183486
MXD1	-0.5176	4.7316E-12	8.06E-10	ENSG00000059728
MXD3	-0.1994	3.1830E-02	1.02E-01	ENSG00000213347
MXD4	-0.1296	3.5948E-02	1.12E-01	ENSG00000123933
MXI1	-0.1662	6.0486E-03	2.90E-02	ENSG00000119950
MXRA7	0.1194	2.1666E-02	7.65E-02	ENSG00000182534
MXRA8	0.0221	8.4755E-01	9.21E-01	ENSG00000162576
MYADM	-0.1028	1.3363E-01	2.87E-01	ENSG00000179820
MYADML2	0.0644	4.3139E-01	6.23E-01	ENSG00000185105
MYB	0.0589	4.5492E-01	6.44E-01	ENSG00000118513
MYBBP1A	0.3280	6.7897E-05	8.21E-04	ENSG00000132382
MYBL1	0.0874	3.4960E-01	5.45E-01	ENSG00000185697
MYBL2	-0.1653	1.1840E-01	2.65E-01	ENSG00000101057
MYBPC1	-0.0092	7.8969E-01		ENSG00000196091
MYBPC2	0.2823	3.9268E-02	1.19E-01	ENSG00000086967
MYBPC3	0.0669	1.1743E-01		ENSG00000134571
MYBPH	0.0341	5.6871E-02		ENSG00000133055
MYBPHL	0.1426	1.5237E-01	3.14E-01	ENSG00000221986
MYC	-0.1140	2.1884E-01	4.00E-01	ENSG00000136997

MYCBP	0.2540	6.1297E-03	2.93E-02	ENSG00000214114
MYCBP2	0.0591	4.2673E-01	6.19E-01	ENSG00000005810
MYCBP2-AS1	0.1370	7.4894E-02	1.91E-01	ENSG00000236051
MYCBPAP	-0.0459	6.3360E-01	7.86E-01	ENSG00000136449
MYCL	0.3144	1.0666E-03	7.47E-03	ENSG00000116990
MYCL-AS1	0.0003	8.8732E-01		ENSG00000236546
MYCN	-0.0975	2.8409E-01	4.76E-01	ENSG00000134323
MYCNOS	-0.0103	9.0731E-01	9.54E-01	ENSG00000233718
MYCT1	0.0071	6.6102E-01		ENSG00000120279
MYD88	0.1399	1.3922E-01	2.95E-01	ENSG00000172936
MYDGF	-0.0844	1.3808E-01	2.93E-01	ENSG00000074842
MYEF2	-0.0600	2.6181E-01	4.51E-01	ENSG00000104177
MYF6	0.0109	6.4868E-01		ENSG00000111046
MYG1	-0.3650	1.7686E-02	6.56E-02	ENSG00000139637
MYH10	0.0597	3.0256E-01	4.95E-01	ENSG00000133026
MYH11	-0.2060	8.7321E-02	2.14E-01	ENSG00000133392
MYH14	-0.2623	1.4617E-02	5.69E-02	ENSG00000105357
MYH15	0.1212	2.3600E-01	4.20E-01	ENSG00000144821
MYH16	0.1520	2.2125E-02		ENSG00000002079
MYH2	-0.0121	7.5976E-01		ENSG00000125414
MYH3	0.0129	8.5405E-01	9.25E-01	ENSG00000109063
MYH6	-0.0973	9.3021E-02		ENSG00000197616
MYH7	0.0742	4.4750E-01	6.37E-01	ENSG00000092054
MYH7B	0.2904	3.5791E-02	1.11E-01	ENSG00000078814
MYH9	-0.2764	3.0792E-05	4.32E-04	ENSG00000100345
MYL1	0.0287	5.1238E-02	1.45E-01	ENSG00000168530
MYL10	0.0019	8.1431E-01		ENSG00000106436
MYL11	0.1841	9.5031E-02	2.27E-01	ENSG00000180209
MYL12-AS1	-0.0867	3.4846E-01	5.43E-01	ENSG00000264235
MYL12A	-0.1554	3.8415E-02	1.17E-01	ENSG00000101608
MYL12B	-0.2744	7.4132E-04	5.63E-03	ENSG00000118680
MYL2	-0.0244	4.1626E-01		ENSG00000111245
MYL3	0.1412	1.8436E-01	3.57E-01	ENSG00000160808
MYL4	-0.0458	3.7512E-01	5.71E-01	ENSG00000198336
MYL5	-0.1043	1.2385E-01	2.73E-01	ENSG00000215375
MYL6	-0.3100	1.4005E-06	3.53E-05	ENSG00000092841
MYL6B	-0.2211	4.4022E-06	9.08E-05	ENSG00000196465
MYL7	-0.0236	6.8498E-01		ENSG00000106631
MYL9	-0.0492	5.9166E-01	7.55E-01	ENSG00000101335
MYLIP	-0.2412	1.2090E-03	8.25E-03	ENSG00000007944
MYLK	0.0294	7.4501E-01	8.60E-01	ENSG00000065534
MYLK-AS1	-0.0784	3.0473E-01	4.97E-01	ENSG00000239523
MYLK-AS2	0.0468	4.5293E-01	6.42E-01	ENSG00000250174
MYLK2	-0.0616	4.3387E-01	6.25E-01	ENSG00000101306
MYLK3	0.1319	2.1175E-01	3.92E-01	ENSG00000140795
MYLK4	-0.0412	6.4174E-01	7.92E-01	ENSG00000145949
MYLKP1	0.0412	1.7304E-01	3.42E-01	ENSG00000228868
MYMK	-0.0065	8.4134E-01		ENSG00000187616
MYMX	-0.2021	8.4095E-02	2.08E-01	ENSG00000262179

MYNN	0.0800	2.0987E-01	3.89E-01	ENSG00000085274
MYO10	-0.2999	9.1723E-06	1.64E-04	ENSG00000145555
MYO15A	0.3371	7.9924E-04	5.98E-03	ENSG00000091536
MYO15B	0.1234	2.2000E-01	4.01E-01	ENSG00000266714
MYO16	-0.3912	2.4251E-04	2.29E-03	ENSG00000041515
MYO16-AS1	0.1016	3.0730E-02		ENSG00000236242
MYO18A	-0.0326	5.9525E-01	7.57E-01	ENSG00000196535
MYO18B	0.1970	8.2600E-02	2.05E-01	ENSG00000133454
MYO19	0.2328	3.2161E-03	1.77E-02	ENSG00000278259
MYO1A	0.0076	6.8604E-01		ENSG00000166866
MYO1B	0.0635	3.6290E-01	5.59E-01	ENSG00000128641
MYO1C	0.0851	3.6461E-01	5.61E-01	ENSG00000197879
MYO1D	0.0889	2.1394E-01	3.94E-01	ENSG00000176658
MYO1D-DT	-0.0160	9.5090E-01		ENSG00000236377
MYO1E	-0.2973	1.4357E-03	9.48E-03	ENSG00000157483
MYO1G	-0.0166	8.4859E-01	9.22E-01	ENSG00000136286
MYO1H	-0.0523	5.0473E-01	6.86E-01	ENSG00000174527
MYO3A	-0.1927	8.2127E-02	2.04E-01	ENSG00000095777
MYO3B	0.0176	8.4320E-01	9.18E-01	ENSG00000071909
MYO3B-AS1	0.0082	4.2786E-01		ENSG00000231898
MYO5A	0.0691	4.1646E-01	6.09E-01	ENSG00000197535
MYO5B	-0.0390	6.6841E-01	8.09E-01	ENSG00000167306
MYO5C	0.0324	6.9911E-01	8.32E-01	ENSG00000128833
MYO6	0.1886	1.9096E-03	1.18E-02	ENSG00000196586
MYO7A	0.5102	1.4016E-05	2.31E-04	ENSG00000137474
MYO7B	0.0032	6.6483E-01	8.07E-01	ENSG00000169994
MYO9A	0.2467	5.9566E-03	2.87E-02	ENSG00000066933
MYO9B	-0.0359	6.1838E-01	7.75E-01	ENSG00000099331
MYOCD	0.0163	3.4714E-01		ENSG00000141052
MYOCOS	-0.0071	8.7756E-01		ENSG00000283683
MYOD1	0.2585	4.2113E-02	1.26E-01	ENSG00000129152
MYOF	-0.4416	4.8206E-05	6.27E-04	ENSG00000138119
MYOG	0.0611	3.8626E-01	5.81E-01	ENSG00000122180
MYOM1	-0.0122	8.9871E-01	9.49E-01	ENSG00000101605
MYOM2	-0.1406	1.8961E-01	3.64E-01	ENSG00000036448
MYOM3	0.0420	8.4755E-02		ENSG00000142661
MYORG	-0.0334	7.0748E-01	8.36E-01	ENSG00000164976
MYOSLID	0.0355	4.4983E-01		ENSG00000229647
MYOT	-0.0810	3.9837E-01	5.93E-01	ENSG00000120729
MYOZ1	-0.0772	4.3327E-01	6.25E-01	ENSG00000177791
MYOZ2	-0.1450	1.5239E-01	3.14E-01	ENSG00000172399
MYPN	-0.0388	6.7726E-01		ENSG00000138347
MYPOP	0.1281	1.9852E-01	3.75E-01	ENSG00000176182
MYRF	0.5253	6.2158E-03	2.96E-02	ENSG00000124920
MYRFL	-0.0451	5.4545E-01	7.17E-01	ENSG00000166268
MYRIP	0.1918	5.8570E-02	1.61E-01	ENSG00000170011
MYSM1	0.0453	5.0570E-01	6.87E-01	ENSG00000162601
MYT1	0.3246	1.2342E-04	1.32E-03	ENSG00000196132
MYT1L	0.3103	3.0342E-03	1.70E-02	ENSG00000186487

MYT1L-AS1	0.0376	4.3886E-01	6.30E-01	ENSG00000225619
MYZAP	0.1721	1.2685E-01	2.77E-01	ENSG00000263155
MZB1	0.0595	2.7959E-01		ENSG00000170476
MZF1	0.2512	1.3387E-04	1.41E-03	ENSG00000099326
MZF1-AS1	0.3534	2.3189E-03	1.38E-02	ENSG00000267858
MZT1	0.0983	2.0022E-01	3.77E-01	ENSG00000204899
MZT2A	-0.1748	4.1347E-03	2.17E-02	ENSG00000173272
MZT2B	-0.2530	2.1929E-06	5.09E-05	ENSG00000152082
N4BP1	-0.4308	6.9259E-14	1.90E-11	ENSG00000102921
N4BP2	0.1048	1.6298E-01	3.29E-01	ENSG00000078177
N4BP2L1	-0.1242	2.0271E-01	3.80E-01	ENSG00000139597
N4BP2L2	-0.0144	7.0534E-01	8.35E-01	ENSG00000244754
N4BP3	0.1391	8.7145E-02	2.14E-01	ENSG00000145911
N6AMT1	-0.2195	8.2480E-03	3.68E-02	ENSG00000156239
NA	0.3867	1.0362E-13	2.70E-11	ENSG00000259943
NA	0.5388	3.9683E-13	9.30E-11	ENSG00000287733
NA	0.4621	2.8238E-12	5.31E-10	ENSG00000174353
NA	0.4561	3.2174E-12	5.87E-10	ENSG00000205583
NA	0.5930	1.5807E-10	1.76E-08	ENSG00000249637
NA	0.4283	6.9776E-10	6.32E-08	ENSG00000259877
NA	0.5852	1.2486E-09	1.03E-07	ENSG00000173727
NA	0.5936	1.3094E-09	1.07E-07	ENSG00000185495
NA	0.5326	1.6236E-09	1.27E-07	ENSG00000277072
NA	0.4529	4.7153E-09	3.13E-07	ENSG00000287760
NA	0.5344	5.5165E-09	3.56E-07	ENSG00000227518
NA	0.4834	6.7233E-09	4.12E-07	ENSG00000127957
NA	0.5802	8.7386E-09	5.11E-07	ENSG00000277369
NA	0.4542	1.2546E-08	6.88E-07	ENSG00000131797
NA	0.5583	1.3437E-08	7.21E-07	ENSG00000204177
NA	0.3988	3.1854E-08	1.52E-06	ENSG00000244879
NA	0.4987	4.1909E-08	1.90E-06	ENSG00000289370
NA	0.5926	7.3199E-08	3.01E-06	ENSG00000272894
NA	0.4216	9.3706E-08	3.71E-06	ENSG00000263004
NA	0.3174	9.7244E-08	3.82E-06	ENSG00000221990
NA	0.5611	1.4943E-07	5.43E-06	ENSG00000258789
NA	0.3185	2.3962E-07	8.14E-06	ENSG00000259366
NA	0.4386	3.9522E-07	1.22E-05	ENSG00000215014
NA	0.5681	6.9816E-07	1.98E-05	ENSG00000260236
NA	0.3830	7.1567E-07	2.02E-05	ENSG00000247121
NA	0.5400	1.4116E-06	3.55E-05	ENSG00000275445
NA	0.5588	1.4190E-06	3.56E-05	ENSG00000257543
NA	0.3600	2.2619E-06	5.23E-05	ENSG00000215908
NA	0.5404	2.3257E-06	5.37E-05	ENSG00000213888
NA	0.4649	3.4466E-06	7.43E-05	ENSG00000289021
NA	0.5469	4.7308E-06	9.60E-05	ENSG00000244257
NA	0.4865	5.3200E-06	1.05E-04	ENSG00000272829
NA	0.5064	5.4400E-06	1.07E-04	ENSG00000287190
NA	0.4221	5.5812E-06	1.10E-04	ENSG00000289872
NA	0.5944	6.2765E-06	1.21E-04	ENSG00000278200

NA	0.5134	6.5163E-06	1.25E-04	ENSG00000257470
NA	0.4313	7.7364E-06	1.44E-04	ENSG00000226200
NA	0.4065	7.7965E-06	1.45E-04	ENSG00000260000
NA	0.4978	7.9255E-06	1.46E-04	ENSG00000288075
NA	0.5439	8.1246E-06	1.49E-04	ENSG00000268883
NA	0.4578	9.0254E-06	1.62E-04	ENSG00000289311
NA	0.4552	9.4791E-06	1.68E-04	ENSG00000228393
NA	0.4332	9.4998E-06	1.68E-04	ENSG00000288765
NA	0.5435	9.9341E-06	1.74E-04	ENSG00000196302
NA	0.3905	1.0397E-05	1.81E-04	ENSG00000237978
NA	0.4638	1.1375E-05	1.95E-04	ENSG00000272092
NA	0.4308	1.3943E-05	2.30E-04	ENSG00000289414
NA	0.4135	1.4890E-05	2.43E-04	ENSG00000253203
NA	0.3526	1.5436E-05	2.50E-04	ENSG00000189229
NA	0.0779	1.6353E-05	2.61E-04	ENSG00000262094
NA	0.2699	1.8461E-05	2.87E-04	ENSG00000187534
NA	0.5368	1.8705E-05	2.90E-04	ENSG00000180229
NA	0.5279	1.8733E-05	2.90E-04	ENSG00000215158
NA	0.5344	2.0220E-05	3.08E-04	ENSG00000269514
NA	0.3934	2.2309E-05	3.34E-04	ENSG00000228232
NA	0.3562	2.2948E-05	3.42E-04	ENSG00000227232
NA	0.5993	2.4821E-05	3.63E-04	ENSG00000290051
NA	0.5552	2.5664E-05	3.74E-04	ENSG00000287600
NA	0.3544	2.6566E-05	3.84E-04	ENSG00000287104
NA	0.2649	2.7896E-05	4.00E-04	ENSG00000196295
NA	0.2915	3.0384E-05	4.28E-04	ENSG00000264575
NA	0.4622	3.1634E-05	4.42E-04	ENSG00000279692
NA	0.5163	3.2655E-05	4.51E-04	ENSG00000271780
NA	0.4699	3.7084E-05	5.01E-04	ENSG00000274422
NA	0.3911	3.7514E-05	5.06E-04	ENSG00000185485
NA	0.5629	4.0352E-05	5.39E-04	ENSG00000289244
NA	0.0553	4.4129E-05		ENSG00000260126
NA	0.3963	4.5530E-05	5.97E-04	ENSG00000213062
NA	0.5759	4.9339E-05	6.39E-04	ENSG00000246250
NA	0.4529	4.9633E-05	6.42E-04	ENSG00000280164
NA	0.0939	5.1534E-05	6.59E-04	ENSG00000280397
NA	0.5078	5.3862E-05	6.81E-04	ENSG00000272716
NA	0.5654	5.4434E-05	6.86E-04	ENSG00000229337
NA	0.5146	6.6436E-05	8.08E-04	ENSG00000286527
NA	0.3856	7.6482E-05	9.03E-04	ENSG00000260025
NA	0.4762	7.7386E-05	9.11E-04	ENSG00000289074
NA	0.5703	7.8155E-05	9.17E-04	ENSG00000288896
NA	0.4322	7.8721E-05	9.23E-04	ENSG00000280254
NA	0.3517	8.0746E-05	9.41E-04	ENSG00000271576
NA	0.4344	9.4373E-05	1.07E-03	ENSG00000197588
NA	0.5372	9.9416E-05	1.11E-03	ENSG00000230454
NA	0.1185	1.0675E-04	1.18E-03	ENSG00000187870
NA	0.4020	1.0802E-04	1.19E-03	ENSG00000225889
NA	0.5359	1.2933E-04	1.37E-03	ENSG00000289537

NA	0.4130	1.3637E-04	1.43E-03	ENSG00000262074
NA	0.3239	1.4866E-04	1.54E-03	ENSG00000253816
NA	0.2848	1.4894E-04	1.55E-03	ENSG00000273456
NA	0.2862	1.5069E-04	1.56E-03	ENSG00000146556
NA	0.5443	1.5825E-04	1.63E-03	ENSG00000278017
NA	0.4232	1.7100E-04	1.73E-03	ENSG00000265185
NA	0.4665	1.7105E-04	1.73E-03	ENSG00000254740
NA	0.0865	1.7966E-04	1.81E-03	ENSG00000213232
NA	0.5812	1.8868E-04	1.87E-03	ENSG00000259553
NA	0.5524	1.9301E-04	1.91E-03	ENSG00000270638
NA	0.3409	2.0203E-04	1.98E-03	ENSG00000246877
NA	0.5775	2.0331E-04	1.99E-03	ENSG00000261420
NA	0.5211	2.0510E-04	2.00E-03	ENSG00000289585
NA	0.3059	2.1711E-04	2.08E-03	ENSG00000263934
NA	0.5692	2.2688E-04	2.17E-03	ENSG00000261447
NA	0.3990	2.4012E-04	2.27E-03	ENSG00000253596
NA	0.5806	2.4288E-04	2.29E-03	ENSG00000214248
NA	0.3951	2.4381E-04	2.30E-03	ENSG00000232931
NA	0.4492	2.4919E-04	2.34E-03	ENSG00000264940
NA	0.4462	2.5332E-04	2.37E-03	ENSG00000288744
NA	0.2964	2.5605E-04	2.39E-03	ENSG00000274828
NA	0.2125	2.6084E-04	2.43E-03	ENSG00000290033
NA	0.5738	2.6424E-04	2.45E-03	ENSG00000260853
NA	0.5709	2.7334E-04	2.52E-03	ENSG00000260475
NA	0.2525	3.0192E-04	2.74E-03	ENSG00000100058
NA	0.2482	3.1365E-04	2.84E-03	ENSG00000170089
NA	0.0661	3.3007E-04		ENSG00000225249
NA	0.3891	3.3010E-04	2.96E-03	ENSG00000259577
NA	0.3575	3.3015E-04	2.96E-03	ENSG00000273355
NA	0.2943	3.3290E-04	2.97E-03	ENSG00000198237
NA	0.5450	3.4140E-04	3.04E-03	ENSG00000229178
NA	0.4357	3.5814E-04	3.17E-03	ENSG00000272853
NA	0.4591	3.7001E-04	3.25E-03	ENSG00000255406
NA	0.4042	3.7871E-04	3.30E-03	ENSG00000288586
NA	0.5813	3.8456E-04	3.34E-03	ENSG00000176289
NA	0.2846	3.9209E-04	3.39E-03	ENSG00000228506
NA	0.4147	3.9501E-04	3.41E-03	ENSG00000272734
NA	0.4320	3.9838E-04	3.43E-03	ENSG00000272525
NA	0.3768	3.9913E-04	3.43E-03	ENSG00000280160
NA	0.4624	4.4105E-04	3.72E-03	ENSG00000230658
NA	0.5584	4.5365E-04	3.80E-03	ENSG00000257556
NA	0.5633	4.7545E-04	3.93E-03	ENSG00000284740
NA	0.2884	5.0108E-04	4.11E-03	ENSG00000268584
NA	0.2313	5.0155E-04	4.11E-03	ENSG00000272994
NA	0.4692	5.3575E-04	4.35E-03	ENSG00000251034
NA	0.3630	5.4483E-04	4.42E-03	ENSG00000289015
NA	0.3253	5.8662E-04	4.69E-03	ENSG00000273117
NA	0.2682	5.9229E-04	4.72E-03	ENSG00000253366
NA	0.2430	6.5154E-04	5.09E-03	ENSG00000228801

NA	0.4175	6.5829E-04	5.13E-03	ENSG00000284707
NA	0.5059	6.6645E-04	5.18E-03	ENSG00000288577
NA	0.5051	6.7057E-04	5.21E-03	ENSG00000289043
NA	0.2627	6.9386E-04	5.35E-03	ENSG00000205534
NA	0.5586	7.0257E-04	5.40E-03	ENSG00000290058
NA	0.4597	7.0281E-04	5.40E-03	ENSG00000260261
NA	0.4324	7.0867E-04	5.43E-03	ENSG00000228084
NA	0.4180	7.4984E-04	5.68E-03	ENSG00000178631
NA	0.5468	7.5803E-04	5.73E-03	ENSG00000259436
NA	0.3343	7.6887E-04	5.79E-03	ENSG00000289532
NA	0.3976	7.7236E-04	5.81E-03	ENSG00000287431
NA	0.3430	8.1168E-04	6.04E-03	ENSG00000203761
NA	0.2853	8.3075E-04	6.16E-03	ENSG00000267278
NA	0.5116	8.3345E-04	6.17E-03	ENSG00000232648
NA	0.2092	8.4066E-04	6.21E-03	ENSG00000246695
NA	0.0835	8.5230E-04		ENSG00000226074
NA	0.4297	8.9078E-04	6.50E-03	ENSG00000260806
NA	0.4304	9.0511E-04	6.59E-03	ENSG00000269176
NA	0.5195	9.4447E-04	6.81E-03	ENSG00000230832
NA	0.4346	9.4679E-04	6.83E-03	ENSG00000232093
NA	0.0997	9.5447E-04	6.88E-03	ENSG00000262558
NA	0.4442	9.5568E-04	6.88E-03	ENSG00000244055
NA	0.4976	9.6184E-04	6.91E-03	ENSG00000259345
NA	0.4785	9.7198E-04	6.96E-03	ENSG00000272631
NA	0.3543	9.7413E-04	6.97E-03	ENSG00000285053
NA	0.1263	9.8741E-04	7.04E-03	ENSG00000286448
NA	0.4708	9.9899E-04	7.11E-03	ENSG00000279491
NA	0.3817	1.0031E-03	7.13E-03	ENSG00000273066
NA	0.5081	1.0128E-03	7.18E-03	ENSG00000287244
NA	0.5414	1.0229E-03	7.23E-03	ENSG00000290117
NA	0.3996	1.0262E-03	7.25E-03	ENSG00000188185
NA	0.0862	1.0292E-03		ENSG00000279181
NA	0.3839	1.0374E-03	7.32E-03	ENSG00000214439
NA	0.5414	1.0395E-03	7.33E-03	ENSG00000269937
NA	0.4535	1.0704E-03	7.48E-03	ENSG00000260563
NA	0.2782	1.0795E-03	7.53E-03	ENSG00000206149
NA	0.5799	1.1171E-03	7.74E-03	ENSG00000227269
NA	0.5320	1.1272E-03	7.79E-03	ENSG00000261324
NA	0.3121	1.2905E-03	8.70E-03	ENSG00000216285
NA	0.5350	1.3505E-03	9.03E-03	ENSG00000285966
NA	0.0912	1.4291E-03	9.45E-03	ENSG00000214546
NA	0.4177	1.4906E-03	9.76E-03	ENSG00000286964
NA	0.5950	1.6389E-03	1.05E-02	ENSG00000214433
NA	0.3624	1.6605E-03	1.06E-02	ENSG00000285804
NA	0.2761	1.6724E-03	1.07E-02	ENSG00000223820
NA	0.5343	1.7338E-03	1.10E-02	ENSG00000250448
NA	0.4152	1.7352E-03	1.10E-02	ENSG00000248769
NA	0.5542	1.7732E-03	1.11E-02	ENSG00000230699
NA	0.4013	1.8240E-03	1.14E-02	ENSG00000288093

NA	0.2830	1.8349E-03	1.14E-02	ENSG00000228327
NA	0.5214	1.8406E-03	1.15E-02	ENSG00000257135
NA	1.6727	1.8530E-03		ENSG00000287088
NA	0.3537	1.8683E-03	1.16E-02	ENSG00000279901
NA	0.4593	1.8802E-03	1.17E-02	ENSG00000278206
NA	0.1430	1.8894E-03	1.17E-02	ENSG00000286586
NA	0.5083	1.9217E-03	1.19E-02	ENSG00000289970
NA	0.5073	1.9309E-03	1.19E-02	ENSG00000289334
NA	0.2860	1.9636E-03	1.21E-02	ENSG00000227855
NA	0.0482	1.9672E-03		ENSG00000259907
NA	0.2170	1.9858E-03	1.22E-02	ENSG00000289901
NA	0.2740	1.9940E-03	1.22E-02	ENSG00000228409
NA	0.3423	2.0014E-03	1.23E-02	ENSG00000288778
NA	0.2655	2.0857E-03	1.27E-02	ENSG00000249572
NA	0.3220	2.1134E-03	1.28E-02	ENSG00000235726
NA	2.0254	2.1182E-03		ENSG00000267360
NA	0.3858	2.1913E-03	1.32E-02	ENSG00000289000
NA	0.5426	2.1991E-03	1.32E-02	ENSG00000256925
NA	0.2535	2.2183E-03	1.33E-02	ENSG00000215154
NA	0.5969	2.2374E-03	1.34E-02	ENSG00000279700
NA	0.4710	2.2474E-03	1.35E-02	ENSG00000269978
NA	0.0988	2.2831E-03	1.36E-02	ENSG00000286620
NA	0.2738	2.3150E-03	1.38E-02	ENSG00000288820
NA	0.2633	2.3644E-03	1.40E-02	ENSG00000198221
NA	0.2875	2.3839E-03	1.41E-02	ENSG00000244119
NA	0.5901	2.4020E-03	1.42E-02	ENSG00000288663
NA	0.4020	2.4313E-03	1.43E-02	ENSG00000263272
NA	0.4937	2.4620E-03	1.44E-02	ENSG00000233175
NA	0.1565	2.5155E-03	1.47E-02	ENSG00000241490
NA	0.3096	2.5407E-03	1.48E-02	ENSG00000255046
NA	0.3187	2.5916E-03	1.50E-02	ENSG00000240225
NA	0.2482	2.6879E-03	1.55E-02	ENSG00000183666
NA	0.2341	2.7000E-03	1.55E-02	ENSG00000270659
NA	0.3810	2.7112E-03	1.56E-02	ENSG00000289340
NA	0.0566	2.7405E-03		ENSG00000290022
NA	0.4384	2.7538E-03	1.58E-02	ENSG00000271918
NA	0.2462	2.8227E-03	1.61E-02	ENSG00000243970
NA	0.3176	2.9355E-03	1.66E-02	ENSG00000237491
NA	0.3888	2.9676E-03	1.67E-02	ENSG00000287576
NA	0.3723	3.0100E-03	1.69E-02	ENSG00000289048
NA	0.5320	3.0510E-03	1.70E-02	ENSG00000233143
NA	1.1729	3.0544E-03		ENSG00000260848
NA	0.4558	3.0886E-03	1.72E-02	ENSG00000241547
NA	0.5457	3.0888E-03	1.72E-02	ENSG00000259556
NA	0.5366	3.0977E-03	1.72E-02	ENSG00000264727
NA	0.5961	3.1633E-03	1.75E-02	ENSG00000289304
NA	0.3584	3.2089E-03	1.77E-02	ENSG00000268628
NA	0.3229	3.2613E-03	1.79E-02	ENSG00000290072
NA	0.5570	3.3728E-03	1.84E-02	ENSG00000270871

NA	0.3564	3.4471E-03	1.88E-02	ENSG00000289055
NA	0.3533	3.6403E-03	1.96E-02	ENSG00000164669
NA	0.5430	3.6525E-03	1.97E-02	ENSG00000279759
NA	0.2576	3.6742E-03	1.97E-02	ENSG00000232320
NA	0.3396	3.6972E-03	1.98E-02	ENSG00000289277
NA	0.2139	3.7345E-03	2.00E-02	ENSG00000279457
NA	0.2808	3.8101E-03	2.03E-02	ENSG00000241549
NA	0.2132	3.8382E-03	2.04E-02	ENSG00000183604
NA	0.2958	3.8761E-03	2.06E-02	ENSG00000250994
NA	0.2103	3.8916E-03	2.07E-02	ENSG00000214135
NA	0.2751	3.8981E-03	2.07E-02	ENSG00000267519
NA	0.5868	4.1243E-03	2.16E-02	ENSG00000273064
NA	0.5900	4.1609E-03	2.18E-02	ENSG00000187686
NA	0.4724	4.2001E-03	2.19E-02	ENSG00000257391
NA	0.3088	4.2429E-03	2.21E-02	ENSG00000283050
NA	0.0772	4.4031E-03	2.27E-02	ENSG00000251250
NA	0.5309	4.4616E-03	2.29E-02	ENSG00000264775
NA	0.4454	4.5739E-03	2.34E-02	ENSG00000272523
NA	0.5036	4.5744E-03	2.34E-02	ENSG00000224702
NA	0.0378	4.5786E-03		ENSG00000288803
NA	0.3804	4.5998E-03	2.35E-02	ENSG00000281091
NA	0.3548	4.6045E-03	2.35E-02	ENSG00000253540
NA	0.3452	4.7133E-03	2.39E-02	ENSG00000213433
NA	0.1711	4.8747E-03	2.45E-02	ENSG00000196312
NA	0.1573	4.8975E-03	2.46E-02	ENSG00000254238
NA	1.0242	4.9578E-03		ENSG00000278908
NA	0.2751	4.9776E-03	2.49E-02	ENSG00000258130
NA	0.3641	4.9908E-03	2.50E-02	ENSG00000289198
NA	0.1094	5.0143E-03		ENSG00000233999
NA	0.2819	5.0499E-03	2.52E-02	ENSG00000257303
NA	0.4737	5.0918E-03	2.54E-02	ENSG00000258001
NA	0.2752	5.0992E-03	2.54E-02	ENSG00000276550
NA	0.5926	5.1076E-03	2.54E-02	ENSG00000289538
NA	0.3761	5.1952E-03	2.58E-02	ENSG00000288917
NA	0.4742	5.3316E-03	2.63E-02	ENSG00000235946
NA	0.5214	5.4405E-03	2.67E-02	ENSG00000268030
NA	0.1231	5.4785E-03	2.69E-02	ENSG00000275540
NA	0.1777	5.4894E-03	2.69E-02	ENSG00000263072
NA	0.5205	5.5124E-03	2.70E-02	ENSG00000289253
NA	0.3689	5.5446E-03	2.71E-02	ENSG00000290015
NA	0.4263	5.5745E-03	2.73E-02	ENSG00000274698
NA	0.0367	5.6122E-03	2.74E-02	ENSG00000285703
NA	0.4991	5.6174E-03	2.74E-02	ENSG00000263126
NA	0.0374	5.6746E-03	2.76E-02	ENSG00000288074
NA	0.0634	5.7548E-03		ENSG00000229276
NA	0.5291	5.7672E-03	2.80E-02	ENSG00000271888
NA	0.5914	5.7699E-03	2.80E-02	ENSG00000290082
NA	0.0613	5.9139E-03	2.85E-02	ENSG00000227542
NA	0.5560	5.9818E-03	2.88E-02	ENSG00000260919

NA	0.2794	6.0809E-03	2.92E-02	ENSG00000258150
NA	0.2253	6.1685E-03	2.95E-02	ENSG00000275964
NA	0.4497	6.2412E-03	2.97E-02	ENSG00000258131
NA	0.5699	6.2559E-03	2.98E-02	ENSG00000218582
NA	0.3449	6.2790E-03	2.98E-02	ENSG00000246596
NA	0.3753	6.4217E-03	3.04E-02	ENSG00000289552
NA	0.2642	6.4382E-03	3.04E-02	ENSG00000228175
NA	0.3951	6.4948E-03	3.06E-02	ENSG00000166763
NA	0.3770	6.6315E-03	3.11E-02	ENSG00000289373
NA	0.4547	6.7291E-03	3.14E-02	ENSG00000289236
NA	0.3487	6.7475E-03	3.15E-02	ENSG00000286659
NA	0.0290	6.7604E-03		ENSG00000234238
NA	0.2716	6.8519E-03	3.19E-02	ENSG00000273080
NA	0.3935	6.8621E-03	3.19E-02	ENSG00000227256
NA	0.4687	6.9071E-03	3.21E-02	ENSG00000239519
NA	0.0766	6.9654E-03	3.23E-02	ENSG00000286328
NA	0.1801	7.0371E-03	3.25E-02	ENSG00000261611
NA	0.5032	7.0602E-03	3.26E-02	ENSG00000247372
NA	0.4519	7.1283E-03	3.28E-02	ENSG00000273384
NA	0.5855	7.1285E-03	3.28E-02	ENSG00000274718
NA	0.5052	7.1915E-03	3.30E-02	ENSG00000271755
NA	0.4119	7.2181E-03	3.31E-02	ENSG00000269352
NA	0.2512	7.2396E-03	3.32E-02	ENSG00000273702
NA	0.5671	7.2684E-03	3.33E-02	ENSG00000267394
NA	0.5273	7.2885E-03	3.34E-02	ENSG00000284052
NA	0.0682	7.3197E-03	3.35E-02	ENSG00000254734
NA	0.0405	7.3330E-03		ENSG00000234156
NA	0.5618	7.4419E-03	3.40E-02	ENSG00000233200
NA	0.0486	7.4541E-03		ENSG00000210191
NA	0.5930	7.5116E-03	3.42E-02	ENSG00000277186
NA	0.2133	7.5784E-03	3.44E-02	ENSG00000254701
NA	0.0458	7.6052E-03	3.46E-02	ENSG00000273124
NA	0.2310	7.6466E-03	3.47E-02	ENSG00000205485
NA	0.5533	7.7238E-03	3.50E-02	ENSG00000287040
NA	0.0543	7.9248E-03		ENSG00000261673
NA	0.2762	7.9502E-03	3.58E-02	ENSG00000288937
NA	0.4795	7.9876E-03	3.59E-02	ENSG00000272791
NA	0.0708	8.0004E-03	3.60E-02	ENSG00000250198
NA	0.4629	8.1193E-03	3.64E-02	ENSG00000286209
NA	0.4027	8.1295E-03	3.64E-02	ENSG00000279750
NA	0.2677	8.1863E-03	3.66E-02	ENSG00000238035
NA	0.4441	8.2013E-03	3.66E-02	ENSG00000276728
NA	0.3124	8.3286E-03	3.70E-02	ENSG00000272449
NA	0.1325	8.3343E-03		ENSG00000261348
NA	0.2990	8.3756E-03	3.72E-02	ENSG00000289881
NA	0.3520	8.3796E-03	3.72E-02	ENSG00000288799
NA	0.8537	8.5523E-03		ENSG00000225555
NA	0.3307	8.5820E-03	3.79E-02	ENSG00000176826
NA	0.0441	8.6822E-03		ENSG00000254033

NA	0.0638	8.7973E-03		ENSG00000277505
NA	0.2061	8.8613E-03	3.89E-02	ENSG00000273270
NA	0.3680	8.8620E-03	3.89E-02	ENSG00000287630
NA	0.3999	8.8880E-03	3.89E-02	ENSG00000265749
NA	0.1074	8.8882E-03	3.89E-02	ENSG00000279190
NA	0.4126	8.8921E-03	3.89E-02	ENSG00000268230
NA	0.3777	8.9679E-03	3.92E-02	ENSG00000280067
NA	0.2896	9.0017E-03	3.93E-02	ENSG00000272583
NA	0.3313	9.1398E-03	3.98E-02	ENSG00000287837
NA	0.5595	9.1799E-03	3.99E-02	ENSG00000223722
NA	0.3250	9.2080E-03	4.00E-02	ENSG00000259683
NA	0.4707	9.2456E-03	4.01E-02	ENSG00000234233
NA	0.0484	9.2493E-03		ENSG00000279613
NA	0.5944	9.2579E-03	4.01E-02	ENSG00000181355
NA	0.4687	9.2585E-03	4.01E-02	ENSG00000254768
NA	0.5118	9.3871E-03	4.06E-02	ENSG00000273576
NA	0.3964	9.4121E-03	4.06E-02	ENSG00000272692
NA	0.4121	9.4342E-03	4.07E-02	ENSG00000289496
NA	0.3461	9.4377E-03	4.07E-02	ENSG00000259135
NA	0.3775	9.5155E-03	4.10E-02	ENSG00000278993
NA	0.2616	9.5249E-03	4.10E-02	ENSG00000258704
NA	0.3422	9.5515E-03	4.11E-02	ENSG00000286885
NA	0.4666	9.5783E-03	4.11E-02	ENSG00000279789
NA	0.3407	9.5903E-03	4.12E-02	ENSG00000289059
NA	0.4938	9.6153E-03	4.13E-02	ENSG00000279243
NA	0.2524	9.6222E-03	4.13E-02	ENSG00000228878
NA	0.5516	9.6630E-03	4.14E-02	ENSG00000287450
NA	0.2409	9.8045E-03	4.19E-02	ENSG00000260565
NA	0.4736	9.8621E-03	4.21E-02	ENSG00000287055
NA	0.2248	9.8918E-03	4.22E-02	ENSG00000225465
NA	0.0708	9.9670E-03	4.24E-02	ENSG00000287384
NA	0.4902	9.9804E-03	4.24E-02	ENSG00000289050
NA	0.5631	1.0032E-02	4.26E-02	ENSG00000227355
NA	0.5260	1.0080E-02	4.27E-02	ENSG00000283162
NA	0.2767	1.0104E-02	4.28E-02	ENSG00000233297
NA	0.5681	1.0147E-02	4.29E-02	ENSG00000267334
NA	0.0394	1.0156E-02	4.30E-02	ENSG00000282863
NA	0.1747	1.0246E-02	4.32E-02	ENSG00000248124
NA	0.4794	1.0246E-02	4.32E-02	ENSG00000257527
NA	0.0412	1.0252E-02	4.33E-02	ENSG00000259235
NA	0.4818	1.0302E-02	4.34E-02	ENSG00000239402
NA	0.4425	1.0397E-02	4.37E-02	ENSG00000290047
NA	0.0511	1.0448E-02	4.39E-02	ENSG00000289840
NA	0.0443	1.0463E-02		ENSG00000279653
NA	0.4157	1.0531E-02	4.41E-02	ENSG00000285979
NA	0.5632	1.0562E-02	4.42E-02	ENSG00000286591
NA	0.2285	1.0583E-02	4.43E-02	ENSG00000288764
NA	0.5325	1.0602E-02	4.43E-02	ENSG00000287677
NA	0.4674	1.0608E-02	4.43E-02	ENSG00000275709

NA	0.4454	1.0610E-02	4.43E-02	ENSG00000210195
NA	0.0333	1.0776E-02	4.49E-02	ENSG00000282952
NA	0.5602	1.0779E-02	4.49E-02	ENSG00000270133
NA	0.2181	1.0859E-02	4.52E-02	ENSG00000210154
NA	0.0416	1.0882E-02		ENSG00000279421
NA	0.5020	1.1034E-02	4.58E-02	ENSG00000288737
NA	0.0452	1.1086E-02		ENSG00000278829
NA	0.0418	1.1099E-02		ENSG00000277308
NA	0.2811	1.1107E-02	4.60E-02	ENSG00000133624
NA	0.3405	1.1212E-02	4.63E-02	ENSG00000272432
NA	0.0590	1.1231E-02		ENSG00000268635
NA	0.3774	1.1251E-02	4.64E-02	ENSG00000259775
NA	0.4478	1.1299E-02	4.66E-02	ENSG00000287862
NA	0.0594	1.1338E-02		ENSG00000270956
NA	0.1555	1.1442E-02	4.70E-02	ENSG00000274114
NA	0.0499	1.1566E-02	4.74E-02	ENSG00000228683
NA	0.3662	1.1623E-02	4.76E-02	ENSG00000272556
NA	0.3788	1.1796E-02	4.82E-02	ENSG00000272622
NA	0.5017	1.1822E-02	4.82E-02	ENSG00000270035
NA	0.1170	1.1822E-02	4.82E-02	ENSG00000210174
NA	0.3543	1.1854E-02	4.83E-02	ENSG00000273188
NA	0.1970	1.1960E-02		ENSG00000287821
NA	0.2396	1.2079E-02	4.91E-02	ENSG00000213189
NA	0.0575	1.2131E-02		ENSG00000261296
NA	0.0136	1.2209E-02	4.95E-02	ENSG00000288745
NA	0.3966	1.2231E-02	4.95E-02	ENSG00000277687
NA	0.4839	1.2291E-02	4.97E-02	ENSG00000214770
NA	0.2578	1.2307E-02	4.98E-02	ENSG00000261377
NA	0.2864	1.2342E-02	4.99E-02	ENSG00000273669
NA	0.3513	1.2391E-02	5.00E-02	ENSG00000227227
NA	0.2493	1.2425E-02	5.01E-02	ENSG00000248415
NA	0.5069	1.2492E-02	5.03E-02	ENSG00000289911
NA	0.3077	1.2494E-02	5.03E-02	ENSG00000270022
NA	0.0729	1.2501E-02	5.04E-02	ENSG00000234267
NA	0.2891	1.2549E-02	5.05E-02	ENSG00000288852
NA	0.0407	1.2634E-02		ENSG00000275927
NA	0.5430	1.2715E-02	5.11E-02	ENSG00000287608
NA	0.2977	1.2731E-02	5.11E-02	ENSG00000253736
NA	0.3120	1.2765E-02	5.12E-02	ENSG00000237436
NA	0.4030	1.2799E-02	5.13E-02	ENSG00000277382
NA	0.1894	1.2832E-02	5.14E-02	ENSG00000279088
NA	0.0860	1.2897E-02		ENSG00000234743
NA	0.4597	1.2931E-02	5.17E-02	ENSG00000226239
NA	0.3047	1.2958E-02	5.18E-02	ENSG00000272163
NA	0.3765	1.3014E-02	5.20E-02	ENSG00000197585
NA	0.2858	1.3025E-02	5.20E-02	ENSG00000287222
NA	0.0551	1.3096E-02		ENSG00000287611
NA	0.4544	1.3099E-02	5.22E-02	ENSG00000255136
NA	0.4173	1.3161E-02	5.24E-02	ENSG00000282022

NA	0.5087	1.3180E-02	5.25E-02	ENSG00000288065
NA	0.3712	1.3231E-02	5.26E-02	ENSG00000237402
NA	0.4387	1.3377E-02	5.31E-02	ENSG00000285871
NA	0.5041	1.3430E-02	5.33E-02	ENSG00000259205
NA	0.3724	1.3642E-02	5.39E-02	ENSG00000261570
NA	0.4262	1.3756E-02	5.43E-02	ENSG00000289375
NA	0.0279	1.3878E-02		ENSG00000248215
NA	0.0674	1.3926E-02	5.48E-02	ENSG00000230233
NA	0.0479	1.4064E-02		ENSG00000255101
NA	0.3093	1.4081E-02	5.53E-02	ENSG00000253653
NA	0.3363	1.4144E-02	5.55E-02	ENSG00000276302
NA	0.0364	1.4148E-02		ENSG00000254575
NA	0.4954	1.4225E-02	5.57E-02	ENSG00000273443
NA	0.2866	1.4284E-02	5.59E-02	ENSG00000154608
NA	0.1326	1.4426E-02	5.63E-02	ENSG00000235587
NA	0.4243	1.4531E-02	5.66E-02	ENSG00000174912
NA	0.4259	1.4631E-02	5.70E-02	ENSG00000230587
NA	0.0689	1.4658E-02	5.70E-02	ENSG00000231764
NA	0.0263	1.4852E-02	5.76E-02	ENSG00000272128
NA	0.2739	1.5150E-02	5.84E-02	ENSG00000268575
NA	0.3970	1.5163E-02	5.85E-02	ENSG00000229953
NA	0.4560	1.5375E-02	5.91E-02	ENSG00000278477
NA	0.4455	1.5397E-02	5.91E-02	ENSG00000285018
NA	0.4144	1.5425E-02	5.92E-02	ENSG00000176933
NA	0.3226	1.5442E-02	5.92E-02	ENSG00000236308
NA	0.3502	1.5476E-02	5.93E-02	ENSG00000230848
NA	0.2422	1.5545E-02	5.95E-02	ENSG00000288818
NA	0.2134	1.5664E-02	5.99E-02	ENSG00000250543
NA	0.4719	1.5695E-02	6.00E-02	ENSG00000280269
NA	0.2581	1.5787E-02	6.03E-02	ENSG00000289410
NA	0.0407	1.5817E-02		ENSG00000258536
NA	0.4363	1.5873E-02	6.05E-02	ENSG00000280087
NA	0.0495	1.5905E-02		ENSG00000287583
NA	0.2712	1.5944E-02	6.07E-02	ENSG00000268107
NA	0.0816	1.5945E-02	6.07E-02	ENSG00000287695
NA	0.1285	1.6023E-02	6.10E-02	ENSG00000289214
NA	0.0975	1.6178E-02		ENSG00000278864
NA	0.4222	1.6206E-02	6.15E-02	ENSG00000280248
NA	0.1678	1.6264E-02	6.16E-02	ENSG00000249335
NA	0.3081	1.6285E-02	6.17E-02	ENSG00000285697
NA	0.0315	1.6312E-02		ENSG00000231405
NA	0.2717	1.6513E-02	6.24E-02	ENSG00000261131
NA	0.2656	1.6557E-02	6.25E-02	ENSG00000260233
NA	0.3687	1.6583E-02	6.26E-02	ENSG00000267737
NA	0.2351	1.6746E-02	6.30E-02	ENSG00000106133
NA	0.2072	1.6775E-02	6.31E-02	ENSG00000235749
NA	0.0316	1.6823E-02		ENSG00000286756
NA	0.3365	1.6879E-02	6.33E-02	ENSG00000261996
NA	0.3869	1.6938E-02	6.35E-02	ENSG00000228467

NA	0.2205	1.6977E-02	6.36E-02	ENSG00000213997
NA	0.0585	1.7087E-02	6.40E-02	ENSG00000286525
NA	0.0470	1.7254E-02		ENSG00000284430
NA	0.3494	1.7314E-02	6.47E-02	ENSG00000287957
NA	0.2444	1.7424E-02	6.49E-02	ENSG00000214796
NA	0.3383	1.7428E-02	6.49E-02	ENSG00000289929
NA	0.4078	1.7589E-02	6.54E-02	ENSG00000255410
NA	0.2461	1.7613E-02	6.55E-02	ENSG00000285596
NA	0.1603	1.7617E-02	6.55E-02	ENSG00000226578
NA	0.3237	1.7631E-02	6.55E-02	ENSG00000275367
NA	0.3383	1.7663E-02	6.56E-02	ENSG00000279529
NA	0.3783	1.7683E-02	6.56E-02	ENSG00000260177
NA	0.3611	1.7976E-02	6.65E-02	ENSG00000283128
NA	0.3151	1.8011E-02	6.66E-02	ENSG00000258101
NA	0.3600	1.8035E-02	6.67E-02	ENSG00000283635
NA	0.3761	1.8295E-02	6.74E-02	ENSG00000267342
NA	0.3029	1.8423E-02	6.78E-02	ENSG00000260747
NA	0.4035	1.8424E-02	6.78E-02	ENSG00000185607
NA	0.2603	1.8542E-02	6.81E-02	ENSG00000289119
NA	0.0379	1.8570E-02		ENSG00000256424
NA	0.3263	1.8599E-02	6.83E-02	ENSG00000226310
NA	0.0840	1.8625E-02	6.83E-02	ENSG00000285582
NA	0.3616	1.8668E-02	6.84E-02	ENSG00000272755
NA	0.3416	1.8704E-02	6.85E-02	ENSG00000259660
NA	0.3996	1.8726E-02	6.86E-02	ENSG00000263603
NA	0.4034	1.8822E-02	6.88E-02	ENSG00000261117
NA	0.0348	1.8945E-02		ENSG00000283400
NA	0.2055	1.9035E-02	6.94E-02	ENSG00000233818
NA	0.1330	1.9123E-02	6.96E-02	ENSG00000235501
NA	0.1946	1.9129E-02	6.96E-02	ENSG00000272693
NA	0.3860	1.9136E-02	6.96E-02	ENSG00000286848
NA	0.3299	1.9198E-02	6.98E-02	ENSG00000236166
NA	0.3457	1.9310E-02	7.01E-02	ENSG00000284882
NA	0.1467	1.9377E-02	7.03E-02	ENSG00000286164
NA	0.3042	1.9382E-02	7.03E-02	ENSG00000259687
NA	0.1543	1.9428E-02		ENSG00000276963
NA	0.2219	1.9471E-02	7.05E-02	ENSG00000225510
NA	0.0724	1.9525E-02	7.07E-02	ENSG00000286063
NA	0.0782	1.9529E-02		ENSG00000287192
NA	0.1176	1.9553E-02	7.07E-02	ENSG00000289941
NA	0.1620	1.9553E-02	7.07E-02	ENSG00000287217
NA	0.1797	1.9745E-02	7.13E-02	ENSG00000254563
NA	0.3570	1.9746E-02	7.13E-02	ENSG00000227527
NA	0.2077	1.9784E-02	7.14E-02	ENSG00000249679
NA	0.3992	1.9867E-02	7.16E-02	ENSG00000273172
NA	0.1906	1.9925E-02	7.18E-02	ENSG00000262481
NA	0.3471	2.0085E-02	7.23E-02	ENSG00000205740
NA	0.3653	2.0158E-02	7.25E-02	ENSG00000128262
NA	0.3155	2.0200E-02	7.26E-02	ENSG00000261401

NA	0.3651	2.0222E-02	7.27E-02	ENSG00000250731
NA	0.0312	2.0224E-02	7.27E-02	ENSG00000287683
NA	0.4081	2.0232E-02	7.27E-02	ENSG00000228709
NA	0.2902	2.0236E-02	7.27E-02	ENSG00000272426
NA	0.0101	2.0303E-02		ENSG00000286353
NA	0.3085	2.0319E-02	7.29E-02	ENSG00000288919
NA	0.3428	2.0433E-02	7.32E-02	ENSG00000186493
NA	0.2234	2.0458E-02	7.33E-02	ENSG00000269935
NA	0.3125	2.0466E-02	7.33E-02	ENSG00000264895
NA	0.0403	2.0502E-02		ENSG00000261313
NA	0.3163	2.0515E-02	7.34E-02	ENSG00000258608
NA	0.2417	2.0523E-02	7.34E-02	ENSG00000288926
NA	0.0311	2.0537E-02	7.34E-02	ENSG00000266369
NA	0.3835	2.0540E-02	7.34E-02	ENSG00000282542
NA	0.2874	2.0737E-02	7.39E-02	ENSG00000258561
NA	0.2651	2.0831E-02	7.42E-02	ENSG00000282798
NA	0.0541	2.1174E-02		ENSG00000287003
NA	0.3724	2.1189E-02	7.52E-02	ENSG00000260615
NA	0.0392	2.1195E-02		ENSG00000262171
NA	0.3660	2.1234E-02	7.54E-02	ENSG00000237337
NA	0.3389	2.1361E-02	7.57E-02	ENSG00000204338
NA	0.0548	2.1520E-02		ENSG00000254556
NA	0.1756	2.1530E-02		ENSG00000257095
NA	0.3050	2.1688E-02	7.66E-02	ENSG00000225146
NA	0.1185	2.1688E-02		ENSG00000280379
NA	0.3267	2.1737E-02	7.67E-02	ENSG00000257826
NA	0.3292	2.1771E-02	7.68E-02	ENSG00000287625
NA	0.3784	2.1856E-02	7.70E-02	ENSG00000179342
NA	0.0432	2.1922E-02		ENSG00000218274
NA	0.2807	2.2036E-02	7.75E-02	ENSG00000254966
NA	0.2710	2.2120E-02	7.77E-02	ENSG00000250575
NA	0.3790	2.2217E-02	7.79E-02	ENSG00000228404
NA	0.0979	2.2283E-02		ENSG00000267838
NA	0.0443	2.2305E-02		ENSG00000224727
NA	0.3357	2.2525E-02	7.88E-02	ENSG00000260285
NA	0.2619	2.2582E-02	7.89E-02	ENSG00000277368
NA	0.3797	2.2659E-02	7.91E-02	ENSG00000267092
NA	0.2423	2.2841E-02	7.96E-02	ENSG00000286835
NA	0.1257	2.2958E-02		ENSG00000235497
NA	0.3571	2.2991E-02	8.00E-02	ENSG00000289096
NA	0.3675	2.3057E-02	8.02E-02	ENSG00000288886
NA	0.3081	2.3085E-02	8.02E-02	ENSG00000260051
NA	0.1518	2.3339E-02	8.09E-02	ENSG00000182841
NA	0.1944	2.3367E-02	8.10E-02	ENSG00000264007
NA	0.3507	2.3415E-02	8.11E-02	ENSG00000269883
NA	0.3136	2.3426E-02	8.11E-02	ENSG00000240520
NA	0.3466	2.3445E-02	8.12E-02	ENSG00000272630
NA	0.1478	2.3499E-02		ENSG00000286505
NA	0.3058	2.3502E-02	8.13E-02	ENSG00000188801

NA	0.2919	2.3534E-02	8.14E-02	ENSG00000225492
NA	0.0599	2.3580E-02		ENSG00000286163
NA	0.2885	2.3628E-02	8.16E-02	ENSG00000249839
NA	0.2694	2.3662E-02	8.17E-02	ENSG00000289155
NA	0.0558	2.3720E-02		ENSG00000231345
NA	0.3570	2.3803E-02	8.21E-02	ENSG00000224680
NA	0.1786	2.3855E-02		ENSG00000272219
NA	0.2224	2.3928E-02	8.24E-02	ENSG00000284846
NA	0.3007	2.3962E-02	8.25E-02	ENSG00000270127
NA	0.0742	2.3975E-02		ENSG00000262692
NA	0.0382	2.4060E-02		ENSG00000254187
NA	0.3658	2.4154E-02	8.30E-02	ENSG00000177173
NA	0.1681	2.4231E-02	8.32E-02	ENSG00000215105
NA	0.3411	2.4290E-02	8.33E-02	ENSG00000282564
NA	0.1395	2.4419E-02	8.37E-02	ENSG00000285948
NA	0.2800	2.4693E-02	8.44E-02	ENSG00000280279
NA	0.2876	2.4760E-02	8.46E-02	ENSG00000263887
NA	0.0379	2.4834E-02		ENSG00000233613
NA	0.0570	2.4870E-02		ENSG00000271963
NA	0.2657	2.4908E-02	8.49E-02	ENSG00000237276
NA	0.2213	2.5028E-02	8.52E-02	ENSG00000269921
NA	0.1894	2.5100E-02	8.54E-02	ENSG00000286009
NA	0.2710	2.5218E-02	8.57E-02	ENSG00000249150
NA	0.0308	2.5323E-02		ENSG00000273106
NA	0.2379	2.5363E-02	8.62E-02	ENSG00000271380
NA	0.0175	2.5384E-02	8.62E-02	ENSG00000238262
NA	0.0426	2.5404E-02		ENSG00000286723
NA	0.1618	2.5499E-02	8.65E-02	ENSG00000251474
NA	0.3266	2.5592E-02	8.67E-02	ENSG00000276213
NA	0.2299	2.5599E-02	8.67E-02	ENSG00000286288
NA	0.3325	2.5741E-02	8.70E-02	ENSG00000224541
NA	0.2095	2.5744E-02	8.70E-02	ENSG00000273748
NA	0.3332	2.5773E-02	8.71E-02	ENSG00000290086
NA	0.2751	2.5926E-02	8.75E-02	ENSG00000261251
NA	0.2401	2.5948E-02	8.75E-02	ENSG00000164845
NA	0.3406	2.6010E-02	8.77E-02	ENSG00000284800
NA	0.0403	2.6033E-02		ENSG00000280265
NA	0.3170	2.6038E-02	8.77E-02	ENSG00000259424
NA	0.3110	2.6038E-02	8.77E-02	ENSG00000223396
NA	0.2045	2.6064E-02	8.78E-02	ENSG00000252569
NA	0.2814	2.6180E-02	8.81E-02	ENSG00000263826
NA	0.2717	2.6315E-02	8.83E-02	ENSG00000286931
NA	0.0888	2.6521E-02		ENSG00000215464
NA	0.3379	2.6521E-02	8.89E-02	ENSG00000287756
NA	0.0347	2.6545E-02		ENSG00000231193
NA	0.2536	2.6552E-02	8.90E-02	ENSG00000252211
NA	0.1179	2.6579E-02	8.90E-02	ENSG00000289328
NA	0.3342	2.6642E-02	8.92E-02	ENSG00000274220
NA	0.2925	2.6746E-02	8.95E-02	ENSG00000260992

NA	0.0446	2.6883E-02	8.98E-02	ENSG00000230323
NA	0.2975	2.7041E-02	9.02E-02	ENSG00000215156
NA	0.3285	2.7051E-02	9.02E-02	ENSG00000258598
NA	0.2922	2.7122E-02	9.04E-02	ENSG00000285637
NA	0.0480	2.7377E-02		ENSG00000275363
NA	0.2932	2.7384E-02	9.10E-02	ENSG00000250166
NA	0.0673	2.7484E-02	9.12E-02	ENSG00000265799
NA	0.1915	2.7507E-02	9.12E-02	ENSG00000183055
NA	0.0340	2.7509E-02		ENSG00000282418
NA	0.2880	2.7571E-02	9.14E-02	ENSG00000257194
NA	0.0633	2.7835E-02	9.21E-02	ENSG00000258748
NA	0.3050	2.7863E-02	9.21E-02	ENSG00000286416
NA	0.0382	2.8088E-02		ENSG00000287493
NA	0.0534	2.8118E-02	9.28E-02	ENSG00000236364
NA	0.3249	2.8229E-02	9.31E-02	ENSG00000279047
NA	0.1637	2.8273E-02		ENSG00000253456
NA	0.0404	2.8349E-02		ENSG00000287332
NA	0.0808	2.8568E-02	9.40E-02	ENSG00000255118
NA	0.3095	2.8631E-02	9.41E-02	ENSG00000261220
NA	0.0249	2.9068E-02		ENSG00000248115
NA	0.0525	2.9132E-02		ENSG00000288153
NA	0.2447	2.9176E-02	9.55E-02	ENSG00000239911
NA	0.1344	2.9353E-02		ENSG00000276845
NA	0.3135	2.9494E-02	9.62E-02	ENSG00000231748
NA	0.1939	2.9547E-02	9.64E-02	ENSG00000259002
NA	0.0801	2.9583E-02	9.64E-02	ENSG00000269903
NA	0.1738	2.9703E-02	9.68E-02	ENSG00000286797
NA	0.3198	2.9718E-02	9.68E-02	ENSG00000261033
NA	0.2473	2.9843E-02	9.71E-02	ENSG00000280294
NA	0.1624	3.0012E-02		ENSG00000287843
NA	0.3106	3.0112E-02	9.77E-02	ENSG00000263531
NA	0.1108	3.0150E-02		ENSG00000279041
NA	0.2567	3.0248E-02	9.81E-02	ENSG00000272787
NA	0.2628	3.0427E-02	9.84E-02	ENSG00000238260
NA	0.2792	3.0440E-02	9.85E-02	ENSG00000280378
NA	0.1938	3.0680E-02	9.91E-02	ENSG00000250132
NA	0.2487	3.0768E-02	9.93E-02	ENSG00000184068
NA	0.2843	3.0794E-02	9.94E-02	ENSG00000280381
NA	0.2447	3.0943E-02	9.97E-02	ENSG00000277324
NA	0.0823	3.1011E-02	9.98E-02	ENSG00000261797
NA	0.2976	3.1301E-02	1.00E-01	ENSG00000256967
NA	0.0496	3.1415E-02		ENSG00000183674
NA	0.1704	3.1526E-02	1.01E-01	ENSG00000288983
NA	0.2934	3.1621E-02	1.01E-01	ENSG00000205622
NA	0.2968	3.1756E-02	1.02E-01	ENSG00000287400
NA	0.0909	3.1886E-02	1.02E-01	ENSG00000286688
NA	0.3181	3.2119E-02	1.03E-01	ENSG00000253666
NA	0.0434	3.2425E-02	1.03E-01	ENSG00000289321
NA	0.2506	3.2614E-02	1.04E-01	ENSG00000289407

NA	0.3078	3.2625E-02	1.04E-01	ENSG00000277450
NA	0.3001	3.2663E-02	1.04E-01	ENSG00000230090
NA	0.2920	3.2874E-02	1.04E-01	ENSG00000265179
NA	0.0233	3.3057E-02		ENSG00000280019
NA	0.0494	3.3254E-02	1.05E-01	ENSG00000276898
NA	0.2206	3.3353E-02	1.06E-01	ENSG00000205464
NA	0.0647	3.3374E-02		ENSG00000241720
NA	0.1900	3.3827E-02	1.07E-01	ENSG00000236682
NA	0.0351	3.3842E-02		ENSG00000243307
NA	0.1551	3.3923E-02		ENSG00000255445
NA	0.0782	3.3962E-02	1.07E-01	ENSG00000250974
NA	0.0556	3.3963E-02		ENSG00000224830
NA	0.2453	3.4033E-02	1.07E-01	ENSG00000284642
NA	0.2152	3.4116E-02	1.07E-01	ENSG00000272277
NA	0.0193	3.4255E-02		ENSG00000282097
NA	0.3026	3.4437E-02	1.08E-01	ENSG00000253931
NA	0.3055	3.4466E-02	1.08E-01	ENSG00000262312
NA	0.0698	3.4501E-02		ENSG00000226699
NA	0.2579	3.4552E-02	1.08E-01	ENSG00000272969
NA	0.2603	3.4724E-02	1.09E-01	ENSG00000279048
NA	0.0286	3.4984E-02	1.09E-01	ENSG00000261243
NA	0.3038	3.5007E-02	1.10E-01	ENSG00000285382
NA	0.2622	3.5038E-02	1.10E-01	ENSG00000267904
NA	0.2904	3.5111E-02	1.10E-01	ENSG00000260912
NA	0.2678	3.5121E-02	1.10E-01	ENSG00000272316
NA	0.2889	3.5132E-02	1.10E-01	ENSG00000260492
NA	0.0937	3.5144E-02	1.10E-01	ENSG00000289886
NA	0.1842	3.5403E-02	1.11E-01	ENSG00000236438
NA	0.2982	3.5564E-02	1.11E-01	ENSG00000267934
NA	0.1279	3.5830E-02	1.12E-01	ENSG00000255983
NA	0.1600	3.5865E-02		ENSG00000273004
NA	0.2953	3.5867E-02	1.12E-01	ENSG00000260488
NA	0.2682	3.5891E-02	1.12E-01	ENSG00000289100
NA	0.0316	3.5936E-02		ENSG00000275665
NA	0.2325	3.6040E-02	1.12E-01	ENSG00000204837
NA	0.0349	3.6128E-02		ENSG00000237233
NA	0.0232	3.6238E-02	1.12E-01	ENSG00000260618
NA	0.2859	3.6330E-02	1.13E-01	ENSG00000240449
NA	0.2682	3.6331E-02	1.13E-01	ENSG00000286437
NA	0.0926	3.6416E-02		ENSG00000230790
NA	0.2795	3.6452E-02	1.13E-01	ENSG00000229422
NA	0.2637	3.6466E-02	1.13E-01	ENSG00000288925
NA	0.1214	3.6726E-02		ENSG00000256747
NA	0.0454	3.6951E-02		ENSG00000225411
NA	0.0289	3.6973E-02		ENSG00000289953
NA	0.0415	3.7081E-02	1.14E-01	ENSG00000267724
NA	0.1798	3.7139E-02	1.14E-01	ENSG00000260447
NA	0.1585	3.7362E-02	1.15E-01	ENSG00000275494
NA	0.0443	3.7364E-02		ENSG00000207955

NA	0.2685	3.7364E-02	1.15E-01	ENSG00000261094
NA	0.0632	3.7507E-02		ENSG00000230783
NA	0.2666	3.7666E-02	1.16E-01	ENSG00000289499
NA	0.0461	3.7793E-02		ENSG00000278595
NA	0.2799	3.8037E-02	1.16E-01	ENSG00000289110
NA	0.0709	3.8147E-02	1.17E-01	ENSG00000267372
NA	0.1636	3.8291E-02	1.17E-01	ENSG00000186076
NA	0.1341	3.8655E-02	1.18E-01	ENSG00000266994
NA	0.2849	3.8821E-02	1.18E-01	ENSG00000231443
NA	0.1604	3.8923E-02	1.19E-01	ENSG00000254682
NA	0.2450	3.8966E-02	1.19E-01	ENSG00000229533
NA	0.2390	3.9021E-02	1.19E-01	ENSG00000225945
NA	0.1504	3.9023E-02	1.19E-01	ENSG00000288016
NA	0.2709	3.9028E-02	1.19E-01	ENSG00000288998
NA	0.1085	3.9046E-02		ENSG00000289275
NA	0.1946	3.9102E-02	1.19E-01	ENSG00000285632
NA	0.0281	3.9618E-02		ENSG00000257350
NA	0.2834	3.9642E-02	1.20E-01	ENSG00000272953
NA	0.0322	3.9763E-02		ENSG00000229209
NA	0.1499	3.9776E-02	1.21E-01	ENSG00000225933
NA	0.2006	3.9818E-02	1.21E-01	ENSG00000260708
NA	0.1276	3.9934E-02		ENSG00000285162
NA	0.2778	3.9967E-02	1.21E-01	ENSG00000237846
NA	0.2188	4.0109E-02	1.21E-01	ENSG00000262202
NA	0.0549	4.0157E-02	1.21E-01	ENSG00000288942
NA	0.0388	4.0166E-02		ENSG00000287754
NA	0.0743	4.0308E-02		ENSG00000215296
NA	0.2771	4.0409E-02	1.22E-01	ENSG00000226453
NA	0.2564	4.0570E-02	1.22E-01	ENSG00000235370
NA	0.0070	4.0743E-02		ENSG00000250961
NA	0.2639	4.1287E-02	1.24E-01	ENSG00000234869
NA	0.0400	4.1305E-02		ENSG00000223374
NA	0.0256	4.1512E-02		ENSG00000254630
NA	0.2801	4.1533E-02	1.25E-01	ENSG00000219881
NA	0.1589	4.1573E-02	1.25E-01	ENSG00000286703
NA	0.0252	4.1613E-02		ENSG00000286947
NA	0.2743	4.1634E-02	1.25E-01	ENSG00000231890
NA	0.0479	4.1822E-02	1.25E-01	ENSG00000233099
NA	0.2385	4.1844E-02	1.25E-01	ENSG00000229407
NA	0.2789	4.1898E-02	1.26E-01	ENSG00000288754
NA	0.0151	4.1947E-02		ENSG00000287599
NA	0.2357	4.1973E-02	1.26E-01	ENSG00000285080
NA	0.0649	4.2121E-02	1.26E-01	ENSG00000287176
NA	0.2402	4.2150E-02	1.26E-01	ENSG00000282143
NA	0.2312	4.2300E-02	1.26E-01	ENSG00000267265
NA	0.2254	4.2730E-02	1.27E-01	ENSG00000188707
NA	0.0359	4.2735E-02		ENSG00000269533
NA	0.2401	4.2740E-02	1.27E-01	ENSG00000210077
NA	0.1069	4.2842E-02		ENSG00000273419

NA	0.2522	4.3102E-02	1.28E-01	ENSG00000286810
NA	0.2550	4.3179E-02	1.28E-01	ENSG00000236333
NA	0.2132	4.3431E-02	1.29E-01	ENSG00000250091
NA	0.2777	4.3569E-02	1.29E-01	ENSG00000264290
NA	0.2300	4.3621E-02	1.29E-01	ENSG00000289213
NA	0.1779	4.3675E-02	1.29E-01	ENSG00000286593
NA	0.1884	4.3812E-02	1.30E-01	ENSG00000282508
NA	0.2689	4.3892E-02	1.30E-01	ENSG00000274367
NA	0.1443	4.3938E-02	1.30E-01	ENSG00000253965
NA	0.2513	4.4152E-02	1.30E-01	ENSG00000287670
NA	0.2211	4.4539E-02	1.31E-01	ENSG00000263535
NA	0.2358	4.4786E-02	1.32E-01	ENSG00000275454
NA	0.2421	4.4857E-02	1.32E-01	ENSG00000260179
NA	0.1861	4.4870E-02	1.32E-01	ENSG00000266990
NA	0.2728	4.5029E-02	1.32E-01	ENSG00000279253
NA	0.0175	4.5096E-02	1.33E-01	ENSG00000262352
NA	0.0247	4.5146E-02		ENSG00000226872
NA	0.2112	4.5214E-02	1.33E-01	ENSG00000265907
NA	0.2016	4.5468E-02	1.33E-01	ENSG00000229294
NA	0.2344	4.5475E-02	1.33E-01	ENSG00000244380
NA	0.2520	4.5510E-02	1.33E-01	ENSG00000290044
NA	0.1218	4.5988E-02	1.35E-01	ENSG00000227398
NA	0.2654	4.6141E-02	1.35E-01	ENSG00000227220
NA	0.2734	4.6203E-02	1.35E-01	ENSG00000289267
NA	0.1048	4.6356E-02		ENSG00000265313
NA	0.1552	4.7126E-02	1.37E-01	ENSG00000285541
NA	0.0831	4.7163E-02		ENSG00000271151
NA	0.2434	4.7210E-02	1.37E-01	ENSG00000272341
NA	0.0839	4.7417E-02	1.37E-01	ENSG00000279798
NA	0.0794	4.7440E-02	1.38E-01	ENSG00000171658
NA	0.0402	4.7508E-02		ENSG00000284418
NA	0.0474	4.7593E-02		ENSG00000285361
NA	0.0222	4.7594E-02		ENSG00000279985
NA	0.1385	4.7685E-02		ENSG00000224220
NA	0.0473	4.7751E-02		ENSG00000228507
NA	0.2509	4.7793E-02	1.38E-01	ENSG00000262050
NA	0.2536	4.7988E-02	1.39E-01	ENSG00000231704
NA	0.0714	4.8100E-02		ENSG00000235347
NA	0.2364	4.8131E-02	1.39E-01	ENSG00000263345
NA	0.1846	4.8136E-02	1.39E-01	ENSG00000248564
NA	0.0484	4.8265E-02		ENSG00000236508
NA	0.1547	4.8306E-02	1.40E-01	ENSG00000285980
NA	0.2532	4.8346E-02	1.40E-01	ENSG00000279254
NA	0.0372	4.8492E-02		ENSG00000283698
NA	0.2585	4.8538E-02	1.40E-01	ENSG00000276449
NA	0.1656	4.8573E-02	1.40E-01	ENSG00000239650
NA	0.1425	4.8604E-02	1.40E-01	ENSG00000272667
NA	0.2250	4.8898E-02	1.41E-01	ENSG00000272137
NA	0.2153	4.9189E-02	1.41E-01	ENSG00000230910

NA	0.2590	4.9264E-02	1.42E-01	ENSG00000207751
NA	0.2256	4.9457E-02	1.42E-01	ENSG00000287574
NA	0.0358	4.9512E-02		ENSG00000287964
NA	0.2570	4.9534E-02	1.42E-01	ENSG00000236556
NA	0.2421	4.9546E-02	1.42E-01	ENSG00000261537
NA	0.2546	4.9751E-02	1.43E-01	ENSG00000230333
NA	0.2472	5.0139E-02	1.43E-01	ENSG00000280007
NA	0.0017	5.0189E-02	1.43E-01	ENSG00000289518
NA	0.0973	5.0234E-02		ENSG00000289954
NA	0.1997	5.0349E-02	1.44E-01	ENSG00000289183
NA	0.2508	5.0350E-02	1.44E-01	ENSG00000278991
NA	0.1597	5.0428E-02	1.44E-01	ENSG00000225406
NA	0.2603	5.0669E-02	1.44E-01	ENSG00000226089
NA	0.1386	5.0695E-02	1.44E-01	ENSG00000274093
NA	0.0007	5.0699E-02	1.44E-01	ENSG00000250420
NA	0.0515	5.0720E-02		ENSG00000254705
NA	0.2428	5.0754E-02	1.44E-01	ENSG00000261502
NA	0.0344	5.0759E-02		ENSG00000286694
NA	0.1201	5.0789E-02	1.45E-01	ENSG00000249125
NA	0.2211	5.0882E-02	1.45E-01	ENSG00000288872
NA	0.2504	5.1074E-02	1.45E-01	ENSG00000288892
NA	0.1065	5.1639E-02	1.46E-01	ENSG00000270130
NA	0.0299	5.1719E-02		ENSG00000288029
NA	0.0338	5.1732E-02		ENSG00000256810
NA	0.1858	5.1744E-02	1.46E-01	ENSG00000279255
NA	0.2419	5.1771E-02	1.47E-01	ENSG00000236206
NA	0.2522	5.1822E-02	1.47E-01	ENSG00000284633
NA	0.2223	5.1893E-02	1.47E-01	ENSG00000249492
NA	0.1665	5.1936E-02	1.47E-01	ENSG00000236022
NA	0.1293	5.1953E-02	1.47E-01	ENSG00000235288
NA	0.2399	5.1998E-02	1.47E-01	ENSG00000273763
NA	0.1662	5.2104E-02	1.47E-01	ENSG00000289427
NA	0.0395	5.2337E-02		ENSG00000278897
NA	0.0940	5.2464E-02		ENSG00000254855
NA	0.2243	5.2596E-02	1.48E-01	ENSG00000279227
NA	0.0242	5.2792E-02		ENSG00000280002
NA	0.2338	5.2798E-02	1.49E-01	ENSG00000213600
NA	0.2438	5.2826E-02	1.49E-01	ENSG00000258216
NA	0.2203	5.2856E-02	1.49E-01	ENSG00000259488
NA	0.0284	5.3004E-02		ENSG00000171101
NA	0.0188	5.3279E-02	1.50E-01	ENSG00000280367
NA	0.2248	5.3424E-02	1.50E-01	ENSG00000280136
NA	0.2178	5.3439E-02	1.50E-01	ENSG00000272148
NA	0.1937	5.3457E-02	1.50E-01	ENSG00000277437
NA	0.0296	5.3987E-02		ENSG00000230500
NA	0.2247	5.3991E-02	1.51E-01	ENSG00000272172
NA	0.2259	5.4504E-02	1.52E-01	ENSG00000282740
NA	0.1874	5.4563E-02	1.52E-01	ENSG00000251459
NA	0.2481	5.4755E-02	1.53E-01	ENSG00000232756

NA	0.1891	5.4765E-02	1.53E-01	ENSG00000230724
NA	0.0734	5.4847E-02		ENSG00000259133
NA	0.1410	5.5138E-02	1.54E-01	ENSG00000205771
NA	0.2007	5.5596E-02	1.55E-01	ENSG00000286123
NA	0.2469	5.5665E-02	1.55E-01	ENSG00000266651
NA	0.0548	5.5839E-02		ENSG00000251031
NA	0.0223	5.5948E-02		ENSG00000260209
NA	0.0907	5.5986E-02		ENSG00000256681
NA	0.0801	5.6104E-02	1.56E-01	ENSG00000262663
NA	0.0804	5.6285E-02	1.56E-01	ENSG00000285747
NA	0.0246	5.6578E-02	1.57E-01	ENSG00000228547
NA	0.1508	5.6588E-02	1.57E-01	ENSG00000260744
NA	0.2159	5.6593E-02	1.57E-01	ENSG00000227848
NA	0.1721	5.6741E-02	1.57E-01	ENSG00000236627
NA	0.1752	5.6748E-02	1.57E-01	ENSG00000264273
NA	0.0476	5.6922E-02		ENSG00000255079
NA	0.2419	5.6933E-02	1.57E-01	ENSG00000225742
NA	0.1870	5.7056E-02	1.58E-01	ENSG00000230185
NA	0.0451	5.7328E-02		ENSG00000271732
NA	0.2241	5.7584E-02	1.59E-01	ENSG00000274038
NA	0.1252	5.7654E-02	1.59E-01	ENSG00000280000
NA	0.2449	5.7734E-02	1.59E-01	ENSG00000261172
NA	0.2445	5.8006E-02	1.59E-01	ENSG00000255008
NA	0.2249	5.8032E-02	1.59E-01	ENSG00000272068
NA	0.1775	5.8428E-02	1.60E-01	ENSG00000269559
NA	0.2364	5.8450E-02	1.60E-01	ENSG00000276698
NA	0.0378	5.8552E-02		ENSG00000229988
NA	0.2088	5.8564E-02	1.61E-01	ENSG00000234459
NA	0.0719	5.8582E-02		ENSG00000223475
NA	0.2359	5.8653E-02	1.61E-01	ENSG00000253333
NA	0.0935	5.8763E-02	1.61E-01	ENSG00000286883
NA	0.2437	5.8970E-02	1.61E-01	ENSG00000232702
NA	0.1558	5.9051E-02	1.61E-01	ENSG00000235944
NA	0.2139	5.9061E-02	1.61E-01	ENSG00000268798
NA	0.1325	5.9128E-02		ENSG00000287405
NA	0.0944	5.9129E-02	1.62E-01	ENSG00000289474
NA	0.0158	5.9152E-02		ENSG00000289839
NA	0.0957	5.9261E-02	1.62E-01	ENSG00000244009
NA	0.2352	5.9298E-02	1.62E-01	ENSG00000256139
NA	0.2365	5.9348E-02	1.62E-01	ENSG00000261349
NA	0.0066	5.9450E-02		ENSG00000269540
NA	0.0052	5.9496E-02		ENSG00000286749
NA	0.1991	5.9622E-02	1.63E-01	ENSG00000288868
NA	0.1755	5.9668E-02	1.63E-01	ENSG00000288823
NA	0.0751	5.9675E-02	1.63E-01	ENSG00000269535
NA	0.0242	5.9730E-02		ENSG00000254497
NA	0.0301	5.9805E-02		ENSG00000287139
NA	0.2394	5.9960E-02	1.63E-01	ENSG00000272986
NA	0.2034	6.0187E-02	1.64E-01	ENSG00000214243

NA	0.2382	6.0202E-02	1.64E-01	ENSG00000260924
NA	0.2357	6.0414E-02	1.64E-01	ENSG00000225507
NA	0.2378	6.1057E-02	1.66E-01	ENSG00000254477
NA	0.0415	6.1202E-02		ENSG00000253896
NA	0.0897	6.1335E-02		ENSG00000288871
NA	0.0505	6.1342E-02		ENSG00000267577
NA	0.2038	6.1457E-02	1.66E-01	ENSG00000287202
NA	0.1886	6.1498E-02	1.66E-01	ENSG00000285555
NA	0.0757	6.1554E-02		ENSG00000249514
NA	0.0290	6.1561E-02		ENSG00000258096
NA	0.0626	6.1581E-02	1.66E-01	ENSG00000255639
NA	0.0805	6.1582E-02		ENSG00000260328
NA	0.0237	6.1666E-02		ENSG00000288860
NA	0.2252	6.1962E-02	1.67E-01	ENSG00000217624
NA	0.0914	6.1997E-02	1.67E-01	ENSG00000215006
NA	0.2027	6.2124E-02	1.68E-01	ENSG00000214331
NA	0.1952	6.2432E-02	1.68E-01	ENSG00000287844
NA	0.0322	6.2483E-02		ENSG00000187999
NA	0.0611	6.2845E-02	1.69E-01	ENSG00000286099
NA	0.2250	6.2907E-02	1.69E-01	ENSG00000279791
NA	0.2333	6.2926E-02	1.69E-01	ENSG00000228430
NA	0.1469	6.2930E-02	1.69E-01	ENSG00000249790
NA	0.1993	6.2946E-02	1.69E-01	ENSG00000270820
NA	0.1172	6.2955E-02	1.69E-01	ENSG00000286653
NA	0.2127	6.3166E-02	1.70E-01	ENSG00000224647
NA	0.0385	6.3242E-02		ENSG00000279758
NA	0.1849	6.3248E-02	1.70E-01	ENSG00000284610
NA	0.2233	6.3305E-02	1.70E-01	ENSG00000257557
NA	0.2237	6.3477E-02	1.70E-01	ENSG00000219433
NA	0.0478	6.3507E-02		ENSG00000238164
NA	0.2238	6.3517E-02	1.70E-01	ENSG00000286909
NA	0.0356	6.3725E-02	1.70E-01	ENSG00000288571
NA	0.0296	6.3894E-02		ENSG00000282915
NA	0.2132	6.4088E-02	1.71E-01	ENSG00000285702
NA	0.0242	6.4137E-02	1.71E-01	ENSG00000257869
NA	0.0298	6.4456E-02	1.72E-01	ENSG00000279993
NA	0.1653	6.5594E-02	1.74E-01	ENSG00000227487
NA	0.1771	6.5601E-02	1.74E-01	ENSG00000286408
NA	0.0192	6.5693E-02		ENSG00000243256
NA	0.1902	6.5733E-02	1.74E-01	ENSG00000215548
NA	0.2307	6.5737E-02	1.74E-01	ENSG00000287425
NA	0.2052	6.5750E-02	1.74E-01	ENSG00000213777
NA	0.0108	6.5802E-02	1.74E-01	ENSG00000277831
NA	0.0839	6.5875E-02		ENSG00000279601
NA	0.0290	6.6087E-02		ENSG00000228829
NA	0.2162	6.6095E-02	1.75E-01	ENSG00000286751
NA	0.2167	6.6149E-02	1.75E-01	ENSG00000289943
NA	0.2152	6.6222E-02	1.75E-01	ENSG00000273338
NA	0.0687	6.6272E-02		ENSG00000257512

NA	0.1196	6.6388E-02	1.75E-01	ENSG00000287467
NA	0.1990	6.6457E-02	1.76E-01	ENSG00000272645
NA	0.2149	6.6547E-02	1.76E-01	ENSG00000287401
NA	0.2188	6.6625E-02	1.76E-01	ENSG00000235421
NA	0.1079	6.6633E-02		ENSG00000274383
NA	0.0423	6.6647E-02		ENSG00000213036
NA	0.0389	6.6783E-02		ENSG00000238085
NA	0.2173	6.6880E-02	1.76E-01	ENSG00000254609
NA	0.0614	6.6979E-02		ENSG00000287887
NA	0.2233	6.7049E-02	1.77E-01	ENSG00000251666
NA	0.2077	6.7562E-02	1.78E-01	ENSG00000285863
NA	0.1609	6.7674E-02	1.78E-01	ENSG00000238279
NA	0.2230	6.7703E-02	1.78E-01	ENSG00000214837
NA	0.1226	6.7836E-02	1.78E-01	ENSG00000228293
NA	0.1087	6.7980E-02		ENSG00000278797
NA	0.0414	6.8515E-02		ENSG00000201134
NA	0.0235	6.8548E-02		ENSG00000253690
NA	0.2259	6.8627E-02	1.80E-01	ENSG00000264015
NA	0.0402	6.8821E-02		ENSG00000278901
NA	0.2247	6.8850E-02	1.80E-01	ENSG00000268093
NA	0.0343	6.8955E-02		ENSG00000256658
NA	0.2012	6.9004E-02	1.80E-01	ENSG00000229931
NA	0.1136	6.9021E-02	1.80E-01	ENSG00000227755
NA	0.1660	6.9054E-02	1.81E-01	ENSG00000253515
NA	0.0502	6.9077E-02	1.81E-01	ENSG00000253168
NA	0.2225	6.9138E-02	1.81E-01	ENSG00000260455
NA	0.1883	6.9369E-02	1.81E-01	ENSG00000255966
NA	0.2282	6.9628E-02	1.82E-01	ENSG00000232063
NA	0.1030	6.9704E-02		ENSG00000289303
NA	0.0369	6.9849E-02		ENSG00000251535
NA	0.1471	6.9977E-02	1.82E-01	ENSG00000238290
NA	0.0835	7.0021E-02	1.82E-01	ENSG00000279361
NA	0.0224	7.0021E-02		ENSG00000262343
NA	0.1050	7.0193E-02	1.83E-01	ENSG00000248243
NA	0.2254	7.0283E-02	1.83E-01	ENSG00000230992
NA	0.1689	7.0324E-02	1.83E-01	ENSG00000253944
NA	0.0373	7.0423E-02		ENSG00000249557
NA	0.1743	7.0538E-02	1.83E-01	ENSG00000289393
NA	0.0230	7.0862E-02		ENSG00000218749
NA	0.2006	7.1127E-02	1.84E-01	ENSG00000260498
NA	0.1692	7.1397E-02	1.85E-01	ENSG00000261669
NA	0.1690	7.1553E-02	1.85E-01	ENSG00000289135
NA	0.0205	7.1601E-02		ENSG00000261522
NA	0.0229	7.1657E-02		ENSG00000279895
NA	0.0229	7.1657E-02		ENSG00000279728
NA	0.2025	7.1920E-02	1.86E-01	ENSG00000262583
NA	0.1889	7.2039E-02	1.86E-01	ENSG00000279616
NA	0.0312	7.2081E-02		ENSG00000273335
NA	0.2191	7.2163E-02	1.86E-01	ENSG00000276740

NA	0.2103	7.2386E-02	1.87E-01	ENSG00000226281
NA	0.1823	7.2644E-02	1.87E-01	ENSG00000271789
NA	0.1292	7.2666E-02	1.87E-01	ENSG00000239912
NA	0.2082	7.2806E-02	1.88E-01	ENSG00000277767
NA	0.2110	7.3049E-02	1.88E-01	ENSG00000279706
NA	0.0363	7.3170E-02		ENSG00000279721
NA	0.1962	7.3185E-02	1.88E-01	ENSG00000237719
NA	0.2135	7.3216E-02	1.88E-01	ENSG00000272275
NA	0.2212	7.3298E-02	1.88E-01	ENSG00000277011
NA	0.2157	7.3304E-02	1.88E-01	ENSG00000238039
NA	0.0660	7.3440E-02		ENSG00000286921
NA	0.2163	7.3651E-02	1.89E-01	ENSG00000234911
NA	0.1105	7.3887E-02	1.89E-01	ENSG00000258904
NA	0.2161	7.3903E-02	1.89E-01	ENSG00000273001
NA	0.2124	7.3931E-02	1.90E-01	ENSG00000261529
NA	0.0079	7.4104E-02		ENSG00000214081
NA	0.0726	7.4288E-02		ENSG00000271283
NA	0.0374	7.4460E-02		ENSG00000224728
NA	0.2110	7.4689E-02	1.91E-01	ENSG00000214776
NA	0.0274	7.4728E-02	1.91E-01	ENSG00000286693
NA	0.0416	7.4748E-02		ENSG00000258077
NA	0.1821	7.4899E-02	1.91E-01	ENSG00000267474
NA	0.1999	7.5290E-02	1.92E-01	ENSG00000111788
NA	0.0290	7.5374E-02		ENSG00000285591
NA	0.0564	7.5382E-02	1.92E-01	ENSG00000285416
NA	0.0292	7.5436E-02		ENSG00000261582
NA	0.1920	7.5480E-02	1.92E-01	ENSG00000272702
NA	0.0636	7.5650E-02		ENSG00000285834
NA	0.2013	7.5884E-02	1.93E-01	ENSG00000255310
NA	0.0742	7.5941E-02		ENSG00000228417
NA	0.2016	7.5962E-02	1.93E-01	ENSG00000238009
NA	0.0036	7.6027E-02		ENSG00000261049
NA	0.1590	7.6288E-02	1.94E-01	ENSG00000250535
NA	0.1069	7.6294E-02		ENSG00000290165
NA	0.0389	7.6360E-02		ENSG00000288579
NA	0.0688	7.6471E-02		ENSG00000285519
NA	0.1972	7.6500E-02	1.94E-01	ENSG00000268573
NA	0.0505	7.6544E-02		ENSG00000223908
NA	0.2115	7.6862E-02	1.95E-01	ENSG00000214826
NA	0.0243	7.7246E-02		ENSG00000289630
NA	0.1131	7.7271E-02	1.96E-01	ENSG00000286302
NA	0.0358	7.7287E-02		ENSG00000285586
NA	0.1509	7.7389E-02	1.96E-01	ENSG00000266904
NA	0.2091	7.7746E-02	1.97E-01	ENSG00000288827
NA	0.1764	7.7945E-02	1.97E-01	ENSG00000289875
NA	0.0794	7.8059E-02	1.97E-01	ENSG00000205056
NA	0.1337	7.8192E-02	1.97E-01	ENSG00000260213
NA	0.0410	7.8349E-02		ENSG00000277668
NA	0.0240	7.8647E-02		ENSG00000287391

NA	0.1795	7.8690E-02	1.98E-01	ENSG00000285796
NA	0.0949	7.8963E-02		ENSG00000249894
NA	0.1740	7.9295E-02	1.99E-01	ENSG00000278133
NA	0.1808	7.9383E-02	2.00E-01	ENSG00000231589
NA	0.2057	7.9539E-02	2.00E-01	ENSG00000286000
NA	0.1653	8.0087E-02	2.01E-01	ENSG00000286787
NA	0.1543	8.0091E-02	2.01E-01	ENSG00000288736
NA	0.1437	8.0158E-02	2.01E-01	ENSG00000260645
NA	0.0563	8.0224E-02		ENSG00000289945
NA	0.1905	8.0346E-02	2.01E-01	ENSG00000268947
NA	0.0892	8.0365E-02		ENSG00000279144
NA	0.0292	8.0665E-02		ENSG00000263300
NA	0.1413	8.0741E-02	2.02E-01	ENSG00000265547
NA	0.0393	8.0975E-02	2.03E-01	ENSG00000226243
NA	0.2143	8.1052E-02	2.03E-01	ENSG00000287091
NA	0.2038	8.1185E-02	2.03E-01	ENSG00000214810
NA	0.2120	8.1332E-02	2.03E-01	ENSG00000264914
NA	0.0374	8.1379E-02	2.03E-01	ENSG00000227172
NA	0.2087	8.1427E-02	2.03E-01	ENSG00000276538
NA	0.1738	8.1612E-02	2.04E-01	ENSG00000290085
NA	0.2098	8.1874E-02	2.04E-01	ENSG00000269981
NA	0.0183	8.2120E-02		ENSG00000218475
NA	0.0500	8.2278E-02		ENSG00000242791
NA	0.1683	8.2377E-02	2.05E-01	ENSG00000253408
NA	0.2103	8.2396E-02	2.05E-01	ENSG00000235636
NA	0.1663	8.2408E-02	2.05E-01	ENSG00000273027
NA	0.0513	8.2708E-02	2.05E-01	ENSG00000201428
NA	0.0693	8.2756E-02		ENSG00000287727
NA	0.2075	8.2841E-02	2.06E-01	ENSG00000229782
NA	0.2095	8.2847E-02	2.06E-01	ENSG00000263307
NA	0.1981	8.2951E-02	2.06E-01	ENSG00000251408
NA	0.1925	8.3084E-02	2.06E-01	ENSG00000227888
NA	0.0570	8.3409E-02		ENSG00000254302
NA	0.2030	8.3453E-02	2.07E-01	ENSG00000259985
NA	0.1573	8.3681E-02	2.07E-01	ENSG00000234300
NA	0.1264	8.3762E-02	2.07E-01	ENSG00000230479
NA	0.0576	8.4010E-02	2.08E-01	ENSG00000227432
NA	0.0296	8.4260E-02		ENSG00000286216
NA	0.0483	8.4416E-02		ENSG00000259846
NA	0.0278	8.4768E-02		ENSG00000217314
NA	0.1725	8.4814E-02	2.09E-01	ENSG00000288956
NA	0.0187	8.4859E-02		ENSG00000279439
NA	0.1150	8.4929E-02	2.09E-01	ENSG00000258919
NA	0.1555	8.5422E-02	2.10E-01	ENSG00000287671
NA	0.0889	8.5750E-02		ENSG00000259306
NA	0.2005	8.5894E-02	2.11E-01	ENSG00000225938
NA	0.1789	8.5923E-02	2.11E-01	ENSG00000269044
NA	0.0618	8.6163E-02		ENSG00000254298
NA	0.1946	8.6313E-02	2.12E-01	ENSG00000234019

NA	0.1564	8.6335E-02	2.12E-01	ENSG00000286504
NA	0.0004	8.6375E-02		ENSG00000286897
NA	0.0436	8.6556E-02		ENSG00000232451
NA	0.2053	8.6581E-02	2.12E-01	ENSG00000214832
NA	0.1260	8.6659E-02	2.13E-01	ENSG00000255992
NA	0.1077	8.6909E-02		ENSG00000282840
NA	0.0282	8.6950E-02		ENSG00000275945
NA	0.0279	8.7085E-02		ENSG00000284825
NA	0.1470	8.7213E-02	2.14E-01	ENSG00000260404
NA	0.1471	8.7549E-02	2.14E-01	ENSG00000288015
NA	0.1647	8.7590E-02	2.14E-01	ENSG00000276744
NA	0.1292	8.7655E-02	2.14E-01	ENSG00000272106
NA	0.1029	8.7906E-02	2.15E-01	ENSG00000274602
NA	0.0494	8.7921E-02	2.15E-01	ENSG00000250600
NA	0.0938	8.7929E-02	2.15E-01	ENSG00000123009
NA	0.0711	8.8223E-02		ENSG00000233821
NA	0.0252	8.8268E-02		ENSG00000276396
NA	0.0487	8.8854E-02		ENSG00000228289
NA	0.1677	8.8855E-02	2.16E-01	ENSG00000214919
NA	0.1853	8.9092E-02	2.17E-01	ENSG00000254536
NA	0.2029	8.9143E-02	2.17E-01	ENSG00000260360
NA	0.0275	8.9510E-02		ENSG00000285894
NA	0.1720	8.9600E-02	2.18E-01	ENSG00000265136
NA	0.2004	8.9637E-02	2.18E-01	ENSG00000287969
NA	0.1485	8.9653E-02	2.18E-01	ENSG00000224363
NA	0.1999	8.9757E-02	2.18E-01	ENSG00000287221
NA	0.0310	8.9934E-02		ENSG00000253796
NA	0.0280	8.9945E-02		ENSG00000286419
NA	0.0192	9.0239E-02		ENSG00000279440
NA	0.0288	9.0368E-02		ENSG00000272941
NA	0.0341	9.0457E-02		ENSG00000259701
NA	0.0573	9.0477E-02	2.19E-01	ENSG00000215304
NA	0.1231	9.0507E-02	2.19E-01	ENSG00000263990
NA	0.1199	9.0647E-02	2.20E-01	ENSG00000239917
NA	0.2016	9.0882E-02	2.20E-01	ENSG00000272006
NA	0.0716	9.0925E-02	2.20E-01	ENSG00000274455
NA	0.1913	9.0939E-02	2.20E-01	ENSG00000289543
NA	0.0211	9.1020E-02		ENSG00000201573
NA	0.1735	9.1629E-02	2.21E-01	ENSG00000289868
NA	0.0384	9.1653E-02		ENSG00000223536
NA	0.1293	9.1659E-02	2.21E-01	ENSG00000286372
NA	0.1432	9.1845E-02	2.22E-01	ENSG00000234585
NA	0.2007	9.1911E-02	2.22E-01	ENSG00000289559
NA	0.1619	9.1941E-02	2.22E-01	ENSG00000260265
NA	0.1147	9.2061E-02	2.22E-01	ENSG00000227073
NA	0.0226	9.2153E-02		ENSG00000288098
NA	0.0240	9.2386E-02		ENSG00000262777
NA	0.0149	9.3057E-02		ENSG00000213014
NA	0.0385	9.3125E-02		ENSG00000275898

NA	0.0274	9.3291E-02		ENSG00000286355
NA	0.1595	9.3377E-02	2.24E-01	ENSG00000231992
NA	0.0244	9.3388E-02		ENSG00000267765
NA	0.0477	9.3429E-02	2.24E-01	ENSG00000261101
NA	0.0339	9.3474E-02		ENSG00000230226
NA	0.0523	9.3642E-02		ENSG00000272379
NA	0.1414	9.3752E-02	2.25E-01	ENSG00000289874
NA	0.1933	9.3824E-02	2.25E-01	ENSG00000272157
NA	0.0723	9.3913E-02		ENSG00000262362
NA	0.1149	9.4074E-02	2.25E-01	ENSG00000282304
NA	0.0732	9.4376E-02	2.26E-01	ENSG00000235491
NA	0.0657	9.4454E-02	2.26E-01	ENSG00000261218
NA	0.0228	9.4801E-02		ENSG00000226798
NA	0.0483	9.5013E-02		ENSG00000289567
NA	0.1956	9.5137E-02	2.27E-01	ENSG00000236017
NA	0.0217	9.5137E-02		ENSG00000271947
NA	0.0138	9.5186E-02		ENSG00000234427
NA	0.1416	9.5208E-02	2.27E-01	ENSG00000261592
NA	0.0216	9.5338E-02	2.27E-01	ENSG00000276071
NA	0.1898	9.5388E-02	2.27E-01	ENSG00000288858
NA	0.1307	9.5507E-02	2.28E-01	ENSG00000267327
NA	0.0337	9.5683E-02		ENSG00000234107
NA	0.1906	9.5818E-02	2.28E-01	ENSG00000289506
NA	0.1171	9.5898E-02	2.28E-01	ENSG00000232034
NA	0.0229	9.6110E-02		ENSG00000177338
NA	0.1472	9.6489E-02	2.29E-01	ENSG00000269296
NA	0.1780	9.6578E-02	2.29E-01	ENSG00000222043
NA	0.1926	9.6638E-02	2.29E-01	ENSG00000248479
NA	0.1852	9.6773E-02	2.29E-01	ENSG00000289433
NA	0.0273	9.6810E-02		ENSG00000223602
NA	0.1877	9.6908E-02	2.30E-01	ENSG00000241743
NA	0.1883	9.6950E-02	2.30E-01	ENSG00000256262
NA	0.1541	9.7048E-02	2.30E-01	ENSG00000267481
NA	0.0487	9.7174E-02		ENSG00000203804
NA	0.1506	9.7336E-02	2.30E-01	ENSG00000241269
NA	0.0317	9.7468E-02		ENSG00000286854
NA	0.1732	9.7640E-02	2.31E-01	ENSG00000249896
NA	0.1952	9.7825E-02	2.31E-01	ENSG00000287356
NA	0.0574	9.7937E-02		ENSG00000236318
NA	0.0437	9.8078E-02		ENSG00000287142
NA	0.1686	9.8195E-02	2.32E-01	ENSG00000226415
NA	0.1461	9.8218E-02	2.32E-01	ENSG00000262823
NA	0.1088	9.8558E-02	2.32E-01	ENSG00000280401
NA	0.0362	9.8751E-02		ENSG00000285954
NA	0.1007	9.8816E-02		ENSG00000266973
NA	0.1946	9.8825E-02	2.33E-01	ENSG00000289147
NA	0.1047	9.8914E-02	2.33E-01	ENSG00000229999
NA	0.1933	9.9023E-02	2.33E-01	ENSG00000254851
NA	0.0774	9.9348E-02	2.34E-01	ENSG00000225080

NA	0.0933	9.9721E-02	2.34E-01	ENSG00000210112
NA	0.0277	9.9972E-02		ENSG00000256721
NA	0.1134	1.0001E-01	2.35E-01	ENSG00000237921
NA	0.0548	1.0005E-01		ENSG00000287426
NA	0.1768	1.0018E-01	2.35E-01	ENSG00000189366
NA	0.0843	1.0047E-01	2.35E-01	ENSG00000289505
NA	0.0393	1.0070E-01		ENSG00000257677
NA	0.0710	1.0080E-01	2.36E-01	ENSG00000251487
NA	0.0301	1.0115E-01		ENSG00000237954
NA	0.1879	1.0118E-01	2.37E-01	ENSG00000262877
NA	0.0349	1.0152E-01		ENSG00000250381
NA	0.1667	1.0161E-01	2.37E-01	ENSG00000286093
NA	0.1904	1.0168E-01	2.38E-01	ENSG00000285907
NA	0.0139	1.0200E-01		ENSG00000267295
NA	0.0477	1.0220E-01	2.38E-01	ENSG00000223523
NA	0.1568	1.0286E-01	2.39E-01	ENSG00000289436
NA	0.0336	1.0311E-01		ENSG00000223665
NA	0.1657	1.0325E-01	2.40E-01	ENSG00000261253
NA	0.1326	1.0331E-01	2.40E-01	ENSG00000283897
NA	0.1689	1.0339E-01	2.40E-01	ENSG00000288559
NA	0.1103	1.0340E-01	2.40E-01	ENSG00000267321
NA	0.0232	1.0342E-01		ENSG00000234997
NA	0.0735	1.0345E-01		ENSG00000230710
NA	0.0214	1.0348E-01		ENSG00000287206
NA	0.0803	1.0356E-01	2.41E-01	ENSG00000251185
NA	0.1225	1.0359E-01	2.41E-01	ENSG00000275956
NA	0.0628	1.0425E-01		ENSG00000260664
NA	0.1288	1.0433E-01	2.42E-01	ENSG00000174403
NA	0.0171	1.0480E-01		ENSG00000242156
NA	0.1678	1.0484E-01	2.43E-01	ENSG00000289121
NA	0.1562	1.0502E-01	2.43E-01	ENSG00000283380
NA	0.1009	1.0507E-01		ENSG00000228283
NA	0.1890	1.0512E-01	2.43E-01	ENSG00000272977
NA	0.1529	1.0527E-01	2.44E-01	ENSG00000259562
NA	0.0149	1.0538E-01		ENSG00000280057
NA	0.0101	1.0557E-01		ENSG00000286990
NA	0.0402	1.0579E-01		ENSG00000260008
NA	0.1232	1.0581E-01	2.44E-01	ENSG00000270095
NA	0.1048	1.0606E-01		ENSG00000285737
NA	0.1855	1.0634E-01	2.45E-01	ENSG00000286196
NA	0.0728	1.0647E-01	2.45E-01	ENSG00000290021
NA	0.1857	1.0678E-01	2.46E-01	ENSG00000266313
NA	0.1682	1.0688E-01	2.46E-01	ENSG00000233058
NA	0.0287	1.0701E-01	2.46E-01	ENSG00000251188
NA	0.0115	1.0728E-01	2.47E-01	ENSG00000260369
NA	0.1357	1.0746E-01	2.47E-01	ENSG00000267592
NA	0.0420	1.0759E-01		ENSG00000259124
NA	0.1400	1.0762E-01	2.47E-01	ENSG00000282876
NA	0.0795	1.0774E-01		ENSG00000274775

NA	0.0376	1.0775E-01		ENSG00000289851
NA	0.1633	1.0791E-01	2.48E-01	ENSG00000272843
NA	0.1635	1.0815E-01	2.48E-01	ENSG00000288865
NA	0.1851	1.0819E-01	2.48E-01	ENSG00000246898
NA	0.0149	1.0833E-01		ENSG00000268520
NA	0.0707	1.0862E-01		ENSG00000256006
NA	0.1409	1.0877E-01	2.49E-01	ENSG00000282386
NA	0.0848	1.0908E-01	2.50E-01	ENSG00000279711
NA	0.1854	1.0909E-01	2.50E-01	ENSG00000276116
NA	0.1682	1.0920E-01	2.50E-01	ENSG00000287808
NA	0.1858	1.0923E-01	2.50E-01	ENSG00000289631
NA	0.1269	1.0971E-01	2.51E-01	ENSG00000255052
NA	0.1773	1.0984E-01	2.51E-01	ENSG00000286427
NA	0.1482	1.1018E-01	2.51E-01	ENSG00000220804
NA	0.1795	1.1038E-01	2.52E-01	ENSG00000235888
NA	0.1543	1.1045E-01	2.52E-01	ENSG00000237082
NA	0.1809	1.1105E-01	2.53E-01	ENSG00000289955
NA	0.0981	1.1154E-01		ENSG00000251454
NA	0.0294	1.1162E-01		ENSG00000237321
NA	0.1705	1.1169E-01	2.54E-01	ENSG00000288916
NA	0.1346	1.1193E-01	2.54E-01	ENSG00000257261
NA	0.1843	1.1207E-01	2.55E-01	ENSG00000246334
NA	0.0244	1.1207E-01		ENSG00000272719
NA	0.0744	1.1244E-01	2.55E-01	ENSG00000214313
NA	0.1795	1.1271E-01	2.56E-01	ENSG00000232044
NA	0.0603	1.1281E-01	2.56E-01	ENSG00000270497
NA	0.0929	1.1296E-01	2.56E-01	ENSG00000282393
NA	0.1120	1.1323E-01	2.57E-01	ENSG00000258847
NA	0.0315	1.1339E-01		ENSG00000284651
NA	0.1551	1.1354E-01	2.57E-01	ENSG00000233013
NA	0.0250	1.1375E-01		ENSG00000267646
NA	0.0355	1.1384E-01		ENSG00000253385
NA	0.1657	1.1395E-01	2.58E-01	ENSG00000249413
NA	0.1652	1.1398E-01	2.58E-01	ENSG00000290115
NA	0.1352	1.1411E-01	2.58E-01	ENSG00000260430
NA	0.0899	1.1420E-01		ENSG00000228222
NA	0.0786	1.1422E-01	2.58E-01	ENSG00000234756
NA	0.1620	1.1431E-01	2.58E-01	ENSG00000273176
NA	0.1666	1.1441E-01	2.58E-01	ENSG00000286577
NA	0.1801	1.1476E-01	2.59E-01	ENSG00000243485
NA	0.1803	1.1488E-01	2.59E-01	ENSG00000289555
NA	0.1510	1.1505E-01	2.59E-01	ENSG00000245904
NA	0.1795	1.1507E-01	2.59E-01	ENSG00000240591
NA	0.1815	1.1517E-01	2.59E-01	ENSG00000235545
NA	0.0093	1.1538E-01		ENSG00000286717
NA	0.0773	1.1544E-01		ENSG00000289278
NA	0.1794	1.1594E-01	2.61E-01	ENSG00000223813
NA	0.1635	1.1609E-01	2.61E-01	ENSG00000274173
NA	0.0160	1.1616E-01		ENSG00000286384

NA	0.0191	1.1631E-01		ENSG00000243230
NA	0.0790	1.1642E-01		ENSG00000253824
NA	0.1737	1.1667E-01	2.62E-01	ENSG00000226472
NA	0.1385	1.1692E-01	2.62E-01	ENSG00000234072
NA	0.1797	1.1696E-01	2.62E-01	ENSG00000235450
NA	0.0231	1.1716E-01		ENSG00000237979
NA	0.0456	1.1718E-01		ENSG00000228444
NA	0.0799	1.1725E-01	2.63E-01	ENSG00000067601
NA	0.0483	1.1761E-01		ENSG00000225637
NA	0.1771	1.1788E-01	2.64E-01	ENSG00000275294
NA	0.1749	1.1789E-01	2.64E-01	ENSG00000285856
NA	0.1362	1.1814E-01	2.64E-01	ENSG00000280332
NA	0.1353	1.1822E-01	2.64E-01	ENSG00000225098
NA	0.1747	1.1826E-01	2.64E-01	ENSG00000241489
NA	0.1350	1.1881E-01	2.65E-01	ENSG00000205578
NA	0.1402	1.1883E-01	2.65E-01	ENSG00000267729
NA	0.0199	1.1893E-01		ENSG00000224371
NA	0.0189	1.1919E-01		ENSG00000268100
NA	0.1688	1.1928E-01	2.66E-01	ENSG00000228107
NA	0.1245	1.1928E-01	2.66E-01	ENSG00000228522
NA	0.0156	1.1949E-01	2.66E-01	ENSG00000286004
NA	0.1350	1.1958E-01	2.66E-01	ENSG00000289594
NA	0.0550	1.1970E-01	2.67E-01	ENSG00000272192
NA	0.1740	1.2025E-01	2.67E-01	ENSG00000280660
NA	0.1091	1.2044E-01	2.68E-01	ENSG00000274723
NA	0.0644	1.2047E-01	2.68E-01	ENSG00000249244
NA	0.1610	1.2060E-01	2.68E-01	ENSG00000269516
NA	0.1268	1.2062E-01	2.68E-01	ENSG00000255141
NA	0.1238	1.2064E-01	2.68E-01	ENSG00000250410
NA	0.1782	1.2070E-01	2.68E-01	ENSG00000234134
NA	0.1621	1.2115E-01	2.69E-01	ENSG00000231341
NA	0.0499	1.2121E-01	2.69E-01	ENSG00000269086
NA	0.0328	1.2123E-01		ENSG00000223812
NA	0.0677	1.2134E-01	2.69E-01	ENSG00000231073
NA	0.0220	1.2142E-01		ENSG00000260370
NA	0.1689	1.2156E-01	2.69E-01	ENSG00000272735
NA	0.1471	1.2160E-01	2.69E-01	ENSG00000250986
NA	0.1740	1.2180E-01	2.70E-01	ENSG00000287743
NA	0.0624	1.2192E-01		ENSG00000274238
NA	0.0609	1.2201E-01		ENSG00000267143
NA	0.1495	1.2204E-01	2.70E-01	ENSG00000259238
NA	0.1756	1.2211E-01	2.70E-01	ENSG00000258682
NA	0.0257	1.2220E-01		ENSG00000254730
NA	0.0272	1.2222E-01		ENSG00000266312
NA	0.1524	1.2235E-01	2.71E-01	ENSG00000270964
NA	0.1045	1.2239E-01		ENSG00000178636
NA	0.1533	1.2252E-01	2.71E-01	ENSG00000265752
NA	0.0373	1.2254E-01		ENSG00000237280
NA	0.1045	1.2274E-01	2.71E-01	ENSG00000228862

NA	0.0349	1.2276E-01		ENSG00000265975
NA	0.1372	1.2282E-01	2.71E-01	ENSG00000279500
NA	0.0569	1.2300E-01		ENSG00000270813
NA	0.1112	1.2339E-01	2.72E-01	ENSG00000233766
NA	0.1369	1.2375E-01	2.73E-01	ENSG00000229855
NA	0.0288	1.2383E-01		ENSG00000220256
NA	0.0446	1.2404E-01	2.73E-01	ENSG00000253811
NA	0.0951	1.2413E-01		ENSG00000275719
NA	0.0251	1.2437E-01		ENSG00000287668
NA	0.0547	1.2440E-01		ENSG00000263567
NA	0.0269	1.2441E-01		ENSG00000260441
NA	0.0265	1.2442E-01		ENSG00000260444
NA	0.1647	1.2453E-01	2.74E-01	ENSG00000272077
NA	0.1125	1.2459E-01	2.74E-01	ENSG00000289637
NA	0.1343	1.2464E-01	2.74E-01	ENSG00000267707
NA	0.0239	1.2487E-01		ENSG00000272958
NA	0.1443	1.2495E-01	2.74E-01	ENSG00000269069
NA	0.1713	1.2499E-01	2.74E-01	ENSG00000227543
NA	0.1432	1.2499E-01	2.74E-01	ENSG00000290057
NA	0.0943	1.2538E-01	2.75E-01	ENSG00000179028
NA	0.1178	1.2557E-01	2.75E-01	ENSG00000272168
NA	0.1708	1.2570E-01	2.75E-01	ENSG00000231793
NA	0.1869	1.2589E-01	2.76E-01	ENSG00000270876
NA	0.0536	1.2606E-01		ENSG00000254057
NA	0.1315	1.2613E-01	2.76E-01	ENSG00000269867
NA	0.0655	1.2617E-01		ENSG00000256972
NA	0.0220	1.2630E-01		ENSG00000262703
NA	0.0237	1.2630E-01		ENSG00000264655
NA	0.0613	1.2675E-01		ENSG00000236848
NA	0.0306	1.2685E-01		ENSG00000228328
NA	0.1728	1.2687E-01	2.77E-01	ENSG00000235934
NA	0.1531	1.2700E-01	2.78E-01	ENSG00000261462
NA	0.0407	1.2711E-01	2.78E-01	ENSG00000282807
NA	0.0204	1.2744E-01		ENSG00000258797
NA	0.1683	1.2744E-01	2.78E-01	ENSG00000280327
NA	0.1037	1.2745E-01	2.78E-01	ENSG00000285473
NA	0.1675	1.2756E-01	2.78E-01	ENSG00000233381
NA	0.0583	1.2760E-01		ENSG00000290043
NA	0.1631	1.2803E-01	2.79E-01	ENSG00000288982
NA	0.0752	1.2808E-01		ENSG00000206781
NA	0.1368	1.2821E-01	2.79E-01	ENSG00000223546
NA	0.1522	1.2822E-01	2.79E-01	ENSG00000227987
NA	0.1296	1.2824E-01	2.79E-01	ENSG00000236833
NA	0.0214	1.2846E-01		ENSG00000233876
NA	0.0166	1.2850E-01		ENSG00000288071
NA	0.1064	1.2853E-01	2.80E-01	ENSG00000265625
NA	0.0151	1.2872E-01		ENSG00000223783
NA	0.1400	1.2873E-01	2.80E-01	ENSG00000256843
NA	0.1611	1.2882E-01	2.80E-01	ENSG00000108448

NA	0.0865	1.2883E-01	2.80E-01	ENSG00000286418
NA	0.0304	1.2888E-01		ENSG00000248958
NA	0.0616	1.2889E-01		ENSG00000223914
NA	0.1308	1.2904E-01	2.80E-01	ENSG00000288770
NA	0.0479	1.2906E-01	2.80E-01	ENSG00000248103
NA	0.1106	1.2938E-01	2.81E-01	ENSG00000260549
NA	0.0480	1.2949E-01	2.81E-01	ENSG00000233349
NA	0.0613	1.2949E-01		ENSG00000279814
NA	0.1712	1.2960E-01	2.81E-01	ENSG00000254675
NA	0.1767	1.2974E-01	2.81E-01	ENSG00000228956
NA	0.1594	1.2980E-01	2.81E-01	ENSG00000257800
NA	0.0225	1.2981E-01		ENSG00000280189
NA	0.0493	1.2986E-01	2.81E-01	ENSG00000255489
NA	0.0257	1.3013E-01		ENSG00000279650
NA	0.1008	1.3017E-01	2.82E-01	ENSG00000280013
NA	0.1666	1.3030E-01	2.82E-01	ENSG00000270083
NA	0.1293	1.3039E-01	2.82E-01	ENSG00000229618
NA	0.0479	1.3050E-01		ENSG00000272908
NA	0.0594	1.3064E-01	2.82E-01	ENSG00000279066
NA	0.0098	1.3066E-01		ENSG00000233571
NA	0.0138	1.3068E-01		ENSG00000269148
NA	0.0135	1.3082E-01		ENSG00000254028
NA	0.1543	1.3126E-01	2.83E-01	ENSG00000289614
NA	0.1400	1.3152E-01	2.84E-01	ENSG00000235021
NA	0.1501	1.3158E-01	2.84E-01	ENSG00000264207
NA	0.0249	1.3167E-01	2.84E-01	ENSG00000284664
NA	0.1670	1.3196E-01	2.84E-01	ENSG00000289200
NA	0.1605	1.3212E-01	2.85E-01	ENSG00000254416
NA	0.1148	1.3234E-01	2.85E-01	ENSG00000239569
NA	0.0009	1.3254E-01	2.85E-01	ENSG00000226540
NA	0.1418	1.3254E-01	2.85E-01	ENSG00000179899
NA	0.0365	1.3264E-01		ENSG00000254347
NA	0.1623	1.3279E-01	2.86E-01	ENSG00000279412
NA	0.0217	1.3342E-01		ENSG00000250891
NA	0.0176	1.3345E-01	2.86E-01	ENSG00000270068
NA	0.1643	1.3372E-01	2.87E-01	ENSG00000232445
NA	0.1531	1.3379E-01	2.87E-01	ENSG00000275155
NA	0.1530	1.3382E-01	2.87E-01	ENSG00000262691
NA	0.1098	1.3399E-01	2.87E-01	ENSG00000271869
NA	0.0696	1.3417E-01	2.87E-01	ENSG00000254042
NA	0.0223	1.3444E-01		ENSG00000287394
NA	0.1681	1.3494E-01	2.89E-01	ENSG00000282051
NA	0.0925	1.3499E-01		ENSG00000259727
NA	0.0266	1.3500E-01		ENSG00000277440
NA	0.1665	1.3512E-01	2.89E-01	ENSG00000254208
NA	0.1066	1.3541E-01	2.89E-01	ENSG00000255372
NA	0.1518	1.3547E-01	2.89E-01	ENSG00000270300
NA	0.1694	1.3547E-01	2.89E-01	ENSG00000287262
NA	0.0215	1.3552E-01		ENSG00000259441

NA	0.1269	1.3554E-01	2.89E-01	ENSG00000258317
NA	0.1573	1.3561E-01	2.89E-01	ENSG00000269102
NA	0.0544	1.3562E-01		ENSG00000232347
NA	0.0254	1.3577E-01		ENSG00000274799
NA	0.1655	1.3597E-01	2.90E-01	ENSG00000288993
NA	0.1675	1.3614E-01	2.90E-01	ENSG00000267672
NA	0.0256	1.3668E-01		ENSG00000229368
NA	0.0251	1.3670E-01		ENSG00000273138
NA	0.0220	1.3682E-01		ENSG00000226767
NA	0.0399	1.3697E-01		ENSG00000258698
NA	0.0340	1.3698E-01		ENSG00000235020
NA	0.0574	1.3700E-01	2.91E-01	ENSG00000289177
NA	0.0245	1.3711E-01		ENSG00000267048
NA	0.0198	1.3716E-01		ENSG00000269275
NA	0.1633	1.3748E-01	2.92E-01	ENSG00000286747
NA	0.1474	1.3800E-01	2.93E-01	ENSG00000225057
NA	0.0286	1.3811E-01		ENSG00000226491
NA	0.1659	1.3811E-01	2.93E-01	ENSG00000259467
NA	0.0620	1.3832E-01		ENSG00000249513
NA	0.0289	1.3850E-01		ENSG00000267203
NA	0.1601	1.3872E-01	2.94E-01	ENSG00000234772
NA	0.1279	1.3878E-01	2.94E-01	ENSG00000264546
NA	0.1659	1.3879E-01	2.94E-01	ENSG00000261790
NA	0.1186	1.3880E-01	2.94E-01	ENSG00000226877
NA	0.1121	1.3936E-01	2.95E-01	ENSG00000281909
NA	0.1583	1.3937E-01	2.95E-01	ENSG00000254409
NA	0.0096	1.3942E-01		ENSG00000259528
NA	0.0304	1.3972E-01		ENSG00000285668
NA	0.1158	1.4014E-01	2.96E-01	ENSG00000243004
NA	0.1568	1.4028E-01	2.96E-01	ENSG00000280069
NA	0.1554	1.4030E-01	2.96E-01	ENSG00000229036
NA	0.1338	1.4030E-01	2.96E-01	ENSG00000275265
NA	0.0173	1.4077E-01		ENSG00000227215
NA	0.0332	1.4102E-01	2.98E-01	ENSG00000213352
NA	0.0235	1.4114E-01		ENSG00000236813
NA	0.1461	1.4143E-01	2.98E-01	ENSG00000218226
NA	0.0111	1.4180E-01		ENSG00000279903
NA	0.0309	1.4197E-01	2.99E-01	ENSG00000283689
NA	0.0196	1.4204E-01	2.99E-01	ENSG00000250734
NA	0.0220	1.4233E-01		ENSG00000248764
NA	0.1625	1.4248E-01	3.00E-01	ENSG00000230648
NA	0.0558	1.4259E-01		ENSG00000262768
NA	0.0287	1.4271E-01		ENSG00000272479
NA	0.1618	1.4293E-01	3.01E-01	ENSG00000279532
NA	0.0565	1.4312E-01		ENSG00000249373
NA	0.1576	1.4314E-01	3.01E-01	ENSG00000272944
NA	0.1286	1.4338E-01	3.01E-01	ENSG00000256757
NA	0.0802	1.4372E-01		ENSG00000229278
NA	0.1593	1.4387E-01	3.02E-01	ENSG00000223749

NA	0.0198	1.4389E-01		ENSG00000287182
NA	0.0632	1.4389E-01	3.02E-01	ENSG00000215347
NA	0.1581	1.4392E-01	3.02E-01	ENSG00000251301
NA	0.0237	1.4393E-01		ENSG00000269400
NA	0.0862	1.4402E-01	3.02E-01	ENSG00000250764
NA	0.0000	1.4409E-01		ENSG00000276505
NA	0.1261	1.4445E-01	3.03E-01	ENSG00000279433
NA	0.0764	1.4457E-01		ENSG00000225632
NA	0.0293	1.4485E-01		ENSG00000254182
NA	0.0330	1.4485E-01		ENSG00000245667
NA	0.1503	1.4486E-01	3.03E-01	ENSG00000287083
NA	0.0220	1.4492E-01		ENSG00000259314
NA	0.0716	1.4494E-01	3.03E-01	ENSG00000236409
NA	0.1355	1.4503E-01	3.03E-01	ENSG00000271848
NA	0.0290	1.4511E-01		ENSG00000280103
NA	0.1454	1.4514E-01	3.04E-01	ENSG00000288806
NA	0.1099	1.4517E-01	3.04E-01	ENSG00000289971
NA	0.1537	1.4533E-01	3.04E-01	ENSG00000279495
NA	0.1365	1.4545E-01	3.04E-01	ENSG00000271474
NA	0.1612	1.4564E-01	3.04E-01	ENSG00000213080
NA	0.1422	1.4566E-01	3.04E-01	ENSG00000274525
NA	0.0387	1.4578E-01		ENSG00000288878
NA	0.1590	1.4586E-01	3.05E-01	ENSG00000260588
NA	0.1035	1.4605E-01	3.05E-01	ENSG00000271009
NA	0.1191	1.4608E-01	3.05E-01	ENSG00000255355
NA	0.0280	1.4621E-01		ENSG00000205488
NA	0.1599	1.4623E-01	3.05E-01	ENSG00000257004
NA	0.0186	1.4623E-01		ENSG00000237031
NA	0.0405	1.4645E-01		ENSG00000258868
NA	0.0804	1.4665E-01		ENSG00000235965
NA	0.1594	1.4677E-01	3.06E-01	ENSG00000290011
NA	0.0341	1.4684E-01		ENSG00000267992
NA	0.1300	1.4687E-01	3.06E-01	ENSG00000223660
NA	0.1559	1.4698E-01	3.06E-01	ENSG00000278291
NA	0.0287	1.4721E-01		ENSG00000287529
NA	0.1325	1.4725E-01	3.07E-01	ENSG00000279476
NA	0.0042	1.4728E-01	3.07E-01	ENSG00000290041
NA	0.0061	1.4735E-01		ENSG00000200488
NA	0.0932	1.4742E-01		ENSG00000286167
NA	0.0293	1.4759E-01		ENSG00000277695
NA	0.0184	1.4832E-01	3.08E-01	ENSG00000289445
NA	0.1300	1.4835E-01	3.08E-01	ENSG00000289877
NA	0.0255	1.4846E-01	3.09E-01	ENSG00000278935
NA	0.1513	1.4853E-01	3.09E-01	ENSG00000256995
NA	0.1149	1.4853E-01	3.09E-01	ENSG00000228782
NA	0.1589	1.4891E-01	3.09E-01	ENSG00000276007
NA	0.0181	1.4903E-01		ENSG00000213376
NA	0.0767	1.4904E-01	3.09E-01	ENSG00000202260
NA	0.1586	1.4930E-01	3.10E-01	ENSG00000267886

NA	0.1414	1.4938E-01	3.10E-01	ENSG00000289013
NA	0.1252	1.4940E-01	3.10E-01	ENSG00000278700
NA	0.1614	1.4970E-01	3.10E-01	ENSG00000256008
NA	0.1405	1.4974E-01	3.10E-01	ENSG00000259594
NA	0.0319	1.4976E-01	3.10E-01	ENSG00000226468
NA	0.0275	1.4981E-01		ENSG00000231128
NA	0.0101	1.5020E-01		ENSG00000286097
NA	0.0799	1.5063E-01	3.11E-01	ENSG00000223697
NA	0.0480	1.5083E-01		ENSG00000275674
NA	0.1509	1.5088E-01	3.12E-01	ENSG00000289829
NA	0.0369	1.5112E-01	3.12E-01	ENSG00000273582
NA	0.0258	1.5126E-01		ENSG00000267677
NA	0.1219	1.5129E-01	3.12E-01	ENSG00000239883
NA	0.1508	1.5130E-01	3.12E-01	ENSG00000272462
NA	0.0861	1.5136E-01	3.12E-01	ENSG00000224452
NA	0.0247	1.5144E-01		ENSG00000286933
NA	0.0144	1.5157E-01		ENSG00000287458
NA	0.1524	1.5174E-01	3.13E-01	ENSG00000287784
NA	0.0272	1.5184E-01		ENSG00000271412
NA	0.0210	1.5207E-01		ENSG00000290096
NA	0.0515	1.5214E-01		ENSG00000280406
NA	0.1274	1.5218E-01	3.14E-01	ENSG00000234509
NA	0.0570	1.5238E-01		ENSG00000255503
NA	0.0242	1.5245E-01	3.14E-01	ENSG00000210184
NA	0.0174	1.5263E-01		ENSG00000253992
NA	0.0337	1.5284E-01		ENSG00000285987
NA	0.0404	1.5310E-01		ENSG00000249741
NA	0.0786	1.5311E-01		ENSG00000226779
NA	0.0215	1.5318E-01		ENSG00000241754
NA	0.1515	1.5372E-01	3.16E-01	ENSG00000272195
NA	0.1485	1.5387E-01	3.16E-01	ENSG00000250159
NA	0.1390	1.5397E-01	3.16E-01	ENSG00000198580
NA	0.0956	1.5414E-01	3.16E-01	ENSG00000279860
NA	0.0081	1.5474E-01		ENSG00000258897
NA	0.0189	1.5488E-01	3.17E-01	ENSG00000258820
NA	0.1544	1.5503E-01	3.18E-01	ENSG00000273084
NA	0.0669	1.5514E-01		ENSG00000225767
NA	0.1549	1.5524E-01	3.18E-01	ENSG00000288748
NA	0.1221	1.5532E-01	3.18E-01	ENSG00000257935
NA	0.0765	1.5560E-01	3.18E-01	ENSG00000288923
NA	0.0717	1.5589E-01		ENSG00000271259
NA	0.0268	1.5594E-01		ENSG00000274769
NA	0.0633	1.5594E-01		ENSG00000272627
NA	0.1533	1.5631E-01	3.19E-01	ENSG00000272619
NA	0.0237	1.5634E-01		ENSG00000287289
NA	0.0241	1.5690E-01		ENSG00000224099
NA	0.0713	1.5708E-01		ENSG00000275005
NA	0.1329	1.5710E-01	3.20E-01	ENSG00000253284
NA	0.0271	1.5712E-01		ENSG00000228509

NA	0.1475	1.5720E-01	3.21E-01	ENSG00000286681
NA	0.1254	1.5724E-01	3.21E-01	ENSG00000273058
NA	0.0222	1.5739E-01		ENSG00000288810
NA	0.0268	1.5797E-01		ENSG00000167798
NA	0.0616	1.5806E-01		ENSG00000270522
NA	0.0063	1.5842E-01		ENSG00000266803
NA	0.0057	1.5855E-01	3.23E-01	ENSG00000231340
NA	0.0220	1.5902E-01		ENSG00000237514
NA	0.0469	1.5907E-01		ENSG00000258881
NA	0.0396	1.5911E-01		ENSG00000259466
NA	0.0219	1.5925E-01		ENSG00000229550
NA	0.1480	1.5937E-01	3.24E-01	ENSG00000255229
NA	0.0266	1.5953E-01		ENSG00000259668
NA	0.0876	1.5981E-01	3.25E-01	ENSG00000258359
NA	0.0140	1.5987E-01		ENSG00000213735
NA	0.1235	1.6003E-01	3.25E-01	ENSG00000175841
NA	0.1168	1.6016E-01	3.25E-01	ENSG00000227236
NA	0.0207	1.6037E-01		ENSG00000251527
NA	0.1334	1.6039E-01	3.25E-01	ENSG00000270175
NA	0.1149	1.6046E-01	3.25E-01	ENSG00000237094
NA	0.0495	1.6055E-01	3.26E-01	ENSG00000248626
NA	0.0703	1.6064E-01	3.26E-01	ENSG00000214593
NA	0.0689	1.6078E-01		ENSG00000223727
NA	0.1534	1.6092E-01	3.26E-01	ENSG00000262333
NA	0.0249	1.6099E-01		ENSG00000289431
NA	0.1521	1.6113E-01	3.26E-01	ENSG00000229932
NA	0.1180	1.6114E-01	3.26E-01	ENSG00000279103
NA	0.0011	1.6129E-01	3.26E-01	ENSG00000286861
NA	0.0240	1.6136E-01		ENSG00000244378
NA	0.0687	1.6137E-01		ENSG00000223511
NA	0.1055	1.6150E-01	3.27E-01	ENSG00000259405
NA	0.0749	1.6152E-01	3.27E-01	ENSG00000285554
NA	0.0156	1.6156E-01	3.27E-01	ENSG00000258428
NA	0.0246	1.6167E-01		ENSG00000242683
NA	0.0667	1.6174E-01		ENSG00000281008
NA	0.0164	1.6208E-01		ENSG00000270598
NA	0.0772	1.6229E-01	3.28E-01	ENSG00000289192
NA	0.0391	1.6234E-01	3.28E-01	ENSG00000289470
NA	0.0718	1.6234E-01		ENSG00000263368
NA	0.1526	1.6259E-01	3.28E-01	ENSG00000274667
NA	0.0682	1.6267E-01	3.28E-01	ENSG00000254777
NA	0.0549	1.6275E-01		ENSG00000279852
NA	0.0286	1.6291E-01		ENSG00000248734
NA	0.0240	1.6305E-01		ENSG00000273243
NA	0.1492	1.6306E-01	3.29E-01	ENSG00000250397
NA	0.0348	1.6316E-01		ENSG00000141028
NA	0.0772	1.6322E-01		ENSG00000203648
NA	0.0322	1.6341E-01		ENSG00000260156
NA	0.1524	1.6345E-01	3.29E-01	ENSG00000222019

NA	0.1489	1.6352E-01	3.30E-01	ENSG00000254528
NA	0.1146	1.6359E-01	3.30E-01	ENSG00000289521
NA	0.0631	1.6360E-01		ENSG00000225213
NA	0.0194	1.6367E-01		ENSG00000235993
NA	0.0846	1.6368E-01		ENSG00000286982
NA	0.0748	1.6407E-01		ENSG00000217241
NA	0.0902	1.6415E-01	3.31E-01	ENSG00000256116
NA	0.1367	1.6425E-01	3.31E-01	ENSG00000289511
NA	0.0651	1.6428E-01	3.31E-01	ENSG00000279905
NA	0.0283	1.6436E-01		ENSG00000223513
NA	0.0950	1.6442E-01	3.31E-01	ENSG00000266311
NA	0.0829	1.6455E-01		ENSG00000229204
NA	0.0332	1.6458E-01		ENSG00000254066
NA	0.1484	1.6462E-01	3.31E-01	ENSG00000278917
NA	0.1000	1.6483E-01	3.31E-01	ENSG00000213204
NA	0.0917	1.6487E-01	3.31E-01	ENSG00000226647
NA	0.1494	1.6515E-01	3.32E-01	ENSG00000215146
NA	0.0945	1.6539E-01	3.32E-01	ENSG00000232807
NA	0.0601	1.6549E-01	3.32E-01	ENSG00000200397
NA	0.0322	1.6558E-01	3.32E-01	ENSG00000250488
NA	0.1463	1.6569E-01	3.32E-01	ENSG00000258302
NA	0.0202	1.6582E-01		ENSG00000286792
NA	0.1383	1.6591E-01	3.33E-01	ENSG00000288674
NA	0.1516	1.6610E-01	3.33E-01	ENSG00000246477
NA	0.0663	1.6637E-01	3.33E-01	ENSG00000288929
NA	0.0277	1.6639E-01		ENSG00000225208
NA	0.1521	1.6647E-01	3.33E-01	ENSG00000256417
NA	0.0964	1.6656E-01	3.34E-01	ENSG00000235038
NA	0.0119	1.6664E-01	3.34E-01	ENSG00000254044
NA	0.1235	1.6679E-01	3.34E-01	ENSG00000237493
NA	0.1434	1.6680E-01	3.34E-01	ENSG00000230583
NA	0.0784	1.6725E-01		ENSG00000267136
NA	0.0224	1.6727E-01		ENSG00000261561
NA	0.0666	1.6757E-01	3.35E-01	ENSG00000226532
NA	0.0306	1.6790E-01		ENSG00000235010
NA	0.0344	1.6793E-01		ENSG00000287594
NA	0.0306	1.6794E-01		ENSG00000289508
NA	0.1465	1.6800E-01	3.35E-01	ENSG00000234678
NA	0.1528	1.6811E-01	3.36E-01	ENSG00000230641
NA	0.1121	1.6814E-01	3.36E-01	ENSG00000244932
NA	0.1494	1.6814E-01	3.36E-01	ENSG00000278784
NA	0.0202	1.6820E-01		ENSG00000253263
NA	0.1044	1.6862E-01	3.36E-01	ENSG00000217261
NA	0.1092	1.6864E-01	3.36E-01	ENSG00000229893
NA	0.1207	1.6891E-01	3.37E-01	ENSG00000260398
NA	0.0640	1.6915E-01		ENSG00000275236
NA	0.1212	1.6921E-01	3.37E-01	ENSG00000214425
NA	0.0158	1.6921E-01		ENSG00000269387
NA	0.0175	1.6948E-01		ENSG00000233189

NA	0.0529	1.6956E-01	3.38E-01	ENSG00000233673
NA	0.0188	1.6981E-01		ENSG00000258290
NA	0.1305	1.7019E-01	3.38E-01	ENSG00000240036
NA	0.1183	1.7022E-01	3.38E-01	ENSG00000259726
NA	0.1455	1.7025E-01	3.38E-01	ENSG00000280273
NA	0.0851	1.7038E-01	3.39E-01	ENSG00000274001
NA	0.0778	1.7044E-01	3.39E-01	ENSG00000273724
NA	0.0708	1.7057E-01	3.39E-01	ENSG00000271917
NA	0.1459	1.7067E-01	3.39E-01	ENSG00000242337
NA	0.0192	1.7081E-01		ENSG00000286348
NA	0.0178	1.7087E-01		ENSG00000285762
NA	0.0173	1.7109E-01		ENSG00000248744
NA	0.0205	1.7124E-01		ENSG00000240793
NA	0.1062	1.7128E-01	3.40E-01	ENSG00000267443
NA	0.1034	1.7145E-01	3.40E-01	ENSG00000264304
NA	0.1464	1.7160E-01	3.40E-01	ENSG00000262089
NA	0.0512	1.7200E-01	3.41E-01	ENSG00000275025
NA	0.1265	1.7206E-01	3.41E-01	ENSG00000259642
NA	0.1475	1.7213E-01	3.41E-01	ENSG00000285728
NA	0.1395	1.7215E-01	3.41E-01	ENSG00000260329
NA	0.1469	1.7233E-01	3.41E-01	ENSG00000285679
NA	0.1462	1.7233E-01	3.41E-01	ENSG00000249019
NA	0.0380	1.7253E-01		ENSG00000234699
NA	0.0316	1.7254E-01		ENSG00000229925
NA	0.1484	1.7260E-01	3.42E-01	ENSG00000273783
NA	0.0265	1.7260E-01		ENSG00000215065
NA	0.1264	1.7287E-01	3.42E-01	ENSG00000289602
NA	0.0967	1.7293E-01	3.42E-01	ENSG00000272788
NA	0.0751	1.7309E-01	3.42E-01	ENSG00000237351
NA	0.0386	1.7311E-01	3.42E-01	ENSG00000222017
NA	0.0172	1.7311E-01		ENSG00000286429
NA	0.1448	1.7317E-01	3.42E-01	ENSG00000236255
NA	0.1001	1.7329E-01	3.43E-01	ENSG00000207342
NA	0.0142	1.7341E-01		ENSG00000223460
NA	0.0490	1.7351E-01		ENSG00000241884
NA	0.1293	1.7377E-01	3.43E-01	ENSG00000073905
NA	0.0541	1.7378E-01	3.43E-01	ENSG00000288976
NA	0.1409	1.7412E-01	3.44E-01	ENSG00000234026
NA	0.1358	1.7418E-01	3.44E-01	ENSG00000244560
NA	0.0440	1.7437E-01		ENSG00000280078
NA	0.1449	1.7447E-01	3.44E-01	ENSG00000259867
NA	0.1405	1.7469E-01	3.45E-01	ENSG00000260163
NA	0.0854	1.7471E-01	3.45E-01	ENSG00000248785
NA	0.1279	1.7473E-01	3.45E-01	ENSG00000225335
NA	0.0200	1.7478E-01		ENSG00000254557
NA	0.0109	1.7495E-01	3.45E-01	ENSG00000289148
NA	0.1486	1.7515E-01	3.45E-01	ENSG00000189014
NA	0.0283	1.7531E-01		ENSG00000248965
NA	0.0892	1.7535E-01	3.45E-01	ENSG00000287242

NA	0.1390	1.7537E-01	3.45E-01	ENSG00000289960
NA	0.0039	1.7537E-01		ENSG00000244723
NA	0.1453	1.7559E-01	3.46E-01	ENSG00000235493
NA	0.0924	1.7577E-01	3.46E-01	ENSG00000174384
NA	0.0286	1.7585E-01		ENSG00000288808
NA	0.0855	1.7591E-01	3.46E-01	ENSG00000253300
NA	0.0190	1.7602E-01		ENSG00000229704
NA	0.1246	1.7630E-01	3.47E-01	ENSG00000286379
NA	0.1204	1.7658E-01	3.47E-01	ENSG00000248268
NA	0.0302	1.7660E-01		ENSG00000289332
NA	0.0287	1.7668E-01		ENSG00000286318
NA	0.0035	1.7684E-01	3.47E-01	ENSG00000279042
NA	0.0050	1.7693E-01	3.47E-01	ENSG00000233817
NA	0.1066	1.7697E-01	3.47E-01	ENSG00000250536
NA	0.0315	1.7760E-01		ENSG00000257515
NA	0.0149	1.7807E-01		ENSG00000229015
NA	0.1398	1.7813E-01	3.49E-01	ENSG00000290094
NA	0.0608	1.7829E-01		ENSG00000279835
NA	0.0969	1.7838E-01	3.49E-01	ENSG00000189136
NA	0.0172	1.7840E-01		ENSG00000253523
NA	0.0276	1.7841E-01		ENSG00000281469
NA	0.1450	1.7853E-01	3.50E-01	ENSG00000205106
NA	0.0335	1.7873E-01		ENSG00000286506
NA	0.0619	1.7907E-01		ENSG00000278668
NA	0.0399	1.7916E-01		ENSG00000253235
NA	0.0203	1.7981E-01		ENSG00000240970
NA	0.0213	1.7984E-01		ENSG00000256499
NA	0.1223	1.7995E-01	3.51E-01	ENSG00000265008
NA	0.0508	1.8014E-01		ENSG00000253737
NA	0.0034	1.8020E-01	3.52E-01	ENSG00000287613
NA	0.1366	1.8056E-01	3.52E-01	ENSG00000116652
NA	0.0126	1.8059E-01		ENSG00000269480
NA	0.1288	1.8075E-01	3.53E-01	ENSG00000290090
NA	0.0232	1.8092E-01		ENSG00000279509
NA	0.0768	1.8098E-01	3.53E-01	ENSG00000235505
NA	0.1424	1.8104E-01	3.53E-01	ENSG00000289317
NA	0.1259	1.8121E-01	3.53E-01	ENSG00000184441
NA	0.1458	1.8148E-01	3.53E-01	ENSG00000289438
NA	0.0177	1.8198E-01		ENSG00000218676
NA	0.0211	1.8230E-01		ENSG00000278890
NA	0.0236	1.8252E-01		ENSG00000237373
NA	0.0214	1.8258E-01		ENSG00000258521
NA	0.1445	1.8280E-01	3.55E-01	ENSG00000230303
NA	0.1407	1.8290E-01	3.55E-01	ENSG00000283355
NA	0.0299	1.8304E-01		ENSG00000229786
NA	0.0207	1.8364E-01		ENSG00000275328
NA	0.1288	1.8373E-01	3.57E-01	ENSG00000232987
NA	0.0963	1.8386E-01	3.57E-01	ENSG00000280190
NA	0.0196	1.8390E-01		ENSG00000260173

NA	0.0238	1.8392E-01		ENSG00000267243
NA	0.1111	1.8423E-01	3.57E-01	ENSG00000188365
NA	0.1338	1.8435E-01	3.57E-01	ENSG00000283538
NA	0.0825	1.8438E-01		ENSG00000242307
NA	0.0119	1.8469E-01		ENSG00000247324
NA	0.1124	1.8483E-01	3.58E-01	ENSG00000271938
NA	0.1437	1.8507E-01	3.58E-01	ENSG00000289117
NA	0.1412	1.8550E-01	3.59E-01	ENSG00000285589
NA	0.1386	1.8552E-01	3.59E-01	ENSG00000286214
NA	0.0218	1.8588E-01		ENSG00000261238
NA	0.0564	1.8606E-01		ENSG00000286194
NA	0.0186	1.8615E-01	3.60E-01	ENSG00000289462
NA	0.0175	1.8624E-01		ENSG00000253911
NA	0.0199	1.8643E-01		ENSG00000286485
NA	0.0736	1.8680E-01	3.61E-01	ENSG00000287229
NA	0.1363	1.8707E-01	3.61E-01	ENSG00000287559
NA	0.0452	1.8711E-01		ENSG00000254691
NA	0.0328	1.8714E-01		ENSG00000285581
NA	0.0214	1.8763E-01	3.62E-01	ENSG00000287385
NA	0.0469	1.8771E-01	3.62E-01	ENSG00000244675
NA	0.0230	1.8772E-01		ENSG00000231292
NA	0.0302	1.8788E-01		ENSG00000236896
NA	0.1381	1.8864E-01	3.63E-01	ENSG00000278952
NA	0.0078	1.8868E-01		ENSG00000255342
NA	0.0135	1.8882E-01		ENSG00000254964
NA	0.1274	1.8883E-01	3.63E-01	ENSG00000243433
NA	0.1258	1.8912E-01	3.64E-01	ENSG00000288459
NA	0.0529	1.8913E-01		ENSG00000276533
NA	0.0403	1.8942E-01	3.64E-01	ENSG00000276282
NA	0.1121	1.8944E-01	3.64E-01	ENSG00000269481
NA	0.0230	1.8950E-01		ENSG00000230666
NA	0.1043	1.8961E-01	3.64E-01	ENSG00000280149
NA	0.1407	1.8972E-01	3.64E-01	ENSG00000289308
NA	0.1326	1.8996E-01	3.65E-01	ENSG00000286234
NA	0.1048	1.9011E-01	3.65E-01	ENSG00000267002
NA	0.1015	1.9017E-01	3.65E-01	ENSG00000254370
NA	0.0534	1.9041E-01		ENSG00000257509
NA	0.1286	1.9063E-01	3.66E-01	ENSG00000224038
NA	0.0529	1.9067E-01		ENSG00000267741
NA	0.0223	1.9068E-01		ENSG00000274711
NA	0.0256	1.9090E-01		ENSG00000232710
NA	0.1210	1.9096E-01	3.66E-01	ENSG00000267666
NA	0.1059	1.9104E-01	3.66E-01	ENSG00000207294
NA	0.1364	1.9105E-01	3.66E-01	ENSG00000279827
NA	0.1411	1.9108E-01	3.66E-01	ENSG00000280287
NA	0.1368	1.9110E-01	3.66E-01	ENSG00000279425
NA	0.0372	1.9118E-01		ENSG00000235077
NA	0.0105	1.9132E-01		ENSG00000227887
NA	0.0288	1.9132E-01		ENSG00000237669

NA	0.0136	1.9139E-01		ENSG00000261792
NA	0.1404	1.9177E-01	3.67E-01	ENSG00000267198
NA	0.0023	1.9184E-01		ENSG00000287842
NA	0.1247	1.9215E-01	3.68E-01	ENSG00000286732
NA	0.0353	1.9220E-01	3.68E-01	ENSG00000259001
NA	0.1277	1.9222E-01	3.68E-01	ENSG00000286198
NA	0.0759	1.9232E-01	3.68E-01	ENSG00000226849
NA	0.1351	1.9239E-01	3.68E-01	ENSG00000236200
NA	0.0758	1.9245E-01	3.68E-01	ENSG00000280182
NA	0.1406	1.9281E-01	3.68E-01	ENSG00000269038
NA	0.0739	1.9283E-01	3.68E-01	ENSG00000286277
NA	0.0329	1.9298E-01		ENSG00000237864
NA	0.0421	1.9302E-01	3.69E-01	ENSG00000272085
NA	0.1357	1.9321E-01	3.69E-01	ENSG00000289307
NA	0.0201	1.9403E-01	3.70E-01	ENSG00000286426
NA	0.1386	1.9454E-01	3.71E-01	ENSG00000273320
NA	0.1226	1.9488E-01	3.71E-01	ENSG00000278066
NA	0.0169	1.9497E-01		ENSG00000289158
NA	0.0268	1.9507E-01	3.71E-01	ENSG00000250064
NA	0.0369	1.9518E-01		ENSG00000289406
NA	0.1095	1.9625E-01	3.73E-01	ENSG00000263120
NA	0.0197	1.9627E-01		ENSG00000223732
NA	0.0130	1.9631E-01		ENSG00000262267
NA	0.0265	1.9657E-01		ENSG00000283765
NA	0.0077	1.9658E-01		ENSG00000260765
NA	0.0443	1.9681E-01		ENSG00000278891
NA	0.1377	1.9688E-01	3.73E-01	ENSG00000289415
NA	0.0349	1.9696E-01	3.73E-01	ENSG00000227179
NA	0.1221	1.9729E-01	3.74E-01	ENSG00000289444
NA	0.1333	1.9734E-01	3.74E-01	ENSG00000237359
NA	0.1305	1.9783E-01	3.75E-01	ENSG00000247970
NA	0.0071	1.9800E-01		ENSG00000253141
NA	0.0146	1.9821E-01		ENSG00000181819
NA	0.0524	1.9832E-01	3.75E-01	ENSG00000280233
NA	0.0138	1.9837E-01		ENSG00000269172
NA	0.1094	1.9858E-01	3.76E-01	ENSG00000200164
NA	0.0975	1.9866E-01	3.76E-01	ENSG00000249881
NA	0.0377	1.9891E-01	3.76E-01	ENSG00000232912
NA	0.1369	1.9962E-01	3.77E-01	ENSG00000287509
NA	0.1208	1.9965E-01	3.77E-01	ENSG00000228917
NA	0.1258	1.9970E-01	3.77E-01	ENSG00000253620
NA	0.0555	1.9980E-01		ENSG00000279340
NA	0.0206	2.0002E-01		ENSG00000187812
NA	0.0411	2.0005E-01	3.77E-01	ENSG00000233928
NA	0.1019	2.0013E-01	3.77E-01	ENSG00000260100
NA	0.1204	2.0031E-01	3.78E-01	ENSG00000267571
NA	0.0132	2.0046E-01		ENSG00000285741
NA	0.0217	2.0080E-01		ENSG00000279220
NA	0.1377	2.0101E-01	3.78E-01	ENSG00000261535

NA	0.0545	2.0102E-01		ENSG00000224885
NA	0.1103	2.0115E-01	3.78E-01	ENSG00000237004
NA	0.0473	2.0115E-01	3.78E-01	ENSG00000289075
NA	0.0957	2.0121E-01	3.79E-01	ENSG00000287691
NA	0.0240	2.0125E-01		ENSG00000283003
NA	0.0279	2.0144E-01		ENSG00000216906
NA	0.0996	2.0159E-01	3.79E-01	ENSG00000269427
NA	0.0212	2.0213E-01		ENSG00000288035
NA	0.0483	2.0245E-01		ENSG00000224635
NA	0.1363	2.0254E-01	3.80E-01	ENSG00000228172
NA	0.0920	2.0255E-01	3.80E-01	ENSG00000224883
NA	0.1333	2.0268E-01	3.80E-01	ENSG00000287562
NA	0.1352	2.0276E-01	3.80E-01	ENSG00000225449
NA	0.1160	2.0277E-01	3.80E-01	ENSG00000287287
NA	0.0064	2.0280E-01		ENSG00000236811
NA	0.1364	2.0289E-01	3.81E-01	ENSG00000232876
NA	0.0356	2.0295E-01		ENSG00000231212
NA	0.0270	2.0298E-01		ENSG00000259290
NA	0.0129	2.0335E-01		ENSG00000265194
NA	0.1017	2.0345E-01	3.81E-01	ENSG00000278900
NA	0.0235	2.0351E-01	3.81E-01	ENSG00000275139
NA	0.0137	2.0372E-01		ENSG00000224553
NA	0.0985	2.0394E-01	3.82E-01	ENSG00000275409
NA	0.1298	2.0433E-01	3.82E-01	ENSG00000227370
NA	0.0329	2.0444E-01	3.83E-01	ENSG00000289417
NA	0.1189	2.0481E-01	3.83E-01	ENSG00000272578
NA	0.0254	2.0511E-01		ENSG00000236857
NA	0.0387	2.0515E-01		ENSG00000289531
NA	0.0180	2.0539E-01		ENSG00000212163
NA	0.0294	2.0541E-01		ENSG00000284672
NA	0.0637	2.0593E-01	3.84E-01	ENSG00000276178
NA	0.0181	2.0620E-01		ENSG00000226284
NA	0.1338	2.0625E-01	3.85E-01	ENSG00000196796
NA	0.0524	2.0687E-01		ENSG00000236262
NA	0.0549	2.0831E-01		ENSG00000287631
NA	0.0180	2.0836E-01		ENSG00000227176
NA	0.1291	2.0844E-01	3.88E-01	ENSG00000235314
NA	0.0128	2.0853E-01		ENSG00000274156
NA	0.1307	2.0861E-01	3.88E-01	ENSG00000278971
NA	0.1206	2.0874E-01	3.88E-01	ENSG00000287575
NA	0.1022	2.0890E-01	3.88E-01	ENSG00000279689
NA	0.0143	2.0894E-01		ENSG00000279404
NA	0.0957	2.0905E-01	3.89E-01	ENSG00000287507
NA	0.1147	2.0915E-01	3.89E-01	ENSG00000241596
NA	0.0255	2.0919E-01	3.89E-01	ENSG00000223711
NA	0.1327	2.0939E-01	3.89E-01	ENSG00000255725
NA	0.1281	2.0957E-01	3.89E-01	ENSG00000267262
NA	0.0131	2.0964E-01		ENSG00000236062
NA	0.1342	2.0973E-01	3.89E-01	ENSG00000260537

NA	0.0701	2.0999E-01	3.89E-01	ENSG00000271155
NA	0.0473	2.1016E-01	3.90E-01	ENSG00000265995
NA	0.1271	2.1028E-01	3.90E-01	ENSG00000289347
NA	0.0219	2.1038E-01		ENSG00000235332
NA	0.0237	2.1056E-01		ENSG00000235847
NA	0.0820	2.1056E-01	3.90E-01	ENSG00000289395
NA	0.0256	2.1066E-01		ENSG00000279485
NA	0.0252	2.1079E-01		ENSG00000279304
NA	0.1149	2.1086E-01	3.90E-01	ENSG00000253669
NA	0.0146	2.1137E-01		ENSG00000184795
NA	0.0259	2.1162E-01		ENSG00000235548
NA	0.0537	2.1190E-01		ENSG00000278071
NA	0.0174	2.1194E-01	3.92E-01	ENSG00000273254
NA	0.1203	2.1197E-01	3.92E-01	ENSG00000276266
NA	0.0430	2.1211E-01	3.92E-01	ENSG00000210127
NA	0.0099	2.1212E-01		ENSG00000274281
NA	0.0695	2.1213E-01		ENSG00000272914
NA	0.0690	2.1219E-01	3.92E-01	ENSG00000287092
NA	0.0789	2.1228E-01	3.92E-01	ENSG00000201217
NA	0.0175	2.1240E-01		ENSG00000228779
NA	0.0143	2.1248E-01		ENSG00000279422
NA	0.0328	2.1335E-01		ENSG00000287203
NA	0.0196	2.1356E-01		ENSG00000281961
NA	0.0435	2.1389E-01		ENSG00000229887
NA	0.1305	2.1397E-01	3.94E-01	ENSG00000232611
NA	0.1137	2.1403E-01	3.94E-01	ENSG00000289343
NA	0.0182	2.1450E-01		ENSG00000234626
NA	0.1298	2.1453E-01	3.95E-01	ENSG00000210196
NA	0.0086	2.1467E-01	3.95E-01	ENSG00000231606
NA	0.0673	2.1521E-01		ENSG00000232454
NA	0.1312	2.1523E-01	3.96E-01	ENSG00000276408
NA	0.0835	2.1524E-01	3.96E-01	ENSG00000268279
NA	0.0050	2.1536E-01		ENSG00000213121
NA	0.0130	2.1558E-01		ENSG00000278866
NA	0.1115	2.1580E-01	3.97E-01	ENSG00000260335
NA	0.0095	2.1585E-01	3.97E-01	ENSG00000279595
NA	0.1199	2.1601E-01	3.97E-01	ENSG00000287241
NA	0.0193	2.1602E-01		ENSG00000232969
NA	0.0094	2.1605E-01		ENSG00000269392
NA	0.0332	2.1606E-01	3.97E-01	ENSG00000277453
NA	0.0883	2.1615E-01	3.97E-01	ENSG00000197813
NA	0.0727	2.1629E-01	3.97E-01	ENSG00000234283
NA	0.0393	2.1634E-01		ENSG00000271396
NA	0.1060	2.1644E-01	3.97E-01	ENSG00000254757
NA	0.0720	2.1645E-01	3.97E-01	ENSG00000260290
NA	0.0178	2.1647E-01		ENSG00000228848
NA	0.0237	2.1650E-01		ENSG00000268744
NA	0.0128	2.1653E-01		ENSG00000213201
NA	0.1162	2.1667E-01	3.97E-01	ENSG00000280721

NA	0.0730	2.1680E-01	3.97E-01	ENSG00000286672
NA	0.0155	2.1688E-01		ENSG00000234262
NA	0.0032	2.1721E-01		ENSG00000229853
NA	0.0134	2.1737E-01	3.98E-01	ENSG00000289837
NA	0.0401	2.1753E-01		ENSG00000232682
NA	0.0779	2.1758E-01	3.98E-01	ENSG00000285841
NA	0.0116	2.1774E-01		ENSG00000236528
NA	0.0139	2.1782E-01		ENSG00000287899
NA	0.0159	2.1810E-01	3.99E-01	ENSG00000259341
NA	0.1292	2.1817E-01	3.99E-01	ENSG00000258702
NA	0.1243	2.1865E-01	4.00E-01	ENSG00000235724
NA	0.0103	2.1867E-01		ENSG00000258683
NA	0.0158	2.1872E-01		ENSG00000214041
NA	0.0172	2.1874E-01		ENSG00000286954
NA	0.0239	2.1895E-01	4.00E-01	ENSG00000283058
NA	0.1312	2.1896E-01	4.00E-01	ENSG00000289394
NA	0.0234	2.1906E-01		ENSG00000267692
NA	0.0289	2.1909E-01	4.00E-01	ENSG00000271138
NA	0.1016	2.1923E-01	4.00E-01	ENSG00000132204
NA	0.0754	2.1952E-01	4.00E-01	ENSG00000271623
NA	0.0264	2.1959E-01		ENSG00000287144
NA	0.0544	2.1960E-01		ENSG00000254538
NA	0.0842	2.1997E-01	4.01E-01	ENSG00000261369
NA	0.1257	2.2022E-01	4.01E-01	ENSG00000227627
NA	0.1287	2.2044E-01	4.02E-01	ENSG00000264666
NA	0.0268	2.2050E-01		ENSG00000223754
NA	0.1049	2.2063E-01	4.02E-01	ENSG00000230311
NA	0.0886	2.2065E-01	4.02E-01	ENSG00000254454
NA	0.0209	2.2133E-01		ENSG00000286811
NA	0.0478	2.2133E-01	4.03E-01	ENSG00000287290
NA	0.1241	2.2141E-01	4.03E-01	ENSG00000243406
NA	0.0610	2.2155E-01	4.03E-01	ENSG00000267197
NA	0.1237	2.2173E-01	4.03E-01	ENSG00000224376
NA	0.0298	2.2249E-01		ENSG00000282048
NA	0.0893	2.2261E-01	4.04E-01	ENSG00000279544
NA	0.1258	2.2287E-01	4.05E-01	ENSG00000286549
NA	0.0602	2.2303E-01		ENSG00000273082
NA	0.0853	2.2372E-01	4.06E-01	ENSG00000248749
NA	0.1132	2.2386E-01	4.06E-01	ENSG00000251639
NA	0.1033	2.2403E-01	4.06E-01	ENSG00000280321
NA	0.0271	2.2419E-01		ENSG00000228056
NA	0.0462	2.2462E-01		ENSG00000230947
NA	0.0897	2.2464E-01	4.07E-01	ENSG00000241057
NA	0.0773	2.2465E-01	4.07E-01	ENSG00000265982
NA	0.0617	2.2466E-01	4.07E-01	ENSG00000261357
NA	0.0244	2.2499E-01	4.07E-01	ENSG00000278987
NA	0.0237	2.2536E-01		ENSG00000223624
NA	0.0625	2.2545E-01	4.08E-01	ENSG00000257838
NA	0.0437	2.2558E-01	4.08E-01	ENSG00000286212

NA	0.0191	2.2578E-01		ENSG00000277690
NA	0.0155	2.2593E-01		ENSG00000285897
NA	0.1126	2.2598E-01	4.09E-01	ENSG00000234764
NA	0.0247	2.2605E-01		ENSG00000270104
NA	0.1177	2.2609E-01	4.09E-01	ENSG00000286588
NA	0.1063	2.2634E-01	4.09E-01	ENSG00000272154
NA	0.0275	2.2643E-01	4.09E-01	ENSG00000186831
NA	0.0957	2.2646E-01	4.09E-01	ENSG00000289912
NA	0.1279	2.2649E-01	4.09E-01	ENSG00000278926
NA	0.1001	2.2654E-01	4.09E-01	ENSG00000261526
NA	0.0211	2.2665E-01		ENSG00000232328
NA	0.0446	2.2673E-01		ENSG00000285783
NA	0.0337	2.2677E-01		ENSG00000233581
NA	0.0082	2.2680E-01		ENSG00000207091
NA	0.0191	2.2688E-01		ENSG00000241886
NA	0.0843	2.2706E-01	4.10E-01	ENSG00000285280
NA	0.0212	2.2720E-01		ENSG00000275691
NA	0.0023	2.2722E-01	4.10E-01	ENSG00000287697
NA	0.1161	2.2723E-01	4.10E-01	ENSG00000275263
NA	0.0483	2.2804E-01	4.11E-01	ENSG00000238021
NA	0.1267	2.2804E-01	4.11E-01	ENSG00000286092
NA	0.1260	2.2813E-01	4.11E-01	ENSG00000231466
NA	0.0267	2.2823E-01		ENSG00000267793
NA	0.1253	2.2827E-01	4.11E-01	ENSG00000230869
NA	0.0419	2.2834E-01		ENSG00000270236
NA	0.1026	2.2841E-01	4.11E-01	ENSG00000271119
NA	0.1258	2.2855E-01	4.11E-01	ENSG00000230730
NA	0.1088	2.2862E-01	4.11E-01	ENSG00000224903
NA	0.0340	2.2905E-01	4.12E-01	ENSG00000233069
NA	0.0123	2.2911E-01		ENSG00000227240
NA	0.1252	2.2921E-01	4.12E-01	ENSG00000261485
NA	0.1227	2.2936E-01	4.12E-01	ENSG00000277597
NA	0.0175	2.2942E-01		ENSG00000250267
NA	0.0094	2.2949E-01		ENSG00000289411
NA	0.1280	2.2956E-01	4.13E-01	ENSG00000283886
NA	0.0834	2.3053E-01	4.14E-01	ENSG00000288105
NA	0.0726	2.3085E-01	4.14E-01	ENSG00000262772
NA	0.1237	2.3105E-01	4.14E-01	ENSG00000261512
NA	0.0185	2.3106E-01		ENSG00000248546
NA	0.1248	2.3109E-01	4.15E-01	ENSG00000239827
NA	0.0043	2.3122E-01		ENSG00000288588
NA	0.0330	2.3135E-01		ENSG00000234428
NA	0.0179	2.3140E-01		ENSG00000287820
NA	0.0647	2.3145E-01	4.15E-01	ENSG00000280186
NA	0.1098	2.3148E-01	4.15E-01	ENSG00000279339
NA	0.0058	2.3199E-01		ENSG00000225099
NA	0.0388	2.3210E-01		ENSG00000273481
NA	0.1230	2.3215E-01	4.16E-01	ENSG00000287655
NA	0.0926	2.3221E-01	4.16E-01	ENSG00000256540

NA	0.1255	2.3235E-01	4.16E-01	ENSG00000288988
NA	0.0798	2.3240E-01	4.16E-01	ENSG00000207497
NA	0.0254	2.3246E-01		ENSG00000271018
NA	0.0722	2.3246E-01	4.16E-01	ENSG00000273015
NA	0.0039	2.3258E-01		ENSG00000286089
NA	0.0398	2.3263E-01		ENSG00000275910
NA	0.0150	2.3313E-01		ENSG00000262339
NA	0.0306	2.3332E-01		ENSG00000234946
NA	0.0161	2.3352E-01		ENSG00000230197
NA	0.1227	2.3356E-01	4.17E-01	ENSG00000253475
NA	0.0635	2.3379E-01	4.18E-01	ENSG00000286443
NA	0.1067	2.3389E-01	4.18E-01	ENSG00000287561
NA	0.0248	2.3404E-01		ENSG00000267682
NA	0.0155	2.3446E-01		ENSG00000240959
NA	0.0220	2.3452E-01		ENSG00000275834
NA	0.0808	2.3456E-01	4.19E-01	ENSG00000256176
NA	0.1075	2.3483E-01	4.19E-01	ENSG00000265496
NA	0.0146	2.3504E-01	4.19E-01	ENSG00000276223
NA	0.0456	2.3509E-01	4.19E-01	ENSG00000273245
NA	0.0911	2.3513E-01	4.19E-01	ENSG00000234782
NA	0.0212	2.3534E-01		ENSG00000210117
NA	0.0742	2.3563E-01	4.20E-01	ENSG00000170919
NA	0.0144	2.3566E-01		ENSG00000231531
NA	0.1190	2.3567E-01	4.20E-01	ENSG00000254348
NA	0.1246	2.3582E-01	4.20E-01	ENSG00000251417
NA	0.1230	2.3588E-01	4.20E-01	ENSG00000289443
NA	0.0486	2.3613E-01		ENSG00000274340
NA	0.0931	2.3640E-01	4.21E-01	ENSG00000257539
NA	0.0213	2.3642E-01	4.21E-01	ENSG00000219951
NA	0.0294	2.3649E-01		ENSG00000215002
NA	0.0861	2.3670E-01	4.21E-01	ENSG00000257513
NA	0.0465	2.3677E-01		ENSG00000234208
NA	0.0546	2.3678E-01		ENSG00000257398
NA	0.0194	2.3684E-01		ENSG00000230849
NA	0.0123	2.3685E-01		ENSG00000287043
NA	0.0219	2.3711E-01		ENSG00000261497
NA	0.1006	2.3717E-01	4.21E-01	ENSG00000146722
NA	0.1237	2.3740E-01	4.22E-01	ENSG00000282416
NA	0.0193	2.3741E-01		ENSG00000279579
NA	0.0961	2.3782E-01	4.22E-01	ENSG00000271500
NA	0.1162	2.3842E-01	4.23E-01	ENSG00000245025
NA	0.0944	2.3859E-01	4.23E-01	ENSG00000215237
NA	0.1091	2.3875E-01	4.23E-01	ENSG00000247011
NA	0.1202	2.3881E-01	4.23E-01	ENSG00000246451
NA	0.0188	2.3899E-01		ENSG00000262096
NA	0.1199	2.3906E-01	4.24E-01	ENSG00000242294
NA	0.1230	2.3939E-01	4.24E-01	ENSG00000289883
NA	0.0264	2.3947E-01		ENSG00000268105
NA	0.0937	2.3949E-01	4.24E-01	ENSG00000234383

NA	0.1011	2.3964E-01	4.24E-01	ENSG00000288794
NA	0.0206	2.3974E-01		ENSG00000257940
NA	0.0877	2.3979E-01	4.24E-01	ENSG00000287473
NA	0.0193	2.3987E-01		ENSG00000236437
NA	0.0699	2.3991E-01	4.25E-01	ENSG00000254038
NA	0.0257	2.4018E-01		ENSG00000287455
NA	0.0519	2.4092E-01		ENSG00000280423
NA	0.0366	2.4116E-01	4.26E-01	ENSG00000251488
NA	0.0146	2.4142E-01		ENSG00000258454
NA	0.0248	2.4176E-01		ENSG00000224677
NA	0.0075	2.4251E-01		ENSG00000250238
NA	0.0207	2.4261E-01		ENSG00000280009
NA	0.0343	2.4298E-01		ENSG00000264750
NA	0.0426	2.4305E-01	4.28E-01	ENSG00000289865
NA	0.0421	2.4307E-01		ENSG00000280169
NA	0.1217	2.4341E-01	4.29E-01	ENSG00000250896
NA	0.1191	2.4376E-01	4.29E-01	ENSG00000230490
NA	0.0741	2.4437E-01	4.30E-01	ENSG00000225979
NA	0.0230	2.4471E-01		ENSG00000270689
NA	0.1160	2.4490E-01	4.31E-01	ENSG00000245059
NA	0.0144	2.4497E-01		ENSG00000230665
NA	0.1205	2.4519E-01	4.31E-01	ENSG00000287555
NA	0.1020	2.4543E-01	4.31E-01	ENSG00000267543
NA	0.0096	2.4575E-01		ENSG00000227253
NA	0.0163	2.4613E-01		ENSG00000228487
NA	0.0253	2.4638E-01		ENSG00000266373
NA	0.0214	2.4676E-01		ENSG00000254060
NA	0.0204	2.4705E-01		ENSG00000222032
NA	0.0230	2.4709E-01		ENSG00000231401
NA	0.1202	2.4718E-01	4.33E-01	ENSG00000253671
NA	0.0330	2.4726E-01		ENSG00000213981
NA	0.0283	2.4756E-01		ENSG00000267152
NA	0.1208	2.4775E-01	4.34E-01	ENSG00000273204
NA	0.0132	2.4782E-01		ENSG00000154198
NA	0.0465	2.4782E-01		ENSG00000284309
NA	0.1113	2.4815E-01	4.34E-01	ENSG00000176912
NA	0.0384	2.4821E-01		ENSG00000230312
NA	0.1200	2.4870E-01	4.35E-01	ENSG00000236519
NA	0.0175	2.4871E-01		ENSG00000272015
NA	0.0539	2.4884E-01		ENSG00000225117
NA	0.1086	2.4885E-01	4.35E-01	ENSG00000237092
NA	0.1202	2.4887E-01	4.35E-01	ENSG00000228925
NA	0.0022	2.4888E-01	4.35E-01	ENSG00000232545
NA	0.1191	2.4925E-01	4.36E-01	ENSG00000250461
NA	0.1210	2.5002E-01	4.37E-01	ENSG00000267649
NA	0.1168	2.5006E-01	4.37E-01	ENSG00000289841
NA	0.0172	2.5022E-01		ENSG00000258352
NA	0.0443	2.5026E-01		ENSG00000287795
NA	0.0760	2.5027E-01	4.37E-01	ENSG00000249602

NA	0.0329	2.5027E-01	4.37E-01	ENSG00000279212
NA	0.0034	2.5040E-01		ENSG00000256633
NA	0.1068	2.5053E-01	4.37E-01	ENSG00000231407
NA	0.0409	2.5070E-01	4.37E-01	ENSG00000225399
NA	0.0331	2.5091E-01	4.38E-01	ENSG00000288850
NA	0.0871	2.5092E-01	4.38E-01	ENSG00000289788
NA	0.1054	2.5120E-01	4.38E-01	ENSG00000244490
NA	0.0874	2.5127E-01	4.38E-01	ENSG00000279345
NA	0.0179	2.5154E-01		ENSG00000256385
NA	0.0431	2.5158E-01		ENSG00000289314
NA	0.0252	2.5165E-01		ENSG00000280415
NA	0.0387	2.5197E-01		ENSG00000278972
NA	0.1038	2.5206E-01	4.39E-01	ENSG00000289339
NA	0.0636	2.5212E-01	4.39E-01	ENSG00000288583
NA	0.0225	2.5235E-01		ENSG00000279575
NA	0.0851	2.5243E-01	4.39E-01	ENSG00000290093
NA	0.0797	2.5246E-01	4.39E-01	ENSG00000233340
NA	0.0068	2.5322E-01		ENSG00000270001
NA	0.0029	2.5381E-01		ENSG00000254695
NA	0.0267	2.5399E-01		ENSG00000269487
NA	0.0480	2.5423E-01	4.41E-01	ENSG00000259045
NA	0.0117	2.5427E-01		ENSG00000287171
NA	0.1194	2.5435E-01	4.42E-01	ENSG00000289689
NA	0.0915	2.5489E-01	4.42E-01	ENSG00000272512
NA	0.0604	2.5630E-01		ENSG00000286489
NA	0.1050	2.5725E-01	4.45E-01	ENSG00000284634
NA	0.0190	2.5813E-01		ENSG00000249238
NA	0.0834	2.5814E-01	4.46E-01	ENSG00000279428
NA	0.0097	2.5841E-01		ENSG00000286466
NA	0.0070	2.5846E-01	4.47E-01	ENSG00000289388
NA	0.1124	2.5860E-01	4.47E-01	ENSG00000290065
NA	0.1014	2.5890E-01	4.47E-01	ENSG00000280422
NA	0.0920	2.5906E-01	4.47E-01	ENSG00000225913
NA	0.0537	2.5909E-01		ENSG00000231210
NA	0.0879	2.5911E-01	4.47E-01	ENSG00000274341
NA	0.0677	2.5915E-01		ENSG00000176761
NA	0.0345	2.5925E-01		ENSG00000269752
NA	0.1028	2.5995E-01	4.48E-01	ENSG00000274528
NA	0.1013	2.6036E-01	4.49E-01	ENSG00000286230
NA	0.0206	2.6048E-01	4.49E-01	ENSG00000131885
NA	0.1046	2.6050E-01	4.49E-01	ENSG00000231711
NA	0.0070	2.6069E-01		ENSG00000251135
NA	0.0466	2.6076E-01		ENSG00000259447
NA	0.0332	2.6084E-01	4.49E-01	ENSG00000272954
NA	0.0114	2.6143E-01	4.50E-01	ENSG00000226609
NA	0.0204	2.6149E-01		ENSG00000237453
NA	0.1152	2.6160E-01	4.50E-01	ENSG00000124593
NA	0.0088	2.6160E-01		ENSG00000280758
NA	0.0460	2.6176E-01		ENSG00000282852

NA	0.0909	2.6180E-01	4.51E-01	ENSG00000211459
NA	0.0105	2.6189E-01	4.51E-01	ENSG00000255200
NA	0.0961	2.6199E-01	4.51E-01	ENSG00000289247
NA	0.0020	2.6199E-01		ENSG00000238199
NA	0.1064	2.6205E-01	4.51E-01	ENSG00000284966
NA	0.0729	2.6272E-01	4.52E-01	ENSG00000234297
NA	0.0044	2.6276E-01		ENSG00000260685
NA	0.0543	2.6277E-01	4.52E-01	ENSG00000180178
NA	0.1135	2.6280E-01	4.52E-01	ENSG00000259377
NA	0.1172	2.6288E-01	4.52E-01	ENSG00000225880
NA	0.0027	2.6307E-01		ENSG00000258303
NA	0.0542	2.6342E-01	4.52E-01	ENSG00000275930
NA	0.0148	2.6343E-01		ENSG00000213082
NA	0.0449	2.6364E-01		ENSG00000199331
NA	0.0135	2.6368E-01	4.52E-01	ENSG00000234925
NA	0.0171	2.6370E-01		ENSG00000279924
NA	0.0151	2.6386E-01		ENSG00000260012
NA	0.0704	2.6406E-01	4.53E-01	ENSG00000277425
NA	0.1151	2.6444E-01	4.53E-01	ENSG00000203709
NA	0.0899	2.6483E-01	4.54E-01	ENSG00000273448
NA	0.0359	2.6504E-01		ENSG00000203647
NA	0.0219	2.6530E-01		ENSG00000233025
NA	0.0010	2.6551E-01		ENSG00000249210
NA	0.0264	2.6564E-01		ENSG00000244712
NA	0.0718	2.6581E-01	4.55E-01	ENSG00000285803
NA	0.0135	2.6582E-01		ENSG00000289002
NA	0.0680	2.6603E-01	4.55E-01	ENSG00000221930
NA	0.0023	2.6606E-01		ENSG00000270048
NA	0.0851	2.6626E-01	4.55E-01	ENSG00000234500
NA	0.0065	2.6648E-01	4.56E-01	ENSG00000290108
NA	0.0838	2.6665E-01	4.56E-01	ENSG00000255216
NA	0.1132	2.6713E-01	4.56E-01	ENSG00000279114
NA	0.0176	2.6719E-01		ENSG00000234622
NA	0.0425	2.6735E-01		ENSG00000267054
NA	0.0943	2.6750E-01	4.57E-01	ENSG00000235436
NA	0.0163	2.6765E-01		ENSG00000278894
NA	0.0149	2.6802E-01		ENSG00000260005
NA	0.0968	2.6807E-01	4.57E-01	ENSG00000218418
NA	0.0736	2.6825E-01	4.57E-01	ENSG00000167807
NA	0.0392	2.6837E-01		ENSG00000278112
NA	0.0933	2.6839E-01	4.58E-01	ENSG00000273151
NA	0.0404	2.6851E-01	4.58E-01	ENSG00000267364
NA	0.0350	2.6871E-01		ENSG00000267393
NA	0.0917	2.6882E-01	4.58E-01	ENSG00000283662
NA	0.0132	2.6888E-01		ENSG00000287963
NA	0.0946	2.6896E-01	4.58E-01	ENSG00000237058
NA	0.1143	2.6918E-01	4.58E-01	ENSG00000261098
NA	0.0273	2.6930E-01		ENSG00000257962
NA	0.0214	2.6940E-01		ENSG00000228686

NA	0.1142	2.6962E-01	4.59E-01	ENSG00000257443
NA	0.0194	2.6966E-01		ENSG00000257542
NA	0.0199	2.6967E-01		ENSG00000283525
NA	0.0715	2.6984E-01	4.59E-01	ENSG00000248514
NA	0.0097	2.6989E-01		ENSG00000280037
NA	0.0081	2.7037E-01		ENSG00000285698
NA	0.0630	2.7049E-01		ENSG00000273261
NA	0.0179	2.7078E-01		ENSG00000232963
NA	0.0206	2.7086E-01		ENSG00000286629
NA	0.0213	2.7086E-01	4.61E-01	ENSG00000226191
NA	0.0289	2.7111E-01		ENSG00000286571
NA	0.0379	2.7132E-01	4.61E-01	ENSG00000228217
NA	0.0826	2.7132E-01	4.61E-01	ENSG00000267446
NA	0.0194	2.7133E-01		ENSG00000290066
NA	0.0830	2.7137E-01	4.61E-01	ENSG00000267287
NA	0.0076	2.7152E-01		ENSG00000289452
NA	0.0257	2.7172E-01	4.62E-01	ENSG00000289082
NA	0.0065	2.7183E-01	4.62E-01	ENSG00000290101
NA	0.1047	2.7185E-01	4.62E-01	ENSG00000279196
NA	0.0761	2.7235E-01	4.62E-01	ENSG00000273493
NA	0.1035	2.7263E-01	4.63E-01	ENSG00000239213
NA	0.1102	2.7264E-01	4.63E-01	ENSG00000270019
NA	0.0359	2.7281E-01		ENSG00000226205
NA	0.1114	2.7292E-01	4.63E-01	ENSG00000198658
NA	0.1070	2.7302E-01	4.63E-01	ENSG00000241860
NA	0.0253	2.7304E-01		ENSG00000258672
NA	0.0248	2.7364E-01		ENSG00000257835
NA	0.0758	2.7395E-01		ENSG00000232457
NA	0.0008	2.7400E-01		ENSG00000227113
NA	0.0520	2.7443E-01	4.65E-01	ENSG00000273252
NA	0.1124	2.7460E-01	4.65E-01	ENSG00000245322
NA	0.0066	2.7484E-01		ENSG00000227295
NA	0.1126	2.7491E-01	4.66E-01	ENSG00000268412
NA	0.0142	2.7497E-01		ENSG00000279512
NA	0.0541	2.7498E-01		ENSG00000287663
NA	0.0957	2.7539E-01	4.66E-01	ENSG00000230530
NA	0.0049	2.7541E-01		ENSG00000271778
NA	0.0066	2.7557E-01		ENSG00000254447
NA	0.1130	2.7579E-01	4.66E-01	ENSG00000258741
NA	0.1055	2.7580E-01	4.66E-01	ENSG00000261123
NA	0.0128	2.7582E-01		ENSG00000268864
NA	0.0700	2.7585E-01		ENSG00000288786
NA	0.0194	2.7592E-01		ENSG00000260042
NA	0.0704	2.7603E-01	4.67E-01	ENSG00000262296
NA	0.0140	2.7606E-01		ENSG00000235518
NA	0.0370	2.7609E-01		ENSG00000184617
NA	0.0985	2.7625E-01	4.67E-01	ENSG00000270218
NA	0.0211	2.7706E-01		ENSG00000261402
NA	0.0023	2.7737E-01		ENSG00000275191

NA	0.1038	2.7753E-01	4.68E-01	ENSG00000204685
NA	0.0585	2.7761E-01		ENSG00000280135
NA	0.1110	2.7773E-01	4.68E-01	ENSG00000278922
NA	0.0216	2.7781E-01		ENSG00000260417
NA	0.0127	2.7878E-01		ENSG00000259890
NA	0.0037	2.7888E-01		ENSG00000226356
NA	0.0840	2.7894E-01	4.70E-01	ENSG00000261916
NA	0.0543	2.7902E-01	4.70E-01	ENSG00000260450
NA	0.1119	2.7905E-01	4.70E-01	ENSG00000289126
NA	0.0776	2.7907E-01	4.70E-01	ENSG00000237343
NA	0.0528	2.7915E-01		ENSG00000275799
NA	0.0424	2.7922E-01	4.70E-01	ENSG00000273821
NA	0.0235	2.7938E-01		ENSG00000248873
NA	0.0651	2.7946E-01	4.71E-01	ENSG00000259347
NA	0.0142	2.7946E-01		ENSG00000270846
NA	0.0206	2.7952E-01		ENSG00000270111
NA	0.0326	2.7969E-01		ENSG00000261303
NA	0.0854	2.7990E-01	4.71E-01	ENSG00000289152
NA	0.0535	2.8004E-01		ENSG00000289289
NA	0.0171	2.8010E-01		ENSG00000199476
NA	0.0983	2.8043E-01	4.71E-01	ENSG00000259869
NA	0.0236	2.8057E-01		ENSG00000206797
NA	0.1076	2.8106E-01	4.72E-01	ENSG00000258768
NA	0.0898	2.8112E-01	4.72E-01	ENSG00000271533
NA	0.0324	2.8120E-01		ENSG00000267114
NA	0.0145	2.8122E-01		ENSG00000279886
NA	0.0200	2.8136E-01		ENSG00000250015
NA	0.0975	2.8138E-01	4.72E-01	ENSG00000231856
NA	0.1093	2.8177E-01	4.73E-01	ENSG00000272519
NA	0.0417	2.8195E-01	4.73E-01	ENSG00000289287
NA	0.0137	2.8199E-01		ENSG00000236740
NA	0.1086	2.8243E-01	4.74E-01	ENSG00000274929
NA	0.0769	2.8283E-01	4.74E-01	ENSG00000120555
NA	0.0724	2.8296E-01	4.74E-01	ENSG00000259318
NA	0.0248	2.8305E-01		ENSG00000230585
NA	0.0914	2.8347E-01	4.75E-01	ENSG00000257390
NA	0.0139	2.8361E-01		ENSG00000237307
NA	0.0132	2.8364E-01		ENSG00000260395
NA	0.0739	2.8374E-01	4.75E-01	ENSG00000271350
NA	0.0402	2.8396E-01		ENSG00000225559
NA	0.1090	2.8422E-01	4.76E-01	ENSG00000240291
NA	0.0316	2.8428E-01		ENSG00000213234
NA	0.0041	2.8440E-01		ENSG00000213076
NA	0.1085	2.8452E-01	4.76E-01	ENSG00000230223
NA	0.0149	2.8461E-01		ENSG00000204011
NA	0.0767	2.8485E-01	4.76E-01	ENSG00000267505
NA	0.1042	2.8490E-01	4.76E-01	ENSG00000231160
NA	0.0022	2.8499E-01	4.76E-01	ENSG00000224066
NA	0.0129	2.8542E-01		ENSG00000287936

NA	0.0613	2.8544E-01		ENSG00000287534
NA	0.0478	2.8554E-01	4.77E-01	ENSG00000260167
NA	0.0227	2.8560E-01		ENSG00000266111
NA	0.0418	2.8569E-01		ENSG00000282944
NA	0.0308	2.8577E-01		ENSG00000271795
NA	0.0637	2.8605E-01	4.77E-01	ENSG00000254272
NA	0.1080	2.8608E-01	4.77E-01	ENSG00000287780
NA	0.0608	2.8609E-01	4.77E-01	ENSG00000274591
NA	0.0123	2.8611E-01		ENSG00000269761
NA	0.1078	2.8626E-01	4.77E-01	ENSG00000250790
NA	0.0219	2.8647E-01		ENSG00000230836
NA	0.0007	2.8656E-01		ENSG00000248476
NA	0.0297	2.8659E-01		ENSG00000228559
NA	0.1096	2.8701E-01	4.78E-01	ENSG00000260641
NA	0.0274	2.8763E-01	4.79E-01	ENSG00000272153
NA	0.0391	2.8788E-01		ENSG00000286918
NA	0.1067	2.8811E-01	4.79E-01	ENSG00000272795
NA	0.0578	2.8815E-01	4.79E-01	ENSG00000288091
NA	0.0762	2.8820E-01	4.79E-01	ENSG00000279085
NA	0.0116	2.8827E-01		ENSG00000285844
NA	0.0120	2.8841E-01		ENSG00000256694
NA	0.0491	2.8846E-01	4.80E-01	ENSG00000228527
NA	0.0170	2.8884E-01		ENSG00000230146
NA	0.0051	2.8889E-01		ENSG00000248773
NA	0.0964	2.8899E-01	4.80E-01	ENSG00000262943
NA	0.0694	2.8914E-01	4.81E-01	ENSG00000179766
NA	0.0933	2.8933E-01	4.81E-01	ENSG00000265460
NA	0.0184	2.8942E-01		ENSG00000279499
NA	0.0273	2.8967E-01	4.81E-01	ENSG00000258450
NA	0.1091	2.8981E-01	4.81E-01	ENSG00000258017
NA	0.0287	2.9005E-01		ENSG00000286494
NA	0.1078	2.9017E-01	4.82E-01	ENSG00000260367
NA	0.1092	2.9021E-01	4.82E-01	ENSG00000278983
NA	0.0216	2.9036E-01		ENSG00000227535
NA	0.0979	2.9038E-01	4.82E-01	ENSG00000270074
NA	0.0217	2.9045E-01		ENSG00000242017
NA	0.0147	2.9050E-01		ENSG00000271806
NA	0.0905	2.9082E-01	4.82E-01	ENSG00000266824
NA	0.0271	2.9093E-01		ENSG00000254710
NA	0.0181	2.9117E-01		ENSG00000288083
NA	0.0364	2.9119E-01		ENSG00000260509
NA	0.1024	2.9148E-01	4.83E-01	ENSG00000277738
NA	0.0939	2.9202E-01	4.84E-01	ENSG00000175741
NA	0.0672	2.9253E-01	4.84E-01	ENSG00000258230
NA	0.1050	2.9268E-01	4.85E-01	ENSG00000284630
NA	0.0072	2.9290E-01		ENSG00000203286
NA	0.0450	2.9299E-01		ENSG00000286121
NA	0.1055	2.9306E-01	4.85E-01	ENSG00000224550
NA	0.0179	2.9362E-01		ENSG00000248830

NA	0.0167	2.9365E-01		ENSG00000255050
NA	0.0095	2.9393E-01		ENSG00000280232
NA	0.0821	2.9427E-01	4.86E-01	ENSG00000223760
NA	0.0541	2.9464E-01	4.87E-01	ENSG00000228629
NA	0.0447	2.9497E-01		ENSG00000269256
NA	0.0752	2.9523E-01	4.87E-01	ENSG00000223745
NA	0.0152	2.9534E-01		ENSG00000250697
NA	0.0989	2.9574E-01	4.88E-01	ENSG00000247934
NA	0.1016	2.9596E-01	4.88E-01	ENSG00000233247
NA	0.0241	2.9638E-01		ENSG00000270789
NA	0.0134	2.9640E-01		ENSG00000255176
NA	0.0747	2.9654E-01	4.89E-01	ENSG00000224032
NA	0.0472	2.9672E-01		ENSG00000278733
NA	0.0467	2.9692E-01		ENSG00000276054
NA	0.0965	2.9732E-01	4.89E-01	ENSG00000285184
NA	0.0122	2.9732E-01		ENSG00000224689
NA	0.0659	2.9790E-01		ENSG00000233708
NA	0.0338	2.9792E-01		ENSG00000282772
NA	0.0242	2.9800E-01		ENSG00000227852
NA	0.0109	2.9839E-01		ENSG00000241473
NA	0.0381	2.9840E-01		ENSG00000269153
NA	0.1010	2.9844E-01	4.91E-01	ENSG00000231154
NA	0.1002	2.9867E-01	4.91E-01	ENSG00000268670
NA	0.0203	2.9868E-01		ENSG00000255496
NA	0.0238	2.9926E-01		ENSG00000259015
NA	0.0061	2.9943E-01		ENSG00000250027
NA	0.1023	2.9945E-01	4.92E-01	ENSG00000226644
NA	0.0858	2.9975E-01	4.92E-01	ENSG00000261499
NA	0.0185	2.9979E-01		ENSG00000251182
NA	0.0292	2.9984E-01		ENSG00000273920
NA	0.1020	2.9987E-01	4.92E-01	ENSG00000260948
NA	0.0805	3.0013E-01	4.93E-01	ENSG00000287124
NA	0.0089	3.0048E-01		ENSG00000269859
NA	0.0188	3.0057E-01		ENSG00000279325
NA	0.0153	3.0068E-01		ENSG00000256670
NA	0.1010	3.0099E-01	4.94E-01	ENSG00000271369
NA	0.0685	3.0110E-01	4.94E-01	ENSG00000213411
NA	0.0976	3.0110E-01	4.94E-01	ENSG00000289492
NA	0.0283	3.0111E-01		ENSG00000203635
NA	0.1013	3.0157E-01	4.94E-01	ENSG00000254461
NA	0.0496	3.0159E-01	4.94E-01	ENSG00000279880
NA	0.0078	3.0217E-01		ENSG00000179038
NA	0.0923	3.0220E-01	4.95E-01	ENSG00000286282
NA	0.0204	3.0228E-01		ENSG00000256312
NA	0.0168	3.0252E-01		ENSG00000229601
NA	0.0965	3.0257E-01	4.95E-01	ENSG00000289216
NA	0.0216	3.0270E-01		ENSG00000251224
NA	0.0129	3.0300E-01		ENSG00000274603
NA	0.0272	3.0305E-01	4.95E-01	ENSG00000288721

NA	0.0161	3.0327E-01		ENSG00000255572
NA	0.0139	3.0333E-01		ENSG00000198555
NA	0.0204	3.0351E-01		ENSG00000289729
NA	0.0158	3.0363E-01		ENSG00000235829
NA	0.0062	3.0372E-01		ENSG00000254456
NA	0.0179	3.0373E-01		ENSG00000287873
NA	0.0368	3.0393E-01		ENSG00000267920
NA	0.0952	3.0417E-01	4.97E-01	ENSG00000226797
NA	0.0561	3.0418E-01		ENSG00000254932
NA	0.0838	3.0436E-01	4.97E-01	ENSG00000259677
NA	0.0106	3.0444E-01		ENSG00000268288
NA	0.1048	3.0465E-01	4.97E-01	ENSG00000229109
NA	0.0692	3.0512E-01	4.98E-01	ENSG00000272405
NA	0.0986	3.0516E-01	4.98E-01	ENSG00000261888
NA	0.0142	3.0549E-01		ENSG00000261044
NA	0.0154	3.0549E-01		ENSG00000236474
NA	0.0330	3.0553E-01		ENSG00000223890
NA	0.0144	3.0614E-01		ENSG00000230010
NA	0.1046	3.0628E-01	4.99E-01	ENSG00000276334
NA	0.0188	3.0644E-01		ENSG00000285850
NA	0.0940	3.0648E-01	4.99E-01	ENSG00000271100
NA	0.1006	3.0658E-01	4.99E-01	ENSG00000270953
NA	0.0766	3.0676E-01	4.99E-01	ENSG00000270108
NA	0.0122	3.0692E-01		ENSG00000261430
NA	0.0196	3.0696E-01		ENSG00000231173
NA	0.0873	3.0708E-01	5.00E-01	ENSG00000269486
NA	0.0280	3.0720E-01		ENSG00000275995
NA	0.0630	3.0734E-01	5.00E-01	ENSG00000255409
NA	0.0877	3.0742E-01	5.00E-01	ENSG00000248898
NA	0.0722	3.0754E-01	5.00E-01	ENSG00000234139
NA	0.0060	3.0768E-01		ENSG00000232489
NA	0.0643	3.0777E-01	5.00E-01	ENSG00000271420
NA	0.0293	3.0792E-01		ENSG00000206582
NA	0.0064	3.0839E-01		ENSG00000233511
NA	0.0312	3.0855E-01	5.01E-01	ENSG00000280334
NA	0.0925	3.0897E-01	5.02E-01	ENSG00000243193
NA	0.0215	3.0932E-01		ENSG00000288882
NA	0.0696	3.0990E-01	5.03E-01	ENSG00000225762
NA	0.0351	3.0997E-01	5.03E-01	ENSG00000229349
NA	0.0983	3.1016E-01	5.03E-01	ENSG00000247345
NA	0.0377	3.1041E-01	5.03E-01	ENSG00000272825
NA	0.0147	3.1048E-01		ENSG00000283991
NA	0.0114	3.1073E-01		ENSG00000277618
NA	0.0100	3.1097E-01		ENSG00000261559
NA	0.0105	3.1099E-01		ENSG00000286343
NA	0.0216	3.1148E-01		ENSG00000286290
NA	0.0107	3.1152E-01		ENSG00000234471
NA	0.0956	3.1192E-01	5.05E-01	ENSG00000234477
NA	0.0686	3.1200E-01	5.05E-01	ENSG00000228415

NA	0.0210	3.1219E-01		ENSG00000289500
NA	0.0981	3.1248E-01	5.06E-01	ENSG00000247679
NA	0.0313	3.1266E-01		ENSG00000288031
NA	0.0152	3.1274E-01		ENSG00000280205
NA	0.0087	3.1275E-01		ENSG00000222788
NA	0.0085	3.1279E-01	5.06E-01	ENSG00000258380
NA	0.0223	3.1295E-01		ENSG00000278665
NA	0.0130	3.1301E-01		ENSG00000275040
NA	0.0798	3.1362E-01	5.07E-01	ENSG00000285816
NA	0.0129	3.1371E-01	5.07E-01	ENSG00000248664
NA	0.0138	3.1416E-01		ENSG00000256824
NA	0.0048	3.1425E-01		ENSG00000240435
NA	0.0125	3.1434E-01		ENSG00000214460
NA	0.1016	3.1470E-01	5.08E-01	ENSG00000262619
NA	0.0145	3.1488E-01		ENSG00000289547
NA	0.0243	3.1518E-01	5.09E-01	ENSG00000264057
NA	0.0933	3.1533E-01	5.09E-01	ENSG00000269993
NA	0.0179	3.1558E-01		ENSG00000227607
NA	0.1023	3.1584E-01	5.09E-01	ENSG00000266473
NA	0.0971	3.1586E-01	5.09E-01	ENSG00000270460
NA	0.0301	3.1587E-01		ENSG00000173867
NA	0.0120	3.1588E-01		ENSG00000236389
NA	0.0089	3.1603E-01		ENSG00000239040
NA	0.1025	3.1622E-01	5.10E-01	ENSG00000235100
NA	0.0221	3.1626E-01		ENSG00000253474
NA	0.0304	3.1633E-01		ENSG00000236466
NA	0.0648	3.1672E-01		ENSG00000279479
NA	0.1000	3.1712E-01	5.11E-01	ENSG00000289378
NA	0.0427	3.1765E-01	5.11E-01	ENSG00000256984
NA	0.0448	3.1781E-01	5.11E-01	ENSG00000280194
NA	0.0124	3.1799E-01		ENSG00000286953
NA	0.0862	3.1841E-01	5.12E-01	ENSG00000241135
NA	0.0132	3.1844E-01		ENSG00000254526
NA	0.1010	3.1873E-01	5.12E-01	ENSG00000262198
NA	0.0167	3.1889E-01		ENSG00000260302
NA	0.0015	3.1918E-01		ENSG00000264834
NA	0.0095	3.1936E-01		ENSG00000280336
NA	0.0909	3.1943E-01	5.13E-01	ENSG00000279434
NA	0.0624	3.1955E-01	5.13E-01	ENSG00000277957
NA	0.0217	3.1983E-01		ENSG00000274918
NA	0.1004	3.2026E-01	5.14E-01	ENSG00000247735
NA	0.0511	3.2040E-01	5.14E-01	ENSG00000268945
NA	0.0810	3.2059E-01	5.14E-01	ENSG00000260669
NA	0.0385	3.2060E-01		ENSG00000279875
NA	0.0298	3.2126E-01		ENSG00000249022
NA	0.1010	3.2150E-01	5.15E-01	ENSG00000289858
NA	0.0116	3.2166E-01		ENSG00000226677
NA	0.0047	3.2176E-01	5.16E-01	ENSG00000226928
NA	0.0914	3.2179E-01	5.16E-01	ENSG00000280310

NA	0.0232	3.2206E-01	5.16E-01	ENSG00000289295
NA	0.0986	3.2247E-01	5.16E-01	ENSG00000289154
NA	0.0236	3.2247E-01		ENSG00000225963
NA	0.0136	3.2258E-01		ENSG00000254317
NA	0.0172	3.2324E-01	5.17E-01	ENSG00000287453
NA	0.0221	3.2364E-01	5.18E-01	ENSG00000236021
NA	0.0134	3.2373E-01		ENSG00000290106
NA	0.0033	3.2400E-01		ENSG00000289949
NA	0.0054	3.2458E-01		ENSG00000277340
NA	0.0054	3.2458E-01		ENSG00000232798
NA	0.0158	3.2480E-01	5.19E-01	ENSG00000259137
NA	0.0581	3.2482E-01		ENSG00000286904
NA	0.0941	3.2498E-01	5.19E-01	ENSG00000261235
NA	0.0114	3.2516E-01		ENSG00000253576
NA	0.0159	3.2518E-01		ENSG00000275481
NA	0.1000	3.2535E-01	5.20E-01	ENSG00000279283
NA	0.0401	3.2540E-01		ENSG00000270228
NA	0.0275	3.2551E-01		ENSG00000289368
NA	0.0648	3.2561E-01	5.20E-01	ENSG00000225108
NA	0.0003	3.2577E-01		ENSG00000273257
NA	0.0040	3.2584E-01		ENSG00000240652
NA	0.0160	3.2595E-01		ENSG00000284772
NA	0.0178	3.2596E-01		ENSG00000254288
NA	0.1000	3.2635E-01	5.21E-01	ENSG00000258311
NA	0.0199	3.2649E-01		ENSG00000238156
NA	0.0040	3.2655E-01		ENSG00000286644
NA	0.0153	3.2661E-01		ENSG00000241889
NA	0.0077	3.2661E-01		ENSG00000273786
NA	0.0107	3.2683E-01		ENSG00000266599
NA	0.0122	3.2702E-01		ENSG00000275011
NA	0.0376	3.2723E-01		ENSG00000203392
NA	0.0203	3.2733E-01		ENSG00000248641
NA	0.0121	3.2786E-01		ENSG00000287882
NA	0.0790	3.2791E-01	5.22E-01	ENSG00000283761
NA	0.0416	3.2799E-01		ENSG00000273554
NA	0.0089	3.2804E-01		ENSG00000290100
NA	0.0197	3.2854E-01		ENSG00000235454
NA	0.0070	3.2855E-01		ENSG00000270016
NA	0.0369	3.2862E-01		ENSG00000280890
NA	0.0910	3.2872E-01	5.23E-01	ENSG00000286976
NA	0.0291	3.2884E-01		ENSG00000251456
NA	0.0179	3.2936E-01		ENSG00000248613
NA	0.0334	3.2947E-01		ENSG00000258759
NA	0.0240	3.2994E-01		ENSG00000224159
NA	0.0404	3.2994E-01		ENSG00000273870
NA	0.0127	3.2995E-01		ENSG00000226599
NA	0.0154	3.3016E-01		ENSG00000237934
NA	0.0246	3.3017E-01		ENSG00000228554
NA	0.0131	3.3057E-01		ENSG00000279684

NA	0.0355	3.3074E-01	5.26E-01	ENSG00000269376
NA	0.0393	3.3082E-01		ENSG00000225506
NA	0.0856	3.3121E-01	5.26E-01	ENSG00000287348
NA	0.0099	3.3138E-01		ENSG00000237090
NA	0.0894	3.3164E-01	5.27E-01	ENSG00000260528
NA	0.0218	3.3189E-01		ENSG00000259669
NA	0.0401	3.3197E-01		ENSG00000237617
NA	0.0173	3.3204E-01		ENSG00000234534
NA	0.0076	3.3205E-01		ENSG00000176183
NA	0.0188	3.3209E-01		ENSG00000273549
NA	0.0154	3.3244E-01		ENSG00000207069
NA	0.0204	3.3244E-01		ENSG00000280936
NA	0.0164	3.3253E-01		ENSG00000286073
NA	0.0229	3.3260E-01	5.28E-01	ENSG00000265413
NA	0.0125	3.3279E-01		ENSG00000254873
NA	0.0218	3.3283E-01		ENSG00000207009
NA	0.0781	3.3290E-01	5.28E-01	ENSG00000283973
NA	0.0110	3.3291E-01		ENSG00000250039
NA	0.0229	3.3306E-01		ENSG00000254739
NA	0.0121	3.3337E-01		ENSG00000227107
NA	0.0254	3.3343E-01		ENSG00000279061
NA	0.0747	3.3386E-01	5.29E-01	ENSG00000263366
NA	0.0434	3.3495E-01	5.30E-01	ENSG00000237281
NA	0.0142	3.3501E-01		ENSG00000225355
NA	0.0222	3.3506E-01		ENSG00000287195
NA	0.0771	3.3557E-01	5.31E-01	ENSG00000260271
NA	0.0285	3.3582E-01	5.31E-01	ENSG00000289077
NA	0.0572	3.3612E-01		ENSG00000264924
NA	0.0846	3.3618E-01	5.31E-01	ENSG00000272541
NA	0.0095	3.3638E-01		ENSG00000215007
NA	0.0067	3.3657E-01		ENSG00000280836
NA	0.0541	3.3669E-01		ENSG00000256407
NA	0.0201	3.3671E-01		ENSG00000270385
NA	0.0471	3.3686E-01	5.32E-01	ENSG00000279199
NA	0.0278	3.3723E-01		ENSG00000248459
NA	0.0533	3.3740E-01	5.33E-01	ENSG00000214141
NA	0.0971	3.3747E-01	5.33E-01	ENSG00000272476
NA	0.0112	3.3748E-01		ENSG00000254732
NA	0.0050	3.3748E-01		ENSG00000217527
NA	0.0812	3.3756E-01	5.33E-01	ENSG00000217801
NA	0.0964	3.3768E-01	5.33E-01	ENSG00000260267
NA	0.0959	3.3779E-01	5.33E-01	ENSG00000225511
NA	0.0535	3.3822E-01	5.33E-01	ENSG00000213144
NA	0.0091	3.3855E-01		ENSG00000180610
NA	0.0700	3.3857E-01	5.34E-01	ENSG00000287624
NA	0.0050	3.3867E-01		ENSG00000289620
NA	0.0081	3.3875E-01		ENSG00000254164
NA	0.0218	3.3890E-01		ENSG00000253915
NA	0.0876	3.3892E-01	5.34E-01	ENSG00000286360

NA	0.0163	3.3928E-01		ENSG00000258539
NA	0.0096	3.3950E-01		ENSG00000202279
NA	0.0105	3.3954E-01		ENSG00000213594
NA	0.0493	3.3983E-01		ENSG00000261326
NA	0.0604	3.3991E-01		ENSG00000289060
NA	0.0172	3.4006E-01		ENSG00000237285
NA	0.0103	3.4070E-01		ENSG00000223608
NA	0.0109	3.4108E-01		ENSG00000229259
NA	0.0885	3.4120E-01	5.36E-01	ENSG00000223838
NA	0.0878	3.4124E-01	5.36E-01	ENSG00000287587
NA	0.0956	3.4142E-01	5.37E-01	ENSG00000261079
NA	0.0107	3.4269E-01		ENSG00000230107
NA	0.0178	3.4269E-01		ENSG00000264016
NA	0.0944	3.4276E-01	5.38E-01	ENSG00000279591
NA	0.0155	3.4284E-01		ENSG00000270578
NA	0.0369	3.4289E-01	5.38E-01	ENSG00000279930
NA	0.0608	3.4297E-01		ENSG00000255556
NA	0.0102	3.4325E-01		ENSG00000289178
NA	0.0438	3.4348E-01	5.39E-01	ENSG00000285952
NA	0.0157	3.4429E-01		ENSG00000286043
NA	0.0103	3.4438E-01		ENSG00000231295
NA	0.0874	3.4472E-01	5.40E-01	ENSG00000235381
NA	0.0740	3.4489E-01	5.40E-01	ENSG00000228302
NA	0.0848	3.4522E-01	5.40E-01	ENSG00000236708
NA	0.0532	3.4548E-01	5.41E-01	ENSG00000289346
NA	0.0855	3.4581E-01	5.41E-01	ENSG00000282978
NA	0.0946	3.4604E-01	5.41E-01	ENSG00000261575
NA	0.0847	3.4605E-01	5.41E-01	ENSG00000287336
NA	0.0204	3.4607E-01		ENSG00000203435
NA	0.0076	3.4624E-01		ENSG00000260690
NA	0.0143	3.4629E-01		ENSG00000266378
NA	0.0517	3.4645E-01	5.42E-01	ENSG00000250686
NA	0.0549	3.4646E-01	5.42E-01	ENSG00000286786
NA	0.0160	3.4677E-01		ENSG00000286276
NA	0.0136	3.4731E-01		ENSG00000267070
NA	0.0947	3.4756E-01	5.43E-01	ENSG00000227775
NA	0.0097	3.4760E-01	5.43E-01	ENSG00000226038
NA	0.0756	3.4784E-01	5.43E-01	ENSG00000289983
NA	0.0199	3.4844E-01		ENSG00000214264
NA	0.0208	3.4890E-01		ENSG00000227170
NA	0.0211	3.4932E-01		ENSG00000232362
NA	0.0133	3.4967E-01		ENSG00000249212
NA	0.0721	3.5011E-01	5.45E-01	ENSG00000253116
NA	0.0431	3.5049E-01		ENSG00000270210
NA	0.0949	3.5081E-01	5.46E-01	ENSG00000286889
NA	0.0181	3.5100E-01		ENSG00000288986
NA	0.0317	3.5118E-01		ENSG00000237154
NA	0.0472	3.5128E-01	5.46E-01	ENSG00000259039
NA	0.0058	3.5140E-01		ENSG00000224142

NA	0.0291	3.5178E-01		ENSG00000272002
NA	0.0127	3.5237E-01		ENSG00000232524
NA	0.0497	3.5253E-01		ENSG00000261019
NA	0.0884	3.5273E-01	5.48E-01	ENSG00000237017
NA	0.0385	3.5286E-01		ENSG00000284626
NA	0.0882	3.5286E-01	5.48E-01	ENSG00000269399
NA	0.0060	3.5297E-01		ENSG00000285898
NA	0.0085	3.5320E-01		ENSG00000266469
NA	0.0147	3.5401E-01		ENSG00000255035
NA	0.0299	3.5404E-01	5.49E-01	ENSG00000213574
NA	0.0135	3.5416E-01		ENSG00000275097
NA	0.0784	3.5443E-01	5.50E-01	ENSG00000255159
NA	0.0908	3.5445E-01	5.50E-01	ENSG00000242861
NA	0.0204	3.5475E-01		ENSG00000243495
NA	0.0171	3.5525E-01		ENSG00000259244
NA	0.0181	3.5541E-01		ENSG00000271314
NA	0.0578	3.5554E-01		ENSG00000286082
NA	0.0050	3.5556E-01		ENSG00000253766
NA	0.0169	3.5602E-01		ENSG00000228876
NA	0.0112	3.5604E-01		ENSG00000273591
NA	0.0329	3.5615E-01		ENSG00000283384
NA	0.0103	3.5637E-01		ENSG00000237418
NA	0.0033	3.5644E-01		ENSG00000274680
NA	0.0078	3.5645E-01		ENSG00000180019
NA	0.0758	3.5645E-01	5.52E-01	ENSG00000289086
NA	0.0745	3.5751E-01	5.53E-01	ENSG00000288526
NA	0.0947	3.5753E-01	5.53E-01	ENSG00000229587
NA	0.0922	3.5769E-01	5.53E-01	ENSG00000288750
NA	0.0685	3.5830E-01	5.54E-01	ENSG00000243659
NA	0.0028	3.5845E-01	5.54E-01	ENSG00000214553
NA	0.0145	3.5849E-01		ENSG00000227338
NA	0.0169	3.5896E-01		ENSG00000279794
NA	0.0090	3.5913E-01		ENSG00000231877
NA	0.0471	3.5919E-01		ENSG00000239528
NA	0.0099	3.5929E-01		ENSG00000253976
NA	0.0168	3.5955E-01		ENSG00000231724
NA	0.0873	3.5960E-01	5.56E-01	ENSG00000286837
NA	0.0874	3.5972E-01	5.56E-01	ENSG00000289018
NA	0.0719	3.5979E-01	5.56E-01	ENSG00000251333
NA	0.0112	3.5991E-01		ENSG00000267786
NA	0.0883	3.6011E-01	5.56E-01	ENSG00000261242
NA	0.0520	3.6035E-01	5.56E-01	ENSG00000263503
NA	0.0570	3.6058E-01	5.57E-01	ENSG00000231208
NA	0.0518	3.6064E-01	5.57E-01	ENSG00000231414
NA	0.0897	3.6087E-01	5.57E-01	ENSG00000277534
NA	0.0191	3.6091E-01		ENSG00000267185
NA	0.0774	3.6092E-01	5.57E-01	ENSG00000261654
NA	0.0174	3.6112E-01	5.57E-01	ENSG00000273432
NA	0.0889	3.6134E-01	5.57E-01	ENSG00000288612

NA	0.0293	3.6138E-01		ENSG00000258670
NA	0.0141	3.6145E-01		ENSG00000286561
NA	0.0924	3.6160E-01	5.58E-01	ENSG00000289486
NA	0.0033	3.6170E-01		ENSG00000269463
NA	0.0621	3.6171E-01	5.58E-01	ENSG00000270755
NA	0.0639	3.6192E-01	5.58E-01	ENSG00000279632
NA	0.0277	3.6227E-01		ENSG00000289545
NA	0.0098	3.6272E-01		ENSG00000265096
NA	0.0886	3.6285E-01	5.59E-01	ENSG00000210140
NA	0.0911	3.6290E-01	5.59E-01	ENSG00000100068
NA	0.0268	3.6298E-01	5.59E-01	ENSG00000251000
NA	0.0856	3.6302E-01	5.59E-01	ENSG00000266910
NA	0.0661	3.6340E-01	5.59E-01	ENSG00000280217
NA	0.0351	3.6341E-01		ENSG00000235489
NA	0.0022	3.6352E-01		ENSG00000277977
NA	0.0077	3.6363E-01		ENSG00000235461
NA	0.0125	3.6371E-01		ENSG00000279200
NA	0.0294	3.6388E-01		ENSG00000279936
NA	0.0023	3.6438E-01		ENSG00000286970
NA	0.0336	3.6453E-01		ENSG00000236327
NA	0.0843	3.6470E-01	5.61E-01	ENSG00000276805
NA	0.0597	3.6490E-01	5.61E-01	ENSG00000239480
NA	0.0395	3.6527E-01		ENSG00000242104
NA	0.0118	3.6625E-01		ENSG00000279845
NA	0.0167	3.6637E-01		ENSG00000219700
NA	0.0093	3.6642E-01		ENSG00000228901
NA	0.0173	3.6668E-01		ENSG00000240048
NA	0.0771	3.6688E-01	5.63E-01	ENSG00000265888
NA	0.0408	3.6708E-01	5.63E-01	ENSG00000289910
NA	0.0133	3.6764E-01		ENSG00000253166
NA	0.0756	3.6799E-01	5.64E-01	ENSG00000289376
NA	0.0116	3.6813E-01		ENSG00000237645
NA	0.0097	3.6828E-01		ENSG00000286838
NA	0.0335	3.6858E-01	5.64E-01	ENSG00000289986
NA	0.0838	3.6858E-01	5.64E-01	ENSG00000286682
NA	0.0898	3.6915E-01	5.65E-01	ENSG00000233264
NA	0.0795	3.6942E-01	5.65E-01	ENSG00000279416
NA	0.0501	3.6996E-01	5.66E-01	ENSG00000185684
NA	0.0894	3.7046E-01	5.66E-01	ENSG00000279198
NA	0.0058	3.7066E-01		ENSG00000274501
NA	0.0143	3.7092E-01		ENSG00000255892
NA	0.0123	3.7102E-01		ENSG00000275070
NA	0.0403	3.7120E-01		ENSG00000182722
NA	0.0886	3.7121E-01	5.67E-01	ENSG00000176700
NA	0.0356	3.7122E-01	5.67E-01	ENSG00000273343
NA	0.0144	3.7188E-01		ENSG00000252202
NA	0.0223	3.7191E-01		ENSG00000201600
NA	0.0139	3.7212E-01		ENSG00000250971
NA	0.0241	3.7213E-01		ENSG00000288690

NA	0.0842	3.7214E-01	5.67E-01	ENSG00000283491
NA	0.0636	3.7273E-01	5.68E-01	ENSG00000289990
NA	0.0902	3.7288E-01	5.68E-01	ENSG00000289390
NA	0.0745	3.7304E-01	5.68E-01	ENSG00000270681
NA	0.0824	3.7316E-01	5.68E-01	ENSG00000234688
NA	0.0097	3.7319E-01		ENSG00000272134
NA	0.0174	3.7349E-01		ENSG00000279954
NA	0.0183	3.7358E-01		ENSG00000286922
NA	0.0766	3.7372E-01	5.69E-01	ENSG00000263466
NA	0.0671	3.7385E-01	5.69E-01	ENSG00000217653
NA	0.0717	3.7512E-01	5.71E-01	ENSG00000250170
NA	0.0215	3.7524E-01		ENSG00000286886
NA	0.0871	3.7551E-01	5.71E-01	ENSG00000224043
NA	0.0208	3.7585E-01		ENSG00000223254
NA	0.0815	3.7594E-01	5.71E-01	ENSG00000258175
NA	0.0873	3.7596E-01	5.71E-01	ENSG00000273674
NA	0.0135	3.7609E-01		ENSG00000235047
NA	0.0458	3.7643E-01	5.72E-01	ENSG00000284677
NA	0.0820	3.7645E-01	5.72E-01	ENSG00000271538
NA	0.0856	3.7646E-01	5.72E-01	ENSG00000270872
NA	0.0132	3.7656E-01		ENSG00000255519
NA	0.0175	3.7674E-01		ENSG00000240216
NA	0.0123	3.7676E-01		ENSG00000232654
NA	0.0130	3.7684E-01		ENSG00000228928
NA	0.0123	3.7709E-01		ENSG00000260159
NA	0.0705	3.7718E-01	5.73E-01	ENSG00000268204
NA	0.0211	3.7733E-01		ENSG00000248423
NA	0.0220	3.7740E-01		ENSG00000242352
NA	0.0298	3.7742E-01		ENSG00000287439
NA	0.0662	3.7742E-01	5.73E-01	ENSG00000226571
NA	0.0088	3.7788E-01		ENSG00000254444
NA	0.0869	3.7800E-01	5.73E-01	ENSG00000275769
NA	0.0181	3.7812E-01		ENSG00000248416
NA	0.0095	3.7828E-01		ENSG00000230637
NA	0.0546	3.7837E-01	5.73E-01	ENSG00000273350
NA	0.0722	3.7839E-01	5.73E-01	ENSG00000260132
NA	0.0855	3.7888E-01	5.74E-01	ENSG00000275180
NA	0.0869	3.7890E-01	5.74E-01	ENSG00000290048
NA	0.0756	3.7936E-01	5.74E-01	ENSG00000272854
NA	0.0192	3.7937E-01		ENSG00000271199
NA	0.0125	3.7940E-01		ENSG00000279940
NA	0.0436	3.8020E-01		ENSG00000285680
NA	0.0638	3.8032E-01	5.75E-01	ENSG00000235688
NA	0.0886	3.8034E-01	5.75E-01	ENSG00000205702
NA	0.0632	3.8038E-01	5.75E-01	ENSG00000279912
NA	0.0136	3.8042E-01		ENSG00000289342
NA	0.0573	3.8100E-01	5.76E-01	ENSG00000246851
NA	0.0537	3.8110E-01	5.76E-01	ENSG00000254092
NA	0.0062	3.8120E-01		ENSG00000266217

NA	0.0878	3.8131E-01	5.76E-01	ENSG00000280046
NA	0.0096	3.8136E-01		ENSG00000287479
NA	0.0133	3.8158E-01		ENSG00000285734
NA	0.0122	3.8159E-01		ENSG00000234967
NA	0.0471	3.8171E-01	5.77E-01	ENSG00000253582
NA	0.0650	3.8178E-01	5.77E-01	ENSG00000274425
NA	0.0135	3.8183E-01		ENSG00000222439
NA	0.0500	3.8186E-01	5.77E-01	ENSG00000257740
NA	0.0186	3.8200E-01		ENSG00000250995
NA	0.0871	3.8201E-01	5.77E-01	ENSG00000270996
NA	0.0464	3.8203E-01	5.77E-01	ENSG00000223349
NA	0.0841	3.8205E-01	5.77E-01	ENSG00000276248
NA	0.0160	3.8215E-01		ENSG00000223039
NA	0.0150	3.8231E-01		ENSG00000287020
NA	0.0328	3.8246E-01		ENSG00000226352
NA	0.0835	3.8262E-01	5.77E-01	ENSG00000279474
NA	0.0308	3.8313E-01		ENSG00000274892
NA	0.0284	3.8346E-01	5.78E-01	ENSG00000270050
NA	0.0027	3.8355E-01		ENSG00000217078
NA	0.0125	3.8368E-01	5.78E-01	ENSG00000273375
NA	0.0423	3.8374E-01		ENSG00000225671
NA	0.0876	3.8381E-01	5.78E-01	ENSG00000224358
NA	0.0108	3.8388E-01		ENSG00000258593
NA	0.0157	3.8389E-01		ENSG00000260725
NA	0.0363	3.8402E-01		ENSG00000278028
NA	0.0877	3.8440E-01	5.79E-01	ENSG00000242338
NA	0.0829	3.8441E-01	5.79E-01	ENSG00000279233
NA	0.0609	3.8444E-01	5.79E-01	ENSG00000288085
NA	0.0308	3.8453E-01		ENSG00000277127
NA	0.0514	3.8473E-01	5.79E-01	ENSG00000287036
NA	0.0221	3.8482E-01		ENSG00000225328
NA	0.0249	3.8497E-01		ENSG00000237868
NA	0.0085	3.8537E-01		ENSG00000265019
NA	0.0156	3.8539E-01		ENSG00000236505
NA	0.0461	3.8541E-01		ENSG00000188755
NA	0.0707	3.8543E-01	5.80E-01	ENSG00000254275
NA	0.0864	3.8545E-01	5.80E-01	ENSG00000203593
NA	0.0025	3.8598E-01	5.81E-01	ENSG00000288042
NA	0.0849	3.8661E-01	5.81E-01	ENSG00000227704
NA	0.0112	3.8666E-01		ENSG00000257966
NA	0.0104	3.8698E-01		ENSG00000241357
NA	0.0308	3.8722E-01		ENSG00000279410
NA	0.0119	3.8747E-01		ENSG00000253108
NA	0.0203	3.8753E-01		ENSG00000287537
NA	0.0794	3.8806E-01	5.83E-01	ENSG00000289574
NA	0.0115	3.8843E-01	5.83E-01	ENSG00000260600
NA	0.0234	3.8865E-01		ENSG00000253899
NA	0.0651	3.8867E-01	5.83E-01	ENSG00000227486
NA	0.0141	3.8898E-01		ENSG00000287432

NA	0.0854	3.8922E-01	5.84E-01	ENSG00000267113
NA	0.0026	3.8925E-01		ENSG00000273965
NA	0.0090	3.8944E-01		ENSG00000277825
NA	0.0806	3.8954E-01	5.84E-01	ENSG00000263571
NA	0.0828	3.8967E-01	5.84E-01	ENSG00000226803
NA	0.0803	3.8973E-01	5.84E-01	ENSG00000279583
NA	0.0269	3.9039E-01		ENSG00000282980
NA	0.0312	3.9044E-01		ENSG00000258524
NA	0.0046	3.9059E-01		ENSG00000236450
NA	0.0448	3.9072E-01	5.85E-01	ENSG00000289122
NA	0.0230	3.9123E-01		ENSG00000225703
NA	0.0850	3.9141E-01	5.86E-01	ENSG00000288095
NA	0.0648	3.9169E-01	5.86E-01	ENSG00000283208
NA	0.0132	3.9186E-01		ENSG00000259442
NA	0.0365	3.9206E-01	5.87E-01	ENSG00000228028
NA	0.0062	3.9220E-01		ENSG00000254258
NA	0.0484	3.9228E-01		ENSG00000248750
NA	0.0178	3.9241E-01	5.87E-01	ENSG00000286903
NA	0.0109	3.9353E-01		ENSG00000260677
NA	0.0349	3.9359E-01		ENSG00000251446
NA	0.0066	3.9363E-01		ENSG00000262172
NA	0.0463	3.9369E-01		ENSG00000286890
NA	0.0575	3.9374E-01	5.89E-01	ENSG00000274414
NA	0.0784	3.9377E-01	5.89E-01	ENSG00000233396
NA	0.0675	3.9380E-01	5.89E-01	ENSG00000287031
NA	0.0157	3.9393E-01		ENSG00000231802
NA	0.0311	3.9433E-01		ENSG00000277290
NA	0.0768	3.9460E-01	5.89E-01	ENSG00000260038
NA	0.0171	3.9466E-01		ENSG00000279497
NA	0.0083	3.9486E-01	5.89E-01	ENSG00000231496
NA	0.0177	3.9511E-01		ENSG00000270987
NA	0.0209	3.9518E-01		ENSG00000286241
NA	0.0157	3.9553E-01		ENSG00000289477
NA	0.0038	3.9556E-01		ENSG00000229312
NA	0.0159	3.9559E-01		ENSG00000214807
NA	0.0347	3.9560E-01		ENSG00000229582
NA	0.0084	3.9568E-01		ENSG00000242390
NA	0.0137	3.9573E-01		ENSG00000255730
NA	0.0703	3.9576E-01	5.90E-01	ENSG00000237877
NA	0.0062	3.9629E-01		ENSG00000267560
NA	0.0155	3.9658E-01		ENSG00000262067
NA	0.0573	3.9672E-01	5.91E-01	ENSG00000250271
NA	0.0256	3.9699E-01		ENSG00000249771
NA	0.0068	3.9701E-01		ENSG00000267612
NA	0.0110	3.9707E-01		ENSG00000227673
NA	0.0147	3.9732E-01		ENSG00000286512
NA	0.0063	3.9746E-01		ENSG00000286090
NA	0.0162	3.9755E-01		ENSG00000226666
NA	0.0126	3.9755E-01		ENSG00000266733

NA	0.0082	3.9757E-01		ENSG00000263235
NA	0.0148	3.9759E-01		ENSG00000279415
NA	0.0329	3.9774E-01		ENSG00000258981
NA	0.0438	3.9788E-01	5.92E-01	ENSG00000249485
NA	0.0070	3.9806E-01		ENSG00000224834
NA	0.0467	3.9822E-01	5.93E-01	ENSG00000286442
NA	0.0422	3.9862E-01		ENSG00000285900
NA	0.0193	3.9885E-01		ENSG00000243797
NA	0.0070	3.9923E-01	5.93E-01	ENSG00000286144
NA	0.0044	3.9939E-01		ENSG00000258925
NA	0.0037	3.9986E-01		ENSG00000287447
NA	0.0832	3.9989E-01	5.94E-01	ENSG00000255121
NA	0.0284	3.9997E-01		ENSG00000263301
NA	0.0816	4.0067E-01	5.95E-01	ENSG00000266602
NA	0.0758	4.0091E-01	5.95E-01	ENSG00000281332
NA	0.0280	4.0132E-01		ENSG00000274996
NA	0.0103	4.0152E-01		ENSG00000289957
NA	0.0814	4.0171E-01	5.96E-01	ENSG00000279641
NA	0.0167	4.0172E-01		ENSG00000273957
NA	0.0187	4.0194E-01		ENSG00000199667
NA	0.0536	4.0210E-01	5.96E-01	ENSG00000272347
NA	0.0570	4.0212E-01		ENSG00000285887
NA	0.0158	4.0228E-01		ENSG00000228639
NA	0.0078	4.0239E-01		ENSG00000229522
NA	0.0536	4.0246E-01	5.96E-01	ENSG00000278905
NA	0.0084	4.0256E-01		ENSG00000287990
NA	0.0106	4.0259E-01		ENSG00000288972
NA	0.0808	4.0268E-01	5.97E-01	ENSG00000280120
NA	0.0333	4.0277E-01		ENSG00000286568
NA	0.0819	4.0317E-01	5.97E-01	ENSG00000237748
NA	0.0799	4.0335E-01	5.97E-01	ENSG00000288904
NA	0.0351	4.0339E-01	5.97E-01	ENSG00000219693
NA	0.0107	4.0347E-01		ENSG00000278001
NA	0.0470	4.0354E-01	5.98E-01	ENSG00000269815
NA	0.0095	4.0363E-01		ENSG00000287269
NA	0.0790	4.0375E-01	5.98E-01	ENSG00000224086
NA	0.0190	4.0459E-01		ENSG00000279330
NA	0.0301	4.0465E-01		ENSG00000201078
NA	0.0098	4.0499E-01		ENSG00000250431
NA	0.0078	4.0501E-01		ENSG00000268027
NA	0.0762	4.0552E-01	5.99E-01	ENSG00000279673
NA	0.0269	4.0633E-01		ENSG00000241535
NA	0.0087	4.0671E-01		ENSG00000231378
NA	0.0070	4.0686E-01		ENSG00000263477
NA	0.0194	4.0709E-01		ENSG00000260070
NA	0.0471	4.0728E-01	6.01E-01	ENSG00000266501
NA	0.0762	4.0733E-01	6.01E-01	ENSG00000248780
NA	0.0314	4.0746E-01	6.01E-01	ENSG00000290001
NA	0.0091	4.0748E-01		ENSG00000257654

NA	0.0830	4.0783E-01	6.01E-01	ENSG00000257839
NA	0.0713	4.0790E-01	6.01E-01	ENSG00000251577
NA	0.0012	4.0822E-01		ENSG00000206739
NA	0.0046	4.0856E-01		ENSG00000267207
NA	0.0139	4.0884E-01		ENSG00000287421
NA	0.0445	4.0885E-01	6.02E-01	ENSG00000289063
NA	0.0117	4.0890E-01		ENSG00000213452
NA	0.0806	4.0892E-01	6.02E-01	ENSG00000270021
NA	0.0169	4.0897E-01		ENSG00000276620
NA	0.0568	4.0923E-01	6.02E-01	ENSG00000231245
NA	0.0301	4.0931E-01	6.02E-01	ENSG00000279349
NA	0.0479	4.0938E-01	6.03E-01	ENSG00000258859
NA	0.0208	4.0942E-01		ENSG00000290164
NA	0.0394	4.0947E-01		ENSG00000263846
NA	0.0171	4.0959E-01		ENSG00000227702
NA	0.0036	4.0961E-01		ENSG00000269980
NA	0.0038	4.0986E-01		ENSG00000280425
NA	0.0334	4.1005E-01	6.03E-01	ENSG00000260466
NA	0.0106	4.1029E-01		ENSG00000286965
NA	0.0029	4.1030E-01		ENSG00000259498
NA	0.0719	4.1049E-01	6.04E-01	ENSG00000207525
NA	0.0102	4.1049E-01		ENSG00000228423
NA	0.0095	4.1078E-01		ENSG00000285570
NA	0.0229	4.1083E-01		ENSG00000257221
NA	0.0216	4.1130E-01		ENSG00000289864
NA	0.0169	4.1170E-01		ENSG00000167774
NA	0.0093	4.1176E-01		ENSG00000260815
NA	0.0159	4.1195E-01		ENSG00000203363
NA	0.0739	4.1202E-01	6.05E-01	ENSG00000234518
NA	0.0208	4.1268E-01		ENSG00000273853
NA	0.0081	4.1286E-01		ENSG00000236741
NA	0.0086	4.1289E-01		ENSG00000242140
NA	0.0525	4.1291E-01	6.06E-01	ENSG00000258983
NA	0.0084	4.1294E-01		ENSG00000205879
NA	0.0631	4.1306E-01	6.06E-01	ENSG00000230612
NA	0.0083	4.1323E-01		ENSG00000259196
NA	0.0504	4.1340E-01	6.06E-01	ENSG00000287777
NA	0.0763	4.1353E-01	6.06E-01	ENSG00000261342
NA	0.0467	4.1368E-01	6.07E-01	ENSG00000286722
NA	0.0810	4.1376E-01	6.07E-01	ENSG00000227586
NA	0.0514	4.1381E-01	6.07E-01	ENSG00000250959
NA	0.0754	4.1382E-01	6.07E-01	ENSG00000259840
NA	0.0773	4.1382E-01	6.07E-01	ENSG00000286584
NA	0.0537	4.1396E-01	6.07E-01	ENSG00000289068
NA	0.0581	4.1397E-01	6.07E-01	ENSG00000259322
NA	0.0761	4.1493E-01	6.08E-01	ENSG00000225313
NA	0.0014	4.1498E-01		ENSG00000279271
NA	0.0057	4.1509E-01		ENSG00000286916
NA	0.0098	4.1523E-01		ENSG00000287563

NA	0.0271	4.1542E-01		ENSG00000260410
NA	0.0149	4.1547E-01		ENSG00000261997
NA	0.0111	4.1571E-01		ENSG00000280244
NA	0.0469	4.1600E-01	6.09E-01	ENSG00000212856
NA	0.0423	4.1606E-01		ENSG00000130612
NA	0.0155	4.1617E-01		ENSG00000253347
NA	0.0376	4.1632E-01	6.09E-01	ENSG00000288782
NA	0.0200	4.1634E-01		ENSG00000279314
NA	0.0569	4.1668E-01	6.09E-01	ENSG00000268543
NA	0.0202	4.1723E-01		ENSG00000241738
NA	0.0418	4.1746E-01	6.10E-01	ENSG00000270282
NA	0.0300	4.1772E-01	6.10E-01	ENSG00000290023
NA	0.0469	4.1812E-01	6.11E-01	ENSG00000227619
NA	0.0090	4.1812E-01	6.11E-01	ENSG00000288828
NA	0.0182	4.1814E-01		ENSG00000229403
NA	0.0716	4.1856E-01	6.11E-01	ENSG00000277867
NA	0.0152	4.1877E-01		ENSG00000285549
NA	0.0145	4.1878E-01		ENSG00000290013
NA	0.0002	4.1907E-01		ENSG00000287079
NA	0.0048	4.1909E-01		ENSG00000279611
NA	0.0274	4.1934E-01	6.12E-01	ENSG00000237415
NA	0.0616	4.1960E-01	6.12E-01	ENSG00000254929
NA	0.0016	4.1970E-01		ENSG00000271267
NA	0.0748	4.2000E-01	6.12E-01	ENSG00000236875
NA	0.0152	4.2019E-01		ENSG00000286769
NA	0.0181	4.2030E-01		ENSG00000267691
NA	0.0388	4.2044E-01	6.13E-01	ENSG00000248245
NA	0.0112	4.2067E-01		ENSG00000225940
NA	0.0507	4.2106E-01		ENSG00000288046
NA	0.0102	4.2124E-01		ENSG00000287776
NA	0.0201	4.2137E-01	6.14E-01	ENSG00000262920
NA	0.0063	4.2138E-01		ENSG00000258168
NA	0.0686	4.2143E-01	6.14E-01	ENSG00000233334
NA	0.0694	4.2151E-01	6.14E-01	ENSG00000228903
NA	0.0062	4.2152E-01		ENSG00000287438
NA	0.0141	4.2154E-01		ENSG00000249112
NA	0.0085	4.2179E-01		ENSG00000273687
NA	0.0674	4.2216E-01	6.15E-01	ENSG00000285780
NA	0.0667	4.2217E-01	6.15E-01	ENSG00000239763
NA	0.0169	4.2252E-01		ENSG00000280436
NA	0.0119	4.2290E-01		ENSG00000229409
NA	0.0078	4.2299E-01		ENSG00000256209
NA	0.0207	4.2308E-01		ENSG00000249006
NA	0.0473	4.2318E-01	6.15E-01	ENSG00000236274
NA	0.0289	4.2318E-01		ENSG00000254921
NA	0.0786	4.2343E-01	6.16E-01	ENSG00000272732
NA	0.0790	4.2387E-01	6.16E-01	ENSG00000270804
NA	0.0252	4.2406E-01		ENSG00000254644
NA	0.0428	4.2412E-01		ENSG00000276831

NA	0.0783	4.2413E-01	6.16E-01	ENSG00000285593
NA	0.0143	4.2421E-01		ENSG00000289988
NA	0.0495	4.2433E-01	6.16E-01	ENSG00000259648
NA	0.0117	4.2449E-01	6.16E-01	ENSG00000230967
NA	0.0093	4.2458E-01		ENSG00000278239
NA	0.0791	4.2485E-01	6.17E-01	ENSG00000232300
NA	0.0681	4.2486E-01	6.17E-01	ENSG00000239906
NA	0.0193	4.2487E-01		ENSG00000279347
NA	0.0218	4.2493E-01		ENSG00000255028
NA	0.0726	4.2512E-01	6.17E-01	ENSG00000213236
NA	0.0687	4.2535E-01	6.17E-01	ENSG00000244265
NA	0.0760	4.2541E-01	6.17E-01	ENSG00000235957
NA	0.0020	4.2548E-01		ENSG00000206549
NA	0.0149	4.2552E-01		ENSG00000269954
NA	0.0207	4.2554E-01		ENSG00000274560
NA	0.0294	4.2563E-01		ENSG00000288081
NA	0.0804	4.2570E-01	6.17E-01	ENSG00000224094
NA	0.0209	4.2615E-01		ENSG00000260954
NA	0.0152	4.2621E-01		ENSG00000226429
NA	0.0162	4.2626E-01		ENSG00000276269
NA	0.0047	4.2661E-01		ENSG00000236018
NA	0.0782	4.2671E-01	6.19E-01	ENSG00000225156
NA	0.0237	4.2673E-01		ENSG00000261635
NA	0.0108	4.2683E-01		ENSG00000248458
NA	0.0663	4.2723E-01	6.19E-01	ENSG00000277692
NA	0.0537	4.2724E-01	6.19E-01	ENSG00000244479
NA	0.0806	4.2775E-01	6.20E-01	ENSG00000279203
NA	0.0772	4.2779E-01	6.20E-01	ENSG00000287009
NA	0.0364	4.2814E-01		ENSG00000286326
NA	0.0180	4.2821E-01		ENSG00000255523
NA	0.0171	4.2832E-01		ENSG00000272537
NA	0.0678	4.2855E-01	6.20E-01	ENSG00000279026
NA	0.0130	4.2860E-01		ENSG00000263338
NA	0.0109	4.2876E-01		ENSG00000249334
NA	0.0083	4.2883E-01		ENSG00000255361
NA	0.0777	4.2893E-01	6.21E-01	ENSG00000229766
NA	0.0238	4.2905E-01		ENSG00000271366
NA	0.0151	4.2914E-01		ENSG00000264421
NA	0.0178	4.2932E-01		ENSG00000204850
NA	0.0062	4.2971E-01		ENSG00000259316
NA	0.0765	4.2983E-01	6.22E-01	ENSG00000254539
NA	0.0143	4.3111E-01		ENSG00000223373
NA	0.0316	4.3119E-01	6.23E-01	ENSG00000288548
NA	0.0708	4.3142E-01	6.23E-01	ENSG00000279342
NA	0.0071	4.3176E-01		ENSG00000244157
NA	0.0648	4.3176E-01	6.23E-01	ENSG00000228158
NA	0.0010	4.3178E-01		ENSG00000253171
NA	0.0154	4.3185E-01		ENSG00000234476
NA	0.0716	4.3243E-01	6.24E-01	ENSG00000287979

NA	0.0201	4.3247E-01		ENSG00000259103
NA	0.0360	4.3251E-01		ENSG00000223528
NA	0.0037	4.3257E-01		ENSG00000281772
NA	0.0470	4.3316E-01	6.25E-01	ENSG00000286409
NA	0.0136	4.3327E-01		ENSG00000278893
NA	0.0175	4.3342E-01		ENSG00000263278
NA	0.0325	4.3346E-01		ENSG00000236998
NA	0.0062	4.3347E-01		ENSG00000274893
NA	0.0718	4.3349E-01	6.25E-01	ENSG00000248475
NA	0.0276	4.3353E-01		ENSG00000223795
NA	0.0081	4.3360E-01		ENSG00000248428
NA	0.0597	4.3423E-01	6.25E-01	ENSG00000269694
NA	0.0059	4.3426E-01		ENSG00000235522
NA	0.0108	4.3446E-01		ENSG00000267036
NA	0.0200	4.3500E-01		ENSG00000224875
NA	0.0131	4.3502E-01		ENSG00000213344
NA	0.0103	4.3504E-01		ENSG00000213449
NA	0.0122	4.3535E-01		ENSG00000261789
NA	0.0081	4.3595E-01		ENSG00000279699
NA	0.0153	4.3605E-01		ENSG00000228873
NA	0.0763	4.3619E-01	6.27E-01	ENSG00000228010
NA	0.0103	4.3623E-01		ENSG00000249664
NA	0.0776	4.3641E-01	6.28E-01	ENSG00000240207
NA	0.0132	4.3656E-01		ENSG00000267402
NA	0.0777	4.3669E-01	6.28E-01	ENSG00000262482
NA	0.0133	4.3669E-01		ENSG00000258210
NA	0.0764	4.3695E-01	6.28E-01	ENSG00000287299
NA	0.0523	4.3708E-01		ENSG00000273387
NA	0.0735	4.3711E-01	6.28E-01	ENSG00000285770
NA	0.0049	4.3770E-01		ENSG00000287061
NA	0.0206	4.3775E-01		ENSG00000288574
NA	0.0064	4.3782E-01		ENSG00000287738
NA	0.0219	4.3790E-01		ENSG00000275649
NA	0.0181	4.3802E-01		ENSG00000277837
NA	0.0408	4.3816E-01		ENSG00000224969
NA	0.0244	4.3855E-01	6.29E-01	ENSG00000280407
NA	0.0232	4.3867E-01		ENSG00000287626
NA	0.0525	4.3918E-01	6.30E-01	ENSG00000260160
NA	0.0178	4.3924E-01		ENSG00000287535
NA	0.0063	4.3925E-01		ENSG00000253852
NA	0.0288	4.3949E-01	6.30E-01	ENSG00000220908
NA	0.0559	4.3955E-01	6.30E-01	ENSG00000263923
NA	0.0093	4.3959E-01		ENSG00000250060
NA	0.0114	4.3962E-01		ENSG00000280179
NA	0.0056	4.4022E-01		ENSG00000234479
NA	0.0191	4.4031E-01		ENSG00000257657
NA	0.0104	4.4037E-01		ENSG00000259725
NA	0.0251	4.4113E-01		ENSG00000267062
NA	0.0563	4.4114E-01	6.32E-01	ENSG00000257246

NA	0.0710	4.4130E-01	6.32E-01	ENSG00000290112
NA	0.0762	4.4148E-01	6.32E-01	ENSG00000184906
NA	0.0353	4.4150E-01		ENSG00000279839
NA	0.0094	4.4165E-01		ENSG00000279586
NA	0.0645	4.4179E-01	6.32E-01	ENSG00000289687
NA	0.0230	4.4187E-01		ENSG00000277463
NA	0.0078	4.4197E-01		ENSG00000240521
NA	0.0093	4.4228E-01		ENSG00000224237
NA	0.0140	4.4273E-01		ENSG00000234504
NA	0.0275	4.4282E-01		ENSG00000275881
NA	0.0165	4.4300E-01		ENSG00000237019
NA	0.0178	4.4311E-01		ENSG00000268366
NA	0.0606	4.4311E-01	6.34E-01	ENSG00000228791
NA	0.0635	4.4312E-01	6.34E-01	ENSG00000275672
NA	0.0111	4.4312E-01		ENSG00000267420
NA	0.0258	4.4316E-01		ENSG00000286544
NA	0.0138	4.4357E-01		ENSG00000260740
NA	0.0139	4.4357E-01		ENSG00000238132
NA	0.0097	4.4360E-01		ENSG00000223429
NA	0.0137	4.4369E-01		ENSG00000287379
NA	0.0394	4.4384E-01	6.34E-01	ENSG00000253341
NA	0.0095	4.4392E-01	6.34E-01	ENSG00000266036
NA	0.0037	4.4411E-01		ENSG00000288055
NA	0.0609	4.4457E-01	6.35E-01	ENSG00000273306
NA	0.0025	4.4470E-01		ENSG00000280095
NA	0.0075	4.4479E-01		ENSG00000279659
NA	0.0071	4.4489E-01		ENSG00000222343
NA	0.0771	4.4497E-01	6.35E-01	ENSG00000261474
NA	0.0632	4.4507E-01	6.35E-01	ENSG00000248752
NA	0.0266	4.4510E-01		ENSG00000233651
NA	0.0089	4.4511E-01	6.35E-01	ENSG00000236449
NA	0.0068	4.4538E-01		ENSG00000267004
NA	0.0130	4.4576E-01		ENSG00000279164
NA	0.0392	4.4588E-01		ENSG00000286493
NA	0.0191	4.4597E-01		ENSG00000254984
NA	0.0140	4.4666E-01		ENSG00000224817
NA	0.0120	4.4698E-01	6.37E-01	ENSG00000279694
NA	0.0687	4.4698E-01	6.37E-01	ENSG00000230457
NA	0.0123	4.4705E-01		ENSG00000283005
NA	0.0430	4.4738E-01		ENSG00000228280
NA	0.0655	4.4755E-01	6.37E-01	ENSG00000260922
NA	0.0106	4.4792E-01		ENSG00000259280
NA	0.0297	4.4824E-01	6.38E-01	ENSG00000266236
NA	0.0323	4.4842E-01		ENSG00000266202
NA	0.0029	4.4853E-01	6.38E-01	ENSG00000283031
NA	0.0474	4.4862E-01		ENSG00000229458
NA	0.0112	4.4921E-01		ENSG00000236928
NA	0.0010	4.4939E-01		ENSG00000260405
NA	0.0167	4.4956E-01		ENSG00000283457

NA	0.0609	4.4971E-01	6.40E-01	ENSG00000234185
NA	0.0680	4.5015E-01	6.40E-01	ENSG00000260796
NA	0.0107	4.5042E-01		ENSG00000240419
NA	0.0157	4.5060E-01		ENSG00000258399
NA	0.0356	4.5064E-01	6.40E-01	ENSG00000250982
NA	0.0303	4.5065E-01		ENSG00000249140
NA	0.0417	4.5072E-01	6.40E-01	ENSG00000234577
NA	0.0670	4.5086E-01	6.41E-01	ENSG00000237863
NA	0.0131	4.5105E-01		ENSG00000276868
NA	0.0006	4.5105E-01		ENSG00000286488
NA	0.0750	4.5141E-01	6.41E-01	ENSG00000226432
NA	0.0121	4.5156E-01		ENSG00000250522
NA	0.0579	4.5159E-01	6.41E-01	ENSG00000223825
NA	0.0145	4.5162E-01		ENSG00000257475
NA	0.0094	4.5176E-01		ENSG00000228176
NA	0.0002	4.5183E-01		ENSG00000256226
NA	0.0122	4.5193E-01		ENSG00000253625
NA	0.0714	4.5210E-01	6.42E-01	ENSG00000289915
NA	0.0183	4.5211E-01		ENSG00000287457
NA	0.0085	4.5228E-01		ENSG00000237947
NA	0.0116	4.5275E-01		ENSG00000279269
NA	0.0190	4.5278E-01	6.42E-01	ENSG00000232998
NA	0.0054	4.5285E-01		ENSG00000285082
NA	0.0078	4.5313E-01		ENSG00000287934
NA	0.0564	4.5326E-01	6.43E-01	ENSG00000234773
NA	0.0142	4.5353E-01		ENSG00000288054
NA	0.0226	4.5380E-01		ENSG00000248213
NA	0.0178	4.5397E-01		ENSG00000236921
NA	0.0411	4.5404E-01		ENSG00000267385
NA	0.0635	4.5413E-01	6.44E-01	ENSG00000284708
NA	0.0160	4.5416E-01		ENSG00000218459
NA	0.0316	4.5473E-01		ENSG00000277087
NA	0.0122	4.5516E-01		ENSG00000285650
NA	0.0463	4.5526E-01	6.45E-01	ENSG00000271754
NA	0.0525	4.5547E-01	6.45E-01	ENSG00000229018
NA	0.0095	4.5549E-01		ENSG00000270427
NA	0.0082	4.5555E-01		ENSG00000226455
NA	0.0075	4.5605E-01		ENSG00000239465
NA	0.0144	4.5617E-01		ENSG00000262445
NA	0.0087	4.5659E-01		ENSG00000266717
NA	0.0632	4.5666E-01	6.46E-01	ENSG00000258355
NA	0.0216	4.5686E-01		ENSG00000242009
NA	0.0087	4.5696E-01		ENSG00000230011
NA	0.0255	4.5709E-01	6.46E-01	ENSG00000286850
NA	0.0126	4.5731E-01		ENSG00000255522
NA	0.0099	4.5754E-01		ENSG00000289465
NA	0.0737	4.5766E-01	6.47E-01	ENSG00000269918
NA	0.0650	4.5778E-01	6.47E-01	ENSG00000269473
NA	0.0173	4.5790E-01		ENSG00000288010

NA	0.0041	4.5799E-01		ENSG00000254339
NA	0.0159	4.5808E-01		ENSG00000290056
NA	0.0155	4.5823E-01		ENSG00000271983
NA	0.0031	4.5834E-01	6.47E-01	ENSG00000258256
NA	0.0692	4.5842E-01	6.47E-01	ENSG00000198134
NA	0.0044	4.5855E-01		ENSG00000271803
NA	0.0656	4.5894E-01	6.48E-01	ENSG00000273141
NA	0.0039	4.5910E-01	6.48E-01	ENSG00000286667
NA	0.0572	4.5910E-01	6.48E-01	ENSG00000279382
NA	0.0115	4.5921E-01		ENSG00000228540
NA	0.0727	4.5924E-01	6.48E-01	ENSG00000237753
NA	0.0219	4.5928E-01		ENSG00000233367
NA	0.0021	4.5928E-01		ENSG00000231421
NA	0.0067	4.5940E-01		ENSG00000247570
NA	0.0477	4.5976E-01	6.48E-01	ENSG00000256564
NA	0.0477	4.6003E-01	6.49E-01	ENSG00000266946
NA	0.0119	4.6009E-01		ENSG00000277883
NA	0.0076	4.6013E-01	6.49E-01	ENSG00000277350
NA	0.0594	4.6029E-01	6.49E-01	ENSG00000242628
NA	0.0487	4.6038E-01	6.49E-01	ENSG00000236212
NA	0.0267	4.6064E-01		ENSG00000286259
NA	0.0049	4.6108E-01		ENSG00000236390
NA	0.0678	4.6133E-01	6.50E-01	ENSG00000231521
NA	0.0176	4.6157E-01		ENSG00000231892
NA	0.0228	4.6158E-01		ENSG00000267033
NA	0.0247	4.6169E-01		ENSG00000266268
NA	0.0114	4.6190E-01		ENSG00000279998
NA	0.0016	4.6200E-01		ENSG00000289457
NA	0.0372	4.6246E-01		ENSG00000254373
NA	0.0014	4.6247E-01		ENSG00000233653
NA	0.0076	4.6257E-01		ENSG00000227279
NA	0.0725	4.6258E-01	6.51E-01	ENSG00000284602
NA	0.0025	4.6282E-01		ENSG00000286825
NA	0.0177	4.6301E-01		ENSG00000200059
NA	0.0591	4.6304E-01	6.51E-01	ENSG00000214773
NA	0.0135	4.6304E-01		ENSG00000233980
NA	0.0109	4.6326E-01		ENSG00000286533
NA	0.0096	4.6331E-01		ENSG00000282975
NA	0.0208	4.6360E-01		ENSG00000285849
NA	0.0526	4.6396E-01	6.52E-01	ENSG00000244346
NA	0.0110	4.6399E-01		ENSG00000237417
NA	0.0288	4.6408E-01	6.52E-01	ENSG00000260518
NA	0.0093	4.6409E-01		ENSG00000280231
NA	0.0241	4.6425E-01		ENSG00000258215
NA	0.0510	4.6477E-01	6.53E-01	ENSG00000267892
NA	0.0144	4.6492E-01		ENSG00000272866
NA	0.0079	4.6527E-01		ENSG00000280104
NA	0.0375	4.6555E-01	6.54E-01	ENSG00000222448
NA	0.0163	4.6556E-01		ENSG00000232259

NA	0.0058	4.6568E-01		ENSG00000225066
NA	0.0269	4.6598E-01		ENSG00000257496
NA	0.0258	4.6604E-01		ENSG00000251276
NA	0.0503	4.6606E-01		ENSG00000275091
NA	0.0077	4.6629E-01		ENSG00000243225
NA	0.0016	4.6633E-01		ENSG00000283563
NA	0.0700	4.6649E-01	6.54E-01	ENSG00000287623
NA	0.0398	4.6663E-01	6.55E-01	ENSG00000231628
NA	0.0086	4.6666E-01		ENSG00000251205
NA	0.0615	4.6690E-01	6.55E-01	ENSG00000267491
NA	0.0107	4.6698E-01		ENSG00000277001
NA	0.0665	4.6745E-01	6.55E-01	ENSG00000289727
NA	0.0119	4.6745E-01		ENSG00000230113
NA	0.0386	4.6792E-01	6.56E-01	ENSG00000278009
NA	0.0092	4.6824E-01		ENSG00000259308
NA	0.0131	4.6842E-01		ENSG00000204055
NA	0.0281	4.6879E-01		ENSG00000280440
NA	0.0124	4.6881E-01		ENSG00000256717
NA	0.0062	4.6909E-01		ENSG00000270521
NA	0.0701	4.6952E-01	6.57E-01	ENSG00000272455
NA	0.0351	4.6954E-01		ENSG00000183929
NA	0.0092	4.6970E-01		ENSG00000251374
NA	0.0211	4.6984E-01		ENSG00000250778
NA	0.0208	4.6989E-01		ENSG00000236457
NA	0.0368	4.6991E-01	6.57E-01	ENSG00000284902
NA	0.0712	4.6996E-01	6.57E-01	ENSG00000290008
NA	0.0135	4.7021E-01	6.58E-01	ENSG00000288963
NA	0.0121	4.7024E-01		ENSG00000286948
NA	0.0310	4.7038E-01	6.58E-01	ENSG00000268375
NA	0.0089	4.7052E-01		ENSG00000228195
NA	0.0036	4.7060E-01		ENSG00000280062
NA	0.0047	4.7071E-01		ENSG00000286217
NA	0.0191	4.7071E-01		ENSG00000230747
NA	0.0364	4.7094E-01	6.58E-01	ENSG00000289029
NA	0.0350	4.7096E-01		ENSG00000285851
NA	0.0109	4.7115E-01		ENSG00000253567
NA	0.0086	4.7121E-01		ENSG00000249084
NA	0.0121	4.7135E-01		ENSG00000289628
NA	0.0105	4.7147E-01		ENSG00000287113
NA	0.0043	4.7158E-01		ENSG00000285577
NA	0.0625	4.7167E-01	6.59E-01	ENSG00000270076
NA	0.0025	4.7172E-01		ENSG00000260648
NA	0.0536	4.7195E-01	6.59E-01	ENSG00000253519
NA	0.0116	4.7198E-01		ENSG00000253236
NA	0.0069	4.7198E-01		ENSG00000279996
NA	0.0679	4.7217E-01	6.60E-01	ENSG00000287168
NA	0.0334	4.7226E-01	6.60E-01	ENSG00000214772
NA	0.0090	4.7236E-01		ENSG00000222881
NA	0.0111	4.7237E-01		ENSG00000264324

NA	0.0717	4.7253E-01	6.60E-01	ENSG00000244300
NA	0.0153	4.7259E-01		ENSG00000261305
NA	0.0039	4.7260E-01		ENSG00000287316
NA	0.0204	4.7268E-01		ENSG00000229391
NA	0.0157	4.7289E-01		ENSG00000286341
NA	0.0524	4.7340E-01	6.60E-01	ENSG00000274898
NA	0.0510	4.7348E-01	6.60E-01	ENSG00000260095
NA	0.0693	4.7363E-01	6.60E-01	ENSG00000277741
NA	0.0389	4.7366E-01	6.60E-01	ENSG00000272369
NA	0.0121	4.7389E-01		ENSG00000286080
NA	0.0097	4.7401E-01		ENSG00000254978
NA	0.0070	4.7402E-01		ENSG00000237438
NA	0.0495	4.7406E-01	6.61E-01	ENSG00000224228
NA	0.0102	4.7410E-01		ENSG00000274269
NA	0.0415	4.7418E-01	6.61E-01	ENSG00000289365
NA	0.0600	4.7443E-01	6.61E-01	ENSG00000286482
NA	0.0259	4.7478E-01		ENSG00000287252
NA	0.0494	4.7482E-01	6.62E-01	ENSG00000259817
NA	0.0606	4.7513E-01	6.62E-01	ENSG00000286460
NA	0.0064	4.7540E-01		ENSG00000289904
NA	0.0676	4.7544E-01	6.62E-01	ENSG00000263081
NA	0.0136	4.7556E-01		ENSG00000227718
NA	0.0278	4.7559E-01		ENSG00000287183
NA	0.0569	4.7582E-01	6.63E-01	ENSG00000285970
NA	0.0275	4.7582E-01		ENSG00000253218
NA	0.0468	4.7622E-01	6.63E-01	ENSG00000272115
NA	0.0027	4.7630E-01		ENSG00000259198
NA	0.0623	4.7642E-01	6.63E-01	ENSG00000271200
NA	0.0682	4.7680E-01	6.64E-01	ENSG00000283156
NA	0.0047	4.7708E-01	6.64E-01	ENSG00000278396
NA	0.0583	4.7750E-01	6.64E-01	ENSG00000262332
NA	0.0093	4.7754E-01		ENSG00000250596
NA	0.0113	4.7759E-01		ENSG00000271490
NA	0.0152	4.7798E-01		ENSG00000285727
NA	0.0165	4.7819E-01		ENSG00000284292
NA	0.0474	4.7835E-01	6.64E-01	ENSG00000254263
NA	0.0133	4.7854E-01		ENSG00000266527
NA	0.0022	4.7858E-01		ENSG00000286481
NA	0.0477	4.7897E-01	6.65E-01	ENSG00000254350
NA	0.0648	4.7916E-01	6.65E-01	ENSG00000257568
NA	0.0688	4.7916E-01	6.65E-01	ENSG00000272247
NA	0.0481	4.7919E-01	6.65E-01	ENSG00000229917
NA	0.0511	4.7920E-01	6.65E-01	ENSG00000226287
NA	0.0622	4.7925E-01	6.65E-01	ENSG00000242659
NA	0.0050	4.7958E-01	6.65E-01	ENSG00000265451
NA	0.0585	4.7995E-01	6.65E-01	ENSG00000206706
NA	0.0085	4.7999E-01		ENSG00000254909
NA	0.0313	4.8024E-01	6.66E-01	ENSG00000289272
NA	0.0177	4.8040E-01		ENSG00000218428

NA	0.0140	4.8041E-01		ENSG00000287134
NA	0.0082	4.8065E-01		ENSG00000284716
NA	0.0186	4.8072E-01		ENSG00000202479
NA	0.0112	4.8085E-01		ENSG00000259595
NA	0.0088	4.8102E-01		ENSG00000235911
NA	0.0349	4.8103E-01	6.66E-01	ENSG00000272689
NA	0.0238	4.8140E-01		ENSG00000232389
NA	0.0130	4.8171E-01		ENSG00000260487
NA	0.0391	4.8184E-01	6.67E-01	ENSG00000229990
NA	0.0073	4.8209E-01		ENSG00000224950
NA	0.0281	4.8221E-01		ENSG00000243280
NA	0.0655	4.8225E-01	6.67E-01	ENSG00000231769
NA	0.0194	4.8246E-01		ENSG00000265863
NA	0.0021	4.8259E-01		ENSG00000240759
NA	0.0077	4.8296E-01		ENSG00000256616
NA	0.0265	4.8362E-01	6.68E-01	ENSG00000285608
NA	0.0089	4.8401E-01		ENSG00000253573
NA	0.0192	4.8411E-01	6.69E-01	ENSG00000267375
NA	0.0471	4.8443E-01	6.69E-01	ENSG00000200788
NA	0.0676	4.8447E-01	6.69E-01	ENSG00000273565
NA	0.0623	4.8458E-01	6.69E-01	ENSG00000279070
NA	0.0302	4.8463E-01	6.69E-01	ENSG00000264932
NA	0.0683	4.8470E-01	6.69E-01	ENSG00000258515
NA	0.0073	4.8508E-01	6.70E-01	ENSG00000263657
NA	0.0139	4.8513E-01		ENSG00000234361
NA	0.0600	4.8530E-01	6.70E-01	ENSG00000271781
NA	0.0038	4.8548E-01		ENSG00000230912
NA	0.0190	4.8577E-01		ENSG00000227406
NA	0.0640	4.8592E-01	6.70E-01	ENSG00000245662
NA	0.0077	4.8594E-01		ENSG00000271077
NA	0.0067	4.8607E-01		ENSG00000268750
NA	0.0036	4.8637E-01		ENSG00000235042
NA	0.0060	4.8642E-01		ENSG00000185390
NA	0.0327	4.8662E-01		ENSG00000263878
NA	0.0465	4.8663E-01	6.71E-01	ENSG00000273247
NA	0.0050	4.8686E-01		ENSG00000232896
NA	0.0090	4.8691E-01		ENSG00000269009
NA	0.0652	4.8697E-01	6.71E-01	ENSG00000279058
NA	0.0144	4.8702E-01		ENSG00000268278
NA	0.0681	4.8705E-01	6.71E-01	ENSG00000271991
NA	0.0182	4.8737E-01		ENSG00000287680
NA	0.0093	4.8753E-01	6.72E-01	ENSG00000272948
NA	0.0672	4.8765E-01	6.72E-01	ENSG00000272173
NA	0.0166	4.8788E-01		ENSG00000230170
NA	0.0077	4.8807E-01		ENSG00000236760
NA	0.0650	4.8807E-01	6.72E-01	ENSG00000275552
NA	0.0090	4.8814E-01		ENSG00000256293
NA	0.0129	4.8849E-01		ENSG00000218965
NA	0.0230	4.8862E-01		ENSG00000248880

NA	0.0177	4.8892E-01		ENSG00000271788
NA	0.0663	4.8906E-01	6.73E-01	ENSG00000260249
NA	0.0270	4.8960E-01	6.74E-01	ENSG00000262678
NA	0.0039	4.8979E-01		ENSG00000276417
NA	0.0086	4.8980E-01	6.74E-01	ENSG00000278725
NA	0.0075	4.8981E-01		ENSG00000224888
NA	0.0496	4.8990E-01	6.74E-01	ENSG00000289073
NA	0.0141	4.8996E-01		ENSG00000254659
NA	0.0198	4.9023E-01		ENSG00000286773
NA	0.0222	4.9024E-01		ENSG00000207484
NA	0.0111	4.9031E-01		ENSG00000240935
NA	0.0176	4.9034E-01		ENSG00000224342
NA	0.0077	4.9069E-01		ENSG00000226822
NA	0.0339	4.9069E-01	6.75E-01	ENSG00000200874
NA	0.0115	4.9098E-01		ENSG00000212533
NA	0.0045	4.9123E-01		ENSG00000253406
NA	0.0735	4.9132E-01	6.75E-01	ENSG00000286736
NA	0.0065	4.9163E-01		ENSG00000279764
NA	0.0651	4.9187E-01	6.75E-01	ENSG00000280061
NA	0.0110	4.9190E-01		ENSG00000257885
NA	0.0566	4.9194E-01	6.75E-01	ENSG00000272654
NA	0.0224	4.9207E-01		ENSG00000226780
NA	0.0618	4.9233E-01	6.76E-01	ENSG00000279923
NA	0.0047	4.9238E-01		ENSG00000254638
NA	0.0175	4.9258E-01		ENSG00000285830
NA	0.0272	4.9271E-01		ENSG00000271265
NA	0.0277	4.9278E-01		ENSG00000273154
NA	0.0419	4.9315E-01	6.77E-01	ENSG00000285928
NA	0.0406	4.9334E-01	6.77E-01	ENSG00000274677
NA	0.0323	4.9398E-01		ENSG00000262693
NA	0.0478	4.9408E-01	6.78E-01	ENSG00000229839
NA	0.0126	4.9410E-01		ENSG00000260123
NA	0.0261	4.9419E-01		ENSG00000288694
NA	0.0648	4.9423E-01	6.78E-01	ENSG00000260176
NA	0.0359	4.9432E-01	6.78E-01	ENSG00000242088
NA	0.0258	4.9443E-01		ENSG00000255968
NA	0.0151	4.9447E-01		ENSG00000226040
NA	0.0478	4.9454E-01	6.78E-01	ENSG00000273253
NA	0.0486	4.9501E-01	6.78E-01	ENSG00000273295
NA	0.0363	4.9516E-01		ENSG00000289027
NA	0.0075	4.9580E-01		ENSG00000259545
NA	0.0432	4.9620E-01	6.79E-01	ENSG00000247699
NA	0.0184	4.9631E-01		ENSG00000237273
NA	0.0459	4.9662E-01		ENSG00000232546
NA	0.0571	4.9665E-01	6.80E-01	ENSG00000231705
NA	0.0098	4.9675E-01		ENSG00000231403
NA	0.0495	4.9690E-01	6.80E-01	ENSG00000286583
NA	0.0374	4.9692E-01	6.80E-01	ENSG00000226361
NA	0.0128	4.9706E-01		ENSG00000262115

NA	0.0107	4.9718E-01		ENSG00000263924
NA	0.0153	4.9736E-01		ENSG00000283259
NA	0.0198	4.9740E-01		ENSG00000248112
NA	0.0083	4.9746E-01		ENSG00000236090
NA	0.0123	4.9759E-01		ENSG00000253660
NA	0.0411	4.9764E-01		ENSG00000279619
NA	0.0054	4.9788E-01		ENSG00000227834
NA	0.0647	4.9790E-01	6.81E-01	ENSG00000233170
NA	0.0184	4.9794E-01		ENSG00000232118
NA	0.0003	4.9805E-01		ENSG00000287913
NA	0.0242	4.9816E-01	6.81E-01	ENSG00000231697
NA	0.0194	4.9834E-01		ENSG00000227969
NA	0.0413	4.9844E-01	6.81E-01	ENSG00000269843
NA	0.0254	4.9845E-01		ENSG00000278616
NA	0.0509	4.9849E-01	6.81E-01	ENSG00000229751
NA	0.0091	4.9891E-01		ENSG00000258284
NA	0.0162	4.9920E-01		ENSG00000286763
NA	0.0001	4.9955E-01		ENSG00000286446
NA	0.0527	4.9966E-01	6.82E-01	ENSG00000280239
NA	0.0168	4.9993E-01	6.82E-01	ENSG00000287633
NA	0.0136	4.9999E-01		ENSG00000258154
NA	0.0608	5.0041E-01	6.83E-01	ENSG00000270171
NA	0.0601	5.0058E-01	6.83E-01	ENSG00000277782
NA	0.0492	5.0077E-01	6.83E-01	ENSG00000275632
NA	0.0647	5.0095E-01	6.83E-01	ENSG00000267264
NA	0.0225	5.0101E-01	6.83E-01	ENSG00000203321
NA	0.0079	5.0116E-01		ENSG00000254678
NA	0.0306	5.0164E-01	6.84E-01	ENSG00000267546
NA	0.0531	5.0165E-01	6.84E-01	ENSG00000250299
NA	0.0297	5.0198E-01		ENSG00000258181
NA	0.0202	5.0217E-01		ENSG00000267160
NA	0.0329	5.0220E-01		ENSG00000278861
NA	0.0642	5.0241E-01	6.84E-01	ENSG00000229097
NA	0.0195	5.0241E-01	6.84E-01	ENSG00000250053
NA	0.0659	5.0273E-01	6.84E-01	ENSG00000289860
NA	0.0182	5.0327E-01		ENSG00000289938
NA	0.0067	5.0334E-01		ENSG00000279090
NA	0.0246	5.0347E-01		ENSG00000236758
NA	0.0647	5.0352E-01	6.85E-01	ENSG00000256745
NA	0.0444	5.0352E-01	6.85E-01	ENSG00000280198
NA	0.0654	5.0373E-01	6.85E-01	ENSG00000255139
NA	0.0189	5.0416E-01		ENSG00000259602
NA	0.0144	5.0419E-01		ENSG00000287108
NA	0.0380	5.0510E-01		ENSG00000234902
NA	0.0288	5.0513E-01		ENSG00000212855
NA	0.0348	5.0536E-01		ENSG00000261707
NA	0.0141	5.0570E-01		ENSG00000290063
NA	0.0457	5.0584E-01	6.87E-01	ENSG00000268362
NA	0.0139	5.0589E-01		ENSG00000236230

NA	0.0071	5.0613E-01		ENSG00000271707
NA	0.0080	5.0614E-01		ENSG00000287586
NA	0.0291	5.0618E-01		ENSG00000288835
NA	0.0594	5.0638E-01	6.87E-01	ENSG00000245330
NA	0.0272	5.0639E-01		ENSG00000280035
NA	0.0162	5.0686E-01		ENSG00000285205
NA	0.0151	5.0727E-01		ENSG00000226447
NA	0.0621	5.0736E-01	6.88E-01	ENSG00000243926
NA	0.0313	5.0745E-01		ENSG00000279594
NA	0.0057	5.0804E-01		ENSG00000229384
NA	0.0104	5.0826E-01		ENSG00000250310
NA	0.0135	5.0935E-01		ENSG00000231046
NA	0.0454	5.0937E-01	6.89E-01	ENSG00000215482
NA	0.0159	5.0944E-01		ENSG00000228156
NA	0.0149	5.0998E-01		ENSG00000280348
NA	0.0268	5.1012E-01	6.90E-01	ENSG00000289603
NA	0.0212	5.1064E-01		ENSG00000214121
NA	0.0067	5.1071E-01		ENSG00000203307
NA	0.0206	5.1078E-01		ENSG00000213543
NA	0.0622	5.1109E-01	6.91E-01	ENSG00000289466
NA	0.0149	5.1115E-01		ENSG00000237604
NA	0.0386	5.1156E-01		ENSG00000233203
NA	0.0040	5.1166E-01		ENSG00000238168
NA	0.0114	5.1169E-01		ENSG00000286877
NA	0.0039	5.1193E-01		ENSG00000264754
NA	0.0453	5.1217E-01	6.92E-01	ENSG00000242190
NA	0.0447	5.1221E-01	6.92E-01	ENSG00000235932
NA	0.0357	5.1244E-01	6.92E-01	ENSG00000289482
NA	0.0189	5.1261E-01		ENSG00000256211
NA	0.0591	5.1286E-01	6.92E-01	ENSG00000198406
NA	0.0488	5.1313E-01	6.93E-01	ENSG00000229127
NA	0.0096	5.1322E-01		ENSG00000248578
NA	0.0117	5.1326E-01		ENSG00000231206
NA	0.0629	5.1344E-01	6.93E-01	ENSG00000289301
NA	0.0096	5.1348E-01		ENSG00000271454
NA	0.0588	5.1369E-01	6.93E-01	ENSG00000220785
NA	0.0220	5.1403E-01		ENSG00000257426
NA	0.0007	5.1411E-01		ENSG00000256234
NA	0.0191	5.1431E-01		ENSG00000251584
NA	0.0380	5.1461E-01		ENSG00000228702
NA	0.0543	5.1462E-01	6.94E-01	ENSG00000278012
NA	0.0090	5.1466E-01		ENSG00000251583
NA	0.0640	5.1488E-01	6.94E-01	ENSG00000224418
NA	0.0624	5.1493E-01	6.94E-01	ENSG00000230091
NA	0.0634	5.1543E-01	6.94E-01	ENSG00000278126
NA	0.0457	5.1552E-01	6.94E-01	ENSG00000237668
NA	0.0095	5.1569E-01		ENSG00000225380
NA	0.0714	5.1573E-01	6.94E-01	ENSG00000236816
NA	0.0328	5.1583E-01		ENSG00000228680

NA	0.0638	5.1604E-01	6.95E-01	ENSG00000274021
NA	0.0359	5.1607E-01		ENSG00000286307
NA	0.0118	5.1609E-01		ENSG00000261250
NA	0.0100	5.1620E-01		ENSG00000276790
NA	0.0180	5.1655E-01		ENSG00000207032
NA	0.0469	5.1659E-01	6.95E-01	ENSG00000240499
NA	0.0449	5.1660E-01	6.95E-01	ENSG00000288753
NA	0.0010	5.1667E-01		ENSG00000288036
NA	0.0295	5.1691E-01	6.95E-01	ENSG00000287580
NA	0.0142	5.1695E-01		ENSG00000278389
NA	0.0000	5.1726E-01		ENSG00000228933
NA	0.0439	5.1735E-01	6.95E-01	ENSG00000250698
NA	0.0480	5.1781E-01	6.96E-01	ENSG00000244036
NA	0.0136	5.1785E-01		ENSG00000287087
NA	0.0106	5.1791E-01		ENSG00000250140
NA	0.0533	5.1798E-01	6.96E-01	ENSG00000233848
NA	0.0058	5.1800E-01		ENSG00000274019
NA	0.0052	5.1851E-01		ENSG00000266179
NA	0.0092	5.1868E-01		ENSG00000277327
NA	0.0140	5.1902E-01		ENSG00000249259
NA	0.0354	5.1970E-01	6.97E-01	ENSG00000259109
NA	0.0593	5.1987E-01	6.97E-01	ENSG00000280195
NA	0.0365	5.2019E-01	6.98E-01	ENSG00000249725
NA	0.0070	5.2058E-01		ENSG00000181511
NA	0.0112	5.2132E-01		ENSG00000264843
NA	0.0540	5.2142E-01	6.99E-01	ENSG00000175604
NA	0.0077	5.2143E-01		ENSG00000284685
NA	0.0609	5.2178E-01	6.99E-01	ENSG00000279168
NA	0.0272	5.2192E-01	6.99E-01	ENSG00000279463
NA	0.0024	5.2209E-01		ENSG00000272566
NA	0.0535	5.2213E-01	6.99E-01	ENSG00000287408
NA	0.0172	5.2234E-01		ENSG00000177197
NA	0.0447	5.2249E-01	6.99E-01	ENSG00000259209
NA	0.0614	5.2258E-01	6.99E-01	ENSG00000228242
NA	0.0618	5.2292E-01	7.00E-01	ENSG00000254186
NA	0.0188	5.2295E-01		ENSG00000242488
NA	0.0007	5.2329E-01		ENSG00000237446
NA	0.0148	5.2330E-01		ENSG00000289526
NA	0.0003	5.2340E-01		ENSG00000249876
NA	0.0094	5.2367E-01		ENSG00000229227
NA	0.0103	5.2374E-01		ENSG00000267432
NA	0.0596	5.2401E-01	7.01E-01	ENSG00000228192
NA	0.0354	5.2405E-01	7.01E-01	ENSG00000213216
NA	0.0590	5.2434E-01	7.01E-01	ENSG00000273062
NA	0.0318	5.2471E-01		ENSG00000259201
NA	0.0491	5.2517E-01	7.01E-01	ENSG00000230630
NA	0.0151	5.2520E-01		ENSG00000205584
NA	0.0043	5.2523E-01		ENSG00000288761
NA	0.0191	5.2566E-01		ENSG00000253369

NA	0.0378	5.2574E-01	7.02E-01	ENSG00000229828
NA	0.0622	5.2586E-01	7.02E-01	ENSG00000241859
NA	0.0081	5.2616E-01		ENSG00000249856
NA	0.0059	5.2617E-01		ENSG00000230993
NA	0.0015	5.2621E-01		ENSG00000239593
NA	0.0055	5.2633E-01		ENSG00000224132
NA	0.0076	5.2641E-01		ENSG00000250541
NA	0.0503	5.2642E-01	7.02E-01	ENSG00000288888
NA	0.0082	5.2666E-01		ENSG00000254001
NA	0.0177	5.2715E-01		ENSG00000279548
NA	0.0157	5.2717E-01		ENSG00000232474
NA	0.0640	5.2726E-01	7.03E-01	ENSG00000284985
NA	0.0483	5.2734E-01	7.03E-01	ENSG00000272933
NA	0.0179	5.2738E-01	7.03E-01	ENSG00000278573
NA	0.0027	5.2750E-01		ENSG00000227716
NA	0.0075	5.2753E-01		ENSG00000230990
NA	0.0101	5.2757E-01		ENSG00000279202
NA	0.0399	5.2784E-01	7.03E-01	ENSG00000233974
NA	0.0598	5.2846E-01	7.04E-01	ENSG00000270039
NA	0.0033	5.2883E-01		ENSG00000287052
NA	0.0446	5.2886E-01	7.04E-01	ENSG00000250243
NA	0.0086	5.2901E-01		ENSG00000236792
NA	0.0587	5.2903E-01	7.04E-01	ENSG00000274964
NA	0.0393	5.2911E-01	7.04E-01	ENSG00000224356
NA	0.0039	5.2914E-01		ENSG00000278546
NA	0.0533	5.2921E-01	7.04E-01	ENSG00000260651
NA	0.0182	5.2947E-01		ENSG00000267141
NA	0.0617	5.2972E-01	7.05E-01	ENSG00000231728
NA	0.0172	5.2980E-01		ENSG00000269189
NA	0.0104	5.2988E-01		ENSG00000277198
NA	0.0274	5.3005E-01		ENSG00000258699
NA	0.0032	5.3021E-01		ENSG00000222511
NA	0.0310	5.3022E-01	7.05E-01	ENSG00000282886
NA	0.0131	5.3030E-01	7.05E-01	ENSG00000243167
NA	0.0167	5.3040E-01		ENSG00000210100
NA	0.0294	5.3056E-01		ENSG00000226669
NA	0.0099	5.3091E-01		ENSG00000251412
NA	0.0283	5.3093E-01	7.05E-01	ENSG00000259925
NA	0.0084	5.3096E-01		ENSG00000284646
NA	0.0288	5.3104E-01		ENSG00000278887
NA	0.0118	5.3131E-01		ENSG00000218347
NA	0.0357	5.3134E-01	7.06E-01	ENSG00000287807
NA	0.0075	5.3136E-01	7.06E-01	ENSG00000286817
NA	0.0504	5.3141E-01	7.06E-01	ENSG00000257918
NA	0.0183	5.3175E-01	7.06E-01	ENSG00000286808
NA	0.0027	5.3232E-01		ENSG00000286407
NA	0.0098	5.3251E-01		ENSG00000259278
NA	0.0015	5.3259E-01		ENSG00000224040
NA	0.0208	5.3259E-01		ENSG00000281849

NA	0.0584	5.3271E-01	7.07E-01	ENSG00000273391
NA	0.0112	5.3310E-01		ENSG00000269118
NA	0.0642	5.3336E-01	7.07E-01	ENSG00000257252
NA	0.0180	5.3345E-01		ENSG00000214880
NA	0.0039	5.3345E-01		ENSG00000288030
NA	0.0189	5.3353E-01		ENSG00000205100
NA	0.0014	5.3354E-01		ENSG00000259453
NA	0.0471	5.3358E-01	7.08E-01	ENSG00000273654
NA	0.0034	5.3361E-01		ENSG00000287792
NA	0.0595	5.3395E-01	7.08E-01	ENSG00000235078
NA	0.0040	5.3432E-01		ENSG00000249852
NA	0.0182	5.3434E-01		ENSG00000285521
NA	0.0142	5.3438E-01		ENSG00000258171
NA	0.0056	5.3454E-01		ENSG00000266950
NA	0.0149	5.3463E-01	7.09E-01	ENSG00000248554
NA	0.0574	5.3478E-01	7.09E-01	ENSG00000205236
NA	0.0260	5.3516E-01		ENSG00000280157
NA	0.0434	5.3518E-01	7.09E-01	ENSG00000206168
NA	0.0163	5.3528E-01		ENSG00000225726
NA	0.0107	5.3550E-01		ENSG00000285840
NA	0.0390	5.3575E-01	7.10E-01	ENSG00000283529
NA	0.0381	5.3600E-01		ENSG00000272626
NA	0.0068	5.3666E-01		ENSG00000287433
NA	0.0074	5.3666E-01		ENSG00000260932
NA	0.0239	5.3700E-01		ENSG00000267316
NA	0.0585	5.3707E-01	7.11E-01	ENSG00000239677
NA	0.0069	5.3727E-01		ENSG00000256898
NA	0.0143	5.3740E-01		ENSG00000254802
NA	0.0493	5.3761E-01	7.11E-01	ENSG00000287146
NA	0.0577	5.3765E-01	7.11E-01	ENSG00000231863
NA	0.0135	5.3766E-01		ENSG00000253307
NA	0.0592	5.3776E-01	7.11E-01	ENSG00000258526
NA	0.0176	5.3780E-01		ENSG00000249234
NA	0.0022	5.3789E-01		ENSG00000242396
NA	0.0083	5.3791E-01		ENSG00000257663
NA	0.0426	5.3804E-01	7.12E-01	ENSG00000279602
NA	0.0059	5.3817E-01		ENSG00000225282
NA	0.0570	5.3824E-01	7.12E-01	ENSG00000259404
NA	0.0110	5.3827E-01		ENSG00000254844
NA	0.0088	5.3849E-01		ENSG00000250796
NA	0.0134	5.3854E-01	7.12E-01	ENSG00000234136
NA	0.0248	5.3905E-01		ENSG00000231069
NA	0.0162	5.3928E-01		ENSG00000285802
NA	0.0113	5.3938E-01		ENSG00000270433
NA	0.0212	5.3948E-01		ENSG00000261187
NA	0.0596	5.3964E-01	7.13E-01	ENSG00000128692
NA	0.0087	5.3971E-01		ENSG00000261435
NA	0.0509	5.4008E-01	7.14E-01	ENSG00000254973
NA	0.0322	5.4018E-01		ENSG00000233330

NA	0.0552	5.4035E-01	7.14E-01	ENSG00000289903
NA	0.0495	5.4117E-01	7.14E-01	ENSG00000214176
NA	0.0136	5.4122E-01		ENSG00000272240
NA	0.0181	5.4127E-01		ENSG00000236491
NA	0.0108	5.4150E-01		ENSG00000118976
NA	0.0098	5.4193E-01	7.15E-01	ENSG00000279077
NA	0.0167	5.4195E-01	7.15E-01	ENSG00000201882
NA	0.0183	5.4199E-01		ENSG00000241059
NA	0.0592	5.4215E-01	7.15E-01	ENSG00000272750
NA	0.0204	5.4246E-01		ENSG00000259425
NA	0.0024	5.4272E-01		ENSG00000237027
NA	0.0560	5.4322E-01	7.15E-01	ENSG00000279792
NA	0.0085	5.4365E-01		ENSG00000274092
NA	0.0594	5.4371E-01	7.16E-01	ENSG00000261351
NA	0.0502	5.4411E-01	7.16E-01	ENSG00000276846
NA	0.0518	5.4426E-01	7.16E-01	ENSG00000279113
NA	0.0078	5.4429E-01		ENSG00000259181
NA	0.0547	5.4441E-01	7.17E-01	ENSG00000285813
NA	0.0047	5.4442E-01		ENSG00000249255
NA	0.0176	5.4454E-01		ENSG00000223419
NA	0.0069	5.4462E-01		ENSG00000232970
NA	0.0127	5.4475E-01		ENSG00000289391
NA	0.0451	5.4501E-01	7.17E-01	ENSG00000255182
NA	0.0590	5.4502E-01	7.17E-01	ENSG00000235618
NA	0.0306	5.4519E-01		ENSG00000284621
NA	0.0493	5.4563E-01	7.18E-01	ENSG00000223704
NA	0.0256	5.4577E-01		ENSG00000270955
NA	0.0011	5.4590E-01	7.18E-01	ENSG00000259564
NA	0.0557	5.4591E-01	7.18E-01	ENSG00000233783
NA	0.0289	5.4628E-01		ENSG00000274944
NA	0.0219	5.4631E-01	7.18E-01	ENSG00000287916
NA	0.0569	5.4639E-01	7.18E-01	ENSG00000225339
NA	0.0206	5.4659E-01		ENSG00000270977
NA	0.0064	5.4669E-01		ENSG00000253399
NA	0.0064	5.4683E-01		ENSG00000287498
NA	0.0098	5.4686E-01		ENSG00000255187
NA	0.0077	5.4717E-01		ENSG00000249678
NA	0.0155	5.4761E-01		ENSG00000286975
NA	0.0079	5.4761E-01		ENSG00000214269
NA	0.0421	5.4765E-01	7.19E-01	ENSG00000234405
NA	0.0034	5.4808E-01		ENSG00000228799
NA	0.0587	5.4892E-01	7.21E-01	ENSG00000250938
NA	0.0049	5.4894E-01		ENSG00000278957
NA	0.0078	5.4909E-01		ENSG00000277950
NA	0.0093	5.4915E-01		ENSG00000269997
NA	0.0543	5.4958E-01	7.21E-01	ENSG00000233290
NA	0.0577	5.5032E-01	7.22E-01	ENSG00000260643
NA	0.0564	5.5080E-01	7.22E-01	ENSG00000255237
NA	0.0564	5.5090E-01	7.22E-01	ENSG00000247317

NA	0.0064	5.5090E-01		ENSG00000286937
NA	0.0093	5.5110E-01		ENSG00000227519
NA	0.0063	5.5162E-01		ENSG00000256650
NA	0.0240	5.5190E-01	7.23E-01	ENSG00000268231
NA	0.0229	5.5237E-01		ENSG00000287437
NA	0.0044	5.5315E-01		ENSG00000253917
NA	0.0496	5.5331E-01	7.24E-01	ENSG00000277406
NA	0.0065	5.5429E-01		ENSG00000286538
NA	0.0021	5.5492E-01		ENSG00000225656
NA	0.0106	5.5494E-01		ENSG00000285561
NA	0.0026	5.5506E-01		ENSG00000273445
NA	0.0112	5.5513E-01		ENSG00000266980
NA	0.0070	5.5523E-01		ENSG00000266830
NA	0.0482	5.5531E-01	7.26E-01	ENSG00000261026
NA	0.0004	5.5613E-01		ENSG00000286741
NA	0.0005	5.5626E-01		ENSG00000233936
NA	0.0187	5.5640E-01		ENSG00000227175
NA	0.0585	5.5672E-01	7.27E-01	ENSG00000279520
NA	0.0167	5.5690E-01		ENSG00000259560
NA	0.0163	5.5707E-01		ENSG00000235852
NA	0.0055	5.5727E-01		ENSG00000228718
NA	0.0079	5.5736E-01		ENSG00000273989
NA	0.0007	5.5749E-01	7.28E-01	ENSG00000263325
NA	0.0168	5.5773E-01	7.28E-01	ENSG00000285215
NA	0.0142	5.5876E-01		ENSG00000232762
NA	0.0570	5.5878E-01	7.29E-01	ENSG00000273156
NA	0.0056	5.5885E-01	7.29E-01	ENSG00000234650
NA	0.0259	5.5892E-01		ENSG00000238232
NA	0.0564	5.5909E-01	7.29E-01	ENSG00000240914
NA	0.0067	5.5930E-01		ENSG00000278384
NA	0.0114	5.5939E-01		ENSG00000287155
NA	0.0561	5.5956E-01	7.29E-01	ENSG00000273199
NA	0.0455	5.5956E-01	7.29E-01	ENSG00000240497
NA	0.0495	5.5961E-01	7.29E-01	ENSG00000290114
NA	0.0076	5.5989E-01		ENSG00000285557
NA	0.0031	5.6010E-01		ENSG00000234551
NA	0.0540	5.6029E-01	7.30E-01	ENSG00000242622
NA	0.0503	5.6038E-01	7.30E-01	ENSG00000265800
NA	0.0363	5.6042E-01	7.30E-01	ENSG00000181097
NA	0.0197	5.6055E-01		ENSG00000234279
NA	0.0130	5.6062E-01		ENSG00000216352
NA	0.0509	5.6066E-01	7.30E-01	ENSG00000277879
NA	0.0081	5.6143E-01		ENSG00000249216
NA	0.0101	5.6151E-01		ENSG00000233578
NA	0.0183	5.6162E-01	7.31E-01	ENSG00000269365
NA	0.0182	5.6175E-01		ENSG00000276673
NA	0.0097	5.6210E-01		ENSG00000259821
NA	0.0137	5.6212E-01		ENSG00000236510
NA	0.0064	5.6232E-01		ENSG00000262052

NA	0.0042	5.6235E-01		ENSG00000237939
NA	0.0045	5.6258E-01		ENSG00000256030
NA	0.0227	5.6311E-01		ENSG00000255886
NA	0.0151	5.6340E-01	7.32E-01	ENSG00000230536
NA	0.0104	5.6346E-01		ENSG00000285210
NA	0.0097	5.6421E-01		ENSG00000287866
NA	0.0036	5.6423E-01		ENSG00000260834
NA	0.0161	5.6437E-01		ENSG00000262434
NA	0.0106	5.6442E-01		ENSG00000275287
NA	0.0371	5.6443E-01	7.33E-01	ENSG00000188460
NA	0.0408	5.6479E-01	7.34E-01	ENSG00000232815
NA	0.0166	5.6484E-01		ENSG00000285854
NA	0.0548	5.6515E-01	7.34E-01	ENSG00000272777
NA	0.0104	5.6531E-01		ENSG00000228352
NA	0.0143	5.6539E-01		ENSG00000289188
NA	0.0205	5.6540E-01		ENSG00000256695
NA	0.0445	5.6569E-01	7.34E-01	ENSG00000286118
NA	0.0414	5.6587E-01	7.34E-01	ENSG00000287248
NA	0.0085	5.6592E-01	7.34E-01	ENSG00000272849
NA	0.0054	5.6614E-01		ENSG00000237077
NA	0.0374	5.6618E-01		ENSG00000229023
NA	0.0343	5.6636E-01		ENSG00000230470
NA	0.0305	5.6646E-01		ENSG00000271078
NA	0.0352	5.6663E-01	7.35E-01	ENSG00000235908
NA	0.0473	5.6770E-01	7.36E-01	ENSG00000289114
NA	0.0343	5.6858E-01	7.37E-01	ENSG00000198106
NA	0.0020	5.6883E-01		ENSG00000259876
NA	0.0446	5.6884E-01	7.37E-01	ENSG00000248544
NA	0.0052	5.6900E-01		ENSG00000271394
NA	0.0120	5.6926E-01		ENSG00000250948
NA	0.0198	5.6927E-01		ENSG00000266120
NA	0.0257	5.6947E-01		ENSG00000279289
NA	0.0537	5.6989E-01	7.38E-01	ENSG00000236603
NA	0.0454	5.7031E-01	7.38E-01	ENSG00000262119
NA	0.0559	5.7034E-01	7.38E-01	ENSG00000259479
NA	0.0098	5.7045E-01		ENSG00000258461
NA	0.0099	5.7061E-01		ENSG00000260981
NA	0.0140	5.7079E-01		ENSG00000232294
NA	0.0000	5.7087E-01	7.39E-01	ENSG00000267275
NA	0.0443	5.7121E-01	7.39E-01	ENSG00000233747
NA	0.0484	5.7176E-01	7.39E-01	ENSG00000278962
NA	0.0107	5.7191E-01		ENSG00000289424
NA	0.0038	5.7194E-01		ENSG00000289704
NA	0.0486	5.7203E-01	7.39E-01	ENSG00000236266
NA	0.0132	5.7235E-01		ENSG00000270894
NA	0.0078	5.7250E-01		ENSG00000259130
NA	0.0177	5.7259E-01		ENSG00000282160
NA	0.0104	5.7265E-01		ENSG00000274297
NA	0.0284	5.7302E-01		ENSG00000271253

NA	0.0428	5.7306E-01	7.41E-01	ENSG00000272374
NA	0.0093	5.7312E-01		ENSG00000196970
NA	0.0537	5.7321E-01	7.41E-01	ENSG00000285644
NA	0.0072	5.7366E-01		ENSG00000272748
NA	0.0190	5.7368E-01		ENSG00000274326
NA	0.0078	5.7393E-01		ENSG00000233347
NA	0.0414	5.7419E-01	7.41E-01	ENSG00000271784
NA	0.0438	5.7434E-01	7.41E-01	ENSG00000288833
NA	0.0348	5.7435E-01	7.41E-01	ENSG00000248322
NA	0.0043	5.7452E-01		ENSG00000215399
NA	0.0083	5.7461E-01		ENSG00000259639
NA	0.0532	5.7465E-01	7.42E-01	ENSG00000285130
NA	0.0066	5.7512E-01		ENSG00000234084
NA	0.0178	5.7523E-01		ENSG00000286180
NA	0.0444	5.7559E-01	7.42E-01	ENSG00000289269
NA	0.0168	5.7595E-01		ENSG00000258088
NA	0.0171	5.7600E-01		ENSG00000273402
NA	0.0174	5.7617E-01		ENSG00000253170
NA	0.0301	5.7646E-01		ENSG00000287413
NA	0.0199	5.7653E-01		ENSG00000259370
NA	0.0063	5.7661E-01		ENSG00000249829
NA	0.0049	5.7672E-01	7.43E-01	ENSG00000274292
NA	0.0121	5.7765E-01		ENSG00000225774
NA	0.0528	5.7781E-01	7.44E-01	ENSG00000263551
NA	0.0104	5.7814E-01		ENSG00000271065
NA	0.0503	5.7832E-01	7.45E-01	ENSG00000289005
NA	0.0011	5.7843E-01	7.45E-01	ENSG00000253190
NA	0.0005	5.7882E-01		ENSG00000271525
NA	0.0047	5.7882E-01		ENSG00000155070
NA	0.0148	5.7892E-01		ENSG00000259483
NA	0.0074	5.7896E-01		ENSG00000214144
NA	0.0102	5.7907E-01		ENSG00000240632
NA	0.0411	5.7925E-01	7.45E-01	ENSG00000284116
NA	0.0002	5.7933E-01		ENSG00000200421
NA	0.0536	5.7955E-01	7.46E-01	ENSG00000285090
NA	0.0172	5.7956E-01		ENSG00000256029
NA	0.0076	5.7965E-01		ENSG00000242163
NA	0.0228	5.7971E-01		ENSG00000232768
NA	0.0225	5.8001E-01		ENSG00000254887
NA	0.0006	5.8014E-01		ENSG00000229808
NA	0.0021	5.8037E-01		ENSG00000259793
NA	0.0527	5.8049E-01	7.47E-01	ENSG00000273014
NA	0.0358	5.8075E-01	7.47E-01	ENSG00000271892
NA	0.0067	5.8096E-01		ENSG00000288563
NA	0.0301	5.8109E-01		ENSG00000290040
NA	0.0265	5.8127E-01	7.47E-01	ENSG00000253720
NA	0.0303	5.8140E-01		ENSG00000251271
NA	0.0047	5.8142E-01		ENSG00000267472
NA	0.0024	5.8161E-01		ENSG00000259789

NA	0.0255	5.8164E-01		ENSG00000277651
NA	0.0084	5.8213E-01		ENSG00000236354
NA	0.0131	5.8214E-01		ENSG00000225886
NA	0.0099	5.8233E-01	7.48E-01	ENSG00000287175
NA	0.0078	5.8241E-01		ENSG00000278630
NA	0.0526	5.8293E-01	7.49E-01	ENSG00000227681
NA	0.0095	5.8308E-01		ENSG00000286882
NA	0.0051	5.8317E-01		ENSG00000270413
NA	0.0088	5.8359E-01		ENSG00000267780
NA	0.0105	5.8378E-01		ENSG00000289081
NA	0.0081	5.8391E-01		ENSG00000224079
NA	0.0089	5.8414E-01		ENSG00000258422
NA	0.0125	5.8419E-01		ENSG00000264772
NA	0.0465	5.8461E-01	7.50E-01	ENSG00000237757
NA	0.0050	5.8480E-01		ENSG00000257729
NA	0.0137	5.8485E-01		ENSG00000265656
NA	0.0272	5.8512E-01	7.50E-01	ENSG00000225916
NA	0.0286	5.8587E-01		ENSG00000232295
NA	0.0057	5.8622E-01		ENSG00000223430
NA	0.0137	5.8718E-01	7.52E-01	ENSG00000255506
NA	0.0049	5.8722E-01	7.52E-01	ENSG00000224971
NA	0.0310	5.8725E-01	7.52E-01	ENSG00000280604
NA	0.0497	5.8804E-01	7.52E-01	ENSG00000233220
NA	0.0404	5.8849E-01	7.52E-01	ENSG00000214106
NA	0.0199	5.8852E-01	7.52E-01	ENSG00000287949
NA	0.0072	5.8866E-01		ENSG00000288735
NA	0.0370	5.8908E-01	7.53E-01	ENSG00000286444
NA	0.0392	5.8922E-01	7.53E-01	ENSG00000268163
NA	0.0528	5.8941E-01	7.53E-01	ENSG00000287853
NA	0.0509	5.8941E-01	7.53E-01	ENSG00000253570
NA	0.0270	5.8958E-01	7.53E-01	ENSG00000249330
NA	0.0081	5.8985E-01		ENSG00000287891
NA	0.0460	5.9012E-01	7.53E-01	ENSG00000213058
NA	0.0477	5.9015E-01	7.53E-01	ENSG00000264548
NA	0.0117	5.9031E-01	7.54E-01	ENSG00000286868
NA	0.0115	5.9039E-01		ENSG00000266802
NA	0.0156	5.9041E-01		ENSG00000287434
NA	0.0303	5.9122E-01		ENSG00000285618
NA	0.0506	5.9143E-01	7.54E-01	ENSG00000236514
NA	0.0041	5.9166E-01		ENSG00000289286
NA	0.0311	5.9179E-01	7.55E-01	ENSG00000287279
NA	0.0477	5.9183E-01	7.55E-01	ENSG00000253200
NA	0.0022	5.9245E-01		ENSG00000286524
NA	0.0217	5.9250E-01	7.55E-01	ENSG00000272205
NA	0.0114	5.9252E-01		ENSG00000225039
NA	0.0016	5.9267E-01		ENSG00000223828
NA	0.0191	5.9272E-01		ENSG00000260585
NA	0.0088	5.9278E-01		ENSG00000270921
NA	0.0488	5.9302E-01	7.55E-01	ENSG00000159860

NA	0.0076	5.9305E-01		ENSG00000272945
NA	0.0416	5.9338E-01	7.56E-01	ENSG00000289551
NA	0.0166	5.9353E-01		ENSG00000286470
NA	0.0457	5.9384E-01	7.56E-01	ENSG00000236453
NA	0.0057	5.9387E-01		ENSG00000288862
NA	0.0515	5.9395E-01	7.56E-01	ENSG00000260838
NA	0.0128	5.9422E-01		ENSG00000286122
NA	0.0428	5.9440E-01	7.57E-01	ENSG00000279838
NA	0.0445	5.9456E-01	7.57E-01	ENSG00000236540
NA	0.0448	5.9476E-01	7.57E-01	ENSG00000286311
NA	0.0184	5.9503E-01		ENSG00000287370
NA	0.0373	5.9512E-01	7.57E-01	ENSG00000261630
NA	0.0377	5.9587E-01	7.58E-01	ENSG00000235731
NA	0.0083	5.9621E-01		ENSG00000202019
NA	0.0460	5.9631E-01	7.58E-01	ENSG00000287209
NA	0.0323	5.9634E-01	7.58E-01	ENSG00000286115
NA	0.0473	5.9637E-01	7.58E-01	ENSG00000258752
NA	0.0104	5.9671E-01		ENSG00000260612
NA	0.0059	5.9686E-01		ENSG00000260495
NA	0.0105	5.9700E-01		ENSG00000267088
NA	0.0022	5.9703E-01		ENSG00000250362
NA	0.0138	5.9708E-01		ENSG00000259740
NA	0.0515	5.9726E-01	7.59E-01	ENSG00000258072
NA	0.0491	5.9744E-01	7.59E-01	ENSG00000225647
NA	0.0055	5.9758E-01		ENSG00000277566
NA	0.0221	5.9781E-01		ENSG00000230997
NA	0.0474	5.9806E-01	7.59E-01	ENSG00000289003
NA	0.0041	5.9814E-01	7.59E-01	ENSG00000283427
NA	0.0036	5.9841E-01		ENSG00000236459
NA	0.0399	5.9888E-01	7.60E-01	ENSG00000230626
NA	0.0463	5.9899E-01	7.60E-01	ENSG00000288885
NA	0.0040	5.9927E-01		ENSG00000288771
NA	0.0298	5.9951E-01	7.60E-01	ENSG00000272858
NA	0.0350	5.9956E-01	7.60E-01	ENSG00000223947
NA	0.0076	5.9957E-01		ENSG00000231394
NA	0.0495	5.9960E-01	7.60E-01	ENSG00000286729
NA	0.0096	5.9993E-01		ENSG00000283669
NA	0.0430	6.0005E-01	7.61E-01	ENSG00000267582
NA	0.0320	6.0012E-01	7.61E-01	ENSG00000255021
NA	0.0100	6.0025E-01		ENSG00000289429
NA	0.0054	6.0048E-01		ENSG00000254458
NA	0.0422	6.0085E-01	7.61E-01	ENSG00000279726
NA	0.0345	6.0120E-01	7.62E-01	ENSG00000288909
NA	0.0028	6.0134E-01		ENSG00000289893
NA	0.0217	6.0138E-01		ENSG00000273123
NA	0.0086	6.0139E-01		ENSG00000207302
NA	0.0100	6.0160E-01	7.62E-01	ENSG00000285151
NA	0.0100	6.0160E-01	7.62E-01	ENSG00000285204
NA	0.0500	6.0182E-01	7.62E-01	ENSG00000242375

NA	0.0498	6.0184E-01	7.62E-01	ENSG00000228857
NA	0.0021	6.0234E-01		ENSG00000255292
NA	0.0227	6.0293E-01		ENSG00000224856
NA	0.0062	6.0336E-01		ENSG00000267919
NA	0.0101	6.0346E-01		ENSG00000261765
NA	0.0161	6.0389E-01		ENSG00000285647
NA	0.0009	6.0411E-01		ENSG00000266744
NA	0.0345	6.0427E-01	7.64E-01	ENSG00000261600
NA	0.0056	6.0461E-01	7.64E-01	ENSG00000282998
NA	0.0495	6.0473E-01	7.64E-01	ENSG00000272990
NA	0.0032	6.0480E-01		ENSG00000233565
NA	0.0106	6.0480E-01		ENSG00000258628
NA	0.0047	6.0483E-01		ENSG00000280280
NA	0.0380	6.0487E-01	7.64E-01	ENSG00000258711
NA	0.0036	6.0509E-01		ENSG00000275162
NA	0.0067	6.0511E-01		ENSG00000289584
NA	0.0362	6.0528E-01	7.65E-01	ENSG00000287998
NA	0.0157	6.0531E-01	7.65E-01	ENSG00000250699
NA	0.0500	6.0533E-01	7.65E-01	ENSG00000267009
NA	0.0065	6.0547E-01		ENSG00000275948
NA	0.0246	6.0591E-01		ENSG00000274949
NA	0.0025	6.0599E-01		ENSG00000258740
NA	0.0432	6.0602E-01	7.65E-01	ENSG00000236498
NA	0.0118	6.0614E-01		ENSG00000270020
NA	0.0077	6.0632E-01		ENSG00000253449
NA	0.0285	6.0678E-01		ENSG00000288700
NA	0.0042	6.0695E-01		ENSG00000263154
NA	0.0245	6.0701E-01		ENSG00000275936
NA	0.0005	6.0751E-01	7.67E-01	ENSG00000242440
NA	0.0043	6.0846E-01		ENSG00000228411
NA	0.0081	6.0858E-01		ENSG00000279819
NA	0.0464	6.0893E-01	7.68E-01	ENSG00000289890
NA	0.0355	6.0921E-01	7.68E-01	ENSG00000254343
NA	0.0144	6.0943E-01		ENSG00000225416
NA	0.0079	6.0990E-01		ENSG00000259241
NA	0.0051	6.1018E-01		ENSG00000285520
NA	0.0107	6.1038E-01	7.69E-01	ENSG00000213871
NA	0.0061	6.1045E-01		ENSG00000288853
NA	0.0120	6.1086E-01		ENSG00000260646
NA	0.0062	6.1110E-01		ENSG00000227206
NA	0.0073	6.1122E-01		ENSG00000258870
NA	0.0496	6.1130E-01	7.70E-01	ENSG00000278867
NA	0.0059	6.1133E-01		ENSG00000235550
NA	0.0102	6.1144E-01		ENSG00000287739
NA	0.0022	6.1151E-01		ENSG00000236772
NA	0.0158	6.1174E-01		ENSG00000229673
NA	0.0259	6.1221E-01		ENSG00000256268
NA	0.0241	6.1275E-01	7.71E-01	ENSG00000279080
NA	0.0119	6.1287E-01		ENSG00000235924

NA	0.0125	6.1311E-01		ENSG00000286562
NA	0.0161	6.1317E-01		ENSG00000237971
NA	0.0093	6.1325E-01		ENSG00000286108
NA	0.0138	6.1404E-01		ENSG00000254295
NA	0.0420	6.1416E-01	7.72E-01	ENSG00000181126
NA	0.0113	6.1451E-01		ENSG00000248113
NA	0.0491	6.1489E-01	7.73E-01	ENSG00000273108
NA	0.0112	6.1490E-01		ENSG00000267749
NA	0.0178	6.1502E-01		ENSG00000261332
NA	0.0043	6.1529E-01		ENSG00000261367
NA	0.0347	6.1566E-01	7.73E-01	ENSG00000279028
NA	0.0088	6.1582E-01		ENSG00000201451
NA	0.0411	6.1595E-01	7.74E-01	ENSG00000234675
NA	0.0490	6.1610E-01	7.74E-01	ENSG00000284727
NA	0.0138	6.1628E-01		ENSG00000196364
NA	0.0076	6.1639E-01		ENSG00000267567
NA	0.0287	6.1694E-01	7.74E-01	ENSG00000289468
NA	0.0154	6.1709E-01		ENSG00000187904
NA	0.0192	6.1713E-01		ENSG00000283317
NA	0.0274	6.1722E-01		ENSG00000225400
NA	0.0310	6.1752E-01	7.75E-01	ENSG00000226772
NA	0.0411	6.1786E-01	7.75E-01	ENSG00000250049
NA	0.0474	6.1800E-01	7.75E-01	ENSG00000280734
NA	0.0218	6.1850E-01	7.75E-01	ENSG00000278931
NA	0.0040	6.1871E-01		ENSG00000231654
NA	0.0065	6.1875E-01	7.75E-01	ENSG00000259358
NA	0.0166	6.1877E-01		ENSG00000261610
NA	0.0217	6.1899E-01		ENSG00000278985
NA	0.0429	6.1903E-01	7.75E-01	ENSG00000289956
NA	0.0325	6.1907E-01	7.75E-01	ENSG00000200737
NA	0.0416	6.1918E-01	7.75E-01	ENSG00000266777
NA	0.0128	6.1935E-01		ENSG00000279769
NA	0.0062	6.1943E-01		ENSG00000287062
NA	0.0012	6.1947E-01		ENSG00000213194
NA	0.0078	6.1978E-01		ENSG00000264705
NA	0.0091	6.1981E-01		ENSG00000188662
NA	0.0330	6.2003E-01	7.76E-01	ENSG00000231240
NA	0.0435	6.2028E-01	7.76E-01	ENSG00000283236
NA	0.0116	6.2038E-01		ENSG00000262402
NA	0.0046	6.2047E-01		ENSG00000275348
NA	0.0451	6.2083E-01	7.77E-01	ENSG00000198416
NA	0.0197	6.2120E-01		ENSG00000258394
NA	0.0325	6.2133E-01	7.77E-01	ENSG00000265630
NA	0.0428	6.2156E-01	7.77E-01	ENSG00000287923
NA	0.0453	6.2185E-01	7.78E-01	ENSG00000272717
NA	0.0411	6.2187E-01	7.78E-01	ENSG00000228532
NA	0.0437	6.2226E-01	7.78E-01	ENSG00000289554
NA	0.0008	6.2251E-01	7.78E-01	ENSG00000258162
NA	0.0160	6.2255E-01	7.78E-01	ENSG00000254676

NA	0.0383	6.2268E-01	7.78E-01	ENSG00000259856
NA	0.0439	6.2293E-01	7.79E-01	ENSG00000260577
NA	0.0086	6.2299E-01		ENSG00000251075
NA	0.0063	6.2305E-01		ENSG00000244585
NA	0.0058	6.2328E-01		ENSG00000249849
NA	0.0081	6.2339E-01		ENSG00000289226
NA	0.0474	6.2343E-01	7.79E-01	ENSG00000285715
NA	0.0108	6.2348E-01		ENSG00000289302
NA	0.0104	6.2358E-01		ENSG00000229742
NA	0.0085	6.2419E-01		ENSG00000254990
NA	0.0174	6.2484E-01	7.80E-01	ENSG00000254632
NA	0.0474	6.2508E-01	7.80E-01	ENSG00000260296
NA	0.0018	6.2509E-01		ENSG00000257268
NA	0.0057	6.2511E-01		ENSG00000213770
NA	0.0466	6.2538E-01	7.80E-01	ENSG00000279133
NA	0.0391	6.2569E-01	7.81E-01	ENSG00000280155
NA	0.0039	6.2596E-01		ENSG00000236567
NA	0.0471	6.2606E-01	7.81E-01	ENSG00000251495
NA	0.0378	6.2611E-01	7.81E-01	ENSG00000273691
NA	0.0208	6.2614E-01	7.81E-01	ENSG00000287250
NA	0.0019	6.2618E-01		ENSG00000227516
NA	0.0005	6.2698E-01		ENSG00000287143
NA	0.0038	6.2737E-01		ENSG00000229779
NA	0.0290	6.2762E-01		ENSG00000234311
NA	0.0194	6.2804E-01		ENSG00000276087
NA	0.0099	6.2865E-01		ENSG00000288615
NA	0.0124	6.2866E-01		ENSG00000289384
NA	0.0416	6.2867E-01	7.83E-01	ENSG00000273013
NA	0.0342	6.2868E-01	7.83E-01	ENSG00000210176
NA	0.0214	6.2902E-01		ENSG00000224031
NA	0.0433	6.2908E-01	7.83E-01	ENSG00000261884
NA	0.0466	6.2911E-01	7.83E-01	ENSG00000286638
NA	0.0047	6.2912E-01		ENSG00000225770
NA	0.0038	6.2928E-01		ENSG00000282206
NA	0.0152	6.2971E-01		ENSG00000250820
NA	0.0471	6.2980E-01	7.84E-01	ENSG00000268942
NA	0.0048	6.3026E-01		ENSG00000276633
NA	0.0328	6.3039E-01	7.84E-01	ENSG00000250945
NA	0.0163	6.3040E-01	7.84E-01	ENSG00000261469
NA	0.0119	6.3054E-01		ENSG00000268140
NA	0.0179	6.3060E-01		ENSG00000146001
NA	0.0068	6.3061E-01		ENSG00000267764
NA	0.0291	6.3062E-01	7.84E-01	ENSG00000288855
NA	0.0103	6.3074E-01		ENSG00000282527
NA	0.0030	6.3110E-01		ENSG00000244528
NA	0.0145	6.3133E-01		ENSG00000237138
NA	0.0126	6.3173E-01		ENSG00000212205
NA	0.0301	6.3173E-01	7.85E-01	ENSG00000260830
NA	0.0298	6.3173E-01	7.85E-01	ENSG00000236861

NA	0.0075	6.3190E-01		ENSG00000258203
NA	0.0196	6.3207E-01		ENSG00000259917
NA	0.0454	6.3250E-01	7.86E-01	ENSG00000287763
NA	0.0378	6.3270E-01	7.86E-01	ENSG00000259236
NA	0.0368	6.3352E-01	7.86E-01	ENSG00000258016
NA	0.0210	6.3357E-01		ENSG00000232228
NA	0.0132	6.3362E-01		ENSG00000287291
NA	0.0023	6.3366E-01		ENSG00000233707
NA	0.0353	6.3375E-01	7.86E-01	ENSG00000185986
NA	0.0065	6.3396E-01		ENSG00000289405
NA	0.0044	6.3403E-01		ENSG00000267681
NA	0.0004	6.3429E-01		ENSG00000286130
NA	0.0445	6.3433E-01	7.87E-01	ENSG00000287404
NA	0.0064	6.3439E-01		ENSG00000260135
NA	0.0066	6.3465E-01		ENSG00000251294
NA	0.0272	6.3473E-01	7.87E-01	ENSG00000272084
NA	0.0087	6.3476E-01		ENSG00000229282
NA	0.0105	6.3492E-01		ENSG00000286979
NA	0.0258	6.3513E-01		ENSG00000232699
NA	0.0051	6.3516E-01		ENSG00000280114
NA	0.0144	6.3516E-01		ENSG00000223634
NA	0.0238	6.3526E-01		ENSG00000229915
NA	0.0043	6.3546E-01		ENSG00000270951
NA	0.0210	6.3549E-01	7.88E-01	ENSG00000229962
NA	0.0056	6.3575E-01		ENSG00000263096
NA	0.0392	6.3576E-01	7.88E-01	ENSG00000250385
NA	0.0218	6.3596E-01	7.88E-01	ENSG00000271252
NA	0.0179	6.3609E-01		ENSG00000226881
NA	0.0053	6.3616E-01		ENSG00000286887
NA	0.0066	6.3634E-01		ENSG00000231888
NA	0.0360	6.3650E-01	7.88E-01	ENSG00000273598
NA	0.0091	6.3652E-01		ENSG00000267733
NA	0.0136	6.3663E-01		ENSG00000283618
NA	0.0456	6.3666E-01	7.89E-01	ENSG00000289701
NA	0.0314	6.3669E-01	7.89E-01	ENSG00000270299
NA	0.0110	6.3677E-01	7.89E-01	ENSG00000250874
NA	0.0071	6.3721E-01		ENSG00000248222
NA	0.0055	6.3742E-01		ENSG00000249920
NA	0.0087	6.3745E-01		ENSG00000287519
NA	0.0461	6.3766E-01	7.89E-01	ENSG00000272562
NA	0.0039	6.3773E-01		ENSG00000286269
NA	0.0033	6.3804E-01		ENSG00000236646
NA	0.0131	6.3832E-01		ENSG00000283040
NA	0.0058	6.3854E-01		ENSG00000234977
NA	0.0118	6.3875E-01		ENSG00000260905
NA	0.0167	6.3884E-01		ENSG00000289292
NA	0.0111	6.3922E-01	7.90E-01	ENSG00000249085
NA	0.0120	6.3965E-01		ENSG00000259691
NA	0.0063	6.3969E-01		ENSG00000287867

NA	0.0084	6.3978E-01	7.91E-01	ENSG00000237382
NA	0.0392	6.3987E-01	7.91E-01	ENSG00000269584
NA	0.0349	6.4009E-01		ENSG00000272855
NA	0.0118	6.4015E-01		ENSG00000271553
NA	0.0414	6.4041E-01	7.91E-01	ENSG00000231079
NA	0.0053	6.4063E-01		ENSG00000279036
NA	0.0445	6.4078E-01	7.91E-01	ENSG00000226445
NA	0.0418	6.4119E-01	7.92E-01	ENSG00000225067
NA	0.0124	6.4128E-01		ENSG00000256906
NA	0.0061	6.4152E-01		ENSG00000223657
NA	0.0073	6.4175E-01		ENSG00000267246
NA	0.0036	6.4180E-01		ENSG00000236325
NA	0.0022	6.4203E-01		ENSG00000250909
NA	0.0065	6.4212E-01		ENSG00000278044
NA	0.0017	6.4245E-01		ENSG00000267312
NA	0.0188	6.4252E-01	7.93E-01	ENSG00000244151
NA	0.0083	6.4288E-01		ENSG00000226253
NA	0.0448	6.4328E-01	7.93E-01	ENSG00000224093
NA	0.0113	6.4335E-01		ENSG00000287007
NA	0.0423	6.4383E-01	7.93E-01	ENSG00000276517
NA	0.0231	6.4448E-01	7.94E-01	ENSG00000226709
NA	0.0327	6.4472E-01	7.94E-01	ENSG00000237904
NA	0.0421	6.4541E-01	7.94E-01	ENSG00000225442
NA	0.0083	6.4549E-01		ENSG00000229593
NA	0.0300	6.4552E-01	7.94E-01	ENSG00000243696
NA	0.0143	6.4615E-01		ENSG00000253213
NA	0.0143	6.4618E-01		ENSG00000234816
NA	0.0087	6.4628E-01		ENSG00000254114
NA	0.0040	6.4641E-01		ENSG00000241825
NA	0.0235	6.4643E-01	7.95E-01	ENSG00000286113
NA	0.0046	6.4645E-01		ENSG00000263305
NA	0.0392	6.4723E-01	7.96E-01	ENSG00000240063
NA	0.0060	6.4730E-01		ENSG00000288927
NA	0.0021	6.4732E-01		ENSG00000259529
NA	0.0051	6.4747E-01	7.96E-01	ENSG00000211513
NA	0.0045	6.4748E-01		ENSG00000289887
NA	0.0440	6.4754E-01	7.96E-01	ENSG00000286373
NA	0.0032	6.4773E-01		ENSG00000260289
NA	0.0129	6.4816E-01		ENSG00000286393
NA	0.0089	6.4820E-01	7.96E-01	ENSG00000215283
NA	0.0553	6.4858E-01	7.96E-01	ENSG00000223635
NA	0.0068	6.4869E-01		ENSG00000215692
NA	0.0140	6.4879E-01		ENSG00000228852
NA	0.0396	6.4906E-01	7.97E-01	ENSG00000255928
NA	0.0130	6.4918E-01	7.97E-01	ENSG00000253106
NA	0.0267	6.4920E-01	7.97E-01	ENSG00000260436
NA	0.0046	6.4957E-01		ENSG00000280284
NA	0.0020	6.4960E-01		ENSG00000287920
NA	0.0231	6.4961E-01		ENSG00000287494

NA	0.0436	6.4987E-01	7.97E-01	ENSG00000237886
NA	0.0137	6.4988E-01		ENSG00000287121
NA	0.0270	6.4998E-01	7.97E-01	ENSG00000227817
NA	0.0035	6.4999E-01		ENSG00000232252
NA	0.0043	6.5028E-01		ENSG00000225018
NA	0.0312	6.5075E-01	7.98E-01	ENSG00000231927
NA	0.0311	6.5078E-01	7.98E-01	ENSG00000227492
NA	0.0376	6.5093E-01	7.98E-01	ENSG00000272360
NA	0.0022	6.5192E-01		ENSG00000278075
NA	0.0084	6.5194E-01		ENSG00000264243
NA	0.0055	6.5195E-01		ENSG00000277297
NA	0.0114	6.5203E-01		ENSG00000269138
NA	0.0102	6.5208E-01		ENSG00000285611
NA	0.0145	6.5223E-01		ENSG00000260721
NA	0.0163	6.5241E-01		ENSG00000237025
NA	0.0233	6.5253E-01	7.99E-01	ENSG00000243368
NA	0.0433	6.5274E-01	7.99E-01	ENSG00000253553
NA	0.0227	6.5279E-01		ENSG00000276968
NA	0.0218	6.5293E-01		ENSG00000279953
NA	0.0134	6.5297E-01		ENSG00000251434
NA	0.0199	6.5308E-01		ENSG00000280711
NA	0.0073	6.5315E-01		ENSG00000273998
NA	0.0344	6.5377E-01	8.00E-01	ENSG00000256481
NA	0.0380	6.5395E-01	8.00E-01	ENSG00000231806
NA	0.0324	6.5404E-01	8.00E-01	ENSG00000261786
NA	0.0143	6.5415E-01	8.00E-01	ENSG00000202079
NA	0.0136	6.5483E-01		ENSG00000217455
NA	0.0049	6.5513E-01		ENSG00000272254
NA	0.0644	6.5519E-01	8.01E-01	ENSG00000230623
NA	0.0132	6.5548E-01	8.01E-01	ENSG00000204622
NA	0.0410	6.5554E-01	8.01E-01	ENSG00000273175
NA	0.0425	6.5572E-01	8.01E-01	ENSG00000272918
NA	0.0082	6.5585E-01		ENSG00000270612
NA	0.0377	6.5658E-01	8.02E-01	ENSG00000266903
NA	0.0013	6.5677E-01		ENSG00000237756
NA	0.0233	6.5704E-01		ENSG00000189089
NA	0.0353	6.5721E-01	8.02E-01	ENSG00000273329
NA	0.0150	6.5731E-01		ENSG00000285800
NA	0.0037	6.5733E-01		ENSG00000235081
NA	0.0422	6.5742E-01	8.02E-01	ENSG00000269906
NA	0.0326	6.5752E-01	8.02E-01	ENSG00000228137
NA	0.0050	6.5778E-01		ENSG00000260871
NA	0.0355	6.5783E-01	8.02E-01	ENSG00000288838
NA	0.0075	6.5848E-01		ENSG00000289221
NA	0.0172	6.5864E-01		ENSG00000227083
NA	0.0058	6.5887E-01		ENSG00000253384
NA	0.0122	6.5893E-01		ENSG00000225779
NA	0.0196	6.5917E-01	8.03E-01	ENSG00000276814
NA	0.0180	6.5936E-01		ENSG00000254094

NA	0.0024	6.5945E-01	8.04E-01	ENSG00000276115
NA	0.0090	6.5966E-01		ENSG00000237806
NA	0.0194	6.5983E-01		ENSG00000207512
NA	0.0420	6.5984E-01	8.04E-01	ENSG00000287358
NA	0.0094	6.5993E-01		ENSG00000286616
NA	0.0333	6.6026E-01	8.04E-01	ENSG00000287180
NA	0.0313	6.6033E-01	8.04E-01	ENSG00000279821
NA	0.0258	6.6046E-01		ENSG00000286132
NA	0.0293	6.6054E-01	8.04E-01	ENSG00000237640
NA	0.0281	6.6101E-01	8.05E-01	ENSG00000232909
NA	0.0129	6.6126E-01		ENSG00000234766
NA	0.0002	6.6138E-01		ENSG00000217783
NA	0.0428	6.6145E-01	8.05E-01	ENSG00000124549
NA	0.0045	6.6158E-01		ENSG00000232889
NA	0.0418	6.6168E-01	8.05E-01	ENSG00000275234
NA	0.0055	6.6191E-01	8.05E-01	ENSG00000234917
NA	0.0002	6.6206E-01		ENSG00000213331
NA	0.0093	6.6206E-01		ENSG00000227962
NA	0.0060	6.6250E-01		ENSG00000283285
NA	0.0038	6.6274E-01		ENSG00000255109
NA	0.0398	6.6298E-01	8.06E-01	ENSG00000273472
NA	0.0261	6.6312E-01		ENSG00000249228
NA	0.0023	6.6317E-01		ENSG00000269119
NA	0.0387	6.6355E-01	8.07E-01	ENSG00000233067
NA	0.0215	6.6373E-01		ENSG00000276121
NA	0.0197	6.6383E-01		ENSG00000289573
NA	0.0336	6.6383E-01	8.07E-01	ENSG00000235586
NA	0.0198	6.6386E-01		ENSG00000271427
NA	0.0009	6.6408E-01		ENSG00000224525
NA	0.0119	6.6415E-01	8.07E-01	ENSG00000272140
NA	0.0042	6.6467E-01		ENSG00000280004
NA	0.0333	6.6494E-01		ENSG00000289896
NA	0.0410	6.6549E-01	8.08E-01	ENSG00000178082
NA	0.0131	6.6551E-01	8.08E-01	ENSG00000276651
NA	0.0103	6.6552E-01		ENSG00000240571
NA	0.0051	6.6561E-01		ENSG00000231534
NA	0.0395	6.6600E-01	8.08E-01	ENSG00000277476
NA	0.0009	6.6603E-01		ENSG00000261588
NA	0.0073	6.6617E-01		ENSG00000244199
NA	0.0272	6.6628E-01	8.08E-01	ENSG00000289562
NA	0.0286	6.6629E-01	8.08E-01	ENSG00000232460
NA	0.0403	6.6631E-01	8.08E-01	ENSG00000286599
NA	0.0088	6.6632E-01		ENSG00000287976
NA	0.0090	6.6639E-01		ENSG00000227045
NA	0.0021	6.6666E-01		ENSG00000276107
NA	0.0081	6.6719E-01		ENSG00000249169
NA	0.0274	6.6739E-01	8.09E-01	ENSG00000289069
NA	0.0299	6.6785E-01	8.09E-01	ENSG00000279879
NA	0.0420	6.6791E-01	8.09E-01	ENSG00000257802

NA	0.0236	6.6812E-01	8.09E-01	ENSG00000289031
NA	0.0348	6.6821E-01	8.09E-01	ENSG00000285600
NA	0.0047	6.6833E-01		ENSG00000158482
NA	0.0322	6.6836E-01	8.09E-01	ENSG00000253636
NA	0.0409	6.6855E-01	8.09E-01	ENSG00000242686
NA	0.0132	6.6860E-01		ENSG00000233133
NA	0.0162	6.6870E-01	8.09E-01	ENSG00000287860
NA	0.0075	6.6885E-01		ENSG00000228019
NA	0.0410	6.6988E-01	8.10E-01	ENSG00000288957
NA	0.0098	6.6994E-01		ENSG00000228153
NA	0.0135	6.7008E-01		ENSG00000288048
NA	0.0017	6.7035E-01		ENSG00000269910
NA	0.0028	6.7044E-01		ENSG00000271215
NA	0.0069	6.7070E-01		ENSG00000224972
NA	0.0285	6.7106E-01	8.11E-01	ENSG00000258717
NA	0.0411	6.7182E-01	8.12E-01	ENSG00000196741
NA	0.0035	6.7193E-01		ENSG00000286581
NA	0.0339	6.7201E-01	8.12E-01	ENSG00000285763
NA	0.0100	6.7215E-01		ENSG00000267828
NA	0.0100	6.7215E-01		ENSG00000202021
NA	0.0026	6.7305E-01		ENSG00000274499
NA	0.0322	6.7308E-01	8.13E-01	ENSG00000289210
NA	0.0211	6.7311E-01		ENSG00000280828
NA	0.0037	6.7323E-01		ENSG00000229672
NA	0.0281	6.7326E-01	8.13E-01	ENSG00000287539
NA	0.0021	6.7333E-01		ENSG00000229072
NA	0.0105	6.7347E-01		ENSG00000237324
NA	0.0273	6.7461E-01	8.14E-01	ENSG00000286692
NA	0.0389	6.7466E-01	8.14E-01	ENSG00000259343
NA	0.0146	6.7467E-01		ENSG00000224839
NA	0.0025	6.7483E-01		ENSG00000224822
NA	0.0060	6.7543E-01		ENSG00000270540
NA	0.0066	6.7550E-01		ENSG00000276667
NA	0.0101	6.7559E-01		ENSG00000278703
NA	0.0259	6.7567E-01	8.15E-01	ENSG00000275390
NA	0.0077	6.7590E-01		ENSG00000256879
NA	0.0348	6.7607E-01	8.15E-01	ENSG00000282885
NA	0.0390	6.7636E-01	8.15E-01	ENSG00000226530
NA	0.0376	6.7668E-01	8.15E-01	ENSG00000261135
NA	0.0050	6.7711E-01		ENSG00000239774
NA	0.0377	6.7732E-01	8.16E-01	ENSG00000273142
NA	0.0078	6.7783E-01		ENSG00000254694
NA	0.0088	6.7804E-01		ENSG00000227080
NA	0.0352	6.7815E-01	8.16E-01	ENSG00000272343
NA	0.0066	6.7888E-01		ENSG00000289601
NA	0.0056	6.7921E-01		ENSG00000281477
NA	0.0348	6.7923E-01	8.17E-01	ENSG00000277342
NA	0.0077	6.7925E-01		ENSG00000226904
NA	0.0028	6.7939E-01		ENSG00000254270

NA	0.0364	6.7942E-01	8.18E-01	ENSG00000159239
NA	0.0006	6.8089E-01		ENSG00000250869
NA	0.0089	6.8093E-01		ENSG00000254455
NA	0.0387	6.8134E-01	8.19E-01	ENSG00000210049
NA	0.0002	6.8182E-01		ENSG00000289206
NA	0.0005	6.8187E-01		ENSG00000257534
NA	0.0038	6.8231E-01		ENSG00000237832
NA	0.0144	6.8242E-01		ENSG00000229905
NA	0.0270	6.8243E-01	8.20E-01	ENSG00000232053
NA	0.0149	6.8253E-01		ENSG00000253205
NA	0.0102	6.8253E-01		ENSG00000254537
NA	0.0006	6.8263E-01		ENSG00000286818
NA	0.0391	6.8266E-01	8.20E-01	ENSG00000289038
NA	0.0153	6.8274E-01		ENSG00000271973
NA	0.0337	6.8286E-01	8.20E-01	ENSG00000259321
NA	0.0030	6.8296E-01		ENSG00000279971
NA	0.0122	6.8348E-01		ENSG00000229263
NA	0.0375	6.8356E-01	8.21E-01	ENSG00000236397
NA	0.0016	6.8358E-01		ENSG00000287983
NA	0.0264	6.8363E-01	8.21E-01	ENSG00000235023
NA	0.0079	6.8384E-01		ENSG00000283360
NA	0.0027	6.8446E-01		ENSG00000289557
NA	0.0386	6.8475E-01	8.21E-01	ENSG00000287038
NA	0.0314	6.8495E-01	8.22E-01	ENSG00000287759
NA	0.0216	6.8499E-01		ENSG00000268603
NA	0.0362	6.8567E-01	8.22E-01	ENSG00000272906
NA	0.0301	6.8575E-01	8.22E-01	ENSG00000215481
NA	0.0025	6.8582E-01		ENSG00000216809
NA	0.0057	6.8609E-01		ENSG00000287041
NA	0.0039	6.8661E-01		ENSG00000230945
NA	0.0343	6.8662E-01	8.23E-01	ENSG00000287550
NA	0.0304	6.8663E-01	8.23E-01	ENSG00000260635
NA	0.0162	6.8666E-01		ENSG00000223725
NA	0.0021	6.8685E-01		ENSG00000265174
NA	0.0219	6.8687E-01	8.23E-01	ENSG00000270822
NA	0.0077	6.8751E-01		ENSG00000268499
NA	0.0237	6.8770E-01		ENSG00000285969
NA	0.0098	6.8780E-01		ENSG00000254165
NA	0.0065	6.8827E-01		ENSG00000260859
NA	0.0051	6.8837E-01		ENSG00000273165
NA	0.0045	6.8861E-01		ENSG00000255239
NA	0.0082	6.8885E-01	8.25E-01	ENSG00000273812
NA	0.0100	6.8887E-01		ENSG00000250348
NA	0.0359	6.8915E-01	8.25E-01	ENSG00000267734
NA	0.0062	6.8921E-01		ENSG00000287621
NA	0.0049	6.8961E-01		ENSG00000237130
NA	0.0304	6.8984E-01	8.26E-01	ENSG00000248740
NA	0.0057	6.8984E-01		ENSG00000227417
NA	0.0017	6.8985E-01		ENSG00000286855

NA	0.0125	6.9021E-01		ENSG00000271137
NA	0.0150	6.9021E-01		ENSG00000241211
NA	0.0010	6.9039E-01		ENSG00000287197
NA	0.0117	6.9073E-01		ENSG00000287970
NA	0.0238	6.9076E-01	8.26E-01	ENSG00000268746
NA	0.0109	6.9083E-01		ENSG00000280068
NA	0.0088	6.9213E-01		ENSG00000275476
NA	0.0150	6.9226E-01		ENSG00000287311
NA	0.0249	6.9230E-01	8.28E-01	ENSG00000257769
NA	0.0117	6.9320E-01		ENSG00000272799
NA	0.0072	6.9353E-01		ENSG00000250167
NA	0.0388	6.9361E-01	8.28E-01	ENSG00000278576
NA	0.0045	6.9370E-01		ENSG00000249031
NA	0.0015	6.9378E-01		ENSG00000278966
NA	0.0044	6.9395E-01		ENSG00000273693
NA	0.0350	6.9395E-01	8.29E-01	ENSG00000124224
NA	0.0312	6.9442E-01	8.29E-01	ENSG00000276136
NA	0.0012	6.9461E-01		ENSG00000279771
NA	0.0034	6.9488E-01	8.29E-01	ENSG00000244461
NA	0.0385	6.9506E-01	8.29E-01	ENSG00000259959
NA	0.0095	6.9515E-01		ENSG00000285909
NA	0.0094	6.9523E-01		ENSG00000279572
NA	0.0102	6.9526E-01		ENSG00000237629
NA	0.0072	6.9558E-01		ENSG00000276471
NA	0.0226	6.9560E-01	8.30E-01	ENSG00000259921
NA	0.0046	6.9603E-01		ENSG00000286201
NA	0.0057	6.9615E-01		ENSG00000259505
NA	0.0285	6.9645E-01	8.30E-01	ENSG00000266918
NA	0.0253	6.9669E-01	8.30E-01	ENSG00000253238
NA	0.0380	6.9703E-01	8.31E-01	ENSG00000180015
NA	0.0093	6.9719E-01		ENSG00000228748
NA	0.0418	6.9723E-01	8.31E-01	ENSG00000276934
NA	0.0343	6.9726E-01	8.31E-01	ENSG00000270457
NA	0.0156	6.9788E-01		ENSG00000266968
NA	0.0162	6.9801E-01	8.31E-01	ENSG00000261089
NA	0.0187	6.9807E-01	8.31E-01	ENSG00000225077
NA	0.0358	6.9847E-01	8.31E-01	ENSG00000251279
NA	0.0056	6.9882E-01		ENSG00000273233
NA	0.0076	6.9888E-01		ENSG00000276092
NA	0.0108	6.9904E-01		ENSG00000249020
NA	0.0037	6.9919E-01		ENSG00000228204
NA	0.0226	6.9944E-01	8.32E-01	ENSG00000259707
NA	0.0123	6.9974E-01	8.32E-01	ENSG00000100181
NA	0.0236	7.0009E-01	8.32E-01	ENSG00000274895
NA	0.0029	7.0022E-01		ENSG00000240015
NA	0.0148	7.0035E-01		ENSG00000254035
NA	0.0194	7.0058E-01	8.33E-01	ENSG00000288845
NA	0.0249	7.0071E-01	8.33E-01	ENSG00000227203
NA	0.0318	7.0090E-01	8.33E-01	ENSG00000289611

NA	0.0284	7.0110E-01	8.33E-01	ENSG00000280187
NA	0.0024	7.0118E-01		ENSG00000218682
NA	0.0368	7.0136E-01	8.33E-01	ENSG00000244513
NA	0.0338	7.0137E-01	8.33E-01	ENSG00000255062
NA	0.0136	7.0149E-01	8.33E-01	ENSG00000257023
NA	0.0241	7.0150E-01	8.33E-01	ENSG00000280850
NA	0.0087	7.0183E-01		ENSG00000269976
NA	0.0238	7.0204E-01	8.33E-01	ENSG00000285847
NA	0.0307	7.0243E-01	8.33E-01	ENSG00000287527
NA	0.0282	7.0262E-01	8.34E-01	ENSG00000232891
NA	0.0274	7.0330E-01	8.34E-01	ENSG00000279021
NA	0.0317	7.0330E-01	8.34E-01	ENSG00000224167
NA	0.0050	7.0332E-01		ENSG00000268879
NA	0.0158	7.0354E-01	8.34E-01	ENSG00000279029
NA	0.0336	7.0366E-01	8.34E-01	ENSG00000241231
NA	0.0041	7.0388E-01		ENSG00000232241
NA	0.0290	7.0410E-01	8.34E-01	ENSG00000261513
NA	0.0103	7.0425E-01		ENSG00000284653
NA	0.0364	7.0444E-01	8.35E-01	ENSG00000224126
NA	0.0060	7.0450E-01	8.35E-01	ENSG00000281538
NA	0.0110	7.0456E-01		ENSG00000203855
NA	0.0205	7.0467E-01		ENSG00000279995
NA	0.0329	7.0480E-01	8.35E-01	ENSG00000242553
NA	0.0051	7.0484E-01		ENSG00000229386
NA	0.0144	7.0488E-01		ENSG00000277007
NA	0.0002	7.0509E-01		ENSG00000261708
NA	0.0181	7.0532E-01	8.35E-01	ENSG00000206660
NA	0.0216	7.0559E-01		ENSG00000285661
NA	0.0237	7.0561E-01	8.35E-01	ENSG00000248576
NA	0.0368	7.0567E-01	8.35E-01	ENSG00000278847
NA	0.0003	7.0569E-01		ENSG00000235795
NA	0.0007	7.0587E-01		ENSG00000286645
NA	0.0305	7.0588E-01		ENSG00000286631
NA	0.0178	7.0642E-01	8.36E-01	ENSG00000251414
NA	0.0338	7.0648E-01	8.36E-01	ENSG00000279908
NA	0.0022	7.0657E-01		ENSG00000257376
NA	0.0079	7.0690E-01		ENSG00000223839
NA	0.0004	7.0694E-01		ENSG00000260037
NA	0.0048	7.0700E-01		ENSG00000207151
NA	0.0324	7.0706E-01	8.36E-01	ENSG00000271204
NA	0.0020	7.0713E-01		ENSG00000253356
NA	0.0212	7.0721E-01		ENSG00000267366
NA	0.0118	7.0771E-01		ENSG00000230537
NA	0.0024	7.0776E-01		ENSG00000269534
NA	0.0130	7.0790E-01	8.37E-01	ENSG00000288560
NA	0.0074	7.0795E-01		ENSG00000273618
NA	0.0014	7.0801E-01		ENSG00000236493
NA	0.0052	7.0815E-01		ENSG00000279878
NA	0.0044	7.0816E-01	8.37E-01	ENSG00000202415

NA	0.0035	7.0834E-01		ENSG00000285547
NA	0.0023	7.0834E-01		ENSG00000235832
NA	0.0045	7.0842E-01		ENSG00000255487
NA	0.0044	7.0868E-01		ENSG00000227016
NA	0.0124	7.0909E-01		ENSG00000220643
NA	0.0035	7.0915E-01		ENSG00000267223
NA	0.0039	7.0928E-01		ENSG00000218902
NA	0.0143	7.0933E-01		ENSG00000248356
NA	0.0117	7.0993E-01		ENSG00000283405
NA	0.0242	7.1017E-01	8.38E-01	ENSG00000235689
NA	0.0078	7.1019E-01		ENSG00000224906
NA	0.0008	7.1062E-01		ENSG00000287525
NA	0.0343	7.1088E-01	8.39E-01	ENSG00000279099
NA	0.0050	7.1091E-01		ENSG00000227087
NA	0.0082	7.1119E-01		ENSG00000271401
NA	0.0137	7.1134E-01		ENSG00000233213
NA	0.0039	7.1172E-01		ENSG00000233492
NA	0.0016	7.1176E-01		ENSG00000279511
NA	0.0337	7.1237E-01	8.40E-01	ENSG00000226519
NA	0.0081	7.1245E-01		ENSG00000197644
NA	0.0319	7.1257E-01	8.40E-01	ENSG00000278493
NA	0.0260	7.1261E-01	8.40E-01	ENSG00000240996
NA	0.0030	7.1297E-01		ENSG00000279146
NA	0.0152	7.1315E-01		ENSG00000229207
NA	0.0353	7.1323E-01	8.40E-01	ENSG00000279863
NA	0.0175	7.1332E-01		ENSG00000248696
NA	0.0054	7.1351E-01		ENSG00000237689
NA	0.0289	7.1358E-01	8.40E-01	ENSG00000265554
NA	0.0237	7.1362E-01		ENSG00000261315
NA	0.0008	7.1390E-01		ENSG00000253924
NA	0.0034	7.1400E-01		ENSG00000235619
NA	0.0234	7.1416E-01		ENSG00000218313
NA	0.0080	7.1427E-01		ENSG00000230240
NA	0.0044	7.1433E-01		ENSG00000278041
NA	0.0049	7.1455E-01		ENSG00000042304
NA	0.0067	7.1517E-01		ENSG00000227920
NA	0.0344	7.1529E-01	8.41E-01	ENSG00000237883
NA	0.0028	7.1541E-01	8.42E-01	ENSG00000280063
NA	0.0110	7.1564E-01		ENSG00000241679
NA	0.0082	7.1576E-01		ENSG00000256452
NA	0.0004	7.1578E-01		ENSG00000289134
NA	0.0012	7.1590E-01		ENSG00000269242
NA	0.0098	7.1591E-01		ENSG00000224172
NA	0.0330	7.1593E-01	8.42E-01	ENSG00000189423
NA	0.0070	7.1603E-01	8.42E-01	ENSG00000259058
NA	0.0334	7.1615E-01	8.42E-01	ENSG00000267868
NA	0.0002	7.1627E-01		ENSG00000289237
NA	0.0025	7.1628E-01		ENSG00000279406
NA	0.0136	7.1632E-01		ENSG00000231004

NA	0.0071	7.1671E-01		ENSG00000267063
NA	0.0044	7.1684E-01		ENSG00000237705
NA	0.0149	7.1703E-01	8.43E-01	ENSG00000230524
NA	0.0048	7.1713E-01		ENSG00000226206
NA	0.0359	7.1743E-01	8.43E-01	ENSG00000286542
NA	0.0200	7.1748E-01		ENSG00000255286
NA	0.0329	7.1774E-01	8.43E-01	ENSG00000288766
NA	0.0298	7.1781E-01	8.43E-01	ENSG00000285081
NA	0.0337	7.1825E-01	8.44E-01	ENSG00000289855
NA	0.0262	7.1833E-01	8.44E-01	ENSG00000258739
NA	0.0012	7.1855E-01		ENSG00000265775
NA	0.0250	7.1867E-01	8.44E-01	ENSG00000272218
NA	0.0313	7.1884E-01	8.44E-01	ENSG00000261560
NA	0.0216	7.1894E-01		ENSG00000285739
NA	0.0185	7.1929E-01	8.44E-01	ENSG00000272950
NA	0.0002	7.1943E-01		ENSG00000227666
NA	0.0191	7.1944E-01	8.44E-01	ENSG00000275383
NA	0.0168	7.1982E-01		ENSG00000224478
NA	0.0173	7.2022E-01		ENSG00000236942
NA	0.0054	7.2023E-01		ENSG00000257964
NA	0.0019	7.2036E-01		ENSG00000282865
NA	0.0064	7.2037E-01		ENSG00000268660
NA	0.0181	7.2052E-01		ENSG00000223503
NA	0.0039	7.2061E-01		ENSG00000274135
NA	0.0294	7.2101E-01	8.45E-01	ENSG00000288948
NA	0.0094	7.2120E-01		ENSG00000259587
NA	0.0069	7.2154E-01		ENSG00000257703
NA	0.0064	7.2156E-01		ENSG00000272745
NA	0.0084	7.2192E-01		ENSG00000237844
NA	0.0049	7.2242E-01		ENSG00000256596
NA	0.0269	7.2278E-01	8.47E-01	ENSG00000225284
NA	0.0170	7.2290E-01	8.47E-01	ENSG00000207425
NA	0.0048	7.2335E-01		ENSG00000287802
NA	0.0339	7.2359E-01	8.47E-01	ENSG00000263884
NA	0.0316	7.2367E-01	8.47E-01	ENSG00000268564
NA	0.0024	7.2397E-01		ENSG00000258571
NA	0.0188	7.2424E-01	8.47E-01	ENSG00000285701
NA	0.0008	7.2438E-01		ENSG00000259113
NA	0.0109	7.2452E-01		ENSG00000280486
NA	0.0021	7.2466E-01	8.48E-01	ENSG00000286385
NA	0.0283	7.2491E-01	8.48E-01	ENSG00000227001
NA	0.0103	7.2500E-01	8.48E-01	ENSG00000232823
NA	0.0115	7.2501E-01		ENSG00000231758
NA	0.0034	7.2526E-01		ENSG00000253181
NA	0.0018	7.2526E-01		ENSG00000289984
NA	0.0164	7.2560E-01		ENSG00000239944
NA	0.0055	7.2560E-01		ENSG00000234261
NA	0.0333	7.2568E-01	8.48E-01	ENSG00000287811
NA	0.0338	7.2589E-01	8.49E-01	ENSG00000267284

NA	0.0185	7.2636E-01		ENSG00000288549
NA	0.0243	7.2636E-01	8.49E-01	ENSG00000284946
NA	0.0191	7.2764E-01	8.50E-01	ENSG00000254332
NA	0.0027	7.2787E-01		ENSG00000241529
NA	0.0176	7.2797E-01		ENSG00000269792
NA	0.0234	7.2840E-01	8.50E-01	ENSG00000260988
NA	0.0340	7.2881E-01	8.51E-01	ENSG00000227077
NA	0.0065	7.2897E-01	8.51E-01	ENSG00000264660
NA	0.0076	7.2916E-01		ENSG00000153923
NA	0.0235	7.2932E-01		ENSG00000264125
NA	0.0239	7.2934E-01		ENSG00000261207
NA	0.0026	7.2985E-01		ENSG00000240375
NA	0.0025	7.2994E-01		ENSG00000286333
NA	0.0089	7.3024E-01		ENSG00000199870
NA	0.0066	7.3083E-01		ENSG00000279735
NA	0.0079	7.3128E-01		ENSG00000203395
NA	0.0160	7.3174E-01		ENSG00000250046
NA	0.0207	7.3202E-01	8.53E-01	ENSG00000239920
NA	0.0308	7.3213E-01	8.53E-01	ENSG00000220848
NA	0.0052	7.3215E-01		ENSG00000267298
NA	0.0297	7.3218E-01	8.53E-01	ENSG00000283696
NA	0.0034	7.3227E-01		ENSG00000289276
NA	0.0115	7.3235E-01		ENSG00000236975
NA	0.0054	7.3236E-01		ENSG00000285573
NA	0.0208	7.3238E-01	8.53E-01	ENSG00000224269
NA	0.0010	7.3254E-01		ENSG00000273189
NA	0.0034	7.3268E-01		ENSG00000264187
NA	0.0019	7.3275E-01		ENSG00000260277
NA	0.0026	7.3289E-01		ENSG00000285565
NA	0.0301	7.3310E-01	8.53E-01	ENSG00000235092
NA	0.0321	7.3320E-01	8.53E-01	ENSG00000289419
NA	0.0086	7.3347E-01		ENSG00000273747
NA	0.0028	7.3371E-01		ENSG00000238242
NA	0.0121	7.3413E-01		ENSG00000248155
NA	0.0038	7.3441E-01		ENSG00000258599
NA	0.0327	7.3454E-01	8.54E-01	ENSG00000286191
NA	0.0091	7.3462E-01		ENSG00000229370
NA	0.0049	7.3521E-01		ENSG00000287424
NA	0.0002	7.3526E-01		ENSG00000254987
NA	0.0066	7.3564E-01		ENSG00000254088
NA	0.0126	7.3626E-01		ENSG00000287729
NA	0.0313	7.3632E-01	8.55E-01	ENSG00000178162
NA	0.0053	7.3675E-01		ENSG00000259848
NA	0.0037	7.3690E-01		ENSG00000225964
NA	0.0006	7.3692E-01		ENSG00000280443
NA	0.0066	7.3700E-01		ENSG00000188029
NA	0.0109	7.3700E-01		ENSG00000258732
NA	0.0099	7.3704E-01		ENSG00000273710
NA	0.0242	7.3706E-01	8.56E-01	ENSG00000244081

NA	0.0008	7.3713E-01		ENSG00000241219
NA	0.0078	7.3723E-01		ENSG00000225303
NA	0.0259	7.3774E-01	8.56E-01	ENSG00000278416
NA	0.0306	7.3785E-01	8.56E-01	ENSG00000288398
NA	0.0069	7.3834E-01		ENSG00000270702
NA	0.0087	7.3846E-01		ENSG00000234999
NA	0.0156	7.3849E-01		ENSG00000244556
NA	0.0291	7.3887E-01	8.57E-01	ENSG00000244733
NA	0.0059	7.3892E-01		ENSG00000227382
NA	0.0095	7.3925E-01		ENSG00000228539
NA	0.0033	7.3934E-01		ENSG00000250500
NA	0.0104	7.3992E-01		ENSG00000262380
NA	0.0062	7.4008E-01		ENSG00000253424
NA	0.0011	7.4026E-01		ENSG00000288749
NA	0.0095	7.4033E-01	8.57E-01	ENSG00000265015
NA	0.0310	7.4068E-01	8.57E-01	ENSG00000243289
NA	0.0024	7.4090E-01		ENSG00000224330
NA	0.0031	7.4169E-01		ENSG00000259999
NA	0.0260	7.4186E-01	8.58E-01	ENSG00000261714
NA	0.0236	7.4223E-01	8.59E-01	ENSG00000228988
NA	0.0099	7.4241E-01		ENSG00000255429
NA	0.0075	7.4250E-01		ENSG00000230572
NA	0.0111	7.4319E-01		ENSG00000234174
NA	0.0003	7.4334E-01	8.59E-01	ENSG00000272647
NA	0.0110	7.4344E-01		ENSG00000236478
NA	0.0258	7.4345E-01	8.59E-01	ENSG00000223930
NA	0.0124	7.4369E-01	8.59E-01	ENSG00000278212
NA	0.0075	7.4450E-01		ENSG00000255986
NA	0.0060	7.4453E-01		ENSG00000279075
NA	0.0112	7.4461E-01		ENSG00000229224
NA	0.0140	7.4476E-01	8.60E-01	ENSG00000260500
NA	0.0017	7.4477E-01		ENSG00000255334
NA	0.0024	7.4482E-01		ENSG00000273181
NA	0.0071	7.4509E-01		ENSG00000279900
NA	0.0073	7.4533E-01		ENSG00000259013
NA	0.0317	7.4536E-01	8.61E-01	ENSG00000232519
NA	0.0254	7.4571E-01	8.61E-01	ENSG00000277159
NA	0.0291	7.4612E-01	8.61E-01	ENSG00000271335
NA	0.0214	7.4616E-01	8.61E-01	ENSG00000287069
NA	0.0112	7.4616E-01		ENSG00000259481
NA	0.0069	7.4635E-01		ENSG00000253273
NA	0.0287	7.4666E-01	8.61E-01	ENSG00000266947
NA	0.0002	7.4669E-01		ENSG00000176857
NA	0.0087	7.4671E-01		ENSG00000226885
NA	0.0064	7.4674E-01		ENSG00000255340
NA	0.0292	7.4680E-01	8.61E-01	ENSG00000288924
NA	0.0277	7.4710E-01	8.61E-01	ENSG00000284669
NA	0.0032	7.4734E-01		ENSG00000287306
NA	0.0144	7.4759E-01	8.62E-01	ENSG00000225264

NA	0.0017	7.4767E-01		ENSG00000260661
NA	0.0111	7.4783E-01	8.62E-01	ENSG00000253893
NA	0.0000	7.4795E-01		ENSG00000218233
NA	0.0055	7.4821E-01		ENSG00000254843
NA	0.0060	7.4850E-01		ENSG00000237631
NA	0.0048	7.4882E-01		ENSG00000287647
NA	0.0076	7.4903E-01	8.63E-01	ENSG00000251510
NA	0.0011	7.4906E-01		ENSG00000272033
NA	0.0118	7.4912E-01		ENSG00000225728
NA	0.0218	7.4938E-01	8.63E-01	ENSG00000267299
NA	0.0258	7.4941E-01	8.63E-01	ENSG00000287475
NA	0.0028	7.4967E-01		ENSG00000225580
NA	0.0280	7.4997E-01	8.63E-01	ENSG00000250069
NA	0.0026	7.5015E-01		ENSG00000228466
NA	0.0305	7.5027E-01	8.63E-01	ENSG00000274225
NA	0.0053	7.5052E-01		ENSG00000230801
NA	0.0034	7.5057E-01		ENSG00000280143
NA	0.0045	7.5099E-01		ENSG00000288162
NA	0.0034	7.5106E-01		ENSG00000275772
NA	0.0028	7.5148E-01		ENSG00000182048
NA	0.0023	7.5215E-01		ENSG00000243501
NA	0.0041	7.5270E-01		ENSG00000277541
NA	0.0029	7.5283E-01	8.65E-01	ENSG00000287698
NA	0.0119	7.5307E-01		ENSG00000235224
NA	0.0275	7.5316E-01	8.65E-01	ENSG00000218996
NA	0.0282	7.5352E-01	8.66E-01	ENSG00000250999
NA	0.0106	7.5366E-01		ENSG00000273551
NA	0.0007	7.5391E-01	8.66E-01	ENSG00000288940
NA	0.0292	7.5397E-01	8.66E-01	ENSG00000289046
NA	0.0037	7.5423E-01		ENSG00000274584
NA	0.0310	7.5461E-01	8.66E-01	ENSG00000261799
NA	0.0027	7.5506E-01		ENSG00000270993
NA	0.0242	7.5578E-01	8.67E-01	ENSG00000232504
NA	0.0024	7.5607E-01		ENSG00000272995
NA	0.0031	7.5665E-01		ENSG00000239413
NA	0.0169	7.5674E-01	8.68E-01	ENSG00000258066
NA	0.0279	7.5699E-01	8.68E-01	ENSG00000205847
NA	0.0244	7.5738E-01	8.68E-01	ENSG00000239405
NA	0.0028	7.5740E-01		ENSG00000274378
NA	0.0032	7.5813E-01		ENSG00000288955
NA	0.0054	7.5841E-01		ENSG00000268081
NA	0.0080	7.5851E-01		ENSG00000287362
NA	0.0289	7.5864E-01	8.69E-01	ENSG00000275807
NA	0.0273	7.5866E-01	8.69E-01	ENSG00000284735
NA	0.0062	7.5936E-01		ENSG00000272159
NA	0.0284	7.5940E-01	8.69E-01	ENSG00000273363
NA	0.0078	7.5987E-01		ENSG00000287329
NA	0.0032	7.6013E-01		ENSG00000259121
NA	0.0232	7.6016E-01	8.70E-01	ENSG00000254551

NA	0.0006	7.6101E-01		ENSG00000278370
NA	0.0166	7.6123E-01		ENSG00000285588
NA	0.0079	7.6137E-01	8.70E-01	ENSG00000249149
NA	0.0111	7.6220E-01		ENSG00000246095
NA	0.0139	7.6249E-01		ENSG00000267523
NA	0.0072	7.6316E-01		ENSG00000270988
NA	0.0092	7.6318E-01		ENSG00000254154
NA	0.0253	7.6366E-01	8.72E-01	ENSG00000232234
NA	0.0099	7.6368E-01	8.72E-01	ENSG00000237781
NA	0.0146	7.6369E-01		ENSG00000229912
NA	0.0020	7.6379E-01		ENSG00000286849
NA	0.0009	7.6383E-01		ENSG00000276241
NA	0.0081	7.6412E-01		ENSG00000187536
NA	0.0286	7.6444E-01	8.72E-01	ENSG00000229152
NA	0.0010	7.6462E-01		ENSG00000267808
NA	0.0134	7.6510E-01	8.72E-01	ENSG00000287894
NA	0.0107	7.6535E-01		ENSG00000286639
NA	0.0042	7.6572E-01		ENSG00000258902
NA	0.0077	7.6597E-01		ENSG00000274993
NA	0.0232	7.6605E-01	8.73E-01	ENSG00000234004
NA	0.0243	7.6622E-01	8.73E-01	ENSG00000229358
NA	0.0279	7.6625E-01	8.73E-01	ENSG00000247728
NA	0.0033	7.6702E-01		ENSG00000286555
NA	0.0078	7.6734E-01		ENSG00000275064
NA	0.0267	7.6787E-01	8.74E-01	ENSG00000228386
NA	0.0002	7.6823E-01		ENSG00000279606
NA	0.0134	7.6848E-01	8.74E-01	ENSG00000228421
NA	0.0270	7.6851E-01	8.74E-01	ENSG00000239521
NA	0.0061	7.6851E-01		ENSG00000176343
NA	0.0116	7.6853E-01	8.74E-01	ENSG00000234604
NA	0.0258	7.6897E-01	8.75E-01	ENSG00000289102
NA	0.0157	7.6911E-01	8.75E-01	ENSG00000286232
NA	0.0033	7.6931E-01		ENSG00000278635
NA	0.0172	7.6957E-01		ENSG00000255581
NA	0.0255	7.6974E-01	8.75E-01	ENSG00000251526
NA	0.0241	7.6978E-01	8.75E-01	ENSG00000286320
NA	0.0081	7.6997E-01		ENSG00000270739
NA	0.0256	7.7046E-01	8.76E-01	ENSG00000287101
NA	0.0090	7.7082E-01	8.76E-01	ENSG00000248131
NA	0.0119	7.7113E-01	8.76E-01	ENSG00000274373
NA	0.0274	7.7133E-01	8.76E-01	ENSG00000227953
NA	0.0053	7.7157E-01		ENSG00000262898
NA	0.0002	7.7167E-01		ENSG00000286905
NA	0.0052	7.7180E-01		ENSG00000204121
NA	0.0046	7.7226E-01		ENSG00000286637
NA	0.0024	7.7303E-01		ENSG00000289608
NA	0.0077	7.7316E-01		ENSG00000231362
NA	0.0019	7.7327E-01		ENSG00000288107
NA	0.0217	7.7348E-01	8.77E-01	ENSG00000213260

NA	0.0015	7.7360E-01		ENSG00000286942
NA	0.0026	7.7532E-01		ENSG00000239622
NA	0.0268	7.7569E-01	8.79E-01	ENSG00000287402
NA	0.0191	7.7588E-01	8.79E-01	ENSG00000236501
NA	0.0073	7.7704E-01		ENSG00000261476
NA	0.0043	7.7716E-01		ENSG00000235028
NA	0.0054	7.7732E-01	8.80E-01	ENSG00000287712
NA	0.0083	7.7732E-01		ENSG00000228877
NA	0.0023	7.7760E-01		ENSG00000275345
NA	0.0270	7.7771E-01	8.80E-01	ENSG00000286875
NA	0.0262	7.7795E-01	8.80E-01	ENSG00000204860
NA	0.0049	7.7800E-01		ENSG00000260650
NA	0.0237	7.7829E-01	8.80E-01	ENSG00000286813
NA	0.0183	7.7835E-01	8.80E-01	ENSG00000276103
NA	0.0153	7.7847E-01		ENSG00000285710
NA	0.0188	7.7884E-01	8.80E-01	ENSG00000259654
NA	0.0033	7.7911E-01		ENSG00000270933
NA	0.0036	7.7926E-01		ENSG00000253389
NA	0.0171	7.7937E-01	8.81E-01	ENSG00000274487
NA	0.0286	7.7938E-01	8.81E-01	ENSG00000287644
NA	0.0267	7.7957E-01	8.81E-01	ENSG00000210135
NA	0.0268	7.7962E-01	8.81E-01	ENSG00000249459
NA	0.0267	7.7964E-01	8.81E-01	ENSG00000286834
NA	0.0100	7.7974E-01		ENSG00000289392
NA	0.0084	7.8012E-01		ENSG00000271653
NA	0.0227	7.8065E-01	8.82E-01	ENSG00000233614
NA	0.0234	7.8073E-01	8.82E-01	ENSG00000250829
NA	0.0088	7.8104E-01		ENSG00000256420
NA	0.0183	7.8165E-01	8.82E-01	ENSG00000269947
NA	0.0004	7.8168E-01	8.82E-01	ENSG00000250508
NA	0.0098	7.8202E-01	8.82E-01	ENSG00000273373
NA	0.0059	7.8294E-01		ENSG00000289702
NA	0.0219	7.8298E-01	8.83E-01	ENSG00000278385
NA	0.0084	7.8321E-01		ENSG00000237584
NA	0.0259	7.8347E-01	8.83E-01	ENSG00000278766
NA	0.0049	7.8361E-01		ENSG00000176654
NA	0.0025	7.8369E-01		ENSG00000257666
NA	0.0253	7.8378E-01	8.84E-01	ENSG00000289004
NA	0.0004	7.8427E-01		ENSG00000250567
NA	0.0064	7.8432E-01		ENSG00000257941
NA	0.0142	7.8451E-01		ENSG00000283930
NA	0.0012	7.8452E-01		ENSG00000254417
NA	0.0061	7.8459E-01		ENSG00000290166
NA	0.0266	7.8486E-01	8.84E-01	ENSG00000289111
NA	0.0191	7.8526E-01	8.85E-01	ENSG00000283303
NA	0.0186	7.8532E-01	8.85E-01	ENSG00000276842
NA	0.0017	7.8536E-01		ENSG00000204872
NA	0.0235	7.8570E-01	8.85E-01	ENSG00000261663
NA	0.0215	7.8586E-01	8.85E-01	ENSG00000237768

NA	0.0156	7.8591E-01		ENSG00000254898
NA	0.0067	7.8603E-01		ENSG00000289919
NA	0.0211	7.8619E-01	8.85E-01	ENSG00000250045
NA	0.0132	7.8625E-01	8.85E-01	ENSG00000259982
NA	0.0353	7.8679E-01		ENSG00000262228
NA	0.0039	7.8684E-01		ENSG00000259079
NA	0.0077	7.8713E-01	8.86E-01	ENSG00000279187
NA	0.0152	7.8715E-01		ENSG00000206995
NA	0.0103	7.8723E-01	8.86E-01	ENSG00000273321
NA	0.0042	7.8727E-01		ENSG00000221042
NA	0.0037	7.8758E-01		ENSG00000237585
NA	0.0019	7.8760E-01		ENSG00000275343
NA	0.0257	7.8816E-01	8.86E-01	ENSG00000289810
NA	0.0061	7.8846E-01		ENSG00000229012
NA	0.0107	7.8888E-01		ENSG00000273449
NA	0.0224	7.8897E-01	8.87E-01	ENSG00000214223
NA	0.0101	7.8921E-01		ENSG00000224700
NA	0.0029	7.8935E-01		ENSG00000228918
NA	0.0078	7.8973E-01	8.87E-01	ENSG00000259909
NA	0.0253	7.8985E-01	8.87E-01	ENSG00000284879
NA	0.0013	7.8995E-01		ENSG00000272362
NA	0.0068	7.9026E-01		ENSG00000279405
NA	0.0267	7.9034E-01	8.87E-01	ENSG00000230732
NA	0.0031	7.9043E-01		ENSG00000231550
NA	0.0037	7.9068E-01	8.88E-01	ENSG00000280184
NA	0.0032	7.9101E-01		ENSG00000256748
NA	0.0022	7.9129E-01		ENSG00000288817
NA	0.0240	7.9132E-01	8.88E-01	ENSG00000228434
NA	0.0004	7.9145E-01		ENSG00000266450
NA	0.0252	7.9155E-01	8.88E-01	ENSG00000267383
NA	0.0221	7.9236E-01	8.89E-01	ENSG00000268873
NA	0.0239	7.9319E-01	8.89E-01	ENSG00000228305
NA	0.0101	7.9367E-01		ENSG00000283994
NA	0.0068	7.9373E-01		ENSG00000255315
NA	0.0246	7.9429E-01	8.89E-01	ENSG00000225822
NA	0.0056	7.9460E-01		ENSG00000231510
NA	0.0006	7.9489E-01		ENSG00000287154
NA	0.0030	7.9494E-01		ENSG00000213355
NA	0.0257	7.9530E-01		ENSG00000289534
NA	0.0057	7.9532E-01		ENSG00000184423
NA	0.0043	7.9577E-01		ENSG00000260851
NA	0.0217	7.9579E-01	8.90E-01	ENSG00000226007
NA	0.0061	7.9613E-01		ENSG00000231564
NA	0.0029	7.9614E-01		ENSG00000279729
NA	0.0127	7.9642E-01		ENSG00000231539
NA	0.0135	7.9663E-01		ENSG00000289434
NA	0.0027	7.9674E-01		ENSG00000243295
NA	0.0226	7.9678E-01	8.91E-01	ENSG00000288949
NA	0.0057	7.9791E-01		ENSG00000231916

NA	0.0243	7.9807E-01	8.92E-01	ENSG00000267632
NA	0.0088	7.9814E-01		ENSG00000253516
NA	0.0006	7.9818E-01		ENSG00000235095
NA	0.0008	7.9868E-01		ENSG00000258122
NA	0.0016	7.9918E-01		ENSG00000225181
NA	0.0075	7.9926E-01		ENSG00000273927
NA	0.0075	7.9926E-01		ENSG00000274984
NA	0.0053	7.9940E-01		ENSG00000288047
NA	0.0244	7.9948E-01	8.93E-01	ENSG00000226756
NA	0.0241	7.9981E-01	8.93E-01	ENSG00000219392
NA	0.0242	7.9983E-01	8.93E-01	ENSG00000171084
NA	0.0206	7.9999E-01		ENSG00000279752
NA	0.0060	8.0000E-01		ENSG00000229832
NA	0.0065	8.0015E-01		ENSG00000263698
NA	0.0065	8.0016E-01		ENSG00000271318
NA	0.0234	8.0046E-01	8.93E-01	ENSG00000250421
NA	0.0004	8.0066E-01		ENSG00000251062
NA	0.0004	8.0104E-01		ENSG00000236388
NA	0.0058	8.0114E-01		ENSG00000204049
NA	0.0044	8.0114E-01		ENSG00000258649
NA	0.0024	8.0154E-01		ENSG00000231261
NA	0.0023	8.0180E-01		ENSG00000240579
NA	0.0086	8.0250E-01		ENSG00000199938
NA	0.0023	8.0274E-01		ENSG00000286949
NA	0.0025	8.0275E-01		ENSG00000258858
NA	0.0226	8.0379E-01	8.96E-01	ENSG00000289319
NA	0.0030	8.0386E-01		ENSG00000286791
NA	0.0138	8.0407E-01		ENSG00000234584
NA	0.0239	8.0474E-01	8.96E-01	ENSG00000187952
NA	0.0175	8.0493E-01	8.96E-01	ENSG00000229970
NA	0.0044	8.0529E-01		ENSG00000235066
NA	0.0021	8.0562E-01		ENSG00000254112
NA	0.0044	8.0569E-01		ENSG00000262870
NA	0.0006	8.0597E-01		ENSG00000230022
NA	0.0035	8.0643E-01	8.97E-01	ENSG00000204791
NA	0.0158	8.0665E-01	8.97E-01	ENSG00000275897
NA	0.0032	8.0665E-01		ENSG00000202190
NA	0.0107	8.0695E-01		ENSG00000219653
NA	0.0043	8.0698E-01	8.97E-01	ENSG00000276952
NA	0.0034	8.0707E-01		ENSG00000225718
NA	0.0104	8.0720E-01		ENSG00000258378
NA	0.0023	8.0722E-01		ENSG00000261029
NA	0.0143	8.0828E-01		ENSG00000286458
NA	0.0078	8.0848E-01		ENSG00000286648
NA	0.0034	8.0852E-01		ENSG00000205325
NA	0.0033	8.0860E-01		ENSG00000249409
NA	0.0109	8.0892E-01	8.99E-01	ENSG00000270823
NA	0.0031	8.0907E-01		ENSG00000224029
NA	0.0094	8.0911E-01		ENSG00000269907

NA	0.0007	8.0953E-01		ENSG00000255128
NA	0.0003	8.0990E-01		ENSG00000266936
NA	0.0008	8.1022E-01		ENSG00000277112
NA	0.0011	8.1057E-01		ENSG00000277299
NA	0.0187	8.1071E-01	9.00E-01	ENSG00000248121
NA	0.0026	8.1094E-01		ENSG00000259585
NA	0.0036	8.1119E-01		ENSG00000271715
NA	0.0196	8.1120E-01	9.00E-01	ENSG00000267011
NA	0.0312	8.1120E-01	9.00E-01	ENSG00000260142
NA	0.0055	8.1132E-01		ENSG00000250237
NA	0.0082	8.1149E-01	9.00E-01	ENSG00000260093
NA	0.0132	8.1151E-01	9.00E-01	ENSG00000281344
NA	0.0023	8.1256E-01		ENSG00000271919
NA	0.0034	8.1274E-01	9.01E-01	ENSG00000278013
NA	0.0047	8.1285E-01		ENSG00000246323
NA	0.0129	8.1305E-01		ENSG00000282440
NA	0.0075	8.1308E-01		ENSG00000267439
NA	0.0028	8.1337E-01		ENSG00000233586
NA	0.0163	8.1388E-01	9.02E-01	ENSG00000285627
NA	0.0197	8.1404E-01	9.02E-01	ENSG00000235660
NA	0.0062	8.1420E-01		ENSG00000286263
NA	0.0075	8.1500E-01		ENSG00000237663
NA	0.0005	8.1514E-01		ENSG00000227505
NA	0.0074	8.1520E-01		ENSG00000285820
NA	0.0026	8.1547E-01		ENSG00000235277
NA	0.0165	8.1576E-01	9.03E-01	ENSG00000286168
NA	0.0083	8.1639E-01		ENSG00000267758
NA	0.0059	8.1642E-01		ENSG00000267703
NA	0.0010	8.1733E-01		ENSG00000200953
NA	0.0189	8.1760E-01	9.04E-01	ENSG00000226065
NA	0.0009	8.1765E-01		ENSG00000260601
NA	0.0076	8.1771E-01		ENSG00000267740
NA	0.0092	8.1772E-01		ENSG00000224751
NA	0.0018	8.1779E-01		ENSG00000227705
NA	0.0092	8.1834E-01		ENSG00000235546
NA	0.0184	8.1881E-01	9.04E-01	ENSG00000261770
NA	0.0173	8.1969E-01	9.05E-01	ENSG00000236953
NA	0.0040	8.2011E-01		ENSG00000285797
NA	0.0007	8.2012E-01	9.05E-01	ENSG00000287294
NA	0.0189	8.2017E-01	9.05E-01	ENSG00000237672
NA	0.0135	8.2028E-01		ENSG00000279071
NA	0.0189	8.2064E-01	9.05E-01	ENSG00000260193
NA	0.0206	8.2120E-01	9.06E-01	ENSG00000288877
NA	0.0207	8.2140E-01	9.06E-01	ENSG00000260448
NA	0.0040	8.2159E-01		ENSG00000231620
NA	0.0044	8.2257E-01		ENSG00000224394
NA	0.0060	8.2275E-01		ENSG00000237919
NA	0.0044	8.2283E-01		ENSG00000214100
NA	0.0162	8.2300E-01	9.07E-01	ENSG00000234281

NA	0.0020	8.2301E-01		ENSG00000284739
NA	0.0092	8.2330E-01		ENSG00000254431
NA	0.0058	8.2348E-01		ENSG00000236115
NA	0.0030	8.2460E-01	9.08E-01	ENSG00000289057
NA	0.0231	8.2508E-01	9.08E-01	ENSG00000287939
NA	0.0017	8.2563E-01		ENSG00000285838
NA	0.0026	8.2582E-01		ENSG00000289264
NA	0.0204	8.2592E-01	9.09E-01	ENSG00000276900
NA	0.0038	8.2628E-01		ENSG00000286878
NA	0.0024	8.2671E-01		ENSG00000264254
NA	0.0188	8.2705E-01	9.09E-01	ENSG00000266680
NA	0.0200	8.2717E-01	9.09E-01	ENSG00000234618
NA	0.0041	8.2719E-01		ENSG00000272555
NA	0.0040	8.2726E-01		ENSG00000289814
NA	0.0209	8.2726E-01	9.09E-01	ENSG00000286358
NA	0.0196	8.2740E-01	9.09E-01	ENSG00000236993
NA	0.0069	8.2910E-01		ENSG00000170409
NA	0.0017	8.2956E-01		ENSG00000286961
NA	0.0032	8.2966E-01		ENSG00000259478
NA	0.0186	8.2984E-01	9.11E-01	ENSG00000123965
NA	0.0207	8.3021E-01	9.11E-01	ENSG00000256667
NA	0.0057	8.3046E-01		ENSG00000270781
NA	0.0221	8.3091E-01	9.12E-01	ENSG00000272330
NA	0.0038	8.3099E-01		ENSG00000290002
NA	0.0044	8.3106E-01		ENSG00000287912
NA	0.0078	8.3110E-01		ENSG00000271046
NA	0.0106	8.3125E-01	9.12E-01	ENSG00000225871
NA	0.0055	8.3155E-01		ENSG00000239831
NA	0.0035	8.3183E-01		ENSG00000254345
NA	0.0009	8.3191E-01		ENSG00000280227
NA	0.0034	8.3192E-01		ENSG00000259515
NA	0.0153	8.3243E-01	9.12E-01	ENSG00000287809
NA	0.0191	8.3293E-01	9.13E-01	ENSG00000283849
NA	0.0249	8.3302E-01	9.13E-01	ENSG00000283913
NA	0.0198	8.3355E-01	9.13E-01	ENSG00000268903
NA	0.0007	8.3403E-01		ENSG00000261104
NA	0.0081	8.3449E-01		ENSG00000289965
NA	0.0153	8.3504E-01	9.14E-01	ENSG00000233609
NA	0.0176	8.3532E-01	9.14E-01	ENSG00000260526
NA	0.0001	8.3536E-01		ENSG00000255300
NA	0.0198	8.3550E-01	9.14E-01	ENSG00000239415
NA	0.0181	8.3579E-01	9.14E-01	ENSG00000259363
NA	0.0008	8.3589E-01		ENSG00000256403
NA	0.0193	8.3618E-01	9.14E-01	ENSG00000227014
NA	0.0001	8.3653E-01		ENSG00000273568
NA	0.0100	8.3675E-01		ENSG00000267784
NA	0.0153	8.3759E-01	9.15E-01	ENSG00000253320
NA	0.0191	8.3778E-01	9.15E-01	ENSG00000237301
NA	0.0172	8.3789E-01	9.15E-01	ENSG00000154874

NA	0.0185	8.3792E-01	9.15E-01	ENSG00000279765
NA	0.0189	8.3854E-01	9.16E-01	ENSG00000267251
NA	0.0177	8.3874E-01	9.16E-01	ENSG00000241962
NA	0.0191	8.3884E-01	9.16E-01	ENSG00000261118
NA	0.0193	8.3897E-01	9.16E-01	ENSG00000275297
NA	0.0027	8.3996E-01		ENSG00000280116
NA	0.0129	8.3998E-01	9.16E-01	ENSG00000257773
NA	0.0015	8.4027E-01		ENSG00000284659
NA	0.0017	8.4027E-01		ENSG00000281904
NA	0.0066	8.4068E-01		ENSG00000219993
NA	0.0010	8.4082E-01		ENSG00000227579
NA	0.0160	8.4094E-01	9.17E-01	ENSG00000228039
NA	0.0177	8.4096E-01	9.17E-01	ENSG00000253559
NA	0.0079	8.4125E-01		ENSG00000280309
NA	0.0027	8.4136E-01		ENSG00000231772
NA	0.0173	8.4165E-01	9.17E-01	ENSG00000188002
NA	0.0159	8.4177E-01	9.17E-01	ENSG00000219201
NA	0.0004	8.4215E-01		ENSG00000264647
NA	0.0003	8.4220E-01		ENSG00000227017
NA	0.0191	8.4229E-01	9.18E-01	ENSG00000275198
NA	0.0134	8.4259E-01	9.18E-01	ENSG00000290124
NA	0.0146	8.4261E-01	9.18E-01	ENSG00000235051
NA	0.0063	8.4263E-01		ENSG00000224688
NA	0.0060	8.4319E-01		ENSG00000270265
NA	0.0030	8.4348E-01	9.18E-01	ENSG00000232448
NA	0.0165	8.4410E-01	9.19E-01	ENSG00000227589
NA	0.0074	8.4478E-01		ENSG00000214533
NA	0.0026	8.4525E-01		ENSG00000228403
NA	0.0009	8.4536E-01		ENSG00000288007
NA	0.0069	8.4664E-01		ENSG00000276851
NA	0.0093	8.4803E-01		ENSG00000235150
NA	0.0140	8.4804E-01	9.22E-01	ENSG00000254864
NA	0.0044	8.4806E-01		ENSG00000289517
NA	0.0086	8.4814E-01		ENSG00000250882
NA	0.0004	8.4841E-01		ENSG00000259929
NA	0.0003	8.4889E-01		ENSG00000287938
NA	0.0017	8.4919E-01		ENSG00000231699
NA	0.0062	8.4921E-01		ENSG00000230304
NA	0.0009	8.4944E-01		ENSG00000270147
NA	0.0171	8.4978E-01	9.23E-01	ENSG00000286242
NA	0.0006	8.4978E-01		ENSG00000259359
NA	0.0026	8.4985E-01		ENSG00000224661
NA	0.0073	8.4996E-01		ENSG00000228983
NA	0.0067	8.5016E-01		ENSG00000267412
NA	0.0028	8.5054E-01		ENSG00000228923
NA	0.0177	8.5078E-01	9.23E-01	ENSG00000231829
NA	0.0010	8.5112E-01		ENSG00000248159
NA	0.0008	8.5125E-01		ENSG00000215049
NA	0.0184	8.5187E-01	9.24E-01	ENSG00000227671

NA	0.0107	8.5194E-01		ENSG00000279375
NA	0.0003	8.5212E-01	9.24E-01	ENSG00000235045
NA	0.0175	8.5222E-01	9.24E-01	ENSG00000234710
NA	0.0179	8.5231E-01	9.24E-01	ENSG00000177855
NA	0.0047	8.5305E-01		ENSG00000252230
NA	0.0175	8.5353E-01	9.25E-01	ENSG00000290121
NA	0.0096	8.5360E-01		ENSG00000276746
NA	0.0127	8.5413E-01	9.25E-01	ENSG00000213212
NA	0.0006	8.5432E-01		ENSG00000280140
NA	0.0159	8.5450E-01	9.25E-01	ENSG00000290091
NA	0.0005	8.5476E-01		ENSG00000234801
NA	0.0159	8.5553E-01	9.26E-01	ENSG00000265218
NA	0.0175	8.5577E-01	9.26E-01	ENSG00000277287
NA	0.0042	8.5586E-01		ENSG00000266794
NA	0.0032	8.5587E-01		ENSG00000288857
NA	0.0132	8.5628E-01	9.26E-01	ENSG00000280426
NA	0.0005	8.5635E-01		ENSG00000270118
NA	0.0086	8.5651E-01		ENSG00000290088
NA	0.0021	8.5653E-01		ENSG00000202169
NA	0.0009	8.5653E-01	9.27E-01	ENSG00000215097
NA	0.0193	8.5655E-01	9.27E-01	ENSG00000281641
NA	0.0119	8.5670E-01		ENSG00000259782
NA	0.0148	8.5722E-01		ENSG00000272979
NA	0.0015	8.5730E-01		ENSG00000229976
NA	0.0063	8.5753E-01	9.27E-01	ENSG00000230415
NA	0.0029	8.5843E-01		ENSG00000253344
NA	0.0073	8.5890E-01		ENSG00000176970
NA	0.0047	8.5974E-01		ENSG00000273312
NA	0.0044	8.6030E-01		ENSG00000242136
NA	0.0058	8.6035E-01		ENSG00000253784
NA	0.0084	8.6050E-01	9.29E-01	ENSG00000287245
NA	0.0096	8.6055E-01		ENSG00000232821
NA	0.0031	8.6079E-01		ENSG00000289615
NA	0.0028	8.6093E-01	9.29E-01	ENSG00000261736
NA	0.0223	8.6138E-01	9.29E-01	ENSG00000276351
NA	0.0050	8.6141E-01		ENSG00000253154
NA	0.0056	8.6188E-01	9.30E-01	ENSG00000259694
NA	0.0174	8.6193E-01	9.30E-01	ENSG00000269054
NA	0.0011	8.6204E-01		ENSG00000269012
NA	0.0138	8.6252E-01	9.30E-01	ENSG00000288863
NA	0.0074	8.6259E-01		ENSG00000246790
NA	0.0115	8.6291E-01	9.30E-01	ENSG00000241464
NA	0.0156	8.6386E-01	9.31E-01	ENSG00000257122
NA	0.0163	8.6441E-01	9.31E-01	ENSG00000237357
NA	0.0026	8.6459E-01		ENSG00000228108
NA	0.0104	8.6465E-01	9.31E-01	ENSG00000262313
NA	0.0032	8.6474E-01		ENSG00000253339
NA	0.0121	8.6477E-01	9.31E-01	ENSG00000279668
NA	0.0090	8.6481E-01		ENSG00000219491

NA	0.0152	8.6549E-01	9.31E-01	ENSG00000228050
NA	0.0094	8.6573E-01		ENSG00000228700
NA	0.0117	8.6576E-01	9.32E-01	ENSG00000271751
NA	0.0155	8.6581E-01	9.32E-01	ENSG00000255158
NA	0.0047	8.6666E-01	9.32E-01	ENSG00000230964
NA	0.0228	8.6740E-01		ENSG00000224251
NA	0.0053	8.6756E-01	9.33E-01	ENSG00000249772
NA	0.0030	8.6762E-01		ENSG00000287701
NA	0.0004	8.6888E-01		ENSG00000273183
NA	0.0146	8.6896E-01	9.34E-01	ENSG00000254639
NA	0.0085	8.6974E-01	9.34E-01	ENSG00000289421
NA	0.0034	8.6976E-01		ENSG00000287496
NA	0.0037	8.6978E-01		ENSG00000280097
NA	0.0141	8.6983E-01	9.34E-01	ENSG00000287234
NA	0.0007	8.7019E-01		ENSG00000268583
NA	0.0144	8.7034E-01	9.34E-01	ENSG00000227959
NA	0.0021	8.7054E-01		ENSG00000279493
NA	0.0152	8.7067E-01	9.34E-01	ENSG00000286652
NA	0.0132	8.7074E-01	9.34E-01	ENSG00000289297
NA	0.0025	8.7104E-01		ENSG00000241416
NA	0.0039	8.7154E-01		ENSG00000236948
NA	0.0024	8.7162E-01		ENSG00000272372
NA	0.0015	8.7181E-01		ENSG00000223220
NA	0.0148	8.7187E-01		ENSG00000260351
NA	0.0000	8.7227E-01		ENSG00000223492
NA	0.0150	8.7244E-01	9.35E-01	ENSG00000227896
NA	0.0157	8.7284E-01	9.36E-01	ENSG00000273162
NA	0.0018	8.7347E-01		ENSG00000251511
NA	0.0145	8.7413E-01	9.36E-01	ENSG00000276386
NA	0.0156	8.7422E-01	9.36E-01	ENSG00000257951
NA	0.0011	8.7445E-01		ENSG00000260078
NA	0.0131	8.7446E-01	9.37E-01	ENSG00000255449
NA	0.0134	8.7468E-01	9.37E-01	ENSG00000260459
NA	0.0023	8.7564E-01		ENSG00000237380
NA	0.0127	8.7583E-01	9.37E-01	ENSG00000255234
NA	0.0021	8.7710E-01		ENSG00000228380
NA	0.0142	8.7715E-01	9.38E-01	ENSG00000280234
NA	0.0159	8.7743E-01	9.38E-01	ENSG00000239922
NA	0.0047	8.7794E-01		ENSG00000279609
NA	0.0113	8.7806E-01		ENSG00000285201
NA	0.0089	8.7842E-01	9.38E-01	ENSG00000287352
NA	0.0126	8.7900E-01	9.39E-01	ENSG00000261758
NA	0.0120	8.7904E-01	9.39E-01	ENSG00000234160
NA	0.0021	8.7942E-01		ENSG00000287106
NA	0.0029	8.7968E-01		ENSG00000258437
NA	0.0041	8.7977E-01		ENSG00000279189
NA	0.0012	8.7992E-01		ENSG00000279629
NA	0.0002	8.8085E-01		ENSG00000235843
NA	0.0008	8.8187E-01		ENSG00000212939

NA	0.0144	8.8189E-01	9.40E-01	ENSG00000184612
NA	0.0025	8.8210E-01		ENSG00000287908
NA	0.0131	8.8311E-01	9.41E-01	ENSG00000258581
NA	0.0030	8.8404E-01		ENSG00000228941
NA	0.0117	8.8437E-01	9.42E-01	ENSG00000187653
NA	0.0021	8.8491E-01		ENSG00000277764
NA	0.0025	8.8608E-01		ENSG00000241146
NA	0.0117	8.8640E-01		ENSG00000267463
NA	0.0097	8.8680E-01	9.43E-01	ENSG00000259711
NA	0.0066	8.8784E-01		ENSG00000256706
NA	0.0034	8.8785E-01		ENSG00000233021
NA	0.0096	8.8820E-01		ENSG00000234921
NA	0.0136	8.8821E-01	9.44E-01	ENSG00000260782
NA	0.0057	8.8822E-01		ENSG00000224447
NA	0.0044	8.8825E-01		ENSG00000261487
NA	0.0030	8.8835E-01		ENSG00000259066
NA	0.0127	8.8917E-01	9.44E-01	ENSG00000278949
NA	0.0007	8.8924E-01		ENSG00000268533
NA	0.0020	8.8924E-01		ENSG00000279570
NA	0.0044	8.8933E-01	9.44E-01	ENSG00000204529
NA	0.0138	8.9025E-01	9.45E-01	ENSG00000234129
NA	0.0052	8.9051E-01		ENSG00000258294
NA	0.0037	8.9078E-01		ENSG00000226801
NA	0.0084	8.9079E-01	9.45E-01	ENSG00000233978
NA	0.0041	8.9086E-01		ENSG00000286021
NA	0.0002	8.9086E-01		ENSG00000287027
NA	0.0031	8.9103E-01		ENSG00000203496
NA	0.0004	8.9113E-01		ENSG00000250038
NA	0.0029	8.9121E-01		ENSG00000207417
NA	0.0060	8.9186E-01		ENSG00000235494
NA	0.0135	8.9232E-01	9.46E-01	ENSG00000223612
NA	0.0031	8.9237E-01		ENSG00000248766
NA	0.0008	8.9246E-01		ENSG00000279833
NA	0.0019	8.9329E-01		ENSG00000232750
NA	0.0091	8.9347E-01	9.47E-01	ENSG00000240024
NA	0.0013	8.9360E-01	9.47E-01	ENSG00000259512
NA	0.0117	8.9362E-01	9.47E-01	ENSG00000269653
NA	0.0018	8.9383E-01		ENSG00000232332
NA	0.0021	8.9404E-01		ENSG00000224309
NA	0.0005	8.9405E-01		ENSG00000230618
NA	0.0109	8.9410E-01	9.47E-01	ENSG00000272812
NA	0.0116	8.9414E-01	9.47E-01	ENSG00000231752
NA	0.0081	8.9429E-01		ENSG00000250651
NA	0.0080	8.9493E-01	9.48E-01	ENSG00000261211
NA	0.0034	8.9562E-01		ENSG00000273901
NA	0.0106	8.9642E-01	9.48E-01	ENSG00000285925
NA	0.0127	8.9671E-01	9.49E-01	ENSG00000154898
NA	0.0006	8.9754E-01		ENSG00000255397
NA	0.0014	8.9760E-01		ENSG00000283972

NA	0.0112	8.9810E-01	9.49E-01	ENSG00000287265
NA	0.0034	8.9881E-01		ENSG00000279104
NA	0.0121	8.9934E-01	9.50E-01	ENSG00000225643
NA	0.0053	8.9948E-01		ENSG00000226134
NA	0.0004	8.9970E-01		ENSG00000237927
NA	0.0063	9.0019E-01	9.50E-01	ENSG00000287878
NA	0.0111	9.0039E-01	9.50E-01	ENSG00000287778
NA	0.0012	9.0080E-01		ENSG00000259158
NA	0.0018	9.0114E-01		ENSG00000253735
NA	0.0057	9.0124E-01	9.51E-01	ENSG00000223901
NA	0.0175	9.0135E-01		ENSG00000253105
NA	0.0016	9.0143E-01		ENSG00000225574
NA	0.0016	9.0160E-01		ENSG00000277358
NA	0.0061	9.0223E-01		ENSG00000260392
NA	0.0102	9.0225E-01	9.51E-01	ENSG00000280417
NA	0.0013	9.0237E-01		ENSG00000224334
NA	0.0107	9.0244E-01	9.51E-01	ENSG00000233862
NA	0.0021	9.0275E-01		ENSG00000260255
NA	0.0009	9.0321E-01		ENSG00000203616
NA	0.0111	9.0330E-01	9.52E-01	ENSG00000289171
NA	0.0108	9.0334E-01		ENSG00000273369
NA	0.0105	9.0386E-01	9.52E-01	ENSG00000263050
NA	0.0094	9.0410E-01	9.52E-01	ENSG00000248656
NA	0.0090	9.0420E-01	9.52E-01	ENSG00000229107
NA	0.0011	9.0433E-01		ENSG00000271590
NA	0.0050	9.0434E-01		ENSG00000235681
NA	0.0118	9.0444E-01	9.52E-01	ENSG00000226900
NA	0.0038	9.0484E-01	9.52E-01	ENSG00000254481
NA	0.0111	9.0484E-01	9.52E-01	ENSG00000236264
NA	0.0018	9.0519E-01		ENSG00000268486
NA	0.0107	9.0519E-01	9.52E-01	ENSG00000289151
NA	0.0078	9.0525E-01		ENSG00000238280
NA	0.0093	9.0542E-01	9.53E-01	ENSG00000277151
NA	0.0131	9.0550E-01		ENSG00000227067
NA	0.0112	9.0551E-01	9.53E-01	ENSG00000289085
NA	0.0097	9.0570E-01	9.53E-01	ENSG00000250838
NA	0.0033	9.0616E-01	9.53E-01	ENSG00000275374
NA	0.0097	9.0641E-01		ENSG00000266896
NA	0.0089	9.0669E-01	9.53E-01	ENSG00000270402
NA	0.0074	9.0688E-01		ENSG00000287931
NA	0.0014	9.0717E-01		ENSG00000224829
NA	0.0054	9.0769E-01		ENSG00000273174
NA	0.0001	9.0781E-01		ENSG00000257288
NA	0.0031	9.0839E-01		ENSG00000227606
NA	0.0009	9.0860E-01		ENSG00000267589
NA	0.0066	9.0883E-01	9.54E-01	ENSG00000259519
NA	0.0103	9.1025E-01	9.55E-01	ENSG00000259242
NA	0.0099	9.1030E-01	9.55E-01	ENSG00000289042
NA	0.0023	9.1045E-01		ENSG00000225794

NA	0.0003	9.1046E-01		ENSG00000286934
NA	0.0038	9.1052E-01	9.55E-01	ENSG00000230438
NA	0.0078	9.1084E-01	9.55E-01	ENSG00000230310
NA	0.0006	9.1100E-01		ENSG00000235808
NA	0.0101	9.1125E-01	9.55E-01	ENSG00000270605
NA	0.0045	9.1153E-01		ENSG00000248242
NA	0.0027	9.1165E-01		ENSG00000236341
NA	0.0101	9.1174E-01	9.55E-01	ENSG00000187229
NA	0.0082	9.1203E-01		ENSG00000257830
NA	0.0095	9.1261E-01	9.56E-01	ENSG00000225695
NA	0.0459	9.1309E-01	9.56E-01	ENSG00000236842
NA	0.0035	9.1374E-01		ENSG00000261056
NA	0.0021	9.1408E-01		ENSG00000288691
NA	0.0098	9.1465E-01	9.57E-01	ENSG00000223461
NA	0.0100	9.1481E-01	9.57E-01	ENSG00000229119
NA	0.0011	9.1490E-01		ENSG00000285848
NA	0.0049	9.1537E-01		ENSG00000271725
NA	0.0060	9.1555E-01		ENSG00000265912
NA	0.0002	9.1555E-01		ENSG00000213177
NA	0.0079	9.1578E-01		ENSG00000270077
NA	0.0026	9.1768E-01		ENSG00000287856
NA	0.0027	9.1781E-01		ENSG00000253282
NA	0.0084	9.1816E-01	9.59E-01	ENSG00000263164
NA	0.0063	9.1828E-01		ENSG00000273186
NA	0.0068	9.1879E-01	9.60E-01	ENSG00000235410
NA	0.0084	9.1904E-01	9.60E-01	ENSG00000251611
NA	0.0002	9.1908E-01		ENSG00000250264
NA	0.0089	9.1981E-01	9.60E-01	ENSG00000213713
NA	0.0071	9.1986E-01	9.60E-01	ENSG00000286951
NA	0.0064	9.1988E-01	9.60E-01	ENSG00000232978
NA	0.0045	9.2008E-01	9.60E-01	ENSG00000233304
NA	0.0026	9.2011E-01		ENSG00000289299
NA	0.0085	9.2014E-01	9.60E-01	ENSG00000254034
NA	0.0010	9.2024E-01		ENSG00000259202
NA	0.0085	9.2038E-01	9.60E-01	ENSG00000223416
NA	0.0069	9.2099E-01	9.61E-01	ENSG00000286457
NA	0.0077	9.2138E-01	9.61E-01	ENSG00000255026
NA	0.0097	9.2173E-01	9.61E-01	ENSG00000288913
NA	0.0015	9.2266E-01		ENSG00000270243
NA	0.0053	9.2266E-01		ENSG00000269304
NA	0.0070	9.2277E-01	9.61E-01	ENSG00000270482
NA	0.0110	9.2280E-01	9.61E-01	ENSG00000289254
NA	0.0011	9.2290E-01		ENSG00000263606
NA	0.0031	9.2314E-01		ENSG00000289008
NA	0.0082	9.2345E-01		ENSG00000264736
NA	0.0024	9.2348E-01		ENSG00000236658
NA	0.0013	9.2386E-01		ENSG00000267016
NA	0.0074	9.2418E-01		ENSG00000244159
NA	0.0002	9.2462E-01		ENSG00000280286

NA	0.0007	9.2490E-01		ENSG00000257272
NA	0.0077	9.2491E-01	9.63E-01	ENSG00000280376
NA	0.0086	9.2543E-01	9.63E-01	ENSG00000240401
NA	0.0065	9.2554E-01	9.63E-01	ENSG00000236526
NA	0.0065	9.2582E-01		ENSG00000225463
NA	0.0073	9.2603E-01	9.63E-01	ENSG00000289052
NA	0.0032	9.2645E-01	9.64E-01	ENSG00000228395
NA	0.0049	9.2652E-01	9.64E-01	ENSG00000254648
NA	0.0031	9.2678E-01	9.64E-01	ENSG00000286351
NA	0.0081	9.2687E-01	9.64E-01	ENSG00000185065
NA	0.0055	9.2754E-01		ENSG00000225710
NA	0.0093	9.2824E-01	9.65E-01	ENSG00000223345
NA	0.0082	9.2832E-01	9.65E-01	ENSG00000289480
NA	0.0006	9.2877E-01		ENSG00000218073
NA	0.0017	9.2908E-01		ENSG00000229032
NA	0.0062	9.2912E-01	9.65E-01	ENSG00000270062
NA	0.0011	9.3015E-01		ENSG00000272226
NA	0.0074	9.3019E-01	9.66E-01	ENSG00000225292
NA	0.0052	9.3035E-01		ENSG00000287661
NA	0.0057	9.3164E-01	9.66E-01	ENSG00000227159
NA	0.0109	9.3179E-01	9.66E-01	ENSG00000258404
NA	0.0088	9.3279E-01	9.67E-01	ENSG00000233221
NA	0.0085	9.3291E-01	9.67E-01	ENSG00000213885
NA	0.0111	9.3300E-01	9.67E-01	ENSG00000229567
NA	0.0049	9.3316E-01		ENSG00000224771
NA	0.0027	9.3349E-01		ENSG00000272463
NA	0.0073	9.3397E-01	9.67E-01	ENSG00000272754
NA	0.0066	9.3527E-01	9.68E-01	ENSG00000196922
NA	0.0029	9.3561E-01		ENSG00000227300
NA	0.0070	9.3575E-01	9.68E-01	ENSG00000273403
NA	0.0060	9.3617E-01	9.68E-01	ENSG00000287023
NA	0.0071	9.3622E-01	9.68E-01	ENSG00000242588
NA	0.0067	9.3691E-01	9.69E-01	ENSG00000280355
NA	0.0032	9.3740E-01	9.69E-01	ENSG00000256458
NA	0.0120	9.3748E-01		ENSG00000257125
NA	0.0019	9.3789E-01		ENSG00000260240
NA	0.0032	9.3790E-01		ENSG00000267706
NA	0.0023	9.3791E-01		ENSG00000220924
NA	0.0022	9.3820E-01		ENSG00000289320
NA	0.0012	9.3822E-01	9.69E-01	ENSG00000276384
NA	0.0085	9.3832E-01	9.69E-01	ENSG00000233577
NA	0.0066	9.3860E-01	9.69E-01	ENSG00000272696
NA	0.0043	9.3879E-01		ENSG00000274105
NA	0.0065	9.3890E-01	9.69E-01	ENSG00000286669
NA	0.0174	9.3918E-01		ENSG00000248629
NA	0.0079	9.3965E-01	9.70E-01	ENSG00000196933
NA	0.0061	9.3986E-01	9.70E-01	ENSG00000233627
NA	0.0066	9.4011E-01		ENSG00000276542
NA	0.0010	9.4101E-01		ENSG00000164616

NA	0.0035	9.4101E-01		ENSG00000224060
NA	0.0073	9.4130E-01	9.71E-01	ENSG00000226478
NA	0.0056	9.4162E-01	9.71E-01	ENSG00000237118
NA	0.0085	9.4162E-01	9.71E-01	ENSG00000229017
NA	0.0034	9.4184E-01		ENSG00000238034
NA	0.0014	9.4211E-01		ENSG00000235371
NA	0.0021	9.4229E-01	9.72E-01	ENSG00000236751
NA	0.0019	9.4244E-01		ENSG00000226238
NA	0.0011	9.4267E-01		ENSG00000272203
NA	0.0069	9.4395E-01	9.72E-01	ENSG00000264063
NA	0.0072	9.4405E-01	9.72E-01	ENSG00000228007
NA	0.0032	9.4409E-01	9.72E-01	ENSG00000269737
NA	0.0048	9.4416E-01		ENSG00000288965
NA	0.0067	9.4440E-01	9.72E-01	ENSG00000235313
NA	0.0041	9.4504E-01		ENSG00000257818
NA	0.0057	9.4526E-01	9.73E-01	ENSG00000289504
NA	0.0065	9.4527E-01	9.73E-01	ENSG00000289225
NA	0.0071	9.4553E-01	9.73E-01	ENSG00000239559
NA	0.0017	9.4554E-01		ENSG00000285742
NA	0.0036	9.4625E-01	9.73E-01	ENSG00000205763
NA	0.0012	9.4642E-01	9.73E-01	ENSG00000287133
NA	0.0013	9.4643E-01		ENSG00000260823
NA	0.0048	9.4679E-01		ENSG00000272181
NA	0.0014	9.4688E-01		ENSG00000236591
NA	0.0044	9.4704E-01	9.73E-01	ENSG00000287673
NA	0.0063	9.4729E-01	9.73E-01	ENSG00000288543
NA	0.0066	9.4772E-01		ENSG00000256103
NA	0.0010	9.4831E-01		ENSG00000255403
NA	0.0047	9.4838E-01	9.74E-01	ENSG00000290146
NA	0.0050	9.4848E-01	9.74E-01	ENSG00000273356
NA	0.0104	9.4860E-01		ENSG00000238749
NA	0.0104	9.4860E-01		ENSG00000275904
NA	0.0043	9.4879E-01		ENSG00000232358
NA	0.0061	9.4884E-01	9.74E-01	ENSG00000272971
NA	0.0030	9.4887E-01		ENSG00000236636
NA	0.0059	9.4890E-01	9.74E-01	ENSG00000280161
NA	0.0061	9.4923E-01	9.74E-01	ENSG00000260278
NA	0.0047	9.4926E-01	9.74E-01	ENSG00000279407
NA	0.0042	9.5062E-01		ENSG00000206676
NA	0.0076	9.5102E-01	9.75E-01	ENSG00000288741
NA	0.0016	9.5126E-01		ENSG00000279960
NA	0.0012	9.5228E-01		ENSG00000269570
NA	0.0000	9.5248E-01		ENSG00000287805
NA	0.0249	9.5312E-01		ENSG00000224881
NA	0.0062	9.5330E-01	9.76E-01	ENSG00000286977
NA	0.0054	9.5344E-01	9.76E-01	ENSG00000206567
NA	0.0020	9.5411E-01		ENSG00000227193
NA	0.0098	9.5438E-01		ENSG00000267708
NA	0.0055	9.5537E-01	9.77E-01	ENSG00000226889

NA	0.0064	9.5566E-01		ENSG00000269807
NA	0.0040	9.5580E-01		ENSG00000228236
NA	0.0045	9.5611E-01	9.78E-01	ENSG00000256552
NA	0.0011	9.5668E-01		ENSG00000271428
NA	0.0034	9.5675E-01	9.78E-01	ENSG00000254366
NA	0.0021	9.5785E-01		ENSG00000279633
NA	0.0004	9.5950E-01		ENSG00000201282
NA	0.0004	9.5950E-01		ENSG00000199913
NA	0.0041	9.5956E-01	9.80E-01	ENSG00000287516
NA	0.0040	9.5959E-01	9.80E-01	ENSG00000269151
NA	0.0041	9.5980E-01	9.80E-01	ENSG00000276573
NA	0.0047	9.6003E-01	9.80E-01	ENSG00000279549
NA	0.0021	9.6057E-01		ENSG00000225076
NA	0.0040	9.6176E-01	9.81E-01	ENSG00000273797
NA	0.0050	9.6213E-01	9.81E-01	ENSG00000263847
NA	0.0005	9.6252E-01		ENSG00000259592
NA	0.0014	9.6316E-01		ENSG00000255395
NA	0.0011	9.6324E-01	9.81E-01	ENSG00000186466
NA	0.0076	9.6336E-01		ENSG00000284721
NA	0.0086	9.6343E-01		ENSG00000235559
NA	0.0020	9.6351E-01		ENSG00000267077
NA	0.0000	9.6365E-01	9.81E-01	ENSG00000226840
NA	0.0050	9.6401E-01	9.82E-01	ENSG00000260625
NA	0.0173	9.6414E-01		ENSG00000287374
NA	0.0044	9.6591E-01	9.83E-01	ENSG00000245156
NA	0.0035	9.6617E-01	9.83E-01	ENSG00000269621
NA	0.0019	9.6656E-01		ENSG00000253897
NA	0.0006	9.6663E-01	9.83E-01	ENSG00000281379
NA	0.0043	9.6668E-01		ENSG00000223800
NA	0.0001	9.6668E-01		ENSG00000265334
NA	0.0028	9.6684E-01	9.83E-01	ENSG00000277013
NA	0.0041	9.6685E-01	9.83E-01	ENSG00000286872
NA	0.0040	9.6716E-01	9.83E-01	ENSG00000279965
NA	0.0033	9.6786E-01	9.83E-01	ENSG00000232150
NA	0.0024	9.6811E-01	9.84E-01	ENSG00000266941
NA	0.0035	9.6862E-01	9.84E-01	ENSG00000266538
NA	0.0050	9.6874E-01		ENSG00000228670
NA	0.0045	9.6965E-01		ENSG00000276756
NA	0.0037	9.6967E-01	9.84E-01	ENSG00000232098
NA	0.0006	9.7062E-01		ENSG00000253476
NA	0.0024	9.7183E-01	9.85E-01	ENSG00000230067
NA	0.0006	9.7187E-01		ENSG00000253509
NA	0.0010	9.7213E-01		ENSG00000201365
NA	0.0061	9.7215E-01		ENSG00000251552
NA	0.0026	9.7237E-01		ENSG00000260858
NA	0.0035	9.7242E-01	9.86E-01	ENSG00000205959
NA	0.0047	9.7268E-01		ENSG00000214552
NA	0.0033	9.7295E-01	9.86E-01	ENSG00000280247
NA	0.0030	9.7300E-01	9.86E-01	ENSG00000267939

NA	0.0014	9.7322E-01	9.86E-01	ENSG00000287330
NA	0.0019	9.7369E-01		ENSG00000289924
NA	0.0030	9.7380E-01	9.87E-01	ENSG00000272054
NA	0.0030	9.7401E-01	9.87E-01	ENSG00000287343
NA	0.0029	9.7404E-01	9.87E-01	ENSG00000259926
NA	0.0033	9.7462E-01	9.87E-01	ENSG00000240057
NA	0.0030	9.7509E-01	9.87E-01	ENSG00000230555
NA	0.0019	9.7577E-01	9.88E-01	ENSG00000271971
NA	0.0003	9.7652E-01		ENSG00000227308
NA	0.0033	9.7703E-01	9.88E-01	ENSG00000248714
NA	0.0017	9.7754E-01	9.88E-01	ENSG00000248585
NA	0.0010	9.7771E-01		ENSG00000250409
NA	0.0022	9.7790E-01	9.88E-01	ENSG00000287855
NA	0.0008	9.7869E-01	9.89E-01	ENSG00000272912
NA	0.0024	9.7879E-01	9.89E-01	ENSG00000288538
NA	0.0015	9.7914E-01	9.89E-01	ENSG00000226989
NA	0.0022	9.8009E-01	9.89E-01	ENSG00000229206
NA	0.0024	9.8032E-01	9.89E-01	ENSG00000226121
NA	0.0026	9.8046E-01	9.89E-01	ENSG00000281383
NA	0.0017	9.8097E-01	9.90E-01	ENSG00000213569
NA	0.0022	9.8110E-01		ENSG00000231755
NA	0.0013	9.8156E-01		ENSG00000278973
NA	0.0025	9.8199E-01	9.90E-01	ENSG00000276957
NA	0.0023	9.8224E-01	9.90E-01	ENSG00000287865
NA	0.0014	9.8227E-01		ENSG00000270114
NA	0.0012	9.8232E-01		ENSG00000230450
NA	0.0020	9.8245E-01		ENSG00000227189
NA	0.0011	9.8268E-01	9.91E-01	ENSG00000266708
NA	0.0041	9.8281E-01	9.91E-01	ENSG00000251161
NA	0.0106	9.8292E-01	9.91E-01	ENSG00000260519
NA	0.0015	9.8409E-01	9.91E-01	ENSG00000245910
NA	0.0022	9.8443E-01	9.92E-01	ENSG00000256968
NA	0.0035	9.8502E-01	9.92E-01	ENSG00000289294
NA	0.0065	9.8523E-01		ENSG00000248373
NA	0.0036	9.8657E-01		ENSG00000280183
NA	0.0039	9.8709E-01		ENSG00000248588
NA	0.0016	9.8728E-01	9.93E-01	ENSG00000267419
NA	0.0016	9.8764E-01	9.93E-01	ENSG00000273374
NA	0.0012	9.8773E-01	9.93E-01	ENSG00000225891
NA	0.0009	9.8780E-01		ENSG00000215102
NA	0.0003	9.8825E-01		ENSG00000257270
NA	0.0026	9.8842E-01	9.94E-01	ENSG00000228031
NA	0.0003	9.8856E-01	9.94E-01	ENSG00000271964
NA	0.0035	9.8871E-01		ENSG00000252615
NA	0.0058	9.8961E-01		ENSG00000248510
NA	0.0016	9.8972E-01		ENSG00000287905
NA	0.0011	9.8975E-01	9.94E-01	ENSG00000244198
NA	0.0013	9.9025E-01	9.95E-01	ENSG00000285531
NA	0.0063	9.9032E-01		ENSG00000275830

NA	0.0016	9.9044E-01		ENSG00000252824
NA	0.0015	9.9180E-01		ENSG00000234724
NA	0.0019	9.9203E-01		ENSG00000288056
NA	0.0016	9.9205E-01		ENSG00000283061
NA	0.0009	9.9206E-01	9.96E-01	ENSG00000254846
NA	0.0005	9.9216E-01	9.96E-01	ENSG00000226054
NA	0.0010	9.9428E-01	9.97E-01	ENSG00000226970
NA	0.0007	9.9464E-01	9.97E-01	ENSG00000280047
NA	0.0043	9.9472E-01		ENSG00000263729
NA	0.0003	9.9538E-01		ENSG00000254331
NA	0.0070	9.9738E-01		ENSG00000258751
NA	0.0029	9.9824E-01		ENSG00000273198
NA	0.0002	9.9840E-01	9.99E-01	ENSG00000267504
NA	0.0002	9.9847E-01		ENSG00000179994
NA	0.0021	9.9849E-01		ENSG00000214268
NA	0.0002	9.9889E-01	1.00E+00	ENSG00000258553
NA	0.0001	9.9920E-01	1.00E+00	ENSG00000204352
NA	0.0002	9.9925E-01		ENSG00000273407
NA	0.0002	9.9939E-01	1.00E+00	ENSG00000238186
NA	0.0001	9.9963E-01	1.00E+00	ENSG00000240005
NA	-0.0378	9.9994E-01	1.00E+00	ENSG00000287004
NA	-0.0036	9.9983E-01		ENSG00000183022
NA	-0.0029	9.9979E-01		ENSG00000259296
NA	-0.0089	9.9976E-01	1.00E+00	ENSG00000225107
NA	-0.0011	9.9974E-01		ENSG00000287508
NA	-0.0070	9.9973E-01		ENSG00000213985
NA	-0.0193	9.9973E-01		ENSG00000236285
NA	-0.0062	9.9960E-01		ENSG00000235279
NA	-0.0061	9.9941E-01	1.00E+00	ENSG00000235354
NA	-0.0136	9.9929E-01		ENSG00000236217
NA	-0.0020	9.9882E-01		ENSG00000288059
NA	-0.0016	9.9881E-01	1.00E+00	ENSG00000286635
NA	-0.0060	9.9870E-01		ENSG00000227743
NA	-0.0241	9.9868E-01		ENSG00000277504
NA	-0.0025	9.9865E-01		ENSG00000251126
NA	-0.0222	9.9843E-01		ENSG00000202512
NA	-0.0022	9.9839E-01	9.99E-01	ENSG00000273979
NA	-0.0032	9.9801E-01		ENSG00000267353
NA	-0.0485	9.9801E-01	9.99E-01	ENSG00000254936
NA	-0.0034	9.9799E-01		ENSG00000199490
NA	-0.0087	9.9796E-01		ENSG00000265908
NA	-0.0238	9.9794E-01		ENSG00000188280
NA	-0.0055	9.9788E-01		ENSG00000268870
NA	-0.0038	9.9767E-01		ENSG00000223462
NA	-0.0007	9.9761E-01		ENSG00000258376
NA	-0.0050	9.9749E-01		ENSG00000283907
NA	-0.0017	9.9727E-01		ENSG00000240002
NA	-0.0099	9.9725E-01		ENSG00000251001
NA	-0.0020	9.9689E-01		ENSG00000226945

NA	-0.0001	9.9677E-01		ENSG00000273971
NA	-0.0011	9.9673E-01		ENSG00000269543
NA	-0.0010	9.9672E-01		ENSG00000205361
NA	-0.0177	9.9667E-01	9.98E-01	ENSG00000274423
NA	-0.0089	9.9632E-01		ENSG00000236015
NA	-0.0239	9.9625E-01	9.98E-01	ENSG00000261431
NA	-0.0326	9.9605E-01	9.98E-01	ENSG00000253869
NA	-0.0307	9.9600E-01		ENSG00000272807
NA	-0.0046	9.9583E-01		ENSG00000225867
NA	-0.0175	9.9566E-01		ENSG00000244124
NA	-0.0002	9.9563E-01	9.98E-01	ENSG00000225279
NA	-0.0113	9.9554E-01	9.98E-01	ENSG00000280073
NA	-0.0275	9.9553E-01	9.98E-01	ENSG00000237612
NA	-0.0138	9.9546E-01		ENSG00000274624
NA	-0.0028	9.9537E-01		ENSG00000228541
NA	-0.0041	9.9535E-01		ENSG00000287972
NA	-0.0054	9.9530E-01		ENSG00000119660
NA	-0.0022	9.9519E-01		ENSG00000253667
NA	-0.0054	9.9508E-01		ENSG00000272910
NA	-0.0086	9.9478E-01		ENSG00000213303
NA	-0.0002	9.9467E-01		ENSG00000278266
NA	-0.0017	9.9439E-01		ENSG00000258896
NA	-0.0021	9.9403E-01		ENSG00000241030
NA	-0.0086	9.9400E-01	9.97E-01	ENSG00000283341
NA	-0.0076	9.9393E-01		ENSG00000279620
NA	-0.0100	9.9348E-01		ENSG00000229385
NA	-0.0014	9.9341E-01		ENSG00000249012
NA	-0.0693	9.9341E-01	9.96E-01	ENSG00000290074
NA	-0.0515	9.9338E-01	9.96E-01	ENSG00000277930
NA	-0.0086	9.9333E-01		ENSG00000240156
NA	-0.0006	9.9310E-01	9.96E-01	ENSG00000224593
NA	-0.0224	9.9295E-01	9.96E-01	ENSG00000279970
NA	-0.0090	9.9284E-01		ENSG00000285777
NA	-0.0521	9.9267E-01	9.96E-01	ENSG00000268416
NA	-0.0079	9.9265E-01		ENSG00000248783
NA	-0.0027	9.9260E-01		ENSG00000286245
NA	-0.0009	9.9242E-01		ENSG00000262408
NA	-0.0256	9.9229E-01	9.96E-01	ENSG00000280274
NA	-0.0038	9.9197E-01		ENSG00000261653
NA	-0.0847	9.9159E-01	9.95E-01	ENSG00000230080
NA	-0.0048	9.9158E-01		ENSG00000220695
NA	-0.0221	9.9152E-01	9.95E-01	ENSG00000289635
NA	-0.0130	9.9149E-01		ENSG00000277152
NA	-0.0131	9.9123E-01		ENSG00000289357
NA	-0.0242	9.9115E-01	9.95E-01	ENSG00000257086
NA	-0.0081	9.9103E-01		ENSG00000248751
NA	-0.0089	9.9101E-01		ENSG00000243415
NA	-0.0218	9.9098E-01		ENSG00000215311
NA	-0.0459	9.9084E-01	9.95E-01	ENSG00000215478

NA	-0.0022	9.9062E-01	9.95E-01	ENSG00000249763
NA	-0.0049	9.9033E-01		ENSG00000253849
NA	-0.0037	9.9032E-01		ENSG00000269707
NA	-0.0018	9.9026E-01		ENSG00000230695
NA	-0.0041	9.9009E-01		ENSG00000255520
NA	-0.0068	9.9005E-01		ENSG00000234952
NA	-0.0080	9.9004E-01		ENSG00000259380
NA	-0.0047	9.8982E-01		ENSG00000238231
NA	-0.0155	9.8982E-01	9.94E-01	ENSG00000288962
NA	-0.0039	9.8979E-01		ENSG00000201084
NA	-0.0070	9.8955E-01		ENSG00000260996
NA	-0.0025	9.8916E-01		ENSG00000289175
NA	-0.0025	9.8913E-01	9.94E-01	ENSG00000231305
NA	-0.0055	9.8861E-01		ENSG00000255325
NA	-0.0022	9.8830E-01		ENSG00000220326
NA	-0.0003	9.8804E-01	9.93E-01	ENSG00000224790
NA	-0.0112	9.8790E-01		ENSG00000218358
NA	-0.0084	9.8781E-01		ENSG00000215319
NA	-0.0033	9.8762E-01		ENSG00000215512
NA	-0.0228	9.8742E-01		ENSG00000270139
NA	-0.0097	9.8712E-01	9.93E-01	ENSG00000250765
NA	-0.0328	9.8671E-01	9.93E-01	ENSG00000278607
NA	-0.0230	9.8666E-01		ENSG00000251118
NA	-0.0087	9.8646E-01		ENSG00000260473
NA	-0.0201	9.8616E-01		ENSG00000286044
NA	-0.0243	9.8612E-01		ENSG00000255074
NA	-0.0144	9.8591E-01		ENSG00000225341
NA	-0.0073	9.8582E-01	9.92E-01	ENSG00000261557
NA	-0.0051	9.8580E-01		ENSG00000236075
NA	-0.0031	9.8567E-01		ENSG00000201555
NA	-0.0028	9.8556E-01		ENSG00000261265
NA	-0.0051	9.8541E-01	9.92E-01	ENSG00000259798
NA	-0.0220	9.8502E-01		ENSG00000289952
NA	-0.0061	9.8492E-01		ENSG00000122043
NA	-0.0268	9.8492E-01	9.92E-01	ENSG00000260036
NA	-0.0063	9.8457E-01		ENSG00000268892
NA	-0.0071	9.8454E-01		ENSG00000228792
NA	-0.0012	9.8445E-01		ENSG00000288172
NA	-0.0122	9.8439E-01		ENSG00000263427
NA	-0.0071	9.8415E-01		ENSG00000257900
NA	-0.0084	9.8414E-01		ENSG00000223452
NA	-0.0004	9.8386E-01		ENSG00000267550
NA	-0.0089	9.8372E-01		ENSG00000286998
NA	-0.0009	9.8362E-01	9.91E-01	ENSG00000200502
NA	-0.0036	9.8355E-01		ENSG00000244063
NA	-0.0290	9.8347E-01		ENSG00000234397
NA	-0.0094	9.8318E-01		ENSG00000249258
NA	-0.0601	9.8315E-01		ENSG00000272599
NA	-0.0017	9.8313E-01	9.91E-01	ENSG00000248569

NA	-0.0020	9.8303E-01		ENSG00000287985
NA	-0.0165	9.8300E-01		ENSG00000288025
NA	-0.0551	9.8263E-01	9.91E-01	ENSG00000284471
NA	-0.0327	9.8253E-01		ENSG00000286788
NA	-0.0020	9.8251E-01		ENSG00000230138
NA	-0.0237	9.8247E-01	9.91E-01	ENSG00000234685
NA	-0.0052	9.8231E-01		ENSG00000240350
NA	-0.0025	9.8228E-01	9.90E-01	ENSG00000263624
NA	-0.0014	9.8221E-01	9.90E-01	ENSG00000176349
NA	-0.0171	9.8207E-01		ENSG00000258199
NA	-0.0129	9.8204E-01		ENSG00000207317
NA	-0.0210	9.8202E-01		ENSG00000264714
NA	-0.0129	9.8199E-01		ENSG00000288637
NA	-0.0223	9.8199E-01		ENSG00000289862
NA	-0.0017	9.8197E-01	9.90E-01	ENSG00000283528
NA	-0.0089	9.8194E-01	9.90E-01	ENSG00000235090
NA	-0.0009	9.8163E-01	9.90E-01	ENSG00000236671
NA	-0.0094	9.8152E-01		ENSG00000265262
NA	-0.0011	9.8143E-01		ENSG00000225751
NA	-0.0028	9.8143E-01		ENSG00000286610
NA	-0.0012	9.8141E-01	9.90E-01	ENSG00000257489
NA	-0.0016	9.8114E-01		ENSG00000233850
NA	-0.0011	9.8109E-01	9.90E-01	ENSG00000230092
NA	-0.0087	9.8055E-01		ENSG00000271916
NA	-0.0148	9.8049E-01		ENSG00000254468
NA	-0.0112	9.8047E-01		ENSG00000271711
NA	-0.0022	9.8047E-01	9.89E-01	ENSG00000262728
NA	-0.0019	9.8043E-01	9.89E-01	ENSG00000273784
NA	-0.0086	9.8038E-01		ENSG00000256897
NA	-0.0004	9.8036E-01		ENSG00000214305
NA	-0.0021	9.8033E-01	9.89E-01	ENSG00000279453
NA	-0.0202	9.8007E-01		ENSG00000288553
NA	-0.0023	9.7997E-01	9.89E-01	ENSG00000272420
NA	-0.0076	9.7971E-01		ENSG00000225498
NA	-0.0014	9.7947E-01	9.89E-01	ENSG00000271214
NA	-0.0303	9.7929E-01	9.89E-01	ENSG00000271029
NA	-0.0099	9.7918E-01		ENSG00000254388
NA	-0.0125	9.7909E-01		ENSG00000286299
NA	-0.0199	9.7905E-01		ENSG00000200091
NA	-0.0017	9.7904E-01	9.89E-01	ENSG00000272263
NA	-0.0140	9.7899E-01		ENSG00000289163
NA	-0.0050	9.7878E-01	9.89E-01	ENSG00000287193
NA	-0.0128	9.7872E-01		ENSG00000230614
NA	-0.0020	9.7870E-01		ENSG00000269680
NA	-0.0023	9.7837E-01	9.89E-01	ENSG00000260206
NA	-0.0022	9.7810E-01		ENSG00000253111
NA	-0.0142	9.7785E-01		ENSG00000289501
NA	-0.0029	9.7760E-01	9.88E-01	ENSG00000289428
NA	-0.0007	9.7755E-01	9.88E-01	ENSG00000228510

NA	-0.0139	9.7747E-01		ENSG00000288873
NA	-0.0281	9.7746E-01	9.88E-01	ENSG00000263648
NA	-0.0152	9.7734E-01	9.88E-01	ENSG00000275140
NA	-0.0020	9.7730E-01	9.88E-01	ENSG00000257910
NA	-0.0481	9.7697E-01		ENSG00000285745
NA	-0.0169	9.7691E-01		ENSG00000219375
NA	-0.0026	9.7658E-01	9.88E-01	ENSG00000243176
NA	-0.0021	9.7656E-01	9.88E-01	ENSG00000273532
NA	-0.0074	9.7640E-01		ENSG00000279366
NA	-0.0354	9.7630E-01	9.88E-01	ENSG00000242660
NA	-0.0027	9.7594E-01	9.88E-01	ENSG00000232692
NA	-0.0165	9.7585E-01		ENSG00000227128
NA	-0.0016	9.7579E-01	9.88E-01	ENSG00000260541
NA	-0.0199	9.7572E-01		ENSG00000286127
NA	-0.0016	9.7522E-01	9.87E-01	ENSG00000106610
NA	-0.0028	9.7518E-01		ENSG00000250761
NA	-0.0183	9.7506E-01		ENSG00000262492
NA	-0.0301	9.7489E-01		ENSG00000288854
NA	-0.0121	9.7489E-01		ENSG00000277022
NA	-0.0021	9.7489E-01		ENSG00000213816
NA	-0.0113	9.7473E-01		ENSG00000280166
NA	-0.0415	9.7452E-01	9.87E-01	ENSG00000248180
NA	-0.0046	9.7445E-01		ENSG00000275516
NA	-0.0029	9.7429E-01	9.87E-01	ENSG00000227008
NA	-0.0023	9.7416E-01	9.87E-01	ENSG00000230205
NA	-0.0022	9.7405E-01		ENSG00000254460
NA	-0.0036	9.7404E-01		ENSG00000259584
NA	-0.0009	9.7385E-01		ENSG00000259833
NA	-0.0044	9.7355E-01		ENSG00000224219
NA	-0.0008	9.7334E-01		ENSG00000243025
NA	-0.0057	9.7316E-01	9.86E-01	ENSG00000233073
NA	-0.0063	9.7267E-01		ENSG00000289426
NA	-0.0072	9.7260E-01		ENSG00000288807
NA	-0.0097	9.7252E-01		ENSG00000273325
NA	-0.0501	9.7215E-01		ENSG00000273368
NA	-0.0323	9.7204E-01		ENSG00000248583
NA	-0.0100	9.7202E-01		ENSG00000254756
NA	-0.0090	9.7186E-01		ENSG00000233859
NA	-0.0157	9.7167E-01	9.85E-01	ENSG00000250900
NA	-0.0099	9.7119E-01		ENSG00000219797
NA	-0.0122	9.7114E-01		ENSG00000213548
NA	-0.0161	9.7106E-01	9.85E-01	ENSG00000276412
NA	-0.0206	9.7093E-01		ENSG00000230807
NA	-0.0011	9.7077E-01		ENSG00000289723
NA	-0.0323	9.7058E-01		ENSG00000259161
NA	-0.0262	9.7051E-01		ENSG00000279091
NA	0.0000	9.7048E-01		ENSG00000261633
NA	-0.0169	9.7027E-01		ENSG00000255641
NA	-0.0058	9.7004E-01		ENSG00000286017

NA	-0.0076	9.6998E-01		ENSG00000184303
NA	-0.0188	9.6988E-01	9.84E-01	ENSG00000223537
NA	-0.0022	9.6983E-01		ENSG00000227352
NA	-0.0071	9.6963E-01		ENSG00000285016
NA	-0.0007	9.6963E-01		ENSG00000217769
NA	-0.0034	9.6955E-01		ENSG00000231040
NA	-0.0048	9.6948E-01	9.84E-01	ENSG00000260422
NA	-0.0061	9.6940E-01		ENSG00000274860
NA	-0.0019	9.6934E-01		ENSG00000233478
NA	-0.0136	9.6914E-01	9.84E-01	ENSG00000280162
NA	-0.0069	9.6895E-01		ENSG00000237263
NA	-0.0105	9.6884E-01		ENSG00000234645
NA	-0.0050	9.6883E-01		ENSG00000289418
NA	-0.0011	9.6881E-01		ENSG00000259500
NA	-0.0151	9.6878E-01		ENSG00000229046
NA	-0.0005	9.6860E-01		ENSG00000280092
NA	-0.0054	9.6831E-01	9.84E-01	ENSG00000224928
NA	-0.0254	9.6828E-01		ENSG00000261595
NA	-0.0037	9.6821E-01	9.84E-01	ENSG00000232862
NA	-0.0137	9.6819E-01	9.84E-01	ENSG00000279461
NA	-0.0016	9.6814E-01		ENSG00000279072
NA	-0.0004	9.6806E-01		ENSG00000229052
NA	-0.0055	9.6793E-01		ENSG00000257754
NA	-0.0029	9.6714E-01	9.83E-01	ENSG00000236044
NA	-0.0064	9.6711E-01		ENSG00000253539
NA	-0.0037	9.6681E-01	9.83E-01	ENSG00000239801
NA	-0.0045	9.6678E-01		ENSG00000263065
NA	-0.0305	9.6650E-01	9.83E-01	ENSG00000259723
NA	-0.0013	9.6645E-01	9.83E-01	ENSG00000274080
NA	-0.0014	9.6627E-01		ENSG00000271931
NA	-0.0034	9.6616E-01		ENSG00000254698
NA	-0.0021	9.6594E-01	9.83E-01	ENSG00000233825
NA	-0.0048	9.6593E-01		ENSG00000233674
NA	-0.0137	9.6570E-01		ENSG00000228973
NA	-0.0088	9.6536E-01		ENSG00000289514
NA	-0.0273	9.6520E-01		ENSG00000284654
NA	-0.0009	9.6520E-01		ENSG00000277744
NA	-0.0051	9.6517E-01		ENSG00000283631
NA	-0.0007	9.6505E-01		ENSG00000222421
NA	-0.0261	9.6468E-01		ENSG00000251484
NA	-0.0036	9.6462E-01	9.82E-01	ENSG00000223773
NA	-0.0088	9.6452E-01		ENSG00000238150
NA	-0.0482	9.6435E-01		ENSG00000233845
NA	-0.0089	9.6428E-01		ENSG00000236695
NA	-0.0145	9.6412E-01		ENSG00000287268
NA	-0.0012	9.6412E-01		ENSG00000231942
NA	-0.0050	9.6366E-01	9.81E-01	ENSG00000260196
NA	-0.0089	9.6366E-01		ENSG00000236723
NA	-0.0029	9.6360E-01		ENSG00000274322

NA	-0.0318	9.6358E-01	9.81E-01	ENSG00000176134
NA	-0.0122	9.6352E-01		ENSG00000254952
NA	-0.0485	9.6342E-01	9.81E-01	ENSG00000279225
NA	-0.0008	9.6340E-01	9.81E-01	ENSG00000229944
NA	-0.0102	9.6332E-01		ENSG00000251330
NA	-0.0081	9.6282E-01		ENSG00000260457
NA	-0.0029	9.6282E-01	9.81E-01	ENSG00000279145
NA	-0.0030	9.6274E-01	9.81E-01	ENSG00000287276
NA	-0.0430	9.6272E-01	9.81E-01	ENSG00000185168
NA	-0.0276	9.6269E-01	9.81E-01	ENSG00000270714
NA	-0.0065	9.6265E-01		ENSG00000266289
NA	-0.0037	9.6259E-01	9.81E-01	ENSG00000289463
NA	-0.0018	9.6248E-01	9.81E-01	ENSG00000205930
NA	-0.0068	9.6230E-01		ENSG00000268731
NA	-0.0033	9.6216E-01		ENSG00000270780
NA	-0.0102	9.6211E-01	9.81E-01	ENSG00000261002
NA	-0.0087	9.6197E-01		ENSG00000223379
NA	-0.0048	9.6173E-01	9.81E-01	ENSG00000288991
NA	-0.0013	9.6160E-01		ENSG00000286671
NA	-0.0018	9.6146E-01		ENSG00000239628
NA	-0.0439	9.6127E-01	9.80E-01	ENSG00000248790
NA	-0.0070	9.6116E-01		ENSG00000267212
NA	-0.0077	9.6101E-01		ENSG00000255648
NA	-0.0313	9.6078E-01		ENSG00000225169
NA	-0.0329	9.6070E-01	9.80E-01	ENSG00000288809
NA	-0.0046	9.6066E-01		ENSG00000269637
NA	-0.0052	9.6038E-01		ENSG00000278727
NA	-0.0061	9.6035E-01		ENSG00000278177
NA	-0.0041	9.6033E-01	9.80E-01	ENSG00000235082
NA	-0.0026	9.6027E-01		ENSG00000235286
NA	-0.0020	9.6022E-01		ENSG00000228082
NA	-0.0102	9.5998E-01		ENSG00000253125
NA	-0.0146	9.5995E-01		ENSG00000289972
NA	-0.0050	9.5986E-01	9.80E-01	ENSG00000259744
NA	-0.0227	9.5973E-01		ENSG00000284196
NA	-0.0048	9.5972E-01		ENSG00000287114
NA	-0.0157	9.5951E-01		ENSG00000237953
NA	-0.0362	9.5947E-01		ENSG00000237586
NA	-0.0430	9.5942E-01	9.80E-01	ENSG00000287067
NA	-0.0037	9.5937E-01		ENSG00000234962
NA	-0.0102	9.5923E-01		ENSG00000260681
NA	-0.0163	9.5908E-01		ENSG00000204183
NA	-0.0057	9.5904E-01		ENSG00000250383
NA	-0.0069	9.5849E-01		ENSG00000215480
NA	-0.0069	9.5838E-01		ENSG00000238783
NA	-0.0033	9.5833E-01	9.79E-01	ENSG00000272186
NA	-0.0048	9.5826E-01		ENSG00000201916
NA	-0.0036	9.5818E-01	9.79E-01	ENSG00000240731
NA	-0.0094	9.5817E-01		ENSG00000274628

NA	-0.0079	9.5811E-01		ENSG00000257548
NA	-0.0091	9.5807E-01		ENSG00000261612
NA	-0.0336	9.5805E-01	9.79E-01	ENSG00000260608
NA	-0.0069	9.5791E-01		ENSG00000238755
NA	-0.0020	9.5788E-01		ENSG00000270052
NA	-0.0163	9.5785E-01		ENSG00000232542
NA	0.0000	9.5785E-01		ENSG00000272508
NA	-0.0095	9.5785E-01		ENSG00000235005
NA	-0.0028	9.5755E-01		ENSG00000269967
NA	-0.0052	9.5745E-01		ENSG00000257759
NA	-0.0084	9.5726E-01		ENSG00000280302
NA	-0.0136	9.5724E-01		ENSG00000290111
NA	-0.0154	9.5721E-01		ENSG00000226008
NA	-0.0044	9.5704E-01	9.78E-01	ENSG00000236452
NA	-0.0046	9.5692E-01		ENSG00000278472
NA	-0.0048	9.5687E-01	9.78E-01	ENSG00000262133
NA	-0.0106	9.5686E-01		ENSG00000267406
NA	-0.0053	9.5668E-01		ENSG00000279352
NA	-0.0160	9.5659E-01	9.78E-01	ENSG00000286576
NA	-0.0026	9.5657E-01		ENSG00000237679
NA	-0.0025	9.5652E-01		ENSG00000257681
NA	-0.0079	9.5641E-01		ENSG00000282080
NA	-0.0051	9.5619E-01	9.78E-01	ENSG00000280222
NA	-0.0131	9.5615E-01		ENSG00000238007
NA	-0.0034	9.5595E-01		ENSG00000256756
NA	-0.0408	9.5586E-01	9.77E-01	ENSG00000259690
NA	-0.0379	9.5567E-01	9.77E-01	ENSG00000272650
NA	-0.0106	9.5555E-01		ENSG00000267557
NA	-0.0010	9.5547E-01		ENSG00000272457
NA	-0.0045	9.5544E-01	9.77E-01	ENSG00000254510
NA	-0.0039	9.5510E-01		ENSG00000235211
NA	-0.0215	9.5501E-01		ENSG00000250658
NA	-0.0030	9.5497E-01		ENSG00000289202
NA	-0.0155	9.5480E-01	9.77E-01	ENSG00000275389
NA	-0.0055	9.5467E-01	9.77E-01	ENSG00000271040
NA	-0.0066	9.5466E-01	9.77E-01	ENSG00000267042
NA	-0.0025	9.5464E-01		ENSG00000232568
NA	-0.0119	9.5459E-01		ENSG00000259402
NA	-0.0023	9.5440E-01		ENSG00000225258
NA	-0.0020	9.5438E-01		ENSG00000286186
NA	-0.0402	9.5391E-01	9.77E-01	ENSG00000228674
NA	-0.0165	9.5377E-01		ENSG00000288707
NA	-0.0179	9.5369E-01		ENSG00000267192
NA	-0.0378	9.5362E-01		ENSG00000199906
NA	-0.0467	9.5359E-01	9.76E-01	ENSG00000250240
NA	-0.0107	9.5324E-01		ENSG00000288234
NA	-0.0039	9.5281E-01		ENSG00000278492
NA	-0.0048	9.5280E-01	9.76E-01	ENSG00000267549
NA	-0.0038	9.5276E-01	9.76E-01	ENSG00000224078

NA	-0.0078	9.5275E-01		ENSG00000261025
NA	-0.0044	9.5267E-01	9.76E-01	ENSG00000285865
NA	-0.0052	9.5245E-01	9.76E-01	ENSG00000286084
NA	-0.0167	9.5244E-01		ENSG00000274191
NA	-0.0024	9.5232E-01		ENSG00000289880
NA	-0.0145	9.5194E-01		ENSG00000201012
NA	-0.0049	9.5186E-01	9.76E-01	ENSG00000288762
NA	-0.0375	9.5153E-01		ENSG00000279568
NA	-0.0067	9.5144E-01		ENSG00000219529
NA	-0.0111	9.5142E-01		ENSG00000279130
NA	-0.0106	9.5127E-01		ENSG00000235933
NA	-0.0024	9.5125E-01		ENSG00000263635
NA	-0.0111	9.5121E-01		ENSG00000248794
NA	-0.0027	9.5113E-01		ENSG00000285675
NA	-0.0001	9.5092E-01		ENSG00000083622
NA	-0.0036	9.5076E-01		ENSG00000272477
NA	-0.0056	9.5053E-01	9.75E-01	ENSG00000279539
NA	-0.0041	9.5029E-01	9.75E-01	ENSG00000275131
NA	-0.0078	9.5022E-01		ENSG00000233775
NA	-0.0069	9.5015E-01	9.75E-01	ENSG00000254305
NA	-0.0120	9.5013E-01		ENSG00000263708
NA	-0.0068	9.5010E-01		ENSG00000278040
NA	-0.0097	9.5001E-01	9.75E-01	ENSG00000272831
NA	-0.0028	9.4964E-01		ENSG00000253252
NA	-0.0019	9.4958E-01		ENSG00000285982
NA	-0.0043	9.4917E-01		ENSG00000224138
NA	-0.0035	9.4891E-01		ENSG00000215734
NA	-0.0025	9.4884E-01		ENSG00000264644
NA	-0.0381	9.4868E-01	9.74E-01	ENSG00000288952
NA	-0.0182	9.4868E-01		ENSG00000225387
NA	-0.0332	9.4843E-01		ENSG00000262020
NA	-0.0076	9.4804E-01		ENSG00000285597
NA	-0.0023	9.4802E-01		ENSG00000279365
NA	-0.0060	9.4799E-01		ENSG00000287593
NA	-0.0020	9.4794E-01		ENSG00000251379
NA	-0.0036	9.4764E-01		ENSG00000286211
NA	-0.0039	9.4764E-01	9.74E-01	ENSG00000288897
NA	-0.0041	9.4759E-01	9.74E-01	ENSG00000288739
NA	-0.0069	9.4757E-01	9.74E-01	ENSG00000234936
NA	-0.0127	9.4753E-01		ENSG00000286134
NA	-0.0033	9.4723E-01	9.73E-01	ENSG00000264538
NA	-0.0065	9.4722E-01	9.73E-01	ENSG00000249621
NA	-0.0018	9.4715E-01		ENSG00000224409
NA	-0.0051	9.4705E-01		ENSG00000228124
NA	-0.0017	9.4700E-01		ENSG00000266786
NA	-0.0024	9.4689E-01		ENSG00000255421
NA	-0.0088	9.4683E-01		ENSG00000268683
NA	-0.0063	9.4681E-01	9.73E-01	ENSG00000226160
NA	-0.0013	9.4677E-01		ENSG00000272078

NA	-0.0027	9.4653E-01		ENSG00000241438
NA	-0.0096	9.4647E-01		ENSG00000217950
NA	-0.0062	9.4624E-01	9.73E-01	ENSG00000288253
NA	-0.0063	9.4620E-01	9.73E-01	ENSG00000275029
NA	-0.0117	9.4618E-01	9.73E-01	ENSG00000280408
NA	-0.0290	9.4615E-01	9.73E-01	ENSG00000266171
NA	-0.0054	9.4593E-01		ENSG00000226163
NA	-0.0049	9.4592E-01	9.73E-01	ENSG00000253447
NA	-0.0363	9.4589E-01		ENSG00000229399
NA	-0.0032	9.4546E-01		ENSG00000288067
NA	-0.0089	9.4545E-01		ENSG00000285524
NA	-0.0122	9.4544E-01	9.73E-01	ENSG00000205955
NA	-0.0062	9.4543E-01	9.73E-01	ENSG00000259088
NA	-0.0119	9.4543E-01		ENSG00000213731
NA	-0.0022	9.4540E-01		ENSG00000273489
NA	-0.0094	9.4530E-01		ENSG00000202357
NA	-0.0044	9.4526E-01		ENSG00000182625
NA	-0.0063	9.4519E-01	9.73E-01	ENSG00000255946
NA	-0.0806	9.4516E-01	9.73E-01	ENSG00000286406
NA	-0.0100	9.4507E-01		ENSG00000227813
NA	-0.0171	9.4502E-01		ENSG00000288921
NA	-0.0076	9.4494E-01		ENSG00000216316
NA	-0.0349	9.4475E-01	9.72E-01	ENSG00000236762
NA	-0.0067	9.4475E-01	9.72E-01	ENSG00000237350
NA	-0.0041	9.4471E-01		ENSG00000284652
NA	-0.0058	9.4470E-01	9.72E-01	ENSG00000289209
NA	-0.0032	9.4458E-01		ENSG00000278273
NA	-0.0206	9.4430E-01		ENSG00000275468
NA	-0.0046	9.4401E-01	9.72E-01	ENSG00000272502
NA	-0.0188	9.4391E-01		ENSG00000258086
NA	-0.0050	9.4379E-01	9.72E-01	ENSG00000253642
NA	-0.0281	9.4361E-01		ENSG00000286480
NA	-0.0296	9.4337E-01		ENSG00000249930
NA	-0.0170	9.4335E-01		ENSG00000235267
NA	-0.0084	9.4331E-01		ENSG00000235271
NA	-0.0481	9.4325E-01	9.72E-01	ENSG00000254387
NA	-0.0002	9.4312E-01		ENSG00000287029
NA	-0.0125	9.4310E-01	9.72E-01	ENSG00000204709
NA	-0.0120	9.4305E-01		ENSG00000285532
NA	-0.0315	9.4300E-01		ENSG00000206728
NA	-0.0199	9.4295E-01	9.72E-01	ENSG00000267279
NA	-0.0027	9.4293E-01		ENSG00000257359
NA	-0.0042	9.4256E-01		ENSG00000260997
NA	-0.0120	9.4249E-01		ENSG00000227933
NA	-0.0044	9.4222E-01		ENSG00000267304
NA	-0.0091	9.4192E-01		ENSG00000274227
NA	-0.0215	9.4190E-01		ENSG00000258631
NA	-0.0192	9.4187E-01		ENSG00000230498
NA	-0.0034	9.4177E-01		ENSG00000229376

NA	-0.0076	9.4175E-01		ENSG00000250740
NA	-0.0011	9.4150E-01	9.71E-01	ENSG00000287035
NA	-0.0031	9.4144E-01	9.71E-01	ENSG00000266718
NA	-0.0210	9.4121E-01	9.71E-01	ENSG00000214077
NA	-0.0057	9.4121E-01		ENSG00000257341
NA	-0.0063	9.4101E-01		ENSG00000233351
NA	-0.0023	9.4047E-01		ENSG00000287338
NA	-0.0296	9.4040E-01		ENSG00000287921
NA	-0.0195	9.4021E-01		ENSG00000264635
NA	-0.0007	9.4003E-01		ENSG00000230952
NA	-0.0004	9.3995E-01		ENSG00000226131
NA	-0.0065	9.3976E-01		ENSG00000270839
NA	-0.0064	9.3955E-01	9.70E-01	ENSG00000275120
NA	-0.0135	9.3955E-01		ENSG00000227230
NA	-0.0010	9.3953E-01		ENSG00000216657
NA	-0.0041	9.3948E-01		ENSG00000237063
NA	-0.0019	9.3931E-01		ENSG00000227018
NA	-0.0041	9.3928E-01	9.69E-01	ENSG00000133519
NA	-0.0030	9.3906E-01		ENSG00000226825
NA	-0.0019	9.3900E-01		ENSG00000254664
NA	-0.0420	9.3884E-01	9.69E-01	ENSG00000223298
NA	-0.0005	9.3871E-01		ENSG00000287456
NA	-0.0129	9.3857E-01		ENSG00000228604
NA	-0.0047	9.3849E-01		ENSG00000284820
NA	-0.0024	9.3825E-01		ENSG00000240143
NA	-0.0038	9.3824E-01		ENSG00000283304
NA	-0.0146	9.3822E-01	9.69E-01	ENSG00000229939
NA	-0.0317	9.3805E-01		ENSG00000258857
NA	-0.0008	9.3797E-01		ENSG00000248359
NA	-0.0005	9.3792E-01	9.69E-01	ENSG00000227400
NA	-0.0478	9.3780E-01	9.69E-01	ENSG00000289402
NA	-0.0127	9.3769E-01		ENSG00000236184
NA	-0.0093	9.3763E-01		ENSG00000257921
NA	-0.0125	9.3761E-01		ENSG00000231961
NA	-0.0143	9.3750E-01		ENSG00000232600
NA	-0.0074	9.3723E-01	9.69E-01	ENSG00000226981
NA	-0.0079	9.3722E-01		ENSG00000287129
NA	-0.0039	9.3703E-01		ENSG00000277349
NA	-0.0126	9.3703E-01	9.69E-01	ENSG00000182397
NA	-0.0065	9.3696E-01		ENSG00000232848
NA	-0.0178	9.3680E-01		ENSG00000264083
NA	-0.0070	9.3645E-01	9.69E-01	ENSG00000271943
NA	-0.0026	9.3609E-01		ENSG00000270837
NA	-0.0035	9.3602E-01		ENSG00000286174
NA	-0.0118	9.3600E-01		ENSG00000273017
NA	-0.0068	9.3600E-01	9.68E-01	ENSG00000238117
NA	-0.0132	9.3595E-01		ENSG00000268087
NA	-0.0032	9.3559E-01		ENSG00000239767
NA	-0.0050	9.3557E-01		ENSG00000285681

NA	-0.0030	9.3549E-01		ENSG00000232386
NA	-0.0069	9.3510E-01	9.68E-01	ENSG00000258675
NA	-0.0059	9.3509E-01		ENSG00000287735
NA	-0.0161	9.3498E-01		ENSG00000199875
NA	-0.0079	9.3488E-01		ENSG00000229161
NA	-0.0007	9.3460E-01		ENSG00000277003
NA	-0.0086	9.3448E-01		ENSG00000277039
NA	-0.0118	9.3448E-01		ENSG00000224121
NA	-0.0104	9.3445E-01		ENSG00000280295
NA	-0.0176	9.3443E-01		ENSG00000286708
NA	-0.0046	9.3413E-01		ENSG00000273712
NA	-0.0061	9.3407E-01	9.67E-01	ENSG00000229447
NA	-0.0019	9.3372E-01		ENSG00000224238
NA	-0.0025	9.3364E-01		ENSG00000258648
NA	-0.0145	9.3338E-01		ENSG00000286579
NA	-0.0045	9.3317E-01		ENSG00000256904
NA	-0.0057	9.3312E-01	9.67E-01	ENSG00000277895
NA	-0.0095	9.3311E-01		ENSG00000215326
NA	-0.0108	9.3309E-01		ENSG00000257043
NA	-0.0014	9.3306E-01	9.67E-01	ENSG00000229431
NA	-0.0105	9.3301E-01	9.67E-01	ENSG00000231731
NA	-0.0036	9.3291E-01		ENSG00000236480
NA	-0.0135	9.3266E-01		ENSG00000275371
NA	-0.0066	9.3258E-01		ENSG00000168122
NA	-0.0285	9.3256E-01		ENSG00000275576
NA	-0.0114	9.3252E-01	9.66E-01	ENSG00000231985
NA	-0.0085	9.3251E-01		ENSG00000236779
NA	-0.0077	9.3224E-01		ENSG00000237478
NA	-0.0488	9.3194E-01	9.66E-01	ENSG00000262712
NA	-0.0078	9.3193E-01		ENSG00000231120
NA	-0.0498	9.3165E-01	9.66E-01	ENSG00000272942
NA	-0.0084	9.3158E-01		ENSG00000227089
NA	-0.0039	9.3145E-01		ENSG00000279679
NA	-0.0056	9.3144E-01	9.66E-01	ENSG00000286553
NA	-0.0080	9.3136E-01		ENSG00000253512
NA	-0.0011	9.3132E-01		ENSG00000273342
NA	-0.0079	9.3115E-01	9.66E-01	ENSG00000288066
NA	-0.0383	9.3114E-01		ENSG00000273557
NA	-0.0049	9.3084E-01		ENSG00000260460
NA	-0.0042	9.3082E-01		ENSG00000288718
NA	-0.0114	9.3080E-01	9.66E-01	ENSG00000249359
NA	-0.0034	9.3079E-01		ENSG00000260033
NA	-0.0016	9.3076E-01		ENSG00000249460
NA	-0.0206	9.3069E-01		ENSG00000231334
NA	-0.0011	9.3048E-01		ENSG00000259792
NA	-0.0062	9.3036E-01		ENSG00000229336
NA	-0.0063	9.3033E-01	9.66E-01	ENSG00000274598
NA	-0.0080	9.3031E-01		ENSG00000286860
NA	-0.0004	9.3026E-01	9.66E-01	ENSG00000249947

NA	-0.0077	9.3015E-01		ENSG00000238022
NA	-0.0132	9.3013E-01	9.66E-01	ENSG00000235833
NA	-0.0082	9.3009E-01		ENSG00000260318
NA	-0.0035	9.2980E-01		ENSG00000289830
NA	-0.0050	9.2968E-01		ENSG00000278818
NA	-0.0014	9.2967E-01	9.66E-01	ENSG00000279414
NA	-0.0046	9.2940E-01		ENSG00000285660
NA	-0.0051	9.2932E-01		ENSG00000253256
NA	-0.0049	9.2926E-01	9.65E-01	ENSG00000286413
NA	-0.0351	9.2918E-01		ENSG00000267476
NA	-0.0142	9.2914E-01		ENSG00000259937
NA	-0.0106	9.2911E-01		ENSG00000287528
NA	-0.0345	9.2908E-01	9.65E-01	ENSG00000255059
NA	-0.0033	9.2849E-01		ENSG00000281460
NA	-0.0026	9.2846E-01		ENSG00000263612
NA	-0.0067	9.2846E-01		ENSG00000289553
NA	-0.0055	9.2833E-01		ENSG00000274051
NA	-0.0116	9.2826E-01		ENSG00000235420
NA	-0.0024	9.2823E-01	9.65E-01	ENSG00000253586
NA	-0.0250	9.2810E-01		ENSG00000233010
NA	-0.0114	9.2796E-01		ENSG00000256400
NA	-0.0079	9.2777E-01	9.64E-01	ENSG00000261167
NA	-0.0002	9.2774E-01		ENSG00000201885
NA	-0.0002	9.2774E-01		ENSG00000201959
NA	-0.0087	9.2755E-01		ENSG00000255226
NA	-0.0045	9.2754E-01		ENSG00000272848
NA	-0.0108	9.2745E-01		ENSG00000250726
NA	-0.0052	9.2699E-01		ENSG00000093100
NA	-0.0046	9.2695E-01		ENSG00000229609
NA	-0.0070	9.2683E-01		ENSG00000257943
NA	-0.0074	9.2676E-01		ENSG00000267336
NA	-0.0032	9.2663E-01		ENSG00000272030
NA	-0.0067	9.2644E-01		ENSG00000236119
NA	-0.0064	9.2641E-01	9.64E-01	ENSG00000237980
NA	-0.0074	9.2641E-01		ENSG00000280016
NA	-0.0092	9.2619E-01		ENSG00000289290
NA	-0.0264	9.2609E-01		ENSG00000268987
NA	-0.0006	9.2600E-01	9.63E-01	ENSG00000280365
NA	-0.0167	9.2527E-01		ENSG00000275223
NA	-0.0085	9.2503E-01	9.63E-01	ENSG00000260352
NA	-0.0082	9.2499E-01		ENSG00000287901
NA	-0.0239	9.2498E-01		ENSG00000235296
NA	-0.0062	9.2479E-01	9.63E-01	ENSG00000288088
NA	-0.0107	9.2471E-01	9.63E-01	ENSG00000261519
NA	-0.0034	9.2469E-01		ENSG00000270690
NA	-0.0350	9.2462E-01		ENSG00000286154
NA	-0.0109	9.2455E-01	9.63E-01	ENSG00000236312
NA	-0.0030	9.2453E-01		ENSG00000255105
NA	-0.0017	9.2403E-01		ENSG00000275497

NA	-0.0058	9.2378E-01		ENSG00000215467
NA	-0.0168	9.2354E-01		ENSG00000229913
NA	-0.0226	9.2320E-01	9.62E-01	ENSG00000230305
NA	-0.0065	9.2297E-01		ENSG00000237670
NA	-0.0032	9.2294E-01		ENSG00000274719
NA	-0.0092	9.2277E-01	9.61E-01	ENSG00000248568
NA	-0.0145	9.2259E-01		ENSG00000280113
NA	-0.0030	9.2255E-01		ENSG00000271926
NA	-0.0235	9.2253E-01	9.61E-01	ENSG00000259250
NA	-0.0031	9.2252E-01		ENSG00000267590
NA	-0.0099	9.2241E-01		ENSG00000266304
NA	-0.0101	9.2224E-01	9.61E-01	ENSG00000260366
NA	-0.0076	9.2201E-01		ENSG00000226548
NA	-0.0092	9.2172E-01	9.61E-01	ENSG00000226781
NA	-0.0290	9.2160E-01	9.61E-01	ENSG00000232739
NA	-0.0054	9.2159E-01	9.61E-01	ENSG00000278816
NA	-0.0020	9.2157E-01		ENSG00000286962
NA	-0.0041	9.2150E-01		ENSG00000204776
NA	-0.0024	9.2129E-01		ENSG00000286865
NA	-0.0127	9.2115E-01		ENSG00000288889
NA	-0.0287	9.2108E-01		ENSG00000230534
NA	-0.0036	9.2096E-01		ENSG00000254900
NA	-0.0072	9.2072E-01		ENSG00000285543
NA	-0.0127	9.2061E-01		ENSG00000287283
NA	-0.0071	9.2060E-01	9.60E-01	ENSG00000251023
NA	-0.0061	9.2051E-01		ENSG00000289828
NA	-0.0125	9.2043E-01	9.60E-01	ENSG00000279494
NA	-0.0353	9.2033E-01	9.60E-01	ENSG00000277938
NA	-0.0246	9.2028E-01	9.60E-01	ENSG00000229122
NA	-0.0059	9.1990E-01		ENSG00000223831
NA	-0.0082	9.1985E-01	9.60E-01	ENSG00000271367
NA	-0.0002	9.1982E-01		ENSG00000178146
NA	-0.0095	9.1947E-01		ENSG00000251614
NA	-0.0012	9.1943E-01		ENSG00000211683
NA	-0.0197	9.1942E-01	9.60E-01	ENSG00000277482
NA	-0.0137	9.1915E-01	9.60E-01	ENSG00000231754
NA	-0.0055	9.1907E-01		ENSG00000254244
NA	-0.0190	9.1903E-01		ENSG00000269924
NA	-0.0142	9.1895E-01	9.60E-01	ENSG00000249959
NA	-0.0097	9.1891E-01		ENSG00000213126
NA	-0.0084	9.1868E-01	9.60E-01	ENSG00000284747
NA	-0.0383	9.1864E-01	9.60E-01	ENSG00000235510
NA	-0.0290	9.1853E-01	9.60E-01	ENSG00000239445
NA	-0.0054	9.1828E-01		ENSG00000286046
NA	-0.0005	9.1798E-01		ENSG00000254050
NA	-0.0086	9.1752E-01		ENSG00000269425
NA	-0.0093	9.1681E-01	9.58E-01	ENSG00000237950
NA	-0.0100	9.1668E-01		ENSG00000261722
NA	-0.0097	9.1668E-01	9.58E-01	ENSG00000226377

NA	-0.0094	9.1643E-01	9.58E-01	ENSG00000273958
NA	-0.0117	9.1638E-01		ENSG00000261103
NA	-0.0173	9.1619E-01		ENSG00000223723
NA	-0.0013	9.1616E-01		ENSG00000272832
NA	-0.0054	9.1594E-01		ENSG00000226986
NA	-0.0322	9.1590E-01	9.58E-01	ENSG00000231549
NA	-0.0292	9.1578E-01	9.58E-01	ENSG00000188681
NA	-0.0109	9.1573E-01		ENSG00000258479
NA	-0.0016	9.1569E-01		ENSG00000259100
NA	-0.0085	9.1543E-01	9.58E-01	ENSG00000284070
NA	-0.0550	9.1533E-01	9.57E-01	ENSG00000260633
NA	-0.0080	9.1519E-01		ENSG00000280117
NA	-0.0186	9.1515E-01		ENSG00000229831
NA	-0.0088	9.1501E-01	9.57E-01	ENSG00000289281
NA	-0.0240	9.1476E-01		ENSG00000286880
NA	-0.0064	9.1476E-01		ENSG00000267735
NA	-0.0006	9.1468E-01		ENSG00000239323
NA	-0.0031	9.1444E-01		ENSG00000286607
NA	-0.0097	9.1437E-01	9.57E-01	ENSG00000227376
NA	-0.0095	9.1433E-01	9.57E-01	ENSG00000277728
NA	-0.0051	9.1427E-01		ENSG00000225458
NA	-0.0091	9.1426E-01	9.57E-01	ENSG00000258153
NA	-0.0083	9.1416E-01	9.57E-01	ENSG00000231424
NA	-0.0009	9.1407E-01		ENSG00000250341
NA	-0.0170	9.1406E-01		ENSG00000229487
NA	-0.0191	9.1396E-01	9.57E-01	ENSG00000268201
NA	-0.0232	9.1379E-01		ENSG00000249406
NA	-0.0212	9.1377E-01		ENSG00000214821
NA	-0.0174	9.1357E-01		ENSG00000261334
NA	-0.0115	9.1333E-01	9.56E-01	ENSG00000270091
NA	-0.0029	9.1332E-01	9.56E-01	ENSG00000261544
NA	-0.0553	9.1326E-01	9.56E-01	ENSG00000225026
NA	-0.0072	9.1320E-01	9.56E-01	ENSG00000280353
NA	-0.0033	9.1311E-01		ENSG00000228721
NA	-0.0034	9.1294E-01		ENSG00000249916
NA	-0.0089	9.1289E-01	9.56E-01	ENSG00000228665
NA	-0.0102	9.1285E-01	9.56E-01	ENSG00000157021
NA	-0.0037	9.1275E-01		ENSG00000267547
NA	-0.0055	9.1267E-01		ENSG00000225402
NA	-0.0091	9.1203E-01		ENSG00000224490
NA	-0.0049	9.1203E-01		ENSG00000229357
NA	-0.0419	9.1191E-01		ENSG00000288530
NA	-0.0236	9.1177E-01	9.55E-01	ENSG00000288746
NA	-0.0253	9.1149E-01		ENSG00000279472
NA	-0.0028	9.1123E-01		ENSG00000225885
NA	-0.0063	9.1101E-01	9.55E-01	ENSG00000203335
NA	-0.0082	9.1093E-01		ENSG00000274023
NA	-0.0066	9.1084E-01		ENSG00000261650
NA	-0.0062	9.1075E-01		ENSG00000271717

NA	-0.0253	9.1048E-01	9.55E-01	ENSG00000202222
NA	-0.0813	9.1040E-01	9.55E-01	ENSG00000231369
NA	-0.0077	9.1040E-01	9.55E-01	ENSG00000225978
NA	-0.0067	9.1037E-01		ENSG00000259598
NA	-0.0242	9.1000E-01	9.55E-01	ENSG00000227914
NA	-0.0098	9.0962E-01	9.55E-01	ENSG00000256148
NA	-0.0023	9.0949E-01		ENSG00000238221
NA	-0.0440	9.0949E-01	9.55E-01	ENSG00000272056
NA	-0.0098	9.0944E-01	9.55E-01	ENSG00000259083
NA	-0.0074	9.0922E-01		ENSG00000249617
NA	-0.0117	9.0913E-01	9.55E-01	ENSG00000239462
NA	-0.0084	9.0864E-01		ENSG00000272515
NA	-0.0244	9.0860E-01		ENSG00000270116
NA	-0.0046	9.0840E-01		ENSG00000282059
NA	-0.0054	9.0830E-01		ENSG00000229044
NA	-0.0056	9.0819E-01		ENSG00000263394
NA	-0.0120	9.0819E-01	9.54E-01	ENSG00000228648
NA	-0.0122	9.0806E-01		ENSG00000269489
NA	-0.0111	9.0795E-01		ENSG00000288936
NA	-0.0187	9.0793E-01	9.54E-01	ENSG00000253454
NA	-0.0140	9.0753E-01	9.54E-01	ENSG00000264458
NA	-0.0096	9.0750E-01	9.54E-01	ENSG00000265100
NA	-0.0106	9.0750E-01	9.54E-01	ENSG00000239407
NA	-0.0062	9.0748E-01		ENSG00000213026
NA	-0.0090	9.0746E-01		ENSG00000206679
NA	-0.0041	9.0734E-01		ENSG00000227653
NA	-0.0021	9.0729E-01		ENSG00000258753
NA	-0.0039	9.0717E-01		ENSG00000229241
NA	-0.0663	9.0717E-01	9.53E-01	ENSG00000262248
NA	-0.0756	9.0714E-01	9.53E-01	ENSG00000279129
NA	-0.0123	9.0668E-01		ENSG00000255557
NA	-0.0153	9.0642E-01		ENSG00000258445
NA	-0.0024	9.0625E-01		ENSG00000235013
NA	-0.0013	9.0608E-01		ENSG00000203334
NA	-0.0020	9.0594E-01		ENSG00000266989
NA	-0.0146	9.0587E-01		ENSG00000267521
NA	-0.0078	9.0579E-01		ENSG00000257169
NA	-0.0026	9.0572E-01		ENSG00000285836
NA	-0.0114	9.0563E-01	9.53E-01	ENSG00000267466
NA	-0.0099	9.0553E-01		ENSG00000274554
NA	-0.0191	9.0551E-01	9.53E-01	ENSG00000280420
NA	-0.0373	9.0533E-01		ENSG00000268070
NA	-0.0105	9.0496E-01	9.52E-01	ENSG00000223984
NA	-0.0396	9.0494E-01	9.52E-01	ENSG00000271874
NA	-0.0381	9.0468E-01	9.52E-01	ENSG00000233952
NA	-0.0263	9.0439E-01		ENSG00000235079
NA	-0.0126	9.0430E-01	9.52E-01	ENSG00000213683
NA	-0.0139	9.0427E-01		ENSG00000260103
NA	-0.0158	9.0404E-01		ENSG00000286074

NA	-0.0093	9.0385E-01	9.52E-01	ENSG00000187984
NA	-0.0002	9.0383E-01		ENSG00000254605
NA	-0.0151	9.0371E-01	9.52E-01	ENSG00000286006
NA	-0.0242	9.0356E-01		ENSG00000267986
NA	-0.0106	9.0354E-01	9.52E-01	ENSG00000232368
NA	-0.0092	9.0339E-01	9.52E-01	ENSG00000246350
NA	-0.0271	9.0321E-01	9.52E-01	ENSG00000289260
NA	-0.0102	9.0305E-01		ENSG00000251009
NA	-0.0281	9.0302E-01	9.52E-01	ENSG00000289034
NA	-0.0181	9.0298E-01	9.52E-01	ENSG00000287806
NA	-0.0048	9.0289E-01		ENSG00000276166
NA	-0.0131	9.0285E-01		ENSG00000282995
NA	-0.0172	9.0277E-01		ENSG00000284299
NA	-0.0069	9.0277E-01		ENSG00000288695
NA	-0.0082	9.0266E-01		ENSG00000270110
NA	-0.0131	9.0262E-01		ENSG00000260185
NA	-0.0028	9.0258E-01		ENSG00000272239
NA	-0.0107	9.0257E-01		ENSG00000242021
NA	-0.0038	9.0242E-01		ENSG00000232887
NA	-0.0048	9.0238E-01		ENSG00000229559
NA	-0.0090	9.0224E-01		ENSG00000162840
NA	-0.0158	9.0208E-01		ENSG00000286812
NA	-0.0040	9.0202E-01		ENSG00000238516
NA	-0.0078	9.0196E-01		ENSG00000271049
NA	-0.0272	9.0195E-01	9.51E-01	ENSG00000236516
NA	-0.0136	9.0190E-01		ENSG00000264659
NA	-0.0107	9.0185E-01	9.51E-01	ENSG00000279069
NA	-0.0071	9.0164E-01		ENSG00000287859
NA	-0.0051	9.0160E-01		ENSG00000259287
NA	-0.0181	9.0159E-01		ENSG00000228816
NA	-0.0081	9.0154E-01	9.51E-01	ENSG00000240122
NA	-0.0034	9.0114E-01		ENSG00000289700
NA	-0.0116	9.0102E-01	9.51E-01	ENSG00000170629
NA	-0.0096	9.0093E-01		ENSG00000280444
NA	-0.0031	9.0089E-01		ENSG00000224981
NA	-0.0098	9.0068E-01		ENSG00000275812
NA	-0.0127	9.0046E-01	9.50E-01	ENSG00000272023
NA	-0.0042	8.9989E-01		ENSG00000253122
NA	-0.0080	8.9984E-01	9.50E-01	ENSG00000262147
NA	-0.0925	8.9978E-01	9.50E-01	ENSG00000288866
NA	-0.0117	8.9955E-01	9.50E-01	ENSG00000280132
NA	-0.0144	8.9948E-01		ENSG00000232015
NA	-0.0166	8.9943E-01		ENSG00000225739
NA	-0.0064	8.9936E-01		ENSG00000287767
NA	-0.0132	8.9897E-01	9.49E-01	ENSG00000287564
NA	-0.0101	8.9897E-01	9.49E-01	ENSG00000269303
NA	-0.0069	8.9868E-01		ENSG00000288681
NA	-0.0120	8.9861E-01		ENSG00000108785
NA	-0.0135	8.9854E-01	9.49E-01	ENSG00000236440

NA	0.0000	8.9853E-01		ENSG00000222395
NA	-0.0084	8.9853E-01		ENSG00000230320
NA	-0.0300	8.9833E-01		ENSG00000270462
NA	-0.0114	8.9814E-01		ENSG00000286463
NA	-0.0032	8.9801E-01		ENSG00000271752
NA	-0.0016	8.9797E-01		ENSG00000226842
NA	-0.0205	8.9784E-01		ENSG00000255045
NA	-0.0254	8.9778E-01		ENSG00000270966
NA	-0.0027	8.9751E-01	9.49E-01	ENSG00000259499
NA	-0.0070	8.9744E-01		ENSG00000251812
NA	-0.0004	8.9742E-01		ENSG00000277677
NA	-0.0066	8.9738E-01		ENSG00000271327
NA	-0.0051	8.9735E-01		ENSG00000230155
NA	-0.0175	8.9723E-01		ENSG00000259771
NA	-0.0177	8.9719E-01		ENSG00000235085
NA	-0.0258	8.9711E-01		ENSG00000271002
NA	-0.0089	8.9697E-01		ENSG00000264812
NA	-0.0019	8.9677E-01		ENSG00000267408
NA	-0.0131	8.9671E-01		ENSG00000236162
NA	-0.0146	8.9667E-01		ENSG00000280916
NA	-0.0114	8.9651E-01		ENSG00000269950
NA	-0.0019	8.9651E-01	9.48E-01	ENSG00000285749
NA	-0.0043	8.9649E-01		ENSG00000229661
NA	-0.0011	8.9644E-01		ENSG00000289061
NA	-0.0094	8.9637E-01		ENSG00000278011
NA	-0.1015	8.9626E-01	9.48E-01	ENSG00000269246
NA	-0.0040	8.9620E-01		ENSG00000224795
NA	-0.0134	8.9615E-01		ENSG00000251513
NA	-0.0098	8.9611E-01	9.48E-01	ENSG00000286207
NA	-0.0237	8.9600E-01		ENSG00000280089
NA	-0.0039	8.9595E-01		ENSG00000228620
NA	-0.0030	8.9583E-01		ENSG00000228139
NA	-0.0014	8.9583E-01		ENSG00000226661
NA	-0.0132	8.9555E-01		ENSG00000278831
NA	-0.0009	8.9547E-01		ENSG00000263717
NA	-0.0078	8.9531E-01	9.48E-01	ENSG00000232533
NA	-0.0112	8.9528E-01	9.48E-01	ENSG00000264112
NA	-0.0100	8.9523E-01		ENSG00000286365
NA	-0.0017	8.9522E-01		ENSG00000227214
NA	-0.0115	8.9521E-01	9.48E-01	ENSG00000289182
NA	-0.0024	8.9520E-01		ENSG00000266340
NA	-0.0118	8.9506E-01		ENSG00000278250
NA	-0.0105	8.9501E-01	9.48E-01	ENSG00000235236
NA	-0.0031	8.9498E-01		ENSG00000280387
NA	-0.0111	8.9476E-01		ENSG00000260338
NA	-0.0093	8.9463E-01		ENSG00000266711
NA	-0.0138	8.9459E-01	9.47E-01	ENSG00000282390
NA	-0.0531	8.9452E-01		ENSG00000274776
NA	-0.0045	8.9439E-01		ENSG00000278704

NA	-0.0126	8.9426E-01	9.47E-01	ENSG00000289854
NA	-0.0043	8.9404E-01		ENSG00000253792
NA	-0.0004	8.9396E-01		ENSG00000261523
NA	-0.0056	8.9369E-01		ENSG00000280202
NA	-0.0242	8.9362E-01	9.47E-01	ENSG00000255703
NA	-0.0041	8.9353E-01		ENSG00000255256
NA	-0.0236	8.9342E-01		ENSG00000253104
NA	-0.0059	8.9341E-01	9.47E-01	ENSG00000275075
NA	-0.0059	8.9341E-01	9.47E-01	ENSG00000287607
NA	-0.0193	8.9327E-01		ENSG00000243680
NA	-0.0017	8.9321E-01		ENSG00000227304
NA	-0.0014	8.9310E-01		ENSG00000227076
NA	-0.0564	8.9281E-01	9.47E-01	ENSG00000210156
NA	-0.0229	8.9280E-01	9.47E-01	ENSG00000267651
NA	-0.0124	8.9269E-01		ENSG00000267314
NA	-0.0227	8.9264E-01		ENSG00000234521
NA	-0.0017	8.9255E-01		ENSG00000256341
NA	-0.0109	8.9253E-01	9.47E-01	ENSG00000289241
NA	-0.0125	8.9230E-01	9.46E-01	ENSG00000238105
NA	-0.0278	8.9227E-01		ENSG00000179141
NA	-0.0170	8.9227E-01		ENSG00000273063
NA	-0.0022	8.9222E-01		ENSG00000274356
NA	-0.0009	8.9215E-01		ENSG00000255910
NA	-0.0097	8.9191E-01		ENSG00000213104
NA	-0.0035	8.9190E-01		ENSG00000255552
NA	-0.0070	8.9177E-01		ENSG00000289215
NA	-0.0063	8.9176E-01		ENSG00000251844
NA	-0.0111	8.9166E-01		ENSG00000285960
NA	-0.0115	8.9149E-01	9.46E-01	ENSG00000234614
NA	-0.0266	8.9146E-01		ENSG00000273056
NA	-0.0419	8.9146E-01	9.46E-01	ENSG00000210164
NA	-0.0068	8.9145E-01		ENSG00000255882
NA	-0.0115	8.9136E-01		ENSG00000273248
NA	-0.0053	8.9073E-01		ENSG00000290083
NA	-0.0025	8.9038E-01		ENSG00000223724
NA	-0.0047	8.9037E-01		ENSG00000241985
NA	-0.0114	8.9024E-01	9.45E-01	ENSG00000286011
NA	-0.0007	8.8993E-01		ENSG00000225715
NA	-0.0059	8.8979E-01		ENSG00000228838
NA	-0.0338	8.8979E-01		ENSG00000243944
NA	-0.0168	8.8968E-01		ENSG00000235997
NA	-0.0081	8.8968E-01		ENSG00000286487
NA	-0.0113	8.8937E-01	9.44E-01	ENSG00000268678
NA	-0.0100	8.8904E-01		ENSG00000271382
NA	-0.0177	8.8889E-01		ENSG00000287532
NA	-0.0015	8.8883E-01		ENSG00000226068
NA	-0.0117	8.8879E-01	9.44E-01	ENSG00000260490
NA	-0.0044	8.8850E-01		ENSG00000224331
NA	-0.0070	8.8844E-01		ENSG00000288970

NA	-0.0096	8.8843E-01		ENSG00000231010
NA	-0.0091	8.8840E-01		ENSG00000287063
NA	-0.0028	8.8831E-01		ENSG00000234814
NA	-0.0018	8.8823E-01		ENSG00000202470
NA	-0.0100	8.8810E-01		ENSG00000287731
NA	-0.0033	8.8785E-01		ENSG00000229116
NA	-0.0075	8.8785E-01	9.44E-01	ENSG00000270249
NA	-0.0021	8.8761E-01		ENSG00000184274
NA	-0.0002	8.8739E-01		ENSG00000213205
NA	-0.0070	8.8737E-01		ENSG00000267282
NA	-0.0093	8.8731E-01	9.43E-01	ENSG00000237588
NA	-0.0015	8.8728E-01	9.43E-01	ENSG00000279862
NA	-0.0149	8.8711E-01		ENSG00000237682
NA	-0.0071	8.8680E-01		ENSG00000237167
NA	-0.0031	8.8669E-01		ENSG00000220614
NA	-0.0074	8.8664E-01		ENSG00000289143
NA	-0.0043	8.8632E-01	9.43E-01	ENSG00000280122
NA	-0.0119	8.8571E-01	9.42E-01	ENSG00000235105
NA	-0.0009	8.8566E-01		ENSG00000255146
NA	-0.0084	8.8566E-01		ENSG00000217862
NA	-0.0194	8.8553E-01		ENSG00000288684
NA	-0.0354	8.8477E-01	9.42E-01	ENSG00000227237
NA	-0.0027	8.8451E-01		ENSG00000214273
NA	-0.0109	8.8411E-01		ENSG00000242411
NA	-0.0010	8.8403E-01		ENSG00000248266
NA	-0.0135	8.8400E-01	9.41E-01	ENSG00000289349
NA	-0.0049	8.8397E-01		ENSG00000278969
NA	-0.0014	8.8363E-01		ENSG00000286748
NA	-0.0116	8.8362E-01	9.41E-01	ENSG00000271933
NA	-0.0092	8.8350E-01		ENSG00000274303
NA	-0.0214	8.8346E-01	9.41E-01	ENSG00000283390
NA	-0.0162	8.8339E-01		ENSG00000251687
NA	-0.0093	8.8326E-01		ENSG00000289479
NA	-0.0108	8.8319E-01	9.41E-01	ENSG00000274270
NA	-0.0037	8.8317E-01		ENSG00000286518
NA	-0.0132	8.8308E-01	9.41E-01	ENSG00000224307
NA	-0.0036	8.8289E-01		ENSG00000232455
NA	-0.0033	8.8286E-01		ENSG00000276548
NA	-0.0138	8.8274E-01	9.41E-01	ENSG00000284705
NA	-0.0089	8.8261E-01		ENSG00000280060
NA	-0.0196	8.8241E-01		ENSG00000287500
NA	-0.0028	8.8241E-01		ENSG00000263494
NA	-0.0092	8.8222E-01		ENSG00000207488
NA	-0.0139	8.8214E-01		ENSG00000230444
NA	-0.0024	8.8205E-01		ENSG00000250583
NA	-0.0113	8.8198E-01		ENSG00000258084
NA	-0.0077	8.8189E-01		ENSG00000276931
NA	-0.0006	8.8176E-01		ENSG00000254897
NA	-0.0134	8.8166E-01	9.40E-01	ENSG00000196096

NA	-0.0109	8.8144E-01		ENSG00000249245
NA	-0.0372	8.8137E-01	9.40E-01	ENSG00000223393
NA	-0.0061	8.8132E-01		ENSG00000226542
NA	-0.0031	8.8113E-01		ENSG00000199466
NA	-0.0129	8.8099E-01	9.40E-01	ENSG00000269318
NA	-0.0052	8.8099E-01		ENSG00000275550
NA	-0.0014	8.8077E-01		ENSG00000259797
NA	-0.0105	8.8065E-01	9.39E-01	ENSG00000262848
NA	-0.0106	8.8057E-01	9.39E-01	ENSG00000259986
NA	-0.0178	8.8053E-01	9.39E-01	ENSG00000269243
NA	-0.0117	8.8042E-01		ENSG00000285998
NA	-0.0274	8.8030E-01	9.39E-01	ENSG00000251171
NA	-0.0263	8.8024E-01		ENSG00000279274
NA	-0.0263	8.8024E-01		ENSG00000279115
NA	-0.0181	8.7993E-01		ENSG00000224003
NA	-0.0416	8.7990E-01	9.39E-01	ENSG00000249494
NA	-0.0150	8.7987E-01	9.39E-01	ENSG00000288728
NA	-0.0440	8.7967E-01		ENSG00000235554
NA	-0.0101	8.7967E-01	9.39E-01	ENSG00000223519
NA	-0.0116	8.7957E-01		ENSG00000287185
NA	-0.0134	8.7956E-01	9.39E-01	ENSG00000244086
NA	-0.0352	8.7914E-01		ENSG00000288804
NA	-0.0047	8.7902E-01		ENSG00000283518
NA	-0.0044	8.7880E-01	9.39E-01	ENSG00000219159
NA	-0.0094	8.7826E-01		ENSG00000279713
NA	-0.0230	8.7814E-01		ENSG00000265671
NA	-0.0176	8.7797E-01		ENSG00000280211
NA	-0.0145	8.7794E-01	9.38E-01	ENSG00000254428
NA	-0.0083	8.7785E-01		ENSG00000227945
NA	-0.0019	8.7759E-01		ENSG00000276375
NA	-0.0114	8.7759E-01		ENSG00000272417
NA	-0.0024	8.7752E-01		ENSG00000255475
NA	-0.0367	8.7751E-01		ENSG00000273212
NA	-0.0079	8.7737E-01		ENSG00000255599
NA	-0.0124	8.7728E-01	9.38E-01	ENSG00000260403
NA	-0.0152	8.7723E-01		ENSG00000224936
NA	-0.0380	8.7684E-01	9.37E-01	ENSG00000248966
NA	-0.0063	8.7669E-01		ENSG00000241169
NA	-0.0287	8.7667E-01	9.37E-01	ENSG00000227782
NA	-0.0083	8.7661E-01		ENSG00000251986
NA	-0.0543	8.7650E-01	9.37E-01	ENSG00000289690
NA	-0.0127	8.7624E-01	9.37E-01	ENSG00000230006
NA	-0.0028	8.7602E-01		ENSG00000275933
NA	-0.0149	8.7601E-01	9.37E-01	ENSG00000273897
NA	-0.0139	8.7596E-01	9.37E-01	ENSG00000287310
NA	-0.0119	8.7584E-01		ENSG00000224063
NA	-0.0093	8.7584E-01		ENSG00000272529
NA	-0.0125	8.7577E-01		ENSG00000267234
NA	-0.0049	8.7559E-01		ENSG00000273381

NA	-0.0031	8.7520E-01		ENSG00000260743
NA	-0.0284	8.7487E-01		ENSG00000259570
NA	-0.0330	8.7481E-01	9.37E-01	ENSG00000279329
NA	-0.0223	8.7470E-01		ENSG00000267096
NA	-0.0070	8.7468E-01		ENSG00000224713
NA	-0.0053	8.7468E-01		ENSG00000226158
NA	-0.0143	8.7467E-01		ENSG00000251461
NA	-0.0206	8.7415E-01		ENSG00000256612
NA	-0.0161	8.7414E-01		ENSG00000211491
NA	-0.0037	8.7404E-01		ENSG00000249780
NA	-0.0024	8.7385E-01		ENSG00000230432
NA	-0.0074	8.7381E-01		ENSG00000265545
NA	-0.0146	8.7377E-01	9.36E-01	ENSG00000225285
NA	-0.0047	8.7373E-01		ENSG00000203327
NA	-0.0106	8.7347E-01	9.36E-01	ENSG00000259920
NA	-0.0095	8.7322E-01		ENSG00000258693
NA	-0.0154	8.7314E-01	9.36E-01	ENSG00000265298
NA	-0.0223	8.7300E-01	9.36E-01	ENSG00000253357
NA	-0.0052	8.7295E-01		ENSG00000244332
NA	-0.0126	8.7293E-01		ENSG00000229497
NA	-0.0314	8.7292E-01	9.36E-01	ENSG00000275163
NA	-0.0176	8.7279E-01		ENSG00000275322
NA	-0.0226	8.7226E-01		ENSG00000224685
NA	-0.0131	8.7204E-01	9.35E-01	ENSG00000157306
NA	-0.0044	8.7184E-01		ENSG00000229271
NA	0.0000	8.7183E-01		ENSG00000286799
NA	-0.0143	8.7183E-01		ENSG00000259682
NA	-0.0110	8.7145E-01	9.35E-01	ENSG00000279319
NA	-0.0062	8.7120E-01		ENSG00000231894
NA	-0.0149	8.7062E-01	9.34E-01	ENSG00000264070
NA	-0.0100	8.7044E-01		ENSG00000237234
NA	-0.0256	8.7035E-01		ENSG00000214286
NA	-0.0519	8.7031E-01	9.34E-01	ENSG00000261096
NA	-0.0610	8.7020E-01	9.34E-01	ENSG00000260029
NA	-0.0086	8.7005E-01		ENSG00000206645
NA	-0.0134	8.7003E-01		ENSG00000287085
NA	-0.0055	8.6988E-01		ENSG00000256568
NA	-0.0128	8.6971E-01	9.34E-01	ENSG00000233846
NA	-0.0150	8.6964E-01	9.34E-01	ENSG00000187979
NA	-0.0039	8.6958E-01		ENSG00000279872
NA	-0.0118	8.6942E-01	9.34E-01	ENSG00000239736
NA	-0.0136	8.6938E-01		ENSG00000270874
NA	-0.0065	8.6911E-01		ENSG00000214366
NA	-0.1342	8.6909E-01	9.34E-01	ENSG00000279693
NA	-0.0178	8.6908E-01		ENSG00000225022
NA	-0.0225	8.6905E-01		ENSG00000257302
NA	-0.0201	8.6903E-01	9.34E-01	ENSG00000131484
NA	-0.0011	8.6900E-01		ENSG00000261060
NA	-0.0128	8.6899E-01	9.34E-01	ENSG00000242078

NA	-0.0122	8.6874E-01		ENSG00000233052
NA	-0.0129	8.6866E-01	9.34E-01	ENSG00000232713
NA	-0.0137	8.6854E-01		ENSG00000260440
NA	-0.0157	8.6838E-01	9.33E-01	ENSG00000289238
NA	-0.0165	8.6828E-01	9.33E-01	ENSG00000276390
NA	-0.0017	8.6824E-01		ENSG00000279147
NA	-0.0062	8.6806E-01		ENSG00000224664
NA	-0.0104	8.6790E-01		ENSG00000289116
NA	-0.0033	8.6769E-01		ENSG00000273232
NA	-0.0056	8.6763E-01		ENSG00000237828
NA	-0.0364	8.6756E-01		ENSG00000230694
NA	-0.0157	8.6738E-01	9.33E-01	ENSG00000261872
NA	-0.0126	8.6734E-01		ENSG00000232057
NA	-0.0018	8.6715E-01		ENSG00000266521
NA	-0.0102	8.6694E-01		ENSG00000284675
NA	-0.0107	8.6639E-01		ENSG00000279732
NA	-0.0100	8.6614E-01		ENSG00000242267
NA	-0.0303	8.6609E-01		ENSG00000259563
NA	-0.0160	8.6594E-01	9.32E-01	ENSG00000289092
NA	-0.0012	8.6563E-01	9.32E-01	ENSG00000289453
NA	-0.0121	8.6545E-01	9.31E-01	ENSG00000268205
NA	-0.0027	8.6544E-01		ENSG00000285974
NA	-0.0014	8.6539E-01		ENSG00000257271
NA	-0.0139	8.6519E-01	9.31E-01	ENSG00000131401
NA	-0.0127	8.6464E-01	9.31E-01	ENSG00000258998
NA	-0.0018	8.6460E-01		ENSG00000232342
NA	-0.0469	8.6456E-01		ENSG00000231255
NA	-0.0135	8.6421E-01		ENSG00000266490
NA	-0.0081	8.6396E-01		ENSG00000265316
NA	-0.0112	8.6395E-01		ENSG00000236662
NA	-0.0049	8.6395E-01		ENSG00000286294
NA	-0.0067	8.6385E-01		ENSG00000249417
NA	-0.0057	8.6370E-01		ENSG00000278785
NA	-0.0164	8.6352E-01		ENSG00000279932
NA	-0.0122	8.6340E-01	9.30E-01	ENSG00000259536
NA	-0.0129	8.6334E-01		ENSG00000279982
NA	-0.0161	8.6321E-01	9.30E-01	ENSG00000261584
NA	-0.0054	8.6317E-01		ENSG00000273160
NA	-0.0056	8.6307E-01		ENSG00000279987
NA	-0.0526	8.6290E-01	9.30E-01	ENSG00000238286
NA	-0.0158	8.6275E-01		ENSG00000277545
NA	-0.0135	8.6271E-01		ENSG00000277558
NA	-0.0181	8.6261E-01		ENSG00000230521
NA	-0.0189	8.6260E-01		ENSG00000231188
NA	-0.0301	8.6257E-01	9.30E-01	ENSG00000287879
NA	-0.0121	8.6251E-01	9.30E-01	ENSG00000258844
NA	-0.0353	8.6241E-01	9.30E-01	ENSG00000289752
NA	-0.0071	8.6239E-01		ENSG00000227748
NA	-0.0054	8.6216E-01		ENSG00000263098

NA	-0.0066	8.6212E-01		ENSG00000241054
NA	-0.0039	8.6210E-01		ENSG00000259162
NA	-0.0158	8.6199E-01	9.30E-01	ENSG00000289261
NA	-0.0012	8.6189E-01		ENSG00000255464
NA	-0.0159	8.6177E-01	9.30E-01	ENSG00000235859
NA	-0.0032	8.6152E-01		ENSG00000284513
NA	0.0000	8.6121E-01		ENSG00000279273
NA	-0.0127	8.6120E-01		ENSG00000277385
NA	-0.0143	8.6006E-01	9.29E-01	ENSG00000272356
NA	-0.0166	8.5999E-01	9.29E-01	ENSG00000237729
NA	-0.0100	8.5963E-01		ENSG00000271228
NA	-0.0133	8.5942E-01	9.28E-01	ENSG00000260281
NA	-0.0090	8.5937E-01	9.28E-01	ENSG00000266288
NA	-0.0004	8.5902E-01		ENSG00000237251
NA	-0.0054	8.5898E-01		ENSG00000219553
NA	-0.0113	8.5890E-01		ENSG00000200883
NA	-0.0226	8.5847E-01		ENSG00000272382
NA	-0.0165	8.5832E-01		ENSG00000261426
NA	-0.0081	8.5825E-01		ENSG00000259575
NA	-0.0186	8.5819E-01		ENSG00000183148
NA	-0.0104	8.5775E-01		ENSG00000267762
NA	-0.0123	8.5770E-01	9.27E-01	ENSG00000227836
NA	-0.0165	8.5742E-01	9.27E-01	ENSG00000244245
NA	-0.0066	8.5740E-01	9.27E-01	ENSG00000225234
NA	-0.0176	8.5737E-01		ENSG00000273973
NA	-0.0395	8.5732E-01	9.27E-01	ENSG00000286468
NA	-0.0033	8.5706E-01		ENSG00000214190
NA	-0.0302	8.5706E-01	9.27E-01	ENSG00000271398
NA	-0.0097	8.5697E-01		ENSG00000243792
NA	-0.0341	8.5679E-01	9.27E-01	ENSG00000275833
NA	-0.0029	8.5663E-01	9.27E-01	ENSG00000232354
NA	-0.0025	8.5615E-01		ENSG00000257897
NA	-0.0214	8.5602E-01		ENSG00000231762
NA	-0.0107	8.5559E-01		ENSG00000256844
NA	-0.0087	8.5551E-01		ENSG00000255439
NA	-0.0052	8.5550E-01	9.26E-01	ENSG00000271771
NA	-0.0043	8.5549E-01		ENSG00000274514
NA	-0.0341	8.5537E-01	9.26E-01	ENSG00000227638
NA	-0.0169	8.5529E-01	9.26E-01	ENSG00000288996
NA	-0.0062	8.5487E-01		ENSG00000287692
NA	-0.0141	8.5472E-01	9.25E-01	ENSG00000271746
NA	-0.0158	8.5467E-01	9.25E-01	ENSG00000288751
NA	-0.0058	8.5464E-01		ENSG00000272248
NA	-0.0098	8.5464E-01		ENSG00000215199
NA	-0.0378	8.5445E-01	9.25E-01	ENSG00000267199
NA	-0.0131	8.5434E-01		ENSG00000258696
NA	-0.0516	8.5424E-01	9.25E-01	ENSG00000228366
NA	-0.0117	8.5389E-01	9.25E-01	ENSG00000275854
NA	-0.0165	8.5384E-01	9.25E-01	ENSG00000261596

NA	-0.0141	8.5373E-01		ENSG00000277589
NA	-0.0093	8.5372E-01		ENSG00000286268
NA	-0.0125	8.5356E-01	9.25E-01	ENSG00000215840
NA	-0.0553	8.5347E-01	9.25E-01	ENSG00000287766
NA	-0.0080	8.5304E-01		ENSG00000258068
NA	-0.0198	8.5302E-01	9.25E-01	ENSG00000285652
NA	-0.0152	8.5280E-01		ENSG00000223505
NA	-0.0338	8.5274E-01	9.25E-01	ENSG00000227698
NA	-0.0174	8.5272E-01	9.25E-01	ENSG00000289196
NA	-0.0107	8.5247E-01		ENSG00000231840
NA	-0.0151	8.5246E-01	9.24E-01	ENSG00000215790
NA	-0.0120	8.5236E-01		ENSG00000267507
NA	-0.0169	8.5213E-01		ENSG00000258658
NA	-0.0138	8.5201E-01	9.24E-01	ENSG00000259746
NA	-0.0062	8.5182E-01		ENSG00000237903
NA	-0.0644	8.5180E-01		ENSG00000267938
NA	-0.0373	8.5156E-01		ENSG00000264078
NA	-0.0074	8.5134E-01		ENSG00000207105
NA	-0.0046	8.5114E-01		ENSG00000237775
NA	-0.0148	8.5111E-01		ENSG00000250375
NA	-0.0050	8.5080E-01		ENSG00000204031
NA	-0.0101	8.5052E-01		ENSG00000251152
NA	-0.0205	8.5038E-01	9.23E-01	ENSG00000235036
NA	-0.0462	8.5024E-01	9.23E-01	ENSG00000276997
NA	-0.0051	8.5004E-01		ENSG00000254627
NA	-0.0081	8.4997E-01		ENSG00000234835
NA	-0.0020	8.4997E-01		ENSG00000229820
NA	-0.0058	8.4952E-01		ENSG00000260593
NA	-0.0128	8.4932E-01		ENSG00000288032
NA	-0.0164	8.4920E-01	9.22E-01	ENSG00000267506
NA	-0.0124	8.4908E-01	9.22E-01	ENSG00000257624
NA	-0.0033	8.4879E-01		ENSG00000254449
NA	-0.0060	8.4879E-01		ENSG00000254552
NA	-0.0129	8.4866E-01	9.22E-01	ENSG00000286070
NA	-0.0077	8.4862E-01		ENSG00000286893
NA	-0.0106	8.4860E-01		ENSG00000260621
NA	-0.0202	8.4851E-01	9.22E-01	ENSG00000255933
NA	-0.0071	8.4831E-01		ENSG00000279640
NA	-0.0177	8.4820E-01		ENSG00000285545
NA	-0.0125	8.4809E-01	9.22E-01	ENSG00000275678
NA	-0.0082	8.4788E-01		ENSG00000278594
NA	-0.0015	8.4768E-01		ENSG00000220702
NA	-0.0029	8.4753E-01		ENSG00000266987
NA	-0.0093	8.4745E-01	9.21E-01	ENSG00000239503
NA	-0.0051	8.4744E-01		ENSG00000250993
NA	-0.0114	8.4742E-01		ENSG00000274578
NA	-0.0088	8.4739E-01		ENSG00000249055
NA	-0.0071	8.4720E-01		ENSG00000263781
NA	-0.0057	8.4716E-01	9.21E-01	ENSG00000287378

NA	-0.0083	8.4688E-01		ENSG00000231464
NA	-0.0214	8.4641E-01		ENSG00000254450
NA	-0.0176	8.4623E-01		ENSG00000231691
NA	-0.0159	8.4619E-01	9.20E-01	ENSG00000259367
NA	-0.0136	8.4607E-01	9.20E-01	ENSG00000274400
NA	-0.0066	8.4606E-01		ENSG00000237666
NA	-0.0058	8.4605E-01		ENSG00000278633
NA	-0.0206	8.4598E-01		ENSG00000226427
NA	-0.0112	8.4538E-01		ENSG00000286415
NA	-0.0096	8.4511E-01		ENSG00000271904
NA	-0.0082	8.4508E-01		ENSG00000258886
NA	-0.0181	8.4486E-01	9.19E-01	ENSG00000205105
NA	-0.0015	8.4483E-01		ENSG00000286594
NA	-0.0098	8.4462E-01		ENSG00000231529
NA	-0.0096	8.4447E-01		ENSG00000274765
NA	-0.0180	8.4415E-01	9.19E-01	ENSG00000289324
NA	-0.0169	8.4412E-01	9.19E-01	ENSG00000280216
NA	-0.0036	8.4404E-01		ENSG00000276058
NA	-0.0072	8.4393E-01	9.19E-01	ENSG00000272182
NA	-0.0059	8.4391E-01		ENSG00000248122
NA	-0.0129	8.4387E-01	9.19E-01	ENSG00000251363
NA	-0.0151	8.4387E-01		ENSG00000272473
NA	-0.0003	8.4385E-01		ENSG00000213155
NA	-0.0016	8.4364E-01		ENSG00000234937
NA	-0.0045	8.4326E-01		ENSG00000248692
NA	-0.0076	8.4313E-01	9.18E-01	ENSG00000288041
NA	-0.0100	8.4306E-01		ENSG00000227992
NA	-0.0007	8.4295E-01		ENSG00000261501
NA	-0.0136	8.4275E-01	9.18E-01	ENSG00000272281
NA	-0.0159	8.4269E-01	9.18E-01	ENSG00000260317
NA	-0.0131	8.4252E-01		ENSG00000271992
NA	-0.0070	8.4252E-01		ENSG00000276067
NA	-0.0080	8.4234E-01		ENSG00000257696
NA	-0.0021	8.4231E-01		ENSG00000224124
NA	-0.0150	8.4190E-01		ENSG00000267333
NA	-0.0122	8.4173E-01	9.17E-01	ENSG00000215284
NA	-0.0110	8.4125E-01		ENSG00000287881
NA	-0.0388	8.4111E-01		ENSG00000261216
NA	-0.0146	8.4107E-01	9.17E-01	ENSG00000289639
NA	-0.0097	8.4107E-01		ENSG00000235736
NA	-0.0008	8.4107E-01		ENSG00000266079
NA	-0.0155	8.4098E-01		ENSG00000248511
NA	-0.0138	8.4088E-01		ENSG00000275981
NA	-0.1478	8.4080E-01	9.17E-01	ENSG00000230393
NA	-0.0047	8.4080E-01		ENSG00000261310
NA	-0.0070	8.4066E-01		ENSG00000289014
NA	-0.0046	8.4060E-01		ENSG00000249966
NA	-0.0037	8.4055E-01		ENSG00000226937
NA	-0.0263	8.4045E-01		ENSG00000226526

NA	-0.0073	8.4042E-01		ENSG00000271181
NA	-0.0158	8.4031E-01	9.17E-01	ENSG00000206927
NA	-0.0188	8.4029E-01	9.17E-01	ENSG00000289296
NA	-0.0068	8.4021E-01		ENSG00000236355
NA	-0.0152	8.4013E-01	9.16E-01	ENSG00000255670
NA	-0.0193	8.4002E-01	9.16E-01	ENSG00000267852
NA	-0.0003	8.3987E-01		ENSG00000224256
NA	-0.0127	8.3973E-01		ENSG00000234162
NA	-0.0159	8.3968E-01	9.16E-01	ENSG00000279443
NA	-0.0313	8.3956E-01	9.16E-01	ENSG00000272328
NA	-0.0139	8.3932E-01	9.16E-01	ENSG00000223188
NA	-0.0201	8.3917E-01		ENSG00000283438
NA	-0.0205	8.3904E-01	9.16E-01	ENSG00000254604
NA	-0.0108	8.3902E-01		ENSG00000275997
NA	-0.0152	8.3888E-01		ENSG00000286984
NA	-0.0058	8.3887E-01		ENSG00000253180
NA	-0.0064	8.3845E-01		ENSG00000233005
NA	-0.0160	8.3817E-01		ENSG00000237015
NA	-0.0191	8.3803E-01		ENSG00000285932
NA	-0.0297	8.3802E-01		ENSG00000261467
NA	-0.0146	8.3798E-01	9.15E-01	ENSG00000253395
NA	-0.0107	8.3792E-01	9.15E-01	ENSG00000287477
NA	-0.0261	8.3791E-01	9.15E-01	ENSG00000246016
NA	-0.0172	8.3766E-01	9.15E-01	ENSG00000267723
NA	-0.0163	8.3740E-01	9.15E-01	ENSG00000229956
NA	-0.0031	8.3721E-01		ENSG00000231990
NA	-0.0174	8.3718E-01		ENSG00000253215
NA	-0.0132	8.3715E-01	9.15E-01	ENSG00000233117
NA	-0.0136	8.3706E-01		ENSG00000256218
NA	-0.0226	8.3671E-01	9.15E-01	ENSG00000234929
NA	-0.0044	8.3639E-01		ENSG00000267405
NA	-0.0193	8.3632E-01	9.14E-01	ENSG00000233830
NA	-0.0227	8.3628E-01		ENSG00000225187
NA	-0.0341	8.3619E-01		ENSG00000224725
NA	-0.0136	8.3599E-01		ENSG00000259735
NA	-0.0181	8.3574E-01	9.14E-01	ENSG00000225447
NA	-0.0097	8.3564E-01		ENSG00000250855
NA	-0.0337	8.3561E-01	9.14E-01	ENSG00000235555
NA	-0.0244	8.3531E-01		ENSG00000283769
NA	-0.0147	8.3494E-01	9.14E-01	ENSG00000279110
NA	-0.0215	8.3494E-01		ENSG00000285791
NA	-0.0019	8.3482E-01		ENSG00000286438
NA	-0.0167	8.3481E-01	9.14E-01	ENSG00000273619
NA	-0.0075	8.3480E-01		ENSG00000235246
NA	-0.0154	8.3461E-01		ENSG00000261000
NA	-0.0004	8.3461E-01		ENSG00000270614
NA	-0.0180	8.3459E-01		ENSG00000289379
NA	-0.0189	8.3450E-01	9.14E-01	ENSG00000272518
NA	-0.0173	8.3448E-01	9.14E-01	ENSG00000257524

NA	-0.0234	8.3417E-01		ENSG00000263904
NA	-0.0160	8.3411E-01	9.13E-01	ENSG00000228169
NA	-0.0250	8.3390E-01		ENSG00000271996
NA	-0.0142	8.3385E-01	9.13E-01	ENSG00000253439
NA	-0.0143	8.3366E-01	9.13E-01	ENSG00000286613
NA	-0.0146	8.3357E-01	9.13E-01	ENSG00000259984
NA	-0.0325	8.3355E-01	9.13E-01	ENSG00000255909
NA	-0.0024	8.3307E-01		ENSG00000251244
NA	-0.0169	8.3270E-01	9.13E-01	ENSG00000288973
NA	-0.0143	8.3267E-01	9.13E-01	ENSG00000253372
NA	-0.0193	8.3251E-01	9.13E-01	ENSG00000268266
NA	-0.0034	8.3240E-01		ENSG00000254297
NA	-0.0309	8.3219E-01	9.12E-01	ENSG00000254180
NA	-0.0232	8.3212E-01		ENSG00000275759
NA	-0.0011	8.3207E-01		ENSG00000277041
NA	-0.0006	8.3197E-01		ENSG00000236417
NA	-0.0244	8.3162E-01	9.12E-01	ENSG00000250950
NA	-0.0172	8.3144E-01		ENSG00000265094
NA	-0.0127	8.3143E-01		ENSG00000201371
NA	-0.0683	8.3139E-01	9.12E-01	ENSG00000265293
NA	-0.0237	8.3133E-01	9.12E-01	ENSG00000235072
NA	-0.0017	8.3133E-01		ENSG00000203588
NA	-0.0524	8.3119E-01		ENSG00000257139
NA	-0.0026	8.3097E-01		ENSG00000201565
NA	-0.0103	8.3073E-01		ENSG00000286280
NA	-0.0023	8.3068E-01		ENSG00000283132
NA	-0.0169	8.3043E-01	9.11E-01	ENSG00000260545
NA	-0.0066	8.2986E-01		ENSG00000264630
NA	-0.0128	8.2973E-01		ENSG00000277498
NA	-0.0402	8.2963E-01	9.11E-01	ENSG00000225300
NA	-0.0017	8.2950E-01		ENSG00000287589
NA	-0.0615	8.2944E-01		ENSG00000230831
NA	-0.0072	8.2918E-01		ENSG00000225417
NA	-0.0073	8.2917E-01		ENSG00000230728
NA	-0.0079	8.2913E-01		ENSG00000235062
NA	-0.0007	8.2908E-01		ENSG00000272324
NA	-0.0139	8.2881E-01		ENSG00000238273
NA	-0.0043	8.2859E-01		ENSG00000226291
NA	-0.0191	8.2842E-01	9.10E-01	ENSG00000260855
NA	-0.0054	8.2828E-01		ENSG00000283914
NA	-0.0318	8.2825E-01	9.10E-01	ENSG00000278068
NA	-0.0115	8.2822E-01		ENSG00000280032
NA	-0.0012	8.2807E-01		ENSG00000289344
NA	-0.0090	8.2804E-01		ENSG00000272057
NA	-0.0047	8.2788E-01		ENSG00000228459
NA	-0.0193	8.2782E-01	9.09E-01	ENSG00000225138
NA	-0.0160	8.2768E-01		ENSG00000223571
NA	-0.0126	8.2758E-01		ENSG00000261670
NA	-0.0186	8.2753E-01	9.09E-01	ENSG00000286045

NA	-0.0086	8.2752E-01	9.09E-01	ENSG00000279678
NA	-0.0228	8.2735E-01	9.09E-01	ENSG00000238251
NA	-0.0509	8.2734E-01	9.09E-01	ENSG00000289141
NA	-0.0284	8.2684E-01		ENSG00000286796
NA	-0.0035	8.2649E-01		ENSG00000259719
NA	-0.0124	8.2602E-01	9.09E-01	ENSG00000260084
NA	-0.0110	8.2601E-01		ENSG00000241493
NA	-0.0384	8.2600E-01	9.09E-01	ENSG00000224479
NA	-0.0115	8.2579E-01		ENSG00000284676
NA	-0.0209	8.2539E-01	9.08E-01	ENSG00000289372
NA	-0.0221	8.2535E-01		ENSG00000250210
NA	-0.0118	8.2531E-01		ENSG00000215270
NA	-0.0116	8.2528E-01		ENSG00000226161
NA	-0.0187	8.2524E-01	9.08E-01	ENSG00000248587
NA	-0.0318	8.2513E-01		ENSG00000258357
NA	-0.0049	8.2512E-01		ENSG00000279739
NA	-0.0051	8.2502E-01	9.08E-01	ENSG00000278467
NA	-0.0458	8.2486E-01		ENSG00000289632
NA	-0.0716	8.2481E-01	9.08E-01	ENSG00000204434
NA	-0.0106	8.2466E-01		ENSG00000279972
NA	-0.0417	8.2459E-01		ENSG00000271662
NA	-0.0102	8.2428E-01		ENSG00000258011
NA	-0.0200	8.2427E-01		ENSG00000255040
NA	-0.0171	8.2416E-01		ENSG00000279459
NA	-0.0409	8.2402E-01		ENSG00000251079
NA	-0.0109	8.2397E-01		ENSG00000215812
NA	-0.0043	8.2386E-01	9.07E-01	ENSG00000224731
NA	-0.0150	8.2371E-01		ENSG00000274515
NA	-0.0175	8.2351E-01	9.07E-01	ENSG00000249565
NA	-0.0047	8.2311E-01		ENSG00000231297
NA	-0.0412	8.2301E-01		ENSG00000262470
NA	-0.0208	8.2285E-01	9.07E-01	ENSG00000286650
NA	-0.0003	8.2277E-01		ENSG00000289128
NA	-0.0252	8.2270E-01		ENSG00000289902
NA	-0.0068	8.2229E-01		ENSG00000279378
NA	-0.0209	8.2221E-01	9.06E-01	ENSG00000261407
NA	-0.0220	8.2220E-01		ENSG00000284722
NA	-0.0221	8.2203E-01	9.06E-01	ENSG00000289291
NA	-0.0041	8.2199E-01		ENSG00000279557
NA	-0.0177	8.2189E-01	9.06E-01	ENSG00000271967
NA	-0.0198	8.2189E-01	9.06E-01	ENSG00000287804
NA	-0.0051	8.2180E-01		ENSG00000270421
NA	-0.0103	8.2179E-01		ENSG00000201554
NA	-0.0237	8.2174E-01	9.06E-01	ENSG00000257953
NA	-0.0095	8.2171E-01		ENSG00000187893
NA	-0.0001	8.2137E-01		ENSG00000287971
NA	-0.0139	8.2115E-01		ENSG00000224016
NA	-0.0021	8.2106E-01		ENSG00000274346
NA	-0.0384	8.2104E-01	9.06E-01	ENSG00000230773

NA	-0.0115	8.2086E-01		ENSG00000267583
NA	-0.0136	8.2063E-01	9.05E-01	ENSG00000232396
NA	-0.0099	8.2055E-01	9.05E-01	ENSG00000289331
NA	-0.0061	8.2042E-01		ENSG00000261158
NA	-0.0022	8.2040E-01		ENSG00000286062
NA	-0.0599	8.2040E-01	9.05E-01	ENSG00000273416
NA	-0.0031	8.2036E-01		ENSG00000213484
NA	-0.0006	8.2024E-01	9.05E-01	ENSG00000230863
NA	-0.0169	8.1998E-01	9.05E-01	ENSG00000279031
NA	-0.0149	8.1988E-01		ENSG00000289058
NA	-0.0087	8.1935E-01		ENSG00000225365
NA	-0.0194	8.1879E-01	9.04E-01	ENSG00000275457
NA	-0.0193	8.1858E-01	9.04E-01	ENSG00000239828
NA	-0.0235	8.1848E-01	9.04E-01	ENSG00000290045
NA	-0.0324	8.1832E-01		ENSG00000267834
NA	-0.0174	8.1815E-01	9.04E-01	ENSG00000280038
NA	-0.0088	8.1814E-01		ENSG00000285610
NA	-0.0066	8.1813E-01		ENSG00000229919
NA	-0.0099	8.1808E-01		ENSG00000285486
NA	-0.0103	8.1790E-01		ENSG00000227712
NA	-0.0306	8.1756E-01		ENSG00000237990
NA	-0.0101	8.1735E-01		ENSG00000279231
NA	-0.0230	8.1724E-01		ENSG00000223313
NA	-0.0124	8.1723E-01		ENSG00000290107
NA	-0.0086	8.1705E-01		ENSG00000201207
NA	-0.0033	8.1695E-01		ENSG00000277488
NA	-0.0483	8.1669E-01	9.03E-01	ENSG00000228118
NA	-0.0205	8.1666E-01	9.03E-01	ENSG00000273987
NA	-0.0210	8.1658E-01	9.03E-01	ENSG00000264204
NA	-0.0533	8.1629E-01	9.03E-01	ENSG00000225905
NA	-0.0428	8.1605E-01	9.03E-01	ENSG00000273143
NA	-0.0145	8.1602E-01	9.03E-01	ENSG00000264881
NA	-0.0092	8.1600E-01		ENSG00000280326
NA	-0.0404	8.1596E-01		ENSG00000288698
NA	-0.0176	8.1594E-01	9.03E-01	ENSG00000231584
NA	-0.0352	8.1592E-01	9.03E-01	ENSG00000287941
NA	-0.0089	8.1589E-01		ENSG00000258225
NA	-0.0272	8.1565E-01	9.03E-01	ENSG00000286018
NA	-0.0140	8.1547E-01		ENSG00000278603
NA	-0.0635	8.1521E-01	9.02E-01	ENSG00000272004
NA	-0.0112	8.1515E-01		ENSG00000228655
NA	-0.0130	8.1511E-01		ENSG00000276406
NA	-0.0162	8.1510E-01		ENSG00000272682
NA	-0.0166	8.1504E-01		ENSG00000261145
NA	-0.0218	8.1500E-01	9.02E-01	ENSG00000288772
NA	-0.0561	8.1497E-01	9.02E-01	ENSG00000289968
NA	-0.0326	8.1488E-01	9.02E-01	ENSG00000276603
NA	-0.0013	8.1487E-01		ENSG00000232518
NA	-0.0328	8.1468E-01		ENSG00000287944

NA	-0.0120	8.1460E-01		ENSG00000269802
NA	-0.0404	8.1435E-01	9.02E-01	ENSG00000266998
NA	-0.0037	8.1431E-01		ENSG00000241475
NA	-0.0222	8.1419E-01	9.02E-01	ENSG00000279691
NA	-0.0030	8.1406E-01		ENSG00000182000
NA	-0.0081	8.1405E-01		ENSG00000225527
NA	-0.0046	8.1388E-01		ENSG00000287427
NA	-0.0010	8.1386E-01		ENSG00000251730
NA	-0.0263	8.1384E-01	9.02E-01	ENSG00000250933
NA	-0.0099	8.1349E-01		ENSG00000276768
NA	-0.0046	8.1334E-01		ENSG00000227141
NA	-0.0091	8.1326E-01		ENSG00000231530
NA	-0.0140	8.1289E-01		ENSG00000281112
NA	-0.0106	8.1274E-01		ENSG00000260041
NA	-0.0034	8.1267E-01		ENSG00000271234
NA	-0.0101	8.1249E-01		ENSG00000228115
NA	-0.0155	8.1229E-01		ENSG00000280384
NA	-0.0230	8.1225E-01		ENSG00000232608
NA	-0.0387	8.1225E-01	9.01E-01	ENSG00000218189
NA	-0.0213	8.1219E-01		ENSG00000224077
NA	-0.0390	8.1218E-01		ENSG00000268896
NA	-0.0449	8.1209E-01	9.01E-01	ENSG00000285732
NA	-0.0102	8.1190E-01		ENSG00000282199
NA	-0.0194	8.1187E-01	9.00E-01	ENSG00000270343
NA	-0.0398	8.1186E-01	9.00E-01	ENSG00000261868
NA	-0.0176	8.1182E-01	9.00E-01	ENSG00000234332
NA	-0.0094	8.1180E-01		ENSG00000214067
NA	-0.0457	8.1160E-01	9.00E-01	ENSG00000288939
NA	-0.0136	8.1114E-01		ENSG00000224128
NA	-0.0157	8.1069E-01		ENSG00000237398
NA	-0.0229	8.1065E-01	9.00E-01	ENSG00000278740
NA	-0.0216	8.1065E-01	9.00E-01	ENSG00000272489
NA	-0.0074	8.1056E-01		ENSG00000266839
NA	-0.0244	8.1027E-01		ENSG00000267652
NA	-0.0229	8.1014E-01	8.99E-01	ENSG00000253706
NA	-0.0630	8.1008E-01		ENSG00000273137
NA	-0.0214	8.1007E-01	8.99E-01	ENSG00000275110
NA	-0.0143	8.0998E-01		ENSG00000237327
NA	-0.0205	8.0997E-01	8.99E-01	ENSG00000258815
NA	-0.0187	8.0983E-01	8.99E-01	ENSG00000232934
NA	-0.0130	8.0975E-01	8.99E-01	ENSG00000232811
NA	-0.0015	8.0971E-01		ENSG00000225385
NA	-0.0035	8.0947E-01		ENSG00000230830
NA	-0.0118	8.0945E-01		ENSG00000255542
NA	-0.0069	8.0939E-01		ENSG00000278967
NA	-0.0226	8.0917E-01	8.99E-01	ENSG00000243802
NA	-0.0074	8.0910E-01		ENSG00000226868
NA	-0.0226	8.0892E-01	8.99E-01	ENSG00000286938
NA	-0.0214	8.0886E-01	8.99E-01	ENSG00000281571

NA	-0.0296	8.0879E-01		ENSG00000289569
NA	-0.0148	8.0798E-01		ENSG00000227747
NA	-0.0168	8.0769E-01		ENSG00000287925
NA	-0.0135	8.0759E-01		ENSG00000255031
NA	-0.0132	8.0718E-01	8.97E-01	ENSG00000230526
NA	-0.0098	8.0714E-01		ENSG00000269938
NA	-0.0143	8.0713E-01		ENSG00000275569
NA	-0.0194	8.0713E-01		ENSG00000256427
NA	0.0000	8.0705E-01		ENSG00000253204
NA	-0.0013	8.0679E-01		ENSG00000287933
NA	-0.0180	8.0669E-01		ENSG00000285602
NA	-0.0227	8.0593E-01		ENSG00000278775
NA	-0.0267	8.0587E-01		ENSG00000267073
NA	-0.0156	8.0585E-01	8.96E-01	ENSG00000231841
NA	-0.0034	8.0570E-01		ENSG00000255448
NA	-0.0231	8.0569E-01	8.96E-01	ENSG00000281741
NA	-0.0126	8.0551E-01		ENSG00000284702
NA	-0.0211	8.0549E-01		ENSG00000234336
NA	-0.0200	8.0545E-01		ENSG00000287451
NA	-0.0243	8.0538E-01	8.96E-01	ENSG00000286340
NA	-0.0178	8.0534E-01		ENSG00000234036
NA	-0.0214	8.0528E-01	8.96E-01	ENSG00000261140
NA	-0.0006	8.0523E-01		ENSG00000282951
NA	-0.0204	8.0509E-01	8.96E-01	ENSG00000265205
NA	-0.0208	8.0480E-01		ENSG00000206734
NA	-0.0218	8.0454E-01		ENSG00000232028
NA	-0.0345	8.0435E-01	8.96E-01	ENSG00000254791
NA	-0.0150	8.0409E-01		ENSG00000236796
NA	-0.0116	8.0408E-01		ENSG00000284618
NA	-0.0121	8.0391E-01		ENSG00000279841
NA	-0.0434	8.0376E-01	8.96E-01	ENSG00000180389
NA	-0.0203	8.0374E-01	8.96E-01	ENSG00000165121
NA	-0.0182	8.0314E-01		ENSG00000272264
NA	-0.0028	8.0275E-01		ENSG00000213642
NA	-0.0202	8.0257E-01	8.95E-01	ENSG00000274492
NA	-0.0057	8.0252E-01		ENSG00000285454
NA	-0.0114	8.0234E-01		ENSG00000223822
NA	-0.0062	8.0213E-01		ENSG00000281133
NA	-0.0045	8.0210E-01		ENSG00000225359
NA	-0.0437	8.0177E-01	8.94E-01	ENSG00000248802
NA	-0.0119	8.0176E-01		ENSG00000255038
NA	-0.0219	8.0156E-01	8.94E-01	ENSG00000289037
NA	-0.0218	8.0148E-01		ENSG00000214855
NA	-0.0089	8.0146E-01		ENSG00000259609
NA	-0.0219	8.0128E-01	8.94E-01	ENSG00000287910
NA	-0.0265	8.0117E-01	8.94E-01	ENSG00000264164
NA	-0.0192	8.0112E-01	8.94E-01	ENSG00000213839
NA	-0.0084	8.0090E-01		ENSG00000225083
NA	-0.0141	8.0088E-01		ENSG00000243960

NA	-0.0065	8.0065E-01		ENSG00000283141
NA	-0.0097	8.0029E-01		ENSG00000228739
NA	-0.0028	8.0009E-01		ENSG00000285878
NA	-0.0088	8.0000E-01		ENSG00000215905
NA	-0.0419	7.9977E-01	8.93E-01	ENSG00000273328
NA	-0.0033	7.9975E-01		ENSG00000289974
NA	-0.0164	7.9974E-01	8.93E-01	ENSG00000269986
NA	-0.0126	7.9972E-01		ENSG00000230186
NA	-0.0301	7.9965E-01	8.93E-01	ENSG00000225082
NA	-0.0290	7.9965E-01		ENSG00000224415
NA	-0.0226	7.9949E-01		ENSG00000260252
NA	-0.0194	7.9946E-01	8.93E-01	ENSG00000276570
NA	-0.0235	7.9938E-01		ENSG00000277435
NA	-0.0140	7.9935E-01		ENSG00000282890
NA	-0.0205	7.9935E-01	8.93E-01	ENSG00000269397
NA	-0.0018	7.9926E-01		ENSG00000228036
NA	-0.0009	7.9915E-01		ENSG00000287850
NA	-0.0146	7.9914E-01		ENSG00000229057
NA	-0.0237	7.9908E-01	8.92E-01	ENSG00000180385
NA	-0.0214	7.9907E-01	8.92E-01	ENSG00000275601
NA	-0.0322	7.9888E-01	8.92E-01	ENSG00000285888
NA	-0.0400	7.9871E-01		ENSG00000259403
NA	-0.0348	7.9861E-01	8.92E-01	ENSG00000254352
NA	-0.0374	7.9858E-01	8.92E-01	ENSG00000253503
NA	-0.0180	7.9853E-01		ENSG00000213131
NA	-0.0189	7.9850E-01		ENSG00000243384
NA	-0.0154	7.9848E-01		ENSG00000281856
NA	-0.0461	7.9846E-01	8.92E-01	ENSG00000244371
NA	-0.0073	7.9836E-01		ENSG00000261102
NA	-0.0106	7.9835E-01		ENSG00000212961
NA	-0.0164	7.9829E-01		ENSG00000225297
NA	-0.0263	7.9823E-01		ENSG00000285870
NA	-0.0200	7.9820E-01	8.92E-01	ENSG00000263786
NA	-0.0121	7.9810E-01		ENSG00000256783
NA	-0.0096	7.9810E-01		ENSG00000234567
NA	-0.0014	7.9801E-01		ENSG00000225790
NA	-0.0075	7.9780E-01		ENSG00000260773
NA	-0.0020	7.9774E-01		ENSG00000196274
NA	-0.0047	7.9773E-01		ENSG00000225106
NA	-0.0262	7.9771E-01		ENSG00000289644
NA	-0.0233	7.9771E-01	8.92E-01	ENSG00000270441
NA	-0.0033	7.9763E-01	8.92E-01	ENSG00000230027
NA	-0.0122	7.9738E-01		ENSG00000220343
NA	-0.0056	7.9709E-01		ENSG00000204894
NA	-0.0286	7.9705E-01		ENSG00000274868
NA	-0.0286	7.9705E-01		ENSG00000275664
NA	-0.0173	7.9688E-01	8.91E-01	ENSG00000204801
NA	-0.0292	7.9669E-01		ENSG00000257322
NA	-0.0171	7.9634E-01		ENSG00000228485

NA	-0.0204	7.9628E-01	8.91E-01	ENSG00000256955
NA	-0.0167	7.9573E-01		ENSG00000259699
NA	-0.0158	7.9570E-01	8.90E-01	ENSG00000213740
NA	-0.0235	7.9567E-01		ENSG00000289067
NA	-0.0054	7.9561E-01		ENSG00000227212
NA	-0.0171	7.9538E-01	8.90E-01	ENSG00000234322
NA	-0.0051	7.9509E-01		ENSG00000288631
NA	-0.0066	7.9499E-01		ENSG00000253244
NA	-0.0417	7.9495E-01		ENSG00000290076
NA	-0.0328	7.9488E-01	8.90E-01	ENSG00000286619
NA	-0.0217	7.9463E-01	8.90E-01	ENSG00000272822
NA	-0.0100	7.9423E-01	8.89E-01	ENSG00000279208
NA	-0.0224	7.9411E-01		ENSG00000254686
NA	-0.0287	7.9410E-01		ENSG00000241641
NA	-0.0187	7.9398E-01	8.89E-01	ENSG00000225193
NA	-0.0251	7.9387E-01	8.89E-01	ENSG00000261248
NA	-0.0128	7.9347E-01		ENSG00000277458
NA	-0.0105	7.9344E-01		ENSG00000229928
NA	-0.0189	7.9334E-01		ENSG00000276631
NA	-0.0271	7.9329E-01	8.89E-01	ENSG00000259635
NA	-0.0041	7.9323E-01		ENSG00000225311
NA	-0.0196	7.9301E-01	8.89E-01	ENSG00000258568
NA	-0.0169	7.9300E-01	8.89E-01	ENSG00000289040
NA	-0.0224	7.9297E-01	8.89E-01	ENSG00000233108
NA	-0.0156	7.9275E-01		ENSG00000268222
NA	-0.0212	7.9273E-01		ENSG00000286778
NA	-0.0188	7.9268E-01	8.89E-01	ENSG00000267424
NA	-0.0245	7.9263E-01	8.89E-01	ENSG00000287419
NA	-0.0179	7.9262E-01	8.89E-01	ENSG00000174715
NA	-0.0330	7.9262E-01	8.89E-01	ENSG00000272768
NA	-0.0061	7.9231E-01		ENSG00000232777
NA	-0.0061	7.9231E-01		ENSG00000280346
NA	-0.0152	7.9225E-01		ENSG00000225900
NA	-0.0813	7.9224E-01	8.88E-01	ENSG00000273716
NA	-0.0124	7.9220E-01	8.88E-01	ENSG00000267215
NA	-0.0019	7.9216E-01		ENSG00000257195
NA	-0.0093	7.9213E-01		ENSG00000272711
NA	-0.0186	7.9205E-01		ENSG00000282041
NA	-0.0271	7.9205E-01		ENSG00000237065
NA	-0.0153	7.9202E-01		ENSG00000258232
NA	-0.0222	7.9202E-01	8.88E-01	ENSG00000273394
NA	-0.0330	7.9196E-01		ENSG00000260310
NA	-0.0084	7.9176E-01		ENSG00000282221
NA	-0.0093	7.9173E-01		ENSG00000241282
NA	-0.0119	7.9164E-01		ENSG00000285559
NA	-0.0168	7.9111E-01		ENSG00000241095
NA	-0.0062	7.9091E-01		ENSG00000273406
NA	-0.0338	7.9081E-01		ENSG00000183562
NA	-0.0790	7.9080E-01	8.88E-01	ENSG00000288992

NA	-0.0056	7.9079E-01		ENSG00000286100
NA	-0.0144	7.9043E-01		ENSG00000260194
NA	-0.0040	7.9034E-01		ENSG00000255790
NA	-0.0219	7.9021E-01	8.87E-01	ENSG00000223896
NA	-0.0236	7.9019E-01	8.87E-01	ENSG00000276337
NA	-0.0173	7.8996E-01		ENSG00000258048
NA	-0.0221	7.8946E-01	8.87E-01	ENSG00000283098
NA	-0.0247	7.8898E-01	8.87E-01	ENSG00000279089
NA	-0.0202	7.8877E-01	8.87E-01	ENSG00000249779
NA	-0.0392	7.8871E-01		ENSG00000272037
NA	-0.0087	7.8862E-01		ENSG00000279927
NA	-0.0117	7.8859E-01	8.87E-01	ENSG00000239791
NA	-0.0160	7.8848E-01	8.87E-01	ENSG00000258384
NA	-0.0103	7.8838E-01		ENSG00000213667
NA	-0.0011	7.8831E-01		ENSG00000225165
NA	-0.0025	7.8830E-01		ENSG00000282849
NA	-0.0132	7.8807E-01		ENSG00000257222
NA	-0.0146	7.8801E-01		ENSG00000223965
NA	-0.0154	7.8795E-01		ENSG00000285619
NA	-0.0190	7.8789E-01		ENSG00000274825
NA	-0.0170	7.8779E-01	8.86E-01	ENSG00000277423
NA	-0.0161	7.8760E-01	8.86E-01	ENSG00000230286
NA	-0.0089	7.8757E-01		ENSG00000232646
NA	-0.0173	7.8754E-01		ENSG00000272980
NA	0.0000	7.8750E-01		ENSG00000290016
NA	-0.0243	7.8744E-01	8.86E-01	ENSG00000267259
NA	-0.0262	7.8737E-01		ENSG00000272088
NA	-0.0155	7.8733E-01		ENSG00000271440
NA	-0.0229	7.8676E-01		ENSG00000221879
NA	-0.0493	7.8673E-01	8.85E-01	ENSG00000278487
NA	-0.0100	7.8672E-01		ENSG00000234919
NA	-0.0135	7.8660E-01		ENSG00000254290
NA	-0.0180	7.8659E-01		ENSG00000280181
NA	-0.0020	7.8659E-01		ENSG00000253836
NA	-0.0010	7.8632E-01		ENSG00000277250
NA	-0.0160	7.8623E-01		ENSG00000234338
NA	-0.0175	7.8621E-01	8.85E-01	ENSG00000260647
NA	-0.0648	7.8607E-01	8.85E-01	ENSG00000288785
NA	-0.0089	7.8584E-01		ENSG00000286720
NA	-0.0028	7.8571E-01		ENSG00000282836
NA	-0.0124	7.8571E-01		ENSG00000258179
NA	-0.0437	7.8568E-01	8.85E-01	ENSG00000262529
NA	-0.0387	7.8556E-01		ENSG00000235837
NA	-0.0126	7.8555E-01		ENSG00000286684
NA	-0.0072	7.8549E-01		ENSG00000229083
NA	-0.0567	7.8545E-01	8.85E-01	ENSG00000279946
NA	-0.0103	7.8532E-01		ENSG00000289914
NA	-0.0122	7.8512E-01		ENSG00000286459
NA	-0.0156	7.8501E-01	8.85E-01	ENSG00000235781

NA	-0.0113	7.8499E-01		ENSG00000278716
NA	-0.0005	7.8428E-01		ENSG00000223870
NA	-0.0183	7.8417E-01		ENSG00000231167
NA	-0.0257	7.8416E-01	8.84E-01	ENSG00000287710
NA	-0.0102	7.8414E-01		ENSG00000227359
NA	-0.0244	7.8407E-01	8.84E-01	ENSG00000254916
NA	-0.0049	7.8386E-01		ENSG00000231808
NA	-0.0043	7.8371E-01		ENSG00000216624
NA	-0.0218	7.8358E-01		ENSG00000263393
NA	-0.0089	7.8341E-01		ENSG00000279235
NA	-0.0201	7.8330E-01		ENSG00000231720
NA	-0.0220	7.8309E-01		ENSG00000269583
NA	-0.0195	7.8287E-01	8.83E-01	ENSG00000272842
NA	-0.0383	7.8272E-01	8.83E-01	ENSG00000261886
NA	-0.0215	7.8270E-01		ENSG00000250906
NA	-0.0002	7.8251E-01		ENSG00000287771
NA	-0.0078	7.8226E-01		ENSG00000285155
NA	-0.0083	7.8221E-01		ENSG00000269729
NA	-0.0012	7.8193E-01		ENSG00000254631
NA	-0.0360	7.8186E-01		ENSG00000230295
NA	-0.0191	7.8160E-01	8.82E-01	ENSG00000287929
NA	-0.0177	7.8148E-01		ENSG00000259931
NA	-0.0157	7.8126E-01		ENSG00000253981
NA	-0.0259	7.8121E-01	8.82E-01	ENSG00000269600
NA	-0.0060	7.8079E-01		ENSG00000213244
NA	-0.0029	7.8072E-01		ENSG00000260198
NA	-0.0012	7.8072E-01		ENSG00000257458
NA	-0.0188	7.8055E-01		ENSG00000286627
NA	-0.0215	7.8036E-01	8.81E-01	ENSG00000224321
NA	-0.0062	7.8035E-01		ENSG00000263321
NA	-0.0068	7.8032E-01		ENSG00000256973
NA	-0.0014	7.8011E-01		ENSG00000237158
NA	-0.0001	7.8002E-01		ENSG00000288648
NA	-0.0028	7.7983E-01		ENSG00000289560
NA	-0.0075	7.7955E-01		ENSG00000287932
NA	-0.0094	7.7953E-01		ENSG00000270983
NA	-0.0049	7.7928E-01		ENSG00000231612
NA	-0.0133	7.7911E-01		ENSG00000277351
NA	-0.0221	7.7891E-01	8.80E-01	ENSG00000280388
NA	-0.0249	7.7864E-01	8.80E-01	ENSG00000186481
NA	-0.0030	7.7852E-01		ENSG00000236073
NA	-0.0190	7.7819E-01	8.80E-01	ENSG00000267397
NA	-0.0191	7.7761E-01		ENSG00000226194
NA	-0.0732	7.7725E-01	8.80E-01	ENSG00000230199
NA	-0.0103	7.7724E-01		ENSG00000288964
NA	-0.0008	7.7716E-01		ENSG00000201034
NA	-0.0052	7.7665E-01	8.79E-01	ENSG00000264116
NA	-0.0254	7.7647E-01	8.79E-01	ENSG00000238278
NA	-0.0223	7.7645E-01	8.79E-01	ENSG00000226055

NA	-0.0140	7.7629E-01		ENSG00000287089
NA	-0.0247	7.7608E-01		ENSG00000266368
NA	-0.0152	7.7607E-01	8.79E-01	ENSG00000223576
NA	-0.0265	7.7587E-01		ENSG00000270140
NA	-0.0265	7.7570E-01	8.79E-01	ENSG00000231993
NA	-0.0241	7.7559E-01	8.79E-01	ENSG00000272973
NA	-0.0232	7.7553E-01	8.79E-01	ENSG00000236814
NA	-0.0189	7.7525E-01	8.78E-01	ENSG00000280214
NA	-0.0044	7.7522E-01		ENSG00000285030
NA	-0.0255	7.7508E-01	8.78E-01	ENSG00000272663
NA	-0.0405	7.7503E-01		ENSG00000225693
NA	-0.0301	7.7494E-01		ENSG00000225787
NA	-0.0056	7.7441E-01		ENSG00000253500
NA	-0.0084	7.7424E-01		ENSG00000220377
NA	-0.0294	7.7422E-01		ENSG00000258788
NA	-0.0023	7.7418E-01		ENSG00000287758
NA	-0.0114	7.7380E-01		ENSG00000271993
NA	-0.0354	7.7376E-01	8.78E-01	ENSG00000286293
NA	-0.0047	7.7371E-01		ENSG00000285931
NA	-0.0028	7.7362E-01		ENSG00000201343
NA	-0.0106	7.7338E-01		ENSG00000287274
NA	-0.0087	7.7331E-01		ENSG00000228408
NA	-0.0276	7.7311E-01		ENSG00000284697
NA	-0.0189	7.7305E-01	8.77E-01	ENSG00000287078
NA	-0.0116	7.7300E-01		ENSG00000233894
NA	-0.0096	7.7291E-01		ENSG00000270815
NA	-0.0179	7.7290E-01		ENSG00000231830
NA	-0.0021	7.7286E-01		ENSG00000277199
NA	-0.0118	7.7249E-01		ENSG00000201208
NA	-0.0276	7.7230E-01	8.77E-01	ENSG00000273271
NA	-0.0080	7.7202E-01		ENSG00000255680
NA	-0.0065	7.7199E-01		ENSG00000286633
NA	-0.0276	7.7190E-01	8.76E-01	ENSG00000262801
NA	-0.0227	7.7173E-01	8.76E-01	ENSG00000236562
NA	-0.0046	7.7149E-01		ENSG00000289166
NA	-0.0256	7.7136E-01		ENSG00000273711
NA	-0.0125	7.7135E-01		ENSG00000257643
NA	-0.0022	7.7125E-01		ENSG00000234182
NA	-0.0168	7.7109E-01	8.76E-01	ENSG00000224810
NA	-0.0083	7.7096E-01		ENSG00000277795
NA	-0.0205	7.7081E-01		ENSG00000265394
NA	-0.0183	7.7064E-01	8.76E-01	ENSG00000159904
NA	-0.0264	7.6987E-01		ENSG00000225063
NA	-0.0096	7.6971E-01		ENSG00000263916
NA	-0.0282	7.6969E-01		ENSG00000268743
NA	-0.0188	7.6966E-01		ENSG00000214702
NA	-0.0002	7.6959E-01		ENSG00000203411
NA	-0.0041	7.6946E-01		ENSG00000284540
NA	-0.0154	7.6937E-01		ENSG00000233068

NA	-0.0100	7.6935E-01		ENSG00000228384
NA	-0.0164	7.6921E-01		ENSG00000287109
NA	-0.0221	7.6892E-01	8.75E-01	ENSG00000264270
NA	-0.0073	7.6875E-01		ENSG00000288946
NA	-0.0213	7.6871E-01	8.74E-01	ENSG00000215859
NA	-0.0194	7.6868E-01		ENSG00000233635
NA	-0.0258	7.6845E-01		ENSG00000284748
NA	-0.0252	7.6822E-01	8.74E-01	ENSG00000215414
NA	-0.0053	7.6810E-01		ENSG00000237481
NA	-0.0248	7.6780E-01	8.74E-01	ENSG00000260711
NA	-0.0141	7.6773E-01		ENSG00000231827
NA	-0.0355	7.6764E-01	8.74E-01	ENSG00000235297
NA	-0.0248	7.6746E-01	8.74E-01	ENSG00000269837
NA	-0.0251	7.6743E-01	8.74E-01	ENSG00000262766
NA	-0.0093	7.6726E-01		ENSG00000225505
NA	-0.0182	7.6725E-01		ENSG00000276988
NA	-0.0094	7.6722E-01		ENSG00000236098
NA	-0.0054	7.6712E-01		ENSG00000259581
NA	-0.0037	7.6699E-01		ENSG00000289889
NA	-0.0034	7.6692E-01		ENSG00000278464
NA	-0.0141	7.6675E-01		ENSG00000280134
NA	-0.0241	7.6657E-01	8.73E-01	ENSG00000289581
NA	-0.0222	7.6615E-01	8.73E-01	ENSG00000234589
NA	-0.0180	7.6610E-01	8.73E-01	ENSG00000267637
NA	-0.0235	7.6609E-01	8.73E-01	ENSG00000180581
NA	-0.0270	7.6604E-01	8.73E-01	ENSG00000260908
NA	-0.0082	7.6595E-01		ENSG00000258676
NA	-0.0117	7.6526E-01		ENSG00000287503
NA	-0.0203	7.6511E-01		ENSG00000237170
NA	-0.0174	7.6485E-01		ENSG00000266076
NA	-0.0065	7.6478E-01	8.72E-01	ENSG00000259709
NA	-0.0179	7.6449E-01	8.72E-01	ENSG00000278330
NA	-0.0185	7.6446E-01		ENSG00000270808
NA	-0.0170	7.6441E-01		ENSG00000213608
NA	-0.0151	7.6439E-01		ENSG00000236797
NA	-0.0135	7.6409E-01		ENSG00000275484
NA	-0.0239	7.6402E-01	8.72E-01	ENSG00000260973
NA	-0.0471	7.6400E-01	8.72E-01	ENSG00000259221
NA	-0.0225	7.6399E-01	8.72E-01	ENSG00000254687
NA	-0.0090	7.6379E-01		ENSG00000261199
NA	-0.0309	7.6373E-01		ENSG00000205414
NA	-0.0037	7.6357E-01		ENSG00000224442
NA	-0.0229	7.6354E-01		ENSG00000274124
NA	-0.0148	7.6326E-01		ENSG00000222515
NA	-0.0152	7.6325E-01		ENSG00000279006
NA	-0.0130	7.6292E-01		ENSG00000233044
NA	-0.0345	7.6254E-01		ENSG00000286999
NA	-0.0086	7.6244E-01		ENSG00000224427
NA	-0.0126	7.6217E-01		ENSG00000234083

NA	-0.0172	7.6204E-01		ENSG00000224988
NA	-0.0073	7.6197E-01		ENSG00000217824
NA	-0.0136	7.6194E-01		ENSG00000226056
NA	-0.0224	7.6153E-01	8.70E-01	ENSG00000288792
NA	-0.0103	7.6150E-01		ENSG00000218350
NA	-0.0236	7.6142E-01		ENSG00000279121
NA	-0.0123	7.6134E-01		ENSG00000287220
NA	-0.0108	7.6119E-01		ENSG00000258469
NA	-0.0102	7.6119E-01		ENSG00000279743
NA	-0.0138	7.6087E-01		ENSG00000270190
NA	-0.0099	7.6084E-01	8.70E-01	ENSG00000227663
NA	-0.0018	7.6083E-01		ENSG00000289895
NA	-0.0347	7.6077E-01	8.70E-01	ENSG00000272588
NA	-0.0105	7.6068E-01		ENSG00000273474
NA	-0.0124	7.6057E-01		ENSG00000251206
NA	-0.0223	7.6047E-01	8.70E-01	ENSG00000267898
NA	-0.0344	7.6046E-01	8.70E-01	ENSG00000289367
NA	-0.0808	7.6018E-01	8.70E-01	ENSG00000224746
NA	-0.0123	7.6008E-01		ENSG00000266944
NA	-0.0074	7.5980E-01		ENSG00000233733
NA	-0.0011	7.5975E-01		ENSG00000265964
NA	-0.0103	7.5951E-01		ENSG00000279100
NA	-0.0279	7.5935E-01		ENSG00000285908
NA	-0.0204	7.5902E-01	8.69E-01	ENSG00000279605
NA	-0.0120	7.5869E-01		ENSG00000228157
NA	-0.0193	7.5854E-01	8.69E-01	ENSG00000259364
NA	-0.0031	7.5827E-01		ENSG00000261816
NA	-0.0287	7.5817E-01	8.69E-01	ENSG00000250415
NA	-0.0529	7.5806E-01		ENSG00000229930
NA	-0.0197	7.5802E-01		ENSG00000226942
NA	-0.0210	7.5801E-01		ENSG00000231393
NA	-0.0271	7.5798E-01	8.69E-01	ENSG00000285693
NA	-0.0081	7.5793E-01		ENSG00000263219
NA	-0.0269	7.5789E-01	8.68E-01	ENSG00000286830
NA	-0.0220	7.5781E-01	8.68E-01	ENSG00000278238
NA	-0.0345	7.5779E-01	8.68E-01	ENSG00000228218
NA	-0.0078	7.5757E-01		ENSG00000251003
NA	-0.0244	7.5743E-01	8.68E-01	ENSG00000230606
NA	-0.0159	7.5725E-01		ENSG00000230946
NA	-0.0131	7.5711E-01		ENSG00000234466
NA	-0.0374	7.5687E-01	8.68E-01	ENSG00000289472
NA	-0.0103	7.5675E-01		ENSG00000289229
NA	-0.0246	7.5666E-01	8.68E-01	ENSG00000236213
NA	-0.0040	7.5665E-01		ENSG00000275201
NA	-0.0615	7.5657E-01	8.68E-01	ENSG00000226396
NA	-0.0120	7.5627E-01		ENSG00000280173
NA	-0.0143	7.5586E-01		ENSG00000287236
NA	-0.0674	7.5534E-01	8.67E-01	ENSG00000252690
NA	-0.0142	7.5533E-01		ENSG00000288779

NA	-0.0134	7.5522E-01		ENSG00000198019
NA	-0.0367	7.5520E-01	8.67E-01	ENSG00000279294
NA	-0.0146	7.5519E-01		ENSG00000223356
NA	-0.0266	7.5513E-01	8.67E-01	ENSG00000260816
NA	-0.0152	7.5507E-01		ENSG00000231296
NA	-0.0206	7.5500E-01		ENSG00000285895
NA	-0.0180	7.5478E-01		ENSG00000249240
NA	-0.0558	7.5459E-01	8.66E-01	ENSG00000286742
NA	-0.0094	7.5441E-01		ENSG00000250762
NA	-0.0418	7.5407E-01		ENSG00000271936
NA	-0.0168	7.5400E-01		ENSG00000266598
NA	-0.0126	7.5396E-01		ENSG00000287334
NA	-0.0166	7.5392E-01		ENSG00000287141
NA	-0.0021	7.5376E-01		ENSG00000206914
NA	-0.0088	7.5361E-01		ENSG00000214973
NA	-0.0261	7.5346E-01		ENSG00000228974
NA	-0.0002	7.5316E-01		ENSG00000254205
NA	-0.0159	7.5312E-01		ENSG00000260377
NA	-0.0375	7.5305E-01		ENSG00000270367
NA	-0.0456	7.5305E-01		ENSG00000237194
NA	-0.0263	7.5289E-01		ENSG00000231977
NA	-0.0122	7.5288E-01		ENSG00000215198
NA	-0.0902	7.5283E-01	8.65E-01	ENSG00000231201
NA	-0.0156	7.5192E-01		ENSG00000274370
NA	-0.0101	7.5182E-01		ENSG00000233038
NA	-0.0232	7.5178E-01	8.64E-01	ENSG00000253744
NA	-0.0283	7.5174E-01	8.64E-01	ENSG00000289507
NA	-0.0036	7.5122E-01		ENSG00000236426
NA	-0.0093	7.5089E-01		ENSG00000272161
NA	-0.0169	7.5089E-01		ENSG00000259632
NA	-0.0214	7.5068E-01		ENSG00000259006
NA	-0.0603	7.5037E-01	8.63E-01	ENSG00000279148
NA	-0.0238	7.5013E-01		ENSG00000225974
NA	-0.0165	7.5013E-01		ENSG00000225170
NA	-0.0005	7.5004E-01	8.63E-01	ENSG00000248968
NA	-0.0527	7.4990E-01		ENSG00000253390
NA	-0.0306	7.4976E-01	8.63E-01	ENSG00000260464
NA	-0.0267	7.4973E-01	8.63E-01	ENSG00000253683
NA	-0.0170	7.4953E-01		ENSG00000285530
NA	-0.0303	7.4940E-01		ENSG00000230433
NA	-0.0039	7.4931E-01		ENSG00000268938
NA	-0.0190	7.4913E-01	8.63E-01	ENSG00000202514
NA	-0.0120	7.4904E-01		ENSG00000257175
NA	-0.0196	7.4891E-01		ENSG00000213478
NA	-0.0205	7.4883E-01		ENSG00000237169
NA	-0.0294	7.4871E-01	8.63E-01	ENSG00000288692
NA	-0.0146	7.4869E-01		ENSG00000237387
NA	-0.0303	7.4823E-01		ENSG00000214195
NA	-0.0088	7.4817E-01	8.62E-01	ENSG00000275426

NA	-0.0101	7.4808E-01		ENSG00000255872
NA	-0.0001	7.4786E-01		ENSG00000273450
NA	-0.0056	7.4773E-01		ENSG00000273367
NA	-0.0252	7.4762E-01		ENSG00000260331
NA	-0.0178	7.4753E-01		ENSG00000260282
NA	-0.0170	7.4729E-01		ENSG00000244510
NA	-0.0113	7.4716E-01		ENSG00000279373
NA	-0.0187	7.4712E-01		ENSG00000266601
NA	-0.0070	7.4698E-01		ENSG00000279300
NA	-0.0276	7.4662E-01	8.61E-01	ENSG00000272345
NA	-0.0103	7.4657E-01		ENSG00000225331
NA	-0.0236	7.4584E-01	8.61E-01	ENSG00000239524
NA	-0.0113	7.4580E-01		ENSG00000230911
NA	-0.0259	7.4538E-01	8.61E-01	ENSG00000226149
NA	-0.0141	7.4536E-01		ENSG00000182257
NA	-0.0292	7.4528E-01		ENSG00000254847
NA	-0.0104	7.4527E-01		ENSG00000229498
NA	-0.0201	7.4488E-01		ENSG00000258465
NA	-0.0301	7.4487E-01	8.60E-01	ENSG00000269001
NA	-0.0106	7.4471E-01		ENSG00000262732
NA	-0.0018	7.4451E-01		ENSG00000172799
NA	-0.0259	7.4406E-01		ENSG00000258712
NA	-0.0120	7.4373E-01		ENSG00000286227
NA	-0.0154	7.4371E-01		ENSG00000289026
NA	-0.0002	7.4364E-01		ENSG00000234091
NA	-0.0246	7.4363E-01	8.59E-01	ENSG00000270165
NA	-0.0607	7.4345E-01	8.59E-01	ENSG00000287726
NA	-0.0085	7.4338E-01		ENSG00000286869
NA	-0.0188	7.4335E-01		ENSG00000231086
NA	-0.0100	7.4331E-01		ENSG00000240093
NA	-0.0157	7.4325E-01		ENSG00000233427
NA	-0.0139	7.4323E-01		ENSG00000279221
NA	-0.0111	7.4310E-01		ENSG00000258943
NA	-0.0221	7.4296E-01		ENSG00000249776
NA	-0.0349	7.4294E-01	8.59E-01	ENSG00000287506
NA	-0.0069	7.4293E-01		ENSG00000284808
NA	-0.0024	7.4283E-01		ENSG00000285684
NA	-0.0105	7.4274E-01		ENSG00000235772
NA	-0.0015	7.4272E-01		ENSG00000250603
NA	-0.0263	7.4262E-01	8.59E-01	ENSG00000214881
NA	-0.0019	7.4233E-01		ENSG00000257680
NA	-0.0288	7.4228E-01	8.59E-01	ENSG00000277701
NA	-0.0087	7.4228E-01		ENSG00000255446
NA	-0.0096	7.4209E-01		ENSG00000232136
NA	-0.0235	7.4185E-01		ENSG00000289131
NA	-0.0686	7.4143E-01	8.58E-01	ENSG00000277561
NA	-0.0233	7.4135E-01		ENSG00000253693
NA	0.0000	7.4115E-01		ENSG00000216867
NA	-0.0113	7.4112E-01		ENSG00000232208

NA	-0.0286	7.4110E-01	8.58E-01	ENSG00000279796
NA	-0.0217	7.4107E-01		ENSG00000285882
NA	-0.0290	7.4104E-01	8.58E-01	ENSG00000288156
NA	-0.0317	7.4084E-01	8.57E-01	ENSG00000289745
NA	-0.0176	7.4080E-01		ENSG00000278182
NA	-0.0152	7.4061E-01		ENSG00000231967
NA	-0.0257	7.4059E-01	8.57E-01	ENSG00000277968
NA	-0.0283	7.4046E-01	8.57E-01	ENSG00000289871
NA	-0.0525	7.4026E-01	8.57E-01	ENSG00000267231
NA	-0.0250	7.3970E-01	8.57E-01	ENSG00000254484
NA	-0.0305	7.3964E-01	8.57E-01	ENSG00000255384
NA	-0.0073	7.3924E-01		ENSG00000257067
NA	-0.0035	7.3917E-01		ENSG00000268458
NA	-0.0024	7.3914E-01		ENSG00000260594
NA	-0.0082	7.3899E-01		ENSG00000272660
NA	-0.0101	7.3897E-01		ENSG00000260015
NA	-0.0349	7.3892E-01	8.57E-01	ENSG00000273447
NA	-0.0093	7.3882E-01		ENSG00000289937
NA	-0.0048	7.3878E-01		ENSG00000269940
NA	-0.0224	7.3871E-01		ENSG00000289243
NA	-0.0313	7.3829E-01	8.56E-01	ENSG00000264589
NA	-0.0130	7.3827E-01		ENSG00000252965
NA	-0.0499	7.3821E-01	8.56E-01	ENSG00000226009
NA	-0.0369	7.3807E-01		ENSG00000256500
NA	-0.0149	7.3795E-01		ENSG00000274284
NA	-0.0149	7.3795E-01		ENSG00000265889
NA	-0.0316	7.3785E-01	8.56E-01	ENSG00000228150
NA	-0.0282	7.3783E-01	8.56E-01	ENSG00000244604
NA	-0.0330	7.3771E-01		ENSG00000278254
NA	-0.0147	7.3756E-01		ENSG00000252254
NA	-0.0086	7.3754E-01		ENSG00000268949
NA	-0.0387	7.3754E-01		ENSG00000237842
NA	-0.0137	7.3751E-01		ENSG00000255327
NA	-0.0275	7.3748E-01		ENSG00000234232
NA	-0.0396	7.3704E-01	8.56E-01	ENSG00000262410
NA	-0.0074	7.3704E-01		ENSG00000252874
NA	-0.0230	7.3683E-01	8.56E-01	ENSG00000217027
NA	-0.0155	7.3682E-01		ENSG00000204990
NA	-0.0040	7.3678E-01	8.56E-01	ENSG00000222024
NA	-0.0064	7.3672E-01		ENSG00000279554
NA	-0.0228	7.3611E-01	8.55E-01	ENSG00000257449
NA	-0.0185	7.3592E-01	8.55E-01	ENSG00000243404
NA	-0.0076	7.3586E-01		ENSG00000214265
NA	-0.0389	7.3568E-01	8.55E-01	ENSG00000271730
NA	-0.0447	7.3560E-01		ENSG00000251189
NA	-0.0049	7.3556E-01		ENSG00000288985
NA	-0.0047	7.3549E-01		ENSG00000287997
NA	-0.0045	7.3548E-01		ENSG00000288984
NA	-0.0201	7.3534E-01	8.55E-01	ENSG00000260750

NA	-0.0252	7.3531E-01	8.55E-01	ENSG00000289550
NA	-0.0297	7.3528E-01	8.55E-01	ENSG00000267714
NA	-0.0094	7.3513E-01		ENSG00000280392
NA	-0.0083	7.3492E-01		ENSG00000227242
NA	-0.0237	7.3483E-01	8.54E-01	ENSG00000286928
NA	-0.0203	7.3470E-01		ENSG00000272576
NA	-0.0337	7.3453E-01	8.54E-01	ENSG00000287868
NA	-0.0346	7.3449E-01	8.54E-01	ENSG00000287643
NA	-0.0081	7.3445E-01		ENSG00000249755
NA	-0.0217	7.3433E-01		ENSG00000271989
NA	-0.0265	7.3392E-01		ENSG00000287551
NA	-0.0381	7.3390E-01	8.54E-01	ENSG00000260874
NA	-0.0127	7.3353E-01		ENSG00000232793
NA	-0.0157	7.3339E-01		ENSG00000255119
NA	-0.0237	7.3327E-01	8.53E-01	ENSG00000276251
NA	-0.0013	7.3326E-01		ENSG00000249426
NA	-0.0226	7.3320E-01	8.53E-01	ENSG00000244730
NA	-0.0371	7.3256E-01		ENSG00000215589
NA	-0.0186	7.3240E-01		ENSG00000279803
NA	-0.0219	7.3233E-01		ENSG00000248927
NA	-0.0015	7.3189E-01		ENSG00000241143
NA	-0.0398	7.3179E-01		ENSG00000286819
NA	-0.0288	7.3154E-01		ENSG00000285642
NA	-0.0072	7.3144E-01		ENSG00000288812
NA	-0.0003	7.3141E-01	8.52E-01	ENSG00000249116
NA	-0.0328	7.3140E-01	8.52E-01	ENSG00000288953
NA	-0.0293	7.3133E-01		ENSG00000255308
NA	-0.0041	7.3121E-01		ENSG00000226774
NA	-0.0229	7.3113E-01	8.52E-01	ENSG00000287249
NA	-0.0075	7.3061E-01		ENSG00000229557
NA	-0.0229	7.3037E-01		ENSG00000229087
NA	-0.0225	7.3005E-01	8.51E-01	ENSG00000264876
NA	-0.0168	7.3001E-01		ENSG00000273792
NA	-0.0083	7.2988E-01		ENSG00000255057
NA	-0.0309	7.2980E-01	8.51E-01	ENSG00000218175
NA	-0.0154	7.2973E-01		ENSG00000197665
NA	-0.0320	7.2965E-01	8.51E-01	ENSG00000260232
NA	-0.0231	7.2961E-01		ENSG00000229750
NA	-0.0249	7.2949E-01	8.51E-01	ENSG00000229539
NA	-0.0072	7.2944E-01		ENSG00000262681
NA	-0.0273	7.2937E-01		ENSG00000232882
NA	-0.1208	7.2896E-01	8.51E-01	ENSG00000241838
NA	-0.0119	7.2871E-01		ENSG00000237861
NA	-0.0324	7.2861E-01	8.51E-01	ENSG00000277235
NA	-0.0180	7.2832E-01		ENSG00000266389
NA	-0.0197	7.2831E-01		ENSG00000261481
NA	-0.0191	7.2802E-01	8.50E-01	ENSG00000227201
NA	-0.0159	7.2793E-01		ENSG00000247853
NA	-0.0162	7.2790E-01		ENSG00000257048

NA	-0.0003	7.2781E-01	8.50E-01	ENSG00000224658
NA	-0.0146	7.2767E-01		ENSG00000286048
NA	-0.0017	7.2732E-01		ENSG00000144785
NA	-0.0245	7.2729E-01	8.50E-01	ENSG00000277020
NA	-0.0241	7.2727E-01		ENSG00000255311
NA	-0.0044	7.2696E-01		ENSG00000286281
NA	-0.0323	7.2692E-01	8.49E-01	ENSG00000269889
NA	-0.0047	7.2666E-01		ENSG00000232787
NA	-0.0101	7.2648E-01		ENSG00000273904
NA	-0.0201	7.2629E-01		ENSG00000263220
NA	-0.0198	7.2605E-01		ENSG00000267533
NA	-0.0565	7.2598E-01	8.49E-01	ENSG00000258837
NA	-0.0130	7.2587E-01		ENSG00000231983
NA	-0.0170	7.2549E-01		ENSG00000275017
NA	-0.0505	7.2534E-01	8.48E-01	ENSG00000231169
NA	-0.0312	7.2525E-01	8.48E-01	ENSG00000279865
NA	-0.0295	7.2466E-01	8.48E-01	ENSG00000278986
NA	-0.0033	7.2450E-01		ENSG00000272321
NA	-0.0215	7.2423E-01		ENSG00000279469
NA	-0.0228	7.2417E-01	8.47E-01	ENSG00000276291
NA	-0.0268	7.2390E-01	8.47E-01	ENSG00000275202
NA	-0.0338	7.2382E-01	8.47E-01	ENSG00000264148
NA	-0.0281	7.2381E-01		ENSG00000218757
NA	-0.0324	7.2375E-01	8.47E-01	ENSG00000287110
NA	-0.0178	7.2373E-01		ENSG00000256361
NA	-0.0328	7.2368E-01	8.47E-01	ENSG00000225489
NA	-0.0191	7.2341E-01		ENSG00000263952
NA	-0.0258	7.2340E-01	8.47E-01	ENSG00000261766
NA	-0.0128	7.2335E-01		ENSG00000281365
NA	-0.0226	7.2332E-01		ENSG00000278543
NA	-0.0032	7.2315E-01		ENSG00000236472
NA	-0.0012	7.2314E-01		ENSG00000232667
NA	-0.0429	7.2304E-01	8.47E-01	ENSG00000254561
NA	-0.0329	7.2266E-01	8.47E-01	ENSG00000267787
NA	-0.0585	7.2255E-01		ENSG00000287688
NA	-0.0345	7.2221E-01		ENSG00000279344
NA	-0.0226	7.2211E-01	8.46E-01	ENSG00000289528
NA	-0.0027	7.2139E-01		ENSG00000213409
NA	-0.0298	7.2137E-01	8.46E-01	ENSG00000228938
NA	-0.0301	7.2105E-01	8.45E-01	ENSG00000286895
NA	-0.0107	7.2091E-01		ENSG00000236908
NA	-0.0215	7.2020E-01		ENSG00000223779
NA	-0.0247	7.2008E-01		ENSG00000250472
NA	-0.0218	7.1993E-01		ENSG00000234702
NA	-0.0286	7.1990E-01	8.45E-01	ENSG00000272461
NA	-0.0296	7.1989E-01	8.45E-01	ENSG00000234181
NA	-0.0204	7.1980E-01		ENSG00000251567
NA	-0.0241	7.1967E-01	8.44E-01	ENSG00000231231
NA	-0.0089	7.1963E-01		ENSG00000226340

NA	-0.0120	7.1948E-01		ENSG00000228120
NA	-0.0278	7.1932E-01		ENSG00000289049
NA	-0.0331	7.1920E-01		ENSG00000275989
NA	-0.0274	7.1913E-01	8.44E-01	ENSG00000286509
NA	-0.0160	7.1909E-01		ENSG00000254306
NA	-0.0345	7.1895E-01	8.44E-01	ENSG00000284657
NA	-0.0085	7.1881E-01		ENSG00000254579
NA	-0.0197	7.1861E-01	8.44E-01	ENSG00000269836
NA	-0.0141	7.1858E-01		ENSG00000288569
NA	-0.0719	7.1846E-01	8.44E-01	ENSG00000267927
NA	-0.0218	7.1845E-01	8.44E-01	ENSG00000225230
NA	-0.0229	7.1832E-01	8.44E-01	ENSG00000288536
NA	-0.0322	7.1828E-01	8.44E-01	ENSG00000283045
NA	-0.0449	7.1816E-01		ENSG00000276488
NA	-0.0043	7.1767E-01		ENSG00000286534
NA	-0.0042	7.1766E-01		ENSG00000213587
NA	-0.0151	7.1759E-01		ENSG00000278601
NA	-0.0069	7.1750E-01		ENSG00000254048
NA	-0.0045	7.1749E-01		ENSG00000240298
NA	-0.0358	7.1739E-01		ENSG00000260971
NA	-0.0318	7.1721E-01	8.43E-01	ENSG00000286195
NA	-0.0252	7.1700E-01	8.43E-01	ENSG00000229873
NA	-0.0189	7.1688E-01		ENSG00000286888
NA	-0.0227	7.1682E-01	8.43E-01	ENSG00000256944
NA	-0.0004	7.1648E-01	8.42E-01	ENSG00000239705
NA	-0.0343	7.1647E-01	8.42E-01	ENSG00000234206
NA	-0.0002	7.1629E-01		ENSG00000229700
NA	-0.0150	7.1616E-01		ENSG00000250614
NA	-0.0111	7.1616E-01		ENSG00000275485
NA	-0.0023	7.1602E-01		ENSG00000239674
NA	-0.0194	7.1589E-01		ENSG00000257829
NA	-0.0905	7.1588E-01	8.42E-01	ENSG00000172186
NA	-0.0105	7.1584E-01		ENSG00000232736
NA	-0.0108	7.1568E-01	8.42E-01	ENSG00000256064
NA	-0.0300	7.1562E-01	8.42E-01	ENSG00000278963
NA	-0.0117	7.1556E-01		ENSG00000280025
NA	-0.0010	7.1524E-01	8.41E-01	ENSG00000285755
NA	-0.0247	7.1497E-01	8.41E-01	ENSG00000272202
NA	-0.0194	7.1482E-01		ENSG00000286309
NA	-0.0045	7.1482E-01		ENSG00000256343
NA	-0.0054	7.1456E-01		ENSG00000251309
NA	-0.0294	7.1449E-01	8.41E-01	ENSG00000144158
NA	-0.0082	7.1439E-01		ENSG00000269161
NA	-0.0349	7.1427E-01	8.41E-01	ENSG00000235254
NA	-0.0111	7.1423E-01		ENSG00000255364
NA	-0.0311	7.1393E-01		ENSG00000219487
NA	-0.0311	7.1391E-01		ENSG00000199530
NA	-0.0062	7.1387E-01		ENSG00000286256
NA	-0.0421	7.1382E-01		ENSG00000280053

NA	-0.0117	7.1382E-01		ENSG00000284726
NA	-0.0079	7.1362E-01		ENSG00000267642
NA	-0.0351	7.1359E-01	8.40E-01	ENSG00000248503
NA	-0.0300	7.1329E-01	8.40E-01	ENSG00000258875
NA	-0.0039	7.1314E-01	8.40E-01	ENSG00000262881
NA	-0.0028	7.1303E-01	8.40E-01	ENSG00000235526
NA	-0.0323	7.1300E-01	8.40E-01	ENSG00000253394
NA	-0.0684	7.1297E-01	8.40E-01	ENSG00000267529
NA	-0.0071	7.1269E-01		ENSG00000274654
NA	-0.0096	7.1230E-01		ENSG00000263893
NA	-0.0126	7.1226E-01		ENSG00000269091
NA	-0.0236	7.1212E-01	8.40E-01	ENSG00000289852
NA	-0.0149	7.1179E-01		ENSG00000232097
NA	-0.0343	7.1054E-01	8.39E-01	ENSG00000257179
NA	-0.0215	7.1049E-01		ENSG00000224825
NA	-0.0345	7.1012E-01	8.38E-01	ENSG00000251348
NA	-0.0286	7.1008E-01		ENSG00000258045
NA	-0.0034	7.1005E-01		ENSG00000272666
NA	-0.0206	7.1002E-01		ENSG00000259052
NA	-0.0250	7.0990E-01	8.38E-01	ENSG00000240163
NA	-0.0265	7.0966E-01		ENSG00000256356
NA	-0.0266	7.0957E-01		ENSG00000256381
NA	-0.0157	7.0951E-01		ENSG00000279464
NA	-0.0093	7.0913E-01		ENSG00000287169
NA	-0.0240	7.0881E-01	8.37E-01	ENSG00000270184
NA	-0.0051	7.0874E-01		ENSG00000263017
NA	-0.0187	7.0851E-01		ENSG00000254099
NA	-0.0148	7.0845E-01		ENSG00000268931
NA	-0.0028	7.0817E-01		ENSG00000260385
NA	-0.0182	7.0815E-01		ENSG00000289095
NA	-0.0278	7.0814E-01		ENSG00000261067
NA	-0.0083	7.0794E-01		ENSG00000244642
NA	-0.0286	7.0784E-01	8.37E-01	ENSG00000289396
NA	-0.0039	7.0753E-01		ENSG00000218672
NA	-0.0133	7.0749E-01		ENSG00000222044
NA	-0.0310	7.0745E-01	8.36E-01	ENSG00000272764
NA	-0.0346	7.0716E-01	8.36E-01	ENSG00000270344
NA	-0.0160	7.0713E-01		ENSG00000232529
NA	-0.0325	7.0687E-01		ENSG00000280118
NA	-0.0355	7.0680E-01	8.36E-01	ENSG00000231441
NA	-0.0083	7.0668E-01		ENSG00000278927
NA	-0.0325	7.0665E-01	8.36E-01	ENSG00000287226
NA	-0.0354	7.0653E-01	8.36E-01	ENSG00000233854
NA	-0.0100	7.0652E-01		ENSG00000213755
NA	-0.0112	7.0646E-01		ENSG00000251194
NA	-0.0266	7.0646E-01		ENSG00000223944
NA	-0.0095	7.0641E-01		ENSG00000248996
NA	-0.0127	7.0606E-01		ENSG00000272506
NA	-0.0172	7.0599E-01		ENSG00000247381

NA	-0.0157	7.0568E-01		ENSG00000270574
NA	-0.0106	7.0566E-01		ENSG00000259649
NA	-0.0024	7.0557E-01		ENSG00000253355
NA	-0.0092	7.0502E-01		ENSG00000236165
NA	-0.0005	7.0454E-01		ENSG00000266963
NA	-0.0281	7.0448E-01	8.35E-01	ENSG00000184188
NA	-0.0374	7.0443E-01	8.35E-01	ENSG00000213857
NA	-0.0228	7.0412E-01	8.34E-01	ENSG00000277095
NA	-0.0331	7.0402E-01	8.34E-01	ENSG00000160172
NA	-0.0298	7.0399E-01	8.34E-01	ENSG00000269378
NA	-0.0266	7.0367E-01	8.34E-01	ENSG00000270072
NA	-0.0568	7.0344E-01	8.34E-01	ENSG00000259020
NA	-0.0007	7.0326E-01	8.34E-01	ENSG00000275092
NA	-0.0112	7.0320E-01		ENSG00000279482
NA	-0.0483	7.0287E-01	8.34E-01	ENSG00000277634
NA	-0.0155	7.0285E-01		ENSG00000257456
NA	-0.0036	7.0269E-01		ENSG00000271198
NA	-0.0133	7.0261E-01		ENSG00000287084
NA	-0.0205	7.0254E-01	8.34E-01	ENSG00000273240
NA	-0.0156	7.0214E-01		ENSG00000287676
NA	-0.0133	7.0204E-01		ENSG00000228847
NA	-0.0216	7.0186E-01		ENSG00000278737
NA	-0.0159	7.0184E-01		ENSG00000229291
NA	-0.0335	7.0169E-01	8.33E-01	ENSG00000215878
NA	-0.0488	7.0164E-01		ENSG00000277223
NA	-0.0321	7.0155E-01	8.33E-01	ENSG00000213763
NA	-0.0277	7.0115E-01	8.33E-01	ENSG00000233325
NA	-0.0125	7.0089E-01		ENSG00000201363
NA	-0.0068	7.0086E-01	8.33E-01	ENSG00000276436
NA	-0.0527	7.0063E-01	8.33E-01	ENSG00000266920
NA	-0.0295	7.0055E-01	8.33E-01	ENSG00000227946
NA	-0.0116	7.0052E-01		ENSG00000283000
NA	-0.0220	7.0047E-01		ENSG00000285560
NA	-0.0139	7.0046E-01		ENSG00000258535
NA	-0.0179	7.0045E-01		ENSG00000276110
NA	-0.0155	7.0032E-01		ENSG00000229468
NA	-0.0282	7.0025E-01	8.32E-01	ENSG00000225051
NA	-0.0002	7.0022E-01	8.32E-01	ENSG00000277010
NA	-0.0281	7.0003E-01		ENSG00000236281
NA	-0.0268	6.9958E-01	8.32E-01	ENSG00000270426
NA	-0.0294	6.9952E-01	8.32E-01	ENSG00000251011
NA	-0.0058	6.9947E-01		ENSG00000237963
NA	-0.0078	6.9937E-01		ENSG00000286363
NA	-0.0099	6.9904E-01		ENSG00000207142
NA	-0.0313	6.9889E-01		ENSG00000227734
NA	-0.0367	6.9829E-01	8.31E-01	ENSG00000231113
NA	-0.0455	6.9816E-01		ENSG00000218502
NA	-0.0034	6.9805E-01		ENSG00000249129
NA	-0.0390	6.9710E-01	8.31E-01	ENSG00000234665

NA	-0.0137	6.9685E-01		ENSG00000254850
NA	-0.0232	6.9655E-01		ENSG00000285752
NA	-0.0186	6.9646E-01		ENSG00000235166
NA	-0.0261	6.9645E-01	8.30E-01	ENSG00000228330
NA	-0.0159	6.9635E-01		ENSG00000276718
NA	-0.0349	6.9599E-01	8.30E-01	ENSG00000267500
NA	-0.0050	6.9585E-01		ENSG00000235024
NA	-0.0535	6.9579E-01	8.30E-01	ENSG00000285041
NA	-0.0291	6.9563E-01		ENSG00000283752
NA	-0.0099	6.9529E-01		ENSG00000225094
NA	-0.0165	6.9525E-01		ENSG00000201668
NA	-0.0280	6.9515E-01	8.29E-01	ENSG00000286656
NA	-0.0012	6.9510E-01		ENSG00000200419
NA	-0.0326	6.9500E-01		ENSG00000233644
NA	-0.0354	6.9496E-01		ENSG00000289361
NA	-0.0217	6.9496E-01		ENSG00000254548
NA	-0.0094	6.9477E-01		ENSG00000250908
NA	-0.0129	6.9460E-01		ENSG00000249786
NA	-0.0079	6.9449E-01		ENSG00000218586
NA	-0.0288	6.9445E-01	8.29E-01	ENSG00000229519
NA	-0.0106	6.9420E-01		ENSG00000284698
NA	-0.0440	6.9407E-01	8.29E-01	ENSG00000227678
NA	-0.0028	6.9341E-01		ENSG00000279214
NA	-0.0372	6.9333E-01	8.28E-01	ENSG00000267640
NA	-0.0346	6.9332E-01		ENSG00000225521
NA	-0.0161	6.9321E-01		ENSG00000267699
NA	-0.0132	6.9321E-01		ENSG00000256720
NA	-0.0103	6.9291E-01		ENSG00000289859
NA	-0.0036	6.9251E-01		ENSG00000277233
NA	-0.0042	6.9251E-01		ENSG00000276063
NA	-0.0075	6.9218E-01	8.27E-01	ENSG00000267731
NA	-0.0369	6.9215E-01	8.27E-01	ENSG00000279861
NA	-0.0152	6.9203E-01		ENSG00000251360
NA	-0.0374	6.9176E-01		ENSG00000274979
NA	-0.0307	6.9174E-01	8.27E-01	ENSG00000255321
NA	-0.0143	6.9164E-01		ENSG00000258260
NA	-0.0156	6.9163E-01		ENSG00000279246
NA	-0.0363	6.9152E-01	8.27E-01	ENSG00000269983
NA	-0.0473	6.9143E-01	8.27E-01	ENSG00000269901
NA	-0.0077	6.9118E-01		ENSG00000250106
NA	-0.0185	6.9060E-01		ENSG00000277156
NA	-0.0160	6.9051E-01		ENSG00000283945
NA	-0.0049	6.9028E-01		ENSG00000236900
NA	-0.0258	6.9024E-01	8.26E-01	ENSG00000261215
NA	-0.0377	6.9006E-01	8.26E-01	ENSG00000223653
NA	-0.0119	6.9001E-01		ENSG00000285807
NA	-0.0227	6.8990E-01	8.26E-01	ENSG00000256673
NA	-0.0069	6.8979E-01		ENSG00000249986
NA	-0.0361	6.8974E-01	8.26E-01	ENSG00000220506

NA	-0.0056	6.8962E-01	8.26E-01	ENSG00000227854
NA	-0.0403	6.8941E-01	8.26E-01	ENSG00000289123
NA	-0.0108	6.8938E-01		ENSG00000255042
NA	-0.0108	6.8938E-01		ENSG00000250332
NA	-0.0125	6.8936E-01		ENSG00000228737
NA	-0.0375	6.8930E-01	8.25E-01	ENSG00000266677
NA	-0.0204	6.8915E-01		ENSG00000262031
NA	-0.0276	6.8897E-01	8.25E-01	ENSG00000250644
NA	-0.0179	6.8884E-01		ENSG00000226148
NA	-0.0656	6.8882E-01	8.25E-01	ENSG00000289987
NA	-0.0305	6.8875E-01		ENSG00000227726
NA	-0.0332	6.8871E-01		ENSG00000270060
NA	-0.0129	6.8863E-01		ENSG00000214125
NA	-0.0055	6.8861E-01		ENSG00000241362
NA	-0.0150	6.8854E-01		ENSG00000250371
NA	-0.0359	6.8843E-01	8.25E-01	ENSG00000259924
NA	-0.0809	6.8819E-01		ENSG00000279083
NA	-0.0222	6.8801E-01		ENSG00000225112
NA	-0.0081	6.8798E-01		ENSG00000270154
NA	-0.0167	6.8769E-01		ENSG00000250081
NA	-0.0042	6.8740E-01		ENSG00000229081
NA	-0.0193	6.8717E-01		ENSG00000233055
NA	-0.0018	6.8717E-01		ENSG00000275401
NA	-0.0103	6.8692E-01		ENSG00000285778
NA	-0.0156	6.8672E-01		ENSG00000254762
NA	-0.0057	6.8661E-01	8.23E-01	ENSG00000253712
NA	-0.0393	6.8659E-01	8.23E-01	ENSG00000256594
NA	-0.0010	6.8652E-01		ENSG00000186940
NA	-0.0157	6.8651E-01		ENSG00000283417
NA	-0.0129	6.8641E-01		ENSG00000289187
NA	-0.0309	6.8638E-01		ENSG00000229325
NA	-0.0114	6.8591E-01		ENSG00000226349
NA	-0.0251	6.8589E-01		ENSG00000288900
NA	-0.0153	6.8576E-01		ENSG00000229275
NA	-0.0096	6.8547E-01		ENSG00000272772
NA	-0.0120	6.8532E-01		ENSG00000255893
NA	-0.0203	6.8510E-01		ENSG00000289975
NA	-0.0150	6.8505E-01		ENSG00000237531
NA	-0.0429	6.8449E-01	8.21E-01	ENSG00000225334
NA	-0.0426	6.8449E-01	8.21E-01	ENSG00000259275
NA	-0.0109	6.8446E-01		ENSG00000250202
NA	-0.0037	6.8427E-01		ENSG00000222649
NA	-0.0443	6.8405E-01		ENSG00000214559
NA	-0.0156	6.8402E-01		ENSG00000250129
NA	-0.0240	6.8397E-01	8.21E-01	ENSG00000269800
NA	-0.0146	6.8384E-01		ENSG00000272211
NA	-0.0156	6.8378E-01		ENSG00000284431
NA	-0.0009	6.8378E-01		ENSG00000289844
NA	-0.0100	6.8376E-01		ENSG00000287303

NA	-0.0381	6.8334E-01	8.21E-01	ENSG00000267890
NA	-0.0168	6.8329E-01		ENSG00000271742
NA	-0.0044	6.8319E-01		ENSG00000287349
NA	-0.0077	6.8317E-01		ENSG00000133475
NA	-0.0130	6.8261E-01		ENSG00000241656
NA	-0.0153	6.8241E-01		ENSG00000286511
NA	-0.0382	6.8234E-01	8.20E-01	ENSG00000272072
NA	-0.0407	6.8206E-01	8.20E-01	ENSG00000233785
NA	-0.0007	6.8195E-01		ENSG00000240535
NA	-0.0053	6.8194E-01		ENSG00000258034
NA	-0.0056	6.8180E-01		ENSG00000236347
NA	-0.0338	6.8172E-01	8.20E-01	ENSG00000287045
NA	-0.0009	6.8162E-01		ENSG00000235979
NA	-0.0004	6.8154E-01		ENSG00000243730
NA	-0.0113	6.8145E-01		ENSG00000240490
NA	-0.0384	6.8137E-01	8.19E-01	ENSG00000280383
NA	-0.0389	6.8136E-01	8.19E-01	ENSG00000249353
NA	-0.0362	6.8135E-01		ENSG00000285548
NA	-0.0293	6.8124E-01		ENSG00000227383
NA	-0.0558	6.8099E-01		ENSG00000239435
NA	-0.0194	6.8083E-01		ENSG00000249685
NA	0.0000	6.8040E-01		ENSG00000249604
NA	-0.0051	6.8029E-01		ENSG00000259843
NA	-0.0226	6.8026E-01		ENSG00000257605
NA	-0.0396	6.7979E-01		ENSG00000258466
NA	-0.0076	6.7976E-01		ENSG00000236229
NA	-0.0072	6.7948E-01		ENSG00000290035
NA	-0.0166	6.7931E-01		ENSG00000279981
NA	-0.0139	6.7906E-01		ENSG00000236133
NA	-0.0149	6.7879E-01		ENSG00000284095
NA	-0.0319	6.7878E-01	8.17E-01	ENSG00000290123
NA	-0.0332	6.7836E-01		ENSG00000278213
NA	-0.0383	6.7834E-01		ENSG00000273340
NA	-0.0296	6.7825E-01	8.16E-01	ENSG00000228347
NA	-0.0198	6.7822E-01		ENSG00000253317
NA	-0.0005	6.7812E-01		ENSG00000278344
NA	-0.0377	6.7769E-01	8.16E-01	ENSG00000240553
NA	-0.0035	6.7767E-01		ENSG00000278770
NA	-0.0150	6.7767E-01		ENSG00000232491
NA	-0.0414	6.7764E-01	8.16E-01	ENSG00000234648
NA	-0.0354	6.7762E-01		ENSG00000259720
NA	-0.0114	6.7727E-01		ENSG00000280028
NA	-0.0044	6.7716E-01		ENSG00000230298
NA	-0.0196	6.7708E-01		ENSG00000236257
NA	-0.0190	6.7689E-01		ENSG00000286866
NA	-0.0209	6.7646E-01		ENSG00000237085
NA	-0.0107	6.7642E-01		ENSG00000261293
NA	-0.0091	6.7635E-01		ENSG00000226994
NA	-0.0400	6.7630E-01	8.15E-01	ENSG00000286724

NA	-0.0404	6.7590E-01	8.15E-01	ENSG00000286020
NA	-0.0403	6.7589E-01	8.15E-01	ENSG00000246889
NA	-0.0330	6.7568E-01		ENSG00000267526
NA	-0.0314	6.7545E-01	8.14E-01	ENSG00000266588
NA	-0.0316	6.7542E-01	8.14E-01	ENSG00000234740
NA	-0.0353	6.7536E-01	8.14E-01	ENSG00000251288
NA	-0.0128	6.7518E-01		ENSG00000283118
NA	-0.0004	6.7511E-01		ENSG00000277548
NA	-0.0305	6.7509E-01	8.14E-01	ENSG00000258604
NA	-0.0016	6.7507E-01		ENSG00000232081
NA	-0.0347	6.7491E-01		ENSG00000252759
NA	-0.0104	6.7463E-01		ENSG00000250501
NA	-0.0402	6.7454E-01	8.14E-01	ENSG00000256167
NA	-0.0211	6.7451E-01		ENSG00000279744
NA	-0.0071	6.7438E-01		ENSG00000249047
NA	-0.0146	6.7412E-01		ENSG00000226652
NA	-0.0403	6.7399E-01	8.13E-01	ENSG00000278107
NA	-0.0366	6.7389E-01	8.13E-01	ENSG00000272509
NA	-0.0137	6.7370E-01		ENSG00000288089
NA	-0.0009	6.7366E-01		ENSG00000251357
NA	-0.0105	6.7314E-01		ENSG00000225807
NA	-0.0230	6.7305E-01		ENSG00000233998
NA	-0.0099	6.7267E-01		ENSG00000289832
NA	-0.0109	6.7260E-01		ENSG00000212663
NA	-0.0337	6.7252E-01	8.12E-01	ENSG00000258917
NA	-0.0153	6.7239E-01		ENSG00000238285
NA	-0.0351	6.7229E-01	8.12E-01	ENSG00000288187
NA	-0.0373	6.7226E-01	8.12E-01	ENSG00000206659
NA	-0.0338	6.7214E-01		ENSG00000225218
NA	-0.0233	6.7210E-01		ENSG00000278083
NA	-0.0150	6.7177E-01		ENSG00000254246
NA	-0.0363	6.7119E-01	8.11E-01	ENSG00000289440
NA	-0.0178	6.7073E-01		ENSG00000256433
NA	-0.0177	6.7032E-01		ENSG00000151303
NA	-0.0282	6.7026E-01	8.11E-01	ENSG00000285639
NA	-0.0264	6.6941E-01		ENSG00000213522
NA	-0.0189	6.6927E-01		ENSG00000278341
NA	-0.0097	6.6876E-01		ENSG00000225670
NA	-0.0158	6.6866E-01		ENSG00000249360
NA	-0.0419	6.6849E-01	8.09E-01	ENSG00000272430
NA	-0.0199	6.6841E-01		ENSG00000241921
NA	-0.0437	6.6834E-01		ENSG00000235358
NA	-0.0357	6.6833E-01	8.09E-01	ENSG00000234141
NA	-0.0211	6.6820E-01		ENSG00000274995
NA	-0.0024	6.6804E-01		ENSG00000200922
NA	-0.0336	6.6788E-01		ENSG00000289726
NA	-0.0171	6.6780E-01		ENSG00000274737
NA	-0.0259	6.6776E-01		ENSG00000219992
NA	-0.0163	6.6774E-01		ENSG00000285314

NA	-0.0266	6.6773E-01	8.09E-01	ENSG00000249096
NA	-0.0052	6.6769E-01		ENSG00000228072
NA	-0.0394	6.6747E-01	8.09E-01	ENSG00000273521
NA	-0.0401	6.6706E-01		ENSG00000271980
NA	-0.0003	6.6686E-01		ENSG00000258637
NA	-0.0158	6.6670E-01		ENSG00000269952
NA	-0.0032	6.6661E-01		ENSG00000225579
NA	-0.0099	6.6636E-01		ENSG00000271192
NA	-0.0091	6.6634E-01		ENSG00000286932
NA	-0.0507	6.6617E-01		ENSG00000290122
NA	-0.0414	6.6599E-01	8.08E-01	ENSG00000223695
NA	-0.0377	6.6566E-01	8.08E-01	ENSG00000237758
NA	-0.0137	6.6565E-01		ENSG00000283057
NA	-0.0355	6.6560E-01	8.08E-01	ENSG00000277501
NA	-0.0225	6.6560E-01	8.08E-01	ENSG00000283312
NA	-0.0089	6.6551E-01		ENSG00000254459
NA	-0.0066	6.6542E-01		ENSG00000275329
NA	-0.0255	6.6516E-01		ENSG00000213063
NA	-0.0340	6.6502E-01	8.07E-01	ENSG00000260018
NA	-0.0328	6.6501E-01		ENSG00000260362
NA	-0.0311	6.6467E-01		ENSG00000213370
NA	-0.0119	6.6409E-01		ENSG00000272428
NA	-0.0167	6.6402E-01		ENSG00000225948
NA	-0.0572	6.6392E-01	8.07E-01	ENSG00000258633
NA	-0.0045	6.6367E-01		ENSG00000220884
NA	-0.0404	6.6361E-01	8.07E-01	ENSG00000283757
NA	-0.0570	6.6359E-01		ENSG00000251411
NA	-0.0416	6.6357E-01	8.07E-01	ENSG00000288935
NA	-0.0010	6.6342E-01		ENSG00000271275
NA	-0.0066	6.6316E-01	8.06E-01	ENSG00000264813
NA	-0.0114	6.6265E-01		ENSG00000288533
NA	-0.0258	6.6248E-01		ENSG00000234607
NA	-0.0091	6.6193E-01		ENSG00000254372
NA	-0.0144	6.6183E-01		ENSG00000214822
NA	-0.0055	6.6154E-01		ENSG00000239511
NA	-0.0325	6.6153E-01	8.05E-01	ENSG00000272468
NA	-0.0059	6.6133E-01		ENSG00000218069
NA	-0.0302	6.6132E-01		ENSG00000286941
NA	-0.0318	6.6121E-01		ENSG00000228312
NA	-0.0138	6.6117E-01		ENSG00000251996
NA	-0.0340	6.6113E-01	8.05E-01	ENSG00000284606
NA	-0.0283	6.6101E-01		ENSG00000278484
NA	-0.0454	6.6093E-01	8.05E-01	ENSG00000288551
NA	-0.0390	6.6088E-01	8.05E-01	ENSG00000234949
NA	-0.0353	6.6086E-01	8.05E-01	ENSG00000268047
NA	-0.0033	6.6077E-01		ENSG00000274553
NA	-0.0398	6.6076E-01	8.05E-01	ENSG00000243403
NA	-0.0028	6.6064E-01		ENSG00000274127
NA	-0.0055	6.6046E-01		ENSG00000226375

NA	-0.0208	6.6031E-01		ENSG00000289617
NA	-0.0117	6.6031E-01	8.04E-01	ENSG00000259378
NA	-0.0127	6.6022E-01		ENSG00000215268
NA	-0.0064	6.6015E-01		ENSG00000229457
NA	-0.0215	6.5999E-01		ENSG00000225216
NA	-0.0166	6.5998E-01		ENSG00000225356
NA	-0.0185	6.5994E-01		ENSG00000262098
NA	-0.0165	6.5971E-01		ENSG00000262652
NA	-0.0018	6.5968E-01		ENSG00000266821
NA	-0.0015	6.5966E-01		ENSG00000213650
NA	-0.0135	6.5941E-01		ENSG00000287267
NA	-0.0404	6.5940E-01	8.03E-01	ENSG00000275056
NA	-0.0260	6.5932E-01		ENSG00000256146
NA	-0.0253	6.5890E-01		ENSG00000261693
NA	-0.0403	6.5881E-01	8.03E-01	ENSG00000215271
NA	-0.0319	6.5861E-01	8.03E-01	ENSG00000253392
NA	-0.0206	6.5850E-01		ENSG00000279250
NA	-0.0224	6.5847E-01		ENSG00000248283
NA	-0.0038	6.5843E-01		ENSG00000233594
NA	-0.0396	6.5830E-01	8.03E-01	ENSG00000186369
NA	-0.0228	6.5828E-01		ENSG00000188828
NA	-0.0107	6.5788E-01		ENSG00000269444
NA	-0.0386	6.5778E-01		ENSG00000267144
NA	-0.0417	6.5778E-01	8.02E-01	ENSG00000289412
NA	-0.0395	6.5773E-01	8.02E-01	ENSG00000273628
NA	-0.0409	6.5769E-01	8.02E-01	ENSG00000226608
NA	-0.0134	6.5764E-01		ENSG00000237223
NA	-0.0421	6.5733E-01	8.02E-01	ENSG00000228323
NA	-0.0035	6.5730E-01		ENSG00000279600
NA	-0.0354	6.5714E-01	8.02E-01	ENSG00000260742
NA	-0.0617	6.5687E-01	8.02E-01	ENSG00000286412
NA	-0.0336	6.5671E-01	8.02E-01	ENSG00000279897
NA	-0.0279	6.5621E-01	8.02E-01	ENSG00000286362
NA	-0.0426	6.5621E-01	8.02E-01	ENSG00000259319
NA	-0.0090	6.5596E-01		ENSG00000285730
NA	-0.0175	6.5590E-01		ENSG00000255780
NA	-0.0279	6.5463E-01		ENSG00000264937
NA	-0.0331	6.5456E-01	8.00E-01	ENSG00000274798
NA	-0.0167	6.5451E-01		ENSG00000271156
NA	-0.0423	6.5449E-01	8.00E-01	ENSG00000274642
NA	-0.0152	6.5426E-01		ENSG00000260555
NA	-0.0138	6.5408E-01		ENSG00000287238
NA	-0.0286	6.5396E-01		ENSG00000202417
NA	-0.0412	6.5383E-01		ENSG00000287721
NA	-0.0094	6.5380E-01		ENSG00000215023
NA	-0.0432	6.5373E-01	8.00E-01	ENSG00000233270
NA	-0.0435	6.5368E-01	8.00E-01	ENSG00000260469
NA	-0.0338	6.5356E-01	8.00E-01	ENSG00000276118
NA	-0.0073	6.5347E-01		ENSG00000276916

NA	-0.0398	6.5328E-01	8.00E-01	ENSG00000249302
NA	-0.0214	6.5327E-01		ENSG00000243494
NA	-0.0053	6.5311E-01		ENSG00000270277
NA	-0.0098	6.5287E-01		ENSG00000288686
NA	-0.0388	6.5286E-01	7.99E-01	ENSG00000288802
NA	-0.0328	6.5248E-01		ENSG00000259932
NA	-0.0434	6.5193E-01	7.99E-01	ENSG00000235887
NA	-0.0377	6.5184E-01		ENSG00000277595
NA	-0.0181	6.5177E-01		ENSG00000267634
NA	-0.0407	6.5171E-01	7.98E-01	ENSG00000203546
NA	-0.0237	6.5171E-01		ENSG00000272983
NA	-0.0264	6.5165E-01		ENSG00000260657
NA	-0.0102	6.5163E-01		ENSG00000286781
NA	-0.0342	6.5148E-01	7.98E-01	ENSG00000286771
NA	-0.0201	6.5139E-01		ENSG00000260479
NA	-0.0370	6.5078E-01	7.98E-01	ENSG00000271737
NA	-0.0144	6.5052E-01		ENSG00000260668
NA	-0.0386	6.5042E-01	7.98E-01	ENSG00000284128
NA	-0.0143	6.5040E-01		ENSG00000231741
NA	-0.0267	6.5039E-01		ENSG00000250990
NA	-0.0394	6.5028E-01		ENSG00000265204
NA	-0.0085	6.5025E-01		ENSG00000253335
NA	-0.0206	6.5006E-01	7.97E-01	ENSG00000240919
NA	-0.0082	6.4980E-01		ENSG00000206028
NA	-0.0196	6.4976E-01		ENSG00000286068
NA	-0.0054	6.4951E-01		ENSG00000224629
NA	-0.0151	6.4950E-01	7.97E-01	ENSG00000180189
NA	-0.0294	6.4941E-01	7.97E-01	ENSG00000259543
NA	-0.0310	6.4937E-01	7.97E-01	ENSG00000232486
NA	-0.0760	6.4933E-01	7.97E-01	ENSG00000255085
NA	-0.0055	6.4927E-01		ENSG00000275661
NA	-0.0078	6.4901E-01		ENSG00000242790
NA	-0.0187	6.4821E-01		ENSG00000289888
NA	-0.0093	6.4816E-01		ENSG00000286052
NA	-0.0321	6.4815E-01		ENSG00000226138
NA	-0.0399	6.4806E-01	7.96E-01	ENSG00000240766
NA	-0.0438	6.4775E-01	7.96E-01	ENSG00000213866
NA	-0.0341	6.4753E-01	7.96E-01	ENSG00000286974
NA	-0.0129	6.4732E-01		ENSG00000286240
NA	-0.0104	6.4713E-01		ENSG00000200888
NA	-0.0668	6.4711E-01	7.96E-01	ENSG00000286529
NA	-0.0171	6.4711E-01		ENSG00000207922
NA	-0.0231	6.4697E-01		ENSG00000261451
NA	-0.0279	6.4673E-01		ENSG00000273069
NA	-0.0439	6.4647E-01	7.95E-01	ENSG00000286220
NA	-0.0149	6.4589E-01		ENSG00000244050
NA	-0.0437	6.4574E-01	7.94E-01	ENSG00000237575
NA	-0.0081	6.4566E-01		ENSG00000230131
NA	-0.0388	6.4564E-01		ENSG00000264000

NA	-0.0128	6.4552E-01		ENSG00000217897
NA	-0.0229	6.4534E-01		ENSG00000279807
NA	-0.0406	6.4532E-01	7.94E-01	ENSG00000260947
NA	-0.0414	6.4527E-01	7.94E-01	ENSG00000203362
NA	-0.0123	6.4523E-01	7.94E-01	ENSG00000229816
NA	-0.0107	6.4504E-01		ENSG00000229642
NA	-0.0190	6.4503E-01		ENSG00000276030
NA	-0.0108	6.4502E-01		ENSG00000230628
NA	-0.0180	6.4470E-01		ENSG00000254080
NA	-0.0418	6.4427E-01	7.94E-01	ENSG00000278768
NA	-0.0277	6.4380E-01		ENSG00000289467
NA	-0.0440	6.4379E-01	7.93E-01	ENSG00000289149
NA	-0.0716	6.4368E-01	7.93E-01	ENSG00000288061
NA	-0.0212	6.4365E-01		ENSG00000287857
NA	-0.0033	6.4362E-01		ENSG00000277647
NA	-0.0328	6.4343E-01	7.93E-01	ENSG00000201592
NA	-0.0206	6.4317E-01		ENSG00000248254
NA	-0.0315	6.4310E-01	7.93E-01	ENSG00000267340
NA	-0.0174	6.4307E-01		ENSG00000250234
NA	-0.0229	6.4258E-01	7.93E-01	ENSG00000267382
NA	-0.0441	6.4251E-01	7.93E-01	ENSG00000279672
NA	-0.0035	6.4246E-01		ENSG00000279315
NA	-0.0181	6.4207E-01		ENSG00000272109
NA	-0.0068	6.4194E-01	7.92E-01	ENSG00000278999
NA	-0.0298	6.4142E-01		ENSG00000233522
NA	-0.0164	6.4113E-01		ENSG00000270909
NA	-0.0436	6.4102E-01	7.92E-01	ENSG00000274561
NA	-0.0047	6.4076E-01		ENSG00000286823
NA	-0.0077	6.4065E-01		ENSG00000258485
NA	-0.0012	6.3983E-01		ENSG00000176043
NA	-0.0454	6.3973E-01	7.91E-01	ENSG00000249249
NA	-0.0444	6.3971E-01		ENSG00000272123
NA	-0.0441	6.3960E-01	7.91E-01	ENSG00000289112
NA	-0.0088	6.3953E-01		ENSG00000213091
NA	-0.0344	6.3948E-01	7.90E-01	ENSG00000271064
NA	-0.0226	6.3928E-01		ENSG00000267905
NA	-0.0444	6.3915E-01	7.90E-01	ENSG00000253676
NA	-0.0209	6.3912E-01		ENSG00000272885
NA	-0.0074	6.3892E-01		ENSG00000261038
NA	-0.0343	6.3885E-01	7.90E-01	ENSG00000279786
NA	-0.0554	6.3852E-01	7.90E-01	ENSG00000287858
NA	-0.0170	6.3835E-01		ENSG00000252096
NA	-0.0236	6.3804E-01	7.90E-01	ENSG00000286957
NA	-0.0291	6.3800E-01		ENSG00000258603
NA	-0.0449	6.3799E-01	7.90E-01	ENSG00000242242
NA	-0.0129	6.3797E-01		ENSG00000258026
NA	-0.0238	6.3776E-01		ENSG00000226790
NA	-0.0279	6.3770E-01		ENSG00000263316
NA	-0.0011	6.3743E-01		ENSG00000224100

NA	-0.0036	6.3738E-01		ENSG00000236471
NA	-0.0418	6.3734E-01	7.89E-01	ENSG00000257576
NA	-0.0133	6.3725E-01		ENSG00000278892
NA	-0.0455	6.3713E-01	7.89E-01	ENSG00000272779
NA	-0.0163	6.3677E-01		ENSG00000229992
NA	-0.0726	6.3650E-01		ENSG00000289481
NA	-0.0479	6.3636E-01	7.88E-01	ENSG00000232031
NA	-0.0156	6.3624E-01		ENSG00000276593
NA	-0.0320	6.3622E-01	7.88E-01	ENSG00000215223
NA	-0.0449	6.3602E-01	7.88E-01	ENSG00000263006
NA	-0.0165	6.3600E-01		ENSG00000258653
NA	-0.0836	6.3599E-01	7.88E-01	ENSG00000282989
NA	-0.0506	6.3598E-01	7.88E-01	ENSG00000224993
NA	-0.0313	6.3564E-01	7.88E-01	ENSG00000289570
NA	-0.0067	6.3554E-01		ENSG00000237646
NA	-0.0138	6.3542E-01		ENSG00000259773
NA	-0.0520	6.3535E-01	7.88E-01	ENSG00000279228
NA	-0.0102	6.3522E-01		ENSG00000240244
NA	-0.0132	6.3415E-01		ENSG00000275965
NA	-0.0255	6.3411E-01		ENSG00000103832
NA	-0.0477	6.3398E-01		ENSG00000266385
NA	-0.0511	6.3391E-01		ENSG00000249352
NA	-0.0161	6.3384E-01		ENSG00000253923
NA	-0.0367	6.3383E-01	7.87E-01	ENSG00000287900
NA	-0.0458	6.3379E-01	7.86E-01	ENSG00000248126
NA	-0.0152	6.3375E-01		ENSG00000261614
NA	-0.0414	6.3368E-01	7.86E-01	ENSG00000272070
NA	-0.0102	6.3368E-01		ENSG00000277373
NA	-0.0183	6.3331E-01		ENSG00000285550
NA	-0.0185	6.3324E-01		ENSG00000289140
NA	-0.0189	6.3294E-01		ENSG00000226549
NA	-0.0442	6.3271E-01	7.86E-01	ENSG00000249626
NA	-0.0183	6.3270E-01		ENSG00000225857
NA	-0.0164	6.3265E-01		ENSG00000257239
NA	-0.0374	6.3240E-01		ENSG00000248165
NA	-0.0339	6.3202E-01		ENSG00000286233
NA	-0.0292	6.3178E-01	7.85E-01	ENSG00000272158
NA	-0.0133	6.3160E-01		ENSG00000236445
NA	-0.0076	6.3116E-01		ENSG00000276359
NA	-0.0230	6.3115E-01		ENSG00000279951
NA	-0.0424	6.3088E-01	7.84E-01	ENSG00000285973
NA	-0.0294	6.3042E-01		ENSG00000238713
NA	-0.0455	6.3023E-01	7.84E-01	ENSG00000228519
NA	-0.0168	6.3009E-01		ENSG00000281348
NA	-0.0204	6.3003E-01		ENSG00000231140
NA	-0.0155	6.3002E-01		ENSG00000237379
NA	-0.0024	6.3000E-01		ENSG00000264031
NA	-0.0366	6.2933E-01	7.83E-01	ENSG00000233870
NA	-0.0056	6.2903E-01		ENSG00000256237

NA	-0.0017	6.2875E-01		ENSG00000287678
NA	-0.0085	6.2818E-01		ENSG00000261395
NA	-0.0411	6.2806E-01	7.82E-01	ENSG00000260273
NA	-0.0420	6.2804E-01	7.82E-01	ENSG00000256705
NA	-0.0381	6.2803E-01	7.82E-01	ENSG00000261659
NA	-0.0086	6.2752E-01		ENSG00000213440
NA	-0.0394	6.2740E-01	7.82E-01	ENSG00000285417
NA	-0.0014	6.2703E-01		ENSG00000251032
NA	-0.0199	6.2691E-01		ENSG00000213684
NA	-0.0426	6.2689E-01		ENSG00000279057
NA	-0.0166	6.2659E-01		ENSG00000289982
NA	-0.0112	6.2638E-01		ENSG00000223811
NA	-0.0120	6.2633E-01		ENSG00000290067
NA	-0.0164	6.2578E-01		ENSG00000277948
NA	-0.0237	6.2574E-01	7.81E-01	ENSG00000246225
NA	-0.0008	6.2519E-01		ENSG00000263946
NA	-0.0093	6.2518E-01		ENSG00000255642
NA	-0.0016	6.2501E-01		ENSG00000260578
NA	-0.0266	6.2493E-01		ENSG00000255434
NA	-0.0149	6.2488E-01		ENSG00000260272
NA	-0.0267	6.2483E-01		ENSG00000234698
NA	-0.0290	6.2480E-01	7.80E-01	ENSG00000259910
NA	-0.0177	6.2478E-01		ENSG00000225415
NA	-0.0017	6.2475E-01		ENSG00000166104
NA	-0.0366	6.2470E-01	7.80E-01	ENSG00000280474
NA	-0.0236	6.2442E-01		ENSG00000266667
NA	-0.0193	6.2436E-01		ENSG00000273956
NA	-0.0190	6.2422E-01		ENSG00000267179
NA	-0.0156	6.2420E-01		ENSG00000277851
NA	-0.0246	6.2372E-01	7.79E-01	ENSG00000286503
NA	-0.0179	6.2361E-01		ENSG00000217746
NA	-0.0247	6.2356E-01		ENSG00000231313
NA	-0.0386	6.2340E-01	7.79E-01	ENSG00000283217
NA	-0.0038	6.2323E-01		ENSG00000253673
NA	-0.0195	6.2311E-01		ENSG00000227484
NA	-0.0183	6.2305E-01		ENSG00000236411
NA	-0.0479	6.2286E-01	7.78E-01	ENSG00000283213
NA	-0.0338	6.2269E-01	7.78E-01	ENSG00000197670
NA	-0.0149	6.2263E-01		ENSG00000255219
NA	-0.0113	6.2258E-01		ENSG00000288894
NA	-0.0084	6.2250E-01		ENSG00000229019
NA	-0.0305	6.2236E-01		ENSG00000238250
NA	-0.0132	6.2223E-01		ENSG00000187472
NA	-0.0451	6.2210E-01	7.78E-01	ENSG00000269066
NA	-0.0331	6.2157E-01	7.77E-01	ENSG00000260269
NA	-0.0188	6.2117E-01		ENSG00000225956
NA	-0.0100	6.2052E-01		ENSG00000285774
NA	-0.0416	6.2045E-01	7.76E-01	ENSG00000232043
NA	-0.0250	6.2036E-01	7.76E-01	ENSG00000184106

NA	-0.0092	6.1997E-01		ENSG00000285553
NA	-0.0164	6.1986E-01		ENSG00000224218
NA	-0.0340	6.1981E-01	7.76E-01	ENSG00000290009
NA	-0.0301	6.1966E-01		ENSG00000287390
NA	-0.0261	6.1963E-01		ENSG00000234175
NA	-0.0335	6.1955E-01		ENSG00000276048
NA	-0.0005	6.1944E-01		ENSG00000231794
NA	-0.0456	6.1940E-01	7.75E-01	ENSG00000253477
NA	-0.0351	6.1934E-01		ENSG00000257119
NA	-0.0785	6.1929E-01	7.75E-01	ENSG00000289115
NA	-0.0209	6.1929E-01		ENSG00000251155
NA	-0.0197	6.1900E-01		ENSG00000280023
NA	-0.0027	6.1898E-01		ENSG00000259845
NA	-0.0350	6.1894E-01	7.75E-01	ENSG00000249870
NA	-0.0409	6.1858E-01	7.75E-01	ENSG00000280010
NA	-0.0399	6.1845E-01	7.75E-01	ENSG00000280106
NA	-0.0460	6.1830E-01	7.75E-01	ENSG00000286129
NA	-0.0228	6.1827E-01		ENSG00000267373
NA	-0.0456	6.1824E-01	7.75E-01	ENSG00000210194
NA	-0.0187	6.1764E-01		ENSG00000278642
NA	-0.0230	6.1764E-01		ENSG00000271032
NA	-0.0087	6.1758E-01		ENSG00000215349
NA	-0.0433	6.1750E-01	7.75E-01	ENSG00000279675
NA	-0.0083	6.1749E-01		ENSG00000229201
NA	-0.0150	6.1748E-01		ENSG00000268615
NA	-0.0657	6.1735E-01	7.75E-01	ENSG00000287502
NA	-0.0140	6.1615E-01		ENSG00000255418
NA	-0.0317	6.1571E-01		ENSG00000272235
NA	-0.0127	6.1567E-01		ENSG00000232830
NA	-0.0469	6.1547E-01	7.73E-01	ENSG00000273026
NA	-0.0369	6.1537E-01	7.73E-01	ENSG00000234042
NA	-0.0286	6.1535E-01		ENSG00000257298
NA	-0.0513	6.1521E-01	7.73E-01	ENSG00000287100
NA	-0.0189	6.1507E-01		ENSG00000289130
NA	-0.0260	6.1480E-01		ENSG00000285579
NA	-0.0262	6.1477E-01		ENSG00000287919
NA	-0.0240	6.1470E-01		ENSG00000286712
NA	-0.0474	6.1458E-01	7.73E-01	ENSG00000254910
NA	-0.0068	6.1449E-01		ENSG00000250403
NA	-0.0467	6.1421E-01	7.72E-01	ENSG00000267413
NA	-0.0007	6.1415E-01		ENSG00000254089
NA	-0.0426	6.1408E-01		ENSG00000260368
NA	-0.0180	6.1403E-01		ENSG00000250256
NA	-0.0245	6.1392E-01		ENSG00000279681
NA	-0.0292	6.1376E-01		ENSG00000237407
NA	-0.0165	6.1353E-01		ENSG00000260532
NA	-0.0095	6.1344E-01		ENSG00000254790
NA	-0.0114	6.1334E-01		ENSG00000267943
NA	-0.0308	6.1300E-01		ENSG00000232855

NA	-0.0301	6.1296E-01		ENSG00000265091
NA	-0.0194	6.1281E-01		ENSG00000237249
NA	-0.0250	6.1274E-01		ENSG00000224417
NA	-0.0010	6.1274E-01	7.71E-01	ENSG00000281195
NA	-0.0030	6.1270E-01		ENSG00000287066
NA	-0.0408	6.1232E-01	7.71E-01	ENSG00000277978
NA	-0.0026	6.1200E-01		ENSG00000228817
NA	-0.0269	6.1178E-01	7.70E-01	ENSG00000270255
NA	-0.0200	6.1177E-01		ENSG00000235848
NA	-0.0272	6.1171E-01		ENSG00000255213
NA	-0.0095	6.1170E-01		ENSG00000237676
NA	-0.0811	6.1157E-01	7.70E-01	ENSG00000288814
NA	-0.0316	6.1116E-01		ENSG00000276261
NA	-0.0196	6.1111E-01		ENSG00000227336
NA	-0.0586	6.1090E-01	7.69E-01	ENSG00000237882
NA	-0.0046	6.1076E-01		ENSG00000255741
NA	-0.0311	6.1073E-01		ENSG00000207195
NA	-0.0081	6.1068E-01		ENSG00000286739
NA	-0.0776	6.1058E-01	7.69E-01	ENSG00000254328
NA	-0.0528	6.1056E-01	7.69E-01	ENSG00000273804
NA	-0.0111	6.1026E-01		ENSG00000268926
NA	-0.0283	6.1019E-01		ENSG00000234496
NA	-0.0219	6.0973E-01		ENSG00000236527
NA	-0.0872	6.0971E-01	7.68E-01	ENSG00000285190
NA	-0.0358	6.0966E-01	7.68E-01	ENSG00000278627
NA	-0.0148	6.0949E-01		ENSG00000264958
NA	-0.0179	6.0945E-01		ENSG00000285575
NA	-0.0307	6.0944E-01		ENSG00000255027
NA	-0.0244	6.0944E-01		ENSG00000286606
NA	-0.0147	6.0936E-01		ENSG00000237784
NA	-0.0496	6.0935E-01	7.68E-01	ENSG00000248559
NA	-0.0161	6.0933E-01		ENSG00000269191
NA	-0.0190	6.0900E-01		ENSG00000261760
NA	-0.0239	6.0893E-01		ENSG00000279817
NA	-0.0373	6.0890E-01	7.68E-01	ENSG00000224846
NA	-0.0553	6.0879E-01	7.68E-01	ENSG00000261003
NA	-0.0087	6.0863E-01		ENSG00000269806
NA	-0.0139	6.0818E-01		ENSG00000254321
NA	-0.0288	6.0783E-01		ENSG00000260698
NA	-0.0016	6.0756E-01		ENSG00000206682
NA	-0.0437	6.0734E-01	7.67E-01	ENSG00000286451
NA	-0.0086	6.0732E-01		ENSG00000228372
NA	-0.0246	6.0725E-01		ENSG00000254991
NA	-0.0217	6.0716E-01		ENSG00000248484
NA	-0.0301	6.0699E-01		ENSG00000224595
NA	-0.0342	6.0698E-01	7.66E-01	ENSG00000231566
NA	-0.0138	6.0692E-01		ENSG00000258373
NA	-0.0078	6.0689E-01		ENSG00000275987
NA	-0.0152	6.0677E-01		ENSG00000263167

NA	-0.0176	6.0658E-01		ENSG00000240915
NA	-0.0100	6.0653E-01		ENSG00000234170
NA	-0.0580	6.0652E-01	7.66E-01	ENSG00000261647
NA	-0.0472	6.0645E-01	7.66E-01	ENSG00000287513
NA	-0.0342	6.0638E-01		ENSG00000279149
NA	-0.0061	6.0636E-01		ENSG00000213579
NA	-0.0191	6.0592E-01		ENSG00000214761
NA	-0.0280	6.0592E-01		ENSG00000287974
NA	-0.0398	6.0588E-01		ENSG00000214027
NA	-0.0209	6.0572E-01		ENSG00000271659
NA	-0.0165	6.0557E-01		ENSG00000273267
NA	-0.0165	6.0554E-01		ENSG00000279356
NA	-0.0378	6.0542E-01		ENSG00000228076
NA	-0.0031	6.0515E-01		ENSG00000244468
NA	-0.0362	6.0510E-01		ENSG00000289079
NA	-0.0159	6.0508E-01		ENSG00000266969
NA	-0.0378	6.0496E-01		ENSG00000286453
NA	-0.0151	6.0461E-01		ENSG00000238061
NA	-0.0092	6.0423E-01		ENSG00000250290
NA	-0.0496	6.0414E-01	7.64E-01	ENSG00000288756
NA	-0.0708	6.0409E-01		ENSG00000232027
NA	-0.0840	6.0372E-01	7.64E-01	ENSG00000283515
NA	-0.0061	6.0371E-01	7.64E-01	ENSG00000280129
NA	-0.0506	6.0335E-01	7.63E-01	ENSG00000214857
NA	-0.0685	6.0306E-01	7.63E-01	ENSG00000224908
NA	-0.0085	6.0303E-01		ENSG00000254593
NA	-0.0273	6.0297E-01		ENSG00000236069
NA	-0.0469	6.0296E-01		ENSG00000266897
NA	-0.0061	6.0293E-01		ENSG00000230435
NA	-0.0111	6.0277E-01		ENSG00000260723
NA	-0.0424	6.0270E-01	7.63E-01	ENSG00000270814
NA	-0.0505	6.0266E-01	7.63E-01	ENSG00000272009
NA	-0.0421	6.0251E-01	7.63E-01	ENSG00000228551
NA	-0.0166	6.0226E-01		ENSG00000267552
NA	-0.0520	6.0204E-01	7.62E-01	ENSG00000246982
NA	-0.0038	6.0167E-01		ENSG00000272707
NA	-0.0277	6.0159E-01		ENSG00000259463
NA	-0.0324	6.0133E-01		ENSG00000230663
NA	-0.0853	6.0130E-01	7.62E-01	ENSG00000289030
NA	-0.0191	6.0124E-01		ENSG00000238010
NA	-0.0548	6.0083E-01	7.61E-01	ENSG00000262539
NA	-0.0150	6.0070E-01		ENSG00000233435
NA	-0.0001	6.0057E-01		ENSG00000251298
NA	-0.0303	6.0045E-01		ENSG00000287383
NA	-0.0153	6.0042E-01		ENSG00000212440
NA	-0.0011	6.0039E-01	7.61E-01	ENSG00000247287
NA	-0.0152	6.0032E-01		ENSG00000261147
NA	-0.0460	6.0030E-01	7.61E-01	ENSG00000132832
NA	-0.0508	6.0012E-01	7.61E-01	ENSG00000175170

NA	-0.0156	5.9996E-01		ENSG00000268992
NA	-0.0357	5.9987E-01	7.61E-01	ENSG00000260891
NA	-0.0315	5.9979E-01		ENSG00000244196
NA	-0.0636	5.9957E-01	7.60E-01	ENSG00000245768
NA	-0.0090	5.9946E-01		ENSG00000286784
NA	-0.0438	5.9944E-01		ENSG00000201071
NA	-0.0288	5.9927E-01		ENSG00000240477
NA	-0.0415	5.9917E-01	7.60E-01	ENSG00000285812
NA	-0.0419	5.9890E-01	7.60E-01	ENSG00000269886
NA	-0.0463	5.9878E-01	7.60E-01	ENSG00000278920
NA	-0.0484	5.9873E-01	7.60E-01	ENSG00000288045
NA	-0.0054	5.9863E-01		ENSG00000286966
NA	-0.0527	5.9857E-01	7.60E-01	ENSG00000272305
NA	-0.0383	5.9841E-01	7.60E-01	ENSG00000261829
NA	-0.0371	5.9829E-01		ENSG00000236936
NA	-0.0610	5.9803E-01	7.59E-01	ENSG00000242094
NA	-0.0137	5.9789E-01		ENSG00000232827
NA	-0.0469	5.9744E-01	7.59E-01	ENSG00000253746
NA	-0.0774	5.9740E-01	7.59E-01	ENSG00000257531
NA	-0.1187	5.9692E-01	7.59E-01	ENSG00000228335
NA	-0.0114	5.9676E-01		ENSG00000231980
NA	-0.0003	5.9663E-01		ENSG00000237848
NA	-0.0073	5.9649E-01		ENSG00000289691
NA	-0.0496	5.9642E-01	7.58E-01	ENSG00000223834
NA	-0.1157	5.9628E-01	7.58E-01	ENSG00000272434
NA	-0.0139	5.9600E-01		ENSG00000285629
NA	-0.0336	5.9590E-01	7.58E-01	ENSG00000175730
NA	-0.0029	5.9582E-01		ENSG00000285955
NA	-0.0242	5.9580E-01	7.58E-01	ENSG00000220157
NA	-0.0017	5.9570E-01	7.58E-01	ENSG00000259788
NA	-0.0286	5.9564E-01		ENSG00000264520
NA	-0.0158	5.9563E-01		ENSG00000285934
NA	-0.0294	5.9562E-01		ENSG00000288945
NA	-0.0305	5.9554E-01		ENSG00000257604
NA	-0.0282	5.9547E-01		ENSG00000223642
NA	-0.0245	5.9546E-01	7.58E-01	ENSG00000258111
NA	-0.0017	5.9540E-01	7.58E-01	ENSG00000256894
NA	-0.0301	5.9526E-01	7.57E-01	ENSG00000279785
NA	-0.0190	5.9479E-01		ENSG00000251613
NA	-0.0153	5.9458E-01		ENSG00000266088
NA	-0.0168	5.9419E-01		ENSG00000283258
NA	-0.0250	5.9413E-01		ENSG00000233820
NA	-0.0331	5.9411E-01	7.57E-01	ENSG00000227835
NA	-0.0265	5.9408E-01		ENSG00000269356
NA	-0.0081	5.9363E-01		ENSG00000284490
NA	-0.0147	5.9354E-01		ENSG00000249984
NA	-0.0205	5.9339E-01		ENSG00000261037
NA	-0.0513	5.9325E-01	7.56E-01	ENSG00000271966
NA	-0.0508	5.9302E-01	7.55E-01	ENSG00000237875

NA	-0.0115	5.9274E-01		ENSG00000287333
NA	-0.0440	5.9268E-01	7.55E-01	ENSG00000184844
NA	-0.0281	5.9268E-01		ENSG00000289293
NA	-0.0049	5.9231E-01		ENSG00000229129
NA	-0.0049	5.9231E-01		ENSG00000229465
NA	-0.0434	5.9194E-01	7.55E-01	ENSG00000256196
NA	-0.0497	5.9186E-01	7.55E-01	ENSG00000238165
NA	-0.0264	5.9150E-01		ENSG00000261461
NA	-0.0289	5.9142E-01	7.54E-01	ENSG00000261051
NA	-0.0093	5.9105E-01		ENSG00000286370
NA	-0.0258	5.9103E-01		ENSG00000200741
NA	-0.0147	5.9094E-01		ENSG00000279599
NA	-0.0258	5.9089E-01		ENSG00000237329
NA	-0.0238	5.9069E-01		ENSG00000264456
NA	-0.0489	5.9065E-01	7.54E-01	ENSG00000229334
NA	-0.0099	5.9045E-01		ENSG00000271266
NA	-0.0486	5.9038E-01		ENSG00000267606
NA	-0.0310	5.9014E-01		ENSG00000230118
NA	-0.0357	5.9007E-01		ENSG00000279674
NA	-0.0136	5.9004E-01		ENSG00000252192
NA	-0.0390	5.8992E-01	7.53E-01	ENSG00000276571
NA	-0.0360	5.8989E-01	7.53E-01	ENSG00000236773
NA	-0.0119	5.8968E-01		ENSG00000275703
NA	-0.0493	5.8961E-01	7.53E-01	ENSG00000217416
NA	-0.0487	5.8942E-01	7.53E-01	ENSG00000261159
NA	-0.0044	5.8934E-01		ENSG00000287658
NA	-0.0480	5.8918E-01	7.53E-01	ENSG00000288033
NA	-0.0161	5.8916E-01		ENSG00000270591
NA	-0.0867	5.8841E-01	7.52E-01	ENSG00000279955
NA	-0.0308	5.8826E-01	7.52E-01	ENSG00000267085
NA	-0.0372	5.8802E-01		ENSG00000226956
NA	-0.0432	5.8796E-01		ENSG00000249094
NA	-0.0362	5.8759E-01		ENSG00000276312
NA	-0.0485	5.8737E-01	7.52E-01	ENSG00000278918
NA	-0.0301	5.8730E-01		ENSG00000255663
NA	-0.0346	5.8715E-01	7.52E-01	ENSG00000263276
NA	-0.0222	5.8693E-01		ENSG00000264145
NA	-0.0415	5.8660E-01	7.51E-01	ENSG00000261386
NA	-0.0112	5.8658E-01		ENSG00000261392
NA	-0.0746	5.8638E-01	7.51E-01	ENSG00000223525
NA	-0.0293	5.8629E-01		ENSG00000278965
NA	-0.0134	5.8615E-01		ENSG00000260174
NA	-0.0358	5.8611E-01		ENSG00000278090
NA	-0.0378	5.8606E-01		ENSG00000231665
NA	-0.0143	5.8591E-01		ENSG00000285578
NA	-0.0476	5.8570E-01	7.50E-01	ENSG00000233860
NA	-0.0226	5.8570E-01		ENSG00000275228
NA	-0.0079	5.8566E-01		ENSG00000272040
NA	-0.0335	5.8566E-01	7.50E-01	ENSG00000275413

NA	-0.0273	5.8528E-01		ENSG00000237212
NA	-0.0120	5.8524E-01		ENSG00000255750
NA	-0.0199	5.8513E-01		ENSG00000226972
NA	-0.0514	5.8507E-01	7.50E-01	ENSG00000241556
NA	-0.0578	5.8450E-01	7.50E-01	ENSG00000256603
NA	-0.0341	5.8431E-01	7.49E-01	ENSG00000254162
NA	-0.0332	5.8425E-01	7.49E-01	ENSG00000233588
NA	-0.0397	5.8407E-01	7.49E-01	ENSG00000230183
NA	-0.0257	5.8385E-01		ENSG00000232060
NA	-0.0030	5.8382E-01		ENSG00000224431
NA	-0.0447	5.8358E-01	7.49E-01	ENSG00000225766
NA	-0.0109	5.8334E-01		ENSG00000219682
NA	-0.0500	5.8306E-01	7.49E-01	ENSG00000275055
NA	-0.0505	5.8287E-01	7.49E-01	ENSG00000266933
NA	-0.0167	5.8287E-01		ENSG00000266379
NA	-0.0361	5.8285E-01		ENSG00000287799
NA	-0.0010	5.8257E-01		ENSG00000205682
NA	-0.0269	5.8245E-01		ENSG00000267426
NA	-0.0107	5.8226E-01	7.48E-01	ENSG00000281903
NA	-0.0178	5.8176E-01		ENSG00000258345
NA	-0.0311	5.8157E-01		ENSG00000214875
NA	-0.0022	5.8127E-01	7.47E-01	ENSG00000231934
NA	-0.0252	5.8108E-01		ENSG00000275512
NA	-0.0392	5.8089E-01		ENSG00000226886
NA	-0.0126	5.8079E-01		ENSG00000253536
NA	-0.0223	5.8067E-01		ENSG00000213872
NA	-0.0181	5.8052E-01		ENSG00000226658
NA	-0.0475	5.8050E-01	7.47E-01	ENSG00000268218
NA	-0.0479	5.8041E-01	7.47E-01	ENSG00000276809
NA	-0.0237	5.7981E-01		ENSG00000213297
NA	-0.0312	5.7966E-01	7.46E-01	ENSG00000288971
NA	-0.0407	5.7965E-01	7.46E-01	ENSG00000268015
NA	-0.0010	5.7929E-01		ENSG00000258446
NA	-0.0304	5.7918E-01		ENSG00000287466
NA	-0.0077	5.7840E-01		ENSG00000279484
NA	-0.0298	5.7827E-01		ENSG00000258798
NA	-0.0012	5.7805E-01		ENSG00000236963
NA	-0.0212	5.7804E-01		ENSG00000259360
NA	-0.0853	5.7763E-01	7.44E-01	ENSG00000259838
NA	-0.0011	5.7757E-01		ENSG00000231561
NA	-0.0129	5.7755E-01		ENSG00000227064
NA	-0.0714	5.7739E-01	7.44E-01	ENSG00000214018
NA	-0.0175	5.7716E-01		ENSG00000258615
NA	-0.0460	5.7685E-01	7.44E-01	ENSG00000223742
NA	-0.0337	5.7676E-01	7.43E-01	ENSG00000257180
NA	-0.0722	5.7675E-01	7.43E-01	ENSG00000279541
NA	-0.0472	5.7673E-01	7.43E-01	ENSG00000251586
NA	-0.0435	5.7659E-01		ENSG00000230140
NA	-0.0082	5.7604E-01		ENSG00000225192

NA	-0.0171	5.7604E-01		ENSG00000231905
NA	-0.0194	5.7584E-01		ENSG00000235672
NA	-0.0280	5.7578E-01		ENSG00000225345
NA	-0.0506	5.7565E-01	7.42E-01	ENSG00000285933
NA	-0.0529	5.7561E-01		ENSG00000284461
NA	-0.0452	5.7547E-01	7.42E-01	ENSG00000270533
NA	-0.0081	5.7535E-01		ENSG00000288800
NA	-0.0184	5.7533E-01		ENSG00000268157
NA	-0.0271	5.7506E-01		ENSG00000183239
NA	-0.0399	5.7505E-01	7.42E-01	ENSG00000287896
NA	-0.0057	5.7489E-01		ENSG00000272672
NA	-0.0327	5.7484E-01		ENSG00000275710
NA	-0.0508	5.7479E-01	7.42E-01	ENSG00000180458
NA	-0.0132	5.7467E-01		ENSG00000230313
NA	-0.0167	5.7458E-01		ENSG00000232433
NA	-0.0190	5.7452E-01		ENSG00000285614
NA	-0.0250	5.7434E-01		ENSG00000233979
NA	-0.0215	5.7422E-01		ENSG00000263020
NA	-0.0395	5.7388E-01		ENSG00000277671
NA	-0.0395	5.7388E-01		ENSG00000273739
NA	-0.0395	5.7388E-01		ENSG00000276197
NA	-0.0395	5.7388E-01		ENSG00000273937
NA	-0.0269	5.7377E-01		ENSG00000236535
NA	-0.0498	5.7368E-01	7.41E-01	ENSG00000229251
NA	-0.0525	5.7351E-01	7.41E-01	ENSG00000206341
NA	-0.0157	5.7349E-01		ENSG00000286755
NA	-0.0196	5.7348E-01		ENSG00000260337
NA	-0.0175	5.7317E-01		ENSG00000259064
NA	-0.0151	5.7285E-01		ENSG00000236152
NA	-0.0431	5.7280E-01	7.40E-01	ENSG00000240219
NA	-0.0071	5.7280E-01		ENSG00000246792
NA	-0.0230	5.7268E-01		ENSG00000270487
NA	-0.0075	5.7239E-01	7.40E-01	ENSG00000286223
NA	-0.0112	5.7227E-01		ENSG00000265490
NA	-0.0501	5.7186E-01	7.39E-01	ENSG00000250568
NA	-0.0546	5.7180E-01	7.39E-01	ENSG00000228043
NA	-0.0072	5.7178E-01		ENSG00000236194
NA	-0.0539	5.7162E-01	7.39E-01	ENSG00000275493
NA	-0.0166	5.7158E-01		ENSG00000261489
NA	-0.0167	5.7150E-01		ENSG00000236654
NA	-0.0016	5.7125E-01		ENSG00000267146
NA	-0.0314	5.7096E-01		ENSG00000271862
NA	-0.0542	5.7060E-01		ENSG00000270492
NA	-0.0150	5.7040E-01		ENSG00000224014
NA	-0.0543	5.7032E-01	7.38E-01	ENSG00000287839
NA	-0.0551	5.7025E-01	7.38E-01	ENSG00000288759
NA	-0.0608	5.7016E-01	7.38E-01	ENSG00000231760
NA	-0.0360	5.6995E-01	7.38E-01	ENSG00000239899
NA	-0.0419	5.6985E-01	7.38E-01	ENSG00000280351

NA	-0.0492	5.6982E-01		ENSG00000289741
NA	-0.0123	5.6982E-01		ENSG00000260907
NA	-0.0339	5.6951E-01		ENSG00000265743
NA	-0.0554	5.6947E-01	7.37E-01	ENSG00000288839
NA	-0.0304	5.6928E-01		ENSG00000233611
NA	-0.0530	5.6918E-01	7.37E-01	ENSG00000274002
NA	-0.0264	5.6873E-01		ENSG00000287870
NA	-0.0326	5.6864E-01		ENSG00000250705
NA	-0.0208	5.6808E-01		ENSG00000289409
NA	-0.0446	5.6791E-01		ENSG00000286252
NA	-0.0259	5.6788E-01		ENSG00000287549
NA	-0.0421	5.6770E-01	7.36E-01	ENSG00000260148
NA	-0.0112	5.6761E-01		ENSG00000271871
NA	-0.0380	5.6745E-01	7.36E-01	ENSG00000266869
NA	-0.0319	5.6715E-01	7.35E-01	ENSG00000289036
NA	-0.0360	5.6697E-01		ENSG00000262810
NA	-0.0204	5.6677E-01		ENSG00000274099
NA	-0.0674	5.6675E-01	7.35E-01	ENSG00000253945
NA	-0.0566	5.6675E-01	7.35E-01	ENSG00000231884
NA	-0.0199	5.6668E-01		ENSG00000271218
NA	-0.0451	5.6630E-01	7.35E-01	ENSG00000271185
NA	-0.0199	5.6627E-01		ENSG00000279942
NA	-0.0529	5.6621E-01	7.35E-01	ENSG00000289380
NA	-0.0581	5.6608E-01	7.35E-01	ENSG00000223715
NA	-0.0402	5.6551E-01	7.34E-01	ENSG00000259692
NA	-0.0287	5.6458E-01		ENSG00000258443
NA	-0.0212	5.6457E-01		ENSG00000228810
NA	-0.0210	5.6435E-01		ENSG00000214455
NA	-0.0128	5.6427E-01		ENSG00000286205
NA	-0.0258	5.6425E-01		ENSG00000233420
NA	-0.0513	5.6402E-01	7.33E-01	ENSG00000278943
NA	-0.0191	5.6381E-01		ENSG00000227161
NA	-0.0532	5.6380E-01	7.33E-01	ENSG00000279337
NA	-0.0354	5.6305E-01		ENSG00000234022
NA	-0.0152	5.6285E-01		ENSG00000233776
NA	-0.0660	5.6278E-01	7.32E-01	ENSG00000229886
NA	-0.0466	5.6276E-01		ENSG00000272103
NA	-0.0274	5.6269E-01		ENSG00000256589
NA	-0.0147	5.6251E-01		ENSG00000269846
NA	-0.0251	5.6236E-01	7.31E-01	ENSG00000123870
NA	-0.0406	5.6202E-01		ENSG00000255440
NA	-0.0563	5.6196E-01	7.31E-01	ENSG00000279179
NA	-0.0298	5.6193E-01	7.31E-01	ENSG00000288876
NA	-0.0180	5.6192E-01		ENSG00000271644
NA	-0.0162	5.6192E-01		ENSG00000258594
NA	-0.0424	5.6188E-01		ENSG00000223799
NA	-0.0206	5.6178E-01		ENSG00000219186
NA	-0.0144	5.6177E-01		ENSG00000287399
NA	-0.0003	5.6156E-01		ENSG00000286401

NA	-0.0059	5.6129E-01		ENSG00000289610
NA	-0.0012	5.6125E-01		ENSG00000279781
NA	-0.0279	5.6120E-01		ENSG00000279589
NA	-0.0130	5.6113E-01		ENSG00000289377
NA	-0.0093	5.6102E-01		ENSG00000253605
NA	-0.0536	5.6091E-01	7.30E-01	ENSG00000258044
NA	-0.0430	5.6087E-01		ENSG00000273816
NA	-0.0543	5.6054E-01	7.30E-01	ENSG00000256443
NA	-0.0559	5.6053E-01	7.30E-01	ENSG00000289306
NA	-0.0310	5.6041E-01	7.30E-01	ENSG00000203258
NA	-0.0160	5.6002E-01		ENSG00000273011
NA	-0.0123	5.5990E-01		ENSG00000288922
NA	-0.0280	5.5962E-01		ENSG00000206976
NA	-0.0370	5.5937E-01	7.29E-01	ENSG00000231703
NA	-0.0549	5.5905E-01	7.29E-01	ENSG00000256712
NA	-0.0140	5.5842E-01		ENSG00000257403
NA	-0.0071	5.5841E-01		ENSG00000269421
NA	-0.0200	5.5840E-01		ENSG00000282943
NA	-0.0112	5.5838E-01		ENSG00000270945
NA	-0.0041	5.5794E-01	7.28E-01	ENSG00000272366
NA	-0.0917	5.5772E-01	7.28E-01	ENSG00000254907
NA	-0.0120	5.5751E-01		ENSG00000279039
NA	-0.0157	5.5729E-01		ENSG00000257042
NA	-0.0706	5.5726E-01	7.28E-01	ENSG00000223508
NA	-0.0336	5.5677E-01		ENSG00000272209
NA	-0.0633	5.5661E-01	7.27E-01	ENSG00000219149
NA	-0.0281	5.5655E-01	7.27E-01	ENSG00000232693
NA	-0.0363	5.5650E-01		ENSG00000271833
NA	-0.0348	5.5631E-01	7.27E-01	ENSG00000269652
NA	-0.0156	5.5623E-01		ENSG00000237836
NA	-0.0081	5.5607E-01		ENSG00000249875
NA	-0.0197	5.5586E-01		ENSG00000286109
NA	-0.0343	5.5544E-01	7.26E-01	ENSG00000285128
NA	-0.0549	5.5522E-01	7.26E-01	ENSG00000259685
NA	-0.0441	5.5514E-01	7.26E-01	ENSG00000234337
NA	-0.0319	5.5512E-01	7.26E-01	ENSG00000231770
NA	-0.0217	5.5509E-01		ENSG00000262921
NA	-0.0168	5.5495E-01		ENSG00000277737
NA	-0.0380	5.5476E-01		ENSG00000231054
NA	-0.0364	5.5475E-01		ENSG00000237740
NA	-0.0365	5.5469E-01		ENSG00000228060
NA	-0.0477	5.5460E-01	7.26E-01	ENSG00000233064
NA	-0.0040	5.5421E-01		ENSG00000267783
NA	-0.0308	5.5393E-01		ENSG00000180172
NA	-0.0186	5.5351E-01		ENSG00000267102
NA	-0.0548	5.5325E-01	7.24E-01	ENSG00000215452
NA	-0.0173	5.5294E-01		ENSG00000231162
NA	-0.0047	5.5274E-01		ENSG00000222007
NA	-0.0494	5.5234E-01	7.24E-01	ENSG00000235790

NA	-0.0352	5.5214E-01		ENSG00000267626
NA	-0.0087	5.5205E-01		ENSG00000278462
NA	-0.0211	5.5193E-01		ENSG00000261723
NA	-0.1166	5.5163E-01	7.23E-01	ENSG00000226701
NA	-0.0633	5.5117E-01	7.23E-01	ENSG00000263938
NA	-0.0389	5.5110E-01		ENSG00000213514
NA	-0.0152	5.5106E-01		ENSG00000232537
NA	-0.0322	5.5069E-01		ENSG00000249669
NA	-0.0327	5.5053E-01		ENSG00000199415
NA	-0.0528	5.5034E-01	7.22E-01	ENSG00000225850
NA	-0.0179	5.4994E-01	7.22E-01	ENSG00000256069
NA	-0.0163	5.4987E-01		ENSG00000259081
NA	-0.0323	5.4946E-01		ENSG00000283511
NA	-0.0430	5.4898E-01	7.21E-01	ENSG00000288819
NA	-0.0316	5.4897E-01		ENSG00000236456
NA	-0.0578	5.4888E-01	7.21E-01	ENSG00000226308
NA	-0.0140	5.4866E-01		ENSG00000261546
NA	-0.0320	5.4861E-01		ENSG00000277806
NA	-0.0177	5.4848E-01		ENSG00000255260
NA	-0.0325	5.4834E-01		ENSG00000258177
NA	-0.0021	5.4829E-01		ENSG00000258657
NA	-0.0138	5.4759E-01		ENSG00000244060
NA	-0.0572	5.4734E-01	7.19E-01	ENSG00000289235
NA	-0.0570	5.4733E-01	7.19E-01	ENSG00000277675
NA	-0.0174	5.4727E-01		ENSG00000234491
NA	-0.0291	5.4717E-01	7.19E-01	ENSG00000285509
NA	-0.0546	5.4692E-01	7.19E-01	ENSG00000260060
NA	-0.0580	5.4657E-01	7.18E-01	ENSG00000279140
NA	-0.0125	5.4641E-01		ENSG00000213197
NA	-0.0100	5.4636E-01		ENSG00000276122
NA	-0.0454	5.4630E-01		ENSG00000275437
NA	-0.0503	5.4614E-01	7.18E-01	ENSG00000269949
NA	-0.0188	5.4542E-01		ENSG00000273115
NA	-0.0481	5.4538E-01	7.17E-01	ENSG00000212769
NA	-0.0044	5.4537E-01		ENSG00000213368
NA	-0.0458	5.4534E-01		ENSG00000236095
NA	-0.0195	5.4483E-01		ENSG00000259215
NA	-0.0150	5.4473E-01		ENSG00000227602
NA	-0.0338	5.4433E-01		ENSG00000280432
NA	-0.0488	5.4430E-01	7.16E-01	ENSG00000214189
NA	-0.0054	5.4421E-01		ENSG00000234663
NA	-0.0120	5.4379E-01		ENSG00000267274
NA	-0.0108	5.4333E-01		ENSG00000239893
NA	-0.1018	5.4314E-01	7.15E-01	ENSG00000287430
NA	-0.0588	5.4294E-01	7.15E-01	ENSG00000229400
NA	-0.0107	5.4291E-01		ENSG00000283084
NA	-0.0055	5.4276E-01	7.15E-01	ENSG00000273167
NA	-0.0132	5.4263E-01		ENSG00000268754
NA	-0.0354	5.4258E-01	7.15E-01	ENSG00000286375

NA	-0.0234	5.4200E-01	7.15E-01	ENSG00000272800
NA	-0.1313	5.4198E-01	7.15E-01	ENSG00000251361
NA	-0.0212	5.4187E-01		ENSG00000260695
NA	-0.0004	5.4164E-01		ENSG00000274751
NA	-0.0484	5.4163E-01	7.15E-01	ENSG00000267345
NA	-0.0457	5.4154E-01	7.14E-01	ENSG00000261226
NA	-0.0577	5.4150E-01	7.14E-01	ENSG00000249815
NA	-0.0099	5.4149E-01		ENSG00000272431
NA	-0.0230	5.4131E-01		ENSG00000219163
NA	-0.0001	5.4119E-01		ENSG00000250325
NA	-0.0594	5.4110E-01	7.14E-01	ENSG00000225518
NA	-0.0334	5.4110E-01		ENSG00000229520
NA	-0.0272	5.4095E-01		ENSG00000267745
NA	-0.0342	5.4066E-01		ENSG00000277840
NA	-0.0598	5.4047E-01	7.14E-01	ENSG00000250130
NA	-0.0599	5.4046E-01	7.14E-01	ENSG00000283537
NA	-0.0688	5.4038E-01	7.14E-01	ENSG00000261505
NA	-0.0358	5.4034E-01	7.14E-01	ENSG00000272836
NA	-0.0095	5.3893E-01		ENSG00000243095
NA	-0.0170	5.3892E-01		ENSG00000279662
NA	-0.0461	5.3887E-01	7.12E-01	ENSG00000228251
NA	-0.1097	5.3867E-01	7.12E-01	ENSG00000258285
NA	-0.0224	5.3844E-01		ENSG00000214976
NA	-0.0735	5.3831E-01	7.12E-01	ENSG00000276529
NA	-0.0119	5.3824E-01		ENSG00000274874
NA	-0.0576	5.3812E-01	7.12E-01	ENSG00000287264
NA	-0.0251	5.3812E-01		ENSG00000258007
NA	-0.0149	5.3780E-01		ENSG00000236940
NA	-0.0283	5.3764E-01		ENSG00000271868
NA	-0.0024	5.3762E-01		ENSG00000260017
NA	-0.0023	5.3702E-01		ENSG00000263787
NA	-0.0355	5.3661E-01		ENSG00000242198
NA	-0.0168	5.3636E-01		ENSG00000262097
NA	-0.0218	5.3632E-01		ENSG00000277017
NA	-0.0367	5.3627E-01	7.10E-01	ENSG00000285676
NA	-0.0246	5.3606E-01		ENSG00000229758
NA	-0.0218	5.3593E-01		ENSG00000224387
NA	-0.0433	5.3576E-01		ENSG00000284776
NA	-0.0289	5.3555E-01		ENSG00000289978
NA	-0.0590	5.3543E-01	7.09E-01	ENSG00000235663
NA	-0.0456	5.3539E-01	7.09E-01	ENSG00000269892
NA	-0.0532	5.3538E-01	7.09E-01	ENSG00000260805
NA	-0.0480	5.3478E-01	7.09E-01	ENSG00000285651
NA	-0.0373	5.3472E-01	7.09E-01	ENSG00000277991
NA	-0.0048	5.3471E-01		ENSG00000285881
NA	-0.0261	5.3452E-01		ENSG00000254006
NA	-0.0264	5.3451E-01		ENSG00000231236
NA	-0.0403	5.3442E-01	7.09E-01	ENSG00000235996
NA	-0.0181	5.3420E-01		ENSG00000280242

NA	-0.0385	5.3380E-01		ENSG00000213033
NA	-0.0068	5.3373E-01		ENSG00000286395
NA	-0.0278	5.3371E-01		ENSG00000265282
NA	-0.0277	5.3369E-01		ENSG00000266907
NA	-0.0103	5.3362E-01		ENSG00000268981
NA	-0.0048	5.3351E-01		ENSG00000267581
NA	-0.0517	5.3330E-01	7.07E-01	ENSG00000215190
NA	-0.0325	5.3284E-01		ENSG00000230696
NA	-0.0758	5.3223E-01	7.06E-01	ENSG00000273893
NA	-0.0392	5.3181E-01	7.06E-01	ENSG00000288557
NA	-0.0667	5.3159E-01	7.06E-01	ENSG00000286145
NA	-0.0511	5.3143E-01	7.06E-01	ENSG00000254153
NA	-0.0440	5.3121E-01	7.06E-01	ENSG00000279812
NA	-0.0023	5.3083E-01	7.05E-01	ENSG00000279894
NA	-0.0169	5.3054E-01	7.05E-01	ENSG00000260978
NA	-0.0554	5.3023E-01	7.05E-01	ENSG00000272720
NA	-0.0618	5.3005E-01	7.05E-01	ENSG00000279738
NA	-0.0233	5.2998E-01		ENSG00000248676
NA	-0.0185	5.2993E-01		ENSG00000288581
NA	-0.0411	5.2993E-01	7.05E-01	ENSG00000257494
NA	-0.0581	5.2955E-01		ENSG00000229256
NA	-0.0490	5.2939E-01		ENSG00000268836
NA	-0.0278	5.2920E-01		ENSG00000280767
NA	-0.0614	5.2914E-01	7.04E-01	ENSG00000231258
NA	-0.0119	5.2907E-01	7.04E-01	ENSG00000274184
NA	-0.0377	5.2904E-01	7.04E-01	ENSG00000249673
NA	-0.0297	5.2903E-01		ENSG00000289572
NA	-0.0279	5.2865E-01		ENSG00000256588
NA	-0.0169	5.2864E-01		ENSG00000232715
NA	-0.0614	5.2852E-01	7.04E-01	ENSG00000233929
NA	-0.0577	5.2844E-01	7.04E-01	ENSG00000284773
NA	-0.0222	5.2813E-01		ENSG00000238140
NA	-0.0514	5.2797E-01		ENSG00000217929
NA	-0.0303	5.2792E-01		ENSG00000222604
NA	-0.0297	5.2788E-01		ENSG00000257155
NA	-0.0449	5.2778E-01	7.03E-01	ENSG00000233514
NA	-0.0319	5.2765E-01		ENSG00000280485
NA	-0.0153	5.2762E-01		ENSG00000251170
NA	-0.0235	5.2752E-01		ENSG00000261970
NA	-0.0430	5.2750E-01		ENSG00000260550
NA	-0.0256	5.2742E-01		ENSG00000134612
NA	-0.0310	5.2723E-01		ENSG00000273855
NA	-0.0515	5.2680E-01	7.03E-01	ENSG00000275741
NA	-0.0090	5.2649E-01	7.02E-01	ENSG00000272540
NA	-0.0234	5.2631E-01		ENSG00000279265
NA	-0.0202	5.2590E-01		ENSG00000289969
NA	-0.0790	5.2555E-01	7.01E-01	ENSG00000254054
NA	-0.0320	5.2550E-01		ENSG00000228998
NA	-0.0488	5.2549E-01	7.01E-01	ENSG00000223599

NA	-0.0461	5.2542E-01	7.01E-01	ENSG00000286943
NA	-0.0543	5.2496E-01	7.01E-01	ENSG00000287637
NA	-0.0221	5.2480E-01		ENSG00000236153
NA	-0.0212	5.2447E-01		ENSG00000232408
NA	-0.0180	5.2437E-01		ENSG00000251675
NA	-0.0317	5.2403E-01	7.01E-01	ENSG00000273287
NA	-0.0538	5.2374E-01		ENSG00000239265
NA	-0.0251	5.2372E-01		ENSG00000276863
NA	-0.0192	5.2353E-01		ENSG00000279630
NA	-0.0464	5.2335E-01	7.00E-01	ENSG00000267422
NA	-0.0352	5.2329E-01		ENSG00000286501
NA	-0.0309	5.2326E-01		ENSG00000266922
NA	-0.0548	5.2321E-01	7.00E-01	ENSG00000249519
NA	-0.0532	5.2312E-01	7.00E-01	ENSG00000256464
NA	-0.0434	5.2291E-01	7.00E-01	ENSG00000237352
NA	-0.0031	5.2287E-01		ENSG00000231698
NA	-0.0281	5.2183E-01		ENSG00000234020
NA	-0.0256	5.2149E-01		ENSG00000271361
NA	-0.0464	5.2132E-01		ENSG00000286181
NA	-0.0057	5.2119E-01		ENSG00000286676
NA	-0.0176	5.2066E-01		ENSG00000279354
NA	-0.0237	5.2035E-01		ENSG00000276002
NA	-0.0592	5.2023E-01	6.98E-01	ENSG00000205424
NA	-0.0184	5.1981E-01		ENSG00000261687
NA	-0.0626	5.1959E-01	6.97E-01	ENSG00000279259
NA	-0.0003	5.1938E-01		ENSG00000241532
NA	-0.0416	5.1936E-01	6.97E-01	ENSG00000285868
NA	-0.0008	5.1917E-01		ENSG00000277313
NA	-0.0623	5.1901E-01	6.96E-01	ENSG00000227615
NA	-0.0268	5.1874E-01		ENSG00000267387
NA	-0.0194	5.1872E-01		ENSG00000273464
NA	-0.0455	5.1844E-01	6.96E-01	ENSG00000278869
NA	-0.0599	5.1803E-01	6.96E-01	ENSG00000258592
NA	-0.0642	5.1800E-01	6.96E-01	ENSG00000275479
NA	-0.0483	5.1791E-01	6.96E-01	ENSG00000241599
NA	-0.0449	5.1784E-01	6.96E-01	ENSG00000233110
NA	-0.0517	5.1694E-01	6.95E-01	ENSG00000230202
NA	-0.0510	5.1685E-01	6.95E-01	ENSG00000279236
NA	-0.0582	5.1679E-01	6.95E-01	ENSG00000250068
NA	-0.0547	5.1666E-01	6.95E-01	ENSG00000286971
NA	-0.0519	5.1665E-01	6.95E-01	ENSG00000261804
NA	-0.0178	5.1664E-01		ENSG00000268510
NA	-0.0005	5.1658E-01		ENSG00000230191
NA	-0.0163	5.1646E-01		ENSG00000276691
NA	-0.0253	5.1642E-01		ENSG00000280079
NA	-0.0481	5.1625E-01		ENSG00000243304
NA	-0.0114	5.1616E-01	6.95E-01	ENSG00000283047
NA	-0.0496	5.1603E-01	6.95E-01	ENSG00000213790
NA	-0.0652	5.1600E-01	6.95E-01	ENSG00000285879

NA	-0.0479	5.1588E-01		ENSG00000267717
NA	-0.0345	5.1583E-01		ENSG00000254271
NA	-0.0160	5.1559E-01		ENSG00000224485
NA	-0.0151	5.1528E-01	6.94E-01	ENSG00000215559
NA	-0.0189	5.1498E-01		ENSG00000287053
NA	-0.0388	5.1484E-01	6.94E-01	ENSG00000264943
NA	-0.0817	5.1471E-01	6.94E-01	ENSG00000234345
NA	-0.0041	5.1460E-01	6.94E-01	ENSG00000273372
NA	-0.0322	5.1458E-01		ENSG00000270321
NA	-0.0296	5.1449E-01		ENSG00000272167
NA	-0.0150	5.1421E-01		ENSG00000274272
NA	-0.0433	5.1408E-01	6.93E-01	ENSG00000244573
NA	-0.0194	5.1388E-01		ENSG00000214244
NA	-0.0606	5.1373E-01	6.93E-01	ENSG00000288704
NA	-0.0218	5.1341E-01		ENSG00000287714
NA	-0.0394	5.1334E-01	6.93E-01	ENSG00000233885
NA	-0.0464	5.1325E-01	6.93E-01	ENSG00000255580
NA	-0.0194	5.1307E-01		ENSG00000283286
NA	-0.0797	5.1299E-01	6.92E-01	ENSG00000249131
NA	-0.0375	5.1274E-01		ENSG00000279019
NA	-0.0417	5.1270E-01		ENSG00000260776
NA	-0.0385	5.1257E-01		ENSG00000263206
NA	-0.0232	5.1252E-01		ENSG00000233535
NA	-0.0186	5.1251E-01		ENSG00000229569
NA	-0.0153	5.1219E-01		ENSG00000279562
NA	-0.0211	5.1175E-01		ENSG00000261775
NA	-0.0433	5.1170E-01	6.92E-01	ENSG00000286104
NA	-0.0213	5.1167E-01		ENSG00000232032
NA	-0.0234	5.1159E-01		ENSG00000283273
NA	-0.0434	5.1140E-01		ENSG00000174680
NA	-0.0187	5.1122E-01		ENSG00000227958
NA	-0.0250	5.1118E-01		ENSG00000236559
NA	-0.0107	5.1111E-01		ENSG00000286449
NA	-0.0279	5.1111E-01		ENSG00000261842
NA	-0.0593	5.1101E-01	6.91E-01	ENSG00000249207
NA	-0.0309	5.1088E-01		ENSG00000286249
NA	-0.0161	5.1080E-01		ENSG00000271761
NA	-0.0022	5.1078E-01		ENSG00000224273
NA	-0.0101	5.1055E-01	6.90E-01	ENSG00000288755
NA	-0.0533	5.1051E-01	6.90E-01	ENSG00000108958
NA	-0.0988	5.1037E-01	6.90E-01	ENSG00000228499
NA	-0.0186	5.1016E-01		ENSG00000229543
NA	-0.0579	5.1015E-01	6.90E-01	ENSG00000214578
NA	-0.0246	5.0971E-01		ENSG00000279437
NA	-0.0186	5.0970E-01		ENSG00000226789
NA	-0.0594	5.0964E-01	6.90E-01	ENSG00000287584
NA	-0.0352	5.0950E-01		ENSG00000261837
NA	-0.0640	5.0945E-01	6.89E-01	ENSG00000283064
NA	-0.0351	5.0889E-01		ENSG00000225761

NA	-0.0095	5.0795E-01		ENSG00000235656
NA	-0.0403	5.0769E-01	6.88E-01	ENSG00000286210
NA	-0.0584	5.0759E-01		ENSG00000253535
NA	-0.1187	5.0718E-01	6.88E-01	ENSG00000273319
NA	-0.0646	5.0689E-01	6.87E-01	ENSG00000230084
NA	-0.0249	5.0681E-01		ENSG00000281780
NA	-0.0641	5.0674E-01	6.87E-01	ENSG00000268751
NA	-0.0002	5.0661E-01		ENSG00000243107
NA	-0.0398	5.0604E-01	6.87E-01	ENSG00000202399
NA	-0.0610	5.0601E-01	6.87E-01	ENSG00000260400
NA	-0.0608	5.0590E-01	6.87E-01	ENSG00000289024
NA	-0.0515	5.0589E-01	6.87E-01	ENSG00000276298
NA	-0.0649	5.0568E-01	6.87E-01	ENSG00000289626
NA	-0.0178	5.0565E-01		ENSG00000231907
NA	-0.0016	5.0561E-01		ENSG00000254401
NA	-0.0485	5.0501E-01	6.86E-01	ENSG00000286634
NA	-0.0582	5.0497E-01	6.86E-01	ENSG00000214514
NA	-0.0227	5.0486E-01		ENSG00000227455
NA	-0.0004	5.0478E-01	6.86E-01	ENSG00000289593
NA	-0.0490	5.0452E-01		ENSG00000237522
NA	-0.0700	5.0438E-01	6.86E-01	ENSG00000276445
NA	-0.0410	5.0393E-01		ENSG00000278572
NA	-0.0897	5.0387E-01	6.85E-01	ENSG00000274364
NA	-0.0635	5.0371E-01	6.85E-01	ENSG00000249684
NA	-0.0847	5.0315E-01	6.84E-01	ENSG00000270792
NA	-0.0587	5.0299E-01	6.84E-01	ENSG00000188383
NA	-0.0251	5.0280E-01		ENSG00000275929
NA	-0.0466	5.0270E-01		ENSG00000279565
NA	-0.0088	5.0260E-01	6.84E-01	ENSG00000231084
NA	-0.0470	5.0221E-01	6.84E-01	ENSG00000257894
NA	-0.0096	5.0215E-01		ENSG00000203520
NA	-0.0015	5.0194E-01		ENSG00000287813
NA	-0.0455	5.0153E-01	6.83E-01	ENSG00000278383
NA	-0.0350	5.0120E-01		ENSG00000280054
NA	-0.0183	5.0116E-01		ENSG00000224666
NA	-0.0035	5.0079E-01		ENSG00000261821
NA	-0.0170	5.0075E-01		ENSG00000264215
NA	-0.0239	5.0052E-01		ENSG00000253374
NA	-0.0297	5.0047E-01	6.83E-01	ENSG00000287679
NA	-0.0444	5.0010E-01		ENSG00000226862
NA	-0.0196	4.9960E-01		ENSG00000255819
NA	-0.0324	4.9958E-01		ENSG00000224566
NA	-0.0609	4.9958E-01	6.82E-01	ENSG00000179082
NA	-0.1352	4.9951E-01	6.82E-01	ENSG00000282996
NA	-0.0164	4.9936E-01		ENSG00000229118
NA	-0.0174	4.9857E-01		ENSG00000218748
NA	-0.0155	4.9845E-01		ENSG00000257956
NA	-0.0523	4.9821E-01	6.81E-01	ENSG00000234043
NA	-0.0639	4.9800E-01	6.81E-01	ENSG00000271646

NA	-0.0571	4.9780E-01	6.81E-01	ENSG00000287702
NA	-0.0426	4.9764E-01		ENSG00000238137
NA	-0.0037	4.9764E-01		ENSG00000288879
NA	-0.0664	4.9764E-01	6.81E-01	ENSG00000253161
NA	-0.0658	4.9749E-01	6.80E-01	ENSG00000249502
NA	-0.0024	4.9692E-01	6.80E-01	ENSG00000256682
NA	-0.0244	4.9663E-01		ENSG00000263829
NA	-0.0377	4.9644E-01	6.80E-01	ENSG00000231156
NA	-0.0279	4.9631E-01		ENSG00000253875
NA	-0.0238	4.9628E-01		ENSG00000274701
NA	-0.0228	4.9584E-01		ENSG00000278886
NA	-0.0265	4.9575E-01		ENSG00000234886
NA	-0.0526	4.9561E-01		ENSG00000274315
NA	-0.0419	4.9559E-01	6.79E-01	ENSG00000235677
NA	-0.0493	4.9555E-01	6.79E-01	ENSG00000189149
NA	-0.0003	4.9553E-01		ENSG00000276017
NA	-0.0445	4.9539E-01		ENSG00000275676
NA	-0.0236	4.9521E-01		ENSG00000227330
NA	-0.0255	4.9517E-01		ENSG00000231662
NA	-0.0199	4.9500E-01		ENSG00000282742
NA	-0.0165	4.9497E-01		ENSG00000289164
NA	-0.0518	4.9489E-01	6.78E-01	ENSG00000254972
NA	-0.0622	4.9479E-01	6.78E-01	ENSG00000248240
NA	-0.0424	4.9465E-01	6.78E-01	ENSG00000289144
NA	-0.0178	4.9448E-01		ENSG00000285731
NA	-0.0011	4.9438E-01		ENSG00000283415
NA	-0.0034	4.9424E-01	6.78E-01	ENSG00000289228
NA	-0.1365	4.9382E-01	6.77E-01	ENSG00000269139
NA	-0.0202	4.9376E-01		ENSG00000268970
NA	-0.0646	4.9369E-01	6.77E-01	ENSG00000247373
NA	-0.0430	4.9345E-01	6.77E-01	ENSG00000277118
NA	-0.0016	4.9343E-01		ENSG00000262979
NA	-0.0259	4.9329E-01		ENSG00000237331
NA	-0.0360	4.9311E-01		ENSG00000215210
NA	-0.0337	4.9311E-01		ENSG00000230777
NA	-0.0007	4.9286E-01		ENSG00000286417
NA	-0.0663	4.9269E-01	6.76E-01	ENSG00000258982
NA	-0.0812	4.9241E-01	6.76E-01	ENSG00000223803
NA	-0.0673	4.9241E-01	6.76E-01	ENSG00000236876
NA	-0.0121	4.9226E-01		ENSG00000243498
NA	-0.0065	4.9206E-01		ENSG00000288773
NA	-0.0338	4.9204E-01		ENSG00000260733
NA	-0.0555	4.9157E-01	6.75E-01	ENSG00000243491
NA	-0.0290	4.9134E-01		ENSG00000213237
NA	-0.0509	4.9076E-01		ENSG00000254315
NA	-0.0403	4.9072E-01		ENSG00000234617
NA	-0.0579	4.9064E-01	6.75E-01	ENSG00000260077
NA	-0.0491	4.9055E-01		ENSG00000250432
NA	-0.0192	4.9051E-01		ENSG00000235149

NA	-0.0650	4.9035E-01	6.74E-01	ENSG00000234832
NA	-0.0423	4.9013E-01		ENSG00000188850
NA	-0.0052	4.9010E-01	6.74E-01	ENSG00000233716
NA	-0.0246	4.8994E-01		ENSG00000253772
NA	-0.0557	4.8962E-01	6.74E-01	ENSG00000234595
NA	-0.0144	4.8961E-01		ENSG00000269944
NA	-0.0432	4.8890E-01		ENSG00000215086
NA	-0.0658	4.8869E-01	6.73E-01	ENSG00000238043
NA	-0.0636	4.8867E-01	6.73E-01	ENSG00000289223
NA	-0.0184	4.8851E-01		ENSG00000266446
NA	-0.0286	4.8845E-01		ENSG00000231461
NA	-0.0137	4.8823E-01		ENSG00000272164
NA	-0.0528	4.8780E-01		ENSG00000235361
NA	-0.0506	4.8773E-01		ENSG00000259874
NA	-0.0213	4.8710E-01		ENSG00000286303
NA	-0.0456	4.8703E-01	6.71E-01	ENSG00000236140
NA	-0.0224	4.8693E-01		ENSG00000287975
NA	-0.0204	4.8680E-01		ENSG00000213875
NA	-0.0265	4.8653E-01		ENSG00000268764
NA	-0.0284	4.8614E-01		ENSG00000270409
NA	-0.0255	4.8610E-01		ENSG00000259637
NA	-0.0499	4.8607E-01	6.71E-01	ENSG00000272402
NA	-0.0202	4.8602E-01		ENSG00000203416
NA	-0.0478	4.8597E-01		ENSG00000226072
NA	-0.0522	4.8566E-01	6.70E-01	ENSG00000254325
NA	-0.0196	4.8482E-01		ENSG00000237262
NA	-0.0184	4.8439E-01		ENSG00000254988
NA	-0.0467	4.8425E-01	6.69E-01	ENSG00000287884
NA	-0.0441	4.8377E-01		ENSG00000258580
NA	-0.0152	4.8348E-01		ENSG00000270665
NA	-0.0517	4.8333E-01	6.68E-01	ENSG00000286618
NA	-0.0573	4.8307E-01	6.68E-01	ENSG00000288719
NA	-0.0222	4.8288E-01		ENSG00000266371
NA	-0.0972	4.8247E-01	6.68E-01	ENSG00000273437
NA	-0.0958	4.8244E-01	6.68E-01	ENSG00000248015
NA	-0.0245	4.8240E-01		ENSG00000224430
NA	-0.0571	4.8214E-01	6.67E-01	ENSG00000258611
NA	-0.0188	4.8181E-01		ENSG00000266959
NA	-0.0466	4.8175E-01	6.67E-01	ENSG00000259429
NA	-0.0638	4.8160E-01	6.67E-01	ENSG00000241520
NA	-0.0751	4.8160E-01		ENSG00000260483
NA	-0.0479	4.8103E-01	6.66E-01	ENSG00000267480
NA	-0.0600	4.8082E-01	6.66E-01	ENSG00000261141
NA	-0.0541	4.8070E-01	6.66E-01	ENSG00000258342
NA	-0.0462	4.8035E-01		ENSG00000264300
NA	-0.0328	4.8005E-01		ENSG00000274682
NA	-0.0139	4.7957E-01		ENSG00000234896
NA	-0.0183	4.7950E-01		ENSG00000254433
NA	-0.0517	4.7948E-01	6.65E-01	ENSG00000260714

NA	-0.0308	4.7915E-01		ENSG00000277958
NA	-0.0240	4.7888E-01		ENSG00000227291
NA	-0.0580	4.7832E-01	6.64E-01	ENSG00000233799
NA	-0.0392	4.7830E-01		ENSG00000259767
NA	-0.0649	4.7817E-01	6.64E-01	ENSG00000266573
NA	-0.0401	4.7814E-01	6.64E-01	ENSG00000277270
NA	-0.0485	4.7811E-01	6.64E-01	ENSG00000257052
NA	-0.0518	4.7798E-01	6.64E-01	ENSG00000232949
NA	-0.0028	4.7774E-01		ENSG00000206702
NA	-0.0617	4.7726E-01	6.64E-01	ENSG00000259398
NA	-0.0643	4.7713E-01	6.64E-01	ENSG00000284636
NA	-0.0467	4.7708E-01	6.64E-01	ENSG00000231799
NA	-0.0778	4.7703E-01	6.64E-01	ENSG00000287160
NA	-0.0235	4.7687E-01		ENSG00000227303
NA	-0.0176	4.7666E-01		ENSG00000286085
NA	-0.0286	4.7655E-01		ENSG00000259212
NA	-0.0478	4.7652E-01	6.63E-01	ENSG00000272010
NA	-0.0285	4.7648E-01		ENSG00000280215
NA	-0.0366	4.7619E-01		ENSG00000199357
NA	-0.0662	4.7608E-01	6.63E-01	ENSG00000217094
NA	-0.0687	4.7589E-01	6.63E-01	ENSG00000287645
NA	-0.0170	4.7570E-01		ENSG00000281162
NA	-0.0406	4.7533E-01		ENSG00000241462
NA	-0.0429	4.7533E-01		ENSG00000267940
NA	-0.0694	4.7502E-01		ENSG00000267149
NA	-0.0219	4.7491E-01		ENSG00000265739
NA	-0.0355	4.7464E-01		ENSG00000216676
NA	-0.0509	4.7448E-01	6.61E-01	ENSG00000261757
NA	-0.0095	4.7397E-01		ENSG00000256280
NA	-0.0371	4.7388E-01		ENSG00000274678
NA	-0.0607	4.7368E-01	6.60E-01	ENSG00000224579
NA	-0.0094	4.7352E-01	6.60E-01	ENSG00000253832
NA	-0.0238	4.7327E-01		ENSG00000258558
NA	-0.0458	4.7317E-01	6.60E-01	ENSG00000200309
NA	-0.0064	4.7311E-01	6.60E-01	ENSG00000278434
NA	-0.0590	4.7304E-01	6.60E-01	ENSG00000237892
NA	-0.0349	4.7298E-01		ENSG00000270002
NA	-0.0733	4.7270E-01	6.60E-01	ENSG00000230593
NA	-0.0150	4.7233E-01		ENSG00000267509
NA	-0.0681	4.7232E-01	6.60E-01	ENSG00000283078
NA	-0.0562	4.7222E-01	6.60E-01	ENSG00000274883
NA	-0.0136	4.7181E-01		ENSG00000172912
NA	-0.0693	4.7163E-01	6.59E-01	ENSG00000258728
NA	-0.0704	4.7155E-01	6.59E-01	ENSG00000278376
NA	-0.0703	4.7150E-01	6.59E-01	ENSG00000286067
NA	-0.0239	4.7148E-01		ENSG00000271452
NA	-0.0604	4.7123E-01	6.59E-01	ENSG00000255689
NA	-0.0515	4.7086E-01		ENSG00000261810
NA	-0.0534	4.7055E-01	6.58E-01	ENSG00000289529

NA	-0.0706	4.7034E-01	6.58E-01	ENSG00000289084
NA	-0.0686	4.7021E-01	6.58E-01	ENSG00000289437
NA	-0.0718	4.7018E-01	6.58E-01	ENSG00000235253
NA	-0.0340	4.6984E-01		ENSG00000289966
NA	-0.0458	4.6971E-01	6.57E-01	ENSG00000289224
NA	-0.0572	4.6969E-01	6.57E-01	ENSG00000279288
NA	-0.0212	4.6948E-01		ENSG00000217718
NA	-0.0164	4.6921E-01		ENSG00000217330
NA	-0.0244	4.6860E-01		ENSG00000256538
NA	-0.1040	4.6854E-01	6.56E-01	ENSG00000253194
NA	-0.0112	4.6851E-01		ENSG00000225642
NA	-0.0291	4.6813E-01		ENSG00000260944
NA	-0.0545	4.6773E-01	6.55E-01	ENSG00000270087
NA	-0.0336	4.6771E-01		ENSG00000255339
NA	-0.0694	4.6755E-01	6.55E-01	ENSG00000224905
NA	-0.0692	4.6731E-01	6.55E-01	ENSG00000280800
NA	-0.0643	4.6721E-01		ENSG00000277447
NA	-0.0696	4.6713E-01	6.55E-01	ENSG00000227038
NA	-0.0181	4.6709E-01		ENSG00000259055
NA	-0.0452	4.6694E-01	6.55E-01	ENSG00000232260
NA	-0.0525	4.6681E-01		ENSG00000226971
NA	-0.0590	4.6641E-01	6.54E-01	ENSG00000287150
NA	-0.0912	4.6637E-01	6.54E-01	ENSG00000253112
NA	-0.0008	4.6601E-01		ENSG00000267319
NA	-0.0574	4.6592E-01	6.54E-01	ENSG00000251139
NA	-0.0136	4.6577E-01		ENSG00000254170
NA	-0.0546	4.6573E-01	6.54E-01	ENSG00000269887
NA	-0.0154	4.6568E-01		ENSG00000275438
NA	-0.0226	4.6562E-01		ENSG00000258646
NA	-0.0229	4.6554E-01		ENSG00000235432
NA	-0.0497	4.6542E-01	6.54E-01	ENSG00000247925
NA	-0.0136	4.6515E-01	6.53E-01	ENSG00000279135
NA	-0.0281	4.6513E-01		ENSG00000251187
NA	-0.0276	4.6494E-01		ENSG00000275006
NA	-0.0215	4.6449E-01		ENSG00000285771
NA	-0.0716	4.6393E-01	6.52E-01	ENSG00000235513
NA	-0.0162	4.6358E-01		ENSG00000238059
NA	-0.0777	4.6342E-01	6.52E-01	ENSG00000289136
NA	-0.0714	4.6335E-01	6.52E-01	ENSG00000273218
NA	-0.0511	4.6331E-01	6.52E-01	ENSG00000219608
NA	-0.0369	4.6323E-01		ENSG00000253172
NA	-0.0275	4.6303E-01		ENSG00000260844
NA	-0.0236	4.6241E-01		ENSG00000234223
NA	-0.0247	4.6234E-01		ENSG00000258662
NA	-0.0115	4.6211E-01		ENSG00000224493
NA	-0.0677	4.6204E-01	6.50E-01	ENSG00000282021
NA	-0.0526	4.6181E-01	6.50E-01	ENSG00000274372
NA	-0.0365	4.6177E-01		ENSG00000286874
NA	-0.0242	4.6148E-01		ENSG00000251665

NA	-0.0587	4.6133E-01	6.50E-01	ENSG00000204652
NA	-0.0666	4.6128E-01		ENSG00000225721
NA	-0.0425	4.6093E-01		ENSG00000286602
NA	-0.0134	4.6086E-01		ENSG00000286572
NA	-0.0253	4.6074E-01		ENSG00000233363
NA	-0.0345	4.6029E-01	6.49E-01	ENSG00000233778
NA	-0.0584	4.6014E-01	6.49E-01	ENSG00000246575
NA	-0.0367	4.6011E-01		ENSG00000202078
NA	-0.0679	4.6007E-01	6.49E-01	ENSG00000273226
NA	-0.0528	4.5973E-01	6.48E-01	ENSG00000237994
NA	-0.0435	4.5966E-01		ENSG00000218561
NA	-0.0362	4.5966E-01		ENSG00000286411
NA	-0.0343	4.5962E-01		ENSG00000260536
NA	-0.0199	4.5956E-01		ENSG00000213498
NA	-0.0263	4.5942E-01		ENSG00000232202
NA	-0.0505	4.5933E-01	6.48E-01	ENSG00000279175
NA	-0.0682	4.5931E-01	6.48E-01	ENSG00000238287
NA	-0.0178	4.5927E-01		ENSG00000229010
NA	-0.1032	4.5919E-01	6.48E-01	ENSG00000289827
NA	-0.0729	4.5907E-01	6.48E-01	ENSG00000230578
NA	-0.0321	4.5904E-01		ENSG00000264196
NA	-0.0574	4.5888E-01	6.48E-01	ENSG00000253133
NA	-0.0645	4.5882E-01		ENSG00000261553
NA	-0.0371	4.5870E-01		ENSG00000233877
NA	-0.0590	4.5855E-01	6.48E-01	ENSG00000251521
NA	-0.0232	4.5850E-01		ENSG00000237464
NA	-0.0211	4.5818E-01		ENSG00000177776
NA	-0.0149	4.5799E-01		ENSG00000234515
NA	-0.0214	4.5782E-01		ENSG00000255097
NA	-0.0173	4.5755E-01		ENSG00000231625
NA	-0.0669	4.5751E-01	6.47E-01	ENSG00000279670
NA	-0.0363	4.5743E-01		ENSG00000286686
NA	-0.0431	4.5727E-01		ENSG00000251402
NA	-0.0592	4.5718E-01	6.46E-01	ENSG00000215492
NA	-0.0156	4.5709E-01		ENSG00000224061
NA	-0.0666	4.5708E-01	6.46E-01	ENSG00000286816
NA	-0.0669	4.5704E-01	6.46E-01	ENSG00000259065
NA	-0.0548	4.5689E-01	6.46E-01	ENSG00000268707
NA	-0.0521	4.5688E-01	6.46E-01	ENSG00000200834
NA	-0.0638	4.5657E-01	6.46E-01	ENSG00000254467
NA	-0.0697	4.5656E-01	6.46E-01	ENSG00000270760
NA	-0.0684	4.5653E-01	6.46E-01	ENSG00000286997
NA	-0.0375	4.5640E-01		ENSG00000257464
NA	-0.1447	4.5636E-01	6.46E-01	ENSG00000227088
NA	-0.0135	4.5593E-01		ENSG00000253383
NA	-0.0145	4.5565E-01		ENSG00000231092
NA	-0.0336	4.5548E-01		ENSG00000286806
NA	-0.0312	4.5530E-01		ENSG00000279481
NA	-0.0415	4.5523E-01	6.45E-01	ENSG00000249526

NA	-0.0496	4.5522E-01	6.45E-01	ENSG00000288769
NA	-0.0406	4.5514E-01		ENSG00000217159
NA	-0.0244	4.5490E-01		ENSG00000244701
NA	-0.0499	4.5469E-01		ENSG00000268051
NA	-0.0731	4.5439E-01	6.44E-01	ENSG00000245248
NA	-0.0422	4.5417E-01		ENSG00000258473
NA	-0.0047	4.5381E-01	6.43E-01	ENSG00000263089
NA	-0.0271	4.5365E-01		ENSG00000217835
NA	-0.0332	4.5362E-01		ENSG00000263370
NA	-0.0661	4.5339E-01	6.43E-01	ENSG00000272721
NA	-0.0244	4.5278E-01		ENSG00000248634
NA	-0.0276	4.5234E-01		ENSG00000287869
NA	-0.0092	4.5226E-01		ENSG00000232470
NA	-0.0188	4.5217E-01		ENSG00000271811
NA	-0.0247	4.5201E-01		ENSG00000248912
NA	-0.0245	4.5151E-01		ENSG00000287903
NA	-0.0123	4.5149E-01	6.41E-01	ENSG00000221184
NA	-0.0507	4.5149E-01	6.41E-01	ENSG00000287051
NA	-0.0887	4.5140E-01		ENSG00000226632
NA	-0.0575	4.5024E-01		ENSG00000290003
NA	-0.0726	4.5007E-01	6.40E-01	ENSG00000228960
NA	-0.0719	4.5006E-01	6.40E-01	ENSG00000246203
NA	-0.0738	4.5005E-01	6.40E-01	ENSG00000261613
NA	-0.0400	4.4944E-01		ENSG00000233090
NA	-0.0539	4.4914E-01	6.39E-01	ENSG00000213386
NA	-0.0247	4.4907E-01		ENSG00000288843
NA	-0.0708	4.4905E-01	6.39E-01	ENSG00000269961
NA	-0.0431	4.4902E-01		ENSG00000258846
NA	-0.0164	4.4888E-01		ENSG00000260021
NA	-0.0708	4.4841E-01	6.38E-01	ENSG00000253377
NA	-0.0324	4.4818E-01		ENSG00000257880
NA	-0.0833	4.4797E-01	6.38E-01	ENSG00000238189
NA	-0.0558	4.4748E-01	6.37E-01	ENSG00000262136
NA	-0.0127	4.4733E-01		ENSG00000287872
NA	-0.0144	4.4712E-01		ENSG00000234451
NA	-0.0040	4.4704E-01		ENSG00000232928
NA	-0.0445	4.4668E-01		ENSG00000286338
NA	-0.0949	4.4636E-01	6.36E-01	ENSG00000289326
NA	-0.0157	4.4618E-01		ENSG00000289473
NA	-0.0356	4.4609E-01	6.36E-01	ENSG00000232431
NA	-0.0660	4.4596E-01	6.36E-01	ENSG00000225591
NA	-0.0724	4.4571E-01	6.36E-01	ENSG00000189212
NA	-0.0457	4.4559E-01		ENSG00000284986
NA	-0.0359	4.4512E-01		ENSG00000279742
NA	-0.0041	4.4501E-01		ENSG00000266696
NA	-0.0157	4.4498E-01		ENSG00000272909
NA	-0.0643	4.4482E-01	6.35E-01	ENSG00000230322
NA	-0.0733	4.4443E-01	6.35E-01	ENSG00000281181
NA	-0.0747	4.4416E-01	6.35E-01	ENSG00000286863

NA	-0.0490	4.4415E-01		ENSG00000200742
NA	-0.0612	4.4323E-01	6.34E-01	ENSG00000289065
NA	-0.0308	4.4295E-01	6.34E-01	ENSG00000287988
NA	-0.0500	4.4292E-01		ENSG00000232218
NA	-0.0350	4.4285E-01		ENSG00000235802
NA	-0.0863	4.4272E-01		ENSG00000236852
NA	-0.0748	4.4247E-01	6.33E-01	ENSG00000224861
NA	-0.0242	4.4244E-01		ENSG00000271897
NA	-0.0745	4.4210E-01	6.33E-01	ENSG00000289255
NA	-0.0422	4.4193E-01		ENSG00000228834
NA	-0.0275	4.4183E-01		ENSG00000287270
NA	-0.0755	4.4178E-01	6.32E-01	ENSG00000259728
NA	-0.0545	4.4134E-01	6.32E-01	ENSG00000253697
NA	-0.0206	4.4073E-01		ENSG00000269933
NA	-0.0733	4.4068E-01	6.31E-01	ENSG00000206228
NA	-0.0264	4.4037E-01		ENSG00000222042
NA	-0.0738	4.4016E-01	6.31E-01	ENSG00000241622
NA	-0.0767	4.4014E-01	6.31E-01	ENSG00000260443
NA	-0.0525	4.3991E-01		ENSG00000259338
NA	-0.0227	4.3989E-01		ENSG00000287798
NA	-0.0636	4.3984E-01	6.31E-01	ENSG00000276672
NA	-0.0015	4.3965E-01		ENSG00000275024
NA	-0.0471	4.3948E-01		ENSG00000270890
NA	-0.0380	4.3921E-01		ENSG00000272076
NA	-0.0518	4.3898E-01		ENSG00000285918
NA	-0.0473	4.3875E-01	6.30E-01	ENSG00000287989
NA	-0.0754	4.3874E-01	6.30E-01	ENSG00000270728
NA	-0.0244	4.3861E-01		ENSG00000275129
NA	-0.0245	4.3837E-01		ENSG00000260031
NA	-0.0031	4.3811E-01		ENSG00000237271
NA	-0.0724	4.3785E-01	6.29E-01	ENSG00000273284
NA	-0.0546	4.3775E-01		ENSG00000201384
NA	-0.0144	4.3761E-01		ENSG00000290163
NA	-0.0545	4.3757E-01	6.28E-01	ENSG00000270589
NA	-0.0432	4.3734E-01	6.28E-01	ENSG00000260118
NA	-0.0743	4.3704E-01	6.28E-01	ENSG00000289909
NA	-0.0316	4.3687E-01		ENSG00000250126
NA	-0.0556	4.3678E-01	6.28E-01	ENSG00000285999
NA	-0.0471	4.3666E-01	6.28E-01	ENSG00000237001
NA	-0.0270	4.3657E-01		ENSG00000289403
NA	-0.0588	4.3657E-01	6.28E-01	ENSG00000237713
NA	-0.0418	4.3637E-01		ENSG00000214089
NA	-0.0282	4.3617E-01		ENSG00000253299
NA	-0.0206	4.3611E-01		ENSG00000200714
NA	-0.0136	4.3592E-01		ENSG00000253197
NA	-0.0722	4.3582E-01	6.27E-01	ENSG00000261754
NA	-0.0068	4.3572E-01		ENSG00000282870
NA	-0.0313	4.3538E-01		ENSG00000272144
NA	-0.0652	4.3531E-01	6.27E-01	ENSG00000274818

NA	-0.0061	4.3510E-01	6.26E-01	ENSG00000272864
NA	-0.0228	4.3498E-01		ENSG00000268790
NA	-0.0311	4.3487E-01		ENSG00000271554
NA	-0.0702	4.3439E-01	6.26E-01	ENSG00000285257
NA	-0.0720	4.3396E-01		ENSG00000266993
NA	-0.0742	4.3395E-01	6.25E-01	ENSG00000258938
NA	-0.0197	4.3382E-01		ENSG00000286678
NA	-0.0734	4.3371E-01	6.25E-01	ENSG00000225446
NA	-0.0099	4.3366E-01		ENSG00000213540
NA	-0.0472	4.3357E-01		ENSG00000286805
NA	-0.0741	4.3347E-01	6.25E-01	ENSG00000257191
NA	-0.0786	4.3345E-01	6.25E-01	ENSG00000185275
NA	-0.0282	4.3341E-01		ENSG00000286072
NA	-0.0394	4.3340E-01		ENSG00000271882
NA	-0.0788	4.3296E-01	6.24E-01	ENSG00000272129
NA	-0.0682	4.3272E-01	6.24E-01	ENSG00000254653
NA	-0.0032	4.3254E-01		ENSG00000274653
NA	-0.0772	4.3246E-01	6.24E-01	ENSG00000259172
NA	-0.0242	4.3223E-01		ENSG00000254865
NA	-0.0683	4.3180E-01	6.23E-01	ENSG00000251026
NA	-0.0290	4.3160E-01		ENSG00000214856
NA	-0.0680	4.3140E-01	6.23E-01	ENSG00000204816
NA	-0.0320	4.3122E-01	6.23E-01	ENSG00000253919
NA	-0.0368	4.3111E-01		ENSG00000236031
NA	-0.0125	4.3109E-01		ENSG00000271825
NA	-0.0001	4.3102E-01		ENSG00000228561
NA	-0.0465	4.3080E-01		ENSG00000234503
NA	-0.0883	4.3029E-01	6.22E-01	ENSG00000289985
NA	-0.0683	4.3019E-01	6.22E-01	ENSG00000213430
NA	-0.0301	4.3014E-01		ENSG00000251689
NA	-0.0435	4.3011E-01	6.22E-01	ENSG00000214765
NA	-0.0462	4.2990E-01		ENSG00000285692
NA	-0.0272	4.2974E-01		ENSG00000227719
NA	-0.0733	4.2962E-01	6.21E-01	ENSG00000279598
NA	-0.0776	4.2958E-01	6.21E-01	ENSG00000267676
NA	-0.0280	4.2929E-01		ENSG00000255358
NA	-0.0772	4.2892E-01	6.21E-01	ENSG00000275734
NA	-0.0277	4.2877E-01	6.21E-01	ENSG00000271937
NA	-0.0291	4.2827E-01		ENSG00000224944
NA	-0.0690	4.2808E-01	6.20E-01	ENSG00000243609
NA	-0.0527	4.2784E-01	6.20E-01	ENSG00000259762
NA	-0.0535	4.2781E-01	6.20E-01	ENSG00000239300
NA	-0.0415	4.2767E-01		ENSG00000234076
NA	-0.0758	4.2766E-01	6.20E-01	ENSG00000233328
NA	-0.0667	4.2623E-01	6.18E-01	ENSG00000272150
NA	-0.0199	4.2603E-01		ENSG00000267005
NA	-0.0588	4.2592E-01	6.18E-01	ENSG00000289054
NA	-0.0283	4.2577E-01		ENSG00000228625
NA	-0.0337	4.2543E-01		ENSG00000250282

NA	-0.1557	4.2463E-01	6.16E-01	ENSG00000271025
NA	-0.0857	4.2458E-01	6.16E-01	ENSG00000260306
NA	-0.0611	4.2408E-01	6.16E-01	ENSG00000284617
NA	-0.0578	4.2390E-01		ENSG00000261668
NA	-0.0683	4.2314E-01	6.15E-01	ENSG00000251550
NA	-0.0181	4.2297E-01		ENSG00000265574
NA	-0.1000	4.2293E-01	6.15E-01	ENSG00000287292
NA	-0.0742	4.2276E-01	6.15E-01	ENSG00000234268
NA	-0.0752	4.2262E-01	6.15E-01	ENSG00000270059
NA	-0.0788	4.2255E-01	6.15E-01	ENSG00000289353
NA	-0.0583	4.2254E-01	6.15E-01	ENSG00000272223
NA	-0.0637	4.2233E-01	6.15E-01	ENSG00000260057
NA	-0.0172	4.2206E-01	6.15E-01	ENSG00000286844
NA	-0.0566	4.2200E-01	6.15E-01	ENSG00000241975
NA	-0.0166	4.2164E-01		ENSG00000250969
NA	-0.0372	4.2156E-01		ENSG00000238041
NA	-0.0356	4.2153E-01		ENSG00000289180
NA	-0.0126	4.2143E-01		ENSG00000231114
NA	-0.0359	4.2082E-01		ENSG00000287247
NA	-0.0102	4.2068E-01		ENSG00000289527
NA	-0.0719	4.2031E-01	6.13E-01	ENSG00000235084
NA	-0.0365	4.2025E-01		ENSG00000223843
NA	-0.0762	4.2015E-01	6.13E-01	ENSG00000234769
NA	-0.0522	4.1971E-01	6.12E-01	ENSG00000229316
NA	-0.0719	4.1969E-01	6.12E-01	ENSG00000255769
NA	-0.0792	4.1868E-01	6.11E-01	ENSG00000274922
NA	-0.0143	4.1843E-01		ENSG00000287836
NA	-0.0323	4.1843E-01		ENSG00000233542
NA	-0.0217	4.1840E-01		ENSG00000286087
NA	-0.0478	4.1839E-01		ENSG00000272371
NA	-0.0887	4.1823E-01	6.11E-01	ENSG00000223864
NA	-0.0477	4.1814E-01		ENSG00000268951
NA	-0.0454	4.1802E-01	6.11E-01	ENSG00000272911
NA	-0.0763	4.1802E-01	6.11E-01	ENSG00000279317
NA	-0.0413	4.1791E-01	6.10E-01	ENSG00000231881
NA	-0.0503	4.1788E-01	6.10E-01	ENSG00000259138
NA	-0.0568	4.1724E-01	6.10E-01	ENSG00000231585
NA	-0.0353	4.1715E-01		ENSG00000267255
NA	-0.0748	4.1708E-01		ENSG00000278000
NA	-0.0177	4.1695E-01		ENSG00000259521
NA	-0.0794	4.1685E-01	6.09E-01	ENSG00000279059
NA	-0.0472	4.1684E-01		ENSG00000287454
NA	-0.0107	4.1674E-01		ENSG00000234558
NA	-0.0601	4.1613E-01	6.09E-01	ENSG00000244586
NA	-0.0451	4.1611E-01	6.09E-01	ENSG00000256951
NA	-0.0568	4.1606E-01		ENSG00000241627
NA	-0.0138	4.1589E-01		ENSG00000266335
NA	-0.0784	4.1571E-01	6.08E-01	ENSG00000223972
NA	-0.0227	4.1548E-01		ENSG00000232917

NA	-0.0491	4.1544E-01	6.08E-01	ENSG00000274251
NA	-0.0476	4.1469E-01	6.07E-01	ENSG00000278997
NA	-0.0790	4.1464E-01	6.07E-01	ENSG00000233967
NA	-0.0302	4.1439E-01		ENSG00000223849
NA	-0.0182	4.1423E-01		ENSG00000213383
NA	-0.0395	4.1390E-01		ENSG00000225028
NA	-0.0312	4.1367E-01		ENSG00000260072
NA	-0.0483	4.1321E-01		ENSG00000256278
NA	-0.0201	4.1303E-01		ENSG00000279784
NA	-0.0487	4.1250E-01	6.05E-01	ENSG00000286596
NA	-0.0638	4.1182E-01	6.05E-01	ENSG00000267044
NA	-0.0142	4.1175E-01		ENSG00000229971
NA	-0.0486	4.1168E-01	6.05E-01	ENSG00000242034
NA	-0.0506	4.1163E-01	6.05E-01	ENSG00000264365
NA	-0.0010	4.1159E-01		ENSG00000243974
NA	-0.0549	4.1120E-01	6.04E-01	ENSG00000287818
NA	-0.0636	4.1107E-01	6.04E-01	ENSG00000278231
NA	-0.0443	4.1103E-01		ENSG00000271716
NA	-0.0675	4.1100E-01	6.04E-01	ENSG00000287617
NA	-0.0489	4.1097E-01		ENSG00000253708
NA	-0.0537	4.1092E-01	6.04E-01	ENSG00000286158
NA	-0.0275	4.1089E-01		ENSG00000277247
NA	-0.0797	4.1063E-01	6.04E-01	ENSG00000279267
NA	-0.0216	4.1061E-01		ENSG00000243018
NA	-0.0614	4.1018E-01	6.03E-01	ENSG00000269984
NA	-0.0466	4.1013E-01		ENSG00000277411
NA	-0.0365	4.0980E-01		ENSG00000258690
NA	-0.1008	4.0973E-01	6.03E-01	ENSG00000270640
NA	-0.0231	4.0935E-01		ENSG00000219410
NA	-0.0822	4.0930E-01	6.02E-01	ENSG00000248787
NA	-0.0247	4.0919E-01		ENSG00000226363
NA	-0.0755	4.0907E-01		ENSG00000227288
NA	-0.0693	4.0893E-01	6.02E-01	ENSG00000286640
NA	-0.0496	4.0875E-01		ENSG00000240673
NA	-0.0197	4.0864E-01		ENSG00000271278
NA	-0.0014	4.0835E-01		ENSG00000231072
NA	-0.0682	4.0826E-01	6.01E-01	ENSG00000231104
NA	-0.0739	4.0812E-01	6.01E-01	ENSG00000255621
NA	-0.0232	4.0806E-01		ENSG00000232194
NA	-0.0634	4.0787E-01	6.01E-01	ENSG00000259232
NA	-0.0515	4.0784E-01		ENSG00000272375
NA	-0.0113	4.0771E-01	6.01E-01	ENSG00000282787
NA	-0.0311	4.0767E-01		ENSG00000234848
NA	-0.1175	4.0767E-01	6.01E-01	ENSG00000260729
NA	-0.0003	4.0715E-01	6.01E-01	ENSG00000285819
NA	-0.0215	4.0701E-01		ENSG00000289109
NA	-0.0249	4.0661E-01		ENSG00000201228
NA	-0.0572	4.0648E-01		ENSG00000233229
NA	-0.0901	4.0647E-01	6.00E-01	ENSG00000235209

NA	-0.0276	4.0593E-01		ENSG00000255817
NA	-0.0211	4.0591E-01		ENSG00000230668
NA	-0.0232	4.0581E-01		ENSG00000235881
NA	-0.0752	4.0571E-01	5.99E-01	ENSG00000267475
NA	-0.0828	4.0570E-01	5.99E-01	ENSG00000265366
NA	-0.0307	4.0558E-01		ENSG00000230290
NA	-0.0310	4.0549E-01	5.99E-01	ENSG00000253496
NA	-0.0672	4.0547E-01	5.99E-01	ENSG00000286337
NA	-0.0829	4.0527E-01	5.99E-01	ENSG00000287817
NA	-0.0414	4.0471E-01		ENSG00000285911
NA	-0.0725	4.0455E-01	5.98E-01	ENSG00000258605
NA	-0.0823	4.0452E-01	5.98E-01	ENSG00000206159
NA	-0.0551	4.0422E-01		ENSG00000285367
NA	-0.0472	4.0418E-01		ENSG00000269888
NA	-0.0831	4.0409E-01	5.98E-01	ENSG00000267277
NA	-0.0815	4.0377E-01	5.98E-01	ENSG00000205579
NA	-0.0823	4.0374E-01	5.98E-01	ENSG00000235893
NA	-0.0845	4.0370E-01	5.98E-01	ENSG00000254649
NA	-0.0724	4.0365E-01	5.98E-01	ENSG00000229585
NA	-0.0712	4.0282E-01	5.97E-01	ENSG00000259430
NA	-0.0432	4.0233E-01		ENSG00000229509
NA	-0.0261	4.0202E-01		ENSG00000262304
NA	-0.0254	4.0201E-01		ENSG00000253778
NA	-0.0212	4.0199E-01		ENSG00000266846
NA	-0.0262	4.0192E-01		ENSG00000235298
NA	-0.0704	4.0112E-01	5.95E-01	ENSG00000214867
NA	-0.0235	4.0068E-01		ENSG00000233278
NA	-0.0648	4.0065E-01	5.95E-01	ENSG00000289476
NA	-0.0278	4.0055E-01		ENSG00000241350
NA	-0.0286	4.0023E-01		ENSG00000250261
NA	-0.0234	4.0011E-01		ENSG00000254197
NA	-0.0260	4.0010E-01		ENSG00000251485
NA	-0.0693	4.0005E-01	5.94E-01	ENSG00000229948
NA	-0.0498	3.9974E-01		ENSG00000236180
NA	-0.0647	3.9947E-01	5.94E-01	ENSG00000285517
NA	-0.0607	3.9934E-01		ENSG00000235674
NA	-0.0741	3.9909E-01	5.93E-01	ENSG00000236972
NA	-0.0609	3.9897E-01		ENSG00000266541
NA	-0.0115	3.9889E-01		ENSG00000243014
NA	-0.0715	3.9881E-01	5.93E-01	ENSG00000223935
NA	-0.0199	3.9839E-01		ENSG00000289076
NA	-0.0761	3.9835E-01	5.93E-01	ENSG00000255162
NA	-0.0790	3.9721E-01	5.91E-01	ENSG00000259863
NA	-0.0822	3.9703E-01	5.91E-01	ENSG00000231503
NA	-0.0386	3.9634E-01		ENSG00000212541
NA	-0.0157	3.9620E-01		ENSG00000267799
NA	-0.0269	3.9611E-01		ENSG00000256789
NA	-0.0845	3.9605E-01	5.90E-01	ENSG00000159247
NA	-0.0825	3.9603E-01	5.90E-01	ENSG00000287117

NA	-0.0673	3.9601E-01	5.90E-01	ENSG00000285567
NA	-0.0901	3.9572E-01	5.90E-01	ENSG00000286899
NA	-0.0028	3.9501E-01		ENSG00000250914
NA	-0.0695	3.9473E-01	5.89E-01	ENSG00000273650
NA	-0.0019	3.9469E-01		ENSG00000225760
NA	-0.0877	3.9456E-01	5.89E-01	ENSG00000279086
NA	-0.0386	3.9422E-01		ENSG00000269476
NA	-0.0820	3.9396E-01	5.89E-01	ENSG00000286757
NA	-0.0428	3.9318E-01		ENSG00000279957
NA	-0.0649	3.9317E-01	5.88E-01	ENSG00000229502
NA	-0.0313	3.9305E-01		ENSG00000283294
NA	-0.0529	3.9278E-01	5.87E-01	ENSG00000280399
NA	-0.0566	3.9275E-01	5.87E-01	ENSG00000236643
NA	-0.0482	3.9270E-01	5.87E-01	ENSG00000260884
NA	-0.0399	3.9250E-01		ENSG00000262959
NA	-0.0454	3.9231E-01		ENSG00000290017
NA	-0.0551	3.9209E-01	5.87E-01	ENSG00000275805
NA	-0.0273	3.9196E-01		ENSG00000227934
NA	-0.0373	3.9170E-01		ENSG00000280027
NA	-0.0305	3.9113E-01		ENSG00000224857
NA	-0.0728	3.9112E-01	5.85E-01	ENSG00000287875
NA	-0.0822	3.9052E-01	5.85E-01	ENSG00000260274
NA	-0.0269	3.9049E-01		ENSG00000255447
NA	-0.0027	3.9040E-01		ENSG00000234860
NA	-0.0858	3.9010E-01	5.84E-01	ENSG00000237529
NA	-0.0856	3.8999E-01	5.84E-01	ENSG00000274213
NA	-0.0203	3.8996E-01		ENSG00000249577
NA	-0.0578	3.8988E-01	5.84E-01	ENSG00000288979
NA	-0.0353	3.8960E-01		ENSG00000273341
NA	-0.0145	3.8953E-01		ENSG00000265791
NA	-0.0828	3.8912E-01	5.84E-01	ENSG00000290127
NA	-0.0422	3.8872E-01		ENSG00000258811
NA	-0.0750	3.8860E-01	5.83E-01	ENSG00000212789
NA	-0.0510	3.8842E-01		ENSG00000249773
NA	-0.0553	3.8817E-01	5.83E-01	ENSG00000286035
NA	-0.0039	3.8782E-01		ENSG00000285769
NA	-0.0853	3.8755E-01	5.82E-01	ENSG00000287650
NA	-0.0074	3.8688E-01		ENSG00000273216
NA	-0.0456	3.8665E-01		ENSG00000289208
NA	-0.1097	3.8606E-01	5.81E-01	ENSG00000283371
NA	-0.0597	3.8581E-01	5.80E-01	ENSG00000249007
NA	-0.0810	3.8574E-01	5.80E-01	ENSG00000259969
NA	-0.0270	3.8557E-01		ENSG00000214549
NA	-0.0242	3.8550E-01		ENSG00000285945
NA	-0.0268	3.8528E-01		ENSG00000228643
NA	-0.0203	3.8502E-01		ENSG00000285748
NA	-0.0317	3.8483E-01		ENSG00000287319
NA	-0.0882	3.8467E-01	5.79E-01	ENSG00000271851
NA	-0.0393	3.8436E-01		ENSG00000258312

NA	-0.0784	3.8430E-01	5.79E-01	ENSG00000279064
NA	-0.0002	3.8423E-01		ENSG00000286428
NA	-0.0419	3.8361E-01		ENSG00000276527
NA	-0.0266	3.8341E-01		ENSG00000289338
NA	-0.0516	3.8289E-01	5.77E-01	ENSG00000280040
NA	-0.0669	3.8264E-01	5.77E-01	ENSG00000279206
NA	-0.0150	3.8259E-01		ENSG00000224391
NA	-0.0311	3.8244E-01		ENSG00000259277
NA	-0.0479	3.8233E-01		ENSG00000238246
NA	-0.0729	3.8208E-01	5.77E-01	ENSG00000196114
NA	-0.0261	3.8185E-01		ENSG00000258760
NA	-0.0521	3.8105E-01	5.76E-01	ENSG00000231713
NA	-0.0853	3.8092E-01	5.76E-01	ENSG00000270157
NA	-0.0943	3.8087E-01		ENSG00000228113
NA	-0.0443	3.8072E-01		ENSG00000246820
NA	-0.0120	3.8067E-01		ENSG00000278881
NA	-0.0382	3.8026E-01		ENSG00000260810
NA	-0.0117	3.7993E-01		ENSG00000254645
NA	-0.0345	3.7974E-01		ENSG00000258527
NA	-0.0756	3.7965E-01	5.75E-01	ENSG00000225868
NA	-0.0861	3.7958E-01	5.75E-01	ENSG00000202310
NA	-0.0374	3.7917E-01		ENSG00000286050
NA	-0.0465	3.7900E-01		ENSG00000236054
NA	-0.0602	3.7888E-01	5.74E-01	ENSG00000284719
NA	-0.0465	3.7881E-01		ENSG00000241907
NA	-0.0696	3.7874E-01	5.74E-01	ENSG00000229989
NA	-0.0349	3.7874E-01		ENSG00000237347
NA	-0.0156	3.7856E-01		ENSG00000287081
NA	-0.0257	3.7844E-01		ENSG00000234943
NA	-0.0862	3.7843E-01	5.73E-01	ENSG00000249593
NA	-0.0309	3.7827E-01		ENSG00000236244
NA	-0.0684	3.7825E-01	5.73E-01	ENSG00000277959
NA	-0.0621	3.7813E-01	5.73E-01	ENSG00000279277
NA	-0.0857	3.7745E-01	5.73E-01	ENSG00000274712
NA	-0.0330	3.7729E-01		ENSG00000279631
NA	-0.0864	3.7706E-01	5.72E-01	ENSG00000262265
NA	-0.0234	3.7687E-01		ENSG00000225183
NA	-0.0215	3.7675E-01		ENSG00000233299
NA	-0.0884	3.7659E-01	5.72E-01	ENSG00000285805
NA	-0.0401	3.7647E-01	5.72E-01	ENSG00000285857
NA	-0.1312	3.7641E-01	5.72E-01	ENSG00000288720
NA	-0.0724	3.7605E-01	5.71E-01	ENSG00000286153
NA	-0.0855	3.7587E-01	5.71E-01	ENSG00000178660
NA	-0.0893	3.7506E-01	5.71E-01	ENSG00000259349
NA	-0.0355	3.7486E-01		ENSG00000261616
NA	-0.0556	3.7472E-01	5.70E-01	ENSG00000213071
NA	-0.0232	3.7471E-01		ENSG00000286124
NA	-0.0430	3.7440E-01		ENSG00000213239
NA	-0.0502	3.7408E-01		ENSG00000286141

NA	-0.0870	3.7379E-01	5.69E-01	ENSG00000257074
NA	-0.0245	3.7368E-01		ENSG00000275654
NA	-0.0410	3.7357E-01		ENSG00000285978
NA	-0.0195	3.7349E-01		ENSG00000231655
NA	-0.0325	3.7316E-01		ENSG00000285584
NA	-0.0195	3.7286E-01		ENSG00000200209
NA	-0.0884	3.7280E-01	5.68E-01	ENSG00000256923
NA	-0.0895	3.7275E-01	5.68E-01	ENSG00000280614
NA	-0.0371	3.7244E-01		ENSG00000216753
NA	-0.0878	3.7227E-01	5.68E-01	ENSG00000213881
NA	-0.0399	3.7217E-01		ENSG00000213513
NA	-0.0767	3.7209E-01	5.67E-01	ENSG00000279649
NA	-0.0869	3.7205E-01	5.67E-01	ENSG00000235238
NA	-0.0802	3.7140E-01	5.67E-01	ENSG00000243753
NA	-0.0719	3.7107E-01	5.67E-01	ENSG00000266456
NA	-0.0277	3.7102E-01		ENSG00000258736
NA	-0.0697	3.7061E-01	5.66E-01	ENSG00000249478
NA	-0.0204	3.7030E-01	5.66E-01	ENSG00000273091
NA	-0.0696	3.7020E-01	5.66E-01	ENSG00000260927
NA	-0.0463	3.7018E-01		ENSG00000286950
NA	-0.0647	3.7004E-01	5.66E-01	ENSG00000286425
NA	-0.0239	3.6950E-01		ENSG00000226522
NA	-0.0357	3.6945E-01		ENSG00000280375
NA	-0.0931	3.6939E-01	5.65E-01	ENSG00000267980
NA	-0.0245	3.6829E-01		ENSG00000227094
NA	-0.0216	3.6803E-01		ENSG00000228086
NA	-0.0755	3.6794E-01	5.64E-01	ENSG00000182057
NA	-0.0753	3.6791E-01	5.64E-01	ENSG00000273139
NA	-0.0216	3.6762E-01		ENSG00000269068
NA	-0.1045	3.6748E-01	5.63E-01	ENSG00000268912
NA	-0.0859	3.6704E-01	5.63E-01	ENSG00000265342
NA	-0.0852	3.6685E-01	5.63E-01	ENSG00000213300
NA	-0.0616	3.6684E-01	5.63E-01	ENSG00000287422
NA	-0.0743	3.6611E-01	5.62E-01	ENSG00000255306
NA	-0.0244	3.6609E-01		ENSG00000243655
NA	-0.0665	3.6596E-01	5.62E-01	ENSG00000259270
NA	-0.0242	3.6547E-01		ENSG00000287707
NA	-0.0280	3.6544E-01		ENSG00000199890
NA	-0.1007	3.6544E-01	5.61E-01	ENSG00000210107
NA	-0.0714	3.6522E-01	5.61E-01	ENSG00000271843
NA	-0.0364	3.6367E-01		ENSG00000273483
NA	-0.0219	3.6358E-01		ENSG00000248545
NA	-0.0230	3.6353E-01		ENSG00000287138
NA	-0.0708	3.6347E-01	5.59E-01	ENSG00000269388
NA	-0.0823	3.6345E-01	5.59E-01	ENSG00000237938
NA	-0.0328	3.6333E-01		ENSG00000250540
NA	-0.0290	3.6332E-01		ENSG00000224222
NA	-0.0869	3.6289E-01	5.59E-01	ENSG00000288002
NA	-0.0381	3.6281E-01		ENSG00000261375

NA	-0.0290	3.6253E-01		ENSG00000280036
NA	-0.0739	3.6223E-01	5.58E-01	ENSG00000243187
NA	-0.0889	3.6189E-01	5.58E-01	ENSG00000281333
NA	-0.0765	3.6119E-01	5.57E-01	ENSG00000243094
NA	-0.0253	3.6100E-01		ENSG00000267587
NA	-0.1148	3.6098E-01	5.57E-01	ENSG00000234789
NA	-0.0470	3.6098E-01		ENSG00000259820
NA	-0.0464	3.6076E-01		ENSG00000275811
NA	-0.0808	3.6024E-01	5.56E-01	ENSG00000230982
NA	-0.0877	3.6021E-01	5.56E-01	ENSG00000228634
NA	-0.0173	3.5995E-01		ENSG00000287037
NA	-0.0189	3.5984E-01		ENSG00000279815
NA	-0.0467	3.5964E-01		ENSG00000283023
NA	-0.0421	3.5963E-01		ENSG00000213703
NA	-0.0857	3.5959E-01	5.56E-01	ENSG00000267429
NA	-0.0347	3.5952E-01		ENSG00000242142
NA	-0.0761	3.5943E-01	5.55E-01	ENSG00000272922
NA	-0.0380	3.5942E-01		ENSG00000277228
NA	-0.0529	3.5923E-01		ENSG00000235408
NA	-0.0375	3.5911E-01		ENSG00000261987
NA	-0.0375	3.5911E-01		ENSG00000265746
NA	-0.0277	3.5884E-01		ENSG00000256084
NA	-0.0226	3.5881E-01		ENSG00000234419
NA	-0.0540	3.5881E-01	5.55E-01	ENSG00000253286
NA	-0.0343	3.5869E-01		ENSG00000259192
NA	-0.0761	3.5864E-01	5.55E-01	ENSG00000234771
NA	-0.0587	3.5863E-01		ENSG00000254786
NA	-0.0718	3.5858E-01	5.54E-01	ENSG00000289826
NA	-0.0343	3.5835E-01		ENSG00000255444
NA	-0.0531	3.5781E-01		ENSG00000267662
NA	-0.0282	3.5778E-01		ENSG00000199899
NA	-0.0589	3.5746E-01		ENSG00000258623
NA	-0.0055	3.5698E-01	5.52E-01	ENSG00000290019
NA	-0.0916	3.5698E-01	5.52E-01	ENSG00000235052
NA	-0.0923	3.5691E-01	5.52E-01	ENSG00000289494
NA	-0.0183	3.5676E-01		ENSG00000279096
NA	-0.0584	3.5652E-01	5.52E-01	ENSG00000261166
NA	-0.0014	3.5518E-01		ENSG00000231272
NA	-0.0863	3.5500E-01	5.50E-01	ENSG00000275630
NA	-0.0143	3.5483E-01		ENSG00000248725
NA	-0.0634	3.5374E-01		ENSG00000285736
NA	-0.0538	3.5358E-01		ENSG00000213856
NA	-0.0916	3.5353E-01	5.49E-01	ENSG00000289878
NA	-0.0254	3.5344E-01		ENSG00000230980
NA	-0.0742	3.5333E-01	5.49E-01	ENSG00000286758
NA	-0.0684	3.5304E-01	5.48E-01	ENSG00000242353
NA	-0.0634	3.5288E-01	5.48E-01	ENSG00000215388
NA	-0.0463	3.5274E-01		ENSG00000276822
NA	-0.0247	3.5255E-01		ENSG00000223552

NA	-0.0337	3.5250E-01		ENSG00000234810
NA	-0.0021	3.5224E-01	5.47E-01	ENSG00000280163
NA	-0.0558	3.5201E-01		ENSG00000214820
NA	-0.0640	3.5165E-01		ENSG00000226141
NA	-0.0398	3.5157E-01		ENSG00000288788
NA	-0.0851	3.5149E-01	5.47E-01	ENSG00000209082
NA	-0.0488	3.5132E-01		ENSG00000177803
NA	-0.0932	3.5115E-01	5.46E-01	ENSG00000289318
NA	-0.0788	3.5096E-01	5.46E-01	ENSG00000230953
NA	-0.0518	3.5091E-01		ENSG00000239254
NA	-0.0204	3.5071E-01		ENSG00000271587
NA	-0.0715	3.5070E-01	5.46E-01	ENSG00000289094
NA	-0.0025	3.5054E-01	5.46E-01	ENSG00000288076
NA	-0.0201	3.5049E-01		ENSG00000255396
NA	-0.0285	3.5040E-01		ENSG00000228586
NA	-0.1289	3.5011E-01	5.45E-01	ENSG00000251307
NA	-0.0406	3.5005E-01		ENSG00000272824
NA	-0.0408	3.5004E-01		ENSG00000279197
NA	-0.0436	3.4988E-01		ENSG00000244676
NA	-0.0658	3.4968E-01		ENSG00000257325
NA	-0.0278	3.4965E-01		ENSG00000230428
NA	-0.0375	3.4958E-01		ENSG00000274507
NA	-0.0067	3.4936E-01	5.44E-01	ENSG00000268189
NA	-0.0835	3.4922E-01	5.44E-01	ENSG00000238058
NA	-0.0607	3.4893E-01	5.44E-01	ENSG00000260710
NA	-0.0441	3.4861E-01		ENSG00000286378
NA	-0.0140	3.4859E-01	5.44E-01	ENSG00000234277
NA	-0.0880	3.4840E-01	5.43E-01	ENSG00000289385
NA	-0.0933	3.4839E-01	5.43E-01	ENSG00000267811
NA	-0.0665	3.4825E-01	5.43E-01	ENSG00000227742
NA	-0.0540	3.4816E-01		ENSG00000261832
NA	-0.0520	3.4785E-01		ENSG00000276523
NA	-0.0021	3.4750E-01		ENSG00000266654
NA	-0.0379	3.4737E-01		ENSG00000236324
NA	-0.0770	3.4727E-01	5.42E-01	ENSG00000262412
NA	-0.0784	3.4688E-01	5.42E-01	ENSG00000287965
NA	-0.0235	3.4686E-01	5.42E-01	ENSG00000270947
NA	-0.0812	3.4683E-01	5.42E-01	ENSG00000273451
NA	-0.0262	3.4680E-01		ENSG00000229237
NA	-0.0628	3.4679E-01	5.42E-01	ENSG00000279737
NA	-0.0405	3.4674E-01		ENSG00000233242
NA	-0.0571	3.4643E-01		ENSG00000286262
NA	-0.0673	3.4615E-01	5.41E-01	ENSG00000244480
NA	-0.0936	3.4593E-01	5.41E-01	ENSG00000204110
NA	-0.0757	3.4581E-01	5.41E-01	ENSG00000285971
NA	-0.0487	3.4564E-01		ENSG00000255669
NA	-0.0600	3.4533E-01		ENSG00000278995
NA	-0.0254	3.4532E-01		ENSG00000286846
NA	-0.0019	3.4518E-01		ENSG00000226390

NA	-0.0741	3.4516E-01	5.40E-01	ENSG00000197083
NA	-0.0889	3.4512E-01	5.40E-01	ENSG00000213830
NA	-0.0306	3.4501E-01		ENSG00000277575
NA	-0.1312	3.4487E-01	5.40E-01	ENSG00000240622
NA	-0.0193	3.4453E-01		ENSG00000259182
NA	-0.1138	3.4396E-01	5.39E-01	ENSG00000279537
NA	-0.0507	3.4390E-01		ENSG00000249319
NA	-0.0291	3.4384E-01		ENSG00000279962
NA	-0.0486	3.4358E-01		ENSG00000259408
NA	-0.0204	3.4354E-01		ENSG00000280778
NA	-0.0503	3.4352E-01		ENSG00000227069
NA	-0.0634	3.4327E-01		ENSG00000279372
NA	-0.0624	3.4311E-01	5.38E-01	ENSG00000286540
NA	-0.0543	3.4305E-01		ENSG00000218890
NA	-0.0580	3.4295E-01		ENSG00000232437
NA	-0.0778	3.4266E-01	5.38E-01	ENSG00000105988
NA	-0.0918	3.4245E-01	5.38E-01	ENSG00000273599
NA	-0.0015	3.4243E-01		ENSG00000250031
NA	-0.0126	3.4191E-01	5.37E-01	ENSG00000251405
NA	-0.0753	3.4181E-01	5.37E-01	ENSG00000255495
NA	-0.0671	3.4177E-01	5.37E-01	ENSG00000240132
NA	-0.0672	3.4169E-01	5.37E-01	ENSG00000288910
NA	-0.0286	3.4143E-01		ENSG00000234818
NA	-0.0192	3.4106E-01		ENSG00000287722
NA	-0.0007	3.4103E-01		ENSG00000289245
NA	-0.0495	3.4029E-01		ENSG00000243388
NA	-0.0166	3.4017E-01		ENSG00000228051
NA	-0.0622	3.3973E-01		ENSG00000250643
NA	-0.0395	3.3944E-01		ENSG00000177725
NA	-0.0953	3.3892E-01	5.34E-01	ENSG00000233971
NA	-0.0952	3.3886E-01	5.34E-01	ENSG00000268154
NA	-0.0355	3.3865E-01		ENSG00000233851
NA	-0.0935	3.3835E-01	5.33E-01	ENSG00000277579
NA	-0.0551	3.3828E-01		ENSG00000255348
NA	-0.0666	3.3827E-01	5.33E-01	ENSG00000231125
NA	-0.0679	3.3818E-01	5.33E-01	ENSG00000286305
NA	-0.0605	3.3743E-01	5.33E-01	ENSG00000234493
NA	-0.0421	3.3738E-01		ENSG00000286992
NA	-0.0347	3.3702E-01		ENSG00000262519
NA	-0.1077	3.3692E-01	5.32E-01	ENSG00000224614
NA	-0.0649	3.3687E-01	5.32E-01	ENSG00000258531
NA	-0.0335	3.3687E-01		ENSG00000267449
NA	-0.0841	3.3681E-01	5.32E-01	ENSG00000285366
NA	-0.0433	3.3657E-01		ENSG00000172746
NA	-0.0396	3.3657E-01		ENSG00000231878
NA	-0.0525	3.3654E-01		ENSG00000286632
NA	-0.0121	3.3636E-01		ENSG00000259302
NA	-0.0952	3.3626E-01	5.31E-01	ENSG00000232748
NA	-0.0491	3.3626E-01	5.31E-01	ENSG00000285622

NA	-0.0235	3.3550E-01		ENSG00000260784
NA	-0.0318	3.3544E-01		ENSG00000224594
NA	-0.0206	3.3544E-01		ENSG00000270705
NA	-0.0300	3.3539E-01		ENSG00000233836
NA	-0.0962	3.3499E-01	5.30E-01	ENSG00000259551
NA	-0.0905	3.3483E-01	5.30E-01	ENSG00000236536
NA	-0.0927	3.3458E-01	5.30E-01	ENSG00000287742
NA	-0.0536	3.3409E-01	5.29E-01	ENSG00000283633
NA	-0.0561	3.3329E-01		ENSG00000285921
NA	-0.0573	3.3328E-01	5.28E-01	ENSG00000259342
NA	-0.0670	3.3308E-01	5.28E-01	ENSG00000269793
NA	-0.0940	3.3295E-01	5.28E-01	ENSG00000288966
NA	-0.0603	3.3278E-01		ENSG00000237188
NA	-0.0433	3.3268E-01		ENSG00000256826
NA	-0.0709	3.3212E-01	5.27E-01	ENSG00000218537
NA	-0.0832	3.3211E-01	5.27E-01	ENSG00000224183
NA	-0.0545	3.3164E-01		ENSG00000279336
NA	-0.0795	3.3150E-01	5.26E-01	ENSG00000258081
NA	-0.0197	3.3146E-01		ENSG00000279754
NA	-0.0655	3.3142E-01	5.26E-01	ENSG00000287961
NA	-0.0910	3.2991E-01	5.24E-01	ENSG00000287569
NA	-0.0739	3.2966E-01	5.24E-01	ENSG00000289425
NA	-0.0215	3.2949E-01		ENSG00000239207
NA	-0.0131	3.2918E-01		ENSG00000236209
NA	-0.1194	3.2887E-01	5.23E-01	ENSG00000263105
NA	-0.0970	3.2885E-01	5.23E-01	ENSG00000231563
NA	-0.0305	3.2865E-01		ENSG00000269873
NA	-0.0355	3.2854E-01		ENSG00000278936
NA	-0.0438	3.2841E-01		ENSG00000238018
NA	-0.0967	3.2829E-01	5.23E-01	ENSG00000273077
NA	-0.0370	3.2808E-01		ENSG00000200241
NA	-0.0551	3.2805E-01	5.23E-01	ENSG00000285930
NA	-0.0677	3.2766E-01		ENSG00000282879
NA	-0.0561	3.2764E-01		ENSG00000286012
NA	-0.0531	3.2733E-01		ENSG00000289725
NA	-0.0794	3.2730E-01	5.22E-01	ENSG00000271727
NA	-0.0987	3.2729E-01	5.22E-01	ENSG00000289045
NA	-0.0164	3.2726E-01	5.22E-01	ENSG00000267696
NA	-0.0212	3.2725E-01		ENSG00000279394
NA	-0.0757	3.2710E-01	5.21E-01	ENSG00000267769
NA	-0.0745	3.2696E-01		ENSG00000280330
NA	-0.0745	3.2696E-01		ENSG00000279177
NA	-0.0373	3.2680E-01		ENSG00000269560
NA	-0.0215	3.2668E-01		ENSG00000279302
NA	-0.0173	3.2659E-01		ENSG00000226859
NA	-0.0077	3.2647E-01	5.21E-01	ENSG00000256609
NA	-0.1002	3.2643E-01	5.21E-01	ENSG00000272572
NA	-0.0954	3.2642E-01	5.21E-01	ENSG00000251396
NA	-0.0233	3.2641E-01		ENSG00000250592

NA	-0.0985	3.2633E-01	5.21E-01	ENSG00000257060
NA	-0.0480	3.2625E-01		ENSG00000268496
NA	-0.0984	3.2621E-01	5.20E-01	ENSG00000245970
NA	-0.0536	3.2601E-01		ENSG00000253622
NA	-0.0764	3.2596E-01	5.20E-01	ENSG00000235060
NA	-0.1056	3.2576E-01	5.20E-01	ENSG00000176320
NA	-0.0541	3.2573E-01		ENSG00000286178
NA	-0.0902	3.2571E-01	5.20E-01	ENSG00000280128
NA	-0.0466	3.2563E-01		ENSG00000261442
NA	-0.1004	3.2510E-01	5.19E-01	ENSG00000272655
NA	-0.0329	3.2488E-01	5.19E-01	ENSG00000287757
NA	-0.0425	3.2482E-01		ENSG00000236838
NA	-0.0375	3.2446E-01		ENSG00000262903
NA	-0.0679	3.2443E-01	5.19E-01	ENSG00000226976
NA	-0.0891	3.2432E-01	5.18E-01	ENSG00000279035
NA	-0.0236	3.2411E-01		ENSG00000251127
NA	0.0000	3.2375E-01		ENSG00000213108
NA	-0.1031	3.2355E-01	5.18E-01	ENSG00000285649
NA	-0.0017	3.2351E-01		ENSG00000228285
NA	-0.0955	3.2339E-01	5.17E-01	ENSG00000257497
NA	-0.0830	3.2304E-01	5.17E-01	ENSG00000224208
NA	-0.1026	3.2268E-01	5.16E-01	ENSG00000265936
NA	-0.0020	3.2250E-01	5.16E-01	ENSG00000197882
NA	-0.0536	3.2230E-01		ENSG00000280212
NA	-0.0665	3.2223E-01		ENSG00000232630
NA	-0.0520	3.2215E-01	5.16E-01	ENSG00000245958
NA	-0.0766	3.2196E-01	5.16E-01	ENSG00000279423
NA	-0.0346	3.2151E-01		ENSG00000272657
NA	-0.0654	3.2150E-01	5.15E-01	ENSG00000279108
NA	-0.0692	3.2134E-01	5.15E-01	ENSG00000255883
NA	-0.0985	3.2117E-01	5.15E-01	ENSG00000262227
NA	-0.0620	3.2109E-01	5.15E-01	ENSG00000286456
NA	-0.0880	3.2101E-01	5.15E-01	ENSG00000217702
NA	-0.1097	3.2079E-01	5.15E-01	ENSG00000283235
NA	-0.0421	3.2062E-01		ENSG00000234709
NA	-0.0963	3.2034E-01	5.14E-01	ENSG00000259658
NA	-0.1004	3.1969E-01	5.13E-01	ENSG00000279967
NA	-0.0479	3.1894E-01		ENSG00000259993
NA	-0.0708	3.1884E-01	5.12E-01	ENSG00000234219
NA	-0.0006	3.1828E-01		ENSG00000250407
NA	-0.0232	3.1802E-01		ENSG00000228054
NA	-0.0459	3.1798E-01		ENSG00000229163
NA	-0.0634	3.1780E-01	5.11E-01	ENSG00000149531
NA	-0.1021	3.1780E-01	5.11E-01	ENSG00000231409
NA	-0.0211	3.1720E-01		ENSG00000207382
NA	-0.0904	3.1655E-01		ENSG00000278514
NA	-0.0295	3.1635E-01		ENSG00000248916
NA	-0.1154	3.1634E-01	5.10E-01	ENSG00000286349
NA	-0.0604	3.1622E-01		ENSG00000235070

NA	-0.0796	3.1569E-01	5.09E-01	ENSG00000259555
NA	-0.0199	3.1565E-01		ENSG00000275392
NA	-0.0822	3.1562E-01	5.09E-01	ENSG00000236986
NA	-0.0912	3.1522E-01	5.09E-01	ENSG00000232273
NA	-0.0639	3.1497E-01	5.08E-01	ENSG00000231903
NA	-0.0469	3.1477E-01		ENSG00000229595
NA	-0.0746	3.1463E-01		ENSG00000277130
NA	-0.0192	3.1453E-01		ENSG00000248774
NA	-0.0311	3.1446E-01		ENSG00000207189
NA	-0.0408	3.1421E-01		ENSG00000267530
NA	-0.0647	3.1387E-01	5.07E-01	ENSG00000276867
NA	-0.1141	3.1339E-01	5.07E-01	ENSG00000272170
NA	-0.0255	3.1337E-01		ENSG00000271618
NA	-0.0353	3.1312E-01		ENSG00000286076
NA	-0.0944	3.1278E-01	5.06E-01	ENSG00000260949
NA	-0.1017	3.1236E-01	5.06E-01	ENSG00000242992
NA	-0.1002	3.1225E-01	5.05E-01	ENSG00000280385
NA	-0.0295	3.1216E-01		ENSG00000214992
NA	-0.0475	3.1148E-01		ENSG00000288813
NA	-0.1012	3.1139E-01	5.04E-01	ENSG00000213362
NA	-0.0643	3.1139E-01		ENSG00000270149
NA	-0.0633	3.1133E-01		ENSG00000248399
NA	-0.0288	3.1125E-01		ENSG00000254692
NA	-0.0798	3.1118E-01	5.04E-01	ENSG00000280347
NA	-0.1107	3.1056E-01	5.03E-01	ENSG00000234183
NA	-0.0608	3.1048E-01		ENSG00000279286
NA	-0.0691	3.1023E-01	5.03E-01	ENSG00000288101
NA	-0.0306	3.1002E-01		ENSG00000265798
NA	-0.1036	3.0964E-01	5.02E-01	ENSG00000233045
NA	-0.0297	3.0963E-01		ENSG00000224808
NA	-0.0878	3.0939E-01		ENSG00000272905
NA	-0.0271	3.0919E-01		ENSG00000257379
NA	-0.0892	3.0893E-01	5.02E-01	ENSG00000259038
NA	-0.0091	3.0842E-01		ENSG00000267595
NA	-0.0241	3.0808E-01		ENSG00000235126
NA	-0.0318	3.0803E-01		ENSG00000259531
NA	-0.0281	3.0798E-01		ENSG00000225092
NA	-0.0796	3.0797E-01	5.01E-01	ENSG00000288815
NA	-0.0908	3.0772E-01	5.00E-01	ENSG00000244625
NA	-0.0749	3.0761E-01		ENSG00000200579
NA	-0.0931	3.0749E-01	5.00E-01	ENSG00000258891
NA	-0.0353	3.0698E-01		ENSG00000250030
NA	-0.1232	3.0694E-01	5.00E-01	ENSG00000272505
NA	-0.0219	3.0686E-01		ENSG00000235782
NA	-0.0834	3.0674E-01	4.99E-01	ENSG00000279489
NA	-0.1024	3.0642E-01	4.99E-01	ENSG00000224505
NA	-0.0293	3.0601E-01		ENSG00000254706
NA	-0.0993	3.0543E-01	4.98E-01	ENSG00000286445
NA	-0.0426	3.0542E-01		ENSG00000223716

NA	-0.0491	3.0504E-01	4.98E-01	ENSG00000255366
NA	-0.0748	3.0496E-01	4.98E-01	ENSG00000287787
NA	-0.0163	3.0488E-01		ENSG00000224295
NA	-0.0375	3.0480E-01		ENSG00000285656
NA	-0.0264	3.0471E-01		ENSG00000231867
NA	-0.0603	3.0471E-01	4.97E-01	ENSG00000234826
NA	-0.0726	3.0459E-01		ENSG00000228360
NA	-0.0249	3.0405E-01		ENSG00000268069
NA	-0.0321	3.0400E-01		ENSG00000232875
NA	-0.0747	3.0366E-01		ENSG00000227157
NA	-0.0457	3.0358E-01		ENSG00000241358
NA	-0.0470	3.0353E-01		ENSG00000253047
NA	-0.0386	3.0349E-01		ENSG00000284728
NA	-0.0307	3.0329E-01		ENSG00000255270
NA	-0.0891	3.0286E-01	4.95E-01	ENSG00000278879
NA	-0.0720	3.0270E-01	4.95E-01	ENSG00000271664
NA	-0.1407	3.0255E-01	4.95E-01	ENSG00000289001
NA	-0.0828	3.0254E-01	4.95E-01	ENSG00000272217
NA	-0.1051	3.0241E-01	4.95E-01	ENSG00000285535
NA	-0.0698	3.0204E-01	4.95E-01	ENSG00000289544
NA	-0.0953	3.0179E-01	4.94E-01	ENSG00000240463
NA	-0.0736	3.0174E-01		ENSG00000267658
NA	-0.0786	3.0173E-01	4.94E-01	ENSG00000288890
NA	-0.0528	3.0166E-01		ENSG00000272940
NA	-0.0949	3.0157E-01	4.94E-01	ENSG00000286530
NA	-0.0967	3.0156E-01	4.94E-01	ENSG00000232352
NA	-0.0888	3.0128E-01	4.94E-01	ENSG00000241185
NA	-0.1036	3.0118E-01	4.94E-01	ENSG00000203325
NA	-0.0964	3.0117E-01	4.94E-01	ENSG00000289935
NA	-0.0857	3.0055E-01		ENSG00000256101
NA	-0.1064	3.0055E-01	4.93E-01	ENSG00000254884
NA	-0.0979	3.0040E-01	4.93E-01	ENSG00000229666
NA	-0.0746	3.0035E-01	4.93E-01	ENSG00000227973
NA	-0.1040	3.0031E-01	4.93E-01	ENSG00000289520
NA	-0.0853	3.0025E-01	4.93E-01	ENSG00000254879
NA	-0.0801	3.0012E-01	4.93E-01	ENSG00000257097
NA	-0.0289	3.0008E-01		ENSG00000259301
NA	-0.0678	3.0005E-01		ENSG00000259274
NA	-0.0449	2.9965E-01		ENSG00000257225
NA	-0.0810	2.9964E-01	4.92E-01	ENSG00000229525
NA	-0.1010	2.9935E-01	4.92E-01	ENSG00000289519
NA	-0.0023	2.9929E-01	4.92E-01	ENSG00000182912
NA	-0.0596	2.9929E-01		ENSG00000271392
NA	-0.0582	2.9905E-01		ENSG00000289931
NA	-0.0614	2.9857E-01		ENSG00000273113
NA	-0.0702	2.9846E-01	4.91E-01	ENSG00000286856
NA	-0.0623	2.9830E-01		ENSG00000260082
NA	-0.1057	2.9798E-01	4.90E-01	ENSG00000286707
NA	-0.1040	2.9743E-01	4.89E-01	ENSG00000231747

NA	-0.0468	2.9719E-01		ENSG00000267079
NA	-0.1053	2.9719E-01	4.89E-01	ENSG00000287967
NA	-0.0630	2.9696E-01	4.89E-01	ENSG00000225648
NA	-0.0607	2.9679E-01		ENSG00000273007
NA	-0.0544	2.9667E-01		ENSG00000253785
NA	-0.0546	2.9667E-01		ENSG00000274841
NA	-0.1000	2.9663E-01	4.89E-01	ENSG00000289194
NA	-0.0238	2.9649E-01		ENSG00000237057
NA	-0.1020	2.9626E-01	4.88E-01	ENSG00000235044
NA	-0.0995	2.9608E-01	4.88E-01	ENSG00000285725
NA	-0.0263	2.9594E-01		ENSG00000280176
NA	-0.0572	2.9585E-01	4.88E-01	ENSG00000261632
NA	-0.0890	2.9579E-01	4.88E-01	ENSG00000224411
NA	-0.0340	2.9555E-01		ENSG00000238024
NA	-0.0728	2.9552E-01		ENSG00000216412
NA	-0.1075	2.9540E-01	4.87E-01	ENSG00000279303
NA	-0.0983	2.9537E-01	4.87E-01	ENSG00000280033
NA	-0.0691	2.9516E-01	4.87E-01	ENSG00000238151
NA	-0.0668	2.9511E-01	4.87E-01	ENSG00000238072
NA	-0.0961	2.9500E-01	4.87E-01	ENSG00000260602
NA	-0.0827	2.9497E-01	4.87E-01	ENSG00000274721
NA	-0.0360	2.9492E-01		ENSG00000285665
NA	-0.0809	2.9484E-01	4.87E-01	ENSG00000285672
NA	-0.0226	2.9481E-01		ENSG00000233547
NA	-0.0105	2.9379E-01	4.86E-01	ENSG00000237166
NA	-0.1054	2.9369E-01	4.86E-01	ENSG00000284959
NA	-0.0881	2.9344E-01	4.85E-01	ENSG00000273424
NA	-0.0871	2.9339E-01	4.85E-01	ENSG00000280096
NA	-0.0728	2.9316E-01		ENSG00000240373
NA	-0.0792	2.9292E-01	4.85E-01	ENSG00000229598
NA	-0.0880	2.9283E-01	4.85E-01	ENSG00000283196
NA	-0.0604	2.9254E-01		ENSG00000278878
NA	-0.0339	2.9253E-01		ENSG00000287603
NA	-0.0448	2.9211E-01		ENSG00000274717
NA	-0.0518	2.9185E-01		ENSG00000287302
NA	-0.1052	2.9175E-01	4.83E-01	ENSG00000231533
NA	-0.0690	2.9162E-01	4.83E-01	ENSG00000279123
NA	-0.0863	2.9111E-01	4.83E-01	ENSG00000282602
NA	-0.0281	2.9072E-01		ENSG00000258090
NA	-0.0207	2.9071E-01		ENSG00000231207
NA	-0.1257	2.9053E-01	4.82E-01	ENSG00000227765
NA	-0.1329	2.9030E-01	4.82E-01	ENSG00000286032
NA	-0.1058	2.9019E-01	4.82E-01	ENSG00000280441
NA	-0.0608	2.9018E-01		ENSG00000232828
NA	-0.0312	2.8985E-01		ENSG00000279853
NA	-0.0638	2.8920E-01		ENSG00000284543
NA	-0.0872	2.8919E-01	4.81E-01	ENSG00000235994
NA	-0.0219	2.8901E-01		ENSG00000290020
NA	-0.0539	2.8894E-01		ENSG00000287011

NA	-0.0510	2.8868E-01		ENSG00000251680
NA	-0.0228	2.8824E-01		ENSG00000268955
NA	-0.0935	2.8810E-01	4.79E-01	ENSG00000261542
NA	-0.1008	2.8765E-01	4.79E-01	ENSG00000280022
NA	-0.0376	2.8764E-01		ENSG00000255108
NA	-0.1109	2.8744E-01	4.79E-01	ENSG00000243538
NA	-0.0916	2.8732E-01	4.78E-01	ENSG00000228247
NA	-0.0914	2.8707E-01	4.78E-01	ENSG00000270532
NA	-0.1087	2.8703E-01	4.78E-01	ENSG00000289592
NA	-0.0312	2.8630E-01		ENSG00000224207
NA	-0.0688	2.8623E-01	4.77E-01	ENSG00000228196
NA	-0.0491	2.8573E-01		ENSG00000224185
NA	-0.0387	2.8497E-01	4.76E-01	ENSG00000285103
NA	-0.0971	2.8492E-01	4.76E-01	ENSG00000198155
NA	-0.0543	2.8484E-01		ENSG00000265445
NA	-0.1091	2.8475E-01	4.76E-01	ENSG00000275636
NA	-0.0834	2.8468E-01	4.76E-01	ENSG00000260496
NA	-0.1093	2.8454E-01	4.76E-01	ENSG00000250101
NA	-0.0020	2.8451E-01		ENSG00000239377
NA	-0.0987	2.8433E-01	4.76E-01	ENSG00000215093
NA	-0.0474	2.8431E-01		ENSG00000216802
NA	-0.0899	2.8424E-01	4.76E-01	ENSG00000282100
NA	-0.0825	2.8418E-01	4.76E-01	ENSG00000285595
NA	-0.0331	2.8403E-01		ENSG00000239455
NA	-0.0844	2.8401E-01	4.75E-01	ENSG00000257438
NA	-0.0887	2.8394E-01	4.75E-01	ENSG00000289270
NA	-0.0068	2.8377E-01		ENSG00000229591
NA	-0.1102	2.8376E-01	4.75E-01	ENSG00000256591
NA	-0.0998	2.8364E-01	4.75E-01	ENSG00000278876
NA	-0.0855	2.8337E-01	4.75E-01	ENSG00000231707
NA	-0.0506	2.8322E-01		ENSG00000230482
NA	-0.0640	2.8314E-01	4.74E-01	ENSG00000248863
NA	-0.1042	2.8306E-01	4.74E-01	ENSG00000254027
NA	-0.0627	2.8298E-01	4.74E-01	ENSG00000258591
NA	-0.0386	2.8292E-01		ENSG00000255959
NA	-0.0502	2.8266E-01		ENSG00000250899
NA	-0.0498	2.8262E-01		ENSG00000267142
NA	-0.0028	2.8258E-01		ENSG00000257346
NA	-0.0251	2.8258E-01		ENSG00000280171
NA	-0.0835	2.8224E-01	4.73E-01	ENSG00000212994
NA	-0.0737	2.8202E-01	4.73E-01	ENSG00000283167
NA	-0.1224	2.8194E-01	4.73E-01	ENSG00000224192
NA	-0.0675	2.8184E-01		ENSG00000250329
NA	-0.1060	2.8146E-01	4.73E-01	ENSG00000282988
NA	-0.0716	2.8140E-01	4.72E-01	ENSG00000259407
NA	-0.0647	2.8111E-01	4.72E-01	ENSG00000249412
NA	-0.0653	2.8103E-01	4.72E-01	ENSG00000232901
NA	-0.0272	2.8101E-01		ENSG00000267417
NA	-0.0473	2.8084E-01		ENSG00000228002

NA	-0.1279	2.8081E-01	4.72E-01	ENSG00000243915
NA	-0.0494	2.8044E-01		ENSG00000258626
NA	-0.0232	2.8044E-01		ENSG00000289104
NA	-0.0452	2.8030E-01		ENSG00000258565
NA	-0.0390	2.8022E-01		ENSG00000236989
NA	-0.0689	2.8015E-01	4.71E-01	ENSG00000289564
NA	-0.0414	2.7969E-01		ENSG00000284969
NA	-0.1087	2.7919E-01	4.70E-01	ENSG00000227248
NA	-0.0780	2.7894E-01		ENSG00000259554
NA	-0.0644	2.7821E-01		ENSG00000259475
NA	-0.0902	2.7800E-01	4.69E-01	ENSG00000241409
NA	-0.0530	2.7760E-01		ENSG00000236234
NA	-0.1054	2.7733E-01	4.68E-01	ENSG00000235411
NA	-0.0527	2.7675E-01		ENSG00000282381
NA	-0.0050	2.7663E-01		ENSG00000258620
NA	-0.1117	2.7646E-01	4.67E-01	ENSG00000264577
NA	-0.1221	2.7639E-01	4.67E-01	ENSG00000213956
NA	-0.0326	2.7625E-01		ENSG00000233448
NA	-0.0566	2.7614E-01		ENSG00000266783
NA	-0.2233	2.7612E-01	4.67E-01	ENSG00000237749
NA	-0.1008	2.7588E-01	4.66E-01	ENSG00000225544
NA	-0.0724	2.7586E-01	4.66E-01	ENSG00000232162
NA	-0.0043	2.7566E-01		ENSG00000230729
NA	-0.1164	2.7554E-01	4.66E-01	ENSG00000277954
NA	-0.0729	2.7504E-01		ENSG00000237477
NA	-0.0999	2.7501E-01	4.66E-01	ENSG00000268034
NA	-0.1071	2.7484E-01	4.65E-01	ENSG00000258645
NA	-0.0903	2.7483E-01	4.65E-01	ENSG00000256988
NA	-0.0562	2.7472E-01		ENSG00000289322
NA	-0.0997	2.7472E-01	4.65E-01	ENSG00000287601
NA	-0.1117	2.7456E-01	4.65E-01	ENSG00000228034
NA	-0.0543	2.7424E-01		ENSG00000283010
NA	-0.0282	2.7403E-01		ENSG00000174325
NA	-0.0395	2.7394E-01		ENSG00000279074
NA	-0.0645	2.7391E-01	4.64E-01	ENSG00000160766
NA	-0.1060	2.7344E-01	4.64E-01	ENSG00000238084
NA	-0.0601	2.7261E-01		ENSG00000259177
NA	-0.0682	2.7247E-01	4.62E-01	ENSG00000234367
NA	-0.0938	2.7239E-01	4.62E-01	ENSG00000227632
NA	-0.0094	2.7236E-01		ENSG00000257228
NA	-0.0776	2.7221E-01	4.62E-01	ENSG00000234819
NA	-0.0256	2.7216E-01		ENSG00000259152
NA	-0.0863	2.7213E-01	4.62E-01	ENSG00000286912
NA	-0.0298	2.7122E-01		ENSG00000167046
NA	-0.1097	2.7067E-01	4.60E-01	ENSG00000240489
NA	-0.0230	2.6928E-01		ENSG00000254938
NA	-0.1086	2.6905E-01	4.58E-01	ENSG00000197744
NA	-0.0808	2.6902E-01		ENSG00000271857
NA	-0.0287	2.6876E-01		ENSG00000289284

NA	-0.1094	2.6875E-01	4.58E-01	ENSG00000230882
NA	-0.0948	2.6873E-01	4.58E-01	ENSG00000280776
NA	-0.0447	2.6872E-01		ENSG00000249286
NA	-0.0383	2.6849E-01		ENSG00000287845
NA	-0.0618	2.6791E-01		ENSG00000266805
NA	-0.0954	2.6745E-01	4.57E-01	ENSG00000261460
NA	-0.1026	2.6734E-01	4.56E-01	ENSG00000206768
NA	-0.0544	2.6731E-01		ENSG00000260910
NA	-0.0614	2.6714E-01		ENSG00000286433
NA	-0.0692	2.6712E-01		ENSG00000232742
NA	-0.0499	2.6702E-01		ENSG00000285704
NA	-0.0918	2.6701E-01	4.56E-01	ENSG00000285744
NA	-0.0290	2.6672E-01		ENSG00000283413
NA	-0.0659	2.6662E-01	4.56E-01	ENSG00000223784
NA	-0.0361	2.6656E-01		ENSG00000265010
NA	-0.1140	2.6643E-01	4.55E-01	ENSG00000225969
NA	-0.1142	2.6629E-01	4.55E-01	ENSG00000267605
NA	-0.0446	2.6567E-01		ENSG00000249605
NA	-0.0410	2.6565E-01		ENSG00000276418
NA	-0.0393	2.6542E-01		ENSG00000273264
NA	-0.0831	2.6532E-01	4.54E-01	ENSG00000273308
NA	-0.1095	2.6513E-01	4.54E-01	ENSG00000285536
NA	-0.0397	2.6502E-01		ENSG00000259354
NA	-0.0345	2.6484E-01		ENSG00000259276
NA	-0.0325	2.6472E-01		ENSG00000249049
NA	-0.0959	2.6468E-01	4.53E-01	ENSG00000227123
NA	-0.0937	2.6446E-01	4.53E-01	ENSG00000231822
NA	-0.0034	2.6409E-01	4.53E-01	ENSG00000285671
NA	-0.0317	2.6406E-01		ENSG00000230046
NA	-0.0612	2.6386E-01		ENSG00000237851
NA	-0.0067	2.6355E-01		ENSG00000289071
NA	-0.0867	2.6354E-01	4.52E-01	ENSG00000283148
NA	-0.1155	2.6354E-01	4.52E-01	ENSG00000279186
NA	-0.0634	2.6346E-01	4.52E-01	ENSG00000286563
NA	-0.1242	2.6312E-01	4.52E-01	ENSG00000250917
NA	-0.1045	2.6300E-01	4.52E-01	ENSG00000286110
NA	-0.0048	2.6291E-01	4.52E-01	ENSG00000286622
NA	-0.0845	2.6274E-01		ENSG00000272989
NA	-0.0457	2.6265E-01		ENSG00000238160
NA	-0.1120	2.6203E-01	4.51E-01	ENSG00000271714
NA	-0.0431	2.6116E-01		ENSG00000250260
NA	-0.0381	2.6105E-01		ENSG00000226987
NA	-0.1452	2.6098E-01	4.50E-01	ENSG00000099251
NA	-0.0110	2.6069E-01		ENSG00000232615
NA	-0.1030	2.6039E-01	4.49E-01	ENSG00000224592
NA	-0.1150	2.6020E-01	4.49E-01	ENSG00000272871
NA	-0.0828	2.6010E-01		ENSG00000272049
NA	-0.0781	2.5986E-01	4.48E-01	ENSG00000287666
NA	-0.0384	2.5978E-01		ENSG00000274064

NA	-0.1165	2.5873E-01	4.47E-01	ENSG00000253227
NA	-0.0735	2.5800E-01	4.46E-01	ENSG00000240687
NA	-0.0394	2.5718E-01		ENSG00000176115
NA	-0.0295	2.5688E-01		ENSG00000283648
NA	-0.0931	2.5677E-01		ENSG00000276250
NA	-0.1182	2.5634E-01	4.44E-01	ENSG00000277245
NA	-0.0754	2.5604E-01	4.44E-01	ENSG00000236888
NA	-0.0863	2.5603E-01		ENSG00000225971
NA	-0.0084	2.5586E-01		ENSG00000264693
NA	-0.0786	2.5572E-01	4.43E-01	ENSG00000238082
NA	-0.0386	2.5559E-01		ENSG00000283977
NA	-0.0305	2.5547E-01		ENSG00000280382
NA	-0.1148	2.5530E-01	4.43E-01	ENSG00000280649
NA	-0.1144	2.5514E-01	4.43E-01	ENSG00000289539
NA	-0.0296	2.5505E-01		ENSG00000289908
NA	-0.1060	2.5470E-01	4.42E-01	ENSG00000283108
NA	-0.1158	2.5449E-01	4.42E-01	ENSG00000248491
NA	-0.1111	2.5421E-01	4.41E-01	ENSG00000253361
NA	-0.0732	2.5419E-01	4.41E-01	ENSG00000258365
NA	-0.0401	2.5406E-01		ENSG00000237528
NA	-0.1122	2.5382E-01	4.41E-01	ENSG00000231991
NA	-0.0654	2.5362E-01	4.41E-01	ENSG00000267117
NA	-0.0963	2.5361E-01	4.41E-01	ENSG00000253829
NA	-0.1273	2.5320E-01	4.40E-01	ENSG00000282057
NA	-0.0561	2.5296E-01		ENSG00000267108
NA	-0.1162	2.5235E-01	4.39E-01	ENSG00000264443
NA	-0.0794	2.5206E-01	4.39E-01	ENSG00000286924
NA	-0.0936	2.5176E-01	4.39E-01	ENSG00000272081
NA	-0.0236	2.5165E-01		ENSG00000249463
NA	-0.1194	2.5161E-01	4.38E-01	ENSG00000283443
NA	-0.0823	2.5156E-01		ENSG00000266282
NA	-0.1183	2.5135E-01	4.38E-01	ENSG00000214719
NA	-0.0595	2.5080E-01		ENSG00000254787
NA	-0.1194	2.5056E-01	4.37E-01	ENSG00000272189
NA	-0.0054	2.5005E-01	4.37E-01	ENSG00000228657
NA	-0.0639	2.4984E-01		ENSG00000264829
NA	-0.0534	2.4964E-01		ENSG00000232696
NA	-0.0664	2.4959E-01		ENSG00000250920
NA	-0.0559	2.4943E-01		ENSG00000224232
NA	-0.0726	2.4913E-01		ENSG00000250934
NA	-0.0626	2.4857E-01		ENSG00000206844
NA	-0.0024	2.4840E-01		ENSG00000241478
NA	-0.0379	2.4829E-01		ENSG00000231147
NA	-0.1205	2.4814E-01	4.34E-01	ENSG00000172971
NA	-0.0475	2.4812E-01		ENSG00000285621
NA	-0.0986	2.4761E-01	4.34E-01	ENSG00000275441
NA	-0.0754	2.4759E-01		ENSG00000290000
NA	-0.0518	2.4739E-01		ENSG00000287840
NA	-0.0504	2.4739E-01		ENSG00000272381

NA	-0.0926	2.4731E-01	4.33E-01	ENSG00000255224
NA	-0.0855	2.4730E-01	4.33E-01	ENSG00000288079
NA	-0.0901	2.4704E-01	4.33E-01	ENSG00000248840
NA	-0.1279	2.4690E-01	4.33E-01	ENSG00000261173
NA	-0.0918	2.4665E-01	4.33E-01	ENSG00000224892
NA	-0.0301	2.4634E-01		ENSG00000257674
NA	-0.0437	2.4623E-01		ENSG00000283737
NA	-0.0864	2.4613E-01	4.32E-01	ENSG00000287218
NA	-0.0573	2.4596E-01		ENSG00000229151
NA	-0.0486	2.4550E-01		ENSG00000270917
NA	-0.1571	2.4532E-01	4.31E-01	ENSG00000286036
NA	-0.0436	2.4527E-01		ENSG00000254236
NA	-0.0082	2.4519E-01		ENSG00000273302
NA	-0.0919	2.4508E-01	4.31E-01	ENSG00000174028
NA	-0.1037	2.4498E-01	4.31E-01	ENSG00000228065
NA	-0.0299	2.4491E-01		ENSG00000287368
NA	-0.1215	2.4472E-01	4.30E-01	ENSG00000237356
NA	-0.0470	2.4469E-01		ENSG00000229393
NA	-0.0462	2.4447E-01		ENSG00000231355
NA	-0.0376	2.4404E-01		ENSG00000251455
NA	-0.0444	2.4393E-01		ENSG00000235816
NA	-0.0867	2.4376E-01	4.29E-01	ENSG00000214975
NA	-0.1221	2.4347E-01	4.29E-01	ENSG00000236958
NA	-0.0440	2.4329E-01		ENSG00000261219
NA	-0.1065	2.4297E-01	4.28E-01	ENSG00000253549
NA	-0.0674	2.4265E-01		ENSG00000265750
NA	-0.0654	2.4235E-01		ENSG00000287449
NA	-0.1502	2.4199E-01	4.27E-01	ENSG00000289447
NA	-0.1167	2.4157E-01	4.27E-01	ENSG00000254610
NA	-0.0235	2.4107E-01		ENSG00000232467
NA	-0.0927	2.4033E-01	4.25E-01	ENSG00000280003
NA	-0.0937	2.3998E-01	4.25E-01	ENSG00000229272
NA	-0.0560	2.3958E-01		ENSG00000277621
NA	-0.0060	2.3953E-01		ENSG00000259591
NA	-0.0865	2.3945E-01	4.24E-01	ENSG00000287619
NA	-0.0610	2.3874E-01		ENSG00000287044
NA	-0.0987	2.3873E-01	4.23E-01	ENSG00000249721
NA	-0.0500	2.3866E-01		ENSG00000281100
NA	-0.0753	2.3757E-01	4.22E-01	ENSG00000224965
NA	-0.0584	2.3744E-01		ENSG00000230404
NA	-0.0574	2.3730E-01		ENSG00000259755
NA	-0.0606	2.3721E-01		ENSG00000213761
NA	-0.0441	2.3715E-01		ENSG00000286171
NA	-0.0522	2.3703E-01	4.21E-01	ENSG00000270666
NA	-0.1179	2.3640E-01	4.21E-01	ENSG00000289613
NA	-0.0381	2.3632E-01		ENSG00000178107
NA	-0.1105	2.3598E-01	4.20E-01	ENSG00000199366
NA	-0.0890	2.3590E-01	4.20E-01	ENSG00000288685
NA	-0.1226	2.3586E-01	4.20E-01	ENSG00000248472

NA	-0.1240	2.3581E-01	4.20E-01	ENSG00000249661
NA	-0.0566	2.3570E-01		ENSG00000254420
NA	-0.0652	2.3539E-01	4.20E-01	ENSG00000275963
NA	-0.0469	2.3531E-01		ENSG00000230833
NA	-0.0947	2.3529E-01	4.20E-01	ENSG00000231460
NA	-0.1141	2.3519E-01	4.19E-01	ENSG00000284512
NA	-0.1210	2.3514E-01	4.19E-01	ENSG00000287828
NA	-0.0520	2.3499E-01		ENSG00000237174
NA	-0.1122	2.3497E-01	4.19E-01	ENSG00000260693
NA	-0.0706	2.3481E-01		ENSG00000240710
NA	-0.1063	2.3413E-01	4.18E-01	ENSG00000254893
NA	-0.0656	2.3367E-01	4.18E-01	ENSG00000282024
NA	-0.0834	2.3329E-01		ENSG00000261419
NA	-0.0396	2.3310E-01		ENSG00000207092
NA	-0.0801	2.3278E-01		ENSG00000256325
NA	-0.0523	2.3269E-01		ENSG00000279958
NA	-0.0449	2.3256E-01		ENSG00000288109
NA	-0.0526	2.3227E-01		ENSG00000272425
NA	-0.0700	2.3205E-01		ENSG00000251609
NA	-0.0019	2.3202E-01		ENSG00000228886
NA	-0.0988	2.3117E-01	4.15E-01	ENSG00000230551
NA	-0.1748	2.3070E-01	4.14E-01	ENSG00000289159
NA	-0.1241	2.3034E-01	4.14E-01	ENSG00000289330
NA	-0.1246	2.3017E-01	4.13E-01	ENSG00000285873
NA	-0.0399	2.3011E-01		ENSG00000232894
NA	-0.0298	2.3005E-01		ENSG00000242001
NA	-0.0894	2.2987E-01	4.13E-01	ENSG00000259732
NA	-0.0364	2.2917E-01		ENSG00000268297
NA	-0.0030	2.2915E-01		ENSG00000233920
NA	-0.0756	2.2905E-01		ENSG00000259126
NA	-0.1222	2.2902E-01	4.12E-01	ENSG00000288995
NA	-0.1012	2.2851E-01	4.11E-01	ENSG00000233695
NA	-0.1191	2.2808E-01	4.11E-01	ENSG00000225031
NA	-0.0798	2.2772E-01	4.10E-01	ENSG00000283283
NA	-0.0667	2.2769E-01	4.10E-01	ENSG00000230068
NA	-0.0364	2.2766E-01		ENSG00000261393
NA	-0.0329	2.2738E-01		ENSG00000279569
NA	-0.0235	2.2735E-01		ENSG00000259950
NA	-0.0953	2.2724E-01	4.10E-01	ENSG00000267809
NA	-0.1131	2.2714E-01	4.10E-01	ENSG00000236924
NA	-0.0835	2.2710E-01		ENSG00000281420
NA	-0.0347	2.2707E-01		ENSG00000233040
NA	-0.0595	2.2673E-01	4.09E-01	ENSG00000249379
NA	-0.1050	2.2657E-01	4.09E-01	ENSG00000272574
NA	-0.0999	2.2632E-01	4.09E-01	ENSG00000233871
NA	-0.0424	2.2542E-01		ENSG00000256994
NA	-0.0481	2.2527E-01		ENSG00000207368
NA	-0.0693	2.2524E-01		ENSG00000275106
NA	-0.1175	2.2498E-01	4.07E-01	ENSG00000289231

NA	-0.1235	2.2495E-01	4.07E-01	ENSG00000234705
NA	-0.1271	2.2470E-01	4.07E-01	ENSG00000248593
NA	-0.0635	2.2459E-01		ENSG00000286148
NA	-0.1213	2.2443E-01	4.07E-01	ENSG00000237264
NA	-0.1250	2.2425E-01	4.07E-01	ENSG00000269688
NA	-0.0890	2.2421E-01	4.06E-01	ENSG00000273133
NA	-0.0474	2.2420E-01		ENSG00000258827
NA	-0.0009	2.2412E-01		ENSG00000290061
NA	-0.1079	2.2398E-01	4.06E-01	ENSG00000254485
NA	-0.0592	2.2393E-01		ENSG00000223390
NA	-0.0467	2.2390E-01		ENSG00000286611
NA	-0.0425	2.2386E-01		ENSG00000283549
NA	-0.1288	2.2369E-01	4.06E-01	ENSG00000254024
NA	-0.0394	2.2365E-01		ENSG00000202318
NA	-0.1253	2.2351E-01	4.06E-01	ENSG00000224597
NA	-0.0384	2.2342E-01		ENSG00000200135
NA	-0.0877	2.2322E-01		ENSG00000227590
NA	-0.0324	2.2319E-01		ENSG00000260113
NA	-0.1332	2.2300E-01	4.05E-01	ENSG00000219747
NA	-0.1157	2.2266E-01	4.05E-01	ENSG00000204959
NA	-0.0522	2.2260E-01		ENSG00000272744
NA	-0.0903	2.2231E-01		ENSG00000286828
NA	-0.0721	2.2179E-01		ENSG00000288605
NA	-0.1143	2.2141E-01	4.03E-01	ENSG00000288848
NA	-0.0953	2.2132E-01	4.03E-01	ENSG00000270720
NA	-0.0345	2.2117E-01		ENSG00000274928
NA	-0.1278	2.2069E-01	4.02E-01	ENSG00000233797
NA	-0.0459	2.2065E-01		ENSG00000268818
NA	-0.0085	2.2043E-01	4.02E-01	ENSG00000227769
NA	-0.1188	2.2012E-01	4.01E-01	ENSG00000270503
NA	-0.0311	2.1989E-01		ENSG00000281974
NA	-0.0696	2.1984E-01	4.01E-01	ENSG00000213590
NA	-0.0652	2.1983E-01		ENSG00000226438
NA	-0.0036	2.1976E-01		ENSG00000289577
NA	-0.0607	2.1971E-01	4.01E-01	ENSG00000258647
NA	-0.1274	2.1967E-01	4.01E-01	ENSG00000159712
NA	-0.0701	2.1947E-01	4.00E-01	ENSG00000279166
NA	-0.0440	2.1936E-01		ENSG00000249465
NA	-0.1120	2.1935E-01	4.00E-01	ENSG00000261114
NA	-0.0740	2.1921E-01	4.00E-01	ENSG00000226499
NA	-0.0466	2.1889E-01		ENSG00000272279
NA	-0.1186	2.1859E-01	4.00E-01	ENSG00000270084
NA	-0.0545	2.1835E-01		ENSG00000267503
NA	-0.0163	2.1826E-01	3.99E-01	ENSG00000248646
NA	-0.0846	2.1798E-01	3.99E-01	ENSG00000236337
NA	-0.1284	2.1789E-01	3.99E-01	ENSG00000284625
NA	-0.1004	2.1747E-01	3.98E-01	ENSG00000278022
NA	-0.0748	2.1745E-01	3.98E-01	ENSG00000225920
NA	-0.0333	2.1739E-01		ENSG00000287093

NA	-0.0336	2.1716E-01		ENSG00000267177
NA	-0.0509	2.1716E-01		ENSG00000266445
NA	-0.1117	2.1698E-01	3.98E-01	ENSG00000254532
NA	-0.1240	2.1643E-01	3.97E-01	ENSG00000251081
NA	-0.0934	2.1637E-01	3.97E-01	ENSG00000281468
NA	-0.0715	2.1618E-01	3.97E-01	ENSG00000263033
NA	-0.1986	2.1598E-01	3.97E-01	ENSG00000220130
NA	-0.0013	2.1590E-01		ENSG00000286310
NA	-0.0541	2.1557E-01		ENSG00000288849
NA	-0.1327	2.1530E-01	3.96E-01	ENSG00000279369
NA	-0.1562	2.1501E-01	3.95E-01	ENSG00000219928
NA	-0.0648	2.1496E-01		ENSG00000235659
NA	-0.1303	2.1492E-01	3.95E-01	ENSG00000288908
NA	-0.0703	2.1486E-01	3.95E-01	ENSG00000252367
NA	-0.1021	2.1467E-01	3.95E-01	ENSG00000266644
NA	-0.0623	2.1457E-01		ENSG00000289540
NA	-0.0608	2.1436E-01		ENSG00000287224
NA	-0.0432	2.1430E-01		ENSG00000272416
NA	-0.1411	2.1406E-01	3.94E-01	ENSG00000249072
NA	-0.1173	2.1403E-01	3.94E-01	ENSG00000235720
NA	-0.0921	2.1402E-01		ENSG00000253819
NA	-0.1306	2.1373E-01	3.94E-01	ENSG00000226580
NA	-0.0425	2.1359E-01		ENSG00000233661
NA	-0.1204	2.1356E-01	3.94E-01	ENSG00000273230
NA	-0.1243	2.1312E-01	3.93E-01	ENSG00000279928
NA	-0.1323	2.1260E-01	3.92E-01	ENSG00000237732
NA	-0.1334	2.1251E-01	3.92E-01	ENSG00000239381
NA	-0.0129	2.1244E-01		ENSG00000264491
NA	-0.1277	2.1242E-01	3.92E-01	ENSG00000284648
NA	-0.1135	2.1231E-01	3.92E-01	ENSG00000216829
NA	-0.1334	2.1230E-01	3.92E-01	ENSG00000278840
NA	-0.0648	2.1225E-01		ENSG00000254842
NA	-0.1151	2.1220E-01	3.92E-01	ENSG00000287705
NA	-0.0926	2.1160E-01	3.91E-01	ENSG00000262668
NA	-0.1253	2.1150E-01	3.91E-01	ENSG00000206417
NA	-0.0428	2.1149E-01		ENSG00000287474
NA	-0.1035	2.1096E-01	3.91E-01	ENSG00000267249
NA	-0.1127	2.1091E-01	3.91E-01	ENSG00000260751
NA	-0.0370	2.1079E-01		ENSG00000229759
NA	-0.1153	2.1055E-01	3.90E-01	ENSG00000286735
NA	-0.0436	2.1039E-01		ENSG00000286189
NA	-0.0646	2.1028E-01		ENSG00000286881
NA	-0.1274	2.0974E-01	3.89E-01	ENSG00000242593
NA	-0.1313	2.0941E-01	3.89E-01	ENSG00000179362
NA	-0.0771	2.0879E-01	3.88E-01	ENSG00000214955
NA	-0.0287	2.0869E-01		ENSG00000273866
NA	-0.1252	2.0853E-01	3.88E-01	ENSG00000282855
NA	-0.0570	2.0847E-01		ENSG00000264769
NA	-0.0866	2.0774E-01	3.87E-01	ENSG00000277873

NA	-0.2127	2.0764E-01	3.87E-01	ENSG00000276500
NA	-0.1232	2.0732E-01	3.86E-01	ENSG00000232536
NA	-0.0286	2.0718E-01		ENSG00000254502
NA	-0.1331	2.0676E-01	3.85E-01	ENSG00000261338
NA	-0.1326	2.0657E-01	3.85E-01	ENSG00000260007
NA	-0.1016	2.0644E-01	3.85E-01	ENSG00000230506
NA	-0.0469	2.0619E-01		ENSG00000264853
NA	-0.1213	2.0592E-01	3.84E-01	ENSG00000279799
NA	-0.0305	2.0578E-01		ENSG00000254500
NA	-0.0409	2.0523E-01		ENSG00000251393
NA	-0.1297	2.0474E-01	3.83E-01	ENSG00000250387
NA	-0.1280	2.0466E-01	3.83E-01	ENSG00000227482
NA	-0.0409	2.0455E-01		ENSG00000279521
NA	-0.1228	2.0434E-01	3.82E-01	ENSG00000286913
NA	-0.0283	2.0390E-01		ENSG00000287511
NA	-0.0937	2.0390E-01		ENSG00000274849
NA	-0.0502	2.0376E-01		ENSG00000285859
NA	-0.1188	2.0340E-01	3.81E-01	ENSG00000289305
NA	-0.1376	2.0321E-01	3.81E-01	ENSG00000256083
NA	-0.0389	2.0320E-01		ENSG00000235698
NA	-0.0771	2.0308E-01		ENSG00000236744
NA	-0.0808	2.0298E-01		ENSG00000287657
NA	-0.1313	2.0280E-01	3.80E-01	ENSG00000219507
NA	-0.1002	2.0242E-01	3.80E-01	ENSG00000280402
NA	-0.1358	2.0204E-01	3.80E-01	ENSG00000242970
NA	-0.1372	2.0185E-01	3.79E-01	ENSG00000242154
NA	-0.1757	2.0173E-01	3.79E-01	ENSG00000239719
NA	-0.0622	2.0047E-01		ENSG00000236086
NA	-0.0865	1.9945E-01		ENSG00000272582
NA	-0.1228	1.9916E-01	3.76E-01	ENSG00000205041
NA	-0.1253	1.9916E-01	3.76E-01	ENSG00000276832
NA	-0.1240	1.9885E-01	3.76E-01	ENSG00000203497
NA	-0.0500	1.9881E-01		ENSG00000259935
NA	-0.1207	1.9788E-01	3.75E-01	ENSG00000288060
NA	-0.1017	1.9756E-01	3.74E-01	ENSG00000233081
NA	-0.1343	1.9743E-01	3.74E-01	ENSG00000242960
NA	-0.0984	1.9727E-01	3.74E-01	ENSG00000279530
NA	-0.0798	1.9719E-01		ENSG00000237470
NA	-0.1370	1.9718E-01	3.74E-01	ENSG00000279652
NA	-0.0538	1.9715E-01		ENSG00000226330
NA	-0.0776	1.9702E-01		ENSG00000251532
NA	-0.1260	1.9675E-01	3.73E-01	ENSG00000255727
NA	-0.1819	1.9632E-01	3.73E-01	ENSG00000286146
NA	-0.1027	1.9630E-01	3.73E-01	ENSG00000287275
NA	-0.0752	1.9620E-01		ENSG00000228950
NA	-0.0527	1.9607E-01		ENSG00000257137
NA	-0.1363	1.9606E-01	3.72E-01	ENSG00000289162
NA	-0.0790	1.9584E-01		ENSG00000230299
NA	-0.1226	1.9493E-01	3.71E-01	ENSG00000268869

NA	-0.0336	1.9481E-01		ENSG00000262039
NA	-0.1192	1.9408E-01	3.70E-01	ENSG00000288106
NA	-0.0544	1.9393E-01		ENSG00000286700
NA	-0.1357	1.9384E-01	3.69E-01	ENSG00000237429
NA	-0.0560	1.9378E-01		ENSG00000235151
NA	-0.0517	1.9363E-01		ENSG00000260211
NA	-0.0983	1.9357E-01	3.69E-01	ENSG00000276188
NA	-0.1274	1.9345E-01	3.69E-01	ENSG00000259865
NA	-0.1333	1.9327E-01	3.69E-01	ENSG00000230896
NA	-0.0281	1.9295E-01		ENSG00000256637
NA	-0.0028	1.9292E-01		ENSG00000149656
NA	-0.1341	1.9290E-01	3.68E-01	ENSG00000289088
NA	-0.1323	1.9285E-01	3.68E-01	ENSG00000018607
NA	-0.1163	1.9264E-01	3.68E-01	ENSG00000220925
NA	-0.0758	1.9234E-01		ENSG00000227034
NA	-0.0956	1.9179E-01	3.67E-01	ENSG00000285525
NA	-0.0823	1.9155E-01		ENSG00000280435
NA	-0.1393	1.9129E-01	3.66E-01	ENSG00000267222
NA	-0.1384	1.9090E-01	3.66E-01	ENSG00000188765
NA	-0.0619	1.9087E-01		ENSG00000214646
NA	-0.0933	1.9080E-01	3.66E-01	ENSG00000273980
NA	-0.1002	1.9031E-01	3.65E-01	ENSG00000233868
NA	-0.1375	1.9017E-01	3.65E-01	ENSG00000233554
NA	-0.1051	1.9011E-01	3.65E-01	ENSG00000287825
NA	-0.0136	1.9005E-01	3.65E-01	ENSG00000255856
NA	-0.0728	1.8998E-01		ENSG00000253721
NA	-0.0518	1.8973E-01		ENSG00000269403
NA	-0.0252	1.8967E-01		ENSG00000214659
NA	-0.1297	1.8957E-01	3.64E-01	ENSG00000226963
NA	-0.1071	1.8951E-01	3.64E-01	ENSG00000273055
NA	-0.1090	1.8918E-01	3.64E-01	ENSG00000254708
NA	-0.1136	1.8878E-01	3.63E-01	ENSG00000204789
NA	-0.1334	1.8855E-01	3.63E-01	ENSG00000227598
NA	-0.1742	1.8854E-01	3.63E-01	ENSG00000235121
NA	-0.1101	1.8851E-01	3.63E-01	ENSG00000255198
NA	-0.0848	1.8815E-01		ENSG00000272733
NA	-0.0415	1.8805E-01		ENSG00000254744
NA	-0.0484	1.8797E-01		ENSG00000254185
NA	-0.2140	1.8765E-01	3.62E-01	ENSG00000229596
NA	-0.1387	1.8742E-01	3.62E-01	ENSG00000270607
NA	-0.1169	1.8729E-01	3.61E-01	ENSG00000286039
NA	-0.0357	1.8696E-01		ENSG00000279887
NA	-0.1396	1.8696E-01	3.61E-01	ENSG00000231519
NA	-0.0015	1.8686E-01		ENSG00000279162
NA	-0.1157	1.8628E-01	3.60E-01	ENSG00000279513
NA	-0.0620	1.8557E-01		ENSG00000278954
NA	-0.0604	1.8555E-01		ENSG00000225342
NA	-0.0565	1.8545E-01		ENSG00000228322
NA	-0.1312	1.8536E-01	3.59E-01	ENSG00000286989

NA	-0.0570	1.8531E-01		ENSG00000213149
NA	-0.0357	1.8530E-01		ENSG00000237141
NA	-0.0856	1.8508E-01	3.58E-01	ENSG00000286728
NA	-0.0317	1.8488E-01		ENSG00000287444
NA	-0.0352	1.8474E-01		ENSG00000272646
NA	-0.1426	1.8437E-01	3.57E-01	ENSG00000213592
NA	-0.1352	1.8413E-01	3.57E-01	ENSG00000248494
NA	-0.1239	1.8395E-01	3.57E-01	ENSG00000212829
NA	-0.0314	1.8281E-01		ENSG00000254207
NA	-0.0347	1.8260E-01		ENSG00000255142
NA	-0.1289	1.8255E-01	3.55E-01	ENSG00000235280
NA	-0.0899	1.8246E-01		ENSG00000277911
NA	-0.0537	1.8215E-01		ENSG00000263680
NA	-0.1432	1.8167E-01	3.54E-01	ENSG00000278996
NA	-0.0834	1.8146E-01		ENSG00000276742
NA	-0.0956	1.8144E-01		ENSG00000231468
NA	-0.0424	1.8135E-01		ENSG00000269839
NA	-0.1577	1.8133E-01	3.53E-01	ENSG00000265943
NA	-0.0423	1.8103E-01		ENSG00000253445
NA	-0.0472	1.8083E-01		ENSG00000213050
NA	-0.0756	1.8056E-01		ENSG00000289173
NA	-0.0408	1.8045E-01		ENSG00000267686
NA	-0.0448	1.8029E-01		ENSG00000237759
NA	-0.0931	1.7982E-01	3.51E-01	ENSG00000273041
NA	-0.0939	1.7971E-01		ENSG00000259616
NA	-0.0875	1.7958E-01		ENSG00000288087
NA	-0.2429	1.7944E-01	3.51E-01	ENSG00000182021
NA	-0.1102	1.7943E-01	3.51E-01	ENSG00000231324
NA	-0.0350	1.7937E-01		ENSG00000262298
NA	-0.0965	1.7926E-01		ENSG00000273765
NA	-0.1451	1.7854E-01	3.50E-01	ENSG00000197550
NA	-0.0895	1.7833E-01	3.49E-01	ENSG00000279392
NA	-0.0769	1.7831E-01		ENSG00000248909
NA	-0.1212	1.7820E-01	3.49E-01	ENSG00000273733
NA	-0.1448	1.7804E-01	3.49E-01	ENSG00000255389
NA	-0.1102	1.7803E-01	3.49E-01	ENSG00000289401
NA	-0.0309	1.7790E-01	3.49E-01	ENSG00000289876
NA	-0.1454	1.7694E-01	3.47E-01	ENSG00000245468
NA	-0.1397	1.7676E-01	3.47E-01	ENSG00000248206
NA	-0.0132	1.7643E-01	3.47E-01	ENSG00000275888
NA	-0.1014	1.7623E-01	3.47E-01	ENSG00000228719
NA	-0.1151	1.7583E-01	3.46E-01	ENSG00000280107
NA	-0.1472	1.7564E-01	3.46E-01	ENSG00000267023
NA	-0.1088	1.7538E-01	3.45E-01	ENSG00000261457
NA	-0.0923	1.7530E-01		ENSG00000277491
NA	-0.1465	1.7514E-01	3.45E-01	ENSG00000286873
NA	-0.0354	1.7425E-01		ENSG00000277191
NA	-0.1060	1.7376E-01	3.43E-01	ENSG00000260052
NA	-0.1085	1.7373E-01	3.43E-01	ENSG00000267317

NA	-0.1435	1.7358E-01	3.43E-01	ENSG00000270177
NA	-0.1475	1.7326E-01	3.43E-01	ENSG00000279722
NA	-0.0682	1.7326E-01		ENSG00000226890
NA	-0.1337	1.7302E-01	3.42E-01	ENSG00000286500
NA	-0.1337	1.7301E-01	3.42E-01	ENSG00000234513
NA	-0.1362	1.7267E-01	3.42E-01	ENSG00000270504
NA	-0.1376	1.7258E-01	3.42E-01	ENSG00000250979
NA	-0.0411	1.7250E-01		ENSG00000233589
NA	-0.1466	1.7245E-01	3.42E-01	ENSG00000250321
NA	-0.0534	1.7236E-01		ENSG00000273118
NA	-0.1214	1.7187E-01	3.41E-01	ENSG00000289698
NA	-0.1639	1.7149E-01	3.40E-01	ENSG00000289879
NA	-0.1349	1.7129E-01	3.40E-01	ENSG00000267750
NA	-0.0574	1.7119E-01		ENSG00000225330
NA	-0.0001	1.7119E-01	3.40E-01	ENSG00000251445
NA	-0.0787	1.7066E-01		ENSG00000232754
NA	-0.0511	1.7048E-01		ENSG00000259915
NA	-0.0583	1.7040E-01		ENSG00000273487
NA	-0.1036	1.7022E-01	3.38E-01	ENSG00000273825
NA	-0.0411	1.7013E-01		ENSG00000255548
NA	-0.1002	1.6991E-01		ENSG00000278979
NA	-0.0938	1.6963E-01	3.38E-01	ENSG00000274265
NA	-0.1290	1.6949E-01	3.38E-01	ENSG00000265916
NA	-0.0323	1.6896E-01		ENSG00000255250
NA	-0.0373	1.6896E-01		ENSG00000207293
NA	-0.1272	1.6858E-01	3.36E-01	ENSG00000273951
NA	-0.0957	1.6795E-01	3.35E-01	ENSG00000235734
NA	-0.1465	1.6778E-01	3.35E-01	ENSG00000230847
NA	-0.1473	1.6774E-01	3.35E-01	ENSG00000272927
NA	-0.1261	1.6768E-01	3.35E-01	ENSG00000236307
NA	-0.0728	1.6760E-01		ENSG00000285159
NA	-0.1071	1.6756E-01	3.35E-01	ENSG00000286804
NA	-0.0793	1.6714E-01	3.34E-01	ENSG00000172974
NA	-0.1353	1.6665E-01	3.34E-01	ENSG00000225259
NA	-0.0381	1.6664E-01		ENSG00000215887
NA	-0.0629	1.6611E-01		ENSG00000287952
NA	-0.1498	1.6608E-01	3.33E-01	ENSG00000242262
NA	-0.0518	1.6595E-01		ENSG00000227200
NA	-0.0978	1.6555E-01	3.32E-01	ENSG00000234913
NA	-0.1467	1.6541E-01	3.32E-01	ENSG00000239486
NA	-0.0439	1.6529E-01		ENSG00000237410
NA	-0.0521	1.6520E-01		ENSG00000251648
NA	-0.1497	1.6511E-01	3.32E-01	ENSG00000242540
NA	-0.1440	1.6476E-01	3.31E-01	ENSG00000254919
NA	-0.1532	1.6464E-01	3.31E-01	ENSG00000225475
NA	-0.1415	1.6464E-01	3.31E-01	ENSG00000232187
NA	-0.0693	1.6395E-01		ENSG00000229190
NA	-0.1507	1.6367E-01	3.30E-01	ENSG00000229212
NA	-0.0373	1.6355E-01		ENSG00000225450

NA	-0.1191	1.6351E-01	3.30E-01	ENSG00000286319
NA	-0.0764	1.6344E-01	3.29E-01	ENSG00000255555
NA	-0.1010	1.6332E-01	3.29E-01	ENSG00000250377
NA	-0.1315	1.6277E-01	3.29E-01	ENSG00000268056
NA	-0.1061	1.6266E-01	3.28E-01	ENSG00000275767
NA	-0.1415	1.6265E-01	3.28E-01	ENSG00000250966
NA	-0.1006	1.6233E-01	3.28E-01	ENSG00000276649
NA	-0.0732	1.6156E-01		ENSG00000268889
NA	-0.1419	1.6154E-01	3.27E-01	ENSG00000266498
NA	-0.1501	1.6120E-01	3.26E-01	ENSG00000249159
NA	-0.1485	1.6090E-01	3.26E-01	ENSG00000264808
NA	-0.0832	1.6088E-01	3.26E-01	ENSG00000228663
NA	-0.1502	1.6073E-01	3.26E-01	ENSG00000223343
NA	-0.1143	1.6072E-01	3.26E-01	ENSG00000288103
NA	-0.0777	1.6065E-01		ENSG00000266900
NA	-0.0436	1.6038E-01		ENSG00000260011
NA	-0.1240	1.6031E-01	3.25E-01	ENSG00000241411
NA	-0.0621	1.6022E-01		ENSG00000235872
NA	-0.0078	1.6004E-01		ENSG00000265511
NA	-0.1155	1.6002E-01	3.25E-01	ENSG00000254662
NA	-0.0699	1.5936E-01		ENSG00000278153
NA	-0.1562	1.5924E-01	3.24E-01	ENSG00000204620
NA	-0.0942	1.5917E-01		ENSG00000275532
NA	-0.0752	1.5902E-01		ENSG00000272293
NA	-0.1455	1.5814E-01	3.22E-01	ENSG00000269755
NA	-0.0250	1.5809E-01		ENSG00000285624
NA	-0.1565	1.5809E-01	3.22E-01	ENSG00000285658
NA	-0.1139	1.5806E-01	3.22E-01	ENSG00000184523
NA	-0.1514	1.5803E-01	3.22E-01	ENSG00000280255
NA	-0.1176	1.5756E-01	3.21E-01	ENSG00000272368
NA	-0.1584	1.5703E-01	3.20E-01	ENSG00000240231
NA	-0.1025	1.5667E-01	3.20E-01	ENSG00000233664
NA	-0.0887	1.5589E-01		ENSG00000258323
NA	-0.0693	1.5571E-01		ENSG00000239263
NA	-0.0804	1.5570E-01		ENSG00000223675
NA	-0.1557	1.5564E-01	3.18E-01	ENSG00000228809
NA	-0.1212	1.5560E-01	3.18E-01	ENSG00000236439
NA	-0.1439	1.5554E-01	3.18E-01	ENSG00000197332
NA	-0.0693	1.5530E-01		ENSG00000289103
NA	-0.1486	1.5529E-01	3.18E-01	ENSG00000272438
NA	-0.1205	1.5476E-01	3.17E-01	ENSG00000231579
NA	-0.1462	1.5456E-01	3.17E-01	ENSG00000176268
NA	-0.0502	1.5447E-01		ENSG00000289478
NA	-0.1326	1.5446E-01	3.17E-01	ENSG00000257027
NA	-0.0794	1.5433E-01		ENSG00000257199
NA	-0.1489	1.5427E-01	3.17E-01	ENSG00000237039
NA	-0.0555	1.5410E-01		ENSG00000237451
NA	-0.1442	1.5384E-01	3.16E-01	ENSG00000273314
NA	-0.0342	1.5353E-01		ENSG00000286603

NA	-0.0438	1.5351E-01		ENSG00000287512
NA	-0.0388	1.5344E-01		ENSG00000259805
NA	-0.1501	1.5336E-01	3.15E-01	ENSG00000205940
NA	-0.0978	1.5316E-01		ENSG00000231434
NA	-0.0987	1.5286E-01		ENSG00000213338
NA	-0.1194	1.5218E-01	3.14E-01	ENSG00000213250
NA	-0.1568	1.5185E-01	3.13E-01	ENSG00000288829
NA	-0.1278	1.5181E-01	3.13E-01	ENSG00000231329
NA	-0.1055	1.5170E-01	3.13E-01	ENSG00000259326
NA	-0.1316	1.5169E-01	3.13E-01	ENSG00000251669
NA	-0.1567	1.5163E-01	3.13E-01	ENSG00000250343
NA	-0.0558	1.5114E-01		ENSG00000270313
NA	-0.1443	1.5105E-01	3.12E-01	ENSG00000267742
NA	-0.1203	1.5097E-01	3.12E-01	ENSG00000236434
NA	-0.1591	1.5023E-01	3.11E-01	ENSG00000289397
NA	-0.0966	1.4990E-01		ENSG00000287284
NA	-0.1515	1.4976E-01	3.10E-01	ENSG00000225393
NA	-0.1159	1.4967E-01	3.10E-01	ENSG00000236283
NA	-0.0625	1.4946E-01		ENSG00000255201
NA	-0.1508	1.4946E-01	3.10E-01	ENSG00000287906
NA	-0.1358	1.4923E-01	3.10E-01	ENSG00000233225
NA	-0.0367	1.4902E-01		ENSG00000263326
NA	-0.0885	1.4874E-01	3.09E-01	ENSG00000213018
NA	-0.1555	1.4864E-01	3.09E-01	ENSG00000258477
NA	-0.1592	1.4810E-01	3.08E-01	ENSG00000229927
NA	-0.1015	1.4809E-01	3.08E-01	ENSG00000271959
NA	-0.0784	1.4802E-01	3.08E-01	ENSG00000286321
NA	-0.1302	1.4779E-01	3.08E-01	ENSG00000270558
NA	-0.0519	1.4774E-01		ENSG00000272482
NA	-0.0668	1.4727E-01		ENSG00000239351
NA	-0.6784	1.4695E-01	3.06E-01	ENSG00000228027
NA	-0.0051	1.4681E-01	3.06E-01	ENSG00000257264
NA	-0.0328	1.4625E-01		ENSG00000203620
NA	-0.1112	1.4544E-01	3.04E-01	ENSG00000276674
NA	-0.0982	1.4501E-01		ENSG00000267346
NA	-0.1222	1.4496E-01	3.03E-01	ENSG00000267565
NA	-0.1327	1.4490E-01	3.03E-01	ENSG00000237977
NA	-0.0673	1.4483E-01		ENSG00000231102
NA	-0.0613	1.4472E-01		ENSG00000285996
NA	-0.1616	1.4444E-01	3.03E-01	ENSG00000272844
NA	-0.0182	1.4417E-01	3.02E-01	ENSG00000272662
NA	-0.0873	1.4407E-01	3.02E-01	ENSG00000287830
NA	-0.1609	1.4400E-01	3.02E-01	ENSG00000230204
NA	-0.1610	1.4350E-01	3.01E-01	ENSG00000271901
NA	-0.1460	1.4349E-01	3.01E-01	ENSG00000288012
NA	-0.0421	1.4346E-01		ENSG00000242507
NA	-0.0692	1.4344E-01		ENSG00000258412
NA	-0.1311	1.4337E-01	3.01E-01	ENSG00000258490
NA	-0.0406	1.4330E-01		ENSG00000258722

NA	-0.1611	1.4316E-01	3.01E-01	ENSG00000235319
NA	-0.1093	1.4306E-01	3.01E-01	ENSG00000229848
NA	-0.0765	1.4304E-01		ENSG00000232874
NA	-0.0692	1.4279E-01		ENSG00000237899
NA	-0.1061	1.4260E-01	3.00E-01	ENSG00000234062
NA	-0.1617	1.4243E-01	3.00E-01	ENSG00000278763
NA	-0.1597	1.4223E-01	2.99E-01	ENSG00000287744
NA	-0.0952	1.4220E-01		ENSG00000258674
NA	-0.1008	1.4210E-01	2.99E-01	ENSG00000214192
NA	-0.1514	1.4199E-01	2.99E-01	ENSG00000224599
NA	-0.0892	1.4173E-01		ENSG00000289950
NA	-0.6316	1.4148E-01	2.98E-01	ENSG00000235449
NA	-0.0579	1.4099E-01		ENSG00000286424
NA	-0.0363	1.4098E-01		ENSG00000224239
NA	-0.1136	1.4097E-01	2.97E-01	ENSG00000277945
NA	-0.1649	1.4096E-01	2.97E-01	ENSG00000279281
NA	-0.1457	1.4080E-01	2.97E-01	ENSG00000274565
NA	-0.1142	1.4051E-01	2.97E-01	ENSG00000267388
NA	-0.0739	1.4046E-01		ENSG00000230756
NA	-0.1656	1.3989E-01	2.96E-01	ENSG00000229021
NA	-0.0830	1.3985E-01		ENSG00000257954
NA	-0.1180	1.3954E-01	2.95E-01	ENSG00000279159
NA	-0.0343	1.3947E-01		ENSG00000223631
NA	-0.1355	1.3882E-01	2.94E-01	ENSG00000280077
NA	-0.1558	1.3865E-01	2.94E-01	ENSG00000233912
NA	-0.1104	1.3800E-01	2.93E-01	ENSG00000273179
NA	-0.1073	1.3752E-01	2.92E-01	ENSG00000224992
NA	-0.0951	1.3741E-01		ENSG00000283647
NA	-0.0440	1.3709E-01		ENSG00000235576
NA	-0.0542	1.3700E-01		ENSG00000263677
NA	-0.0475	1.3661E-01		ENSG00000280042
NA	-0.0716	1.3654E-01		ENSG00000257345
NA	-0.1234	1.3627E-01	2.90E-01	ENSG00000287125
NA	-0.1623	1.3609E-01	2.90E-01	ENSG00000218018
NA	-0.1364	1.3596E-01	2.90E-01	ENSG00000277310
NA	-0.1621	1.3543E-01	2.89E-01	ENSG00000271830
NA	-0.3187	1.3525E-01	2.89E-01	ENSG00000232480
NA	-0.1006	1.3523E-01		ENSG00000286803
NA	-0.1803	1.3505E-01	2.89E-01	ENSG00000287897
NA	-0.1657	1.3499E-01	2.89E-01	ENSG00000272079
NA	-0.0545	1.3494E-01		ENSG00000224961
NA	-0.1481	1.3471E-01	2.88E-01	ENSG00000278934
NA	-0.0728	1.3420E-01		ENSG00000228692
NA	-0.1214	1.3416E-01	2.87E-01	ENSG00000289833
NA	-0.0607	1.3410E-01		ENSG00000289524
NA	-0.1282	1.3373E-01	2.87E-01	ENSG00000214999
NA	-0.1660	1.3337E-01	2.86E-01	ENSG00000262873
NA	-0.1636	1.3305E-01	2.86E-01	ENSG00000258427
NA	-0.0523	1.3300E-01		ENSG00000180066

NA	-0.0644	1.3296E-01		ENSG00000244381
NA	-0.1085	1.3267E-01	2.85E-01	ENSG00000253330
NA	-0.0732	1.3252E-01		ENSG00000243007
NA	-0.1569	1.3183E-01	2.84E-01	ENSG00000251615
NA	-0.1114	1.3178E-01		ENSG00000255291
NA	-0.1215	1.3176E-01	2.84E-01	ENSG00000227449
NA	-0.1878	1.3166E-01	2.84E-01	ENSG00000272024
NA	-0.1590	1.3158E-01	2.84E-01	ENSG00000276840
NA	-0.0524	1.3157E-01		ENSG00000287685
NA	-0.1163	1.3154E-01	2.84E-01	ENSG00000237821
NA	-0.0782	1.3152E-01		ENSG00000255092
NA	-0.1507	1.3131E-01	2.83E-01	ENSG00000203706
NA	-0.1683	1.3051E-01	2.82E-01	ENSG00000244538
NA	-0.1638	1.3048E-01	2.82E-01	ENSG00000280088
NA	-0.0682	1.3012E-01		ENSG00000289688
NA	-0.0852	1.2982E-01	2.81E-01	ENSG00000226259
NA	-0.0780	1.2966E-01		ENSG00000226957
NA	-0.0364	1.2963E-01		ENSG00000241651
NA	-0.1474	1.2944E-01	2.81E-01	ENSG00000267127
NA	-0.1010	1.2914E-01	2.80E-01	ENSG00000226899
NA	-0.1603	1.2909E-01	2.80E-01	ENSG00000277383
NA	-0.1692	1.2894E-01	2.80E-01	ENSG00000280206
NA	-0.1195	1.2843E-01	2.79E-01	ENSG00000226965
NA	-0.1569	1.2834E-01	2.79E-01	ENSG00000250220
NA	-0.1197	1.2812E-01		ENSG00000273509
NA	-0.1551	1.2745E-01	2.78E-01	ENSG00000289629
NA	-0.0631	1.2742E-01		ENSG00000285641
NA	-0.1679	1.2723E-01	2.78E-01	ENSG00000279762
NA	-0.1204	1.2709E-01	2.78E-01	ENSG00000236035
NA	-0.1127	1.2668E-01	2.77E-01	ENSG00000215184
NA	-0.0760	1.2645E-01		ENSG00000270986
NA	-0.1416	1.2640E-01	2.77E-01	ENSG00000235578
NA	-0.0676	1.2615E-01		ENSG00000260105
NA	-0.0699	1.2558E-01		ENSG00000276704
NA	-0.0684	1.2514E-01		ENSG00000274967
NA	-0.1714	1.2510E-01	2.75E-01	ENSG00000236939
NA	-0.1580	1.2496E-01	2.74E-01	ENSG00000280285
NA	-0.1537	1.2481E-01	2.74E-01	ENSG00000261270
NA	-0.1728	1.2467E-01	2.74E-01	ENSG00000246100
NA	-0.0460	1.2466E-01		ENSG00000262623
NA	-0.1435	1.2412E-01	2.73E-01	ENSG00000250182
NA	-0.1731	1.2407E-01	2.73E-01	ENSG00000236060
NA	-0.1732	1.2379E-01	2.73E-01	ENSG00000287665
NA	-0.0652	1.2375E-01		ENSG00000235290
NA	-0.0923	1.2372E-01		ENSG00000214289
NA	-0.0925	1.2362E-01		ENSG00000286495
NA	-0.1626	1.2356E-01	2.72E-01	ENSG00000263982
NA	-0.0835	1.2278E-01		ENSG00000253956
NA	-0.0437	1.2271E-01		ENSG00000249278

NA	-0.0883	1.2268E-01		ENSG00000273507
NA	-0.1723	1.2234E-01	2.71E-01	ENSG00000260558
NA	-0.0526	1.2225E-01		ENSG00000275381
NA	-0.1448	1.2223E-01	2.70E-01	ENSG00000213400
NA	-0.1581	1.2199E-01	2.70E-01	ENSG00000279092
NA	-0.0863	1.2196E-01	2.70E-01	ENSG00000250597
NA	-0.0445	1.2196E-01	2.70E-01	ENSG00000259474
NA	-0.1149	1.2188E-01	2.70E-01	ENSG00000229473
NA	-0.1065	1.2113E-01	2.69E-01	ENSG00000273972
NA	-0.0746	1.2065E-01		ENSG00000271828
NA	-0.1438	1.2004E-01	2.67E-01	ENSG00000200385
NA	-0.0698	1.2002E-01		ENSG00000236549
NA	-0.1255	1.1992E-01	2.67E-01	ENSG00000213406
NA	-0.1381	1.1897E-01	2.65E-01	ENSG00000277702
NA	-0.1214	1.1860E-01	2.65E-01	ENSG00000279730
NA	-0.0908	1.1848E-01	2.65E-01	ENSG00000271855
NA	-0.0897	1.1805E-01		ENSG00000274937
NA	-0.1796	1.1799E-01	2.64E-01	ENSG00000279384
NA	-0.0995	1.1773E-01	2.64E-01	ENSG00000288918
NA	-0.1638	1.1756E-01	2.63E-01	ENSG00000288670
NA	-0.1770	1.1707E-01	2.63E-01	ENSG00000272892
NA	-0.1125	1.1675E-01	2.62E-01	ENSG00000240211
NA	-0.0761	1.1650E-01		ENSG00000287801
NA	-0.1463	1.1629E-01	2.61E-01	ENSG00000286689
NA	-0.1323	1.1599E-01	2.61E-01	ENSG00000259918
NA	-0.1286	1.1596E-01	2.61E-01	ENSG00000227192
NA	-0.1707	1.1571E-01	2.60E-01	ENSG00000262049
NA	-0.0145	1.1501E-01	2.59E-01	ENSG00000279917
NA	-0.1251	1.1493E-01	2.59E-01	ENSG00000271849
NA	-0.1798	1.1491E-01	2.59E-01	ENSG00000258458
NA	-0.1676	1.1487E-01	2.59E-01	ENSG00000233538
NA	-0.1215	1.1480E-01	2.59E-01	ENSG00000285827
NA	-0.0494	1.1452E-01		ENSG00000267051
NA	-0.0955	1.1415E-01		ENSG00000283709
NA	-0.1293	1.1394E-01	2.58E-01	ENSG00000273096
NA	-0.0337	1.1377E-01		ENSG00000277386
NA	-0.1753	1.1347E-01	2.57E-01	ENSG00000213453
NA	-0.1641	1.1289E-01	2.56E-01	ENSG00000228261
NA	-0.1309	1.1238E-01	2.55E-01	ENSG00000273209
NA	-0.0691	1.1220E-01		ENSG00000275613
NA	-0.0440	1.1206E-01		ENSG00000279663
NA	-0.0459	1.1182E-01	2.54E-01	ENSG00000273119
NA	-0.0469	1.1175E-01		ENSG00000276772
NA	-0.1802	1.1162E-01	2.54E-01	ENSG00000272108
NA	-0.0960	1.1151E-01		ENSG00000260186
NA	-0.1239	1.1101E-01	2.53E-01	ENSG00000185641
NA	-0.1237	1.1097E-01	2.53E-01	ENSG00000231205
NA	-0.1773	1.1084E-01	2.52E-01	ENSG00000229335
NA	-0.1157	1.1064E-01		ENSG00000254780

NA	-0.1639	1.1061E-01	2.52E-01	ENSG00000228201
NA	-0.0442	1.1038E-01		ENSG00000288793
NA	-0.1682	1.1019E-01	2.51E-01	ENSG00000213025
NA	-0.1508	1.0982E-01	2.51E-01	ENSG00000223756
NA	-0.1752	1.0962E-01	2.50E-01	ENSG00000260701
NA	-0.1110	1.0950E-01	2.50E-01	ENSG00000279413
NA	-0.0660	1.0926E-01		ENSG00000223427
NA	-0.1516	1.0921E-01	2.50E-01	ENSG00000228502
NA	-0.0719	1.0893E-01		ENSG00000260112
NA	-0.1874	1.0888E-01	2.49E-01	ENSG00000242797
NA	-0.0786	1.0795E-01		ENSG00000275393
NA	-0.1001	1.0782E-01		ENSG00000288393
NA	-0.1069	1.0767E-01	2.47E-01	ENSG00000243550
NA	-0.0499	1.0694E-01		ENSG00000234998
NA	-0.1449	1.0690E-01	2.46E-01	ENSG00000279138
NA	-0.1082	1.0659E-01	2.46E-01	ENSG00000250280
NA	-0.1729	1.0659E-01	2.46E-01	ENSG00000236539
NA	-0.0514	1.0650E-01		ENSG00000225125
NA	-0.0348	1.0647E-01		ENSG00000230294
NA	-0.1937	1.0646E-01	2.45E-01	ENSG00000232618
NA	-0.1849	1.0602E-01	2.45E-01	ENSG00000289483
NA	-0.1446	1.0591E-01	2.45E-01	ENSG00000258033
NA	-0.1550	1.0577E-01	2.44E-01	ENSG00000248994
NA	-0.0797	1.0522E-01		ENSG00000242602
NA	-0.0792	1.0487E-01		ENSG00000287616
NA	-0.1798	1.0426E-01	2.42E-01	ENSG00000268555
NA	-0.1106	1.0426E-01	2.42E-01	ENSG00000254912
NA	-0.0348	1.0405E-01		ENSG00000274330
NA	-0.1416	1.0403E-01	2.41E-01	ENSG00000261168
NA	-0.0342	1.0395E-01		ENSG00000266767
NA	-0.1745	1.0344E-01	2.40E-01	ENSG00000259223
NA	-0.0423	1.0316E-01		ENSG00000288528
NA	-0.1450	1.0308E-01	2.40E-01	ENSG00000278948
NA	-0.1151	1.0307E-01		ENSG00000169253
NA	-0.0443	1.0302E-01		ENSG00000278192
NA	-0.1409	1.0299E-01	2.40E-01	ENSG00000279656
NA	-0.1845	1.0230E-01	2.39E-01	ENSG00000257433
NA	-0.0549	1.0226E-01		ENSG00000267225
NA	-0.1893	1.0201E-01	2.38E-01	ENSG00000287737
NA	-0.0857	1.0183E-01		ENSG00000248349
NA	-0.0353	1.0138E-01		ENSG00000272861
NA	-0.1823	1.0130E-01	2.37E-01	ENSG00000279306
NA	-0.0359	1.0129E-01		ENSG00000276292
NA	-0.0584	1.0099E-01		ENSG00000259048
NA	-0.1033	1.0081E-01	2.36E-01	ENSG00000287032
NA	-0.1779	1.0056E-01	2.36E-01	ENSG00000205018
NA	-0.1656	1.0050E-01	2.36E-01	ENSG00000289017
NA	-0.1007	1.0044E-01	2.35E-01	ENSG00000221883
NA	-0.1479	1.0034E-01	2.35E-01	ENSG00000168852

NA	-0.1430	1.0014E-01	2.35E-01	ENSG00000272256
NA	-0.1886	1.0008E-01	2.35E-01	ENSG00000231952
NA	-0.0824	9.9808E-02	2.34E-01	ENSG00000262714
NA	-0.1402	9.9575E-02	2.34E-01	ENSG00000235363
NA	-0.0705	9.9477E-02		ENSG00000226622
NA	-0.1767	9.8980E-02	2.33E-01	ENSG00000267778
NA	-0.0568	9.8192E-02	2.32E-01	ENSG00000228314
NA	-0.0890	9.8100E-02		ENSG00000231344
NA	-0.1862	9.8004E-02	2.31E-01	ENSG00000285799
NA	-0.0486	9.7839E-02		ENSG00000259631
NA	-0.2371	9.7531E-02	2.31E-01	ENSG00000289028
NA	-0.1984	9.7076E-02	2.30E-01	ENSG00000256742
NA	-0.2052	9.6909E-02	2.30E-01	ENSG00000230751
NA	-0.1629	9.6904E-02	2.30E-01	ENSG00000275557
NA	-0.0960	9.6802E-02		ENSG00000285766
NA	-0.1740	9.6789E-02	2.29E-01	ENSG00000226564
NA	-0.1753	9.6295E-02	2.29E-01	ENSG00000241772
NA	-0.0486	9.6109E-02		ENSG00000287018
NA	-0.1516	9.6046E-02	2.28E-01	ENSG00000231132
NA	-0.1220	9.5717E-02	2.28E-01	ENSG00000272597
NA	-0.2088	9.5355E-02	2.27E-01	ENSG00000261329
NA	-0.1348	9.5014E-02	2.27E-01	ENSG00000229048
NA	-0.1859	9.4850E-02	2.27E-01	ENSG00000276148
NA	-0.0426	9.4714E-02		ENSG00000232193
NA	-0.0819	9.4688E-02		ENSG00000259416
NA	-0.1878	9.4628E-02	2.26E-01	ENSG00000286592
NA	-0.0845	9.4498E-02		ENSG00000253925
NA	-0.2275	9.4426E-02	2.26E-01	ENSG00000285605
NA	-0.0536	9.4420E-02		ENSG00000254522
NA	-0.1147	9.4058E-02	2.25E-01	ENSG00000181741
NA	-0.1995	9.4003E-02	2.25E-01	ENSG00000258457
NA	-0.1988	9.3787E-02	2.25E-01	ENSG00000233672
NA	-0.0989	9.3701E-02		ENSG00000236003
NA	-0.0950	9.3667E-02		ENSG00000254336
NA	-0.1269	9.3650E-02	2.25E-01	ENSG00000235192
NA	-0.1782	9.3491E-02	2.24E-01	ENSG00000260774
NA	-0.1962	9.3224E-02	2.24E-01	ENSG00000229728
NA	-0.1210	9.3149E-02		ENSG00000224367
NA	-0.0541	9.3105E-02		ENSG00000197099
NA	-0.0616	9.3064E-02		ENSG00000267694
NA	-0.3008	9.2855E-02	2.23E-01	ENSG00000289627
NA	-0.1950	9.2758E-02	2.23E-01	ENSG00000289435
NA	-0.0204	9.2610E-02	2.23E-01	ENSG00000272444
NA	-0.1378	9.2606E-02	2.23E-01	ENSG00000279526
NA	-0.1865	9.2512E-02	2.23E-01	ENSG00000213467
NA	-0.1465	9.1724E-02	2.21E-01	ENSG00000251661
NA	-0.1456	9.1502E-02	2.21E-01	ENSG00000235043
NA	-0.1828	9.1428E-02	2.21E-01	ENSG00000285904
NA	-0.0686	9.1094E-02		ENSG00000285712

NA	-0.1853	9.0789E-02	2.20E-01	ENSG00000231170
NA	-0.0827	9.0314E-02		ENSG00000234120
NA	-0.1369	8.9760E-02	2.18E-01	ENSG00000234571
NA	-0.1424	8.9742E-02	2.18E-01	ENSG00000227512
NA	-0.1963	8.9580E-02	2.18E-01	ENSG00000248290
NA	-0.1303	8.9109E-02	2.17E-01	ENSG00000272141
NA	-0.1869	8.8861E-02	2.16E-01	ENSG00000234292
NA	-0.0698	8.8751E-02		ENSG00000256913
NA	-0.1307	8.8697E-02		ENSG00000289204
NA	-0.1972	8.8463E-02	2.16E-01	ENSG00000272459
NA	-0.1851	8.8367E-02	2.16E-01	ENSG00000228274
NA	-0.1639	8.7870E-02	2.15E-01	ENSG00000272817
NA	-0.0445	8.7690E-02		ENSG00000257906
NA	-0.0566	8.7625E-02		ENSG00000279665
NA	-0.1114	8.7536E-02	2.14E-01	ENSG00000286870
NA	-0.1700	8.7503E-02	2.14E-01	ENSG00000273906
NA	-0.1974	8.7448E-02	2.14E-01	ENSG00000254719
NA	-0.0494	8.7378E-02		ENSG00000233541
NA	-0.0421	8.7288E-02		ENSG00000272443
NA	-0.2259	8.7264E-02	2.14E-01	ENSG00000250659
NA	-0.1213	8.7240E-02	2.14E-01	ENSG00000223542
NA	-0.1442	8.6978E-02	2.13E-01	ENSG00000259712
NA	-0.0748	8.6948E-02		ENSG00000286836
NA	-0.1152	8.6722E-02		ENSG00000273950
NA	-0.1438	8.6020E-02		ENSG00000286721
NA	-0.2449	8.5816E-02	2.11E-01	ENSG00000260197
NA	-0.1953	8.5414E-02	2.10E-01	ENSG00000289190
NA	-0.1323	8.5404E-02	2.10E-01	ENSG00000274987
NA	-0.1838	8.5278E-02	2.10E-01	ENSG00000280339
NA	-0.1265	8.5177E-02		ENSG00000287033
NA	-0.1938	8.4946E-02	2.09E-01	ENSG00000248127
NA	-0.1474	8.4875E-02	2.09E-01	ENSG00000250258
NA	-0.1897	8.4723E-02	2.09E-01	ENSG00000271978
NA	-0.1685	8.4566E-02	2.09E-01	ENSG00000251186
NA	-0.2053	8.3820E-02	2.07E-01	ENSG00000258824
NA	-0.1404	8.3141E-02	2.06E-01	ENSG00000279502
NA	-0.0411	8.3111E-02		ENSG00000242951
NA	-0.1637	8.3095E-02	2.06E-01	ENSG00000280011
NA	-0.1674	8.2579E-02	2.05E-01	ENSG00000286900
NA	-0.1859	8.2566E-02	2.05E-01	ENSG00000224738
NA	-0.1993	8.2311E-02	2.05E-01	ENSG00000289056
NA	-0.0389	8.2250E-02		ENSG00000275216
NA	-0.0599	8.1835E-02		ENSG00000225401
NA	-0.1665	8.1796E-02	2.04E-01	ENSG00000258520
NA	-0.1732	8.1556E-02	2.04E-01	ENSG00000258851
NA	-0.1656	8.0578E-02	2.02E-01	ENSG00000255565
NA	-0.1586	8.0536E-02	2.02E-01	ENSG00000260798
NA	-0.1506	8.0519E-02	2.02E-01	ENSG00000278974
NA	-0.1680	8.0494E-02	2.02E-01	ENSG00000163016

NA	-0.2149	8.0072E-02	2.01E-01	ENSG00000289831
NA	-0.1688	7.9964E-02	2.00E-01	ENSG00000277662
NA	-0.1839	7.9905E-02	2.00E-01	ENSG00000224858
NA	-0.1119	7.9876E-02		ENSG00000265678
NA	-0.1591	7.9816E-02	2.00E-01	ENSG00000273035
NA	-0.1543	7.9713E-02	2.00E-01	ENSG00000272472
NA	-0.2132	7.9575E-02	2.00E-01	ENSG00000286342
NA	-0.2040	7.9538E-02	2.00E-01	ENSG00000250519
NA	-0.2083	7.9429E-02	2.00E-01	ENSG00000261620
NA	-0.1668	7.9025E-02	1.99E-01	ENSG00000284655
NA	-0.1838	7.8983E-02	1.99E-01	ENSG00000263590
NA	-0.0975	7.8654E-02		ENSG00000250551
NA	-0.1909	7.8389E-02	1.98E-01	ENSG00000233405
NA	-0.2076	7.7777E-02	1.97E-01	ENSG00000124097
NA	-0.2045	7.7730E-02	1.97E-01	ENSG00000288096
NA	-0.1326	7.7519E-02	1.96E-01	ENSG00000260467
NA	-0.1821	7.7434E-02	1.96E-01	ENSG00000276075
NA	-0.0780	7.7312E-02		ENSG00000251467
NA	-0.2061	7.7187E-02	1.96E-01	ENSG00000279207
NA	-0.0909	7.7020E-02		ENSG00000272564
NA	-0.0891	7.6957E-02	1.95E-01	ENSG00000214174
NA	-0.1131	7.6564E-02		ENSG00000225984
NA	-0.1647	7.6447E-02	1.94E-01	ENSG00000270179
NA	-0.0898	7.6154E-02		ENSG00000285696
NA	-0.2038	7.6143E-02	1.94E-01	ENSG00000240661
NA	-0.1659	7.6037E-02	1.93E-01	ENSG00000288831
NA	-0.2128	7.5894E-02	1.93E-01	ENSG00000248079
NA	-0.1122	7.5649E-02		ENSG00000256377
NA	-0.0026	7.5586E-02	1.93E-01	ENSG00000186743
NA	-0.2113	7.5072E-02	1.92E-01	ENSG00000288994
NA	-0.2113	7.4481E-02	1.90E-01	ENSG00000237976
NA	-0.1892	7.4399E-02	1.90E-01	ENSG00000286001
NA	-0.2159	7.4337E-02	1.90E-01	ENSG00000279573
NA	-0.1885	7.3976E-02	1.90E-01	ENSG00000276663
NA	-0.1331	7.3851E-02	1.89E-01	ENSG00000288723
NA	-0.2191	7.3664E-02	1.89E-01	ENSG00000241954
NA	-0.2161	7.3661E-02	1.89E-01	ENSG00000272338
NA	-0.1982	7.3549E-02	1.89E-01	ENSG00000281920
NA	-0.1381	7.3442E-02	1.89E-01	ENSG00000287173
NA	-0.1799	7.3425E-02	1.89E-01	ENSG00000270055
NA	-0.0493	7.3078E-02		ENSG00000256351
NA	-0.1128	7.2974E-02	1.88E-01	ENSG00000267069
NA	-0.1495	7.2718E-02	1.87E-01	ENSG00000288021
NA	-0.1795	7.2466E-02	1.87E-01	ENSG00000285761
NA	-0.1171	7.2222E-02		ENSG00000284380
NA	-0.1999	7.2107E-02	1.86E-01	ENSG00000277778
NA	-0.0368	7.2096E-02	1.86E-01	ENSG00000251598
NA	-0.1864	7.1971E-02	1.86E-01	ENSG00000251634
NA	-0.1134	7.1969E-02	1.86E-01	ENSG00000289274

NA	-0.1227	7.1945E-02	1.86E-01	ENSG00000284661
NA	-0.1412	7.1699E-02	1.85E-01	ENSG00000287351
NA	-0.0785	7.1570E-02		ENSG00000278202
NA	-0.1340	7.0818E-02	1.84E-01	ENSG00000216775
NA	-0.1242	7.0812E-02	1.84E-01	ENSG00000287214
NA	-0.1945	7.0806E-02	1.84E-01	ENSG00000273073
NA	-0.1988	7.0630E-02	1.83E-01	ENSG00000285636
NA	-0.2209	7.0499E-02	1.83E-01	ENSG00000279555
NA	-0.1733	7.0349E-02	1.83E-01	ENSG00000169662
NA	-0.2191	7.0094E-02	1.83E-01	ENSG00000218596
NA	-0.1400	7.0058E-02	1.82E-01	ENSG00000253695
NA	-0.0951	6.9841E-02		ENSG00000273473
NA	-0.2209	6.9821E-02	1.82E-01	ENSG00000249456
NA	-0.2018	6.9704E-02	1.82E-01	ENSG00000271976
NA	-0.2051	6.9156E-02	1.81E-01	ENSG00000172014
NA	-0.1766	6.9014E-02	1.80E-01	ENSG00000235262
NA	-0.1106	6.9002E-02		ENSG00000260899
NA	-0.2199	6.8383E-02	1.79E-01	ENSG00000233293
NA	-0.4165	6.8379E-02	1.79E-01	ENSG00000286548
NA	-0.1060	6.8129E-02		ENSG00000270403
NA	-0.0265	6.8125E-02	1.79E-01	ENSG00000226939
NA	-0.0878	6.7624E-02		ENSG00000277672
NA	-0.2170	6.7306E-02	1.77E-01	ENSG00000281566
NA	-0.2266	6.7221E-02	1.77E-01	ENSG00000254602
NA	-0.2798	6.7087E-02	1.77E-01	ENSG00000285943
NA	-0.2494	6.6616E-02	1.76E-01	ENSG00000236710
NA	-0.0889	6.6320E-02		ENSG00000256220
NA	-0.1369	6.5869E-02	1.74E-01	ENSG00000277581
NA	-0.1489	6.5276E-02	1.73E-01	ENSG00000289523
NA	-0.1213	6.5048E-02	1.73E-01	ENSG00000289265
NA	-0.2102	6.5035E-02	1.73E-01	ENSG00000287446
NA	-0.1734	6.4710E-02	1.72E-01	ENSG00000179101
NA	-0.0682	6.4288E-02		ENSG00000224565
NA	-0.2522	6.4278E-02	1.71E-01	ENSG00000201501
NA	-0.0983	6.4249E-02		ENSG00000275339
NA	-0.2353	6.4172E-02	1.71E-01	ENSG00000278834
NA	-0.0666	6.4125E-02		ENSG00000274403
NA	-0.1599	6.3920E-02	1.71E-01	ENSG00000233144
NA	-0.2286	6.3808E-02	1.71E-01	ENSG00000279467
NA	-0.1593	6.3556E-02	1.70E-01	ENSG00000289589
NA	-0.1844	6.3351E-02	1.70E-01	ENSG00000262420
NA	-0.2038	6.3324E-02	1.70E-01	ENSG00000238249
NA	-0.2214	6.3130E-02	1.69E-01	ENSG00000288632
NA	-0.0858	6.2836E-02		ENSG00000289606
NA	-0.1631	6.2636E-02	1.69E-01	ENSG00000225071
NA	-0.0015	6.2368E-02	1.68E-01	ENSG00000279240
NA	-0.2143	6.2267E-02	1.68E-01	ENSG00000267053
NA	-0.2481	6.2071E-02	1.67E-01	ENSG00000235912
NA	-0.0632	6.1870E-02		ENSG00000279525

NA	-0.1653	6.1530E-02	1.66E-01	ENSG00000288911
NA	-0.0826	6.1512E-02		ENSG00000261543
NA	-0.1579	6.1394E-02	1.66E-01	ENSG00000234287
NA	-0.0609	6.1259E-02		ENSG00000289371
NA	-0.2110	6.1243E-02	1.66E-01	ENSG00000281021
NA	-0.2291	6.1227E-02	1.66E-01	ENSG00000269951
NA	-0.1945	6.1148E-02	1.66E-01	ENSG00000233765
NA	-0.1502	6.0922E-02	1.65E-01	ENSG00000257735
NA	-0.1235	6.0836E-02	1.65E-01	ENSG00000286795
NA	-0.2306	6.0803E-02	1.65E-01	ENSG00000258813
NA	-0.2349	6.0784E-02	1.65E-01	ENSG00000227012
NA	-0.1594	6.0633E-02	1.65E-01	ENSG00000271437
NA	-0.2308	6.0187E-02	1.64E-01	ENSG00000282933
NA	-0.2283	6.0085E-02	1.64E-01	ENSG00000263731
NA	-0.2339	6.0052E-02	1.64E-01	ENSG00000287558
NA	-0.2344	5.8880E-02	1.61E-01	ENSG00000272769
NA	-0.0993	5.8706E-02		ENSG00000254731
NA	-0.2231	5.8333E-02	1.60E-01	ENSG00000254670
NA	-0.2418	5.8057E-02	1.59E-01	ENSG00000255471
NA	-0.1893	5.8014E-02	1.59E-01	ENSG00000286064
NA	-0.2145	5.7992E-02	1.59E-01	ENSG00000237126
NA	-0.1934	5.7189E-02	1.58E-01	ENSG00000260604
NA	-0.0527	5.7117E-02		ENSG00000241661
NA	-0.1992	5.7005E-02	1.58E-01	ENSG00000288830
NA	-0.0953	5.6965E-02		ENSG00000278925
NA	-0.2346	5.6328E-02	1.56E-01	ENSG00000257058
NA	-0.2092	5.6190E-02	1.56E-01	ENSG00000230562
NA	-0.2136	5.6009E-02	1.56E-01	ENSG00000241048
NA	-0.1961	5.5914E-02	1.55E-01	ENSG00000265478
NA	-0.1472	5.5867E-02	1.55E-01	ENSG00000241151
NA	-0.2516	5.5599E-02	1.55E-01	ENSG00000286907
NA	-0.2384	5.5290E-02	1.54E-01	ENSG00000267219
NA	-0.1669	5.5263E-02	1.54E-01	ENSG00000258960
NA	-0.2440	5.5234E-02	1.54E-01	ENSG00000219085
NA	-0.2499	5.5020E-02	1.53E-01	ENSG00000259972
NA	-0.2477	5.4995E-02	1.53E-01	ENSG00000290095
NA	-0.2508	5.4925E-02	1.53E-01	ENSG00000237457
NA	-0.2441	5.4438E-02	1.52E-01	ENSG00000226332
NA	-0.2275	5.4039E-02	1.51E-01	ENSG00000275580
NA	-0.1837	5.3958E-02	1.51E-01	ENSG00000261064
NA	-0.2254	5.3740E-02	1.51E-01	ENSG00000289515
NA	-0.2183	5.3547E-02	1.50E-01	ENSG00000253854
NA	-0.2032	5.3408E-02	1.50E-01	ENSG00000285667
NA	-0.1548	5.3403E-02	1.50E-01	ENSG00000274317
NA	-0.1326	5.3265E-02	1.50E-01	ENSG00000251131
NA	-0.1058	5.3097E-02		ENSG00000270031
NA	-0.2440	5.2639E-02	1.48E-01	ENSG00000286334
NA	-0.1586	5.2464E-02	1.48E-01	ENSG00000288891
NA	-0.2420	5.2345E-02	1.48E-01	ENSG00000279432

NA	-0.0633	5.2057E-02		ENSG00000265018
NA	-0.2561	5.1973E-02	1.47E-01	ENSG00000264964
NA	-0.2129	5.1963E-02	1.47E-01	ENSG00000277511
NA	-0.1562	5.1782E-02	1.47E-01	ENSG00000276724
NA	-0.2284	5.1610E-02	1.46E-01	ENSG00000265519
NA	-0.1316	5.1602E-02		ENSG00000284071
NA	-0.2427	5.1566E-02	1.46E-01	ENSG00000273759
NA	-0.1192	5.1393E-02		ENSG00000270069
NA	-0.2039	5.1158E-02	1.45E-01	ENSG00000289513
NA	-0.0727	5.0857E-02		ENSG00000287579
NA	-0.1736	5.0675E-02	1.44E-01	ENSG00000226471
NA	-0.2558	5.0627E-02	1.44E-01	ENSG00000278276
NA	-0.2505	5.0547E-02	1.44E-01	ENSG00000286864
NA	-0.2551	5.0428E-02	1.44E-01	ENSG00000235945
NA	-0.2407	5.0399E-02	1.44E-01	ENSG00000232709
NA	-0.1913	5.0360E-02	1.44E-01	ENSG00000250853
NA	-0.2473	5.0327E-02	1.44E-01	ENSG00000272156
NA	-0.1622	5.0186E-02	1.43E-01	ENSG00000285892
NA	-0.1061	5.0173E-02		ENSG00000240634
NA	-0.1964	4.9990E-02	1.43E-01	ENSG00000273492
NA	-0.1732	4.9925E-02	1.43E-01	ENSG00000258661
NA	-0.1750	4.9838E-02	1.43E-01	ENSG00000273365
NA	-0.2167	4.9722E-02	1.43E-01	ENSG00000232022
NA	-0.0739	4.9718E-02		ENSG00000258569
NA	-0.1228	4.9459E-02		ENSG00000201800
NA	-0.2118	4.9459E-02	1.42E-01	ENSG00000256663
NA	-0.0943	4.9087E-02		ENSG00000258777
NA	-0.2605	4.8990E-02	1.41E-01	ENSG00000289283
NA	-0.1402	4.8699E-02		ENSG00000243155
NA	-0.1758	4.8422E-02	1.40E-01	ENSG00000259087
NA	-0.2672	4.8166E-02	1.39E-01	ENSG00000259086
NA	-0.1525	4.7518E-02	1.38E-01	ENSG00000226520
NA	-0.2530	4.7250E-02	1.37E-01	ENSG00000226465
NA	-0.2568	4.6991E-02	1.37E-01	ENSG00000234446
NA	-0.1319	4.6939E-02	1.37E-01	ENSG00000287469
NA	-0.2167	4.6777E-02	1.36E-01	ENSG00000256056
NA	-0.1590	4.6759E-02	1.36E-01	ENSG00000224621
NA	-0.1338	4.6728E-02	1.36E-01	ENSG00000224750
NA	-0.1521	4.6675E-02	1.36E-01	ENSG00000233966
NA	-0.2641	4.6627E-02	1.36E-01	ENSG00000273249
NA	-0.2598	4.6516E-02	1.36E-01	ENSG00000260918
NA	-0.1977	4.6295E-02	1.35E-01	ENSG00000226851
NA	-0.1815	4.6030E-02	1.35E-01	ENSG00000234624
NA	-0.1961	4.6022E-02	1.35E-01	ENSG00000264860
NA	-0.1842	4.5975E-02	1.35E-01	ENSG00000263563
NA	-0.2091	4.5954E-02	1.34E-01	ENSG00000272669
NA	-0.4143	4.5620E-02	1.34E-01	ENSG00000213228
NA	-0.2658	4.5513E-02	1.33E-01	ENSG00000286519
NA	-0.2685	4.5442E-02	1.33E-01	ENSG00000272267

NA	-0.2692	4.5328E-02	1.33E-01	ENSG00000279977
NA	-0.2826	4.5110E-02	1.33E-01	ENSG00000287076
NA	-0.2744	4.5109E-02	1.33E-01	ENSG00000259146
NA	-0.0445	4.4785E-02		ENSG00000278959
NA	-0.2660	4.4504E-02	1.31E-01	ENSG00000224216
NA	-0.2528	4.4453E-02	1.31E-01	ENSG00000274767
NA	-0.2326	4.4449E-02	1.31E-01	ENSG00000188459
NA	-0.2427	4.4154E-02	1.30E-01	ENSG00000227388
NA	-0.1938	4.4109E-02	1.30E-01	ENSG00000286314
NA	-0.0566	4.3966E-02		ENSG00000286719
NA	-0.1647	4.3937E-02	1.30E-01	ENSG00000215580
NA	-0.0100	4.3933E-02	1.30E-01	ENSG00000285961
NA	-0.2686	4.3581E-02	1.29E-01	ENSG00000278765
NA	-0.2150	4.3563E-02	1.29E-01	ENSG00000259459
NA	-0.1875	4.3434E-02	1.29E-01	ENSG00000228446
NA	-0.0640	4.3087E-02		ENSG00000231390
NA	-0.1508	4.3055E-02		ENSG00000237456
NA	-0.1719	4.2835E-02	1.28E-01	ENSG00000272384
NA	-0.2134	4.2554E-02	1.27E-01	ENSG00000289455
NA	-0.0932	4.2458E-02		ENSG00000286807
NA	-0.2029	4.2192E-02	1.26E-01	ENSG00000286536
NA	-0.0751	4.1763E-02		ENSG00000225674
NA	-0.1576	4.1509E-02	1.25E-01	ENSG00000273890
NA	-0.4108	4.1040E-02	1.23E-01	ENSG00000250538
NA	-0.1477	4.0666E-02		ENSG00000265261
NA	-0.2646	4.0651E-02	1.23E-01	ENSG00000251682
NA	-0.2618	4.0596E-02	1.22E-01	ENSG00000234636
NA	-0.1899	4.0381E-02	1.22E-01	ENSG00000255122
NA	-0.2197	4.0101E-02	1.21E-01	ENSG00000226268
NA	-0.1979	3.9995E-02	1.21E-01	ENSG00000258337
NA	-0.2697	3.9833E-02	1.21E-01	ENSG00000285867
NA	-0.2187	3.9824E-02	1.21E-01	ENSG00000278743
NA	-0.2849	3.9822E-02	1.21E-01	ENSG00000253954
NA	-0.2211	3.9752E-02	1.21E-01	ENSG00000288717
NA	-0.2814	3.9502E-02	1.20E-01	ENSG00000278909
NA	-0.2532	3.9349E-02	1.20E-01	ENSG00000279390
NA	-0.1702	3.9308E-02	1.20E-01	ENSG00000218283
NA	-0.2284	3.9277E-02	1.19E-01	ENSG00000274594
NA	-0.2370	3.9194E-02	1.19E-01	ENSG00000276216
NA	-0.2769	3.9138E-02	1.19E-01	ENSG00000285877
NA	-0.2166	3.8922E-02	1.19E-01	ENSG00000253771
NA	-0.2649	3.8523E-02	1.18E-01	ENSG00000249609
NA	-0.2873	3.8447E-02	1.17E-01	ENSG00000272715
NA	-0.1780	3.8274E-02	1.17E-01	ENSG00000220660
NA	-0.0919	3.8220E-02		ENSG00000239969
NA	-0.2531	3.8180E-02	1.17E-01	ENSG00000287460
NA	-1.1600	3.8169E-02	1.17E-01	ENSG00000255330
NA	-0.2808	3.7790E-02	1.16E-01	ENSG00000241255
NA	-0.2130	3.7741E-02	1.16E-01	ENSG00000233705

NA	-0.2757	3.7651E-02	1.16E-01	ENSG00000280099
NA	-0.2790	3.7610E-02	1.15E-01	ENSG00000247311
NA	-0.2173	3.7512E-02	1.15E-01	ENSG00000289201
NA	-0.2771	3.7078E-02	1.14E-01	ENSG00000273076
NA	-0.1712	3.6937E-02	1.14E-01	ENSG00000268364
NA	-0.2920	3.6792E-02	1.14E-01	ENSG00000287207
NA	-0.2421	3.6649E-02	1.13E-01	ENSG00000259659
NA	-0.2819	3.6368E-02	1.13E-01	ENSG00000229321
NA	-0.0883	3.6278E-02		ENSG00000244076
NA	-0.2823	3.6217E-02	1.12E-01	ENSG00000279332
NA	-0.2705	3.6078E-02	1.12E-01	ENSG00000280152
NA	-0.1174	3.6065E-02	1.12E-01	ENSG00000244459
NA	-0.3035	3.5886E-02	1.12E-01	ENSG00000274447
NA	-0.7723	3.5700E-02	1.11E-01	ENSG00000267325
NA	-0.3085	3.5608E-02	1.11E-01	ENSG00000286472
NA	-0.3270	3.4941E-02	1.09E-01	ENSG00000233451
NA	-0.2833	3.4890E-02	1.09E-01	ENSG00000288928
NA	-0.2984	3.4632E-02	1.09E-01	ENSG00000235904
NA	-0.1861	3.4538E-02		ENSG00000279543
NA	-0.1798	3.4222E-02	1.08E-01	ENSG00000207370
NA	-0.2287	3.4142E-02	1.07E-01	ENSG00000225808
NA	-0.2571	3.4106E-02	1.07E-01	ENSG00000273010
NA	-0.0971	3.4077E-02		ENSG00000105694
NA	-0.1091	3.3955E-02		ENSG00000288768
NA	-0.2550	3.3758E-02	1.07E-01	ENSG00000226702
NA	-0.1735	3.3696E-02		ENSG00000289981
NA	-0.2207	3.3648E-02	1.06E-01	ENSG00000288899
NA	-0.2164	3.3618E-02	1.06E-01	ENSG00000261279
NA	-0.2103	3.3604E-02	1.06E-01	ENSG00000277763
NA	-0.2813	3.3079E-02	1.05E-01	ENSG00000284624
NA	-0.0588	3.2931E-02		ENSG00000289184
NA	-0.1728	3.2824E-02	1.04E-01	ENSG00000278532
NA	-0.2340	3.2802E-02	1.04E-01	ENSG00000269019
NA	-0.0744	3.2664E-02		ENSG00000229146
NA	-0.3007	3.2439E-02	1.03E-01	ENSG00000185834
NA	-0.1057	3.2222E-02		ENSG00000267055
NA	-0.0540	3.2025E-02		ENSG00000277628
NA	-0.0744	3.1012E-02		ENSG00000288805
NA	-0.2340	3.0942E-02	9.97E-02	ENSG00000277715
NA	-0.3194	3.0909E-02	9.96E-02	ENSG00000200090
NA	-0.2887	3.0724E-02	9.92E-02	ENSG00000239804
NA	-0.2620	3.0609E-02	9.89E-02	ENSG00000287042
NA	-0.2886	3.0416E-02	9.84E-02	ENSG00000250770
NA	-0.3009	3.0235E-02	9.80E-02	ENSG00000288881
NA	-0.3123	3.0156E-02	9.78E-02	ENSG00000204584
NA	-0.2777	3.0042E-02	9.76E-02	ENSG00000249650
NA	-0.3571	3.0001E-02	9.75E-02	ENSG00000285669
NA	-0.1547	2.9949E-02		ENSG00000255081
NA	-0.2652	2.9875E-02	9.72E-02	ENSG00000276855

NA	-0.2996	2.9785E-02	9.69E-02	ENSG00000287406
NA	-0.2785	2.9712E-02	9.68E-02	ENSG00000269973
NA	-0.3213	2.9702E-02	9.68E-02	ENSG00000251442
NA	-0.1948	2.9452E-02	9.61E-02	ENSG00000255145
NA	-0.3343	2.9388E-02	9.60E-02	ENSG00000241170
NA	-0.3156	2.9313E-02	9.58E-02	ENSG00000259251
NA	-0.1841	2.9093E-02	9.53E-02	ENSG00000234354
NA	-0.2137	2.9046E-02	9.52E-02	ENSG00000237852
NA	-0.3314	2.8864E-02	9.47E-02	ENSG00000285571
NA	-0.2405	2.8855E-02	9.47E-02	ENSG00000226025
NA	-0.1799	2.8772E-02	9.45E-02	ENSG00000260190
NA	-0.1015	2.8510E-02		ENSG00000279022
NA	-0.1578	2.8288E-02	9.32E-02	ENSG00000272086
NA	-0.2261	2.8219E-02	9.30E-02	ENSG00000284968
NA	-0.2017	2.8189E-02		ENSG00000213253
NA	-0.3402	2.7836E-02	9.21E-02	ENSG00000286608
NA	-0.2937	2.7695E-02	9.17E-02	ENSG00000188985
NA	-0.3329	2.7511E-02	9.12E-02	ENSG00000225976
NA	-0.1741	2.7496E-02		ENSG00000274833
NA	-0.1019	2.7462E-02		ENSG00000223575
NA	-0.2196	2.7336E-02	9.09E-02	ENSG00000230037
NA	-0.2258	2.7297E-02	9.08E-02	ENSG00000179978
NA	-0.3328	2.7058E-02	9.02E-02	ENSG00000279660
NA	-0.1341	2.6845E-02		ENSG00000260342
NA	-0.3036	2.6703E-02	8.94E-02	ENSG00000277459
NA	-0.3320	2.6636E-02	8.92E-02	ENSG00000250602
NA	-0.3343	2.6512E-02	8.89E-02	ENSG00000289546
NA	-0.2497	2.6434E-02	8.87E-02	ENSG00000269925
NA	-0.1134	2.6260E-02	8.82E-02	ENSG00000204072
NA	-0.3370	2.6054E-02	8.77E-02	ENSG00000261268
NA	-1.0423	2.5961E-02	8.75E-02	ENSG00000214222
NA	-0.2995	2.5947E-02	8.75E-02	ENSG00000229896
NA	-0.2397	2.5840E-02	8.73E-02	ENSG00000210082
NA	-0.2685	2.5831E-02	8.73E-02	ENSG00000289146
NA	-0.3276	2.5645E-02	8.68E-02	ENSG00000266578
NA	-0.2568	2.5636E-02	8.68E-02	ENSG00000282572
NA	-0.2917	2.5588E-02	8.67E-02	ENSG00000279456
NA	-0.3144	2.5379E-02	8.62E-02	ENSG00000255328
NA	-0.3419	2.5211E-02	8.57E-02	ENSG00000271743
NA	-0.3395	2.5107E-02	8.54E-02	ENSG00000259644
NA	-0.3547	2.4908E-02	8.49E-02	ENSG00000225806
NA	-0.3369	2.4719E-02	8.45E-02	ENSG00000230804
NA	-0.3087	2.4709E-02	8.45E-02	ENSG00000272483
NA	-0.3613	2.4480E-02	8.39E-02	ENSG00000230216
NA	-0.1725	2.4473E-02		ENSG00000286782
NA	-0.4597	2.4325E-02	8.34E-02	ENSG00000286994
NA	-0.2677	2.3722E-02	8.18E-02	ENSG00000273345
NA	-0.1934	2.3704E-02	8.18E-02	ENSG00000215861
NA	-0.3416	2.3526E-02	8.14E-02	ENSG00000280369

NA	-0.1710	2.3396E-02	8.11E-02	ENSG00000231536
NA	-0.6615	2.3380E-02	8.10E-02	ENSG00000218980
NA	-0.2970	2.3281E-02		ENSG00000267698
NA	-0.2897	2.3266E-02	8.07E-02	ENSG00000290073
NA	-0.2999	2.3251E-02	8.07E-02	ENSG00000287024
NA	-0.3640	2.3237E-02	8.07E-02	ENSG00000279623
NA	-0.8954	2.3172E-02	8.05E-02	ENSG00000266441
NA	-0.3578	2.3048E-02	8.02E-02	ENSG00000290012
NA	-0.2143	2.3016E-02	8.01E-02	ENSG00000259511
NA	-0.3427	2.2827E-02	7.95E-02	ENSG00000278058
NA	-0.3479	2.2522E-02	7.88E-02	ENSG00000253821
NA	-0.3593	2.2462E-02	7.86E-02	ENSG00000286215
NA	-0.3441	2.2303E-02	7.82E-02	ENSG00000289993
NA	-0.2482	2.2286E-02	7.81E-02	ENSG00000288584
NA	-0.1491	2.2249E-02	7.80E-02	ENSG00000288040
NA	-0.3156	2.2222E-02	7.79E-02	ENSG00000264968
NA	-0.3743	2.2143E-02	7.77E-02	ENSG00000219274
NA	-0.3224	2.2134E-02	7.77E-02	ENSG00000270587
NA	-0.3144	2.1958E-02	7.73E-02	ENSG00000232065
NA	-0.2240	2.1931E-02	7.72E-02	ENSG00000276975
NA	-0.2698	2.1928E-02	7.72E-02	ENSG00000161912
NA	-0.3363	2.1787E-02	7.69E-02	ENSG00000239556
NA	-0.3645	2.1691E-02	7.66E-02	ENSG00000287445
NA	-0.1065	2.1571E-02		ENSG00000267748
NA	-0.6257	2.1554E-02	7.62E-02	ENSG00000280193
NA	-0.3045	2.1328E-02	7.56E-02	ENSG00000288851
NA	-0.3375	2.1304E-02	7.56E-02	ENSG00000244953
NA	-0.3409	2.1111E-02	7.50E-02	ENSG00000256263
NA	-0.2812	2.0793E-02	7.41E-02	ENSG00000288760
NA	-0.2198	2.0760E-02	7.40E-02	ENSG00000226964
NA	-0.2482	2.0722E-02	7.39E-02	ENSG00000277855
NA	-0.8210	2.0632E-02	7.37E-02	ENSG00000225111
NA	-0.3540	2.0405E-02	7.31E-02	ENSG00000260259
NA	-0.2451	2.0359E-02	7.30E-02	ENSG00000228536
NA	-0.3366	2.0269E-02	7.28E-02	ENSG00000258634
NA	-0.0826	2.0242E-02		ENSG00000288895
NA	-0.4995	1.9938E-02	7.18E-02	ENSG00000280303
NA	-0.3294	1.9712E-02	7.12E-02	ENSG00000286331
NA	-0.1848	1.9707E-02	7.12E-02	ENSG00000266865
NA	-0.0035	1.9446E-02	7.05E-02	ENSG00000226521
NA	-0.4035	1.9318E-02	7.01E-02	ENSG00000279217
NA	-0.0864	1.9305E-02		ENSG00000228802
NA	-0.2338	1.9285E-02	7.01E-02	ENSG00000269982
NA	-0.4001	1.9203E-02	6.98E-02	ENSG00000275894
NA	-0.1653	1.9159E-02	6.97E-02	ENSG00000288999
NA	-0.3917	1.9137E-02	6.96E-02	ENSG00000283692
NA	-0.2428	1.8994E-02	6.93E-02	ENSG00000270802
NA	-0.3723	1.8950E-02	6.91E-02	ENSG00000283240
NA	-0.1362	1.8921E-02	6.91E-02	ENSG00000254202

NA	-0.4675	1.8759E-02	6.86E-02	ENSG00000261124
NA	-0.4062	1.8735E-02	6.86E-02	ENSG00000240695
NA	-0.3712	1.8732E-02	6.86E-02	ENSG00000234197
NA	-0.3999	1.8605E-02	6.83E-02	ENSG00000284620
NA	-0.2598	1.8552E-02	6.81E-02	ENSG00000289138
NA	-0.1438	1.8531E-02	6.81E-02	ENSG00000259434
NA	-0.1467	1.8398E-02		ENSG00000279696
NA	-0.4011	1.8393E-02	6.77E-02	ENSG00000267801
NA	-0.1246	1.8184E-02		ENSG00000270207
NA	-0.1449	1.8107E-02		ENSG00000274943
NA	-0.3008	1.8077E-02	6.68E-02	ENSG00000249264
NA	-0.2744	1.8016E-02	6.66E-02	ENSG00000219891
NA	-0.4094	1.8016E-02	6.66E-02	ENSG00000267645
NA	-0.3923	1.7944E-02	6.64E-02	ENSG00000226823
NA	-0.3428	1.7734E-02	6.58E-02	ENSG00000287829
NA	-0.3957	1.7725E-02	6.58E-02	ENSG00000285633
NA	-0.2952	1.7593E-02	6.54E-02	ENSG00000264107
NA	-0.2912	1.7330E-02	6.47E-02	ENSG00000286156
NA	-0.3979	1.7166E-02	6.42E-02	ENSG00000235609
NA	-0.3704	1.7101E-02	6.40E-02	ENSG00000274204
NA	-0.1236	1.6985E-02		ENSG00000198857
NA	-0.4082	1.6761E-02	6.30E-02	ENSG00000275645
NA	-0.3936	1.6712E-02	6.29E-02	ENSG00000279118
NA	-0.2890	1.6500E-02	6.23E-02	ENSG00000272335
NA	-0.2638	1.6441E-02	6.22E-02	ENSG00000255277
NA	-0.3060	1.6427E-02	6.22E-02	ENSG00000255129
NA	-0.3713	1.6248E-02	6.16E-02	ENSG00000268049
NA	-0.2843	1.6191E-02	6.14E-02	ENSG00000289268
NA	-0.3843	1.6100E-02	6.12E-02	ENSG00000278989
NA	-0.3449	1.6096E-02	6.12E-02	ENSG00000268061
NA	-0.2135	1.6070E-02	6.11E-02	ENSG00000244627
NA	-0.3618	1.5851E-02	6.05E-02	ENSG00000288934
NA	-0.2295	1.5809E-02	6.03E-02	ENSG00000249937
NA	-0.5352	1.5433E-02	5.92E-02	ENSG00000270124
NA	-0.4090	1.5421E-02	5.92E-02	ENSG00000213509
NA	-0.2943	1.5393E-02	5.91E-02	ENSG00000288597
NA	-0.7539	1.5048E-02	5.81E-02	ENSG00000287077
NA	-0.3406	1.4986E-02	5.79E-02	ENSG00000243024
NA	-0.3685	1.4919E-02	5.78E-02	ENSG00000285991
NA	-0.3159	1.4809E-02	5.75E-02	ENSG00000251867
NA	-0.2633	1.4743E-02	5.73E-02	ENSG00000250462
NA	-0.4090	1.4741E-02	5.73E-02	ENSG00000287915
NA	-0.4266	1.4726E-02	5.73E-02	ENSG00000279766
NA	-0.4077	1.4642E-02	5.70E-02	ENSG00000175658
NA	-0.3234	1.4585E-02	5.68E-02	ENSG00000281501
NA	-0.1285	1.4350E-02		ENSG00000251229
NA	-0.1438	1.4336E-02	5.61E-02	ENSG00000184319
NA	-0.4242	1.4181E-02	5.56E-02	ENSG00000241112
NA	-0.4231	1.4021E-02	5.51E-02	ENSG00000232721

NA	-0.2349	1.3964E-02		ENSG00000259032
NA	-0.1777	1.3863E-02		ENSG00000249610
NA	-0.4043	1.3777E-02	5.43E-02	ENSG00000228477
NA	-0.2718	1.3753E-02	5.43E-02	ENSG00000248925
NA	-0.3752	1.3702E-02	5.41E-02	ENSG00000289032
NA	-0.4926	1.3661E-02	5.40E-02	ENSG00000260911
NA	-0.4899	1.3564E-02	5.37E-02	ENSG00000229492
NA	-0.2357	1.3560E-02	5.37E-02	ENSG00000226085
NA	-0.2111	1.3406E-02	5.32E-02	ENSG00000248334
NA	-0.3679	1.3265E-02	5.27E-02	ENSG00000276203
NA	-0.5213	1.2765E-02	5.12E-02	ENSG00000279748
NA	-0.0995	1.2669E-02		ENSG00000286980
NA	-0.4742	1.2638E-02	5.08E-02	ENSG00000259508
NA	-0.1150	1.2438E-02	5.02E-02	ENSG00000214318
NA	-0.4927	1.2041E-02	4.89E-02	ENSG00000231351
NA	-0.3712	1.1997E-02	4.88E-02	ENSG00000271344
NA	-0.5127	1.1924E-02	4.86E-02	ENSG00000272361
NA	-0.2173	1.1853E-02	4.83E-02	ENSG00000289579
NA	-0.2252	1.1828E-02	4.83E-02	ENSG00000246560
NA	-0.2162	1.1352E-02	4.68E-02	ENSG00000230409
NA	-0.2009	1.1336E-02		ENSG00000277144
NA	-0.4890	1.1295E-02	4.66E-02	ENSG00000259523
NA	-0.5312	1.1232E-02	4.64E-02	ENSG00000287021
NA	-0.3785	1.1218E-02	4.63E-02	ENSG00000288710
NA	-0.1765	1.1137E-02		ENSG00000279536
NA	-0.4463	1.1132E-02	4.61E-02	ENSG00000289212
NA	-0.4601	1.1070E-02	4.59E-02	ENSG00000276509
NA	-0.3311	1.1032E-02	4.58E-02	ENSG00000276564
NA	-0.4760	1.1010E-02	4.57E-02	ENSG00000287704
NA	-0.3955	1.0806E-02	4.50E-02	ENSG00000284237
NA	-0.4721	1.0795E-02	4.50E-02	ENSG00000248100
NA	-0.4643	1.0773E-02	4.49E-02	ENSG00000289106
NA	-0.1827	1.0718E-02	4.47E-02	ENSG00000236698
NA	-0.3251	1.0596E-02	4.43E-02	ENSG00000224842
NA	-0.2073	1.0575E-02	4.43E-02	ENSG00000225678
NA	-0.3129	1.0504E-02	4.40E-02	ENSG00000273796
NA	-0.1366	1.0348E-02		ENSG00000278095
NA	-0.2341	1.0190E-02	4.31E-02	ENSG00000213178
NA	-0.3245	9.9339E-03	4.22E-02	ENSG00000260418
NA	-0.3607	9.7948E-03	4.18E-02	ENSG00000224848
NA	-0.1716	9.7701E-03		ENSG00000261618
NA	-0.3333	9.6531E-03	4.14E-02	ENSG00000262468
NA	-0.2341	9.5915E-03	4.12E-02	ENSG00000228835
NA	-0.4966	9.3494E-03	4.04E-02	ENSG00000233205
NA	-0.1938	9.1118E-03	3.97E-02	ENSG00000259781
NA	-0.3188	8.9169E-03	3.90E-02	ENSG00000249014
NA	-0.4279	8.9015E-03	3.90E-02	ENSG00000286675
NA	-0.2446	8.6844E-03	3.83E-02	ENSG00000235939
NA	-0.4395	8.5089E-03	3.77E-02	ENSG00000227725

NA	-0.5097	8.3838E-03	3.72E-02	ENSG00000213089
NA	-0.1017	8.1720E-03		ENSG00000244641
NA	-0.3703	8.0024E-03	3.60E-02	ENSG00000251050
NA	-0.5079	7.9463E-03	3.58E-02	ENSG00000228252
NA	-0.1590	7.7081E-03	3.49E-02	ENSG00000254635
NA	-0.2532	7.7035E-03	3.49E-02	ENSG00000287978
NA	-0.5540	7.6108E-03	3.46E-02	ENSG00000227379
NA	-0.3917	7.5708E-03	3.44E-02	ENSG00000267174
NA	-0.4885	7.5679E-03	3.44E-02	ENSG00000279137
NA	-0.3893	7.2512E-03	3.33E-02	ENSG00000270071
NA	-0.4979	7.1391E-03	3.28E-02	ENSG00000264513
NA	-0.5793	7.1090E-03	3.28E-02	ENSG00000257920
NA	-1.3594	7.0707E-03		ENSG00000286492
NA	-0.3693	6.9705E-03	3.23E-02	ENSG00000263011
NA	-0.4027	6.9344E-03	3.22E-02	ENSG00000259448
NA	-0.2157	6.9213E-03		ENSG00000232493
NA	-0.2180	6.8591E-03	3.19E-02	ENSG00000230510
NA	-0.4278	6.6416E-03	3.11E-02	ENSG00000287886
NA	-0.5851	6.5912E-03	3.10E-02	ENSG00000261578
NA	-0.1463	6.4841E-03	3.06E-02	ENSG00000277053
NA	-0.3637	6.4180E-03	3.04E-02	ENSG00000251259
NA	-0.2515	6.3480E-03	3.01E-02	ENSG00000203279
NA	-0.3911	6.2700E-03	2.98E-02	ENSG00000197258
NA	-0.3330	6.2507E-03	2.97E-02	ENSG00000271121
NA	-0.3369	6.1627E-03	2.94E-02	ENSG00000214199
NA	-0.2227	6.0496E-03	2.90E-02	ENSG00000180867
NA	-0.3626	5.9250E-03	2.85E-02	ENSG00000169668
NA	-0.1618	5.7347E-03	2.78E-02	ENSG00000229180
NA	-0.4786	5.4531E-03	2.68E-02	ENSG00000273891
NA	-0.4362	5.4299E-03	2.67E-02	ENSG00000267191
NA	-0.2474	5.3119E-03	2.62E-02	ENSG00000289843
NA	-0.4687	5.2147E-03	2.59E-02	ENSG00000280832
NA	-0.3148	4.8198E-03	2.43E-02	ENSG00000286833
NA	-0.4688	4.8046E-03	2.43E-02	ENSG00000251637
NA	-0.4315	4.7745E-03	2.42E-02	ENSG00000286431
NA	-0.1828	4.6643E-03	2.37E-02	ENSG00000240356
NA	-0.4392	4.6220E-03	2.36E-02	ENSG00000287463
NA	-0.3149	4.6180E-03	2.36E-02	ENSG00000286388
NA	-0.2327	4.5095E-03	2.31E-02	ENSG00000178458
NA	-0.2609	4.2189E-03	2.20E-02	ENSG00000235386
NA	-0.4437	4.1133E-03	2.16E-02	ENSG00000242615
NA	-0.2889	4.0209E-03	2.12E-02	ENSG00000179277
NA	-0.5436	3.8506E-03	2.05E-02	ENSG00000287127
NA	-0.5990	3.8184E-03	2.03E-02	ENSG00000265735
NA	-0.3588	3.8059E-03	2.03E-02	ENSG00000231686
NA	-0.2642	3.8038E-03	2.03E-02	ENSG00000234664
NA	-0.1785	3.6824E-03	1.98E-02	ENSG00000131002
NA	-0.3216	3.5855E-03	1.94E-02	ENSG00000227799
NA	-0.2458	3.5484E-03	1.92E-02	ENSG00000179967

NA	-0.5030	3.4049E-03	1.86E-02	ENSG00000259994
NA	-0.5985	3.2503E-03	1.79E-02	ENSG00000286366
NA	-1.5552	3.1495E-03		ENSG00000254985
NA	-0.2669	3.1300E-03	1.74E-02	ENSG00000290032
NA	-0.2989	3.0293E-03	1.70E-02	ENSG00000215302
NA	-0.2818	2.8934E-03	1.64E-02	ENSG00000227081
NA	-1.4332	2.8682E-03		ENSG00000286347
NA	-0.4479	2.8655E-03	1.63E-02	ENSG00000243389
NA	-0.4275	2.8497E-03	1.62E-02	ENSG00000260086
NA	-0.3081	2.6414E-03	1.53E-02	ENSG00000256340
NA	-0.3832	2.5956E-03	1.50E-02	ENSG00000260293
NA	-0.3467	2.5151E-03	1.47E-02	ENSG00000251095
NA	-0.3711	2.4187E-03	1.42E-02	ENSG00000224786
NA	-1.3598	2.3133E-03		ENSG00000234393
NA	-0.3074	2.1594E-03	1.30E-02	ENSG00000196205
NA	-0.3738	2.1071E-03	1.28E-02	ENSG00000184809
NA	-0.4450	1.8213E-03	1.14E-02	ENSG00000287271
NA	-0.4133	1.6424E-03	1.05E-02	ENSG00000275764
NA	-0.4849	1.6321E-03	1.05E-02	ENSG00000287387
NA	-0.2753	1.5867E-03	1.02E-02	ENSG00000249936
NA	-0.3985	1.5626E-03	1.01E-02	ENSG00000244471
NA	-0.4694	1.5590E-03	1.01E-02	ENSG00000234692
NA	-0.1856	1.5246E-03	9.94E-03	ENSG00000224616
NA	-0.5095	1.4758E-03	9.69E-03	ENSG00000286705
NA	-0.2147	1.4704E-03	9.66E-03	ENSG00000225151
NA	-0.2894	1.3652E-03	9.11E-03	ENSG00000244313
NA	-0.3901	1.3305E-03	8.92E-03	ENSG00000246263
NA	-0.2921	1.2666E-03	8.58E-03	ENSG00000290018
NA	-0.5631	1.2641E-03	8.57E-03	ENSG00000244125
NA	-0.5898	1.2310E-03	8.37E-03	ENSG00000230105
NA	-0.4373	1.1962E-03	8.17E-03	ENSG00000262001
NA	-0.4103	1.0998E-03	7.65E-03	ENSG00000188013
NA	-0.5795	9.6714E-04	6.94E-03	ENSG00000287115
NA	-0.3822	9.6692E-04	6.94E-03	ENSG00000244558
NA	-0.3867	8.7977E-04	6.44E-03	ENSG00000226853
NA	-0.4336	8.2439E-04	6.11E-03	ENSG00000234286
NA	-0.2861	7.7365E-04	5.82E-03	ENSG00000242759
NA	-0.4738	7.6197E-04	5.75E-03	ENSG00000279608
NA	-0.3403	7.3322E-04	5.58E-03	ENSG00000213707
NA	-0.5092	7.0951E-04	5.43E-03	ENSG00000269958
NA	-0.3613	6.6332E-04	5.17E-03	ENSG00000267390
NA	-0.5452	6.2542E-04	4.92E-03	ENSG00000239881
NA	-0.4316	5.6270E-04	4.53E-03	ENSG00000225398
NA	-0.3982	4.7542E-04	3.93E-03	ENSG00000204253
NA	-0.3883	4.5929E-04	3.84E-03	ENSG00000264350
NA	-0.5425	4.4927E-04	3.78E-03	ENSG00000196381
NA	-0.4281	4.4484E-04	3.75E-03	ENSG00000175772
NA	-0.3818	4.1508E-04	3.55E-03	ENSG00000270761
NA	-0.3073	3.8983E-04	3.37E-03	ENSG00000277969

NA	-0.3368	3.6577E-04	3.22E-03	ENSG00000227097
NA	-0.4119	3.2336E-04	2.91E-03	ENSG00000231551
NA	-0.2905	2.6700E-04	2.47E-03	ENSG00000264281
NA	-0.5289	2.4972E-04	2.34E-03	ENSG00000260136
NA	-0.5434	2.4571E-04	2.31E-03	ENSG00000226334
NA	-0.4494	2.3178E-04	2.20E-03	ENSG00000258744
NA	-0.5143	2.3108E-04	2.20E-03	ENSG00000274840
NA	-0.3411	2.2385E-04	2.14E-03	ENSG00000272419
NA	-0.4106	2.0367E-04	1.99E-03	ENSG00000250511
NA	-2.4447	2.0193E-04		ENSG00000272865
NA	-0.5183	1.9179E-04	1.89E-03	ENSG00000232485
NA	-0.2089	1.6371E-04	1.67E-03	ENSG00000237854
NA	-0.2311	1.1891E-04	1.29E-03	ENSG00000224837
NA	-0.3040	1.0970E-04	1.21E-03	ENSG00000235174
NA	-0.2462	1.0548E-04	1.17E-03	ENSG00000272888
NA	-0.3958	9.7327E-05	1.09E-03	ENSG00000249898
NA	-0.3516	9.5195E-05	1.07E-03	ENSG00000249042
NA	-0.5294	8.7682E-05	1.01E-03	ENSG00000272710
NA	-0.4867	7.7967E-05	9.16E-04	ENSG00000268516
NA	-0.2677	7.3094E-05	8.71E-04	ENSG00000277147
NA	-0.3230	5.3768E-05	6.80E-04	ENSG00000258655
NA	-0.3363	5.2787E-05	6.72E-04	ENSG00000234964
NA	-0.3271	5.0090E-05	6.46E-04	ENSG00000276141
NA	-0.5010	2.4222E-05	3.57E-04	ENSG00000224543
NA	-0.2997	2.0325E-05	3.10E-04	ENSG00000262879
NA	-0.3625	2.0213E-05	3.08E-04	ENSG00000246067
NA	-0.3970	1.8646E-05	2.89E-04	ENSG00000233476
NA	-0.3400	1.8062E-05	2.82E-04	ENSG00000226067
NA	-0.2963	1.7898E-05	2.81E-04	ENSG00000112096
NA	-0.2377	1.7395E-05	2.75E-04	ENSG00000078319
NA	-0.3311	7.8357E-06	1.45E-04	ENSG00000274104
NA	-0.5240	7.6076E-06	1.42E-04	ENSG00000248161
NA	-0.3580	4.4234E-06	9.11E-05	ENSG00000227063
NA	-0.4813	3.7555E-06	7.98E-05	ENSG00000278730
NA	-0.4209	2.1636E-06	5.04E-05	ENSG00000223509
NA	-0.3626	1.8579E-06	4.44E-05	ENSG00000174977
NA	-0.3715	1.3963E-06	3.53E-05	ENSG00000235655
NA	-0.5427	1.1647E-06	3.04E-05	ENSG00000186312
NA	-0.4620	8.1627E-07	2.27E-05	ENSG00000284428
NA	-0.2972	6.6169E-07	1.88E-05	ENSG00000232024
NA	-0.3560	4.3268E-07	1.32E-05	ENSG00000279483
NA	-0.4201	3.2261E-07	1.04E-05	ENSG00000248092
NA	-0.4758	1.3374E-07	4.93E-06	ENSG00000230330
NA	-0.3476	1.0782E-07	4.14E-06	ENSG00000223804
NA	-0.3911	1.0599E-07	4.09E-06	ENSG00000228589
NA	-0.3879	2.0029E-08	1.03E-06	ENSG00000196656
NA	-0.5118	9.8678E-09	5.62E-07	ENSG00000283103
NA	-0.4998	8.6310E-10	7.58E-08	ENSG00000197582
NA	-0.5303	6.3793E-10	5.86E-08	ENSG00000203644

NA	-0.4514	1.8208E-10	1.95E-08	ENSG00000198618
NA	-0.5179	1.1411E-10	1.34E-08	ENSG00000256393
NA	-0.5623	3.1680E-11	4.45E-09	ENSG00000280138
NAA10	-0.0563	2.8587E-01	4.77E-01	ENSG00000102030
NAA15	0.0633	3.4776E-01	5.43E-01	ENSG00000164134
NAA16	-0.0191	7.1551E-01	8.42E-01	ENSG00000172766
NAA20	-0.1197	7.2997E-02	1.88E-01	ENSG00000173418
NAA25	0.1326	5.3122E-02	1.49E-01	ENSG00000111300
NAA30	0.0233	7.6398E-01	8.72E-01	ENSG00000139977
NAA35	0.2138	4.6190E-04	3.86E-03	ENSG00000135040
NAA38	-0.0836	1.1737E-01	2.63E-01	ENSG00000183011
NAA40	0.2307	6.5510E-04	5.11E-03	ENSG00000110583
NAA50	-0.0685	2.6871E-01	4.58E-01	ENSG00000121579
NAA60	-0.0061	8.9790E-01	9.49E-01	ENSG00000122390
NAA80	-0.1067	7.5667E-02	1.93E-01	ENSG00000243477
NAAA	-0.0847	2.7654E-01	4.67E-01	ENSG00000138744
NAALAD2	-0.0317	7.2573E-01	8.48E-01	ENSG00000077616
NAALADL1	0.1768	9.4255E-02	2.26E-01	ENSG00000168060
NAALADL2	-0.0963	2.9911E-01	4.91E-01	ENSG00000177694
NAB1	-0.3919	2.0293E-06	4.79E-05	ENSG00000138386
NAB2	-0.1406	1.0532E-01	2.44E-01	ENSG00000166886
NABP1	0.3117	1.8774E-02	6.87E-02	ENSG00000173559
NABP2	-0.0327	6.6604E-01	8.08E-01	ENSG00000139579
NACA	-0.2236	7.0333E-05	8.44E-04	ENSG00000196531
NACA2	-0.0727	5.6219E-01	7.31E-01	ENSG00000253506
NACA3P	-0.0464	6.2786E-01	7.82E-01	ENSG00000121089
NACA4P	-0.0403	6.8031E-01	8.18E-01	ENSG00000228224
NACAD	0.0865	3.0772E-01	5.00E-01	ENSG00000136274
NACC1	-0.0896	3.2733E-01	5.22E-01	ENSG00000160877
NACC2	-0.0297	7.2929E-01	8.51E-01	ENSG00000148411
NADK	0.0034	9.6257E-01	9.81E-01	ENSG00000008130
NADK2	-0.1143	7.6150E-02	1.94E-01	ENSG00000152620
NADSYN1	0.0945	5.7773E-02	1.59E-01	ENSG00000172890
NAE1	0.0235	7.4216E-01	8.59E-01	ENSG00000159593
NAF1	-0.2265	1.3755E-02	5.43E-02	ENSG00000145414
NAGA	-0.0588	3.4455E-01	5.40E-01	ENSG00000198951
NAGK	0.1790	1.5113E-02	5.83E-02	ENSG00000124357
NAGLU	0.0327	6.2778E-01	7.82E-01	ENSG00000108784
NAGPA	0.0020	9.9749E-01	9.99E-01	ENSG00000103174
NAIF1	0.0009	9.8825E-01	9.94E-01	ENSG00000171169
NAIP	-0.0939	2.7000E-01	4.60E-01	ENSG00000249437
NAIPP1	-0.1598	1.4233E-01	3.00E-01	ENSG00000250687
NAIPP3	-0.2335	6.3390E-02	1.70E-01	ENSG00000248477
NALCN	0.2711	3.2131E-03	1.77E-02	ENSG00000102452
NALCN-AS1	1.5174	3.2351E-03		ENSG00000233009
NALF1	0.1862	1.4348E-02	5.61E-02	ENSG00000204442
NALF2	-0.0606	5.0312E-01	6.84E-01	ENSG00000130054
NAMA	0.0045	7.4551E-01		ENSG00000271086
NAMPT	-0.3804	3.8591E-05	5.18E-04	ENSG00000105835

NAMPTP1	-0.2671	1.2233E-02	4.96E-02	ENSG00000229644
NANOG	0.0781	3.0115E-01	4.94E-01	ENSG00000111704
NANOGP8	0.0248	4.7207E-02		ENSG00000255192
NANOS1	-0.1369	1.2383E-01	2.73E-01	ENSG00000188613
NANOS2	-0.0001	9.8328E-01		ENSG00000188425
NANOS3	-0.3820	7.8147E-04	5.88E-03	ENSG00000187556
NANP	0.0995	2.4451E-01	4.30E-01	ENSG00000170191
NANS	-0.0689	2.1451E-01	3.95E-01	ENSG00000095380
NAP1L1	-0.1265	7.9076E-02	1.99E-01	ENSG00000187109
NAP1L1P1	-0.0233	7.4050E-01	8.57E-01	ENSG00000254759
NAP1L2	0.0184	8.1940E-01	9.05E-01	ENSG00000186462
NAP1L3	-0.0921	2.9827E-01	4.90E-01	ENSG00000186310
NAP1L4	0.0058	9.0519E-01	9.52E-01	ENSG00000205531
NAP1L4P3	-0.0021	5.0841E-01		ENSG00000234145
NAP1L5	0.0020	9.7971E-01	9.89E-01	ENSG00000177432
NAP1L6P	-0.0489	6.0364E-01	7.64E-01	ENSG00000204118
NAPA	-0.0277	5.5319E-01	7.24E-01	ENSG00000105402
NAPB	0.4025	1.8365E-04	1.84E-03	ENSG00000125814
NAPEPLD	-0.0664	4.2201E-01	6.15E-01	ENSG00000161048
NAPG	0.0782	2.0306E-01	3.81E-01	ENSG00000134265
NAPRT	-0.1172	1.8303E-01	3.56E-01	ENSG00000147813
NAPSA	0.5075	1.4690E-02	5.71E-02	ENSG00000131400
NARF	0.5366	1.5395E-12	3.03E-10	ENSG00000141562
NARF-AS2	0.1823	1.1390E-02	4.69E-02	ENSG00000265458
NARS1	0.0121	8.9047E-01	9.45E-01	ENSG00000134440
NARS2	-0.0040	9.4635E-01	9.73E-01	ENSG00000137513
NASP	-0.4179	6.8365E-08	2.86E-06	ENSG00000132780
NAT10	0.2237	4.8082E-03	2.43E-02	ENSG00000135372
NAT14	-0.0236	6.9224E-01	8.27E-01	ENSG00000090971
NAT16	0.1963	2.7113E-02	9.03E-02	ENSG00000167011
NAT2	0.0690	4.9005E-01	6.74E-01	ENSG00000156006
NAT8L	-0.1591	1.0552E-01	2.44E-01	ENSG00000185818
NAT9	0.2838	4.1475E-04	3.54E-03	ENSG00000109065
NATD1	-0.0675	4.2711E-01	6.19E-01	ENSG00000274180
NAV1	-0.1457	2.8203E-02	9.30E-02	ENSG00000134369
NAV2	-0.0725	3.1829E-01	5.12E-01	ENSG00000166833
NAV2-AS2	0.0186	6.4768E-01		ENSG00000254453
NAV2-AS3	-0.0174	6.8402E-01		ENSG00000254542
NAV2-AS4	-0.0115	8.4333E-01		ENSG00000254622
NAV2-AS5	-0.0076	9.5044E-01		ENSG00000255043
NAV3	0.0840	3.5415E-01	5.49E-01	ENSG00000067798
NAXD	0.0180	7.4004E-01	8.57E-01	ENSG00000213995
NAXD-AS1	0.4555	3.7594E-03	2.01E-02	ENSG00000275880
NAXE	-0.1322	1.2882E-01	2.80E-01	ENSG00000163382
NBAS	-0.1538	3.1183E-02	1.00E-01	ENSG00000151779
NBDY	-0.3485	3.9698E-06	8.32E-05	ENSG00000204272
NBEA	-0.1438	2.3609E-02	8.16E-02	ENSG00000172915
NBEAL1	-0.0261	7.1408E-01	8.41E-01	ENSG00000144426
NBEAL2	-0.0876	2.4097E-01	4.26E-01	ENSG00000160796

NBEAP2	-0.0048	9.0992E-01		ENSG00000197927
NBL1	-0.0479	5.1886E-01	6.96E-01	ENSG00000158747
NBN	-0.2892	1.1185E-04	1.23E-03	ENSG00000104320
NBPF1	0.1488	4.4562E-02	1.31E-01	ENSG00000219481
NBPF10	-0.3651	6.6604E-04	5.18E-03	ENSG00000271425
NBPF11	0.1299	9.0277E-02	2.19E-01	ENSG00000263956
NBPF12	0.1172	1.3441E-01	2.88E-01	ENSG00000268043
NBPF14	-0.4913	1.2484E-04	1.33E-03	ENSG00000270629
NBPF15	-0.0903	1.7596E-01	3.46E-01	ENSG00000266338
NBPF19	-0.1991	7.1397E-02	1.85E-01	ENSG00000271383
NBPF20	0.0058	9.4790E-01	9.74E-01	ENSG00000162825
NBPF26	-0.3916	8.8219E-05	1.01E-03	ENSG00000273136
NBPF3	0.2811	6.0717E-03	2.91E-02	ENSG00000142794
NBPF8	-0.0123	8.6327E-01	9.30E-01	ENSG00000270231
NBPF9	0.0138	8.3894E-01	9.16E-01	ENSG00000269713
NBR1	0.1385	9.2536E-03	4.01E-02	ENSG00000188554
NBR2	-0.0653	3.5398E-01	5.49E-01	ENSG00000198496
NCALD	-0.4892	3.6942E-10	3.64E-08	ENSG00000104490
NCAM1	0.0298	7.0560E-01	8.35E-01	ENSG00000149294
NCAM2	0.2418	8.2582E-03	3.68E-02	ENSG00000154654
NCAN	0.0556	5.5662E-01	7.27E-01	ENSG00000130287
NCAPD2	-0.0433	5.4421E-01	7.16E-01	ENSG00000010292
NCAPD3	-0.1566	4.4225E-02	1.31E-01	ENSG00000151503
NCAPG	-0.2911	2.2274E-02	7.81E-02	ENSG00000109805
NCAPG2	-0.0828	3.8388E-01	5.78E-01	ENSG00000146918
NCAPH	-0.3430	1.1417E-02	4.70E-02	ENSG00000121152
NCAPH2	0.1111	1.2483E-01	2.74E-01	ENSG00000025770
NCBP1	0.0357	6.2032E-01	7.76E-01	ENSG00000136937
NCBP2	0.1623	1.5985E-02	6.08E-02	ENSG00000114503
NCBP2-AS1	-0.0272	6.3553E-01		ENSG00000225578
NCBP2AS2	-0.0049	9.0657E-01	9.53E-01	ENSG00000270170
NCBP3	-0.1388	5.6206E-03	2.74E-02	ENSG00000074356
NCDN	0.0586	4.6585E-01	6.54E-01	ENSG00000020129
NCEH1	-0.0671	4.1839E-01	6.11E-01	ENSG00000144959
NCF1C	0.5934	3.2148E-03	1.77E-02	ENSG00000165178
NCF2	-0.0289	7.2274E-01	8.47E-01	ENSG00000116701
NCF4	0.0118	6.5549E-01		ENSG00000100365
NCK1	-0.1329	3.1177E-02	1.00E-01	ENSG00000158092
NCK2	0.0875	7.3276E-02	1.88E-01	ENSG00000071051
NCKAP1	-0.2888	2.4130E-05	3.56E-04	ENSG00000061676
NCKAP1L	-0.0219	3.6380E-01	5.59E-01	ENSG00000123338
NCKAP5	-0.0407	5.8432E-01	7.49E-01	ENSG00000176771
NCKAP5-AS1	0.0451	4.0739E-02		ENSG00000233729
NCKAP5-AS2	-0.4159	6.6424E-03	3.11E-02	ENSG00000226953
NCKAP5L	-0.0184	7.4778E-01	8.62E-01	ENSG00000167566
NCKIPSD	0.3056	3.7387E-04	3.28E-03	ENSG00000213672
NCL	-0.2274	8.8264E-04	6.46E-03	ENSG00000115053
NCLN	0.0605	3.8195E-01	5.77E-01	ENSG00000125912
NCMAP	-0.4243	1.2338E-03	8.38E-03	ENSG00000184454

NCOA1	-0.0118	8.3854E-01	9.16E-01	ENSG00000084676
NCOA2	-0.0959	2.0368E-01	3.82E-01	ENSG00000140396
NCOA3	0.0454	5.4816E-01	7.20E-01	ENSG00000124151
NCOA4	0.0213	7.0596E-01	8.36E-01	ENSG00000266412
NCOA5	0.3007	1.1861E-04	1.28E-03	ENSG00000124160
NCOA6	-0.1999	4.5475E-03	2.33E-02	ENSG00000198646
NCOA7	-0.0081	8.8934E-01	9.44E-01	ENSG00000111912
NCOR1	-0.0060	8.3676E-01	9.15E-01	ENSG00000141027
NCOR2	-0.1155	3.5169E-02	1.10E-01	ENSG00000196498
NCR2	0.0027	9.8459E-01		ENSG00000096264
NCR3	-0.0073	8.7542E-01		ENSG00000204475
NCR3LG1	0.1305	2.1630E-01	3.97E-01	ENSG00000188211
NCRNA00250	-0.0595	3.2904E-01		ENSG00000253433
NCS1	-0.0644	3.7829E-01	5.73E-01	ENSG00000107130
NCSTN	-0.0845	2.0451E-01	3.83E-01	ENSG00000162736
ND1	0.0254	7.5518E-01	8.67E-01	ENSG00000198888
ND2	-0.1453	1.1353E-01	2.57E-01	ENSG00000198763
ND3	-0.1105	1.5903E-01	3.23E-01	ENSG00000198840
ND4	-0.0651	5.0031E-01	6.83E-01	ENSG00000198886
ND4L	-0.1308	1.6794E-01	3.35E-01	ENSG00000212907
ND5	-0.0998	2.5779E-01	4.46E-01	ENSG00000198786
ND6	-0.2747	3.2508E-02	1.04E-01	ENSG00000198695
NDC1	0.0201	7.7146E-01	8.76E-01	ENSG00000058804
NDC80	-0.3309	1.5181E-02	5.85E-02	ENSG00000080986
NDE1	-0.2690	6.3975E-04	5.01E-03	ENSG00000072864
NDEL1	0.1602	4.2828E-02	1.28E-01	ENSG00000166579
NDFIP1	-0.2196	3.0133E-05	4.25E-04	ENSG00000131507
NDFIP2	-0.0110	8.3448E-01	9.14E-01	ENSG00000102471
NDN	-0.0628	3.1514E-01	5.09E-01	ENSG00000182636
NDNF	0.0051	9.5484E-01	9.77E-01	ENSG00000173376
NDOR1	0.1469	1.1357E-01	2.57E-01	ENSG00000188566
NDP	-0.0366	5.6215E-01	7.31E-01	ENSG00000124479
NDP-AS1	0.0803	2.9389E-01	4.86E-01	ENSG00000236276
NDRG1	0.3084	1.2845E-03	8.67E-03	ENSG00000104419
NDRG2	-0.0950	2.4204E-01	4.27E-01	ENSG00000165795
NDRG3	-0.0788	2.1033E-01	3.90E-01	ENSG00000101079
NDRG4	0.4264	2.1443E-06	5.00E-05	ENSG00000103034
NDST1	-0.0829	2.4175E-01	4.27E-01	ENSG00000070614
NDST1-AS1	-0.0056	9.4706E-01	9.73E-01	ENSG00000254333
NDST2	-0.0485	8.3163E-01	9.12E-01	ENSG00000166507
NDST3	-0.2131	6.3947E-02	1.71E-01	ENSG00000164100
NDST4	-0.1779	8.4245E-02	2.08E-01	ENSG00000138653
NDUFA1	-0.4063	1.6006E-07	5.75E-06	ENSG00000125356
NDUFA10	-0.0507	4.6548E-01	6.54E-01	ENSG00000130414
NDUFA11	-0.4036	2.7724E-06	6.25E-05	ENSG00000174886
NDUFA12	-0.2778	9.5880E-07	2.58E-05	ENSG00000184752
NDUFA13	-0.0503	5.4765E-01	7.19E-01	ENSG00000186010
NDUFA2	-0.3112	3.6676E-07	1.15E-05	ENSG00000131495
NDUFA3	-0.4287	3.2656E-08	1.55E-06	ENSG00000170906

NDUFA3P2	0.0074	5.7021E-01		ENSG00000254856
NDUFA3P4	0.0616	3.2455E-01	5.19E-01	ENSG00000259262
NDUFA4	-0.1845	2.2646E-02	7.91E-02	ENSG00000189043
NDUFA5	-0.0411	4.8266E-01	6.68E-01	ENSG00000128609
NDUFA6	-0.2423	1.5014E-05	2.44E-04	ENSG00000184983
NDUFA6-DT	-0.0768	2.6726E-01	4.56E-01	ENSG00000237037
NDUFA7	-0.0414	6.2339E-01	7.79E-01	ENSG00000267855
NDUFA8	-0.1837	3.9278E-03	2.08E-02	ENSG00000119421
NDUFA9	-0.2267	2.2555E-03	1.35E-02	ENSG00000139180
NDUFA9P1	0.0394	6.4798E-01	7.96E-01	ENSG00000237406
NDUFAB1	-0.0944	1.3550E-01	2.89E-01	ENSG00000004779
NDUFAB1P1	-0.0752	3.3470E-01	5.30E-01	ENSG00000217767
NDUFAF1	-0.0264	6.6601E-01	8.08E-01	ENSG00000137806
NDUFAF2	0.0221	7.0985E-01	8.38E-01	ENSG00000164182
NDUFAF2P1	0.0186	7.6571E-01	8.73E-01	ENSG00000279307
NDUFAF3	-0.2595	8.8598E-04	6.47E-03	ENSG00000178057
NDUFAF4	-0.1563	9.1154E-03	3.97E-02	ENSG00000123545
NDUFAF5	0.0038	9.4512E-01	9.73E-01	ENSG00000101247
NDUFAF6	0.0474	4.0677E-01	6.00E-01	ENSG00000156170
NDUFAF7	0.1047	9.4333E-02	2.26E-01	ENSG00000003509
NDUFAF8	-0.4622	8.8641E-15	3.24E-12	ENSG00000224877
NDUFB1	-0.2503	7.1434E-05	8.55E-04	ENSG00000183648
NDUFB10	-0.1169	2.0582E-02	7.35E-02	ENSG00000140990
NDUFB10P2	0.0024	8.7538E-01		ENSG00000261624
NDUFB11	-0.4034	1.4122E-12	2.89E-10	ENSG00000147123
NDUFB1P2	-0.2788	1.8050E-02	6.67E-02	ENSG00000234523
NDUFB2	-0.4746	1.1088E-14	3.81E-12	ENSG00000090266
NDUFB2-AS1	0.2779	3.6145E-02	1.12E-01	ENSG00000240889
NDUFB3	-0.2204	3.5885E-03	1.94E-02	ENSG00000119013
NDUFB4	-0.1699	6.9237E-03	3.21E-02	ENSG00000065518
NDUFB4P11	-0.0121	8.8309E-01		ENSG00000259374
NDUFB4P12	0.2008	8.5150E-02	2.10E-01	ENSG00000124399
NDUFB4P2	-0.0017	9.6867E-01		ENSG00000251306
NDUFB4P8	0.0195	3.5669E-01		ENSG00000223663
NDUFB5	-0.1785	3.9001E-02	1.19E-01	ENSG00000136521
NDUFB6	-0.1960	6.8509E-04	5.30E-03	ENSG00000165264
NDUFB7	-0.1458	6.2257E-03	2.97E-02	ENSG00000099795
NDUFB8	-0.2229	4.5573E-04	3.82E-03	ENSG00000166136
NDUFB8P2	0.0061	5.6103E-01	7.30E-01	ENSG00000270264
NDUFB9	-0.1462	4.3470E-02	1.29E-01	ENSG00000147684
NDUFC1	-0.3857	7.7087E-11	9.61E-09	ENSG00000109390
NDUFC2	-0.5448	3.4049E-15	1.34E-12	ENSG00000151366
NDUFC2-KCTD14	0.0055	8.0739E-01		ENSG00000259112
NDUFS1	-0.0008	9.8791E-01	9.93E-01	ENSG00000023228
NDUFS2	0.1205	1.0225E-01	2.38E-01	ENSG00000158864
NDUFS3	-0.2204	1.1812E-04	1.28E-03	ENSG00000213619
NDUFS4	-0.1454	9.1997E-03	3.99E-02	ENSG00000164258
NDUFS5	-0.3503	5.8828E-09	3.75E-07	ENSG00000168653
NDUFS5P1	-0.0509	9.4355E-01	9.72E-01	ENSG00000218424

NDUFS6	-0.2693	9.0500E-07	2.47E-05	ENSG00000145494
NDUFS7	-0.2422	1.4260E-03	9.43E-03	ENSG00000115286
NDUFS8	-0.1359	2.8068E-02	9.27E-02	ENSG00000110717
NDUFV1	0.1378	3.5895E-02	1.12E-01	ENSG00000167792
NDUFV1-DT	0.0427	6.1645E-01	7.74E-01	ENSG00000184224
NDUFV2	-0.2167	9.7144E-03	4.16E-02	ENSG00000178127
NDUFV2-AS1	0.2105	8.5689E-03	3.79E-02	ENSG00000266053
NDUFV3	-0.0738	2.1995E-01	4.01E-01	ENSG00000160194
NEAT1	-0.1352	1.3732E-01	2.92E-01	ENSG00000245532
NEB	0.1269	1.8692E-01	3.61E-01	ENSG00000183091
NEBL	-0.2176	1.0530E-05	1.83E-04	ENSG00000078114
NEBL-AS1	-0.3273	1.1198E-03	7.75E-03	ENSG00000231920
NECAB1	-0.5486	4.0811E-07	1.26E-05	ENSG00000123119
NECAB2	0.0700	3.1794E-01	5.12E-01	ENSG00000103154
NECAB3	0.0113	9.2018E-01	9.60E-01	ENSG00000125967
NECAP1	0.2020	7.9804E-03	3.59E-02	ENSG00000089818
NECAP1P2	-0.0517	3.2577E-01		ENSG00000234632
NECAP2	0.0396	4.6943E-01	6.57E-01	ENSG00000157191
NECTIN1	-0.0708	4.2842E-01	6.20E-01	ENSG00000110400
NECTIN1-AS1	0.0142	7.0827E-01		ENSG00000255247
NECTIN1-DT	0.0137	8.8880E-01	9.44E-01	ENSG00000254854
NECTIN2	-0.1891	2.4201E-03	1.43E-02	ENSG00000130202
NECTIN3	-0.4488	2.3691E-05	3.51E-04	ENSG00000177707
NECTIN4	0.1085	2.6635E-01	4.55E-01	ENSG00000143217
NEDD1	0.0419	5.9202E-01	7.55E-01	ENSG00000139350
NEDD4	-0.1473	9.7645E-02	2.31E-01	ENSG00000069869
NEDD4L	-0.0353	4.8540E-01	6.70E-01	ENSG00000049759
NEDD8	-0.1236	3.4615E-02	1.09E-01	ENSG00000129559
NEDD8-MDP1	-0.1507	3.0289E-02		ENSG00000255526
NEFH	0.3263	2.2514E-02	7.88E-02	ENSG00000100285
NEFL	0.2903	3.9326E-03	2.08E-02	ENSG00000277586
NEFM	0.3598	1.1103E-03	7.70E-03	ENSG00000104722
NEGR1	-0.4684	2.7963E-08	1.37E-06	ENSG00000172260
NEIL1	0.0731	3.8762E-01	5.82E-01	ENSG00000140398
NEIL2	0.1499	4.7755E-02	1.38E-01	ENSG00000154328
NEIL3	-0.1020	2.8153E-01	4.73E-01	ENSG00000109674
NEK1	0.0209	7.4246E-01	8.59E-01	ENSG00000137601
NEK10	0.5122	1.2244E-04	1.32E-03	ENSG00000163491
NEK2	-0.4628	7.6045E-04	5.74E-03	ENSG00000117650
NEK3	0.0162	7.9616E-01	8.91E-01	ENSG00000136098
NEK4	0.1568	6.7337E-02	1.77E-01	ENSG00000114904
NEK4P2	0.0200	1.1782E-01		ENSG00000264359
NEK5	-0.4432	6.9978E-04	5.38E-03	ENSG00000197168
NEK6	-0.2988	3.1018E-03	1.72E-02	ENSG00000119408
NEK7	-0.1483	8.8473E-02	2.16E-01	ENSG00000151414
NEK8	0.0327	6.8943E-01	8.26E-01	ENSG00000160602
NEK9	-0.1531	4.9171E-02	1.41E-01	ENSG00000119638
NELFA	-0.0461	4.4945E-01	6.39E-01	ENSG00000185049
NELFB	0.0987	2.2832E-01	4.11E-01	ENSG00000188986

NELFCD	0.0910	6.4171E-02		ENSG00000101158
NELFE	0.1299	4.4766E-03	2.30E-02	ENSG00000204356
NELL1	0.3308	2.5717E-02	8.70E-02	ENSG00000165973
NELL2	-0.1651	1.2216E-01	2.70E-01	ENSG00000184613
NEMF	-0.1059	1.4500E-02	5.66E-02	ENSG00000165525
NEMP1	-0.1246	1.9891E-01	3.76E-01	ENSG00000166881
NEMP2	-0.2142	4.5939E-02	1.34E-01	ENSG00000189362
NEMP2-DT	-0.4737	3.9394E-03	2.09E-02	ENSG00000233654
NENF	-0.3770	3.2231E-08	1.54E-06	ENSG00000117691
NENFP1	-0.0235	9.7665E-01		ENSG00000233647
NEO1	-0.1589	2.4759E-02	8.46E-02	ENSG00000067141
NEPRO	0.1368	6.3212E-02	1.70E-01	ENSG00000163608
NES	0.0134	8.6211E-01	9.30E-01	ENSG00000132688
NET1	-0.4441	3.0584E-07	9.97E-06	ENSG00000173848
NETO1	0.2188	6.6912E-02	1.76E-01	ENSG00000166342
NETO1-DT	-0.0432	6.5303E-01	7.99E-01	ENSG00000263958
NETO2	-0.1106	1.8931E-01	3.64E-01	ENSG00000171208
NEU1	0.0072	9.3529E-01	9.68E-01	ENSG00000204386
NEU3	0.0152	8.5441E-01	9.25E-01	ENSG00000162139
NEU4	-0.0609	3.1473E-01		ENSG00000204099
NEURL1	0.2074	1.2435E-02	5.02E-02	ENSG00000107954
NEURL1-AS1	0.0474	2.6778E-01	4.57E-01	ENSG00000235470
NEURL1B	-0.3485	2.1369E-04	2.06E-03	ENSG00000214357
NEURL2	0.0185	7.9873E-01	8.92E-01	ENSG00000124257
NEURL4	0.1889	7.4852E-02	1.91E-01	ENSG00000215041
NEUROD2	-0.0871	1.6885E-01	3.37E-01	ENSG00000171532
NEUROD6	-0.0834	4.2094E-02		ENSG00000164600
NEUROG1	0.0458	4.1588E-01	6.09E-01	ENSG00000181965
NEUROG3	-0.0027	9.1579E-01		ENSG00000122859
NEXMIF	-0.1888	5.7904E-02	1.59E-01	ENSG00000050030
NEXN	-0.3366	1.3126E-02	5.23E-02	ENSG00000162614
NEXN-AS1	-0.0838	3.3566E-01	5.31E-01	ENSG00000235927
NF1	-0.0592	4.8361E-01	6.68E-01	ENSG00000196712
NF2	0.1072	3.7380E-02	1.15E-01	ENSG00000186575
NFAM1	0.0699	4.4378E-01	6.34E-01	ENSG00000235568
NFASC	0.1899	8.9187E-03	3.90E-02	ENSG00000163531
NFAT5	-0.1331	4.1843E-02	1.25E-01	ENSG00000102908
NFATC1	-0.3019	4.4608E-03	2.29E-02	ENSG00000131196
NFATC2	-0.4246	1.3254E-03	8.89E-03	ENSG00000101096
NFATC2IP	0.1371	4.5455E-02	1.33E-01	ENSG00000176953
NFATC3	0.0501	5.1408E-01	6.93E-01	ENSG00000072736
NFATC4	-0.2476	6.5518E-03	3.08E-02	ENSG00000100968
NFE2	-0.0080	9.2997E-01	9.66E-01	ENSG00000123405
NFE2L1	-0.2515	1.8204E-04	1.82E-03	ENSG00000082641
NFE2L1-DT	-0.1558	1.4946E-01	3.10E-01	ENSG00000263412
NFE2L2	-0.0910	2.3579E-01	4.20E-01	ENSG00000116044
NFE2L3	0.2006	3.4158E-02	1.07E-01	ENSG00000050344
NFE4	-0.0074	8.0040E-01		ENSG00000230257
NFIA	-0.4793	1.4132E-05	2.32E-04	ENSG00000162599

NFIA-AS1	0.0222	7.9068E-01		ENSG00000237853
NFIB	-0.5224	7.3032E-06	1.37E-04	ENSG00000147862
NFIL3	-0.4993	1.7876E-09	1.36E-07	ENSG00000165030
NFILZ	-0.0636	2.4191E-01		ENSG00000268480
NFKB2	-0.5130	8.6833E-06	1.57E-04	ENSG00000077150
NFKBIB	-0.0590	3.6730E-01	5.63E-01	ENSG00000104825
NFKBID	0.0628	5.1728E-01	6.95E-01	ENSG00000167604
NFKBIL1	0.0827	1.9471E-01	3.71E-01	ENSG00000204498
NFKBIZ	-0.3495	2.2653E-03	1.35E-02	ENSG00000144802
NFRKB	-0.0030	9.3440E-01	9.67E-01	ENSG00000170322
NFS1	0.1693	3.6224E-02	1.12E-01	ENSG00000244005
NFU1	-0.0910	1.0414E-01	2.42E-01	ENSG00000169599
NFX1	0.3887	4.8643E-06	9.82E-05	ENSG00000086102
NFXL1	0.0628	4.3312E-01	6.24E-01	ENSG00000170448
NFYA	0.1833	3.7351E-02	1.15E-01	ENSG00000001167
NFYB	0.0309	6.8996E-01	8.26E-01	ENSG00000120837
NFYC	-0.0862	4.2298E-02	1.26E-01	ENSG00000066136
NGDN	-0.2092	5.0439E-05	6.49E-04	ENSG00000129460
NGEF	-0.1085	2.6205E-01	4.51E-01	ENSG00000066248
NGF	0.0037	9.6923E-01	9.84E-01	ENSG00000134259
NGFR	0.1920	9.4158E-02	2.26E-01	ENSG00000064300
NGLY1	0.2174	5.8441E-03	2.82E-02	ENSG00000151092
NGRN	-0.0614	3.5263E-01	5.48E-01	ENSG00000182768
NHEG1	0.0456	7.1912E-02		ENSG00000225391
NHEJ1	-0.1571	2.2057E-01	4.02E-01	ENSG00000187736
NHERF1	-0.1825	1.5521E-02	5.94E-02	ENSG00000109062
NHERF2	0.0573	4.3541E-01	6.27E-01	ENSG00000065054
NHERF4	0.0090	6.2742E-01		ENSG00000172367
NHLH1	0.1191	1.9872E-01	3.76E-01	ENSG00000171786
NHLRC1	0.4090	1.1463E-02	4.71E-02	ENSG00000187566
NHLRC2	-0.0341	5.5986E-01	7.30E-01	ENSG00000196865
NHLRC4	-0.0368	6.8877E-01	8.25E-01	ENSG00000257108
NHP2	-0.1700	7.9920E-04	5.98E-03	ENSG00000145912
NHS	-0.2709	1.1365E-02	4.68E-02	ENSG00000188158
NHSL1	-0.1735	4.7617E-02	1.38E-01	ENSG00000135540
NHSL2	0.0834	3.8052E-01	5.75E-01	ENSG00000204131
NHSL3	-0.0511	5.2922E-01	7.04E-01	ENSG00000162522
NIBAN1	-0.2382	5.6361E-02	1.56E-01	ENSG00000135842
NIBAN2	-0.0808	3.6162E-01	5.58E-01	ENSG00000136830
NICOL1	-0.3675	8.9096E-11	1.08E-08	ENSG00000243449
NID1	0.3832	7.2029E-03	3.31E-02	ENSG00000116962
NID2	-0.3302	1.6485E-02	6.23E-02	ENSG00000087303
NIF3L1	0.1103	4.4880E-02	1.32E-01	ENSG00000196290
NIFK	-0.1021	1.1685E-01	2.62E-01	ENSG00000155438
NIFK-AS1	-0.0502	5.1560E-01	6.94E-01	ENSG00000236859
NIFKP8	-0.0138	4.4744E-01		ENSG00000259449
NIM1K	0.0446	6.3013E-01	7.84E-01	ENSG00000177453
NIN	0.4087	2.7605E-04	2.54E-03	ENSG00000100503
NINJ1	-0.4539	7.6372E-08	3.13E-06	ENSG00000131669

NINJ2	0.1559	3.3473E-02	1.06E-01	ENSG00000171840
NINJ2-AS1	0.0307	7.4507E-01	8.60E-01	ENSG00000177406
NINL	0.3275	3.7686E-05	5.08E-04	ENSG00000101004
NIP7	0.2885	3.0173E-05	4.25E-04	ENSG00000132603
NIPA1	0.0577	3.8554E-01	5.80E-01	ENSG00000170113
NIPA2	0.0833	2.7239E-01	4.62E-01	ENSG00000140157
NIPAL1	0.4902	2.8300E-05	4.05E-04	ENSG00000163293
NIPAL2	-0.0325	7.3209E-01	8.53E-01	ENSG00000104361
NIPAL3	0.1380	1.5717E-02	6.01E-02	ENSG00000001461
NIPAL4	-0.4250	1.5139E-02	5.84E-02	ENSG00000172548
NIPBL	-0.2523	8.2198E-04	6.10E-03	ENSG00000164190
NIPBL-DT	0.1909	1.6512E-02	6.24E-02	ENSG00000285967
NIPSNAP1	0.0324	6.0017E-01	7.61E-01	ENSG00000184117
NIPSNAP2	-0.0607	3.5638E-01	5.52E-01	ENSG00000146729
NIPSNAP3A	-0.1934	3.0288E-03	1.70E-02	ENSG00000136783
NIPSNAP3B	0.0991	2.7420E-01	4.65E-01	ENSG00000165028
NISCH	-0.1567	6.1161E-03	2.93E-02	ENSG00000010322
NIT1	-0.1841	5.7796E-03	2.80E-02	ENSG00000158793
NIT2	0.2731	5.9430E-04	4.73E-03	ENSG00000114021
NKAIN1	0.1658	7.7880E-02	1.97E-01	ENSG00000084628
NKAIN2	0.3385	1.0452E-02	4.39E-02	ENSG00000188580
NKAIN3	-0.5012	1.7982E-03	1.13E-02	ENSG00000185942
NKAIN4	-0.3068	3.7180E-03	1.99E-02	ENSG00000101198
NKAP	-0.0464	5.2367E-01	7.00E-01	ENSG00000101882
NKAPD1	-0.0683	2.2536E-01	4.08E-01	ENSG00000150776
NKAPL	-0.1652	9.6856E-02	2.30E-01	ENSG00000189134
NKAPP1	-0.2966	4.8027E-03	2.43E-02	ENSG00000233382
NKD1	-0.2299	1.4825E-02	5.75E-02	ENSG00000140807
NKD2	0.3497	4.4276E-03	2.28E-02	ENSG00000145506
NKILA	-0.1018	2.6517E-01	4.54E-01	ENSG00000278709
NKIRAS1	0.0796	1.9052E-01	3.66E-01	ENSG00000197885
NKIRAS2	0.1005	4.2248E-02	1.26E-01	ENSG00000168256
NKPD1	0.2041	6.5182E-02	1.73E-01	ENSG00000179846
NKRF	-0.2206	9.2825E-03	4.02E-02	ENSG00000186416
NKTR	0.0922	2.1079E-01	3.90E-01	ENSG00000114857
NKX2-1	-0.5277	8.5475E-04	6.31E-03	ENSG00000136352
NKX2-2	-0.0043	9.7566E-01		ENSG00000125820
NKX2-3	0.0179	3.4536E-01		ENSG00000119919
NKX2-5	0.0343	7.0323E-01	8.34E-01	ENSG00000183072
NKX2-8	-0.0337	3.2485E-01		ENSG00000136327
NKX3-1	0.0079	9.3065E-01	9.66E-01	ENSG00000167034
NKX3-2	-0.5100	3.6740E-03	1.97E-02	ENSG00000109705
NKX6-1	0.5590	3.5574E-06	7.63E-05	ENSG00000163623
NKX6-2	0.4046	1.2072E-02	4.90E-02	ENSG00000148826
NKX6-3	0.0077	8.2618E-01		ENSG00000165066
NLE1	0.1782	1.0982E-01	2.51E-01	ENSG00000073536
NLGN1	0.1485	1.0682E-01	2.46E-01	ENSG00000169760
NLGN1-AS1	-0.0059	9.6258E-01		ENSG00000228213
NLGN2	-0.3311	3.1216E-03	1.73E-02	ENSG00000169992

NLGN3	-0.2302	3.6235E-02	1.12E-01	ENSG00000196338
NLGN4X	-0.0887	3.2495E-01	5.19E-01	ENSG00000146938
NLGN4Y	-0.2204	1.4377E-02	5.62E-02	ENSG00000165246
NLGN4Y-AS1	0.0041	7.9979E-01		ENSG00000228787
NLK	-0.0531	5.0357E-01	6.85E-01	ENSG00000087095
NLN	0.1391	1.1572E-02	4.74E-02	ENSG00000123213
NLRC3	0.1032	2.9969E-01	4.92E-01	ENSG00000167984
NLRC4	-0.0060	4.4018E-01		ENSG00000091106
NLRC5	-0.1640	1.3877E-01	2.94E-01	ENSG00000140853
NLRP1	0.5119	9.4104E-04	6.79E-03	ENSG00000091592
NLRP12	0.0032	4.2166E-01		ENSG00000142405
NLRP14	0.1604	1.4531E-01	3.04E-01	ENSG00000158077
NLRP2	0.0835	2.4626E-01	4.32E-01	ENSG00000022556
NLRP3	-0.0184	7.6929E-01		ENSG00000162711
NLRP6	0.0106	4.6550E-01		ENSG00000174885
NLRP9	-0.0957	6.1341E-02		ENSG00000185792
NLRX1	-0.0691	4.2578E-01	6.17E-01	ENSG00000160703
NMB	-0.1109	1.2529E-01	2.75E-01	ENSG00000197696
NMBR	-0.1040	1.9150E-01	3.67E-01	ENSG00000135577
NMD3	-0.0237	6.9416E-01	8.29E-01	ENSG00000169251
NME1	0.1271	8.9847E-02	2.18E-01	ENSG00000239672
NME1-NME2	-0.5953	1.3376E-04	1.41E-03	ENSG00000011052
NME2	0.0792	4.1599E-01	6.09E-01	ENSG00000243678
NME3	-0.4285	1.5393E-05	2.49E-04	ENSG00000103024
NME5	-0.3195	4.3105E-05	5.70E-04	ENSG00000112981
NME6	-0.0371	5.5711E-01	7.28E-01	ENSG00000172113
NME7	0.0553	2.8974E-01	4.81E-01	ENSG00000143156
NME8	-0.0447	6.9419E-01	8.29E-01	ENSG00000086288
NME9	-0.0652	4.4480E-01	6.35E-01	ENSG00000181322
NMNAT1	-0.1995	1.7326E-02	6.47E-02	ENSG00000173614
NMNAT2	-0.0891	2.2763E-01	4.10E-01	ENSG00000157064
NMNAT3	0.0038	9.7512E-01	9.87E-01	ENSG00000163864
NMRAL1	-0.0877	2.3097E-01	4.14E-01	ENSG00000153406
NMRK1	-0.1774	7.4275E-02	1.90E-01	ENSG00000106733
NMRK2	-0.4121	1.2388E-02	5.00E-02	ENSG00000077009
NMS	-0.0885	9.0373E-02	2.19E-01	ENSG00000204640
NMT1	0.0132	8.1834E-01	9.04E-01	ENSG00000136448
NMT2	0.0148	8.0795E-01	8.98E-01	ENSG00000152465
NMU	0.0598	4.5895E-01	6.48E-01	ENSG00000109255
NMUR1	0.0041	5.4343E-01		ENSG00000171596
NNAT	0.3892	5.1491E-04	4.21E-03	ENSG00000053438
NNMT	-0.4309	8.3047E-03	3.70E-02	ENSG00000166741
NNT	-0.1961	9.6971E-04	6.94E-03	ENSG00000112992
NOA1	-0.0196	7.3237E-01	8.53E-01	ENSG00000084092
NOB1	-0.0146	8.3554E-01	9.14E-01	ENSG00000141101
NOC2L	0.1714	2.2809E-02	7.95E-02	ENSG00000188976
NOC2LP1	0.0049	4.0774E-01		ENSG00000213225
NOC3L	0.0001	9.9881E-01	1.00E+00	ENSG00000173145
NOC4L	0.1984	2.5721E-03	1.49E-02	ENSG00000184967

NOCT	-0.5637	1.9532E-07	6.82E-06	ENSG00000151014
NOD1	-0.0477	5.9181E-01	7.55E-01	ENSG00000106100
NOD2	-0.0361	6.6997E-01	8.10E-01	ENSG00000167207
NODAL	-0.0305	5.1520E-01		ENSG00000156574
NOG	-0.5995	7.5257E-04	5.69E-03	ENSG00000183691
NOL10	0.0522	4.0518E-01	5.99E-01	ENSG00000115761
NOL11	0.0884	1.2844E-01	2.79E-01	ENSG00000130935
NOL12	0.3669	8.5166E-03	3.77E-02	ENSG00000273899
NOL3	0.2364	1.9543E-02	7.07E-02	ENSG00000140939
NOL4	-0.0492	5.5543E-01	7.26E-01	ENSG00000101746
NOL4L	0.2058	5.0565E-04	4.14E-03	ENSG00000197183
NOL4L-DT	-0.0055	9.8894E-01		ENSG00000204393
NOL6	0.3753	8.3435E-04	6.17E-03	ENSG00000165271
NOL7	-0.0085	8.6315E-01	9.30E-01	ENSG00000225921
NOL8	0.1387	5.1039E-02	1.45E-01	ENSG00000198000
NOL9	0.0051	9.4687E-01	9.73E-01	ENSG00000162408
NOLC1	0.1080	1.1746E-01	2.63E-01	ENSG00000166197
NOM1	0.0872	3.1217E-01	5.05E-01	ENSG00000146909
NOMO1	0.2652	2.9562E-04	2.70E-03	ENSG00000103512
NOMO2	0.1556	1.1940E-02	4.86E-02	ENSG00000185164
NOMO3	0.2141	7.4910E-04	5.68E-03	ENSG00000103226
NONO	-0.0886	2.9782E-02	9.69E-02	ENSG00000147140
NOP10	-0.3856	9.1525E-10	7.87E-08	ENSG00000182117
NOP14	-0.0470	5.2187E-01	6.99E-01	ENSG00000087269
NOP16	0.1884	9.4162E-03	4.06E-02	ENSG00000048162
NOP2	0.3842	7.1865E-04	5.49E-03	ENSG00000111641
NOP53	-0.0879	2.4477E-01	4.30E-01	ENSG00000105373
NOP53-AS1	0.0017	6.3208E-01		ENSG00000269656
NOP56	-0.1622	6.0009E-05	7.44E-04	ENSG00000101361
NOP58	-0.1233	3.1688E-02	1.01E-01	ENSG00000055044
NOP9	-0.3790	8.0349E-04	6.00E-03	ENSG00000196943
NOPCHAP1	-0.1930	6.6601E-03	3.12E-02	ENSG00000151131
NORAD	-0.1631	1.4444E-02	5.64E-02	ENSG00000260032
NOS1	-0.3333	2.2506E-02	7.88E-02	ENSG00000089250
NOS1AP	-0.4765	1.0410E-09	8.84E-08	ENSG00000198929
NOS2	0.1741	1.0312E-01	2.40E-01	ENSG00000007171
NOSIP	0.0524	2.2773E-01	4.10E-01	ENSG00000142546
NOSTRIN	0.1721	7.9733E-02	2.00E-01	ENSG00000163072
NOTCH1	-0.3389	1.8741E-03	1.16E-02	ENSG00000148400
NOTCH2	-0.2427	4.1736E-03	2.18E-02	ENSG00000134250
NOTCH2NLA	-0.3232	2.8669E-04	2.63E-03	ENSG00000264343
NOTCH2NLB	-0.2772	9.5698E-04	6.89E-03	ENSG00000286019
NOTCH2NLC	-0.0905	2.7449E-01	4.65E-01	ENSG00000286219
NOTCH2NLR	-0.2538	2.7306E-02	9.08E-02	ENSG00000286106
NOTCH3	-0.1442	2.4102E-02	8.28E-02	ENSG00000074181
NOTCH4	0.1767	5.2943E-02	1.49E-01	ENSG00000204301
NOTO	-0.0099	9.4864E-01		ENSG00000214513
NOTUM	0.1660	1.2515E-01	2.75E-01	ENSG00000185269
NOVA1	-0.2684	7.1401E-05	8.55E-04	ENSG00000139910

NOVA1-DT	-0.0245	7.8796E-01	8.86E-01	ENSG00000257842
NOVA2	-0.0555	5.1703E-01	6.95E-01	ENSG00000104967
NOX1	-0.1202	1.3533E-01	2.89E-01	ENSG00000007952
NOX3	0.0200	1.3653E-01	2.91E-01	ENSG00000074771
NOX4	-0.1427	1.6924E-01	3.37E-01	ENSG00000086991
NOX4P1	-0.0116	7.7607E-01		ENSG00000255532
NOX5	0.0163	3.3226E-01		ENSG00000255346
NOXA1	0.0282	7.6410E-01	8.72E-01	ENSG00000188747
NOXO1	0.0017	7.3881E-01		ENSG00000196408
NOXRED1	-0.0997	1.6849E-01	3.36E-01	ENSG00000165555
NPAP1	0.0068	7.3744E-01		ENSG00000185823
NPAS1	-0.0990	2.4124E-01	4.26E-01	ENSG00000130751
NPAS2	-0.1150	2.2527E-01	4.08E-01	ENSG00000170485
NPAS3	-0.0597	4.1057E-01	6.04E-01	ENSG00000151322
NPAS4	0.5602	1.1862E-03	8.12E-03	ENSG00000174576
NPAT	0.4032	1.7189E-04	1.74E-03	ENSG00000149308
NPBWR1	0.1217	2.0617E-01	3.85E-01	ENSG00000288611
NPBWR2	0.0059	1.3535E-01		ENSG00000125522
NPC1	0.1479	5.2102E-02	1.47E-01	ENSG00000141458
NPC1L1	0.1146	2.5361E-01	4.41E-01	ENSG00000015520
NPC2	-0.2863	3.3262E-05	4.58E-04	ENSG00000119655
NPDC1	-0.3156	1.5222E-05	2.47E-04	ENSG00000107281
NPEPL1	0.4246	2.3075E-05	3.44E-04	ENSG00000215440
NPEPPS	-0.1074	6.5348E-04	5.10E-03	ENSG00000141279
NPEPPSP1	0.0045	9.5015E-01	9.75E-01	ENSG00000274615
NPFF	-0.0131	8.6508E-01	9.31E-01	ENSG00000139574
NPFFR1	-0.0504	3.4144E-01	5.37E-01	ENSG00000148734
NPFFR2	-0.0706	4.7800E-01	6.64E-01	ENSG00000056291
NPHP1	-0.2411	1.6105E-04	1.65E-03	ENSG00000144061
NPHP3	0.0711	4.6967E-01	6.57E-01	ENSG00000113971
NPHP4	0.1558	7.7825E-02	1.97E-01	ENSG00000131697
NPHS1	0.1100	2.6261E-01	4.51E-01	ENSG00000161270
NPHS2	2.9995	8.0181E-05		ENSG00000116218
NPIPA1	0.0174	8.4305E-01	9.18E-01	ENSG00000183426
NPIPA2	0.1272	2.0610E-01	3.85E-01	ENSG00000254852
NPIPA3	0.1952	6.9452E-02	1.81E-01	ENSG00000224712
NPIPA5	0.0359	6.8842E-01	8.25E-01	ENSG00000183793
NPIPA7	0.0217	8.1251E-01	9.01E-01	ENSG00000214967
NPIPA8	0.1610	1.4926E-01	3.10E-01	ENSG00000214940
NPIPA9	0.0047	9.5670E-01	9.78E-01	ENSG00000233024
NPIPA9	-0.0007	9.9279E-01	9.96E-01	ENSG00000183889
NPIPB11	0.3759	3.6614E-03	1.97E-02	ENSG00000254206
NPIPB12	0.5224	6.0374E-06	1.17E-04	ENSG00000169203
NPIPB13	0.4301	3.5953E-05	4.88E-04	ENSG00000198064
NPIPB15	0.1239	1.9812E-01	3.75E-01	ENSG00000196436
NPIPB2	0.0439	5.4214E-01	7.15E-01	ENSG00000234719
NPIPB3	0.2876	6.8142E-03	3.17E-02	ENSG00000169246
NPIPB4	0.4771	8.5039E-05	9.82E-04	ENSG00000185864
NPIPB5	0.4073	1.5069E-04	1.56E-03	ENSG00000243716

NPIPB6	0.0335	7.2290E-01	8.47E-01	ENSG00000198156
NPIPB7	0.0389	5.5862E-01	7.29E-01	ENSG00000233232
NPIPB8	0.0974	3.3296E-01	5.28E-01	ENSG00000255524
NPIPB9	0.0330	7.1925E-01	8.44E-01	ENSG00000196993
NPIPP1	0.0406	6.7320E-01	8.13E-01	ENSG00000188599
NPL	0.0268	7.9064E-01	8.88E-01	ENSG00000135838
NPLOC4	-0.0330	4.7780E-01	6.64E-01	ENSG00000182446
NPM1	-0.3018	1.1663E-05	1.98E-04	ENSG00000181163
NPM1P30	-0.0016	7.1276E-01	8.40E-01	ENSG00000228219
NPM1P38	0.0112	4.8826E-01		ENSG00000219257
NPM1P39	-0.1803	1.0836E-01	2.49E-01	ENSG00000225159
NPM1P40	0.0163	4.9186E-01		ENSG00000236523
NPM1P48	-0.0088	8.4167E-01		ENSG00000231821
NPM2	-0.1575	1.2471E-01	2.74E-01	ENSG00000158806
NPM3	0.0509	5.5021E-01	7.22E-01	ENSG00000107833
NPNT	-0.0159	8.5621E-01	9.26E-01	ENSG00000168743
NPPC	0.2888	8.8611E-03	3.89E-02	ENSG00000163273
NPR1	0.0304	4.3222E-01		ENSG00000169418
NPR2	-0.1622	1.0628E-01	2.45E-01	ENSG00000159899
NPR3	-0.2198	7.1044E-02	1.84E-01	ENSG00000113389
NPRL2	0.2550	2.3517E-03	1.39E-02	ENSG00000114388
NPRL3	-0.2639	1.7562E-03	1.11E-02	ENSG00000103148
NPS	0.0092	4.5282E-01	6.42E-01	ENSG00000214285
NPSR1	0.0008	9.6504E-01		ENSG00000187258
NPSR1-AS1	-0.0066	9.7888E-01		ENSG00000197085
NPTN	-0.1119	5.3939E-02	1.51E-01	ENSG00000156642
NPTN-IT1	0.4004	2.2534E-03	1.35E-02	ENSG00000281183
NPTX2	-0.1300	6.7051E-02	1.77E-01	ENSG00000106236
NPTXR	-0.1231	1.2383E-01	2.73E-01	ENSG00000221890
NPW	0.1999	7.0959E-02	1.84E-01	ENSG00000183971
NPY	-0.0559	3.8934E-01	5.84E-01	ENSG00000122585
NPY1R	-0.1244	2.3388E-01	4.18E-01	ENSG00000164128
NPY2R	0.0779	3.8933E-02	1.19E-01	ENSG00000185149
NPY5R	0.0379	6.8015E-01	8.18E-01	ENSG00000164129
NPY6R	-0.0146	7.9423E-01		ENSG00000226306
NQO1	-0.0216	7.3924E-01	8.57E-01	ENSG00000181019
NQO2	0.1746	3.2199E-03	1.77E-02	ENSG00000124588
NR0B1	0.5983	6.1069E-04	4.83E-03	ENSG00000169297
NR0B2	-0.0083	8.9489E-01	9.48E-01	ENSG00000131910
NR1D1	-0.3584	5.0064E-05	6.46E-04	ENSG00000126368
NR1D2	-0.3827	1.8323E-10	1.96E-08	ENSG00000174738
NR1H2	0.0695	2.4891E-01	4.35E-01	ENSG00000131408
NR1H3	-0.3227	4.0613E-05	5.42E-04	ENSG00000025434
NR1H4	-0.0070	9.1143E-01		ENSG00000012504
NR1I2	-0.0108	4.2464E-01	6.16E-01	ENSG00000144852
NR1I3	-0.0528	4.3577E-01	6.27E-01	ENSG00000143257
NR2C1	0.2952	1.6733E-05	2.67E-04	ENSG00000120798
NR2C2	-0.0613	5.1573E-01	6.94E-01	ENSG00000177463
NR2C2AP	0.0306	6.9525E-01	8.29E-01	ENSG00000184162

NR2E1	-0.0320	5.4392E-01		ENSG00000112333
NR2E3	-0.0636	2.0618E-01	3.85E-01	ENSG00000278570
NR2F1	-0.2379	9.1724E-03	3.99E-02	ENSG00000175745
NR2F1-AS1	0.0569	4.5231E-01	6.42E-01	ENSG00000237187
NR2F2	0.1786	2.6472E-02	8.88E-02	ENSG00000185551
NR2F2-AS1	-0.0157	8.5399E-01	9.25E-01	ENSG00000247809
NR2F6	-0.1635	8.2603E-03	3.68E-02	ENSG00000160113
NR3C1	0.1562	5.3691E-02	1.51E-01	ENSG00000113580
NR3C1P1	-0.0464	6.0800E-01		ENSG00000276393
NR3C2	-0.4502	8.7938E-05	1.01E-03	ENSG00000151623
NR4A1	-0.5282	1.7763E-06	4.27E-05	ENSG00000123358
NR4A1AS	0.0303	8.2455E-02	2.05E-01	ENSG00000259884
NR4A2	0.0286	6.9892E-01	8.32E-01	ENSG00000153234
NR4A3	0.2055	8.1757E-02	2.04E-01	ENSG00000119508
NR5A2	0.1493	1.4431E-01	3.02E-01	ENSG00000116833
NR6A1	0.0684	4.5287E-01	6.42E-01	ENSG00000148200
NRAD1	-0.0400	2.7015E-01		ENSG00000233725
NRAP	0.0460	5.4412E-01		ENSG00000197893
NRARP	0.0758	3.6590E-01	5.62E-01	ENSG00000198435
NRAS	-0.0557	2.2355E-01	4.06E-01	ENSG00000213281
NRAV	-0.0423	5.5911E-01	7.29E-01	ENSG00000248008
NRBF2	0.0430	4.8649E-01	6.71E-01	ENSG00000148572
NRBP1	-0.0221	6.6824E-01	8.09E-01	ENSG00000115216
NRBP2	0.0700	4.0028E-01	5.94E-01	ENSG00000185189
NRCAM	-0.1284	9.9074E-03	4.22E-02	ENSG00000091129
NRDC	-0.0230	6.1960E-01	7.76E-01	ENSG00000078618
NRDE2	-0.0837	2.5585E-01	4.43E-01	ENSG00000119720
NREP	-0.0155	8.5738E-01	9.27E-01	ENSG00000134986
NRF1	0.0503	5.0904E-01	6.89E-01	ENSG00000106459
NRG1	0.0696	4.0005E-01	5.94E-01	ENSG00000157168
NRG1-IT1	-0.1727	1.2383E-01	2.73E-01	ENSG00000253974
NRG1-IT3	0.0062	3.9426E-01		ENSG00000254049
NRG2	-0.0542	5.3511E-01	7.09E-01	ENSG00000158458
NRG3	0.0238	8.0064E-01	8.93E-01	ENSG00000185737
NRG3-AS1	0.0236	4.1955E-01		ENSG00000225738
NRG4	0.0750	4.1944E-01	6.12E-01	ENSG00000169752
NRGN	-0.0530	5.2878E-01	7.04E-01	ENSG00000154146
NRIP1	-0.0051	9.3850E-01	9.69E-01	ENSG00000180530
NRIP3	-0.0398	5.5373E-01	7.25E-01	ENSG00000175352
NRIP3-DT	0.1839	1.0436E-01	2.42E-01	ENSG00000253973
NRK	-0.0241	5.1241E-01		ENSG00000123572
NRL	0.1420	6.7049E-02	1.77E-01	ENSG00000129535
NRM	0.0952	6.6493E-02	1.76E-01	ENSG00000137404
NRN1	0.1380	1.4422E-01	3.02E-01	ENSG00000124785
NRN1L	0.0288	3.3110E-01		ENSG00000188038
NRP2	-0.0032	9.6646E-01	9.83E-01	ENSG00000118257
NRSN1	0.2460	6.6881E-03	3.13E-02	ENSG00000152954
NRSN2	0.1024	7.0880E-02	1.84E-01	ENSG00000125841
NRSN2-AS1	-0.1762	2.0404E-02	7.31E-02	ENSG00000225377

NRTN	0.3129	1.5409E-02	5.92E-02	ENSG00000171119
NRXN1	-0.1535	1.5354E-02	5.90E-02	ENSG00000179915
NRXN1-DT	-0.0335	6.9293E-01	8.28E-01	ENSG00000231918
NRXN2	0.0295	7.3055E-01	8.52E-01	ENSG00000110076
NRXN3	-0.1246	2.1124E-01	3.91E-01	ENSG00000021645
NSA2	-0.0015	9.8054E-01	9.89E-01	ENSG00000164346
NSD1	-0.0641	2.4133E-01	4.26E-01	ENSG00000165671
NSD2	-0.0554	2.9410E-01	4.86E-01	ENSG00000109685
NSD3	-0.1419	4.1041E-03	2.16E-02	ENSG00000147548
NSDHL	-0.2089	6.2682E-03	2.98E-02	ENSG00000147383
NSF	0.1503	2.6562E-02	8.90E-02	ENSG00000073969
NSFL1C	-0.1251	1.0228E-03	7.23E-03	ENSG00000088833
NSG1	0.0644	4.7020E-01	6.58E-01	ENSG00000168824
NSG2	-0.0301	6.8426E-01	8.21E-01	ENSG00000170091
NSL1	-0.0503	2.9895E-01	4.91E-01	ENSG00000117697
NSMAF	0.0729	3.1823E-01	5.12E-01	ENSG00000035681
NSMCE1	-0.1989	1.9465E-02	7.05E-02	ENSG00000169189
NSMCE1-DT	-0.0683	5.7048E-01	7.38E-01	ENSG00000245888
NSMCE2	0.0076	9.4290E-01	9.72E-01	ENSG00000156831
NSMCE3	-0.1325	1.9718E-02	7.12E-02	ENSG00000185115
NSMCE4A	-0.0994	4.3854E-02	1.30E-01	ENSG00000107672
NSMF	0.0577	3.9874E-01	5.93E-01	ENSG00000165802
NSRP1	0.0228	7.2461E-01	8.48E-01	ENSG00000126653
NSRP1P1	0.2411	5.5359E-02	1.54E-01	ENSG00000235613
NSUN2	0.2568	1.1213E-02	4.63E-02	ENSG00000037474
NSUN3	0.1131	7.7567E-02	1.96E-01	ENSG00000178694
NSUN4	0.0504	4.8059E-01	6.66E-01	ENSG00000117481
NSUN5	0.1054	9.1262E-02	2.21E-01	ENSG00000130305
NSUN5P1	0.3344	3.0626E-08	1.47E-06	ENSG00000223705
NSUN6	0.3295	8.2413E-05	9.58E-04	ENSG00000241058
NSUN7	0.0120	8.8305E-01	9.41E-01	ENSG00000179299
NT5C	0.0816	2.0247E-01	3.80E-01	ENSG00000125458
NT5C1A	0.1432	1.7729E-01	3.48E-01	ENSG00000116981
NT5C1B	-0.0973	7.3811E-02		ENSG00000185013
NT5C2	0.2070	6.2662E-02	1.69E-01	ENSG00000076685
NT5C3A	-0.0146	7.5139E-01	8.64E-01	ENSG00000122643
NT5C3AP1	-0.0455	5.4307E-01	7.15E-01	ENSG00000213492
NT5C3B	0.0642	3.8272E-01	5.77E-01	ENSG00000141698
NT5DC1	-0.1880	1.0966E-02	4.56E-02	ENSG00000178425
NT5DC2	-0.2866	2.0023E-04	1.96E-03	ENSG00000168268
NT5DC3	-0.1194	2.1202E-01	3.92E-01	ENSG00000111696
NT5DC4	0.1635	1.1877E-01	2.65E-01	ENSG00000144130
NT5M	0.0852	2.3710E-01	4.21E-01	ENSG00000205309
NTAN1	-0.2177	1.1000E-04	1.21E-03	ENSG00000157045
NTAN1P2	0.0689	4.3200E-02		ENSG00000250569
NTAQ1	0.0758	2.8241E-01	4.74E-01	ENSG00000156795
NTF3	-0.1248	2.2635E-01	4.09E-01	ENSG00000185652
NTF4	-0.5791	2.5383E-03	1.48E-02	ENSG00000225950
NTHL1	-0.1054	9.4978E-02	2.27E-01	ENSG00000065057

NTM	0.4027	1.6468E-03	1.05E-02	ENSG00000182667
NTMT1	-0.0743	2.0343E-01	3.81E-01	ENSG00000148335
NTN1	-0.5057	2.1056E-04	2.03E-03	ENSG00000065320
NTN3	0.1372	1.7629E-01	3.47E-01	ENSG00000162068
NTN4	0.0821	3.9537E-01	5.90E-01	ENSG00000074527
NTN5	-0.1108	2.7895E-01	4.70E-01	ENSG00000142233
NTNG2	0.0386	6.4833E-01	7.96E-01	ENSG00000196358
NTPCR	-0.1727	1.5555E-03	1.01E-02	ENSG00000135778
NTRK1	-0.0525	4.4668E-01	6.37E-01	ENSG00000198400
NTRK3	0.2491	6.2947E-03	2.99E-02	ENSG00000140538
NTSR1	0.0004	6.4641E-01		ENSG00000101188
NTSR2	-0.0837	5.4771E-02		ENSG00000169006
NUAK1	-0.0408	6.2565E-01	7.81E-01	ENSG00000074590
NUAK2	-0.3348	3.7061E-04	3.26E-03	ENSG00000163545
NUB1	0.2801	2.4331E-06	5.59E-05	ENSG00000013374
NUBP1	0.0650	3.4437E-01	5.40E-01	ENSG00000103274
NUBP2	0.1792	4.0776E-03	2.14E-02	ENSG00000095906
NUBPL	0.1393	4.1014E-02	1.23E-01	ENSG00000151413
NUCB1	-0.1450	6.2109E-02	1.68E-01	ENSG00000104805
NUCB2	-0.2090	1.2524E-05	2.10E-04	ENSG00000070081
NUCKS1	-0.3362	1.4600E-04	1.52E-03	ENSG00000069275
NUDC	-0.1905	1.1810E-03	8.09E-03	ENSG00000090273
NUDCD1	0.1530	2.2803E-02	7.95E-02	ENSG00000120526
NUDCD2	-0.1854	2.8634E-03	1.63E-02	ENSG00000170584
NUDCD3	0.0555	3.1374E-01	5.07E-01	ENSG00000015676
NUDT1	-0.0308	6.1804E-01	7.75E-01	ENSG00000106268
NUDT10	0.0449	6.3159E-01	7.85E-01	ENSG00000122824
NUDT11	0.0286	7.6112E-01	8.70E-01	ENSG00000196368
NUDT12	0.2729	3.8807E-04	3.36E-03	ENSG00000112874
NUDT13	-0.0387	6.8604E-01	8.23E-01	ENSG00000166321
NUDT14	-0.1660	3.6725E-02	1.13E-01	ENSG00000183828
NUDT15	-0.0256	7.2166E-01	8.46E-01	ENSG00000136159
NUDT16	-0.1649	2.9958E-03	1.68E-02	ENSG00000198585
NUDT16L1	-0.2265	8.3590E-04	6.18E-03	ENSG00000168101
NUDT16L2P	0.1249	2.2715E-01	4.10E-01	ENSG00000246082
NUDT17	-0.0829	3.7367E-01	5.69E-01	ENSG00000186364
NUDT18	0.1079	2.3774E-01	4.22E-01	ENSG00000275074
NUDT19	-0.0525	4.6793E-01	6.56E-01	ENSG00000213965
NUDT2	-0.0623	2.6045E-01	4.49E-01	ENSG00000164978
NUDT21	-0.0948	1.3672E-01	2.91E-01	ENSG00000167005
NUDT22	-0.0163	8.0022E-01	8.93E-01	ENSG00000149761
NUDT3	-0.4867	1.0192E-08	5.76E-07	ENSG00000272325
NUDT4	-0.4246	4.7450E-08	2.12E-06	ENSG00000173598
NUDT4B	-0.4929	3.9110E-12	6.84E-10	ENSG00000177144
NUDT5	-0.3256	4.7267E-06	9.60E-05	ENSG00000165609
NUDT6	-0.0248	7.5921E-01	8.69E-01	ENSG00000170917
NUDT7	0.1846	4.5130E-02	1.33E-01	ENSG00000140876
NUDT8	-0.1032	2.0394E-01	3.82E-01	ENSG00000167799
NUDT9	-0.2452	2.1250E-05	3.21E-04	ENSG00000170502

NUF2	-0.2815	2.1243E-02	7.54E-02	ENSG00000143228
NUFIP1	-0.1396	3.2765E-02	1.04E-01	ENSG00000083635
NUFIP2	-0.0893	1.3437E-01	2.88E-01	ENSG00000108256
NUMA1	-0.0569	3.9679E-01	5.91E-01	ENSG00000137497
NUMB	0.0761	2.6543E-01	4.54E-01	ENSG00000133961
NUMBL	0.0365	5.3116E-01	7.06E-01	ENSG00000105245
NUP107	0.4275	9.1594E-08	3.63E-06	ENSG00000111581
NUP133	0.1782	3.5504E-03	1.92E-02	ENSG00000069248
NUP153	-0.2210	3.1725E-03	1.76E-02	ENSG00000124789
NUP155	0.2368	4.1666E-04	3.56E-03	ENSG00000113569
NUP160	0.5144	6.9578E-11	8.80E-09	ENSG00000030066
NUP188	0.0376	5.2220E-01	6.99E-01	ENSG00000095319
NUP205	0.0429	5.4302E-01	7.15E-01	ENSG00000155561
NUP210	-0.1006	1.1695E-01	2.62E-01	ENSG00000132182
NUP210L	-0.0128	8.5458E-01	9.25E-01	ENSG00000143552
NUP214	-0.0137	8.2507E-01	9.08E-01	ENSG00000126883
NUP35	0.0980	1.7880E-01	3.50E-01	ENSG00000163002
NUP37	0.0796	3.6663E-01	5.63E-01	ENSG00000075188
NUP42	0.1616	2.5549E-03	1.48E-02	ENSG00000136243
NUP43	0.1183	1.4751E-01	3.07E-01	ENSG00000120253
NUP50	-0.0002	9.6270E-01	9.81E-01	ENSG00000093000
NUP50-DT	-0.1208	1.6859E-01	3.36E-01	ENSG00000226328
NUP54	0.1034	4.6168E-02	1.35E-01	ENSG00000138750
NUP58	0.1086	1.3701E-01	2.91E-01	ENSG00000139496
NUP62	-0.0703	2.2935E-01	4.12E-01	ENSG00000213024
NUP62CL	-0.2573	5.5140E-03	2.70E-02	ENSG00000198088
NUP85	0.3289	6.2494E-09	3.91E-07	ENSG00000125450
NUP88	0.1030	2.1581E-01	3.97E-01	ENSG00000108559
NUP93	0.0999	1.4643E-01	3.05E-01	ENSG00000102900
NUP93-DT	0.1084	2.3096E-01	4.14E-01	ENSG00000261302
NUP98	-0.0866	1.9454E-01	3.71E-01	ENSG00000110713
NUPR1	-0.5326	5.8525E-04	4.68E-03	ENSG00000176046
NUS1	-0.1003	1.1341E-01	2.57E-01	ENSG00000153989
NUSAP1	0.1353	1.6163E-01	3.27E-01	ENSG00000137804
NUTF2	-0.3024	2.1088E-07	7.31E-06	ENSG00000102898
NUTF2P6	-0.0142	4.9247E-01		ENSG00000259710
NUTM1	-0.0101	7.4161E-01		ENSG00000184507
NUTM2A	0.0082	9.4062E-01	9.70E-01	ENSG00000184923
NUTM2A-AS1	-0.1106	2.1988E-02	7.74E-02	ENSG00000223482
NUTM2B	-0.0051	9.4711E-01	9.73E-01	ENSG00000188199
NUTM2B-AS1	-0.0568	2.4770E-01	4.34E-01	ENSG00000225484
NUTM2D	-0.1668	1.3307E-01	2.86E-01	ENSG00000214562
NUTM2E	-0.0483	5.8150E-01	7.47E-01	ENSG00000228570
NUTM2F	0.0792	1.2053E-02	4.90E-02	ENSG00000130950
NUTM2G	0.3283	2.7888E-02	9.22E-02	ENSG00000188152
NVL	0.0715	2.6901E-01	4.58E-01	ENSG00000143748
NWD1	0.0835	3.3781E-01	5.33E-01	ENSG00000188039
NWD2	0.0306	7.4582E-01	8.61E-01	ENSG00000174145
NXF1	-0.2260	9.7874E-04	6.99E-03	ENSG00000162231

NXF3	0.0074	7.3579E-01		ENSG00000147206
NXN	-0.2270	9.8303E-04	7.01E-03	ENSG00000167693
NXNL2	-0.1418	1.8688E-01	3.61E-01	ENSG00000130045
NXPE1	-0.0160	4.8316E-01		ENSG00000095110
NXPE2	-0.0049	7.1028E-01		ENSG00000204361
NXPE3	-0.0341	6.3368E-01	7.86E-01	ENSG00000144815
NXPH1	-0.5545	6.4085E-05	7.86E-04	ENSG00000122584
NXPH2	-0.0078	9.3771E-01	9.69E-01	ENSG00000144227
NXPH3	0.0046	9.6103E-01	9.80E-01	ENSG00000182575
NXT1	-0.2957	1.5016E-07	5.45E-06	ENSG00000132661
NXT2	-0.0780	3.1143E-01	5.04E-01	ENSG00000101888
NYAP1	0.0319	7.3919E-01	8.57E-01	ENSG00000166924
NYAP2	0.3613	5.0799E-03	2.53E-02	ENSG00000144460
NYNRIN	-0.2672	3.9081E-03	2.07E-02	ENSG00000205978
NYX	-0.0031	8.6902E-01		ENSG00000188937
OAF	0.0141	8.8266E-01	9.41E-01	ENSG00000184232
OARD1	0.0536	3.1069E-01	5.04E-01	ENSG00000124596
OAS1	-0.2515	5.1653E-02	1.46E-01	ENSG00000089127
OAS2	0.0049	8.0553E-01		ENSG00000111335
OAT	-0.1217	7.9621E-02	2.00E-01	ENSG00000065154
OAZ1	-0.2166	2.1356E-04	2.06E-03	ENSG00000104904
OAZ2	-0.0273	6.3509E-01	7.87E-01	ENSG00000180304
OAZ3	-0.1165	2.6186E-01	4.51E-01	ENSG00000143450
OBI1	0.4563	4.8940E-09	3.22E-07	ENSG00000152193
OBI1-AS1	0.0057	9.9554E-01		ENSG00000234377
OBSCN	-0.0275	7.6298E-01	8.71E-01	ENSG00000154358
OBSCN-AS1	0.0296	7.5704E-01	8.68E-01	ENSG00000162913
OBSL1	-0.0957	1.8326E-01	3.56E-01	ENSG00000124006
OCA2	-0.1967	7.7321E-02	1.96E-01	ENSG00000104044
OCEL1	-0.1432	8.2923E-02	2.06E-01	ENSG00000099330
OCIAD1	-0.1678	4.6050E-03	2.35E-02	ENSG00000109180
OCIAD1-AS1	-0.1253	1.8130E-01	3.53E-01	ENSG00000248256
OCIAD2	-0.1816	7.4597E-03	3.40E-02	ENSG00000145247
OCLN	-0.0624	3.9060E-01	5.85E-01	ENSG00000197822
OCM	0.3211	3.1129E-02	1.00E-01	ENSG00000122543
OCM2	-0.0110	9.4071E-01		ENSG00000135175
OCRL	-0.0699	3.2984E-01	5.24E-01	ENSG00000122126
OCSTAMP	-0.0589	4.6744E-01	6.55E-01	ENSG00000149635
ODAD1	-0.0760	4.1408E-01	6.07E-01	ENSG00000105479
ODAD2	-0.0387	6.8717E-01	8.24E-01	ENSG00000169126
ODAD3	0.4214	9.6762E-04	6.94E-03	ENSG00000198003
ODAD4	-0.1202	1.4109E-01	2.98E-01	ENSG00000204815
ODAM	-0.1934	9.5387E-02	2.27E-01	ENSG00000109205
ODAPH	-0.0952	3.1673E-01	5.10E-01	ENSG00000174792
ODC1	0.0652	3.5512E-01	5.50E-01	ENSG00000115758
ODF1	-0.0420	5.0055E-01		ENSG00000155087
ODF2	0.4542	1.1951E-07	4.48E-06	ENSG00000136811
ODF2-AS1	0.0172	4.7520E-01		ENSG00000225951
ODF2L	-0.0023	9.6710E-01	9.83E-01	ENSG00000122417

ODF4	0.0250	8.8651E-02		ENSG00000184650
ODR4	0.0376	5.2463E-01	7.01E-01	ENSG00000157181
OFD1	-0.0383	5.5672E-01	7.27E-01	ENSG00000046651
OFD1P17	0.3012	4.0503E-02	1.22E-01	ENSG00000228212
OFD1P1Y	0.0035	7.1475E-01		ENSG00000226011
OFD1P2Y	-0.0022	8.4291E-01		ENSG00000226611
OFD1P4Y	0.0152	4.6229E-01		ENSG00000229406
OFD1P5Y	-0.0154	9.6546E-01		ENSG00000240438
OFD1P6Y	0.0057	7.1252E-01		ENSG00000242153
OFD1P7Y	0.0246	4.5784E-01		ENSG00000238088
OGA	0.0154	7.7574E-01	8.79E-01	ENSG00000198408
OGDH	-0.2126	9.9101E-04	7.06E-03	ENSG00000105953
OGDHL	-0.2258	4.2391E-02	1.27E-01	ENSG00000197444
OGFOD1	0.3235	2.8205E-05	4.04E-04	ENSG00000087263
OGFOD2	0.1258	1.2726E-01	2.78E-01	ENSG00000111325
OGFOD3	-0.1235	7.4045E-02	1.90E-01	ENSG00000181396
OGFR	0.1039	1.6312E-01	3.29E-01	ENSG00000060491
OGFRL1	-0.0369	4.9406E-01	6.78E-01	ENSG00000119900
OGG1	0.0041	9.5592E-01	9.77E-01	ENSG00000114026
OGT	0.1786	2.1077E-02	7.49E-02	ENSG00000147162
OIP5	-0.3918	9.1442E-03	3.98E-02	ENSG00000104147
OIP5-AS1	-0.1360	2.7807E-02	9.20E-02	ENSG00000247556
OLA1	0.0087	8.2184E-01	9.06E-01	ENSG00000138430
OLA1P2	-0.0039	9.7998E-01		ENSG00000213671
OLAH	0.0923	1.7024E-01	3.38E-01	ENSG00000152463
OLFM1	-0.1480	1.8934E-03	1.17E-02	ENSG00000130558
OLFM2	-0.2207	1.3651E-03	9.11E-03	ENSG00000105088
OLFM3	0.3054	7.6257E-05	9.02E-04	ENSG00000118733
OLFM4	0.2787	3.6000E-02	1.12E-01	ENSG00000102837
OLFML1	-0.0211	6.9503E-01	8.29E-01	ENSG00000183801
OLFML2A	-0.1398	1.6984E-01	3.38E-01	ENSG00000185585
OLFML3	0.1335	1.8652E-01	3.60E-01	ENSG00000116774
OLIG1	0.0785	3.2151E-01	5.15E-01	ENSG00000184221
OLIG2	0.0412	4.3113E-01		ENSG00000205927
OLIG3	-0.0038	7.1315E-01		ENSG00000177468
OMA1	-0.5822	1.2278E-05	2.06E-04	ENSG00000162600
OMD	-0.1353	2.4599E-02	8.42E-02	ENSG00000127083
OMG	-0.2289	1.7468E-02	6.50E-02	ENSG00000126861
OOSP3	-0.0263	4.1144E-01		ENSG00000285231
OPA1	-0.0080	8.7138E-01	9.35E-01	ENSG00000198836
OPA1-AS1	0.0459	3.7117E-01	5.67E-01	ENSG00000224855
OPA3	0.1045	8.3467E-02	2.07E-01	ENSG00000125741
OPCML	0.2881	3.2583E-03	1.79E-02	ENSG00000183715
OPCML-IT1	0.0129	4.2261E-01		ENSG00000254896
OPHN1	-0.0505	5.2644E-01	7.02E-01	ENSG00000079482
OPLAH	-0.0038	9.6288E-01	9.81E-01	ENSG00000178814
OPRD1	-0.0196	7.1365E-01	8.40E-01	ENSG00000116329
OPRK1	0.2548	2.9284E-02	9.58E-02	ENSG00000082556
OPRL1	-0.3252	1.4950E-02	5.79E-02	ENSG00000125510

OPRM1	-0.0918	3.2183E-01	5.16E-01	ENSG00000112038
OPTN	-0.0029	9.2200E-01	9.61E-01	ENSG00000123240
OR10A2	-0.0208	7.9283E-01		ENSG00000170790
OR10G3	0.0069	5.3115E-01		ENSG00000169208
OR11G2	-0.0018	8.9728E-01		ENSG00000196832
OR11H7	-0.0081	9.5737E-01		ENSG00000258806
OR11N1P	-0.0014	9.9166E-01		ENSG00000185903
OR11Q1P	0.0017	7.3527E-01		ENSG00000237650
OR13A1	-0.0127	8.6630E-01		ENSG00000256574
OR13C5	-0.0393	3.5670E-01		ENSG00000277556
OR13J1	-0.0055	8.8814E-01		ENSG00000168828
OR1D2	-0.0244	3.8186E-01		ENSG00000184166
OR1F1	-0.0324	8.1765E-01		ENSG00000168124
OR1I1	-0.0047	8.5020E-01		ENSG00000094661
OR1L8	0.0059	8.0695E-01		ENSG00000171496
OR2A1	0.0374	3.0723E-01		ENSG00000221970
OR2A20P	0.0930	2.5609E-01	4.44E-01	ENSG00000170356
OR2A4	-0.0993	2.4508E-01	4.31E-01	ENSG00000180658
OR2A42	0.0593	1.7916E-01		ENSG00000212807
OR2A5	-0.0305	8.5480E-01		ENSG00000221836
OR2A7	-0.0826	4.0102E-01	5.95E-01	ENSG00000243896
OR2AG2	0.0037	3.8720E-01		ENSG00000188124
OR2AK2	-0.4379	1.2405E-02	5.01E-02	ENSG00000187080
OR2B4P	0.0219	4.3470E-01		ENSG00000197171
OR2B6	-0.0283	1.3756E-01		ENSG00000124657
OR2C1	-0.0098	6.7011E-01		ENSG00000168158
OR2D3	0.0016	8.6681E-01		ENSG00000178358
OR2F1	-0.0135	8.4223E-01		ENSG00000213215
OR2H2	-0.0007	9.1911E-01		ENSG00000204657
OR2I1P	0.0488	1.9460E-01	3.71E-01	ENSG00000237988
OR2K2	-0.0135	8.7157E-01		ENSG00000171133
OR2L1P	0.0333	1.5065E-01		ENSG00000224227
OR2L2	0.0180	8.3568E-01	9.14E-01	ENSG00000203663
OR2L3	0.1080	1.6278E-01	3.29E-01	ENSG00000198128
OR2L8	0.0070	7.4867E-01		ENSG00000279263
OR2T32P	0.0051	8.7391E-01		ENSG00000197067
OR2T33	0.0009	9.9631E-01		ENSG00000177212
OR2T8	0.0014	9.7222E-01	9.86E-01	ENSG00000177462
OR2W3	0.1316	1.4692E-01	3.06E-01	ENSG00000238243
OR2W6P	-0.0302	2.4763E-01		ENSG00000168126
OR4D1	0.0589	4.1782E-01	6.10E-01	ENSG00000141194
OR4F5	0.0191	2.7771E-01		ENSG00000186092
OR4G3P	0.0208	2.2067E-01		ENSG00000282137
OR4G6P	0.0247	6.3438E-02		ENSG00000275771
OR51A9P	-0.0080	8.9300E-01		ENSG00000180723
OR51B5	0.0076	5.3047E-01		ENSG00000167355
OR51E1	-0.1188	8.5038E-02	2.10E-01	ENSG00000180785
OR51E2	0.0335	3.8111E-01	5.76E-01	ENSG00000167332
OR51F5P	-0.0192	4.2264E-01		ENSG00000272634

OR51G2	0.0072	7.7428E-01		ENSG00000176893
OR51H2P	-0.0020	6.9242E-01		ENSG00000198217
OR51L1	-0.0003	9.3712E-01		ENSG00000176798
OR51M1	0.0345	2.1509E-01		ENSG00000184698
OR52E4	0.0045	6.2975E-01		ENSG00000180974
OR52K3P	0.1101	1.9679E-01	3.73E-01	ENSG00000225101
OR52N2	0.0137	4.3540E-01		ENSG00000180988
OR52T1P	-0.0292	2.2193E-01		ENSG00000233646
OR56A1	-0.0133	7.6432E-01		ENSG00000180934
OR56A3	0.0130	6.3917E-01		ENSG00000184478
OR56A5	-0.0066	7.0472E-01		ENSG00000188691
OR56A7P	-0.0991	1.5672E-02		ENSG00000255481
OR5BA1P	-0.0074	8.5336E-01		ENSG00000255303
OR5BH1P	-0.0022	7.3610E-01		ENSG00000242610
OR6V1	0.0041	8.5243E-01		ENSG00000225781
OR7A17	-0.0068	8.4369E-01		ENSG00000185385
OR7C1	-0.0571	3.4402E-01		ENSG00000127530
OR7D2	-0.0300	2.1748E-01		ENSG00000188000
OR7E121P	-0.2264	3.0233E-02	9.80E-02	ENSG00000244222
OR7E122P	0.0147	6.4293E-01		ENSG00000215160
OR7E125P	-0.0850	2.5436E-01	4.42E-01	ENSG00000177306
OR7E128P	-0.0071	9.9790E-01		ENSG00000228915
OR7E12P	0.0535	5.4769E-01	7.19E-01	ENSG00000189398
OR7E145P	0.0000	6.1124E-01		ENSG00000235855
OR7E14P	-0.0937	2.5441E-01	4.42E-01	ENSG00000184669
OR7E154P	-0.0846	2.5429E-01	4.42E-01	ENSG00000254715
OR7E157P	0.0184	3.4567E-01		ENSG00000233176
OR7E162P	0.0067	4.0517E-01		ENSG00000234386
OR7E22P	0.1469	1.5102E-01	3.12E-01	ENSG00000179799
OR7E25P	0.0039	8.2601E-01		ENSG00000187847
OR7E28P	0.0599	2.3756E-01	4.22E-01	ENSG00000251491
OR7E36P	0.0120	6.9473E-01		ENSG00000205240
OR7E38P	0.0122	3.8831E-01		ENSG00000183444
OR7E4P	-0.0499	2.6123E-01		ENSG00000255261
OR7E7P	-0.0348	6.9960E-01	8.32E-01	ENSG00000238228
OR7E83P	0.0049	8.2779E-01		ENSG00000249866
OR7E96P	0.0090	5.6961E-01		ENSG00000227013
OR7E99P	0.0078	6.1211E-01		ENSG00000250710
OR8A1	0.1299	4.6560E-02	1.36E-01	ENSG00000196119
OR8B5P	0.0021	5.7395E-01		ENSG00000255030
OR8D1	0.0115	4.6801E-01		ENSG00000196341
OR8G1	0.0193	3.0559E-01		ENSG00000197849
OR8G3P	0.0196	2.7505E-01	4.66E-01	ENSG00000255425
OR8G5	0.1087	2.2894E-01	4.12E-01	ENSG00000255298
OR8T1P	-0.0321	3.6859E-01		ENSG00000226413
OR9H1P	-0.0105	6.4901E-01		ENSG00000228336
OR9K2	0.0228	1.7418E-01		ENSG00000170605
OR9R1P	0.0543	1.1979E-02		ENSG00000224622
ORAI1	-0.1004	1.0009E-01	2.35E-01	ENSG00000276045

ORAI2	0.0478	4.3570E-01	6.27E-01	ENSG00000160991
ORAI3	-0.2456	2.4419E-03	1.43E-02	ENSG00000175938
ORC1	-0.1697	1.2786E-01	2.79E-01	ENSG00000085840
ORC2	0.5797	1.3562E-06	3.45E-05	ENSG00000115942
ORC3	-0.0185	7.4067E-01	8.57E-01	ENSG00000135336
ORC4	-0.0287	6.2009E-01	7.76E-01	ENSG00000115947
ORC5	0.1415	4.8662E-02	1.40E-01	ENSG00000164815
ORC6	0.1118	1.9893E-01	3.76E-01	ENSG00000091651
ORM2	0.0018	9.9979E-01		ENSG00000228278
ORMDL1	0.1218	3.8180E-02	1.17E-01	ENSG00000128699
ORMDL2	-0.0695	3.1702E-01	5.11E-01	ENSG00000123353
ORMDL3	-0.1022	1.1651E-01	2.62E-01	ENSG00000172057
OS9	0.0904	3.0605E-01	4.99E-01	ENSG00000135506
OSBP	-0.0122	8.0573E-01	8.96E-01	ENSG00000110048
OSBPL10	0.2381	2.4235E-03	1.43E-02	ENSG00000144645
OSBPL10-AS1	0.0022	8.7054E-01	9.34E-01	ENSG00000232490
OSBPL11	-0.1172	1.5441E-01	3.17E-01	ENSG00000144909
OSBPL1A	0.0010	9.8389E-01	9.91E-01	ENSG00000141447
OSBPL2	0.0861	5.3878E-02	1.51E-01	ENSG00000130703
OSBPL3	-0.0365	6.9845E-01	8.31E-01	ENSG00000070882
OSBPL5	0.1384	9.3641E-02	2.25E-01	ENSG00000021762
OSBPL6	0.0643	3.5627E-01	5.52E-01	ENSG00000079156
OSBPL7	0.0645	4.1001E-01	6.03E-01	ENSG00000006025
OSBPL8	-0.0698	2.4519E-01	4.31E-01	ENSG00000091039
OSBPL9	0.0030	9.9400E-01	9.97E-01	ENSG00000117859
OSCAR	-0.1341	1.1392E-01	2.58E-01	ENSG00000170909
OSCP1	-0.1710	1.1945E-02	4.86E-02	ENSG00000116885
OSER1	-0.1296	1.4095E-02	5.54E-02	ENSG00000132823
OSER1-DT	0.0628	3.1103E-01	5.04E-01	ENSG00000223891
OSGEP	-0.0518	2.8639E-01	4.77E-01	ENSG00000092094
OSGEPL1	0.1686	8.9550E-02	2.18E-01	ENSG00000128694
OSGIN1	0.2042	2.7527E-02	9.13E-02	ENSG00000140961
OSGIN2	-0.1507	2.8791E-02	9.45E-02	ENSG00000164823
OSM	-0.0426	6.5099E-01	7.98E-01	ENSG00000099985
OSMR	-0.2444	5.1633E-02	1.46E-01	ENSG00000145623
OSR1	-0.0133	6.2431E-01		ENSG00000143867
OSR2	0.3623	1.9034E-02	6.94E-02	ENSG00000164920
OST4	-0.5541	8.3700E-14	2.25E-11	ENSG00000228474
OSTC	-0.2076	1.3354E-02	5.30E-02	ENSG00000198856
OSTCP2	-0.0179	8.4115E-01		ENSG00000225294
OSTCP3	-0.0090	6.4116E-01		ENSG00000269608
OSTF1	-0.0613	3.3345E-01	5.28E-01	ENSG00000134996
OSTM1	0.2348	8.6588E-03	3.82E-02	ENSG00000081087
OSTN	0.0379	4.5362E-02		ENSG00000188729
OTC	0.1045	2.0217E-01	3.80E-01	ENSG00000036473
OTOA	-0.0039	9.4327E-01	9.72E-01	ENSG00000155719
OTOF	0.1673	1.0881E-01	2.49E-01	ENSG00000115155
OTOG	0.0389	5.6138E-01	7.31E-01	ENSG00000188162
OTOGL	-0.0007	9.8100E-01	9.90E-01	ENSG00000165899

OTOL1	0.0062	8.1319E-01	9.01E-01	ENSG00000182447
OTOP2	0.0233	7.2580E-02		ENSG00000183034
OTOP3	0.0638	5.2633E-01	7.02E-01	ENSG00000182938
OTOR	0.3632	1.4410E-02	5.63E-02	ENSG00000125879
OTOS	-0.0166	8.2544E-01		ENSG00000178602
OTP	-0.0228	7.9216E-01	8.88E-01	ENSG00000171540
OTUB1	-0.0935	6.4785E-02	1.73E-01	ENSG00000167770
OTUB2	-0.0301	7.3697E-01	8.56E-01	ENSG00000089723
OTUD1	0.0766	3.4721E-01	5.42E-01	ENSG00000165312
OTUD3	-0.0487	5.5465E-01	7.26E-01	ENSG00000169914
OTUD4	-0.0967	1.3635E-01	2.90E-01	ENSG00000164164
OTUD5	-0.0539	2.3193E-01	4.15E-01	ENSG00000068308
OTUD6A	0.0098	4.7406E-01		ENSG00000189401
OTUD6B	0.1297	8.6117E-02	2.12E-01	ENSG00000155100
OTUD6B-AS1	0.0888	2.3996E-02	8.26E-02	ENSG00000253738
OTUD7A	0.3149	6.9869E-03	3.23E-02	ENSG00000169918
OTUD7B	0.3930	7.0541E-04	5.41E-03	ENSG00000264522
OTULIN	-0.1504	4.2465E-02	1.27E-01	ENSG00000154124
OTULIN-DT	0.2409	5.6762E-02	1.57E-01	ENSG00000261360
OTULINL	0.1805	6.9141E-02	1.81E-01	ENSG00000145569
OTX1	0.0246	7.9253E-01	8.89E-01	ENSG00000115507
OTX2	-0.0724	3.3636E-01	5.32E-01	ENSG00000165588
OTX2-AS1	-0.3747	7.2672E-04	5.54E-03	ENSG00000248550
OVCH1	0.0241	4.9984E-01		ENSG00000187950
OVCH1-AS1	0.0213	8.1589E-01	9.03E-01	ENSG00000257599
OVGP1	0.3777	1.0627E-02	4.44E-02	ENSG00000085465
OVOL1	0.0831	3.9766E-01	5.92E-01	ENSG00000172818
OVOL1-AS1	0.0606	4.7534E-01	6.62E-01	ENSG00000255120
OVOL2	-0.0136	8.6525E-01	9.31E-01	ENSG00000125850
OVOL3	0.1646	1.2817E-01	2.79E-01	ENSG00000105261
OVOS2	-0.0844	3.5059E-01	5.46E-01	ENSG00000177359
OXA1L	0.1072	9.2453E-02	2.23E-01	ENSG00000155463
OXCT1	-0.1811	1.7748E-02	6.58E-02	ENSG00000083720
OXCT1-AS1	0.4563	2.5599E-03	1.49E-02	ENSG00000248668
OXCT2	0.1597	1.2105E-01	2.69E-01	ENSG00000198754
OXCT2P1	0.0624	4.8731E-01	6.71E-01	ENSG00000237624
OXER1	0.2671	4.4822E-02	1.32E-01	ENSG00000162881
OXGR1	-0.0472	7.8501E-01	8.85E-01	ENSG00000165621
OXLD1	0.0633	2.8250E-01	4.74E-01	ENSG00000204237
OXNAD1	0.2458	3.7382E-03	2.00E-02	ENSG00000154814
OXR1	0.0630	3.3186E-01	5.27E-01	ENSG00000164830
OXSM	0.4222	3.3549E-06	7.25E-05	ENSG00000151093
OXSR1	-0.1438	5.1548E-03	2.56E-02	ENSG00000172939
OXT	0.0280	6.0769E-01	7.67E-01	ENSG00000101405
OXTR	-0.2204	4.2888E-02	1.28E-01	ENSG00000180914
P2RX1	0.0377	1.5140E-01	3.12E-01	ENSG00000108405
P2RX3	0.5912	5.2422E-03	2.60E-02	ENSG00000109991
P2RX4	-0.1962	7.0400E-03	3.25E-02	ENSG00000135124
P2RX5	-0.0272	7.5082E-01	8.64E-01	ENSG00000083454

P2RX5-TAX1BP3	-0.0245	8.0289E-01	8.95E-01	ENSG00000257950
P2RX6	-0.1848	9.4388E-02	2.26E-01	ENSG00000099957
P2RX7	0.0855	3.8981E-01	5.84E-01	ENSG00000089041
P2RY1	-0.5197	5.0045E-05	6.46E-04	ENSG00000169860
P2RY11	-0.0175	9.0721E-01	9.53E-01	ENSG00000244165
P2RY12	-0.0654	6.5247E-01	7.99E-01	ENSG00000169313
P2RY13	-0.0192	9.3320E-01	9.67E-01	ENSG00000181631
P2RY14	0.0409	5.0518E-01	6.86E-01	ENSG00000174944
P2RY2	0.0361	7.0652E-01	8.36E-01	ENSG00000175591
P2RY6	0.1177	1.4484E-01	3.03E-01	ENSG00000171631
P2RY8	0.0264	2.8119E-01		ENSG00000182162
P3H1	0.3698	1.4607E-10	1.64E-08	ENSG00000117385
P3H2	-0.1387	1.2966E-01	2.81E-01	ENSG00000090530
P3H2-AS1	-0.0284	7.6501E-01	8.72E-01	ENSG00000225764
P3H3	-0.0535	4.1084E-01	6.04E-01	ENSG00000110811
P3H4	-0.0657	3.0312E-01	4.95E-01	ENSG00000141696
P4HA1	0.1438	1.7478E-01	3.45E-01	ENSG00000122884
P4HA2	-0.2157	7.5037E-03	3.42E-02	ENSG00000072682
P4HA2-AS1	-0.0172	6.9686E-01		ENSG00000237714
P4HA3	-0.1234	2.3639E-01	4.21E-01	ENSG00000149380
P4HB	0.2739	5.9252E-05	7.37E-04	ENSG00000185624
P4HTM	-0.0806	1.7363E-01	3.43E-01	ENSG00000178467
PA2G4	-0.0172	6.8737E-01	8.24E-01	ENSG00000170515
PAAF1	0.3343	2.8482E-06	6.36E-05	ENSG00000175575
PABIR1	0.0180	7.7357E-01	8.77E-01	ENSG00000187866
PABIR2	0.3046	9.7288E-05	1.09E-03	ENSG00000156504
PABIR3	-0.0126	8.6748E-01	9.33E-01	ENSG00000156500
PABPC1	-0.1648	7.9033E-02	1.99E-01	ENSG00000070756
PABPC1L	-0.4501	3.9426E-06	8.27E-05	ENSG00000101104
PABPC1L2A	-0.0730	4.2293E-01	6.15E-01	ENSG00000186288
PABPC1L2B	0.1223	2.3694E-01	4.21E-01	ENSG00000184388
PABPC1L2B-AS1	0.1109	2.7166E-01	4.62E-01	ENSG00000226725
PABPC4	-0.2190	2.7356E-02	9.09E-02	ENSG00000090621
PABPC4L	-0.3808	2.0390E-02	7.31E-02	ENSG00000254535
PABPC5	-0.0281	7.6146E-01	8.70E-01	ENSG00000174740
PABPC5-AS1	-0.1347	1.7814E-01	3.49E-01	ENSG00000234161
PABPN1	0.1287	6.0010E-02	1.63E-01	ENSG00000100836
PABPN1L	0.0563	5.3642E-01	7.10E-01	ENSG00000205022
PACC1	-0.0663	2.5539E-01	4.43E-01	ENSG00000065600
PACRG	0.0245	6.9715E-01	8.31E-01	ENSG00000112530
PACRG-AS1	0.0755	2.0786E-01	3.87E-01	ENSG00000281692
PACRG-AS3	-0.1763	4.0772E-01	6.01E-01	ENSG00000225683
PACRGL	0.1576	2.8271E-02	9.32E-02	ENSG00000163138
PACS1	0.0619	4.4345E-01	6.34E-01	ENSG00000175115
PACS2	-0.0658	2.5635E-01	4.44E-01	ENSG00000179364
PACSIN1	0.0858	2.8525E-01	4.76E-01	ENSG00000124507
PACSIN2	0.0968	2.0780E-01	3.87E-01	ENSG00000100266
PACSIN3	-0.1032	1.9460E-01	3.71E-01	ENSG00000165912
PADI1	0.0145	5.3680E-01		ENSG00000142623

PADI2	0.2722	3.0755E-02	9.93E-02	ENSG00000117115
PADI4	-0.0010	8.0905E-01		ENSG00000159339
PAEP	0.0326	1.1835E-01		ENSG00000122133
PAF1	-0.0793	2.3416E-01	4.18E-01	ENSG00000006712
PAFAH1B1	-0.2681	6.8251E-05	8.23E-04	ENSG00000007168
PAFAH1B2	-0.2414	1.0772E-03	7.52E-03	ENSG00000168092
PAFAH1B2P2	0.0537	5.4098E-01	7.14E-01	ENSG00000257501
PAFAH1B3	-0.0120	8.2461E-01	9.08E-01	ENSG00000079462
PAFAH2	0.2172	1.3277E-02	5.28E-02	ENSG00000158006
PAG1	0.0119	8.7732E-01	9.38E-01	ENSG00000076641
PAGE2B	-0.0751	1.2547E-01		ENSG00000238269
PAGE4	0.0897	4.5166E-02		ENSG00000101951
PAGR1	-0.0129	8.5458E-01	9.25E-01	ENSG00000280789
PAH	0.3677	8.5921E-03	3.79E-02	ENSG00000171759
PAICS	-0.1527	9.5705E-03	4.11E-02	ENSG00000128050
PAIP1	-0.0803	1.9335E-01	3.69E-01	ENSG00000172239
PAIP1P1	0.0098	8.3274E-01		ENSG00000233892
PAIP2	-0.1610	5.7231E-03	2.78E-02	ENSG00000120727
PAIP2B	0.0338	6.2423E-01	7.80E-01	ENSG00000124374
PAK1	-0.1583	1.6601E-03	1.06E-02	ENSG00000149269
PAK1IP1	0.3078	3.7061E-04	3.26E-03	ENSG00000111845
PAK2	-0.2296	4.7181E-04	3.92E-03	ENSG00000180370
PAK3	-0.0562	4.3753E-01	6.28E-01	ENSG00000077264
PAK4	-0.1325	9.5922E-02	2.28E-01	ENSG00000130669
PAK5	-0.1517	1.2150E-01	2.69E-01	ENSG00000101349
PAK6	-0.0104	8.6416E-01	9.31E-01	ENSG00000137843
PAK6-AS1	-0.0734	4.0481E-01	5.99E-01	ENSG00000176753
PALB2	0.2771	5.3007E-04	4.31E-03	ENSG00000083093
PALD1	-0.3306	2.9888E-03	1.68E-02	ENSG00000107719
PALLD	-0.1250	1.3000E-01	2.82E-01	ENSG00000129116
PALM	-0.2590	1.2414E-02	5.01E-02	ENSG00000099864
PALM2AKAP2	-0.3228	6.9571E-06	1.31E-04	ENSG00000157654
PALM3	-0.1091	2.8871E-01	4.80E-01	ENSG00000187867
PALMD	-0.3041	3.9781E-03	2.10E-02	ENSG00000099260
PALS1	-0.2498	2.9652E-03	1.67E-02	ENSG00000072415
PALS2	-0.2817	4.6677E-04	3.88E-03	ENSG00000105926
PAM	0.0169	8.2524E-01	9.08E-01	ENSG00000145730
PAM16	-0.1011	3.0302E-01	4.95E-01	ENSG00000217930
PAMR1	-0.1852	7.0135E-02	1.83E-01	ENSG00000149090
PAN2	0.0068	9.1981E-01	9.60E-01	ENSG00000135473
PAN3	-0.1455	1.1256E-01	2.55E-01	ENSG00000152520
PANCR	-0.2833	1.0113E-02	4.28E-02	ENSG00000250103
PANK1	-0.0590	5.1161E-01	6.92E-01	ENSG00000152782
PANK1-AS1	-0.0883	1.5312E-01	3.15E-01	ENSG00000232936
PANK2	-0.4035	1.2437E-09	1.03E-07	ENSG00000125779
PANK3	-0.1008	1.1979E-01	2.67E-01	ENSG00000120137
PANK4	0.1126	1.4145E-01	2.98E-01	ENSG00000157881
PANO1	0.0715	4.4113E-01	6.32E-01	ENSG00000288675
PANTR1	-0.5050	4.1608E-03	2.18E-02	ENSG00000233639

PANX1	-0.0371	6.2147E-01	7.77E-01	ENSG00000110218
PANX2	0.0677	4.7692E-01	6.64E-01	ENSG00000073150
PANX3	-0.0117	8.2464E-01		ENSG00000154143
PAOX	-0.0838	3.9773E-01	5.92E-01	ENSG00000148832
PAPLN	-0.1963	9.1614E-02	2.21E-01	ENSG00000100767
PAPOLA	-0.1753	1.2061E-03	8.23E-03	ENSG00000090060
PAPOLB	0.0074	6.9274E-01		ENSG00000218823
PAPOLG	0.2833	3.5947E-04	3.18E-03	ENSG00000115421
PAPPA	-0.0309	7.4237E-01	8.59E-01	ENSG00000182752
PAPPA2	-0.3155	2.0174E-02	7.25E-02	ENSG00000116183
PAPSS1	0.0229	6.7399E-01	8.13E-01	ENSG00000138801
PAPSS2	0.1353	9.4676E-02	2.26E-01	ENSG00000198682
PAQR3	0.1345	9.8082E-02	2.32E-01	ENSG00000163291
PAQR4	-0.0497	5.5637E-01	7.27E-01	ENSG00000162073
PAQR5	-0.3838	1.9662E-02	7.11E-02	ENSG00000137819
PAQR6	0.0065	9.4540E-01	9.73E-01	ENSG00000160781
PAQR7	-0.0668	4.9617E-01	6.79E-01	ENSG00000182749
PAQR8	-0.5414	4.6844E-07	1.41E-05	ENSG00000170915
PAQR9	0.0571	4.9812E-01	6.81E-01	ENSG00000188582
PAQR9-AS1	0.1884	1.0211E-01	2.38E-01	ENSG00000241570
PARAIL	-0.0681	3.5979E-01	5.56E-01	ENSG00000251136
PARD3	-0.1063	6.9382E-02	1.81E-01	ENSG00000148498
PARD3-DT	-0.0512	9.5074E-01	9.75E-01	ENSG00000226386
PARD3B	-0.3945	1.1325E-05	1.95E-04	ENSG00000116117
PARD6A	0.1719	3.2404E-02	1.03E-01	ENSG00000102981
PARD6B	-0.0681	3.9190E-01	5.86E-01	ENSG00000124171
PARD6G	-0.0802	2.4751E-01	4.34E-01	ENSG00000178184
PARD6G-AS1	0.4321	1.4229E-04	1.49E-03	ENSG00000267270
PARG	0.0809	2.3322E-01	4.17E-01	ENSG00000227345
PARK7	-0.1994	7.8529E-06	1.45E-04	ENSG00000116288
PARK7P1	0.0025	6.1171E-01		ENSG00000257243
PARL	0.0738	2.1762E-01	3.98E-01	ENSG00000175193
PARLP1	0.0036	7.2894E-01		ENSG00000239835
PARLP2	-0.4219	4.7348E-03	2.40E-02	ENSG00000217648
PARM1	-0.2204	1.8813E-03	1.17E-02	ENSG00000169116
PARM1-AS1	-0.0550	4.4251E-01	6.33E-01	ENSG00000249717
PARN	0.1365	2.5856E-02	8.73E-02	ENSG00000140694
PARP1	-0.2151	2.0548E-05	3.12E-04	ENSG00000143799
PARP10	-0.0527	5.5453E-01	7.26E-01	ENSG00000178685
PARP11	0.0954	3.1887E-01	5.12E-01	ENSG00000111224
PARP11-AS1	-0.0150	7.5937E-01	8.69E-01	ENSG00000256862
PARP12	-0.4329	5.7271E-03	2.78E-02	ENSG00000059378
PARP2	0.2212	3.6521E-03	1.97E-02	ENSG00000129484
PARP3	0.2651	8.3046E-03	3.70E-02	ENSG00000041880
PARP4	-0.1962	9.6256E-03	4.13E-02	ENSG00000102699
PARP4P2	-0.0324	6.3402E-01		ENSG00000224976
PARP6	0.2043	1.4913E-02	5.78E-02	ENSG00000137817
PARP8	-0.1842	4.1354E-02	1.24E-01	ENSG00000151883
PARPBP	-0.2810	5.8171E-03	2.81E-02	ENSG00000185480

PARS2	0.2396	5.8975E-02	1.61E-01	ENSG00000162396
PART1	-0.4862	1.6890E-06	4.11E-05	ENSG00000152931
PARTICL	0.3031	5.8013E-05	7.24E-04	ENSG00000286532
PARVA	-0.1690	9.1169E-03	3.97E-02	ENSG00000197702
PARVB	-0.0269	7.6727E-01	8.74E-01	ENSG00000188677
PARVG	-0.0268	7.3161E-01	8.52E-01	ENSG00000138964
PASD1	0.0508	2.1885E-01		ENSG00000166049
PASK	0.0972	2.4921E-01	4.36E-01	ENSG00000115687
PATE2	-0.0100	6.5162E-01		ENSG00000196844
PATJ	0.0343	4.8806E-01	6.72E-01	ENSG00000132849
PATL1	-0.1285	1.3166E-01	2.84E-01	ENSG00000166889
PATL2	-0.0144	8.8698E-01	9.43E-01	ENSG00000229474
PATZ1	-0.1492	2.1612E-02	7.64E-02	ENSG00000100105
PAWR	-0.3835	1.9758E-09	1.48E-07	ENSG00000177425
PAWRP2	0.1383	1.0201E-01	2.38E-01	ENSG00000270424
PAX1	0.0692	4.0553E-01	5.99E-01	ENSG00000125813
PAX2	-0.0041	8.2640E-01		ENSG00000075891
PAX5	-0.1016	1.3750E-01	2.92E-01	ENSG00000196092
PAX6-AS1	-0.0744	2.3568E-01	4.20E-01	ENSG00000281880
PAX7	0.0086	7.3957E-01	8.57E-01	ENSG00000009709
PAX8	-0.0623	2.4192E-01		ENSG00000125618
PAX8-AS1	-0.0105	5.4192E-01		ENSG00000189223
PAX9	0.0044	9.5929E-01	9.79E-01	ENSG00000198807
PAXBP1	-0.2805	1.4728E-04	1.53E-03	ENSG00000159086
PAXBP1-AS1	0.0079	9.7356E-01	9.86E-01	ENSG00000238197
PAXIP1	-0.1600	7.8521E-02	1.98E-01	ENSG00000157212
PAXIP1-DT	0.1667	9.7839E-03	4.18E-02	ENSG00000273344
PAXX	-0.2819	3.0090E-04	2.74E-03	ENSG00000148362
PBDC1	-0.1974	1.3319E-05	2.21E-04	ENSG00000102390
PBK	-0.4649	1.9321E-03	1.19E-02	ENSG00000168078
PBLD	0.2137	1.9423E-02	7.04E-02	ENSG00000108187
PBOV1	-0.0327	4.1487E-01		ENSG00000254440
PBRM1	0.0312	6.2125E-01	7.77E-01	ENSG00000163939
PBX1	-0.2132	2.1106E-02	7.50E-02	ENSG00000185630
PBX1-AS1	0.4719	1.5268E-02	5.88E-02	ENSG00000233693
PBX2	-0.4029	1.3021E-03	8.76E-03	ENSG00000204304
PBX3	-0.2333	4.3624E-03	2.26E-02	ENSG00000167081
PBX4	0.2595	1.2765E-03	8.63E-03	ENSG00000105717
PBXIP1	0.0276	7.3500E-01	8.54E-01	ENSG00000163346
PC	-0.0580	4.3027E-01	6.22E-01	ENSG00000173599
PCA3	0.0439	6.1646E-01	7.74E-01	ENSG00000225937
PCARE	0.1179	1.6596E-01	3.33E-01	ENSG00000179270
PCAT1	0.0218	8.1087E-01	9.00E-01	ENSG00000253438
PCAT18	0.0280	3.9320E-01		ENSG00000265369
PCAT19	-0.0183	9.0551E-01	9.53E-01	ENSG00000267107
PCAT2	0.0081	6.4621E-01	7.95E-01	ENSG00000254166
PCAT4	-0.1312	3.4654E-02	1.09E-01	ENSG00000251321
PCAT6	0.3237	3.2769E-05	4.52E-04	ENSG00000228288
PCBD1	-0.5545	6.4317E-09	3.98E-07	ENSG00000166228

PCBD2	-0.0639	3.1811E-01	5.12E-01	ENSG00000132570
PCBP1	-0.1916	7.5191E-02	1.92E-01	ENSG00000169564
PCBP1-AS1	0.2245	1.1477E-05	1.96E-04	ENSG00000179818
PCBP2	-0.3860	7.9945E-04	5.98E-03	ENSG00000197111
PCBP2-OT1	-0.1276	4.4461E-02		ENSG00000282977
PCBP3	0.2050	4.5261E-02	1.33E-01	ENSG00000183570
PCBP4	0.2499	1.8000E-03	1.13E-02	ENSG00000090097
PCCA	0.0459	5.7184E-01	7.39E-01	ENSG00000175198
PCCA-DT	-0.2995	2.2860E-03	1.36E-02	ENSG00000274605
PCCB	-0.1940	1.2965E-02	5.18E-02	ENSG00000114054
PCDH1	0.2798	9.2602E-03	4.01E-02	ENSG00000156453
PCDH10	0.0398	6.5928E-01	8.03E-01	ENSG00000138650
PCDH10-DT	0.1430	1.7442E-01	3.44E-01	ENSG00000250241
PCDH11Y	0.4290	1.5946E-03	1.03E-02	ENSG00000099715
PCDH12	0.0495	6.0790E-01	7.67E-01	ENSG00000113555
PCDH15	0.1917	9.1103E-02	2.20E-01	ENSG00000150275
PCDH17	0.2735	4.1593E-03	2.18E-02	ENSG00000118946
PCDH18	0.0673	4.7878E-01	6.65E-01	ENSG00000189184
PCDH19	-0.1789	9.5963E-02	2.28E-01	ENSG00000165194
PCDH20	-0.0718	2.3217E-01		ENSG00000280165
PCDH7	-0.0185	8.1464E-01	9.02E-01	ENSG00000169851
PCDH8	0.3945	1.5516E-03	1.01E-02	ENSG00000136099
PCDH9	0.1061	1.7852E-01	3.50E-01	ENSG00000184226
PCDH9-AS2	0.0219	2.5277E-01		ENSG00000228842
PCDH9-AS3	-0.0049	9.1656E-01		ENSG00000225263
PCDHA10	-0.0115	8.8381E-01	9.41E-01	ENSG00000250120
PCDHA11	0.0232	8.1092E-01	9.00E-01	ENSG00000249158
PCDHA12	-0.1565	1.2869E-01	2.80E-01	ENSG00000251664
PCDHA13	0.0303	6.8034E-01	8.18E-01	ENSG00000239389
PCDHA2	-0.3266	1.9224E-02	6.99E-02	ENSG00000204969
PCDHA3	0.1419	1.7777E-01	3.49E-01	ENSG00000255408
PCDHA4	-0.0786	3.4788E-01	5.43E-01	ENSG00000204967
PCDHA5	0.1684	1.3232E-01	2.85E-01	ENSG00000204965
PCDHA6	0.0716	4.1954E-01	6.12E-01	ENSG00000081842
PCDHA7	0.0538	5.7531E-01	7.42E-01	ENSG00000204963
PCDHA8	0.0763	3.0465E-01	4.97E-01	ENSG00000204962
PCDHA9	0.2088	6.9752E-02	1.82E-01	ENSG00000204961
PCDHAC1	0.0778	4.3666E-01	6.28E-01	ENSG00000248383
PCDHAC2	0.0043	9.6503E-01	9.82E-01	ENSG00000243232
PCDHB1	-0.0632	5.2068E-01	6.98E-01	ENSG00000171815
PCDHB10	-0.1051	1.6859E-01	3.36E-01	ENSG00000120324
PCDHB11	0.1565	1.2240E-01	2.71E-01	ENSG00000197479
PCDHB12	0.4094	9.1398E-03	3.98E-02	ENSG00000120328
PCDHB13	0.1858	3.1789E-02	1.02E-01	ENSG00000187372
PCDHB14	0.1146	1.1447E-01	2.58E-01	ENSG00000120327
PCDHB15	-0.1587	9.7472E-02	2.31E-01	ENSG00000113248
PCDHB16	0.1808	6.1558E-02	1.66E-01	ENSG00000272674
PCDHB17P	0.0026	9.7805E-01	9.89E-01	ENSG00000255622
PCDHB2	-0.1199	3.5402E-02	1.11E-01	ENSG00000112852

PCDHB3	-0.1336	1.6462E-01	3.31E-01	ENSG00000113205
PCDHB4	0.4322	6.8048E-03	3.17E-02	ENSG00000081818
PCDHB5	-0.3456	1.0849E-03	7.56E-03	ENSG00000113209
PCDHB6	-0.0247	8.9949E-01	9.50E-01	ENSG00000113211
PCDHB7	0.1221	2.2135E-01	4.03E-01	ENSG00000113212
PCDHB8	0.2757	3.7255E-02	1.15E-01	ENSG00000120322
PCDHB9	0.2637	4.5640E-03	2.34E-02	ENSG00000177839
PCDHGA1	-0.0563	6.1048E-01	7.69E-01	ENSG00000204956
PCDHGA10	0.0663	5.0300E-01	6.84E-01	ENSG00000253846
PCDHGA11	-0.0145	8.6863E-01	9.34E-01	ENSG00000253873
PCDHGA12	0.0318	7.3747E-01	8.56E-01	ENSG00000253159
PCDHGA2	-0.0206	7.0282E-01		ENSG00000081853
PCDHGA3	-0.1408	1.3991E-01	2.96E-01	ENSG00000254245
PCDHGA4	0.1194	1.2269E-01	2.71E-01	ENSG00000262576
PCDHGA5	0.0083	8.9513E-01	9.48E-01	ENSG00000253485
PCDHGA6	-0.0489	9.1858E-01	9.60E-01	ENSG00000253731
PCDHGA7	-0.0164	9.4237E-01	9.72E-01	ENSG00000253537
PCDHGA8	0.1677	1.3300E-01	2.86E-01	ENSG00000253767
PCDHGA9	-0.0124	8.9782E-01	9.49E-01	ENSG00000261934
PCDHGB1	-0.0645	5.9421E-01	7.57E-01	ENSG00000254221
PCDHGB2	0.0589	4.4423E-01	6.35E-01	ENSG00000253910
PCDHGB3	0.0079	5.6759E-01		ENSG00000262209
PCDHGB4	-0.0068	9.3115E-01	9.66E-01	ENSG00000253953
PCDHGB5	-0.0006	9.9368E-01	9.97E-01	ENSG00000276547
PCDHGB6	-0.0468	5.7188E-01	7.39E-01	ENSG00000253305
PCDHGB7	-0.0513	5.9259E-01	7.55E-01	ENSG00000254122
PCDHGB8P	0.1157	2.5456E-01	4.42E-01	ENSG00000248449
PCDHGB9P	0.0565	5.3084E-01	7.05E-01	ENSG00000276545
PCDHGC3	-0.1185	2.5009E-01	4.37E-01	ENSG00000240184
PCDHGC4	0.2371	5.4045E-02	1.51E-01	ENSG00000242419
PCDHGC5	0.0115	8.5289E-01	9.25E-01	ENSG00000240764
PCED1A	-0.1484	4.9040E-02	1.41E-01	ENSG00000132635
PCED1B	0.0226	8.2781E-01	9.09E-01	ENSG00000179715
PCED1B-AS1	-0.0045	9.2124E-01	9.61E-01	ENSG00000247774
PCF11	0.0253	7.4840E-01	8.62E-01	ENSG00000165494
PCGEM1	0.0028	5.7540E-01		ENSG00000227418
PCGF1	-0.3201	2.1251E-06	4.98E-05	ENSG00000115289
PCGF2	-0.5268	2.3364E-05	3.47E-04	ENSG00000277258
PCGF3	0.1253	7.5274E-02	1.92E-01	ENSG00000185619
PCGF3-AS1	-0.0185	8.1554E-01	9.03E-01	ENSG00000249592
PCGF5	-0.3462	6.4749E-07	1.85E-05	ENSG00000180628
PCGF6	-0.1633	4.7993E-03	2.42E-02	ENSG00000156374
PCID2	-0.1566	6.8202E-03	3.18E-02	ENSG00000126226
PCIF1	-0.1989	4.4176E-05	5.83E-04	ENSG00000100982
PCK1	0.4064	8.6958E-03	3.83E-02	ENSG00000124253
PCK2	0.1138	1.7237E-01	3.41E-01	ENSG00000100889
PCLO	0.1568	3.1049E-02	9.99E-02	ENSG00000186472
PCM1	-0.1137	2.6484E-02	8.88E-02	ENSG00000078674
PCMT1	0.1005	1.6210E-01	3.28E-01	ENSG00000120265

PCMTD1	-0.2081	3.0899E-03	1.72E-02	ENSG00000168300
PCMTD2	-0.0469	4.3280E-01	6.24E-01	ENSG00000203880
PCNA	-0.2537	5.4606E-04	4.42E-03	ENSG00000132646
PCNP	-0.0895	1.3478E-01	2.88E-01	ENSG00000081154
PCNT	0.3445	9.0433E-05	1.03E-03	ENSG00000160299
PCNX1	0.1283	6.2274E-02	1.68E-01	ENSG00000100731
PCNX2	0.1464	4.3893E-02	1.30E-01	ENSG00000135749
PCNX3	-0.0647	4.4962E-01	6.39E-01	ENSG00000197136
PCNX4	0.0026	9.7868E-01	9.89E-01	ENSG00000126773
PCOLCE	0.3998	2.2544E-05	3.37E-04	ENSG00000106333
PCOLCE-AS1	0.1876	7.6657E-02	1.95E-01	ENSG00000224729
PCOLCE2	-0.1606	1.1645E-01	2.62E-01	ENSG00000163710
PCOTH	0.0297	2.7564E-01	4.66E-01	ENSG00000205861
PCP2	0.2816	3.5769E-02	1.11E-01	ENSG00000174788
PCP4L1	0.0545	5.5799E-01	7.28E-01	ENSG00000248485
PCSK1N	-0.0498	4.1478E-01	6.08E-01	ENSG00000102109
PCSK2	0.1995	3.7866E-02	1.16E-01	ENSG00000125851
PCSK4	0.2362	2.2860E-02	7.96E-02	ENSG00000115257
PCSK5	-0.3361	1.9334E-03	1.19E-02	ENSG00000099139
PCSK6	0.0216	7.8686E-01	8.86E-01	ENSG00000140479
PCSK6-AS1	0.0028	6.3773E-01		ENSG00000259764
PCSK7	0.3745	8.3064E-05	9.63E-04	ENSG00000160613
PCSK9	-0.0751	9.2377E-02		ENSG00000169174
PCTP	0.1189	1.8312E-01	3.56E-01	ENSG00000141179
PCYOX1	-0.0992	9.5400E-03	4.10E-02	ENSG00000116005
PCYOX1L	0.3110	5.3629E-04	4.36E-03	ENSG00000145882
PCYT1A	0.0732	2.4154E-01	4.27E-01	ENSG00000161217
PCYT1B	-0.5818	1.2851E-05	2.14E-04	ENSG00000102230
PCYT2	-0.1923	5.7817E-03	2.80E-02	ENSG00000185813
PDAP1	0.0126	8.7788E-01	9.38E-01	ENSG00000106244
PDC	0.0014	9.4286E-01		ENSG00000116703
PDC-AS1	0.1144	2.3179E-01	4.15E-01	ENSG00000229739
PDCD1	-0.0024	8.7137E-01	9.35E-01	ENSG00000188389
PDCD10	0.0920	2.8452E-01	4.76E-01	ENSG00000114209
PDCD11	0.1332	9.8395E-02	2.32E-01	ENSG00000148843
PDCD1LG2	-0.0208	7.1375E-01		ENSG00000197646
PDCD2	-0.0520	2.7834E-01	4.69E-01	ENSG00000071994
PDCD2L	0.0678	3.4098E-01	5.36E-01	ENSG00000126249
PDCD4	-0.0750	3.5135E-01	5.47E-01	ENSG00000150593
PDCD5	-0.0844	1.1981E-01	2.67E-01	ENSG00000105185
PDCD6	0.2928	1.1864E-02	4.83E-02	ENSG00000249915
PDCD6IP	0.1422	1.6835E-02	6.32E-02	ENSG00000170248
PDCD6IP-DT	-0.1313	1.8396E-01	3.57E-01	ENSG00000271643
PDCD6IPP1	0.0329	7.3171E-01	8.52E-01	ENSG00000275325
PDCD7	0.2126	2.5457E-03	1.48E-02	ENSG00000090470
PDCL	-0.0408	4.4262E-01	6.33E-01	ENSG00000136940
PDCL2	-0.0310	8.9937E-01		ENSG00000163440
PDCL3	-0.1532	3.1172E-03	1.73E-02	ENSG00000115539
PDCL3P6	0.0411	8.3967E-02		ENSG00000224255

PDE10A	0.2584	1.7415E-04	1.76E-03	ENSG00000112541
PDE11A	0.1506	1.5450E-01	3.17E-01	ENSG00000128655
PDE11A-AS1	0.0057	6.1327E-01		ENSG00000229941
PDE12	0.0654	3.5499E-01	5.50E-01	ENSG00000174840
PDE1A	-0.0950	2.9652E-01	4.89E-01	ENSG00000115252
PDE1B	0.3608	3.3612E-03	1.84E-02	ENSG00000123360
PDE1C	-0.3267	1.2642E-02	5.08E-02	ENSG00000154678
PDE2A	-0.0672	4.7927E-01	6.65E-01	ENSG00000186642
PDE3A	-0.1496	1.0202E-01	2.38E-01	ENSG00000172572
PDE4A	0.1050	1.2985E-01	2.81E-01	ENSG00000065989
PDE4B	-0.5145	1.0670E-07	4.11E-06	ENSG00000184588
PDE4B-AS1	-0.0063	8.1601E-01		ENSG00000227466
PDE4C	0.0916	1.0751E-01	2.47E-01	ENSG00000105650
PDE4C	-0.0291	3.7918E-01		ENSG00000285188
PDE4D	-0.0997	1.2528E-01	2.75E-01	ENSG00000113448
PDE4DIP	-0.0597	3.5277E-01	5.48E-01	ENSG00000178104
PDE5A	0.1369	1.5556E-01	3.18E-01	ENSG00000138735
PDE6A	0.0674	3.5914E-01	5.55E-01	ENSG00000132915
PDE6B	-0.1639	6.3139E-02	1.69E-01	ENSG00000133256
PDE6C	0.0911	1.0046E-01		ENSG00000095464
PDE6D	-0.0771	9.3078E-02	2.24E-01	ENSG00000156973
PDE6G	0.1089	2.5915E-01	4.47E-01	ENSG00000185527
PDE6H	0.3468	2.5659E-02	8.68E-02	ENSG00000139053
PDE7A	0.1636	9.6238E-02	2.29E-01	ENSG00000205268
PDE7B	-0.0016	9.8505E-01	9.92E-01	ENSG00000171408
PDE7B-AS1	0.0247	3.9821E-01	5.93E-01	ENSG00000237596
PDE8A	0.0815	2.9292E-01	4.85E-01	ENSG00000073417
PDE8B	-0.2837	1.7917E-02	6.63E-02	ENSG00000113231
PDE9A	-0.1361	7.2068E-02	1.86E-01	ENSG00000160191
PDE9A-AS1	0.0283	4.9109E-01		ENSG00000225731
PDF	-0.1193	1.5305E-01	3.15E-01	ENSG00000258429
PDGFA	-0.3440	2.9830E-04	2.72E-03	ENSG00000197461
PDGFA-DT	-0.3096	2.7844E-02	9.21E-02	ENSG00000223855
PDGFB	-0.2065	4.0627E-02	1.23E-01	ENSG00000100311
PDGFC	-0.3800	1.0983E-04	1.21E-03	ENSG00000145431
PDGFD	-0.3985	1.1596E-03	7.97E-03	ENSG00000170962
PDGFRA	0.0054	9.3901E-01	9.69E-01	ENSG00000134853
PDGFRL	-0.1270	1.1375E-01	2.57E-01	ENSG00000104213
PDHA1	-0.0130	8.1991E-01	9.05E-01	ENSG00000131828
PDHA1P1	-0.0045	9.1730E-01		ENSG00000270772
PDHB	0.1518	4.5811E-02	1.34E-01	ENSG00000168291
PDHX	0.3186	1.7338E-05	2.74E-04	ENSG00000110435
PDIA2	0.1708	8.8580E-02	2.16E-01	ENSG00000185615
PDIA3	-0.1567	5.1531E-02	1.46E-01	ENSG00000167004
PDIA4	-0.1063	2.4234E-01	4.27E-01	ENSG00000155660
PDIA5	0.2163	2.0033E-03	1.23E-02	ENSG00000065485
PDIA6	-0.2892	5.3057E-03	2.62E-02	ENSG00000143870
PDIK1L	0.2341	1.5214E-03	9.93E-03	ENSG00000175087
PDK1-AS1	0.0851	3.3497E-01	5.30E-01	ENSG00000225205

PDK1P1	-0.0508	4.2264E-01	6.15E-01	ENSG00000277646
PDK2	-0.1070	9.3783E-02	2.25E-01	ENSG00000005882
PDK3	0.1965	1.0700E-02	4.47E-02	ENSG00000067992
PDK4	-0.4296	6.3993E-03	3.03E-02	ENSG00000004799
PDLIM1	-0.1802	5.4213E-02	1.52E-01	ENSG00000107438
PDLIM2	-0.1549	9.7112E-02	2.30E-01	ENSG00000120913
PDLIM3	-0.0873	3.0925E-01	5.02E-01	ENSG00000154553
PDLIM4	-0.0213	7.6718E-01	8.74E-01	ENSG00000131435
PDLIM5	-0.2678	6.9944E-03	3.24E-02	ENSG00000163110
PDLIM7	-0.2309	3.9421E-06	8.27E-05	ENSG00000196923
PDP1	0.1184	1.3869E-01	2.94E-01	ENSG00000164951
PDP2	0.1434	1.4528E-01	3.04E-01	ENSG00000172840
PDPK1	0.0755	2.3931E-01	4.24E-01	ENSG00000140992
PDPK2P	0.2144	8.2968E-03	3.69E-02	ENSG00000205918
PDPN	-0.2026	7.4460E-02	1.90E-01	ENSG00000162493
PDPR	0.0807	3.0695E-01	5.00E-01	ENSG00000090857
PDRG1	-0.1709	6.7029E-05	8.13E-04	ENSG00000088356
PDS5A	-0.2105	1.5682E-04	1.61E-03	ENSG00000121892
PDS5B	-0.0959	1.6685E-01	3.34E-01	ENSG00000083642
PDSS1	0.0624	3.7575E-01	5.71E-01	ENSG00000148459
PDSS2	-0.0433	5.4109E-01	7.14E-01	ENSG00000164494
PDX1	-0.0305	5.6604E-01		ENSG00000139515
PDXDC1	0.3089	7.4303E-05	8.83E-04	ENSG00000179889
PDXDC2P	-0.0480	5.4645E-01	7.18E-01	ENSG00000255185
PDXDC2P-NPIPB14P	0.3124	7.4449E-03	3.40E-02	ENSG00000196696
PDXK	0.0103	8.6340E-01	9.30E-01	ENSG00000160209
PDXP	0.2423	1.2296E-02	4.98E-02	ENSG00000241360
PDXP-DT	-0.0104	5.3496E-01	7.09E-01	ENSG00000233360
PDYN	0.0939	3.4870E-01	5.44E-01	ENSG00000101327
PDYN-AS1	0.0088	5.6608E-01		ENSG00000233896
PDZD11	-0.1521	4.8593E-03	2.45E-02	ENSG00000120509
PDZD2	0.0207	8.2470E-01	9.08E-01	ENSG00000133401
PDZD4	-0.0097	8.9958E-01	9.50E-01	ENSG00000067840
PDZD7	0.5836	3.9106E-17	2.38E-14	ENSG00000186862
PDZD8	-0.3108	5.7359E-08	2.49E-06	ENSG00000165650
PDZD9	-0.0708	4.1221E-01	6.05E-01	ENSG00000155714
PDZK1	-0.0005	9.9734E-01	9.99E-01	ENSG00000174827
PDZK1IP1	-0.0103	8.4505E-01		ENSG00000162366
PDZPH1P	-0.0232	7.4877E-01	8.63E-01	ENSG00000226926
PDZRN3	-0.1198	6.5901E-02	1.75E-01	ENSG00000121440
PDZRN4	0.0408	6.3972E-01	7.91E-01	ENSG00000165966
PEA15	-0.4158	1.9122E-07	6.69E-06	ENSG00000162734
PEAK1	-0.1402	4.6991E-02	1.37E-01	ENSG00000173517
PEAK3	-0.1341	2.0564E-01	3.84E-01	ENSG00000188305
PEBP1	-0.1668	2.2004E-04	2.11E-03	ENSG00000089220
PEBP4	-0.1187	1.8092E-01	3.53E-01	ENSG00000134020
PECAM1	0.0116	4.7435E-01		ENSG00000261371
PECR	0.0420	5.8172E-01	7.48E-01	ENSG00000115425
PEDS1	-0.1412	2.3427E-02	8.11E-02	ENSG00000240849

PEF1	-0.0169	7.2815E-01	8.50E-01	ENSG00000162517
PEG10	-0.1360	4.8721E-02	1.40E-01	ENSG00000242265
PEG13	0.3812	9.8271E-03	4.19E-02	ENSG00000282164
PEG3	0.0092	4.9140E-01	6.75E-01	ENSG00000198300
PELATON	-0.0091	8.1320E-01		ENSG00000224397
PELI1	-0.4129	1.1456E-04	1.25E-03	ENSG00000197329
PELI2	0.0792	3.5877E-01	5.55E-01	ENSG00000139946
PELI3	-0.0442	5.6615E-01	7.35E-01	ENSG00000174516
PELO	-0.2400	2.8794E-04	2.64E-03	ENSG00000152684
PELP1	-0.0446	5.3062E-01	7.05E-01	ENSG00000141456
PELP1-DT	0.1705	1.3142E-01	2.84E-01	ENSG00000244184
PEMT	-0.0965	6.6842E-02	1.76E-01	ENSG00000133027
PENK	-0.0456	5.5537E-01	7.26E-01	ENSG00000181195
PENK-AS1	0.0175	3.5131E-01		ENSG00000254254
PEPD	-0.1562	1.8201E-02	6.71E-02	ENSG00000124299
PER1	-0.3301	4.8645E-04	4.01E-03	ENSG00000179094
PER2	-0.5050	2.2452E-14	7.00E-12	ENSG00000132326
PER3	-0.0511	5.0702E-01	6.87E-01	ENSG00000049246
PERCC1	0.0079	7.7768E-01		ENSG00000284395
PERM1	0.0051	6.3584E-01		ENSG00000187642
PERP	-0.2053	1.1554E-02	4.74E-02	ENSG00000112378
PES1	0.1678	4.2995E-03	2.23E-02	ENSG00000100029
PET100	-0.2043	1.5169E-03	9.90E-03	ENSG00000229833
PET117	-0.2629	8.6352E-03	3.81E-02	ENSG00000232838
PEX1	0.2419	1.4131E-03	9.36E-03	ENSG00000127980
PEX10	-0.0197	7.4482E-01	8.60E-01	ENSG00000157911
PEX11A	-0.1281	1.5201E-01	3.13E-01	ENSG00000166821
PEX11B	0.0978	1.5244E-01	3.14E-01	ENSG00000131779
PEX11G	0.1932	3.8746E-02	1.18E-01	ENSG00000104883
PEX12	0.3056	2.3147E-03	1.38E-02	ENSG00000108733
PEX13	-0.0918	1.1171E-01	2.54E-01	ENSG00000162928
PEX14	0.0117	8.4359E-01	9.19E-01	ENSG00000142655
PEX16	0.2539	1.6032E-05	2.57E-04	ENSG00000121680
PEX19	-0.0850	1.6854E-01	3.36E-01	ENSG00000162735
PEX2	0.1447	3.8107E-02	1.17E-01	ENSG00000164751
PEX26	-0.2700	7.6133E-03	3.46E-02	ENSG00000215193
PEX3	0.1065	1.8206E-01	3.54E-01	ENSG00000034693
PEX5	0.0774	2.4446E-01	4.30E-01	ENSG00000139197
PEX5L	-0.0962	3.1805E-01	5.12E-01	ENSG00000114757
PEX5L-AS2	-0.0446	6.5549E-01		ENSG00000244302
PEX6	0.0027	9.7088E-01	9.85E-01	ENSG00000124587
PEX7	-0.0473	5.4498E-01	7.17E-01	ENSG00000112357
PF4V1	0.0129	6.4191E-01	7.92E-01	ENSG00000109272
PFAS	0.0763	2.8926E-01	4.81E-01	ENSG00000178921
PFDN1	-0.3151	8.1705E-10	7.25E-08	ENSG00000113068
PFDN2	-0.1906	3.4917E-03	1.90E-02	ENSG00000143256
PFDN4	-0.1832	1.4152E-02	5.55E-02	ENSG00000101132
PFDN5	0.0883	3.4629E-01	5.41E-01	ENSG00000123349
PFDN6	0.0172	8.1365E-01	9.02E-01	ENSG00000204220

PFKFB1	0.0899	3.4691E-01	5.42E-01	ENSG00000158571
PFKFB2	-0.0593	5.0155E-01	6.83E-01	ENSG00000123836
PFKFB3	0.2648	6.2902E-03	2.99E-02	ENSG00000170525
PFKFB4	0.4825	6.8170E-05	8.23E-04	ENSG00000114268
PFKL	0.2056	6.2462E-03	2.97E-02	ENSG00000141959
PFKM	-0.2302	3.8505E-04	3.34E-03	ENSG00000152556
PFKP	0.2824	1.3000E-03	8.75E-03	ENSG00000067057
PFN1	-0.3700	5.0019E-09	3.27E-07	ENSG00000108518
PFN1P2	-0.0948	2.8587E-01	4.77E-01	ENSG00000270392
PFN2	-0.0619	2.9219E-01	4.84E-01	ENSG00000070087
PFN3	0.0094	5.8332E-01		ENSG00000196570
PFN4	0.4364	8.8446E-04	6.47E-03	ENSG00000176732
PGA4	-0.2115	9.1844E-03	3.99E-02	ENSG00000229183
PGAM1	0.1630	1.1190E-02	4.62E-02	ENSG00000171314
PGAM1P11	-0.0038	9.7308E-01		ENSG00000233623
PGAM1P3	0.0119	4.8451E-01		ENSG00000230274
PGAM1P6	-0.0048	4.4856E-01		ENSG00000224464
PGAM4	0.2332	1.3128E-02	5.23E-02	ENSG00000226784
PGAM5	0.0201	7.2489E-01	8.48E-01	ENSG00000247077
PGAP1	0.3915	1.7431E-04	1.76E-03	ENSG00000197121
PGAP2	-0.1348	4.2964E-02	1.28E-01	ENSG00000148985
PGAP3	0.2959	5.6535E-04	4.54E-03	ENSG00000161395
PGAP4	-0.2457	2.5435E-03	1.48E-02	ENSG00000165152
PGAP6	0.0477	5.2726E-01	7.03E-01	ENSG00000129925
PGBD1	0.0815	2.5137E-01	4.38E-01	ENSG00000137338
PGBD2	0.4840	4.7471E-03	2.40E-02	ENSG00000185220
PGBD4	0.0536	5.7278E-01	7.40E-01	ENSG00000182405
PGBD5	0.0855	3.3403E-01	5.29E-01	ENSG00000177614
PGC	0.0041	7.8597E-01		ENSG00000096088
PGD	-0.0125	8.8576E-01	9.42E-01	ENSG00000142657
PGDP1	-0.0032	9.5607E-01		ENSG00000265273
PGF	0.2341	3.7473E-02	1.15E-01	ENSG00000119630
PGGHG	-0.0623	4.9145E-01	6.75E-01	ENSG00000142102
PGGT1B	0.1078	6.4501E-02	1.72E-01	ENSG00000164219
PGK1	0.3917	2.9148E-05	4.14E-04	ENSG00000102144
PGK1P2	-0.0031	8.0068E-01		ENSG00000213290
PGLS	-0.1825	3.0784E-04	2.79E-03	ENSG00000130313
PGLS-DT	0.1724	7.7555E-02	1.96E-01	ENSG00000269439
PGLYRP2	-0.0039	8.8583E-01		ENSG00000161031
PGM1	0.0471	5.8441E-01	7.49E-01	ENSG00000079739
PGM2	-0.1626	6.3214E-02	1.70E-01	ENSG00000169299
PGM2L1	0.1587	1.5464E-02	5.93E-02	ENSG00000165434
PGM3	0.1383	4.3990E-02	1.30E-01	ENSG00000013375
PGM5	-0.0492	5.9251E-01	7.55E-01	ENSG00000154330
PGP	-0.0232	6.5657E-01	8.02E-01	ENSG00000184207
PGPEP1	-0.3181	1.8423E-04	1.84E-03	ENSG00000130517
PGPEP1L	0.0139	5.1722E-01		ENSG00000183571
PGR	-0.0388	5.4595E-01		ENSG00000082175
PGRMC1	-0.0348	6.4862E-01	7.97E-01	ENSG00000101856

PGRMC2	-0.1629	2.5220E-02	8.57E-02	ENSG00000164040
PGS1	-0.0484	4.7827E-01	6.64E-01	ENSG00000087157
PHACTR1	0.1043	2.3381E-01	4.18E-01	ENSG00000112137
PHACTR2	-0.1902	2.9603E-02	9.65E-02	ENSG00000112419
PHACTR2-AS1	-0.0236	7.9081E-01		ENSG00000235740
PHACTR3	0.1627	4.6091E-02	1.35E-01	ENSG00000087495
PHACTR4	0.0167	8.1683E-01	9.03E-01	ENSG00000204138
PHAF1	0.4159	2.3386E-05	3.48E-04	ENSG00000125149
PHAX	-0.3167	3.5817E-09	2.47E-07	ENSG00000164902
PHB1	-0.1870	8.4697E-03	3.75E-02	ENSG00000167085
PHB1P15	0.0616	2.9547E-01	4.87E-01	ENSG00000262648
PHB1P4	0.0212	7.5890E-01	8.69E-01	ENSG00000230251
PHB1P5	0.0838	1.9224E-01	3.68E-01	ENSG00000233523
PHB1P6	0.0517	3.7513E-01	5.71E-01	ENSG00000276592
PHB1P9	-0.2895	3.9331E-02	1.20E-01	ENSG00000230224
PHB2	0.0692	2.1983E-01	4.01E-01	ENSG00000215021
PHC1	-0.0415	4.7943E-01	6.65E-01	ENSG00000111752
PHC2	0.1099	1.3963E-01	2.95E-01	ENSG00000134686
PHC2-AS1	0.0101	5.5713E-01	7.28E-01	ENSG00000233246
PHC3	-0.0310	5.7885E-01	7.45E-01	ENSG00000173889
PHETA1	0.1057	2.4527E-01	4.31E-01	ENSG00000198324
PHEX	-0.0739	4.4463E-01	6.35E-01	ENSG00000102174
PHEX-AS1	0.0911	3.5886E-01	5.55E-01	ENSG00000224204
PHF1	0.0574	3.1674E-01	5.10E-01	ENSG00000112511
PHF10	-0.2249	2.5024E-03	1.46E-02	ENSG00000130024
PHF11	-0.3671	3.7790E-04	3.30E-03	ENSG00000136147
PHF12	-0.1609	5.6607E-02	1.57E-01	ENSG00000109118
PHF13	0.0053	9.4555E-01	9.73E-01	ENSG00000116273
PHF14	-0.1240	5.1579E-02	1.46E-01	ENSG00000106443
PHF19	-0.3617	2.8015E-04	2.58E-03	ENSG00000119403
PHF2	-0.1511	6.3695E-02	1.70E-01	ENSG00000197724
PHF20	0.0149	8.4477E-01	9.19E-01	ENSG00000025293
PHF20L1	0.1429	5.0010E-02	1.43E-01	ENSG00000129292
PHF21A	-0.0677	3.0969E-01	5.03E-01	ENSG00000135365
PHF21B	-0.0671	4.9817E-01	6.81E-01	ENSG00000056487
PHF23	0.0633	3.5201E-01	5.47E-01	ENSG00000040633
PHF24	0.0033	9.7189E-01	9.85E-01	ENSG00000122733
PHF3	-0.1742	1.3017E-03	8.76E-03	ENSG00000118482
PHF5A	-0.1121	4.7955E-02	1.39E-01	ENSG00000100410
PHF6	-0.1914	2.6622E-03	1.54E-02	ENSG00000156531
PHF7	0.5579	1.7612E-09	1.35E-07	ENSG00000010318
PHF8	0.0092	8.8370E-01	9.41E-01	ENSG00000172943
PHGDH	-0.1639	3.2666E-02	1.04E-01	ENSG00000092621
PHGR1	-0.2300	3.1473E-02	1.01E-01	ENSG00000233041
PHIP	0.0515	4.1487E-01	6.08E-01	ENSG00000146247
PHKA1	-0.1435	8.3058E-02	2.06E-01	ENSG00000067177
PHKA1-AS1	-0.0313	8.7267E-01	9.36E-01	ENSG00000231944
PHKA2	0.0160	8.4791E-01	9.22E-01	ENSG00000044446
PHKB	-0.1351	4.2704E-02	1.27E-01	ENSG00000102893

PHKG1	0.0994	2.0984E-01	3.89E-01	ENSG00000164776
PHKG2	-0.0098	7.9698E-01	8.91E-01	ENSG00000156873
PHLDA1	-0.1990	1.1292E-02	4.66E-02	ENSG00000139289
PHLDA2	-0.2236	3.6031E-02	1.12E-01	ENSG00000181649
PHLDA3	-0.0193	7.6222E-01	8.71E-01	ENSG00000174307
PHLDB1	0.0975	1.4225E-01	2.99E-01	ENSG00000019144
PHLDB2	-0.2012	1.7473E-02	6.50E-02	ENSG00000144824
PHLDB3	0.2116	4.5308E-02	1.33E-01	ENSG00000176531
PHLPP1	-0.0904	3.2642E-01	5.21E-01	ENSG00000081913
PHLPP2	0.1422	8.1789E-02	2.04E-01	ENSG00000040199
PHOSPHO1	0.1017	2.2314E-01	4.05E-01	ENSG00000173868
PHOSPHO2	0.2131	3.7058E-02	1.14E-01	ENSG00000144362
PHOX2A	0.0254	2.0996E-01		ENSG00000165462
PHOX2B	0.0310	1.5302E-02		ENSG00000109132
PHPT1	-0.2989	5.3611E-08	2.35E-06	ENSG00000054148
PHRF1	0.2788	1.4068E-03	9.34E-03	ENSG00000070047
PHTF1	0.0578	3.0658E-01	4.99E-01	ENSG00000116793
PHTF2	0.3521	1.1921E-05	2.02E-04	ENSG00000006576
PHYH	-0.4236	7.4994E-05	8.89E-04	ENSG00000107537
PHYHD1	-0.3432	1.7336E-03	1.10E-02	ENSG00000175287
PHYHIP	0.0407	6.0442E-01	7.64E-01	ENSG00000168490
PHYHIPL	0.1805	7.6174E-02	1.94E-01	ENSG00000165443
PHYKPL	-0.2351	3.0127E-03	1.69E-02	ENSG00000175309
PI15	0.0365	6.4281E-01	7.93E-01	ENSG00000137558
PI16	-0.0080	9.1970E-01	9.60E-01	ENSG00000164530
PI3	-0.0246	9.7967E-01		ENSG00000124102
PI4K2A	-0.1315	5.4229E-02	1.52E-01	ENSG00000155252
PI4K2B	0.3728	2.7639E-03	1.58E-02	ENSG00000038210
PI4KA	-0.0786	8.5257E-02	2.10E-01	ENSG00000241973
PI4KAP2	0.2012	3.6624E-04	3.22E-03	ENSG00000183506
PI4KB	-0.0557	1.7283E-01	3.42E-01	ENSG00000143393
PIANP	-0.1234	9.8978E-02	2.33E-01	ENSG00000139200
PIAS1	0.1666	6.4099E-03	3.04E-02	ENSG00000033800
PIAS2	0.0386	5.0408E-01	6.85E-01	ENSG00000078043
PIAS3	0.0279	6.6730E-01	8.09E-01	ENSG00000131788
PIAS4	0.0323	6.7221E-01	8.12E-01	ENSG00000105229
PIBF1	0.0216	7.4653E-01	8.61E-01	ENSG00000083535
PICALM	-0.1528	3.3893E-02	1.07E-01	ENSG00000073921
PICART1	0.1297	1.9075E-01	3.66E-01	ENSG00000246640
PICK1	0.2080	3.5716E-03	1.93E-02	ENSG00000100151
PICSAR	-0.0051	9.3681E-01		ENSG00000275874
PID1	0.0299	7.4475E-01	8.60E-01	ENSG00000153823
PIDD1	0.2656	3.4351E-03	1.87E-02	ENSG00000177595
PIERCE1	-0.0200	7.9987E-01	8.93E-01	ENSG00000160345
PIERCE2	-0.1843	4.9749E-02	1.43E-01	ENSG00000261652
PIEZO1	0.0970	3.2614E-01	5.20E-01	ENSG00000103335
PIEZO2	0.2322	1.6980E-02	6.36E-02	ENSG00000154864
PIF1	0.0614	5.2186E-01	6.99E-01	ENSG00000140451
PIGA	-0.0587	4.8594E-01	6.70E-01	ENSG00000165195

PIGB	-0.2628	6.5428E-03	3.08E-02	ENSG00000069943
PIGBOS1	-0.1319	2.9441E-02	9.61E-02	ENSG00000225973
PIGC	-0.0716	1.8814E-01	3.63E-01	ENSG00000135845
PIGF	-0.2270	1.0440E-03	7.35E-03	ENSG00000151665
PIGG	0.1130	1.2666E-01	2.77E-01	ENSG00000174227
PIGH	0.2077	1.1492E-02	4.72E-02	ENSG00000100564
PIGK	0.0560	4.4343E-01	6.34E-01	ENSG00000142892
PIGL	0.4378	5.5460E-05	6.98E-04	ENSG00000108474
PIGM	0.0276	6.0051E-01	7.61E-01	ENSG00000143315
PIGN	-0.0011	9.8821E-01	9.94E-01	ENSG00000197563
PIGO	-0.0517	4.8936E-01	6.73E-01	ENSG00000165282
PIGP	-0.1219	9.5233E-02	2.27E-01	ENSG00000185808
PIGQ	-0.2332	9.7232E-06	1.71E-04	ENSG00000007541
PIGR	-0.0162	7.6897E-01		ENSG00000162896
PIGS	0.1215	5.5116E-02	1.54E-01	ENSG00000087111
PIGT	-0.0046	9.4751E-01	9.73E-01	ENSG00000124155
PIGU	-0.1370	1.8874E-02	6.90E-02	ENSG00000101464
PIGV	-0.0200	7.6425E-01	8.72E-01	ENSG00000060642
PIGW	0.4982	2.5503E-05	3.72E-04	ENSG00000277161
PIGX	-0.0588	2.1691E-01	3.98E-01	ENSG00000163964
PIGY-DT	-0.0149	8.8806E-01	9.44E-01	ENSG00000285122
PIGZ	0.1504	4.2671E-02	1.27E-01	ENSG00000119227
PIH1D1	0.0633	2.9644E-01	4.88E-01	ENSG00000104872
PIH1D2	0.0161	8.0855E-01	8.98E-01	ENSG00000150773
PIK3AP1	0.5274	2.4643E-03	1.45E-02	ENSG00000155629
PIK3C2A	-0.1090	1.6240E-01	3.28E-01	ENSG00000011405
PIK3C2B	0.1021	2.6082E-01	4.49E-01	ENSG00000133056
PIK3C2G	-0.0052	9.5553E-01	9.77E-01	ENSG00000139144
PIK3C3	0.0985	7.4298E-02	1.90E-01	ENSG00000078142
PIK3CA	0.1298	1.5297E-01	3.15E-01	ENSG00000121879
PIK3CA-DT	0.0286	1.5404E-01		ENSG00000229102
PIK3CB	0.0960	1.4701E-01	3.06E-01	ENSG00000051382
PIK3CD	0.1734	8.4232E-02	2.08E-01	ENSG00000171608
PIK3CD-AS2	0.1085	1.4811E-01	3.08E-01	ENSG00000231789
PIK3CG	-0.0003	9.7395E-01		ENSG00000105851
PIK3IP1	-0.1272	4.8602E-02	1.40E-01	ENSG00000100100
PIK3IP1-DT	0.1520	1.4911E-01	3.09E-01	ENSG00000228839
PIK3R1	0.0283	7.0618E-01	8.36E-01	ENSG00000145675
PIK3R2	-0.0119	8.8052E-01	9.39E-01	ENSG00000105647
PIK3R3	-0.1738	2.4748E-02	8.46E-02	ENSG00000117461
PIK3R4	0.1745	2.3554E-02	8.14E-02	ENSG00000196455
PIK3R5	0.2804	2.4206E-02	8.31E-02	ENSG00000141506
PIKFYVE	-0.0003	9.9559E-01	9.98E-01	ENSG00000115020
PILRA	0.0691	4.7845E-01	6.64E-01	ENSG00000085514
PILRB	0.0462	6.3812E-01	7.90E-01	ENSG00000121716
PIM1	-0.4257	4.0292E-09	2.74E-07	ENSG00000137193
PIM2	0.1015	1.8758E-01	3.62E-01	ENSG00000102096
PIM3	0.0456	5.7361E-01	7.41E-01	ENSG00000198355
PIMREG	-0.3912	3.9825E-03	2.10E-02	ENSG00000129195

PIN1	-0.0689	1.4892E-01	3.09E-01	ENSG00000127445
PIN1-DT	-0.1461	1.7145E-01	3.40E-01	ENSG00000267289
PIN1P1	0.0016	7.0144E-01		ENSG00000229359
PIN4	-0.1686	6.7472E-03	3.15E-02	ENSG00000102309
PINCR	-0.0039	8.7287E-01		ENSG00000224294
PINK1	0.1301	9.0715E-02	2.20E-01	ENSG00000158828
PINK1-AS	0.1510	1.3283E-01	2.86E-01	ENSG00000117242
PINLYP	-0.1318	1.9626E-01	3.73E-01	ENSG00000234465
PINX1	0.0273	7.2751E-01	8.50E-01	ENSG00000254093
PIP	-0.0662	1.4766E-02		ENSG00000159763
PIP4K2A	-0.0568	4.8488E-01	6.69E-01	ENSG00000150867
PIP4K2B	-0.0638	4.0034E-01	5.94E-01	ENSG00000276293
PIP4K2C	0.2254	9.1226E-03	3.97E-02	ENSG00000166908
PIP4P1	0.1439	5.7101E-02	1.58E-01	ENSG00000165782
PIP4P2	-0.0689	1.9864E-01	3.76E-01	ENSG00000155099
PIP5K1A	0.3724	3.7166E-06	7.91E-05	ENSG00000143398
PIP5K1B	-0.0564	5.5401E-01	7.25E-01	ENSG00000107242
PIP5K1C	-0.0614	3.0151E-01	4.94E-01	ENSG00000186111
PIP5KL1	0.0591	4.5735E-01	6.46E-01	ENSG00000167103
PIPOX	0.2052	1.6863E-02	6.33E-02	ENSG00000179761
PIPSL	0.2186	7.2622E-02	1.87E-01	ENSG00000180764
PIR	-0.0132	8.6949E-01	9.34E-01	ENSG00000087842
PIRAT1	0.0045	6.8581E-01		ENSG00000237803
PIRT	-0.2730	2.4195E-02	8.31E-02	ENSG00000233670
PISD	0.2665	2.7389E-04	2.53E-03	ENSG00000241878
PITHD1	-0.0037	8.5027E-01	9.23E-01	ENSG00000057757
PITPNA	0.1616	1.6502E-02	6.23E-02	ENSG00000174238
PITPNA-AS1	-0.2396	2.1967E-02	7.73E-02	ENSG00000236618
PITPNB	0.2457	2.1091E-04	2.04E-03	ENSG00000180957
PITPNC1	0.0933	9.2906E-02	2.23E-01	ENSG00000154217
PITPNM1	0.1656	4.3430E-02	1.29E-01	ENSG00000110697
PITPNM2	0.3885	1.9147E-03	1.18E-02	ENSG00000090975
PITPNM2-AS1	0.0241	3.0263E-01		ENSG00000251497
PITPNM3	-0.0338	6.8195E-01	8.20E-01	ENSG00000091622
PITRM1	0.0758	2.1670E-01	3.97E-01	ENSG00000107959
PITRM1-AS1	0.0377	6.1275E-01	7.71E-01	ENSG00000237399
PITX1	-0.0237	7.8654E-01	8.85E-01	ENSG00000069011
PITX1-AS1	-0.0162	8.6483E-01	9.31E-01	ENSG00000224186
PITX2	0.3180	1.2245E-06	3.16E-05	ENSG00000164093
PITX3	0.1984	6.1447E-02	1.66E-01	ENSG00000107859
PIWIL2	0.0796	4.2129E-01	6.14E-01	ENSG00000197181
PIWIL2-DT	0.0649	3.6720E-01	5.63E-01	ENSG00000254064
PIWIL4	0.0078	9.2818E-01	9.65E-01	ENSG00000134627
PIWIL4-AS1	0.0050	6.4802E-01		ENSG00000255929
PJA1	-0.0533	5.1857E-01	6.96E-01	ENSG00000181191
PJA2	0.0674	3.1428E-01	5.08E-01	ENSG00000198961
PJVK	0.3557	2.6445E-05	3.83E-04	ENSG00000204311
PKD1	0.4118	5.3271E-08	2.35E-06	ENSG00000008710
PKD1-AS1	0.1129	1.7987E-01	3.51E-01	ENSG00000259933

PKD1L1	0.0543	2.5187E-01	4.39E-01	ENSG00000158683
PKD1L2	0.3043	2.8212E-02	9.30E-02	ENSG00000166473
PKD1L3	-0.2439	4.8176E-02	1.39E-01	ENSG00000277481
PKD1P2	0.5771	1.5051E-07	5.45E-06	ENSG00000227827
PKD1P3	0.5687	7.5439E-06	1.41E-04	ENSG00000183458
PKD1P4	0.4565	2.2230E-05	3.34E-04	ENSG00000205746
PKD1P5	0.4529	3.0142E-06	6.63E-05	ENSG00000254681
PKD1P6	0.1949	2.1126E-02	7.50E-02	ENSG00000250251
PKD1P6-NPIPP1	0.2408	4.3740E-02	1.30E-01	ENSG00000270580
PKD2	-0.0783	2.4270E-01	4.28E-01	ENSG00000118762
PKD2L2	0.2490	5.3950E-02	1.51E-01	ENSG00000078795
PKDCC	0.5069	4.8261E-06	9.76E-05	ENSG00000162878
PKDREJ	0.0320	4.6187E-01		ENSG00000130943
PKHD1L1	0.0081	9.1071E-01	9.55E-01	ENSG00000205038
PKIA	0.1656	7.0517E-02	1.83E-01	ENSG00000171033
PKIA-AS1	-0.1825	8.5099E-02	2.10E-01	ENSG00000254266
PKIB	-0.0702	2.9380E-01	4.86E-01	ENSG00000135549
PKIG	-0.1126	1.6611E-02	6.27E-02	ENSG00000168734
PKLR	0.0123	4.1795E-01	6.11E-01	ENSG00000143627
PKM	0.2656	8.9513E-03	3.91E-02	ENSG00000067225
PKMYT1	-0.0163	8.5217E-01	9.24E-01	ENSG00000127564
PKN1	0.1139	9.4281E-02	2.26E-01	ENSG00000123143
PKN2	0.0041	9.8597E-01	9.92E-01	ENSG00000065243
PKN2-AS1	-0.1606	1.1145E-01	2.53E-01	ENSG00000237505
PKN3	-0.1503	1.5269E-01	3.14E-01	ENSG00000160447
PKNOX1	-0.2625	5.3687E-03	2.65E-02	ENSG00000160199
PKNOX2	-0.1488	7.6472E-02	1.94E-01	ENSG00000165495
PKNOX2-DT	0.0918	2.4953E-01	4.36E-01	ENSG00000254880
PKP1	0.1232	4.2012E-02	1.26E-01	ENSG00000081277
PKP2	0.0181	7.7790E-01	8.80E-01	ENSG00000057294
PKP3	0.4564	2.0914E-03	1.27E-02	ENSG00000184363
PKP4	-0.1117	1.8372E-01	3.57E-01	ENSG00000144283
PKP4-AS1	0.0217	5.2246E-01		ENSG00000204380
PLA1A	-0.0315	6.8567E-01	8.22E-01	ENSG00000144837
PLA2G10	0.2484	5.3517E-02	1.50E-01	ENSG00000069764
PLA2G10EP	-0.0154	8.5350E-01	9.25E-01	ENSG00000255037
PLA2G10FP	0.0499	4.5300E-02		ENSG00000261181
PLA2G10GP	0.0350	2.4568E-01		ENSG00000277770
PLA2G10IP	0.0233	1.9742E-01		ENSG00000276484
PLA2G12A	-0.0429	5.2430E-01	7.01E-01	ENSG00000123739
PLA2G12B	0.0485	5.6170E-01	7.31E-01	ENSG00000138308
PLA2G15	0.0106	8.9936E-01	9.50E-01	ENSG00000103066
PLA2G1B	-0.1598	1.0959E-01	2.50E-01	ENSG00000170890
PLA2G2A	-0.0471	1.8290E-01	3.55E-01	ENSG00000188257
PLA2G2C	-0.2927	3.6077E-02	1.12E-01	ENSG00000187980
PLA2G3	0.0366	6.7811E-01	8.16E-01	ENSG00000100078
PLA2G4A	-0.0736	4.5187E-01	6.41E-01	ENSG00000116711
PLA2G4C	0.0862	2.7615E-01	4.67E-01	ENSG00000105499
PLA2G4D	0.0850	9.7465E-02		ENSG00000159337

PLA2G4E	-0.0137	8.7416E-01		ENSG00000188089
PLA2G4E-AS1	0.0946	1.4582E-01		ENSG00000246740
PLA2G4F	-0.1614	1.3119E-01	2.83E-01	ENSG00000168907
PLA2G5	-0.2126	6.9504E-02	1.81E-01	ENSG00000127472
PLA2G6	-0.2007	2.9131E-02	9.54E-02	ENSG00000184381
PLA2G7	-0.0682	4.4148E-01	6.32E-01	ENSG00000146070
PLA2R1	-0.0930	3.2756E-01	5.22E-01	ENSG00000153246
PLAA	0.0781	1.1619E-01	2.61E-01	ENSG00000137055
PLAAT1	0.4026	1.7249E-04	1.74E-03	ENSG00000127252
PLAAT2	-0.0880	1.0926E-01	2.50E-01	ENSG00000133328
PLAAT3	-0.4568	8.2196E-09	4.86E-07	ENSG00000176485
PLAAT5	-0.1012	4.1421E-02		ENSG00000168004
PLAC4	-0.0056	7.5555E-01		ENSG00000280109
PLAC8	-0.4163	8.4540E-03	3.75E-02	ENSG00000145287
PLAC8L1	0.1026	1.4120E-02		ENSG00000173261
PLAGL1	0.4143	2.9441E-03	1.66E-02	ENSG00000118495
PLAGL2	0.1901	7.3708E-03	3.37E-02	ENSG00000126003
PLAUR	-0.3730	1.9128E-02	6.96E-02	ENSG00000011422
PLB1	0.5582	1.7175E-06	4.16E-05	ENSG00000163803
PLBD1	-0.0941	2.7963E-01	4.71E-01	ENSG00000121316
PLBD1-AS1	-0.0499	4.9394E-01	6.77E-01	ENSG00000256751
PLBD2	0.3658	6.5696E-05	8.01E-04	ENSG00000151176
PLCB1	0.1980	1.8283E-03	1.14E-02	ENSG00000182621
PLCB1-IT1	-0.0150	7.7178E-01		ENSG00000225479
PLCB2	0.0893	3.4354E-01	5.39E-01	ENSG00000137841
PLCB3	-0.1126	2.3008E-01	4.13E-01	ENSG00000149782
PLCB4	0.1244	2.7230E-02	9.06E-02	ENSG00000101333
PLCD1	-0.1974	2.8979E-02	9.50E-02	ENSG00000187091
PLCD3	-0.0075	9.1691E-01	9.58E-01	ENSG00000161714
PLCD4	-0.1384	1.3501E-01	2.89E-01	ENSG00000115556
PLCE1	-0.0143	8.3164E-01	9.12E-01	ENSG00000138193
PLCE1-AS1	-0.0079	9.3183E-01		ENSG00000268894
PLCE1-AS2	-0.0284	4.8345E-01		ENSG00000232913
PLCG1	0.1580	5.1056E-02	1.45E-01	ENSG00000124181
PLCG1-AS1	0.3795	1.0680E-03	7.47E-03	ENSG00000226648
PLCG2	0.0456	6.9106E-02		ENSG00000197943
PLCH1	-0.1051	2.4418E-01	4.30E-01	ENSG00000114805
PLCH2	0.0253	7.7581E-01	8.79E-01	ENSG00000149527
PLCL1	0.4067	5.3550E-05	6.80E-04	ENSG00000115896
PLCL2	0.3633	6.7174E-04	5.21E-03	ENSG00000154822
PLCXD1	-0.0436	5.1174E-01	6.92E-01	ENSG00000182378
PLCXD2	-0.1492	4.3170E-02	1.28E-01	ENSG00000240891
PLCXD3	0.0678	4.8731E-01	6.71E-01	ENSG00000182836
PLCZ1	0.1230	1.9520E-01	3.71E-01	ENSG00000139151
PLD1	-0.0509	5.6774E-01	7.36E-01	ENSG00000075651
PLD2	0.1907	4.5964E-03	2.35E-02	ENSG00000129219
PLD3	0.1718	4.4335E-03	2.28E-02	ENSG00000105223
PLD4	0.1060	1.1401E-01	2.58E-01	ENSG00000166428
PLD5	-0.0310	7.2670E-01	8.49E-01	ENSG00000180287

PLD6	-0.5460	1.9976E-03	1.22E-02	ENSG00000179598
PLEC	-0.3518	1.0913E-04	1.20E-03	ENSG00000178209
PLEK	0.0115	2.5882E-01		ENSG00000115956
PLEK2	-0.0057	9.3506E-01	9.68E-01	ENSG00000100558
PLEKHA1	0.2657	8.0852E-06	1.49E-04	ENSG00000107679
PLEKHA2	-0.3636	1.5381E-03	1.00E-02	ENSG00000169499
PLEKHA3	-0.1251	1.0049E-02	4.27E-02	ENSG00000116095
PLEKHA4	-0.5440	1.7165E-09	1.33E-07	ENSG00000105559
PLEKHA5	0.0909	2.9667E-02	9.67E-02	ENSG00000052126
PLEKHA6	0.0325	6.3824E-01	7.90E-01	ENSG00000143850
PLEKHA7	-0.0784	3.1944E-01	5.13E-01	ENSG00000166689
PLEKHA8	0.2986	4.6514E-04	3.87E-03	ENSG00000106086
PLEKHA8P1	-0.0978	3.2137E-01	5.15E-01	ENSG00000134297
PLEKHB1	-0.2688	1.6174E-02	6.14E-02	ENSG00000021300
PLEKHB2	0.0393	5.1342E-01	6.93E-01	ENSG00000115762
PLEKHD1	-0.0476	5.9080E-01	7.54E-01	ENSG00000175985
PLEKHF1	-0.3668	1.7809E-05	2.79E-04	ENSG00000166289
PLEKHF2	-0.0442	6.3009E-01	7.84E-01	ENSG00000175895
PLEKHG1	-0.2646	9.1738E-03	3.99E-02	ENSG00000120278
PLEKHG2	0.0300	7.1657E-01	8.43E-01	ENSG00000090924
PLEKHG3	-0.0104	9.1725E-01	9.59E-01	ENSG00000126822
PLEKHG4	0.3387	1.9588E-02	7.08E-02	ENSG00000196155
PLEKHG4B	-0.0619	4.8784E-01	6.72E-01	ENSG00000153404
PLEKHG5	-0.1398	7.6108E-02	1.94E-01	ENSG00000171680
PLEKHG6	0.3493	6.9019E-03	3.20E-02	ENSG00000008323
PLEKHG7	-0.0234	7.8129E-01		ENSG00000187510
PLEKHH1	0.1036	1.6472E-01	3.31E-01	ENSG00000054690
PLEKHH2	0.0771	3.9464E-01	5.89E-01	ENSG00000152527
PLEKHH3	0.0062	9.3254E-01	9.66E-01	ENSG00000068137
PLEKHJ1	0.0574	1.8806E-01	3.63E-01	ENSG00000104886
PLEKHM1	-0.1931	1.4377E-02	5.62E-02	ENSG00000225190
PLEKHM2	-0.2036	1.8666E-04	1.86E-03	ENSG00000116786
PLEKHM3	0.0318	6.6140E-01	8.05E-01	ENSG00000178385
PLEKHN1	0.0611	3.7937E-01	5.74E-01	ENSG00000187583
PLEKHO1	0.0031	9.7761E-01	9.88E-01	ENSG00000023902
PLEKHO2	0.0622	4.6914E-01	6.57E-01	ENSG00000241839
PLET1	0.0584	4.8641E-01	6.71E-01	ENSG00000188771
PLG	0.0052	7.9858E-01		ENSG00000122194
PLGLB1	0.1054	3.0168E-01	4.94E-01	ENSG00000183281
PLGLB2	0.1116	2.6198E-01	4.51E-01	ENSG00000125551
PLGRKT	-0.1061	1.2475E-01	2.74E-01	ENSG00000107020
PLIN1	0.0313	7.1315E-01	8.40E-01	ENSG00000166819
PLIN2	0.0816	3.8123E-01	5.76E-01	ENSG00000147872
PLIN3	-0.2075	1.2201E-02	4.95E-02	ENSG00000105355
PLIN4	0.2619	4.2956E-02	1.28E-01	ENSG00000167676
PLIN5	0.0514	5.9109E-01	7.54E-01	ENSG00000214456
PLK1	-0.1699	9.7500E-02	2.31E-01	ENSG00000166851
PLK2	-0.0689	3.5580E-01	5.51E-01	ENSG00000145632
PLK3	-0.1799	1.4962E-02	5.79E-02	ENSG00000173846

PLK4	-0.0832	3.8034E-01	5.75E-01	ENSG00000142731
PLK5	0.1361	1.8358E-01	3.56E-01	ENSG00000185988
PLLP	-0.5788	4.4535E-04	3.75E-03	ENSG00000102934
PLN	-0.0425	4.9151E-01	6.75E-01	ENSG00000198523
PLOD1	0.0620	5.0584E-01	6.87E-01	ENSG00000083444
PLOD2	0.0481	6.0197E-01	7.62E-01	ENSG00000152952
PLOD3	-0.0544	3.1420E-01	5.07E-01	ENSG00000106397
PLP2	-0.3466	1.0086E-04	1.13E-03	ENSG00000102007
PLPBP	-0.1850	1.0574E-04	1.17E-03	ENSG00000147471
PLPP1	-0.1500	5.0549E-02	1.44E-01	ENSG00000067113
PLPP2	-0.2507	3.1709E-02	1.02E-01	ENSG00000141934
PLPP3	-0.2584	1.2949E-03	8.73E-03	ENSG00000162407
PLPP4	0.0009	9.9185E-01	9.96E-01	ENSG00000203805
PLPP5	-0.2061	1.8786E-03	1.17E-02	ENSG00000147535
PLPP7	0.2085	3.4480E-02	1.08E-01	ENSG00000160539
PLPPR1	0.0373	7.1410E-01	8.41E-01	ENSG00000148123
PLPPR2	-0.0037	9.3727E-01	9.69E-01	ENSG00000105520
PLPPR3	0.1831	6.7767E-02	1.78E-01	ENSG00000129951
PLPPR4	0.1228	2.2534E-01	4.08E-01	ENSG00000117600
PLPPR5	-0.0440	6.4448E-01	7.94E-01	ENSG00000117598
PLPPR5-AS1	0.0017	8.7392E-01		ENSG00000232825
PLRG1	0.2427	1.2458E-04	1.33E-03	ENSG00000171566
PLS1	-0.0802	4.0307E-01	5.97E-01	ENSG00000120756
PLS3	0.1000	2.4829E-01	4.35E-01	ENSG00000102024
PLS3-AS1	0.0575	5.5778E-01	7.28E-01	ENSG00000271826
PLSCR1	-0.2758	9.6870E-04	6.94E-03	ENSG00000188313
PLSCR2	-0.2251	6.8817E-02	1.80E-01	ENSG00000163746
PLSCR3	0.0291	3.6835E-01		ENSG00000187838
PLTP	-0.4781	6.8940E-08	2.87E-06	ENSG00000100979
PLVAP	0.0235	1.0015E-01		ENSG00000130300
PLXDC1	0.1171	2.0469E-01	3.83E-01	ENSG00000161381
PLXDC2	-0.2359	1.3863E-04	1.45E-03	ENSG00000120594
PLXNA1	0.1555	5.5476E-02	1.54E-01	ENSG00000114554
PLXNA2	0.0199	7.9150E-01	8.88E-01	ENSG00000076356
PLXNA3	-0.0357	6.0390E-01	7.64E-01	ENSG00000130827
PLXNA4	-0.0031	9.7714E-01	9.88E-01	ENSG00000221866
PLXNB1	-0.2121	6.4951E-03	3.06E-02	ENSG00000164050
PLXNB2	-0.0205	7.4589E-01	8.61E-01	ENSG00000196576
PLXNB3	-0.0439	6.4150E-01	7.92E-01	ENSG00000198753
PLXNC1	-0.3930	8.0728E-04	6.02E-03	ENSG00000136040
PLXND1	-0.0975	2.9842E-01	4.91E-01	ENSG00000004399
PM20D1	0.0280	2.4575E-01	4.32E-01	ENSG00000162877
PM20D2	-0.1909	1.7495E-02	6.51E-02	ENSG00000146281
PMAIP1	-0.3380	2.5413E-03	1.48E-02	ENSG00000141682
PMCH	-0.0773	3.9637E-01	5.91E-01	ENSG00000183395
PMCHL2	-0.0373	4.8981E-01	6.74E-01	ENSG00000169040
PMEPA1	-0.2588	1.5827E-03	1.02E-02	ENSG00000124225
PMF1	-0.2322	6.3118E-04	4.95E-03	ENSG00000160783
PML	0.0751	2.9870E-01	4.91E-01	ENSG00000140464

PMM1	-0.0599	3.3172E-01	5.27E-01	ENSG00000100417
PMM2	0.1402	1.1011E-01	2.51E-01	ENSG00000140650
PMP2	-0.0570	2.1585E-01	3.97E-01	ENSG00000147588
PMP22	-0.3756	2.9965E-03	1.68E-02	ENSG00000109099
PMPCA	0.1569	1.7302E-02	6.46E-02	ENSG00000165688
PMPCB	0.0457	4.3032E-01	6.22E-01	ENSG00000105819
PMS1	-0.2010	4.9890E-03	2.50E-02	ENSG00000064933
PMS2	0.0325	6.5301E-01	7.99E-01	ENSG00000122512
PMS2CL	-0.1262	9.3454E-02	2.24E-01	ENSG00000187953
PMS2P12	-0.0087	9.7707E-01		ENSG00000228546
PMS2P14	-0.0222	8.0907E-01	8.99E-01	ENSG00000277125
PMVK	0.0721	1.8523E-01	3.59E-01	ENSG00000163344
PNCK	0.3464	2.9271E-04	2.67E-03	ENSG00000130822
PNISR	-0.2737	5.0743E-06	1.01E-04	ENSG00000132424
PNKD	-0.0184	7.4291E-01	8.59E-01	ENSG00000127838
PNKP	0.2803	6.4505E-08	2.73E-06	ENSG00000039650
PNLDC1	0.0302	4.8743E-01	6.72E-01	ENSG00000146453
PNLIP	0.0012	7.6873E-01		ENSG00000175535
PNLIPRP1	0.0136	5.0919E-01		ENSG00000187021
PNLIPRP3	-0.0033	8.9946E-01		ENSG00000203837
PNMA1	-0.0737	2.9284E-01	4.85E-01	ENSG00000176903
PNMA2	0.1225	1.5990E-01	3.25E-01	ENSG00000240694
PNMA3	0.4136	3.7078E-05	5.01E-04	ENSG00000183837
PNMA5	0.3119	1.9197E-02	6.98E-02	ENSG00000198883
PNMA6A	0.3575	3.9082E-04	3.38E-03	ENSG00000235961
PNMA6F	0.0454	5.8403E-01	7.49E-01	ENSG00000225110
PNMA8A	-0.0404	4.6775E-01	6.55E-01	ENSG00000182013
PNMA8B	0.1558	1.2678E-01	2.77E-01	ENSG00000204851
PNMA8C	0.3720	1.4977E-06	3.72E-05	ENSG00000277531
PNMT	0.0545	5.0092E-01	6.83E-01	ENSG00000141744
PNN	0.1812	2.0974E-02	7.46E-02	ENSG00000100941
PNO1	0.2954	2.0726E-04	2.01E-03	ENSG00000115946
PNOC	0.0737	3.9937E-01	5.94E-01	ENSG00000168081
PNP	0.1845	1.4393E-02	5.63E-02	ENSG00000198805
PNPLA1	0.0398	2.9661E-02		ENSG00000180316
PNPLA2	-0.0673	3.4197E-01	5.37E-01	ENSG00000177666
PNPLA3	-0.2247	4.3117E-03	2.24E-02	ENSG00000100344
PNPLA4	0.1221	6.8866E-02	1.80E-01	ENSG00000006757
PNPLA5	0.0335	5.8821E-01	7.52E-01	ENSG00000100341
PNPLA6	-0.0362	6.7430E-01	8.14E-01	ENSG00000032444
PNPLA7	-0.1813	6.0311E-02	1.64E-01	ENSG00000130653
PNPLA8	-0.0566	4.2411E-01	6.16E-01	ENSG00000135241
PNPO	-0.2873	4.8420E-04	3.99E-03	ENSG00000108439
PNPT1	0.1613	2.8533E-02	9.39E-02	ENSG00000138035
PNRC1	-0.2771	5.6557E-04	4.54E-03	ENSG00000146278
PNRC2	0.0834	2.6443E-01	4.53E-01	ENSG00000189266
POC1A	-0.1286	2.0339E-01	3.81E-01	ENSG00000164087
POC1B	-0.0753	3.8700E-01	5.82E-01	ENSG00000139323
POC1B-GALNT4	-0.0424	4.3411E-01		ENSG00000259075

POC5	0.3288	1.3831E-05	2.28E-04	ENSG00000152359
PODN	0.0621	1.7768E-01		ENSG00000174348
PODNL1	-0.4120	3.2703E-03	1.79E-02	ENSG00000132000
PODXL	-0.1726	6.5482E-02	1.74E-01	ENSG00000128567
PODXL2	0.2785	4.1048E-03	2.16E-02	ENSG00000114631
POF1B	-0.1121	2.7451E-01	4.65E-01	ENSG00000124429
POFUT1	0.0122	8.4066E-01	9.17E-01	ENSG00000101346
POFUT2	0.1238	1.1055E-01	2.52E-01	ENSG00000186866
POGK	0.0737	1.9676E-01	3.73E-01	ENSG00000143157
POGLUT1	-0.0116	8.4913E-01	9.22E-01	ENSG00000163389
POGLUT2	0.0076	9.4780E-01	9.74E-01	ENSG00000134901
POGLUT3	-0.1045	1.9642E-01	3.73E-01	ENSG00000178202
POGZ	-0.1481	3.6818E-02	1.14E-01	ENSG00000143442
POLA1	-0.2049	3.2505E-02	1.04E-01	ENSG00000101868
POLA2	0.0557	5.6881E-01	7.37E-01	ENSG00000014138
POLB	0.0767	2.1134E-01	3.91E-01	ENSG00000070501
POLD1	0.0479	6.1559E-01	7.73E-01	ENSG00000062822
POLD2	-0.0406	4.6290E-01	6.51E-01	ENSG00000106628
POLD3	-0.0053	9.3938E-01	9.70E-01	ENSG00000077514
POLD4	0.0422	6.2644E-01	7.81E-01	ENSG00000175482
POLDIP2	0.1050	8.3351E-02	2.07E-01	ENSG00000004142
POLDIP3	-0.0406	4.0288E-01	5.97E-01	ENSG00000100227
POLE	0.0659	5.0483E-01	6.86E-01	ENSG00000177084
POLE2	-0.2695	1.6916E-02	6.35E-02	ENSG00000100479
POLE3	0.0524	4.5767E-01	6.47E-01	ENSG00000148229
POLE4	-0.2094	3.1951E-03	1.77E-02	ENSG00000115350
POLG	0.2522	3.7244E-03	1.99E-02	ENSG00000140521
POLG2	0.0839	3.3064E-01	5.25E-01	ENSG00000256525
POLH	0.3319	4.5137E-04	3.79E-03	ENSG00000170734
POLI	0.2761	1.2482E-04	1.33E-03	ENSG00000101751
POLK	0.0129	8.9598E-01	9.48E-01	ENSG00000122008
POLL	0.1190	9.5825E-02	2.28E-01	ENSG00000166169
POLM	0.1599	7.6258E-02	1.94E-01	ENSG00000122678
POLN	-0.0143	8.5267E-01	9.25E-01	ENSG00000130997
POLQ	-0.0511	5.9264E-01	7.55E-01	ENSG00000051341
POLR1A	0.2821	2.6998E-04	2.50E-03	ENSG00000068654
POLR1B	0.2395	7.3848E-03	3.37E-02	ENSG00000125630
POLR1C	0.2317	1.5922E-05	2.56E-04	ENSG00000171453
POLR1D	-0.2553	4.1030E-05	5.46E-04	ENSG00000186184
POLR1E	-0.1380	3.3073E-02	1.05E-01	ENSG00000137054
POLR1F	-0.3135	5.5612E-07	1.63E-05	ENSG00000105849
POLR1G	-0.5397	9.1936E-04	6.67E-03	ENSG00000117877
POLR1H	0.0841	2.3216E-01	4.16E-01	ENSG00000066379
POLR1HASP	0.2024	5.1170E-02	1.45E-01	ENSG00000204623
POLR2A	-0.0725	1.7144E-01	3.40E-01	ENSG00000181222
POLR2B	0.0576	2.6499E-01	4.54E-01	ENSG00000047315
POLR2C	-0.0408	3.8023E-01	5.75E-01	ENSG00000102978
POLR2D	-0.0939	1.1543E-01	2.60E-01	ENSG00000144231
POLR2E	-0.0715	2.8564E-02	9.40E-02	ENSG00000099817

POLR2F	-0.1853	1.8730E-04	1.86E-03	ENSG00000100142
POLR2G	-0.1366	3.5590E-02	1.11E-01	ENSG00000168002
POLR2H	-0.0863	5.7833E-02	1.59E-01	ENSG00000163882
POLR2I	-0.3024	2.4233E-06	5.57E-05	ENSG00000105258
POLR2J	-0.1880	1.7554E-04	1.77E-03	ENSG00000005075
POLR2J2	0.4473	1.1035E-06	2.91E-05	ENSG00000228049
POLR2J3	0.3053	7.3497E-04	5.59E-03	ENSG00000285437
POLR2J3-UPK3BL2	0.1049	3.0029E-01	4.93E-01	ENSG00000168255
POLR2J4	-0.1684	1.1893E-01	2.65E-01	ENSG00000214783
POLR2K	-0.0638	3.1308E-01	5.06E-01	ENSG00000147669
POLR2L	-0.3194	1.6001E-06	3.93E-05	ENSG00000177700
POLR2M	-0.2937	5.1790E-07	1.53E-05	ENSG00000255529
POLR3A	0.2747	1.5924E-04	1.63E-03	ENSG00000148606
POLR3B	0.0294	7.0346E-01	8.34E-01	ENSG00000013503
POLR3C	0.2058	4.7186E-03	2.39E-02	ENSG00000186141
POLR3D	-0.1189	1.0588E-01	2.45E-01	ENSG00000168495
POLR3E	0.2779	3.6539E-05	4.94E-04	ENSG00000058600
POLR3F	0.3082	8.5348E-07	2.35E-05	ENSG00000132664
POLR3G	0.1400	9.9027E-02	2.33E-01	ENSG00000113356
POLR3GL	-0.0042	9.3285E-01	9.67E-01	ENSG00000121851
POLR3H	-0.1502	4.0197E-02	1.22E-01	ENSG00000100413
POLR3K	-0.1286	7.0408E-02	1.83E-01	ENSG00000161980
POLRMT	0.2345	6.2263E-03	2.97E-02	ENSG00000099821
POLRMTP1	0.0712	2.9301E-01	4.85E-01	ENSG00000266066
POM121	0.0876	1.7704E-01	3.47E-01	ENSG00000196313
POM121C	0.0401	5.1841E-01	6.96E-01	ENSG00000272391
POM121L15P	-0.0817	3.8079E-01	5.76E-01	ENSG00000161103
POMGNT1	-0.0017	9.5013E-01	9.75E-01	ENSG00000085998
POMGNT2	-0.2041	3.5097E-04	3.11E-03	ENSG00000144647
POMK	-0.2162	3.7737E-03	2.02E-02	ENSG00000185900
POMP	-0.1760	3.1886E-03	1.76E-02	ENSG00000132963
POMT1	0.2297	1.7127E-03	1.09E-02	ENSG00000130714
POMT2	0.1076	1.6683E-01	3.34E-01	ENSG00000009830
POMZP3	-0.0888	2.8060E-01	4.72E-01	ENSG00000146707
PON1	0.5610	4.9655E-03	2.49E-02	ENSG00000005421
PON2	-0.3856	7.6616E-05	9.04E-04	ENSG00000105854
PON3	0.1341	1.0137E-01	2.37E-01	ENSG00000105852
POP1	-0.0625	5.0611E-01	6.87E-01	ENSG00000104356
POP4	-0.0425	3.7075E-01	5.66E-01	ENSG00000105171
POP5	-0.1543	2.6721E-03	1.54E-02	ENSG00000167272
POP7	-0.0723	1.3309E-01	2.86E-01	ENSG00000172336
POPDC2	0.1722	1.0638E-01	2.45E-01	ENSG00000121577
POPDC3	-0.2386	3.0849E-02	9.95E-02	ENSG00000132429
POR	0.0485	4.9067E-01	6.75E-01	ENSG00000127948
PORCN	-0.1194	7.0921E-02	1.84E-01	ENSG00000102312
PORCN-DT	-0.3052	3.1674E-02	1.01E-01	ENSG00000224292
POT1	0.0612	2.3473E-01	4.19E-01	ENSG00000128513
POT1-AS1	-0.0603	5.2883E-01	7.04E-01	ENSG00000224897
POTEC	-0.0178	8.6324E-01		ENSG00000183206

POTEE	0.0260	7.0975E-01	8.38E-01	ENSG00000188219
POTEF	0.1253	1.6778E-01	3.35E-01	ENSG00000196604
POTEG	-0.0876	9.6328E-01	9.81E-01	ENSG00000187537
POTEH	0.0096	4.4579E-01		ENSG00000198062
POTEI	0.5383	3.7845E-04	3.30E-03	ENSG00000196834
POTEJ	0.4393	3.7534E-03	2.01E-02	ENSG00000222038
POTEM	-0.1096	7.6736E-01	8.74E-01	ENSG00000222036
POU1F1	0.0276	2.2870E-01		ENSG00000064835
POU2AF1	-0.0350	3.8053E-01		ENSG00000110777
POU2AF3	-0.0182	8.3364E-01	9.13E-01	ENSG00000214290
POU2F1	0.2609	2.0337E-04	1.99E-03	ENSG00000143190
POU2F2	0.2554	1.0316E-02	4.35E-02	ENSG00000028277
POU2F3	-0.0466	7.2100E-01	8.45E-01	ENSG00000137709
POU3F1	0.0153	8.7483E-01	9.37E-01	ENSG00000185668
POU3F2	0.2002	7.0117E-02	1.83E-01	ENSG00000184486
POU3F3	-0.0241	7.9880E-01	8.92E-01	ENSG00000198914
POU3F4	-0.1858	1.0752E-01	2.47E-01	ENSG00000196767
POU4F1	0.3642	1.3284E-02	5.28E-02	ENSG00000152192
POU4F2	-0.0075	8.2892E-01		ENSG00000151615
POU4F3	-0.0703	3.6128E-01	5.57E-01	ENSG00000091010
POU5F1	-0.0184	8.0602E-01		ENSG00000204531
POU5F1B	0.0055	8.0750E-01		ENSG00000212993
POU5F1P3	-0.0095	8.7752E-01		ENSG00000235602
POU5F1P4	0.0428	2.6406E-02		ENSG00000237872
POU5F2	-0.0383	6.5869E-01	8.03E-01	ENSG00000248483
POU6F1	-0.0556	5.3203E-01	7.06E-01	ENSG00000184271
POU6F2	0.4078	2.7523E-06	6.22E-05	ENSG00000106536
PP12613	-0.0064	9.0794E-01		ENSG00000226757
PP2D1	-0.2159	7.8041E-02	1.97E-01	ENSG00000183977
PPA1	-0.1744	5.2678E-03	2.61E-02	ENSG00000180817
PPA2	-0.2118	2.0528E-02	7.34E-02	ENSG00000138777
PPAN	0.1520	1.6512E-01	3.32E-01	ENSG00000130810
PPARA	0.0806	3.3743E-01	5.33E-01	ENSG00000186951
PPARD	0.0094	9.0249E-01	9.51E-01	ENSG00000112033
PPARG	-0.2288	6.2546E-02	1.68E-01	ENSG00000132170
PPARGC1A	-0.0652	3.9728E-01	5.92E-01	ENSG00000109819
PPARGC1B	0.0785	4.2912E-01	6.21E-01	ENSG00000155846
PPAT	-0.0005	9.7769E-01	9.88E-01	ENSG00000128059
PPATP1	-0.0187	4.4559E-01		ENSG00000241293
PPCDC	-0.1321	1.3083E-01	2.83E-01	ENSG00000138621
PPCS	0.0263	7.1817E-01	8.44E-01	ENSG00000127125
PPDPF	-0.1944	5.6229E-03	2.74E-02	ENSG00000125534
PPDPFL	0.0028	7.3870E-01		ENSG00000168333
PPEF1	-0.0399	6.7431E-01	8.14E-01	ENSG00000086717
PPEF2	0.0129	4.3150E-01		ENSG00000156194
PPFIA1	-0.0027	9.2793E-01	9.64E-01	ENSG00000131626
PPFIA2	0.1276	1.6775E-01	3.35E-01	ENSG00000139220
PPFIA2-AS1	0.1309	1.0811E-01	2.48E-01	ENSG00000257467
PPFIA3	0.1310	8.7707E-02	2.14E-01	ENSG00000177380

PPFIA4	0.3539	4.2228E-03	2.20E-02	ENSG00000143847
PPFIBP1	-0.3830	4.4483E-05	5.86E-04	ENSG00000110841
PPFIBP2	0.0036	9.4908E-01	9.74E-01	ENSG00000166387
PPHLN1	-0.0363	3.2688E-01	5.21E-01	ENSG00000134283
PPIA	-0.3602	7.9622E-09	4.74E-07	ENSG00000196262
PPIAL4C	-0.1453	2.1272E-01	3.93E-01	ENSG00000288867
PPIAL4G	-0.1240	3.0192E-01	4.94E-01	ENSG00000236334
PPIAP20	-0.0001	9.9674E-01		ENSG00000235686
PPIAP30	-0.0098	9.0625E-01	9.53E-01	ENSG00000206448
PPIAP36	-0.0127	9.1651E-01		ENSG00000236872
PPIAP41	-0.0379	6.3901E-01	7.90E-01	ENSG00000254463
PPIAP45	-0.0016	9.6465E-01		ENSG00000258116
PPIAP46	-0.0032	7.3566E-01	8.55E-01	ENSG00000260266
PPIAP49	0.0067	6.2308E-01		ENSG00000260471
PPIAP50	-0.0097	7.6790E-01		ENSG00000261330
PPIAP51	-0.0873	6.1855E-01	7.75E-01	ENSG00000260229
PPIAP52	0.0211	7.3086E-01	8.52E-01	ENSG00000270606
PPIAP68	-0.0405	6.0271E-01		ENSG00000213739
PPIAP69	-0.0124	9.6204E-01		ENSG00000228337
PPIAP75	-0.0083	8.3729E-01		ENSG00000271131
PPIAP76	-0.0139	7.3964E-01		ENSG00000250484
PPIAP91	-0.0107	8.7357E-01		ENSG00000228728
PPIB	-0.3652	4.7191E-11	6.25E-09	ENSG00000166794
PPIC	-0.3680	1.6051E-03	1.03E-02	ENSG00000168938
PPID	-0.2812	3.2793E-04	2.94E-03	ENSG00000171497
PPIE	-0.0454	4.5840E-01	6.47E-01	ENSG00000084072
PPIF	-0.2192	2.6235E-02	8.82E-02	ENSG00000108179
PPIG	-0.2329	1.5997E-04	1.64E-03	ENSG00000138398
PPIGP1	-0.0221	5.0353E-01		ENSG00000270442
PPIH	-0.0853	1.1522E-01	2.59E-01	ENSG00000171960
PPIL1	0.1231	5.5023E-02	1.53E-01	ENSG00000137168
PPIL2	0.3347	2.7312E-04	2.52E-03	ENSG00000100023
PPIL3	0.0746	1.6745E-01	3.35E-01	ENSG00000240344
PPIL4	-0.2017	6.1719E-03	2.95E-02	ENSG00000131013
PPIL6	-0.3141	3.4457E-07	1.10E-05	ENSG00000185250
PPIP5K1	0.2421	5.1876E-03	2.58E-02	ENSG00000168781
PPIP5K2	0.2811	2.5116E-03	1.47E-02	ENSG00000145725
PPL	-0.1132	1.4884E-01	3.09E-01	ENSG00000118898
PPM1A	-0.1467	3.2183E-02	1.03E-01	ENSG00000100614
PPM1AP1	-0.0577	9.2266E-01	9.61E-01	ENSG00000250483
PPM1B	0.1713	1.1428E-02	4.70E-02	ENSG00000138032
PPM1D	-0.1203	1.3787E-01	2.93E-01	ENSG00000170836
PPM1E	0.0449	6.3009E-01	7.84E-01	ENSG00000175175
PPM1F	-0.0231	7.7988E-01	8.81E-01	ENSG00000100034
PPM1G	0.1105	1.9650E-02	7.10E-02	ENSG00000115241
PPM1H	-0.0697	4.3727E-01	6.28E-01	ENSG00000111110
PPM1J	0.4772	3.8982E-05	5.22E-04	ENSG00000155367
PPM1K	0.0379	6.4915E-01	7.97E-01	ENSG00000163644
PPM1K-DT	-0.0376	6.6724E-01	8.09E-01	ENSG00000246375

PPM1L	-0.0131	8.2927E-01	9.11E-01	ENSG00000163590
PPM1M	-0.4495	5.6776E-03	2.76E-02	ENSG00000164088
PPM1N	0.1592	1.5367E-01	3.16E-01	ENSG00000213889
PPME1	0.1188	8.8226E-02	2.15E-01	ENSG00000214517
PPOX	0.1228	2.2622E-02	7.90E-02	ENSG00000143224
PPP1CA	-0.0352	5.1842E-01	6.96E-01	ENSG00000172531
PPP1CB	-0.1331	3.5836E-03	1.94E-02	ENSG00000213639
PPP1CC	0.0047	9.3157E-01	9.66E-01	ENSG00000186298
PPP1R10	0.1718	5.8500E-02	1.60E-01	ENSG00000204569
PPP1R11	0.1064	1.3128E-01	2.83E-01	ENSG00000204619
PPP1R12A	0.0611	2.0438E-01	3.83E-01	ENSG00000058272
PPP1R12B	0.0147	8.3704E-01	9.15E-01	ENSG00000077157
PPP1R12BP1	0.0048	8.7796E-01		ENSG00000229238
PPP1R12C	0.0651	4.6022E-01	6.49E-01	ENSG00000125503
PPP1R13B	0.1654	1.9527E-02	7.07E-02	ENSG00000088808
PPP1R13B-DT	-0.0429	3.2710E-01		ENSG00000258735
PPP1R13L	-0.0325	7.1933E-01	8.44E-01	ENSG00000104881
PPP1R14A	0.0266	7.2691E-01	8.49E-01	ENSG00000167641
PPP1R14B	-0.2948	4.1857E-08	1.90E-06	ENSG00000173457
PPP1R14B-AS1	-0.1610	8.1578E-02	2.04E-01	ENSG00000256940
PPP1R14C	-0.1123	2.0035E-01	3.78E-01	ENSG00000198729
PPP1R14D	-0.0360	2.6297E-01		ENSG00000166143
PPP1R15A	-0.4416	4.5465E-09	3.03E-07	ENSG00000087074
PPP1R15B	-0.1821	3.3213E-03	1.82E-02	ENSG00000158615
PPP1R16A	-0.1141	9.7757E-02	2.31E-01	ENSG00000160972
PPP1R16B	-0.0896	3.6584E-01	5.62E-01	ENSG00000101445
PPP1R17	-0.0970	3.3648E-01	5.32E-01	ENSG00000106341
PPP1R18	-0.0568	5.0221E-01	6.84E-01	ENSG00000146112
PPP1R1A	0.2529	1.6135E-03	1.04E-02	ENSG00000135447
PPP1R1B	-0.1135	1.7073E-01	3.39E-01	ENSG00000131771
PPP1R1C	-0.0493	4.9840E-01	6.81E-01	ENSG00000150722
PPP1R2	-0.0388	4.0782E-01	6.01E-01	ENSG00000184203
PPP1R21	-0.0947	9.9052E-02	2.33E-01	ENSG00000162869
PPP1R26	-0.0347	6.0780E-01	7.67E-01	ENSG00000196422
PPP1R26-AS1	-0.1484	1.7317E-01	3.42E-01	ENSG00000225361
PPP1R26P1	0.0367	5.2201E-01	6.99E-01	ENSG00000238086
PPP1R27	0.0209	7.6453E-01	8.72E-01	ENSG00000182676
PPP1R2B	0.0035	7.1386E-01		ENSG00000231989
PPP1R35	0.1400	2.3516E-02	8.13E-02	ENSG00000160813
PPP1R37	-0.3077	7.8617E-04	5.91E-03	ENSG00000104866
PPP1R3B	0.3102	4.0739E-04	3.50E-03	ENSG00000173281
PPP1R3C	0.3672	6.5026E-03	3.07E-02	ENSG00000119938
PPP1R3D	0.2145	1.2335E-02	4.99E-02	ENSG00000132825
PPP1R3E	0.0014	9.6365E-01	9.81E-01	ENSG00000235194
PPP1R3F	-0.0222	7.5424E-01	8.66E-01	ENSG00000049769
PPP1R3G	-0.0362	6.7728E-01	8.16E-01	ENSG00000219607
PPP1R42	-0.2661	2.6492E-03	1.53E-02	ENSG00000178125
PPP1R7	-0.0618	1.9286E-01	3.68E-01	ENSG00000115685
PPP1R8	-0.1299	5.4331E-02	1.52E-01	ENSG00000117751

PPP1R8P1	-0.0529	5.3655E-01	7.10E-01	ENSG00000224986
PPP1R9A	-0.3043	3.3539E-06	7.25E-05	ENSG00000158528
PPP1R9A-AS1	-0.2670	2.0183E-02	7.26E-02	ENSG00000236197
PPP1R9B	-0.2798	1.4815E-02	5.75E-02	ENSG00000108819
PPP2CA	-0.2644	1.9114E-04	1.89E-03	ENSG00000113575
PPP2CB	-0.5553	7.9147E-08	3.23E-06	ENSG00000104695
PPP2R1A	-0.0383	4.7499E-01	6.62E-01	ENSG00000105568
PPP2R1B	0.2143	1.9451E-02	7.05E-02	ENSG00000137713
PPP2R2A	-0.1533	2.5453E-02	8.64E-02	ENSG00000221914
PPP2R2B	0.1713	1.4146E-02	5.55E-02	ENSG00000156475
PPP2R2B-IT1	-0.0297	5.6380E-01		ENSG00000249553
PPP2R2C	-0.1959	5.0314E-02	1.44E-01	ENSG00000074211
PPP2R2D	-0.1670	2.1079E-04	2.03E-03	ENSG00000175470
PPP2R3A	0.0410	5.9610E-01	7.58E-01	ENSG00000073711
PPP2R3B	-0.1921	9.9274E-03	4.22E-02	ENSG00000167393
PPP2R3C	-0.0144	8.1488E-01	9.02E-01	ENSG00000092020
PPP2R5A	-0.1594	2.7916E-02	9.23E-02	ENSG00000066027
PPP2R5B	0.1372	8.8570E-02	2.16E-01	ENSG00000068971
PPP2R5C	0.0043	9.2636E-01	9.64E-01	ENSG00000078304
PPP2R5CP	-0.1204	1.4700E-01	3.06E-01	ENSG00000239557
PPP2R5D	0.1679	7.7162E-02	1.96E-01	ENSG00000112640
PPP2R5E	-0.1312	1.3148E-02	5.24E-02	ENSG00000154001
PPP3CA	-0.3184	1.6747E-03	1.07E-02	ENSG00000138814
PPP3CB	-0.1617	4.7287E-02	1.37E-01	ENSG00000107758
PPP3CB-AS1	-0.2358	4.1180E-02	1.24E-01	ENSG00000221817
PPP3CC	0.2357	7.4645E-03	3.40E-02	ENSG00000120910
PPP3R1	-0.1756	2.2135E-02	7.77E-02	ENSG00000221823
PPP3R2	0.0227	8.6992E-02		ENSG00000188386
PPP4C	-0.1584	5.3937E-03	2.65E-02	ENSG00000149923
PPP4R1	0.0440	5.4639E-01	7.18E-01	ENSG00000154845
PPP4R1-AS1	0.1162	2.0070E-01	3.78E-01	ENSG00000263627
PPP4R2	-0.2720	8.7069E-04	6.39E-03	ENSG00000163605
PPP4R3A	-0.0006	9.9158E-01	9.95E-01	ENSG00000100796
PPP4R3B	0.1513	1.9852E-02	7.16E-02	ENSG00000275052
PPP4R4	0.5462	9.7568E-05	1.10E-03	ENSG00000119698
PPP5C	-0.0120	7.8653E-01	8.85E-01	ENSG00000011485
PPP6C	0.0207	6.9708E-01	8.31E-01	ENSG00000119414
PPP6R1	0.3212	8.5025E-04	6.28E-03	ENSG00000105063
PPP6R2	0.1192	6.3419E-02	1.70E-01	ENSG00000100239
PPP6R3	-0.0896	1.7467E-01	3.45E-01	ENSG00000110075
PPRC1	0.0538	4.6234E-01	6.51E-01	ENSG00000148840
PPT1	-0.0870	2.5750E-01	4.46E-01	ENSG00000131238
PPT2	0.0416	6.2938E-01	7.83E-01	ENSG00000221988
PPT2-EGFL8	-0.0246	7.4949E-01	8.63E-01	ENSG00000258388
PPTC7	-0.2841	1.0639E-04	1.18E-03	ENSG00000196850
PPWD1	0.0416	5.8003E-01	7.46E-01	ENSG00000113593
PQBP1	-0.0440	3.6182E-01	5.58E-01	ENSG00000102103
PRAC2	-0.0053	6.3181E-01		ENSG00000229637
PRADC1	0.1397	7.3467E-02	1.89E-01	ENSG00000135617

PRADX	-0.0030	9.7764E-01		ENSG00000235027
PRAF2	-0.1136	1.1172E-01	2.54E-01	ENSG00000243279
PRAG1	-0.1344	2.0281E-01	3.80E-01	ENSG00000275342
PRAM1	0.0980	2.0633E-01	3.85E-01	ENSG00000133246
PRAMEF12	-0.0311	3.2862E-01		ENSG00000116726
PRANCR	-0.0509	4.7411E-01	6.61E-01	ENSG00000257815
PRAP1	0.0262	2.8472E-01	4.76E-01	ENSG00000165828
PRB1	0.0064	6.9534E-01		ENSG00000251655
PRB3	0.3772	1.8804E-02	6.88E-02	ENSG00000197870
PRC1	-0.2689	2.9201E-02	9.56E-02	ENSG00000198901
PRC1-AS1	-0.0091	9.1969E-01	9.60E-01	ENSG00000258725
PRCC	-0.0690	2.6613E-01	4.55E-01	ENSG00000143294
PRCD	-0.5206	6.3012E-04	4.95E-03	ENSG00000214140
PRCP	-0.2686	4.6821E-04	3.89E-03	ENSG00000137509
PRDM1	0.1657	8.5539E-02	2.10E-01	ENSG00000057657
PRDM10	0.0649	4.7695E-01	6.64E-01	ENSG00000170325
PRDM11	0.1002	2.7772E-01	4.68E-01	ENSG00000019485
PRDM13	0.0136	3.1231E-01		ENSG00000112238
PRDM14	0.0000	7.9154E-01		ENSG00000147596
PRDM15	0.1906	6.3418E-02	1.70E-01	ENSG00000141956
PRDM16	-0.1229	2.1911E-01	4.00E-01	ENSG00000142611
PRDM16-DT	-0.1296	2.1445E-01	3.95E-01	ENSG00000177133
PRDM2	0.0552	4.8330E-01	6.68E-01	ENSG00000116731
PRDM4	0.0230	7.3534E-01	8.55E-01	ENSG00000110851
PRDM4-AS1	0.2822	6.4553E-03	3.05E-02	ENSG00000258136
PRDM5	-0.4145	2.7592E-04	2.54E-03	ENSG00000138738
PRDM6	0.0003	9.6779E-01		ENSG00000061455
PRDM7	-0.0201	7.6282E-01		ENSG00000126856
PRDM8	0.0042	9.0423E-01	9.52E-01	ENSG00000152784
PRDM9	0.0037	5.3009E-01		ENSG00000164256
PRDX1	0.0446	6.4460E-01	7.94E-01	ENSG00000117450
PRDX2	-0.2541	2.5807E-05	3.76E-04	ENSG00000167815
PRDX3	-0.2001	1.6691E-02	6.29E-02	ENSG00000165672
PRDX4	-0.0688	2.7632E-01	4.67E-01	ENSG00000123131
PRDX5	-0.1837	7.2168E-04	5.51E-03	ENSG00000126432
PRDX6	-0.1528	1.7017E-02	6.38E-02	ENSG00000117592
PRDX6-AS1	-0.0107	8.9584E-01	9.48E-01	ENSG00000203739
PREB	0.2033	9.8111E-03	4.19E-02	ENSG00000138073
PRECSIT	0.0788	2.8914E-01	4.81E-01	ENSG00000255874
PRELID1	-0.0691	2.1914E-01	4.00E-01	ENSG00000169230
PRELID1P1	0.0222	7.8736E-01	8.86E-01	ENSG00000217325
PRELID2	0.1397	1.5717E-01	3.21E-01	ENSG00000186314
PRELID3A	-0.0528	5.4619E-01	7.18E-01	ENSG00000141391
PRELID3B	-0.0127	7.9790E-01	8.92E-01	ENSG00000101166
PRELID3BP5	-0.0111	7.0105E-01		ENSG00000251656
PREP	0.0344	6.4412E-01	7.94E-01	ENSG00000085377
PREPL	-0.0316	5.6518E-01	7.34E-01	ENSG00000138078
PREX1	-0.1977	1.1180E-02	4.62E-02	ENSG00000124126
PREX2	-0.2320	6.2616E-02	1.69E-01	ENSG00000046889

PRF1	0.0203	2.6194E-01		ENSG00000180644
PRG4	-0.0976	2.4043E-01	4.25E-01	ENSG00000116690
PRH1	0.2972	4.2004E-03	2.19E-02	ENSG00000231887
PRH1	-0.0003	7.7736E-01		ENSG00000111215
PRH1-PRR4	-0.2623	4.1491E-02	1.25E-01	ENSG00000275778
PRH2	-0.0486	5.9222E-01	7.55E-01	ENSG00000134551
PRICKLE1	0.0542	5.2504E-01	7.01E-01	ENSG00000139174
PRICKLE2	0.1036	1.3690E-01	2.91E-01	ENSG00000163637
PRICKLE2-AS1	0.0048	6.8497E-01		ENSG00000241111
PRICKLE2-AS3	0.0168	8.5492E-01	9.25E-01	ENSG00000226017
PRICKLE2-DT	0.0115	9.0516E-01	9.52E-01	ENSG00000244564
PRICKLE3	0.0150	8.7530E-01	9.37E-01	ENSG00000012211
PRICKLE4	0.0393	3.8107E-01		ENSG00000278224
PRIM1	-0.3534	1.2038E-05	2.04E-04	ENSG00000198056
PRIM2	0.0097	9.1065E-01	9.55E-01	ENSG00000146143
PRIM2BP	0.0525	8.1140E-02		ENSG00000283453
PRIMA1	0.0030	7.7127E-01		ENSG00000175785
PRIMPOL	-0.2309	2.6149E-02	8.80E-02	ENSG00000164306
PRKAA1	-0.1667	2.6618E-03	1.54E-02	ENSG00000132356
PRKAA2	0.0186	8.1614E-01	9.03E-01	ENSG00000162409
PRKAB1	0.2163	7.2295E-04	5.52E-03	ENSG00000111725
PRKAB2	-0.0123	8.5140E-01	9.24E-01	ENSG00000131791
PRKACA	0.0232	7.9094E-01	8.88E-01	ENSG00000072062
PRKACB	-0.0269	7.4587E-01	8.61E-01	ENSG00000142875
PRKAG1	-0.0278	7.6902E-01	8.75E-01	ENSG00000181929
PRKAG2	0.2135	2.7330E-03	1.57E-02	ENSG00000106617
PRKAR1A	0.0917	1.8239E-01	3.55E-01	ENSG00000108946
PRKAR1B	-0.0851	2.4786E-01	4.34E-01	ENSG00000188191
PRKAR1B-AS1	-0.0296	2.9519E-01		ENSG00000237181
PRKAR2A	-0.1028	1.4646E-01	3.05E-01	ENSG00000114302
PRKAR2A-AS1	0.4756	2.0450E-04	1.99E-03	ENSG00000224424
PRKAR2B	0.2095	3.6598E-02	1.13E-01	ENSG00000005249
PRKCA	-0.4188	4.6235E-04	3.86E-03	ENSG00000154229
PRKCB	0.3131	7.2756E-03	3.34E-02	ENSG00000166501
PRKCD	0.0244	7.6811E-01	8.74E-01	ENSG00000163932
PRKCE	-0.0994	1.5393E-01	3.16E-01	ENSG00000171132
PRKCG	0.1246	1.8850E-01	3.63E-01	ENSG00000126583
PRKCH	-0.1110	2.6310E-01	4.52E-01	ENSG00000027075
PRKCI	-0.1880	3.4113E-04	3.04E-03	ENSG00000163558
PRKCQ	0.0514	5.6522E-01	7.34E-01	ENSG00000065675
PRKCQ-AS1	-0.0511	5.3667E-01	7.10E-01	ENSG00000237943
PRKCSH	0.0629	3.3028E-01	5.25E-01	ENSG00000130175
PRKCZ	0.1425	7.0362E-02	1.83E-01	ENSG00000067606
PRKCZ-AS1	-0.0834	3.9741E-01	5.92E-01	ENSG00000182873
PRKCZ-DT	0.0207	7.6613E-01	8.73E-01	ENSG00000226969
PRKD1	-0.0146	8.7808E-01	9.38E-01	ENSG00000184304
PRKD2	0.0711	3.1127E-01	5.04E-01	ENSG00000105287
PRKD3	-0.0005	9.9455E-01	9.97E-01	ENSG00000115825
PRKDC	-0.2969	8.4402E-06	1.54E-04	ENSG00000253729

PRKG1	-0.5090	2.6744E-09	1.93E-07	ENSG00000185532
PRKG2	0.5047	2.0301E-03	1.24E-02	ENSG00000138669
PRKG2-AS1	-0.0162	5.6184E-01		ENSG00000251059
PRKN	0.4198	1.8425E-08	9.53E-07	ENSG00000185345
PRKRA	-0.0172	7.3921E-01	8.57E-01	ENSG00000180228
PRKRIP1	0.1934	2.2732E-03	1.36E-02	ENSG00000128563
PRKX	-0.0813	2.3822E-01	4.23E-01	ENSG00000183943
PRKX-AS1	0.0074	6.7909E-01		ENSG00000236188
PRKY	-0.0365	6.9590E-01	8.30E-01	ENSG00000099725
PRL	0.1772	5.8075E-02	1.60E-01	ENSG00000172179
PRLHR	0.5252	8.6090E-03	3.80E-02	ENSG00000119973
PRLR	-0.0011	9.8894E-01	9.94E-01	ENSG00000113494
PRMT1	-0.0251	5.9680E-01	7.58E-01	ENSG00000126457
PRMT2	-0.1164	9.3358E-02	2.24E-01	ENSG00000160310
PRMT3	0.0609	4.7781E-01	6.64E-01	ENSG00000185238
PRMT5	0.1393	1.1208E-01	2.55E-01	ENSG00000100462
PRMT5-AS1	0.1174	2.5805E-01	4.46E-01	ENSG00000237054
PRMT5-DT	-0.3060	2.6554E-02	8.90E-02	ENSG00000257285
PRMT6	0.1525	2.5185E-02	8.57E-02	ENSG00000198890
PRMT7	0.0090	9.6173E-01	9.81E-01	ENSG00000132600
PRMT8	-0.0052	9.7058E-01	9.85E-01	ENSG00000111218
PRMT9	0.0289	6.7511E-01	8.14E-01	ENSG00000164169
PRNCR1	0.0305	7.3918E-01	8.57E-01	ENSG00000282961
PRNP	-0.5593	1.4797E-05	2.42E-04	ENSG00000171867
PRO1804	-0.0175	4.4496E-01		ENSG00000278873
PROB1	0.0652	4.6127E-01	6.50E-01	ENSG00000228672
PROC	0.1441	1.7807E-01	3.49E-01	ENSG00000115718
PROCA1	0.1436	1.5455E-01	3.17E-01	ENSG00000167525
PROCR	-0.2503	3.0149E-02	9.78E-02	ENSG00000101000
PRODH	-0.1946	9.5156E-02	2.27E-01	ENSG00000100033
PRODH	-0.2756	2.2841E-02	7.96E-02	ENSG00000277196
PRODH2	-0.0145	8.7565E-01		ENSG00000250799
PRODHLP	0.1330	1.9322E-01	3.69E-01	ENSG00000161132
PROK1	-0.0401	7.1814E-01		ENSG00000143125
PROK2	-0.4845	3.9662E-03	2.10E-02	ENSG00000163421
PROKR1	-0.2352	6.1239E-02	1.66E-01	ENSG00000169618
PROM1	0.3282	2.5879E-02	8.74E-02	ENSG00000007062
PROM2	0.1385	1.9932E-01	3.76E-01	ENSG00000155066
PRORP	0.0015	9.8211E-01	9.90E-01	ENSG00000100890
PRORP	-0.0860	9.0422E-02		ENSG00000258790
PRORSD1P	0.5731	3.0986E-06	6.78E-05	ENSG00000162997
PROS1	-0.1092	2.0848E-01	3.88E-01	ENSG00000184500
PROSER1	0.1375	7.1085E-02	1.84E-01	ENSG00000120685
PROSER2	-0.1551	1.1495E-01	2.59E-01	ENSG00000148426
PROSER2-AS1	0.0374	5.7650E-01	7.43E-01	ENSG00000225778
PROSER3	0.0215	7.1079E-01	8.39E-01	ENSG00000167595
PROX1	0.0248	7.6323E-01	8.72E-01	ENSG00000117707
PROX1-AS1	-0.0282	7.5047E-01	8.63E-01	ENSG00000230461
PROX2	-0.0019	8.3004E-01		ENSG00000119608

PROZ	0.0436	1.0073E-01		ENSG00000126231
PRPF18	0.1825	2.1888E-03	1.32E-02	ENSG00000165630
PRPF19	-0.2769	6.0624E-04	4.81E-03	ENSG00000110107
PRPF3	0.0650	3.7913E-01	5.74E-01	ENSG00000117360
PRPF31	0.0254	6.2512E-01	7.80E-01	ENSG00000105618
PRPF38A	-0.0099	8.4970E-01	9.23E-01	ENSG00000134748
PRPF38B	-0.1501	5.7458E-03	2.79E-02	ENSG00000134186
PRPF39	0.0590	3.5156E-01	5.47E-01	ENSG00000185246
PRPF39-DT	-0.0481	4.7769E-01		ENSG00000249163
PRPF4	0.2926	1.1945E-04	1.29E-03	ENSG00000136875
PRPF40A	0.0925	3.5718E-02	1.11E-01	ENSG00000196504
PRPF40B	-0.0211	8.0226E-01	8.94E-01	ENSG00000110844
PRPF4B	-0.1329	2.1648E-02	7.65E-02	ENSG00000112739
PRPF6	0.1870	1.0230E-03	7.23E-03	ENSG00000101161
PRPF8	-0.0043	9.4123E-01	9.71E-01	ENSG00000174231
PRPH	0.0078	9.2718E-01	9.64E-01	ENSG00000135406
PRPH2	0.1421	8.2144E-02	2.04E-01	ENSG00000112619
PRPS1	0.0453	5.8316E-01	7.49E-01	ENSG00000147224
PRPS2	-0.1531	9.9275E-02	2.34E-01	ENSG00000101911
PRPSAP1	-0.0001	9.5576E-01	9.77E-01	ENSG00000161542
PRPSAP2	-0.0094	8.5777E-01	9.27E-01	ENSG00000141127
PRR11	-0.5645	6.6222E-05	8.06E-04	ENSG00000068489
PRR12	0.0515	5.7642E-01	7.43E-01	ENSG00000126464
PRR13	0.0582	3.7788E-01	5.73E-01	ENSG00000205352
PRR14	-0.2400	2.1240E-03	1.28E-02	ENSG00000156858
PRR14L	0.2633	2.6401E-04	2.45E-03	ENSG00000183530
PRR15	0.2738	6.0622E-03	2.91E-02	ENSG00000176532
PRR15L	-0.0550	5.2930E-01	7.04E-01	ENSG00000167183
PRR16	0.1026	3.0730E-01	5.00E-01	ENSG00000184838
PRR18	0.1222	2.3158E-01	4.15E-01	ENSG00000176381
PRR19	-0.3591	5.8407E-03	2.82E-02	ENSG00000188368
PRR22	-0.0438	6.5746E-01	8.02E-01	ENSG00000212123
PRR23D1	-0.0541	1.8454E-01		ENSG00000255251
PRR23D2	-0.0541	1.8454E-01		ENSG00000255378
PRR27	-0.5869	3.0902E-03	1.72E-02	ENSG00000187533
PRR29	-0.4922	1.1045E-04	1.21E-03	ENSG00000224383
PRR29-AS1	-0.0374	5.5079E-01	7.22E-01	ENSG00000264954
PRR3	0.1435	9.6059E-03	4.12E-02	ENSG00000204576
PRR32	-0.0664	3.5434E-01	5.50E-01	ENSG00000183631
PRR35	0.0055	6.0340E-01		ENSG00000161992
PRR36	0.0530	2.9420E-01	4.86E-01	ENSG00000183248
PRR5	-0.2705	3.8804E-03	2.06E-02	ENSG00000186654
PRR5-ARHGAP8	-0.0004	9.5000E-01		ENSG00000248405
PRR5L	-0.1242	2.2816E-01	4.11E-01	ENSG00000135362
PRR7	-0.0151	7.3523E-01	8.55E-01	ENSG00000131188
PRRC1	0.0587	2.7581E-01	4.66E-01	ENSG00000164244
PRRC2A	-0.4970	2.8699E-08	1.40E-06	ENSG00000204469
PRRC2B	-0.0617	3.1572E-01	5.09E-01	ENSG00000288701
PRRC2C	-0.0311	5.8759E-01	7.52E-01	ENSG00000117523

PRRG1	0.0096	9.0744E-01	9.54E-01	ENSG00000130962
PRRG2	0.0926	3.4200E-01	5.37E-01	ENSG00000126460
PRRG3	-0.1443	1.7733E-01	3.48E-01	ENSG00000130032
PRRG4	-0.2281	3.7874E-02	1.16E-01	ENSG00000135378
PRRT1	-0.0198	7.7804E-01	8.80E-01	ENSG00000204314
PRRT1B	-0.0384	6.4507E-01	7.94E-01	ENSG00000283526
PRRT2	0.3798	2.3794E-05	3.52E-04	ENSG00000167371
PRRT3	-0.0639	3.8806E-01	5.83E-01	ENSG00000163704
PRRT3-AS1	0.0159	7.9983E-01	8.93E-01	ENSG00000230082
PRRT4	-0.2360	2.8373E-02	9.34E-02	ENSG00000224940
PRRX2	-0.1175	1.9862E-01	3.76E-01	ENSG00000167157
PRSS1	-0.0119	7.6009E-01		ENSG00000204983
PRSS12	-0.0686	4.9125E-01	6.75E-01	ENSG00000164099
PRSS16	-0.1079	2.4208E-01	4.27E-01	ENSG00000112812
PRSS2	-0.0497	7.7471E-01	8.78E-01	ENSG00000275896
PRSS22	0.1439	1.7219E-01	3.41E-01	ENSG00000005001
PRSS23	-0.1977	8.5265E-02	2.10E-01	ENSG00000150687
PRSS3	0.1153	2.3452E-01	4.19E-01	ENSG00000010438
PRSS30P	0.0045	9.4551E-01		ENSG00000172460
PRSS33	0.0453	6.2444E-01	7.80E-01	ENSG00000103355
PRSS35	-0.1710	6.6731E-02	1.76E-01	ENSG00000146250
PRSS36	0.0141	8.8850E-01	9.44E-01	ENSG00000178226
PRSS37	0.0176	6.3985E-01		ENSG00000165076
PRSS43P	0.0004	5.7426E-01	7.41E-01	ENSG00000286651
PRSS46P	0.0379	2.4265E-01		ENSG00000261603
PRSS48	0.0176	1.2895E-01		ENSG00000189099
PRSS51	-0.0467	3.8332E-01		ENSG00000253649
PRSS53	0.0089	9.0491E-01	9.52E-01	ENSG00000151006
PRSS54	-0.0080	9.2423E-01		ENSG00000103023
PRSS55	-0.0082	7.1968E-01		ENSG00000184647
PRSS56	0.0921	3.0940E-01	5.02E-01	ENSG00000237412
PRSS57	0.0387	7.5919E-02		ENSG00000185198
PRSS58	-0.0423	2.7820E-01		ENSG00000258223
PRSS59P	0.0722	5.1110E-02	1.45E-01	ENSG00000186163
PRSS8	0.1383	1.5366E-01	3.16E-01	ENSG00000052344
PRTFDC1	-0.2690	1.3109E-02	5.23E-02	ENSG00000099256
PRTG	0.0414	6.2752E-01	7.82E-01	ENSG00000166450
PRTN3	0.0276	7.6273E-01	8.71E-01	ENSG00000196415
PRUNE1	0.0061	9.2426E-01	9.62E-01	ENSG00000143363
PRUNE2	0.0357	5.5756E-01	7.28E-01	ENSG00000106772
PRX	-0.0869	3.8731E-01	5.82E-01	ENSG00000105227
PRXL2A	-0.2237	1.8550E-03	1.16E-02	ENSG00000122378
PRXL2B	0.1793	2.4676E-02	8.44E-02	ENSG00000157870
PRXL2C	-0.1774	8.8396E-03	3.88E-02	ENSG00000158122
PSAP	-0.0904	2.3593E-01	4.20E-01	ENSG00000197746
PSAPL1	0.0002	4.9689E-01		ENSG00000178597
PSAT1	-0.3919	6.1023E-04	4.83E-03	ENSG00000135069
PSCA	0.2327	5.9342E-02	1.62E-01	ENSG00000167653
PSD	0.2847	6.6362E-04	5.17E-03	ENSG00000059915

PSD2	0.0749	3.9881E-01	5.93E-01	ENSG00000146005
PSD3	0.1124	1.3580E-01	2.90E-01	ENSG00000156011
PSD4	-0.1342	1.7783E-01	3.49E-01	ENSG00000125637
PSEN1	0.1491	1.3894E-02	5.47E-02	ENSG00000080815
PSEN2	0.0396	6.7279E-01	8.13E-01	ENSG00000143801
PSENEN	-0.2692	2.2309E-05	3.34E-04	ENSG00000205155
PSG3	0.0033	9.8034E-01		ENSG00000221826
PSG8-AS1	-0.0262	4.2575E-01		ENSG00000225877
PSIP1	-0.1925	7.1090E-04	5.44E-03	ENSG00000164985
PSKH1	0.2507	2.8927E-03	1.64E-02	ENSG00000159792
PSMA1	-0.0969	6.7575E-02	1.78E-01	ENSG00000129084
PSMA2	0.2911	2.7287E-02	9.08E-02	ENSG00000106588
PSMA3	-0.1049	8.2920E-02	2.06E-01	ENSG00000100567
PSMA3-AS1	-0.2036	4.9359E-04	4.05E-03	ENSG00000257621
PSMA4	0.1005	6.2133E-02	1.68E-01	ENSG00000041357
PSMA5	0.0565	2.8611E-01	4.77E-01	ENSG00000143106
PSMA6	-0.2642	2.8500E-05	4.07E-04	ENSG00000100902
PSMA7	-0.0414	4.3075E-01	6.22E-01	ENSG00000101182
PSMB1	-0.1347	1.4554E-02	5.67E-02	ENSG00000008018
PSMB10	-0.4455	3.1140E-03	1.73E-02	ENSG00000205220
PSMB2	-0.2543	8.7273E-05	1.00E-03	ENSG00000126067
PSMB3	-0.1458	9.8714E-03	4.21E-02	ENSG00000277791
PSMB4	-0.1732	1.6030E-02	6.10E-02	ENSG00000159377
PSMB5	-0.0952	1.1713E-01	2.63E-01	ENSG00000100804
PSMB6	-0.2665	1.4505E-05	2.38E-04	ENSG00000142507
PSMB7	-0.0294	3.6696E-01	5.63E-01	ENSG00000136930
PSMB8	-0.4527	9.2119E-06	1.64E-04	ENSG00000204264
PSMB8-AS1	-0.3530	2.3231E-02	8.06E-02	ENSG00000204261
PSMC1	0.0391	5.7857E-01	7.45E-01	ENSG00000100764
PSMC1P1	-0.0202	7.3879E-01	8.57E-01	ENSG00000241506
PSMC1P10	0.0078	5.7508E-01		ENSG00000236348
PSMC1P11	-0.0155	9.2579E-01		ENSG00000217385
PSMC1P12	-0.0361	6.4524E-01		ENSG00000226126
PSMC1P3	0.0044	5.6567E-01		ENSG00000231244
PSMC1P4	0.0066	5.8933E-01		ENSG00000248781
PSMC1P5	0.0397	5.9571E-01	7.58E-01	ENSG00000250273
PSMC1P9	0.0225	7.5151E-02	1.92E-01	ENSG00000255993
PSMC2	0.0369	5.6035E-01	7.30E-01	ENSG00000161057
PSMC2P1	-0.0271	7.2127E-01		ENSG00000240854
PSMC3	0.0137	8.7703E-01	9.38E-01	ENSG00000165916
PSMC3IP	0.0077	9.3782E-01	9.69E-01	ENSG00000131470
PSMC3P1	-0.0150	8.9480E-01		ENSG00000236680
PSMC4	0.0394	3.8851E-01	5.83E-01	ENSG00000013275
PSMC5	-0.0386	3.7977E-01	5.75E-01	ENSG00000087191
PSMC6	-0.0523	1.9038E-01	3.65E-01	ENSG00000100519
PSMD1	0.1002	9.3213E-02	2.24E-01	ENSG00000173692
PSMD10	-0.1017	1.0544E-01	2.44E-01	ENSG00000101843
PSMD11	0.0933	4.2371E-02	1.27E-01	ENSG00000108671
PSMD12	0.0877	6.8020E-02	1.79E-01	ENSG00000197170

PSMD13	0.0572	2.1195E-01	3.92E-01	ENSG00000185627
PSMD14	0.0713	2.0200E-01	3.80E-01	ENSG00000115233
PSMD2	0.1143	1.0253E-01	2.39E-01	ENSG00000175166
PSMD2P1	0.1422	6.4951E-02	1.73E-01	ENSG00000228818
PSMD3	0.2172	9.8804E-03	4.21E-02	ENSG00000108344
PSMD4	-0.0157	6.5701E-01	8.02E-01	ENSG00000159352
PSMD5	0.0056	9.5269E-01	9.76E-01	ENSG00000095261
PSMD6	-0.0011	9.7961E-01	9.89E-01	ENSG00000163636
PSMD6-AS2	0.0401	6.6256E-01	8.06E-01	ENSG00000239653
PSMD7	-0.1518	2.3030E-02	8.01E-02	ENSG00000103035
PSMD7-DT	0.0087	9.2866E-01	9.65E-01	ENSG00000261404
PSMD8	-0.0411	3.0779E-01	5.00E-01	ENSG00000099341
PSMD8P1	0.0010	8.0072E-01		ENSG00000228264
PSMD9	-0.0357	4.9399E-01	6.78E-01	ENSG00000110801
PSME1	-0.1489	2.0337E-02	7.29E-02	ENSG00000092010
PSME2	-0.0993	2.0990E-02	7.47E-02	ENSG00000100911
PSME2P1	-0.1018	4.7399E-01	6.61E-01	ENSG00000238000
PSME2P2	0.2059	6.6369E-02	1.75E-01	ENSG00000225131
PSME2P3	0.0041	6.9343E-01		ENSG00000248988
PSME2P5	0.0735	9.0112E-02	2.19E-01	ENSG00000253208
PSME3	0.0721	2.0168E-01	3.79E-01	ENSG00000131467
PSME3IP1	0.0285	5.2466E-01	7.01E-01	ENSG00000172775
PSME4	-0.1026	1.9835E-01	3.75E-01	ENSG00000068878
PSMF1	-0.1278	3.7847E-02	1.16E-01	ENSG00000125818
PSMG1	-0.1168	1.3058E-01	2.82E-01	ENSG00000183527
PSMG2	-0.0265	6.2764E-01	7.82E-01	ENSG00000128789
PSMG3	0.0092	8.6441E-01	9.31E-01	ENSG00000157778
PSMG3-AS1	-0.0627	4.1759E-01	6.10E-01	ENSG00000230487
PSMG4	0.3277	6.4115E-05	7.86E-04	ENSG00000180822
PSORS1C1	-0.1496	1.3226E-01	2.85E-01	ENSG00000204540
PSPC1	0.0087	9.5139E-01	9.75E-01	ENSG00000121390
PSPC1P1	-0.1049	2.8539E-01	4.76E-01	ENSG00000227879
PSPH	-0.2809	1.2388E-03	8.42E-03	ENSG00000146733
PSPHP1	-0.0537	9.1776E-01	9.59E-01	ENSG00000226278
PSPN	-0.1662	9.0974E-02	2.20E-01	ENSG00000125650
PSRC1	0.0620	4.8315E-01	6.68E-01	ENSG00000134222
PSTK	-0.0859	1.8488E-01	3.58E-01	ENSG00000179988
PSTPIP1	0.2155	6.9702E-02	1.82E-01	ENSG00000140368
PSTPIP2	0.1723	6.3674E-02	1.70E-01	ENSG00000152229
PTAFR	0.1567	1.5520E-01	3.18E-01	ENSG00000169403
PTBP1	-0.0934	8.1705E-02	2.04E-01	ENSG00000011304
PTBP2	0.3957	1.1187E-03	7.75E-03	ENSG00000117569
PTBP3	-0.2087	4.2029E-03	2.19E-02	ENSG00000119314
PTCD1	0.3510	1.3162E-03	8.84E-03	ENSG00000106246
PTCD2	-0.0522	5.1181E-01	6.92E-01	ENSG00000049883
PTCD3	-0.0007	9.8864E-01	9.94E-01	ENSG00000132300
PTCH1	0.3252	1.7845E-03	1.12E-02	ENSG00000185920
PTCHD1	-0.2594	1.5467E-03	1.00E-02	ENSG00000165186
PTCHD3	0.0259	3.4735E-01		ENSG00000182077

PTCHD4	0.3438	8.9696E-04	6.54E-03	ENSG00000244694
PTCSC2	-0.0103	8.3293E-01	9.13E-01	ENSG00000236130
PTDSS1	0.0183	7.8759E-01	8.86E-01	ENSG00000156471
PTDSS2	-0.0350	5.4621E-01	7.18E-01	ENSG00000174915
PTEN	-0.0501	3.6291E-01	5.59E-01	ENSG00000171862
PTENP1	0.1141	2.4516E-01	4.31E-01	ENSG00000237984
PTENP1-AS	0.0226	7.9891E-01	8.92E-01	ENSG00000281128
PTER	0.5244	5.0708E-05	6.51E-04	ENSG00000165983
PTF1A	-0.0638	2.5845E-01		ENSG00000168267
PTGDR	0.0010	9.6053E-01		ENSG00000168229
PTGDR2	-0.0448	5.8365E-01		ENSG00000183134
PTGDS	-0.1897	1.0244E-01	2.39E-01	ENSG00000107317
PTGER1	0.1094	2.8346E-01	4.75E-01	ENSG00000160951
PTGER2	0.0357	6.5960E-01	8.04E-01	ENSG00000125384
PTGER4	-0.0258	7.9170E-01	8.88E-01	ENSG00000171522
PTGER4P2-CDK2AP2P2	0.0826	3.1405E-01	5.07E-01	ENSG00000275450
PTGES	0.0622	4.6201E-01	6.50E-01	ENSG00000148344
PTGES2	0.1120	1.5181E-01	3.13E-01	ENSG00000148334
PTGES2-AS1	0.4341	1.5527E-02	5.95E-02	ENSG00000232850
PTGES3	-0.1117	2.5544E-02	8.66E-02	ENSG00000110958
PTGES3L	0.2763	1.6115E-02	6.12E-02	ENSG00000267060
PTGES3L-AARSD1	-0.0026	6.8276E-01		ENSG00000108825
PTGFR	-0.3365	1.5487E-02	5.94E-02	ENSG00000122420
PTGFRN	-0.2885	3.1662E-04	2.86E-03	ENSG00000134247
PTGIR	0.0754	2.5689E-02		ENSG00000160013
PTGIS	-0.1530	1.6053E-01	3.26E-01	ENSG00000124212
PTGR1	-0.0067	9.1619E-01	9.58E-01	ENSG00000106853
PTGR2	-0.0396	6.4827E-01	7.96E-01	ENSG00000140043
PTGR3	-0.0269	7.1153E-01	8.39E-01	ENSG00000180011
PTGS1	-0.1813	1.0794E-01	2.48E-01	ENSG00000095303
PTH	0.0337	8.2499E-03		ENSG00000152266
PTH1R	0.0021	9.7953E-01	9.89E-01	ENSG00000160801
PTH2	0.1665	6.2728E-02	1.69E-01	ENSG00000142538
PTH2R	-0.0511	5.2595E-01		ENSG00000144407
PTK2	0.1678	5.1332E-03	2.55E-02	ENSG00000169398
PTK2B	0.3241	5.1412E-04	4.20E-03	ENSG00000120899
PTK6	0.0963	3.0545E-01	4.98E-01	ENSG00000101213
PTK7	0.0479	5.1220E-01	6.92E-01	ENSG00000112655
PTMA	-0.0708	3.0917E-01	5.02E-01	ENSG00000187514
PTMAP5	-0.0316	7.0755E-01	8.36E-01	ENSG00000214182
PTMS	-0.1912	6.8468E-03	3.18E-02	ENSG00000159335
PTOV1	-0.4537	1.3681E-12	2.82E-10	ENSG00000104960
PTOV1-AS1	-0.0062	9.5102E-01	9.75E-01	ENSG00000268006
PTOV1P1	-0.0008	7.8355E-01		ENSG00000240882
PTP4A1	0.0814	3.1403E-01	5.07E-01	ENSG00000112245
PTP4A1P3	0.0227	5.8909E-01		ENSG00000226254
PTP4A2	-0.2587	3.1129E-08	1.50E-06	ENSG00000184007
PTP4A3	0.0915	1.9725E-01	3.74E-01	ENSG00000184489
PTPA	-0.2490	7.3299E-03	3.35E-02	ENSG00000119383

PTPDC1	0.0689	3.2912E-01	5.23E-01	ENSG00000158079
PTPMT1	-0.1820	1.1768E-02	4.81E-02	ENSG00000110536
PTPN1	-0.0344	5.9301E-01	7.55E-01	ENSG00000196396
PTPN11	-0.3229	3.3765E-11	4.69E-09	ENSG00000179295
PTPN12	-0.1826	4.3932E-02	1.30E-01	ENSG00000127947
PTPN13	-0.1975	1.2798E-02	5.13E-02	ENSG00000163629
PTPN14	-0.4962	7.9563E-05	9.30E-04	ENSG00000152104
PTPN18	-0.1722	1.9625E-02	7.10E-02	ENSG00000072135
PTPN2	-0.1894	4.6964E-03	2.39E-02	ENSG00000175354
PTPN20	0.1813	6.3955E-02	1.71E-01	ENSG00000204179
PTPN21	-0.1773	1.9684E-02	7.11E-02	ENSG00000070778
PTPN22	-0.0129	9.2778E-01		ENSG00000134242
PTPN23	-0.1202	3.5177E-02	1.10E-01	ENSG00000076201
PTPN3	0.0004	9.9779E-01	9.99E-01	ENSG00000070159
PTPN4	0.3121	1.0222E-06	2.72E-05	ENSG00000088179
PTPN5	0.2791	1.6317E-02	6.18E-02	ENSG00000110786
PTPN6	0.1813	4.6493E-02	1.36E-01	ENSG00000111679
PTPN7	0.1713	5.9578E-02	1.63E-01	ENSG00000143851
PTPN9	0.0255	6.6830E-01	8.09E-01	ENSG00000169410
PTPRA	-0.2465	3.2210E-05	4.48E-04	ENSG00000132670
PTPRB	0.0238	7.8673E-01	8.85E-01	ENSG00000127329
PTPRD	-0.0086	9.1501E-01	9.57E-01	ENSG00000153707
PTPRD-AS1	0.0100	9.2603E-01	9.63E-01	ENSG00000225706
PTPRD-DT	0.1683	7.0639E-02	1.83E-01	ENSG00000226717
PTPRF	-0.1026	1.2468E-01	2.74E-01	ENSG00000142949
PTPRG	-0.4124	7.8467E-06	1.45E-04	ENSG00000144724
PTPRG-AS1	0.1847	1.2129E-02	4.92E-02	ENSG00000241472
PTPRJ	0.0190	8.2219E-01	9.06E-01	ENSG00000149177
PTPRK	-0.0790	2.2203E-01	4.04E-01	ENSG00000152894
PTPRM	-0.0068	8.9340E-01	9.47E-01	ENSG00000173482
PTPRN	0.1361	1.1139E-01	2.53E-01	ENSG00000054356
PTPRN2	-0.0452	4.6855E-01	6.56E-01	ENSG00000155093
PTPRO	-0.0894	1.2780E-01	2.79E-01	ENSG00000151490
PTPRQ	0.1024	1.3546E-01	2.89E-01	ENSG00000139304
PTPRR	-0.1309	1.9447E-01	3.70E-01	ENSG00000153233
PTPRS	-0.0443	5.3764E-01	7.11E-01	ENSG00000105426
PTPRT	-0.1477	1.3902E-01	2.95E-01	ENSG00000196090
PTPRT-DT	-0.1118	2.3848E-01	4.23E-01	ENSG00000233508
PTPRU	0.1519	6.5746E-02	1.74E-01	ENSG00000060656
PTPRVP	0.0046	6.7272E-01	8.13E-01	ENSG00000243323
PTRH1	0.0094	9.1142E-01	9.55E-01	ENSG00000187024
PTRH2	-0.0587	3.2574E-01	5.20E-01	ENSG00000141378
PTRHD1	-0.1891	8.2066E-03	3.66E-02	ENSG00000184924
PTS	-0.1819	2.5189E-03	1.47E-02	ENSG00000150787
PTTG1	-0.2548	1.4912E-02	5.78E-02	ENSG00000164611
PTTG1IP	-0.0397	3.9136E-01	5.86E-01	ENSG00000183255
PUDP	0.4503	8.9323E-05	1.02E-03	ENSG00000130021
PUF60	-0.0191	7.1902E-01	8.44E-01	ENSG00000179950
PUM1	-0.1532	1.0304E-04	1.15E-03	ENSG00000134644

PUM2	-0.0397	6.0776E-01	7.67E-01	ENSG00000055917
PUM3	0.1613	2.9336E-02	9.59E-02	ENSG00000080608
PURA	-0.0114	8.8529E-01	9.42E-01	ENSG00000185129
PURB	-0.2991	2.8924E-05	4.11E-04	ENSG00000146676
PURG	-0.3510	1.7219E-03	1.09E-02	ENSG00000172733
PURPL	0.5469	8.1873E-05	9.53E-04	ENSG00000250337
PUS1	0.0846	3.8211E-01	5.77E-01	ENSG00000177192
PUS10	0.0572	4.2398E-01	6.16E-01	ENSG00000162927
PUS3	0.3716	6.3307E-09	3.94E-07	ENSG00000110060
PUS7	0.2921	4.4971E-04	3.78E-03	ENSG00000091127
PUS7L	0.0560	5.4797E-01	7.20E-01	ENSG00000129317
PUSL1	0.3690	9.6414E-05	1.09E-03	ENSG00000169972
PVALB	-0.0847	2.3708E-01	4.21E-01	ENSG00000100362
PVALEF	-0.0394	5.9892E-01	7.60E-01	ENSG00000225180
PVR	0.0710	4.1186E-01	6.05E-01	ENSG00000073008
PVRIG	-0.0002	6.2823E-01		ENSG00000213413
PVT1	-0.3283	3.3832E-03	1.84E-02	ENSG00000249859
PWP1	-0.0846	1.8537E-02	6.81E-02	ENSG00000136045
PWP2	0.2435	3.2211E-03	1.77E-02	ENSG00000241945
PWRN1	-0.0372	6.6977E-01	8.10E-01	ENSG00000259905
PWWP2A	-0.1279	6.1278E-02	1.66E-01	ENSG00000170234
PWWP2B	-0.1026	2.6130E-01	4.50E-01	ENSG00000171813
PWWP3A	0.2703	3.7932E-04	3.31E-03	ENSG00000160953
PWWP3B	0.0877	2.9264E-01	4.85E-01	ENSG00000157502
PWWP4	0.1798	6.5467E-02	1.74E-01	ENSG00000278803
PXDC1	-0.5900	6.5679E-09	4.05E-07	ENSG00000168994
PXDN	-0.0646	3.5259E-01	5.48E-01	ENSG00000130508
PXDNL	-0.1270	1.9183E-01	3.67E-01	ENSG00000147485
PXK	0.1318	3.3605E-02	1.06E-01	ENSG00000168297
PXMP2	-0.5697	1.3744E-07	5.05E-06	ENSG00000176894
PXMP4	0.0837	2.0501E-01	3.83E-01	ENSG00000101417
PXN	0.0417	4.8836E-01	6.72E-01	ENSG00000089159
PXN-AS1	0.1369	1.1785E-01	2.64E-01	ENSG00000255857
PXT1	-0.0680	2.8539E-01	4.76E-01	ENSG00000179165
PXYLP1	0.0477	5.4614E-01	7.18E-01	ENSG00000155893
PYCARD	-0.2861	1.3720E-02	5.42E-02	ENSG00000103490
PYCARD-AS1	-0.0296	3.6584E-01		ENSG00000261359
PYCR1	0.0705	4.0683E-01	6.00E-01	ENSG00000183010
PYCR2	-0.0367	6.1839E-01	7.75E-01	ENSG00000143811
PYCR3	-0.0095	8.9029E-01	9.45E-01	ENSG00000104524
PYDC1	-0.0815	3.9563E-01	5.90E-01	ENSG00000169900
PYDC5	0.0120	5.0158E-01		ENSG00000289721
PYGB	0.2523	7.4799E-05	8.88E-04	ENSG00000100994
PYGL	-0.2867	4.1349E-03	2.17E-02	ENSG00000100504
PYGM	0.0536	5.7338E-01	7.41E-01	ENSG00000068976
PYGO1	-0.4571	1.2087E-07	4.52E-06	ENSG00000171016
PYGO2	0.1940	3.9846E-03	2.10E-02	ENSG00000163348
PYM1	0.0374	5.5438E-01	7.26E-01	ENSG00000170473
PYROXD1	-0.0341	5.8941E-01	7.53E-01	ENSG00000121350

PYROXD2	-0.3311	1.1096E-02	4.60E-02	ENSG00000119943
PYY	0.0082	8.3969E-01		ENSG00000131096
PZP	0.4398	1.6484E-02	6.23E-02	ENSG00000126838
QARS1	0.1863	2.5223E-03	1.47E-02	ENSG00000172053
QDPR	-0.0328	4.7528E-01	6.62E-01	ENSG00000151552
QKI	-0.5423	1.9113E-07	6.69E-06	ENSG00000112531
QNG1	0.0705	2.3185E-01	4.15E-01	ENSG00000165118
QPCT	-0.0402	6.2985E-01	7.84E-01	ENSG00000115828
QPCTL	0.2923	2.5963E-03	1.50E-02	ENSG00000011478
QPRT	-0.1470	9.8325E-02	2.32E-01	ENSG00000103485
QRFP	-0.0255	4.3659E-01		ENSG00000188710
QRFPR	0.0664	3.5242E-01	5.48E-01	ENSG00000186867
QRICH1	-0.0671	1.6814E-01	3.36E-01	ENSG00000198218
QRICH2	-0.1818	9.5493E-02	2.28E-01	ENSG00000129646
QRSL1	0.1054	1.0435E-01	2.42E-01	ENSG00000130348
QRSL1P3	0.0003	9.9675E-01	9.98E-01	ENSG00000257957
QSER1	-0.1775	2.4729E-02	8.45E-02	ENSG00000060749
QSOX1	0.0151	8.2930E-01	9.11E-01	ENSG00000116260
QSOX2	-0.2595	1.0471E-02	4.39E-02	ENSG00000165661
QTRT1	0.0629	4.5228E-01	6.42E-01	ENSG00000213339
QTRT2	0.0998	1.7677E-01	3.47E-01	ENSG00000151576
R3HCC1	0.1248	3.0489E-02	9.86E-02	ENSG00000104679
R3HCC1L	0.2308	1.8203E-03	1.14E-02	ENSG00000166024
R3HDM1	-0.1814	2.8528E-03	1.62E-02	ENSG00000048991
R3HDM2	-0.1455	4.4044E-03	2.27E-02	ENSG00000179912
R3HDM4	0.1832	7.4857E-05	8.88E-04	ENSG00000198858
R3HDML	-0.2135	7.7967E-02	1.97E-01	ENSG00000101074
R3HDML-AS1	-0.0739	5.1688E-01	6.95E-01	ENSG00000226812
RAB10	-0.3576	2.4938E-08	1.24E-06	ENSG00000084733
RAB11A	-0.1876	1.0574E-03	7.42E-03	ENSG00000103769
RAB11B	-0.0357	5.2170E-01	6.99E-01	ENSG00000185236
RAB11B-AS1	-0.5087	1.1346E-05	1.95E-04	ENSG00000269386
RAB11FIP1	-0.1674	1.4175E-02	5.56E-02	ENSG00000156675
RAB11FIP2	-0.0328	5.9909E-01	7.60E-01	ENSG00000107560
RAB11FIP3	0.0693	2.9874E-01	4.91E-01	ENSG00000090565
RAB11FIP4	-0.0491	3.7501E-01	5.70E-01	ENSG00000131242
RAB11FIP5	-0.1515	3.2404E-02	1.03E-01	ENSG00000135631
RAB12	0.0035	9.6665E-01	9.83E-01	ENSG00000206418
RAB13	-0.3798	1.6147E-05	2.59E-04	ENSG00000143545
RAB14	-0.0041	9.3623E-01	9.68E-01	ENSG00000119396
RAB15	-0.0513	4.7986E-01	6.65E-01	ENSG00000139998
RAB17	0.1204	2.3702E-01	4.21E-01	ENSG00000124839
RAB18	-0.0310	6.7738E-01	8.16E-01	ENSG00000099246
RAB19	0.0057	9.5313E-01	9.76E-01	ENSG00000146955
RAB1A	0.0302	6.0187E-01	7.62E-01	ENSG00000138069
RAB1B	-0.0170	8.4950E-01	9.23E-01	ENSG00000174903
RAB20	-0.1997	2.4996E-02	8.51E-02	ENSG00000139832
RAB21	-0.0853	7.9471E-02	2.00E-01	ENSG00000080371
RAB22A	0.1290	2.0498E-02	7.34E-02	ENSG00000124209

RAB23	-0.1157	2.2308E-01	4.05E-01	ENSG00000112210
RAB24	-0.1143	1.3563E-01	2.89E-01	ENSG00000169228
RAB25	-0.0060	9.5472E-01	9.77E-01	ENSG00000132698
RAB26	0.1142	2.0282E-01	3.80E-01	ENSG00000167964
RAB27A	0.0823	3.6669E-01	5.63E-01	ENSG00000069974
RAB27B	-0.4107	4.3910E-04	3.71E-03	ENSG00000041353
RAB28	0.2720	1.1744E-04	1.27E-03	ENSG00000157869
RAB29	-0.2133	1.5056E-03	9.84E-03	ENSG00000117280
RAB2A	-0.0459	3.6595E-01	5.62E-01	ENSG00000104388
RAB2B	-0.0968	8.3680E-02	2.07E-01	ENSG00000129472
RAB30	-0.0939	1.5982E-01	3.25E-01	ENSG00000137502
RAB31	-0.3560	4.6935E-05	6.12E-04	ENSG00000168461
RAB32	-0.5019	5.4676E-08	2.39E-06	ENSG00000118508
RAB33A	0.2002	3.7991E-02	1.16E-01	ENSG00000134594
RAB33B	-0.1841	4.2037E-02	1.26E-01	ENSG00000172007
RAB34	-0.1578	4.2521E-02	1.27E-01	ENSG00000109113
RAB35	0.1829	2.8959E-02	9.50E-02	ENSG00000111737
RAB36	-0.2880	5.1075E-04	4.18E-03	ENSG00000100228
RAB37	0.4538	2.2580E-03	1.35E-02	ENSG00000172794
RAB38	-0.1265	1.9640E-01	3.73E-01	ENSG00000123892
RAB39B	0.1371	1.2406E-02	5.01E-02	ENSG00000155961
RAB3A	0.2186	5.5941E-03	2.73E-02	ENSG00000105649
RAB3B	0.3559	9.7183E-07	2.61E-05	ENSG00000169213
RAB3C	0.1366	2.0019E-02	7.21E-02	ENSG00000152932
RAB3D	-0.0554	5.1287E-01	6.92E-01	ENSG00000105514
RAB3GAP1	-0.2313	6.2831E-07	1.81E-05	ENSG00000115839
RAB3GAP2	-0.0882	1.1119E-01	2.53E-01	ENSG00000118873
RAB3IL1	-0.2161	1.4505E-02	5.66E-02	ENSG00000167994
RAB3IP	-0.0217	6.6481E-01	8.07E-01	ENSG00000127328
RAB40AL	-0.0140	7.2937E-01		ENSG00000102128
RAB40B	0.5399	5.1886E-14	1.46E-11	ENSG00000141542
RAB40C	0.1279	5.8190E-02	1.60E-01	ENSG00000197562
RAB41	0.0284	6.8371E-01	8.21E-01	ENSG00000147127
RAB42	-0.0318	7.2912E-01	8.51E-01	ENSG00000188060
RAB43	0.0404	5.8440E-01	7.49E-01	ENSG00000172780
RAB44	0.0070	5.3233E-01		ENSG00000255587
RAB4A	-0.0387	3.5602E-01	5.51E-01	ENSG00000168118
RAB4A-AS1	0.0563	5.5778E-01	7.28E-01	ENSG00000177788
RAB4B	-0.0272	7.6518E-01	8.73E-01	ENSG00000167578
RAB5A	0.0075	9.7355E-01	9.86E-01	ENSG00000144566
RAB5B	-0.0375	3.8656E-01	5.81E-01	ENSG00000111540
RAB5C	0.0795	3.9866E-01	5.93E-01	ENSG00000108774
RAB5IF	-0.3297	2.2130E-05	3.33E-04	ENSG00000101084
RAB6A	0.1515	6.4080E-02	1.71E-01	ENSG00000175582
RAB6B	-0.2185	4.0990E-03	2.15E-02	ENSG00000154917
RAB6C	0.0425	6.4889E-01	7.97E-01	ENSG00000222014
RAB6D	0.0574	5.4914E-01	7.21E-01	ENSG00000233087
RAB7A	-0.0880	6.0474E-02	1.64E-01	ENSG00000075785
RAB7B	0.0265	7.5114E-01	8.64E-01	ENSG00000276600

RAB8A	-0.0239	6.8960E-01	8.26E-01	ENSG00000167461
RAB8B	-0.0838	2.7378E-01	4.64E-01	ENSG00000166128
RAB9A	-0.3800	1.0909E-07	4.17E-06	ENSG00000123595
RAB9B	0.0614	5.2010E-01	6.97E-01	ENSG00000123570
RABAC1	-0.2429	3.3456E-04	2.98E-03	ENSG00000105404
RABEP1	-0.0640	3.0101E-01	4.94E-01	ENSG00000029725
RABEP2	0.0637	4.4864E-01	6.39E-01	ENSG00000177548
RABEPK	-0.0458	3.8277E-01	5.77E-01	ENSG00000136933
RABGAP1	-0.2107	2.9272E-05	4.15E-04	ENSG00000011454
RABGAP1L	-0.0217	6.2876E-01	7.83E-01	ENSG00000152061
RABGAP1L-AS1	0.0080	6.5892E-01	8.03E-01	ENSG00000229531
RABGAP1L-DT	0.0014	9.8775E-01	9.93E-01	ENSG00000227373
RABGEF1	-0.0722	4.0820E-01	6.01E-01	ENSG00000154710
RABGEF1P2	-0.0505	6.5462E-01	8.00E-01	ENSG00000230189
RABGGTA	0.2508	2.6541E-04	2.46E-03	ENSG00000100949
RABGGTB	-0.0988	1.3619E-01	2.90E-01	ENSG00000137955
RABIF	0.0198	7.7051E-01	8.76E-01	ENSG00000183155
RABL2A	0.0662	4.4478E-01	6.35E-01	ENSG00000144134
RABL2B	0.0439	6.3852E-01	7.90E-01	ENSG00000079974
RABL3	-0.0777	2.6186E-01	4.51E-01	ENSG00000144840
RABL6	-0.1332	1.2215E-01	2.70E-01	ENSG00000196642
RAC1	-0.2844	1.6834E-07	6.00E-06	ENSG00000136238
RAC2	0.2490	3.3383E-02	1.06E-01	ENSG00000128340
RAC3	0.2543	5.0649E-02	1.44E-01	ENSG00000169750
RACGAP1	-0.1907	5.7535E-02	1.59E-01	ENSG00000161800
RACK1	-0.2094	5.2826E-03	2.61E-02	ENSG00000204628
RAD1	0.1360	3.0951E-02	9.97E-02	ENSG00000113456
RAD17	0.0986	9.6163E-02	2.28E-01	ENSG00000152942
RAD17P1	-0.0353	3.1110E-01		ENSG00000232400
RAD18	-0.0172	8.3640E-01	9.14E-01	ENSG00000070950
RAD21	-0.1014	1.2402E-01	2.73E-01	ENSG00000164754
RAD21-AS1	0.1578	1.3442E-01	2.88E-01	ENSG00000253327
RAD21L1	0.0337	5.5950E-01	7.29E-01	ENSG00000244588
RAD23A	-0.1369	4.6033E-02	1.35E-01	ENSG00000179262
RAD23B	-0.3193	3.5886E-06	7.68E-05	ENSG00000119318
RAD50	-0.0399	6.3088E-01	7.84E-01	ENSG00000113522
RAD51	-0.2073	7.7389E-02	1.96E-01	ENSG00000051180
RAD51-AS1	0.3926	4.1004E-04	3.51E-03	ENSG00000245849
RAD51AP1	-0.3309	3.7798E-03	2.02E-02	ENSG00000111247
RAD51AP2	-0.0406	1.6408E-01		ENSG00000214842
RAD51B	0.0125	9.0861E-01	9.54E-01	ENSG00000182185
RAD51C	-0.1427	2.5385E-02	8.62E-02	ENSG00000108384
RAD51D	0.1484	1.5103E-01	3.12E-01	ENSG00000185379
RAD52	0.2362	1.4742E-02	5.73E-02	ENSG00000002016
RAD54B	-0.0634	4.9837E-01	6.81E-01	ENSG00000197275
RAD54L	0.0298	7.4308E-01	8.59E-01	ENSG00000085999
RAD54L2	0.2027	3.6768E-03	1.98E-02	ENSG00000164080
RAD9A	0.0383	5.9252E-01	7.55E-01	ENSG00000172613
RAD9B	-0.0175	8.2673E-01	9.09E-01	ENSG00000151164

RADIL	0.2290	7.9705E-03	3.58E-02	ENSG00000157927
RADX	0.0760	3.6604E-01	5.62E-01	ENSG00000147231
RAE1	-0.1187	1.0271E-02	4.33E-02	ENSG00000101146
RAET1E	0.0552	4.1631E-01	6.09E-01	ENSG00000164520
RAET1E-AS1	-0.2087	3.0344E-02	9.83E-02	ENSG00000268592
RAET1G	-0.1190	1.9678E-01	3.73E-01	ENSG00000203722
RAET1L	-0.0104	8.7602E-01	9.37E-01	ENSG00000155918
RAF1	0.0737	3.8191E-01	5.77E-01	ENSG00000132155
RAG1	0.0362	6.7547E-01	8.14E-01	ENSG00000166349
RAG2	-0.0250	7.8143E-01	8.82E-01	ENSG00000175097
RAI1	0.1429	1.6209E-02	6.15E-02	ENSG00000108557
RAI1-AS1	-0.0555	4.5408E-01	6.44E-01	ENSG00000237328
RAI14	-0.4034	4.3938E-05	5.80E-04	ENSG00000039560
RAI2	-0.0822	2.9861E-01	4.91E-01	ENSG00000131831
RALA	-0.0137	6.9792E-01	8.31E-01	ENSG00000006451
RALB	0.2056	1.6806E-02	6.32E-02	ENSG00000144118
RALBP1	-0.0243	7.1492E-01	8.41E-01	ENSG00000017797
RALGAPA1	0.0399	4.4328E-01	6.34E-01	ENSG00000174373
RALGAPA1P1	0.1306	1.1124E-01	2.53E-01	ENSG00000229419
RALGAPA2	0.0835	2.0474E-01	3.83E-01	ENSG00000188559
RALGAPB	0.0616	3.5235E-01	5.47E-01	ENSG00000170471
RALGDS	-0.0178	7.9379E-01	8.89E-01	ENSG00000160271
RALGPS1	0.1463	5.3505E-02	1.50E-01	ENSG00000136828
RALGPS2	-0.3543	2.8899E-05	4.11E-04	ENSG00000116191
RALY	0.0928	1.4170E-01	2.99E-01	ENSG00000125970
RALY-AS1	-0.0721	4.5314E-01	6.43E-01	ENSG00000285230
RALYL	-0.1858	5.4546E-02	1.52E-01	ENSG00000184672
RAMAC	-0.1176	2.5506E-02	8.65E-02	ENSG00000169612
RAMACL	-0.0580	2.0335E-01	3.81E-01	ENSG00000235272
RAMP1	-0.3557	6.0115E-04	4.77E-03	ENSG00000132329
RAMP2	0.1057	1.5804E-01	3.22E-01	ENSG00000131477
RAMP2-AS1	0.0300	7.3871E-01	8.57E-01	ENSG00000197291
RAN	-0.2706	2.7467E-06	6.21E-05	ENSG00000132341
RANBP1	-0.1460	1.9773E-03	1.21E-02	ENSG00000099901
RANBP10	-0.0210	7.7224E-01	8.77E-01	ENSG00000141084
RANBP17	0.3149	9.1310E-04	6.63E-03	ENSG00000204764
RANBP2	-0.3309	1.2148E-08	6.73E-07	ENSG00000153201
RANBP3	-0.2371	1.1538E-02	4.73E-02	ENSG00000031823
RANBP3-DT	-0.1062	2.2992E-01	4.13E-01	ENSG00000266983
RANBP3L	-0.2879	3.6825E-02	1.14E-01	ENSG00000164188
RANBP6	-0.3204	9.1418E-05	1.04E-03	ENSG00000137040
RANBP9	-0.2232	1.5872E-03	1.02E-02	ENSG00000010017
RANGAP1	0.1574	6.6736E-03	3.12E-02	ENSG00000100401
RANGRF	0.1152	2.6452E-01	4.53E-01	ENSG00000108961
RAP1A	-0.2404	6.6453E-04	5.17E-03	ENSG00000116473
RAP1B	-0.0878	2.8791E-01	4.79E-01	ENSG00000127314
RAP1GAP	0.0027	9.7146E-01	9.85E-01	ENSG00000076864
RAP1GAP2	0.0639	4.0365E-01	5.98E-01	ENSG00000132359
RAP1GDS1	-0.1059	8.8582E-02	2.16E-01	ENSG00000138698

RAP2A	-0.1019	1.7960E-01	3.51E-01	ENSG00000125249
RAP2B	-0.3227	6.7239E-05	8.15E-04	ENSG00000181467
RAP2C	-0.1481	5.9336E-02	1.62E-01	ENSG00000123728
RAP2C-AS1	0.0750	3.4669E-01	5.42E-01	ENSG00000232160
RAPGEF1	0.0424	5.7364E-01	7.41E-01	ENSG00000107263
RAPGEF2	-0.0715	3.4461E-01	5.40E-01	ENSG00000109756
RAPGEF3	0.1435	1.8365E-01	3.56E-01	ENSG00000079337
RAPGEF4	0.1466	8.8562E-02	2.16E-01	ENSG00000091428
RAPGEF4-AS1	0.0087	7.4630E-01	8.61E-01	ENSG00000228016
RAPGEF5	0.4024	8.7381E-06	1.57E-04	ENSG00000136237
RAPGEFL1	-0.1054	2.2581E-01	4.08E-01	ENSG00000108352
RAPH1	-0.4658	1.1866E-07	4.47E-06	ENSG00000173166
RAPSN	0.1774	3.0861E-02	9.95E-02	ENSG00000165917
RARA	-0.1791	2.1690E-02	7.66E-02	ENSG00000131759
RARA-AS1	-0.2271	1.7464E-02	6.50E-02	ENSG00000265666
RARB	0.1732	1.1695E-01	2.62E-01	ENSG00000077092
RARG	-0.3352	2.0709E-02	7.39E-02	ENSG00000172819
RARRES1	0.0275	7.7164E-01	8.76E-01	ENSG00000118849
RARRES2	0.0227	7.9801E-01	8.92E-01	ENSG00000106538
RARRES2P3	-0.0061	8.1921E-01		ENSG00000231913
RARS1	0.1690	1.7980E-02	6.65E-02	ENSG00000113643
RARS2	0.2627	1.2240E-05	2.06E-04	ENSG00000146282
RASA1	0.1533	3.3770E-02	1.07E-01	ENSG00000145715
RASA2	0.0577	4.7903E-01	6.65E-01	ENSG00000155903
RASA3	0.0245	7.7182E-01	8.76E-01	ENSG00000185989
RASA4	0.1067	1.3268E-01	2.85E-01	ENSG00000170667
RASA4	0.0607	4.4613E-01	6.36E-01	ENSG00000105808
RASAL1	-0.0181	8.5813E-01	9.27E-01	ENSG00000111344
RASAL2	-0.0611	3.8596E-01	5.81E-01	ENSG00000075391
RASAL2-AS1	0.2778	4.1234E-02	1.24E-01	ENSG00000224687
RASAL3	-0.0894	3.5451E-01	5.50E-01	ENSG00000105122
RASD1	0.1347	1.1041E-01	2.52E-01	ENSG00000108551
RASD2	0.0903	3.6515E-01	5.61E-01	ENSG00000100302
RASEF	-0.2458	4.2163E-03	2.20E-02	ENSG00000165105
RASGEF1A	0.1224	2.2850E-01	4.11E-01	ENSG00000198915
RASGEF1B	0.0750	4.3064E-01	6.22E-01	ENSG00000138670
RASGEF1C	-0.1237	2.1058E-01	3.90E-01	ENSG00000146090
RASGRF1	0.0514	5.6207E-01	7.31E-01	ENSG00000058335
RASGRF2	-0.2104	1.7373E-02	6.48E-02	ENSG00000113319
RASGRF2-AS1	0.1097	2.5702E-01	4.45E-01	ENSG00000251450
RASGRP1	-0.1188	2.1647E-01	3.97E-01	ENSG00000172575
RASGRP2	0.0909	3.4768E-01	5.43E-01	ENSG00000068831
RASGRP3	0.3675	1.0218E-02	4.32E-02	ENSG00000152689
RASGRP4	-0.0112	9.0544E-01	9.53E-01	ENSG00000171777
RASL10A	-0.0568	5.4258E-01	7.15E-01	ENSG00000100276
RASL10B	-0.1011	1.2907E-01	2.80E-01	ENSG00000270885
RASL11A	0.0316	7.3921E-01	8.57E-01	ENSG00000122035
RASL11B	-0.0006	5.0870E-01	6.89E-01	ENSG00000128045
RASL12	-0.0028	9.9436E-01		ENSG00000103710

RASSF1	0.3515	5.7682E-04	4.62E-03	ENSG00000068028
RASSF10	-0.0298	7.3162E-01	8.52E-01	ENSG00000189431
RASSF2	0.0068	9.4781E-01	9.74E-01	ENSG00000101265
RASSF3	-0.3742	1.5343E-02	5.90E-02	ENSG00000153179
RASSF4	-0.1182	9.9638E-02	2.34E-01	ENSG00000107551
RASSF5	0.1451	4.9433E-02	1.42E-01	ENSG00000266094
RASSF6	-0.1157	2.4755E-01	4.34E-01	ENSG00000169435
RASSF7	0.5521	3.5588E-07	1.12E-05	ENSG00000099849
RASSF8	-0.0621	4.4671E-01	6.37E-01	ENSG00000123094
RASSF9	0.1173	1.7628E-01	3.47E-01	ENSG00000198774
RAVER1	0.0135	8.8066E-01	9.39E-01	ENSG00000161847
RAVER2	-0.1167	1.8629E-01	3.60E-01	ENSG00000162437
RAX2	0.0182	4.8188E-01		ENSG00000173976
RB1	-0.0679	2.1004E-01	3.89E-01	ENSG00000139687
RB1CC1	0.0987	1.3785E-01	2.93E-01	ENSG00000023287
RBAK	0.1290	8.1573E-02	2.04E-01	ENSG00000146587
RBAK-RBAKDN	0.0129	6.3975E-01		ENSG00000272968
RBBP4	-0.0143	7.9173E-01	8.88E-01	ENSG00000162521
RBBP4P2	-0.0477	4.9472E-01	6.78E-01	ENSG00000242457
RBBP5	-0.0100	8.9672E-01	9.49E-01	ENSG00000117222
RBBP6	-0.1177	3.3994E-02	1.07E-01	ENSG00000122257
RBBP7	0.0261	7.2968E-01	8.51E-01	ENSG00000102054
RBBP8	-0.2084	1.1035E-03	7.67E-03	ENSG00000101773
RBBP8NL	-0.0987	1.9079E-01	3.66E-01	ENSG00000130701
RBBP9	0.1218	6.7631E-02	1.78E-01	ENSG00000089050
RBCK1	0.0031	9.9932E-01	1.00E+00	ENSG00000125826
RBFA	0.1453	5.7198E-02	1.58E-01	ENSG00000101546
RBFADN	0.0376	5.8730E-01	7.52E-01	ENSG00000261126
RBFOX1	0.5090	1.2076E-05	2.04E-04	ENSG00000078328
RBFOX2	0.0186	7.6075E-01	8.70E-01	ENSG00000100320
RBFOX3	0.1374	1.5110E-01	3.12E-01	ENSG00000167281
RBIS	-0.2001	2.2180E-03	1.33E-02	ENSG00000176731
RBKS	0.1262	7.1795E-02	1.86E-01	ENSG00000171174
RBL1	0.3548	2.4120E-03	1.42E-02	ENSG00000080839
RBL2	0.1420	5.3077E-02	1.49E-01	ENSG00000103479
RBM10	-0.4088	1.5677E-09	1.24E-07	ENSG00000182872
RBM11	0.0091	8.9515E-01	9.48E-01	ENSG00000185272
RBM12	0.1409	1.7377E-03	1.10E-02	ENSG00000244462
RBM12B	-0.0232	7.8033E-01	8.81E-01	ENSG00000183808
RBM12B-AS1	0.1316	1.8345E-01	3.56E-01	ENSG00000279331
RBM12B-DT	-0.0166	8.2465E-01	9.08E-01	ENSG00000253848
RBM14	-0.0537	4.4670E-01	6.37E-01	ENSG00000239306
RBM14-RBM4	-0.1037	2.5862E-01	4.47E-01	ENSG00000248643
RBM15	-0.0701	3.5488E-01	5.50E-01	ENSG00000162775
RBM15-AS1	0.0817	3.7208E-01	5.67E-01	ENSG00000227963
RBM15B	-0.0761	1.7839E-01	3.49E-01	ENSG00000259956
RBM17	-0.1032	1.2598E-02	5.07E-02	ENSG00000134453
RBM18	-0.0302	6.1572E-01	7.73E-01	ENSG00000119446
RBM19	-0.1123	1.7981E-01	3.51E-01	ENSG00000122965

RBM20	-0.0145	8.7494E-01	9.37E-01	ENSG00000203867
RBM22	0.0309	6.4464E-01	7.94E-01	ENSG00000086589
RBM23	0.1904	8.1780E-03	3.66E-02	ENSG00000100461
RBM24	-0.3431	8.2107E-05	9.55E-04	ENSG00000112183
RBM25	-0.1003	6.1191E-02	1.66E-01	ENSG00000119707
RBM26	-0.0284	6.6728E-01	8.09E-01	ENSG00000139746
RBM26-AS1	0.5974	1.9328E-09	1.46E-07	ENSG00000227354
RBM27	-0.2390	2.9780E-02	9.69E-02	ENSG00000091009
RBM28	0.0865	2.3573E-01	4.20E-01	ENSG00000106344
RBM3	0.2923	2.5002E-03	1.46E-02	ENSG00000102317
RBM33	0.0879	2.2442E-01	4.07E-01	ENSG00000184863
RBM33-DT	-0.0142	8.6185E-01	9.30E-01	ENSG00000216895
RBM34	-0.2129	3.2963E-02	1.05E-01	ENSG00000188739
RBM38	-0.4924	2.5954E-07	8.71E-06	ENSG00000132819
RBM39	-0.4341	7.2768E-15	2.73E-12	ENSG00000131051
RBM39P1	-0.0109	8.7418E-01		ENSG00000214019
RBM4	0.3752	1.2652E-09	1.04E-07	ENSG00000173933
RBM41	-0.0743	2.1108E-01	3.91E-01	ENSG00000089682
RBM42	0.0033	9.5428E-01	9.77E-01	ENSG00000126254
RBM43	-0.1564	9.2389E-02	2.23E-01	ENSG00000184898
RBM44	-0.1310	2.0947E-01	3.89E-01	ENSG00000177483
RBM45	-0.0633	3.5103E-01	5.46E-01	ENSG00000155636
RBM46	-0.0070	8.5082E-01		ENSG00000151962
RBM47	-0.1126	1.2791E-01	2.79E-01	ENSG00000163694
RBM48	-0.1233	6.6561E-02	1.76E-01	ENSG00000127993
RBM4B	-0.0451	4.9852E-01	6.81E-01	ENSG00000173914
RBM5	0.2117	7.7672E-04	5.84E-03	ENSG00000003756
RBM5-AS1	0.0953	1.9989E-01	3.77E-01	ENSG00000281691
RBM6	0.0146	7.8867E-01	8.87E-01	ENSG00000004534
RBM7	-0.4709	1.4330E-08	7.68E-07	ENSG00000076053
RBM8A	-0.2182	6.9882E-04	5.38E-03	ENSG00000265241
RBMS1	0.0354	5.5941E-01	7.29E-01	ENSG00000153250
RBMS2	-0.1109	1.2112E-01	2.69E-01	ENSG00000076067
RBMS3	-0.0262	6.9212E-01	8.27E-01	ENSG00000144642
RBMS3-AS2	0.1132	7.4933E-02		ENSG00000203506
RBMX	-0.2952	3.3264E-06	7.20E-05	ENSG00000147274
RBMX2	-0.2214	3.7266E-04	3.27E-03	ENSG00000134597
RBMXL1	-0.2956	2.4526E-04	2.31E-03	ENSG00000213516
RBP1	-0.1204	1.3033E-01	2.82E-01	ENSG00000114115
RBP2	-0.0390	6.0485E-01	7.64E-01	ENSG00000114113
RBP3	-0.0163	6.2496E-01		ENSG00000265203
RBP4	-0.3034	7.7188E-03	3.49E-02	ENSG00000138207
RBP5	-0.0107	9.0483E-01	9.52E-01	ENSG00000139194
RBP7	-0.0601	5.2794E-01	7.03E-01	ENSG00000162444
RBPJ	0.0831	9.6608E-02	2.29E-01	ENSG00000168214
RBPMS	0.2523	5.5488E-03	2.72E-02	ENSG00000157110
RBPMS-AS1	-0.5250	1.0148E-02	4.29E-02	ENSG00000254109
RBPMS2	0.0379	6.9915E-01	8.32E-01	ENSG00000166831
RBSN	0.1142	1.1623E-01	2.61E-01	ENSG00000131381

RBX1	-0.2595	5.3792E-05	6.80E-04	ENSG00000100387
RC3H1	-0.1096	5.2586E-02	1.48E-01	ENSG00000135870
RC3H1-DT	0.4661	5.4830E-04	4.44E-03	ENSG00000224977
RC3H2	0.0166	7.2221E-01	8.46E-01	ENSG00000056586
RCAN1	0.0768	4.0751E-01	6.01E-01	ENSG00000159200
RCAN2	0.1945	6.2051E-02	1.67E-01	ENSG00000172348
RCAN3	-0.1838	7.5777E-04	5.72E-03	ENSG00000117602
RCAN3AS	-0.0432	6.4774E-01	7.96E-01	ENSG00000286061
RCBTB1	0.0338	6.1966E-01	7.76E-01	ENSG00000136144
RCBTB2	-0.5051	5.3714E-05	6.80E-04	ENSG00000136161
RCC1	-0.1174	1.7764E-01	3.48E-01	ENSG00000180198
RCC1L	-0.0431	5.3746E-01	7.11E-01	ENSG00000274523
RCC2	-0.0792	2.3013E-01	4.13E-01	ENSG00000179051
RCCD1	0.0102	8.9305E-01	9.47E-01	ENSG00000166965
RCE1	0.0658	3.5319E-01	5.48E-01	ENSG00000173653
RCHY1	-0.0924	1.8524E-01	3.59E-01	ENSG00000163743
RCL1	-0.1135	6.3653E-02	1.70E-01	ENSG00000120158
RCN1	-0.2290	2.7017E-02	9.01E-02	ENSG00000049449
RCN2	0.0271	6.7898E-01	8.17E-01	ENSG00000117906
RCOR1	-0.0245	7.6144E-01	8.70E-01	ENSG00000089902
RCOR2	0.2294	2.7016E-02	9.01E-02	ENSG00000167771
RCOR3	-0.0607	2.0562E-01	3.84E-01	ENSG00000117625
RCSD1	0.0006	9.0916E-01		ENSG00000198771
RCVRN	-0.0607	2.4052E-01		ENSG00000109047
RD3	0.1453	1.8108E-01	3.53E-01	ENSG00000198570
RDH10	0.0322	7.0055E-01	8.33E-01	ENSG00000121039
RDH10-AS1	-0.0436	5.8610E-01	7.51E-01	ENSG00000250295
RDH11	-0.1673	8.6144E-03	3.80E-02	ENSG00000072042
RDH12	-0.0896	3.4860E-01	5.44E-01	ENSG00000139988
RDH13	-0.0088	8.9777E-01	9.49E-01	ENSG00000160439
RDH14	-0.0785	2.4868E-01	4.35E-01	ENSG00000240857
RDH16	-0.0242	6.9582E-01	8.30E-01	ENSG00000139547
RDH5	-0.1716	1.2446E-01	2.74E-01	ENSG00000135437
RDH8	0.0112	4.2658E-01		ENSG00000080511
RDM1P1	0.0197	7.7474E-01	8.78E-01	ENSG00000266504
RDM1P2	-0.0937	3.4388E-01	5.39E-01	ENSG00000266497
RDM1P4	-0.0232	7.9828E-01		ENSG00000271974
RDM1P5	-0.1124	2.6315E-01	4.52E-01	ENSG00000263818
RDX	-0.4219	8.7503E-11	1.06E-08	ENSG00000137710
RDXP1	-0.0418	4.1800E-01		ENSG00000255387
RDXP3	-0.0167	9.2295E-01		ENSG00000238247
REC114	-0.0379	2.8503E-01		ENSG00000183324
REC8	0.0163	8.5017E-01	9.23E-01	ENSG00000100918
RECK	0.2886	5.7944E-04	4.64E-03	ENSG00000122707
RECQL	0.2954	6.1613E-03	2.94E-02	ENSG00000004700
RECQL4	-0.3675	1.0359E-02	4.36E-02	ENSG00000160957
RECQL5	0.1654	8.5760E-02	2.11E-01	ENSG00000108469
REELD1	-0.0221	6.3429E-01	7.87E-01	ENSG00000250673
REEP1	0.0501	4.4813E-01	6.38E-01	ENSG00000068615

REEP2	0.0657	3.2890E-01	5.23E-01	ENSG00000132563
REEP3	-0.3911	1.2807E-09	1.05E-07	ENSG00000165476
REEP4	0.0057	9.6413E-01	9.82E-01	ENSG00000168476
REEP5	-0.2200	3.1585E-05	4.41E-04	ENSG00000129625
REEP6	0.2210	2.4850E-03	1.45E-02	ENSG00000115255
REG4	0.0109	3.6651E-01		ENSG00000134193
REL	-0.2945	2.6468E-05	3.83E-04	ENSG00000162924
REL-DT	-0.1276	1.9695E-01	3.73E-01	ENSG00000228414
RELA	-0.1264	3.0376E-02	9.83E-02	ENSG00000173039
RELA-DT	0.2409	8.7897E-03	3.86E-02	ENSG00000285533
RELCH	0.3658	1.8590E-06	4.44E-05	ENSG00000134444
RELL1	-0.0229	7.5322E-01	8.66E-01	ENSG00000181826
RELL2	0.5161	6.3644E-06	1.23E-04	ENSG00000164620
RELN	-0.3342	6.7366E-03	3.14E-02	ENSG00000189056
RELT	0.3562	5.7167E-04	4.58E-03	ENSG00000054967
REM1	-0.0139	9.0417E-01		ENSG00000088320
REM2	-0.0012	9.7374E-01	9.87E-01	ENSG00000139890
REN	-0.0508	2.3842E-01		ENSG00000143839
REP15	-0.0390	1.2132E-01		ENSG00000174236
REPIN1	-0.1545	3.4363E-03	1.87E-02	ENSG00000214022
REPS1	-0.1057	2.5219E-02	8.57E-02	ENSG00000135597
REPS2	-0.1349	1.5235E-01	3.14E-01	ENSG00000169891
RER1	0.0807	2.2686E-01	4.09E-01	ENSG00000157916
RERE	-0.0550	4.3284E-01	6.24E-01	ENSG00000142599
RERG	-0.0600	4.8628E-01	6.71E-01	ENSG00000134533
RERG-AS1	-0.0002	9.0727E-01		ENSG00000255660
RERGL	0.4674	1.0003E-03	7.11E-03	ENSG00000111404
RESF1	0.0679	3.1027E-01	5.03E-01	ENSG00000174718
RESP18	0.1732	8.5595E-02	2.11E-01	ENSG00000182698
REST	-0.2591	4.7364E-04	3.92E-03	ENSG00000084093
RET	-0.1599	3.7419E-02	1.15E-01	ENSG00000165731
RETNLB	-0.0151	5.1406E-01		ENSG00000163515
RETREG1	-0.0899	2.1963E-02	7.73E-02	ENSG00000154153
RETREG1-AS1	-0.0484	4.9931E-01	6.82E-01	ENSG00000246214
RETREG2	0.0026	9.5789E-01	9.79E-01	ENSG00000144567
RETREG3	0.2416	1.5156E-04	1.57E-03	ENSG00000141699
RETSAT	-0.0113	8.2091E-01	9.06E-01	ENSG00000042445
REV1	0.0564	3.5142E-01	5.47E-01	ENSG00000135945
REV3L	0.1000	1.0628E-01	2.45E-01	ENSG00000009413
REX1BD	0.1015	6.1433E-02	1.66E-01	ENSG00000006015
REXO1	0.1209	1.2529E-01	2.75E-01	ENSG00000079313
REXO2	-0.4893	1.7134E-08	8.95E-07	ENSG00000076043
REXO4	-0.1618	5.5514E-03	2.72E-02	ENSG00000148300
REXO5	0.2374	1.1926E-02	4.86E-02	ENSG00000005189
RFC1	-0.1625	1.1329E-02	4.67E-02	ENSG00000035928
RFC2	-0.0572	3.1457E-01	5.08E-01	ENSG00000049541
RFC3	0.1320	9.5776E-02	2.28E-01	ENSG00000133119
RFC4	0.1641	5.6329E-03	2.75E-02	ENSG00000163918
RFC5	0.0764	2.5583E-01	4.43E-01	ENSG00000111445

RFESD	0.3242	3.2190E-03	1.77E-02	ENSG00000175449
RFFL	-0.0986	2.0638E-01	3.85E-01	ENSG00000092871
RFK	-0.0863	1.1757E-01	2.63E-01	ENSG00000135002
RFLNA	-0.0298	7.4693E-01	8.61E-01	ENSG00000178882
RFLNB	-0.0056	9.5068E-01	9.75E-01	ENSG00000183688
RFNG	-0.3644	2.3112E-07	7.91E-06	ENSG00000169733
RFPL1	-0.0367	3.2691E-01		ENSG00000128250
RFPL2	0.0537	5.6294E-01	7.32E-01	ENSG00000128253
RFPL3	-0.0225	4.4843E-01		ENSG00000128276
RFPL3S	-0.2495	5.4414E-02	1.52E-01	ENSG00000205853
RFT1	0.0663	3.8320E-01	5.78E-01	ENSG00000163933
RFTN1	0.0563	5.2578E-01	7.02E-01	ENSG00000131378
RFTN2	-0.1542	1.4436E-01	3.02E-01	ENSG00000162944
RFWD3	-0.0598	4.1711E-01	6.10E-01	ENSG00000168411
RFX1	-0.1037	2.8392E-01	4.75E-01	ENSG00000132005
RFX2	-0.1674	1.5292E-02	5.88E-02	ENSG00000087903
RFX3	-0.3661	1.1698E-04	1.27E-03	ENSG00000080298
RFX3-DT	0.0653	3.9670E-01	5.91E-01	ENSG00000232104
RFX4	-0.1285	2.0652E-01	3.85E-01	ENSG00000111783
RFX5	0.0297	6.4891E-01	7.97E-01	ENSG00000143390
RFX6	-0.0163	8.5699E-01		ENSG00000185002
RFX7	0.2480	8.1409E-03	3.65E-02	ENSG00000181827
RFX8	0.1838	1.1076E-01	2.52E-01	ENSG00000196460
RFXANK	-0.5076	1.2763E-08	6.95E-07	ENSG00000064490
RFXAP	0.1496	8.7256E-02	2.14E-01	ENSG00000133111
RGCC	-0.3457	1.3452E-02	5.33E-02	ENSG00000102760
RGL1	-0.0428	4.9972E-01	6.82E-01	ENSG00000143344
RGL2	-0.2385	2.3553E-04	2.24E-03	ENSG00000237441
RGL3	0.1053	2.5348E-01	4.41E-01	ENSG00000205517
RGL4	-0.0246	9.9038E-01		ENSG00000159496
RGMA	-0.3725	4.9499E-05	6.40E-04	ENSG00000182175
RGMB	-0.2909	1.9710E-05	3.02E-04	ENSG00000174136
RGMB-AS1	0.0824	4.0003E-01	5.94E-01	ENSG00000246763
RGN	-0.2029	4.7686E-02	1.38E-01	ENSG00000130988
RGP1	-0.0018	9.6570E-01	9.83E-01	ENSG00000107185
RGPD1	0.1746	3.5759E-02	1.11E-01	ENSG00000187627
RGPD2	0.1809	3.0081E-02	9.77E-02	ENSG00000185304
RGPD3	0.0320	7.1498E-01	8.41E-01	ENSG00000153165
RGPD4	0.1466	1.4093E-01	2.97E-01	ENSG00000196862
RGPD5	-0.1522	1.3688E-02	5.41E-02	ENSG00000015568
RGPD6	-0.1624	9.3762E-03	4.05E-02	ENSG00000183054
RGPD8	-0.1499	1.5179E-02	5.85E-02	ENSG00000169629
RGS1	-0.4017	8.0987E-03	3.63E-02	ENSG00000090104
RGS11	-0.3778	3.4896E-03	1.90E-02	ENSG00000076344
RGS12	-0.1455	3.2617E-02	1.04E-01	ENSG00000159788
RGS13	0.0084	6.1073E-01		ENSG00000127074
RGS14	0.3503	4.2144E-05	5.59E-04	ENSG00000169220
RGS16	-0.1027	2.3097E-01	4.14E-01	ENSG00000143333
RGS17	-0.0881	1.4117E-01	2.98E-01	ENSG00000091844

RGS19	-0.2717	8.6577E-03	3.82E-02	ENSG00000171700
RGS2	0.2353	1.6085E-03	1.03E-02	ENSG00000116741
RGS20	-0.1830	1.0211E-01	2.38E-01	ENSG00000147509
RGS22	-0.3787	3.2598E-03	1.79E-02	ENSG00000132554
RGS3	0.0669	4.0579E-01	5.99E-01	ENSG00000138835
RGS4	-0.0393	6.3017E-01	7.84E-01	ENSG00000117152
RGS5	0.1227	2.3578E-01	4.20E-01	ENSG00000143248
RGS5-AS1	-0.0165	9.7121E-01		ENSG00000232892
RGS6	0.5004	1.7477E-03	1.10E-02	ENSG00000182732
RGS7	0.4161	2.0669E-03	1.26E-02	ENSG00000182901
RGS7BP	0.2663	4.5395E-02	1.33E-01	ENSG00000186479
RGS8	-0.0554	5.3190E-01	7.06E-01	ENSG00000135824
RGS9	-0.2323	3.6438E-04	3.21E-03	ENSG00000108370
RGS9BP	0.0166	8.6082E-01	9.29E-01	ENSG00000186326
RHAG	0.0507	5.2051E-01	6.98E-01	ENSG00000112077
RHBDD1	-0.1546	6.0220E-02	1.64E-01	ENSG00000144468
RHBDD2	-0.1340	5.2915E-03	2.62E-02	ENSG00000005486
RHBDD3	-0.3531	9.7946E-07	2.62E-05	ENSG00000100263
RHBDF1	-0.1429	4.0638E-02	1.23E-01	ENSG00000007384
RHBDF1P1	-0.0001	9.8281E-01		ENSG00000234123
RHBDF2	0.0467	6.2700E-01	7.82E-01	ENSG00000129667
RHBDL1	-0.0501	5.1431E-01	6.93E-01	ENSG00000103269
RHBDL2	-0.1455	1.7621E-01	3.47E-01	ENSG00000158315
RHBDL3	-0.1834	6.7387E-02	1.77E-01	ENSG00000141314
RHBG	-0.1006	8.0869E-02		ENSG00000132677
RHCE	0.5898	2.4123E-07	8.19E-06	ENSG00000188672
RHCG	0.0696	4.1044E-01	6.04E-01	ENSG00000140519
RHD	-0.0762	5.0302E-01	6.84E-01	ENSG00000187010
RHEB	-0.2494	7.0757E-05	8.48E-04	ENSG00000106615
RHEBL1	0.1774	7.8975E-02	1.99E-01	ENSG00000167550
RHEX	0.0026	5.1270E-01		ENSG00000263961
RHNO1	-0.1926	7.9073E-03	3.56E-02	ENSG00000171792
RHOA	-0.1148	6.5596E-02	1.74E-01	ENSG00000067560
RHOB	-0.3626	2.7698E-07	9.19E-06	ENSG00000143878
RHOBTB1	0.4272	2.5969E-05	3.78E-04	ENSG00000072422
RHOBTB2	-0.0365	6.2442E-01	7.80E-01	ENSG00000008853
RHOBTB3	-0.1644	2.4454E-02	8.38E-02	ENSG00000164292
RHOC	-0.1711	5.6959E-03	2.77E-02	ENSG00000155366
RHOF	-0.0021	9.7212E-01	9.86E-01	ENSG00000139725
RHOG	-0.1821	1.8090E-02	6.68E-02	ENSG00000177105
RHOG2P	0.0373	1.5181E-01		ENSG00000213706
RHOQ	-0.1297	8.1240E-02	2.03E-01	ENSG00000119729
RHOQ-AS1	0.1639	2.2657E-02	7.91E-02	ENSG00000250116
RHOT1	0.1198	5.4998E-02	1.53E-01	ENSG00000126858
RHOT2	0.3424	8.2096E-05	9.55E-04	ENSG00000140983
RHOU	-0.0954	2.4088E-01	4.26E-01	ENSG00000116574
RHOV	-0.1157	2.3121E-01	4.15E-01	ENSG00000104140
RHOXF1	-0.0058	4.1282E-01	6.06E-01	ENSG00000101883
RHOXF2	0.0774	2.5334E-01	4.41E-01	ENSG00000131721

RHOXF2B	0.0113	2.0111E-01		ENSG00000203989
RHPN1	-0.0791	3.0871E-01	5.01E-01	ENSG00000158106
RHPN1-AS1	-0.0259	7.7746E-01	8.80E-01	ENSG00000254389
RHPN2	-0.3747	2.4322E-05	3.58E-04	ENSG00000131941
RIBC1	-0.3267	2.4963E-04	2.34E-03	ENSG00000158423
RIBC2	-0.1417	1.6420E-01	3.31E-01	ENSG00000128408
RIC1	0.0424	5.2320E-01	7.00E-01	ENSG00000107036
RIC3	-0.0516	3.2831E-01	5.23E-01	ENSG00000166405
RIC8A	0.0030	9.5622E-01	9.78E-01	ENSG00000177963
RIC8B	0.0318	6.1967E-01	7.76E-01	ENSG00000111785
RICTOR	0.0296	6.9943E-01	8.32E-01	ENSG00000164327
RIDA	-0.5737	1.1071E-06	2.91E-05	ENSG00000132541
RIF1	0.0671	2.3663E-01	4.21E-01	ENSG00000080345
RIGI	0.0622	5.2349E-01	7.00E-01	ENSG00000107201
RIIAD1	0.0438	5.7519E-01	7.42E-01	ENSG00000178796
RILP	0.1425	9.0268E-02	2.19E-01	ENSG00000167705
RILPL1	-0.0255	7.3737E-01	8.56E-01	ENSG00000188026
RILPL2	-0.2549	1.0490E-03	7.37E-03	ENSG00000150977
RIMBP2	-0.0231	7.8700E-01	8.86E-01	ENSG00000060709
RIMBP3	-0.0710	4.5310E-01	6.43E-01	ENSG00000275793
RIMBP3B	-0.0428	6.3628E-01	7.88E-01	ENSG00000274600
RIMBP3C	-0.0844	3.5527E-01	5.51E-01	ENSG00000183246
RIMKLA	0.1191	1.4238E-01	3.00E-01	ENSG00000177181
RIMKLB	-0.2141	7.4897E-04	5.68E-03	ENSG00000166532
RIMOC1	-0.1292	9.4437E-02	2.26E-01	ENSG00000205765
RIMS1	-0.1740	5.6247E-02	1.56E-01	ENSG00000079841
RIMS2	-0.0775	2.9194E-01	4.84E-01	ENSG00000176406
RIMS3	0.1658	6.2851E-02	1.69E-01	ENSG00000117016
RIMS4	-0.1172	1.4290E-01	3.00E-01	ENSG00000101098
RIN1	0.0082	9.1127E-01	9.55E-01	ENSG00000174791
RIN2	-0.1018	2.6698E-01	4.56E-01	ENSG00000132669
RIN3	-0.2538	4.9110E-02	1.41E-01	ENSG00000100599
RING1	0.0941	1.6925E-01	3.37E-01	ENSG00000204227
RINL	0.3192	1.4091E-03	9.35E-03	ENSG00000187994
RINT1	0.3620	4.2735E-05	5.65E-04	ENSG00000135249
RIOK1	0.3653	1.7184E-05	2.72E-04	ENSG00000124784
RIOK2	0.2151	6.8987E-03	3.20E-02	ENSG00000058729
RIOK3	0.0447	3.6779E-01	5.64E-01	ENSG00000101782
RIOX1	0.1237	9.8495E-02	2.32E-01	ENSG00000170468
RIOX2	-0.2325	5.8720E-03	2.83E-02	ENSG00000170854
RIPK1	0.2607	1.1827E-04	1.28E-03	ENSG00000137275
RIPK3	0.0539	3.9306E-01	5.88E-01	ENSG00000129465
RIPK4	-0.1683	1.0093E-01	2.36E-01	ENSG00000183421
RIPOR1	-0.2558	3.4179E-04	3.04E-03	ENSG00000039523
RIPOR2	0.0950	3.2044E-01	5.14E-01	ENSG00000111913
RIPOR3	-0.1248	2.1626E-01	3.97E-01	ENSG00000042062
RIPOR3-AS1	0.0203	4.3619E-01		ENSG00000234693
RIPPLY2	-0.5946	2.3646E-04	2.24E-03	ENSG00000203877
RIPPLY3	0.0498	5.7004E-01	7.38E-01	ENSG00000183145

RIT1	-0.4053	4.0487E-06	8.47E-05	ENSG00000143622
RIT2	0.0574	5.5608E-01	7.27E-01	ENSG00000152214
RITA1	-0.0163	7.3446E-01	8.54E-01	ENSG00000139405
RLBP1	-0.0562	3.1951E-01		ENSG00000140522
RLF	-0.1704	1.5415E-02	5.92E-02	ENSG00000117000
RLIG1	-0.0297	6.3875E-01	7.90E-01	ENSG00000133641
RLIM	-0.1479	7.1844E-03	3.30E-02	ENSG00000131263
RLIMP1	-0.0101	9.9187E-01		ENSG00000229456
RLIMP3	-0.0399	3.2662E-01		ENSG00000259388
RLN1	-0.1853	7.9440E-02	2.00E-01	ENSG00000107018
RLN2	-0.4651	1.9878E-04	1.95E-03	ENSG00000107014
RLN3	0.0487	4.6306E-01	6.51E-01	ENSG00000171136
RMC1	0.0641	3.4531E-01	5.40E-01	ENSG00000141452
RMDN1	0.0895	1.3213E-01	2.85E-01	ENSG00000176623
RMDN2	-0.2577	3.1011E-03	1.72E-02	ENSG00000115841
RMDN3	0.1546	8.8948E-03	3.89E-02	ENSG00000137824
RMI1	0.2456	4.8272E-03	2.43E-02	ENSG00000178966
RMI2	-0.0905	2.9177E-01	4.83E-01	ENSG00000175643
RMND1	0.2373	6.3184E-04	4.96E-03	ENSG00000155906
RMND5A	-0.1483	2.4852E-03	1.45E-02	ENSG00000153561
RMND5B	0.1299	5.8853E-02	1.61E-01	ENSG00000145916
RMST	0.1280	2.0343E-01	3.81E-01	ENSG00000255794
RN7SKP11	0.0042	5.0505E-01		ENSG00000252827
RN7SKP110	0.0541	3.1050E-02		ENSG00000222078
RN7SKP112	0.0104	7.9639E-01		ENSG00000223145
RN7SKP114	0.0295	4.0718E-01		ENSG00000222259
RN7SKP151	-0.0596	2.3902E-01		ENSG00000222162
RN7SKP154	-0.0197	6.0075E-01		ENSG00000222068
RN7SKP16	-0.0543	4.2423E-01	6.16E-01	ENSG00000222112
RN7SKP160	0.0025	9.1428E-01		ENSG00000200674
RN7SKP197	-0.0004	9.8151E-01		ENSG00000252886
RN7SKP225	0.0142	1.7895E-01		ENSG00000222337
RN7SKP23	-0.1150	3.4678E-01	5.42E-01	ENSG00000280039
RN7SKP239	-0.0083	9.3166E-01		ENSG00000201684
RN7SKP253	-0.0022	9.8759E-01		ENSG00000252233
RN7SKP266	-0.0609	1.0124E-01		ENSG00000222164
RN7SKP272	-0.0315	3.8479E-01		ENSG00000252797
RN7SKP276	0.0172	6.2063E-01		ENSG00000252051
RN7SKP292	-0.0233	7.5873E-01	8.69E-01	ENSG00000202392
RN7SKP299	-0.0012	2.4637E-01		ENSG00000271765
RN7SKP30	0.0047	6.6890E-01		ENSG00000223305
RN7SKP38	0.0161	6.9463E-01		ENSG00000222714
RN7SKP4	-0.0457	5.9205E-01		ENSG00000271818
RN7SKP56	0.0269	2.2827E-01		ENSG00000222445
RN7SKP57	-0.0038	9.7519E-01		ENSG00000253057
RN7SKP64	-0.0061	8.3463E-01		ENSG00000253015
RN7SKP76	0.0093	6.0627E-01		ENSG00000201289
RN7SKP80	0.3504	2.1957E-02	7.73E-02	ENSG00000202058
RN7SKP83	-0.0477	1.3037E-01		ENSG00000252321

RN7SKP88	0.0112	3.5604E-01		ENSG00000252656
RN7SKP97	0.1038	1.2028E-02		ENSG00000222898
RN7SL105P	-0.0408	4.8760E-01		ENSG00000243704
RN7SL11P	-0.0616	9.6721E-02		ENSG00000239316
RN7SL130P	-0.0027	9.2259E-01		ENSG00000244256
RN7SL138P	-0.0114	6.4181E-01		ENSG00000266274
RN7SL141P	-0.0048	9.9980E-01		ENSG00000243398
RN7SL146P	-0.0390	8.9069E-01		ENSG00000240106
RN7SL149P	-0.0058	5.8262E-01		ENSG00000241834
RN7SL166P	0.0138	3.6942E-01		ENSG00000275014
RN7SL180P	-0.0270	5.9671E-01		ENSG00000242860
RN7SL181P	0.0100	5.6725E-01		ENSG00000243738
RN7SL192P	0.0192	4.2052E-01		ENSG00000276757
RN7SL196P	0.0085	5.3841E-01		ENSG00000274424
RN7SL199P	0.1440	1.5326E-01	3.15E-01	ENSG00000265315
RN7SL200P	0.1188	8.1082E-02		ENSG00000265123
RN7SL202P	0.0112	4.5504E-01		ENSG00000266627
RN7SL208P	0.0031	7.5191E-01		ENSG00000264391
RN7SL20P	-0.0020	8.3983E-01		ENSG00000242251
RN7SL217P	0.0218	1.3070E-01		ENSG00000264706
RN7SL220P	0.0068	4.6013E-01		ENSG00000266467
RN7SL221P	-0.0275	2.7121E-01		ENSG00000239472
RN7SL230P	0.0018	9.3696E-01		ENSG00000264916
RN7SL233P	-0.0453	7.8104E-01		ENSG00000242971
RN7SL239P	-0.0337	5.5451E-01		ENSG00000242999
RN7SL23P	0.0066	6.2331E-01		ENSG00000240823
RN7SL242P	-0.0297	5.3711E-01		ENSG00000244389
RN7SL246P	-0.0664	5.1632E-01		ENSG00000265093
RN7SL258P	0.0598	1.3816E-01		ENSG00000240589
RN7SL263P	0.0067	4.3943E-01		ENSG00000240160
RN7SL265P	0.0121	7.7735E-01		ENSG00000241709
RN7SL268P	0.2369	6.4795E-02	1.73E-01	ENSG00000244425
RN7SL269P	-0.0008	4.5193E-01		ENSG00000243051
RN7SL273P	-0.0891	2.3099E-01	4.14E-01	ENSG00000239953
RN7SL280P	0.0160	2.0867E-01		ENSG00000244671
RN7SL284P	-0.0017	8.1193E-01		ENSG00000243446
RN7SL285P	-0.0787	3.6973E-01	5.65E-01	ENSG00000243313
RN7SL286P	0.0085	5.3841E-01		ENSG00000277464
RN7SL297P	0.0015	9.8471E-01		ENSG00000240183
RN7SL3	0.0093	5.0518E-01		ENSG00000278771
RN7SL306P	0.0295	4.4107E-01		ENSG00000242241
RN7SL308P	0.0011	5.0661E-01		ENSG00000243951
RN7SL328P	0.0042	6.9715E-01		ENSG00000240853
RN7SL329P	-0.0112	8.9771E-01	9.49E-01	ENSG00000242170
RN7SL331P	0.0044	6.8030E-01		ENSG00000278422
RN7SL336P	0.0444	5.5327E-01	7.24E-01	ENSG00000264017
RN7SL338P	0.0237	3.9200E-01		ENSG00000241568
RN7SL351P	0.0001	9.8278E-01		ENSG00000240098
RN7SL353P	0.0007	8.2057E-01		ENSG00000263608

RN7SL359P	-0.0090	7.2281E-01		ENSG00000244384
RN7SL364P	-0.0263	7.0452E-01	8.35E-01	ENSG00000243560
RN7SL370P	0.0003	8.4811E-01		ENSG00000243437
RN7SL378P	-0.0121	5.6812E-01		ENSG00000265684
RN7SL37P	0.0291	6.3936E-01	7.90E-01	ENSG00000242493
RN7SL381P	-0.0563	5.7351E-01		ENSG00000263968
RN7SL382P	-0.0020	9.7622E-01	9.88E-01	ENSG00000240723
RN7SL384P	-0.0617	3.2309E-01		ENSG00000242912
RN7SL385P	-0.0265	1.1514E-01		ENSG00000241333
RN7SL388P	-0.0045	7.8967E-01		ENSG00000239577
RN7SL393P	0.0156	2.8641E-01		ENSG00000243723
RN7SL394P	0.0206	4.2825E-01		ENSG00000239942
RN7SL395P	-0.0140	8.2654E-01		ENSG00000244307
RN7SL403P	-0.0282	3.6863E-01		ENSG00000240625
RN7SL40P	0.0040	6.8151E-01		ENSG00000242696
RN7SL413P	0.0079	4.6614E-01		ENSG00000242893
RN7SL417P	-0.0090	7.4492E-01		ENSG00000244056
RN7SL42P	-0.0074	7.0399E-01		ENSG00000263999
RN7SL430P	0.0055	7.7378E-01		ENSG00000243911
RN7SL441P	-0.0158	9.8099E-01		ENSG00000241413
RN7SL443P	0.0779	4.2902E-01	6.21E-01	ENSG00000241229
RN7SL444P	-0.2309	4.1580E-02	1.25E-01	ENSG00000265753
RN7SL449P	0.0311	6.4612E-01	7.95E-01	ENSG00000242889
RN7SL452P	-0.0347	4.6358E-01		ENSG00000243103
RN7SL456P	-0.0062	8.6708E-01		ENSG00000242266
RN7SL459P	0.0068	5.6942E-01		ENSG00000240993
RN7SL45P	0.0058	6.5339E-01		ENSG00000264862
RN7SL461P	-0.0272	6.9312E-01	8.28E-01	ENSG00000243801
RN7SL469P	0.0015	9.4621E-01		ENSG00000277637
RN7SL470P	0.0329	1.9650E-01		ENSG00000263669
RN7SL473P	-0.0809	4.2128E-01	6.14E-01	ENSG00000277452
RN7SL477P	0.0159	7.7294E-01		ENSG00000239367
RN7SL47P	0.0309	5.1303E-01		ENSG00000244710
RN7SL481P	-0.0156	8.9369E-01		ENSG00000240322
RN7SL49P	-0.0017	7.2370E-01		ENSG00000265802
RN7SL502P	0.0034	9.4351E-01		ENSG00000240733
RN7SL505P	-0.0130	8.4779E-01		ENSG00000241420
RN7SL508P	-0.0530	7.2298E-01	8.47E-01	ENSG00000244112
RN7SL516P	0.0078	5.1522E-01		ENSG00000242699
RN7SL520P	0.0128	3.7253E-01		ENSG00000240199
RN7SL524P	-0.3981	2.0951E-02	7.46E-02	ENSG00000266754
RN7SL525P	0.0115	5.2525E-01		ENSG00000264910
RN7SL526P	-0.0158	5.3011E-01		ENSG00000243642
RN7SL530P	0.0007	6.2376E-01		ENSG00000239910
RN7SL539P	0.0085	5.3841E-01		ENSG00000274076
RN7SL544P	0.0083	5.5719E-01		ENSG00000275230
RN7SL547P	0.0715	3.6859E-02		ENSG00000240584
RN7SL558P	0.0270	3.3800E-01		ENSG00000243260
RN7SL559P	0.0080	3.3347E-01		ENSG00000240750

RN7SL55P	-0.0049	6.9573E-01		ENSG00000243227
RN7SL565P	0.0085	7.0579E-01		ENSG00000276562
RN7SL566P	-0.1374	7.7032E-02	1.95E-01	ENSG00000241983
RN7SL574P	-0.0077	9.7661E-01	9.88E-01	ENSG00000266075
RN7SL577P	-0.1455	7.2970E-02	1.88E-01	ENSG00000265942
RN7SL582P	0.0037	5.3446E-01		ENSG00000241156
RN7SL587P	0.0324	7.5259E-01	8.65E-01	ENSG00000240233
RN7SL589P	0.0029	6.6777E-01		ENSG00000239247
RN7SL600P	-0.0043	9.5733E-01	9.78E-01	ENSG00000274963
RN7SL606P	-0.0079	9.4295E-01		ENSG00000239932
RN7SL608P	0.0206	2.1374E-01		ENSG00000239884
RN7SL610P	-0.0005	9.9556E-01		ENSG00000243847
RN7SL612P	-0.0161	8.6960E-01		ENSG00000266160
RN7SL615P	-0.0411	3.6172E-01		ENSG00000263989
RN7SL622P	0.0008	8.2454E-01		ENSG00000265052
RN7SL628P	-0.0004	8.8430E-01		ENSG00000277467
RN7SL635P	-0.0097	6.4063E-01		ENSG00000243029
RN7SL638P	-0.0756	1.5999E-01		ENSG00000243702
RN7SL649P	-0.0076	8.8005E-01		ENSG00000243539
RN7SL653P	0.0097	4.5274E-01		ENSG00000239794
RN7SL655P	0.0434	3.4812E-01	5.43E-01	ENSG00000241963
RN7SL656P	0.2070	7.6237E-02	1.94E-01	ENSG00000265411
RN7SL660P	0.0186	4.8639E-01	6.71E-01	ENSG00000278746
RN7SL663P	0.0492	2.4879E-01		ENSG00000275132
RN7SL672P	-0.0547	4.6514E-01	6.53E-01	ENSG00000239742
RN7SL673P	0.0017	8.7288E-01		ENSG00000273818
RN7SL677P	0.0175	4.3147E-01		ENSG00000242863
RN7SL678P	0.0016	7.3785E-01		ENSG00000263969
RN7SL67P	0.0236	7.8192E-02		ENSG00000243854
RN7SL683P	-0.0042	6.6752E-01		ENSG00000242330
RN7SL684P	-0.0149	8.0461E-01		ENSG00000243959
RN7SL688P	-0.0004	9.8876E-01		ENSG00000239726
RN7SL689P	0.0132	8.4892E-01	9.22E-01	ENSG00000263432
RN7SL68P	-0.0750	3.5339E-01	5.49E-01	ENSG00000242020
RN7SL693P	0.0193	3.8072E-01		ENSG00000265272
RN7SL697P	0.0258	2.8612E-01		ENSG00000264484
RN7SL698P	-0.0230	5.1004E-01		ENSG00000244232
RN7SL704P	0.0082	3.8886E-01		ENSG00000241693
RN7SL722P	0.0085	7.0579E-01		ENSG00000273940
RN7SL724P	0.0090	7.3098E-01		ENSG00000244692
RN7SL726P	-0.0517	2.6028E-01		ENSG00000272232
RN7SL736P	0.0329	1.8038E-01		ENSG00000275803
RN7SL738P	-0.1695	1.0434E-01	2.42E-01	ENSG00000243339
RN7SL73P	0.0124	2.0951E-01		ENSG00000265882
RN7SL743P	-0.0150	6.3886E-01	7.90E-01	ENSG00000243954
RN7SL749P	0.1542	7.4837E-02	1.91E-01	ENSG00000242853
RN7SL750P	0.0058	5.8468E-01		ENSG00000263672
RN7SL751P	-0.0375	6.5644E-01	8.02E-01	ENSG00000240964
RN7SL757P	-0.0021	8.1143E-01		ENSG00000239249

RN7SL763P	0.0083	5.5719E-01		ENSG00000277774
RN7SL76P	-0.0055	8.0833E-01		ENSG00000241959
RN7SL771P	0.0094	7.7837E-01		ENSG00000240173
RN7SL775P	0.0135	5.9411E-01		ENSG00000243370
RN7SL788P	-0.0242	4.2113E-01		ENSG00000241745
RN7SL789P	-0.0296	5.5632E-01		ENSG00000242436
RN7SL791P	-0.0447	7.8646E-01	8.85E-01	ENSG00000241291
RN7SL794P	-0.0194	9.9682E-01		ENSG00000240235
RN7SL796P	-0.0032	8.3843E-01		ENSG00000277031
RN7SL809P	-0.0083	6.4842E-01		ENSG00000241217
RN7SL812P	0.0370	4.7862E-01		ENSG00000242876
RN7SL814P	-0.0191	5.1327E-01		ENSG00000244326
RN7SL815P	-0.0194	9.1854E-01		ENSG00000243359
RN7SL81P	0.0390	4.3846E-01		ENSG00000244218
RN7SL827P	-0.0056	9.1596E-01		ENSG00000271986
RN7SL82P	0.0019	8.4325E-01		ENSG00000278696
RN7SL832P	0.4681	9.7566E-04	6.98E-03	ENSG00000243819
RN7SL833P	-0.0421	1.9450E-01		ENSG00000244080
RN7SL846P	0.0096	5.7474E-01		ENSG00000242818
RN7SL851P	0.0296	2.0079E-01		ENSG00000240718
RN7SL856P	-0.0555	8.5248E-01		ENSG00000276653
RNA5-8SN1	-0.0206	8.0667E-01	8.97E-01	ENSG00000278189
RNA5-8SN2	-0.0172	8.5419E-01	9.25E-01	ENSG00000278233
RNA5-8SN3	-0.0329	7.2596E-01	8.49E-01	ENSG00000275215
RNA5-8SN4	0.0517	5.5103E-01	7.22E-01	ENSG00000276700
RNA5-8SN5	0.0176	8.5474E-01	9.25E-01	ENSG00000274917
RNA5-8SP10	0.0222	8.0450E-01	8.96E-01	ENSG00000277739
RNA5-8SP6	-0.0003	9.4736E-01		ENSG00000251705
RNA5SP118	0.0168	6.7207E-01		ENSG00000222317
RNA5SP122	0.0177	2.3620E-01		ENSG00000207186
RNA5SP123	0.0261	1.2629E-01		ENSG00000200114
RNA5SP124	0.0077	5.4703E-01		ENSG00000199609
RNA5SP130	0.0227	1.9836E-01		ENSG00000201301
RNA5SP144	-0.0032	9.6762E-01		ENSG00000222778
RNA5SP151	-0.0961	1.2870E-01		ENSG00000202502
RNA5SP159	-0.0127	7.0441E-01		ENSG00000252970
RNA5SP179	0.0157	5.6789E-01		ENSG00000253093
RNA5SP18	0.0082	5.8248E-01		ENSG00000212237
RNA5SP180	0.0090	7.1391E-01		ENSG00000251990
RNA5SP194	-0.0002	8.1425E-01		ENSG00000201532
RNA5SP195	0.0159	4.5010E-01		ENSG00000199545
RNA5SP206	-0.0010	9.0237E-01		ENSG00000252512
RNA5SP210	-0.0075	9.5804E-01		ENSG00000212336
RNA5SP218	-0.0151	5.8364E-01		ENSG00000200058
RNA5SP221	0.0285	3.6336E-02	1.13E-01	ENSG00000223203
RNA5SP233	-0.0203	3.9320E-01		ENSG00000238391
RNA5SP247	0.0141	9.4899E-01		ENSG00000223113
RNA5SP262	0.0036	8.2355E-01		ENSG00000252261
RNA5SP278	-0.0091	4.6225E-01		ENSG00000252864

RNA5SP282	-0.0895	2.6166E-01		ENSG00000252164
RNA5SP289	-0.0221	5.5482E-01		ENSG00000199202
RNA5SP295	0.0177	1.3598E-01		ENSG00000199525
RNA5SP301	-0.0438	2.8089E-01		ENSG00000199407
RNA5SP304	0.0417	6.9312E-03	3.21E-02	ENSG00000223131
RNA5SP310	-0.0058	9.9108E-01		ENSG00000239152
RNA5SP311	-0.0046	7.2775E-01		ENSG00000238405
RNA5SP312	-0.0531	7.8590E-02		ENSG00000252877
RNA5SP315	-0.0531	7.8590E-02		ENSG00000252149
RNA5SP317	0.0361	6.0559E-01		ENSG00000222108
RNA5SP319	-0.0127	8.1217E-01		ENSG00000199638
RNA5SP343	0.0255	4.8091E-02	1.39E-01	ENSG00000284736
RNA5SP351	0.0626	2.4994E-02	8.51E-02	ENSG00000238965
RNA5SP363	-0.0303	2.7301E-01		ENSG00000201942
RNA5SP37	0.0162	8.3017E-01	9.11E-01	ENSG00000202290
RNA5SP371	-0.0127	9.4768E-01		ENSG00000222302
RNA5SP372	0.0162	4.3302E-01		ENSG00000212138
RNA5SP425	-0.0013	8.6941E-01		ENSG00000222268
RNA5SP429	0.0128	5.3703E-01		ENSG00000200558
RNA5SP437	0.1997	8.5153E-02	2.10E-01	ENSG00000253058
RNA5SP44	0.0056	7.5981E-01		ENSG00000222378
RNA5SP443	0.0073	8.4304E-01		ENSG00000199953
RNA5SP450	-0.0736	1.5502E-01		ENSG00000223138
RNA5SP452	-0.0236	8.2489E-01		ENSG00000199874
RNA5SP453	-0.0108	8.9611E-01		ENSG00000199373
RNA5SP462	-0.0246	8.7211E-01		ENSG00000252539
RNA5SP463	0.0119	2.7515E-01		ENSG00000285674
RNA5SP464	0.0026	8.7899E-01		ENSG00000252060
RNA5SP465	-0.0208	5.5824E-01		ENSG00000212497
RNA5SP466	0.0081	6.9260E-01		ENSG00000252546
RNA5SP469	0.0096	6.5543E-01		ENSG00000201035
RNA5SP474	0.0232	6.9898E-01	8.32E-01	ENSG00000212536
RNA5SP477	0.0814	2.7065E-01	4.60E-01	ENSG00000199509
RNA5SP479	0.0157	2.4946E-01		ENSG00000201728
RNA5SP48	-0.0852	1.4764E-01		ENSG00000200839
RNA5SP481	0.0090	4.6429E-01		ENSG00000252623
RNA5SP492	-0.2031	8.8261E-02	2.15E-01	ENSG00000223262
RNA5SP494	-0.0139	6.2075E-01		ENSG00000222585
RNA5SP495	0.0130	4.7404E-01		ENSG00000252267
RNA5SP496	0.0002	8.6726E-01		ENSG00000212542
RNA5SP497	0.0252	3.6950E-01		ENSG00000251890
RNA5SP508	0.0235	1.9916E-01		ENSG00000223259
RNA5SP515	-0.0102	9.8460E-01		ENSG00000201440
RNA5SP525	-0.0001	7.4710E-01		ENSG00000212595
RNA5SP530	-0.0041	8.9652E-01		ENSG00000277049
RNA5SP68	0.0128	6.4274E-01		ENSG00000200755
RNA5SP78	0.0757	2.1225E-01		ENSG00000201492
RNA5SP84	0.0099	4.4108E-01		ENSG00000201610
RNA5SP85	0.0496	1.0661E-03		ENSG00000285626

RNA5SP88	-0.0093	4.4588E-01		ENSG00000202430
RNA5SP93	0.0110	4.2977E-01		ENSG00000199395
RNASE1	0.1485	1.7307E-01	3.42E-01	ENSG00000129538
RNASE10	-0.0060	8.5364E-01		ENSG00000182545
RNASE4	0.1378	1.3652E-01	2.91E-01	ENSG00000258818
RNASE6	0.0011	5.6076E-01		ENSG00000169413
RNASE7	-0.0010	8.3613E-01		ENSG00000165799
RNASEH1	0.3622	1.8580E-08	9.59E-07	ENSG00000171865
RNASEH1-DT	0.0938	1.8857E-01	3.63E-01	ENSG00000234171
RNASEH1P1	-0.0385	5.8824E-01	7.52E-01	ENSG00000265790
RNASEH1P2	-0.0103	7.9915E-01		ENSG00000231458
RNASEH2A	0.4627	1.3244E-02	5.27E-02	ENSG00000104889
RNASEH2B	-0.2063	7.6802E-03	3.48E-02	ENSG00000136104
RNASEH2C	-0.4120	2.7271E-06	6.18E-05	ENSG00000172922
RNASEK	0.0051	9.5669E-01	9.78E-01	ENSG00000219200
RNASEK-C17orf49	-0.0931	2.4003E-01	4.25E-01	ENSG00000161939
RNASEL	-0.0343	7.1093E-01	8.39E-01	ENSG00000135828
RNASET2	-0.0216	7.4385E-01	8.60E-01	ENSG00000026297
RND2	-0.1111	8.4487E-02	2.09E-01	ENSG00000108830
RND3	0.1393	6.6419E-02	1.75E-01	ENSG00000115963
RNF10	-0.0810	1.5161E-01	3.13E-01	ENSG00000022840
RNF103	0.1100	9.3985E-02	2.25E-01	ENSG00000239305
RNF103-CHMP3	0.0074	9.3181E-01	9.66E-01	ENSG00000249884
RNF11	-0.2559	3.5588E-06	7.63E-05	ENSG00000123091
RNF111	0.1617	1.1950E-02	4.86E-02	ENSG00000157450
RNF112	0.0511	5.9059E-01	7.54E-01	ENSG00000128482
RNF113A	0.0424	3.8364E-01	5.78E-01	ENSG00000125352
RNF113B	-0.0365	6.5980E-01		ENSG00000139797
RNF114	-0.0910	7.5128E-02	1.92E-01	ENSG00000124226
RNF115	-0.0545	3.2662E-01	5.21E-01	ENSG00000265491
RNF121	0.0976	2.2605E-01	4.09E-01	ENSG00000137522
RNF122	-0.0349	6.8053E-01	8.19E-01	ENSG00000133874
RNF123	0.1912	2.5649E-02	8.68E-02	ENSG00000164068
RNF125	-0.0005	9.9463E-01	9.97E-01	ENSG00000101695
RNF126	0.2700	4.4385E-07	1.35E-05	ENSG00000070423
RNF128	0.2257	1.6753E-02	6.30E-02	ENSG00000133135
RNF13	-0.0330	5.5538E-01	7.26E-01	ENSG00000082996
RNF130	0.0669	2.7930E-01	4.70E-01	ENSG00000113269
RNF133	-0.0411	1.9225E-01		ENSG00000188050
RNF135	-0.0457	6.3773E-01	7.89E-01	ENSG00000181481
RNF138	-0.1376	6.4524E-02	1.72E-01	ENSG00000134758
RNF139	-0.3752	1.2769E-04	1.36E-03	ENSG00000170881
RNF14	0.1310	5.1212E-02	1.45E-01	ENSG00000013561
RNF141	0.0338	5.2526E-01	7.01E-01	ENSG00000110315
RNF144A	0.1087	1.6764E-01	3.35E-01	ENSG00000151692
RNF144B	0.0058	9.4489E-01	9.72E-01	ENSG00000137393
RNF145	-0.3491	7.5843E-06	1.42E-04	ENSG00000145860
RNF146	0.1131	1.3962E-01	2.95E-01	ENSG00000118518
RNF148	0.0182	7.3441E-01		ENSG00000235631

RNF149	0.1325	2.7680E-02	9.17E-02	ENSG00000163162
RNF14P3	-0.0500	7.3510E-01		ENSG00000238109
RNF150	-0.0098	8.8019E-01	9.39E-01	ENSG00000170153
RNF151	-0.0178	8.1391E-01		ENSG00000179580
RNF152	0.2341	1.2628E-02	5.08E-02	ENSG00000176641
RNF157	0.2020	1.7363E-02	6.48E-02	ENSG00000141576
RNF157-AS1	-0.1341	2.0680E-01	3.85E-01	ENSG00000267128
RNF166	0.0480	4.5444E-01	6.44E-01	ENSG00000158717
RNF167	0.0596	3.0285E-01	4.95E-01	ENSG00000108523
RNF168	-0.1747	1.3414E-02	5.32E-02	ENSG00000163961
RNF169	-0.1189	2.1106E-01	3.91E-01	ENSG00000166439
RNF17	0.0839	3.0538E-01	4.98E-01	ENSG00000132972
RNF170	0.0806	1.0975E-01	2.51E-01	ENSG00000120925
RNF180	-0.0927	1.7778E-01	3.49E-01	ENSG00000164197
RNF181	-0.0649	2.3659E-01	4.21E-01	ENSG00000168894
RNF182	-0.2499	9.2375E-03	4.01E-02	ENSG00000180537
RNF183	0.5514	8.7022E-04	6.39E-03	ENSG00000165188
RNF185	0.0371	5.8743E-01	7.52E-01	ENSG00000138942
RNF187	-0.1530	1.5856E-02	6.05E-02	ENSG00000168159
RNF19A	-0.2166	8.3819E-04	6.20E-03	ENSG00000034677
RNF19B	-0.0087	8.9150E-01	9.46E-01	ENSG00000116514
RNF2	-0.0904	2.4934E-01	4.36E-01	ENSG00000121481
RNF20	-0.1899	6.1767E-04	4.88E-03	ENSG00000155827
RNF207	0.2988	2.4418E-02	8.37E-02	ENSG00000158286
RNF207-AS1	0.0842	3.4244E-01	5.38E-01	ENSG00000226944
RNF208	-0.3437	1.3161E-02	5.24E-02	ENSG00000212864
RNF212	0.4518	2.0859E-05	3.16E-04	ENSG00000178222
RNF212B	0.0425	5.2433E-01	7.01E-01	ENSG00000215277
RNF213	-0.2438	6.6583E-03	3.12E-02	ENSG00000173821
RNF213-AS1	0.2545	2.9898E-02	9.72E-02	ENSG00000263069
RNF214	0.0048	9.4670E-01	9.73E-01	ENSG00000167257
RNF215	-0.0204	8.0131E-01	8.94E-01	ENSG00000099999
RNF216	0.0632	2.7384E-01	4.64E-01	ENSG00000011275
RNF216-IT1	-0.0627	2.3831E-01		ENSG00000237738
RNF216P1	0.1934	1.6942E-03	1.08E-02	ENSG00000196204
RNF217	-0.3440	8.6520E-06	1.56E-04	ENSG00000146373
RNF217-AS1	0.0691	4.6871E-01	6.56E-01	ENSG00000236548
RNF220	0.2490	3.6155E-04	3.19E-03	ENSG00000187147
RNF222	-0.0083	9.2604E-01		ENSG00000189051
RNF223	-0.0632	5.0241E-01	6.84E-01	ENSG00000237330
RNF224	-0.3174	3.0801E-02	9.94E-02	ENSG00000233198
RNF225	0.0150	8.2053E-01	9.05E-01	ENSG00000269855
RNF227	0.4819	5.0149E-05	6.46E-04	ENSG00000179859
RNF24	0.3195	1.3727E-04	1.44E-03	ENSG00000101236
RNF25	0.4373	2.4279E-15	9.85E-13	ENSG00000163481
RNF26	-0.0574	4.1507E-01	6.08E-01	ENSG00000173456
RNF31	-0.0590	5.4064E-01	7.14E-01	ENSG00000092098
RNF32	-0.1765	5.4403E-02	1.52E-01	ENSG00000105982
RNF32-DT	0.2096	1.4508E-03	9.56E-03	ENSG00000182648

RNF34	0.2897	7.2943E-05	8.70E-04	ENSG00000170633
RNF38	-0.0266	6.8068E-01	8.19E-01	ENSG00000137075
RNF39	0.1006	3.2394E-01	5.18E-01	ENSG00000204618
RNF4	0.2952	8.7249E-08	3.49E-06	ENSG00000063978
RNF40	0.3889	1.4460E-03	9.54E-03	ENSG00000103549
RNF41	0.3267	6.5300E-06	1.25E-04	ENSG00000181852
RNF43	-0.0619	5.1740E-01	6.95E-01	ENSG00000108375
RNF44	0.1178	2.1794E-01	3.99E-01	ENSG00000146083
RNF5	0.1203	4.7122E-02	1.37E-01	ENSG00000204308
RNF6	-0.0863	2.0538E-01	3.84E-01	ENSG00000127870
RNF7	-0.1195	2.4386E-02	8.36E-02	ENSG00000114125
RNF8	-0.1116	7.3459E-02	1.89E-01	ENSG00000112130
RNFT1	-0.2231	2.5833E-03	1.50E-02	ENSG00000189050
RNFT1-DT	0.0477	6.0039E-01	7.61E-01	ENSG00000267302
RNFT2	-0.1542	2.7233E-02	9.06E-02	ENSG00000135119
RNGTT	0.1287	9.1141E-02	2.20E-01	ENSG00000111880
RNH1	0.0942	1.8429E-01	3.57E-01	ENSG00000023191
RNLS	-0.4438	2.6433E-05	3.83E-04	ENSG00000184719
RNMT	-0.1197	4.4020E-02	1.30E-01	ENSG00000101654
RNPC3	0.4419	6.2900E-09	3.93E-07	ENSG00000185946
RNPC3-DT	0.0415	4.2781E-01	6.20E-01	ENSG00000224613
RNPEP	-0.2370	1.7927E-05	2.81E-04	ENSG00000176393
RNPEPL1	0.0061	9.2585E-01	9.63E-01	ENSG00000142327
RNPS1	-0.0417	3.2059E-01	5.14E-01	ENSG00000205937
RNU1-1	0.4164	1.7247E-03	1.09E-02	ENSG00000206652
RNU1-100P	-0.0184	7.0589E-01		ENSG00000202125
RNU1-124P	0.0381	2.5502E-01		ENSG00000200731
RNU1-134P	-0.0175	7.6810E-01		ENSG00000199805
RNU1-16P	0.0157	6.9579E-01	8.30E-01	ENSG00000202347
RNU1-2	0.3391	1.3127E-02	5.23E-02	ENSG00000207005
RNU1-27P	0.4042	2.8454E-04	2.61E-03	ENSG00000206596
RNU1-28P	0.4035	3.3138E-04	2.96E-03	ENSG00000206588
RNU1-3	0.1020	2.6478E-01	4.54E-01	ENSG00000207513
RNU1-4	0.2530	3.3604E-02	1.06E-01	ENSG00000207389
RNU1-47P	0.0003	6.1321E-01		ENSG00000199836
RNU1-83P	-0.0004	8.9745E-01		ENSG00000200296
RNU1-8P	-0.0054	8.2138E-01		ENSG00000207056
RNU1-91P	-0.0040	9.6741E-01		ENSG00000201616
RNU1-94P	-0.0085	9.9540E-01		ENSG00000199497
RNU105B	0.0119	3.6319E-01		ENSG00000201348
RNU11	0.0083	6.6552E-01		ENSG00000274978
RNU11-2P	-0.0250	3.3544E-01		ENSG00000252707
RNU2-1	0.0101	4.0214E-01		ENSG00000274585
RNU2-11P	0.0201	2.4005E-01		ENSG00000239122
RNU2-17P	-0.0199	6.2684E-01	7.81E-01	ENSG00000222222
RNU2-22P	0.0135	6.8015E-01		ENSG00000223198
RNU2-26P	0.0069	4.5950E-01		ENSG00000222440
RNU2-28P	-0.0108	6.8538E-01		ENSG00000222389
RNU2-31P	-0.0111	7.1938E-01		ENSG00000252763

RNU2-33P	-0.0399	1.8486E-01		ENSG00000222276
RNU2-39P	-0.0339	7.2464E-01		ENSG00000222536
RNU2-42P	-0.0049	9.2619E-01		ENSG00000222629
RNU2-57P	0.0023	2.6382E-01		ENSG00000252468
RNU2-59P	-0.0158	3.7176E-01		ENSG00000222414
RNU2-5P	-0.0326	3.8553E-01		ENSG00000222465
RNU2-63P	0.4912	3.7039E-03	1.99E-02	ENSG00000222724
RNU2-68P	0.0199	3.6014E-01		ENSG00000222810
RNU2-7P	0.0629	4.4935E-01	6.39E-01	ENSG00000222726
RNU4-1	0.2142	1.2891E-02	5.16E-02	ENSG00000200795
RNU4-2	0.3557	1.0180E-03	7.21E-03	ENSG00000202538
RNU4-22P	-0.0285	1.4659E-01		ENSG00000222990
RNU4-23P	-0.0071	7.8285E-01		ENSG00000199709
RNU4-36P	-0.0159	8.1197E-01	9.01E-01	ENSG00000201164
RNU4-40P	-0.1745	8.3471E-03		ENSG00000201221
RNU4-42P	-0.0063	9.6610E-01		ENSG00000201608
RNU4-47P	-0.0211	9.6207E-01		ENSG00000222808
RNU4-51P	-0.0158	6.1390E-01		ENSG00000201076
RNU4-68P	-0.0394	3.5950E-01		ENSG00000201184
RNU4-78P	-0.0456	1.6508E-01		ENSG00000222872
RNU4-86P	-0.0097	9.0976E-01		ENSG00000222067
RNU4ATAC16P	0.0381	1.9909E-01		ENSG00000221439
RNU4ATAC18P	-0.0211	5.1414E-01		ENSG00000251988
RNU5A-1	0.5893	8.3831E-05	9.71E-04	ENSG00000199568
RNU5A-8P	-0.0237	3.8470E-01	5.79E-01	ENSG00000200972
RNU5D-1	0.2144	7.3513E-02	1.89E-01	ENSG00000200169
RNU5E-10P	-0.0125	5.5943E-01		ENSG00000200376
RNU5E-4P	-0.0230	3.9654E-01		ENSG00000201801
RNU5E-8P	0.0502	4.0212E-01	5.96E-01	ENSG00000200372
RNU6-1	-0.0115	9.1152E-01		ENSG00000206625
RNU6-1004P	-0.0072	9.6301E-01		ENSG00000252393
RNU6-1005P	-0.0136	7.3941E-01		ENSG00000207248
RNU6-1011P	0.0007	9.0882E-01		ENSG00000207399
RNU6-1016P	-0.0232	3.8049E-01	5.75E-01	ENSG00000252498
RNU6-101P	0.0166	5.8756E-01		ENSG00000222255
RNU6-1023P	0.0254	1.4389E-01		ENSG00000251946
RNU6-1024P	-0.0132	8.3013E-01		ENSG00000206926
RNU6-1025P	-0.0163	7.9252E-01		ENSG00000222561
RNU6-1026P	0.0048	7.7703E-01		ENSG00000207194
RNU6-103P	0.0985	1.5064E-01	3.11E-01	ENSG00000200556
RNU6-1053P	-0.0120	3.7956E-01		ENSG00000251739
RNU6-1061P	0.0668	3.7259E-01	5.68E-01	ENSG00000252339
RNU6-106P	-0.0026	9.7103E-01		ENSG00000207134
RNU6-1076P	-0.0033	7.2690E-01		ENSG00000222329
RNU6-1093P	0.0051	8.9474E-01		ENSG00000212496
RNU6-1099P	0.1222	2.1907E-01	4.00E-01	ENSG00000200403
RNU6-10P	-0.0013	9.4830E-01		ENSG00000206763
RNU6-1100P	0.0075	3.6925E-01		ENSG00000222623
RNU6-1107P	-0.0256	5.7690E-01		ENSG00000201687

RNU6-1111P	0.0040	6.7694E-01		ENSG00000212420
RNU6-1123P	-0.0023	9.2183E-01		ENSG00000199217
RNU6-1136P	0.0032	5.8154E-01		ENSG00000252643
RNU6-1138P	0.0035	5.5079E-01		ENSG00000202229
RNU6-1157P	0.0038	5.3833E-01		ENSG00000207185
RNU6-1158P	-0.0073	8.9924E-01		ENSG00000212469
RNU6-1165P	0.0262	1.2427E-01		ENSG00000222051
RNU6-116P	0.0300	3.6843E-02		ENSG00000206769
RNU6-1178P	-0.0035	9.6956E-01	9.84E-01	ENSG00000252483
RNU6-1181P	-0.0139	7.7266E-01		ENSG00000206918
RNU6-1187P	-0.0124	7.0725E-01		ENSG00000201641
RNU6-1188P	-0.0170	6.4320E-01		ENSG00000200665
RNU6-118P	-0.1196	1.9872E-01	3.76E-01	ENSG00000252361
RNU6-1196P	-0.0207	4.8311E-01		ENSG00000252030
RNU6-1200P	-0.0198	7.0549E-01		ENSG00000206889
RNU6-1204P	-0.0393	3.8093E-01		ENSG00000200818
RNU6-1208P	-0.0001	9.6902E-01		ENSG00000238482
RNU6-1222P	-0.0021	6.9788E-01		ENSG00000207345
RNU6-1254P	-0.0026	9.0779E-01		ENSG00000212568
RNU6-125P	0.1266	1.0876E-01	2.49E-01	ENSG00000207234
RNU6-1262P	-0.0014	7.4579E-01		ENSG00000252026
RNU6-1263P	-0.0077	7.5278E-01		ENSG00000207331
RNU6-1272P	-0.0018	8.8125E-01		ENSG00000199646
RNU6-1285P	-0.1239	3.6180E-01	5.58E-01	ENSG00000200350
RNU6-1294P	-0.0100	9.3771E-01		ENSG00000251804
RNU6-1301P	-0.0152	6.7896E-01		ENSG00000199594
RNU6-130P	0.0024	9.3918E-01		ENSG00000223044
RNU6-1316P	-0.0094	5.5840E-01		ENSG00000206969
RNU6-1318P	0.0285	1.8472E-01		ENSG00000202259
RNU6-131P	0.0367	7.3703E-02		ENSG00000212446
RNU6-1333P	-0.0259	7.4627E-01		ENSG00000251971
RNU6-1337P	0.0671	2.5925E-01	4.47E-01	ENSG00000252334
RNU6-137P	-0.0115	8.7870E-01	9.38E-01	ENSG00000200550
RNU6-151P	0.0529	3.1026E-01		ENSG00000201028
RNU6-171P	-0.0267	7.7990E-01	8.81E-01	ENSG00000207082
RNU6-177P	-0.0232	4.5735E-01		ENSG00000223189
RNU6-188P	-0.0282	4.3632E-01		ENSG00000201077
RNU6-190P	0.0027	9.4580E-01		ENSG00000206881
RNU6-197P	-0.0030	5.8202E-01		ENSG00000252489
RNU6-199P	-0.0101	8.1918E-01		ENSG00000199824
RNU6-218P	0.0571	2.5883E-01		ENSG00000252929
RNU6-223P	-0.0014	9.2112E-01		ENSG00000199700
RNU6-228P	-0.0018	9.0174E-01		ENSG00000199570
RNU6-230P	0.0106	6.0351E-01		ENSG00000199327
RNU6-236P	0.0053	7.4918E-01		ENSG00000200756
RNU6-238P	0.0079	4.5713E-01		ENSG00000200183
RNU6-245P	-0.0221	4.5581E-01		ENSG00000206747
RNU6-247P	-0.0093	9.5659E-01		ENSG00000199506
RNU6-250P	0.0033	8.6923E-01		ENSG00000252767

RNU6-254P	-0.0195	6.6079E-01		ENSG00000200247
RNU6-262P	-0.0026	7.8605E-01		ENSG00000222249
RNU6-268P	-0.0283	3.7019E-01		ENSG00000201044
RNU6-26P	0.0301	2.7226E-02		ENSG00000206712
RNU6-288P	-0.0343	4.7084E-01		ENSG00000200560
RNU6-28P	-0.0124	7.5890E-01		ENSG00000199248
RNU6-29P	0.0088	6.4007E-01		ENSG00000207367
RNU6-306P	0.0268	9.6026E-02		ENSG00000207347
RNU6-307P	0.0145	2.9760E-01		ENSG00000252552
RNU6-30P	0.0936	9.4668E-02	2.26E-01	ENSG00000207291
RNU6-312P	-0.0126	6.5619E-01		ENSG00000201499
RNU6-313P	-0.0476	2.1341E-01		ENSG00000252126
RNU6-319P	0.0006	8.4388E-01		ENSG00000251834
RNU6-31P	-0.0034	9.2048E-01		ENSG00000207116
RNU6-321P	-0.0734	7.0104E-01	8.33E-01	ENSG00000222844
RNU6-322P	-0.0105	6.1653E-01		ENSG00000251819
RNU6-32P	-1.0997	2.4870E-02	8.48E-02	ENSG00000206675
RNU6-335P	0.0334	4.9324E-01		ENSG00000201433
RNU6-353P	0.0090	9.2442E-01		ENSG00000201136
RNU6-354P	0.0428	3.9072E-01		ENSG00000206589
RNU6-355P	-0.0033	7.2690E-01		ENSG00000222359
RNU6-379P	-0.0842	1.7562E-01	3.46E-01	ENSG00000200683
RNU6-37P	-0.1645	2.5297E-01	4.40E-01	ENSG00000199562
RNU6-387P	0.0300	3.4061E-01		ENSG00000223263
RNU6-388P	-0.0019	9.8845E-01		ENSG00000252821
RNU6-396P	-0.0236	7.1955E-01		ENSG00000202239
RNU6-405P	-0.0107	6.9800E-01		ENSG00000252446
RNU6-407P	-0.0430	5.1529E-01		ENSG00000202150
RNU6-415P	-0.3740	2.2050E-02	7.75E-02	ENSG00000252061
RNU6-418P	0.0110	4.4072E-01		ENSG00000206762
RNU6-430P	-0.0560	1.3749E-01		ENSG00000252887
RNU6-431P	0.0235	6.5111E-01		ENSG00000206601
RNU6-433P	-0.0459	2.4314E-01		ENSG00000200086
RNU6-446P	0.0458	2.7863E-01		ENSG00000199697
RNU6-447P	-0.0019	7.0728E-01		ENSG00000222225
RNU6-450P	0.0097	5.8536E-01		ENSG00000201524
RNU6-455P	0.0067	3.8749E-01		ENSG00000207412
RNU6-457P	0.0066	6.3342E-01		ENSG00000200869
RNU6-460P	-0.0155	7.6723E-01		ENSG00000212460
RNU6-469P	0.0547	4.6983E-01	6.57E-01	ENSG00000252062
RNU6-476P	0.1342	1.6655E-01	3.34E-01	ENSG00000207457
RNU6-482P	-0.0018	8.1913E-01		ENSG00000212370
RNU6-48P	0.0355	2.1799E-01		ENSG00000206888
RNU6-503P	-0.0346	8.7510E-01		ENSG00000252338
RNU6-514P	-0.0098	9.2036E-01		ENSG00000206935
RNU6-527P	-0.0157	4.4594E-01		ENSG00000200295
RNU6-530P	-0.0030	7.4140E-01		ENSG00000212482
RNU6-531P	-0.0415	4.7718E-01		ENSG00000252503
RNU6-539P	0.0279	4.0981E-01		ENSG00000252474

RNU6-554P	0.0043	6.9287E-01		ENSG00000222398
RNU6-564P	0.0049	7.3334E-01		ENSG00000222915
RNU6-574P	0.0844	3.7070E-01	5.66E-01	ENSG00000206992
RNU6-577P	-0.0394	3.3689E-01		ENSG00000252756
RNU6-593P	-0.0636	3.2986E-02		ENSG00000201586
RNU6-606P	-0.0198	6.0366E-01		ENSG00000207452
RNU6-60P	0.0163	8.0568E-01	8.96E-01	ENSG00000201662
RNU6-610P	0.1014	1.0840E-01		ENSG00000206991
RNU6-611P	-0.0695	6.5901E-01	8.03E-01	ENSG00000207003
RNU6-613P	-0.0319	6.4370E-01		ENSG00000222344
RNU6-625P	-0.0087	7.8424E-01		ENSG00000238658
RNU6-628P	0.1470	1.1916E-01	2.66E-01	ENSG00000222607
RNU6-638P	-0.0079	9.6131E-01		ENSG00000252391
RNU6-644P	-0.0055	9.3869E-01		ENSG00000212457
RNU6-645P	-0.0274	5.9261E-01		ENSG00000201519
RNU6-652P	0.0015	2.6470E-01	4.53E-01	ENSG00000202358
RNU6-661P	0.0087	6.7365E-01		ENSG00000199279
RNU6-678P	-0.0057	8.7656E-01		ENSG00000252641
RNU6-681P	-0.0082	8.5580E-01		ENSG00000200882
RNU6-695P	-0.0895	1.2278E-01		ENSG00000212459
RNU6-696P	0.0151	5.0921E-01		ENSG00000212136
RNU6-7	-0.0421	3.3951E-01		ENSG00000201654
RNU6-701P	-0.0032	8.7415E-01		ENSG00000206786
RNU6-703P	0.0345	4.0302E-02		ENSG00000252755
RNU6-705P	0.0075	7.8712E-01		ENSG00000222533
RNU6-711P	-0.0128	5.4923E-01		ENSG00000253064
RNU6-723P	0.0043	7.7542E-01		ENSG00000206700
RNU6-729P	-0.0287	3.5740E-01		ENSG00000207128
RNU6-731P	0.0123	3.1858E-01		ENSG00000253022
RNU6-757P	0.0467	3.7019E-01	5.66E-01	ENSG00000222266
RNU6-758P	-0.0046	9.9116E-01		ENSG00000252101
RNU6-781P	0.0203	2.6449E-01		ENSG00000252186
RNU6-784P	-0.0160	7.8536E-01		ENSG00000207058
RNU6-785P	-0.0033	7.2690E-01		ENSG00000223256
RNU6-786P	-0.0710	1.9856E-01		ENSG00000252658
RNU6-789P	-0.0422	2.1397E-01		ENSG00000252914
RNU6-790P	-0.0215	7.1598E-01		ENSG00000207208
RNU6-795P	0.0039	7.0527E-01		ENSG00000252132
RNU6-79P	0.0091	5.3287E-01		ENSG00000199381
RNU6-8	-0.0390	5.6904E-01	7.37E-01	ENSG00000202337
RNU6-807P	-0.0975	2.1892E-01	4.00E-01	ENSG00000252614
RNU6-808P	-0.0289	3.5835E-01		ENSG00000212535
RNU6-824P	0.0270	5.9873E-01		ENSG00000200594
RNU6-826P	0.0032	8.2853E-01		ENSG00000252636
RNU6-828P	0.0013	8.0640E-01		ENSG00000201746
RNU6-82P	-0.0216	3.3445E-01		ENSG00000200840
RNU6-833P	-0.0048	7.7524E-01		ENSG00000200356
RNU6-834P	0.0198	2.8838E-01		ENSG00000199237
RNU6-844P	0.0070	6.6639E-01		ENSG00000252984

RNU6-850P	-0.0154	7.9020E-01	8.87E-01	ENSG00000252743
RNU6-853P	0.0008	9.0313E-01		ENSG00000201176
RNU6-859P	-0.0007	9.4305E-01		ENSG00000199598
RNU6-860P	-0.0099	4.6773E-01		ENSG00000222792
RNU6-862P	-0.0212	4.6600E-01		ENSG00000199674
RNU6-866P	0.0283	4.3818E-01		ENSG00000274607
RNU6-881P	0.0052	8.6792E-01		ENSG00000252622
RNU6-882P	0.0110	7.0246E-01		ENSG00000212327
RNU6-883P	-0.0573	3.7920E-01	5.74E-01	ENSG00000207327
RNU6-886P	0.0010	8.8104E-01		ENSG00000207046
RNU6-890P	0.0005	5.7544E-01		ENSG00000206848
RNU6-892P	0.1089	1.4261E-01	3.00E-01	ENSG00000222267
RNU6-904P	-0.0510	4.4207E-01	6.33E-01	ENSG00000252015
RNU6-91P	-0.0532	2.4973E-01		ENSG00000272439
RNU6-923P	-0.0083	6.4601E-01		ENSG00000201623
RNU6-924P	0.0260	1.8492E-01		ENSG00000199796
RNU6-925P	0.0327	6.4706E-01	7.96E-01	ENSG00000207359
RNU6-930P	-0.0043	7.4419E-01		ENSG00000212240
RNU6-936P	0.0039	7.4315E-01		ENSG00000206732
RNU6-942P	-0.0280	4.0875E-01		ENSG00000199872
RNU6-945P	-0.0168	8.7056E-01		ENSG00000206674
RNU6-946P	-0.0241	6.1930E-01		ENSG00000206605
RNU6-94P	0.0203	2.5629E-01	4.44E-01	ENSG00000271819
RNU6-969P	0.0029	9.4189E-01		ENSG00000206627
RNU6-975P	0.0095	5.0846E-01		ENSG00000207023
RNU6-97P	-0.0327	9.6850E-01		ENSG00000200257
RNU6-988P	0.0127	6.5612E-01		ENSG00000253437
RNU6ATAC	0.0577	3.7838E-02		ENSG00000221676
RNU6ATAC12P	0.0098	6.8728E-01		ENSG00000252351
RNU6ATAC24P	-0.0265	9.4360E-01		ENSG00000252620
RNU6ATAC26P	-0.0117	8.5371E-01		ENSG00000210841
RNU6ATAC27P	0.0440	4.2210E-01		ENSG00000221216
RNU6ATAC39P	-0.0488	2.2037E-01		ENSG00000252118
RNU6ATAC9P	0.0178	5.3275E-01		ENSG00000252019
RNU7-107P	-0.0134	8.3654E-01		ENSG00000238523
RNU7-115P	-0.0221	4.4393E-01		ENSG00000252242
RNU7-123P	0.0547	3.4885E-01	5.44E-01	ENSG00000251720
RNU7-133P	-0.0005	9.9775E-01		ENSG00000238987
RNU7-140P	-0.0924	2.7194E-01	4.62E-01	ENSG00000238364
RNU7-143P	0.0239	5.2656E-01		ENSG00000252590
RNU7-169P	-0.0152	6.2844E-01		ENSG00000238457
RNU7-171P	0.0098	4.8097E-01		ENSG00000238406
RNU7-181P	0.0599	3.0536E-01		ENSG00000253043
RNU7-187P	0.0094	3.1516E-01		ENSG00000238998
RNU7-194P	-0.0122	7.8979E-01		ENSG00000238721
RNU7-195P	0.0221	7.4453E-01	8.60E-01	ENSG00000239151
RNU7-3P	0.0941	1.0363E-01		ENSG00000252244
RNU7-40P	0.0004	8.2106E-01		ENSG00000252206
RNU7-43P	-0.0084	9.4478E-01		ENSG00000252363

RNU7-49P	0.0061	8.7784E-01		ENSG00000251991
RNU7-57P	0.0259	1.5375E-01		ENSG00000238365
RNU7-59P	0.0311	4.5979E-01		ENSG00000238880
RNU7-63P	-0.0361	2.8276E-01		ENSG00000238417
RNU7-75P	0.0584	9.0538E-02		ENSG00000251880
RNU7-77P	-0.0463	5.2781E-01		ENSG00000253054
RNU7-79P	-0.0262	4.0139E-01		ENSG00000251891
RNU7-81P	0.0153	4.2347E-01		ENSG00000252507
RNVU1-1	-0.0055	8.4218E-01		ENSG00000207340
RNVU1-15	-0.0935	3.4429E-01	5.39E-01	ENSG00000207205
RNVU1-18	0.3232	2.0713E-02	7.39E-02	ENSG00000206737
RNVU1-2	0.0973	3.5276E-02	1.10E-01	ENSG00000238825
RNVU1-21	0.0095	4.8138E-01		ENSG00000202408
RNVU1-22	-0.0291	8.0799E-01	8.98E-01	ENSG00000199879
RNVU1-23	0.0314	4.0165E-02		ENSG00000252826
RNVU1-24	0.5402	2.0138E-04	1.97E-03	ENSG00000201699
RNVU1-2A	0.1359	1.5627E-01	3.19E-01	ENSG00000278099
RNVU1-3	0.0944	2.1742E-01	3.98E-01	ENSG00000201183
RNVU1-33	-0.0115	6.8625E-01		ENSG00000200597
RNVU1-34	0.2512	3.3664E-02	1.06E-01	ENSG00000200997
RNVU1-4	-0.0214	7.8465E-01	8.84E-01	ENSG00000277610
RNVU1-7	0.0658	3.2402E-01	5.18E-01	ENSG00000206585
RNVU1-8	0.0531	1.2461E-01	2.74E-01	ENSG00000286172
RNY1	-0.0448	5.0320E-01		ENSG00000201098
RNY1P12	-0.0269	8.5968E-01		ENSG00000201121
RNY1P13	-0.0086	8.4349E-01		ENSG00000201900
RNY1P14	-0.0149	7.4860E-01		ENSG00000207155
RNY1P15	0.0218	3.1465E-01		ENSG00000222351
RNY1P16	-0.0140	8.8995E-01		ENSG00000199933
RNY1P2	0.0020	5.4127E-01		ENSG00000201690
RNY1P4	-0.0126	6.9953E-01		ENSG00000207325
RNY1P5	0.0596	2.0869E-01		ENSG00000206617
RNY1P9	-0.0070	8.4513E-01		ENSG00000255156
RNY3	-0.0011	7.7461E-01		ENSG00000202354
RNY3P13	0.0033	8.2061E-01		ENSG00000202412
RNY3P14	-0.0298	8.2277E-01		ENSG00000207036
RNY4P10	0.0207	5.2469E-01		ENSG00000202441
RNY4P19	-0.0529	2.8703E-01		ENSG00000199400
RNY4P25	-0.0836	2.3062E-01		ENSG00000238711
RNY4P34	-0.0126	7.4457E-01		ENSG00000201649
RNY4P36	-0.0071	8.4665E-01		ENSG00000212418
RNY4P6	-0.0001	8.8373E-01		ENSG00000200537
RO60	0.0013	9.8344E-01	9.91E-01	ENSG00000116747
ROBO1	-0.0806	2.8804E-01	4.79E-01	ENSG00000169855
ROBO2	0.1504	1.0577E-01	2.44E-01	ENSG00000185008
ROBO3	0.0464	5.9535E-01	7.57E-01	ENSG00000154134
ROBO4	0.0380	8.1101E-02		ENSG00000154133
ROCK1	-0.0050	8.8650E-01	9.43E-01	ENSG00000067900
ROCK2	0.2626	8.3253E-04	6.16E-03	ENSG00000134318

ROGDI	0.0162	8.3257E-01	9.13E-01	ENSG00000067836
ROM1	-0.0147	8.5421E-01	9.25E-01	ENSG00000149489
ROMO1	-0.1736	6.8201E-03	3.18E-02	ENSG00000125995
ROPN1	0.0807	3.5382E-01	5.49E-01	ENSG00000065371
ROPN1B	0.0645	4.9513E-01	6.78E-01	ENSG00000114547
ROPN1L	-0.3630	2.4757E-03	1.45E-02	ENSG00000145491
ROR1	-0.0644	4.7920E-01	6.65E-01	ENSG00000185483
ROR1-AS1	0.0259	2.7057E-01		ENSG00000223949
ROR2	0.1470	1.1413E-01	2.58E-01	ENSG00000169071
RORA	0.0285	7.5810E-01	8.69E-01	ENSG00000069667
RORB	-0.1401	1.8908E-01	3.64E-01	ENSG00000198963
RORC	0.4994	6.4645E-03	3.05E-02	ENSG00000143365
RP1	-0.0713	2.2832E-01		ENSG00000104237
RP1L1	-0.0050	6.8671E-01		ENSG00000183638
RP2	-0.1158	2.1711E-01	3.98E-01	ENSG00000102218
RP9	-0.1272	2.5868E-02	8.73E-02	ENSG00000164610
RPA1	0.0202	7.3943E-01	8.57E-01	ENSG00000132383
RPA2	0.0957	1.1560E-01	2.60E-01	ENSG00000117748
RPA3	-0.0477	5.0698E-01	6.87E-01	ENSG00000106399
RPA4	0.0195	1.8342E-01		ENSG00000204086
RPAIN	-0.0998	5.1671E-02	1.46E-01	ENSG00000129197
RPAP1	-0.0323	6.0227E-01	7.62E-01	ENSG00000103932
RPAP2	-0.1771	4.1912E-03	2.19E-02	ENSG00000122484
RPAP3	0.0487	4.6116E-01	6.50E-01	ENSG00000005175
RPE	-0.1542	6.1108E-02	1.66E-01	ENSG00000197713
RPE65	-0.3004	3.2068E-02	1.02E-01	ENSG00000116745
RPEL1	-0.0078	8.8605E-01		ENSG00000235376
RPF1	-0.1247	3.1288E-02	1.00E-01	ENSG00000117133
RPF2	0.1255	7.5582E-02	1.93E-01	ENSG00000197498
RPF2P1	0.0141	4.2811E-01		ENSG00000225357
RPGR	-0.0838	3.1133E-01	5.04E-01	ENSG00000156313
RPGRIP1	-0.0370	6.9734E-01	8.31E-01	ENSG00000092200
RPGRIP1L	-0.1061	1.0446E-01	2.42E-01	ENSG00000103494
RPH3A	-0.0528	5.7622E-01	7.43E-01	ENSG00000089169
RPH3AL	0.2614	4.3977E-03	2.27E-02	ENSG00000181031
RPH3AL-AS1	-0.0082	8.5235E-01		ENSG00000262061
RPIA	0.1923	1.8147E-02	6.69E-02	ENSG00000153574
RPL10	-0.2410	6.3727E-04	4.99E-03	ENSG00000147403
RPL10A	-0.2451	1.6234E-03	1.04E-02	ENSG00000198755
RPL10AP1	0.0012	8.9785E-01		ENSG00000244691
RPL10AP2	-0.1059	3.7009E-01	5.66E-01	ENSG00000188873
RPL10AP6	-0.1411	1.9070E-01	3.66E-01	ENSG00000226360
RPL10AP9	-0.0062	8.8392E-01	9.41E-01	ENSG00000242477
RPL10P1	-0.0340	3.7850E-01		ENSG00000217026
RPL10P12	0.0139	8.5341E-01	9.25E-01	ENSG00000234040
RPL10P13	-0.0253	7.7859E-01		ENSG00000258245
RPL10P15	0.0470	6.2621E-01	7.81E-01	ENSG00000267119
RPL10P16	-0.1129	2.1391E-01	3.94E-01	ENSG00000178464
RPL10P18	0.0026	7.4370E-01		ENSG00000253995

RPL10P3	-0.0933	3.0709E-01	5.00E-01	ENSG00000230734
RPL10P4	-0.0295	5.6996E-01		ENSG00000238003
RPL10P5	-0.0073	8.0585E-01		ENSG00000235644
RPL10P6	-0.1094	2.3748E-01	4.22E-01	ENSG00000230076
RPL10P7	-0.0050	9.8453E-01		ENSG00000242052
RPL10P8	-0.0245	9.4062E-01	9.70E-01	ENSG00000254012
RPL10P9	-0.2155	2.2433E-02	7.85E-02	ENSG00000233913
RPL11	-0.2066	2.7691E-03	1.58E-02	ENSG00000142676
RPL11P3	0.0378	6.4495E-01	7.94E-01	ENSG00000213613
RPL12	-0.2834	1.7239E-03	1.09E-02	ENSG00000197958
RPL12P10	-0.0182	6.0627E-01		ENSG00000226279
RPL12P12	-0.0268	7.8096E-01		ENSG00000236992
RPL12P20	-0.0362	5.5672E-01		ENSG00000239627
RPL12P25	-0.0074	9.1050E-01		ENSG00000235330
RPL12P37	-0.0221	8.6914E-01		ENSG00000240270
RPL12P6	-0.0955	2.2417E-01	4.06E-01	ENSG00000243824
RPL12P8	-0.0386	6.2292E-01		ENSG00000219932
RPL13	-0.3662	6.4065E-06	1.23E-04	ENSG00000167526
RPL13A	-0.2272	5.3387E-03	2.63E-02	ENSG00000142541
RPL13AP11	0.0030	7.0257E-01		ENSG00000229657
RPL13AP12	0.2408	5.8968E-02	1.61E-01	ENSG00000236047
RPL13AP14	-0.0194	7.7894E-01		ENSG00000242858
RPL13AP16	0.0249	1.8701E-01		ENSG00000226624
RPL13AP17	-0.0075	9.0486E-01		ENSG00000231322
RPL13AP19	0.0047	8.0472E-01		ENSG00000228754
RPL13AP2	0.0518	5.6149E-02		ENSG00000244053
RPL13AP20	-0.0489	6.1410E-01	7.72E-01	ENSG00000234498
RPL13AP22	0.0205	6.7598E-01	8.15E-01	ENSG00000243517
RPL13AP25	-0.0198	8.2503E-01	9.08E-01	ENSG00000136149
RPL13AP26	-0.0272	7.4553E-01	8.61E-01	ENSG00000250318
RPL13AP3	0.0090	6.5135E-01	7.98E-01	ENSG00000177350
RPL13AP5	-0.1943	5.0459E-02	1.44E-01	ENSG00000236552
RPL13AP6	-0.2385	1.2032E-01	2.68E-01	ENSG00000234118
RPL13P12	-0.4167	9.3451E-06	1.66E-04	ENSG00000215030
RPL13P2	-0.0259	2.6910E-01		ENSG00000213820
RPL13P4	-0.0130	5.4965E-01		ENSG00000241890
RPL13P5	0.1667	1.2132E-01	2.69E-01	ENSG00000240370
RPL13P6	0.0089	7.4458E-01		ENSG00000213176
RPL13P8	0.0174	7.2009E-01	8.45E-01	ENSG00000241634
RPL14	-0.2477	6.5502E-04	5.11E-03	ENSG00000188846
RPL14P1	-0.2989	8.1243E-03	3.64E-02	ENSG00000139239
RPL14P2	0.0182	3.2629E-01		ENSG00000225662
RPL14P3	-0.1015	1.4429E-01	3.02E-01	ENSG00000241923
RPL14P4	-0.0012	5.6171E-01		ENSG00000237774
RPL14P6	0.0134	3.7187E-01		ENSG00000185631
RPL15	-0.2150	1.0703E-03	7.48E-03	ENSG00000174748
RPL15P11	-0.0008	7.8789E-01		ENSG00000223718
RPL15P16	0.0246	3.3352E-01		ENSG00000234751
RPL15P17	-0.0663	8.5426E-01	9.25E-01	ENSG00000243830

RPL15P18	0.0111	9.3689E-01	9.69E-01	ENSG00000228501
RPL15P20	-0.1879	1.7174E-01	3.41E-01	ENSG00000215003
RPL15P3	-0.2240	1.5444E-03	1.00E-02	ENSG00000212802
RPL17	-0.5047	1.6195E-06	3.97E-05	ENSG00000265681
RPL17-C18orf32	-0.0153	7.2392E-01		ENSG00000215472
RPL17P16	-0.0152	8.2114E-01		ENSG00000236768
RPL17P18	0.0017	9.8382E-01	9.91E-01	ENSG00000234742
RPL17P19	-0.0227	7.4194E-01		ENSG00000241261
RPL17P22	0.1085	1.0152E-01	2.37E-01	ENSG00000243592
RPL17P23	-0.0562	2.1941E-01		ENSG00000220694
RPL17P25	-0.0047	8.4985E-01		ENSG00000219547
RPL17P26	0.0430	1.5609E-01	3.19E-01	ENSG00000216854
RPL17P27	-0.0353	5.3396E-01		ENSG00000243099
RPL17P28	-0.0036	6.8893E-01		ENSG00000229677
RPL17P29	-0.0041	9.8877E-01		ENSG00000231965
RPL17P34	-0.0266	7.9663E-01	8.91E-01	ENSG00000213432
RPL17P36	-0.0175	9.1040E-01	9.55E-01	ENSG00000236058
RPL17P38	-0.0150	9.9997E-01		ENSG00000240441
RPL17P39	-0.2123	4.9146E-02	1.41E-01	ENSG00000212664
RPL17P41	-0.0286	2.6417E-01		ENSG00000213179
RPL17P43	0.0068	9.0995E-01	9.55E-01	ENSG00000228331
RPL17P44	0.0423	3.5673E-02		ENSG00000241088
RPL17P5	-0.0241	7.0390E-01		ENSG00000230721
RPL17P50	-0.0719	3.8889E-01	5.83E-01	ENSG00000213700
RPL17P6	-0.1897	5.2886E-02	1.49E-01	ENSG00000226084
RPL17P7	-0.1112	2.4444E-01	4.30E-01	ENSG00000244716
RPL17P8	-0.0942	1.9218E-01	3.68E-01	ENSG00000213041
RPL17P9	0.0143	8.2832E-01		ENSG00000223583
RPL18	-0.1989	8.7387E-03	3.85E-02	ENSG00000063177
RPL18A	-0.2205	1.0579E-03	7.42E-03	ENSG00000105640
RPL18AP1	0.0053	8.8845E-01		ENSG00000240096
RPL18AP10	-0.0005	7.6207E-01		ENSG00000213717
RPL18AP11	-0.0116	9.5850E-01		ENSG00000212695
RPL18AP12	0.0061	5.3880E-01		ENSG00000276810
RPL18AP13	0.0016	3.9517E-01		ENSG00000213304
RPL18AP15	0.0059	7.7758E-01		ENSG00000236189
RPL18AP3	-0.1005	1.1786E-01	2.64E-01	ENSG00000213442
RPL18AP6	0.0723	4.6032E-01	6.49E-01	ENSG00000230979
RPL18AP7	0.0006	8.9119E-01		ENSG00000232439
RPL18AP9	0.0033	5.3399E-01		ENSG00000223916
RPL18P10	-0.0097	7.9360E-01		ENSG00000214359
RPL18P13	-0.0037	8.2297E-01		ENSG00000244485
RPL19	-0.2737	1.1320E-04	1.24E-03	ENSG00000108298
RPL19P14	-0.0098	8.4880E-01		ENSG00000240870
RPL19P19	-0.0037	9.4444E-01		ENSG00000243234
RPL19P21	-0.0852	1.8940E-01	3.64E-01	ENSG00000230508
RPL19P5	0.0051	6.5556E-01		ENSG00000230580
RPL19P6	-0.0102	9.6905E-01		ENSG00000233597
RPL19P8	0.0132	4.6943E-01		ENSG00000241877

RPL21	-0.2341	1.7232E-03	1.09E-02	ENSG00000122026
RPL21P1	0.0227	8.1334E-01	9.01E-01	ENSG00000214760
RPL21P10	-0.0208	8.0784E-01	8.98E-01	ENSG00000239272
RPL21P100	-0.0934	2.7501E-01	4.66E-01	ENSG00000242405
RPL21P105	0.0095	3.9679E-01		ENSG00000239374
RPL21P11	-0.0116	8.5245E-01		ENSG00000242571
RPL21P116	0.0328	2.9812E-01		ENSG00000243711
RPL21P119	-0.1633	5.8589E-02	1.61E-01	ENSG00000220793
RPL21P120	-0.0443	6.1448E-01	7.73E-01	ENSG00000244582
RPL21P123	0.0099	6.9380E-01		ENSG00000240531
RPL21P125	-0.0074	7.8854E-01	8.87E-01	ENSG00000240898
RPL21P128	0.0162	4.4166E-01		ENSG00000244527
RPL21P134	-0.0443	7.0708E-01	8.36E-01	ENSG00000233254
RPL21P137	0.0131	2.4900E-01		ENSG00000236732
RPL21P14	0.0258	7.3255E-01	8.53E-01	ENSG00000242747
RPL21P16	-0.2211	1.2562E-02	5.06E-02	ENSG00000220842
RPL21P18	-0.1580	5.2183E-02		ENSG00000213343
RPL21P19	-0.0026	9.7385E-01	9.87E-01	ENSG00000219133
RPL21P2	-0.0210	3.5370E-01		ENSG00000215063
RPL21P20	-0.0518	1.4942E-01		ENSG00000227311
RPL21P28	-0.1840	3.5244E-02	1.10E-01	ENSG00000220749
RPL21P29	-0.0082	8.7685E-01		ENSG00000232037
RPL21P3	-0.0459	3.6934E-01		ENSG00000225419
RPL21P32	-0.0518	4.6808E-01	6.56E-01	ENSG00000224019
RPL21P37	0.3060	1.8086E-02	6.68E-02	ENSG00000229503
RPL21P39	0.2633	2.2815E-02	7.95E-02	ENSG00000239797
RPL21P4	0.0063	8.8648E-01		ENSG00000240828
RPL21P40	-0.0394	2.1024E-01		ENSG00000235670
RPL21P45	0.0034	7.3032E-01		ENSG00000239532
RPL21P46	-0.1601	1.4520E-01	3.04E-01	ENSG00000241612
RPL21P48	-0.0361	2.3673E-01		ENSG00000242318
RPL21P5	0.0238	6.3336E-01		ENSG00000258640
RPL21P51	0.0569	1.1999E-01		ENSG00000240674
RPL21P52	0.0049	6.1331E-01		ENSG00000242431
RPL21P53	0.0002	9.8975E-01	9.94E-01	ENSG00000244021
RPL21P54	-0.0303	6.6928E-01	8.10E-01	ENSG00000241829
RPL21P57	-0.0103	7.2186E-01		ENSG00000241187
RPL21P6	-0.0290	2.9622E-01		ENSG00000239199
RPL21P60	0.0031	9.7242E-01	9.86E-01	ENSG00000240729
RPL21P69	-0.1386	2.3879E-02		ENSG00000216480
RPL21P7	0.0331	1.1760E-01		ENSG00000258613
RPL21P75	-0.2955	2.0665E-04	2.01E-03	ENSG00000213860
RPL21P76	-0.0031	8.0544E-01		ENSG00000228030
RPL21P80	-0.0881	3.6091E-01	5.57E-01	ENSG00000243181
RPL21P83	-0.0254	4.9551E-01		ENSG00000230188
RPL21P87	-0.0613	6.1689E-01	7.74E-01	ENSG00000228053
RPL21P89	-0.0287	6.8319E-01	8.21E-01	ENSG00000229870
RPL21P90	-0.0085	6.3800E-01		ENSG00000227805
RPL21P93	0.0566	5.0851E-01	6.89E-01	ENSG00000229605

RPL21P95	-0.0977	1.9683E-01	3.73E-01	ENSG00000241782
RPL21P96	0.0142	5.5008E-01		ENSG00000239861
RPL21P97	0.0534	5.7946E-01	7.46E-01	ENSG00000239470
RPL21P98	-0.0189	8.3668E-01	9.15E-01	ENSG00000243071
RPL21P99	-0.0065	9.8337E-01		ENSG00000242986
RPL22	-0.3359	2.5342E-04	2.37E-03	ENSG00000116251
RPL22L1	-0.5148	5.6333E-07	1.65E-05	ENSG00000163584
RPL22P16	-0.0112	9.3553E-01		ENSG00000213935
RPL22P19	-0.0043	7.8092E-01		ENSG00000241129
RPL22P2	0.0262	4.0166E-01	5.96E-01	ENSG00000241081
RPL22P3	0.1234	1.5284E-01	3.14E-01	ENSG00000226457
RPL23	-0.3032	1.8969E-04	1.88E-03	ENSG00000125691
RPL23A	-0.4540	1.3184E-09	1.07E-07	ENSG00000198242
RPL23AP12	-0.0304	7.2650E-01		ENSG00000228861
RPL23AP15	-0.0824	2.2966E-01		ENSG00000230841
RPL23AP18	-0.0414	6.7990E-01	8.18E-01	ENSG00000225338
RPL23AP21	0.0029	9.7548E-01	9.87E-01	ENSG00000228463
RPL23AP24	-0.0575	5.4989E-01	7.22E-01	ENSG00000236679
RPL23AP25	-0.3987	3.0086E-04	2.74E-03	ENSG00000233084
RPL23AP3	-0.0423	8.5574E-01		ENSG00000214914
RPL23AP36	0.0303	6.5587E-02		ENSG00000225124
RPL23AP37	-0.0455	1.9450E-01		ENSG00000236148
RPL23AP4	-0.2944	1.0487E-03	7.37E-03	ENSG00000212932
RPL23AP41	-0.0032	8.3939E-01		ENSG00000224085
RPL23AP43	-0.0486	5.8554E-01	7.50E-01	ENSG00000227337
RPL23AP45	-0.0839	4.0562E-01	5.99E-01	ENSG00000213285
RPL23AP47	-0.0807	4.1279E-01	6.06E-01	ENSG00000219814
RPL23AP48	-0.0022	8.8219E-01		ENSG00000219135
RPL23AP49	-0.5298	3.1512E-05	4.41E-04	ENSG00000243422
RPL23AP5	0.0610	5.3365E-01	7.08E-01	ENSG00000236829
RPL23AP50	-0.0023	9.5372E-01		ENSG00000232778
RPL23AP51	-0.0302	6.9686E-01		ENSG00000234718
RPL23AP52	0.0112	5.5338E-01		ENSG00000230370
RPL23AP57	0.0008	4.8882E-01		ENSG00000206147
RPL23AP60	-0.0739	4.1134E-01	6.04E-01	ENSG00000213147
RPL23AP61	0.0742	2.1606E-01	3.97E-01	ENSG00000280012
RPL23AP65	-0.1660	1.3299E-01	2.86E-01	ENSG00000243964
RPL23AP67	-0.0260	7.5181E-01		ENSG00000240991
RPL23AP74	-0.0627	1.8284E-01		ENSG00000227694
RPL23AP75	0.0099	6.0745E-01		ENSG00000240125
RPL23AP78	-0.0239	9.3091E-01		ENSG00000269244
RPL23AP80	-0.0096	8.5870E-01		ENSG00000213035
RPL23AP81	-0.0247	3.6991E-01		ENSG00000242748
RPL23AP84	-0.0807	3.8833E-01	5.83E-01	ENSG00000261914
RPL23AP87	0.4771	9.5681E-04	6.89E-03	ENSG00000232938
RPL23AP92	-0.0210	5.0053E-01		ENSG00000270723
RPL23P2	0.1692	1.3380E-01	2.87E-01	ENSG00000176054
RPL23P6	-0.0253	7.7091E-01		ENSG00000180211
RPL23P7	-0.0234	6.8648E-01		ENSG00000232383

RPL23P8	-0.0570	4.6041E-01	6.49E-01	ENSG00000219451
RPL24	-0.2571	1.4401E-03	9.51E-03	ENSG00000114391
RPL24P2	0.1080	2.1389E-01	3.94E-01	ENSG00000235065
RPL24P4	-0.0150	8.7237E-01	9.35E-01	ENSG00000181524
RPL24P5	-0.0210	8.3146E-01		ENSG00000235582
RPL24P6	0.0196	3.6070E-01		ENSG00000236844
RPL24P7	0.0038	5.5570E-01		ENSG00000215016
RPL24P8	-0.4389	1.0553E-02	4.42E-02	ENSG00000236801
RPL26	-0.2522	1.3306E-03	8.92E-03	ENSG00000161970
RPL26L1	-0.1331	3.4140E-02	1.07E-01	ENSG00000037241
RPL26L1-AS1	0.0627	5.1546E-01	6.94E-01	ENSG00000204758
RPL26P10	-0.0010	7.6416E-01		ENSG00000226984
RPL26P19	-0.1937	8.6087E-02	2.11E-01	ENSG00000226221
RPL26P2	-0.0189	5.3385E-01		ENSG00000242061
RPL26P22	-0.0174	6.5539E-01		ENSG00000229977
RPL26P23	0.0039	3.6335E-01		ENSG00000234450
RPL26P24	-0.0174	6.5539E-01		ENSG00000232145
RPL26P27	-0.0496	1.7095E-01		ENSG00000226493
RPL26P29	-0.0470	2.9712E-01		ENSG00000234806
RPL26P32	0.0228	3.4221E-01		ENSG00000230291
RPL26P33	-0.0056	9.8071E-01		ENSG00000244266
RPL26P36	0.0123	7.2496E-01	8.48E-01	ENSG00000225912
RPL26P37	0.0156	3.9716E-01		ENSG00000235175
RPL26P4	-0.1936	1.1171E-01	2.54E-01	ENSG00000241494
RPL26P6	0.0296	6.8486E-01	8.22E-01	ENSG00000229659
RPL26P8	0.0026	6.8279E-01		ENSG00000227312
RPL27	-0.1640	1.6325E-02	6.18E-02	ENSG00000131469
RPL27A	-0.3581	2.0336E-05	3.10E-04	ENSG00000166441
RPL27AP5	-0.0125	9.1110E-01		ENSG00000182383
RPL27AP6	-0.0200	8.1960E-01	9.05E-01	ENSG00000218426
RPL27AP8	-0.0092	9.3228E-01		ENSG00000229423
RPL27P11	0.0018	6.8030E-01		ENSG00000233287
RPL27P3	0.0463	3.1702E-01		ENSG00000226545
RPL27P4	0.0882	1.3309E-01		ENSG00000225616
RPL27P5	-0.0005	8.2286E-01		ENSG00000232403
RPL28	-0.1930	3.5364E-03	1.92E-02	ENSG00000108107
RPL28P2	0.0716	7.0636E-02	1.83E-01	ENSG00000237641
RPL28P5	0.0070	3.3715E-01		ENSG00000213293
RPL29	-0.3080	1.0736E-05	1.86E-04	ENSG00000162244
RPL29P2	-0.0043	6.5246E-01		ENSG00000240480
RPL29P21	-0.0539	1.3903E-01		ENSG00000213779
RPL29P34	0.0020	7.5076E-01		ENSG00000239255
RPL3	-0.1772	6.8320E-03	3.18E-02	ENSG00000100316
RPL30	-0.3948	3.1391E-08	1.50E-06	ENSG00000156482
RPL30P13	-0.1234	9.6718E-02	2.29E-01	ENSG00000229014
RPL31	-0.2536	1.5421E-03	1.00E-02	ENSG00000071082
RPL31P10	0.0027	5.2355E-01		ENSG00000213942
RPL31P12	0.0024	5.1632E-01		ENSG00000227207
RPL31P17	-0.0382	5.0888E-01	6.89E-01	ENSG00000235225

RPL31P18	0.0454	1.8975E-01		ENSG00000213849
RPL31P19	0.0069	4.7479E-01		ENSG00000227309
RPL31P2	-0.0057	6.9758E-01		ENSG00000230837
RPL31P21	-0.0232	5.2385E-01		ENSG00000241400
RPL31P28	-0.0210	9.4737E-01		ENSG00000219863
RPL31P30	0.0709	4.1236E-01	6.05E-01	ENSG00000230702
RPL31P31	-0.0058	7.6814E-01		ENSG00000239983
RPL31P34	-0.0885	2.6154E-01	4.50E-01	ENSG00000231359
RPL31P36	-0.0041	8.4940E-01		ENSG00000224545
RPL31P4	0.1046	2.7642E-01	4.67E-01	ENSG00000213598
RPL31P43	-0.0147	5.1345E-01		ENSG00000213557
RPL31P49	-0.0031	6.4397E-01		ENSG00000241680
RPL31P52	-0.0050	3.2090E-01	5.15E-01	ENSG00000219355
RPL31P55	0.0020	8.5062E-01		ENSG00000242295
RPL31P58	-0.0255	8.2946E-01		ENSG00000243053
RPL31P61	0.0179	7.3456E-01	8.54E-01	ENSG00000243297
RPL31P62	0.0007	7.3714E-01		ENSG00000235328
RPL31P63	-0.0369	6.5025E-01	7.97E-01	ENSG00000236735
RPL31P7	-0.0845	3.0640E-01	4.99E-01	ENSG00000232083
RPL31P8	-0.0296	9.2526E-01		ENSG00000213896
RPL32	-0.2755	2.4638E-04	2.32E-03	ENSG00000144713
RPL32P13	0.0024	5.4181E-01		ENSG00000241002
RPL32P18	-0.1499	1.6746E-01	3.35E-01	ENSG00000146677
RPL32P25	0.0091	7.1474E-01		ENSG00000242993
RPL32P26	-0.0226	5.6065E-01		ENSG00000241941
RPL32P27	-0.0121	5.4060E-01	7.14E-01	ENSG00000239617
RPL32P32	0.0137	1.6392E-01		ENSG00000241157
RPL32P6	-0.0189	4.0940E-01		ENSG00000224064
RPL34	-0.1820	7.6995E-03	3.49E-02	ENSG00000109475
RPL34-DT	0.2194	7.1439E-02	1.85E-01	ENSG00000234492
RPL34P10	-0.0291	2.6726E-01		ENSG00000236489
RPL34P16	-0.0060	6.5420E-01		ENSG00000217239
RPL34P18	-0.0958	3.6088E-01	5.57E-01	ENSG00000240509
RPL34P22	-0.0202	5.8384E-01		ENSG00000244267
RPL34P24	-0.0363	6.6453E-01	8.07E-01	ENSG00000240808
RPL34P26	-0.0197	5.9671E-01		ENSG00000223676
RPL34P27	-0.0558	7.7886E-01	8.80E-01	ENSG00000232858
RPL34P28	-0.0197	5.6883E-01		ENSG00000239686
RPL34P4	0.0070	9.1954E-01		ENSG00000225854
RPL34P5	0.0028	2.6086E-01		ENSG00000231416
RPL34P6	0.0517	4.7706E-01	6.64E-01	ENSG00000231333
RPL35	-0.2528	1.6819E-04	1.71E-03	ENSG00000136942
RPL35A	-0.2894	1.6061E-04	1.64E-03	ENSG00000182899
RPL35AP14	-0.0050	9.8134E-01		ENSG00000240436
RPL35AP6	-0.0153	9.1295E-01		ENSG00000228879
RPL35P1	-0.1573	1.4227E-01	2.99E-01	ENSG00000237991
RPL35P5	-0.0306	7.0257E-01	8.34E-01	ENSG00000225573
RPL35P6	-0.0584	3.2783E-01		ENSG00000244018
RPL36	-0.2694	4.4371E-05	5.85E-04	ENSG00000130255

RPL36A	0.2761	1.4195E-02	5.56E-02	ENSG00000241343
RPL36AL	-0.3180	1.0575E-07	4.09E-06	ENSG00000165502
RPL36AP1	0.0075	8.8453E-01		ENSG00000242995
RPL36AP11	0.0046	5.7418E-01		ENSG00000237003
RPL36AP21	-0.1630	1.3270E-01	2.85E-01	ENSG00000240785
RPL36AP23	-0.0049	9.2150E-01		ENSG00000241722
RPL36AP26	-0.1065	3.7832E-01	5.73E-01	ENSG00000235828
RPL36AP37	-0.1980	4.2284E-02	1.26E-01	ENSG00000244398
RPL36AP41	-0.0017	6.0999E-01		ENSG00000242087
RPL36AP45	-0.0679	6.1790E-01	7.75E-01	ENSG00000240366
RPL36AP46	-0.2950	6.9956E-02	1.82E-01	ENSG00000224011
RPL36AP48	-0.0244	7.2183E-01		ENSG00000213180
RPL36AP49	0.0131	8.3376E-01	9.13E-01	ENSG00000243779
RPL36AP51	-0.0053	7.5811E-01		ENSG00000242291
RPL36AP52	-0.0283	8.1271E-01		ENSG00000214925
RPL36P15	-0.0062	8.5132E-01		ENSG00000240100
RPL36P20	0.0034	7.8782E-01	8.86E-01	ENSG00000234270
RPL36P6	0.1283	1.2284E-01	2.71E-01	ENSG00000224348
RPL36P7	0.0227	5.5119E-01		ENSG00000213519
RPL37	-0.2659	4.9453E-04	4.06E-03	ENSG00000145592
RPL37A	-0.3430	2.3525E-06	5.42E-05	ENSG00000197756
RPL37AP6	-0.0040	7.6404E-01		ENSG00000228341
RPL37P10	-0.0252	5.8630E-01		ENSG00000224627
RPL37P15	-0.0248	3.6021E-01		ENSG00000216365
RPL37P20	0.0019	9.2558E-01		ENSG00000239701
RPL37P6	-0.0347	7.6791E-01	8.74E-01	ENSG00000241431
RPL38	-0.3250	1.0122E-05	1.77E-04	ENSG00000172809
RPL39	-0.3127	1.1119E-05	1.91E-04	ENSG00000198918
RPL39L	-0.1034	1.1645E-01	2.62E-01	ENSG00000163923
RPL39P16	-0.0664	3.7990E-01		ENSG00000227470
RPL3L	-0.0130	8.7236E-01	9.35E-01	ENSG00000140986
RPL3P1	-0.0113	8.6250E-01	9.30E-01	ENSG00000228149
RPL3P2	-0.0101	9.2318E-01	9.62E-01	ENSG00000227939
RPL3P4	-0.4200	6.0249E-05	7.47E-04	ENSG00000232573
RPL3P6	-0.0106	8.6382E-01		ENSG00000213891
RPL3P7	-0.0905	2.2460E-01	4.07E-01	ENSG00000225093
RPL3P9	-0.0236	8.4713E-01	9.21E-01	ENSG00000240540
RPL4	-0.1555	3.2793E-02	1.04E-01	ENSG00000174444
RPL41	-0.3334	1.6892E-05	2.68E-04	ENSG00000229117
RPL41P2	-0.1514	6.3579E-02	1.70E-01	ENSG00000256338
RPL4P2	-0.0105	9.0556E-01		ENSG00000232341
RPL4P3	-0.0050	8.8483E-01	9.42E-01	ENSG00000230364
RPL4P4	-0.0387	6.7521E-01	8.14E-01	ENSG00000229638
RPL4P5	-0.0297	7.5520E-01	8.67E-01	ENSG00000230207
RPL4P6	-0.0783	9.1330E-02	2.21E-01	ENSG00000230071
RPL5	-0.1276	7.0954E-02	1.84E-01	ENSG00000122406
RPL5P1	-0.0526	6.3258E-01	7.86E-01	ENSG00000241061
RPL5P12	-0.0102	8.7016E-01	9.34E-01	ENSG00000242697
RPL5P17	-0.0229	5.6022E-01		ENSG00000243859

RPL5P18	-0.0109	9.4465E-01		ENSG00000220744
RPL5P19	-0.0059	7.6814E-01		ENSG00000220553
RPL5P22	-0.0693	4.2924E-01	6.21E-01	ENSG00000239198
RPL5P23	-0.0154	8.7500E-01	9.37E-01	ENSG00000240395
RPL5P24	-0.0274	7.0791E-01		ENSG00000244052
RPL5P28	-0.1934	2.1916E-01	4.00E-01	ENSG00000235264
RPL5P29	-0.0784	4.3568E-01		ENSG00000242208
RPL5P3	-0.1454	9.1132E-02	2.20E-01	ENSG00000242276
RPL5P30	-0.0707	7.6095E-01	8.70E-01	ENSG00000243431
RPL5P34	0.0424	6.6387E-01	8.07E-01	ENSG00000234009
RPL5P4	-0.0223	8.1189E-01	9.00E-01	ENSG00000229994
RPL5P5	0.0034	2.8357E-01		ENSG00000213051
RPL5P6	0.0077	1.0558E-01		ENSG00000232393
RPL5P8	0.0337	8.6090E-02		ENSG00000213917
RPL5P9	-0.1044	3.0400E-01	4.96E-01	ENSG00000236330
RPL6	-0.2944	1.4397E-03	9.51E-03	ENSG00000089009
RPL6P1	-0.0216	4.0762E-01		ENSG00000236111
RPL6P10	-0.1304	2.0933E-01	3.89E-01	ENSG00000243199
RPL6P13	0.0211	1.1088E-01		ENSG00000243312
RPL6P19	-0.0843	3.9486E-01	5.89E-01	ENSG00000230383
RPL6P20	0.0306	5.6767E-01	7.36E-01	ENSG00000236976
RPL6P22	-0.0034	8.8576E-01		ENSG00000240738
RPL6P24	-0.0564	1.8744E-01		ENSG00000227474
RPL6P25	0.0154	3.3795E-01		ENSG00000213270
RPL6P26	-0.0545	2.9045E-01		ENSG00000240305
RPL6P27	-0.1932	5.1792E-02	1.47E-01	ENSG00000235552
RPL6P28	-0.0197	8.5880E-01	9.28E-01	ENSG00000223730
RPL6P3	-0.0220	5.0164E-01		ENSG00000229022
RPL6P31	0.0102	3.7694E-01		ENSG00000223945
RPL7	-0.2608	3.5152E-03	1.91E-02	ENSG00000147604
RPL7A	-0.2769	2.8345E-03	1.61E-02	ENSG00000148303
RPL7AP10	0.0261	2.3086E-01		ENSG00000240522
RPL7AP11	-0.0656	5.1655E-01	6.95E-01	ENSG00000242445
RPL7AP14	-0.0104	6.4382E-01	7.93E-01	ENSG00000213979
RPL7AP16	-0.0241	9.3092E-01	9.66E-01	ENSG00000234329
RPL7AP19	0.0155	3.2735E-01		ENSG00000227777
RPL7AP20	-0.0472	5.6033E-01	7.30E-01	ENSG00000234981
RPL7AP21	-0.0266	4.9718E-01		ENSG00000229757
RPL7AP24	-0.0238	3.1164E-01		ENSG00000241307
RPL7AP25	-0.0031	8.6609E-01		ENSG00000235294
RPL7AP26	0.0028	7.8105E-01		ENSG00000214988
RPL7AP27	0.0060	6.9021E-01		ENSG00000242169
RPL7AP29	-0.0144	5.4965E-01		ENSG00000248843
RPL7AP3	-0.0051	9.9548E-01		ENSG00000256243
RPL7AP30	-0.0134	8.3001E-01	9.11E-01	ENSG00000241741
RPL7AP31	0.0041	4.9761E-01		ENSG00000242083
RPL7AP34	-0.0049	7.5748E-01		ENSG00000213312
RPL7AP35	0.0105	6.4269E-01		ENSG00000216639
RPL7AP37	0.0155	4.0854E-01		ENSG00000213109

RPL7AP39	0.4336	8.9092E-03	3.90E-02	ENSG00000213385
RPL7AP4	0.0043	8.1915E-01		ENSG00000213641
RPL7AP43	0.2193	6.7622E-02	1.78E-01	ENSG00000213542
RPL7AP45	-0.1190	2.5081E-01	4.37E-01	ENSG00000275160
RPL7AP46	0.0008	6.4002E-01		ENSG00000233555
RPL7AP47	-0.0266	4.0073E-01		ENSG00000234982
RPL7AP48	-0.0486	1.7753E-01		ENSG00000237088
RPL7AP50	-0.0911	4.9700E-01	6.80E-01	ENSG00000213609
RPL7AP53	-0.0105	9.9606E-01		ENSG00000235962
RPL7AP55	0.0025	8.7776E-01		ENSG00000241008
RPL7AP57	-0.0118	5.1901E-01		ENSG00000242248
RPL7AP6	-0.1751	6.8912E-02	1.80E-01	ENSG00000242071
RPL7AP62	-0.0513	5.7561E-01	7.42E-01	ENSG00000213862
RPL7AP63	0.0067	2.8840E-01		ENSG00000243697
RPL7AP64	0.0138	5.5421E-01		ENSG00000213876
RPL7AP65	-0.0181	9.0898E-01	9.54E-01	ENSG00000228000
RPL7AP66	-0.0260	7.8461E-01	8.84E-01	ENSG00000175886
RPL7AP70	-0.0875	3.8302E-01	5.78E-01	ENSG00000235776
RPL7AP71	-0.0565	2.7137E-01		ENSG00000234566
RPL7AP73	0.0148	3.6794E-01		ENSG00000237952
RPL7AP79	0.0054	7.0875E-01		ENSG00000255106
RPL7AP8	-0.0015	7.1266E-01		ENSG00000230507
RPL7AP82	-0.0319	8.2736E-01	9.09E-01	ENSG00000228955
RPL7AP83	-0.0038	8.6846E-01		ENSG00000229339
RPL7AP9	0.0131	6.6136E-01		ENSG00000213272
RPL7L1	-0.0726	1.4306E-01	3.01E-01	ENSG00000146223
RPL7P1	-0.1480	1.0832E-01	2.48E-01	ENSG00000214485
RPL7P10	-0.0091	9.1440E-01		ENSG00000229752
RPL7P11	-0.0095	8.4674E-01		ENSG00000234619
RPL7P12	-0.0137	8.9128E-01		ENSG00000227770
RPL7P13	0.0794	1.6237E-01		ENSG00000234253
RPL7P16	-0.0084	9.4298E-01		ENSG00000242899
RPL7P18	0.0156	7.1615E-01		ENSG00000243806
RPL7P19	-0.0878	3.8081E-01	5.76E-01	ENSG00000241458
RPL7P20	-0.0208	5.4620E-01		ENSG00000254252
RPL7P22	0.0542	4.9522E-02		ENSG00000226544
RPL7P23	-0.0005	9.8428E-01	9.91E-01	ENSG00000244363
RPL7P24	-0.0215	5.4952E-01	7.21E-01	ENSG00000240003
RPL7P28	-0.0091	9.1888E-01		ENSG00000218632
RPL7P29	-0.0105	7.6999E-01		ENSG00000214457
RPL7P32	-0.0430	6.2673E-01	7.81E-01	ENSG00000231006
RPL7P38	0.0463	3.8649E-01		ENSG00000239473
RPL7P4	-0.0877	2.3386E-01	4.18E-01	ENSG00000230449
RPL7P43	0.0030	6.6480E-01		ENSG00000241045
RPL7P44	-0.0443	5.2163E-01	6.99E-01	ENSG00000233873
RPL7P47	-0.0501	6.9597E-01	8.30E-01	ENSG00000228071
RPL7P48	-0.0080	6.7859E-01		ENSG00000187504
RPL7P49	-0.0629	4.3517E-01	6.26E-01	ENSG00000242931
RPL7P52	0.0086	2.3095E-01		ENSG00000228599

RPL7P56	-0.0234	9.4351E-01	9.72E-01	ENSG00000231164
RPL7P57	-0.0099	8.5418E-01		ENSG00000224401
RPL7P6	-0.0170	8.4120E-01	9.17E-01	ENSG00000227525
RPL7P60	0.0004	2.1949E-01	4.00E-01	ENSG00000214142
RPL7P7	-0.0023	9.7597E-01		ENSG00000224315
RPL7P9	-0.1677	5.4636E-02	1.53E-01	ENSG00000137970
RPL8	-0.2610	3.0368E-04	2.76E-03	ENSG00000161016
RPL8P3	-0.0152	5.9877E-01		ENSG00000240950
RPL8P4	-0.0310	8.9826E-01	9.49E-01	ENSG00000213169
RPL9	-0.2412	1.4652E-03	9.64E-03	ENSG00000163682
RPL9P11	-0.1240	2.2312E-01	4.05E-01	ENSG00000231181
RPL9P17	0.0276	5.9214E-01		ENSG00000224282
RPL9P18	-0.0127	9.9465E-01		ENSG00000213309
RPL9P2	0.0126	5.7900E-01		ENSG00000234324
RPL9P22	-0.0218	4.5338E-01		ENSG00000240174
RPL9P27	0.0665	3.1263E-01		ENSG00000244251
RPL9P3	-0.0118	9.4186E-01	9.71E-01	ENSG00000240392
RPL9P30	0.0276	3.8433E-01		ENSG00000240074
RPL9P32	-0.1019	4.8181E-01	6.67E-01	ENSG00000242100
RPL9P7	-0.2671	1.5921E-03	1.03E-02	ENSG00000238103
RPL9P8	-0.2890	2.8728E-04	2.63E-03	ENSG00000237550
RPLP0	-0.1082	1.3518E-01	2.89E-01	ENSG00000089157
RPLP0P2	-0.1817	9.0820E-02	2.20E-01	ENSG00000243742
RPLP0P6	-0.0011	9.8846E-01	9.94E-01	ENSG00000213553
RPLP1	-0.2630	9.9219E-05	1.11E-03	ENSG00000137818
RPLP1P3	-0.0131	6.1933E-01		ENSG00000224600
RPLP2	-0.2216	6.9346E-04	5.35E-03	ENSG00000177600
RPN1	-0.0639	3.8627E-01	5.81E-01	ENSG00000163902
RPN2	-0.0292	6.2409E-01	7.79E-01	ENSG00000118705
RPP14	0.1600	1.2902E-01	2.80E-01	ENSG00000163684
RPP21	-0.0709	4.5671E-01	6.46E-01	ENSG00000241370
RPP25	-0.1296	1.5855E-01	3.23E-01	ENSG00000178718
RPP25L	-0.3345	2.1782E-03	1.31E-02	ENSG00000164967
RPP30	0.0639	2.5411E-01	4.41E-01	ENSG00000148688
RPP38	0.0443	4.6464E-01	6.53E-01	ENSG00000152464
RPP38-DT	0.2013	7.2426E-02	1.87E-01	ENSG00000176236
RPP40	-0.3376	2.2041E-03	1.32E-02	ENSG00000124787
RPRD1A	0.0507	3.3935E-01	5.34E-01	ENSG00000141425
RPRD1B	0.1524	1.5774E-02	6.02E-02	ENSG00000101413
RPRD2	-0.1181	4.5516E-02	1.33E-01	ENSG00000163125
RPRM	0.0635	4.8962E-01	6.74E-01	ENSG00000177519
RPRML	0.5194	2.3596E-03	1.40E-02	ENSG00000179673
RPS10	-0.3778	4.3989E-04	3.72E-03	ENSG00000124614
RPS10P1	-0.1448	1.6147E-01	3.27E-01	ENSG00000217275
RPS10P10	0.0041	8.8222E-01		ENSG00000226082
RPS10P11	-0.0478	9.4584E-01	9.73E-01	ENSG00000243648
RPS10P12	-0.0102	9.3078E-01		ENSG00000240627
RPS10P13	0.1194	2.3143E-01	4.15E-01	ENSG00000217130
RPS10P15	-0.0535	5.7838E-01	7.45E-01	ENSG00000213280

RPS10P2	0.0781	1.6520E-01	3.32E-01	ENSG00000213950
RPS10P22	-0.0139	8.7661E-01		ENSG00000242667
RPS10P23	0.0164	7.8307E-01	8.83E-01	ENSG00000240861
RPS10P26	0.1073	2.9313E-01	4.85E-01	ENSG00000239246
RPS10P27	0.0053	5.1994E-01		ENSG00000267752
RPS10P3	0.1694	1.1012E-01	2.51E-01	ENSG00000217716
RPS10P4	-0.0419	8.9821E-01	9.49E-01	ENSG00000240776
RPS10P5	0.1922	9.8050E-02	2.32E-01	ENSG00000101278
RPS10P6	-0.0053	9.5225E-01	9.76E-01	ENSG00000223861
RPS10P8	-0.0100	8.6947E-01		ENSG00000229914
RPS11	-0.2283	1.3770E-03	9.18E-03	ENSG00000142534
RPS11P7	-0.0114	9.6959E-01		ENSG00000214047
RPS12	-0.1622	3.7421E-02	1.15E-01	ENSG00000112306
RPS12P17	0.0016	9.3200E-01		ENSG00000233163
RPS12P20	-0.0149	4.6402E-01		ENSG00000244134
RPS12P29	0.5414	8.3195E-04	6.16E-03	ENSG00000213939
RPS12P31	-0.0120	6.5300E-01		ENSG00000242325
RPS12P9	-0.0543	5.5214E-01	7.23E-01	ENSG00000226439
RPS13	-0.1279	3.3832E-02	1.07E-01	ENSG00000110700
RPS13P2	0.0721	4.4494E-01	6.35E-01	ENSG00000228929
RPS13P4	0.0427	5.0850E-01	6.89E-01	ENSG00000238111
RPS13P6	-0.0141	8.2682E-01		ENSG00000239615
RPS14	-0.1805	1.2941E-02	5.18E-02	ENSG00000164587
RPS14P1	0.0841	3.8373E-01	5.78E-01	ENSG00000224114
RPS15	-0.4361	1.0319E-10	1.22E-08	ENSG00000115268
RPS15A	-0.3947	2.9707E-07	9.74E-06	ENSG00000134419
RPS15AP1	-0.1480	1.7526E-01	3.45E-01	ENSG00000214535
RPS15AP11	-0.0155	9.6869E-01		ENSG00000234093
RPS15AP12	-0.0762	6.9877E-01	8.32E-01	ENSG00000232134
RPS15AP14	-0.0242	8.2077E-01		ENSG00000233131
RPS15AP16	0.0001	9.6595E-01	9.83E-01	ENSG00000239483
RPS15AP17	0.0212	8.4681E-01	9.21E-01	ENSG00000225405
RPS15AP19	-0.1199	2.1896E-01	4.00E-01	ENSG00000219023
RPS15AP24	-0.0488	5.0734E-01		ENSG00000241511
RPS15AP25	0.0161	4.2481E-01		ENSG00000236191
RPS15AP29	-0.0271	4.5985E-01		ENSG00000237827
RPS15AP30	-1.4717	2.2628E-02	7.91E-02	ENSG00000227560
RPS15AP35	-0.0051	9.5600E-01		ENSG00000214917
RPS15AP36	-0.0076	9.0079E-01		ENSG00000213013
RPS15AP40	-0.0647	1.1433E-01		ENSG00000233921
RPS15AP6	-0.0414	6.2807E-01	7.82E-01	ENSG00000233072
RPS15AP8	-0.0479	3.8203E-01		ENSG00000235002
RPS15P1	-0.0067	9.6420E-01		ENSG00000236887
RPS15P2	-0.0675	3.8818E-01	5.83E-01	ENSG00000235605
RPS15P4	-0.5385	4.0819E-11	5.52E-09	ENSG00000233762
RPS15P5	0.0041	6.0641E-01	7.66E-01	ENSG00000229806
RPS15P7	0.0564	3.6090E-01	5.57E-01	ENSG00000213970
RPS16	-0.2102	1.1310E-03	7.81E-03	ENSG00000105193
RPS16P1	0.0504	1.2521E-01		ENSG00000178715

RPS16P4	-0.0814	2.4362E-01		ENSG00000231449
RPS16P7	0.0014	5.4771E-01		ENSG00000243445
RPS17	-0.1936	3.4035E-02	1.07E-01	ENSG00000182774
RPS17P16	-0.0340	6.8968E-01	8.26E-01	ENSG00000232346
RPS17P3	-0.0073	9.9139E-01		ENSG00000242439
RPS18	-0.0814	2.4867E-01	4.35E-01	ENSG00000231500
RPS18P12	0.0341	5.2482E-01	7.01E-01	ENSG00000230897
RPS18P14	0.0126	8.5159E-01		ENSG00000214093
RPS18P2	0.3244	2.1496E-02	7.61E-02	ENSG00000213315
RPS18P5	0.1153	1.8861E-01	3.63E-01	ENSG00000242299
RPS18P6	-0.0064	5.9234E-01		ENSG00000244048
RPS18P7	-0.0030	8.0409E-01		ENSG00000240388
RPS19	-0.3740	2.9528E-06	6.53E-05	ENSG00000105372
RPS19BP1	0.0120	8.8066E-01	9.39E-01	ENSG00000187051
RPS19P1	-0.0363	5.1461E-01		ENSG00000214612
RPS19P5	-0.0125	8.5681E-01		ENSG00000219559
RPS19P7	-0.0624	3.6959E-01	5.65E-01	ENSG00000228462
RPS2	-0.2417	1.8458E-03	1.15E-02	ENSG00000140988
RPS20	-0.1588	2.2520E-02	7.88E-02	ENSG00000008988
RPS20P15	-0.0268	6.6449E-01		ENSG00000227523
RPS20P17	-0.0263	8.4024E-01	9.17E-01	ENSG00000239393
RPS20P28	-0.0248	3.9037E-01		ENSG00000240739
RPS20P31	0.0013	9.5601E-01		ENSG00000240925
RPS20P33	0.0131	8.6835E-01	9.33E-01	ENSG00000242085
RPS20P6	-0.0080	7.6663E-01		ENSG00000228399
RPS21	-0.1128	9.1489E-02	2.21E-01	ENSG00000171858
RPS23	-0.3923	2.2185E-09	1.63E-07	ENSG00000186468
RPS23P1	-0.0486	5.8549E-01	7.50E-01	ENSG00000243504
RPS23P2	-0.0016	9.5205E-01		ENSG00000213448
RPS23P8	-0.1435	1.3325E-01	2.86E-01	ENSG00000230629
RPS24	-0.4269	8.1916E-08	3.31E-06	ENSG00000138326
RPS25	-0.1534	6.5234E-03	3.07E-02	ENSG00000118181
RPS25P10	-0.0462	9.2722E-01	9.64E-01	ENSG00000234735
RPS25P3	-0.0173	8.7793E-01		ENSG00000233416
RPS25P7	0.0192	3.0074E-01		ENSG00000218472
RPS26	-0.2557	9.4732E-05	1.07E-03	ENSG00000197728
RPS26P12	-0.0249	5.7920E-01		ENSG00000235869
RPS26P21	-0.0075	9.4765E-01		ENSG00000242829
RPS26P22	0.0227	3.1922E-01		ENSG00000242199
RPS26P23	-0.1061	1.9860E-01	3.76E-01	ENSG00000240775
RPS26P24	0.0077	6.0170E-01		ENSG00000243920
RPS26P31	-0.0246	7.9413E-01	8.89E-01	ENSG00000235459
RPS27	-0.1406	3.2274E-02	1.03E-01	ENSG00000177954
RPS27A	-0.2861	5.9892E-05	7.43E-04	ENSG00000143947
RPS27AP1	-0.0556	5.4118E-01	7.14E-01	ENSG00000242692
RPS27AP11	-0.1301	2.1631E-01	3.97E-01	ENSG00000218208
RPS27AP12	0.4893	1.0347E-02	4.36E-02	ENSG00000225224
RPS27AP16	-0.1736	4.9682E-02	1.42E-01	ENSG00000224631
RPS27AP2	-0.0279	8.1573E-01	9.03E-01	ENSG00000232333

RPS27AP20	-0.0046	7.1850E-01		ENSG00000182888
RPS27AP5	-0.2232	6.1180E-02	1.66E-01	ENSG00000231767
RPS27AP6	0.0100	4.6581E-01	6.54E-01	ENSG00000224800
RPS27AP8	-0.0593	4.8485E-01	6.69E-01	ENSG00000237483
RPS27AP9	0.0095	5.7343E-01		ENSG00000242706
RPS27L	-0.3406	3.2979E-05	4.54E-04	ENSG00000185088
RPS27P30	0.0307	3.0539E-01		ENSG00000270285
RPS28	-0.3127	9.1106E-07	2.47E-05	ENSG00000233927
RPS28P2	-0.0157	8.4871E-01		ENSG00000236317
RPS29	-0.1598	2.3679E-02	8.17E-02	ENSG00000213741
RPS29P14	0.0611	1.2300E-01		ENSG00000229054
RPS29P19	0.0174	2.7798E-01		ENSG00000243353
RPS29P20	-0.0156	5.8816E-01		ENSG00000240385
RPS2P1	-0.0651	3.7867E-01	5.74E-01	ENSG00000225246
RPS2P11	-0.1893	2.8319E-01	4.74E-01	ENSG00000234080
RPS2P14	0.0877	2.6197E-01	4.51E-01	ENSG00000232450
RPS2P15	-0.0270	2.7028E-01		ENSG00000235440
RPS2P17	-0.1264	1.9906E-01	3.76E-01	ENSG00000213399
RPS2P19	-0.0123	8.5567E-01		ENSG00000244249
RPS2P20	0.0435	6.4926E-01	7.97E-01	ENSG00000228981
RPS2P21	-0.0238	9.9187E-01		ENSG00000213851
RPS2P23	-0.0090	9.8649E-01		ENSG00000205644
RPS2P24	-0.0281	8.6513E-01		ENSG00000244019
RPS2P28	-0.0765	5.5837E-01	7.28E-01	ENSG00000179157
RPS2P29	0.1156	2.6731E-01	4.56E-01	ENSG00000219470
RPS2P3	-0.0201	3.5517E-01		ENSG00000242888
RPS2P31	-0.0281	4.8466E-01		ENSG00000213296
RPS2P32	0.0901	3.6801E-01	5.64E-01	ENSG00000232818
RPS2P33	-0.1406	3.0220E-01	4.95E-01	ENSG00000213750
RPS2P34	-0.0083	9.8929E-01		ENSG00000235377
RPS2P35	-0.2185	8.3770E-02	2.07E-01	ENSG00000238172
RPS2P4	-0.1654	1.4056E-01	2.97E-01	ENSG00000196183
RPS2P40	-0.0476	7.1726E-01	8.43E-01	ENSG00000197149
RPS2P42	0.2385	6.0185E-02	1.64E-01	ENSG00000243916
RPS2P46	-0.2192	1.4620E-03	9.63E-03	ENSG00000189343
RPS2P47	-0.0016	9.8789E-01	9.93E-01	ENSG00000239291
RPS2P48	-0.2006	2.8272E-01	4.74E-01	ENSG00000233380
RPS2P49	-0.1083	2.3562E-01	4.20E-01	ENSG00000274308
RPS2P5	-0.2179	4.8135E-03	2.43E-02	ENSG00000240342
RPS2P50	-0.0783	4.0320E-01	5.97E-01	ENSG00000274756
RPS2P51	-0.0902	3.1700E-01	5.11E-01	ENSG00000240418
RPS2P53	0.0415	5.9285E-01	7.55E-01	ENSG00000234750
RPS2P54	-0.0315	8.9802E-01	9.49E-01	ENSG00000215450
RPS2P55	-0.1107	2.0869E-01	3.88E-01	ENSG00000216866
RPS2P6	-0.0139	7.2699E-01		ENSG00000214255
RPS2P7	-0.1032	2.8515E-01	4.76E-01	ENSG00000235508
RPS2P8	0.1812	9.0021E-02	2.19E-01	ENSG00000233406
RPS3	-0.2111	8.3935E-03	3.72E-02	ENSG00000149273
RPS3A	-0.3148	1.3648E-04	1.43E-03	ENSG00000145425

RPS3AP12	-0.0444	4.3278E-01	6.24E-01	ENSG00000236804
RPS3AP14	-0.0443	3.4355E-01		ENSG00000242229
RPS3AP15	-0.0705	3.0839E-01	5.01E-01	ENSG00000244674
RPS3AP16	-0.0515	4.3480E-01	6.26E-01	ENSG00000243894
RPS3AP18	0.0111	5.2561E-01		ENSG00000243417
RPS3AP19	0.0163	5.9018E-01		ENSG00000244004
RPS3AP20	0.0607	5.2747E-01	7.03E-01	ENSG00000213757
RPS3AP21	-0.0570	5.3868E-01	7.12E-01	ENSG00000214784
RPS3AP25	-0.0775	4.3360E-01	6.25E-01	ENSG00000232385
RPS3AP26	-0.0843	3.4060E-01	5.36E-01	ENSG00000214389
RPS3AP28	-0.0035	7.1630E-01		ENSG00000234995
RPS3AP29	-0.1061	6.9175E-02		ENSG00000237818
RPS3AP3	-0.0135	8.3457E-01		ENSG00000231241
RPS3AP30	0.0086	7.0565E-01		ENSG00000228744
RPS3AP31	-0.0031	8.6255E-01		ENSG00000244427
RPS3AP34	-0.1727	1.0464E-01	2.42E-01	ENSG00000242607
RPS3AP35	0.0230	2.8135E-01		ENSG00000244289
RPS3AP36	-0.0043	8.1730E-01		ENSG00000214362
RPS3AP39	-0.0741	2.0258E-01		ENSG00000237226
RPS3AP40	-0.0290	4.8091E-01		ENSG00000243672
RPS3AP41	-0.0081	7.2974E-01		ENSG00000239481
RPS3AP43	-0.0450	3.0156E-01	4.94E-01	ENSG00000242661
RPS3AP44	-0.4053	1.6757E-02	6.30E-02	ENSG00000234031
RPS3AP47	-0.1047	2.9326E-01	4.85E-01	ENSG00000205871
RPS3AP48	-0.0398	4.1715E-01		ENSG00000242278
RPS3AP49	-0.0078	9.2893E-01	9.65E-01	ENSG00000242060
RPS3AP5	0.0567	5.5405E-01	7.25E-01	ENSG00000178429
RPS3AP6	-0.1371	1.1706E-01	2.63E-01	ENSG00000234797
RPS3AP7	-0.2091	2.8573E-02	9.40E-02	ENSG00000229965
RPS3AP8	0.0322	2.9677E-01		ENSG00000233562
RPS3P1	0.0053	3.6155E-01		ENSG00000230935
RPS3P2	-0.0325	4.7980E-01		ENSG00000231307
RPS3P3	-0.2416	5.6148E-02	1.56E-01	ENSG00000228205
RPS3P4	-0.0801	5.4390E-01	7.16E-01	ENSG00000220472
RPS3P6	-0.0419	2.6635E-01		ENSG00000213363
RPS3P7	0.0386	4.5793E-01		ENSG00000243101
RPS4X	-0.3475	5.0775E-05	6.52E-04	ENSG00000198034
RPS4XP1	-0.0269	6.7342E-01		ENSG00000214203
RPS4XP11	0.0077	5.8605E-01		ENSG00000234335
RPS4XP13	0.0056	3.6341E-01		ENSG00000240371
RPS4XP2	0.0225	6.6531E-01	8.08E-01	ENSG00000226948
RPS4XP20	-0.0175	6.7885E-01		ENSG00000249987
RPS4XP22	-0.0326	4.0978E-01		ENSG00000239830
RPS4XP3	-0.0788	2.0674E-01		ENSG00000214541
RPS4XP4	0.0136	2.4528E-01		ENSG00000215520
RPS4XP5	-0.0063	7.2547E-01		ENSG00000229920
RPS4XP6	-0.0825	1.0132E-01		ENSG00000244073
RPS4XP7	0.0022	7.2933E-01		ENSG00000218265
RPS4XP8	0.0019	6.4467E-01		ENSG00000219146

RPS4Y1	-0.2377	2.2918E-03	1.37E-02	ENSG00000129824
RPS4Y1P1	-0.0184	9.6821E-01		ENSG00000213318
RPS5	-0.0296	6.3096E-01	7.84E-01	ENSG00000083845
RPS5P3	0.0159	4.2295E-01		ENSG00000215088
RPS5P4	-0.0102	8.6202E-01	9.30E-01	ENSG00000236905
RPS5P6	-0.0059	6.6810E-01		ENSG00000220105
RPS6	-0.3112	4.9686E-04	4.08E-03	ENSG00000137154
RPS6KA1	0.1909	3.5280E-02	1.10E-01	ENSG00000117676
RPS6KA2	0.4588	1.3046E-05	2.17E-04	ENSG00000071242
RPS6KA3	0.1536	4.1188E-02	1.24E-01	ENSG00000177189
RPS6KA4	0.0290	7.6798E-01	8.74E-01	ENSG00000162302
RPS6KA5	0.2951	9.1843E-04	6.66E-03	ENSG00000100784
RPS6KA6	-0.0635	4.3074E-01	6.22E-01	ENSG00000072133
RPS6KB1	0.3459	6.6407E-06	1.27E-04	ENSG00000108443
RPS6KB2	0.2363	3.6085E-03	1.95E-02	ENSG00000175634
RPS6KC1	0.1696	1.7535E-02	6.52E-02	ENSG00000136643
RPS6KL1	0.3952	1.0148E-05	1.77E-04	ENSG00000198208
RPS6P1	-0.0608	3.9602E-01	5.90E-01	ENSG00000241352
RPS6P10	0.0379	2.0021E-01	3.77E-01	ENSG00000232176
RPS6P16	0.0861	3.8605E-02		ENSG00000243250
RPS6P25	0.0467	6.1917E-01	7.75E-01	ENSG00000240616
RPS6P9	0.0112	4.9904E-01	6.81E-01	ENSG00000239344
RPS7	-0.1991	1.6393E-02	6.21E-02	ENSG00000171863
RPS7P1	-0.0254	7.7148E-01	8.76E-01	ENSG00000263266
RPS7P10	-0.0001	9.9689E-01	9.98E-01	ENSG00000226525
RPS7P11	0.0155	8.6470E-01	9.31E-01	ENSG00000213326
RPS7P14	-0.0051	8.3478E-01		ENSG00000213695
RPS7P3	-0.0742	4.4502E-01	6.35E-01	ENSG00000231940
RPS7P4	0.0232	3.6979E-01		ENSG00000229133
RPS7P8	-0.0034	9.8340E-01		ENSG00000213183
RPS8	-0.3133	3.5243E-04	3.12E-03	ENSG00000142937
RPS8P1	-0.1072	2.8019E-01	4.71E-01	ENSG00000225200
RPS8P10	-0.0552	2.9108E-01		ENSG00000223877
RPS8P4	-0.0285	2.6243E-01		ENSG00000227437
RPS8P8	-0.1031	3.7389E-01	5.69E-01	ENSG00000240376
RPS8P9	0.0069	7.0444E-01		ENSG00000233668
RPS9	-0.2698	4.4110E-04	3.72E-03	ENSG00000170889
RPS9P2	-0.1340	1.7281E-01	3.42E-01	ENSG00000237214
RPS9P4	0.0105	6.3378E-01	7.86E-01	ENSG00000243829
RPSA	-0.2240	4.4348E-03	2.28E-02	ENSG00000168028
RPSA2	-0.2065	1.8455E-02	6.78E-02	ENSG00000288920
RPSAP11	-0.0138	6.9910E-01		ENSG00000237433
RPSAP12	0.0042	9.6302E-01	9.81E-01	ENSG00000240087
RPSAP13	-0.0006	9.1768E-01		ENSG00000233924
RPSAP15	-0.1607	1.0396E-01	2.41E-01	ENSG00000237506
RPSAP16	-0.0377	7.2253E-01		ENSG00000235145
RPSAP17	-0.1132	2.2169E-01	4.03E-01	ENSG00000226855
RPSAP19	-0.0974	3.2911E-01	5.23E-01	ENSG00000183298
RPSAP21	0.0164	1.8275E-01		ENSG00000226498

RPSAP23	-0.0240	5.7083E-01		ENSG00000230391
RPSAP29	-0.0144	9.4452E-01		ENSG00000244722
RPSAP3	0.0067	6.7081E-01		ENSG00000242952
RPSAP31	0.0140	6.9545E-01		ENSG00000234371
RPSAP36	-0.1429	9.8789E-02	2.33E-01	ENSG00000243175
RPSAP4	-0.1565	1.5993E-01	3.25E-01	ENSG00000239269
RPSAP43	0.0138	7.0655E-01	8.36E-01	ENSG00000216471
RPSAP44	0.3296	2.6241E-02	8.82E-02	ENSG00000220773
RPSAP45	-0.0263	9.1738E-01	9.59E-01	ENSG00000219773
RPSAP46	-0.0016	7.7243E-01		ENSG00000213394
RPSAP47	-0.0098	9.0868E-01	9.54E-01	ENSG00000188856
RPSAP49	-0.0126	9.7949E-01		ENSG00000226327
RPSAP5	-0.0015	9.3971E-01		ENSG00000243904
RPSAP51	-0.0410	5.1940E-01		ENSG00000244131
RPSAP52	-0.0778	3.8980E-01	5.84E-01	ENSG00000241749
RPSAP53	0.0014	6.9203E-01		ENSG00000214263
RPSAP54	-0.0967	3.3228E-01	5.27E-01	ENSG00000213621
RPSAP55	0.0079	5.1355E-01		ENSG00000214896
RPSAP56	0.0111	5.2658E-01	7.02E-01	ENSG00000284670
RPSAP57	0.0020	8.0610E-01		ENSG00000267524
RPSAP6	0.0156	4.4263E-01		ENSG00000214629
RPSAP61	-0.0759	4.9098E-01	6.75E-01	ENSG00000214016
RPSAP70	-0.0027	9.7075E-01	9.85E-01	ENSG00000250327
RPSAP71	-0.0054	6.7087E-01		ENSG00000254256
RPSAP74	0.0012	5.6670E-01		ENSG00000253621
RPSAP8	-0.0522	5.8886E-01		ENSG00000230592
RPTOR	0.0349	6.8438E-01	8.21E-01	ENSG00000141564
RPUSD1	0.2690	3.2546E-03	1.79E-02	ENSG00000007376
RPUSD2	0.0473	3.5560E-01	5.51E-01	ENSG00000166133
RPUSD3	-0.0989	8.8980E-02	2.17E-01	ENSG00000156990
RPUSD4	0.2943	6.9893E-04	5.38E-03	ENSG00000165526
RRAGA	-0.1013	1.0611E-01	2.45E-01	ENSG00000155876
RRAGB	-0.1803	3.9064E-03	2.07E-02	ENSG00000083750
RRAGC	-0.2600	1.6155E-02	6.13E-02	ENSG00000116954
RRAGC-DT	-0.0855	3.3335E-01	5.28E-01	ENSG00000228436
RRAGD	-0.2886	2.5026E-05	3.65E-04	ENSG00000025039
RRAS	-0.3864	2.2863E-04	2.18E-03	ENSG00000126458
RRAS2	0.2107	1.2830E-03	8.66E-03	ENSG00000133818
RRBP1	0.0009	9.9150E-01	9.95E-01	ENSG00000125844
RREB1	-0.2816	2.0056E-03	1.23E-02	ENSG00000124782
RRH	-0.0140	8.0406E-01		ENSG00000180245
RRM1	0.0023	9.8789E-01	9.93E-01	ENSG00000167325
RRM2	-0.2040	8.2570E-02	2.05E-01	ENSG00000171848
RRM2B	0.1069	4.8223E-02	1.39E-01	ENSG00000048392
RRM2P2	-0.0141	8.6874E-01		ENSG00000236484
RRN3	0.0290	6.8043E-01	8.19E-01	ENSG00000085721
RRN3P2	-0.0235	6.2025E-01		ENSG00000103472
RRP1	0.1512	5.9171E-02	1.62E-01	ENSG00000160214
RRP12	0.0903	2.4041E-01	4.25E-01	ENSG00000052749

RRP15	0.0754	3.1468E-01	5.08E-01	ENSG00000067533
RRP1B	0.1950	4.9890E-03	2.50E-02	ENSG00000160208
RRP36	0.0885	7.3988E-02	1.90E-01	ENSG00000124541
RRP7A	0.0091	8.5112E-01	9.24E-01	ENSG00000189306
RRP8	0.2140	1.3171E-02	5.24E-02	ENSG00000132275
RRP9	-0.0298	6.3653E-01	7.88E-01	ENSG00000114767
RRS1	0.1367	9.8561E-02	2.32E-01	ENSG00000179041
RRS1-DT	0.0297	7.3796E-01	8.56E-01	ENSG00000246145
RS1	0.0133	2.7230E-01		ENSG00000102104
RSAD1	0.3419	1.0826E-05	1.87E-04	ENSG00000136444
RSAD2	-0.0446	6.0801E-01	7.67E-01	ENSG00000134321
RSBN1	-0.0180	7.5526E-01	8.67E-01	ENSG00000081019
RSBN1L	0.2104	7.8092E-03	3.52E-02	ENSG00000187257
RSC1A1	0.0034	7.0715E-01		ENSG00000215695
RSF1	-0.2518	4.2370E-05	5.61E-04	ENSG00000048649
RSKR	0.2907	2.2206E-02	7.79E-02	ENSG00000167524
RSL1D1	-0.1789	3.3468E-04	2.98E-03	ENSG00000171490
RSL24D1	-0.1106	2.4851E-02	8.48E-02	ENSG00000137876
RSPH1	-0.4409	2.1879E-05	3.29E-04	ENSG00000160188
RSPH1-DT	-0.0437	5.9072E-01	7.54E-01	ENSG00000239930
RSPH10B	0.0615	5.2879E-01	7.04E-01	ENSG00000155026
RSPH10B2	0.2958	2.8550E-02	9.39E-02	ENSG00000169402
RSPH14	0.1466	7.5185E-02	1.92E-01	ENSG00000100218
RSPH3	0.2301	1.3849E-04	1.45E-03	ENSG00000130363
RSPH4A	-0.3302	6.5620E-05	8.01E-04	ENSG00000111834
RSPH6A	-0.0229	5.8809E-01		ENSG00000104941
RSPH9	-0.0057	9.4867E-01	9.74E-01	ENSG00000172426
RSPO1	0.1312	1.8823E-01	3.63E-01	ENSG00000169218
RSPO2	-0.5135	1.7007E-04	1.72E-03	ENSG00000147655
RSPO3	-0.3584	4.3371E-03	2.25E-02	ENSG00000146374
RSPO4	-0.0190	8.1618E-01	9.03E-01	ENSG00000101282
RSPRY1	0.2711	1.1629E-05	1.98E-04	ENSG00000159579
RSRC1	-0.1616	1.2227E-02	4.95E-02	ENSG00000174891
RSRC2	-0.3744	2.3664E-16	1.19E-13	ENSG00000111011
RSRP1	0.3291	3.2129E-05	4.47E-04	ENSG00000117616
RSU1	-0.0257	6.5770E-01	8.02E-01	ENSG00000148484
RSU1P3	-0.0089	9.7574E-01		ENSG00000213659
RTBDN	0.0557	5.4299E-01	7.15E-01	ENSG00000132026
RTCA	0.0428	5.0792E-01	6.88E-01	ENSG00000137996
RTCB	0.1638	1.1637E-02	4.76E-02	ENSG00000100220
RTEL1	0.3050	3.4785E-02	1.09E-01	ENSG00000258366
RTEL1-TNFRSF6B	0.0493	5.8097E-01	7.47E-01	ENSG00000026036
RTEL1P1	0.0095	6.9807E-01		ENSG00000251441
RTF1	-0.0268	6.8133E-01	8.19E-01	ENSG00000137815
RTF2	-0.0206	6.1211E-01	7.70E-01	ENSG00000022277
RTKN	-0.0882	3.3170E-01	5.27E-01	ENSG00000114993
RTKN2	-0.2157	4.4516E-02	1.31E-01	ENSG00000182010
RTL1	0.5880	5.0525E-05	6.50E-04	ENSG00000254656
RTL10	0.1912	2.4043E-02	8.27E-02	ENSG00000215012

RTL4	-0.0207	4.1002E-01		ENSG00000187823
RTL5	-0.0720	3.9062E-01	5.85E-01	ENSG00000242732
RTL6	-0.2799	1.0665E-05	1.85E-04	ENSG00000188636
RTL8A	-0.0635	1.5410E-01	3.16E-01	ENSG00000203950
RTL8B	0.0995	1.0276E-01	2.39E-01	ENSG00000212747
RTL8C	-0.0985	3.6429E-02	1.13E-01	ENSG00000134590
RTL9	-0.2799	3.3883E-02	1.07E-01	ENSG00000243978
RTN1	-0.0905	3.0497E-01	4.98E-01	ENSG00000139970
RTN2	0.0218	7.9413E-01	8.89E-01	ENSG00000125744
RTN3	-0.0249	7.5960E-01	8.69E-01	ENSG00000133318
RTN4	-0.1662	1.0830E-03	7.55E-03	ENSG00000115310
RTN4IP1	0.1497	9.6957E-02	2.30E-01	ENSG00000130347
RTN4R	-0.0115	8.7463E-01	9.37E-01	ENSG00000040608
RTN4RL1	0.2705	1.2142E-02	4.93E-02	ENSG00000185924
RTN4RL2	-0.0947	3.4493E-01	5.40E-01	ENSG00000186907
RTP1	0.1448	1.5806E-01	3.22E-01	ENSG00000175077
RTP2	-0.0058	7.7844E-01		ENSG00000198471
RTP4	0.0045	9.5339E-01	9.76E-01	ENSG00000136514
RTP5	-0.0095	7.6534E-01		ENSG00000188011
RTRAF	-0.0945	5.7370E-02	1.58E-01	ENSG00000087302
RTTN	0.3306	3.0533E-03	1.70E-02	ENSG00000176225
RUBCN	0.1955	2.5322E-02	8.61E-02	ENSG00000145016
RUBCNL	0.1490	1.6963E-01	3.38E-01	ENSG00000102445
RUFY1	0.0046	9.9849E-01	9.99E-01	ENSG00000176783
RUFY1-AS1	-0.0134	8.9440E-01	9.47E-01	ENSG00000244945
RUFY2	0.2471	8.0505E-04	6.01E-03	ENSG00000204130
RUFY3	0.0063	9.9799E-01	9.99E-01	ENSG00000018189
RUFY4	-0.0150	9.5726E-01	9.78E-01	ENSG00000188282
RUNDC1	0.1846	3.5995E-03	1.94E-02	ENSG00000198863
RUNDC3A	0.0981	1.2520E-01	2.75E-01	ENSG00000108309
RUNDC3B	0.1598	1.1679E-01	2.62E-01	ENSG00000105784
RUNX1	-0.3461	7.6849E-03	3.48E-02	ENSG00000159216
RUNX1T1	0.5583	7.3144E-06	1.37E-04	ENSG00000079102
RUNX2	-0.0517	5.8672E-01	7.51E-01	ENSG00000124813
RUNX3	0.2455	2.1096E-02	7.50E-02	ENSG00000020633
RUSC1	-0.0794	1.9506E-01	3.71E-01	ENSG00000160753
RUSC1-AS1	0.0562	5.6099E-01	7.30E-01	ENSG00000225855
RUSC2	0.1857	1.8950E-02	6.91E-02	ENSG00000198853
RUSF1	0.3597	5.1876E-04	4.23E-03	ENSG00000140688
RUVBL1	-0.0611	2.5913E-01	4.47E-01	ENSG00000175792
RUVBL1-AS1	0.0269	3.0382E-01		ENSG00000239608
RUVBL2	0.1364	3.5846E-03	1.94E-02	ENSG00000183207
RWDD1	-0.1669	6.4963E-04	5.08E-03	ENSG00000111832
RWDD2A	0.0937	2.1544E-01	3.96E-01	ENSG00000013392
RWDD2B	-0.0216	8.0056E-01	8.93E-01	ENSG00000156253
RWDD3	-0.3401	8.6015E-04	6.33E-03	ENSG00000122481
RWDD3-DT	-0.2077	8.1486E-02	2.03E-01	ENSG00000226026
RWDD4	0.0350	5.9483E-01	7.57E-01	ENSG00000182552
RXFP3	-0.0324	2.8531E-01		ENSG00000182631

RXFP4	-0.1235	1.3243E-01	2.85E-01	ENSG00000173080
RXRA	-0.1587	7.9423E-02	2.00E-01	ENSG00000186350
RXRB	-0.0677	4.3114E-01	6.23E-01	ENSG00000204231
RXRG	0.4416	4.8536E-03	2.44E-02	ENSG00000143171
RXYLT1	0.0923	1.5241E-01	3.14E-01	ENSG00000118600
RYBP	-0.3605	2.4239E-04	2.29E-03	ENSG00000163602
RYK	-0.1833	2.5309E-03	1.47E-02	ENSG00000163785
RYR1	0.2562	1.9296E-02	7.01E-02	ENSG00000196218
RYR3	0.0509	5.7096E-01	7.39E-01	ENSG00000198838
RYR3-DT	-0.0180	7.2889E-01	8.51E-01	ENSG00000259446
S100A1	-0.0068	8.5741E-01		ENSG00000160678
S100A10	-0.2619	8.8768E-03	3.89E-02	ENSG00000197747
S100A11	-0.3336	5.9191E-05	7.37E-04	ENSG00000163191
S100A13	-0.0344	6.3117E-01		ENSG00000271853
S100A13	-0.2757	5.3159E-05	6.76E-04	ENSG00000189171
S100A14	0.0607	5.2653E-01	7.02E-01	ENSG00000189334
S100A16	-0.3590	1.1904E-04	1.29E-03	ENSG00000188643
S100A2	-0.1853	1.0257E-01	2.39E-01	ENSG00000196754
S100A3	0.0033	6.9873E-01	8.32E-01	ENSG00000188015
S100A5	0.0211	3.1126E-01		ENSG00000196420
S100A6	-0.2079	6.1019E-03	2.92E-02	ENSG00000197956
S100A7	-0.0760	5.8405E-02		ENSG00000143556
S100A8	0.0005	8.1629E-01		ENSG00000143546
S100A9	-0.0191	3.7051E-01	5.66E-01	ENSG00000163220
S100B	-0.5064	1.0522E-05	1.83E-04	ENSG00000160307
S100G	0.0029	6.4639E-01		ENSG00000169906
S100P	0.0903	3.2171E-01	5.16E-01	ENSG00000163993
S100PBP	-0.1556	2.6920E-02	8.99E-02	ENSG00000116497
S100Z	0.0334	6.2282E-01		ENSG00000171643
S1PR1	-0.2108	5.0830E-02	1.45E-01	ENSG00000170989
S1PR2	-0.3085	2.0773E-03	1.26E-02	ENSG00000267534
S1PR3	-0.4541	3.7806E-04	3.30E-03	ENSG00000213694
S1PR4	-0.0574	1.7921E-01		ENSG00000125910
S1PR5	-0.0821	2.7472E-01	4.65E-01	ENSG00000180739
SAA1	0.0119	6.1109E-01		ENSG00000173432
SAA2	0.0237	6.2045E-01	7.76E-01	ENSG00000134339
SAAL1	0.1732	4.5746E-02	1.34E-01	ENSG00000166788
SAC3D1	-0.0733	1.5740E-01	3.21E-01	ENSG00000168061
SACM1L	-0.0254	6.6986E-01	8.10E-01	ENSG00000211456
SACS	-0.0149	8.4249E-01	9.18E-01	ENSG00000151835
SACS-AS1	0.0046	8.2235E-01		ENSG00000229558
SAE1	-0.0332	5.8392E-01	7.49E-01	ENSG00000142230
SAFB	0.0108	8.7266E-01	9.36E-01	ENSG00000160633
SAFB2	-0.0574	3.2311E-01	5.17E-01	ENSG00000130254
SAG	-0.0068	6.8231E-01		ENSG00000130561
SALL1	0.0250	7.9380E-01	8.89E-01	ENSG00000103449
SALL2	-0.4890	1.2485E-08	6.87E-07	ENSG00000165821
SALL4	0.1370	1.8255E-01	3.55E-01	ENSG00000101115
SAMD1	-0.1907	9.1421E-03	3.98E-02	ENSG00000141858

SAMD10	0.3166	3.1460E-03	1.74E-02	ENSG00000130590
SAMD11	-0.1794	3.0960E-02	9.97E-02	ENSG00000187634
SAMD12	-0.3135	4.0198E-03	2.12E-02	ENSG00000177570
SAMD13	-0.3283	2.7529E-03	1.58E-02	ENSG00000203943
SAMD14	0.2299	6.9814E-03	3.23E-02	ENSG00000167100
SAMD15	-0.0783	3.3890E-01	5.34E-01	ENSG00000100583
SAMD3	0.5607	5.2200E-06	1.04E-04	ENSG00000164483
SAMD4A	0.1689	1.1365E-01	2.57E-01	ENSG00000020577
SAMD4B	-0.0961	1.2408E-01	2.73E-01	ENSG00000179134
SAMD5	-0.0921	3.2020E-01	5.14E-01	ENSG00000203727
SAMD8	-0.0911	5.0700E-02	1.44E-01	ENSG00000156671
SAMD9	-0.4317	1.8926E-03	1.17E-02	ENSG00000205413
SAMD9L	-0.4442	4.2032E-03	2.19E-02	ENSG00000177409
SAMHD1	-0.2154	3.7428E-03	2.00E-02	ENSG00000101347
SAMM50	0.0743	1.1910E-01	2.66E-01	ENSG00000100347
SAMMSON	0.0281	2.1671E-02		ENSG00000240405
SANBR	0.1270	6.2300E-02	1.68E-01	ENSG00000162929
SAP130	-0.0268	6.7622E-01	8.15E-01	ENSG00000136715
SAP18	-0.2999	5.8947E-08	2.55E-06	ENSG00000150459
SAP30	-0.2390	4.2736E-03	2.22E-02	ENSG00000164105
SAP30-DT	-0.0303	7.3389E-01	8.54E-01	ENSG00000272870
SAP30BP	-0.2215	2.4720E-05	3.62E-04	ENSG00000161526
SAP30L	0.0304	6.8966E-01	8.26E-01	ENSG00000164576
SAP30L-AS1	0.1175	2.2771E-01	4.10E-01	ENSG00000245275
SAPCD1	0.0127	8.7785E-01	9.38E-01	ENSG00000228727
SAPCD2	0.0785	3.3801E-01	5.33E-01	ENSG00000186193
SAR1A	-0.1805	1.6588E-03	1.06E-02	ENSG00000079332
SAR1B	-0.2075	3.8413E-05	5.16E-04	ENSG00000152700
SARAF	-0.1203	1.3978E-01	2.96E-01	ENSG00000133872
SARDH	-0.0318	7.3833E-01	8.56E-01	ENSG00000123453
SARM1	0.0531	5.3483E-01	7.09E-01	ENSG00000004139
SARNP	0.0779	4.3094E-01	6.23E-01	ENSG00000205323
SARS1	0.0538	4.0859E-01	6.02E-01	ENSG00000031698
SARS2	0.2528	1.5906E-02	6.06E-02	ENSG00000104835
SART1	0.3061	9.0802E-04	6.61E-03	ENSG00000175467
SART3	0.2208	1.0086E-03	7.16E-03	ENSG00000075856
SASH1	-0.0986	1.7142E-01	3.40E-01	ENSG00000111961
SASH3	0.0068	3.8872E-01		ENSG00000122122
SASS6	0.0442	5.6999E-01	7.38E-01	ENSG00000156876
SAT1	0.0841	3.3631E-01	5.31E-01	ENSG00000130066
SAT2	-0.2079	2.7977E-06	6.28E-05	ENSG00000141504
SATB1	-0.1354	1.0687E-01	2.46E-01	ENSG00000182568
SATB2	0.1704	1.0948E-01	2.50E-01	ENSG00000119042
SATB2-AS1	0.0799	3.9463E-01	5.89E-01	ENSG00000225953
SATL1	0.0012	9.8420E-01	9.91E-01	ENSG00000184788
SAV1	-0.1061	1.0709E-01	2.46E-01	ENSG00000151748
SAXO1	0.5831	1.7408E-03	1.10E-02	ENSG00000155875
SAXO2	0.0032	9.8461E-01	9.92E-01	ENSG00000188659
SAXO4	-0.5061	1.8558E-06	4.44E-05	ENSG00000162148

SAXO5	0.1345	1.7236E-01	3.41E-01	ENSG00000198723
SAYSD1	-0.2355	2.5135E-03	1.47E-02	ENSG00000112167
SBDS	-0.2305	4.1894E-04	3.57E-03	ENSG00000126524
SBF1	0.0876	1.9092E-01	3.66E-01	ENSG00000100241
SBF1P1	0.0809	2.9044E-01	4.82E-01	ENSG00000248522
SBF2	-0.0009	9.8807E-01	9.93E-01	ENSG00000133812
SBF2-AS1	-0.0987	2.9078E-01	4.82E-01	ENSG00000246273
SBK1	0.1082	1.1961E-01	2.67E-01	ENSG00000188322
SBK2	0.0069	9.3406E-01	9.67E-01	ENSG00000187550
SBK3	-0.0167	9.0305E-01		ENSG00000231274
SBNO1	0.2040	2.7159E-03	1.56E-02	ENSG00000139697
SBNO1-AS1	-0.1464	1.6022E-01	3.25E-01	ENSG00000256092
SBNO2	0.0605	5.2834E-01	7.04E-01	ENSG00000064932
SBSN	-0.0019	8.0696E-01		ENSG00000189001
SBSPON	-0.2824	1.2212E-02	4.95E-02	ENSG00000164764
SC5D	-0.0716	3.4456E-01	5.40E-01	ENSG00000109929
SCAANT1	0.0191	7.5673E-01	8.68E-01	ENSG00000280620
SCAF1	-0.2938	1.8942E-03	1.17E-02	ENSG00000126461
SCAF11	-0.1725	3.9129E-05	5.24E-04	ENSG00000139218
SCAF4	0.0861	1.8248E-01	3.55E-01	ENSG00000156304
SCAF8	-0.2016	2.7864E-03	1.59E-02	ENSG00000213079
SCAI	0.0288	6.6713E-01	8.09E-01	ENSG00000173611
SCAMP1	-0.0446	5.3453E-01	7.09E-01	ENSG00000085365
SCAMP1-AS1	-0.0782	2.7354E-01	4.64E-01	ENSG00000245556
SCAMP2	-0.0989	1.5410E-01	3.16E-01	ENSG00000140497
SCAMP3	0.0196	7.5130E-01	8.64E-01	ENSG00000116521
SCAMP4	-0.2134	6.4322E-03	3.04E-02	ENSG00000227500
SCAMP5	-0.0361	6.3265E-01	7.86E-01	ENSG00000198794
SCAND1	-0.1869	4.6637E-04	3.88E-03	ENSG00000171222
SCAND3	0.4090	2.8437E-05	4.06E-04	ENSG00000232040
SCAND3P1	-0.0132	6.7548E-01		ENSG00000232795
SCAP	-0.0351	4.4340E-01	6.34E-01	ENSG00000114650
SCAPER	-0.0399	5.0946E-01	6.89E-01	ENSG00000140386
SCARA3	-0.1132	2.4174E-01	4.27E-01	ENSG00000168077
SCARA5	-0.0577	3.1994E-01		ENSG00000168079
SCARB1	-0.0853	2.2423E-01	4.06E-01	ENSG00000073060
SCARB2	-0.1519	6.2397E-04	4.91E-03	ENSG00000138760
SCARF1	0.0041	9.6669E-01	9.83E-01	ENSG00000074660
SCARF2	-0.0154	8.1777E-01	9.04E-01	ENSG00000244486
SCARNA1	-0.0343	8.3687E-01	9.15E-01	ENSG00000252947
SCARNA14	-0.0120	7.4644E-01		ENSG00000252712
SCARNA17	-0.2117	9.5711E-02	2.28E-01	ENSG00000267322
SCARNA18	-0.0417	4.0752E-01		ENSG00000238835
SCARNA21	-0.0356	6.6209E-01		ENSG00000252835
SCARNA23	0.0182	5.2224E-01		ENSG00000251869
SCARNA3	0.0425	4.0795E-01		ENSG00000252906
SCARNA5	-0.0097	8.9772E-01		ENSG00000252010
SCARNA7	-0.0123	6.1165E-01	7.70E-01	ENSG00000238741
SCARNA8	-0.0335	7.6940E-01		ENSG00000251733

SCARNA9	-0.0529	6.5040E-01	7.98E-01	ENSG00000254911
SCAT8	0.2777	3.8270E-02	1.17E-01	ENSG00000236345
SCCPDH	-0.0045	8.8980E-01	9.45E-01	ENSG00000143653
SCD5	-0.4635	2.3545E-05	3.50E-04	ENSG00000145284
SCEL	0.3443	2.4622E-02	8.43E-02	ENSG00000136155
SCFD1	0.0846	6.2530E-02	1.68E-01	ENSG00000092108
SCFD2	0.3010	5.6585E-03	2.76E-02	ENSG00000184178
SCG2	-0.0953	2.3360E-01	4.17E-01	ENSG00000171951
SCG3	-0.1364	5.2427E-02	1.48E-01	ENSG00000104112
SCG5	0.0659	4.9553E-01	6.79E-01	ENSG00000166922
SCG5-AS1	0.0119	6.4473E-01		ENSG00000241818
SCGB1A1	0.0456	5.8974E-01	7.53E-01	ENSG00000149021
SCGB1D2	-0.0960	4.0636E-02	1.23E-01	ENSG00000124935
SCGB2A1	0.0337	7.1338E-01	8.40E-01	ENSG00000124939
SCGB2B2	0.0494	5.8692E-01	7.51E-01	ENSG00000205209
SCGB3A1	-0.2658	3.9590E-02	1.20E-01	ENSG00000161055
SCGB3A2	-0.0219	9.9253E-01		ENSG00000164265
SCHIP1	-0.0597	4.7922E-01	6.65E-01	ENSG00000283154
SCHIP1	-0.2934	4.4604E-03	2.29E-02	ENSG00000151967
SCHLAP1	0.1010	9.5500E-03	4.11E-02	ENSG00000281131
SCIMP	0.0797	2.8788E-01	4.79E-01	ENSG00000161929
SCIN	-0.0294	7.8870E-01	8.87E-01	ENSG00000006747
SCLT1	-0.1517	2.7954E-02	9.24E-02	ENSG00000151466
SCLY	0.2887	2.6764E-02	8.95E-02	ENSG00000132330
SCMH1	-0.0148	7.8117E-01	8.82E-01	ENSG00000010803
SCML1	-0.0222	7.4876E-01	8.63E-01	ENSG00000047634
SCML2	0.1334	1.1361E-01	2.57E-01	ENSG00000102098
SCML4	0.1449	1.7445E-01	3.44E-01	ENSG00000146285
SCN10A	-0.0060	7.2223E-01		ENSG00000185313
SCN11A	0.0843	3.8251E-01	5.77E-01	ENSG00000168356
SCN1A	-0.0122	8.9725E-01	9.49E-01	ENSG00000144285
SCN1A-AS1	0.1067	2.1832E-01	3.99E-01	ENSG00000236107
SCN1B	0.2691	1.8082E-02	6.68E-02	ENSG00000105711
SCN2A	0.4434	3.3308E-04	2.97E-03	ENSG00000136531
SCN2B	-0.4705	5.4466E-03	2.68E-02	ENSG00000149575
SCN3A	0.0768	3.8733E-01	5.82E-01	ENSG00000153253
SCN3B	0.0258	7.0310E-01	8.34E-01	ENSG00000166257
SCN4A	0.0198	5.7422E-01		ENSG00000007314
SCN4B	0.0845	3.9691E-01	5.91E-01	ENSG00000177098
SCN5A	-0.0546	5.7017E-01	7.38E-01	ENSG00000183873
SCN7A	-0.2057	6.4728E-02	1.72E-01	ENSG00000136546
SCN8A	0.2364	1.2217E-02	4.95E-02	ENSG00000196876
SCN9A	0.0794	3.0387E-01	4.96E-01	ENSG00000169432
SCNM1	-0.0752	1.4317E-01	3.01E-01	ENSG00000163156
SCNN1A	0.2018	6.0466E-02	1.64E-01	ENSG00000111319
SCNN1B	-0.2085	8.4872E-02	2.09E-01	ENSG00000168447
SCNN1D	0.0694	4.6413E-01	6.52E-01	ENSG00000162572
SCNN1G	-0.1377	1.2668E-01	2.77E-01	ENSG00000166828
SCO1	0.1350	3.7129E-02	1.14E-01	ENSG00000133028

SCO2	0.1157	2.1454E-01	3.95E-01	ENSG00000284194
SCOC	-0.1093	1.1677E-01	2.62E-01	ENSG00000153130
SCOC-AS1	0.1796	5.5494E-02	1.54E-01	ENSG00000196951
SCP2	-0.1101	4.4509E-02	1.31E-01	ENSG00000116171
SCPEP1	-0.1517	4.2414E-02	1.27E-01	ENSG00000121064
SCRIB	-0.3076	1.6829E-03	1.07E-02	ENSG00000180900
SCRN1	-0.1331	6.8777E-04	5.31E-03	ENSG00000136193
SCRN2	-0.1515	3.1009E-02	9.98E-02	ENSG00000141295
SCRN3	0.0728	2.5750E-01	4.46E-01	ENSG00000144306
SCRT1	0.0591	4.8314E-01	6.68E-01	ENSG00000261678
SCRT2	0.2189	6.6229E-02	1.75E-01	ENSG00000215397
SCT	0.0133	6.7461E-01		ENSG00000070031
SCTR	0.0099	3.4664E-01		ENSG00000080293
SCTR-AS1	0.0190	3.1556E-01		ENSG00000231013
SCUBE1	0.1910	2.2189E-03	1.33E-02	ENSG00000159307
SCUBE1-AS1	0.0975	2.8703E-01	4.78E-01	ENSG00000203527
SCUBE1-AS2	-0.0172	8.1063E-01		ENSG00000236272
SCUBE2	-0.5308	3.3150E-05	4.56E-04	ENSG00000175356
SCUBE3	-0.4021	8.1112E-05	9.45E-04	ENSG00000146197
SCX	0.0305	7.3016E-01	8.51E-01	ENSG00000260428
SCYL1	0.0784	1.1331E-01	2.57E-01	ENSG00000142186
SCYL2	-0.2007	1.4124E-03	9.36E-03	ENSG00000136021
SCYL2P1	-0.4579	1.6622E-02	6.27E-02	ENSG00000213090
SCYL3	0.3523	1.3613E-04	1.43E-03	ENSG00000000457
SDAD1	-0.2140	2.7205E-03	1.56E-02	ENSG00000198301
SDAD1-AS1	0.0308	3.0778E-01		ENSG00000245928
SDAD1P1	0.1899	1.1831E-02	4.83E-02	ENSG00000228451
SDC1	0.1748	2.7243E-02	9.06E-02	ENSG00000115884
SDC2	0.0047	9.3714E-01	9.69E-01	ENSG00000169439
SDC3	-0.1377	1.2231E-01	2.71E-01	ENSG00000162512
SDC4	-0.3908	4.9777E-05	6.43E-04	ENSG00000124145
SDCBP	-0.1787	2.8822E-02	9.46E-02	ENSG00000137575
SDCBP2	0.0462	6.2229E-01	7.78E-01	ENSG00000125775
SDCBP2-AS1	-0.3042	1.1839E-03	8.11E-03	ENSG00000234684
SDCBPP3	-0.0203	5.0975E-01		ENSG00000232801
SDCCAG8	-0.0679	1.8809E-01	3.63E-01	ENSG00000054282
SDE2	-0.1361	1.7678E-02	6.56E-02	ENSG00000143751
SDF2	-0.3602	2.5174E-08	1.24E-06	ENSG00000132581
SDF2L1	-0.2184	4.7223E-03	2.39E-02	ENSG00000128228
SDF4	-0.0393	5.4049E-01	7.14E-01	ENSG00000078808
SDHA	0.0241	7.0510E-01	8.35E-01	ENSG00000073578
SDHAF1	0.2554	1.3672E-04	1.44E-03	ENSG00000205138
SDHAF2	0.0372	5.1876E-01	6.96E-01	ENSG00000167985
SDHAF3	0.1840	7.3293E-03	3.35E-02	ENSG00000196636
SDHAF4	-0.0870	2.1766E-01	3.98E-01	ENSG00000154079
SDHAP2	0.3975	1.4973E-04	1.55E-03	ENSG00000242086
SDHB	-0.0217	7.3625E-01	8.55E-01	ENSG00000117118
SDHC	-0.1867	4.7554E-03	2.41E-02	ENSG00000143252
SDHD	-0.1009	2.4347E-01	4.29E-01	ENSG00000204370

SDK1	0.0470	4.6688E-01	6.55E-01	ENSG00000146555
SDK2	-0.0580	5.1350E-01	6.93E-01	ENSG00000069188
SDR16C5	-0.0557	5.6493E-01	7.34E-01	ENSG00000170786
SDR39U1	0.0762	2.9494E-01	4.87E-01	ENSG00000100445
SDR42E1	0.0974	1.2375E-01	2.73E-01	ENSG00000184860
SDR42E2	0.3660	1.3007E-02	5.20E-02	ENSG00000183921
SDR9C7	0.0408	2.2636E-01		ENSG00000170426
SDS	0.0733	3.8276E-01	5.77E-01	ENSG00000135094
SDSL	-0.4261	2.0865E-04	2.02E-03	ENSG00000139410
SEBOX	-0.0254	2.1408E-01		ENSG00000274529
SEC11A	-0.1253	3.2821E-02	1.04E-01	ENSG00000140612
SEC11C	-0.0883	2.8126E-01	4.72E-01	ENSG00000166562
SEC13	0.0458	4.4833E-01	6.38E-01	ENSG00000157020
SEC14L1	-0.3397	7.7402E-10	6.92E-08	ENSG00000129657
SEC14L1P1	0.0697	4.7358E-01	6.60E-01	ENSG00000213693
SEC14L2	-0.2582	1.4000E-02	5.50E-02	ENSG00000100003
SEC14L3	-0.0278	2.4232E-01		ENSG00000100012
SEC14L4	-0.0243	5.4034E-01		ENSG00000133488
SEC14L5	-0.0236	7.9957E-01	8.93E-01	ENSG00000103184
SEC14L6	-0.2899	2.9842E-02	9.71E-02	ENSG00000214491
SEC16A	-0.0947	1.9939E-01	3.76E-01	ENSG00000148396
SEC16B	-0.0022	9.7798E-01		ENSG00000120341
SEC1P	-0.1025	1.0188E-01	2.38E-01	ENSG00000232871
SEC22A	0.4428	3.8512E-06	8.12E-05	ENSG00000121542
SEC22B	0.0924	1.3972E-01	2.96E-01	ENSG00000265808
SEC22C	0.0404	4.8014E-01	6.66E-01	ENSG00000093183
SEC23A	0.1050	1.7250E-01	3.42E-01	ENSG00000100934
SEC23A-AS1	0.0142	2.9834E-01		ENSG00000258651
SEC23B	0.0009	9.7884E-01	9.89E-01	ENSG00000101310
SEC23IP	0.1264	2.0532E-02	7.34E-02	ENSG00000107651
SEC24A	0.0556	3.8367E-01	5.78E-01	ENSG00000113615
SEC24B	0.1279	3.3437E-02	1.06E-01	ENSG00000138802
SEC24B-AS1	0.0516	5.2661E-01	7.02E-01	ENSG00000247950
SEC24C	0.3476	2.5627E-03	1.49E-02	ENSG00000176986
SEC24D	0.0292	6.7359E-01	8.13E-01	ENSG00000150961
SEC31A	0.0770	1.5928E-01	3.24E-01	ENSG00000138674
SEC31B	0.1689	3.2765E-02	1.04E-01	ENSG00000075826
SEC61A1	0.1005	1.8821E-01	3.63E-01	ENSG00000058262
SEC61A2	0.1058	2.1061E-01	3.90E-01	ENSG00000065665
SEC61B	-0.3248	5.1478E-07	1.53E-05	ENSG00000106803
SEC61G	-0.0691	3.6883E-01	5.65E-01	ENSG00000132432
SEC61G-DT	0.0030	9.6599E-01	9.83E-01	ENSG00000234707
SEC62	-0.2167	6.1241E-04	4.84E-03	ENSG00000008952
SEC63	0.0455	3.7606E-01	5.71E-01	ENSG00000025796
SEC63P1	-0.0793	5.2443E-01	7.01E-01	ENSG00000228057
SECISBP2	0.1920	7.3126E-04	5.57E-03	ENSG00000187742
SECISBP2L	-0.0050	9.3187E-01	9.66E-01	ENSG00000138593
SECTM1	0.0201	7.2367E-01	8.47E-01	ENSG00000141574
SEH1L	-0.0694	3.2924E-01	5.24E-01	ENSG00000085415

SEL1L	0.0172	7.9803E-01	8.92E-01	ENSG00000071537
SEL1L2	-0.0492	5.8174E-01	7.48E-01	ENSG00000101251
SEL1L3	0.2143	1.7300E-02	6.46E-02	ENSG00000091490
SELE	0.0137	4.8790E-01		ENSG00000007908
SELENBP1	-0.3276	3.8279E-03	2.04E-02	ENSG00000143416
SELENOF	-0.3306	1.1122E-05	1.91E-04	ENSG00000183291
SELENOH	-0.2669	4.9789E-06	1.00E-04	ENSG00000211450
SELENOI	0.1956	1.7922E-02	6.64E-02	ENSG00000138018
SELENOK	-0.1068	3.8689E-02	1.18E-01	ENSG00000113811
SELENOM	-0.1712	9.3568E-03	4.05E-02	ENSG00000198832
SELENON	-0.2136	1.0201E-03	7.22E-03	ENSG00000162430
SELENOO	0.0164	8.3074E-01	9.11E-01	ENSG00000073169
SELENOP	-0.1291	8.7125E-02	2.13E-01	ENSG00000250722
SELENOS	-0.0944	7.1030E-02	1.84E-01	ENSG00000131871
SELENOT	-0.2494	2.6181E-04	2.44E-03	ENSG00000198843
SELENOV	0.0302	7.5142E-01	8.64E-01	ENSG00000186838
SELENOW	-0.4126	1.1464E-07	4.35E-06	ENSG00000178980
SELL	0.0611	3.1412E-01	5.07E-01	ENSG00000188404
SELP	0.0428	3.6787E-01		ENSG00000174175
SELPLG	-0.1417	1.6870E-01	3.36E-01	ENSG00000110876
SEM1	-0.1742	2.8908E-03	1.64E-02	ENSG00000127922
SEMA3A	0.0438	6.1669E-01	7.74E-01	ENSG00000075213
SEMA3B	0.2287	6.5797E-03	3.09E-02	ENSG00000012171
SEMA3C	-0.2161	1.7272E-02	6.45E-02	ENSG00000075223
SEMA3D	-0.1082	2.6317E-01	4.52E-01	ENSG00000153993
SEMA3E	-0.1529	9.9577E-02	2.34E-01	ENSG00000170381
SEMA3F	0.1443	7.4609E-02	1.91E-01	ENSG00000001617
SEMA3F-AS1	0.0202	8.3942E-01	9.16E-01	ENSG00000235016
SEMA3G	-0.1322	1.0918E-01	2.50E-01	ENSG00000010319
SEMA4A	-0.1788	5.8831E-02	1.61E-01	ENSG00000196189
SEMA4B	0.0475	4.8511E-01	6.70E-01	ENSG00000185033
SEMA4C	0.0744	4.2993E-01	6.22E-01	ENSG00000168758
SEMA4D	0.2214	3.8873E-03	2.07E-02	ENSG00000187764
SEMA4F	-0.0534	4.0665E-01	6.00E-01	ENSG00000135622
SEMA4G	0.0078	9.2221E-01	9.61E-01	ENSG00000095539
SEMA5A	0.0436	6.4969E-01	7.97E-01	ENSG00000112902
SEMA5B	-0.1176	2.0363E-01	3.81E-01	ENSG00000082684
SEMA6A	-0.1461	6.6949E-02	1.76E-01	ENSG00000092421
SEMA6A-AS1	0.0291	7.3997E-01	8.57E-01	ENSG00000248445
SEMA6A-AS2	-0.0359	6.7603E-01	8.15E-01	ENSG00000249167
SEMA6B	-0.4256	1.0335E-07	4.01E-06	ENSG00000167680
SEMA6C	0.1959	2.3934E-02	8.24E-02	ENSG00000143434
SEMA6D	-0.0600	4.6450E-01	6.53E-01	ENSG00000137872
SEMA7A	0.0192	8.3715E-01	9.15E-01	ENSG00000138623
SEMG2	0.1773	8.9306E-03	3.90E-02	ENSG00000124157
SENP1	0.1059	1.2370E-01	2.73E-01	ENSG00000079387
SENP2	0.2067	5.6670E-04	4.55E-03	ENSG00000163904
SENP3	-0.1792	4.0676E-02	1.23E-01	ENSG00000161956
SENP5	0.1336	2.4751E-02	8.46E-02	ENSG00000119231

SENP6	-0.0111	7.5022E-01	8.63E-01	ENSG00000112701
SENP7	0.2386	4.3263E-03	2.24E-02	ENSG00000138468
SENP8	0.1998	4.8745E-02	1.41E-01	ENSG00000166192
SEPHS1	0.0663	3.3525E-01	5.31E-01	ENSG00000086475
SEPHS1P6	0.0073	4.7053E-01		ENSG00000213938
SEPHS2	0.0554	2.8466E-01	4.76E-01	ENSG00000179918
SEPSECS	0.3555	5.5218E-04	4.46E-03	ENSG00000109618
SEPT5-GP1BB	-0.0014	3.8581E-01		ENSG00000284874
SEPTIN1	0.0581	5.2706E-01	7.03E-01	ENSG00000180096
SEPTIN10	-0.3307	1.9974E-05	3.05E-04	ENSG00000186522
SEPTIN11	-0.2058	4.9324E-07	1.48E-05	ENSG00000138758
SEPTIN12	-0.0138	4.3252E-01	6.24E-01	ENSG00000140623
SEPTIN2	-0.1912	1.6610E-02	6.27E-02	ENSG00000168385
SEPTIN3	-0.1097	2.3848E-01	4.23E-01	ENSG00000100167
SEPTIN4	0.2554	1.3874E-04	1.45E-03	ENSG00000108387
SEPTIN4-AS1	-0.0481	2.1515E-01		ENSG00000264672
SEPTIN5	0.0146	8.7169E-01	9.35E-01	ENSG00000184702
SEPTIN6	0.1398	3.2585E-02	1.04E-01	ENSG00000125354
SEPTIN7	-0.1459	2.0769E-02	7.40E-02	ENSG00000122545
SEPTIN7P1	-0.0153	7.4012E-01		ENSG00000259090
SEPTIN7P10	-0.0038	5.1800E-01		ENSG00000248885
SEPTIN7P15	-0.0125	7.8989E-01		ENSG00000275061
SEPTIN7P3	0.0054	9.3754E-01	9.69E-01	ENSG00000226102
SEPTIN7P4	-0.0087	8.8559E-01		ENSG00000240651
SEPTIN7P5	-0.0087	8.8559E-01		ENSG00000164740
SEPTIN7P6	-0.0564	2.4169E-01		ENSG00000241007
SEPTIN7P7	-0.0557	6.0155E-01	7.62E-01	ENSG00000229897
SEPTIN8	-0.3207	2.7214E-03	1.56E-02	ENSG00000164402
SEPTIN9	-0.1787	2.7227E-02	9.06E-02	ENSG00000184640
SEPTIN9-DT	-0.0158	9.9968E-01	1.00E+00	ENSG00000263718
SERAC1	0.0263	6.8428E-01	8.21E-01	ENSG00000122335
SERBP1	-0.1854	2.2691E-03	1.36E-02	ENSG00000142864
SERF1A	-0.4085	2.1911E-12	4.24E-10	ENSG00000205572
SERF1A	-0.4174	1.4697E-12	2.98E-10	ENSG00000172058
SERF1AP1	0.0021	6.3857E-01		ENSG00000250813
SERGEF	-0.3456	4.5603E-05	5.97E-04	ENSG00000129158
SERHL	0.0311	6.3836E-01	7.90E-01	ENSG00000172250
SERHL2	0.0983	3.0443E-01	4.97E-01	ENSG00000183569
SERINC1	0.0019	9.8214E-01	9.90E-01	ENSG00000111897
SERINC2	0.1345	1.1273E-01	2.56E-01	ENSG00000168528
SERINC3	0.1808	1.1698E-02	4.78E-02	ENSG00000132824
SERINC5	-0.3451	7.4278E-06	1.39E-04	ENSG00000164300
SERP1	-0.1205	2.5868E-02	8.73E-02	ENSG00000120742
SERP2	-0.4510	8.1238E-07	2.26E-05	ENSG00000151778
SERPINA1	-0.3367	2.4081E-02	8.28E-02	ENSG00000197249
SERPINA10	-0.0241	6.8313E-01	8.21E-01	ENSG00000140093
SERPINA11	0.3096	3.2865E-02	1.04E-01	ENSG00000186910
SERPINA3	-0.0383	7.4367E-01	8.59E-01	ENSG00000196136
SERPINB1	0.3141	9.9247E-03	4.22E-02	ENSG00000021355

SERPINB10	-0.0082	6.5947E-01		ENSG00000242550
SERPINB11	-0.0092	7.6154E-01	8.70E-01	ENSG00000206072
SERPINB12	-0.0688	6.4782E-02		ENSG00000166634
SERPINB13	-0.0407	1.1285E-01		ENSG00000197641
SERPINB2	-0.1135	4.0817E-02	1.23E-01	ENSG00000197632
SERPINB3	-0.1030	2.3280E-02	8.08E-02	ENSG00000057149
SERPINB4	-0.0334	5.8325E-01	7.49E-01	ENSG00000206073
SERPINB5	0.0248	4.9002E-01		ENSG00000206075
SERPINB6	-0.2233	2.9350E-02	9.59E-02	ENSG00000124570
SERPINB7	-0.0723	7.7986E-02	1.97E-01	ENSG00000166396
SERPINB8	-0.0285	7.2665E-01	8.49E-01	ENSG00000166401
SERPINB9	-0.0747	3.9975E-01	5.94E-01	ENSG00000170542
SERPINC1	-0.0296	9.3748E-01		ENSG00000117601
SERPIND1	-0.0825	3.9526E-01	5.90E-01	ENSG00000099937
SERPINE1	-0.2748	3.6599E-02	1.13E-01	ENSG00000106366
SERPINE2	0.1541	1.0461E-01	2.42E-01	ENSG00000135919
SERPINE3	0.0100	3.7518E-01		ENSG00000253309
SERPINF1	-0.1792	1.3809E-02	5.44E-02	ENSG00000132386
SERPINF2	0.5435	3.4115E-04	3.04E-03	ENSG00000167711
SERPING1	-0.4035	8.7369E-03	3.85E-02	ENSG00000149131
SERPINI1	-0.1571	7.2151E-02	1.86E-01	ENSG00000163536
SERPINI2	0.0167	6.5486E-01	8.01E-01	ENSG00000114204
SERTAD1	-0.3439	2.1657E-09	1.61E-07	ENSG00000197019
SERTAD2	-0.3489	4.7708E-05	6.21E-04	ENSG00000179833
SERTAD3	0.0289	6.5841E-01	8.03E-01	ENSG00000167565
SERTAD4	0.1455	1.6102E-01	3.26E-01	ENSG00000082497
SERTM1	-0.3543	5.4743E-03	2.69E-02	ENSG00000180440
SERTM2	0.0425	3.6368E-01		ENSG00000260802
SESN1	-0.0323	6.3170E-01	7.85E-01	ENSG00000080546
SESN2	-0.5976	9.9353E-17	5.29E-14	ENSG00000130766
SESN3	-0.1800	3.6872E-02	1.14E-01	ENSG00000149212
SESTD1	0.2357	2.6396E-04	2.45E-03	ENSG00000187231
SET	-0.3124	1.5767E-09	1.24E-07	ENSG00000119335
SETBP1	-0.1475	8.7365E-02	2.14E-01	ENSG00000152217
SETBP1-DT	0.3362	4.8044E-03	2.43E-02	ENSG00000267414
SETD1A	-0.3235	5.0593E-04	4.14E-03	ENSG00000099381
SETD1B	-0.0376	6.6356E-01	8.07E-01	ENSG00000139718
SETD2	-0.2175	5.1390E-05	6.58E-04	ENSG00000181555
SETD3	0.1348	4.6656E-02	1.36E-01	ENSG00000183576
SETD4	0.3264	2.6711E-06	6.06E-05	ENSG00000185917
SETD5	0.1233	4.4697E-02	1.32E-01	ENSG00000168137
SETD6	0.2809	1.4191E-04	1.48E-03	ENSG00000103037
SETD9	-0.3116	1.0569E-04	1.17E-03	ENSG00000155542
SETDB1	-0.0357	5.2431E-01	7.01E-01	ENSG00000143379
SETDB2	0.1200	1.4456E-01	3.03E-01	ENSG00000136169
SETMAR	-0.0813	2.1914E-01	4.00E-01	ENSG00000170364
SETP11	-0.0138	9.5721E-01		ENSG00000239570
SETSIP	-0.0030	8.3129E-01		ENSG00000230667
SETX	-0.0808	2.1899E-01	4.00E-01	ENSG00000107290

SEZ6	0.1118	2.4247E-01	4.28E-01	ENSG00000063015
SEZ6L	-0.3068	4.7093E-06	9.57E-05	ENSG00000100095
SEZ6L2	-0.1106	1.0948E-01	2.50E-01	ENSG00000174938
SF1	-0.0282	4.8650E-01	6.71E-01	ENSG00000168066
SF3A1	-0.0503	4.0485E-01	5.99E-01	ENSG00000099995
SF3A2	0.1603	2.1014E-02	7.47E-02	ENSG00000104897
SF3A3	0.0457	4.9473E-01	6.78E-01	ENSG00000183431
SF3A3P1	-0.0087	7.1623E-01		ENSG00000230568
SF3B1	-0.2319	2.6692E-09	1.93E-07	ENSG00000115524
SF3B2	-0.0069	8.7140E-01	9.35E-01	ENSG00000087365
SF3B3	-0.2449	1.5641E-05	2.52E-04	ENSG00000189091
SF3B4	-0.0137	8.1554E-01	9.03E-01	ENSG00000143368
SF3B5	-0.1577	6.1977E-03	2.96E-02	ENSG00000169976
SF3B6	-0.2492	6.3494E-05	7.81E-04	ENSG00000115128
SFI1	0.2597	1.7520E-03	1.10E-02	ENSG00000198089
SFMBT1	0.1824	1.8360E-02	6.76E-02	ENSG00000163935
SFMBT2	0.3612	5.5283E-04	4.47E-03	ENSG00000198879
SFN	0.1100	5.1222E-02	1.45E-01	ENSG00000175793
SFPQ	0.1336	1.4909E-02	5.78E-02	ENSG00000116560
SFR1	0.0371	5.8454E-01	7.50E-01	ENSG00000156384
SFRP1	0.1691	1.0910E-01	2.50E-01	ENSG00000104332
SFRP2	-0.0749	2.4211E-01	4.27E-01	ENSG00000145423
SFRP4	-0.3014	2.9551E-02	9.64E-02	ENSG00000106483
SFRP5	-0.5980	1.9417E-03	1.20E-02	ENSG00000120057
SFSWAP	-0.1365	5.4076E-02	1.51E-01	ENSG00000061936
SFT2D1	-0.2035	1.2929E-03	8.72E-03	ENSG00000198818
SFT2D2	0.0614	4.6191E-01	6.50E-01	ENSG00000213064
SFTA1P	0.5751	1.0406E-02	4.37E-02	ENSG00000225383
SFTA2	0.1550	1.5872E-02	6.05E-02	ENSG00000196260
SFTPA1	0.0208	3.1156E-01		ENSG00000122852
SFTPA2	0.0265	6.5262E-01	7.99E-01	ENSG00000185303
SFTPB	-0.0135	7.6202E-01		ENSG00000168878
SFTPC	-0.0947	2.7942E-01	4.70E-01	ENSG00000168484
SFTPD	-0.2551	4.5751E-02	1.34E-01	ENSG00000133661
SFXN1	-0.0041	9.1935E-01	9.60E-01	ENSG00000164466
SFXN2	0.0106	9.1149E-01	9.55E-01	ENSG00000156398
SFXN3	0.3207	1.1893E-03	8.13E-03	ENSG00000107819
SFXN4	-0.0713	3.3678E-01	5.32E-01	ENSG00000183605
SFXN5	-0.1445	9.4368E-02	2.26E-01	ENSG00000144040
SGCA	0.0202	7.7632E-01	8.79E-01	ENSG00000108823
SGCB	-0.2865	1.3785E-04	1.45E-03	ENSG00000163069
SGCD	0.0640	5.1071E-01	6.91E-01	ENSG00000170624
SGCE	-0.0435	5.0869E-01	6.89E-01	ENSG00000127990
SGCG	0.0038	5.8904E-01		ENSG00000102683
SGCZ	-0.0116	8.9831E-01	9.49E-01	ENSG00000185053
SGF29	-0.0225	7.6445E-01	8.72E-01	ENSG00000176476
SGIP1	0.2391	9.1789E-03	3.99E-02	ENSG00000118473
SGK1	-0.4225	2.0502E-04	2.00E-03	ENSG00000118515
SGK2	0.0990	2.9514E-01	4.87E-01	ENSG00000101049

SGK3	-0.2996	3.1876E-02	1.02E-01	ENSG00000104205
SGMS1	-0.1233	4.6201E-02	1.35E-01	ENSG00000198964
SGMS2	-0.1855	6.2696E-02	1.69E-01	ENSG00000164023
SGO1	-0.1426	1.7890E-01	3.50E-01	ENSG00000129810
SGO1-AS1	0.1487	1.0112E-01	2.37E-01	ENSG00000231304
SGO1P1	0.0146	5.1066E-01		ENSG00000270615
SGO2	-0.2049	4.5518E-02	1.33E-01	ENSG00000163535
SGPL1	-0.1149	1.1140E-01	2.53E-01	ENSG00000166224
SGPP1	-0.0169	8.2527E-01	9.08E-01	ENSG00000126821
SGPP2	-0.1241	2.1197E-01	3.92E-01	ENSG00000163082
SGSH	0.0482	4.6252E-01	6.51E-01	ENSG00000181523
SGSM1	-0.0402	5.6946E-01	7.37E-01	ENSG00000167037
SGSM2	-0.0028	9.4050E-01	9.70E-01	ENSG00000141258
SGSM3	0.0367	7.0054E-01	8.33E-01	ENSG00000100359
SGTA	0.1492	5.2073E-03	2.58E-02	ENSG00000104969
SGTB	-0.0742	2.6726E-01	4.56E-01	ENSG00000197860
SH2B1	-0.1086	1.8494E-01	3.58E-01	ENSG00000178188
SH2B2	0.1497	8.6642E-02	2.12E-01	ENSG00000160999
SH2B3	0.1222	1.2692E-01	2.77E-01	ENSG00000111252
SH2D1A	-0.0070	9.1863E-01		ENSG00000183918
SH2D2A	0.0591	4.9948E-01	6.82E-01	ENSG00000027869
SH2D3A	0.2192	6.4249E-02	1.71E-01	ENSG00000125731
SH2D3C	-0.0071	9.1763E-01	9.59E-01	ENSG00000095370
SH2D4A	-0.1840	1.0501E-01	2.43E-01	ENSG00000104611
SH2D4B	-0.0827	2.6308E-01	4.52E-01	ENSG00000178217
SH2D5	0.1288	2.2165E-01	4.03E-01	ENSG00000189410
SH2D6	-0.0817	4.0964E-01	6.03E-01	ENSG00000152292
SH2D7	-0.1353	1.6066E-01	3.26E-01	ENSG00000183476
SH3BGR	-0.5314	5.2219E-05	6.66E-04	ENSG00000185437
SH3BGRL	-0.2030	1.5097E-03	9.86E-03	ENSG00000131171
SH3BGRL2	-0.0422	4.5908E-01	6.48E-01	ENSG00000198478
SH3BGRL3	-0.2011	1.0486E-03	7.37E-03	ENSG00000142669
SH3BP1	-0.2141	7.6185E-02	1.94E-01	ENSG00000100092
SH3BP2	0.0060	9.2632E-01	9.64E-01	ENSG00000087266
SH3BP4	0.0587	3.1934E-01	5.13E-01	ENSG00000130147
SH3BP5	0.0915	3.3458E-01	5.30E-01	ENSG00000131370
SH3BP5-AS1	0.0220	8.0066E-01	8.93E-01	ENSG00000224660
SH3BP5L	0.1170	2.0251E-01	3.80E-01	ENSG00000175137
SH3D19	-0.0338	6.6028E-01	8.04E-01	ENSG00000109686
SH3D21	0.2742	8.5867E-03	3.79E-02	ENSG00000214193
SH3GL1	-0.0444	4.8341E-01	6.68E-01	ENSG00000141985
SH3GL2	-0.3287	1.0117E-03	7.17E-03	ENSG00000107295
SH3GL3	0.3560	5.2533E-03	2.60E-02	ENSG00000140600
SH3GLB1	-0.3360	9.4515E-16	4.24E-13	ENSG00000097033
SH3GLB2	0.0657	3.5873E-01	5.55E-01	ENSG00000148341
SH3KBP1	-0.1245	1.1897E-01	2.65E-01	ENSG00000147010
SH3PXD2A	-0.1164	1.3866E-01	2.94E-01	ENSG00000107957
SH3PXD2A-AS1	-0.1904	1.5154E-01	3.13E-01	ENSG00000280693
SH3PXD2B	-0.2240	1.0530E-02	4.41E-02	ENSG00000174705

SH3RF1	-0.2011	5.7074E-03	2.78E-02	ENSG00000154447
SH3RF2	-0.2726	4.3593E-02	1.29E-01	ENSG00000156463
SH3RF3	0.0805	3.8309E-01	5.78E-01	ENSG00000172985
SH3TC1	-0.0165	8.7024E-01	9.34E-01	ENSG00000125089
SH3TC2	-0.0355	7.2034E-01		ENSG00000169247
SH3YL1	-0.1728	1.0086E-02	4.28E-02	ENSG00000035115
SHANK1	-0.1873	5.5657E-02	1.55E-01	ENSG00000161681
SHANK2	-0.1747	2.8706E-02	9.43E-02	ENSG00000162105
SHANK2-AS1	0.0101	7.9800E-01	8.92E-01	ENSG00000226627
SHANK3	0.0139	8.8441E-01	9.42E-01	ENSG00000251322
SHARPIN	-0.0008	9.3172E-01	9.66E-01	ENSG00000179526
SHB	-0.1241	6.7556E-02	1.78E-01	ENSG00000107338
SHBG	0.0306	7.4985E-01	8.63E-01	ENSG00000129214
SHC1	0.1006	1.8227E-01	3.54E-01	ENSG00000160691
SHC2	-0.0628	2.8962E-01	4.81E-01	ENSG00000129946
SHC3	0.1453	1.1898E-01	2.65E-01	ENSG00000148082
SHC4	0.1974	2.3609E-02	8.16E-02	ENSG00000185634
SHCBP1	-0.3393	1.4482E-02	5.65E-02	ENSG00000171241
SHD	0.0466	5.6583E-01	7.34E-01	ENSG00000105251
SHE	-0.0292	7.5351E-01	8.66E-01	ENSG00000169291
SHF	0.2948	5.6718E-05	7.11E-04	ENSG00000138606
SHFL	-0.2062	9.1352E-03	3.98E-02	ENSG00000130813
SHH	-0.4090	9.4090E-03	4.06E-02	ENSG00000164690
SHISA2	-0.3824	3.4691E-03	1.89E-02	ENSG00000180730
SHISA3	-0.0508	2.0256E-01		ENSG00000178343
SHISA4	-0.3695	7.1743E-07	2.02E-05	ENSG00000198892
SHISA5	-0.0788	2.2020E-01	4.01E-01	ENSG00000164054
SHISA6	-0.1519	9.5620E-02	2.28E-01	ENSG00000188803
SHISA7	0.1530	1.4896E-01	3.09E-01	ENSG00000187902
SHISA8	0.1178	2.5649E-01	4.44E-01	ENSG00000234965
SHISA9	-0.0299	7.3283E-01	8.53E-01	ENSG00000237515
SHISAL1	-0.1489	9.6046E-02	2.28E-01	ENSG00000138944
SHISAL2A	0.2873	2.0385E-02	7.31E-02	ENSG00000182183
SHISAL2B	-0.0649	5.8571E-01	7.50E-01	ENSG00000145642
SHKBP1	0.2735	1.1336E-04	1.24E-03	ENSG00000160410
SHLD1	-0.0664	3.7417E-01	5.70E-01	ENSG00000171984
SHLD2	0.0597	3.2881E-01	5.23E-01	ENSG00000122376
SHLD2P1	0.0012	9.9478E-01	9.97E-01	ENSG00000165874
SHLD3	-0.4085	1.1050E-07	4.21E-06	ENSG00000253251
SHMT1	0.0659	4.4239E-01	6.33E-01	ENSG00000176974
SHMT2	0.0039	9.6542E-01	9.82E-01	ENSG00000182199
SHOC1	-0.0334	6.2972E-01	7.84E-01	ENSG00000165181
SHOC2	-0.0669	2.0110E-01	3.78E-01	ENSG00000108061
SHOX	-0.0441	5.5831E-01	7.28E-01	ENSG00000185960
SHOX2	0.0009	9.8410E-01	9.91E-01	ENSG00000168779
SHPK	-0.0515	5.6861E-01	7.37E-01	ENSG00000197417
SHPRH	0.2728	5.5732E-04	4.49E-03	ENSG00000146414
SHQ1	0.1527	3.3872E-02	1.07E-01	ENSG00000144736
SHROOM1	-0.1252	2.1723E-01	3.98E-01	ENSG00000164403

SHROOM2	-0.0780	2.7046E-01	4.60E-01	ENSG00000146950
SHROOM3	-0.2074	3.1915E-03	1.76E-02	ENSG00000138771
SHROOM4	-0.5914	2.9336E-04	2.68E-03	ENSG00000158352
SHTN1	-0.2378	1.0499E-05	1.83E-04	ENSG00000187164
SIAH1	0.0626	3.1413E-01	5.07E-01	ENSG00000196470
SIAH2	0.0894	2.2647E-01	4.09E-01	ENSG00000181788
SIAH3	0.2048	3.0665E-02	9.90E-02	ENSG00000215475
SIDT2	-0.2496	8.7890E-03	3.86E-02	ENSG00000149577
SIGIRR	-0.0126	8.7665E-01	9.37E-01	ENSG00000185187
SIGLEC1	0.4054	1.7214E-02	6.44E-02	ENSG00000088827
SIGLEC10	-0.0445	1.9707E-01		ENSG00000142512
SIGLEC11	-0.0315	3.1304E-01		ENSG00000161640
SIGLEC14	-0.0120	6.5715E-01		ENSG00000254415
SIGLEC15	0.0276	5.5158E-01		ENSG00000197046
SIGLEC16	-0.0276	9.1922E-01		ENSG00000161643
SIGLEC5	-0.0483	1.3401E-01		ENSG00000268500
SIGMAR1	-0.2056	2.4791E-03	1.45E-02	ENSG00000147955
SIK1	-0.3573	6.6438E-04	5.17E-03	ENSG00000142178
SIK2	-0.0610	4.4938E-01	6.39E-01	ENSG00000170145
SIK3	-0.0586	2.9326E-01	4.85E-01	ENSG00000160584
SIKE1	0.0286	6.4596E-01	7.95E-01	ENSG00000052723
SIL1	0.0089	8.7029E-01	9.34E-01	ENSG00000120725
SIM1	0.0154	6.6139E-01		ENSG00000112246
SIM2	-0.2307	1.8876E-02	6.90E-02	ENSG00000159263
SIMC1	0.3133	2.2296E-05	3.34E-04	ENSG00000170085
SIN3A	-0.1413	7.7790E-02	1.97E-01	ENSG00000169375
SIN3B	-0.1381	4.6742E-02	1.36E-01	ENSG00000127511
SINHCAF	-0.0493	5.8249E-01	7.48E-01	ENSG00000139146
SIPA1	0.0819	4.0359E-01	5.98E-01	ENSG00000213445
SIPA1L1	-0.4333	1.1090E-07	4.22E-06	ENSG00000197555
SIPA1L2	-0.2724	3.6517E-05	4.94E-04	ENSG00000116991
SIPA1L3	0.0712	3.2563E-01	5.20E-01	ENSG00000105738
SIRLNT	0.0311	8.7734E-01	9.38E-01	ENSG00000253802
SIRPA	-0.4605	1.2193E-05	2.06E-04	ENSG00000198053
SIRPB1	-0.0137	8.7363E-01		ENSG00000101307
SIRT1	0.0577	4.4011E-01	6.31E-01	ENSG00000096717
SIRT2	-0.0481	2.9021E-01	4.82E-01	ENSG00000068903
SIRT3	-0.0036	9.1728E-01	9.59E-01	ENSG00000142082
SIRT4	0.0372	6.9791E-01	8.31E-01	ENSG00000089163
SIRT5	-0.2889	2.0021E-03	1.23E-02	ENSG00000124523
SIRT6	0.1072	1.9592E-01	3.72E-01	ENSG00000077463
SIRT7	0.0207	7.7597E-01	8.79E-01	ENSG00000187531
SIT1	0.0147	1.2746E-01		ENSG00000137078
SIVA1	-0.1143	4.6695E-02	1.36E-01	ENSG00000184990
SIX1	-0.0327	7.2719E-01	8.50E-01	ENSG00000126778
SIX2	-0.2968	2.4912E-02	8.49E-02	ENSG00000170577
SIX3	0.1807	1.8191E-02	6.71E-02	ENSG00000138083
SIX3-AS1	0.0368	6.9795E-01	8.31E-01	ENSG00000236502
SIX4	-0.1870	4.7746E-02	1.38E-01	ENSG00000100625

SIX5	-0.0645	4.9311E-01	6.77E-01	ENSG00000177045
SIX6	-0.0262	4.6395E-01		ENSG00000184302
SKA1	-0.5224	9.7524E-04	6.98E-03	ENSG00000154839
SKA2	-0.2506	9.4814E-04	6.84E-03	ENSG00000182628
SKAP1-AS2	0.0348	5.0689E-02		ENSG00000235300
SKAP2	-0.1231	7.3368E-02	1.89E-01	ENSG00000005020
SKI	-0.1001	3.1599E-01	5.09E-01	ENSG00000157933
SKIC2	0.0004	9.9973E-01	1.00E+00	ENSG00000204351
SKIC3	-0.0658	1.9367E-01	3.69E-01	ENSG00000198677
SKIC8	0.1478	3.3646E-02	1.06E-01	ENSG00000140395
SKIDA1	0.1219	1.8186E-01	3.54E-01	ENSG00000180592
SKIL	-0.2813	1.9342E-04	1.91E-03	ENSG00000136603
SKOR1	-0.0696	3.8871E-01	5.83E-01	ENSG00000188779
SKOR1-AS1	0.0069	7.9011E-01		ENSG00000245719
SKOR2	-0.0097	9.5546E-01		ENSG00000215474
SKP1	-0.1443	9.4335E-03	4.07E-02	ENSG00000113558
SKP2	0.1072	1.3670E-01	2.91E-01	ENSG00000145604
SLA	0.0390	1.5201E-01		ENSG00000155926
SLA2	-0.0393	9.0285E-01		ENSG00000101082
SLAIN1	0.0107	8.9755E-01	9.49E-01	ENSG00000139737
SLAIN2	-0.0292	5.1994E-01	6.97E-01	ENSG00000109171
SLAMF8	0.0219	2.6588E-01		ENSG00000158714
SLAMF9	0.3502	2.1440E-02	7.59E-02	ENSG00000162723
SLBP	-0.0100	8.0616E-01	8.97E-01	ENSG00000163950
SLC10A1	-0.0276	2.4876E-01		ENSG00000100652
SLC10A3	-0.0911	1.2792E-01	2.79E-01	ENSG00000126903
SLC10A4	-0.3669	1.2452E-02	5.02E-02	ENSG00000145248
SLC10A5	-0.0932	2.3054E-01	4.14E-01	ENSG00000253598
SLC10A6	0.0057	5.1891E-01		ENSG00000145283
SLC10A7	-0.0128	8.7686E-01	9.37E-01	ENSG00000120519
SLC11A1	0.4040	7.2458E-03	3.32E-02	ENSG00000018280
SLC11A2	0.1686	6.3859E-03	3.03E-02	ENSG00000110911
SLC12A1	0.3683	2.3194E-02	8.05E-02	ENSG00000074803
SLC12A2	-0.3799	5.0181E-07	1.49E-05	ENSG00000064651
SLC12A2-DT	-0.1442	7.3160E-03	3.35E-02	ENSG00000245937
SLC12A5	-0.1225	1.1254E-01	2.55E-01	ENSG00000124140
SLC12A5-AS1	0.0716	4.4349E-01	6.34E-01	ENSG00000204044
SLC12A6	-0.1646	3.4588E-02	1.09E-01	ENSG00000140199
SLC12A7	-0.1327	1.4343E-01	3.01E-01	ENSG00000113504
SLC12A8	-0.1352	1.8674E-01	3.61E-01	ENSG00000221955
SLC12A9	-0.0063	9.3808E-01	9.69E-01	ENSG00000146828
SLC12A9-AS1	-0.0181	9.5807E-01		ENSG00000236305
SLC13A2	0.0247	4.0762E-01		ENSG00000007216
SLC13A3	0.4763	6.8664E-06	1.30E-04	ENSG00000158296
SLC13A4	0.1764	4.0194E-02	1.22E-01	ENSG00000164707
SLC13A5	0.0394	6.8455E-01	8.21E-01	ENSG00000141485
SLC14A1	-0.0409	1.6994E-01		ENSG00000141469
SLC14A2	-0.0469	5.6167E-01		ENSG00000132874
SLC15A1	-0.0019	6.8433E-01		ENSG00000088386

SLC15A3	-0.0160	8.4938E-01	9.23E-01	ENSG00000110446
SLC15A4	-0.0876	3.6254E-01	5.58E-01	ENSG00000139370
SLC15A5	-0.0061	6.3270E-01		ENSG00000188991
SLC16A1	-0.2925	5.5985E-03	2.74E-02	ENSG00000155380
SLC16A1-AS1	0.3287	4.4310E-03	2.28E-02	ENSG00000226419
SLC16A10	-0.5643	9.5572E-05	1.08E-03	ENSG00000112394
SLC16A11	0.1083	2.8637E-01	4.77E-01	ENSG00000174326
SLC16A12	-0.0767	2.8292E-01	4.74E-01	ENSG00000152779
SLC16A13	0.0834	3.9087E-01	5.85E-01	ENSG00000174327
SLC16A14	-0.1151	2.1063E-01	3.90E-01	ENSG00000163053
SLC16A2	-0.0085	9.0623E-01	9.53E-01	ENSG00000147100
SLC16A3	0.5539	5.9713E-03	2.87E-02	ENSG00000141526
SLC16A5	0.2420	5.7707E-02	1.59E-01	ENSG00000170190
SLC16A6	-0.0281	7.3421E-01	8.54E-01	ENSG00000108932
SLC16A7	0.0391	6.7543E-01	8.14E-01	ENSG00000118596
SLC16A8	0.4850	1.0644E-02	4.45E-02	ENSG00000100156
SLC16A9	-0.2277	1.5873E-02	6.05E-02	ENSG00000165449
SLC17A3	0.0010	7.5556E-01		ENSG00000124564
SLC17A4	0.0190	1.6355E-01		ENSG00000146039
SLC17A5	0.0792	2.3237E-01	4.16E-01	ENSG00000119899
SLC17A7	0.1915	8.5107E-02	2.10E-01	ENSG00000104888
SLC18A1	0.0216	8.2453E-01	9.08E-01	ENSG00000036565
SLC18A2	-0.2382	4.8519E-02	1.40E-01	ENSG00000165646
SLC18A2-AS1	-0.0036	7.9638E-01		ENSG00000225936
SLC18B1	-0.2699	1.5355E-03	1.00E-02	ENSG00000146409
SLC19A1	-0.0806	2.1118E-01	3.91E-01	ENSG00000173638
SLC19A2	-0.0148	8.6411E-01	9.31E-01	ENSG00000117479
SLC19A3	-0.0479	5.6785E-01	7.36E-01	ENSG00000135917
SLC1A1	0.1435	1.2916E-01	2.80E-01	ENSG00000106688
SLC1A2	0.0645	5.0578E-01	6.87E-01	ENSG00000110436
SLC1A3-AS1	0.0062	6.0772E-01		ENSG00000250155
SLC1A4	-0.2168	6.3287E-03	3.00E-02	ENSG00000115902
SLC1A5	0.2575	1.0827E-02	4.51E-02	ENSG00000105281
SLC1A6	0.1574	1.4945E-01	3.10E-01	ENSG00000105143
SLC20A1	-0.0188	7.8357E-01	8.83E-01	ENSG00000144136
SLC20A2	-0.1255	7.4053E-02	1.90E-01	ENSG00000168575
SLC22A1	-0.0424	5.9786E-01	7.59E-01	ENSG00000175003
SLC22A11	0.0350	3.0776E-01	5.00E-01	ENSG00000168065
SLC22A12	0.0021	4.1797E-01	6.11E-01	ENSG00000197891
SLC22A13	0.0067	6.0650E-01		ENSG00000172940
SLC22A14	0.0108	2.0781E-01		ENSG00000144671
SLC22A15	-0.0618	4.8397E-01	6.69E-01	ENSG00000163393
SLC22A16	-0.0021	9.6232E-01		ENSG00000004809
SLC22A17	-0.0013	9.8512E-01	9.92E-01	ENSG00000092096
SLC22A18	0.0835	3.3438E-01	5.30E-01	ENSG00000110628
SLC22A18AS	-0.0425	6.4457E-01	7.94E-01	ENSG00000254827
SLC22A2	-0.0850	2.4958E-01		ENSG00000112499
SLC22A20P	0.1443	1.6147E-01	3.27E-01	ENSG00000197847
SLC22A23	0.0692	3.3880E-01	5.34E-01	ENSG00000137266

SLC22A4	0.3733	6.6426E-03	3.11E-02	ENSG00000197208
SLC22A6	-0.1023	6.0423E-02	1.64E-01	ENSG00000197901
SLC22A7	-0.0604	3.4461E-01	5.40E-01	ENSG00000137204
SLC22A8	-0.0235	7.9135E-01	8.88E-01	ENSG00000149452
SLC23A1	-0.0574	5.5856E-01	7.29E-01	ENSG00000170482
SLC23A2	0.0468	4.6701E-01	6.55E-01	ENSG00000089057
SLC23A3	0.0050	4.3854E-01		ENSG00000213901
SLC24A1	-0.1263	1.4991E-01	3.10E-01	ENSG00000074621
SLC24A2	0.1529	1.0228E-01	2.38E-01	ENSG00000155886
SLC24A3-AS1	0.0450	3.8962E-01	5.84E-01	ENSG00000179447
SLC24A4	-0.0302	6.5673E-01	8.02E-01	ENSG00000140090
SLC25A1	-0.1476	2.9270E-02	9.57E-02	ENSG00000100075
SLC25A10	-0.0415	5.4348E-01	7.16E-01	ENSG00000183048
SLC25A11	-0.0450	5.0693E-01	6.87E-01	ENSG00000108528
SLC25A12	0.0111	8.7568E-01	9.37E-01	ENSG00000115840
SLC25A13	-0.0275	7.1981E-01	8.45E-01	ENSG00000004864
SLC25A14	0.1434	8.7853E-02	2.15E-01	ENSG00000102078
SLC25A15	0.0353	6.8417E-01	8.21E-01	ENSG00000102743
SLC25A16	0.0623	3.8879E-01	5.83E-01	ENSG00000122912
SLC25A17	-0.0734	2.3715E-01	4.21E-01	ENSG00000100372
SLC25A18	-0.2060	8.3837E-02	2.07E-01	ENSG00000182902
SLC25A19	-0.0294	7.5781E-01	8.68E-01	ENSG00000125454
SLC25A20	0.1832	8.8836E-03	3.89E-02	ENSG00000178537
SLC25A21	-0.1087	2.9342E-01	4.85E-01	ENSG00000183032
SLC25A21-AS1	-0.0160	8.3582E-01	9.14E-01	ENSG00000258708
SLC25A22	0.0958	2.0921E-01	3.89E-01	ENSG00000177542
SLC25A23	-0.0914	1.2058E-01	2.68E-01	ENSG00000125648
SLC25A24	0.0196	7.5280E-01	8.65E-01	ENSG00000085491
SLC25A25	-0.3352	6.2649E-04	4.93E-03	ENSG00000148339
SLC25A26	-0.0683	2.4638E-01	4.32E-01	ENSG00000144741
SLC25A27	0.4665	6.7941E-05	8.21E-04	ENSG00000153291
SLC25A28	0.2125	6.7605E-03	3.15E-02	ENSG00000155287
SLC25A29	0.0317	6.6391E-01	8.07E-01	ENSG00000197119
SLC25A3	-0.0803	1.2987E-01	2.81E-01	ENSG00000075415
SLC25A30	0.0233	8.0577E-01	8.96E-01	ENSG00000174032
SLC25A30-AS1	0.0217	7.3743E-01	8.56E-01	ENSG00000251015
SLC25A32	0.0370	6.4017E-01	7.91E-01	ENSG00000164933
SLC25A33	-0.4103	3.6962E-12	6.60E-10	ENSG00000171612
SLC25A34	0.0810	3.9943E-01	5.94E-01	ENSG00000162461
SLC25A35	-0.4265	1.2499E-04	1.34E-03	ENSG00000125434
SLC25A36	-0.0283	5.4006E-01	7.14E-01	ENSG00000114120
SLC25A36P1	-0.0091	9.1402E-01		ENSG00000267781
SLC25A37	-0.0920	2.0435E-01	3.82E-01	ENSG00000147454
SLC25A38	-0.1582	2.1690E-03	1.31E-02	ENSG00000144659
SLC25A39	-0.1860	1.0110E-02	4.28E-02	ENSG00000013306
SLC25A3P1	-0.1530	1.2306E-01	2.72E-01	ENSG00000236253
SLC25A4	-0.0913	1.4585E-01	3.05E-01	ENSG00000151729
SLC25A40	0.1333	1.1091E-01	2.53E-01	ENSG00000075303
SLC25A41	-0.0889	7.6135E-01	8.70E-01	ENSG00000181240

SLC25A42	0.2995	2.0725E-04	2.01E-03	ENSG00000181035
SLC25A43	-0.3273	2.2899E-03	1.37E-02	ENSG00000077713
SLC25A44	0.2605	2.9454E-03	1.66E-02	ENSG00000160785
SLC25A45	0.3709	9.6335E-04	6.92E-03	ENSG00000162241
SLC25A46	0.0839	2.2506E-01	4.07E-01	ENSG00000164209
SLC25A47	0.0315	2.5708E-01		ENSG00000140107
SLC25A48	-0.4360	1.2713E-02	5.11E-02	ENSG00000145832
SLC25A5	-0.0510	5.5043E-01	7.22E-01	ENSG00000005022
SLC25A5-AS1	0.4779	6.5015E-06	1.25E-04	ENSG00000224281
SLC25A51	-0.1453	8.9060E-02	2.17E-01	ENSG00000122696
SLC25A53	-0.5774	1.6684E-09	1.30E-07	ENSG00000269743
SLC25A5P5	-0.0149	7.1000E-01		ENSG00000226421
SLC25A6	-0.0070	9.1660E-01	9.58E-01	ENSG00000169100
SLC26A1	0.1937	9.8733E-02	2.33E-01	ENSG00000145217
SLC26A10P	-0.0170	7.7469E-01		ENSG00000135502
SLC26A11	0.0605	3.1748E-01	5.11E-01	ENSG00000181045
SLC26A2	0.1215	8.8114E-02	2.15E-01	ENSG00000155850
SLC26A3	-0.0180	6.9388E-01		ENSG00000091138
SLC26A4	0.2233	6.2915E-02	1.69E-01	ENSG00000091137
SLC26A5	-0.0867	4.2688E-01	6.19E-01	ENSG00000170615
SLC26A5-AS1	-0.0403	6.7291E-01	8.13E-01	ENSG00000234715
SLC26A6	-0.0127	8.7405E-01	9.36E-01	ENSG00000225697
SLC26A7	-0.4870	4.3551E-04	3.69E-03	ENSG00000147606
SLC26A8	-0.0102	8.7437E-01	9.37E-01	ENSG00000112053
SLC26A9	0.0082	9.2119E-01	9.61E-01	ENSG00000174502
SLC27A1	-0.2153	9.3935E-03	4.06E-02	ENSG00000130304
SLC27A2	-0.1576	1.3800E-01	2.93E-01	ENSG00000140284
SLC27A3	-0.0616	3.9694E-01	5.91E-01	ENSG00000143554
SLC27A4	-0.1625	4.0379E-02	1.22E-01	ENSG00000167114
SLC27A5	-0.1496	3.2582E-02	1.04E-01	ENSG00000083807
SLC28A1	0.0090	4.2758E-01	6.20E-01	ENSG00000156222
SLC28A2	0.0765	3.1860E-01	5.12E-01	ENSG00000137860
SLC28A2-AS1	0.0203	8.3127E-01	9.12E-01	ENSG00000259520
SLC28A3	0.2459	5.2933E-02	1.49E-01	ENSG00000197506
SLC28A3-AS1	0.0427	2.7528E-01		ENSG00000233262
SLC29A1	0.2667	4.8739E-03	2.45E-02	ENSG00000112759
SLC29A2	-0.3402	3.7019E-03	1.99E-02	ENSG00000174669
SLC29A3	-0.0732	4.3507E-01	6.26E-01	ENSG00000198246
SLC29A4	0.2352	3.5944E-04	3.18E-03	ENSG00000164638
SLC2A1	0.2389	2.8006E-02	9.25E-02	ENSG00000117394
SLC2A1-DT	0.2206	5.4427E-02	1.52E-01	ENSG00000227533
SLC2A10	-0.2270	4.3621E-02	1.29E-01	ENSG00000197496
SLC2A11	-0.0645	3.9620E-01	5.90E-01	ENSG00000133460
SLC2A12	-0.0849	3.6294E-01	5.59E-01	ENSG00000146411
SLC2A13	-0.0993	1.6660E-01	3.34E-01	ENSG00000151229
SLC2A3	0.5436	5.6959E-09	3.64E-07	ENSG00000059804
SLC2A4	-0.2734	2.7392E-02	9.10E-02	ENSG00000181856
SLC2A4RG	-0.0391	6.6533E-01	8.08E-01	ENSG00000125520
SLC2A6	-0.1212	9.4594E-02	2.26E-01	ENSG00000160326

SLC2A7	-0.0016	8.2326E-01		ENSG00000197241
SLC2A8	0.2034	1.7153E-02	6.42E-02	ENSG00000136856
SLC2A9	-0.1126	2.0997E-01	3.89E-01	ENSG00000109667
SLC30A1	-0.0168	7.6664E-01	8.73E-01	ENSG00000170385
SLC30A10	-0.2268	4.9530E-02	1.42E-01	ENSG00000196660
SLC30A2	-0.0544	5.5798E-01	7.28E-01	ENSG00000158014
SLC30A3	-0.1139	2.5703E-01	4.45E-01	ENSG00000115194
SLC30A4	0.1073	2.2502E-01	4.07E-01	ENSG00000104154
SLC30A5	-0.0461	4.4557E-01	6.36E-01	ENSG00000145740
SLC30A6	0.2012	6.4978E-03	3.06E-02	ENSG00000152683
SLC30A7	0.0676	3.6345E-01	5.59E-01	ENSG00000162695
SLC30A8	0.1369	1.9487E-01	3.71E-01	ENSG00000164756
SLC30A9	0.0591	4.5532E-01	6.45E-01	ENSG00000014824
SLC31A1	-0.0546	2.8034E-01	4.71E-01	ENSG00000136868
SLC31A2	0.1407	1.8472E-01	3.58E-01	ENSG00000136867
SLC32A1	0.0474	4.9115E-01	6.75E-01	ENSG00000101438
SLC33A1	0.1246	1.4952E-01	3.10E-01	ENSG00000169359
SLC34A1	0.0164	4.8350E-01		ENSG00000131183
SLC34A2	-0.1215	1.5437E-01	3.17E-01	ENSG00000157765
SLC34A3	0.0877	2.7011E-01	4.60E-01	ENSG00000198569
SLC35A1	-0.2902	2.2286E-04	2.14E-03	ENSG00000164414
SLC35A2	0.4396	8.4054E-08	3.38E-06	ENSG00000102100
SLC35A3	-0.1026	1.1589E-01	2.61E-01	ENSG00000117620
SLC35A4	0.1327	7.6381E-02	1.94E-01	ENSG00000176087
SLC35A5	-0.0087	8.5906E-01	9.28E-01	ENSG00000138459
SLC35B1	0.0003	9.4418E-01	9.72E-01	ENSG00000121073
SLC35B2	-0.0300	6.3699E-01	7.89E-01	ENSG00000157593
SLC35B3	0.1301	7.2115E-02	1.86E-01	ENSG00000124786
SLC35B4	0.1315	1.2348E-01	2.72E-01	ENSG00000205060
SLC35C1	-0.1933	4.6386E-03	2.36E-02	ENSG00000181830
SLC35C2	0.0196	7.3989E-01	8.57E-01	ENSG00000080189
SLC35D1	-0.1117	1.8147E-01	3.53E-01	ENSG00000116704
SLC35D2	0.0255	2.7388E-01		ENSG00000285269
SLC35D2	-0.0336	6.5449E-01	8.00E-01	ENSG00000130958
SLC35D3	0.1686	1.2444E-01	2.74E-01	ENSG00000182747
SLC35E1	0.0656	3.6198E-01	5.58E-01	ENSG00000127526
SLC35E2B	-0.0668	3.0883E-01	5.02E-01	ENSG00000189339
SLC35E3	0.2051	2.7641E-04	2.54E-03	ENSG00000175782
SLC35E4	0.0068	9.6075E-01	9.80E-01	ENSG00000100036
SLC35F1	-0.3460	3.7609E-04	3.29E-03	ENSG00000196376
SLC35F2	-0.3350	5.9981E-03	2.88E-02	ENSG00000110660
SLC35F3	0.0840	3.4172E-01	5.37E-01	ENSG00000183780
SLC35F4	-0.1200	2.4972E-01	4.36E-01	ENSG00000151812
SLC35F5	-0.2222	4.3473E-04	3.68E-03	ENSG00000115084
SLC35F6	-0.1685	1.4409E-02	5.63E-02	ENSG00000213699
SLC35G1	0.0361	6.3404E-01	7.87E-01	ENSG00000176273
SLC35G2	-0.0881	1.4709E-01	3.06E-01	ENSG00000168917
SLC35G5	0.0086	4.3540E-01		ENSG00000177710
SLC35G6	0.0675	6.6920E-02	1.76E-01	ENSG00000259224

SLC36A1	0.1501	1.0592E-01	2.45E-01	ENSG00000123643
SLC36A2	0.0052	8.4371E-01		ENSG00000186335
SLC36A3	0.0562	1.6639E-02		ENSG00000186334
SLC36A4	0.3504	8.9048E-05	1.02E-03	ENSG00000180773
SLC37A1	-0.0023	9.7338E-01	9.86E-01	ENSG00000160190
SLC37A2	0.2237	5.3098E-02	1.49E-01	ENSG00000134955
SLC37A3	0.1488	3.5099E-02	1.10E-01	ENSG00000157800
SLC37A4	0.1150	9.0973E-02	2.20E-01	ENSG00000137700
SLC38A1	0.1777	4.6821E-02	1.36E-01	ENSG00000111371
SLC38A10	-0.1300	3.9996E-02	1.21E-01	ENSG00000157637
SLC38A11	-0.0953	8.1711E-02	2.04E-01	ENSG00000169507
SLC38A2	0.1781	5.6678E-02	1.57E-01	ENSG00000134294
SLC38A3	0.0786	3.1395E-01	5.07E-01	ENSG00000188338
SLC38A4	0.0569	3.1082E-01		ENSG00000139209
SLC38A6	-0.3790	1.6236E-05	2.60E-04	ENSG00000139974
SLC38A7	0.0421	5.5902E-01	7.29E-01	ENSG00000103042
SLC38A8	-0.0992	2.0091E-01	3.78E-01	ENSG00000166558
SLC38A9	0.0765	3.7586E-01	5.71E-01	ENSG00000177058
SLC39A1	0.0032	9.7422E-01	9.87E-01	ENSG00000143570
SLC39A10	-0.1067	8.5174E-02	2.10E-01	ENSG00000196950
SLC39A11	-0.0861	3.1483E-01	5.08E-01	ENSG00000133195
SLC39A13	0.2614	8.9101E-05	1.02E-03	ENSG00000165915
SLC39A13-AS1	0.1191	1.6204E-01	3.27E-01	ENSG00000255197
SLC39A14	-0.1451	5.8198E-02	1.60E-01	ENSG00000104635
SLC39A2	-0.1321	1.9518E-01	3.71E-01	ENSG00000165794
SLC39A3	-0.0091	8.3462E-01	9.14E-01	ENSG00000141873
SLC39A4	0.2991	1.1801E-02	4.82E-02	ENSG00000147804
SLC39A5	0.1157	2.0925E-01	3.89E-01	ENSG00000139540
SLC39A6	0.0206	7.6824E-01	8.74E-01	ENSG00000141424
SLC39A7	0.1570	9.5881E-02	2.28E-01	ENSG00000112473
SLC39A8	0.1435	2.2637E-02	7.91E-02	ENSG00000138821
SLC39A9	0.0426	4.2349E-01	6.16E-01	ENSG00000029364
SLC3A1	0.0048	9.1789E-01	9.59E-01	ENSG00000138079
SLC3A2	-0.2929	8.0496E-06	1.48E-04	ENSG00000168003
SLC40A1	-0.1749	1.0140E-01	2.37E-01	ENSG00000138449
SLC41A1	-0.3392	1.9956E-05	3.05E-04	ENSG00000133065
SLC41A2	-0.0638	4.4696E-01	6.37E-01	ENSG00000136052
SLC41A3	-0.2158	2.1223E-04	2.04E-03	ENSG00000114544
SLC43A1	0.0616	5.3557E-01	7.09E-01	ENSG00000149150
SLC43A2	-0.3008	9.5883E-06	1.70E-04	ENSG00000167703
SLC43A3	0.1503	1.5601E-01	3.19E-01	ENSG00000134802
SLC44A1	-0.1887	5.9627E-04	4.74E-03	ENSG00000070214
SLC44A2	-0.2994	3.0675E-05	4.31E-04	ENSG00000129353
SLC44A3	0.0495	5.3209E-01	7.06E-01	ENSG00000143036
SLC44A3-AS1	-0.2142	1.2335E-02	4.99E-02	ENSG00000224081
SLC44A4	0.2285	2.5026E-02	8.52E-02	ENSG00000204385
SLC44A5	0.2427	2.3296E-03	1.38E-02	ENSG00000137968
SLC45A1	-0.0721	2.4881E-01	4.35E-01	ENSG00000162426
SLC45A2	0.0702	3.8234E-01	5.77E-01	ENSG00000164175

SLC45A3	-0.1046	3.0215E-01	4.95E-01	ENSG00000158715
SLC45A4	0.0207	7.9557E-01	8.90E-01	ENSG00000022567
SLC46A1	-0.1848	3.5663E-02	1.11E-01	ENSG00000076351
SLC46A2	-0.0156	6.8954E-01		ENSG00000119457
SLC46A3	0.3701	1.6051E-04	1.64E-03	ENSG00000139508
SLC47A1	-0.3384	2.3434E-02	8.12E-02	ENSG00000142494
SLC47A2	-0.2972	3.1869E-02	1.02E-01	ENSG00000180638
SLC48A1	0.0863	2.1647E-01	3.97E-01	ENSG00000211584
SLC49A3	0.1335	2.0907E-01	3.89E-01	ENSG00000169026
SLC49A4	0.1366	7.9465E-02	2.00E-01	ENSG00000138463
SLC4A1	0.0184	2.4056E-01		ENSG00000004939
SLC4A10	-0.0600	5.3403E-01	7.08E-01	ENSG00000144290
SLC4A11	0.0209	8.2805E-01	9.10E-01	ENSG00000088836
SLC4A1AP	0.0267	5.6852E-01	7.37E-01	ENSG00000163798
SLC4A2	-0.1575	5.5250E-02	1.54E-01	ENSG00000164889
SLC4A3	-0.0818	1.8099E-01	3.53E-01	ENSG00000114923
SLC4A4	-0.4107	8.1009E-03	3.63E-02	ENSG00000080493
SLC4A5	-0.0326	6.9501E-01	8.29E-01	ENSG00000188687
SLC4A7	0.2462	2.6929E-02	8.99E-02	ENSG00000033867
SLC4A8	0.1924	1.6118E-02	6.12E-02	ENSG00000050438
SLC4A9	-0.0358	9.6945E-01		ENSG00000113073
SLC50A1	-0.1463	3.6912E-02	1.14E-01	ENSG00000169241
SLC51A	0.0788	4.1506E-01	6.08E-01	ENSG00000163959
SLC51B	0.1036	2.6334E-01	4.52E-01	ENSG00000186198
SLC52A1	0.0188	8.8362E-01	9.41E-01	ENSG00000132517
SLC52A2	0.0054	9.4480E-01	9.72E-01	ENSG00000185803
SLC52A3	0.2228	6.0365E-02	1.64E-01	ENSG00000101276
SLC5A1	0.0317	7.9130E-02		ENSG00000100170
SLC5A10	-0.0032	8.0586E-01		ENSG00000154025
SLC5A11	0.0004	9.5985E-01		ENSG00000158865
SLC5A12	0.1905	7.0313E-02	1.83E-01	ENSG00000148942
SLC5A2	0.0874	3.5364E-01	5.49E-01	ENSG00000140675
SLC5A3	-0.3780	9.2401E-04	6.69E-03	ENSG00000198743
SLC5A4	-0.0354	5.6462E-01	7.33E-01	ENSG00000100191
SLC5A4-AS1	-0.0054	5.0781E-01	6.88E-01	ENSG00000242082
SLC5A5	0.0392	3.1477E-02		ENSG00000105641
SLC5A6	-0.0178	7.9530E-01	8.90E-01	ENSG00000138074
SLC5A9	-0.0264	6.4033E-01		ENSG00000117834
SLC66A1	0.0879	1.7850E-01	3.50E-01	ENSG00000040487
SLC66A1L	-0.0634	5.0814E-01	6.88E-01	ENSG00000174899
SLC66A2	0.0979	1.4984E-01	3.10E-01	ENSG00000122490
SLC66A2P2	-0.0270	8.1770E-01		ENSG00000270480
SLC66A3	-0.0438	5.7332E-01	7.41E-01	ENSG00000162976
SLC6A1	0.4948	3.7250E-04	3.27E-03	ENSG00000157103
SLC6A11	-0.1306	1.8618E-01	3.60E-01	ENSG00000132164
SLC6A12	0.2087	8.4534E-02	2.09E-01	ENSG00000111181
SLC6A12-AS1	0.0655	2.9094E-01	4.83E-01	ENSG00000256577
SLC6A13	-0.0094	5.2469E-01	7.01E-01	ENSG00000010379
SLC6A14	-0.1543	1.0367E-01	2.41E-01	ENSG00000268104

SLC6A15	0.0268	7.4989E-01	8.63E-01	ENSG00000072041
SLC6A16	-0.0037	9.6816E-01	9.84E-01	ENSG00000063127
SLC6A17	-0.2098	2.5397E-02	8.62E-02	ENSG00000197106
SLC6A17-AS1	0.0036	8.2504E-01		ENSG00000227091
SLC6A19	-0.0143	6.4551E-01		ENSG00000174358
SLC6A3	-0.0489	5.6401E-01	7.33E-01	ENSG00000142319
SLC6A4	-0.0178	8.7413E-01	9.36E-01	ENSG00000108576
SLC6A5	0.0185	4.7758E-01		ENSG00000165970
SLC6A6	-0.0406	6.5701E-01	8.02E-01	ENSG00000131389
SLC6A7	0.0045	9.5464E-01	9.77E-01	ENSG00000011083
SLC6A8	-0.0310	7.1504E-01	8.41E-01	ENSG00000130821
SLC6A9	-0.1841	7.9090E-02	1.99E-01	ENSG00000196517
SLC7A1	0.0969	2.8708E-01	4.78E-01	ENSG00000139514
SLC7A10	-0.0410	5.3065E-01		ENSG00000130876
SLC7A11	0.0742	4.4176E-01	6.32E-01	ENSG00000151012
SLC7A11-AS1	0.0729	3.9014E-01	5.85E-01	ENSG00000250033
SLC7A14	-0.0594	4.5161E-01	6.41E-01	ENSG00000013293
SLC7A14-AS1	0.0350	7.9803E-02		ENSG00000285051
SLC7A2	-0.2187	5.1429E-02	1.46E-01	ENSG00000003989
SLC7A3	-0.1809	7.6817E-02	1.95E-01	ENSG00000165349
SLC7A4	-0.0953	2.9650E-01	4.89E-01	ENSG00000099960
SLC7A5	0.0401	6.1279E-01	7.71E-01	ENSG00000103257
SLC7A6	-0.1711	5.0388E-02	1.44E-01	ENSG00000103064
SLC7A6OS	-0.0630	3.0958E-01	5.02E-01	ENSG00000103061
SLC7A7	-0.0396	6.8410E-01	8.21E-01	ENSG00000155465
SLC7A8	-0.3121	3.4057E-05	4.67E-04	ENSG00000092068
SLC7A9	-0.0625	4.4830E-01		ENSG00000021488
SLC8A1	-0.1755	1.5013E-02	5.80E-02	ENSG00000183023
SLC8A1-AS1	0.0820	3.4478E-01	5.40E-01	ENSG00000227028
SLC8A2	-0.0912	3.2834E-01	5.23E-01	ENSG00000118160
SLC8A3	0.1147	2.6784E-01	4.57E-01	ENSG00000100678
SLC8B1	0.1169	2.4883E-01	4.35E-01	ENSG00000089060
SLC9A1	-0.2142	1.8290E-03	1.14E-02	ENSG00000090020
SLC9A2	-0.0264	7.2366E-01	8.47E-01	ENSG00000115616
SLC9A3	0.0482	6.0962E-01	7.68E-01	ENSG00000066230
SLC9A3-OT1	0.0196	3.6735E-01		ENSG00000188242
SLC9A4	-0.0300	3.1238E-01		ENSG00000180251
SLC9A5	0.1286	1.4558E-01	3.04E-01	ENSG00000135740
SLC9A6	0.0756	3.6225E-01	5.58E-01	ENSG00000198689
SLC9A7	0.0110	8.9267E-01	9.47E-01	ENSG00000065923
SLC9A7P1	0.1117	2.0777E-01	3.87E-01	ENSG00000227825
SLC9A8	0.0704	2.2549E-01	4.08E-01	ENSG00000197818
SLC9A9	-0.0096	9.0098E-01	9.51E-01	ENSG00000181804
SLC9B1	0.0976	3.3397E-01	5.29E-01	ENSG00000164037
SLC9B2	-0.2936	1.0084E-03	7.16E-03	ENSG00000164038
SLC9C1	-0.0933	2.9142E-01	4.83E-01	ENSG00000172139
SLC9C2	0.0122	7.2243E-01		ENSG00000162753
SLCO1A2	0.0283	7.4066E-01	8.57E-01	ENSG00000084453
SLCO1B1	0.1080	2.9355E-01	4.86E-01	ENSG00000134538

SLCO1C1	-0.5713	4.9814E-03	2.49E-02	ENSG00000139155
SLCO2A1	-0.0822	3.5060E-01	5.46E-01	ENSG00000174640
SLCO2B1	-0.0089	5.1204E-01		ENSG00000137491
SLCO3A1	0.0436	6.4452E-01	7.94E-01	ENSG00000176463
SLCO4A1	0.0999	3.2380E-01	5.18E-01	ENSG00000101187
SLCO4C1	-0.1786	1.0985E-01	2.51E-01	ENSG00000173930
SLCO5A1	0.3921	2.0911E-04	2.02E-03	ENSG00000137571
SLCO5A1-AS1	0.5253	1.2304E-04	1.32E-03	ENSG00000246528
SLCO6A1	-0.0256	9.9377E-01	9.97E-01	ENSG00000205359
SLED1	-0.0275	4.6849E-01		ENSG00000249138
SLF1	-0.1569	4.8223E-02	1.39E-01	ENSG00000133302
SLF2	0.0648	3.5573E-01	5.51E-01	ENSG00000119906
SLFN11	0.0033	9.5030E-01	9.75E-01	ENSG00000172716
SLFN12	0.0462	6.0996E-01	7.69E-01	ENSG00000172123
SLFN12L	-0.0465	4.5332E-01		ENSG00000205045
SLFN13	0.1989	8.7516E-02	2.14E-01	ENSG00000154760
SLFN5	-0.2018	3.8721E-02	1.18E-01	ENSG00000166750
SLFNL1	0.0138	5.6743E-01		ENSG00000171790
SLFNL1-AS1	0.1910	3.2932E-02	1.05E-01	ENSG00000281207
SLIRP	-0.1448	2.2679E-02	7.92E-02	ENSG00000119705
SLIT1	-0.1877	7.1948E-02	1.86E-01	ENSG00000187122
SLIT2	-0.2306	2.6921E-02	8.99E-02	ENSG00000145147
SLIT2-IT1	-0.0008	8.1394E-01	9.02E-01	ENSG00000248228
SLIT3	-0.2962	1.2665E-04	1.35E-03	ENSG00000184347
SLITRK1	0.2573	4.8789E-02	1.41E-01	ENSG00000178235
SLITRK2	0.1722	4.6872E-02	1.36E-01	ENSG00000185985
SLITRK3	0.2397	2.3447E-02	8.12E-02	ENSG00000121871
SLITRK4	-0.0847	2.1778E-01	3.99E-01	ENSG00000179542
SLITRK5	0.1888	3.2369E-02	1.03E-01	ENSG00000165300
SLK	0.0838	3.1344E-01	5.07E-01	ENSG00000065613
SLMAP	-0.1291	3.4895E-02	1.09E-01	ENSG00000163681
SLN	0.0164	8.2657E-01	9.09E-01	ENSG00000170290
SLPI	-0.5877	3.4031E-03	1.85E-02	ENSG00000124107
SLTM	-0.1809	1.4784E-06	3.68E-05	ENSG00000137776
SLU7	0.0210	6.5207E-01	7.99E-01	ENSG00000164609
SLX1A	0.1118	2.7952E-01	4.71E-01	ENSG00000132207
SLX1A-SULT1A3	0.1791	1.1965E-01	2.67E-01	ENSG00000213599
SLX1B	0.2171	1.7769E-02	6.59E-02	ENSG00000181625
SLX1B-SULT1A4	0.2002	9.2423E-02	2.23E-01	ENSG00000260280
SLX4	0.0735	3.8534E-01	5.80E-01	ENSG00000188827
SLX4IP	-0.2485	1.3776E-03	9.18E-03	ENSG00000149346
SLX9	-0.3122	6.4251E-03	3.04E-02	ENSG00000160256
SMAD1	-0.2100	4.4987E-04	3.78E-03	ENSG00000170365
SMAD1-AS1	0.0006	7.9476E-01		ENSG00000250902
SMAD1-AS2	0.0194	1.6973E-01		ENSG00000250582
SMAD2	-0.0986	1.4126E-02	5.55E-02	ENSG00000175387
SMAD3	-0.1492	4.9613E-02	1.42E-01	ENSG00000166949
SMAD4	0.0931	9.8898E-02	2.33E-01	ENSG00000141646
SMAD5	0.0159	7.7484E-01	8.78E-01	ENSG00000113658

SMAD5-AS1	-0.0667	4.9219E-01		ENSG00000164621
SMAD6	-0.0066	9.5061E-01	9.75E-01	ENSG00000137834
SMAD7	-0.4828	4.2961E-08	1.94E-06	ENSG00000101665
SMAD9	-0.2506	3.5892E-03	1.94E-02	ENSG00000120693
SMAGP	-0.0980	2.7549E-01	4.66E-01	ENSG00000170545
SMAP1	-0.1815	3.9328E-03	2.08E-02	ENSG00000112305
SMAP2	0.2012	1.0837E-02	4.51E-02	ENSG00000084070
SMARCA1	-0.0633	2.8462E-01	4.76E-01	ENSG00000102038
SMARCA2	-0.1286	9.6838E-02	2.30E-01	ENSG00000080503
SMARCA4	0.0438	4.0690E-01	6.00E-01	ENSG00000127616
SMARCA5	-0.4876	1.8152E-12	3.54E-10	ENSG00000153147
SMARCA5-AS1	-0.5797	1.8971E-04	1.88E-03	ENSG00000245112
SMARCAD1	0.0335	6.3880E-01	7.90E-01	ENSG00000163104
SMARCAL1	-0.0164	8.4765E-01	9.21E-01	ENSG00000138375
SMARCB1	0.0544	4.3460E-01	6.26E-01	ENSG00000099956
SMARCC1	-0.1154	1.4298E-02	5.60E-02	ENSG00000173473
SMARCC2	0.0039	9.7813E-01	9.89E-01	ENSG00000139613
SMARCD1	0.0491	4.0608E-01	6.00E-01	ENSG00000066117
SMARCD2	0.2545	3.9884E-03	2.11E-02	ENSG00000108604
SMARCD3	-0.0080	8.5903E-01	9.28E-01	ENSG00000082014
SMARCE1	-0.0404	5.5958E-01	7.29E-01	ENSG00000073584
SMARCE1P1	-0.0317	3.7190E-01		ENSG00000225215
SMARCE1P2	0.0147	3.0562E-01		ENSG00000226309
SMARCE1P5	0.0176	2.6879E-01		ENSG00000230793
SMC1A	-0.2141	2.1540E-04	2.07E-03	ENSG00000072501
SMC1B	-0.0051	9.7061E-01	9.85E-01	ENSG00000077935
SMC2	-0.1012	1.7688E-01	3.47E-01	ENSG00000136824
SMC3	-0.0093	7.9540E-01	8.90E-01	ENSG00000108055
SMC4	-0.5411	2.0409E-05	3.10E-04	ENSG00000113810
SMC5	-0.1066	9.9627E-02	2.34E-01	ENSG00000198887
SMC6	0.1029	2.2881E-01	4.12E-01	ENSG00000163029
SMCHD1	-0.1684	2.1816E-03	1.31E-02	ENSG00000101596
SMCO1	-0.0010	8.1888E-01		ENSG00000214097
SMCO2	-0.0335	6.1596E-01		ENSG00000165935
SMCO3	-0.0405	6.3483E-01	7.87E-01	ENSG00000179256
SMCO4	0.3053	5.7823E-03	2.80E-02	ENSG00000166002
SMCR2	-0.0750	1.6497E-01		ENSG00000223979
SMCR5	-0.0472	8.3635E-01	9.14E-01	ENSG00000226746
SMCR8	0.0167	8.4333E-01	9.18E-01	ENSG00000176994
SMDT1	-0.1993	9.2621E-04	6.71E-03	ENSG00000183172
SMG1	0.1876	6.2801E-03	2.98E-02	ENSG00000157106
SMG1P1	0.3692	2.2790E-04	2.17E-03	ENSG00000237296
SMG1P3	0.3341	4.2520E-04	3.62E-03	ENSG00000180747
SMG1P4	0.3900	8.7011E-05	1.00E-03	ENSG00000185710
SMG1P6	0.2109	8.2645E-02	2.05E-01	ENSG00000254634
SMG1P7	0.3475	1.2005E-02	4.88E-02	ENSG00000261556
SMG5	0.2998	3.1686E-04	2.86E-03	ENSG00000198952
SMG6	-0.0380	6.0442E-01	7.64E-01	ENSG00000070366
SMG7	-0.1171	6.8307E-02	1.79E-01	ENSG00000116698

SMG7-AS1	-0.0426	6.4415E-01	7.94E-01	ENSG00000232860
SMG8	-0.0371	6.4205E-01	7.92E-01	ENSG00000167447
SMG9	0.0589	4.4599E-01	6.36E-01	ENSG00000105771
SMIM1	-0.1256	1.7609E-01	3.46E-01	ENSG00000235169
SMIM10	0.0680	4.6359E-01	6.52E-01	ENSG00000184785
SMIM10L1	-0.1737	4.8564E-05	6.31E-04	ENSG00000256537
SMIM10L2A	0.0122	8.9136E-01	9.46E-01	ENSG00000178947
SMIM10L2B	0.3862	3.4693E-07	1.10E-05	ENSG00000196972
SMIM11	-0.1228	5.7202E-02	1.58E-01	ENSG00000205670
SMIM12	0.0016	9.9914E-01	1.00E+00	ENSG00000163866
SMIM13	-0.3124	5.6937E-07	1.66E-05	ENSG00000224531
SMIM14	-0.0292	5.9926E-01	7.60E-01	ENSG00000163683
SMIM14-DT	0.2786	1.2542E-03	8.51E-03	ENSG00000255458
SMIM15	-0.0642	2.1490E-01	3.95E-01	ENSG00000188725
SMIM17	-0.0136	8.8378E-01	9.41E-01	ENSG00000268182
SMIM18	-0.4237	5.6298E-06	1.11E-04	ENSG00000253457
SMIM19	-0.0307	5.4772E-01	7.19E-01	ENSG00000176209
SMIM2-AS1	-0.0736	4.9906E-01	6.81E-01	ENSG00000227258
SMIM2-IT1	-0.0654	2.0049E-01		ENSG00000235285
SMIM20	-0.1276	1.1235E-01	2.55E-01	ENSG00000250317
SMIM22	0.0422	6.4918E-01	7.97E-01	ENSG00000267795
SMIM26	-0.3210	1.2361E-06	3.18E-05	ENSG00000232388
SMIM27	-0.1023	1.3280E-01	2.86E-01	ENSG00000235453
SMIM28	-0.1768	4.8636E-02	1.40E-01	ENSG00000262543
SMIM29	-0.1858	1.6021E-03	1.03E-02	ENSG00000186577
SMIM3	0.1935	7.0277E-02	1.83E-01	ENSG00000256235
SMIM30	-0.1621	2.6145E-02	8.80E-02	ENSG00000214194
SMIM31	0.0097	9.0839E-01	9.54E-01	ENSG00000248771
SMIM32	0.1030	3.0980E-01	5.03E-01	ENSG00000271824
SMIM34	0.0051	9.3094E-01	9.66E-01	ENSG00000243627
SMIM35	0.0969	7.9214E-02		ENSG00000255274
SMIM38	0.0910	4.8143E-02	1.39E-01	ENSG00000284713
SMIM40	0.0971	2.2165E-01	4.03E-01	ENSG00000286920
SMIM41	0.1553	2.6275E-02	8.83E-02	ENSG00000284791
SMIM43	0.0277	3.5042E-01	5.46E-01	ENSG00000164112
SMIM45	0.1577	1.5176E-01	3.13E-01	ENSG00000205704
SMIM47	0.2149	7.6442E-02	1.94E-01	ENSG00000261341
SMIM6	0.1324	1.4812E-01	3.08E-01	ENSG00000259120
SMIM7	-0.0092	8.7682E-01	9.37E-01	ENSG00000214046
SMIM8	0.1118	9.1069E-02	2.20E-01	ENSG00000111850
SMKR1	-0.3823	7.4270E-05	8.83E-04	ENSG00000240204
SMN1	-0.1066	4.6388E-02	1.35E-01	ENSG00000172062
SMN1	-0.1343	1.2932E-02	5.17E-02	ENSG00000205571
SMNDC1	-0.2344	1.1562E-03	7.96E-03	ENSG00000119953
SMO	-0.5759	1.6428E-05	2.63E-04	ENSG00000128602
SMOC2	0.1247	2.2048E-01	4.02E-01	ENSG00000112562
SMOX	-0.0769	3.3184E-01	5.27E-01	ENSG00000088826
SMPD1	-0.2197	2.3489E-02	8.13E-02	ENSG00000166311
SMPD2	-0.1168	1.1446E-01	2.58E-01	ENSG00000135587

SMPD3	0.0092	9.1427E-01	9.57E-01	ENSG00000103056
SMPD4	0.0786	2.2397E-01	4.06E-01	ENSG00000136699
SMPD4BP	0.1169	1.3235E-01	2.85E-01	ENSG00000152117
SMPD4P1	0.0049	9.9330E-01	9.96E-01	ENSG00000223553
SMPDL3A	-0.3616	5.8389E-04	4.67E-03	ENSG00000172594
SMPDL3B	-0.0402	6.5982E-01	8.04E-01	ENSG00000130768
SMS	0.0511	3.7808E-01	5.73E-01	ENSG00000102172
SMTN	0.1601	9.0569E-02	2.20E-01	ENSG00000183963
SMTNL1	0.2106	6.3908E-02	1.71E-01	ENSG00000214872
SMTNL2	0.3018	2.3382E-03	1.39E-02	ENSG00000188176
SMU1	-0.0305	5.2407E-01	7.01E-01	ENSG00000122692
SMUG1	0.0186	7.9372E-01	8.89E-01	ENSG00000123415
SMURF1	0.0774	3.3844E-01	5.34E-01	ENSG00000198742
SMURF2	-0.0966	2.3447E-01	4.19E-01	ENSG00000108854
SMYD1	0.0000	7.5390E-01		ENSG00000115593
SMYD2	-0.1040	1.0883E-01	2.49E-01	ENSG00000143499
SMYD3	0.0529	3.7118E-01	5.67E-01	ENSG00000185420
SMYD3-AS1	-0.1190	2.1757E-01	3.98E-01	ENSG00000226876
SMYD4	0.3739	6.0492E-06	1.18E-04	ENSG00000186532
SMYD5	0.1682	3.1220E-02	1.00E-01	ENSG00000135632
SNAI1	-0.2683	3.2280E-03	1.78E-02	ENSG00000124216
SNAI2	-0.0225	8.0116E-01	8.94E-01	ENSG00000019549
SNAI3	-0.4398	7.3513E-03	3.36E-02	ENSG00000185669
SNAI3-AS1	-0.3146	7.7663E-03	3.51E-02	ENSG00000260630
SNAP23	-0.0190	8.6010E-01	9.29E-01	ENSG00000092531
SNAP25	0.0786	2.8723E-01	4.78E-01	ENSG00000132639
SNAP25-AS1	-0.0600	5.0890E-01	6.89E-01	ENSG00000227906
SNAP29	-0.0479	2.7750E-01	4.68E-01	ENSG00000099940
SNAP47	0.0804	2.3967E-01	4.24E-01	ENSG00000143740
SNAP91	0.0159	8.4849E-01	9.22E-01	ENSG00000065609
SNAPC1	-0.0661	4.3103E-01	6.23E-01	ENSG00000023608
SNAPC2	0.0330	6.2342E-01	7.79E-01	ENSG00000104976
SNAPC3	-0.1523	3.7153E-02	1.14E-01	ENSG00000164975
SNAPC4	0.1484	1.0090E-01	2.36E-01	ENSG00000165684
SNAPC5	0.1016	6.7402E-02	1.77E-01	ENSG00000174446
SNAPIN	-0.0616	2.2823E-01	4.11E-01	ENSG00000143553
SNCA	0.0416	6.3917E-01	7.90E-01	ENSG00000145335
SNCA-AS1	0.0794	3.4849E-02		ENSG00000247775
SNCAIP	-0.0014	9.8689E-01	9.93E-01	ENSG00000064692
SNCB	0.2395	3.6211E-03	1.95E-02	ENSG00000074317
SNCG	0.5773	5.4568E-11	7.04E-09	ENSG00000173267
SND1	0.0861	2.0647E-01	3.85E-01	ENSG00000197157
SND1-IT1	0.0995	3.1551E-01	5.09E-01	ENSG00000279078
SNED1	0.0181	8.4565E-01	9.20E-01	ENSG00000162804
SNF8	0.0066	8.9850E-01	9.49E-01	ENSG00000159210
SNHG1	0.1546	7.9707E-02	2.00E-01	ENSG00000255717
SNHG10	0.2072	5.2401E-03	2.60E-02	ENSG00000247092
SNHG11	0.2382	7.6451E-04	5.76E-03	ENSG00000174365
SNHG12	0.1011	2.7543E-01	4.66E-01	ENSG00000197989

SNHG15	0.0383	5.7201E-01	7.39E-01	ENSG00000232956
SNHG16	0.0573	2.3765E-01	4.22E-01	ENSG00000163597
SNHG17	-0.0777	1.2726E-01	2.78E-01	ENSG00000196756
SNHG18	-0.1971	4.9460E-02	1.42E-01	ENSG00000250786
SNHG19	-0.2212	3.1131E-02	1.00E-01	ENSG00000260260
SNHG20	0.4292	1.9583E-03	1.20E-02	ENSG00000234912
SNHG21	0.0031	9.6889E-01	9.84E-01	ENSG00000250988
SNHG25	-0.0266	7.5991E-01	8.69E-01	ENSG00000266402
SNHG26	-0.0296	8.4129E-01	9.17E-01	ENSG00000228649
SNHG27	-0.0053	9.9762E-01		ENSG00000251676
SNHG29	-0.2659	4.6110E-06	9.41E-05	ENSG00000175061
SNHG3	-0.0881	1.3014E-01	2.82E-01	ENSG00000242125
SNHG31	0.1040	3.0701E-01	5.00E-01	ENSG00000229267
SNHG32	-0.3392	4.1731E-07	1.28E-05	ENSG00000204387
SNHG4	0.5435	3.1696E-04	2.86E-03	ENSG00000281398
SNHG5	0.0528	5.2975E-01	7.05E-01	ENSG00000203875
SNHG7	-0.0506	5.4254E-01	7.15E-01	ENSG00000233016
SNHG8	-0.1379	3.0073E-02	9.76E-02	ENSG00000269893
SNIP1	-0.1381	2.7929E-02	9.23E-02	ENSG00000163877
SNN	0.0583	4.5149E-01	6.41E-01	ENSG00000184602
SNORA11	0.0343	6.9260E-01	8.28E-01	ENSG00000221716
SNORA11F	-0.0323	4.4976E-01		ENSG00000221164
SNORA12	-0.2457	1.6853E-01	3.36E-01	ENSG00000212464
SNORA14A	0.0175	5.5822E-01		ENSG00000201643
SNORA14B	-0.0047	9.8971E-01		ENSG00000207181
SNORA15B-1	-0.0014	3.3112E-01		ENSG00000207062
SNORA19	0.0067	3.0442E-01		ENSG00000207468
SNORA1B	0.0060	6.6761E-01		ENSG00000199405
SNORA20	-0.0073	8.9501E-01		ENSG00000207392
SNORA22	0.0083	5.4806E-01		ENSG00000206634
SNORA22B	-0.0078	9.9489E-01		ENSG00000206603
SNORA28	-0.4071	1.3737E-01	2.92E-01	ENSG00000272533
SNORA2A	0.0053	7.6752E-01		ENSG00000206612
SNORA2B	-0.0508	3.5621E-01		ENSG00000207313
SNORA2C	-0.0363	1.8206E-01		ENSG00000221491
SNORA31	-0.1372	1.2363E-01	2.73E-01	ENSG00000199477
SNORA33	0.5503	1.1589E-02	4.75E-02	ENSG00000200534
SNORA35	0.0192	3.9193E-01	5.86E-01	ENSG00000208839
SNORA38	0.0029	8.7335E-01		ENSG00000200816
SNORA3B	0.0642	3.2609E-01	5.20E-01	ENSG00000212607
SNORA40B	0.0229	4.2070E-01		ENSG00000208308
SNORA53	0.0033	8.9626E-01		ENSG00000212443
SNORA54	0.0148	3.1189E-01		ENSG00000207008
SNORA59B	-0.0075	7.9414E-01	8.89E-01	ENSG00000239149
SNORA5C	-0.0116	5.6792E-01		ENSG00000201772
SNORA63C	-0.0297	4.7955E-01		ENSG00000201448
SNORA65	-0.0008	7.3828E-01		ENSG00000201302
SNORA66	-0.0356	6.9247E-01		ENSG00000207523
SNORA68B	0.0208	2.1435E-01		ENSG00000201388

SNORA70B	-0.0305	6.5403E-01		ENSG00000206937
SNORA70D	-0.0060	7.9902E-01		ENSG00000263666
SNORA70F	-0.0099	9.8330E-01		ENSG00000206869
SNORA70G	-0.1129	2.1152E-01	3.91E-01	ENSG00000206650
SNORA73B	0.0223	6.3280E-01	7.86E-01	ENSG00000200087
SNORA74C-1	-0.0086	8.3551E-01		ENSG00000265733
SNORA74C-2	-0.0593	1.3509E-01		ENSG00000288603
SNORA74D	-0.0059	8.8808E-01		ENSG00000252213
SNORA77	-0.0321	6.9087E-01		ENSG00000221643
SNORA7B	-0.0022	9.7442E-01	9.87E-01	ENSG00000207088
SNORA80A	-0.0924	5.8676E-02		ENSG00000200792
SNORA80B	0.2835	1.5326E-02	5.89E-02	ENSG00000206633
SNORA80C	-0.0128	7.8569E-01		ENSG00000199787
SNORA80D	-0.1763	4.9817E-02	1.43E-01	ENSG00000207217
SNORA80E	0.0487	2.6527E-01	4.54E-01	ENSG00000207475
SNORA84	0.1015	2.7315E-01	4.63E-01	ENSG00000239183
SNORA9B	0.0492	3.9425E-02		ENSG00000206897
SNORC	-0.5717	4.1273E-04	3.53E-03	ENSG00000182600
SNORD100	0.0019	9.3984E-01		ENSG00000221500
SNORD101	-0.0033	8.2493E-01		ENSG00000206754
SNORD104	0.0417	2.8412E-01	4.76E-01	ENSG00000199753
SNORD11	-0.0911	2.9361E-01	4.86E-01	ENSG00000238317
SNORD114-20	-0.0051	6.6670E-01		ENSG00000202048
SNORD114-3	0.0098	4.2093E-01		ENSG00000201839
SNORD117	-0.0210	7.3464E-01		ENSG00000201785
SNORD11B	-0.0693	1.7863E-01		ENSG00000271852
SNORD121A	-0.0025	7.5396E-01		ENSG00000238886
SNORD124	0.0335	5.3437E-02		ENSG00000238793
SNORD12C	0.2748	4.0787E-02	1.23E-01	ENSG00000209042
SNORD13	-0.0283	8.9542E-01	9.48E-01	ENSG00000239039
SNORD13E	-0.1058	2.6818E-01	4.57E-01	ENSG00000238311
SNORD14A	-0.0924	3.3185E-01	5.27E-01	ENSG00000272034
SNORD14E	0.0056	8.7524E-01		ENSG00000200879
SNORD15B	-0.0130	9.1221E-01		ENSG00000207445
SNORD17	-0.0278	4.2366E-01		ENSG00000212232
SNORD19	0.0742	4.2324E-01	6.15E-01	ENSG00000212493
SNORD19C	0.0118	3.6823E-01		ENSG00000222345
SNORD37	0.0164	3.8445E-01		ENSG00000206775
SNORD38C	-0.0076	4.6133E-01		ENSG00000207109
SNORD3G	-0.1796	2.9420E-02	9.61E-02	ENSG00000199666
SNORD46	-0.1198	1.7702E-01	3.47E-01	ENSG00000200913
SNORD59A	0.4325	1.0342E-02	4.35E-02	ENSG00000207031
SNORD6	-0.0052	8.8481E-01		ENSG00000202314
SNORD62B	-0.0167	7.8277E-01	8.83E-01	ENSG00000231587
SNORD65C	-0.0337	3.9358E-01		ENSG00000212264
SNORD69	-0.0019	8.8204E-01		ENSG00000212452
SNORD70	-0.0527	5.9927E-01		ENSG00000212534
SNORD71	-0.0103	7.3955E-01		ENSG00000223224
SNORD72	-0.0043	8.6533E-01		ENSG00000212296

SNORD83A	0.0777	2.4088E-01	4.26E-01	ENSG00000209482
SNORD89	-0.0443	4.0620E-01		ENSG00000212283
SNORD99	0.1162	6.5551E-02		ENSG00000221539
SNPH	-0.0872	1.9399E-01	3.70E-01	ENSG00000101298
SNRK	-0.2149	1.2672E-03	8.58E-03	ENSG00000163788
SNRNP200	0.0454	4.3773E-01	6.29E-01	ENSG00000144028
SNRNP25	-0.1324	6.0417E-03	2.90E-02	ENSG00000161981
SNRNP27	-0.0940	5.3232E-02	1.50E-01	ENSG00000124380
SNRNP35	-0.1296	9.2065E-02	2.22E-01	ENSG00000184209
SNRNP40	0.1692	5.6031E-03	2.74E-02	ENSG00000060688
SNRNP48	-0.0639	4.0368E-01	5.98E-01	ENSG00000168566
SNRNP70	-0.1006	1.2187E-01	2.70E-01	ENSG00000104852
SNRPA	-0.1502	9.5076E-03	4.09E-02	ENSG00000077312
SNRPA1	0.1770	2.4425E-03	1.44E-02	ENSG00000131876
SNRPB	-0.1333	2.2332E-02	7.83E-02	ENSG00000125835
SNRPB2	-0.2153	4.3231E-04	3.67E-03	ENSG00000125870
SNRPC	-0.2225	1.0546E-04	1.17E-03	ENSG00000124562
SNRPCP10	0.0067	7.7871E-01		ENSG00000243794
SNRPCP11	-0.0126	5.5265E-01		ENSG00000241346
SNRPCP14	0.0048	3.3682E-01		ENSG00000254805
SNRPCP16	-0.0300	4.1251E-01		ENSG00000250342
SNRPCP6	-0.0115	7.9839E-01		ENSG00000254683
SNRPCP7	-0.0038	4.2681E-01		ENSG00000277952
SNRPD1	-0.1163	1.8098E-02	6.68E-02	ENSG00000167088
SNRPD2	-0.2106	1.2868E-04	1.37E-03	ENSG00000125743
SNRPD3	-0.0319	5.8334E-01	7.49E-01	ENSG00000100028
SNRPE	-0.1945	4.7177E-03	2.39E-02	ENSG00000182004
SNRPF	-0.0410	3.6574E-01	5.62E-01	ENSG00000139343
SNRPF-DT	-0.0551	7.5592E-01	8.67E-01	ENSG00000258343
SNRPG	-0.2004	7.4046E-04	5.62E-03	ENSG00000143977
SNRPGP14	0.0431	3.9714E-02		ENSG00000236577
SNRPGP18	-0.0642	1.5922E-01		ENSG00000257979
SNRPN	-0.3045	2.1153E-06	4.96E-05	ENSG00000128739
SNTA1	-0.1466	5.6330E-03	2.75E-02	ENSG00000101400
SNTB1	-0.4109	5.7298E-05	7.17E-04	ENSG00000172164
SNTB2	0.3602	2.2944E-06	5.31E-05	ENSG00000168807
SNTG1	-0.1087	2.6191E-01	4.51E-01	ENSG00000147481
SNTG2	0.0252	7.8007E-01	8.81E-01	ENSG00000172554
SNTN	-0.4958	9.2497E-03	4.01E-02	ENSG00000188817
SNU13	-0.1518	3.9641E-04	3.42E-03	ENSG00000100138
SNUPN	-0.3892	1.8001E-04	1.81E-03	ENSG00000169371
SNURF	0.0547	5.7340E-01	7.41E-01	ENSG00000273173
SNW1	-0.2191	1.9148E-04	1.89E-03	ENSG00000100603
SNX1	-0.0767	2.2456E-01	4.07E-01	ENSG00000028528
SNX10	-0.0216	7.2665E-01	8.49E-01	ENSG00000086300
SNX10-AS1	0.0645	4.9778E-01	6.81E-01	ENSG00000225792
SNX11	0.1313	6.5294E-02	1.73E-01	ENSG00000002919
SNX12	-0.2340	2.5860E-02	8.73E-02	ENSG00000147164
SNX13	-0.0694	3.2744E-01	5.22E-01	ENSG00000071189

SNX14	0.2221	8.6381E-03	3.81E-02	ENSG00000135317
SNX15	0.0079	3.5456E-01		ENSG00000110025
SNX16	0.0433	6.1687E-01	7.74E-01	ENSG00000104497
SNX17	-0.0114	7.3054E-01	8.52E-01	ENSG00000115234
SNX18	-0.3699	9.7994E-05	1.10E-03	ENSG00000178996
SNX19	0.1165	1.8331E-02	6.75E-02	ENSG00000120451
SNX2	0.0799	2.7535E-01	4.66E-01	ENSG00000205302
SNX20	0.0031	9.3054E-01		ENSG00000167208
SNX21	-0.2559	1.4650E-03	9.64E-03	ENSG00000124104
SNX22	-0.0260	7.7129E-01	8.76E-01	ENSG00000157734
SNX24	-0.2195	2.5193E-04	2.36E-03	ENSG00000064652
SNX25	0.3378	6.2348E-04	4.91E-03	ENSG00000109762
SNX25P1	-0.0935	2.1056E-01	3.90E-01	ENSG00000236809
SNX27	-0.1236	1.1883E-01	2.65E-01	ENSG00000143376
SNX29	0.0657	3.4937E-01	5.44E-01	ENSG00000048471
SNX3	-0.3658	4.6181E-08	2.07E-06	ENSG00000112335
SNX30	0.1271	1.6793E-01	3.35E-01	ENSG00000148158
SNX31	0.0093	7.1620E-01		ENSG00000174226
SNX32	0.0808	3.1827E-01	5.12E-01	ENSG00000172803
SNX33	-0.1264	1.3047E-01	2.82E-01	ENSG00000173548
SNX4	0.0635	4.1885E-01	6.11E-01	ENSG00000114520
SNX5	-0.0215	7.0795E-01	8.37E-01	ENSG00000089006
SNX5P1	0.0015	9.0728E-01		ENSG00000249858
SNX6	-0.2687	1.4837E-06	3.69E-05	ENSG00000129515
SNX7	-0.2837	4.8530E-03	2.44E-02	ENSG00000162627
SNX8	0.1198	4.0009E-02	1.21E-01	ENSG00000106266
SNX9	0.0230	7.2142E-01	8.46E-01	ENSG00000130340
SOAT1	-0.5426	8.0358E-07	2.24E-05	ENSG00000057252
SOAT2	0.0016	7.0311E-01		ENSG00000167780
SOBP	-0.1000	9.4683E-02	2.26E-01	ENSG00000112320
SOCS1	0.2650	4.2347E-03	2.21E-02	ENSG00000185338
SOCS2	-0.2178	1.3912E-02	5.48E-02	ENSG00000120833
SOCS2-AS1	-0.0684	4.6773E-01	6.55E-01	ENSG00000246985
SOCS3	-0.0313	7.2440E-01	8.48E-01	ENSG00000184557
SOCS3-DT	0.2219	6.9097E-02	1.81E-01	ENSG00000266970
SOCS4	-0.0636	3.4954E-01	5.45E-01	ENSG00000180008
SOCS5	-0.2316	1.7061E-03	1.08E-02	ENSG00000171150
SOCS5P2	-0.0340	8.3886E-01		ENSG00000224682
SOCS5P4	-0.0080	6.9892E-01		ENSG00000227536
SOCS6	-0.1394	6.3861E-02	1.71E-01	ENSG00000170677
SOCS7	-0.0467	5.6510E-01	7.34E-01	ENSG00000274211
SOD1	-0.3343	1.4661E-05	2.40E-04	ENSG00000142168
SOD2-OT1	-0.0198	8.3150E-01	9.12E-01	ENSG00000285427
SOHLH1	0.0182	2.7156E-01		ENSG00000165643
SOHLH2	-0.2203	6.8718E-02	1.80E-01	ENSG00000120669
SON	-0.1795	2.6635E-04	2.47E-03	ENSG00000159140
SORBS1	0.1464	1.2358E-01	2.72E-01	ENSG00000095637
SORBS2	0.0790	3.2521E-01	5.19E-01	ENSG00000154556
SORBS3	0.0788	2.1288E-01	3.93E-01	ENSG00000120896

SORCS1	-0.0036	9.6266E-01	9.81E-01	ENSG00000108018
SORCS2	0.0165	8.2973E-01	9.11E-01	ENSG00000184985
SORCS3-AS1	-0.0003	9.6140E-01		ENSG00000226387
SORD	0.0765	2.8742E-01	4.79E-01	ENSG00000140263
SORL1	-0.0282	7.0540E-01	8.35E-01	ENSG00000137642
SORT1	-0.0092	9.0497E-01	9.52E-01	ENSG00000134243
SOS1	0.0329	5.8145E-01	7.47E-01	ENSG00000115904
SOS2	-0.0788	2.3142E-01	4.15E-01	ENSG00000100485
SOST	0.0183	3.7088E-01		ENSG00000167941
SOWAHA	-0.0931	3.0998E-01	5.03E-01	ENSG00000198944
SOWAHB	0.1566	1.5280E-01	3.14E-01	ENSG00000186212
SOWAHC	-0.0680	2.8283E-01	4.74E-01	ENSG00000198142
SOWAHD	-0.1104	1.7912E-01	3.50E-01	ENSG00000187808
SOX1	0.1661	1.3571E-01	2.90E-01	ENSG00000182968
SOX1-OT	-0.5096	6.2938E-03	2.99E-02	ENSG00000224243
SOX10	0.0012	9.0704E-01		ENSG00000100146
SOX11	0.0770	3.8449E-01	5.79E-01	ENSG00000176887
SOX12	-0.0880	1.8425E-01	3.57E-01	ENSG00000177732
SOX13	0.1209	1.6852E-01	3.36E-01	ENSG00000143842
SOX14	0.0097	2.7278E-01		ENSG00000168875
SOX15	-0.2130	6.1397E-04	4.85E-03	ENSG00000129194
SOX17	-0.0466	1.2217E-01		ENSG00000164736
SOX18	-0.0061	9.3624E-01	9.68E-01	ENSG00000203883
SOX2	-0.3838	2.1460E-04	2.07E-03	ENSG00000181449
SOX2-OT	-0.0532	5.1805E-01	6.96E-01	ENSG00000242808
SOX21	0.1815	8.5217E-02	2.10E-01	ENSG00000125285
SOX21-AS1	0.0409	6.4215E-01	7.92E-01	ENSG00000227640
SOX3	-0.3057	2.8551E-02	9.39E-02	ENSG00000134595
SOX30	0.1283	4.3107E-02	1.28E-01	ENSG00000039600
SOX4	-0.3345	1.0652E-05	1.85E-04	ENSG00000124766
SOX5	-0.0947	3.1092E-01	5.04E-01	ENSG00000134532
SOX5-AS1	0.0459	4.3295E-02		ENSG00000256120
SOX6	0.1767	2.3734E-02	8.19E-02	ENSG00000110693
SOX7	-0.0499	3.9497E-01	5.89E-01	ENSG00000171056
SOX8	-0.0711	4.0672E-01	6.00E-01	ENSG00000005513
SOX9-AS1	-0.0893	3.6479E-01	5.61E-01	ENSG00000234899
SP1	-0.0618	5.0138E-01	6.83E-01	ENSG00000185591
SP100	-0.1038	3.0277E-01	4.95E-01	ENSG00000067066
SP110	-0.5529	1.6237E-06	3.98E-05	ENSG00000135899
SP140	-0.0063	9.1849E-01	9.60E-01	ENSG00000079263
SP140L	-0.1158	2.5832E-01	4.46E-01	ENSG00000185404
SP2	-0.0968	1.8480E-01	3.58E-01	ENSG00000167182
SP2-AS1	-0.0501	6.0519E-01	7.65E-01	ENSG00000234494
SP2-DT	0.2374	5.6448E-02	1.56E-01	ENSG00000264920
SP3	-0.0419	4.8400E-01	6.69E-01	ENSG00000172845
SP4	-0.0605	4.4705E-01	6.37E-01	ENSG00000105866
SP5	0.1422	1.6148E-01	3.27E-01	ENSG00000204335
SP6	0.3413	1.9040E-02	6.94E-02	ENSG00000189120
SP7	-0.0252	7.0949E-01		ENSG00000170374

SP8	-0.0040	7.9260E-01		ENSG00000164651
SP9	-0.0811	1.9667E-01	3.73E-01	ENSG00000217236
SPA17	-0.5686	8.3226E-11	1.02E-08	ENSG00000064199
SPAAR	-0.0003	5.4002E-01		ENSG00000235387
SPACA4	0.0202	2.6975E-01		ENSG00000177202
SPACA5	-0.0789	1.7846E-01		ENSG00000171489
SPACA5B	-0.1292	3.0039E-02		ENSG00000171478
SPACA6	-0.0037	9.6460E-01	9.82E-01	ENSG00000182310
SPACA6-AS1	-0.0365	7.1236E-01	8.40E-01	ENSG00000269959
SPACA9	-0.4154	2.3056E-08	1.16E-06	ENSG00000165698
SPACDR	-0.4912	1.3387E-02	5.31E-02	ENSG00000185955
SPAG1	-0.3776	9.2003E-07	2.50E-05	ENSG00000104450
SPAG16	-0.1957	9.2338E-04	6.69E-03	ENSG00000144451
SPAG17	0.0106	9.1011E-01	9.55E-01	ENSG00000155761
SPAG5	-0.0427	6.5420E-01	8.00E-01	ENSG00000076382
SPAG6	-0.2210	2.6425E-02	8.87E-02	ENSG00000077327
SPAG7	-0.1689	2.7435E-05	3.95E-04	ENSG00000091640
SPAG8	-0.3260	6.1796E-03	2.95E-02	ENSG00000137098
SPAG9	-0.1344	7.1627E-02	1.85E-01	ENSG00000008294
SPANXA2	-0.0158	6.9876E-01		ENSG00000203926
SPANXA2-OT1	-0.0035	9.6928E-01	9.84E-01	ENSG00000277215
SPANXN3	0.0336	9.7456E-02		ENSG00000189252
SPARC	-0.4616	1.1722E-04	1.27E-03	ENSG00000113140
SPART	-0.4456	2.9014E-06	6.45E-05	ENSG00000133104
SPART-AS1	-0.4528	1.2619E-02	5.08E-02	ENSG00000120664
SPAST	-0.0206	7.6852E-01	8.74E-01	ENSG00000021574
SPATA1	-0.0116	8.8527E-01	9.42E-01	ENSG00000122432
SPATA12	0.0153	8.7322E-01	9.36E-01	ENSG00000186451
SPATA13	-0.0126	8.9769E-01	9.49E-01	ENSG00000228741
SPATA13	-0.3585	3.7495E-05	5.06E-04	ENSG00000182957
SPATA17	-0.1813	6.1545E-03	2.94E-02	ENSG00000162814
SPATA18	-0.5015	7.5170E-04	5.69E-03	ENSG00000163071
SPATA19	-0.0392	1.7390E-01		ENSG00000166118
SPATA2	-0.4611	1.7569E-10	1.90E-08	ENSG00000158480
SPATA20	0.0154	8.5074E-01	9.23E-01	ENSG00000006282
SPATA21	-0.3063	3.5664E-02	1.11E-01	ENSG00000187144
SPATA22	-0.0674	1.6377E-01		ENSG00000141255
SPATA24	-0.1267	1.2379E-01	2.73E-01	ENSG00000170469
SPATA25	-0.0064	8.4592E-01		ENSG00000149634
SPATA2L	0.1627	5.8450E-02	1.60E-01	ENSG00000158792
SPATA2P1	-0.0277	3.4429E-01		ENSG00000228319
SPATA3	0.0093	4.5915E-01		ENSG00000173699
SPATA3-AS1	0.0170	2.3695E-01		ENSG00000238062
SPATA31C1	0.0185	2.6825E-01		ENSG00000230246
SPATA31C2	0.0075	5.2624E-01		ENSG00000177910
SPATA31G1	-0.0275	8.6827E-01		ENSG00000174038
SPATA31H1	0.0513	6.0203E-01	7.62E-01	ENSG00000221843
SPATA32	-0.0368	5.1385E-01		ENSG00000184361
SPATA33	-0.0344	6.4521E-01	7.94E-01	ENSG00000167523

SPATA4	0.0677	4.4378E-01	6.34E-01	ENSG00000150628
SPATA41	0.0856	2.8617E-01	4.77E-01	ENSG00000189419
SPATA42	-0.0084	8.8102E-01		ENSG00000203897
SPATA45	-0.0731	4.3267E-01	6.24E-01	ENSG00000185523
SPATA46	0.0103	2.8118E-01		ENSG00000171722
SPATA6	-0.2380	3.8240E-04	3.33E-03	ENSG00000132122
SPATA6L	0.1288	9.7599E-02	2.31E-01	ENSG00000106686
SPATA7	0.2473	6.0538E-05	7.49E-04	ENSG00000042317
SPATA8	0.0176	3.2146E-01		ENSG00000185594
SPATA9	0.0833	2.7967E-01	4.71E-01	ENSG00000145757
SPATC1	-0.0054	6.1687E-01		ENSG00000186583
SPATC1L	0.1263	1.7603E-02	6.54E-02	ENSG00000160284
SPATS1	0.1359	4.5245E-02		ENSG00000249481
SPATS2	-0.1793	3.8413E-04	3.34E-03	ENSG00000123352
SPATS2L	-0.3845	8.0471E-08	3.26E-06	ENSG00000196141
SPCS1	-0.1555	2.0230E-02	7.27E-02	ENSG00000114902
SPCS2	-0.2991	4.4412E-06	9.14E-05	ENSG00000118363
SPCS2P1	-0.0050	7.8943E-01		ENSG00000260928
SPCS3	-0.3042	5.8455E-10	5.45E-08	ENSG00000129128
SPDL1	0.2244	1.0180E-02	4.30E-02	ENSG00000040275
SPDYA	0.0156	8.5994E-01	9.29E-01	ENSG00000163806
SPDYC	-0.0202	4.5941E-01		ENSG00000204710
SPDYE1	0.0119	8.9941E-01	9.50E-01	ENSG00000136206
SPDYE10	0.1393	1.4108E-01	2.98E-01	ENSG00000274570
SPDYE11	0.1818	6.4590E-02	1.72E-01	ENSG00000275976
SPDYE12	0.0467	5.6033E-01	7.30E-01	ENSG00000184616
SPDYE13	0.1790	1.0504E-01	2.43E-01	ENSG00000286038
SPDYE14	0.2530	3.5111E-02	1.10E-01	ENSG00000286137
SPDYE15	0.2482	2.7334E-02	9.09E-02	ENSG00000286014
SPDYE16	0.1140	2.3930E-01	4.24E-01	ENSG00000185040
SPDYE17	0.2431	5.6222E-02	1.56E-01	ENSG00000186645
SPDYE18	0.1415	1.7363E-01	3.43E-01	ENSG00000205482
SPDYE2	0.1493	9.8640E-02	2.32E-01	ENSG00000205238
SPDYE21	0.2207	6.4518E-02	1.72E-01	ENSG00000230358
SPDYE2B	0.0834	3.5733E-01	5.53E-01	ENSG00000173678
SPDYE3	-0.0474	6.2784E-01	7.82E-01	ENSG00000214300
SPDYE5	0.0845	3.9854E-01	5.93E-01	ENSG00000170092
SPDYE6	0.0341	7.1795E-01	8.44E-01	ENSG00000260097
SPDYE8	0.1706	7.9270E-02	1.99E-01	ENSG00000273520
SPDYE9	0.1673	8.4485E-02	2.09E-01	ENSG00000262461
SPECC1	0.0197	7.8649E-01	8.85E-01	ENSG00000128487
SPECC1L	-0.0197	8.1694E-01	9.03E-01	ENSG00000100014
SPECC1L-ADORA2A	-0.0048	9.4458E-01		ENSG00000258555
SPECC1P2	-0.0135	8.1376E-01		ENSG00000244456
SPEF1	-0.3828	1.0474E-03	7.37E-03	ENSG00000101222
SPEF2	-0.0887	2.2544E-01	4.08E-01	ENSG00000152582
SPEG	-0.0239	7.8036E-01	8.81E-01	ENSG00000072195
SPEM1	0.0027	8.3835E-01		ENSG00000181323
SPEM2	-0.0110	9.8793E-01		ENSG00000184560

SPEM3	-0.0034	9.9828E-01		ENSG00000283439
SPEN	-0.1193	3.1419E-02	1.01E-01	ENSG00000065526
SPEN-AS1	0.0737	2.0236E-01	3.80E-01	ENSG00000179743
SPESP1	-0.0325	5.1661E-01		ENSG00000258484
SPG11	-0.0827	1.4443E-01	3.03E-01	ENSG00000104133
SPG21	-0.2239	2.7267E-04	2.52E-03	ENSG00000090487
SPG7	0.1433	2.1204E-02	7.53E-02	ENSG00000197912
SPHK1	-0.5000	3.2333E-04	2.91E-03	ENSG00000176170
SPHK2	0.1599	5.0661E-02	1.44E-01	ENSG00000063176
SPHKAP	0.4813	5.6740E-03	2.76E-02	ENSG00000153820
SPI1	0.0005	9.3732E-01		ENSG00000066336
SPIB	0.0172	8.4526E-01		ENSG00000269404
SPICE1	0.1228	2.2304E-01	4.05E-01	ENSG00000163611
SPIDR	-0.2849	4.7370E-04	3.92E-03	ENSG00000164808
SPIN1	-0.1591	7.7163E-03	3.49E-02	ENSG00000106723
SPIN2A	0.2278	2.3449E-03	1.39E-02	ENSG00000147059
SPIN2B	-0.0789	1.2660E-01	2.77E-01	ENSG00000186787
SPIN2P1	0.0044	6.5809E-01		ENSG00000237708
SPIN3	0.1702	9.5243E-02	2.27E-01	ENSG00000204271
SPIN4	-0.2041	5.0025E-02	1.43E-01	ENSG00000186767
SPINDOC	0.1006	2.8910E-01	4.81E-01	ENSG00000168005
SPINK1	-0.1524	1.2921E-01	2.80E-01	ENSG00000164266
SPINK13	0.0394	7.3585E-01	8.55E-01	ENSG00000214510
SPINK14	0.0535	2.9256E-01	4.84E-01	ENSG00000196800
SPINK4	-0.0776	3.7035E-01	5.66E-01	ENSG00000122711
SPINK5	-0.1046	2.2980E-01	4.13E-01	ENSG00000133710
SPINK6	0.0256	4.8127E-01	6.66E-01	ENSG00000178172
SPINK7	-0.0119	8.4145E-01		ENSG00000145879
SPINK8	0.0701	4.5119E-01	6.41E-01	ENSG00000229453
SPINK9	-0.0370	3.6427E-01		ENSG00000204909
SPINT1	0.1884	6.0908E-02	1.65E-01	ENSG00000166145
SPINT1-AS1	-0.0762	4.1090E-01	6.04E-01	ENSG00000261183
SPINT2	0.0443	6.0117E-01	7.62E-01	ENSG00000167642
SPIRE1	0.3043	8.9699E-04	6.54E-03	ENSG00000134278
SPIRE2	0.1848	5.3180E-03	2.63E-02	ENSG00000204991
SPMAP1	0.0302	2.7480E-01		ENSG00000275489
SPMAP2	0.0101	2.7566E-01		ENSG00000105549
SPMAP2L	0.1577	1.4996E-01	3.10E-01	ENSG00000249693
SPMIP1	-0.1443	1.8189E-01	3.54E-01	ENSG00000272899
SPMIP10	-0.0270	4.5153E-01		ENSG00000196900
SPMIP11	-0.0246	5.4266E-01		ENSG00000257987
SPMIP2	0.1588	7.8819E-02	1.99E-01	ENSG00000164123
SPMIP3	0.0656	1.3723E-01		ENSG00000173728
SPMIP3P1	-0.0037	9.3918E-01		ENSG00000276925
SPMIP4	0.1603	1.3283E-01	2.86E-01	ENSG00000153790
SPMIP5	0.0079	9.1964E-01	9.60E-01	ENSG00000165863
SPMIP7	-0.0307	4.7677E-01		ENSG00000164500
SPMIP8	-0.1384	1.8615E-01	3.60E-01	ENSG00000159648
SPNS1	0.2448	5.4571E-02	1.52E-01	ENSG00000169682

SPNS2	0.3230	5.0294E-03	2.51E-02	ENSG00000183018
SPNS3	0.5934	2.9830E-03	1.68E-02	ENSG00000182557
SPOCD1	0.2767	3.7827E-02	1.16E-01	ENSG00000134668
SPOCK1	-0.3065	1.1618E-04	1.26E-03	ENSG00000152377
SPOCK2	-0.0750	4.3844E-01	6.29E-01	ENSG00000107742
SPOCK3	0.0661	4.3001E-01	6.22E-01	ENSG00000196104
SPON1	-0.4376	1.4976E-03	9.80E-03	ENSG00000262655
SPON2	-0.3357	2.4700E-02	8.45E-02	ENSG00000159674
SPOP	-0.0573	3.7160E-01	5.67E-01	ENSG00000121067
SPOPL	-0.0112	8.8387E-01	9.41E-01	ENSG00000144228
SPOUT1	0.0643	4.9434E-02	1.42E-01	ENSG00000198917
SPP1	-0.1832	9.5257E-02	2.27E-01	ENSG00000118785
SPPL2A	-0.2786	6.9227E-06	1.31E-04	ENSG00000138600
SPPL2B	-0.1381	2.1447E-02	7.59E-02	ENSG00000005206
SPPL2C	0.0054	5.7738E-01		ENSG00000185294
SPPL3	-0.1130	1.4941E-02	5.78E-02	ENSG00000157837
SPRED1	-0.2884	7.0061E-05	8.42E-04	ENSG00000166068
SPRED2	-0.0866	1.6632E-01	3.33E-01	ENSG00000198369
SPRED3	-0.0355	6.5410E-01	8.00E-01	ENSG00000188766
SPRING1	-0.0393	5.5455E-01	7.26E-01	ENSG00000111412
SPRN	-0.1811	1.0160E-01	2.37E-01	ENSG00000203772
SPRR1A	-0.0336	6.2369E-01	7.79E-01	ENSG00000169474
SPRR1B	-0.1014	1.3447E-01	2.88E-01	ENSG00000169469
SPRR2A	-0.0369	9.7058E-02	2.30E-01	ENSG00000241794
SPRR2F	-0.0812	1.1350E-01		ENSG00000244094
SPRR2G	0.0008	6.6301E-01		ENSG00000159516
SPRR3	0.0597	4.2193E-01	6.15E-01	ENSG00000163209
SPRTN	0.2506	3.1943E-04	2.88E-03	ENSG00000010072
SPRY1	0.0300	6.9034E-01	8.26E-01	ENSG00000164056
SPRY3	0.0836	3.5934E-01	5.55E-01	ENSG00000168939
SPRY4	-0.4692	4.5892E-08	2.06E-06	ENSG00000187678
SPRY4-AS1	-0.0678	4.7798E-01	6.64E-01	ENSG00000231185
SPRYD3	0.1309	9.0240E-02	2.19E-01	ENSG00000167778
SPRYD4	0.0420	5.9159E-01	7.55E-01	ENSG00000176422
SPRYD7	0.1160	6.6590E-02	1.76E-01	ENSG00000123178
SPSB1	-0.0316	7.3196E-01	8.53E-01	ENSG00000171621
SPSB2	0.1012	1.3923E-01	2.95E-01	ENSG00000111671
SPSB3	0.0375	3.7390E-01	5.69E-01	ENSG00000162032
SPSB4	0.0284	7.1923E-01	8.44E-01	ENSG00000175093
SPTAN1	-0.0014	9.8391E-01	9.91E-01	ENSG00000197694
SPTB	-0.0820	4.0511E-01	5.99E-01	ENSG00000070182
SPTBN1	-0.1869	6.1907E-03	2.95E-02	ENSG00000115306
SPTBN2	0.0682	4.3257E-01	6.24E-01	ENSG00000173898
SPTBN4	0.1766	5.4667E-02	1.53E-01	ENSG00000160460
SPTBN5	0.2087	7.2304E-02	1.87E-01	ENSG00000137877
SPTLC1	-0.1137	6.6067E-02	1.75E-01	ENSG00000090054
SPTLC1P1	-0.0748	2.3359E-01		ENSG00000230397
SPTLC1P4	0.0280	3.7287E-01		ENSG00000232678
SPTLC2	0.0850	2.3803E-01	4.22E-01	ENSG00000100596

SPTLC3	-0.1447	1.6993E-01	3.38E-01	ENSG00000172296
SPTSSA	-0.1340	7.8378E-02	1.98E-01	ENSG00000165389
SPTSSB	-0.3162	2.5472E-02	8.64E-02	ENSG00000196542
SPTY2D1	-0.1557	4.3513E-02	1.29E-01	ENSG00000179119
SPX	-0.4996	3.4155E-04	3.04E-03	ENSG00000134548
SPZ1	-0.0265	8.7821E-01		ENSG00000164299
SQLE	-0.2893	9.6125E-05	1.08E-03	ENSG00000104549
SQLE-DT	0.0463	3.9966E-01	5.94E-01	ENSG00000253513
SQOR	-0.1518	1.2801E-01	2.79E-01	ENSG00000137767
SQSTM1	-0.1720	6.5516E-02	1.74E-01	ENSG00000161011
SRA1	0.0336	5.1420E-01	6.93E-01	ENSG00000213523
SRARP	-0.0385	5.2762E-01	7.03E-01	ENSG00000183888
SRBD1	-0.3481	1.7284E-04	1.75E-03	ENSG00000068784
SRC	-0.0931	1.8711E-01	3.61E-01	ENSG00000197122
SRCAP	0.1042	2.9782E-01	4.90E-01	ENSG00000080603
SRCIN1	-0.0726	4.1585E-01	6.09E-01	ENSG00000277363
SRD5A1	-0.0260	6.7074E-01	8.11E-01	ENSG00000145545
SRD5A2	-0.1267	7.7504E-02	1.96E-01	ENSG00000277893
SRD5A3	-0.0104	9.0767E-01	9.54E-01	ENSG00000128039
SRD5A3-AS1	-0.0131	8.6734E-01	9.33E-01	ENSG00000249700
SREBF1	-0.5764	8.0970E-09	4.80E-07	ENSG00000072310
SREBF2	-0.3673	1.8605E-09	1.41E-07	ENSG00000198911
SREK1	-0.2656	1.6910E-06	4.11E-05	ENSG00000153914
SREK1IP1	-0.2451	6.7119E-05	8.14E-04	ENSG00000153006
SREK1IP1P2	-0.0057	9.1135E-01		ENSG00000271227
SRF	-0.1504	1.0376E-01	2.41E-01	ENSG00000112658
SRFBP1	-0.0452	4.6624E-01	6.54E-01	ENSG00000151304
SRGAP1	0.0526	4.7884E-01	6.65E-01	ENSG00000196935
SRGAP2	-0.0783	2.7047E-01	4.60E-01	ENSG00000266028
SRGAP2-AS1	-0.0495	6.4298E-01	7.93E-01	ENSG00000230806
SRGAP2B	-0.1466	7.4458E-02	1.90E-01	ENSG00000196369
SRGAP2C	-0.1358	6.5260E-02	1.73E-01	ENSG00000171943
SRGAP3	-0.2356	1.0085E-03	7.16E-03	ENSG00000196220
SRGAP3-AS2	-0.0074	3.8659E-02	1.18E-01	ENSG00000228723
SRGAP3-AS3	-0.0706	3.7378E-01	5.69E-01	ENSG00000227929
SRGN	-0.0171	6.6179E-01		ENSG00000122862
SRI	-0.2245	9.2131E-04	6.68E-03	ENSG00000075142
SRI-AS1	0.0160	4.7057E-01	6.58E-01	ENSG00000254003
SRM	0.0699	2.6959E-01	4.59E-01	ENSG00000116649
SRMS	0.1198	2.0883E-01	3.88E-01	ENSG00000125508
SRP14	-0.4028	3.8512E-10	3.74E-08	ENSG00000140319
SRP14-DT	-0.1983	6.5913E-03	3.10E-02	ENSG00000248508
SRP14P2	-0.1026	2.8588E-01	4.77E-01	ENSG00000234106
SRP14P3	-0.2111	2.8892E-02	9.48E-02	ENSG00000235183
SRP14P4	-0.0055	9.4797E-01		ENSG00000268062
SRP19	-0.2258	4.2412E-04	3.61E-03	ENSG00000153037
SRP54	0.3094	4.8245E-05	6.27E-04	ENSG00000100883
SRP68	0.0493	5.0550E-01	6.87E-01	ENSG00000167881
SRP72	-0.1141	1.2783E-02	5.13E-02	ENSG00000174780

SRP72P1	0.0183	2.9472E-01		ENSG00000265182
SRP72P2	-0.1870	7.1150E-02	1.84E-01	ENSG00000188451
SRP9	-0.1001	1.5990E-01	3.25E-01	ENSG00000143742
SRPK1	-0.1694	4.8888E-03	2.46E-02	ENSG00000096063
SRPK2	-0.1766	1.0685E-04	1.18E-03	ENSG00000135250
SRPK2P	-0.0424	3.7335E-01		ENSG00000253431
SRPK3	0.3976	1.1499E-02	4.72E-02	ENSG00000184343
SRPRA	0.2276	9.2599E-04	6.71E-03	ENSG00000182934
SRPRB	-0.0951	1.5728E-01	3.21E-01	ENSG00000144867
SRPX	0.0149	8.6684E-01	9.32E-01	ENSG00000101955
SRPX2	0.0921	3.5601E-01	5.51E-01	ENSG00000102359
SRR	-0.0524	4.2859E-01	6.20E-01	ENSG00000167720
SRRD	0.1452	3.0054E-02	9.76E-02	ENSG00000100104
SRRM1	-0.2954	2.5456E-07	8.59E-06	ENSG00000133226
SRRM2	-0.2307	4.5881E-06	9.37E-05	ENSG00000167978
SRRM2-AS1	-0.0152	8.5133E-01	9.24E-01	ENSG00000205913
SRRM3	0.2547	5.1091E-03	2.54E-02	ENSG00000177679
SRRM4	0.2718	8.8994E-04	6.49E-03	ENSG00000139767
SRRM5	-0.0293	7.1460E-01	8.41E-01	ENSG00000226763
SRRT	-0.1319	1.5573E-02	5.96E-02	ENSG00000087087
SRSF1	-0.3044	1.1070E-06	2.91E-05	ENSG00000136450
SRSF10	-0.2154	3.9989E-06	8.38E-05	ENSG00000188529
SRSF11	-0.1548	4.0721E-03	2.14E-02	ENSG00000116754
SRSF12	0.1045	1.2842E-01	2.79E-01	ENSG00000154548
SRSF2	-0.1236	2.0838E-02	7.42E-02	ENSG00000161547
SRSF3	-0.3150	7.7565E-07	2.16E-05	ENSG00000112081
SRSF4	-0.0279	5.6528E-01	7.34E-01	ENSG00000116350
SRSF5	-0.2753	3.0105E-04	2.74E-03	ENSG00000100650
SRSF6	-0.2278	1.1514E-02	4.73E-02	ENSG00000124193
SRSF7	-0.0129	8.5055E-01	9.23E-01	ENSG00000115875
SRSF8	0.0393	4.4075E-01	6.31E-01	ENSG00000263465
SRSF9	-0.2559	5.5285E-06	1.09E-04	ENSG00000111786
SRXN1	-0.0166	8.0622E-01	8.97E-01	ENSG00000271303
SRY	-0.0021	9.9681E-01		ENSG00000184895
SS18	0.1534	7.5330E-02	1.92E-01	ENSG00000141380
SS18L1	0.1164	2.1835E-01	3.99E-01	ENSG00000184402
SS18L2	-0.2725	4.9148E-05	6.37E-04	ENSG00000008324
SSB	-0.1906	3.2206E-05	4.48E-04	ENSG00000138385
SSBL5P	-0.0167	6.2627E-01		ENSG00000236048
SSBP1	-0.1164	4.4100E-02	1.30E-01	ENSG00000106028
SSBP2	0.0680	2.4389E-01	4.29E-01	ENSG00000145687
SSBP3	-0.1276	1.2752E-01	2.78E-01	ENSG00000157216
SSBP3-AS1	-0.0267	7.7813E-01	8.80E-01	ENSG00000198711
SSBP4	-0.0550	3.6404E-01	5.60E-01	ENSG00000130511
SSC4D	0.0227	8.1114E-01	9.00E-01	ENSG00000146700
SSC5D	-0.1307	2.0589E-01	3.84E-01	ENSG00000179954
SSH1	-0.1098	6.5523E-02	1.74E-01	ENSG00000084112
SSH2	0.0969	1.6751E-01	3.35E-01	ENSG00000141298
SSH3	0.1862	2.9888E-02	9.72E-02	ENSG00000172830

SSMEM1	-0.0193	7.5019E-01		ENSG00000165120
SSNA1	-0.1143	2.9306E-02	9.58E-02	ENSG00000176101
SSPN	-0.3076	1.4659E-02	5.70E-02	ENSG00000123096
SSR1	-0.0502	2.4520E-01	4.31E-01	ENSG00000124783
SSR2	0.1205	7.1312E-02	1.85E-01	ENSG00000163479
SSR3	-0.3279	6.7465E-05	8.17E-04	ENSG00000114850
SSR4	-0.2273	4.5981E-04	3.85E-03	ENSG00000180879
SSR4P1	0.0225	7.7578E-01	8.79E-01	ENSG00000235374
SSRP1	-0.1635	5.6963E-03	2.77E-02	ENSG00000149136
SST	-0.1736	1.2349E-01	2.72E-01	ENSG00000157005
SSTR1	-0.1640	6.8624E-02	1.80E-01	ENSG00000139874
SSTR2	-0.0393	6.6860E-01	8.09E-01	ENSG00000180616
SSTR3	-0.0192	5.4204E-01		ENSG00000278195
SSTR4	0.0310	2.0177E-01		ENSG00000132671
SSTR5	0.0232	7.4870E-01	8.63E-01	ENSG00000162009
SSTR5-AS1	-0.0052	9.8951E-01		ENSG00000261713
SSU72	-0.0770	9.7524E-02	2.31E-01	ENSG00000160075
SSUH2	-0.0272	7.4464E-01	8.60E-01	ENSG00000125046
SSX2IP	0.0650	4.4226E-01	6.33E-01	ENSG00000117155
ST13	-0.1392	2.1470E-04	2.07E-03	ENSG00000100380
ST13P12	-0.0155	8.7407E-01		ENSG00000248400
ST13P15	-0.0428	7.2948E-01		ENSG00000243759
ST13P17	0.0172	2.5469E-01		ENSG00000253183
ST13P19	-0.0426	2.9950E-01		ENSG00000228110
ST14	0.0465	6.1738E-01	7.75E-01	ENSG00000149418
ST20	-0.5820	1.1235E-03	7.77E-03	ENSG00000180953
ST20-MTHFS	-0.2174	9.4191E-03		ENSG00000259332
ST3GAL2	-0.0583	3.7814E-01	5.73E-01	ENSG00000157350
ST3GAL3	-0.0003	9.9996E-01	1.00E+00	ENSG00000126091
ST3GAL3-AS1	0.0038	9.4533E-01		ENSG00000229444
ST3GAL4	0.2257	8.7754E-03	3.86E-02	ENSG00000110080
ST3GAL5	0.0748	3.7013E-01	5.66E-01	ENSG00000115525
ST3GAL6	0.4794	6.4286E-03	3.04E-02	ENSG00000064225
ST6GAL1	-0.2037	6.5297E-02	1.73E-01	ENSG00000073849
ST6GAL2	-0.1404	1.8504E-01	3.58E-01	ENSG00000144057
ST6GALNAC1	0.0238	7.6858E-01	8.74E-01	ENSG00000070526
ST6GALNAC2	0.0242	8.0397E-01	8.96E-01	ENSG00000070731
ST6GALNAC3	0.0723	3.9884E-01	5.93E-01	ENSG00000184005
ST6GALNAC4	0.0875	3.4587E-01	5.41E-01	ENSG00000136840
ST6GALNAC5	0.0226	8.3074E-01	9.11E-01	ENSG00000117069
ST6GALNAC6	0.0821	2.3406E-01	4.18E-01	ENSG00000160408
ST7	0.0712	3.3586E-01	5.31E-01	ENSG00000004866
ST7-AS1	0.0945	2.1232E-01	3.92E-01	ENSG00000227199
ST7-AS2	-0.1222	2.3438E-01	4.18E-01	ENSG00000226367
ST7-OT4	-0.0478	5.6311E-01		ENSG00000214188
ST7L	0.0774	1.2550E-01	2.75E-01	ENSG00000007341
ST8SIA1	0.0284	7.5804E-01	8.69E-01	ENSG00000111728
ST8SIA2	-0.0179	8.4286E-01	9.18E-01	ENSG00000140557
ST8SIA3	-0.0537	4.3278E-01	6.24E-01	ENSG00000177511

ST8SIA4	-0.0748	3.1294E-01	5.06E-01	ENSG00000113532
ST8SIA5	-0.0499	6.0452E-01	7.64E-01	ENSG00000101638
ST8SIA5-DT	-0.0269	7.6233E-01	8.71E-01	ENSG00000270112
ST8SIA6	-0.0374	6.9503E-01	8.29E-01	ENSG00000148488
ST8SIA6-AS1	0.1108	2.2639E-01	4.09E-01	ENSG00000204832
STAB1	0.3965	2.0143E-02	7.24E-02	ENSG00000010327
STAC	0.5630	1.6364E-04	1.67E-03	ENSG00000144681
STAC2	0.0125	8.7263E-01	9.36E-01	ENSG00000141750
STAC3	-0.0102	8.6408E-01	9.31E-01	ENSG00000185482
STAG1	0.0062	9.3376E-01	9.67E-01	ENSG00000118007
STAG2	-0.1687	3.6914E-03	1.98E-02	ENSG00000101972
STAG3	0.2356	3.0011E-02	9.75E-02	ENSG00000066923
STAG3L5P-PVRIG2P-PIL	0.0072	7.4120E-01		ENSG00000272752
STAM	0.0206	7.7265E-01	8.77E-01	ENSG00000136738
STAM-DT	0.5738	4.6280E-06	9.44E-05	ENSG00000260589
STAM2	0.1990	9.2137E-03	4.00E-02	ENSG00000115145
STAMBP	0.0603	1.9809E-01	3.75E-01	ENSG00000124356
STAMBPL1	-0.0141	8.6574E-01	9.32E-01	ENSG00000138134
STAP1	0.1860	9.8668E-02	2.33E-01	ENSG00000035720
STAP2	-0.0863	2.4839E-01	4.35E-01	ENSG00000178078
STAR	-0.0330	7.2047E-01	8.45E-01	ENSG00000147465
STARD10	-0.0210	7.9089E-01	8.88E-01	ENSG00000214530
STARD13	-0.3792	1.8915E-03	1.17E-02	ENSG00000133121
STARD13-AS	-0.4253	1.4798E-02	5.75E-02	ENSG00000236581
STARD3	0.0370	5.7776E-01	7.44E-01	ENSG00000131748
STARD3NL	0.0098	8.8458E-01	9.42E-01	ENSG00000010270
STARD4	-0.5387	1.4475E-10	1.64E-08	ENSG00000164211
STARD5	0.1262	2.3088E-01	4.14E-01	ENSG00000172345
STARD6	-0.0126	8.9240E-01	9.46E-01	ENSG00000174448
STARD7	0.0030	9.6350E-01	9.81E-01	ENSG00000084090
STARD8	0.5215	6.1407E-03	2.94E-02	ENSG00000130052
STARD9	0.1920	5.4467E-02	1.52E-01	ENSG00000159433
STAT1	-0.2784	8.8147E-05	1.01E-03	ENSG00000115415
STAT2	-0.0228	7.0081E-01	8.33E-01	ENSG00000170581
STAT3	0.1201	1.2151E-02	4.93E-02	ENSG00000168610
STAT4	0.0741	4.5245E-01	6.42E-01	ENSG00000138378
STAT4-AS1	0.0225	3.8807E-01		ENSG00000231858
STAT5A	0.1366	9.9451E-02	2.34E-01	ENSG00000126561
STAT5B	0.1697	1.4827E-02	5.75E-02	ENSG00000173757
STAT6	-0.1598	5.0128E-03	2.50E-02	ENSG00000166888
STATH	-0.0053	7.7196E-01		ENSG00000126549
STAU1	-0.0996	2.7058E-02	9.02E-02	ENSG00000124214
STAU2	-0.0708	1.9892E-01	3.76E-01	ENSG00000040341
STAU2-AS1	-0.0126	9.6225E-01	9.81E-01	ENSG00000253302
STBD1	-0.0412	6.3515E-01	7.87E-01	ENSG00000118804
STC1	0.5602	1.0744E-05	1.86E-04	ENSG00000159167
STC2	0.4850	4.9354E-04	4.05E-03	ENSG00000113739
STEAP1B-AS1	-0.0427	5.2350E-01		ENSG00000232759
STEAP2	-0.0862	3.6389E-01	5.60E-01	ENSG00000157214

STEAP2-AS1	0.0044	7.7269E-01	8.77E-01	ENSG00000227646
STEAP3	-0.5892	1.1961E-04	1.29E-03	ENSG00000115107
STEAP3-AS1	-0.0659	2.8528E-01	4.76E-01	ENSG00000229867
STEAP4	0.0444	2.6555E-01	4.54E-01	ENSG00000127954
STEEP1	-0.1210	4.2302E-02	1.26E-01	ENSG00000018610
STH	0.0466	4.7720E-01	6.64E-01	ENSG00000256762
STIL	0.0333	7.2411E-01	8.47E-01	ENSG00000123473
STIM1	-0.1233	8.3380E-02	2.07E-01	ENSG00000167323
STIM1-AS1	-0.1013	1.9353E-01	3.69E-01	ENSG00000228661
STIM2	0.0572	3.9429E-01	5.89E-01	ENSG00000109689
STIMATE	0.1119	2.8038E-01	4.71E-01	ENSG00000213533
STIMATE-MUSTN1	0.0170	4.3028E-01		ENSG00000248592
STING1	0.0466	6.1617E-01	7.74E-01	ENSG00000184584
STIP1	-0.0632	3.4050E-01	5.36E-01	ENSG00000168439
STK10	0.2613	2.5197E-03	1.47E-02	ENSG00000072786
STK11	0.0511	4.3063E-01	6.22E-01	ENSG00000118046
STK11IP	0.2362	8.7335E-04	6.40E-03	ENSG00000144589
STK16	-0.0943	1.6396E-01	3.30E-01	ENSG00000115661
STK17A	-0.1960	2.5558E-02	8.66E-02	ENSG00000164543
STK17B	-0.0740	4.1984E-01	6.12E-01	ENSG00000081320
STK19	0.2204	1.6549E-04	1.69E-03	ENSG00000204344
STK24	-0.0954	2.5903E-01	4.47E-01	ENSG00000102572
STK25	-0.0555	3.7655E-01	5.72E-01	ENSG00000115694
STK26	0.0837	3.4329E-01	5.38E-01	ENSG00000134602
STK3	-0.1587	4.9111E-02	1.41E-01	ENSG00000104375
STK31	0.0130	1.9691E-01		ENSG00000196335
STK32A	-0.1218	1.1036E-01	2.52E-01	ENSG00000169302
STK32B	0.0041	9.6700E-01	9.83E-01	ENSG00000152953
STK32C	0.2655	1.8734E-04	1.86E-03	ENSG00000165752
STK33	-0.4686	3.0026E-07	9.84E-06	ENSG00000130413
STK35	-0.1992	8.1095E-04	6.04E-03	ENSG00000125834
STK36	0.0904	2.3183E-01	4.15E-01	ENSG00000163482
STK38	0.2886	1.3949E-03	9.27E-03	ENSG00000112079
STK38L	-0.0054	8.7500E-01	9.37E-01	ENSG00000211455
STK39	0.1537	6.0111E-02	1.64E-01	ENSG00000198648
STK4	0.1328	7.1510E-02	1.85E-01	ENSG00000101109
STK4-DT	-0.0315	8.5381E-01	9.25E-01	ENSG00000227477
STK40	0.3565	8.4641E-05	9.78E-04	ENSG00000196182
STKLD1	0.1064	2.7156E-01	4.61E-01	ENSG00000198870
STMN1	-0.2450	1.8875E-03	1.17E-02	ENSG00000117632
STMN2	-0.1123	1.0598E-01	2.45E-01	ENSG00000104435
STMN3	0.0691	2.8244E-01	4.74E-01	ENSG00000197457
STMN4	0.2544	2.2601E-02	7.90E-02	ENSG00000015592
STMND1	-0.0058	9.5039E-01	9.75E-01	ENSG00000230873
STMP1	-0.1810	8.6827E-03	3.83E-02	ENSG00000243317
STN1	0.3407	5.8402E-03	2.82E-02	ENSG00000107960
STOM	-0.1929	5.5345E-02	1.54E-01	ENSG00000148175
STOML1	0.1618	3.7776E-02	1.16E-01	ENSG00000067221
STOML2	-0.0434	4.6060E-01	6.49E-01	ENSG00000165283

STOML3	-0.0743	4.3646E-01	6.28E-01	ENSG00000133115
STON1-GTF2A1L	0.0068	6.4491E-01		ENSG00000068781
STON2	-0.3319	1.4137E-06	3.55E-05	ENSG00000140022
STOX1	-0.1509	8.7372E-02	2.14E-01	ENSG00000165730
STOX2	0.1667	1.6321E-02	6.18E-02	ENSG00000173320
STPG1	-0.1636	3.7484E-02	1.15E-01	ENSG00000001460
STPG2	-0.1418	1.8605E-01	3.60E-01	ENSG00000163116
STPG2-AS1	-0.1468	1.6811E-01	3.36E-01	ENSG00000251620
STPG3	-0.0537	3.2469E-01		ENSG00000197768
STPG3-AS1	0.0038	9.8464E-01	9.92E-01	ENSG00000275549
STPG4	-0.0049	8.3108E-01		ENSG00000239605
STRA6	-0.0218	7.9741E-01	8.92E-01	ENSG00000137868
STRA8	-0.0020	8.0116E-01		ENSG00000146857
STRADB	0.0971	1.8909E-01	3.64E-01	ENSG00000082146
STRAP	0.1636	7.5088E-03	3.42E-02	ENSG00000023734
STRBP	0.0681	2.6948E-01	4.59E-01	ENSG00000165209
STRC	0.4810	8.5120E-04	6.28E-03	ENSG00000242866
STRIP1	0.1655	4.8864E-02	1.41E-01	ENSG00000143093
STRIP2	-0.1736	6.7562E-02	1.78E-01	ENSG00000128578
STRN	-0.0605	4.2387E-01	6.16E-01	ENSG00000115808
STRN3	0.0294	7.0786E-01	8.37E-01	ENSG00000196792
STRN4	0.1253	4.2847E-02	1.28E-01	ENSG00000090372
STS	0.3319	1.5681E-03	1.02E-02	ENSG00000101846
STT3A	-0.1045	2.0312E-01	3.81E-01	ENSG00000134910
STT3B	-0.1988	8.7844E-05	1.01E-03	ENSG00000163527
STUB1	-0.0199	6.7511E-01	8.14E-01	ENSG00000103266
STUB1-DT	0.5317	9.1388E-03	3.98E-02	ENSG00000260394
STUM	0.0210	8.1512E-01	9.02E-01	ENSG00000203685
STX10	0.1157	3.6327E-02	1.13E-01	ENSG00000104915
STX11	-0.1515	1.4019E-01	2.96E-01	ENSG00000135604
STX12	0.2484	2.6709E-03	1.54E-02	ENSG00000117758
STX16	0.0299	7.1698E-01	8.43E-01	ENSG00000124222
STX17	-0.0508	3.6171E-01	5.58E-01	ENSG00000136874
STX18	-0.0802	1.3033E-01	2.82E-01	ENSG00000168818
STX18-AS1	0.0076	9.2144E-01	9.61E-01	ENSG00000247708
STX18-IT1	-0.1424	5.1037E-02		ENSG00000248221
STX18P1	0.0285	4.2482E-01		ENSG00000266876
STX19	-0.1107	2.8449E-01	4.76E-01	ENSG00000178750
STX1A	0.2351	8.1928E-03	3.66E-02	ENSG00000106089
STX1B	-0.1132	1.4866E-01	3.09E-01	ENSG00000099365
STX2	0.1460	7.7964E-02	1.97E-01	ENSG00000111450
STX3	-0.0815	2.0151E-01	3.79E-01	ENSG00000166900
STX4	0.0496	4.1211E-01	6.05E-01	ENSG00000103496
STX5	0.0663	3.5165E-01	5.47E-01	ENSG00000162236
STX5-DT	0.0192	5.5307E-01		ENSG00000256690
STX6	0.2323	1.1403E-04	1.25E-03	ENSG00000135823
STX7	-0.0247	6.7811E-01	8.16E-01	ENSG00000079950
STX8	-0.2042	1.8717E-04	1.86E-03	ENSG00000170310
STXBP1	0.0299	6.6823E-01	8.09E-01	ENSG00000136854

STXBP2	-0.0337	7.1278E-01	8.40E-01	ENSG00000076944
STXBP3	-0.0941	1.1427E-01	2.58E-01	ENSG00000116266
STXBP4	-0.0709	3.8414E-01	5.79E-01	ENSG00000166263
STXBP5	0.2855	5.2455E-03	2.60E-02	ENSG00000164506
STXBP5-AS1	0.5030	5.5281E-09	3.56E-07	ENSG00000233452
STXBP5L	0.2556	1.4317E-02	5.60E-02	ENSG00000145087
STXBP6	0.2058	6.6000E-02	1.75E-01	ENSG00000168952
STYK1	0.0540	5.7103E-01	7.39E-01	ENSG00000060140
STYX	-0.1087	1.0095E-01	2.36E-01	ENSG00000198252
STYXL1	-0.2934	7.7132E-03	3.49E-02	ENSG00000127952
SUB1	-0.2967	8.5382E-06	1.55E-04	ENSG00000113387
SUCLA2	-0.0151	8.2857E-01	9.10E-01	ENSG00000136143
SUCLG1	-0.0950	1.3649E-01	2.91E-01	ENSG00000163541
SUCLG2	-0.1359	1.0561E-01	2.44E-01	ENSG00000172340
SUCLG2-DT	-0.2825	1.7926E-02	6.64E-02	ENSG00000241316
SUCNR1	0.0243	6.0061E-01		ENSG00000198829
SUCO	-0.0853	1.1050E-01	2.52E-01	ENSG00000094975
SUDS3	-0.1560	3.4907E-02	1.09E-01	ENSG00000111707
SUFU	-0.0582	4.3960E-01	6.30E-01	ENSG00000107882
SUGCT	-0.2566	1.3527E-02	5.36E-02	ENSG00000175600
SUGCT-AS1	-0.0094	7.0988E-01		ENSG00000203446
SUGP1	0.1334	4.9589E-02	1.42E-01	ENSG00000105705
SUGP2	0.1577	3.3806E-02	1.07E-01	ENSG00000064607
SUGT1	-0.1621	4.3752E-03	2.26E-02	ENSG00000165416
SUGT1-DT	0.3257	2.5063E-03	1.46E-02	ENSG00000273723
SUGT1P2	0.0403	6.0852E-01	7.68E-01	ENSG00000213842
SUGT1P4-STRA6LP	-0.0090	8.2320E-01	9.07E-01	ENSG00000254876
SUGT1P4-STRA6LP-CCD	-0.0196	7.3906E-01		ENSG00000255036
SULF1	-0.4470	2.8818E-03	1.63E-02	ENSG00000137573
SULF2	0.1184	1.7620E-01	3.47E-01	ENSG00000196562
SULT1A1	0.1778	1.1994E-01	2.67E-01	ENSG00000196502
SULT1A2	0.0658	4.6296E-01	6.51E-01	ENSG00000197165
SULT1A3	0.3923	1.1104E-03	7.70E-03	ENSG00000261052
SULT1A4	0.2832	3.9870E-02	1.21E-01	ENSG00000213648
SULT1B1	0.0899	2.3503E-01	4.19E-01	ENSG00000173597
SULT1C2	0.1401	1.5697E-01	3.20E-01	ENSG00000198203
SULT1C4	-0.1218	2.0471E-01	3.83E-01	ENSG00000198075
SULT1D1P2	0.0057	6.1997E-01		ENSG00000242968
SULT1E1	-0.2068	3.1330E-02	1.01E-01	ENSG00000109193
SULT2A1	0.0303	6.1926E-01	7.75E-01	ENSG00000105398
SULT2B1	-0.0353	7.0815E-01	8.37E-01	ENSG00000088002
SULT4A1	-0.0457	4.5017E-01	6.40E-01	ENSG00000130540
SULT6B1	-0.0507	2.7836E-01		ENSG00000138068
SUMF1	-0.1483	5.8351E-02	1.60E-01	ENSG00000144455
SUMF2	-0.1523	7.2201E-02	1.86E-01	ENSG00000129103
SUMO1	-0.2730	3.2352E-06	7.02E-05	ENSG00000116030
SUMO2	-0.3194	1.3773E-06	3.49E-05	ENSG00000188612
SUMO2P15	0.0171	3.3377E-01		ENSG00000271236
SUMO3	-0.1485	2.3373E-03	1.39E-02	ENSG00000184900

SUMO4	-0.1777	1.1930E-01	2.66E-01	ENSG00000177688
SUN1	-0.2458	5.1185E-07	1.52E-05	ENSG00000164828
SUN2	0.2498	6.4196E-03	3.04E-02	ENSG00000100242
SUN3	0.0075	3.9760E-01		ENSG00000164744
SUOX	0.2134	2.1327E-02	7.56E-02	ENSG00000139531
SUPT16H	-0.0134	7.9202E-01	8.88E-01	ENSG00000092201
SUPT16HP1	-0.0048	9.1495E-01		ENSG00000256238
SUPT20H	0.0231	7.6379E-01	8.72E-01	ENSG00000102710
SUPT20HL1	0.0010	6.5525E-01		ENSG00000223731
SUPT20HL2	-0.0017	8.6226E-01		ENSG00000223611
SUPT3H	-0.4884	6.0635E-05	7.50E-04	ENSG00000196284
SUPT4H1	-0.0933	2.1220E-01	3.92E-01	ENSG00000213246
SUPT5H	-0.0267	5.9767E-01	7.59E-01	ENSG00000196235
SUPT6H	0.0002	9.9868E-01	1.00E+00	ENSG00000109111
SUPT7L	-0.2540	1.7262E-05	2.73E-04	ENSG00000119760
SUPV3L1	-0.0877	1.9716E-01	3.74E-01	ENSG00000156502
SURF1	0.0795	1.9786E-01	3.75E-01	ENSG00000148290
SURF2	0.0880	2.6184E-01	4.51E-01	ENSG00000148291
SURF4	-0.0289	6.2112E-01	7.77E-01	ENSG00000148248
SURF6	0.1754	1.5002E-02	5.80E-02	ENSG00000148296
SUSD1	-0.1670	7.9506E-02	2.00E-01	ENSG00000106868
SUSD2	0.0238	8.0410E-01	8.96E-01	ENSG00000099994
SUSD3	0.2300	5.5210E-02	1.54E-01	ENSG00000157303
SUSD4	0.3361	1.1652E-06	3.04E-05	ENSG00000143502
SUSD6	-0.4364	3.6245E-08	1.68E-06	ENSG00000100647
SUV39H1	-0.0303	7.3353E-01	8.54E-01	ENSG00000101945
SUV39H2	0.0857	2.4916E-01	4.35E-01	ENSG00000152455
SUZ12	-0.3141	6.8009E-05	8.21E-04	ENSG00000178691
SV2A	-0.1035	1.7140E-01	3.40E-01	ENSG00000159164
SV2B	0.2877	2.4251E-03	1.43E-02	ENSG00000185518
SV2C	0.0125	8.9358E-01	9.47E-01	ENSG00000122012
SVBP	-0.0800	1.3192E-01	2.84E-01	ENSG00000177868
SVEP1	0.1526	1.1219E-01	2.55E-01	ENSG00000165124
SVIL	-0.0392	6.2798E-01	7.82E-01	ENSG00000197321
SVIP	-0.0163	7.9323E-01	8.89E-01	ENSG00000198168
SVOP	0.1686	6.3068E-02	1.69E-01	ENSG00000166111
SVOPL	-0.0172	8.1739E-01	9.03E-01	ENSG00000157703
SWAP70	0.0538	4.1867E-01	6.11E-01	ENSG00000133789
SWI5	-0.1039	4.5327E-02	1.33E-01	ENSG00000175854
SWSAP1	0.0528	4.4944E-01	6.39E-01	ENSG00000173928
SWT1	0.1882	6.2467E-02	1.68E-01	ENSG00000116668
SYAP1	0.0312	5.8150E-01	7.47E-01	ENSG00000169895
SYBU	-0.1453	1.4795E-02	5.75E-02	ENSG00000147642
SYCE1	-0.0053	9.0030E-01		ENSG00000171772
SYCE1L	-0.3905	3.5683E-03	1.93E-02	ENSG00000205078
SYCE2	-0.0056	9.4361E-01	9.72E-01	ENSG00000161860
SYCE3	-0.2128	7.5202E-02	1.92E-01	ENSG00000217442
SYCP2	-0.4003	1.3848E-02	5.46E-02	ENSG00000196074
SYCP2L	0.1351	2.0106E-01	3.78E-01	ENSG00000153157

SYCP3	0.0129	4.8107E-01		ENSG00000139351
SYDE1	-0.3055	2.7200E-02	9.06E-02	ENSG00000105137
SYDE2	0.1091	2.0631E-01	3.85E-01	ENSG00000097096
SYF2	-0.1915	7.9407E-04	5.96E-03	ENSG00000117614
SYF2P2	-0.0209	7.3078E-01		ENSG00000233148
SYK	0.1340	1.4383E-01	3.02E-01	ENSG00000165025
SYMPK	0.2962	2.0660E-03	1.26E-02	ENSG00000125755
SYN1	0.1036	1.1740E-01	2.63E-01	ENSG00000008056
SYN2	-0.0899	3.1464E-01	5.08E-01	ENSG00000157152
SYN3	0.1239	1.6909E-01	3.37E-01	ENSG00000185666
SYNC	-0.4648	5.4684E-03	2.69E-02	ENSG00000162520
SYNCRIP	-0.2386	7.0703E-03	3.26E-02	ENSG00000135316
SYNDIG1	0.0306	7.4768E-01	8.62E-01	ENSG00000101463
SYNDIG1L	0.1347	2.1025E-01	3.90E-01	ENSG00000183379
SYNE1	0.0286	7.2095E-01	8.45E-01	ENSG00000131018
SYNE2	-0.1362	7.6224E-02	1.94E-01	ENSG00000054654
SYNE3	0.0314	7.4286E-01	8.59E-01	ENSG00000176438
SYNE4	-0.1131	2.3502E-01	4.19E-01	ENSG00000181392
SYNGAP1	0.0049	9.5590E-01	9.77E-01	ENSG00000197283
SYNGAP1-AS1	0.0075	9.0264E-01	9.52E-01	ENSG00000274259
SYNGR1	0.1570	1.9384E-02	7.03E-02	ENSG00000100321
SYNGR2	-0.0362	6.2325E-01	7.79E-01	ENSG00000108639
SYNGR3	0.1392	6.5646E-02	1.74E-01	ENSG00000127561
SYNGR4	-0.5155	1.8504E-04	1.85E-03	ENSG00000105467
SYNJ1	0.1265	1.5070E-01	3.12E-01	ENSG00000159082
SYNJ2	0.1552	1.0051E-01	2.36E-01	ENSG00000078269
SYNJ2-IT1	-0.0383	2.3244E-01		ENSG00000233496
SYNJ2BP	0.0127	8.4903E-01	9.22E-01	ENSG00000213463
SYNJ2BP-COX16	0.0077	9.6716E-01		ENSG00000258644
SYNM	-0.2196	1.6596E-02	6.26E-02	ENSG00000182253
SYNPO	-0.1543	1.5626E-01	3.19E-01	ENSG00000171992
SYNPO2	0.0247	7.8997E-01	8.87E-01	ENSG00000172403
SYNPO2L	0.2968	3.5081E-02	1.10E-01	ENSG00000166317
SYNPR	0.3517	3.1536E-03	1.75E-02	ENSG00000163630
SYNPR-AS1	0.1198	2.4880E-01	4.35E-01	ENSG00000241359
SYNRG	-0.0028	9.5305E-01	9.76E-01	ENSG00000275066
SYP	0.3920	8.1181E-05	9.45E-04	ENSG00000102003
SYP-AS1	-0.0954	2.4911E-01	4.35E-01	ENSG00000237341
SYPL1	-0.3662	6.6749E-05	8.11E-04	ENSG00000008282
SYPL1P2	-0.0630	5.0278E-01	6.84E-01	ENSG00000265683
SYPL2	-0.1288	1.8685E-01	3.61E-01	ENSG00000143028
SYS1	-0.2334	2.8733E-04	2.63E-03	ENSG00000204070
SYS1-DBNDD2	-0.1074	2.9467E-01	4.87E-01	ENSG00000254806
SYT1	-0.1405	6.5945E-02	1.75E-01	ENSG00000067715
SYT10	-0.0571	5.5366E-01	7.25E-01	ENSG00000110975
SYT11	-0.0485	4.9997E-01	6.82E-01	ENSG00000132718
SYT12	0.4581	1.0323E-03	7.29E-03	ENSG00000173227
SYT13	0.2343	1.8679E-02	6.85E-02	ENSG00000019505
SYT14	-0.2725	1.6853E-03	1.07E-02	ENSG00000143469

SYT15	-0.0133	6.1747E-01	7.75E-01	ENSG00000204176
SYT15B	0.1261	1.4982E-01	3.10E-01	ENSG00000277758
SYT16	-0.1999	6.4266E-02	1.71E-01	ENSG00000139973
SYT17	-0.0715	2.7769E-01	4.68E-01	ENSG00000103528
SYT2	0.1449	1.8148E-01	3.53E-01	ENSG00000143858
SYT3	0.1158	1.5163E-01	3.13E-01	ENSG00000213023
SYT4	0.2605	1.5036E-02	5.81E-02	ENSG00000132872
SYT5	0.1713	5.0683E-02	1.44E-01	ENSG00000129990
SYT6	0.3815	5.0383E-06	1.01E-04	ENSG00000134207
SYT7	0.0074	9.2876E-01	9.65E-01	ENSG00000011347
SYT8	0.0919	1.3479E-01	2.88E-01	ENSG00000149043
SYT9	-0.0602	4.2921E-01	6.21E-01	ENSG00000170743
SYT9-AS1	0.0835	3.6583E-01	5.62E-01	ENSG00000251364
SYTL1	0.4492	3.7978E-04	3.31E-03	ENSG00000142765
SYTL2	0.0694	3.8100E-01	5.76E-01	ENSG00000137501
SYTL3	-0.0891	3.7153E-01	5.67E-01	ENSG00000164674
SYTL4	0.2996	1.9395E-02	7.04E-02	ENSG00000102362
SYTL5	0.5244	7.1438E-04	5.47E-03	ENSG00000147041
SYVN1	0.0354	6.0347E-01	7.63E-01	ENSG00000162298
SZRD1	-0.3090	1.2418E-06	3.19E-05	ENSG00000055070
SZT2	0.4975	5.0157E-08	2.23E-06	ENSG00000198198
TAB1	-0.0468	5.5736E-01	7.28E-01	ENSG00000100324
TAB2	-0.4139	5.9215E-06	1.15E-04	ENSG00000055208
TAB2-AS1	-0.0058	9.1093E-01		ENSG00000225924
TAB3	0.1621	5.2650E-02	1.48E-01	ENSG00000157625
TAC1	0.5771	1.5019E-03	9.82E-03	ENSG00000006128
TAC3	0.0103	9.1332E-01	9.56E-01	ENSG00000166863
TACC1	-0.3874	1.2550E-08	6.88E-07	ENSG00000147526
TACC2	0.1466	2.8756E-02	9.45E-02	ENSG00000138162
TACC3	-0.3472	8.1748E-03	3.66E-02	ENSG00000013810
TACO1	0.0203	7.8573E-01	8.85E-01	ENSG00000136463
TACR1	-0.1851	9.9651E-02	2.34E-01	ENSG00000115353
TACR2	-0.0962	2.3612E-01	4.20E-01	ENSG00000075073
TACR3	0.0571	5.5273E-01	7.24E-01	ENSG00000169836
TACSTD2	0.5031	4.0350E-03	2.12E-02	ENSG00000184292
TADA1	-0.1648	3.2725E-02	1.04E-01	ENSG00000152382
TADA2A	0.0017	9.7976E-01	9.89E-01	ENSG00000276234
TADA2B	-0.1555	1.9886E-02	7.17E-02	ENSG00000173011
TADA3	-0.1107	5.2578E-02	1.48E-01	ENSG00000171148
TAF1	0.1832	4.6542E-02	1.36E-01	ENSG00000147133
TAF10	-0.1447	1.5246E-02	5.87E-02	ENSG00000166337
TAF11	-0.0990	4.4480E-02	1.31E-01	ENSG00000064995
TAF12	0.0938	7.9726E-02	2.00E-01	ENSG00000120656
TAF12-DT	0.0483	6.1578E-01	7.74E-01	ENSG00000229388
TAF13	-0.2242	1.3545E-02	5.36E-02	ENSG00000197780
TAF15	0.5055	7.2082E-09	4.36E-07	ENSG00000270647
TAF1A	0.1869	7.4607E-02	1.91E-01	ENSG00000143498
TAF1A-AS1	0.0361	6.1076E-01	7.69E-01	ENSG00000225265
TAF1B	-0.1102	1.2597E-01	2.76E-01	ENSG00000115750

TAF1C	0.2939	7.3357E-04	5.58E-03	ENSG00000103168
TAF1D	-0.1507	4.4475E-02	1.31E-01	ENSG00000166012
TAF2	0.0307	6.3745E-01	7.89E-01	ENSG00000064313
TAF3	0.1187	8.2989E-02	2.06E-01	ENSG00000165632
TAF4	-0.0433	6.2673E-01	7.81E-01	ENSG00000130699
TAF4B	-0.0397	6.5251E-01	7.99E-01	ENSG00000141384
TAF5	0.3326	1.9542E-03	1.20E-02	ENSG00000148835
TAF5L	0.0812	3.2515E-01	5.19E-01	ENSG00000135801
TAF6	0.0583	4.2240E-01	6.15E-01	ENSG00000106290
TAF6L	0.1045	7.8036E-02	1.97E-01	ENSG00000162227
TAF7	-0.3443	6.7401E-08	2.83E-06	ENSG00000178913
TAF7L	0.0899	2.7576E-01	4.66E-01	ENSG00000102387
TAF8	0.0541	5.2434E-01	7.01E-01	ENSG00000137413
TAF9	0.0381	4.5188E-01	6.41E-01	ENSG00000273841
TAF9B	0.1595	5.7736E-02	1.59E-01	ENSG00000187325
TAFA1	0.0158	9.6808E-01	9.84E-01	ENSG00000183662
TAFA2	-0.1123	1.8810E-01	3.63E-01	ENSG00000198673
TAFA3	0.3582	2.3831E-02	8.21E-02	ENSG00000184599
TAFA4	-0.4607	1.7341E-03	1.10E-02	ENSG00000163377
TAFA5	-0.1676	5.0824E-02	1.45E-01	ENSG00000219438
TAFAZZIN	0.1859	3.5888E-03	1.94E-02	ENSG00000102125
TAGAP	-0.0015	9.8256E-01		ENSG00000164691
TAGAP-AS1	0.1448	1.0082E-01	2.36E-01	ENSG00000271913
TAGLN	-0.2943	5.8817E-03	2.84E-02	ENSG00000149591
TAGLN2	-0.0073	9.1683E-01	9.58E-01	ENSG00000158710
TAGLN3	-0.0657	2.9877E-01	4.91E-01	ENSG00000144834
TAL2	0.0075	9.2927E-01	9.65E-01	ENSG00000186051
TALAM1	-0.0365	6.9975E-01	8.32E-01	ENSG00000289740
TALDO1	-0.2035	4.9113E-02	1.41E-01	ENSG00000177156
TAMALIN	0.3603	8.0941E-04	6.03E-03	ENSG00000161835
TAMM41	0.1285	2.1810E-02	7.69E-02	ENSG00000144559
TANC1	0.1414	1.1797E-01	2.64E-01	ENSG00000115183
TANC2	0.0678	2.8117E-01	4.72E-01	ENSG00000170921
TANGO2	0.0994	1.7567E-01	3.46E-01	ENSG00000183597
TANGO6	0.2527	6.3938E-03	3.03E-02	ENSG00000103047
TANK	0.0472	4.6054E-01	6.49E-01	ENSG00000136560
TAOK1	-0.0302	6.9903E-01	8.32E-01	ENSG00000160551
TAOK2	0.3021	2.1850E-04	2.10E-03	ENSG00000149930
TAOK3	0.3261	2.8373E-05	4.05E-04	ENSG00000135090
TAP1	-0.1267	1.4694E-01	3.06E-01	ENSG00000168394
TAP2	-0.0682	5.0809E-01	6.88E-01	ENSG00000204267
TAPBP	-0.5246	5.4271E-05	6.85E-04	ENSG00000231925
TAPBPL	-0.0584	5.0293E-01	6.84E-01	ENSG00000139192
TAPT1	-0.0981	1.8620E-01	3.60E-01	ENSG00000169762
TAPT1-AS1	-0.0285	7.4720E-01	8.61E-01	ENSG00000263327
TARBP1	0.1312	1.1727E-01	2.63E-01	ENSG00000059588
TARBP2	0.0744	1.6551E-01	3.32E-01	ENSG00000139546
TARDBP	-0.0038	9.4305E-01	9.72E-01	ENSG00000120948
TARID	-0.0021	9.3435E-01	9.67E-01	ENSG00000227954

TARS1	-0.2346	6.3706E-04	4.99E-03	ENSG00000113407
TARS2	0.1697	1.8735E-02	6.86E-02	ENSG00000143374
TARS3	0.1465	2.3624E-02	8.16E-02	ENSG00000185418
TAS1R1	0.0003	3.9863E-01	5.93E-01	ENSG00000173662
TAS1R3	-0.1707	1.2567E-01	2.75E-01	ENSG00000169962
TAS2R10	-0.0006	9.4049E-01		ENSG00000121318
TAS2R13	0.0125	2.4271E-01		ENSG00000212128
TAS2R14	-0.5747	2.8265E-04	2.60E-03	ENSG00000212127
TAS2R15P	0.0151	8.7466E-01	9.37E-01	ENSG00000212125
TAS2R18P	-0.0048	9.4541E-01	9.73E-01	ENSG00000256981
TAS2R19	0.0235	7.6544E-01	8.73E-01	ENSG00000212124
TAS2R20	0.3215	4.3124E-03	2.24E-02	ENSG00000255837
TAS2R2P	-0.0004	4.3822E-01		ENSG00000203523
TAS2R30	-0.0591	2.7509E-01		ENSG00000256188
TAS2R31	0.0031	7.1156E-01		ENSG00000256436
TAS2R4	0.0005	8.2863E-01		ENSG00000127364
TAS2R5	-0.0379	7.0489E-01		ENSG00000127366
TAS2R60	0.0096	5.1354E-01		ENSG00000185899
TAS2R64P	-0.0020	7.7053E-01		ENSG00000256274
TASOR	-0.1176	5.8971E-02	1.61E-01	ENSG00000163946
TASOR2	-0.0497	5.1270E-01	6.92E-01	ENSG00000108021
TASP1	0.2041	2.8162E-03	1.61E-02	ENSG00000089123
TAT	0.5915	9.5357E-03	4.10E-02	ENSG00000198650
TAT-AS1	0.0751	4.4172E-01	6.32E-01	ENSG00000260886
TATDN1	0.0124	8.6815E-01	9.33E-01	ENSG00000147687
TATDN1P1	-0.0579	4.7934E-01	6.65E-01	ENSG00000234369
TATDN2	-0.0593	5.0419E-01	6.85E-01	ENSG00000157014
TATDN3	0.1181	8.4515E-02	2.09E-01	ENSG00000203705
TAX1BP1	-0.1342	1.2570E-04	1.34E-03	ENSG00000106052
TAX1BP3	-0.4622	6.9828E-03	3.23E-02	ENSG00000213977
TBC1D1	-0.1732	4.5250E-02	1.33E-01	ENSG00000065882
TBC1D10A	-0.0157	8.2714E-01	9.09E-01	ENSG00000099992
TBC1D10B	-0.0527	5.1497E-01	6.94E-01	ENSG00000169221
TBC1D12	0.0028	9.8163E-01	9.90E-01	ENSG00000108239
TBC1D13	0.1708	4.6680E-03	2.37E-02	ENSG00000107021
TBC1D14	0.1387	1.6348E-01	3.30E-01	ENSG00000132405
TBC1D15	0.0738	2.1214E-01	3.92E-01	ENSG00000121749
TBC1D16	-0.1302	5.1884E-02	1.47E-01	ENSG00000167291
TBC1D17	0.0759	2.0310E-01	3.81E-01	ENSG00000104946
TBC1D19	0.0009	9.9448E-01	9.97E-01	ENSG00000109680
TBC1D2	0.1016	2.7657E-01	4.67E-01	ENSG00000095383
TBC1D20	-0.0020	9.5941E-01	9.80E-01	ENSG00000125875
TBC1D22A	0.1425	1.6791E-02	6.31E-02	ENSG00000054611
TBC1D22A-AS1	0.1114	1.4017E-01	2.96E-01	ENSG00000280080
TBC1D22B	-0.0561	4.2964E-01	6.21E-01	ENSG00000065491
TBC1D23	0.1905	8.6142E-03	3.80E-02	ENSG00000036054
TBC1D24	-0.0501	5.3383E-01	7.08E-01	ENSG00000162065
TBC1D25	0.0835	2.9755E-01	4.90E-01	ENSG00000068354
TBC1D2B	-0.0781	2.5711E-01	4.45E-01	ENSG00000167202

TBC1D3	0.0096	9.1892E-01	9.60E-01	ENSG00000274512
TBC1D3	0.0079	9.3153E-01	9.66E-01	ENSG00000274611
TBC1D30	-0.0288	7.4023E-01	8.57E-01	ENSG00000111490
TBC1D31	-0.0648	4.6171E-01	6.50E-01	ENSG00000156787
TBC1D32	0.2513	7.2846E-03	3.34E-02	ENSG00000146350
TBC1D3B	-0.0022	9.8127E-01	9.90E-01	ENSG00000274808
TBC1D3C	0.0130	8.8849E-01	9.44E-01	ENSG00000278299
TBC1D3D	-0.0345	7.1835E-01	8.44E-01	ENSG00000274419
TBC1D3E	0.0154	8.6950E-01	9.34E-01	ENSG00000278599
TBC1D3F	-0.0165	8.6283E-01	9.30E-01	ENSG00000275954
TBC1D3G	-0.0106	9.0543E-01	9.53E-01	ENSG00000260287
TBC1D3H	-0.0296	7.4963E-01	8.63E-01	ENSG00000274226
TBC1D3I	0.0075	9.3715E-01	9.69E-01	ENSG00000274933
TBC1D3JP	0.0021	5.1925E-01		ENSG00000274658
TBC1D3K	0.0409	6.5900E-01	8.03E-01	ENSG00000273513
TBC1D3P1	-0.0167	8.3830E-01	9.15E-01	ENSG00000238283
TBC1D4	-0.0381	6.4048E-01	7.91E-01	ENSG00000136111
TBC1D5	-0.1737	3.0355E-03	1.70E-02	ENSG00000131374
TBC1D7	0.1100	1.5378E-02	5.91E-02	ENSG00000145979
TBC1D8	0.5176	2.1737E-06	5.06E-05	ENSG00000204634
TBC1D8-AS1	0.1311	2.1916E-01	4.00E-01	ENSG00000272902
TBC1D8B	-0.1933	8.3411E-02	2.07E-01	ENSG00000133138
TBC1D9	-0.0609	3.3933E-01	5.34E-01	ENSG00000109436
TBC1D9B	0.0373	4.9751E-01	6.80E-01	ENSG00000197226
TBCA	-0.3973	2.5386E-08	1.25E-06	ENSG00000171530
TBCB	-0.1481	1.7109E-03	1.09E-02	ENSG00000105254
TBCC	-0.1317	1.8294E-02	6.74E-02	ENSG00000124659
TBCCD1	0.1215	1.4857E-01	3.09E-01	ENSG00000113838
TBCD	0.1011	1.3086E-01	2.83E-01	ENSG00000141556
TBCEL	0.2083	3.3184E-02	1.05E-01	ENSG00000154114
TBCK	-0.4976	2.9426E-05	4.17E-04	ENSG00000145348
TBILA	-0.0951	2.1175E-01	3.92E-01	ENSG00000261488
TBK1	0.1974	1.1820E-02	4.82E-02	ENSG00000183735
TBKBP1	-0.1355	1.6923E-01	3.37E-01	ENSG00000198933
TBL1X	0.0489	5.0295E-01	6.84E-01	ENSG00000101849
TBL1XR1	-0.0492	4.9163E-01	6.75E-01	ENSG00000177565
TBL1XR1-AS1	-0.0065	7.2140E-01		ENSG00000231310
TBL1Y	-0.2831	2.3285E-02	8.08E-02	ENSG00000092377
TBL2	0.0328	6.5410E-01	8.00E-01	ENSG00000106638
TBL3	0.0905	1.7528E-01	3.45E-01	ENSG00000183751
TBP	0.3546	7.7192E-07	2.16E-05	ENSG00000112592
TBPL1	0.0075	9.8631E-01	9.93E-01	ENSG00000028839
TBPL2	0.1126	1.7456E-02	6.50E-02	ENSG00000182521
TBR1	-0.0193	7.9598E-01	8.91E-01	ENSG00000136535
TBRG1	0.1969	2.7693E-03	1.58E-02	ENSG00000154144
TBRG4	-0.0247	7.3497E-01	8.54E-01	ENSG00000136270
TBX1	-0.5385	6.1223E-04	4.84E-03	ENSG00000184058
TBX15	0.0108	5.2740E-01		ENSG00000092607
TBX18	-0.2279	6.4164E-02	1.71E-01	ENSG00000112837

TBX21	-0.0074	9.1794E-01		ENSG00000073861
TBX22	0.0023	8.6701E-01		ENSG00000122145
TBX3	-0.0465	6.3190E-01	7.85E-01	ENSG00000135111
TBX4	-0.0032	6.5891E-01		ENSG00000121075
TBX6	0.0597	5.0117E-01	6.83E-01	ENSG00000149922
TBXA2R	-0.0957	1.3586E-01	2.90E-01	ENSG00000006638
TBXAS1	-0.1062	2.2051E-01	4.02E-01	ENSG00000059377
TC2N	-0.2313	9.5451E-03	4.11E-02	ENSG00000165929
TCAF1	-0.1403	1.0354E-02	4.36E-02	ENSG00000198420
TCAF1P1	0.1877	7.6169E-02	1.94E-01	ENSG00000223459
TCAF2	0.0411	6.6750E-01	8.09E-01	ENSG00000170379
TCAIM	-0.0895	2.0220E-01	3.80E-01	ENSG00000179152
TCAM1P	-0.0214	5.2103E-01	6.98E-01	ENSG00000240280
TCAP	0.0538	5.8141E-01	7.47E-01	ENSG00000173991
TCEA1	-0.2222	3.9014E-07	1.21E-05	ENSG00000187735
TCEA2	-0.0731	1.2186E-01	2.70E-01	ENSG00000171703
TCEA3	0.4104	4.0169E-03	2.12E-02	ENSG00000204219
TCEAL1	-0.2643	5.8217E-07	1.70E-05	ENSG00000172465
TCEAL2	-0.1245	6.8902E-02	1.80E-01	ENSG00000184905
TCEAL3	-0.1234	2.1017E-02	7.47E-02	ENSG00000196507
TCEAL4	-0.2740	3.5603E-08	1.66E-06	ENSG00000133142
TCEAL5	0.1431	1.1570E-01	2.60E-01	ENSG00000204065
TCEAL6	-0.0602	5.3238E-01	7.07E-01	ENSG00000204071
TCEAL7	-0.0616	4.0189E-01	5.96E-01	ENSG00000182916
TCEAL8	-0.2220	1.6756E-04	1.70E-03	ENSG00000180964
TCEAL9	-0.3347	1.2030E-07	4.51E-06	ENSG00000185222
TCEANC	0.0811	3.7291E-01	5.68E-01	ENSG00000176896
TCEANC2	-0.0889	2.0024E-01	3.77E-01	ENSG00000116205
TCERG1	0.2015	1.1385E-02	4.69E-02	ENSG00000113649
TCERG1L-AS1	0.1578	6.3788E-02	1.70E-01	ENSG00000230098
TCF12	-0.1351	1.7561E-02	6.53E-02	ENSG00000140262
TCF12-DT	-0.0932	3.4940E-01	5.44E-01	ENSG00000285331
TCF15	-0.0045	9.5347E-01	9.76E-01	ENSG00000125878
TCF19	-0.0260	7.8191E-01	8.82E-01	ENSG00000137310
TCF20	-0.0538	5.4268E-01	7.15E-01	ENSG00000100207
TCF23	0.0090	2.9553E-01		ENSG00000163792
TCF24	0.0022	8.4093E-01		ENSG00000261787
TCF25	-0.2635	5.1643E-08	2.28E-06	ENSG00000141002
TCF3	-0.1030	1.5363E-01	3.16E-01	ENSG00000071564
TCF4	-0.3876	1.7743E-04	1.79E-03	ENSG00000196628
TCF4-AS1	-0.0360	3.5464E-01		ENSG00000267028
TCF7	-0.2694	1.5987E-03	1.03E-02	ENSG00000081059
TCF7L1	-0.3089	1.3085E-04	1.38E-03	ENSG00000152284
TCF7L1-IT1	-0.0236	7.5085E-01		ENSG00000231134
TCF7L2	-0.4455	4.0710E-06	8.50E-05	ENSG00000148737
TCFL5	-0.0729	3.8055E-01	5.75E-01	ENSG00000101190
TCHP	-0.0299	6.8578E-01	8.22E-01	ENSG00000139437
TCIM	-0.0201	7.9784E-01	8.92E-01	ENSG00000176907
TCIRG1	-0.0678	4.8053E-01	6.66E-01	ENSG00000110719

TCN1	0.0265	2.4944E-01	4.36E-01	ENSG00000134827
TCN2	-0.0874	1.6225E-01	3.28E-01	ENSG00000185339
TCOF1	0.1290	4.5794E-02	1.34E-01	ENSG00000070814
TCP1	0.1086	8.1517E-02	2.03E-01	ENSG00000120438
TCP10L	-0.0315	4.2901E-01		ENSG00000242220
TCP11	0.1514	1.3147E-01	2.84E-01	ENSG00000124678
TCP11L1	0.1580	6.7437E-02	1.77E-01	ENSG00000176148
TCP11L2	0.1820	2.5608E-02	8.67E-02	ENSG00000166046
TCP11X1	0.4363	6.8572E-03	3.19E-02	ENSG00000268235
TCP11X2	0.1661	4.3770E-02	1.30E-01	ENSG00000215029
TCP1P3	-0.0161	9.4427E-01		ENSG00000256849
TCTA	0.0741	2.7796E-01	4.69E-01	ENSG00000145022
TCTE1	0.1299	1.6487E-01	3.31E-01	ENSG00000146221
TCTN1	-0.3226	6.2517E-04	4.92E-03	ENSG00000204852
TCTN2	-0.0934	3.0068E-01	4.93E-01	ENSG00000168778
TCTN3	-0.0284	6.5606E-01	8.02E-01	ENSG00000119977
TDG	0.1557	7.4114E-03	3.38E-02	ENSG00000139372
TDH	0.0399	6.3776E-01	7.89E-01	ENSG00000154316
TDH-AS1	0.1856	1.0413E-01	2.42E-01	ENSG00000255020
TDO2	0.3399	1.7429E-02	6.49E-02	ENSG00000151790
TDP1	0.5257	4.8853E-09	3.22E-07	ENSG00000042088
TDP2	0.0421	6.4524E-01	7.94E-01	ENSG00000111802
TDRD1	-0.0015	7.6193E-01		ENSG00000095627
TDRD10	-0.0533	2.3361E-01		ENSG00000163239
TDRD12	-0.1733	4.7829E-01	6.64E-01	ENSG00000173809
TDRD3	-0.2258	1.8627E-03	1.16E-02	ENSG00000083544
TDRD5	0.0445	3.9578E-01		ENSG00000162782
TDRD6	0.0800	3.4065E-01	5.36E-01	ENSG00000180113
TDRD7	-0.0875	2.7084E-01	4.61E-01	ENSG00000196116
TDRD9	-0.0023	9.7910E-01	9.89E-01	ENSG00000156414
TDRKH	0.2547	9.8527E-03	4.20E-02	ENSG00000182134
TDRKH-AS1	0.2719	3.1780E-02	1.02E-01	ENSG00000203288
TDRP	-0.0159	8.2380E-01	9.07E-01	ENSG00000180190
TEAD1	-0.3007	6.0403E-05	7.48E-04	ENSG00000187079
TEAD2	-0.5136	4.8819E-05	6.34E-04	ENSG00000074219
TEAD3	0.0703	3.9546E-01	5.90E-01	ENSG00000007866
TEAD4	-0.0883	3.6783E-01	5.64E-01	ENSG00000197905
TEC	-0.1501	1.2933E-01	2.81E-01	ENSG00000135605
TECPR1	0.0539	4.9183E-01	6.75E-01	ENSG00000205356
TECPR2	0.0603	4.3175E-01	6.23E-01	ENSG00000196663
TECR	-0.3007	9.3851E-08	3.71E-06	ENSG00000099797
TECRL	-0.0011	6.1198E-01		ENSG00000205678
TECTA	-0.2189	7.6541E-02	1.94E-01	ENSG00000109927
TEDC1	0.0107	8.8906E-01	9.44E-01	ENSG00000185347
TEDC2	0.0461	5.9231E-01	7.55E-01	ENSG00000162062
TEDC2-AS1	-0.0038	9.5735E-01	9.78E-01	ENSG00000259895
TEF	0.0440	6.4257E-01	7.93E-01	ENSG00000167074
TEFM	0.2166	2.4647E-03	1.45E-02	ENSG00000172171
TEK	-0.0675	2.6408E-01		ENSG00000120156

TEKT1	-0.5780	3.5812E-05	4.86E-04	ENSG00000167858
TEKT2	-0.0370	6.6372E-01	8.07E-01	ENSG00000092850
TEKT3	-0.2768	2.2794E-02	7.95E-02	ENSG00000125409
TEKT4	-0.1650	7.8522E-02	1.98E-01	ENSG00000163060
TEKT4P3	-0.0282	8.7079E-01		ENSG00000271435
TEKT5	0.1298	1.1999E-01	2.67E-01	ENSG00000153060
TEKTIP1	-0.0252	8.2253E-01	9.07E-01	ENSG00000183397
TEKTL1	0.0982	5.1911E-02	1.47E-01	ENSG00000160994
TELO2	0.0492	5.3894E-01	7.12E-01	ENSG00000100726
TEN1	-0.0087	8.4960E-01		ENSG00000257949
TEN1-CDK3	0.5850	6.8578E-03	3.19E-02	ENSG00000261408
TENM1	0.5554	5.9710E-05	7.41E-04	ENSG00000009694
TENM2	-0.0157	8.6676E-01	9.32E-01	ENSG00000145934
TENM2-AS1	-0.0004	9.8975E-01	9.94E-01	ENSG00000253978
TENM3	-0.3777	8.3585E-06	1.53E-04	ENSG00000218336
TENM3-AS2	-0.0496	1.4965E-01		ENSG00000251336
TENM4	-0.5188	8.7375E-11	1.06E-08	ENSG00000149256
TENT2	0.0738	2.4976E-01	4.36E-01	ENSG00000164329
TENT4A	0.0023	9.7831E-01	9.89E-01	ENSG00000112941
TENT4B	-0.3857	8.9649E-06	1.61E-04	ENSG00000121274
TENT5A	0.5220	4.7494E-06	9.62E-05	ENSG00000112773
TENT5B	-0.3945	3.6831E-03	1.98E-02	ENSG00000158246
TENT5C	-0.5385	9.8687E-07	2.63E-05	ENSG00000183508
TENT5C-DT	-0.0118	9.5273E-01		ENSG00000236866
TEP1	0.0656	4.6376E-01	6.52E-01	ENSG00000129566
TEPSIN	0.0218	8.3211E-01	9.12E-01	ENSG00000167302
TERB1	0.0262	2.0154E-01		ENSG00000249961
TERF1	-0.2108	1.2662E-04	1.35E-03	ENSG00000147601
TERF1P7	0.0273	7.1887E-01	8.44E-01	ENSG00000232186
TERF2	0.0134	8.6541E-01	9.31E-01	ENSG00000132604
TERF2IP	-0.1538	3.0624E-03	1.71E-02	ENSG00000166848
TERT	0.0385	4.7817E-01	6.64E-01	ENSG00000164362
TES	-0.1606	6.3217E-02	1.70E-01	ENSG00000135269
TESC	0.0143	8.7355E-01	9.36E-01	ENSG00000088992
TESK1	0.0216	7.3896E-01	8.57E-01	ENSG00000107140
TESK2	-0.0541	5.0260E-01	6.84E-01	ENSG00000070759
TESMIN	0.3995	1.8132E-03	1.13E-02	ENSG00000132749
TET1	-0.0636	4.3973E-01	6.31E-01	ENSG00000138336
TET2	-0.0901	2.8417E-01	4.76E-01	ENSG00000168769
TET3	-0.0677	4.1432E-01	6.07E-01	ENSG00000187605
TEX10	0.1022	7.8765E-02	1.98E-01	ENSG00000136891
TEX101	-0.0201	6.2982E-01		ENSG00000131126
TEX11	0.0024	8.9050E-01		ENSG00000120498
TEX12	0.1181	2.5629E-01	4.44E-01	ENSG00000150783
TEX14	0.0118	9.0218E-01	9.51E-01	ENSG00000121101
TEX15	-0.4609	2.3995E-04	2.27E-03	ENSG00000133863
TEX19	0.0212	5.4914E-01		ENSG00000182459
TEX2	0.0605	4.7788E-01	6.64E-01	ENSG00000136478
TEX22	-0.1689	8.4927E-02	2.09E-01	ENSG00000226174

TEX26	-0.2051	7.0783E-02	1.84E-01	ENSG00000175664
TEX26-AS1	0.0014	9.8660E-01	9.93E-01	ENSG00000224743
TEX261	-0.1931	3.8343E-03	2.04E-02	ENSG00000144043
TEX264	-0.1111	5.7044E-02	1.58E-01	ENSG00000164081
TEX29	-0.0593	3.6624E-01	5.62E-01	ENSG00000153495
TEX30	0.0013	9.9716E-01	9.99E-01	ENSG00000151287
TEX35	-0.0084	9.7512E-01		ENSG00000240021
TEX38	0.0111	8.9587E-01	9.48E-01	ENSG00000186118
TEX41	0.0722	4.4844E-01	6.38E-01	ENSG00000226674
TEX46	0.0075	1.1260E-01	2.55E-01	ENSG00000227868
TEX52	0.0125	3.0039E-01		ENSG00000283297
TEX56P	0.0985	2.4222E-01	4.27E-01	ENSG00000185689
TEX9	-0.3485	4.6586E-06	9.48E-05	ENSG00000151575
TF	0.0773	4.0993E-01	6.03E-01	ENSG00000091513
TFAM	-0.1471	2.1474E-03	1.30E-02	ENSG00000108064
TFAP2A	0.0074	9.1576E-01	9.58E-01	ENSG00000137203
TFAP2A-AS2	0.0106	9.0404E-01	9.52E-01	ENSG00000285278
TFAP2B	-0.0184	8.3155E-01	9.12E-01	ENSG00000008196
TFAP2C	0.3515	2.3329E-02	8.09E-02	ENSG00000087510
TFAP2E	0.1517	1.6732E-01	3.35E-01	ENSG00000116819
TFAP2E-AS1	-0.0961	3.2606E-01	5.20E-01	ENSG00000239636
TFAP4	0.3003	3.5922E-02	1.12E-01	ENSG00000090447
TFB1M	0.3065	2.3855E-03	1.41E-02	ENSG00000029639
TFB2M	0.2047	1.3618E-03	9.09E-03	ENSG00000162851
TFCP2	-0.0205	7.2624E-01	8.49E-01	ENSG00000135457
TFCP2L1	-0.0336	7.2367E-01	8.47E-01	ENSG00000115112
TFDP1	-0.0274	6.4015E-01	7.91E-01	ENSG00000198176
TFDP2	-0.1422	4.7125E-03	2.39E-02	ENSG00000114126
TFE3	-0.1442	6.1800E-03	2.95E-02	ENSG00000068323
TFEB	0.0213	8.1923E-01	9.05E-01	ENSG00000112561
TFF1	-0.0909	3.2304E-01	5.17E-01	ENSG00000160182
TFF3	-0.4050	1.4532E-05	2.38E-04	ENSG00000160180
TFG	-0.0836	1.5125E-01	3.12E-01	ENSG00000114354
TFIP11	0.3459	6.7032E-05	8.13E-04	ENSG00000100109
TFIP11-DT	0.4754	1.0118E-08	5.73E-07	ENSG00000261188
TFPI2	-0.2778	2.7270E-02	9.07E-02	ENSG00000105825
TFPI2-DT	-0.0749	4.0330E-01	5.97E-01	ENSG00000234695
TFPT	-0.3445	1.7894E-06	4.30E-05	ENSG00000105619
TFR2	0.4427	5.6481E-03	2.75E-02	ENSG00000106327
TFRC	-0.0073	8.9723E-01	9.49E-01	ENSG00000072274
TG	-0.0602	9.2598E-01	9.63E-01	ENSG00000042832
TGDS	0.3117	1.1314E-03	7.82E-03	ENSG00000088451
TGFA	-0.1633	8.3166E-02	2.06E-01	ENSG00000163235
TGFB1	0.0932	3.1313E-01	5.06E-01	ENSG00000105329
TGFB1I1	0.0047	9.6111E-01	9.80E-01	ENSG00000140682
TGFB2	-0.5239	6.6890E-06	1.28E-04	ENSG00000092969
TGFB3	-0.5776	5.8975E-07	1.71E-05	ENSG00000119699
TGFBI	-0.1351	1.9483E-01	3.71E-01	ENSG00000120708
TGFBR1	0.0074	9.2859E-01	9.65E-01	ENSG00000106799

TGFBR2	-0.2915	1.1836E-02	4.83E-02	ENSG00000163513
TGFBR3	-0.2333	1.4519E-02	5.66E-02	ENSG00000069702
TGFBR3L	-0.0234	7.8137E-01	8.82E-01	ENSG00000260001
TGFBRAP1	0.0958	1.8024E-01	3.52E-01	ENSG00000135966
TGIF1	-0.4273	2.5094E-08	1.24E-06	ENSG00000177426
TGIF2	0.0244	7.6707E-01	8.74E-01	ENSG00000118707
TGIF2-RAB5IF	-0.1067	1.9103E-01		ENSG00000259399
TGM1	-0.0142	9.8632E-01		ENSG00000092295
TGM2	-0.0620	4.6891E-01	6.56E-01	ENSG00000198959
TGM4	-0.1294	1.2757E-01	2.78E-01	ENSG00000163810
TGM5	-1.4017	5.1404E-03		ENSG00000104055
TGM7	0.0297	1.1038E-01		ENSG00000159495
TGOLN2	-0.1220	1.1138E-02	4.61E-02	ENSG00000152291
TGS1	0.1144	4.3709E-02	1.30E-01	ENSG00000137574
TH	0.4654	1.9525E-05	3.00E-04	ENSG00000180176
TH2LCRR	0.0010	8.7137E-01		ENSG00000223442
THADA	0.0882	2.0513E-01	3.83E-01	ENSG00000115970
THAP1	-0.2029	1.0621E-02	4.44E-02	ENSG00000131931
THAP10	0.4291	7.6027E-06	1.42E-04	ENSG00000129028
THAP11	-0.0858	6.6215E-02	1.75E-01	ENSG00000168286
THAP12	-0.0466	3.7624E-01	5.72E-01	ENSG00000137492
THAP12P10	0.0068	8.6607E-01		ENSG00000274351
THAP2	-0.2525	4.5821E-03	2.34E-02	ENSG00000173451
THAP3	-0.0065	8.9719E-01	9.49E-01	ENSG00000041988
THAP4	-0.1177	4.0084E-02	1.21E-01	ENSG00000176946
THAP5	0.0312	6.2672E-01	7.81E-01	ENSG00000177683
THAP6	0.3885	2.5018E-04	2.35E-03	ENSG00000174796
THAP7	-0.0061	9.0204E-01	9.51E-01	ENSG00000184436
THAP7-AS1	0.2386	1.6892E-02	6.34E-02	ENSG00000230513
THAP8	-0.2650	3.1856E-03	1.76E-02	ENSG00000161277
THAP9	0.0015	9.6902E-01	9.84E-01	ENSG00000168152
THAP9-AS1	0.0714	1.8115E-01	3.53E-01	ENSG00000251022
THBD	0.0060	9.4982E-01	9.75E-01	ENSG00000178726
THBS1	-0.4549	2.1543E-03	1.30E-02	ENSG00000137801
THBS2	0.0173	8.5680E-01	9.27E-01	ENSG00000186340
THBS3	-0.1819	1.1312E-02	4.66E-02	ENSG00000169231
THBS3-AS1	-0.0543	5.7572E-01	7.43E-01	ENSG00000231064
THBS4	0.0201	8.3009E-01	9.11E-01	ENSG00000113296
THBS4-AS1	-0.1613	1.2818E-01	2.79E-01	ENSG00000249825
THCAT155	0.0745	3.9530E-01	5.90E-01	ENSG00000287243
THEM4	0.1615	3.0448E-03	1.70E-02	ENSG00000159445
THEM5	-0.0103	9.9327E-01	9.96E-01	ENSG00000196407
THEM6	0.0282	7.3101E-01	8.52E-01	ENSG00000130193
THEMIS2	-0.0435	6.3213E-01	7.85E-01	ENSG00000130775
THG1L	-0.0657	3.2862E-01	5.23E-01	ENSG00000113272
THNSL1	-0.1623	1.3059E-01	2.82E-01	ENSG00000185875
THNSL2	-0.1764	8.9283E-02	2.17E-01	ENSG00000144115
THOC1	0.0725	3.5559E-01	5.51E-01	ENSG00000079134
THOC2	0.0447	3.7516E-01	5.71E-01	ENSG00000125676

THOC3	0.1761	1.3270E-02	5.28E-02	ENSG00000051596
THOC5	0.3262	8.0650E-06	1.48E-04	ENSG00000100296
THOC6	-0.2804	1.7805E-03	1.12E-02	ENSG00000131652
THOC7	-0.0545	4.5192E-01	6.41E-01	ENSG00000163634
THOP1	0.2250	1.6926E-03	1.08E-02	ENSG00000172009
THORLNC	-0.0442	9.9569E-01	9.98E-01	ENSG00000226856
THPO	0.0095	9.1784E-01	9.59E-01	ENSG00000090534
THRA	-0.2110	8.2178E-03	3.67E-02	ENSG00000126351
THRAP3	-0.1075	5.6521E-02	1.57E-01	ENSG00000054118
THRAP3P1	-0.0337	6.1487E-01		ENSG00000227339
THRSP	-0.2184	7.5520E-02	1.92E-01	ENSG00000151365
THSD1	0.4495	2.9066E-03	1.64E-02	ENSG00000136114
THSD4	0.0273	7.7676E-01	8.79E-01	ENSG00000187720
THSD4-AS1	0.1330	1.8769E-02	6.87E-02	ENSG00000259964
THSD7A	-0.0120	8.8868E-01	9.44E-01	ENSG00000005108
THSD7B	0.0442	4.3754E-01	6.28E-01	ENSG00000144229
THSD8	0.0496	1.3595E-01	2.90E-01	ENSG00000284491
THTPA	-0.1114	1.5637E-01	3.19E-01	ENSG00000259431
THUMPD1	-0.1332	9.9164E-03	4.22E-02	ENSG00000066654
THUMPD2	0.2147	3.2943E-03	1.80E-02	ENSG00000138050
THUMPD3	0.0439	3.9450E-01	5.89E-01	ENSG00000134077
THUMPD3-AS1	0.2429	6.8387E-04	5.29E-03	ENSG00000206573
THY1	-0.0990	2.1513E-01	3.96E-01	ENSG00000154096
THYN1	-0.0909	1.3833E-01	2.93E-01	ENSG00000151500
TIA1	-0.0069	8.7585E-01	9.37E-01	ENSG00000116001
TIAL1	-0.2574	9.9028E-03	4.22E-02	ENSG00000151923
TIAM1	0.2313	4.7207E-03	2.39E-02	ENSG00000156299
TIAM1-AS1	-0.0416	6.6726E-01	8.09E-01	ENSG00000237594
TIAM2	-0.0235	8.0160E-01	8.94E-01	ENSG00000146426
TICAM2	-0.0558	4.2559E-01		ENSG00000243414
TICRR	-0.0640	5.0695E-01	6.87E-01	ENSG00000140534
TIE1	1.3727	3.1920E-03		ENSG00000066056
TIFA	-0.3168	1.0577E-03	7.42E-03	ENSG00000145365
TIGAR	-0.1666	1.1079E-01	2.52E-01	ENSG00000078237
TIGD1	0.1305	7.3653E-02	1.89E-01	ENSG00000221944
TIGD2	0.1580	2.4829E-02	8.48E-02	ENSG00000180346
TIGD3	0.2686	3.6680E-03	1.97E-02	ENSG00000173825
TIGD4	-0.1605	1.3213E-01	2.85E-01	ENSG00000169989
TIGD5	-0.3559	5.7152E-03	2.78E-02	ENSG00000179886
TIGD6	0.0631	4.0590E-01	6.00E-01	ENSG00000164296
TIGD7	0.1687	7.2857E-02	1.88E-01	ENSG00000140993
TIMELESS	-0.0581	4.7305E-01	6.60E-01	ENSG00000111602
TIMM10	0.0129	8.5610E-01	9.26E-01	ENSG00000134809
TIMM10B	0.5632	8.9044E-08	3.54E-06	ENSG00000132286
TIMM13	-0.1152	2.7087E-02	9.03E-02	ENSG00000099800
TIMM17A	-0.0579	2.8759E-01	4.79E-01	ENSG00000134375
TIMM17B	0.0067	9.0839E-01	9.54E-01	ENSG00000126768
TIMM21	0.1647	3.9331E-03	2.08E-02	ENSG00000075336
TIMM22	-0.0242	6.4901E-01	7.97E-01	ENSG00000177370

TIMM23	-0.0858	4.7370E-02	1.37E-01	ENSG00000265354
TIMM23B	-0.0288	7.0164E-01	8.33E-01	ENSG00000204152
TIMM23B-AGAP6	-0.0121	8.7825E-01	9.38E-01	ENSG00000178440
TIMM29	-0.2468	2.9190E-04	2.67E-03	ENSG00000142444
TIMM44	-0.0071	9.0874E-01	9.54E-01	ENSG00000104980
TIMM50	-0.1231	1.8607E-02	6.83E-02	ENSG00000105197
TIMM8A	0.0025	9.8570E-01	9.92E-01	ENSG00000126953
TIMM8B	-0.1242	2.1159E-02	7.52E-02	ENSG00000150779
TIMM9	0.0103	9.1399E-01	9.57E-01	ENSG00000100575
TIMMDC1	-0.1088	1.0651E-01	2.45E-01	ENSG00000113845
TIMP1	-0.5607	6.0379E-09	3.84E-07	ENSG00000102265
TIMP2	-0.4774	8.4501E-05	9.77E-04	ENSG00000035862
TIMP3	-0.5720	3.8698E-06	8.15E-05	ENSG00000100234
TIMP4	-0.5841	1.1042E-03	7.68E-03	ENSG00000157150
TINAG	0.0099	2.7719E-01		ENSG00000137251
TINAGL1	0.0347	6.8244E-01	8.20E-01	ENSG00000142910
TINCR	-0.4069	8.1639E-04	6.06E-03	ENSG00000223573
TINF2	0.1552	9.2443E-03	4.01E-02	ENSG00000092330
TIPIN	0.0498	4.9466E-01	6.78E-01	ENSG00000075131
TIPRL	-0.0186	7.1486E-01	8.41E-01	ENSG00000143155
TIRAP	-0.0417	6.5924E-01	8.03E-01	ENSG00000150455
TJAP1	0.3624	3.9016E-04	3.37E-03	ENSG00000137221
TJP1	-0.1348	4.9852E-02	1.43E-01	ENSG00000104067
TJP2	-0.0663	4.6953E-01	6.57E-01	ENSG00000119139
TJP3	0.0435	6.3853E-01	7.90E-01	ENSG00000105289
TK1	-0.3380	7.1405E-03	3.28E-02	ENSG00000167900
TK2	-0.0202	7.8310E-01	8.83E-01	ENSG00000166548
TKFC	-0.0833	3.1004E-01	5.03E-01	ENSG00000149476
TKT	-0.1998	5.0010E-02	1.43E-01	ENSG00000163931
TKTL1	0.1138	2.2939E-01	4.12E-01	ENSG00000007350
TKTL2	-0.0113	7.3218E-01		ENSG00000151005
TLCD1	-0.0957	2.0814E-01	3.87E-01	ENSG00000160606
TLCD2	0.0779	3.3998E-01	5.35E-01	ENSG00000185561
TLCD3A	-0.1554	6.4571E-02	1.72E-01	ENSG00000167695
TLCD3B	-0.0989	2.7650E-01	4.67E-01	ENSG00000149926
TLCD4	-0.1837	1.8447E-02	6.78E-02	ENSG00000152078
TLCD4-RWDD3	0.1418	9.5845E-02	2.28E-01	ENSG00000271092
TLCD5	-0.3871	2.5587E-04	2.39E-03	ENSG00000181264
TLDC2	0.0467	4.9103E-01	6.75E-01	ENSG00000101342
TLE1	-0.0447	4.1723E-01	6.10E-01	ENSG00000196781
TLE1-DT	-0.1074	2.5063E-01	4.37E-01	ENSG00000233926
TLE2	-0.2209	2.0193E-03	1.23E-02	ENSG00000065717
TLE3	-0.4157	1.1501E-06	3.01E-05	ENSG00000140332
TLE4	-0.0769	2.5443E-01	4.42E-01	ENSG00000106829
TLE5	-0.1966	7.9847E-04	5.98E-03	ENSG00000104964
TLE6	0.1877	8.3829E-03	3.72E-02	ENSG00000104953
TLK1	-0.3147	1.8224E-05	2.84E-04	ENSG00000198586
TLK2	-0.0419	4.1729E-01	6.10E-01	ENSG00000146872
TLK2P1	-0.0472	5.5694E-01	7.27E-01	ENSG00000226049

TLK2P2	0.2817	3.3967E-02	1.07E-01	ENSG00000228828
TLL1	0.0420	6.8464E-01	8.21E-01	ENSG00000038295
TLL2	-0.0083	9.2717E-01	9.64E-01	ENSG00000095587
TLN1	-0.3076	1.2285E-11	1.93E-09	ENSG00000137076
TLN2	0.0885	3.0205E-01	4.95E-01	ENSG00000171914
TLNRD1	-0.1058	2.1593E-01	3.97E-01	ENSG00000140406
TLR1	0.0225	8.1316E-01	9.01E-01	ENSG00000174125
TLR10	0.0236	3.0584E-01		ENSG00000174123
TLR2	-0.0691	3.7336E-01	5.69E-01	ENSG00000137462
TLR3	-0.4024	1.6779E-02	6.31E-02	ENSG00000164342
TLR4	-0.0544	1.9556E-01		ENSG00000136869
TLR5	-0.3346	2.4770E-02	8.46E-02	ENSG00000187554
TLR6	-0.1002	1.0732E-01		ENSG00000174130
TLX1	-0.0142	9.6996E-01	9.84E-01	ENSG00000107807
TLX2	-0.0017	7.0523E-01		ENSG00000115297
TLX3	0.0034	9.5254E-01		ENSG00000164438
TM2D1	-0.3112	9.1512E-06	1.63E-04	ENSG00000162604
TM2D2	-0.1130	7.2071E-02	1.86E-01	ENSG00000169490
TM2D3	-0.0505	2.5673E-01	4.45E-01	ENSG00000184277
TM4SF1	-0.1701	1.2318E-01	2.72E-01	ENSG00000169908
TM4SF1-AS1	0.0062	8.3940E-01	9.16E-01	ENSG00000240541
TM4SF18	-0.0294	7.4667E-01	8.61E-01	ENSG00000163762
TM4SF19	0.0711	2.9634E-01	4.88E-01	ENSG00000145107
TM4SF19-AS1	-0.0597	4.1433E-01	6.07E-01	ENSG00000235897
TM6SF1	-0.3268	2.1524E-02	7.61E-02	ENSG00000136404
TM6SF2	-0.1249	1.4130E-01	2.98E-01	ENSG00000213996
TM7SF2	-0.2988	9.0639E-07	2.47E-05	ENSG00000149809
TM7SF3	-0.1516	8.0856E-02	2.02E-01	ENSG00000064115
TM9SF1	-0.0675	4.5041E-01	6.40E-01	ENSG00000100926
TM9SF2	-0.0082	9.0615E-01	9.53E-01	ENSG00000125304
TM9SF3	-0.1094	1.2493E-02	5.03E-02	ENSG00000077147
TM9SF4	-0.1852	4.9911E-03	2.50E-02	ENSG00000101337
TMA16	0.1556	5.2272E-02	1.48E-01	ENSG00000198498
TMA7	-0.3424	2.8035E-06	6.29E-05	ENSG00000232112
TMA7B	-0.0438	8.8740E-01	9.43E-01	ENSG00000225528
TMBIM1	0.1600	7.3046E-02	1.88E-01	ENSG00000135926
TMBIM4	-0.0448	5.1257E-01	6.92E-01	ENSG00000155957
TMBIM6	-0.3447	1.5506E-05	2.51E-04	ENSG00000139644
TMBIM7P	-0.0160	6.1056E-01		ENSG00000230927
TMC1	-0.0419	5.2895E-01	7.04E-01	ENSG00000165091
TMC2	-0.2942	2.6215E-02	8.81E-02	ENSG00000149488
TMC3	0.1307	1.7357E-01	3.43E-01	ENSG00000188869
TMC4	0.0541	5.6227E-01	7.31E-01	ENSG00000167608
TMC5	0.3004	4.2327E-03	2.21E-02	ENSG00000103534
TMC6	0.0958	3.1920E-01	5.13E-01	ENSG00000141524
TMC7	-0.0544	4.9727E-01	6.80E-01	ENSG00000170537
TMC8	-0.4818	3.5857E-03	1.94E-02	ENSG00000167895
TMCC1	-0.2516	4.6281E-04	3.86E-03	ENSG00000172765
TMCC1-DT	-0.2588	3.2718E-02	1.04E-01	ENSG00000271270

TMCC2	0.1202	7.7070E-02	1.95E-01	ENSG00000133069
TMCC3	-0.1834	4.2637E-02	1.27E-01	ENSG00000057704
TMCO1	-0.1965	2.6403E-04	2.45E-03	ENSG00000143183
TMCO2	0.0020	6.7568E-01		ENSG00000188800
TMCO3	-0.2114	1.2957E-03	8.73E-03	ENSG00000150403
TMCO4	-0.1071	2.8725E-01	4.78E-01	ENSG00000162542
TMCO6	-0.0180	8.5300E-01	9.25E-01	ENSG00000113119
TMDD1	0.0551	3.0906E-01	5.02E-01	ENSG00000284730
TMED1	-0.1948	3.3545E-02	1.06E-01	ENSG00000099203
TMED10	-0.2566	1.9476E-05	2.99E-04	ENSG00000170348
TMED11P	0.0187	5.5801E-01		ENSG00000215367
TMED2	0.0149	8.2373E-01	9.07E-01	ENSG00000086598
TMED3	0.2681	3.4026E-05	4.67E-04	ENSG00000166557
TMED4	-0.0904	1.2080E-01	2.68E-01	ENSG00000158604
TMED5	-0.1686	7.7351E-03	3.50E-02	ENSG00000117500
TMED6	-0.0686	3.3727E-01	5.32E-01	ENSG00000157315
TMED7	-0.0691	3.1939E-01	5.13E-01	ENSG00000134970
TMED7-TICAM2	-0.0257	7.0532E-01		ENSG00000251201
TMED8	-0.0031	9.4260E-01	9.72E-01	ENSG00000100580
TMED9	-0.0124	8.5816E-01	9.27E-01	ENSG00000184840
TMEFF1	0.1064	2.7345E-01	4.64E-01	ENSG00000241697
TMEFF2	0.0719	4.3562E-01	6.27E-01	ENSG00000144339
TMEM100	-0.1077	2.8077E-01	4.72E-01	ENSG00000166292
TMEM101	-0.0476	3.3968E-01	5.35E-01	ENSG00000091947
TMEM102	0.2005	6.1216E-02	1.66E-01	ENSG00000181284
TMEM104	0.0316	7.0713E-01	8.36E-01	ENSG00000109066
TMEM105	-0.0369	6.8629E-01	8.23E-01	ENSG00000185332
TMEM106A	0.0071	9.3049E-01	9.66E-01	ENSG00000184988
TMEM106B	0.0185	7.7530E-01	8.78E-01	ENSG00000106460
TMEM106C	-0.2132	2.1121E-03	1.28E-02	ENSG00000134291
TMEM107	-0.2553	1.2045E-03	8.22E-03	ENSG00000179029
TMEM108	-0.0320	7.0481E-01	8.35E-01	ENSG00000144868
TMEM109	-0.2191	3.8094E-03	2.03E-02	ENSG00000110108
TMEM11	-0.1579	7.6234E-04	5.75E-03	ENSG00000178307
TMEM11-DT	0.1298	2.0784E-01	3.87E-01	ENSG00000235530
TMEM114	-0.0292	6.0101E-01		ENSG00000232258
TMEM115	-0.0604	3.5352E-01	5.49E-01	ENSG00000126062
TMEM116	-0.0950	2.4412E-01	4.30E-01	ENSG00000198270
TMEM117	-0.4547	1.4298E-03	9.45E-03	ENSG00000139173
TMEM119	0.0652	5.0854E-01	6.89E-01	ENSG00000183160
TMEM120A	-0.1751	2.7066E-03	1.56E-02	ENSG00000189077
TMEM120B	0.1281	1.1251E-01	2.55E-01	ENSG00000188735
TMEM121	0.0000	9.8388E-01	9.91E-01	ENSG00000184986
TMEM121B	0.1204	1.6682E-01	3.34E-01	ENSG00000183307
TMEM123	-0.1076	1.7025E-01	3.38E-01	ENSG00000152558
TMEM123-DT	-0.2252	6.3608E-02	1.70E-01	ENSG00000255337
TMEM125	-0.0363	6.9362E-01	8.28E-01	ENSG00000179178
TMEM126A	-0.3352	1.1297E-04	1.24E-03	ENSG00000171202
TMEM126B	-0.0425	4.3922E-01	6.30E-01	ENSG00000171204

TMEM127	0.0323	5.5663E-01	7.27E-01	ENSG00000135956
TMEM128	-0.1902	1.9718E-03	1.21E-02	ENSG00000132406
TMEM129	0.0718	3.1655E-01	5.10E-01	ENSG00000168936
TMEM130	0.0721	2.0662E-01	3.85E-01	ENSG00000166448
TMEM131	-0.1139	1.3624E-01	2.90E-01	ENSG00000075568
TMEM131L	0.0986	2.5930E-01	4.47E-01	ENSG00000121210
TMEM132A	-0.0412	5.0340E-01	6.85E-01	ENSG00000006118
TMEM132B	0.3610	2.4753E-04	2.33E-03	ENSG00000139364
TMEM132C	-0.0268	8.7005E-01	9.34E-01	ENSG00000181234
TMEM132D	-0.0789	2.2455E-01	4.07E-01	ENSG00000151952
TMEM132E	0.3929	6.0978E-03	2.92E-02	ENSG00000181291
TMEM132E-DT	0.3764	2.0769E-02	7.40E-02	ENSG00000197322
TMEM134	-0.1713	2.7495E-02	9.12E-02	ENSG00000172663
TMEM135	-0.1533	5.5943E-02	1.55E-01	ENSG00000166575
TMEM138	-0.0818	1.4294E-01	3.01E-01	ENSG00000149483
TMEM139	0.0755	4.4063E-01	6.31E-01	ENSG00000178826
TMEM140	0.3903	1.0059E-02	4.27E-02	ENSG00000146859
TMEM141	-0.2103	3.1193E-02	1.00E-01	ENSG00000244187
TMEM144	0.1592	7.9039E-02	1.99E-01	ENSG00000164124
TMEM145	-0.0018	9.7560E-01	9.88E-01	ENSG00000167619
TMEM147	-0.2508	1.0352E-04	1.15E-03	ENSG00000105677
TMEM147-AS1	0.0524	5.2142E-01	6.99E-01	ENSG00000236144
TMEM14A	-0.2712	2.0835E-04	2.02E-03	ENSG00000096092
TMEM14B	-0.0489	3.9693E-01	5.91E-01	ENSG00000137210
TMEM14B-DT	-0.1379	1.5458E-01	3.17E-01	ENSG00000272097
TMEM14C	-0.1356	1.1872E-02	4.84E-02	ENSG00000111843
TMEM150A	0.0243	7.5762E-01	8.68E-01	ENSG00000168890
TMEM150B	-0.0073	7.5318E-01		ENSG00000180061
TMEM150C	-0.2176	3.6727E-02	1.13E-01	ENSG00000249242
TMEM151A	-0.0805	3.5177E-01	5.47E-01	ENSG00000179292
TMEM151B	-0.2654	4.7419E-03	2.40E-02	ENSG00000178233
TMEM154	-0.2328	6.3248E-02	1.70E-01	ENSG00000170006
TMEM156	-0.0004	9.9262E-01	9.96E-01	ENSG00000121895
TMEM158	0.0845	2.9459E-01	4.87E-01	ENSG00000249992
TMEM160	0.0622	3.1782E-01	5.11E-01	ENSG00000130748
TMEM161A	-0.1674	5.4011E-02	1.51E-01	ENSG00000064545
TMEM161B	0.3924	1.6189E-06	3.97E-05	ENSG00000164180
TMEM161B-DT	-0.1397	2.4889E-02	8.49E-02	ENSG00000247828
TMEM163	0.1810	4.8836E-02	1.41E-01	ENSG00000152128
TMEM164	-0.3127	4.1690E-03	2.18E-02	ENSG00000157600
TMEM165	-0.1342	2.0675E-02	7.38E-02	ENSG00000134851
TMEM167A	-0.1613	1.3877E-02	5.47E-02	ENSG00000174695
TMEM167B	0.1202	2.6962E-02	9.00E-02	ENSG00000215717
TMEM168	0.2682	8.0653E-03	3.62E-02	ENSG00000146802
TMEM169	-0.2018	1.0537E-02	4.41E-02	ENSG00000163449
TMEM17	-0.2454	1.7750E-03	1.11E-02	ENSG00000186889
TMEM170A	-0.4453	6.7730E-12	1.10E-09	ENSG00000166822
TMEM170B	0.0607	4.9964E-01	6.82E-01	ENSG00000205269
TMEM175	0.2812	3.6484E-09	2.51E-07	ENSG00000127419

TMEM176A	-0.0644	4.5415E-01	6.44E-01	ENSG00000002933
TMEM176B	-0.1117	2.4383E-01	4.29E-01	ENSG00000106565
TMEM177	0.3760	2.4096E-04	2.28E-03	ENSG00000144120
TMEM178A	0.0635	4.3699E-01	6.28E-01	ENSG00000152154
TMEM178B	-0.0516	4.6890E-01	6.56E-01	ENSG00000261115
TMEM179	0.1018	1.9351E-01	3.69E-01	ENSG00000258986
TMEM179B	-0.2494	3.5044E-03	1.90E-02	ENSG00000185475
TMEM18	-0.2191	5.6706E-04	4.55E-03	ENSG00000151353
TMEM18-DT	0.0011	5.9952E-01		ENSG00000233296
TMEM181	0.0127	8.5811E-01	9.27E-01	ENSG00000146433
TMEM182	0.0095	9.0128E-01	9.51E-01	ENSG00000170417
TMEM183A	-0.2436	1.5590E-07	5.62E-06	ENSG00000163444
TMEM183AP2	-0.0035	9.9908E-01		ENSG00000249142
TMEM183BP	-0.2403	1.0351E-03	7.31E-03	ENSG00000224831
TMEM184A	-0.1984	5.3563E-02	1.50E-01	ENSG00000164855
TMEM184B	0.1714	1.1026E-02	4.58E-02	ENSG00000198792
TMEM184C	0.0829	2.3601E-01	4.20E-01	ENSG00000164168
TMEM185A	-0.0135	7.6982E-01	8.75E-01	ENSG00000269556
TMEM185B	-0.2986	3.7436E-04	3.28E-03	ENSG00000226479
TMEM186	0.1657	7.3711E-02	1.89E-01	ENSG00000184857
TMEM187	-0.1616	9.2254E-02	2.22E-01	ENSG00000177854
TMEM19	-0.2825	2.0424E-05	3.10E-04	ENSG00000139291
TMEM190	0.1976	9.0005E-02	2.19E-01	ENSG00000160472
TMEM191B	0.1383	1.2801E-01	2.79E-01	ENSG00000278558
TMEM191C	0.1672	2.6295E-02	8.83E-02	ENSG00000206140
TMEM192	0.0829	1.5298E-01	3.15E-01	ENSG00000170088
TMEM196	0.1419	1.7125E-01	3.40E-01	ENSG00000173452
TMEM198	0.1718	4.9816E-02	1.43E-01	ENSG00000188760
TMEM198B	-0.1026	1.6137E-01	3.27E-01	ENSG00000182796
TMEM199	0.1289	1.1040E-01	2.52E-01	ENSG00000244045
TMEM200A	0.0894	2.3867E-01	4.23E-01	ENSG00000164484
TMEM200B	-0.1925	1.0070E-01	2.36E-01	ENSG00000253304
TMEM200C	-0.4038	6.9100E-06	1.31E-04	ENSG00000206432
TMEM201	-0.0860	2.4781E-01	4.34E-01	ENSG00000188807
TMEM202-AS1	-0.0276	7.7430E-01	8.78E-01	ENSG00000261423
TMEM203	-0.1117	8.3667E-02	2.07E-01	ENSG00000187713
TMEM204	0.0037	8.1269E-01		ENSG00000131634
TMEM205	-0.1472	3.9436E-02	1.20E-01	ENSG00000105518
TMEM208	-0.0516	3.9472E-01	5.89E-01	ENSG00000168701
TMEM209	0.1004	1.6080E-01	3.26E-01	ENSG00000146842
TMEM212	-0.0271	9.5821E-01		ENSG00000186329
TMEM213	-0.0361	6.3544E-01	7.88E-01	ENSG00000214128
TMEM214	-0.0275	5.8947E-01	7.53E-01	ENSG00000119777
TMEM215	-0.0253	4.7650E-01		ENSG00000188133
TMEM216	0.1952	3.8571E-03	2.05E-02	ENSG00000187049
TMEM217	-0.2718	3.1991E-02	1.02E-01	ENSG00000172738
TMEM218	-0.5391	2.2928E-07	7.85E-06	ENSG00000150433
TMEM219	-0.2027	4.6398E-04	3.87E-03	ENSG00000149932
TMEM220	0.0207	8.2027E-01	9.05E-01	ENSG00000187824

TMEM220-AS1	-0.1627	1.0344E-01	2.40E-01	ENSG00000263400
TMEM222	0.0749	1.2372E-01	2.73E-01	ENSG00000186501
TMEM223	-0.0494	3.9453E-01	5.89E-01	ENSG00000168569
TMEM225B	0.5009	1.3108E-04	1.39E-03	ENSG00000244219
TMEM229A	0.0371	6.2045E-01	7.76E-01	ENSG00000234224
TMEM229B	-0.2498	2.0863E-02	7.43E-02	ENSG00000198133
TMEM230	-0.2539	2.4335E-04	2.30E-03	ENSG00000089063
TMEM231	-0.3348	7.6277E-06	1.42E-04	ENSG00000205084
TMEM232	-0.5013	3.5036E-07	1.11E-05	ENSG00000186952
TMEM234	-0.2620	4.6327E-05	6.06E-04	ENSG00000160055
TMEM235	-0.0004	6.7263E-01		ENSG00000204278
TMEM236	-0.0111	8.2517E-01		ENSG00000148483
TMEM237	0.0577	3.7315E-01	5.68E-01	ENSG00000155755
TMEM238	-0.4288	9.6842E-04	6.94E-03	ENSG00000233493
TMEM240	-0.1326	2.1023E-01	3.90E-01	ENSG00000205090
TMEM241	0.1992	2.3897E-02	8.23E-02	ENSG00000134490
TMEM242	-0.2548	6.9427E-05	8.36E-04	ENSG00000215712
TMEM243	0.0052	9.8011E-01	9.89E-01	ENSG00000135185
TMEM244	-0.0089	9.1258E-01		ENSG00000203756
TMEM245	-0.1594	1.7363E-02	6.48E-02	ENSG00000106771
TMEM246-AS1	0.3184	1.3567E-02	5.37E-02	ENSG00000225376
TMEM248	-0.1070	1.1020E-02	4.57E-02	ENSG00000106609
TMEM249	0.1322	1.4908E-01	3.09E-01	ENSG00000261587
TMEM25	0.0253	6.8884E-01	8.25E-01	ENSG00000149582
TMEM250	0.4819	2.9025E-08	1.41E-06	ENSG00000238227
TMEM252	-0.0965	1.7908E-01	3.50E-01	ENSG00000181778
TMEM253	-0.0449	4.7988E-01	6.65E-01	ENSG00000232070
TMEM254	-0.1945	1.2580E-02	5.06E-02	ENSG00000133678
TMEM255A	0.2512	1.9350E-02	7.02E-02	ENSG00000125355
TMEM255B	-0.0006	9.9570E-01	9.98E-01	ENSG00000184497
TMEM256	-0.2964	1.2636E-03	8.56E-03	ENSG00000205544
TMEM256P2	0.0809	7.1659E-02	1.85E-01	ENSG00000226726
TMEM258	-0.2644	1.4284E-04	1.49E-03	ENSG00000134825
TMEM259	0.2246	8.8108E-04	6.45E-03	ENSG00000182087
TMEM26	0.0450	6.3557E-01	7.88E-01	ENSG00000196932
TMEM260	-0.0512	4.3699E-01	6.28E-01	ENSG00000070269
TMEM262	0.0248	7.0172E-01	8.33E-01	ENSG00000187066
TMEM263	0.1232	8.1181E-02	2.03E-01	ENSG00000151135
TMEM266	0.0852	3.8883E-01	5.83E-01	ENSG00000169758
TMEM267	0.0345	6.5244E-01	7.99E-01	ENSG00000151881
TMEM268	-0.0239	7.2579E-01	8.49E-01	ENSG00000157693
TMEM269	0.3321	2.0258E-02	7.27E-02	ENSG00000274386
TMEM270	0.0795	4.1560E-01	6.08E-01	ENSG00000175877
TMEM271	-0.0538	5.4007E-01	7.14E-01	ENSG00000273238
TMEM272	-0.0782	1.6840E-01		ENSG00000281106
TMEM274P	0.0081	5.9062E-01		ENSG00000283611
TMEM275	-0.0257	7.4691E-01		ENSG00000282881
TMEM30A	0.0878	9.3391E-02	2.24E-01	ENSG00000112697
TMEM30A-DT	0.0567	5.2410E-01	7.01E-01	ENSG00000225793

TMEM30B	0.0884	3.7159E-01	5.67E-01	ENSG00000182107
TMEM31	0.0192	8.2370E-01	9.07E-01	ENSG00000179363
TMEM33	-0.1613	1.6031E-02	6.10E-02	ENSG00000109133
TMEM35A	0.0283	7.6184E-01	8.71E-01	ENSG00000126950
TMEM37	-0.0283	7.7403E-01	8.78E-01	ENSG00000171227
TMEM38A	-0.0066	8.9657E-01	9.48E-01	ENSG00000072954
TMEM38B	-0.2188	5.8584E-03	2.83E-02	ENSG00000095209
TMEM39A	-0.0288	6.7885E-01	8.17E-01	ENSG00000176142
TMEM39B	-0.2629	2.1730E-02	7.67E-02	ENSG00000121775
TMEM40	0.1609	1.3722E-01	2.92E-01	ENSG00000088726
TMEM41A	0.0322	5.7543E-01	7.42E-01	ENSG00000163900
TMEM41B	0.2236	6.1145E-03	2.93E-02	ENSG00000166471
TMEM42	-0.0262	7.0903E-01	8.37E-01	ENSG00000169964
TMEM43	-0.0397	6.1903E-01	7.75E-01	ENSG00000170876
TMEM44	-0.0007	9.9224E-01	9.96E-01	ENSG00000145014
TMEM45A	-0.1164	1.5558E-01	3.18E-01	ENSG00000181458
TMEM47	-0.2617	1.6684E-02	6.28E-02	ENSG00000147027
TMEM50A	-0.1594	4.5277E-04	3.80E-03	ENSG00000183726
TMEM50B	-0.0316	6.1837E-01	7.75E-01	ENSG00000142188
TMEM51	-0.3376	3.1796E-03	1.76E-02	ENSG00000171729
TMEM51-AS1	0.0769	3.6096E-01	5.57E-01	ENSG00000175147
TMEM52	0.1357	1.5629E-01	3.19E-01	ENSG00000178821
TMEM53	-0.0269	6.7509E-01	8.14E-01	ENSG00000126106
TMEM54	-0.4418	1.8108E-05	2.83E-04	ENSG00000121900
TMEM59	-0.1844	1.4671E-03	9.65E-03	ENSG00000116209
TMEM59L	-0.1510	2.7879E-02	9.22E-02	ENSG00000105696
TMEM60	0.1449	1.7469E-02	6.50E-02	ENSG00000135211
[truncated: 146,372 more chars]
