# Supplementary material for: Effects of microgravity on human iPSC-derived neural organoids on the International Space Station
Source: Stem Cells Transl Med. 2024 Oct 23;13(12):1186–97. doi: 10.1093/stcltm/szae070 (PMC11631337; doi:10.1093/stcltm/szae070)
Supplement: szae070_suppl_Supplementary_Materials [file szae070_suppl_supplementary_materials.zip › R1Table S3a Cortical-Gene ontology labels for differentiatlly expressed transcripts.pdf]

P-value' is the enrichment p-value computed according to the mHG or HG model; 'FDR q-value' is the correction of the above p-value for multiple testing using the Benjamini and Hochberg (1995) method. Namely, for the ith term (ranked according to p-value) the FDR q-value is (p-value * number of GO terms) / i; Enrichment (N, B, n, b) is defined as follows: N - is the total number of genes, B - is the total number of genes associated with a specific GO term, n - is the number of genes in the top of the user's input list or in the target set when appropriate,b - is the number of genes in the intersection; Enrichment = (b/n) / (B/N)

CORTICAL ORGANOIDS: GENE ONTOLOGY PROCESS TERMS FOR TRANSCRIPTS WITH HIGHER EXPRESSION IN LEO

GO Term	Description	P-value	FDR q-value	Enrich-ment	N	B	n	b	Genes
GO:0032501	multicellular organismal process	1.72E-06	2.62E-02	1.64	16220	2899	276	81	[GUCA1C - guanylate cyclase activator 1c, ASIC3 - acid-sensing (proton-gated) ion channel 3, ADAMTS16 - adam metallopeptidase with thrombospondin type 1 motif, 16, TRPV1 - transient receptor potential cation channel, subfamily v, member 1, LRP2 - low density lipoprotein receptor-related protein 2, ZFHX3 - zinc finger homeobox 3, RPE65 - retinal pigment epithelium-specific protein 65kda, CACNB2 - calcium channel, voltage-dependent, beta 2 subunit, RSPO2 - r-spondin 2, RELN - reelin, MMRN1 - multimerin 1, ID3 - inhibitor of dna binding 3, dominant negative helix-loop-helix protein, EBF1 - early b-cell factor 1, FCER1G - fc fragment of ige, high affinity i, receptor for; gamma polypeptide, DCDC2 - doublecortin domain containing 2, SIAH3 - siah e3 ubiquitin protein ligase family member 3, NPY1R - neuropeptide y receptor y1, SLITRK6 - slit and ntrk-like family, member 6, CYP4A11 - cytochrome p450, family 4, subfamily a, polypeptide 11, CRYGS - crystallin, gamma s, HTR3B - 5-hydroxytryptamine (serotonin) receptor 3b, ionotropic, IFITM1 - interferon induced transmembrane protein 1, NPS - neuropeptide s, PNOC - prepronociceptin, AIF1 - allograft inflammatory factor 1, IL15 - interleukin 15, DAB2 - dab, mitogen-responsive phosphoprotein, homolog 2 (drosophila), ESPNL - espin-like, SCN2A - sodium channel, voltage-gated, type ii, alpha subunit, TH - tyrosine hydroxylase, C9orf116 - chromosome 9 open reading frame 116, HES1 - hairy and enhancer of split 1, (drosophila), ALB - albumin, COL13A1 - collagen, type xiii, alpha 1, ALDH1A2 - aldehyde dehydrogenase 1 family, member a2, CATSPERG - catsper channel auxiliary subunit gamma, IDO1 - indoleamine 2,3-dioxygenase 1, MYH7 - myosin, heavy chain 7, cardiac muscle, beta, LGI4 - leucine-rich repeat lgi family, member 4, OTOF - otoferlin, KCNK2 - potassium channel, subfamily k, member 2, CDH6 - cadherin 6, type 2, k-cadherin (fetal kidney), CDH9 - cadherin 9, type 2 (t1-cadherin), ALK - anaplastic lymphoma receptor tyrosine kinase, AVP - arginine vasopressin, UTS2 - urotensin 2, NOX4 - nadph oxidase 4, CHRNA9 - cholinergic receptor, nicotinic, alpha 9 (neuronal), DNAAF1 - dynein, axonemal, assembly factor 1, CDH18 - cadherin 18, type 2, ARHGDIB - rho gdp dissociation inhibitor (gdi) beta, SHROOM4 - shroom family member 4, PDC - phosducin, CALHM1 - calcium homeostasis modulator 1, GREB1L - growth regulation by estrogen in breast cancer-like, SEMA3E - sema domain, immunoglobulin domain (ig), short basic domain, secreted, (semaphorin) 3e, ZIC1 - zic family member 1, C6 - complement component 6, F5 coagulation factor v (proaccelerin, labile factor), OPRM1 - opioid receptor, mu 1, CLDN4 - claudin 4, SLC26A2 - solute carrier family 26 (anion exchanger), member 2, PDGFRA - platelet-derived growth factor receptor, alpha polypeptide, TWIST2 - twist basic helix-loop-helix transcription factor 2, AMY2A - amylase, alpha 2a (pancreatic), CFC1 - cripto, frl-1, cryptic family 1, CD109 - cd109 molecule, KRT75 - keratin 75, MOV10L1 - mov10l1, moloney leukemia virus 10-like 1, homolog (mouse), NXF2 - nuclear rna export factor 2, GRM1 - glutamate receptor, metabotropic 1, PRKCDBP - protein kinase c, delta binding protein, SLC11A1 - solute carrier family 11 (proton-coupled divalent metal ion transporter), member 1, TTN - titin, CNTN5 - contactin 5, CFC1B - cripto, frl-1, cryptic family 1b, ENPEP - glutamyl aminopeptidase (aminopeptidase a), TNNI1 - troponin i type 1 (skeletal, slow), VIP - vasoactive intestinal peptide, CXCR4 - chemokine (c-x-c motif) receptor 4, BBS12 - bardet-biedl syndrome 12]
GO:0009653	anatomical structure morphogenesis	9.62E-06	7.32E-02	2	16220	1265	276	43	[ADAMTS16 - adam metallopeptidase with thrombospondin type 1 motif, 16, FRZB - frizzled-related protein, CDH6 - cadherin 6, type 2, k-cadherin (fetal kidney), LRP2 - low density lipoprotein receptor-related protein 2, RPE65 - retinal pigment epithelium-specific protein 65kda, CDH9 - cadherin 9, type 2 (t1-cadherin), RSPO2 - r-spondin 2, RELN - reelin, ID3 - inhibitor of dna binding 3, dominant negative helix-loop-helix protein, DCDC2 - doublecortin domain containing 2, NOX4 - nadph oxidase 4, CHRNA9 - cholinergic receptor, nicotinic, alpha 9 (neuronal), DNAAF1 - dynein, axonemal, assembly factor 1, CDH18 - cadherin 18, type 2, ONECUT2 - one cut homeobox 2, B4GALT6 - udp-gal:betaglcnac beta 1,4-galactosyltransferase, polypeptide 6, NPY1R - neuropeptide y receptor y1, ONECUT1 - one cut homeobox 1, SLITRK6 - slit and ntrk-like family, member 6, SEMA3E - sema domain, immunoglobulin domain (ig), short basic domain, secreted, (semaphorin) 3e, ZIC1 - zic family member 1, CRYGS - crystallin, gamma s, ETV7 - ets variant 7, SPEF2 - sperm flagellar 2, DMRT1 - doublesex and mab-3 related transcription factor 1, CSF3R - colony stimulating factor 3 receptor (granulocyte), LAMA2 - laminin, alpha 2, PDGFRA - platelet-derived growth factor receptor, alpha polypeptide, TGFBR3 - transforming growth factor, beta receptor iii, CFC1 - cripto, frl-1, cryptic family 1, TH - tyrosine hydroxylase, HES1 - hairy and enhancer of split 1, (drosophila), COL13A1 - collagen, type xiii, alpha 1, ALDH1A2 - aldehyde dehydrogenase 1 family, member a2, TENM3 - teneurin transmembrane protein 3, TTN - titin, PHEX - phosphate regulating endopeptidase homolog, x-linked, CFC1B - cripto, frl-1, cryptic family 1b, MYH7 - myosin, heavy chain 7, cardiac muscle, beta, CXCR4 - chemokine (c-x-c motif) receptor 4, FN1 - fibronectin 1, NCKAP1L - nck-associated protein 1-like, FOXI3 - forkhead box i3]
GO:0007267	cell-cell signaling	4.10E-05	2.08E-01	2.55	16220	531	276	23	[SEMA3B - sema domain, immunoglobulin domain (ig), short basic domain, secreted, (semaphorin) 3b, LHX5 - lim homeobox 5, PNOC - prepronociceptin, IL1RAP - interleukin 1 receptor accessory protein, HTR1E - 5-hydroxytryptamine (serotonin) receptor 1e, g protein-coupled, CACNB2 - calcium channel, voltage-dependent, beta 2 subunit, WISP2 - wnt1 inducible signaling pathway protein 2, GHRH - growth hormone releasing hormone, AVP - arginine vasopressin, UTS2 - urotensin 2, IL15 - interleukin 15, PCSK1 - proprotein convertase subtilisin/kexin type 1, FGFBP2 - fibroblast growth factor binding protein 2, CHRNA9 - cholinergic receptor, nicotinic, alpha 9 (neuronal), GRM1 - glutamate receptor, metabotropic 1, TH - tyrosine hydroxylase, HES1 - hairy and enhancer of split 1, (drosophila), PHEX - phosphate regulating endopeptidase homolog, x-linked, BMP3 - bone morphogenetic protein 3, ENPEP - glutamyl aminopeptidase (aminopeptidase a), HTR3B - 5-hydroxytryptamine (serotonin) receptor 3b, ionotropic, INHA - inhibin, alpha, GJD2 - gap junction protein, delta 2, 36kda]
GO:0050766	positive regulation of phagocytosis	6.84E-05	2.60E-01	6.86	16220	60	276	7	[C4A - complement component 4a (rodgers blood group), C4B - complement component 4b (chido blood group), AHSG - alpha-2-hs-glycoprotein, SLC11A1 - solute carrier family 11 (proton-coupled divalent metal ion transporter), member 1, IL15 - interleukin 15, FCER1G - fc fragment of ige, high affinity i, receptor for; gamma polypeptide, NCKAP1L - nck-associated protein 1-like]
GO:0007600	sensory perception	8.85E-05	2.70E-01	2.7	16220	413	276	19	[GUCA1C - guanylate cyclase activator 1c, ASIC3 - acid-sensing (proton-gated) ion channel 3, TRPV1 - transient receptor potential cation channel, subfamily v, member 1, OPRM1 - opioid receptor, mu 1, LRP2 - low density lipoprotein receptor-related protein 2, PNOC - prepronociceptin, RPE65 - retinal pigment epithelium-specific protein 65kda, CACNB2 - calcium channel, voltage-dependent, beta 2 subunit, DCDC2 - doublecortin domain containing 2, ESPNL - espin-like, GRM1 - glutamate receptor, metabotropic 1, TH - tyrosine hydroxylase, PDC - phosducin, NPY1R - neuropeptide y receptor y1, CALHM1 - calcium homeostasis modulator 1, SLITRK6 - slit and ntrk-like family, member 6, CNTN5 - contactin 5, CRYGS - crystallin, gamma s, OTOF - otoferlin]
GO:0050764	regulation of phagocytosis	9.85E-05	2.50E-01	5.53	16220	85	276	8	[FER1L5 - fer-1-like 5 (c. elegans), C4A - complement component 4a (rodgers blood group), C4B - complement component 4b (chido blood group), AHSG - alpha-2-hs-glycoprotein, SLC11A1 - solute carrier family 11 (proton-coupled divalent metal ion transporter), member 1, IL15 - interleukin 15, FCER1G - fc fragment of ige, high affinity i, receptor for; gamma polypeptide, NCKAP1L - nck-associated protein 1-like]
GO:0048856	anatomical structure development	9.89E-05	2.15E-01	1.5	16220	3024	276	77	[FRZB - frizzled-related protein, GNGT1 - guanine nucleotide binding protein (g protein), gamma transducing activity polypeptide 1, LRP2 - low density lipoprotein receptor-related protein 2, ZFHX3 - zinc finger homeobox 3, RPE65 - retinal pigment epithelium-specific protein 65kda, RSPO2 - r-spondin 2, RELN - reelin, ID3 - inhibitor of dna binding 3, dominant negative helix-loop-helix protein, AK8 - adenylate kinase 8, EBF1 - early b-cell factor 1, ONECUT2 - one cut homeobox 2, DMRTA1 - dmrt-like family a1, SIAH3 - siah e3 ubiquitin protein ligase family member 3, TP73 - tumor protein p73, SLITRK6 - slit and ntrk-like family, member 6, MSLN - mesothelin, LDB3 - lim domain binding 3, BMP3 - bone morphogenetic protein 3, RCN3 - reticulocalbin 3, ef-hand calcium binding domain, CYP4A11 - cytochrome p450, family 4, subfamily a, polypeptide 11, CRYGS - crystallin, gamma s, CHODL - chondrolectin, ZNF750 - zinc finger protein 750, LGR6 - leucine-rich repeat containing g protein-coupled receptor 6, SPEF2 - sperm flagellar 2, AHSG - alpha-2-hs-glycoprotein, DMRT1 - doublesex and mab-3 related transcription factor 1, LAMA2 - laminin, alpha 2, TGFBR3 - transforming growth factor, beta receptor iii, GHRH - growth hormone releasing hormone, IL15 - interleukin 15, DAB2 - dab, mitogen-responsive phosphoprotein, homolog 2 (drosophila), FAM9A - family with sequence similarity 9, member a, SCN2A - sodium channel, voltage-gated, type ii, alpha subunit, TH - tyrosine hydroxylase, HES1 - hairy and enhancer of split 1, (drosophila), COL13A1 - collagen, type xiii, alpha 1, TENM3 - teneurin transmembrane protein 3, ALDH1A2 - aldehyde dehydrogenase 1 family, member a2, PHEX - phosphate regulating endopeptidase homolog, x-linked, COL19A1 - collagen, type xix, alpha 1, CATSPERG - catsper channel auxiliary subunit gamma, MYH7 - myosin, heavy chain 7, cardiac muscle, beta, INHA - inhibin, alpha, KCNK2 - potassium channel, subfamily k, member 2, LCP1 - lymphocyte cytosolic protein 1 (l-plastin), CDH6 - cadherin 6, type 2, k-cadherin (fetal kidney), CDH9 - cadherin 9, type 2 (t1-cadherin), ALK - anaplastic lymphoma receptor tyrosine kinase, DNAAF1 - dynein, axonemal, assembly factor 1, CDH18 - cadherin 18, type 2, ARHGDIB - rho gdp dissociation inhibitor (gdi) beta, SHROOM4 - shroom family member 4, TLL1 - tolloid-like 1, ONECUT1 - one cut homeobox 1, GREB1L - growth regulation by estrogen in breast cancer-like, ZIC1 - zic family member 1, C6 - complement component 6, LHX5 - lim homeobox 5, POSTN - periostin, osteoblast specific factor, PDGFRA - platelet-derived growth factor receptor, alpha polypeptide, TWIST2 - twist basic helix-loop-helix transcription factor 2, CFC1 - cripto, frl-1, cryptic family 1, ZIC4 - zic family member 4, CD109 - cd109 molecule, MOV10L1 - mov10l1, moloney leukemia virus 10-like 1, homolog (mouse), NXF2 - nuclear rna export factor 2, STC2 - stanniocalcin 2, AGBL4 - atp/gtp binding protein-like 4, TTN - titin, OTX2 - orthodenticle homeobox 2, CFC1B - cripto, frl-1, cryptic family 1b, CALCRL - calcitonin receptor-like, ENPEP - glutamyl aminopeptidase (aminopeptidase a), CXCR4 - chemokine (c-x-c motif) receptor 4, FN1 - fibronectin 1, NCKAP1L - nck-associated protein 1-like]
GO:0003008	system process	1.45E-04	2.77E-01	1.86	16220	1198	276	38	[GUCA1C - guanylate cyclase activator 1c, ASIC3 - acid-sensing (proton-gated) ion channel 3, TRPV1 - transient receptor potential cation channel, subfamily v, member 1, LRP2 - low density lipoprotein receptor-related protein 2, RPE65 - retinal pigment epithelium-specific protein 65kda, CACNB2 - calcium channel, voltage-dependent, beta 2 subunit, AVP - arginine vasopressin, UTS2 - urotensin 2, DCDC2 - doublecortin domain containing 2, NOX4 - nadph oxidase 4, CHRNA9 - cholinergic receptor, nicotinic, alpha 9 (neuronal), SHROOM4 - shroom family member 4, PDC - phosducin, NPY1R - neuropeptide y receptor y1, CALHM1 - calcium homeostasis modulator 1, SLITRK6 - slit and ntrk-like family, member 6, CYP4A11 - cytochrome p450, family 4, subfamily a, polypeptide 11, CRYGS - crystallin, gamma s, HTR3B - 5-hydroxytryptamine (serotonin) receptor 3b, ionotropic, F5 - coagulation factor v (proaccelerin, labile factor), NPS - neuropeptide s, OPRM1 - opioid receptor, mu 1, PNOC - prepronociceptin, CLDN4 - claudin 4, IL15 - interleukin 15, ESPNL - espin-like, SCN2A - sodium channel, voltage-gated, type ii, alpha subunit, GRM1 - glutamate receptor, metabotropic 1, TH - tyrosine hydroxylase, TTN - titin, CNTN5 - contactin 5, ENPEP - glutamyl aminopeptidase (aminopeptidase a), MYH7 - myosin, heavy chain 7, cardiac muscle, beta, CXCR4 - chemokine (c-x-c motif) receptor 4, TNNI1 - troponin i type 1 (skeletal, slow), VIP - vasoactive intestinal peptide, OTOF - otoferlin, KCNK2 - potassium channel, subfamily k, member 2]
GO:0006776	vitamin A metabolic process	2.56E-04	4.33E-01	22.04	16220	8	276	3	[RPE65 - retinal pigment epithelium-specific protein 65kda, ALDH1A2 - aldehyde dehydrogenase 1 family, member a2, BCMO1 - beta-carotene 15,15'-monooxygenase 1]
GO:0003351	epithelial cilium movement	2.60E-04	3.96E-01	12.37	16220	19	276	4	[SPEF2 - sperm flagellar 2, DNAAF1 - dynein, axonemal, assembly factor 1, ROPN1L - rhophilin associated tail protein 1-like, SPAG17 - sperm associated antigen 17]
GO:0036269	swimming behavior	2.89E-04	3.99E-01	58.77	16220	2	276	2	[ALK - anaplastic lymphoma receptor tyrosine kinase, IDO1 - indoleamine 2,3-dioxygenase 1]

GO:0023052	signaling	2.97E-04	3.76E-01	2.23	16220	607	276	23	[SEMA3B - sema domain, immunoglobulin domain (ig), short basic domain, secreted, (semaphorin) 3b, LHX5 - lim homeobox 5, PNOC - prepronociceptin, IL1RAP - interleukin 1 receptor accessory protein, HTR1E - 5-hydroxytryptamine (serotonin) receptor 1e, g protein-coupled, CACNB2 - calcium channel, voltage-dependent, beta 2 subunit, WISP2 - wnt1 inducible signaling pathway protein 2, GHRH - growth hormone releasing hormone, AVP - arginine vasopressin, UTS2 - urotensin 2, IL15 - interleukin 15, PCSK1 - proprotein convertase subtilisin/kexin type 1, FGFBP2 - fibroblast growth factor binding protein 2, CHRNA9 - cholinergic receptor, nicotinic, alpha 9 (neuronal), GRM1 - glutamate receptor, metabotropic 1, TH - tyrosine hydroxylase, HES1 - hairy and enhancer of split 1, (drosophila), PHEX - phosphate regulating endopeptidase homolog, x-linked, BMP3 - bone morphogenetic protein 3, ENPEP - glutamyl aminopeptidase (aminopeptidase a), HTR3B - 5-hydroxytryptamine (serotonin) receptor 3b, ionotropic, INHA - inhibin, alpha, GJD2 - gap junction protein, delta 2, 36kda]
GO:0007154	cell communication	3.41E-04	4.00E-01	2.05	16220	775	276	27	[SEMA3B - sema domain, immunoglobulin domain (ig), short basic domain, secreted, (semaphorin) 3b, TRPV1 - transient receptor potential cation channel, subfamily v, member 1, IL1RAP - interleukin 1 receptor accessory protein, CACNB2 - calcium channel, voltage-dependent, beta 2 subunit, DNAJC15 - dnaj (hsp40) homolog, subfamily c, member 15, AVP - arginine vasopressin, UTS2 - urotensin 2, PCSK1 - proprotein convertase subtilisin/kexin type 1, CHRNA9 - cholinergic receptor, nicotinic, alpha 9 (neuronal), FGFBP2 - fibroblast growth factor binding protein 2, BMP3 - bone morphogenetic protein 3, HTR3B - 5-hydroxytryptamine (serotonin) receptor 3b, ionotropic, GJD2 - gap junction protein, delta 2, 36kda, LHX5 - lim homeobox 5, PNOC - prepronociceptin, HTR1E - 5-hydroxytryptamine (serotonin) receptor 1e, g protein-coupled, POSTN - periostin, osteoblast specific factor, WISP2 - wnt1 inducible signaling pathway protein 2, GHRH - growth hormone releasing hormone, IL15 - interleukin 15, GRM1 - glutamate receptor, metabotropic 1, TH - tyrosine hydroxylase, HES1 - hairy and enhancer of split 1, (drosophila), ALB - albumin, PHEX - phosphate regulating endopeptidase homolog, x-linked, ENPEP - glutamyl aminopeptidase (aminopeptidase a), INHA - inhibin, alpha]
GO:0035456	response to interferon-beta	4.71E-04	5.12E-01	10.69	16220	22	276	4	[IRGM - immunity-related gtpase family, m, IFITM3 - interferon induced transmembrane protein 3, IFITM1 - interferon induced transmembrane protein 1, IFITM2 - interferon induced transmembrane protein 2]
GO:0050877	nervous system process	4.74E-04	4.81E-01	2.04	16220	750	276	26	[GUCA1C - guanylate cyclase activator 1c, ASIC3 - acid-sensing (proton-gated) ion channel 3, TRPV1 - transient receptor potential cation channel, subfamily v, member 1, LRP2 - low density lipoprotein receptor-related protein 2, RPE65 - retinal pigment epithelium-specific protein 65kda, CACNB2 - calcium channel, voltage-dependent, beta 2 subunit, DCDC2 - doublecortin domain containing 2, CHRNA9 - cholinergic receptor, nicotinic, alpha 9 (neuronal), SHROOM4 - shroom family member 4, PDC - phosducin, NPY1R - neuropeptide y receptor y1, CALHM1 - calcium homeostasis modulator 1, SLITRK6 - slit and ntrk-like family, member 6, CRYGS - crystallin, gamma s, HTR3B - 5-hydroxytryptamine (serotonin) receptor 3b, ionotropic, NPS - neuropeptide s, OPRM1 - opioid receptor, mu 1, PNOC - prepronociceptin, ESPNL - espin-like, SCN2A - sodium channel, voltage-gated, type ii, alpha subunit, GRM1 - glutamate receptor, metabotropic 1, TH - tyrosine hydroxylase, CNTN5 - contactin 5, VIP - vasoactive intestinal peptide, OTOF - otoferlin, KCNK2 - potassium channel, subfamily k, member 2]
GO:0002576	platelet degranulation	7.81E-04	7.43E-01	4.09	16220	115	276	8	[ALB - albumin, TTN - titin, AHSG - alpha-2-hs-glycoprotein, MMRN1 - multimerin 1, CD109 - cd109 molecule, FN1 - fibronectin 1, F5 - coagulation factor v (proaccelerin, labile factor), CTSW - cathepsin w]
GO:0016108	tetraterpenoid metabolic process	8.56E-04	7.66E-01	39.18	16220	3	276	2	[RPE65 - retinal pigment epithelium-specific protein 65kda, BCMO1 - beta-carotene 15,15'-monooxygenase 1]
GO:0016116	carotenoid metabolic process	8.56E-04	7.24E-01	39.18	16220	3	276	2	[RPE65 - retinal pigment epithelium-specific protein 65kda, BCMO1 - beta-carotene 15,15'-monooxygenase 1]
GO:1905447	negative regulation of mitochondrial ATP synthesis coupled electron transport	8.56E-04	6.86E-01	39.18	16220	3	276	2	[MIR210 - microrna 210, DNAJC15 - dnaj (hsp40) homolog, subfamily c, member 15]
GO:0042214	terpene metabolic process	8.56E-04	6.51E-01	39.18	16220	3	276	2	[BCMO1 - beta-carotene 15,15'-monooxygenase 1, TH - tyrosine hydroxylase]
GO:1902957	negative regulation of mitochondrial electron transport, NADH to ubiquinone	8.56E-04	6.20E-01	39.18	16220	3	276	2	[MIR210 - microrna 210, DNAJC15 - dnaj (hsp40) homolog, subfamily c, member 15]

CORTICAL ORGANOIDS: GENE ONTOLOGY PROCESS TERMS FOR TRANSCRIPTS WITH LOWER EXPRESSION IN LEO

GO Term	Description	P-value	FDR q-value	Enrichment	N	B	n	b	Genes
GO:0048523	negative regulation of cellular process	7.09E-08	1.08E-03	1.78	16219	4299	144	68	[SFRP1 - secreted frizzled-related protein 1, H1FX - h1 histone family, member x, SLC18A2 - solute carrier family 18 (vesicular monoamine transporter), member 2, ATF3 - activating transcription factor 3, MDFI - myod family inhibitor, FTH1 - ferritin, heavy polypeptide 1, AES - amino-terminal enhancer of split, ARRDC3 - arrestin domain containing 3, IL8 - interleukin 8, UBB - ubiquitin b, UBC - ubiquitin c, CRYM - crystallin, mu, OPN3 - opsin 3, HIST1H4K - histone cluster 1, h4k, TBX1 - t-box 1, HIST1H4C - histone cluster 1, h4c, RGS10 - regulator of g-protein signaling 10, CFLAR - casp8 and fadd-like apoptosis regulator, CAPG - capping protein (actin filament), gelsolin-like, KCNIP3 - kv channel interacting protein 3, calsenilin, MYC - v-myc avian myelocytomatosis viral oncogene homolog, MXD1 - max dimerization protein 1, GPR21 - g protein-coupled receptor 21, RND1 - rho family gtpase 1, TBXA2R - thromboxane a2 receptor, STMN1 - stathmin 1, NR4A2 - nuclear receptor subfamily 4, group a, member 2, KLF9 - kruppel-like factor 9, ACVR1C - activin a receptor, type ic, TICAM1 - toll-like receptor adaptor molecule 1, NFIX - nuclear factor i/x (ccaat-binding transcription factor), ARHGAP42 - rho gtpase activating protein 42, DUSP16 - dual specificity phosphatase 16, H1F0 - h1 histone family, member 0, HSPA5 - heat shock 70kda protein 5 (glucose-regulated protein, 78kda), NFKBIA - nuclear factor of kappa light polypeptide gene enhancer in b-cells inhibitor, alpha, HIST1H1C - histone cluster 1, h1c, HIST1H1E - histone cluster 1, h1e, ALOX5 - arachidonate 5-lipoxygenase, HSPB1 - heat shock 27kda protein 1, HIST1H2AD - histone cluster 1, h2ad, LEPR - leptin receptor, YWHAE - tyrosine 3-monooxygenase/tryptophan 5-monooxygenase activation protein, epsilon polypeptide, HMX1 - h6 family homeobox 1, INSM1 - insulinoma-associated 1, DUSP10 - dual specificity phosphatase 10, TLE2 - transducin-like enhancer of split 2 (e(sp1) homolog, drosophila), H3F3B - h3 histone, family 3b (h3.3b), CRLF1 - cytokine receptor-like factor 1, FKBP4 - fk506 binding protein 4, 59kda, SOX4 - sry (sex determining region y)-box 4, PDE2A - phosphodiesterase 2a, cgmp-stimulated, ARMCX5-GPRASP2 - armcx5-gprasp2 readthrough, NR1D1 - nuclear receptor subfamily 1, group d, member 1, FOXD1 - forkhead box d1, HNRNPC - heterogeneous nuclear ribonucleoprotein c (c1/c2), HES2 - hairy and enhancer of split 2 (drosophila), CDKN2A - cyclin-dependent kinase inhibitor 2a, CCND2 - cyclin d2, PPP1R14A - protein phosphatase 1, regulatory (inhibitor) subunit 14a, PIM1 - pim-1 oncogene, WNT16 - wingless-type mmtv integration site family, member 16, IKZF1 - ikaros family zinc finger 1 (ikaros), OSTN - osteocrin, NBL1 - neuroblastoma 1, dan family bmp antagonist, CHRDL1 - chordin-like 1, SPOCK1 - sparc/osteonectin, cwcv and kazal-like domains proteoglycan (testican) 1, HIST1H2AI - histone cluster 1, h2ai]
GO:0031497	chromatin assembly	1.42E-07	1.08E-03	24.14	16219	28	144	6	[H3F3B - h3 histone, family 3b (h3.3b), H1FX - h1 histone family, member x, H1F0 - h1 histone family, member 0, HIST1H1C - histone cluster 1, h1c, HIST1H1E - histone cluster 1, h1e, CDKN2A - cyclin-dependent kinase inhibitor 2a]
GO:0006333	chromatin assembly or disassembly	2.35E-07	1.19E-03	12.51	16219	72	144	8	[H3F3B - h3 histone, family 3b (h3.3b), HIST1H4C - histone cluster 1, h4c, H1FX - h1 histone family, member x, H1F0 - h1 histone family, member 0, HIST1H1C - histone cluster 1, h1c, HIST1H1E - histone cluster 1, h1e, CDKN2A - cyclin-dependent kinase inhibitor 2a, HIST1H4K - histone cluster 1, h4k]
GO:0048519	negative regulation of biological process	2.45E-07	9.34E-04	1.68	16219	4824	144	72	[ADRB1 - adrenoceptor beta 1, SFRP1 - secreted frizzled-related protein 1, H1FX - h1 histone family, member x, SLC18A2 - solute carrier family 18 (vesicular monoamine transporter), member 2, ATF3 - activating transcription factor 3, MDFI - myod family inhibitor, FTH1 - ferritin, heavy polypeptide 1, AES - amino-terminal enhancer of split, ZC3HAV1 - zinc finger ccch-type, antiviral 1, ARRDC3 - arrestin domain containing 3, IL8 - interleukin 8, UBB - ubiquitin b, UBC - ubiquitin c, CRYM - crystallin, mu, OPN3 - opsin 3, HIST1H4K - histone cluster 1, h4k, TBX1 - t-box 1, HIST1H4C - histone cluster 1, h4c, RGS10 - regulator of g-protein signaling 10, CFLAR - casp8 and fadd-like apoptosis regulator, CAPG - capping protein (actin filament), gelsolin-like, KCNIP3 - kv channel interacting protein 3, calsenilin, MYC - v-myc avian myelocytomatosis viral oncogene homolog, MXD1 - max dimerization protein 1, GPR21 - g protein-coupled receptor 21, RND1 - rho family gtpase 1, TBXA2R - thromboxane a2 receptor, STMN1 - stathmin 1, NR4A2 - nuclear receptor subfamily 4, group a, member 2, KLF9 - kruppel-like factor 9, ACVR1C - activin a receptor, type ic, TICAM1 - toll-like receptor adaptor molecule 1, NFIX - nuclear factor i/x (ccaat-binding transcription factor), ARHGAP42 - rho gtpase activating protein 42, DUSP16 - dual specificity phosphatase 16, H1F0 - h1 histone family, member 0, HSPA5 - heat shock 70kda protein 5 (glucose-regulated protein, 78kda), NFKBIA - nuclear factor of kappa light polypeptide gene enhancer in b-cells inhibitor, alpha, HIST1H1C - histone cluster 1, h1c, HIST1H1E - histone cluster 1, h1e, MICB - mhc class i polypeptide-related sequence b, ALOX5 - arachidonate 5-lipoxygenase, TIPARP - tcdd-inducible poly(adp-ribose) polymerase, HSPB1 - heat shock 27kda protein 1, HIST1H2AD - histone cluster 1, h2ad, LEPR - leptin receptor, YWHAE - tyrosine 3-monooxygenase/tryptophan 5-monooxygenase activation protein, epsilon polypeptide, HMX1 - h6 family homeobox 1, INSM1 - insulinoma-associated 1, DUSP10 - dual specificity phosphatase 10, TLE2 - transducin-like enhancer of split 2 (e(sp1) homolog, drosophila), H3F3B - h3 histone, family 3b (h3.3b), CRLF1 - cytokine receptor-like factor 1, FKBP4 - fk506 binding protein 4, 59kda, SOX4 - sry (sex determining region y)-box 4, PDE2A - phosphodiesterase 2a, cgmp-stimulated, ARMCX5-GPRASP2 - armcx5-gprasp2 readthrough, NR1D1 - nuclear receptor subfamily 1, group d, member 1, FOXD1 - forkhead box d1, HNRNPC - heterogeneous nuclear ribonucleoprotein c (c1/c2), HES2 - hairy and enhancer of split 2 (drosophila), CDKN2A - cyclin-dependent kinase inhibitor 2a, CCND2 - cyclin d2, PPP1R14A - protein phosphatase 1, regulatory (inhibitor) subunit 14a, PIM1 - pim-1 oncogene, WNT16 - wingless-type mmtv integration site family, member 16, IKZF1 - ikaros family zinc finger 1 (ikaros), OSTN - osteocrin, NBL1 - neuroblastoma 1, dan family bmp antagonist, CHRDL1 - chordin-like 1, SPOCK1 - sparc/osteonectin, cwcv and kazal-like domains proteoglycan (testican) 1, HIST1H2AI - histone cluster 1, h2ai]
GO:0006334	nucleosome assembly	4.41E-07	1.34E-03	11.55	16219	78	144	8	[HIST1H2BC - histone cluster 1, h2bc, H3F3B - h3 histone, family 3b (h3.3b), HIST1H4C - histone cluster 1, h4c, H1FX - h1 histone family, member x, H1F0 - h1 histone family, member 0, HIST1H1C - histone cluster 1, h1c, HIST1H1E - histone cluster 1, h1e, HIST1H4K - histone cluster 1, h4k]

GO:0016584	nucleosome positioning	7.25E-07	1.84E-03	50.06	16219	9	144	4	[H1FX - h1 histone family, member x, H1F0 - h1 histone family, member 0, HIST1H1C - histone cluster 1, h1c, HIST1H1E - histone cluster 1, h1e]
GO:0071824	protein-DNA complex subunit organization	7.50E-07	1.63E-03	6.73	16219	184	144	11	[HIST1H2BC - histone cluster 1, h2bc, H3F3B - h3 histone, family 3b (h3.3b), HIST1H4C - histone cluster 1, h4c, H1FX - h1 histone family, member x, H1F0 - h1 histone family, member 0, HIST1H1C - histone cluster 1, h1c, HIST1H1E - histone cluster 1, h1e, UBB - ubiquitin b, UBC - ubiquitin c, MYC - v-myc avian myelocytomatosis viral oncogene homolog, HIST1H4K - histone cluster 1, h4k]
GO:0065004	protein-DNA complex assembly	7.98E-07	1.52E-03	7.61	16219	148	144	10	[HIST1H2BC - histone cluster 1, h2bc, H3F3B - h3 histone, family 3b (h3.3b), HIST1H4C - histone cluster 1, h4c, H1FX - h1 histone family, member x, H1F0 - h1 histone family, member 0, HIST1H1C - histone cluster 1, h1c, HIST1H1E - histone cluster 1, h1e, UBB - ubiquitin b, UBC - ubiquitin c, HIST1H4K - histone cluster 1, h4k]
GO:0045934	negative regulation of nucleobase-containing compound metabolic process	9.80E-07	1.66E-03	2.55	16219	1371	144	31	[NFIX - nuclear factor i/x (ccaat-binding transcription factor), H1FX - h1 histone family, member x, SFRP1 - secreted frizzled-related protein 1, H1F0 - h1 histone family, member 0, HIST1H1C - histone cluster 1, h1c, HIST1H1E - histone cluster 1, h1e, ATF3 - activating transcription factor 3, MDFI - myod family inhibitor, HIST1H2AD - histone cluster 1, h2ad, HMX1 - h6 family homeobox 1, INSM1 - insulinoma-associated 1, AES - amino-terminal enhancer of split, TLE2 - transducin-like enhancer of split 2 (e(sp1) homolog, drosophila), H3F3B - h3 histone, family 3b (h3.3b), PDE2A - phosphodiesterase 2a, cgmp-stimulated, NR1D1 - nuclear receptor subfamily 1, group d, member 1, UBB - ubiquitin b, HNRNPC - heterogeneous nuclear ribonucleoprotein c (c1/c2), FOXD1 - forkhead box d1, HES2 - hairy and enhancer of split 2 (drosophila), UBC - ubiquitin c, CDKN2A - cyclin-dependent kinase inhibitor 2a, CRYM - crystallin, mu, HIST1H4K - histone cluster 1, h4k, HIST1H4C - histone cluster 1, h4c, IKZF1 - ikaros family zinc finger 1 (ikaros), KCNIP3 - kv channel interacting protein 3, calsenilin, MYC - v-myc avian myelocytomatosis viral oncogene homolog, MXD1 - max dimerization protein 1, NR4A2 - nuclear receptor subfamily 4, group a, member 2, HIST1H2AI - histone cluster 1, h2ai]
GO:0009890	negative regulation of biosynthetic process	1.14E-06	1.73E-03	2.48	16219	1454	144	32	[NFIX - nuclear factor i/x (ccaat-binding transcription factor), SFRP1 - secreted frizzled-related protein 1, H1F0 - h1 histone family, member 0, SLC18A2 - solute carrier family 18 (vesicular monoamine transporter), member 2, ATF3 - activating transcription factor 3, MDFI - myod family inhibitor, HIST1H2AD - histone cluster 1, h2ad, LEPR - leptin receptor, HMX1 - h6 family homeobox 1, INSM1 - insulinoma-associated 1, AES - amino-terminal enhancer of split, TLE2 - transducin-like enhancer of split 2 (e(sp1) homolog, drosophila), H3F3B - h3 histone, family 3b (h3.3b), PDE2A - phosphodiesterase 2a, cgmp-stimulated, NR1D1 - nuclear receptor subfamily 1, group d, member 1, UBB - ubiquitin b, HNRNPC - heterogeneous nuclear ribonucleoprotein c (c1/c2), FOXD1 - forkhead box d1, HES2 - hairy and enhancer of split 2 (drosophila), UBC - ubiquitin c, CDKN2A - cyclin-dependent kinase inhibitor 2a, CRYM - crystallin, mu, OPN3 - opsin 3, HIST1H4K - histone cluster 1, h4k, HIST1H4C - histone cluster 1, h4c, IKZF1 - ikaros family zinc finger 1 (ikaros), CFLAR - casp8 and fadd-like apoptosis regulator, KCNIP3 - kv channel interacting protein 3, calsenilin, MYC - v-myc avian myelocytomatosis viral oncogene homolog, MXD1 - max dimerization protein 1, NR4A2 - nuclear receptor subfamily 4, group a, member 2, HIST1H2AI - histone cluster 1, h2ai]
GO:0065008	regulation of biological quality	1.52E-06	2.11E-03	1.82	16219	3398	144	55	[ADRB1 - adrenoceptor beta 1, SFRP1 - secreted frizzled-related protein 1, CDC42EP1 - cdc42 effector protein (rho gtpase binding) 1, SLC18A2 - solute carrier family 18 (vesicular monoamine transporter), member 2, SEZ6 - seizure related 6 homolog (mouse), MDFI - myod family inhibitor, BHMT - betaine–homocysteine s-methyltransferase, FTH1 - ferritin, heavy polypeptide 1, ARRDC3 - arrestin domain containing 3, UBB - ubiquitin b, UBC - ubiquitin c, CRYM - crystallin, mu, HIST1H4K - histone cluster 1, h4k, HIST1H4C - histone cluster 1, h4c, CAPG - capping protein (actin filament), gelsolin-like, HRH1 - histamine receptor h1, MYC - v-myc avian myelocytomatosis viral oncogene homolog, CHRM1 - cholinergic receptor, muscarinic 1, GPR21 - g protein-coupled receptor 21, RND1 - rho family gtpase 1, TBXA2R - thromboxane a2 receptor, ACTB - actin, beta, STMN1 - stathmin 1, RAC3 - ras-related c3 botulinum toxin substrate 3 (rho family, small gtp binding protein rac3), ACVR1C - activin a receptor, type ic, TICAM1 - toll-like receptor adaptor molecule 1, ARHGAP42 - rho gtpase activating protein 42, HSPA5 - heat shock 70kda protein 5 (glucose-regulated protein, 78kda), NFKBIA - nuclear factor of kappa light polypeptide gene enhancer in b-cells inhibitor, alpha, SCGN - secretagogin, ef-hand calcium binding protein, ALOX5 - arachidonate 5-lipoxygenase, TIPARP - tcdd-inducible poly(adp-ribose) polymerase, HSPB1 - heat shock 27kda protein 1, GADD45G - growth arrest and dna-damage-inducible, gamma, LEPR - leptin receptor, YWHAE - tyrosine 3-monooxygenase/tryptophan 5-monooxygenase activation protein, epsilon polypeptide, H3F3B - h3 histone, family 3b (h3.3b), SOX4 - sry (sex determining region y)-box 4, SLC30A3 - solute carrier family 30 (zinc transporter), member 3, PDE2A - phosphodiesterase 2a, cgmp-stimulated, ARMCX5-GPRASP2 - armcx5-gprasp2 readthrough, NR1D1 - nuclear receptor subfamily 1, group d, member 1, FOXD1 - forkhead box d1, HNRNPC - heterogeneous nuclear ribonucleoprotein c (c1/c2), PIM1 - pim-1 oncogene, KCNK6 - potassium channel, subfamily k, member 6, GRASP - grp1 (general receptor for phosphoinositides 1)-associated scaffold protein, NBL1 - neuroblastoma 1, dan family bmp antagonist, CCSAP - centriole, cilia and spindle-associated protein, SLC9A3 - solute carrier family 9, subfamily a (nhe3, cation proton antiporter 3), member 3, CRABP1 - cellular retinoic acid binding protein 1, SPR - sepiapterin reductase (7,8-dihydrobiopterin:nadp+ oxidoreductase), ANO4 - anoctamin 4, KCNK12 - potassium channel, subfamily k, member 12, TAC3 - tachykinin 3]
GO:0050794	regulation of cellular process	2.15E-06	2.73E-03	1.32	16219	9374	144	110	[H1FX - h1 histone family, member x, CDC42EP1 - cdc42 effector protein (rho gtpase binding) 1, SLC18A2 - solute carrier family 18 (vesicular monoamine transporter), member 2, SEZ6 - seizure related 6 homolog (mouse), BHMT - betaine–homocysteine s-methyltransferase, FTH1 - ferritin, heavy polypeptide 1, JUND - jun d proto-oncogene, ZC3HAV1 - zinc finger ccch-type, antiviral 1, GPD1 - glycerol-3-phosphate dehydrogenase 1 (soluble), OPN3 - opsin 3, HIST1H4K - histone cluster 1, h4k, TBX1 - t-box 1, MGARP - mitochondria-localized glutamic acid-rich protein, HIST1H4C - histone cluster 1, h4c, EEF1A2 - eukaryotic translation elongation factor 1 alpha 2, RGS10 - regulator of g-protein signaling 10, TCERG1L - transcription elongation regulator 1-like, KCNIP3 - kv channel interacting protein 3, calsenilin, MXD1 - max dimerization protein 1, GPR21 - g protein-coupled receptor 21, TBXA2R - thromboxane a2 receptor, ACTB - actin, beta, TNFAIP8L3 - tumor necrosis factor, alpha-induced protein 8-like 3, KLF9 - kruppel-like factor 9, ACVR1C - activin a receptor, type ic, NET1 - neuroepithelial cell transforming 1, TICAM1 - toll-like receptor adaptor molecule 1, EFS - embryonal fyn-associated substrate, NFIX - nuclear factor i/x (ccaat-binding transcription factor), ARHGAP42 - rho gtpase activating protein 42, NFKBIA - nuclear factor of kappa light polypeptide gene enhancer in b-cells inhibitor, alpha, TCF15 - transcription factor 15 (basic helix-loop-helix), SCGN - secretagogin, ef-hand calcium binding protein, TIPARP - tcdd-inducible poly(adp-ribose) polymerase, GADD45G - growth arrest and dna-damage-inducible, gamma, YWHAE - tyrosine 3-monooxygenase/tryptophan 5-monooxygenase activation protein, epsilon polypeptide, HMX1 - h6 family homeobox 1, DUSP10 - dual specificity phosphatase 10, FKBP4 - fk506 binding protein 4, 59kda, SOX4 - sry (sex determining region y)-box 4, PDE2A - phosphodiesterase 2a, cgmp-stimulated, FOXD1 - forkhead box d1, HNRNPC - heterogeneous nuclear ribonucleoprotein c (c1/c2), CDKN2A - cyclin-dependent kinase inhibitor 2a, PPP1R14A - protein phosphatase 1, regulatory (inhibitor) subunit 14a, TUBB2B - tubulin, beta 2b class iib, IKZF1 - ikaros family zinc finger 1 (ikaros), GRASP - grp1 (general receptor for phosphoinositides 1)-associated scaffold protein, CHRDL1 - chordin-like 1, CFD - complement factor d (adipsin), CCSAP - centriole, cilia and spindle-associated protein, SPOCK1 - sparc/osteonectin, cwcv and kazal-like domains proteoglycan (testican) 1, PDLIM1 - pdz and lim domain 1, CRABP1 - cellular retinoic acid binding protein 1, CXCL2 - chemokine (c-x-c motif) ligand 2, CXCL3 - chemokine (c-x-c motif) ligand 3, ADRB1 - adrenoceptor beta 1, STMN4 - stathmin-like 4, SFRP1 - secreted frizzled-related protein 1, ATF3 - activating transcription factor 3, MDFI - myod family inhibitor, AES - amino-terminal enhancer of split, ARRDC3 - arrestin domain containing 3, IL8 - interleukin 8, UBB - ubiquitin b, UBC - ubiquitin c, CRYM - crystallin, mu, CFLAR - casp8 and fadd-like apoptosis regulator, CAPG - capping protein (actin filament), gelsolin-like, HRH1 - histamine receptor h1, MYC - v-myc avian myelocytomatosis viral oncogene homolog, CHRM1 - cholinergic receptor, muscarinic 1, RND1 - rho family gtpase 1, NTS - neurotensin, STMN1 - stathmin 1, NR4A2 - nuclear receptor subfamily 4, group a, member 2, RAC3 - ras-related c3 botulinum toxin substrate 3 (rho family, small gtp binding protein rac3), DUSP16 - dual specificity phosphatase 16, H1F0 - h1 histone family, member 0, HSPA5 - heat shock 70kda protein 5 (glucose-regulated protein, 78kda), CSRNP1 - cysteine-serine-rich nuclear protein 1, HIST1H1C - histone cluster 1, h1c, HIST1H1E - histone cluster 1, h1e, MICB - mhc class i polypeptide-related sequence b, ALOX5 - arachidonate 5-lipoxygenase, HSPB1 - heat shock 27kda protein 1, HIST1H2AD - histone cluster 1, h2ad, LEPR - leptin receptor, INSM1 - insulinoma-associated 1, TLE2 - transducin-like enhancer of split 2 (e(sp1) homolog, drosophila), OR2AK2 - olfactory receptor, family 2, subfamily ak, member 2, H3F3B - h3 histone, family 3b (h3.3b), CRLF1 - cytokine receptor-like factor 1, SLC30A3 - solute carrier family 30 (zinc transporter), member 3, ARMCX5-GPRASP2 - armcx5-gprasp2 readthrough, NR1D1 - nuclear receptor subfamily 1, group d, member 1, HES2 - hairy and enhancer of split 2 (drosophila), FOXN2 - forkhead box n2, CCND2 - cyclin d2, PIM1 - pim-1 oncogene, WNT16 - wingless-type mmtv integration site family, member 16, OSTN - osteocrin, PROKR2 - prokineticin receptor 2, LMNB1 - lamin b1, NBL1 - neuroblastoma 1, dan family bmp antagonist, NDP - norrie disease (pseudoglioma), H3F3C - h3 histone, family 3c, HIST1H2AI - histone cluster 1, h2ai, EPHA6 - eph receptor a6, TAC3 - tachykinin 3]
GO:0032501	multicellular organismal process	2.28E-06	2.66E-03	1.9	16219	2899	144	49	[ADRB1 - adrenoceptor beta 1, SFRP1 - secreted frizzled-related protein 1, SLC25A25 - solute carrier family 25 (mitochondrial carrier; phosphate carrier), member 25, SLC18A2 - solute carrier family 18 (vesicular monoamine transporter), member 2, SEZ6 - seizure related 6 homolog (mouse), MDFI - myod family inhibitor, AES - amino-terminal enhancer of split, ARRDC3 - arrestin domain containing 3, UBB - ubiquitin b, CRYM - crystallin, mu, TBX1 - t-box 1, CFLAR - casp8 and fadd-like apoptosis regulator, HRH1 - histamine receptor h1, KCNIP3 - kv channel interacting protein 3, calsenilin, MYC - v-myc avian myelocytomatosis viral oncogene homolog, CHRM1 - cholinergic receptor, muscarinic 1, TBXA2R - thromboxane a2 receptor, ACTA1 - actin, alpha 1, skeletal muscle, STMN1 - stathmin 1, NR4A2 - nuclear receptor subfamily 4, group a, member 2, RAC3 - ras-related c3 botulinum toxin substrate 3 (rho family, small gtp binding protein rac3), ACVR1C - activin a receptor, type ic, ARHGAP42 - rho gtpase activating protein 42, TCF15 - transcription factor 15 (basic helix-loop-helix), CSRNP1 - cysteine-serine-rich nuclear protein 1, ALOX5 - arachidonate 5-lipoxygenase, TIPARP - tcdd-inducible poly(adp-ribose) polymerase, GADD45G - growth arrest and dna-damage-inducible, gamma, LEPR - leptin receptor, YWHAE - tyrosine 3-monooxygenase/tryptophan 5-monooxygenase activation protein, epsilon polypeptide, HMX1 - h6 family homeobox 1, H3F3B - h3 histone, family 3b (h3.3b), SOX4 - sry (sex determining region y)-box 4, PDE2A - phosphodiesterase 2a, cgmp-stimulated, ARMCX5-GPRASP2 - armcx5-gprasp2 readthrough, NR1D1 - nuclear receptor subfamily 1, group d, member 1, FOXD1 - forkhead box d1, HES2 - hairy and enhancer of split 2 (drosophila), PIM1 - pim-1 oncogene, KCNK6 - potassium channel, subfamily k, member 6, WNT16 - wingless-type mmtv integration site family, member 16, OSTN - osteocrin, NBL1 - neuroblastoma 1, dan family bmp antagonist, CHRDL1 - chordin-like 1, CCSAP - centriole, cilia and spindle-associated protein, CRABP1 - cellular retinoic acid binding protein 1, NDP - norrie disease (pseudoglioma), EPHA6 - eph receptor a6, TAC3 - tachykinin 3]

GO:0007165	signal transduction	2.36E-06	2.56E-03	1.71	16219	4007	144	61	[ADRB1 - adrenoceptor beta 1, SFRP1 - secreted frizzled-related protein 1, CDC42EP1 - cdc42 effector protein (rho gtpase binding) 1, ATF3 - activating transcription factor 3, AES - amino-terminal enhancer of split, IL8 - interleukin 8, UBB - ubiquitin b, UBC - ubiquitin c, OPN3 - opsin 3, TBX1 - t-box 1, RGS10 - regulator of g-protein signaling 10, CFLAR - casp8 and fadd-like apoptosis regulator, HRH1 - histamine receptor h1, KCNIP3 - kv channel interacting protein 3, calsenilin, MYC - v-myc avian myelocytomatosis viral oncogene homolog, CHRM1 - cholinergic receptor, muscarinic 1, GPR21 - g protein-coupled receptor 21, RND1 - rho family gtpase 1, TBXA2R - thromboxane a2 receptor, NTS - neurotensin, ACTB - actin, beta, STMN1 - stathmin 1, NR4A2 - nuclear receptor subfamily 4, group a, member 2, TNFAIP8L3 - tumor necrosis factor, alpha-induced protein 8-like 3, RAC3 - ras-related c3 botulinum toxin substrate 3 (rho family, small gtp binding protein rac3), NET1 - neuroepithelial cell transforming 1, ACVR1C - activin a receptor, type ic, EFS - embryonal fyn-associated substrate, TICAM1 - toll-like receptor adaptor molecule 1, ARHGAP42 - rho gtpase activating protein 42, HSPA5 - heat shock 70kda protein 5 (glucose-regulated protein, 78kda), NFKBIA - nuclear factor of kappa light polypeptide gene enhancer in b-cells inhibitor, alpha, CSRNP1 - cysteine-serine-rich nuclear protein 1, MICB - mhc class i polypeptide-related sequence b, ALOX5 - arachidonate 5-lipoxygenase, TIPARP - tcdd-inducible poly(adp-ribose) polymerase, HSPB1 - heat shock 27kda protein 1, LEPR - leptin receptor, YWHAE - tyrosine 3-monooxygenase/tryptophan 5-monooxygenase activation protein, epsilon polypeptide, TLE2 - transducin-like enhancer of split 2 (e(sp1) homolog, drosophila), OR2AK2 - olfactory receptor, family 2, subfamily ak, member 2, CRLF1 - cytokine receptor-like factor 1, SOX4 - sry (sex determining region y)-box 4, PDE2A - phosphodiesterase 2a, cgmp-stimulated, NR1D1 - nuclear receptor subfamily 1, group d, member 1, CDKN2A - cyclin-dependent kinase inhibitor 2a, PIM1 - pim-1 oncogene, WNT16 - wingless-type mmtv integration site family, member 16, OSTN - osteocrin, GRASP - grp1 (general receptor for phosphoinositides 1)-associated scaffold protein, PROKR2 - prokineticin receptor 2, LMNB1 - lamin b1, NBL1 - neuroblastoma 1, dan family bmp antagonist, CHRDL1 - chordin-like 1, CFD - complement factor d (adipsin), CRABP1 - cellular retinoic acid binding protein 1, CXCL2 - chemokine (c-x-c motif) ligand 2, NDP - norrie disease (pseudoglioma), CXCL3 - chemokine (c-x-c motif) ligand 3, EPHA6 - eph receptor a6, TAC3 - tachykinin 3]
GO:0050789	regulation of biological process	2.36E-06	2.40E-03	1.29	16219	10030	144	115	[H1FX - h1 histone family, member x, CDC42EP1 - cdc42 effector protein (rho gtpase binding) 1, SLC18A2 - solute carrier family 18 (vesicular monoamine transporter), member 2, SEZ6 - seizure related 6 homolog (mouse), BHMT - betaine–homocysteine s-methyltransferase, FTH1 - ferritin, heavy polypeptide 1, JUND - jun d proto-oncogene, ZC3HAV1 - zinc finger ccch-type, antiviral 1, GPD1 - glycerol-3-phosphate dehydrogenase 1 (soluble), OPN3 - opsin 3, HIST1H4K - histone cluster 1, h4k, TBX1 - t-box 1, MGARP - mitochondria-localized glutamic acid-rich protein, HIST1H4C - histone cluster 1, h4c, EEF1A2 - eukaryotic translation elongation factor 1 alpha 2, RGS10 - regulator of g-protein signaling 10, TCERG1L - transcription elongation regulator 1-like, KCNIP3 - kv channel interacting protein 3, calsenilin, MXD1 - max dimerization protein 1, GPR21 - g protein-coupled receptor 21, TBXA2R - thromboxane a2 receptor, ACTA1 - actin, alpha 1, skeletal muscle, ACTB - actin, beta, TNFAIP8L3 - tumor necrosis factor, alpha-induced protein 8-like 3, KLF9 - kruppel-like factor 9, ACVR1C - activin a receptor, type ic, NET1 - neuroepithelial cell transforming 1, TICAM1 - toll-like receptor adaptor molecule 1, EFS - embryonal fyn-associated substrate, NFIX - nuclear factor i/x (ccaat-binding transcription factor), ARHGAP42 - rho gtpase activating protein 42, NFKBIA - nuclear factor of kappa light polypeptide gene enhancer in b-cells inhibitor, alpha, TCF15 - transcription factor 15 (basic helix-loop-helix), SCGN - secretagogin, ef-hand calcium binding protein, TIPARP - tcdd-inducible poly(adp-ribose) polymerase, GADD45G - growth arrest and dna-damage-inducible, gamma, YWHAE - tyrosine 3-monooxygenase/tryptophan 5-monooxygenase activation protein, epsilon polypeptide, HMX1 - h6 family homeobox 1, DUSP10 - dual specificity phosphatase 10, FKBP4 - fk506 binding protein 4, 59kda, SOX4 - sry (sex determining region y)-box 4, PDE2A - phosphodiesterase 2a, cgmp-stimulated, FOXD1 - forkhead box d1, HNRNPC - heterogeneous nuclear ribonucleoprotein c (c1/c2), CDKN2A - cyclin-dependent kinase inhibitor 2a, PPP1R14A - protein phosphatase 1, regulatory (inhibitor) subunit 14a, TUBB2B - tubulin, beta 2b class iib, KCNK6 - potassium channel, subfamily k, member 6, IKZF1 - ikaros family zinc finger 1 (ikaros), GRASP - grp1 (general receptor for phosphoinositides 1)-associated scaffold protein, CHRDL1 - chordin-like 1, CFD - complement factor d (adipsin), CCSAP - centriole, cilia and spindle-associated protein, SPOCK1 - sparc/osteonectin, cwcv and kazal-like domains proteoglycan (testican) 1, PDLIM1 - pdz and lim domain 1, CRABP1 - cellular retinoic acid binding protein 1, SPR - sepiapterin reductase (7,8-dihydrobiopterin:nadp+ oxidoreductase), CXCL2 - chemokine (c-x-c motif) ligand 2, CXCL3 - chemokine (c-x-c motif) ligand 3, ADRB1 - adrenoceptor beta 1, STMN4 - stathmin-like 4, SFRP1 - secreted frizzled-related protein 1, ATF3 - activating transcription factor 3, MDFI - myod family inhibitor, AES - amino-terminal enhancer of split, ARRDC3 - arrestin domain containing 3, IL8 - interleukin 8, UBB - ubiquitin b, UBC - ubiquitin c, CRYM - crystallin, mu, ZEB2-AS1 - zeb2 antisense rna 1, CFLAR - casp8 and fadd-like apoptosis regulator, CAPG - capping protein (actin filament), gelsolin-like, HRH1 - histamine receptor h1, MYC - v-myc avian myelocytomatosis viral oncogene homolog, CHRM1 - cholinergic receptor, muscarinic 1, RND1 - rho family gtpase 1, NTS - neurotensin, STMN1 - stathmin 1, NR4A2 - nuclear receptor subfamily 4, group a, member 2, RAC3 - ras-related c3 botulinum toxin substrate 3 (rho family, small gtp binding protein rac3), DUSP16 - dual specificity phosphatase 16, H1F0 - h1 histone family, member 0, HSPA5 - heat shock 70kda protein 5 (glucose-regulated protein, 78kda), CSRNP1 - cysteine-serine-rich nuclear protein 1, HIST1H1C - histone cluster 1, h1c, HIST1H1E - histone cluster 1, h1e, MICB - mhc class i polypeptide-related sequence b, ALOX5 - arachidonate 5-lipoxygenase, HSPB1 - heat shock 27kda protein 1, HIST1H2AD - histone cluster 1, h2ad, LEPR - leptin receptor, INSM1 - insulinoma-associated 1, TLE2 - transducin-like enhancer of split 2 (e(sp1) homolog, drosophila), OR2AK2 - olfactory receptor, family 2, subfamily ak, member 2, H3F3B - h3 histone, family 3b (h3.3b), CRLF1 - cytokine receptor-like factor 1, SLC30A3 - solute carrier family 30 (zinc transporter), member 3, ARMCX5-GPRASP2 - armcx5-gprasp2 readthrough, NR1D1 - nuclear receptor subfamily 1, group d, member 1, HES2 - hairy and enhancer of split 2 (drosophila), FOXN2 - forkhead box n2, CCND2 - cyclin d2, PIM1 - pim-1 oncogene, WNT16 - wingless-type mmtv integration site family, member 16, OSTN - osteocrin, PROKR2 - prokineticin receptor 2, LMNB1 - lamin b1, NBL1 - neuroblastoma 1, dan family bmp antagonist, NDP - norrie disease (pseudoglioma), H3F3C - h3 histone, family 3c, KCNK12 - potassium channel, subfamily k, member 12, HIST1H2AI - histone cluster 1, h2ai, EPHA6 - eph receptor a6, TAC3 - tachykinin 3]
GO:0031936	negative regulation of chromatin silencing	2.79E-06	2.66E-03	37.54	16219	12	144	4	[H1FX - h1 histone family, member x, H1F0 - h1 histone family, member 0, HIST1H1C - histone cluster 1, h1c, HIST1H1E - histone cluster 1, h1e]
GO:0045892	negative regulation of transcription, DNA-templated	2.84E-06	2.55E-03	2.64	16219	1151	144	27	[NFIX - nuclear factor i/x (ccaat-binding transcription factor), SFRP1 - secreted frizzled-related protein 1, H1F0 - h1 histone family, member 0, ATF3 - activating transcription factor 3, MDFI - myod family inhibitor, HIST1H2AD - histone cluster 1, h2ad, HMX1 - h6 family homeobox 1, INSM1 - insulinoma-associated 1, AES - amino-terminal enhancer of split, TLE2 - transducin-like enhancer of split 2 (e(sp1) homolog, drosophila), H3F3B - h3 histone, family 3b (h3.3b), PDE2A - phosphodiesterase 2a, cgmp-stimulated, NR1D1 - nuclear receptor subfamily 1, group d, member 1, UBB - ubiquitin b, FOXD1 - forkhead box d1, HES2 - hairy and enhancer of split 2 (drosophila), UBC - ubiquitin c, CDKN2A - cyclin-dependent kinase inhibitor 2a, CRYM - crystallin, mu, HIST1H4K - histone cluster 1, h4k, HIST1H4C - histone cluster 1, h4c, IKZF1 - ikaros family zinc finger 1 (ikaros), KCNIP3 - kv channel interacting protein 3, calsenilin, MYC - v-myc avian myelocytomatosis viral oncogene homolog, MXD1 - max dimerization protein 1, NR4A2 - nuclear receptor subfamily 4, group a, member 2, HIST1H2AI - histone cluster 1, h2ai]
GO:1903507	negative regulation of nucleic acid-templated transcription	2.94E-06	2.48E-03	2.64	16219	1153	144	27	[NFIX - nuclear factor i/x (ccaat-binding transcription factor), SFRP1 - secreted frizzled-related protein 1, H1F0 - h1 histone family, member 0, ATF3 - activating transcription factor 3, MDFI - myod family inhibitor, HIST1H2AD - histone cluster 1, h2ad, HMX1 - h6 family homeobox 1, INSM1 - insulinoma-associated 1, AES - amino-terminal enhancer of split, TLE2 - transducin-like enhancer of split 2 (e(sp1) homolog, drosophila), H3F3B - h3 histone, family 3b (h3.3b), PDE2A - phosphodiesterase 2a, cgmp-stimulated, NR1D1 - nuclear receptor subfamily 1, group d, member 1, UBB - ubiquitin b, FOXD1 - forkhead box d1, HES2 - hairy and enhancer of split 2 (drosophila), UBC - ubiquitin c, CDKN2A - cyclin-dependent kinase inhibitor 2a, CRYM - crystallin, mu, HIST1H4K - histone cluster 1, h4k, HIST1H4C - histone cluster 1, h4c, IKZF1 - ikaros family zinc finger 1 (ikaros), KCNIP3 - kv channel interacting protein 3, calsenilin, MYC - v-myc avian myelocytomatosis viral oncogene homolog, MXD1 - max dimerization protein 1, NR4A2 - nuclear receptor subfamily 4, group a, member 2, HIST1H2AI - histone cluster 1, h2ai]
GO:1902679	negative regulation of RNA biosynthetic process	3.03E-06	2.43E-03	2.63	16219	1155	144	27	[NFIX - nuclear factor i/x (ccaat-binding transcription factor), SFRP1 - secreted frizzled-related protein 1, H1F0 - h1 histone family, member 0, ATF3 - activating transcription factor 3, MDFI - myod family inhibitor, HIST1H2AD - histone cluster 1, h2ad, HMX1 - h6 family homeobox 1, INSM1 - insulinoma-associated 1, AES - amino-terminal enhancer of split, TLE2 - transducin-like enhancer of split 2 (e(sp1) homolog, drosophila), H3F3B - h3 histone, family 3b (h3.3b), PDE2A - phosphodiesterase 2a, cgmp-stimulated, NR1D1 - nuclear receptor subfamily 1, group d, member 1, UBB - ubiquitin b, FOXD1 - forkhead box d1, HES2 - hairy and enhancer of split 2 (drosophila), UBC - ubiquitin c, CDKN2A - cyclin-dependent kinase inhibitor 2a, CRYM - crystallin, mu, HIST1H4K - histone cluster 1, h4k, HIST1H4C - histone cluster 1, h4c, IKZF1 - ikaros family zinc finger 1 (ikaros), KCNIP3 - kv channel interacting protein 3, calsenilin, MYC - v-myc avian myelocytomatosis viral oncogene homolog, MXD1 - max dimerization protein 1, NR4A2 - nuclear receptor subfamily 4, group a, member 2, HIST1H2AI - histone cluster 1, h2ai]
GO:0051253	negative regulation of RNA metabolic process	3.81E-06	2.90E-03	2.54	16219	1241	144	28	[NFIX - nuclear factor i/x (ccaat-binding transcription factor), SFRP1 - secreted frizzled-related protein 1, H1F0 - h1 histone family, member 0, ATF3 - activating transcription factor 3, MDFI - myod family inhibitor, HIST1H2AD - histone cluster 1, h2ad, HMX1 - h6 family homeobox 1, INSM1 - insulinoma-associated 1, AES - amino-terminal enhancer of split, TLE2 - transducin-like enhancer of split 2 (e(sp1) homolog, drosophila), H3F3B - h3 histone, family 3b (h3.3b), PDE2A - phosphodiesterase 2a, cgmp-stimulated, NR1D1 - nuclear receptor subfamily 1, group d, member 1, UBB - ubiquitin b, HNRNPC - heterogeneous nuclear ribonucleoprotein c (c1/c2), FOXD1 - forkhead box d1, HES2 - hairy and enhancer of split 2 (drosophila), UBC - ubiquitin c, CDKN2A - cyclin-dependent kinase inhibitor 2a, CRYM - crystallin, mu, HIST1H4K - histone cluster 1, h4k, HIST1H4C - histone cluster 1, h4c, IKZF1 - ikaros family zinc finger 1 (ikaros), KCNIP3 - kv channel interacting protein 3, calsenilin, MYC - v-myc avian myelocytomatosis viral oncogene homolog, MXD1 - max dimerization protein 1, NR4A2 - nuclear receptor subfamily 4, group a, member 2, HIST1H2AI - histone cluster 1, h2ai]
GO:0031324	negative regulation of cellular metabolic process	3.88E-06	2.81E-03	2.02	16219	2345	144	42	[NFIX - nuclear factor i/x (ccaat-binding transcription factor), SFRP1 - secreted frizzled-related protein 1, H1FX - h1 histone family, member x, DUSP16 - dual specificity phosphatase 16, H1F0 - h1 histone family, member 0, SLC18A2 - solute carrier family 18 (vesicular monoamine transporter), member 2, HIST1H1C - histone cluster 1, h1c, HIST1H1E - histone cluster 1, h1e, ATF3 - activating transcription factor 3, MDFI - myod family inhibitor, HSPB1 - heat shock 27kda protein 1, HIST1H2AD - histone cluster 1, h2ad, LEPR - leptin receptor, YWHAE - tyrosine 3-monooxygenase/tryptophan 5-monooxygenase activation protein, epsilon polypeptide, HMX1 - h6 family homeobox 1, INSM1 - insulinoma-associated 1, DUSP10 - dual specificity phosphatase 10, AES - amino-terminal enhancer of split, TLE2 - transducin-like enhancer of split 2 (e(sp1) homolog, drosophila), H3F3B - h3 histone, family 3b (h3.3b), SOX4 - sry (sex determining region y)-box 4, PDE2A - phosphodiesterase 2a, cgmp-stimulated, UBB - ubiquitin b, NR1D1 - nuclear receptor subfamily 1, group d, member 1, FOXD1 - forkhead box d1, HNRNPC - heterogeneous nuclear ribonucleoprotein c (c1/c2), HES2 - hairy and enhancer of split 2 (drosophila), UBC - ubiquitin c, CDKN2A - cyclin-dependent kinase inhibitor 2a, CRYM - crystallin, mu, PPP1R14A - protein phosphatase 1, regulatory (inhibitor) subunit 14a, OPN3 - opsin 3, HIST1H4K - histone cluster 1, h4k, HIST1H4C - histone cluster 1, h4c, IKZF1 - ikaros family zinc finger 1 (ikaros), CFLAR - casp8 and fadd-like apoptosis regulator, KCNIP3 - kv channel interacting protein 3, calsenilin, MYC - v-myc avian myelocytomatosis viral oncogene homolog, MXD1 - max dimerization protein 1, SPOCK1 - sparc/osteonectin, cwcv and kazal-like domains proteoglycan (testican) 1, NR4A2 - nuclear receptor subfamily 4, group a, member 2, HIST1H2AI - histone cluster 1, h2ai]
GO:0006342	chromatin silencing	4.51E-06	3.12E-03	13.79	16219	49	144	6	[H3F3B - h3 histone, family 3b (h3.3b), HIST1H4C - histone cluster 1, h4c, H1F0 - h1 histone family, member 0, HIST1H2AD - histone cluster 1, h2ad, HIST1H2AI - histone cluster 1, h2ai, HIST1H4K - histone cluster 1, h4k]
GO:0034728	nucleosome organization	6.94E-06	4.59E-03	8.05	16219	112	144	8	[HIST1H2BC - histone cluster 1, h2bc, H3F3B - h3 histone, family 3b (h3.3b), HIST1H4C - histone cluster 1, h4c, H1FX - h1 histone family, member x, H1F0 - h1 histone family, member 0, HIST1H1C - histone cluster 1, h1c, HIST1H1E - histone cluster 1, h1e, HIST1H4K - histone cluster 1, h4k]

GO:0031327	negative regulation of cellular biosynthetic process	6.94E-06	4.41E-03	2.36	16219	1430	144	30	[NFIX - nuclear factor i/x (ccaat-binding transcription factor), SFRP1 - secreted frizzled-related protein 1, H1F0 - h1 histone family, member 0, ATF3 - activating transcription factor 3, MDFI - myod family inhibitor, HIST1H2AD - histone cluster 1, h2ad, LEPR - leptin receptor, HMX1 - h6 family homeobox 1, INSM1 - insulinoma-associated 1, AES - amino-terminal enhancer of split, TLE2 - transducin-like enhancer of split 2 (e(sp1) homolog, drosophila), H3F3B - h3 histone, family 3b (h3.3b), PDE2A - phosphodiesterase 2a, cgmp-stimulated, NR1D1 - nuclear receptor subfamily 1, group d, member 1, UBB - ubiquitin b, HNRNPC - heterogeneous nuclear ribonucleoprotein c (c1/c2), FOXD1 - forkhead box d1, HES2 - hairy and enhancer of split 2 (drosophila), UBC - ubiquitin c, CDKN2A - cyclin-dependent kinase inhibitor 2a, CRYM - crystallin, mu, OPN3 - opsin 3, HIST1H4K - histone cluster 1, h4k, HIST1H4C - histone cluster 1, h4c, IKZF1 - ikaros family zinc finger 1 (ikaros), KCNIP3 - kv channel interacting protein 3, calsenilin, MYC - v-myc avian myelocytomatosis viral oncogene homolog, MXD1 - max dimerization protein 1, NR4A2 - nuclear receptor subfamily 4, group a, member 2, HIST1H2AI - histone cluster 1, h2ai]
GO:0009892	negative regulation of metabolic process	1.07E-05	6.54E-03	1.88	16219	2702	144	45	[NFIX - nuclear factor i/x (ccaat-binding transcription factor), SFRP1 - secreted frizzled-related protein 1, H1FX - h1 histone family, member x, DUSP16 - dual specificity phosphatase 16, H1F0 - h1 histone family, member 0, SLC18A2 - solute carrier family 18 (vesicular monoamine transporter), member 2, HIST1H1C - histone cluster 1, h1c, HIST1H1E - histone cluster 1, h1e, ATF3 - activating transcription factor 3, MDFI - myod family inhibitor, TIPARP - tcdd-inducible poly(adp-ribose) polymerase, HSPB1 - heat shock 27kda protein 1, HIST1H2AD - histone cluster 1, h2ad, LEPR - leptin receptor, YWHAE - tyrosine 3-monooxygenase/tryptophan 5-monooxygenase activation protein, epsilon polypeptide, HMX1 - h6 family homeobox 1, INSM1 - insulinoma-associated 1, DUSP10 - dual specificity phosphatase 10, AES - amino-terminal enhancer of split, TLE2 - transducin-like enhancer of split 2 (e(sp1) homolog, drosophila), H3F3B - h3 histone, family 3b (h3.3b), SOX4 - sry (sex determining region y)-box 4, ARRDC3 - arrestin domain containing 3, IL8 - interleukin 8, PDE2A - phosphodiesterase 2a, cgmp-stimulated, UBB - ubiquitin b, NR1D1 - nuclear receptor subfamily 1, group d, member 1, FOXD1 - forkhead box d1, HNRNPC - heterogeneous nuclear ribonucleoprotein c (c1/c2), HES2 - hairy and enhancer of split 2 (drosophila), UBC - ubiquitin c, CDKN2A - cyclin-dependent kinase inhibitor 2a, CRYM - crystallin, mu, PPP1R14A - protein phosphatase 1, regulatory (inhibitor) subunit 14a, OPN3 - opsin 3, HIST1H4K - histone cluster 1, h4k, HIST1H4C - histone cluster 1, h4c, IKZF1 - ikaros family zinc finger 1 (ikaros), CFLAR - casp8 and fadd-like apoptosis regulator, KCNIP3 - kv channel interacting protein 3, calsenilin, MYC - v-myc avian myelocytomatosis viral oncogene homolog, MXD1 - max dimerization protein 1, SPOCK1 - sparc/osteonectin, cwcv and kazal-like domains proteoglycan (testican) 1, NR4A2 - nuclear receptor subfamily 4, group a, member 2, HIST1H2AI - histone cluster 1, h2ai]
GO:0061844	antimicrobial humoral immune response mediated by antimicrobial peptide	1.16E-05	6.81E-03	16.56	16219	34	144	5	[HIST1H2BC - histone cluster 1, h2bc, IL8 - interleukin 8, CXCL2 - chemokine (c-x-c motif) ligand 2, CXCL3 - chemokine (c-x-c motif) ligand 3, HMGN2 - high mobility group nucleosomal binding domain 2]
GO:0009968	negative regulation of signal transduction	1.37E-05	7.71E-03	2.48	16219	1180	144	26	[ARHGAP42 - rho gtpase activating protein 42, SFRP1 - secreted frizzled-related protein 1, DUSP16 - dual specificity phosphatase 16, HSPA5 - heat shock 70kda protein 5 (glucose-regulated protein, 78kda), NFKBIA - nuclear factor of kappa light polypeptide gene enhancer in b-cells inhibitor, alpha, ATF3 - activating transcription factor 3, MDFI - myod family inhibitor, HSPB1 - heat shock 27kda protein 1, DUSP10 - dual specificity phosphatase 10, AES - amino-terminal enhancer of split, TLE2 - transducin-like enhancer of split 2 (e(sp1) homolog, drosophila), ARRDC3 - arrestin domain containing 3, IL8 - interleukin 8, PDE2A - phosphodiesterase 2a, cgmp-stimulated, NR1D1 - nuclear receptor subfamily 1, group d, member 1, UBB - ubiquitin b, UBC - ubiquitin c, RGS10 - regulator of g-protein signaling 10, CFLAR - casp8 and fadd-like apoptosis regulator, MYC - v-myc avian myelocytomatosis viral oncogene homolog, NBL1 - neuroblastoma 1, dan family bmp antagonist, CHRDL1 - chordin-like 1, GPR21 - g protein-coupled receptor 21, STMN1 - stathmin 1, NR4A2 - nuclear receptor subfamily 4, group a, member 2, TICAM1 - toll-like receptor adaptor molecule 1]
GO:0010648	negative regulation of cell communication	1.63E-05	8.88E-03	2.4	16219	1266	144	27	[ARHGAP42 - rho gtpase activating protein 42, SFRP1 - secreted frizzled-related protein 1, DUSP16 - dual specificity phosphatase 16, HSPA5 - heat shock 70kda protein 5 (glucose-regulated protein, 78kda), NFKBIA - nuclear factor of kappa light polypeptide gene enhancer in b-cells inhibitor, alpha, ATF3 - activating transcription factor 3, MDFI - myod family inhibitor, HSPB1 - heat shock 27kda protein 1, DUSP10 - dual specificity phosphatase 10, AES - amino-terminal enhancer of split, TLE2 - transducin-like enhancer of split 2 (e(sp1) homolog, drosophila), ARRDC3 - arrestin domain containing 3, IL8 - interleukin 8, PDE2A - phosphodiesterase 2a, cgmp-stimulated, NR1D1 - nuclear receptor subfamily 1, group d, member 1, UBB - ubiquitin b, UBC - ubiquitin c, RGS10 - regulator of g-protein signaling 10, CFLAR - casp8 and fadd-like apoptosis regulator, MYC - v-myc avian myelocytomatosis viral oncogene homolog, NBL1 - neuroblastoma 1, dan family bmp antagonist, CHRDL1 - chordin-like 1, GPR21 - g protein-coupled receptor 21, STMN1 - stathmin 1, NR4A2 - nuclear receptor subfamily 4, group a, member 2, ACVR1C - activin a receptor, type ic, TICAM1 - toll-like receptor adaptor molecule 1]
GO:0023057	negative regulation of signaling	1.70E-05	8.95E-03	2.4	16219	1269	144	27	[ARHGAP42 - rho gtpase activating protein 42, SFRP1 - secreted frizzled-related protein 1, DUSP16 - dual specificity phosphatase 16, HSPA5 - heat shock 70kda protein 5 (glucose-regulated protein, 78kda), NFKBIA - nuclear factor of kappa light polypeptide gene enhancer in b-cells inhibitor, alpha, ATF3 - activating transcription factor 3, MDFI - myod family inhibitor, HSPB1 - heat shock 27kda protein 1, DUSP10 - dual specificity phosphatase 10, AES - amino-terminal enhancer of split, TLE2 - transducin-like enhancer of split 2 (e(sp1) homolog, drosophila), ARRDC3 - arrestin domain containing 3, IL8 - interleukin 8, PDE2A - phosphodiesterase 2a, cgmp-stimulated, NR1D1 - nuclear receptor subfamily 1, group d, member 1, UBB - ubiquitin b, UBC - ubiquitin c, RGS10 - regulator of g-protein signaling 10, CFLAR - casp8 and fadd-like apoptosis regulator, MYC - v-myc avian myelocytomatosis viral oncogene homolog, NBL1 - neuroblastoma 1, dan family bmp antagonist, CHRDL1 - chordin-like 1, GPR21 - g protein-coupled receptor 21, STMN1 - stathmin 1, NR4A2 - nuclear receptor subfamily 4, group a, member 2, ACVR1C - activin a receptor, type ic, TICAM1 - toll-like receptor adaptor molecule 1]
GO:2000113	negative regulation of cellular macromolecule biosynthetic process	1.95E-05	9.89E-03	2.33	16219	1354	144	28	[NFIX - nuclear factor i/x (ccaat-binding transcription factor), SFRP1 - secreted frizzled-related protein 1, H1F0 - h1 histone family, member 0, ATF3 - activating transcription factor 3, MDFI - myod family inhibitor, HIST1H2AD - histone cluster 1, h2ad, HMX1 - h6 family homeobox 1, INSM1 - insulinoma-associated 1, AES - amino-terminal enhancer of split, TLE2 - transducin-like enhancer of split 2 (e(sp1) homolog, drosophila), H3F3B - h3 histone, family 3b (h3.3b), PDE2A - phosphodiesterase 2a, cgmp-stimulated, NR1D1 - nuclear receptor subfamily 1, group d, member 1, UBB - ubiquitin b, HNRNPC - heterogeneous nuclear ribonucleoprotein c (c1/c2), FOXD1 - forkhead box d1, HES2 - hairy and enhancer of split 2 (drosophila), UBC - ubiquitin c, CDKN2A - cyclin-dependent kinase inhibitor 2a, CRYM - crystallin, mu, HIST1H4K - histone cluster 1, h4k, HIST1H4C - histone cluster 1, h4c, IKZF1 - ikaros family zinc finger 1 (ikaros), KCNIP3 - kv channel interacting protein 3, calsenilin, MYC - v-myc avian myelocytomatosis viral oncogene homolog, MXD1 - max dimerization protein 1, NR4A2 - nuclear receptor subfamily 4, group a, member 2, HIST1H2AI - histone cluster 1, h2ai]
GO:0010558	negative regulation of macromolecule biosynthetic process	2.23E-05	1.09E-02	2.31	16219	1364	144	28	[NFIX - nuclear factor i/x (ccaat-binding transcription factor), SFRP1 - secreted frizzled-related protein 1, H1F0 - h1 histone family, member 0, ATF3 - activating transcription factor 3, MDFI - myod family inhibitor, HIST1H2AD - histone cluster 1, h2ad, HMX1 - h6 family homeobox 1, INSM1 - insulinoma-associated 1, AES - amino-terminal enhancer of split, TLE2 - transducin-like enhancer of split 2 (e(sp1) homolog, drosophila), H3F3B - h3 histone, family 3b (h3.3b), PDE2A - phosphodiesterase 2a, cgmp-stimulated, NR1D1 - nuclear receptor subfamily 1, group d, member 1, UBB - ubiquitin b, HNRNPC - heterogeneous nuclear ribonucleoprotein c (c1/c2), FOXD1 - forkhead box d1, HES2 - hairy and enhancer of split 2 (drosophila), UBC - ubiquitin c, CDKN2A - cyclin-dependent kinase inhibitor 2a, CRYM - crystallin, mu, HIST1H4K - histone cluster 1, h4k, HIST1H4C - histone cluster 1, h4c, IKZF1 - ikaros family zinc finger 1 (ikaros), KCNIP3 - kv channel interacting protein 3, calsenilin, MYC - v-myc avian myelocytomatosis viral oncogene homolog, MXD1 - max dimerization protein 1, NR4A2 - nuclear receptor subfamily 4, group a, member 2, HIST1H2AI - histone cluster 1, h2ai]
GO:0045814	negative regulation of gene expression, epigenetic	2.59E-05	1.23E-02	10.24	16219	66	144	6	[H3F3B - h3 histone, family 3b (h3.3b), HIST1H4C - histone cluster 1, h4c, H1F0 - h1 histone family, member 0, HIST1H2AD - histone cluster 1, h2ad, HIST1H2AI - histone cluster 1, h2ai, HIST1H4K - histone cluster 1, h4k]
GO:0051172	negative regulation of nitrogen compound metabolic process	2.77E-05	1.28E-02	1.95	16219	2191	144	38	[NFIX - nuclear factor i/x (ccaat-binding transcription factor), SFRP1 - secreted frizzled-related protein 1, H1FX - h1 histone family, member x, DUSP16 - dual specificity phosphatase 16, H1F0 - h1 histone family, member 0, HIST1H1C - histone cluster 1, h1c, HIST1H1E - histone cluster 1, h1e, ATF3 - activating transcription factor 3, MDFI - myod family inhibitor, HSPB1 - heat shock 27kda protein 1, HIST1H2AD - histone cluster 1, h2ad, YWHAE - tyrosine 3-monooxygenase/tryptophan 5-monooxygenase activation protein, epsilon polypeptide, HMX1 - h6 family homeobox 1, INSM1 - insulinoma-associated 1, DUSP10 - dual specificity phosphatase 10, AES - amino-terminal enhancer of split, TLE2 - transducin-like enhancer of split 2 (e(sp1) homolog, drosophila), H3F3B - h3 histone, family 3b (h3.3b), SOX4 - sry (sex determining region y)-box 4, PDE2A - phosphodiesterase 2a, cgmp-stimulated, UBB - ubiquitin b, NR1D1 - nuclear receptor subfamily 1, group d, member 1, FOXD1 - forkhead box d1, HNRNPC - heterogeneous nuclear ribonucleoprotein c (c1/c2), HES2 - hairy and enhancer of split 2 (drosophila), UBC - ubiquitin c, CDKN2A - cyclin-dependent kinase inhibitor 2a, CRYM - crystallin, mu, PPP1R14A - protein phosphatase 1, regulatory (inhibitor) subunit 14a, HIST1H4K - histone cluster 1, h4k, HIST1H4C - histone cluster 1, h4c, IKZF1 - ikaros family zinc finger 1 (ikaros), KCNIP3 - kv channel interacting protein 3, calsenilin, MYC - v-myc avian myelocytomatosis viral oncogene homolog, MXD1 - max dimerization protein 1, SPOCK1 - sparc/osteonectin, cwcv and kazal-like domains proteoglycan (testican) 1, NR4A2 - nuclear receptor subfamily 4, group a, member 2, HIST1H2AI - histone cluster 1, h2ai]

GO:0065007	biological regulation	2.96E-05	1.32E-02	1.23	16219	10672	144	117	[H1FX - h1 histone family, member x, CDC42EP1 - cdc42 effector protein (rho gtpase binding) 1, SLC18A2 - solute carrier family 18 (vesicular monoamine transporter), member 2, SEZ6 - seizure related 6 homolog (mouse), BHMT - betaine–homocysteine s-methyltransferase, FTH1 - ferritin, heavy polypeptide 1, JUND - jun d proto-oncogene, ZC3HAV1 - zinc finger ccch-type, antiviral 1, GPD1 - glycerol-3-phosphate dehydrogenase 1 (soluble), OPN3 - opsin 3, HIST1H4K - histone cluster 1, h4k, TBX1 - t-box 1, MGARP - mitochondria-localized glutamic acid-rich protein, HIST1H4C - histone cluster 1, h4c, EEF1A2 - eukaryotic translation elongation factor 1 alpha 2, RGS10 - regulator of g-protein signaling 10, TCERG1L - transcription elongation regulator 1-like, KCNIP3 - kv channel interacting protein 3, calsenilin, MXD1 - max dimerization protein 1, GPR21 - g protein-coupled receptor 21, TBXA2R - thromboxane a2 receptor, ACTA1 - actin, alpha 1, skeletal muscle, ACTB - actin, beta, TNFAIP8L3 - tumor necrosis factor, alpha-induced protein 8-like 3, KLF9 - kruppel-like factor 9, ACVR1C - activin a receptor, type ic, NET1 - neuroepithelial cell transforming 1, TICAM1 - toll-like receptor adaptor molecule 1, EFS - embryonal fyn-associated substrate, NFIX - nuclear factor i/x (ccaat-binding transcription factor), ARHGAP42 - rho gtpase activating protein 42, NFKBIA - nuclear factor of kappa light polypeptide gene enhancer in b-cells inhibitor, alpha, TCF15 - transcription factor 15 (basic helix-loop-helix), SCGN - secretagogin, ef-hand calcium binding protein, TIPARP - tcdd-inducible poly(adp-ribose) polymerase, GADD45G - growth arrest and dna-damage-inducible, gamma, YWHAE - tyrosine 3-monooxygenase/tryptophan 5-monooxygenase activation protein, epsilon polypeptide, HMX1 - h6 family homeobox 1, DUSP10 - dual specificity phosphatase 10, FKBP4 - fk506 binding protein 4, 59kda, SOX4 - sry (sex determining region y)-box 4, PDE2A - phosphodiesterase 2a, cgmp-stimulated, FOXD1 - forkhead box d1, HNRNPC - heterogeneous nuclear ribonucleoprotein c (c1/c2), CDKN2A - cyclin-dependent kinase inhibitor 2a, PPP1R14A - protein phosphatase 1, regulatory (inhibitor) subunit 14a, TUBB2B - tubulin, beta 2b class iib, KCNK6 - potassium channel, subfamily k, member 6, IKZF1 - ikaros family zinc finger 1 (ikaros), GRASP - grp1 (general receptor for phosphoinositides 1)-associated scaffold protein, CHRDL1 - chordin-like 1, CFD - complement factor d (adipsin), CCSAP - centriole, cilia and spindle-associated protein, SPOCK1 - sparc/osteonectin, cwcv and kazal-like domains proteoglycan (testican) 1, PDLIM1 - pdz and lim domain 1, CRABP1 - cellular retinoic acid binding protein 1, SPR - sepiapterin reductase (7,8-dihydrobiopterin:nadp+ oxidoreductase), ANO4 - anoctamin 4, CXCL2 - chemokine (c-x-c motif) ligand 2, CXCL3 - chemokine (c-x-c motif) ligand 3, ADRB1 - adrenoceptor beta 1, STMN4 - stathmin-like 4, SFRP1 - secreted frizzled-related protein 1, ATF3 - activating transcription factor 3, MDFI - myod family inhibitor, AES - amino-terminal enhancer of split, ARRDC3 - arrestin domain containing 3, IL8 - interleukin 8, UBB - ubiquitin b, UBC - ubiquitin c, CRYM - crystallin, mu, ZEB2-AS1 - zeb2 antisense rna 1, CFLAR - casp8 and fadd-like apoptosis regulator, CAPG - capping protein (actin filament), gelsolin-like, HRH1 - histamine receptor h1, MYC - v-myc avian myelocytomatosis viral oncogene homolog, CHRM1 - cholinergic receptor, muscarinic 1, RND1 - rho family gtpase 1, NTS - neurotensin, STMN1 - stathmin 1, NR4A2 - nuclear receptor subfamily 4, group a, member 2, RAC3 - ras-related c3 botulinum toxin substrate 3 (rho family, small gtp binding protein rac3), DUSP16 - dual specificity phosphatase 16, H1F0 - h1 histone family, member 0, HSPA5 - heat shock 70kda protein 5 (glucose-regulated protein, 78kda), CSRNP1 - cysteine-serine-rich nuclear protein 1, HIST1H1C - histone cluster 1, h1c, HIST1H1E - histone cluster 1, h1e, MICB - mhc class i polypeptide-related sequence b, ALOX5 - arachidonate 5-lipoxygenase, HSPB1 - heat shock 27kda protein 1, HIST1H2AD - histone cluster 1, h2ad, LEPR - leptin receptor, INSM1 - insulinoma-associated 1, TLE2 - transducin-like enhancer of split 2 (e(sp1) homolog, drosophila), OR2AK2 - olfactory receptor, family 2, subfamily ak, member 2, H3F3B - h3 histone, family 3b (h3.3b), CRLF1 - cytokine receptor-like factor 1, SLC30A3 - solute carrier family 30 (zinc transporter), member 3, ARMCX5-GPRASP2 - armcx5-gprasp2 readthrough, NR1D1 - nuclear receptor subfamily 1, group d, member 1, HES2 - hairy and enhancer of split 2 (drosophila), FOXN2 - forkhead box n2, CCND2 - cyclin d2, PIM1 - pim-1 oncogene, WNT16 - wingless-type mmtv integration site family, member 16, OSTN - osteocrin, PROKR2 - prokineticin receptor 2, LMNB1 - lamin b1, NBL1 - neuroblastoma 1, dan family bmp antagonist, SLC9A3 - solute carrier family 9, subfamily a (nhe3, cation proton antiporter 3), member 3, NDP - norrie disease (pseudoglioma), H3F3C - h3 histone, family 3c, KCNK12 - potassium channel, subfamily k, member 12, HIST1H2AI - histone cluster 1, h2ai, EPHA6 - eph receptor a6, TAC3 - tachykinin 3]
GO:1901701	cellular response to oxygen-containing compound	3.55E-05	1.54E-02	2.86	16219	749	144	19	[TBX1 - t-box 1, MGARP - mitochondria-localized glutamic acid-rich protein, SFRP1 - secreted frizzled-related protein 1, HSPA5 - heat shock 70kda protein 5 (glucose-regulated protein, 78kda), CFLAR - casp8 and fadd-like apoptosis regulator, FBXO32 - f-box protein 32, TBXA2R - thromboxane a2 receptor, ACTB - actin, beta, SOX4 - sry (sex determining region y)-box 4, IL8 - interleukin 8, PDE2A - phosphodiesterase 2a, cgmp-stimulated, CXCL2 - chemokine (c-x-c motif) ligand 2, NR4A2 - nuclear receptor subfamily 4, group a, member 2, GPD1 - glycerol-3-phosphate dehydrogenase 1 (soluble), CXCL3 - chemokine (c-x-c motif) ligand 3, NR1D1 - nuclear receptor subfamily 1, group d, member 1, KLF9 - kruppel-like factor 9, NET1 - neuroepithelial cell transforming 1, TICAM1 - toll-like receptor adaptor molecule 1]
GO:0009889	regulation of biosynthetic process	4.84E-05	2.05E-02	1.62	16219	3896	144	56	[SFRP1 - secreted frizzled-related protein 1, H1FX - h1 histone family, member x, SLC18A2 - solute carrier family 18 (vesicular monoamine transporter), member 2, ATF3 - activating transcription factor 3, MDFI - myod family inhibitor, AES - amino-terminal enhancer of split, JUND - jun d proto-oncogene, IL8 - interleukin 8, GPD1 - glycerol-3-phosphate dehydrogenase 1 (soluble), UBB - ubiquitin b, UBC - ubiquitin c, CRYM - crystallin, mu, OPN3 - opsin 3, HIST1H4K - histone cluster 1, h4k, TBX1 - t-box 1, HIST1H4C - histone cluster 1, h4c, CFLAR - casp8 and fadd-like apoptosis regulator, HRH1 - histamine receptor h1, KCNIP3 - kv channel interacting protein 3, calsenilin, TCERG1L - transcription elongation regulator 1-like, MYC - v-myc avian myelocytomatosis viral oncogene homolog, MXD1 - max dimerization protein 1, NR4A2 - nuclear receptor subfamily 4, group a, member 2, KLF9 - kruppel-like factor 9, TICAM1 - toll-like receptor adaptor molecule 1, NFIX - nuclear factor i/x (ccaat-binding transcription factor), H1F0 - h1 histone family, member 0, HSPA5 - heat shock 70kda protein 5 (glucose-regulated protein, 78kda), NFKBIA - nuclear factor of kappa light polypeptide gene enhancer in b-cells inhibitor, alpha, TCF15 - transcription factor 15 (basic helix-loop-helix), CSRNP1 - cysteine-serine-rich nuclear protein 1, HIST1H1C - histone cluster 1, h1c, HIST1H1E - histone cluster 1, h1e, ALOX5 - arachidonate 5-lipoxygenase, HSPB1 - heat shock 27kda protein 1, HIST1H2AD - histone cluster 1, h2ad, LEPR - leptin receptor, HMX1 - h6 family homeobox 1, INSM1 - insulinoma-associated 1, TLE2 - transducin-like enhancer of split 2 (e(sp1) homolog, drosophila), H3F3B - h3 histone, family 3b (h3.3b), SOX4 - sry (sex determining region y)-box 4, PDE2A - phosphodiesterase 2a, cgmp-stimulated, NR1D1 - nuclear receptor subfamily 1, group d, member 1, FOXD1 - forkhead box d1, HNRNPC - heterogeneous nuclear ribonucleoprotein c (c1/c2), HES2 - hairy and enhancer of split 2 (drosophila), CDKN2A - cyclin-dependent kinase inhibitor 2a, FOXN2 - forkhead box n2, PIM1 - pim-1 oncogene, IKZF1 - ikaros family zinc finger 1 (ikaros), NBL1 - neuroblastoma 1, dan family bmp antagonist, CHRDL1 - chordin-like 1, PDLIM1 - pdz and lim domain 1, NDP - norrie disease (pseudoglioma), HIST1H2AI - histone cluster 1, h2ai]
GO:0009410	response to xenobiotic stimulus	5.52E-05	2.27E-02	6.05	16219	149	144	8	[HSPA5 - heat shock 70kda protein 5 (glucose-regulated protein, 78kda), SLC18A2 - solute carrier family 18 (vesicular monoamine transporter), member 2, RGS10 - regulator of g-protein signaling 10, CFLAR - casp8 and fadd-like apoptosis regulator, PDE2A - phosphodiesterase 2a, cgmp-stimulated, NR4A2 - nuclear receptor subfamily 4, group a, member 2, TIPARP - tcdd-inducible poly(adp-ribose) polymerase, FBXO32 - f-box protein 32]
GO:0019222	regulation of metabolic process	5.83E-05	2.34E-02	1.42	16219	6171	144	78	[ADRB1 - adrenoceptor beta 1, SFRP1 - secreted frizzled-related protein 1, H1FX - h1 histone family, member x, SLC18A2 - solute carrier family 18 (vesicular monoamine transporter), member 2, ATF3 - activating transcription factor 3, MDFI - myod family inhibitor, BHMT - betaine–homocysteine s-methyltransferase, AES - amino-terminal enhancer of split, JUND - jun d proto-oncogene, ZC3HAV1 - zinc finger ccch-type, antiviral 1, ARRDC3 - arrestin domain containing 3, IL8 - interleukin 8, GPD1 - glycerol-3-phosphate dehydrogenase 1 (soluble), UBB - ubiquitin b, UBC - ubiquitin c, CRYM - crystallin, mu, OPN3 - opsin 3, ZEB2-AS1 - zeb2 antisense rna 1, HIST1H4K - histone cluster 1, h4k, TBX1 - t-box 1, HIST1H4C - histone cluster 1, h4c, EEF1A2 - eukaryotic translation elongation factor 1 alpha 2, CFLAR - casp8 and fadd-like apoptosis regulator, HRH1 - histamine receptor h1, TCERG1L - transcription elongation regulator 1-like, KCNIP3 - kv channel interacting protein 3, calsenilin, MYC - v-myc avian myelocytomatosis viral oncogene homolog, MXD1 - max dimerization protein 1, ACTA1 - actin, alpha 1, skeletal muscle, ACTB - actin, beta, NR4A2 - nuclear receptor subfamily 4, group a, member 2, TNFAIP8L3 - tumor necrosis factor, alpha-induced protein 8-like 3, KLF9 - kruppel-like factor 9, ACVR1C - activin a receptor, type ic, TICAM1 - toll-like receptor adaptor molecule 1, NFIX - nuclear factor i/x (ccaat-binding transcription factor), DUSP16 - dual specificity phosphatase 16, H1F0 - h1 histone family, member 0, HSPA5 - heat shock 70kda protein 5 (glucose-regulated protein, 78kda), NFKBIA - nuclear factor of kappa light polypeptide gene enhancer in b-cells inhibitor, alpha, TCF15 - transcription factor 15 (basic helix-loop-helix), CSRNP1 - cysteine-serine-rich nuclear protein 1, HIST1H1C - histone cluster 1, h1c, HIST1H1E - histone cluster 1, h1e, ALOX5 - arachidonate 5-lipoxygenase, TIPARP - tcdd-inducible poly(adp-ribose) polymerase, HSPB1 - heat shock 27kda protein 1, HIST1H2AD - histone cluster 1, h2ad, LEPR - leptin receptor, GADD45G - growth arrest and dna-damage-inducible, gamma, YWHAE - tyrosine 3-monooxygenase/tryptophan 5-monooxygenase activation protein, epsilon polypeptide, HMX1 - h6 family homeobox 1, INSM1 - insulinoma-associated 1, DUSP10 - dual specificity phosphatase 10, TLE2 - transducin-like enhancer of split 2 (e(sp1) homolog, drosophila), H3F3B - h3 histone, family 3b (h3.3b), CRLF1 - cytokine receptor-like factor 1, SOX4 - sry (sex determining region y)-box 4, PDE2A - phosphodiesterase 2a, cgmp-stimulated, NR1D1 - nuclear receptor subfamily 1, group d, member 1, FOXD1 - forkhead box d1, HNRNPC - heterogeneous nuclear ribonucleoprotein c (c1/c2), HES2 - hairy and enhancer of split 2 (drosophila), CDKN2A - cyclin-dependent kinase inhibitor 2a, FOXN2 - forkhead box n2, CCND2 - cyclin d2, PPP1R14A - protein phosphatase 1, regulatory (inhibitor) subunit 14a, PIM1 - pim-1 oncogene, WNT16 - wingless-type mmtv integration site family, member 16, IKZF1 - ikaros family zinc finger 1 (ikaros), NBL1 - neuroblastoma 1, dan family bmp antagonist, CHRDL1 - chordin-like 1, SPOCK1 - sparc/osteonectin, cwcv and kazal-like domains proteoglycan (testican) 1, PDLIM1 - pdz and lim domain 1, SPR - sepiapterin reductase (7,8-dihydrobiopterin:nadp+ oxidoreductase), NDP - norrie disease (pseudoglioma), HIST1H2AI - histone cluster 1, h2ai, EPHA6 - eph receptor a6]
GO:0031323	regulation of cellular metabolic process	6.42E-05	2.51E-02	1.45	16219	5654	144	73	[SFRP1 - secreted frizzled-related protein 1, H1FX - h1 histone family, member x, SLC18A2 - solute carrier family 18 (vesicular monoamine transporter), member 2, ATF3 - activating transcription factor 3, MDFI - myod family inhibitor, BHMT - betaine–homocysteine s-methyltransferase, AES - amino-terminal enhancer of split, JUND - jun d proto-oncogene, ZC3HAV1 - zinc finger ccch-type, antiviral 1, ARRDC3 - arrestin domain containing 3, IL8 - interleukin 8, GPD1 - glycerol-3-phosphate dehydrogenase 1 (soluble), UBB - ubiquitin b, UBC - ubiquitin c, CRYM - crystallin, mu, OPN3 - opsin 3, HIST1H4K - histone cluster 1, h4k, TBX1 - t-box 1, HIST1H4C - histone cluster 1, h4c, EEF1A2 - eukaryotic translation elongation factor 1 alpha 2, CFLAR - casp8 and fadd-like apoptosis regulator, HRH1 - histamine receptor h1, KCNIP3 - kv channel interacting protein 3, calsenilin, TCERG1L - transcription elongation regulator 1-like, MYC - v-myc avian myelocytomatosis viral oncogene homolog, MXD1 - max dimerization protein 1, ACTB - actin, beta, NR4A2 - nuclear receptor subfamily 4, group a, member 2, TNFAIP8L3 - tumor necrosis factor, alpha-induced protein 8-like 3, KLF9 - kruppel-like factor 9, ACVR1C - activin a receptor, type ic, TICAM1 - toll-like receptor adaptor molecule 1, NFIX - nuclear factor i/x (ccaat-binding transcription factor), DUSP16 - dual specificity phosphatase 16, H1F0 - h1 histone family, member 0, HSPA5 - heat shock 70kda protein 5 (glucose-regulated protein, 78kda), NFKBIA - nuclear factor of kappa light polypeptide gene enhancer in b-cells inhibitor, alpha, TCF15 - transcription factor 15 (basic helix-loop-helix), CSRNP1 - cysteine-serine-rich nuclear protein 1, HIST1H1C - histone cluster 1, h1c, HIST1H1E - histone cluster 1, h1e, ALOX5 - arachidonate 5-lipoxygenase, HSPB1 - heat shock 27kda protein 1, GADD45G - growth arrest and dna-damage-inducible, gamma, HIST1H2AD - histone cluster 1, h2ad, LEPR - leptin receptor, YWHAE - tyrosine 3-monooxygenase/tryptophan 5-monooxygenase activation protein, epsilon polypeptide, HMX1 - h6 family homeobox 1, INSM1 - insulinoma-associated 1, DUSP10 - dual specificity phosphatase 10, TLE2 - transducin-like enhancer of split 2 (e(sp1) homolog, drosophila), H3F3B - h3 histone, family 3b (h3.3b), CRLF1 - cytokine receptor-like factor 1, SOX4 - sry (sex determining region y)-box 4, PDE2A - phosphodiesterase 2a, cgmp-stimulated, NR1D1 - nuclear receptor subfamily 1, group d, member 1, FOXD1 - forkhead box d1, HNRNPC - heterogeneous nuclear ribonucleoprotein c (c1/c2), HES2 - hairy and enhancer of split 2 (drosophila), CDKN2A - cyclin-dependent kinase inhibitor 2a, FOXN2 - forkhead box n2, CCND2 - cyclin d2, PPP1R14A - protein phosphatase 1, regulatory (inhibitor) subunit 14a, PIM1 - pim-1 oncogene, WNT16 - wingless-type mmtv integration site family, member 16, IKZF1 - ikaros family zinc finger 1 (ikaros), NBL1 - neuroblastoma 1, dan family bmp antagonist, CHRDL1 - chordin-like 1, SPOCK1 - sparc/osteonectin, cwcv and kazal-like domains proteoglycan (testican) 1, PDLIM1 - pdz and lim domain 1, NDP - norrie disease (pseudoglioma), HIST1H2AI - histone cluster 1, h2ai, EPHA6 - eph receptor a6]
GO:0031935	regulation of chromatin silencing	6.52E-05	2.48E-02	18.02	16219	25	144	4	[H1FX - h1 histone family, member x, H1F0 - h1 histone family, member 0, HIST1H1C - histone cluster 1, h1c, HIST1H1E - histone cluster 1, h1e]

GO:0040029	regulation of gene expression, epigenetic	7.20E-05	2.68E-02	4.56	16219	247	144	10	[H3F3B - h3 histone, family 3b (h3.3b), HIST1H4C - histone cluster 1, h4c, H1FX - h1 histone family, member x, ACTB - actin, beta, H1F0 - h1 histone family, member 0, HIST1H1C - histone cluster 1, h1c, HIST1H1E - histone cluster 1, h1e, HIST1H2AD - histone cluster 1, h2ad, HIST1H2AI - histone cluster 1, h2ai, HIST1H4K - histone cluster 1, h4k]
GO:0010605	negative regulation of macromolecule metabolic process	7.90E-05	2.86E-02	1.82	16219	2473	144	40	[NFIX - nuclear factor i/x (ccaat-binding transcription factor), SFRP1 - secreted frizzled-related protein 1, H1FX - h1 histone family, member x, DUSP16 - dual specificity phosphatase 16, H1F0 - h1 histone family, member 0, HIST1H1C - histone cluster 1, h1c, HIST1H1E - histone cluster 1, h1e, ATF3 - activating transcription factor 3, MDFI - myod family inhibitor, TIPARP - tcdd-inducible poly(adp-ribose) polymerase, HSPB1 - heat shock 27kda protein 1, HIST1H2AD - histone cluster 1, h2ad, YWHAE - tyrosine 3-monooxygenase/tryptophan 5-monooxygenase activation protein, epsilon polypeptide, HMX1 - h6 family homeobox 1, INSM1 - insulinoma-associated 1, DUSP10 - dual specificity phosphatase 10, AES - amino-terminal enhancer of split, TLE2 - transducin-like enhancer of split 2 (e(sp1) homolog, drosophila), H3F3B - h3 histone, family 3b (h3.3b), SOX4 - sry (sex determining region y)-box 4, IL8 - interleukin 8, PDE2A - phosphodiesterase 2a, cgmp-stimulated, UBB - ubiquitin b, NR1D1 - nuclear receptor subfamily 1, group d, member 1, FOXD1 - forkhead box d1, HNRNPC - heterogeneous nuclear ribonucleoprotein c (c1/c2), HES2 - hairy and enhancer of split 2 (drosophila), UBC - ubiquitin c, CDKN2A - cyclin-dependent kinase inhibitor 2a, CRYM - crystallin, mu, PPP1R14A - protein phosphatase 1, regulatory (inhibitor) subunit 14a, HIST1H4K - histone cluster 1, h4k, HIST1H4C - histone cluster 1, h4c, IKZF1 - ikaros family zinc finger 1 (ikaros), KCNIP3 - kv channel interacting protein 3, calsenilin, MYC - v-myc avian myelocytomatosis viral oncogene homolog, MXD1 - max dimerization protein 1, SPOCK1 - sparc/osteonectin, cwcv and kazal-like domains proteoglycan (testican) 1, NR4A2 - nuclear receptor subfamily 4, group a, member 2, HIST1H2AI - histone cluster 1, h2ai]
GO:0071396	cellular response to lipid	8.24E-05	2.92E-02	3.79	16219	357	144	12	[TBX1 - t-box 1, MGARP - mitochondria-localized glutamic acid-rich protein, SFRP1 - secreted frizzled-related protein 1, IL8 - interleukin 8, CFLAR - casp8 and fadd-like apoptosis regulator, CXCL2 - chemokine (c-x-c motif) ligand 2, KLF9 - kruppel-like factor 9, CXCL3 - chemokine (c-x-c motif) ligand 3, NR1D1 - nuclear receptor subfamily 1, group d, member 1, TICAM1 - toll-like receptor adaptor molecule 1, FBXO32 - f-box protein 32, TBXA2R - thromboxane a2 receptor]
GO:0045815	positive regulation of gene expression, epigenetic	9.56E-05	3.31E-02	10.83	16219	52	144	5	[H1FX - h1 histone family, member x, ACTB - actin, beta, H1F0 - h1 histone family, member 0, HIST1H1C - histone cluster 1, h1c, HIST1H1E - histone cluster 1, h1e]
GO:0060968	regulation of gene silencing	9.69E-05	3.28E-02	6.57	16219	120	144	7	[H3F3B - h3 histone, family 3b (h3.3b), HIST1H4C - histone cluster 1, h4c, H1FX - h1 histone family, member x, H1F0 - h1 histone family, member 0, HIST1H1C - histone cluster 1, h1c, HIST1H1E - histone cluster 1, h1e, HIST1H4K - histone cluster 1, h4k]
GO:0006325	chromatin organization	1.04E-04	3.46E-02	2.95	16219	610	144	16	[HIST1H4C - histone cluster 1, h4c, H1FX - h1 histone family, member x, IKZF1 - ikaros family zinc finger 1 (ikaros), H1F0 - h1 histone family, member 0, HIST1H1C - histone cluster 1, h1c, HIST1H1E - histone cluster 1, h1e, HIST1H2AD - histone cluster 1, h2ad, MYC - v-myc avian myelocytomatosis viral oncogene homolog, H3F3B - h3 histone, family 3b (h3.3b), HIST1H2BC - histone cluster 1, h2bc, ACTB - actin, beta, HNRNPC - heterogeneous nuclear ribonucleoprotein c (c1/c2), CDKN2A - cyclin-dependent kinase inhibitor 2a, HIST1H2AI - histone cluster 1, h2ai, HIST1H4K - histone cluster 1, h4k, HMGN2 - high mobility group nucleosomal binding domain 2]
GO:0051252	regulation of RNA metabolic process	1.17E-04	3.80E-02	1.64	16219	3438	144	50	[SFRP1 - secreted frizzled-related protein 1, H1FX - h1 histone family, member x, ATF3 - activating transcription factor 3, MDFI - myod family inhibitor, AES - amino-terminal enhancer of split, JUND - jun d proto-oncogene, ZC3HAV1 - zinc finger ccch-type, antiviral 1, UBB - ubiquitin b, UBC - ubiquitin c, CRYM - crystallin, mu, HIST1H4K - histone cluster 1, h4k, TBX1 - t-box 1, HIST1H4C - histone cluster 1, h4c, CFLAR - casp8 and fadd-like apoptosis regulator, KCNIP3 - kv channel interacting protein 3, calsenilin, TCERG1L - transcription elongation regulator 1-like, MYC - v-myc avian myelocytomatosis viral oncogene homolog, MXD1 - max dimerization protein 1, NR4A2 - nuclear receptor subfamily 4, group a, member 2, KLF9 - kruppel-like factor 9, TICAM1 - toll-like receptor adaptor molecule 1, NFIX - nuclear factor i/x (ccaat-binding transcription factor), H1F0 - h1 histone family, member 0, HSPA5 - heat shock 70kda protein 5 (glucose-regulated protein, 78kda), NFKBIA - nuclear factor of kappa light polypeptide gene enhancer in b-cells inhibitor, alpha, TCF15 - transcription factor 15 (basic helix-loop-helix), CSRNP1 - cysteine-serine-rich nuclear protein 1, HIST1H1C - histone cluster 1, h1c, HIST1H1E - histone cluster 1, h1e, HSPB1 - heat shock 27kda protein 1, HIST1H2AD - histone cluster 1, h2ad, HMX1 - h6 family homeobox 1, INSM1 - insulinoma-associated 1, TLE2 - transducin-like enhancer of split 2 (e(sp1) homolog, drosophila), H3F3B - h3 histone, family 3b (h3.3b), SOX4 - sry (sex determining region y)-box 4, PDE2A - phosphodiesterase 2a, cgmp-stimulated, NR1D1 - nuclear receptor subfamily 1, group d, member 1, FOXD1 - forkhead box d1, HNRNPC - heterogeneous nuclear ribonucleoprotein c (c1/c2), HES2 - hairy and enhancer of split 2 (drosophila), CDKN2A - cyclin-dependent kinase inhibitor 2a, FOXN2 - forkhead box n2, PIM1 - pim-1 oncogene, IKZF1 - ikaros family zinc finger 1 (ikaros), NBL1 - neuroblastoma 1, dan family bmp antagonist, CHRDL1 - chordin-like 1, PDLIM1 - pdz and lim domain 1, NDP - norrie disease (pseudoglioma), HIST1H2AI - histone cluster 1, h2ai]
GO:0031326	regulation of cellular biosynthetic process	1.18E-04	3.74E-02	1.59	16219	3822	144	54	[SFRP1 - secreted frizzled-related protein 1, H1FX - h1 histone family, member x, ATF3 - activating transcription factor 3, MDFI - myod family inhibitor, AES - amino-terminal enhancer of split, JUND - jun d proto-oncogene, IL8 - interleukin 8, GPD1 - glycerol-3-phosphate dehydrogenase 1 (soluble), UBB - ubiquitin b, UBC - ubiquitin c, CRYM - crystallin, mu, OPN3 - opsin 3, HIST1H4K - histone cluster 1, h4k, TBX1 - t-box 1, HIST1H4C - histone cluster 1, h4c, CFLAR - casp8 and fadd-like apoptosis regulator, HRH1 - histamine receptor h1, KCNIP3 - kv channel interacting protein 3, calsenilin, TCERG1L - transcription elongation regulator 1-like, MYC - v-myc avian myelocytomatosis viral oncogene homolog, MXD1 - max dimerization protein 1, NR4A2 - nuclear receptor subfamily 4, group a, member 2, KLF9 - kruppel-like factor 9, TICAM1 - toll-like receptor adaptor molecule 1, NFIX - nuclear factor i/x (ccaat-binding transcription factor), H1F0 - h1 histone family, member 0, HSPA5 - heat shock 70kda protein 5 (glucose-regulated protein, 78kda), NFKBIA - nuclear factor of kappa light polypeptide gene enhancer in b-cells inhibitor, alpha, TCF15 - transcription factor 15 (basic helix-loop-helix), CSRNP1 - cysteine-serine-rich nuclear protein 1, HIST1H1C - histone cluster 1, h1c, HIST1H1E - histone cluster 1, h1e, HSPB1 - heat shock 27kda protein 1, HIST1H2AD - histone cluster 1, h2ad, LEPR - leptin receptor, HMX1 - h6 family homeobox 1, INSM1 - insulinoma-associated 1, TLE2 - transducin-like enhancer of split 2 (e(sp1) homolog, drosophila), H3F3B - h3 histone, family 3b (h3.3b), SOX4 - sry (sex determining region y)-box 4, PDE2A - phosphodiesterase 2a, cgmp-stimulated, NR1D1 - nuclear receptor subfamily 1, group d, member 1, FOXD1 - forkhead box d1, HNRNPC - heterogeneous nuclear ribonucleoprotein c (c1/c2), HES2 - hairy and enhancer of split 2 (drosophila), CDKN2A - cyclin-dependent kinase inhibitor 2a, FOXN2 - forkhead box n2, PIM1 - pim-1 oncogene, IKZF1 - ikaros family zinc finger 1 (ikaros), NBL1 - neuroblastoma 1, dan family bmp antagonist, CHRDL1 - chordin-like 1, PDLIM1 - pdz and lim domain 1, NDP - norrie disease (pseudoglioma), HIST1H2AI - histone cluster 1, h2ai]
GO:0030261	chromosome condensation	1.19E-04	3.70E-02	15.54	16219	29	144	4	[H1FX - h1 histone family, member x, H1F0 - h1 histone family, member 0, HIST1H1C - histone cluster 1, h1c, HIST1H1E - histone cluster 1, h1e]
GO:0010033	response to organic substance	1.26E-04	3.83E-02	1.94	16219	1917	144	33	[SFRP1 - secreted frizzled-related protein 1, HSPA5 - heat shock 70kda protein 5 (glucose-regulated protein, 78kda), NFKBIA - nuclear factor of kappa light polypeptide gene enhancer in b-cells inhibitor, alpha, SLC18A2 - solute carrier family 18 (vesicular monoamine transporter), member 2, MICB - mhc class i polypeptide-related sequence b, TIPARP - tcdd-inducible poly(adp-ribose) polymerase, HSPB1 - heat shock 27kda protein 1, LEPR - leptin receptor, TUBA1B - tubulin, alpha 1b, DUSP10 - dual specificity phosphatase 10, AES - amino-terminal enhancer of split, JUND - jun d proto-oncogene, SOX4 - sry (sex determining region y)-box 4, IL8 - interleukin 8, PDE2A - phosphodiesterase 2a, cgmp-stimulated, GPD1 - glycerol-3-phosphate dehydrogenase 1 (soluble), NR1D1 - nuclear receptor subfamily 1, group d, member 1, TBX1 - t-box 1, MGARP - mitochondria-localized glutamic acid-rich protein, RGS10 - regulator of g-protein signaling 10, CFLAR - casp8 and fadd-like apoptosis regulator, HRH1 - histamine receptor h1, MYC - v-myc avian myelocytomatosis viral oncogene homolog, FBXO32 - f-box protein 32, TBXA2R - thromboxane a2 receptor, ACTA1 - actin, alpha 1, skeletal muscle, ACTB - actin, beta, CXCL2 - chemokine (c-x-c motif) ligand 2, NR4A2 - nuclear receptor subfamily 4, group a, member 2, KLF9 - kruppel-like factor 9, CXCL3 - chemokine (c-x-c motif) ligand 3, ACVR1C - activin a receptor, type ic, TICAM1 - toll-like receptor adaptor molecule 1]
GO:0048585	negative regulation of response to stimulus	1.40E-04	4.17E-02	2.08	16219	1514	144	28	[ARHGAP42 - rho gtpase activating protein 42, SFRP1 - secreted frizzled-related protein 1, DUSP16 - dual specificity phosphatase 16, HSPA5 - heat shock 70kda protein 5 (glucose-regulated protein, 78kda), NFKBIA - nuclear factor of kappa light polypeptide gene enhancer in b-cells inhibitor, alpha, ATF3 - activating transcription factor 3, MDFI - myod family inhibitor, MICB - mhc class i polypeptide-related sequence b, ALOX5 - arachidonate 5-lipoxygenase, HSPB1 - heat shock 27kda protein 1, DUSP10 - dual specificity phosphatase 10, AES - amino-terminal enhancer of split, TLE2 - transducin-like enhancer of split 2 (e(sp1) homolog, drosophila), ARRDC3 - arrestin domain containing 3, IL8 - interleukin 8, PDE2A - phosphodiesterase 2a, cgmp-stimulated, NR1D1 - nuclear receptor subfamily 1, group d, member 1, UBB - ubiquitin b, UBC - ubiquitin c, RGS10 - regulator of g-protein signaling 10, CFLAR - casp8 and fadd-like apoptosis regulator, MYC - v-myc avian myelocytomatosis viral oncogene homolog, NBL1 - neuroblastoma 1, dan family bmp antagonist, CHRDL1 - chordin-like 1, GPR21 - g protein-coupled receptor 21, STMN1 - stathmin 1, NR4A2 - nuclear receptor subfamily 4, group a, member 2, TICAM1 - toll-like receptor adaptor molecule 1]
GO:0000183	chromatin silencing at rDNA	1.42E-04	4.16E-02	28.16	16219	12	144	3	[H3F3B - h3 histone, family 3b (h3.3b), HIST1H4C - histone cluster 1, h4c, HIST1H4K - histone cluster 1, h4k]
GO:0006355	regulation of transcription, DNA-templated	1.44E-04	4.14E-02	1.66	16219	3182	144	47	[NFIX - nuclear factor i/x (ccaat-binding transcription factor), SFRP1 - secreted frizzled-related protein 1, H1FX - h1 histone family, member x, H1F0 - h1 histone family, member 0, HSPA5 - heat shock 70kda protein 5 (glucose-regulated protein, 78kda), NFKBIA - nuclear factor of kappa light polypeptide gene enhancer in b-cells inhibitor, alpha, TCF15 - transcription factor 15 (basic helix-loop-helix), CSRNP1 - cysteine-serine-rich nuclear protein 1, HIST1H1C - histone cluster 1, h1c, HIST1H1E - histone cluster 1, h1e, ATF3 - activating transcription factor 3, MDFI - myod family inhibitor, HIST1H2AD - histone cluster 1, h2ad, HMX1 - h6 family homeobox 1, INSM1 - insulinoma-associated 1, AES - amino-terminal enhancer of split, TLE2 - transducin-like enhancer of split 2 (e(sp1) homolog, drosophila), H3F3B - h3 histone, family 3b (h3.3b), JUND - jun d proto-oncogene, SOX4 - sry (sex determining region y)-box 4, PDE2A - phosphodiesterase 2a, cgmp-stimulated, UBB - ubiquitin b, NR1D1 - nuclear receptor subfamily 1, group d, member 1, FOXD1 - forkhead box d1, HES2 - hairy and enhancer of split 2 (drosophila), UBC - ubiquitin c, CDKN2A - cyclin-dependent kinase inhibitor 2a, CRYM - crystallin, mu, FOXN2 - forkhead box n2, PIM1 - pim-1 oncogene, HIST1H4K - histone cluster 1, h4k, TBX1 - t-box 1, HIST1H4C - histone cluster 1, h4c, IKZF1 - ikaros family zinc finger 1 (ikaros), CFLAR - casp8 and fadd-like apoptosis regulator, KCNIP3 - kv channel interacting protein 3, calsenilin, TCERG1L - transcription elongation regulator 1-like, MYC - v-myc avian myelocytomatosis viral oncogene homolog, MXD1 - max dimerization protein 1, NBL1 - neuroblastoma 1, dan family bmp antagonist, CHRDL1 - chordin-like 1, PDLIM1 - pdz and lim domain 1, NDP - norrie disease (pseudoglioma), NR4A2 - nuclear receptor subfamily 4, group a, member 2, KLF9 - kruppel-like factor 9, TICAM1 - toll-like receptor adaptor molecule 1, HIST1H2AI - histone cluster 1, h2ai]

GO:1903506	regulation of nucleic acid-templated transcription	1.45E-04	4.09E-02	1.66	16219	3183	144	47	[NFIX - nuclear factor i/x (ccaat-binding transcription factor), SFRP1 - secreted frizzled-related protein 1, H1FX - h1 histone family, member x, H1F0 - h1 histone family, member 0, HSPA5 - heat shock 70kda protein 5 (glucose-regulated protein, 78kda), NFKBIA - nuclear factor of kappa light polypeptide gene enhancer in b-cells inhibitor, alpha, TCF15 - transcription factor 15 (basic helix-loop-helix), CSRNP1 - cysteine-serine-rich nuclear protein 1, HIST1H1C - histone cluster 1, h1c, HIST1H1E - histone cluster 1, h1e, ATF3 - activating transcription factor 3, MDFI - myod family inhibitor, HIST1H2AD - histone cluster 1, h2ad, HMX1 - h6 family homeobox 1, INSM1 - insulinoma-associated 1, AES - amino-terminal enhancer of split, TLE2 - transducin-like enhancer of split 2 (e(sp1) homolog, drosophila), H3F3B - h3 histone, family 3b (h3.3b), JUND - jun d proto-oncogene, SOX4 - sry (sex determining region y)-box 4, PDE2A - phosphodiesterase 2a, cgmp-stimulated, UBB - ubiquitin b, NR1D1 - nuclear receptor subfamily 1, group d, member 1, FOXD1 - forkhead box d1, HES2 - hairy and enhancer of split 2 (drosophila), UBC - ubiquitin c, CDKN2A - cyclin-dependent kinase inhibitor 2a, CRYM - crystallin, mu, FOXN2 - forkhead box n2, PIM1 - pim-1 oncogene, HIST1H4K - histone cluster 1, h4k, TBX1 - t-box 1, HIST1H4C - histone cluster 1, h4c, IKZF1 - ikaros family zinc finger 1 (ikaros), CFLAR - casp8 and fadd-like apoptosis regulator, KCNIP3 - kv channel interacting protein 3, calsenilin, TCERG1L - transcription elongation regulator 1-like, MYC - v-myc avian myelocytomatosis viral oncogene homolog, MXD1 - max dimerization protein 1, NBL1 - neuroblastoma 1, dan family bmp antagonist, CHRDL1 - chordin-like 1, PDLIM1 - pdz and lim domain 1, NDP - norrie disease (pseudoglioma), NR4A2 - nuclear receptor subfamily 4, group a, member 2, KLF9 - kruppel-like factor 9, TICAM1 - toll-like receptor adaptor molecule 1, HIST1H2AI - histone cluster 1, h2ai]
GO:0070887	cellular response to chemical stimulus	1.53E-04	4.23E-02	2.07	16219	1522	144	28	[SFRP1 - secreted frizzled-related protein 1, HSPA5 - heat shock 70kda protein 5 (glucose-regulated protein, 78kda), TIPARP - tcdd-inducible poly(adp-ribose) polymerase, HSPB1 - heat shock 27kda protein 1, MSRA - methionine sulfoxide reductase a, TUBA1B - tubulin, alpha 1b, JUND - jun d proto-oncogene, SOX4 - sry (sex determining region y)-box 4, IL8 - interleukin 8, PDE2A - phosphodiesterase 2a, cgmp-stimulated, GPD1 - glycerol-3-phosphate dehydrogenase 1 (soluble), NR1D1 - nuclear receptor subfamily 1, group d, member 1, TBX1 - t-box 1, MGARP - mitochondria-localized glutamic acid-rich protein, CFLAR - casp8 and fadd-like apoptosis regulator, HRH1 - histamine receptor h1, MYC - v-myc avian myelocytomatosis viral oncogene homolog, FBXO32 - f-box protein 32, TBXA2R - thromboxane a2 receptor, ACTA1 - actin, alpha 1, skeletal muscle, ACTB - actin, beta, CXCL2 - chemokine (c-x-c motif) ligand 2, NR4A2 - nuclear receptor subfamily 4, group a, member 2, CXCL3 - chemokine (c-x-c motif) ligand 3, KLF9 - kruppel-like factor 9, NET1 - neuroepithelial cell transforming 1, ACVR1C - activin a receptor, type ic, TICAM1 - toll-like receptor adaptor molecule 1]
GO:2001141	regulation of RNA biosynthetic process	1.56E-04	4.23E-02	1.66	16219	3192	144	47	[NFIX - nuclear factor i/x (ccaat-binding transcription factor), SFRP1 - secreted frizzled-related protein 1, H1FX - h1 histone family, member x, H1F0 - h1 histone family, member 0, HSPA5 - heat shock 70kda protein 5 (glucose-regulated protein, 78kda), NFKBIA - nuclear factor of kappa light polypeptide gene enhancer in b-cells inhibitor, alpha, TCF15 - transcription factor 15 (basic helix-loop-helix), CSRNP1 - cysteine-serine-rich nuclear protein 1, HIST1H1C - histone cluster 1, h1c, HIST1H1E - histone cluster 1, h1e, ATF3 - activating transcription factor 3, MDFI - myod family inhibitor, HIST1H2AD - histone cluster 1, h2ad, HMX1 - h6 family homeobox 1, INSM1 - insulinoma-associated 1, AES - amino-terminal enhancer of split, TLE2 - transducin-like enhancer of split 2 (e(sp1) homolog, drosophila), H3F3B - h3 histone, family 3b (h3.3b), JUND - jun d proto-oncogene, SOX4 - sry (sex determining region y)-box 4, PDE2A - phosphodiesterase 2a, cgmp-stimulated, UBB - ubiquitin b, NR1D1 - nuclear receptor subfamily 1, group d, member 1, FOXD1 - forkhead box d1, HES2 - hairy and enhancer of split 2 (drosophila), UBC - ubiquitin c, CDKN2A - cyclin-dependent kinase inhibitor 2a, CRYM - crystallin, mu, FOXN2 - forkhead box n2, PIM1 - pim-1 oncogene, HIST1H4K - histone cluster 1, h4k, TBX1 - t-box 1, HIST1H4C - histone cluster 1, h4c, IKZF1 - ikaros family zinc finger 1 (ikaros), CFLAR - casp8 and fadd-like apoptosis regulator, KCNIP3 - kv channel interacting protein 3, calsenilin, TCERG1L - transcription elongation regulator 1-like, MYC - v-myc avian myelocytomatosis viral oncogene homolog, MXD1 - max dimerization protein 1, NBL1 - neuroblastoma 1, dan family bmp antagonist, CHRDL1 - chordin-like 1, PDLIM1 - pdz and lim domain 1, NDP - norrie disease (pseudoglioma), NR4A2 - nuclear receptor subfamily 4, group a, member 2, KLF9 - kruppel-like factor 9, TICAM1 - toll-like receptor adaptor molecule 1, HIST1H2AI - histone cluster 1, h2ai]
GO:0042221	response to chemical	1.70E-04	4.54E-02	1.8	16219	2382	144	38	[SFRP1 - secreted frizzled-related protein 1, SLC25A25 - solute carrier family 25 (mitochondrial carrier; phosphate carrier), member 25, HSPA5 - heat shock 70kda protein 5 (glucose-regulated protein, 78kda), NFKBIA - nuclear factor of kappa light polypeptide gene enhancer in b-cells inhibitor, alpha, SLC18A2 - solute carrier family 18 (vesicular monoamine transporter), member 2, MICB - mhc class i polypeptide-related sequence b, TIPARP - tcdd-inducible poly(adp-ribose) polymerase, HSPB1 - heat shock 27kda protein 1, LEPR - leptin receptor, MSRA - methionine sulfoxide reductase a, TUBA1B - tubulin, alpha 1b, DUSP10 - dual specificity phosphatase 10, AES - amino-terminal enhancer of split, JUND - jun d proto-oncogene, SOX4 - sry (sex determining region y)-box 4, SLC30A3 - solute carrier family 30 (zinc transporter), member 3, IL8 - interleukin 8, PDE2A - phosphodiesterase 2a, cgmp-stimulated, GPD1 - glycerol-3-phosphate dehydrogenase 1 (soluble), NR1D1 - nuclear receptor subfamily 1, group d, member 1, TBX1 - t-box 1, MGARP - mitochondria-localized glutamic acid-rich protein, RGS10 - regulator of g-protein signaling 10, EEF1A2 - eukaryotic translation elongation factor 1 alpha 2, CFLAR - casp8 and fadd-like apoptosis regulator, HRH1 - histamine receptor h1, MYC - v-myc avian myelocytomatosis viral oncogene homolog, FBXO32 - f-box protein 32, TBXA2R - thromboxane a2 receptor, ACTA1 - actin, alpha 1, skeletal muscle, ACTB - actin, beta, CXCL2 - chemokine (c-x-c motif) ligand 2, NR4A2 - nuclear receptor subfamily 4, group a, member 2, KLF9 - kruppel-like factor 9, CXCL3 - chemokine (c-x-c motif) ligand 3, NET1 neuroepithelial cell transforming 1, ACVR1C - activin a receptor, type ic, TICAM1 - toll-like receptor adaptor molecule 1]
GO:0048518	positive regulation of biological process	1.72E-04	4.51E-02	1.44	16219	5494	144	70	[ADRB1 - adrenoceptor beta 1, SFRP1 - secreted frizzled-related protein 1, H1FX - h1 histone family, member x, CDC42EP1 - cdc42 effector protein (rho gtpase binding) 1, ATF3 - activating transcription factor 3, AES - amino-terminal enhancer of split, JUND - jun d proto-oncogene, ZC3HAV1 - zinc finger ccch-type, antiviral 1, ARRDC3 - arrestin domain containing 3, IL8 - interleukin 8, GPD1 - glycerol-3-phosphate dehydrogenase 1 (soluble), UBB - ubiquitin b, UBC - ubiquitin c, OPN3 - opsin 3, ZEB2-AS1 - zeb2 antisense rna 1, TBX1 - t-box 1, MGARP - mitochondria-localized glutamic acid-rich protein, EEF1A2 - eukaryotic translation elongation factor 1 alpha 2, CFLAR - casp8 and fadd-like apoptosis regulator, HRH1 - histamine receptor h1, MYC - v-myc avian myelocytomatosis viral oncogene homolog, CHRM1 - cholinergic receptor, muscarinic 1, GPR21 - g protein-coupled receptor 21, TBXA2R - thromboxane a2 receptor, ACTA1 - actin, alpha 1, skeletal muscle, ACTB - actin, beta, STMN1 - stathmin 1, NR4A2 - nuclear receptor subfamily 4, group a, member 2, TNFAIP8L3 - tumor necrosis factor, alpha-induced protein 8-like 3, RAC3 - ras-related c3 botulinum toxin substrate 3 (rho family, small gtp binding protein rac3), NET1 - neuroepithelial cell transforming 1, ACVR1C - activin a receptor, type ic, TICAM1 - toll-like receptor adaptor molecule 1, NFIX - nuclear factor i/x (ccaat-binding transcription factor), H1F0 - h1 histone family, member 0, HSPA5 - heat shock 70kda protein 5 (glucose-regulated protein, 78kda), NFKBIA - nuclear factor of kappa light polypeptide gene enhancer in b-cells inhibitor, alpha, TCF15 - transcription factor 15 (basic helix-loop-helix), CSRNP1 - cysteine-serine-rich nuclear protein 1, HIST1H1C - histone cluster 1, h1c, HIST1H1E - histone cluster 1, h1e, MICB - mhc class i polypeptide-related sequence b, ALOX5 - arachidonate 5-lipoxygenase, TIPARP - tcdd-inducible poly(adp-ribose) polymerase, HSPB1 - heat shock 27kda protein 1, GADD45G - growth arrest and dna-damage-inducible, gamma, LEPR - leptin receptor, YWHAE - tyrosine 3-monooxygenase/tryptophan 5-monooxygenase activation protein, epsilon polypeptide, INSM1 - insulinoma-associated 1, DUSP10 - dual specificity phosphatase 10, H3F3B - h3 histone, family 3b (h3.3b), CRLF1 - cytokine receptor-like factor 1, SOX4 - sry (sex determining region y)-box 4, SLC30A3 - solute carrier family 30 (zinc transporter), member 3, PDE2A - phosphodiesterase 2a, cgmp-stimulated, ARMCX5-GPRASP2 - armcx5-gprasp2 readthrough, NR1D1 - nuclear receptor subfamily 1, group d, member 1, FOXD1 - forkhead box d1, HNRNPC - heterogeneous nuclear ribonucleoprotein c (c1/c2), CDKN2A - cyclin-dependent kinase inhibitor 2a, CCND2 - cyclin d2, PIM1 - pim-1 oncogene, TUBB2B - tubulin, beta 2b class iib, WNT16 - wingless-type mmtv integration site family, member 16, NBL1 - neuroblastoma 1, dan family bmp antagonist, CFD - complement factor d (adipsin), PDLIM1 - pdz and lim domain 1, NDP - norrie disease (pseudoglioma), H3F3C - h3 histone, family 3c, EPHA6 - eph receptor a6]
GO:0071310	cellular response to organic substance	1.77E-04	4.56E-02	2.22	16219	1218	144	24	[TBX1 - t-box 1, MGARP - mitochondria-localized glutamic acid-rich protein, SFRP1 - secreted frizzled-related protein 1, HSPA5 - heat shock 70kda protein 5 (glucose-regulated protein, 78kda), CFLAR - casp8 and fadd-like apoptosis regulator, HRH1 - histamine receptor h1, TIPARP - tcdd-inducible poly(adp-ribose) polymerase, HSPB1 - heat shock 27kda protein 1, TUBA1B - tubulin, alpha 1b, FBXO32 - f-box protein 32, TBXA2R - thromboxane a2 receptor, ACTA1 - actin, alpha 1, skeletal muscle, ACTB - actin, beta, SOX4 - sry (sex determining region y)-box 4, IL8 - interleukin 8, PDE2A - phosphodiesterase 2a, cgmp-stimulated, CXCL2 - chemokine (c-x-c motif) ligand 2, NR4A2 - nuclear receptor subfamily 4, group a, member 2, GPD1 - glycerol-3-phosphate dehydrogenase 1 (soluble), CXCL3 - chemokine (c-x-c motif) ligand 3, KLF9 - kruppel-like factor 9, NR1D1 - nuclear receptor subfamily 1, group d, member 1, ACVR1C - activin a receptor, type ic, TICAM1 - toll-like receptor adaptor molecule 1]
GO:0060969	negative regulation of gene silencing	1.77E-04	4.48E-02	14.08	16219	32	144	4	[H1FX - h1 histone family, member x, H1F0 - h1 histone family, member 0, HIST1H1C - histone cluster 1, h1c, HIST1H1E - histone cluster 1, h1e]
GO:0048856	anatomical structure development	1.80E-04	4.48E-02	1.68	16219	3024	144	45	[SFRP1 - secreted frizzled-related protein 1, SLC25A25 - solute carrier family 25 (mitochondrial carrier; phosphate carrier), member 25, TCF15 - transcription factor 15 (basic helix-loop-helix), CSRNP1 - cysteine-serine-rich nuclear protein 1, HSPA5 - heat shock 70kda protein 5 (glucose-regulated protein, 78kda), SEZ6 - seizure related 6 homolog (mouse), TIPARP - tcdd-inducible poly(adp-ribose) polymerase, GADD45G - growth arrest and dna-damage-inducible, gamma, LEPR - leptin receptor, YWHAE - tyrosine 3-monooxygenase/tryptophan 5-monooxygenase activation protein, epsilon polypeptide, HMX1 - h6 family homeobox 1, INSM1 - insulinoma-associated 1, AES - amino-terminal enhancer of split, H3F3B - h3 histone, family 3b (h3.3b), CRLF1 - cytokine receptor-like factor 1, JUND - jun d proto-oncogene, SOX4 - sry (sex determining region y)-box 4, ARRDC3 - arrestin domain containing 3, IL8 - interleukin 8, PDE2A - phosphodiesterase 2a, cgmp-stimulated, UBB - ubiquitin b, FOXD1 - forkhead box d1, PCDHAC2 - protocadherin alpha subfamily c, 2, PIM1 - pim-1 oncogene, TUBB2B - tubulin, beta 2b class iib, TBX1 - t-box 1, IKZF1 - ikaros family zinc finger 1 (ikaros), OSTN - osteocrin, CFLAR - casp8 and fadd-like apoptosis regulator, CAPG - capping protein (actin filament), gelsolin-like, NBL1 - neuroblastoma 1, dan family bmp antagonist, CHRDL1 - chordin-like 1, CHRM1 - cholinergic receptor, muscarinic 1, CCSAP - centriole, cilia and spindle-associated protein, ACTA1 - actin, alpha 1, skeletal muscle, SPOCK1 - sparc/osteonectin, cwcv and kazal-like domains proteoglycan (testican) 1, PDLIM1 - pdz and lim domain 1, ACTB - actin, beta, CRABP1 - cellular retinoic acid binding protein 1, STMN1 - stathmin 1, NDP - norrie disease (pseudoglioma), NR4A2 - nuclear receptor subfamily 4, group a, member 2, RAC3 - ras-related c3 botulinum toxin substrate 3 (rho family, small gtp binding protein rac3), ACVR1C - activin a receptor, type ic, EPHA6 - eph receptor a6]
GO:0003085	negative regulation of systemic arterial blood pressure	1.84E-04	4.51E-02	25.99	16219	13	144	3	[ADRB1 - adrenoceptor beta 1, ARHGAP42 - rho gtpase activating protein 42, KCNK6 - potassium channel, subfamily k, member 6]
GO:0036499	PERK-mediated unfolded protein response	1.84E-04	4.44E-02	25.99	16219	13	144	3	[HSPA5 - heat shock 70kda protein 5 (glucose-regulated protein, 78kda), IL8 - interleukin 8, ATF3 - activating transcription factor 3]
GO:1901700	response to oxygen-containing compound	2.00E-04	4.75E-02	2.2	16219	1228	144	24	[TBX1 - t-box 1, MGARP - mitochondria-localized glutamic acid-rich protein, SFRP1 - secreted frizzled-related protein 1, HSPA5 - heat shock 70kda protein 5 (glucose-regulated protein, 78kda), NFKBIA - nuclear factor of kappa light polypeptide gene enhancer in b-cells inhibitor, alpha, CFLAR - casp8 and fadd-like apoptosis regulator, MICB - mhc class i polypeptide-related sequence b, DUSP10 - dual specificity phosphatase 10, FBXO32 - f-box protein 32, TBXA2R - thromboxane a2 receptor, ACTB - actin, beta, JUND - jun d proto-oncogene, SOX4 - sry (sex determining region y)-box 4, IL8 - interleukin 8, PDE2A - phosphodiesterase 2a, cgmp-stimulated, CXCL2 - chemokine (c-x-c motif) ligand 2, NR4A2 - nuclear receptor subfamily 4, group a, member 2, GPD1 - glycerol-3-phosphate dehydrogenase 1 (soluble), CXCL3 - chemokine (c-x-c motif) ligand 3, KLF9 - kruppel-like factor 9, NR1D1 - nuclear receptor subfamily 1, group d, member 1, NET1 - neuroepithelial cell transforming 1, ACVR1C - activin a receptor, type ic, TICAM1 - toll-like receptor adaptor molecule 1]

GO:0045910	negative regulation of DNA recombination	2.25E-04	5.26E-02	13.25	16219	34	144	4	[H1FX - h1 histone family, member x, H1F0 - h1 histone family, member 0, HIST1H1C - histone cluster 1, h1c, HIST1H1E - histone cluster 1, h1e]
GO:0051239	regulation of multicellular organismal process	2.27E-04	5.25E-02	1.69	16219	2869	144	43	[ADRB1 - adrenoceptor beta 1, SFRP1 - secreted frizzled-related protein 1, ARHGAP42 - rho gtpase activating protein 42, HSPA5 - heat shock 70kda protein 5 (glucose-regulated protein, 78kda), NFKBIA - nuclear factor of kappa light polypeptide gene enhancer in b-cells inhibitor, alpha, SEZ6 - seizure related 6 homolog (mouse), ALOX5 - arachidonate 5-lipoxygenase, HSPB1 - heat shock 27kda protein 1, GADD45G - growth arrest and dna-damage-inducible, gamma, LEPR - leptin receptor, YWHAE - tyrosine 3-monooxygenase/tryptophan 5-monooxygenase activation protein, epsilon polypeptide, DUSP10 - dual specificity phosphatase 10, H3F3B - h3 histone, family 3b (h3.3b), FKBP4 - fk506 binding protein 4, 59kda, JUND - jun d proto-oncogene, SOX4 - sry (sex determining region y)-box 4, ARRDC3 - arrestin domain containing 3, IL8 - interleukin 8, ARMCX5-GPRASP2 - armcx5-gprasp2 readthrough, NR1D1 - nuclear receptor subfamily 1, group d, member 1, FOXD1 - forkhead box d1, HES2 - hairy and enhancer of split 2 (drosophila), PIM1 - pim-1 oncogene, TUBB2B - tubulin, beta 2b class iib, HIST1H4K - histone cluster 1, h4k, KCNK6 - potassium channel, subfamily k, member 6, TBX1 - t-box 1, HIST1H4C - histone cluster 1, h4c, OSTN - osteocrin, CFLAR - casp8 and fadd-like apoptosis regulator, HRH1 - histamine receptor h1, KCNIP3 - kv channel interacting protein 3, calsenilin, MYC - v-myc avian myelocytomatosis viral oncogene homolog, NBL1 - neuroblastoma 1, dan family bmp antagonist, CHRM1 - cholinergic receptor, muscarinic 1, TBXA2R - thromboxane a2 receptor, CCSAP - centriole, cilia and spindle-associated protein, GPR21 - g protein-coupled receptor 21, SPOCK1 - sparc/osteonectin, cwcv and kazal-like domains proteoglycan (testican) 1, NR4A2 - nuclear receptor subfamily 4, group a, member 2, RAC3 - ras-related c3 botulinum toxin substrate 3 (rho family, small gtp binding protein rac3), ACVR1C - activin a receptor, type ic, TICAM1 - toll-like receptor adaptor molecule 1]
GO:0051171	regulation of nitrogen compound metabolic process	2.41E-04	5.48E-02	1.43	16219	5340	144	68	[SFRP1 - secreted frizzled-related protein 1, H1FX - h1 histone family, member x, ATF3 - activating transcription factor 3, MDFI - myod family inhibitor, BHMT - betaine–homocysteine s-methyltransferase, AES - amino-terminal enhancer of split, JUND - jun d proto-oncogene, ZC3HAV1 - zinc finger ccch-type, antiviral 1, ARRDC3 - arrestin domain containing 3, GPD1 - glycerol-3-phosphate dehydrogenase 1 (soluble), UBB - ubiquitin b, UBC - ubiquitin c, CRYM - crystallin, mu, HIST1H4K - histone cluster 1, h4k, TBX1 - t-box 1, HIST1H4C - histone cluster 1, h4c, EEF1A2 - eukaryotic translation elongation factor 1 alpha 2, CFLAR - casp8 and fadd-like apoptosis regulator, KCNIP3 - kv channel interacting protein 3, calsenilin, TCERG1L - transcription elongation regulator 1-like, MYC - v-myc avian myelocytomatosis viral oncogene homolog, MXD1 - max dimerization protein 1, ACTB - actin, beta, NR4A2 - nuclear receptor subfamily 4, group a, member 2, TNFAIP8L3 - tumor necrosis factor, alpha-induced protein 8-like 3, KLF9 - kruppel-like factor 9, ACVR1C - activin a receptor, type ic, TICAM1 - toll-like receptor adaptor molecule 1, NFIX - nuclear factor i/x (ccaat-binding transcription factor), DUSP16 - dual specificity phosphatase 16, H1F0 - h1 histone family, member 0, HSPA5 - heat shock 70kda protein 5 (glucose-regulated protein, 78kda), NFKBIA - nuclear factor of kappa light polypeptide gene enhancer in b-cells inhibitor, alpha, TCF15 - transcription factor 15 (basic helix-loop-helix), CSRNP1 - cysteine-serine-rich nuclear protein 1, HIST1H1C - histone cluster 1, h1c, HIST1H1E - histone cluster 1, h1e, TIPARP - tcdd-inducible poly(adp-ribose) polymerase, HSPB1 - heat shock 27kda protein 1, GADD45G - growth arrest and dna-damage-inducible, gamma, HIST1H2AD - histone cluster 1, h2ad, LEPR - leptin receptor, YWHAE - tyrosine 3-monooxygenase/tryptophan 5-monooxygenase activation protein, epsilon polypeptide, HMX1 - h6 family homeobox 1, INSM1 - insulinoma-associated 1, DUSP10 - dual specificity phosphatase 10, TLE2 - transducin-like enhancer of split 2 (e(sp1) homolog, drosophila), H3F3B - h3 histone, family 3b (h3.3b), CRLF1 - cytokine receptor-like factor 1, SOX4 - sry (sex determining region y)-box 4, PDE2A - phosphodiesterase 2a, cgmp-stimulated, NR1D1 - nuclear receptor subfamily 1, group d, member 1, FOXD1 - forkhead box d1, HNRNPC - heterogeneous nuclear ribonucleoprotein c (c1/c2), HES2 - hairy and enhancer of split 2 (drosophila), CDKN2A - cyclin-dependent kinase inhibitor 2a, FOXN2 - forkhead box n2, CCND2 - cyclin d2, PPP1R14A - protein phosphatase 1, regulatory (inhibitor) subunit 14a, PIM1 - pim-1 oncogene, WNT16 - wingless-type mmtv integration site family, member 16, IKZF1 - ikaros family zinc finger 1 (ikaros), NBL1 - neuroblastoma 1, dan family bmp antagonist, CHRDL1 - chordin-like 1, SPOCK1 - sparc/osteonectin, cwcv and kazal-like domains proteoglycan (testican) 1, PDLIM1 - pdz and lim domain 1, NDP - norrie disease (pseudoglioma), HIST1H2AI - histone cluster 1, h2ai]
GO:0051235	maintenance of location	2.42E-04	5.41E-02	5.67	16219	139	144	7	[NFKBIA - nuclear factor of kappa light polypeptide gene enhancer in b-cells inhibitor, alpha, HSPA5 - heat shock 70kda protein 5 (glucose-regulated protein, 78kda), SLC18A2 - solute carrier family 18 (vesicular monoamine transporter), member 2, MDFI - myod family inhibitor, FTH1 - ferritin, heavy polypeptide 1, ACVR1C - activin a receptor, type ic, NBL1 - neuroblastoma 1, dan family bmp antagonist]
GO:0071407	cellular response to organic cyclic compound	2.47E-04	5.44E-02	3.61	16219	343	144	11	[MGARP - mitochondria-localized glutamic acid-rich protein, ACTB - actin, beta, SFRP1 - secreted frizzled-related protein 1, HSPA5 - heat shock 70kda protein 5 (glucose-regulated protein, 78kda), CFLAR - casp8 and fadd-like apoptosis regulator, PDE2A - phosphodiesterase 2a, cgmp-stimulated, HRH1 - histamine receptor h1, KLF9 - kruppel-like factor 9, GPD1 - glycerol-3-phosphate dehydrogenase 1 (soluble), TIPARP - tcdd-inducible poly(adp-ribose) polymerase, FBXO32 - f-box protein 32]
GO:0032502	developmental process	2.49E-04	5.41E-02	1.5	16219	4424	144	59	[ADRB1 - adrenoceptor beta 1, SFRP1 - secreted frizzled-related protein 1, SLC25A25 - solute carrier family 25 (mitochondrial carrier; phosphate carrier), member 25, SEZ6 - seizure related 6 homolog (mouse), ATF3 - activating transcription factor 3, MDFI - myod family inhibitor, AES - amino-terminal enhancer of split, JUND - jun d proto-oncogene, ARRDC3 - arrestin domain containing 3, IL8 - interleukin 8, UBB - ubiquitin b, PCDHAC2 - protocadherin alpha subfamily c, 2, OPN3 - opsin 3, TBX1 - t-box 1, CFLAR - casp8 and fadd-like apoptosis regulator, CAPG - capping protein (actin filament), gelsolin-like, MYC - v-myc avian myelocytomatosis viral oncogene homolog, CHRM1 - cholinergic receptor, muscarinic 1, RND1 - rho family gtpase 1, ACTA1 - actin, alpha 1, skeletal muscle, ACTB - actin, beta, STMN1 - stathmin 1, NR4A2 - nuclear receptor subfamily 4, group a, member 2, RAC3 - ras-related c3 botulinum toxin substrate 3 (rho family, small gtp binding protein rac3), ACVR1C - activin a receptor, type ic, HSPA5 - heat shock 70kda protein 5 (glucose-regulated protein, 78kda), TCF15 - transcription factor 15 (basic helix-loop-helix), CSRNP1 - cysteine-serine-rich nuclear protein 1, TIPARP - tcdd-inducible poly(adp-ribose) polymerase, GADD45G - growth arrest and dna-damage-inducible, gamma, LEPR - leptin receptor, YWHAE - tyrosine 3-monooxygenase/tryptophan 5-monooxygenase activation protein, epsilon polypeptide, HMX1 - h6 family homeobox 1, INSM1 - insulinoma-associated 1, DUSP10 - dual specificity phosphatase 10, TLE2 - transducin-like enhancer of split 2 (e(sp1) homolog, drosophila), H3F3B - h3 histone, family 3b (h3.3b), CRLF1 - cytokine receptor-like factor 1, SOX4 - sry (sex determining region y)-box 4, PDE2A - phosphodiesterase 2a, cgmp-stimulated, NR1D1 - nuclear receptor subfamily 1, group d, member 1, FOXD1 - forkhead box d1, HNRNPC - heterogeneous nuclear ribonucleoprotein c (c1/c2), CDKN2A - cyclin-dependent kinase inhibitor 2a, FOXN2 - forkhead box n2, PIM1 - pim-1 oncogene, TUBB2B - tubulin, beta 2b class iib, WNT16 - wingless-type mmtv integration site family, member 16, IKZF1 - ikaros family zinc finger 1 (ikaros), OSTN - osteocrin, BCAP29 - b-cell receptor-associated protein 29, NBL1 - neuroblastoma 1, dan family bmp antagonist, CHRDL1 - chordin-like 1, CCSAP - centriole, cilia and spindle-associated protein, SPOCK1 - sparc/osteonectin, cwcv and kazal-like domains proteoglycan (testican) 1, PDLIM1 - pdz and lim domain 1, CRABP1 - cellular retinoic acid binding protein 1, NDP - norrie disease (pseudoglioma), EPHA6 - eph receptor a6]
GO:0006323	DNA packaging	2.52E-04	5.40E-02	12.87	16219	35	144	4	[H1FX - h1 histone family, member x, H1F0 - h1 histone family, member 0, HIST1H1C - histone cluster 1, h1c, HIST1H1E - histone cluster 1, h1e]
GO:0061448	connective tissue development	2.52E-04	5.33E-02	12.87	16219	35	144	4	[SLC25A25 - solute carrier family 25 (mitochondrial carrier; phosphate carrier), member 25, ARRDC3 - arrestin domain containing 3, UBB - ubiquitin b, FOXD1 - forkhead box d1]
GO:0019730	antimicrobial humoral response	2.77E-04	5.78E-02	8.66	16219	65	144	5	[HIST1H2BC - histone cluster 1, h2bc, IL8 - interleukin 8, CXCL2 - chemokine (c-x-c motif) ligand 2, CXCL3 - chemokine (c-x-c motif) ligand 3, HMGN2 - high mobility group nucleosomal binding domain 2]
GO:0030522	intracellular receptor signaling pathway	2.87E-04	5.91E-02	5.51	16219	143	144	7	[TBX1 - t-box 1, NFKBIA - nuclear factor of kappa light polypeptide gene enhancer in b-cells inhibitor, alpha, NR4A2 - nuclear receptor subfamily 4, group a, member 2, UBB - ubiquitin b, NR1D1 - nuclear receptor subfamily 1, group d, member 1, UBC - ubiquitin c, PIM1 - pim-1 oncogene]
GO:0009719	response to endogenous stimulus	2.94E-04	5.96E-02	2.36	16219	954	144	20	[TBX1 - t-box 1, MGARP - mitochondria-localized glutamic acid-rich protein, SFRP1 - secreted frizzled-related protein 1, HSPA5 - heat shock 70kda protein 5 (glucose-regulated protein, 78kda), CFLAR - casp8 and fadd-like apoptosis regulator, HRH1 - histamine receptor h1, LEPR - leptin receptor, FBXO32 - f-box protein 32, TBXA2R - thromboxane a2 receptor, ACTA1 - actin, alpha 1, skeletal muscle, ACTB - actin, beta, JUND - jun d proto-oncogene, IL8 - interleukin 8, PDE2A - phosphodiesterase 2a, cgmp-stimulated, NR4A2 - nuclear receptor subfamily 4, group a, member 2, GPD1 - glycerol-3-phosphate dehydrogenase 1 (soluble), KLF9 - kruppel-like factor 9, NR1D1 - nuclear receptor subfamily 1, group d, member 1, ACVR1C - activin a receptor, type ic, TICAM1 - toll-like receptor adaptor molecule 1]
GO:0050793	regulation of developmental process	3.11E-04	6.22E-02	1.76	16219	2363	144	37	[ADRB1 - adrenoceptor beta 1, SFRP1 - secreted frizzled-related protein 1, CDC42EP1 - cdc42 effector protein (rho gtpase binding) 1, HSPA5 - heat shock 70kda protein 5 (glucose-regulated protein, 78kda), NFKBIA - nuclear factor of kappa light polypeptide gene enhancer in b-cells inhibitor, alpha, SEZ6 - seizure related 6 homolog (mouse), ALOX5 - arachidonate 5-lipoxygenase, HSPB1 - heat shock 27kda protein 1, INSM1 - insulinoma-associated 1, DUSP10 - dual specificity phosphatase 10, H3F3B - h3 histone, family 3b (h3.3b), FKBP4 - fk506 binding protein 4, 59kda, JUND - jun d proto-oncogene, SOX4 - sry (sex determining region y)-box 4, IL8 - interleukin 8, ARMCX5-GPRASP2 - armcx5-gprasp2 readthrough, NR1D1 - nuclear receptor subfamily 1, group d, member 1, FOXD1 - forkhead box d1, HES2 - hairy and enhancer of split 2 (drosophila), CDKN2A - cyclin-dependent kinase inhibitor 2a, PIM1 - pim-1 oncogene, TUBB2B - tubulin, beta 2b class iib, HIST1H4K - histone cluster 1, h4k, TBX1 - t-box 1, HIST1H4C - histone cluster 1, h4c, OSTN - osteocrin, CFLAR - casp8 and fadd-like apoptosis regulator, MYC - v-myc avian myelocytomatosis viral oncogene homolog, NBL1 - neuroblastoma 1, dan family bmp antagonist, CHRM1 - cholinergic receptor, muscarinic 1, TBXA2R - thromboxane a2 receptor, CCSAP - centriole, cilia and spindle-associated protein, GPR21 - g protein-coupled receptor 21, RND1 - rho family gtpase 1, SPOCK1 - sparc/osteonectin, cwcv and kazal-like domains proteoglycan (testican) 1, RAC3 - ras-related c3 botulinum toxin substrate 3 (rho family, small gtp binding protein rac3), ACVR1C - activin a receptor, type ic]
GO:0006959	humoral immune response	3.13E-04	6.19E-02	5.44	16219	145	144	7	[HIST1H2BC - histone cluster 1, h2bc, IL8 - interleukin 8, CXCL2 - chemokine (c-x-c motif) ligand 2, CXCL3 - chemokine (c-x-c motif) ligand 3, ALOX5 - arachidonate 5-lipoxygenase, CFD - complement factor d (adipsin), HMGN2 - high mobility group nucleosomal binding domain 2]
GO:0033993	response to lipid	3.24E-04	6.31E-02	2.67	16219	674	144	16	[TBX1 - t-box 1, MGARP - mitochondria-localized glutamic acid-rich protein, SFRP1 - secreted frizzled-related protein 1, CFLAR - casp8 and fadd-like apoptosis regulator, MICB - mhc class i polypeptide-related sequence b, DUSP10 - dual specificity phosphatase 10, FBXO32 - f-box protein 32, TBXA2R - thromboxane a2 receptor, ACTA1 - actin, alpha 1, skeletal muscle, JUND - jun d proto-oncogene, IL8 - interleukin 8, CXCL2 - chemokine (c-x-c motif) ligand 2, CXCL3 - chemokine (c-x-c motif) ligand 3, NR1D1 - nuclear receptor subfamily 1, group d, member 1, KLF9 - kruppel-like factor 9, TICAM1 - toll-like receptor adaptor molecule 1]
GO:0007019	microtubule depolymerization	3.53E-04	6.80E-02	21.12	16219	16	144	3	[STMN4 - stathmin-like 4, STMN1 - stathmin 1, CCSAP - centriole, cilia and spindle-associated protein]

GO:0080090	regulation of primary metabolic process	3.56E-04	6.78E-02	1.41	16219	5510	144	69	[SFRP1 - secreted frizzled-related protein 1, H1FX - h1 histone family, member x, ATF3 - activating transcription factor 3, MDFI - myod family inhibitor, BHMT - betaine–homocysteine s-methyltransferase, AES - amino-terminal enhancer of split, JUND - jun d proto-oncogene, ZC3HAV1 - zinc finger ccch-type, antiviral 1, ARRDC3 - arrestin domain containing 3, GPD1 - glycerol-3-phosphate dehydrogenase 1 (soluble), UBB - ubiquitin b, UBC - ubiquitin c, CRYM - crystallin, mu, HIST1H4K - histone cluster 1, h4k, TBX1 - t-box 1, HIST1H4C - histone cluster 1, h4c, EEF1A2 - eukaryotic translation elongation factor 1 alpha 2, CFLAR - casp8 and fadd-like apoptosis regulator, HRH1 - histamine receptor h1, KCNIP3 - kv channel interacting protein 3, calsenilin, TCERG1L - transcription elongation regulator 1-like, MYC - v-myc avian myelocytomatosis viral oncogene homolog, MXD1 - max dimerization protein 1, ACTB - actin, beta, NR4A2 - nuclear receptor subfamily 4, group a, member 2, TNFAIP8L3 - tumor necrosis factor, alpha-induced protein 8-like 3, KLF9 - kruppel-like factor 9, ACVR1C - activin a receptor, type ic, TICAM1 - toll-like receptor adaptor molecule 1, NFIX - nuclear factor i/x (ccaat-binding transcription factor), DUSP16 - dual specificity phosphatase 16, H1F0 - h1 histone family, member 0, HSPA5 - heat shock 70kda protein 5 (glucose-regulated protein, 78kda), NFKBIA - nuclear factor of kappa light polypeptide gene enhancer in b-cells inhibitor, alpha, TCF15 - transcription factor 15 (basic helix-loop-helix), CSRNP1 - cysteine-serine-rich nuclear protein 1, HIST1H1C - histone cluster 1, h1c, HIST1H1E - histone cluster 1, h1e, TIPARP - tcdd-inducible poly(adp-ribose) polymerase, HSPB1 - heat shock 27kda protein 1, GADD45G - growth arrest and dna-damage-inducible, gamma, HIST1H2AD - histone cluster 1, h2ad, LEPR - leptin receptor, YWHAE - tyrosine 3-monooxygenase/tryptophan 5-monooxygenase activation protein, epsilon polypeptide, HMX1 - h6 family homeobox 1, INSM1 - insulinoma-associated 1, DUSP10 - dual specificity phosphatase 10, TLE2 - transducin-like enhancer of split 2 (e(sp1) homolog, drosophila), H3F3B - h3 histone, family 3b (h3.3b), CRLF1 - cytokine receptor-like factor 1, SOX4 - sry (sex determining region y)-box 4, PDE2A - phosphodiesterase 2a, cgmp-stimulated, NR1D1 - nuclear receptor subfamily 1, group d, member 1, FOXD1 - forkhead box d1, HNRNPC - heterogeneous nuclear ribonucleoprotein c (c1/c2), HES2 - hairy and enhancer of split 2 (drosophila), CDKN2A - cyclin-dependent kinase inhibitor 2a, FOXN2 - forkhead box n2, CCND2 - cyclin d2, PPP1R14A - protein phosphatase 1, regulatory (inhibitor) subunit 14a, PIM1 - pim-1 oncogene, WNT16 - wingless-type mmtv integration site family, member 16, IKZF1 - ikaros family zinc finger 1 (ikaros), NBL1 - neuroblastoma 1, dan family bmp antagonist, CHRDL1 - chordin-like 1, SPOCK1 - sparc/osteonectin, cwcv and kazal-like domains proteoglycan (testican) 1, PDLIM1 - pdz and lim domain 1, NDP - norrie disease (pseudoglioma), HIST1H2AI - histone cluster 1, h2ai]
GO:0042981	regulation of apoptotic process	3.61E-04	6.79E-02	2.07	16219	1358	144	25	[SFRP1 - secreted frizzled-related protein 1, HSPA5 - heat shock 70kda protein 5 (glucose-regulated protein, 78kda), NFKBIA - nuclear factor of kappa light polypeptide gene enhancer in b-cells inhibitor, alpha, ATF3 - activating transcription factor 3, HSPB1 - heat shock 27kda protein 1, GADD45G - growth arrest and dna-damage-inducible, gamma, YWHAE - tyrosine 3-monooxygenase/tryptophan 5-monooxygenase activation protein, epsilon polypeptide, AES - amino-terminal enhancer of split, CRLF1 - cytokine receptor-like factor 1, SOX4 - sry (sex determining region y)-box 4, ARMCX5-GPRASP2 - armcx5-gprasp2 readthrough, UBB - ubiquitin b, UBC - ubiquitin c, CDKN2A - cyclin-dependent kinase inhibitor 2a, PIM1 - pim-1 oncogene, CCND2 - cyclin d2, OPN3 - opsin 3, TBX1 - t-box 1, EEF1A2 - eukaryotic translation elongation factor 1 alpha 2, CFLAR - casp8 and fadd-like apoptosis regulator, MYC - v-myc avian myelocytomatosis viral oncogene homolog, TNFAIP8L3 - tumor necrosis factor, alpha-induced protein 8-like 3, NR4A2 - nuclear receptor subfamily 4, group a, member 2, NET1 - neuroepithelial cell transforming 1, ACVR1C - activin a receptor, type ic]
GO:0014070	response to organic cyclic compound	3.63E-04	6.73E-02	2.65	16219	681	144	16	[MGARP - mitochondria-localized glutamic acid-rich protein, SFRP1 - secreted frizzled-related protein 1, HSPA5 - heat shock 70kda protein 5 (glucose-regulated protein, 78kda), NFKBIA - nuclear factor of kappa light polypeptide gene enhancer in b-cells inhibitor, alpha, CFLAR - casp8 and fadd-like apoptosis regulator, HRH1 - histamine receptor h1, TIPARP - tcdd-inducible poly(adp-ribose) polymerase, FBXO32 - f-box protein 32, TBXA2R - thromboxane a2 receptor, ACTA1 - actin, alpha 1, skeletal muscle, ACTB - actin, beta, JUND - jun d proto-oncogene, PDE2A - phosphodiesterase 2a, cgmp-stimulated, GPD1 - glycerol-3-phosphate dehydrogenase 1 (soluble), KLF9 - kruppel-like factor 9, TICAM1 - toll-like receptor adaptor molecule 1]
GO:0009605	response to external stimulus	4.09E-04	7.49E-02	2.1	16219	1289	144	24	[SFRP1 - secreted frizzled-related protein 1, HSPA5 - heat shock 70kda protein 5 (glucose-regulated protein, 78kda), SLC25A25 - solute carrier family 25 (mitochondrial carrier; phosphate carrier), member 25, NFKBIA - nuclear factor of kappa light polypeptide gene enhancer in b-cells inhibitor, alpha, ATF3 - activating transcription factor 3, HSPB1 - heat shock 27kda protein 1, DUSP10 - dual specificity phosphatase 10, AES - amino-terminal enhancer of split, TBXA2R - thromboxane a2 receptor, ACTA1 - actin, alpha 1, skeletal muscle, HIST1H2BC - histone cluster 1, h2bc, JUND - jun d proto-oncogene, ZC3HAV1 - zinc finger ccch-type, antiviral 1, IL8 - interleukin 8, STMN1 - stathmin 1, PDE2A - phosphodiesterase 2a, cgmp-stimulated, CXCL2 - chemokine (c-x-c motif) ligand 2, NR4A2 - nuclear receptor subfamily 4, group a, member 2, CXCL3 - chemokine (c-x-c motif) ligand 3, NR1D1 - nuclear receptor subfamily 1, group d, member 1, ACVR1C - activin a receptor, type ic, TICAM1 - toll-like receptor adaptor molecule 1, OPN3 - opsin 3, HMGN2 - high mobility group nucleosomal binding domain 2]
GO:0010629	negative regulation of gene expression	4.12E-04	7.47E-02	1.92	16219	1700	144	29	[NFIX - nuclear factor i/x (ccaat-binding transcription factor), SFRP1 - secreted frizzled-related protein 1, H1F0 - h1 histone family, member 0, ATF3 - activating transcription factor 3, MDFI - myod family inhibitor, TIPARP - tcdd-inducible poly(adp-ribose) polymerase, HIST1H2AD - histone cluster 1, h2ad, HMX1 - h6 family homeobox 1, INSM1 - insulinoma-associated 1, AES - amino-terminal enhancer of split, TLE2 - transducin-like enhancer of split 2 (e(sp1) homolog, drosophila), H3F3B - h3 histone, family 3b (h3.3b), IL8 - interleukin 8, PDE2A - phosphodiesterase 2a, cgmp-stimulated, UBB - ubiquitin b, NR1D1 - nuclear receptor subfamily 1, group d, member 1, FOXD1 - forkhead box d1, HES2 - hairy and enhancer of split 2 (drosophila), UBC - ubiquitin c, CDKN2A - cyclin-dependent kinase inhibitor 2a, CRYM - crystallin, mu, HIST1H4K - histone cluster 1, h4k, HIST1H4C - histone cluster 1, h4c, IKZF1 - ikaros family zinc finger 1 (ikaros), KCNIP3 - kv channel interacting protein 3, calsenilin, MYC - v-myc avian myelocytomatosis viral oncogene homolog, MXD1 - max dimerization protein 1, NR4A2 - nuclear receptor subfamily 4, group a, member 2, HIST1H2AI - histone cluster 1, h2ai]
GO:0008585	female gonad development	4.25E-04	7.62E-02	19.88	16219	17	144	3	[SFRP1 - secreted frizzled-related protein 1, TIPARP - tcdd-inducible poly(adp-ribose) polymerase, UBB - ubiquitin b]
GO:0043067	regulation of programmed cell death	4.30E-04	7.62E-02	2.05	16219	1374	144	25	[SFRP1 - secreted frizzled-related protein 1, HSPA5 - heat shock 70kda protein 5 (glucose-regulated protein, 78kda), NFKBIA - nuclear factor of kappa light polypeptide gene enhancer in b-cells inhibitor, alpha, ATF3 - activating transcription factor 3, HSPB1 - heat shock 27kda protein 1, GADD45G - growth arrest and dna-damage-inducible, gamma, YWHAE - tyrosine 3-monooxygenase/tryptophan 5-monooxygenase activation protein, epsilon polypeptide, AES - amino-terminal enhancer of split, CRLF1 - cytokine receptor-like factor 1, SOX4 - sry (sex determining region y)-box 4, ARMCX5-GPRASP2 - armcx5-gprasp2 readthrough, UBB - ubiquitin b, UBC - ubiquitin c, CDKN2A - cyclin-dependent kinase inhibitor 2a, PIM1 - pim-1 oncogene, CCND2 - cyclin d2, OPN3 - opsin 3, TBX1 - t-box 1, EEF1A2 - eukaryotic translation elongation factor 1 alpha 2, CFLAR - casp8 and fadd-like apoptosis regulator, MYC - v-myc avian myelocytomatosis viral oncogene homolog, TNFAIP8L3 - tumor necrosis factor, alpha-induced protein 8-like 3, NR4A2 - nuclear receptor subfamily 4, group a, member 2, NET1 - neuroepithelial cell transforming 1, ACVR1C - activin a receptor, type ic]
GO:0010468	regulation of gene expression	4.47E-04	7.82E-02	1.5	16219	4215	144	56	[SFRP1 - secreted frizzled-related protein 1, H1FX - h1 histone family, member x, ATF3 - activating transcription factor 3, MDFI - myod family inhibitor, AES - amino-terminal enhancer of split, JUND - jun d proto-oncogene, ZC3HAV1 - zinc finger ccch-type, antiviral 1, IL8 - interleukin 8, UBB - ubiquitin b, UBC - ubiquitin c, CRYM - crystallin, mu, ZEB2-AS1 - zeb2 antisense rna 1, HIST1H4K - histone cluster 1, h4k, TBX1 - t-box 1, HIST1H4C - histone cluster 1, h4c, CFLAR - casp8 and fadd-like apoptosis regulator, KCNIP3 - kv channel interacting protein 3, calsenilin, TCERG1L - transcription elongation regulator 1-like, MYC - v-myc avian myelocytomatosis viral oncogene homolog, MXD1 - max dimerization protein 1, ACTA1 - actin, alpha 1, skeletal muscle, ACTB - actin, beta, NR4A2 - nuclear receptor subfamily 4, group a, member 2, KLF9 - kruppel-like factor 9, TICAM1 - toll-like receptor adaptor molecule 1, NFIX - nuclear factor i/x (ccaat-binding transcription factor), H1F0 - h1 histone family, member 0, HSPA5 - heat shock 70kda protein 5 (glucose-regulated protein, 78kda), NFKBIA - nuclear factor of kappa light polypeptide gene enhancer in b-cells inhibitor, alpha, TCF15 - transcription factor 15 (basic helix-loop-helix), CSRNP1 - cysteine-serine-rich nuclear protein 1, HIST1H1C - histone cluster 1, h1c, HIST1H1E - histone cluster 1, h1e, TIPARP - tcdd-inducible poly(adp-ribose) polymerase, HSPB1 - heat shock 27kda protein 1, HIST1H2AD - histone cluster 1, h2ad, HMX1 - h6 family homeobox 1, INSM1 - insulinoma-associated 1, TLE2 - transducin-like enhancer of split 2 (e(sp1) homolog, drosophila), H3F3B - h3 histone, family 3b (h3.3b), SOX4 - sry (sex determining region y)-box 4, PDE2A - phosphodiesterase 2a, cgmp-stimulated, NR1D1 - nuclear receptor subfamily 1, group d, member 1, FOXD1 - forkhead box d1, HNRNPC - heterogeneous nuclear ribonucleoprotein c (c1/c2), HES2 - hairy and enhancer of split 2 (drosophila), CDKN2A - cyclin-dependent kinase inhibitor 2a, FOXN2 - forkhead box n2, PIM1 - pim-1 oncogene, WNT16 - wingless-type mmtv integration site family, member 16, IKZF1 - ikaros family zinc finger 1 (ikaros), NBL1 - neuroblastoma 1, dan family bmp antagonist, CHRDL1 - chordin-like 1, PDLIM1 - pdz and lim domain 1, NDP - norrie disease (pseudoglioma), HIST1H2AI - histone cluster 1, h2ai]
GO:0007166	cell surface receptor signaling pathway	4.55E-04	7.87E-02	1.83	16219	1967	144	32	[SFRP1 - secreted frizzled-related protein 1, CSRNP1 - cysteine-serine-rich nuclear protein 1, NFKBIA - nuclear factor of kappa light polypeptide gene enhancer in b-cells inhibitor, alpha, MICB - mhc class i polypeptide-related sequence b, ALOX5 - arachidonate 5-lipoxygenase, TIPARP - tcdd-inducible poly(adp-ribose) polymerase, HSPB1 - heat shock 27kda protein 1, LEPR - leptin receptor, AES - amino-terminal enhancer of split, TLE2 - transducin-like enhancer of split 2 (e(sp1) homolog, drosophila), CRLF1 - cytokine receptor-like factor 1, IL8 - interleukin 8, UBB - ubiquitin b, UBC - ubiquitin c, PIM1 - pim-1 oncogene, WNT16 - wingless-type mmtv integration site family, member 16, OSTN - osteocrin, MYC - v-myc avian myelocytomatosis viral oncogene homolog, LMNB1 - lamin b1, CHRDL1 - chordin-like 1, CFD - complement factor d (adipsin), ACTB - actin, beta, STMN1 - stathmin 1, CXCL2 - chemokine (c-x-c motif) ligand 2, NDP - norrie disease (pseudoglioma), NR4A2 - nuclear receptor subfamily 4, group a, member 2, CXCL3 - chemokine (c-x-c motif) ligand 3, RAC3 - ras-related c3 botulinum toxin substrate 3 (rho family, small gtp binding protein rac3), ACVR1C - activin a receptor, type ic, EFS - embryonal fyn-associated substrate, TICAM1 - toll-like receptor adaptor molecule 1, EPHA6 - eph receptor a6]
GO:0009893	positive regulation of metabolic process	4.61E-04	7.88E-02	1.58	16219	3340	144	47	[NFIX - nuclear factor i/x (ccaat-binding transcription factor), ADRB1 - adrenoceptor beta 1, SFRP1 - secreted frizzled-related protein 1, H1FX - h1 histone family, member x, H1F0 - h1 histone family, member 0, HSPA5 - heat shock 70kda protein 5 (glucose-regulated protein, 78kda), NFKBIA - nuclear factor of kappa light polypeptide gene enhancer in b-cells inhibitor, alpha, TCF15 - transcription factor 15 (basic helix-loop-helix), CSRNP1 - cysteine-serine-rich nuclear protein 1, HIST1H1C - histone cluster 1, h1c, HIST1H1E - histone cluster 1, h1e, ATF3 - activating transcription factor 3, TIPARP - tcdd-inducible poly(adp-ribose) polymerase, GADD45G - growth arrest and dna-damage-inducible, gamma, LEPR - leptin receptor, CRLF1 - cytokine receptor-like factor 1, JUND - jun d proto-oncogene, ZC3HAV1 - zinc finger ccch-type, antiviral 1, SOX4 - sry (sex determining region y)-box 4, ARRDC3 - arrestin domain containing 3, IL8 - interleukin 8, PDE2A - phosphodiesterase 2a, cgmp-stimulated, GPD1 - glycerol-3-phosphate dehydrogenase 1 (soluble), UBB - ubiquitin b, NR1D1 - nuclear receptor subfamily 1, group d, member 1, FOXD1 - forkhead box d1, HNRNPC - heterogeneous nuclear ribonucleoprotein c (c1/c2), UBC - ubiquitin c, CCND2 - cyclin d2, OPN3 - opsin 3, PIM1 - pim-1 oncogene, ZEB2-AS1 - zeb2 antisense rna 1, WNT16 - wingless-type mmtv integration site family, member 16, TBX1 - t-box 1, EEF1A2 - eukaryotic translation elongation factor 1 alpha 2, CFLAR - casp8 and fadd-like apoptosis regulator, HRH1 - histamine receptor h1, MYC - v-myc avian myelocytomatosis viral oncogene homolog, ACTA1 - actin, alpha 1, skeletal muscle, PDLIM1 - pdz and lim domain 1, ACTB - actin, beta, NDP - norrie disease (pseudoglioma), NR4A2 - nuclear receptor subfamily 4, group a, member 2, TNFAIP8L3 - tumor necrosis factor, alpha-induced protein 8-like 3, ACVR1C - activin a receptor, type ic, TICAM1 - toll-like receptor adaptor molecule 1, EPHA6 - eph receptor a6]
GO:1904612	response to 2,3,7,8-tetrachlorodibenzodioxine	4.64E-04	7.85E-02	56.32	16219	4	144	2	[PDE2A - phosphodiesterase 2a, cgmp-stimulated, TIPARP - tcdd-inducible poly(adp-ribose) polymerase]
GO:0045638	negative regulation of myeloid cell differentiation	4.76E-04	7.96E-02	7.71	16219	73	144	5	[HIST1H4C - histone cluster 1, h4c, SFRP1 - secreted frizzled-related protein 1, NFKBIA - nuclear factor of kappa light polypeptide gene enhancer in b-cells inhibitor, alpha, MYC - v-myc avian myelocytomatosis viral oncogene homolog, HIST1H4K - histone cluster 1, h4k]

GO:0090288	negative regulation of cellular response to growth factor stimulus	4.87E-04	8.05E-02	5.05	16219	156	144	7	[SFRP1 - secreted frizzled-related protein 1, HSPA5 - heat shock 70kda protein 5 (glucose-regulated protein, 78kda), CFLAR - casp8 and fadd-like apoptosis regulator, UBB - ubiquitin b, UBC - ubiquitin c, NBL1 - neuroblastoma 1, dan family bmp antagonist, CHRDL1 - chordin-like 1]
GO:0019219	regulation of nucleobase-containing compound metabolic process	4.91E-04	8.04E-02	1.54	16219	3736	144	51	[SFRP1 - secreted frizzled-related protein 1, H1FX - h1 histone family, member x, ATF3 - activating transcription factor 3, MDFI - myod family inhibitor, AES - amino-terminal enhancer of split, JUND - jun d proto-oncogene, ZC3HAV1 - zinc finger ccch-type, antiviral 1, GPD1 - glycerol-3-phosphate dehydrogenase 1 (soluble), UBB - ubiquitin b, UBC - ubiquitin c, CRYM - crystallin, mu, HIST1H4K - histone cluster 1, h4k, TBX1 - t-box 1, HIST1H4C - histone cluster 1, h4c, CFLAR - casp8 and fadd-like apoptosis regulator, KCNIP3 - kv channel interacting protein 3, calsenilin, TCERG1L - transcription elongation regulator 1-like, MYC - v-myc avian myelocytomatosis viral oncogene homolog, MXD1 - max dimerization protein 1, NR4A2 - nuclear receptor subfamily 4, group a, member 2, KLF9 - kruppel-like factor 9, TICAM1 - toll-like receptor adaptor molecule 1, NFIX - nuclear factor i/x (ccaat-binding transcription factor), H1F0 - h1 histone family, member 0, HSPA5 - heat shock 70kda protein 5 (glucose-regulated protein, 78kda), NFKBIA - nuclear factor of kappa light polypeptide gene enhancer in b-cells inhibitor, alpha, TCF15 - transcription factor 15 (basic helix-loop-helix), CSRNP1 - cysteine-serine-rich nuclear protein 1, HIST1H1C - histone cluster 1, h1c, HIST1H1E - histone cluster 1, h1e, HSPB1 - heat shock 27kda protein 1, HIST1H2AD - histone cluster 1, h2ad, HMX1 - h6 family homeobox 1, INSM1 - insulinoma-associated 1, TLE2 - transducin-like enhancer of split 2 (e(sp1) homolog, drosophila), H3F3B - h3 histone, family 3b (h3.3b), SOX4 - sry (sex determining region y)-box 4, PDE2A - phosphodiesterase 2a, cgmp-stimulated, NR1D1 - nuclear receptor subfamily 1, group d, member 1, FOXD1 - forkhead box d1, HNRNPC - heterogeneous nuclear ribonucleoprotein c (c1/c2), HES2 - hairy and enhancer of split 2 (drosophila), CDKN2A - cyclin-dependent kinase inhibitor 2a, FOXN2 - forkhead box n2, PIM1 - pim-1 oncogene, IKZF1 - ikaros family zinc finger 1 (ikaros), NBL1 - neuroblastoma 1, dan family bmp antagonist, CHRDL1 - chordin-like 1, PDLIM1 - pdz and lim domain 1, NDP - norrie disease (pseudoglioma), HIST1H2AI - histone cluster 1, h2ai]
GO:0071216	cellular response to biotic stimulus	5.06E-04	8.19E-02	5.02	16219	157	144	7	[HSPA5 - heat shock 70kda protein 5 (glucose-regulated protein, 78kda), IL8 - interleukin 8, CXCL2 - chemokine (c-x-c motif) ligand 2, CXCL3 - chemokine (c-x-c motif) ligand 3, NR1D1 - nuclear receptor subfamily 1, group d, member 1, TICAM1 - toll-like receptor adaptor molecule 1, TBXA2R - thromboxane a2 receptor]
GO:0006338	chromatin remodeling	5.45E-04	8.74E-02	4.96	16219	159	144	7	[H3F3B - h3 histone, family 3b (h3.3b), HIST1H4C - histone cluster 1, h4c, ACTB - actin, beta, HNRNPC - heterogeneous nuclear ribonucleoprotein c (c1/c2), CDKN2A - cyclin-dependent kinase inhibitor 2a, MYC - v-myc avian myelocytomatosis viral oncogene homolog, HIST1H4K - histone cluster 1, h4k]
GO:0051716	cellular response to stimulus	6.08E-04	9.65E-02	1.67	16219	2628	144	39	[SFRP1 - secreted frizzled-related protein 1, HSPA5 - heat shock 70kda protein 5 (glucose-regulated protein, 78kda), NFKBIA - nuclear factor of kappa light polypeptide gene enhancer in b-cells inhibitor, alpha, ATF3 - activating transcription factor 3, TIPARP - tcdd-inducible poly(adp-ribose) polymerase, HSPB1 - heat shock 27kda protein 1, MSRA - methionine sulfoxide reductase a, YWHAE - tyrosine 3-monooxygenase/tryptophan 5-monooxygenase activation protein, epsilon polypeptide, TUBA1B - tubulin, alpha 1b, AES - amino-terminal enhancer of split, JUND - jun d proto-oncogene, SOX4 - sry (sex determining region y)-box 4, IL8 - interleukin 8, PDE2A - phosphodiesterase 2a, cgmp-stimulated, UBB - ubiquitin b, GPD1 - glycerol-3-phosphate dehydrogenase 1 (soluble), NR1D1 - nuclear receptor subfamily 1, group d, member 1, UBC - ubiquitin c, CDKN2A - cyclin-dependent kinase inhibitor 2a, OPN3 - opsin 3, HIST1H4K - histone cluster 1, h4k, TBX1 - t-box 1, WNT16 - wingless-type mmtv integration site family, member 16, MGARP - mitochondria-localized glutamic acid-rich protein, HIST1H4C - histone cluster 1, h4c, CFLAR - casp8 and fadd-like apoptosis regulator, HRH1 - histamine receptor h1, MYC - v-myc avian myelocytomatosis viral oncogene homolog, FBXO32 - f-box protein 32, TBXA2R - thromboxane a2 receptor, ACTA1 - actin, alpha 1, skeletal muscle, ACTB - actin, beta, CXCL2 - chemokine (c-x-c motif) ligand 2, NR4A2 - nuclear receptor subfamily 4, group a, member 2, KLF9 - kruppel-like factor 9, CXCL3 - chemokine (c-x-c motif) ligand 3, NET1 - neuroepithelial cell transforming 1, ACVR1C - activin a receptor, type ic, TICAM1 - toll-like receptor adaptor molecule 1]
GO:0010941	regulation of cell death	6.47E-04	1.02E-01	1.96	16219	1495	144	26	[SFRP1 - secreted frizzled-related protein 1, HSPA5 - heat shock 70kda protein 5 (glucose-regulated protein, 78kda), NFKBIA - nuclear factor of kappa light polypeptide gene enhancer in b-cells inhibitor, alpha, ATF3 - activating transcription factor 3, HSPB1 - heat shock 27kda protein 1, GADD45G - growth arrest and dna-damage-inducible, gamma, YWHAE - tyrosine 3-monooxygenase/tryptophan 5-monooxygenase activation protein, epsilon polypeptide, AES - amino-terminal enhancer of split, CRLF1 - cytokine receptor-like factor 1, SOX4 - sry (sex determining region y)-box 4, ARMCX5-GPRASP2 - armcx5-gprasp2 readthrough, UBB - ubiquitin b, UBC - ubiquitin c, CDKN2A - cyclin-dependent kinase inhibitor 2a, PIM1 - pim-1 oncogene, CCND2 - cyclin d2, OPN3 - opsin 3, TBX1 - t-box 1, WNT16 - wingless-type mmtv integration site family, member 16, EEF1A2 - eukaryotic translation elongation factor 1 alpha 2, CFLAR - casp8 and fadd-like apoptosis regulator, MYC - v-myc avian myelocytomatosis viral oncogene homolog, TNFAIP8L3 - tumor necrosis factor, alpha-induced protein 8-like 3, NR4A2 - nuclear receptor subfamily 4, group a, member 2, NET1 - neuroepithelial cell transforming 1, ACVR1C - activin a receptor, type ic]
GO:0010628	positive regulation of gene expression	6.77E-04	1.05E-01	1.81	16219	1925	144	31	[NFIX - nuclear factor i/x (ccaat-binding transcription factor), SFRP1 - secreted frizzled-related protein 1, H1FX - h1 histone family, member x, TCF15 - transcription factor 15 (basic helix-loop-helix), CSRNP1 - cysteine-serine-rich nuclear protein 1, H1F0 - h1 histone family, member 0, HSPA5 - heat shock 70kda protein 5 (glucose-regulated protein, 78kda), NFKBIA - nuclear factor of kappa light polypeptide gene enhancer in b-cells inhibitor, alpha, HIST1H1C - histone cluster 1, h1c, HIST1H1E - histone cluster 1, h1e, ATF3 - activating transcription factor 3, JUND - jun d proto-oncogene, SOX4 - sry (sex determining region y)-box 4, IL8 - interleukin 8, PDE2A - phosphodiesterase 2a, cgmp-stimulated, UBB - ubiquitin b, NR1D1 - nuclear receptor subfamily 1, group d, member 1, FOXD1 - forkhead box d1, HNRNPC - heterogeneous nuclear ribonucleoprotein c (c1/c2), UBC - ubiquitin c, PIM1 - pim-1 oncogene, ZEB2-AS1 - zeb2 antisense rna 1, TBX1 - t-box 1, WNT16 - wingless-type mmtv integration site family, member 16, MYC - v-myc avian myelocytomatosis viral oncogene homolog, ACTA1 - actin, alpha 1, skeletal muscle, PDLIM1 - pdz and lim domain 1, ACTB - actin, beta, NDP - norrie disease (pseudoglioma), NR4A2 - nuclear receptor subfamily 4, group a, member 2, TICAM1 - toll-like receptor adaptor molecule 1]
GO:0048869	cellular developmental process	6.82E-04	1.05E-01	1.69	16219	2460	144	37	[ADRB1 - adrenoceptor beta 1, SFRP1 - secreted frizzled-related protein 1, TCF15 - transcription factor 15 (basic helix-loop-helix), HSPA5 - heat shock 70kda protein 5 (glucose-regulated protein, 78kda), ATF3 - activating transcription factor 3, MDFI - myod family inhibitor, TIPARP - tcdd-inducible poly(adp-ribose) polymerase, GADD45G - growth arrest and dna-damage-inducible, gamma, LEPR - leptin receptor, INSM1 - insulinoma-associated 1, DUSP10 - dual specificity phosphatase 10, H3F3B - h3 histone, family 3b (h3.3b), JUND - jun d proto-oncogene, SOX4 - sry (sex determining region y)-box 4, PDE2A - phosphodiesterase 2a, cgmp-stimulated, UBB - ubiquitin b, NR1D1 - nuclear receptor subfamily 1, group d, member 1, FOXD1 - forkhead box d1, HNRNPC - heterogeneous nuclear ribonucleoprotein c (c1/c2), CDKN2A - cyclin-dependent kinase inhibitor 2a, FOXN2 - forkhead box n2, OPN3 - opsin 3, TBX1 - t-box 1, WNT16 - wingless-type mmtv integration site family, member 16, IKZF1 - ikaros family zinc finger 1 (ikaros), OSTN - osteocrin, BCAP29 - b-cell receptor-associated protein 29, NBL1 - neuroblastoma 1, dan family bmp antagonist, CHRDL1 - chordin-like 1, RND1 - rho family gtpase 1, ACTA1 - actin, alpha 1, skeletal muscle, SPOCK1 - sparc/osteonectin, cwcv and kazal-like domains proteoglycan (testican) 1, ACTB - actin, beta, STMN1 - stathmin 1, NR4A2 - nuclear receptor subfamily 4, group a, member 2, RAC3 - ras-related c3 botulinum toxin substrate 3 (rho family, small gtp binding protein rac3), ACVR1C - activin a receptor, type ic]
GO:0060548	negative regulation of cell death	6.93E-04	1.06E-01	2.33	16219	869	144	18	[TBX1 - t-box 1, WNT16 - wingless-type mmtv integration site family, member 16, SFRP1 - secreted frizzled-related protein 1, HSPA5 - heat shock 70kda protein 5 (glucose-regulated protein, 78kda), NFKBIA - nuclear factor of kappa light polypeptide gene enhancer in b-cells inhibitor, alpha, CFLAR - casp8 and fadd-like apoptosis regulator, HSPB1 - heat shock 27kda protein 1, YWHAE - tyrosine 3-monooxygenase/tryptophan 5-monooxygenase activation protein, epsilon polypeptide, MYC - v-myc avian myelocytomatosis viral oncogene homolog, CRLF1 - cytokine receptor-like factor 1, SOX4 - sry (sex determining region y)-box 4, ARMCX5-GPRASP2 - armcx5-gprasp2 readthrough, NR4A2 - nuclear receptor subfamily 4, group a, member 2, UBB - ubiquitin b, UBC - ubiquitin c, PIM1 - pim-1 oncogene, CCND2 - cyclin d2, OPN3 - opsin 3]
GO:0050896	response to stimulus	7.21E-04	1.09E-01	1.45	16219	4494	144	58	[ADRB1 - adrenoceptor beta 1, SFRP1 - secreted frizzled-related protein 1, SLC25A25 - solute carrier family 25 (mitochondrial carrier; phosphate carrier), member 25, SLC18A2 - solute carrier family 18 (vesicular monoamine transporter), member 2, ATF3 - activating transcription factor 3, FTH1 - ferritin, heavy polypeptide 1, TUBA1B - tubulin, alpha 1b, AES - amino-terminal enhancer of split, CERS6 - ceramide synthase 6, HIST1H2BC - histone cluster 1, h2bc, JUND - jun d proto-oncogene, ZC3HAV1 - zinc finger ccch-type, antiviral 1, IL8 - interleukin 8, GPD1 - glycerol-3-phosphate dehydrogenase 1 (soluble), UBB - ubiquitin b, UBC - ubiquitin c, OPN3 - opsin 3, HIST1H4K - histone cluster 1, h4k, TBX1 - t-box 1, MGARP - mitochondria-localized glutamic acid-rich protein, HIST1H4C - histone cluster 1, h4c, EEF1A2 - eukaryotic translation elongation factor 1 alpha 2, RGS10 - regulator of g-protein signaling 10, CFLAR - casp8 and fadd-like apoptosis regulator, HRH1 - histamine receptor h1, MYC - v-myc avian myelocytomatosis viral oncogene homolog, FBXO32 - f-box protein 32, TBXA2R - thromboxane a2 receptor, ACTA1 - actin, alpha 1, skeletal muscle, ACTB - actin, beta, STMN1 - stathmin 1, NR4A2 - nuclear receptor subfamily 4, group a, member 2, KLF9 - kruppel-like factor 9, NET1 - neuroepithelial cell transforming 1, ACVR1C - activin a receptor, type ic, TICAM1 - toll-like receptor adaptor molecule 1, HMGN2 - high mobility group nucleosomal binding domain 2, HSPA5 - heat shock 70kda protein 5 (glucose-regulated protein, 78kda), NFKBIA - nuclear factor of kappa light polypeptide gene enhancer in b-cells inhibitor, alpha, MICB - mhc class i polypeptide-related sequence b, ALOX5 - arachidonate 5-lipoxygenase, TIPARP - tcdd-inducible poly(adp-ribose) polymerase, HSPB1 - heat shock 27kda protein 1, LEPR - leptin receptor, MSRA - methionine sulfoxide reductase a, YWHAE - tyrosine 3-monooxygenase/tryptophan 5-monooxygenase activation protein, epsilon polypeptide, DUSP10 - dual specificity phosphatase 10, OR2AK2 - olfactory receptor, family 2, subfamily ak, member 2, SOX4 - sry (sex determining region y)-box 4, SLC30A3 - solute carrier family 30 (zinc transporter), member 3, PDE2A - phosphodiesterase 2a, cgmp-stimulated, NR1D1 - nuclear receptor subfamily 1, group d, member 1, CDKN2A - cyclin-dependent kinase inhibitor 2a, WNT16 - wingless-type mmtv integration site family, member 16, CFD - complement factor d (adipsin), PDLIM1 - pdz and lim domain 1, CXCL2 - chemokine (c-x-c motif) ligand 2, CXCL3 - chemokine (c-x-c motif) ligand 3]
GO:0060613	fat pad development	7.69E-04	1.15E-01	45.05	16219	5	144	2	[ARRDC3 - arrestin domain containing 3, UBB - ubiquitin b]

GO:0060255	regulation of macromolecule metabolic process	8.02E-04	1.18E-01	1.37	16219	5653	144	69	[SFRP1 - secreted frizzled-related protein 1, H1FX - h1 histone family, member x, ATF3 - activating transcription factor 3, MDFI - myod family inhibitor, AES - amino-terminal enhancer of split, JUND - jun d proto-oncogene, ZC3HAV1 - zinc finger ccch-type, antiviral 1, ARRDC3 - arrestin domain containing 3, IL8 - interleukin 8, UBB - ubiquitin b, UBC - ubiquitin c, CRYM - crystallin, mu, ZEB2-AS1 - zeb2 antisense rna 1, HIST1H4K - histone cluster 1, h4k, TBX1 - t-box 1, HIST1H4C - histone cluster 1, h4c, EEF1A2 - eukaryotic translation elongation factor 1 alpha 2, CFLAR - casp8 and fadd-like apoptosis regulator, KCNIP3 - kv channel interacting protein 3, calsenilin, TCERG1L - transcription elongation regulator 1-like, MYC - v-myc avian myelocytomatosis viral oncogene homolog, MXD1 - max dimerization protein 1, ACTA1 - actin, alpha 1, skeletal muscle, ACTB - actin, beta, NR4A2 - nuclear receptor subfamily 4, group a, member 2, TNFAIP8L3 - tumor necrosis factor, alpha-induced protein 8-like 3, KLF9 - kruppel-like factor 9, ACVR1C - activin a receptor, type ic, TICAM1 - toll-like receptor adaptor molecule 1, NFIX - nuclear factor i/x (ccaat-binding transcription factor), DUSP16 - dual specificity phosphatase 16, H1F0 - h1 histone family, member 0, HSPA5 - heat shock 70kda protein 5 (glucose-regulated protein, 78kda), NFKBIA - nuclear factor of kappa light polypeptide gene enhancer in b-cells inhibitor, alpha, TCF15 - transcription factor 15 (basic helix-loop-helix), CSRNP1 - cysteine-serine-rich nuclear protein 1, HIST1H1C - histone cluster 1, h1c, HIST1H1E - histone cluster 1, h1e, TIPARP - tcdd-inducible poly(adp-ribose) polymerase, HSPB1 - heat shock 27kda protein 1, GADD45G - growth arrest and dna-damage-inducible, gamma, HIST1H2AD - histone cluster 1, h2ad, LEPR - leptin receptor, YWHAE - tyrosine 3-monooxygenase/tryptophan 5-monooxygenase activation protein, epsilon polypeptide, HMX1 - h6 family homeobox 1, INSM1 - insulinoma-associated 1, DUSP10 - dual specificity phosphatase 10, TLE2 - transducin-like enhancer of split 2 (e(sp1) homolog, drosophila), H3F3B - h3 histone, family 3b (h3.3b), CRLF1 - cytokine receptor-like factor 1, SOX4 - sry (sex determining region y)-box 4, PDE2A - phosphodiesterase 2a, cgmp-stimulated, NR1D1 - nuclear receptor subfamily 1, group d, member 1, FOXD1 - forkhead box d1, HNRNPC - heterogeneous nuclear ribonucleoprotein c (c1/c2), HES2 - hairy and enhancer of split 2 (drosophila), CDKN2A - cyclin-dependent kinase inhibitor 2a, FOXN2 - forkhead box n2, CCND2 - cyclin d2, PPP1R14A - protein phosphatase 1, regulatory (inhibitor) subunit 14a, PIM1 - pim-1 oncogene, WNT16 - wingless-type mmtv integration site family, member 16, IKZF1 - ikaros family zinc finger 1 (ikaros), NBL1 - neuroblastoma 1, dan family bmp antagonist, CHRDL1 - chordin-like 1, SPOCK1 - sparc/osteonectin, cwcv and kazal-like domains proteoglycan (testican) 1, PDLIM1 - pdz and lim domain 1, NDP - norrie disease (pseudoglioma), HIST1H2AI - histone cluster 1, h2ai]
GO:2000112	regulation of cellular macromolecule biosynthetic process	8.03E-04	1.18E-01	1.53	16219	3616	144	49	[SFRP1 - secreted frizzled-related protein 1, H1FX - h1 histone family, member x, ATF3 - activating transcription factor 3, MDFI - myod family inhibitor, AES - amino-terminal enhancer of split, JUND - jun d proto-oncogene, UBB - ubiquitin b, UBC - ubiquitin c, CRYM - crystallin, mu, HIST1H4K - histone cluster 1, h4k, TBX1 - t-box 1, HIST1H4C - histone cluster 1, h4c, CFLAR - casp8 and fadd-like apoptosis regulator, KCNIP3 - kv channel interacting protein 3, calsenilin, TCERG1L - transcription elongation regulator 1-like, MYC - v-myc avian myelocytomatosis viral oncogene homolog, MXD1 - max dimerization protein 1, NR4A2 - nuclear receptor subfamily 4, group a, member 2, KLF9 - kruppel-like factor 9, TICAM1 - toll-like receptor adaptor molecule 1, NFIX - nuclear factor i/x (ccaat-binding transcription factor), H1F0 - h1 histone family, member 0, HSPA5 - heat shock 70kda protein 5 (glucose-regulated protein, 78kda), NFKBIA - nuclear factor of kappa light polypeptide gene enhancer in b-cells inhibitor, alpha, TCF15 - transcription factor 15 (basic helix-loop-helix), CSRNP1 - cysteine-serine-rich nuclear protein 1, HIST1H1C - histone cluster 1, h1c, HIST1H1E - histone cluster 1, h1e, HSPB1 - heat shock 27kda protein 1, HIST1H2AD - histone cluster 1, h2ad, HMX1 - h6 family homeobox 1, INSM1 - insulinoma-associated 1, TLE2 - transducin-like enhancer of split 2 (e(sp1) homolog, drosophila), H3F3B - h3 histone, family 3b (h3.3b), SOX4 - sry (sex determining region y)-box 4, PDE2A - phosphodiesterase 2a, cgmp-stimulated, NR1D1 - nuclear receptor subfamily 1, group d, member 1, FOXD1 - forkhead box d1, HNRNPC - heterogeneous nuclear ribonucleoprotein c (c1/c2), HES2 - hairy and enhancer of split 2 (drosophila), CDKN2A - cyclin-dependent kinase inhibitor 2a, FOXN2 - forkhead box n2, PIM1 - pim-1 oncogene, IKZF1 - ikaros family zinc finger 1 (ikaros), NBL1 - neuroblastoma 1, dan family bmp antagonist, CHRDL1 - chordin-like 1, PDLIM1 - pdz and lim domain 1, NDP - norrie disease (pseudoglioma), HIST1H2AI - histone cluster 1, h2ai]
GO:2001251	negative regulation of chromosome organization	8.35E-04	1.21E-01	5.45	16219	124	144	6	[H3F3B - h3 histone, family 3b (h3.3b), H1FX - h1 histone family, member x, H1F0 - h1 histone family, member 0, HIST1H1C - histone cluster 1, h1c, HIST1H1E - histone cluster 1, h1e, HNRNPC - heterogeneous nuclear ribonucleoprotein c (c1/c2)]
GO:0043065	positive regulation of apoptotic process	8.44E-04	1.21E-01	2.65	16219	595	144	14	[SFRP1 - secreted frizzled-related protein 1, EEF1A2 - eukaryotic translation elongation factor 1 alpha 2, CFLAR - casp8 and fadd-like apoptosis regulator, ATF3 - activating transcription factor 3, GADD45G - growth arrest and dna-damage-inducible, gamma, YWHAE - tyrosine 3-monooxygenase/tryptophan 5-monooxygenase activation protein, epsilon polypeptide, MYC - v-myc avian myelocytomatosis viral oncogene homolog, AES - amino-terminal enhancer of split, SOX4 - sry (sex determining region y)-box 4, UBB - ubiquitin b, UBC - ubiquitin c, NET1 - neuroepithelial cell transforming 1, ACVR1C - activin a receptor, type ic, CDKN2A - cyclin-dependent kinase inhibitor 2a]
GO:0097366	response to bronchodilator	8.56E-04	1.22E-01	9.39	16219	48	144	4	[SLC18A2 - solute carrier family 18 (vesicular monoamine transporter), member 2, RGS10 - regulator of g-protein signaling 10, CFLAR - casp8 and fadd-like apoptosis regulator, NR4A2 - nuclear receptor subfamily 4, group a, member 2]
GO:0071222	cellular response to lipopolysaccharide	8.71E-04	1.23E-01	5.41	16219	125	144	6	[IL8 - interleukin 8, CXCL2 - chemokine (c-x-c motif) ligand 2, CXCL3 - chemokine (c-x-c motif) ligand 3, NR1D1 - nuclear receptor subfamily 1, group d, member 1, TICAM1 - toll-like receptor adaptor molecule 1, TBXA2R - thromboxane a2 receptor]
GO:0090101	negative regulation of transmembrane receptor protein serine/threonine kinase signaling pathway	9.08E-04	1.27E-01	5.36	16219	126	144	6	[SFRP1 - secreted frizzled-related protein 1, HSPA5 - heat shock 70kda protein 5 (glucose-regulated protein, 78kda), UBB - ubiquitin b, UBC - ubiquitin c, NBL1 - neuroblastoma 1, dan family bmp antagonist, CHRDL1 - chordin-like 1]
GO:0048871	multicellular organismal homeostasis	9.08E-04	1.26E-01	5.36	16219	126	144	6	[ADRB1 - adrenoceptor beta 1, ARRDC3 - arrestin domain containing 3, STMN1 - stathmin 1, UBB - ubiquitin b, NR1D1 - nuclear receptor subfamily 1, group d, member 1, LEPR - leptin receptor]
GO:0043068	positive regulation of programmed cell death	9.29E-04	1.27E-01	2.62	16219	601	144	14	[SFRP1 - secreted frizzled-related protein 1, EEF1A2 - eukaryotic translation elongation factor 1 alpha 2, CFLAR - casp8 and fadd-like apoptosis regulator, ATF3 - activating transcription factor 3, GADD45G - growth arrest and dna-damage-inducible, gamma, YWHAE - tyrosine 3-monooxygenase/tryptophan 5-monooxygenase activation protein, epsilon polypeptide, MYC - v-myc avian myelocytomatosis viral oncogene homolog, AES - amino-terminal enhancer of split, SOX4 - sry (sex determining region y)-box 4, UBB - ubiquitin b, UBC - ubiquitin c, NET1 - neuroepithelial cell transforming 1, ACVR1C - activin a receptor, type ic, CDKN2A - cyclin-dependent kinase inhibitor 2a]
GO:0010556	regulation of macromolecule biosynthetic process	9.79E-04	1.33E-01	1.51	16219	3647	144	49	[SFRP1 - secreted frizzled-related protein 1, H1FX - h1 histone family, member x, ATF3 - activating transcription factor 3, MDFI - myod family inhibitor, AES - amino-terminal enhancer of split, JUND - jun d proto-oncogene, UBB - ubiquitin b, UBC - ubiquitin c, CRYM - crystallin, mu, HIST1H4K - histone cluster 1, h4k, TBX1 - t-box 1, HIST1H4C - histone cluster 1, h4c, CFLAR - casp8 and fadd-like apoptosis regulator, KCNIP3 - kv channel interacting protein 3, calsenilin, TCERG1L - transcription elongation regulator 1-like, MYC - v-myc avian myelocytomatosis viral oncogene homolog, MXD1 - max dimerization protein 1, NR4A2 - nuclear receptor subfamily 4, group a, member 2, KLF9 - kruppel-like factor 9, TICAM1 - toll-like receptor adaptor molecule 1, NFIX - nuclear factor i/x (ccaat-binding transcription factor), H1F0 - h1 histone family, member 0, HSPA5 - heat shock 70kda protein 5 (glucose-regulated protein, 78kda), NFKBIA - nuclear factor of kappa light polypeptide gene enhancer in b-cells inhibitor, alpha, TCF15 - transcription factor 15 (basic helix-loop-helix), CSRNP1 - cysteine-serine-rich nuclear protein 1, HIST1H1C - histone cluster 1, h1c, HIST1H1E - histone cluster 1, h1e, HSPB1 - heat shock 27kda protein 1, HIST1H2AD - histone cluster 1, h2ad, HMX1 - h6 family homeobox 1, INSM1 - insulinoma-associated 1, TLE2 - transducin-like enhancer of split 2 (e(sp1) homolog, drosophila), H3F3B - h3 histone, family 3b (h3.3b), SOX4 - sry (sex determining region y)-box 4, PDE2A - phosphodiesterase 2a, cgmp-stimulated, NR1D1 - nuclear receptor subfamily 1, group d, member 1, FOXD1 - forkhead box d1, HNRNPC - heterogeneous nuclear ribonucleoprotein c (c1/c2), HES2 - hairy and enhancer of split 2 (drosophila), CDKN2A - cyclin-dependent kinase inhibitor 2a, FOXN2 - forkhead box n2, PIM1 - pim-1 oncogene, IKZF1 - ikaros family zinc finger 1 (ikaros), NBL1 - neuroblastoma 1, dan family bmp antagonist, CHRDL1 - chordin-like 1, PDLIM1 - pdz and lim domain 1, NDP - norrie disease (pseudoglioma), HIST1H2AI - histone cluster 1, h2ai]
