# Supplementary material for: Effects of microgravity on human iPSC-derived neural organoids on the International Space Station
Source: Stem Cells Transl Med. 2024 Oct 23;13(12):1186–97. doi: 10.1093/stcltm/szae070 (PMC11631337; doi:10.1093/stcltm/szae070)
Supplement: szae070_suppl_Supplementary_Materials [file szae070_suppl_supplementary_materials.zip › R1Table S3b Dopaminergic-Gene ontology labels for differentiatlly expressed transcripts.pdf]

P-value' is the enrichment p-value computed according to the mHG or HG model; 'FDR q-value' is the correction of the above p-value for multiple testing using the Benjamini and Hochberg (1995) method. Namely, for the ith term (ranked according to p-value) the FDR q-value is (p-value * number of GO terms) / i; Enrichment (N, B, n, b) is defined as follows: N - is the total number of genes, B - is the total number of genes associated with a specific GO term, n - is the number of genes in the top of the user's input list or in the target set when appropriate,b - is the number of genes in the intersection; Enrichment = (b/n) / (B/N)

DOPAMINERGIC ORGANOIDS: GENE ONTOLOGY PROCESS TERMS FOR TRANSCRIPTS WITH HIGHER EXPRESSION IN LEO

GO Term	Description	P-value	FDR q-value	Enrich-ment	N	B	n	b	Genes
GO:0007267	cell-cell signaling	1.23E-06	1.88E-02	2.97	16227	531	257	25	[NMUR2 - neuromedin u receptor 2, EGR3 - early growth response 3, DLGAP2 - discs, large (drosophila) homolog-associated protein 2, SLC18A3 - solute carrier family 18 (vesicular acetylcholine transporter), member 3, AVP - arginine vasopressin, PCSK1 - proprotein convertase subtilisin/kexin type 1, GLRA3 - glycine receptor, alpha 3, DRD2 - dopamine receptor d2, NPTX1 - neuronal pentraxin i, HAP1 - huntingtin-associated protein 1, POMC - proopiomelanocortin, GJC3 - gap junction protein, gamma 3, 30.2kda, NRP1 - neuropilin 1, SLC17A6 - solute carrier family 17 (vesicular glutamate transporter), member 6, P2RX2 - purinergic receptor p2x, ligand-gated ion channel, 2, GRIN2C - glutamate receptor, ionotropic, n-methyl d-aspartate 2c, GHRH - growth hormone releasing hormone, GUCA1B - guanylate cyclase activator 1b (retina), SLC5A7 - solute carrier family 5 (sodium/choline cotransporter), member 7, CHRNA5 - cholinergic receptor, nicotinic, alpha 5 (neuronal), RYR2 - ryanodine receptor 2 (cardiac), FGFR3 - fibroblast growth factor receptor 3, CHRNB3 - cholinergic receptor, nicotinic, beta 3 (neuronal), INHA - inhibin, alpha, CHRNE - cholinergic receptor, nicotinic, epsilon (muscle)]
GO:0023052	signaling	1.29E-05	9.79E-02	2.6	16227	607	257	25	[NMUR2 - neuromedin u receptor 2, EGR3 - early growth response 3, DLGAP2 - discs, large (drosophila) homolog-associated protein 2, SLC18A3 - solute carrier family 18 (vesicular acetylcholine transporter), member 3, AVP - arginine vasopressin, PCSK1 - proprotein convertase subtilisin/kexin type 1, GLRA3 - glycine receptor, alpha 3, DRD2 - dopamine receptor d2, NPTX1 - neuronal pentraxin i, HAP1 - huntingtin-associated protein 1, POMC - proopiomelanocortin, GJC3 - gap junction protein, gamma 3, 30.2kda, NRP1 - neuropilin 1, SLC17A6 - solute carrier family 17 (vesicular glutamate transporter), member 6, P2RX2 - purinergic receptor p2x, ligand-gated ion channel, 2, GRIN2C - glutamate receptor, ionotropic, n-methyl d-aspartate 2c, GHRH - growth hormone releasing hormone, GUCA1B - guanylate cyclase activator 1b (retina), SLC5A7 - solute carrier family 5 (sodium/choline cotransporter), member 7, CHRNA5 - cholinergic receptor, nicotinic, alpha 5 (neuronal), RYR2 - ryanodine receptor 2 (cardiac), FGFR3 - fibroblast growth factor receptor 3, CHRNB3 - cholinergic receptor, nicotinic, beta 3 (neuronal), INHA - inhibin, alpha, CHRNE - cholinergic receptor, nicotinic, epsilon (muscle)]
GO:0031018	endocrine pancreas development	1.58E-05	8.04E-02	15.03	16227	21	257	5	[ONECUT1 - one cut homeobox 1, MNX1 - motor neuron and pancreas homeobox 1, GIP - gastric inhibitory polypeptide, NEUROD1 - neuronal differentiation 1, ONECUT2 - one cut homeobox 2]
GO:0007218	neuropeptide signaling pathway	2.31E-05	8.81E-02	5.86	16227	97	257	9	[NMUR2 - neuromedin u receptor 2, NXPH4 - neurexophilin 4, NTS - neurotensin, POMC - proopiomelanocortin, PPY - pancreatic polypeptide, SORCS3 - sortilin-related vps10 domain containing receptor 3, NPPA - natriuretic peptide a, NPPB - natriuretic peptide b, GLRA3 - glycine receptor, alpha 3]
GO:0060079	excitatory postsynaptic potential	3.48E-05	1.06E-01	7.62	16227	58	257	7	[DRD2 - dopamine receptor d2, P2RX2 - purinergic receptor p2x, ligand-gated ion channel, 2, CHRNA5 - cholinergic receptor, nicotinic, alpha 5 (neuronal), GRIN2C - glutamate receptor, ionotropic, n-methyl d-aspartate 2c, CHRNB3 - cholinergic receptor, nicotinic, beta 3 (neuronal), GLRA3 - glycine receptor, alpha 3, CHRNE - cholinergic receptor, nicotinic, epsilon (muscle)]
GO:0008217	regulation of blood pressure	6.17E-05	1.56E-01	4.64	16227	136	257	10	[NOS3 - nitric oxide synthase 3 (endothelial cell), DRD2 - dopamine receptor d2, POMC - proopiomelanocortin, ANPEP - alanyl (membrane) aminopeptidase, POSTN - periostin, osteoblast specific factor, AVP - arginine vasopressin, RENBP - renin binding protein, NPPA - natriuretic peptide a, NPPB - natriuretic peptide b, AVPR1B - arginine vasopressin receptor 1b]
GO:0003008	system process	6.89E-05	1.50E-01	1.95	16227	1198	257	37	[AVP - arginine vasopressin, NPPA - natriuretic peptide a, AVPR1B - arginine vasopressin receptor 1b, NPPB - natriuretic peptide b, GLRA3 - glycine receptor, alpha 3, MIP - major intrinsic protein of lens fiber, PRDM12 - pr domain containing 12, DRD2 - dopamine receptor d2, USH1C - usher syndrome 1c (autosomal recessive, severe), ABCA4 - atp-binding cassette, sub-family a (abc1), member 4, MYOC - myocilin, trabecular meshwork inducible glucocorticoid response, SLITRK6 - slit and ntrk-like family, member 6, CRYGD - crystallin, gamma d, MUC4 - mucin 4, cell surface associated, SMPX - small muscle protein, x-linked, ADCY8 - adenylate cyclase 8 (brain), RAX - retina and anterior neural fold homeobox, P2RX2 - purinergic receptor p2x, ligand-gated ion channel, 2, PAX3 - paired box 3, GRIN2C - glutamate receptor, ionotropic, n-methyl d-aspartate 2c, HRC - histidine rich calcium binding protein, PKD2L1 - polycystic kidney disease 2-like 1, GHSR - growth hormone secretagogue receptor, GUCA1B - guanylate cyclase activator 1b (retina), ESPNL - espin-like, GIP - gastric inhibitory polypeptide, NOS3 - nitric oxide synthase 3 (endothelial cell), CASP3 - caspase 3, apoptosis-related cysteine peptidase, CHRNA5 - cholinergic receptor, nicotinic, alpha 5 (neuronal), SORCS3 - sortilin-related vps10 domain containing receptor 3, RYR2 - ryanodine receptor 2 (cardiac), C2CD4B - c2 calcium-dependent domain containing 4b, THRB - thyroid hormone receptor, beta, TNNI1 - troponin i type 1 (skeletal, slow), VIP - vasoactive intestinal peptide, CHRNB3 - cholinergic receptor, nicotinic, beta 3 (neuronal), CHRNE - cholinergic receptor, nicotinic, epsilon (muscle)]
GO:0030252	growth hormone secretion	7.58E-05	1.44E-01	31.57	16227	6	257	3	[LTBP4 - latent transforming growth factor beta binding protein 4, GHRH - growth hormone releasing hormone, GHSR - growth hormone secretagogue receptor]
GO:0001952	regulation of cell-matrix adhesion	8.89E-05	1.50E-01	4.94	16227	115	257	9	[ONECUT1 - one cut homeobox 1, MYOC - myocilin, trabecular meshwork inducible glucocorticoid response, BST1 - bone marrow stromal cell antigen 1, APOD - apolipoprotein d, POSTN - periostin, osteoblast specific factor, SKAP1 - src kinase associated phosphoprotein 1, ACER2 - alkaline ceramidase 2, ONECUT2 - one cut homeobox 2, NRP1 - neuropilin 1]
GO:0007154	cell communication	1.05E-04	1.60E-01	2.2	16227	775	257	27	[NMUR2 - neuromedin u receptor 2, EGR3 - early growth response 3, DLGAP2 - discs, large (drosophila) homolog-associated protein 2, SLC18A3 - solute carrier family 18 (vesicular acetylcholine transporter), member 3, AVP - arginine vasopressin, PCSK1 - proprotein convertase subtilisin/kexin type 1, GLRA3 - glycine receptor, alpha 3, DRD2 - dopamine receptor d2, NPTX1 - neuronal pentraxin i, POMC - proopiomelanocortin, HAP1 - huntingtin-associated protein 1, GJC3 - gap junction protein, gamma 3, 30.2kda, NRP1 - neuropilin 1, SLC17A6 - solute carrier family 17 (vesicular glutamate transporter), member 6, IFI16 - interferon, gamma-inducible protein 16, P2RX2 - purinergic receptor p2x, ligand-gated ion channel, 2, GRIN2C - glutamate receptor, ionotropic, n-methyl d-aspartate 2c, POSTN - periostin, osteoblast specific factor, GHRH - growth hormone releasing hormone, GUCA1B - guanylate cyclase activator 1b (retina), SLC5A7 - solute carrier family 5 (sodium/choline cotransporter), member 7, CHRNA5 - cholinergic receptor, nicotinic, alpha 5 (neuronal), RYR2 - ryanodine receptor 2 (cardiac), CHRNB3 - cholinergic receptor, nicotinic, beta 3 (neuronal), FGFR3 - fibroblast growth factor receptor 3, INHA - inhibin, alpha, CHRNE - cholinergic receptor, nicotinic, epsilon (muscle)]
GO:0030072	peptide hormone secretion	1.48E-04	2.04E-01	7.43	16227	51	257	6	[LTBP4 - latent transforming growth factor beta binding protein 4, GHRH - growth hormone releasing hormone, VIP - vasoactive intestinal peptide, GHSR - growth hormone secretagogue receptor, CYB5R4 - cytochrome b5 reductase 4, NEUROD1 - neuronal differentiation 1]

GO:0098916	anterograde trans-synaptic signaling	2.09E-04	2.66E-01	3.07	16227	288	257	14	[SLC17A6 - solute carrier family 17 (vesicular glutamate transporter), member 6, EGR3 - early growth response 3, P2RX2 - purinergic receptor p2x, ligand-gated ion channel, 2, DLGAP2 - discs, large (drosophila) homolog-associated protein 2, SLC18A3 - solute carrier family 18 (vesicular acetylcholine transporter), member 3, GRIN2C - glutamate receptor, ionotropic, n-methyl d-aspartate 2c, GLRA3 - glycine receptor, alpha 3, NPTX1 - neuronal pentraxin i, DRD2 - dopamine receptor d2, SLC5A7 - solute carrier family 5 (sodium/choline cotransporter), member 7, HAP1 - huntingtin-associated protein 1, CHRNA5 - cholinergic receptor, nicotinic, alpha 5 (neuronal), CHRNB3 - cholinergic receptor, nicotinic, beta 3 (neuronal), CHRNE - cholinergic receptor, nicotinic, epsilon (muscle)]
GO:0007268	chemical synaptic transmission	2.09E-04	2.45E-01	3.07	16227	288	257	14	[SLC17A6 - solute carrier family 17 (vesicular glutamate transporter), member 6, EGR3 - early growth response 3, P2RX2 - purinergic receptor p2x, ligand-gated ion channel, 2, DLGAP2 - discs, large (drosophila) homolog-associated protein 2, SLC18A3 - solute carrier family 18 (vesicular acetylcholine transporter), member 3, GRIN2C - glutamate receptor, ionotropic, n-methyl d-aspartate 2c, GLRA3 - glycine receptor, alpha 3, NPTX1 - neuronal pentraxin i, DRD2 - dopamine receptor d2, SLC5A7 - solute carrier family 5 (sodium/choline cotransporter), member 7, HAP1 - huntingtin-associated protein 1, CHRNA5 - cholinergic receptor, nicotinic, alpha 5 (neuronal), CHRNB3 - cholinergic receptor, nicotinic, beta 3 (neuronal), CHRNE - cholinergic receptor, nicotinic, epsilon (muscle)]
GO:0044057	regulation of system process	2.30E-04	2.50E-01	2.44	16227	518	257	20	[NMUR2 - neuromedin u receptor 2, DLGAP2 - discs, large (drosophila) homolog-associated protein 2, HRC - histidine rich calcium binding protein, AVP - arginine vasopressin, NPPA - natriuretic peptide a, GHSR - growth hormone secretagogue receptor, AVPR1B - arginine vasopressin receptor 1b, NPPB - natriuretic peptide b, NEUROD1 - neuronal differentiation 1, DRD2 - dopamine receptor d2, NOS3 - nitric oxide synthase 3 (endothelial cell), NPTX1 - neuronal pentraxin i, ITGAX - integrin, alpha x (complement component 3 receptor 4 subunit), POMC - proopiomelanocortin, RYR2 - ryanodine receptor 2 (cardiac), MLIP - muscular lmna-interacting protein, THRB - thyroid hormone receptor, beta, TNNI1 - troponin i type 1 (skeletal, slow), VIP - vasoactive intestinal peptide, INHA - inhibin, alpha]
GO:0006801	superoxide metabolic process	2.76E-04	2.80E-01	8.53	16227	37	257	5	[NOS3 - nitric oxide synthase 3 (endothelial cell), NCF1 - neutrophil cytosolic factor 1, MPO - myeloperoxidase, NCF1B - neutrophil cytosolic factor 1b pseudogene, CYB5R4 - cytochrome b5 reductase 4]
GO:0002790	peptide secretion	3.19E-04	3.04E-01	4.68	16227	108	257	8	[LTBP4 - latent transforming growth factor beta binding protein 4, PPY - pancreatic polypeptide, RCN3 - reticulocalbin 3, ef-hand calcium binding domain, GHRH - growth hormone releasing hormone, VIP - vasoactive intestinal peptide, CYB5R4 - cytochrome b5 reductase 4, GHSR - growth hormone secretagogue receptor, NEUROD1 - neuronal differentiation 1]
GO:0007271	synaptic transmission, cholinergic	3.59E-04	3.22E-01	11.48	16227	22	257	4	[SLC5A7 - solute carrier family 5 (sodium/choline cotransporter), member 7, CHRNA5 - cholinergic receptor, nicotinic, alpha 5 (neuronal), CHRNB3 - cholinergic receptor, nicotinic, beta 3 (neuronal), CHRNE - cholinergic receptor, nicotinic, epsilon (muscle)]
GO:0032501	multicellular organismal process	3.59E-04	3.04E-01	1.48	16227	2899	257	68	[MPO - myeloperoxidase, PTCH2 - patched 2, RASIP1 - ras interacting protein 1, NPPA - natriuretic peptide a, NPPB - natriuretic peptide b, PRDM12 - pr domain containing 12, USH1C - usher syndrome 1c (autosomal recessive, severe), ABCA4 - atp-binding cassette, sub-family a (abc1), member 4, SLITRK6 - slit and ntrk-like family, member 6, KRT40 - keratin 40, LTK - leukocyte receptor tyrosine kinase, CRYGD - crystallin, gamma d, MUC4 - mucin 4, cell surface associated, NRP1 - neuropilin 1, RAX - retina and anterior neural fold homeobox, P2RX2 - purinergic receptor p2x, ligand-gated ion channel, 2, PAX3 - paired box 3, HRC - histidine rich calcium binding protein, PKD2L1 - polycystic kidney disease 2-like 1, GHSR - growth hormone secretagogue receptor, GUCA1B - guanylate cyclase activator 1b (retina), ESPNL - espin-like, GIP - gastric inhibitory polypeptide, NEUROD1 - neuronal differentiation 1, SLC5A7 - solute carrier family 5 (sodium/choline cotransporter), member 7, CASP3 - caspase 3, apoptosis-related cysteine peptidase, ALDH1A2 - aldehyde dehydrogenase 1 family, member a2, CHRNA5 - cholinergic receptor, nicotinic, alpha 5 (neuronal), SORCS3 - sortilin-related vps10 domain containing receptor 3, C2CD4B - c2 calcium-dependent domain containing 4b, THRB - thyroid hormone receptor, beta, CHRNB3 - cholinergic receptor, nicotinic, beta 3 (neuronal), FGFR3 - fibroblast growth factor receptor 3, CHRNE - cholinergic receptor, nicotinic, epsilon (muscle), HEYL - hairy/enhancer-of-split related with yrpw motif-like, SERPINA5 - serpin peptidase inhibitor, clade a (alpha-1 antiproteinase, antitrypsin), member 5, GPC3 - glypican 3, NMUR2 - neuromedin u receptor 2, ALK - anaplastic lymphoma receptor tyrosine kinase, CDH10 - cadherin 10, type 2 (t2-cadherin), CDH12 - cadherin 12, type 2 (n-cadherin 2), AVP - arginine vasopressin, AVPR1B - arginine vasopressin receptor 1b, GLRA3 - glycine receptor, alpha 3, MIP - major intrinsic protein of lens fiber, MST1R - macrophage stimulating 1 receptor (c-met-related tyrosine kinase), ARHGDIB - rho gdp dissociation inhibitor (gdi) beta, DRD2 - dopamine receptor d2, LTBP4 - latent transforming growth factor beta binding protein 4, DDC - dopa decarboxylase (aromatic l-amino acid decarboxylase), HINFP - histone h4 transcription factor, SLC2A14 - solute carrier family 2 (facilitated glucose transporter), member 14, MYOC - myocilin, trabecular meshwork inducible glucocorticoid response, SMPX - small muscle protein, x-linked, TBX19 - t-box 19, ADCY8 - adenylate cyclase 8 (brain), GRIN2C - glutamate receptor, ionotropic, n-methyl d-aspartate 2c, MOV10L1 - mov10l1, moloney leukemia virus 10-like 1, homolog (mouse), LEFTY1 - left-right determination factor 1, NXF2 - nuclear rna export factor 2, SPN - sialophorin, NOS3 - nitric oxide synthase 3 (endothelial cell), PRKCDBP - protein kinase c, delta binding protein, RYR2 - ryanodine receptor 2 (cardiac), TNNI1 - troponin i type 1 (skeletal, slow), VIP - vasoactive intestinal peptide, PLAG1 - pleiomorphic adenoma gene 1, PLAT - plasminogen activator, tissue]
GO:0099537	trans-synaptic signaling	3.63E-04	2.91E-01	2.91	16227	304	257	14	[SLC17A6 - solute carrier family 17 (vesicular glutamate transporter), member 6, EGR3 - early growth response 3, P2RX2 - purinergic receptor p2x, ligand-gated ion channel, 2, DLGAP2 - discs, large (drosophila) homolog-associated protein 2, SLC18A3 - solute carrier family 18 (vesicular acetylcholine transporter), member 3, GRIN2C - glutamate receptor, ionotropic, n-methyl d-aspartate 2c, GLRA3 - glycine receptor, alpha 3, NPTX1 - neuronal pentraxin i, DRD2 - dopamine receptor d2, SLC5A7 - solute carrier family 5 (sodium/choline cotransporter), member 7, HAP1 - huntingtin-associated protein 1, CHRNA5 - cholinergic receptor, nicotinic, alpha 5 (neuronal), CHRNB3 - cholinergic receptor, nicotinic, beta 3 (neuronal), CHRNE - cholinergic receptor, nicotinic, epsilon (muscle)]
GO:0050877	nervous system process	3.73E-04	2.84E-01	2.1	16227	750	257	25	[GLRA3 - glycine receptor, alpha 3, MIP - major intrinsic protein of lens fiber, PRDM12 - pr domain containing 12, DRD2 - dopamine receptor d2, USH1C - usher syndrome 1c (autosomal recessive, severe), ABCA4 - atp-binding cassette, sub-family a (abc1), member 4, SLITRK6 - slit and ntrk-like family, member 6, CRYGD - crystallin, gamma d, ADCY8 - adenylate cyclase 8 (brain), RAX - retina and anterior neural fold homeobox, P2RX2 - purinergic receptor p2x, ligand-gated ion channel, 2, PKD2L1 - polycystic kidney disease 2-like 1, PAX3 - paired box 3, GRIN2C - glutamate receptor, ionotropic, n-methyl d-aspartate 2c, GHSR - growth hormone secretagogue receptor, GUCA1B - guanylate cyclase activator 1b (retina), ESPNL - espin-like, GIP - gastric inhibitory polypeptide, CASP3 - caspase 3, apoptosis-related cysteine peptidase, SORCS3 - sortilin-related vps10 domain containing receptor 3, CHRNA5 - cholinergic receptor, nicotinic, alpha 5 (neuronal), THRB - thyroid hormone receptor, beta, VIP - vasoactive intestinal peptide, CHRNB3 - cholinergic receptor, nicotinic, beta 3 (neuronal), CHRNE - cholinergic receptor, nicotinic, epsilon (muscle)]
GO:0035094	response to nicotine	4.01E-04	2.91E-01	7.89	16227	40	257	5	[DRD2 - dopamine receptor d2, CASP3 - caspase 3, apoptosis-related cysteine peptidase, CHRNA5 - cholinergic receptor, nicotinic, alpha 5 (neuronal), AVP - arginine vasopressin, CHRNB3 - cholinergic receptor, nicotinic, beta 3 (neuronal)]
GO:0099536	synaptic signaling	4.14E-04	2.86E-01	2.87	16227	308	257	14	[SLC17A6 - solute carrier family 17 (vesicular glutamate transporter), member 6, EGR3 - early growth response 3, P2RX2 - purinergic receptor p2x, ligand-gated ion channel, 2, DLGAP2 - discs, large (drosophila) homolog-associated protein 2, SLC18A3 - solute carrier family 18 (vesicular acetylcholine transporter), member 3, GRIN2C - glutamate receptor, ionotropic, n-methyl d-aspartate 2c, GLRA3 - glycine receptor, alpha 3, NPTX1 - neuronal pentraxin i, DRD2 - dopamine receptor d2, SLC5A7 - solute carrier family 5 (sodium/choline cotransporter), member 7, HAP1 - huntingtin-associated protein 1, CHRNA5 - cholinergic receptor, nicotinic, alpha 5 (neuronal), CHRNB3 - cholinergic receptor, nicotinic, beta 3 (neuronal), CHRNE - cholinergic receptor, nicotinic, epsilon (muscle)]
GO:0060123	regulation of growth hormone secretion	4.34E-04	2.87E-01	18.94	16227	10	257	3	[DRD2 - dopamine receptor d2, GHRH - growth hormone releasing hormone, GHSR - growth hormone secretagogue receptor]

GO:0007168	receptor guanylyl cyclase signaling pathway	4.34E-04	2.75E-01	18.94	16227	10	257	3	[NPPA - natriuretic peptide a, GUCA1B - guanylate cyclase activator 1b (retina), NPPB - natriuretic peptide b]
GO:0046903	secretion	4.39E-04	2.67E-01	1.95	16227	938	257	29	[NMUR2 - neuromedin u receptor 2, ALDOC - aldolase c, fructose-bisphosphate, MPO - myeloperoxidase, SLC18A3 - solute carrier family 18 (vesicular acetylcholine transporter), member 3, NPPB - natriuretic peptide b, LTBP4 - latent transforming growth factor beta binding protein 4, DRD2 - dopamine receptor d2, HK2 - hexokinase 2, HAP1 - huntingtin-associated protein 1, RCN3 - reticulocalbin 3, ef-hand calcium binding domain, NHLRC3 - nhl repeat containing 3, PPY - pancreatic polypeptide, DOK3 - docking protein 3, ITGA2B - integrin, alpha 2b (platelet glycoprotein iib of iib/iiia complex, antigen cd41), GHRH - growth hormone releasing hormone, GHSR - growth hormone secretagogue receptor, GUCA1B - guanylate cyclase activator 1b (retina), CDA - cytidine deaminase, NEUROD1 - neuronal differentiation 1, TBC1D10C - tbc1 domain family, member 10c, ITGAX - integrin, alpha x (complement component 3 receptor 4 subunit), SLC5A7 - solute carrier family 5 (sodium/choline cotransporter), member 7, BST1 - bone marrow stromal cell antigen 1, SLC17A9 - solute carrier family 17 (vesicular nucleotide transporter), member 9, ANPEP - alanyl (membrane) aminopeptidase, VIP - vasoactive intestinal peptide, CYB5R4 - cytochrome b5 reductase 4, HSPA1A - heat shock 70kda protein 1a, HSPA1B - heat shock 70kda protein 1b]
GO:0046879	hormone secretion	4.75E-04	2.78E-01	6.01	16227	63	257	6	[LTBP4 - latent transforming growth factor beta binding protein 4, GHRH - growth hormone releasing hormone, VIP - vasoactive intestinal peptide, GHSR - growth hormone secretagogue receptor, CYB5R4 - cytochrome b5 reductase 4, NEUROD1 - neuronal differentiation 1]
GO:0007625	grooming behavior	5.90E-04	3.33E-01	17.22	16227	11	257	3	[DRD2 - dopamine receptor d2, NMUR2 - neuromedin u receptor 2, AVP - arginine vasopressin]
GO:0019932	second-messenger-mediated signaling	6.00E-04	3.26E-01	2.9	16227	283	257	13	[NMUR2 - neuromedin u receptor 2, HRC - histidine rich calcium binding protein, GRIN2C - glutamate receptor, ionotropic, n-methyl d-aspartate 2c, GHRH - growth hormone releasing hormone, NPPA - natriuretic peptide a, NPPB - natriuretic peptide b, NEUROD1 - neuronal differentiation 1, NOS3 - nitric oxide synthase 3 (endothelial cell), DRD2 - dopamine receptor d2, RYR2 - ryanodine receptor 2 (cardiac), IRGM - immunity-related gtpase family, m, VIP - vasoactive intestinal peptide, ADCY8 - adenylate cyclase 8 (brain)]
GO:0060078	regulation of postsynaptic membrane potential	6.01E-04	3.16E-01	4.86	16227	91	257	7	[DRD2 - dopamine receptor d2, P2RX2 - purinergic receptor p2x, ligand-gated ion channel, 2, CHRNA5 - cholinergic receptor, nicotinic, alpha 5 (neuronal), GRIN2C - glutamate receptor, ionotropic, n-methyl d-aspartate 2c, CHRNB3 - cholinergic receptor, nicotinic, beta 3 (neuronal), GLRA3 - glycine receptor, alpha 3, CHRNE - cholinergic receptor, nicotinic, epsilon (muscle)]
GO:0051480	regulation of cytosolic calcium ion concentratio n	6.41E-04	3.25E-01	2.88	16227	285	257	13	[NMUR2 - neuromedin u receptor 2, P2RX2 - purinergic receptor p2x, ligand-gated ion channel, 2, SCGN - secretagogin, ef-hand calcium binding protein, HRC - histidine rich calcium binding protein, GRIN2C - glutamate receptor, ionotropic, n-methyl d-aspartate 2c, AVP - arginine vasopressin, CAPN3 - calpain 3, (p94), AVPR1B - arginine vasopressin receptor 1b, DRD2 - dopamine receptor d2, HAP1 - huntingtin-associated protein 1, RYR2 - ryanodine receptor 2 (cardiac), ADCY8 - adenylate cyclase 8 (brain), CD19 - cd19 molecule]
GO:0010817	regulation of hormone levels	7.31E-04	3.59E-01	2.35	16227	484	257	18	[CYP21A2 - cytochrome p450, family 21, subfamily a, polypeptide 2, FFAR2 - free fatty acid receptor 2, CYP27B1 - cytochrome p450, family 27, subfamily b, polypeptide 1, GHRH - growth hormone releasing hormone, PCSK1 - proprotein convertase subtilisin/kexin type 1, GHSR - growth hormone secretagogue receptor, GIP - gastric inhibitory polypeptide, NEUROD1 - neuronal differentiation 1, DRD2 - dopamine receptor d2, LTBP4 - latent transforming growth factor beta binding protein 4, SLC5A7 - solute carrier family 5 (sodium/choline cotransporter), member 7, POMC - proopiomelanocortin, ALDH1A2 - aldehyde dehydrogenase 1 family, member a2, DHRS2 - dehydrogenase/reductase (sdr family) member 2, VIP - vasoactive intestinal peptide, CYB5R4 - cytochrome b5 reductase 4, INHA - inhibin, alpha, ADCY8 - adenylate cyclase 8 (brain)]
GO:0007600	sensory perception	9.35E-04	4.45E-01	2.45	16227	413	257	16	[RAX - retina and anterior neural fold homeobox, P2RX2 - purinergic receptor p2x, ligand-gated ion channel, 2, PKD2L1 - polycystic kidney disease 2-like 1, PAX3 - paired box 3, GUCA1B - guanylate cyclase activator 1b (retina), ESPNL - espin-like, GIP - gastric inhibitory polypeptide, MIP - major intrinsic protein of lens fiber, PRDM12 - pr domain containing 12, DRD2 - dopamine receptor d2, USH1C - usher syndrome 1c (autosomal recessive, severe), ABCA4 - atp-binding cassette, sub-family a (abc1), member 4, SLITRK6 - slit and ntrk-like family, member 6, CASP3 - caspase 3, apoptosis-related cysteine peptidase, CRYGD - crystallin, gamma d, THRB - thyroid hormone receptor, beta]
GO:0031644	regulation of neurological system process	9.90E-04	4.57E-01	3.95	16227	128	257	8	[NMUR2 - neuromedin u receptor 2, NOS3 - nitric oxide synthase 3 (endothelial cell), NPTX1 - neuronal pentraxin i, ITGAX - integrin, alpha x (complement component 3 receptor 4 subunit), DLGAP2 - discs, large (drosophila) homolog-associated protein 2, AVP - arginine vasopressin, VIP - vasoactive intestinal peptide, GHSR - growth hormone secretagogue receptor]
GO:0023061	signal release	9.90E-04	4.43E-01	3.95	16227	128	257	8	[LTBP4 - latent transforming growth factor beta binding protein 4, SLC5A7 - solute carrier family 5 (sodium/choline cotransporter), member 7, SLC18A3 - solute carrier family 18 (vesicular acetylcholine transporter), member 3, GHRH - growth hormone releasing hormone, VIP - vasoactive intestinal peptide, CYB5R4 - cytochrome b5 reductase 4, GHSR - growth hormone secretagogue receptor, NEUROD1 - neuronal differentiation 1]
**DOPAMINERGIC ORGANOIDS: GENE ONTOLOGY PROCESS TERMS FOR TRANSCRIPTS WITH LOWER EXPRESSION IN LEO**										
GO Term	Description	P-value	FDR q-value	nrichme	N	B	n	b	Genes

GO:0009605	response to external stimulus	5.22E-11	7.94E-07	2.34	16231	1289	355	66	[HFE - hemochromatosis, OASL - 2'-5'-oligoadenylate synthetase-like, FOLR1 - folate receptor 1 (adult), ATF3 - activating transcription factor 3, PTX3 - pentraxin 3, long, SLC22A5 - solute carrier family 22 (organic cation/carnitine transporter), member 5, ATP1A2 - atpase, na+/k+ transporting, alpha 2 polypeptide, TRPV4 - transient receptor potential cation channel, subfamily v, member 4, IL8 - interleukin 8, APOA1 - apolipoprotein a-i, KCNA1 - potassium voltage-gated channel, shaker-related subfamily, member 1 (episodic ataxia with myokymia), ZC3H12A - zinc finger ccch-type containing 12a, TFPI - tissue factor pathway inhibitor (lipoprotein-associated coagulation inhibitor), OPN3 - opsin 3, RGR - retinal g protein coupled receptor, PLSCR4 - phospholipid scramblase 4, IFIT2 - interferon-induced protein with tetratricopeptide repeats 2, IFIT1 - interferon-induced protein with tetratricopeptide repeats 1, IFIT3 - interferon-induced protein with tetratricopeptide repeats 3, FAS - fas cell surface death receptor, CXCL13 - chemokine (c-x-c motif) ligand 13, ACTA2 - actin, alpha 2, smooth muscle, aorta, KCNJ8 - potassium inwardly-rectifying channel, subfamily j, member 8, UCP3 - uncoupling protein 3 (mitochondrial, proton carrier), TICAM1 - toll-like receptor adaptor molecule 1, MGST1 - microsomal glutathione s-transferase 1, KLF10 - kruppel-like factor 10, INHBB - inhibin, beta b, OAS3 - 2'-5'-oligoadenylate synthetase 3, 100kda, CCL2 - chemokine (c-c motif) ligand 2, NFKB1 - nuclear factor of kappa light polypeptide gene enhancer in b-cells 1, PARP9 - poly (adp-ribose) polymerase family, member 9, HSPA5 - heat shock 70kda protein 5 (glucose-regulated protein, 78kda), NFKBIA - nuclear factor of kappa light polypeptide gene enhancer in b-cells inhibitor, alpha, CAV3 - caveolin 3, DCN - decorin, HSPB1 - heat shock 27kda protein 1, HERC5 - hect and rld domain containing e3 ubiquitin protein ligase 5, DUSP10 - dual specificity phosphatase 10, CCL20 - chemokine (c-c motif) ligand 20, GADD45A - growth arrest and dna-damage-inducible, alpha, DDIT3 - dna-damage-inducible transcript 3, MAOB - monoamine oxidase b, SOX9 - sry (sex determining region y)-box 9, DDX60 - dead (asp-glu-ala-asp) box polypeptide 60, IRF1 - interferon regulatory factor 1, TNFRSF11B - tumor necrosis factor receptor superfamily, member 11b, USP2 - ubiquitin specific peptidase 2, COLEC12 - collectin sub-family member 12, CXCL12 - chemokine (c-x-c motif) ligand 12, ITGA2 - integrin, alpha 2 (cd49b, alpha 2 subunit of vla-2 receptor), FOSL1 - fos-like antigen 1, NMI - n-myc (and stat) interactor, PTN - pleiotrophin, RIPK2 - receptor-interacting serine-threonine kinase 2, VCAM1 - vascular cell adhesion molecule 1, CPS1 - carbamoyl-phosphate synthase 1, mitochondrial, ADORA1 - adenosine a1 receptor, TNC - tenascin c, TNFAIP3 - tumor necrosis factor, alpha-induced protein 3, CXCL1 - chemokine (c-x-c motif) ligand 1 (melanoma growth stimulating activity, alpha), OPN1SW - opsin 1 (cone pigments), short-wave-sensitive, CXCL2 - chemokine (c-x-c motif) ligand 2, CXCL3 - chemokine (c-x-c motif) ligand 3, PTPRC - protein tyrosine phosphatase, receptor type, c, HMCN1 - hemicentin 1]
GO:0071310	cellular response to organic substance	7.75E-10	5.90E-06	2.29	16231	1218	355	61	[ARID5B - at rich interactive domain 5b (mrf1-like), FOLR1 - folate receptor 1 (adult), ID1 - inhibitor of dna binding 1, dominant negative helix-loop-helix protein, ID3 - inhibitor of dna binding 3, dominant negative helix-loop-helix protein, MSTN - myostatin, ATP1A2 - atpase, na+/k+ transporting, alpha 2 polypeptide, IL8 - interleukin 8, ZC3H12A - zinc finger ccch-type containing 12a, TFPI - tissue factor pathway inhibitor (lipoprotein-associated coagulation inhibitor), COL4A1 - collagen, type iv, alpha 1, CD58 - cd58 molecule, WNT8B - wingless-type mmtv integration site family, member 8b, PLSCR4 - phospholipid scramblase 4, IFIT2 - interferon-induced protein with tetratricopeptide repeats 2, IFIT1 - interferon-induced protein with tetratricopeptide repeats 1, IFIT3 - interferon-induced protein with tetratricopeptide repeats 3, GHR - growth hormone receptor, SPRY2 - sprouty homolog 2 (drosophila), NTRK2 - neurotrophic tyrosine kinase, receptor, type 2, CXCL13 - chemokine (c-x-c motif) ligand 13, YAP1 - yes-associated protein 1, ZFP36L1 - zfp36 ring finger protein-like 1, AQP4 - aquaporin 4, RAMP3 - receptor (g protein-coupled) activity modifying protein 3, KLF9 - kruppel-like factor 9, UCP3 - uncoupling protein 3 (mitochondrial, proton carrier), TICAM1 - toll-like receptor adaptor molecule 1, KLF10 - kruppel-like factor 10, INHBB - inhibin, beta b, CCL2 - chemokine (c-c motif) ligand 2, NFKB1 - nuclear factor of kappa light polypeptide gene enhancer in b-cells 1, KCNMB1 - potassium large conductance calcium-activated channel, subfamily m, beta member 1, HSPA5 - heat shock 70kda protein 5 (glucose-regulated protein, 78kda), CAV3 - caveolin 3, IQGAP3 - iq motif containing gtpase activating protein 3, TIPARP - tcdd-inducible poly(adp-ribose) polymerase, HSPB1 - heat shock 27kda protein 1, IGFBP7 - insulin-like growth factor binding protein 7, CCL20 - chemokine (c-c motif) ligand 20, SLC2A5 - solute carrier family 2 (facilitated glucose/fructose transporter), member 5, SOX9 - sry (sex determining region y)-box 9, IRF1 - interferon regulatory factor 1, KLF15 - kruppel-like factor 15, COLEC12 - collectin sub-family member 12, CXCL12 - chemokine (c-x-c motif) ligand 12, MME - membrane metallo-endopeptidase, ITGA2 - integrin, alpha 2 (cd49b, alpha 2 subunit of vla-2 receptor), PTN - pleiotrophin, RIPK2 - receptor-interacting serine-threonine kinase 2, VCAM1 - vascular cell adhesion molecule 1, MLC1 - megalencephalic leukoencephalopathy with subcortical cysts 1, CPS1 - carbamoyl-phosphate synthase 1, mitochondrial, PDGFRB - platelet-derived growth factor receptor, beta polypeptide, TNC - tenascin c, TNFAIP3 - tumor necrosis factor, alpha-induced protein 3, CXCL1 - chemokine (c-x-c motif) ligand 1 (melanoma growth stimulating activity, alpha), CXCL2 - chemokine (c-x-c motif) ligand 2, CXCL3 - chemokine (c-x-c motif) ligand 3, CALCRL - calcitonin receptor-like, ANXA1 - annexin a1, IBSP - integrin-binding sialoprotein]
GO:0010033	response to organic substance	1.23E-09	6.22E-06	1.96	16231	1917	355	82	[ARID5B - at rich interactive domain 5b (mrf1-like), FOLR1 - folate receptor 1 (adult), MXRA5 - matrix-remodelling associated 5, ID1 - inhibitor of dna binding 1, dominant negative helix-loop-helix protein, ID3 - inhibitor of dna binding 3, dominant negative helix-loop-helix protein, RELB - v-rel avian reticuloendotheliosis viral oncogene homolog b, MSTN - myostatin, ATP1A2 - atpase, na+/k+ transporting, alpha 2 polypeptide, IL8 - interleukin 8, TFPI - tissue factor pathway inhibitor (lipoprotein-associated coagulation inhibitor), ZC3H12A - zinc finger ccch-type containing 12a, COL4A1 - collagen, type iv, alpha 1, CD58 - cd58 molecule, PLSCR4 - phospholipid scramblase 4, WNT8B - wingless-type mmtv integration site family, member 8b, RGS10 - regulator of g-protein signaling 10, IFIT2 - interferon-induced protein with tetratricopeptide repeats 2, IFIT1 - interferon-induced protein with tetratricopeptide repeats 1, IFIT3 - interferon-induced protein with tetratricopeptide repeats 3, SCD - stearoyl-coa desaturase (delta-9-desaturase), GHR - growth hormone receptor, SPRY2 - sprouty homolog 2 (drosophila), NTRK2 - neurotrophic tyrosine kinase, receptor, type 2, CXCL13 - chemokine (c-x-c motif) ligand 13, YAP1 - yes-associated protein 1, ZFP36L1 - zfp36 ring finger protein-like 1, AQP4 - aquaporin 4, RAMP3 - receptor (g protein-coupled) activity modifying protein 3, KCNJ8 - potassium inwardly-rectifying channel, subfamily j, member 8, GAB1 - grb2-associated binding protein 1, HSD17B2 - hydroxysteroid (17-beta) dehydrogenase 2, KLF9 - kruppel-like factor 9, UCP3 - uncoupling protein 3 (mitochondrial, proton carrier), KLF10 - kruppel-like factor 10, TICAM1 - toll-like receptor adaptor molecule 1, MGST1 - microsomal glutathione s-transferase 1, INHBB - inhibin, beta b, CCL2 - chemokine (c-c motif) ligand 2, NFKB1 - nuclear factor of kappa light polypeptide gene enhancer in b-cells 1, RNF175 - ring finger protein 175, KCNMB1 - potassium large conductance calcium-activated channel, subfamily m, beta member 1, HSPA5 - heat shock 70kda protein 5 (glucose-regulated protein, 78kda), NFKBIA - nuclear factor of kappa light polypeptide gene enhancer in b-cells inhibitor, alpha, CAV3 - caveolin 3, IQGAP3 - iq motif containing gtpase activating protein 3, DCN - decorin, TIPARP - tcdd-inducible poly(adp-ribose) polymerase, HSPB1 - heat shock 27kda protein 1, LEPR - leptin receptor, HERPUD1 - homocysteine-inducible, endoplasmic reticulum stress-inducible, ubiquitin-like domain member 1, DUSP10 - dual specificity phosphatase 10, SETD7 - set domain containing (lysine methyltransferase) 7, SERPINH1 - serpin peptidase inhibitor, clade h (heat shock protein 47), member 1, (collagen binding protein 1), CCL20 - chemokine (c-c motif) ligand 20, IGFBP7 - insulin-like growth factor binding protein 7, SLC2A5 - solute carrier family 2 (facilitated glucose/fructose transporter), member 5, DDIT3 - dna-damage-inducible transcript 3, MAOB - monoamine oxidase b, SOX9 - sry (sex determining region y)-box 9, IRF1 - interferon regulatory factor 1, KLF15 - kruppel-like factor 15, COLEC12 - collectin sub-family member 12, CXCL12 - chemokine (c-x-c motif) ligand 12, CHAC1 - chac, cation transport regulator homolog 1 (e. coli), MME - membrane metallo-endopeptidase, ITGA2 - integrin, alpha 2 (cd49b, alpha 2 subunit of vla-2 receptor), FOSL1 - fos-like antigen 1, PTN - pleiotrophin, VCAM1 - vascular cell adhesion molecule 1, RIPK2 - receptor-interacting serine-threonine kinase 2, MLC1 - megalencephalic leukoencephalopathy with subcortical cysts 1, CPS1 - carbamoyl-phosphate synthase 1, mitochondrial, PDGFRB - platelet-derived growth factor receptor, beta polypeptide, TNC - tenascin c, TNFAIP3 - tumor necrosis factor, alpha-induced protein 3, CXCL1 - chemokine (c-x-c motif) ligand 1 (melanoma growth stimulating activity, alpha), CXCL2 - chemokine (c-x-c motif) ligand 2, CXCL3 - chemokine (c-x-c motif) ligand 3, CALCRL - calcitonin receptor-like, ANXA1 - annexin a1, IBSP - integrin-binding sialoprotein, CD83 - cd83 molecule]

GO:0071396	cellular response to lipid	5.55E-09	2.11E-05	3.59	16231	357	355	28	[CCL2 - chemokine (c-c motif) ligand 2, NFKB1 - nuclear factor of kappa light polypeptide gene enhancer in b-cells 1, KCNMB1 - potassium large conductance calcium-activated channel, subfamily m, beta member 1, ID3 - inhibitor of dna binding 3, dominant negative helix-loop-helix protein, MSTN - myostatin, ATP1A2 - atpase, na+/k+ transporting, alpha 2 polypeptide, IL8 - interleukin 8, SOX9 - sry (sex determining region y)-box 9, ZC3H12A - zinc finger ccch-type containing 12a, TFPI - tissue factor pathway inhibitor (lipoprotein-associated coagulation inhibitor), WNT8B - wingless-type mmtv integration site family, member 8b, PLSCR4 - phospholipid scramblase 4, ITGA2 - integrin, alpha 2 (cd49b, alpha 2 subunit of vla-2 receptor), PTN - pleiotrophin, MLC1 - megalencephalic leukoencephalopathy with subcortical cysts 1, CPS1 - carbamoyl-phosphate synthase 1, mitochondrial, CXCL13 - chemokine (c-x-c motif) ligand 13, YAP1 - yes-associated protein 1, ZFP36L1 - zfp36 ring finger protein-like 1, TNC - tenascin c, RAMP3 - receptor (g protein-coupled) activity modifying protein 3, TNFAIP3 - tumor necrosis factor, alpha-induced protein 3, CXCL1 - chemokine (c-x-c motif) ligand 1 (melanoma growth stimulating activity, alpha), CXCL2 - chemokine (c-x-c motif) ligand 2, KLF9 - kruppel-like factor 9, CXCL3 - chemokine (c-x-c motif) ligand 3, ANXA1 - annexin a1, TICAM1 - toll-like receptor adaptor molecule 1]
GO:0009607	response to biotic stimulus	7.87E-09	2.40E-05	2.65	16231	724	355	42	[OAS3 - 2'-5'-oligoadenylate synthetase 3, 100kda, CCL2 - chemokine (c-c motif) ligand 2, OASL - 2'-5'-oligoadenylate synthetase-like, NFKB1 - nuclear factor of kappa light polypeptide gene enhancer in b-cells 1, PARP9 - poly (adp-ribose) polymerase family, member 9, HSPA5 - heat shock 70kda protein 5 (glucose-regulated protein, 78kda), DCN - decorin, HSPB1 - heat shock 27kda protein 1, PTX3 - pentraxin 3, long, HERC5 - hect and rld domain containing e3 ubiquitin protein ligase 5, DUSP10 - dual specificity phosphatase 10, CCL20 - chemokine (c-c motif) ligand 20, SLC22A5 - solute carrier family 22 (organic cation/carnitine transporter), member 5, MAOB - monoamine oxidase b, DDIT3 - dna-damage-inducible transcript 3, IL8 - interleukin 8, DDX60 - dead (asp-glu-ala-asp) box polypeptide 60, IRF1 - interferon regulatory factor 1, ZC3H12A - zinc finger ccch-type containing 12a, TFPI - tissue factor pathway inhibitor (lipoprotein-associated coagulation inhibitor), COLEC12 - collectin sub-family member 12, CXCL12 - chemokine (c-x-c motif) ligand 12, PLSCR4 - phospholipid scramblase 4, IFIT2 - interferon-induced protein with tetratricopeptide repeats 2, NMI - n-myc (and stat) interactor, FOSL1 - fos-like antigen 1, IFIT1 - interferon-induced protein with tetratricopeptide repeats 1, IFIT3 - interferon-induced protein with tetratricopeptide repeats 3, RIPK2 - receptor-interacting serine-threonine kinase 2, VCAM1 - vascular cell adhesion molecule 1, CPS1 - carbamoyl-phosphate synthase 1, mitochondrial, CXCL13 - chemokine (c-x-c motif) ligand 13, ACTA2 - actin, alpha 2, smooth muscle, aorta, KCNJ8 - potassium inwardly-rectifying channel, subfamily j, member 8, TNFAIP3 - tumor necrosis factor, alpha-induced protein 3, CXCL1 - chemokine (c-x-c motif) ligand 1 (melanoma growth stimulating activity, alpha), CXCL2 - chemokine (c-x-c motif) ligand 2, CXCL3 - chemokine (c-x-c motif) ligand 3, PTPRC - protein tyrosine phosphatase, receptor type, c, MGST1 - microsomal glutathione s-transferase 1, TICAM1 - toll-like receptor adaptor molecule 1, HMCN1 - hemicentin 1]
GO:0070887	cellular response to chemical stimulus	8.18E-09	2.08E-05	2.04	16231	1522	355	68	[HFE - hemochromatosis, ARID5B - at rich interactive domain 5b (mrf1-like), FOLR1 - folate receptor 1 (adult), ID1 - inhibitor of dna binding 1, dominant negative helix-loop-helix protein, ITPKB - inositol-trisphosphate 3-kinase b, ID3 - inhibitor of dna binding 3, dominant negative helix-loop-helix protein, MSTN - myostatin, ATP1A2 - atpase, na+/k+ transporting, alpha 2 polypeptide, IL8 - interleukin 8, KCNA1 - potassium voltage-gated channel, shaker-related subfamily, member 1 (episodic ataxia with myokymia), ZC3H12A - zinc finger ccch-type containing 12a, TFPI - tissue factor pathway inhibitor (lipoprotein-associated coagulation inhibitor), COL4A1 - collagen, type iv, alpha 1, CD58 - cd58 molecule, WNT8B - wingless-type mmtv integration site family, member 8b, PLSCR4 - phospholipid scramblase 4, IFIT2 - interferon-induced protein with tetratricopeptide repeats 2, IFIT1 - interferon-induced protein with tetratricopeptide repeats 1, IFIT3 - interferon-induced protein with tetratricopeptide repeats 3, GHR - growth hormone receptor, SPRY2 - sprouty homolog 2 (drosophila), NTRK2 - neurotrophic tyrosine kinase, receptor, type 2, FAS - fas cell surface death receptor, CXCL13 - chemokine (c-x-c motif) ligand 13, YAP1 - yes-associated protein 1, ZFP36L1 - zfp36 ring finger protein-like 1, AQP4 - aquaporin 4, RAMP3 - receptor (g protein-coupled) activity modifying protein 3, KLF9 - kruppel-like factor 9, UCP3 - uncoupling protein 3 (mitochondrial, proton carrier), TICAM1 - toll-like receptor adaptor molecule 1, MGST1 - microsomal glutathione s-transferase 1, KLF10 - kruppel-like factor 10, INHBB - inhibin, beta b, CCL2 - chemokine (c-c motif) ligand 2, NFKB1 - nuclear factor of kappa light polypeptide gene enhancer in b-cells 1, KCNMB1 - potassium large conductance calcium-activated channel, subfamily m, beta member 1, HSPA5 - heat shock 70kda protein 5 (glucose-regulated protein, 78kda), CAV3 - caveolin 3, IQGAP3 - iq motif containing gtpase activating protein 3, TIPARP - tcdd-inducible poly(adp-ribose) polymerase, HSPB1 - heat shock 27kda protein 1, SOD3 - superoxide dismutase 3, extracellular, IGFBP7 - insulin-like growth factor binding protein 7, CCL20 - chemokine (c-c motif) ligand 20, SLC2A5 - solute carrier family 2 (facilitated glucose/fructose transporter), member 5, SOX9 - sry (sex determining region y)-box 9, PTGS2 - prostaglandin-endoperoxide synthase 2 (prostaglandin g/h synthase and cyclooxygenase), IRF1 - interferon regulatory factor 1, KLF15 - kruppel-like factor 15, COLEC12 - collectin sub-family member 12, CXCL12 - chemokine (c-x-c motif) ligand 12, MME - membrane metallo-endopeptidase, ITGA2 - integrin, alpha 2 (cd49b, alpha 2 subunit of vla-2 receptor), PTN - pleiotrophin, RIPK2 - receptor-interacting serine-threonine kinase 2, VCAM1 - vascular cell adhesion molecule 1, MLC1 - megalencephalic leukoencephalopathy with subcortical cysts 1, CPS1 - carbamoyl-phosphate synthase 1, mitochondrial, PDGFRB - platelet-derived growth factor receptor, beta polypeptide, TNC - tenascin c, TNFAIP3 - tumor necrosis factor, alpha-induced protein 3, CXCL1 - chemokine (c-x-c motif) ligand 1 (melanoma growth stimulating activity, alpha), CXCL2 - chemokine (c-x-c motif) ligand 2, CXCL3 - chemokine (c-x-c motif) ligand 3, CALCRL - calcitonin receptor-like, ANXA1 - annexin a1, IBSP - integrin-binding sialoprotein]
GO:0033993	response to lipid	9.81E-09	2.13E-05	2.71	16231	674	355	40	[CCL2 - chemokine (c-c motif) ligand 2, NFKB1 - nuclear factor of kappa light polypeptide gene enhancer in b-cells 1, KCNMB1 - potassium large conductance calcium-activated channel, subfamily m, beta member 1, ID3 - inhibitor of dna binding 3, dominant negative helix-loop-helix protein, DCN - decorin, DUSP10 - dual specificity phosphatase 10, MSTN - myostatin, IGFBP7 - insulin-like growth factor binding protein 7, ATP1A2 - atpase, na+/k+ transporting, alpha 2 polypeptide, MAOB - monoamine oxidase b, IL8 - interleukin 8, SOX9 - sry (sex determining region y)-box 9, ZC3H12A - zinc finger ccch-type containing 12a, TFPI - tissue factor pathway inhibitor (lipoprotein-associated coagulation inhibitor), WNT8B - wingless-type mmtv integration site family, member 8b, PLSCR4 - phospholipid scramblase 4, ITGA2 - integrin, alpha 2 (cd49b, alpha 2 subunit of vla-2 receptor), FOSL1 - fos-like antigen 1, PTN - pleiotrophin, SCD - stearoyl-coa desaturase (delta-9-desaturase), VCAM1 - vascular cell adhesion molecule 1, MLC1 - megalencephalic leukoencephalopathy with subcortical cysts 1, CPS1 - carbamoyl-phosphate synthase 1, mitochondrial, PDGFRB - platelet-derived growth factor receptor, beta polypeptide, CXCL13 - chemokine (c-x-c motif) ligand 13, YAP1 - yes-associated protein 1, ZFP36L1 - zfp36 ring finger protein-like 1, TNC - tenascin c, RAMP3 - receptor (g protein-coupled) activity modifying protein 3, KCNJ8 - potassium inwardly-rectifying channel, subfamily j, member 8, TNFAIP3 - tumor necrosis factor, alpha-induced protein 3, CXCL1 - chemokine (c-x-c motif) ligand 1 (melanoma growth stimulating activity, alpha), CXCL2 - chemokine (c-x-c motif) ligand 2, HSD17B2 - hydroxysteroid (17-beta) dehydrogenase 2, CXCL3 - chemokine (c-x-c motif) ligand 3, KLF9 - kruppel-like factor 9, ANXA1 - annexin a1, UCP3 - uncoupling protein 3 (mitochondrial, proton carrier), MGST1 - microsomal glutathione s-transferase 1, TICAM1 - toll-like receptor adaptor molecule 1]

GO:0042221	response to chemical	1.61E-08	3.06E-05	1.77	16231	2382	355	92	[HFE - hemochromatosis, ARID5B - at rich interactive domain 5b (mrf1-like), FOLR1 - folate receptor 1 (adult), MXRA5 - matrix-remodelling associated 5, ID1 - inhibitor of dna binding 1, dominant negative helix-loop-helix protein, ITPKB - inositol-trisphosphate 3-kinase b, ID3 - inhibitor of dna binding 3, dominant negative helix-loop-helix protein, RELB - v-rel avian reticuloendotheliosis viral oncogene homolog b, MSTN - myostatin, ATP1A2 - atpase, na+/k+ transporting, alpha 2 polypeptide, IL8 - interleukin 8, APOA1 - apolipoprotein a-i, KCNA1 - potassium voltage-gated channel, shaker-related subfamily, member 1 (episodic ataxia with myokymia), TFPI - tissue factor pathway inhibitor (lipoprotein-associated coagulation inhibitor), ZC3H12A - zinc finger ccch-type containing 12a, COL4A1 - collagen, type iv, alpha 1, CD58 - cd58 molecule, PLSCR4 - phospholipid scramblase 4, WNT8B - wingless-type mmtv integration site family, member 8b, RGS10 - regulator of g-protein signaling 10, IFIT2 - interferon-induced protein with tetratricopeptide repeats 2, IFIT1 - interferon-induced protein with tetratricopeptide repeats 1, IFIT3 - interferon-induced protein with tetratricopeptide repeats 3, SCD - stearoyl-coa desaturase (delta-9-desaturase), GHR - growth hormone receptor, SPRY2 - sprouty homolog 2 (drosophila), NTRK2 - neurotrophic tyrosine kinase, receptor, type 2, FAS - fas cell surface death receptor, CXCL13 - chemokine (c-x-c motif) ligand 13, YAP1 - yes-associated protein 1, ZFP36L1 - zfp36 ring finger protein-like 1, AQP4 - aquaporin 4, RAMP3 - receptor (g protein-coupled) activity modifying protein 3, KCNJ8 - potassium inwardly-rectifying channel, subfamily j, member 8, GAB1 - grb2-associated binding protein 1, HSD17B2 - hydroxysteroid (17-beta) dehydrogenase 2, KLF9 - kruppel-like factor 9, UCP3 - uncoupling protein 3 (mitochondrial, proton carrier), KLF10 - kruppel-like factor 10, TICAM1 - toll-like receptor adaptor molecule 1, MGST1 - microsomal glutathione s-transferase 1, INHBB - inhibin, beta b, CCL2 - chemokine (c-c motif) ligand 2, NFKB1 - nuclear factor of kappa light polypeptide gene enhancer in b-cells 1, RNF175 - ring finger protein 175, KCNMB1 - potassium large conductance calcium-activated channel, subfamily m, beta member 1, HSPA5 - heat shock 70kda protein 5 (glucose-regulated protein, 78kda), NFKBIA - nuclear factor of kappa light polypeptide gene enhancer in b-cells inhibitor, alpha, CAV3 - caveolin 3, IQGAP3 - iq motif containing gtpase activating protein 3, DCN - decorin, TIPARP - tcdd-inducible poly(adp-ribose) polymerase, HSPB1 - heat shock 27kda protein 1, SOD3 - superoxide dismutase 3, extracellular, LEPR - leptin receptor, HERPUD1 - homocysteine-inducible, endoplasmic reticulum stress-inducible, ubiquitin-like domain member 1, DUSP10 - dual specificity phosphatase 10, SETD7 - set domain containing (lysine methyltransferase) 7, SERPINH1 - serpin peptidase inhibitor, clade h (heat shock protein 47), member 1, (collagen binding protein 1), CCL20 - chemokine (c-c motif) ligand 20, IGFBP7 - insulin-like growth factor binding protein 7, SLC2A5 - solute carrier family 2 (facilitated glucose/fructose transporter), member 5, DDIT3 - dna-damage-inducible transcript 3, MAOB - monoamine oxidase b, SOX9 - sry (sex determining region y)-box 9, PTGS2 - prostaglandin-endoperoxide synthase 2 (prostaglandin g/h synthase and cyclooxygenase), IRF1 - interferon regulatory factor 1, KLF15 - kruppel-like factor 15, TNFRSF11B - tumor necrosis factor receptor superfamily, member 11b, HBA2 - hemoglobin, alpha 2, COLEC12 - collectin sub-family member 12, GJC2 - gap junction protein, gamma 2, 47kda, CXCL12 - chemokine (c-x-c motif) ligand 12, CHAC1 - chac, cation transport regulator homolog 1 (e. coli), MME - membrane metallo-endopeptidase, ITGA2 - integrin, alpha 2 (cd49b, alpha 2 subunit of vla-2 receptor), FOSL1 - fos-like antigen 1, PTN - pleiotrophin, VCAM1 - vascular cell adhesion molecule 1, RIPK2 - receptor-interacting serine-threonine kinase 2, MLC1 - megalencephalic leukoencephalopathy with subcortical cysts 1, CPS1 - carbamoyl-phosphate synthase 1, mitochondrial, PDGFRB - platelet-derived growth factor receptor, beta polypeptide, TNC - tenascin c, TNFAIP3 - tumor necrosis factor, alpha-induced protein 3, CXCL1 - chemokine (c-x-c motif) ligand 1 (melanoma growth stimulating activity, alpha), CXCL2 - chemokine (c-x-c motif) ligand 2, CXCL3 - chemokine (c-x-c motif) ligand 3, CALCRL - calcitonin receptor-like, ANXA1 - annexin a1, IBSP - integrin-binding sialoprotein, CD83 - cd83 molecule]
GO:0006952	defense response	1.88E-08	3.19E-05	2.38	16231	921	355	48	[GZMB - granzyme b (granzyme 2, cytotoxic t-lymphocyte-associated serine esterase 1), CFH - complement factor h, OAS3 - 2'-5'-oligoadenylate synthetase 3, 100kda, HFE - hemochromatosis, OASL - 2'-5'-oligoadenylate synthetase-like, CCL2 - chemokine (c-c motif) ligand 2, NFKB1 - nuclear factor of kappa light polypeptide gene enhancer in b-cells 1, PARP9 - poly (adp-ribose) polymerase family, member 9, RELB - v-rel avian reticuloendotheliosis viral oncogene homolog b, PTX3 - pentraxin 3, long, HERC5 - hect and rld domain containing e3 ubiquitin protein ligase 5, PTGER3 - prostaglandin e receptor 3 (subtype ep3), CCL20 - chemokine (c-c motif) ligand 20, ATP1A2 - atpase, na+/k+ transporting, alpha 2 polypeptide, IL8 - interleukin 8, PLP1 - proteolipid protein 1, C5 - complement component 5, DDX60 - dead (asp-glu-ala-asp) box polypeptide 60, PARP14 - poly (adp-ribose) polymerase family, member 14, PTGS2 - prostaglandin-endoperoxide synthase 2 (prostaglandin g/h synthase and cyclooxygenase), IRF1 - interferon regulatory factor 1, TAC4 - tachykinin 4 (hemokinin), TRIM17 - tripartite motif containing 17, C7 - complement component 7, ZC3H12A - zinc finger ccch-type containing 12a, COLEC12 - collectin sub-family member 12, HTR1A - 5-hydroxytryptamine (serotonin) receptor 1a, g protein-coupled, CXCL12 - chemokine (c-x-c motif) ligand 12, CSF1 - colony stimulating factor 1 (macrophage), IFIT2 - interferon-induced protein with tetratricopeptide repeats 2, FOSL1 - fos-like antigen 1, IFIT1 - interferon-induced protein with tetratricopeptide repeats 1, IFIT3 - interferon-induced protein with tetratricopeptide repeats 3, RIPK2 - receptor-interacting serine-threonine kinase 2, VCAM1 - vascular cell adhesion molecule 1, CXCL13 - chemokine (c-x-c motif) ligand 13, ADORA1 - adenosine a1 receptor, KCNJ8 - potassium inwardly-rectifying channel, subfamily j, member 8, TNFAIP3 - tumor necrosis factor, alpha-induced protein 3, CXCL1 - chemokine (c-x-c motif) ligand 1 (melanoma growth stimulating activity, alpha), CXCL2 - chemokine (c-x-c motif) ligand 2, CXCL3 - chemokine (c-x-c motif) ligand 3, ANXA1 - annexin a1, PTPRC - protein tyrosine phosphatase, receptor type, c, TICAM1 - toll-like receptor adaptor molecule 1, INHBB - inhibin, beta b, SLC15A2 - solute carrier family 15 (oligopeptide transporter), member 2, CD83 - cd83 molecule]
GO:0043207	response to external biotic stimulus	2.48E-08	3.78E-05	2.62	16231	697	355	40	[OAS3 - 2'-5'-oligoadenylate synthetase 3, 100kda, CCL2 - chemokine (c-c motif) ligand 2, OASL - 2'-5'-oligoadenylate synthetase-like, NFKB1 - nuclear factor of kappa light polypeptide gene enhancer in b-cells 1, PARP9 - poly (adp-ribose) polymerase family, member 9, DCN - decorin, HSPB1 - heat shock 27kda protein 1, PTX3 - pentraxin 3, long, HERC5 - hect and rld domain containing e3 ubiquitin protein ligase 5, DUSP10 - dual specificity phosphatase 10, CCL20 - chemokine (c-c motif) ligand 20, SLC22A5 - solute carrier family 22 (organic cation/carnitine transporter), member 5, MAOB - monoamine oxidase b, IL8 - interleukin 8, DDX60 - dead (asp-glu-ala-asp) box polypeptide 60, IRF1 - interferon regulatory factor 1, ZC3H12A - zinc finger ccch-type containing 12a, TFPI - tissue factor pathway inhibitor (lipoprotein-associated coagulation inhibitor), COLEC12 - collectin sub-family member 12, CXCL12 - chemokine (c-x-c motif) ligand 12, PLSCR4 - phospholipid scramblase 4, IFIT2 - interferon-induced protein with tetratricopeptide repeats 2, NMI - n-myc (and stat) interactor, FOSL1 - fos-like antigen 1, IFIT1 - interferon-induced protein with tetratricopeptide repeats 1, IFIT3 - interferon-induced protein with tetratricopeptide repeats 3, RIPK2 - receptor-interacting serine-threonine kinase 2, VCAM1 - vascular cell adhesion molecule 1, CPS1 - carbamoyl-phosphate synthase 1, mitochondrial, CXCL13 - chemokine (c-x-c motif) ligand 13, ACTA2 - actin, alpha 2, smooth muscle, aorta, KCNJ8 - potassium inwardly-rectifying channel, subfamily j, member 8, TNFAIP3 - tumor necrosis factor, alpha-induced protein 3, CXCL1 - chemokine (c-x-c motif) ligand 1 (melanoma growth stimulating activity, alpha), CXCL2 - chemokine (c-x-c motif) ligand 2, CXCL3 - chemokine (c-x-c motif) ligand 3, PTPRC - protein tyrosine phosphatase, receptor type, c, MGST1 - microsomal glutathione s-transferase 1, TICAM1 - toll-like receptor adaptor molecule 1, HMCN1 - hemicentin 1]
GO:0060326	cell chemotaxis	8.22E-08	1.14E-04	4.86	16231	160	355	17	[CCL2 - chemokine (c-c motif) ligand 2, CXCL12 - chemokine (c-x-c motif) ligand 12, PTN - pleiotrophin, VCAM1 - vascular cell adhesion molecule 1, HBEGF - heparin-binding egf-like growth factor, PDGFRB - platelet-derived growth factor receptor, beta polypeptide, CXCL13 - chemokine (c-x-c motif) ligand 13, CKLF - chemokine-like factor, CCL20 - chemokine (c-c motif) ligand 20, GAB1 - grb2-associated binding protein 1, CXCL1 - chemokine (c-x-c motif) ligand 1 (melanoma growth stimulating activity, alpha), IL8 - interleukin 8, CXCL2 - chemokine (c-x-c motif) ligand 2, C5 - complement component 5, CXCL3 - chemokine (c-x-c motif) ligand 3, ANXA1 - annexin a1, CYP7B1 - cytochrome p450, family 7, subfamily b, polypeptide 1]
GO:0043062	extracellular structure organization	1.16E-07	1.48E-04	3.28	16231	362	355	26	[FBLN5 - fibulin 5, MMP21 - matrix metallopeptidase 21, DCN - decorin, PTX3 - pentraxin 3, long, SERPINH1 - serpin peptidase inhibitor, clade h (heat shock protein 47), member 1, (collagen binding protein 1), CD34 - cd34 molecule, LTBP3 - latent transforming growth factor beta binding protein 3, ACAN - aggrecan, SOX9 - sry (sex determining region y)-box 9, APOA1 - apolipoprotein a-i, TNFRSF11B - tumor necrosis factor receptor superfamily, member 11b, COL4A1 - collagen, type iv, alpha 1, ITGA2 - integrin, alpha 2 (cd49b, alpha 2 subunit of vla-2 receptor), ELN - elastin, CTSV - cathepsin v, COL22A1 - collagen, type xxii, alpha 1, VCAM1 - vascular cell adhesion molecule 1, COL8A1 - collagen, type viii, alpha 1, ECM2 - extracellular matrix protein 2, female organ and adipocyte specific, COL12A1 - collagen, type xii, alpha 1, TNC - tenascin c, SMOC1 - sparc related modular calcium binding 1, IBSP - integrin-binding sialoprotein, TTR - transthyretin, LCAT - lecithin-cholesterol acyltransferase, ITGB7 - integrin, beta 7]

GO:0036499	PERK-mediated unfolded protein response	1.58E-07	1.85E-04	21.1	16231	13	355	6	[CCL2 - chemokine (c-c motif) ligand 2, HSPA5 - heat shock 70kda protein 5 (glucose-regulated protein, 78kda), DDIT3 - dna-damage-inducible transcript 3, IL8 - interleukin 8, ATF3 - activating transcription factor 3, HERPUD1 - homocysteine-inducible, endoplasmic reticulum stress-inducible, ubiquitin-like domain member 1]
GO:1901701	cellular response to oxygen-containing compound	1.71E-07	1.86E-04	2.44	16231	749	355	40	[CCL2 - chemokine (c-c motif) ligand 2, NFKB1 - nuclear factor of kappa light polypeptide gene enhancer in b-cells 1, KCNMB1 - potassium large conductance calcium-activated channel, subfamily m, beta member 1, FOLR1 - folate receptor 1 (adult), HSPA5 - heat shock 70kda protein 5 (glucose-regulated protein, 78kda), ID1 - inhibitor of dna binding 1, dominant negative helix-loop-helix protein, ID3 - inhibitor of dna binding 3, dominant negative helix-loop-helix protein, MSTN - myostatin, SLC2A5 - solute carrier family 2 (facilitated glucose/fructose transporter), member 5, IL8 - interleukin 8, SOX9 - sry (sex determining region y)-box 9, ZC3H12A - zinc finger ccch-type containing 12a, TFPI - tissue factor pathway inhibitor (lipoprotein-associated coagulation inhibitor), KLF15 - kruppel-like factor 15, COL4A1 - collagen, type iv, alpha 1, WNT8B - wingless-type mmtv integration site family, member 8b, PLSCR4 - phospholipid scramblase 4, ITGA2 - integrin, alpha 2 (cd49b, alpha 2 subunit of vla-2 receptor), PTN - pleiotrophin, RIPK2 - receptor-interacting serine-threonine kinase 2, VCAM1 - vascular cell adhesion molecule 1, MLC1 - megalencephalic leukoencephalopathy with subcortical cysts 1, NTRK2 - neurotrophic tyrosine kinase, receptor, type 2, CPS1 - carbamoyl-phosphate synthase 1, mitochondrial, CXCL13 - chemokine (c-x-c motif) ligand 13, YAP1 - yes-associated protein 1, ZFP36L1 - zfp36 ring finger protein-like 1, TNC - tenascin c, RAMP3 - receptor (g protein-coupled) activity modifying protein 3, TNFAIP3 - tumor necrosis factor, alpha-induced protein 3, CXCL1 - chemokine (c-x-c motif) ligand 1 (melanoma growth stimulating activity, alpha), CXCL2 - chemokine (c-x-c motif) ligand 2, CXCL3 - chemokine (c-x-c motif) ligand 3, KLF9 - kruppel-like factor 9, CALCRL - calcitonin receptor-like, ANXA1 - annexin a1, MGST1 - microsomal glutathione s-transferase 1, TICAM1 - toll-like receptor adaptor molecule 1, KLF10 - kruppel-like factor 10, INHBB - inhibin, beta b]
GO:0007166	cell surface receptor signaling pathway	2.10E-07	2.14E-04	1.79	16231	1969	355	77	[HFE - hemochromatosis, OASL - 2'-5'-oligoadenylate synthetase-like, ARID5B - at rich interactive domain 5b (mrf1-like), ID1 - inhibitor of dna binding 1, dominant negative helix-loop-helix protein, ITPKB - inositol-trisphosphate 3-kinase b, PTPRZ1 - protein tyrosine phosphatase, receptor-type, z polypeptide 1, RELB - v-rel avian reticuloendotheliosis viral oncogene homolog b, MSTN - myostatin, LTBP3 - latent transforming growth factor beta binding protein 3, BTN3A3 - butyrophilin, subfamily 3, member a3, BIRC3 - baculoviral iap repeat containing 3, IL8 - interleukin 8, PLP1 - proteolipid protein 1, APOA1 - apolipoprotein a-i, ZC3H12A - zinc finger ccch-type containing 12a, COL4A1 - collagen, type iv, alpha 1, NEDD9 - neural precursor cell expressed, developmentally down-regulated 9, ATP6V1C2 - atpase, h+ transporting, lysosomal 42kda, v1 subunit c2, CSF1 - colony stimulating factor 1 (macrophage), WNT8B - wingless-type mmtv integration site family, member 8b, IFI35 - interferon-induced protein 35, IFIT2 - interferon-induced protein with tetratricopeptide repeats 2, IFIT1 - interferon-induced protein with tetratricopeptide repeats 1, IFIT3 - interferon-induced protein with tetratricopeptide repeats 3, SOSTDC1 - sclerostin domain containing 1, GHR - growth hormone receptor, TBX2 - t-box 2, NTRK2 - neurotrophic tyrosine kinase, receptor, type 2, FAS - fas cell surface death receptor, FGF1 - fibroblast growth factor 1 (acidic), CXCL13 - chemokine (c-x-c motif) ligand 13, YAP1 - yes-associated protein 1, PSMB9 - proteasome (prosome, macropain) subunit, beta type, 9, BTC - betacellulin, GAB1 - grb2-associated binding protein 1, MUC15 - mucin 15, cell surface associated, GPR35 - g protein-coupled receptor 35, TICAM1 - toll-like receptor adaptor molecule 1, INHBB - inhibin, beta b, OAS3 - 2'-5'-oligoadenylate synthetase 3, 100kda, CCL2 - chemokine (c-c motif) ligand 2, NFKB1 - nuclear factor of kappa light polypeptide gene enhancer in b-cells 1, CSRNP1 - cysteine-serine-rich nuclear protein 1, ANGPTL1 - angiopoietin-like 1, NFKBIA - nuclear factor of kappa light polypeptide gene enhancer in b-cells inhibitor, alpha, TIPARP - tcdd-inducible poly(adp-ribose) polymerase, HSPB1 - heat shock 27kda protein 1, LEPR - leptin receptor, CCL20 - chemokine (c-c motif) ligand 20, DDIT3 - dna-damage-inducible transcript 3, SOX9 - sry (sex determining region y)-box 9, C5 - complement component 5, PTGS2 - prostaglandin-endoperoxide synthase 2 (prostaglandin g/h synthase and cyclooxygenase), IRF1 - interferon regulatory factor 1, TNFRSF11B - tumor necrosis factor receptor superfamily, member 11b, CXCL12 - chemokine (c-x-c motif) ligand 12, GRIK4 - glutamate receptor, ionotropic, kainate 4, CHAC1 - chac, cation transport regulator homolog 1 (e. coli), ITGA2 - integrin, alpha 2 (cd49b, alpha 2 subunit of vla-2 receptor), NMI - n-myc (and stat) interactor, PTN - pleiotrophin, VCAM1 - vascular cell adhesion molecule 1, RIPK2 - receptor-interacting serine-threonine kinase 2, GPR87 - g protein-coupled receptor 87, HBEGF - heparin-binding egf-like growth factor, PDGFRB - platelet-derived growth factor receptor, beta polypeptide, SUSD5 - sushi domain containing 5, ADORA1 - adenosine a1 receptor, GPHA2 - glycoprotein hormone alpha 2, CXCL1 - chemokine (c-x-c motif) ligand 1 (melanoma growth stimulating activity, alpha), CXCL2 - chemokine (c-x-c motif) ligand 2, CXCL3 - chemokine (c-x-c motif) ligand 3, CALCRL - calcitonin receptor-like, ANXA1 - annexin a1, PTPRC - protein tyrosine phosphatase, receptor type, c, ITGB7 - integrin, beta 7, ANXA4 - annexin a4]
GO:1901700	response to oxygen-containing compound	2.62E-07	2.50E-04	2.05	16231	1228	355	55	[FOLR1 - folate receptor 1 (adult), ID1 - inhibitor of dna binding 1, dominant negative helix-loop-helix protein, ID3 - inhibitor of dna binding 3, dominant negative helix-loop-helix protein, MSTN - myostatin, ATP1A2 - atpase, na+/k+ transporting, alpha 2 polypeptide, IL8 - interleukin 8, ZC3H12A - zinc finger ccch-type containing 12a, TFPI - tissue factor pathway inhibitor (lipoprotein-associated coagulation inhibitor), COL4A1 - collagen, type iv, alpha 1, WNT8B - wingless-type mmtv integration site family, member 8b, PLSCR4 - phospholipid scramblase 4, SCD - stearoyl-coa desaturase (delta-9-desaturase), NTRK2 - neurotrophic tyrosine kinase, receptor, type 2, CXCL13 - chemokine (c-x-c motif) ligand 13, YAP1 - yes-associated protein 1, ZFP36L1 - zfp36 ring finger protein-like 1, RAMP3 - receptor (g protein-coupled) activity modifying protein 3, KCNJ8 - potassium inwardly-rectifying channel, subfamily j, member 8, HSD17B2 - hydroxysteroid (17-beta) dehydrogenase 2, KLF9 - kruppel-like factor 9, UCP3 - uncoupling protein 3 (mitochondrial, proton carrier), TICAM1 - toll-like receptor adaptor molecule 1, MGST1 - microsomal glutathione s-transferase 1, KLF10 - kruppel-like factor 10, INHBB - inhibin, beta b, CCL2 - chemokine (c-c motif) ligand 2, NFKB1 - nuclear factor of kappa light polypeptide gene enhancer in b-cells 1, KCNMB1 - potassium large conductance calcium-activated channel, subfamily m, beta member 1, HSPA5 - heat shock 70kda protein 5 (glucose-regulated protein, 78kda), NFKBIA - nuclear factor of kappa light polypeptide gene enhancer in b-cells inhibitor, alpha, DCN - decorin, DUSP10 - dual specificity phosphatase 10, SETD7 - set domain containing (lysine methyltransferase) 7, IGFBP7 - insulin-like growth factor binding protein 7, SLC2A5 - solute carrier family 2 (facilitated glucose/fructose transporter), member 5, MAOB - monoamine oxidase b, SOX9 - sry (sex determining region y)-box 9, KLF15 - kruppel-like factor 15, HBA2 - hemoglobin, alpha 2, CXCL12 - chemokine (c-x-c motif) ligand 12, ITGA2 - integrin, alpha 2 (cd49b, alpha 2 subunit of vla-2 receptor), FOSL1 - fos-like antigen 1, PTN - pleiotrophin, RIPK2 - receptor-interacting serine-threonine kinase 2, VCAM1 - vascular cell adhesion molecule 1, MLC1 - megalencephalic leukoencephalopathy with subcortical cysts 1, CPS1 - carbamoyl-phosphate synthase 1, mitochondrial, PDGFRB - platelet-derived growth factor receptor, beta polypeptide, TNC - tenascin c, TNFAIP3 - tumor necrosis factor, alpha-induced protein 3, CXCL1 - chemokine (c-x-c motif) ligand 1 (melanoma growth stimulating activity, alpha), CXCL2 - chemokine (c-x-c motif) ligand 2, CXCL3 - chemokine (c-x-c motif) ligand 3, CALCRL - calcitonin receptor-like, ANXA1 - annexin a1]
GO:0032101	regulation of response to external stimulus	3.18E-07	2.85E-04	2.38	16231	767	355	40	[CFH - complement factor h, CCL2 - chemokine (c-c motif) ligand 2, NFKB1 - nuclear factor of kappa light polypeptide gene enhancer in b-cells 1, PARP9 - poly (adp-ribose) polymerase family, member 9, NFKBIA - nuclear factor of kappa light polypeptide gene enhancer in b-cells inhibitor, alpha, HSPB1 - heat shock 27kda protein 1, HERC5 - hect and rld domain containing e3 ubiquitin protein ligase 5, DUSP10 - dual specificity phosphatase 10, CD34 - cd34 molecule, MSTN - myostatin, PTGER3 - prostaglandin e receptor 3 (subtype ep3), BIRC3 - baculoviral iap repeat containing 3, IL8 - interleukin 8, C5 - complement component 5, DDX60 - dead (asp-glu-ala-asp) box polypeptide 60, PTGS2 - prostaglandin-endoperoxide synthase 2 (prostaglandin g/h synthase and cyclooxygenase), APOA1 - apolipoprotein a-i, ZC3H12A - zinc finger ccch-type containing 12a, C7 - complement component 7, TFPI - tissue factor pathway inhibitor (lipoprotein-associated coagulation inhibitor), CXCL12 - chemokine (c-x-c motif) ligand 12, CSF1 - colony stimulating factor 1 (macrophage), NT5E - 5'-nucleotidase, ecto (cd73), IFI35 - interferon-induced protein 35, ITGA2 - integrin, alpha 2 (cd49b, alpha 2 subunit of vla-2 receptor), NMI - n-myc (and stat) interactor, IFIT1 - interferon-induced protein with tetratricopeptide repeats 1, PTN - pleiotrophin, FGL2 - fibrinogen-like 2, RIPK2 - receptor-interacting serine-threonine kinase 2, PDGFRB - platelet-derived growth factor receptor, beta polypeptide, FGF1 - fibroblast growth factor 1 (acidic), CXCL13 - chemokine (c-x-c motif) ligand 13, ADORA1 - adenosine a1 receptor, TNFAIP3 - tumor necrosis factor, alpha-induced protein 3, CALCRL - calcitonin receptor-like, ANXA1 - annexin a1, PTPRC - protein tyrosine phosphatase, receptor type, c, TICAM1 - toll-like receptor adaptor molecule 1, PLAU - plasminogen activator, urokinase]

GO:0051707	response to other organism	6.12E-07	5.17E-04	2.74	16231	500	355	30	[OAS3 - 2'-5'-oligoadenylate synthetase 3, 100kda, CCL2 - chemokine (c-c motif) ligand 2, OASL - 2'-5'-oligoadenylate synthetase-like, PARP9 - poly (adp-ribose) polymerase family, member 9, HSPB1 - heat shock 27kda protein 1, PTX3 - pentraxin 3, long, HERC5 - hect and rld domain containing e3 ubiquitin protein ligase 5, CCL20 - chemokine (c-c motif) ligand 20, SLC22A5 - solute carrier family 22 (organic cation/carnitine transporter), member 5, IL8 - interleukin 8, DDX60 - dead (asp-glu-ala-asp) box polypeptide 60, IRF1 - interferon regulatory factor 1, ZC3H12A - zinc finger ccch-type containing 12a, COLEC12 - collectin sub-family member 12, CXCL12 - chemokine (c-x-c motif) ligand 12, IFIT2 - interferon-induced protein with tetratricopeptide repeats 2, NMI - n-myc (and stat) interactor, FOSL1 - fos-like antigen 1, IFIT1 - interferon-induced protein with tetratricopeptide repeats 1, IFIT3 - interferon-induced protein with tetratricopeptide repeats 3, RIPK2 - receptor-interacting serine-threonine kinase 2, CXCL13 - chemokine (c-x-c motif) ligand 13, ACTA2 - actin, alpha 2, smooth muscle, aorta, KCNJ8 - potassium inwardly-rectifying channel, subfamily j, member 8, CXCL1 - chemokine (c-x-c motif) ligand 1 (melanoma growth stimulating activity, alpha), CXCL2 - chemokine (c-x-c motif) ligand 2, CXCL3 - chemokine (c-x-c motif) ligand 3, PTPRC - protein tyrosine phosphatase, receptor type, c, TICAM1 - toll-like receptor adaptor molecule 1, HMCN1 - hemicentin 1]
GO:0030198	extracellular matrix organization	6.63E-07	5.32E-04	3.28	16231	321	355	23	[FBLN5 - fibulin 5, ITGA2 - integrin, alpha 2 (cd49b, alpha 2 subunit of vla-2 receptor), ELN - elastin, CTSV - cathepsin v, MMP21 - matrix metallopeptidase 21, COL22A1 - collagen, type xxii, alpha 1, DCN - decorin, VCAM1 - vascular cell adhesion molecule 1, COL8A1 - collagen, type viii, alpha 1, PTX3 - pentraxin 3, long, ECM2 - extracellular matrix protein 2, female organ and adipocyte specific, SERPINH1 - serpin peptidase inhibitor, clade h (heat shock protein 47), member 1, (collagen binding protein 1), LTBP3 - latent transforming growth factor beta binding protein 3, COL12A1 - collagen, type xii, alpha 1, TNC - tenascin c, SMOC1 - sparc related modular calcium binding 1, ACAN - aggrecan, SOX9 - sry (sex determining region y)-box 9, IBSP - integrin-binding sialoprotein, TTR - transthyretin, ITGB7 - integrin, beta 7, TNFRSF11B - tumor necrosis factor receptor superfamily, member 11b, COL4A1 - collagen, type iv, alpha 1]
GO:0050896	response to stimulus	9.01E-07	6.86E-04	1.42	16231	4497	355	140	[CFH - complement factor h, HFE - hemochromatosis, OASL - 2'-5'-oligoadenylate synthetase-like, ARID5B - at rich interactive domain 5b (mrf1-like), MXRA5 - matrix-remodelling associated 5, ID1 - inhibitor of dna binding 1, dominant negative helix-loop-helix protein, ITPKB - inositol-trisphosphate 3-kinase b, ID3 - inhibitor of dna binding 3, dominant negative helix-loop-helix protein, RELB - v-rel avian reticuloendotheliosis viral oncogene homolog b, SLC22A5 - solute carrier family 22 (organic cation/carnitine transporter), member 5, GP9 - glycoprotein ix (platelet), PLP1 - proteolipid protein 1, HLA-DMA - major histocompatibility complex, class ii, dm alpha, APOA1 - apolipoprotein a-i, KCNA1 - potassium voltage-gated channel, shaker-related subfamily, member 1 (episodic ataxia with myokymia), TRIM17 - tripartite motif containing 17, ZC3H12A - zinc finger ccch-type containing 12a, COL4A1 - collagen, type iv, alpha 1, OPN3 - opsin 3, RGR - retinal g protein coupled receptor, CD58 - cd58 molecule, WNT8B - wingless-type mmtv integration site family, member 8b, IFIT2 - interferon-induced protein with tetratricopeptide repeats 2, RGS10 - regulator of g-protein signaling 10, IFIT1 - interferon-induced protein with tetratricopeptide repeats 1, IFIT3 - interferon-induced protein with tetratricopeptide repeats 3, SCD - stearoyl-coa desaturase (delta-9-desaturase), SPRY2 - sprouty homolog 2 (drosophila), TBX2 - t-box 2, FAS - fas cell surface death receptor, PAX6 - paired box 6, LRRC8C - leucine rich repeat containing 8 family, member c, FGF1 - fibroblast growth factor 1 (acidic), CXCL13 - chemokine (c-x-c motif) ligand 13, PSMB9 - proteasome (prosome, macropain) subunit, beta type, 9, ZFP36L1 - zfp36 ring finger protein-like 1, ACTA2 - actin, alpha 2, smooth muscle, aorta, AQP4 - aquaporin 4, RAMP3 - receptor (g protein-coupled) activity modifying protein 3, KCNJ8 - potassium inwardly-rectifying channel, subfamily j, member 8, GAB1 - grb2-associated binding protein 1, KLF9 - kruppel-like factor 9, TICAM1 - toll-like receptor adaptor molecule 1, NFKB1 - nuclear factor of kappa light polypeptide gene enhancer in b-cells 1, CCL2 - chemokine (c-c motif) ligand 2, KCNMB1 - potassium large conductance calcium-activated channel, subfamily m, beta member 1, RNF175 - ring finger protein 175, PARP9 - poly (adp-ribose) polymerase family, member 9, NFKBIA - nuclear factor of kappa light polypeptide gene enhancer in b-cells inhibitor, alpha, DCN - decorin, TIPARP - tcdd-inducible poly(adp-ribose) polymerase, SOD3 - superoxide dismutase 3, extracellular, HERPUD1 - homocysteine-inducible, endoplasmic reticulum stress-inducible, ubiquitin-like domain member 1, DCT - dopachrome tautomerase, HERC5 - hect and rld domain containing e3 ubiquitin protein ligase 5, DUSP10 - dual specificity phosphatase 10, IGFBP7 - insulin-like growth factor binding protein 7, PTGER3 - prostaglandin e receptor 3 (subtype ep3), CCL20 - chemokine (c-c motif) ligand 20, GADD45A - growth arrest and dna-damage-inducible, alpha, DDIT3 - dna-damage-inducible transcript 3, MAOB - monoamine oxidase b, SOX9 - sry (sex determining region y)-box 9, ERAP2 - endoplasmic reticulum aminopeptidase 2, C5 - complement component 5, DDX60 - dead (asp-glu-ala-asp) box polypeptide 60, PTGS2 - prostaglandin-endoperoxide synthase 2 (prostaglandin g/h synthase and cyclooxygenase), C7 - complement component 7, KLF15 - kruppel-like factor 15, USP2 - ubiquitin specific peptidase 2, CXCL12 - chemokine (c-x-c motif) ligand 12, FOSL1 - fos-like antigen 1, NMI - n-myc (and stat) interactor, PTN - pleiotrophin, RIPK2 - receptor-interacting serine-threonine kinase 2, CPS1 - carbamoyl-phosphate synthase 1, mitochondrial, PDGFRB - platelet-derived growth factor receptor, beta polypeptide, NEK11 - nima-related kinase 11, ADORA1 - adenosine a1 receptor, CXCL1 - chemokine (c-x-c motif) ligand 1 (melanoma growth stimulating activity, alpha), CXCL2 - chemokine (c-x-c motif) ligand 2, CXCL3 - chemokine (c-x-c motif) ligand 3, PTPRC - protein tyrosine phosphatase, receptor type, c, HMCN1 - hemicentin 1, FOLR1 - folate receptor 1 (adult), SLC39A12 - solute carrier family 39 (zinc transporter), member 12, ATF3 - activating transcription factor 3, OR2L13 - olfactory receptor, family 2, subfamily l, member 13, PTX3 - pentraxin 3, long, MSTN - myostatin, ATP1A2 - atpase, na+/k+ transporting, alpha 2 polypeptide, BTN3A3 - butyrophilin, subfamily 3, member a3, TRPV4 - transient receptor potential cation channel, subfamily v, member 4, IL8 - interleukin 8, SFTA3 - surfactant associated 3, TFPI - tissue factor pathway inhibitor (lipoprotein-associated coagulation inhibitor), CSF1 - colony stimulating factor 1 (macrophage), PLSCR4 - phospholipid scramblase 4, GHR - growth hormone receptor, NTRK2 - neurotrophic tyrosine kinase, receptor, type 2, YAP1 - yes-associated protein 1, SLC24A5 - solute carrier family 24 (sodium/potassium/calcium exchanger), member 5, HSD17B2 - hydroxysteroid (17-beta) dehydrogenase 2, UCP3 - uncoupling protein 3 (mitochondrial, proton carrier), MGST1 - microsomal glutathione s-transferase 1, KLF10 - kruppel-like factor 10, INHBB - inhibin, beta b, GZMB - granzyme b (granzyme 2, cytotoxic t-lymphocyte-associated serine esterase 1), OAS3 - 2'-5'-oligoadenylate synthetase 3, 100kda, HSPA5 - heat shock 70kda protein 5 (glucose-regulated protein, 78kda), CAV3 - caveolin 3, IQGAP3 - iq motif containing gtpase activating protein 3, HSPB1 - heat shock 27kda protein 1, LEPR - leptin receptor, SETD7 - set domain containing (lysine methyltransferase) 7, SERPINH1 - serpin peptidase inhibitor, clade h (heat shock protein 47), member 1, (collagen binding protein 1), SLC2A5 - solute carrier family 2 (facilitated glucose/fructose transporter), member 5, IRF1 - interferon regulatory factor 1, PARP14 - poly (adp-ribose) polymerase family, member 14, TAC4 - tachykinin 4 (hemokinin), TNFRSF11B - tumor necrosis factor receptor superfamily, member 11b, HBA2 - hemoglobin, alpha 2, COLEC12 - collectin sub-family member 12, GJC2 - gap junction protein, gamma 2, 47kda, HTR1A - 5-hydroxytryptamine (serotonin) receptor 1a, g protein-coupled, MME - membrane metallo-endopeptidase, CHAC1 - chac, cation transport regulator homolog 1 (e. coli), ITGA2 - integrin, alpha 2 (cd49b, alpha 2 subunit of vla-2 receptor), CTSV - cathepsin v, VCAM1 - vascular cell adhesion molecule 1, MLC1 - megalencephalic leukoencephalopathy with subcortical cysts 1, TNC - tenascin c, TNFAIP3 - tumor necrosis factor, alpha-induced protein 3, OPN1SW - opsin 1 (cone pigments), short-wave-sensitive, CALCRL - calcitonin receptor-like, ANXA1 -
GO:0002237	response to molecule of bacterial origin	1.28E-06	9.26E-04	3.5	16231	261	355	20	[CCL2 - chemokine (c-c motif) ligand 2, NFKB1 - nuclear factor of kappa light polypeptide gene enhancer in b-cells 1, PLSCR4 - phospholipid scramblase 4, DCN - decorin, RIPK2 - receptor-interacting serine-threonine kinase 2, VCAM1 - vascular cell adhesion molecule 1, CPS1 - carbamoyl-phosphate synthase 1, mitochondrial, CXCL13 - chemokine (c-x-c motif) ligand 13, DUSP10 - dual specificity phosphatase 10, KCNJ8 - potassium inwardly-rectifying channel, subfamily j, member 8, TNFAIP3 - tumor necrosis factor, alpha-induced protein 3, MAOB - monoamine oxidase b, CXCL1 - chemokine (c-x-c motif) ligand 1 (melanoma growth stimulating activity, alpha), IL8 - interleukin 8, CXCL2 - chemokine (c-x-c motif) ligand 2, CXCL3 - chemokine (c-x-c motif) ligand 3, ZC3H12A - zinc finger ccch-type containing 12a, TFPI - tissue factor pathway inhibitor (lipoprotein-associated coagulation inhibitor), TICAM1 - toll-like receptor adaptor molecule 1, MGST1 - microsomal glutathione s-transferase 1]
GO:0019221	cytokine-mediated signaling pathway	1.32E-06	9.14E-04	2.54	16231	575	355	32	[OAS3 - 2'-5'-oligoadenylate synthetase 3, 100kda, CCL2 - chemokine (c-c motif) ligand 2, OASL - 2'-5'-oligoadenylate synthetase-like, NFKB1 - nuclear factor of kappa light polypeptide gene enhancer in b-cells 1, NFKBIA - nuclear factor of kappa light polypeptide gene enhancer in b-cells inhibitor, alpha, PTPRZ1 - protein tyrosine phosphatase, receptor-type, z polypeptide 1, LEPR - leptin receptor, CCL20 - chemokine (c-c motif) ligand 20, BIRC3 - baculoviral iap repeat containing 3, IL8 - interleukin 8, PTGS2 - prostaglandin-endoperoxide synthase 2 (prostaglandin g/h synthase and cyclooxygenase), IRF1 - interferon regulatory factor 1, TNFRSF11B - tumor necrosis factor receptor superfamily, member 11b, CXCL12 - chemokine (c-x-c motif) ligand 12, CSF1 - colony stimulating factor 1 (macrophage), IFI35 - interferon-induced protein 35, IFIT2 - interferon-induced protein with tetratricopeptide repeats 2, NMI - n-myc (and stat) interactor, IFIT1 - interferon-induced protein with tetratricopeptide repeats 1, IFIT3 - interferon-induced protein with tetratricopeptide repeats 3, GHR - growth hormone receptor, RIPK2 - receptor-interacting serine-threonine kinase 2, VCAM1 - vascular cell adhesion molecule 1, FAS - fas cell surface death receptor, CXCL13 - chemokine (c-x-c motif) ligand 13, YAP1 - yes-associated protein 1, PSMB9 - proteasome (prosome, macropain) subunit, beta type, 9, CXCL1 - chemokine (c-x-c motif) ligand 1 (melanoma growth stimulating activity, alpha), CXCL2 - chemokine (c-x-c motif) ligand 2, CXCL3 - chemokine (c-x-c motif) ligand 3, ANXA1 - annexin a1, GPR35 - g protein-coupled receptor 35]

GO:0007165	signal transduction	1.67E-06	1.10E-03	1.45	16231	4009	355	127	[HFE - hemochromatosis, OASL - 2'-5'-oligoadenylate synthetase-like, ARID5B - at rich interactive domain 5b (mrf1-like), CDC42EP1 - cdc42 effector protein (rho gtpase binding) 1, E2F2 - e2f transcription factor 2, ID1 - inhibitor of dna binding 1, dominant negative helix-loop-helix protein, ITPKB - inositol-trisphosphate 3-kinase b, RELB - v-rel avian reticuloendotheliosis viral oncogene homolog b, RHOD - ras homolog family member d, CD34 - cd34 molecule, LTBP3 - latent transforming growth factor beta binding protein 3, BIRC3 - baculoviral iap repeat containing 3, PLP1 - proteolipid protein 1, APOA1 - apolipoprotein a-i, KCNA1 - potassium voltage-gated channel, shaker-related subfamily, member 1 (episodic ataxia with myokymia), ZC3H12A - zinc finger ccch-type containing 12a, DAND5 - dan domain family member 5, bmp antagonist, COL4A1 - collagen, type iv, alpha 1, OPN3 - opsin 3, RGR - retinal g protein coupled receptor, NEDD9 - neural precursor cell expressed, developmentally down-regulated 9, ATP6V1C2 - atpase, h+ transporting, lysosomal 42kda, v1 subunit c2, RHOJ - ras homolog family member j, WNT8B - wingless-type mmtv integration site family, member 8b, IFI35 - interferon-induced protein 35, IFIT2 - interferon-induced protein with tetratricopeptide repeats 2, RGS10 - regulator of g-protein signaling 10, IFIT1 - interferon-induced protein with tetratricopeptide repeats 1, IFIT3 - interferon-induced protein with tetratricopeptide repeats 3, SOSTDC1 - sclerostin domain containing 1, KCNIP3 - kv channel interacting protein 3, calsenilin, TBX2 - t-box 2, FAS - fas cell surface death receptor, FGF1 - fibroblast growth factor 1 (acidic), CXCL13 - chemokine (c-x-c motif) ligand 13, PSMB9 - proteasome (prosome, macropain) subunit, beta type, 9, ZFP36L1 - zfp36 ring finger protein-like 1, LPAR4 - lysophosphatidic acid receptor 4, RAMP3 - receptor (g protein-coupled) activity modifying protein 3, BTC - betacellulin, GAB1 - grb2-associated binding protein 1, GPR35 - g protein-coupled receptor 35, MUC15 - mucin 15, cell surface associated, TICAM1 - toll-like receptor adaptor molecule 1, SPARCL1 - sparc-like 1 (hevin), NFKB1 - nuclear factor of kappa light polypeptide gene enhancer in b-cells 1, CCL2 - chemokine (c-c motif) ligand 2, RNF175 - ring finger protein 175, NFKBIA - nuclear factor of kappa light polypeptide gene enhancer in b-cells inhibitor, alpha, ANGPTL1 - angiopoietin-like 1, TIPARP - tcdd-inducible poly(adp-ribose) polymerase, PCP4 - purkinje cell protein 4, HERPUD1 - homocysteine-inducible, endoplasmic reticulum stress-inducible, ubiquitin-like domain member 1, CKLF - chemokine-like factor, PTGER3 - prostaglandin e receptor 3 (subtype ep3), CCL20 - chemokine (c-c motif) ligand 20, RHOH - ras homolog family member h, GADD45A - growth arrest and dna-damage-inducible, alpha, DDIT3 - dna-damage-inducible transcript 3, SOX9 - sry (sex determining region y)-box 9, ERAP2 - endoplasmic reticulum aminopeptidase 2, C5 - complement component 5, PTGS2 - prostaglandin-endoperoxide synthase 2 (prostaglandin g/h synthase and cyclooxygenase), LRRC2 - leucine rich repeat containing 2, PTHLH - parathyroid hormone-like hormone, CXCL12 - chemokine (c-x-c motif) ligand 12, GRIK4 - glutamate receptor, ionotropic, kainate 4, NMI - n-myc (and stat) interactor, PTN - pleiotrophin, RIPK2 - receptor-interacting serine-threonine kinase 2, PDGFRB - platelet-derived growth factor receptor, beta polypeptide, NEK11 - nima-related kinase 11, SUSD5 - sushi domain containing 5, ADORA1 - adenosine a1 receptor, GPHA2 - glycoprotein hormone alpha 2, CXCL1 - chemokine (c-x-c motif) ligand 1 (melanoma growth stimulating activity, alpha), CA8 - carbonic anhydrase viii, CXCL2 - chemokine (c-x-c motif) ligand 2, CXCL3 - chemokine (c-x-c motif) ligand 3, PTPRC - protein tyrosine phosphatase, receptor type, c, TTR - transthyretin, GSC - goosecoid homeobox, SLC39A12 - solute carrier family 39 (zinc transporter), member 12, ATF3 - activating transcription factor 3, PTPRZ1 - protein tyrosine phosphatase, receptor-type, z polypeptide 1, OR2L13 - olfactory receptor, family 2, subfamily l, member 13, MSTN - myostatin, ATP1A2 - atpase, na+/k+ transporting, alpha 2 polypeptide, ATOH8 - atonal homolog 8 (drosophila), BTN3A3 - butyrophilin, subfamily 3, member a3, TRPV4 - transient receptor potential cation channel, subfamily v, member 4, IL8 - interleukin 8, CSF1 - colony stimulating factor 1 (macrophage), GHR - growth hormone receptor, NTRK2 - neurotrophic tyrosine kinase, receptor, type 2, YAP1 - yes-associated protein 1, RND1 - rho family gtpase 1, INHBB - inhibin, beta b, OAS3 - 2'-5'-oligoadenylate synthetase 3, 100kda, HSPA5 - heat shock 70kda protein 5 (glucose-regulated protein, 78kda), CSRNP1 - cysteine-serine-rich nuclear protein 1, IQGAP3 - iq motif containing gtpase activating protein 3, HSPB1 - heat shock 27kda protein 1, LEPR - leptin receptor, OGN - osteoglycin, IRF1 - interferon regulatory factor 1, TAC4 - tachykinin 4 (hemokinin), TNFRSF11B - tumor necrosis factor receptor superfamily, member 11b, COLEC12 - collectin sub-family member 12, HTR1A - 5-hydroxytryptamine (serotonin) receptor 1a, g protein-coupled, RRAD - ras-related associated with diabetes, CHAC1 - chac, cation transport regulator homolog 1 (e. coli), HTR1D - 5-hydroxytryptamine (serotonin) receptor 1d, g protein-coupled, ITGA2 - integrin, alpha 2 (cd49b, alpha 2 subunit of vla-2 receptor), GPR87 - g protein-coupled receptor 87, VCAM1 - vascular cell adhesion molecule 1, HBEGF - heparin-binding egf-like growth factor, PROKR2 - prokineticin receptor 2, TNFAIP3 - tumor necrosis factor, alpha-induced protein 3, OPN1SW - opsin 1 (cone pigments), short-wave-sensitive, CALCRL - calcitonin receptor-like, ANXA1 - annexin a1, ITGB7 - integrin, beta 7, RAB39A - rab39a, member ras oncogene family, ANXA4 - annexin a4, PLAU - plasminogen activator, urokinase, CD83 - cd83 molecule]
GO:0071216	cellular response to biotic stimulus	1.87E-06	1.18E-03	4.37	16231	157	355	15	[NFKB1 - nuclear factor of kappa light polypeptide gene enhancer in b-cells 1, CCL2 - chemokine (c-c motif) ligand 2, PLSCR4 - phospholipid scramblase 4, HSPA5 - heat shock 70kda protein 5 (glucose-regulated protein, 78kda), RIPK2 - receptor-interacting serine-threonine kinase 2, CXCL13 - chemokine (c-x-c motif) ligand 13, TNFAIP3 - tumor necrosis factor, alpha-induced protein 3, DDIT3 - dna-damage-inducible transcript 3, CXCL1 - chemokine (c-x-c motif) ligand 1 (melanoma growth stimulating activity, alpha), IL8 - interleukin 8, CXCL2 - chemokine (c-x-c motif) ligand 2, CXCL3 - chemokine (c-x-c motif) ligand 3, ZC3H12A - zinc finger ccch-type containing 12a, TFPI - tissue factor pathway inhibitor (lipoprotein-associated coagulation inhibitor), TICAM1 - toll-like receptor adaptor molecule 1]
GO:0051239	regulation of multicellular organismal process	1.94E-06	1.18E-03	1.56	16231	2871	355	98	[HFE - hemochromatosis, E2F2 - e2f transcription factor 2, ID1 - inhibitor of dna binding 1, dominant negative helix-loop-helix protein, ITPKB - inositol-trisphosphate 3-kinase b, ID3 - inhibitor of dna binding 3, dominant negative helix-loop-helix protein, RELB - v-rel avian reticuloendotheliosis viral oncogene homolog b, CD34 - cd34 molecule, SLC22A5 - solute carrier family 22 (organic cation/carnitine transporter), member 5, LTBP3 - latent transforming growth factor beta binding protein 3, BIRC3 - baculoviral iap repeat containing 3, APOA1 - apolipoprotein a-i, KCNA1 - potassium voltage-gated channel, shaker-related subfamily, member 1 (episodic ataxia with myokymia), ZC3H12A - zinc finger ccch-type containing 12a, DAND5 - dan domain family member 5, bmp antagonist, CD58 - cd58 molecule, RHOJ - ras homolog family member j, FGL2 - fibrinogen-like 2, SCD - stearoyl-coa desaturase (delta-9-desaturase), SOSTDC1 - sclerostin domain containing 1, SPRY2 - sprouty homolog 2 (drosophila), TBX2 - t-box 2, KCNIP3 - kv channel interacting protein 3, calsenilin, PAX6 - paired box 6, SMR3B - submaxillary gland androgen regulated protein 3b, FGF1 - fibroblast growth factor 1 (acidic), CXCL13 - chemokine (c-x-c motif) ligand 13, PSMB9 - proteasome (prosome, macropain) subunit, beta type, 9, ZFP36L1 - zfp36 ring finger protein-like 1, SMOC1 - sparc related modular calcium binding 1, PRRX1 - paired related homeobox 1, GAB1 - grb2-associated binding protein 1, GPR35 - g protein-coupled receptor 35, TICAM1 - toll-like receptor adaptor molecule 1, NFKB1 - nuclear factor of kappa light polypeptide gene enhancer in b-cells 1, NFKBIA - nuclear factor of kappa light polypeptide gene enhancer in b-cells inhibitor, alpha, DCN - decorin, PCP4 - purkinje cell protein 4, DCT - dopachrome tautomerase, HERC5 - hect and rld domain containing e3 ubiquitin protein ligase 5, DUSP10 - dual specificity phosphatase 10, PTGER3 - prostaglandin e receptor 3 (subtype ep3), RHOH - ras homolog family member h, GADD45A - growth arrest and dna-damage-inducible, alpha, DDIT3 - dna-damage-inducible transcript 3, SOX9 - sry (sex determining region y)-box 9, C5 - complement component 5, DDX60 - dead (asp-glu-ala-asp) box polypeptide 60, PTGS2 - prostaglandin-endoperoxide synthase 2 (prostaglandin g/h synthase and cyclooxygenase), PTHLH - parathyroid hormone-like hormone, CXCL12 - chemokine (c-x-c motif) ligand 12, NMI - n-myc (and stat) interactor, PTN - pleiotrophin, RIPK2 - receptor-interacting serine-threonine kinase 2, PDGFRB - platelet-derived growth factor receptor, beta polypeptide, ADORA1 - adenosine a1 receptor, PTPRC - protein tyrosine phosphatase, receptor type, c, SLC39A12 - solute carrier family 39 (zinc transporter), member 12, PTPRZ1 - protein tyrosine phosphatase, receptor-type, z polypeptide 1, MAFF - v-maf avian musculoaponeurotic fibrosarcoma oncogene homolog f, MSTN - myostatin, ATP1A2 - atpase, na+/k+ transporting, alpha 2 polypeptide, ATOH8 - atonal homolog 8 (drosophila), BTN3A3 - butyrophilin, subfamily 3, member a3, TRPV4 - transient receptor potential cation channel, subfamily v, member 4, IL8 - interleukin 8, TFPI - tissue factor pathway inhibitor (lipoprotein-associated coagulation inhibitor), CSF1 - colony stimulating factor 1 (macrophage), MBOAT1 - membrane bound o-acyltransferase domain containing 1, BARHL2 - barh-like homeobox 2, GHR - growth hormone receptor, NTRK2 - neurotrophic tyrosine kinase, receptor, type 2, YAP1 - yes-associated protein 1, ALDH1A1 - aldehyde dehydrogenase 1 family, member a1, LCAT - lecithin-cholesterol acyltransferase, MGP - matrix gla protein, KLF10 - kruppel-like factor 10, INHBB - inhibin, beta b, HSPA5 - heat shock 70kda protein 5 (glucose-regulated protein, 78kda), CAV3 - caveolin 3, IQGAP3 - iq motif containing gtpase activating protein 3, HSPB1 - heat shock 27kda protein 1, LEPR - leptin receptor, ARMCX5-GPRASP2 - armcx5-gprasp2 readthrough, IRF1 - interferon regulatory factor 1, TNFRSF11B - tumor necrosis factor receptor superfamily, member 11b, GJC2 - gap junction protein, gamma 2, 47kda, HTR1A - 5-hydroxytryptamine (serotonin) receptor 1a, g protein-coupled, MME - membrane metallo-endopeptidase, ITGA2 - integrin, alpha 2 (cd49b, alpha 2 subunit of vla-2 receptor), CTSV - cathepsin v, HBEGF - heparin-binding egf-like growth factor, TNFAIP3 - tumor necrosis factor, alpha-induced protein 3, CALCRL - calcitonin receptor-like, ANXA1 - annexin a1, SMR3A - submaxillary gland androgen regulated protein 3a, PLAU - plasminogen activator, urokinase, ANXA4 - annexin a4, CD83 - cd83 molecule]

GO:0071407	cellular response to organic cyclic compound	2.07E-06	1.21E-03	3.07	16231	343	355	23	[CCL2 - chemokine (c-c motif) ligand 2, NFKB1 - nuclear factor of kappa light polypeptide gene enhancer in b-cells 1, KCNMB1 - potassium large conductance calcium-activated channel, subfamily m, beta member 1, FOLR1 - folate receptor 1 (adult), HSPA5 - heat shock 70kda protein 5 (glucose-regulated protein, 78kda), ID1 - inhibitor of dna binding 1, dominant negative helix-loop-helix protein, ITGA2 - integrin, alpha 2 (cd49b, alpha 2 subunit of vla-2 receptor), IFIT1 - interferon-induced protein with tetratricopeptide repeats 1, PTN - pleiotrophin, ID3 - inhibitor of dna binding 3, dominant negative helix-loop-helix protein, TIPARP - tcdd-inducible poly(adp-ribose) polymerase, MLC1 - megalencephalic leukoencephalopathy with subcortical cysts 1, CPS1 - carbamoyl-phosphate synthase 1, mitochondrial, ZFP36L1 - zfp36 ring finger protein-like 1, MSTN - myostatin, ATP1A2 - atpase, na+/k+ transporting, alpha 2 polypeptide, TNC - tenascin c, RAMP3 - receptor (g protein-coupled) activity modifying protein 3, KLF9 - kruppel-like factor 9, ANXA1 - annexin a1, ZC3H12A - zinc finger ccch-type containing 12a, TFPI - tissue factor pathway inhibitor (lipoprotein-associated coagulation inhibitor), COLEC12 - collectin sub-family member 12]
GO:0051241	negative regulation of multicellular organismal process	2.08E-06	1.17E-03	2.03	16231	1080	355	48	[HFE - hemochromatosis, NFKB1 - nuclear factor of kappa light polypeptide gene enhancer in b-cells 1, E2F2 - e2f transcription factor 2, NFKBIA - nuclear factor of kappa light polypeptide gene enhancer in b-cells inhibitor, alpha, CAV3 - caveolin 3, ID1 - inhibitor of dna binding 1, dominant negative helix-loop-helix protein, ITPKB - inositol-trisphosphate 3-kinase b, ID3 - inhibitor of dna binding 3, dominant negative helix-loop-helix protein, RELB - v-rel avian reticuloendotheliosis viral oncogene homolog b, DCN - decorin, HERC5 - hect and rld domain containing e3 ubiquitin protein ligase 5, DUSP10 - dual specificity phosphatase 10, CD34 - cd34 molecule, MSTN - myostatin, PTGER3 - prostaglandin e receptor 3 (subtype ep3), ATP1A2 - atpase, na+/k+ transporting, alpha 2 polypeptide, LTBP3 - latent transforming growth factor beta binding protein 3, GADD45A - growth arrest and dna-damage-inducible, alpha, TRPV4 - transient receptor potential cation channel, subfamily v, member 4, DDIT3 - dna-damage-inducible transcript 3, SOX9 - sry (sex determining region y)-box 9, IRF1 - interferon regulatory factor 1, APOA1 - apolipoprotein a-i, PTHLH - parathyroid hormone-like hormone, ZC3H12A - zinc finger ccch-type containing 12a, TFPI - tissue factor pathway inhibitor (lipoprotein-associated coagulation inhibitor), DAND5 - dan domain family member 5, bmp antagonist, TNFRSF11B - tumor necrosis factor receptor superfamily, member 11b, NMI - n-myc (and stat) interactor, PTN - pleiotrophin, FGL2 - fibrinogen-like 2, SOSTDC1 - sclerostin domain containing 1, SPRY2 - sprouty homolog 2 (drosophila), TBX2 - t-box 2, PAX6 - paired box 6, CXCL13 - chemokine (c-x-c motif) ligand 13, ZFP36L1 - zfp36 ring finger protein-like 1, ADORA1 - adenosine a1 receptor, TNFAIP3 - tumor necrosis factor, alpha-induced protein 3, ALDH1A1 - aldehyde dehydrogenase 1 family, member a1, CALCRL - calcitonin receptor-like, ANXA1 - annexin a1, PTPRC - protein tyrosine phosphatase, receptor type, c, GPR35 - g protein-coupled receptor 35, INHBB - inhibin, beta b, PLAU - plasminogen activator, urokinase, ANXA4 - annexin a4, CD83 - cd83 molecule]
GO:0032496	response to lipopolysacc haride	2.37E-06	1.29E-03	3.5	16231	248	355	19	[CCL2 - chemokine (c-c motif) ligand 2, NFKB1 - nuclear factor of kappa light polypeptide gene enhancer in b-cells 1, PLSCR4 - phospholipid scramblase 4, DCN - decorin, VCAM1 - vascular cell adhesion molecule 1, CPS1 - carbamoyl-phosphate synthase 1, mitochondrial, CXCL13 - chemokine (c-x-c motif) ligand 13, DUSP10 - dual specificity phosphatase 10, KCNJ8 - potassium inwardly-rectifying channel, subfamily j, member 8, TNFAIP3 - tumor necrosis factor, alpha-induced protein 3, MAOB - monoamine oxidase b, CXCL1 - chemokine (c-x-c motif) ligand 1 (melanoma growth stimulating activity, alpha), IL8 - interleukin 8, CXCL2 - chemokine (c-x-c motif) ligand 2, CXCL3 - chemokine (c-x-c motif) ligand 3, ZC3H12A - zinc finger ccch-type containing 12a, TFPI - tissue factor pathway inhibitor (lipoprotein-associated coagulation inhibitor), TICAM1 - toll-like receptor adaptor molecule 1, MGST1 - microsomal glutathione s-transferase 1]
GO:0009612	response to mechanical stimulus	2.38E-06	1.25E-03	4.04	16231	181	355	16	[NFKB1 - nuclear factor of kappa light polypeptide gene enhancer in b-cells 1, CXCL12 - chemokine (c-x-c motif) ligand 12, NFKBIA - nuclear factor of kappa light polypeptide gene enhancer in b-cells inhibitor, alpha, CAV3 - caveolin 3, ITGA2 - integrin, alpha 2 (cd49b, alpha 2 subunit of vla-2 receptor), FOSL1 - fos-like antigen 1, PTN - pleiotrophin, DCN - decorin, FAS - fas cell surface death receptor, ATP1A2 - atpase, na+/k+ transporting, alpha 2 polypeptide, TNC - tenascin c, GADD45A - growth arrest and dna-damage-inducible, alpha, TRPV4 - transient receptor potential cation channel, subfamily v, member 4, SOX9 - sry (sex determining region y)-box 9, IRF1 - interferon regulatory factor 1, KCNA1 - potassium voltage-gated channel, shaker-related subfamily, member 1 (episodic ataxia with myokymia)]
GO:0014070	response to organic cyclic compound	2.54E-06	1.29E-03	2.35	16231	681	355	35	[CCL2 - chemokine (c-c motif) ligand 2, NFKB1 - nuclear factor of kappa light polypeptide gene enhancer in b-cells 1, KCNMB1 - potassium large conductance calcium-activated channel, subfamily m, beta member 1, FOLR1 - folate receptor 1 (adult), HSPA5 - heat shock 70kda protein 5 (glucose-regulated protein, 78kda), NFKBIA - nuclear factor of kappa light polypeptide gene enhancer in b-cells inhibitor, alpha, ID1 - inhibitor of dna binding 1, dominant negative helix-loop-helix protein, ID3 - inhibitor of dna binding 3, dominant negative helix-loop-helix protein, TIPARP - tcdd-inducible poly(adp-ribose) polymerase, MSTN - myostatin, IGFBP7 - insulin-like growth factor binding protein 7, ATP1A2 - atpase, na+/k+ transporting, alpha 2 polypeptide, MAOB - monoamine oxidase b, ZC3H12A - zinc finger ccch-type containing 12a, TFPI - tissue factor pathway inhibitor (lipoprotein-associated coagulation inhibitor), COLEC12 - collectin sub-family member 12, WNT8B - wingless-type mmtv integration site family, member 8b, ITGA2 - integrin, alpha 2 (cd49b, alpha 2 subunit of vla-2 receptor), FOSL1 - fos-like antigen 1, IFIT1 - interferon-induced protein with tetratricopeptide repeats 1, PTN - pleiotrophin, RIPK2 - receptor-interacting serine-threonine kinase 2, MLC1 - megalencephalic leukoencephalopathy with subcortical cysts 1, CPS1 - carbamoyl-phosphate synthase 1, mitochondrial, PDGFRB - platelet-derived growth factor receptor, beta polypeptide, YAP1 - yes-associated protein 1, ZFP36L1 - zfp36 ring finger protein-like 1, TNC - tenascin c, KCNJ8 - potassium inwardly-rectifying channel, subfamily j, member 8, RAMP3 - receptor (g protein-coupled) activity modifying protein 3, KLF9 - kruppel-like factor 9, ANXA1 - annexin a1, UCP3 - uncoupling protein 3 (mitochondrial, proton carrier), TICAM1 - toll-like receptor adaptor molecule 1, CD83 - cd83 molecule]
GO:0006935	chemotaxis	3.02E-06	1.48E-03	3.31	16231	276	355	20	[CCL2 - chemokine (c-c motif) ligand 2, CXCL12 - chemokine (c-x-c motif) ligand 12, FOSL1 - fos-like antigen 1, PTN - pleiotrophin, VCAM1 - vascular cell adhesion molecule 1, HBEGF - heparin-binding egf-like growth factor, PDGFRB - platelet-derived growth factor receptor, beta polypeptide, CKLF - chemokine-like factor, CXCL13 - chemokine (c-x-c motif) ligand 13, CCL20 - chemokine (c-c motif) ligand 20, GAB1 - grb2-associated binding protein 1, CXCL1 - chemokine (c-x-c motif) ligand 1 (melanoma growth stimulating activity, alpha), IL8 - interleukin 8, CXCL2 - chemokine (c-x-c motif) ligand 2, C5 - complement component 5, CXCL3 - chemokine (c-x-c motif) ligand 3, ANXA1 - annexin a1, APOA1 - apolipoprotein a-i, CYP7B1 - cytochrome p450, family 7, subfamily b, polypeptide 1, PLAU - plasminogen activator, urokinase]
GO:0070098	chemokine-mediated signaling pathway	3.17E-06	1.51E-03	7.35	16231	56	355	9	[CCL20 - chemokine (c-c motif) ligand 20, CCL2 - chemokine (c-c motif) ligand 2, CXCL12 - chemokine (c-x-c motif) ligand 12, IL8 - interleukin 8, CXCL1 - chemokine (c-x-c motif) ligand 1 (melanoma growth stimulating activity, alpha), CXCL2 - chemokine (c-x-c motif) ligand 2, CXCL3 - chemokine (c-x-c motif) ligand 3, GPR35 - g protein-coupled receptor 35, CXCL13 - chemokine (c-x-c motif) ligand 13]
GO:0042330	taxis	3.37E-06	1.56E-03	3.29	16231	278	355	20	[CCL2 - chemokine (c-c motif) ligand 2, CXCL12 - chemokine (c-x-c motif) ligand 12, FOSL1 - fos-like antigen 1, PTN - pleiotrophin, VCAM1 - vascular cell adhesion molecule 1, HBEGF - heparin-binding egf-like growth factor, PDGFRB - platelet-derived growth factor receptor, beta polypeptide, CKLF - chemokine-like factor, CXCL13 - chemokine (c-x-c motif) ligand 13, CCL20 - chemokine (c-c motif) ligand 20, GAB1 - grb2-associated binding protein 1, CXCL1 - chemokine (c-x-c motif) ligand 1 (melanoma growth stimulating activity, alpha), IL8 - interleukin 8, CXCL2 - chemokine (c-x-c motif) ligand 2, C5 - complement component 5, CXCL3 - chemokine (c-x-c motif) ligand 3, ANXA1 - annexin a1, APOA1 - apolipoprotein a-i, CYP7B1 - cytochrome p450, family 7, subfamily b, polypeptide 1, PLAU - plasminogen activator, urokinase]

GO:0051716	cellular response to stimulus	5.30E-06	2.37E-03	1.57	16231	2628	355	90	[HFE - hemochromatosis, ARID5B - at rich interactive domain 5b (mrf1-like), FOLR1 - folate receptor 1 (adult), ID1 - inhibitor of dna binding 1, dominant negative helix-loop-helix protein, ITPKB - inositol-trisphosphate 3-kinase b, ATF3 - activating transcription factor 3, ID3 - inhibitor of dna binding 3, dominant negative helix-loop-helix protein, RELB - v-rel avian reticuloendotheliosis viral oncogene homolog b, MSTN - myostatin, ATP1A2 - atpase, na+/k+ transporting, alpha 2 polypeptide, TRPV4 - transient receptor potential cation channel, subfamily v, member 4, IL8 - interleukin 8, APOA1 - apolipoprotein a-i, KCNA1 - potassium voltage-gated channel, shaker-related subfamily, member 1 (episodic ataxia with myokymia), TFPI - tissue factor pathway inhibitor (lipoprotein-associated coagulation inhibitor), ZC3H12A - zinc finger ccch-type containing 12a, COL4A1 - collagen, type iv, alpha 1, OPN3 - opsin 3, RGR - retinal g protein coupled receptor, CD58 - cd58 molecule, PLSCR4 - phospholipid scramblase 4, WNT8B - wingless-type mmtv integration site family, member 8b, IFIT2 - interferon-induced protein with tetratricopeptide repeats 2, IFIT1 - interferon-induced protein with tetratricopeptide repeats 1, IFIT3 - interferon-induced protein with tetratricopeptide repeats 3, GHR - growth hormone receptor, SPRY2 - sprouty homolog 2 (drosophila), TBX2 - t-box 2, NTRK2 - neurotrophic tyrosine kinase, receptor, type 2, FAS - fas cell surface death receptor, LRRC8C - leucine rich repeat containing 8 family, member c, FGF1 - fibroblast growth factor 1 (acidic), CXCL13 - chemokine (c-x-c motif) ligand 13, YAP1 - yes-associated protein 1, PSMB9 - proteasome (prosome, macropain) subunit, beta type, 9, ZFP36L1 - zfp36 ring finger protein-like 1, AQP4 - aquaporin 4, RAMP3 - receptor (g protein-coupled) activity modifying protein 3, KLF9 - kruppel-like factor 9, UCP3 - uncoupling protein 3 (mitochondrial, proton carrier), KLF10 - kruppel-like factor 10, TICAM1 - toll-like receptor adaptor molecule 1, MGST1 - microsomal glutathione s-transferase 1, INHBB - inhibin, beta b, CCL2 - chemokine (c-c motif) ligand 2, NFKB1 - nuclear factor of kappa light polypeptide gene enhancer in b-cells 1, RNF175 - ring finger protein 175, KCNMB1 - potassium large conductance calcium-activated channel, subfamily m, beta member 1, PARP9 - poly (adp-ribose) polymerase family, member 9, HSPA5 - heat shock 70kda protein 5 (glucose-regulated protein, 78kda), NFKBIA - nuclear factor of kappa light polypeptide gene enhancer in b-cells inhibitor, alpha, CAV3 - caveolin 3, IQGAP3 - iq motif containing gtpase activating protein 3, TIPARP - tcdd-inducible poly(adp-ribose) polymerase, HSPB1 - heat shock 27kda protein 1, SOD3 - superoxide dismutase 3, extracellular, HERPUD1 - homocysteine-inducible, endoplasmic reticulum stress-inducible, ubiquitin-like domain member 1, SETD7 - set domain containing (lysine methyltransferase) 7, CCL20 - chemokine (c-c motif) ligand 20, IGFBP7 - insulin-like growth factor binding protein 7, SLC2A5 - solute carrier family 2 (facilitated glucose/fructose transporter), member 5, GADD45A - growth arrest and dna-damage-inducible, alpha, DDIT3 - dna-damage-inducible transcript 3, SOX9 - sry (sex determining region y)-box 9, PTGS2 - prostaglandin-endoperoxide synthase 2 (prostaglandin g/h synthase and cyclooxygenase), IRF1 - interferon regulatory factor 1, KLF15 - kruppel-like factor 15, COLEC12 - collectin sub-family member 12, CXCL12 - chemokine (c-x-c motif) ligand 12, CHAC1 - chac, cation transport regulator homolog 1 (e. coli), MME - membrane metallo-endopeptidase, ITGA2 - integrin, alpha 2 (cd49b, alpha 2 subunit of vla-2 receptor), FOSL1 - fos-like antigen 1, PTN - pleiotrophin, VCAM1 - vascular cell adhesion molecule 1, RIPK2 - receptor-interacting serine-threonine kinase 2, MLC1 - megalencephalic leukoencephalopathy with subcortical cysts 1, CPS1 - carbamoyl-phosphate synthase 1, mitochondrial, PDGFRB - platelet-derived growth factor receptor, beta polypeptide, NEK11 - nima-related kinase 11, TNC - tenascin c, TNFAIP3 - tumor necrosis factor, alpha-induced protein 3, OPN1SW - opsin 1 (cone pigments), short-wave-sensitive, CXCL1 - chemokine (c-x-c motif) ligand 1 (melanoma growth stimulating activity, alpha), CXCL2 - chemokine (c-x-c motif) ligand 2, CXCL3 - chemokine (c-x-c motif) ligand 3, CALCRL - calcitonin receptor-like, ANXA1 - annexin a1, PTPRC - protein tyrosine phosphatase, receptor type, c, IBSP - integrin-binding sialoprotein]
GO:0006954	inflammatory response	5.49E-06	2.39E-03	2.81	16231	390	355	24	[CCL2 - chemokine (c-c motif) ligand 2, HFE - hemochromatosis, NFKB1 - nuclear factor of kappa light polypeptide gene enhancer in b-cells 1, CSF1 - colony stimulating factor 1 (macrophage), RELB - v-rel avian reticuloendotheliosis viral oncogene homolog b, RIPK2 - receptor-interacting serine-threonine kinase 2, VCAM1 - vascular cell adhesion molecule 1, PTX3 - pentraxin 3, long, CXCL13 - chemokine (c-x-c motif) ligand 13, CCL20 - chemokine (c-c motif) ligand 20, PTGER3 - prostaglandin e receptor 3 (subtype ep3), ADORA1 - adenosine a1 receptor, TNFAIP3 - tumor necrosis factor, alpha-induced protein 3, CXCL1 - chemokine (c-x-c motif) ligand 1 (melanoma growth stimulating activity, alpha), IL8 - interleukin 8, PLP1 - proteolipid protein 1, CXCL2 - chemokine (c-x-c motif) ligand 2, CXCL3 - chemokine (c-x-c motif) ligand 3, C5 - complement component 5, ANXA1 - annexin a1, PTGS2 - prostaglandin-endoperoxide synthase 2 (prostaglandin g/h synthase and cyclooxygenase), TAC4 - tachykinin 4 (hemokinin), ZC3H12A - zinc finger ccch-type containing 12a, TICAM1 - toll-like receptor adaptor molecule 1]
GO:0071219	cellular response to molecule of bacterial origin	7.07E-06	2.99E-03	4.47	16231	133	355	13	[NFKB1 - nuclear factor of kappa light polypeptide gene enhancer in b-cells 1, CCL2 - chemokine (c-c motif) ligand 2, PLSCR4 - phospholipid scramblase 4, RIPK2 - receptor-interacting serine-threonine kinase 2, CXCL13 - chemokine (c-x-c motif) ligand 13, TNFAIP3 - tumor necrosis factor, alpha-induced protein 3, CXCL1 - chemokine (c-x-c motif) ligand 1 (melanoma growth stimulating activity, alpha), IL8 - interleukin 8, CXCL2 - chemokine (c-x-c motif) ligand 2, CXCL3 - chemokine (c-x-c motif) ligand 3, ZC3H12A - zinc finger ccch-type containing 12a, TFPI - tissue factor pathway inhibitor (lipoprotein-associated coagulation inhibitor), TICAM1 - toll-like receptor adaptor molecule 1]
GO:0071621	granulocyte chemotaxis	9.87E-06	4.06E-03	6.43	16231	64	355	9	[CCL20 - chemokine (c-c motif) ligand 20, CCL2 - chemokine (c-c motif) ligand 2, IL8 - interleukin 8, CXCL1 - chemokine (c-x-c motif) ligand 1 (melanoma growth stimulating activity, alpha), CXCL2 - chemokine (c-x-c motif) ligand 2, CXCL3 - chemokine (c-x-c motif) ligand 3, ANXA1 - annexin a1, CXCL13 - chemokine (c-x-c motif) ligand 13, CKLF - chemokine-like factor]
GO:0043331	response to dsRNA	1.32E-05	5.29E-03	8.65	16231	37	355	7	[NFKB1 - nuclear factor of kappa light polypeptide gene enhancer in b-cells 1, NFKBIA - nuclear factor of kappa light polypeptide gene enhancer in b-cells inhibitor, alpha, KCNJ8 - potassium inwardly-rectifying channel, subfamily j, member 8, IFIT1 - interferon-induced protein with tetratricopeptide repeats 1, RIPK2 - receptor-interacting serine-threonine kinase 2, TICAM1 - toll-like receptor adaptor molecule 1, COLEC12 - collectin sub-family member 12]
GO:0009615	response to virus	1.76E-05	6.86E-03	3.17	16231	260	355	18	[OAS3 - 2'-5'-oligoadenylate synthetase 3, 100kda, OASL - 2'-5'-oligoadenylate synthetase-like, PARP9 - poly (adp-ribose) polymerase family, member 9, CXCL12 - chemokine (c-x-c motif) ligand 12, IFIT2 - interferon-induced protein with tetratricopeptide repeats 2, NMI - n-myc (and stat) interactor, IFIT1 - interferon-induced protein with tetratricopeptide repeats 1, FOSL1 - fos-like antigen 1, IFIT3 - interferon-induced protein with tetratricopeptide repeats 3, HSPB1 - heat shock 27kda protein 1, HERC5 - hect and rld domain containing e3 ubiquitin protein ligase 5, ACTA2 - actin, alpha 2, smooth muscle, aorta, KCNJ8 - potassium inwardly-rectifying channel, subfamily j, member 8, DDX60 - dead (asp-glu-ala-asp) box polypeptide 60, PTPRC - protein tyrosine phosphatase, receptor type, c, IRF1 - interferon regulatory factor 1, ZC3H12A - zinc finger ccch-type containing 12a, TICAM1 - toll-like receptor adaptor molecule 1]
GO:0071222	cellular response to lipopolysaccharide	1.91E-05	7.27E-03	4.39	16231	125	355	12	[NFKB1 - nuclear factor of kappa light polypeptide gene enhancer in b-cells 1, CCL2 - chemokine (c-c motif) ligand 2, PLSCR4 - phospholipid scramblase 4, TNFAIP3 - tumor necrosis factor, alpha-induced protein 3, CXCL1 - chemokine (c-x-c motif) ligand 1 (melanoma growth stimulating activity, alpha), IL8 - interleukin 8, CXCL2 - chemokine (c-x-c motif) ligand 2, CXCL3 - chemokine (c-x-c motif) ligand 3, ZC3H12A - zinc finger ccch-type containing 12a, TFPI - tissue factor pathway inhibitor (lipoprotein-associated coagulation inhibitor), CXCL13 - chemokine (c-x-c motif) ligand 13, TICAM1 - toll-like receptor adaptor molecule 1]
GO:2000147	positive regulation of cell motility	2.22E-05	8.26E-03	2.42	16231	510	355	27	[HSPA5 - heat shock 70kda protein 5 (glucose-regulated protein, 78kda), HSPB1 - heat shock 27kda protein 1, RHOD - ras homolog family member d, CCL20 - chemokine (c-c motif) ligand 20, MSTN - myostatin, ATOH8 - atonal homolog 8 (drosophila), IL8 - interleukin 8, PLP1 - proteolipid protein 1, SOX9 - sry (sex determining region y)-box 9, PTGS2 - prostaglandin-endoperoxide synthase 2 (prostaglandin g/h synthase and cyclooxygenase), TAC4 - tachykinin 4 (hemokinin), ZC3H12A - zinc finger ccch-type containing 12a, NEDD9 - neural precursor cell expressed, developmentally down-regulated 9, CXCL12 - chemokine (c-x-c motif) ligand 12, CSF1 - colony stimulating factor 1 (macrophage), RHOJ - ras homolog family member j, ITGA2 - integrin, alpha 2 (cd49b, alpha 2 subunit of vla-2 receptor), PTN - pleiotrophin, SPRY2 - sprouty homolog 2 (drosophila), HBEGF - heparin-binding egf-like growth factor, PDGFRB - platelet-derived growth factor receptor, beta polypeptide, FGF1 - fibroblast growth factor 1 (acidic), CXCL13 - chemokine (c-x-c motif) ligand 13, GAB1 - grb2-associated binding protein 1, ANXA1 - annexin a1, PTPRC - protein tyrosine phosphatase, receptor type, c, PLAU - plasminogen activator, urokinase]

GO:0032502	developmental process	2.40E-05	8.71E-03	1.36	16231	4425	355	132	[HFE - hemochromatosis, ARID5B - at rich interactive domain 5b (mrf1-like), ID1 - inhibitor of dna binding 1, dominant negative helix-loop-helix protein, ID3 - inhibitor of dna binding 3, dominant negative helix-loop-helix protein, MMP21 - matrix metallopeptidase 21, RELB - v-rel avian reticuloendotheliosis viral oncogene homolog b, CD34 - cd34 molecule, LTBP3 - latent transforming growth factor beta binding protein 3, NEUROG2 - neurogenin 2, PLP1 - proteolipid protein 1, APOA1 - apolipoprotein a-i, KCNA1 - potassium voltage-gated channel, shaker-related subfamily, member 1 (episodic ataxia with myokymia), ZC3H12A - zinc finger ccch-type containing 12a, DAND5 - dan domain family member 5, bmp antagonist, COL4A1 - collagen, type iv, alpha 1, OPN3 - opsin 3, MFRP - membrane frizzled-related protein, RHOJ - ras homolog family member j, WNT8B - wingless-type mmtv integration site family, member 8b, SOSTDC1 - sclerostin domain containing 1, COL22A1 - collagen, type xxii, alpha 1, SPRY2 - sprouty homolog 2 (drosophila), TBX2 - t-box 2, COL8A1 - collagen, type viii, alpha 1, PAX6 - paired box 6, FGF1 - fibroblast growth factor 1 (acidic), CXCL13 - chemokine (c-x-c motif) ligand 13, ZFP36L1 - zfp36 ring finger protein-like 1, ACTA2 - actin, alpha 2, smooth muscle, aorta, SLC17A8 - solute carrier family 17 (vesicular glutamate transporter), member 8, COL12A1 - collagen, type xii, alpha 1, SLC24A3 - solute carrier family 24 (sodium/potassium/calcium exchanger), member 3, SMOC1 - sparc related modular calcium binding 1, RAMP3 - receptor (g protein-coupled) activity modifying protein 3, KCNJ8 - potassium inwardly-rectifying channel, subfamily j, member 8, GAB1 - grb2-associated binding protein 1, PRRX1 - paired related homeobox 1, SPARCL1 - sparc-like 1 (hevin), CCL2 - chemokine (c-c motif) ligand 2, KCNMB1 - potassium large conductance calcium-activated channel, subfamily m, beta member 1, ANGPTL1 - angiopoietin-like 1, DCN - decorin, TIPARP - tcdd-inducible poly(adp-ribose) polymerase, DCT - dopachrome tautomerase, IGFBP3 - insulin-like growth factor binding protein 3, DUSP10 - dual specificity phosphatase 10, MSX2 - msh homeobox 2, CEP55 - centrosomal protein 55kda, RHOH - ras homolog family member h, DDIT3 - dna-damage-inducible transcript 3, MAOB - monoamine oxidase b, SOX9 - sry (sex determining region y)-box 9, C5 - complement component 5, ZIC2 - zic family member 2, PTHLH - parathyroid hormone-like hormone, CYP7B1 - cytochrome p450, family 7, subfamily b, polypeptide 1, KLF15 - kruppel-like factor 15, IRF2BP2 - interferon regulatory factor 2 binding protein 2, USP2 - ubiquitin specific peptidase 2, CXCL12 - chemokine (c-x-c motif) ligand 12, ELN - elastin, FOSL1 - fos-like antigen 1, PTN - pleiotrophin, BCAP29 - b-cell receptor-associated protein 29, CPS1 - carbamoyl-phosphate synthase 1, mitochondrial, PDGFRB - platelet-derived growth factor receptor, beta polypeptide, KRT75 - keratin 75, SPINK2 - serine peptidase inhibitor, kazal type 2 (acrosin-trypsin inhibitor), ADORA1 - adenosine a1 receptor, CXCL1 - chemokine (c-x-c motif) ligand 1 (melanoma growth stimulating activity, alpha), PTPRC - protein tyrosine phosphatase, receptor type, c, KCNQ4 - potassium voltage-gated channel, kqt-like subfamily, member 4, FOLR1 - folate receptor 1 (adult), GSC - goosecoid homeobox, ATF3 - activating transcription factor 3, PTPRZ1 - protein tyrosine phosphatase, receptor-type, z polypeptide 1, MAFF - v-maf avian musculoaponeurotic fibrosarcoma oncogene homolog f, MSTN - myostatin, ATP1A2 - atpase, na+/k+ transporting, alpha 2 polypeptide, ATOH8 - atonal homolog 8 (drosophila), TRPV4 - transient receptor potential cation channel, subfamily v, member 4, IL8 - interleukin 8, ACAN - aggrecan, DLX3 - distal-less homeobox 3, SCRG1 - stimulator of chondrogenesis 1, CSF1 - colony stimulating factor 1 (macrophage), GCNT4 - glucosaminyl (n-acetyl) transferase 4, core 2, FJX1 - four jointed box 1 (drosophila), BARHL2 - barh-like homeobox 2, NTRK2 - neurotrophic tyrosine kinase, receptor, type 2, YAP1 - yes-associated protein 1, ANGPTL2 - angiopoietin-like 2, BARX2 - barx homeobox 2, RND1 - rho family gtpase 1, PRELP - proline/arginine-rich end leucine-rich repeat protein, SLC24A5 - solute carrier family 24 (sodium/potassium/calcium exchanger), member 5, HSD17B2 - hydroxysteroid (17-beta) dehydrogenase 2, UCP3 - uncoupling protein 3 (mitochondrial, proton carrier), B3GNT5 - udp-glcnac:betagal beta-1,3-n-acetylglucosaminyltransferase 5, MGP - matrix gla protein, MGST1 - microsomal glutathione s-transferase 1, KLF10 - kruppel-like factor 10, INHBB - inhibin, beta b, HOPX - hop homeobox, IER3 - immediate early response 3, HSPA5 - heat shock 70kda protein 5 (glucose-regulated protein, 78kda), CSRNP1 - cysteine-serine-rich nuclear protein 1, CAV3 - caveolin 3, CLEC3A - c-type lectin domain family 3, member a, MECOM - mds1 and evi1 complex locus, LEPR - leptin receptor, SPDEF - sam pointed domain containing ets transcription factor, SERPINH1 - serpin peptidase inhibitor, clade h (heat shock protein 47), member 1, (collagen binding protein 1), WFIKKN1 - wap, follistatin/kazal, immunoglobulin, kunitz and netrin domain containing 1, IRF1 - interferon regulatory factor 1, TNFRSF11B - tumor necrosis factor receptor superfamily, member 11b, NKX2-4 - nk2 homeobox 4, GJC2 - gap junction protein, gamma 2, 47kda, VAMP5 - vesicle-associated membrane protein 5, DAW1 - dynein assembly factor with wdr repeat domains 1, MME - membrane metallo-endopeptidase, CHAC1 - chac, cation transport regulator homolog 1 (e. coli), ITGA2 - integrin, alpha 2 (cd49b, alpha 2 subunit of vla-2 receptor), VCAM1 - vascular cell adhesion molecule 1, HBEGF - heparin-binding egf-like growth factor, FABP7 - fatty acid binding protein 7, brain, TNC - tenascin c, CALCRL - calcitonin receptor-like, ANXA1 - annexin a1, IBSP - integrin-binding sialoprotein, ITGB7 - integrin, beta 7, ANXA4 - annexin a4]
GO:0030595	leukocyte chemotaxis	2.66E-05	9.41E-03	4.61	16231	109	355	11	[CCL20 - chemokine (c-c motif) ligand 20, CCL2 - chemokine (c-c motif) ligand 2, CXCL1 - chemokine (c-x-c motif) ligand 1 (melanoma growth stimulating activity, alpha), IL8 - interleukin 8, PTN - pleiotrophin, CXCL2 - chemokine (c-x-c motif) ligand 2, CXCL3 - chemokine (c-x-c motif) ligand 3, ANXA1 - annexin a1, CYP7B1 - cytochrome p450, family 7, subfamily b, polypeptide 1, CXCL13 - chemokine (c-x-c motif) ligand 13, CKLF - chemokine-like factor]
GO:0031347	regulation of defense response	2.74E-05	9.50E-03	2.13	16231	729	355	34	[CFH - complement factor h, NFKB1 - nuclear factor of kappa light polypeptide gene enhancer in b-cells 1, PARP9 - poly (adp-ribose) polymerase family, member 9, NFKBIA - nuclear factor of kappa light polypeptide gene enhancer in b-cells inhibitor, alpha, RELB - v-rel avian reticuloendotheliosis viral oncogene homolog b, HERC5 - hect and rld domain containing e3 ubiquitin protein ligase 5, DUSP10 - dual specificity phosphatase 10, PTGER3 - prostaglandin e receptor 3 (subtype ep3), BIRC3 - baculoviral iap repeat containing 3, C5 - complement component 5, DDX60 - dead (asp-glu-ala-asp) box polypeptide 60, PARP14 - poly (adp-ribose) polymerase family, member 14, PTGS2 - prostaglandin-endoperoxide synthase 2 (prostaglandin g/h synthase and cyclooxygenase), IRF1 - interferon regulatory factor 1, APOA1 - apolipoprotein a-i, ZC3H12A - zinc finger ccch-type containing 12a, C7 - complement component 7, COLEC12 - collectin sub-family member 12, NT5E - 5'-nucleotidase, ecto (cd73), IFI35 - interferon-induced protein 35, ITGA2 - integrin, alpha 2 (cd49b, alpha 2 subunit of vla-2 receptor), NMI - n-myc (and stat) interactor, IFIT1 - interferon-induced protein with tetratricopeptide repeats 1, FGL2 - fibrinogen-like 2, RIPK2 - receptor-interacting serine-threonine kinase 2, PSMB9 - proteasome (prosome, macropain) subunit, beta type, 9, ADORA1 - adenosine a1 receptor, TNFAIP3 - tumor necrosis factor, alpha-induced protein 3, CALCRL - calcitonin receptor-like, ANXA1 - annexin a1, PTPRC - protein tyrosine phosphatase, receptor type, c, MUC15 - mucin 15, cell surface associated, TICAM1 - toll-like receptor adaptor molecule 1, SLC15A2 - solute carrier family 15 (oligopeptide transporter), member 2]

GO:0006950	response to stress	2.81E-05	9.52E-03	1.51	16231	2701	355	89	[CFH - complement factor h, HFE - hemochromatosis, OASL - 2'-5'-oligoadenylate synthetase-like, FOLR1 - folate receptor 1 (adult), ATF3 - activating transcription factor 3, ID3 - inhibitor of dna binding 3, dominant negative helix-loop-helix protein, RELB - v-rel avian reticuloendotheliosis viral oncogene homolog b, PTX3 - pentraxin 3, long, MSTN - myostatin, ATP1A2 - atpase, na+/k+ transporting, alpha 2 polypeptide, TRPV4 - transient receptor potential cation channel, subfamily v, member 4, IL8 - interleukin 8, PLP1 - proteolipid protein 1, SFTA3 - surfactant associated 3, APOA1 - apolipoprotein a-i, TRIM17 - tripartite motif containing 17, ZC3H12A - zinc finger ccch-type containing 12a, CSF1 - colony stimulating factor 1 (macrophage), IFIT2 - interferon-induced protein with tetratricopeptide repeats 2, IFIT1 - interferon-induced protein with tetratricopeptide repeats 1, IFIT3 - interferon-induced protein with tetratricopeptide repeats 3, TBX2 - t-box 2, PAX6 - paired box 6, FAS - fas cell surface death receptor, LRRC8C - leucine rich repeat containing 8 family, member c, FGF1 - fibroblast growth factor 1 (acidic), CXCL13 - chemokine (c-x-c motif) ligand 13, YAP1 - yes-associated protein 1, PSMB9 - proteasome (prosome, macropain) subunit, beta type, 9, ZFP36L1 - zfp36 ring finger protein-like 1, KCNJ8 - potassium inwardly-rectifying channel, subfamily j, member 8, UCP3 - uncoupling protein 3 (mitochondrial, proton carrier), KLF10 - kruppel-like factor 10, TICAM1 - toll-like receptor adaptor molecule 1, MGST1 - microsomal glutathione s-transferase 1, INHBB - inhibin, beta b, GZMB - granzyme b (granzyme 2, cytotoxic t-lymphocyte-associated serine esterase 1), OAS3 - 2'-5'-oligoadenylate synthetase 3, 100kda, CCL2 - chemokine (c-c motif) ligand 2, NFKB1 - nuclear factor of kappa light polypeptide gene enhancer in b-cells 1, RNF175 - ring finger protein 175, KCNMB1 - potassium large conductance calcium-activated channel, subfamily m, beta member 1, PARP9 - poly (adp-ribose) polymerase family, member 9, HSPA5 - heat shock 70kda protein 5 (glucose-regulated protein, 78kda), NFKBIA - nuclear factor of kappa light polypeptide gene enhancer in b-cells inhibitor, alpha, DCN - decorin, HSPB1 - heat shock 27kda protein 1, SOD3 - superoxide dismutase 3, extracellular, HERPUD1 - homocysteine-inducible, endoplasmic reticulum stress-inducible, ubiquitin-like domain member 1, HERC5 - hect and rld domain containing e3 ubiquitin protein ligase 5, SETD7 - set domain containing (lysine methyltransferase) 7, SERPINH1 - serpin peptidase inhibitor, clade h (heat shock protein 47), member 1, (collagen binding protein 1), PTGER3 - prostaglandin e receptor 3 (subtype ep3), CCL20 - chemokine (c-c motif) ligand 20, IGFBP7 - insulin-like growth factor binding protein 7, GADD45A - growth arrest and dna-damage-inducible, alpha, DDIT3 - dna-damage-inducible transcript 3, C5 - complement component 5, DDX60 - dead (asp-glu-ala-asp) box polypeptide 60, PARP14 - poly (adp-ribose) polymerase family, member 14, PTGS2 - prostaglandin-endoperoxide synthase 2 (prostaglandin g/h synthase and cyclooxygenase), IRF1 - interferon regulatory factor 1, TAC4 - tachykinin 4 (hemokinin), C7 - complement component 7, KLF15 - kruppel-like factor 15, HBA2 - hemoglobin, alpha 2, COLEC12 - collectin sub-family member 12, CXCL12 - chemokine (c-x-c motif) ligand 12, HTR1A - 5-hydroxytryptamine (serotonin) receptor 1a, g protein-coupled, CHAC1 - chac, cation transport regulator homolog 1 (e. coli), ITGA2 - integrin, alpha 2 (cd49b, alpha 2 subunit of vla-2 receptor), FOSL1 - fos-like antigen 1, PTN - pleiotrophin, VCAM1 - vascular cell adhesion molecule 1, RIPK2 - receptor-interacting serine-threonine kinase 2, CPS1 - carbamoyl-phosphate synthase 1, mitochondrial, PDGFRB - platelet-derived growth factor receptor, beta polypeptide, NEK11 - nima-related kinase 11, ADORA1 - adenosine a1 receptor, TNC - tenascin c, TNFAIP3 - tumor necrosis factor, alpha-induced protein 3, CXCL1 - chemokine (c-x-c motif) ligand 1 (melanoma growth stimulating activity, alpha), CXCL2 - chemokine (c-x-c motif) ligand 2, CXCL3 - chemokine (c-x-c motif) ligand 3, ANXA1 - annexin a1, PTPRC - protein tyrosine phosphatase, receptor type, c, SLC15A2 - solute carrier family 15 (oligopeptide transporter), member 2, PLAU - plasminogen activator, urokinase, CD83 - cd83 molecule]
GO:0030335	positive regulation of cell migration	2.94E-05	9.73E-03	2.43	16231	489	355	26	[HSPA5 - heat shock 70kda protein 5 (glucose-regulated protein, 78kda), HSPB1 - heat shock 27kda protein 1, RHOD - ras homolog family member d, CCL20 - chemokine (c-c motif) ligand 20, MSTN - myostatin, ATOH8 - atonal homolog 8 (drosophila), IL8 - interleukin 8, PLP1 - proteolipid protein 1, SOX9 - sry (sex determining region y)-box 9, PTGS2 - prostaglandin-endoperoxide synthase 2 (prostaglandin g/h synthase and cyclooxygenase), ZC3H12A - zinc finger ccch-type containing 12a, NEDD9 - neural precursor cell expressed, developmentally down-regulated 9, CXCL12 - chemokine (c-x-c motif) ligand 12, CSF1 - colony stimulating factor 1 (macrophage), RHOJ - ras homolog family member j, ITGA2 - integrin, alpha 2 (cd49b, alpha 2 subunit of vla-2 receptor), PTN - pleiotrophin, SPRY2 - sprouty homolog 2 (drosophila), HBEGF - heparin-binding egf-like growth factor, PDGFRB - platelet-derived growth factor receptor, beta polypeptide, FGF1 - fibroblast growth factor 1 (acidic), CXCL13 - chemokine (c-x-c motif) ligand 13, GAB1 - grb2-associated binding protein 1, ANXA1 - annexin a1, PTPRC - protein tyrosine phosphatase, receptor type, c, PLAU - plasminogen activator, urokinase]
GO:0097530	granulocyte migration	3.27E-05	1.06E-02	5.56	16231	74	355	9	[CCL20 - chemokine (c-c motif) ligand 20, CCL2 - chemokine (c-c motif) ligand 2, IL8 - interleukin 8, CXCL1 - chemokine (c-x-c motif) ligand 1 (melanoma growth stimulating activity, alpha), CXCL2 - chemokine (c-x-c motif) ligand 2, CXCL3 - chemokine (c-x-c motif) ligand 3, ANXA1 - annexin a1, CXCL13 - chemokine (c-x-c motif) ligand 13, CKLF - chemokine-like factor]
GO:0042127	regulation of cell proliferation	3.71E-05	1.18E-02	1.74	16231	1449	355	55	[ID1 - inhibitor of dna binding 1, dominant negative helix-loop-helix protein, ATF3 - activating transcription factor 3, PTPRZ1 - protein tyrosine phosphatase, receptor-type, z polypeptide 1, MSTN - myostatin, ATOH8 - atonal homolog 8 (drosophila), LTBP3 - latent transforming growth factor beta binding protein 3, IL8 - interleukin 8, CSF1 - colony stimulating factor 1 (macrophage), IFI35 - interferon-induced protein 35, IFIT3 - interferon-induced protein with tetratricopeptide repeats 3, SPRY2 - sprouty homolog 2 (drosophila), TBX2 - t-box 2, NTRK2 - neurotrophic tyrosine kinase, receptor, type 2, FGF1 - fibroblast growth factor 1 (acidic), YAP1 - yes-associated protein 1, ZFP36L1 - zfp36 ring finger protein-like 1, PRRX1 - paired related homeobox 1, BTC - betacellulin, KLF9 - kruppel-like factor 9, TICAM1 - toll-like receptor adaptor molecule 1, KLF10 - kruppel-like factor 10, CCL2 - chemokine (c-c motif) ligand 2, NFKBIA - nuclear factor of kappa light polypeptide gene enhancer in b-cells inhibitor, alpha, CAV3 - caveolin 3, IQGAP3 - iq motif containing gtpase activating protein 3, IGFBP3 - insulin-like growth factor binding protein 3, DCT - dopachrome tautomerase, DUSP10 - dual specificity phosphatase 10, IGFBP7 - insulin-like growth factor binding protein 7, OGN - osteoglycin, SOX9 - sry (sex determining region y)-box 9, IRF1 - interferon regulatory factor 1, PTHLH - parathyroid hormone-like hormone, CYP7B1 - cytochrome p450, family 7, subfamily b, polypeptide 1, GJC2 - gap junction protein, gamma 2, 47kda, HTR1A - 5-hydroxytryptamine (serotonin) receptor 1a, g protein-coupled, CXCL12 - chemokine (c-x-c motif) ligand 12, ITGA2 - integrin, alpha 2 (cd49b, alpha 2 subunit of vla-2 receptor), FOSL1 - fos-like antigen 1, NMI - n-myc (and stat) interactor, ELN - elastin, PTN - pleiotrophin, RIPK2 - receptor-interacting serine-threonine kinase 2, VCAM1 - vascular cell adhesion molecule 1, HBEGF - heparin-binding egf-like growth factor, PDGFRB - platelet-derived growth factor receptor, beta polypeptide, FABP7 - fatty acid binding protein 7, brain, ADORA1 - adenosine a1 receptor, TNC - tenascin c, TNFAIP3 - tumor necrosis factor, alpha-induced protein 3, CXCL1 - chemokine (c-x-c motif) ligand 1 (melanoma growth stimulating activity, alpha), CALCRL - calcitonin receptor-like, ANXA1 - annexin a1, PTPRC - protein tyrosine phosphatase, receptor type, c, PLAU - plasminogen activator, urokinase]
GO:0051272	positive regulation of cellular component movement	3.81E-05	1.18E-02	2.35	16231	526	355	27	[HSPA5 - heat shock 70kda protein 5 (glucose-regulated protein, 78kda), HSPB1 - heat shock 27kda protein 1, RHOD - ras homolog family member d, CCL20 - chemokine (c-c motif) ligand 20, MSTN - myostatin, ATOH8 - atonal homolog 8 (drosophila), IL8 - interleukin 8, PLP1 - proteolipid protein 1, SOX9 - sry (sex determining region y)-box 9, PTGS2 - prostaglandin-endoperoxide synthase 2 (prostaglandin g/h synthase and cyclooxygenase), TAC4 - tachykinin 4 (hemokinin), ZC3H12A - zinc finger ccch-type containing 12a, NEDD9 - neural precursor cell expressed, developmentally down-regulated 9, CXCL12 - chemokine (c-x-c motif) ligand 12, CSF1 - colony stimulating factor 1 (macrophage), RHOJ - ras homolog family member j, ITGA2 - integrin, alpha 2 (cd49b, alpha 2 subunit of vla-2 receptor), PTN - pleiotrophin, SPRY2 - sprouty homolog 2 (drosophila), HBEGF - heparin-binding egf-like growth factor, PDGFRB - platelet-derived growth factor receptor, beta polypeptide, FGF1 - fibroblast growth factor 1 (acidic), CXCL13 - chemokine (c-x-c motif) ligand 13, GAB1 - grb2-associated binding protein 1, ANXA1 - annexin a1, PTPRC - protein tyrosine phosphatase, receptor type, c, PLAU - plasminogen activator, urokinase]
GO:0045765	regulation of angiogenesis	4.08E-05	1.24E-02	2.97	16231	277	355	18	[RHOJ - ras homolog family member j, E2F2 - e2f transcription factor 2, SLC39A12 - solute carrier family 39 (zinc transporter), member 12, PTN - pleiotrophin, DCN - decorin, SPRY2 - sprouty homolog 2 (drosophila), HSPB1 - heat shock 27kda protein 1, FGF1 - fibroblast growth factor 1 (acidic), CXCL13 - chemokine (c-x-c motif) ligand 13, CD34 - cd34 molecule, GADD45A - growth arrest and dna-damage-inducible, alpha, TNFAIP3 - tumor necrosis factor, alpha-induced protein 3, GAB1 - grb2-associated binding protein 1, IL8 - interleukin 8, C5 - complement component 5, PTGS2 - prostaglandin-endoperoxide synthase 2 (prostaglandin g/h synthase and cyclooxygenase), ANXA1 - annexin a1, ZC3H12A - zinc finger ccch-type containing 12a]
GO:0045661	regulation of myoblast differentiation	4.31E-05	1.29E-02	7.27	16231	44	355	7	[MSTN - myostatin, ZFP36L1 - zfp36 ring finger protein-like 1, DDIT3 - dna-damage-inducible transcript 3, ID3 - inhibitor of dna binding 3, dominant negative helix-loop-helix protein, SOX9 - sry (sex determining region y)-box 9, SOSTDC1 - sclerostin domain containing 1, IGFBP3 - insulin-like growth factor binding protein 3]

GO:0050727	regulation of inflammatory response	4.48E-05	1.31E-02	2.67	16231	359	355	21	[CFH - complement factor h, NFKB1 - nuclear factor of kappa light polypeptide gene enhancer in b-cells 1, NT5E - 5'-nucleotidase, ecto (cd73), IFI35 - interferon-induced protein 35, NFKBIA - nuclear factor of kappa light polypeptide gene enhancer in b-cells inhibitor, alpha, ITGA2 - integrin, alpha 2 (cd49b, alpha 2 subunit of vla-2 receptor), NMI - n-myc (and stat) interactor, DUSP10 - dual specificity phosphatase 10, PTGER3 - prostaglandin e receptor 3 (subtype ep3), ADORA1 - adenosine a1 receptor, TNFAIP3 - tumor necrosis factor, alpha-induced protein 3, BIRC3 - baculoviral iap repeat containing 3, C5 - complement component 5, CALCRL - calcitonin receptor-like, PTGS2 - prostaglandin-endoperoxide synthase 2 (prostaglandin g/h synthase and cyclooxygenase), PTPRC - protein tyrosine phosphatase, receptor type, c, ANXA1 - annexin a1, APOA1 - apolipoprotein a-i, ZC3H12A - zinc finger ccch-type containing 12a, C7 - complement component 7, TICAM1 - toll-like receptor adaptor molecule 1]
GO:0030593	neutrophil chemotaxis	4.59E-05	1.32E-02	6.1	16231	60	355	8	[CCL20 - chemokine (c-c motif) ligand 20, CCL2 - chemokine (c-c motif) ligand 2, IL8 - interleukin 8, CXCL1 - chemokine (c-x-c motif) ligand 1 (melanoma growth stimulating activity, alpha), CXCL2 - chemokine (c-x-c motif) ligand 2, CXCL3 - chemokine (c-x-c motif) ligand 3, CXCL13 - chemokine (c-x-c motif) ligand 13, CKLF - chemokine-like factor]
GO:0002376	immune system process	4.83E-05	1.36E-02	1.62	16231	1863	355	66	[CFH - complement factor h, OASL - 2'-5'-oligoadenylate synthetase-like, ITPKB - inositol-trisphosphate 3-kinase b, RELB - v-rel avian reticuloendotheliosis viral oncogene homolog b, PTX3 - pentraxin 3, long, CD34 - cd34 molecule, BTN3A3 - butyrophilin, subfamily 3, member a3, BIRC3 - baculoviral iap repeat containing 3, IL8 - interleukin 8, HLA-DMA - major histocompatibility complex, class ii, dm alpha, TRIM17 - tripartite motif containing 17, ZC3H12A - zinc finger ccch-type containing 12a, CD58 - cd58 molecule, CSF1 - colony stimulating factor 1 (macrophage), IFI35 - interferon-induced protein 35, IFIT2 - interferon-induced protein with tetratricopeptide repeats 2, IFIT1 - interferon-induced protein with tetratricopeptide repeats 1, IFIT3 - interferon-induced protein with tetratricopeptide repeats 3, FGL2 - fibrinogen-like 2, FAS - fas cell surface death receptor, CXCL13 - chemokine (c-x-c motif) ligand 13, PSMB9 - proteasome (prosome, macropain) subunit, beta type, 9, ZFP36L1 - zfp36 ring finger protein-like 1, KCNJ8 - potassium inwardly-rectifying channel, subfamily j, member 8, MUC15 - mucin 15, cell surface associated, TICAM1 - toll-like receptor adaptor molecule 1, MGST1 - microsomal glutathione s-transferase 1, GZMB - granzyme b (granzyme 2, cytotoxic t-lymphocyte-associated serine esterase 1), OAS3 - 2'-5'-oligoadenylate synthetase 3, 100kda, CCL2 - chemokine (c-c motif) ligand 2, NFKB1 - nuclear factor of kappa light polypeptide gene enhancer in b-cells 1, PARP9 - poly (adp-ribose) polymerase family, member 9, NFKBIA - nuclear factor of kappa light polypeptide gene enhancer in b-cells inhibitor, alpha, LEPR - leptin receptor, HERC5 - hect and rld domain containing e3 ubiquitin protein ligase 5, CKLF - chemokine-like factor, CCL20 - chemokine (c-c motif) ligand 20, RHOH - ras homolog family member h, SLC2A5 - solute carrier family 2 (facilitated glucose/fructose transporter), member 5, ERAP2 - endoplasmic reticulum aminopeptidase 2, C5 - complement component 5, DDX60 - dead (asp-glu-ala-asp) box polypeptide 60, PARP14 - poly (adp-ribose) polymerase family, member 14, IRF1 - interferon regulatory factor 1, C7 - complement component 7, CYP7B1 - cytochrome p450, family 7, subfamily b, polypeptide 1, IRF2BP2 - interferon regulatory factor 2 binding protein 2, COLEC12 - collectin sub-family member 12, CXCL12 - chemokine (c-x-c motif) ligand 12, MME - membrane metallo-endopeptidase, NMI - n-myc (and stat) interactor, PTN - pleiotrophin, CTSV - cathepsin v, VCAM1 - vascular cell adhesion molecule 1, RIPK2 - receptor-interacting serine-threonine kinase 2, TNFAIP3 - tumor necrosis factor, alpha-induced protein 3, CXCL1 - chemokine (c-x-c motif) ligand 1 (melanoma growth stimulating activity, alpha), CXCL2 - chemokine (c-x-c motif) ligand 2, CXCL3 - chemokine (c-x-c motif) ligand 3, ANXA1 - annexin a1, PTPRC - protein tyrosine phosphatase, receptor type, c, TTR - transthyretin, ITGB7 - integrin, beta 7, SLC15A2 - solute carrier family 15 (oligopeptide transporter), member 2, PLAU - plasminogen activator, urokinase, CD83 - cd83 molecule]
GO:0016477	cell migration	4.85E-05	1.34E-02	2.05	16231	782	355	35	[CCL2 - chemokine (c-c motif) ligand 2, ARID5B - at rich interactive domain 5b (mrf1-like), PARP9 - poly (adp-ribose) polymerase family, member 9, FOLR1 - folate receptor 1 (adult), ID1 - inhibitor of dna binding 1, dominant negative helix-loop-helix protein, RHOD - ras homolog family member d, CKLF - chemokine-like factor, CD34 - cd34 molecule, MSTN - myostatin, CCL20 - chemokine (c-c motif) ligand 20, IL8 - interleukin 8, C5 - complement component 5, APOA1 - apolipoprotein a-i, CYP7B1 - cytochrome p450, family 7, subfamily b, polypeptide 1, CD58 - cd58 molecule, NEDD9 - neural precursor cell expressed, developmentally down-regulated 9, CXCL12 - chemokine (c-x-c motif) ligand 12, RHOJ - ras homolog family member j, ITGA2 - integrin, alpha 2 (cd49b, alpha 2 subunit of vla-2 receptor), PTN - pleiotrophin, BARHL2 - barh-like homeobox 2, VCAM1 - vascular cell adhesion molecule 1, HBEGF - heparin-binding egf-like growth factor, NTRK2 - neurotrophic tyrosine kinase, receptor, type 2, PDGFRB - platelet-derived growth factor receptor, beta polypeptide, CXCL13 - chemokine (c-x-c motif) ligand 13, RND1 - rho family gtpase 1, TNFAIP3 - tumor necrosis factor, alpha-induced protein 3, GAB1 - grb2-associated binding protein 1, CXCL1 - chemokine (c-x-c motif) ligand 1 (melanoma growth stimulating activity, alpha), CXCL2 - chemokine (c-x-c motif) ligand 2, CXCL3 - chemokine (c-x-c motif) ligand 3, ANXA1 - annexin a1, ITGB7 - integrin, beta 7, PLAU - plasminogen activator, urokinase]
GO:0104004	cellular response to environmental stimulus	4.92E-05	1.34E-02	2.93	16231	281	355	18	[NFKB1 - nuclear factor of kappa light polypeptide gene enhancer in b-cells 1, HSPA5 - heat shock 70kda protein 5 (glucose-regulated protein, 78kda), MME - membrane metallo-endopeptidase, ITGA2 - integrin, alpha 2 (cd49b, alpha 2 subunit of vla-2 receptor), PTN - pleiotrophin, RELB - v-rel avian reticuloendotheliosis viral oncogene homolog b, FAS - fas cell surface death receptor, LRRC8C - leucine rich repeat containing 8 family, member c, YAP1 - yes-associated protein 1, ZFP36L1 - zfp36 ring finger protein-like 1, ATP1A2 - atpase, na+/k+ transporting, alpha 2 polypeptide, GADD45A - growth arrest and dna-damage-inducible, alpha, TRPV4 - transient receptor potential cation channel, subfamily v, member 4, OPN1SW - opsin 1 (cone pigments), short-wave-sensitive, SOX9 - sry (sex determining region y)-box 9, IRF1 - interferon regulatory factor 1, OPN3 - opsin 3, RGR - retinal g protein coupled receptor]
GO:0071214	cellular response to abiotic stimulus	4.92E-05	1.31E-02	2.93	16231	281	355	18	[NFKB1 - nuclear factor of kappa light polypeptide gene enhancer in b-cells 1, HSPA5 - heat shock 70kda protein 5 (glucose-regulated protein, 78kda), MME - membrane metallo-endopeptidase, ITGA2 - integrin, alpha 2 (cd49b, alpha 2 subunit of vla-2 receptor), PTN - pleiotrophin, RELB - v-rel avian reticuloendotheliosis viral oncogene homolog b, FAS - fas cell surface death receptor, LRRC8C - leucine rich repeat containing 8 family, member c, YAP1 - yes-associated protein 1, ZFP36L1 - zfp36 ring finger protein-like 1, ATP1A2 - atpase, na+/k+ transporting, alpha 2 polypeptide, GADD45A - growth arrest and dna-damage-inducible, alpha, TRPV4 - transient receptor potential cation channel, subfamily v, member 4, OPN1SW - opsin 1 (cone pigments), short-wave-sensitive, SOX9 - sry (sex determining region y)-box 9, IRF1 - interferon regulatory factor 1, OPN3 - opsin 3, RGR - retinal g protein coupled receptor]
GO:0080134	regulation of response to stress	4.97E-05	1.30E-02	1.73	16231	1429	355	54	[CFH - complement factor h, FBLN5 - fibulin 5, MDFIC - myod family inhibitor domain containing, RELB - v-rel avian reticuloendotheliosis viral oncogene homolog b, CD34 - cd34 molecule, BIRC3 - baculoviral iap repeat containing 3, APOA1 - apolipoprotein a-i, ZC3H12A - zinc finger ccch-type containing 12a, TFPI - tissue factor pathway inhibitor (lipoprotein-associated coagulation inhibitor), NT5E - 5'-nucleotidase, ecto (cd73), IFI35 - interferon-induced protein 35, IFIT1 - interferon-induced protein with tetratricopeptide repeats 1, FGL2 - fibrinogen-like 2, FAS - fas cell surface death receptor, PSMB9 - proteasome (prosome, macropain) subunit, beta type, 9, PRRX1 - paired related homeobox 1, MUC15 - mucin 15, cell surface associated, TICAM1 - toll-like receptor adaptor molecule 1, IER3 - immediate early response 3, NFKB1 - nuclear factor of kappa light polypeptide gene enhancer in b-cells 1, PARP9 - poly (adp-ribose) polymerase family, member 9, HSPA5 - heat shock 70kda protein 5 (glucose-regulated protein, 78kda), NFKBIA - nuclear factor of kappa light polypeptide gene enhancer in b-cells inhibitor, alpha, CAV3 - caveolin 3, MECOM - mds1 and evi1 complex locus, HSPB1 - heat shock 27kda protein 1, HERPUD1 - homocysteine-inducible, endoplasmic reticulum stress-inducible, ubiquitin-like domain member 1, HERC5 - hect and rld domain containing e3 ubiquitin protein ligase 5, DUSP10 - dual specificity phosphatase 10, PTGER3 - prostaglandin e receptor 3 (subtype ep3), GADD45A - growth arrest and dna-damage-inducible, alpha, DDIT3 - dna-damage-inducible transcript 3, C5 - complement component 5, DDX60 - dead (asp-glu-ala-asp) box polypeptide 60, PARP14 - poly (adp-ribose) polymerase family, member 14, PTGS2 - prostaglandin-endoperoxide synthase 2 (prostaglandin g/h synthase and cyclooxygenase), IRF1 - interferon regulatory factor 1, C7 - complement component 7, COLEC12 - collectin sub-family member 12, EFHD1 - ef-hand domain family, member d1, CXCL12 - chemokine (c-x-c motif) ligand 12, ITGA2 - integrin, alpha 2 (cd49b, alpha 2 subunit of vla-2 receptor), NMI - n-myc (and stat) interactor, PTN - pleiotrophin, RIPK2 - receptor-interacting serine-threonine kinase 2, MLC1 - megalencephalic leukoencephalopathy with subcortical cysts 1, HBEGF - heparin-binding egf-like growth factor, ADORA1 - adenosine a1 receptor, TNFAIP3 - tumor necrosis factor, alpha-induced protein 3, CALCRL - calcitonin receptor-like, ANXA1 - annexin a1, PTPRC - protein tyrosine phosphatase, receptor type, c, SLC15A2 - solute carrier family 15 (oligopeptide transporter), member 2, PLAU - plasminogen activator, urokinase]

GO:0009628	response to abiotic stimulus	5.22E-05	1.35E-02	1.91	16231	982	355	41	[NFKB1 - nuclear factor of kappa light polypeptide gene enhancer in b-cells 1, KCNMB1 - potassium large conductance calcium-activated channel, subfamily m, beta member 1, HSPA5 - heat shock 70kda protein 5 (glucose-regulated protein, 78kda), NFKBIA - nuclear factor of kappa light polypeptide gene enhancer in b-cells inhibitor, alpha, CAV3 - caveolin 3, SLC39A12 - solute carrier family 39 (zinc transporter), member 12, RELB - v-rel avian reticuloendotheliosis viral oncogene homolog b, DCN - decorin, SOD3 - superoxide dismutase 3, extracellular, DCT - dopachrome tautomerase, MSTN - myostatin, IGFBP7 - insulin-like growth factor binding protein 7, ATP1A2 - atpase, na+/k+ transporting, alpha 2 polypeptide, GADD45A - growth arrest and dna-damage-inducible, alpha, TRPV4 - transient receptor potential cation channel, subfamily v, member 4, SOX9 - sry (sex determining region y)-box 9, PTGS2 - prostaglandin-endoperoxide synthase 2 (prostaglandin g/h synthase and cyclooxygenase), IRF1 - interferon regulatory factor 1, KCNA1 - potassium voltage-gated channel, shaker-related subfamily, member 1 (episodic ataxia with myokymia), USP2 - ubiquitin specific peptidase 2, OPN3 - opsin 3, RGR - retinal g protein coupled receptor, CXCL12 - chemokine (c-x-c motif) ligand 12, MME - membrane metallo-endopeptidase, ITGA2 - integrin, alpha 2 (cd49b, alpha 2 subunit of vla-2 receptor), FOSL1 - fos-like antigen 1, PTN - pleiotrophin, VCAM1 - vascular cell adhesion molecule 1, FAS - fas cell surface death receptor, LRRC8C - leucine rich repeat containing 8 family, member c, PDGFRB - platelet-derived growth factor receptor, beta polypeptide, FGF1 - fibroblast growth factor 1 (acidic), YAP1 - yes-associated protein 1, ZFP36L1 - zfp36 ring finger protein-like 1, ADORA1 - adenosine a1 receptor, TNC - tenascin c, OPN1SW - opsin 1 (cone pigments), short-wave-sensitive, ANXA1 - annexin a1, PTPRC - protein tyrosine phosphatase, receptor type, c, UCP3 - uncoupling protein 3 (mitochondrial, proton carrier), PLAU - plasminogen activator, urokinase]
GO:0040017	positive regulation of locomotion	5.42E-05	1.38E-02	2.3	16231	537	355	27	[HSPA5 - heat shock 70kda protein 5 (glucose-regulated protein, 78kda), HSPB1 - heat shock 27kda protein 1, RHOD - ras homolog family member d, CCL20 - chemokine (c-c motif) ligand 20, MSTN - myostatin, ATOH8 - atonal homolog 8 (drosophila), IL8 - interleukin 8, PLP1 - proteolipid protein 1, SOX9 - sry (sex determining region y)-box 9, PTGS2 - prostaglandin-endoperoxide synthase 2 (prostaglandin g/h synthase and cyclooxygenase), TAC4 - tachykinin 4 (hemokinin), ZC3H12A - zinc finger ccch-type containing 12a, NEDD9 - neural precursor cell expressed, developmentally down-regulated 9, CXCL12 - chemokine (c-x-c motif) ligand 12, CSF1 - colony stimulating factor 1 (macrophage), RHOJ - ras homolog family member j, ITGA2 - integrin, alpha 2 (cd49b, alpha 2 subunit of vla-2 receptor), PTN - pleiotrophin, SPRY2 - sprouty homolog 2 (drosophila), HBEGF - heparin-binding egf-like growth factor, PDGFRB - platelet-derived growth factor receptor, beta polypeptide, FGF1 - fibroblast growth factor 1 (acidic), CXCL13 - chemokine (c-x-c motif) ligand 13, GAB1 - grb2-associated binding protein 1, ANXA1 - annexin a1, PTPRC - protein tyrosine phosphatase, receptor type, c, PLAU - plasminogen activator, urokinase]
GO:1901342	regulation of vasculature development	5.54E-05	1.38E-02	2.8	16231	310	355	19	[RHOJ - ras homolog family member j, E2F2 - e2f transcription factor 2, ID1 - inhibitor of dna binding 1, dominant negative helix-loop-helix protein, SLC39A12 - solute carrier family 39 (zinc transporter), member 12, PTN - pleiotrophin, DCN - decorin, SPRY2 - sprouty homolog 2 (drosophila), HSPB1 - heat shock 27kda protein 1, FGF1 - fibroblast growth factor 1 (acidic), CXCL13 - chemokine (c-x-c motif) ligand 13, CD34 - cd34 molecule, GADD45A - growth arrest and dna-damage-inducible, alpha, TNFAIP3 - tumor necrosis factor, alpha-induced protein 3, GAB1 - grb2-associated binding protein 1, IL8 - interleukin 8, C5 - complement component 5, PTGS2 - prostaglandin-endoperoxide synthase 2 (prostaglandin g/h synthase and cyclooxygenase), ANXA1 - annexin a1, ZC3H12A - zinc finger ccch-type containing 12a]
GO:0043330	response to exogenous dsRNA	5.88E-05	1.44E-02	8.57	16231	32	355	6	[NFKBIA - nuclear factor of kappa light polypeptide gene enhancer in b-cells inhibitor, alpha, KCNJ8 - potassium inwardly-rectifying channel, subfamily j, member 8, IFIT1 - interferon-induced protein with tetratricopeptide repeats 1, RIPK2 - receptor-interacting serine-threonine kinase 2, TICAM1 - toll-like receptor adaptor molecule 1, COLEC12 - collectin sub-family member 12]
GO:0070848	response to growth factor	5.90E-05	1.43E-02	2.89	16231	285	355	18	[CCL2 - chemokine (c-c motif) ligand 2, HSPA5 - heat shock 70kda protein 5 (glucose-regulated protein, 78kda), MXRA5 - matrix-remodelling associated 5, ID1 - inhibitor of dna binding 1, dominant negative helix-loop-helix protein, PTN - pleiotrophin, VCAM1 - vascular cell adhesion molecule 1, SPRY2 - sprouty homolog 2 (drosophila), HSPB1 - heat shock 27kda protein 1, NTRK2 - neurotrophic tyrosine kinase, receptor, type 2, CPS1 - carbamoyl-phosphate synthase 1, mitochondrial, PDGFRB - platelet-derived growth factor receptor, beta polypeptide, ZFP36L1 - zfp36 ring finger protein-like 1, TNC - tenascin c, GAB1 - grb2-associated binding protein 1, IL8 - interleukin 8, SOX9 - sry (sex determining region y)-box 9, ANXA1 - annexin a1, IBSP - integrin-binding sialoprotein]
GO:0051607	defense response to virus	6.60E-05	1.57E-02	3.62	16231	164	355	13	[OAS3 - 2'-5'-oligoadenylate synthetase 3, 100kda, OASL - 2'-5'-oligoadenylate synthetase-like, PARP9 - poly (adp-ribose) polymerase family, member 9, IFIT2 - interferon-induced protein with tetratricopeptide repeats 2, IFIT1 - interferon-induced protein with tetratricopeptide repeats 1, IFIT3 - interferon-induced protein with tetratricopeptide repeats 3, HERC5 - hect and rld domain containing e3 ubiquitin protein ligase 5, KCNJ8 - potassium inwardly-rectifying channel, subfamily j, member 8, DDX60 - dead (asp-glu-ala-asp) box polypeptide 60, PTPRC - protein tyrosine phosphatase, receptor type, c, IRF1 - interferon regulatory factor 1, ZC3H12A - zinc finger ccch-type containing 12a, TICAM1 - toll-like receptor adaptor molecule 1]

GO:0065008	regulation of biological quality	6.65E-05	1.56E-02	1.41	16231	3401	355	105	[HFE - hemochromatosis, CDC42EP1 - cdc42 effector protein (rho gtpase binding) 1, ID1 - inhibitor of dna binding 1, dominant negative helix-loop-helix protein, ITPKB - inositol-trisphosphate 3-kinase b, RHOD - ras homolog family member d, SLC22A3 - solute carrier family 22 (organic cation transporter), member 3, CD34 - cd34 molecule, SLC22A5 - solute carrier family 22 (organic cation/carnitine transporter), member 5, LTBP3 - latent transforming growth factor beta binding protein 3, GP9 - glycoprotein ix (platelet), APOA1 - apolipoprotein a-i, KCNA1 - potassium voltage-gated channel, shaker-related subfamily, member 1 (episodic ataxia with myokymia), ZC3H12A - zinc finger ccch-type containing 12a, DAND5 - dan domain family member 5, bmp antagonist, ATP6V1C2 - atpase, h+ transporting, lysosomal 42kda, v1 subunit c2, RHOJ - ras homolog family member j, SCD - stearoyl-coa desaturase (delta-9-desaturase), PAX6 - paired box 6, CXCL13 - chemokine (c-x-c motif) ligand 13, PSMB9 - proteasome (prosome, macropain) subunit, beta type, 9, ZFP36L1 - zfp36 ring finger protein-like 1, ACTA2 - actin, alpha 2, smooth muscle, aorta, AQP4 - aquaporin 4, LPAR4 - lysophosphatidic acid receptor 4, SLC17A8 - solute carrier family 17 (vesicular glutamate transporter), member 8, SLC24A3 - solute carrier family 24 (sodium/potassium/calcium exchanger), member 3, RAMP3 - receptor (g protein-coupled) activity modifying protein 3, KCNJ8 - potassium inwardly-rectifying channel, subfamily j, member 8, BTC - betacellulin, PRND - prion protein 2 (dublet), GPR35 - g protein-coupled receptor 35, TICAM1 - toll-like receptor adaptor molecule 1, NFKB1 - nuclear factor of kappa light polypeptide gene enhancer in b-cells 1, CCL2 - chemokine (c-c motif) ligand 2, NFKBIA - nuclear factor of kappa light polypeptide gene enhancer in b-cells inhibitor, alpha, NFKBIE - nuclear factor of kappa light polypeptide gene enhancer in b-cells inhibitor, epsilon, DCN - decorin, TIPARP - tcdd-inducible poly(adp-ribose) polymerase, HERPUD1 - homocysteine-inducible, endoplasmic reticulum stress-inducible, ubiquitin-like domain member 1, PTGER3 - prostaglandin e receptor 3 (subtype ep3), RHOH - ras homolog family member h, DDIT3 - dna-damage-inducible transcript 3, MAOB - monoamine oxidase b, SOX9 - sry (sex determining region y)-box 9, ERAP2 - endoplasmic reticulum aminopeptidase 2, PTGS2 - prostaglandin-endoperoxide synthase 2 (prostaglandin g/h synthase and cyclooxygenase), C7 - complement component 7, CYP7B1 - cytochrome p450, family 7, subfamily b, polypeptide 1, KLF15 - kruppel-like factor 15, USP2 - ubiquitin specific peptidase 2, CXCL12 - chemokine (c-x-c motif) ligand 12, GRIK4 - glutamate receptor, ionotropic, kainate 4, ELN - elastin, PTN - pleiotrophin, CPS1 - carbamoyl-phosphate synthase 1, mitochondrial, ADORA1 - adenosine a1 receptor, SPR - sepiapterin reductase (7,8-dihydrobiopterin:nadp+ oxidoreductase), PTPRC - protein tyrosine phosphatase, receptor type, c, TTR - transthyretin, JMJD1C - jumonji domain containing 1c, SLC39A12 - solute carrier family 39 (zinc transporter), member 12, MAFF - v-maf avian musculoaponeurotic fibrosarcoma oncogene homolog f, PTX3 - pentraxin 3, long, EMCN - endomucin, MSTN - myostatin, ATP1A2 - atpase, na+/k+ transporting, alpha 2 polypeptide, TRPV4 - transient receptor potential cation channel, subfamily v, member 4, TFPI - tissue factor pathway inhibitor (lipoprotein-associated coagulation inhibitor), CSF1 - colony stimulating factor 1 (macrophage), GCNT4 - glucosaminyl (n-acetyl) transferase 4, core 2, PLSCR4 - phospholipid scramblase 4, GHR - growth hormone receptor, NTRK2 - neurotrophic tyrosine kinase, receptor, type 2, YAP1 - yes-associated protein 1, CSN3 - casein kappa, RND1 - rho family gtpase 1, SLC24A5 - solute carrier family 24 (sodium/potassium/calcium exchanger), member 5, ALDH1A1 - aldehyde dehydrogenase 1 family, member a1, HSD17B2 - hydroxysteroid (17-beta) dehydrogenase 2, LCAT - lecithin-cholesterol acyltransferase, INHBB - inhibin, beta b, GZMB - granzyme b (granzyme 2, cytotoxic t-lymphocyte-associated serine esterase 1), HSPA5 - heat shock 70kda protein 5 (glucose-regulated protein, 78kda), CAV3 - caveolin 3, IQGAP3 - iq motif containing gtpase activating protein 3, HSPB1 - heat shock 27kda protein 1, SLC1A3 - solute carrier family 1 (glial high affinity glutamate transporter), member 3, LEPR - leptin receptor, SLC2A5 - solute carrier family 2 (facilitated glucose/fructose transporter), member 5, ARMCX5-GPRASP2 - armcx5-gprasp2 readthrough, IRF1 - interferon regulatory factor 1, TAC4 - tachykinin 4 (hemokinin), TNFRSF11B - tumor necrosis factor receptor superfamily, member 11b, SERF2 - small edrk-rich factor 2, HTR1A - 5-hydroxytryptamine (serotonin) receptor 1a, g protein-coupled, MME - membrane metallo-endopeptidase, HTR1D - 5-hydroxytryptamine (serotonin) receptor 1d, g protein-coupled, ITGA2 - integrin, alpha 2 (cd49b, alpha 2 subunit of vla-2 receptor), PLSCR5 - phospholipid scramblase family, member 5, TNFAIP3 - tumor necrosis factor, alpha-induced protein 3, ANXA1 - annexin a1, RAB39A - rab39a, member ras oncogene family, SLC12A4 - solute carrier family 12 (potassium/chloride transporter), member 4, STEAP1 - six transmembrane epithelial antigen of the prostate 1, PLAU - plasminogen activator, urokinase]
GO:0044092	negative regulation of molecular function	6.69E-05	1.54E-02	1.89	16231	993	355	41	[HFE - hemochromatosis, NFKB1 - nuclear factor of kappa light polypeptide gene enhancer in b-cells 1, PARP9 - poly (adp-ribose) polymerase family, member 9, NFKBIA - nuclear factor of kappa light polypeptide gene enhancer in b-cells inhibitor, alpha, CAV3 - caveolin 3, ID1 - inhibitor of dna binding 1, dominant negative helix-loop-helix protein, NFKBIE - nuclear factor of kappa light polypeptide gene enhancer in b-cells inhibitor, epsilon, ID3 - inhibitor of dna binding 3, dominant negative helix-loop-helix protein, HSPB1 - heat shock 27kda protein 1, LEPR - leptin receptor, HERPUD1 - homocysteine-inducible, endoplasmic reticulum stress-inducible, ubiquitin-like domain member 1, PTX3 - pentraxin 3, long, DUSP10 - dual specificity phosphatase 10, SERPINH1 - serpin peptidase inhibitor, clade h (heat shock protein 47), member 1, (collagen binding protein 1), MSX2 - msh homeobox 2, MSTN - myostatin, WFIKKN1 - wap, follistatin/kazal, immunoglobulin, kunitz and netrin domain containing 1, RHOH - ras homolog family member h, ATP1A2 - atpase, na+/k+ transporting, alpha 2 polypeptide, GADD45A - growth arrest and dna-damage-inducible, alpha, DDIT3 - dna-damage-inducible transcript 3, C5 - complement component 5, APOA1 - apolipoprotein a-i, ZC3H12A - zinc finger ccch-type containing 12a, TFPI - tissue factor pathway inhibitor (lipoprotein-associated coagulation inhibitor), RRAD - ras-related associated with diabetes, IFIT2 - interferon-induced protein with tetratricopeptide repeats 2, IFIT1 - interferon-induced protein with tetratricopeptide repeats 1, PTN - pleiotrophin, GPR87 - g protein-coupled receptor 87, SPRY2 - sprouty homolog 2 (drosophila), SMR3B - submaxillary gland androgen regulated protein 3b, SPINK2 - serine peptidase inhibitor, kazal type 2 (acrosin-trypsin inhibitor), ITPRIP - inositol 1,4,5-trisphosphate receptor interacting protein, RAMP3 - receptor (g protein-coupled) activity modifying protein 3, TNFAIP3 - tumor necrosis factor, alpha-induced protein 3, ANXA1 - annexin a1, PTPRC - protein tyrosine phosphatase, receptor type, c, GPR35 - g protein-coupled receptor 35, SMR3A - submaxillary gland androgen regulated protein 3a, ANXA4 - annexin a4]
GO:0030154	cell differentiatio n	7.10E-05	1.61E-02	1.64	16231	1702	355	61	[ARID5B - at rich interactive domain 5b (mrf1-like), ID1 - inhibitor of dna binding 1, dominant negative helix-loop-helix protein, ATF3 - activating transcription factor 3, ID3 - inhibitor of dna binding 3, dominant negative helix-loop-helix protein, PTPRZ1 - protein tyrosine phosphatase, receptor-type, z polypeptide 1, RELB - v-rel avian reticuloendotheliosis viral oncogene homolog b, MMP21 - matrix metallopeptidase 21, MAFF - v-maf avian musculoaponeurotic fibrosarcoma oncogene homolog f, CD34 - cd34 molecule, ATOH8 - atonal homolog 8 (drosophila), NEUROG2 - neurogenin 2, ZC3H12A - zinc finger ccch-type containing 12a, DLX3 - distal-less homeobox 3, COL4A1 - collagen, type iv, alpha 1, OPN3 - opsin 3, CSF1 - colony stimulating factor 1 (macrophage), WNT8B - wingless-type mmtv integration site family, member 8b, BARHL2 - barh-like homeobox 2, TBX2 - t-box 2, NTRK2 - neurotrophic tyrosine kinase, receptor, type 2, COL8A1 - collagen, type viii, alpha 1, FGF1 - fibroblast growth factor 1 (acidic), YAP1 - yes-associated protein 1, BARX2 - barx homeobox 2, ZFP36L1 - zfp36 ring finger protein-like 1, COL12A1 - collagen, type xii, alpha 1, SLC24A5 - solute carrier family 24 (sodium/potassium/calcium exchanger), member 5, SMOC1 - sparc related modular calcium binding 1, MGP - matrix gla protein, MGST1 - microsomal glutathione s-transferase 1, INHBB - inhibin, beta b, HOPX - hop homeobox, HSPA5 - heat shock 70kda protein 5 (glucose-regulated protein, 78kda), CAV3 - caveolin 3, MECOM - mds1 and evi1 complex locus, TIPARP - tcdd-inducible poly(adp-ribose) polymerase, LEPR - leptin receptor, SPDEF - sam pointed domain containing ets transcription factor, IGFBP3 - insulin-like growth factor binding protein 3, DUSP10 - dual specificity phosphatase 10, MSX2 - msh homeobox 2, RHOH - ras homolog family member h, SOX9 - sry (sex determining region y)-box 9, ZIC2 - zic family member 2, IRF1 - interferon regulatory factor 1, KLF15 - kruppel-like factor 15, IRF2BP2 - interferon regulatory factor 2 binding protein 2, NKX2-4 - nk2 homeobox 4, VAMP5 - vesicle-associated membrane protein 5, CHAC1 - chac, cation transport regulator homolog 1 (e. coli), ITGA2 - integrin, alpha 2 (cd49b, alpha 2 subunit of vla-2 receptor), PTN - pleiotrophin, BCAP29 - b-cell receptor-associated protein 29, VCAM1 - vascular cell adhesion molecule 1, CPS1 - carbamoyl-phosphate synthase 1, mitochondrial, KRT75 - keratin 75, TNC - tenascin c, ANXA1 - annexin a1, PTPRC - protein tyrosine phosphatase, receptor type, c, IBSP - integrin-binding sialoprotein, ANXA4 - annexin a4]
GO:0071496	cellular response to external stimulus	7.38E-05	1.65E-02	2.84	16231	290	355	18	[HFE - hemochromatosis, NFKB1 - nuclear factor of kappa light polypeptide gene enhancer in b-cells 1, FOLR1 - folate receptor 1 (adult), HSPA5 - heat shock 70kda protein 5 (glucose-regulated protein, 78kda), ITGA2 - integrin, alpha 2 (cd49b, alpha 2 subunit of vla-2 receptor), FOSL1 - fos-like antigen 1, PTN - pleiotrophin, ATF3 - activating transcription factor 3, FAS - fas cell surface death receptor, ATP1A2 - atpase, na+/k+ transporting, alpha 2 polypeptide, TNC - tenascin c, GADD45A - growth arrest and dna-damage-inducible, alpha, SOX9 - sry (sex determining region y)-box 9, PTPRC - protein tyrosine phosphatase, receptor type, c, IRF1 - interferon regulatory factor 1, ZC3H12A - zinc finger ccch-type containing 12a, KLF10 - kruppel-like factor 10, INHBB - inhibin, beta b]

GO:0032501	multicellular organismal process	8.58E-05	1.89E-02	1.45	16231	2901	355	92	[HFE - hemochromatosis, ARID5B - at rich interactive domain 5b (mrf1-like), JMJD1C - jumonji domain containing 1c, GSC - goosecoid homeobox, ID1 - inhibitor of dna binding 1, dominant negative helix-loop-helix protein, ID3 - inhibitor of dna binding 3, dominant negative helix-loop-helix protein, PTPRZ1 - protein tyrosine phosphatase, receptor-type, z polypeptide 1, RELB - v-rel avian reticuloendotheliosis viral oncogene homolog b, MMP21 - matrix metallopeptidase 21, MAFF - v-maf avian musculoaponeurotic fibrosarcoma oncogene homolog f, CD34 - cd34 molecule, MSTN - myostatin, ATP1A2 - atpase, na+/k+ transporting, alpha 2 polypeptide, LTBP3 - latent transforming growth factor beta binding protein 3, TRPV4 - transient receptor potential cation channel, subfamily v, member 4, KRTAP3-1 - keratin associated protein 3-1, GP9 - glycoprotein ix (platelet), ENKUR - enkurin, trpc channel interacting protein, APOA1 - apolipoprotein a-i, KCNA1 - potassium voltage-gated channel, shaker-related subfamily, member 1 (episodic ataxia with myokymia), TFPI - tissue factor pathway inhibitor (lipoprotein-associated coagulation inhibitor), DLX3 - distal-less homeobox 3, DAND5 - dan domain family member 5, bmp antagonist, COL4A1 - collagen, type iv, alpha 1, RGR - retinal g protein coupled receptor, PGA3 - pepsinogen 3, group i (pepsinogen a), MFRP - membrane frizzled-related protein, PGA5 - pepsinogen 5, group i (pepsinogen a), CSF1 - colony stimulating factor 1 (macrophage), WNT8B - wingless-type mmtv integration site family, member 8b, SOSTDC1 - sclerostin domain containing 1, SPRY2 - sprouty homolog 2 (drosophila), TBX2 - t-box 2, KCNIP3 - kv channel interacting protein 3, calsenilin, NTRK2 - neurotrophic tyrosine kinase, receptor, type 2, PAX6 - paired box 6, FGF1 - fibroblast growth factor 1 (acidic), YAP1 - yes-associated protein 1, ANGPTL2 - angiopoietin-like 2, ZFP36L1 - zfp36 ring finger protein-like 1, SLC17A8 - solute carrier family 17 (vesicular glutamate transporter), member 8, ACTA2 - actin, alpha 2, smooth muscle, aorta, AQP4 - aquaporin 4, PRRX1 - paired related homeobox 1, BTC - betacellulin, HSD17B2 - hydroxysteroid (17-beta) dehydrogenase 2, UCP3 - uncoupling protein 3 (mitochondrial, proton carrier), LCAT - lecithin-cholesterol acyltransferase, MGP - matrix gla protein, KLF10 - kruppel-like factor 10, CCL2 - chemokine (c-c motif) ligand 2, CSRNP1 - cysteine-serine-rich nuclear protein 1, CAV3 - caveolin 3, CLEC3A - c-type lectin domain family 3, member a, TIPARP - tcdd-inducible poly(adp-ribose) polymerase, LEPR - leptin receptor, SPDEF - sam pointed domain containing ets transcription factor, CEP55 - centrosomal protein 55kda, PTGER3 - prostaglandin e receptor 3 (subtype ep3), SLC2A5 - solute carrier family 2 (facilitated glucose/fructose transporter), member 5, DDIT3 - dna-damage-inducible transcript 3, SOX9 - sry (sex determining region y)-box 9, ARMCX5-GPRASP2 - armcx5-gprasp2 readthrough, C5 - complement component 5, ZIC2 - zic family member 2, PTGS2 - prostaglandin-endoperoxide synthase 2 (prostaglandin g/h synthase and cyclooxygenase), IRF1 - interferon regulatory factor 1, PTHLH - parathyroid hormone-like hormone, KLF15 - kruppel-like factor 15, MNS1 - meiosis-specific nuclear structural 1, USP2 - ubiquitin specific peptidase 2, NKX2-4 - nk2 homeobox 4, CXCL12 - chemokine (c-x-c motif) ligand 12, HTR1A - 5-hydroxytryptamine (serotonin) receptor 1a, g protein-coupled, DAW1 - dynein assembly factor with wdr repeat domains 1, MME - membrane metallo-endopeptidase, HTR1D - 5-hydroxytryptamine (serotonin) receptor 1d, g protein-coupled, ITGA2 - integrin, alpha 2 (cd49b, alpha 2 subunit of vla-2 receptor), ELN - elastin, FOSL1 - fos-like antigen 1, PTN - pleiotrophin, VCAM1 - vascular cell adhesion molecule 1, CPS1 - carbamoyl-phosphate synthase 1, mitochondrial, PDGFRB - platelet-derived growth factor receptor, beta polypeptide, KRT75 - keratin 75, ADORA1 - adenosine a1 receptor, OPN1SW - opsin 1 (cone pigments), short-wave-sensitive, ANXA1 - annexin a1, KCNQ4 - potassium voltage-gated channel, kqt-like subfamily, member 4, SLC15A2 - solute carrier family 15 (oligopeptide transporter), member 2, PLAU - plasminogen activator, urokinase, HMCN1 - hemicentin 1]
GO:0098739	import across plasma membrane	9.42E-05	2.05E-02	4.02	16231	125	355	11	[HFE - hemochromatosis, ATP1A2 - atpase, na+/k+ transporting, alpha 2 polypeptide, FOLR1 - folate receptor 1 (adult), SLC2A5 - solute carrier family 2 (facilitated glucose/fructose transporter), member 5, SLC6A20 - solute carrier family 6 (proline imino transporter), member 20, TRPV4 - transient receptor potential cation channel, subfamily v, member 4, KCNJ8 - potassium inwardly-rectifying channel, subfamily j, member 8, SLC39A12 - solute carrier family 39 (zinc transporter), member 12, SLC1A3 - solute carrier family 1 (glial high affinity glutamate transporter), member 3, SLC12A4 - solute carrier family 12 (potassium/chloride transporter), member 4, SLC15A2 - solute carrier family 15 (oligopeptide transporter), member 2]
GO:0060602	branch elongation of an epithelium	9.69E-05	2.08E-02	15.24	16231	12	355	4	[TNC - tenascin c, SPRY2 - sprouty homolog 2 (drosophila), FGF1 - fibroblast growth factor 1 (acidic), YAP1 - yes-associated protein 1]
GO:0050793	regulation of development al process	9.74E-05	2.06E-02	1.51	16231	2363	355	78	[CDC42EP1 - cdc42 effector protein (rho gtpase binding) 1, E2F2 - e2f transcription factor 2, ID1 - inhibitor of dna binding 1, dominant negative helix-loop-helix protein, ITPKB - inositol-trisphosphate 3-kinase b, SLC39A12 - solute carrier family 39 (zinc transporter), member 12, ID3 - inhibitor of dna binding 3, dominant negative helix-loop-helix protein, PTPRZ1 - protein tyrosine phosphatase, receptor-type, z polypeptide 1, MAFF - v-maf avian musculoaponeurotic fibrosarcoma oncogene homolog f, RHOD - ras homolog family member d, CD34 - cd34 molecule, MSTN - myostatin, ATOH8 - atonal homolog 8 (drosophila), LTBP3 - latent transforming growth factor beta binding protein 3, TRPV4 - transient receptor potential cation channel, subfamily v, member 4, IL8 - interleukin 8, APOA1 - apolipoprotein a-i, ZC3H12A - zinc finger ccch-type containing 12a, DAND5 - dan domain family member 5, bmp antagonist, NEDD9 - neural precursor cell expressed, developmentally down-regulated 9, CSF1 - colony stimulating factor 1 (macrophage), RHOJ - ras homolog family member j, MBOAT1 - membrane bound o-acyltransferase domain containing 1, BARHL2 - barh-like homeobox 2, FGL2 - fibrinogen-like 2, SOSTDC1 - sclerostin domain containing 1, GHR - growth hormone receptor, SPRY2 - sprouty homolog 2 (drosophila), TBX2 - t-box 2, NTRK2 - neurotrophic tyrosine kinase, receptor, type 2, PAX6 - paired box 6, FGF1 - fibroblast growth factor 1 (acidic), CXCL13 - chemokine (c-x-c motif) ligand 13, YAP1 - yes-associated protein 1, PSMB9 - proteasome (prosome, macropain) subunit, beta type, 9, RND1 - rho family gtpase 1, ZFP36L1 - zfp36 ring finger protein-like 1, SMOC1 - sparc related modular calcium binding 1, PRRX1 - paired related homeobox 1, BTC - betacellulin, GAB1 - grb2-associated binding protein 1, MGP - matrix gla protein, KLF10 - kruppel-like factor 10, HOPX - hop homeobox, CCL2 - chemokine (c-c motif) ligand 2, NFKB1 - nuclear factor of kappa light polypeptide gene enhancer in b-cells 1, HSPA5 - heat shock 70kda protein 5 (glucose-regulated protein, 78kda), NFKBIA - nuclear factor of kappa light polypeptide gene enhancer in b-cells inhibitor, alpha, CAV3 - caveolin 3, IQGAP3 - iq motif containing gtpase activating protein 3, AAMDC - adipogenesis associated, mth938 domain containing, DCN - decorin, PCP4 - purkinje cell protein 4, HSPB1 - heat shock 27kda protein 1, SPDEF - sam pointed domain containing ets transcription factor, DCT - dopachrome tautomerase, IGFBP3 - insulin-like growth factor binding protein 3, DUSP10 - dual specificity phosphatase 10, RHOH - ras homolog family member h, GADD45A - growth arrest and dna-damage-inducible, alpha, DDIT3 - dna-damage-inducible transcript 3, SOX9 - sry (sex determining region y)-box 9, ARMCX5-GPRASP2 - armcx5-gprasp2 readthrough, C5 - complement component 5, PTGS2 - prostaglandin-endoperoxide synthase 2 (prostaglandin g/h synthase and cyclooxygenase), IRF1 - interferon regulatory factor 1, PTHLH - parathyroid hormone-like hormone, TNFRSF11B - tumor necrosis factor receptor superfamily, member 11b, GJC2 - gap junction protein, gamma 2, 47kda, CXCL12 - chemokine (c-x-c motif) ligand 12, MME - membrane metallo-endopeptidase, PTN - pleiotrophin, CTSV - cathepsin v, RIPK2 - receptor-interacting serine-threonine kinase 2, PDGFRB - platelet-derived growth factor receptor, beta polypeptide, TNFAIP3 - tumor necrosis factor, alpha-induced protein 3, ANXA1 - annexin a1, PTPRC - protein tyrosine phosphatase, receptor type, c, CD83 - cd83 molecule]
GO:0050688	regulation of defense response to virus	1.03E-04	2.14E-02	5.46	16231	67	355	8	[PARP9 - poly (adp-ribose) polymerase family, member 9, BIRC3 - baculoviral iap repeat containing 3, TNFAIP3 - tumor necrosis factor, alpha-induced protein 3, IFIT1 - interferon-induced protein with tetratricopeptide repeats 1, FGL2 - fibrinogen-like 2, DDX60 - dead (asp-glu-ala-asp) box polypeptide 60, ZC3H12A - zinc finger ccch-type containing 12a, HERC5 - hect and rld domain containing e3 ubiquitin protein ligase 5]

GO:0002682	regulation of immune system process	1.06E-04	2.18E-02	1.71	16231	1363	355	51	[CFH - complement factor h, HFE - hemochromatosis, ITPKB - inositol-trisphosphate 3-kinase b, RELB - v-rel avian reticuloendotheliosis viral oncogene homolog b, CD34 - cd34 molecule, MSTN - myostatin, BTN3A3 - butyrophilin, subfamily 3, member a3, BIRC3 - baculoviral iap repeat containing 3, IL8 - interleukin 8, APOA1 - apolipoprotein a-i, ZC3H12A - zinc finger ccch-type containing 12a, CSF1 - colony stimulating factor 1 (macrophage), IFI35 - interferon-induced protein 35, IFIT1 - interferon-induced protein with tetratricopeptide repeats 1, FGL2 - fibrinogen-like 2, CXCL13 - chemokine (c-x-c motif) ligand 13, YAP1 - yes-associated protein 1, PSMB9 - proteasome (prosome, macropain) subunit, beta type, 9, ZFP36L1 - zfp36 ring finger protein-like 1, MUC15 - mucin 15, cell surface associated, TICAM1 - toll-like receptor adaptor molecule 1, KLF10 - kruppel-like factor 10, CCL2 - chemokine (c-c motif) ligand 2, NFKB1 - nuclear factor of kappa light polypeptide gene enhancer in b-cells 1, PARP9 - poly (adp-ribose) polymerase family, member 9, NFKBIA - nuclear factor of kappa light polypeptide gene enhancer in b-cells inhibitor, alpha, HERC5 - hect and rld domain containing e3 ubiquitin protein ligase 5, DUSP10 - dual specificity phosphatase 10, CCL20 - chemokine (c-c motif) ligand 20, RHOH - ras homolog family member h, SOX9 - sry (sex determining region y)-box 9, C5 - complement component 5, DDX60 - dead (asp-glu-ala-asp) box polypeptide 60, PARP14 - poly (adp-ribose) polymerase family, member 14, IRF1 - interferon regulatory factor 1, SIAE - sialic acid acetylesterase, C7 - complement component 7, COLEC12 - collectin sub-family member 12, CXCL12 - chemokine (c-x-c motif) ligand 12, ITGA2 - integrin, alpha 2 (cd49b, alpha 2 subunit of vla-2 receptor), NMI - n-myc (and stat) interactor, PTN - pleiotrophin, RIPK2 - receptor-interacting serine-threonine kinase 2, VCAM1 - vascular cell adhesion molecule 1, ADORA1 - adenosine a1 receptor, TNFAIP3 - tumor necrosis factor, alpha-induced protein 3, ANXA1 - annexin a1, PTPRC - protein tyrosine phosphatase, receptor type, c, ITGB7 - integrin, beta 7, SLC15A2 - solute carrier family 15 (oligopeptide transporter), member 2, CD83 - cd83 molecule]
GO:0030155	regulation of cell adhesion	1.08E-04	2.19E-02	2.1	16231	653	355	30	[CCL2 - chemokine (c-c motif) ligand 2, HFE - hemochromatosis, ITPKB - inositol-trisphosphate 3-kinase b, RHOD - ras homolog family member d, DUSP10 - dual specificity phosphatase 10, RHOH - ras homolog family member h, TRPV4 - transient receptor potential cation channel, subfamily v, member 4, IL8 - interleukin 8, SOX9 - sry (sex determining region y)-box 9, IRF1 - interferon regulatory factor 1, APOA1 - apolipoprotein a-i, ZC3H12A - zinc finger ccch-type containing 12a, NEDD9 - neural precursor cell expressed, developmentally down-regulated 9, CXCL12 - chemokine (c-x-c motif) ligand 12, CSF1 - colony stimulating factor 1 (macrophage), ITGA2 - integrin, alpha 2 (cd49b, alpha 2 subunit of vla-2 receptor), PTN - pleiotrophin, FGL2 - fibrinogen-like 2, RIPK2 - receptor-interacting serine-threonine kinase 2, VCAM1 - vascular cell adhesion molecule 1, COL8A1 - collagen, type viii, alpha 1, ECM2 - extracellular matrix protein 2, female organ and adipocyte specific, CXCL13 - chemokine (c-x-c motif) ligand 13, RND1 - rho family gtpase 1, TNC - tenascin c, ANXA1 - annexin a1, PTPRC - protein tyrosine phosphatase, receptor type, c, IBSP - integrin-binding sialoprotein, PLAU - plasminogen activator, urokinase, CD83 - cd83 molecule]
GO:0051094	positive regulation of developmental process	1.09E-04	2.18E-02	1.74	16231	1258	355	48	[NFKB1 - nuclear factor of kappa light polypeptide gene enhancer in b-cells 1, HSPA5 - heat shock 70kda protein 5 (glucose-regulated protein, 78kda), CAV3 - caveolin 3, IQGAP3 - iq motif containing gtpase activating protein 3, ITPKB - inositol-trisphosphate 3-kinase b, AAMDC - adipogenesis associated, mth938 domain containing, SLC39A12 - solute carrier family 39 (zinc transporter), member 12, PTPRZ1 - protein tyrosine phosphatase, receptor-type, z polypeptide 1, DCN - decorin, PCP4 - purkinje cell protein 4, HSPB1 - heat shock 27kda protein 1, SPDEF - sam pointed domain containing ets transcription factor, IGFBP3 - insulin-like growth factor binding protein 3, DCT - dopachrome tautomerase, DUSP10 - dual specificity phosphatase 10, CD34 - cd34 molecule, RHOH - ras homolog family member h, ATOH8 - atonal homolog 8 (drosophila), LTBP3 - latent transforming growth factor beta binding protein 3, IL8 - interleukin 8, ARMCX5-GPRASP2 - armcx5-gprasp2 readthrough, SOX9 - sry (sex determining region y)-box 9, C5 - complement component 5, PTGS2 - prostaglandin-endoperoxide synthase 2 (prostaglandin g/h synthase and cyclooxygenase), APOA1 - apolipoprotein a-i, ZC3H12A - zinc finger ccch-type containing 12a, GJC2 - gap junction protein, gamma 2, 47kda, NEDD9 - neural precursor cell expressed, developmentally down-regulated 9, CXCL12 - chemokine (c-x-c motif) ligand 12, CSF1 - colony stimulating factor 1 (macrophage), RHOJ - ras homolog family member j, MME - membrane metallo-endopeptidase, PTN - pleiotrophin, GHR - growth hormone receptor, RIPK2 - receptor-interacting serine-threonine kinase 2, TBX2 - t-box 2, NTRK2 - neurotrophic tyrosine kinase, receptor, type 2, PDGFRB - platelet-derived growth factor receptor, beta polypeptide, FGF1 - fibroblast growth factor 1 (acidic), YAP1 - yes-associated protein 1, ZFP36L1 - zfp36 ring finger protein-like 1, TNFAIP3 - tumor necrosis factor, alpha-induced protein 3, GAB1 - grb2-associated binding protein 1, BTC - betacellulin, ANXA1 - annexin a1, PTPRC - protein tyrosine phosphatase, receptor type, c, KLF10 - kruppel-like factor 10, CD83 - cd83 molecule]
GO:1990266	neutrophil migration	1.14E-04	2.25E-02	5.38	16231	68	355	8	[CCL20 - chemokine (c-c motif) ligand 20, CCL2 - chemokine (c-c motif) ligand 2, IL8 - interleukin 8, CXCL1 - chemokine (c-x-c motif) ligand 1 (melanoma growth stimulating activity, alpha), CXCL2 - chemokine (c-x-c motif) ligand 2, CXCL3 - chemokine (c-x-c motif) ligand 3, CXCL13 - chemokine (c-x-c motif) ligand 13, CKLF - chemokine-like factor]
GO:0060337	type I interferon signaling pathway	1.14E-04	2.23E-02	6.28	16231	51	355	7	[OAS3 - 2'-5'-oligoadenylate synthetase 3, 100kda, OASL - 2'-5'-oligoadenylate synthetase-like, IFI35 - interferon-induced protein 35, IFIT2 - interferon-induced protein with tetratricopeptide repeats 2, IFIT1 - interferon-induced protein with tetratricopeptide repeats 1, IFIT3 - interferon-induced protein with tetratricopeptide repeats 3, IRF1 - interferon regulatory factor 1]
GO:0045662	negative regulation of myoblast differentiation	1.18E-04	2.28E-02	9.94	16231	23	355	5	[MSTN - myostatin, DDIT3 - dna-damage-inducible transcript 3, ID3 - inhibitor of dna binding 3, dominant negative helix-loop-helix protein, SOX9 - sry (sex determining region y)-box 9, SOSTDC1 - sclerostin domain containing 1]
GO:0010632	regulation of epithelial cell migration	1.27E-04	2.42E-02	3.06	16231	224	355	15	[RHOJ - ras homolog family member j, ITGA2 - integrin, alpha 2 (cd49b, alpha 2 subunit of vla-2 receptor), PTN - pleiotrophin, DCN - decorin, HSPB1 - heat shock 27kda protein 1, HBEGF - heparin-binding egf-like growth factor, FGF1 - fibroblast growth factor 1 (acidic), CXCL13 - chemokine (c-x-c motif) ligand 13, DUSP10 - dual specificity phosphatase 10, ATOH8 - atonal homolog 8 (drosophila), GADD45A - growth arrest and dna-damage-inducible, alpha, SOX9 - sry (sex determining region y)-box 9, PTGS2 - prostaglandin-endoperoxide synthase 2 (prostaglandin g/h synthase and cyclooxygenase), ANXA1 - annexin a1, ZC3H12A - zinc finger ccch-type containing 12a]
GO:0071495	cellular response to endogenous stimulus	1.30E-04	2.45E-02	2.08	16231	660	355	30	[CCL2 - chemokine (c-c motif) ligand 2, NFKB1 - nuclear factor of kappa light polypeptide gene enhancer in b-cells 1, FOLR1 - folate receptor 1 (adult), HSPA5 - heat shock 70kda protein 5 (glucose-regulated protein, 78kda), CAV3 - caveolin 3, ID1 - inhibitor of dna binding 1, dominant negative helix-loop-helix protein, MSTN - myostatin, IGFBP7 - insulin-like growth factor binding protein 7, ATP1A2 - atpase, na+/k+ transporting, alpha 2 polypeptide, IL8 - interleukin 8, SOX9 - sry (sex determining region y)-box 9, TFPI - tissue factor pathway inhibitor (lipoprotein-associated coagulation inhibitor), KLF15 - kruppel-like factor 15, COL4A1 - collagen, type iv, alpha 1, ITGA2 - integrin, alpha 2 (cd49b, alpha 2 subunit of vla-2 receptor), PTN - pleiotrophin, GHR - growth hormone receptor, RIPK2 - receptor-interacting serine-threonine kinase 2, VCAM1 - vascular cell adhesion molecule 1, NTRK2 - neurotrophic tyrosine kinase, receptor, type 2, CPS1 - carbamoyl-phosphate synthase 1, mitochondrial, PDGFRB - platelet-derived growth factor receptor, beta polypeptide, ZFP36L1 - zfp36 ring finger protein-like 1, TNC - tenascin c, RAMP3 - receptor (g protein-coupled) activity modifying protein 3, KLF9 - kruppel-like factor 9, ANXA1 - annexin a1, UCP3 - uncoupling protein 3 (mitochondrial, proton carrier), KLF10 - kruppel-like factor 10, INHBB - inhibin, beta b]
GO:0001525	angiogenesis	1.36E-04	2.52E-02	2.91	16231	251	355	16	[CCL2 - chemokine (c-c motif) ligand 2, RHOJ - ras homolog family member j, ANGPTL1 - angiopoietin-like 1, ID1 - inhibitor of dna binding 1, dominant negative helix-loop-helix protein, COL22A1 - collagen, type xxii, alpha 1, LEPR - leptin receptor, COL8A1 - collagen, type viii, alpha 1, PDGFRB - platelet-derived growth factor receptor, beta polypeptide, FGF1 - fibroblast growth factor 1 (acidic), ANGPTL2 - angiopoietin-like 2, CD34 - cd34 molecule, RAMP3 - receptor (g protein-coupled) activity modifying protein 3, GAB1 - grb2-associated binding protein 1, IL8 - interleukin 8, CALCRL - calcitonin receptor-like, ZC3H12A - zinc finger ccch-type containing 12a]

GO:0006984	ER-nucleus signaling pathway	1.60E-04	2.94E-02	7.22	16231	38	355	6	[CCL2 - chemokine (c-c motif) ligand 2, HSPA5 - heat shock 70kda protein 5 (glucose-regulated protein, 78kda), DDIT3 - dna-damage-inducible transcript 3, IL8 - interleukin 8, ATF3 - activating transcription factor 3, HERPUD1 - homocysteine-inducible, endoplasmic reticulum stress-inducible, ubiquitin-like domain member 1]
GO:2000145	regulation of cell motility	1.78E-04	3.23E-02	1.89	16231	869	355	36	[CCL2 - chemokine (c-c motif) ligand 2, HSPA5 - heat shock 70kda protein 5 (glucose-regulated protein, 78kda), DCN - decorin, HSPB1 - heat shock 27kda protein 1, RHOD - ras homolog family member d, IGFBP3 - insulin-like growth factor binding protein 3, DUSP10 - dual specificity phosphatase 10, MSTN - myostatin, CCL20 - chemokine (c-c motif) ligand 20, RHOH - ras homolog family member h, ATOH8 - atonal homolog 8 (drosophila), GADD45A - growth arrest and dna-damage-inducible, alpha, IL8 - interleukin 8, PLP1 - proteolipid protein 1, SOX9 - sry (sex determining region y)-box 9, C5 - complement component 5, PTGS2 - prostaglandin-endoperoxide synthase 2 (prostaglandin g/h synthase and cyclooxygenase), TAC4 - tachykinin 4 (hemokinin), ZC3H12A - zinc finger ccch-type containing 12a, NEDD9 - neural precursor cell expressed, developmentally down-regulated 9, CXCL12 - chemokine (c-x-c motif) ligand 12, CSF1 - colony stimulating factor 1 (macrophage), RHOJ - ras homolog family member j, ITGA2 - integrin, alpha 2 (cd49b, alpha 2 subunit of vla-2 receptor), PTN - pleiotrophin, SPRY2 - sprouty homolog 2 (drosophila), HBEGF - heparin-binding egf-like growth factor, PDGFRB - platelet-derived growth factor receptor, beta polypeptide, FGF1 - fibroblast growth factor 1 (acidic), CXCL13 - chemokine (c-x-c motif) ligand 13, ADORA1 - adenosine a1 receptor, GAB1 - grb2-associated binding protein 1, BTC - betacellulin, ANXA1 - annexin a1, PTPRC - protein tyrosine phosphatase, receptor type, c, PLAU - plasminogen activator, urokinase]
GO:0048870	cell motility	1.78E-04	3.20E-02	1.89	16231	869	355	36	[CCL2 - chemokine (c-c motif) ligand 2, ARID5B - at rich interactive domain 5b (mrf1-like), PARP9 - poly (adp-ribose) polymerase family, member 9, FOLR1 - folate receptor 1 (adult), ID1 - inhibitor of dna binding 1, dominant negative helix-loop-helix protein, RHOD - ras homolog family member d, CKLF - chemokine-like factor, CD34 - cd34 molecule, MSTN - myostatin, CCL20 - chemokine (c-c motif) ligand 20, IL8 - interleukin 8, C5 - complement component 5, ENKUR - enkurin, trpc channel interacting protein, APOA1 - apolipoprotein a-i, CYP7B1 - cytochrome p450, family 7, subfamily b, polypeptide 1, CD58 - cd58 molecule, NEDD9 - neural precursor cell expressed, developmentally down-regulated 9, CXCL12 - chemokine (c-x-c motif) ligand 12, RHOJ - ras homolog family member j, ITGA2 - integrin, alpha 2 (cd49b, alpha 2 subunit of vla-2 receptor), PTN - pleiotrophin, BARHL2 - barh-like homeobox 2, VCAM1 - vascular cell adhesion molecule 1, HBEGF - heparin-binding egf-like growth factor, NTRK2 - neurotrophic tyrosine kinase, receptor, type 2, PDGFRB - platelet-derived growth factor receptor, beta polypeptide, CXCL13 - chemokine (c-x-c motif) ligand 13, RND1 - rho family gtpase 1, TNFAIP3 - tumor necrosis factor, alpha-induced protein 3, GAB1 - grb2-associated binding protein 1, CXCL1 - chemokine (c-x-c motif) ligand 1 (melanoma growth stimulating activity, alpha), CXCL2 - chemokine (c-x-c motif) ligand 2, CXCL3 - chemokine (c-x-c motif) ligand 3, ANXA1 - annexin a1, ITGB7 - integrin, beta 7, PLAU - plasminogen activator, urokinase]
GO:0002684	positive regulation of immune system process	2.05E-04	3.63E-02	1.86	16231	909	355	37	[CFH - complement factor h, CCL2 - chemokine (c-c motif) ligand 2, NFKB1 - nuclear factor of kappa light polypeptide gene enhancer in b-cells 1, PARP9 - poly (adp-ribose) polymerase family, member 9, NFKBIA - nuclear factor of kappa light polypeptide gene enhancer in b-cells inhibitor, alpha, ITPKB - inositol-trisphosphate 3-kinase b, RELB - v-rel avian reticuloendotheliosis viral oncogene homolog b, DUSP10 - dual specificity phosphatase 10, MSTN - myostatin, CCL20 - chemokine (c-c motif) ligand 20, RHOH - ras homolog family member h, BTN3A3 - butyrophilin, subfamily 3, member a3, BIRC3 - baculoviral iap repeat containing 3, IL8 - interleukin 8, C5 - complement component 5, DDX60 - dead (asp-glu-ala-asp) box polypeptide 60, C7 - complement component 7, ZC3H12A - zinc finger ccch-type containing 12a, COLEC12 - collectin sub-family member 12, CXCL12 - chemokine (c-x-c motif) ligand 12, CSF1 - colony stimulating factor 1 (macrophage), IFI35 - interferon-induced protein 35, ITGA2 - integrin, alpha 2 (cd49b, alpha 2 subunit of vla-2 receptor), NMI - n-myc (and stat) interactor, PTN - pleiotrophin, RIPK2 - receptor-interacting serine-threonine kinase 2, VCAM1 - vascular cell adhesion molecule 1, CXCL13 - chemokine (c-x-c motif) ligand 13, PSMB9 - proteasome (prosome, macropain) subunit, beta type, 9, ZFP36L1 - zfp36 ring finger protein-like 1, TNFAIP3 - tumor necrosis factor, alpha-induced protein 3, ANXA1 - annexin a1, PTPRC - protein tyrosine phosphatase, receptor type, c, MUC15 - mucin 15, cell surface associated, TICAM1 - toll-like receptor adaptor molecule 1, KLF10 - kruppel-like factor 10, CD83 - cd83 molecule]
GO:2000026	regulation of multicellular organismal development	2.12E-04	3.72E-02	1.55	16231	1883	355	64	[E2F2 - e2f transcription factor 2, ID1 - inhibitor of dna binding 1, dominant negative helix-loop-helix protein, ITPKB - inositol-trisphosphate 3-kinase b, SLC39A12 - solute carrier family 39 (zinc transporter), member 12, PTPRZ1 - protein tyrosine phosphatase, receptor-type, z polypeptide 1, MAFF - v-maf avian musculoaponeurotic fibrosarcoma oncogene homolog f, CD34 - cd34 molecule, MSTN - myostatin, ATOH8 - atonal homolog 8 (drosophila), LTBP3 - latent transforming growth factor beta binding protein 3, TRPV4 - transient receptor potential cation channel, subfamily v, member 4, IL8 - interleukin 8, APOA1 - apolipoprotein a-i, ZC3H12A - zinc finger ccch-type containing 12a, DAND5 - dan domain family member 5, bmp antagonist, CSF1 - colony stimulating factor 1 (macrophage), RHOJ - ras homolog family member j, MBOAT1 - membrane bound o-acyltransferase domain containing 1, BARHL2 - barh-like homeobox 2, FGL2 - fibrinogen-like 2, SOSTDC1 - sclerostin domain containing 1, SPRY2 - sprouty homolog 2 (drosophila), TBX2 - t-box 2, NTRK2 - neurotrophic tyrosine kinase, receptor, type 2, PAX6 - paired box 6, FGF1 - fibroblast growth factor 1 (acidic), CXCL13 - chemokine (c-x-c motif) ligand 13, YAP1 - yes-associated protein 1, PSMB9 - proteasome (prosome, macropain) subunit, beta type, 9, ZFP36L1 - zfp36 ring finger protein-like 1, PRRX1 - paired related homeobox 1, GAB1 - grb2-associated binding protein 1, MGP - matrix gla protein, KLF10 - kruppel-like factor 10, HSPA5 - heat shock 70kda protein 5 (glucose-regulated protein, 78kda), NFKBIA - nuclear factor of kappa light polypeptide gene enhancer in b-cells inhibitor, alpha, CAV3 - caveolin 3, IQGAP3 - iq motif containing gtpase activating protein 3, DCN - decorin, PCP4 - purkinje cell protein 4, HSPB1 - heat shock 27kda protein 1, DCT - dopachrome tautomerase, DUSP10 - dual specificity phosphatase 10, RHOH - ras homolog family member h, GADD45A - growth arrest and dna-damage-inducible, alpha, DDIT3 - dna-damage-inducible transcript 3, SOX9 - sry (sex determining region y)-box 9, ARMCX5-GPRASP2 - armcx5-gprasp2 readthrough, C5 - complement component 5, PTGS2 - prostaglandin-endoperoxide synthase 2 (prostaglandin g/h synthase and cyclooxygenase), IRF1 - interferon regulatory factor 1, PTHLH - parathyroid hormone-like hormone, TNFRSF11B - tumor necrosis factor receptor superfamily, member 11b, GJC2 - gap junction protein, gamma 2, 47kda, CXCL12 - chemokine (c-x-c motif) ligand 12, MME - membrane metallo-endopeptidase, PTN - pleiotrophin, CTSV - cathepsin v, RIPK2 - receptor-interacting serine-threonine kinase 2, PDGFRB - platelet-derived growth factor receptor, beta polypeptide, TNFAIP3 - tumor necrosis factor, alpha-induced protein 3, ANXA1 - annexin a1, PTPRC - protein tyrosine phosphatase, receptor type, c, CD83 - cd83 molecule]
GO:0002685	regulation of leukocyte migration	2.22E-04	3.84E-02	3.21	16231	185	355	13	[CCL2 - chemokine (c-c motif) ligand 2, CXCL12 - chemokine (c-x-c motif) ligand 12, CSF1 - colony stimulating factor 1 (macrophage), ITGA2 - integrin, alpha 2 (cd49b, alpha 2 subunit of vla-2 receptor), PTN - pleiotrophin, CXCL13 - chemokine (c-x-c motif) ligand 13, CCL20 - chemokine (c-c motif) ligand 20, MSTN - myostatin, RHOH - ras homolog family member h, ADORA1 - adenosine a1 receptor, IL8 - interleukin 8, C5 - complement component 5, ANXA1 - annexin a1]
GO:0030334	regulation of cell migration	2.23E-04	3.81E-02	1.91	16231	812	355	34	[CCL2 - chemokine (c-c motif) ligand 2, HSPA5 - heat shock 70kda protein 5 (glucose-regulated protein, 78kda), DCN - decorin, HSPB1 - heat shock 27kda protein 1, RHOD - ras homolog family member d, IGFBP3 - insulin-like growth factor binding protein 3, DUSP10 - dual specificity phosphatase 10, MSTN - myostatin, CCL20 - chemokine (c-c motif) ligand 20, RHOH - ras homolog family member h, ATOH8 - atonal homolog 8 (drosophila), GADD45A - growth arrest and dna-damage-inducible, alpha, IL8 - interleukin 8, PLP1 - proteolipid protein 1, SOX9 - sry (sex determining region y)-box 9, C5 - complement component 5, PTGS2 - prostaglandin-endoperoxide synthase 2 (prostaglandin g/h synthase and cyclooxygenase), ZC3H12A - zinc finger ccch-type containing 12a, NEDD9 - neural precursor cell expressed, developmentally down-regulated 9, CXCL12 - chemokine (c-x-c motif) ligand 12, CSF1 - colony stimulating factor 1 (macrophage), RHOJ - ras homolog family member j, ITGA2 - integrin, alpha 2 (cd49b, alpha 2 subunit of vla-2 receptor), PTN - pleiotrophin, SPRY2 - sprouty homolog 2 (drosophila), HBEGF - heparin-binding egf-like growth factor, PDGFRB - platelet-derived growth factor receptor, beta polypeptide, FGF1 - fibroblast growth factor 1 (acidic), CXCL13 - chemokine (c-x-c motif) ligand 13, ADORA1 - adenosine a1 receptor, GAB1 - grb2-associated binding protein 1, ANXA1 - annexin a1, PTPRC - protein tyrosine phosphatase, receptor type, c, PLAU - plasminogen activator, urokinase]

GO:0034097	response to cytokine	2.28E-04	3.86E-02	2.23	16231	493	355	24	[CD58 - cd58 molecule, CCL2 - chemokine (c-c motif) ligand 2, ARID5B - at rich interactive domain 5b (mrf1-like), NFKB1 - nuclear factor of kappa light polypeptide gene enhancer in b-cells 1, CXCL12 - chemokine (c-x-c motif) ligand 12, MME - membrane metallo-endopeptidase, HSPA5 - heat shock 70kda protein 5 (glucose-regulated protein, 78kda), IFIT2 - interferon-induced protein with tetratricopeptide repeats 2, FOSL1 - fos-like antigen 1, IFIT1 - interferon-induced protein with tetratricopeptide repeats 1, IFIT3 - interferon-induced protein with tetratricopeptide repeats 3, RELB - v-rel avian reticuloendotheliosis viral oncogene homolog b, RIPK2 - receptor-interacting serine-threonine kinase 2, VCAM1 - vascular cell adhesion molecule 1, SPRY2 - sprouty homolog 2 (drosophila), CCL20 - chemokine (c-c motif) ligand 20, ZFP36L1 - zfp36 ring finger protein-like 1, AQP4 - aquaporin 4, IL8 - interleukin 8, SOX9 - sry (sex determining region y)-box 9, ANXA1 - annexin a1, IRF1 - interferon regulatory factor 1, ZC3H12A - zinc finger ccch-type containing 12a, TFPI - tissue factor pathway inhibitor (lipoprotein-associated coagulation inhibitor)]
GO:0032103	positive regulation of response to external stimulus	2.40E-04	4.02E-02	2.67	16231	291	355	17	[CXCL12 - chemokine (c-x-c motif) ligand 12, CSF1 - colony stimulating factor 1 (macrophage), IFI35 - interferon-induced protein 35, NFKBIA - nuclear factor of kappa light polypeptide gene enhancer in b-cells inhibitor, alpha, ITGA2 - integrin, alpha 2 (cd49b, alpha 2 subunit of vla-2 receptor), NMI - n-myc (and stat) interactor, PTN - pleiotrophin, RIPK2 - receptor-interacting serine-threonine kinase 2, HSPB1 - heat shock 27kda protein 1, PDGFRB - platelet-derived growth factor receptor, beta polypeptide, CXCL13 - chemokine (c-x-c motif) ligand 13, PTGER3 - prostaglandin e receptor 3 (subtype ep3), MSTN - myostatin, IL8 - interleukin 8, DDX60 - dead (asp-glu-ala-asp) box polypeptide 60, PTGS2 - prostaglandin-endoperoxide synthase 2 (prostaglandin g/h synthase and cyclooxygenase), TICAM1 - toll-like receptor adaptor molecule 1]
GO:0071345	cellular response to cytokine stimulus	2.44E-04	4.03E-02	2.43	16231	376	355	20	[CD58 - cd58 molecule, CCL2 - chemokine (c-c motif) ligand 2, ARID5B - at rich interactive domain 5b (mrf1-like), NFKB1 - nuclear factor of kappa light polypeptide gene enhancer in b-cells 1, CXCL12 - chemokine (c-x-c motif) ligand 12, HSPA5 - heat shock 70kda protein 5 (glucose-regulated protein, 78kda), MME - membrane metallo-endopeptidase, IFIT2 - interferon-induced protein with tetratricopeptide repeats 2, IFIT1 - interferon-induced protein with tetratricopeptide repeats 1, IFIT3 - interferon-induced protein with tetratricopeptide repeats 3, VCAM1 - vascular cell adhesion molecule 1, SPRY2 - sprouty homolog 2 (drosophila), ZFP36L1 - zfp36 ring finger protein-like 1, CCL20 - chemokine (c-c motif) ligand 20, AQP4 - aquaporin 4, IL8 - interleukin 8, SOX9 - sry (sex determining region y)-box 9, IRF1 - interferon regulatory factor 1, ZC3H12A - zinc finger ccch-type containing 12a, TFPI - tissue factor pathway inhibitor (lipoprotein-associated coagulation inhibitor)]
GO:0098657	import into cell	2.58E-04	4.22E-02	2.13	16231	559	355	26	[HFE - hemochromatosis, FOLR1 - folate receptor 1 (adult), CAV3 - caveolin 3, SLC39A12 - solute carrier family 39 (zinc transporter), member 12, SLC1A3 - solute carrier family 1 (glial high affinity glutamate transporter), member 3, LEPR - leptin receptor, SLC22A3 - solute carrier family 22 (organic cation transporter), member 3, ATP1A2 - atpase, na+/k+ transporting, alpha 2 polypeptide, SLC2A5 - solute carrier family 2 (facilitated glucose/fructose transporter), member 5, TRPV4 - transient receptor potential cation channel, subfamily v, member 4, IL8 - interleukin 8, APOA1 - apolipoprotein a-i, COLEC12 - collectin sub-family member 12, HBA2 - hemoglobin, alpha 2, RHOJ - ras homolog family member j, GHR - growth hormone receptor, MLC1 - megalencephalic leukoencephalopathy with subcortical cysts 1, ADORA1 - adenosine a1 receptor, SLC6A20 - solute carrier family 6 (proline imino transporter), member 20, RAMP3 - receptor (g protein-coupled) activity modifying protein 3, KCNJ8 - potassium inwardly-rectifying channel, subfamily j, member 8, STON1 - stonin 1, CALCRL - calcitonin receptor-like, ANXA1 - annexin a1, SLC12A4 - solute carrier family 12 (potassium/chloride transporter), member 4, SLC15A2 - solute carrier family 15 (oligopeptide transporter), member 2]
GO:0009719	response to endogenous stimulus	2.58E-04	4.19E-02	1.82	16231	954	355	38	[CCL2 - chemokine (c-c motif) ligand 2, NFKB1 - nuclear factor of kappa light polypeptide gene enhancer in b-cells 1, FOLR1 - folate receptor 1 (adult), HSPA5 - heat shock 70kda protein 5 (glucose-regulated protein, 78kda), CAV3 - caveolin 3, MXRA5 - matrix-remodelling associated 5, ID1 - inhibitor of dna binding 1, dominant negative helix-loop-helix protein, LEPR - leptin receptor, MSTN - myostatin, IGFBP7 - insulin-like growth factor binding protein 7, ATP1A2 - atpase, na+/k+ transporting, alpha 2 polypeptide, IL8 - interleukin 8, MAOB - monoamine oxidase b, SOX9 - sry (sex determining region y)-box 9, TFPI - tissue factor pathway inhibitor (lipoprotein-associated coagulation inhibitor), KLF15 - kruppel-like factor 15, COL4A1 - collagen, type iv, alpha 1, WNT8B - wingless-type mmtv integration site family, member 8b, CXCL12 - chemokine (c-x-c motif) ligand 12, ITGA2 - integrin, alpha 2 (cd49b, alpha 2 subunit of vla-2 receptor), FOSL1 - fos-like antigen 1, PTN - pleiotrophin, GHR - growth hormone receptor, RIPK2 - receptor-interacting serine-threonine kinase 2, VCAM1 - vascular cell adhesion molecule 1, NTRK2 - neurotrophic tyrosine kinase, receptor, type 2, CPS1 - carbamoyl-phosphate synthase 1, mitochondrial, PDGFRB - platelet-derived growth factor receptor, beta polypeptide, YAP1 - yes-associated protein 1, ZFP36L1 - zfp36 ring finger protein-like 1, TNC - tenascin c, RAMP3 - receptor (g protein-coupled) activity modifying protein 3, KLF9 - kruppel-like factor 9, ANXA1 - annexin a1, UCP3 - uncoupling protein 3 (mitochondrial, proton carrier), TICAM1 - toll-like receptor adaptor molecule 1, KLF10 - kruppel-like factor 10, INHBB - inhibin, beta b]
GO:0040011	locomotion	2.64E-04	4.23E-02	1.82	16231	955	355	38	[CCL2 - chemokine (c-c motif) ligand 2, ARID5B - at rich interactive domain 5b (mrf1-like), PARP9 - poly (adp-ribose) polymerase family, member 9, FOLR1 - folate receptor 1 (adult), ID1 - inhibitor of dna binding 1, dominant negative helix-loop-helix protein, RHOD - ras homolog family member d, CKLF - chemokine-like factor, CD34 - cd34 molecule, MSTN - myostatin, CCL20 - chemokine (c-c motif) ligand 20, ATP1A2 - atpase, na+/k+ transporting, alpha 2 polypeptide, IL8 - interleukin 8, C5 - complement component 5, ENKUR - enkurin, trpc channel interacting protein, APOA1 - apolipoprotein a-i, CYP7B1 - cytochrome p450, family 7, subfamily b, polypeptide 1, CD58 - cd58 molecule, NEDD9 - neural precursor cell expressed, developmentally down-regulated 9, CXCL12 - chemokine (c-x-c motif) ligand 12, RHOJ - ras homolog family member j, ITGA2 - integrin, alpha 2 (cd49b, alpha 2 subunit of vla-2 receptor), FOSL1 - fos-like antigen 1, PTN - pleiotrophin, BARHL2 - barh-like homeobox 2, VCAM1 - vascular cell adhesion molecule 1, HBEGF - heparin-binding egf-like growth factor, NTRK2 - neurotrophic tyrosine kinase, receptor, type 2, PDGFRB - platelet-derived growth factor receptor, beta polypeptide, CXCL13 - chemokine (c-x-c motif) ligand 13, RND1 - rho family gtpase 1, TNFAIP3 - tumor necrosis factor, alpha-induced protein 3, GAB1 - grb2-associated binding protein 1, CXCL1 - chemokine (c-x-c motif) ligand 1 (melanoma growth stimulating activity, alpha), CXCL2 - chemokine (c-x-c motif) ligand 2, CXCL3 - chemokine (c-x-c motif) ligand 3, ANXA1 - annexin a1, ITGB7 - integrin, beta 7, PLAU - plasminogen activator, urokinase]
GO:0042493	response to drug	2.73E-04	4.34E-02	1.99	16231	689	355	30	[NFKB1 - nuclear factor of kappa light polypeptide gene enhancer in b-cells 1, KCNMB1 - potassium large conductance calcium-activated channel, subfamily m, beta member 1, FOLR1 - folate receptor 1 (adult), HSPA5 - heat shock 70kda protein 5 (glucose-regulated protein, 78kda), NFKBIA - nuclear factor of kappa light polypeptide gene enhancer in b-cells inhibitor, alpha, ID1 - inhibitor of dna binding 1, dominant negative helix-loop-helix protein, SETD7 - set domain containing (lysine methyltransferase) 7, MSTN - myostatin, IGFBP7 - insulin-like growth factor binding protein 7, ATP1A2 - atpase, na+/k+ transporting, alpha 2 polypeptide, MAOB - monoamine oxidase b, SOX9 - sry (sex determining region y)-box 9, APOA1 - apolipoprotein a-i, ZC3H12A - zinc finger ccch-type containing 12a, TNFRSF11B - tumor necrosis factor receptor superfamily, member 11b, HBA2 - hemoglobin, alpha 2, RGS10 - regulator of g-protein signaling 10, ITGA2 - integrin, alpha 2 (cd49b, alpha 2 subunit of vla-2 receptor), FOSL1 - fos-like antigen 1, PTN - pleiotrophin, RIPK2 - receptor-interacting serine-threonine kinase 2, VCAM1 - vascular cell adhesion molecule 1, CPS1 - carbamoyl-phosphate synthase 1, mitochondrial, PDGFRB - platelet-derived growth factor receptor, beta polypeptide, YAP1 - yes-associated protein 1, TNC - tenascin c, TNFAIP3 - tumor necrosis factor, alpha-induced protein 3, KLF9 - kruppel-like factor 9, ANXA1 - annexin a1, MGST1 - microsomal glutathione s-transferase 1]
GO:0045785	positive regulation of cell adhesion	2.99E-04	4.70E-02	2.39	16231	382	355	20	[CCL2 - chemokine (c-c motif) ligand 2, NEDD9 - neural precursor cell expressed, developmentally down-regulated 9, CXCL12 - chemokine (c-x-c motif) ligand 12, CSF1 - colony stimulating factor 1 (macrophage), ITPKB - inositol-trisphosphate 3-kinase b, ITGA2 - integrin, alpha 2 (cd49b, alpha 2 subunit of vla-2 receptor), PTN - pleiotrophin, RIPK2 - receptor-interacting serine-threonine kinase 2, VCAM1 - vascular cell adhesion molecule 1, COL8A1 - collagen, type viii, alpha 1, RHOD - ras homolog family member d, ECM2 - extracellular matrix protein 2, female organ and adipocyte specific, CXCL13 - chemokine (c-x-c motif) ligand 13, DUSP10 - dual specificity phosphatase 10, RHOH - ras homolog family member h, PTPRC - protein tyrosine phosphatase, receptor type, c, ANXA1 - annexin a1, IBSP - integrin-binding sialoprotein, APOA1 - apolipoprotein a-i, CD83 - cd83 molecule]
GO:0003401	axis elongation	3.32E-04	5.17E-02	11.43	16231	16	355	4	[TNC - tenascin c, SPRY2 - sprouty homolog 2 (drosophila), FGF1 - fibroblast growth factor 1 (acidic), YAP1 - yes-associated protein 1]

GO:0048869	cellular developmental process	3.59E-04	5.52E-02	1.45	16231	2461	355	78	[ARID5B - at rich interactive domain 5b (mrf1-like), GSC - goosecoid homeobox, ID1 - inhibitor of dna binding 1, dominant negative helix-loop-helix protein, ATF3 - activating transcription factor 3, ID3 - inhibitor of dna binding 3, dominant negative helix-loop-helix protein, PTPRZ1 - protein tyrosine phosphatase, receptor-type, z polypeptide 1, RELB - v-rel avian reticuloendotheliosis viral oncogene homolog b, MMP21 - matrix metallopeptidase 21, MAFF - v-maf avian musculoaponeurotic fibrosarcoma oncogene homolog f, CD34 - cd34 molecule, ATOH8 - atonal homolog 8 (drosophila), NEUROG2 - neurogenin 2, PLP1 - proteolipid protein 1, ZC3H12A - zinc finger ccch-type containing 12a, DLX3 - distal-less homeobox 3, COL4A1 - collagen, type iv, alpha 1, OPN3 - opsin 3, MFRP - membrane frizzled-related protein, CSF1 - colony stimulating factor 1 (macrophage), WNT8B - wingless-type mmtv integration site family, member 8b, BARHL2 - barh-like homeobox 2, COL22A1 - collagen, type xxii, alpha 1, SPRY2 - sprouty homolog 2 (drosophila), TBX2 - t-box 2, NTRK2 - neurotrophic tyrosine kinase, receptor, type 2, PAX6 - paired box 6, COL8A1 - collagen, type viii, alpha 1, FGF1 - fibroblast growth factor 1 (acidic), YAP1 - yes-associated protein 1, BARX2 - barx homeobox 2, PRELP - proline/arginine-rich end leucine-rich repeat protein, RND1 - rho family gtpase 1, ZFP36L1 - zfp36 ring finger protein-like 1, ACTA2 - actin, alpha 2, smooth muscle, aorta, COL12A1 - collagen, type xii, alpha 1, SLC24A5 - solute carrier family 24 (sodium/potassium/calcium exchanger), member 5, SMOC1 - sparc related modular calcium binding 1, PRRX1 - paired related homeobox 1, MGP - matrix gla protein, MGST1 - microsomal glutathione s-transferase 1, INHBB - inhibin, beta b, HOPX - hop homeobox, HSPA5 - heat shock 70kda protein 5 (glucose-regulated protein, 78kda), CAV3 - caveolin 3, MECOM - mds1 and evi1 complex locus, TIPARP - tcdd-inducible poly(adp-ribose) polymerase, LEPR - leptin receptor, SPDEF - sam pointed domain containing ets transcription factor, DCT - dopachrome tautomerase, IGFBP3 - insulin-like growth factor binding protein 3, DUSP10 - dual specificity phosphatase 10, SERPINH1 - serpin peptidase inhibitor, clade h (heat shock protein 47), member 1, (collagen binding protein 1), MSX2 - msh homeobox 2, WFIKKN1 - wap, follistatin/kazal, immunoglobulin, kunitz and netrin domain containing 1, RHOH - ras homolog family member h, SOX9 - sry (sex determining region y)-box 9, ZIC2 - zic family member 2, IRF1 - interferon regulatory factor 1, PTHLH - parathyroid hormone-like hormone, KLF15 - kruppel-like factor 15, IRF2BP2 - interferon regulatory factor 2 binding protein 2, NKX2-4 - nk2 homeobox 4, VAMP5 - vesicle-associated membrane protein 5, CHAC1 - chac, cation transport regulator homolog 1 (e. coli), MME - membrane metallo-endopeptidase, ITGA2 - integrin, alpha 2 (cd49b, alpha 2 subunit of vla-2 receptor), PTN - pleiotrophin, BCAP29 - b-cell receptor-associated protein 29, VCAM1 - vascular cell adhesion molecule 1, CPS1 - carbamoyl-phosphate synthase 1, mitochondrial, KRT75 - keratin 75, SPINK2 - serine peptidase inhibitor, kazal type 2 (acrosin-trypsin inhibitor), TNC - tenascin c, ANXA1 - annexin a1, PTPRC - protein tyrosine phosphatase, receptor type, c, IBSP - integrin-binding sialoprotein, ITGB7 - integrin, beta 7, ANXA4 - annexin a4]
GO:0040012	regulation of locomotion	3.61E-04	5.50E-02	1.81	16231	936	355	37	[CCL2 - chemokine (c-c motif) ligand 2, HSPA5 - heat shock 70kda protein 5 (glucose-regulated protein, 78kda), DCN - decorin, HSPB1 - heat shock 27kda protein 1, RHOD - ras homolog family member d, IGFBP3 - insulin-like growth factor binding protein 3, DUSP10 - dual specificity phosphatase 10, MSTN - myostatin, CCL20 - chemokine (c-c motif) ligand 20, RHOH - ras homolog family member h, ATOH8 - atonal homolog 8 (drosophila), GADD45A - growth arrest and dna-damage-inducible, alpha, IL8 - interleukin 8, PLP1 - proteolipid protein 1, SOX9 - sry (sex determining region y)-box 9, C5 - complement component 5, PTGS2 - prostaglandin-endoperoxide synthase 2 (prostaglandin g/h synthase and cyclooxygenase), TAC4 - tachykinin 4 (hemokinin), ZC3H12A - zinc finger ccch-type containing 12a, NEDD9 - neural precursor cell expressed, developmentally down-regulated 9, CXCL12 - chemokine (c-x-c motif) ligand 12, CSF1 - colony stimulating factor 1 (macrophage), RHOJ - ras homolog family member j, HTR1D - 5-hydroxytryptamine (serotonin) receptor 1d, g protein-coupled, ITGA2 - integrin, alpha 2 (cd49b, alpha 2 subunit of vla-2 receptor), PTN - pleiotrophin, SPRY2 - sprouty homolog 2 (drosophila), HBEGF - heparin-binding egf-like growth factor, PDGFRB - platelet-derived growth factor receptor, beta polypeptide, FGF1 - fibroblast growth factor 1 (acidic), CXCL13 - chemokine (c-x-c motif) ligand 13, ADORA1 - adenosine a1 receptor, GAB1 - grb2-associated binding protein 1, BTC - betacellulin, ANXA1 - annexin a1, PTPRC - protein tyrosine phosphatase, receptor type, c, PLAU - plasminogen activator, urokinase]
GO:0097529	myeloid leukocyte migration	3.69E-04	5.56E-02	4.07	16231	101	355	9	[CCL20 - chemokine (c-c motif) ligand 20, CCL2 - chemokine (c-c motif) ligand 2, CXCL1 - chemokine (c-x-c motif) ligand 1 (melanoma growth stimulating activity, alpha), IL8 - interleukin 8, CXCL2 - chemokine (c-x-c motif) ligand 2, CXCL3 - chemokine (c-x-c motif) ligand 3, ANXA1 - annexin a1, CXCL13 - chemokine (c-x-c motif) ligand 13, CKLF - chemokine-like factor]
GO:0051270	regulation of cellular component movement	3.84E-04	5.73E-02	1.8	16231	939	355	37	[CCL2 - chemokine (c-c motif) ligand 2, HSPA5 - heat shock 70kda protein 5 (glucose-regulated protein, 78kda), DCN - decorin, HSPB1 - heat shock 27kda protein 1, RHOD - ras homolog family member d, IGFBP3 - insulin-like growth factor binding protein 3, DUSP10 - dual specificity phosphatase 10, MSTN - myostatin, CCL20 - chemokine (c-c motif) ligand 20, RHOH - ras homolog family member h, ATP1A2 - atpase, na+/k+ transporting, alpha 2 polypeptide, ATOH8 - atonal homolog 8 (drosophila), GADD45A - growth arrest and dna-damage-inducible, alpha, IL8 - interleukin 8, PLP1 - proteolipid protein 1, SOX9 - sry (sex determining region y)-box 9, C5 - complement component 5, PTGS2 - prostaglandin-endoperoxide synthase 2 (prostaglandin g/h synthase and cyclooxygenase), TAC4 - tachykinin 4 (hemokinin), ZC3H12A - zinc finger ccch-type containing 12a, NEDD9 - neural precursor cell expressed, developmentally down-regulated 9, CXCL12 - chemokine (c-x-c motif) ligand 12, CSF1 - colony stimulating factor 1 (macrophage), RHOJ - ras homolog family member j, ITGA2 - integrin, alpha 2 (cd49b, alpha 2 subunit of vla-2 receptor), PTN - pleiotrophin, SPRY2 - sprouty homolog 2 (drosophila), HBEGF - heparin-binding egf-like growth factor, PDGFRB - platelet-derived growth factor receptor, beta polypeptide, FGF1 - fibroblast growth factor 1 (acidic), CXCL13 - chemokine (c-x-c motif) ligand 13, ADORA1 - adenosine a1 receptor, GAB1 - grb2-associated binding protein 1, BTC - betacellulin, ANXA1 - annexin a1, PTPRC - protein tyrosine phosphatase, receptor type, c, PLAU - plasminogen activator, urokinase]
GO:0001101	response to acid chemical	3.84E-04	5.68E-02	2.57	16231	303	355	17	[WNT8B - wingless-type mmtv integration site family, member 8b, KCNMB1 - potassium large conductance calcium-activated channel, subfamily m, beta member 1, FOLR1 - folate receptor 1 (adult), ITGA2 - integrin, alpha 2 (cd49b, alpha 2 subunit of vla-2 receptor), PTN - pleiotrophin, ID3 - inhibitor of dna binding 3, dominant negative helix-loop-helix protein, SCD - stearoyl-coa desaturase (delta-9-desaturase), NTRK2 - neurotrophic tyrosine kinase, receptor, type 2, CPS1 - carbamoyl-phosphate synthase 1, mitochondrial, PDGFRB - platelet-derived growth factor receptor, beta polypeptide, YAP1 - yes-associated protein 1, IGFBP7 - insulin-like growth factor binding protein 7, TNC - tenascin c, HSD17B2 - hydroxysteroid (17-beta) dehydrogenase 2, SOX9 - sry (sex determining region y)-box 9, ZC3H12A - zinc finger ccch-type containing 12a, COL4A1 - collagen, type iv, alpha 1]
GO:0071804	cellular potassium ion transport	4.16E-04	6.08E-02	3.4	16231	148	355	11	[ATP1A2 - atpase, na+/k+ transporting, alpha 2 polypeptide, KCNMB1 - potassium large conductance calcium-activated channel, subfamily m, beta member 1, SLC24A3 - solute carrier family 24 (sodium/potassium/calcium exchanger), member 3, SLC24A5 - solute carrier family 24 (sodium/potassium/calcium exchanger), member 5, KCNJ8 - potassium inwardly-rectifying channel, subfamily j, member 8, LRRC52 - leucine rich repeat containing 52, KCNQ4 - potassium voltage-gated channel, kqt-like subfamily, member 4, SLC1A3 - solute carrier family 1 (glial high affinity glutamate transporter), member 3, KCNA1 - potassium voltage-gated channel, shaker-related subfamily, member 1 (episodic ataxia with myokymia), KCNIP3 - kv channel interacting protein 3, calsenilin, SLC12A4 - solute carrier family 12 (potassium/chloride transporter), member 4]
GO:0071805	potassium ion transmembrane transport	4.16E-04	6.03E-02	3.4	16231	148	355	11	[ATP1A2 - atpase, na+/k+ transporting, alpha 2 polypeptide, KCNMB1 - potassium large conductance calcium-activated channel, subfamily m, beta member 1, SLC24A3 - solute carrier family 24 (sodium/potassium/calcium exchanger), member 3, SLC24A5 - solute carrier family 24 (sodium/potassium/calcium exchanger), member 5, KCNJ8 - potassium inwardly-rectifying channel, subfamily j, member 8, LRRC52 - leucine rich repeat containing 52, KCNQ4 - potassium voltage-gated channel, kqt-like subfamily, member 4, SLC1A3 - solute carrier family 1 (glial high affinity glutamate transporter), member 3, KCNA1 - potassium voltage-gated channel, shaker-related subfamily, member 1 (episodic ataxia with myokymia), KCNIP3 - kv channel interacting protein 3, calsenilin, SLC12A4 - solute carrier family 12 (potassium/chloride transporter), member 4]

GO:0048519	negative regulation of biological process	4.42E-04	6.34E-02	1.28	16231	4828	355	135	[HFE - hemochromatosis, OASL - 2'-5'-oligoadenylate synthetase-like, ARID5B - at rich interactive domain 5b (mrf1-like), E2F2 - e2f transcription factor 2, MDFIC - myod family inhibitor domain containing, ID1 - inhibitor of dna binding 1, dominant negative helix-loop-helix protein, ITPKB - inositol-trisphosphate 3-kinase b, ID3 - inhibitor of dna binding 3, dominant negative helix-loop-helix protein, RELB - v-rel avian reticuloendotheliosis viral oncogene homolog b, CD34 - cd34 molecule, LTBP3 - latent transforming growth factor beta binding protein 3, BIRC3 - baculoviral iap repeat containing 3, APOA1 - apolipoprotein a-i, ZC3H12A - zinc finger ccch-type containing 12a, DAND5 - dan domain family member 5, bmp antagonist, OPN3 - opsin 3, IFI35 - interferon-induced protein 35, RGS10 - regulator of g-protein signaling 10, IFIT1 - interferon-induced protein with tetratricopeptide repeats 1, IFIT3 - interferon-induced protein with tetratricopeptide repeats 3, FGL2 - fibrinogen-like 2, SOSTDC1 - sclerostin domain containing 1, SPRY2 - sprouty homolog 2 (drosophila), KCNIP3 - kv channel interacting protein 3, calsenilin, TBX2 - t-box 2, FAS - fas cell surface death receptor, PAX6 - paired box 6, SMR3B - submaxillary gland androgen regulated protein 3b, TPX2 - tpx2, microtubule-associated, CXCL13 - chemokine (c-x-c motif) ligand 13, PSMB9 - proteasome (prosome, macropain) subunit, beta type, 9, ITPRIP - inositol 1,4,5-trisphosphate receptor interacting protein, ZFP36L1 - zfp36 ring finger protein-like 1, SLC24A3 - solute carrier family 24 (sodium/potassium/calcium exchanger), member 3, BTC - betacellulin, PRRX1 - paired related homeobox 1, KLF9 - kruppel-like factor 9, GPR35 - g protein-coupled receptor 35, TICAM1 - toll-like receptor adaptor molecule 1, NFIC - nuclear factor i/c (ccaat-binding transcription factor), NFIX - nuclear factor i/x (ccaat-binding transcription factor), NFKB1 - nuclear factor of kappa light polypeptide gene enhancer in b-cells 1, CCL2 - chemokine (c-c motif) ligand 2, PARP9 - poly (adp-ribose) polymerase family, member 9, NFKBIA - nuclear factor of kappa light polypeptide gene enhancer in b-cells inhibitor, alpha, NFKBIE - nuclear factor of kappa light polypeptide gene enhancer in b-cells inhibitor, epsilon, DCN - decorin, TIPARP - tcdd-inducible poly(adp-ribose) polymerase, HERPUD1 - homocysteine-inducible, endoplasmic reticulum stress-inducible, ubiquitin-like domain member 1, IGFBP3 - insulin-like growth factor binding protein 3, HERC5 - hect and rld domain containing e3 ubiquitin protein ligase 5, DUSP10 - dual specificity phosphatase 10, MSX2 - msh homeobox 2, IGFBP7 - insulin-like growth factor binding protein 7, PTGER3 - prostaglandin e receptor 3 (subtype ep3), RHOH - ras homolog family member h, GADD45A - growth arrest and dna-damage-inducible, alpha, DDIT3 - dna-damage-inducible transcript 3, MAOB - monoamine oxidase b, SOX9 - sry (sex determining region y)-box 9, C5 - complement component 5, ZIC2 - zic family member 2, PTHLH - parathyroid hormone-like hormone, CYP7B1 - cytochrome p450, family 7, subfamily b, polypeptide 1, KLF15 - kruppel-like factor 15, IRF2BP2 - interferon regulatory factor 2 binding protein 2, USP2 - ubiquitin specific peptidase 2, CXCL12 - chemokine (c-x-c motif) ligand 12, FOSL1 - fos-like antigen 1, NMI - n-myc (and stat) interactor, PTN - pleiotrophin, RIPK2 - receptor-interacting serine-threonine kinase 2, PDGFRB - platelet-derived growth factor receptor, beta polypeptide, SPINK2 - serine peptidase inhibitor, kazal type 2 (acrosin-trypsin inhibitor), NEK11 - nima-related kinase 11, ADORA1 - adenosine a1 receptor, CXCL1 - chemokine (c-x-c motif) ligand 1 (melanoma growth stimulating activity, alpha), PTPRC - protein tyrosine phosphatase, receptor type, c, ZNF404 - zinc finger protein 404, DYDC2 - dpy30 domain containing 2, GSC - goosecoid homeobox, ATF3 - activating transcription factor 3, PTPRZ1 - protein tyrosine phosphatase, receptor-type, z polypeptide 1, PTX3 - pentraxin 3, long, AEBP1 - ae binding protein 1, MSTN - myostatin, ATP1A2 - atpase, na+/k+ transporting, alpha 2 polypeptide, ATOH8 - atonal homolog 8 (drosophila), TRPV4 - transient receptor potential cation channel, subfamily v, member 4, IL8 - interleukin 8, TFPI - tissue factor pathway inhibitor (lipoprotein-associated coagulation inhibitor), CSF1 - colony stimulating factor 1 (macrophage), NT5E - 5'-nucleotidase, ecto (cd73), NTRK2 - neurotrophic tyrosine kinase, receptor, type 2, YAP1 - yes-associated protein 1, BARX2 - barx homeobox 2, RND1 - rho family gtpase 1, SLC24A5 - solute carrier family 24 (sodium/potassium/calcium exchanger), member 5, ALDH1A1 - aldehyde dehydrogenase 1 family, member a1, KLF10 - kruppel-like factor 10, INHBB - inhibin, beta b, HOPX - hop homeobox, GZMB - granzyme b (granzyme 2, cytotoxic t-lymphocyte-associated serine esterase 1), OAS3 - 2'-5'-oligoadenylate synthetase 3, 100kda, IER3 - immediate early response 3, HSPA5 - heat shock 70kda protein 5 (glucose-regulated protein, 78kda), CAV3 - caveolin 3, IQGAP3 - iq motif containing gtpase activating protein 3, MECOM - mds1 and evi1 complex locus, ZBTB2 - zinc finger and btb domain containing 2, HSPB1 - heat shock 27kda protein 1, LEPR - leptin receptor, SPDEF - sam pointed domain containing ets transcription factor, SERPINH1 - serpin peptidase inhibitor, clade h (heat shock protein 47), member 1, (collagen binding protein 1), WFIKKN1 - wap, follistatin/kazal, immunoglobulin, kunitz and netrin domain containing 1, OGN - osteoglycin, ARMCX5-GPRASP2 - armcx5-gprasp2 readthrough, IRF1 - interferon regulatory factor 1, PARP14 - poly (adp-ribose) polymerase family, member 14, VEPH1 - ventricular zone expressed ph domain-containing 1, FBXL7 - f-box and leucine-rich repeat protein 7, TNFRSF11B - tumor necrosis factor receptor superfamily, member 11b, GJC2 - gap junction protein, gamma 2, 47kda, RRAD - ras-related associated with diabetes, CHAC1 - chac, cation transport regulator homolog 1 (e. coli), HBEGF - heparin-binding egf-like growth factor, FABP7 - fatty acid binding protein 7, brain, TNC - tenascin c, TNFAIP3 - tumor necrosis factor, alpha-induced protein 3, CALCRL - calcitonin receptor-like, ANXA1 - annexin a1, SMR3A - submaxillary gland androgen regulated protein 3a, ANXA4 - annexin a4, PLAU - plasminogen activator, urokinase, CD83 - cd83 molecule]
GO:0014036	neural crest cell fate specification	4.77E-04	6.79E-02	45.72	16231	2	355	2	[GSC - goosecoid homeobox, SOX9 - sry (sex determining region y)-box 9]
GO:2000016	negative regulation of determination of dorsal identity	4.77E-04	6.72E-02	45.72	16231	2	355	2	[DDIT3 - dna-damage-inducible transcript 3, SOSTDC1 - sclerostin domain containing 1]
GO:0070427	nucleotide-binding oligomerization domain containing 1 signaling pathway	4.77E-04	6.66E-02	45.72	16231	2	355	2	[NFKBIA - nuclear factor of kappa light polypeptide gene enhancer in b-cells inhibitor, alpha, RIPK2 - receptor-interacting serine-threonine kinase 2]
GO:0045580	regulation of T cell differentiation	4.79E-04	6.63E-02	3.6	16231	127	355	10	[RHOH - ras homolog family member h, ITPKB - inositol-trisphosphate 3-kinase b, FGL2 - fibrinogen-like 2, RIPK2 - receptor-interacting serine-threonine kinase 2, PTPRC - protein tyrosine phosphatase, receptor type, c, IRF1 - interferon regulatory factor 1, ANXA1 - annexin a1, ZC3H12A - zinc finger ccch-type containing 12a, DUSP10 - dual specificity phosphatase 10, CD83 - cd83 molecule]
GO:0071363	cellular response to growth factor stimulus	4.93E-04	6.76E-02	2.7	16231	254	355	15	[CCL2 - chemokine (c-c motif) ligand 2, HSPA5 - heat shock 70kda protein 5 (glucose-regulated protein, 78kda), ID1 - inhibitor of dna binding 1, dominant negative helix-loop-helix protein, PTN - pleiotrophin, VCAM1 - vascular cell adhesion molecule 1, SPRY2 - sprouty homolog 2 (drosophila), HSPB1 - heat shock 27kda protein 1, NTRK2 - neurotrophic tyrosine kinase, receptor, type 2, CPS1 - carbamoyl-phosphate synthase 1, mitochondrial, PDGFRB - platelet-derived growth factor receptor, beta polypeptide, ZFP36L1 - zfp36 ring finger protein-like 1, IL8 - interleukin 8, SOX9 - sry (sex determining region y)-box 9, ANXA1 - annexin a1, IBSP - integrin-binding sialoprotein]
GO:0048545	response to steroid hormone	4.95E-04	6.72E-02	2.96	16231	201	355	13	[FOSL1 - fos-like antigen 1, PTN - pleiotrophin, CPS1 - carbamoyl-phosphate synthase 1, mitochondrial, YAP1 - yes-associated protein 1, ZFP36L1 - zfp36 ring finger protein-like 1, MSTN - myostatin, IGFBP7 - insulin-like growth factor binding protein 7, ATP1A2 - atpase, na+/k+ transporting, alpha 2 polypeptide, MAOB - monoamine oxidase b, KLF9 - kruppel-like factor 9, ANXA1 - annexin a1, UCP3 - uncoupling protein 3 (mitochondrial, proton carrier), TFPI - tissue factor pathway inhibitor (lipoprotein-associated coagulation inhibitor)]

GO:0002697	regulation of immune effector process	5.22E-04	7.03E-02	2.42	16231	340	355	18	[CFH - complement factor h, HFE - hemochromatosis, PARP9 - poly (adp-ribose) polymerase family, member 9, IFIT1 - interferon-induced protein with tetratricopeptide repeats 1, FGL2 - fibrinogen-like 2, RIPK2 - receptor-interacting serine-threonine kinase 2, HERC5 - hect and rld domain containing e3 ubiquitin protein ligase 5, DUSP10 - dual specificity phosphatase 10, TNFAIP3 - tumor necrosis factor, alpha-induced protein 3, BIRC3 - baculoviral iap repeat containing 3, C5 - complement component 5, DDX60 - dead (asp-glu-ala-asp) box polypeptide 60, PTPRC - protein tyrosine phosphatase, receptor type, c, ANXA1 - annexin a1, APOA1 - apolipoprotein a-i, ZC3H12A - zinc finger ccch-type containing 12a, C7 - complement component 7, TICAM1 - toll-like receptor adaptor molecule 1]
GO:0045619	regulation of lymphocyte differentiation	5.51E-04	7.36E-02	3.29	16231	153	355	11	[ZFP36L1 - zfp36 ring finger protein-like 1, RHOH - ras homolog family member h, ITPKB - inositol-trisphosphate 3-kinase b, FGL2 - fibrinogen-like 2, RIPK2 - receptor-interacting serine-threonine kinase 2, PTPRC - protein tyrosine phosphatase, receptor type, c, IRF1 - interferon regulatory factor 1, ANXA1 - annexin a1, ZC3H12A - zinc finger ccch-type containing 12a, DUSP10 - dual specificity phosphatase 10, CD83 - cd83 molecule]
GO:0071347	cellular response to interleukin-1	5.80E-04	7.68E-02	4.85	16231	66	355	7	[CCL20 - chemokine (c-c motif) ligand 20, NFKB1 - nuclear factor of kappa light polypeptide gene enhancer in b-cells 1, CCL2 - chemokine (c-c motif) ligand 2, IL8 - interleukin 8, SOX9 - sry (sex determining region y)-box 9, TFPI - tissue factor pathway inhibitor (lipoprotein-associated coagulation inhibitor), ZC3H12A - zinc finger ccch-type containing 12a]
GO:0046634	regulation of alpha-beta T cell activation	5.82E-04	7.64E-02	4.25	16231	86	355	8	[HFE - hemochromatosis, ITPKB - inositol-trisphosphate 3-kinase b, RIPK2 - receptor-interacting serine-threonine kinase 2, IRF1 - interferon regulatory factor 1, PTPRC - protein tyrosine phosphatase, receptor type, c, ANXA1 - annexin a1, ZC3H12A - zinc finger ccch-type containing 12a, CD83 - cd83 molecule]
GO:0002683	negative regulation of immune system process	5.92E-04	7.70E-02	2.27	16231	403	355	20	[CCL2 - chemokine (c-c motif) ligand 2, HFE - hemochromatosis, CXCL12 - chemokine (c-x-c motif) ligand 12, NFKBIA - nuclear factor of kappa light polypeptide gene enhancer in b-cells inhibitor, alpha, ITPKB - inositol-trisphosphate 3-kinase b, NMI - n-myc (and stat) interactor, FGL2 - fibrinogen-like 2, DUSP10 - dual specificity phosphatase 10, ZFP36L1 - zfp36 ring finger protein-like 1, ADORA1 - adenosine a1 receptor, TNFAIP3 - tumor necrosis factor, alpha-induced protein 3, SOX9 - sry (sex determining region y)-box 9, C5 - complement component 5, PARP14 - poly (adp-ribose) polymerase family, member 14, ANXA1 - annexin a1, PTPRC - protein tyrosine phosphatase, receptor type, c, IRF1 - interferon regulatory factor 1, APOA1 - apolipoprotein a-i, ZC3H12A - zinc finger ccch-type containing 12a, TICAM1 - toll-like receptor adaptor molecule 1]
GO:0006813	potassium ion transport	6.14E-04	7.93E-02	3.24	16231	155	355	11	[ATP1A2 - atpase, na+/k+ transporting, alpha 2 polypeptide, KCNMB1 - potassium large conductance calcium-activated channel, subfamily m, beta member 1, SLC24A3 - solute carrier family 24 (sodium/potassium/calcium exchanger), member 3, SLC24A5 - solute carrier family 24 (sodium/potassium/calcium exchanger), member 5, KCNJ8 - potassium inwardly-rectifying channel, subfamily j, member 8, LRRC52 - leucine rich repeat containing 52, KCNQ4 - potassium voltage-gated channel, kqt-like subfamily, member 4, SLC1A3 - solute carrier family 1 (glial high affinity glutamate transporter), member 3, KCNA1 - potassium voltage-gated channel, shaker-related subfamily, member 1 (episodic ataxia with myokymia), KCNIP3 - kv channel interacting protein 3, calsenilin, SLC12A4 - solute carrier family 12 (potassium/chloride transporter), member 4]
GO:0009611	response to wounding	6.17E-04	7.90E-02	3.05	16231	180	355	12	[ZFP36L1 - zfp36 ring finger protein-like 1, FOLR1 - folate receptor 1 (adult), TNC - tenascin c, ID3 - inhibitor of dna binding 3, dominant negative helix-loop-helix protein, DCN - decorin, APOA1 - apolipoprotein a-i, SFTA3 - surfactant associated 3, PAX6 - paired box 6, FGF1 - fibroblast growth factor 1 (acidic), PDGFRB - platelet-derived growth factor receptor, beta polypeptide, YAP1 - yes-associated protein 1, INHBB - inhibin, beta b]
GO:0001818	negative regulation of cytokine production	6.28E-04	7.97E-02	2.64	16231	260	355	15	[HFE - hemochromatosis, NFKB1 - nuclear factor of kappa light polypeptide gene enhancer in b-cells 1, NMI - n-myc (and stat) interactor, RELB - v-rel avian reticuloendotheliosis viral oncogene homolog b, HERC5 - hect and rld domain containing e3 ubiquitin protein ligase 5, CD34 - cd34 molecule, TNFAIP3 - tumor necrosis factor, alpha-induced protein 3, DDIT3 - dna-damage-inducible transcript 3, PTPRC - protein tyrosine phosphatase, receptor type, c, ANXA1 - annexin a1, APOA1 - apolipoprotein a-i, ZC3H12A - zinc finger ccch-type containing 12a, INHBB - inhibin, beta b, ANXA4 - annexin a4, CD83 - cd83 molecule]
GO:0050777	negative regulation of immune response	6.49E-04	8.17E-02	3.46	16231	132	355	10	[HFE - hemochromatosis, TNFAIP3 - tumor necrosis factor, alpha-induced protein 3, NMI - n-myc (and stat) interactor, FGL2 - fibrinogen-like 2, PTPRC - protein tyrosine phosphatase, receptor type, c, PARP14 - poly (adp-ribose) polymerase family, member 14, ANXA1 - annexin a1, APOA1 - apolipoprotein a-i, ZC3H12A - zinc finger ccch-type containing 12a, DUSP10 - dual specificity phosphatase 10]
GO:0090280	positive regulation of calcium ion import	6.72E-04	8.39E-02	9.63	16231	19	355	4	[CCL2 - chemokine (c-c motif) ligand 2, CXCL12 - chemokine (c-x-c motif) ligand 12, RAMP3 - receptor (g protein-coupled) activity modifying protein 3, PDGFRB - platelet-derived growth factor receptor, beta polypeptide]
GO:0048585	negative regulation of response to stimulus	7.11E-04	8.80E-02	1.57	16231	1514	355	52	[HFE - hemochromatosis, GSC - goosecoid homeobox, ATF3 - activating transcription factor 3, CD34 - cd34 molecule, MSTN - myostatin, IL8 - interleukin 8, APOA1 - apolipoprotein a-i, ZC3H12A - zinc finger ccch-type containing 12a, TFPI - tissue factor pathway inhibitor (lipoprotein-associated coagulation inhibitor), DAND5 - dan domain family member 5, bmp antagonist, NT5E - 5'-nucleotidase, ecto (cd73), IFI35 - interferon-induced protein 35, RGS10 - regulator of g-protein signaling 10, FGL2 - fibrinogen-like 2, SOSTDC1 - sclerostin domain containing 1, SPRY2 - sprouty homolog 2 (drosophila), FAS - fas cell surface death receptor, CXCL13 - chemokine (c-x-c motif) ligand 13, YAP1 - yes-associated protein 1, PSMB9 - proteasome (prosome, macropain) subunit, beta type, 9, ITPRIP - inositol 1,4,5-trisphosphate receptor interacting protein, BTC - betacellulin, TICAM1 - toll-like receptor adaptor molecule 1, CCL2 - chemokine (c-c motif) ligand 2, NFKB1 - nuclear factor of kappa light polypeptide gene enhancer in b-cells 1, HSPA5 - heat shock 70kda protein 5 (glucose-regulated protein, 78kda), NFKBIA - nuclear factor of kappa light polypeptide gene enhancer in b-cells inhibitor, alpha, CAV3 - caveolin 3, MECOM - mds1 and evi1 complex locus, DCN - decorin, HSPB1 - heat shock 27kda protein 1, HERPUD1 - homocysteine-inducible, endoplasmic reticulum stress-inducible, ubiquitin-like domain member 1, IGFBP3 - insulin-like growth factor binding protein 3, DUSP10 - dual specificity phosphatase 10, WFIKKN1 - wap, follistatin/kazal, immunoglobulin, kunitz and netrin domain containing 1, RHOH - ras homolog family member h, DDIT3 - dna-damage-inducible transcript 3, SOX9 - sry (sex determining region y)-box 9, C5 - complement component 5, PARP14 - poly (adp-ribose) polymerase family, member 14, VEPH1 - ventricular zone expressed ph domain-containing 1, CYP7B1 - cytochrome p450, family 7, subfamily b, polypeptide 1, CXCL12 - chemokine (c-x-c motif) ligand 12, CHAC1 - chac, cation transport regulator homolog 1 (e. coli), NMI - n-myc (and stat) interactor, HBEGF - heparin-binding egf-like growth factor, ADORA1 - adenosine a1 receptor, TNFAIP3 - tumor necrosis factor, alpha-induced protein 3, CALCRL - calcitonin receptor-like, ANXA1 - annexin a1, PTPRC - protein tyrosine phosphatase, receptor type, c, PLAU - plasminogen activator, urokinase]

GO:0022610	biological adhesion	7.58E-04	9.31E-02	1.81	16231	835	355	33	[SPARCL1 - sparc-like 1 (hevin), CCL2 - chemokine (c-c motif) ligand 2, FOLR1 - folate receptor 1 (adult), FBLN5 - fibulin 5, CDC42EP1 - cdc42 effector protein (rho gtpase binding) 1, ID1 - inhibitor of dna binding 1, dominant negative helix-loop-helix protein, HSPB1 - heat shock 27kda protein 1, CD34 - cd34 molecule, IGFBP7 - insulin-like growth factor binding protein 7, GP9 - glycoprotein ix (platelet), ACAN - aggrecan, SOX9 - sry (sex determining region y)-box 9, CD58 - cd58 molecule, TROAP - trophinin associated protein, NEDD9 - neural precursor cell expressed, developmentally down-regulated 9, CXCL12 - chemokine (c-x-c motif) ligand 12, NT5E - 5'-nucleotidase, ecto (cd73), ITGA2 - integrin, alpha 2 (cd49b, alpha 2 subunit of vla-2 receptor), VCAM1 - vascular cell adhesion molecule 1, COL8A1 - collagen, type viii, alpha 1, ECM2 - extracellular matrix protein 2, female organ and adipocyte specific, SUSD5 - sushi domain containing 5, ZFP36L1 - zfp36 ring finger protein-like 1, TNC - tenascin c, COL12A1 - collagen, type xii, alpha 1, IGSF5 - immunoglobulin superfamily, member 5, ANXA1 - annexin a1, PTPRC - protein tyrosine phosphatase, receptor type, c, IBSP - integrin-binding sialoprotein, LRRC4 - leucine rich repeat containing 4, ITGB7 - integrin, beta 7, INHBB - inhibin, beta b, HMCN1 - hemicentin 1]
GO:0071260	cellular response to mechanical stimulus	7.60E-04	9.26E-02	4.64	16231	69	355	7	[ATP1A2 - atpase, na+/k+ transporting, alpha 2 polypeptide, NFKB1 - nuclear factor of kappa light polypeptide gene enhancer in b-cells 1, GADD45A - growth arrest and dna-damage-inducible, alpha, ITGA2 - integrin, alpha 2 (cd49b, alpha 2 subunit of vla-2 receptor), SOX9 - sry (sex determining region y)-box 9, IRF1 - interferon regulatory factor 1, FAS - fas cell surface death receptor]
GO:2000347	positive regulation of hepatocyte proliferation	7.90E-04	9.55E-02	15.24	16231	9	355	3	[TNFAIP3 - tumor necrosis factor, alpha-induced protein 3, PTN - pleiotrophin, FGF1 - fibroblast growth factor 1 (acidic)]
GO:0018298	protein-chromophore linkage	7.90E-04	9.47E-02	15.24	16231	9	355	3	[OPN1SW - opsin 1 (cone pigments), short-wave-sensitive, OPN3 - opsin 3, RGR - retinal g protein coupled receptor]
GO:0030903	notochord development	7.90E-04	9.40E-02	15.24	16231	9	355	3	[ID3 - inhibitor of dna binding 3, dominant negative helix-loop-helix protein, SOX9 - sry (sex determining region y)-box 9, YAP1 - yes-associated protein 1]
GO:0061844	antimicrobial humoral immune response mediated by antimicrobial peptide	8.03E-04	9.48E-02	6.72	16231	34	355	5	[IL8 - interleukin 8, CXCL1 - chemokine (c-x-c motif) ligand 1 (melanoma growth stimulating activity, alpha), CXCL2 - chemokine (c-x-c motif) ligand 2, CXCL3 - chemokine (c-x-c motif) ligand 3, CXCL13 - chemokine (c-x-c motif) ligand 13]
GO:0045595	regulation of cell differentiation	8.09E-04	9.47E-02	1.53	16231	1673	355	56	[ID1 - inhibitor of dna binding 1, dominant negative helix-loop-helix protein, ITPKB - inositol-trisphosphate 3-kinase b, SLC39A12 - solute carrier family 39 (zinc transporter), member 12, ID3 - inhibitor of dna binding 3, dominant negative helix-loop-helix protein, PTPRZ1 - protein tyrosine phosphatase, receptor-type, z polypeptide 1, MAFF - v-maf avian musculoaponeurotic fibrosarcoma oncogene homolog f, CD34 - cd34 molecule, MSTN - myostatin, ATOH8 - atonal homolog 8 (drosophila), LTBP3 - latent transforming growth factor beta binding protein 3, TRPV4 - transient receptor potential cation channel, subfamily v, member 4, APOA1 - apolipoprotein a-i, ZC3H12A - zinc finger ccch-type containing 12a, NEDD9 - neural precursor cell expressed, developmentally down-regulated 9, CSF1 - colony stimulating factor 1 (macrophage), MBOAT1 - membrane bound o-acyltransferase domain containing 1, BARHL2 - barh-like homeobox 2, FGL2 - fibrinogen-like 2, SOSTDC1 - sclerostin domain containing 1, SPRY2 - sprouty homolog 2 (drosophila), NTRK2 - neurotrophic tyrosine kinase, receptor, type 2, PAX6 - paired box 6, YAP1 - yes-associated protein 1, PSMB9 - proteasome (prosome, macropain) subunit, beta type, 9, ZFP36L1 - zfp36 ring finger protein-like 1, SMOC1 - sparc related modular calcium binding 1, PRRX1 - paired related homeobox 1, BTC - betacellulin, KLF10 - kruppel-like factor 10, HOPX - hop homeobox, NFKB1 - nuclear factor of kappa light polypeptide gene enhancer in b-cells 1, HSPA5 - heat shock 70kda protein 5 (glucose-regulated protein, 78kda), NFKBIA - nuclear factor of kappa light polypeptide gene enhancer in b-cells inhibitor, alpha, CAV3 - caveolin 3, AAMDC - adipogenesis associated, mth938 domain containing, PCP4 - purkinje cell protein 4, SPDEF - sam pointed domain containing ets transcription factor, DCT - dopachrome tautomerase, IGFBP3 - insulin-like growth factor binding protein 3, DUSP10 - dual specificity phosphatase 10, RHOH - ras homolog family member h, DDIT3 - dna-damage-inducible transcript 3, SOX9 - sry (sex determining region y)-box 9, ARMCX5-GPRASP2 - armcx5-gprasp2 readthrough, PTGS2 - prostaglandin-endoperoxide synthase 2 (prostaglandin g/h synthase and cyclooxygenase), IRF1 - interferon regulatory factor 1, PTHLH - parathyroid hormone-like hormone, GJC2 - gap junction protein, gamma 2, 47kda, CXCL12 - chemokine (c-x-c motif) ligand 12, MME - membrane metallo-endopeptidase, PTN - pleiotrophin, CTSV - cathepsin v, RIPK2 - receptor-interacting serine-threonine kinase 2, ANXA1 - annexin a1, PTPRC - protein tyrosine phosphatase, receptor type, c, CD83 - cd83 molecule]
GO:0098542	defense response to other organism	8.18E-04	9.50E-02	2.4	16231	324	355	17	[OAS3 - 2'-5'-oligoadenylate synthetase 3, 100kda, OASL - 2'-5'-oligoadenylate synthetase-like, PARP9 - poly (adp-ribose) polymerase family, member 9, IFIT2 - interferon-induced protein with tetratricopeptide repeats 2, IFIT1 - interferon-induced protein with tetratricopeptide repeats 1, IFIT3 - interferon-induced protein with tetratricopeptide repeats 3, RIPK2 - receptor-interacting serine-threonine kinase 2, HERC5 - hect and rld domain containing e3 ubiquitin protein ligase 5, CXCL13 - chemokine (c-x-c motif) ligand 13, CCL20 - chemokine (c-c motif) ligand 20, KCNJ8 - potassium inwardly-rectifying channel, subfamily j, member 8, DDX60 - dead (asp-glu-ala-asp) box polypeptide 60, PTPRC - protein tyrosine phosphatase, receptor type, c, IRF1 - interferon regulatory factor 1, ZC3H12A - zinc finger ccch-type containing 12a, TICAM1 - toll-like receptor adaptor molecule 1, COLEC12 - collectin sub-family member 12]
GO:0045766	positive regulation of angiogenesis	8.42E-04	9.72E-02	3.12	16231	161	355	11	[RHOJ - ras homolog family member j, GAB1 - grb2-associated binding protein 1, SLC39A12 - solute carrier family 39 (zinc transporter), member 12, IL8 - interleukin 8, C5 - complement component 5, PTGS2 - prostaglandin-endoperoxide synthase 2 (prostaglandin g/h synthase and cyclooxygenase), HSPB1 - heat shock 27kda protein 1, ANXA1 - annexin a1, ZC3H12A - zinc finger ccch-type containing 12a, FGF1 - fibroblast growth factor 1 (acidic), CD34 - cd34 molecule]

GO:0045597	positive regulation of cell differentiation	8.51E-04	9.74E-02	1.76	16231	910	355	35	[NFKB1 - nuclear factor of kappa light polypeptide gene enhancer in b-cells 1, HSPA5 - heat shock 70kda protein 5 (glucose-regulated protein, 78kda), CAV3 - caveolin 3, ITPKB - inositol-trisphosphate 3-kinase b, AAMDC - adipogenesis associated, mth938 domain containing, PTPRZ1 - protein tyrosine phosphatase, receptor-type, z polypeptide 1, PCP4 - purkinje cell protein 4, SPDEF - sam pointed domain containing ets transcription factor, IGFBP3 - insulin-like growth factor binding protein 3, DCT - dopachrome tautomerase, DUSP10 - dual specificity phosphatase 10, CD34 - cd34 molecule, RHOH - ras homolog family member h, ATOH8 - atonal homolog 8 (drosophila), LTBP3 - latent transforming growth factor beta binding protein 3, ARMCX5-GPRASP2 - armcx5-gprasp2 readthrough, SOX9 - sry (sex determining region y)-box 9, PTGS2 - prostaglandin-endoperoxide synthase 2 (prostaglandin g/h synthase and cyclooxygenase), APOA1 - apolipoprotein a-i, ZC3H12A - zinc finger ccch-type containing 12a, GJC2 - gap junction protein, gamma 2, 47kda, NEDD9 - neural precursor cell expressed, developmentally down-regulated 9, CXCL12 - chemokine (c-x-c motif) ligand 12, CSF1 - colony stimulating factor 1 (macrophage), MME - membrane metallo-endopeptidase, PTN - pleiotrophin, RIPK2 - receptor-interacting serine-threonine kinase 2, NTRK2 - neurotrophic tyrosine kinase, receptor, type 2, YAP1 - yes-associated protein 1, ZFP36L1 - zfp36 ring finger protein-like 1, BTC - betacellulin, ANXA1 - annexin a1, PTPRC - protein tyrosine phosphatase, receptor type, c, KLF10 - kruppel-like factor 10, CD83 - cd83 molecule]
GO:0007154	cell communication	9.02E-04	1.02E-01	1.83	16231	775	355	31	[HFE - hemochromatosis, KCNMB1 - potassium large conductance calcium-activated channel, subfamily m, beta member 1, FOLR1 - folate receptor 1 (adult), HSPA5 - heat shock 70kda protein 5 (glucose-regulated protein, 78kda), ATF3 - activating transcription factor 3, SLC1A3 - solute carrier family 1 (glial high affinity glutamate transporter), member 3, CD34 - cd34 molecule, CCL20 - chemokine (c-c motif) ligand 20, ATP1A2 - atpase, na+/k+ transporting, alpha 2 polypeptide, PLP1 - proteolipid protein 1, PTHLH - parathyroid hormone-like hormone, KCNA1 - potassium voltage-gated channel, shaker-related subfamily, member 1 (episodic ataxia with myokymia), ZC3H12A - zinc finger ccch-type containing 12a, GJD2 - gap junction protein, delta 2, 36kda, GJC2 - gap junction protein, gamma 2, 47kda, HTR1A - 5-hydroxytryptamine (serotonin) receptor 1a, g protein-coupled, GRIK4 - glutamate receptor, ionotropic, kainate 4, HTR1D - 5-hydroxytryptamine (serotonin) receptor 1d, g protein-coupled, FOSL1 - fos-like antigen 1, PTN - pleiotrophin, FJX1 - four jointed box 1 (drosophila), NTRK2 - neurotrophic tyrosine kinase, receptor, type 2, FAS - fas cell surface death receptor, CXCL13 - chemokine (c-x-c motif) ligand 13, SLC17A8 - solute carrier family 17 (vesicular glutamate transporter), member 8, ADORA1 - adenosine a1 receptor, TNC - tenascin c, PTPRC - protein tyrosine phosphatase, receptor type, c, SLC12A4 - solute carrier family 12 (potassium/chloride transporter), member 4, KLF10 - kruppel-like factor 10, INHBB - inhibin, beta b]
GO:1902107	positive regulation of leukocyte differentiation	9.17E-04	1.03E-01	3.31	16231	138	355	10	[ZFP36L1 - zfp36 ring finger protein-like 1, RHOH - ras homolog family member h, CSF1 - colony stimulating factor 1 (macrophage), ITPKB - inositol-trisphosphate 3-kinase b, RIPK2 - receptor-interacting serine-threonine kinase 2, PTPRC - protein tyrosine phosphatase, receptor type, c, ANXA1 - annexin a1, DUSP10 - dual specificity phosphatase 10, KLF10 - kruppel-like factor 10, CD83 - cd83 molecule]
GO:0090279	regulation of calcium ion import	9.21E-04	1.03E-01	6.53	16231	35	355	5	[CCL2 - chemokine (c-c motif) ligand 2, CXCL12 - chemokine (c-x-c motif) ligand 12, CAV3 - caveolin 3, RAMP3 - receptor (g protein-coupled) activity modifying protein 3, PDGFRB - platelet-derived growth factor receptor, beta polypeptide]
GO:0048247	lymphocyte chemotaxis	9.21E-04	1.02E-01	6.53	16231	35	355	5	[CCL20 - chemokine (c-c motif) ligand 20, CCL2 - chemokine (c-c motif) ligand 2, CYP7B1 - cytochrome p450, family 7, subfamily b, polypeptide 1, CKLF - chemokine-like factor, CXCL13 - chemokine (c-x-c motif) ligand 13]
GO:0071774	response to fibroblast growth factor	9.21E-04	1.02E-01	6.53	16231	35	355	5	[ZFP36L1 - zfp36 ring finger protein-like 1, CCL2 - chemokine (c-c motif) ligand 2, TNC - tenascin c, IL8 - interleukin 8, CPS1 - carbamoyl-phosphate synthase 1, mitochondrial]
GO:1901698	response to nitrogen compound	9.55E-04	1.05E-01	1.8	16231	812	355	32	[NFKB1 - nuclear factor of kappa light polypeptide gene enhancer in b-cells 1, RNF175 - ring finger protein 175, FOLR1 - folate receptor 1 (adult), HSPA5 - heat shock 70kda protein 5 (glucose-regulated protein, 78kda), NFKBIA - nuclear factor of kappa light polypeptide gene enhancer in b-cells inhibitor, alpha, CAV3 - caveolin 3, ID1 - inhibitor of dna binding 1, dominant negative helix-loop-helix protein, HERPUD1 - homocysteine-inducible, endoplasmic reticulum stress-inducible, ubiquitin-like domain member 1, SOX9 - sry (sex determining region y)-box 9, KLF15 - kruppel-like factor 15, COL4A1 - collagen, type iv, alpha 1, COLEC12 - collectin sub-family member 12, CXCL12 - chemokine (c-x-c motif) ligand 12, RGS10 - regulator of g-protein signaling 10, ITGA2 - integrin, alpha 2 (cd49b, alpha 2 subunit of vla-2 receptor), FOSL1 - fos-like antigen 1, IFIT1 - interferon-induced protein with tetratricopeptide repeats 1, PTN - pleiotrophin, RIPK2 - receptor-interacting serine-threonine kinase 2, VCAM1 - vascular cell adhesion molecule 1, NTRK2 - neurotrophic tyrosine kinase, receptor, type 2, CPS1 - carbamoyl-phosphate synthase 1, mitochondrial, ZFP36L1 - zfp36 ring finger protein-like 1, RAMP3 - receptor (g protein-coupled) activity modifying protein 3, KCNJ8 - potassium inwardly-rectifying channel, subfamily j, member 8, TNFAIP3 - tumor necrosis factor, alpha-induced protein 3, ANXA1 - annexin a1, UCP3 - uncoupling protein 3 (mitochondrial, proton carrier), TICAM1 - toll-like receptor adaptor molecule 1, MGST1 - microsomal glutathione s-transferase 1, KLF10 - kruppel-like factor 10, INHBB - inhibin, beta b]
GO:0006955	immune response	9.60E-04	1.04E-01	1.82	16231	778	355	31	[GZMB - granzyme b (granzyme 2, cytotoxic t-lymphocyte-associated serine esterase 1), CFH - complement factor h, CCL2 - chemokine (c-c motif) ligand 2, PARP9 - poly (adp-ribose) polymerase family, member 9, RELB - v-rel avian reticuloendotheliosis viral oncogene homolog b, PTX3 - pentraxin 3, long, HERC5 - hect and rld domain containing e3 ubiquitin protein ligase 5, CCL20 - chemokine (c-c motif) ligand 20, BTN3A3 - butyrophilin, subfamily 3, member a3, IL8 - interleukin 8, ERAP2 - endoplasmic reticulum aminopeptidase 2, HLA-DMA - major histocompatibility complex, class ii, dm alpha, C5 - complement component 5, DDX60 - dead (asp-glu-ala-asp) box polypeptide 60, PARP14 - poly (adp-ribose) polymerase family, member 14, TRIM17 - tripartite motif containing 17, C7 - complement component 7, COLEC12 - collectin sub-family member 12, CXCL12 - chemokine (c-x-c motif) ligand 12, CSF1 - colony stimulating factor 1 (macrophage), RIPK2 - receptor-interacting serine-threonine kinase 2, CTSV - cathepsin v, FAS - fas cell surface death receptor, CXCL13 - chemokine (c-x-c motif) ligand 13, CXCL1 - chemokine (c-x-c motif) ligand 1 (melanoma growth stimulating activity, alpha), CXCL2 - chemokine (c-x-c motif) ligand 2, CXCL3 - chemokine (c-x-c motif) ligand 3, ANXA1 - annexin a1, TICAM1 - toll-like receptor adaptor molecule 1, SLC15A2 - solute carrier family 15 (oligopeptide transporter), member 2, CD83 - cd83 molecule]
GO:0010594	regulation of endothelial cell migration	9.80E-04	1.06E-01	3.07	16231	164	355	11	[RHOJ - ras homolog family member j, ATOH8 - atonal homolog 8 (drosophila), GADD45A - growth arrest and dna-damage-inducible, alpha, PTN - pleiotrophin, DCN - decorin, PTGS2 - prostaglandin-endoperoxide synthase 2 (prostaglandin g/h synthase and cyclooxygenase), HSPB1 - heat shock 27kda protein 1, ANXA1 - annexin a1, ZC3H12A - zinc finger ccch-type containing 12a, FGF1 - fibroblast growth factor 1 (acidic), CXCL13 - chemokine (c-x-c motif) ligand 13]
