# Supplementary material for: Effects of microgravity on human iPSC-derived neural organoids on the International Space Station
Source: Stem Cells Transl Med. 2024 Oct 23;13(12):1186–97. doi: 10.1093/stcltm/szae070 (PMC11631337; doi:10.1093/stcltm/szae070)
Supplement: szae070_suppl_Supplementary_Materials [file szae070_suppl_supplementary_materials.zip › R1Table S4 Transcription Factor Enrichment Analysis.pdf]

Table S4. Transcription Factor Enrichment Analysis

The most significant shared transcription binding sites among differentially expressed genes, suggesting co-regulation of groups of genes. In some cases, such as the REST binding sites, the transcription factor is repressive. The highest ranked 25 binding sites are listed, with the exception of the genes upregulated in dopaminergic organoids in LEO, which had only 14 significant sites.

**Cortical organoids: Transcription factor binding site enrichment: Genes that are higher in LEO than ground**		
**Rank**	**Transcription Factor**	**Hypergeometric p-value**
1	SALL4	0.003052
2	NFE2L2	0.02373
3	SUZ12	0.02696
4	REST	0.03635
5	ESR1	0.03924
6	FOXA1	0.0519
7	SUZ12	0.06062
8	NFIC	0.07004
9	FOXA2	0.07901
10	BHLHE40	0.08672
11	REST	0.09509
12	RELA	0.1189
13	EGR1	0.1313
14	MYC	0.1396
15	TCF7L2	0.1418
16	SP1	0.1699
17	ZNF384	0.175
18	GATA2	0.1843
19	SOX2	0.185
20	GATA1	0.192
21	TCF3	0.1992
22	E2F1	0.2034
23	AR	0.2533
24	SIN3A	0.2607
25	SMC3	0.2709

**Cortical organoids: Transcription factor binding site enrichment: Genes that are lower in LEO than ground**		
**Rank**	**Transcription Factor**	**Hypergeometric p-value**
1	CEBPD	0.000002643
2	KLF4	0.00001281
3	TCF3	0.0002542
4	NELFE	0.0002565
5	SALL4	0.0003687
6	BCL3	0.0005716
7	ZBTB7A	0.002032
8	SUZ12	0.003086
9	RFX5	0.004793
10	UBTF	0.006349
11	HNF4A	0.007056
12	GATA2	0.007825
13	SIN3A	0.008691
14	TAF7	0.009739
15	GATA1	0.01007
16	TCF3	0.01195
17	CEBPB	0.01728
18	ZMIZ1	0.01994
19	ESR1	0.02054
20	RUNX1	0.02074
21	NFIC	0.02083
22	E2F6	0.02355
23	MYOD1	0.02487
24	SOX2	0.02509
25	ATF2	0.02836

**Dopaminergic organoids: Transcription factor binding site enrichment : Genes that are higher in LEO than ground**		
**Rank**	**Transcription Factor**	**Hypergeometric p-value**
1	REST	0.004137
2	REST	0.01957
3	SUZ12	0.06471
4	TCF3	0.1113
5	AR	0.1284
6	EZH2	0.1798
7	NANOG	0.2963
8	GATA2	0.3677
9	KLF4	0.4458
10	NFE2L2	0.4576
11	RAD21	0.5339
12	UBTF	0.6309
13	E2F6	0.8781
14	TAF1	0.8868

**Dopaminergic organoids: Transcription factor binding site enrichment : Genes that are lower in LEO than ground**		
**Rank**	**Transcription Factor**	**Hypergeometric p-value**
1	RELA	5.060e-7
2	AR	0.00002807
3	CEBPB	0.0001756
4	FOSL2	0.0008571
5	TP63	0.001605
6	ESR1	0.001827
7	SALL4	0.003759
8	NFIC	0.004819
9	TRIM28	0.006616
10	TP53	0.008973
11	NELFE	0.01019
12	MYOD1	0.01433
13	SMAD4	0.016
14	HDAC2	0.017
15	STAT3	0.01764
16	STAT3	0.01923
17	PPARD	0.02177
18	TCF3	0.02317
19	NFE2L2	0.02564
20	TRIM28	0.03026
21	FOXA2	0.03186
22	TCF3	0.04318
23	UBTF	0.06425
24	SUZ12	0.0791
25	HNF4A	0.09398
