# Supplementary material for: Effects of microgravity on human iPSC-derived neural organoids on the International Space Station
Source: Stem Cells Transl Med. 2024 Oct 23;13(12):1186–97. doi: 10.1093/stcltm/szae070 (PMC11631337; doi:10.1093/stcltm/szae070)
Supplement: szae070_suppl_Supplementary_Materials [file szae070_suppl_supplementary_materials.zip › R1Table S5 CT Radiation dose.pdf]

**Table S6:** Radiation Doses for Common CT Scans		
**Anatomical Location**	**Dose**	**Approximate Equivalent Background Radiation Exposure**
Belly and pelvis	10 mSv	3 years of background radiation
Colonography	6 mSv	2 years of background radiation
Head	2 mSv	8 months of background radiation
Spine	6 mSv	2 years of background radiation
Chest	7 mSv	2 years of background radiation
Lung cancer screening	1.5 mSv	6 months of background radiation
Coronary Angiography (CTA)	12 mSv	4 years of background radiation
Cardiac (calcium scoring)	1 mSv	1 year of background radiation
Source: https://www.webmd.com/cancer/radiation-doses-ct-scans		
