# Supplementary figures and images for: Effects of microgravity on human iPSC-derived neural organoids on the International Space Station
Source: Stem Cells Transl Med. 2024 Oct 23;13(12):1186–97. doi: 10.1093/stcltm/szae070 (PMC11631337; doi:10.1093/stcltm/szae070)

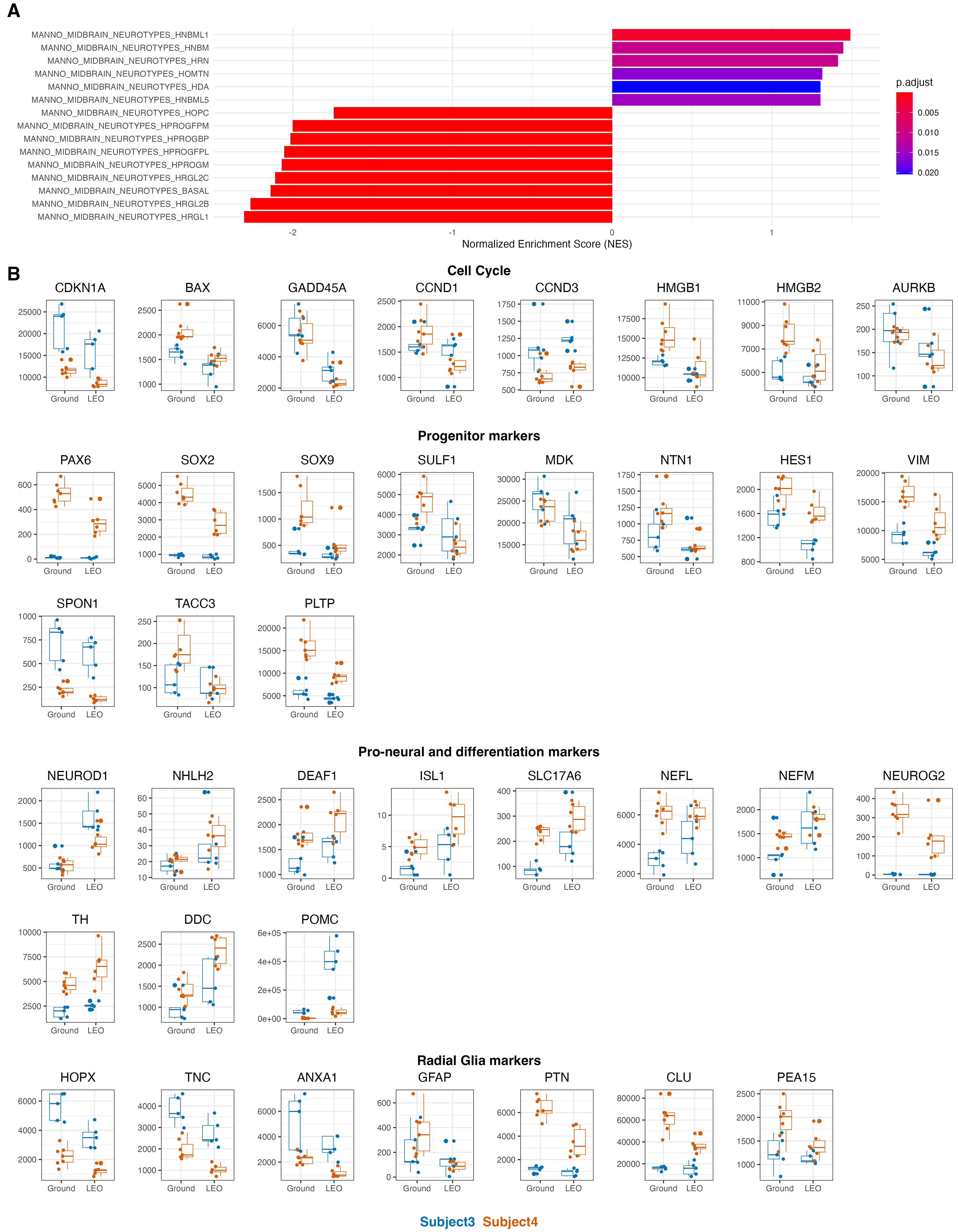

Supplement: szae070_suppl_Supplementary_Materials [file szae070_suppl_supplementary_materials.zip › R1FigureS3 Dopaminergic organoid gene set analysis and individual genes.tiff]

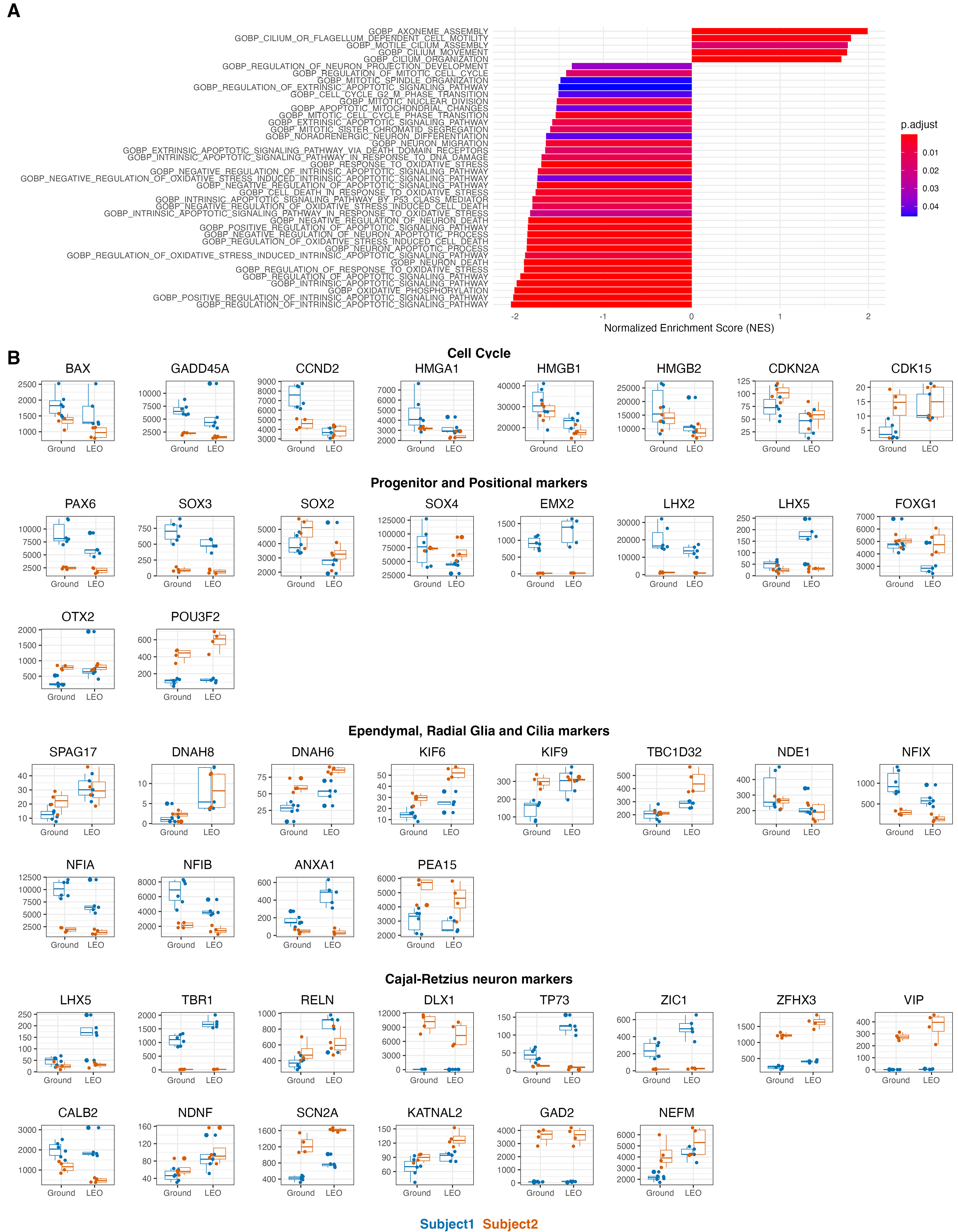

Supplement: szae070_suppl_Supplementary_Materials [file szae070_suppl_supplementary_materials.zip › R1FigureS4 Cortical organoid gene set analysis and individual genes.tiff]

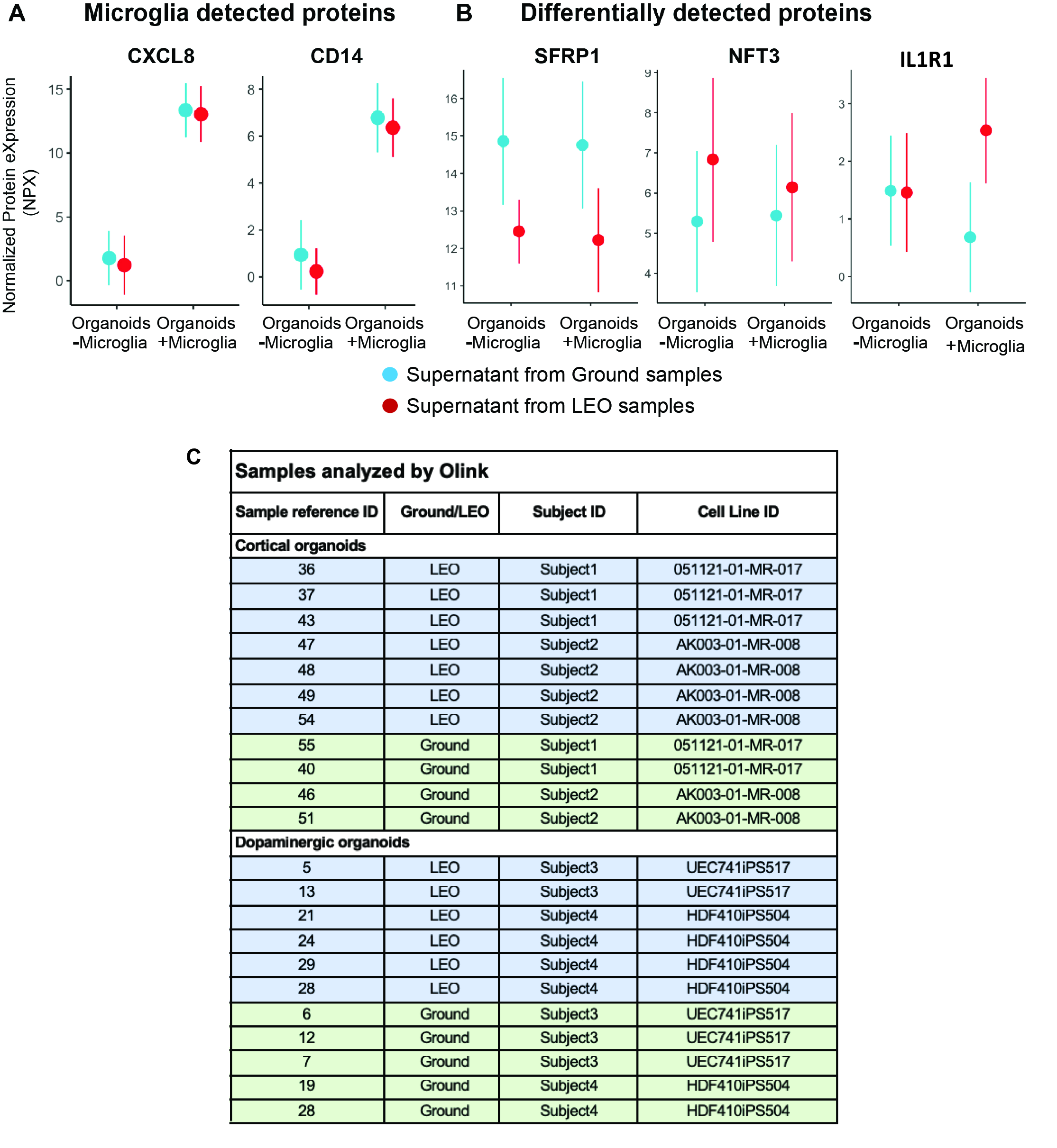

Supplement: szae070_suppl_Supplementary_Materials [file szae070_suppl_supplementary_materials.zip › R1FigureS5 Olink proteomic analysis.tif]

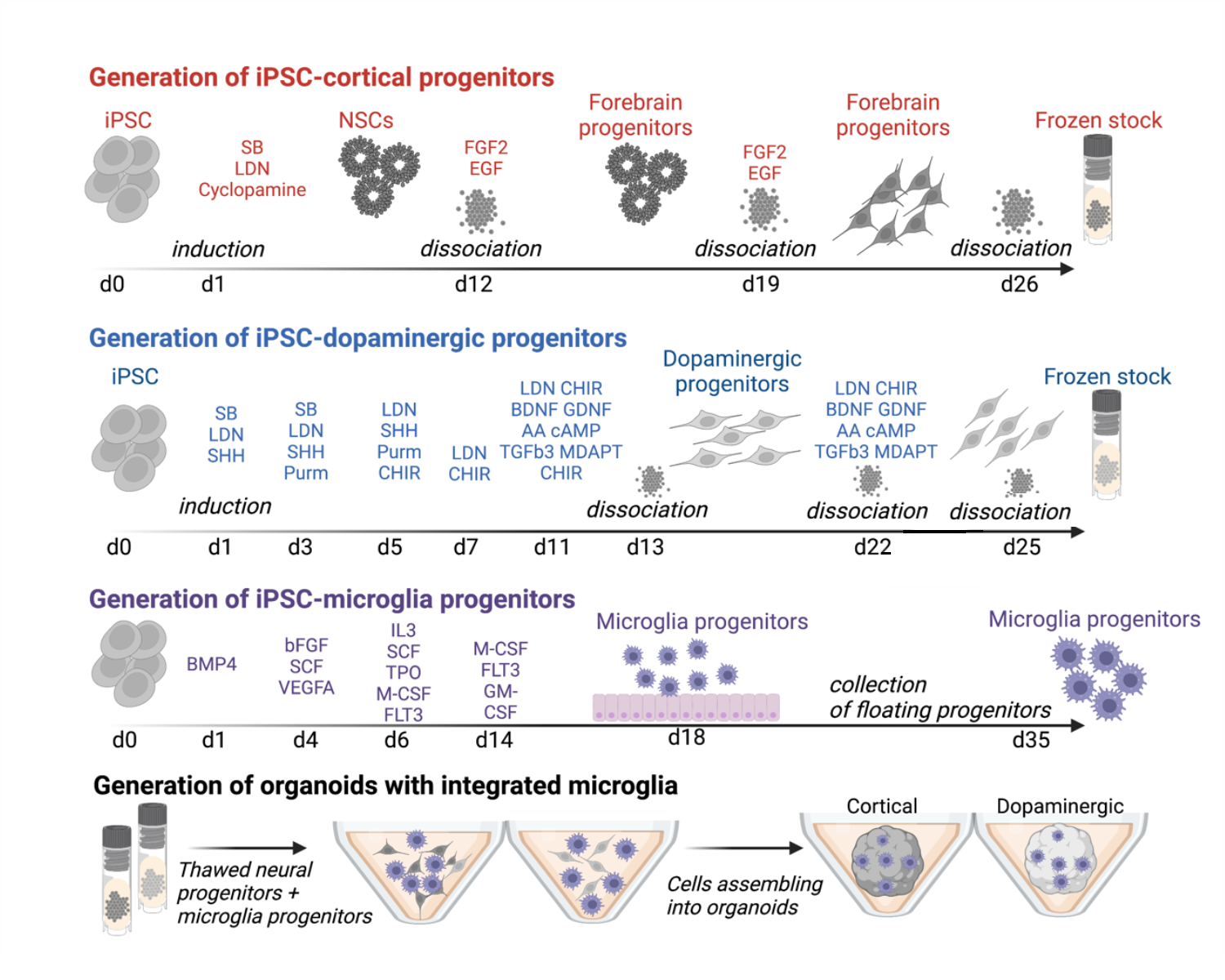

Supplement: szae070_suppl_Supplementary_Materials [file szae070_suppl_supplementary_materials.zip › R1FigureS1 Culture methods.tif]

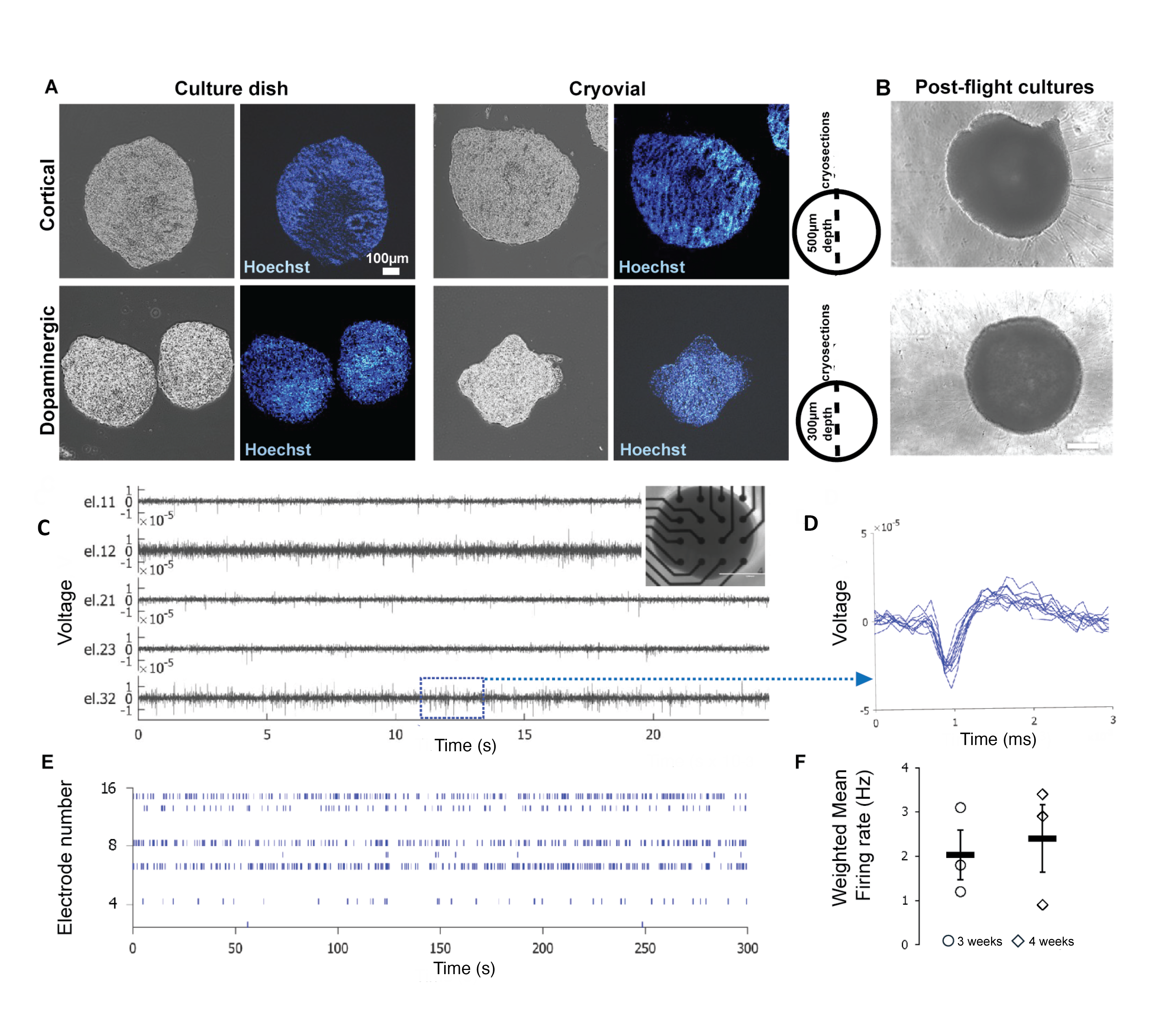

Supplement: szae070_suppl_Supplementary_Materials [file szae070_suppl_supplementary_materials.zip › R1FigureS2 Histology and electrophysiology of organoids.tif]
